# Holistic genome assembly and analysis of the *Tremella fuciformis* interaction community uncovers intergenomic insights beyond dual genomes

**DOI:** 10.3897/imafungus.17.185345

**Published:** 2026-06-15

**Authors:** Fengjiao Lin, Hualian Chen, Jingjing Ye, Huaizhen Xu, Hang Lin, Linyan Cen, Dingping Luo, Xiangzhen Chen, Huiying Hu, Ziyan Wang, Youjin Deng, Liping Deng

**Affiliations:** 1 College of Life Sciences, Fujian Agriculture and Forestry University, Fuzhou 350002, China College of Life Sciences, Fujian Agriculture and Forestry University Fuzhou China; 2 Tongjiang Tremella Research Institute, Bazhong 636713, China Tongjiang Tremella Research Institute Bazhong China; 3 Fujian Xiangyun Biotechnology Development Co., Ltd., Sanming 365100, China Fujian Xiangyun Biotechnology Development Co., Ltd. Sanming China

**Keywords:** Genome validation, genome-wide molecular marker, locus polymorphism, symbiotic fungi

## Abstract

*Tremella fuciformis* (*T. fuciformis*) is consistently found in association with *Annulohypoxylon stygium* (*A. stygium*) in natural environments. However, their interaction remains largely cryptic and requires a dedicated in situ sequencing approach for elucidation. Traditional genome sequencing and assembly yield genetic information for only one species at a time. In this study, the interacting community of *T. fuciformis* was sequenced as an integrated unit, obtaining three complete genomes in a single run, specifically two heterokaryotic genomes of *T. fuciformis* and one of *A. stygium*. Validated across four dimensions, these genomes showed excellent continuity, completeness, and accuracy. Interspecifically, the cell ratio of *T. fuciformis* to *A. stygium* was estimated at 1:1.09, and no genomic evidence supported DNA exchange through long-term symbiosis. Heterokaryotically, distinct chromosomal structural variations were observed between the core and accessory chromosomes of *T. fuciformis*, while internal transcribed spacer (ITS) fragment polymorphism indicated that single-locus ITS data may inadequately reflect genetic complexity. Using the community genome as molecular markers enabled strain identification and confirmed interactions. Overall, this study provides methods for studying interactive community genomes and their interspecific and internuclear connections.

## Introduction

Organisms coexisting for extended periods within a microecosystem frequently evolve unique symbiotic relationships (Bleiker and Six 2009). These ecological interactions can take the form of mutualism, commensalism, parasitism, and competition, ultimately giving rise to a distinctive interaction community (Mougi and Kondoh 2012). Such associations are found in a wide array of inter-kingdom combinations, including animal–plant, animal–microbe, and plant–microbe interactions, as well as intra-kingdom pairings (Bell and Adams 2011; Brunetti et al. 2018). These interactions are not only fundamental to organism survival, growth, and evolution (Stachowicz 2001) but also play a key role in maintaining stability and functionality (Raes and Bork 2008; Wagg et al. 2019). Consequently, understanding these complex interactions is essential for predicting ecological responses to environmental changes and for developing effective strategies for ecosystem management and conservation (Tylianakis et al. 2008; Peck 2011).

In recent years, rapid advances in genome sequencing and assembly technologies have revolutionized the field of genomics. The quality of individual genomes has progressively reached the chromosome level (Li and Durbin 2024; Liang et al. 2024; Xia et al. 2025), enabling more detailed and accurate genetic studies. Beyond facilitating a deeper understanding of genetic architecture and function, these advancements offer novel insights into evolutionary processes and organismal adaptation (Liao et al. 2022; Voorhies et al. 2022; Liang et al. 2024). Sequencing has emerged as a powerful tool for characterizing microbial communities from human and animal hosts, as well as a broad range of environmental niches (Dai et al. 2025). The application of high-throughput sequencing in these investigations has established metagenomics, a distinct field focused on analyzing the collective genomic repertoire of organisms living in a shared community (Goussarov et al. 2022). Metagenomic sequencing enables the analysis of entire microbial communities without the need for culturing individual species. This approach provides insights into the diversity, functions, and interactions of microorganisms across diverse environments, especially unculturable taxa that cannot be studied by traditional methods (Lu et al. 2025). However, assembling complete genomes from metagenomic sequencing data remains challenging, primarily due to the unknown complexity of microbial communities, including species number, composition, and interrelationships (Wei et al. 2021). Additionally, the low abundance of many microbial species in environmental samples prevents their whole-genome sequencing and assembly (Kim et al. 2025). These low-abundance species can also interfere with the assembly of genomes from more abundant species, affecting the accuracy and completeness of the assembled genomes (Yorki et al. 2023).

In an interaction community, when the participating individuals are present at relatively high cellular abundance, they can be reliably identified and quantified (Khadempour et al. 2012). As a result, such a community can serve as an experimental model, enabling the simultaneous assembly of complete genomes for all members (Wu et al. 2022). This approach could reduce the cost of genome sequencing and assembly. Moreover, it would enable the analysis of genome architecture interactions, both among homologous chromosomes within a species and between different individuals (Wang et al. 2020). To date, there have been no reports of simultaneous complete genome sequencing and assembly of all individuals within an entire interaction community *in situ*.

*Tremella fuciformis*, a basidiomycete fungus from the *Tremellaceae*, typically forms chrysanthemum-like or cockscomb-shaped fruiting bodies (Huang 1993; Li et al. 2014). These soft, white, translucent, and elastic structures are rich in tremella polysaccharides and bioactive compounds and nutrients, conferring significant culinary and medicinal value (Rui et al. 2025). In natural habitats, *T. fuciformis* primarily grows on decaying wood of broadleaf trees such as oaks, chestnuts, and maples in warm and humid subtropical to temperate regions. It is commonly found in forested areas of Sichuan, Yunnan, and Fujian in China, as well as in other parts of Asia, America, and Europe (Cheung 1996; Wu et al. 2019). Wild *T. fuciformis* fruiting bodies are frequently accompanied by the fungus *A. stygium* (Deng et al. 2016). This filamentous ascomycete, classified in the *Hypoxylaceae*, is renowned for its strong ability to decompose lignin and cellulose (Hsieh et al. 2005; Wingfield et al. 2018). While it has been suggested that *A. stygium* may provide nutritional support for *T. fuciformis*, the precise nature and extent of this interaction remain unclear (Liu et al. 2019). The genomes of the main cultivated variety of *T. fuciformis*, Tr01, and its associated *A. stygium* TJAS01 have been successfully sequenced. Notably, the heterokaryotic genome of *Tremella* has achieved a telomere-to-telomere chromosome-level assembly (Deng et al. 2023), providing an excellent reference resource for subsequent genomic and functional studies.

The primary objective of this study is to develop novel methodologies for studying interaction communities and their interrelationships, with a specific focus on the *T. fuciformis* interaction community. To achieve this goal, wild *T. fuciformis* YN01 collected from Yunnan was utilized as the initial strain, and a mixed substrate of *T. fuciformis* and *A. stygium* was employed for *in situ* PacBio circular consensus sequencing (CCS) and chromosome conformation capture (Hi-C) sequencing. Specifically, the aim was to assemble two complete genome sequences of the heterokaryotic *T. fuciformis* YN01 and a complete genome sequence of *A. stygium* YNAS01, which would be validated based on multiple criteria: completeness, base accuracy, heterokaryotic chromosome typing accuracy, and chromosome architecture correctness. Furthermore, the principal genetic organization interactions were analyzed both between the heterokaryotic genomes of *T. fuciformis* and between *T. fuciformis* and *A. stygium*. Ultimately, the entire *T. fuciformis* interaction community was considered a whole, and a comprehensive genome-wide molecular marker set was constructed, which facilitates the most accurate identification of the interaction community and its individual members. The findings are expected to fulfill the study objectives and provide a new technical framework for investigating interaction communities in similar research systems.

## Materials and methods

### Strain isolation and identification

The strains of *T. fuciformis* YN01 and its associated fungus *A. stygium* YNAS01 were isolated from natural habitats in Yunnan Province, China. The growth conditions of the samples were documented, and both the *T. fuciformis* fruiting bodies and their substrates were placed in sterile plastic boxes and promptly transported to the laboratory for further isolation and identification.

To mitigate potential contamination by exogenous microorganisms, mycelial isolation was performed on the day of sample collection. First, in a sterile laminar flow hood, the surface of the *T. fuciformis* fruiting bodies was disinfected by wiping with 75% ethanol. Following disinfection, the fruiting bodies were rinsed 2–3 times with sterile water and blotted dry with sterile filter paper. Using a sterile scalpel, tissue blocks (approximately 3 × 3 mm) were excised from the medullary mycelial region. Care was taken to exclude the surface and outer mycelium to minimize the risk of exogenous contamination. The tissue blocks were then inoculated onto Potato Dextrose Agar (PDA) medium and incubated at 25 °C in the dark. Upon the emergence of white mycelia from the tissue margins, the growing mycelial tips were transferred to fresh PDA plates. This serial subculturing was repeated 2–3 times until a stable pure culture of *T. fuciformis* mycelium was established.

For *A. stygium* isolation, pieces of wood containing mycelia were excised from the interface between the *T. fuciformis* fruiting body and the woody substrate. The wood pieces were cut into small blocks of approximately 3 × 3 mm and inoculated onto PDA plates. These were then incubated at 28 °C in the dark. Once white mycelium emerged from the wood block edges, mycelial tips were transferred to fresh PDA plates, with 2–3 subculturing rounds to obtain a pure culture of *A. stygium* mycelium.

To verify the symbiotic relationship between *T. fuciformis* and *A. stygium*, the pure culture mycelium of *A. stygium* was first inoculated onto a sawdust-based medium (formula: 76% sawdust, 20% wheat bran, 2% gypsum, 1.3% sucrose, 0.4% magnesium sulfate, and 0.3% urea) and incubated at 25 °C in the dark. After the *A. stygium* mycelium evenly covered the surface of the medium, the pure culture mycelium of *T. fuciformis* was inoculated under sterile conditions. Cultivation was maintained under identical conditions for 15 days to monitor the characteristic white clump structure (*i.e*., mixed mycelium, a structure formed via the symbiotic growth of *T. fuciformis* and *A. stygium*). Subsequently, the mixed mycelium obtained from the co-cultivation was inoculated onto the *T. fuciformis* cultivation medium already inoculated with *A. stygium* (formula: 10.0% wheat bran, 27.9% broadleaf sawdust, 1.0% corn powder, 0.5% sucrose, 0.2% MgSO_4_·7H_2_O, 0.4% CaSO_4_·2H_2_O, and the moisture content of the medium was adjusted to 60.0% with distilled water). The inoculated medium was placed in cultivation bottles with covers and incubated at 25 °C. After 20 days, the bottle covers were removed for fruiting induction. The bottles were maintained at 25 °C and 95% humidity for an additional 20 days to observe the formation of fruiting bodies, thereby verifying whether the co-cultivation of the two fungi could induce fruiting body formation. The mixed mycelial culture and its preserved strain are stored at the Fujian Edible Fungus Germplasm Resource Sharing Platform, maintained by the Mycological Research Center of Fujian Agriculture and Forestry University.

### DNA extraction and sequencing

The mixed mycelium samples of *T. fuciformis* and *A. stygium* were divided into six portions, rapidly frozen in liquid nitrogen, and ground into a fine powder. One sample underwent PacBio CCS and Hi-C sequencing, with all processes conducted by Novogene Bioinformatics Technology Co., Ltd. in Beijing, China. The PacBio CCS generated approximately 10 Gb of data, while the Hi-C sequencing produced approximately 4 Gb of data. The remaining five samples were subjected to Illumina HiSeq paired-end sequencing. Genomic DNA was extracted using the TIANGEN plant genomic DNA extraction kit. Novogene also managed the library construction and Illumina HiSeq sequencing, with each library yielding approximately 4 Gb of paired-end reads per sample.

### Assembly of holistic genome

To separate the mixed HiFi reads into species-specific datasets, a preliminary assembly of all reads was first performed using HIFIASM v0.25.0 (Cheng et al. 2021). The resulting contigs were then aligned using local BLAST v2.11.0 against the published genomes of *T. fuciformis* (GCA_032464415.1, GCA_032767855.1) and *A. stygium* (GCA_040126005.1) from NCBI. This alignment allowed for the classification of contigs into reference-guided clusters associated with either *T. fuciformis* or *A. stygium*. Based on these clusters, HiFi reads were sorted into species-specific sets for the genome assembly of each species. For *T. fuciformis*, HIFIASM v0.25.0 in Hi-C mode was used to generate two sets of contigs. The Hi-C data provided long-range interaction information, aiding in scaffold construction and validating chromosomal conformation. Telomeres were identified by manually searching for tandem repeat sequences TTA(G)_3–5_ or AAT(C)_3–5_ at the ends of contigs, which helped assess chromosome number and completeness. Contigs with telomeres at both ends were considered complete chromosomes. The ITS sequence of *T. fuciformis* CBS 6970 (GenBank accession: NR_155936.1) was retrieved from the nt database on the NCBI website and compared with the remaining contigs using local BLAST v2.11.0 to identify contigs carrying the rDNA region and exclude contigs that fall within the rDNA region. When two contigs carrying the rDNA region are found, they are considered connected, forming a new scaffold. If only one contig with the rDNA region is identified, the presence of rDNA at the chromosome end is determined by searching for HiFi reads that contain both rDNA repeat units and telomere repeat sequences. In addition to the nuclear genome assembly, circular contigs and low GC content contigs (significantly lower than the nuclear genome) were generated during the initial HIFIASM assembly process. Given that the GC content of mitochondrial genomes is significantly lower than that of nuclear genomes (typically < 30%), contigs with a GC content < 30% (including some circular contigs and other low GC linear contigs) were preselected as potential mitochondrial sequences. To confirm their mitochondrial origin and integrity, a BLASTn v2.11.0 alignment against the NCBI nucleotide database was performed to assess their homology with mitochondrial genes from closely related species. Subsequently, MITOS2 (Sheffield et al. 2010) online annotation was used to evaluate the completeness of protein-coding genes, rRNAs, and tRNAs, as well as to detect potential gene deletions, truncations, or frameshifts. The genome of *A. stygium* was assembled using the same methodology. The reference sequence for rDNA identification is *A. stygium* isolate TJAS01-V1 (GenBank accession: PP140388.1).

### Construction of phylogenetic tree

Protein sequences from *T. fuciformis* and *A. stygium*, as well as their closely related species, were downloaded from the NCBI database. Gene annotation was performed on *T. fuciformis* YN01 and *A. stygium* YNAS01. After annotation, single-copy orthologous genes were identified from all protein sequences of the tested strains using OrthoFinder v2.5.5 software. The protein sequences of each orthologous group were aligned using MAFFT v7.525 with the L-INS-i strategy. High-quality alignments were concatenated into a supermatrix, which was used to construct a phylogenetic tree via maximum likelihood (ML) using IQ-TREE v3.0.1. The ModelFinder Plus (MFP) module was used to automatically select the best-fit nucleotide or amino acid substitution models. *Cryptococcus neoformans* H99 and *Xylaria hypoxylon* DSM 108379 were used as outgroups for tree rooting, and branch support was evaluated using 1000 non-parametric bootstrap replicates.

### Estimation and comparative analysis of rDNA repeat copy number

The nuclear-encoded rDNA repeat unit in fungal genomes comprises the 18S, 5.8S, and 28S rRNA coding sequences, along with the intergenic spacer sequences ITS1 and ITS2. To clarify the structural composition of the rDNA repeat unit, the analysis was performed as previously described (Deng et al. 2023). Briefly, the rDNA repeat units of *T. fuciformis* and *A. stygium* were identified by first localizing the rDNA-bearing chromosomes using the ITS sequence of *T. fuciformis* CBS 6970 (GenBank accession: NR_155936.1) and *A. stygium* isolate TJAS01-V1 (GenBank accession: PP140388.1), followed by a self-BLAST search to extract sequences of uniformly sized fragments that occur in tandem repeats, from which the repeat units were ultimately obtained. Subsequently, the repeat unit was divided into 500 bp bins and subjected to BLAST searches against the NCBI rRNA/ITS database to confirm the functional regions. The copy number of the rDNA repeat unit was estimated by comparing its sequencing depth to that of single-copy sequences. The heterokaryotic genome rDNA repeat unit of *T. fuciformis* was analyzed comparatively using CLUSTAL OMEGA (https://www.ebi.ac.uk/Tools/msa/clustalo/) to investigate their overall differences. The ITS region within the rDNA repeat unit, defined by the universal primers ITS1 (5’-TCCGTAGGTGAACCTGCGG-3’) and ITS4 (5’-GCATATCAATAAGCGGAGGA-3’), is a molecular marker for fungal identification (Schoch et al. 2012). To confirm the accuracy of ITS region differences in the heterokaryotic genome of *T. fuciformis*, polymerase chain reaction (PCR) amplification was conducted with ITS1/ITS4 universal primers. The amplification products were sent to Sangon Biotech in Shanghai for Sanger sequencing, where the accuracy of variable sites was verified by examining the overlap of base signals in the sequencing chromatograms.

### Identification of mating type loci in *T. fuciformis*

In the *Tremellaceae* family, the SXI1 and SXI2 genes are crucial components located at the Homeodomain (HD) locus, while the pheromone/pheromone receptor (PR) locus comprises at least the *STE3*, *STE12*, *MFA1/2*, *CNB00600*, and *CNG04540* genes (Metin et al. 2010). Proteins encoded by these genes in *Tremella mesenterica* strain DSM 1558 were utilized as queries to identify the HD and PR loci in *T. fuciformis* through tBLASTn searches against the hapA and hapB genomes, respectively.

### Validation of genome assembly quality

The genome assemblies of *T. fuciformis* and its symbiotic partner *A. stygium* were evaluated in four key properties: genome completeness, chromosomal conformation, base-level assembly accuracy, and dikaryotic genome phasing. Genome completeness was assessed using two parallel methods. First, the BUSCO v. 5.2.2 (Simão et al. 2015) evaluation was conducted using *Tremellomycetes* (tremellomycetes_odb10) and *Sordariomycetes* (sordariomycetes_odb10) databases for *T. fuciformis* and *A. stygium*, respectively. Second, HiFi reads were aligned to the assembled genome, and the number of unmapped reads was calculated to evaluate the completeness of the genome assembly. For chromosomal conformation analysis, Hi-C data were processed using HIC-PRO v. 2.10.0 (Servant et al. 2015), and chromatin interaction matrices were visualized with HICPLOTTER (Akdemir and Chin 2015) to verify scaffold continuity and identify potential misjoins through overlapping interaction domains. Base-level accuracy of the corrected assemblies was assessed with Inspector to quantify base errors and estimate overall assembly precision. In the heterokaryotic cells of *T. fuciformis*, the nuclei are physically separated, resulting in each chromosome exhibiting significantly stronger Hi-C contact signals with chromosomes from the same nucleus compared to those from a different nucleus (Deng et al. 2023). The NUCLEARPHASER (Duan et al. 2022) program was utilized to assign all chromosomes to two distinct haploid genomes, thereby enabling the validation of dikaryotic genome phasing.

### Single nucleotide polymorphism (SNP) and structural variant (SV) detection

HiFi reads of *T. fuciformis* were aligned to the hapA genome using PBMM2 (available at https://github.com/PacificBiosciences/pbmm2), with the reference genome concurrently indexed using SAMTOOLS (Li et al. 2009). Variant calling was performed using DeepVariant, adhering to best-practice workflows, and variants with quality scores below 30 (QUAL < 30) and depth of coverage below 10 (DP < 10) were filtered out to ensure accuracy. SNP density was calculated using the ‘vcftools –SNPdensity’ command (Danecek et al. 2011), and visualizations were generated using an online platform (https://www.bioinformatics.com.cn). Whole-genome alignments between hapA and hapB of *T. fuciformis* were conducted using MUMMER v. 4.x (Delcher et al. 2003). Low-quality alignments were excluded using the ‘delta-filter’ command with parameters -m -i 90 -l 1000 to maintain high-quality data. SVs were identified using the ‘show-diff’ command, and their lengths and chromosomal distributions were statistically analyzed to provide insights into genomic variations.

### Analysis of TE-mediated SVs

To explore the TE-mediated SVs in the *T. fuciformis* genome, an integrated, multi-step annotation workflow was implemented following widely accepted TE annotation standards. First, de novo TE detection was performed using a combination of EDTA (Su et al. 2021) and REPEATMODELER2 (Flynn et al. 2020) with the -LTRStruct option. For EDTA, in addition to using the data of *T. fuciformis* strain YN01, genome data from another 16 *T. fuciformis* strains were also utilized to construct a more comprehensive and species-specific TE library (Zhang et al. 2026), enabling accurate identification of LTR retrotransposons and DNA transposons.

To adhere to the 80-80-80 rule for TE classification (Wicker et al. 2007), the identified TE sequences were clustered at 80% sequence identity using CD-HIT-EST to remove redundancy while preserving biologically meaningful TE families. The resulting species-specific library was used as the custom library for homology-based whole-genome annotation by REPEATMASKER (Chen 2004) for whole-genome TE annotation. Finally, BEDTOOLS v. 2.27.1 was used to identify overlapping regions between SVs and annotated TEs, allowing quantification of TE contributions to SV formation.

### Validation of mosaic sequences in *T. fuciformis* and *A. stygium*

Mosaic sequences refer to chimeric reads containing fragments from both *T. fuciformis* and *A. stygium* within a single read, as identified by screening HiFi reads against both reference genomes. These sequences of 500 bp upstream and downstream of the junction regions were extracted for primer design. Specific primers were designed using NCBI Primer-BLAST with the following settings: primer length of 18–20 bp, annealing temperature of 58–62 °C, and GC content of 40–60%. The forward primer was positioned in the 500 bp region upstream of the junction site, while the reverse primer was located in the downstream region (Suppl. material 2: table S1). PCR products were analyzed by 1% agarose gel electrophoresis. If a distinct band was observed, the PCR product was subjected to Sanger sequencing to verify the presence of a mosaic sequence. Conversely, the absence of amplification indicated that the mosaic sequence was not present.

### Construction of whole-genome molecular markers for *T. fuciformis* interaction communities

To establish a systematic molecular marker framework for the *T. fuciformis* interaction community, the high-quality genomes of *T. fuciformis* (hapA and hapB) and its symbiotic partner *A. stygium* were integrated. First, the reference genomes of *T. fuciformis* YN01 and *A. stygium* YNAS01 were divided into contiguous, non-overlapping 100 bp sliding windows. To eliminate windows with abnormal depth caused by highly repetitive sequences or sequencing randomness, windows with read depths more than 50% above or below the genome-wide median were excluded, resulting in a set of highly reliable, effective windows.

Next, three independent Illumina resequencing datasets generated in this study from the same *T. fuciformis* YN01 and *A. stygium* YNAS01 community were aligned to the effective window set using BWA-MEM v. 0.7.17. Windows that were completely consistent with the resequencing data were marked as “Pass,” while those with any nucleotide discrepancies were marked as “Fail.” For the “Fail” windows, variant detection was performed using GATK HAPLOTYPECALLER v. 4.x (do Valle et al. 2016), followed by strict quality control filtering (QUAL > 30, DP ≥ 10) to identify reliable variant sites and their types.

## Results

### *Tremella fuciformis* interaction community as a study system for holo-genome construction

**Figure 1.**
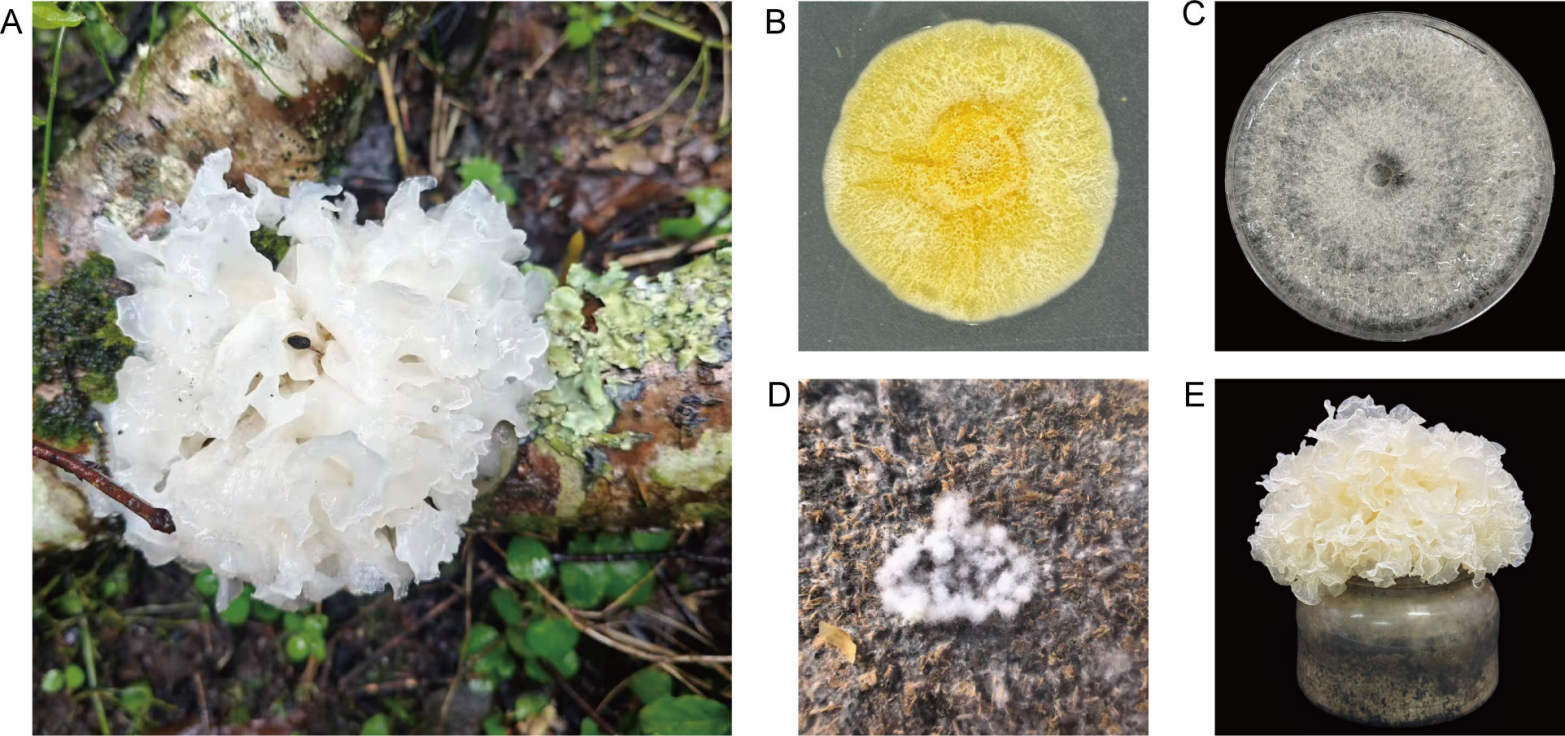
Morphological and cultural characteristics of *T. fuciformis* YN01 and its symbiotic partner *A. stygium* YNAS01. **A** Wild basidiocarp of *T. fuciformis* growing on fallen hardwood logs in its natural habitat. **B** Front view of the colony of *T. fuciformis* strain on PDA medium (25 °C for 18 days). **C** Colony morphology of *A. stygium* strain on PDA medium (28 °C for 7 days). **D** Mycelial growth status of *T. fuciformis* when co-cultured with its symbiotic partner *A. stygium*. **E** Basidiocarp of *T. fuciformis* obtained through artificial domestication and cultivation.

*T. fuciformis* YN01 and its symbiotic fungus *A. stygium* YNAS01 were isolated from natural habitats in Yunnan Province, China. The wild fruiting bodies of *T. fuciformis* YN01 were milky white to translucent and gelatinous, possessing a soft and resilient texture. Mature specimens exhibited a multi-lobed, caespitose structure, with a surface featuring wrinkles and a reticulate pattern. The fruiting bodies were thin and adhered to the surface of branches, with their edges spreading outward. The *A. stygium* specimen was also collected from this region, at the junction between the *T. fuciformis* fruiting body and its woody substrate (Fig. 1A).

The morphological characterization revealed that the *T. fuciformis* YN01 mycelium was fluffy and yellowish-white. The colony exhibited a bright pale yellow center that gradually faded toward the margins (Fig. 1B). The mycelium grew densely and uniformly, exhibiting a dense, woolly texture typical of this species. These observations are consistent with the documented phenotypic traits of *T. fuciformis*, specifically its characteristic centrifugal color gradient and robust aerial hyphae (Wang et al. 2026). In contrast, the mycelium of *A. stygium* YNAS01 was initially white but subsequently secreted melanin, which pigmented the medium to a deep black (Fig. 1C). *A. stygium* YNAS01 also emitted a slight, characteristic rose-like aroma during cultivation, which is consistent with previous reports on the species (Tong et al. 2024). These morphological and biochemical traits confirmed the identification of both species. Subsequent co-culture experiments further validated the symbiotic relationship between *T. fuciformis* YN01 and *A. stygium* YNAS01 mycelia (Fig. 1D). The two mycelia formed characteristic white clump structures, and successful fruiting body development was observed under controlled cultivation conditions (Fig. 1E).

**Figure 2.**
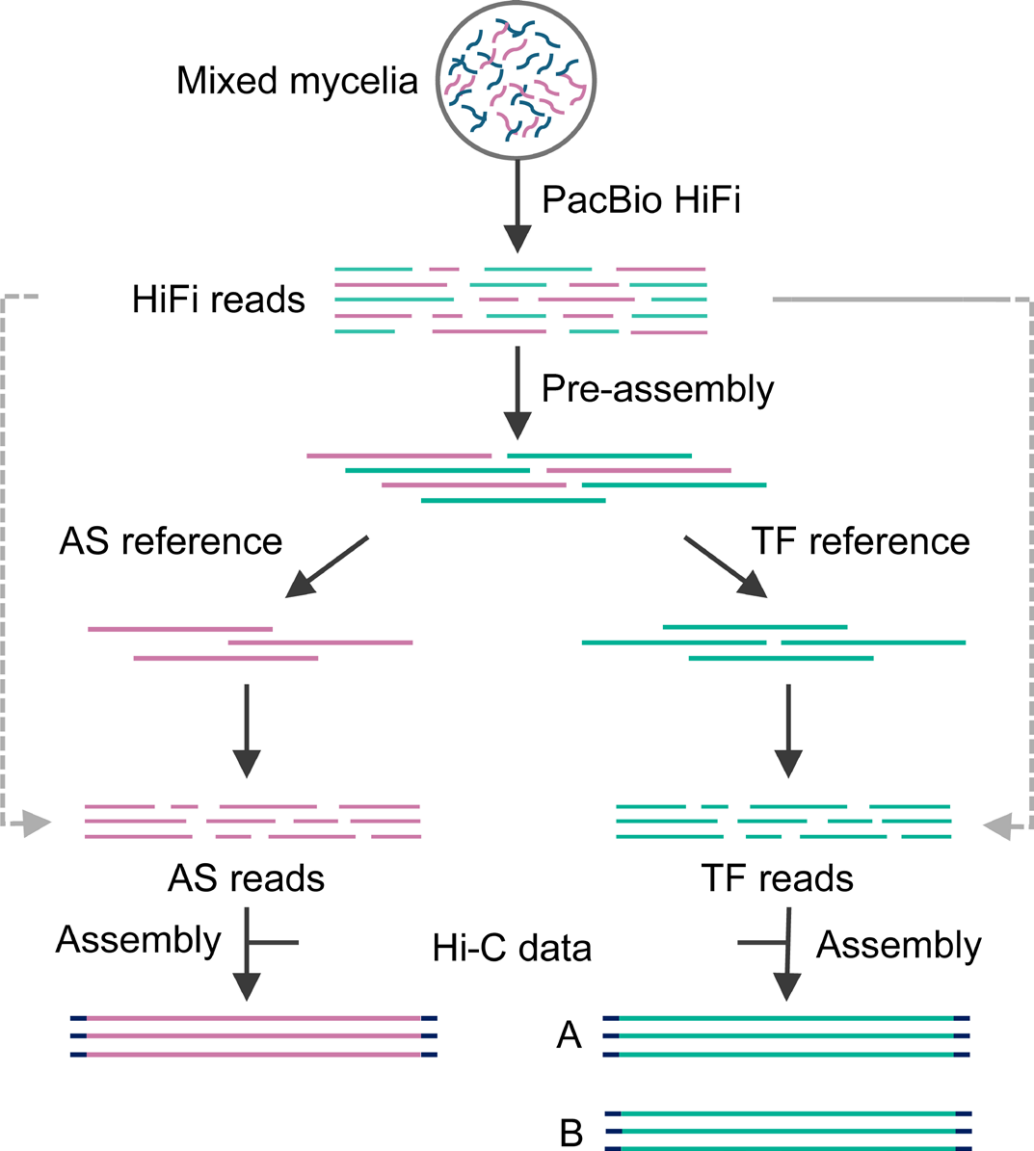
Holistic genome assembly strategy for the *T. fuciformis* interaction community. The TF for *T. fuciformis* and AS for *A. stygium*.

*A. stygium* is a symbiotic fungus of *T. fuciformis*, and they belong to distinct taxonomic groups, specifically the phyla *Ascomycota* and *Basidiomycota*, respectively. This substantial taxonomic divergence results in a limited overlap of sequences between their genomes. In an innovative approach, whole-genome sequencing and assembly were performed on their mixed mycelia, successfully obtaining the complete genome sequences of *A. stygium* and *T. fuciformis* in a single experiment. The mixed mycelia of *A. stygium* and *T. fuciformis* from an interaction community were used as the sample, and PacBio CCS long-read sequencing was employed to obtain high-quality HiFi reads. Initially, these reads were pre-assembled using HIFIASM to generate mixed contigs. The published genomes of *A. stygium* and *T. fuciformis* were used as references for local BLAST comparisons to distinguish the contigs belonging to each species. Subsequently, species-specific reads for *A. stygium* and *T. fuciformis* were extracted from the original sequencing database through local BLAST comparisons, using these contigs as references. These specific reads were then independently assembled, ultimately resulting in the construction of a complete genome for *A. stygium* and two complete haploid genomes for *T. fuciformis* (Fig. 2).

A total of 1,097,604 HiFi reads were generated. The initial assembly produced 322 contigs. Of these, 52 contigs were assigned to *T. fuciformis* YN01 and 248 contigs to *A. stygium* YNAS01 based on alignment to reference genomes (Suppl. material 3). These two sets of contigs were then used as species-specific references to partition the raw reads, resulting in 426,414 reads (38.85%) for *T. fuciformis* and 671,190 reads (61.15%) for *A. stygium*. *T. fuciformis* reads were assembled into two haplotype assemblies, hapA with 36 contigs and hapB with 25 contigs. Within hapA, three contigs exhibited a distinctly low GC content below 40% compared with the remaining contigs, which had an average GC content of 54%. BLAST searches confirmed these three contigs were of mitochondrial origin and were further assembled into a single circular mitochondrial DNA molecule. Nine contigs carried telomeric sequences at both ends and were considered complete chromosomes. Contigs (h1tg000004l and h1tg000007l) contain a telomere at one end and the rDNA sequences at the other. Since each haplotype genome harbors one rDNA region, these two contigs were considered sequences flanking the rDNA region and were joined into a chromosome. Twenty-one short contigs (<60 kb) were composed of tandem arrays of more than two rDNA repeat units or their fragments, indicating that they represent portions of the rDNA region. The remaining contig (h1tg000001l) carried a telomeric sequence only at its 3’ end. Sequence reads mapped to the 5’ end of the contig were extended twice (overlaps > 10 kb), ultimately extending to the telomere. As a result, hapA of YN01 comprises 11 telomere-to-telomere (T2T) chromosomes and a circular mitochondrial sequence. hapB was assembled into 11 chromosomes from 25 contigs using hapA as a reference. A similar assembly strategy was applied to *A. stygium* YNAS01, where HIFIASM in Hi-C mode generated 16 contigs that were organized into 10 T2T chromosomes and a circular mitochondrial sequence.

To further accurately verify the taxonomic status of the wild-collected Tremella fuciformis and its symbiotic fungus, after complete genome assembly, a phylogenetic tree was constructed using single-copy orthologous genes from the whole genome. The results showed that *T. fuciformis* YN01 clustered with *T. fuciformis* (Suppl. material 1: fig. S1), while *A. stygium* YNAS01 clustered with *A. stygium*, showing clear divergence from closely related species (Suppl. material 1: fig. S2). Combining morphological characteristics, *T. fuciformis* YN01 and *A. stygium* YNAS01 were confirmed as *T. fuciformis* and *A. stygium*, respectively.

**Figure 3.**
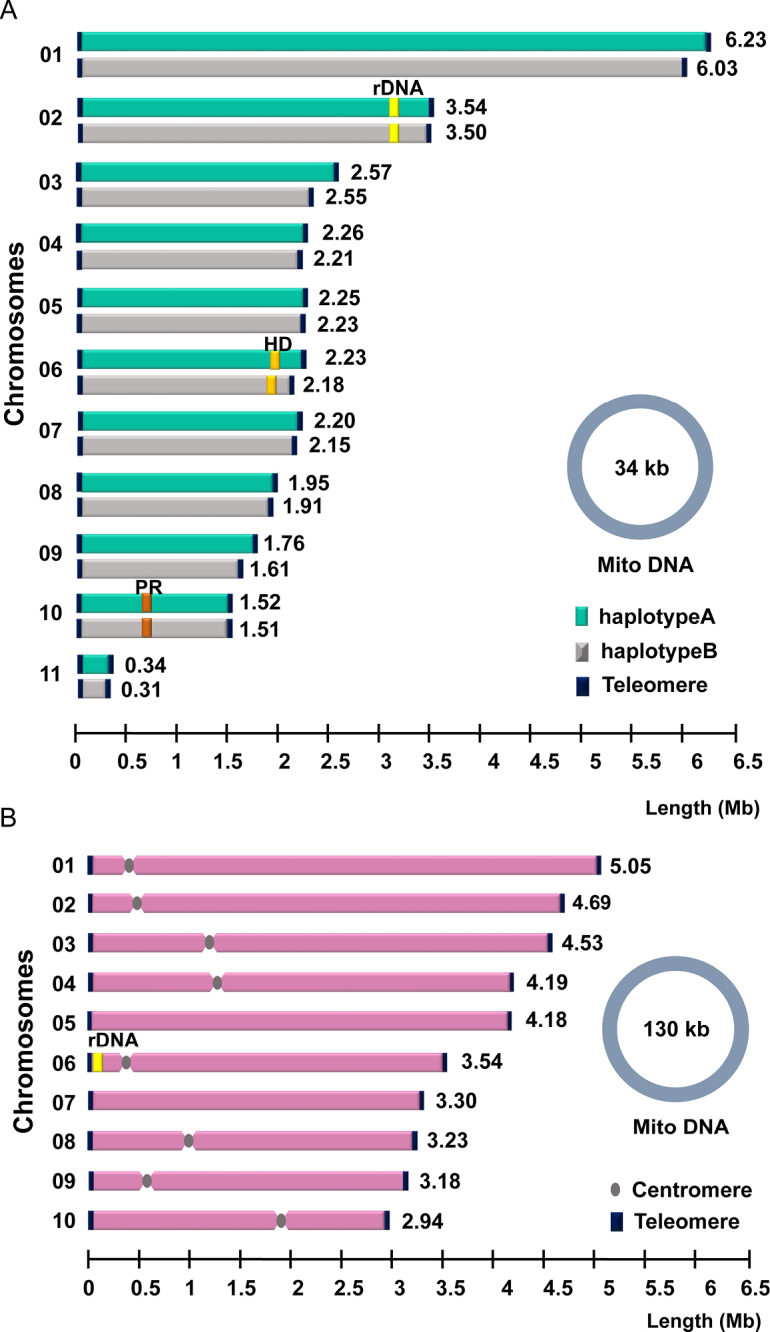
Genomic structures of *T. fuciformis* YN01 and *A. stygium* YNAS01. **A** Chromosomes 01–11 of *T. fuciformis* are shown for haplotype A (hapA, teal) and haplotype B (hapB, gray), with telomeric regions indicated in navy blue. The rDNA region, homeodomain (HD), and pheromone/pheromone receptor (PR) loci are highlighted in yellow, orange, and dark orange, respectively. The circular structure represents the mitochondrial DNA sequence. **B** Chromosomes 01–10 of *A. stygium* are shown in pink, with telomeres in navy blue and centromeres as gray dots. The rDNA region is highlighted in yellow. The circular structure represents the mitochondrial DNA sequence.

The hapA genome of *T. fuciformis* YN01 spans 26.75 Mb, with chromosome sizes ranging from 0.34 to 6.23 Mb (average 2.43 Mb), whereas the hapB genome spans 26.30 Mb, with chromosome sizes from 0.31 to 6.03 Mb (average 2.39 Mb). Each chromosome terminus contains 20–40 tandem telomeric repeats (TTAGGGGG), and the two chromosome sets exhibit a high degree of overall symmetry. The mating-type A (homeodomain, HD) locus was located on Chr06A (1,966,725–1,968,956 bp) and Chr06B (1,968,510–1,970,855 bp), each spanning approximately 2 kb. The mating-type B (pheromone/receptor, P/R) locus was located on Chr10A (752,919–772,435 bp) and Chr10B (777,380–794,939 bp), with a size of approximately 20 kb. Neither type of the mating locus showed genetic linkage between hapA and hapB, consistent with findings in fungal sexual reproduction and mating-type loci (Sun et al. 2025), indicating that *T. fuciformis* is a tetrapolar species. In addition, the mitochondrial genome of *T. fuciformis* YN01 is 34 kb, comprising 14 conserved protein-coding genes, two rRNA genes (rns and rnl), and 26 tRNA genes (Fig. 3A). The genome of *A. stygium* YNAS01 spans 38.83 Mb and comprises 10 chromosomes, with lengths ranging from 2.94 to 5.05 Mb. Each chromosome terminus contains 20–40 tandem telomeric repeats (TTAGGG). While centromere positions could not be inferred for *T. fuciformis* by Hi-C signals, strong intra-centromeric interactions were observed for all chromosomes of *A. stygium*. Based on these interaction patterns, centromere locations were predicted; the centromere of Chr07 is situated near the telomeric region, whereas the centromeres of the remaining chromosomes are all positioned toward one side of the chromosomes (Suppl. material 1: fig. S3). The mitochondrial genome is a 130 kb circular molecule, comprising 14 conserved protein-coding genes, two rRNA genes (rns and rnl), and 29 tRNA genes (Fig. 3B).

A single *T. fuciformis* cell contains two nuclear genomes and one mitochondrial genome, with a total genomic size of 53.10 Mb. In contrast, a single *A. stygium* cell harbors one nuclear genome and one mitochondrial genome, amounting to a total genomic size of 38.43 Mb. PacBio HiFi sequencing of the mixed mycelia generated 1.84 × 10^10^ bp of data, of which 7.13 × 10^9^ bp originated from *T. fuciformis* and 1.12 × 10^10^ bp from *A. stygium*. The viable cell ratio of *T. fuciformis* to *A. stygium* in the mixed sample was estimated at approximately 1:1.09 based on the ratio of sequencing yield to genome size.

**Figure 4.**
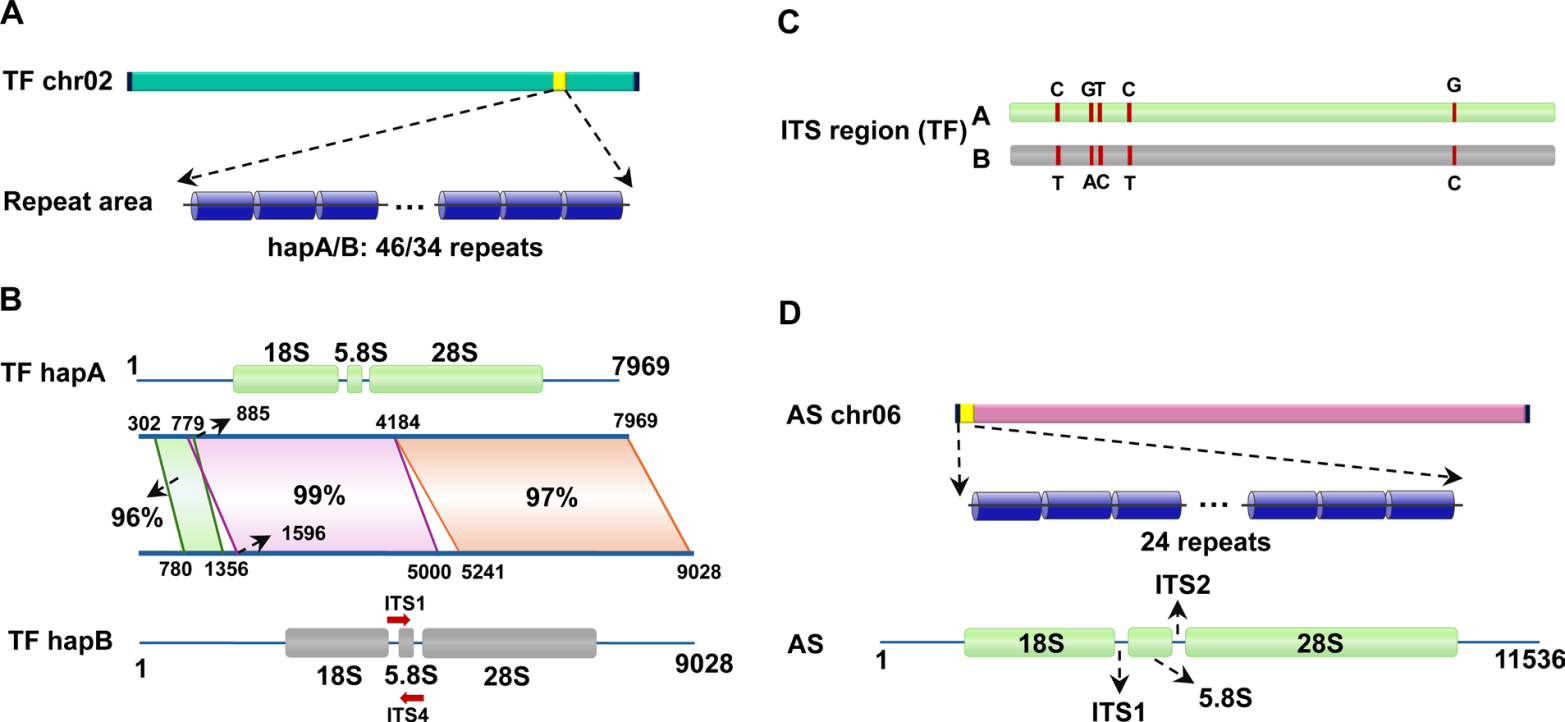
Schematic representation of the rDNA region in *T. fuciformis* YN01 (TF) and *A. stygium* YNAS01 (AS). **A** Organization of the rDNA region in the *T. fuciformis* genome. The rDNA repeat unit is shown in dark blue. **B** Structural comparison of rDNA repeat units between *T. fuciformis* hapA and hapB. **C** Nucleotide polymorphisms within the universal ITS1/ITS4 primer region between *T. fuciformis* hapA and hapB. **D** Organization and structural comparison of the rDNA region in the *A. stygium* genome. The rDNA repeat unit is shown in dark blue.

*T. fuciformis* contains two nuclei, each with a single rDNA region composed of tandemly repeated rDNA repeat units. In hapA and hapB, these regions were located on Chr02A (2.71–3.01 Mb) and Chr02B (2.67–2.98 Mb), positioned 781 kb and 787 kb from the 3’ end, respectively. Due to the excessive length of the rDNA regions, which precluded full assembly using PacBio HiFi reads, their copy number was estimated by calculating the sequencing depth ratio between the rDNA repeat unit and single-copy genomic regions, enabling the inference of complete sequence information. Analysis of the assembled sequences revealed that the rDNA region of hapA consisted of 46 tandem repeats of a 7,969 bp unit, while hapB contained 34 repeats of 9,028 bp (Fig. 4A, Suppl. material 4). Each repeat unit in both haplotypes harbored the 18S, 5.8S, and 28S rRNA genes, as well as the ITS1 and ITS2 spacer regions. Within the hapB repeat unit, three segments (780–1356 bp, 1596–5000 bp, and 5241–9028 bp) exhibited high similarity to the corresponding regions in hapA, with sequence identity of 96%, 99%, and 97%, respectively (Suppl. material 5), and three insertions (478 bp, 240 bp, and 241 bp) accounted for its greater length (Fig. 4B). The fragment within the rDNA repeat unit, defined by the universal primers ITS1 and ITS4, is a molecular marker for fungal identification. This fragment is 474 bp in length in both hapA and hapB (Suppl. material 6), with five single-nucleotide differences located at positions 17, 30, 35, 68, and 365, accounting for 1.1% of the total sequence (Fig. 4C, Suppl. materials 1: fig. S4, 7). In contrast to *T. fuciformis*, the rDNA region of *A. stygium* was located at the 5’ end of Chr06, adjacent to the telomere, and consists of 24 tandem repeats of an 11,536 bp unit (Fig. 4D).

### Validation of the final assembly in the *T. fuciformis* interaction community

**Figure 5.**
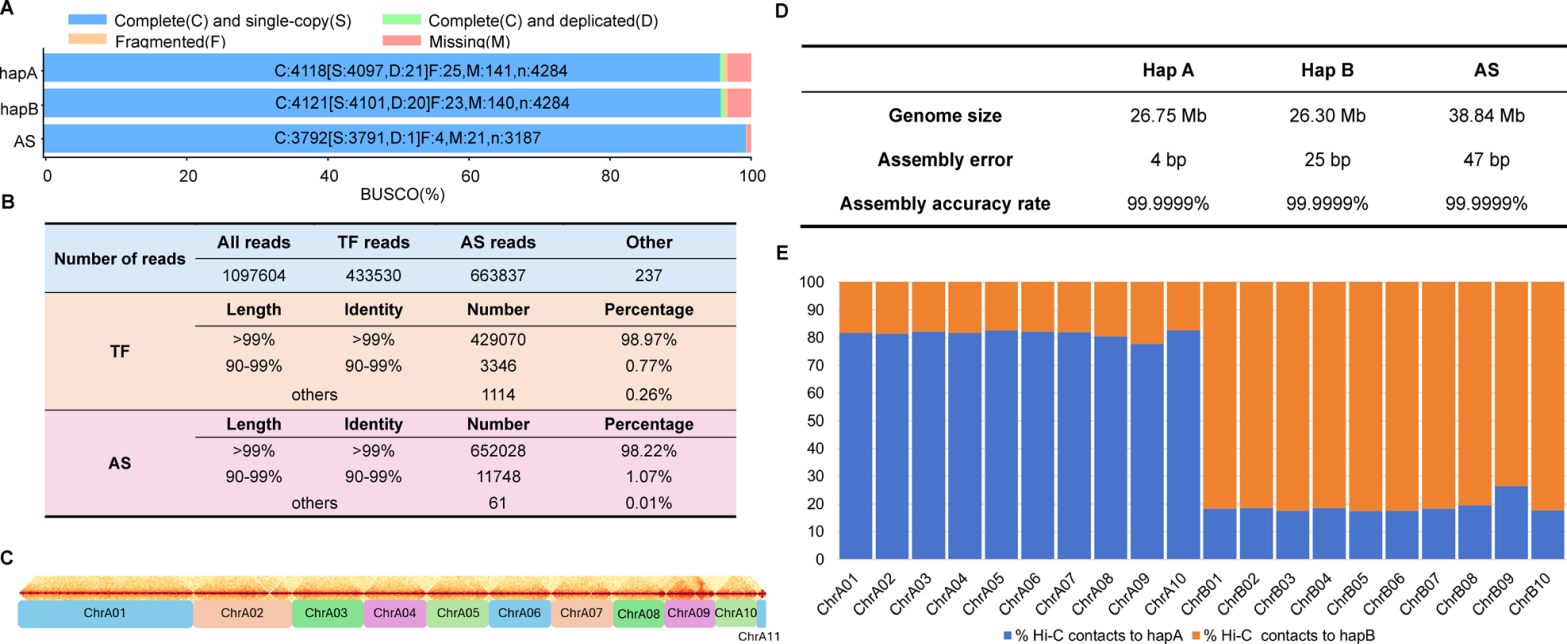
Evaluation of the final genome assemblies. **A** BUSCO assessment. **B** Read mapping statistics, showing the number of reads and their length–identity distribution for TF and AS assemblies. **C** The Hi-C contact heatmap across chromosomes (Chr01A–Chr11A) for hapA of TF. **D** Assembly metrics, including genome size, number of base-pair errors, and overall accuracy for hapA, hapB (TF), and AS. **E** Genome assembly of TF was supported by Hi-C analyses. *Y*-axis, percentage of Hi-C contacts to haplotype A (dark orange) and B (dark blue) of each assembled chromosome. The pair of Chr11 is almost identical except for some structural variations (SVs), resulting in irregular Hi-C contacts. The data of this chromosome pair were not included in this plot. The TF for *T. fuciformis* YN01 and AS for *A. stygium* YNAS01.

To comprehensively validate the assembly quality of *T. fuciformis* and its symbiotic partner *A. stygium*, genome completeness, chromosomal architecture, base-level accuracy, and nuclear haplotype phasing were assessed. For genome completeness, both *Tremellomycetes* (tremellomycetes_odb10) and *Sordariomycetes* (sordariomycetes_odb10) BUSCO databases were employed. The two nuclear genomes of *T. fuciformis* contained 95.6% and 95.7% complete single-copy genes, whereas *A. stygium* achieved 99.3%, values comparable to those of other high-quality fungal genomes (Fig. 5A) (Simão et al. 2015). Consistently, read mapping revealed that 1,081,098 reads (98.5%) exhibited both coverage and concordance above 99%, and an additional 15,094 reads (1.38%) exceeded 90%, with one or both metrics falling between 90% and 99%. Manual inspection confirmed that these deviations were attributable to the CCS correction process. A total of 1,175 reads (0.10%) mapped to multiple genomic loci, yet no breakpoint-associated duplications were detected, suggesting sequencing artifacts. Only 237 reads (0.02%) aligned to *Drosophila* or bacterial genomes, likely resulting from minor contamination or residual adapter sequences (Fig. 5B).

For chromosomal architecture, Hi-C interaction maps demonstrated continuous and intact contact patterns across all chromosomes of *T. fuciformis* haplotypes (hapA and hapB), as well as *A. stygium*, without detectable structural breaks, confirming the correctness of the chromosome-level assemblies (Fig. 5C, Suppl. material 1: fig. S5).

Base-level accuracy was evaluated using Inspector. After correction, the numbers of erroneous bases in the genomes of *T. fuciformis* hapA, hapB, and *A. stygium* were reduced to just 4, 25, and 47, respectively, corresponding to an overall assembly accuracy exceeding 99.9999% (Fig. 5D).

Finally, in the heterokaryotic genome of *T. fuciformis*, the two nuclei are physically separated, and Hi-C crosslinking occurs only between chromatin regions within the same nucleus, as it depends on spatial proximity. Consequently, interaction signals were markedly stronger between chromosomes of the same nucleus than between those of different nuclei. Using NUCLEARPHASER (Duan et al. 2022), the 22 chromosomes were partitioned into two groups, each containing 11 chromosomes that corresponded precisely to the hapA and hapB karyotypes, indicating accurate separation of the dikaryotic genome (Fig. 5E).

### Comprehensive analysis of *T. fuciformis* heterokaryotic genome variations and absence of interspecific gene flow

**Figure 6.**
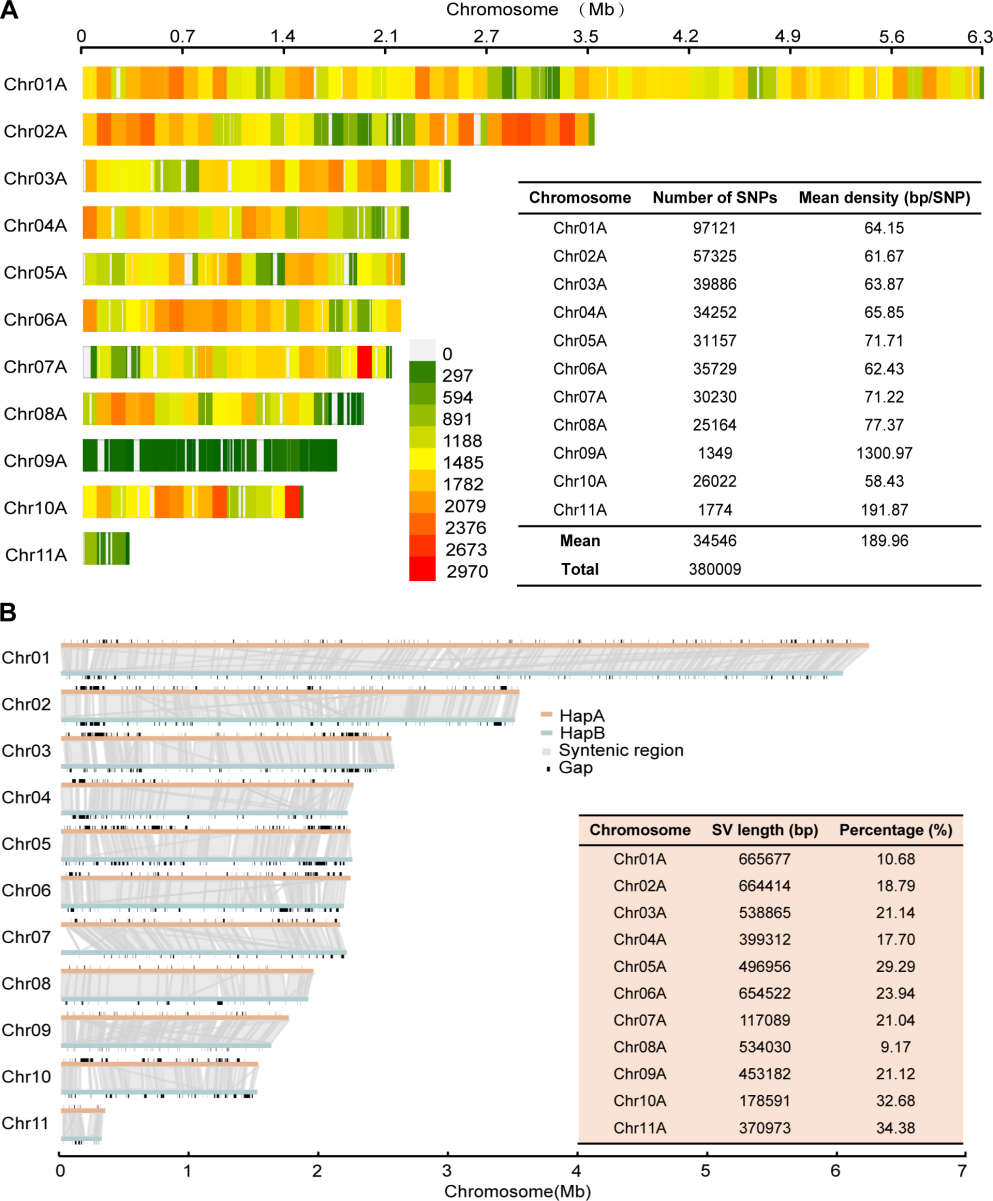
Genome variation analyses of the heterokaryotic genome in *T. fuciformis* YN01. **A** Distribution and density of SNPs across chromosomes (Chr01A–Chr11A) of *T. fuciformis* hapA. Colors indicate SNP density per 100 kb window, ranging from low (green) to high (red). The table summarizes the total number of SNPs and their mean densities (bp/SNP) for each chromosome. **B** Comparison of hapA and hapB assemblies showing syntenic regions and gaps across homologous chromosomes. The table lists total SV length and their proportion relative to chromosome size.

Using the hapA genome of *T. fuciformis* as a reference, total PacBio HiFi reads were aligned to detect single nucleotide polymorphisms (SNPs), insertions/deletions (InDels), and structural variations (SVs), thereby characterizing genomic differences between the two nuclei. In total, 380,009 SNPs/InDels were identified, corresponding to an average density of 1 per 189.96 bp. The density varied markedly across chromosomes, ranging from 1 per 1,300.97 bp on Chr09A to 1 per 58.43 bp on Chr10A (Fig. 6A). Among the SNPs, transitions (A↔G, C↔T) accounted for 62.70%, whereas transversions (A↔C, A↔T, G↔C, G↔T) comprised 37.30%. A total of 1,146 SVs were detected, ranging from 52 to 75,125 bp in size, with a combined length of 5,073,611 bp, representing 18.96% of the hapA genome. Among them, 928 SVs (80.98%) were associated with transposable elements (TEs), predominantly long terminal repeat (LTR) retrotransposons (Suppl. material 2: table S2). The distribution of SVs was highly heterogeneous among chromosomes. Chr01A harbored the largest number of SVs (219), with a cumulative length of 665,445 bp (10.68% of its length), whereas Chr11A contained the fewest (37) but exhibited the highest proportional coverage, with 117,089 bp accounting for 34.38% of its length (Fig. 6B). These results indicate that TEs are the primary drivers of genomic divergence between the two nuclei of *T. fuciformis*, as evidenced by the predominance of TE-associated SVs (80.98%) and the distinct SV distribution patterns across chromosomes.

In the hapA genome of *T. fuciformis* YN01, nine chromosomes (Chr01A–Chr08A and Chr10A) exhibited strong synteny with the core chromosomes of the Tr01 genome and were therefore equally classified as core chromosomes. In contrast, no syntenic segments were detected for Chr09A (1.76 Mb) or Chr11A (0.34 Mb) in comparison with the Tr01 genome (Suppl. material 1: fig. S6). Analysis of molecular features revealed that Chr09A and Chr11A had GC contents of 55.8% and 54.9%, respectively, significantly lower than the average observed for the core chromosomes (57.0%, *p* = 0.040) (Suppl. material 2: table S3). Gene densities were also markedly reduced, with 32.8 and 29.7 genes per 100 kb, respectively, compared with an average of 37.3 genes per 100 kb in the core chromosomes (*p* = 0.036) (Suppl. material 2: table S4). Conversely, repetitive sequences accounted for 8.6% and 7.1% of Chr09A and Chr11A, respectively, significantly higher than the 3.3% average of the core set (*p* = 0.036) (Suppl. material 2: table S5). Notably, neither chromosome contained any *Basidiomycota* BUSCO genes. Collectively, these features are consistent with the typical characteristics of accessory chromosomes, and thus Chr09A and Chr11A were classified accordingly. It is noteworthy that SNP density on Chr09A (1 per 1,300.97 bp) and Chr11A (1 per 191.87 bp) was significantly lower than that of the other chromosomes (1 per 66.3 bp, *p* = 0.036) (Fig. 6A). In contrast, structural variations accounted for 21.12% and 34.38% of Chr09A and Chr11A, respectively, both exceeding the average proportion observed in the core chromosomes (20.21%) (Fig. 6B). The identification of core and accessory chromosomes aligns well with recent genome-wide analyses in *T. fuciformis* (Zhang et al. 2026), demonstrating that core chromosomes maintain conserved essential functions, whereas accessory chromosomes are enriched with transposable elements and exhibit extensive structural variation, consistent with a rapidly evolving ‘accessome’ involved in adaptive evolution.

**Figure 7.**
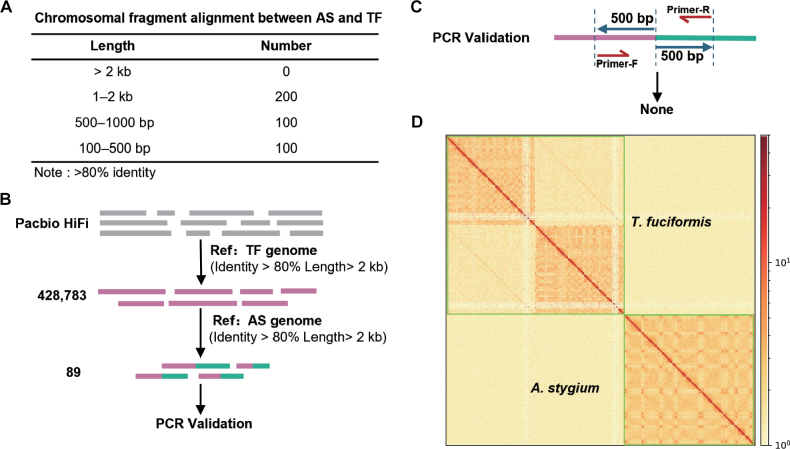
Chromosomal alignment and genomic similarity analysis indicating the absence of genetic exchange between *T. fuciformis* YN01 and *A. stygium* YNAS01. **A** Size–distribution of aligned chromosomal fragments between *A. stygium* (AS) and *T. fuciformis* (TF). **B** Workflow of chromosomal fragment alignment validation. **C** Schematic diagram of primer design and the result of PCR validation. **D** Genomic similarity heatmap between *T. fuciformis* and *A. stygium*.

In the natural environment, *T. fuciformis* and *A. stygium* coexist in a symbiotic relationship. To investigate whether there is any genetic material exchange between their genomes, a comparative genomic analysis was conducted, and segments with sequence identity greater than 80% were identified. In total, 200 segments in the 1–2 kb range were identified, along with 100 segments each in the 500–1000 bp and 100–500 bp ranges (Fig. 7A). These segments showed 82.1–94.4% identity and were confined to the rDNA regions of both species. Thus, they represent universally conserved ribosomal sequences, not interspecific genetic exchange (Suppl. material 8). Given the absence of long high-similarity segments (>2 kb), the genomes of *T. fuciformis* and *A. stygium* were used as reference sequences to screen PacBio HiFi reads, identifying 89 putative interspecies chimeric reads (Fig. 7B, Suppl. material 9). These reads had different breakpoints and were composed of sequences from both species (Suppl. material 10). To verify the authenticity of these chimeric signals, eight sequences were randomly selected for PCR validation using primers designed upstream and downstream of the breakpoints (Fig. 7C), but all results were false positives (Suppl. material 1: fig. S7), likely due to sequencing errors, indicating no horizontal genetic material exchange between *T. fuciformis* and *A. stygium*. Additionally, Hi-C collinearity heatmaps showed no significant interaction hotspots between the two genomes (Fig. 7D), suggesting that there is no stable physical connection or interaction of genetic material.

### Genome-wide construction of effective molecular markers for the *T. fuciformis* interaction community

**Figure 8.**
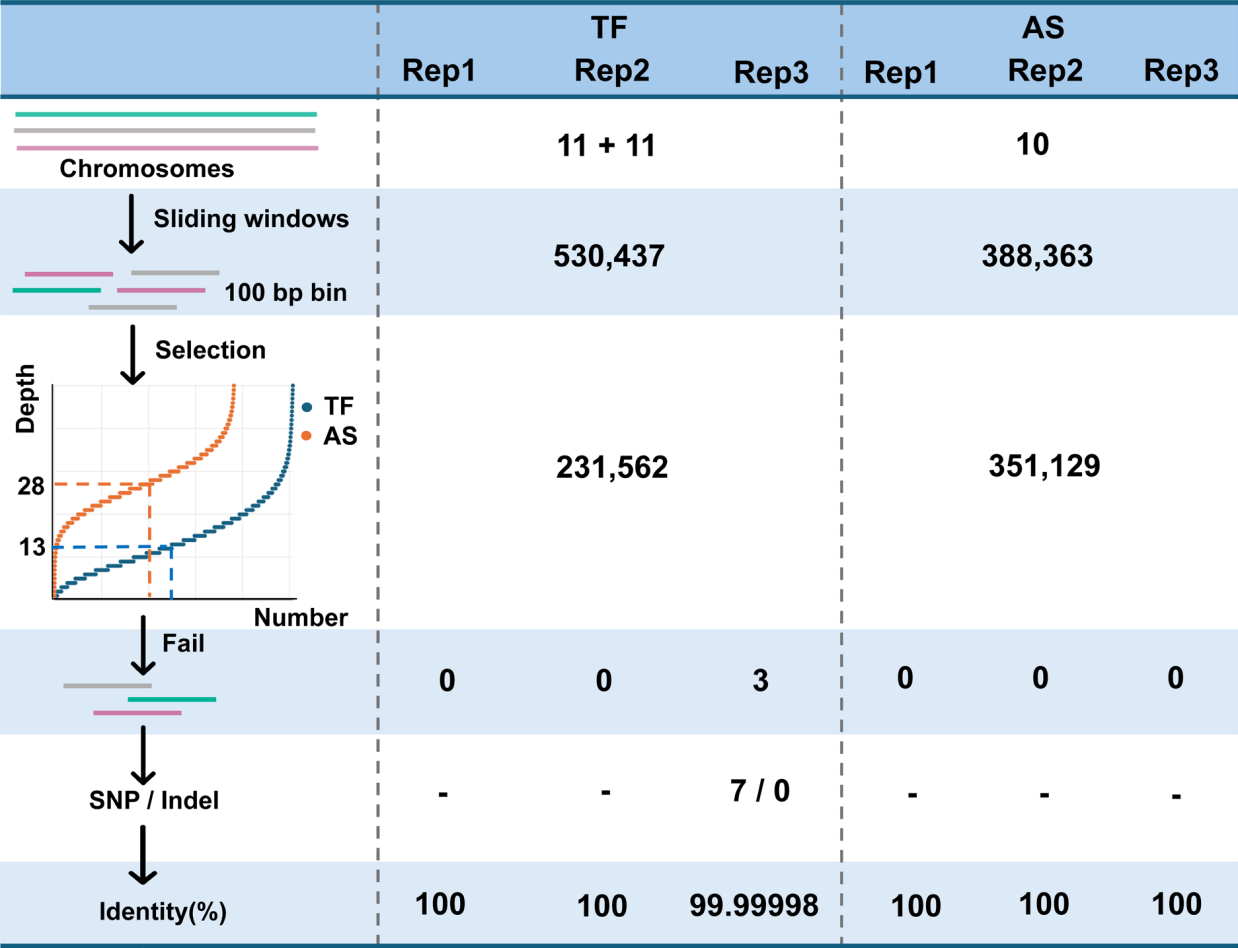
Workflow for interaction community genome molecular marker construction.

A comprehensive genome-wide molecular marker for the *T. fuciformis* interaction community was established. The three assembled genomes were partitioned into non-overlapping windows, and unreliable regions were filtered based on sequencing depth. To validate the marker reliability, three independent Illumina resequencing datasets generated from the same interaction community of *T. fuciformis* YN01 and *A. stygium* YNAS01 were employed for validation. The results showed that the *T. fuciformis* genome yielded 530,437 initial windows, of which 231,562 were effective. In the three validation datasets, only three windows in the third dataset were marked as “Fail” (with a total of seven SNPs detected), maintaining an overall consistency of 99.9999% with the effective windows. The *A. stygium* genome produced 388,363 initial windows, with 351,129 being effective, and all effective windows were marked as “Pass” (Fig. 8). A series of high-quality alignment sets were generated. Based on these results, the study proposes using a 99.9% sequence similarity threshold as an empirical cutoff to differentiate between different *T. fuciformis* interaction communities. Consequently, a comprehensive genome-wide molecular marker set was constructed, covering 231,562 effective windows for *T. fuciformis* and 351,129 for *A. stygium*. This marker system can be used not only for the precise identification of target strains but also to further determine whether *T. fuciformis* and *A. stygium* form an interaction pair.

The construction strategy of this genome-wide molecular marker system provides a methodological reference for developing similar markers in other fungal species. For strains with pre-constructed markers, evaluating the genetic relatedness of unknown strains to the reference strains requires only low-cost Illumina resequencing, avoiding costly *de novo* genome assembly and significantly reducing costs for large-scale applications. Furthermore, these markers enabled precise discrimination of compatibility groups within the *T. fuciformis* symbiotic system. Analysis of 16 strains revealed three distinct pairing groups based on specificity with *A. stygium*, with successful pairing occurring within, but not between, groups (Zhang et al. 2026). These markers allow simultaneous identification of both partners and determination of their interaction compatibility, facilitating targeted screening of compatible strain pairs for breeding. Furthermore, crossing strains from different groups and analyzing marker inheritance in offspring enables localization of pairing control loci, which can identify accessory chromosomes associated with symbiotic specificity, laying a foundation for understanding the molecular mechanisms of *T. fuciformis* and *A. stygium* interactions.

## Discussion

Metagenomic sequencing circumvents the limitations of traditional cultivation methods, allowing for comprehensive recovery and analysis of genetic material from entire microbial communities. This approach significantly enhances understanding of their diversity, functions, and ecological interactions (Zhou et al. 2022; Dai et al. 2025). In this study, a single PacBio CCS and Hi-C sequencing run of the *T. fuciformis* interaction community yielded three complete, high-quality genome assemblies. Bulk community sequencing preserved the natural composition of the heterokaryotic *T. fuciformis* (hapA and hapB) and co-occurring *A. stygium* genomes, enabling reliable quantification of both intra- and interspecific genomic variation. By maintaining the natural genomic context, this approach provided an efficient means of assessing interspecific genetic relationships, efficiently detecting short homologous regions between partners and simplifying downstream validation. Compared with multi-step isolate-based workflows, it minimized technical artifacts such as DNA loss or cross-contamination, particularly critical for fragile fungal tissues. These findings highlight the utility of a rapid, cost-effective strategy for generating high-resolution genomic profiles of symbiotic communities.

Conventional metagenomic analysis typically relies on *de novo* assembly and binning to reconstruct metagenome-assembled genomes (MAGs). However, these approaches are designed for highly diverse communities containing thousands of species. In contrast, this study focused on a simplified symbiotic system comprising only *T. fuciformis* and *A. stygium*, allowing the use of high-quality reference genomes for accurate read assignment and variant detection. Inaccurate taxonomic binning of assembled contigs may result in chimeric MAGs, which contain contigs derived from multiple species (Kayani et al. 2021; Zhou et al. 2022). To improve the accuracy of assembly, the published genomes were used as templates during the binning process. Subsequently, the reads extracted after comparing the binned sequences with the original reads were used for final assembly. For given interaction communities, reference-dependent binning is more effective in recovering taxon bins from hybrid read data RE (Beghini et al. 2021; Blanco-Míguez et al. 2023; Heumos et al. 2024). Another crucial aspect of the metagenomic assembly strategy was the use of a hybrid assembly method combining HiFi and Hi-C sequencing. Metagenomic studies primarily reliant on second-generation short-read sequencing (SRS) face inherent limitations in resolving identical or highly similar genomic regions (Maguire et al. 2020; Gehrig et al. 2022; Heidrich and Beule 2022). Third-generation sequencing technologies, notably PacBio HiFi (Circular Consensus Sequencing) and Oxford Nanopore Technologies (ONT), overcome these constraints by generating long reads that unambiguously span complex genomic architectures (Gehrig et al. 2022; Nurk et al. 2022; Albertsen 2023). While ONT can produce ultra-long reads (>2 Mb), its traditionally higher error rates have been mitigated by recent advancements (e.g., R10.4.1 flow cells achieving ~99% raw accuracy) (Sereika et al. 2022). Nevertheless, cost barriers currently restrict ONT’s large-scale application in metagenomics. The results demonstrate that HiFi- and Hi-C-based metagenomic assembly provides accurate and complete genomes for the *T. fuciformis* interaction community. A recent comparative analysis of MAG assemblies utilizing Illumina, ONT, and HiFi sequencing technologies revealed that HiFi-based metagenomic assembly outperforms the others (Deng et al. 2025). HiFi metagenomic sequencing represents an effective approach for obtaining high-quality genomes (Nurk et al. 2022). When combined with emerging technologies, such as Hi-C employed in this study, its utility in metagenomics research can be further amplified (Regmi et al. 2025).

Beyond achieving a T2T chromosome-level assembly, a comprehensive and systematic framework for evaluating fungal genome assembly quality was established using *T. fuciformis* and its symbiotic partner *A. stygium* as a model. This framework integrates four complementary dimensions, including genome completeness, chromosomal architecture, base-level accuracy, and nuclear haplotype phasing, to provide an unbiased and quantitative assessment of assembly integrity. Compared with conventional completeness- or mapping-based evaluations (Cissé and Stajich 2019), this approach offers a higher-resolution, multilayered validation pipeline that distinguishes genuine biological heterogeneity from sequencing or assembly artifacts (Tam et al. 2025). The near-complete BUSCO recovery, ultra-high mapping concordance, and base-level accuracy exceeding 99.9999% collectively confirm that the assemblies of both *T. fuciformis* and *A. stygium* represent some of the highest-quality fungal genomes currently available (Liang et al. 2023). In addition, Hi-C-based nuclear phasing successfully resolved the dikaryotic genome of *T. fuciformis* into two physically independent haplotypes, providing a robust reference for future studies on nuclear differentiation and inter-nuclear interactions in dikaryotic fungi. Collectively, these results establish, for the first time, a generalized and reproducible quality validation method for complex fungal genomes, setting a benchmark for future fungal genomics research.

At the genomic structural level, this study reveals marked divergence in rDNA regions between haplotypes, underscoring their potential biological significance. As a multicopy tandem repeat, rDNA displays extensive variability in fungal genomes, with copy numbers differing by tens to thousands across species and even strains of the same species (Lofgren et al. 2019). In *T. fuciformis*, the rDNA array of hapA is substantially larger than that of hapB, primarily due to differences in ITS unit copy number and structure: hapA contains 46 copies of a 7.97 kb repeat, whereas hapB harbors 34 copies of a 9.03 kb repeat unit, with multiple insertions present in variable regions. This observation aligns with the prevalent endogenous rDNA heterogeneity seen in multinucleate and heterokaryotic fungi (Paloi et al. 2022). Although classical models propose intragenomic homogenization of rDNA via concerted evolution, such as gene conversion (Sergeeva et al. 2017), accumulating evidence indicates significant inter-copy sequence polymorphisms within heterokaryotic fungi (Paloi et al. 2022). These variations likely stem from copy number fluctuations, mutation accumulation, or reduced gene conversion efficiency, contributing to environmental adaptation and population divergence (Kiss 2012). Comparative analyses of ITS regions from the two haplotypes further revealed lower-than-expected sequence concordance: despite identical amplicon lengths (474 bp) amplified with universal primers ITS1 and ITS4, five nucleotide polymorphisms were detected, resulting in an overall similarity of 98.9%. This finding underscores that even the widely used fungal barcode, ITS, can exhibit significant nuclear divergence within heterokaryons (Kiss 2012; Schoch et al. 2012), suggesting that single-locus ITS data alone may not adequately capture the genetic complexity of heterokaryotic fungi. Therefore, multilocus or whole-genome approaches are crucial for accurate phylogenetic inference, species delimitation, and population structure analysis. Furthermore, this observation has broader implications for fungal metabarcoding studies. The 1.1% ITS sequence divergence between the two haplotypes falls below the conventional 97% identity threshold for operational taxonomic unit (OTU) clustering. Consequently, intra-individual polymorphism within a heterokaryon could be misinterpreted as interspecific diversity in environmental sequencing datasets. This supports the growing consensus to replace OTU clustering with amplicon sequence variant (ASV) approaches, which retain true biological sequence resolution and avoid arbitrary similarity thresholds (Callahan et al. 2017).

Putative interspecies chimeric reads were detected by aligning PacBio HiFi reads against the reference genomes of *T. fuciformis* and *A. stygium*. Several features suggest these reads represent technical artifacts rather than genuine interspecific genetic exchange. The chimeras lacked sequence homology at breakpoints, occurred at extremely low abundance, and showed no recurrence across independent reads or sequencing runs. In PCR-free preparations, artifactual chimeras primarily arise from random ligation of heterologous DNA fragments during library construction (Griffith et al. 2018). Mechanical shearing and blunt-end ligation can fuse distinct genomic sequences when mixed templates are present, generating non-homologous junctions at low frequency with poor replicability. PacBio CCS technology itself may also produce spurious chimeras via rare consensus errors (Wenger et al. 2019), misalignments during read processing, or intramolecular collisions in zero-mode waveguides (Iizuka et al. 2022). Such stochastic events generate rare, non-repetitive artifacts that are not supported by PCR validation. Consistent with this, targeted PCR across predicted breakpoints failed to validate any chimeric junctions, indicating that these are technical false positives. *T. fuciformis* and *A. stygium* maintain strict genomic isolation without interspecific genetic exchange. Despite this complete genomic separation, symbiotic fungi and their partners can communicate efficiently via non-genetic mechanisms. A recent study on the symbiotic relationship between the mycoheterotrophic plant *Gastrodia elata* (*G. elata*) and its symbiotic fungus *Mycena purpureofusca* (*M. purpureofusca*) demonstrates that, during long-term coevolution, *G. elata* has lost key genes for nitrogen utilization and auxin biosynthesis, while *M. purpureofusca* has retained these pathways and upregulates them during symbiosis to supply nitrogen and indole-3-acetic acid, thereby initiating seed germination (Yuan et al. 2025). The observed “no gene flow” symbiosis likely relies on metabolic coordination and signaling network coupling rather than genomic integration. Overall, this study provides robust genetic evidence for species delimitation and strain identification within this interaction system, laying a foundation for investigating non-genetic interaction mechanisms in fungal symbioses. Future integration of transcriptomic, metabolomic, proteomic, and spatial multi-omics data will further elucidate the dynamic regulation and ecological roles underpinning this symbiotic association.

In *Basidiomycota*, the heterokaryotic stage of aerial hyphae constitutes the predominant duration of the life cycle. The heterokaryons of fungi maintain genetic diversity through nuclear phase separation, and their genomic variations are closely related to functional differentiation (Ángeles-Argáiz et al. 2024). Single-library sequencing of the natural symbiotic community avoids karyotype disturbances caused by mononuclear isolation or pure culture and can truly reflect the native ratio of hapA to hapB and the state of genomic interactions. The analysis revealed striking differences in SNP density and SVs between the hapA and hapB genomes of heterokaryotic *T. fuciformis*. The average SNP density of 1 per 189.96 bp across the hapA chromosomes, with pronounced variation among chromosomes (for example, a 21-fold higher density in Chr10A compared to Chr09A), is consistent with previous findings that heterokaryotic fungi often display substantial genetic divergence between nuclei (James et al. 2008). The identification of Chr09 and Chr11 as accessory chromosomes, consistent with their distinct SNP distribution patterns, further supports the view that accessory chromosomes play an important role in promoting genome plasticity in fungi (Yang et al. 2020; Ayhan et al. 2025). These chromosomes, typically enriched in non-core genes, may enable *T. fuciformis* to adapt to fluctuating environmental conditions, such as nutrient availability in *A. stygium* (Croll et al. 2013; Grandaubert et al. 2014; van Dam et al. 2017). The uneven distribution of SVs (e.g., 34.38% of Chr11A vs. 9.17% of Chr08A) further highlights the dynamic nature of the *T. fuciformis* heterokaryon. The pronounced SV divergence between hapA and hapB regions of Chr09 suggests that accessory chromosomes are hotspots for structural rearrangements, potentially driven by transposable element activity or meiotic instability (Vlaardingerbroek et al. 2016). This pattern mirrors findings in *Cryptococcus neoformans*, where heterokaryotic nuclei accumulate SVs preferentially in non-essential genomic regions (Samarasinghe and Xu 2018; Fan and Lin 2020), reinforcing the role of SVs in shaping fungal genome evolution.

## Conclusion

This study transcends the limitations of traditional methods by employing *in situ* sequencing and assembly of the mixed mycelial interaction community formed by *T. fuciformis* and its associated fungus *A. stygium*, successfully achieving T2T genomes. Multiple validation techniques confirmed that the three genome sets obtained exhibit exceptional continuity, completeness, and accuracy. The analyses uncovered significant structural variations between the chromosomes of the two nuclei in *T. fuciformis*. Notably, the ITS region, a traditional marker for fungal species identification, showed substantial polymorphisms, suggesting that it is insufficient for reliable delimitation when used alone. Despite their long-term coexistence in natural environments, no genomic evidence of genetic material exchange was detected between *T. fuciformis* and *A. stygium*. Furthermore, genome-wide molecular markers enable precise strain identification, determination of interaction compatibility between *T. fuciformis* and *A. stygium*, and screening of compatible strain pairs for breeding programs, thereby improving cultivation efficiency.

## Additional information

### Artificial Intelligence (AI) use

The authors accept full responsibility for the content of the manuscript, including the disclosure of any use of AI.

No AI tools were used in the preparation of this manuscript.

### Adherence to national and international regulations

All the fungal strains used in this study have been legally obtained, respecting the Convention on Biological Diversity (Rio Convention).

### Data availability

Raw sequencing reads of the *T. fuciformis* interaction community are available in NCBI’s Sequence Read Archive (SRA) under PRJNA1354668 and PRJNA1354665. The assembled genome sequences are available in the National Genomics Data Center under PRJCA050678 and PRJCA050682 (https://ngdc.cncb.ac.cn/).

## Supplementary materials

Supplementary material 1 Supplementary images Data type pdf Explanation note A supplementary file containing additional figures in the main text. https://binary.pensoft.net/file/1678623This dataset is made available under the Open Database License (http://opendatacommons.org/licenses/odbl/1.0/). The Open Database License (ODbL) is a license agreement intended to allow users to freely share, modify, and use this Dataset while maintaining this same freedom for others, provided that the original source and author(s) are credited. Fengjiao Lin, Hualian Chen, Jingjing Ye, Huaizhen Xu, Hang Lin, Linyan Cen, Dingping Luo, Xiangzhen Chen, Huiying Hu, Ziyan Wang, Youjin Deng, Liping Deng

Supplementary material 2 Supplementary tables Data type pdf Explanation note A supplementary file containing additional tables in the main text. https://binary.pensoft.net/file/1678624This dataset is made available under the Open Database License (http://opendatacommons.org/licenses/odbl/1.0/). The Open Database License (ODbL) is a license agreement intended to allow users to freely share, modify, and use this Dataset while maintaining this same freedom for others, provided that the original source and author(s) are credited. Fengjiao Lin, Hualian Chen, Jingjing Ye, Huaizhen Xu, Hang Lin, Linyan Cen, Dingping Luo, Xiangzhen Chen, Huiying Hu, Ziyan Wang, Youjin Deng, Liping Deng

Supplementary material 3 Summary of contigs alignment information Data type xlsx Explanation note BLAST-based assignment of contigs from the mixed mycelial assembly to *T. fuciformis* YN01 and its symbiotic *A. stygium* YNAS01 genome. https://binary.pensoft.net/file/1678625This dataset is made available under the Open Database License (http://opendatacommons.org/licenses/odbl/1.0/). The Open Database License (ODbL) is a license agreement intended to allow users to freely share, modify, and use this Dataset while maintaining this same freedom for others, provided that the original source and author(s) are credited. Fengjiao Lin, Hualian Chen, Jingjing Ye, Huaizhen Xu, Hang Lin, Linyan Cen, Dingping Luo, Xiangzhen Chen, Huiying Hu, Ziyan Wang, Youjin Deng, Liping Deng

Supplementary material 4 *T. fuciformis* rDNA Data type pdf Explanation note The rDNA sequence information of *T. fuciformis*. https://binary.pensoft.net/file/1678626This dataset is made available under the Open Database License (http://opendatacommons.org/licenses/odbl/1.0/). The Open Database License (ODbL) is a license agreement intended to allow users to freely share, modify, and use this Dataset while maintaining this same freedom for others, provided that the original source and author(s) are credited. Fengjiao Lin, Hualian Chen, Jingjing Ye, Huaizhen Xu, Hang Lin, Linyan Cen, Dingping Luo, Xiangzhen Chen, Huiying Hu, Ziyan Wang, Youjin Deng, Liping Deng

Supplementary material 5 Alignment of *T. fuciformis* rDNA Data type pdf Explanation note Alignment of rDNA sequences between *T. fuciformis* hapA and hapB. https://binary.pensoft.net/file/1678627This dataset is made available under the Open Database License (http://opendatacommons.org/licenses/odbl/1.0/). The Open Database License (ODbL) is a license agreement intended to allow users to freely share, modify, and use this Dataset while maintaining this same freedom for others, provided that the original source and author(s) are credited. Fengjiao Lin, Hualian Chen, Jingjing Ye, Huaizhen Xu, Hang Lin, Linyan Cen, Dingping Luo, Xiangzhen Chen, Huiying Hu, Ziyan Wang, Youjin Deng, Liping Deng

Supplementary material 6
*T. fuciformis* ITS
Data type pdf Explanation note The sequence information of *T. fuciformis*ITS. https://binary.pensoft.net/file/1678628This dataset is made available under the Open Database License (http://opendatacommons.org/licenses/odbl/1.0/). The Open Database License (ODbL) is a license agreement intended to allow users to freely share, modify, and use this Dataset while maintaining this same freedom for others, provided that the original source and author(s) are credited. Fengjiao Lin, Hualian Chen, Jingjing Ye, Huaizhen Xu, Hang Lin, Linyan Cen, Dingping Luo, Xiangzhen Chen, Huiying Hu, Ziyan Wang, Youjin Deng, Liping Deng

Supplementary material 7 Alignment of *T. fuciformis*ITS Data type pdf Explanation note Alignment of ITS sequences between *T. fuciformis* hapA and hapB. https://binary.pensoft.net/file/1678629This dataset is made available under the Open Database License (http://opendatacommons.org/licenses/odbl/1.0/). The Open Database License (ODbL) is a license agreement intended to allow users to freely share, modify, and use this Dataset while maintaining this same freedom for others, provided that the original source and author(s) are credited. Fengjiao Lin, Hualian Chen, Jingjing Ye, Huaizhen Xu, Hang Lin, Linyan Cen, Dingping Luo, Xiangzhen Chen, Huiying Hu, Ziyan Wang, Youjin Deng, Liping Deng

Supplementary material 8 Summary of similar sequences between *T. fuciformis* and *A. stygium* Data type xlsx Explanation note Comparative genomic analysis identified segments with > 80% sequence identity between *T. fuciformis* YN01 and *A. stygium* YNAS01. These highly similar segments (82.1%–94.4%) were localized primarily in rDNA regions and are not indicative of intergenomic sequence exchange. https://binary.pensoft.net/file/1678630This dataset is made available under the Open Database License (http://opendatacommons.org/licenses/odbl/1.0/). The Open Database License (ODbL) is a license agreement intended to allow users to freely share, modify, and use this Dataset while maintaining this same freedom for others, provided that the original source and author(s) are credited. Fengjiao Lin, Hualian Chen, Jingjing Ye, Huaizhen Xu, Hang Lin, Linyan Cen, Dingping Luo, Xiangzhen Chen, Huiying Hu, Ziyan Wang, Youjin Deng, Liping Deng

Supplementary material 9 Interspecies chimeric reads from raw sequencing data Data type pdf Explanation note Sequence information for 89 putative interspecies chimeric reads from raw sequencing data. https://binary.pensoft.net/file/1678631This dataset is made available under the Open Database License (http://opendatacommons.org/licenses/odbl/1.0/). The Open Database License (ODbL) is a license agreement intended to allow users to freely share, modify, and use this Dataset while maintaining this same freedom for others, provided that the original source and author(s) are credited. Fengjiao Lin, Hualian Chen, Jingjing Ye, Huaizhen Xu, Hang Lin, Linyan Cen, Dingping Luo, Xiangzhen Chen, Huiying Hu, Ziyan Wang, Youjin Deng, Liping Deng

Supplementary material 10 Mosaic sequence information Data type xlsx Explanation note All mosaic sequence information. https://binary.pensoft.net/file/1678632This dataset is made available under the Open Database License (http://opendatacommons.org/licenses/odbl/1.0/). The Open Database License (ODbL) is a license agreement intended to allow users to freely share, modify, and use this Dataset while maintaining this same freedom for others, provided that the original source and author(s) are credited. Fengjiao Lin, Hualian Chen, Jingjing Ye, Huaizhen Xu, Hang Lin, Linyan Cen, Dingping Luo, Xiangzhen Chen, Huiying Hu, Ziyan Wang, Youjin Deng, Liping Deng
