# Supplementary material for: Holistic genome assembly and analysis of the Tremella fuciformis interaction community uncovers intergenomic insights beyond dual genomes
Source: IMA Fungus. 2026 Jun 15;17:e185345. doi: 10.3897/imafungus.17.185345 (PMC13288022; doi:10.3897/imafungus.17.185345)
Supplement: Supplementary material 1 — Supplementary images [file imafungus-17-e185345-s001.pdf]

Gene Annotation

Gene annotation of the *T. fuciformis* genome assembly was performed using FUNANNOTATE (v1.8.16) (https://doi.org/10.5281/zenodo.4054262). The software integrated homologous protein sequences from the *Tremella* reference genome Tr01 with full-length transcript evidence. The annotation parameters were set to specify *T. fuciformis* as the species, using the closely related species *Cryptococcus neoformans* JEC21 as the training set for the Augustus software. A similar workflow was applied to annotate the genome of *A. stygium*, integrating homologous protein sequences from the *A.* reference genome TJAS01 with full-length transcript evidence. The annotation parameters were set to specify *A. stygium* as the species, with *Xylaria hypoxylon* DSM 108379 as the training set for Augustus.

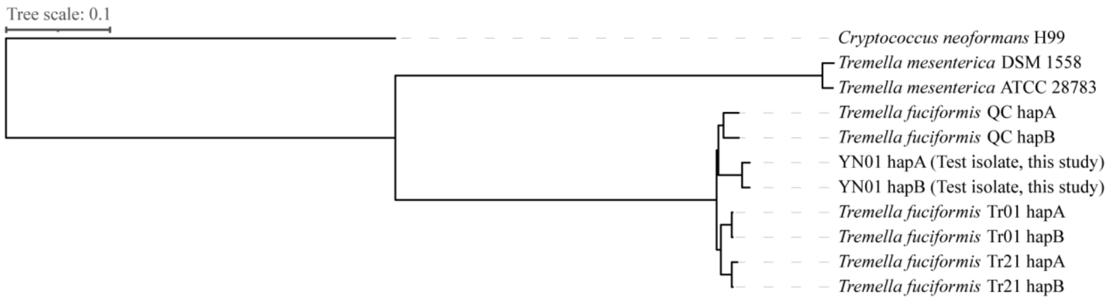

Supplementary Figure S1. Phylogenetic tree of *Tremella* species constructed from single-copy orthologous genes.

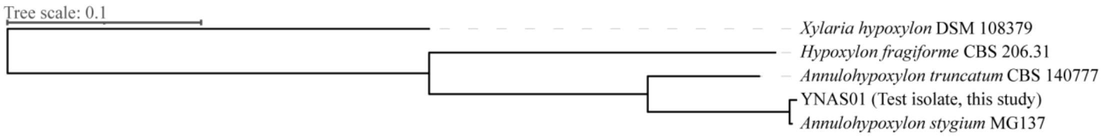

Supplementary Figure S2. Phylogenetic tree of *Xylariaceae* species inferred from single-copy orthologous genes.

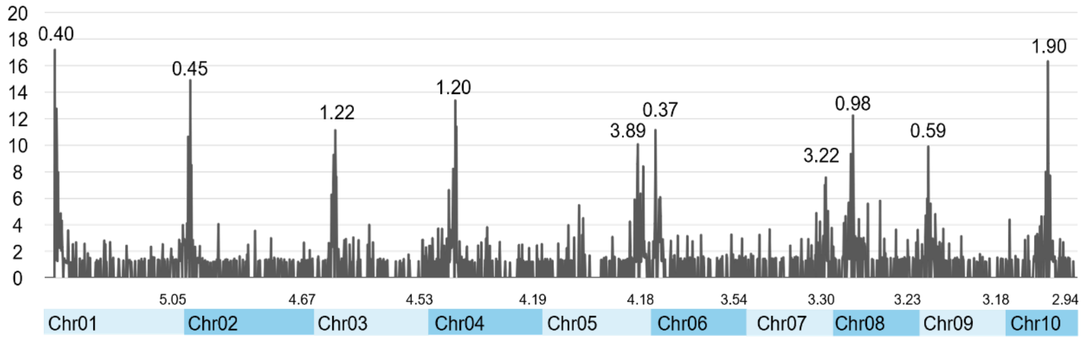

Supplementary Figure S3. Location information of the centromere of each chromosome in the genome of *A. stygium*. Abscissa: Length of each chromosome (Mb); Ordinate: Hi-C interaction value, higher value indicates stronger cross-link signal.

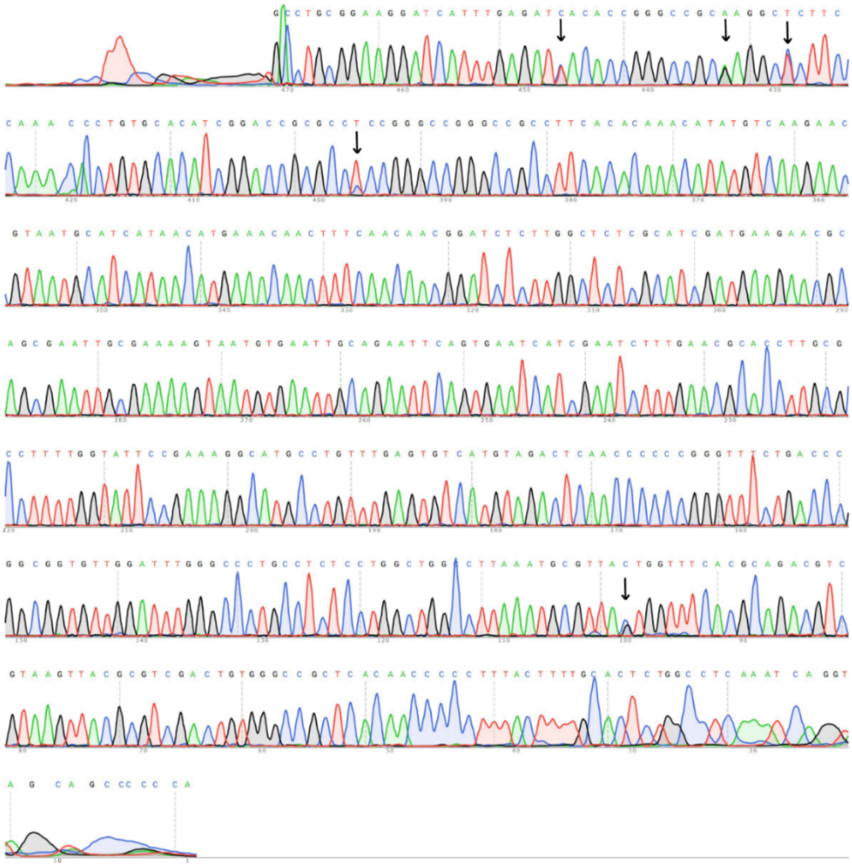

Supplementary Figure S4. Sanger sequencing electropherogram of the ITS region from *T. fuciformis*. The electropherogram shows four-color traces (A, green; T, red; C, blue; G, black) corresponding to nucleotide bases. Black arrows indicate variable sites within the ITS1–ITS4 region, where overlapping peaks suggest sequence heterogeneity.

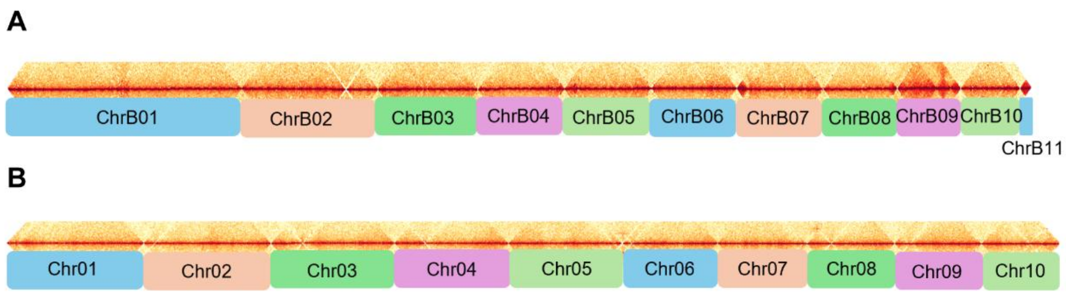

Supplementary Figure S5. Hi-C interaction maps demonstrated chromosomal architecture. **A** Hi-C contact heatmap across chromosomes (Chr01–Chr11) for hapB of *T. fuciformis*. **B** Hi-C contact heatmap across chromosomes (Chr01–Chr10) for *A. stygium*.

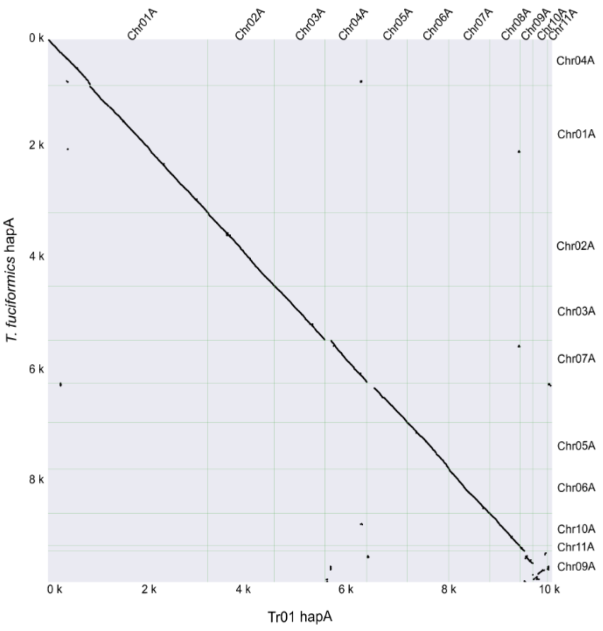

Supplementary Figure S6. Genome-wide synteny dot plot reveals extensive collinearity between *T. fuciformis* hapA Tr01 and YN01. The x-axis denotes the genomic position of Tr01 hapA, and the y-axis indicates that of YN01.

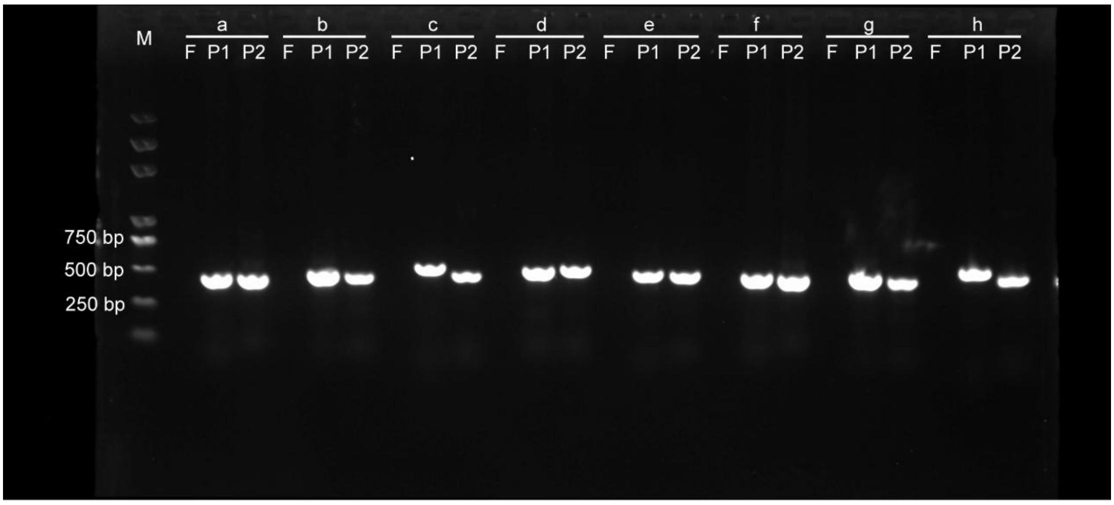

Supplementary Figure S7. PCR analysis to check the DNA breakpoint. Agarose gel electrophoresis showing PCR amplification products for DNA breakpoint verification using different primers. The gel contains the following lanes: Lane M: DNA molecular weight marker (750, 500, and 250 bp bands). Lanes a–h: PCR products amplified using: F: Primer for verifying the DNA breakpoint. P1: Positive control primer targeting the region before the breakpoint. P2: Positive control primer targeting the region after the breakpoint. The bands observed represent successful amplification, with product sizes indicated by the molecular weight marker.
