# Supplementary material for: Holistic genome assembly and analysis of the Tremella fuciformis interaction community uncovers intergenomic insights beyond dual genomes
Source: IMA Fungus. 2026 Jun 15;17:e185345. doi: 10.3897/imafungus.17.185345 (PMC13288022; doi:10.3897/imafungus.17.185345)
Supplement: Supplementary material 2 — Supplementary tables [file imafungus-17-e185345-s002.pdf]

Supplementary Table 1. Primer sequences and product sizes for DNA breakpoint verification across different sequences in *T. fuciformis*.

Group	Primer ID	Forward primer (5'→3')	Reverse primer (5'→3')	Product size (bp)
	F	GTTGCGACCAAGACTAGGCA	GGAACGAGAGCAGCTGAAGT	894
**a**	P_1	GTTGCGACCAAGACTAGGCA	ATAATCGCTTGGCACTTGAGC	401
	P_2	ACTCGCTACAACCCTTGACG	CGATCCCGAGCTGTTCGTTA	408
	F	CGTCCATGCCAAGGTAAGGT	TACCTGGACAGGGGAAGTCA	809
**b**	P_1	GGAGCTTGAAGCGGGATGTT	TGGAATCCGAGAACACGACC	435
	P_2	TCTACAAGTCTGCATCGCCT	TCACTACCTGGACAGGGGAA	406
	F	AGCCAGGCTTGGCAAAGTAG	ATGAACCTACCCAATCGCCC	802
**c**	P_1	TTGAAAACGACAACGGAGCC	AAGCACTTTGGAAGGCGGAT	476
	P_2	GTGCGGCATTCGCCATATTT	AAACGATGACGTCAATACCCAT	408
	F	TGAACGCCCATGATCCCATC	ATTCAGGACCTTCGTTGCGT	859
**d**	P_1	TGAACGCCCATGATCCCATC	AGATACCAGCCAGACGCCTA	449
	P_2	TCGAAATCGATAGCGGGTCG	TGCCAACGAGATCTTCCAGT	459
	F_e	CTTTCCCGCATTGCACTCAG	ACTTGCAACTCGGAGGACAG	844
**e**	P_1	GCGACGCTCTACACTCTAGC	TCCTGTGAGGTGCGAAGAAC	412
	P_2	CGAGTCCGGAGTCCTCGATA	TGGCAAACAAGTGTACCGGA	409
	F	CCCAAACTTGGAACACTGCC	TGATGAACGATAGGCGCGAG	904
**f**	P_1	ATGCTCTACCGACACGCAG	GGTAGTAGCTAGCGGCGAAA	403
	P_2	ATCTCAGTCCCAGCTCAACC	ATGATGAACGATAGGCGCGA	402
	F	TGCTCAGGAAGAAAGCCTCG	ATGAACCAGAGAAGCTCGGC	859
**g**	P_1	GACGCTCGTTCTCTTCTTGC	GATCGCCAGGGGTAGTCTTC	454
	P_2	GCCTTCCGAATTAACCGTCAT	AGGAGGCTAGGCGGTAACTA	420
	F	TGGCTCTTCCGATTCGTCTC	CGCAACATCTTGGTTGGTCG	912
**h**	P_1	TGCTCAGGAAGAAAGCCTCG	TCCTTGGTCTCTTTGCGTCC	463
	P_2	CCTTAGCACAGCACGAGGAA	CCGTACACTGGCCCTAAGTC	422

Supplementary Table S2. SVs and TE-mediated SVs across chromosomes in *T. fuciformis* YN01

Chromosome	SV number	TE-mediated SV number
Chr01A	219	160
Chr02A	175	148
Chr03A	91	70
Chr04A	101	80
Chr05A	99	73
Chr06A	105	86
Chr07A	108	93
Chr08A	55	45
Chr09A	79	75
Chr10A	77	62
Chr11A	37	36
**Total**	1146	928

Supplementary Table S3. GC Content (%) across chromosomes in *T. fuciformis* YN01

Chromosome	GC content (%)
Chr01A	56.8
Chr02A	57.2
Chr03A	56.7
Chr04A	57.0
Chr05A	56.8
Chr06A	56.8
Chr07A	56.8
Chr08A	57.5
Chr09A	55.8
Chr10A	57.0
Chr11A	54.9

Supplementary Table S4. Gene density (per 100 kb) across chromosomes in *T. fuciformis* YN01

Chromosome	Gene density (per 100 kb)
Chr01A	37.60
Chr02A	37.11
Chr03A	37.59
Chr04A	36.36
Chr05A	38.27
Chr06A	37.55
Chr07A	36.28
Chr08A	36.88
Chr09A	29.67
Chr10A	38.28
Chr11A	32.82

Supplementary Table S5. Repeat sequence length (bp) and proportion (%) across chromosomes in *T. fuciformis* YN01

Chromosome	Repeat sequence length (bp)	Repeat sequence proportion (%)
Chr01A	179,806	2.89
Chr02A	131,043	3.71
Chr03A	69,166	2.72
Chr04A	81,279	3.60
Chr05A	55,059	2.46
Chr06A	65,218	2.92
Chr07A	84,745	3.94
Chr08A	73,510	3.78
Chr09A	151,297	8.62
Chr10A	57,351	3.77
Chr11A	24,100	7.08
