# Supplementary material for: Holistic genome assembly and analysis of the Tremella fuciformis interaction community uncovers intergenomic insights beyond dual genomes
Source: IMA Fungus. 2026 Jun 15;17:e185345. doi: 10.3897/imafungus.17.185345 (PMC13288022; doi:10.3897/imafungus.17.185345)
Supplement: Supplementary material 4 — T. fuciformis rDNA [file imafungus-17-e185345-s004.pdf]

>YN01_hapA_rDNA

ACCCATCCCAAGTGCTGGGGGCTCTGGTCTTGCCGGGGACTTGGTGAAATC
ACCACCCAAGAGTTAAACAGCACGTGAAATTGTTGAAAGGGAAACGATTG
AAGTCAGTCGTGTCCGGGGGGTTCAGCCGGTTCTGCCGGTGCATTCCTCTC
GGACGGGTCAACATCGGTTTTGCCCGGCGGAAAAGGGCGTGAGGAATGTG
GCACCTCCGGGTGTGTTATAGCCTCGCGTCGCATACGTCGGGCGGGACCGA
GGAACGCAGCTCGCCTTCACGGCCGGGGTTCGCCCACGTACGAGCTTACC
ACACAAGCACCACCAAGCCCTCACCGGTCTGCGGGCCGCCCCTCCGTCCC
CGAGCGGTCCCGAGCCGTCCTGGCCGCCCAGGACACCGTCCCGGCCAAGA
CCGTGGCCTCTCGGGTCGTCCTCGGGCCGCCCACAGGCCCCGAGAGCAAA
ACTGCCAAAAACACCTATTTCGAACCGGACGATTCGTCGGCCAAAATCCCC
CAAAAATCCCATTTTGGCTGGTAAAACATATTTTTTTTCGTTTTTGTCCAGTT
TTTGCTTGTACGGTTTTTTTAAGATACATGTTTTGGCCCAGGGCCCTCCCTG
CCACCTATATGGGGAAGGGGTGGTCCTAGGGGACTGTCAATGGGCACTTTA
CGGGTCTGTTGGTAGGAACTGAAAGGCTATTCTGGAACCTGTGTCTGTTGG
TAATGGGTTGATGGCGGTGCGTGAGTGGGCCATGCGGGAGGTCGAAGGGT
CTTGATGTGAGCTGCCTAGCAGTTCGTCGGAGCGTGAGCGGTTTGCGGGA
GGGAGTCCGAGTCGGTCTAGAACGTTGAGTCATCCGTTCTATTACTGCCGT
TCCTCTCGTAAATGGCCGTTTCGGGTCGAGTTACCCCTTCCTCTCGGTGCGT
CGACCGTACTGTGGTCCTCATTTCCCTTCGGCCACGTTCGGTTGGGACTCT
GCTGGCGGGTCGGTGGTATTCTCTCGGGCGTCTCCGGATGTCTGTTGGGTC
ACGCCTCTGTCTCGCTTGCGGCGGTTCCGGTTGGTATCGCTGTCGATTTTCC
TTCCGCTGAGTCGAGCACCCAGGTGCTCCTCTCGGGAGCGGGTCGACGGT
GCCCGTGTTGGTGACGTGTCGGCTCTTGCCGTCGTGGCTCATCGTCCGGGT
CTGGTCGGGTCGAGGTTGGCTTCGGCCTCCTTGGCCCGCACCTTAGGAATT
GAGGGTTCATTACTGTTGGTTACACTTTTCCTCGAGCCATTTGGAGGTGCG
AGCGGGTCCTGCTCCGGCGGTTCCCGCTCCAGTCTCCACGATGGTCCTGAT
TCCCGGCTCCGGCCGGGATGACTGGACCGAGGACTTGTGCGGACGGAAAC
TGGGAGCCCGATGGGGCTTCTGGACGGGCGTGTCCCTCGGGACGCTAGAT
GCACCGGCTGGGAGCCTCTTGGGATCTGTTCTTCCTATGTGACAAACTTCG
TCTCGCTGGACAAGGCGGGTCTCTCCCTCGGGAGCGGCCGGCCCCCGGCG
GGCGCCGTCGTGGATGCCTGTCGTCCCCTCGGTGGTAAGATAGTTACCTGG
TTGATCCTGCCAGTAGTCATATGCTTGTCTCAAAGATTAAGCCATGCATGTC
TAAGTATAAACGGATTCATACTGTGAAACTGCGAATGGCTCATTAAATCAGT
TATAGTTTATTTGACGGTACCTTGCTACATGGATAACTGTGGTAATTCTAGAG
CTAATACATGCCGAAAAGCCCCGACTTCTGGAAGGGGTGTATTTATTAGATA
AAAAACCAATGCGGGCAACCGCTCTTTGGTGATTCATGATAACTTCTCGAA
TCGCATGGCCTTGCGCCGGCGATGCTTCATTCAAATATCTGCCCTATCAACT

TTCGATGGTAGGATAGAGGCCTACCATGGTATCAACGGGTAACGGGGAATT
AGGGTTCGATTCCGGAGAGGGAGCCTGAGAAACGGCTACCACATCCAAGG
AAGGCAGCAGGCGCGCAAATTACCCAATCCCGACACGGGGAGGTAGTGAC
AATAAATAACAATACAGGGCTCTATTGGGTCTTGTAATTGGAATGAGTACAA
TTTAAATCCCTTAACGAGGAACAACTGGAGGGCAAGTCTGGTGCCAGCAG
CCGCGGTAATTCCAGCTCCAGTAGCGTATATTAAAGTTGTTGCAGTTAAAAC
GCTCGTAGTCGAAACTCGGGCCCGGCGGGCCGGTCCGCCTCACGGTGTGC
ACTGTCCGGCCGGGCCTTACCTCTTGGTGGCCGCGTTCCTCTCACGGGGTG
CGTGGTGTAACCAGGAACTTTACCTTGAGAAAATTAGAGTGTTCAAAGCAG
GCAAACGCCCGGATACATTAGCATGGAATAATAGAATAGGACGTGCGGTTC
TATTTTGTTGGTTTCTAGGATCGCCGTAATGATCAATGGGGACGGTCGGGGG
CATTGGTATTCCTTTGCTAGAGGTGAAATTCTTAGATTACAGGAAGACCGAC
AACTGCGAAAGCATTTGCCAAGGACGTTCTCGTCGATCAAGAACGAAGGT
TAGGGGATCAAAAACGATTAGATACCGTTGTAGTCTTAACAGTAAACTATGC
CGACTAGGGATCGGGCCACGTCATCCTCTGACTGGCTCGGCACCTTACGAG
AAATCAAAGTCTTTGGGTTCTGGGGGGAGTATGGTCGCAAGGCTGAAACTT
AAAGGAATTGACGGAAGGGCACCACCAGGTGTGGAGCCTGCGGCTTAATT
TGACTCAACACGGGGAAACTCACCAGGTCCAGACATAGTGAGGATTGACA
GATTGATAGCTCTTTCTTGATTCTATGGGTGGTGGTGCATGGCCGTTCTTAGT
TGGTGGAGTGATTTGTCTGGTTAATTCCGATAACGAACGAGACCTTAACCT
GCTAAATAGCCAGGCCGGCTTTTGCTGGTCGTGGGCTTCTTAGAGGGACTG
TCGGCGTCTAGTCGACGGAAGTTTGAGGCAATAACAGGTCTGTGATGCCCT
TAGATGTTCTGGGCCGCACGCGCGCTACACTGACCGAGCCAGCGAGTTCAT
CGCCTTGCCCGAGAGGGTTGGGTAATCTTGTGAAACTCGGTCGTGCTGGGG
ATAGAGCATTGCAATTATTGCTCTTCAACGAGGAATACCTAGTAAGCGTGAG
TCACCAGCTCGCGTTGATTACGTCCCTGCCCTTTGTACACACCGCCTGTCGC
TACTACCGATTGAATGGCTTAGTGAGATCTCCGGATTGGCGTTGGGGAGCC
GGCAACGGCACCCCTTGGCCGAGAAGCTGATCAAACTTGGTCATTTAGAG
GAAGTAAAAGTCATAACAAGGTTTCCGTAGGTGAACCTGCGGAAGGATCAT
TTGAGATTACACCGGGCCGCAAGGCCCTTCCAAACACCTGTGCACATCGGA
CCGCGCCTCCGGGCCGGGCCGCCTTCACACAAACATATGTCAAGAACGTA
ATGCATCATAACATGAAACAACTTTCAACAACGGATCTCTTGGCTCTCGCAT
CGATGAAGAACGCAGCGAATTGCGAAAAGTAATGTGAATTGCAGAATTCA
GTGAATCATCGAATCTTTGAACGCACCTTGCGCCTTTTGGTATTCCGAAAGG
CATGCCTGTTTGAGTGTCATGTAGACTCAACCCCCCGGGTTTCTGACCCGG
CGGTGTTGGATTTGGGCCCTGCCTCTCCTGGCTGGCCTTAAATGCGTTACTG
GTTTCACGCAGACGTCGTAAGTTACGCGTCGACTGTGGGCCGCTCACAACC
CCCTTTACTTTTGCACTCTGGCCTCAAATCAGGTAGGGCTACCCGCTGAACT

TAAGCATATCAATAAGCGGAGGAAAAGAAACTAACAAGGATTCCCCTAGTA
ACGGCGAGCGAACCGGGAAGAGCTCAAATTTGAAATCTGGCGTCCTCCGG
GCGTCCGAGTTGTAATCTACAGAGGCGTTTTCCGCGCCGGTCCGTGTCCAA
GTCCCTTGGAACAGGGTATCAAAGAGGGTGACAATCCCGTACTTGACACG
ACCGCCGGTGCTTTGTGATACGTCTTCTAAGAGTCGAGTTGTTTGGGAATG
CAGCTCAAAACGGGTGGTAAATTCCATCTAAGGCTAAATATTGGCGAGAGA
CCGATAGCGAACAAGTACCGTGAGGGAAAGATGAAAAGCACTTTGGAAAG
AGGGATGTTGACATAATGGCTTTAAACGACCCGTCTTGAAACACGGACCAA
GGAGTCTAACATATCTGCGAGTGTTTGGGTGTCAAACCCGAGCGCGTAATG
AAAGTGAACGTAGGAGGGATCCGCAAGGAGCACCTTCGACCGATCCGGAT
CTTCTGTGATGGATTTGAGTAAGAGCATATATGCTGGGACCCGAAAGATGGT
GAACTATGCCTGAATAGGGCGAAGCCAGGGGAAACTCTGGTGGAGGCTCG
TAGCGATTCTGACGTGCAAATCGATCGTCAAATTTGGGTATAGGGGCGAAA
GACTAATCGAACCATCTAATGGCTGGTTCCCGCCGAAGTTTCCCTCAGGATA
GCAGAAGCTCGCATCAGTTTTATGAGGTAAAGCGAATGATTAGAGGCCTTG
GGGACGAAACGTCCTTAACCTATTCTCAAACTTTAAATGTGTAAGAAGCCA
CCGTCGCTTGATTGGACGGTCGGCGTGCGAATGAGAGCTTCTAGTGGGCCA
TTTTTGGTAAGCAGAACTGGCGATGCGGGATGAACCGATCGTGAGGTTAAG
GTGCCGGAATACACGCTCATCAGACACCACAAAAGGTGTTAGTTCATCTAG
ACAGCAGGACGGTGGCCATGGAAGTCGGAATCCGCTAAGGAGTGTGTAAC
AACTCACCTGCCGAATGAACTAGCCCTGAAAATGGATGGCGCTCAAGCGT
GTTACCCATACCTCACCGTTGGCGTTTCAGTGACGCGCCAACGAGTAGGCG
GGCGTGGGGGTCCGTGCAGAAGCCTTGGCAGTGATGCCGGGTGGAACGGC
CCCTAGTGCAGATCTTGGTGGTAGTAGCAAATATTCAAGTGAGAACCTTGA
AGACTGAAGTGGAGAAAGGTTCCATGGTAACAGCAGTTGGACATGGGTCA
GTCGATCCTAAGAGATAGGGAAGCTCCGTTTCAAAGTGCGCGATTTTCCGT
GCCGCCTATCGAAAGGGAATCCGGTTAAGATTCCGGAACCAGGATGTGGAT
CTTTGACGGCGACGTAAGTGAAGTTGGAGACGTTGGCAAGGGCCCCGGGA
AGAGTTCTCTTTTCTCCTTGACCGCCTACGACCCTGAAATCGGATTATCCGG
AGCTAGGGTTAAATGGCGGGTAGAGCACGACACCTCTGTCGTGTCCGGTGC
GTCCTTGACAGCCCTTGAAAATCCGACGGAACGTATAAGTCTCACGCCTGG
TCGTACTCATAACCGCAGCAGGTCTCCAAGGTGAACAGCCTCTAGTTGATA
GAACAATGTAGATAAGGGAAGTCGGCAAAATAGATCCGTAACTTCGGGATA
AGGATTGGCTCTAAGGGTTGGGTGCGTCGGGCCGTTGGTGGAAGAGAGCT
GGACCGGGCCGGACTGGGCGGGGCGACCCGTCTGGACTGGCTCGGACCG
GCGATCGGACGCCTCTGGCAGCCCTCGGGCGTCTGGCGCACGGTTAACAA
CCGACTTAGAACTGGTACGGACAAGGGGAATCTGACTGTCTAATTAAAACA
TAGCATTGCGATGGCCAGAAAGTGGTGTTGACGCAATGTGATTTCTGCCCA

GTGCTCTGAATGTCAAAGTGAAGAAATTCAACCAAGCGCGGGTAAACGGC
GGGAGTAACTATGACTCTCTTAAGGTAGCCAAATGCCTCGTCATCTAATTAG
TGACGCGCATGAATGGATTAACGAGATTCCCACTGTCCCTATCTACTATCTA
GCGAAACCACAGCCAAGGGAACGGGCTTGGCAGAATCAGCGGGGAAAGA
AGACCCTGTTGAGCTTGACTCTAGTTTGACATTGTGAAAAGACATGGAGGG
TGTAGAATAAGTGGGAGCTTCGGCGCCGGTGAAATACCACTACCTCCATCG
TTTTTTTACTTATTCAATGAGGCGGAGCTGGGATTAACGTCCCACCTTTTGG
CTTCAAGGTCCTTCGCGGGCCGATCCGGGTTGAAGACATTGTCAGGTGGG
GAGTTTGGCTGGGGCGGCACATCTGTTAAAAGATAACGCAGGTGTCCTAAG
GGGGACTCATGGAGAACAGAAATCTCCAGTGGAACAAAAGGGTAAAAGTC
CCCTTGATTTTGATTTTCAGTGTGAATACAAACCATGAAAGTGTGGCCTATC
GATCCTTTAGTCCCTCGGAATTTGAGGCTAGAGGTGCCAGAAAAGTTACCA
CAGGGATAACTGGCTTGTGGCAGCCAAGCGTTCATAGCGACGTTGCTTTTT
GATCCTTCGATGTCGGCTCTTCCTATCATACCGAAGCAGAATTCGGTAAGCG
TTGGATTGTTCACCCACTAATAGGGAACGTGAGCTGGGTTTAGACCGTCGT
GAGACAGGTTAGTTTTACCCTACTGATGGAGTGACGTCGTGACAGCAATTG
AGGGTAGTACGAGAGGAACTGCTCATTCGGATAATTGGTATTTGCGCCTGT
CCGATCGGGCAATGGCGCGAAGCTATCATCCGTCAGATTATGGCTGAACGC
CTCTAAGTCAGAATCTGTACTGGAAACGACGTTGTTGGTCCCGCACGTGTT
AGTCGCGTTGGAATAGGCTTCGGCTGTGAACCATACCTGGGTAGGGCGTGT
CGGCGGAAATGCCGGCCCGTCCCCCTCTATGAAACGAATATGGGCGGGGGT
GAATCTCTTGCAGACGACTTGACTGGAACGGGGTGCTGTAAGCGGTAGAG
TAGCCTTGTTGCTACGATCCGCTGAGGCTAAGCCTTTGTTCCTCGATTTGTC
CCTCGTGTGTTGACGTGCTGGGGGCTCTGATGCGCCTGGTGCGGGACGTGC
GGGCGACTTGGTTGTGTTTTTGTGTGGGTGTGGGTGCGAGATGTGCTGGGG
GCTCTGGTTTGTGGGTTGCGGGAGTGGTGGGGGACTCGGGTGAGAGGGGA
CGGGATGAGACGGGACGGAACTGGGACGGCCGGGCGGAACCTGGGACAT
GCCTCCGGGACGACGGTGACGGGCCCAAGAGGTGTGGCGGGTGGCTGGC
GTCGGGGGGAGATGTTGCGGGCGTGCAGGCAAAGGGGTGGCGGGGGCGA
GATGGGAGGGGTTCAACTACTGGGGGCTCTGGTCTTGTTGGTCGGGGGAC
GGCGGGGACTTGAGGTTTCTGGGAGTAGGTGTGATGTGCTGGGGGCTCTG
GTCCGGGGAGACGGGAGACGTTGGGGACTTGTCAGTCAGCATAAGTGATC
ATCCCAAGTGCTGGGGGCTCTGCTTTGTTGGCTCGGGGGATGGGGAGGAC
TTGTGATTTGGGCCGGGAGGTGGTGCGGGTGGTGACGTGCCGGGGGCTCT
GGTCGGGAGACGTTTTTGGCCGAGGACGTAGCCCCGGCCGGCCCGATCCC
GGGACCGGTCTTGGGACTCTTGTCGGCACAACGCTCGGCCGGGAAAACGG
CCCGGAAGTTCCGGGTATCGACGATTTTTGGGCGACCGAGCCTCCCTGGGG
TGGGACCGGTGACGTGCTGGGGGCTCTGGTCCGAGAACGTTTTTGGCCGA

GGACGTAGCCCGGGGCGGCCCGATCCCGGGAGCGGTCTCGGGATTCGTGT
CGGCACGACGGTTGACCGGGAAAACGGCCCGGAAGTTCCGGGACCCGAC
GATTTTTGACCCCCTGGGCCTACAGGGGGTGGGCAATGACGACGTGCTGG
GGGCTCTGGTTGTCAGACGTTTTGGGCCGAGGACGTAGCCCCGGTCGGCC
CGATCTCGGGACCGGTCTCGGGACTCTTGTCGGCTCGACGGTTGACCGGG
AACCGGGAAAACGGCCCGGAAGTTTCGAGACCCGACGATTTTC

>YN01_hapB_rDNA

GAGCCACCAAGCCGTCCCGAGCCGTCCCGAGCCAAGACGCTGACGTGCTG
GGGGCTCTGGTCGGAAAACGTTTTCGGCCGAGGACGTAGCCCCGATCGGC
CCGATCCTGGGACCGGTCTCGGGACTCGTGTCGGCTCGACGGTCGGCCGG
GAAAACGGCCCGGAAGTTCCGAGACCCGACGATTTTCGAGCCACCGAGCC
CTCCCGGGCCGAGACGGTGACGTGCTGGGGGCTCTGGTCCGAGAACGTTT
TTGGCCGAGGACGTAGCCCCGGTCGGCCCGATCCCGGGACCGGTCTCGGG
ACTCGTGTCGGCTCGACGGTCGGCCGGGAAAACGGCCCGGAAGTTCCGAG
ACCCGACGATTTTCGAGCCACCGAGCCCTCCCGGGCCGAGACGGTGACGT
GCTGGGGGCTCTGGTCCGAGAACGTTTTTGGCCGAGGACGTAGCCCCGGT
CGGCCCGATCCCGGGACCGGTCTCGGGACTCGTGTCGGCTCGACGGTCGG
CCGGGAAAACGGCCCGGAAGTTCCGAGACCCGACGATTTTCGAGCCACCG
AGCCCTCCCGGGCCGAGACGGTGACGTGCTGGGGGCTCTGGTCCGAGAAC
GTTTTTGGCCGAGGACGTAGCCCCGGTCGGCCCGATCCCGGGACCGGTCTC
GGGACTCGTGTCGGCTCGACGGTCGGCCGGGAAAACGGCCCGGAAGTTCC
GAGACCCGACGATTTTCACCCATCCCAAGTGCTGGGGGCTCTGGTCTTGCC
GGGGACTTGGTGAAATCACCACCCAAGCCGCACAAGCACCACCAAGCCCT
AGCCCGTCTCCGGGCCGCCCCTCCGTCCCCGAGCGGTCCCGAGCCATCCTG
GCCGCCCAGGACACCGTCCCGGCCGAGACCGTGGCCTCTCGGGCCGTCCT
CGGGCCGCCCACAGGCCCCAAGAGCAAAACTGCCAAAAACACCTATTTCG
AACCGGACGATTCGTCGGCCAAAATCCCCCAAAAATCCCATTTTGGCTGGT
AAAACATATTTTTTTTCGTTTTTGTCCAGTTTTTGCTTGTACGGTTTTTTTAA
GATACATGTTTTGGTCCAGGGCCCTCCCTGCCACCTATATGGGGAAGGGGT
GGTCCTAGGGGACTGTCAATGGGCACTTTACGGGTCTATTGGTAGGAACTG
AAAGGCTATTCTGGAACCTGTGTCTGTTGGTAATGGGTTGACTGCGGTGCG
CGAGTGGGCCATCGGGAGGTCGAAGGGTCATGATGTGAGCTGCGTTTGCA
GGAGCGTGAGCGGTTTGCGGGAGGGAGTCCGAGTCGGTCTAGAACGTCGA
GTCATCCGTTCTATTACTGCCGTTCCTCTCGTAAATGGCCGTTTCGAGCAGT
TCGTCGGAGCGTGAGCGGTTTGCGGGAGGGAGTCCGAGTCGGTCTAGAAC
GTCGAGTCATCCGTTCTATTACTGCCGTTCCTCTTGTAAATGGCCGTTTCGG
GTCTAGTTAGAGTCTTGATGTGAGCTGCGTTTGCAGGAGCGTGAGCGGTTT

GCGGGAGGGAGTCCGAGTCGGTCTAGAACGTCGAGTCATCCGTTCTATTAC
TGCCGTTCCTCTCGTAAATGGCCGTTTCGAGCAGTTCGTCGGAGCGTGAGC
GGTTTGCGGGAGGGAGTCCGAGTCGGTCTAGAACGTCGAGTCATCCGTTCT
ATTACTGCCGTTCCTCTTGTAAATGGCCGTTTCGGGTCGAGTTACCCCTTCC
TCTTGGTGCGTCGACCGTACTGTGGACCTCATTTCCCTTCGGCCACGTTCG
GTTGGGACTCTGCTGGCGGGTCGGTGGTATTCCCTCGGGCGTCTCCGGACG
TCTGCTGGGTCACGCCTCTGTCTCGCTTGCGGCGGTCCCGGTTGGTATCGC
CGTCGATTTTCCTTCCGCTGAGTCGAGCACCCAGGTGCTCCTCTCGGGAGC
GGGTCGACGGTGCCCGTGTTGGTGACGTGTCGGCTCTTGCCGTCATGGCTC
ATCGTCCGGGTCTGGTCGGGTCGAGGTTGGCTTCGGCCTCCTTGGCCCGCA
CCTTAGGAATTGAGGGTTCATTACTGTTGGTTACACTTTTCCTCGAGCCATT
TGGAGGTGCGAGCGGGTCCTGCTCCGGCGGTTCCCGCTCCAGTCTCCACG
ATGGTCCTGATTCCCGGCTCCGGCCGGGATGACTGGACCGAGGACTTGTGC
GGACGGAAACTGGGAGCCCGATGGGGTTTCTGGACGGGCGTGTCTCTCGG
GACGCTAGATGCACCGGCTGGGAGCCTCTCGGGATCTGTTCTTCCTATGTG
ACAAACTTCGTCTCGCTGGACAAGGCGGGTCTCTCCCTCGGGAGCGGCCG
GCCCCCGGCGGGCGCCGTCGTGGATGCCTGTCGTCCCCTCGGTGGTAAGAT
AGTTACCTGGTTGATCCTGCCAGTAGTCATATGCTTGTCTCAAAGATTAAGC
CATGCATGTCTAAGTATAAATGGATTCATACTGTGAAACTGCGAATGGCTCA
TTAAATCAGTTATAGTTTATTTGACGGTACCTTGCTACATGGATAACTGTGGT
AATTCTAGAGCTAATACATGCCGAAAAGCCCCGACTTCTGGAAGGGGTGTA
TTTATTAGATAAAAAACCAATGCGGGCAACCGCTCTTTGGTGATTCATGATA
ACTTCTCGAATCGCATGGCCTTGCGCCGGCGATGCTTCATTCAAATATCTGC
CCTATCAACTTTCGATGGTAGGATAGAGGCCTACCATGGTATCAACGGGTAA
CGGGGAATTAGGGTTCGATTCCGGAGAGGGAGCCTGAGAAACGGCTACCA
CATCCAAGGAAGGCAGCAGGCGCGCAAATTACCCAATCCCGACACGGGGA
GGTAGTGACAATAAATAACAATACAGGGCTCTATTGGGTCTTGTAATTGGAA
TGAGTACAATTTAAATCCCTTAACGAGGAACAACTGGAGGGCAAGTCTGGT
GCCAGCAGCCGCGGTAATTCCAGCTCCAGTAGCGTATATTAAAGTTGTTGC
AGTTAAAACGCTCGTAGTCGAAACTCGGGCCCGGCGGGCCGGTCCGCCTC
ACGGTGTGCACTGTCCGGCCGGGCCTTACCTCTTGGTGGCCGCGTTCCTCT
CACGGGGTGCGTGGTGTAACCAGGAACTTTACCTTGAGAAAATTAGAGTGT
TCAAAGCAGGCAAACGCCCGGATACATTAGCATGGAATAATAGAATAGGAC
GTGCGGTTCTATTTTGTTGGTTTCTAGGATCGCCGTAATGATCAATGGGGAC
GGTCGGGGGCATTGGTATTCCTTTGCTAGAGGTGAAATTCTTAGATTACAGG
AAGACCGACAACTGCGAAAGCATTTGCCAAGGACGTTCTCGTCGATCAAG
AACGAAGGTTAGGGGATCAAAAACGATTAGATACCGTTGTAGTCTTAACAG
TAAACTATGCCGACTAGGGATCGGGCCACGTCATCCTCTGACTGGCTCGGC

ACCTTACGAGAAATCAAAGTCTTTGGGTTCTGGGGGGAGTATGGTCGCAAG
GCTGAAACTTAAAGGAATTGACGGAAGGGCACCACCAGGTGTGGAGCCTG
CGGCTTAATTTGACTCAACACGGGGAAACTCACCAGGTCCAGACATAGTG
AGGATTGACAGATTGATAGCTCTTTCTTGATTCTATGGGTGGTGGTGCATGG
CCGTTCTTAGTTGGTGGAGTGATTTGTCTGGTTAATTCCGATAACGAACGAG
ACCTTAACCTGCTAAATAGCCAGGCCGGCTTTTGCTGGTCGTGGGCTTCTTA
GAGGGACTGTCGGCGTCTAGTCGACGGAAGTTTGAGGCAATAACAGGTCT
GTGATGCCCTTAGATGTTCTGGGCCGCACGCGCGCTACACTGACCGAGCCA
GCGAGTTCATCGCCTTGCCCGAGAGGGTTGGGTAATCTTGTGAAACTCGGT
CGTGCTGGGGATAGAGCATTGCAATTATTGCTCTTCAACGAGGAATACCTAG
TAAGCGTGAGTCACCAGCTCGCGTTGATTACGTCCCTGCCCTTTGTACACA
CCGCCTGTCGCTACTACCGATTGAATGGCTTAGTGAGATCTCCGGATTGGCG
TTGGGGAGCCGGCAACGGCACCCCTTGGCCGAGAAGCTGATCAAACTTGG
TCATTTAGAGGAAGTAAAAGTCATAACAAGGTTTCCGTAGGTGAACCTGCG
GAAGGATCATTTGAGATCACACCGGGCCGCGAGGCTCTTCCAAACACCTGT
GCACATCGGACCGCGCCCCCGGGCCGGGCCGCCTTCACACAAACATATGTC
AAGAACGTAATGCATCATAACATGAAACAACTTTCAACAACGGATCTCTTG
GCTCTCGCATCGATGAAGAACGCAGCGAATTGCGAAAAGTAATGTGAATTG
CAGAATTCAGTGAATCATCGAATCTTTGAACGCACCTTGCGCCTTTTGGTAT
TCCGAAAGGCATGCCTGTTTGAGTGTCATGTAGACTCAACCCCCCGGGTTT
CTGACCCGGCGGTGTTGGATTTGGGCCCTGCCTCTCCTGGCTGGCCTTAAA
TGCGTTAGTGGTTTCACGCAGACGTCGTAAGTTACGCGTCGACTGTGGGCC
GCTCACAACCCCCTTTACTTTTGCACTCTGGCCTCAAATCAGGTAGGGCTA
CCCGCTGAACTTAAGCATATCAATAAGCGGAGGAAAAGAAACTAACAAGG
ATTCCCCTAGTAACGGCGAGCGAACCGGGAAGAGCTCAAATTTGAAATCTG
GCGTCCTCCGGGCGTCCGAGTTGTAATCTACAGAGGCGTTTTCCGCGCCGG
TCCGTGTCCAAGTCCCTTGGAACAGGGTATCAAAGAGGGTGACAATCCCGT
ACTTGACACGACCGCCGGTGCTTTGTGATACGTCTTCTAAGAGTCGAGTTG
TTTGGGAATGCAGCTCAAAACGGGTGGTAAATTCCATCTAAGGCTAAATATT
GGCGAGAGACCGATAGCGAACAAGTACCGTGAGGGAAAGATGAAAAGCA
CTTTGGAAAGAGAGTTAAACAGCACGTGAAATTGTTGAAAGGGAAACGAT
TGAAGTCAGTCGTGTCCGGGGGGTTCAGCCGGTTCAGCCGGTGCATTCCTC
TCGGATGGGTCAACATCGGTTTTGCCCGGCGGAAAAGGGCGTGAGGAATG
TGGCACCTCCGGGTGTGTTATAGCCTCGCGTCGCATACGTCGGGCGGACC
GAGGAACGCAGCTCGCCTTCACGGCCGGGGTTCGCCCACGTACGAGCTTA
GGATGTTGACATAATGGCTTTAAACGACCCGTCTTGAAACACGGACCAAGG
AGTCTAACATATCTGCGAGTGTTTGGGTGTCAAACCCGAGCGCGTAATGAA
AGTGAACGTAGGAGGGATCCGCAAGGAGCACCTTCGACCGATCCGGATCT

TCTGTGATGGATTTGAGTAAGAGCATATATGCTGGGACCCGAAAGATGGTG
AACTATGCCTGAATAGGGCGAAGCCAGGGGAAACTCTGGTGGAGGCTCGT
AGCGATTCTGACGTGCAAATCGATCGTCAAATTTGGGTATAGGGGCGAAAG
ACTAATCGAACCATCTAATGGCTGGTTCCCGCCGAAGTTTCCCTCAGGATAG
CAGAAGCTCGCATCAGTTTTATGAGGTAAAGCGAATGATTAGAGGCCTTGG
GGACGAAACGTCCTTAACCTATTCTCAAACTTTAAATGTGTAAGAAGCCAC
CGTCGCTTGATTGGACGGTCGGCGTGCGAATGAGAGCTTCTAGTGGGCCAT
TTTTGGTAAGCAGAACTGGCGATGCGGGATGAACCGATCGTGAGGTTAAG
GTGCCGGAATACACGCTCATCAGACACCACAAAAGGTGTTAGTTCATCTAG
ACAGCAGGACGGTGGCCATGGAAGTCGGAATCCGCTAAGGAGTGTGTAAC
AACTCACCTGCCGAATGAACTAGCCCTGAAAATGGATGGCGCTCAAGCGT
GTTACCCATACCTCACCGTTGGCGTTTCAGTGACGCGCCAACGAGTAGGCG
GGCGTGGGGGTCCGTGCAGAAGCCTTGGCAGTGATGCCGGGTGGAACGGC
CCCTAGTGCAGATCTTGGTGGTAGTAGCAAATATTCAAGTGAGAACCTTGA
AGACTGAAGTGGAGAAAGGTTCCATGGTAACAGCAGTTGGACATGGGTCA
GTCGATCCTAAGAGATAGGGAAGCTCCGTTTCAAAGTGCGCGATTTTCCGT
GCCGCCTATCGAAAGGGAATCCGGTTAAGATTCCGGAACCAGGATGTGGAT
CTTTGACGGCGACGTAAGTGAAGTTGGAGACGTTGGCAAGGGCCCCGGGA
AGAGTTCTCTTTTCTCCTTGACCGCCTACGACCCTGAAATCGGATTATCCGG
AGCTAGGGTTAAATGGCGGGTAGAGCACGACACCTCTGTCGTGTCCGGTGC
GTCCTTGACAGCCCTTGAAAATCCGACGGAACGTATAAGTCTCACGCCTGG
TCGTACTCATAACCGCAGCAGGTCTCCAAGGTGAACAGCCTCTAGTTGATA
GAACAATGTAGATAAGGGAAGTCGGCAAAATAGATCCGTAACTTCGGGATA
AGGATTGGCTCTAAGGGTTGGGTGCGTCGGGCCGTTGGTGGAAGAGAGCT
GGACCGGGCCGGACTGGGCGGGGCGACCCGTCTGGACTGGCTCGGACCG
GCGATCGGACGCCTCTGGCAGCCCTCGGGCGTCTGGCGCACGGTTAACAA
CCGACTTAGAACTGGTACGGACAAGGGGAATCTGACTGTCTAATTAAAACA
TAGCATTGCGATGGCCAGAAAGTGGTGTTGACGCAATGTGATTTCTGCCCA
GTGCTCTGAATGTCAAAGTGAAGAAATTCAACCAAGCGCGGGTAAACGGC
GGGAGTAACTATGACTCTCTTAAGGTAGCCAAATGCCTCGTCATCTAATTAG
TGACGCGCATGAATGGATTAACGAGATTCCCACTGTCCCTATCTACTATCTA
GCGAAACCACAGCCAAGGGAACGGGCTTGGCAGAATCAGCGGGGAAAGA
AGACCCTGTTGAGCTTGACTCTAGTTTGACATTGTGAAAAGACATGGAGGG
TGTAGAATAAGTGGGAGCTTCGGCGCCGGTGAAATACCACTACCTCCATCG
TTTTTTTACTTATTCAATGAGGCGGAGCTGGGATTAACGTCCCACCTTTTGG
CTTCAAGGTCCTTCGCGGGCCGATCCGGGTTGAAGACATTGTCAGGTGGG
GAGTTTGGCTGGGGCGGCACATCTGTTAAAAGATAACGCAGGTGTCCTAAG
GGGGACTCATGGAGAACAGAAATCTCCAGTGGAACAAAAGGTAAAAGTC

CCCTTGATTTTGATTTTCAGTGTGAATACAAACCATGAAAGTGTGGCCTATC
GATCCTTTAGTCCCTCGGAATTTGAGGCTAGAGGTGCCAGAAAAGTTACCA
CAGGGATAACTGGCTTGTGGCAGCCAAGCGTTCATAGCGACGTTGCTTTTT
GATCCTTCGATGTCGGCTCTTCCTATCATACCGAAGCAGAATTCGGTAAGCG
TTGGATTGTTCACCCACTAATAGGGAACGTGAGCTGGGTTTAGACCGTCGT
GAGACAGGTTAGTTTTACCCTACTGATGGAGTGACGTCGTGACAGCAATTG
AGGGTAGTACGAGAGGAACTGCTCATTCGGATAATTGGTATTTGCGCCTGT
CCGATCGGGCAATGGCGCGAAGCTATCATCCGTCAGATTATGGCTGAACGC
CTCTAAGTCAGAATCTGTACTGGAAACGACGTTGTTGGTCCCGCACGTGTT
AGTCGCGTTGGAATAGGCTTCGGCTGTGAACCATACCTGGGTAGGGCGTGG
CGGCGGAAATGCCGGCCCGTCCCCCTCTATGAAACGAATATGGGCGGGGGT
GAATCTCTTGCAGACGACTTGACTGGAACGGGGTGCTGTAAGCGGTAGAG
TAGCCTTGTTGCTACGATCCGCTGAGGCTAAGCCTTTGTTCCTCGATTTGTC
CCTCGTGTGTTGACGTGCTGGGGGCTCTGATGCGCCTGGTGCGGGACGTGC
GGGCGACTTGGTTGTGTTTTTGTGTGGGTGTCGGTGCGAGATGTGCTGGGG
GCTCTGGTTTGTGGGTTGCGGGAGCGGCAGGGGACTTGGTGAGGCGGGAC
GGGACAGGACGAGACCGAACGGGGACGGCCGGGCGGAACCTGGGACACG
CCTCCGGGACGACGGTGACGGGCCCGAGAGGTGTGGCGGGTGGCTGGTGT
CGGGGGGAGACGTTGCGGGCGCGGAGGCGTATGGGTGGCGGGGGTGAGA
TGGGAGGGGTTCAACTACTGGGGGCTCTGGTCTCGTTGGTCGGGGGACGG
CGGGGACTTGAGGTTTCTGGGTGTAGGTGTGATGTGCTGGGGGCTCTGGTG
CGGGGAGACGGGAGACGGTGGGGACTTGTCATAAGTGATCATCCCAAGTG
CTGGGGGCTCTGCTTTGTTGGCTCGGGGGGTGGGGAGGACTTGCGATTTG
GGCCGGGAGGTGGTGCGGGTGGTGACGTGCTGGGGGCTCTGGTCGTCAGA
CGTTTTTGGCCGAGGATGTAGCCCCGGTCGACCCGATCCCGGGACCGGTCT
CGGGACTCGTGTCGGCTCGACGGTCGGCCGGGAAAACGGCCCGGAAGTTC
CGAGACCCGACGATTTTCGAGCCACCAAGCCGTCCCGAGCCGTCCCGAGC
CAAGACGCTGACGTGCTGGGGGCTCTGGTCGGAAAACGTTTTCGGCCGAG
GACGTAGCCCCGATCGGCCCGATCCTGGGACCGGTCTCGGGACTCGTGTCG
GCTCGACGGTCGGCCGGGAAAACGGCCCGGAAGTTCCGAGACCCGACGAT
TTTCGAGCCACCAAGCCGTCCCGAGCCGTCCCGAGCCAAGACGCTGACGT
GCTGGGGGCTCTGGTCGGAAAACGTTTTCGGCCGAGGACGTAGCCCCGAT
CGGCCCGATCCTGGGACCGGTCTCGGGACTCGTGTCGGCTCGACGGTCGG
CCGGGAAAACGGCCCGGAAGTTCCGAGACCCGACGATTTTC
