# Supplementary material for: Holistic genome assembly and analysis of the Tremella fuciformis interaction community uncovers intergenomic insights beyond dual genomes
Source: IMA Fungus. 2026 Jun 15;17:e185345. doi: 10.3897/imafungus.17.185345 (PMC13288022; doi:10.3897/imafungus.17.185345)
Supplement: Supplementary material 5 — Alignment of T. fuciformis rDNA [file imafungus-17-e185345-s005.pdf]

```
YN01_hapA_rDNA  1     ACCCATCCC--------------AAGTGCTGGGGGCTCTGGTC------------TTGCCGGGGACTT--  42
                      | || ||||              | ||||||||||||||||||            | |||| |||| |
YN01_hapB_rDNA  374   AGCCCTCCCGGGCCGAGACGGTGACGTGCTGGGGGCTCTGGTCCGAGAACGTTTTTGGCCGAGGACGTAG  443

YN01_hapA_rDNA  42    ----GGT------GAAATC-----ACC---------AC---------------------CCAAGAGTTAA  67
                          |||      |  |||     |||         ||                     ||  ||   ||
YN01_hapB_rDNA  444   CCCCGGTCGGCCCG--ATCCCGGGACCGGTCTCGGGACTCGTGTCGGCTCGACGGTCGGCCGGGA---AA  508

YN01_hapA_rDNA  68    ACAGCACGTGAAATTGTT--GAAAGGGA---AACGATT---GAAGTCAGTCGTG---TCCGGGG--GGTT  124
                      || || || |||   |||  ||    ||    ||||||    | || ||  || |   ||| |||  |
YN01_hapB_rDNA  509   ACGGCCCG-GAA---GTTCCGA----GACCCGACGATTTTCG-AGCCA-CCGAGCCCTCCCGGGCCG---  565

YN01_hapA_rDNA  125   CAGCCGGT--TCTGCCGGTGCATTCCTCTCGGACGGGT-CAACATCGGTTTTGCCCGGCGGAAAAGGGCG  191
                       || ||||    ||| || |     ||||      ||| | | | || ||||| |||       ||| ||
YN01_hapB_rDNA  565   -AGACGGTGACGTGCTGGGG----GCTCT------GGTCCGAGAACGTTTTTGGCCG-------AGGACG  617

YN01_hapA_rDNA  192   TGAG----GAATGT-GGCACC---TCCGGGTGTGTTAT--AGCCTCGCGTCGCAT--AC-GTCGGGCGGG  248
                      | ||    |   || ||| ||    |||||   | | |   | |||| ||||  |  || ||||| ||||
YN01_hapB_rDNA  618   T-AGCCCCG---GTCGGC-CCGATCCCGGGACCGGTCTCGGGACTCGTGTCGGCTCGACGGTCGGCCGGG  682

YN01_hapA_rDNA  249   A----------------CCGAG----GA--------ACGCA---------------GCTC--GCCTTCAC  273
                      |                |||||    ||        || ||               ||||  | |||
YN01_hapB_rDNA  683   AAAACGGCCCGGAAGTTCCGAGACCCGACGATTTTCACCCATCCCAAGTGCTGGGGGCTCTGGTCTT---  749

YN01_hapA_rDNA  274   GGCCGGGG--TTCG-------CCCACGTACGAGCTTACCACACAAGCACCACCAAGCCCTCACCGGTCTG  334
                       |||||||  || |        ||||  | |      || ||||||||||||||||||||  || ||||
YN01_hapB_rDNA  749   -GCCGGGGACTTGGTGAAATCACCACCCAAG------CCGCACAAGCACCACCAAGCCCTAGCCCGTCTC  812

YN01_hapA_rDNA  335   CGGGCCGCCCCTCCGTCCCCGAGCGGTCCCGAGCCGTCCTGGCCGCCCAGGACACCGTCCCGGCCAAGAC  404
                      ||||||||||||||||||||||||||||||||||| ||||||||||||||||||||||||||||| ||||
YN01_hapB_rDNA  813   CGGGCCGCCCCTCCGTCCCCGAGCGGTCCCGAGCCATCCTGGCCGCCCAGGACACCGTCCCGGCCGAGAC  882

YN01_hapA_rDNA  405   CGTGGCCTCTCGGGTCGTCCTCGGGCCGCCCACAGGCCCCGAGAGCAAAACTGCCAAAAACACCTATTTC  474
                      |||||||||||||| ||||||||||||||||||||||||| |||||||||||||||||||||||||||||
YN01_hapB_rDNA  883   CGTGGCCTCTCGGGCCGTCCTCGGGCCGCCCACAGGCCCCAAGAGCAAAACTGCCAAAAACACCTATTTC  952

YN01_hapA_rDNA  475   GAACCGGACGATTCGTCGGCCAAAATCCCCCAAAAATCCCATTTTGGCTGGTAAAACATATTTTTTTTCG  544
                      ||||||||||||||||||||||||||||||||||||||||||||||||||||||||||||||||||||||
YN01_hapB_rDNA  953   GAACCGGACGATTCGTCGGCCAAAATCCCCCAAAAATCCCATTTTGGCTGGTAAAACATATTTTTTTTCG  1022

YN01_hapA_rDNA  545   TTTTTGTCCAGTTTTTGCTTGTACGGTTTTTTTAAGATACATGTTTTGGCCCAGGGCCCTCCCTGCCACC  614
                      |||||||||||||||||||||||||||||||||||||||||||||||||| |||||||||||||||||||
YN01_hapB_rDNA  1023  TTTTTGTCCAGTTTTTGCTTGTACGGTTTTTTTAAGATACATGTTTTGGTCCAGGGCCCTCCCTGCCACC  1092

YN01_hapA_rDNA  615   TATATGGGGAAGGGGTGGTCCTAGGGGACTGTCAATGGGCACTTTACGGGTCTGTTGGTAGGAACTGAAA  684
                      |||||||||||||||||||||||||||||||||||||||||||||||||||| |||||||||||||||||
YN01_hapB_rDNA  1093  TATATGGGGAAGGGGTGGTCCTAGGGGACTGTCAATGGGCACTTTACGGGTCTATTGGTAGGAACTGAAA  1162

YN01_hapA_rDNA  685   GGCTATTCTGGAACCTGTGTCTGTTGGTAATGGGTTGATGGCGGTGCGTGAGTGGGCCATGCGGGAGGTC  754
                      ||||||||||||||||||||||||||||||||||||||  |||||||| ||||||||||| |||||||||
YN01_hapB_rDNA  1163  GGCTATTCTGGAACCTGTGTCTGTTGGTAATGGGTTGACTGCGGTGCGCGAGTGGGCCAT-CGGGAGGTC  1231

YN01_hapA_rDNA  755   GAAGGGTCTTGATGTGAGCTGC------------------------------------------------  776
                      |||||||| |||||||||||||
YN01_hapB_rDNA  1232  GAAGGGTCATGATGTGAGCTGCGTTTGCAGGAGCGTGAGCGGTTTGCGGGAGGGAGTCCGAGTCGGTCTA  1301
```

```
YN01_hapA_rDNA  776  ----------------------------------------------------------------------  776

YN01_hapB_rDNA 1302  GAACGTCGAGTCATCCGTTCTATTACTGCCGTTCCTCTCGTAAATGGCCGTTTCGAGCAGTTCGTCGGAG  1371

YN01_hapA_rDNA  776  ----------------------------------------------------------------------  776

YN01_hapB_rDNA 1372  CGTGAGCGGTTTGCGGGAGGGAGTCCGAGTCGGTCTAGAACGTCGAGTCATCCGTTCTATTACTGCCGTT  1441

YN01_hapA_rDNA  776  ----------------------------------------------------------------------  776

YN01_hapB_rDNA 1442  CCTCTTGTAAATGGCCGTTTCGGGTCTAGTTAGAGTCTTGATGTGAGCTGCGTTTGCAGGAGCGTGAGCG  1511

YN01_hapA_rDNA  776  ----------------------------------------------------------------------  776

YN01_hapB_rDNA 1512  GTTTGCGGGAGGGAGTCCGAGTCGGTCTAGAACGTCGAGTCATCCGTTCTATTACTGCCGTTCCTCTCGT  1581

YN01_hapA_rDNA  776  ------------CTAGCAGTTCGTCGGAGCGTGAGCGGTTTGCGGGAGGGAGTCCGAGTCGGTCTAGAAC  834
                                 | ||||||||||||||||||||||||||||||||||||||||||||||||||||||||
YN01_hapB_rDNA 1582  AAATGGCCGTTTCGAGCAGTTCGTCGGAGCGTGAGCGGTTTGCGGGAGGGAGTCCGAGTCGGTCTAGAAC  1651

YN01_hapA_rDNA  835  GTTGAGTCATCCGTTCTATTACTGCCGTTCCTCTCGTAAATGGCCGTTTCGGGTCGAGTTACCCCTTCCT  904
                     || ||||||||||||||||||||||||||||||| |||||||||||||||||||||||||||||||||||
YN01_hapB_rDNA 1652  GTCGAGTCATCCGTTCTATTACTGCCGTTCCTCTTGTAAATGGCCGTTTCGGGTCGAGTTACCCCTTCCT  1721

YN01_hapA_rDNA  905  CTCGGTGCGTCGACCGTACTGTGGTCCTCATTTCCCTTCGGCCACGTTCGGTTGGGACTCTGCTGGCGGG  974
                     || ||||||||||||||||||||| |||||||||||||||||||||||||||||||||||||||||||||
YN01_hapB_rDNA 1722  CTTGGTGCGTCGACCGTACTGTGGACCTCATTTCCCTTCGGCCACGTTCGGTTGGGACTCTGCTGGCGGG  1791

YN01_hapA_rDNA  975  TCGGTGGTATTCTCTCGGGCGTCTCCGGATGTCTGTTGGGTCACGCCTCTGTCTCGCTTGCGGCGGTTCC  1044
                     |||||||||||| |||||||||||||||| ||||| ||||||||||||||||||||||||||||||| ||
YN01_hapB_rDNA 1792  TCGGTGGTATTCCCTCGGGCGTCTCCGGACGTCTGCTGGGTCACGCCTCTGTCTCGCTTGCGGCGGTCCC  1861

YN01_hapA_rDNA 1045  GGTTGGTATCGCTGTCGATTTTCCTTCCGCTGAGTCGAGCACCCAGGTGCTCCTCTCGGGAGCGGGTCGA  1114
                     |||||||||||| |||||||||||||||||||||||||||||||||||||||||||||||||||||||||
YN01_hapB_rDNA 1862  GGTTGGTATCGCCGTCGATTTTCCTTCCGCTGAGTCGAGCACCCAGGTGCTCCTCTCGGGAGCGGGTCGA  1931

YN01_hapA_rDNA 1115  CGGTGCCCGTGTTGGTGACGTGTCGGCTCTTGCCGTCGTGGCTCATCGTCCGGGTCTGGTCGGGTCGAGG  1184
                     ||||||||||||||||||||||||||||||||||||| ||||||||||||||||||||||||||||||||
YN01_hapB_rDNA 1932  CGGTGCCCGTGTTGGTGACGTGTCGGCTCTTGCCGTCATGGCTCATCGTCCGGGTCTGGTCGGGTCGAGG  2001

YN01_hapA_rDNA 1185  TTGGCTTCGGCCTCCTTGGCCCGCACCTTAGGAATTGAGGGTTCATTACTGTTGGTTACACTTTTCCTCG  1254
                     ||||||||||||||||||||||||||||||||||||||||||||||||||||||||||||||||||||||
YN01_hapB_rDNA 2002  TTGGCTTCGGCCTCCTTGGCCCGCACCTTAGGAATTGAGGGTTCATTACTGTTGGTTACACTTTTCCTCG  2071

YN01_hapA_rDNA 1255  AGCCATTTGGAGGTGCGAGCGGGTCCTGCTCCGGCGGTTCCCGCTCCAGTCTCCACGATGGTCCTGATTC  1324
                     ||||||||||||||||||||||||||||||||||||||||||||||||||||||||||||||||||||||
YN01_hapB_rDNA 2072  AGCCATTTGGAGGTGCGAGCGGGTCCTGCTCCGGCGGTTCCCGCTCCAGTCTCCACGATGGTCCTGATTC  2141

YN01_hapA_rDNA 1325  CCGGCTCCGGCCGGGATGACTGGACCGAGGACTTGTGCGGACGGAAACTGGGAGCCCGATGGGGCTTCTG  1394
                     |||||||||||||||||||||||||||||||||||||||||||||||||||||||||||||||| |||||
YN01_hapB_rDNA 2142  CCGGCTCCGGCCGGGATGACTGGACCGAGGACTTGTGCGGACGGAAACTGGGAGCCCGATGGGGTTTCTG  2211

YN01_hapA_rDNA 1395  GACGGGCGTGTCCCTCGGGACGCTAGATGCACCGGCTGGGAGCCTCTTGGGATCTGTTCTTCCTATGTGA  1464
                     |||||||||||| |||||||||||||||||||||||||||||||||| ||||||||||||||||||||||
YN01_hapB_rDNA 2212  GACGGGCGTGTCTCTCGGGACGCTAGATGCACCGGCTGGGAGCCTCTCGGGATCTGTTCTTCCTATGTGA  2281
```

```
YN01_hapA_rDNA  1465  CAAACTTCGTCTCGCTGGACAAGGCGGGTCTCTCCCTCGGGAGCGGCCGGCCCCCGGCGGGCGCCGTCGT  1534
                      ||||||||||||||||||||||||||||||||||||||||||||||||||||||||||||||||||||||
YN01_hapB_rDNA  2282  CAAACTTCGTCTCGCTGGACAAGGCGGGTCTCTCCCTCGGGAGCGGCCGGCCCCCGGCGGGCGCCGTCGT  2351

YN01_hapA_rDNA  1535  GGATGCCTGTCGTCCCCTCGGTGGTAAGATAGTTACCTGGTTGATCCTGCCAGTAGTCATATGCTTGTCT  1604
                      ||||||||||||||||||||||||||||||||||||||||||||||||||||||||||||||||||||||
YN01_hapB_rDNA  2352  GGATGCCTGTCGTCCCCTCGGTGGTAAGATAGTTACCTGGTTGATCCTGCCAGTAGTCATATGCTTGTCT  2421

YN01_hapA_rDNA  1605  CAAAGATTAAGCCATGCATGTCTAAGTATAAACGGATTCATACTGTGAAACTGCGAATGGCTCATTAAAT  1674
                      |||||||||||||||||||||||||||||||| |||||||||||||||||||||||||||||||||||||
YN01_hapB_rDNA  2422  CAAAGATTAAGCCATGCATGTCTAAGTATAAATGGATTCATACTGTGAAACTGCGAATGGCTCATTAAAT  2491

YN01_hapA_rDNA  1675  CAGTTATAGTTTATTTGACGGTACCTTGCTACATGGATAACTGTGGTAATTCTAGAGCTAATACATGCCG  1744
                      ||||||||||||||||||||||||||||||||||||||||||||||||||||||||||||||||||||||
YN01_hapB_rDNA  2492  CAGTTATAGTTTATTTGACGGTACCTTGCTACATGGATAACTGTGGTAATTCTAGAGCTAATACATGCCG  2561

YN01_hapA_rDNA  1745  AAAAGCCCCGACTTCTGGAAGGGGTGTATTTATTAGATAAAAAACCAATGCGGGCAACCGCTCTTTGGTG  1814
                      ||||||||||||||||||||||||||||||||||||||||||||||||||||||||||||||||||||||
YN01_hapB_rDNA  2562  AAAAGCCCCGACTTCTGGAAGGGGTGTATTTATTAGATAAAAAACCAATGCGGGCAACCGCTCTTTGGTG  2631

YN01_hapA_rDNA  1815  ATTCATGATAACTTCTCGAATCGCATGGCCTTGCGCCGGCGATGCTTCATTCAAATATCTGCCCTATCAA  1884
                      ||||||||||||||||||||||||||||||||||||||||||||||||||||||||||||||||||||||
YN01_hapB_rDNA  2632  ATTCATGATAACTTCTCGAATCGCATGGCCTTGCGCCGGCGATGCTTCATTCAAATATCTGCCCTATCAA  2701

YN01_hapA_rDNA  1885  CTTTCGATGGTAGGATAGAGGCCTACCATGGTATCAACGGGTAACGGGGAATTAGGGTTCGATTCCGGAG  1954
                      ||||||||||||||||||||||||||||||||||||||||||||||||||||||||||||||||||||||
YN01_hapB_rDNA  2702  CTTTCGATGGTAGGATAGAGGCCTACCATGGTATCAACGGGTAACGGGGAATTAGGGTTCGATTCCGGAG  2771

YN01_hapA_rDNA  1955  AGGGAGCCTGAGAAACGGCTACCACATCCAAGGAAGGCAGCAGGCGCGCAAATTACCCAATCCCGACACG  2024
                      ||||||||||||||||||||||||||||||||||||||||||||||||||||||||||||||||||||||
YN01_hapB_rDNA  2772  AGGGAGCCTGAGAAACGGCTACCACATCCAAGGAAGGCAGCAGGCGCGCAAATTACCCAATCCCGACACG  2841

YN01_hapA_rDNA  2025  GGGAGGTAGTGACAATAAATAACAATACAGGGCTCTATTGGGTCTTGTAATTGGAATGAGTACAATTTAA  2094
                      ||||||||||||||||||||||||||||||||||||||||||||||||||||||||||||||||||||||
YN01_hapB_rDNA  2842  GGGAGGTAGTGACAATAAATAACAATACAGGGCTCTATTGGGTCTTGTAATTGGAATGAGTACAATTTAA  2911

YN01_hapA_rDNA  2095  ATCCCTTAACGAGGAACAACTGGAGGGCAAGTCTGGTGCCAGCAGCCGCGGTAATTCCAGCTCCAGTAGC  2164
                      ||||||||||||||||||||||||||||||||||||||||||||||||||||||||||||||||||||||
YN01_hapB_rDNA  2912  ATCCCTTAACGAGGAACAACTGGAGGGCAAGTCTGGTGCCAGCAGCCGCGGTAATTCCAGCTCCAGTAGC  2981

YN01_hapA_rDNA  2165  GTATATTAAAGTTGTTGCAGTTAAAACGCTCGTAGTCGAAACTCGGGCCCGGCGGGCCGGTCCGCCTCAC  2234
                      ||||||||||||||||||||||||||||||||||||||||||||||||||||||||||||||||||||||
YN01_hapB_rDNA  2982  GTATATTAAAGTTGTTGCAGTTAAAACGCTCGTAGTCGAAACTCGGGCCCGGCGGGCCGGTCCGCCTCAC  3051

YN01_hapA_rDNA  2235  GGTGTGCACTGTCCGGCCGGGCCTTACCTCTTGGTGGCCGCGTTCCTCTCACGGGGTGCGTGGTGTAACC  2304
                      ||||||||||||||||||||||||||||||||||||||||||||||||||||||||||||||||||||||
YN01_hapB_rDNA  3052  GGTGTGCACTGTCCGGCCGGGCCTTACCTCTTGGTGGCCGCGTTCCTCTCACGGGGTGCGTGGTGTAACC  3121

YN01_hapA_rDNA  2305  AGGAACTTTACCTTGAGAAAATTAGAGTGTTCAAAGCAGGCAAACGCCCGGATACATTAGCATGGAATAA  2374
                      ||||||||||||||||||||||||||||||||||||||||||||||||||||||||||||||||||||||
YN01_hapB_rDNA  3122  AGGAACTTTACCTTGAGAAAATTAGAGTGTTCAAAGCAGGCAAACGCCCGGATACATTAGCATGGAATAA  3191

YN01_hapA_rDNA  2375  TAGAATAGGACGTGCGGTTCTATTTTGTTGGTTTCTAGGATCGCCGTAATGATCAATGGGGACGGTCGGG  2444
                      ||||||||||||||||||||||||||||||||||||||||||||||||||||||||||||||||||||||
YN01_hapB_rDNA  3192  TAGAATAGGACGTGCGGTTCTATTTTGTTGGTTTCTAGGATCGCCGTAATGATCAATGGGGACGGTCGGG  3261
```

```
YN01_hapA_rDNA  2445  GGCATTGGTATTCCTTTGCTAGAGGTGAAATTCTTAGATTACAGGAAGACCGACAACTGCGAAAGCATTT  2514
                      ||||||||||||||||||||||||||||||||||||||||||||||||||||||||||||||||||||||
YN01_hapB_rDNA  3262  GGCATTGGTATTCCTTTGCTAGAGGTGAAATTCTTAGATTACAGGAAGACCGACAACTGCGAAAGCATTT  3331

YN01_hapA_rDNA  2515  GCCAAGGACGTTCTCGTCGATCAAGAACGAAGGTTAGGGGATCAAAAACGATTAGATACCGTTGTAGTCT  2584
                      ||||||||||||||||||||||||||||||||||||||||||||||||||||||||||||||||||||||
YN01_hapB_rDNA  3332  GCCAAGGACGTTCTCGTCGATCAAGAACGAAGGTTAGGGGATCAAAAACGATTAGATACCGTTGTAGTCT  3401

YN01_hapA_rDNA  2585  TAACAGTAAACTATGCCGACTAGGGATCGGGCCACGTCATCCTCTGACTGGCTCGGCACCTTACGAGAAA  2654
                      ||||||||||||||||||||||||||||||||||||||||||||||||||||||||||||||||||||||
YN01_hapB_rDNA  3402  TAACAGTAAACTATGCCGACTAGGGATCGGGCCACGTCATCCTCTGACTGGCTCGGCACCTTACGAGAAA  3471

YN01_hapA_rDNA  2655  TCAAAGTCTTTGGGTTCTGGGGGGAGTATGGTCGCAAGGCTGAAACTTAAAGGAATTGACGGAAGGGCAC  2724
                      ||||||||||||||||||||||||||||||||||||||||||||||||||||||||||||||||||||||
YN01_hapB_rDNA  3472  TCAAAGTCTTTGGGTTCTGGGGGGAGTATGGTCGCAAGGCTGAAACTTAAAGGAATTGACGGAAGGGCAC  3541

YN01_hapA_rDNA  2725  CACCAGGTGTGGAGCCTGCGGCTTAATTTGACTCAACACGGGGAAACTCACCAGGTCCAGACATAGTGAG  2794
                      ||||||||||||||||||||||||||||||||||||||||||||||||||||||||||||||||||||||
YN01_hapB_rDNA  3542  CACCAGGTGTGGAGCCTGCGGCTTAATTTGACTCAACACGGGGAAACTCACCAGGTCCAGACATAGTGAG  3611

YN01_hapA_rDNA  2795  GATTGACAGATTGATAGCTCTTTCTTGATTCTATGGGTGGTGGTGCATGGCCGTTCTTAGTTGGTGGAGT  2864
                      ||||||||||||||||||||||||||||||||||||||||||||||||||||||||||||||||||||||
YN01_hapB_rDNA  3612  GATTGACAGATTGATAGCTCTTTCTTGATTCTATGGGTGGTGGTGCATGGCCGTTCTTAGTTGGTGGAGT  3681

YN01_hapA_rDNA  2865  GATTTGTCTGGTTAATTCCGATAACGAACGAGACCTTAACCTGCTAAATAGCCAGGCCGGCTTTTGCTGG  2934
                      ||||||||||||||||||||||||||||||||||||||||||||||||||||||||||||||||||||||
YN01_hapB_rDNA  3682  GATTTGTCTGGTTAATTCCGATAACGAACGAGACCTTAACCTGCTAAATAGCCAGGCCGGCTTTTGCTGG  3751

YN01_hapA_rDNA  2935  TCGTGGGCTTCTTAGAGGGACTGTCGGCGTCTAGTCGACGGAAGTTTGAGGCAATAACAGGTCTGTGATG  3004
                      ||||||||||||||||||||||||||||||||||||||||||||||||||||||||||||||||||||||
YN01_hapB_rDNA  3752  TCGTGGGCTTCTTAGAGGGACTGTCGGCGTCTAGTCGACGGAAGTTTGAGGCAATAACAGGTCTGTGATG  3821

YN01_hapA_rDNA  3005  CCCTTAGATGTTCTGGGCCGCACGCGCGCTACACTGACCGAGCCAGCGAGTTCATCGCCTTGCCCGAGAG  3074
                      ||||||||||||||||||||||||||||||||||||||||||||||||||||||||||||||||||||||
YN01_hapB_rDNA  3822  CCCTTAGATGTTCTGGGCCGCACGCGCGCTACACTGACCGAGCCAGCGAGTTCATCGCCTTGCCCGAGAG  3891

YN01_hapA_rDNA  3075  GGTTGGGTAATCTTGTGAAACTCGGTCGTGCTGGGGATAGAGCATTGCAATTATTGCTCTTCAACGAGGA  3144
                      ||||||||||||||||||||||||||||||||||||||||||||||||||||||||||||||||||||||
YN01_hapB_rDNA  3892  GGTTGGGTAATCTTGTGAAACTCGGTCGTGCTGGGGATAGAGCATTGCAATTATTGCTCTTCAACGAGGA  3961

YN01_hapA_rDNA  3145  ATACCTAGTAAGCGTGAGTCACCAGCTCGCGTTGATTACGTCCCTGCCCTTTGTACACACCGCCTGTCGC  3214
                      ||||||||||||||||||||||||||||||||||||||||||||||||||||||||||||||||||||||
YN01_hapB_rDNA  3962  ATACCTAGTAAGCGTGAGTCACCAGCTCGCGTTGATTACGTCCCTGCCCTTTGTACACACCGCCTGTCGC  4031

YN01_hapA_rDNA  3215  TACTACCGATTGAATGGCTTAGTGAGATCTCCGGATTGGCGTTGGGGAGCCGGCAACGGCACCCCTTGGC  3284
                      ||||||||||||||||||||||||||||||||||||||||||||||||||||||||||||||||||||||
YN01_hapB_rDNA  4032  TACTACCGATTGAATGGCTTAGTGAGATCTCCGGATTGGCGTTGGGGAGCCGGCAACGGCACCCCTTGGC  4101

YN01_hapA_rDNA  3285  CGAGAAGCTGATCAAACTTGGTCATTTAGAGGAAGTAAAAGTCATAACAAGGTTTCCGTAGGTGAACCTG  3354
                      ||||||||||||||||||||||||||||||||||||||||||||||||||||||||||||||||||||||
YN01_hapB_rDNA  4102  CGAGAAGCTGATCAAACTTGGTCATTTAGAGGAAGTAAAAGTCATAACAAGGTTTCCGTAGGTGAACCTG  4171

YN01_hapA_rDNA  3355  CGGAAGGATCATTTGAGATTACACCGGGCCGCAAGGCCCTTCCAAACACCTGTGCACATCGGACCGCGCC  3424
                      ||||||||||||||||||| |||||||||||| |||| ||||||||||||||||||||||||||||||||
YN01_hapB_rDNA  4172  CGGAAGGATCATTTGAGATCACACCGGGCCGCGAGGCTCTTCCAAACACCTGTGCACATCGGACCGCGCC  4241
```

```
YN01_hapA_rDNA  3425  TCCGGGCCGGGCCGCCTTCACACAAACATATGTCAAGAACGTAATGCATCATAACATGAAACAACTTTCA  3494
                       |||||||||||||||||||||||||||||||||||||||||||||||||||||||||||||||||||||
YN01_hapB_rDNA  4242  CCCGGGCCGGGCCGCCTTCACACAAACATATGTCAAGAACGTAATGCATCATAACATGAAACAACTTTCA  4311

YN01_hapA_rDNA  3495  ACAACGGATCTCTTGGCTCTCGCATCGATGAAGAACGCAGCGAATTGCGAAAAGTAATGTGAATTGCAGA  3564
                      ||||||||||||||||||||||||||||||||||||||||||||||||||||||||||||||||||||||
YN01_hapB_rDNA  4312  ACAACGGATCTCTTGGCTCTCGCATCGATGAAGAACGCAGCGAATTGCGAAAAGTAATGTGAATTGCAGA  4381

YN01_hapA_rDNA  3565  ATTCAGTGAATCATCGAATCTTTGAACGCACCTTGCGCCTTTTGGTATTCCGAAAGGCATGCCTGTTTGA  3634
                      ||||||||||||||||||||||||||||||||||||||||||||||||||||||||||||||||||||||
YN01_hapB_rDNA  4382  ATTCAGTGAATCATCGAATCTTTGAACGCACCTTGCGCCTTTTGGTATTCCGAAAGGCATGCCTGTTTGA  4451

YN01_hapA_rDNA  3635  GTGTCATGTAGACTCAACCCCCCGGGTTTCTGACCCGGCGGTGTTGGATTTGGGCCCTGCCTCTCCTGGC  3704
                      ||||||||||||||||||||||||||||||||||||||||||||||||||||||||||||||||||||||
YN01_hapB_rDNA  4452  GTGTCATGTAGACTCAACCCCCCGGGTTTCTGACCCGGCGGTGTTGGATTTGGGCCCTGCCTCTCCTGGC  4521

YN01_hapA_rDNA  3705  TGGCCTTAAATGCGTTACTGGTTTCACGCAGACGTCGTAAGTTACGCGTCGACTGTGGGCCGCTCACAAC  3774
                      ||||||||||||||||| ||||||||||||||||||||||||||||||||||||||||||||||||||||
YN01_hapB_rDNA  4522  TGGCCTTAAATGCGTTAGTGGTTTCACGCAGACGTCGTAAGTTACGCGTCGACTGTGGGCCGCTCACAAC  4591

YN01_hapA_rDNA  3775  CCCCTTTACTTTTGCACTCTGGCCTCAAATCAGGTAGGGCTACCCGCTGAACTTAAGCATATCAATAAGC  3844
                      ||||||||||||||||||||||||||||||||||||||||||||||||||||||||||||||||||||||
YN01_hapB_rDNA  4592  CCCCTTTACTTTTGCACTCTGGCCTCAAATCAGGTAGGGCTACCCGCTGAACTTAAGCATATCAATAAGC  4661

YN01_hapA_rDNA  3845  GGAGGAAAAGAAACTAACAAGGATTCCCCTAGTAACGGCGAGCGAACCGGGAAGAGCTCAAATTTGAAAT  3914
                      ||||||||||||||||||||||||||||||||||||||||||||||||||||||||||||||||||||||
YN01_hapB_rDNA  4662  GGAGGAAAAGAAACTAACAAGGATTCCCCTAGTAACGGCGAGCGAACCGGGAAGAGCTCAAATTTGAAAT  4731

YN01_hapA_rDNA  3915  CTGGCGTCCTCCGGGCGTCCGAGTTGTAATCTACAGAGGCGTTTTCCGCGCCGGTCCGTGTCCAAGTCCC  3984
                      ||||||||||||||||||||||||||||||||||||||||||||||||||||||||||||||||||||||
YN01_hapB_rDNA  4732  CTGGCGTCCTCCGGGCGTCCGAGTTGTAATCTACAGAGGCGTTTTCCGCGCCGGTCCGTGTCCAAGTCCC  4801

YN01_hapA_rDNA  3985  TTGGAACAGGGTATCAAAGAGGGTGACAATCCCGTACTTGACACGACCGCCGGTGCTTTGTGATACGTCT  4054
                      ||||||||||||||||||||||||||||||||||||||||||||||||||||||||||||||||||||||
YN01_hapB_rDNA  4802  TTGGAACAGGGTATCAAAGAGGGTGACAATCCCGTACTTGACACGACCGCCGGTGCTTTGTGATACGTCT  4871

YN01_hapA_rDNA  4055  TCTAAGAGTCGAGTTGTTTGGGAATGCAGCTCAAAACGGGTGGTAAATTCCATCTAAGGCTAAATATTGG  4124
                      ||||||||||||||||||||||||||||||||||||||||||||||||||||||||||||||||||||||
YN01_hapB_rDNA  4872  TCTAAGAGTCGAGTTGTTTGGGAATGCAGCTCAAAACGGGTGGTAAATTCCATCTAAGGCTAAATATTGG  4941

YN01_hapA_rDNA  4125  CGAGAGACCGATAGCGAACAAGTACCGTGAGGGAAAGATGAAAAGCACTTTGGAAAGAG-----------  4183
                      |||||||||||||||||||||||||||||||||||||||||||||||||||||||||||
YN01_hapB_rDNA  4942  CGAGAGACCGATAGCGAACAAGTACCGTGAGGGAAAGATGAAAAGCACTTTGGAAAGAGAGTTAAACAGC  5011

YN01_hapA_rDNA  4183  ----------------------------------------------------------------------  4183

YN01_hapB_rDNA  5012  ACGTGAAATTGTTGAAAGGGAAACGATTGAAGTCAGTCGTGTCCGGGGGGTTCAGCCGGTTCAGCCGGTG  5081

YN01_hapA_rDNA  4183  ----------------------------------------------------------------------  4183

YN01_hapB_rDNA  5082  CATTCCTCTCGGATGGGTCAACATCGGTTTTGCCCGGCGGAAAAGGGCGTGAGGAATGTGGCACCTCCGG  5151

YN01_hapA_rDNA  4183  ----------------------------------------------------------------------  4183

YN01_hapB_rDNA  5152  GTGTGTTATAGCCTCGCGTCGCATACGTCGGGCGGGACCGAGGAACGCAGCTCGCCTTCACGGCCGGGGT  5221
```

```
YN01_hapA_rDNA 4183 -------------------GGATGTTGACATAATGGCTTTAAACGACCCGTCTTGAAACACGGACCAAGG 4234
                                       |||||||||||||||||||||||||||||||||||||||||||||||||||
YN01_hapB_rDNA 5222 TCGCCCACGTACGAGCTTAGGATGTTGACATAATGGCTTTAAACGACCCGTCTTGAAACACGGACCAAGG 5291

YN01_hapA_rDNA 4235 AGTCTAACATATCTGCGAGTGTTTGGGTGTCAAACCCGAGCGCGTAATGAAAGTGAACGTAGGAGGGATC 4304
                    ||||||||||||||||||||||||||||||||||||||||||||||||||||||||||||||||||||||
YN01_hapB_rDNA 5292 AGTCTAACATATCTGCGAGTGTTTGGGTGTCAAACCCGAGCGCGTAATGAAAGTGAACGTAGGAGGGATC 5361

YN01_hapA_rDNA 4305 CGCAAGGAGCACCTTCGACCGATCCGGATCTTCTGTGATGGATTTGAGTAAGAGCATATATGCTGGGACC 4374
                    ||||||||||||||||||||||||||||||||||||||||||||||||||||||||||||||||||||||
YN01_hapB_rDNA 5362 CGCAAGGAGCACCTTCGACCGATCCGGATCTTCTGTGATGGATTTGAGTAAGAGCATATATGCTGGGACC 5431

YN01_hapA_rDNA 4375 CGAAAGATGGTGAACTATGCCTGAATAGGGCGAAGCCAGGGGAAACTCTGGTGGAGGCTCGTAGCGATTC 4444
                    ||||||||||||||||||||||||||||||||||||||||||||||||||||||||||||||||||||||
YN01_hapB_rDNA 5432 CGAAAGATGGTGAACTATGCCTGAATAGGGCGAAGCCAGGGGAAACTCTGGTGGAGGCTCGTAGCGATTC 5501

YN01_hapA_rDNA 4445 TGACGTGCAAATCGATCGTCAAATTTGGGTATAGGGGCGAAAGACTAATCGAACCATCTAATGGCTGGTT 4514
                    ||||||||||||||||||||||||||||||||||||||||||||||||||||||||||||||||||||||
YN01_hapB_rDNA 5502 TGACGTGCAAATCGATCGTCAAATTTGGGTATAGGGGCGAAAGACTAATCGAACCATCTAATGGCTGGTT 5571

YN01_hapA_rDNA 4515 CCCGCCGAAGTTTCCCTCAGGATAGCAGAAGCTCGCATCAGTTTTATGAGGTAAAGCGAATGATTAGAGG 4584
                    ||||||||||||||||||||||||||||||||||||||||||||||||||||||||||||||||||||||
YN01_hapB_rDNA 5572 CCCGCCGAAGTTTCCCTCAGGATAGCAGAAGCTCGCATCAGTTTTATGAGGTAAAGCGAATGATTAGAGG 5641

YN01_hapA_rDNA 4585 CCTTGGGGACGAAACGTCCTTAACCTATTCTCAAACTTTAAATGTGTAAGAAGCCACCGTCGCTTGATTG 4654
                    ||||||||||||||||||||||||||||||||||||||||||||||||||||||||||||||||||||||
YN01_hapB_rDNA 5642 CCTTGGGGACGAAACGTCCTTAACCTATTCTCAAACTTTAAATGTGTAAGAAGCCACCGTCGCTTGATTG 5711

YN01_hapA_rDNA 4655 GACGGTCGGCGTGCGAATGAGAGCTTCTAGTGGGCCATTTTTGGTAAGCAGAACTGGCGATGCGGGATGA 4724
                    ||||||||||||||||||||||||||||||||||||||||||||||||||||||||||||||||||||||
YN01_hapB_rDNA 5712 GACGGTCGGCGTGCGAATGAGAGCTTCTAGTGGGCCATTTTTGGTAAGCAGAACTGGCGATGCGGGATGA 5781

YN01_hapA_rDNA 4725 ACCGATCGTGAGGTTAAGGTGCCGGAATACACGCTCATCAGACACCACAAAAGGTGTTAGTTCATCTAGA 4794
                    ||||||||||||||||||||||||||||||||||||||||||||||||||||||||||||||||||||||
YN01_hapB_rDNA 5782 ACCGATCGTGAGGTTAAGGTGCCGGAATACACGCTCATCAGACACCACAAAAGGTGTTAGTTCATCTAGA 5851

YN01_hapA_rDNA 4795 CAGCAGGACGGTGGCCATGGAAGTCGGAATCCGCTAAGGAGTGTGTAACAACTCACCTGCCGAATGAACT 4864
                    ||||||||||||||||||||||||||||||||||||||||||||||||||||||||||||||||||||||
YN01_hapB_rDNA 5852 CAGCAGGACGGTGGCCATGGAAGTCGGAATCCGCTAAGGAGTGTGTAACAACTCACCTGCCGAATGAACT 5921

YN01_hapA_rDNA 4865 AGCCCTGAAAATGGATGGCGCTCAAGCGTGTTACCCATACCTCACCGTTGGCGTTTCAGTGACGCGCCAA 4934
                    ||||||||||||||||||||||||||||||||||||||||||||||||||||||||||||||||||||||
YN01_hapB_rDNA 5922 AGCCCTGAAAATGGATGGCGCTCAAGCGTGTTACCCATACCTCACCGTTGGCGTTTCAGTGACGCGCCAA 5991

YN01_hapA_rDNA 4935 CGAGTAGGCGGGCGTGGGGGTCCGTGCAGAAGCCTTGGCAGTGATGCCGGGTGGAACGGCCCCTAGTGCA 5004
                    ||||||||||||||||||||||||||||||||||||||||||||||||||||||||||||||||||||||
YN01_hapB_rDNA 5992 CGAGTAGGCGGGCGTGGGGGTCCGTGCAGAAGCCTTGGCAGTGATGCCGGGTGGAACGGCCCCTAGTGCA 6061

YN01_hapA_rDNA 5005 GATCTTGGTGGTAGTAGCAAATATTCAAGTGAGAACCTTGAAGACTGAAGTGGAGAAAGGTTCCATGGTA 5074
                    ||||||||||||||||||||||||||||||||||||||||||||||||||||||||||||||||||||||
YN01_hapB_rDNA 6062 GATCTTGGTGGTAGTAGCAAATATTCAAGTGAGAACCTTGAAGACTGAAGTGGAGAAAGGTTCCATGGTA 6131

YN01_hapA_rDNA 5075 ACAGCAGTTGGACATGGGTCAGTCGATCCTAAGAGATAGGGAAGCTCCGTTTCAAAGTGCGCGATTTTCC 5144
                    ||||||||||||||||||||||||||||||||||||||||||||||||||||||||||||||||||||||
YN01_hapB_rDNA 6132 ACAGCAGTTGGACATGGGTCAGTCGATCCTAAGAGATAGGGAAGCTCCGTTTCAAAGTGCGCGATTTTCC 6201
```

```
YN01_hapA_rDNA  5145  GTGCCGCCTATCGAAAGGGAATCCGGTTAAGATTCCGGAACCAGGATGTGGATCTTTGACGGCGACGTAA  5214
                      ||||||||||||||||||||||||||||||||||||||||||||||||||||||||||||||||||||||
YN01_hapB_rDNA  6202  GTGCCGCCTATCGAAAGGGAATCCGGTTAAGATTCCGGAACCAGGATGTGGATCTTTGACGGCGACGTAA  6271

YN01_hapA_rDNA  5215  GTGAAGTTGGAGACGTTGGCAAGGGCCCCGGGAAGAGTTCTCTTTTCTCCTTGACCGCCTACGACCCTGA  5284
                      ||||||||||||||||||||||||||||||||||||||||||||||||||||||||||||||||||||||
YN01_hapB_rDNA  6272  GTGAAGTTGGAGACGTTGGCAAGGGCCCCGGGAAGAGTTCTCTTTTCTCCTTGACCGCCTACGACCCTGA  6341

YN01_hapA_rDNA  5285  AATCGGATTATCCGGAGCTAGGGTTAAATGGCGGGTAGAGCACGACACCTCTGTCGTGTCCGGTGCGTCC  5354
                      ||||||||||||||||||||||||||||||||||||||||||||||||||||||||||||||||||||||
YN01_hapB_rDNA  6342  AATCGGATTATCCGGAGCTAGGGTTAAATGGCGGGTAGAGCACGACACCTCTGTCGTGTCCGGTGCGTCC  6411

YN01_hapA_rDNA  5355  TTGACAGCCCTTGAAAATCCGACGGAACGTATAAGTCTCACGCCTGGTCGTACTCATAACCGCAGCAGGT  5424
                      ||||||||||||||||||||||||||||||||||||||||||||||||||||||||||||||||||||||
YN01_hapB_rDNA  6412  TTGACAGCCCTTGAAAATCCGACGGAACGTATAAGTCTCACGCCTGGTCGTACTCATAACCGCAGCAGGT  6481

YN01_hapA_rDNA  5425  CTCCAAGGTGAACAGCCTCTAGTTGATAGAACAATGTAGATAAGGGAAGTCGGCAAAATAGATCCGTAAC  5494
                      ||||||||||||||||||||||||||||||||||||||||||||||||||||||||||||||||||||||
YN01_hapB_rDNA  6482  CTCCAAGGTGAACAGCCTCTAGTTGATAGAACAATGTAGATAAGGGAAGTCGGCAAAATAGATCCGTAAC  6551

YN01_hapA_rDNA  5495  TTCGGGATAAGGATTGGCTCTAAGGGTTGGGTGCGTCGGGCCGTTGGTGGAAGAGAGCTGGACCGGGCCG  5564
                      ||||||||||||||||||||||||||||||||||||||||||||||||||||||||||||||||||||||
YN01_hapB_rDNA  6552  TTCGGGATAAGGATTGGCTCTAAGGGTTGGGTGCGTCGGGCCGTTGGTGGAAGAGAGCTGGACCGGGCCG  6621

YN01_hapA_rDNA  5565  GACTGGGCGGGGCGACCCGTCTGGACTGGCTCGGACCGGCGATCGGACGCCTCTGGCAGCCCTCGGGCGT  5634
                      ||||||||||||||||||||||||||||||||||||||||||||||||||||||||||||||||||||||
YN01_hapB_rDNA  6622  GACTGGGCGGGGCGACCCGTCTGGACTGGCTCGGACCGGCGATCGGACGCCTCTGGCAGCCCTCGGGCGT  6691

YN01_hapA_rDNA  5635  CTGGCGCACGGTTAACAACCGACTTAGAACTGGTACGGACAAGGGGAATCTGACTGTCTAATTAAAACAT  5704
                      ||||||||||||||||||||||||||||||||||||||||||||||||||||||||||||||||||||||
YN01_hapB_rDNA  6692  CTGGCGCACGGTTAACAACCGACTTAGAACTGGTACGGACAAGGGGAATCTGACTGTCTAATTAAAACAT  6761

YN01_hapA_rDNA  5705  AGCATTGCGATGGCCAGAAAGTGGTGTTGACGCAATGTGATTTCTGCCCAGTGCTCTGAATGTCAAAGTG  5774
                      ||||||||||||||||||||||||||||||||||||||||||||||||||||||||||||||||||||||
YN01_hapB_rDNA  6762  AGCATTGCGATGGCCAGAAAGTGGTGTTGACGCAATGTGATTTCTGCCCAGTGCTCTGAATGTCAAAGTG  6831

YN01_hapA_rDNA  5775  AAGAAATTCAACCAAGCGCGGGTAAACGGCGGGAGTAACTATGACTCTCTTAAGGTAGCCAAATGCCTCG  5844
                      ||||||||||||||||||||||||||||||||||||||||||||||||||||||||||||||||||||||
YN01_hapB_rDNA  6832  AAGAAATTCAACCAAGCGCGGGTAAACGGCGGGAGTAACTATGACTCTCTTAAGGTAGCCAAATGCCTCG  6901

YN01_hapA_rDNA  5845  TCATCTAATTAGTGACGCGCATGAATGGATTAACGAGATTCCCACTGTCCCTATCTACTATCTAGCGAAA  5914
                      ||||||||||||||||||||||||||||||||||||||||||||||||||||||||||||||||||||||
YN01_hapB_rDNA  6902  TCATCTAATTAGTGACGCGCATGAATGGATTAACGAGATTCCCACTGTCCCTATCTACTATCTAGCGAAA  6971

YN01_hapA_rDNA  5915  CCACAGCCAAGGGAACGGGCTTGGCAGAATCAGCGGGGAAAGAAGACCCTGTTGAGCTTGACTCTAGTTT  5984
                      ||||||||||||||||||||||||||||||||||||||||||||||||||||||||||||||||||||||
YN01_hapB_rDNA  6972  CCACAGCCAAGGGAACGGGCTTGGCAGAATCAGCGGGGAAAGAAGACCCTGTTGAGCTTGACTCTAGTTT  7041

YN01_hapA_rDNA  5985  GACATTGTGAAAAGACATGGAGGGTGTAGAATAAGTGGGAGCTTCGGCGCCGGTGAAATACCACTACCTC  6054
                      ||||||||||||||||||||||||||||||||||||||||||||||||||||||||||||||||||||||
YN01_hapB_rDNA  7042  GACATTGTGAAAAGACATGGAGGGTGTAGAATAAGTGGGAGCTTCGGCGCCGGTGAAATACCACTACCTC  7111

YN01_hapA_rDNA  6055  CATCGTTTTTTTACTTATTCAATGAGGCGGAGCTGGGATTAACGTCCCACCTTTTGGCTTCAAGGTCCTT  6124
                      ||||||||||||||||||||||||||||||||||||||||||||||||||||||||||||||||||||||
YN01_hapB_rDNA  7112  CATCGTTTTTTTACTTATTCAATGAGGCGGAGCTGGGATTAACGTCCCACCTTTTGGCTTCAAGGTCCTT  7181
```

```
YN01_hapA_rDNA  6125  CGCGGGCCGATCCGGGTTGAAGACATTGTCAGGTGGGGAGTTTGGCTGGGGCGGCACATCTGTTAAAAGA  6194
                      ||||||||||||||||||||||||||||||||||||||||||||||||||||||||||||||||||||||
YN01_hapB_rDNA  7182  CGCGGGCCGATCCGGGTTGAAGACATTGTCAGGTGGGGAGTTTGGCTGGGGCGGCACATCTGTTAAAAGA  7251

YN01_hapA_rDNA  6195  TAACGCAGGTGTCCTAAGGGGGACTCATGGAGAACAGAAATCTCCAGTGGAACAAAAGGGTAAAAGTCCC  6264
                      ||||||||||||||||||||||||||||||||||||||||||||||||||||||||||||||||||||||
YN01_hapB_rDNA  7252  TAACGCAGGTGTCCTAAGGGGGACTCATGGAGAACAGAAATCTCCAGTGGAACAAAAGGGTAAAAGTCCC  7321

YN01_hapA_rDNA  6265  CTTGATTTTGATTTTCAGTGTGAATACAAACCATGAAAGTGTGGCCTATCGATCCTTTAGTCCCTCGGAA  6334
                      ||||||||||||||||||||||||||||||||||||||||||||||||||||||||||||||||||||||
YN01_hapB_rDNA  7322  CTTGATTTTGATTTTCAGTGTGAATACAAACCATGAAAGTGTGGCCTATCGATCCTTTAGTCCCTCGGAA  7391

YN01_hapA_rDNA  6335  TTTGAGGCTAGAGGTGCCAGAAAAGTTACCACAGGGATAACTGGCTTGTGGCAGCCAAGCGTTCATAGCG  6404
                      ||||||||||||||||||||||||||||||||||||||||||||||||||||||||||||||||||||||
YN01_hapB_rDNA  7392  TTTGAGGCTAGAGGTGCCAGAAAAGTTACCACAGGGATAACTGGCTTGTGGCAGCCAAGCGTTCATAGCG  7461

YN01_hapA_rDNA  6405  ACGTTGCTTTTTGATCCTTCGATGTCGGCTCTTCCTATCATACCGAAGCAGAATTCGGTAAGCGTTGGAT  6474
                      ||||||||||||||||||||||||||||||||||||||||||||||||||||||||||||||||||||||
YN01_hapB_rDNA  7462  ACGTTGCTTTTTGATCCTTCGATGTCGGCTCTTCCTATCATACCGAAGCAGAATTCGGTAAGCGTTGGAT  7531

YN01_hapA_rDNA  6475  TGTTCACCCACTAATAGGGAACGTGAGCTGGGTTTAGACCGTCGTGAGACAGGTTAGTTTTACCCTACTG  6544
                      ||||||||||||||||||||||||||||||||||||||||||||||||||||||||||||||||||||||
YN01_hapB_rDNA  7532  TGTTCACCCACTAATAGGGAACGTGAGCTGGGTTTAGACCGTCGTGAGACAGGTTAGTTTTACCCTACTG  7601

YN01_hapA_rDNA  6545  ATGGAGTGACGTCGTGACAGCAATTGAGGGTAGTACGAGAGGAACTGCTCATTCGGATAATTGGTATTTG  6614
                      ||||||||||||||||||||||||||||||||||||||||||||||||||||||||||||||||||||||
YN01_hapB_rDNA  7602  ATGGAGTGACGTCGTGACAGCAATTGAGGGTAGTACGAGAGGAACTGCTCATTCGGATAATTGGTATTTG  7671

YN01_hapA_rDNA  6615  CGCCTGTCCGATCGGGCAATGGCGCGAAGCTATCATCCGTCAGATTATGGCTGAACGCCTCTAAGTCAGA  6684
                      ||||||||||||||||||||||||||||||||||||||||||||||||||||||||||||||||||||||
YN01_hapB_rDNA  7672  CGCCTGTCCGATCGGGCAATGGCGCGAAGCTATCATCCGTCAGATTATGGCTGAACGCCTCTAAGTCAGA  7741

YN01_hapA_rDNA  6685  ATCTGTACTGGAAACGACGTTGTTGGTCCCGCACGTGTTAGTCGCGTTGGAATAGGCTTCGGCTGTGAAC  6754
                      ||||||||||||||||||||||||||||||||||||||||||||||||||||||||||||||||||||||
YN01_hapB_rDNA  7742  ATCTGTACTGGAAACGACGTTGTTGGTCCCGCACGTGTTAGTCGCGTTGGAATAGGCTTCGGCTGTGAAC  7811

YN01_hapA_rDNA  6755  CATACCTGGGTAGGGCGTGTCGGCGGAAATGCCGGCCCGTCCCCCTCTATGAAACGAATATGGGCGGGGG  6824
                      |||||||||||||||||||| |||||||||||||||||||||||||||||||||||||||||||||||||
YN01_hapB_rDNA  7812  CATACCTGGGTAGGGCGTGGCGGCGGAAATGCCGGCCCGTCCCCCTCTATGAAACGAATATGGGCGGGGG  7881

YN01_hapA_rDNA  6825  TGAATCTCTTGCAGACGACTTGACTGGAACGGGGTGCTGTAAGCGGTAGAGTAGCCTTGTTGCTACGATC  6894
                      ||||||||||||||||||||||||||||||||||||||||||||||||||||||||||||||||||||||
YN01_hapB_rDNA  7882  TGAATCTCTTGCAGACGACTTGACTGGAACGGGGTGCTGTAAGCGGTAGAGTAGCCTTGTTGCTACGATC  7951

YN01_hapA_rDNA  6895  CGCTGAGGCTAAGCCTTTGTTCCTCGATTTGTCCCTCGTGTGTTGACGTGCTGGGGGCTCTGATGCGCCT  6964
                      ||||||||||||||||||||||||||||||||||||||||||||||||||||||||||||||||||||||
YN01_hapB_rDNA  7952  CGCTGAGGCTAAGCCTTTGTTCCTCGATTTGTCCCTCGTGTGTTGACGTGCTGGGGGCTCTGATGCGCCT  8021

YN01_hapA_rDNA  6965  GGTGCGGGACGTGCGGGCGACTTGGTTGTGTTTTTGTGTGGGTGTGGGTGCGAGATGTGCTGGGGGCTCT  7034
                      |||||||||||||||||||||||||||||||||||||||||||||| |||||||||||||||||||||||
YN01_hapB_rDNA  8022  GGTGCGGGACGTGCGGGCGACTTGGTTGTGTTTTTGTGTGGGTGTCGGTGCGAGATGTGCTGGGGGCTCT  8091

YN01_hapA_rDNA  7035  GGTTTGTGGGTTGCGGGAGTGGTGGGGGACTCGGGTGAGAGGGGACGGGATGAGACGGGACGGAACTGGG  7104
                      ||||||||||||||||||| ||  |||||||  ||||||  |||||||||   |||| ||| |||| |||
YN01_hapB_rDNA  8092  GGTTTGTGGGTTGCGGGAGCGGCAGGGGACT-TGGTGAGGCGGGACGGGACAGGACGAGACCGAACGGGG  8160
```

```
YN01_hapA_rDNA  7105  ACGGCCGGGCGGAACCTGGGACATGCCTCCGGGACGACGGTGACGGGCCCAAGAGGTGTGGCGGGTGGCT  7174
                      ||||||||||||||||||||||| |||||||||||||||||||||||| |||||||||||||||||||||
YN01_hapB_rDNA  8161  ACGGCCGGGCGGAACCTGGGACACGCCTCCGGGACGACGGTGACGGGCCCGAGAGGTGTGGCGGGTGGCT  8230

YN01_hapA_rDNA  7175  GGCGTCGGGGGGAGATGTTGCGGGCGTGCAGGCAAAGGGGTGGCGGGGGCGAGATGGGAGGGGTTCAACT  7244
                      || |||||||||||| |||||||||| | ||||  | |||||||||||| ||||||||||||||||||||
YN01_hapB_rDNA  8231  GGTGTCGGGGGGAGACGTTGCGGGCGCGGAGGCGTATGGGTGGCGGGGGTGAGATGGGAGGGGTTCAACT  8300

YN01_hapA_rDNA  7245  ACTGGGGGCTCTGGTCTTGTTGGTCGGGGGACGGCGGGGACTTGAGGTTTCTGGGAGTAGGTGTGATGTG  7314
                      ||||||||||||||||| ||||||||||||||||||||||||||||||||||||| ||||||||||||||
YN01_hapB_rDNA  8301  ACTGGGGGCTCTGGTCTCGTTGGTCGGGGGACGGCGGGGACTTGAGGTTTCTGGGTGTAGGTGTGATGTG  8370

YN01_hapA_rDNA  7315  CTGGGGGCTCTGGTCCGGGGAGACGGGAGACGTTGGGGACTTGTCAGTCAGCATAAGTGATCATCCCAAG  7384
                      |||||||||||||| ||||||||||||||||| |||||||||||       |||||||||||||||||||
YN01_hapB_rDNA  8371  CTGGGGGCTCTGGTGCGGGGAGACGGGAGACGGTGGGGACTTGT-------CATAAGTGATCATCCCAAG  8433

YN01_hapA_rDNA  7385  TGCTGGGGGCTCTGCTTTGTTGGCTCGGGGGATGGGGAGGACTTGTGATTTGGGCCGGGAGGTGGTGCGG  7454
                      ||||||||||||||||||||||||||||||| ||||||||||||| ||||||||||||||||||||||||
YN01_hapB_rDNA  8434  TGCTGGGGGCTCTGCTTTGTTGGCTCGGGGGGTGGGGAGGACTTGCGATTTGGGCCGGGAGGTGGTGCGG  8503

YN01_hapA_rDNA  7455  GTGGTGACGTGCCGGGGGCTCTGGTCGGGAGACGTTTTTGGCCGAGGACGTAGCCCCGGCCGGCCCGATC  7524
                      |||||||||||| ||||||||||||||  ||||||||||||||||||| |||||||||| || |||||||
YN01_hapB_rDNA  8504  GTGGTGACGTGCTGGGGGCTCTGGTCGTCAGACGTTTTTGGCCGAGGATGTAGCCCCGGTCGACCCGATC  8573

YN01_hapA_rDNA  7525  CCGGGACCGGTCTTGGGACTCTTGTCGGCACAACGCTCGGCCGGGAAAACGGCCCGGAAGTTCCGGGTAT  7594
                      ||||||||||||| ||||||| ||||||| | ||| ||||||||||||||||||||||||||||| |
YN01_hapB_rDNA  8574  CCGGGACCGGTCTCGGGACTCGTGTCGGCTCGACGGTCGGCCGGGAAAACGGCCCGGAAGTTCCGAGACC  8643

YN01_hapA_rDNA  7595  CGACGATTTTTGGGCGACCGAGCC-TCCCTGGG--GT---GGGAC----CGGTGACGTGCTGGGGGCTCT  7654
                      |||||||||| | || ||| |||| |||| | |  ||   | | |    || ||||||||||||||||||
YN01_hapB_rDNA  8644  CGACGATTTTCGAGCCACCAAGCCGTCCC-GAGCCGTCCCGAGCCAAGACGCTGACGTGCTGGGGGCTCT  8712

YN01_hapA_rDNA  7655  GGTCCGAGAACGTTTTTGGCCGAGGACGTAGCCCGGGGCGGCCCGATCCCGGGAGCGGTCTCGGGATTCG  7724
                      |||| || |||||||| ||||||||||||||||| |  ||||||||||| |||| ||||||||||| |||
YN01_hapB_rDNA  8713  GGTCGGAAAACGTTTTCGGCCGAGGACGTAGCCCCGATCGGCCCGATCCTGGGACCGGTCTCGGGACTCG  8782

YN01_hapA_rDNA  7725  TGTCGGCACGACGGTTGACCGGGAAAACGGCCCGGAAGTTCCGGGACCCGACGATTTTTGA-CCCCCTGG  7793
                      ||||||| ||||||| | ||||||||||||||||||||||||| |||||||||||||| || || ||  |
YN01_hapB_rDNA  8783  TGTCGGCTCGACGGTCGGCCGGGAAAACGGCCCGGAAGTTCCGAGACCCGACGATTTTCGAGCCACCAAG  8852

YN01_hapA_rDNA  7794  GCCTACAG-GGGGT---GGGCAATGAC---GACGTGCTGGGGGCTCTGGTTGTCAGACGTTTTGGGCCGA  7856
                       | | | | |  ||   | || | |||   |||||||||||||||||||| |    ||||||| ||||||
YN01_hapB_rDNA  8853  CCGTCCCGAGCCGTCCCGAGCCAAGACGCTGACGTGCTGGGGGCTCTGGTCGGAAAACGTTTTCGGCCGA  8922

YN01_hapA_rDNA  7857  GGACGTAGCCCCGGTCGGCCCGATCTCGGGACCGGTCTCGGGACTCTTGTCGGCTCGACGGTTGACCGGG  7926
                      ||||||||||||| |||||||||||  ||||||||||||||||||| |||||||||||||||    |||
YN01_hapB_rDNA  8923  GGACGTAGCCCCGATCGGCCCGATCCTGGGACCGGTCTCGGGACTCGTGTCGGCTCGACGGT----CGG-  8987

YN01_hapA_rDNA  7927  AACCGGGAAAACGGCCCGGAAGTTTCGAGACCCGACGATTTTC  7969
                        |||||||||||||||||||||| ||||||||||||||||||
YN01_hapB_rDNA  8987  --CCGGGAAAACGGCCCGGAAGTTCCGAGACCCGACGATTTTC  9028
```
