# Supplementary material for: Holistic genome assembly and analysis of the Tremella fuciformis interaction community uncovers intergenomic insights beyond dual genomes
Source: IMA Fungus. 2026 Jun 15;17:e185345. doi: 10.3897/imafungus.17.185345 (PMC13288022; doi:10.3897/imafungus.17.185345)
Supplement: Supplementary material 6 — T. fuciformis ITS [file imafungus-17-e185345-s006.pdf]

>YN01_hapA_ITS

AAGGATCATTTGAGATTACACCGGGCCGCAAGGCCCTTCCAAACACCTGTGCACA
TCGGACCGCGCCTCCGGGCCGGGCCGCCTTCACACAAACATATGTCAAGAACGTA
ATGCATCATAACATGAAACAACTTTCAACAACGGATCTCTTGGCTCTCGCATCGAT
GAAGAACGCAGCGAATTGCGAAAAGTAATGTGAATTGCAGAATTCAGTGAATCAT
CGAATCTTTGAACGCACCTTGCGCCTTTTGGTATTCCGAAAGGCATGCCTGTTTGA
GTGTCATGTAGACTCAACCCCCCGGGTTTCTGACCCGGCGGTGTTGGATTTGGGCC
CTGCCTCTCCTGGCTGGCCTTAAATGCGTTACTGGTTTCACGCAGACGTCGTAAGT
TACGCGTCGACTGTGGGCCGCTCACAACCCCCTTTACTTTTGCACTCTGGCCTCAA
ATCAGGTAGGGCTACCCGCTGAACTTAA

>YN01_hapB_ITS

AAGGATCATTTGAGATCACACCGGGCCGCGAGGCTCTTCCAAACACCTGTGCACA
TCGGACCGCGCCCCCGGGCCGGGCCGCCTTCACACAAACATATGTCAAGAACGTA
ATGCATCATAACATGAAACAACTTTCAACAACGGATCTCTTGGCTCTCGCATCGAT
GAAGAACGCAGCGAATTGCGAAAAGTAATGTGAATTGCAGAATTCAGTGAATCAT
CGAATCTTTGAACGCACCTTGCGCCTTTTGGTATTCCGAAAGGCATGCCTGTTTGA
GTGTCATGTAGACTCAACCCCCCGGGTTTCTGACCCGGCGGTGTTGGATTTGGGCC
CTGCCTCTCCTGGCTGGCCTTAAATGCGTTAGTGGTTTCACGCAGACGTCGTAAGT
TACGCGTCGACTGTGGGCCGCTCACAACCCCCTTTACTTTTGCACTCTGGCCTCAA
ATCAGGTAGGGCTACCCGCTGAACTTAA
