# Supplementary material for: Holistic genome assembly and analysis of the Tremella fuciformis interaction community uncovers intergenomic insights beyond dual genomes
Source: IMA Fungus. 2026 Jun 15;17:e185345. doi: 10.3897/imafungus.17.185345 (PMC13288022; doi:10.3897/imafungus.17.185345)
Supplement: Supplementary material 7 — Alignment of T. fuciformisITS [file imafungus-17-e185345-s007.pdf]

```
YN01_hapA_ITS  1    AAGGATCATTTGAGATTACACCGGGCCGCAAGGCCCTTCCAAACACCTGTGCACATCGGACCGCGCCTCC  70
                    |||||||||||||||| |||||||||||| |||| |||||||||||||||||||||||||||||||| ||
YN01_hapB_ITS  1    AAGGATCATTTGAGATCACACCGGGCCGCGAGGCTCTTCCAAACACCTGTGCACATCGGACCGCGCCCCC  70

YN01_hapA_ITS  71   GGGCCGGGCCGCCTTCACACAAACATATGTCAAGAACGTAATGCATCATAACATGAAACAACTTTCAACA  140
                    ||||||||||||||||||||||||||||||||||||||||||||||||||||||||||||||||||||||
YN01_hapB_ITS  71   GGGCCGGGCCGCCTTCACACAAACATATGTCAAGAACGTAATGCATCATAACATGAAACAACTTTCAACA  140

YN01_hapA_ITS  141  ACGGATCTCTTGGCTCTCGCATCGATGAAGAACGCAGCGAATTGCGAAAAGTAATGTGAATTGCAGAATT  210
                    ||||||||||||||||||||||||||||||||||||||||||||||||||||||||||||||||||||||
YN01_hapB_ITS  141  ACGGATCTCTTGGCTCTCGCATCGATGAAGAACGCAGCGAATTGCGAAAAGTAATGTGAATTGCAGAATT  210

YN01_hapA_ITS  211  CAGTGAATCATCGAATCTTTGAACGCACCTTGCGCCTTTTGGTATTCCGAAAGGCATGCCTGTTTGAGTG  280
                    ||||||||||||||||||||||||||||||||||||||||||||||||||||||||||||||||||||||
YN01_hapB_ITS  211  CAGTGAATCATCGAATCTTTGAACGCACCTTGCGCCTTTTGGTATTCCGAAAGGCATGCCTGTTTGAGTG  280

YN01_hapA_ITS  281  TCATGTAGACTCAACCCCCCGGGTTTCTGACCCGGCGGTGTTGGATTTGGGCCCTGCCTCTCCTGGCTGG  350
                    ||||||||||||||||||||||||||||||||||||||||||||||||||||||||||||||||||||||
YN01_hapB_ITS  281  TCATGTAGACTCAACCCCCCGGGTTTCTGACCCGGCGGTGTTGGATTTGGGCCCTGCCTCTCCTGGCTGG  350

YN01_hapA_ITS  351  CCTTAAATGCGTTACTGGTTTCACGCAGACGTCGTAAGTTACGCGTCGACTGTGGGCCGCTCACAACCCC  420
                    |||||||||||||| |||||||||||||||||||||||||||||||||||||||||||||||||||||||
YN01_hapB_ITS  351  CCTTAAATGCGTTAGTGGTTTCACGCAGACGTCGTAAGTTACGCGTCGACTGTGGGCCGCTCACAACCCC  420

YN01_hapA_ITS  421  CTTTACTTTTGCACTCTGGCCTCAAATCAGGTAGGGCTACCCGCTGAACTTAA  473
                    |||||||||||||||||||||||||||||||||||||||||||||||||||||
YN01_hapB_ITS  421  CTTTACTTTTGCACTCTGGCCTCAAATCAGGTAGGGCTACCCGCTGAACTTAA  473
```
