# Supplementary material for: Holistic genome assembly and analysis of the Tremella fuciformis interaction community uncovers intergenomic insights beyond dual genomes
Source: IMA Fungus. 2026 Jun 15;17:e185345. doi: 10.3897/imafungus.17.185345 (PMC13288022; doi:10.3897/imafungus.17.185345)
Supplement: Supplementary material 9 — Interspecies chimeric reads from raw sequencing data [file imafungus-17-e185345-s009.pdf]

>Mosaic_sequence_01

CTCCGTGGAATAAGCCGCTATTCCTGCCACCATAAGCGATCATCTGAAGACT
GATGCTAGCGGCGCCGGCAGAATCTCCGCCTAAAACGACATGGCTCGGATC
GCCACCAAATGCTGAAATATACCGATTTACCCATTGGAGAGCTTTCCACTGA
TCGCGCAGCCCGTTGTTGGCTTTAATTCGGTTGCCATTGGTAATAAAACCAT
ATGGACCAACGCGATAGTTGAAAGTAACAACGACTATATTGTAGTCGCTAG
CTTCGATTAAGCCAGCCCCATTCAGATTCGGGTTACTATTGACGCTGAACCC
CCCACCTTGGATGAAGAAAAATACCGGCAACCTGGAGTTCCGCGTCGCATT
ACTTGGCGCGTACACGTCAAGAAACAAACAATCTTCCGAGTTTCCATTACC
ACCCGCCCCACCAACTGGTATACAGATAGGCCCGTGCTAATATTAGGAAAA
GGATGTGTTAGCAAGGATTTAGAGATCGGAAATAATCTAGGGAACATTCGC
ACCTGGGTGGCGTCTTGTGTGGCGGTAAAATTGCAAGGGGGGTCTTGCGG
AGGTGCGAATCTTAAATCACCGAGAGGCGCGGCGGCGTACCGAATGCCAA
GCCATTGTGTGATGCCGTTCGGAGTCGAGTAGCCTGTATACTTGTTACATAT
TAAGTTGACTGTAGGATCTACAGCTAATGCAAGGCGTAATAGCTGGCAAAG
CAAAAAGGCAAGCTTGGTCGGAAGCATTGGGAAGCCTGGATTCTCGGATT
GCTCCGAGCGGGAGGCCGGCGAGATGTCGCTTTGATTAAGGGGTCGCGTG
GGAGAGCAGCATTTTGCAACGCGGGCATCGCGGCCTTCATATATCTGCAGG
CCTGGGATGGCTACTGTAACGGTGTGTAGAGCTGGCTATTAGGATTCCACG
ACATCATCTGACGAAGATTCCATTCCCCTACTCCCCTACTCCTCTACTCCCC
CGTCCCGCGTCTAGACCCGATAGTGTTTTACTTCCCTGCATCTACCGTCCTT
GCTCAAGGCTGTGTGACCAATGCCGAACCATGCGAGCTGACCACATTCCA
GTCTTCGAGCGTGTTGGTCAACAGTTAGTTATCTCATCTTCCTTGTAGATAC
CTAAGCATGTATGTATGTAGGTAGGTACTTGGTTAGGGTAGTGTAGTAGTGT
AAACAACACCGCGTGATCACGACGTAAAGAATCCGGGTGATAAATGTCTG
ACCGCCGCCGTCAGCAAGCGGCACTGTCTGCACGGCACATCTGGGCACAT
CTCAAGGGATATGCAAAGATTGGAGAATTATCGGCATGAGCGTCGCGGGTG
TGGGGGAATAAGGCTCTAAGGCTCCAAGGTTCTACTCTATCTCCCTCTATCG
TCTATCGTAGTCCATCGTAGTCTCCCAGCTCCAGCTCCAGTCTTCAGAGTTA
CTTTGTTACATACCTACTCCATTTTTCTCCATTTCCTTCTATGGAACTCATAA
ACCGTAAACCTGATTTGATCAGTTATCCAATGTGGGGAACCCCGGGCGATG
CTCGCTCCATCAATGCCGAATCGGTACATGACCCCTGGTTCCTTTCCCTCCC
CTTGCATGGGGCCGTCGGGGCGTCATCAATACGAGATTTCTGCATATTTCCA
CGCTCACATTGAAAGTGTAGTTTTGTGGTAAACGTTACAACTCGTCAGCAA
ATTTTAAAAAATTTTATTTTGACGGCGGCTTGATGAGAAAAGGGGGATATTA
ATTCCAACTATCTGGTAAATGTTGCGACCAAGACTAGGCATCGTTGGCGTC
GTTAGGATCAATAATCCTACTCAAAAAGTCCCTCGATGTGCATCTACGGATG
TACGCGTGTTAAGACTAATGTACGTACTGTGTAGGTGATATTATATGTACATG

GAAAAGTATGAGGTTGAAACCAATGGCAAATTGCACCGTCCGAGTGCCGT
CTATGCTTGATTTTCTAGATGCCGCCTCGCCAAGTCCCGTCAATACTTACCG
CCTGGCTTGTACTCTGGCCAATCTAGATCCCTCAAAAAGAAGCAAAGTCCG
TGCCATCGATCAAGAACCCGTACGTTGCTACCAATAGAAACAATCCTGTTC
CTGGCCGTATTATGAACTAGGGGCGCGCCATAATAAACTAATCTCCCGCTCA
AGTGCCAAGCGATTATTAAAAGAAAGCTGAAAAGATGCAGAATAACATCAT
TCAGATTTCTCAGATACCCGACATCCAAATCTCAAAAGTTGCGTTCCGTCTG
ACCCCAGGGGTCTGCTAGCGAATTCATCGATCGCTCGGAAAAAATGGCGAC
GCAGACAGGCTTCCAGGTAATGCACTCGCTACAACCCTTGACGATATGGTG
CGACAAATCCGGCACCCGCGGTGCGGTAGTATGTCCCGCCATCATCGCCGT
CAGGCAGACCGTGTCGGACGATGCTGGCAAGGACGTCGAGATCGACCTCG
AGAGCCACACGCGGATCTTATATAGAACGGTACGCCGTCCGGAAGGCTGGA
AGCTGTACAGCCTGGACGTAGTGTACGTAAAAGACTGCCTCCGCACGGCC
ATACCGGGAGAATCGCTGCAAATTGATGAAGCGGGGGTGAGAGACACCTC
GTGCCCGGCTTTGAGGAACACGCTTACTTCAGCTGCTCTCGTTCCGATCCC
CTTACCGCCCTGCTCTCCTGGTCGTTCACCCGTAAGGGCATACCCGTCAGTA
ACGAACAGCTCGGGATCGATCGGCCAGGCGATATGGGGAAAGCAATAACA
GATCTGTTGTCATGGGTGGATCTTGTCCCCGCATAGAAGGCATACGGCTCA
GTAGAGGGCGATGGCCGTGTCACACCGTCGTCAGTCCGCTAGCAACAGCC
GTGACTGCATGTAAACAAAAGAGTGCCGCTAGAATAGTGAACAAACCGGC
GTGAGCCCGGGCACCACCGGCTGTCCTCTCTGAGGTCTCTGCTACTCCTCC
CAAGACCTCACAGTCCGTCTCCGACGAGCCAGACCGACATCGGCCTGGGA
CGCACACCCGTTCGTACGATAGGGATGTCTCCCGCGCTTCAGAAGGCCATA
AGTCATTCATACCGAGAGAAATGTCGAAAGAAGGCTCGGCATAAATCCCAT
ACGTATTCCATACCTATTCTGCAACCGCCCCCACCACTTTTCAGCAGACCGT
GAACACCCTCAACGTCCTTGGATCACTTGCCGATTTGCTTGCGTCGGGGCA
ATTCTTGCCGACTCACTTCGTCGAGGCCTTCTCATCCTCTTCGTGGTTGATC
TCGGCTTTCTCTGCCTCTTGCCCGGCGATGACACCTGCCTTCTCCCCGTCAA
AGAGGATGGCAATCTCCTCCAGAGTAAGTCCCTTTGTTTCGACCACCAAGA
AGTAGGCGAGGAGAACAGAGACGTAATTCCCGATGAGGAAGATCAAGTAG
TACTTCCATCCGAGCGCCTTGAGGGCTACAGGATTGACCAGACTGTTGTAC
AGCGCACCGCAGTTCCCGGCGAGCGCGTAGGTCGCCATACCTTGGGTTCGC
AGGTGGAATGGAAGGATTTCGGGCAGGTCTAAGATTATAAGCAGTGGACG
GCGTGCGTCAGAGAGGATCTGTACGCACACAAGAAGGTGAACGGCCAGA
ATCCAAAGACATACACATCGACGAAGATGTAGAGGAACGCCACTGCAGCC
CCGGCAACGGCTGTACTGCCTGTATGGACAAATGTAGCCTACAATACATGA
GCTTGATCCCAATAAAGCGAGTGAGAATACTCACGGTCAGAGCTGTGAAG
AGTGTGAAACTGACAGCGGTCGCCAGTCCCGAACTCAGAACCAGCTTACG

CCTTCCGATACGGTCTGCCAGCTGCGCACCGGCCAATCCGAAAAACAGCA
GGCTGACGAACTTGCCGACAAGTAGACCTTGCACTTTGAGGGGATCGTGG
AAGCCCAAGGTACCGCTACGCCCTCAGTAAAGAACGGTTCACAGCACGTC
TCTGCGTACATGATCACTGGCCAGTAGTATCCCACGAGGTTATTCTGGTGAC
TATCAGCGAGGGGTCGACTGCCAACGGGGAGTAACACTCACACCGGCCAT
CGAGATGGTCCACCCGAAGAGCATGAGAGAGTAGAACCTTTTGCGGTTGC
CAGGTGTGACGAACCACTCCTTGATCGTAATCGCGGATGCTTCATCTCCGC
GTCGAACTTTACGCCGTTTATGATCTCCTTAAGTTCGTGCTGAACAAGCTCG
TCATCCTTGTCACCATTCGCGTGGAGCTTGGCGAGCACGTCGAGCGCCTCG
TCTGGCCTGTCTTGCTTGACCAGCCACCGGGGGGATTCGACCATTATCGGT
GTGACACTGAAGATCAAAAGCGGGGATTGCACCACTGAACTGCATGAGCG
TAAGGACACGCCAGGCCCAGTTCGAATTCCAGTTTGCACATCCGAATCCGA
TCCACCCGACAATGATGGCACCGATAGAGAACATGCAGTAGGTACTGCCCT
GAACGACGGCACGGAATCGAGGGTGGGCAATCTCCGCGATGAGGGGAGCC
GCCACGACCTGATAGAATGACGTAGAGATTCTGGGCTCGTCAGACTGGGCA
TTCAAGCGCCAACCCAGCTTCACTCACCCGGTGACAACCCTTCCAACGCA
GAACGTAGCAGTCGAATTACCGAACCCAGAGATCATAGGACCTGCCACACC
GATCAATGACGCCACAAAGATGATTGTCTTTCGGCCGTAGCGGTCCCCAAG
CCAAGCCCCAAGATAGGCAAATGGGAGCTGCGGAAGTAGGTAAGAGGCGG
CAATGAGACCAAGCTTGATCCCAGTGGGAGCCCCGTAAAACCTGATGAGT
CAATCTTGGCGTCGGCCATGGAGATGCTCTCACTTCTGCCATGGAGGCAGT
CCTTGTACTGTCGAGATATACGCTCCATCGTAGCCGTAGGCGATGATTGCGC
AGTAGAGCAATAGAATGTTGATCATGCACTTGCGGAGGCCGGCATCTTTCC
ACCAGCAAGGTGCAGTGTTGTTACGGAGATTCGACCAATGAACGATCGAG
TCGGCGAGTTCGGCTTTGGCATTCGAAGACATTGTCGATTGGACGTCTGAG
GTCGAGAGTGCGAGTTCAACAATATCAGGGTTTATATACAGTCCCACGTCA
AGAAAGCGCAGAGAGCTGGGCGTCCGGTGCTCCACGGAAAGTCGAGGAC
ATCCCACGGAGAGTCGGAAAAGTCGGGTGGGGCCGATAATTCTGCGGGTG
CCCCGGCGTGTCCCGCTTGACTTGCCGCGGGGCCATACAACAGCTGCTCAC
TACCTTCGCAATCTGGAATGAGCATTCACTCGACATGACTTCCGTACACTCT
CCGCCAACCAGCGGAATGTCCTTAGCGGGGAGCCCATCTGCTATCCTGGCT
CGAAGGAGCCGTTCGGCGGAGTCAGCAGAAGACCCGCCTGGTGAAACGC
CCTCGCAACCGACCGTGGGGCAAGAGGGGAGCCCCTAGTTGTACGGAATG
TCAGCGCCGCAAGGTCAGATGCGATCACGGGTAAGTGATGATTCACTAGGA
CGGTAGTCGTCACGGTGCTCACAGCTTCTTAGAATACCCTGCGGTGAGTCA
CCCCAAGTGACTGGCCAAGCTCGGACTAAAGATGATCAGGACAATGCCTG
TTGAGGAATGCTGGCGACGCTTGTAAACGAGCTACTGGTATCGACTCTGAC
GTGTCGGGGCCAAAAGCAGCAGTGTCTTCTGCGAGTATCAAGGCATCGTCT

TTTGACGCCATCCTGGAGCGCGTTAGTGTCCTCGAGCGAACGATAAGTAGC
ATACCGTCCGAACGGGTCTCAGTGGGACCGGGCAGACAAGATAATGCTGC
GCTACGCAAACGCGAGCAGTCTCCAGAGGATCCTATCCGTACCGCAGCCCT
CGAGCTGCTCGTTCTGGGTCGGCGTGCCGAGGGAAATGCCAGCGACACGG
CTATCTCGCCCTTGCAGCAGGAGCAGGACGACGATTCGCCCCGCCTGGACA
TGCCAGTGGTCCCGGACAACACCACGCAAGACCAGACTAGACAATGCAAG
GTGCAAGAGCTGTTGGCGTTATTGGGGCCGGATGACATGTTTGACATGTTT
TACCGACGGACCGGTGTTGGATTGAGATTCATCTCGGCTCTGCTTCCCACTC
GGCAACAGGGGGAAGTGTTGATCGGAGCAGTGAGCGAATTGATACTGCCA
GTTTGCTGATGCACTGACCCATGGCAGTATGTCGATAAAGCGGACTGGCTC
CATCGATGCGTTCATATGCCCACCTTCCTCCAACAGTGCAATGTGCTCTGGC
AGTATCCGCGCGATACCGTCCTACGCCATGTTTCCCTACCATTTGTAGGCGT
CTATGTCTCGGCTTGCCTACTCGGGTTACAATTTATGGATGACCAAGACCGA
CGGCGTCTTCGAAACCTAGAGATGCCAGGCAAGATGCCGGACTTGCTGCG
AAAAGCTCTAAGATCCGCAATGTGGGGATCCGATTTCATGGGTCATCACAA
CATGGAGCATTTGCAAACAATCATCAACTACACGGTGTTCATGGTGAGCCT
TGCTACAAGCCCACTAACGCAGCTGAATCATGCCAGAACAACCAAGATCG
GTCCGATGCGGCATGGGCTTACCTTGGTTCAGCCATGAAGATCGCTCAGGC
TCTTGGACTCGGCGAGTTGGTTGACGAGGCCTCAGCAACAGGCTCCTGGC
CACCTCGCTGGTCGAGCCGTGTTCAACGCGAAGTTGGCAGACGCATTTGG
TGGAACCTACTATACCTCGATCTCAACCTGGCCCCGAAACCCAGATACAGT
TGTGCGAGTCAGCTCCCTCATTTTCGGACCGCTCTGCCAGCGAACATCGAT
GATGACGATCTGTCCGACAGCGGTCCCTTCTGCCCGCAGCCTGCAAATATT
CGGACCAAACTTTCGTACCAGATCGCCCGATTCAAACTGGCTCGAGCGTTC
TATCTGGAGCAAGGGGCGACGCCTTTCGAGCGAGACTCGACGCATGCATAC
GAACGCCTTTTGAAAGCTGACACTACGTTGGGAGATATCGCTACGGAAGTG
CTCGGTCAGTTTCGTGATTCCCCGTCAAGCGGTCGTCCCGAGGAGAACCTC
GAAAACGCTATGATTCAGCTGGCCGTCCGATCGTATACAATAAAGCTACATC
GGCCTTATCTTTTCAAGGGCTATCAAGAGGAGGATTTCGCGTACTCGAAGG
AGCGATGCCTACATGCCGCCAGAGAGACCCTACGTCTGGCCAATGACGATA
AGGCCAGTGCTTTGGAAACATGGTGGATTCCGGTAATGTACGTCTCGGTCG
CCGCTTGGATTCTCATGTTCGATCTGTTGCGCCTGTCCCCGGTGGCGTCTCG
CCTCAGTAGAACGCAAGAGACTCTCGGCGAACTCAGACGAGCGGCAAAG
CAATTGCGAAGGGTATCTCACATTTCCCGACCGTCTCGCATGGTTGTTAAAG
TCCTCGATGCTCTCCTGGCCGAATACAACGGACGCCAGCAACTCGCATCAT
CCTCCTCACGCTCGACCCCCGAGGCAGATTTGCGGTCTGTCGTTGATCGAG
TTATGCGAAAGGCTCTGAAAGAGCCGGTCACTGGTGATGGCACTCACCAG
GACGCTTCCCATCGACAAAATCTGCACCCCACCGATCCGGCAACGGCATTG

CAGTCGTCAGACTTCGACAACCTTGTCTACGGGAATGAGACATCTACTATG
GGGTTCAACCAGGATCTTCCAGTCACCATGATGTATGCTTCCCCATCGTGGC
CGCCACACACCGAAGCTATTCCTGTCAACAATGTTTCGGACCTCGTAGGCA
CGCCAAGCAACCAAGCCACAACCGAGACTATCTCTAACATCCTGGCAGTC
AGCGATTGGGACCGCTTATTCGATCAACTTGACATGGGCTCAATTGTGCCA
GAGTACAGTTGGGATAGCGAGACGGCGAACATCAGCACAACGGCATGCGC
AAGTACGGGAGATTTATTTGCGGATTGGCGCGTATGAGGATTCAGCAACCG
AGAAAATCTCAACAGAACAACAATGCACATTTGTGATCCGGGCTCTAGAAC
GCCTATCTGTTGGTAATGGCTTCCAATTCTTTGTGAATGCCTGAAACACCTC
AGTGCACGTCTTTCCGGATAATGTGACTCACCACGCCAGAAACATTTATCG
CCAGAGCGGGTGTCGATCAGTTCCGTCTGGAGTTCCGCCTGCGCATCGAAG
ACCAGGATCTTGGGATCATGAGCCGTCCACGCTGGCCAATCTGTCGCGACT
CAGCTAGGTCTATTCTCAGTGCGACCCGCTCACTGTCTTTGAATCCTGAAG
CTGTTGAGCCCGTGTGAGCAAACTTGGCGACATAATCCGTGACATTCTCAG
CAGTCCTGCCTTCCACGGAGTGCCCATCGCAATCCCACATGATGTTGATATT
GAATAAGTACGCCAGTGACATGGAATGACCGCCTGTCAGTCGATATACGTT
TACATGAAAGTCACTCACGTCGCTCCCACTTTCAGAACCCTATCGACCCGA
CTGATCGGCGCGTCGATACGATAGATGTGGACCATTTTTTTCTGCTCATTCA
AACTTTCGGCCAATTCGTCGATCGCAGGGTAGAAAAGAAAGTCGCCAACG
AAAGTGACGGGGTCGACCTCATCCCAGGACTTATCTTGCGAGTCGCCAAG
ATAGTATCGTGTGATCTTCCCAGCGATTTCTGTCGGAAATCTTTGTAGCCAA
AGCTCTTGGGGCACGGGGCTACGTTCTCGGGCCGTGACTTCCATGATACCT
CCCTCTCGCTTCATATAGCCGATAAGGACTTCTTGGACAGAAGAGCTCATCT
GACCCCGCTGTATAAGCGTGATGGGATGAGCTGTGAAGATTGCTCCATCCC
CGGCCTCAATCACATGCCTGCACCAAAATGGAGGCAGACTTGCGTTGCAG
GAGGCCCAGAACGGTGTAAGGGAGTTCCCGTAGTGCCTGGAGTCGACTTG
CCGCGGTTGGGTGCGTGTCCGGCGTGATGCCATGGTGCTGTAGAACTCCCT
GATAAATCGCCTCAGCAGTTTCTCGATTGTGGGGGTGCTGCCATATTAGCCA
GAATCTCCTTGGGGCTGGACTCACAATCGAGCTCGGTGCCCCAGACTGCA
GTATCGCCCGCTTGAAAAGATTCTCGGGGTGAACTAGCAGGTACGACGTCA
AGATCGAGCCTGACATGATCAGACTGAAAAGCATTATTGTCTAATGGGCAC
ACTTACCAGCGCTCTCACCGTACGCAGTGACGTTGTCTGGATCACCTCCGA
AGGCGGCAATGTTCCTCTGAACCCATCGGAGCCATGCGACGTTGTCATAGA
GGCCTGACCATGAGATGTACACGATAGGAAAAGAGACCATGCGAACCAAA
ATTGCCGGTGCTGTGACCGCCAGCCTCTTCTTGCATGTCACTGCCTGCCAA
GAATCCCAAAATGTTCAATCGATACGCTCCTTGAACGCAAATGACTCGACG
ACCTTTTTTAGCTTGATGACGAATGAAGATGGTCGGATCGTTCGGAATGTCT
CCTGGATAACCAAATTGGAAGCCTCCGCCATGGATCCAAGCGAGGACTGGC

AGCCTCTCCCCGGGTTGAATGTCGCCTGGAGCATAGACATTGAGGGTGAAA
CCCTTCTCCTCGTCCATGTACACCCTGTTTCGGCGATAGGGAACTCCATGGT
CCCCGAAGAGAAGATGTGCGTTGGCATCCTTGGGGGCTGGGAAGGCCGGA
CTAATGGCTCGTTAGTTCCTGAGGAACGTCACGAATATGAGAGCGAGCCCA
CCCATAGTCGTAGCATTTCAAGGGGTCTGTCCATGGCACCGGTCTCTCACA
AGGTCTGAATCGCTGCGCGCGACCATATGGAATACCCTGTATGGTGACTCA
GCAAGCTCCAAAGCCAGTCCTTGTCTGCGTGGCTCACTAGCCATTTGAGCA
CTGTCGGATGTCCTCCATCTTCCCCTCTCTCCGCAGCAGGAGCCGGAGAAG
CGTCCCAAAGCGGATACGTGTCGCGGAAGCCGAGCACGGGACCATCATCC
GTTTGTACGAGTACACAGTTGCTCATCGTCGTTGCAGCGTCGAAAAGAATG
TGATAGTCTCCAGCCTGTCCTTCGCTTACTTGCTCTTCGATTGAAAGCGCAC
GAAGTGAAGCTCGGCCTGTTTGCGAAATCCCACGGAGAGTTGCGTTTTCG
GGGGTAATCAGCGACGCTTTTGCCGTAGTTGTTCCGGACTCCAAGATGGAG
GCCGCCGCTTATGCTATGGACTTGGTCGCCGAGTCAAGCTTGGGGTGGTTT
ACTCCTCTGATTGCGATCTCACGCGATGTCCTTCCATCCCGAACCTAATGTG
ACTCTCCGCGGACTTGCTTGTTCCAGCAGGCGATGAGCCCCACAGTTGCCG
AGAGGCGCTGGTATTTGAGCCTCTCGAAGCAATCGCAAACTCTTTCCCCCA
CCCAGTTTTACTGCCATGTCCGAGCAAATCGCCTTAGAAAGCCTCAACGTG
CACTGGTCGGCTTTGCCGAACAACACGGCCAAGAACTGGTGGCAGGACGC
AGGTCTTCGACGCTGCATCTTGAATGTCATTCTGCTTTACGCAAGTATCGTT
GCCTACGGATACGATGGTGCCTTCATCTCTGGTGTTCAAGGTTTACCTCTGT
GGGGAACGTAAGCTTTCCACCAATGTAAAATCAAGCTGATGGGGAGTTACT
TTGGGCACCCAGCTGGTATCAAGTTGGGGCTCATCACCTCGTCTTATCTTAT
CCCGCAACTGCCCTTCGCCTACTTGGGCTCCTGGTTGTCCGACAGATACGG
CCGAAAGGCCGTCATCTTCGTCGCCTCACTGATCGGGGTTCTTGGACCGCT
GGTATCAGGTTTCGGGAAGAATTTGGCAGCATTCTGTGCAGGGAGGGTTAT
CACCGGGTCAGTAGTCAAATCCTCGACCGCTACTATGCTTAATATTCCAGCA
TATCAACCTCGTTCTACCAGGTGGTCACTCCTCCCCTCATCGCAGAAATCGC
CCACCCTCGTTTTCGTGCAGCTGCATCGGGGAGTACGTACTGCATCTATGCG
GTTGGTGCTCTCGTCTGCGGCTGGATCGGCTTTGGGATGGCCCATTGGCATT
CGAATTGGGCTTGGAGGGTTCTCACTTTCCTACAATGCTTTGGATCGCTTCC
TCTGCTCGTCTGCAGCGTGACCCCCCTTATGGTCGAATCGCCCCGCTGGCT
CGTTAAACAAGAGCGGCCAGAGGAGGCTCTCGCTATACTCGCCAAACTAC
ATGCGAATGGGGACTTGACCGACCCCTTGGTCCAGTACGAGCTCCAAGAG
ATCACCGGAGCCATCAACAACGACATCCTCGGACGCCAGTGGCAATTCAA
AAATTGGTTCACCACTCCAGGGAATCGCAGGCGCTTCGCGACGCTCATATA
CTTTGGATGGTCAATCTCTATGGCAGGGGTGAGTCGCACTTTAGTCGCAAG
CTCTCCACTCATCTCAGAGCAACGTCATCGCGTACTACTTCCCTAGCGTGCT

GCGAGTGCTTGGCTACACAAATGCCGTCCAGGTTGAAGGGATGTTAGCGG
GAGACTTTGGCGTCCTGCTCGTGTTTGGTCTTGCTGGCGCCCAGCTTGCGG
ATCGCATTGGACGGAGGAAGTTGGTACTATTTTCGAGTACGCTCACAGCTAT
CACTTTGACGATTTTCACCGCCCTCATGGTCAGTCTGGATCTCATCTGCTCT
CCACGAAACTGATTCAAAGGCCGTTTTTGTCAATCGGGGCGGAAAGGCGG
TGGCCGGTGCCAGCCTGTCCATGCTGTATCTCCACGCTGGTCTGTACGTTTT
CGGCTATTGGCCTCTGACTTTCCTGTGTAAGCATTCAGCTCGCAAATCAACA
GTGTTACTGACATTCAGATCTTCCCGAGATCCTTCCGTTCCATATGCGGACC
CAAGGCATGGCGACCTTCTCCCTGTTCGCCAACCTCGGGGCTCTATACAGC
GCGTTGCTGACTCCGATCGCACTGGCTGGTATAGGCTGGAAATACTTCCTG
GTATTTGCGGTCGGAAACTACATCATGATAGTGGCCGCCTACTTCCTCATTC
TGGAGACGAAGGGACTCTCCCTGGAAGAGATAGCAGTTCTCTTCGACGGC
GACGATGCGGCGCTTGTCCGTGCGCAGCACAGCGCTAAGGCTGATGCGAT
GAACGAGGAAGACAAAACAGTTGATCAGCAATTCGAGTTGACTCAGCTAC
CTGCCTGATCGACTTGGGAGCAATTGCGGGGTTTATGCATAATGTAAAGGTT
GCAATGGCGCCTGAGGTATGCAATTGCTGCTTCGTAGCGAATCAGTCCAAA
TTTTCTCGGACGGGTCGGTCATGTCAAAGAGCAAGCTTCCGTTACGTGTCT
CGTCATGCGAATGGCTCGTCGCGCGCAAAAGAAGCGCTCGAATCCGTGGG
GACATGTTTAGTGGGCCGAGGCGACGTAGAAGGTGCCGAACATCCCGAGA
CTACAAGTTGCCGCGGACTGTACACAATTTCGGGAATTCCCCTCCCTCTTTC
TTTCCGCCAGTAACTTACTGAGTGATGAATCAAAGCTTTTCCTGCTTCCTTA
CTCAAGCCAGCATCGCGTACGATGGACGACAAGGAGGCCAGTCCACTTGA
GCCGGCAATTGACATCGAACACGTCCCATGCGATGACGATCCACGGAAATG
GAGTTATGTGAAGAAGCACGTTGTCTTGGCTATGGTGACTTCGGCAAGTGT
AGGTTGCGGGCATATGTAGGGAGTACAATCCTTGATATCGAACAGATAGGTC
CATTGCTCGCCCCTTCTATATATAATCCCGTCATCGATCAAATCAAAGCCGAC
CTTGACGCCAGCGACACGCAAATAGCGTTGAGTCTGAGTATCTATATCCTGT
GAGGTTCTTTCGGAAGCGGAAATCCGCTTACGGGCAGCTTCCAGGGATGGT
TTCCAATAATGTGGGCGACCGCGTCGGAGGTGAGCCGCCCCAGATCAGCTG
GACGCGTAGCTGACAAGTGCAGGTCTTCGGACGCAAGGTAAATCCTGCCT
ATCGGAATACGAATATATCGCTGACCAACAGTCGATATACATCTTGGCGTATT
CCGTGAGCCGGTCTCATTGACCGAAGCTGTCTCGCTGACACCCAGTTCTAC
ACAGTCGCTACTGTCATTGCATCGAGATCGACCAATATGCCAACTCTGATCG
CGATGCGGGCGATCAGTGCAATGGGCTCTGCTGCTGTCTTCCCAATCGGAG
CGGGCACCCTAGCAGAGATCTTCGATCCCGAAGAAAGAGGACGCAAGGTG
GGTTGCTACTGCGTACGCTGTGAGATTGTTGACTGGGAGCTTGGCTTCTAC
TATGGTACTGGTCTGGCTGGGCCGGCAGTGGGTCCATTCATAGGCGGTCTT
CTTGGCCAGGTGAGCAGTCCTATGGTCGACCCATCGACCCAAGCTCATAGT

CGGGCATTTGGTTGGAGAGCACCGTTGTATTTCTTGGCCGCCTTTGCAGCT
GTAGTAGACGTTTCTTTCCTGTTCTATCCGGATTCTTGGCGGAAAGAGGTGA
GACTCAAACGTCGGAAATTCTGATCCATAGCGGTCGCCGTTGTATCAAAAG
GCACTAAACAGAGCCCTGAAAGAGACGCCGCGATCCAGCACGACATCGTT
GCAACATGCGCGTCCTCAAGAGCAATCAGATGTAGCAGATGTCATCGTTCA
GGGGACTCTATCGACATCAACGACGCCTCACGCCACGCAGAGTGTTTCACC
GGGCTCGAGCATCCTACCCAATTTTAAGCCAAGCCTGCGTGATGCGAATCC
TCTCCCGACCATGTGGGCGATCATGAAGCAGCACAATAACTCGCTTATCATA
TTGTGTTCTGGTCAGTGTCTCTGATCTTAGAGGAGGCGGCTCATAAGCAGG
CTGCTTCTTCGGCGCCCAGTATTGTCTCCCCTACACGACCAGTCTCACACTA
GCTAGGTGCATACCTCTTCCCGCCTTATTCCATGGTCGCTAAAGAAGCTCAG
ATTGCCGTATCATTACGACGCCTTGAAGATCGGACTCGTGCTCCTATCTCTA
GGGAGCGGGGCTTTGCTGGGGAGTATATTTGGAGGACGAGCGTCAGACAT
GACCCTAAAAAGGATGAAGGCAAGAAACGGCGGAATCCTCATACCTGAGG
TGGGTGACACATCGACCGGGGCGTGCGCTCTGATTTTGCAGTTCCGTTTGA
GAAGTGTTCTTCCCTTCCTGCCGATACTTTTCGCTTCTTTCCTCACCTATGG
GTGGATAGCGAATCAGCACGTTCACGTAGCTGGATTGGTCGTTTGCCTATTC
TTCGCAGGCCTTTCGTTGATGTTTGTGTCTCCGTGCTGGCATCTGCACAAA
GCCCCCCAGCTGATCAGTTCCAGGCTCATCTACTCGAGTACCCTGGCTTACC
TGGTCGATGCGAATCCAGTGAGCCTCGAATACTCTGCATATGTCGGTTGACC
AACTAAAACATCAGGGCCACAGCGCCGGTGCCGTCGCATGCAATTCGGCG
GCTCGGGGAACTATCGGTTGCGTACTGTCTCAAGTCGTGGTGTACATCTTG
AATGGCATTGGCGACGGGGGGTTGTACACGATCATCACTGCGATTCTGGCA
TTCGCCTCGCTCGGCATGATCTTGGTCATGAGTGAGCTGCGTTTGCCGTAA
GCCAAGGCTCAACTGACGATCAATTTAGAGAAAGGGCCAAGATGGGGCAT
GGCCAACCGGCATACAGCACAATAGAGTCACGAAGATAGCATGAGTGCATC
ATTCTCTGATGCGCCACGGACTGAAAGAAGCTCTCGACTGTTCCCGGCAAT
CCTTCTTCTAAAGTCTAAGTCCTCCGGTGTAAGGGACGCGATGTTCACTCA
CTGCCCCCTGCCCTCAACTTCCCATGCTAGGCGTATGCTTCGATAACACTGA
TTGGCTTGATGTACGTGCCGATGGGTTTGTAGGGTATGTCATACCCGAAGGC
AGGCAGAGCCCCAATATCTAATCCTCTTTGTGTGTCGGTCCATTGGGGCGCT
GCAGTGATGCCGAGAACAAGCACCTTCAATCCGTACTTGTAATCCGGCGTC
CCTAGAGCATATCCGCTTCCCGCATCGATGACCGAGATCAGATCAGGAACC
CCTGCGATTATGGTAGATTTGTCGCCGAGGCGATGTTCAGCATAGAGGTTCT
CGTTCTTGAATGGGATGACGAGAGATCCCTCGAAGTGCTCCCGCGGATGCT
GCGGATCCTCTTCCTCCTCCTCATCCATCGCCAGAGCTTGAATGACTACCTC
GCCGACCGTGTGCCCCTTGTACATCCGCCGAACAACGTCAACAATTTTGCC
TTCGAAGAGTACCTTTGCTGCTCCACGACCTCCCATCGATTCGACCAGGAT

GGACCCGAGGCTGCCCAGCCGACCTTGCCTCTGCGCTAGGGCGACGGCGC
GGCCGATTCTCCAGGACTGCGAGACGGTGTTCTTCACCATGGAAAGCTCGC
ACTCCTTCTTAGTCAACGGACGCTGAGCTGCACCAGCATGAGTTCCCATAT
CGACACAGGCGGCCCGCATAACCGAGTCGCAGTCGAGGTAATGTTTGGCT
GAGGTCATGAAAACGGTATTTCCATCACCACTGCACATCGCCGCCGGCAAC
AACATTTCCCCTCGGTCTCCCTTGTCGTAGACGTTCGGGGTAGTCTGCCATC
CGGTTGGGTATGCCCGACCCATGAAATCGCCGTCGATGATTGGCAAGTCCA
GCAAGTTGCTGGCACCCGCAATCATGTTGATTAGTCCATTTCCACCGCCAAT
CTCGAGTGCTGCCATTGCGACAAGATTATCGATCCTGAGGAATTTGATAAGC
TCAAGTACAGCATCGTTGTAGCTTGCAATCAGCGTGGATGAGCATGTCGTAT
CCAGAAAACACCCACTCCCCGCTGGTCAAGCGCTCTGCGGAGACTTCGGG
GGACCCAATTCCTCCTCCCCAGATACACATCCCGCCGTCCTGCATCTTCGCG
AGGTCGATGACGCGTACGACTGCACCTGACCGGATGAGGTTTCGCAAGGC
CAAGAATGTTGCATGAGGCGAGCCTCCACCACCACAGCCAAGGATGTAAC
AACCCGTAGCCATCCATTCAACGTCGATCTCCGAAAGAATCCACTCTCCAG
CCTTGACGATCGGCTTGTACGCTTGCACTGCGGAGGCCGTCCACTCGATGT
CTTTGGTTGGTAGAGTGAGCTCCTGGTTGCACGGCTTGGCCGTACTTGACG
GTAAGCTTGGTGATGGCGACCGTTCACGGAGGACTCGTACATAGCCTCGCT
GGTCGACTTCGCTATGGACGGGTCAGCCGTCCACTCGCCGGCCGCTTTAAC
GTAGAATCGGCATCGGCCAGCAGTGTCTGATGTCAGCTGAAGTGAGACGA
CGCGAGTGCTCACTCACACGCGATCGGGATGGCCTCGCTCTCCACTACCTT
GCAGTCGCTCGGGTCAGCCCCGGCCGCGATGGCCTTCTTGATGGCACGCTG
CTCAACCATGTGCCGCATCTCGGCCATGGACACCTTGGTCGTGTCTTCAAA
GGTGTCAATCACGGCCGAGATCTGCGCACATGCTGCACCGACCGCGTTCGC
AGCATCGTAATAAGGAAATCGGTGGACCTTGCTGATCCCCTTCAGAGTATCC
GGAGCCAATATTGCGCCGCCTCCAACCAAGTACACGGGTATATCTTGAGTG
CTGGTCTTCATCGAGTCTAGGGCCAGCTCGAGCATGACTTTCATCCTGGCCT
GTGCAGCTTCCAGCATTGACGGATCGACCTGATTGACCAAGGTAGGATCAC
CGATGTCCATAGCTCGACCAGCAGCTACGACAATATCGGTCGCTGTGAGAA
CGCTTCCGCCAAATGCTCGCGACTGGTCCATACGGTATCCCACGGAGTCAG
GGCCAACTGTGGTCTTCCCGTCCTCATCGATGCGCACCTTGGAGCCACCGC
CGAGACCGATGCTCAGCACGTGAGGCATGGAAAAGTTTAGAGAAACACCG
CAGAGCTCATGCGACGCACCCGCCTGTCGTGGAAACCCGGTCGGCAGCAA
GGCTCCGATTTCGGTTGTCGTGCCGCCAATGTCCACCACTAAAGCCGTCTC
TTTCCGTTGTCCTCCACTGGCAAGACTGGCCAAAAGTTGGCACCACGCATC
GAGTTGGTCGGTCCGCTGGAGAAGGTCTTGATTGGGAACCTCTCTGCTTGC
TCGCATGTCATGAGCGTTCCGTCGTTGGAAGTGAGGAACAAGGGACACGT
CAAGTCGAGGGCTGTGGTCGAAGCTTGGAAACCCTTGATGGTGGACGAAG

CAAAGGAGAGGAGAGCGGCATTGAGAATGGTGGCATTCTCTCGTTGCAAC
AGACCTGCCTTTCATTGACCCGCCTATCCAGTGGATTCCACTACCAACCTAG
ATTGGCAACCTCCTTGCTGATGGTCACCTTTACCTCAGGAAGATAGCTCTTG
ATGAGTCCCCGGGCGGTTTCCTCCTGTTTGTGGTCGTGATCGATAGGCGCAT
ATATGGACGAGACTGCCACTGTCCTGATCCCCTGTCTGCGTATTTCGGCGCA
GATTTCGCCAATCTCTTTGGGATCCACCTTAAGTTTCAGCCAACACACCAA
ACTTGGTCGTTCCTCACTCACGCTCTCTATTTCTCGTCCGTCCACTTGGAGT
CCCCCGGCCGCCAAAAATGCAGGTCCCTCAAGGATCGCTCGGAGCTCGTAT
GGGAAGCTGGAGAAGGGCGGTGATAGTCGACTGTGCGGCCCGCATAGGCG
CACAACGGCCACCCTCTCAAGTTTCTCCGCATTGCGTTCGATCAAGCTATTT
ACGAAAGAGGTCGTACCGATAGCGATGGCTTGCAGATGACTACGATCGATA
CTGGCTTTCTGCAGGACAGCCTCGACCGCCTGTTGAATGCCCGTTGTCACA
TCTGCGGTTGTTGGGGCCTTATGAGCAGCGAGCACGGGGTTGAGGGCTCC
AGAAGACAAGTCAAGTATGACCGCATCGCTATACAGCTTCAGTGATCCCCA
CTCAAAGCAGCGCGACTCACGTATTGGTACCGCCAACATCGACACCTATTC
GCAAGGCTTTCGCTGCTGCACCCATCGCGAGAATGGATCGAAGTACGGTAG
ATTGGGAACACGGAAGACCGATAACGAGGTGTTTTGTTGCAATGCCTTTTG
GCACCGGAGGAGCCCGACAGGCTGCGCAAGTAGCTGTACCTAACTATGTAT
CTCTGAATAGCACATGTCTGTCCCGATAGTATCGGGATCTGAGGGGTATGAG
GGGTCCCAATTCCGCTCCCCTGCCGGCAGACTATCTGGTCGACCAGACTGC
CCACCACACAGGCCCTGGGCCGCTCCCCGCCGGCCGTTGCCTGCCCACTG
CCCACCTGCAGAACCGATAGTACCTGACGCTTAGCCGGAAGCCCGCAACTC
GCTAACGTCGTGACATGAGCTTCGATTGCCAGCGATCTCTCCTCCCACGCC
TTGTCATACAGACAGCCAAAGTCGGAAGCAATCTGGACTCGCGAGCAACT
TTCTCCCACGCTTCCCCACGCCAATCAGTAGAGATAGGAGCGGATGTCACA
TTTGCGCTATATGCCCGGGGCTTATATTGCACACCTGGTCAGGACCTACGCA
TTCACATCCCCTCCCTTCAGAATAGCTACCAGACAGTCGAAGGTATCCGTG
AAGATGGTGTGGAGCAAGTCGGGGATCCTGAAGAAGCTCGAAGTCTCTCA
GCGAGACGAGGAGAAGGACGTGTGGTCCAACAACGCCTGGGTGAGCGAT
AATGAGACTACTTGTCTCGTTCCAGCCTGACCAAAAGCTCAACCGGGACA
ATGTCCCCCTGCCCCCCTCTCGCCGAACGTGGGGGGCCTGGTCTTTCGTCG
GGGTACTGGTGCGCGACTGGCATCAACATCAGTGGCTGGTCTGGCGGATCA
TCTTTGCTGTCGTTAGGACTGAGTGTCGGTAAGCCATCATGGGAATGCAAA
TAATGCTTAGCTTACGGCAAACAGGTCAAGCGATGGGGGTCGTGGTAGTCG
GGCAGTTCATCGTCGCCGGGGCGGTCATACTTACCGGACTCGTAGGTGCCA
AGTGGCACGTCGGGTTTCCTATGTGGAACCGCATGACCTGGGGTATGCGGG
CGGCGTGGTTTCCTGTGAGCATTCTGGGGAGTGGGTCATCGCGTGTGACTG
ATGAGTTAAGTTGCTCAACCGGATTATCTTGTCCTTCACCTGGTACGCTAGT

GAGTCTACAACACATATGTGTCAGTACAAAAGTTTATCCCATAGCCCAAGC
ATGGTTCGGAGGGCAGGTCGTCAAGACACTTCTTGGGTCGATCTGGCCGTC
CATATACAGTCAGTTCACCGTGAAAATATCGCTAGGGACATACTGTTGACTT
CTATAGCCATGAAGAATATCATGCCCGCATCCACGGACATGACCTCAGCCGA
TTTCTTGTGCTTTTGGATCTTTCTCATCATCTCATTCCCTCTCGTGTGGATTC
CGCCTGAGCACTACCGCAAGCCTTTCCTCGCCACGTCAGTCATCAGCACGA
TCTGTTGCTTCTCGCTCTTCATCTGGTCTCTGGCTCGCGCCCATGGTGCAGG
GCCTCTTGTCAATGGCGACGAAACGACTCTGGTAGGGGTGGCCCCTGTCA
AGGGATCAGCGCTGGCCTGGGCGTTCTTCTATGGCATCAGCAGCCAGATTG
GGGGGATTTGTGCCGGTGTGAGTGCCCTCGTCTCAATCTGACTGACAATGA
TCTTCTCGACTCATGGCAGATTTTGAACATGTCGGACTACACTCGATTCGCA
GTCAAGCCGGGTGATCAGATCCTCTCCCAGGCTATTTGCGTTCCAGTCATG
ACGACTCTGACCTGCCTGATAGGCATTGTCTGCACCTCGGTCGGTGCGCAA
TACTATCCTGACGAGGGTCTGCTCTGGAATCCGTACAGCCTCTTGACTGCG
ATCCAGAAGTATGGCGGGAACGGTGCGCGCGGCGGTGTTCTTCTGCTGT
ATGTCCTGCATGACTCAATCTCGATTTCGTGCCATGTCTGATTGTTGCAGCT
GTCGCATTCCTCATCAGTCAGCTCGGGATCAACATCGCCGGAAATGCCATC
GCGGGAGGCATCGATTTGACCTCTCTGTTCCCTAGGTACCTCAACATCCGC
CGCGGTGCTATCATCACCACTGTAATGGCTTTGCCTTTGTGCCCATGGGCGC
TGGTGAGCGGAGCGACTGTCTACATCAGCGTGATGGGAGGCTACGCGACG
TTCCTTGGACCCATGACGGGTCTGATGGTATTCGACTACATGTTCATCCGCA
AGCAGAAGATGAAA

>Mosaic_sequence_02

GACTATATATGAGCTTTGCAGGATCATCTCGCCGTCGTCAGCGGGGCACTGC
GAGCCATCTCTCCCGCCGTGCACATCGGACGGGTCATGTATTCGGGGTCGT
ACACAACCGTCAGACGGGTCATCTCACCTCACCACGGGTCCACGTGTTTCA
GTGCAAGGCAAGTGTATGCTCGAGCCGATGCATGGGCCAGATGCCGATCTA
CTTCTATACCTTGGTGGTGTGGTTTACCTAAAGCAACATCTAACTAGGGATA
CAGTCGAACAATCTCGGGAGAAGAGCGGAATAACTAGTGCAATACAGTAA
AGGACAGAGTGCCCGAGGGGTTCTGCGAGACCGAGACCTGCTCCATGGTC
TCAACAATGTAGTGGGCGCCGAGGCCCTCGATCTGCTCGCGCTGGTGCGAG
GTGCAGACGGCGATGACAGTCGAGCCAGCCGCGACGGCAGCCTTGATGCC
GGACGGCGAGTCTTCAAAGATAACGGCACGCTTGGGGTCGATGCCGAGAT
CCTTGGCGGCGAGGAGGAACGGGTCGGGGAACGGTTTGCCCCGCTCGAG
GCGGGCGTCGTCGGCAGTCACGCACACGTCGGGGATGGCGATCCTGCACC
CGTCAGCGAGGCATCATTACGGATGACTTTTGCTCACTCGGTGCGCGTGAG
GCAGCCGTGGCAGTAGGTCTTTGCGCCGGAAGTGGCGACGGCGTATTTCTC

CTTTGGCAGCGAGTTGATCATCTTGCGCACGCCGGGCAGGATCCGCACGCT
CATGTCGATCACGTCCTCCTCGTCCTCAAAGGGCGAGTCGTGGAACCCCCG
GGCGGGCTCCTCGGTCTGCACCTTGGTCAGCGCCATGGACAGCGCGGGGC
CGAGCGAGTTGGACGACTGGCGCCGCGACTTGGGCGTGGCGGGGTCGAG
CGGCGTCATGGGGCCCATCGAGGAGAGCGTCGAGCCCGACTGCGAGCTGC
GCATCGACGAGGAGCGCGGGGTGGACGAGATTGAGTTTTTGCGCGACTGA
GGGGGCGTGTCTGCAAAGTCGAGAATGGAGCGTTCAAAGCCTGTTCAAGT
TAGGACGGTGCAGGGCTTTCTGGGAGGGGGGTGACTCACGGTCCACCTCG
CGGTCGACGTGCTCCTTCTTGAGCCCGGGCACGAGGTCCATGAGGTTGTCG
CTGGCCCGCCGGCCGTGGGTGGCCTTGATGACGGCTTTGGGCTCGAGGCC
GAGCTCGTGCGCCTTGGCAGTCCACGCCGCCTCCACGGCGGCGATCGAGT
CGGTGAGGGTACCGTCCTGAACGGTGGTCAGCGTAGGCACATACACATTGC
GTCAACTAGAGCAGGGAGGGGGATGTGGGGCTCACCATGTCGAACAGCAC
ACCGTCAGCCTCGAATGTGGCGCGCTGGAGCGACGCGAGGTCGGGGCCGC
TGCTGGGCTCTGAGAGCTGGGTGCTGGCGGTCGAGGTCATGGCGAGGGAC
GACATCATATTGCGTGTATAGTATGCGCGTCGACGATAATCAACAAGAGAAT
AGAAATGCAGAATTAGAAGTGGCGACGGGGTCCATGGGTCCCCAGCGCCT
GTTAATATATGTTGATGGCTCGGTCACGGAGACCATACTTTTTAGGGTGATG
ACCCTCGGCGTGCAACGCAATCGAGGGTGCGGATCGATAACACCTGCATGA
TCAGACCATGCATTGGTCCGGACGGCGACGGAGCAGAGGTCAACCAATGA
GCAAGCTGTTCTGTCCAGTCCACGCCGAGGTTTTACTCCTCTTTTTTCATCT
TTGGAGCTGTTTCAGGGCTTTCCGACGTCACCAGTTTCCGATCGTCTCCGT
CTCGTTTCGCGCCATTCAAACTACTACTGCTGCTCCTGCAGCTCTTGTTCCT
ACTGTGATCAACAGTGCATGTGTCGACGTAGTAGGCATGATGACCATCAAT
CATCTAGCCATCACTGTGTCAATTCAACAGCTGAACAGACGAGATGATCCG
TGTACATTTACGTGTTTGACCATCAAAAAGTGGAGTCACTGCCGCCGGCAT
TGCAGGAATCATCTCCGGACCCTGAATGCATGGGAATCTGATTGATGAACC
CTGAGCAGGTGACGGTGCACTGACGTGTGTATGTGTGTGTTGACGGTATCA
AAACGAGACACGTGTCTGAGGCTTTATCGAGTCGCAAAGTGGGGGGATGA
AAGAGTACGGTCAAATAGGGAAAGGTGAGTTTGGGTGGGGATGTCCTTTG
GATGTCCGGCGTTTTCGCACACCCGCTCTGACCCACGTCTCTCTCTTTCTCT
TTCTTTCTCTTCTCTTTCTTTCTTCTCTTTCTTTCTCTAGTCTTACTAAGCAAA
GCATCGCCTCGCATCACAGCTACACGGCCTTACCCTAACCCTAACCCTAACC
CTAACCCTAACCCTAACCCTAACCCTAACCCTAACCCTAACCCTAACCCTAA
CCCTAACCCTAACCCTAACCCTAACCCTAACCCTAACCCTAACCCTAACCCT
AACCCTAACCCTAACCCTAACCCTAACCCTAACCCTAACCCTAACCCTAACC
CTAAACCCTAACCCTAACCCTAACCCTAACCCTAACCCTAACCCTAAACACT
GCGTATGCCCGCCTCTTTCCACACCCCAAATACTAGGCCACTTCATCTTTCA

ACATGAGCTACGTATTCAACTCGGTTCTTTTGGGACTCACTCTCCTCGCATT
TTTGATTTTTTGGTGTCAGCCTCGGAGGAATGCTTCTATACAAAGCCATGGA
TGCGAACCCGCGGTCTCCCATCAGACTACGGAGCCCTTTTTCGGATTAGAT
TTTCAGAAGAGCATGTACTCGGACATACCCTTTCTTTATCGGCTTCATCAAC
GTTACGGAACTACATATCAAGTTCGCTCTTGGATCAGCCCGCCAACGATCTG
TACCATTGCAACTGAAAACCTCCAAACAATCAATACGTCAAAGGACTTCGG
TGTCGGCCCTATGAGGCTGCCTGGACTCGAATATTTCTGTGGTCGTGGTTTC
ATTACGACGGACGGCGACACCTGAAATCACTCGCGGAAACTATTGAAGCC
ATCTTTCGACCTGAGCAATATCCGAAACATGAAGACCTTGAAGCGAGAAGT
CGATGGGTTGCTTGAGAAACTGCCAAAGGATAGTTCGAATATCGATCTTCA
GCCTCATCTTTATGTCATGGTAAATTATTCGAGAGAATATTTATTAATAGTTAT
TTTAAAGAAGCTCTCATGCTAAAGATGACTTGAAGTTTCTTAACTCGGCGC
TACATTTCGTGCTGGGTGTTGATCCGTCAGAGCAGTCGCTTGGCGCACCCC
TTACGGCCAACGAATTTGTCGAAGCTTTTCACAACGCTCTTTTCTACAGCAT
GATTCGGGTCGTTCTCGGTAGGACATGGAAACTTCTCCCACAGTCGAAATA
TGTCAATACTTGCGGAAAAGTGCATAATTTCATTGACTATTACATCAGCCAG
GCCTACGAAGAGAACGTACCGAAGCAAAAAGCTTGATCAGAGCTCTTCTC
GTCCAAACGGAAGATCAAAATTTTATCAGGAGTCAGGTAATCCAAGCTATG
ATGGCGGCGCAGGACACAACATCCGAACTTATTACAAATTCGTTGTTTCTG
CTCGCACGTCATCCAAGGTATTGGGAACAGCTTCGTCTTGAGTTTCTTGAC
AAAGCAGAGGATGATCTCTCTATGGAGACTTTAATGAGTTCCAAGTTGATT
GAGAACATCCTGCATGAAAGTAAGTGCCGCATCAGAGTCCTGGTCTATGGA
ATAGACTGGACTAAGACCGAACTCCCCATCCTGCCGTCCTACAGACTAGAA
TTCTGATATGCCGCATCCCTCATATATTCACTTTAAAATTTTTTGCTAATCGTT
AGTCTAGCCTTACGACTTCACCCAATATTCCCTCTGCTTGGGCGAGTAGCTT
TGCGAGACACAAAGCTGCCAGTCGGAGGGGGCCCTCGTCATGACCGTCCC
GTCTTCGTGCCCAAAGGTTCAACGGTAGTCATGGGGAATTATGCACTCTAC
CGTAACCCAGACGTATTTGGTCATGACGTTGAGACCTTTCGGCCTGAGCGC
TGGAATTCGATTAGGCCAACGCAATGGGAGTTTCTTAAATTTAGTAGTAGAA
ACCAGGCATGCTTGGGACAACAGAAGGCGCTAATTGAAGCATCATATGTTC
TTGCATGACTTACTCAGGCAATCGAGAAATTGACGAGCGCGGATAGTCGGG
AGTGTAAGGGAGAATTGAAGTTAACATGCAAGAGCGCGAATGGCTGTAAA
GTGACAGTCCATTAACCTAGGTTAGACATAAATTACCTCATTATCTAAGTAG
ACTAGGTATAGCGAGGTATGACATTATTGATATAGAAAGATAATCAAGCTTT
CTCTTTCGCTTAGGGTAGTAAACAATGAAGAGAAATCGTTTGCATTAGACTT
CATCCAAGCTCAGGCTGAAGTTCGGATGTTGACACTAAGTATAAGGGTGGG
CAAACATTATTATATTGTAAAGTTAATGAGAGGTTTTAATGGGAAACGGGGT
GGCCAGCGAGGGTGTGCCCGCGGGTACATAACGCATGGTATGGCATAATAT

CTCTTCGTAAACTTAACGTATAAAACCCTTATACCTTTAGTAAACAACCTCT
AATTTCTTAAAATCGCGGTAATCCAAGTGCTTCTCTTCTTCCGGTGCTCGCT
GGGTCTAAGTACGGTTTTTCGCCAATTCCGGAGGCTCGCACGCCTGTCCTT
ATCCCTTCCACAAGATGGATATCGGGCTAACATCGTTTACGTTAGCACGAAA
GGTGGTCAAACGCCGAGGAAATGTACCTACGGTAGCAGCGTGAAGAGTAC
ATATATTACTCCAGATAGCACAGGCTCCTCTTGTCCAGTGCATCCGTGCCAC
GCAGTCGTGGTTGCGAGTGATGAGGCGCATGACATAATCTCGAATCATGTA
ATCTTCGTAGGAATAAACGTCGTTGATGCGGCTAACATGCAAGCCGACCCC
TGCAAATAGTGACTTCCAACCTGTTACCGGGTGTGTTCGTATGACCGGATG
AGACGGGGAGAACTCGTAGCCGACGTTAAGAGGTGACCCGCGATGGGAA
GTGACCTCGTATCCACCAGCCTTACACGCAGACTCAAAAACTGGTTGCGCG
CATGTAGCCGTTAAGGTTTCGAGAAATCTCTGGAAAGGGGGTGACAAGCG
ATCGTACAGTTCATAGCCGGATACCCAAAGGGTATCCCCGCCTGCCGTTGG
CGTATCTTGCATTCGTAGGAACGAAAAGTCCGAAGGACAATTCTCGAAAAG
ACCTAGCAGGCTGCAGTCAGATGAAGAACGAAAGAATGGTGAATGGTGAT
CCTCGACATACTATCACTATGCCATTCCCGACCCAGATCTCGGGGCTCCTTG
CTTGAATCGGGACGGTTTTTCATCGTAGCATAAAGCTTTTTTGCAGCCTCGG
AATTAATGACATAGCTAAGAGTCTGTCAGAAACTACTAGGAAGTTCCGACT
GTTTGCCTTTCTCCTTACATGTTCTTATCGGTAGTCCCGTCTGCCATAGGGA
GGTTATTGGGGTCATTATATAAAGGGTGCACGTGTAATCCGTGCTCCGCGGG
TCTTCCAGTCAACTTGCCTAGCATATCGGTAATATGCTTTTGCTCCTCAATAG
ACAGATCATCTTGAGGGGCTCTGAAGAAGACGACACCGCGCTCGCATATCG
TGATGGCAAGGTCTCGCAGCTGCTCGTTAACATTAGGGGCTTGGATCATATC
CTTGAGCTTTGCTGTAGGATACTCTCTACCGATGATGGGGGTACTGTCGACA
TATTCGAGATGATCGATAGATGACCCTTTTTTCAGGGGCTCCCTGATCAGCT
TACTCATAGACAAGGAACCCATATTATTGGATAGCCTGAATAGCGACGTGTT
TTTTGGTTTCTTATATTAACGATTGCCGAACATACGTCCCATTTTTGAACGGT
TATCAGTTATGCAGATTCGACAATAAAGGAGACGGCCACTTATATTAGCATG
ACTACCTCCCCATTATACACCCATAAACTCGAAATCCCTTACGCTACTCCAA
CACACATCCCTGTCCACATGATCCAGTTCTGCCGCTCGAGAATGCGGGGAG
AGCTAAGCGGCCATGTTTTCAATTTGGCTGTCGGCAATGAGGATTCAGACG
AGTTGGAGATATCAAGCGGGGACATCTCTTGATGGCAACCCAAATGGTGGT
CAAATTTGTTTTGGGGTTAGTCTAAGATTATGACGTGGGTAGGCAGTGACG
AACTCACCATGTTTACCAGCATCTCGCCTTATAGGTGAGGAGGTGTCTCGA
ATTCTATGATTTCACTCGCCTTGCACCATTTACCTAATTGTGATCTCAGTTGA
TAGTTGGGTAGCAAGAGATATAATATACCGCAAGCATGGTGGGCACCGTCG
TATCAGCAAATCCTCTCCAGCTAGACATACGAAGTCATCAAAAATGCCTAG
CTATCTGCTTTGTTGTAACGGTTTCGAGTTTCCAATATGGCCTTGACTATGCC

CTCGTCGGTGGCTTCATGGCTATGCCCGGGTTTCTAAGGGTCTTCGGCTACT
ATGACGACGTAGCTGGCAGATGGGCCATAGACCCGACTGTTCAACAGCTG
ATCTCTTCTCTCATGACAATTGGGACATTCGTCGGATCTCTATCGGTAGGAC
CGTTCAGCTCTAAACTCGGAAGACGACACGGGCTATGGGCAGCTTCGATTT
TGAATTACATAGCGACGGCGGTTATGATCGGGACAACTAATCTTGGGGCTCT
TTACTTTGCGCGTTTTCTTCTTGGTAACTGTAAAACTCGACGTTTATGGTAA
CTGTGGCTGACGCGGAAATAGGAATTTCAGTTGGGTGGTTTCTCACCTTCG
CTCAGGTTTATATCAACGAAGTAGCTCCCGCCCATCTACGGGGCATCGTGTT
CGCCGTATACCAAAGTCAACTATGTGTCGGTTCCATCGTAGGGGCAGCGAT
AGACAACGGTACGCACGAGATGCTCGGCAAAGAAGCGTATCAAATCCCTT
TAGCGATATTCTTCGTCGCTCCGACTATTCAATCCGCGTCCCTCTTCTTTTTT
CCGGAAAGTCCGCGATGGCTAATGACGCGTGGAAAAGAAGCGGCGGCCAA
ATCGGCGCTTCGCCGGCTCCGCAATAAGAATATTAGAGAAGCCGAATTTCAT
GCGGAATTCGAAGAGATTAAAGCCTCCATGATGGAGCAATCAGGTCATTTT
AAAGGGAAACATTTATGGATTGAAATGTGGAAAGGCATAAATCGACGTCGT
TCCTTTCTGTCTATAGCTGTCATTTGTTTCCATTCAGCTAATGGTGGGTCCTC
ACAACCCCTTTTTTGGCGGGAAGATAAGTATGTACAGCTACTTCGAATATTA
ATCCGCATAGTTAGGTTCCTCTTGGGTAAGTACTTTTTCATTGCCTTTTAGGG
TCATGTCGTTCGGTAAACTAATGGTAAAGACAAGTTAAACATCTATACAACC
TACTTCCTCACTGTGGCTGGGGTAAAAGAAGCTTTCGCACTTTCCACGATG
ATAACATGCATGGGACTCGTCGGTGTCCTAGCTAGCACTTTAATAGTATTATA
CGTTGGCCGCCGTTTCATTTTACTTATAGGGGTAGGCGCATGTGGTTTGTGT
CAGCTCGCTTTTGCAATTTCTTGGACTGTGGCTCCGGGAACCGAAATTGCG
GCTAAGCTGGTAGTTGCCTTCAACGCGTTGTTCACGTTCTTTTATGTTGCCT
ATGGTAAGAATCTCGCGAAGATTTAATGCCGACCATTTTCTATATTGTTTACG
CTAACGCGGCTAGCCCCTTATGCGTGGCTTTTAGGAAGCGAACTTCCCAAC
AATCATCTTCGGGCTCACACATATGGCTTAGGCACAGCTTTAAATTTCCTCG
GAAATTGGTTAGGCGTTTTCACGGCACCATATTTCATCAACCCAGCCAGCTT
AGGATGGAGCGCTAAGTACGGGTTAGTCTTAAGGAGAATATTCCCGCCGTT
GGTATAATATTGATGGTCTCATAGGTATATCTGGTTTCTTTCTAACGCTATAGT
GTTCGTGTTCACATGGTGCTTCGTACCTGAAACCCGGGATCGAACTTTAGA
AGAGATTGATGAGATATTCGAAGCTAAAGTTCCAGCACAGAAGTTTAAGAG
TTATGTTTGCATAAATACTGAAGCGTTGAAGAGAGCAACAATGGACGAGAC
AGATCAAAACATTGCTCGTATGAAGGATTAACTTTATAAACCTGTTCTACAG
GCACTTAAGTATCTAGATATTTACGTAATTCACTTACGGTCCGAACGTTCCC
GGTCACATCGGTCCCGAAATGATTTCTCCGGAACCGTTGTATATATTTATACG
GACGATCACGCGTCTTTGCGTCTCTCTTTCCATATATGTCCGTAACAATGCG
GATCCCTTCCACCGCTTCTATTACATCCGACAATTCCGTTAGAGTAAGTGTG

GGCGGATCTTATGACTGACAACAACCCCATGTCTAACCCTGCGTATGCTTTT
CCTTATCGCACCAATTGCTTCGGAATTTCGTTCTTAACGCCGAAAACAAAC
CCCCGGTTCCAGATATTCAACATTAGAATATTCACCGTGATGGGATAAGCGT
ATGGCTCAGTAACCTGCCAAGTGTAGATCCCGATCCATTCATGTAAATCGGG
GGCGCGGACGGAGGGTGGGGAGGACCGACGAAGGCGGCGCCGACATTCC
GAGGCGCGTCTCCCGCAATGATAAATGTCTTCTAAATCCCGAACTTCATCAT
GCTCATGTATCACAAGTCGAAAGCGTATACCTAGTTAGCCATCTTCACCACC
AACAAGCACAATGGCAGGCAAACAATTGATGACTCCACCAGATATTCCTGT
CAACCTCGTTCGAGCGCATGCCGGAGAGACCTTCATGTTAGGGACCGTCCG
TACGTAACTTCTCGACCAATTTGCCTTAGAGCCAACGCTGAAAATTATTGTA
GAGATTCGCGTGATGGAAGACGGTAGCAACACTCGTACGTTTATCCCGCCC
TAGCCCAGAAAGTTTCTTTGCACAAATGTCTCGCTTACCTTTGCGGCCGTC
AGATGATCGACTTGGCACTGCCGAGTTTACCGTTCCCCCTAACACAGACGG
GCCGCCGCTACACTGGCATGAGATGGTACGACGCGAAACCCTTTGGACCC
GAGATCTCGTGCTAACGAGAGCCTTGTAGCATGACGAGACATTTCTAGTTC
TTTCCGGTCGAATGCGTTTCTTCGTAAAGGATGGCGGCATCGTCGATTGCAC
CGAGGGTGACTACGTAACAGTCCCCGTCAGATCGGTGCACACTTGGGGAA
CTTTGGGTGAAGGGTGCAAATTTCTCAACACGTTTACGCCCTCTTATTATAT
CAATTATTTCAAGATATTGAGCCAGATGACAGAGGAGGGGAAGCCCATGGA
CAAGGAGATAAATAAAAAGGCCATGTCGTACTTCGCGACCATTGGGGTGGA
CTAGAAGTGGATACTAGCACCGGCTTGTGACACCTCCTCGTGCCTACGGCA
CTGCGCTTTAATTAGGGAATCGGGCAGCGATTGCTACAATACCTTACTTTGT
ATGTGGCATCTTATATAATAAGAAGTATCTCCTTAACAAATACACCAAATTCT
CAAAACGGCAACGATGTTCAAGATGTTAAGGATTACACCAAAATCCAGTAC
ATCGGTTAACCTTCGGAGATGTAGCAATCGCTGCCCGTTTCCTAATTAAAGC
GCAGTGCCGTAGGCACGAGGAGGCATCACAAGCCAGTGCTAGTAATGTGT
CGACCTGCTGTCTTTATTTCAACGCATATACAATGGTCGCCGCCGGGGATAA
GGTGGAATGATAGTCCAAGCATCAAATCTTTCATATAAGCTTTTTAGCTCAT
GTTGAGGTTTCCAAAGATATAGTGTGACGTCAACCCACGTGCTGTTTAAGT
TATTCGCGAATGTTCCTATATATACCTAATACCTGAGCGTTTTGCAGAATAAC
TACAATTAGAGGTACCTAGGTATGTAACTACTAGGTAATACACAATTTAGCC
TCTAGTAAGATCAGACTTCCTTCAGTTGTCCAGATGCCACCAATCAAGACA
GCGACCATTTTTATCGCGATGTATCGATAATTAGATGCCCCGTAGTATAAATG
TTACCATGAAGGCGCATATGGTGTAGAAACTTTAACTGCTGTTACTAACTAG
ACGAATCCGTGCAGACTCGGGAACAGATCACCCGTCAGGGCTTGGTCATTA
CCCCTGACGTGAAAATTCGGGCGGGTGTGGTTACATCTAAGATTGGGTAGC
GCGAAACCCAGGTAGTGACGGGACCTTAATTAATATATGGCTAATGCGGCC
CGGAACCACAGCCCTTTTTCCCTCTTCCCGCTTAAAAACCATCGCATTTCGG

GGAAATCCCATCTTCGCTCTCGCTAGAAGAGGAGATCTTAGACTATTTAGA
GAGCTGGGAGCCGAGGAGTTGCCGTTAGCCAAGATGGGACGAGACCGTCA
ATATATCTCAGGCATGGAAACGCGGCAAGAAAATGCCATTCTTTCCTTTCTA
CGCTTCTCCGTCGATCCCCGGATCGAGTAGGTCCGCGACCCTCGCGACGCC
ATGACATTTGACAGATTTAAAGTTCGAATTACCTACCTAGGAGATATATTGT
GGAGCTGAGTGCTACTGCGAAAGATGAAGCGCTTTCCGGATAGTTCATATA
AGGCCGGCGGGCTACGCCTCGAGATTTCGAAGCGTGCCAGAGGCCTTAAG
CATCTGGCATATTTCAAATCAAGTCGCTGATGATGCGATCCGCGATCCTCTC
TGTAGCGTTTGCCACGGCATCCGCCGCTGCGGTACTCGATTGGGCTGCTGC
TTATGAGAAAGCCAACGCTTCTCTAGCCAAACTATCTCAGAACGACAAGAT
CAACATTGTCACTGGAGTAGGCTGGAATAAAGGTCCCTGCGTTGGGAACA
CAGCGGCAGTTAGCTCTATTAACTATCCCCAGCTATGTTTACAAGACGGACC
TCTGGGTATTCGATTCGCTACCAACGCCAACGCCTTTGTCCCGGGTATCCAT
GTCGCCTCTACCTGGGACCGCGCCCTCATGAGGGAGCGCGGACAGTTCATG
GCTGAGGAGGCAAAAGGCGCTGGCGTTCACGTCCTTCTAGGGCCCGTCGC
CGGTCCCCTAGGTAAAATCCCTACAGGCGGTCGAAATTGGGAGGGATTCGG
TGCCGATCCGTATCTTCAAGGCGTTGCGATGGCGGAAACGATTGAAGGCAT
GCAGAGTGTAGGAGTTCAGGCAAATGCGAAGCATTATATCCTAAACGAGCA
AGAGCTAAATCGCGAGACTTTAAGCGAAAATTTGGACGATCGTTCATTCCA
TGAACTGTATCTATTCCCATTCGCAGAGTCGGTCAGAGCAAATGTGGCATC
GGTCATGTGTTCCTACAATAAGATCAACGGCACCTGGACCTGCGAAAAGTG
AGTTACCTAGGTAGACCCTATCATGCCCAAGGTCTACTCCTTCGCTAATTTG
TTACACAATAGTCCGCATGTGCTAGACACTGTTCTGAAAAAGGAGCTAGGC
TTCAAAGGCTACGTGATGACGGATTGGAACGCGCAGCACTCTACTGCACCG
GCCGCAATTTCAGGATTAGATATGACCATGCCAGGCTCGGATTTCAACGGC
GGGTCCATCTATTGGGGTTCCAAGCTAACCCAAGCCGTGAGCAGCGGTGA
GGTACCGCAGTCACGTCTCGACGACATGGTACTCAGAATCTTGGCAGCGTG
GTATCTGCTAGGTCAAGATGAGGGCTATCCTGCGACCAATCTCAACGCCAA
TGTTCAAGGAACCCACAAGACCAACATCCGCGCTACCGCAAGGGACGGCA
TAGTCTTGCTCAAGAACGAAGGAGGGATACTTCCTCTCAAGAAGCCCGCG
AAGCTTGGGCTGGTCGGCTCGGCTGCTGTTGTTAACCCTCAGGGAATGAAC
TCCTGCACGGATCAAGGATGCAATACCGGCGCTCTCGGCATGGGCTGGGGA
TCGGGTACTGCTTCCTACCCCTATTTCAGCGCTCCAGCCGACGCCATCAAG
GCGCGAGCTCAGACCGACGGTACCGAGATCAGTTTGTCCGCTTCCGATAGC
ACCAGCTCCGTCAGTTCGACAGTCTCTGGCGCCGACGCGGCAATCGTATTC
ATCACTTCCGACAGTGGCGAAGGATATATCACTGTAGAGAATAACGCGGGT
GATCGCAACGACCTCAACCCGTGGCACGGCGGAAACGATCTCGTCAAAGC
GGTAGCGGCAGCGAATGATAATGTAATTGTAGTCATTCACGCTGTTGGTCCC

GTCATTCTCGAGACGATTCTCGCTCAACCTAACGTCAAGGCAATCGTGTGG
GCCGGTCTTCCCTCACAGGAGAATGGGAACTCCCTCGTAGATATTCTTTACG
GAGATGCCAATCCTTCGGGGAAACTGCCTTACACCATTGCCAAATCTGCCG
ATGATTACGGCACGAGTGTCGTGAACGGTAATGACGACTTTAAGGAAGGTC
TATTTATCGACTACCGCAGATTTGATCAGGAAGGTATCGAGCCTCGGTATGA
GTTTGGATATGGGATATGTGAGTTACTTTATAAACTCCAGTCTGATCAGAGA
TGGAGGCATTAGCTAACCCTCTACAGCGTATACCAACTTCACTTATTCGGAC
ATATTCGTCGATGGGCGGCCGACGTCCGGTCCCGCGACGGGGGAAATCGTT
CCGGGAGGCCCCGCCGATTTATGGGAAACGGTTCTCACCGTCACGGTGACT
ATTACTAACACGGGTGAGGTACAAGGTGCCGAGGTAGCGCAATTATATGTA
GGTTTCCCCGAGTCTGCAGGCTCGCCGCCCAAGCAACTACGCGGCTTCGA
AAAATTGGCATTAGAGTCCGGTGCCAAGGACAACGTGGCTTTCGAATTGAA
GAGACGAGATATTAGTATATGGGATACAGCGCAGCAAAGTTGGGTTGTCCC
TGCAGGGACTTTCAATATTAGTGTTGGTTCGAGCAGCCGCGACATTCGCCA
GACAGGCACTCTTGAAGTGCCGTAGGACGGATATAAACATCAGGAAACCAT
TGTACATAGTTCTTCCACCCCCTGAATGTAAAAGGTTTATGGAACCGGACA
CTTGCTCGCTTCCTGCTTATCTAACATATGTGTAACAAGTGCTCAGATCGGA
TACACTGCTATGCAGCATCTACTTAGTAAGCATAATATATATCTGGGACACAG
ATCCTTGATGCTTGCTTGGATGGGGTTGCGAATCGGTAGAATCGGTAGAATA
GATTGAATCGAGATATTGCATCCCAAGTCGGGAATGACGTGCGTGTTACGC
CCAACATAATACTAAGTAATAAGATGAAAAGATGCTTAGCCCTAATCGATAG
TAGTGGTCGCCTTAGCGCGTCAAGTGAGGAAGATTCATCCATGCCTAATAG
TGACGCTATCACGGCTAGTGTTTCTTGTGTTTCTTAGCTACTAACATAAGTA
ATTGGATGAGTGAAGATGTTGAAGCAACAGCAGAACTTGGAAGTGCACCA
GATACGTATGAGGTACATGTATTAGGAATGAACAGTTCGCGTTCTCTTACTA
ATATCGTATCTGATATAGTAACGATGTAATCTGTGTTGTTGAGTTGGCGGCTC
GACCAAGCCCAACATGAACAAAGCCAAGAACGGGGGAACGGTGGAGATT
GGAGAGTGGAGAGTTCATCTTTGAAAAAAAGTTACTTGTACAATTACTGCA
TATCGCCGATACAGTGTGAACACTTGAGCATTAAAGTAGGTACCTACATAAG
CATGATGTAGCACAAGATAGCTCCTAGCTCCAGGTCGGGTTCCTCCGTAGAT
CCCCCGCCATTGACGTCGTTGGCGTCTTTGGCGTCTTTGGAACTCCGGACG
GACTCAACAAATCTTGCACAGGGCTATGGGCTACGGGAATTCTTGCTTACC
ATTCCCCTCCTCGCCTCCGGACTTCATAGACTCGACGTACGACAACTCCGAT
GCTTCACAGAGCACGTTTTGGCTGCGCTCTCTTCTCGTCAGCTAGACAAAC
CCTTTGTTCGAGCCCGAGGCAGATTCCCAAATTTCGACAATTCTCGAGCTC
GACACCGAGCTTTTACGGTTGGCGCGCCATGTCGCAGTCGGAGATTTCCAA
GTATCTTCAGCAGACGCACGACCGCGTCTTCGAGCACAACCGTGCTTGGGC
GGAGGAGCAGACGAGGCGGGATCCCGACTTCTTCGCAAAGCTAGCCGCCG

GGCAGACTCCCGAGTATCTATGGATCGGATGCAGCGACTCGCGCATTCCCG
CCGAGCAGATTACGGGCCTAGGACCGGGAGAGGCGTTCGTCCACCGTAAC
ATCGCTAACCTAGTCATCAACACCGACCTCAACGTCATGAGCGTCATTAACT
ATGCCGTCCGCCACCTGCAGGTCAAGCACATCGTGGTATGCGGGCACTACG
GCTGCGGCGGTGTCAAAGCCGCCATGACGCCGAAGGACATGGGATTGCTC
AACCCCTGGCTTAGGAACATCCGGGACGTGTATCGGCTGCACGAGGCGGA
GCTGGACGCCATCGCGGACGAGCGCGAGCGTTATAACCGCCTGGTGGAAG
TTAGCGTTATCGAGCAGTGCCGGAACGTCATTAAGACTGCGGCGGTACAGC
AATCCTACGCTAAGAACCAGTTCCCCATAGTCCACGGATGGGTATTCGGATT
CGAGGACGGTTTGCTCAAGGACCTCAAAGTGGATCATGAGGCCATGTTGC
ACAGCATCCAGAAGATCTACAATCTCACGGAAGAATGATGTTACACCTGCC
CTGTGGGGAGGATGGTTAAAATTTATCTGTATTATTTGAGTTGGCTGTGATAT
AGCTTCTATCGTATCCAGCCTTGTTCAAAAAGCTCCATCATCCTACAATATAC
CGCATTGATGCATTTGGGTCGAATAGAGTTTATAAGGTCCCGTGACGATCGA
AATCATCGTCGCCTAGAGGTTATATAATCAAACTCCTCGTAGTTCCAACGTC
TTCATCCTATACACCTTGACTATAATTAGTGCCCATGATGAATATTGTATGCCT
ATCGTGATGGCCGAGCCTACGCAGCTCGTTGCCCAGTGTGCGTCTACCTGA
TTACGCGTCTGCGTAGTCGTTAGTGCTATTACGTTCTAGCGACAGCTGCATG
TCTCTCTAACAAGTTGCTGGCCATTGCTGAACGTGCAGGAACCACCTGCTA
CTTCGAGAGGGTAACAAAGCTGGGGTTATAATGTCCCGTCAGCTCGTAAAG
ACTGGCTTGCTATTTATTCCCGCTTAGCGAAGCGTGGGCGATTACTCGGCTT
CGTCATCGCCGCTATCGGGTTCCCAAAATCCATGTTCGAAGCTCTTTTTTTG
TCTAAAGGCGAAACGTTCCCAGCTTACGCCTCTCCTAACCTTGGTGCCGTC
TGGTTTGGTTTCGTAGCGTCGAGGTTCGAAATCCATCACGGCACGCCAGTT
TCGGATCATTTGGCGCCAGTAACAAGCTTCGGCGGCCGGAGTGAGATTGCG
GTCTCGGAATACTCTCGTTGACTCTTGGGCAATCTGCCGTGCTCGATCGGG
GTCTGCTAGAAGGCCGTCCATCTGAGCCTTTAAATCTGACCAATCCCGGTT
CACTTTCACGTAATTCTAGCAAAAATAGGTAGTTAGTCGAATCAAAGTCCTT
GATAGCTGGATAAGGAGGGGTTTGTCTACCTGGTGAGGGCCACTATCCTTT
AGAAGAGGCTGGTAGTGGAATACGTAGTCCAACTTATGAATAACGGGGACG
CTATCGCAGTTGTGCAGATATCGTAACCGTCCGCTCCACGAGTTACCCTCAG
TGTGCGCGACGTACTTAAATCGGCAGAAGTCTCGCATATCTAGAATGTTGG
AACGGTTATGCCAATCTATGGCTTCAACGTCGTTCCAGTCATTTCCCTTGGC
GGCTTCGACGAGGGCAGTCCGCAGCTTCCGGTTCACGGCTAAGCTCCCCC
GCCAGCACAGCTTATCCTCCTTGTCATTCCAACCGTTGGGGTATTCTGTCTC
TAGCTCTTCAACGTCCTCACGGAACTGCGCATACGATCCGACACCGTCGTC
GGGATAGCTCCACCCATCGAAATCGGGCATAACCCATAACCAAGGATTGCT
TCCTGACGCGCCCAAGCCCACCTGATACGCTTCGGGTCCTTTTCGCCCCGG

GCAGCGCGGTCTTCGATGTCGATGATAAATTCGATGTTCGGCAGCGATCGA
GGATCCGGGATGGCGGTTATGGCACGATACATGTTGGCGAGAGCAGCGAG
GCCGCGAGAGCGATCTGGTTCGCCTTTTCTCTCTTTGATGACATACAATTCT
CCGTTAGAAATCATGACGTGGAATTCGCCATGCTGAATACCGGTATAGTCAT
CCTTGGCATCCAGTTTGATGTCAGATTCCTTTATGCGTTTATTTTGGTCCAAG
AGAAAGTCGCGAGCGCGATCAAGTTCTTGGAATAAGTCGGGAAATGCGCT
CTGTAGCAAGATAAACATTAAGTTATTGCCCATTCGTCGTTGGACCATGAGG
GCTTTCTACATGTACTGGCTTACCTTGCACTGGTCATCGGAAAGACCGTAGT
CGAGACTGTCACGTTTCGGGTCGAATTCCCAGACGGCGTCTTCGGGTCGTG
GGGGCGTCGCATAGGTCTGCTGTTCCGTAGTCTCTTTGTATGATTCTTGGGC
AGGTTTATGAAATGGGGACTTTACGCCGCCAAAGAGAGAAAAGGTGGTGA
CGATAAATACGATAGCCAGAAGCAACCATCTAGAGCGGCGCAGGATAAGGG
CCATCGAAGATTCGCCCGGCTGCTGACCGCAGGAAAACAATGACTTGTTG
ACAAACATTTTGAAAACGAAGGTGTGGGCTAACCAGGTAGCTGGTAGACA
GCGGTCGAGCTCCGCCGGGGCGTGGCTAGGCGAGGCGAGGCGAGAAAGG
AAATGCGTACCGACTTACCAACGTAATGGCGGGACGGTGTCTCAAGATATA
ATCGCTGCCATTTGGATCCTGTAAACCGTTGAGACGTTTTTAGTATTAGTAC
AAGAGTCAAGAGTCAAGAGAGAGAGAGAAGGAAGATAACAAAAGGACCC
GCTTCATTTTACTACTGCTAGTTCTAGTATCTACCAGGTGCTAGGTACCTCAA
CTAGGGTCATTACCATGGATGGACTTTGTGTCACCTAATCTCTCTAATCTTTC
TAGTCTGTCCTGGTTTTGCTATGCGCTGATTGCTGATTGCGTTAGTGAAGGG
AATGAATGCGGCGCTGTGCCTGAGATAAGTCCGGAAAATTATAAGGGATGG
TATCATTTTGAAGGAGCTACTGCATAGTACCTATCACCTAAGCACGGACACT
GCGGCACAGAATCCATCCATTGCATCCATTGGACCGTAGTCGGTAGCGGGT
TCCATCCCTAGTAAGAATAGGGAACCTGGACCTGGGATTTAGCTTGTTCTGC
TGCTCAGTAGTTGTATACTGATTATTAGTGCGACAAAGAAATCCATCCAGCA
AGAAATTTTGGACTCCTACACATTCCGATCTCCCACGTGGGCTACTTCAACT
TACGATTAGAACATGTGAAGACCTTCTTTACTTATATCTTACCAAGATATCCA
GATATAGAGATTTGTTTGTTGAATCATACCTAAATATGCTGTCCGAATTCAGA
TGAGACATCCTGGTTACTTCATTTAGGTATTTGTTATAAAGTCGACCTATCAC
GTTCTACGGCTACCATCTCCGATAGGAATACCTTCCCAAGGCTTTGTACCCC
TTACCTACTACCTACCCGTAGTACTACCCTTATTATAATAGGGTGGGTTAGGT
ACCCAGTGAATCCTTCGACGCTATGCATAGCGAAGCGCCTAAAGAAATTTG
CCCATGTAAATACTAAATAGTCGTCTATTTTTACTTTGAGCTGATCTCAACCC
ATACCTCTAACCTAAAATTCCAATAATCAGACCAATTTCAGAAATACCCCTT
GGCTCATGCGCGTCATCAAAGCTTAGTGCTCGAATGTCAACGTGGTGGCAG
GGTCAAAGCTCTAGCAATTCAGGCTAGCTGGCTACCTAGGTGGATATATCCT
CGCACGGCAGTTTGCGGTGAGACAACCATTAAATTTTTATAAATCATTCTAG

GGCTTTATTTTCCGCGAACTTTAACCATGAGGCAAAGAAATTATTAGTCCCA
CGTTAAATCGCGAAGCGCTAAGCATGAGGTAAACTCAAGAACGTGATAGTT
ACATACCTATGTAGGCCTTTATTTACTTGAACAACGTAAGGGGTCGCTTGTG
TGGTTGACGTCGATGACACCCTACCGATCACACAGATACGAAGCTGCACCG
CATATTAGATCATGCCGTCAGCAATAGATTTATATCCTCCTGCCTATAGCATAC
GTCCCAGTATCTATCCGGCCAACCAATTTTTCACAACTCAATCCGGCGCTAT
GGCGAGGGGTTAGATTGGGTGACTTCTTCATGCCATAGTAATCGTTTTCTAT
CATAATAATTGAGCCTAGACCGACCTATTGGTTCTGAGCCTTCTTTGAGGCG
CAAAGCATAACCTTCTAGCTATCTGCGGGCAACAGATTAAGTTGAAACTATT
GGGTGGCGTCGTCGCCCAGCTGCCCTCCCCGAGAACAGCTAAGCTTAGCC
ACGCTCACTTGATTGCAACCCACCTCCATTTGCAATCATTCTAGCTATGGTG
GCAGACATTGCTAGATGCTTTGAGTTTTGTAAGATAAGTAAATAAAGTGAA
AAAAGAAAAGCCATCTCACTCATGGCGTTTCTCAGCTCAACAATCGATATC
TTTTATACCCTTGCCTCTGGGTTCCGCGTCTCAACTATGCTCTCTACTCGCG
AATATTCTGACCACACTTTGGCATTTGACTATATTGTGCTCGCGCGGAAAAG
CCAGGGGACGTTGTATCGATCAAGCAATTAAGGACTTTGAGCAGTGCGTGA
TTTGCGTGATTACAACCTCGTGGAACTTAATCCAAGTCAAATCGAGTCCGA
GTATCACCGCATTCTATCGCTGACAGTCATGCGTCCCTCGTTCGCCGCATTA
ACTGCGGTCGCCTCCCTCGCCGTTGCGCAAACACGATCGTCGAAGCGCGG
CTTGGTTTTTGTCCCGAATCAGGACAGCCCGCAAGATAACAAGATATGGAC
CCAGTCAGGATCTGATCTGACTTGGTACTACAACTACGGCTCGACTCCGTC
GCCCGCTTTTGATGATGTGTCGCAAGACGACTTCGAATTTGTCCCTATGCTG
TGGGGATCCATCGACGACACATCATTCCTCGATTCGGTTCGAGGTCAGATC
GATTCCGGCCGCCGCATCACGCACGTGCTAGGATTCAACGAGCCGGATACC
CAGTACGCCGGCGGTAGCAATATCCAACCTTCGGTAGCAGCAGACGTGTGG
GTGAAGAACATCGCTCCACTCGCGGACGAAGGAGTCAAGCTAGGGTTACC
AGCCTGCACGGGCGGGTGGGGAGGAATACCGTGGTTGCAGCAATTTCTTG
GGAACTGCTCCGAGCTGATCAGCACGGAAGACGAGAAGAAGAACTGCAC
TTATGACTTCGTGGCGATCCATTGGTATGGTAATTTTGAGGGTTTGGCGAGC
CACATGGGCTCTTACGCGGCTGCGTTTCCGAACGTGAGCCAGTGGATTACC
GAGTATAACCTCAACGATCAGGACCTCGAGACAACCCAAGCTTTTTACAAC
ACTTCCGCTGAGTATTTTGATCGCATGGACTCTGTCGGGCGCTACAGTCTCT
TCGGCTCTTTCCGATCTGCTAAGAGCAACGTAGGCCCCAATGCAGCGATGC
TGAACAATGACGGGAAGCTCACGGACATCGGCTCGTGGTACTTGGGCATTA
CGGCCGCAGGTGTTGACCCCAAATCTGGATCCCAGTCCGGATCTCAGTCTA
TATCGAGGAGAGAGAGAGCGCCTATAGTGAGCGCAGTGGTGTTTTTGGTAG
TTTTAGTCGTCTCTATCGTCTTTTAGACGAAATGTTCGACTAGAACATGACA
TATCGAGATTGACTTCCACTCAACACATTAGAATTCCTTGACTTACCAGTTG

AGTATGTTAGTAGAATTAATAGCTTAGTACTTAATTGAGGCTGTATATATTTA
ATCAGCAACCTATGACTATGGATCTATTCCAGATGAAGAGTAGATGCCTACT
TAGTATGCATGTCGAGTCTAGACCGGTTTAAAAGGTCAACATTTTAAGTTTT
GTTGGTAACAGATGTGAAAGTACCCAGTACCTTAATCGGGGAATATCAACC
ATGAATTGAAGCGCTAGCAGAACGTACCAAGTACAGACAACAAGTCCCCA
CCGATACATCTGCCGATGACCGAAGTGACCGAAGCAAGCCTCGGCAAAAT
GCTAGGTAATACACATCCATAGCAGGTGTTACCTCGATTCCTTCTTCTTGGA
AAGCACATTCCATCCTTTATTAACTGTAGACAATGCCGCTCGGAAGTGGCC
AAGATCTAAGGTTTAGTTGATTTAGAGTCTTGGTAATGATTTAAATGCTTAA
ACGATTAAACACTTAAAGATAGCGATCTCCTCATTTCACTTCTTCTACATCTC
GTCTGAACATCCATCTTGACATCCATCTACAATATTCCCGGCCCCTTCTCTGT
TACCACGTCCCGTCAAGATGGCTACCGACAGAGTGAACTCGATCCTGAAGC
ACCTATCTCCAGATTCTCCTCTAGCTACTATGTAAGAAGTGCTCTACCTTTGT
TTTTGTCTCGTCTAGTTTCGCCGATTGAAGACGGTGTAGTACTGTAAACTTC
AATCCATCGGACATGAGATGGGGAAGACTAATATTCTTCTTTCTAGCACCTC
TAAGAACCCAGACGATGTAGTGATCACGCTCGCTGTTCGAACGCCTCTTTG
TAAAGGCAAGAAGGGCGGGTTTAAAGACACCTCCGTCGAGTACCTCATGT
ACGCCATTCTCAAAGCCGCACGCGAGCGATTGGGATTTTCCCCAGATCTTG
TAGAAGATATCTGCATGGGCAATGTATGTGTAAATCCTCCACCCTTCCTGCT
CGCTTTTGTTCGCTTTTGTTCGCGTTTGTAGAAGAGACGATCTCTAACACCT
ACCCTTGGAGTACAGGTATCGGATGGCAAGGCAGCTTACAAAGCCCGTGCC
GCCGCTCTTGCCGCCGGATT

>Mosaic_sequence_03

AAGGAGGCTGGAGATGGTCCGGATGCGAGGGGGTTTGCCCACAAAATACT
AAGGATAGAGCTGTACGGTTTGAGGTTAGTGTTCCGTGCGTCCACGAGCGT
GGTTTTCCTGATTTACAGACTTCTAGCTGTTTGCATGCATGAGCTACATGGA
TCTCGGTGATGGCGGTCGCTAAAACGCACGGAAATTTGGGTGTCCCGTCCG
GTGTGACACCCCACTGTAGCGAGCTTTTCGAAGACGCGAGGTTACATTTAC
GGGCCGGTGCAATTATCGATGCGCTACACCTCGATGGGCCTGCCCGACAAT
GGCTCTGTCTCGAACCATAAGACCCAGTCTAGTGCGTCCAGTCACACAAGG
TTCATGTCGTGTTGGCGGACCTGTACAGATGCTGCGTCAGGTGGACACTCA
GGTCATCTAGACTTTCAACTCACTCGTGAGTACTGCCTGATCCTTATGCCCT
CGGTGATCATGCCTGGGCGTCGCGGCCCGCCAGGTTCCCGGCTGACAGTTA
GAGGTGTCCAGTAACCCTTGTGCTGGCGTCCGGATCACAATCAACGACATC
AGATGCGCTTGAAGACTCTACGCCTCACAGCTTGCCATCAAAGGTGAAGG
GATGCATGGAAGACATCAACATGTTCGAGCCCTCGCTGCGACTGCTTGGAT
CTTGCCCCACTGCACGAATGATAAAGCCTTTGCTCCAAAGCGCAGCCGACT

CCGTCACGACACGTATCCTTCGCGCGGACGCATTCGCTCTTTGCCCTCTGG
CAGGTCAGCACAAAGACATCGCCATCGACCAAGCCCATCACCCCAGTTGG
GCAAGCTTCTGACGCCGGAACTGGTGACCATCAGTTGGAGACTGTCTGTT
GTCGTGACTAGGAGGGATGTCGAAGAAGTCTTCAGACCATCAAAGCCCTT
GGCGCTCCGCGTGGGTTATCTTTCTGCTGCAACTTCCATGCTTTTAGACCGA
ATGGCTGACTTGCAGATGATGCGGCGACCCGCGGGAGCCCAACGGGCAGC
TCCCTAGGATTGTATCGCCAGGACGATGCTTCAAGCAGTTATGCCGCTGTCC
ACTAGGCGGGGCAGTCCCAACACGGTTCGGGATCCACGATATTGAGAGGG
AGCAGTGGGCCGCCAGTGTGGACGGGGAGCGGGACATTGGCGCGACCGC
CATTATTTCGGACTCCAGAGCGGCCACACCAAGGAGAGGGCGGTGCAACA
CGAAGAATGGACCCAGCAGAAAGGCATCGCCAGTTGATGTTGTCCTCTCGT
GGGTGTTTCGAATATGCTGACGGACAGTCGACGCACCCCTCGCTCAACGGC
AATACGCTTGCGATTTCCCGCTTGGCAAATTGTCCGGCAGGCACTCGTCGA
CTACCTATGGTCTCCTTGGTCGCTCAACAGCTCACGAACTGCGCCGAGGAG
AACGACAACTGCATGCCCCGGGACCTTGGCCTTGGACAGCCGCAGAGATG
GAGCTCAAGCAGGGGAGAGGAGAGGGGGCTTGTTCCACCTTTGCATACTC
GGCAACAGCTGCACAAGCCCGTCTCGACCACACCCGCGTTCAGACTGGCT
TCCGCTTCTGCTGTTGTCGGTATGCCATCGGCTTCTAGCCTGTTCCGGCAGG
CAGAAGAGCATCTGGAGAAGTGAACCGACAGACAGCCCCACCTCAGCGTT
AGGATCCACGAATCGACTGAGGCGTCTTCGTGCCCGTCGGCTGCGTTCGGC
ACAAGGGGAGACCTCTCCCTGGCGACCTTGCCGAGCCTCAAGACACGGTC
AGAGACTGTCGTGACGGACGGCACACTCCGGCCTCCTGCCGGGCCCACGA
GGCAGTCAGGGTGAGTGGGACGATCTCTGTCGCCAGTGGTCAAGCTTGTC
GCTGACTTCCAGTTGTCTTCTCGTACGCGTGAGATCGGAAGGCTCGACTGG
GGCATCTGCTCGCTCGCTCGGCACATGTACCGGCCCTCGCTGCACAATTCC
GTCAGCTATCTGCTTCGTGCGGCTATCCGTGCGCCGCTCGCTCATCTTCGAT
GCTACTACGCGACTAGTCTGTGCTTGCCTTGCGCTACCAGGGCAAGACGCG
CTGTGACCGCTGAGGCAGCAGACCTGGAAAGTGCGTCCTGTCGGCATCGA
CTTCGGGTGTCTGTGGCTGACTTTCAGATGACCGCGATAGGATGTCAGAAA
GCGATGCGCCAACTTCGAGTGCTACGACAGAACAAAAACACGACAAAGCT
GTACAGCCCACCGACCAATTTCCTGTAGGAGAGCCGGCCAGTCTTATCCAC
GATGGCCCTGCGGAATATCATCGCCTCTGGCTACCTTCGCCACCGATACGTC
TATCGCTTCGCCCGGTCTCCGCGCAGCCACAGAGCACTCGAGTCCACCTGC
TCGCCGGCATATGTGCTCGACCTCAGGAAGGTGTTGACGCGGGGCTGCGA
CTCGTCAGTCCTTCGCCAGAGCCAAAGTCGGAGATTTCAACTATTTTCAGT
GTTGCAAAATGCTAGATGCCGCGGATCGGATCCCTTGTATGCATACATCTAC
GAATTGTACACTTCATCGTCTGCTTCTCTATGACACAGTTGACACCTCGCTT
GTTCATTGCGGAGCGGCTCTACACCACGTGTCCATGCTCGATGCAGGGTTT

CCAAAATCACACCAGACACGTCATAAGACCGAGAGAATAATGCCTTGTACC
TATGTTCAATCTCTGCTTTGATGAACCTATTGCTCTACAATTTAGAGTCCTTA
TTTATGGTTGCATATGATTTCACATTGAGGAATTGCATTCGAATGTGGACTG
CTGCCCATATCGATCACCTATTCCGTTGTGGCTTTTCCCAAATCCACGGGAT
AATTTACGAATATCCCGTGGATTCGGGTTTGCCAAGACAGCTAGCAACCTTA
CGCACTAATTATACCCGCGAGTAAGGCATGAGCTGAAATTGAATCGGTATGA
TTTTACAATACAAGTATCCTATTATTTCATCATTCTAAGATGCGGTCATGAAG
GAATGCTCCGATTCCGAACTATCCTAGTAGCTAAGCCCCGAAATCAAACCAT
CTCAGGCTAGAATAGGTTAATATTATGGGTAGGCACCTATGAAGTACGTACG
TTATGTAAGACGGAGTGCTCTAGGTACGGAAGTCCTTAACTCCATTTGATAA
AATCCGGCTCCGTCATAGACTTCTTCAGCCCAGCTAGCCCAGGAACAACTG
CATATTTCAGCAACGAACGAAAAGTGCAGAACCAACAATGCAACCTAAGA
AGTCACGCGTCTGGAGTCTATTCCCGTATTATTTAGTCCACGTCTCCTTTCCC
ACCCATTTAAAGGCATCTCTGGCCAACTTATGAGTTCTTACTTTCGAGTTCT
CTTTCGAAGAGGACCCACATTCTCATCCAGCCAATTCACTAAAAGAGCTAC
GATGACAGCCATTCCAGTTGCGCTCTCAGTGGCGGCGTACCACAGCACCGT
CACGGTACGGCCGACGCAAGCCAGCGCCAACGAAGCCCAAGAATTTGAGA
ATGAGGCGCATCATGTCAAGAACCGGTGCGGGAAGACAGTGAAATTTCGG
AATCCGCATCCCTCGTACACTTTCCCCGCGCCTAGAGACCTCTTCGAGTTG
ATGTGGTGCGTATTCGGAAGCTTACATGCTACTACCCTGATTTCTATATATGC
GGGTTGACCAACAACGAAATCCAGGGGCTACTATTCGGGGACGCTCAACT
GGCCAAAGCCCAAACTAGAGCAGAAGCTTGTGCCTGTGGTAGAACCGAAG
TTTCTACCAACGCGCACTGGGTCATCTAAACTACGTGCCACTTGGCTCGGC
CATGCGTGTTACTACGTGGAGTATCCTTCGGGACTGAGGGTGCTATTCGACC
CCGTCTTCGAAGACTGCTGTGCGCCTTTTGGGTTCCTCGGTTTCAAGAGGT
ATACCGAGCCGCCTTGCCAAATCGCAGACTTACCTTTCGTAGACGCCGTGG
TTATCAGCCATAGTCATTATGATCATCTATCCTATCCAACGGTTCAGGATATC
CAGAAACACCATCCTAACGCGCACTTCTTCGTTGGTCTGGGGTTGGAGAAG
TGGTTCAAATCAAGCGGCGTGACGAATGTGACGGAGATGGACTGGTGGCA
GGAGGCGGAAATGAAACTAACCCCGCAAGCCGGCAAAGGCGAAACGGAC
GCGAAAAGCGGCGATAGCTCAGAGCTCCAGGGACCCATCACCGCGCGGTT
TTCTTGCCTGCCCTGCCAGCACGGGTCCGGCAGAACGGGCTTGGACCGGG
ACAAGACGCTCTGGGCGTCATGGGCCGTAACGTCAGGTGAGCCAGCCAAA
TCGGTTTGGTTCGGAGGCGATACAGGGTACCGCTCGGTGCCGAAAGTGGC
CAAGGGCGTAGATGACTACGGCCCGGAGTTCGAACACTTGCCGCGGTGTC
CGCAGTTCAAGCAGATTGGTGATAAGTTCGGCTCCTTCGACGTCGGACTCA
TCCCCATCGGCGCGTACCACCCTCGCCAGCTATTCTCCGGCGTTCACGCGA
ATCCGTTCGACGCCGTGGAGATTTTCAAGGATACGAAGTGTAAGAAGGCTA

TGGGCATACATTGGGGCACTTGGGTTATGACGCCGGAGCCGGTGCTGGAGC
CCCCGCAGCTGTTGAGGGAGGCGCTGAAGAGGAAGGGGTTGCCGGAGAC
GGGCGTTTTTGATATTTGCGATATTGGTGAGACGAGAGAGTTCTAAGGTCTA
AGGTCTCAGGTCTAAGGTGTTGGGGATGTTGGAGACAGGTCTCTCACAGTA
CATATGATATGTCATAGGGGATGCGGAGTGCGGAGTGTCGGAGTTTTGGGC
AGCAGCATGGTTGGGCGTGTGTGATGAGTTATTTACGACCCACATAATGAAT
GTCATCTAGATTCAAGAGATCTCAACTCGAAGTTACAGTAAGATCCGATACC
CAAATATGAGTTATTTTCGGTATGTTCGGCGAGATCTAGATTTTAGGTATCAT
GTAACATGTCACTCGATGCTGCTTTATCTGGGTGTGTAGGCAAGTACCTATG
TACTGTGTCGTCCAATGACGACATCCATGGAACTTTAGCGCTGGTCCGCTA
GCCACAAGACCTTTAGGCGGGCACCTGCACTGTTCTTACCATCTGGACACC
AACAAACTCCCCGACCGCAAATCGTCGCACCAATCAAAATCTAGAAAACC
TGATCGTCTTCCTCAGTCATCTCATCCACATCTTTCCGTCAAAAGAATGAAT
CATTCATCCGATTATATATTAGTCGAACACACTCGTTATCTCAACTTCCACCT
TTATCATGCGTCGACGATATAATCGAGGTTTCTGGCTTTACAGTTCTGGTCTT
TCGCAATTCTTTCCACTATAGAAATTAATTATAGTGTGATTTTGACGACTTAC
TACTGCACATCGACTCCTTAGATTTTTGGGCTTGAATCCATTATGGAAAATC
AAAGCAAGAAGTTAATAGTGAGCCGATATTATTTCATTTCTACAACATAGCG
CATCAACCGGTCTTGCATGTAACTAGACCTTTGTGAATGCAACTCCATCGA
GTCTTTCGTACCGATAGCATTCGTCTAAGTTGCCCAATTCTGTCAAGTCGCA
TGAACAAGGCCGGCACCATGCTATCGTCTGCCGTGATATGTTTCACAAATC
GGATGCAGACGACTAGGTGAACGTCTATGTTGGTTGCAAATCGCATTTTAC
ACCGAAGATAACGTTTCATGCTAACCTGAGGGTGCAGGACGAGCTTCGATC
GCTCCTTGACGAAGCCACCATCCTTTCAATTTCTAGTGATTATAACCTAGAA
AATCCTGAAGAATTCGCTGCTGCGCGGCAAGTTCTGCTAACCATCTCCAGA
GATGTAGTAGCGGAAGAAGCTACCGGCTTCAACCCAAGCGGCCTCGGCCC
CGATGCAGATGCTACCGGAACTACTAGTAGCGATGCTAAGAGCGTGGGAGG
ATTCACTGCCACGACGGAGAATTCCATCCCCCCGAGTCTCGTGTCTTCGGG
ATCCTCCAAAGCCTCCTCCGGTGGGACTCCGGGACTTCTTCATATCGACATC
TTCGACAGCCTAACTGAAGGCGAGAAAGAGCTTCAACTCTCCGAGATGTT
TGTCTCACTCAAGCCCATCGATGTAAAGTTAGCTTTACAGAAGTCGAAGGG
CGATGCAAGCCTTGCCATGGATGAACTCCTTAATCTTCAATGGCTTGAACA
GACAGGCCAGCGCCCTAAAGGCGTTGATGGCTTTTATGTGTCCGACAATGA
TAACGTTGCAAGCAAGAAAAAGAAGAAGGGAAAGAAGAAGAAAAAAGT
CAAGGTAGCGTTACCTAGGCCCAACGCCTCGGAAAATACATCCGAGGAAA
ACAGTAATAGTGACAATGCACAAAACGGTAAGACCTTTGAAACTGAAGAG
TAGACTCGCTAGGTTGGTAAATGACTTACGACTAATATTTCTACAGACCACA
TCGCACTTATATCAGACAGGCTCAACCTAGCCCCCTCGGACGTGATGAGTAT

TTACCAGGGCCACAATGCCTCACCTGGAGCCACTGTAGTCGAAATTCTTGA
AAATTACATTGCAGTAGGCCTGCCATTATCTCCAGACTCCGAACTAGTATCC
GATACTAGAGAAGTGATGAAAGAATTTTCCTGGGTGCCCCAACAATATGCC
AAAGCTACTCTTGAAATTTGTTCTACACGTAATGATGCTGTAGACATTATCC
GAATTTTGGCAGATCACTTCGAGAAGCCGGCATACTTGAAATACGATGTATC
GTACAGTTTGGTCGCGTCTGACTTGGGGGAAATTGAAGGTGATCCCCAAAT
TATCAACGTATCAAAATCGGTACAACTGCCGAAGTCTCCTACAAGCCCCAG
AAGCACGCGAGCAACGGCAGTTGTGTTGCCTCTTCGGACCTCCAACCAAC
CCACGAATTTGGAGGCCGCTGCAGCTGCCTCAAGAGCACTTGCCGAATCA
AGGAACCACTCATTCACATCTGCTGCGGCTGCATTCAGAAAGGGCAAATCT
GATCCCCTGTACAGACAAGCTGGCGCATACTATGCGAACCGTGCCCGCGAA
CAAGCCACAAGCTTCCGACAGGCTAACAGCATCGAGGCCAACTACCTCGT
CAACTCGAAGAGCACGGAAAACATGATCGACCTTCACGGCGTAACGGTGC
AGGATGGAGTCGAAATCGCACTCGACCGGGTAAGGAGGTGGTGGCAAAGC
CTTGGAGAGGACAGGGCGCGAAAGGCAAAGCAGGGATTCACCGTAGTGA
CGGGTCTCGGTCGACACAGCTCAGACGGAAAGTCGCGACTGCGCATCAAC
GTGTTCAAAGCACTTGTCGCTGACGGGTGGAAAGCAGAGGTACTGACTGG
ATCATATCTCATTACGGGGAGGAGATAATGATTGCCACCACAGATTATCTCT
CATGCATCAATCCCTTAATGTGTCTAACGGCGTGCACGAAAATTGGGTCCAT
GATACAGAATACAGAATACAGATCATACAGGAGACCATTGGCGGCGTTTCA
AGGTGGGAATGGCAGGGGCTCTGACGATCCATTGTTTTGGCGGTTTGGTAA
GGGGAATTTCTAAGAATCTATCTAATGCTTCCAACCGGAGTTATTGCTCGAG
ACGACTTATGTCGATATGTGACAAGATGAGGCTCAATTAGTTTGGAGTCTTG
ATAGAGCAACGATTAACTTGGTATACCTGAATAATTGACATTGGTATCTTGAT
TTTAACCCAATTTTATGTGAGGAGAATGGCATGTGGGTTATTTGATATCATAT
AAGCTCCATAGTAGACTTGTGAGTCAATTGAGTATAACTTCTTGCATACATA
GATGTAAGTAAGCCAAGAATAACCTTGGAAATCTCTTCCTAAGCTGCAAAT
GCAATTGGAACTTTTTAGACGGGTTATCGAGTGAGTCCCACAGTCTAGGAC
ATTCTTCGGAATGTAAAGTGTACGTAACCGAATTGCGTGACGGTCTGAGAT
TATTTTACCAACCTCACATTTAGCTCACCCGCGAAATCCACTGCGCCAGATC
CATTAGACCAAACAGAACCGGGGGATCTGAGACGGAGACAGCAAAGTCGT
ATCGTCGTTAGCAATTGTTAACCGAGACTACTTCACGGAAAATAGATCCCAT
TCCGATACAGTTATAGTTACATATAATTATTATTTGTTATAGATGACTGCTAAT
ATTTGACAGTTATGTTCATATGTTATGCCGAATAATGTAACCAATCAATCCCT
AATTATTTACATTGCAAAGTCTCAACTAGAACTCTTAAAAGGTAGCCGATAT
CCGATTGATGATTCGCTTCAATTATGTATTCATAGCCCTGATGATCATCCCCA
ACTCGGAATATAGTAGTAGTACAACCGTACAACCAAGTACAAAACAATGTA
TCTACCTGAATAATGCTCGATACATAAGAGCTAAGAAAGAAGGCAACTATTA

AAAGCCGGGCATCGCCGACCACGGCCGTAGCTAAACTTTACTGGAGCCTTC
TAGGTCGCAATTGTGTAAAGTCACAAGTCACAAGTACAGCAGATATCCTAG
GCATCCTAGGCAGTCCCACTCGGACTTACTTCCAAATGCCTTTGACCAACT
TCCCAGTCAAAACCGTTAAGTACGACTACAAAACTCAAACCGTACATCTTC
TATAACTTAAACTTGCTAATTACACAAAGAAAATCTAGCTGCTAGATCTGCC
CGCATTTGATTGATTTATCTCCACGATCTTGTTGCTAATATATTGAATTCCAA
AACCGCCACAATCTTAAGTGTTTAATAATACGAGCGCCCGATACTACTCAAC
AACGATGACGAACGACGTCGCGGGGCCAGTGCCGCTGGCAGCATCGGCCA
TGACACTAGAAAACTACGCCTTTCCTGCACACCGGCTGCTGCGACAGCAG
ACCGATCCCGAACGCATCCCTTTAGTGCTCGTCGCTTGCGGTAGTTTCTCTC
CAATAACATATCTCCATCTTCGAATGTTCGAGATGGCAAACGACTACGCTCG
CATAAATACTCAATATGAGGTTGTCGGAGGTTGTATGTTATATTGATCCATTG
TAACACATGCCTATGGTCAAGCACAGGATCTAACGGCTCTCTTAGACTTATC
ACCTGTGAGCGACGCTTACAAAAAGGCTGGATTAGCGGAGTCACGCCACA
GGTAAGTTACGAACCACGCCCTTCCTCGGCGCTGTTCGCGGCGTTACTATC
TACATCGGTTCAATTGCTTTTTCTAATACCTGACATATGACAGAGTGCGCAT
GTGCGAATTAGCGGCGGAAGAGACTTCAAGCTGGCTGATGGTTGATCCGTG
GGAGGCTCTTCAACCCGACTATATGCCAACTGCTAAAGTGTTAGATGTGAG
TATACGCTAAATTCCTCTAACCGTTTATATCGCAAAGTTTGCTCTATCACGAA
TAATTTGTCCAATTTATAAAAGTCCCTCATGTTTATTCCCGACATCTATCACT
GCTTATAGTTACTAATCAGAAACGCCACAGCACTTTGAGTACGAAATTAAC
CAAGCCGGCCGTGGCGTTTTGGCTGCAGATGGCGAAACCAGAAGGCCAGT
TAAGATCATGCTTTTAGCAGGTGCAGACCTAATCGGCACCATGTCTACACCT
GGTGTCTGGGCTGAGAAGGATCTTGACCACATCTTAGGCAACTATGGAGCC
ATGATCGTCGAGAGAGACGGCACGGATATGGACGAAGCTCTGGCCAACCT
AAAGCAGTGGGAAGACAATATTCACGTCATCGCACAGATGATACAAAACA
ATGTCAGCAGTACTAAGATCCGTTTATTCCTTAGAAGGGAGTAAGTACATAT
CCGTCTACATCGTCTAATTTCGAGTCTGGTGTGACTAATACGAATTCTGCAG
CATGAGCGTACAATACTTAATTCCGGCATCCGTCATCCAGTATATCGATCAA
AACAGACTTTACACAGACGACGGCGAAGGGAAGTCCAAGGAAAAGGAAA
AGCAACGAGCAACAGTCTGAAGCATCACCAACCAATACATGCGTCCGTGTT
CCGTTTACAGCAACGTCATGGTCGCGAATTGAAGGAAGGACCGGTCTGTTG
GGTTGTGCAAACTGCACGAATTTGCACATGTGCTTCTTCCTTTCCTCCGTAT
CTGTACAAGGACATTGCAGCACGATCGCTGCTCCAAATCCAGGGATGGCCA
ACGAAGATGAGCTTGGCCACGCGGAATTTCCAAATGCGGGGGACTCGAGA
GCGTAATGCCGTCAATGAGGAGCGGCAGGGGTACAGCTCTAGTCAGCCAA
AATACGGATGAGGGGCTGAGTGGTGGGGGAAGGGTTACTGATAGAAACCC
CTCTTACCCTGCCCCCAAATTCGTAATTTCGGGTTGGAGTATGTATGAGTAC

ACGAGTACCGAGTACCTAGGACTGTACCACACGCATTTTAGATGTTTGGAA
ACTTCCAAGAGGCCTCACGACGCACGACGCTAGGAAACATTACCGATTGG
GACCTGCCTCCTTTTCTTTCTTATTATCTTGGAAGCGGGAATTTTCTTGGTAT
AACTATCAGCGATTAGAGATGATATCCATCCTCACATACATATAGAGTGTACA
TAGGTGCAATCTTAAAAACACATTGGGTCGACCGTCGGTCCCCATTGTCCC
ACTGCATATAATGCATGCCAAAAAGGTATCCCGTATTTAGCTAGCTTTGCCG
GCTTCTGAGGTGAGGTGACTTATGGCTTATTGGTTGCTGTAGCATCTTGAAT
CATAAGGAATTACCTACCTCTTTACCTTGAAGTGTAACGTCTTGGAGATGTA
TGGTATGAAGTGGTATCTTGATGTAGGTGTGAACAAAGTGGTAGGAGGGTT
TCAATCTAGATAAGTATAAGTATGTCTGGTGGGCATGCGAGCAGTTTCTAGG
TAAGTAGAAGATGTTCGATGGCATCGTCGGGGATCTCCCCTATGAAGGTATG
TACCTGTAGCAACCGGCCTAAATCCGTTTGCCCGGATATATTTGTATATGTC
TACTAGGTGCTTACGGATACACTGTTTTTCTAAGTATGCGTAGGTATACTTAC
ATAAGTGGCTTGTCAATGACGCTGAAGAAAGGATACCATCCCTAACCAATT
CTGATGACTCAGTATCATTCCGAGAAACTCACCACAAAGTCCGAATGCTCG
CTCAACCAGTTCGAATCTCACCAAGTCTTGAATTCAATGGTTTGATCGGTAA
ATAGGTAATTAGAAATCAAGGCATTTTATCATATGTATCCGACTTTACTTTTG
ATAATCCGAAGCTTCATGCATTTCTCACCGAGCGAGTTTTCATATATCTATAT
GAAATGCATAACTATTATGTAATTTAATGAAACTAATCAGGTTATCTAATGTA
AAGGCGACATTATTCGTTGGTAAAAGTATCGCTAACTTCGATCCACTAACCC
CTTGACTACCTAGGTACCTGGTAAACATATTATACTCAGGACTTCATCGTGAT
GATGGAGTATAACTCTACTGTTGCTTCTGACCCCTTATATCAGCGTTCGTATA
GCCGTTACACAACTCATTGTTGAGAAAGAAGCACCTGAGCGACTATTATAA
GTGCTCATAACATCTCAATAATTAAACTCTATATCGCACTTTTTTAGATAATA
ATTGCCTCTTTAGTATAAACATGGGATGAACCATCATCAGCTATAAGAGCTT
GAAGAGGGTGTAAACAGGATAAAGTTCGAATAAGGTTTTATATAACTCGCT
CATTTACCATCAGTGAGAGATTAACACGGCTACGAGAAGGTTATAAGGTTT
CATAGATTCTAAACTACCTCAGCCCATCTACTGATATATCTAAGGCTTACACC
CGAGTAACTTCTTCTCAACTTTCATCTATCTAAGGCGAACCTATATAGCTGTC
TATCTCTTGTCAGCATTCTTCCAAAATGGAGTTGACGAGGTATATCATTATCT
CTATTCTTGAATTAGAGAGAATGATGAGAGATTATGATATTCTGACAACTATA
CACGCCCCCTTTTTGCTCATACTTGCTCTGCCTCCCATACACAATGTCTTGG
GAAACTTATAAGTTCATTCTAGCAATCTTTCTTACTATTTTCCTCTTACTCTC
AGTTGCTTCTACTCTCTTCCTTGTATTCGTTGTTATATTATTCTATAATAATTTC
CTTAAGAAGGATTAGGTGTAAACGAACTTTTTACGCCCCCAGGTCTCCTGT
ACCGGGCCATTTTTTCTACACAGCTTTAACTACAACTTGGATATCCATTGGG
CCTCTATCGAATATCTTGCTTACCACAAATTCTGGGGTCACATTATGGAATCA
AAGAAGACTGATACGTCTTCTGGCTTGGATAATGACACCGAAACCAGGGTA

GACGTTAATGAGCAGGCACTAGGCATAGATCCCGGTTCATCTTCTGAGACA
AACGATGACCGATATGACAATTCTCTGCCTCAGCTCTCGGCCCAGACACCG
CCTCGGCCTGAACCCGCCGCACCATATCTGGACCCTCAACCACAATCCTAT
CAAGGACTTGATTACCACACTCAGGTCAGACATTTCTGCCCTAAGACTCTT
GTGATGACCGCCGGTCCTTCTTGCGAACATTGTCGTCCTGAATACTTCAAA
CTGGACGAGGATTCCTCTCGCCCAGTTGCCTCTCCCTCATCGCCGTCATCGT
CGCCAGCGGGAAATGACCAATCCTCTGGCAGTTCCGAAGTAGACACTGAC
GGAATCATTACGCGAACAGACGGTCCAGAACGACATTCCCTAGAACTCATG
GAGACCGTCCGTCGTCTGGTACAAGAGCGTGACGCCCTCCGACGACGTAT
CGAGTATCTGGAGAGACGTCTGGGAATTATAGAGGAGCTCGGCTTGTACAG
CCGTTCGTCAAACGGCAACGATCGGGAACTCCTGCTCGGCAACGGGAGTT
GGGAGGACGTCGACCGCGACACGGAGCTGGAGGCTGGATCGGTGAGTGA
CTAGGTACTTCCCAGCGCCTAACCCGTCGTGGGTTTCCTCGCCCCTTTTCTG
CGGCACGGTGGCTGATACTCATGACTAGATGGTGTCTAGTCAGCACACTAA
TGAGTTGGATACGGAGATTGTCCCGATCGACCCGGAGTGGTTCGTTTTGTG
ATCTCTTCTTCACAGACTTAGTTAGACAATCGAGAAAGAGAAAATAGCTCG
GTCACTCGTCGAATGCCTCTAACGCATGGGTAGCATGTGTTACCCATACCAT
AGCCAGAACGGGCGAACAGAGCTTCGTGTCCATGGAAAGCATCTTGACGA
ATGCTAGTCGGTATCTGGATGAAGCTTGAGAGAAAATCAATATTGGGGACG
ATGCGACTTCGATTCGATAGATGATTTCTCTTCCAGGGAGTCCAGCCGAGA
ACGCTAAGGCTAGAAATCAACTCAGCAGCGTGGTCGCAACGCTTGAGCCG
CCATCCGAGGGCGGGACACAGATATCTAAACCCGTCCCTATCTGTCACAAG
AAAAATCTGTTTAATGATGGATATCACACTGAGTGCTTGTCAGAAGCAGGT
TCTGGATTCAGGATCCTTCCCCGAACCCGTCCATAACAACTCTATACCGACT
AATTGTGTCTAAGCCTAATTGCGGACAGCTAAGAGCTTGCTGATGACAGCC
GGATGTTGTCATGAAGAGCCTGTCTGGTTTTACCAAGGCTGCAGGGAAAGC
CTCGTTTGAGAAGTGTATAGCACTAAGGAGTATCTCAACACCCGTCGCTCC
AGAGTCAATGGCAAGAAAATCGTCTGGCTCTTATCCGCCGCGAAGTATCGG
ACCGGTCCGGAGAGGTATACGATGAAATGTACATCGTTATCTAGTAAAACG
GGTGGTTTACCCTACAGGGCCAGATCCAGAGAAGACATAGGAAAAATCGAT
CGCCTCCGGATAATTCTAGATGACGAGATCGGCGAGCAGTACAAGAGCATT
GATATATACCTCCACGATCAACTCATGATTGGTTCTAGGAAGACCACAGAGA
TGACTTGCAGCAATTGTGGCAATTTCCTCAATACCATGCAGGGGAGTCTTTT
GGAGACACTACTATATGTTCCAGTCTCCTGAAACCCTTATTTCGCGGACGCG
CTCAACTGAGCGGCTTGTTCATCGGAGGCGAAGGCCCACTGCTTGGTGATA
ACGATCACTATACTACGAGTTTAAGATGAATACCTATAGGTATCTTCCAAGC
AACGGCAAAGATCCGAGACGCGCACCATTCTAAAGATCTAGTTCTTCGGTG
TATAGATTTCAAAGATTGGGTCAGGAGTGTTGGAAGCATTGGTGTAGGGAT

TTGTGAATGGACCAAGATGCCTAATTCACAACCACCACGCAGTGCAATCGA
CGAACATCTCTGAGCCTAGGATGAACCACCGACAGAGGGATCTTGCGTGG
CTCCTCAATTATAGACATGTGTATCGGACATACGCTCGGTTATCAAGCGAGC
TGAGGCTACTGGATATAAAGACTATGTTTATCGAAGGAACCAGACACCTCG
TCCCATCGAGTACCATATTGGTTGGGAATGACAATGATGGGTTAAAGTAGGT
GGTGCAGTGGATACATACGATGGGGTACGAATTCTGGGACATCTTTCGTCAT
GACTGGCCATGATGATTCCCTGTTTCTCCTCGATGCTCGAGAGACTCTTGTC
TACAGCAACAGCAACTATCCCAGCAACTATCACAGGAACTCTCTCCCAAAC
ATATGGCACTCATCAAGTGGTAGCACCTCTCCGTTAGGATCTGAGGTGGGT
AAGTGTTTTATCTGAAGCCGAAAAGAAAGAATGTCTGGATGTCGATGTGGC
GCTGAAAAAACTCAAAGCCGAGTGGGCTATGCCGACCCACGAGGCCATGG
TGATCATAAGACAAGCCAGATACATACGGGAGGATTGTTTGAAAGGGACCA
TGATCAAGCAATGGGCAATGGCCCTCCTTAGAGCATGTCGAGCTGCAAACT
TCACGGATCCACAGCATCAGATGGCAGGCTTTAGGACGTACTAGCCAAACT
TGACAGTCATACAGAGATCTATTTTGGCGCCATCTAATCACCAACTCGGATT
CCTTCCAGCCGTTCGTCAGCACTGGCACTGGGTCAGCTGCCATACAGCCTA
TGTTATCTGGAATTCAACGGAATGAAATCCTACCATATCGTCGCGGTTCAAG
GATTGGGCTGTGTCGGTCATGAGATAGTGTCGAGATGGTAATGTTCAAGGC
GTTGGCGGTCCGTCGTCAAGTTCGAACAAACCTGATGTTCAAGCGTTTGTT
AATAATTCTGAAAAACCTTGGGGACTTCGTTTCTATACTGGACATAGAGACC
TTGTCAGTATTGTGTTTTGTAGCTAGAAGAAAACGCATCCCATGCTTTCGAC
TGCTCGAAATAGCTAGCTGTCTGGTTGAGCAACTCAAGAGGGGGATAAGC
ACTGGCATATACTTGGGCCGGGAAAACATTTTCGACTGGTCGTGAAACCAC
ACTGGATCCGCACTGTCTGTGTGTTCACCCGCGAAGTTTCGGTGCGGGATC
GGGCAATGGACAGCGCCACCAGTTTATGCAGCCACGAATTACCCTCGATCT
CCCCACCTCCAGTCCGGTGCCCGCTGTACCCTCAGGGTAAATTTATTCAAA
CCTTTCCAGGAGAAGACCGTGGCTCCCTGGTTGGTATGACTCATTTACGAG
TGTTGGTCAGAAAATAATGCCAGACATCGCTAAGGGAATAAGGTCGGCTGT
GTGCTGATTCGGGTGCCAGCAACGGAATGGTGGACAGGAAATAGCTGCAA
GAGTCTATTCAGGACTTTGAAGAATTCACGCTTGTAGAATCAACATATGTGG
TGGGAATACAGGCGAAAGGAGAGAAACTGACCCAGCAATCGTGTGTGTTG
GTCATTGTCCTACTTATGGTCCTCATATAGGGATGGAAGACCCTGAGATAAA
CAGCTAGGTCGGGATAAGGGCATTATAGCATGTGTACTAAAGAGCGCTTAA
AGGCTCATTTCTGGCTAAGACTGGGTGGGAAATCCGACGAACTGACATGAT
TGAGCAGGATTAACATGGCGGGACTTCGATATCAAAAAAGTCTCGACAGCA
ACAACAACTATCTTCCAAAGGTCTCGGCAGTCTACTCGCCATAGCACACCA
TCAGCCTTACTACTAAGTACTGTTTTAGAGGCCTCCTAAGGCCTATCTCGAC
CTGTCAGCAGGCTTCGTCTTGAAAGCCTAGCAGTGAATCAGCAAGGCCTA

GCCGCATTACCTGTAGATCGTCTTCGGGTCGCCTCAGAACCTTTCTCAGATC
TTAGAGGGATACATCAATGCTTTACCGGACACAACAGACCTACCATATGGTA
GCACCGCTCCGTTTCGATCGGAGGACCTCCGGGTTATGAGAGTAGGAGGGT
ATCGTAGGCGAAGGTTAGGCGACCCTACCCCGTCACCTAACTTCGCACCCG
CCCGATACTCGAACAGGCGGTGCACCGAAACGAAGCCTATACCAGTCGAA
CCGTCCCGACGAGTGTTATTGAAAGCTACCTAAGCAAGATCAGCAGTACGG
AACTCTGAAAGCGAGGCAGGAAGAGCATGAGAAGGCATTGATGACTTAGC
ACGATCAGTGTCACGAGACTTCGACGACGGCCTGCTACGATTGCCAGGCGT
GAACATCCTAGGAGCCATGCGACAGCCGTCGATTGAGGCTCACCAAACAG
AAACTAAATGTACCTACATGCTGTGATTTTTAACATCACAAGCTAGTCGACT
CGAGACGCCAGTGCCAATATCGCGGGGGTTCCTGATGCTGCGCCAGTATTC
CAAGACACTTATTGGAAAGCCAGTATATATGTGCTATATATCAAGGCCGCGA
GAACGTCCTGAGTTCGGATGGAAATCATCCGCGGGAAGTCTCCGCCGCCA
CCCATGGGCGCTTGCTCAACGATTGGTAACTTCACTAGCTCCCGCATTCTTT
TGAGAGCAACAATCGGTACCAAGCCCTGCCACTCGTCGGATGGCCATCTAT
ATCTGGTGCCTCCCACATCGGATGACCGGTGGATCTCGGGGTTAGATTTAG
GAACCCAAGCCAAAATTTGCGAGAGTGTTATACACGTACGAGAGAGAATCT
TGCTACTACCCGATCCCGGACATTCCAGCAATCGACTTGTACCAACATCGA
GTCTAGTATGGACAGTTAGAGACAATAAGCTTTTCGAGGCGAAAACAATGG
ACAATCCCACAACATAACTGGCTGGACCGGTTGATCCAGGAAGAGATAGAT
ATAGGCATGTACGGATCCACGAAGGCCGCAAACGGTCATCTGACTAGTTTG
GGGCTTGCCGTTTACTCCGGTGTGGAGGTACTAAACAGGTATATATGGGAG
AAAACCCCCTTCCTCAGATGTAGACCCTATCTCCGAAATTCACGCAAGTGA
CGCATTCCCTACATGTATATAGTAACAGCCAATGGACACCTGTCGGACACGC
AAGGCACATAAAGGATACTGAGAGCATCATCCCATTGCCAATTGATAGCAC
CTCTCCGTACTTGCAAATAAAGGGACCGCAATGAAGACCTCGCTATATTATA
TCAAACTGCTCTCTGTATTGTTCTACATATTAGCTTCAATGTTCTCATCTTCC
AGTGATATATCACGTATCCCGCGTATCCCATCTGATTCCAAGTTAAACCCAAT
TTACTTAGCACCCCTTTCTCTCTCTCTAATACTCCAGAATAAAGCTTAAGAA
AGAAAGAATACGCTTGGCTCCTGGTCCCGTGAATGCTATACTGTGCTGTCT
GTGATCCCGTATTCTTCTATATCATACATTTCGGTCTTAATACCCCTAACACC
ATTCTCTCGTTTTGAAATTCTTTTGCTTTTAACTTCTCTTCTTACGCATCGTC
GTCGTCATCAGACTCGCTATCGTCCGCGCGTAGCGCTCTGAACCGCCTATTT
GCCAAGTCCTCGACGAGCCCTCCCTCGCTCTCGGGGTCCACGTACCCCTCT
CCTCCGACAAGCGACAGCGCTACTGGTAAGAAGATGAGGGCGTGCGTGGC
CGCGAGCACGACGAGTGCGAGCCAGACCCTGAAGTAGTAGATCTCGAAGA
TCTTGGAGCGCGTGAAAGCGAGAACGCACACTCCTAGTAATTTGGTGACG
GTGATGCCGCTGAAGACGGATCCGCCCACGTTGACGAGCGCGGTCCAGGC

CCTCGCGTCGCGGCCGCGGAAGCGGTTTTAGCGCGCTCCATAACGGCTCC
GGAGGGAAACATGAAAGCGCGAGCGATGTGGGCGCAGAATTCGACTGCA
ATACCGGTACATATGATTAGATTCACCAATGAGACAGCGTTTAGAGACACGT
TGAAGGCCGCCATAGCGCCAATGATGTCAACGACTGTCATTACAACAGTCA
GTGTGACAACGAGAGCTGTGACAATGGATCCTAGGAGGATAGATGCGACG
ACGAATATAATTGCCACAGCGGAACCGAGAAGAGTACCAGTTAGGCTGAC
GATGCTAGCATATTGGTCGAAGAAAATGTAGAATACGCTGTACGGGAAGAT
CTCAACCCCAGTGTTAGCACTGACGTCATTGGCAATACGACGGGCTGATGC
GTAGGCATTGATGAAGTCATCTTGACTACGCAAGGGCTTGTGCATGGTTCG
GAAATGACTTGCGGGGACGGAAGTCTTTTCAGCGTCCACGACAACAGCCT
GACTGTAGGAAGCCTGGCCGGCTAGAGGACAATCATCGGTTGTTGGAGCC
TGTAGGTATTTCTCTAGGTAGTGGACGAACTCATCGCCTTCAGGCATACCGT
GAAGCGTGATATTCCAGGATGGGTCTCGGCCGGTGAAGCAGACATGATTGC
TTTCCTTGCAGCACTTTTCGAAGTCAGGGTTCAACCAGAGGAAGAAGTCG
TCAAGCCAGCTAGCCGTCGGGGAAGAGATATAAGAAACATCAGGTCGCTTC
CTCTCTTGTTCGAGTACATTCGTGAGAGATAATTGCTCGCAAGTTGTGAAA
CGCGAGCAGATATCTCGTTGACTATCGCGCTGAGTGAAATTCAAATCCTTG
GTCACGAAATACACAGGTGGGCCGGTATCCATATAACTGTACATGTCGTTGA
AATAATCGATGAGATAAGAATCATCGGGTAAAGCAACACGCTGGTCCA

>Mosaic_sequence_04

GGATGGCCAAAATTGCCACGCACGTTCAGGAATGGCCGGAGATACTGTAGT
ATTTCGATGCTGCGGATTCAGCTCATATGCTACTCGAAGTCTGGCCGGGCCA
ACGTCCACTGCCTGAGTTGCTCAAGAAGGGCCGCGGAGCGCTCTTCGGCG
CCATAAAGCCGTAATATGCTGTCCTCCACCCGTGCCATGCCCCAACTTCGTG
GAGCGGAGTAGGTCAGAGCTCATTCGGTGCGCCTCTTTTTCGAGGATAGAA
TACGCATACTGTACAAAATCTTCCGCCTCTTTCGCCTGCTGTTGTTACAGGA
GTCCAGCCGCTGCGGACGTTCGGGTCCGATAACAAGCAGGGGGAGTGTGG
CGACTTTGGGTCAGCTTTTGGAAAAGCAATCCTCGCCCCCTTCGTATTGGC
AAGACTGGGGAAAGGGCGCCTGCGTCATTTGGGAAAGAAACGATCTATGT
GTCTCACGTCGTTGGAAATGATTCTGCACCTTTGTTTGCAGGAGCATAGCAT
GGCCTCTATGGCTCGCATACATAAGCTGTCGAAAGCGGTGGCGAAAATCTC
ATCACTTGTCCACGATTTCTATCCCATGATGACTGAATCGACTCGAACCCCG
AGCGGCACGAATGAGACTTCTCCCTTGCTGGGAACCGATTCGAACGGTTTG
GGATATTTCATCTCCAACCAGGGAATTACTGCGGAAGGCATTCCGAGAAGC
TTGCTCATCAACGAACCTCGCGACACGGGCCTAACTAACGAGCTATTTTGT
GAGGAATGGGACGCCGCCTTCGGCTCGGCCCATGGGACTCGTGCTGACAC
TCCGTGTCCATGGAACTACTGCCACGAACTCCATCCCAAAGCGGCCGTACA

CATCGAACACTTGCACATTCAGGGCCGGATTGCTGCGTTGAAGTTACAAGA
GGATCGGGATCGTGAGTCGAGGGACACAAACATCACCATGTTAAAGCGAC
ACGGAGTAGAGCCTCTTTTCCCTTCCACCGCTCAGCTCGACGACGCTTTCC
CCTTTGCTACGGATCCCAAAGGAACCGGATACTTTGCCGCCAAATGGGGAA
AGCAGTCAATATGCCTTCGGAACCGGCCTGTCGACTCGGGCATCTCGAACA
CAGACTTGAGTGAGCTCATGGAGGAGGCTTTCCCTGTCGGGTCGATCGGA
AGTGCCAGCACAGCACTTCAAGTCCAATTTCTGCAAGACTACTTGGGCGCC
AACAAAGTTCAGCTCTATGCCCCAGTGGTGAGCAAAAAGCACAGAGAGGA
GGTGGCGTTCGGTACTTCGACCCAGGCCAACGTCCCGACAAGCTTGCATCC
CTAGGGAAAGATGGCTATCTGCGCGCCGATCACAACATCTTGGTCCTTGTC
ACATTGTGCATGACGGCCTGCTCTCCCCGCTTTTTCTGAATACCTCTTCTCT
CTCGCTGTAAATGTATCCTGTCTTTCCGGCAAGAAAAGTTCTGGTCATTTCT
CAAAGTGGATTATTCTATGGCTTCATAGTCTGATACGCTTCAGAGTCTTCTG
TCACGCAATGAGCGTGGTATCCATTCGCCCTCCCGGAGGGGAATCTTCATCT
ATATATTTGTCAGACCGTCTATGCCCATGACGCTGTCCTCCCCATTGGACCC
AAAGAGGCAATTCCGGTGGCTATGGGGCGATCACAGTGCTCATCGTTGAAC
ATCTTCGGTGATCAAGTCTCAGAGAGCCAACGTGTGCGCTTGCCAAGATCA
TGCAGTCTCCTTCTGTAGTGCCAGGCTTCATGATCACGCGCCCGCCGGCTG
TCCATATATATCAAGTCTCCGAGGCATTCCGTCGGCAACAGCCCAAAGCTG
GGCGTTTCCAATCCATTTACTGTGATTGACCTGACTTAGCGGTGGGTCAGGT
GACATTTGCAACGCAGCTTTTGAATTCACGACAGATTTGTACTGGACAAAG
ACACCAGCGGCAGGTGCGAATCGACCACCTCAAACATACCCAAACCATGT
GGCACTTGCTTTGTGACCGTGGGACTAGGAAGAACGTCAGCATGGTCTCA
AGATCTTGACGCGTGAAAGGAGTTTCCCCAAAGGGTTTCCACTTATTCAAA
TAGTATCACTACTCGTAGCCTGGACGATGTAGGATTGGCAAAACCCCTGGT
CGACTTCACTGTATAGCTCCGTGCGCGGAATATAAAGCCTTCGCGTGGGTG
GCGAATCTCCCATCAGTCTCATTGACTTAGCTTTCGACCAACAGTAGCCCGT
CAAGAATGTCTGAATCTACTGCCGTGCCGAGCGGCACGGCGCGTACTCTGC
GCCTTCTTGGCATCGACGACAATGGACTGGGATACTTCTGCGACGACAACG
ATCGGACGGACCAAGGGATTCCAAAGTCCCTGTATATCAACAACCCCTCTA
AAACCTTGTGGGACAACGGCAACTTTGTAGAAGAATTGAAAGCGGCATCT
GGGATGACACCGCAACACTTCATGACCAATCCCGAGATAACAAGAAATCCG
GCAAGAATCTCTTACTCACCTCAAGCTTCAGGAGCATTCGAGTCAGCTTAC
GTTGAACTGGCTGGTTACCAAGGTGCAGACACATGTGGTAGAGTCTCCCGT
TTCCGGCCATGACTACTCCCATATCTCAGACGACACTGGCGGCAGCTATCTC
GCAGCTACTTGGGGGACCGCAGTCGATATGCTTCCGCCACAATCCGGGCGG
AGCCAGCACCAGAAAGCGTGGTGTCCGATGCAATTTCTTTACTCTGACTAT
TAATGTAGACACAACGCACAAGGTGTGAGCGAGTGCCAATGACACCAAAT

TATTAAAGCCCGAAGAGAGGGATCAGGCTATGTCCGTCCCCGTCAAGGGCG
CCCATGGGATTCTTGGCTTGTCAAATCGGCCAATCCCAGGTCTTTGATTTGC
TTCCGTGTTACATAGTAGCTGTCGCTGCGGAAGGCAAAATCGTCGTTGTGC
ATCCGGAGGCTAGACTGACCTAGATGGAAGGTAGCTTTCATTATTGGCTCG
AGAATTGAACGGAAGTCCCGTCAAGTGATCTCTTGATAAGAAAAGATTACA
TTATACGTGGCTAGTGTCAAAATATATTTATGCGAGTATGATCGCAATAGGTC
GTGATATGCCTTCACGAGGAAAACAAGTACTTTACCAAGTAGGTAGATTCC
CGATACATAAATTTTGTGTGCCATACCACGTTTTCTTCACCAACGGGAACGG
GCTGTTCTAGGCTTCCCAATTGACGGATTGACGAGGTCACACAGCAAGTAC
TTATCCGTCGAGCTTTTGCCATACATACCTAAGTACCTACCCAGTAGCAGGT
AAATGTTGTTGGGAGGGAGACGGGAGCAACAGCTAGCCTCGCGCGGCGGG
ATTAGGTAGGTATTACTAGTAGCAGCCACAGGCATTCCAAACTGCCTATTAA
GCTAATCCTCTGAGGCTTAAATCAATATTGACAGACCTTACAACATCCCAGA
ATCGATGTTGGCCATATCCTTCGACAGCCAGGCTTACCGTGTGGGAAGTTG
ATCCTGATGAATGCCAGGGTCTCGCGAAGGGAAATTGCCATTGCCATTGCC
ATACTTCTAGACTAGTTGAAGAAGCTTGACTTATATGCAGGTAAGACTGGCC
ATGTAAGACTGGTCATATATTCGATTCAGGGACCAATTCGAAAGCATATTTG
TGGGCAGGTTTTTTTGAGTGAGCGAACAAAAAATATAGCTTTTTGTGTGTT
GGTACATCCATGGATAGCTAACGACCATCTGCAAGCTGCGATCTTCGGCGG
CTACAGCGCTGTTAAGACCCGTATATTAGGTGGGTCCGGATATAAACTGTAT
ACCTAGGTACTTGGATACCACACACATAGAATTAACCCAGATAGGTACAGAT
TAGCAGTAGCCTGCATCTTTTTACCTTGGCCTGCCATTTTTAGTCTATCGCAT
TAAAGGGGGCCCTTGTTTCTTGAAGCAAACCTCGTCATGGAGGAAATAGTA
ATGGGTAACAAGAACAAGATACATCCGTATCTTCGCCATAGGTACTTACGTG
TTTGTCATACCGAACAGATGAAGGAAGGCATCGTATGAACGTTCAACTACA
AGTATGTCCCTGGGTATTTTTGTTCCAATATAAATCGTATGAGACCCAGGCTT
AAAGATCAGCAACCTCCAGTAATCCACTTATGCGGATTGAATGATTTGCTGT
GCCATTGGATCCGAGACAAGCTTCCTCTTTTGTTCGTCAATACGGGAGTAG
ATGAATCCTAGATTACTATCGTTAACGCGACGTTATATATATCCGGAGAGGGC
TATTGGCCTGGACGGAACCCCTGACGGCGAACGTATAAGAATAGTGTGTAC
TACGTAATTTGTGCTATATGCGCGTGTGTTGCGTGTGGTGTGAAGCTCTAGG
AGTATGCAGCATAGATGATTGAGATGCCTTTCTGTGGGTACGATGCTTAGCT
GGATACGTCTAAGCGCGGGATAGGTACTGCGTAGTAGTCATAGTGTAGAAG
ATGGACCCGATATTAGCCAATGGCAAGACGTTCGTGAGACTCTAACGCTTG
ACGCAAGGCGGCCGGGATCGCAAAGTTCGATCGACTCATCCGGCGCCGTC
GATGGTGATAGTGCATATACTACATACATACACATACATGTACGTACTGTATG
TCTTTTAAAATATAGAAATTCGATTGGGCGAAAGGGGATACGGTGCAGATAT
AGAGAAGACCGGACTGGACGAGGCAAGAGGTGATGATGTGCCTGCAGGC

CGCTAACAGACCGAAAAAGAACCACCAGATTCGCCAGCCAGCCTAGTTAG
CTTAAGGAAGTCGACGGGTTGACGAGTTGGCTTCGAAACAGCTTGCCCCT
CGGTCCCCTTCCAGCCCCTCCAAGGGTCCCCTTTCGCGGCAAGAAGGTTGT
TGGAGCCTTGGGAGGCTTGATTTCAAACCAGAGCAGATCCAAATCGGTTAC
CAAGCTCCATTTCGCCGAATTCGCTCCGTGGGCAGGCTTGTGGAACGGGTA
GCTTCCCTGAAGATCATCTCATTGGTAGGTAAAGAGGCTGACATCAAGTGA
GAACAGCAGAGACCACACACAGTCAAAACTATCAACTGTACAACTGCATA
CCTGTAGGTATTAATGGTATTAATAACTGGAATACATACCTAAGCGAATTTTT
AAAAGAATAAAAAAGTTGATTTAGTCTCGTGATAACATATATTACGAGAAGC
GATAAAGCAATTCAGGTCCAATGAGGGTAATATCCGATTATTGATGATTGAT
ACCATTGATACCATTTGATAGGCAAACAGCTGTCATGGTGCCCTGGTGCCCT
GGGGGTGCCTTTGGTGGTTGAGGAACAGAAAATAAAACCGTTGGTAAAAA
TCAATTCGAGTGTGAACCGGACATTGAATGCAACCCGACCTGGTGAATCAA
ATTGAAAGGCAAGATCTAAGTGATTCCCTGTCAAACGTTAAGGATAACGGT
TACTTAGAATCGATGTATACTATACATCCATTTAGATTTGCATCTTGTGACCA
GAATCCACTCTGAACTGTGGATTTGGCTCCGGCGGTTCTTCCCATATGAGCC
AATTAGATCATAGGAAGGAGAATAAACCCCTGTACGTGGATCCCTTGTCAG
TGAGTGAGATGCCCTGAAACTAACGGGCTGCCCTTGCGCAAGATCAGCCC
ATACGCAATCGCCTAAACGATGTTATAGATACATGGGTTAGCGGTACTTACC
TGGTAATACAAGCAATTTTAGCCATATCAAATGACCTGCTAAGTGATTTCAC
AGCCCACTGCCACTGCCAACCTTAACCTGTCTTTTTCTCTGCCATTACGCTT
GCCCTGAAAAGTGCGATGAAATCACACTGTGGCAATGGACCGGCGTAATTG
CCTTCAAGTAAACTTGAATCCATCCCCAGCGACCCGATCTTGTTCAACCCG
GTTTGGAAATGTTAAGGATCTTCTATTCTGCCAATTGTACTTTTTGAACGGT
TGATCATTTTTTCTTTAATCCTTTTCTGCTTCTCTGTCTTGCAATTTTTAATTC
GTCAAAGTTGCTTTATCTATTTTCCCGACCTTTATAACATCCGTCACATACCT
AGTACTAGATCCCGTCTGACCGCTGGTTCAAACGTTTCCAATTGGAAAAAA
TAATAATTAGTAGTTCTTTATCTTACATACGTACCTATTACCAGACTGCCAGA
CTCTGGACCACATGTAGCGTCCAGGTGATTGAGACCGTATCCAATTGTACCT
AGGTCCTAGGTCTATTTAGCAGGTACGCTACACAGGCTACGTACTTTACACA
GTACGACAACGATTACTGTACGATGTACCGAGGGGTTATTCGATAATCGATA
CGAAAAGGTCCAAGCTCTTCTAGCTCTGTCGAAGCTCTGACGCTCTGCCCC
TCCAAACATTTGAAGGAATCTGAGGTACCCGCCTAACCTTGGAAAACCTAC
CCACTACTGTGCGCCTAACTTCCCACGTCTTAGCCAATCCACCCGTCTAATT
CCTTCCCAGCGTCTTTCTCCCACTCCAAAATCTCCCCACCACACGCAACGC
AGAGGCGCAAAAACACTTTCAACCACAATCAATCAACGGTTCTAATCGCTC
GCTCTGCCTTTTCCTCCCACTTCCACCCAACCTTGGAACCCTCGATTTCCGC
CCGCCGTTGAGTTTATCTTAGGGAATAGGGAAATAACCACAAACTATTTGCT

TTCAATTCCGAAACCTTCAAGAGTTCGACGGTAGTCGACGGTTGGATTTCC
TCTTTCCCCCCCTTCTTCTTTCTTCCGCTTCTCCCACTTCTCAATCAGTCATG
TCCAACCCATCGCCGCCCGCTGCAGTCTCAGGGAACAATGCTGGCAACGAT
GACTCCGGTATGGCTCCTAAATGTCCCCTCCAAGTCCTCCAAGAACGCTCC
CCAATTGCCCCTCCATATTTCCCCATCCATATTGTCTTATCTAGCTTCGGCGA
CCGTCAGCTACGCCGACGTCAGTTTGTTAATACTAAGCTACTACATTGAACG
ATATTGTCCTCGAATCACCACGCCACTAATGTGACCAACTTGTTTTAAATTT
AACTAACCTTGACTGACTTGTAAAGCCTCTTTCTATTACGGTGAATACCACC
GCCGCTGCTGCCGCTCCCGCGCCGTTTCCTCCGCCTAAGACCGACAAGCCT
CGTCCTCATGTCTGTGCTACTTGTCAACGCTCATTTGCCCGATTGGAACACC
TGAAACGTCACGAACGTTCCCACACTAAAGAGAAACCTTTCGAGTGCCCC
GAATGCACACGATGCTTTGCCCGGCGAGATTTGCTCTTGCGCCACCAGCAG
AAGCTTCACCAGACCACGACCCCCTCGTCGCGGCCGAGAAACCGTCGCGA
AAGTACTAGCAGCACTGCCACAGCTCAGAGCCGAGCTCGTAAGAATAGCG
TTGCTAGTGCAAATGCTTCCGCCCAAGCCGCCGCGGCGAATGCCTCTATGA
GGCCCCGCGCAAATACTATTAGCCATGTCGACGGCAATCATATGCATATGAT
TGCGGCTGCCAACGCTCAGGTCGCAAGAAACCAGCAGCATCATATGCCCCC
GACCCACAGCCGCCACCAGAGCCTCGTAGGGCTACCATTGCACAATATGGA
CAATGGTTTCGCCGGCATGTCTTCTGTAATGGGCCACAGAGGCATGAGCCA
TGGGCTGCCAAAATTGGAGACTCACAGCATAAACAACATGGATTTCAACGC
CGGGTTGCGGACTGCACCTCCTCTGCCTTTCCAACCAGAGTACGATTTCGA
GGGGCTGCTATTCGGTCAGCCTGGCTCGACTATCAACCCAAATGCGCTCCA
CTATACAGAATCCCCACACTCGATGTCGTTGGATCCTACATCCCCTTTTGGT
AATGGCATGACAGACATGTCTACTCCGAGCCAGCATATAGATGACAGCTTT
GACTGGCTAACAGGATTCGAGCATCAGATGTCGTTCAACAACACTGGCGA
AAATGCTGTTGACGGCTCGTCCCCATCCGCGATGAGCACCACCAGTCAAAG
TGGCATTAGCGATGTGATGCTTGATGGCTCCAATCCTACGGCTGTGACAACC
GCTTCCAGCTCTATGTGGCAGCCGGCACTAATGGGTCCTCCTCAGATGACG
AACCCTTTTTCCATGGATATGAGCGGGGACAGTGTTTCCGGATCTTTTAAAT
GGAGCACCAGTGTCTCCTCAACCCTCATCCCAGAAGAATATGAGTGACATC
TACTTCTCAACGCCACCTCCATCTATGAGTTCGCTCAGTCCCTCCGTAATGC
CTGGTCTGACGACACACGGTATGAACCAATCGCTCAACTTTGGACCTGGCC
CCGAGACACCTACTTCTATCGGAGGTGGCCATCAGTCACACTCTCCTGTCT
CTACCATTACAGACACTACGCGTAATGCTATCATTGCTGCATTGTCCCAGTG
CGCCCCTTTTGGTGGCCGTAAATACTCTTTCACGTCACCCACTTCACCTATG
TCTCCACAGTTTTCTAGCACCCCAGACAATGCCCCCGACAACATAAAGAAT
TTACCCAGTACTCAGGATCTACAACGATATGTTACAAGTTACTTTCGTTATTT
TCATCCTCATCTCCCATTCTTACATATTCCTACTTTGTCGTTTGATACCCCGTC

CAATTCAGGCAATGGACGACCAAACACTGTTGGTGGAAGTGGGTGCCTGG
TTCTTTCAATGGCAGCTATTGGTGCCTTGTACGAAATGGATCGTACACAATC
GAGAGAAACTTTTTGAGATGGCAAAAAAAGATGATTCAATTATACTTGGAA
GAGCGAAGGAAAGCTGATATGCGAAAGGCTGATTTCAGGCGGACCCCGTC
ATCCGACCAAGGATCACAAGGGACCGGAATCCTCCATCCACACTCCACTAT
GGCTGGTGCAGGCAATGCTTTTGAATGTTGTGTATGGGCATAACTGCGGTG
ACAAGATGCGAGCGATATCGCTTCAACTCATTCTGCTGCTCTTGTCAGCCTC
GCCCAGGCTGCCGGTTTACTTCGGCCCGTGAAGATGGATGCAGCTGAGATT
CGCGAAATTCAGATGAATGATGCCGAAGGAAACTGGAATGGGGGACTGAA
GGCTGAATCAGAGGAACAAGCTGAGTGGTTGAGATGGAAAACAATGGAG
GAACGAAAACGCACTCTCTACGTCATCTTCATTCTCTCCAGTCTCCTGGTTA
CTGCGTATAACCACACTCCGGCGTTGACGAATTCTGAGATAGTACTAGATCT
GCCTAGCGACGAAGAGTCTTCGCAGCCGAGAGTGCCGCTTCATTCCAACT
GAAGGGTGGTGTGCGGGCTGCTGGACACAACCGGACAACTTTCCATGAAG
CTTTAAGCGAATTGCTTCGCACTAATGAGAAACAGCAAAAGTTAGCATTCG
GCAATGGGCAACAGCAGTTAGGTCCAGGAGCTCTGATCAATGACATCCCAC
CGAGTGAGCTTAAACCTAGCACCCTTGGTTGCTTAGTTTTGATCAATGCGCT
CCACAATTACATATGGGAAACGCGTCAGCGCCATCACAACAAGATGTGGAC
AAATGAGGAAACCGAGAAGATGCACCGTCATATTGAACCAGCGCTGAAAG
CATGGCAATCTGCCTGGGCGAGTAACCCACACCACAGCTTAGAACGTCCG
AATCCATTTGGCATGGGTCCTCTATCGGCTGATGCTATCCCCTTACTTGATTT
AGCTTACATCCGATTATTCGTCAATATGTCTAGAACTAAAGAGAAATTTTGG
CAGCGAGACTGGGATGGCCTAGCTGACGAACTCTCCCGCGGAACTGAGAT
TGTGCAACATGCTGAGCATTCTCCCTCCTCGAACGCAGAATCTACGACGGA
CCCGTCTGATGCCTCCGCGACGAGCTCAGTTTTCATCGATTCACCGCCAAC
ACAGTCGTCGTCGCCCGAATTTTCTGCAGTCAAGTTTCCACCACCTATGCA
GGCGCAAGGTACAAACAGCCGAACGATAACTCGACGAGAGAAGCATCTTC
GCAAAGCAGCCTTCTACGCTGCAGACTCGCTCTCTATGTCTGACAAGCTAG
GTGTCACATTTGCCGAGTTCAACAGCGGCGAACTTCCAGTACAATCCGCGA
TGTGCTCCTTTGATTGTGCGCAGGTTCTCGCCGAATGGGTTGCCACGCTTC
AGGATCGTGTTGGAAGATACCTGGGTGTCCTTGGTAAGGATGACGTTGATA
TAGGAGGAGTTCCTGCGATAATGCTCCTTGAGGATGAAGATGTCAAACTCA
TTCACAAGATCCAAGAGCTTCTAGCTAGCGCCGATATGAAGATGAACATGG
ATATCGTGAGCGGCAATGTCAATAGCGGAATTGATTCTCGTCTCCAGATGGA
TAGCCATAGCGGATAATGCGGCCAAGATCTTAAGGGTTACGGCGTATATGCT
TGATAAATCCGCCATATGGCCAGGTACGTTAATCATGCAGAGCTTATTGAAC
TGATCTAAGACTTACTCAAGTTTTCTAGTACTTCACCTGATGGCACACTGCC
TCGAGAGCCATGCCAATTTCATGGCATCGCGAGCCGATAAGTCGATCTTGG

CCAGTGAGTAAGCAACAATTTCAAGGTTTCATCACCATAATCGATACTTGTC
GAAGGCCGGATCCTTCAGACGGTGAAGTCCTAGCGTCAACACCTTTTGAGT
GCCGCTAGCCTCCGCTAGATAGCTACCTGCTCGAGTAAGCCGCGTAAATCC
CACACTTGCGACAGGGATTATCCACAAACTCAATACGCCGACTTCAATCCA
ATCCTATGTTCGAATGGCCCAGATAATTACCCGTTGTTGCCAGGACATTCGT
CCAGACATCCGATTTCGAGACAACAGATTAGTCCTACAAGACGTAATTACC
CCTTGTATATATCTTCAACCCTCAGAGATTACCCCGAAATGTCGATACAACTT
TCAAAAGGAAGGATTCCTGATGATACACGAATGTTCAAAAAAACGGAAAA
TAAAAATAAAGCGCCTAGCCACTTCCACTTAACGCCACACACATCCCCGAC
TATTCTACAGTCTTGACGCTACCATTTACACGAAGGCGATGATAAAATCAAT
GCAGTCACGACATTATGGAATAACAGATGATAACTATTGAGACGACTTTCGT
AACACAGCGATGGGTCGAAGTTCACGTTGGCGATAAGTCGCCGAATCAAA
AGGGGGTTAAAAAGACGCCGGAAAATAGTAGAATATGAGAAAATTCCATTT
AGAATTGTTAGCCATCAAACAAAGCATTTATGATGGCGATGGAGTTGACAA
TCTCAAAACCATTCACTAATGGTCTAGGATCTCATCTGAATCTAACTCCAGC
GATAGATGATTTTACGAGATGTGCTTGACTACTGGGTATGGTTATTTAAGGA
CTGGGGGAAATTTACAAAATCGTGTATATGATGGAATACAGTACATGAGTCT
GTCTTAGCTAGCGCGATCGGGGGTTGTTGGTTTGGTGTTAGAGAGGGTGTT
GAAGGTTTTCCGGAGTTGTAAGGTAGCAAAGGCGTGCTATTATCGAGCTCA
TGTCACCTGTCACATTGTGCTTTACAACTCTAGCACCTATGATTCTCGATGTT
TTAGGTATCGATTTTCCTACTAGGGAGGTTACGAAAGCTTGAGGGTATGACT
TTATTCGTATACCCCTTATCGGGGCTAGGCGCATTCGCTCGGTCCTTTGGGAT
GCACGTGAGGTTCCCTAGGTCTGATATGACGATGTATTTAGGTATAGGTATAT
ATCGAAATGATATGTATTCAAAGTAATCCATGATGTGTACATGCTAAATCAAC
ATCCATGTCCTAAGTTTTGCGTACAAATGAGGCTCGAGTCCTCACCCCACCT
TATCAAAATTGGATGTATTCATTCAATCTGAACACTCTGAAAAACGACATTT
CTTATTTTCTAATTTTAAGAAAACAGTTAGCTCGTATGATACCATGCGAGTTT
AGAAATACTAATATCTCTAGATAAACATTGGTAGGTAACCAGGTCGCTACTA
GGTATGTTTTAATAAGTAGCCCGTTGCAAACAGGACTCAATGACACATACG
GTTAGTAACGGTACAAACAACACAGCAACGCAAAACTCTGTGGTCGCCGT
CGCAACGCCTACGAAGTTGCACCACAGCGTTATGGAATTCATCATAACCAA
GTTTTATTCATTCGTCGAAATTCATCCAGCCACAACTACCTAGTAGCCAATC
CTAGTTTGGTGGGCCAAATTCTTGCACTTTACTCTTCCACCAACGCATCCGT
TCTCCAGACCCAAGGGGCCTTTATGAGTTATGCGATAGCGAATTGCTTCTTT
TACTTCGTTTTCCTGCCTCGTAAGTGTACTTGGCCCCATCGTGACACCACGA
TATGGAAATTTCGCATTACCCGGCACGTCGAAATGTACTAGAGCGTGACCC
TCGCGAAGGTCATCAAGGTGATAATACGTATTGGCGTGTCTAGGAGGGCCA
TATATTCGAATATAGACGAGTAGGTCCCTGACGTTAGGCGAATATCCGACAT

TGTCAATGCAGAAGGATGTACGGCTTTGACGAACTCGAGCAGGTCACTCTC
GAAGATGTAGACACCGCGTAATCTGCATATTTCAAGACATGCAAAATTCGA
AATCGCTAAGCTCGACTGATGAGTCCAGTGGCGCAGTAGAGGTGTCGTAAG
CAATAATATTGTACCAGTGTACGTCCAGGCTTTCCAGTTTCGGCATGATACT
TGACATGTCTAAGAGACCTCGTAGAGCTAGTTTTCCATGCACGGTCTGACT
GAATTGTTCCGGCGTGCTAATCGCTGAATCTGATGTACTCTCTTCGGGATTG
ATGAAAGGCGACGACAGGCTCACCGTCAGTTTCTTTAGCGAGCTGAATACA
GGCATCGACGTAAACCTTTGAGTGAGGGGTAAAAGAGCATCGTATGGAAG
ACTGCACCTTAAAACTCCACCGTAAATGTCTAGATGCTCGTCCACAGGCAG
TTGGGATGCGTGTTAATGCTGCTATGGTGACATTGAAGGTTCGCAGAGCCG
TAGCCCAGATACTTTCTCGCAATGAAGGACTATCTGACTCGGAAGTGCTGT
CGCGGTTGCGCACATCCACACGGAGGTTCAGCGAGGTAAGTCTACCTTTGG
GCGAACGCTGCTTTAGGTTGCTGAAAGCCCTGGCTAGAAGATGTATGTGCT
CGTCGTCATTTCGTGTTCTGCTCATGTCACTTCCCGCGACGCCGGTAATGGT
ACAGTGTTCTAATAGACAGCCTAGGCGGCCTTGGCTAGTTATATATGCCAAG
TTCTCCAGGGGCGCCGGAGCCATCTCGACATGCTTATAGTTGAAGTACTTA
GCGAGATGTTGCTGGGAAACCTTACTCTCGACAGTGCGGCTCGCAAGTCG
AAGTGAGACAATATCCCGAAGATCAATGAGCGTCACTATGTGACCAATCAA
CTCTACAGGGAGAGTTTCGAGGGACGATTTCATACTGTTGTAAGTCGTGGC
CGAAAACAATATTCTTTGTCGGGATTAGGAATTTAGGAATAGATCGATCAGT
TTCTGTTAATGTCTGTTGATGTATGTTGGGGAAATAAACCAAGGTTGCTCCT
ACAAATATACCTAGTGCCTAGGCCTAGTGGGGTTCGGTTACCGGTAGGTACT
GGTACTTAACTATCTTAGAACATAAACTGTGAGCCATTAAGAAGAGTGATAC
TAGGTACAGATTAGTTTTTCGACCTCATTTACAGATTGGTTTTTTAGCCCCAT
TTAATGACAGCTGCAAAACAGTAACACCTCGCTATCCTACACCCCGCCAAT
TTCCTTCACTTAAACTATTACTTTAAAGATACTTACTCAACTCAACACTTTCT
ATTTTAAATTAAATATACAGGAAAAAATAGATTTTTAAAACCTCTATCTAATA
GTATAAATTTTTAATATTTAAAAATAAGGTTTAAAAGTACCAAGGAACTACC
CTTTAAGCCCTAAAACACATCTATAACTTCAAAAACGTAAAAAATACAATC
ACTTATTAACCCACAGTAACCCACCTTAATAAGACATTCGAATATTTTTAAA
ACCCTAACTTAATAAACATTTAAAATCTAAATACAGAAAACTAAGTTTACAA
ATATTAAATCGTTTTAATAACTCGAAGATTAAGTTAAAGAAAATATTAATAAA
TAATACATTAAAATAGGCAACTGTCACCAATTAAGATAAAAACCAATCTGCA
AATGAGGTCAAAAACTAATCTATACCTAATATCACTCATTAAGAACACAGCT
CAAGATTGCATAAATATACACCACTATCATTACTTCTAAGTTATCTACAGAGA
AAATAAATTCTATTAACCATAGATTATAGGCATGGAATACTAAATTCATATAAT
ATTAAATTGTAAAATTAGAAACCCATTTGGCGGAGGGAAATGATATGATCAG
GTAGATACCTACCTACCTACCTAGGCAACTACCTACTATCCTAGAAAGGTTT

CCACGGTTAGATCTAGATATACGGGCGGCTCACGATATCTCAACTATATATAA
TTATAGTATGTGCAACTATTAAAGTAGTTTTGCACCAGTGGCAGTCTGCGAA
GTTTGCTCGTACCAGAGCCGGAAGTTAGGGTTATTATTTCCATCAGCATTCG
CATCATACTCAAACAGTGCCTTCCCTTGATACTTTCTCGTGTACTCCTCAAT
GTTCGTCGCAATGTAATTATAATAAATAACTGATAACCTTGGAACCAAATTG
ATCAGAGTCTGAGAAAGCTGATTCATTCTCCCTCCATAAAGTTGCGTGTCA
GCACTCGGAAACCGGCGGGTTGGGGTTGTCTGGTGTAGGATCAGCCTGATC
TTGCCGAGGCGTCATGATGTAGGCAAAAGCTGGGCCATTATCTCCTGTGAA
CAAGCTTGGATCCAGGCTATCCCCTCTCGCTCTCCATGTCTGGCCCTGTCCT
GTAATTAGGTTGAGGCAGTTCTGCTGGAAGGCAGTATCTGGGCTTTTGCGC
CGTCTGGAGAAAACGCCAGGTTCTCAAAGAAGTGCGCCTTATTCTAGAAG
TTAGAAAGAGGAACTGCTACTTTGATGTGACTTACCCTTTTATTTTCGCTTA
CCATATAAACTGCCGTCTTTGAGACGACGGTCAGAACAGTGCATCCTTCGA
GACCTTCAATGCCAATATTGAGGAAGTTAGGTGCTTGAAAATCCGAGAAAC
CGTTGAGTGTGGCGAATGTATAGTCATTCGTACCCGCTAGGTATTATTGTCA
GCATTGACTCATTAAACGTAGATACCTAGGTGCTTGGGAGGTCTTGATAATG
GAATGGTTTGATTGGGTAGGTACGTATTATTTGATTAAAGTCTTGAAGACTT
ACTAGTATCAGCCAAACTGATAGTATTAGTGTTGGGGTTGTTTCTCAGTAGG
ACCAGGGCATCTGTTTTACGCTGGAGGAAATTTTCGATGCTGTTTTGGCGTA
CGTTCTGAAGCGTTCTCTTAAGCAAGTGGTGAGTTCCCTCCTGCTCAGAAG
CCGCCTCATCCTGAACCGTATCATTGGTCGTTACAAGTTGAAGTAAGCTACG
TTTTGAAACTGTTGAAGGAGAGCATGAGCTCCCACAGAGCCACATATTCGA
CGACAAGTAGGACCATCACTATTGCTCGAGTCCCTAGACTGAATATTGGCG
AATACCTCAGGCAATCTACAGAAAGCTTAGCGACTTTCCTATGAGACTTCA
ACGGAAACATACCCATTGAAGGCCAGATCACGCTGTGCTAGATAGCGAGCA
TTTGCAAAACAACCGAAGAGCACCAGGCTTCCTATAATGCTGCGAGCAAA
GTACATGATGGCTGATGCTCCAAGACCGGGTATAATTCAACAAAATGAGGG
AATTGTTGTTAGGGCTGGGTTATTAGCAACGAAGAAAGCGAAGCTAAGAC
ACTGTTTAT

>Mosaic_sequence_05

GCTCTTTCTGCTTCCTTCGATAAACATTATCTTTGTTGCTCGGTAGCCGCAA
CTCACTAACCTAGTAGATATGCATTCGCCGTACCTGCTGTCAATCGAGGGAC
TATAGGAGCTATAGAGAGGTTATGTGTATAATAAGAGTTTCGGGAATCTGTT
ACATGTGATGAATGACGCCTCCGGAAAACCTTCACGACAGTACGCAGTTCT
GATTGGTAAGTCCAACCAGATATTAATAAACACGGATCCCTCATATAGCGGA
TTGGCACCATGGTTCGCCCCAAACATCTCCGGGTCCATGAACCTGGACGAC
ACGGCTAGCTATGCCCTCGGCGCAGGGACATTCGTAGAGGTAACTCGGAAA

ATGGGTTCCTTCGCACGGGCGATGACCAGATGGTAGGAATTATCCTCTCTCC
GTGAACTGGCCGTATAGTTCGAACCCTAGACAACGGCCGCCACATACCATC
AAAGGTCTCTTCGCGGCCGACCAGGCGACAGACAAGATGCCCTTGGTAGT
GCCCGAAAACGTAGCCAGCAGAGGGCTAATCTGTGTAAGACGTGATGAGA
GGGTCATCTCTATATCGCCAACGTCAGGCAACGAAGCCTTGTCAGAGAGAC
TAGCGTTAGTAAGAGGAAGCCATCGTTAAAGTCTTTTAACGTTGGCAAACC
AGTCGGTTAAGACGTCTTCTTAAGAGCCCGCCACTTATTGAGTGTGACTGG
AATGCCCCTTGATAAGTGCATGCCGAGCTTAGAGGCAGCAGCAGCTATCGC
TTCTTTCTATCAACTCGTTTTATGCCTTGGAAAAAGGCCAATTATAGCATCTT
GGCTGCGGTGTAGGGAAGGTAGCCGTAGGAAGCCAACGGCTTAAGGAGAA
GGACTTTTATGCCCTCTGTACCCGGGCCTTTCCTGCCAGCTTAGCGAATTCG
ATCTTAAAGGTAACGAATGTATCAGACGGTTTCATTCGCAGGTCTCTGGATT
CATCTCAGGCCTTGTCGTATGAATTAGGGTCTGGGTACTGGCTGCATAGATG
TTCAATCATAGCTTTACTGGTAGTCAACTTTTCGGGTAATTGACTATCATCCG
AGACAAACGGCAACAGGTTAGCCGCGGCTGTCTCGCTAGTTCGTGCATCTG
ATCGTTTCGATATTATCGAGCCTTCCGTTCTGTCAATGCTCGAGACTTTGATT
GCTGTGCTCGTGGATTATACAGGTAGATCTCCCTCTTCAGATACTCTTTCCA
CTCTACAGGAAGCCAATCGCTGATGAACAGCTTCGACAGACCACGTTCCCA
AGCCCGTACTTTGGTCTCTATTAGCTTCGCTCGTTCTTTCGGCAGCATGTTG
AGATATTTTAAAGATTATTCGGCAGGATTCGACTCAGTAGGCTCTGTTGTAC
AATCTGCCGGGTCAACCAGAACGAGTCAGGCATCGACCCGTGAGAGACCG
TGATGGCCTCGGGTAGCTCTAAAGTGGAACCTGTTGAGAACTGACCATAGG
GCGGATATGCCACTGTATGGCTTGCGAAGTGGTATGTATGTATGCCGCATTC
CCTAATCAGGAGTAGTTGAGAGCTCTTTGTGAACCAACGTCGGAGTTCCTT
TGCGTCGTAGTTTCGCCTATTAGTGAAAGCTCCATCGATAGGGGGAATGTCG
TCCCTCATAGATAAGATACGATGACCCTGCGTAGTATGCTGTTCCTGAGCTG
AGCTCCAGGAGTCATGGTTCGTTCCTCGTATGTGCCGCATTTATCCATATATG
CTTTCATTCGATAGAAAGACAGCAAGCGGTAGTACGGGTGAGGTGTTGTAA
GTGCTACCTAGTTCCTGGGATGTATCGAACCGATAGATGGTGTTCTGAGTTA
TTCCAGACGCTTAATTATAACATCTACCGTGTTAAATCACACTACCCCCATTT
CTTAGGCCGTTATCTCCCACAGCTCGTGCCGAATGTGCCGTAGGGGAGAAT
ATAGGAGATGCCAGCAATTGTAACCACAGGTCTCGCAGCAATTTTGCGAGG
TTTTCCTGTCCTGTCATCATTGGGGGAGATGACTGGATTGATTAGGATGGAG
CCGACTTGACGACGGAAGTCATCATCTGATTAGATGTAATGAGCTAGAGCC
AAGCCTAGCACAATAGCTTGGCGTAATACTAAGGCCAATCTCCTTAATCCTT
TAGACCCGTCGCCAAAGTGGGCGATGACTTGAACGAAAGCACCAATGCAC
TTCTCTAGCTTCAACGTTGTTCGAAGTATGAGAGAGGCAACGCGGCCACCT
CTCCATCGATTTCCCCGGCGCTCACATGCACACACCCGGCCAGACAGGCCC

AGATAAGCTTGCGGTCTTCTGAAAGAATGATGTGCGACCATAATTTGACAC
AAAATCTGGCCCAGAGACCAGCGTACAGTTATGCCGAGCATCAGGCGTATT
GTTAAAATAGGCCTTCTAGATGACCCGGAATGTGTGATGCCGAAGACAGAT
AGTTCGAGAAGTCGATGAAGTTTTCCGGTCGCCAGAAAGGCTCGAAGGGG
GCTGTGACACCAGAGCCTAAGCCCAAACCTGCTAGCCACTCATGACCATAG
GTAAGACATTCTCTCGGCTTGAACTCAGCCAAATTACTACCCGGCCGTAGT
AAGGCCTCCAGATATCGTTAGCAAGCCTCAACCCCAAGATTACAGTGAGTG
TAAGTAGTGGAATTGAGGCAAATTCTAAAATCGGGTTGGTGGCACTCAGGG
AGTAATCGTTGACGGCGACGTAAGTGAAGTTGGAGACGTTGGCAAGGGCC
CCGGGAAGAGTTCTCTTTTCTCCTTGACCGCCTACGACCCTGAAATCGGAT
TATCCGGAGCTAGGGTTAAATGGCGGGTAGAGCACGACACCTCTGTCGTGT
CCGGTGCGTCCTTGACAGCCCTTGAAAATCCGACGGAACGTATAAGTCTCA
CGCCTGGTCGTACTCATAACCGCAGCAGGTCTCCAAGGTGAACAGCCTCTA
GTTGATAGAACAATGTAGATAAGGGAAGTCGGCAAAATAGATCCGTAACTT
CGGGATAAGGATTGGCTCTAAGGGTTGGGTGCGTCGGGCCGTTGGTGGAA
GAGAGCTGGACCGGGCCGGACTGGGCGGGGCGACCCGTCTGGACTGGCT
CGGACCGGCGATCGGACGCCTCTGGCAGCCCTCGGGCGTCTGGCGCACGG
TTAACAACCGACTTAGAACTGGTACGGACAAGGGGAATCTGACTGTCTAAT
TAAAACATAGCATTGCGATGGCCAGAAAGTGGTGTTGACGCAATGTGATTT
CTGCCCAGTGCTCTGAATGTCAAAGTGAAGAAATTCAACCAAGCGCGGGT
AAACGGCGGAGTAACTATGACTCTCTTAAGGTAGCCAAATGCCTCGTCATC
TAATTAGTGACGCGCATGAATGGATTAACGAGATTCCCACTGTCCCTATCTA
CTATCTAGCGAAACCACAGCCAAGGGAACGGGCTTGGCAGAATCAGCGGG
GAAAGAAGACCCTGTTGAGGCTTGACTCTAGTTTGACATTGTGAAAAGAC
ATGGAGGGTGTAGAATAAGTGGGAGCTTCGGCGCCGGTGAAATACCACTAC
CTCCATCGTTTTTTTACTTATTCAATGAGGCGGAGCTGGGATTAACGTCCCA
CCTTTTGGCTTCAAGGTCCTTCGCGGGCCGATCCGGGTTGAAGACATTGTC
AGGTGGGGAGTTTGGCTGGGGCGGCACATCTGTTAAAAGATAACGCAGGT
GTCCTAAGGGGGACTCATGGAGAACAGAAATCTCCAGTGGAACAAAAGGG
TAAAAGTCCCTTGATTTTGATTTTCAGTGTGAATACAAACCATGAAAGTGTG
GCCTATCGATCCTTTAGTCCCTCGGAATTTGAGGCTAGAGGTGCCAGAAAA
GTTACCACAGGGATAACTGGCTTGTGGCAGCCAAGCGTTCATAGCGACGTT
GCTTTTTGATCCTTCGATGTCGGCTCTTCCTATCATACCGAAGCAGAATTCG
GTAAGCGTTGGATTGTTCACCCACTAATAGGGAACGTGAGCTGGGTTTAGA
CCGTCGTGAGACAGGTTAGTTTTACCCTACTGATGGAGTGACGTCGTGACA
GCAATTGAGGGTAGTACGAGAGGAACTGCTCATTCGGATAATTGGTATTTG
CGCCTGTCCGATCGGGCAATGGCGCGAAGCTATCATCCGTCAGATTATGGCT
GAACGCCTCTAAGTCAGAATCTGTACTGGAAACGACGTTGTTGGTCCCGCA

CGTGTTAGTCGCGTTGGAATAGGCTTCGGCTGTGAACCATACCTGGGTAGG
GCGTGTCGGCGGAAATGCCGGCCCGTCCCCCTCTATGAAACGAATATGGGC
GGGGGTGAATCTCTTGCAGACGACTTGACTGGAACGGGGTGCTGTAAGCG
GTAGAGTAGCCTTGTTGCTACGATCCGCTGAGGCTAAGCCTTTGTTCCTCG
ATTTGTCCCTCGTGTGTTGACGTGCTGGGGGCTCTGATGCGCCTGGTGCGG
GACGTGCGGGCGACTTGGTTGTGTTTTTGTGTGGGTGTGGGTGCGAGATGT
GCTGGGGGCTCTGGTTTGTGGGTTGCGGGAGTGGTGGGGGACTCGGGTGA
GAGGGGACGGGATGAGACGGGACGGAACTGGGGACGGCCGGCGGAACCT
GGGACATGCCTCCGGGACGACGGTGACGGGCCCAAGAGGTGTGGCGGGT
GGCTGGCGTCGGGGGGAGATGTTGCGGGCGTGCAGGCAAAGGGGTGGCG
GGGGCGAGATGGGAGGGGTTCAACTACTGGGGGCTCTGGTCTTGTTGGTC
GGGGGACGGCGGGGACTTGAGGTTTCTGGGAGTAGGTGTGATGTGCTGGG
GGCTCTGGTCCGGGGAGACGGGAGACGTTGGGGACTTGTCAGTCAGCATA
AGTGATCATCCCAAGTGCTGGGGGCTCTGCTTTGTTGGCTCGGGGGATGGG
GAGGACTTGTGATTTGGGCCGGGAGGTGGTGCGGGTGGTGACGTGCCGGG
GGCTCTGGTCGGGAGACGTTTTTGGCCGAGGACGTAGCCCCGGCCGGCCC
GATCCCGGGACCGGTCTTGGGACTCTTGTCGGCACAACGCTCGGCCGGGA
AAACGGCCCGGAAGTTCCGGGTATCGACGATTTTTGGGCGACCGAGCCTCC
CTGGGGTGGGACCGGTGACGTGCTGGGGGCTCTGGTCCGAGAACGTTTTT
GGCCGAGGACGTAGCCCGGGGCGGCCCGATCCCGGGAGCGGTCTCGGGAT
TCGTGTCGGCACGACGGTTGACCGGGAAAACGGCCCGGAAGTTCCGGGAC
CCGACGATTTTTGACCCCCTGGGCCTACAGGGGGTGGGCAATGACGACGT
GCTGGGGGCTCTGGTTGTCAGACGTTTTGGGCCGAGGACGTAGCCCCGGT
CGGCCCGATCTCGGGACCGGTCTCGGGACTCTTGTCGGCTCGACGGTTGAC
CGGGAACCGGGAAAACGGCCCGGAAGTTTCGAGACCCGACGATTTTCACC
CATCCCAAGTGCTGGGGGCTCTGGTCTTGCCGGGGACTTGGTGAAATCACC
ACCCAAGCCACACAAGCACCACCAAGCCCTCACCGGTCTGCGGGCCGCCC
CTCCGTCCCCGAGCGGTCCCGAGCCGTCCTGGCCGCCCAGGACACCGTCC
CGGCCAAGACCGTGGCCTCTCGGGTCGTCCTCGGGCCGCCACAGGCCCCG
AGAGCAAAACTGCCAAAACACCTATTTCGAACCGGACGATTCGTCGGCCA
AAATCCCCCAAAAATCCCATTTTGGCTGGTAAAACATATTTTTTTCGTTTTT
GTCCAGTTTTTGCTTGTACGGTTTTTTTAAGATACATGTTTTGGCCCAGGGC
CCTCCCTGCCACCTATATGGGGAAGGGGTGGTCCTAGGGGACTGTCAATGG
GCACTTTACGGGTCTGTTGGTAGGAACTGAAAGGCTATTCTGGAACCTGTG
TCTGTTGGTAATGGGTTGATGGCGGTGCGTGAGTGGGCCATGCGGGAGGTC
GAAGGGTCTTGATGTGAGCTGCCTAGCAGTTCGTCGGAGCGTGAGCGGTTT
GCGGGAGGGAGTCCGAGTCGGTCTAGAACGTTGAGTCATCCGTTCTATTAC
TGCCGTTCCTCTCGTAAATGGCCGTTCGGGTCGAGTTACCCCTTCCTCTCGG

TGCGTCGACCGTACTGTGGTCCTCATTTCCCTTCGGCCACGTTCGGTTGGG
ACTCTGCTGGCGGGTCGGTGGTATTCTCTCGGGCGTCTCCGGATGTCTGTT
GGGTCACGCCTCTGTCTCGCTTGCGGCGGTTCCGGTTGGTATCGCTGTCGA
TTTTCCTTCCGCTGAGTCGAGCACCCAGGTGCTCCTCTCGGGAGCGGGTCG
ACGGTGCCCGTGTTGGTGACGTGTCGGCTCTTGCCGTCGTGGCTCATCGTC
CGGGTCTGGTCGGGTCGAGGTTGGCTTCGGCCTCCTTGGCCCGCACCTTAG
GAATTGAGGGTTCATTACTGTTGGTTACACTTTTCCTCGAGCCATTTGGAGG
TGCGAGCGGGTCCTGCTCCGGCGGTTCCCGCTCCAGTCTCCACGATGGTCC
TGATTCCCGGCTCCGGCCGGGATGACTGGACCGAGGACTTGTGCGGACGG
AAACTGGGAGCCCGATGGGGCTTCTGGACGGGCGTGTCCCTCGGGACGCT
AGATGCACCGGCTGGGAGCCTCTTGGGATCTGTTCTTCCTATGTGACAAAC
TTCGTCTCGCTGGACAAGGCGGGTCTCTCCCTCGGGAGCGGCCGGCCCCC
GGCGGGCGCCGTCGTGGATGCCTGTCGTCCCCTCGGTGGTAAGATAGTTAC
CTGGTTGATCCTGCCAGTAGTCATATGCTTGTCTCAAAGATTAAGCCATGCA
TGTCTAAGTATAAACGGATTCATACTGTGAAACTGCGAATGGCTCATTAAAT
CAGTTATAGTTTATTTGACGGTACCTTGCTACATGGATAACTGTGGTAATTCT
AGAGCTAATACATGCCGAAAAGCCCGACTTCTGGAAGGGGTGTATTTATTA
GATAAAAAACCAATGCGGGCAACCGCTCTTTGGTGATTCATGATAACTTCT
CGAATCGCATGGCCTTGCGCCGGCGATGCTTCATTCAAATATCTGCCCTATC
AACTTTCGATGGTAGGATAGAGGCCTACCATGGTATCAACGGGTAACGGGG
AATTAGGGTTCGATTCCGGAGAGGGAGCCTGAGAAACGGCTACCACATCA
AGGAAGGCAGCAGGCGCGCAAATTACCCAATCCCGACACGGGGAGGTAGT
GACAATAAATAACAATACAGGGCTCTATTGGGTCTTGTAATTGGAATGAGTA
CAATTTAAATCCCTTAACGAGGAACAACTGGGAGGGCAAGTCTGGTGCCA
GCAGCCGCGGTAATTCCAGCTCCAGTAGCGTATTAAAGTTGTTGCAGTTAA
AACGCTCGTAGTCGAAACTCGGGCCCGGCGGGCCGGTCCGCCTCACGGTG
TGCACTGTCCGGCCGGGCCTTACCTCTTGGTGGCCGCGTTCCTCTCACGGG
GTGCGTGGTGTAACCAGGAACTTTACCTTGAGAAAATTAGAGTGTTCAAAG
GCAGGCAAACGCCCGGATACATTAGCATGGAATAATAGAATAGGACGTGCG
GTTCTATTTTGTTGGTTTCTAGGATCGCCGTAATGATCAATGGGGACGGTCG
GGGGCATTGGTATTCCTTTGCTAGAGGTGAATTCTTAGATTACAGGAAGACC
GACAACTGCGAAAGCATTTGCCAAGGACGTTCTCGTCGATCAAGAACGAA
GGTTAGGGGATCAAAAACGATTAGATACCGTTGTAGTCTTAACAGTAAACT
ATGCCGACTAGGGATCGGGCCACGTCATCCTCTGACTGGCTCGGCACCTTA
CGAGAAATCAAAGTCTTTGGGTTCTGGGGGGAGTATGGTCGCAAGGCTGA
AACTTAAAGGAATTGACGGAAGGGCACCACCAGGTGTGGAGCCTGCGGCT
TAATTTGACTCAACACGGGGAAACTCACCAGGTCCAGACATAGTGAGGATT
GACAGATTGATAGCTCTTTCTTGATTCTATGGGTGGTGGTGCATGGCCGTTC

TTAGTTGGTGGAGTGATTTGTCTGGTTAATTCCGATAACGAACGAGACCTTA
ACCTGCTAAATAGCCAGGCCGGCTTTTGCTGGTCGTGGGCTTCTTAGAGGG
ACTGTCGGCGTCTAGTCGACGGAAGTTTGAGGCAATAACAGGTCTGTGATG
CCCTTAGATGTTCTGGGCCGCACGCGCGCTACACTGACCGAGCCAGCGAGT
TCATCGCCTTGCCCGAGAGGGTTGGGTAATCTTGTGAAACTCGGTCGTGCT
GGGGATAGAGCATTGCAATTATTGCTCTTCAACGAGGAATACCTAGTAAGC
GTGAGTCACCAGCTCGCGTTGATTACGTCCCTGCCCTTTGTACACACCGCC
TGTCGCTACTACCGATTGAATGGCTTAGTGAGATCTCCGGATTGGCGTTGGG
GAGCCGGCAACGGCACCCCTTGGCCGAGAAGCTGATCAAACTTGGTCATT
TAGAGGAAGTAAAAGTCATAACAAGGTTTCCGTAGGTGAACCTGCGGAAG
GATCATTTGAGATTACACCGGGCCGCAAGGCCCTTCCAAACACCTGTGCAC
ATCGGACCGCGCCTCCGGGCCGGGCCGCCTTCACACAAACATATGTCAAGA
ACGTAATGCATCATAACATGAAACAACTTTCAACAACGGATCTCTTGGCTCT
CGCATCGATGAAGAACGCAGCGAATTGCGAAAAGTAATGTGAATTGCAGA
ATTCAGTGAATCATCGAATCTTTGAACGCACCTTGCGCCTTTTGGTATTCCG
AAAGGCATGCCTGTTTGAGTGTCATGTAGACTCAACCCCCCGGGTTTCTGA
CCCGGCGGTGTTGGATTTGGGCCCTGCCTCTCCTGGCTGGCCTTAAATGCG
TTACTGGTTTCACGCAGACGTCGTAAGTTACGCGTCGACTGTGGGCCCGCT
CACAACCCCCTTTACTTTTGCACTCTGGCCTCAAATCAGGTAGGGCTACCC
GCTGAACTTAAGCATATCAATAAGCGGAGGAAAAGAAACTAACAAGGATT
CCCCTAGTAACGGCGAGCGAACCGGGAAGAGCTCAAATTTGAAATCTGGC
GTCCTCCGGGCGTCCGAGTTGTAATCTACAGAGGCGTTTTCCGCGCCGGTC
CGTGTCCAAGTCCCTTGGAACAGGGTATCAAAGAGGGTGACAATCCCGTA
CTTGACACGACCGCCGGTGCTTTGTGATACGTCTTCTAAGAGTCGAGTTGT
TTGGGAATGCAGCTCAAAACGGGTGGTAAATTCCATCTAAGGCTAAATATT
GGCGAGAGACCGATAGCGAACAAGTACCGTGAGGGAAAGATGAAAAGCA
CTTTGGAAAGAGAGTTAAACAGCACGTGAAATTGTTGAAAGGAAACGATT
GAAGTCAGTCGTGTCCGGGGGGTTCAGCCGGTTCTGCCGGTGCATTCCTCT
CGGACGGGTCAACATCGGTTTTGCCCGGCGGAAAAGGGCGTGAGGAATGT
GGCACCTCCGGGTGTGTTATAGCCTCGCGTCGCATACGTCGGGCGGGACCG
AGGAACGCAGCTCGCCTTCACGGCCGGGGTTCGCCCACGTACGAGCTTAG
GATGTTGACATAATGGCTTTAAACGACCCGTCTTGAAACACGGACCAAGGA
GTCTAACATATCTGCGAGTGTTTGGGTGTCAAACCCGAGCGCGTAATGAAA
GTGAACGTAGGAGGGATCCGCAAGGAGCACCTTCGACCGATCCGGATCTT
CTGTGATGGATTTGAGTAAGAGCATATATGCTGGGACCCGAAAGATGGTGA
ACTATGCCTGAATAGGGCGAAGCCAGGGGGAAACTCTGGTGGAGGCTCGT
AGCGATTCTGACGTGCAAATCGATCGTCAAATTTGGGTATAGGGGCGAAAG
ACTAATCGAACCATCTAATGGCTGGTTCCCGCCGAAGTTTCCCTCAGGATAG

CAGAAGCTCGCATCAGTTTTATGAGGTAAAGCGAATGATTAGAGGCCTTGG
GGACGAAACGTCCTTAACCTATTCTCAAACTTTAAATGTGTAAGAAGCCAC
CGTCGCTTGATTGGACGGTCGGCGTGCGAATGAGAGCTTCTAGTGGGCCAT
TTTTGGTAAGCAGAACTGGCGATGCGGGATGAACCGATCGTGAGGTTAAG
GTGCCGGAATACACGCTCATCAGCACCACAAAAGGTGTTAGTTCATCTAGA
CAGCAGGACGGTGGCCATGGAAGTCGGAATCCGCTAAGGAGTGTGTAACA
ACTCACCTGCCGAATGAACTAGCCCTGAAAATGGATGGCGCTCAAGCGTGT
TACCCATACCTCACCGTTGGCGTTTCAGTGACGCGCCAACGAGTAGGCGGG
CGTGGGGGTCCGTGCAGAAGCCTTGGCAGTGATGCCGGGTGGAACGGCCC
CTAGTGCAGATCTTGGTGGTAGTAGCAAATATTCAAGTGAGAACCTTGAAG
ACTGAAGTGGAGAAAGGTTCCATGGTAACAGCAGTTGGACATGGGTCAGT
CGATCCTAAGAGATAGGGAAGCTCCGTTTCAAAGTGCGCGATTTTCCGTGC
CGCCTATCGAAAGGGAATCCGGTTAAGATTCCGGAACCAGGATGTGGATCT
TTGACGGCGACGTAAGTGAAGTTGGAGACGTTGGCAAGGGCCCCGGGAA
GAGTTCTCTTTTCTCCTTGACCGCCTACGACCCTGAAATCGGATTATCCGGA
GCTAGGGTTAAATGGCGGGTAGAGCACGACACCTCTGTCGTGTCCGGTGCG
TCCTTGACAGCCCTTGAAAATCCGACGGAACGTATAAGTCTCACGCCTGGT
CGTACTCATAACCGCAGCAGGTCTCCAAGGTGAACAGCCTCTAGTTGATAG
AACAATGTAGATAAGGGAAGTCGGCAAAATAGATCCGTAACTTCGGGATAA
GGATTGGCTCTAAGGGTTGGGTGCGTCGGGCCGTTGGTGGAAGAGAGCTG
GACCGGGCCGGACTGGGCGGGGCGACCCGTCTGGACTGGCTCGGACCGG
CGATCGGACGCCTCTGGCAGCCCTCGGGCGTCTGGCGCACGGTTAACAAC
CGACTTAGAACTGGTACGGACAAGGGGAATCTGACTGTCTAATTAAAACAT
AGCATTGCGATGGCCAGAAAGTGGTGTTGACGCAATGTGATTTCTGCCCAG
TGCTCTGAATGTCAAAGTGAAGAATTCAACCAAGCGCGGGTAAACGGCGG
GAGTAACTATGACTCTCTTAAGGTAGCCAAATGCCTCGTCATCTAATTAGTG
ACGCGCATGAATGGATTAACGAGATTCCCACTGTCCCTATCTACTATCTAGC
GAAACCACAGCCAAGGGAACGGGCTTGGCAGAATCAGCGGGGAAAGAAG
ACCCTGTTGAGCTTGACTCTAGTTTGACATTGTGAAAAGACATGGAGGGTG
TAGAATAAGTGGGAGCTTCGGCGCCGGTGAAATACCACTACCTCCATCGTT
TTTTTACTTATTCAATGAGGCGGAGCTGGGATTAACGTCCCACCTTTTGGCT
TCAAGGTCCTTCGCGGGCCGATCCGGGTTGAAGACATTGTCAGGTGGGGA
GTTTGGCTGGGGCGGCACATCTGTTAAAAGATAACGCAGGTGTCCTAAGGG
GCTCATGGAGAACAGAAATCTCATGAACAGAACAGTGGAACAAAAGGGTA
AAAGTCCCCTTGATTTTGATTTTCAGTGTGAATACAAACCATGAAAGTGTG
GCCTATCGATCCTTTAGTCCCTCGGAATTTGAGGCTAGAGGTGCCAGAAAA
GTTACCACAGGGATAACTGGCTTGTGGCAGCCAAGCGTTCATAGCGACGTT
GCTTTTTGATCCTTCGATGTCGGCTCTTCCTATCATACCGAAGCAGAATTCG

GTAAGCGTTGGATTGTTCACCCACTAATAGGGAACGTGAGCTGGGTTTAGA
CCGTCGTGAGACAGGTTAGTTTTACCCTACTGATGGAGTGACGTCGTGACA
GCAATTGAGGGTAGTACGAGAGGAACTGCTCATTCGGATAATTGGTATTTG
CGCCTGTCCGATCGGGCAATGGCGCGAAGCTATCATCCGTCAGATTATGGCT
GAACGCCTCTAAGTCAGAATCTGTACTGGAAACGACGTTGTTGGTCCCGCA
CGTGTTAGTCGCGTTGGAATAGGCTTCGGCTGTGAACCATACCTGGGTAGG
GCGTGTCGGCGGAAATGCCGGCCCGTCCCCCTCTATGAAACGAATATGGGC
GGGGGTGAATCTCTTGCAGACGACTTGACTGGAACGGGGTGCTGTAAGCG
GTAGAGTAGCCTTGTTGCTACGATCCGCTGAGGCTAAGCCTTTGTTCCTCG
ATTTGTCCCTCGTGTGTTGACGTGCTGGGGGCTCTGATGCGCCTGGTGCGG
GACGTGCGGGCGACTTGGTTGTGTTTTTGTGTGGGTGTGGGTGCGAGATGT
GCTGGGGGCTCTGGTTTGTGGGTTGCGGGAGTGGTGGGGGACTCGGGTGA
GAGGGGACGGGATGAGACGGGACGGAACTGGGACGGCCGGGCGGAACCT
GGGACATGCCTCCGGGACGACGGGTGACGGCCCAAGAGGTGTGGCGGG
TGGCTGGCGTCGGGGGGAGATGTTGCGGGCGTGCAGGCAAAGGGGTGGC
GGGGGCGAGATGGGAGGGGTTCAACTACTGGGGGCTCTGGTCTTGTTGGT
CGGGGGACGGCGGGGACTTGAGGTTTCTGGGAGTAGGTGTGATGTGCTGG
GGGCTCTGGTCCGGGGAGACGGGAGACGTTGGGGACTTGTCAGTCAGCAT
AAGTGATCATCCCAAGTGCTGGGGGCTCTGCTTTGTTGGCTCGGGGGATGG
GGAGGACTTGTGATTTGGGCCGGGAGGTGGTGCGGGTGGTGACGTGCCGG
GGGCTCTGGTCGGGAGACGTTTTGGCCGAGGACGTAGCCCCGGCCGGCCC
GATCCCGGGACCGGTCTTGGGACTCTTGTCGGCACAACGCTCGGCCGGGA
AAACGGCCCGGAAGTTCCGGGTATCGACGATTTTTGGGCGACCGAGCCTCC
CTGGGGTGGGACCGGTGACGTGCTGGGGGCTCTGGTCCGAGAACGTTTTT
GGCCGAGGACGTAGCCCGGGGCGGCCCGATCCCGGGAGCGGTCTCGGGAT
TCGTGTCGGCACGACGGTTGACCGGGAAAACGGCCCGGAAGTTCCGGGAC
CCGACGATTTTTGACCCCCTGGGCCTACAGGGGGTGGGCAATGACGACGT
GCTGGGGGCTCTGGTTGTCAGACGTTTTGGGCCGAGGACGTAGCCCCGGT
CGGCCCGATCTCGGGACCCGGTCTCGGGACTCTTGTCGGCTCGACGGTTGA
CCGGGAACCGGGAAAACGGCCCGGAAGTTTCGAGACCCCGACGATTTTCA
CCCATCCCAAGTGCTGGGGGCTCTGGTCTTGCCGGGGACTTGGTGAAATCA
CCACCCAAGCCACACAAGCACCACCAAGCCCTCACCGGTCTGCGGGCCGC
CCCTCCGTCCCCGAGCGGTCCCGAGCCGTCCTGGCCGCCCAGGACACCGT
CCCGGCCAAGACCGTGGCCTCTCGGGTCGTCCTCGGGCCGCCCACAGGCC
CCGAGAGCAAAACTGCCAAAAACACCTATTTCGAACCGGACGATTCGTCG
GCCAAAATCCCCCAAAAATCCCATTTTGGCTGGTAAAACATATTTTTTTCGT
TTTTGTCCAGTTTTTGCTTGTACGGTTTTTTAAGATACATGTTTTGGCCCA

>Mosaic_sequence_06

GAGGTATTCGTGATACCGCTCCGGGACAAGATGTTGCACAGCTGCTTCTTC
CTCTGGGCTGAGTTCGTACTCGTCGTAGTCGGGTAGGACGTCGATGTATGC
CTCTTTCTCGCTCAACTCCTCGGTCACTGTGCCGCATAGCTCGACGTATTTC
CCTGGTTCTGCTCCCATCGGTTGAATGGGAGCTCAATGCTGTTCGCCTTCG
ATCGCCTGCTCCTTCGTTTACTTTGTCGCCTTTCCGCCTTGGTCTCCAATGTT
CCTGATAGTCGAGAAACCTCGTCGAGGTTCTGTGGTCCGTTCTCCTGGTTC
GTCTCTTTCCCGCCTGTCCTCTTCCTTGTCGCCTTTTTCCTCCCAGCATCCA
ATATCACACCGTTCTCCTTCAATCCAGTCATATCCCAACACCACGTCCGCTC
CTGCCAATGTTGTCACACCTAACTCTTCCCTTCCTTCACCGCCTAGGACTCT
TACTGTTCCCGTCACTCCCTTTTCTACTTGTCCTGCTGAGCTTGGTTTACCG
TCAAAGAGGAGGAGTTGCCGGGGTCGAGGGAGTGGGTAACAGGGAATGT
GATGCCGTTCAACGAACACAGAGTCGACGAAGTTACAGTGGCCCCGGTGT
CGAGCAGCACCTTCACCCCGTCGACTTGATTGACGCTTCCTGACACGACAG
CCTGCTCTCTGCCTAAACAGCAAACCTCACCACGAATGCATGCCTCCTCAT
CACTAGCTATTCCGTCCGTGGCAACCAGTGCCTTCTCATCTTTCGCTTCCTC
GTCGAGATTCGGGATTAAAGGTGATTGTGATACAGGGGGTTGGGAGGTGA
ATGGGAGGGGAGGTGGCCGAGAGGATGTCTTGATGGGGCCGCTGGACCT
CTCGTATGTTCTCAGCGTCCCTCAAGCTTTTGGGTCCTCCTGCTTGACGATA
GGTCGGCCGGGTGCAGGGCTGCCTCGAGGAACGGGAGGTCGTTGCCCGG
GAACACAGGCATTGGCGAAGTGTCCGACCCCGCCGCAACGGTAGCAGAGA
CGCTGCTTGTTCCTCCTCTGGCGTTCCGCTGGGTCGAGTCCTCCAGGAGGC
GTTGGTCTGTAACCAAGCGGGCCGCTAGGGGCTGGTGAAGGGCGCGCCGA
CGGGGATTGGCGGGAGAGGGCGGTAGGTCGACGAGGCAGGGGGAGGAAC
TTGTCCCTGGGTAGTCGTAGCTGGCCGTCGTCGAGCTTCTTCGTACCGCTTG
ACCTCGGCCTTTTTCTCGGCCTCCCTTGCTCTGAGCCGGTTGTCCAACTTG
ATGACATAGGACTGAAGCTCTCCGAAGGTCTCCAGCTCCACTGTGGTCCGA
GCAACCTCGTCTCGAAGATTGTCTGAGAGGCCTTCGAGCGCCCTAGCTCTA
GCTTGCTCGTTGGCTTCCCATCCCAAGACCGCCAGGTACTCCCTAAGCTTC
GCGAAGTACTCTGCCGCTGATCCGGTCTGCTTCAAGTCGAAGTATCGCCTC
TGAGCGGTGCCCTTGATGTCGGGATCCCCGAAGTTGTCCCTCAAGTATCTC
GATAGCCTCTGAAAGCTACTGATCTTCTCGTCGAGTTCTTCTCCCGACGCG
AGAGGCTGGATAGCGTCTTGAACGAAGCCCTAAGGAAGGAGATGGCGTAG
ATGACTTTGGACTCCTCTGTAAACTTCGAAGGCTGTAGGCGAAAGACGATG
TTGCATTGCATGAGGAATCCTTCGAGTTTCTCCGGCTTAGAGCCATCGAAA
GTGTCGGGCGGGTTGGCCTTGGGCTCGCTCGATGATGCGTTGGTCGAGGG
AGTGGTGAATTGGCTCGACGAGCCTCGATGGCAACGAGTTGTTGCAGGAT
CTGAGCGAGGGAAGCCTGATCCGCCATGTTGTTGGGTGTGCTAGACCGGCT
AGCAGGAGGGAAGACGAAGGGACTGTGGTAAAGCTTGGTGCTCGTGGTTC

GGGTTGAGTGATGTTGAACGTCGAGTCTCGATGGGCATATGCCCCGGGTGT
GCGTGGTTGTGCGAAGAGGTAATAGCACTCTCTGATACTGTCACGATGCTG
CGCTCTCCCGTTGGCAGAGGCCCTCTCGCACAATATCAGAGGCAAGAATAA
AGGATAAAGGGGTAGAAACGAGGATGTCGAGGTAGGATCCTCGTCGAGGT
TGGATCCTCGTCGAGGTTACAAGGTCGTCGAGGTAACAAGGTCGTCGAGG
ATACAGCGTGAGTGCGACTCAGCCACGCTGCGTGCCACGGAGAGAGTGAG
ATCGTCAATGGTGATCCATGAGACTCAATCAGAGAGTGCTGAAGAACTACG
ACGGGCGCCCGGGGCATCCTATATGACTCTGCGCCTGCTTCCCGTCGTCGC
GCGTTCATCCAACGTCTACTCCCTCGTCGAGGTTCTCCAGCCCGAGGTAGG
TCACGTGTCGGCCACGTGAGGGGTGTCGGTCACGTGCGTAGGTCGTCGAG
GGCGTGGCCGTCGTGACAGTCGGCGACGTCGGCGGCCTTGGCCCCGGTGG
ACGAAGAGGCTGTGGCAGCAGCCCCTTCGCCCGCCTGGGGATCCCTGGGC
CGCCCACTGGCTCTATCGGAATCCTCTGCCGATGAGGACGATGTCTTGTCC
GACCATGGCTTGATAACCGAATTTTGGGCCCCGAGCCGCCAGCGAGGTCCA
AACGCCATTGCCTGGTGCGCAGGAGGGACCTGGGGAGCCTTCCACGCCCA
CCCCAAAGGCCAGGAAGACCCCTAAGATGAAGCGGGCTAGGATTGAGTGT
TCACCACTGTTTTCGAACGGCCGCGAAATTCATGCCAGAGCTGGCGGCCA
GATAATCCTAGCCCAATATGACTGTTCGTAAGACCACCTGCTGTCATTGCAC
TGGTTGCTTGTTGTCATGGCCCCACAGGAGCAGATATGCGGAAGCTCAACA
TGTCGGTGTGCGCAGGTGGGACAAGGCAATCCATAATTGACCGCATGAGTC
AACACGTAGGCAATCAGGCACGGATGGTTGGTCGCTCGCGTAGAGACGCA
TCGTGAAAAGCTGGAGGAAGAAAACAATGAGCAAGAACTTACACAAGATA
GCCCTTGAAGAACTCTTTCAACTCCCCCTCGGTTGTCGCGTAGGCGAGTTG
CCGATGTACAGGCGGCGACCCTCGGCAGCGCTGGCGTGACGGCATCCTTGT
TATCATCCTTGCCAGTGATTGAGGCATTGCTCAGAGCATTCGTAAGTCGTTG
ACGGCAGCGTCAGCAACTTTCTCCACGGTGGTGGCCATTGTAATTGATGAT
TATCTAGGTGGGAGATTAGTTGAAAGTAGGTTGATTGACTTGACGGGAATC
GATTGTTAACGTTGAGGACGTTCGGGTTAGGGTTCAGTTATTGAACTCTTG
AACAATTTCGGGGAGGAATAGGGTGGCTAGGTGCCCTAGAGAAGATTGAA
AGATTTGGGGGGTTTGAGTGACTGTGACACAACGAGCTTTGGATTGATGGT
ATTTGAGAGGTGTGAAGAAGAAATGAGAGAGAGACCAGATGTACGGACGC
GGCCAGAATTTTTTAAGGGTTGTTGTTTCTTGTGTGGGTCAAGGAAAAGGA
TAGAATTACGGCAGGTGTGGGAAGTGCATATAGATTGCTTGGCAGGTGCGC
ATGGGCCAATGGGAAGGAAGTGGGTATGTGGGGCAGTATTGTCTCTGTGGG
TACGTACCTTGTCGTCAAAATTGCGTGAGCTAGTACCTATCAACGTAAGATC
GGGAAAGCTTCAGTGCGCGACCTAATGACGTTAATATTGCTCTCGGTAAATT
GTTTATCGCGAAGTGGTTTGCCTATGCGGGCGATAATAAGAAAATAAAATAA
AATAAACGAGAAGCTTACCGAGACTCAAGGATCGTATGTTGTAGAAAAAG

GTCGGTATGTATGGGATGGTACGGACTAGGTTCAATGTTGACTTGACAAGT
GGCGGGTTGTTTGTTTTCGTTTCGTGGCGAAATGGAGGGTAGGGAGTTTGA
GAAGGTGAAGAGAGTAAAAGTGGAGGAAACGTTTATGCGGTCTGTAGAGA
TAAAAAGGTGGAAAGGAAAGAAATCGTCAATCAGTTTGGCAAAGCTGGCA
AGTGCACCTGAAAGAAAATTGATGCGAGTGAACCTGCTTGTTCCGGCTGG
CAACTTCAGGTTGTTCTTCAACATTCAACCTTTTAGAGTTTATAGATCTTGT
GCTTTCCACGACGAACGATGCCTGGAGAAGATTATTAGGAAAGAACCATTT
AATTGATTTAAATAAAGTTAGCAGGTACAATTATATAAGACTTTGCGTATATT
TACTCACCTACTAGAGAGGTAACAACCTTAATCTTCAAAAGAGTCACGCGC
TGTCCACGGTATATCTAACATTTCTTATCGTGGTGTTTTGTATGACTTGGTTT
ATACAAATTTAAATTATCCATTAACCATTGCATCTATCTTATTCGACATCTACC
TAATAGCCTCCCTACCTCATCGAATACATGATTCGTATCACATCTAATTGCTC
CGATTCAATTTTTTTATACTTCTCGTCAATTGAGAGCTTGATGCTCAGGGAG
GTAGGTAGCTACCTATGTCAGCATAACACTCATGATTAAGTATCTATGTAGTT
AATAATTTCTAAGCATATCCGAATTACTGCGCTTAGGCGCAGAGGCAGATCG
GTGCTGACATCTACCTAAGGTCAAGCTCGGTAAGATGCCTGAAGTGTCGAA
ATTGGATGCTGCAGAGAGCTTGGGGTGCATTGAAATAGCAAATTACGCCAC
GTCCAAGCTCCAAGCTTCCCAACTTGCTTGACGCGCCTTTGTCAGCTGAAA
TTCGAAAAAGAAAAAAGAATACGCTTCCAGAAACGCTCGTCGGCTGGTTA
GTCATTTCTGTACACCCACTGTGTTACCGTGCGCACATGCGGTCACTAAAA
AGGAATCATTTCAGGGTCTCGATTCTCATTCATGGTTTTTTTCTTTCCCTAC
GCGAACTAATAGAATCCAGTCAAAAGTCGGTTGGCATTCTGGAAGCTTTCA
AAGAACCTTGTCTCCGGGGGTTTCGATGATCGAGCCACTCAATGGGAACG
AAACGAAGCCCTGCAAGGATTATTCTAAACACACCCCTATCTTTCTTCATCG
TTTAGACGCCTCATTCACCTATGATCCTAGAGAACCCTGAAAGAGACGTCC
CCCTTTTTTGGCCCCTTATAATCGCATCTCTATTAAGAAAACAAAATTTCTAA
TATTCGTCAAATTTTCGCAGGCAGCGCTGAATCCGGCGGATTATTTGAAAAC
CCCGAGGTGTCCTATCACCGTGCTTGACAGATAAAAACCTCCCAGTCTGCC
AATCCAAAATTTCAGTGTCTCTTCAGCGAATAGGTAAACGAGCCTTCTCAA
TATCACATCCAGGCGCCGAATATGCGAGGTACTACATACACTACATACAGCA
CAGTAGCAAAACACGTTGTTAGCTTTGGTCCACATGGTTTTGCAGATGTCT
TCGGCTGGGTTAGGGGTCAGGTAGCTTATTAGCTCTTGTTGCGTGCATGAAT
ATTGCCAAACAAGCAGCCTTACCAGAAATACCGGCACCTCAGAAATGCAG
ACGGCCAGAAAATTCAACCCAACGTTACTTTGCAGCCCGGCTATGGAAGA
GTGAGCGAGGTCGTTAAAACTAGGTACCTAGCCTCCAGCTACTCGTCACAA
TATTTACCGATACATCCATCTGTACATACACACCATATACACACATACAGTAC
ATGCCTACCTACCTAGGTAGGTATGTATCTACCTCCTTAGCCGCGCGCACAC
AGCTAACCCGCCTACTTTTATCTAGCGCCGTGCCCGAAGCTTCAAGCCATTT

GGTCCTGACAGGTGTCCCATCGTGTATGGATTTCGTCCACCTGCTAACCCAT
CTATTATGTAACTTAGGGGAGAACTATGCCTACGAAATGGATCTAGAGGATC
CTAGAACTTCCACATTACGGTACATGCTCTGAACTTTTACTCCAATCATCGT
CTGTGCACGTCACGTGGGGAAAGACTCTACTGTCGTAGCATTTAATATTTTC
CACATTCCACCGAAGGCTCGGAGTTCAGTGTGTTCCCGCAGCGCTCAAATA
TCACTGTATTCCTATCGGGTGTGCCGAAGTTGCTCGGGTCCGGTTTAGTGTC
CTCTCCTTTTTTTAATGTCGGCTGAAAGCAGATATTCGATATTGGAGAAAAC
AAAGTGCTTCAGAGATTGTAGATCTAAGCTTAAGTGCCTAGTATGATCCGTC
ACTACGCTAAGCCCGCTCTGCAAGCCTGTAAATTCTCCACCTCACCACGTC
TCTCCGGTAAATCACCAGTACCCAGGACCTAGATAGGTGCTAACATACTAG
GTAATCTTTGGTGTATTATACATATTACACATACCTAATATAATACTAAGACTT
CTATAGTATGCATATGTAGTATGTTTATAGTAAGCATTCACCCTAATTGAGAA
ATTTTGATGTATTATATCTACTATTATTAGAATCTAAGACTGGGGATCTGATCC
AAAATTTAAGAATACTTGGTAGCCAACATCCTCAGAATTAACCTTTCTCTAT
GCCTTAAATTTCCAGATGTCAGAGTGCCATGCGATAGAACTTCTATGCGTCG
TTCTAGCGTCGTTAAGATGCTTCCAATGAGTATATATTCAGGGGTATAGGTAA
GGTAGTTCTATTATATGGGAATTCTGTCTTGTATCGAATTTTGAAGGTGATAT
AATAGTGGCCGTGTAAGTGTATAAGGACCGGTAACATCTTTACACATCTCTC
TATTGACGAAAGACACTGCTCAGTATATACAAGTAAAGCATTATATTTCATC
GTTAATATTATACCATATGCTTAGGTAGGCAAGCCGTACTAATGTGAGCATTT
CTAGTGTCATGATCAAGAGTTTCATGTATACTCAGTGTGTATATAACAAACA
CCTATACCAAATCAAGGTTAAATGCCGTGTGCTCTATTTCATTGGTAGTGAA
CCGCGACATACAAATACCTACCTACCTACTATATATCTCGATAGCCTCAAAA
AGACTACAGAGTTGTATATGCCTACTAGTAGGTACACATGTAATAGGAGTTC
AGAACAGCCGTAGAGCTTTTCACGTATTAGTTATAGGGGAGTAGCGCTGGA
GGGTTCCAGGTTCCGATCGGACCAGGTTAGTTATGCACGGGCAATGAAGTA
ATAGAGTTCTTACAATTCTTTATCTTATCAATCTCATCGGTCGGGGTTTTATG
ATCTACTGTAATCCTTGTAACAATTCACTTTGTTTCTCCCTTCACTTTAATATT
AAACATTTACGAGATTGGTGATGGCTTCGGGGGCCAGCGAGGACAATCAG
GACGATGGGGATAAGGCCGCGAAGGTTGAGGATAGGCGCATGCTTGTCCTC
TACGGTTCCGAAACAGGGAACAGCCAAGACCTAGCGGGTGATCTGGAAAG
GCTCGGGGAGAGACTGCACTTCAGAACATTTGTCTGCGAGATGAACGACG
TCGAACTTGTAAGTCGAGGGATTCATTGATTCACTCACTTCCAACGCTCCC
AACATTCCCAACAATCACTTGTTCGTCATTGCATTACCACTACCCAAGGCCA
TTATCATTTTATCGTTTTAACCAGTCATCTTTCTTCATTTGCCTTATCCACCCT
TTTTACATGGCCTGTTCTTTAGGTGTGTGTTTTTTACTAGGTGCACGGTGAC
GTACGTCAGTTCCCCTCTAATTCCAGCTCCAGCCCTGGAGATTAAAAAAAA
ATTACCCAACTGAACACTGAAGGTAGAGAAGCAAATACAGCCAAACTAGG

TACCTACATAGCCCTGGAGGTATACCTACTTCAGTATACCGGGTACCTATCCA
GCATTCGATGTTCAATGTTGTTTACTTGCATCCATCGGATCCGCGCGCACTC
TGCACCCTCTGCACTCTCCTACTGCTTATGCATCGATCTTTGACCAACTTAC
CAACGAGATCACTGGTAACAAGAACCTGCCTCATTTAGAACACTTTACTAC
AATACCCCTTGGTCATATTCGTCATTTCTACTACCGGCCAAGGCGAAATTCC
TCGAAACGCGCTTAAATTCTGGAAGAGCCTCCTCCGTAAACGACTACCACC
AAATTGCCTGCAACGAGTCAACTTCACCACATTTGGTCTTGGCGATAGTTC
GTATACCAAGTAAGTATTACATATACATATACATACTTACGTATATGTAAATTC
ACACAAATTCATGATACATACCATGTCTCCTTCCAATTTGATTTCTTTCTGTT
TTCTACTTCCCACATACTTTTCATGTCTATATTCTCGTACCCATATTACATCAT
CACTAACTGACTGACTGACTAACCTCACTTTTTTTTCTTACTTCTGCAGGTA
CAACTGGGCAGTTAGGAAACTTCACAAACGGCTAGAACAGCTAGGTGCGA
CCGAGTTCTTCGCTCGTGGCGAGGCTGACGAGCGTCACCAAGACGGGTAT
GTCTCTCCGATTTCCATATGTTTGCTAATTTTCATTTTGCTTCCATCCTTGCCC
CCCCTTTTTCATTTCTTTATTTGCCCATCTTATTCACGTTTCTCGCTATTTTTG
GAAATACACGGCTACTCGCAGTTTTAGACTAATACTTCCCAGCATCGACGG
CACATTTTATCCTGGTCGTTAAGCCTTCGAGTCATCTTCTAAAGGAATATCCT
TTGCCAGAAGGTGTAATCTCCCATACCTCGGAGGTTCAACTTCCTCCAAAA
TTCACATTAGAGTTAGTATCCAACATGGATACGTCAGATGAGTCAATGCCTG
ATCGGGACACGAGAGAGGCGACAGAAGTCGAGAGCTAAGCACCACAGAC
GTAGCTCTTGCCACTACCACGTTATCAAGTGGCGATCCTCAAACCATCCCTC
AGGAAAAGGCTTTCGACACAACCGCCGCGCTTTTCAGTCATCCGATACGCC
TAACTCCGATGCCTTAGAGCACGCTCTTCGGGCAGAGAGGATGGCCATCAA
CAGCAACTCGTACACCGCTCGTATATCAGAGAATACGGCCAAGTTAATACC
AGGTGGAATAGATACTCTGGACAAGCCCAACGTGTTGAGAGATCACCCATC
AAGATACTCTCTTGAGGATTTGACCGCCAAGGAGGTCCTTCCTCCTGCGGA
CCTTCTTCCTATACCTCGAGCGTGGCCAGCCCTTCTGGTAGAGAATAATAGA
GTCACACCTACTTCTCACTGGCAGGATGTTCGCCATCTAATCTTCGAGATTC
CCGCACAGCCGGACACATCTAGAAAGAGGGACTTTAGTCTCTTCAGTTACG
TACCTGGTGACGCCGTCGTCATCTATCCCAAGAACTTTCCTGTGGATGTGCA
AGCGCTGATCGATCTCATGGGCTGGCAAGATGTCGCCGATTTACCTTTTGAG
CATCGCGCAACCCAGGAGGACTTACTATACTCGCGACCTCGAAATTGTCAC
CCGCTTCCCAACTCCACTTACGACAACTCCTCCTCCACAATTACGACATCA
CCGCCATCCCTAAGCGCATCTTCTTCGAGGAGATCGCATTCTACACCGATGA
TCCCACTCACTCGGAACGACTTAGAGAATTTGCAAACCCTAGCCTGTCAGA
TGAATTCTACGACTACACATCTCGTCCTCGACGATCCATTCTAGATGTTCTA
CAGGATTTCCCAACAGTCCAAAATCCCTTACGAGCACGTGCCATCTATCTTC
CCTGTCATGCGCGGCCGAGAATACAGCGTTGCGAGTGGCGGTATGCTTCTT

AAGAGCGAAGACAATCACCGAGTCACGAGACTCGAGCTCCTAGTGGCGCT
AGTCAAGTACAAGACTGTGTTACGAAAGACCCGGCAGGGCCCTTTGTTCT
CGTTATCTAGCATCCCTTAGTCCCGGCACGATGATCAACCTGACCCACAGA
GAGTATGACACTCTGCAGACGGAGCTCATCCGTAGACCGCTACTAGCCATC
TCCCCCGGCACCGGCGTCGCGCCTATCCGCGCACCTGTGGGATCGCAGC
GAGTACGCCGAAGCCGGCGAGGCCCTGCTTATCTTCGGCGGGCGGAACCG
CAACGCGGATTTCTACTTCAAACACAGATTGGGATAAGCTCGGCACAAGGT
CCCTTACGGCCTTCTCCAGAGACCAGGAAAAGAAGATTTACGTTCAGGAC
CGTATCCGTGAGAACGCGCACTTGATCTGCGAGTTTATTCAGAGGAGATGT
GTTATTTGTATTTGCGGCAGCTCGGGCAAGATGCCGGAGGCTGTGCGCGCC
GCGCTGTACGATGTCATGGTTATAGGCAAGCTGGCTCCTAACCATGAGGTC
GCGAAGAGGTTGTTACATATGCATAATCCCATGTGGGAAGAGGTGTGGTGA
TGGGGGCGAGAGGTGTAAAAAGCTGCTACGAAGACTGATGTGGCTGTGTC
ATGGGACCAGGCGTAATTGATGTCGCTATGGCTAGATAACGAATGCATGAG
GTGCGATAGATAGCATCTCGACGAAAATAATTGAACGCGTGTGATCAACAG
AAATCCCTTCGTACGACTTTTGAGTAAAGCCTAGGGGAAGCTGCTTTCTGT
TCGTCTGTGACAACCCATAGTATGACAGAGAATGAATTCCTGTAACCGTGA
AGTAGTCTGCAGAAGCCCTTTACAATGCTCCCTTTGCACCAACCGTTAAAG
TTTCTGCTCGTTGTCGTTAATATTAGTATTGCTAAGCAAGTCATGCTGTGATA
AAGTTTTATATGATGTTTATAATAAGACAGTTGGCTCGCCGGAGAGTTTCTG
CGGGTGGAGAGGAAAAGGGGTAGAAAGTGATGGCACGCCAAGGACTTTA
CTAAGACCCTTGAAGTTTTTCAGGTATAATACATTATCATATGACAATACAGA
ACTATAATTAAGAGACTATAAGTGATATTTACATTTTTAGGTTTAAGATAAAT
TAGTTGGGTATTGTAGCTGTTCAGTCATCTAAGCAGGCCTGTCTCGCGAAC
AGCTAAGGCGGTTTCATCTCCACGTACAAGAATGAGGGATGACTACCAGGC
GCACTCTATCTATGTGATAATAGTAGATGTCCTGGAAATATATAGGCAATTAT
TTTCGTTAAGTAGGCGTAGCGCGTGGCGTACTCATCCCCATCGGGACTCCG
GATCCAGGAACTAAACTCACGGCAGACACAATACCATAGAGTGAACATTCA
TCAATATTTCTTCAATTTTCCAACATTGGCAGTCTATTGATTTATAATTGATAT
AGCACACATCTGCGTGTATTACGAATACCGGTGTCATATTGTCTTGCCCAAT
CATGACTTCCCTTCTTCCGACCGCTGGGAGATCGGCGGCTGCTGGCAAATA
TTGCTACCCCGTCAAGCAGTGCCGGCATACCGCCGTCACATCGATTCCGGG
TCTATGCCGTCACTAGTAGACAGGTGTAGGGAAGTCCCACATGTCCGTGAG
TTGTTCGACATCTACCGACTACCGTGTGCCCCTATATAGTGTTGCATGAATC
GATATCGGGTAGCCGATGTGAGTGGCGCAATCTCTGGTGTGCTTTTCTAACA
TCTATGAGGAGCAGTATTGCCTGCCTCAAAGGCCTCGGAAAATTTAGGGAT
CAAATCACTATCGGCTTCGTTGGAGTGGATGGTCGAGTACTATAACTAGATG
ATATCTTATTAACAGTCATTATTCAAACAATACTCAGCAACTACATTAGCTAC

CATCCACGTCAGGCACATTCCCGTAAATGATACTTAGAAAACTAGCATCCTC
AGACCAAATCGTCATCCTACGAAGAGAGACGATTCATCTATCCACGACACA
CCAATCTCAAACTGATAAATAGAAAATCGCAAAAACGGCATAGTCCGACTG
CCGCACCCTCTCACATCTAGAGGGAAACGCATAATACCCCAAGGCAGAATC
TCCCAAACCCACAGTCTCGGCTCCGAAGCCTCCCCTGTCTGCGACACAGT
GCCGGAGCATCCCTCTAGGTCCGGCGGAACACCCGGGGCACACCCATCCA
CCCATCCACTCCTATCCAGCTACCTAGAACCCACCTCACGGCATTACCGCAT
CCTGACCCTGCAACCGACAATGCCGTTCGCCACCATGAAACGACCCAAAG
CCAAAAGGTATTCCCATGGTAAACATCTCGGACTATGCCGTTTACGAGCGC
ATGAACCGCGAAAGAAAAAAAGAGGATCTACGCTGCAGGGACCTTCGATC
CCGACCAACCCCGGCACGTTTCAGAACTGATGTGACCGGAGCAAGCCGAC
GATGACGAGTCACAACTCGCAAAACGGGCGCGCATGGCAAATTCTAATATG
CAGAGAACTTCTCTTTTCGGCGATGTGATCCTTTGAAAGTGTTTATACATGC
AAAACTTTTGCCTTCGGTACTTGGTAGGTATTAAGTACATACTTTGTTGGCC
CCTGAACCCGACCCTTGATGATGCGGCTCCAACATTGCATCAGTCGATCATT
TAGTGAATCCTAAGAAGCCGGGTGAAATAATTACATACATTCAATATACATA
GTACAATGTCCCATTATGTGATGTTATCTCCGAATCCTTTGGCCTTCAGCACT
GGTAGATGTAAGATGTTTAAATGTGTATATGCACTTGTGGGTATCATAACGTC
GTCAACTGAACGATTGACTTTTCGTGTCATCTCCTCTTTTCTTCATTCCTGC
CTGCGCCGTGTGGAAGCGAAATCAACCCTTCGATGTGTATTTATGTACGTGG
ATAGCTTGAGCGATAGGGTGGCATTCAAGGCTTGTTTCACAGCAGAGCAAG
ATACCCGATGCTGGCCGAGAAGAGACACTGTAGTCCCCAATCTGGTCTTAG
ACCTTGTCCTCCATTCCCCTTTCGCTCCCCTACGGACCCTGATCCGGTCGCC
GATGGTATTGTCAAGGCAGCATTATTCCCACCGCTACCGCTACCTTGACCCG
GGTTAAGCGTCGTCGTTGACGCCCCTGGTGCCCCCAGGGTCCAGGTCTCCA
TGCCAACACCTTCGGGACCAATCGTCAGCGTGTGTCCCCCAATGACAGTGG
TCAAGGCTTCCGAAGCCCCTATCCCGACGTGGAATGTGATTCCACCTATCTC
GACTGCTGTAAGTCCCATCTGTGTAACCGTAGCCCCGCCCGCAATCGCGTA
CTTAGTGGCTCCGGGTGCCGCTGTAATCCCGCCTATATTCTGACCATGAATT
ATGATACCTGTCGGTGCCACGAGGATTGTGTCCCCATCTACAACTGTTGTGA
GCGTGCTACTGGCCATGGGCCCGTAAGTTAATGTAGTACCCTCGATGACGAT
TATGTCTGATCCGATGGCAGTGATAAGTTCCCCACCCATGACGGCGGTCTGC
GTCGGTCCGGGCCCGCCCGCCGCCACATGAAGAGTTTGTGTGGGGAAGAC
GATGTCTGTCGGCCCAAGGGTTATTATCTGTCCCCTAATGACTACCACTGTA
GATGTGGGTTCGATGGTGAATACTGTGCCGTCTATCGTCGCGATCGATGGGC
CGAATGTTACCCCTAAACCTCCAACCGAGGTTGTTATCGGTGTAGGTGGTG
GTGCAAAACCGCCACCTATGTTGGGCGGAGGTCGAGTTATAGTGGCACCAG
CGCCAATTACTCGGGACGGATTAATGACGAATGAGTCGCCTCCAACGACTA

CTGTAGATGTCTGTTGCCCTGGGTCATCGATAAATGTGTGGTCGCCGATTAC
TACCGCACCGGGTTGAACTATCACGGTAGTCGGAGGCGGGATCGTGCTAGT
CCTGCCGCCATTTCCATTTCCATTTCCGCTATTGCCTCCACCTCCGCCACCG
CTCCCGTTTCCACCTCCATTCCATTGCCGCTTTCACTATTTCCTTCATTATTG
CCTGCACCAGTGTCGCTGACTGGGGTCGGTGTCTGTTTAGCTGAATCGACA
GGTGACGGGTCTATACCATATCCAGGGGTTACCAAATTTCCACCGCTCACTG
CTGTCTCGTGACTATTTGGCCCGGAAGGAGACCCACCATAACCAGGGGGTG
TTTGGGTCGGGAAAACCACAGCAGGGTTTTTGTCTGTCGTAATCAAGGTAA
CTGTGGTAGCTGGATCGATGAAGTTATTCGTCGTAGTAGTATGAATAAATGA
ACAAGTCTCGCCCGTGCTGTCGCACAAGGTTATGGTAAAACGCCCGGTTGG
ACTCTCCCTAGGAGTACATGGTATCGGTGTGCTTATTGGTGGGTAAGGGGG
GATATAGTCGGATGGATCCCCACTCCAAGTTGTCGTCGACGTGTAGATGATG
GAATAAGAAGTGACAGTAACACGCCCGCCGTCTCTCGCCGGACACTCGCT
TGTTGTAGCGGTAGTTGTCGGGGTAGTACTGACAGTCGTGCTAACAGTCGG
CGGAGTCACATTAGGTGACGATAGTGAAGAGGCACTAGAGGTATCGTTCCA
GCCCCATGGTCTCGACGTCGCGTTTAAAGAGGATGACGATGCTATCGGCGG
TAAAGAAGTTCCTACGCCTGGATTGGAAGCAGAGCTTGAACTGCTGTCTAG
TATCCTTGAACTACCTCTTATCGGTGTTGACGTAGGTAATACAAGTGTCGGC
CTCACAATCGTAGTAATGGTTCCTAGGTTCAATGGCTCAAAGAAAGAGTCG
GGCGTTGCTGGAGTAGAACAGTCCCCTGACGATGCTGTAACGACAGACAC
GTCATCATCAGAAGTCGTAATCGCGTGTTTGAAACAGTATCTATAGGCCTAT
GATACGTCTCTGAGTTCGTCACTATGAAACTAGATGCTAGTATATCAGGTGA
GCTTGCGAACTCCGGGATCACCGAGTCGTTGCGAGATGGTACCCTTTTGTT
GAATTGACCAGGACCGTACTCTATAATATCATTAGCATCGAGAATAACTGGA
ACGATCGGTGAGCCTCGACTGACCGGCTCTAACCAATTCTAGGCCATGACT
TGAGGACACCACAACACATAGTACTATTAAAATGAAAATGAGCATTGCTAA
CCCGGGGCGAGCACGTTTCTTCGCCCCTATCTCTCACCCGGTGCTTACACC
GGCGTGTTTACTTCGAGGAGGGCCCAACTCCTCGGCTTTACATCTGGAAGG
GAATGGATATGGTTTTATCAGAAATCGCATCACGTGTAATATGGAAGCGAGA
CAAGACGTCGCAATAACCCCACACCCACTGGCGAGAAAATGCTCGGCTGA
GCCGCGACTTTTTATGTGAAGCACCATGATTCGGAATTGGCAGAAGTCCAG
ACGAGCTAAGACCTGTCGGTAAGTGGCTCGCAAGTCCCGTAACGGGTTGG
AACGCAGACACGGGACGTTTAATGGTGAGTGGTCGTAAGAAAGTGAAATT
GATTTGCAACCGTTTTGGGGGTTCTAAGTGGTGGGCCGATTCATTTGGGAT
GCCGCCTAATAAGGGGAAATATGCATATCCCCGGAAAAGAAGGACCCCATA
CGAATAGGTAACTACTTCGTAAAGTTCTCAAATAGACCTATTTTGTACACAT
AAGCGAGGTCCCCTACGTTGTACTACTTACATATATGCATTCCATATAACCTA
GATGGCTCTACGTTCGTGGATTACTAGCTTTGCCATTGAGCGATCTACCCCG

TAGGCCACTTGCCCGAGAAGCCGTAGTTGAAGATAGATAATAACCGAGACG
TAAATCTACGAGAGCGGAGATCTATGCCTATGGTAGAGTAAGATGGCGAAA
TGGAAGTAACGAAGCGACGATCAATTAGATTTGCCGGATGAAGGGTAGATT
GCGGCATGCTACACTCGTACCGACCGCAGGGTCCAATGGCGCAGAGGTTT
GCAGAGGTTGACACCCAGACCACCTGGACACCATATATGAACACCACCCG
ACTCCAAGTCGATTATACCGAGATTGGACAGTGCAGGTGGGAGGTGTTCGC
TTAGACTACCTATTTTGACTCTTGGTAGCATCTTCGGGGTATTGTTTCGTGGG
CAGGTAGGTCGTTAGCAACTTGATTATCATACGTCCTAGATGTCCCAGATAT
CCGGGGGCGCACTGATAGGTGTCTTTAAGTCCGATGACGCAAACTACCTGG
AGGATGAGTGGGATTGGTATGAAGTAGTTGTAATTGAGAGATTCGGTCATCT
TGAACAAGGCTAGCGATCCTATAGCCTCATAGAATCCTGATCAGATACGTCT
GACGGTCGAGACCAACGTCCAAATGTTGGCTGTACGCTGAGGCGCTGCCC
CTGACGCTAATAAGGTAAAATGCGGGAATGGAATACCTGTATTGTACCTAGG
ATAGGTACCGTAGGTAGGCACCAACATTGACTGGTCACAAGTCCGGGCATA
GTTTTATAGGAGGTAGGTCAGCCAAGAAATCCTTCTGCGCTCCTCTCGGTAT
TGACCTAGATTTCCAGTTGGGGATCCAAACGGAGTGTTTTTTTTCAATTGA
ATGCAGAAAGGCGCAGAGAAATACAGAAGACAAGTGTCTCGGTTGTTATAT
CAATACCGTTGTCTTAGTTGAAGTTGATCGTCGATATTCTAGAAGGTGAGAA
TGACGATCTGGAAGTTTTTAGCAACCAAGAAAGGTAAAGGTGACAGGCAT
TGCTGAGCAAGTAAGACAAGTTTGGAAGATATTGATCTTGTGTCAAATAAT
GAAAAAATAATGAGAAATAATGGGATTAGGTGAGCTCCCGGATAGGTACCT
CCGGCGGGCAACCCCTTCCTTTCTAACCAGGGGCTTGTTCTCGATGTGGAA
GGGACTTTGCATTTGCTCTGGAAGGGGAACTTGGAAGAGCTTGGAACAGG
AAACGCTGAAGGAACGAGGGCGGGGGATAGCCCTGGAGGCAGGTAGAAA
TCGGGAATTATTGACTCGATTATTCAATCTTTGTGCTACCTTCTGGGTAGGC
AATTGACGCAATTGATGACCCGCCGATCTCAAACAAACTTTTCAATTCCCA
CGGTTCCCGCGATTCCTTATCGTGTCACTTTCACTTCCAACTGTTATCCTTCA
TTATGCCCTCAGTTTTGGAGTGTGGAATTGTTCGTGGCTCTTTTAGGCGATC
GACAAATGCAAAAGTGCAAGATTCTTATGAAACCGTCTAAATACCGCAAAT
TCTTAAAAACATACCACACAAATCTCATACCACATACCTTTTCCTACGCGTA
CGTACTACACTACGCGTCGGCCTCGGTCGCTTTTTATTACTCGACGTGTCGG
CAGAAAAGACCTTCGGTGGTCTTATTTAACCAGCCCTGTCTAAGTAATTTAC
CACGTATTAACTAGCTTTGGCCACTGCCCAGATTTTCATGCGCTGGTCATCA
GATACCTAAAACTGCTAGTTGCCGGTTCCATTCCAGATGCTACCTTGTGCCT
CAGTTAGGTAATGGGGGCGCAGGACTGCCGAAAACCGCGAGGGACTGTGT
GTTCAAACAAGATCACCCAAGTCAACAAAAGCCCCAGTGGATCAATCTTT
GGCCCACTACTTCAGTGCTCCAGTGCTCCAGTGCAGCGAAACAGCTAGATA
TCGTCGCAGTAAAACGCCAAAAAGTCCCAGACCACCCTAATTAGTCTACCT

ACTAATACCCGGAGAGGTTCCGGACAGTTAGACCTTTAGACATGATAACAT
GATAGCTACATAACCTACTGAGAGCTACCGTTGATACTGCATACACTACTGT
AAAGGAGGATCGTCTGGGGAACACTAGACCGAAAAGCCACGCGAGAGGA
TGAGGCAACGCTGCTAAAGCCGAAAAAACCAACTTACCAGTCGTCTTCCT
TCAAACTGCTCCTCCGCCGCCTCTCTCCTGCATAGCCAACTTAACTAAGAC
AACACTCATAAAACGATAACACGTAATAGCACTAAAATATAAGACATACGA
GTTACACGAGTGTACCTGATAATATTCAATGTTATCAGCATCACCGACCCCT
AAAACAGATCAAGCCTCGTAGCTCAAAAA

>Mosaic_sequence_07

CAAAGATTGATTTAAGCTGTGTTATTAGTTCAACGGCCGGGAGGGGAGAAT
GTTTTCTTTTTCTTTTCAAAAAATATAGAAAGGGAAAGTTGAAAAACCCGT
CTATTCCTACAGCCTTTAGTTGTGGCGATTTATTAATATCTAGCCGGAAGAAA
GATGGTGGGATAAATCGAATTAGGGGTTTCAAAAACGTTGCAATCGTTAAA
TCGATATCGATTGACAAGGCGCAATTAGAGAGCCCATATGGTTAGAAGCCC
CGAGCTTCCAACTTTGGTGCAGAAGGTTGCAATCTAGGTAATTGGGTATCTT
CGTCTCCTACCCCGTTCAGCCCTCTTGTCTGTGCCGGGGGAGAAAAGAAA
ATGACTGTTTCTTGTACAACACAATGATGGATATAAAAAGCAAGGGGTAA
GTACAATAGGTAGTACAAGAGGCAGGGAGGTTGAGTGGTGATAAGCACCA
ATGATGTTGAGTTGCGGCAATGCAGACGTTAAGGTGTGTAAGAAAACGATG
CCGAGGATTGATTGGGTTGTTTCGCGGTGAGTCTGCGAGTCTGCGAGTCTG
CGAGTCTGTGACTGGAAGACATACACACAGGACATACGCCCGGGCCAATT
CAAACACTTTTCTCTCTCCGTTTTCTGATATATAGACTCCCTGTCAATTAACA
GAGGACCGGGAGGACCAGAGAGCCGGTAGGTGAGTTCCGAGGTTGCGATC
CCGCCTGTAATACCGAGAATACGCAGACGCGAGAGAGCAAAGTATTCCGTG
GACGTATCAACGCAGCTGGCCCCGAGAAGGCGAAGCACATACAACCTTGA
ATCGGATACAATGCAAAGTTCTAAAGTTCCTCCACTCTCTACTCGTCGCAAA
ACAGTACAAAAGGGGGCAAAAGGAGACCAAGAGGGACAAGAGGACAAG
TAGATTGGGTAGATTGGACGTGGTGAAGGTATTATGTATGCATGCACGGATG
TGACCCAGCAATGAAGGAACAATGTAGCAGCATGATGCAGCTCAGTGGAC
GAAGGGGGAAAAAAAGGGTGTTAGATTTGGAAGACGATAGGCGATAGAGG
GGAAGTTAGCCGTTTGTGGGATGGCTGGTCGTCATTACCATTGGACAGCCG
CTGATGTGATGTGTCCACAATTATTTTACCTCACCCCATGCTACATGGATGTG
AAAGATGGGAACTGCACCCCTTCAATATGTCTTCGCCCGTTCCTGCCTACC
ATTCTTTCTATCCTATTTCCTATTTTCTATAACAACAATAGATTGTACTGATTG
TATTGAAGTAGTTGCAAACTAACCTTAAGAGTCTACCAACCTTACTTCTGGC
ATTATCCTTGTCTGTATCGAGAGATTCTTACGTATTTTCTTATGTACACAGAC
ACAATAGACACAATGCCTTCCATTTTGCTCCAATGGATGTTACTTTGATGAC

GGAGCCTTGACCTTGACACACCGAAAGTCCCCGGCTCTTGCCCTCATTCTA
TCCACTTCAGGCCGCGAGACACCAGGTGACATTCCCCGAGTTTATTCTCCT
TTCGTCTAAGTATCTCGGTCTCGAATTGACATGACCGAATAAACTTTACTAC
CTAGTGCTTTCTAACTATACAAACATGACATGGAAATCAACCGGATGAAAA
GTTTAATCAATCAGTCATCAATTGAATGGATAGGTACATATCAAAATTGACTT
GTCAATTTGATTGAGTGTACTTGCCGTCTGGTCCCTGCGTTCGGTGCTTCTA
TTGCAGTAAACGTGCGAACCGATCTTGACCATTCCCGTCTCGATGAAACCC
CGCCACATGAATTTTCACTCCTCTCACTCTCATCCACATTGGCCACACTGGC
CACACCGGCAGCTTACGGTCCCCCTCCCATTCTGTTTGGACCCCTCATCAAT
TTTCCCCCCTTCAGCTTTTCTGTCGCCTAACCAATCCAATACCAAAATGTATT
TTGTGTATCCATCCCCTGCAGCTTATGTATCATGCCCTTATCTATTGTTCGAAT
TGGATGAATTCCAGAGCGACGGCTGAGCTATCTGTTGCTGTCAAGGACGCG
TATGATACTACACACACTTTACGTACGCTGGTAGGGCACGGTCGACCATTTG
ATGACTCGATGTATGGTGTAGTTGAACAAATCGCATCTCCAATCTGGAAAAT
TGCACTTTGCATGCATAAAAAAAGTCATCCCGTTTTTTTCTAAGGCTTGCAC
GAAAGTTACGGAAGCGAGTAATTTTTCCGTTTGCCTTGTCCGCCAATGTCT
GAGACGAATGCCTGTTTACATACGAGCATATGTACAATACCTACCTGGGCAT
TTGTATCCAAATAATCTAAACATTCAAATACGATCCGCGGGCAAGCGTCCAA
TTACCGAAGCATTTGCTCACGCGCAGTCAAAGCGGAACGATCAATGGTTCA
ATGGAATCGAAAGAATCCTCCGAAGTCTGCTCTTCTCGGCTGCAATGGGAA
GATGGGAACACCGTCCAGGTATTGATACATGACGGATGGGCAGCCATGCAC
CTATTAGCTTGTATATGCTCCTTGTCCGGCGCGTCGCCATTGTTCCACGTCGT
CGAGGACCCATTCGAAAGTGGATGGTGTTAAGGCTAAAGCATGCCAGATGC
ATCTTGTACATCTTGTACCGCATCTGACAAACCCATGCTTACCATTTGATCAC
TTGGACTGCTTGATTGCTCGAGGTGTTCAGGGTGTTCAGAGTGTGGTTGCT
ATGGAAATACACTGATCCATTATACACGAGAAATATGGAAAGGCATACATTC
ATAACGAACGCGAGCTGCCATCTGCTACGGTGTCGCTACTGTATCTTGCCGT
CAAACCCTGGCTTCCCGCAACACCCGCTGTTGACCCAGTCAAATCAAATCG
GCAACTATTAATGTCTAATGTACCTAAGTAGGGTCTTGGGTCACGTTGAGGT
GTCAGGATGTGCGATGGTCGTCCCTTTTTTCGAAGTGCACAGGTCATCGTC
AAGATTCGCTCTGGCTCCACCAGCAAAGCTCTGACGCATGGGTACAGAGC
GTCATGCTTCCATTTGCTTGTGAAACACGTTGGTGGGGCTTTTGTGCATCTT
CTCATGCTGAACTCTCCACGAGGTGGAACGCCGAAAGAAGAGCAAGCCCT
AAGCGTCTGAGTCGCTAGATGCTACATATGCTGCGTGTATGCACGTTTCATT
ATACTCGATTTCTGTACTTATGCAGCGTCAATGCCTCTAATTCCCGGCATCTA
AGATTGAGAGAATCTAGTGCTCCCCGATCCAGCGTCCTTTGACAGCCGCCA
CATTCCTTCTCTCTGGCGAGCCGCTGGCGCTGACTGTGGCAGCGGTTGTCG
GCGAGTTGGTGGGGATGCTGATGTTGGTGGGGACCGACGAGAACGACGGA

GGCGCAACGGTGGCGTTGCCGGGCAGGATGGTCATGTTGCCAACCACAAT
GGTGTATGGATATGTCGAGTTGTCTGCGACCTCGGTGATGTTGGTCGGCAA
CGTCCCGTTGGTGGTGATGTTATAGCCTTCGGCGGCGCAGGTGCCGTCGAG
ATACTGGTTCAGGGTGTAATTGCTCGCTATGGAGTAGTTGCCGACGCTGGG
GTATTCCTGTTCGACCAGGTCGAGCGCAGCAAAGATGCACTCGGAGCAAA
GAGCAGTCGGCGGGATCATCTCGAGAGTCTGCAGGGCGGTCGAGTTGGCA
CCGAGAGCGACCGTGAGGACATACGGGACGGTGAGGTTGGCCCCAAGGTA
AGCGGATAGCTCTGTAAGCAGCGATGTGGCGCAGAAGGTTCCGTTGGTCTC
GTTGTAGCCTCCCGAAGAGATGGGGATGGTGGCGTTTGGCGCGTTGAATGG
GTCGCTGGTTTTCAAGCATGCGATCTCCCGTGCGAGAGGGTACAGGCCGAA
GATGTCCTCGATGGTCGCGTTCGATACGCCGGCGTTGGACAGGTCAGACTG
GCAGCCTGTGATGAGGGCCTGCGCTGTCGATGCCAGAGTGGTATTGCTACA
CGGATTTGAGGCGCATGTGTCGGCGAGCCAGGTGTCGATGGGGGCGATGA
CTGAGGAGTTGGAGGTGAAGACGGGCACGAGGGCCGAGAAATCGAGACA
TTGAGCGAGGGATGAGTTGGTGGCGAGACTCAAGACCGTCGACTGGCATG
AAGGTGAAAGGCTGTGCATAAGCTGAATAGCCCGTTTATGGTATCTGCTCTA
GGCAAACTCACTAGGAGAGGACGGTGGAAGCGATGGACTCTGCACTCTGT
GCGTATGCAGCAGATGCGAGGAGAGCTCCGGTAGTGGCGAATCGTGCGGC
ACGCATCTTTGAAATATGAGAGGTGTTCGGACTCGAATAGAGGTAGACAGG
CCTGACCTTATATGCTCGAGTGGCCAACGCCGAGACTTTTGTCTACGGGTTC
ATACGTCACCTTACTCGTACAAGTCGCACCACTCGTGCCTTTCTGCCACTCT
ACTTCTGGCTTCTTTCAAGCAATGACCGGCGGCAGCTGACTGCACAGCCTT
CAAGCGGCGAACGAACCAACCCACGCCGAGCAGAGTATAGCTAGCTGTCC
AGTGACCTGTGCATCCATGCCTGGCAACCACACGCTTGGGACATACAGTAG
CTCATGCTCCACGATAGCCTCGACCCAATGAGCGTCTAGAAGAGAGCAGA
AACTTCGGTGGAGCACGTCAGGCTACATACGTCATGTCCGACGGCGATCTC
CTCACCCTGACCAAACGATACCTTGGGCCCTCGGACCCTTACCAACATAGG
CGTGCAACGCCCCTCGGATCCGAAACAAGTCACCTGCTCCCTTGTCCAACG
AGCGTGTCAAGCAGCTCTTCAGTGTCGCCTTCATCTCCATCGCGCGATGGC
AATCCCACACGTCAATGTCAAGCATTGAAAGTCGCCGAGCTCAAAGAGCA
GCTTGAGCTGAGAGGACTGGAGACGACAGGTCTGAAAAAAGACGTCAGT
CTCCCCTACATGCTGGGCAGTCGGTGCTCACACGCAGCTTGCGGACCGGTT
GCAGAAGGCGCAAGACCTCGACCAGTCGGCAGGGACCGAGCTGGCGCCC
GACGCCAGCGTTGACGATGCACTTCCTCCGAAAGAGGCAGCTGCTGCCAT
CGCGCAAATGGCGCAGGTGATGGATGGGCCGGCAAAAGAGGCAGGCGAA
CAGGAGGCTGACGTGCTCGAGGGCGCTCAAGCTGGGGAGCCTGAGGCGG
CGCCGGCTGATTTGGAGGTGGTGGAGGATGGATCCGGAGGTGTTGGGGAC
GTCATGGTCGACCGCGAGCCTTCCAGTCCGACCAACGCCGAGGTTTACGA

AACCGCCCAGACCGAACCAGTCGCCCTCGATGCCGAAGTAGCCGGGCTCG
TAGCGGAGGCCGAAGCAAAAGACGCGCTCCCACCGTCTCCCAAGGGCTAT
TTCTCCTGCATCTGGCCCAATCGAGTCTCCTGCGGAGGGTGCGTCATCGGC
AGCAGATCAGTCGCGACCGAGATCACCGTCTCTCCCGCCCCCTCCTCCCGC
TGCAGAACAGCCCACTTCCAACGCATCCAAGCGATCTGCATCTCCCTTGGC
AGATGCGGCCTCCGAGGCTGGTCCATCAAAGAGACGCCGACAGCGCTCTT
TCCCACCTCTTCCGGAGGCGCTTTCCCACCTTGTGCACCCACCTACCTCCG
TCCTTTACCTCTCCGATCTTCGCCGGCCTCTCTTACTCCCAACACTGCACAA
ATACGTATTCGTCGACTCCTCGCCTCGGCAAGACTATGCCGATCTTCTTCCT
TCCGCCAAAGCGCCGTTTGCTTCAACCGATTACCCGGGTCTCTGGCTGTCA
GGCATCAAGTCACACGCCTACGCGGTGTTCGAGTCTGTGGAACAGGCTAC
GGCCGCTGCCGAGAGGATAGAAGGGTACAGTGGCCCGAAGATACCGGTGC
AGAAGTCCGAGTCGAGTTTGTTGACGAAGACGTGGTGAAAGGACTGGTGG
AACGAGAAGAGTTCGCGTGGACGAATGGACGACAAAAGATGACTCTGAA
GGTCGCCCAGAAAGGGTTCCGATCCCGTCGAATACGATTTCCACTTTGAGG
GAGTCTCTGGAGGTCAGGTAGCACGACCCGCGCCTGGCGTTCGAGGCGCG
CCATCCGGTCCTGCTGCCGGACCAGGCAGTAGACCGCCGCCACCAGGTGC
GCTGGGTGGAGCGAACGGTATACCTCTGCTTGGTCCGAGAGGCATGCCCAT
CATGGGCCCGTGGAGGTGCGCCGGGCGGCCCAGCTGGAGTGAACATACGG
GGATTCGGGGGCTCGCAATCAGATCGCCCACCTCCGCTGGCAGACGGACG
CGGTGGACCTCTCGGGCGACCTCTTTCGGCAATGACAGCGGGCGAGGCCA
GAGACCACGATACGCGATGAGGGGGACAAAGACTCGTCCGCGGCTGTTCT
GGAAGGAGGGTCCTGGCGCGGCTGCAGCAGCATGACAACATAGGATATGC
ATGGCAAGGGATTCACCGACACAGTGCCTGTGCAGGCTTAACAGTCGCCC
AGAAGATGCAACAAGATGTCGCCTGACTTGCCCTTTTGCCAGCCTTCTTCC
AGCTTGCCGTTATCTGGTGTCTTGCCCTCTAGCAACGGCGACGCAGAAAGC
TCTGAGCGCAAGAAAGAGACCGAGAGGGCGTCTATCGTCTTCAGCAACGG
CAGAGCTGCTCGGCTAGCCAGGAGAGGGATGTCCGAAAAGGTTTGGTCTG
CCATGAGCGCCCCAGCACCAGAGAGAGGAGATAACACACGCGACGCCCCG
AGCAAAGTCATGAAGCTGCCCTTTGCCTCCCGGACCATCCCCAGCAGACG
CCCAAAGTGCGGTGGCCAGTCTGTGAATCCAACAGAGTTGATCACGAGCA
TAGGGGGCATCATCGGGTTCGCCTTGGGCGAGGCCGCCGGCGCGGGAATC
GGCTCGACCCACGGGTCAAGAGCGATGGCTCTTCTGACGGGAAGAGGTCC
AAATCCATCCGGCGGCGCATTTTGTAGCAGGTGAAACTATACATTAGCCAA
GCTGGTGCATTTGGGGCTGTGTTGCTGACCACCGTGCCGCCGCCAAATGAG
TGTCCTGCAAGAGTCAGGTTGTCCGTGTCCACCTTGTCTTTCCAGCTCTCG
ATCCACGTAGACCTGTGCTCGGAAGGTAGGTCGCCTACGTGTGTAGTGACC
GGTGTGCCAGCTCCGCAAAGCTGCTTGAAGGACGCGTAAGCCTCGTAGAC

TTCGCGGACTCGCACGTCCAGCTGCAATGTTCGCAGATCCAGCAGTTTGTG
CTCTTGTTGGCCCTCAAACCTGCTCGTAAGCCGACCGAGCCTCCAGATACC
CTTCGAGAGCCTACTCTAGCTCGTCCATCTTCATGTAGTACTTGACCTCCTG
CTCCCCCGGGATCCCATTCGCGAGAGGAAGGATGACAGCCGGCCCAGATC
CGTCGCGATGTTCGAGGGAGATGACAACATAGCCCTCGCTCGCCAGCGCCG
CACAGTAGTGCGAGTAGGTATGTCGGGTCCCAGCCAGCCCGTGGGAGAAG
ATAACCAGAGGCCACTGGTCCTGGGCATGTGCTTGGCGCCGAAGGGGTATC
AAGCGCGTGAACGGGTATCTTCCGGGTCAGCTTTGGCTTCTCGCCCCCTCA
TGTGAGGCTCACTCGGATGCGAGAAGCAAAGGGTCTGATCGCCGCCACTC
CTGGTCAGCGAAGCAATCTAGGCAAATAGCTAGCTGAAGCTTACGCGCCCA
TGACGCCCATGTGGATCCGACAAACTTGTCGTACCCCTTGCTCGTCTCTGC
AGGATCGGGCAACCACGGTATTCTCCCGCTATGCCTCGACGAGGCCTTTGC
AGCGGGGAAGAAGATGGAGTACGCCGTCTCTCGGATCTCGAGTGCAGGCT
TGGAAGTGCTGCGATACCTTGGGTAAGGCTTAGTGTATGGTATAAGCGGTG
GCGATCGGACGTATGCGTATGAGATGGGGTATGGCCCGGTGGCGAGGGGTA
TGCTTCCAGTGATGAGGGGTACCATGCTGTTCGATTGACTTCTAGAGGTATT
TTGTGGCCAGTGTTGCTTGAGACAGAGTCATGAAGCCATCTACTGCTCTCT
GCCCACTGCGTTGTGAAGGGATGGACCGGTGAACAAGACGGTCTTTGATG
ACGCAGTGATCGGGACGAGCGCGAATGACTTTACTTTGGACCGGCTGAATT
CCACACCGGTCGGTGACTCACCTCTTTCTTGCATCTTCTCTCCTACTCTGGG
TACAGGTAGACAACTACAACTTGCATTCTTGGTACATACTTCTTGCAAAGAC
CAACGCTTCGTACACGTAACAATGGCGGACAACGAGCTGCAAGCGTAAGT
CCTCCGCCCCTCCGAGAACCTCTTGGTCCCGCCCCCTCTCCACCCTTTTCCC
CTGCCGTGTATTGTTGGTGGCAACTCGCTGATGGTCGAGATACCCTCTGAG
CAACTACCAGATGATGTGAGTGTGTCTGCCTCCAACACCCCTCCCTTTTGA
GATGTTTGCAAGACACTTGCAAGATGCTGCCTGGCAAGTGGGCCCCTCGCT
CCCCAGTGTGTGGTCCGGACACACTCAACTTGATCCGACATGTCTCATGGC
TTTCCATATGATGACAGCCCATGCTCTTTGGTCGTCTCGCCACGTCAGGTAT
TGCGCGCGCCTTGAGGTCTCCCTCTGAGGCTCGATCTGAGATGGGCGTATG
TGAGTCGCATGGGCTGATCGGCAGCGAGCGCGAGTCGCAGTCAGAAGAAG
ACTCGTCCGTTCCTGCGCGCGTGACCAGGCTTGAGAAGCAGTACGCCGAG
ACGGGCATGCGTCGCTCAGTCGATGCTTTGATGGTCGTGATGGTCAGCCCC
GCGCGGAGTTCATGGTCAACGAGTTGATGAACAGGACCACGGGTTTCCAC
ACGTGCTCGTTCTTCAGATCGCCAACGCCTTCTTCAAGCTGTGAGATTTCC
CGTGGCTTGTGAGCGCTGACCCATAGACCCGGAGGTTACCTTGACCCTACC
GAATCCGATGCTGAGGGCCTGATGACCCGCTTGAACGAGCAGCTCGGCATC
CCTCTTCCAGATGGCTCTTTTGGTCGTTCCTCCGGCGGCAGGACTGGGAGA
TCGGAGAGTGCTTGAGCATCTGGTGGAGGCCCAACTTCGATACTTTCTTGG

TCGGTCTGAACGTTTCCAAAAGGATGGCTTGGGCTAAGGTTCAGTATCCCT
ACTTGCCTGCTCACGCCTCATTCCCCAAGGAGTGCAAGAAGTTGTATCTTC
GTGCAGCTTCCGGAGAAGAGTGGGTCCATGGCAGTGTCAAAGTTGTCGCT
CATGCCGCAGAGACGTTTGCGGTCCCGGCCAACATGAAGCTGCATGCGATT
CCGATTTTCGAGTTTTACGATAACGCCGCACGTTACGGTCCCCAGGCCGCC
GGCATCCCGTACTTGTTGTCCAAGTGAGTCAAGCTGGGGAGTGCGGCGCCT
ATGGGAACGGCGAAGGCGCATCCCAGCGAGAATCGCCGCTCAATGCCTCG
CTTCGCGCGGGCTGACCGACAGGTCTGGCTGCAGCAGCACTCCGATGTGG
TCAATTATGCCGCCTCTTACGCTGCATACGAGACCAAGCTCAACCAGTTCTA
GTCGACCGAAGCTTGACGCTGGTATGTTCTCCGAGTGAGTCCGCGAGAATG
TGGCCTAGTCAGCGCGATTGAAGGCATGACGGATGGCGGAAGGGATGTGT
CAGGCAATTCGGTGAGCCAGCGCGTCGATTATATATGTGCCCGAGTAGGTAT
TACTAAGCCACTACTTTCCGAGAAACAACGCAGTGGTGCTTAGCAATTTCT
GACCCTGAAAAATAGATCTGACTCCGACACCAGCGCCTAACCGCCAGAGC
CCGGAGGGGGATTAACCAATTCCCTCGCGATGGTCAGCTCAAGCAAGACC
CGCCGGCGAATCCCCGAGACAAGCCTGGCCGGAGAGCATGTCTTGTCCGC
GCGGTAGCTCGGTGCGGTGACTGAACTAACGGCATTGCTTTGGAATCCCCA
CCCCTTTCGTCATCTCGGGCACTCCGATCTGTGTCACCGGCTTGGCGAAAA
TTAACAAGCAGCGGCTTAGTGGAGGCTCGGCGGGGAGGACAGGTTAGTCA
TGGATGGCGACGACTGGGGGCGGTCCGACGTGCCCATGCTGTCTTGCGAG
CCCTGCTGCCCGACGCCGAGATGTCCGGCTGACGCGTGTGATTTGATGGCT
GACTCTCCTGTGATGTTCTCACAGGACTATAAAGACGGTCGCATTGGCGAC
GAGCCTTTGTTCCCCATCCTCTTCTCACTCCGACCGAAGCATTTCTCTGACC
ACAACCGCCGCATAGACACATACACAACAGCTAGATTAGACACCCCTCGAC
AACCTGACAGACACTCGCCATCACCAACCAATCCCACCACCTACGAGCGCT
ACGCCTACCATGTCCGCCGCCGCTCTCGACACCACGACGCCCGCCTCCGGC
TCGACCCCTCTGCCTCGCAACCACAGCATGAGCCGGACAGCTACTTTCTCG
TCTTCCCACTCGGGAACAATTTCTCCCGAAGCCGACGCCGAACGCACGGC
TGTCGACCTCAATGCTGCCGTGGCCAAGGTCGAGAAGACTGAAGACGCGC
CTCATCAGGCTCCTGAATACCACCAGGAGAAGGAGGGGATCATCACTGCTT
CTGGCGCCGAGATCAAATTGAGCCAAAGCCGCAAATGGTTCCTCCTCTTCA
TCTTCTCCATCGCCCAGTATCTCGACATTGCCTCGTACTCGGGTCTCTTCGT
CTTCACCGATGGCGATCTTGTCTGATCTCGGTATCCTGTACGAGTCGTCTTC
GTGGATTATTGTAAGCGCTTCTCCCACAGACGAGTTTCAGCGCTGACCGCC
CAGACCGCGTACTCTGTGACGTTTGCAGCCTTCCTCCTTTTCTGGGGTCCG
GGTCTCGGATCTGTACTCGGCCAAGCCAGTTTTCGCATACGGCTTCCTCGG
TCTTGGCATCACCAACCTTGTCATTTCGTTCATGCCAAATCAATACGCCTAC
TTCGTGTTCCGCGCTATCAGCGGTATCTTCGGTGCGGCCACCATTCCCTCGG

CTTTCCGACTGATCCTCGCGATCTTCGAGCCAGCCGAGCTGAATGTTGCAC
TCACACTTTTCGGTCTTTCCGGCGCCCTCGCCAACGTGACAGGTCTCGTCT
TTGCCGGTTTTTTCGGCTTCATCACCGCCGGTGGGCAGATGGCAGCCTGGA
GGTGGTTCTTCCGGGCCATTACAATCGTCATCGTCCCCTTCGCCTTCCTCGC
GTTTGCGCTCGTGCCCAAGACCCGCGGGACCGCGCCAAAGAGTGCACACA
GACCGACAAGCTCAAGCGCCTGGATATCATTGGCTGCTTCATGATGCTCGT
CTCGATCATCCTGCTCATCCTCGGTCTCACCCTCGGCGCCTCGTATGGCTTC
AAGACGGCCAAGTTCCTCGTGCCATTCCTCCTGTCGTGGCCGATCTTTGTT
GGCTTCTTTATCTGGGAATACCGCCTGCCTCAGGGCTACGCGCTCATTCCCC
CGAGCTTCTGGAAGATCCCCAACATGACTCTGCTCATCGTTTTCGCCTCTCG
CCATCTACCCGTGGTGGGCGGTCAACCAGCTCCCGATCGTCGAGCGCTTCA
TCGCCTTCTTCGGCGAGTCCCCTATCATCGCCGCCGTCCGCATGCTCCCCCA
GGGTATCGCCGCCCTCGCCACCGCCATGGTGATCCCTCCCGCCATCCAGAA
GCTCGGCAGCGCCAGGATCCCCATCGCCATCGGCATGCTCCTCGGCGCCGT
CTCGTACCTCCTCATGATCTTCATCCCGAACGGTGATATTCACCACAACGAG
TACTGGAGGTGGTACTTCCCCGCCTTCATAATCGGTTCCGGTGCCGCCATGA
TGAGCTTCCTCGGCGTCAACATCACCGTCATGACCTCGGTTCCGCCGCAGA
TGTCCGTGTCGCCGGCGCCCTCCTCCAGGTCGCCCTCCAGGTCGGCGCCGT
CATCGGTCTCTCCGTCCAGGCCGGCCTGCTCACCGCCGAGCCCGGATCGTT
TGCCAACTACGCCAACGTCCAGGCTTCCTTCTGGTTCCAGTTCGGATGGTG
TGTCTTCAACCTCATCATCTTCCTCGCGTTCTACCGCGCCGGCCGCGCTGCC
GAGGGCAGCGAGCTGGCACAAGCAGCTGACACGCGGAGAGCCAGAGGAA
GACCAAGGTCGTTGTCGCCGTCTAGACTCCGACTAGATAGATACAGCAATT
ACAAATTAGATGCGCCTATAATAACAACAGTCTCGAGCTTGTGACTCATGTA
GCCTATGCACATATAGCAATACCTGTCGGAGCTCTCCAGAAGTGTGCCACTA
AACTGCATTTCTGTTCCCTCAATTGTACAGATATCCCATCGATCAATGGTATC
ACGTAGTCAGTACACGAGACATATCAGTCGCCTCAAAGCCATAATGCTACT
AGCACGCACGGTGACAGACGTAGCAGCTGCTGCTGCATGGCTTTGTGGCT
ACTACACACCCTCTGCCGCTATTCGTGGAGCAATCGGCCATCCCCCGCTCT
GCGTGTTCAACCGCTCTGCCTCCTCTTCTGCCGCTTCACACAGCTGCTGCA
GCTCCCGCACCTCGTCGTCCCCCGGCTCATGCAGAGGCAGAGCTGTCCGTC
CATATGCGAGCTCGAAGCGCTTGATCAGTCTTTTGGCCGTACTCCGGACGC
TCGGGCTCTGAAGGAGGCATGACCCCCACACTGCTCGCTTGCCTCTCCAGC
AACTGGACGAGCGAGACGGCGCGCACGTCCGCCAGCCCGCACGCCATGAC
GAGGTCGAACCACTTGGTCGGCTCGTCCGTGACGTTGATGGCGAACCCAT
GCGAGGTGATGCGGTGGCGAAGGTGTATGCCTATCGAGGCGACCTGCGTC
AGCACCCTGGAAAACAGCCGGGAGTCTCTCGCCTTCTCCGGCGGCGAGGC
GAACACGCCGACGTGGCCGTCCGGGTGCGGCGCGAGGATATTTCTAGCCC

CAGGTCCGACCGGCAGTGCTCCGCGAGGAGGCGCTGCATGTACTCCACAT
AACCGCGTGGAGAGCTGTGACTCAGCATCGTCCCCCTCTTCCCGCGGATCC
TGTTCCCACGTCCATGTGATTCAAGTCGAAGATGGGGTACCCGACCAGCTG
ACCCGGCCCGTGATACGTCACCTGGCCGCCGCGCTTTGTGATGAAAAAGTC
CGCGCCGACATTCTGCACCTTGCGCTCTTCGGGGTGCAGCTCGTTGGGATT
TGGCGAGTTGTCCCTGCGGCCAGTCGTGTATGTCGGCGTGTGTTCTGAGGC
GTCAGCCACCCAAGTTAAAGGAGGATTAGCGGTGCGCACCTAGGAGAAGA
GCACGTCCTGCCCGCTTCGACGCTCGGGCTCCCGTTCTTTGCGTTTCAGGC
GCCGGTCGATGATGTCGTTCTGCAGATTGAGGCCAGTCGGATATGGCAGCG
GGTTGCGGAAGAGGTGATAGCGTATAGGCGGCAGCGTGGTCTTGGAGGAC
GAGGCCGCCGCGTTGCCGGAGCATGCTGCGCCTATGGCGTTGCGCAAGAG
AGATGTGGGTCTCATGCTGGTTGCTGAGTGAGATGAGATGCAGAATGCAGG
CACGTCAATGGCTCGGACGTAGTGAGGCGCGGGGCTCCGCTTCGAACCTG
AAAGAGGACACACCCTGTGTGTTGAACGCCACTTAGACGGCGACGCTCGA
GCCTTCTTTCCTTCTCATTGTTCCCATCACACAGTCTAGCTACACAAGGAGA
GGACATGCGGCGGACTGACTGCTCACTTCAGCGACTACAACCGCCGTCCG
ACCCAACCTTGTCCTGCCCAAAGGGAGGACATACACAGAGCGCGGCCGAC
ATCGTTGTCATGCACCTCGCTGGCCCTCCAGATCACGCGCAGCGCCTCACG
AGGCAAAGTCCTCCGAACGACACTCGGTCTATGCCACTGGACCGCGATCA
GCTCCGCGACATCTCGGCAATACAGCGCATATGACGACCTTGGATTCTGAG
CACAATTACAACATATGCGTCACCTCCATGTTTCTGGTATTGGACTTTCGTG
GTGGAGTGGTTTTCACGTCCTTATGCCCAACAGATCCGGGACAGACGGCAG
TGATGTGATCGTGGGTTCTAATCCCCGTCGCCGTTCTGTCAGCGCGTGAAC
GGAGATCCCTCTCGCTCTTGGAATTTAACTCGCCGCTTGGGTCAAAGCGGG
AGAACGCCACAAAACATACACAAACCACCAAGAACAGTCCCTAACACGCC
ATTTTTGGAGGTATCACACCGCTCTCAGCGCTGGCGGTGTCTGTTCGACGA
CCGTGCTCGTGCCCTCGAGGATCAACTCCGTGCTGCCCTCGCGTGACAAAG
TCGCCTGGCTGGGCTCACCGACAGAGCAGCCTCGGTCAGCCGCCTGCTGC
TGCTGCTGGCAGCCACCCTGCAACCCATCCGAGGGATACAACAGCCACAG
TGACTTTGCACACACACTCCCCGAGGGGACAGCAAGCCCACACACCCTTC
TGACATGCTCATAGATGGCTGCACACAGAGTCGTCTCCTGGGACGACCAGG
TCAATAGCAATTCCGGCATAAACAAGTCGACCCGCTCAGCGATGAACGTAT
AACTACCAGGCCCGACGGCTCTTAACCCTTCATCCCACTCCCTTCCCAATCC
AGCCTTTTTCCACTTTCCCGCGAAGCAAGCCCGTCGCCCCCGGCTGACGGC
TGTTTCTC

>Mosaic_sequence_08

GGCGGGTTTGGGGATACGACCAAGTCGACATCCGGCTGGGCAAAAGCCAG

CTCGTCCCCATCCCACTCGCCCATCCGGGACTTTGGCGCCGCCGGGCGGTA
TCGCCTCTCGTCCACCTCGCGGGATCCAGTGTACGGTCTGCCCGGCACCAC
CGTCGCGACCGCCTCTCCGCTGCCGAGCTCTGCCACGCCATACCAGGGCAC
AGGCGGACATCGGTACTCCCCGTCGGTGCCCCCTTCCGGCGGGCACGCCA
CGGCCGGCCAAGGACTCGGTGCGGGCCGCACCACCGCCCCGAGCTCGTAT
GCCTATGCGCCCGTCGTCGCTGTCGCTGAAGACGACGCCGTCGCGCACAG
AGGCCGCGCCGGCCGGGTCGCGAGCGAGGGCGGCACCTCGCGGGATCCC
AGCGAGCCCCCCGGTGCCGGTGCCGGTCCCAGCAGAGATCGGACCGTCAC
GCGCGGCGGTCTGGCCGGTCAGAGGCGGAGACACGAGTCGCTCGAAGGC
GGGTTTGGCGAGATGAGCCTCGAGATGGACCGCGGGGAGCATACTGATACT
GTACTGCAATAACCTAATGTCTTTTCTATCTTTCTTTGTCATCTCCCGTGTTG
TGCATACAGTACTCTTAATTGTAGGGACGCTTGCCAATGCTCACCCTCATCC
ACAGAAGTTGTAGATGGGCAGGGTATACGATGCATCTGCATATCCCAATTCC
CAACTCCAATCTGCATGCTGTCTGTCTCTCTCTCTGTATCTGCGTCTGAAAC
TGGATCTGATGGCGGTAGATGCATACACAGGTGGAAGGGTGAACGTGCGG
TGAGGCCCAGGAAACGGAAAGAAGAAAGAAGGTTGGGTTCACTGGGCGA
CGTGCCAGTCGAGCTTGTCGGCCATGTCCTTGTTCTTGAGCGTGAGCAGGA
CTTCCGCAACGTCCTTGGCGCCGTTCCCGGAGAGACAGCTGCGGGTCAGC
GAAGCCCTGCCGTCGCGTAGCGTAGCAGCTCTGCTAAGATCCGGGAGAGA
GAGAGAGGGGTTGAGGAAAAGAGGAGCTCACATCACCACGTCCTTGTCCT
TTGGCAGGGTCTTGGCGAGCTGCATGCCGCCCCAGATCCCGTGCGAGCTCT
CGAGGGCAGGGATAATACCCTCGAGCTCTGTGCACATGCGGAAGGCCGTG
AGGCACTGCACGTCGTCGGCGACGACGTACTCGGCGCGGCCGATGTACTT
CAAGTGCGAGTGCTCGGGGCCGACCGAGTTGTAGTCGAGTCCGGCGGAGA
TCGAGTGCGTGGGGGTGAGCTGGCCCTGCTTGCTCTGGATGATGTAGGACG
AGGCGCCGTGCACGACACCGATGACGCCCTTGGTGAGCGTCGCTGACGGT
CAGCTGGTCTTCTGAGGGACGAGAGGGAAGGGGCCTACCCGAGTGGGCCG
CGGTGTCGATGCCGTGCCCGCCGGCCTCGACGCCGACGAGGCGCACCGAC
TCGTCCTCGATAAAGTCGTAGAATGTGCCGATGGCATTACTGCCCCCGCCCA
CACATGCCACGACGGCGTCGGGGAGCTTGCCGCACCGCTCGGCCATCTGC
GACTTGATCTCGCGGCCGATGACGCGCTGGAAGTCTCGGACGATGGTGGG
GAACGGGTGCGGGCCGATGGCCGAGCCGATCAGGTAATGTGTGGTGTCGA
GCCGCGTGACCCACTCGCGCATCGCCTCGTTGACGGCGTCTTTGAGCGTCT
GCGTACCGGCTGTGACAGGCACGACCTGCGACTTTAGCTGCTGACTCTGA
GACTCCGGCAGCAGACTTACGGTTCCGCCCAGCATCTTGATCCGGAACACG
TTGAGCTCCTGTCTCCGCACGTCCTCGGCGCCCATGTAGATGACACACTCC
ATGCCGAATCGCGCGCAGACAGTGGCGGTGGCCACACCATGCTGTCCCGC
ACCTGTCTCGGCGATGATCCTCCGCTTGCCCAATCGCTTGGCGAGCAGGAT

CTGGCCGACGGCGTTGTTTATCTTGTGCGAACCGGTGTGGTTCAGGTCCTC
GCGCTTCAACCAGATCTTGGCTCCGCCCATCTTCTCGGTCAACCGCTCGGC
GAGGTAGAGCTCGCTGGGCCGGTTCATGTAGCCGTACATGTCCTCAAACTC
CTTCCAGAATGCCGGATCTTTGCTCGCCTCGACATATGCAGCCTCGAGCTCG
TTCAAGCAGTCGACGAGTGATTCGGGAACGTAGGCCCCGCCGAAGATACC
GAAGCGCGATGGCAGCTTGCCCGCGCCGTTCACTGTGAGCGCGTCCTTCTC
CAGCGGGTTGGTAGCCGGGATGGGCAGGGGCGGGGAAGGGTGACCGGCG
GCATCGTCATTGTGATTGCGAGCCTTACGTCCCAGGGGCTTCTTCTCGGACC
CCTTGAGCGAGATCTCGCGGCAGAACGCTTCGAGGCTTGAGGAGCGTTGT
TGACCTTGGCGCCGTCAAGAATGACCTTGATGACCTTGGAACCGACAACG
ACACCATCTGCGCCAGACGAGGTGACATAGTCAAAGTGGGTGCGGTTGTC
CACGCCGAAGCCCACGGCGAGGGGTACGGGCGTGAGCGACTGCACTCGCT
TGACAAGCTCTGGGAGACCGGCAGACATGGCCATGTCGGCGGCCGAGCCG
GTGACACCCATCTTGGACACAACGTAGACGAACGAGTCGGCGATGGAGGT
GAGGAATTTGACTCGGTCGACCGGGGTGGAGGGGGCGATCAGGGGGATGT
AAGACATGCTGCCATGAGCGATCACCCACACAGAACGAGGGCGGCGCCCC
ACCCTTGCCTCGCGCAGATGTTCCGGAATTTGAAAGCCTCCTCTGGCGGCA
GATCAACCATGATGTACCCGTTAGCGCCCGCCGCCCGGGCGTCAATGACAG
CCTTCTCCTCGCCGTAGGCGATGAGCGGGTTGTAGTAGCCCATGAGGACGA
TCGGGGTCTTGAGGCCCTGCGCGCGCGCCTGGCGCACGTACTCGAGGCAG
TTCTCAGCCAGAAACTGAACTCACAATACATTATACCGTCTCTGACACCGCT
GACTGACCACACCGACGTGGACTATTATTCCCCAAGGATCACCATCAACAA
CACTATTCGTAACCATAGACACTTGCGTGAAACACCTTCGACCGAAATTATA
TTGGCTCAAGCCAACGCGCCGCTCGGATCGCATTCTACCAGCAATCCCCGG
AGGCACCGGCCTAAACCTCGAGTAATTCAACCCAACCCACCGCGCATTAGC
CGCACCACTACGTTGGAATCAGACACGATGTGCCACAAGAACATCTTCACC
TACGTTTACCCTGACGGACACAAGGGAGAGCACATCAAGCATGACCTATGC
GACAATTCGCGACATGGCGTACCGTGCAAGCTTACGAAGACCTTCGAACA
CCCGCCTCAGAAGGTCCGTGCTGGTCAATTGAGCCCTCCAGCTTTGACCTA
CGGACAATTCCCGCCGACGCCGCCGCTCTCTTCTCACTCTGCGTCTGCTTC
TGACTCGGAACACTCTACTAAGGAGCGATCGGGCGTCTACATTAATGGCGA
GAGAGTTAGCGACATCAACAGGCGCCAGTCGCGACGCGACAAGAACGATC
GTGAGCGCGACCGCACTGTCTACATCGAAGGCTCGTCGCTATCGCGCACCC
CACCTCGCCGATACAGCGTCTCGCACAGCAGCCCCTCCACTAGCCCGCGCG
AGGAAGGAACTTACATGCGCGAATCTCGCCGGCGCGAACGAACTCCGGAA
TCGCGCGATCGCTCTACATCATCACACTACCGAGGACCGACCATTGAAGTC
AAGATAGTTAATGAGAAACGTGAACGCGACCACAGCACCCACAGTACTCA
CCGTCGACATGGATCTTCTACTAAGTCTAGCTCCCAATCTGGCGACGACGA

AGAGCGCCGCCGACGTCGCCGAGACAGCCACGTCAGATTTCAGGATGACG
AGAAGACTGAAGAGGAAAAGAAGAAGAGATTGCAATCCGAGATCGATCG
CCAGAATGCGACCATTGCCAGCCGCCCCGCTGTCCCGTCCTCCCAGGCGCC
TTCTAATGCACGATACCGCCGAGGCTCAGTAGTTGTCGACCGATCTGAGAC
CGCTTTAATTACCCCTATGGAGACGCTACAACTCGAGCGCGACAGACAACG
ACGGGAGAGACGGAAGTTGGAAAAGCAAGCGCGCGAAGAGGAAGAGGC
GCAACGACAGAGACTGAAGAGTCGACTGGCACCTACTCGTCCCACTGTTG
CTGTCACTCAAACCCAAACTCGTCCGCGACCGATTCTCTACGAGGACACAT
ACTACGCGGAATGATTACGAATAAGCAAACATTAAACTATCAAACACATTA
AAAAAATGGGTTAAAATTTGCATGTAAAGGATATGACAACGGCGTTGGATC
AATGATACCTGGGCACTATGATTACTACATGGTTTGATGTACTTTTTTTTCTT
GCTTATAGACGGACGGCATGCCTATTTTCATGTATTATTGATAAGCCTGTATG
TATTGATATCACTCGTACCGAAGTGGCACACGCCTTGGTCTGAGGGATTTTG
GGGGATCACGAATGCGCAATATCAAGAAAATATTGGAAAGAGAAAATTTTT
GTCTTATCACCTTTTCCCAGTCAATAGTCGTTTCAATACCCCAGTCGAAATA
CGATGTCTACTTTTTTTATTTTATTTCAAAGCATGTAGCCTTTTGTAGATAGA
AACGATACCATTAGAAATATTACACTCAGGTACAAAATAGATCATAGTTGTA
GTTTAGTTGATCCATGGAAATGACCCAATAGATTATCAGCAATGCATAACCA
TTCACCTAGTCGACATGAAAGCATTGTTGTCGATTCCTGTGCGACAAACAA
ATGGATGGCATTTGCCAATGACTGACTCATTGCGCCCCTAGTCAGGTACAAT
TATGTACCTAAGTACTAGACACTTCCCAAGAAGGAAAAAAAAGATCAATTA
AAAAAAGACGAGGGAAAAAAGAAAAAGAAAAAGACGAAGACAATGGCC
CCGCCAAAACGCGGTCAAATCGCGGGGCACGTCCCCCCTATTCCCCTATCC
AGACTTAATCCCAGTCCTCTTTCGACCCAACTCCAATTATATTTATTACTCAC
ACACCGATTGCTGCCTGATTGGCCCCTCCTTGTCTTCCCTTCATATGTTTTTC
CATTTCTCTTCCCATCTTGAACCATCCCGACCATTCATAGTCGGTCTAGGAC
TCGTAGGTACTCTCGTCCAGGTCGTCTTCAACGATCTGTTACTTATCACTTT
CTCTCTAACATCACTCTACATACAGTCCCAAGTTTGCTAGGTTCCAAGGTTG
TGAGGATTGTAGCATTATATTCAACGATCGGTACCCGTCCGTTCCGTAGATC
TCTCACCCTTCGACCAGGTTCAGGATACAAGTTTTCCTAGTAATATAATTAA
CAAACACCATTTGCACCTTCTGCCTTTATTCATATCCGTGCTCTCCGTGCCA
GCGCACTCCCCATCAATTTGTCGCTCAATTTGAAATTCTCCCGTTCCTTACT
ATGTGTAAAAATGCAATACGCGTCCCCATCATCTATTGGCCCGTCCCTCGCC
TCCCTACATTGGACTACATTAGGCAGATCTAGGACTCTTGCAGACGGGAGA
CCCATTTCCCATCGTGGGAGACTAGTAATTAAATCTTGTTTCGTACTACAAG
ACTCATACTTCGTGCATAAGAAGCGTGGGGCTTTACCTGTCTTTACCACGCT
TCAGTGGATACTTACGAACCGATCGTGCGCGAGCTGCCCTACCCGGTCAAG
TGGCTACAGGTAGACGGATACCATAGGCAGCCTAGCAAGCCTAGGAAGGC

AGACAGAACACATGATAATCACTGTGTCGCATTCCGTCGTCGATCCGGTCG
CTCTGGTTGGATTACTTCTTCCTCAGGTCTAGACCAGTTACTGTCATATCGTA
GGACTTAGACTACGTTCATCAATATCATCAACGCTCTGGACGCCCAGTGGC
AAAATGATCAGTAGAACGATCTCCCAATCCTTCCAGTCGGATAGGTTACCTA
TGAAGTATTTGGGTAGCCGTCACATCTCGGCTCAGAGGACCCTTCTTGCAC
TTTACGTACCTACCCACGTTCCAGGCAAACATTTTGGTTCCATCTACTCAGT
CCAGTCATTTGTACTATGTAGGCAGTTACCCGTCCTGTCTTTTCTACATATGT
ACTATCGCAATGGGTTGCGGCATTCCAGCCGGTTGGCTGCATGTCTCACCC
CATCAGCTCAACAAGCTTAGGAATTCGATAAGCATGATGCCATGGGGATTAT
CGATATCATGATTGCCGTCCACCCTCACCTACGACGGGATACGCTACCGTCT
TCTCTGCATCGCACCATCCCACATAGGACACCCAAGACACCCAACCCACGC
TGTGGAAGATGGAGGACTCATCAAGTCCCGTGGCTTGATATTCCACCCACA
CACTCACACAGGCCTTCTTCGAGTGGAGGTAGAAGTGGCTCATACTCGGCG
TAGGAGCGAATCGACCGTCCACCCTGCCGGTCTCGGCTCGGCATCCGGGCT
GTGACGCATGGCTCCATGCAGCACACCTAGGGTATACGACCCTTGCACTCC
CGCCCACAGACCTATATAGCACACACACATTTCGCGGGCAGAGGCAGCGG
AAAATCTGGACCATGCGATCAGGCGAGCACCCGGTCCCCATGAGGGGATG
TGTGGAGACGCTAGACATGAGCTTTTTCGCGAGCTTTTTCCCTCTCAGCATA
ATAACTTAATAAGAGAGTTGGGTGGTTGCCTCTAAGACATGCAGACCCGGC
CGAGTCACGCCGTACGTCTGCACCCATGTTACATACCACACCGAAGATCTG
TCAATCTACCGGTCGGCTCGACGCTATTGGCTATCGCTGGTGAGAATGTGG
AATGCCGTAGAATGGAGCTGGTGAAATGCGTTGATGGTGTCACGCCATACA
AACGTTCGATCAGGCAGCCCAGCTCGTCGGCTTTTGAAGTTGACGGCGAG
GAATATTATTTCGCACGATGTCATCGAGCATATTCTTGGTTTCTTTGAAACAT
TACTGGGGTAAAAGGGGCCGATCTTATTGGCTCCGCGACATTCTAGTAAGTT
CATGACTATCGAAAAATTTTGTTGATCTTGTGGTCGAAATATTTCCATGAGA
GTCTGAGAGTCTGAGAGTCATTCAATGTATCTACAAAGATGGGCATACATGT
GTAGATATTAGCTCATAGAGATAATTCTAGGTAATTTCTAATAAGACACCTTT
GTAGGATTTTCTCTAAGCAAACACGCCCTCTACATGACAGATACTGCAGTTT
ACAATGCCGGTGTAGACATCTATAACTTTAAGTGGAGAAGATGCTTCCCCAT
TCTGCATGTAGTTGCATGTATCGGTATCGTGAGAGTTCTCTATAGATGTTGAG
TAACGTCCGAGGTTACACTGAAGCTTACCAAGGTATACTAGGTGCAGCATC
ACGACCTTATCTAGTTGCCTAGACAGATTCAAAGTAAGGACCTTATCGGCA
GAAAAATCTACGTATTGGTAGACATGGGTTTATTTTCATTTTCATTCGGTACC
CAATCACCTCCCTTGTCTTTCAGTGTTTCGCCCCTCGACGCCAAGCCCAAA
ACGTGAAATTGGTAAAGGAAAGTCAGCCGCTGGTAGAACGATGAGTCTTT
TTCAGCTGCTCTAGGATGATTACTCCAAGAACAGGAATATCGGAATATCTGC
TAGCTCAAAGATTAGATTCGGAAGTTAGTTACTCCTCACCTAGAGTCTAACG

ACATGTCTTCAGGTGACATCGACTAGAACGCACTATCCTACCACTACCTTTA
GGGGACTAGCAAAGGTGCTTTGTGAATATCTACGGCACTCGCAGTTTGCTA
TATGATGTTTTAGTCCGAGAGCTAATCCGAGGACATATTCGCAGCTTCACAT
CGGAATTCATGTCTTATTCTAGAAGCGTTCGCCGAGGGCTGGCTTTGCGGTA
TGAAACCACAACCTACGATCATTACCTAAATGGCTAACTAAACCTTTAGGG
ACCTGCGGGTTGTGTTTGCAACTGGTTTGCAGCTAGATTAATGCCTAGGCC
GGAGGAGTCCAAGATGCTTGGCTTTACCAAGCCCCTGAGTCCGACGGGAA
CGGGCGTTCGCCTTGAAAATACACCAAGACATCTCAGACAATCGGGCGCTC
TGTATGGACGCATCATCCGGGTAGGAAGAGCTACTGTTGTATCGGATTATCC
ATTGCGACGATGGACGTCCGTACTCACCTGAAGTCCGGAGGCGGGTTTATG
CTCGGCGATATGCTTCGGAATAAGACGACTACTAGGAGCTCAATTTTATACC
CCTTTATCGACATGGTTTTGACTATTCGAGCGGAATATCATTCAAGATACCG
GAGAAAGTATCTCACGACTACCCAGGTGGAATGGTAGACTTTTGCCAATCG
AGAATTATACGTCGCGCAACTTTATCGCATTATGGGATAAGCTTACCATGGT
CGCCGATATGGCTTCAGTACCTTGGATGAAGGATTTCAGCTCTGTCTGACTG
TGGGGAAGGCCCTCTTGGGATGCTATCTGAAGCCACCAGAACGCCATGATT
ACCTACTCTTGAGGAGAACAGTTTCTCAGGTACCATTCACCGGTGGGATGG
GCATTAACAGCCAAGCTGGTTGAGAGCTAGGCTCAACGGGCTCGACGGAC
CTTTTTGGGCATATTCCGCTATCCTGTAGCAACCCGAAAATCCGGATCACTC
GGGACTCAGCAGTTGCTATCGAAGAGACCTTTATGAGAGCATTGCGTGTGA
AACCGGACATCAGGTAGGGTAAACTCTGCGATAATCCTACGAGTGGTGTGT
ACCTAGCTAACAAGTATCTTCATGAAGACCGATACAAGAGGCCTGCCTCAC
ATACTAAGAAAAGATTGATCGACATGGCACATCCCGGTAGGAATCAATCTA
GAAGAAGACCACGGCATAGAGGCTGGAAAGTTACGTTTAAATGTGCCGGA
CGAGTTGGTTTCTTATCTGGTAGGTTGGGAGCTATTTGAAATTGATCCTTCA
AAAGCCCCCAATCCTGCGAAGGATGAGACCCAACTCAATAAAAATGGCGT
CTTAGGGCTCAGTTGTCGCATGCTAAAGAACATCTTAATACGAGGTGTCAAT
CGCAGTATTATTAGGTATTTTATTGAGAGATAAAATGCGACGCATTCCAACT
CCTCTCCAACACTATTCACACCAGTGTCGGAGTATTTATTATAGCGACTAAG
GGAGGCTAGGTCAGCTCTAGCCAGGCCAGGGCAAGAGAAGGAACCGAAC
CTATGGGTCGAGGCAAGGCACAGAACTAACATTAGACAAATCCTGCATGTT
TCAACGGTCAAGGGACGGATCTTGGGGGCCGATCACGAAGGATGATCTTT
GGCGTGAGTTGCAATAGCCTAACCACATTATCCCATCTAGCTTTAATTGTAG
GTAGGGTTTACATAATTACTCGGTGCACCATCCCATAGAAAACTCGGTGACG
AATGATACTTGTTTCCCTCGATTAAAGCTAGAGATTTGAACCAACTTTGGCA
AAATGTATCTTCTCTTAGGTGTGGATATTGACGCATTTCACCAACAAGACCA
AACGGTTACAATGAAGACGACTCTGACATCAATAATCAATTACGTCTATTTT
TATTCCTCGGAAACCCTCCAGATAAGCTTCTCTCCGCTCACCCCTTTCCGAA

TTAAACCTAACGCGTTGGTAGCTCTCTCTAGCAGATTGCGCCCTTCGACCA
CGCGAAAGCGATTCGGTGTTACATAGCCCTTAGCAAGTAAAGTGGGCATGA
TCTCTACCTGTAACTTCTCCTTGAAGAGATCATTCTATTTCTTAGTTAGTACA
TATCCATTGTAGCGATGCTTGAAGGGGTTGATTGGTAACGTACCTTCCAGTA
GAAGTGCGTTCGGACTCCACGCACGTTCACACCTTCAGCCCACCGGGCAC
TGGTGCTTGCGTCCATCGAGTACTCAGGTGCTTCTTCCTCTGTCGCATGTTT
AACGATGACAGGCAACATAATCGCGACAGTTGTGCCATTCTGAGCAATCTT
AGAAATAGCACCGATTGAACCAGTCTGAGAGCCGATACAATCGACGATGA
GCGGGAAGGCCGGCGCATTTCCTTGTTGGTATGTCTTTAACAAGTCATCAA
CCACCGTCGGGCTCCTATAGTCAAAGACCTCCTCGGCGCCCGCTGACTTCA
AGTACTCGTGATGTCGCGGCGATGCCGTCGCGATAATGTGACGGTATCCATA
AAATTTGAGGACTGCTATCGTGAGTTGCCCTACCGAAGATGAGGCGCCCCA
GATCAGAATCTTGTCGTCAGCCCTAGGCGGTACGTAGCCTTCAGGCTTCGG
CCACGGGGTAGGCAACTCTAAGTCAGCTGAGATTGTATTGAATGCGGTGAC
AAGGTTCTCGGGAAGCGTGACCACTTGTTCAAACGAGAAGCCCTCGGGAA
CCTATCGATTCATTAGACGCCATGCAATTATTAGCATCAGAGCCCCTTTTCCT
CAAGGCTATCTAATTCCTACCTTGGCAAAGACCCATTCAGGGGCTGTCGCAT
ACTCCTGATGAGCCTTCCACTTCGGCTGTTGGTGCGCGTAGCCGAACACTC
TGTCGCCGACGTTGAAATGCTTCACATCGGGGCCGACTTCAACAACAACA
CCAGCTGAAGTCGACCCAGTTCTATAAGGAGGTTCGACCAGAAGGCCACC
ATCCGCTTGATGGAGATCAAGAGGGGTGGATGCTGTCCATTTTACGTGGAC
TAAAATTTCATCTTCTACTGGTGAAATAGTGGGGTATTGATGGATCTCATAA
GGGGCTCGGGGCGCGACTATGACAAGCGCATCGTGCGTTCGAGCTGCCAT
GGTGAATGCAATGTGCCTGAAAATAACGACGATAGAAGAGTAAAGATATCA
GTATTATCTTGGGTACTCGTGGCTTGTCTTTGGATCGTAACTTTATTGCCTTT
TGACGAGAGCTTCCTTCTTTTTATGCGTATGTGATGATATGCTGACAAATTG
AACGAATTTGTAAGCCGCCCACAATGGTAGACGCATGTTGGCAGGATCCAG
GGATGCCAAAACTGAAATTGGTAAAGTTCATGTCATAGAAAATGGGAGTTT
AGGTAAAATGAAGTCATATCTATGGAATTGTACGCAATATGCTGGTATCTTCT
AGCATAAGCTCAATTGAACGCATACACTCAACAAGTATGAGTCACATTGCT
CTCCTGTCAATCTGCGCTTTGTCATGATGCACTTCAATGTATTCTAGAAGAG
ACAATTCATTTGCGATAATGACATTAGGACTTTAGTCGACCTTGTTACGGTC
TTGCGCGGTCTGTGGTATGGAATCAAAGGTCCAGGTAACCGTCAATCCACA
TACTATCATCTTTATCAATCGAGGACTAGCAAGTATGTGGAGTTACGTTCAA
ACCAACTAAGAAATGATAAAATGCGAAGAATAAGGAGTCGACTGATACTTT
TAAGTGACCTTGGTACCCACCAGACTCGCCAGTCTCTGTTACGTCGACGAC
GTTAAAATAAGTCACGCCGCTATCACCCAACCCTGCCATCATCGTTGAGAA
CTGAGTCTATCACCGCAGCATTACCTATAATTAAACTGCTCATACCTAGGAC

CGCGGCGATACATCCTATCTCCTTGACACTATGTAAATGCCTAGGTGTTGTT
CAGTATGAATGAACTAGGTGATTCCATAGGTAACGATCACTCGCCTTCTTGC
TTCCTCCGGGCTCGCATCCTGTAACTGAGGATCCTCGGTCGGTCTGAATTCG
AAGGTACCCTTTCCAACACCTGGGGACTACTGAGTAATTGACCTGTCCGCG
CGTTGCGCGGAAGTCATGTTGAAATAATTCCCAGCAACCCTCAGAATTCTA
ATAAGTTCGGACAACGACGGATTTCCGTTATAGAAAGTCTACACGTAGGAG
CGTCTGAGCTAGAACGGACAGAATAACATCATATATTTGGTCGCCAGAAGC
TGCTGTCCTGGCCCTTTCGCTTGCTTCATTTCGGGATTCTGTGAATCTCGCA
AAGTCTCGTAATGTCGAGTCAACGACCTTCTATGATGGGCAATAGGCTGGA
TCTCGCGTTTACAACATTCACTAGTCTTCCCTAACAGTTACGATAATAAGTC
GAAGGTAGGACAGATGGCCTATTCTCTTAACCAGCGAGCGGGAATGAAGC
CCGGCAGAGTAGTCGGTCGTTCATTCTGCTTCCGTTCTCTTCAGATATAATT
CGCAATCTGCCTCGAATCCAGGAGATGGCTTTCACATACGGCTAGATTGCCT
CGTTAGCATACCGCGCTTGAATTAACCTATGAGTCTCGAAGTATACTTCGCC
TGATAGCTCAAAATTGAGGCTAAGATTGGTTCTTCTTGCTTTGTCCACTATT
CACTCACGCAATACATATATCAAGGGTGTATGGAGTCGTCCATTGGCTTCCA
GCAGAGCTGTTTCAGGACATTTAGGTTCCGAGAACTTTATATACTCCGAGA
AAGAGGACTTGCGAATGGCGGCCCATAAAGTTTCATGTTGCTTTTCACAAG
GCTAGCGGTGAAAACAAATATAATTCGGTTCGATAAGGTAGATTGACCTCTT
CCGGAATGTGACCGAAGTCATCTAGAGCGAGTACTTGGACTCGCGCCTCCA
GGACGCGGGTCATCTTGTAGTTCTTCCATGTGCGTTTCTGCGTGATAATAGG
TAATTTAGAATTGTTAGCAAGAATATATTGAACAGATGGTATGAAGAGAGGA
ATTCATGAGGCAGAGACTGCTAATGGAGTTCCAATTGAGTTTCTGCTGATGT
AACAAGCTGTCCTTGCCTCTACCCCACATCCTGTTTTACCCCCTTTACCCCT
TTTTTCTTCAATATTATTTCATAATATGGAGCTCCTACTTCAGACCTTATCATT
ATATCAGCTCTAAAGCAGCTCACAATCAGTCTCTGTTAAAGCTACCTAGCCA
GGGAATTGTAATATGCCCCAAGTCAAATCGTGGTAAGGCGTTGGCACAGCA
ATTTTGTCAAGTAAATGGTACTTCTCTAGGTATGGATGATACTTGTACACCCC
TAATTTATGTTGTTGTTCCACGACAGCAACTTATTCATTGTAATACTGTAGCT
ACCCGTAAGAGTACTAGGGACTTGGCACGATATAGTCCTAGACCTCTTTTGC
GTAAAAGTTGGTGGCTTGAAATAGCGCGAGATCACTGTTATTTTTAGGCAC
ACTTTTTGGGGGGGCGGTGGTTTCGACACTATGGTAGTAACTCTCAAGCAA
GAGATGGTATAGAGTGTAAATTGGTATCATGGTTAGTAGGTAAGTAGGCGTA
GCGCGACAATGGCAAACGAGGATATTAACTCCCTCAAGCTTCTGGTAATCT
ACCGAAAGTCATTTCATAGAGGTAGACTGGGATAATAGAGCTCACGGACGA
TATTCTAGAGGTATAGCGCGAATATAGGTAACTACCAAATACTGCTGATTCG
TCGATTCATAAGAAAGCTTATAAGAGTAGCTTTAAGATATCTTGAGCTTGTG
ACCTCCATAGAAACGAGGAATGAAACGCTAATAGACAAGCTTCAGAAGCT

CCTGTTGCAGCTACATGAGTTTAACGCTTTCGGACCAAGATCCAAAGCAAT
CCCACTATCGATGTCAATCCGACGAGCAACTCCCCTGTGTAAACCAGGCTC
TGGCCCCATAATAGGGGCTGGTCTAGGCGCTCTATGCGCTATAGCTGCACAC
TGACGGCAACTGCTCCTAGATGTAAATCAGTTTAAAACAATTTCTCATTGCA
CTATCAGATACTACATCTCTTACCGTTGCTATATATGATGATGATGCTAAATGT
GCATAGAGATGGATTCCTGATGATTATGATTTGAATTCTAGCTGAGCATCGA
GGGGAAAATCTGCTAATGCGGAAAGCCCATTGATAAATGTTACGACCAACT
TAACACTTAGTAGGTATAGGTACTAGGTAGGTACCTTCTATGTAAACAAGCT
TGGATTTCTAGATTCATTTTTCGATATTTCGCAAGGTAATGGTTGGTTGAGTT
AGAAACGAATCAATTTATTTAGTTAAGGCCGTAAGGTAGAGTTTATGCCGG
GCAGGTACATTCATCACGGAACCAACGCTGACGAAAGTTGATAGGCTTTCA
TGGTATTCTAACCTGATCTGGAATCTAATCCATGGAGATTCAGCACCGTATT
CTGTACCCAAACAAGAAACATTCTCGCCAGGTTCTAGCTACCGTGTGCGTT
CGCATTGGAAGGTAAGATTGGGAACTTTTGGGTGGGATTTGAAACACTCTA
TAGGGTATTCTATCAGTACCTGCTCCCGTGAGTTTTATATTTGCTTTTTGCTA
GATACAACTGTATCGGTGTCTACCCGAGACATAAAAAAAATACTCTACAGC
GGGTGGAGATCAGCCCGTTTGTGGCTACCTGACCGGTAGACCGGTAAGCA
GAATTTTACCTCCTTGTAGCGACATAGGCAGTAGATCCTAAAACACACAAA
AATGAAAATCCAGGTCGTTTTCGTTCTTCAAGACATATCATGTGCTCGTATAT
CTGAGTAAGAAGGTTCAAGGAACAATAAGAATATTGCCAATAATCTGAGTG
AGTTATGCTGATCACAAGTGAGATCATGGGCTCTTGATTATTATTCAGCAAG
TTTCGCTAATTTCAGTTATATAGATATCGGGATAATATGTCTCATTAAAAGCAT
TTAGTTGATGAAAATAGAATGGTTACACAGAGTGGCCAGCTAAATGAATGG
TGCCGCGTAAATGTCTTGGCATGGGGGCGTGTTTAATTGATGATTTCTAGCA
CCATTTGACCATTTGGTGAGTTTAAGATTCCCTATTGATGCGAAGAGAAAA
GACATAATATTTTTCGCATGAATAATCAATCAATTGAAAGGAATAACTTTTAT
CTTGTATTCTCAGCATCAGTTGCATGCCAGAATATAGCAAAATTCAAATTGA
TCATCTGTAAACTCCCCTTGGGCGCTGCGAGCATTAGGAACGCCGTCAGAT
TCTAATCGGAAATTAGGACTTGCTGTCTTCAATCTGTCCTAAAATAGAATTA
GACCTGCCTATTTTGGGAATCACGATGTGGGTGCAATTACAAGTAGCAGCA
GCTAAGCAACCTGCAAAATCGATAGCCTTCACCACCTAGGCAGACTAGAAT
GCAAGTAACGTAGCGAGCCCCTTTTAGCTTAGATGCCACTTGCCCGACTAG
ACAATCCCTTACGATATACTTTACCTACCCAGGAACCAGCGAATGGTGGTTA
GGCGTTCAGGCTGTCAGGTAGGCTATAAGATGGCCTATTTTCGTCCCATCGC
CATAATGAATGCTTAATAAACAACTTATTAATTCATATGGGGCCAAGATCCGT
CGCAATATAATATTTGCAGAATGACTTCAGCTGACAATATTCCCATATGCCTG
GCTTCTAGCTACATGTGCTGACTATGGCGGAAAGTGTAAAGGGGGCCATCA
TAGAGGGACGATTGTAAGTTCCAATGCGGCTGTTAAGTTAGGAGGATTGAT

ACAGTAAGAGCCACTACCGAAATAATATTTCGGCTTCAACTGCCGTGACCT
TTCTATACTAGCTAGCCATTCGGATTTACTTCCACTCTTAATATGGCCGAAAC
GGCAGAAAATTCGCCATGAAGTTGAAAATAATCTGACTGCCTGCCTGTAGC
GACTCACGATACTGCTGGCGTCATCATAACCAGCTGTCCTTGCTAGCATGAA
CCCAAGCTCATAAGCTAATTAGAAAATGCTACCAGCCTCGTTCTCGATGTTA
CCCCGCCTGCTTAAATATATATAACACCAAGAGCCCTATACCGTAAGGCCAG
TATTTTGGTTAAGTCTTCGGATATCGCCACGGATTGATGTTTTGACGGAGAA
TCGATATTCAGTATGATCAAATTCGGGCTTCTCGCTGCTGCCCTGCTGGGGG
GAGCGTCGTGCTCGGCAATTCCCAAGGACAATGAAACTGGCGACAACCTC
GGAAAACGGGCTACCAGCTTCTGGTATGCCAATATGGACCATACCGGCACG
GCTCGCGGATATGCTCCAGACTTGGGGACCGACACATACCCAGTGTATATG
GCCGTCAAGGCCGGTGACGGTGCTGGAATACAGAACGCCATCAACGATGC
AGGTGATGGCCAAAAACGACACGGCGAGTGGTTGGCATCTCAACCGAGGG
TATGATTCGTGTAACTAAAATTATCGAGCCTATAACTAAGAACTAAGTAGGT
CGTGTATATACCTCCTGGAACGTACGAAGTCAGCTCGACAATTGAGATGAA
TACGGACACTATTTTGATGGGTGACGCTACAGATGTTAGTGGTGTATAAATG
ACGGTTTGACTCTGCACTCGCTAATACTGTCAATAGCCACCAACCATCAAA
GCTGCCGCTGGTTTCTCAGGAGATCGACTACTTTCTGGTAAATCTCCCTCAC
TCTAAGCTCAGAATCATCGCATCTAACTGTAATTTAGGACAGGATCCAGCGA
CAGGAGAATCTGGAGAACTTTCATTTGCGGTTGGATTAAAGAATTTGATTCT
TGATACCACCAACATCTCAGGGGGTAAGATTTGACATATTATGTATGCCTAG
ATCATCCATATCTTCTAACCTGTGTCAGGCACTGCATTCACTGCACTATGGT
GGGGCGTGGCCCAAGCTGCGCAGCTCCAAAATGTCAAGATTGTGATGGCG
CCATCTAGCAACGGTAATGGACACACGGGTATCCAACTTGGGAGAGGTTCT
ACTCTTGGACTCGCTGATGTCCGCGTTGAACACGGTCAGGTAAGCTTTCAA
ATGTTTCGCATTTTCGACGCGACAGTGGAAATACTAATTCGCAAAAGAATG
GTATCTGGCACAATGGACATCAGCAAGCACTCTACAAGGGAATCTACTTCT
TCCAAAACACCATCGGCATGCTGATCAACGGTGGAAACACAATCAGCATCA
TTGGCGCAACGTTTGACACTGTTGGAACTGCTGTTCGACATGATGGCGGGT
CTCCCTGGATTGCGCTGATTGACGCAACATCTAAGAACTCGGGCGTAACGT
TCGCGACATCAGGTAAGCAAACGTATCGCCCATGACTATGAAGGATCGGAC
CGGACCGACTTGTGCGACTGATCTGGATCCATAAAATCCCGTTCGCCAACC
GCGTCGGTTGATGTCTCACTCAACGGTTTGGAGGGATGGTGGTCTGCGGCT
CGGCTAACCTAGTGACAGGCTATCCCTCATTCATAATCGAGAACCTAACCAA
GGACACCAATTCGGATGTAGCACAAGTTCCAGGGAAAACGGCTCTTGGCG
CCCAGTCTCACGTGGATACCTTCACCTATGGTAATACCGTAGGGAACAACC
CCGTGTACGCTGCCATAACATCTCCGTTGAACCGACCCTCTATCTTAGCGCC
AAACGGCAAGTATCCCATAAATGCTGCGCCTAACTATGCGAACAATCCCGT

GTCGGACTTCATCAACATCAAGGATCCGGCTCAGAACGGCGGTCACACTGT
CTTGGGTGATCACACCAAGGATGAATCAGGCGTTTTGAACCAGGTCTTGCA
ATATGCGGCGAGCAACAACAAGATCGCATATTTTCCATTTGGCAAGTACAG
GGTCGACGATACTCTCGTGATTCCGAAGGGTTCGCGGATTGTCGGTGAAGC
GTGGGCGACTATTACTGGTAATGGCGATAAGTTCAAGGATGCCTCGAATCC
GGTCCCGGTCGTGTCTGTTGGCAAGGACGGTGATGTCGGTGTCGCTCAGAT
TCAGGATATGCGGTTTACAGTTTCCGACGTGCTCCCTGGTGCCATTATCTTG
CAATTTAACATTGCGGGCTCAGCGCCGGGCGACGTGGCATTGTGGAACTCC
CTAGTCACCGTTGGTGGCACGCTTGGGGCATCGGCGCTCGGAAATGCTTGC
GGAGATGCCAGCAACGAGTGTCAAGCAGCTTTTATCGGAATCCATCTCACT
AAAGACTCGTCAGTCTTGATCGAGAACACGTGGGTGTGGGTTGCCGACCA
TTTTACCGAAGGTAGCGGTGGCTGCAACATTGCCGCCAAAGGTGGCGTTCT
CGTCGAATCCCAGAAAGGAACCTGGCTCCACGCGCTCGGAAGCGAGCATT
GGTGGTTGTACCAACTAAATTTGAAAAATGCGGAAAACGTTATGGTTAGCA
TGCTCCAGTCGGAAACCAACTACGACCAAGGTGACAACACGAGACAAAC
GGTGCCGGCCCCTTGGACAGCTGATGTGAACAACTGGGGAGACCCAGATT
TTTCATGGTGCGGAGGTGGAGACAACCGGTGCCGAATGGGATTTGCCAAC
TACATTCAGGGCGGCTCGAACATTTACACTTACGCGTCTGCATCTTGGGCGT
TTTTCAGTGGGCCAGGATACCAAGGCTGCTCGGGAGATTGTCAAGGTGAGT
CGATACAGAAAGAAAAGGGAAAATAGAATCTCGTTCCTAGATCAGATGCTT
ACTATTATGACAGATACCATGCATTGGATTTCCCAGACGCCTACGAATCTCA
AATCGTATGGCTGGTGCTCGAAAAGCACTACAACCACGCTCCACCTCGGGG
ATGGGACCAAAATTAACACCCAACCAGACTTCACTGGGTCCTGGGGAGGG
TTGGTGGGCCGCTACACGCCGTAAGGGCATGGCTTCGGCTCCATTTCCAAC
TCCACGTGCATTTTTATGACGACATTGCTGTACTAGTATGCATTCCTTCAAA
GTAGCGGATAGTTAATAGGATATAGATGTTTCATGATAAGCATGTGATTGGAT
TTGAACCTACATTATCTCCCTGTTGTGCAATGGTTCGTGATACCTTCATACTC
TGTTCTATTGGTGTTTAATAATACCAGATCGGTCTTGCGCGTCGACACGGCA
TACCCAACTTAGAGTGTCCCGCCAGAGTGGTTATTAAGTTTCGAGTATACCC
TTCTCCGCGTAAATCAGGAGTTGCAATGAAATACCCCGTGGAGAAGCCCAT
ATTTTTTTAATATTAATAAAGTGTACCCTCAAAATTCTTGAGTTCCAATCACT
AGTTCATTATAAGTAAGGTGGTCTATACTCTTGGAAAATTCTGTTTGCCTAG
TTTTTTTCCTGTTTCAAGGATTTGAAGGATTTCAACTTTCGAACTAGTATATC
CAATTTTTTTGCTACTAATTTATTTCCCAGGGACGAGAAAAATTCGCAAACA
ACTCAACTCAGTCAGATGCACGATGACCAACATTGCCTTACATGGTACATG
TACTATAATGTACAGGATCCAATGCAAGGTGTCGGCCGAAAGACACGAGGT
ATCAAGCCATCTCCAATCCATCTCCAACACATCCATCCACAATCTTGAAATA
ATTATTCAGCCGCATCAAAATACTAACTCCGGGTTACCTAAAGAGCGTAGG

GCTCGTATTCTCGTGGCTTGAAATTTTCTTGTTTGGCTTTGTTGAAATTGGC
AATTGAAAGCCCACAATCCTTCTCCGGCTCTGCTTACCACCATTTTCTTTTT
TTCTTCAGCCTTCAACAGCAACACGAGTTCTTTATTGGCCCGAGGCGAGTT
GGCGCGTTGCGTGTGTGATTGCGAAATCGCCCCCGACCGGGACTCGAGATT
GGGTCTTTGTCAGTTCTCATGTGATATGTGATACGCTTTGAATAGTTTGTTAC
AGTCGAAAATCTTTTTTGGCTGTGCATTTTCCAACGAGGTAAGACCTTGGA
ATATCTCACCGAACTCTGGCCGTGCAATTGGGTCCCTAAATGACGATAGTGG
GTCTCGGAAGGGGGGAGTGGAAATAAACCTCCACTAAATGACCCATATTTG
AGCCCCACGATATGCCGTTCAGCGGGGCAACGGGGTCAGCGGCTCGAGGC
GAGCTGGGTGTTGAAGGATGCGAATTTTTTGGGGGCTTTGGGGGTGCCCCG
GCGCCGTTGGGGAGACGCCTTGAACACCCTTTGGTCCCCTAATCCCCGCGC
TGATGCCGCGTAGGTGGGAAGAGACTCCCGAAGTACGAGGGTATTGTCGG
GAGCGGATGAGACATTAGATAGCCGGTCCTACCCATCTAGGAAACCCGACA
TATTCCATGTCCTGCATGTCTTACAGGTTATTCTGTTAGGTAAAGATTTACGT
ACTATACATAGGGATGTTATGTAGTATACAAGTATACCGTACTTGAAAACAC
ATGGGAACTGGGAGACCGGACGGCTCAGGGTCTTTGATCCAGCGTGCCAT
CCTGTCTGCCTGTCTTAAATTATTTGTTCCTTAGCTGGCACACGAATCTAAC
GTGCGTGTCTTGGGACCTTTCTTCACTTGCGGATGCTAGTGGTAGTTTTATA
CTAGGTGCATAAGCTTTCTTTGGGATCGTCGACGCCGTCGCCCTTACTTC

>Mosaic_sequence_09

ACCGCAATATAGCCACAAGTCTTCGAACGTTCCCCCTTCATCGGCTACGGC
GTTCTGATGCGTTCTATACAGGTGTTTCACCAAGGGTCTCATCAGTCGGGTT
AGGTCCGGCTGCGACTGAGTGCCGGCGAGATATTCGTCGATCTGAGCGACA
TTTTGCCTGCAGCCTTCAACGTTGCGATAGACCTTGCGCCAAGCGTGGCGT
TGCTCGCGCAAAGCTCGCGAATGACGGATGACATTTTTGCTTGACTCAATC
GTTTGGTTCATCTGTTCGGTGCATTTGTACATCGCACACAAAGTCCTCAACG
CTTCTATGAGGACATCGCCCGTGAGATCGTGGTCGATCGAGAGGGAGTCTT
TCAATTTTAGGCCCTTCTCGCCTCCAGCGGTAGCAGTAATGCGGAGGGACA
TGTTGTGGCTCAGAGGTATGAAGGGGCCAGTGGTGTACTCGGTTGGGGGAT
TGTACGGCTCTTGGCGAGCAAAATCGTCAGTCACAGAGTAGGCGATCGTAG
CCGAGTCAAAGGTCTGGGCAGTGGTGTGTGTCCCAGCGTTGGTCCAGGTC
GCTCCTGTTGTCCAGTCATGCGTATCTAGCGTGCCAGAAGTGCTGAGTGCG
CTTTGTGGTGCAACCGGCTGCACCCAATCCCCCCCGGCAGTCCAGTGCGTG
ACCACGTCATCCTGTGCAGCATTTGAGCCGTCGTTTCCGCCCATGACGTCC
GGGGGATACGCAGCGAAGTCATCCGGTTGCGGTATTCTGTCCAGAAACATG
GCGCTCGTCATTTGCATCTGGTTGGCAAACGGATCGTCCGCAGCGGGATTC
GCCTGAGTGGAATAGTCCAGGAATGCCTCCTCGAACTCACCCTTATCGTCA

AGCGACATGGCTTGCATCGCCTCCGTCGTTTCATTGTACTCCGTGTCGTCCA
TCTTCCAGCAATAGTGCACCTGATCGCTTGAAATAAGATTGCCGGAATGCTC
TTGAAAAGTCAAGCGCAATGAGTCGTTTGCATTTGGAGGACGGGGTCTTAA
GTACTTCAGGCTAAAAGCGCACCGATTGAAATGCGAGGTGCAGATTGGCG
CTCAGAAATCCTCAGAAACACGAAGATGTAGCGGTGCTCGAACGGCTCGG
CAAAGGACGGCCTTGGGCGCATGTGCAGGCCAGTCGCCGTGAGCGTCACA
TATGCCGAGCTGAGATGTGTCATTCAGAGTGCGGTGCGGAAACGTCAAAC
GATGAACGTAAGCGAGGCGACTTTAGCCCCTGTAAGCGATCAGACGGGAA
GATTGGCCCTGCGTTAACATGTCTTACAGATCTTTGCAAATTTCCACTGTTT
TCGAGTATCTGGTTCAACTCGCGAAACTAGTTACCGTCGTGCGGGTCACAG
GAACACCTGAGGCCGTACTGCTTCCACAATACGTCAAACGCCACCTCCTGA
TCGGCAATGACCTGCTGGTGCGTCTCATACAAGTGCTCCACCAGCGGGATC
AACAACGCCCTCGTACTCGCTTCCGAGCGAGAGCTGTTGCCCGTTCGCCGC
AACCGCCTGTAGTTATCACGGCAAGACTTCAGCCCCTGGTACGCGGCGCGC
CAAGAGAAGACGGCTGTCCGGGCGTCGGGGTCGTCCTCGGCCGGCATCCC
GCCAGTGTGGAGGTTGTTCTCGAGCGTCCGGTTCATAAACCGTGCGCAGTC
GTACATTTTCAAGGTAGTGTTCACGGTGTCCGCAAGGTCCTGGCCCGTGAT
GGCGTATTCTTCTTGCTTGTCATCAAACCAGGCCAGGTCGAGCTGCCCGTC
AGGGCCGAGCTTGGTCCGCAGCCAGTAGTATTCGTCTACCGGTACCCATAC
GCCAGGCTTGACGTTGGGTGGCGGCTGATACGTGGTTCGGGAGACCTTGAT
ACCGGTTGTCGAGCCAAGGAGTGTCCCAGTCTCGCTCCTTGAGCCGCCGC
TATACATGGCGTGGTCTGTAGGTGGGATATGAGTATGGTGCCCAGGCTGCTG
TCGACATCACCAGCGTCATCGTCGGCTTGGACCGTGTACGCAGACGTCCAG
TCGCCTCGGATGGTCGACTCGTCATCGCCGTCCCCGCTGTAAGTGCTCTGT
GCCACGACATGTGCGCTCATCTGCTCTGTCCGCGGCCATCCGTTTGGGAAC
ATGGCATTCGCTACGCGGTCCATGGCGTTATCCATCTGCAGCTCATCGCTGT
CGGCATATTGACCTGAGTTATGATACGCGTCCGAGAAGGAGTCCTCGTACC
CGTTTAGGTCCATGACATCCATAGCCTCGGTGAGATCAACAGTATCATGGTC
AGCCATATCGAGGTAGGGTACGTAAGAAGACTCGATGGTATTTCTGTTCGGT
ATGGAGGGGCTACCCTCTGATGCAAAGCACATTTACAGTACTGTACATGCAT
ATCGGTTGTCGACATCGCACGCAATACATGTGTGCTGCTACATACCTTTGTC
CCCGAGGAGATGACCGCTAAATGCAGACCCCATCTGGCGCCATGAATTGGA
AGGCAACCACACACACAGATGATATGGACCCCACTCCAGCCTCAGTGAAACG
GCTTGGTCAAAGGAGGAGTGCCCAGGCGCATTATGAGATGCCGTGCCGGC
CTGACGTGCGGGAAACGGTGGCCACAATGGCCCTTGAGGTTGTCACATCA
AGAGGAAAATTCAGACTGACAGACACGGCCTATATCTGCGCGCCTGCTCAC
GATGGATCAATCTCGGGAGCTTGTGGAAGCATTGCACAAAGTCCCAGAGG
TGTCCAAATCAGAGAGGATTGTATGCTCAACTGATCATCAAATCCAAGCTC

ACACCTCATGTGCCTTCTATGGAATGACGCACGGTTCTACTTGGGTGTCCCA
TTCCTTGTATTTTCGGGACTGACGCACTTCCAGATGAAAGTCGCAATCGAA
AGAGTGCCATCTTATAAACACCCTCTCAGACATGGTCACCACCCTTATGTGA
CCTGCAGCAACTGACATCTTTCGGGTGCTTGGGTCAGACTAGATCCTGCCA
GCAGCTCATATACCTATTCGCCCATCTTCACATATGGCCAGGTGTCTCATATA
GTGATCACGGAAATCGAGAACTTAGACGAGGAGCCTTATTACGCACGTTTG
GTCTGGGCGCCATAATAGGTGAAATGAAGAGGCGATAAAGTGGTGCATTGA
AAAGATGAAAATCATGCTTTTCCCCTGTAAATAGTTACAAGACTCAGGGCA
TCCTGTACCTAAGATATGATACCTGCTAGGACAATGACTTGCCCGCTATGAT
AAAATATAAGTCGCATTGGCAGTTATTGTTCCTTTATCACAGTTAAAGTATCT
CAGCATTGTAAAAAGAGAATGATACCTGCTGAGAAATATATATATCTACCTA
AATTGTCTTCTCCCGGTGCATTGTTCTTTTCGTCTCGTATCCTCCAATGTATT
TCCCCCAATTCCAGAAACCAACCGCCAACTAGCCTCACTACGGTGTACCTG
TAATAATGGGGGTGCTATTCAGCAAACCTAAACGCAGGGGCAGGTATTCAG
GCCGCCCACGGCGCTCAAGGCATCACACACATCATTCACGCCATACAAGAC
CCAGGCATCATGAGCAGTCTCATCGTTCAAGAACAAATAGGCGCATAGAGA
GATGGATAATGTCAACCCCGGGCTAGGCACCCGCCCACTTCTGGATTAGTC
GCAATGACTCGGTGGTTAGGTAGCGATGGTAGCGACAGGAGAAAAGGGAA
TGGCGCGGTCACTCGCTAGTTACTACCTAGCACCTAGGGCCTAGGTAAGAG
AATATAGTACTTGCATGCGGCGAAGCATCAGCATCTTATATAATATGAGCTAG
ATGACCAGAGGAAATCTTCGCGTTCGTCTAGGACTTTTCTTGTGATTAATAT
TTATCAATGTGATCCTTTGAATTTCGATCTATGTTATATCTTAAATGCCCGTAG
GGAATTATCCGCTCTCACAATGTCTATACCAATTCATAAGCTGATCAATCCTG
ACCCGCCTTCAGCGACTATCACAAACTTACCTGTTGAACTTCTCAGCTCCAT
TGTGTCTTACCTACCTAATCGCGATATCAAGAACTTACGGCAAACATGCAAA
TTCTTGCACAGGATAGCTCACCTCCGCTTGAGTAGAATTTTTCTATCCCCCA
ATCCTCGCAACCTCGAGGTCTTCCGCGCCATCGCGGATCATGAAACCTTCC
GCAAGCAAATCGTAGAGATCATCTACGACGACGCGCGCCTGGCTCAACCA
CCCCGTCTCCCACAACCTGGGACTTATGAGTACGACTCCAACGACGCTTAC
GAGGAAGAAGAAAGCCTCGAAACTCACGGAGCTCCGTCGTGGTTTGTAAA
GGCGTGCGAGGAAAATGCGTTTTTGCTGAATTCACGGCGCAATATCTATAG
CGAGATCGAAACTCCTGTGCACGCTGCGCGGGCTAAGCAGCTTGAGGCCC
AATTACCATTTAAGGAGTCGTGGGACCATTACCAAGATTTACTGCTACAGCA
AGACGATATCTTGCGGGTTGGGAGCGATATTGAGGCGTTTAGGTATGGGTTT
AAAACGCTTTCCTGCGCTGAAAAGAGTCACTGTTACGCCGGCGGCTCATG
GCTATTTGTACACTCCATTGTACGAAACGCCGATGATCCGCTCGCTTCCGTA
TGGATTCAATTGCCCGACCCCACGGGGTTGGCCGTGTGTGGGCGACGGAG
AGATAATGCCGGAGGCCGAGGAGTGGGACGATGAAGCGTACAAAAACAAC

TGGCGGGGGTTTCGCGAGATAATTCGCGAACTCGCGCGTGGAGAGCACAA
CGTCATTGAGCTTCTAATCGATGCCCATCTCCTGCAGACTGGACTTAATTGC
CGTATCTTCGACGAGCCGTGTGATGAGTACAACGACTTCATCAACATAATCC
GCAGACCCGGCTTCCGCCGCCTCGATCTCGACTTGCTTGTTGGCGCACAAG
AGCACTTCGGATGGCCTTCTTTCCGCCACGGCTACTTGCGTCCGAGCATTG
ACCGAGGCGGGAGGTTTGGAAAGTATTACTTTGCGGACGAATGTCGTCCAC
GACCCTGATGCCCGGGCTACGGTACGGGGGAGCGCGGGGTCAATCGAACA
TCACATCCCGCTAAACACTATCTTCCCCGTCGAAAAGTGGCCTCGTCTACA
GCACTTTGGACTAAAAAACTTCTTGGTCACGCAAGAGGACGTTCTATCCTT
CCTCGCCTCGCTGCCACCAACTCTCCGAACGGTGGAATTGAGCTTCCTTGG
TTTCTTGGATACCGGTGGGAGCTACAAGACGCTGCTCGAAGGCATGCGGG
ATACGCTAGGATGGCAAGATAGACACGAAGATTTGCGACCGAGAGTCGCG
ATTTGCATCGAAGCGATAAACGATCAATATAGTGGGCGTTTTATAGTCATCG
GCAAGGAGGTTGACGACTTTTTATATTGTGGCGCCGAGAATCCCTTCGGGC
GCGGAGGATGGGCCTCAATCAGATCGCGGAAGGAATAGGGATGGTGCGTG
ATCTGTTCGACCCGGCTTTTGATAGACCTTGGGTTGAGTGGGAGGAGTATC
AGAGGTTAGGGATATATATAATACATCCTTGGAGCATGAAAAAGAACGCGG
TACAGGCTACGGAGTAGATAGAGGTGGGCAAAACGAGGTTGTATTGTGAAT
GATCGATGGATGTCTTGTATTTACTGGAGGTTCACTGGGTTTAGCCCTAGAA
TTCAGGGAGTTTAGCGCGATTCTAGCCCTGGAAATTCTTCCATGTACCCAGT
AATTTAGGGCAAGGCATCACTAAAAAGCACTGAGTGTGCTAAAACAATTGG
TGGATGTTAACATTGAATTAATCTATGTACACACTACCCCTATATAAAAATCG
CCCGTATATATACATCAGAGGCACTACATCTAGCATTTAGTAAAATATTCATT
ATGAATACGGTTAAAATGGTTGCTCACCTGTGAAAATGTTTCATTTTGGTAC
GTGCAGGTCAGCGACGACGCGCACTGGAAGCTGCCCGTCCAATTGACCCC
GCCGCACTGCGCAGATTGCAGTTGCGCCTCCGGCAGCTCCAAGCTAAGGAT
TCTTTCAGACGCGTGCTCGGCGGCGATGACAATGTACGCAGAAGGATTGGT
GGTGATATGACCGGGAAAGATGCTCGCGTCGACAACAAAAAGATTATCCGT
GCCATAGACCTTGGTATCGAGATCTACCACGGCCGAACCGTTTGCTCGGCC
ATCGTCTGTTCCAAGTTTATTGGTTCCTAAAGATAATTAGAGATGTTGCTGG
CGCAATTGAAGAACGGCAGGAACTTACCAATCCAATGGTTTGAGCGCCGG
TTGGTGTACGAAACAAGCATCTGAGATCATTAGCTTGGTAATCCTTCAAAC
ATGTAGTAGTATGCTTTACATCATTAACAAAGTCTTCGACCGTCGTGTTGGC
CGATGGATAAGTCCAAGTAAGGTTCTGAACATTTGAAAGAGCTGCCTGAAG
GTTCTTGATCCCCTGGATTACAGCTTGGACGTCGTTCTCGTCGTGTAGATAG
GGCAGCGTGGAGACCACAGTGGTCAAAGCCTTCGTGATAGTCATTCGACC
CTCGATGTGGCACCGCGTCCAAGATATTGACTCATGGTCATAGCGGCTGTTT
CAGTTAGTGTGAGGTATATATTGTTAAGAATTGGAATGCCTTACTTCCACTA

GGTGTACCATCACTCCCTTCAACCCTGGCAGTCCACTGAAGCTGTCGAACA
ATTCCATCATCGCCCGTGATTTCATCCCAAAACCTACATCCCGTTAGAAAAC
GTGTAATAGCCCCGAGAGTAAATTCTTACATTGGGCCAATGTTTGGTGCTGC
TTGTGCGAGCATACCCGATCGAGAATCTTTCGCCTAAGATTAGTACGTTAAA
GTGTAAAGGGTCGTCTATAGCTTACCTATATATTGGTCCTTGTCCGTAGTGTT
GGAGAGTCCCAAGCTCCGTAATAATCGTAGAAAACCACATCGGGGTGCGTG
ATAACGGTGTCGGTATTTGTATGATCTTCCAGATTGTACCCAACGGGTAAGT
CAATCCAAGAAGTGTTGCCAATCATCGTTGGCCCATCGGTTGATGATTGGA
CGACATCCAACTGGTCTGATGGGCCGATTCCACCTGAAAAAGTTAGAAACT
AATATCTAACAACAGTTCTCGGTACCCTTAGAATGCGAGTCGCTTACTTCTT
AACAATATTTTGGCTGACCCGAACGTTCCTGCAGACAATATGACCCGGCCT
GTAACTGATGTGACATTGACAACACCCGTATAACCACCATCTAGAAACGGT
TCAACCTCAATGCCAGTAATGTGGCCGCCAGTTCGGACAACTCTCTTTACT
GCCGTGTTCATCCACAGGTTGAAATTCTTGCGTCCACTGGCAGACACTAAG
TATGTGGCCATAGGTCCACCTCTTTCTCCGCCGGAGAACATAAACGGCGTG
TGGGAATAAGTGTGGTTTTTCTCATCAGGTACGTCGTTTGCAGTGACTTCA
GTCCATCCGGCGGTTTTTAATCCGCCACTAAGGACGTCAAACCCTTGTTGAT
AATACAGCTTGCCATCCATGGATGGACTGTACGTGCCTGGGATTCGAGGAA
AAAACTCGTGTGGTCGCAGCAGCCATGTCTGCTGCTTGCCACCCTGCAGG
AAAGTTATAGTCCCAGTCCAAAGTATATGGCTACGTCAAATAGTTAGCACTT
TTCAGGAAAACATAGCTGGGGATCGCATACCCTCCACCATAACCCCGAATT
GACAGCCGTACCTCCACCTAGCACGCAACCTGCCATTTGATCCGTGTCGGT
GCAAGTAATGCCAGTTGAGTCTACCCATATTTGGTTACATAGACCGGGAAC
ATCGAATCGAGTCAAGTTAGTGTCATCTAACCACTCTGGCCTAAGCGACCC
ACCCCATCGCCCCGAGGATGGAGGCCCCTTCTCAATTAGCAAGACACTGTT
TCCAGCCTCGCTCAGTTTATCGGCTAGGGGGATCCCGCCTGCTCCTGCGCC
AACTACGACATAATCATAAGTAGCTGTCGGAACGGGTACGCCTGTAGCGCT
GGTAGTAGTAGTTGTGCTTACAGATGTAGTAGTCGTGGGCGTTGCAGTCGA
ACAACTACCAGTAACGGTAGCAGTAGCTTGAGAAACCCATTCAGTATAAGA
TGGGTTCGCAGCATTCTGATCCAGAGTCGCGCTCCACAGACTATCGCCATT
ACTGTGGAATTCGAGGCCAATGTCGGCTGGGCAAGCTGGGTTTGTTGGAG
CTTTGAGCGAACTTGCCCAGCCCAGTAGCAATGCGCCACTACTAGTACCTG
TCGCAGCACCTGTCGCCCCATCTTGGTCCCAGGCAAAAACAATCTTGACAT
CTGAAGATGACACTGAAATGATCCTCATCAATGGTAGACGATATTTGGGTCA
AGGTAGCGTTGCCAGTGTAAACATCTGGAGCGCTGTATCCACTCGCGAAAC
GAAAAGACGTTAATACTTGGTCCTCGTATGGCCAAGCCATGACAAGTAAGC
TGTTTGTCATCGTGCCACCAAAGCTTATGCCACACCACTATAAGAAACAGG
GGTTAGCAACTCCTTATTGAGGGGGGTATATTTGACACATGCTCGGGGTCCA

AGAGAGCGGAGAATATGCTAGAAAAGGGAGAAGGCGACTTTGAATGTGAC
ATACAGCATTAGTGGTTGTACCATTTGAACCGCATTTCTGCTAGATGGTCAG
TATATCGGCGGGGAATGGTCAATGAAGTGCACTGACTAAGTATCCTATCAGC
TCAGTAGCATTGGCTATCAAGGCATCCTCGGGGAGAGCGACGCCGAAGCT
GAGTCCACCCAAAACTGAGACACTGCCGCCTCCATCTGCGCTATCCGGTAC
GGTCCATAAATCGAATGTTATACCAGTGCTGTTGTCTGTAAAAGCGGCCGG
CGTGCTATTCTGGCATAGACACTGCTGGGTAACTAAGCTGAGTTAGCAAGA
ATCTGTGAGACATTTTGAGAATAACTCACGTAAGACTACTAGAAGCAACAA
TGCATTTGCTAGAGTGCTGAAAGGAGCCATGATGAAGGGGTTTATCAACTT
CACTTCACTTGAGTACAGGTACACAGATGGAAAACAAGACACTTTGATTTC
AGAAAGATAAAGTTGGGAACTCGATTTTTATATGATCAAATACCCAAGAAA
CGAAGCTGACAAGCATCACTAGGCTGGTGAAAATGACGCCGAACGTAACT
TAGCTAAACGCTCGAGTATGCACTCATGATGGCCAAAGTCACCCTACGTCA
TATCCCATACATACTTAGTAGAATAGGGGAATGCCATGCTCCCCATGTTCGT
GGTCGTATTATAATGGTTTCCTTGCATTATCACAAGTAGGCTTTCATTAGAAG
CTATACAAAAAAAGGCCGAGTAATCATAAGCTCCATTAGGGGCGGCTCTTAT
TCCTGGAACCGGAGTTGGAGAACTACGCTGGACCCTGGATGTTCGATGTGA
TTCCGGTCCAGCATCGGGCGGGCTGTACTCGGCTCCCGTATATGCTGACAA
ACTAGTAAAAGGAATGATTCCGTGTGGACGGATTGATGAGTTGTTATAGGA
AATATACCCCTCGTGACCATAAGATCATCGAGAAGACCCTGACGTAGATCGT
GCGGACGGACTCATGGGCCTTGAGGTTTGACCGAGTACACCCTGGCCATG
ACCAACCTGCCACTTAGGTCGACGGAGTCTCGGCGGAGTTCTTAAGAAAC
GGATATGTCGATGTGTTTCACGGACCGAAAATGAGCGATCTTATCTGTTTTC
AGGTTATGTTGTGAAGTTTTTGCCCCGGCTCTTCCCCGCATTCAGATGCACC
ACTTGGAGTTGGGAGATTAATAATTTTAGTATTTTGTAGTGAACAGGAAGCG
TCAGGGGTTCACATGGATGTAACCAATGTCATCTCTTAAGCCACAGCCACA
GTGATCATTAGCCACTTGTCTAGTACGTCAAGCCACGACACGTCCCACAAG
TATGATGTAAGCAGAGGACCAGTCCTCATTAATTCACTTTCTTACGACGCAA
GGCCCTCCGACCTTTAACCCTCTACAATTCAATCACTTGGATGCTTGGGTAG
AACTACATCCCCCCATACACGACTATTTTATCATATAATTGATCTATACAGGA
ATTCTCGCCACCAACCGTCGAGTGGATATACTGAAATAGATCCCGGTCGAC
GAGTCATCCGTATCTGACAATCGACCAATCAACGTTGTCTATTCCTATTACAT
AATCATAGGGGTAGATGATACTTAGTACCTTTGATAATGTCTTTAATAGTACA
TTATCCTTAAATGGACTTGATCTTGACTTTAATCGTTACGAACACAGACCTT
TTGGTGCTCGCGAAATCATGGTCAATCGAATTATGGAAGAATTCATGGCACC
AAAACGAGTGGAGGCTGAAGTCTATAATAGGAGAAGTGGTACCTACGAGG
AGCTATGGAAACAAATCAAATCCTTGGACAAGCGCCTAGGCCTTGATGTTC
AAGTTTTATTTAAATTGCTACGGACATACCACTGCTGGCCACCAAGAATAAC

GCTTACACAGGAATACTCTACGTTTCTAAGCGTAATCCTCATCTGGCCCTAC
GTTCAGACTCGAACTGATTTCGACAGTGCAATATTAGGGCTAGTTGAACGA
CACTACCTCGAATGTCACCCTTCCTTGAGAATTATTCTTAGGTTTTTTCAGTC
ATGGTCGACTAATCAAGGACCTAAAGAGCAATCAGGTTGGAAGAAGGACA
TGATCGAGCATTTAAAAGAGTTTCAATCAGCGAAGGAAACACAATATTTAC
ACCACTTATATTCTAAGAGTCACTACCTACCTCCTGGTCCTACTGGTGCCCA
ATTCTCAATCGAGCCTTGTTTTGTCGTTGCATGGAAGTTGAGCTACACATCG
GCTAGGAAAACAATATTTGAGAACATGCCAAAAGTACATCGAGGGAGATCA
CAACGTTCCAAAAGTTTACCTCCAGATGGCGCGTGGGGAACAGCTGCGAT
CAAAAACTACAATCCCGAGGAGAAATTCGTCATATATTTCCCTCGTCGTCGA
TTATCTGAGCTAGACAAGCTTTCACGTCGGAGGAGTCTGAGTCGAACGCAT
ATCAAAGAAATGTTTACAGACTTTGCACCGAAATTATACTACAAAAAGCCG
ACAGGGAAAGATACTTTAAAAAACAAGTGCGAAAACTGCGCCCAGTATAC
TCATCGTACGAAGGATTGCAAAGCGCGCTGCGGCTTTTGTGGGTTGTCATT
TCATAAAGCGAGCGGCTGTAAAGTCAAATCATCTAACCGCTGTAAATGTCA
ACCTTTTCCGCAATTCCATACGGCTCAGGAATGTTACGTGCGATGCTCTCGC
GCCTGCGGAAGTCCCTATCACCCCGGCCATTTTAAACACAAAAACGCCATG
ACTTGTTCTTATCGTTGTTGTATGTGTGGTATCAAAGGACATTCGGGTAGAA
AATGCTCGTTTAAGAAATGTCCTTGCGGGGAGCGACATTTGACCCAAGACT
GCCGTTGGAAGGTGGAATGTCCTGCTAAAGGCTGTCATTACTATCTATGTTC
CTTACATTGCAGAGAATGCGGAAAAAAGAAGGAAAAGGGAACTCAGGAC
AGTTTTGTAGGTAGGACGTGCCAGGATTGCTTGAAGAATGGCAAGCCAGC
CTCGGTCAGAGTTTAGCTAGACCACCAAAAACTGCCAAAAGCTCGATACTA
CATCAGAATTTCCAGGACTTTATCATAGTATGATACACGGTGTTGATAATATC
TCCATATATAAGATCTTCAATCTCAACGAAGAAGTGTGGTCTGTCAAGCGTT
GATTAAAATACACCGCAACCCCTCTTTAGCTCTCAATCAAATTGCGTAGTGT
CATAGTCCAAAACAAGGAGAACAAGCGAGGAACTAGATGATTGACACAAC
CCAATAGAGCCAGAATCATTCTTAAAATTGAAAAAGACGTCTACTTAATTCC
AATATCAAACTGTAATTCGTAATGAATAAGTGGGTTTAAAAAAAGCGCACA
GGGATAGGATGTATGAAAGTGAAAATGGGGGTTAGGGTTAAAGGAAGGTT
AGGGTTAGGTAGGGTCATGTCAAGAGATAACTTCAAATAGAATAGAACTTG
AAAGAAGTAACAAGTTTAGAAACGTCTGATACTCTATCAAATATTCAATAGA
AAAGATGTGCATGTGTTATTATTTCTCAAAAATTGAGGTAGGAACATCTTGT
GGTCGTCTTCAGAAACCCTAACCCTAGCCCCAGACTCTATCACAACCCTAA
CCCTCAATGCACTCTGAGATATGGTACCACAGCCATGAGTACCTAGTGGCC
AGCCTTTTGGGAAACATATCTTAAGCTCAATGAAATACTTTAATCTCCTAGA
AAGCTTCAAATATTTCAAAGAATAATGCTATGCTATTGTATTTTCACAATAAT
TAATTTGTTGTCATCTCAACCCTAACCCCAATTCTCTATTCAACCATTACCCC

ATCCCTTACCCTACCCCTGCCCCTAACCCTGGCCTTAGTCCCCGACGCACTC
CCATTGCGAATTGAGATAACATGAAAGTATAATACTATTATTTCTCTACAGCC
GTATAATACATGAGATACATATGAAAAAAAACTAACATTGTTTTTAAAGCCT
TGTAGAAATTAAACATCATAAATGTAAACCCTAGCTAGGCATTTGAACCCTA
ACCCTCGACCCTAACCCCCAGAACGAGTTTGAGGGTTTTGAAGGAAGCAA
AGGGATCCTTGACGATGAGCTCCGTTCGTTCCGTCTAATATCCCTTTAATTAT
GATCTCGCGATATGTTATACTTCCAATCCTTCTCAAATATCCGTTCTCCATTCT
CGAAAGCCTCAATCTGAAAGTTCAAAAGCCACTCAGTTGCCGAAGCGGTC
ATAGTCGCCACCCCTTCTATGGCCAGATCCAACCCATTACGACCATATTCTA
GACGGAAGTTCTGCTGCGCCAGTCCTGAAAGCGGGTCGTCGGGCTTGATC
TTACTTATAACTTCAGCGCGTTCCGCAAAGCGCCAGCCTGTCTCTTCGATCT
CGTATAACCCGGTATCACTGTAGTAGGTCGCTGTCTTTTCCCCGGAGTCCAG
ATCCTCGGTTATCGTGTTATTACTTTTCCCTTCGCGTAGGTGGATTGCCTTGA
GTGGAGCACCGTTTACAGCGGGCTCGAATGGTCTGAGCTCATCGTCTAGTG
GGTTGACGAGCCGTGTGGGCAGTTCGAGGGAGCTACTCGCGCAGTCGACG
GTCAGTGTAGCAGCTTCTGGTGATGGCCAGGCGGTTGGGAAGTATGAGGTA
GAAAGCGCAAGGCGAATTACGCTTCCTGTTCCGATGCGCTGGCAACATTCG
TTCAGTCTGATAGTGACATCGTAAAACCGGTGCGGTTCGACCGTCTTTAGG
TCAGTGTGACTATCGCGGTGAGTCAGGTTGAGAACGCCGTACGTGATTCTC
GCAGCGGACCCATCGGGCAGAAGTTCGGAAATCACAGCAGCTACAAGAGC
ATTAGGCTTGTCGCTTGACACTCGCAGACGAAGCAAGGCAGCACCGAAGA
TATCCAGTGGTTCGGTTAGAGGCTGGCTATCGAAGGTCAAAGAATCACGCG
CATCTTCGAGTTGATCTCCAGGTAAATCCGCCTCTAAGCCGTAAGGAATCCA
CCTACCGGTACCAAAACCGACAGTTTGGGAAGAGCAGAAAGACAATTTAG
CATCGCTGCATGAGACTTTGTTGACAAGTTGCTTCGGTTCTAAACCCATGA
GTTGGGTAGCAACCCGTGGACTTGGCCATATATTCTCAGCTATCCATCGACC
CGGAACAGGCGAATTGAAATGTCGCTTCGGTGGATTACTGCTTTGCTGATA
AAATCGAAATTTAGGTTCATCCATAATATTGGTCTCGATTCCTTTGAGCCACT
TATCCCACCAGCGCAGCGACTCTTGAAGAAATCCGACTGGTTCAGGCACA
GCCATATTGGGATATTTATGGCCCCAAGGTCCGATGAGTGCCTTCTTGGGAC
ATTCGAGATGCTCCATCATTCGAAAGATTGAGTTGTGATAGGGGTCCTGCCA
TCCGCCGACAAGGTAGACCGGGCATTGGATTGCGCCATAGTCCTCGCATAT
GGATGCATGTTTCCAGAAATCGTCACGACGCTGGTGTTCATGCCATTCTGCC
ACGTACGGCACGCCGTATTCAAGCCGATCCAACCACATTTTCCTCCACTTGT
CACCCACCAAAGCCGGATCTGGGGGTGTGGGGGAGACAGTAAACATGACG
GCGCCCCAGTGGAAGTTCTCAACCAATTGACAGCCGCCCATGTAGTGGAC
ATCGTTGTCATAACGATCGTCGGTTGAGCATACTGTGATAATAGCTTTAAGT
TCAGGCGGACGAAGTGCTGCAACTTGCAATGCATTAAAGCCACCCCAGGA

TATGCCCATCATACCAATAGATCCGGAGCACCACGTCTGTGCGGCGATCCAT
TTCAAGATCTCCAGTGCGTCGTCTTGCTCTTGCTGAAGGTATTCACCCTTCA
AGAGCCCTTCCGAATCACCTGAGCCGCGCATATCCACACGCACACATGCGT
AGCCGTGCGCGGCAAGATACCCATGATTAAGAGCATCTCGATGCGCCGTGC
TGTCGCGTTTGCGGTAGGGTAAATATTCGAGGATGGCCGGCGCGGGATCAG
CGTCGGCCTGAATAGGCATCCAAGTCAGAGCGGAAAGAACAGTGCCATCA
GAAAGCACTATTTTCTCGTTGTGATTGACACGGATTGGGAGTGGAGGGTCT
GACATTTGAAATCTTCCACTGATTTGATTGACGAAGTAGGCACGCTGCAGT
CAATACTACTTGGGATTCGGTGTATTGAGCTCAAGCAACTTAGTCGTCTTCC
AATAAGTCATTTTGCTTATCTGGACCCCTTCATTCGTGTGTCGTTAACCGAG
CGCCCTGCATTATTCTATCCGACCCGTTCGCGCCGGTGCTGTAGTTCTATCTA
GCATGGTTAGAATGGAGATTCCAATAAGCTACTTTTCTCAAAATATTGCCCA
TATTGAGTCTAGCGTCATCTGCGGTAATTGACGCCGCTTATAGAATTGAACT
GGAGAGGACTAACCAATCACAGTTAGGTCGTATCGAGGTTCCAGACCTCAA
TGACGTTTGGGTCAAAGCCCCTCGAGACTCGAGAGGGTGAAATGTGTGTC
TGAAGGTAGTTGATAAGGAATTAGCAATATGAAATCCTTCCTGTCAAAGAAT
AGTACCTAGAAAGCTTTGATCTACTTGTGCTACGAAGCCTAGAAATGTAGG
GTGATGAATCTGGATGGTGAGAAGATAATCGTTGTAAATTACTAGGGGGGT
TGCCTGTCCTGAAAATTCTCTGATGGTCAGGGAATAAATGATCCAGAAAGT
CAAATACACCGTTTAATAGTACGGAGAGGGACAATGTGTTGAATAGAGATG
AATAAAAGAGATGGCTTGGAGTAAATTAAAGTTATAGAAGTAGTTTGGGTA
GAAGGCATCCTGAGAATGGCCTTATTGTGGATTGATAAATCAAGACAAGCC
GCAACTTATCTTAGGCCAACTCCATCCATCAAACTAGTTCATGTCAAACCAA
ATTCTACCTTTATCGCGTCCCCAAATCTTCGTATGCCTTCTACAAGATCCATC
CGCGGTGCTGCCGCGAATGTCAATCGAAAGGTCAAGGTCTCAGCAGAGTT
CCGATCGGCACGAAACCAGCTTCCCTTGGCTACAGCGACGCCGTCTGCAG
CGGCTCGTTTGAATATAGCTTCTTCGATCGTCAATATTGCTCGGATGTTGTCC
CCTGACCCATGGTCTGGGTTTGCCTGTGCACGGGGGTGTTTGTGCCAGTCT
ATGGTGAGGAAAAATAGCATCCCGAATTCTGGTATCTCCCAGGAACAGATG
TCGACCGGAAGATACTGTTCGCAGCCGGCCAGCAGTGCATCTCGGCGAATG
CGATACTGTGAACTCAGTGTCCCCAGCCAGAGTGAAAACCCTTCATAATCC
CAAGCTTCACTCAAGAGTTTATATACCATAATCTGCGACATGCCGCTTGGTG
CCACAACGCTCACCTCGTTATGAGCCACCATCAACCGCGCAATCTGCGCGG
ATGCAGTCATCCAGCCACAGCGTAGTCCCGGACCAAGAATCTTGGACGTCG
AATCTAGCCTAAGAACGCGACCGGATATGTCCATAGAGAGGTACGATGGCG
GTAAGGTTCGTAGGATCTCATCTAGGCGAGGAAATGATATCCTACTCGATTT
GGTCCCTGTGCTATTTTCATACGGGCCAAGAGGAAGAAGATAATAAGGATC
GTCCTCGACAATATATAAGTCGTGTTTCTCAGCCACTGCGTAGATGGCCTTT

CTTCTCTCTGCTGGCTGGGTAATTCCCGTCGGATTATGTCCTGTCGGGACCA
TATATAACAGAAACGGCTTGGGTCCGTGGGACAGATCCCATGTGTCAAGTA
CTCTTTCTAGATCTTCTGGCATCAACCCGTGTTTGTCCATCTTTATAGCCTGT
ATATTTATACCAAGCGGCTAAGAGCCTCGATAGTGCCGGAATAGGTGTTCTC
TTCACAGAGTACGTAGTCGCCTCTGTTGCAAAAGGTACGAAACACCATATC
CAGGGCGGACGTATTCCCGCATGTAAGAGTGCATTCCCAATCCTCGTACGG
TGGATTGTGGAATTGCTCTATATGTCTAGTCAGAAAGTGAACTAGCTGCGGC
GCTCCGGCTGAGTGACAGTAGTTTAATGCCTCTCCAAGCTTAAAAGCGCCG
CCATACTTGTCGGCACGCAAGGAAGACTCTTCCTGCCCTTCTGTGTTCAGT
TTTTTGTGAGACGACGGCATTAGTGCATTAAAAACTACCGAGGTCCAGGGG
AATGCTTCTGCGGTTGGTTTAGCAGTGCCTAGAGCAATAGTCTTGTTTTGAC
TCTGGGTGACTGCATTGCTCTTCAGCGTTGAGCCAGTGCGAGATTTGCTCT
CCACATTCAACCTGTGATCCCATCTTCTTGACAAAGTCTTAGAAGGAATCG
ATTTCAGCATTTTCGATAATGGATATATTGGGAGTCTTCGTTTTGCAAGTGTG
ACTTTTTGTCGATTGAGTATTTGCGACAGATGCCGAGGTCATGGAATTGCAT
TGCGCACCAGGAAAATCTAGGGAAGAGATTTTAGACGCCATGCAAATGTAA
AAGAAACTTACATAGTTGCCCCAAGGATTTATGCGGTAGTTCGTTCCAATGA
ACCTCCAATCCTAAGCCTCTAGCCATCGAGCCAATCATTAGGTGAATGAAG
AAAACGATAATAAGTATGAGAGCAGCTCAGAATCAGAACCACATGCAGCCT
TATCAGAGCTGAATAGTATCAGTCACATAATAGACTGCTCTTCGAGTACCGT
GATCGGAAGTGATTAAGCCTAGGTTATCTCCCGATGATTGATTACTCGATCC
TAGATCGTTTTCAGTGTCGGTAAGATCTTCGTTCTCACTGATATGACCGCGT
TTTCGCTGAGCACGCGACCATAGTGCATATCGCATTCATTTGAATCTGAGCA
ACTTTTATAAGAAGATCAAAGGTTATTATTAACATACGAGGTATCAATCATCA
GGGAAAGGCTATGCCTCTCTATGATGCGTTCACGGTGGCCTCCGAGATCCA
TTACGATACGCATATTCGGCAAGGGCCTGACGAATAAGACCGGCATTTTCCT
TGGTGTTTTCAAAGAAGTCCTGAAGTAGTGATGCTTTATGAACGGTTCTCAT
CTCATGGCGATATATGTTTGCGTCTTATTTGATAATTATGATGGTCTGTTTATG
AAGATTGTTAAAAAGCAAATTGAGACTTCCAGGGTTTTTACAGAATCATGG
AATTACCTAAGACATTCCCAACAAAATTATGTAATCAATTCTAGCTTACTCAT
CTTCAACTTATGTAATAAAGTCTGGATTATATAATCTGACTACATGATTAATA
AGCCGCGGGACCAACCCCTCCCAGCTGATAAGATTCAACAGTGCTGAGTC
ACCTATGCCATTATTCCAGCTTCATTCAACGACTCATCAGCTCAACGTAGCC
CTACCATTCATCGTTTGCTCTGAGTGGGGTCATCAAGTATGAGCGTGGCATA
AAGCACGAAGTTATACCACGATCTCTGATATGGGAACTAAGTCTTCCGAGC
CTCAGACCAAAGGTGCCCTCACTTATCCATAGTTATTTAGAGTATGAACTAA
TCGCGGACCATGGTCCTTGTTAGAATGCCGTAATTGCGTGCTAAGACGTATT
CGTTGTGATGGAACTCTACCACATTGCAAGAAATGTATGAAAAAGGGTACA

CAATGTCCAGGATACGGACAGCACATCCGTTGGGTCAACGCTATTGCAAGT
CGTGGAAGATTCAAGGGTCTCGGTAAACCAGAACAACCAAAGTCGGTTGA
TCATACTCCTAGGCCTACATCTGAATCACCTCGATCACCTACGGGCTTGAGC
CCTCTAGGGTCACCAGAATCGACATGTTCGTCCGGAACTGAATCGGCCCTA
GGTCTTCTACCTGAGTCTGTCCAGGACCAACTTCTGCAATACTATTCATGCA
ATATTGCACCGATTAACGTATGGTTCGATTCCCCTCAGAATGGGTATAAAAG
GCTGGTCATACCTCTAGCTATGAGACAGCCTGTTCTGCGACTTGCAATTCTA
GGGATCTCTGCTGCGCACTCATCAGATGATTTAGAAGTCGACAAGAGTTTC
TCACAGCTCGCATGTCATACAGCAATGACCATCATTACATCGCAGATCAAGA
TTCTAGTCGGCAGAGAGCTTGGGAGGTTCGACACTCAGATTGACCAGGATA
CGGAAATTCTTCCGGCAGTCCTCGCTACTATGCTCGTCCTCTCGAATTACTC
CCTCCTTGAATCTGACATTTCATTAGCACAGATACACTTACAAGCGATCCGC
GTCCTAATCAGGACGCCTACACTCAACAATGAGTGTAATGGCGATCTCACT
CTTTTTCTCCGAAATCAAATTGCGTCGTACGACGTCTTGACCTGTACTACGT
TGTTTGATCGCGAACATATCCGGAATGCCGTAGTACCCGGTGTCAAACCGA
AACCGGTCATTTTTAATGACTATTTAAACATCATACATGACATCACGATGCG
ATCTGTTCAAGAGACAGCCCAAGAACTCTCCCTTTCTGAGATAGAAACACA
ACTAGAGCTCGCGCGAGGCTCTAGTCTGTTAGCAGGGGGTCCACTATCCAC
GCAATGTACTTCATCGTCCTATCGCGATTTCATCCGCCTCACACAATCGTTTC
ATCACGCGGGATTTCTCTACGCATGCAAACGCATACCTAACTTAACCGAGG
CGCGCGAAGTCCAGGAGTATCATGTGTCGAAGCTCTTTAGCGTGTTTTCCC
AATTTGAAGATATGGATGTATCAGTGGTGAGGCTATCATGGCCCATTTTTATC
GGCGGAATCTGTAGCTGTGGCCACAAGGAACGCATGGGTATTATCACTGCC
TTATGTCGAGCATTATCTAAGTCACGCTTTAGATTCTATGATAGCGTATTGGT
GTTTCTGGAAGAGCTTTGGGTGAGCCGAGACCAAGACTGGATCTCCCGCG
CACAAGAATGGGAAAGAAGGGGAACGCCCCTCGTAGTACTGTAGCGAAAA
GCGCATAGCTAAGGAATACTCTCACACGGAGATAAAGGACAGGGCTAGTAA
TTCTTACAATTTTATGCCCCGTTTAGGTAAGGCGTTAATTCTCGCGCTAAATT
TTCGATACGACCTACCACTCGGACCGCTACATTTCCTCCACCATCACTTCCC
CGGCTTTCACGACGAATCGAAGTTTTATTATTTCCAACTTTCCAACCTTGAT
CGCCATGCGGACAGACGAGCTATGTTCTGATAAGTGTAGATTTCTGATAACC
AAGGGGGATTAGTATCCCGCCAATGAGAGGCCCTTTCTTAATCTCAATATGC
CGCCTAATTTGGCATCCATGATATACACCTTGAAGTACGTCTGTTACTTAATG
TGACTTGCGCAATGGGACAATGGTTAGTGAAAATGTTCGCACAGGATCTCA
CACCACATTTCAGCGACATTAGATAGTTTCTCTGCTATCAACCCTTTGCTCA
ATATGTCTAATCCAAAATCCATGGCTGAGGAGATGGTCATCGACGCGAGTC
CCGTGTCCGAGAGTGAGAGCGACGAGAAGCAACATGTCTCAGAGCCCGAC
GGAAGCCCGCGTGCTACCACATTCAGCAAGATCTCAGTATGGTTGACGTTG

AGCTTCATGGCCATTGCCCAGGTGACAGATGGCTAGTAAGTCCTTCTTTGA
CAAACTTCATCTCAAGTGTGAGAGATAATCTCGGATCAAATACTAATACCTC
AAAGCAATGCTTCTGTCATCGGCTACCTGTACTTCTTCCTCGTAGTCCAGTA
TCCTGATGCCATGACGGACTCCATGTATAACCGTCTGTCGAATGCATTTCTC
ATTGGTATGATCGTAGGGACTTTGATATTTGGCTATGTTGCCGATCTTTTTGG
TCGCAAGACTAGCGCGGTTGCGACAACAGGTCTCCTAACCCTGGGAATCG
TGCTCAGTACCGCTGCCAGCGGCACAACGCCCAATGGTCTCATGTGGATGA
TGGTAATTGCGCGTGGCATTGCCGGCGTCGGTGCCGGTGGCGAGTACCCAC
GACCGGTAAGCTCTCTTGGAAAAAAAAAGATTATCTGAACATGGTGCTTAC
TCCCCATAGGCGCCGCCGCCATGGAGGCTACCGACGAGGCCAGCAATTACC
GAAAACACCGCGGCTTCATCTTCGCTACCTTAGGAGAGGTCGCTGCCTCTT
TCGGCTATTGCTTAGGCCCTCTTATTCCACTCATCCTATACGTGATCGTAGGC
CAAAACGAAGCCCATTACGAGTTGATTTGGCGCTTATCCTTTGCGCTGGGC
ATGATCCCAACTCTCTCCATCTTCTACTTCCGCCTTCGCATAGCGGTTTCGA
ACGCGTACCGCAAGTCCGCTCTGCGCAACCAACGTGCCCCCTATCTCCTAA
TCGTCAAGAAGTACTGGCTACGTCTCATCGCCGGCTCGTTGTCATGGTTCCT
CTATAACTGGATTGCTATTCCCTTTGGCATTTTCAGTTCTGTCGTAATCTCTA
AAGCCAATTCCGATAATACTTTACTCCTAAATTTAGCCTGGGGCGTGGTCAT
CAATGCCTTTTACCTCCCCGGACCCTTCCTTGGCGGTTGGTTAGTCGATAGG
ATTGGGCGACGATATACTATGGCACTAGGTTTCGGCTGCCAGGCAGTCTTG
GGATTCATCTTAGGCGGGGCGTATAGCAAGATCGAGACCATATTTCCGTTAT
TCGTCGTCTTATATGGTGTTTTCCTTACATTTGGTGAAATTGGCCCAGGAAG
GTATGTAGAGTATGATATACGGTTCGGTGGAAACCAATACTAACCGATCTTA
GTGTCATCGGTCTGGTGTCGACGGAGCCATTCCCCACATCGGTTCGAGGCC
AAACATCTGGATTAGTTTCGGCAGTGGCTAAAGCCGGAGCTGCCATTGGTA
CACAGGTGGGCGTTTCTACCATCCCCCAGATCTATAACCAAGCTAATTCGAT
ATATAGGTCTTCAACGCCATCCTTAATGCATATGATAGCAGTGATAAGGGGA
ATCAAGTGGTTTTCATGATCGGGTCTGCGATCGCTGTCATCGGCGTTCTAGT
CTCCCTTTTCCTCGTAGAGGAAGTGTCCAGAAACCTGGATGATGAAGATCG
CATCTGGAAGGAGTATTTGGCCGTTCACGGAGTCGAGGTTTCCTGGGGTGA
CAACACCAAAGATCAAGCTGCGGTACTCAAGCAGGCCACGACCCTCACAT
CTTAAATTTCTTCTTCATGTTGATTTATATTGTTTAATATACTGCTAATCGTGC
GAGTGAGAGATGTGGTAGGAATAATAAACGCCACATTTCTAAGATCAGAAC
CCATTCTCCTAACATTGCGATTTGTCGATAGGTAAATACCTAAAAGTCTCAA
AAGAGCGCTCAGTGCCACGGCATAAATGATAGCCCCCACCAAATACAAGCT
TAATAAAAGCAAGAACAATCAGTTCACAGCTACCCGCCCACCTTCGCTTCT
TCCTTGCGTTGCATCTTCTCTCTGTACCCCTCAACGACCATCTTCCTGACCT
GCTGCTGGTAATGGCTTACGCCATTTTCCTTGACGGGATTCAGTATGCCCTG

GCAGTAAACGGCCCGCTCTCAAGATTAGCCTGTGCCTTTTCGCAGAGCTCG
AAATCTTCGAGCGCTACCTTGCGAACAAAGCGAAAGTACTCCTCGAACGG
CTCGCCTTCCTTCAGGTTGAAGTAGTCAAACTCCATACGCGTGACACCGGG
GTTTTCTGTGGGGCAGACCCGAAAGACGGACATGCCGCCTCCGTAGGCGT
TTAGGGTGCATACAGGGAAGAAGTAGAGAAATAACCCGTCATCCGGCTTCT
CTGGGTCCGCGATGTGCTGCGAATAGTTGCCGCAGTTCTGTACGGCATAAA
AGGTAGGTGGGTACAGGGCCGAGAATGACGGATGGGTATAGGAACAATGA
AGGCATTCTGTAGCAGTCTGAGCATAAACTGATAGAGTTATCATACGTAGAC
GAAGGTACCTTGGTATCCATCAACCCTAAGGGAGCAGTCAGTGCGGTGCTG
GGAGGATGATAAGGTGGACGAATAAGAATATGGGGAAGAAAAGATAATGG
GGCTCGAAGGAAATGGTACGTTTCCCCCCAGTGGAGCTAGGTTATCTTGAG
AGATAGAAGAGGGAGCCAGTGCTCACATAGTCTTCCAGTTGAAATTTCCTT
CGTAAGAGATAGTTCTTCTATACGGAAGCTTAGTATAGTCTACCCGAGCCAA
CAGCTCCTCCAACCCTGGAAAGAATGACTCAAAACTCGGCGCCTCATCTG
AAAGAGCTGCAAACAATAGGCCGCTCGAGGTCCGTTTTTGAGGAAGGGGA
TGTTCCTAGTGTCTAGTCAATACTTCTGTG

>Mosaic_sequence_10

CTAGTGGCCTACGTAGCATTTTGGCAGGGGTCGCCCTGCCTGAAACGGTTA
TCGTTGGATTTGGTGAGGTAGTCGAGGACATGCAGACATGCAGACATGTTC
GGCCAAGGTACTGGCTACTAACCAACTAACTCCGACATAACCGACCAAACC
CCCGGATCGGACAGGATGGCTGGATGGATGGATGCAGCTTTCATCGGTATG
AAACTACTTCGGTTCTAAGGGCCTATATCTGGGACGCTATACGTGTATTGTA
CGAAGGCGAATCGTTCGAAATCTAAGGGGGCTGAGAGCAAGATTTGGGGG
TTGTACGACGGATGAGAGCATAAGGAGCGCCATGAGCACATGAGTACGATG
ACTCGCAGCATGGCATCAGCCAATCTTGCGACGATAGGAATGTAACTCCTG
ATCAAACTCGAACCCAATTAGATCTTCGAGGGTGGTTAGGTTGGGCTATATT
TTCGTCTGTACAATTCATCGCCCATGTTGGCTACCTCCAAGCCTTCCGTGGT
GCGACTTTTGACGCCGGAGTGAAGGATGGGGGTTTGATTTCCCAGCAGATT
GGCCTTCCGTGGATGCCGTATTGTCCAGGTGATGCGAGCTGCGTTACTTAG
GACATACCAAGTTTAAGTCTTTTCAAAGGGGGGGCAAATATCTAGGTTATGT
CGTTATGTTGTTATTGGTAGCCTCATATTTGGCTTGACTTATTTATATTTCATC
TGGACTTTTTCTAAAGAGATGTATGGTAGTATTTTGGAATTTTTAGTGTCAGT
GACGTAGAAAGGCTTTCAGTGTTCCAATTGTAAGTTTTGTAAGCGCGCGGG
TGTAAATGTAAATGCAGCTAAGCCACCCCTTCTTAACAAACCCTCCCACAC
TTCTTCGGATGGAGGAGCATATGTGGTTGGTCAGGTACCTGCCACGCCTAA
CAAAAACATACGAAGGTACAGGTACCTACCCCAAATGTCTCCAAATGTTTT
TTTAGAGTGTAAATGTGCTAACCCTCCAGGCTTGGGGGTGCCCAGTGTTCT

CCTTATTCTAAGCCACAGTTCGCAAGCCTCTTGGCCCCACCAAAGCTTTGC
AGCTGTCCATCGAGAGAAAGCAACGTCAATTCTCCCCCACCATCAATCTTC
TAGCTTCAACGTCCACCGACCAAGAGCCTCACTATTTCCGATACACTCATTT
TCGGGATCACATGTCCACGTAGCCGAGCCACGAGAAAGCAGGCAAAGGCG
GCAAAATACACTATACTCACTTATCCTAAACATCCTAAGCCTAGACTGCTTA
GCCCTTAGGGTCTTTAGGCATCAAGAGACGAGCGAAGCCTTCTATACGTTA
CCCGTTACCTCTTCCTCCTCTGTTGGGCAGACTCGTTCTGGCTTTCGGAGCC
GAGTGACCATGTCTATCATCCAGCAGCACACCCAGGCTTCGCTAAGCGATG
CCTGGAGGACCATCAACATCGACGCTCTAGACCCGGACTCCTCCCAGAACT
TCGACACTTCCACCTTGCACCCGCCGTTACCCGAGATCGGCGAGGCGGAC
GTTCGGCAGCTGAATGCCCAGGTGCGGCAGCTGCTTCGAGGCGGCGACTC
CGAAGGCGCTCTGAGAGGCTGTCTGGAGAGCCCGGTGTATAATGGCAATG
AATCTGCTAAGGTAGGTCATGCTCCAACCATAGCCTTGGGTTGTCATGTCAG
GAAATATGAATCAGGGAGTTTGGAGGTCATGAGGAATAGAGTGCTGATGAC
GTCGTCATAATACAGGAGTCTCATATGCAGACCGTGGTTGAGGTTCTACAGT
CGATCAAAGCTAGCGAGATGACTCCGATGCTACAGAGAATCTACGCCTCAG
AGGGCGGCAGTGAGCTGTTAGATGTGTTGATGAAGTATCTGTATGTCTCTTC
TCTACCCTTATCTAAGGTAGTCGTCAGATCTAACCCAGATATACAGATACAA
AGGTATGTCAGGCGGGTCGTCGAGCGGAGGACTAAAGACCCCAACCCGGG
GCTTAACGCCGCAGGCCACGGGGTTTAGCCAGATCGGCTCAAGGCCCCGT
TGGCACAAACGAGTCTACGGGAACGGCTATGAGCGTTTTGCTAAGCTGGC
ACGAGAAGGTCGTCGATGTCGCGGGCTTGGGTAGCATAGAGCGATGCATG
ACTGACTGGGCGAAAAGTCTGAAGCCCGAGGTGGCTTTAAGAGTTGATGT
TCTATAGAAATGGGATTACGAAGTATTTGCTTGCTTTGATATTGATATCTTCA
TTTTGAATCTTCTCCATTGCCAATCCTAAGATACCATCAGTTCCAAACATCG
ATTCTCATTCATTTTGAAAGCCTTTGGTTTCCAGCTCTTCCAATCCACTGTCT
GCCTTATGATCTTTATTAGTATGAATAAGTTCATTCTTTGTCTTGATATATCTC
TCATTTTTAACGTTCTTATATGCTTACATAGATGCTTCAAGATGTTTTCCGTG
TTTGTCTTCACTCATCCTACTTGTATTATATGTAAGCATTCATATCGACTCTAC
ACATATCAACTCGAGTATACGTCGTATGAAGCTGCTTACCCGTTCCTCCCAA
TCATTCATCCTCTTATACACATCGTCCGTTGCTGATGTAACACAACACCATC
GTCTACTCAACCGTAACGCTTCCTATTACATCCTTGATTCGTATATACACAGG
GCAAGTATTCATAGTGTAGGTAATAAGGTCCCAACGATTGATTACACCAAAT
GCTTGGGGCATTGGCACTGGGTAGGCTTAAATGACTAGATATTTCTTTCATT
TACCTTTTCATACTTTTTCCAGAACAACCATCCTTCACTTACAATAATATACG
GGTAGTTTTCAAACTCCGACAATTTGCAAGCCTTGCTCATCATTCTCCATTC
CCAGGTAAGTGAGACCTTCATATTTTCCATCAAACCACCTTTGTGAATTCCC
TTTCTGTGATATTCTCCTCTTGTAGTCATCTCTCAAGTAAGGCCCCCTTCTGT

CTTTGTATCCCCTACTTTCGCTAAAGTATCTGGCTGGCCCGCACGATATTCG
GCCTGCTCGTCCTCGTCAGCCCATACCTCTGGATCACCTCGCCGCTGTGCAT
CTCCGTCGAACGCGGCGCGGACCAGACGGGCCAAGACCAGCCCGTGACC
GTGCTCGGGTATGCCAACGCCTTCGGCTCGACCTACATCCCTCTTCTACCTG
ATCCCATTCGGGCTGTTCTACCTCGTGAGCCAGCCCATGGCGCAAATAACA
ACCATCCTGCTCCTGCTCGCGCTGCTCGTCTACCTCGACGTGGTCGACGCG
CGACGCGACGCCGTGCTCATGCAGGCGCAGTTCGCGTCGGCCTCGACGCC
CGGCGCGTTCGAGCCGGGCCAGAGCACCGCGCTGGTCCAGCCCGCATTCA
CCGACCTCGTCCCGCTCGCGCTGATGGGCTTCGTCCACTTCTTCGCCACGG
GCCACCAGGCCGTCCTCACCTCGATCCAGTGGAAGCGGCCTTTGTCGGGTT
CCCCACCGTCACCTACCCTGGTCGCCCGTCCTCGTCGCGCTCAACGCCTTT
GGCCCGTTCTTTTTCTCGGCCATCGCCGTTCCGCTGCTCGCGCTGTGGAAC
GTCTCGCCCCGCCCGCAAGGCACCGTGCCGGTGCTGGGCCACACGGTGCA
GCTGGCGCTCGGCATGATGATGTACCACGCGACGGTGACGCTGGCCAGTGC
GGCGTGCGCCGCGTGGCTGAGGCGACACCTGATGGTGTGGAAGGTGTTCG
CGCCGCGCTTCATGATGGGCGGCATCACCCTGCTGGTCGTGGACCTGGGTG
TTATCGTGGGCTTGCTGGGTATGAGGGGCACGAGCTGGAAGGTGTGGCGG
ACATTCAGGACGGAGGCTATATAGATCAATCACACCCGTAGCATCGCACAA
CATCGCATTGCATGACATTGCATCGCATCATAGACGCTGACAAGTGATCGGT
GGGTCAAATGCAAAGTTCATACTGATATCGTTCCACTCGTGATTAGTTACAA
TCTATGTACAGTACCTTCCACTTGGTCACTTCGTATATACAGTGATACCGCCC
ACAATGGGCAAGGACAGGCTATGTGAGTCACTCCGCTTGGTTTGGCTACAA
GAGAGCTACTGGCTGCTCTGGCTCATGACGCCTTGCGCAGACTGAGGCGCT
TCTTGCGTTTCTCCTTCTGCGCCTCGGCCGGCGTGGCGGGCCTACTGCCAG
TCTGGCTCTGCCCTTGGTTTGAGATTTGGCAGGAGAGCTAATCGCCACTCT
GGCGCGGCTGGTCTTGCTCCGCGTCCACGGCCTCGCCTTCAGGCGTGTTGA
GAGGCGAAGCGGCGCCTTTGCCGCCGAACGAGTGGCTGCGCTTGACGCCA
CCCTGGTCTGAGATCCGGCGGACAAAGCCTTTGATAGAGGACAAACGCTT
GCGCCTCTTGTCGGCCTTGTCTTTGTCCTTGCCCTCGGGCAGATCACCGAC
CTCGTTGTCGCCGCCAGACGAGGCAGCCGACATGCCCGACATTTGCGAGG
TCGAGTGAGGTAGGCGTGGGGGGATGGGCATGACGCTAGGCGACGTCGAC
GGGAAAACGACGGCACGGGCGTCTGATGATCTCTTCGGGAAGGAAGCTGA
CCTGGCTCAGACGTTGGTGCAGTCTCAACAGGTGGTGCTCGAGGGGTCGG
AGACATAGGCGGAGTAGGGACGACCAAGGGAGACCGCTCCTCGACCGTTT
CCTCATCTTCGACGATGGGCTCGGGCTCTTCGGGCGTTGGAGCGCGCGGTC
GGACTGTTTCGGCGTATGAGGAAGGTTCGGCTCGGACTGCGTCGGCATACG
ATACTTCCTCTGTCACGGGTCGGTCAACGAAATAGCCTTGGCTCTTCGCTAG
GCTGATTGGGCGGAGGCGCATCCGGTTTTGGAGGCTTGTCAGAACCGCCA

GACTTCGCCGATTTTGATGGAAGCTGCCTTCGTGTCCGGCTCAGCCTTGGG
GAGCGCATCCACGTCGACCAGTGCTCGGTGTCCGACGTGGGCGAGGCGAT
GTTTGAATCACCAGCAAGAGTGGTAGCTTTCGAAGAGTCCAGCAAAGGAA
TGGCTGCCGCGATCTCCTCGAGGTTGATGTCGGGATTTGCTTCTTCCAGCA
ACTCCGCCTCCGAGAAGACCTGTTTCTTCCACCTGCAGCTTAGCTGATTCC
AGCATCGAAAATCTTGAGCTCACATTGAAGCGAAGACGCCTTTTTCTCTAA
GCAGCTGCTTGTAGCTGCCCTGCTCGACGATTTTGCCCTCCTTCATAACAGC
GATGATGTCCGAGTTGACGATGGTGGACAAGCGATGGGCGATGGAAAGAC
TAGTCTTGCCTTTAGACTGACGGTTAGCGGGTTCATTAGGTGAATGAGCCA
CAACATGCCAGTTTTTGAAGTGCCCGTTGGATCTCGCGCTCCGTCTCTGTG
TCAAGGGCAGATGTGGCTTCATCCAACAACTACAACGGTCAGCTCAGAAG
ATTAGACTACACTGCTTGATGACCCACGAGAATGGTCGGGTTTTTGACGAA
CATTCTTGCCCAGGCTGACTCTTTGCTTTTCACCGCCGGACAAACGGACCC
CGCGCTCGCCTACGATCGTGCCGTATTCGTCAGTCATAGTCATGATCTTGTC
GTGCAACTGTGCCGCTTTGGCCGCTTCGATGATATCGTCGTCCTCGGCATCG
ACCTTTCCATACGCGATATTGGCGCCAATCGTATCATTCCACAGAACAGAGT
CCTGGGGAACGATACCAATCGCTTGTCGCAAGCTGAGTTGAGTGACTTTGG
CAATGTCCTGACCATCGACCAGGATTTGACCCTCGGACGGATCGTAGAAAC
GGTACAGCAATCGCAGTATCGTTGACTTGCCCGAACCAGTCTGTCCGACCA
AGGCAAGCGACTGGCCTTTCCCAACGGTAAACGAGATGTTGTCAATTGCG
ATTTTCTTGTCGTCGTAGGCAAAAGACACGTTGCGGAACTCGATGACTCCG
TCGGTGATCTCGATGTCTTTGGCATCCGGCTCGTCTTTGATGTCCGATTTCT
CCTCAAGTAGCTTGAACATCCTGTCGGCGTCGGTCATGTCGTCATGGAAGG
CCTTCCACCGTTGGGTGAGGGCGCTGACCGGATAAGTGAGTTGACTGAGG
TACGACTGGAAGATGACATAGCTGGCGGCGTCTGCCTGGCCATTCAATACG
CGAGTCGCAATGGTGAGAGTACCGATCAGGGAAGCGAGGATCATGAGATAT
CCCTGAGCGAGCCAGAATAACTCCCATAGGATGTCCAAGTGTCGGGCAGC
GTCCTGAGACTCGATGAGGGCAGTACGATAGCGATCATTTTCGCGGTTCTC
GGCGGTGAAGTACTTGATCGACTCCAGTTGGTGAGGGCGTCTTGATATATTC
CGCGTTGCCTCGTGTTCTTGTCATTCCGATCTTGGCGCAATGGTCGCAGCGT
CGCTGATCGCCTAACTTGCAGATGTGCGTAGAAGATCATGATGATGTAGATG
ACAACGGTGAGCCACAGCCCATAAAGCCAATAGAAGACGCCGATGGAGAT
GAACATGTCGAGGAAGGTGGGAACGAGCTGGAACAAAATGGTCGAGAAG
AGGCTGTTGACGGCCGATCCCTTGGATATGATCGACTGTACTTCTCCCGTGT
TGCGTTTTGTGTGAAAAGAGAGGGACAGCCAGAGCAGGTGGTGAAACAA
CATGACCGACATCTCTCTATTGGTGTTCTGCTCCACCGACATCCAGAGAATG
GTCCGGAGCAGTTCGATGGGCGAGGTGTAGCCGCCGAACATCAAGCGGGT
GCCAAAGTAGATGGCAAACGGCTTGACAATCGCACCGTATTGTCGGTCGCC

TTGATGTTGAACGAGAGCGCGGACAAATTGACCCAAAGCCAGCGGCATGA
CCGGGTTGGGCGACTCGTTCAAGGAGGACCAGGACAATACAAACCACAAC
GTTAAGCTGGAGACGGCGACTCTGGGACGGCCACAGGTGAGGCGCAATCT
TCGCAAGCCGAGGTCGCAGCTCACTCCATTTGATTTTTTTGTCATCTGGTTC
TTCCTTTTTCTTGCCCTCGCCAAGTTTCTTGGGTATCGGCTTCATGTTGGGC
TTTCGAGGCGGCCCTGACGCCTCGCCCTCGGCATTGGCATCGTCGACAGGT
TTGAACTGCGAGGGGTCGGTCGAGTCGGGCTGGTCGAACGTGCCATAAGC
CTCTCGGGCAGCGGTATCGCCATCGGCGCCTCCGCTGAGCAGACCGGTGGT
CTCTTCAGCCGGCTCAAAGCTGATGCGCGGATTGGATATGGCAGCGAGCAG
GACGAGCAAGAGTAGACTGCGGAGTGCTGAGGGTGGGATGGCAAGGATC
GCATAGAGCTTGTTGCCATCTGATGATCAGCATTGATCCGCGTACTCGCCAC
AGGTCCCTCCTGATCACTCTCGTGGCCAAGGAGGTAGGGTCAAGTCAAGC
TCACGCAAGTGCACCTCTCTCAGGATGAGAAAACGAGATTAGGGACCTCG
CACACGAGCCCCAGGGTGCCAAGCACCACCAGACCCTTCGACCCCCACTT
CTGCCTCCAAATCACGACCAACGCCGCCGCCGACCAAATGACGAAGCCCC
GATGGCATAGACAAGGGCAGCGGCAAGGTTCAGGCTCTTGCCCTTGTAGT
GGTGCTGGCGTAACCAGGCAATGTCCAGCGCTGCCAGTACCGCGTCAGCTA
CCCCCGTGAGGGAGAGGAGGACGAGGCATGCGAGGATGAGGGCGCGGCG
GGGGTAGACTTTGCGGACAGTGATGGGGCGTATGCCTGGGAGCTCGACGG
GCGGTGCGCGGCGGGGTACGAGGAGGTGCGCGAGGGGCAGGAGGAGCAG
CGGGGAGGTGACGAGGTGTAGGTAGGTGAGCGGCGAAGTGGGCGCCATGT
TGCCGGTCTCCTGCAGCCTATAGGCTCATGAGGGGGAGCAGGAGAGAACG
AAGAGAGGAGAGAGGCGAGAGGCGAGAGGAGAGGGACGTTGTCGAAATT
TGTGGTCGATGTTGAGGATGCAGAGTGTTCCTACCCGTGAGCTCGTGCAAC
ATTTGTACTCGTAGACCAAAGGATGTCTGACAAGGGGTGAGGAGGCCTCG
AGGTACTGACAGCGACCCAAGCTGATGTTGATTAACACTGCGTACACCGTG
GCGTGGGTCGTGGAGCGCCCTCACACCTATGATCGTCCGACCGACGCGCA
AACGACGCCCGACGACGCCGGCGATACACTAGACAGAGAAATCAGAGCTG
ATTCAAGAGAGATGTTCATGAAATCACTTTAATCACTTTGGACCGCGTCACT
TGCGCGTTGGTCCGAAAAGTGGAGGCCAAAGCCGCAGCACGCTCCTACAG
CCGCTCGGCACTGTACAGGAGCATTCCAGGTAACACGCGATGATGCATGGA
CAGCATCCTACGACTCGACTGGGTCTGCACAGAATCATTAGATATGGGACC
GCCTTTTCCGATCTGGTCGGGTGAATAGGATGGGACGGAATACAAGAGGTA
CGTGTGAGACGGACGGGGGTAGTGACAGTAAGGTGATGGATGGATGGGAG
GGGCTGTCTAGTTCTTTTTCAATCCGATGGCCAAACCGATGGCCAGACCTG
CAATGACGAGGATGAGCACTGCAACGTCAGCCAAGCGCGATCGAAATGAT
ATGACTGATTACGACGTACCGCAGATCCAGAAGCAGATCCACTTCTTCCTT
CGCGCCTTCCTTGCCGACTCGACAGCACGTTCCGTTTGCACGAGGCTGCTG

ATGAGCTACACGATCACGGGCGCGTACGACCCGATCACTGACCCTTGCTTG
ATGTCGGTGTCCACACCTTGCGCCTGACCCTCCACGTTGACGATCGTCTCG
TCCTGCTGCTCGACGAGCATCGCCATCTCGTTGAACATCTGCGCCAGCTCC
GTAAGAGTCTTTTCGATCTTTTGAATCTCGACATGACGATCTTGCACTTCCT
TGAGTGCTCCTCTTGCCGAAGCGTAATCTTCCCGAGTCATCAACTGGACGG
GTAAGCATTGATCGACAGGGCAGATCGCCAACGTACAGCCTGTTGGAATAC
TTGGGGGTTATCGCTCTCGACCACCTCCCTGACCTCCTCGTCGGTGGCATCC
GGCTTGACAATACGGTACTGCCTCTCGACTCGTCCCCTGACTTTCTGCCTG
AACTCCTTCTCGACAACCTGATGCTCATTGAGCAGGGTGGTGAAGCGACTC
TTGACGACCTCGACCTGGGTCTTCTTCGCCTTGTCCCCCTTGGCTTGCTTGA
ACAAGCGCTTGATCTGGTTTTTGCATTCTTCACGGGTTGCTCGAGCTTGTTC
GTTCAGCTGGTCGGCGTAGGCCCCGGCGTTCGCATCGGTCGATGCCTGAGG
AGTCAGTTAGGGCGCAGCTTGTCGTGACGTGACTACACGGACCAGGCTTT
GCGCATGAGCGCTGCGGACGGCCTTGATTTGCTCGTCCAACTGGGTGAGG
AGGGCCTGAGTAGCGCCAACATCCTCATAAAAGCGGTCTACGCCGCCGCCG
TTGACAGCTCCGCCCCCTGCGGCGTAGCCCTGCTGCTGCCCGTACTGCTGC
TGAGCGTACGGGTTGCCGGCCTGTTGGCCGTAGCCTCCCGTTCGCGGCGTT
CTGCTGCGCGTAGGGGTTGCCGGCCTGCTGACCGTAGCCACCGGGACCTG
CGTTCTGCTGAGCGTACGGGTTGGGTTGTCGGGAGGGAGGGATGACGCCG
TTGGCGGGAGGAGGGTAGGTGGTCTGCTGAGGAGCGGGCGCGTCCCCGTA
GTTGCGTTGCTGATCGTCAGCCGGGGTGTCGGCAAACGTCGAGGGGAACA
CTCACCACGCCTGCAAGACGGTCACGAGCCATGTCGAAGAGGTGATGAAA
AGGCGGACGGCGGGAAGGGGCAGAACCAGCTGCTGGCGCGTCGGAATGG
AGATCCCTGCGTTTCTAGTTGATGAGGAGATTCGAGGGTGTCGCCGACGAG
CACGATGGGCCGCTTCGGGATGGTGAGCAGGGGGTCTGGAGACGAAAAGC
GGGCAGATGTTGTATGTGTATTGAATGTGGTCGGATCGCCGAAGTCGTGCC
GTGCAGCTACCGCCCTTGGCAGGTCGGTGGTGATGACCAGCGTCGAGCTG
AAACAGATGCGGGAGGATGGGCGGATTGGGCGAAAGGCGGGGGTGAGAG
GGTCTTTGTGTCGGATGTAAGGGGGTTGAAAGGAAGAATGTCTTGTGAACC
ACCTTGCAGCTGCAGTGGTGTTGGTGAAGGTGGTGTCTATGGCCGATGCTG
GGAGACGACGACACGGGCACAGTACGAGACGAAAGCCCTTCCTTACCACC
CAGGGTCTGCGTCACTCCCACTCCTCACGTCACACCGCCTCGTACCGCCCG
GACCTCTTCTCCCCATGGTCATCGACCCTGAAAATCCGATAGTGGCACCTC
GGCAAAAACGCGGCTCTTCATCCCCTTCACGTTACCTGGCTGCTGCTTGTC
TTGCATACAGAACCATCATGCAAGTCATGTTACTCTAGCTTCCACCTCTGAG
TAGCACCCGGCAGGACCTGAGCTTGGGCGTCCACCTGAGACGTCTTGGCG
TGTTTCAGCATGGCCGGACTGGGACTCCCTCTCGCTGAGCATAACATTAGA
CTGCGAGAGACCGGCAAGCAACTGCGCCAACTACAACCCAGCATACAGCA

CCATGCATCTCTCGGCCAGGCAACTGCGGCACCATGCACAGAAGCGACTC
GCTCAAACTGGAGTGCAATAGGGTTGGAGAATTTCGGGATTGGCGATCGGT
TATAAACCACTCCTGACTCGATCACCCTCTCAGCGGGGTGTGATACACATTA
TGTGTATATCGCACACAAACCCGCGTTCGAATTTGTTTCCCCACTCATCTTC
ATGAGTCTCTTCCCCATTCTTTTGCCTTCTTTGCCCCTCAATAGCAAACAAC
ATGCATCGCTGCTGCTTTCTCCAGCATGGCGGACGTAGCATCGCTCATGAGC
GGCCACCTACATGTCCAATGCAACGTTTGCTGGTGTCTGATGAATCAAAAA
CAATCTGCTGGATTTTATCGCCGCCACCGAAGGCCACGCGCCGTCGGTCTC
TCACTCCAGTTTCACTCTCTTCCTTTGAATCTCGCCTGGCTCTTGTCATTCCT
TCTCTCTGCTTGTATACAATACTCGGCGGTCACCATCATCTGCTTCGGCCCC
GCGAGCGACACCCTGACACCGTGAGACACCCCGCACCTGCTCCGACGGCG
CTCCCGGGATGTCCAGCCGTCCATTTGAGACCCCGGTCCGCTCTTCTTGCA
CTCAATCACGGGCGTTGGCTGTTTGGGCCAAGAGAATCGCGCCTCGGCTTG
TGAGAGCAGATTTTCCAGCTCACGCAGAGCCGCACGCGATATGGATCCTCC
TCAGAAGTCCGGGCTCGCCAGCTCGTCCTCCCTCTCCATCGCTTTACCCCC
CTCGGTCCCTCATAAGCCCTCGATGCCGCTGTCTGACCGCAACGGAGGTGG
AGGAGGAGTCTTCGATCGTGCAGGCGGGTCGAGCTACTCTGGCTCGCGCA
ATTCGGACTATAAGGACGCACAGCGCCGCCGGCCGTCGCCCTCGAAAGATT
CCCAATATCCGCCTTTCAAGCGCCACGACCATAAGCGGTCGCCCAGCCCGA
ATAGAAGATACGATAGCTCTTCAAGGAGGGAGAGTGGTACTGTGTGGGACC
ACCACCATCTCGATTCCGAAGCCCGGCCGCGGTATGACGACCGGGAGAGG
TATGATAGGGGAAGCTATCGCGACCGACCGCGGGACGACGATCGCCCTCCC
CGACCACGATGGGACGAGCGGCCGGCAAGTGATGCATGGGCAGAGTATCA
CGCCTCAGGACCCTATCATAAGTCACGGCCGCCAGCGTACGATGATGATAA
CGACTATCGTTATCGGCCACGGTATTCGGACAAACGCTCGCCCCCGCCCAG
AGGCAGATCGCGCTCGCCTCGCAGCTCGCGTGACTCGCCGAGGCGGCATC
GCAGTTCCCCAAGCCGCCGCGATTCGTTCGATAGAGGCGCCAAACACCTAC
ACGATCCGAAGCAGGGCGATGATCCAGTGCCATCAAGCACGGCTATGACTG
CGGTAAACTCTGCTGATACCGAGGAAGGAGAGATTCCAGACTCCCCAGAC
GAGCCCCCACCCTCTTCGCCAGACAAAGCGATCGTGGATAAATATCGCCCA
AAGAGAATCATCAGGCCGGCTTACCCCTCCGGTCCTTCCTTCCCACTCGTC
TCTCCTTCTGAGGCCCCCGCGCCGCCTCCAGAACCGAGCGGCGTGCCACC
TCCCCCGCCTGATGATGCTCCGCCGCTCCCTTCCACCTCTCCGTTTGGTGCT
CGCTCTTCTGCTCCAACAGGTCCCCGCCCGTCTCCGTACGGACTGTCTCCC
TCAATTCGGACCTCAAATGGTCACCCGCCAGACCATCCTTCGCCTTATGGG
ACCGCAAATGGCTATGGCCCCGTCACTCCCTATGTGCCTGCAGCAAAACCT
GCAAATCGTAAAGTGCAAGACTTGCAGGGTCACGGTGAACATGCGACGCC
GCACGCGCCCAATCGGGATCTGCTCAACCCGAGCACACGAGCAGCGACTC

CATCTGGCGAGCGGCCGGTCAAGGTTGAAGTATCCCCGAAGCTGTTTACGA
GGCGAACGGCGGACGAGGAGCTTGCCGCGCTTGGCAAGCAGTTCGTTGGC
ACAAACGGCTTGGACGCGTACGATCTCGGGACCAAGCTTGGAGAGGGCAC
CTTTGGGTGAGCATTCAATGATTGGCCCTTGAGCGCTCAGCTTACTGTCCA
GTGTCGTTACGAAGGGCGTAGAACGGGCTACGGGCAAACAGGTCGCGCTG
AAAAAGCTCATCACGCACAACCCGCGTGATGGTGTCTCAGTGACGACCGT
TCGCGAGATCAAAATCATCAAGGCATTACATCATGAGAATGTGGTCGACAT
CATGGACATGATTGTTGAGCGGAGTTGGTATGCTTCCCGTTTAACGTGCTTG
TCACTGATGTGCAGAAAATCCCAACGACCGAGGAAGCGAGGGGATGTCTT
CATGGTTTTCCCATACATGGATCATGATCTCTGCGGGCTCCTCAACAACAGC
GAATGGAAGATGACGCACTCGATTGCCAAAATTGATCTTCCGCCAGACGCT
CGAGGGACTGGCATACATACACGCCAACAACTACATTCACCGGGATATCAA
GACTGCCAACATTCTCGTCGATCGCCGTGGTCAAGTCAAGATTGCCGACTT
TGGTTTGGCCAGAGCCTGGACCGACGGCAAGCTGCTCCCTCCCCACTCAG
CAAAGGAGTACACCAACATGGTGGTGACGCGGTGGTACCGCGCTCCCTGA
ACTCCTTCTCGGAGACAGGAAGTACGGGCCCGCGATTGACATGTGGAGTAT
GGGGTGAGTTGTGTTTCTCCGTCTCAGATGTCATAGCTGATCAGGGAGATG
TGTCCTAGGCGAGATGTATCTCAAGAACCCTATCCTGCAGGGCGATTCGGAT
CGTGATCAGCTTCGACTGATCTGCGCCAAGTGTGGGCCCCTCTGCCAGGCG
AGCTATCCAGACTGGGACAAGCTGCCCGGATTCCCCGACGCTGAGGGGCA
TCCATGGGAGCAGACGCCTCAGGATATATCTCTGCTGGAGGCGTCTAAGAA
CTGGCAGTGAGCCATCTCTTGGCATCGGAGGCAGTCGCTGACGGGCAGTAT
GGAGAGGGGTTGTGCCGACCTGATGATGCAGCTCTTGACTCTCGACCCGTC
AAAGCGCCAACCGCCACTCAGGCCCTAGACCACCGGTGGTTCTGGACACA
GGCGTTTCCAGCTAAGCCGAAAGAGTGAGTCCCACGAGATGATGGGTTTC
GTCACTGACACTTCAGTGCCCTCATCTCTGTCGCTTCTTCCCACGAGATGA
CGTCGAAGCAGCGACGCGACGGGCCCGCCCCTGCCATGCCTGCGCAAGTC
ACTCGTCCCATGGCAGTTCGCGCTGCGCCACAACGTCCACCCCCATTCATT
CCTGGGCCTCAGAGATTTTCTGGTCATCCCGGCCAATCTGGGCCCTCGCGT
CCTCCAATGAACGGACCCAATATGGGCTTCCCCAATCAACGAGACTTTCCG
CCACCCCAACACTTCCAACAGCCGTTCCCGCCGCCTCCCATGTCGCAAGGT
TACAATACCGGCGGCCGGCCCATGCAGCAGCAACAGCCGCCATGGCAACC
GCATCCTCGCGGCGGTGGTCCATACATCCCTCCAATGGGCGGCCCACCCCC
CAACATGCCACAATCGCAACGACAGTCCGGTGGCGGATATGCACCTCCGCC
CCCGCCATTCACGCTCTCCGATTCGAGATCTGGCGGCGGTCCCGTGCCAAC
AGGGCCGGCCAATGCCTCGCAGAAGCAAGGACAACCTTGGAACAACCCCC
GGCCGCGGCCCGATGTACCCAGTACGGGACTGAACTACGGATAAAGAATG
AATACAAGTCGTTGGGCGGTGCCATTTCTTGCATGTTTCTCTGTTATGCATG

CCTGCGATTTGGTTGTTGATTGTGTCATTAGGGTGGGAAAGGCGTCTAAGG
CACCGACCGACTGACAAGCCAGAGATGCCATGTGCACGTTTGTACATCTCT
CCTCCGAGTCTCTCTCTCCATCTCCATCTCTCTCCTTGGCGGCAGCGCGTCA
CTGGCCTGCCAGCTCGTCACAGCCTCGGTCGACAGAATAATATAGCATGGC
TACGTCGCGATAAGCCCCCGCCGCCAGATCGATCACACATCAAGACTCCCG
ACTTCCTCTCAGACGAGCAGCTCGCATCTCGGACATCCGACATCCAGGCAG
GCCGCTTGCATCCTTTCACCACGCCCCTTATTGCGTCTCTTCCGTCGTCTCT
GTCTCTTCTACCGTCTTCACTTCCTTGTCGCTCGCCGCCGCACAGTACAACA
CACGCCTCGAGCACAAGATTAGTCGAGCGGCAGGTGGATGACACCCGAGG
ATGGACCCGTCGTGGCGGCCCACCCTACACACATCTTCGCCCCTGCACCCG
CACCTGCTGCTGCAGCAGCAACAGTACCGCCCACCACGCACGACGCGATG
AAAGCCGAGGACGAGGCGCCCGATGCAGGACCCAGCCGGCCGCGACGGC
CATCGCATCCTCCCAACCTCATGGACAACGACGATGATCCCTCCGTCCCAC
CCCCATCCACCGCTCGCCCCGCCGCCGCCGTCGCAATGTCCCACTCGCGCT
CCGCCTCTTCCTCCCACTCGCATGCGCACGCGCACGCACACTTACCCCCGC
TGCCCGCTTCCCCGCCTCGGCCCCCGACGCCGCATCTCGACCTCGCGACCT
ACCCCACGCCGACGCTGCTGCGCCTCCTCGCCAGCCTCCTGCAGCAGATCG
CGACGTCCAACGACCAGCTGCGCGCCGCCGAACCCGAGGACGAGTACATG
GACGAGGACGCCGAGTCGCACCACCCGCAGCCTCAGCCTGCCCAGCCGCC
GGGCGGCAGCGCGTCTGCTGGCGCTGGCGCGGACGGCGACTCGTCCACGT
CCAACTCGGGCAGGTCGCATGTGTCCCGCTCGTCCGTCCGCCCCTCGCCCA
CCACTGCGCTGTTCGACACGCATCCCCCCGCCTCGCCGCCGGCGAGCAGC
CGGCTCGACCCTGCCCCGCACGTCTTCTCCGCCTCGCGCACCTCGCTCATG
CACCCCTCCTCGCTGCTCTCCTTCCACGCGCGCCACGTGCCGAGCATCACG
ATCGAGGCGTACCTCCTGCGTATACTCAAGTACTGCCCCACGACCAACGAG
GTCTTCCTCAGCCTGCTGGTGTACTTTGACCGCATGAGCCGACTCGGTACG
GCCATGCAGCTTGCGCGCAACGCTGCTGGAGGCGAGGGGGCCAAGGCCGT
TCGAGGCTTGGCGATTGATAGCTTCAACGTGCACCGGCTCGTCATTGCTGG
TGTCACGGTAGCGAGCAAGTTTTTCTCCGGTATGTCGTGACATTTCGATGG
GCATGTGTGGCTGACTCTTTGCAGATGTCTTTTACACCAACTCGCGGTATGC
CAAAGTCGGCGGGCTGCCCTCGCACGAGCTCAACCAGCTGGAATTGCAGT
TCCTCCTCCTGAACGACTTTCGGCTCACGGTGCCGCCCGACGAGATGCAGC
GGTATGCCGATCGCCTGTTGGGGTACTGGGAAGGCAAGGAGGCGGCGGCG
GCCGCAGCGGCATCTACGTCTGCAGCAGACCCACCGCCTGCTGCTGAGCC
CGCTCAGGGGGCGCAGGGCGAGGCGGCTATGCAGGTGGATAGTCCCGCCC
CCAGCCGACCTGCGGCGGAGACATTGACCGAGGGGAGCGGCGGAGGAGG
TCGCGCCGGCCAGGAGGGACCCAGACTGCCTTGCCTGAAATCACGGCGAG
AGGCAAGGAGCGGGATACGGACGATGGGCCGAACGTCCTGCCGCCCGGGA

CGGCGTCGGCTCCTGCCCCGGCGCCAGTCCCAGTGTAGTGTAGTCACATGT
CATCGGTATTTCTCTTTTCATAGACGCATCTCATATCCCGGTTCTGGACCCT
GGACTATTAATCGAGCGGCGAAATCACTCGCTCAATGCCAAGGTCAAGAGC
ACCATATGCATCAGGCATCAAGCTCTTGTTCGTCCTCTTCCTTCTCCCCCTC
CCCCTCCCTTGAGGTATGAGGTAAGGGTCGCCACACTACCCACATGTTGCC
TTTCGGCATGGTCTCCCGGTGTCCAGGGCCGAATCCCCACTGTTTAGCGAC
GAGGTCGATAGAGGCAATAGAGCGCAGACCAAGGAGGGGCGACACGGCC
CGGACGTGCGTCGCGTCAAACTGCCATCAGTCCTCCTTCACTGTGCCTGCT
ACACCCAGGCTGGGCTGGGGATGAGGGTGGGGGACATACCTCGGCGTCCG
CTGGAGAGGCATACGATCCCCCGTCAGTCAAGAACGGGCCGTACGCCGCT
ATCCACCCCTTTGAGGGGTCCAGCATGGCCCTGCCCCCTGCTGAAAGGGG
GGATAGGTGCTGGAATATCTTGGGTGGCAGCGAGGGCGGTATGCAGTGCAG
TATGTTGACTGACCCCATCAGCCAGTGCCTCTCTTCAGACTAAAACTAGAG
GGGGGGGTGGTGACTCACTCAAGAGGACGCCGTCGAACGGGCCTCGTCCT
TCGCCCTGCACGGCACGGGCTAGTTGTGCCCAATCTGCCTCGGAGGCAATG
TCCCTGTATTCTAGGTGGGTCTATGTTAGATGCCAGAGAATCCAGGTCCAGA
CGGCGCTGCACATCTCTACCTCTCAGCTGCACCCTCGTCGCTGCCAACCTC
TGCGAGGTTGGAGATTGAGGGTTGAACTAGGGGGAATGGGGATGACTCAC
GCCAGTTCGGCAGTAGCGCACAGTGCCCGTCCATCTGACATGGGGGAAGG
TCTTGCCCAACGATCGGAGATGGTCGCATGACCCCGACCCGATCTCCAGCA
CTCTCGCCCCCTTGCCATGATGGCTGGAAGAGCGCGGGGTCGAGAGGAGA
GACGAGAGTGCTGTCGAGATGGGCTGGATGTTCCTGCTCGCCGAGCCTTCT
GTCAGTCCACCTCTCGTGGCTTACAGTGGGCCCACTCGCCTTCTTGGGAGA
TGACGGATGGGTAGGGGTCGGACATGTCAGCGAGGCATGGTCAGTAGTAA
CTTGTTGAGATGAAACGAGTGCAGTGATAAATGGAAATCTCCAGCCAGGA
ATTATCTTATCTCAGAGATCTTGTCTCAGGTCCTCTCATCCCGACGTTGAGA
ACCCCAAGATGTCCCCCTCATAACATGCTGCATGACAATATAACAATGAGCC
ACGACCTCTATGAACCACCGCGCAATTACTAACCCCGGCGGATGGAATTATA
CCTGCTCATCAGCCCACCTCCCCACATGACACAGTCAACTCACCCAGGGGA
ATCTCGGACAGACCCTCCTCCTCTTCGTCATCCGGCACGCTGCCCATATCCG
TGTGCTGATACACGGTACTCCTGCCGTCGCTCAAAGCCTGGCTGCTGCGCC
TGTTCACGCCGTCCGCCCCAGCACCCATATCGGGACTTGTGGGCTGCTGAC
CCCGACCCGCTCGGCCCGTCATAGGTCGACATCCGCAGCCTGTTTCGCTCG
GCCTGGGCCTCTCGCTGCTTGGCAATGGCCGCAGCACTCATCCCGGCACCC
GCACCGACTCCCGCACCGCCGACGGCGCTCTGAGGTGGCGACATGGGCGG
GGACGAATGGCTGCCGCTGCTCGTACGCCTGTCATACTGCTCGTACGGGTC
GTTGCCGTAGCCACCCGCCGCGCCATATGCAGCGGCCGCACCCGCACCTGC
GCCAGCGCCAGCGGGCAGCAGCGCCGTATCCGCCCTCGAGGCCGCTCGAG

CCGTATTGCGGATAGCCAGAGGACATGTAGTCGCGCGTCGGGCATGTGC
ATCTGCTGTGGGGCGTGGCCGTACGGGTCGTAGGCGTTCTGAGGCGACTGC
GGCGCCTGGTAAGGGAATGGGTCGACGTGAGACCCGGCACCTGCACCGGT
ACCCGCGGCAGCTCCAACGCCGGCGGCCCCCATGTCGGGAACGGAGGGCG
AGAGGATGTCCAGCGGGTCCGAGGTCGAGTGCCGGGTTGGGTCAAAGGTC
TTCTCGTCAAAGGCATGTTGACGACTGCGTCGACGGCGGAAAGCAGAAGA
ACCATATGAGTGCGGCGAGAAGGGCGAGACCGACAACGCCGCCGACTACA
CCGCCAACGATCGCGCCGGTGTCTGCGGTCAGCTTTGGGACTATGACGTGG
AATGCTATTCAACGCACTGCTGCCTCCGCTCTTGTGGCTGCCGCTGCCCGA
GCCGCCCGACGAGATGGCCGAAGTGGTGGTGTGGGTCTTGCCGCTCGAGT
CGGTGACGGTCTGGGTGCTGTCTGTCAGCGAGGTCGGAGAAGAGGGACAT
AATGAGGCTCACACGTAAGCGGTACTTGTATCCGGGGCCGAGGTGGTGGTA
CTGCTCGACGCCGCGCTGGAAGTGGTAGAGCCAGCCGAGGATGTTGCGCT
GGATGAGGCACTGCTCGAGGTGTTTGAAGCAGACGAGGAGGAGCTGCTCG
ATGTGCCAGAAGCAGAGACCGACTGGCTGCTGGACGACTGCGACGCCGAG
CCGGACGCGCTCGACGAAGGCGGTGCAGACGAGCTGGCGGAGGACGACG
GAGGGACGCTGGAGGCGCTGGACGAGGTAGTGGAGATGGACGAGGAGGA
TGACGTGGGCGGTGTGGACGAGGTGGGGGAGGAGGATGCGGATGAGGTG
GTCGAGGCTGTTGATGATTCGCCGGGGGTAGAGGAGGGTATGAGGGAGGT
GCCGATGGAAGAGGGGTTGATGATGGAGGCCATGGTCAGCGGTTGACTTT
GTGTTGATGCGATGCGATGCGACGTTATCGGGCAGGTGGATTATAGACCGA
GACAGACGTGAGATATGCTACAGACGAAGAAGGTGATGATGATGCAGATG
CAGATGCAATGCCAGTGATGACAGTGATGACAATGAGTGAGAAGGGTGAC
GGGGCAAAGCGGGCAGCAAGAAGACGCTAGCGAATGGGTAACGGGGCAG
AAAAAAGCGAGGAGAACAGGGTAAAACAGGCAGGCTCAGGCTGCTTTGC
CTGCGTCGGTCCTCGGTCTGCGGGTGCAAGTGCGGGGGTCCAATCCAGCA
CGAGGATAGTACAGCACAGCCTAGCTGTGTACTCGTGCAGCCGTCTGGTCA
CGAGTGCAGCGGCCGTGATGGGACGAAACCGGAGGGTAGGGAGGATGTTG
GGTAAGGCAGACGCATGCCAAGATGGGCAGATAGGCAGAGAGATGAGACG
ACCCGACTCACCGGACATGGTTATCGCCTGTAGCTTTTGCTGATGCTCCCTC
GAGGCGACTGATCGAGTGACTTATCGTCTTGCTTTTGCTGTTGCTGCGGATG
GGTGTATATATAGGGAAGAGGTTCGTCTGTAACTGCGAGAGAAGGGTGACG
GAGAAACAGGCGGAGGTTTTGACCCCTGGCTGACTGCCTTTGCTCGGAGT
TTGCCTGTCCACTGCCCACTGCTGAGGGTACCCTCGTGATAAGTTACGTTG
CACGACTGCAGGAACTGGGGCTATGCAAAGAAAGAAGAGAAAAGTGTAC
TCGTAGCGAGCTTGGCATCGGACTCCCTTGCCCTGCCTTGTTGTTCTCCCCG
CTCTCTTCTCTCGAGCAGGCAGGCGGCAGAGAGCATAAGGTTGTGCCATCC
CGGTGAAGTGAGATTACTTTCCCTTTTGGGCAATAACACCCTCCATGCTCG

GGGGTATCGCCGAGACGGACCAAGTTCATCGCACTCCGCCGGCCGCATGAT
CACCTGTCGCAGAGACGGAGCGGACATGCAGCAACCCTATTTAACGAGCG
TCCACGTTCCCTCCATGGCTTGGCGAAATGTACCTGGCCGTCGCCGCAACT
CGCAACCCGTCTGGCAACACACGGTGCTCGTACCGTCTCTGCAGCTCGTAG
CCAGTCATAATCACCACATCCTGCTGTTTAGCCGTCGCTCCATCTTCTCGGA
TGACGAATTATTTACGCCGTGCGACAGTTTCAGAGTGAGACTCGCCTCGGC
GTGCGGGCGATTGGGTCTAGCCGACCCGCCTTGCTCGAAACGCCGTGAGG
TGTCCTTGCGCGGCCCTTTCGGGCGGAAGGACATGATTGCCGTCGATCCCA
AGCTGAGCGAGGCGACAACTGGTCCTAGCCCTGCATCTTCAACAACGCCT
CTCCGAGCAGCAGAAGCCCGCTGACCTTCTTCTCCGTCGTCGTGCCAGCTC
ATCTTCTGCCCTTCGCGGTACAACACCGACAAAACCTCGTTGAATGTCGCC
GGCTCGCCCAATACCGCAGAAGTATAGTGGCGGATGCTGCACCGTCAGCGC
TTGTCATTCGCAACTTGTCAGACTCACTGATTCAGTGCAGAGAGGACACAC
TCGGGCGACTCGTCGAACGGGACCGAGACTGTGTCGACCGCGTACTTGTA
CGCTGCGCCGGAGTTGAGCGCAAAATGTTGAGCAAGGGTAGCTCCCTTCA
CTGCCGATCAGTCGGCTGATCTGGTCCCGGACTGTCGCCTTACCAGTATGG
ATGGACTTCAGCGGCCGCCGTTCGAAGAGACAGCGAGGGGAATACAGTGC
CTCTTCCTGATACTGCTCGAACAAGTCGTCGGCATCGTCAACGACATCGGG
GATGACATGGTAGACCTGACCACCGCGAGGCAGGTGATAGTGCATGACTGT
CGCCCCAGGAGTCGTCATTCTTCGCATTTCCGTGCGCATCTGCTCTGCCACG
CGAGCTTCCCCCAAGAACGCAGGCCGTCGAGGCATTGCTGCGACAGGGAC
GAAGTTCGCCATGGTCAGTTGATGGGTCTGCTTGCCGCACGCTCGGCATTC
CTTCTCGTCAGTCAATCGCCCTAGACCTTTTTGAGTTTTGACCAGTCTAACT
CACCCAACAGTCCCATCGATACCGACAATTCGCCCGGCCACACTCACACAC
CCAGCCTCGACGTCAGAGCGAGCTAGTTACTTTTATGCAGTAGATCTCACC
TGTCTGCAATCGTCTGTCGCCCATATGCGTCTCGAAAAGGTCTTCGCTTGGC
AGAGGCGGACAGACAGAGTAAGGCGGGATAGCATGCGGGGGAGTCGAGA
CTTGCCGCAGAAAGTCTGGCGAGTATCGCAGTCTGAATCCTGGCGGTATAG
GCAAAAGTCCGACTGCGCTGGCCAAAAGCCAGAACGCAGGACATTCGGG
CTGAAGAACAAAACCAGCCTTCCGCATAGACGCGGAGAGACTCTTTGCCG
CACGTCGAGCACACCGAGACTCGTCTTCCGCTCACATGTCAGATAACGCGA
AGAATGACGGATGATGGACGCACTAGATATCAAAGGTGTCATTCTCGGCAC
CCAGGACTGGTACATCTGTCCGGTCGTAAGTCGAGGTCGCCTCGCTGCCCC
TCGACACACTTGACGTAGCCGAAGCGCTGAATGCTGGCTGTGGCGGCGTG
TCAGGCGGAGTGAGGAGACCGTGGGCAGTGACCACAGCGGAGTTGAAGT
CGTTATCCTCGGCTCGATCAGTCTTGATTGCAGGGTTCAGCAAGCACTCCA
CTCGCAATCGCTCTTTGAGAAAAGCATTGGCCGGTGCACCAAAAGCGGCA
GGGATTGGTGGTTCGGGCGGTCCTGGAGCCGCTCCCGACGTGAGTGAAGG

GCTGTCTGCTAGCCACCAAGGTGTTCCCTGTGAGTCGATCCAGTCAAAACG
GATCTTGACTCGTTGAAACCAGTCCCGGTCCGCCGGAGCTGCCACTCCGCT
GGCAACAGGCTCGGCTTCGACCCAAGTGGCGGAGATCCAATACCTGGAAT
CAACACGATGTTGGCAGTAGATCACGAAACACCTACCAACCCAAAACCAC
ATACATGCAGTCTAGCTTCCACGGCAAGAGCTCGTACCCCTCGCCGGCGAT
AAGAACAATCGGAGTTTTCCTCTTCGCTGCCTCGAGCAGTGTCGCCACTCT
TGCGTCCGTAGGGGATTGATCGGCTTGAAGGACAGCTGGGACTTTGTCATT
CGTCGCCGTTGGCGGACGTATACACTGTCCTCCGCCGTGAGAAATGACCAC
TCGCCCTTCATCCGCCCAGACGTCCCGTCTAAGCCAGTCAGCGAAGTGACA
GCACAAGGCGTCAGAAGGGTCCAAGTGGACTGGTCAGGACTGACGGAGC
ACCGAAACCCTCCAGCAGATAACCATATGCGACTTTGCGGTACATGTGCAA
CCCCGCCTGATATGCTCGATAATATGGTAGAGTCTCACAGAGCTCTTGGCGC
GACTCGGCCCACACTGGCGGGCGCATGGGCGGTGGTGCTCGTTCACAGAA
GGCTGGTCGGACCAGGCTCGCCGCACATCACAGACTAGCCGAAGCACCCG
GCAGAAAGTGGAGGAGGGTATACCTTGCAGCCGAGGAGACAACGGTCGAT
TAGGAGGCCGGGCGGCGGTGGAAGGTTGGACGGTGCTCGAATCTCGCAGG
ACGGTTGACTGTTCGTTCCCTTTTGTGTTGGCCAAGGTTGCCTTCTTGCGC
GCGGCAGGTGGCCGCGATGCCGGTGCCATTGCTACAGGTTTGAACTGCCCT
AGGTCCAAGACAGCAAGCATGCCATCCGATACCTTGGAGGCTTTCCATGCC
GATTTACCCGCCCGGGGCCTTGTCTGACTGGCCTCGGGCGCGCCTTCGAGC
CCATCCTTGATCTTCTCTCTCTTCAAACCTTTGTCACTCTTGCGCTTGAGCG
GCTTGACATCCTCCTCTTCATACTCGCTAGATTCTCGGCGTCGCCGTTTGAG
TGCCGCCCTTTCGTGCGGCCGAGCCGCTTTCG

>Mosaic_sequence_11

CTCGGTCGGTGCGTCTCACACGGGTTTGAGCGATAATCTAGCGTAGCGTGG
GGGTGGTGTGTCGGTGTCTGAGGGGGCTGCACCGGAGAGAGTAGGCGAG
GACGTATAAAGAGCAGGAGCAGGAGCAGGCTGCGAGACTCGAGTTGGGC
GAGTGGGTCTGCAGGCTCGGCGATGGGTGATACGACGACGAGCAAAGAAG
AGAAAGAGGTGGGTGGACGAGGAACGACGACGATCCTTCAGGTCCCCCTG
TCATATACCCAGCAACGAGCAGCCAGGGATCCACTGGGTTTTGCACCACCG
CTGCACTGGCGCCGGGACCCGAGATGGCGGACCTGGCCATCGACATAGAG
TGAGTGGGTGGGTGCATCAGAGCGTGCGCCTCCGCCTCGCCGCGAGGCGT
TCTTCTGTGGGCATGGCAGGCAGGTGCTGGGTGGGCGGGGCCGACCGATC
GAGCTAAGCATCGAGGCAAAGACAAAAAGCCTCGTCAACTGCCGCTTGCA
GCGATAGGGTCGCGCGCAAGCTCCGAGCGAGCTGAATTATCGCTGGGGCC
CGCCGACATGTGTCCGGGCCCAGCGAGCGCAGCGCAGCGGGCTGCTGATT
GCCAGCAGCTGGCAGCTGCCAGCTGGAAGAGGATGGCAAGCCGCCCGGC

GGCCCTGACTTGGACCTAGTCTCGCAAGATCGTAGCCGTAGCTTGGCTCTG
CGAGTCGGGCGACGGCCTTTCGCCCAGCACCGCCGAGAGCCAAGTAGGCT
CGCTCGCTCGCCATTAGAGTGCAGGATGTGCGAGAAGAATGTAGACGGGA
AGGTGCGCCATTGTGTCAGTCGAACGCACCATCTTGGCCGGCCGGGGTGC
CGAATCGGGCAATCGCAGAGATAAACCTTCAAGCCACCGGTCCCAAGCTCT
GGAAAAGCGAGATGGCTCGCGGGTAGGAACAGAGAGCTGTTCAGACGAC
GTCGATGGCTTTAAAGTTACCGGCCTGACCCGGGACCGTCAAGGTTATTCT
CCGATCCGTGCACCTCCTTCTCTGGCTGTCCAGAGCTGGAGTATGACTTGC
TCGATCCGTCTCGGGGAATTGGTGACGCAGTGCCTGACGACTCACCATCGA
AGCTTGTTTCCTCCATCTCTTGCGCTGCTGCTTCCTGCCAAGCAGCAGCAG
CAGCAGCAGCACTACAGAGTGGAGTACCGGTGGTGGCGTCGTGGATCATC
GACGAGGCTAATTAACATCGGTGGGCGGGCGTGGCAGTCGCTCAGTGGCA
GAATCGAATGGTGGCAGTGGCAGTGAATTACCACTGAGACCTTTCTGGCTT
TCTGGAGCTCCGAAGCACCGAGCGGGCGTCGTTCGCTGGAACGTCGCATA
GCCGGGACGATAGCGATACCGGTTCGAGCACGATCGGCCCGCCAGCCATTC
GCCCGCCCGACCAATCAGTCGCTGAGCAATGGCGATACGATACGAGCGTCT
CGATCGCTCGTGTGGGCGAGATGAGCACTGACGCAAGCGGCACTTTGCGC
TGTGCAGCGGTGGAATGCGGCCAACACCGTTCATCGACGCCTGCTTTCTCT
ACAATCTAGGTGGAGGGGATATCGTTAAGCATACACGGCGGTCCGCGAGAC
CCACGCCGAGCCCACCTCCCAGTGCGCCCGGCACGCGGCAGCCGCTGTGA
GGTATGAGAGACACGACGGCCACGAGCATTGCCATGCCCATGGCAAAACG
TGCTCAAGCAACTCGCGGCCAAGCGGCGGGGGCCCGCCCGCATGGAAGA
GCAAGAGAGCAAGTATCAGGGACTGTCTCACACGGTGTATCACCTCTTCAT
CCCAGCCGGGGGTCTCAGCCCTTCGCGCCACGCGACAGAGACGATATGTA
CTGAAGTGCTTGTTGATTCCCACTTTCGGAGCGCGCTCGGAGATCGGCGTC
GGCCACCCCGGCAGGTGAGGCCTGGACCTTTACGGATTCGGACCCGTGTA
CAATGTTTGTTCTGCGACGCACAGGTGAAGCAACGTGCTTGCCCTCATCCT
TCTTTTTGTTCGGTAGCGACGGCGTGAGACTCGCGTGGACGGTGACTGCCT
CCTCATACCAGACACTCACACTGATGTGGCATTACCCATGCGTGAAGTGAA
GACGTCGTCGGTCATTGACGTAGCTGCCGAATCCCGACTCCCGACTCGGGA
TTGCGGGGAGCCACGTAAAGAGTCCGCCACAAATGAAAACAATGACATCG
TTAATCGACGCCCAAAAGAGACTTGAGCAGCACTGCATATCGATCCCACTC
TGCCCTTCGAAATCGGCGCGAACAAAGATTTCTCTGCATGCCACCGACAAC
CATAAGCCTCCTACTTGTACTTGTCTTCCTCCCTACCTGAAAGATCTAGTGG
TACACCTATGAGGGGAATCTCGACACGGATATATACCATTCGTCCACTGACC
TGTCCCAAAAGTCCAGAGGGAACCTAGACGAGTGTTCACTATACTTATAAC
CCCAGGACAACATAGATGGTATGCACAAGATCTCCTTCTCAGATTCCGTCC
ACCGCCGGCGCAAGGTCGGACGCGACATCCCCGTTCGGGGCAACACCGTC

GAGCTCTGTAGGCGCGGCTTCGGCTGAGCCACTCGGCATCTCGTCGAAAG
CTTCCGGGACCGCATCGCCACCGAGCGCGGCAGCCTCAAAGACGTCTTCG
GCTTCTGCTGCGGATGCTTCGACAGCAGGCTCCTCGGTCGAAGCCTCGACA
GCCTCCACTTGATCGATGGTGCTCGTGGGAACCTCGTCGACGACGGGCGG
CAGGTCAGCCGGGGGCAGCTCGTCGACCTCCTCGATGGGGACCCCAGATG
CGTCCGCGGACACTTCAGCCTCCACAGGGACAGACTCGTATGCTTCGAAG
GCTTCCGTCGCGACGGGCGGAACAACCGACTCGCCTTCCGCTTGCAAGGC
TACTTCGGCAGCTACCTCGCTCGGTTGTTCGGTTGCAGTCTCGGCACTCGC
AGGGATCTCGGCGGTTTCGGCTACGGCTTCTTCCAGAGCAGGGGCAACAG
AGACTTCCTCGGTCGACATGACTTGGTCGATGACGGCAGGGCATCTCCGTC
TATTGCGAGTCTCGTTGACCCTCCTGTGCCAAATATTATCTCGCCAGTTCCG
TCTCACACATCTTTTACACAGAATGCGACCGCGCATTCTCAATCTCAGTCCG
GTGTTCACAAGGAGTCCGGTCCGATGCCGGAATCGCCGAATCCATTCCGCA
AAGAATTTATTCCGCCTCCAGCGCCGAAACCACCCACAGTTGAGGCAAAG
GAAGCGAAGGATTCGAAAAAGCTAGCGCCGCCTCTTGCACCTACGGCTATG
TCTAAGATCGAAATTCGACCCACGTCCATCACTACCATTGGTGGCGCTGGTT
CTAAGAAGCCCTCTTCTAAGAATCAGAGCAGGGCATCATCTCCTCGTATCA
ATGCGACAAAGGACTCCGTCGCTCCTCTTCCTGCTGGGTCAGATCGTTCTAT
CCTTGACTTTGGTAAAGCAGAGCCAGGATCCGAGACTGTTGCGCCGTCAAT
CATCCTCGATATTCCATTAAACGGAGAAACGAACAAGTATATAAATTTCATG
CGTATGGCTGAAGACCAGTATGGTTGGGACGCCCTTCACCCTCGGCAAGCC
GAGCACAAAGCACGCAAGGCTCGCATTGCAGCTGCGTCCGCCGCATTAGC
TCAGAACGACTCCAGCCGTGAGGGAGATGAGATGTCGGTAGATGAATCTG
ACAACGACGATTCTAACGTAGAGATGGGCGGTACCAGTGGGCCAGATAAG
CCGGCTGATGGTAAGCCGGCAAAGAAGAAGAACACTTCAAGGAAGATGA
ATACGACAAAGACGACGATTTCGTTGACGACTCCGAATTACTCTGGGAAGA
GCAAGCGGCGGCTAGTAGAGATGGCTTCTTCGTTTACTCTGGACCTTTAGT
ACCGGAGGTTGAGAAGCCAGAAGCCGGACGGTATGTACACCAGACACCCC
TGATAATTAAAAGTATACTAATTAAGCATAGCGGTGACGGTCTTCCTAAGAG
AGGACGAGGTTCGCGTGGTGGCCGTGGTGGTACTCGTGGTGCTGCTACCG
GCACAGGACGAGGTCGTGGTGGCGGACCGGGATCTCGTGGAGGAGTGGT
GCGAAAACCCCGAATGACAAAAGCAGACAAGGCACAGATGGACCGCGAG
AAGGCCGAGCGCGAAGCTACCCTATTAAAATCAGCGACCGGCGGAGCCTA
CAATGTTCTGCACCCAGGCAGCCCCCAATTTGCGGGCTTGTAGGACTATGC
TCAGTGGAGGAACAGTTAGCAATGAAGACTTCACGTTTGTAATATATGCAC
ACACAGCATGGGCGTTGGGCAACTTATCTTTGATCGCCAGCGGCAGGCGTG
GGGTTAGAGGGTCTCAAACACTGACTCGATGGATCAGCTCCTATTTATGTCG
ATACCATCATCATCGTTCTGGCGCATTTGAGGAGTTGTGGAATTGTGGAATT

GAGGAATTCGGCGTACAATTGATCGGTGACCTCGAGAGGTTAGATTTGATT
TGGGAGGAGGGAGTGTGCACTTTGTAATATGCCAATAGTAAACATGGTGCT
TCAAGTATAAAGTTGTTGATCTCATGGTGAATGGCTGAAAAGGTGTGAATG
TGGTAGAAGACATAATAGCAACAAATGCGTGCCTAGTACTTAGTAAATACA
ACCGGAACACGCTCAGCTAGCGCTTACCCAGTCACGTAAGCTCAATGTGAT
TGGTCCAAATGTACATACCCACCTTTAATATTGGGACCTGGTTCCTACGCTG
GAAGTTCGGAGTGACCCCTGAAGCCGGACTATGGTCTCCAAGCATGTCGC
AGTGGACTGCGTCTCCAGGTCTTCTTTTCGTTTCTATAGTATTGCCAAAGAG
GCATTATGACTTTGAAAAATGGATGTCTGGTTAGTCACCAATGGAGTTAGCC
GGTATTTGCGGGTATTTATAAAATCCGAGTCGTCCACCCGTTCTACTCGACC
TGCCCCTTTTTCTCCTCTTCTCTTCTCCTTTACTGCTTCCCACGCCATTTTCT
ATTATTTACTCAATCCTCTCATTTACTTTGAATTAGGCTAGTAAATTATAGCTA
GAATCATCTATTCCGCGAAAATGACTTCACTACACCCCCTTTCCGCAGCTGC
GTTGTTTTCGCTCAAGGACTACGTCGCCGTTGGTCAGGCTTCAACCCCCTT
TTCTAGTGTTCCCCTCTTTTGCTAACTAAGGCTGCATAGTCACCGGCGGGGG
AACGGGCACCGGATTGAGTAAGTCACCCTGTTTGTTTATCTGAGATATTACC
CCTTTTCGTAAACCATTTCGGACGGCTTACACGGAGGTTCATAGTGATCGCC
CAAACACTCGCAGCCAACGGGGCTAAGGTCTACATTACTGGACGCCGTGTC
GATGTTCTCGAAACAAGTGCGCGTATCCATGGTTCACCGGAGAAGCTCAGA
GAGAGCGGAGGGAGTATAATCCCGCTGGCTATGGACGTCACGTCGAAGGAT
AGCATCAGAACCGCCGTTGCCGCGATCTCGGAGAAGGAGGAGTTCGTGAA
TATGTGAGTTCCAATCCCCCTTATGAAGCTACTTCACAATTAGCGATTACAA
GGCCGTACCCACACATCTTCTCACAACCCATTTACCAGTCTCGTAAACAAC
GCTGGGATATGGGGCGGCCGACCAGCCGCAACGCCCCAGGACGGGCCGGA
GGTACTAAGCGAGGCCATGTTCGCGGAGGATAAAGTCGGTAATTGGCAGA
AATGTCAGACTACCCCAATATCCCGTCTTACGTCTCGTGATCGCGTCTTCAT
GCTAATTGTCGCACAGCGTTCGACGTCAATGTTACCTCGCAGTACTTCGTCA
CTGCGGCCTTCCTCCACTCTTGGCTAAGGCGGCGTCGGGTTTGACGGGGAA
GATCGGGAGCGTGATTAATAATTGCTCGATGGCTGGCTACTTGCGTATGTGA
GTATATCGATATTCTAAAGCTGTCGGAAGAGGAAAACCTATAGATTGCTAAT
GAGGTAACGGCCGTGATTCTCTAGGTCGCAAAACCGCCAGTTTTCTTACAA
TGTGAGTTTGCAGAATTTGTGCGAGGAGTTAGCATTCGGACAAATAGGGCT
AACAGAGATAGTTGACCAAAGCTGCGCTTATTCACCTTACGCGCCAGCTAG
CGTTTGAATTAAGCCATAAAGAAGTGAATGTGCGCGTCAACGGGCTTGCGT
TGGGTAAGTGTTTCTTACCCCTTTCCTGCCCTCGGAGAGGACTAATGCGGG
CGCGGTAGGATACTTCCCGTCCGAGATGACGACCGGCGAATCGAACGCCG
AAAACGAAAGCACGGTATCTAACGAGGAGTTTGGGAAGTTCTTGGAGAGC
ATGGGTGGGAGGATTTTGAATCGCATGGGGACGCCACAGGAACTTGCTAGC

GTACGTATAAGATTGTTTTCCTCGTTACCCCCCTTTTTTACTGCGCAGTCTA
GTTCGAGTCTGGAGATTGCGCAAAAGACTATAATTAGGGATATTATATTAAC
AGATCTGTTTTTTAGATCGTGTTGACGCTTGCGACGAACGAATACATTTGGG
GTACTGTCACGATTGTCGACGGCGGGTTACGTTGGCTGTTCCGGGGAATAT
GTGAAGTAAGGGAAAGCGCACATTCGGAAAAGATAAGGAAATTAAATTTT
ATCATCTCCGAGGTTTGCTAGCAATAGGGAGAAAAAAATTAGAAAAGGAA
AAAAAAGTTGGATTGTATGCGAATCGAACCTGCGTGGAACAAAAAAGAAA
CTCGCGCAGGTTCGTTCTTATTTTGGATTGTAGTGGAACATCCATAGAGATG
CATCCATAATCATGGTGCCCACAAACATCCGCACTACTCACCAATCTGCGAG
AGTGTTGTTGTTTATCCTTATAAAGCTGGACAGTGTCAGGTGTGAGTCAAG
TGTGCCTGATGACTAACAGCTATGACCACCTAACGGTACACCCCACACCGT
ATGAAATCAATGGATCTTTCTCATTTCGTCCGATATTATAGGTATCGATATAA
GCAAGTACCCAGAGATGAATATGAATTTCATTGTTCCTACGTAGATGCCTCA
CGAATCGCGAAGATAGGTTTGTTTCTCGTAGAGATCCACCGACTCGCACCC
GTAACTTAACATTCAAGCTCCCTCTAGAACTAGAGACTATTAATCTCCCTAG
CATGATCATACACCAGCGTCTTCGCCTCCTCATCATCCAAGTAAATCGTAAA
CCCATCGTTATGATTCCGCCCATAAGAGTACCCCGAAAACCCATACTGCCAG
TACATATCCGACACCCCCTCCTCAACCATAATCCTCTGCCACCCCCCCTCAA
CCTCGACACGCGTCAAGTCGGATGCCGGGATCCCGTTCTGCTCCCGCACTT
CGGCTGTCATCCACCCGTACTCCTCGTGCACGACCGGCTTCCCCGCGTCCC
GTGCCGCGGAGGCATGGTCCCGGATCCATTCGTCGGCCCACGCGACACTGC
GGCCCCACCAATCGGGGTACGTGTGAAACACCCCGAAGTCGATGCTCGGC
AGCGCGATGTTCAGGTCGAAATCACCCCCGTCGCTGCCATTGTACAGCGTG
TCGTTCGACGCAAGGTTGAAGGCGCCTTCGCCGCCCCAGGTCACGAGGTG
GTCCGGGTCCAGGGTCTTGATGTAGGAGCTCATCTCGTCGATCCAGGAGGT
TAGTGTGGAGGCGGTGCAGGAGGGGGAGGTAGGAAGGTTGCGGGTGGCG
TCGCCGCCGCAGCGGGGCTCGTTCGCAAGTTCCCAGGCGAGGATTGTGGG
GGAGCTGGAGTAGCGTGTTATGAACGTGGAGATGTAGCGCTTGTATGCGTC
TTTGATTTGGGGGAGGCGATAGAACTGTGGTCTGGTGTTAGAATTAGAAGT
ATGGAGGGGATGGTATGGAACAAGGAAAGGGGCAGAAGGGTGAAAAGGG
GTGGGGTGAATTTACATCGTCGTGGTACTTCCCGCCAAGATTGACGGTAAA
AACGTCCATGCCTCCATAGTCGGCCCAGTTATTGGTGAAGGTGACAATGAG
CTTAATATCGGCTTTAGTAGCGGCGTCAACCACCTTGTCGAACTCGGTGATG
TTGATGGTGGATGTGCCATTTTCCCACCATTGGAAAATGGCGTTGGTTGCGC
CCGCGCCTTCGCCGCCGTATTGTGGTAGGCCTTGAGGATCATACGTGACGTT
CTTGTCGTTGAAGCCCCAGGTGCGGAATACTTTCAACCCGGCATCCTTGGC
GGCAGCGAGACCAACTTCGACATCAGTCGCATTCTGTGTTCTAGTTAGCGC
TGTATTTGCCCAGGTATAGAATAGTGTTGGGTATTTACGCTATCGAAGGGAA

AATAGTATGCATTGCTCCCAGCAAAATTGAAGTCTTTCCCATCTAATTGGAA
TTTGCCGTTCTCGACAGTGGCGAAGCCCTCAGGGACTGTTTTGCTCTTGGC
CACATTCACAGGGCTGGCATAGATTCTGCTCGCCAGTAGACTGAAAGACAG
AAGGTGAGAAAGCCTAACAACCATATTGGCAATTGACGCAATTGGTAAATT
GGCCGTATAATCTACGATTGAGCTTCTGCGGTTGGGTAGATTATTTATTTATT
TTGTCGTGATGCTATTTCTCCAATTTCCACACTTCCTCTTTCGGTCTACGGA
AGTCCTCGAGCCGCTGTGCTAGGGTTCTACTCATTGAGCCTCCGGGTAGCA
CTAGTAACCCTTGGTCCACCACTAAGAGGCAGCAGTTCAATAGTTGGCGAA
TAAAATCCAAGTTTGGATGCTATTCGGATATATGAGTGGTAACTGATATTTTT
ATCAGTCTCTTAGAGAGAAATACCCAAGATGCCTACCATGTTTCACCTTGAT
CAACGACCTATCTCATCAATCAGAGGATTCACAATCTAGCCAGGGTTGGGG
GTGGACCTGGTGAGGAGATATCATCACTCCGCTTGAGATTTCACTAGTGAC
AGCGATTTCCGTGGTCCTAAGTATCCCTCCATATGCGAGGTCATCCTCTGTG
AATCTATCCCTCACCCCACTGTCCCGAGTTCTCACAGGTCCCAACTCGGCA
ATGTTTACCGCACTATCATCGTCCATCCAAGGGGCAATGACGGGGTCGGTG
CTTCTATAACTTGCTTGAGGGCGTGACAGACGTTGAGCACTACCCTCAGGG
GGACCGAGCGGAAAGAATGTGCCAACCTTTTGTCGGAATCGAACGCCAAT
ATCTGGTATTTGGAATTTATTTCGAAAAGCTGGTATTGCTGGAGCACAGAAT
ATCAACCATCCTGTTGAACATTCGGCCAAACCCCAGATCAAGTATCCAGAG
TAGTCATAAGAAGGATCAGTGCTATATACCAGGCTGATCGAACAAGCAAGG
GGACCTGCAGCGCATACGCAGTTCCTGAAGAAGGTGGGACTGTCAGCATC
TCAACTGAGGTAACCATCGAAAGAGAGGTAGTAACGCACATAATTCCGACT
GAGAAAACAACATAAATAAGGATCTTCTGTCGGAATGATATCGCTAACTTCC
AAATGCGCTTATAGGGGATTAACAACAAGGCAATGTTGAAAATCAAGTGAA
GGGTGTTTATGAGAAGGTTCGAGACGTAGATGTTGATACACGTTCCAGGAA
GATATCGACGCCAGGTTCTTTCTCGGGGATTACAAGCGAGGTTGAAGACAA
ATATTGTCGAAATATATAAACAGCTGCTGGTCCCAATCATGGCGTAACAGGT
CCAGAAGAATTTATTGCGAGTGGCTCTTGGAATGAAAAAGTGAATCCATTC
TAGAATTATAGCTATTTTGACTAATCCAAGCACAACGCAATATAACGTACTG
ACTAAAACGTAAGTCTGTTTTGTATGAGCCGCCCGATCGAGGGTGTGGGCT
TTGTTAGGTATACACTGACATGAAGCCATGCTTCGTAATCAACCACTAGCAT
ATTCCACTGTCTCACGAATAATCCAGGGCGCGAAGAGGTCATGGTGGCGAG
TTTTACCGCAGCCGCTATGAAGAATGGCTAGTCTATATTAGCATAGCTCCGG
GGGTCATACGGCGTACGGCAGTATGGAAAGGGGAAAGGATATCGAATACAA
ATCCAAGGAGGCCGATGTCTGATTTTGTGGTTAGTTTACTGTTAGGGATCAC
CGTGGACTAGGGTCTGAGGGGCTAACAGTCTTCGATCCTAGTTTTCCTGAC
ATAGAATATCCTCGTAATAATTCGGATCAGAAATAAGATGGTAACCAGCGAT
ATACATATAGAGATGACTGCAATAGCGAGCTTATTATTATTAGGCGGATCGAC

GAAGTTGGGTATAACACCATCAGGGGGGCTTAATGCTGGCTGACTTCGGAT
GAATTGGATCTCGGTGTCGTTTAGAGTTGCTCTGGATGCCATCTTCATAAAG
GTGAACAGCTAGCCCCTGTCGGAGAAAAGAAAGAAAAGAACCACACTAG
CAAGATTACCCACTAGGTGGGTGGTAGGTATATATCAATTATTCTACTCTTAT
TGCTTCTTCACGTAACTGACTTCTCCCATATTGGTAGCGAATAAAAGTTACG
GCCCCCATCTTACGGTCTCCATTAGAGAAGGAATTCTCTTTCGACAAGACC
GAGTATTACACCAACGCTTAACCATTGGTGCCATTACTAATGAGAAACGAG
GCTGCTGAAACAGTATAATTCGGGTTGTTATTGCGGCAATCTTTCGTCCAGG
GCAACTGTCAATTTGATACTACGCGTATGCATGGTAGGTAAATATATTGTAGT
TGGTTACACAATGTGTGAGAAAGATGTGACGCTGAACTGGTCGGGAAGGA
AGATAATCGTAACTGATTGGTCAAGCATGCAGAAATATGCCAAAATCTGGG
TGACTTCAAATCATTCCTAGCTAGCCTATTACATGCGATAGCGGTATCACAAT
TGCCAGTCGCCTAATGCGTTCATGTTGCAGCCGATGGAACTCCGTATCGACT
GGTTGGGGGTCTAGATGTCAGCTATCAGACTATATGGCACTACCTTTCGTCA
ATTCCTAGGTACCTACCTAGGTATATAAGCTCCCCGACTTCCCGAGATGGAG
TTGAAGCCTTATGATCTCCTTGGTACACATGTCAAGAAATCATAGCCAATGT
TTGCCATACCGTCAACAGCCAAGAAACCCCCGCCATGTCCAGCCTACCTGC
CTAGTGGTTGCTAATATGCGTTGTGTTCGATGCCTACCTACATATCTGCTTGT
CGCACAGCATTTACGTTGAGTACTAGGAAGGGCAGAAAAAAGCGACGTGC
ATGAAGCAGTGAAAATAGACGATTGTAAGCTGTGAGTACTGGTAATTAGAG
GTTTGACTCGGCCTGATTTTTGGATTGGAGAACGGGTAGCTTAAGGAAATT
TCTTAGACACCTTGATCTCATATAAGGGGGTCTGGTTTAATAGCCTTCCTGG
AAGTGACATTGTTCAGTCTCTGGCCAGTTTGTTCGTATCTAATATAAGTGGC
TTTAAGTGTTAGGGGGTGAGTGATCAAGCACTCACTAAGCCACATACCTAC
CGCACCTAAGTGAGTAAGGTGAGATGGGAATGAGAGTAGCAATATATAAGT
TACCTACCAACGGGCTGCCTAGTACTAAAGCAGGTAGGTAGGTATTCATGTC
CAAGGACTATTTCTAGTACGAGGTTGAGGTAACGGTGCGGATTAGTGGCAC
AGAAGAGAGAAAGTTCCTTGGCCGACGGCCATGAGATCCGCCATTCCGTC
AGGCTATTTTTACTCTCTAGGGCTGTAACAAAATACCCCGTAATTATAGTGG
ATTGAGGCGGTTCAGTGCGCTAAAGTCACGGACGTTTTAGCGCGCTTTAAT
AGTGAATACTGTGACTTTTTGAGCTTTTACTAAGCCATTGCTAAACCGTTGT
TGAAAAGAGTGCGCTCGATTTAGGGTCAGAAATATGGTTTCGATATACGATA
TCAGGATTTCGCCCGACGATGCCGAGGTTGCTCCAATGCTACCTACCTACCT
ACAGCAACATAAGCGTCGTACTCTGAACCATTCCATTAGTTAAGCTGGCCG
GTTTCGTCGGCGTTAACTAACCGATGTAATGAGAAGCAAAGTCAATCCGAG
CGCAGTGTCTAGACAGTGGTTTAATAACCGACTCTATGTACGTCCCGGTAA
GGACATGCCTACTATGCTAAAAGCCAATCTAGGAATATAATATTTCGTGGTTC
TGATATTGTAAAGCTCGTAGAAATCTTCCAATTCCTTGACCACAACTATTTTT

TCCTAACCACCTGAAATTTTACAATATTACAACATGTCGTCCTCGATTGCCTA
TCCTACTGCCTCCGTATATCCCTTAAAACAGTTCAGCGATGGCCTCATAATG
TAATTTGCTTCTTTTTCCACATCAACAAGTTGCAGGGGATTTGCGCCGCCGC
AGAGACACAGAGCACGAGGATTTGGCGATTGTAGCCTTCGGCAAGAACGA
GCCGAACCTGCTCTTGGAGCGCTGGAGCTAACGTAGGCAAAAGCTCGGAT
AAAGCTTCAGTATCTGACATCCCTAGAAGCTTCTGCAGCTCGGATCGTGCG
TAGGTGTTGAAGACCGAAGTAGAGATCGCTAGAATCATGGAACTACCTATC
ATTCTGAACTGGGATCCAGCGCCCATACCAACAGCTAGAATTGATTAGATCG
GTAGATCTGGCGAGAAACACGGAACAGCTTACCGTTATCCATTTCAGCAGT
TACAAGTGGAATCATAAGGAGAACCATGAGGAAGTTAATTCCACAGCCAA
ATCCGGCGAGAACTTCCAGGCCATAGGTTCGTGTAGGTATTGTTAAAGTCTT
CGGTAGAGTACCTAAAAACGCAAAACCGATTATCTGCATGATAGACCCGAT
CAACATGAGATATACGCACGGAATCTTGAACCTTCCCGAGATTCTACTCCCA
AAAGCTGATCCAATCGGGATCAACGCGGTGAAGGGAATTAATCTCACGCCA
GCGTCAAGGCCAGAAAATCCATATACCAGTTGAAATCTTTGCGGAAGGGTA
AATATACAAACGACAGTAGGGCCGCCGAGGAAAATTGTGTTTCTGAGATAT
GATTAGCTGAACCGACGATATACTGGATAACGGCCAGCGGAACTTACAGCA
AGATCCCCACCATCTGGCGATTCTTGAAAAATCGCCAGGGTAGAACAGGCT
CTCGAGCACCATTAGAGAGTGTGACATGGCGTTCCCACAAAGAAAGTGCA
ATCCACAGAAACCCAGATATGGTTAATAATGAAATGACATATCCAGACTTCC
AAGGGAATTTCTTATCGGCTTCTTCAAACCCGGATGTCAAAGCCAGTAAGG
CGAATAAGATGAGAACGGTGCCGAGCATGTCTATCCGAGCGAGTGATTTCT
TCATGCCGGCTTTTCGTAGGTAACTAGGTTTGTCTCCGTATGGAAATCCGTG
GGGAATAGCAAGAAAGGCTACGACAAACGCTGGAACGCAAATAGGAACA
CTGGTTGATCATACATATGTCAGTATAGAAGTAGAATTATAGGTGTTATATAG
GTAGATGGGCGAGATGATATTGGGACTTACTTGATGATAAAAATCCATCTCT
AGGTAGTATTAGTAGCGATAGCCCCGCCGATGATAGGTCCACACAGAAGTG
CTAAAGCGTTCGTCGTAGAAGCATGCAGGAGAAATTTTGCGTATCTCTCAG
GTGGAACCATCTCGATTATATATATGGTCGAGAGAGCCCAGCAACCGCCGC
CTCCAAGACCCTGGAACCCTCGGAATATGCAACTATAAGATCGTAAGTAGG
TGGTCTGAGTAGGGGTAAGTGACGATATTATCTCACAGCTGAGCCATTGTCT
GTGCAGCAGCACAAGCGGCGGAGAAGATTATGAAAACCACAATAGATATC
AAGAGGATGAGTTTTCGGCCGAAGATGTCGCTGAAGTTGGAAAGAATAAC
GATTGCGCCTATTCAAGTTCATGAGAAATGAGAGGGTGGCTAGATAGATAG
TTTAGAATTGGAGTAACTGACCGACGTAACCTAGCAGATATGCCGAGACAA
CCCAACTGACACTGTCAAACCCGCCAACGTCATTTACGATGGCAACCAAG
GTCGTGTTGACAACAGAAAGCTCAAAGTTGACGATGAACATCATTATGGCC
ACGCTACCAAAGACGTAAGCGACCCAAGAATTGAAATATCATTTAAAAACT

AGACCTACGCGGCGAAGATACACCAAAACCTGGCGCCTTCAGTGTAAACT
ATATCGTTTCTGTCGTACATCTTTTCTGCGTGCATTGTTGATGTATAAGTTTC
TCTCTGCTCTATCGACGGGTCGGGCTTCTGTTCTTGAACTTTCTCGAAGCTT
TTACCATCTAGGCTGGAGTTGGTGCTGGACTCAAGTTCCATGATGAGTCGC
GAGGTAAGCGTTATCTACTAGATTAGATATCAAGGATATGTACGAGAAGTAA
GGATAGCCAAAAGAAAGAGATGGAGAGGGGCCGGCTGGTTCAAGGGGGG
ATCGAGTAGGAAAAGTGTTGGCGCCTTATAGATTATATTATATTCTACTGCCA
ATCAGTTCTGCTTAGTAGATAGGTTTCCAGTACAGTCACCGCGACAAGAAC
TGCAACCTTACTATCCACCCTACCATAATATCCTTGTACCCACGGATCGTAA
GGGGGCCATTCGGAAGACCGCAGATAAGTCCGAACTCAAGGCGTGTTGCA
TCAGGCAAAGAAAGGGTTCCTCTAATGTTTTGGAATGAACGAGAGAACCT
TAGTGATCTATATTTTATGTCCTTATGCAGATATATGCACGAGGACGTGTTATA
AGGTACGTGTATTGAAGGCTCAGCGCTTGGGACTTTCGTATCTTTCGGGATT
TTCATAGTTCCTATGCCTATTGTCTCTTTATGTATTCTATAGTTACATTCTTCG
CGTATAAAGCAGGTCAAAGTATTTTTGGAACTCGACTTAATATTGATATTGT
GAGTTGTACATTATGTGTTTCTGTTACGAACCTATACCTAGGTACTTAGGTAT
ATGGGGTGTTTCGTGGGTTTCCTTGTTTGTTAACAGTACTCGTAGAGATCAG
ATCAAAGGTAGTCTGTTCGTAAGAGAGGCTTGGAGTCCTTTCTCTATTAGTT
CGATGCGGTTAAGGTAATAGACTTAGATTGATAGCTGGTGAGCTAAATGAAT
GACTAGATACATAGGGGCCTGCCATGTTTATGTAGATATCCCCCCCTACCTAT
TCTTTATCGTAATCTGATTGATCTAGTCGCGATCATAAAGATCACACTAATCA
ATTGATCTGGTTGATCTGGCTGATCTGGTCGCGATTATAAAGATTACATTGAT
CAATTAATTTGATTGGAGCTCTTTACGGATTAATCAAATTGGTTATTCCAGAG
TTTTTCCGTTGTTGCTCCGTCTTGGGGTCTTATGTTGGGCCTTGGTTATTGG
ATCGTGACAGTTTCTCGAGGTCCTTATACGGTACTCGATGTATTATCCCAGTT
CTCTTTCTTAATATCCAAGCACGTATTTATCTAGTGCATACGTAGTATACTAG
GTACTAGTGTAGTGG

>Mosaic_sequence_12

TGAATGATGATGCACAGTGATGAAAGTGCTGGAATGGCATTACGAGCCGAT
CGACCGGGTCTCTCTTCCTCGTCCTCTCGTCCTCTCCTTACTCTTCTCTTCTC
TGAACATGTAAGGTTCCTCACAAGGTTGTTCAGTCTCATCGGCCAGTCTAG
ACAGGCTGGGCAGACTGGAGCTTGGCGTGCAGCGTATCGACGATCTGCTT
GAGGTACGGGATGTGCTGCATGGCGGCCATCTCGTAGCTCCGGCTGAGCGT
CTGTATGAGCGTGTTGAGCGTCTGCAAGACGCCGGGTGCGATGCGCGAAA
AGTACGTCGAGATCATCGAGTCCTGCAACTGTCAGCGGGGTCATGCTTGAC
TTGATGCAGGATGCTCACTCTTGGTTCCACCAACGTCACCACACGCTTCTC
GAGCATCAGTACCCGTGGGTACATGGGGTGGTTCACGTCGAGCTCGTTGAG

GGTACTTGCGAGCTGCATGATCAGCTTCTCCTCCCAAACGCCCACCGACTG
CTCACCTGGTGCATCATAGTGAAGACCACGGCTTGGGAAACAAGCGGCGG
AGCACCGGGCACAGTCGGGAAACAGCGGTCCATGATCCCCGCATGATCGT
TGATGAGCTGGAGAATCGCTTGGGTGCTCTGGCTAGCCAGCGCAACAGAG
AACATGTCCTCCATTTGCTGGGTTGTAGGCTGCGTAGTTGACTCTCGGCGG
GTGGACGAGGGCATCGTCGAGCCCATTGCAGGAGCCATATTGGAGGTCATC
GTCGGAGCCATTGTAGGAGCCATGCTGGAGGCCATGGTCGGGGGCATCGTT
GGGGCCATCGTGGGAGGCATAGCCGGGGGTAGAGCCGGGGGCATCGAGGA
CTGCCCGCTTGGCACAGCCGGAGCTGGAGGGCTGAAAGCAGCAATTGGTG
CCGGAGCAGGGGCCTGGCGGCGGACCTGGTCCGAAAGCGTGCTGAGCTG
CCTTTGAAGGTCTGCGATAGTCGCCGAGATCTGCCTCAAAGTGGCGTCGTT
GGCCGTAGTAGCTTCGCGGATCGGGAACTCGAAATCTTTGCGTAATTGCAG
CATCTCCGTCTTGAGGTCCCTGGACACTTGATCAGCCATATTGGAGCCAGA
CGTAGACATCGCGGGGATGAGCGAGTTGTGTAGGGCATCGCCAACGACGC
GGTCGAGCTGTGCATGAGCCGAGATGATCGTGCTCGATTACAGCTTGACTC
ACTGAAGATTGCACAGTCGTCGCCACATTGCGCTGTATAGTCGGAGCCAGT
GTCTTCTCGAGCTCCCGAGGAACGGAAGATAACGCATCATGGATCGCACCG
GGTATCTGGCTACTGGTGACTGACCTGATCTCGGACTGTATAGCCAAGTTG
ACTGCTGGCAATACGCTAGTAGCTCAGCAGCTGACACGAGGTCAACGAAC
GTCAAAGCTCACGCCTTCCTGATCTCGTCCTGTATCAAGGTCTGGAGCTGC
GTCTTGATCGAGCGGTCCACCTTTGCCGCAATCTCTGACCCCAGGTCGTGG
GACAGCTTGTCGATTTTGGCCTTGAGATCTTTGTTGGAGGCGGCGTTCTGG
TCCTTGAGCTGCTTGGTCAAGCGGTCTTCGAACTGATCTGTCAGCCCGAGT
CGGGATTGGGTGCTAAGAGGACTGACAGCCTTGAGCTGCATTTTTGCCAGC
CTCGCCTCTCCGCCAAGGTTTTCTTGGCTTTGCATTCAGTGCGAGGTTCTCC
GCCTCGTCATCCGTCAAGTCGGTAGTCGCCTCGCCTTCATCATCCACGAGG
GAGGGTTTCCTGGCCTCGTCCGCGACCGGCGCTCTCGCCGTGATGCTGAAA
GGCGGGGTGCTTGACTGCGGCAATACCGGCTTGACGTCTTCCTGGACTGGC
AGACCAGGTGGGAGCGTGGTGTTGAGTGCGTCAACGACGACTTGGTTGAT
AATCACGGTGCTCAGGCCCGCGGTATGAGCGATGAAGAGCTCGACATCGTC
TGTCATCTTGGGACTGAGGGCCATGCTGAGGACTGGTGACAGGGCAAGTT
CGACACTCGGTCAAAGCCAATGACGGCAGGCGCGTTCTGACTGACGTTCT
TGGCTGGCGGGAGGCTCTTGAGGGCATACTTGAAGCCGTACAGACTCGATC
GAGCATACGAGGCGATCCAGAGGACGCACTTGCTCGGGTCGTAAATGACG
CGGCAGGAGTCGTCTTCAACTGCATGTGGGGCGCGGGAAACGGATACTCG
TCAAGATCGTCAGTTCCGTTGAGAGTTGAACTAGCTCGAAGATGGTGTTGT
CGTTCCGACCAATGATGACGTTGGACTCGATGAATGTCGCAGAGCATGGCC
TGCTCTTCTCGAAGGTGGTAGGCAGTTGGCGATGCCAGACCCGGGTCAGAT

TGGCGATTGAGAAAAGGGAGAAGAAGCCTGTCTCTGAGAGCAGACCGATA
GCCTGATGCGTCTGGTTGAGAGCGAAGCACGCAACAGTCTGGACAGTCAG
ACCTGGAGCCATTGGTGAAAAGACGACTCACTCCTTCTGTCTGCAGCAGG
GTGCTGCGCAGATCGGCGGCCCTTCGTACATTAGGCTGGTTCTCTGGGTCG
ACAATGATGACTCCTTCATTACCACCGATAGCGAGCCGGCCGTCCTTCAGC
CACTCGACCTGGGCGGGCTTGCCGATGTTCTGTCCTTCGGTCACATCCACA
TGGAGGAAGCGAGAGCAAGTCGGGCTCTCCTTGGACCATTGCCTTGGGAC
GTTGTAGGCTGAAACGAACCCGCTTGATCCGAGGACGGCGACAGTCGTGT
CCGCCACCGAGATGTCCAAGATGTGGCCATCCCTCAGGTCTGGGATGTCGA
GTGTGGCCGAATGGGCGGTGACTTTGTCAGCCAGCCGGATCCGGCCTGCG
GATCAGCTCAAGTCTTGGTCCAAAGAGAACAAGCAAACCCTTGGTGGTCG
CGTAAGCGATCCAGTTCGAGGTGATGCCAACAGTACGTCCGATGGTGAAA
GTATTGGTGCGAGCCTTCATGTTCGCCACGGGGTTGATGAAGACCGCGCCT
GTCTTGTTGATCAAAGATTCGAGGTTGGGCTTGGAGATATCAACACAGTAA
ACACGGTAACTTGGTGGCCTGCCTATAGTCTACTGGAAGTGATACTGAAAC
CCAAGCTTGGTATATATACTAGGCGATCATGAAATCCTGGAATGACAGCCGC
TAACCATTACCAAGATCCCTCGTCAATCTTGATAATATATTTTTGTAATTAATT
CATAATTCTTAATACCACTATGCAAGGTATATGATAAACGGTCATTATTCGTA
GGGCTGAGCTGAAATGCGACGATAGACAGACAATGCCGTACGTCTTTGGTG
AGGCAACGAGGCAACCGGAAAACGAGGTAATGTGCAAGAAAAAAGAGCA
GCCATCTTACAATTGAGACGTTTTGCCGTGATCTTGTGCATCTTTCTTTTCTA
ATTATCCTTAGATCTCGGTTTCTCCGTGTGTTCCCATCCCTCGCTGCTGTAGA
CATGCAAGTTGTCCGCTGCAACAAGAGACAAGGAACCTTTGGAATCTTGG
AGAACCCATGAAGACCTTGATTTTGAGCAAGGGGTTTTTTGTATCGATAAG
CTCTTACCAAAGTCTGCAAAGCCTGCAATCTCGCCAGGGGTAAATGGTAAA
TACTAAAAACGTCGCTCTCAAAATACTTACTACCCTTACTACCTAACCTAGT
ACCAGGGTCCTTCTTCCTACCATAGCGTTGGTCGCTGTGCTGCATACCATCG
GCATACTATACTACTAGGTATGTAGGGTATGAGGCATCCATGGATGTATAATG
CCTGTCCATAGGAAATACTTGGTAACCCTTTCTACATGTTTTTTTCGTGCGTA
TCGTACGTATCATCTGTATCATTTTTCTCTCCCTCACAACTTCGCAACGCGCT
AAATCCTTGTTTAGCTTCCTTTTGCTCTTCCTCCCTGCGGTTGATTCCCCCGT
AATTAGACGCTGTCGTCTCCTGTGACATATTGACGACATGTCATGTACGAAA
TCGCTACCCAGTACTTCGCCTCTTTCTCATGCCAAGGGTACCCTCCGTTGCA
CAAAAACAGCCACTTCATTTTCACCCTAGCCTATACCTGGACCGTAACTACG
CTTCTAAGACTGTGGATGCTAGGTCTCGAAAATTTGGATAAGCTCATGCAA
GAAGCCGTCCCGGAGTTCTAGCTGGTGGTCGGGACCCCGAGTTCAGGGAA
CGAGGTGTGGAGGGCGGGAACCGACCTCACCTCACCTACCTACCTACCTA
ACTAGCCTATTGTGAACCCAGAGGCTTGCTCTGGCTTGGGGAGGCGCGAAT

AAAACCAATATGTCGGCTGTCCGAGACTTCAGCCGCGTACGCCAGCCCACT
AAACTGGCGGTCCCGTATTCCATCGTCGTCTTTCCGACGCACATGTCAATAT
CCAAGAGGTGGACTATTTGACACCTTAATATTCCGATTAATCGCCTTGGCGA
TGTTGCCGACGTTCGAGACCGGTCACATCTCGATCTTCATTTCTTCCGCGGC
AGCAACACAGTTTGTGCAGTTTGGGTTGGGGGAAACAAGCTCCTGAATTG
GGCTTCCATTGCCGACGATGCTCCAGGCTCATCTCCGTGCTAGTGTACAAG
GCGGATCCTGGGCTTGCTGGTGGTTTCCATCAGGTCGAATCGTCGAAATTC
GCACTTACACGACACAGAAACGCCTCATGGAACCTACCGATTGGACGGAC
TTGGAACCAACCGTACGCCGGTCATTGGCCGTTGAGCCATACCAGGTAATA
TGTAGATTGTGACTGCTCGACATCTTATTCCCCCCTTGTCTTGCTACGGCAC
ATGGATAACCCTCATAATGAACCCATCTACGCTTTCGTCCAATTAACAGTTT
ACATGGAAGCGTAACTAAAATTCTAACGGCCGAGCGGGAATAGCGCAAGC
CACATTGCGACTTAACTTGCGCAATCATCACCTGATTATTGGGACGCCAGCC
GTCTATGGACTACGATATCTTCGCCCTAGTTAGGACGAGACAGGCTGACGG
CTTTTAGGTGCAATCTTTGTGGTCTATTCTCCCATGTCACTTGGTCTACCACG
ACATTCACCCTAGAGACTACACGAACATACCGCCGCGGCTAAACATTCCAG
TCACGACGATTCGTAGGCAGTACGCTTCAATTTATCGGCGACATGACGCTA
GCAGTGTTCTTCGCCCAGTAATTTTCAGGCAAGATGGACTTCGTGCACACC
CAAGACTCAAGCAATGTGAAGCTGTCTTCGTACAAATTTCCAGATCGTAAC
TAAACGACACACTTGAAGGCATTGATTTTTCTTGTTGCTCAACGAACACAT
CCTGACAACAAAGATCACACGCGTCAAGAAACTCAGCTAGGAGTCTACTA
AGTAGGTACCTACCTACACACTTAGTCCGTGATAGTGCCAAGAGGGCTATCT
ACACTCAGTTGCGGCTACTTTTCCGGCAATAACATCGATAGATTTCTCGCAA
ATTCCTGCATCAACCCAGCTGCTTAAAGCCCGGTTCAAGGACCAGAAATAC
CCTCAGACCTATCTGCCGTGAACCAGATGATCTACAAGGACGTCATGATGA
TTAAATTCCCTTCTCAGCTATAGAAACCAGGCCAAGATGCTGACCAGAATA
AACATTTCAGCCCGCATTTGCATGGATCTCAGTCTCCCCTCCTCTACAACCC
CGGCCTGCATACTTCGTGGATTTATGCGGGAGCCGTCCATTACGTCCCAGTA
TTTTCCTCTCCCGCCTCATGCACGAACCGAAGATGCTTTGCTGCATGAATTC
CAGCACGTGGGCGATCAAGGCAGTATCCGCCCCAAATCAAGGACGTATCGC
TCACGGTATAGCTTGCCACCCTTACACCATCCAACCTTGCCTTTTCTTGATC
CTACGATAGGCCTAGAGCTCTCGTGAACCAAACCAAGCATCATCATCTATAT
CATCGACAGGGTGTAGTTTGATCTAGCATCCTGGTCTCGGAAGATAAGTGAT
GGTCCTCCGTGACCCTAGATGTTGACCCATTTCCGCCTCCGAAAAACGTATT
TTCGGGATGCTATATGAAGATACTTAGTACATTTATCAGCTGGCTCAGCTCA
CTATAATGCACACAATCGTTTCGATCGAAATTTTAGTCAGCGAGGAAAGCA
CTGACGTACAGTTCGTTGCCGTGTCGTACCGGCCTATCTCAATATCAAGAAC
CGTGGATAGAATTCTTGATAATGGTTCCCGAGGTCCCCAATTGTACGGTAGT

CGGCTTATGATTCTGATACCTACTAGGTAAATGGGTAAATTCAAGCTGCTTC
ATTCGCTACCGACGGTAGCTGTCCCGTTAGCTCCAGCCGCTTGGGGAAATA
CCGGACAAGGTTCGATTGCGAGACTGCTACCGGCTAATACGGGTTATCCCC
CAAGCAGAAAGGCGTGGCCACTGGGCAAAGCCCTATGGCATGTATGTAACT
TGCCATGAGATTCTGCGCATTCCCTAATTCCTTATATGGTCCCAAAACCACC
AAGCATCCCGCGTTCCTATTGGCCGGGATCGCACAACACCTATTCGCTCGC
GGAAGCAGCTTTCACGACATCTCTCGCCCTTTGCGGGACTAGCTTGCCCGT
TTTTGACAGCCTGCGGACGTGTGGATGCGACACATTACTTGCCGGGAATAT
AGGGGTAAGCTTATCCGAAGCGCGCCTGTCGGTAAGTCGTCCTCGCTAGGC
GCGCGATGAGGCAATACACCGCGCGGAGAATACGTCTCTGCAAAGAGACA
GAATGGGAATCCAGCTGTCGATAGAAGGGCCGATAATGTCTCGCAAGCGGC
CCCCACGTTCTAGAAAAACTTGTCCGTGGCTAAGGTCGCCATGACCGCGCC
GCCAAGAGCTTCCCCAGGCACTCTAATCTCATCATTGTTATGCGTTCTTGCT
ACAGCGACCGTAACCTTAAATTTCGATACTCATCTTACTTCATCTCGGAACC
GACATGTGCTGACTTTCCCTTTAAGGTATAAATTGGTAAAATTACCAGCGCC
GAAAAATACGTGATTCGTTGAACTCTCAAGCGCCAGTAACCATGACGAAGC
CTTCAAGATCACCAATTTTGGATCGCTAGCTTCGTATTGAAAATTCTACAAT
ACGGCTACGGTACTTTGGATAGAATTACACAGGGGCGTCTCATGCCGCCTC
TCGGGCGACGTGTCTCCGGTTTGACGCGCTCAGAGTGTGGACATGCGTCA
CATTTTGATTTCAGGCGCCAACGTGCCTGTGTTGGCGCCTGTCATCGGGCT
CTTGTTCACCCACGATACGGCGTCTAGAGGTTGATATATTTGCCACGCTCTG
CTCACCAAGCCGAATCCAATCCCGAGCCGCGCTTCGGCAACTTGTTGCGTA
ACGGTTCTGCATAGCGGTAGGTAGGCTAGGAACTCAACAGAACTCGAAAA
CTACGATGTAGAATGTATCAATCATGTCATAGTCCAATACGTATGAATATGTT
AGCCTTATTGTACCATCATAGGCCCTCTATCTTTGAAGGCTGTAGCCTTGCG
AAATGTGATAATGTCGGGCATGCTCTGTGATGAGATCTTGATAGATGGAATG
ACGTATATACACATACGCTTTACTGCCTAGAAGATAAATATTGAATTCTGCGT
AGTATTATTGGCACGAGTAGTTAACTCATTTGAGCATGATCGGCCTAGAAAA
TCTCATGAGCGACTTTCGAGAGCCGCCGAGTTCAAGTATACCCGACTCTCA
TGTCGCTATGCCCGGGCCGATTTGCGCACTAAATATCGTCTTCAATCTTCAA
CTCTTGTAATGAATGACCTGTCAGATAGATCGCTGTAGCGCGAGATCTAAAG
AGTTCGATAAACTTGCAGTCAATAGTTCGTATTGATTGATCTTATTTGCGTCG
ACGGGAAGAGGAAATAGTAGAGTAGGCTACGTCAGCCACGTGTCTCGTTA
CCAACGGGATGCATACCTAGGAGGTAGATAAGTCCTCATCTTCGGTATCTCG
CAGCATCCCCAGAGACGCTGACGCGGTCATTCTGGGGCCCCTCAACAACG
CTATACTCTTCTCGATGGTCGATTCCTCCAAGCCTGCTGCTTTGCGGAGTAC
GGCTCGGGAATACTCTGACATCTGCGACTAGGCAGGCCGAGAGCTCCGATA
TCGATGACTACAAAGACCCGTCGTTACTGATATAGGTAAGGTCTTTCGGGA

ATAGTTACAAGATAGTTAAAACCTGTGGAGACGACCAGAGTTGCGTAAGAA
AAGAGTATCGTAGTGACCATAAAGTGACCGTTCTGCATGAGGCTGTGATAG
ATTATTAGAAATCAAGAATCTCAAGAGGTAAAGAGAAAAGAGTATCGCGTG
CTTTGACGGTTTAAATGAGTAAACGGGATTCCAAATATTTTACTCGAAATGA
GTTCTTATATTCAATGTCCAATACAGGTATTAACGGACTTGGTAATATATACG
GCAGCCAAAGTCTGACTAAAGGCTCATAATAACACATACTACATAGAAAAG
AATAGTCATCTCGCTTCACCAGAGATAATCATAACCGTACACATATCATATAT
GTTAACAGATTTTTGATTCATCTTAAAACTGCATGCCTGCGGTCTATCTAGA
CCTGCCTCCAACGCCGAAGTGATTATGTCTTTATGTCTTATGTCTAGACTTC
ATATTCATTCATACCCAAGGCTCTTCTTGCCCTATAGGTTCACCACAGCATAT
CCATTATGATTACCACACTTAAACCTCCTCCTTGGAGACGTGCTCGTGATGA
GTTCCGGCGTTCTTATCCGTGAAGCCCATCTCGGCAGCATATGTAGAGTGAG
GCACCCACTTGGCTGAGGTTCGGGGGGTAGTCTCCTCCAACATGCGGTCGA
CCTGTTCTAGGGTCAATCCCTTGGTCTCGGGGATCAGGAGGTAGGCGTAGA
CGAAGCAGATGGCGCAGAGACTGCCCCAGATGTAGAAGACCTTGGCACCG
AGGTTGCCCTTGTCCTCGTCGGTCATGTAGGGGGTGATGACGGCGATGATG
CAGTTCCATAGCCAGTTGCTGGCGGTTGAGAGAGCGACACCACGAGCACG
CATGGGCAGGGGGTAGATTTCTCCGATGACAACCCAAGCACCACTGTATGA
ATATTAGTATATGAATAAGTGGCATTTTTGAAGATGAGGATCAACTTACGGG
CCCCAGGTGCTGGCGAAGAAGAAGATGTAGATGCAGATGAAGGCAATCAT
GGTCTTGACGGCAGCGTCGGCGCTGGTGACAGTTCCGATGATGGCAACGA
TGAACTGGCAGATAACCATGCCAAGAGCACCCCAGATGAGCAGAGGTCGT
CGTCCAATCTTCTCCATCGACCAGAAGGAGATGGGGGTGGAGCAGACGTT
GACGAGGGTGGTGACGAGGGAGATCAAGAAGGGGTTGTCGATAGTTCCGA
GCTGCTTGAAGAAGGTAGTTCCTGTAATGAATTAGAACGTGACTCTGTGAA
TTGAGTGATCACCTGGTTTTCTGAAATACGATATTGCAGAAGAATATGAAAT
TGTTCGGTAGGATTTGAAATTTCAAATCTTGCCAAATAACGTCGTATACTCC
GTAAAATTTAATTCCAGAACCGGCAAAGGGATAGAAAATTGTTAATCAGTG
ATTAAAAGCTAAAAAAACTTTTTACTTACCGAAGTAGAACACAAAATTCAC
TCCTGTCCACTGCTGCATCATCTGAAGTGAGGTACCAAGAATAGTACGGCG
GAGGTTGCTGCTGCTCTTCCAGAGAGATCCGCTGAAGCAAGCGCCCCAGG
AGCTCCAGTATCCTGCCTGAGGAACGACGGACAATTCGTATTCGTGGTTGG
CGATAATCTCAGCTAACTCATCCTGAATGTAATCAGATTCGATAGCCTGGCC
ACGTAGACGAGCCAAAACCTTGGCAGCGAGGTCGAGCTTTCCCTTCTTGA
CGTAGTATCGAGGGGATTCGGGGAGGATGAATAGACCGGTTCCGAGGATGA
GGGCCCAGAGCATCTGGATGGCAATGGGGATACGGTAAGAACCGGTGTCAT
TTCGGTCCTGGGTACCGTAGTCGATAGCTGACGCCAGCAGAAGACCGACA
GTGACGCAGAACTGGTAGCCGGAGACGAGGGCACCACGAACTTTTCGGGG

AGCAATCTCTGACATGTACAGAATCAGGATGGCAGAGACAGCACCAACAC
CGAAACCAGCCACGAGACGACCGGCCACGATGAGACCCAAGCCGTAAGA
GGCGGTCTGGAGAGCGACACCGATGAGGAAGACAACGCAGCCGAGAATG
ATAGTGGTTCGTCGACCAATCCAATCAGCGACATCACCAGCCATAAGAGCA
CCTAATCATGGGTTAGTTAGGTAATCACGAGAAAGGGGGCTTTGTGTATCCT
GTTCGCTTACCAAAGAAAGTACCAGCAGAGAGAATAGAAGTGATCAGAGA
CTTGTGGGTTGAGGTAAGAACGGTAGCGTCAGGGCCTTCGATTACGTGAAT
GAAGTAGTCCATTCCCATAACACCGCTGATGTAACCACTGTCATATCCGAAG
AAGATACCACCGAAGGCGGCAAAGGCACACATGAGGTAGGCCTTGAGCGT
GACGGGTGCCTCGACCCGGTTCACGTCCGAGGACCCTTGGGGAACGGCTC
CGCCGGCCATTGTGAAAGTTGTTTTACGTCGACTTATCGGAGGTATCAATGC
TCGACGTGGGATTTCGGATTTCGATTATGGGGAAGAGTGTTGGCGGGATGG
TAAGATATATATATGTATTTGTTTACCTATATATATATATATACCCAATATATAGT
AAGCACTGTGGATTATTGGCCCGAGTGCCTGATGTAGATCTTTTAGGGTTAG
GGGGGTGGTGGGGAGGGTAGTAGTGAAGATATTAGAATATGTGATAGAGAA
GTGAGGAACAAAGTGTATAGGTGAAAAAGGAGGGATCGTCTTTTAAATATA
CCTAGGGTATACACATGCGAGTGTAATTACCAACGCCGCGGTTTTGTTAATT
ACCTCTAGATCTCTTCATCTAGTTCCTGACCCACCTCCGCTCCCCGACAATG
CCTGGGATAGAATGGGTGCCAATTTGATGCCCACAATCACTCCATAAAGTCT
CCATGCACGGGCACGAGGTCAACCCGCAAATACGCCAGAGCACTCCAGAG
CTCCATACTCCGTCCCCTCGGGGTCACCAACTAAAGCTGGCCACCGCTAGC
TCTCTTTTAGTCTAGGAACCTTACGTTTCTGGAACGCCGATGGGATTTTAGA
AGGGTTAGCAGGTTGTATTCATCCCGAGGTTGCTACGCGCGTCATGCAATCC
TACACCTCCCACATTGGAAATCATGGCCTACCCTTGACCCCCACAGTCACC
CACTCTGCTCCAGCTGCTCCAGGAAACAATTCGTTTGACATTGCCAGACTG
AGTTATTCCATGAAGCTATCGTGAGTTTTTCTAAGATCATCCTTGTAAAAATT
CATTGGCCCTAAAAACCATGAATTTGTGACTTCCGTTGCTCCTGTGACTGCT
CGTATCTCTTAGTCATCACAGCCTCTGAGTCTCCGTCAGTCATTTGACAATT
GATATTTTTCGGCCTAATATTTTTATCACCCCATGAAGGATTTCGGGGATATG
TTGCTGCAATGGTCTATGCGAAAGATTCGCTTCTCTATTAACAGCAAGCAAG
AATTCGGTGTAATAGCTCAGCTCGTATTAGGTTGGTAATCCGAAGAGCGAA
GCCAAAACCCCATCTACTTAGTTTCAGGGTCCTTGTGGGAAGAGGGTTTTT
TTTTCTTGCCTCCCCCGTACTCTTCCCCGTCAACTCCAGAATATGAGATGAA
CAGCTCCACCAGCCATTGGGCCGACTTGAAGCTTCAGCTACCTAAACTACC
CACCACATTTCCCCAACTTCCCCCCAGTTTCTCCCCAGCATGGAGTTTGTAG
ACTGATCGGTCCGGTGTCAAGCTGGCGTGAGGAGAAATGGGAAAGACGCA
TTGAAAAAGGGCCTTTAGAAAAGCAAAAGAACAAGGAAGAGAAATCGGC
CGAAATCCAAGCTTCATTCAACTAGAGGATTGGAAAGACTGAACGCAGAG

TTAAGGCGTGGACCTGACTCGGTATATTCGGCCAGATTGTGGGGCCACGGC
GATAGCGTCGGCCGTTTTGTCGTTGGCTTCCAATCTTCCTTGGACTTTATGG
TCCGTCCCCTGGTAAAAAAAAAAAAGGCCAAATGAGATGCGGCTCCGCTGA
CAAGCTCAGTTCTAAACACGGCAATTGGCCAATCTTGGCTAGTAGGCTGTT
TATGTCATGAGAACATAATACCCTAAATTTTCTAGGGGCAAATGAGAAGCTT
AGGAAAAGATAATTACATCCCCGGGGACCAGAAAAATTTGGTTGAAGATG
GATCTAAATTCAATTCTGTAACGGTAGCCGACGTAGTGGAGAGAGGGTGGC
CCGATGCAACGACGCGACGACGGTCAATTGCGCTTCCAGTTCGTCGGTGTC
AATCAATTGGAGACTTCCAAGTTCCATCTTCGATCTCCATTGTTTTAATTATC
TTGCTTCAATTGATTCTTTCTTTCGTTTCCTCTTTCGCGTTCGCGATCAACTT
GCGATCAACGGTGACTTGTTATGTACCTAGCTTCCAGCTTCTCCGGAGGTC
AAGCCGCTAAGGTTACGCATTTGTAAAGAAGAGAGCGAAGACGGTCCGAC
TCGGATGTATTGTCCTCATAAAGCCTCCAAGTTGTTGCAGGTTTTATTGAGC
TGATTGGCTTGCGCCATCTGCAAAGTTATCGATAACGTTGATGATGCGTTGA
CGAAGGTGTAAGAATGTTCGCCGAAACGTTTCTATCTTGTCTTAGAATTGG
AAAGTTGTAAACTGCCATACAACCTTCTCAACCTTGGAACCCTTGCAGCTT
CAAAGGATCTAATTAATTTCTATGGATCCTTGCCACGTGTTGAAGAATTTTA
GATTTCTACAACGTTGGAACGTTGAAACTAAAATGAATGTACCCATGTAGAT
GGGATCATGAAGGCTTAGGTAATAGAAAGAAAAAAAAAGTTTAACGTTCT
GGACTTTCTTCGGTCGCACCGATCCTTACCAACGGCGCCTGCCGCAAAAGA
CGTTGCGCGCGTTGTCAGGTGGAACTTTAGCTTTTCGCACGCCCGAAACTA
AAGCCTGCGCCTACTCTCAGCTGAAATCAAAATTTCTACAGGCCTTACCTA
CCTGGTAATTATGTCCCGTAGCAAATGCGAGAACTTTCGACATTGTGCCATT
TTCACAAACCATCCCACTCACGAGGCCTTCATAGTTCCAAATTGTTGCGCG
GCTTTGAAAGGCCATTCTAAAGCACGTTAGTTTAAAGTCGAGTCACCGTAA
AACAAAGTACAATCACATGGACACAGACATCGCAATGGGGCTACAGACTA
CAGCAAACATCGGATGCCTTTCCACCCTTTCCATCCACCGTCTAACAAAGA
TGGGAGGGACGAGCGGCAACACTAGTAAACGATCACAGCAAACATTTGAA
CAAATTCTCGATCAATTCTCAAGTATTGTGCTATGCGCACAGCATAGCTAGC
ATTTCAATTCCTCAATTCCTATTACACGTACATTTGAATCTGACAACATCAAC
ATCGCACCATCAATTGGTACCTGTGATAACATCCACCTCCTTATCTCATCCAT
GCCACCTGAAAACGCCCTCTCGGTGCGAAAATAAAAGAAGAATCAAGGTG
ATCGAACATCTTACCCATCCAGGCCGTCAGAAATGAAAAGTTATGAATCCA
ACGTTTCCTTCCTGGAACGTTATCTCATCCATTAACGCCGAGTAGATGCAGA
AGAGACGAGAGACAAAGCAAAGGAAATAAGATATACCACAATCCGGTCCG
GCCGACTATTCCACCGGCGTCTCAGTATGAACAGATCTTCGCCCGGTATGTA
CTGTGTTGGTAGGTTGCCCCCATAGTCTCCCCGAATTCCAACCCCCGGGGC
CTCCATTCAATATGTAGTATCACTTTGCAGCGTATGTGGAATCCTGGATCGTA

CATGTACTCTCGTACTAATCGCCGCAACGCACCACCATCACCACTACCACC
ATTACCACCTATGTGGAACCCCCTGCCACTAGTTTAGGAGGTTTTGGGGATT
GGGGAGTGACCAGCGTAGTAGAAAGTTGGGGGACGGACTAGTCTTGACTT
AAAGTTGGGGCGGAGGGCGGAGTTAGGGCAGCATCGAGGAGGTTCTAGAT
GCAAACCCACTCCGCAAAGACACACCACCTGGCCGTGCGGTCCGCGAGCA
CCAAGCCGCGCCAGACCCCGCGCTCCGAACACTTTATGATAGTCTCCCACG
TGCCACCGTCGCTAGGGTCATCCTCGACCGGAGCGCGACCGCCGACTCCG
ATACCGCCTCTGCCACTCCGGTCCTCCTTACGCATGAGTCTGAGGATCTTTC
CTCCGGGTGTCTGGATAGCTAGCTTAGCCGGTTTCTTGCCTGCGATGCTACC
CGTCGCCGAACTTGGATTTGATGCCGCTGCGCTGCTTGACATGCTCATAGC
ACTGCCCGGTCGCGCGAACGGAATCGGACTTGTACCTCCCGGATTACTGGA
TGGTCCCCGAAAGACTAGACGGGTGCTGGCGGATGGCGAACACAAATGTG
TTATTTAGCGCTAGTCGCTGACACTGTGGCTGGACGACATAGATGTCGTGA
CGCTGCGCATGTGGCGTCGGGTGACGAGTGACGAACTCGTAAGGCGGGTT
TTCGCCCGTGTGCATAATTGGCAATGCAAACGCCAGAGGGTGCGGTATATC
TTTGATGCTATGCATAAGCCCACGCTTACCCGCATAGACTTTTAGGATACCA
CTGCCCTCGTCACCCGGAAGGAGGGCTTTCACTGTGTACCGTTTTCCGCCA
TTGTACCAATCTGCTTGTGCGAGCGCACGCTTCTTCACCACCTCCCCGTTTT
CAAACAAGTCACCATCCGCATCGCGTGTAAACCCTTTCACTTCGACCTCAG
CCGCTACTTCGATATCCGGGGGCACGTTGAACTTGATGTGCACCATTTCGA
GATCCTCGATACGAGTTAGGGAGGTATTGTAGTCGACCATAGCAATTGATTG
CTTGAAATACGGAGGACATGCACCAGGTAAGTGGAGCAATGTCTCATGAGC
AACCGGTGGGCAAATATGCTGTTGACCGTGATGTTCAGGGATATGCGTCCA
GCAGAGCTCAGTTGGCCGGGTGAGAAAGTACCAAAATTCCGCGGTACTATT
GCTGGCGCCTGAGTAAAGTCCGCGGCGGGGGTTCGAGGGGCTTGCCAAGC
AGCAATCTATCATGCGCCACTCATTGTCGATGAGCACAGCATTCCACCAATG
ATTTGATCGAGGCATCATCCCGGACTCGGGAACTTCGCCCGGAGTTTTCAG
GTAGCCACGAATCACCTCAGAGGGTATGCCGATCGCTGTGCACATCTCGTG
GACAAGAACCGCGTACTCTTCAGAGCAGGCTCGTTTTGTTGAGATCACCCG
GCGCGTATCTATGTCCCCATCAAAATCTTCCTCCCAATTAACTTTCTCAGCA
ACCCAGGTGAAGATCGCTCGGAGGCGCTGCACTTCGCTTTTGTATGGTCTG
CAGACGTAAGTCGTCGCTAGAGTAACCGCAGTCGTCATTGACGGCAGACT
GCTGACGAAGCGGGAGTTTTTGTCCACTTGACTCAGATTTATGGCTTGGTA
ATTGATCGGTTCGCGCACTGGCATGCCGTCGGCACCTTCATCACCCTCGAC
GCTCTCATAAAGCTCATCGACGGGATCTATTGCCGGATAATCTAGCATTAGA
CAGCGGTCACGTCGCTCGGTTCGCTCGTTCTTGCTCAACGTATTCGAACGG
TTCACATCTCTTCGTACTTGAACCCAGTCCACGCCACTCGTAAGCCCCATAT
CCGTTGCCGCGTCCCGGGCTAGGTTCGTGCTACTAGCACCGCCTGCTAGAG

CCGTGATCCCATTAGGAACTATCGACCGCCTAGTTGGCGAAGCCTGCGAAG
ACCCCGAACCAGTCGCAATACTACTGCGACCACTAGCAACGCCGGTCTTCG
TCGCCTCCAAAATTCTCTTGATGAAATTCTGCTTCTTAGGTTTCGTAGTTCC
TGGCTGTACGAGACCACCTACAGAAGCAATACCCTCTTCATTAAAGCCAGC
TTGCGACTGGGGTCCTCATTGCCATTGCAGATGTAGAGTGACTCGGATGAT
AAGATACACCTGTAAATGGCGTAGTTGGTCGGTTCGGATCCCAATCCTCATC
ATCGCCACCGTCCCTGAATCCCAAAGCACTCCGCGCACGTAAGTTCTTTCT
CGCCAGGCTACCAGCGCTCGTAGCGCTGATTCCGCCGGCCGAGATGCCACT
CATCAGACTTCGCTGAGTGCTGTTCGTCGCGGTACTCCTATTACCGGACGA
AGCATTCGTCGAATTCGTCTTCGTTGTACTTGTCCTATCCAGCAGACCGACC
TCATAAGACGATTTCTGCTTTCGTAGGAACTTGCGTCCTCCGCCTTCGTCGC
GATCACCCATCGAGGACTTCGGTCGACTGTATATGGGGTTCTTAGGCGGTG
GCGGCGGCGGTGGACCCATATCTTCATCATTATCCATGCTCCCCGCATTCGA
GCCCTGGTGATGCATCTTGCGAAAGCGGTTCTCCATCCTCTCCACGTAAGT
ATTCAACTGCGGCAGCGGATGCTCTTGCGGAACGCTGCCGTGGAATGTGTT
CTCTTGCGACGAGCCACCGTGAAACGAGCCGCCGTACCCAGAGTCCTCGA
AATGCATGCTTCTAGAAGCTAGGGGTCGGGTCTGCGCTCTTTTTCGCGAAC
GGCGGTGAACTAGGTCGAAGGACTCGGGTGACCATGGATCTAACGCCGGC
GGTTCGGGGGATTCTGTTGAGCGCGAGCGGGACATGCCTAACGCGTCTAGT
TGCTCGATCACACCGTCCATGGCCTCTCGCAGCGGCGATGGGGTATAGCCA
TCGCCGGCTGGTGATTGGGGTAGGGGTGAAGGTATCCTAGGTGTATGATATC
CTTCTTGCCGTCGAGTCTCAAACGATGCACGGGACATGTGGGATGCATTTG
AGTTTCCTCTCGTGATTACGTGACGATGAGGGGGCGGAGCGGGAGGTGGT
GGTGAGTCGCGCTCTGCTCCGGGACGGTACGGCTTGAAGCTGAATGACGG
AGCCGGCGACGGAGCTCTTGAGCCGTAGTGCTGAGATGGAGGTGGGACTG
GCGACGGACCTCGGCTATAGCTATAGCTTTGCTGAGGAATTGGTGAAGGAC
CCCTGGAATGGGGACGCGAAAGGGATTGCGGCATTGAATGCGAGTGTGAG
TGCATAGGGGGTATTGGGGACGCGCCTCTAGGGGCGTAACTGTTCGCCGTA
GGAGGAGGTACCGGCGACGGACCTCTCGATCCATGTCCCCGAATCTCAGAC
GGCGATGGGCCGCGGTCAGCGCCGCGGCGCGCATCGTTTCTAAACGAGCT
AGCCGAATTCTTTCTAGGAGGTGTATTGGATGTATTTCGTATTATTTCGGGCT
GTTTTGCCGTTGTGTAATGAGGTGCCTTTGCATAAGCCTCGAACGGTTTGC
GGAAGTTTCGCGACTTGGATTTATGCGGCGTGTTTGTAGGACTCGGGAGTG
ACTCGACGTCAACGGACTCGCAGACCTCGTAACAGGTCGAAAATCCTCGG
GCAAGACCTCGACAAAATTGGACGGGAAAACACCAACGGTCTTGTTTCGC
CGCAGTCTTCCCGTCCACCACGATCCATCTCCCGCATTGAGACATTCTATTA
AATCACCCTCGATGAATCCTAAATCGTGTTGTGACTGGTCCGGCGGTTAGC
AGAAGTAAACAAAGTTTAGGCGGAGGCGCAAAAAAATCAACGTTCGAAAT

AAAAAGTCAATCACATACTTCTCCTCCCCAAGAATAAACGGCTCTGCACCA
GCACGGAAACCGCGTGGGTAGCGGCGGTGCTGACCCCATTGTGCCGATATT
CGCAGCGTAAGTAATCGGTTATTCGCCAGCTTTGCAGCAATATGCGGCGATG
GAGGATGTGTGAAAAGTCGGTTCGGCCTAAGAGGATTTCGATTGTTTTACT
CTAAATCCCTTACGGCGCTTCCGGTTCTTGCTGCAAGGCGATGGAGTAAAC
ACGCAGAATACGCAGCAGAGGAAGACGATGGCATCTGAGGCGGTGTGGTA
CACGAATAGTATTGTAGTGTGCGCTCGTGGGTACGTCCCCCGACGGATGGA
TCAACAATGCAATGCCGTCCGATAATACGCTGGATTATTATGATTGTTATCGG
TACTAAGCAGCTAAGCAAGCTTAAGCAGCACCTAGGCGAGCGGGTGGTAA
GGGTCAGGAATTTGGGGACTTGCAGGTTTGTAGGGGAAAGCAGCAAAGTA
TGGGGATTGCCAGCACATCAGGTCAGATCTGTCGATTATGATTGGGATATTG
AGTGCGAAAAGTGCGGTTCAATAGCAGAA

>Mosaic_sequence_13

GCTGAACGCGCCCACCGTTGCGTTCATGCTTTCTGTTTGGGCCAAAGTCGA
CTTGACGGCCAAAGTTGCCACGCCCGCGGCGTTGGTGGTTCCCGATGCTG
GCGAATAGCTGTTGGCCGATCCGGTCGCACCCCAGGTCACGGACTGACCG
CTGATAGGATGGCTCTGCGCGTCGAGCAGCGTCAACGTCAAGGTGATGGTG
CTGCTGTTGTTGGCGACCTGCGTCGTGGGATTGGCTGTCCACGTCGACGTT
GCCGTGCTCGGTGCCCCGGGCGTGAATTGCACCGACGCAGTCATATTGATG
CCAGCGAACGTGGCGGTTGCAGTGACAGTCTCCACGGTCGAATCGGTGAT
TGTGACCGTCACCGTACAGTACCGGCGCTGTTGGTGGTGCCGCTGAGTGCC
GACAAAGTGGCGGAGCCCGACACAGCCAGCGCCACCGTTTGACCCGGCA
CCGCATTGCCCTGTGCGTCTTCCACCAACACCGTGAGCGTGCTGGTGGTGG
CGCCATCGGCAACCACCGACGTTGGGCTGGGTCTCACGGACGAGGTGCCG
CTGTCGTCGGCGCCAGGCGAGAAGACACACGGCCTCCGCCGATGCGCCCC
GACGAGAGTGGTGTTTGCTTTCTGCTGTTGTGGAGGCAAGTGTCGCGGTG
GTGATGAAGACGCCTCCGCCGTTGATATTGCCGCTGGCAGGTGTGAGGGTG
TTGTTGAAACCGCTGATCGCAGGCGATACCGCCTGGCCCGCAATCACATTC
CTCTGACCATCTTTCACCGTGAGCGTCACGGTGGATGTGTTGGTGCCGTTG
GCTGGCACGCTCGAAGGGCTCCTCGAAGCGTGGTGGTGCTGCTGCTCGGC
GGCCGGCGGTTAAAGGTCACTGGGGTGGTCACCGTCACGCTGCCAAAGGC
GGCGGTGATGGTTATGGTTTGGGCCGTCGTTGACGACAGTGTTGCCGTGAA
CACACCCGAGGCGTTGGTCGTACCGCTGCTGGGGTTAAATGTGTTGTTGCT
GCCCTGCTCGTCCAGGGCACGACTTGTCCCGACAGGAGATTGCCACTGGC
ATCCTGCACGATGACCGTTAGGGTCGTGGTGGTTGCGCCATCGGCGACGAG
CGACGCGGGGCTGGCTGCCACCGAGGTGTTGGTGGAACTTGGGGGCCACA
CGCCGGCCACAAATGTCACCACAGCGGTCTCGCTCGCCGCGCCCAAGGTG

GCCGTGACGGTTTTGGCTTGCGCCGTCGTCGATGCAGCGTCGCCGTGAAA
ACGCCCGACGCGTTGGTGGTGCCGCTGCTCGAAGTCAATGTGTTGTTCGTG
CCGGTCGAGGTCAGGGTGACCGCTTGCCCTTGAATGAGGTTGCCTTGCGC
GTCTTTGGCCGCACTGTCAATGTGCTGGTGGTGGAGCCGTTGGCTGTCACG
CACGTCGGGCTGGCAATCAACGTAGAATCGGCTGAACTCGGTGCGCCTGC
CGCGACGGTGAGGCTGGTGGTAGCGTTGATGGAGCCAATGGCAGCGGTGA
TTGTTTCATGCTGCGCCGTGGTCGAACTCACGGTGGCCGTGAACACGCCCG
ACGTGTTGGTGCGCCCACTTGCGGGCGTGAATGTGTTGTTTGGCTCACTGC
TTGACCCGAGATGAGATTGCCTTGCGCGTCTTTCACGGTCACCGTTAGGGT
CGACTTTGCGGAGCCGTTGGCGGTGACAGAGGCAGGGCTGGCGGTCAACG
TGGAGGTGCTGGTGCTTGGGGCGCCCGCGTCGAATGTGACGCCGGTTGTTT
CGGTGCTTCTGCCAATGGTTGCCGTCACGGTTTTTGCTTGAGCCACCGTGG
AGGTGAACGTGCGGTGAACACGCCGAACGTATTGGTGGTGCCGCTCGTGG
GGGAGAAGGTACTGTTCGACCCACTGCTCGCAAGCGATACCGCTTGACCA
GAAATTACGTTGCCACTGGCATCTTTCACCGTGACGGTCACGTTGGAAGTC
GTCGTTCCATCGGCAGTGAGCGACGTGGGGCTGGCGGTGACGCTCGAATT
GGTCGCATTGGGGGATCCCGCTACGAACGTAACGTTCACTGTCTCAGAAAT
GGATCGGATGGTGGCTGTGACCACCTTGCTCTGCGCCACCGTAGACGCGAG
CGTGGCAGAAAAACCACCTGACGCGTTGATGCTGCCCGTTGCTGGGGTGA
AGGTATGGCCGGTGCCCGTTGCGGCGAATGACACGGCTTGGCCGCTCACA
AGGTTGCCCTGCGCGTCTTTGACAGTCACGGTCAGGCTGGTGGTGCTCGA
CCCATTGGCGGTTACGGAGCTCGGACTGGCCGTGACGCGCGATGTGCTCGC
GGCGGGAGCTCCAGCCACGAACGTGACGGTGGTCGTTTCGTTGAACGAGC
CGACCACTGCTGCGATTGTCTTCGTTTCGGCGGTGGTGGACGTGAAGGAG
CTCGTTACGACCCCGGGGGCGTTGGTGGTGCCACTGGCGGGCTGAAGGTG
TTGCTGGAACCGCTGCTGCTCAGTGAAACGGCCTGACCGGACACCAGGTT
GCCGTTGACGTCTTTGACGGTGACCGTCACCGTGGTGTTGCTGGAACCATC
CGCCGTCACCGAATTGGGGCTGACGGCGATGAGGATGTCCCGCTGTTGGC
AGCGCCTTCGGTGAACAACACAATCGCTGAGTCACTCACGCTGCCCAACG
TGGCGGTGATGGTTTTGGACTGCGCCGTGGTGGAGCTGAGCGTTGCGGTG
AAAACGCCCGATGCGTTGGTGCTGCCACTTACCGGCGTGTATGTGTTGTTG
CTCCCGGTGGATGAAAGCGTGACCGCTTGCCCCGCAATGCTGTTGCTCTGA
GCATCGCGGGCCGGTAACCGTCAGGGTTGTCGTGGTGGTGCCGTCCGCCGT
GATGGATGTGGGATTGGCAAGGTGGAGGTGCTGTTGCTCGGCGCGCCCGC
CTAGAACGTGACGTTGGTTGATTCGTTGAAGTCTGCTAACGTTGCAGTGAC
GGTTTTTGTCTCCGCCACCGTCGATGCCAACGTGGCCGTAAACACCCCCGA
AGCGTTCGTTGTGCCGGAAGCCGGTGTGAAGGTGTTGCTGGTGCCGGTTG
CGCCGAACGTCACGGGCTGGCCCGCAGCAAGATTCTCTTGCGCGTCTCGC

GCGGTAACCGTCAGCGTTGTGGTGCTGCTGCCATCGGCCGTGACGAGAGC
GGTGCTTGCCGTGAGCGTCGACTGAGCTTGACTTGGGGCGCCGCCGACGA
ATGAGACCGATGCGGTCTCCACTTATTCATGCCGTCTACCTAAACACCTAAC
ACTATGCCTAATGCCTTTCTGATGGTGTATATGACTGTATCTAAATGTTTCCG
TTTCACAAAGTATTTTCTAGATGGAAATATTATAATATAGATCGGGCGACCCG
TCATGTCAGCCTGGTAGGTGGCATTGTCTGATGTGCATTTCCTTCTAGATCG
CTGTCGATGATACAGACGAAAGATCATGATCGAGATCTGCGGAGGATCTGT
GGATCTGTTTGATACCGGACCGCCGTTACTCTTCTGGTTTGCCGTTTCACAT
GTGCGCTCTCGGGCGGCTTGATCGACGTGTGTTCTCTTGTCGCAGTGCTGG
TGGCGTCCAATCGCATTATCGGCCCTACCTATCACGGGTTCAGCCCGTGTGC
ATTAACCCTTCGTCCCAAATGGTTGATGCGAGTAAGAGTCTAGTAAGTTTG
AGTAGAATGGCATGGCAGCGATCGACGGCATTTCACGTTTGTCTAGGATCC
CCCTAGTTGAGGCTGAACCGTAGAAACGCTCAGCTCTCTCTCTAGTTTCTG
AATCGCACGAGTTTCATCTGCTGTCCAACCACCATCGTCGGCATAGTTGAC
ATAGACTATCGCGACACCCTTCCAACACAACGTCAGGTCATGGATGCAGTA
GCCTGTGTTGCGTGATCTGGGGTAAGACCTATACCAGGGCGATGAGGTGCG
GTGCCAAGACTTGTGATACCGCATTTCGGTAGTTCATCTATTATGGCATAGA
AGACTCAAAGAGCACGTACGCCGCGACCGACGCTTCGAGTTTCGAATACT
TCCACTAGGCCGGACCCAACTGCCTCTTGCAAGATCCAGTCAAATGAAAGT
CCGTAAAATTGCCTCCATTTCCTAGTCTGTCCAGGTAACACACCAGCGAAC
TCTCCTCGCGCGCGCTTTGCTGCATACCGAGCTTCTTCTTTCGCAATGCCAC
CATCCCATCGATCTCGAAGCATCGTTTCTGGCGCTTCGCGGAAGCGTGACC
TGCTTAGCAACGTAACGAGGTGATCGAGTGCCAAAGAGAGAAGCTTTAGA
AACGTGCCGTTCCCAGGCACGGCGAGCTTTAATTCCGCAGCACCAATATCT
ATCCAAGACATACCCAGCGAGCCCGGACCGCTACCGCCAGTTTGGTGTATT
ACACCCTCACCTCCCACGGCATCGAGTGAGCCAGTTGGCTGCCTAGATATA
TTTTCGAGGGACGTTAGTGTCACCTTATCACCAGGTCGCGAATATAGATTCG
CAGCAAGTGACGAACGGCCCGCATGGCCATGGTATATTGCTCCAGGATTGT
GAGATGTTAGAAATCCAGCCTTAAGAAGCTGATCAATCTCAGACGCACTAA
AAGCCTCTAACTTCAATGCTTGAGTTCCATGATTCTCCTTTAAATATGCAGC
CATTTTATCTTTCGTGTTGTCATCTAAACTTGTAGAAGCTTTCACCATTTGTT
CATAGTCCGCCTCGAGCACTAAAATCTCTCCTATATCACCTCGTCGTGGAAC
AACTACCTTGCGAACAAAGCCGGCCCGCGCAAGTTCCGCGATTTCTCGTTC
CACTGCGCTCGGCGACGATAATAAGGCTTGTATGTGGGATATCGTAACGAC
GGGGGGAAGAGACTTCCGATAGTTCAGCACTTCTGCGATTCTTATAGAATT
CATTCCGGCAGCTCGATCGGGCATTGGACAGAACATGTGGTTACGTATATGG
AGAATGGCTTGGGAGGTGTCGCGCAAGTTTAGTCGGTCGCTAGAGCTTTGA
CGAGCCCATAATCGTCAAGTTTGTCGTCAAAGAGGTCATCTTCCTCATCTTC

ATCGCCGGGAAATGACTTCCCACGCTGGATTCCCGGTTTGCGGCGAGGCAG
TGGAGAGACCCATGAGGCTGGCGAAGGCGAAGATGGTTTTGCGCCGGACT
TCTTAGAGGCTTTCTTAACCCGGCTGGAACCTCCTAAGATCTTCCGCAGAC
TCATATCTGATGGAAGATAACATCAGATGAATATTTCAGAATGTGTAAAATA
AGAAATAAATTGGCAATTGGAAGTAGATAGCCGTTAGTAAAGTTTGTCTGA
CTCATAAAGTTAAAACAAGGCTATCCGCACTTAATGAACCGTACCTGCCTTC
AGGCTAGCTACAGTCATTCATAGTGGGTTGTGACGCACGTTTAGCGGGCGA
TAGGGCGAACCACATTGAAATCTGACCTTTTGATTAGATGCCAAGAAACAG
GCTAACCGAATTCGGCTTTTTATGAATAACTGAATGTGACACTATGAAGCGT
CGATAGTTGTGGTCTCTCTTGTTCAATGTTCTCTTGTGTAATGTAATGTTCTG
CTCTTTACGAAGGACAATCTCCGAATTCCTAAATTCAAACTGTTTGAATTCC
TTAAAGTACAAACTAGGAGTCAATCGTGATTCACTTTTATTTAAAGGAAAG
GAAATAAAAGAATGCATCGACCCAGAGAGTTGGCCAAGAGAATACTTGCC
CGTCTCTCAACTGACCCCTTTGCCTAAAGAGAAACACCAAGACATTCAATC
AAGATGCCACCAACCGTCATTTTAGTGCGTCATGGAGAAGCGTTACATAGT
AGGCATCTATCACCGATCTCTCAAACATATCATCTAACTGCTTATAGATATTA
ACAATAAGTTACCTCAAACTTATACTAAGCTACAATAGCTAACACGGCACA
GCACACGACTGGTCACTTGCCGATCCAGGTCTAACTCCCAGAGGTATAGAT
CTTACCAGAAGGTTTCAATGGCAATTTGAGCGCTACTTCCGTATTATCCCAG
AGGCAGAGGACTGCCTTATAGTGGTAAGCCCTCTGAAAAGAACGCTAGAG
ACCGTCAAGTATGGTTTCCAGTGGCTCCGTGATCTCGGCGTTCGTGTTGAA
GTCCGCGCCGAATGGCAGGTACGTGTAACATGTCTCATTAGAAGCAATTCG
CCCGGATGAGTGTTGACCATGTCTAGGAAACTAGCAAAAATCCTTGTGATA
TCGGTAGTCATCCCGATAAGCTCAAATCCGAATGGCCAGATCTTGATTTCTC
AACATTAGATCCTATATATCCCCAGAAGACTGGCTTATATGAGAGTTCAGAA
GATGCTTATCAAAAGAGGGGAGATGTCGCGAAAAGGTGGCTCTACGATCGT
CCTGAAAGGTGTGTCATAGTTGTTACACACGCGGGTTTTATTCGAAGACTT
GTCAAAGGACCAAAATACGATAACCTAGAATTCCACACGTATAATCTTGTCG
AGACACAGGATAATAGGCTTGATTTCGAGGAGATCCGAAGGGTCATGGACT
ATTCTTAATAGGATTGTCGTTAGTGGTCAAGCAAGTTCGGGAATGCTCGGA
CTAATGGGGTAGATTATGAATATGAAAAGACAGCAAAACAATGGGTCTGTA
GATAGGGATTTAAAATGCAACATATTCCTGCGAAGAGTGAAATTCAGATGT
GTCTTTGCCGATGAAATTACTGCATGAAACCATACATGTATGTGGATACTTG
AGGATAGGGATGATACAGTGGCATTCCGTTATATCCCGTGTTAAAACAAGAC
GACGTGAGACAACATAATGCTAGAAACACTTGGTTATAAATTACCTAGTATT
TGAAAGGCAGTTGAACAGACACAATGCAACCTAAATAACCGGCCTACTTAT
CGGAAGCGCAGTGTCCATTGACACGTGTCGCCCGTCGTCCACCAGAACAG
AAAATTCGATTGATTTGAATGAGATTGCGATGTCGCCGAGATTTACCATAGG

AACCCGATGTTTTGAAGACAATATCGGGTTCCCTCAAACGAACGGCATGTT
CTAATCTCGCACTAGACTCGGCCTTGAAGACGGTGAGAGCGCTCGTCTTTG
AGCAATCGTAAACGATTAGGAATCAGATGTGCTGTCGAATGTAAAGATTAA
AAAGAGAAAGTAAAAAGAATCTTCATCCCGCACTGGAAAAATTTAGTGTAC
CTGCTACGTAGGCGACCTTATGTAAGCGTACCTTAGTGTAAGAGTGGAGCA
GACCCCTAAAGGACTTTAAATAACATCATTGGCCAATAGGTCCAGGGGTCT
CGTTACATTCATGTATGTAAGTATATACGTACATATGTATGTCTTCCTTCGTCA
CTGCTAATTTTCAACTATTTCACTTTCTTAGCTCGACACTTATAGACCATTTG
ACATGAAGTGGGTATTCTAAATATTCTAAACCTTGGAAACTTTCCAGGTTCA
CAAACTTCCCCATGGCACAATCTAGATCATGAGAGCATCAATAGAAAGTTC
CCTAATTTTCATCTCGTACGCTCGTACTAGGTGACCGGTCAGCAACGCGGC
AATGAATAGATTCATTTTTAGACTATTAGCAGACTAGTTCCAACACGGAATT
CACGGCTTCGCATCTTACTCATTGTGTTAGGTCTGACGTCACTTCATCCTCG
AAAGGGTCAATTAAAGGATCGCCCGAATCGTCCTATTGACTTAGAATAGCA
CCTTGATGGCCAGTAGAACTTGGCAGCCGGTTAGGTGCAGGAGACGCAGC
AGACGCAGGAGACACGAGAACGGGAAGTCGGCAGAAAGGAGTTTCTTTG
TCTCCATCACGGCCGTGCTTTTTACCTCCACCTGACGACTCGGTCAAGAAC
TTGCAGGACGACTACATTTGCATATGACCTTAGCAAACTCTCGGATATTGAT
CTTTCCATAAATGACTGGACAGGAATTTAGGCAAGTATGAAAAGTGTGTGG
ATCTAATCGATCTGCCCACTGTCGTGTGCCCCCTAAGGTGGCGCTTGGAGG
AGGTCCGCGATGTCTCAGTTGCTCGACTCTCCGCGATTAACCTCCTACACG
TGCGGAATCCTGCGCTGTGATTCGTCGCCTATCATCAGCCTTCAGCCTCGTG
AACAGTAATCCACCCGAAAGTGAGGGAATACTAGATAGCTTAAGAAGCCCG
TAAGCCGCCCCCGATTTGCTTGTGATTTTAGAATTGGAAATTTCTATGTTTTG
TGTTTTGGGAAATTCATCTTGGGCTCAAGCAGTCAGTAGGTACGTAGTGTT
CTAGACCCCATCGTTGGTTATCCACTTGGCACAGAGCCCTTGGCTAAAAAA
AGACGCCTTTTTGGCCTCTACAGCAGATTTTATTGGTCCCTAGACGGATAGT
TAGCCCTGTTTAACGTCCCCGTCGATCTTTAATAACATATTCTCGAATCTACG
GTTGCCAATGACGAAGTTGTCACACTGAACACGACAGCTTTGCAATTCTTT
TCTTGCATTTGCATTTGTCCATAAGCTGATATTTATTCCCTGGCTATGCGACT
TGTTTTCTCCCCCTTTCAGTCATAGCTCTCTTAGAGCACCCAAAAAGGTCA
AATATGGCTCCGAATTGGAGAACAGCTGGTGCCTGTTTATTGGCGGTTCCC
AGCCTCCTGGCGACTTTCGTCATAGGTGCACCAACTACCGGAGGTCAATAC
GTCCTGCAGGCAGGCAAAGATGGCAGACCACTCACATCGCAGCGCAAGTT
GCACGGCAGATTCCTCCATATAACAGGTACTGTACATCGCGATTCATAACAT
CCCACCACACTTCGATCTATAAAACATGGATTTTAAGGCTAACATCTCGTAC
CTTGCGTAGATCTACACCCGGACGAATATTACAAAGTCCACACATCTACGG
AGGAGAACATCGCATGCCACCGAGGCCATGGCCCAGCCGGCACTTATGGC

GCAGAAACTTCTGATTGCGATTCTCCCCTTGCATTGGTAGATGCAACCCTTG
ATTGGGTCGCTGCCAATTTGAAAGACTCGATTGATTTTGTCGTTTGGACAG
GCGATTCTGCCCGCCATGACAGCGACGAGAAGACACCCCGCCACAATAAA
GATATCTTGGCCATGAACCGCAGAATTGCGGATTCATTTGTCAAGACATTTT
CCGACGATAATGGATTGGTAGTTCCTGTCGTTCCAACATTCGGTAACAACG
ATATATATCCGCACAATGTTTTAATGCCAGGTCCCAACGATATCTTGAGAGAT
TATACCGATATTTGGCAGCCATTCATCCCAGAAGACCAGCGTCACTCATTCG
AATTTGGAGGATGGTTATATGTTGAGGTTATCCCCAACAAACTTGCTGTCTT
CAGTCTCAATACATTGTACTTCTTCGATCGCAATGCGGGCATCGATGATTGC
ACGCACCCGAGTGAGCCAGGATACCAGCACTTCGACTGGCTGCGAGTTCA
ACTGCAATTCATGCGAAACCGTGGTATGAAAGCCATCCTTATGGGACATGTT
CCTCCTGCTAGAACCTCCGGCAAGCAGTTGTGGCATGAAACATGCTGGCAG
AAATACACTCTCTGGCTGCAACAGTATCGCGACGTCGTGATTACTGGTCTCT
TTGGTCACATGAACATTGACCATTTTCTTCTGCAAGATACAAAGGAGATAG
ACTTAGCCTATCTCGGAGCTCAGTCTAGTCAATTGGCAAGCATTCGCGAGA
CCATGGAGGATGAGATTTCAATCGAGGGTAGCAAAGATTATCTCATGGAATT
ACGAGAAGACTGGTCGAAATTGCCAGACCCTATCAAGCCCCAAGAATTTG
GAGGAGTTGAAGAAAAAGAAGAAAGGTGGCAAGAAGAAGAAGATTGGG
GGGCCGTGGGCAGAGAGATACCAACTCACTCTCATCAGCCCTAGCGTCGTC
CCAAATTACTTTCCAACTCTTAGAGTCTTTGAGTACAACATCACTAGCATCG
AAGACACTCCCACTTGGCTGGATACCGTGCGCAAGTCAAGTATGCAAAGT
GCATCCGCGCCCGACTTGGAAACGCACTCGCTAGAACTACGTAGCGAGCC
CGATGTGGAGATTGAGAAGAAAAAGAAAGGGAAGCACGGCAAAGACCCC
AACCCTCCCGATCTGCAAATACCAGATGCACCAGCAAAGGGAAGTCTGCC
AGGCCCTGGTTACTATCCCCAACCCTTGACACTAAAGGGTTACGTCCAGTA
CTATGCCAACTTGACCGTTATCAACAATGATATCACTGCGGACAATGTTGGA
GAAGATGAGTTGTCGTCAAACAAGTGGGACGCGGGAAAGCACCACGACA
AGAAGCCCAAACACGACAAACCTCAACCCAAGGAATTCAACTACCAAGTT
GAATACACTACTTTTGACGACAAGATCTACAAGTTGAAGGATCTAACCGTC
AGGAGTTTCCTACGGCTTGCCCACAGAATTGGGGAGACTTCCGCCGAGTCC
ACCGGACTTAGCGAGGAATACGAAGATTACGACGATGAAGAAGTTGCTGAT
AACGAGGTCGAAGATGTAGAGTCCACAGACGAGGAAGAGGACTCAGATA
ACGACCTCGAGGCCGAGAAGAAGAAGAAGAAAAAGAAGAAGGGAGGCC
ACAAGATGAAGAACACGAAGATATGGCTTCACTTCCTCAAACACGCGTTCG
TCAGCACGAAAAGCATCGACGAGCTCAAGAATATCAAGTAAGCATAGCCTG
TAGACGGGGTAGAGGGTTTAGTTCAACTGAGCCTCGACCCAAGGAGTTTTA
TTGTCTTTCGTTTTTATTTGGTTTCGCATGGCATGAGAGCGGCGCATTTTAG
GTACGGGAATTATGACGACGTTGGTT

>Mosaic_sequence_14

GAAATGTTGCACAAGATCAACCACACTGGGTCCGACAATGGTAAGGGAGG
TGTCATGCCACGCTGGACAGGGTGAGCATGAGTAGCCGTCGTGTTGATTTA
CTCACGCATTCGAGTGGTGGACTGGATTGCGAGTGCGTTTCGAGGTGTATT
TCTCGACCGTCTGGAAATCCATAACCCGCGAGTTGTTGTAATCTTGGCCTG
GGAAAAGACTCCTTGAACCACTCTGTCGGATGCACGTCTGCCAGTGGGTG
ATTCCGAGTGTCCCACCGGCCAAAGCATGCGTCCAATCCCCCCATTGCTGC
AATCTTGTTGTCGACCACGCATAACTTTTCGTGGTGCGAGAAATAGTAGAC
AAGTTCTCCACCGCTGTGATCGGGATGCCTCATGACTGCGATGTTTTCATGT
AGGTCTTCTAGGGCGTGCTTGTATCGTCAACATATGAAAGCGGATTTAGACT
ATCTCCTGACCTTGGTGTGCTTGGACGACAGCGCCATGGACGCCGTGACCT
GGTAATGTCAGCGGACACTAATTGGCCAATGGCCAACCGACCTCTTTGTAC
ACCATGACGTATACTCTGACCCCTTGTTCTGCCTTCTTCTTGATGAGCCTGT
CTAGACGCCATTCGGGGAACAATGCAGCTGGACGACGGAGCTGATAAGTC
AGAAGGGGGATTCTAGGCAGCTGTGGAATCCCTCACTTGTAGCTCTGGTGA
CAGCCACCAGTCGAGAATCATGATTGTCTGCCTTTAAGCCTCGCCCAGGAG
GGCGACGGCAACGTACCTCCTTGGCACCGTCGATGAGCTCCGAAAGGGCC
CAGAAGTAATCTGTCGATGAGCTTTCAGTGTGCGACATTGCGGGAGTGACG
GACCATGCCCATCAACGTGCCACTTGACGATATTGCCTGGAGCTCAGCGTC
GCCGCTCACACTCATCGCTATCCTCACCGTCTCGCTCGCCAGCAAAACTCC
TGAACCTGTGGCCATCTCTTATCCGCTCCATCTTGGCATCGACCTCCTGCTC
CATGCCTCATCGTGCCGGTGATTCGGGTTCCATGTGGTGCTGACGGCCACA
CCGAGTCTTGACAAGTCAGCCGAGAGGTGGTGTACCTTGCTCTTGAGCTTG
GAGAACTGCAGGCCCATTAGTTGCTTGCTATCTCTGGTGCACTCGAATGAC
AAAGGGAAGTCGGACTGACAGGGTGACTGGTCGGGTTCGGGGCGTGAGC
CGCGTCGGCTGCGGGGTGGGCCATGTTGTTGCCACCGACGCTTCTCTAGCA
GTGTGCGTGTGCTCTTGGTTGTGCAAGAGAACACGTTGAGACGAAGTCGT
TGTCAGATAGAGAGAGTGTCAATAATGTCTGTGCCGCGTGGTCGTTGGGCA
CGGACATCTTTGTCGCCCTTGACTTGGCGGTCATTGCGTCATGGCCCGAAC
CGTTTCTCCGGCTGATAACCGATCCCGAATCTTCCTTCTTACGTAAGGAAGT
CAGACGCGTTTGCTTTTCGCATCGCGTGTCGAACTGGAAAGCAATTCATCT
GGAAATCATCCCTGGTGATATCGACAGCCTGCTCATCTTGCTCCTCTGTAGC
AGCAGCAACTGGGCTTTGACAACGCTCTATGCCTCTTCTACAACCGCCTTC
ACCCGAGCTTCGTCACGATGAGCTCAGCTACACGCACGCGTACAATTCGAT
AGTCTCGCGAGTCAGGCGGAGTGGAGGATCGGGCTCTAGTGGAGTGGTCA
TCTTTGTTGGCGTGGACGTGGTGAGCAGCCGATCAGCCCGATGCCAGGAG
CAGGGGGAATCTCATTATGCAGGACGGTCTGCTTGCTGCCCGTATCCTCGCT
TCTCTCTTCCGACAAGATGATGTCCCTTACCGCCTCGTGCCGGTTGGAGGTT

ACTCGGAACTGGACAGCGCAGAGACGAAGCGTTTGCGTCTGATGAGGTAA
TTCACCGAAATGAATGGAAAGAATCTTGCAGGGCTCACTTCACAGCTACAC
ACACTGATCCTTCTCTCCCTGGGATCCTTATTGCCCCTGTCGTCCTACTTTAC
TTTGCCAAAAGGTTGTCATTTACACATCATCGATTCGCACCGCCCCTGGAAT
CTGCAAAATCTGTTCGGATTAGACGCAGACTTCAGCGAGGAAGAAGGCGA
AGGCGGCGAAAGACGAGTCTGGGTTTGGGGAGATGGGGACGAAGCGGAC
CTCGGTAACATCAAGAAGAGCTGGGAGGCGTTGGAGGTGAGCAGATCACG
GTAAGCCTCATTCCAGGCTGACTGCCCAGTATGAGCCGTCCGACTCGGGCT
CGGACACCGAAGGCTCGGACGATGAGGCGACCGAAGACGAGGAGGAAGA
TGACGGCTCTGGCAGCGAAGCGGAGAGCTCGGCCATGGCTGGGAGCGGA
AAAGGAATGGCTCAGTCGGGGCCAGGGGAAAACGGCGCAGAGAAGATGA
TGACCGGGTGAGCGAGGAAGGCGCGAAAGCAACATGTGGCTGACGATCC
AGCCGAAACGTCTGCCCCGCGCCGTTCGAGATGCTCACTATGATCGGATAC
AGCGTTACTACGAGGCCGGCACGCATTGGGGCATGTCTGTGGCGCAGACG
ATATATCTCCTGGCTACGGTTCTCGAGCGCGCTGACAATGACCTTCTTTGGC
TCGCGATCCTCGGCATCACCTTCCAGTATGTGTCCGCCAGGATCGATCGAG
AGCGATACGACCTCAATCAAGAAATCTTCATGGACGAGGTCGCTCGCTTGA
ATCACGAGACGTTCGGAGGGGGAGGGAAGAAAGAGCCAAATCCGGATGA
CAGGAGTATAGCAAGAAGTGACGAATTGCGTTTCATGCTCTTTCGGCATTG
GAACCTGTACGACGCGATGCTGCACTCTGGCTATGTAGCCGGCAGAATGGG
TATATGGAAGGAGAAGGGTCGAAAGAGGCTACAAGGCTTGTTGGCAAAGA
TGGGGTGAGTGCGACAATTATTCTGTTCTTGCTCGACCTGACGCCCAGCTT
CTCCCTACAACAATGTCAGCAGGCTTGGGTACATATGGACCGCGATTTGAA
ACGCCAATTGCCCGACAAACTCGATACTATCGCTCCAGAGTACGGATTGGT
GGAGTTGTCATATCCGTCCTTCACGCGCGCGTTCGGATTCCAGCTCGCCTCC
CTGTCGGCGGCAGATGCAGTGGAAGGCTTGTCAGCGCTACTGGAAGCCGC
GAAAGGAGTCAGAATGGAGGTGGACAAGGAGGGTGGCAAGGGTGGCGGC
GAATGGTTTGGCGGTACTCGGCTGTGGAACGTGGGCGGTGGAGTAGGAGG
TCGCGAGAAGAATGGCGGAGACGAAGATAAGGAGAACGTCGATCCTCGTG
GGAGTGGTGCTGCAAACGCGAACGGCGAACCGGCAGATACACAAGAGGG
GAAGAAGAAGGAACAGGCATGGCACGTCGCCAACTTCTGGATCTAACAAA
CCTTGAACATTAGAACTACTAGGTAGGAACCTTGGAAGTTTCCGTCTTCGA
TTTAACATCCCAAGGCTTAGTGCCCAGTACTTCTTCTCCTTCTTAAAACTAC
ATACATATTCCGTTTTCAAGCATCTACACAATTCATCCAATATGTTGAATAAT
TCCCTAGTGTCTACATATGTATATACAGTATATTCCAAGGAACTAAAGAAATA
CAGCACTAGACTTATACCTCAACAATACTAAAACACAAGTATAACCATGTTA
TTTTTAAAGGAGGAACGAAGCCTCGTTTAAATAATGCCATCTATCATGGATC
AGGATTGATAGATAACCATCAAGGTGAACAGGTCCCAGGTCCCAGACCAA

CAAGGGACTCAACTCCGCCAGCCGTCAGTGCACCAGATACCTATATGTATA
CCAGGAATCATAATCCTGAAGGCAGACGCCTTTACTTGATGCTATTAGCATT
AAGCAACACGATCACGATGCCGTACTACATCCATGGATGTCCTATGTCCTAC
ATACACACCTAGCAGTGCCAACTCGCGAGATAGCGGAAGACGTCGTTGGC
GCCGAAGGCCGGGTAGGTTCCCTTACGTTGTGCTACAAAGACGAAGTAAC
GCGGCAGACTCGGTGAGCATGATTTAATCCACACATGCCTATTCCGGACCT
GTAAATATCAGATGTCATACAAGGATGTTGGGATCTCACAAGTCTATAGGAT
CTCCTAACACTTTCCGGCCCACACGTGTAATATATCGACTTTGGAGAACCGC
AAAGGTTGTGGGGATAAAACCTATGTGCTGAACCATAAACTCACAAGACTA
TTTTATTTACGGGCTCTTTCATTTCCCTCGGTCCTTAAGAATTCCTCAGCTTC
CCAGCTAATCCCACAATCTGGTGCTTTCGAGATTGTCTGCTGACGAACTGA
CGCCGGGAGTCTCAAGTTCTCGACACAATGGAGAAAACAGGTAAAGAAAC
ATCTGCCGTTGTCGAGAAAACCGTTGGCAGCGAAACCGAAGAGGGGATAT
CATCGGTTTCTATATCTATGGACAGTACCGAAACCCAGGGGTTTGATGTGAA
GGCCAGAAGACGGTTGCTGAGAAAAATCGACATCCACATCCTTCCCGTCAT
GTGTCTTATCTGCCTGTTAGTTAGAAGCATCTTAATAGTTTCGACGAGGGAG
ACACCTGTTTTTTGGCTAACGTATCTGTACCCAGACTTTGCTTTCTTGATCG
TGCAAATATCGGAAATGCACGACTAGACAATCTAGAGTCAGATCTTCACCT
CGTCGGCCTGCAATATAATGACTGCCTCGCTATCCTGTTCCCGTTCTATATCA
TAGCAGAAATCCCTAGTATTATCATGATGAAGGTCAGATTGCCTTAGACGAT
TACGCTTCATGGCTTTCCTTTCCATACCTTGATACCTTGACATCCTGATCGCT
AACAAATCTGCGTTTAAAGCACACAAAACCTTCTTCGTGGCTAGCATTCAT
CATGTTCGCCTGGTCAGCTTGTATTATCGGTCAGGGTTTCGTGACAAATTAT
CAGGGGCTTATGGCCACGCGGGCGCTCCTTGGTGCCTTTGAGGGGGCTTCT
TCCCTGGTATCAACTTCTACATCACGCAGTGGTATCCCAGAAACGAGTGCG
GGGTGCGTATGGCCATCATATTCTCCTGTGCCACTCTAGCTGGCGCCTTCGG
CGGCATTCTGGCTCGCGGCATCGCCGAGATGAGCGGTGTTGGGGGATTACC
CGCATGGTCTTGGATTTTATAATCGAGGGTCTCGCGAGCGTCGTCGTCTCGG
TCCTAGCGTATTTTGCCATTTATGGCTATCCCGCGAGGTGAGACTCATTTATC
ATAATTGCAAAATTAGATCGATGTTCCAAACATACCTACTTACCTTGTATATA
TGTTAGCGCGCGTTTCCTCAAGCCGGCCGAACGCCACGAAGTAGAAAGGT
ATCTCGCAATAGACTCGGGCTCCCTCCCCGACGACTTTGATGTCAAATACAT
ATGGCAGGCACTCTTAGACTGGAAAATTTATATTCATATGTTAATCTGCCTTG
GTGGCTTTTGCCCTGTCTACTCCTTCTCGCTTTTCCTGCCCACCATCATTCG
GAATATGGGCTACTCGGCCAATAACGCGCAGCTCATGAGCACTCCTCCTTAT
GTTCTCGCCTGCCTTTTCGCAATTGCCGGAAGTCAACTGGCAGACCGGTGC
CGCCAGCGTGGCGTTTTCCTACTAGGCTTCCAACTCATCGCGATATTGGGAC
TTGCAATGCTGGCCGCGAGCGAAAACCCCACTCTACAGTATACGGGGACG

GTACTCGCTGCTATCGGTAAGTTTGTACCCGATCGGACGCTTAATACGCAAG
CGGTACGAAACGCTAATCGACTTCTTTGCGGATAAATACAGGAATCTACCCC
CAGATCCCGCTCGGTCTGGCGTGGAACGGAGCAACATCGGCGGCAGCCTC
AAGCGCGCAACTGGAATCGCTATGCAGGTGATGGGAGGTAACTGTGGGGG
GATTATTGCCTCGTATGTCTACCTAGCGAGGGATGCGCCGCGTTACATCGCC
GGCCACAGCATCTTGATCGGATTCGTTGGGTAAGTCTGCTGAAACACCTCC
TCGCGCCACTGCGTTTCCCCTAACTCGTATTCTTGTTACAGACTCGCCTTTG
TCATGACGCTATTTATGACAATTTGGTGCCGCAAGGAAAATGCCCGGCGTG
AGACTATTGCAAGACAAACGGTCGTTGCAGGATTGACGATAGAGCAGAAG
ACATTGGAACGGGAACTTGCGGACGAGGTTTCGTGGTTTAGATATACCATC
TGACATCAAGACAGACATGTATATTTTATATAGCAACGAAGATCATTGATCA
AGTGATGAACTTGAGACCTACGCCCAGCGATGGGCTTGACACACACCTTTA
CCACTTAATCAATTCCGTTTTGGTGCCATTCATGATCTCTCGCGAATCACTAT
GATACAACACCCATGATTCCAAGATTGAACACATCTACGTAATAAGGTGCA
GAAGCAACTCGTTTTGAATATAGCTAGAAAGTCTCCCACCACAATGATCCC
AATATCAATCCGGGACCAACACAACATCTGTACCGGACCTAAAGCCAACAT
CACTCTCTGCACCTCGCTCCGACAGGGTGCGCCGATAGGCTAGCTCAGATG
TGTCACTCGCGAAGACATGACACACGTTTGCTTCTTGAACGACGAATGATG
ATGCTGGTCTAACAAGTGACCGATGGGAAATAGCAACCAGTGTAGCTATGC
ACTTATATCCCAGCGCATTCGCTGGAGTGCTCCTAGCGTGACATATTCCTTTT
GACTACCTAGATGTTCGTAGCGGGTTGAAAATCGTGGCTTTCGTATACAGA
GCATATCTGATCAAGCCAATGCATACTTCCATCATATATCCCGCCAGGCGGAT
CATAGCCAACCCCTCTTGCGTTCCCGGCACCCAGACCGGCGCTACCGGGAG
TCTCGGCATCCGCTTGTGCCGTGTTCGTTTTTCGTAGCATGAACATAGGCTT
CTTCCCTTCCTTATCCAATTGCTTAAAAAGGATCAATGGTTTTTCATGTAAAC
CTAGGACATCGTTCTTGATCACCGTAGACTATATAGAGCGCGTACTGATCCC
ACGGAGCGTTGATCTGGTATTTCTTCAGGGCCGCGGGGAGGACCTTGTAGC
AAGGATCATCCATACTGACCCTGAACGACTTGAAGATCTCTACCGAAGCTG
AGTTTGAGCCGGGAGTCTCGGGAACGGGCGTCTGCATCCGTCGTGGCGCG
ACGGGCTTCGAACCATAACCGTGTTCGCTTTCCGAGAACGTCGTGCGACTC
GACGGCGTCTGTCGGTCATCGGATCGGTAGGAACGGGAGGCGAGGGTAGC
ACTGCCTAAAGTCGGGGGTGAGGTGGGCGAAGGTATGTTCGGAGAATAAC
CGGGCGACGTGGCCTGGCCGGAGCCGTTCATGGCGGCTAGGTGCGAGGAG
GATAAGACGGCTCTTTCGGCGGCATTCGCGGGATCTAGGTTTGCTCTACCG
AAGGCCGCTCATGAGTAGTCTGCAGATGGGTGGGGGATGTGCTCTTGGGAT
TCGTACCAAGGACGATCTTCTTGGTAGAGTATTGTCGCTTGAGACCACTCG
AGGACTGATCTGACGATGGTGTAGGGGCGGGAGGGGAAACTGTCGAAGCC
TCATAGCCATAACCTCCTTGATTATTGGCTAGGTGAGGCAGAGGCTGCTGG

GCGTCCTTAGCAAGGCGCAAAGCCGGTAGCATATCTTCGCGCAGCCGCCTG
AAATCTTCGGTCATGCGTCGTAGGTCTTGTTCTAGAACCCCATCCTCTCGTC
TCGGAGACGAAGTTGCTCAACCAGATGCTTGATATCTTTCAGGGTTGCAGT
TGCGTACTGGGCCTCGGCGTCGGCGGCTGTTATCGAGGGGGAAAACAAAC
GAGTTAATTATTGGCAACATAAAGGGCGCACTACGAAAAAGAACAAGTAG
AAGAAATTGGACAATACGTACATAATGGCACATAGTGATCGCTTTCTATGGG
AACATCTTGTGCCTTTTTCACATCATAGACGCTCTTTAAGAATGGTCAAGCG
ATGGCCAACACTGGTCACTCCCATAGCCTTGAGGTCATCGTGCAGCAGAGC
CACTAAAGCTTCGCCGACGATCTCATTCTCTAAGACAAGGGGGGCTCAAGT
TAGAACGATGATCATGACGATAACAGGAACGGCAAACAAAATGCGACTGC
GAAAACACACCGATAAACCGATTTGAGTACTGCTCCAACCCTATACTCGCG
ATAAAATCCGCACATTCGTCGGCGTCCCATTCAGATATTATAGTTTCGGGCA
ATCTGTCGGCACTGTTGCGGTGACCATAGGTAGTAGGCGTGTGCTCGGTGG
AGGAACGACCCTCGGAGTCGGTTGGGGATATTTCAGAATCGGTGGCGAGT
ACTGGTGAGCTGGTATGGACACTCCTCTCATATTCGTCGTCGGCGTCGGATT
CCGCGTATGCGGTGCCGGAATCGAAGTTCATCGTCGATTTGTTGGAGTTAA
AAAGAAAGGCCTCGTTTAATCAGGAGTGAAAATAAATTTGAGACTGTGGC
GTTGTTTAGGTTATGTCGTTCCTTGCGTTTCAGAATGATCTTTTCCCTTTTTC
CCCCTCGTTCCTTTTACTGCTTCTTCTTCTCTCGGTCTCGGTGTCTTTTCCTT
CTCTTTCACCCCTCCTTTTGATTTTCTTCTTTCGATAGAACCGTTCAAAGAC
GAAAGTCCAAGAACGACGGGGGACCTACAACGGCCTAAAATCTCGATAAC
TAGCTCCCCCGTAGCGTTCTCTCTTCTCTCCTCGTGAGGTTGTCTCGAAAAG
TCCACCCTGATTGAATTTCGGGAGAATTATTGGGAATACTTTAGATACATTC
ACGGACCGTGTGTCAATTTAGCAAGACGGCGAGCAAAATGGGGTATGTTC
GATAGACGGAAACGTTGTAAAAGGGACGTGAGCGAGCGTGTGATTTCGAA
GAAACAAAAGTTTGGAGACTAAAGTCGCAGTCGTAACCGTGATTGGGGAA
CTGTTAGTTAGGTGTCAGGTATGGATTGTTTGAATGGGAATCTAAGAGCTGG
TTGCTAACTTCCAAGGTTGCTAACGATTGATTTGAGCCCTGGACGATGTTAA
AAAAATTAAAAAATGGATGTAAAAGCCTAGAAGCCAGAAGCCAGGAAGCC
ACAACCTAGCACCCAGAACATTGACGACCACAATACATCCGCATCCACATC
CACACTAGAAACCCAGCAACGCTTCGCAACTGCCCAGTAATCCAGCAGCA
TCAGGCAGCCCCGGGTGCAGGTCTGTGGGACCCGCTTCGCATCCAACTTAA
ACTTGAACATCCTGACAGCCTGAACATATAGGTATCTACGTATGTACTGTAG
TACCTTTTCAATGTTGGAAAGTCCAGTCTGTTCCTGATGGGATGTTCCTTAC
TGGTCCAATGGCGACTTCTCTACTGCGTAGCGATTCCTAACTATGCATACAT
ATCCGTTAGTCCTATTGGCCGGCCGCACTACATAGTACTATGTATTTCTGTAC
CTATCCTTGCCCTTCTAAATTGAAACTGCATAGCATAAAAAGGGGGTACACA
GCCCCAAGACCAGCTGTACATAAACTGCCGGGCGTTTCTGATACTACTGCT

CATGTACCTTAGCGGACCTAGCTGGGTACATTGTAGCCGCTGCTTGGCACCC
CTTCTCTCGCTTTCCTCTATGCAGATGTAGGTGGTGCAGATGTGCAGATGTC
TTGAGCGTCTTTCCACCCTTTTATTTCACCTGTAAAGTGTGTAGTGGACGGA
CCACCATTTTGAACCAGTGGACCTCTCTCCCGGTCCGCCATTCTAGTGGAC
CAATACCCCTGTTTTTCTAACCACCATTTTATGTATATTTGCTAGTGCGCGTC
TACTCGCATATCACTACACATCACCTATGCACATACTAATATGAGTAGTACAT
AATATTATATGTATAGCACATTCGTGTTGCCCCTGGTTTCTACATGGGTACCT
CATTTTGCGAAATCAAGACCAGAGCTCCTAACAGGTAGCTCTTCAGACATA
CTTAGGCAGGTGTGCCACTTACGACCAGAACGCAGTGCCGACGATAGTTG
ATTTGCTTCGCTCGAAAACCGTTGGTCCTTACGTCCAGTCTAAGTTACCCTA
CATAAATCCATAGATGTTACATTCGCTTCTACTGACCTAGCGCTCACCTTTCC
ACCATTCGTCATCTATCAACTCTAACCATTCTCCATATCGTGGACCGAGCGA
TCCCACATGCACATCTTTACTACCATGGATGGTTCTGCGTTGATTAGTAAGT
CATACATGAATACAGTACAATAATATACAAATATCTAGCCCAAGGGACAGAT
ACATCAAACATAGAATGGACGTTGGTGGAAAATTCGAGAGATAGGTACAAA
TACATAGCCATATTCATAGGTATCTGTGAATGTCAGTACTATTCACAAACACG
GTCGCACACATAATAGTGTATCTAACATTAGTTAAGTCCTTTGCTTATTACCC
GAGACTACATCCATCGATATCGCTGGCCTAAATGTCAGGTTAGGCTCTCTTC
AAGTCTACACACCATCAAATCTTGTGTCACCTATCGACCAGCATTGGTTACC
TGCGCCAGTTACGCTGGGGCATGTATAGTAGCGAACGTATTTAATTTCTATTT
GAACATGATTAAGGCCTTGTCCACAAGAGACAAACTCCAGGTCTTTGACCC
AAGGCAAATTTGACAAGGCCGGCAATACAATAAGTACAGCGTCGGGGTATC
TTACTATAGAAGTTGTTGCGCCTCGCTCGTCCTTTTGACATACCAGCCGGTG
TGAAGAAGCGAACCAAGGTTCATCTGTAAAGCGAGTGCGGGCAAAAAACC
CCCCCCGATTCTACAACCCTTACCGGCACACGGCCGTCTATTACCATCACCC
ATCACAACTCCGAGGCGCCCTAGTTAGTTTGACAAATGCGTTCCCGTGCCT
TGATTAAACGCCTTGATGCAAGCTATTCACATTCCGCTGATCGCGGACTAGA
TAGTCTACCGTAACCTTATCTTAGCATCTTCGGCGCCGCGGGCGATCACTTC
TTCAAGCGACCATCGTGGTGGCTCGATGACGCCTTCGAGCCCACTAGCTTT
GCGCCACGCCTTTTCATTCTCCGGCGCAACACCATCCGGCAGAGGCCTGAG
ACCTCCTGCGACGCCCGTGGAGAACTTGAGTTGTAGAATATCTCCGACTTC
GTCGCAAGCAGCTAGAAGACGATCCATGTCGTCCTTGTTGTGGGCCGCTGA
AACACAGAATCGGGCGCGGGAGCTAATCAGAGGAGTTGCGGGGTATCCAA
CAACGACAACGGAAATATTACGCTTAAGCATTTCATGGCTGAAAGCGGAGA
TCTTAGCTGGGTTATATAGAGCGACAGGGATGATAGGCGAGTCATCATGGCC
GTAGACGATCAAACCGAGACGCTTCAGACCGAGACGAAGATATCTAGAGT
TGAAAGCAATGCGTTGTAGCCGTTCTTCGCCTTGACCAGGGACGAGCTCGC
CAGAAATAATTTTCAACGAAGCCATAATTTGCATCAAGACGGCGGGCGTCG

GCGATTCACCAAGCATGGTAGCAGCATTCGAGCTTCGCAGCTTGTCGATGA
TGTGCTTATCGGCCGCAACATAACCACCATTTGCACCAAAGGACTTCGTCA
AAGTGCCCATGAGAATGTCCACCCTACTGGGATCAATGCCAAAGTAATCGC
AAACACCGCGACCGCGGGCGCCAAGGGCTCCAATGCTGTGAGCTTCATCA
ACGTAGAGGGCGAACTTATATCTATCCTTGAGGGCCAGGACACCAGGTAAA
TCGCACATAGTACCTTCCATTGAGTATAATCCTTCGATAGCAACCAAGATCT
TCTTCCAGGGGCGGTGCGTCCGAGGCTGGCCTTGGGCGATGGCTTCACGTA
AACGCCTCTCCAAGTCAACCATATCGTTATGCTTGAATGAACGAATGACAG
CTCCAGAAAGACGGGCTCCGAGTCGAATTGAGGCATGGTTCAACTCATCA
GAGAGGATTAGGCAACCCTTGGAGACAAGAGCTGGGAAAGAGCTAGCATT
TGTAACAAAACCCATCGAGAATACCATGGCATCGGGCTTTCCGACGAAGGA
CGCAACCTCTTTCTCGACTTCACAGACGAGATCCGAAGTACCCGAATCAGC
GCGAGGGCTACAGAAACTGAGGCCATATCTCTTGACGCATTCCTCGACGGC
ATTGGCACAGGGACCTTCAGATTGTGCAAATCCGAGATAGTTGTACGAGCT
CATGTTCAATGTCTCCGTTGTCCCACCGATATATTTGTATGTTTCATTGAAGT
CGTATGAGACACGGTTCAAGAGGTTATATACCGACCCGGAACGCCAGTGCT
TGTCCTCGCGAAACAATCGTCTATACGCATCTTGAGTCTCCTGGTATAGAAG
TTGTCGAAGTCGTCGTTCAAAGGGGCGTACCCATCGTTTGCGCGGATTGTC
TTATAGGTTGGCTTTTTACTGAATCTTTTCCCGAAGAAGTCCCTGATATGGC
CGAGGGCGAGGAGTAATAGGTAGCTAATGTAAGTAGTCAAGACATAATAAT
AAGGCGGCTCATCAATGACGGGGGCTTCCAAAGGTTCTCCTTGGTGCTTGC
TGACGTATCGGTGGCTTGGGTGAACCAATGGGCCGAACTGAGCATCGAATC
TCTTCTGACGCGAAATTTCGGTCGAATCAGGTGGTTGAAATAAGGTAGTGG
GCGGCTCGTCGTTATGAGGCTCGCCGCCAGTGGCCGTAGAGAGAGAAATA
GAGGGCAGTGACAAATTAGGGGTAGGAATAGAAGGAGCGGAATTGTTTTG
CATGGCCATTAGGGCTTGCTGAGCGGTAGGCTGGTTGGCCTTTGCAGACGG
AGAGAAGAATGAGCTGAGGCGATTTCCTCTAGATTGCGAGGGCCTTCTCTC
GCCAGACCTCTCGGAGTGTGGCATATTTGTAGAACTGGTAAAAGGAATCGA
GATACGTCGTGGCATTTCTATAGGTAATGGAAATCGGTAGCGGAGAGGTTAT
ATATTAAGGCAGTGTTAGGAGAGCGCCTGGAAACGAGATTGACTTTTTCGC
GAATCTAGAATTATGTTAATCGACCGTCATGAGTTTATTAGGATCATTCAATT
TGATAGTTGGAAATCGAAGGAGGAAAAGAGGGAATATCAGGAAAGCTAGT
ATTCGTAGTTTGGGAAATAAGGCGCCGGGGTATTTCAGCGAGTGTTGGGCG
GTATTGAGCAACATTTGGGACCAAGACTGGCTTTTCTGAAGGTTTGAAAGA
CCATCCTAAGCTTATGGGAAGGATCGAAGGCGCAGCATGATTTCGATGTTTC
CCAATGACGGAGGCATGGGTATTGTGGCGCCATTGACATAGACAATAAAGG
ACCGATGAATTCGTTTGTGCGAACAATAGGATAACAATGTGCATATAGACCC
GCCTATGCGCAATAGTTTTCGAGTGAAGTAGACGAAGAAAACGATAAATAA

GCTTACCTTAACAACTAGCAAATCCCCAAGCATTCACCACGTAAGTAGTTA
ATTTTCCAAGACGTTGTGTTGTGTTGCGTTGCGTCGCGATGTGTCTGGTCCT
GTCTTGCCTATCGGTCCACGGATGAATCGGTCGGAGAAGCTCGAAGCTATG
TAATACCTTAAGCTGCTGAGCTCGAATGAGGGAGGTTCAATTGGCCGGTGG
CTGATATCGAGAAAACCAAGGAGCGTGGTTAAGGGAAAAGAGAATTCCCG
TCGATAACAACACAACGTCAACAACTGCCCTCGACGCCAACAGCACCTTT
CCGGAGTTGACAAGGAAGAGAGGTGCACAATGTTGAAAAGGAGAGACGA
AGTGAGAAGAGATAAAATGAAAATTAGCAATTCGATTGACGGGAAAGAGT
TCCAAGAGGTGGATCCCAATGAATATGTATTGTACCTTTGTACTCACTACATT
CATATGCACAAACATATGCACATACATGTATAATTATCCCTGATACTTACCTAT
ATATGTACTTAATGCAAAGCATCGTTCATTAAGTTCATATCAAGATAGCGTTT
AGGACCGTCCAAGCCACCGTGTCACGCGAGAGTTTCGAGGCGTTTCGGTA
GATCAATGTGAAAAGGCCAATCAAGTAAGATTAACAAAGGATAATATGGAT
CCCTCCGTTTCCCCCTTAATAAGGTACGGTTATAAATCGACAATGCTATCACT
TTACTAACGAACCCGTACTTTCCGCCAATACCACCACCTCTCACTTTTTCTT
TCTTTCTTTCCGAGCGATCCGTGCTCCACACCGTACAAATTATGGGGCAATT
AATCCTGGAGATCAAATCCCCTTTAGAGGTTCCCAGCTATTACACTAGGTAT
TTCGCGTTCGATTAACGCCCCGTTTCGGCAAGCTGCGTCTTCGCGATACCCC
ATCGTTGGCTATCGAACTTTGGAAGTCACCTAGCACCTAGCTTGAGATTTTG
AGTCCGCACTGAAGCTTCCTACAT

>Mosaic_sequence_15

GTGAGTCTACCGCATATTTCACGAGAAGGAGGGGCTAACGAAGTACGTAAA
GTATGCGTCCACTAGCGCCTTCGTCTCCCTTGCCGGAGGCAGTCTAGGCGG
TCTGGGCGAAGGGTCGACAGGAATGGGCGGAGGGGCGGTATCGACATTCC
AAACCACGCGAGGATGGTGTTGACATCAAACGGGCTCGTCGCAGCCGGGT
TGCTAGTGGGCGTTGGGAGGAATATGTCCTTGTTGCAGAGTCGCCTGATGA
CATTGAGGAAGGTCGCAGCGGAGGACAGACCCATGTAGCTCATTCCCCTGC
TTGACGAATTGATGGGCCGCCCATGCCCGGTCCCTCCTCGGTGGAATCGGG
ATCTCTCGTGAGGTTCTGGTCTTCGTGCCATTCGTACGATCCGGCCTCAAGT
GCATGGTCCACGTATACAGGCTTGAGCGAGAGTCCATTGCCGTTCCCGCTG
GGTGAGCCGGCACGTGTAGGGGGGATCGAGCGCAGGTCGTGCCTCGCAAG
CGGTGACTGATGGTCAGCACGTCCAATTGCTCACAACAATGTACTGGCATT
CACGTTCAAGTGCTGGTGGCTGTGGTTCGGAAGGTGACTTCTTGCCTTGGC
GAGCACGGATGCGATTCCTCCTTTGGGGTCCACCGCATCTCTACATGCCTGC
TCGAGGGGGTATGCGGGCAACAGGGAATCCCACAGCAGCTCATGCAACGA
CAGCCTGTCCTGTCAGCGAGCCTCCCCGACCAGTAAGACCGTCGCTCACG
CCTCCTCCAGCTCGGTCATCCGGACTCGCGTCAGCGCCGAGCGCTCTGGCC

GCGCCTCGTAGATACACTTGGCAGCATCCTCCGCACACCTCAGGCAGGGGA
CCGAGCCATCGCATGCAACCTTGCGCTTTCCTTCGGCGTCAGTATGCTGTAC
CCTGCACGAGAATGGAGAGGAACCCACGGCATCGCTCGCAGGCCGCAGAT
GTCCGTTGTCTCTTCAGGCTGCCCTGCGGCGAGATGTGCGGCGACACGGA
GCGAGCTGACATTGCTAGCATCCCCCTTCCTGGTCTAGCTTGCTACGTTTCA
CGGGTCACAGTTGGAATGTTGCTCGTTCGTACGGTTGACCGTCGACGGCTC
TGAGTGTCCCGTCGACTACGGCTCTTCTGTTTAGCCGGAGTCAGCATCTTTC
CCTATTGACCTCTCTGAAGACCTTCGGTATACTTACTCCCAGGCTGGCAGAG
CATGCCGTCGACAGACGGGCAGAGCCGAAAGAGTCGAAAGTGCATGCGC
GCCCACGGGAAAGTTCATAAGCAAGCTGAAACCCGGGATTCCACCTCACA
AGCTCAACGCCTTCCATTTTGAGATGCTTCAGTGTCATTTGTGACAGCTGG
GGTGGTATATATATGGGCCTCATGCCCATAGATTGTCCTTAGCACGTGCTAGT
CCTATGGATAGAGGCCAACCGCACAGCCATATTGTTGATTCACGAGAGGCT
GTAGGAGACACCAATTGATCCTCAATGATAGAGACACAAGTTGCATGATAC
AGTTCATCAGTTGACACCGGACCGCACCCCATGACAAGTAGCATACTGTCA
CAAATGTCACCAAAGCAACAGCAAGACCGAGCTAAAGGAACGTGAGTTG
GCCACTCGTTGGCGCTTGCCAAAGCTTGCCGCGACTCGCCAAGACCAACC
CATACTGTATGTAACAATGGGTCAAGGTCAAGGTCTTTCAAGGTGAGGAAT
AATATCCCAAAAGGATATTTGCGCCCCAAGTTCTGGTTAATCCCTAACCTGA
ACTCGGTTTATCAAGGGTACCTTGGACGTGTCATGCCCATCATGACACTGTA
TTACCGCATCTGTCAAACAAAAGGATGCATTTTGCACAACTAGGTATCATTG
TCGCCAGATTTCTTGCCAACAACAAAGAGGCCGGCGCTGCTCGCCGTTGCT
TGCCTTGCTTGCCATGCTTGCTGTCAAAGAAGAATAGGCCTCCTTCATGCTT
ACTCAGCGAGTTTGCCAGATGCGGCAAACTCGACAAAGTGAAACGAGAG
AACAATGAGCTTGGCGTAGGTGGACCAAGGCCTTGCTGACGTTGCAGAGA
CAAGCCGCAGAAGCAATGGGACGCGCTGTAGGATACATAAGGCTCGCAGA
GCCCTCGCAGAATTCCAGTACTCTCGCATTTGAGTTGCATGGGACTTATCCC
CTTCACTTACTCTCTCTTGCTCCGTCTTGCAAACAGCTGCTAAGGCCTAGCA
CTTGTCGGCTCACCCTGTCTTGTCTTGCTTTGTCTAGCCAACCTTGGCTACC
CTTGTCAACACTCGTTGAGACTCTAGAGATCCTTCGAGCCCTTGCCCTTGC
CATTTCTCCTGGCCTCGCCCAGCCTCGCCAAAGTCAAAGAGTCTTGGTCAC
CAAGTGATGGGCACTTGGACAGAGACAGAGAGGTATTGTGACAAAAAGCT
TGTTCTTTATGTGGCAAGCGCAATGGAAACAGGAGTCTGGCTCAGATGAGA
TTGACTTGTCCTCAAGTCCCCTTGTCAAGGCACACCCTTGATAAGGTTAGA
GAAAGGCAGAAAGGCTGCTGCCAATGAGAATTCCATCTGTGCCCCCAATAC
CTTAACAACCTTGTAGAATTTGCCACTGTTGCTCTCCTGATCACTCTCAGCA
AGGGCTGAGCAGGAAACAGGTCTTCAACAGAAAGAAAGCCCAGGCTTGG
CAAGGTGGGCCTCCATGTCAAGCCAGTTGGCAACTCCTGTGTGTCTCACAC

CCCCAATTTGGTTTCTGGTATGGAGGGCAACAGTGAATGGATTGTAACCGC
ATCAAAGTTGAAGATATGTATATGCATGAGAAAGAAAAAAACAGAGCAGC
AAAAAATATCAGCAGAGGCTAGTCACTGGACAAGTACTTAGGTCTGACTAT
TGGACCCGGCCTCAGTCCCTGGCATAGTAGGGTGAGTATGTTGGCACTGTT
GGATTTTCTTATGAATTTTGTCCTGAATAGTGGGATGGTTGTCTGTCCATACA
CTCATGCCTTGTAGACCTTGTCATAAGGTGTGGATGTCAATCGAGACACTC
GCTCCGCTTCTATCCTGATGATAGTATCTGTGAACACCTCTGCTTGGTTTATG
GAGGTGAAACTTGACGATTACACTTGTTGTTCATACGTCTCATAAAATCGTC
ACTAACCGTATACTCTGTCTGGCTATTAGCTTTAGTAGAAGAAGCTGTTTTC
AGCAACCCGCCCTGAGGAGACTGGTAGATGGATTCACTGCTCATGTTGAGA
AGCAAGTAGCAAGATTTCTGCTTGAAATTAGATAGTTTGGATACTGTTTGGT
GATACACCCATGCATTTGCTTTATATATCTGGCCACAATCCACCAACTAGTGC
CATTTGCAGTTTTACGCTCAATGATTGCATCTCCTTGCAGATTGCTGTAGGA
AATTGGGTAAGAAAGGAAGGATAAGGTGGCAAAGCTGGGAGAGTGGTAAT
CAATATTGGTAAACCAGCACTAGATTGTTAATAGCAGTTGTGAAGAGGTATG
TCAGACCTGTACCCTGGTCTTGTGTCATTGGCGGAAATGTCGTGACTCGAG
AGCAGGCTGCGAACCTCATTGCTGCTGGTGTCGACGGACTACGAATTGGG
AATGGGTAGTGGAAGTGCTTGTATTACCCAGGAAGTCATGGCCGTCGGTCG
TCCTCAAGCAGCTGCGGTATACTCTGTTTCCGCCTTTGCTGCGAAATTTGGA
GTGCCGTGCATTGCTGATGGCGGTGTTCAAAACGTCGGTCACATCGTCAAG
GGTCTTTCCCTTGGCGCGTCTACTGTCATGATGGGTGGCTTGCTTGCCGGCA
CCACAGAGTCACAGGTACATCGTTTTGTATCGCGGGAAGGCAAGCTTGTCA
AAGCATACCGTGGTATGGGTAGTATCGATGCTATGCAAGATAAGAAGGCTG
GTGCAGGTGGCAAGAACAGCCAGGCTAGTAACGCTGGTACTGCCCGATAC
TTCAGCGAAGGAGACAGCGTCCTAGTCGCTCAGGGTGTATCTGGTGCTGTA
GCACACCGCGGATCCATCAACAAGTTCGTGCCCTACTTGGCTGCGGGTCTC
AAACACTCGATGCAAGATTGTGGCATGCGAAGCTTGAAAGAGCTTCACGA
GTGCGCGGCTAACGGAATACTCCGATTCGAGGTTCGCACCGCGAGCGCTCA
GTTGGAGGGTGGTGTGAACATGGAAAGCTACGAGAAGAAGCTCTACGCTT
AGTAGGCTTTGATCTTCAGGTTAGCGAGTGATTGTACCAATTTGGACAATGA
ACAATTAGGCGGAGGGCAGCAAGTTCTTTGCAAAGACAACGATTGGGAAT
CGGTTAGGACATAACGAATTTCTCAGATAGTCGTTGGTGTGGAAAAACAAA
AACTAAAAGTGATACCTCTTGATAGTTTCTTATGCTTGATGATGAGCAAGTG
GCTTCGTTCCACTCATAGGCGTTTTGCGGCTCATAAGAGGGAAGGTCATGC
CGGTTCGGCAGGAGCAATCTTGGGATGGAGGGAGTTCATGTCTCATTTTCC
ACGGGCGTACGAGACATAAAAATGGGATTTAGCTAGACGCGATGAATGCAA
AAAGCATACAAATCAAAAATTACCTCGACTTCACTTTTGATCATGTTAGTGT
TTCTCAGTGTGATGGCAATGATGAATTTCAATTTTCTATCTATCGTAAGCCAA

GAAATAGTATCTGCTTGCTTCTGAAATGTATCCCAACCCCATTTTCTAGATCC
GGAACGCCACCCATGAAAGTAATGATGTTGATGTCTCGTCTCATGTATCGTT
TATTTTATCACCCTCCAATTCCCCCTATTTTCATATATGTAGCCTAACCGTCCT
TCCATCATGAGACCATTAGTTACTAGCATTAAGTCCGTCGCATCCCACATCT
CGTACTCAATTCGTTACAGTTCCGTAGCCGTTACTATTCCTCGAGCCTTCGG
CGCCCTCTGAAGATGGGGTTTCCTGTGGCTGAGGTTCCACCACGGTAAGTG
TGTCTTCGTCGTCATCAGCAATATCATCATCAACCTCTCCTTCCTCATCCTCG
TCATCGCTGTCGACAGGTGTAACGGCCCCACTTGGTCCATTCCCCCTCAAC
AACCATGTATGCAGGCACAGCGCCCAATGTCGCAATGCACGCCAACAACC
ACCATGGAACGCCAATAATACCGTGCTTAACACCCCAGGTAAACACGCTCC
CCGTAAGCGCCGGTCCCGTCGCTCGTCCTAGTGCGCTAAAAGTTGTCGCAA
ACCCATTCAAGGTACCCAGAATACTCAGACTCGCTGCGCTGTTCGTGAGCA
AGATCGTCGTGCACGGGAACCCCACGATAACGGCAAAAGTCTTAGTCATAA
GAATCACAGCAAGAGCGGCGTATCGTAGCAATTTAGGCTCGATCAGCACGA
CGAAGGGGGTCACGGCGTATACGAGCGGGAAGACGATAACACACGTCTTG
AAGCAGCGAAGCACGCCGAACCGTTTGCAGAGGACCGGGAAGATCAAGA
ACTGAACGAAACCGGATAACACGCCTTCGGCGGCAAAGATGGCTCCGATG
TGCGCGTGGTTCAGCCCGGAAGCCGCCGCTGAAAATGAAAGGGAAGGTTG
TGTTTTCGGGACTGTGGGCTTGGGGTGGGTAGTTCAGAAATACGGGTAGCA
CTTGGTCGTATGCGACAGCGTGGAGCGCAAGGAAGGTATACGCAATTAGGT
TAATGGTTGTTTGCCGGCTGAATACTGCACTGATGGGTAGGGGCTTGGGTTT
CTCGGCTCGCGTCTCGACAACGGTGGCTCGTGCAGTTTTCGAAGGGGCGG
TTCCGCTAAGTAATGGTGCGGTGATCTCGTCATCTTGGAGGGAGTATCGGC
GGAGACGCTGCGCTCGTCGCTTAGGCGAGTGTTCTCGGAATGCCTCCTTGA
TCCGTTCGCCGAGGACTAGACCCCAGTCTCGAGAGTGACGCTTGCTCTGG
AGGGTTTCCTTTAGGAATAGCATGGCAGTAGAGAGCGATATCATAAAAAAG
ATAGAACCTATTAAGTTAGGGAAAGCGAACGGGTATTTCTTGAAAAACGTG
CTGTTGCCGAATACGCCCGGCCATTGCTCTGCAGGCTTAGCGAATAGACCG
CCGAACGCTGGTCCGAATACAGATCCGACGCTCCATACTAGTGGCATGACG
GAGAAGGCCTTCGGTTGAAGTTCTTTTTCTGTTACCATTTCCGCGACCATCG
TTCGAATGATACCAACTGAAAGATGATTAGTCTCGACCTCGAGGGTAGTAG
GAAAGCAGATTAGTCGCAAATACGCACCATTCCCATTACCAGCTCCCATCA
GAGCACGGACAGTGATAGCCATGGGCAAAACTCGTTGATACACCCCACAC
CAAGAAACACAACATCGTACACAAAAGACCGGTCATAATGACGGGCTTTC
TCCCAAATCTATCTGAGGCCCTACCCCAAAATACAGCGGTTAAGCTTTGAG
CGAGGGAAAAGACGGAGCTTGTGATGCCAGCCCATTTTGCGACTTCCTTTC
TCTCAACTCCAAAACTAGAAATCATCTCTGGCAGGTATGGAAGACAGACGT
GTAAGAGAGGGGTTCGGCAAATCGAGCGACGGCTATAAGCAGAGTTAGCT

TTAGTACAAAAGGGGATGGAATACCTGGTTTCTTGTTGTATTGTGTATAATC
CTGCCATATCCTCCATTGAGTTTTCATTTCCATTTAGTTAGAACTATTGTATAT
TCCTTATACTTTACTCTCGTCAAGACACCTTTGACACCAGTTTCAACGAGTC
ACGACGGACAAGTGACAAAATGTAATGATTCTAGCCTCTGGGACTCGTATA
CATTCCACACGTAAAAGCCAGTAATTTATTTGGGACACGATAAGCGTATTAG
ACCGTTTCTGATAGACGTTCACCCGACCGCATCCACAGCCTTTGAATACAC
TGATATTATAGATCCGTCCAAGAGACGATAGCAAAAAACGCTAGCTAAATG
AAGGAGAAGATGAGCTGGTAAGTAGAAGAGAGGTCTGAAGCTCACCCAAT
ATGGCCAACTGCTGGGCCGGCAATTTCGGCGCATGATTCTTAGGCGTCATT
GTTGTCGATATGATCAAATGACAACTCGGAATTGAGGGTTGAAAAGGAAGA
ATATGGGCGTCAGGGCGTCTAGACTCGTGTGACGTCTAAACTTGCGTATAA
GGTTTGCGAAGGCCGTGGGGCAACTCCGAAGAGAAAGAGCAGTATGCAA
AACTTGGATGCAGCTACTTTAATAATATGCAGACTGTGTTTGAGACTCGCAA
GGACGACTGATCTGCGCTGGAAGGGTAGGACTGCCTACTAAGCAGTAGGA
ACGAATGGTAGAGATTACCCAACCAAGACGAAAAAAGAGAGAAACTTTTC
TTTTTTTTCTTTTTTCTCTGAACTGGCGGGGAACAAGGTGATTATAAACAAC
GGAGGACGTTACAAGATACTACGTGACGACACCCATATATCGTACCGAACA
CGCCTAGGCCCTGGATTCATCGGCGTGTCTCTCACCTCGCGCAAACCCTCC
TTGTCCACCTTGCCCACCCTGTCGTATGCAAGACTGCAACTTTCTTGAGGC
TAAGCCGAGATAGCAGCAATCCGGGTCTGAGGACGGATCGTCCGCAGAGT
AGGTCGCTTCTGATTTGCGGCTTACCGCACATAGGCACCCGAATCCTCATCC
AAGCGCGGTAGTAGTGGACCAAGCTATGTCTTTAAAGAGTTACAGCGGTAA
GGCGTGCTGGTTGCAATGCAAAAATTTTCCTACCCTTTCTTTTTTTTTTAAA
CGACGAACTGGAACTTTGTCTCGTAACGGACTATCATCTTAGTTAGTGACC
CGCCTTCAGCTAGAAGGAAACGAATCGGAAAGGTGCAGGAGTTGCAAAGT
GGCTCCCGATGTTGTTGCATGTTGCAGCAACGGATTCTTTCAGTGAAACCC
CAGAACGATCAACCCGGATCATCTCCCAACAGGTGAGTCAAGGTGTGTCAT
GTCCAACATCTCTAATAGTGCAGAATAATGATGCAGGAGGGTGTAAGGAAA
CCTGGAAGGCTCTCTCAATGGCCGGTGTCGGACGTGGTAAACAGAGATTTG
TAGGCGTATGGAAATTAGTTTGATGAGAGAAGCCCAAAATCGAAAGTCACA
CGCCAACGTCCAGCCTCCTGATCATGCAAAGACATGCAAAGACAAGGAAG
AGGTTGAAGGGAGCGGATCTACGCTTGCGCGGATGCGGGCTGGTCGCACT
GCAGCAAGGCTTAAACACGCCCCGTTCTTCTTAGAAATAAAGAGCCACGGT
TGGCTCGCATTCGTCCTTACAGCGTTTGTCTTATGCCCCGCATAATCCACCTT
GAAGCATGGGCCTCAATGTTAGGACCCCGCTGTGCTTAAGCTCCCTTGAGG
TGCGTGATGCAGCTTTCGAGCAAGCTTATAGATTCCCCATATGCCAGGCTAA
GTCGGGATACGGGCATGAAATTTGTTGTGTTACTGCCTTTTTAGGACTTTGG
GAAGCAATCGTCAAATTGTTGACGAAATACGCTGTGAGACAGGATCGCTCG

GAGTTATGATCTATCATAAATGATTTCTTTTGAGGCTTCGATAGTTGACTAGG
TGCATTTTCTAGCACTCGCATAAGTCATTCCCAAAAGAGTAGGTCGAATAGT
CGAGCGGGCAGCCACGATCAAATTCTTAGGCTTCCCAAAGTTTCCCAGAGT
TCCCTAAAGTGTCGGGTAAGACACGGAAGCATAATAATCTCCAGAGTCAAG
TTGATTGGATAGACTGTAAAGGAAAGTGCCAATTAATACCGATTATTATGGG
ACCTTACATTGGATGAACATTGAGTACAAGAACTCGAGCTTAATAATTATAC
TTTAATCCTTCGAAGAAGTTACAAACCTTTAATTGTATTTAATATTTACCAAC
TTCATTTCAAACTGCAAGTCGAACTTTACAATCTTTCTAACAGATCCATAGA
TGTCTTTAAAACAAGAATAATCCCCCAGTCCCCTTAACTTATTTCTCTGGCA
ATATGTGATGACTAACTGGAATTCTCTCTTCTTATCTCGTCAGTGCGAGTGG
CCCCACGTAAGCTAGCTAGGGATGTATGTAGATAATGGCCCGTAGGATGTCT
AGGATTAAGATCGCCGAATCCGATTGCAACATTTTCATGGTTCATTACGTCG
TGGCGGGGAGAAATCAAAACACTTCCCGAACAACTTCATTCATTCATTATC
GCCACTCTCGAGACTCGGCCTCGTCAACGACTTGATCGAGAGTCGAAGGG
TTGTCACAGCTATAGAATCATGAAGCGGTCCGAGCATAGCTTCGGTCGCGA
TGAAAAGATGCCGAATGAACATACACCATTAATCACCACCGTCACCGTCGG
TGAAGTCCGTCGGAGATACCCTCACCAGACACTCCGCCGATTCTGCACATA
GCTTTGAGCTCCTCCCTCATCGCCCTCTTTATCACATTTCTCATCACCGTAGT
CTTTGCTCCACCTCATACCGGCCATCGCGGGTGGCCTGGACATGACCATAA
ACATGTCAGCTTCGAAGAGCTCCAAAAGATTCTTTTAGACACTCCAAGCTC
TGATAAGGCTTCCGAATGGAGCAAGTACTACACAGCCGGTGCGCATTTGGC
AGGAAGAACTTATCTCAGGTAAGGCACATAGGTATAAATTATAATGTAAGAA
TATAATTTCTCATTAACATATAAAAATAGGCCGAGTGGACGCGAGATCGTTG
GACTGAATGGGGAATTAAATCAGAAGTCGTTGCGTATGAGACCTATCTGAA
CTACCCAGTCGATCATCGACTTGCTCTGTTAGAGAAATCAAAGTCGGACTC
TACCGATGCTGGAACATGGAAAGTCTCCTTTGAGGCCACTCTAGAAGAGG
ACGTGTTGGACGAGGACCCCACTACCCAACTAGACGAAAGTATTCCGACAT
TCCACGGCTACTCTGCCAGTGGTAACGTCACCGGTTCATTTGTCTATGTAAA
CTATGGTACGTTCTGGGATTTTGAGGATCTTGTCAAGGCCAACGTCAGTCTC
GAGGGCAAGATCGCCATTGCTCGCTATGGCGGTATCTTCCGAGGCTTGAAA
GTCAAGCGAGCCCAGGAGCTTGGTATGATCGGTATTGTCCTCTACAGTGAC
CCTGGCGATGATGGCAAAATCACCGAGGCCAATGGATATGAGACATATCCT
AACGGCCCTGCACGACATCCAAGTAGCGTCCAGCGAGGTAGTGTCCAGTT
TCTCAGTGTCGCACCAGGAGACCCAACAACACCTGGTTACCCTTCAAAGC
CAGGCGTACCACGAGCCCCAGTCGATGGTTCAATTTCCAGTATACCCTCTTT
GCCAATCTCCTACGTGGAAGCCTTGCCCATATTAAAGGCCACTCAACGGTC
TTGGTCCTAAAGCCAAAGATTTTGGCGAGTACTGGACTAGAAATACTGGCC
TCGACTATAAGGGGATCGAGTACAACGTTGGTCCCTCGCCCGATAACGTTG

TTCTAAATCTTTATAACGAACAGGAATACACCATCACTCCCCAGTGGGACGT
CATTGGTATCATCAATGGTACCATTCCTGACGAAGTCATTGTAGTTGGCAAT
CATCGTGATGCGTGGATTGCTGGCGGTGCAGGCGACCCCAACAGCGGTTCT
GCCGTTATTAACGAGGCAATTCGCAGTTTCGGTCAGGCTTTAGAAAAGGGT
TGGAAACCCCTACGAACGATCGTCTTCGCTAGTTGGGACGGTGAAGAATAC
GGATTGGTAGGAAGCCACCGAATGGGTCGAAGAATATTTGCCGTGGCTATC
GGTCACCAACGTTGCCTATATCAATGTTGACGTTGGTGTAAGGAGTCCGATC
TTCGAGGCTAATGCTGCTCCGTTGCTACACAAGCTCATCTACGACGTGACC
GCCCTAGTACAATCACCTAACCAAACTGTTAAGGGACAGACTGTTCGCGAC
TTGTGGAATGGGTACATCGGTACGATGGGAAGTGGCAGCGACTTTACGGCT
TTCCAAGATTTTGCGGGCGTGCCTAGTCTCGATATGAACTTCGGTGGACAA
ACCGACGACTCTCCCATCTATCATTATCACAGCAATTACGATAGTTATCATTG
GATGGCTGAATTCGGCGACCCTGGATTTGTGTACCACAAGACTATGGCTCA
AATTCTTGGTTTAGCTGTAGCAAAAATAGCCGAGACGCCGCTTATTTCCCTG
AACGCCACCGATTATGCATATGCGCTCAAAGAATATGTCGAGAAGGCCGAA
GCAAGACTGGAATCCTCCCAGGCAGAATTATCTACCGAGGAAGAGATCTTC
GAATTCAGGGCTCGTTCTGTTGGCGCCGAAGTTAAAGGTGACCAGGAAGC
TTTCAAGGTGTCACTAAACCTCCTATACCAGGCCATTAGCGAGTTCAAGGA
GGCGGCGATCGCACTCGACGCTAAATCAGACGAACTAGCCGAGAAAATAA
ACGAGGACATCCCCTGGTGGAAATGGATCACTAAATTGAAGCTATTCCACG
AGATTCGCTTTGTCAACACCAAGTACAAGACGATCGAGCGCGCTTTCCTAT
ATCCAAAAGGATTAGACGAGAGGCCCTGGTTCAAACACGTTGTTTTGCCC
CCGGAATATGGACTGGTTATGCGGGGGCTGTCTTCCCCGGGCTTGTGGAGA
GTATCGACAACCAGGACTTCGAGAATGCTCAGAGATGGGTCTACAATCATC
GAAGACCGCATCAAGACTGCTACAGAGACGATTCGGTAAACCAAAAACGC
ATAATTGGATTCATAGTTTAGTTTTGGATTTAGGGTGCTGCGGCACAGTCGC
TATATTCATAGACCTAGAGGTGTCTCGAAAGACCCAAGGTCGCGTAGGTAG
TTAAGATCGACGAGCGGAATATACCCGCTCTTAGCTAATGTGGATTGGATTC
GAATGTTCAGGCGATGTCAATATATATACCTTACATCATATTTCTAAAAATCAT
CCCAAATTGAATCCTCAAAGCTTGATTTTACGTCCCAAGTGGATTGGGGATT
CCTATTCGAGAAACGACTTGCAAAGACTTAGGATCCTCCAATATCCTCCACC
TCCACATCCTAAGTTTAGCACACCCACAATCCCGCCTCGTGGGATCGGTAA
GGTTTAGCAATTGACGACTATGATTGGCTGTTAAATTTCAGCTCTACGGCAC
TGCGCTATTCATGGTAAAGATATGACACCAATGTGATCGGCGATGGATAGAA
GCGATGTCAAGGTTGGGATATGGCATGTTTAGCACGTTACCGTCGAGTTAAT
GATCTTCAGGCTGTGCTTTTGGAGAAGGCGGTCAAAACTTCAGACGATGA
GTATAGACTATCGTGTACAGTCGTGTGATTTTGAGTATAAAAGGTGAAGAA
ACCGCGACGATAATGAAGATGAAATTCTCAGATCACACTCACACTCACATC

TACATTCACAATCACATTCAAAAATCAAGCGCAAAGCAACAAGTCAACTTC
ACCTATCACTTCTAAATTTCACTTACACACTACCGCCACTATGTAGTAAGTT
GAGCCCAACACGAGTCTTAGAACTCATCAAGAGTTTCTCTAGCTAACGAAG
TTCATCTTCATAGCGCCAACGTTATCACCGTCATCGGCCTTGCCGCAACCCT
CTTCGGCACCCAAGTCATGGGCCGCTCTTGCCACCTCACGACTACTGCTTC
CCACAGACAGAATTCCGCCTGCGGAGCTGGATCTTGCATTCCTGGTGACAC
CTTGTAAGTCCACCACTCCCCTACAACCCTCCCTCTCTTTACCATATCCATAT
ATTGCAAAACTCAAAACCAGTCGCTAACCCACCTCCCCAATAATCCAGCAT
CGGCAGCGGTGTTCTCAAGATCGACAACAAGATCCAGTGGGACGGCAAAG
GCGCCAACTACGACAAGCTGAGCAACGGCAAGGCGTGGACGTCGACGCG
GGCGGCGTCAAGTTCCACATCGACCCCCTGGGCATCCCCGACGCCCAGGA
CAAGCCCAGCTGCAAGGTCCACATCGGCAGCTACACCAACCTCGGCATCG
GCAAGTCCAAGAGCATCGGCCTGCCTGGCGTCGTCGGCAAGGACTTCGAC
TGCACCCACGACTGGAACTGCTAAGCTGGTACCCGAGTGTCTAGGACTCAT
TATGTATATAGTGGTTATCTAGGGGTGGGATAGGATGGTTGGTGTTTATAGAA
GAGGCTTGATGGGCCGTGGATTTCGAGTTGTATATACTTCTTAGATGTACATT
GTTGATACATTCGTTTTTCATTTACTCAACATATTTCTAAATGCTGGCGTTTG
TTTGGGTCTCTGTGAAAATCGATCCGTTCATGTCCATTCGGGAATATGGGAG
TGAAAGTACGACTCAAGCGGTCGGAACTTTATGCTGTTGGCATATCCAGAT
GAGAATTTAATGTGACGCAGTATCTCTCTTCAATTGGCAATATTTCGGAGAT
AAGATTTCAATTTGTGTAGAGCCGGGAGCCGGAAGACATTGTCGAGATCCG
GACTTCGTTCCATTTTCCCCATCTGAAGTCCGGGGCATTTACAGCGATGAGT
TGAAAACAACTTGCTATGTAAAGAATGTCTCTATGATCTCAATGAGGTTATC
GTCTTTTGAGATGTCTAAGTGTCTTTTAAGAGACGTCATATTGGTATAGTTA
GATATTGTTTAGATTTTTGGTATTGTCGTCGATTTATTGTGGTGGGATTGCTT
ACATGTCGTCATTTTATGCAGGTAATATTTCGAGTAACCAGACAAAATCGGG
TCAAAATGCCTTCTAGTGTAAAATCTGAGTTTCTGCGCCGTCGACCAAGAC
GCCCCCTAATTACCGACAGCCGGGGAAGATGGGAAGGATCCATCGATGTGG
CTATGCACGTATTGGCAACCGGGGTTTAGGGCTGATAGTAAACCATCATTCG
ACACTACGACAAATGATCCATGAAACTCATTAAACAGTTTCCATAGAATATT
CTGTACAGCTTCCGTGCTGCCGAGTAGACGATGAATAAAATGTCACGATAG
CGCTGCAACCTTTTTGTGTTATGTTGCGTTCTTTTCCTGGCGGTTGACTCTG
GGCGACGATTCGGGGCAAACGCATGAGAAACGAAACGGAAACGTCTGGT
GTTAATTCAACATGCGGTGGCTACTCAAGCTTACAGCCGGAGCGCTTTTGG
CTGTCTCGACAGCTGCTGCAACGCCCGATAAGCCAGCGCCGCCGAGACTG
CGAACAGACGATATGTAGAGGATCGGAATGGAGTGCACTACAATGTATTCG
AGCACGTCGCCACGCGATCCAAGTTGTCGTTTGTCAAGAACTCGGGTATAT
GCGAAACCACCCTTGGCGTCAACCAGTATTCGGGTTACCTGACGGTGGGC

GAGAATATGAACATGTGGTTTTGGTAGGTCGACACAGTGATGGTTAGGACG
CAAAGTCAAGACGCTGACACATGGCCAAGGTTCTTCGAGGCTCGCGAGAA
TCCCCAAAAGGCTCCATTAGTAGCATATTTAGATGGTGGTCCAGGCGCTGC
GTCAGAGTACGGGCTGTTCACGCAAATTGGACCTTGTCATTTCGTGAATAA
CGAGACAACGCCATCATTGAATCCATATGCGTTTAACGACTATGCCAACATG
CTGTACATTGACCAGCCTATAGGTGTGGGCTTTTCGTATGGTAACGAGAGCA
CCAATTCTACCAAAGCAGCTGCCCCTTTGGTATGGAATTTCCTTCAGGCATT
CTACGACAACTTTCCCGAATATGAGAATCGCGATTTCGGTATTGTTACTGAA
TCATACGGGGGCCATTATATACCAGAATTCGCGCGTTACATCCTAGACAAGA
ATGCCGCAATCGTCCGAGGGGATCTCCAAGGCGAAAAGATCCATATGACAG
GTGTAGCCATTCACAACGGATGGTTCGACGCTGAGATTCAAGAAAAGGCC
AACATTGAGTACATGTATAACAATTCATACAGACAAATCATCAATGAGTCGT
TCCACGACCAGCTTTTGGACAAGTACGACACGACCGTTAAACCTGCGGTG
AACCTTTGCCATACCAATGGTACGAATGAAAACTGCCTTGCCGCTTATAACT
CGTACTCGAATACGGTCGAGCTTCCGTTATTAGCCGCTTTTAGAGAGAAATC
TTCCTAACGGGTACTATCTTGGTGACATCCGCGAACCATATTCAAGGCCCCC
CTCGACACATGTTGCATATCTGCAAAGGGCAGATATCCAGAAGGCTCTTGG
GGTAAAGGCGAATTACACAGACCAGGCAGGACAAATCGGGTTCTATTATTC
AGGCGATGGTATGACCACCTACTAGAGGATTATATGAGATATATTAACGAAA
ACCATAGATGCGCGTTCATTCATCCCTCAGCTCTCGGACGTTGTACAAGCTG
GTATCAAGGTTCTCATCTGGACGGGTGACGCCGACTATGTCTCAAACTGGT
ACGGAACACTTGACGTGGCACACGCTGTTGAATGGAACGGAAAGGATACG
TTCGCGACCAAGAAGCTGGAGCCTTACACCTTAAACGGTGTAGAGAAGGG
AACATTTAAGTCTGTGGATAACTTCACTTTTATGCGAATATATGAAGCAGGA
CACAATGTGCCGTTCTATCGTAAGTTCATAACCGACGAGAAATAAGACTTG
GGGCAGTGAAAACTAACTTGATGTAGAGCCCGAAGTTTCATTGCAGATTTT
CAGGCAGTTCATGGAGACCGGTAGCGTAATTCCAACGTGAGGCCTCAGCCT
TGATTCTGCCTAGAGATATGCCTATTTTATATTACTGATATCTAGCTCACTCCG
TTTCACATTTCTAGACATGATCAGCGCATATGGTATGACCTCATGCAGCATAT
TAACTAGGTATATACGCTTGCCATCCATTGGTTGCCCTCTATGTTTGATTCCG
CTTCCCTCGCCTATGTGATACCTACAGTAGCCCCCACATATATGAGTCGATAA
TGCGTGCAGCTCTATTTCATTTGATAGGTCGATATAAATGCAGTTTGCTAGGT
AGATATGTCTTAATCACGCCCTTTGTATATGTACTTTGAGGGGTTGAAACTC
CATCTTTCTTGTTATCATGAATATTGCCTAGAACCCCGCTGGCTGCCTGCATG
AAGTCTCACTTTATTGGTTGATTTTTCAGCCCTATAGGCTCGTTCTTGATGAC
TGCATATACCGGACAGGTAACTAGTCCGTAGGATTGCCTCATCAATGGTTGT
GAAAGTGACACGGGGTCCTGGAGCTCTCGCCACTCCTCTAGTCGGGCAAC
CTAGGCCTTCAAAATGACTCTGGGCTTCTTCCCATATTCATATAATATCATGT

TCTCTGACTTTCCTCGTCTGATCATTCTTCCCTCTCAGCTTTAACTTCTCATC
ATCTGTATTGTAACTTATCTCACATCTACTCCTGTCCAGTGACATCTTCAAAC
AATGGATGACTCTTGCTCGATTGCTAGTTACGAGGAGATTGGCCCCGCTAA
CAAACCCTTCAGACAAGGCTCTGGGTTTAGTGACGAGGAACAGAATGTCC
GGAAACTGAGAGATCTCGTTGATAAATCCACCATCAATGACGGCGCTGCCG
GTGAACTTGCCAAAGACGAGGTTCCTGACATCTGCTACGTTCTCCAATACA
AGGGGTGGGGTGGTAAGGTTATCGATGGTTGGTCATCCTATTTCCTTCCAGT
CTACGGATATTCAACAGCTTGCTAATATAATATTCAGTGCGTCGTAGTAACG
AACCTATCAATGTTCAGCTAGATGAGATGAATGAAGAGGTTCCGACTTTGA
AAAATAAACCGGTCCTCGAAATTATAACTCAGGTCTCACTGCCGTTGTCCA
CAAATCGCAGCCAAAGGGCCGTCGTCGTCCCCCTCCACATCATAGATGGAA
CCCCAGATATGACTTCGATGAGTTTGATGACTTTCCCTATCAAGGGGGTCGT
ATACCAATGATTACCGATGAGTCTGGGATGAAGGTCACCAAAGTAGAAACA
ACATGCATGGTGATCAACTCCGTTCACCTCATCAATGCCCTCAAGGCTGTG
GTCGAATACTACCCTTCTGTATCTTTCCTTGGAGAGACCGTCACGATTAATG
CCCCGTATCATGTACTAGTTCATCACAAGGATGAACTTGCCCGTCATCAAAT
TGAGCAGGAAAAGTTTCACGACGCGGAATACGCCGCTACAACCTCCAAAC
ACATCGATATTCTGCTCAACTTCCTCGACAATACCCTTGGCGAGCAGATTTT
AGAGGAAGAAAAGCGGCACAAATCCCGCGTCCCATCAGCTACTTTCAACA
AGCTCGGTTTGATATTCAAGCCTGGTGAAGTTGTATATGCCAAACAGGACT
ACAAGTGGACTCCTTTCGTACTGTCCAGCTTTAGAGAAGGCTATACCAACC
TTCACGGGGCAGTTACCTCGCACGAAGTCGTCTGCTGGAATATTTCATATTC
TGCAGAGAGATTCTGTCGCACCATGCGTTCATTCGTCATTGATCCTTTTACC
GGAGAGGAGGCTATTTCGAACTTGCCCGTCATACCAGCCGAATTCTTCAAG
GGTGAAAATGGCGATATGAGCCCCTTCCAGGTAGAAGAAACACAATACCA
ATTGGGTAAGATGGTCTGGAAACTTTCCCAGGAGCCGAGCTACATGTCCCA
CGACGGCAGCCTGGCTGAAAGGGACCCTGAGTTTAACTGGTCTTACCCAG
TCGGTGCAAATGGCTATATGAGCGGCCGTGTCATTGTTGACCAGGCCGGAT
TCGCACGCTATGCCATGGACTGTCCCGGCGAAAGCCGTCGCCAAGGGCGT
AGCCCACCACCACCTAACCGACCTCCGATTTCGAAGGACATCCTACCTTAC
CTTGCTCCCTGCTGTGGATGCAACTCCTGCACCAAGTCGAAACGAGCACA
GTCTGTCGGTCCATTCGCCGATTTTGAAGACCTCGTCCCGACACACAAAAA
GGCGCCATCGGAAGATCTCTACTACATTGTCCTGAACAAAGTCGTCTCCGG
CTTCATCCTAGGCGAGCGGCGCTGGGGCCACTTCAACGTTGAGCACTTGCG
CGAAGTCGAGTTCGACAAGGACGCGTTCAAGTATCTCGTCCTCGACAACG
AGATCAAGCTCACGGTCCGGTCCCTCATTGGCAAGTTCGCCAGCGCGAAC
GGTCAGGTGACCCCGTGGCCAAATGACTTCGTGCAGAATAAGGGCCAGGG
CCGTATCTTCCTGCTGCATGGTAGCCCCGGCGTGGGCAAAACGTGCACAGC

GGAGTGCGTGGCAGAACTTACACATCGACCTCTCCTCTCACTCACGAGCGG
TGACTTGAGCACCAACTCGTACCAGGTGGAAAAGAACCTCGAGTACTTCT
ACAGCTAGGCGAACGCTTCGGGGCCATGGTACTACTCGACGAGGCAGACG
TGTACCTAGAAGCGCGTCGTGCTAAAGACATCGCTCGCAACGGCCTCGTGT
CAATCTTCCTACGCGCGCTAGAGTACTACCGCGGCGTGCTATTCCTAACCAC
GAACCGCGTGCAGACGTTCGACTCGGCGTTCACGTCCAGGATCCACGTCG
CGCTGCACTACAAGGCGCTGAGCAACATCGACCGGGAGAAGATTTGGGTC
AACAGTTTTGAGCGGCTCGAGCGCGATAGCGGTGGGAAGGTCCATGTTGG
TGTCTCGAACGCGCGAGTACGCGTACGAGAGCCGGGACGTGAAGAGCCTG
CGCTGGAACGGACGTGAGATCCGGAATGCGCTGCAGACTGCTGTCGCGCT
GGCTGAGACTGAGGCGCTTGAGGATGGGAATGAGAAAGGTTACTGTTACA
GATAAACACCTCCGGGCCGTAGTCAAGATGAGCCGCGGTTTTAAGAACTTC
CTTCGCAGTCAGAAATTAAAGGGCGATGCTGAGGTCGAAGACGACGATGA
TGATGAGATTGAAGAAGTGATCGATGATGGAAATTCGTCTGATGTGTACGA
CTAGGAGTTGCAGATCCTCCGCTATTGCTTAGGAATCTAGGTTATGAGGGAA
TAGAAGAGAAACTAAGGTGTATTAGTTGGTACCTATAATACATAGGTAGTAT
GCGATATGAATTCACGACGCCTATTCTTATGTCTCCAAGGCAGGGGGAAATA
CACAAGTTTTATTCAGTTTTTTCATAATGATTGAAGCGATAAATAACTGTGA
ATGAAGACGATTCAAACTTCGAATTATTCTGTAAAAAAATACTCCGAACTG
AGAAAAAGTAAATAAACTGTTGAAGGTGTTGTCATTTATCAAGGATGAAGG
TTATCATTGGTATGAATATACTGGGTAAGACCACTTTATACTCTTGATATTCTT
ATAAGGTAGGTCAGTAACGAAATTGATTTATAAGAATGAAAAGAATGATGT
GTACCTAATTATACTCACTATGGACAAGTTGAATACATTTTAGCGACGGTGA
TATGAGGTTTTTGGCGTTGAGTCAAAACCCACTTTATCTGTTGGGTGGGGTT
GGCCAATCGGGTAAAACCTGGGTAAACTAAAGACATATATAGCTAAATAATA
GTCAGTTTGCTGCCTTCGGGCATTTGGTCAATTTGAAACGATACAGAGAAG
ATTAGCATGGCCCCTGCACTAAGGATGACACGCTCAATCAAAGAGAAGCA
AACAATTTTTTTGTCATATTTTTAAAATGCACAGGTTCGAATCACTCCCACG
CTTACTTTTTTTTTTAACTACTTTTTCCATCATTACACAAGTCGCATCAACGT
ATTTCTACATGTAATGGTATGCAAATGCATACGATAATTCTTCTTGTTTTTATC
TTCCTTTCTGATTATTGTAATTCAACATGCGTGAAGATTGGCGATAACCTTG
ATTACTTGCATGTAAGTCTCATTCTACGCTCGATTCATCGGAAATACTCGGG
TGGTTTACTCGTCCCCGTATTGCAGTCTTCATTCGCTCAGATCTGATTACTTG
ATCAACATTACATTTAAAGACTACCTAAGTAGGTATACATGCTTCGATTTCAA
CTTTACCTTGAAGCGAATCAACAATCTCACTAACACTCTATTGACTCTCACC
CAGGGCCAGGGCTAATAACCTCCTACATACTCAAACAAGTCAGATAAGTAT
AGAGTACGGTAGAAGCTCGTATCACAACTATTGTCTTAGTTTGAGGAATGA
AATATACCATGAAAGACGAGCGTTCCCATAGAGGCTGGTGACGGAACCGCC

TCCATGACCAAACGGCATTAATGGACGGCATATATCCTGGTCGTCGAGATGG
CAGGGACTCAGTAAATGCCTGGTAGATGATATATCATATATGGCAATCTTGG
ATTGAGACCTAGGGTGCTGGGGAAAGATAAGACCACAATTCCAGAAGCAA
AGAGACCACGGTTCTCCTTTCAGAGGACTCAAATGGGACTGAGGTTGAAA
ATTTCCAAATGACTGAGTAATAGAGATCTACGAACTGGTCTCAACAATCGG
AAAACCATCCTACGTTCGCTTGGCAAGAGAGATATTAGCACTAACGCTCTT
AAGCACTAGAGCGACGGCATAGATGCCTATCAACAAAACGAGGTAGACTT
CGAGAACATGGGATACTCCTAAAATTCTCACCATACAAGACACACAGACAT
AGTAATCACGGAAGATCCCCCTTTTCTATGCCTCTAAGTCGAATAGTCGAAC
CGCTCTCTCCTGAGATCTCCAGCGCTACTGTGCCTGATCCCGGTCCTGGCAT
TAAACCCGGTCACGTACCCAAGCTGATACGCAAATAGTCTGAGCACGAGCG
TAAAATGATTCTAGAACACCGACGTTTGATCTTGTTTCTGGCTCATACTGCC
GGGATCGTCGTACAAGCATTACTAGGGCTGTATGGTGTCTGCTATTATGTGG
TTGTTTGCGACCACACAATGCTCGAAGGAGACACGCAATCGGAAAAGCTG
GATTCGTCTCGGGATGCCCAGCGATTAATCTCAAGAGATCTCGAATGGGAC
TTGAACACAAGATTAGTAATATCTCAACAACTGCCCCTAAATTCAGCAACA
CATCTCAACATCATGAAGTGAAGGCTTTAAATGGCCACAGAGAAAGAAGA
CCGGCGTTCTCCCCTTTTCATTATTTTAGATACATCAACGGATCATACGTCTC
ATCTCATCTCCACATCGACATGTAAAATCCTCTCTGTATCGTGTACATATTATT
AAGTAAGGTAGGTATCATAAGTTGTTCTCGTCGTCTAGAACGCTGTGCGATC
TATCACCAAGTTAACCACGTAAAACTTTTGCATCACAAAATTGGCCAAAAA
ATCAAGACAGCCAGCCACCCCTTAAGTCCGATCGCCGATTTAAGCGGTCTC
GCGCGCGGTCTTAGTAGCACGGTCCTGTCGTCCCTTCTCAGACCATGGGAA
GTGGAAGACGGGGATGATAAAGAATGAGTTGCTACACTGGTGTTAGTTACC
ATTATACAGTGAGAATGTAGCGAAAGGGATACGAACCTAGAGTCAGCAATA
CCAGCAAAGGCATTGTACCAAGCCAAGAAAGCGGCGAGCAGACCAAACC
CACCACCGGCCTGTCCAAGCTTCAAGGCGGCGGTAGCCGCACTGTTGGCC
GCAGCGTATTCGGAGCAGGCCAACATGAGGAAAGCAAGGTCAAGAGTGA
AGAACAGCATGAAGAACATGACAGTCGATCGCAGCGTGCACAGAAGCAG
GAGGAAGGTGAAGATGAACCAGCCGGTTAGGAAGAAACCGAGAACGGAG
TGTTGCTCAAGCTCGCCATACCCTTCGAGGACGGCAAAACCAGGGGTCAA
GACAATGGCGTAAGAGATCCAGAAACCACCGTAAGAGGACAAGGCAGTAG
CACCGAAAGTGTTGCCAACGGCCATCTCCCTATTACAATTGGTTAGTAACC
ATATATATTTTTCAAAACTTGAAGATAGTTGTGATATCTCCTAATTCCAACAC
TTACCACATGCCGGCCAGTAGTTGAACTAAACCACCGTATCCATAGGCCAG
AGACAGAGCGATGTTGGGCTTGATGATGTCTCGCGCGCCGCAGTTGACGA
GGGATAGGACGAAGGTGGTCAGGGCGAAAGCAGACAGGCCCAGCGGTGC
AGGATTAGCGAACTTGCGGTGGGCGATCGGCTTGTATAAACCAGGTTGGAA

CTCTCCGCCGAAGGCGGGAAGTCTTGCGTCCGGGCCGGAGTGAGCATGGT
ACAGCGGGTGCCGCCGTAGTCGTAGCGATGAGTGGTGTC

>Mosaic_sequence_16

GGGGGGTTTATAATAGAGCGACGGTACAACCAGTGAATGCGCGACAAAGC
AAGGATAAATGCCTAGTTATTTTTGGATTCCGTTATAAATCTAGCTTTTGATG
ACTAACCTATAACATGAGGAGTTAAACAGCCTAGAGATCCATGGTTCTTCTA
GTGTTTTTGGTAGCTCGATCGTGGTTTGGGGGCCGTGACGCAACCTGGACA
TGGAGCTGAATCCTAGCTCGCAATCACGAAGGTTAGCGAACGGATTGTGAA
GTCCGGAATACCAATGGGAATACACGGAGATAATGCTACTAGCTATACTAAG
GTATAATAGGATCAATAAGGACGCTGTTCACTTGGATAATACATTGAGGGGA
AGCTTGATTCAATCTGAGGTTTTATTTTCTTCGGAGGTTATAGAATGGATCG
GTCTAGCCATGTACTATCTAGCTTAGCAAGAAGCGCCAGAAGGGATGTTCG
AGCACTTGGTGCTTGTCTTGCCTCCAGTAACCTTCACATTAGTCCAAGTCC
AGTCGCTGCAGGAGCCGTCACCGCACAGAATGTACACGTCCGTAGCACTT
GAGGCGACACTGCCAGAAATTGTTTCAAGAGTGAGGTCGGTGATGGGAAC
ACCGGTGGTAGGAGTGCCAGTTGGGCTACCGTTCTCATAGTCTTGCTCTATG
ACAATACCGTAGCTGGTAATGTTGCTAAGAGTGATGTCGGAGTAAGTGACG
TTCTTAACGGAACCGGTAGCATCGTACACGGTCTTGATGCGCACACCGTTC
TGAGAGTTGCTAATCTTGGAGTTGGAAATGACAACATTGCTAACGTCATTG
TCACTTCGTCCGCCGACGGAGCCGATGCTCAGACCGTGGCCACCAGAGCA
GGTGCCTCCGGTAAACTCGATGTTGCTGCCCGAGTTGATGGCAAGGCAGTC
ATCCTGGTTCTTGATGTTGGCGTTGATGATCTTCACGCCGTCAGACGAGCC
GACATCGAAAGCGTCTGTGTTGTGGCCACCGGCGGAATCTCCAGCGGAGT
TGTCGATGGTGATGCTATCTAGGGTGAGGTCGGTTGCGCCGTTGACAGAGA
AGCCCTGGACAGGCGTGTTCTTGACGTTGAGACCAGTGATCGTCGAAGAG
GTCAAGCTATGGGCGTAGAAGAACTTGGGCTTGGTCTTGCCTCCGTTGCTG
CCCTTTCCGTCCCACCACGAGGCTCCATTTCCGTCAATGACATGGCCACTA
GCACCCTTGACAGTGATGCTGGTGCCGGAGACGCTAATAAGAGGACCCGA
CCATTCCTTGTATCCGAACGAGGTGGTACCTTGGAACGTAACGGTAGTTCC
CGTGGTAAGCTTCGTGAGGTCGAGGGTCTCGCCCGCGGGAACGGTGATTC
CGTTAAGCACGATGTCCGTACAGGAGCTCTTGCCAGACGCTAAGGCATCGG
TCGTCGTGAAAGTACAGCTGGCTCGCTGCTTGATAGTCTTGCTCGGAGCCG
CGAGGGCTAGGGTAGCGAAGCTGATAGCGAGAATTGGATTGGAATGCATTG
TAGAATAGAACAGGCTCTGCCGAATGAACGAATGTCTGAATGACTTTAGAT
ATATACAAATATGAACGAATGATCAACCTCGAGAGGAAAGAGAGAAACCTG
ATTGAGGTGTTGAGGTATCAAGTCAACACCAAGACTCTGGGCGCCGTCGA
CATCTTATACTTCAGCATTCAGCATTGAGGTCGTCGCTATCCACTTACCTTTT

GCGACTGGTGGCATGATGATCCTCCGAAGATGTCGTCCCCTTAGGAAAATG
CGGAGTACGTGTGAACCCCTGAATTAATATGCGCCTCCTATTCGCGCGGGTG
CTGATCTGCACTGGGAATACTATTAAATGATCGAAACTACGCCCGCCCCGA
AGAGCTGACGAGTTTACCGCACGGATAGGATGCTAACACGGCTAACACCAT
TTGATCAGATCATCTGCAATAGTTAGGGAATAGTTAGGAAACCGCACGGCT
TCCAAAGGAGCAGATTTCCTATAATGGTAGTTTGGTGGATCATTGGTTGCAT
CGTGAGCCCACCGGACCTGTCCTGTCGTTTCATCTGATACATTAGGGGTTAG
ACTAACGACGAACGCGTATACCTGTAATCATCATTGGAGGTAAATATATGCA
TATACATAGCAAAGCTAGCATAAACGGGAACTTCCACTGGTGGAAAACGTG
ATGGATTGCATGGAGGCGTTGTTCTTTTGAAAGACAGTGGATGCGACTGGC
TCTTTCATTCCGGACGAAGACGGACTCCTTCATATAAGCCGGGTCGCGATAC
TATTAAATACCGAACGATCGGAACGCTAAGCTAGGGTCCGTCACGGACGGA
TCACCCGGATTCTCCACTAATCATAAGCTCCCGCTACCTAAGATCAGCAGAA
TCGATCTAGAAGGATCAGCCCCTATCTTGATTAGTGAGATTAAGGAGTCAGT
ATCGCCTTAGCTTACACCGTCGACATGGCCCTATCGGACGACAACACAAGA
AAATGTGTTATGGTTCTATGGTTTCGGTGGAAAGCCGGATGTTGACAACCG
TGTAAATCGCCCAGTTTAAGAATAGAAGAACCATGGATAGAGTTGTGTGCA
CGCTAGCTTTGAATACCTGAATGTTTCCCTATCCTCGATAAATTCCACCATTC
ACGCTATCATACGCCAGAAAAGAGACGGCTTCTAGGGGCTGCAATTTTTGG
ACTTCGGAGGTATAAACTCCTCATCGCCATCATCCTTTTGAGGTCTAGCTCT
CTTCGTACCCTAAAATTTGGCTCTAGCAAGGCGATGACCATTCCCAAGATAA
CATATATCATGGCCTGATGTGGTGACTGCAGCGTCAAACGAAACGTTTGAA
TTAGTTCCGTATTACCCCAGGTCGTCTGCCAAGACCGCTCACGCTCTGTGTC
GAGAGTACGAGTAGAGAGCGGCTCCGATGGATAATTGCTAGACGCTAGTAA
CGGGCGTCTAAAAGATGTAACGTATTGAAGTTGATGTAGTGGAGAATATATC
TGATAGGGCGGAAATATCTGAGTAGAAAATGCAACGGGAGATTGATCCTAC
GGCCAGTCTTCTGAGCAGACTGCGTTCGAGTAGTCGCAATAAGTTGAGGAT
CATCGAGATTTTAGTCGAGCGAGAGTCGAATGTCAATCACGTAATAGATTTA
TGATGACGCACGAATTCTGCTGGAGCAATTCTCTAGCTTAGATTGTGATCAC
AGCCATGTCTTTCATTAGACTGTTTTGACTGTGCTCGGGCTTTGCCCTAAGG
TTAGCTAGACCATTTTAGAAAGGTCGGACGGAATTCTCTGGATTATAACTAC
ATGTCTTCATAATTACAACCTAGATTCATTCGTCAATATCGGTCGTCAGAAGT
ATGGTGTATACGAGAACTTACCAACTAGATCGCTCGGGGTTCCCAGTCTATA
ATCTCCTAGTCAGCAGTTTGCGAGTATGAAAGAAAGGGGACATAGCTACGT
ATCCGTCCCCGAGCGGTTGTTATGCTATTCGGTTTGGTTCTTGGTGCTCTTG
ATTTTATGTCGGACAGCGAGACGAACAAGCAGCGGCGCATTGTGCCCACG
ACCCAAGCCAGAGAGCCTGGTGGCACACAGGCTAGGATGGATGGCAGCCA
AGAATGGGTTAGCCTGTTGGCCTGCATCTGTGCAGATGGCACTGCTCTCAC

ACCCGCAGTCATCTTTTCAGGCAAGCAGGTCGGCGAGGGCTGGATGTCAG
AGCTCTCAGTGGAGGAGAAGTACTGGGTGTCAGTGTCAGAGAAGGGATGG
AAAACGGAGGAACTTGGACTGAGATGGTTGGCGGAGGTCTTTCATCCCGA
GACAAGAGCCAAGGCTGGCAATCGACGTCGACTTCTCATCATGGATGGCC
ATGGCAGCCATGTCAATTGCACCTTCATCGAGAAATGTGACTCTCTTAGAAT
CGACCTTTTGATTCTGCCTCCCCACTCAACCCACCGACTTCAACCTTTGGA
CGTTGGCTTTTTCTCCCCTCTATCTACTGCATACACCACCATCTTTGATCGTC
AATGGCAAGCAGCAGAGGGACGGCTCTATGTCAACAAAGCCCGCTTTTTC
AGCATCTTACTTCAAGCTTGGACGAGAGCTGTCACCTCCGACAACATCAAA
AGTGCTTTCAACAAGGCCGGCATCATTCCTCACGACCGTCAAATGGTCATG
GACAAAGTCTTTCAGCCATTTCTACCTCCTCGGACACCATCCCCTGCACCG
ACTGACACTGCAATTCCCCCAAGCTCTCCACTGCGGGAAGGCTGCTCCGGT
CAGCTTCTGCGAAGGGTCAAAAGCAACAAGAAAAAGGCTCCTGCTGCGGT
GGTGAAGATGAACAAGAGGATTGTCAGACTGGAGACGGAATGCAAGCTGC
ATGTCAATACTGCTGCGGGTCTGAGGGAGGCAATGAAGCAGGCAAACAGC
AAGAGGGCTTTTAGGCCTATTGTGCCCGGACATGTGGAGAGACATGGGGTG
TGGATAGGACCGGAGGAGGTGGAAGCCAATCGTCGGAGGCAGATGGAGG
AGACGGCAGCAAGGGAAGAAGTAGAACAGGAGAGGAGCCTGCGCAAAG
AGGCTGCAGCTGCTAAGCGGTCAGAGAAGGAATCACAGAAGGCAAGAAG
GGCGGAGGTCAGAGCAGAGAAGGAGAAATGGAAGGCAGACTTCATAGCT
GCCCGGCCAGCAAGAGAGCGTGCAGCTCAGTTAGCTCAGTCAACCCGGGC
ACTCATAGAGCTCGGTCCAAACCAAGCAGGACCATCAGACTTCTTCAGATA
GCCTCTAGAATACCTACAGTACGGCCACATAGTGAGTCTATGTGAATAAAAT
GCCCAAAAAGGCCTGGTGATATCATAGGGTGGCATGGACATGGTGGCGGG
AGAGGGAGCTTCTGTGCCGACAAATGTCATTCACTAGATCTCAAGACTGGG
CACAGCAATGCATGCATACATTGCTATGGGACTTCTTTCAGTCCCCTAGAGC
ACATTCTCCTATGTGGGCCAATCAGTAAAATTGAACTGCAAGCTGCGGGAC
AACAACAAGACCATTGCTAGCACGGAAGAGTCAGAAGTCCCGAGGCAGGT
GCTCCATCTCTGTCTGCTACAATTCCTCATTGGCGGCCAGTTGGGTTCTTTG
AGCTTGATTTCCGGTACATGGCATGTACATGCAGGCTTCCGACAGCCTTACA
CCATGCATAAGTGCCTAAGAGTGCTGGACTGGGATACTCTTTGCCCCGGGC
AAATCAGTCACCATACCTCTTTGTCCGTGATTCAGTCCTCAACAACCAGCA
CAGCAATGCATGCATAAATTGCAGGGGACACTCTCTGGTCATCATAGAGCA
ATATGCATGGGGTGGGGCAATCAGTAAAATTAAGATGCAAGCTACAGAGCT
CACTAGAGATACCCATAGCCAGCGAGAGTTGACCGGTCAAAATGCCAAAA
CCGACCATCACGTGCGGGTACCCTTATGGCGACCGGTCAAGCCTGGCCATC
TTGGCCACGCGCCGTTCCCAGATGGGACCGGATCACTTGTCCACATGATAC
CATATCTCCATCGCCTCATCCCTCAGTAGTATCCTACATGACATGTCACATCC

AAAGAACCTAGATAGAGGGACTGCCTATGCCAAGAACTACTACAATGGCAG
ATAGGTGCCAGGCGTCGTTGCGCCTCCATTCTTCACATCTCACGCGGATTT
ACAATCATCGACATTGCGTTGGAAGAGAGACCACCGCACCCAAATCGGAG
CCCCCCGCAGCTGCAACGTCGGATGGGTCTGTGGCAGGATGGGAACGCCA
TGACAATCTAAACCTCAAGCGAGCTACTAAGAAGCCTTGCGACTGGCATGC
TGTCAACACAAGTTGCAGTATCCGGTAAAACACTTCCGCCCATTTGAAGAG
GGGCTCTCCCTGCAACCGTGCGGTCCGCCTAGCCAGATCTGTCATCAAAGC
TGGAACAGCCAGAGAATGGCTGCACCGACAGAGTCATACGGTTTACACCA
TGCTTCTTCATGCCGGCGCTGGCAGCCGTAACACACCATCAGGAGGGAGG
GGAGATTGGACAAAGTCGAGAGAAACAACGAACAAGAGTCAGACGTAGT
GGGTCCAAGTCCTATCGAGCAGTCTTGACGGCGCACAAAGGAAAATACGA
TGCAAACATCGATCAGTGAGCGTTTCTGGACATGCTACGTGTCACGATGCT
CTCCCGTTGGCAGAGGCCCTCTCGCACAATATCAGAGTCAAGAATAAAGGA
TAAAGGGGTAGAAACGAGGATGTCGAGGTAGGATCCTCGTCGAGGTTGAA
TCCTCGTCGAGGTTGCAAGGTCGTCGAGGTAACAAGGTCGTCGAGGATAC
AGCGTGAGTGCGACTCAGCCACGCTGCGTGCCACGGAGAGAGTGAGATCG
TCAATGGTGATCCATGAGACTCAATCAGAGACTGCTGAAGAACTACGACGG
GCGCCCTGGGCATCCTATATGACTCTGCTCCTGCTTCCCGTCGTCGCGCGTT
CATTCAACGTCTACACCCTCGTCGAGGTTCTCCAGCCCGAGGTAGGTCACG
TGTCGGCCACGTGAGAGGTGTCGGTCACGTGTGCGTAGGTCGTCGAGGGC
GTGGCCGTCGTGACAACTCACTTCACTTATCTATCTCTCGTCACTGGTACGG
TCGATTCTCTAAGTAGACGTTCTATCTAGAGGACTAGTTAACTAGATCGCTG
AGGACTTCCTCTGCGATCTTCCTTCTTAAGTATCCGCTTACTCTCGCCCGCT
CTCTCTCTGATCCTTCTAGCATCGTCCTTCATGGATCGTTCCATCGTTCTCCT
AGTCGTTTCTACTCGTTCCCAATACTCTCTAGTATTCTCTCGTCGTCTCTCTT
CTCTTCTATCGCCTTCTCTCCGCGCTGGCTTGCATCTAATCTCTTTGCGTGCC
CGTCGTCGTCCTTATCGTCCTCGGTCTCCGTCCCGTGGCCCTGACTGTGAG
ATAACTCTATCCTCCGTTATCGTGGACCTGACTCTTGTCCTTGCGTGCGTTC
CTATCTCAACTCGGCCGATGGAAGACTGAGACGTATCCTTCTATCGTTCTCC
AGGTTGTTCCAGCGCCTTCAAGTGCAAGTCGTAGTTATTCGTGACTAATAGT
GACGGTTCTACCGTACTCACGTTCTTCGGTTTGTAGGATCCTTTGGATGTTT
CCGTCGGAAATTGCCTCGTGGTAGTCTCGTGGTCAGGATGCCGTGTCGGTT
TCCTGACAGATAGTGGACTCCGTATGGGTGTCAGAGATGGGGGTCGGTCCA
AAATGCCCTTGCAACACTGCCCTGCAAGACTGCATCAGGTGAAACTGGCA
AACTCTGATGCAACAGCCTGGGAAGACCGTCAAGATCGCTTGCCGCTCGG
CCCCGCTCGCATCGATCATGAACATGGCGGGCTTGATGTCACGTCGGCCAC
GCCTCCATGATCACAAGCGCACGCCTACAACCCTCGTTCCAGCCCAGCACG
CCTGTCAGACACGTGAGCCTATCGTCGATCCTCGAGTAGCTTTGACGAGCC

TCAAGTAGCCTCGACGACTCTAGAGCTTGAGCCTCGTCGATGGTGTTTCAA
GGAAAAAGCCCGAGGCGGTGCAGAGGAGGGCACGGGTGATATATATACAG
GTCCTCACCTGTAGACCGTCCTTAGCACCATGGTATTGAGTCCCAGGATAG
AAAGTAACGGCGCAGCCGTGTTGTCGAAGCACGAGAGGCTGTACACCTTC
TCGTGGAGGACAGAGACAGATCGAGAGAGAGCACATGTGAGAGGGCCTAT
CAGTCAACAACCGGCCTGGGCCCGTGACAGGTGATGTACTCTGTCGAGCC
AGAGATCGCTTGCCCATTGCGTGGATGGTTCCGTGGGGCTATGCGGTGTGT
TGTGCGCTTGTAGCAGAGGGAGAAGAATGATTTGTTGACGTTGTAGATGCC
CGTGGGGTCATGCAAGCCGCTGCAATAGAGTTCAGAGACCTAGAATCGTGT
CAGAGCCGGTCTTCACATCGCAGTGGGAAGTTAAGAGTGCTCACAAGGTT
AAATCAGGCTCTGCAAAACTCCTGACAGTCCGCTTGCAAGCGCGCAAGCT
CTCTCTATAGGTGTGAATCTTGACACCAGCCGGTCTGAATGATGATTGATAA
AGCGCCCGACCCAGTTCTCCCCAATTTGCTCACCACAGACGGCCTCTGCAA
GGTTGTGGATGTGCCGCGGGGCCAGGAGCGTCCCTCAAGCCGCAAATTGG
TTGATCTTGTCCACCAGAGCGTCTTCCTGGACGGGTGAGAGGTGTTGCTTG
GCACCATCAGCATGAGAGACGTGGGTGTTGGTCCAGCGGTCATGGAGGGT
TGATTTCGCAGCGTGTTGCTCTTGCGCAACCTGTCACACATTCTTCTCGGG
ATACTCTCGCAGCTCCTGGATGGCCGAGTCAATAGCTGAATGCATGGCTGA
GCAATGACAATATGTAGGCTACAATGAAATCTCAGAGGTATCGTCCGAAAA
TTTGACAGGTCCGAGATTCACGTGGCAGCGAGTAGAGGGTCGAAAATGTT
CGAAGATGCGAAGCTTGCGATCAGAAGGTGATTCATAGAATGCGTAGCTAC
GATACGACTATGTTGAAAATTTCGTATGCGTTATCACGAGGACGTGTCATTA
TTCCTAACAGAAAACCGCATAAGGAGAGTGGGTATTTGGTCACGCTGGTGG
TGGGATGGTGGGGGCAGTAAGCTCAGCGGCGCTTGCCCATGAGATCTGCG
CCAGTTAGCGACGGAGCACGGCACGGCTTGGTCGGGCGCAGGCATTTTGC
CACGGCACAGCGCGTTGGCCGAGGGGTGGGCACTGCCCGCAAGGCAGGG
AGGGCGTGGGTACGACCCAGGCCACGCCGCTAAGGATACTGCCCTGTGAG
GTGGTGGGGAAGTGCAGTACGACCGCGCTGTTCGCAGGTTGTCCGACGGT
AAGGAATTGCGACACGGACGCTCACCTTCATGGGTATTGTCTACGGGTATA
CTCTGCACAGTTGCCGAAGCACTTCCTGCACGCGTACTTGTGCCCAAAGGG
GCAGTCCAGGCGCAGGCAGGATCCTGCATTCCAGTTGGCAAGTTGGCGCA
TGCACGGGCCTGTCGCGACGGGTGTTCGAGGAGCCGGCTGGTGGTTGCGA
GCCGGTTCCCGAGGCCTGACGAGGCCTTTTTTTCGGGACAGAGCCATGTAC
GCGCGCCGTGAGCTGGCCAGAGATGAAGGGAAAGGGAACGACGGACAAG
GATCGGTCAGCTCGATCCGTCGTCGTCTTCGTCGTCGATGTCCTCAGTGCAT
CGTCCATTCATTGTCCATTTGTCACTGTTCCATGCGTATCTGAGCCTCGCCA
CACGTCACTTCCATCAGACCCAGCATCTCCCTCACATTGCTCAACTCGTTGT
CGCACATGGCGCTCCACTCTTCTTCGTCGTCAAAATCTCCCTTCAGCTTTGA

CTGTCCGAAATCGGATATGACGGGGATGCGTATCCCTCGAGGGTGCTGGTG
GGCCCAACGCATCATGCCCAGATCTCCGCGTACACGTGTCGGGCCTGGCTC
GGGGGTGGCAGTCGTCGAAGAGGTCGTCTCATCGTTATAGTAGAAGAGTAG
GTTGGAGAGGCGGACGTCGGAATGTAACACCCCCAACTCGTGGATACGTC
GATAGGTGTCGACGACTTGATGTCTGGATAACGCAAAAGTCGAAAGCGAG
AGCGGGGCAAGTGGGGCACGTGACGGGTCGTCGAGAACGCCCGTGTTAGC
TCATGCGCGCGCAAGAGAATCGATAAAGGGCAGTCCAGAGACCCACTTTT
CATGCCAGAGTAAATCATCCGCGATCGCCACAATGTCCCCTTTGTCCATCAT
GATTCCCCAAACACCCAAGTCGGGACCTCGCCGCTTTCTTACAGCCTCTTT
GCGGGCTAACGTCCGAGCCGACGCGATCAGAATCTGGCGGATCATCGGTAC
GACCGAGCCTTGCAATCTCGCCAAGGGACCGGTAAGTATGTCCAGCTCACG
CTTGATGGATATCCTGACATCGTCTTGCGAAAACTCGACCGAGTCTGGGTC
TGATAGTAGATCGTGTCGCGATGGGATGCAGAGTTTCAGTACCATGGTGTCT
ATCCCGTGCGCACCTTCGATCGGAACGAGAAGACTCTGTTTGTCGGCTTGA
TGATCAGAAAGAGCACTCCGTGATGACGATGCGATGGACACCGAAAAGGT
CGTCCAGACCGCACCGGAGCCAATCTCATCCCCCAATCGGAATCGAAGGGT
AGAGCATGCTTCAGTTGTGATGCTCGTCTCCGTTTGCTGCTGCTGCTGCTGC
TGCTGCCGCTGCGTAAGGATCGGCGTCGAGTGCCATGCTGATAAAGTCACC
TGGGTCCAGGGTATCGGCCCGTGGAATTCCAAGATTCCTTCACGGGCGTAT
TCTTGCAACGGATGGTCGGCAGAAGGGAGCAGCTCGAAAGATTGCAAAGG
GTACTTGAGGTCCATCAGCCTCGACATACTTTCCCAACCAAGTCTCCTCAC
CTGAAAGATGTCGAGCTCTGCGGGGATATCCGACGAAGGTGGGGTATGTGG
TGTATCACATCTGGCGCGTTCGTCCACCTATGATACCGGTCAGCGGCGGGTC
TAGAGGTAGAGGCCAATTGGGGGTGAAGAGTAGGGTGTATGACATGCTCA
CCTCGAAAAGGATTGCTGATTCGATCGACTCCATACTAGAGTCGGACGTGT
CTGCTTGGACCTGCTCAATGTCGTCTACCTCGATGCTGAAATCTGGATCGAC
ATTGTCCCGCGGGTGATGGCCAGATGACAGCCGAAATGCCGAGTTTACGGT
TGGGTGTTGAGCATTGCGTCCTCTCTTGCCCGTATCTCGGTCACGACCACTG
TCGCCCTCTCGCTTTGGACCTTGTCTGGCTTGTCTGGCAGAGCTTCTCGTAC
CCTGTCTTCCCGCTGGAGGCTCATCACGACCGGTTTGATCACCGTCCGCCC
CCTGTGCTCCCCTCAACAGACCGGGAGCCGGGCGAGGAGGGGGATTGTCG
TCAGGTGCAATACAACCTCCCAAGCTGCCGAGGGTGGATGTGGGTGGCGG
GTAGGTCGGTCGGGGGGGAGGAAGAGGTGGATCTTGGGGTCGGGTCACCG
GATCCAACTGACCCTTCGGCATGACTGTGAGCGCGAGCAGCAACATGGCT
GGGCAACTGATGGGATCTTCCGGGCTGATCGCGGCAGCCGTCGTGATCGGG
TTGTCCGCGATGGCAATGTAGTCCGGGTGCACGGTGACGTGTCGATCGAAG
ACGAGCGCGGTCGACAAGCCACCGATTTTTCGGGTACTGGGGTTCGCTGG
ATCAGTCCAGTCTCGGGTGGACTCGCACAGGACCACTGCATAGCGGACGG

ATCCTCTGGCCGGGGCGAGACTGTATTGGGCGTACATCTCGATCGATGAGC
AATCCCCGGGTATGGTCGGGGGTGAATGACTTGCCTGGGTGATGATCTTTG
CAGTGTCCGTGCTGGGTTTCGAGCTGGTCTCGGCTCTGGTAGGTCTCTTGA
GATTGACGCATACGGACGTCATCTTGGAGGGACCGAGGACCTGTCGAGCG
AGTATCAGCGTTCTGACCGATCGAGGGGAAGAGAGGACTCGCGCAGTTGG
GTTTTCAGCTCGACGAGCGCCCGGAGTACACCATCAACAAACAGCCCCCA
GTCCGTCTTGCCGGTCTTGCCTATCCCCACAACTTTCCAATACCTCGTCTGT
CCCGCCCCGACAAAGTAGGCATCCGCCAGATCGTTCAGCCTGTCAAACAG
GCTCATCTTCAGCCAATGGCTCAGGCCCGCCTCGCCAGAAGGGCGGATCCT
GTCGGCCTCCCTGACGCATTTCATCCCGTGGACCCGATAGTACTCCGAGTTC
AGGTAGACCGTGGGGAACCGACCTTGGTCTTGGACAAAGTCGAGCAACGA
TCGTACGATCGGGAGGACGAGTTGACGGGTGGGCGCCGTCTTGAAGGTGA
TCGGTACGTGACCCGGTGTCGAGGAAGGCTGGATTCGCAAGGGATTGATG
CTCGTGGTCGAATGGTTCGAGCTGCTGAACTCGCACTCGATCGTTGGGGGA
GGTGGCAGGGTCGCGTGCAGGGATTCTAACCGTCCAGTGATGAGATCCGA
CGTGTCTTCTGGGTCTGGCTCATGCTGCTGATGCTGCTCTGAATGCGACATG
TCGACAACAAGGTTGATGATGGTGGTACAATGAGAAGTTAAGTGGGGGAC
AGACGGTGGGAAGAGGGGTGGTTGTTGGGTTTTGAAGTTGGCTTAAAAGG
TAATCGGATATTCCGGGAGTCGCACCCAGATTACCGGTGTGTTACTATTACA
CTAAATACCCATTAGGGAGATTAGCTCTCCCGCGGAGGCTATTCCAACAGTG
GTCCGGGCCGGAATGATTACCGATCCGGGCCGGAAAGCGGGATGAGGACT
GGTCACGTGTGCATGTGTCTCTCTGTCTCTTCGCTCTTCTCTATCTCGCCTCT
CTCGTTGTTCTCCTGTTCAGCAGCGCCAACGGGACGACGTGAGGCTTTGAT
GCACTGCGGGTCAGCTGTTTCAATCTCAGTCTGTACCACCACCCACCACCA
AGGGAGGGGAGGCGAGAAGATGGGGATTGAAACCACAGCACATTGTCAC
GTAATGTCTAGAAGCGAAGACTCTCGACCGCTTTGAGGTGCTCTCGATTGG
TCAAAGACATGTCATCGTCCAGCTCTCCCTCGTCCTCCGAGGGCGGAGAGG
AAGCAAACGGTAACAGTCCACTCGCCAGCTGCGAGTTGACAGGGTGAACC
ACGGGCGTCGCTACTTGCCTCAGCGACCTGACTCAAACAGGGAGTGCTAC
TCACAGGCAAAGATACCATGCTCGTAACGGTACATGCGCTCTTGAAGAACT
TTGTTGGCCGCTTTGAGGTCCCGGACGCTCTTCCCGAAGGCCAGCAGGCAT
TCACCTTGGTCTGATGGTGTCCCGCAGCTCGGCAAGCGCCGACTCTTGCGT
CCGGTCCGAAGTGCGGAGCACGGTGATCTCGCCCGTCAGGTTTGACAAGA
GTCCCAAAACTTGGTCATCTCTCTCTTCAATCTTGTCGACACGTTCAACGA
CCTTGGCAACGGTCATATTGACGTCAGTGTCGGCGGCGGCGGCGGCGGCG
GCGGCGGCGGAATCGGTGACGGCGGTGGGCGTGGGCATTGGCGTGGTGGC
GGCGACATCCAGCTTGAACAGGGCGGATGAACGAGTCGAGGCTCAAGGTC
GGGGAATACCGCTCGCAATGGGTTCAGGCCCTTTGCCGAGAGGCGTGCCC

ATTCACGCAAGAGCCGTGAACGAAGACTGATGCGCCTTCCCATGACCTTAG
CCAGCGGGATTGAGTTGCGAGCCGTGTCCTCGATCGCCTTGTAGATCAACT
TTTTTGCGGTCAACGTACATTTGCTGTCGGGAGCTGGGACGAGGACGGATG
GACTGCAGCTGCTGCTCTTGCAGGTCGGTCGGATGAAAGTAGTGCAGGTT
GTCCAAAACCAGACCCAACTTGTGAGGATCAAATGTTGCGACCATCCCAA
AGTATATGTTGGAGGTGGGAGCCGGGGTCAGATGCCAGAAGAAGACGGTC
CTGTACCGAGGTCAGCCCTAGCCATCCATCCTCGCCTCGCAGCCTGAAAAA
GGCAAGACGAAAGACACTGACAGGAGACCGGTCTTGTGACAGGTTTGATC
TTTGAACCACTGGAGGCAATGACCACGGATCTCTTTGCGGTATTGGAACGC
CCTGATCGAGGCAAGATCCGGCAGGCGGCAGTCTGGAGATCCACCGATGT
CTTGTCTTTGTCGGCAACACCCAAGGGTGAGGCAGGGGGGACTCACCCGC
GAATACGACGGTCGACACGCCCTTCTGCTTGGGATGGCGCTCGTTTCCATG
CTCGACATTCTCAAAGGTCAGCTCAAGGATACGAGCAGGGGCTGGATCAG
AGCAGAGGCGAGGCCAGGCGAGGCGAGGCGAGAGGGAGTGGCCAATTCG
ACAACGCTTGTCGGCCGAGTCACGATGATGGTGAGCAAGGGACCTCCCAT
GGCTGGATCTTCCATTCTCCCCTGCTCGCCCCCATCCAGCTCGGCCAGGCG
CTTCCTCGTAGCTCTACCGTCGTTCCACACCTTGCAAGCAAAGTCCGAATC
GATCTTGACCAGCTGGCAACATTGTCCGTCCGCGCCATGCCCAACGGTCAG
GAGATCGTGCACGACTTCCTGACCGATGCCGGGGCTGTATTTCACGGTCTT
GGGACAGATTTTGATCTGAGAGAGCAAGACGTTGACTGCCGGTTTGTGTG
ACTTGGGCACTGATGCGCCGACGCAGGTCTCGATGTGCTCGAGTATATGTA
CCAGGAGTCTGTGATACAAATCGTGTTTGACCATCTGCGATCCGCCGTGCA
GCAACACCTTGCGGCATTGACCAGGGCTGGCGACAACATCTTTTCTCGTCA
GCCCACCCCTCCCTCTCTTTTCTCTCTCTCTGTCTGTCTTTCTCGCGACAGG
GGACATACCCAAGCAAAGTCCCTTGATGACGATGTCAAAGATTGACTGTGA
GTCCTTGACGGTACACGACGTGAGGACGTGATCGAAAGAGTACTTGTTGC
CCGAGTCGACCAATGCGACGACACCTCCTGCCTCACATGATGGCAGGGCG
CCGGCGCCCTCGACGCGCGTGTGAGCGGCTGGAAGCACGTTGACGGCTTG
ACGGTCGGCAAGCGACAGGGTGGTCCTGTAGAGGGTACGTCAGCATTGAT
GACCTCTTGAGGGTAGCAAGACTCACACCAAGGTGGCCTTGGTGTCCCTT
GTCTTCTGAACACTGCCCATGACGCCGGGCCAAGAAGAGGTCGAGGACAG
TCTAGGTCTTACTGCTGATGCTACTGATGCTACTGCTGCCGCCACCAGAAA
AGTTTACAAGTCGTCAACTTGTCAAAAGGGACAAGATAAAGAACGGAAAA
AAAAGTTCAAGTCAACATGTTACGAAAACCGAATTTGGGACGACCTCGAC
CGCACTATACTGCACTGTACTGTGCTGTGCTGTACCTGCATCAACCGTGCAC
GGTCGGTCTCTCTTTCTATCTCTCAACTTTTCTGCATCACAACACAAAGCTC
GCTGCAAGAGTCAAAAGTCAAACAGAAAGAATCACAGAGTCCTCATGGAT
CAGCAAGATGCCATGGCGGTTGCAAAGCGTGCATTTTGTCTGTGGAACCTT

CGTTGCCTACCTGCCTGCTTGCTCGCTCCCGTTAGGGGATTTTCGACTGACT
CTACCCCATAGTCACCGATACCATCTACAGACTCGCCAAGGAGGGCCGTGG
ACTGTCCACGGTCGGCAACGTCCTCATCTCGACCAATATAGGTCGACTGGC
CTCATCACCAACACAACCAGATGTTCCCCCTCGTGGCCATGCGTCTCACGC
TGATGTCATGGGCAAGTGTTATGCCGTGCGCGACCAGCTCTTGGGCAGACT
GGCAGCTACCGGCAAGCCTCAGATGATTCTGCATATCGGAATTTCGTTGGTT
TTCCTCGTCGTGTCGTTGCCTGCTTACGATGTGACAGAGGTGCCGGCAAAT
CGTTCATGACTTGGAAAGAGTTTGCCTACCTCATCGGTTCGAGCCCTACCG
CTGTTCCCACTACCGCTGCCGCCAATGCCAATCTCCCTCCTGGCCCCGATGA
CAATGTGTCGATCGGACTGGCACGACTCTTCAAATGTTGCAAGGCGGGCTC
GACGCTCCAAGAGGCTTTTGGTAAAGCTGCGACCGCCAACAATTCCAACT
CTTCCAGGTTTGGCCGTTTCATCAAGGTGAGCTCTACTCGTAGCGCTCTACA
AGGGAGATGCTGATTCGGGCAAGATCACGTTCATGACCGGCAATGATGGCC
TTGCTCGACCTTGCGGCGCCTCCATCGAGACGTTCGCCTGGGAGGTGAACC
GTAGCTTCATCCCCCGAGAGGACGAGTCCAACTTCGTCATTCTCGAAAGTA
TCCACCGGGTCAAGGAAACATACACCAGAGCAGTCGTGTCCGATCCTGCTT
CCTGCCATACCCCTGCCGATACCGATACCGATACCGCCCTCGAAGAGTGGA
GATCGCTCATGGGGTCTTTGGAGATCTTCTTCGATCCTGACCAAGTCGCCG
AGATCGTCAATGTCCTCCTGGTCATTGTCGACATTGTCAAGGATCCATCCCC
GGTCGACTTTGACTTTGTCCTTGCCGACAAGGCGTTCGGCTTTACAAGAGG
AATGTTTGAGCATGTCAGGCAGGAACACTGGCCAAAGTTGGCAGCATCCG
CGTATGAACGGCTGTTCAATGTGAGTCCGATGAGGGTATCCGTGTCCGCCC
AGCTGACGTTGCCCTCTCTCCTCTCGCAGCTCATCATGGCTCGCGTCAACT
CGGATCTGGCCAACATCGGCGATGCCCTCTTTGAGACATATTCGAGGCAGT
TTGGCAATCCTCGCCATCTCGATGTCAATCACCTGCCCCACATCTGTCTTGT
GGACAACGTGAGCTGGCCCCTGACGCGTCCATAGTATCTGTCAAGGAGCTG
ACCACCTTCAAGCCCGGCCGAGAGGTTGTGGAATACAATCAGCTCGATCAG
CTCGTCTACAACACCTGGAATGGGGTCACTCATGCTTGCGTGCGTTGACGT
CGGCCTTCTTGGTGCAGCTCTGCTGACCAGCACAAGCATTTGGATGCAATG
CAGACAGAGCTGTCTCGTACCGTCCAAGCCCAGCTTGAGGCTCAAGCTAC
ACGAGTCGACGACACGACGACGACGACGACGGGCAAAGATGGTCCCGGT
CCTTGCAACGACCATCTGAGACAAGCAGCCGCTCAATACGGTCCGCTCTTG
AACTGTCACGGTGTCACGGGGGGTTCCACACCTGAAGAGCGGTGTGGCAA
GAATGTACGCGAAGGTACAACGAGGTTGCCCGGGGGCTTAGTCTCTGGCTC
GCAAAAGCCTAGAAGTCTGCCTTAGGACGAGAGTGTTGGGTGAAACCAAC
TGTGTGACTTAACTGTAGAAGAAAGTACAGTAGAGATCCATCGCTAGAGAA
GAAGGGGGTCAGAGGCAAAACCTCTGGGTTTATATCCCCTTCACATGACTA
CAGGAGGGACTAAATAAAGAGTATCGCTTATCAAGCAAGTGGAGGTACAA

GAGCGTGTATCCCGTATATACTTGGTTTGGTACCTTCGTATATCTGAGTACCT
TCATATATCTGAGTACTTCGTATATCTGAGTACCTTCGTCTATCTGAGATGGC
TTGAAGCCTAACTCTGATGTACCTAGATCACGGGAACACAAGAACGCGAG
AATACGAGAACGCGAGAAACTAGAACACGAGAAACTAGAATAAGTGAAGC
GTGCGCTATCAAACCTAAGTGGTTGACCTGAATGACAGGTCTAGGAAGAGT
TGTCGATCATGTCGACTGCCAAGTGGTACATTGTCGACCCTGTCGATTTAGT
GGGAACACTTGGTTGATCATGTCGATCCATTGTCGATTGTGTCGGTTGGCCA
GAGCGACGGAATCTTAGCTCTGAGGCAATGGGATTCCGGTCACCTGTTGCC
CGCTCAGCACTTGCTATGAGTGATGCATTCAGACTCACCTGTTGCCCCGCTC
AGCACTTGCTATGAGTGATGAGAAGGGCAACAGGCGGCGGCATTCTACAA
GGTCGTCGAGGGTGTCGGCGTTATATGCGGAGTTATCGTTGGTGGCGGCTT
CTCTGCCTCGTCGAGGTTGAACCTCGTCGAGGC

>Mosaic_sequence_17

CCTTACCCAGAGCAACCTGAAAATGGACAGCGTCGAGAATGGCGTAACTC
CGGCTGGGGTAGGCCCCCAAACAATAATTCCGTCACGACCGGCACAGGAT
GATTTCTTCGGTTCCTATTTGGTATATATCTCTCCACGTCAGCGCGCTCTTCG
ATTTCGACTTCGACTTCGACTCCTACGAAGTGGGGAGGGATACCCCATCAT
ATTCGCAGGCCTTGATTTTCACCAGGACATAGCATGAGTCGGTTGTAGCGA
AACCAAACAGCATTTCGGCCGGCGTTGCGCATCTCTCGATTTCCCTAAAAT
GGAGGACCTTACGACTTTTGTTTCGCTTTTGATATCTTCGAGGTGAGGTCAC
CTAGGCGAGGCGGTCTTCCAGTGTGCCTATATTCCGTTCTAGAAGCATATTT
GTGTGTTGGGAAGGGGTGTGCCATTATCGAGCGGTTTGGCAGCGCAGCTTG
GAGTAGTAGGAGAGATATACCTTTGCCTTCTTTCTAGCTTGACCAAGCAGGT
CAATTTATTCAAAGGGCCCCGACTTGAGTTAAAAAGTTCGGATTATTAAATT
ATTGTGTTGGAGCTAGGGGGCGGAGGGGTCCAGTGCAGGCGAAGTAGGGA
CAAAGGGGCCGAGACAGAGATATTGGAGATATGGTGCCTAGATGCGTCCGA
TTCAGATAGGCTGAGCCCCTCGGCTTTTCATTTGTGAGGAAGTGCTTGCCA
CATTTCGAAGAGCGGTCGGCCACACGAAGATGGCTACGCCAAGTGGGAAA
GCCATGAAGGGCAAGCTAGATACATCAGATCGTGACTACAACGGAGACAA
CAGAGAAGCATTCCAGGCCTACCTCGTTCAATTGACACCTATATGCAATTAT
GTGATCTCGGCTAGACCCCGGCGTTTATAAGACAGTCTTGGATGGAGGGTA
CGACAAGTTCAACGTTGTATGGTGTTATATCACCATCCAAAATGCAGATATT
ACAAATATAGGTACAAATACATTTTATCGCTTCATATAGGACTATGAGTGTTA
AGATGAGGTATACAAACAATATAATCTCTAGAAAATACACCAAACATTTCAA
AAATCAATCATCGGCGGCAGAATGGTAATAGATGAAAGGTGCAAGCCTAAA
CCTTTCACAGTTGTGATTAATCCAGCTATATTAGTTGCCGTAGATCTAAAAA
CCCAGCAATTTGATATTAAATCCTTCGCTATTTTAAGTTTGATGTTACATGTAT

CGTGAACTTTTGCATCTTTTAATTATACACATTTCAGAAAAATCGTAAGACT
CCTTTACGGCTTCTTGTTCTGTTGTTGGATAAAGTCCATCTCACAGTCTTCT
TGACACCATCACTCAGGCTTACTAGAGGACGGTAACCGAGACGGCGCTTC
GCCTTCGTAATATCGTAGTACCTCGTCATAGAGGCAAGGACAATTCGCTGGC
GATTGAAAGTCGGAGTCTTCCTGATTATCCCGAAGAAGACTTCGCTCAGAA
ATCCTAGGAGAAGACCCACGTCTCTGCTCATAACCCACACGTGATCGGTAC
CCTTTTGCGATCCGGCCTCTCGCCATATGGCACGCATCAGATCCCAAAAGTA
GACAGGGCTATCGTTTGTGATGATGAAGGCTTCTCCGTCCACTTTCTCGTAA
TCGAGCGGTTCCGTGGTCGACGCATGAGTTACTAGCAGAGGCGCGGGCGG
CTAGAAGATGGGCGTGGGCAACGTTTTCGACATATGTGAAATCGAATAAGT
TTTCGTTCAAGCCGACTTGTATCTTGTCCTTCCCTCACGGAAAATATTGACG
GCGTGATAGACTAATTGCATGTCGCCCTCACCGAAGATACCGGATGGTCGG
ATGGCAGCAGTCAGGAATTCGGAACCATCGGCGCGGTTTGCTTCTAGGACG
AGCTGCTCGGCGTGCGCCTGATTAATAGGATTAGTAAATCATGTATGATGCA
ACTATATCATCAGACAGAAAGTACCTTTGTCTCGGAATAATATTCGGTTTGC
ATTTTACCCTGATCACTGGATAATCTTCGTTCGCATTTATTAGGTCGTCCTTAT
TGTTGCTCATAACACTGGCGGAGCTCGTGTATACCAACGCCTTCACGCCGC
TCTTCTGACACGCAGAGATCACAGCGCGAGTACCATCCACGTTCACCTTCT
TGAATACATCCGTTGGCGACGGCGCCGAGACCTTGGGCAGGTGGCGAAGC
CGTATGGATAACAACATCGGGCTTCAGCTTCCCGAAGACCTTAAGTAGACC
ATCGGCATCAGTGATATCGGCCTCGACATATTCGACGCCGTCAGAGTCGGG
TCTCCGGTTGCGCTGGCACTTGAGGTCGGACGACGGAAACGGTACTGGTC
CAGTCGCGCAAGAGCAGATTCACGACGTGGTGGCCGGAGAAAACCACATC
CGCCGATGACGAGGACTCTGCCGAGCAGGGGCTGCGCTACAGTATCGGGC
ATGGTGTAGTATTTTGCGATTTGTGTTTGCACATGTACTTTCGAAGTTTGGTT
GCCGATTGAACCTTAATGCAACGTGGAAGAGGCTCCTAGCTGGGAATATAC
AAGTTAAGCAAGTTGCAATTTAGGACCCTTCACCGCCTGGCAATTAAGCTA
GGTATGGGAAGCTCAAGCGATGACCCATTTCTCGGCGCCCTAGAAGCCCGA
ATTGGTGAAGATCCTAAGGGGAAAAAAAGGAAAATTGGGTGTGGGGCGTT
CCTGGTTTCCTTCCCCCCAGGATGGAGGCAAGGGGGTAAAACTTTAGGGG
AGATAGTACGATAGGTCATCCCAGCATTATCAGAGTTAATACAGTGCTAATA
CCGTATCCGGTTAGGTGTCTTCAGCAGGTAAGCGCCTGGCTTAGCAGGGTG
CTGTTGTGTAGGCATTTAGAGGTACTAGGTATCTTCCAAGTAAACAACATTA
TTACATCATTTCAAGTTATTGAATACTGGGAAGGTACTTGATAGGTACATGTA
GCTGTAGCAGATTCAACTTGGAAATCTGAGATGGATGGAGAATACGTGAAA
TGGCAGCCACAAACATCAAGGCACATACATGATCTGACGTAATAAAGTGTA
TCTGCACCCTGGATCTTCTTTTTTAATGACCAATACCTACAAGAGTTATGTG
ACTGTTTGTAAAGAACGAATTGTGAAAAAGGTACCATACATCCTCAGTCAG

CCCCGCGACGCTCTACACTCTAGCAGACCCCGCCTTCTTCGCTGCCTTGGG
GGACCTCCCCACTTTCCCGCATTGCACTCAGCGCTGCTGCAAGTGCTACCA
TATGTATTTCCTAACATTGAACCAACCTTATTACCTTCACACTATATTCTTCCT
TTGAAGATTGTAACCCAAAAATCTATCAGACGCGCCGAAACCTTCTACACC
AAATCTTACTCAACGACATACAAACTCCTCAAAACTATTACCAACCATCTAC
CCTACCAATCCGATAATCAAACACTAAGCGCCGACCTAGATAGCTGGTAGA
AGCTATGGCGGACCAGTCTATTATCCGCATCACCAAGGTACGCTGCGGGCG
TCATTTAGCTCTTGGAGGGGCAGCGAGGCTGACGAGTTCTTCGCACCTCAC
AGGAAATCGGTGAACTCCAGAGCAATGAAGTTTCATCGAGTCCGGAGTCC
TCGATATTCAAGGCCAGAGGATGCTGTGACTGGACTTGACTATACTGAACA
TACTGTGGCCCACTCTTTATTCGATAGAATCCGAGCTTCCAGCAAAGCTATT
GCGTGTCAACGACTTTTGCCCCCTCTATCGGCTTCTCCCAGTCGGCCATATT
CTTGGCGAAGACGTGACTGGACGGTTTCGGAATGTCACTTGGTTCAAAGA
GACCTGACTATCAGTGGACATAGGGGAAACCTGCGAAGCGGGTCTCACCT
GCCTTGACATATACAATGTCCCAGGTCCTGAACAGTCGTATAGGCCGGACT
GACATTTCAGTGGACCAGCTTCGTAACATGATTTGCCATGACACTTACCACC
CGCATTCGCCGCAAAGTTCCGGTACACTTGTTTGCCAGACAAGCCTTTGTC
GTGATATATCTCAGGCTGTCCTCCGAGTTGCAAGTCTTTTTTGCGGACAGCA
AAGTTGCCCGAGTGACTGCCAGTGAGTTGGACTGGCGACTTGTGCTCAAA
CATACTTACGTTGTGCCGGAGGCCGACGACAAGAGGAGCAGTCTGGCATC
ATGACGCGTTCTCACCATGCGTTCGGAAGAGCGACTCACGACATGTGCCCA
TGGGCAGCCCGCTGGGTATCGTGACTTTGATATTCCCGCAGATACATCTGCC
GGTGATGGTTGCGCCGGCCATGTCGTGAGCTGGAGTATGGTGCAGGACTGT
GTCAAGAAGGCGCCCCTGCTCGTCTTCAGCAGTCAGTGAGTCCAGCTATCC
AGTGGTCGACGTGGAGGTGAGCTGGTGATGAATGCATGGTCCTTTGGGTG
ACGTAATCTTGATCTCTGGCTGCCCTTTGTTTAATTAAAAATGTGATCGAGA
AAGGTGTATCCATGCACAGAAAGAGACGTGGGGAGGAAACAGATTTCATA
TACAACCGTCACATCACCTCTCTCTTGCATGTTCAAAAGTCGAATAAGAAA
CGATGACAAGGAAACAGCGAAGAGTGGAGCGGGAGCACGACAACGACAT
GGCTGCAGAGCTTTCCGGGGCGGGAGAGGCAAGGGGGGAGAGAAAACGG
CAAACAGGCTGCCATATCACGCCGTTTTGCGTGACCGGATGGACAGGCACC
GAGAGCGATGACATCTGATATCGATCGACATTTGGCGACTCACCCCCCGCA
ACTGACAGATAGAAAAGGATGATCAACGAGCAAGAGCAAGAACTGCTTCC
CAGCGCCAGCCCGTCGTCGAAGGGGACTGAATAAAGTCGCTCGCCGCTAC
CCTCCCTCCCACCCCTGGCCGTCTCGGGCCATATCGGAATTTCCCCGTCTTG
CAGGCTTCTCTTCTCAGCCTAGCTCGCCTCGTTTTTGACGGCCGACGCCGT
GTTCGTTGTTGTTTGCTCACTCCCCTCACTGTGCATAGGCGAGATGACGAC
CACGCAAGAACGACATGCATCCCGACGCAGGTAGGCGAGCGAGCGCCGAT

TCTACGCAATGCTGGGGGCTATGGGACTGTACCGACGGTCGACCCCGACCG
CATTCGGGCCGAGCTATCCCGCGCCAGGTCACACGAGTCGGCGCAGTGGG
CGGCGGACGGATGATGAGGACGATGAGCCCCCGAGGACGTTGGGCGTACG
GCGGAACACGTTCTCGGTGCCGCCATCAGTCTACGAAGCTGCAGGAACAT
CATCCAGGAGAATGGGCAGAGTGGGCGAGGGAACTCATCCCTCGACTTCC
CTTGTTGTGCCTCTGCTTGTCATCGGCTTTGCCACCGGGTGAGCCTTCATGC
TTCCTCTTTCTCGGTCGGCGTGCTGATGCGCCAGTATCATCGATGCCATGAC
TTCTGAGGGCGTACAATACCTTTGTCACCAACCAGACCGGCTCGTCATCTG
TTCTAGCCGTTGCAAGTGTCAGTGGGACAGATCGAAATATCCTTCATGCCG
CGCTTGCCCTGCGGGTTTCGTGGTGTTTGCCTTTATATTTGGGCAGCTTGGC
GCCCGCAATGGTGAGCATGGAAAGCGTAACACATCTTGAATGATGTCACTG
ACGATCAGGCCACCGAAACGCTGGTGGCTCGTCCTTTCGCTGAGCTGGCA
ATTCTGTGTATCTTTGTTACCACCATCCTTGTATCACCAATCGGGCCTCATTA
CACTCGGATCTGAGGGGCAGCAGACTGGATAGGCGTGTTGCTGCTGGCGTT
GAATGGCGGACCGCAAGTCACAATGGTCAGTCAGATCAGTCACGGTGCCT
TGGTGGCGTGATGATCTACATACTGACGAGCAGGCTGTCAATGTCGGCATT
CCTGAGATTCCGACCGGCATGATCTCCAACGTCTACGCCACGCTACTTTCA
CATCGCACTTTGCTTCACACGCGCGTGGGCTCAGATACGGTTGGAAGGACC
GGCGAATACTCTTTCTGGCAGCATTCTGGGCGGGAGGATTGATAGGATCTG
CCATCGGCCGCGCTGGGCAGGCGAGTGGCTCACCCTCCCTCCTGGGCGGG
GTGTGCAAGTGTGCTGTCCTCGCATGGACACTTGCGTGCCGTGGGGAGGA
CAGGCTACTTTGCTCGAGATGAGAGCTAGCATACATCAATAGACGATGAGA
TGCATAGATGGACAATCATGGCGGTGTTCCGGGAGGCAGGCACGGTGGTG
ATGGGACCTAGTCTCGTGACAATGACGATATGCGAGATGGTGTCGGATGTC
GCATACATGGCGTGCTCGCGAGAAGCGAAGCTAGTGTAAAGCACACTTGA
CATGTCAAGACTATGGAGTATGTAAAGACTCTCGACAGAGACTTCTAGCGC
TGCTCTGAACACGCCATGGCGCCCATAGCAAATGCGGCGCCGGCCCCGCA
GCTGAAGACCGCCTTATCAGAGGGTCCCGTCGACCTGAACATCTTCCCAGA
CGGTATCAAGACGTCGGGAGCGCACCCTCCTCTCTCAGAGCTCATTGTTGC
CGTACGAAGAGTGGCCCAAGCACGTCGACGGCCCGACGCTGTGGAAGGC
AGAAGACTACGCCGATCATCCCGAGCGATGGACGAAGCCATTCGACGCAG
AGGAGATCGCTGAGCTGAGCACAGCCGCAGATGCGTACATTGATAGTGGC
GCGCCCTCTAACAGGTATCTCCAAGGTATGCTTCTCTGTCAGGTAGCGTGG
ACACCTGGAAGCTGACGAGGGGTAGGAGCTGTTCCCACTACCGACGCTCG
GACCGTACTTGTCAGAATTGCGCAGAGAGATCCTGGACGGCAGGGGCTTT
GTGCTCTTTCGCAATTTGCCGGTGCAGGACTGGGGCAATCACAAGTCCTGC
CGTGGTGTACCTCGGGCTCGGCACGTACTTTGGCAGATTCACCAGCCAGAA
CTCGCGGGGACATATCCTGGGGCACGTCAAGGACCTGGGGAGGACCCGAC

GCAGAAAGACCGCGTGAGGATCTACCGCACCAGAGAGAAGTGCGCCGTCA
CATAATTACTGCACCACAGCGGGCAGGTGCTGACGGTAGGCAATTCTTCCA
TACCGACGGGTCGGACCTCGTCGGGCTGCTCTGTATGGCTCGTGCGCTCGA
AGGCGGTGAGTCGCACATTGTCTCCACACACCACGTCTTCAACACCCTCCA
GGCCGAGCATCCCGAGGCTGTGCGGGCGCTGGTCACTCCCAATTGGTACTA
CGACCGCAAGGGCGAGTGTCCGTGGGCGAAGCTCCCTACTACCGCATAGC
GCCGTTCTACCTCGAGAACAACCCTCTCGCCCTGGCGGCCGCCGCGTGTAT
GCCAACTTTGACCCCAACTACGTGACCTCGCTCGCGCGGTTCAACTCTGGG
CCCGACGCGGTCATCCCGCCCCTGTCCGATGCGCAGAAAGACGCCATGCG
CCTGCTGGAGGAGGACGGCCAACCGCCTGTGCCTGCACATGGTCCTCCGG
CCCGGCGACATACAGCTCGTCGCCAACACACATGTCTTCCATGATCGCACG
GCGTACACCGACTGGCCCGACGGCGCGGTGGACGAGCAGGGCCGCCCGA
GGATCCGCAGACACCTCATGCGTCTGTGGCTGGCGACGAGTGAGGATGAG
GGGGCTGGAAGCTGGTCACGCCCGACAGCCGGCACAAGAAGCGGGGAGG
GATACAGGTTGATGATACGCCGCCCGTTTGTCCGCTGGATGCCGAGTAGCA
ACCATGCGTAATTGTATAGTTTCACTGCGTCCGTCCATGTACAATAGCATCAT
GCCCCACCTGCTGCGCTCTCGGCGAGGACGGAGTTCGGTCAAGTCCGACA
TCGGAGCAACAGGTGATGATCCCGAATCGCACCATGCAAAAAGAGCTTTCT
CAACTTCGACTCGCCTTTCATCTTCCCCCTTGCGTTGTGCTATCATACTGCA
AAGAAGGCGCGTCGTGTCTAGTATCAGCATATCAGGATGACCATCGAGACT
TCCTCTCGACCGCTCGAGATTCTCCTGATCGGTCTCGGCTCGCTCGGCAGC
ATCTACTCGTACTGCTTGGAGAGAGTGAGCTTTTGCTCTCCGTAATCAGGC
GCGTAGTTGATCATGATGAACAGGCAGGTGCGGCACGGGTGACTGCTGTG
GCTCGCTCCAATTACAAGCTCTACACGACTACCGGCGTGACCTTGCGCACA
CAGCGATATGGCTTTATCGAGAACTGGCGACCGCACCGGGGGAGCTCGAA
ATCGTTTCGCCAAAGGATAAGGGCTAATGGGCAGTGGTCAAATCGCAGGCA
GAGGCATTGGAGGGCGACGTGACATATGATTTCTGTCTTATCACCACGAAA
TGCCTGCCGGATATCAATCCCACCCCGAAGCTCGTCCAGGAGGTCATCAAT
AGCGATAAGGTCAAGGCGTTCGCCCTCATCCAGGTATTTTTCCCGATGAGA
GACGGGATTCAGAGGCTGATGGCTAGAACGGGCTCGATGTGGAGCGGGAT
CTCTATGAGGCTGTCAAGGACAAAGACATGCCTATCATCTCCTGTGCCGCC
TGGATCGGCGTCATGACTTCGCCAGACGGGTCCATTGTCAGCTGGAGCGGT
ACTGTACGTGTCTGAGAACCCGTGTTCACTTCAGCCGGGCTGACGCCAGAT
CTCGACCACGGCAATGGGAATCTATCCGCCTTCGGAGAGCCCTCAGCTTCC
GAGACCAAAGCCCTCCAAGCGCTCTATGGGGCCTGGCAAAAAGCAGACCA
GAGGTCTTTCGCCGTGGAGAACATCAGCGCAGTTCGGTTCAACAAGGTGC
GTCCTAAGCTGCGGCTCTGGTCGAGCTTAGCGACTGATGTGCAGAACATCT
GGAACTGTGTCTGGTCCTCCATTCAGGGTCTGACTCGCACAACCAACGAG

GTGTTTGGCGCTCTGGACCCCAGCGTGCAGAAGCACTTCCGAGCATTCATC
GACGAGGTGGTTCAGGTCGGCTTCAAGTCGGGATTGCTCAAAGAGGATCG
ATCTCGTACCCGTCGGGGGAGAAGATGGGGGATGCCAAGAGTGTGGCAGA
GCACGCCTGGACTGTGCGTCTACAACTTCAGACTTGGGTCACAATGAGCTG
ACTTCTAGCCGGTCGACCTTGCCCTGTCGCAGATATCGAAGGGCCTCCATC
CTCACAAGATGTCCCTGCAGATCGACGTGGCTATGGGCAGGCCTTTTGAGG
TCGAAGTGAGGTCGTCCCTGAAGTGTAATCGGCAGCGGCTAACCACAGGT
CATCACTGGTGCGGTGCTCAAGCTCGCCGAGGAACACGGTATCGACACGC
CCCGACTGGCCATGCTTTATGCCATGTTGAAGGGATTGCAGGCCGACTTGA
TCCACAAGCAATCGGCACGGGCTTGAGGAATACTATACATAGAGTACATAG
ATGTCAAAGATTTCGGTGTCCACGTGTAGAATGCATGCATACCACATTCCAC
TTCTGCGGGCACTCTCAGAATAGCCACGTCGACCCGCCTCGCTATCTGATTA
GGTCGGAGAGATTCCAGCGACCCGCGGTGGTCCATCTCTCCCCCAGCACTT
TTCGGCCTCCGACACACCACTCTCCCTTCCAAGGCGGCAGCATGCGCTCAG
CCGTGCTCACGCATCCACCTCTTGACCCGCGAGAACACTCAACACTATGCT
CGAGTGGGACACCGAAGCTGACGAGCCGTACTTGCCCGTCAAAGGCAAGG
AGGGATACCGCTTGACGCCCCTCCGCACCAGCGATATCGACGGCTTTTGGC
AGCTCTATCGCGAGGATGCGATATCGGCCAATAGCATTCGGCACCGCAAGC
CGTCAGTCCAGTCATCGTCCCTCTTACCGAGTCCGACTGATGCCCAGCTTTC
CCCGCGAGGACGCCGAGAAGCGGATGCAGATGATCATGGACCGCACGCAG
GGTGTCCTCGCGAGATACGGGCGGCGCATGCGTCTGGCCGGCTTAGCCCTG
ATCATACTTTTGCCGAATGTCCCTTCGAGGTGATCAAAGATGCTTCCGGGAA
CATTGTCGGTGGGTCTCCATACTTTGTGAGCCTATTCCTGGCTGAAGATCGT
CAAAGGTACGCTCAACCTGCACACTTCCGATGTCGACTTTCAAGACCCCCT
CGACCCGCCTTCGGAACTCGAGCACGTCAACCAGACCTGGGACGTGGGGT
ACAACGTCTCAGCCTCGCAGGCGGGCAAGGGCTTAGCGACTGCCTCTCTA
CGCACGCTGATAGACGATTGGGTCAAGCCGTACATGAAGTTGAGGCGAATG
GGAGCGGCGAGTCTTGTGCGGTGTCATGGCCGTTGCTGACTCTTAGGGGGC
GAGGGCGACCAACGGAGCCTCGCATGCCGTCCTGGCGCAAGTGTGGATAC
GTCGAGATTGCTCGATATGAGCGCACCACTGCGCCTGAGATTGGTGGACAG
ACATTTGCAGCTGTCAAGTTTATGCTTCGGCTGGATTGACAATGTCATGTGT
GTGTGTGTGTGTGTGTCGTCAAGGGGCTTCAGGTACCATCATCGACGTCTG
ACTGGACTCTTTGCACAGACCACTATCAGCTGATGACCCTCTCTACGAGTA
AAAAAAACCTGCATTTGTTCTTTGACATTGACTCGTCACCAGCCTCATTATA
TTACAGCCCCTCACTCCGAGGTCGAGTTCCACGCCACCCGTCTCGTGATGG
TCACCGCTGTCGCTGGCAAAGAGGACAAGGCGCTGCCTTCGGGTAAGCCC
GGTGTTCCGTCCGGGATCTCTCAAGCAATGCATGCTTTCCACGTAAGTTCAT
GCAAGTCGACATGACACCACCTTCTCGCTCAGGGACTCCATGAGGCGGCT

GACTCGCAAGCTCAATCGCGATCAGAAGCCAGTGCCGCTCCCTCCGGTCTT
CAAGAATGATTGGGCATGGGCGTCAGAGCAGCATTTTCTGCGAAAAGACAT
TCCACCTCCCAAAGGGCCACTCTTCGTCTCATACGGTGATGCCACGAGCTG
TTCCAACAGGGACTGGAGGACACTGAGGGACGTCACTGTGAACGATCTCA
AATGTCCTTACGTGCATAGTGGTGCGTATCAGCAGTGCAGCAGTGCAGCAG
TGCAGCAGTGCAGCAGTGCAGCAGTGCAAGAGGTGGTGGCTGACGAGAT
GACAGATCAGGTCTTCATCTGTCGAATTATCACCTCACCTCGTGCGGGTACA
GGTGCGACCTTGTGAGCGGCTCCTGAATTGATTCTTGTTGTTCGTGAGAAA
CTAAAAGTTAGCTTTGTGGAAGACGCCAAGGGCCTCGCAATCATGGTGCAC
GCGCCTCACATCGCTCCGGAGCTTGGACTCGACCTGGGCGAGGACATCCT
GTACGACATGTATCCCGTTGGAGCTGTGCTAGCCATCTGCGAGCCCTACGTC
AAATATTCCGCGATTCATGTGGGCGTGGAGATCTGCGTGGATATCCCCACCG
ATATTACCCGGATCCATACCAGGGATCCACTTATCCGCGCAATAAAATGGGC
AGCTCCCCTCCCAGTAAGCTGAAAACTCCTGGTCCAAGATCCTGCGTTCTG
TGCTGACAGATGGATCGAAGCCGAACCGACAGTCGAGCAACTCAAGACAC
AAGGCAACGAGGCATTCGCCAAGCGGAATATGAGGTCAGACTCCGACCTC
CTCAAGCAGGGAGAATGACTGACTGTCAGTATGGGTACGAGCGATACACG
CTAGCTCTTGCTGCACCGGAGACCGAGCGGGACATCGCTACGACTTTCACA
CTTCTCCTTAACAGAGCGATTCTCAGCATCAAGCAAGGCTGTCCCGGAGCC
GCCTACCGAGACTGCATCGCCGCTGAGGCGCTCGGCCTAGTGTTGAGCGCC
GGTCAGACGCAGAGCTGACTTGGAGGAAAGCGCTTGCTGCGTACGACCTG
CGGATGTGGACTCTTGCCGAGAAACCTTGCGATGATGCGCTCAAGTTTGGA
GTGCAGCTGGCAGCTGGTCTGCAGAAAGACATACGTCAACGTCTTTTGGA
GACTCGGGGGATATTCGACTGGCGCGACCTCTGGACGCGCCTGTGCAAAA
CTGGCTCCGTTGTAATGGCGGACTACATCGGTCCCATCAAGCTCGAGCGAC
TGCCGGGACGCGGACGCGGACTGGTATTGACTGAGGATGTCAAGGCTGGC
CAGCTTCTGCTCGTCGATCAAGCATTGGTGCGATCAGTCATAAAAGGTCGC
CGGGAGTTGTTCGAACTCGACTGCGCCCTCAACGTGGCAGCCAATACAGC
GTCCGTCTCGATTTACTCCAAGAACAAGGCCATCCACGAGCTCATCGACAA
TCCGGGCAAAATGAAGCTATGGACGGGACTGACTTACCTTGATAGTCGCAC
GGGGCGTCCAGCGGAGATCCCAGATCCTATGCTCCGCCCGACTCATTCGCT
CGAAGACCTCTTCGTCCAGGTCAACATCGAACCTTGTCGTCTCAGTGCGAT
CTTGAGGAGCAACGCGTTCGGCACCGAACCTGACGTATGGACTCCTGCAA
CAATTGCGCTTCCGGACGGCACGATGGAAAAAGAAGGCGCCGGAGGGGTC
TGCTACCGTCTAGGCTCCCTTTTCAATCATTCCTGCTGCGCCAACGTGAACC
AGGAACAGTGGCGCGAGGTGCGTGAATCAGCAGAAGAAGACCTGCTCGA
CTGACGCACGACTCAGAGCGCGGTCGTCCGTGCTCGCATGGACTTGCCGA
AAGGTACCGAGTTGGTACTCACCTACTTGCAGGATTCGTATGCCTCGCGGA

AAGAGCGACTCGCTCGCTCTTGGCGCTTCACTTGCGACTGCGAGCTCTGCG
TTGCCGACAGCCAGGACGATCATGCGAAGCGCACGGAAATTATGAGAGCG
TTCGATCCGTACTCGCTCCAGTCCCTACAAGGCTTATCAGATCCCCGGGCAC
ACCAGGCTTCGAGTTTGAAGATCATCAACGAGCTCGAAAAGACGTATGCA
GTAGGCAGGCGTATCAAGCCTGAGGTTTTTGAGCATGCCTCCCTGTTGTGT
CTTTCACCCGACTGGGCGAGAAAGGTCAGTATAGCCAGGCCTGGCTCTGTG
CGGACCGCCGCTGATTCCAGGCTTATCTCAATCTAGGCGCTACTCTTTCCAC
TACAGAATCGCGCAAGATCCACGGACGACAGCTCGACACCTTGGGCCTGG
TCAACCCCATAACCTTCCCAAGATGCTAGTCGCTGCATCCCTTTTTTCATCC
CTGTGAGCATCTTCCCCTGTTATCGCGAAGGGGGTAGATGGAGGCTGACTT
CAGCATCCAGAAGAGGCCGCGAGCTGGATTGCGACCGCCTGTTGGGCTCA
CGACGTGCTGCATGGCGGCGGGCGTTGGCTCTTTGAGCAAAAGTTTGCGG
CTCTGCTGCCGAAGAATATCTCTTGGGACTATAAGAATCCAGTGCAGGATC
ATGGCCCCGCCGAAGACCTGGGCGATGATCTGGACTAGACTAATCGGGTCT
CGATTATGTCTACAAGGGTGTATATAGGTCCTAGCATGCGCAGATGAGGCTC
TTGACCAAGGTTCTTGCATAGTTGGTTTCTGTACATGAACGAATTTGCCACC
CAACCTAAGTGCATGGTTATCAAAGAGGTCCTTTGCATTTCTATCTTAACTC
TGCATCTCCTCATATGCCGGCGTCAAGGGCCATCGCACGAGGTCTACCGGC
TCGAGATGTTCCGTGCGATGTCCATCTCCTTGACCTCGGACCCCTCCCCCGC
TCAAGAACACAGCGGGCTCCGTCCCCATTGGCCCTTCTTCGATCGGCCCCA
ACTCTTTCAAGTATGGCTCCCTTGGTCTTGCCGCCATGCCATGCACGGTCAT
CGTGTACATGACTGATGGGCAGCCCTGCTACCGAACATCCTATCGACGGAC
CCAGACCTTCTCCAGGCCTTGTTGCGCGCTGGCATATCGCCCTATATTGGCG
ATGCGTCGCTCTCTGCGACAATCTCGAGATACATCTGGGAGCTCAGAGGCC
CGAGACACCCCTTGCACCAGATTCCGATTGCTCAAGATCAGGGCTTGCCGT
CCACGCCCGGTACTTTGACATTCCATGTGTCGGTCGGTCATGCGGCTGACG
AGATGAGATGGACTCGAAGTTTCCCCAGGATCCGGCTATCTTCCCTCATGA
CCAGACAGGCGGGTGAGCACACAGACGATCCTTGTGCCCAGGCGAGCGCT
CTTCTGCGACGTTGCTTGCAAGTGACCAGTCTGGATATCGTGGCCGGGGCA
TTGATAGACCACGGATTTTGGAGGGTGCGATAGCTATGACTTTTGGCGAAG
GACGTGCTGATTTCAAGGCGATTGCCAAGCTGCCCCATCTCGAAGAGCTCT
CCATCCGGTCGCCAACGCCGCATGTGCTTCATGAGCCGTCTCTGACCTCTC
CGTCACAGAGAATCAGCCCCGCCCTCCAGCTTCTCATTCCGCCTGGACTTG
CAAGACGGCCACCCTCTCCGCCCCCGAACGATATAGAGGACGCAATCAAG
TTCCGCTCAGCAAGGATCATTGGCGCAGGTGGATATGGTTTGGGACCCGCT
TGGGAGCATTTACAAGTGCTTCATCTCGAAGGCTTGGACCAGACCGGGGTA
CGCCTCGGTCTCTCTTCTGAGTCGGAGCTGAAAGAGCAGTC

>Mosaic_sequence_18

ATAGTTGGGAAGCGAATCGCTGTACGAGCATGCATGATCTGAAGGTGAGAG
ACAGGCAGAAGGAGTGCTGTCAGTGAAGCCGCACCACTACTTGGTGGCGT
GAAGTGAGAGAAAGAAACAGCATAAAAGGTGTAAAGCCGACAACGACCA
CCTCAAGAAGATGGTCGATGTGCCCGTCTCGTCTGTCGACGTTCCGACCGA
GTATCACGGCGACACAGGAGAGTCGGAGTCTAGGTTAGCCAGATCGGACA
GTCCTGGCACAACGCAAACAAGAGACGACGCGAGAAATCAGTCGCGATCG
TTGATCGATCCATCTTACCCCAGATTGACCTCATCCCCGCCATTCTTCGTCAT
CAGGGTGACCACTCTCACCCAGTCGAGCTTGGCGGAGCTGGAACGATTCC
GATGTTACTGTCTGACCGATCCGATCTCATGGCAAGACCACCAGCACCTCC
TCCCGCTGTCCAACACCATACCGGCATCGATGACGATCCTGGAACGTGTTG
ACGCTGGTCCGCTCACCGATCGAGCCATGAGATGACCTCACCACCAGCCA
GGTGCACACCTGCCCATATAACACAAATACCGCTTGATCCTAGACTGCCCCC
CAGCATCCTCTCGCTATCTGACTTCTCCTCAGAGATTAAGAAACAGCACCA
GCCATGTCCGCAAACAACCAGGGCGAGTCCAACCTCGGCGGCGAGCACCT
CAAAGCCGATGGCTCCCCCGACAGGCGATTCAAAGAGGTAAGCTGTGCCC
CATCTTCCCACTCAGCAGAGTGTGTGCTGAGGGGTAGCACGGCGGCGGGC
AGCACGGGAACCAGGGTACCGGCTTCGAGGAGGACGGCGATGGCACTTCC
TCCCTCAGGACCGAGGGCACCGGCGAGGACACGGGCACGTACAAGCCTTC
CGAGCACGGGGGCATGAAGCAGGACGGTACGCCTGACAAGCGTGAGTCCC
ACCTTGGTCCAGAGACCATGTCACCCGGTCAAGTGCTTATGAAATTAGGCG
TCTCATCCGAGCACGGCTTTGGCGGTGATCGCGAGGCGGCGTCGGAGCAG
GGCAAGAAGGGCGGCTCGGCGTCGTATGAGAACGACGGGCTGGAGCACTA
GGCGATCTCCACACAAAGACACCAGGCGAGTATACCAGTGCGGACGAAGA
GTGACAGAAGAGAAGAGAAGCATGTATCTATATCGTCTTGCTAGTCTTGCT
GTCGTTGACCGGTGTCCCCGGCGATCTCGGCATGTTCGCCGTTTTCCCCTCC
CATCTCGCCATCGCTCGTATCTCATCGACAATATACTTGTCAACAACTGTCA
CTACACGGCTCAAACACACCACCGATCCCTCATTACTCCACTCCCAGGCCT
ACCTTTCATCGCCCCGACCGCCTTCGAGGCCTTCTGGGGCCTTCTCCACTC
TGATCGATCAAGTCCACCGGCATTCTCCCCGGCTAAATCGCGATAAGCAAC
GCCTTCATATACACCTCCCCAATAACCCATCGACCATCATCGATCTTCTCTGG
ACAGCACCATCATGGCCCTCATCTACGCGGGAGACCATCGCTACCCCGCCC
CACATGTGCACCCGCCGCCGGCGCAGGTGCGCGGCATACCGAGTCAAGAA
GAGCTGGACGCCTTCCCACGGATGTTCACGTGGGGCGAGCTCAAGGAGAT
CGTGAAGAGCGGGGACCTCGAGCGGCTCGGGAGGAACTATGCTCTCCAGA
GGCGGTATGATATATGGGCAGCGGCGATCAAGAAGAAGTACGGCTCGACGG
GTGCGTCCTAGATCTTCCCAACCGGTTCCACTGTGACTTGTAGCTGACTAA
CAGAGAACTACCTCTCGAGCATACGGCTGCCGTGGCCGAAAGCAGAGGCA
GCTGCGGCTGAACCGACATACAACCCGGATCCGACACTGTCCGTACAGGTC

GAGCCACCCCTCACCTCCCCAGGACCCTCGCCACTGCCTGCAGCTGCCAA
CAGCGGGAAGGGCAAGTCGTCCCCGACATCCGGCACTTCGACACCTCTCT
CAGCGGGTTTCGTGTCCCTCTCCTCGCTCAGACTGGTCAACGGGAAACTGC
GCACAGCCAGCGGGTCCTCCACCCCCGCTGCGCGCAGTCCGACCAAAGGT
CAACCACGCATCAACGGGTTCAGCACGCCGGAAGTCGACGGCGAGCCCGA
GCCGGACGAGGACGAGGACGAGGAGGGAGAAGAGGAGGAAGCCGAGTA
CATGAAGTTCGACCCGGCCCTCGGGAAAGGGTTCGACACGAACAAGTACG
CCGTGCTGGCAAACGACTGGCCGTACAACACGCCCTACGGGGTGCGGCAC
TACTGCGTGTGGTCCAAGGTGCCCATCGCGCACCCCGAGCTGGTGGGCTAC
GACCCCGCCGCATGGGCGCGCGTCGAGCTGGAGGGTCTGTCGGGGTTCAC
GGGCGTGTCGCCCAACGTCCCGGGCAAGCGGGACGCCGATGCGATGCTCG
AGGTGGAGCTGCCCTCGGGTGAGGTGACCACTGTGCCGCTCTACGCCGCC
GGGGCCGGGGCGACCGGCAGGGGCGCCTTGCAGAATGGGAATGGGAGTG
GGAGTCCGGAAGGGGCGGACGTGCCGCTGACGGCGGAACGGCTGGCGGG
CAAGGAGGCGCAGCGGTGGGCCGGGGTGCAGCACGAGACGCCGGGCGGG
CAGCATGTGGGCGCGATGGTGCGCGCGCTGTGGGACGAGCGGGGCTGGGA
GTGTCTGTGGTTCATCAACCCGCCGCACCTGCAGAGCGTCCCTTCGTTCTC
GCACTTTCACGTCTTTGCGCGGCGCAAGACGCCAGAGGAGATCGACAGGG
CCGAGCAGCTGCTCAGCGCGGGCCGATAGCCCGTAGCCGTGGGCAGAGGA
CTCTACCACGTATCTTATTAGACGTACTCGTGACTTAGACTTAATAGCATCAA
CACAGCGCATGTACATATCACGCATCCCCAACCCAGCTAGCTACTGGGACC
TGCACCAAACACATCGCAGGTGCATGCCCGACTGGCAATATCGAAAGAAA
GTATAGGAGACCGGGTACGAAGCAAGTATCGAGGAGGACTGGGTATACCAT
CGCCGTCACCACCGCCGCCCTCGCCAAAATAAGAGACACGCTGTATTGTAC
ACCTTCCCAGGCCGCAGATAGATCTTCTCTCGCCGACCCGCCAGGCAAAAA
CCTAGGCGGCGACCTTGGCGACAGGCTTGACCGGGGCGACCTTACCGACG
AGCGACTGTAAGTCAGCTTCTGAGTGGAGAAGGGCCTGCAGGCCCTGGAC
TGAGAGGTGCGCACCTTGTGGATGTGCGGCAGGACACCACCACCGGCGAT
GGTGGCGCGGATCAAGAGGTCCAGTTCTTCGTCACCACGGATGGCGAGTT
GCAAGTGGCGGGGGGTGATACGCTTCACGCGGAGATCCTTGGCGGCGTTG
CCTGTTTGTCAGCGCTGACTCAGGTCGACTCTGTGTGCTGGCGCTGGGTAT
GAAGCTCCCACTCACCCGCCAGCTCTAGCACCTCGGCGGTGAGGTACTCC
ATGATGGCCGCGACGTAGACGGCGGCCTTGGCGCCTACCCGCACATTGTTG
GCGTTCTTGGATCGGAGGAATCTGGGATCAGGGACATGTCGAGAGGCAGG
ATTGGGATGGCTTACCGGTGGATACGACCGACGGGGAACTGGATGTCAGCT
CAGAAGCAGGTATTCCTCCAGCCGGCCAGCCACGCCATCCTCCAAACACCC
CACGCCTCCCAAAAGCCCGCCCAGCATCTCCATATCCTACTCCTGATACCAT
CCTTCCTCGCCTTGCTCCAGGATGGCATTCCTCCAGCCCATCCTCGAAGGA

AGTTGAAGGGCTGAGACTTCTCCTTCATGGGGGTGGCGACGTTGGGGTCG
GTAGGCATGGAAGGAAAGCCGTTGGCGTTGGAGACTATGGAGGGTTTGAT
AAGCGCGAGAACCGCTGTCTGTGCCGCAGAGGCTGGATCGTCGGGTACTT
CAACATCGTAAGCGTTGTACGAGCGATCGACGAGCTTCCGGCGGCTGCGTA
TTTTCGTCGAGTTGTCGAAGATGGAGGGTTCGTAGAAGCCCATCGGGGCGA
GGATGACTTCGTCGTACGTTTTAAATAGGTACTTCTTCGTCGATTGGTTTGG
TGCGCGGAGGTGAAAATCGCAAGGCTGCACTGATATGTCCTTTTGTACCAT
AGTGCAAAATTTAATCTTCAGCTCCTCCGCTAGGAGGAAATCGTATCGTCTT
CGCAGGTTGATATCTTGGTAAGGGAAGTTGTCGTAAAGCATCATCTTGATGA
ATGTTTCCGTGACATCGTAGCCACCATACTTGAGGTTGACGCGCGAATCTTC
GACGCAGAGCCCATCCTCGACACATGCAATCGACGTGTTTTGGGCTCCAAC
ATCGACGACGCAGGCTTGCGTATAGCCAGCACCAAACGTCGCCGCCATACT
CTCTTGTATGAAACATATCTTGTTGAATTCGAACCAAGTCATACACGATTTC
AGGATCTGCTCCACGTACTTCTTGTCGTATAGATCAGGGATAACAAATACGC
AGCTGTAATTCTTCCACGCGCTGTTCCTGGTCAAACCAAGCTCGTGCCTGA
TGGCCTTGTCTAGGAGGGTCTCGAAGTCGTCGTATAGATGCTCGGCTGTCG
TGTAGTCATCCTCGTTTAACCAGCCATTTTGTATGGGCCACCATAGCTTGAA
CTTGGGGACTGAGTCGTCAGGTATACGCTGGGCGGCGTGGCCAATGAAGC
ACGATGCTGCCGAGTCGGGGTCCTCCAAAGACTTGACGTCTGTCCATTCGA
TCTGTAGGGGGTCGTTATGCAAAGGTATCTCTTCCGACTCTGTTCGTCTGTT
GTAGTTAACTACCAGTTCCTTGGAGTTGGGCAACACTTTTAGCTTCTTCGCT
CTCATGTCCACCTTCAAGTCATTGCATAGCTTCTGATACTTCTTAGACCACT
CCTCTCCATATTGTTGGTCTATGGTCTTGGCTTCGAACTGACGTCGGGGGAG
AGCTTCGTACATCTCGGACTCAGTCTGGGGGAATTTGGTCGCGATAGCCAT
GGGTATGGTTTTAGGCAAGGCATCGCTAGCAAAGCCTATTCTGAGGTTTTG
GCTGCCGGGATGTATTACGATAACCTTGGACGGGTCTAAACCCTCGGTGGC
AGCCGGGGCGCCATCTTCTGGTTGGATATCAGCAACGGGAAGGGGAACCT
CGGCTTGCCCGTTGCGCGCAAGGGCTCGGTCGCGATCTTTAGCACTTTGAA
CAAGTCGATCTCTATTCGCTTCTGCTTGTAGTCGAAGGGCTAATACTTGGTC
ATCGCGTTTCATGTAATCACTATGAAGTAGAAAGCATTAATTAGCCTTCTAG
TTCTACTCTATATGATATAGAGATACAAATATAGTGGGGGGTCGCGTACGTGT
AGTAGTTCTTCTGATTGATCGGTGTGACGTCGGGCCAGCTGGTCTGCTTCAT
GCTATTGTCCGTCCGTTCGAGTCCTTTAGTGATGAATACATGTTAGCTGAAC
AGTCATCACGGTACGGCGCGATTGAACTGACCTTCTCGAAGGAGAACCTTC
TCGCTGACTTTGCCGACCATGATCGCGGCTTAAGATAATTGGCGACGGTGT
TGATGATGTACTAGCGATAGCAGATATTACGATAGTTGCATTTAGATGGATTC
TCGTGCTAGCCTTTGTAGTTGGGCGATTCAATGTTGAGATGTGAAATTTGGC
TAGGGCTTGAGGAGATTACGGATGCATTTTTAGATATGGATTGTTATCAGGT

TTGTGATTGTGAGATTTTAAATTTTTTGTCTACGGCCAATTTTGTCTTCAAGC
TTTCGTCCTTCTTTCCCTGCGGACCATATCAAATCAGTCAGTCGCGGCTGCG
CCAGAATAACTCAATTAGCTACCTGAGTACCTCTCTGAGTACCTCTCATATC
CTTCGTCCTGGATCCCGCTAAGCCCTATTTGAACTAGGGGTAGCGGAGCTC
GGTAGCTCGATTATCCTATCTCACTTTAGGTTAGGACCATGCGTTTGGTAAG
TGGCAGAGTGGCCGGACACATTGACTCAATCCCATTCACGACAACAACTTC
AATATTTGGACTTTAAGATATAAGAACCAACATTTTACTTGCCTATGTAATTC
TTCTTGTGAATCATATCTTTATTTTTCCATTTACACTTGTGAAGAGAAGGTAG
GAGGCGTTCGGATAGCTTGACCAGGTTGTTCTAACGACTTAGGCAATCCTC
TATCTCCGATCTTCCATCTCATAGGACATCAAAAGCACAAAGCACAAAGAG
ATCCAGAAAACAAATCCACAGGATTCTACTTTTGAAATATTTATCAAGCAAA
GTACTAATATACAAACCTATGATCATAATAATTCTCCCTTTTTTTCAATTTATG
AAAAAAGCCTAAAAGTTTTGTTAGTCACCTGATTACATGACGCCTATGACG
TCGCTTTTCAATTTACCTTTTCCCTCCCCCCAGTCGAGTCGTTGAGTCGTAC
AAATTCATCCGACTATTAGAATAGGCCTAGCAGAGACGACACGTAGTAACA
ACTTACGAATATCTTATTTTCCCCAAGTCTTGCTACAAAGACTTAGCTAAAG
AGAGTTGTTTCAAAATAATTACATATCATAATTCTCAGCTGCCTTTACGTCAT
TCTATTTATCCTATTCAGCTGTAGGTCAAGGGTATATTCACATATTCACGATG
GCGCCTATTCCCGTCTATACCGACAGTCCTATTGCTGCTTCGAAGCCATCCG
GGGTAACTCCCCAGACTGCCGAAGCGCCATCTGCTGGAAACCCAGCAAAA
CCAGCGACGAGCACTACATCCCCTACTCCAACTAGCAATACTTCATACCCAC
CCGCACAACCCGGAGCTACGCCTTCACTTCCGGCTCCAACGGCGGCCATAT
CTCAGACATATGTCCCTCAGAATAGTCCTCCAGCGCCACAACCTGGTGCCT
TCCCTACCCCGCCAGAAGCCACCAAGACCAGGATTCCACCACCACCGAAG
GCCGGCGAGAAGTATCAACCACCCCAGCAGCCGTCGACAACGCAGCCTAT
GAGCATTCCGTATCCTCCGCAAATGTCTATCCCCCCTCCTAGCGCACCACAC
GCATCTCAGCAGCGTGGTACGTTCACGGCCTCCGTACCTACTTCGGTATATA
GTGGCCAGGGTCCCACGGCTATAGGTGGCCCACCCACCCAAAGCTTACAG
CATCCTCCGGGATATCACCAAAACGTAAACGCTTCAGAGTTAGACATGCAT
CAAAGGTCAGCTATTCAGCAGAGCGAGTTAGAAGGTCAGAGAGAGGATTC
TGGTGGGGTATGGGATTCGGCGAAGAAATGGGCCCAGCAGACGGGCGAAA
AACTGGCCGCAGCTGAGAATGAAGTATGGAAGAAGCTTAACAAGGACTAA
CGGTGCCACCACGGTCTCCGAGACCTTTCGTAGTCGTCCACATTCCGGATC
AATCCGTCGCGATCTAGGTCCTAGCGTAGGAATTGCCCACCGAGATCCAAG
AAAGACAAATAGTCCTGCGTCGGAGTTTAACAATACGAATAGTCTCTCATG
CTGAACTGGATATTTTGGGATTACGAAGCGTTGCGTTTGGACCGGTTGATGT
CAGCTATACTCGGGTCTTTTGACCTTTTAGCAAGCAGATCTAATACAAACAC
GGCGATAGCTGTTTGAACAACCTGATCGCAAAGGGAAACGAAAATTTTTGC

TTATAGAAGTTACGAGGCTTGATTCATGCTAACCCACGCAGCCTAGGCTTTG
TGGGCTCTGTTCGGAGGACGCGGTACCTACTCGTAAGCGCGGCTCGGACTG
GCTTTCTTTTCCTTCTCTCTTCGCTCTTTCGCCATGAACTTCCGTTTTGTCAT
AGGCGACCTTTTTTACGCATGATATGATCTTCCAATGGGTCTACGCTAGGCA
TCTGGGGAAGTGATCTCGTCATCGATAGTCCGCGATCTTCATGACGAGAGG
TAGGAGTTGCTAAATTTAATAGCAACGGCCTGCGGGTAAAGGCGAGGCATT
GAATAATTTCCCAAAGGAGCCGACAATGTTATGCAGTGGACCGGGCCTACC
CCGGTAGGCTAGCAAGAAAGTAGCAGCAAGCCAGCCTCGGAGTGGGGTGC
ACAAGCGGCCCCCATAGAATGGAACGATTGATAGAACATGTGGAAAGACTA
GAAGCCCATTCCTGTCCACTTTACACGACACGATCTGACAGAAACTAATTA
AAATGCTGCTCTTAAGATGTGACATAGCAGCATGTACGTATGTACCGTCCGA
GGGTATTCGAGGTCAGTTTGTCTCGGTATTGATTCCCATTTCGATAATGCATC
TGATATCAATATGGATCAGCTACCTCACACCTAACCTTTTTCGAGTCCGTCT
GATATGCATAAGTGTTTTGTGCAAGTTTTTCTATGAACCAAATAGGACTATA
CGCGTGTATATACGTATACCTACTTGTAATAGGCAGAATTATACCAGCCAAG
ATACGTGGCGCTGGATGGCACTGGTTGGAGATGGGAAAGAAAGGAAGCAG
GTCGAGCCGTACCGTGCAAGCCGCGTTCCATAAGCTGATCCTTCCAAGACA
TGGAGTTAAGTCAGTTGATACATACTTTTACCGCGTATCGACCTATCTCCATA
CATCTGAGAATCTCATATCGATCAGCGCTTGGCCAACGCTTACGACAGCGG
AGCGTAATGCAGGTACATCTCCAACTAGTAGTATCATGATCTAGCGCTCAGG
CCGCGGCCACAGCCGAGATAGCTAACGACATACCGCGAGGCGCAGGTGAT
TGCTTAGATGCAGGAGATCCAGCTTGGAGGACCGCTGCAGCACGCGGCGT
TTATGCTGAAGTGTGCTCAGGCTCAGGCACCCACAGCGCCCACAACGCCC
ACAACACCCACACATGTAAGCACTATAAAAAATGTACGTCGAGTGGTAACG
CGGGTTGGTAAGGGCTGTACGGTTGGTACGGAGAGTAAGGGAGGGTCCCC
CATAGGGTCCATACGATAATACGACTGACAAATAGATAGATCCATGATACAA
GGGCAATGTCGCCGAGTATATCGATAAGAATCTTGGAAAGAGAAGAGAAA
GCCCTGTTTACCCGATCAGGGATCGTCAGATCCTCTCGACCATACCCCCCGC
GCCATAATATGACGATTGCTATTACCTACATAGTACGATTCGGTAAAGCCGTA
GGGCCAAACATATTCCGCAAAGACGGATACGCCAAGATGACGCGACTCGG
TATTATGTCGACTAGCTCGAGTGTCAGTGTTCCGTACGAGATTTCTCGTCTG
GGGCTTTGAAAACTAGGAACTAGGGCTGCTAAGCCAGGTACTAGGTTTGTA
ATGAAAAGTTCAACGTTCACTTTAATAAGTAATAACCACGAGTCCATCTCA
AACCCATCTACCTAACTGTCATAGGTAATAATGTGGCTACCTATCTTAAACG
GAATACGACCTTAACGTCTACCTAATTACCTAGTAATGCCTGTAAGGTCTAC
CTAGGTCAAATGCCCACGTTCAGAGCTAACCCATGGAGGTCAGTCGTCCAA
GGACCTAGGTGACAACCCGTAGGGCTTATCTCTATCGGGAACAAATGTCAG
GTCCCGTTAGTTAGACCCCGTCAGATCCAATGACCACCTCGAAAAATCGGA

TGTTCTATTAAAACTTGGAACTAGTGGGGCGCTCTACCTCGGCCATTTATTT
GTCTCTGACAAGGCAAAACAAAGTTGTTGAACGTTGAGATGAAGGGTTCA
CAAGAAAAACGCCGTACCAAAGAATTTGATAATCTGTATACTCTGCGCTTG
AGCTTTGATAGAACGATATATTTCTCGAATGGTATTGTCGAAACAAGTGAGG
GGTTGAAGGGAACGTTGGGATAATTGCGAAGGAGGAATGGAGATGACAAG
ATGGACCCCGTTGGCCGGGCGTTCGACAGGGCGTTCGACATGGCGTGGAA
CAGGGTCTAGAGGCAGCCCGAGTGATTGCCGTCTTTGGGTCTTTTGGGGGA
GGGGCGAGAAGTTGTTGATGGGGGGTTCTTTTGGAGCACTGCACCTCAGC
ACTGGACTGACTGGAGCACGCACACCTTCTAACCAGTCCTTAGGTAATCTT
GGAGAAATAAAAAGGAAAATATCCATGATGTACATAAGATTATGTACGTAGT
ATGTACTACCAGTAAGTACCTTGTATAAAATTCTCAGTACCTTTTTCTAAACG
TACTCTACCCACCCATATAAGACAGCCCCGGTAAATTGTTTCTAAAAATGAA
CCAGGTATGTGATATGTACCTGGTATATATTATAACAAGACTCGAGATTGATA
TAGACTGACTGAATTTGTCGCGGGGGGGAAATAAAACCTTTTTCTCGAAAC
GGGACGGCTACATTTAAGATCGCGCACTGCTACCGTAAGCTAATGTTTCCA
AAATTTTCTGCCGTAAGCCATGTGCGCAACCTCACCTCATCAAGATGGTGG
AAGAATGGTAGTAGAAGGGTAGAAGGATAGATGTGGATTGGGTTTGAAGA
GGAATGCGGGATGCGGTGTGAAGGCACAGCGAAGTACTACGCGGTACAAA
GCTAGTGGATCTGGACTGCTAGAGTTGCTAAGACTGCACCTTTCTTACTGC
GGACTGGAACGACTGGACCTCTGGACCTTTTACCTTTGCCTTTTTGACTTTC
GGGACTGAGGCAAGTGGGGCATACAAGGATCATGCCTTCTGTTACAGATCC
TGAGACGCGTCAGGGATTAAACCCCCTGGCCCTTACACCCCCTGCCTGCAT
ACTATTTTCCGTAGGACCAATGAGAAGAGTGCCCGAGGCAACTGAAATTCA
ACATCAAACGCGCTCCCGCTGACGAACGAGACGACAACTCCTAATCTTGGT
CGGAATTGTCTAAGTTCCAGAGCCTCTAGCCTTTTTTTACTATACACTATCCC
ACCACTCATCGTCCTCATCGTCGATTAGATGAGCCAACAACAATACCGAAC
GCAGCCGTAACTCTGCATTCTGCGCTGCGCTCTCCATTTTCTCTGCACTTAC
TCTGTGCTTTGCTACCACTCTGCAGTTTGTTTATTCTCACGAACCAGAGCCT
GCCGATTCACTTTTCATTATCTCATTATCTCATTATACCCTCCCTCAAATCGCG
CCAGGGTTTTATCTGAAGCCCCGGCTACACCATTCCACGATGTTAGAGGTC
CAAGTATCGACCAACCCACCATCATCTCCTCCACTTTACCACTCGACCACGT
CGCTCCGCCTCCCCATCCATTAGTCCTCGACGCCGTCATTGGCTACTATCGC
CATTCCGGAGCCATCCATCTCGTGGCTTCGTCAGTCCGTTCTTTGTACCTAG
GTAGTTCTTGGCCATCCGGTCTTTCAGATGAGTTCACTTACGTTTACCTATC
AAAGCCCCTCATCTCACATCTCAGCTTCCAACCATTCAACCATCCAAGCAA
CGCGCCAGTCGGCTAAACTGCTGAGCTTTTTTAGTCATTATGGTTGTTGGAT
CTCAATGGATGTCAGGCTTTAAGTTTTCGTATGCACCTTTCCTGGCTTCTTA
AACAGATGTTGCCTGCTTTGGGCTTCATTCGCCAACCTGATTGGCAATGTC

GCGCATACTACCCAACGCAAGACATCGAAAAATAGATATCAGCTTTTTGCG
AGGTGTGGTCTTCGAACCAGACTAACCGTATTCTTCTCACCGGGCAGAGAG
TAATGTTAGCAGTAGAGATTTCCTCATTTAAACAATTCCACCTCGACCGATT
ACATACTACACCGCAAAGGATGTGTTTCGCAGCAAGATGTATTCGGCCGGC
TGGTTCCACCGGCCCTGGTCTCTACTCCACGAGACCCTAGAGTGTGTTTCC
AAAGAACAAGACCCCTGGTTGGTAATAAGAGATTGTACTTTTCGGAAACCT
GTCTTTCTCCAGATCGACGTATCCGGTCCACGAAAGTGTCATGGTCGTGTC
ATACGACCCTACGGCAGCAGAATCCTAACTATATCCTATATACCCTATTCTGT
ATGGCTCGTGGACCTACGCAGTATGTACGCGCAAAAAGCCAGCAATCCTCC
GAGACCTATTTTCGCGGCTCTCGGTAGTTCGCGAATCGTCCCGTCGTCCCA
GCCTGCTTCTCGACCCGCATGCGCCGTGCCTACTATGTAGGTACGTACGTAC
GAGAGAGTCTGATGAATACCTATTTAGCAACTTGCCACATGGCCCGGACAC
AGATGATGAGACCTGTCGCTTCCCATGAGGCTCAACGGGAGCGGGAAGGG
CTAAAAATAAGTGGATGTCATGTTTATGGTGTTTATGTAGAGCTTCATTTAAC
TTCCAATTGCCGAAGACATATGTTTTATTTGATCTAATGAAATTCTCATCATA
TTTCTCATCTTTCATTTTTCGATATCAAATTACGTCCGTCCTGTTATATCAAGA
CCCCTGCTCGACTATTCCAAAAGAGTTGGAGATTTCATGGATATTTTCTGAC
TGGGGTTGTCCCTAGTCGTCAGTCTAATCACTTTCTGGCTACCTCTTACATG
CTAGGTACCCGAGACCCATCTGCCCTCTCCGCCGCTCCCCCATACGCTCACC
ACTCTCCCCTGCCCTGGAGGTGCCCTGAAGGCTTGAGTATTCCTCGCAACA
CATCATCGACGAACAGATGTAACCCTCCGTACCCTGTATTGCAAATCCTTGC
AGAGCCCTGCCCGCCGGTCCCTGGTCTGCCCTGTCTTGTCTGTCTCTTTCC
AGCAAGCCAAGCAGAACCTCGCACACCACCTAGTCCCTCCTAGTCCCTCCT
CAGTCTCTCCTGTTGAGTTCACAGACGTGGCCAGGTTTGTGTCAAGAACTC
CGGCTTACCTCCTACCGATGTTCCGAGCCTGGTCCAGCCTGACCGTGTTGG
ATTATAATCGGACTGTCCATGGGTCCCTAGGTAGTGCCTGTCACTCGAGAGG
ACCTCTTTATATCTCTCGGTCTTCTGCACCGCCCCTCCATTGACCTGCCCTTT
TACTCCTCTTCTCGCCTCGCCGCTCTCCCTCACACCATTATACTAAGGCATTC
ATACTACTAGTCACTCCTTTTTTCCAAGCCCTTAACAAACTCTATTATATTGC
CACTCTGGTCTATAACACTGGGAGCTGGCTCTCTTCCTTCCAACCTGGACA
CCTCCGTCTATGTGAAGCAAGCATCAACTAGCTTACCTAACGAGTTGTTCTG
TCCCCAGTCAACTCATTACATTCATTAATTAAGACCTTACGCACTCGACCTT
GAGCTCAACGCTGCTCGCCAACAACATATACACTCACTTCCTCCCACCCTC
GACGTGGGACACCCACACTCTACATTCCTTCCCAGCTTAAAATCTCACCAA
CGTCATCGGAGATTATAGTCCATACTACTATTGACCATGTATGTTATGAAAGG
TCACCTACCTGCCGTGACGCCCTCATGCATGGGCCATATAGCTATCCCTTGT
TGATATCATTATATCGACCAGCATCCTCATGTCGTCTCGCTCATTAACTAACC
TTCATGCCTCATGAATAGTATACAGTCTATAATAGATCAAATCACATTCCTTT

CAACAGTCAGATAATCATGGACTCAAGTCAAGCGCAACTGTATGAAAAATT
CGGACCGGCAATATATGACAGAAGCTCACAGTACGACAGGAGGCAGCAAA
TCCAGGCGCCTACTTTTACGCTTACCTCGAACGACAAGTTCATCGGAGAGG
TTCCGTCACCGCATCCGCCTCCACTGAACCCGATGAGCAGGAACGAAATGC
CAGTCAAGACAGAGGCTCATGTGGCACCTACTGGGGTATGGGCGACGCCA
GCACAGGAAACGCGACCAAGGCCAGCTACTATTCACGAAGGCTTCTCATTC
TGCGCTGGAGATGAATTTGGAACGATGCCCTCGTGGGCAACGCCGTCTGG
GTTAACTATTCACACACCAAGCGATACCATGTCACGACCCGTCTCGATGCA
CCAAGACTTTTACCCGGTCACGACGATGGATAGCAGTATGTTTTATTTAAGA
AAGCGTAAGATAACCGCTAACTATGAAATAGGCCCATGGGGACCTAAGCCA
TGTGACGATCATCTCGAGGTTCCTAACCTAGATATGGATATGAGCATTGGTC
AGGCATATACGACAGACGAGGCAATTCCGATCATAGACCTGAGGTACTCTG
GTCCTCAGATGGACCACGAATCGATGGGCTTTGAATCTGGTTTAAACCCTA
GGCGGATGTCTGGATCGTCTTTCACCGTATCAACAACGGGCAATCTCTCCG
AGATGCCTTCATACGAGGATTTCTCGGCCGCTCTATCCGAAGCCCCATCTTT
TACCTCGGACTACCCGCCACCCTCGAACCGCAACTCGCTTATGTCTTCAAC
GCAACTATCTCCCGTGGCCTCCCCTCGCATGACCCCCCAGAGCCGTTCAGA
CCTAGTCCGGACAGCAAGCCGTGGACGTGGTGCTTCGCCGTCTCCTCGACC
AGGAATGAGATCTGCTCCCTACAATGTTGACAGCAGTGCAAGGAATAAGA
GGTGGTCTACTGGGTCTTATGGCACCAACGCTAACCGCCGACCGTCACCTT
TCGTCTACCACCACACGCATGATGCTTTTGGTCCCCGGATGTCTTCAAGGC
ACTCATCTCCTACGATGCCGAACCACCCGCTTCCGTTGAACTTCGGGAATC
TTCAGGCCGTCCAACAACACCCATTCCTTGTTTCGCATCCGCCAGCTTTCC
GAAATAGTATGCTTCTTCCAACGCAATTACCGTCTCAATTGCCTTCGCAGCA
TTTCCACCCTGAGGCTCACCACCATTTCGAAAACCCCCCACCTCTGCTGTC
TCACGGACTCTTTAGGATGCTGCAAAGCAACGCTGACCCACATTCCCTGCA
TGGGCATTACACGGACTTATCCGATCCACCAGATCTATATGCTTCCCTGCAG
GAAGAACAGGTACCGCCACCGCCTGAGGACATGAATCCTTCGGATCCGGAT
CTAGTCCCTCACGAGCAAGAGTTAAGATTTGAAGGCGACTTGTATACTCCG
AAGTGGGTTCGCGGTCATGGAAACAAGAGAGAAGGATGGTGCGGTATTTG
CAAGCCTGGTCGGTGGCTAGTGTTGAAGAATTCCGCTTTCTGGTACGACAA
GAGTTTCACACACGGTATCAGTGCGGCAACCGGCAATCCCTTCCAAGAACC
TCAAGAGACCAGGCGGATGGACGGTAACCCCGATGTCTGGGAAGGTCTTT
GCGGAAGCTGCAACGATTGGATCGCCTTGGTTAGCAGCAAGAAAAAGGGT
ACAACTTGGTTTAGACATGCGTACAAGGTAAGAACCAAAGCGACATTCATC
ATTCTAGCTATATACTAATCCGTCACAGTGCCATACACACCCTAAGATCAAG
GATGCGCCTAAGAGGCGACGCGAAAGTGGAAATACGAGAATTGTTACAGC
TTCTCAAATTTCAAAGCCCAAGATCGAAGCTCAACAACCTTCAACTCCTCA

ACCAAAGACTACGCCTGTTTCGCTAGCGACAACACCAGTTCCTATTCCTATC
CCGGTACCTGGCTCTCTGCCGCACCAGCAACACCCGCAACACATGGTCCA
GCCACAACAACCTACTCCCAAAAAGTCATTCCCTCCACTCGACGGACTAAC
TAACATGATTTAAATCATGTCAATTTACCCGAGTCATATCTTTTCAGACCAAT
CGTTTCTTATTATGTCTGGATAGAAATCTTGGACGAGCAATGAAACATTTCC
TTGGATTCCCCCCGTCATTACGAAGACGGACCTCTATAGACGGACAGCCGG
GCCTTACATCCGGCGCCACCAATCTCACTCATCCCCTCTCTACGGCCAGTCA
CATCGGCATTGGTCCCAACTTTTCATTATTATCTACATCACACTATACCCCAA
CATGAACAGAATCTTCTCCAGCTGAGAATGCTATGACGAATCGAGCAACAA
ACTGGTTGATATTTTTTATGAGGAAATGGGATAGACCGGAGGGTATTTATCG
CGGCGGTTACGACAAAACAAAAAAGATTGCTTCACTTCGTTGTGTGCTATT
TTACTCACCCGTGGTGGCGTGTTCCAACCCGCCTGCCCAGGTGTTACCTTA
CGCACCAGCCGTTGATCTTATAACTTTTTGCGTCTGGCTTTTCAATCCTCTTT
CGATGTCCATATGCGAAACATACAAACTTTTTTTTTTTTTACTACAGCCAA
CTTACTTAGCTTTTTTCTTTTTCGTTCTTTTTTTTACTCTCTAATTAATGTAGA
TAGCTCAAATCAAGGTGATACCCTCGGAAAATATTGGGGGTGATACGGGGG
CTGGGGATCGGTAAGAGGAGGATAGGGACGCGGGTTTTTTCTCACTCGGG
AACGGGGTTGCGCGGGTGGTGGCTCAGTGGTTGGTTCTCTCAGTCTATATA
GCCCTGCTGGGCAGCGGTAGAAATATTTGGGAAAGGGAGGGTGGAAGGGG
GACACGAAACATTGCGATATCCGTAGACTTAGTTTAGATGATGAACGAATG
ATTTCTTAGTTCTGAACAACACAAAACAAATCAGCGATATTTGTTTTTTTTT
CTTCTTCTTCCCTTCATGGATTTCTAGAATTGTGATTTGTAATTTTGTGATTTT
GTGTATGTGTTTCTGTTCGACTCGGCAGACAGGGCTAGAAACAAGTCAACG
TCAACGCCACCAACTTTAGTCAAACAATCCCGTGGATCAGGGGGAACGGC
ATCCTTTCTTCGAACCGCCCGGGCGGACGAAATTCCACGTAAGCCGTCGGA
AACGTTCAGAGCCTGCTTTTTGGAGCTAGGTAGGACCCAGGTAAAGGTAG
ATAGGTATGTTAGATGATATGCTGGCCATCGGATTATCAACCAATGCGAGATA
CAGACGTAGATCGGTCCCTCGAGGGCGTACATACAACTCAGCGACATCAAA
TCTTGGATACGCCCTGGAGATCATATGTGCTCGTGGCCTATGAACCGGGAAC
TACCCAGGAGCTCCCCCACCGGAGAGCCGCCAGCGGAAAGGTATCCTTAC
GCGGTACAGGAACCGACAGGTGGTGTGCTGGAACTTGGAAGGGGAAAGG
GCTCGGGCGTTGGATAAGCCACTCTTAACCGCGGGTCTCTGGTTGCTCTGC
TACGGGTTTCGTGGCGACACAGCCGTAAAACTTAAGGGTCCCGATCCCTGG
CAATAGGATCTGATCCGACAGACCCAGAGCCATGGCTAGGTACCTAGTTGG
ACGGTATTAGCCAATGAATTATTTTTGAGGCATTGGTTTCTTGCTTAAGGCG
CGAGGATTGGAGTTCATTCGTCGCTGGCCTGCATTATTCGTTTTCACAGATC
GCCTCACTAAAAAAGCCATCTCGTCATTAGAAAAGAAAGCGAAAAAAAAGA
AGCGAAACGATAGAATGACGCAAAATAAACTGACAAAAAAATAAAAACGG

AAAAAAGAACATACAACACCACGGATTCGCCGGTCGTCACCGACCCGACT
ACTGATGCGGCTGTAGCTAGCTTATCTATGGGAGAGCGGACGGGATCCCGA
GTTCTCTAGCTCATATGGTCGTATGTGCTAGACTATGGGACTAACGTGCTATA
TATGTGCACTGCAACATTTTTTGCCCCACCTAAATTCCCTTCCCGCAGGACA
TTTCGCAGCACCATTTCAACGGACACAAGTTTGTCGGGTAGATTACCGAGA
TTCGACGAAGCAAATCGAAAAGCTTTCCGCACAATTCGCCAAAAACGAGA
GAAACATCGGATCGCATATTCTCATAATTTGTGCTACCGGAATATAACATGTC
GCAAAATTTTCATCACCTATATTTCTTTAGGTGCCATGCCCGCGTATTCGCAT
CCGCAACCTCCAGCTCATCAACGCCCTAAATCTCCAGCGCTCTCCTCCCTAT
TCCAACACCCCGCCTACTGGTGTCCCGAGATCTGCTTCGGCTGATACAACT
CCTCCAAATACCTCTCCTCCTCCTCCGTCAACGTCTTCCCACCCGCCTCAAT
CGCCTCATCGATCCTCTTCGTGCTCGAGAACCCAATAATCGGGCTGGCAAC
CCTCTGATTAATCCACGCGAGCGCAACATGGCTCATCGGCCATCCCTTCTTC
TGCGCCAACTCCTGCACCCTCGCAATAATACTTAAATCCGGCTCCACGGTG
CCGTGCGAGCCGGGCGAAGTGTCCTTCTCCATCTTACTCCTCTCGGTGGTC
CCGAACTGCGCGGGCGGGCGCGCGAGGTGTCCGCGACACAGCGGCGCCC
ACGGGATAAGCCCTACGCCTGTATCCCTGCAAAAGCGGTTCATCTCGCGTT
CTTCCTCGCGGTAGAGGAGGTTGTAGTGGTTCTGCATGGAGATGAACTTCG
TCCACCCGTTCTTCTCCGCCGCGAACTGGTACCGCGCGAAATTCACCGCCC
ACATGCTGCTCGCGCCGATATACCTAACCTTCCCTAACTGCACCAAGTCGTG
CAACGCTTTCATGGTCTCTTCTATGGGCGTGTGAGGGTCGTATCGGTGGATT
TGCAACACGTCGACGTAGTCGAGGTCTAGGCGCTTGAGGGACGCTTCCAC
GGCGTTGAAGATGGCGAGGCGGGACAGCCCGTACTGGTTGACGTAGTCTT
TGGACTTGTCGACGGCGGCGCCGTGCATGAAGTGCCGGACTTCGGGGGTC
TCGCCGACGGCCCAGAAGCACTTCGTCATGATGATCACTTTCTCCCGCGGG
ATGTTGTATTTTTTGATCGCTTTGCCGACGATCACTTCGCTTGCTCCTTGCGA
GTAGACGTTGGCTGTGTCCCACGTGTTTAGGCCTTTGTCGTAGGCGTACTT
GAGCAGAGGCAGAGCCTGGGGGCGATGTTAGTGAATGAGTCTAAGGTGAG
TCGTCTGAGGTGAGTTTCCGGAGAGTGTACCTCTTCTTCGTCAACGGCCCA
TGGCTGCGCCTTAGTATCGCCAAAACTCATACACCCGAATATCGGGACTGA
GATGCTCAGACCACTGTTACCAAGTCTACGATACTCGACCTTGGTCGCATCT
AGCGACTCCTGGAGAGACTTGGGGAGACTGGGAGCCATGGCGATTCTAAC
TAGCGATTGAGGTTGAAATTACGTTTCTACTAACAAAGAAGTCTCAATTGA
GTGAAGTTGAGACGAGCCCGGAATGTTACAGTTTATGTACCTGTCAGTAAG
TATAGCAGCTATAGCAGGTACCTTTAGGACTATCCACAATGTGTCTCAGGTT
ACACCCGTTCTGGCTGAACTCTATTCCCCGCAGAAGCCGGGAGATATTTCC
ATTAGCGTTTCCTTAAATCCCTTCCCTAAACTCCAAACTCCAGCGGGGTAGC
AGTTGCTACTTAACATCGAAGTTATCATCACTGTTATGGCCTATGGCCTATGG

CCGATGGCTCGGGTTGCACTAGACGGTCCGCTAGCGTGATATTGCGTTTATG
CTAGCGGATATCGTCACCAATGATGTCTTGTATGCACGATATCGTCGTGTCG
ACGTAGAAGACTATTGGACAATGCTTCGATCACGTCATCATGCGTTATTAGT
AATAAGGTAGGAGATAACTATACACATTTTTCTTTGTAAGCTACCCATCGAA

>Mosaic_sequence_19

GCATTTTTCTTCAATAAGGCTGTGATTGGGTGCGTGATCTTGGAGAATCCCT
TAATGAAGTGTCGATAGAAATTTGCAAAGCCTAGGAAGACTCGACAGTCAT
GGACATTCTTGGGAGTAGGCCAGTCAGCAACGCTAGCCACTTTCTCAGAGT
CCATCTGAGTGCCGTCTTTGGATACAATAAAGCCAAGGTAGCTGACTTTATC
CTTGAAGAATTCCACTTTCTTGGCTTTGCACCACAGGTGAGCTTGAGCAAG
TCTACGCAAGATCTCTCGGACATCTTTGATATGATCTTCTCTTGTCTTAGAGA
AGATGAGGATGTCATCCATGTAAACAATTACACAGACATCCAGAAGGTCTC
GAAGAACATCATTGATGAAATGTTGGAATGATGCTGGAGCATTGGTGAGAC
CAAAAGGCATGACTTGATACTCGAATAGACCATATCGAGTTCTGAAGGCTG
TCTTCCATTCGTCGCCTTCTTTGATCCTAAGCTGATGGTATGCTTGGGCTACA
TCAAGCTTGGAGTAGAATTTGGCAGCTTGGAGCCGATCAAGATTCCTCGGA
TGAGAGGCAAGGGATACCGGTTCTTCTTGGTGATTTTGTTGAGCTGTCTATA
GTCAACACAGAGTCGGAGAGAAACGTCAGATTTCTTGACAAATAGGATTG
GAGATCCAGCAGAGCTTTTGCTGGGCTTGATAAATCCATTTGCCAGATTCTT
CTTGAGATAGTCGTCTAATGCTTCTAACTCGACTGCTGATAGTGGATAGATT
GGCCAAATTTTGGTTGAGTTCCCTCTTCCAAATCAATGGCGTGATCATATTC
AGAATGTTCAGCTAGAGTCTCAGCATTAGACTCTGAGAAGACCTCAATGAA
GTCATGGAATTCCTCAGGACCTTTTTCTTGAGATCTGCAATCTCTTCTGGAG
AGAGGGTCTTCTGAGTAGAATCTTGAATAGGTGGGCCTCGGGAGACTTCTC
CTACACAGACAGAGACAATGGTTTGGGGGGTCTCTTTGGCTGTGAGGATG
AACATGGAGGACACCATGGAGTACTGAATCCTTCTTGAGTCGCTGATATTC
AATGGCTCCTACCATTCGAATGTCAGGTTGTGAAGAGTGATGTGAGCGACT
GAGAGGAACAGATCGAGAACTAGATTGCCTAGAATGGGGATTTTGCTGATT
ATCCCTATAGGAGCTAGGCACATGACTGGACTTGACTAGATGACCGGATGC
GGTCTGACTGGAGACATGACTTGTCTTCCTGGAGCTTGATTTACTAGACTG
TTCTGATTTGACCACCTTGGTCCCAGAACTCTGGATAGTTCTTGAAGAACTT
GCGTGATTGTGAGACTCTTCAGAGTACTCTTCCTTGGTTGCCCTAAGAGGC
ACACGAGAGCGCATGGAAGCGCGAGCAGGATGACTCACAGCCTGATGATA
TGACTGGTCTTGTCTAGGTTCTGCTGAATAGAACCTTGGAACATCGCGACG
CACAGCTGGAGGTCTTAACTGATCTTGTCGGCGGACGAGGTGTTTTGGAGG
TGGTACTGAGTAGTAGCTGTTGTGCTTGACCCTTTTTGAATCTCTGCTGGAA
GAAGGAGATTCACGGGCAGTCCAATAAGGAGTGCTTGTGAGTTGGTTTTGC

TCTTTCCAGTTTGATCCTTTAGGCCATAAGCAGTGGGCTTCACAATACCGAT
GAGGAAACAAGACTGAGGGTTCGTCACCAAAGTCAAGTTGTACTTTGTGA
CGTTCAATCCATGGGAGACCTAGGATTAGGTCTAAGCCTCGGAGTTTTGCA
ACATCCAGGCACTTTTCTCGAGATGATCTCCCATCATGATTGAGAGACCAG
ACACATTATGAGTGATTGGGTTGAGATATCCATCTCGGTGACCAGCCACGCC
TGTCATATAGAGAGGTTCTTCGCGTGCTGTCAGAGGCGCTTGAAGTTTGTT
GACTATGTCGTAGTCAATAAGACTGAACTCTGCACCAGAGTCAATCAGAGC
ATCGACAGTAACGGGGTGTCGAGCTTCAGGGTGATCAAGGTGAACTTGAA
CTTTAAGCAGGGTTTGGGGTTGTCAGGGCGGATGATCTTTTCAACCTTGTA
CATTAGAATCCGAGCTGTATCTGAGAACTTGTTTTTATATTTTTCAAATTTTG
TAGTGTTTTGGGTTTCTTTGTTTTCTTTGAAAACGTACCAGAAGGAGACTTA
ATACAGTTAATACCCCAGGAGTCCCAAGCGGGGGCTGGATTTATGCTTGGG
ACTTGGAGTTTCCCCAGCCATCATTGTCTTTCTCCTCATGAATGACATTGAG
GCGGGCCTTATTGGTGGGCTGGCGAGAAGCTTTCTTCTTGTCAGCTTCCTT
CTTCTTGGGACAAGCATTTGCCCAATGTTCAGGAGAGCCACAATAGTCGCA
GAGGTTGTTCTCACGGCGACGCTTCTTTTCAGCATCAGAGAGCTTGAAATT
CCGGATGGTGTCAACCTCCATAGGCTCATAGAAGCGAGGTTGACTTGGTGG
GCCTGGAACTGGGCGATGAGCCTGTCTGAAGGTAGTGTTCTGAATCTGCTT
GACAGGCTGGCGAGATTGGAAAGGCTATCAGGAGTAGTCTTTGACCTTTGT
CTCTGCCATAGGAGTGCGTCCCAGGAGTTGGCTAGCTGAGCCAGCTCATCA
AGACTTGAGGCTTTGTTCTGGGGAGAGGATGTGATCCTTGATATTGTCCTCA
GACCATCAAAGAAGAAGTTGCGAAGAGCCTTATCACGTAATCAGTGAGGT
ATGCTGCCAATGCCTGAACTCTGATTCATAGGCAATGGCTGAACCTGTCTGC
TTGAGGTTCTTGACTTTGTCATGGGCTTGGATTGCAAAGTTGGTTTGACCA
AAAGCTTTCTGGAACTCTTGACGGAAGAGGTCCAAGTCTTCATCCAAGGA
ACAACCCTTTCTGGGTCATTGAGGTGGAAGCAAAGGGAGCGATCATCTGTA
GGCATCTCCCTTGATCCTGAAATGGCATACCAAATTTTGGCTTCTTCTGAGG
TATAGGTGAGTGCTGGGAAGCGGAATACTGCTTCACACTTGACAAGAACTC
CAAGACATTCTGCTCTTACTCTCAAAGTTGTCAGGTCCTTCATCTTAGGTTC
CCTGGTGTTTGGAGCATGCATGAGAAGGTTGTCTCGAACAGGCTCTTGGCT
TTGAGGCTGTTGGAGAGATAGCTCTTGAAGCAGATGTTGGTTTTGGACTGC
AAGCTCTTCATTCTGTTGGTGAAGAGTTTGCTGGCTTTGTTGCAAAGAGCA
ACTTGTAGAGATAGCTCGTGCATTTGAGCCAAGAAGTGACCAGAGGTTTCT
TCCTCAATAGGAAGCAGGGTCTGGTTTGACATGTCGATTAGAGGAGTGTAG
AGGGTGACAAACAAGGACTTGTTTGGATCTTCCAGATACTGTCAAGAACG
GGACAACCGGGGATACCGAGAGTCCCTAGAGCACCGATAGAACAAGACAA
GGAGTCTCTGGTTTGTCAAGAGAGCAAGATGCAAGACAAGATAGCACAAA
GGCTAGACTAGATCAAGAAGTAGTCAGAGGATTGACTACAAGAAAGAATG

GATTATGAGAATGACTTGTGAAGAATGACTTGCGATGAATGACTTGCGATCT
GGAAGATGGGGATCCCCTCCATCTATCCAGCTCCTATTTATAGACAGAACTC
CAGGCTAGTCGTCCAATGTCATTGGCAATGCCTCCACCAATCCTTTCTTCAG
ATGAGAAGGCTCCTCGGCCACATATTGCCAATCTTGCACATATTGCCAATAA
GCTGGGAAGGCAAGTGGGGTGATGAGGGAAGGCCGGTGGTGTAGAGTGG
GGGAAGGAGGAAGGCTTGCTAAATGAACCTGGTGTAACACCAGCCAATAT
AATACAACATCGCAGAGATCAGGGCTCACTATTGGCAATGATATATATCGCC
TAATATTGCTGTCTTTGCTGGAGAGTAGTGTTAATTGAAGCTTTTCATTTGC
AATACAGAACTCCGGAGCACTATATCCTCGGATTCTATGGTCTATACCAAAT
GCGTTCTCAATTCCTCGATACCCTTACTACAGTCTTACGAACATGATATCGG
CTTTAGTCCATGAGTCTAACCTGATAAGTCCAGCCGGTGAATTACTATAATA
GACGGAAAGACAGAAGTCTTGCTAATACTCTCCATGAGGCTCGAGCTGATG
ACTATACTTAACGGGCCCCCAACTATGGACATCCTACCAAGATAGCACCCTC
CTAGGGTGAAGATCGCCATGAGAGGGGCGTGACAGGGGAATTTGCATGGA
GAGACACCTCGGTGGAGTTGCGAAGCAAAATCTCCTCAGTCTTATCCTTTC
GATAAGGGAGTATTTGAGTAGTTATACGTAACCCATATTTGGATGTCGATCG
GCGATGGCGTCTATTTATTGAGCTGGCGGACATCCGTACGCGCTGCAGAAG
CTATTCTAAAATGGGACGAGACACTAATACGAATATAATCAAGTAAACATTA
GTACCACGTAATTATGAAATGTGACGCATCCTGTGGTGGTCATCTGGCAGTA
GGGTCTTTCAGCTCTCCGCGCTCTAGCTGAACCAGCCAGCCTACCGACATC
TTCACATATGATATAAGATTTCCCTTGTCACTACGCGGCAATTATATAGAATA
CCCTCTTTTCGTTCTAATATCTGTGTAAGCAAAGCGATGTGAAGTTTTCTTAT
GTATGTTTCTTGCTTTGAAGGTCAAGCCTTTAACAAGCATTTGGATTGGAGA
CAATGACAGGCAAGACACCTACTGAAACTAAGTAAGTCTTCCATACTTATC
TATTTGTTTCAAGTCAAAATTAATATGAGAAGTGCTGTCGAAAACGCGGTTC
AATCTAGGGGTGGATCTGTAGTATCTACGGAGACTACGGACATCAGTTCATT
GCCTGAGGGGTCGATAAAATTCTCACAGCGAAGGTCCTGGACAACTAATAG
GAGAGGAGAGCTTTCGTGCTAGTCGGTTCGCAAAGAGAGCGCACTCTCCC
AATGGTTCCCCCAGATTAAAGACGACGAGAACAACAGCAATCGTATATACT
TCTCGCGGAGGAGCCGCGCCCTCTTATTACAAATTCTTCTCATAGGCATCAT
ATTCACGGCTAATCTTGGTTTAACGATCTTTGCGATTGTCCATTACGAGAGC
CAGAATGGCGTGGGAACCATCTACGAAGGCGACTGCAAACAAGGTCAAGA
CCCTTGACCAATGGCTGCATCTCTTAATCAACTTCCTTGGGACCGGCATGCT
ATCGGCGTCGAACTACTGTATGCAACTACAAGCTGCTCCTACTCGACAAGA
TATTGATCGTGCCCACGAAGCCGAGGCTTATAGGCGGGACCCAAATCATTC
AGAGGGCCGGTGGCTGGATATTGGCGTACCGAGTTTACGAAACCTCCGATA
TATTAGCAACTGGAGGCGAGTTGGATGGGTTCTCTTGCTATAAGTTCTATTC
CTATTCACCTAATGTAGGTTGCTTTTCTTTGCTACGCTGAGTTTAAATACGAG

TCTGTTTGAACTGGCTTACAAATCGATAGGTATAATTCTGCCGTGTTTCAAT
CGCTCGCATCAAACGACTATACTATCGCAGTAGTCAAAGATTCCTTTGTCAA
TGGGAGTTCCTGGAACCCTCACCACGGCCGAAAACAACCGACGAGGAGA
CTACGGGTGGGACGAAAGTAGGGTTAATCCGTCGAACCGCAATATACAGAA
ATTATTCAGGACATGCAGGATAATGTCATGGGCGTCGGCTGGGAGGCGTTG
AATGTCTCCGCCTGTTACGCACTATACAACGATTACTTCACAGCCCAAGGC
AACGGCGTCATATTTGTAAAGAACGATACCGGATCAGAAGACGACAGCTTT
TAATGTACGTCTCCATCATACCACGGTCGACGATTGGGCGAAGAATATGTGG
GCGGCTATGAACGGGTCCGACAATTTCATAGCTAGAGGCACCGACGATACG
GTAACCACGTGGTACCTCGGCCTGAAACAATACGAAGTAGCCTCATGCCGA
GTACAATCTCCCGATACGATCGTAAACCACTGCCGATTCGAGTACTCGTCGG
GAATAATGCTTACTGTCTGCGTGCTCAACTTCGCGAAGGCATCTGTGATGTT
GTTTATTTGGACACTGAGGAAGTGGCAGGAGAGGGAGCGCAGTGACGAGG
ATAAGTGATCTTATATACTGTTGGAGACGCTATTGCCTCTTTCATGCGGAAA
CCAGATCCAACGACTGCCAACATGGGTCTTGCCACTAAAGATGAATTTATA
CGTCGACGTCCGTGGAAGAATCACCTCGTGAAACAACCCCTTGTGCTGAAT
CGCGAAGCTCGAAAATGGAGAAAGAAAGACAAGTTTTGGTTCACTGCTGC
TAGCATCCGACGATGGTTTATATTACTATTCTTGTAAGTTGATCAGAATTTAT
ATCCCGTGTACTGAGTCAATATTCCTAACTCGTTCTCAGGTGCTGTTTGACC
ATCTTGGTAGCAGCAATCCTATTAGGGTATCTTTCACGAGTCTACACAACGA
AACTTCTCCACGTCCATACCAGCGCTCTACGAACTAGGATTCGGCCAACTA
ACCCCTTACACTTATCTCGTCGTTGGGCTGCCGCGAGAGGACCCGAAAGGC
CTTATCTTGAATGTTCTCTTGGCGAACCTACCTCAACTGATCGTATCCATTCT
CTACATATTCTATAATGCCATGCTCAGCACTTTTCTTGTTCAGCGAGAGTTCA
GCCGGATGTATCTAAATCAGCGGAAAACCACTTCGAGTCTCAGAGCCTATA
GGGATTCAGCGGTCTAGTTACTTCATATCCCTCCCGCTTCGCTATGGAATAC
CACTTTATGCGACATCCGGCCTGATGCACTGGTTGATATCCCAGAGCTTGTT
TCTGGCTCGGATCACGGCGATATGTGGGGACGGTGATCCGCAGGTATGTCC
CGGAGGTCAAGTAGACCTTCCTAACTCTTCTCAACTTGCGGGTATAGCCCT
TTCGCCGTCTTTATCAGTAAGTCTAGACGGCATCCTATGACGCATTCAAATC
TTCTATCTAACGAATAATAGCACTGCTCGTCGGCGCCTTCATCGTCCTATCA
ATTGTAGCTATAGGCTTCCGCAAATACGACGGCACCATGAGCATGGTCTCTT
ACCAACAGCATGGCAATCAGCGCCGCATGTCACGTACTCAGAGAAGATCA
AAAGGACGGTTACCTTCTCCCGATCCAGTGGGGAGTGGTAGAGATTAATGA
TGGAATTGGAAAGTGCGCATTCACGACAGCTCCTGCGGACAGGATAAAGA
ATGCCGGAGGCTGGGTTAGAGTATATTTGATTACATTTCAAAGCGATGCATC
TAAATAACCCCCAATACGATTAACAGTTAATGTGTTACATTCCTAGCCGACA
TCAATTCCATAATCATGCAGTCATTGCCCAATCCTAATTGCTTGCCCCATGTA

TAGCGAGTACAGTAACCTGTAGGTGTCTTTTCCCTAGCCATAGCCTTTAGAA
CCTAGTGAATGGATGCGCCCGGAGGATAACTCGGGAATAAATCGTGTAAGT
CATCAATCATAAAATAATAACATCCAAAATCATCTCGCTCAAATCCTCGCAG
CTCGACTCCCCTTCCGTCCGTCCCGCGCATCTGCCGCGTTCCAGCGGCCTT
CTTTCCCAGGCCTGCGCGTCAGCTTGCCATTTAGCTTCTTAGCTGCGCCCTC
GATCTTGTCTTTAGATGCGGCTTGCGGTGGTGGTGCGTCGGCGGGCTGCGT
GGTACCCGTGATACCGTGGCCACGCGGCGACCGGTGGTCGTGGTTGTGGT
GTGTTTGGTGTTTCGCTTGCGCTCCGGCTCTGTCGAGATGTTCAATGGATGC
GCGGCTTTGAATGCCCTGTTGACGAGCGGGTGAATGCCATTTCTGCAGTTT
CAATTGTGTTATATTTCAATGACACAGACAGTAGTCGTGATGCGACAATGTT
TCTGGATTTAAGATGGAATATCTAGAGGTAACTTCGTGTGGCAGTCGAGCG
TGCTACCAGCTCTCCTTTAAAAGGGGGGATCGAGGTCATACCCCGAGTCCA
TGGTTCTTTGACATGAGACACGTACGGGGTATACTGTCATCTCATTAGTTGC
CGCGTGGAGGAAGTGACGTCATGCTAGTTGTCAAGCAGGTTTGTCCTTTTA
CGTCGATCTAGTCTCGTGAATGACGTGGCGAATGTTTTGAGCAGAGAGGCG
TCGGATGGAATAATACGCGTGCTAATAGACTGTGACGTACTGTACAGGGCTA
TCAATTACCCCTTACATCACGTAGTCAAGATCCCTGTGTCAGCCACCACACG
TGTAACCTATCACGGTTTATTTCAGAGTATGATATTAGCTCAAATTCGATGCT
TATATTGTATTTAAGCTAACCATTGAATCAGATTGACCAGGAAATATCTTAGC
GCGCGTTATCGGACTGGGCGCTCGTATCTTTCGTGAGTATAATTTGTAGGTC
TAAGATGCATGGGTTTTTCTCTCGATTTATTTATATATCTTATCGCCTCTTCGA
AGTACTCAGCGCTTCTTTCCTTCTAATCGCAAGCGAGTCATTAGGCGTTTCA
TTACCCGCTGTGTTAAGCGGATCAGGAGTGACTTGTTGGATCCAGGAGCAT
ATATCTCTATACTCTAATCCTTCACGGTCACGACGACCGCTAGACCCCGCGT
TGTGCGTTATTACATCCGAGTCTCGACGTTTATGACTCGACAGAGCCGCCTC
GACAGTCTTATACATTGGAGTATACGAGTCGCCGGCCTCTTCATCTGACTTA
ATATCCTCGTTATAATCATCTCGTTCAGGCTCGACGTCGTATCGCGTCGCGTA
TTATATGAGTTGTTCGACGGATAGTAATATCTTTTCCGAACAGTTGGTTTGTA
TAGAGTATCCGTATCGGTGGCGTCTTCCGATGAAGAATTCTCGGAAGCACTT
CGTTTCGGAGCCACATTCGCAGGCTAGCAATATTCGATATATGCTTTGGTTT
CTCCCTCGGCCCGCGAATACGAATGCGTGGCTGGTCGAAGATAAGGCCGCT
ATAGTTTTGAATGCGTTTTTTGATCGTAGAATTCCTGAGCACCCCGTTTTCAT
ACAAGTACATAATAAAATTCTTGACATCGAGCCGATCCTCGTATGTCGATCC
TTGCTAAATCTAGTAGACCAGAGTTGGTTGAGTTTGTGGTCAATCAGAATTC
TGTTGAATAATGTTAGATTGATATCCTTAGAACAGCATCGTAATCTGAATCTT
TTCCTGAGATAGAATTTCCGACGAGCAGATTCAACGCGAAACGTTCTACGA
GGCAGTAGTGACATATCCAATGTCCGCATGGGAAGAAGTCGAATCTCGTGG
TCATTAATACGGTATTCGGATGAGCAGAAAGCGCACGGTATAGTTCTAAATC

TCCTTCTGCTGATGAGTTAGTAATGGTAGGGGGAGAGGAAGTAAACCACTC
ACGCGTAAAGGAGCTTTCTTTGGTACTCTTCAGGATCTTTTATATATTCGGCT
TCTGGATTCCTTTCTCTTCCCGGACCACTCTTTCGTCCTGTGTAGAGCACGA
GGGCTTTGCAATCGGCATCTGCCTCGTTTTTCTCTCCTTCGTCACCTGCCAT
GCTTGTTTCACTGACTTGATGTTCACTTGTAACACTATCGAGGCTTTGAACT
ACTGAGTTTTTGTCAACGTCTTCAGCATTAACGATAGACGGTACGAGTGTG
GCGGGAGATTGGTGCGCTTCTCTGACACTGCTTCTTCTGTAATGCTGTGAC
GGTGATTCGACACCGTCTGGGGTAGGCGAAATATCTAGTTTATCTCCAACAT
GGACAAAATAAGGGGACCTTGGGCGCGAGATCAGGTTTGGTGCTAAAATT
TCCCTTGAGATACTAATTGGAGAATTTGATGTCTCCTCGCGGGACATAATCG
GAGACCCTTCGTTTGGTTTAAGGGGGGTGTTAGAGATTTATGAGAGGATGA
ATCCTCAGATGTAATTATATCTGGTGTATCAGCATCTTCTAATACGTAAGGAC
AGTCAGAAGGTAGCATCTTTCCATTGGTCTCCTCATATTCATCATCGCTGTC
GACATAAGGGAGAGCGATATATAGGGGTTGAAGTATTTCTCGGCTCTTCTAG
GCGCATTGTCGTCTTTGTTTCAGAAGGTTCCTCGACTTGCTTCTCTGTTCTT
GATTCAATTGGAATTTCGGGTTGCTCCCCAGGTTCGTCAGCGATGGAGCTC
CAGGCAGCCGAAGCTACCGACAACATCGTGTTGCTGCTTTTATTGTTAGAC
TACGCGTATAGATACAGATTTCAGGCAATGGCATGTCTCTTACCCAGAATTA
AAGTCATCCGAATGAGCAGGCAAACTCGTCGTTTATCCACGGATGTCTATCT
GCGACTTCGGAGGAGGGACGTGCCGCAGGGTCAGGAGACATTAGGGTTAA
TAATAAATTGTTGGCCTTCGTCAGTAATAGACCGTGACTGAAGACTACCCCT
GGGAAACTTGAGCATCCCATTGACGTATTGAAACAATTCGCCAATATTCGA
GAATGGGAGGTCGTTTATGAGCATCATGTGAACACATGCGCCAAGTGACCA
GAGATCTACAGCGGCAGTGTAAGATCTCGATTCATTGCCCAGGCTTAACCC
CATCGTCTCAGGCGCCGCGAAGCCTAAGGTCCCCATCTGTAAAGTGTGTAG
GCTCGTGACGTCTTGCTGTCTTCTCTTGCTAATTCCGAAGTCCGCAATTTTG
ACAAACCACGATGGTCCTGTCTCGACGACCATGATGTTCTGATATCAAATAA
GCAGGTCAACGAGTGAGCTAATGGCTATGTAGATAGTAGAGAACAACTTAC
CCGGGCTTGAGATCTCGATGCACAAAGCATTGTCATGCATGAATTTTAGACC
CTCTAAAACTTGGGAGGTGATGGTTCTAGCTTCCGATTCTGGCAATGGTTTG
GTTAAATGCTTGTGTAAGTCGCCCTTTCTCGAGGTATTCCATGGTGATAAAC
ACCGATTCAGTGTTCTCGAACCAGCCATCTGAGCGAACAAAACAATGCGA
GTACTTCAAACAGTTCAGCAAGAAATAATTGACAGGGTCGACTCTACATTG
TTTACATACCCGCTTGTGAGAGAATTTAGAAATGGCTTCAAGTTCTCTCATG
TATTCTAACTCTCTTCCCGCGACATTATATTTCTTAATCTGCTTAACCGCTCAT
AATTTCGCACTACCGTCCCTCTGTTCGCATCGTTCTAAGTGTACAGTTCCGT
AGGCTCCCTGGCCGAGGAATTGTTTCGCACCCATCGTTCTTCCTTCGGTAC
ATGCCGTTCGCGAGCGGAATAACCGGGATACGTAAACGATGTGTTGCGTGT

AGGCGTTGAAGAATTCGGTCTCGAGCCTCGAATCACGGACGATGTCGGGTA
ACGATTGGATTAGTTGTCGTACTGACATACTTGTGAAAAGTAGATTTCGCAC
ATCGGTCGACAACACATTATCGAATTAGTATATGATCATATTCGCTAGGTGTA
CTTGGATTTGGCTTCGACAGACGGTGATGAATCAGACTTGGATTGAATGTG
TTGTTCAAGTTCATAGGAGTGTGACTGAAGAGAACCTGGGCTTCTTGGAAA
TCACGAGAGCCATGAGGGGCGTGGGGGCTGAGCCACCATCCGCATACCCT
ACTAGCTATACTGGGCGAATCCAGGTAGTACGCCTAGGTAGCTGCCTTACAC
AGCCTACAGCTTTCCAAGGTTCGCATCCTTCAGCTACAATTACTAACCCAA
GATCTTACCTTACCATTGAAAAAAACTGGTCTCATGTCGTTTACCTACTGCA
ACTCGTTGAGAACGAAAAGTCGTATGCTGGAAGGCAGAGGAGGAATACTT
CAATGTCAGCGGAATTGATTCTAGCCGCTGTTGGGGCTGCAGATCTGTGCC
TAAAGTCCGTTTCCTCACAGCGACACTCAACGTTACAATGACATCTCCAGC
TAAAATATCGCAGATACGGACAACATCTCTTAAAGATCTATCAAGCTTTCAA
ACAGGCAGATGAAGATGTGAGAGCTAAAACTCTACTTATCGAATCAAGATG
GAGCCAAATGGCCGTACAGGTGGAATTTGTCCAACGTATTGCCCATACCAT
GAGCCCGGATCATTGTCGTATCCACTTTGAGGTGCTAGAGATGTTGCAGGC
CAAGTTTCAGGTCGCCATTAGAAAATTGAACCGCTATTGAAGAAACTCGGA
GAATACGCTCGGAAGTAGGGGTTAAGAGGTGGAAGTATCTTCTCATTCGTG
AGTCGCTCGACAAGATAATATCGGACCTAGAGCAATGGCAGCGCATCTTCG
ACCCTACGTGGTATCTCATCATCTTGATGAAAGACAAGGCAGTTGATTCAA
GACTGCTGGAGGAATTCGGGATACCCAAAGGCGATCTTTCTGATGTAAGCT
CGGGACGAATGAGCCTATTGTCTAATTCCACTTTGGCCACGATACGGAATTT
GCGCAATACTCTCAAAAATGAGTCGATACCTAATACCCATATTACACTCCCT
GCAGAAGGGTTGGCCTGGGAAGGTGCACGGAAAATCCACCACTCAACTAC
ACGGCTTGTTCCCCGGACGGGGTCTAACAAATTGTTTCTCGTAGACTCGAT
GCTCTGCGATTCGAATCTCGATATTCCGAGGGCGAGGGCCGACGCAGAGAT
TCTGGCAAGGAAACTCAAGCAGGTCGAAACTAGCAATTTCGGCCTCCTCTC
ATGTCATGGACTCGTCAAACGTAAAGCACAACCAACAGGGACCCTATTTTC
CATTGATATTGTCTTCCGACTCCCGAACGGAAAGACAACGCCTGCCAGCCT
ACGATCAGAACTCCTGCAACACCCTATAGTTAGTTTGAGCAACAGAATAGA
TATCGCGAGGCAGTTGGCGATGGCGGTTAGCTTTGTCCACACTTGCGATTT
CGTCCATAAGAATATTCGACCCGAGACGATATTACTCCTAGCTAATTCGGAT
ATGGAACAAACGGAGGGAAGAGCGGTTCCCGAATCGCTTTATCTGTTAGGC
TTCGATCGATTCCGTAGTGTTAATTTCTCTACCATGCGTAAGGGCGATACGG
CTTGGGAGCGCAACCTATATCGACACCCTCTACGGCAAGGCTCGCGCGCGC
AGGAAGATTATGTTATGCAGCACGACGTGTATAGCCTTGGAGTCTGCCTACT
TGAAGTTGGACTTTGGGAGTCATTCGTAGTTGACGAGGGAGGCAAGGATG
CCCATGCTGAGGATGAGGATCGGGTTAAGGTCCCATCGTCGACCCTAGGAT

TGACATTGGGTGATTTCAATCCAGATGCAAGTTTCTCAGCAGAATCAGCAG
AACCAGCAACAAAGATAAAAGACCATCTCATCCAACTTGCTAGATCTCAAC
TACCGCAGCGTATGGGTGACAAGTACTACAGCTGTCGTCATTACGTGCCTC
ACCTGCTTGGACAAGGAAAATGAGGATTTTAGAGATGAAGACGATATGCAT
GACGAAGACGGAATTTTGATTGGTGTAAGATTTATTGAAAAGATACTGTTTA
AACTAAGTGAGATTTCAATATGAACAATTAAAGAGACAAAAAATAAAAATA
TAAACTGAGACGAAAGAAGGTCAAAAGTCAAAAAGGGACTCGGGGCCTG
GAAATTTAGGTACCATATCTGCCTTAGTGTCTTATCCAAACACGCTGTGGAG
AACCGCCAAAGCTTAAACGTGGCGGGTATCGAACCCTTCAGGTGGGTCTC
GTGACGCCGGAGCTGCGGAGGAAGCCACACCCATATCTGACACAGATCTA
ATTAACATTACCAATCGGGATTAGCGTCTTGAATTTTGCATATCGTTAGTGCA
CTAAGGTGGGACTAGCCGGTAACTACCGCTATTTGGTGGTGGTACGGCTGA
AAGTTGCTAAAGTTGCCTTCGTCCATCTTCAAACATCGTCGCAATCAACAA
CAATTTCATCAAATTCAAATTTAGGTCTTTCAGGGTTCAAACTACTTGTCTC
TTTTTGATATTGATGATCTGTCTTTAATATCCTCACTTATTAGACCGCAAGGC
GTTTTGACTGACGACAGACGACGCGTTCTATACTTCTGACGATTTTTTTGA
CGTCTTCCTAATCTAATCCACCCAGTTCAACGCTGCATTCGAGCTATCATCC
AGAAAATTCAATATTATCACAATTGTCGCACGGGTGAATCGTTGCAAATGCG
GGTTCTTACCCTTAGCAGCTTCATCGCCTACACCTTCGGACACCTTTGTGCC
TCGCCTGATTGATCCTCATTAATCAACCGCGGCTCCTTCGCCGGTTCTCACG
ATGGAAATAGATCCGGAGAAGAATTATTCGCTGCGGCTACCAAAACCTAAC
TATCTAGCCCACGCGGTCTGCGTTGATGAACCATTGGTCAAGAAGTTCACA
AAACCTTTAGATCGCCCAAGCGACTTAGAAAATGCCGCCTATCTTAATAAG
ATCAAACGCTTCGCTCACGAACATGGCATCACCAAACCTAAGGGTTACACA
GTCTCATTTCACTACAACCCTGAAATGGAGAGACACCACTTCGGCCAAACG
CATCCTATGAAACCTTGGCGCTTAACACTTACCAAGAGCCTGGTATCATCGT
ACGGCATGAATTTCGCCATGGACAACTATACTGCCCGTGCGGCTACTTATGA
GGAGCTCACGGCTTTTCACACAGACGACTACGTTAAATTCCTTGCCAGTGT
CGAGCCTCAACCGCTCCCACTTGACTTTGACAATTCTAGTACAGATCTCAA
ATTCAACTTGGGGGGTAGTGACTGCCCACTATTTGAGGGATTGTATGATTAT
TGTTCGATGTCTGCTGGTGGTTCCCTAGATGCTGCTAGGAAATTATGTGCAG
GACAATCTGACATCGCTATTCTTGGGGTGGTGGACTGCATCACGCCAAGAA
ATCGGAGGCCTCAGGTTTCTGTTATATCAACGATATTGTCCTTGGTATACTAC
AACTATTACGCGTTCATCCTCGAGTTCTTTATATCGATATCGACGTACATCAC
GGCGACGGCGTAGAGGAAGCATTCTTCTCTACTGACCGTGTCATGACAGTA
TCCTTCCACAAGTTCGACCCCGTGCTCTTTTTCCCTGGTACTGGCGGCCTAG
ATGAACACGTCCTAAAAGCGAACATAATCCAGGAGCCCATCATGCTATCAA
TGTGCCTTTGAATGATGGGAATTACCGATGAACAATACGAGTGGCTTTTCA

AGAGTGTCATTTCGAGGTGCATCGAAAAATTTAGACCTGGCGCGATTGCTC
TGCAATGTGGCGCAGACTCTCTCGCGGGAGACAGATTAGGTAAATTCAATC
TGCTCGTCCAAGGTCACGCGTCGTGTGTGGAGTACTGCAAAAGCCTCAATA
TCCCCCTGCTCATGTTGTGTGGGGGTGGGTATACGCCACGAAATGTCGCCC
GCGCCTGGGCGTATGAAACAGCTGTCGCTATCGGACACAAAGAAGACATA
CCTCCGGAGATCCCCCTTCACACTCCGTGGCGTCAGCGCTTCCGTCAAGAC
ACGCTTTTGCCCACCCTACAACAGATCTTGGGCGAGCCGCGCCAGAACAA
GAACACTAAAAAGAAGCTGGACGATATTATCGAGCACGTGAGCGAGCAGC
TGCGATTCGTCGAGTCTGCGCCCAGCGTGCAATTCTCTACTATACCACCAGA
TCTCGGACCTATTAGAGACGAGGTGGAGCAACGGATCAAGGAAGAGGTAG
ACGAGAAAGATGAATTGGGGCGAAAGCTTCGGGAAGAAGGCGTTGG

>Mosaic_sequence_20

CCCCCTCCGATAGATCTCCGAATGGCGCACCTGCTGACACCAGCATGTCCG
GCACTACGTACAACGCACCTCAGCGATCCGGTTCGAACACCTCAGTGGCC
AGGGATCAGTACGGCAATCCGATAGATCAACCTCAGGATAATTATAGTATGC
GACGAGGGCCATCTTTCGAAAGTATGAACTCGAGAGGTCGTGGTGGACCT
CCAAGTGGTATAGAGGGCGAGGAGGTTACGGCAGGGGCGGTTATGGTGGT
CCGTACGGTCCCCGGGTGGACGAGGTGGAAGAGGTGGATATGGCCCACCA
CCGGGGAGAGGCGGGTATGGCCCACCTGGCCGTGGTGGATACGGGCCACC
ACCACCAAATGGACGAGGAGGTTACGGTCCGCGTGGGGGTTATGGACCTC
CCGGAATGATGAGGGGCGGAAGAACACCACCGCCAGCTTACCAAGGCGGT
GGTGGTCCTTACGATCGACGACCATCCCCAGCCAATGCTTATGGGGACTAC
GGTCCTGCTCGACAAATATCTCCAGCACCTCCAGGCGGCCCGTATGCGATG
GATGCACCCCCTATGCCAAATGCTCCGATGGGTGCGGCGGAAGCAGGTGGT
TTCCAAGCCTACAAGCCCGATCGTGTCAGCGAATTGCCTAGAGCAGAATCA
CCACCTCCGCTACCTGGTATCGATGATGCCCATGAGACACCAGTCGGCCAG
GCAGTGGAAATGGATGCCGTTACAGGAAGTCCTGCTCATCCTCCTCAAGGT
TTTGGCCAGTTCAACAACCAATTACGAGATAGTGATGCCGATGTAGCCGGC
ATGTTAGCGCTACAACAGGCGAGGACGTCTGGGGCAAATAGACATGATACC
ATAATGAGTGAAGGAAGCAGATATAGTCAAGAAGAGTAAGTCAAATATTGC
GTCAAATAAATCTTAGCACTAACAATTTGGTGTGTAGTGGTTCATTTGTTCC
GGCTCGGCAAGCATGGAACCAAGGAAATATGCGGCCCTCCGCAGTACCGT
CGCCTCTTCAGATCCCTACTAATCCGCAAGATGTGGGTAACAGGACACCAC
CGCCACCTATGCCTACACAACAACAACCAGCGAATGGCGATAGAGGCGAC
TACTACGAGGATGTAGATCCTCGCTTCACTGAACCAGCACCCAGGCCGCCG
CAACAGCCAATCCACGAAGAACCAATAAGTTATGACGATATACCACCGCCT
AGGAGTCCAGCGATATCCGAACAGTCAGGTTTTACGTCAGTCTCACAACGA

GGTATCAACCCTCGGTGGAACCCACCTCCCGGCCAAGGGTTCAATCCAGG
AATGCCACCACGCAAACCGGTTGGCCGAAATGAAGTGAATGTTCTTAATAG
TAACCCGGACTTTCAGCTACCATCGACGCGTGGAGGGCCTAGTTCTGGAAG
GGGAGGAGGCATGATTCCCGGGAGCGCATACCCGGGGCATATAAAACCTTG
CATTCATTAATATCCTTACTTAGGGCTATTGGGGAATAATAAAATTCTCTGGG
TGGCGTTATGTGTGGATTTGAATCTTCGGTGCCGCATATGATTCTTTAATCAC
AGATGCTGTTCAAGCACTTTTCGTATGGACATTGTCCAACTCGATCATACAA
CTGACGTCTCTCCAATTTTCCTTTATCATAAATTCCACTATACCCTCATATCTT
CACTTCTTTTCGACCTACGACTTATTCGGCTCGTTACCACAACCCTGCGGGC
GTCAGATACACGTACCTTCTCCTTGAACAGCATACTACGGGTTATGGGCGG
ATAATCTATTATCGCCATCCACGACCGTTAACCTCGGAACAAGCGGGGTTCA
TCAATCGCCATTTTATCTATCTTTCTAAACACTCAAGGCAACGAGCAAATTA
CCAGTCTCTACCGCCATTGATTGGACAGGAGCTTGGAGACCGAACATGTAG
TAACCTAGCTACATGGGATGATGAGGCAGACGTGCTCGTCTTTGTCAAAAG
CTATCTAGGGAATGTATTCGGAGGCATTTATCTTTTCTTTTTTTTGCCTTGCG
CGTTAGGGTAATGCAAAAAATAACTACCACTACAACTACGTTTGAGTATGTA
AGCTCGGCTTGGCTGATATACGGGGGCTACGTGCTTGGGTTACATATGAAG
ATAGTTGGGTAGCTGAGCGATGTGGTGTGTCCCTTGCCAATGCGGAGGATA
GCAGAGCATGAGCATGTTCTCGCCGGATGCGCTGAGCGGAGCTAGGCATG
GCATGGCAAGGCAGAGCTGAGCAGGTAGTGCTGAATGCTAAGAATGAACA
AAAAAGATCTTTGATTCTGATGGTGATTGTGATTGTAAATGTCACGTAGAGT
ACGTTGCGGAAAGGGAGGATCCCAACTACTTGGGTGAGTTTACATCGTAAA
ATCGTTAGTAGTGCTTGGTTCAAGACGTTCTGCTAGCATTGTATTAACCTCG
AAATGCTATTCTGGGTCGAATATGATCTCACAGCAAGAAAATCTATTAGACG
GCTCGGACTGTAGTCCTCTTGGAGATGTCGATGACTTCAAATCTCGGTCTAT
TAGGTGAGGCCAAACTATTTCCTGGCACTCATGGAAACTGAAGACGTGTCA
CGTTCAAAAAGGATCCGTCGTTGGCATTTGATATATTTTTGTCATTCCTCTAG
GCAGTGCCTATGTACCTCCATGCCTTGCCATGCACAGAAGATTGAGATTCCT
AGCCACGCGAAAAACCCCTAATGCAATGAGAAAGAGCAACCGGCAGAAG
AACCCAGTAACACCACGCGACCGCCACATCCGTACACCACCTTTTCCCTTC
CTTAGATGGTCGTCTCGTACGCGATAGGAACCACCGTCTCGGGTTGGTTGT
CCTTCATGGACACGGTGTGCGCCTCACGGGGAGGGTAGGTAGTCGCTTAAC
GGTGAGAGTGGTGCCCTCGGCCTTGGCCTTCTTCTTGAGCTCCGCGTTTTG
CTTGACACGTCGGAGGAAGTCCTCTCGTGATCGGGATCGGTTGATGTGCTC
AATGCGAACGTTGATTCGCTTCTCGATGTACCGGTGCTTGACCTTCTTGTAG
ATGATTACGCCGACGGCGGTCTTTGGTGACGTTGTAGACGACACCGGTCTT
TCCGTGGTAGACCTTGTGAGGCATACTATTCCTCCAGTCAGCTCGGGTTCCC
TAATAACAATTGTGCTCTTGTCCAAATTCGCAGTATTCGTGTGTCAGGATTC

GGATCGATCGCATTTTCGTAGGATTGGAATTTGCCTTACCCCTTCTGAACAG
CACCATTTGCCTTGATGTCAACGATGTCGCCAACCCTATCCAATTGTCAATA
TTTTCCCTTCCCGCGTCAGATATTCGAGTCGGAACATACTTGTACTGTTGTA
GGTATGTAGAGAGGTGGATCATACCCTTCTGTCGGAAGTTCCGAGAGAAGG
CATACTATAGAATTTGTTAGACTCGCTTCTCCCTCATAATCAATCAAAAATTG
ATTGTAATCGCATTTAACGTACTCGAGTGCCAGCGCGAAGACCTGCGGCGT
GACCCATCTTGTCGGTGTTGAGGTGTGGCGAGAGAAAGTTGTTATGGTGAT
GGGAGTTGTTTGATTTCTCTCGATGGTCGCTACTCAAGAAATTCGCTTAGGA
ATTTTTTCGGGGCTTGGATTGCGGTTGTGGACGTTAGTAAGTCACTACCCGT
ATGTGCACTGGCCAGCAGCCGCCAATTCTACCAATTAAGCGAGGGGTACTT
TGTCCAACTTGGGTTTAGCTCCACCCATGCCCTACTCCATTCCACAAATCCA
CTTCTTTTCATTTCAAACTTTGACCTCTAACTTTTCTGATTCGCTTTTTCTAC
TTTGCGAATTCACACGACAACTTCAATTTCTTCATTCCCTCCCCCCCTCGAC
AATCATCTCAAAAGACAATTGAGTCGTCGCAACCATGGCGCCTTTGTCTTA
CTCCAAGACCGCAAAGGTCCCCCGTCGTCCCTTCGAGGCCGCTCGTCTGTC
AGTAATCAACGGCCACTGAGCTAGAGCTTGACTCTACTTAAGTCGTGGAAA
AAGAAACTGACATCGTCGCCCTTATAGTGATTCCGAGTTGAAGCTCGTCGG
AGAGTATGGTCTACGCAACAAGCGTGAGGTCTGGCGTGTCCAGCTCACCCT
TTCTAGATTCTGACGTGCGCAGTGGGTCAAGGACAACAAGCCCAAATTTGA
TGTCAACGTCGTCCTGCCCGTCTTCATCATCGGCCCCACCGTGCTGCCCGT
CAAGGGCGTCTCCTCCCTCCCGCCTCGCTCGAGTCGATCGGCAAGTACTTC
CACAGCAAGTCGCTCCCGTCCGAGCCGGTCGCCCCGCCAATGGTCGCCCT
CGCCGACATTGTCTCGGCGCACATTGCGTGCCAGGAGACGCCCACAGCCG
GCGGTCAGCGGTTCCTCATTGGCGGATATGGTCACACCCCCGGGCTGGTCG
CTTCGTGGTTGCGCAAGGGCATCCCCGAGGCTGTCGACCGGATACCGGAG
GGCGACGAGACGCCTGTGGGCGGCGACTTCAAGTGGGACGCGTCGAAGT
GCGTCAGCATCCTCTAGAGTACTAGGTGCAGCTGATATTGCAGAGCTGAGC
GCGAGCTGGGCATCAAGTACACCCCATTGAATGTCGCGGTCGTCGCAGCGG
GTAAGCAGTACCTCGAGCTGGAGAAAGCAGCATAATGTTCGTTCAAGAAG
CGAAAGAGAGCCCACTGCCCAACATCCCAGCCAGACTGAACATCGCCCGC
TCTTTCCTCGAGAAGGGCGCTGACGCCAATAAGAGGGAAGACACATCATG
CATATCGTTCATCGAGGATTGCTACAGTCTTGATGTCGTGTTGCAATGTATCC
TCGACTCGACTCAGAATATCGAATCGGCTGTGGCCCCTTCGGCAGCGCAAG
TATCTTCACATTGATCCACGATCCAGTCTGACATAGTAATTGTACAGACTGA
AGATGCCGAAGACGCAGACTGAGTGAAGACCTGCGCTGAATCGCCTGAAA
TGGGTATTTCGAGCGTGTCAGCGGTTTGTATGTAAGGCTGGGTGGCCTTATA
CAATGTGCAGGCCCACTCCGCCAGAGCGCGACCATCGATCTCCGCATGGAC
TGTGTCAGCCCAGTCGGTGGAGTCGAGCGAACTCCTTGCCATACCAGTGAT

CTCTTAACTCGGGATGCTGCTTCTCAGCCCCACTCCACCGCTTCACGAAAG
GGCGCATGATGTCTTCCCAAAGTCTCGAGCTTCTGGCGGAAGTTCACCTAT
CGGATACTGTTGCGCTATCGAGTCGAGAGACTCGTCATCGTTCTGAGGTGT
AGCCTCCAGCGTGTCGAGGTTGTGACGGTAACAGCAGAGACTCTCGTGAT
GCAACTCGCAGAAGCGGTTCCACTTATGGATGTCGACATCGACCCCGTTAT
CGAGCAGAAGAGAGGGCTCTGCTGTGGTGTCTGTGTCTTCATACTCCATCA
TCTTGTCCATGTCGATGAGCGGGGTCCAGTCGTCTTCAGGAATAAGAGTCG
AGTCTTCCGACATAGCAGCATCGTCCAGCAGCTCGGGTTGTGGCAACACCG
AGTCGTCGATAAATTCTGGCCCTTGCTCTTCCATCCAATTGCTCGAGGGCCA
CGAAAGGGGAAGATCCTGCGATAGGCCGTCCATTTTCGGTGAGTCACGGTG
CTGAGTTGCGGTGTCACTCGATGCATGCGAGGAGCTGACTCGGGGTCATAA
AGCATTTGTGAAGGTCGCATTCTGATGGGGAGCATTCGAAATGGGACGAAA
CACCTGTCAAGTATGCGCTCCTTAGTCTCGCTCAGCTCTGCTACATAGGCCT
TTCAAAACGACAAATGACGTGGACGGCTTGACGCCATTGCAATGGGAGTG
CATGAGAGAGGGTACTTGAAACTGGCAGCGCCGTTTCAATGCCGTGCCGC
GTTTGGGCTGAGCTGAGTGCCGCCCAGGCTCACAAGACACACTCCTGACT
TGCCCAATTCTGCGGGGCACGAGCTGGGATATCTCGGAAGGAGTGAAATGC
AGCCTTTCGAGTACAATACATACTTCCAACGTACATCCAGTCAAATGTCCGG
CTGAAAGCATGCCCCATATGTACACCTTTTCGCTGGTAAACACTTGTCGAGT
ATACAGTACTGTCACAAGATCCACCTTGGGAGTTCATTGACCCCGAGCGGC
CCTTTCAGTATCCAGAGCTGTACTTCGTTCAGCTGAACCTGATCTGTTGGCG
TGGTCGCTTCTCACTGAACTTTCCTCCTATGCGAGCTTCCGTGCGCGGCTGC
ATTATTGTTGTTGAAGCAATAAGCCCCGAGGACGCCTCATAGGGTGAAGAT
CACCTCTTCGGACAGGGTTCGTGCAGAACAGTAAGACTAAGACTGAGCAG
TCGTGCACAAACCGCCAGATTCACAATACACATATGTACACGGTGCTGCGA
GAAACAACGTGTTCAGGGAGGTAGAAAGGGCAGGACTTCTTGGATCTTCG
AGCGACCCAGTCAAGAAAGGAGATTATGCAGCCTTTCACATCGTCTCTTTA
TTCGGTAGGGGGCATGTTCGCGGAGAAGTCCTCTGAGGACGCTTTGTGCCC
CGTAGTCGCTTGCACGATACCAACAAAGGAACCCGGCGGGATACGCTTCGC
CAGCGGGTTGGCAGACCTTGGAGTGGTATGTGTGGCTGTTGTGCTGGAAC
GGAGTACGTTGAGTTCCGCCATCGTCGCAATGAGCAAGCGGTACCCTAGTT
CGAGAAGGACTCATAAACATACATAAGACAGAGGATCAGCAAACAAGGGC
GTATGGCCTCCTGATGCTGTGCACGCTATGTGCACAGCAAACGGAAAGCGT
TGTCCCGTCTGTTGCTTCATGATGAAAGTACCATGAAGTCTATTTCGTCGAC
TTGGCGGTCGGCGGCGATGCGCCACCGGACTTGGTGGCCACTTCAAAGCA
GTCTGGCAAGATCTCCTCGACCTGCTTAGTCGATTCGATGTCAGCTCGTGTC
GGCGCATGGTCCAGTACTCCATGCCCATCCCATTCCTCCTTCAGTTTTCTGT
CTTCTTCGTCGGTGTGGGTGACCACGAAAGGCTTCGCGGAAGACTTCAGA

TCCTCGAGCCTGCTTTTCGCCTCGTGTAAAGTGGCGCTCCCCGCTAGAAGT
GACCCGAGTCTTTCCGAGTATCGCGAGATTCGCGCGGACGCCCTCAGAT
AAGGCATTCCATGCGGTGATATACCACATGTCGTCTGCGCTGGGCTGCACTC
CCTCAGACGGCGTGGCAAGTCTCTCCGCGGGGGGTCTTCCGTGCACGGCT
CGATGGGATACCCATTCTCCTCGCACAGTCGTTCGTAGAACGCATCGAAATT
GGACATGCTAAAATAATCCGGTCCCACCATTATCGCACTGCCTGCTCATGTT
GGCTCTTCTCTGAACTGGGGTGAGGAAGTTGAGGGTGATCGTGGTTGCCAT
ACCTGCTGTAACAAGCTGAGAAGTCTCAACTCCTGAGGAAAATACCAGAA
AAAGGAGGTCTTCCAGCCTGTGCGTGACAATTCCTCGAGCGCAACCCACT
GAGGACCCTTTTTCCCAAGGCTGGCAGCAATGCTGTCATACCGAAGAGACT
GGTTTCAGTTGAACCATTGTGTTCCAAACCATAAGGGCTAAAGATCAACAA
ATGTCATGCGAGAGACGGATTTGTTACGAGTCAATGACAGGCATGATTTCG
TTGGGCGTGCGCCTTGGGGTGTCTCGGGTTTCCTCAAGATGATCACATTTCT
CATCGCCAATCCCTGGAACCCAGATGCTTTCCATCGCATCGACTTCCGAGG
TGCATCTGTTGCTTTACAATGAGGAGTGGGGTATGTAGTGCTGACGCGTTTC
TTATACCAGCTTCCTTTGAACACTTTGGCTGATCCTACGAGGGCCTGCTCGA
AGGCTTAGCATAGCAGATGCGGGGCACAGGTGGCATCAGCTACTGCCACTC
GGTCAGAGCGTACTCTTGCAATTCAAAGTTGCGCGGAGAACTCTGCATGCA
TCTGCCCCCCCATGACATGTAGTATATGCGGGGGGCTAGGATAGCACGGTAG
GCAGAAAGTTCGGCCCGCGGTTTTCGTGATACTATTTGGCTAATGTGACGA
ACATCTTTTAGGTCCCTAGACGACATTGTAATCGAGAGTGCCCTCGATGAGC
AAGGTCAGAGGCTTGCAGAAGGGGGAAGGAACGGGGGAGTATCCCGTGC
TACTGTAACGTCCACTGCGAAGCCCCTGGCGCTCTAGCAGAGGACTCCTCA
CCCCATATGTGACTGCTCCCCTGAATGTTCGCGCAAGTGTCATATATGACAG
CATGTCGTGCACCTTCTGCAGTGCGCTTTCGGTCTCACCGAAGCCATGCTC
TCTGCCAGGAATTGAAGATCGTCCCCGAAGACTACGATTAGCAGCTTGTCT
CTGCGCCCGATAGTTAGACGACTGAGGCTGCAAGTATTGCTCCATTCGTCC
CTGACCCGCCAACAGAGCAGGGCAGTCTCTGCTCAGCTGGGTGCTTATCCT
GTGTACCCCTTGTTTGGTAGCGTCACCTCGCTGGTTAGAGTGTTCATTCCGT
TGCCAATGGTCGCCAAGGCGTAGAGGGCGTTGTTGAGTTGAGTAGTTGTTT
CCATTGCTCTATCCCGCCACTGGATTGCCAGCTTCCGGTGCTTGCATGTTCT
CTTACAGCTGGCTTTGCCTTTCCCTTTTTCGGTCTCAGTATCTTCAGCCGAC
TCAGCCTCAACACTTGGCATGCAGGAGCCACTGGCTCTGGACGTCGAGGA
TCCAGGCTGGAAGGCTGATTCGTCGAGTGTATCCGGCAGGGTGCTTGGATT
GAAGTAGTTGTCATTCGGGGCGACCCTACCGGCTGCGCGAGCGATCCCTGC
TCCCTCAACTCTGGGTCGAGAGAAGCATATGCGTCGTAAGTGGGGCCATCT
CCGTTGGAATGGCGGTCGCGATCCATCGGGACGTGGTGTGGATAGTTGGAG
AACATTTTCCAAGAGCGCTCTTGGGGAAGAGTTATTCACTGGTACCGCCGA

TTGTCCACTGACTTTTGTAGCTGTGTGTTGTCGGTGTGTACGTCGGAGCGG
ATAACAACACGTCTTCCATTACTATCCACCAGCGTTCTCCATACGGTGGCGG
GGTGCTGATCGCCTCCTCTGTCCTTTGTTAGACTAGACGAAACGTGTAACG
GGATTCACGTACAATCTGCGGAGCACGCCCCTTTGTTCGGAAGAGCGGGC
GGACTCGCTTTCTGAGGGGGCGCGCACATATTGCATCATAGGCGGCGAGAA
GATCCGCCCCGCTCTTTTTCTTTGTACAAAGCTGACAGATGTCTCGCTTGGC
CGGTCACCGCTAAACAGAACAGACGATCTCGACGTCCCTCCGTTGGAAGG
AGAAGACAACAAACGGATACATACTTCTACACAATGCCGAAGTACGCGGG
TTCATCTGCACGAGCAGGCAGGTCTGTGGCCACTGAACCGCGCGCACGCC
ACCGCATGAACGGTACAGCTCGTGAAGTCCAATTCTGAAGCGAAGTAGTG
AGGCGGAAATTCCGTGCGTCAAGCACTCCGGACAAAGGTGCAGCTGCTTT
GGACAAAGAAGTGCAGGGAAAGGTCATGCATCATATCATTATATTCACCTG
CAGAGTCTGTCTTTTCACTCTTTTCACTCAAAAGGTTCGTCGTAGTCGTCAT
CGACGTCGTAGGTCTTGCTTACTGGAGGTATGATCAATCGATGGGGAGCGA
TGGCATCGAGCAGTACTTGATCCACCTATTCAGGGTGTCAAGGGACTGGAG
AACGGTTTGCATATCTTGCTCCGTGGGTTGATCCACCAGGAAGCCTTTCCC
GTACCACCGCTCCCTGTCCTCGTCAGTCTCTCTTTGCCCAAATGGCCCCCCG
TAATAAGGCCGAGCCTTGTTCTCAAGAGTGGACACCTGGTACCGCATATTA
GGCCATTCAGGTTGCTCGCCCCCAGCCCATGGCTCGTGGCCCAATACCAAC
CGCGCGGCCTTGATGTTGTCTAGGTTGGCTGTGCAGACCCTGTTCCACGTA
CTGGTCTCCGCGGAATATCTCGCACCGTCTGAGACCTTGAGCGGGCTTTGT
GCGTGGCAGAACTTCGCGTCTTCGACGGGGCAGGTTGCTCATATGTCGGCC
TAGGATCGGGGATTTGACTTTGTGACGAGTCGCGTATTGCAAGGTATCCGA
AGGACGTGTGCGCTTTCTTTGTGCCTGTACATTCTCTGGCGATTGGAGACT
GAACAACGAGTCACCGTTCGTTGGCGTAGTCTCATCAACAATGGTGTCGGG
ATTGACTGAAGCGTCCAAGCCGAACAGATCCCCATGATCGTCGGTATCGAG
AAGTCCATCATGGAGTCAGATCCCTCCGGCCACGTTAGCTCGTCTTCTGAC
GGAGATTGAAGGAAGGCGTAAGGGTCCGCGGAATTGGACCCAAGCCAATC
GTACTCATTGTCCATCTTGCTATTGCACTCTTGCGGGGTGAAGGTGCGTCTC
TTGACAAGTGCGGATCTCCAGGAGACAGGAAAAGGACCAGAAGTCTTTAT
ATCCTCCACAATACACTCCTGATCCTCGCCCTTTCCTGACCAGGGCCCGTTC
GACTCTGCAATCTCTCCCAATATTGGGTCCCCGACTCATAATGTGGCTCAAT
CTGATTCTCGATACCCGTTTCAGGCTCCGCTACAGCAAGCACACAGCGGAA
GGACGGGCCGCTGACCATAAATGGAGCGTCGCAACCCCGTGCCCCATGCA
GTACATCGATACATGTCGGAGAGGACAGAATATATATATTGTGTGTGACGGA
GACCCTTTGGGCCATATCGCTCTCAGCGATAGCTGATGCAGCAATGGCCGA
ATCATTGCAGCTCCTCTGACCTGCTGTGACCATTGTGTTCATTCATTGACGG
CCAGTACCTGGGGTCTTCGGGCAAGTCGAGAGCGGTGTTGCGGATAGCGG

AGCACCGATCCATGTACGAAAGCATCTCTTGCACTTTGCTCAGTCCTGCCTC
GGAGTCACCGTTCTCGAGGCTCGAACGTATGAAACTCAGGTCCTGATAAAG
ATGTTGATTGGCAGCCTGCGTCTCTTTCCGCTCGTATGCAACCCCCTCGGTC
TGATGCAGGTGTGTTTGGGGAGCTCGCACAACGCCCGCTTGTTCAGCTAAA
ACAGCTGCAGGCTTGTCTCGATCCGAGCCATACTTCGTCTAAGGGCAGTTT
GCCCTTGACGTAGCGCTTCCATCTCGCTCTGCATACTGGGAACGGATGCTG
CGGTCCGTTGAGCGATCTCAAGATCGATGCGTGTATGACGGGCCACGCTTT
CCCAGTGATACGCGACAGCCTGGTAGTGTTGTGCCAAGGGTCGGTGCTTGC
AATCCGTGGGCTGCGAGATTGTCCCCATACGCTTGGCATTTGCAATGTTGTC
AGTCTGTACGCCTTGAAAAGCAGTGGGAGATGCAATAGTCTGCATTGGGTC
CATGTAAGGCATGAAGGAGGGTGTCATCGATGGAAAGGCCATGTTTGAACC
AAATGCGACAGTGGGATCATAGGCACTAGTGGTCGCCACCACCGGTTCGA
GGTCGGGGTAGAACGGTTGACCCATCGAGTCGCGTGCTTCGTACTCAGGTT
GCCCGCCAGAAGTCGGCTTGCATAGAGGGTTCGGGTGCATTGTGCTGAGA
CATGATCTGCCTCGCCAAGCTTAGGATATAGCTCGATCTGTTGTTAGGCTGG
GGGGACCCCCTGCCGCTTTACATCTGGCTCAGGCGGGCGCCCAGCTCGGTA
AAACGCGCTGAAGATCTGTCTGTCCTTTCAAGCTCAACGACGAACAGGGG
AGCGGCTGAGAAAGGTCATCCAAGTGTGGCAAAGCGGGTTCGCAGAATTT
CGAAGGCCAGTCTTATGTGCTGCAGCAAATATACCAGAACGCTCCAGCTTC
CCGCGCGCGCAAAGACCCGATTCCGCACACCTCGAATGCGCCACTTTCGG
ACACGCCCATCATCTCGAGACAAAGGATCAGTGATATTCGTCGATCATCTCA
TGCTCGCATGCTTGAGAGGAGGGGAAAGGGGCGGACCGACAGGCTCCCG
CACGCGTTTCAGCGTCGCATACATGCGCTAGACAGCTTGTCTTCGCGCCCG
AGCTCACGCCATCACCGGTACGCCGAGGCGAGATCCCCTGGAGACAGCTC
TCCCGTTTGTTCGCGCCTTCTCGTATCTGACGAGATATCGCTATGACGGGCT
CGGGCACTGCTGCAATTCCGCTCGATACGCGCAAATCTCGCCGTGGGCTGT
CGAGTCCTCGCGCCAAAACCATCACCCCTACGCTGGAATCGGCTCGGACGT
CGCGGAGCCCTTCGCCAAACACACCTGAAGAGGTGGACGAGGTGGACCA
GCTTGAGATTCGACATGACACGAGCGAGCCCAATTTGGCGGGGAGCGGAG
ACGACAGACAGCTCCTGTTGGGCAAAGGAGTGAAGGAAGGCCCGTCAAA
GCAGGCCAGCCGCTCGACATGGGTGACTGTCCTGGGTGTCAAGGTACATG
CCCCCCGTCCTGTCACTTTCTTGAACTGATCATCGACTGGCAGATACCTACG
GCACTCATGTACCTCCTCGCGCTACCCCTCCTCATCGCACTTCTGGGCGTGT
ATCGACTCTCAGGGAACATACGCTACCCGCCAAAGGTCCTCCTGTCCAATG
GTACCCACCCTTATCATCCCACAACGATACTTGCTTTCGCTCGATGGCTTTC
GCCCGTCGTACTTGCGTCCCATCCGGACTTGCTGCCGAACATGCTGGCTTT
GGCGAACTCGTCTCTGGGACTCCGAGCTGAATATATGACACCTGTATTCCCT
ACGTTGACTTTCCCTAATCACTGGGCACTGATGACCGGGCTCTACCCGGCA

TGGAGCGGGATTGTCGCGAACGACTTCTGGGCACCCGATATGTCTAGAGGG
GACGAGTTCACACGGCCACCGCCTGTGAAGGCTACCGATAAGCCTGACGG
GTCGGAAGAGCCCGAGGCGGGTGACGGCGAGTGGGGTCACGGAGAGAAG
AGCGGGAAACAGTTCATTTACACCAATCCCGACAAGAGCTGGGATAGCGG
TGGTGGTGGGGCGAGCCGTTGTGGGGTGTCGCGGAACGAGGCGGTCGTAA
AGCCGCCAACCTCATGTGGTGCGCCTCCCCATCCAGCTCTCCCGAAATTGG
GCATAAGCTGACCACGCAGGCCCGGTCCTCCAGTCACGTTGACAGGCGCT
GCCCCGAGCTTCTTCGTGCCGTACCGCGTTGGTCGTTTCTCGTCCGACGAT
CGTCTGACTGACAACGTAGGGGAAAATCGACCCGTCCGCAAAACTATCAC
AAATACTCGCTTGGCTCGACCACCCCTTCGACAACCGACCAGAGCTCATCA
CCGCCTACTTCCCCGACGTCGACCAGTCTGGTCATTCCGGCGGTCCTGTCT
CGCCTCAGGTTGATGCGGCCTTGAGCCGGGTGGACAAAATGGTCGGCGGC
CTTATCTCTGGCCTTCAAGAGCGGAACCTCACCAGTGTCGTCGACGTAGTC
ATCGTCTCTGACCACGTAAGCCATCCGTGCGTGTGCGATACCCTGAGCTGA
TCAGCAGGAATGGCAAGTACCTCCAACGAGCGTCTTGTTTATCTGGACGAC
ATCCTGCAGGAAGACGGATACGCCGCTATTGCGCATCGCGACGGCTGGCCC
TGCGTCGGACTGCGTTTCAAGCCAGGATCCGACGTTTCCACGTACCATCGG
CGCCTCATCGACGCTGCGGCCGCCTCTGACGGAGCCTTCGCGGTGTACACG
CACGAGACCATGCTTGATCGGTGGCACTTTGGCGGAAACGACCGTATCGCT
CCAATATACGTCGTGCCGGCTCTCGGATGGGCCCTGACAGATCACCACGAG
CACGAAGTCGAGCATGAAGGCGATTATCGGCCGAAAGGGAATCATGGGTA
CGACAACAAGTACGAGGAGATGCGTGCTATCTTCATGGCTCATGGACCGTT
CGCCGCTGGGCTCAAATCGGGCAAAGGCGCTGATGAGGCGTTCGTCCAGA
CTGAGCCGCACGTCATGAAGAGTGAGTCTCTTTCTTTTCTCGGGAGAAGGC
AGAAGTACGGGCTGATACCGACCAGACTTCATGAACTCGAGGTCTACTCGC
TTGTACTGCGCTTGCTGGGCTTGCAGTCACTTGAGCCCCTTCACAACGGCA
CAGTCGGTTTTTGGGACACTTACCTGAACTAGACATCCTAGAATTTAGCGG
AGACGCAATGTATATGCATCGTTGTCACTGGTTTGGATGATTGGTACAACAG
TGGGTCTAATGACATGGTGTTCACAGCAACACAATCTGATGATGAATTCTG
GCATCCCCGTCTCCCATCCCTCACGTTAAGCTCAGCAGGCTGAGACGCGCG
GGGCGGGCAGAGGAACAGCAGCCATGGCCAAACTACTCTTGCGCCTCTTG
ACGGGCAACGCGGGATCCGGGACACTCAACAGCCGACGACTGTCCCATGC
TCCGCATACATGCCGTTTGCCGTCTCCCGCCTTGCGCTCGTCCACCCTACAT
GCCCTCGAGCAGAACGTCAAGCCGCACTTCCTGCACTCGGGAAAATTGAC
GCCCTGCGCGCCGCACACGCCGCAGGTAACGTGTTTCGTCAGCGCCGATCT
GAGCTCCGCAGCGGCCAAGTCGGGATCTGTGCCCTGTATGGCCGAATCTGC
ATTGGGTTTCGAAGGGCAGCTTGCGCCGCGGCGTAGAGGCGGCCGAGTGA
TTTGATGCGCGACGATCGCGATCGCGTTGCTCAAGATAGGCGAGGTGGGAC

GGGAAATTTGGCGAGGAAGCGACTGGACGGAGCGAGTCGGTGAAAGATG
CGAGAGGTGCGCGGACCGCTTTCTCGTGATGCATACTCGGGCCGTTGGTGA
CGATGGGCTCTGGCTGTTGTCGGACTGTCGAGTCAGACACGACAGAGCTT
GGTTCCACAGAGCATTCGAGTAATGGACTTGGCCGCATTTGCCGCCAAATA
TGCTCGATGGCTGCCACCACGTCGCCTGCGTATTGCGGAGCTCTGGCAGGT
CTACAGCCGATTGAGCATGTATGCGCCGAAGGCTGTCGGTGGCCCCTGTCG
CTGTCGACTCAGCGTGTACCGGCGACAAGGTAGCTTGCACTGCTTCCTGGA
CCAGCGAGGCCGTCGACTTGACCGGCGAAGATAAGATTCGAACGCTGGCG
GTAGACATGAAAGATGCGTTGCTTGCCTTCCTTCGCAAGTTGGGTCGGCCG
AATATCGAAACCCCGCATGCACTCAACCTGCTTGCTGTGCTCAATTGTTTGG
CGTGGCGAGGACGGAATTTCTCAGGAGAGACGTGAGGAAATGGAAACCC
GTCGGGTTTGCCCTTTGCCGCGGGGCAAGGGGTGCTGACATGAAAGGAGG
TGAGCCAACGCCTGGAGTACGGCGAATGATATCCAGTCGCTGCACCGCCTG
GGACACCAACTGATCAGGCAGACTCAAGCCGCACTGTCCAAGCGTGACGA
CGTTGCGGAAGTATTGTTCTGCGAGATGCGAAGTTGTGGTCATGCCTCTCTT
TTGGGTGTCAGCTATGAATTGTGAGACGTAGGAATGGCCTAGCCTCACCAT
ATAGTTGGCCATAATGCCCACGCCGGCACGAAACTGGGAAGCCTTTCTATAT
CTTTTGTCGGGGATGACCTTCGGCTCGAGGAGGTCGAGAGCCAAGATGCG
ACTTGCCACGACACCACCGAGATGCCAGACGTTCCGACCTTTCTCCAAATC
GCCTTGACGCCGAAGGACTTTAGGGAGTTCAGATGAGGCAATAGGCGGCA
AAGCGACCGGATCGTGCTGCTGTATGTGCCCGTTGCGATACAGGTGCATGA
GCGGTAGGAGGAGGTCGTACTGGGCTATCAGCGTTGGTCAGCCGGTGCTC
AACAAAACTCACAAGAAGTTTGAGACATTCAAAATCCCTTTCCTGATCTTC
TGTAGTTAAGGTACTCTCCTCAACACTGCCATCCACAGTGAATGGGGTTTT
GACCATTGTCGATTCTTCCGTTTTGCTTGTGCCCGAAGCGCTGGCCAGGGT
CCCAGAGCGGGAGAGGTCGTCTGTCGATGTGGATGTCGAGGCGGAGTCAC
CGGTATGTGGGGCTTCTGTGACATGCTCGTTCGAGGGATGTGGAAAAGGCA
GGCGCCGCGATCTTCCCTTGCCTTTTGAATTGAACATGACCAGGCCATCGC
CTTCGACTTCGGCACCGAACTTGACATAGCTGCCGTCCCCGCTTGGATTCT
GCACGAAATCTCTCTGCGACACCGTCCGAGCGGCTTCTCGTTCAAATTCCC
CGTCTGCGACCCAGCCAAGACCGAGAACGAACCATCCCCCTGCGATCCCC
ATCCCATGCGCATCTCTGACGGCACGCTTGCCGTCCTTTGGGATGATCCACC
CATTCGTTACCAAGTCATAGCGTTTCTCCGGCGGCGACGGCAGCGGCTGTC
CGTCCGGTGGCGAGCTGGACGACGGCTCTGGTGGGCTGGAAGACTGGAA
GAGGCTCAGGAATCTGCTGTAACCTGTCGTCGGTGACGTGGGCGACAGTG
GTGGGCTGCGAGACGATGGCAGGATCGAGGTAGCCTTTCCTTTCCCTTTGC
GATCGTCGGTTGGAGGGTTGTCTAGGGTGGAGCGTGGTTCTGCGAAAGCT
GTCCCACGTGTGAGGAGATTGCCCAGGGCCAAGCATGCAGCGGCTGATCT

ATACGGCGGCAGGGCAGCGATCGTATAGGTCTGGATGGCGATTTGAGGCTG
ATGGGCGATCAGAGCCGCCTGATGTCGTCGGCATGCCCGCACCACACTCGC
TAGGGAGAAAGATTACATACCTGAGCCTGCCACATGAGACTCTCCTCGTCC
ATGGGCTCTTCCTCTGCTTCCGCCGTCTCTGCGGCCTCGACGCTGACCTCAT
CATCGCCGTCCGAGTCCAATTTCGCCTCTTGGTCGACCGGCTGATCTTTGCT
GGTACCTACCCCGAAGAATCGGGCCAGGTTTGCAAACGATCGATTGGCCTT
TGCCGCGGGAGGCGGGGGAAGCGATGATGGGGATGGTGATGCCAGAAACT
GTGGTGGTCGCCTAGGCGACTGTAACGTTGTCATTGCGTGACGAACATGAA
GCAGCCTGTCGATCCCCGGGAGACTCTCTATAGGGATAGACGTAGAGAAGG
GAGGAGATATGAGGAGGAGGAAGAAGTTGAAAGCCAAGCCAAAGAAGGA
GGAATTGAAGAAGCGGGGTAAAGATCCCTGAGCGACAAACATTTTCATGA
CTCATAGCTAACGTTACCTAGACCGCCACCATTTCACTCGAATGCACCGTCG
CACAGAAGGCGAGCGCAGCCCGCTCCAACGCACTGCGAGGCTGACTTCAG
AGATGCATGACCTCTGCAAGAGGGCGACAAGTGGGGTGAAATGCCACGTC
ATTCTCTCCCGAGTGTCGGAGCTGCCCTGGCCGATGCCCGAGAGATGATAA
GATGACTACCCCTCTCCACAACCAGATTCGTGTATCTTTCGCATCGTGCACT
CACAAATTCGTGCGGCCAAGATGCCTGTGCGATCGGCTTCGCCTCCTGTAC
CGTCGGGTGCCATACCATCGAGTGTTCCCCGTGAGACCGACGAGCGAGCC
CCGCTGCTGCCGCCCACAGGCCAGCAATCTTATGATGCGACGTCCGAGCAC
AACGAGCAGGACACGAAGAGCGGTCATTTCAACCTGGCAGGACTGTCTGC
CAGAGATTTCTGGGTACTTGTAAGTTCATGATGGTCCATCATCATCCATGCA
AGCCATGCTGAGACCCAGATACTATCCATGCTCACCGTCCGTGTTCTGCGCC
GCATTCGATGCAACCATCGGCGAGTCATGCAGACGTGCAACTGCTGATTTA
CGCTGATCAACCCAGTCTCCACACTTCTCGGTCCGATCTCGTCTTCCTTCCG
AGCTACCAATCTTGCCTCTTGGCTTGGAACCTCTTAGTAAGCTTGTACTTCT
GGCCTTCATGATCGGTCTCACCAAACCAGCTTGCTGTCTGTGTGCTGCTTC
ACCCCGATCTACGGTAGATTGTGCAATATCATAGGTCGGCAAGCATCCATGT
TGATTGCTCTGAGTTTCTTCTGTGCGTCTTCTGAGGCAAATCGGGTCTGAA
CAATAATGATATCCAGGCATTGGAAACGTCGGCTGCGCCTTGGCGCCCAGC
ATGCCACTTCTTATTGCTAGCAGAGCTGTAGCAGGGGTCGGTGGAGGCGGT
CTGCAATCTGTCAGCAGCACAATCTTCTCCGACCTTGTGCCCATCACCCATC
GGGGTCTGTTTCAAGGTCTTGGCAATATCGCTTTCTCGCTGGGTACCGGTCT
CGGCGCGCCTCTCGGCGGGATGATCAATGATGCTTTCGGTTGGCGATGGGC
ATTCGGAGTGCAGATACCCATGATGCTTGTCGCTATCGTTCTCATCTACTTG
AAGGTGCGATACGAGGTGCATGCGGATCCGTCCAAACCACAAGAGACTGC
TCGACAAAAGCTGGCGCGCATCGACTTTTTGGGCTGCTTCACCTTGGCCGG
ATTTGTCGGAGCGGCCCTGATCGCAGTATCTCTGCCGACGGCCGACACATC
AGAAGATGCTGTCAAATGGACCGACACGCCTGTTCTTGTGCTTTTCGCCAT

CAGCGCGGTTCTTCTGGTCGCGTTCATCATCGTGGAATGGCGGGAGAAGGA
GCCTGTCCTCCCCTTGGAGCTTTTGGTGACCCGGACGCCGATCGCAGTATC
CCTCAACGGCTTTCTATCGTCAATTATCGCATTCATGGTGGTGAGTCAACAA
TTCTGCGGACTGAGATCCGGCAACTCAGATGCAGCTATATACGGTGCCTCTA
TTCTTCACCACTGTCATCTTAATGTCTTCCTCCTCAGCTGGACAACATCTTAT
TCCGAACTCCGTCTGTGTAGCCACTTCCAGCGTCCTGACCGGATTGGTCAT
CAAACGCACGCATCGGTACTACTGGCTTACGGTTGGCCTCAGTATGCTCATC
ATCTTCTCCGCAGTGCTGCTGGCCACCTGGAACCCGAACACACCTGAGTG
AGTGGTCGTGTCTCGAAGCAAAGGGCGACGCTCAAGTCCCAGTTGGATGT
TGTGGACCAACGTAGCCCCCCTCACCTTCGCGGGTGGCGGTGTCTCGACTT
CCACCATTGTTGCCCTCATTGCAGACGTGGGAAGGGAAAATGTGGCTATCG
CGACAAGCTGCGAGTCGCCATGTTATATATTGGTCGTTGTTGAGCTCACGAT
CAGTGTCATACGTTGCTCGTACCACTGGTCAAGTGATCGGAGTAGCCTTGA
GCGGCGCCATAACCCAGGCTATCCTGAACAAAGAACTGCACACCGGATCC
GAGGTCCCAACGCTCCCAAGGTGAGCCCGACTATTTCCTGCTCGAAGGGT
GACTCGTTGGCCTAACATGCAGATTATCGCATCTATTCGCGAGTCCTCTGAC
TCGATACGAAAATTGCCGCCGGATCTGCGCGCGACGGCTCAAGCTTCGTAC
GCGGCAGCCCTGCACGGCGTCTTCGTCACCACCATCGTTTTCACCGTGCTG
GCAGCACTATCCGTGCTGTTCATTCGCGAGGTCGACATGAGCCCGCCAAAG
AAGACATCAGAGGAAAACGAGGAGGCACAGGATGCTTAGACAGCAAGAG
AGAAACGAGATCGATTTTCTATAGAGAATTACATTTCAAGCCGTATGCGACT
GAACCGCCTGTGCTAGTTCTTGCGAGTCGACTTCACCCTTGGCCCGGGCAA
GTACTTCGGCAAAGCGATGATTGTGGAGATGTTGCCGAACCTGCCGCTCGA
GCGCACAATGCGCTTCGCTTCCAGCATCAGCATGGGGGAGATTTACTGTCA
GGGGGCTAACGGATCGTCCACTTTCACCTTTCCACGAAGAAATTCCTCGCA
ATCTTCGACAGTCCAGTTGAGGATCTGGGCTAATCTCCCAACCCATACTTTC
GGCTCGACCGAGGTGGTTGCGTCGACCTTCGATGAGCCAGAGGCTATATTT
CCCATGCAGAATTCTTGACTATGAGAGGTGCCGTGGCTCACAGCGACATCC
AGGGCGACTGCACGGCCAGGAGACTTTCTTTTGAGCCACGAGACCCGGCG
ACCAAAGGAGGCGACGCTGACACCCCGCACATGGGTCAACATTGGGGCGA
ACAGTCAGGCAAAATGGAGCAGTTGATACAGCCTGCACCTCAATGGTGGA
AGATCGCGCTGCCTGCGATCTGGTGAGGCGCTTCTGCCTCAATATACTTGGT
ATGAATGACTCGACAATCTGCCGACGGCGCACATTACCGTACACGCACGGA
CGCCAACAGGCAGGGAGAGGGGCCGAGGTGGACGAGCGCTCTGCCAACC
CATGACGAGCTGTAGCCGTCACAGCATGTGAAGCTGTGCGTGGAAGTGAG
ACATCTCGGGCTAGAGCTCACATCACATCACAGAGGCCCTGAGACGCCGA
GCGGTTTACACTCATTCCCGACCGCCCGCGGCCTTGCCTGACCGGGCGCCT
GACCCGGACAAAGGATCGGCCGTTACTCGCGACACCGCCCGCAGCCGATG

TGCTTGAGACCGGCGTCACACGGGGGTCTATTTGCGGTCAGGTGACACAC
CGAGCGATCCAACCCTCCTTTTCGCAACCAGGCGTAATCGAGATCCGTGCG
CGTGGGCGGTACTGTTCCATCCCTGCTACGATTGCGAGATCCGAATCCATGT
CAGATTGGAAGGAAGAGCTGCGGCAAAGAGCTCAGCTATGGCTCGTCCCG
GTCTGGCGGGCTCGGTCGAGCGATGTGACATGCTGATTTGTTTGTTTCGGT
TCGGCGATCTTGGTCTGACGTGACTCGGGTGTCTGGTCTGTGTGAGGTGAC
GATCATGGGATCATTGCGATTCGGCGTCCAGGCGTCCCGGGCGGCTTGAAC
GACTGAGTGGACGAATGATCGGTGAGCCATGCGCCAGATGGGCGTTGCCAT
GGTATAAATACGGGCTCTGCTCGCTCCACAGTCCCCATCAATTCCCACTTCC
ACA

>Mosaic_sequence_21

GTCCGCGTCGCTCAACTTTGAAGAGCCCGAAGCGCTGCGCCGATGCGTCG
GCTTCAGCCGCGAGAGCGCGCCTTTGCATCGCCTCCAGCTCTTCGACACTG
ATGCGTCGAGCATGGAGATGGGTTTCGACAGAGGTGGTGGCGAGGGAGGG
CAGGAGCGCGGCGAGGAGGAACGGGACCCAGGAAGGGCGCATTGGCTCG
AAGGTCGGCAAGAATGAGAGTGATAATGCTCGGCCAGCGAGCGAGCTGTA
CGAAGCAAAGTTGGAATGTACGAAGAATGCAAAGAGAGAAGTGAGTAGT
GCGCTGGTCGATAGGACCCCTCGCGCATCATCTGGCAGAAAAGACCTTCCC
ACGGCAGACTTGACGACGGTAGTTGCGTTGACGAGGGGCCTAGCGACAAC
CCCGCGAAGCACGGGTTTCATCGTCATGTCCGAGCGACGGAATGGCCGTG
CGCAGCGAGTCCAGATTGAAACTCGTTTTCTGAGATACAGCTCGCAGCGAC
ACCGCACGTCGCGCTCGTGCTCACACCAATGGCCGGTCATAAATCATGCAC
CCTTGCTTGACCAGGCATGACGCGCCTGATTTTTGCCACCCAAATGAGAGT
GATCCCTTCGGATGGTATACCTCCGAGGTCGCGTGGGGTGTCTGTCACCCC
TGAGCCCAACTGCATAATAAACCCCCACAAGCTCCAAGGCGAGCATGCTGT
TGTCTCTTGTACACCGACAAACATACTGCTTGTCCAGACGCACAGCAAGAT
GGTCGCCCGGTCCCCGTCAGCAATCAGCGATTTCAGTCTGGTGTCTTGCTT
CGATGTGCTCTCCCTTGGCGAATCATCCTCGGCTGCCTCCTCCCGACGGCC
CTCGGTTGCGATACCGATGGGCAACATTGCTGCCTCGGATCGCGGGTTCTT
GGAAGTCATGAGACCCGGCGCAACCCCGGAATTCTACAACTGCGACCAGT
CCGCCGCCCGTCGAGCCCGACGGCGCAAACTGGAGACGGCCAATATCTCG
AGACAGAACGACGCCTCAACACCATGCCGAAAGCCGGGCCCTATTGGTGG
GTCGAGACACGACACTCACGATGACCAATCTGGTTCTCGCTTGCCTCTTGG
GCCTTCTCGTGTACAAGCGACAGCGCCGAGTCTGCCGCTCGAGACACCGT
TCCGACCTCAGCTGAAGATACACCTCTGCACACCAACGTCCCAACCTTCGC
CAAACACCACACTTGTCGAGATCAAGCAGCGCAAGAAGACTCGCAGAGC
AGGCAAGAAGATCCGGGCGAGGAAAGATCGGGCGGCGGTGCGGGATCTG

CTCTGCGCTGAGCCTGGTGATGGCGATGACGACGACCACCTCATCACCCCT
CAGATGTTGTCGCGGTGGTCCCCCGCTCCTGATGAGGACGACGACTGGTCT
GAGGATGGCGCATCAAGATTTCTGGATGTAGAAGCGGATACGGAGGAGGA
GCTGGAGGAGAACGAGGTCGATGACTTGAGTGTGTTGGATGCGAGGAGTG
GCAGGAGCAGCCCAGCTATCGAAGGTGGTGTCGCTACAGGTGGTGTGATG
AGCAGAGGAGATGCCAAGTCATCCATCGATAGGTGAGATGCTCCTTCCAAG
GGTGTTTTGGAGCTAATACGAAGCTTCTTGACTGACTCGGACAACTTTATG
CTGTTCAAGGCTAACAAGCTCCGGCTGTGGCAGGCTTTGAGCATTGAGGTG
TGTACGGTCCGCGAAGAAGATGAGGCGTGCTGATTATACAGCTTGGCCTGG
TGGAGTTGCGGCACCCAGATCCTGCTCCCGAAATCGATACAGCTCCTGCTA
CTGAAAGCCTTACGACCGATGATCTCCCGGGTCAGCTGCCACCTGTCCCGC
AGACCCTCAATCAGGCTCGAAATATCTTGAAAACACATGCGCATATCAATCT
CGTCGACTATCTGGAGGAGAGACGAACTCATCCTGCAGCTGGAGCAGGAT
CTTATCGGCACTTGGTCTACGCGAGCGCTGCGGAGATGATGAAGAGCGCCA
GAAAAGACAAGAAGCTCGCCTTGATGGGTGAAGTCAAGCGTGAATGGCTG
GAACCGTTGCTTCGTGATTTCGGTATAAAGAAGTCCAAAGCTGCGGCGTGA
TGCACCAGCTATGGCTGTTCTGCGATGTATGGCCTGCTCACTTCGTTACTTG
CGTCGGCTACACTGTCAGAAAGAAAGCAGTCACCAGATTCAGACCGGTTA
AGCCCATCATCATGATCCGGCCGGACCGTGATCCGATGAGCTACCATAGAG
GAATGACTCTCTCTCTCTTGATCTTGTGTTGCTCATGTGGACAACACACCAT
GTAAAGCCCAGACAAGCACACGCCAAGCCAAAGCAGCAGCAGCGGTCAT
CGTCATACTGCCTTTGGTCTCACAACGATACATGCAGTCACCGTCCATCTCT
TACTTCTCCCTTTATAGAGACGATCGTGCTCGCACAGCACCGTCATGCTCCT
TCCTCGCCGCCTGAGTATAGGTGGGCGCCCCGCGCTTGGATTTCGGCTGAG
TCTGCCGCTTGGAGTTGGCAGAGCATATCATGACGATGCTTGCTTCGGGTA
CAGGGTCCCCTCCAAGTACAAGCTCCCAGACTGTACGTCTAGATCGCAAAT
GCGTAGCAGGAGAAGGAGCGGGCTGATGGCTAGACACACCCGAAGAGCT
GCACAACAGGTGTGGTGGTTTCTTTAGCCTGTGATTGCATCTGACGGCTAG
GAAACGTTAACGCACCCCTGCTGCGATACGTGGAGTCGGTCAGGCGCCAT
GGGCACAGGGCGGCCCAGGTCGACCCTCTCGACCTCATGGTCCGCGAGTG
AGTCTTGAGCGGGCTATACGGTGCAGCGAAATTCCTCGTGAGCCCGAGCTC
ACCTCCAGCTCTGTGGCAGCTCTCGATCCGCAACGCTATGGGCTCAAAGAA
GGCTCGACATACCCCCTGAACGGTATCTTACACCTGCCATCTGCCGCCACA
AGCTTGGCTGCGCCTCCGCTAGCCCAGGCTCAAACCGAACGGGCAGAGAC
AGGAGAGGGCACGTCGGCCTCAGCTTCGCTCGAGACCATCTCAAAGCACC
TCATGGGCGTATATGTCGACAAGATCGGCTACGAATACATGCACTGCCCTGA
GAAAGACGAGCGGCTGTGCGTGATAATATCTGAACCATTTTCAGGCTAACC
GTGCAGCTGGTTCTCACATCAGGTTGAGACAGACGCCACGTCATCTCCCGG

CCATTTACCCCAGCTCGAAAGGAGAGGATCTGGAACCTGTTGTCGAGGAG
CGAGGAGCTCGATCGTTTCTTGGGCAAGAAGTTTCCCAACCTTAAGCGATA
TGGTGAGCTGAACGGCGGCGTGCCGTGCGGGGCGCGAAGCTGAGTGGCA
GGCTGCGAAGGCGCCGAGAGCATGTTGCCGGCAATGGACACCTTGTTCGA
GCTGAGCGCGGATTGTGGGTGACTAAACCAGCTACAGCGAGCTGATTCTCT
TAGCGGATATCCGATCAGTTGTCCTGTCTTTGCCTCATCGCGGCCGGCTGTC
TCTGCTCTGCGATCCCGATCTGCTCAGCTACTCGCCGACACAGCTGTTCGC
CAAGATCAAGGGTCAGCCCGAATTCGACCCCTCCACGGCTCCCGGCGCAA
CGGGCGATGTCATCTCGCACCTTGCTGCTATACGTCGTGTGCCCTACGGCAA
CAAGTCGGTCAAGGTGGAGATGCTGCAGAATCCGAGTCACCTGGAGGTGA
GTCAAAAGTAGTCATCATTGAGAATGCTTGTAAAGTCAGGCCGTCAATCCC
GTTGCCCTTGGTGTGACACGCGCCAAGCAGATGTCCCTACTCAAGTCGTCT
CCGAGGGAGTGCCAACTTGGCGACCGGGTGCTTTGCGTCCAGCTACATGG
TGATGCTGCGTTCGCCGGCCAAGGCGTAGTCGCAGAATCGCTAGGGCTGA
GCGGTTTGCCTCATTACGGTAGCGGCGGCACTGTTCACCTTATTGTGAACA
ACAACATCGGATATACTACGCCGGCCTCATCAGCTCGCTCGAGCATCTACTC
CTCTGACATTGGAAAGATGATCGGCTGCCCCATCTTACATGTCAACGGGGA
CTACCCAGAGGGTGAGAGAGAGTCCGATGATACATGCGCTGGCTGATCAA
AAGCCGTGGTCCGTGCCATGGATGTCGCCTTCCGCTATCGCCAGATGTTCC
GTAAAGATGTGATTGTCGACCTCATCTGTTACCGACGCTGGGGCCACAACG
AGCTTGACGAGCCGGCCTATACGCAGCCGAAGATGTACGAGAAGATTCGTG
CTCGAAAGAGCGTGCCTCAGCTATATGAGGAGAAGCTTCTGGTGAGCCTTC
AGGTCCACCCATTGGATCCCGAGCTGAAGGTCAAGGAAGCAGGGTCACTG
AAAGCCGAGCTGGCAAGCCAGGCGCGCAAGCAGTATGCGGCAGATCTGGA
GGAGAAATTGGCAGCGGTGGGCGACTACAAGGCAAAGTCGGAAATGCTCG
AAGGCCAATGGGCAACATGCGTGTGGCCGGCGGGAGCGAGCGCGGACCA
CGCCCCTTTGACGGGCATCTCAAGAGATGTTCTGATAGACGCCGCTACGAA
GAGCGTGACGCTACCGGACTCTTTCGTTAGTCCTAATTCCTTCAAACATTTC
AAATTTCCCCAACTTCAACAATAAAAAATACATAATACAGGTATGTAATTCC
TCAACAACGTCCAATCCATTGAGACTGTTAAGATGCTCATTATACCTACCTA
TAATATCCCTCAACCACCTGCCTAATACCTCATGTGTGATTTGTATTTGTGAT
GAAAAGGCGTGATGGGTTAGGTGTTCACTTTTTTATACACAAAAGGATCCC
CCAATCCATGATCTGACAGTTTGCGGGGAGCTATATCTCCCTTACATCCTAC
CACAAACCAGCATTCAATTCTTAAAATCTAGGATCGCGTAACTAAGAGGTT
TGCGCTTATCGCAGATTCAACCGCAATGACGACACCTGAAGTATCCCACGG
CCGTGGCGGCGCTGGCAACATCCATGAGGACAACACCAAGTACGTCGACG
GCGAGATCGTGCGCGAGGGCCCGGAAGGCAGCCATGGCGACGGCGCATTT
AGTACTGGACGAGGAGGTACATATCCCGCCTTTCCTAATTGCTTCAACGATA

TTGCGCATTATCTCACCTAATTCTACGAAACCGGCGACGAGTCGCAACCTA
GTCACAGCCTTCTTTACTGACACACAAATAGGTGCAGCCAACATAGCCGAT
GCCACCGTCCCCACGGCTCCAGTGCGCGGACCGCGAGGTCGTGCCTGA
GGAAGCTACGCGCCCCTCGACCGACGACGCCAGCTTCCACGTCGGCCGCG
GCGGCGCCGCCAACGTGCACCTCTCGCCCGAGGACAAAGCCAAGCAAGA
AGCGAAGCACGAGCCCAAGCACGCGCACGCCGCTTCCAAGGTCGTCGAC
AGCCCGACCGGCACCGGCGATGCGAAGGCGAACCAGGGCTTGGCTGATAA
GTTGAAACATAAGATATTTGGTGTTTTTAAGAAGTAGGCGGCAGATGGCAA
TCTGCGCATGAGAGATCGAGGTTGGAGATAGGAACTAGTTTGTGATGGCGA
TGGGGAGAGGGGCAGGGAGGTGGTGGGCTATGTAAGATGGTGTAGTGGAA
ATGGAAGTTGATGAGAATATGAGTACCTTTTTCTTTGCGTTGTGACGGAGA
CAGCGAAAAAGGAGGAGAGAAAAGTTAGTCTCCTGATTTTAACTTAATATC
CCGGGCTGGTAGCGTAACGCTGCTGGTTTTTTATTTGAATTTACATTACGAT
GATATCCTGTCAATCAATCGCATTTATGAGGTGGTGTCGCTGTGGTTAGTCC
ATGATACTCAGGTTGTCAGATCGCTTATATGCAAATTTCACATTCGGGTTCA
GCGGTGATATTTATATATCTGGTTGAGCAATATATACAATTAGATCATGAGGT
AGTCTTAGCGAACTCCAAATAGTCTTCTCCCCTCTTAGGTCAGATAAGAGTC
GCTAGCTTATTTCAGGGCGTTCACGACAGCAGTGTACGCGGGCTTAGGGTT
AAAGCCACTGTCGAACAGGAGGGGGTTGGTGCTAGCTCTCCAGGAGTCCT
ATCACGTCTTCAGTCAGCTGATTTCCTCCAGATCATTCATAATAGTCAAGAG
GGTGGTTCTTACGGGATCCCGGACACCCCAGACGGTGATACCGACACACTT
GGGCTCATCGAGACAAGCCTTGGTGACCTGAGCGTAGTCGTTGGCGGGGG
CGGTTTGGATGTCTACTTCGGTGAAAGCGACCTCACTGACACCACTGGCGG
CAAGGGCCTTGATGGTGGCGCCGGCTTGGGCGGCGAGACCGCTCGTGAGG
TGGGCTTGCGATCCGATGCCGTCGATGGGGACACCGGCTTCGAGCCACTTC
TTGACGTGGTTGATCATGCCAGTGGTGGTTTTAGCACTGTTTGGTACAGTAA
GTAACTATTAATTCTAGAAGTCTCAACAGGGTGTTCTAACGTACTAGTTAGG
CTGGTCGAGGTTGTAGTCGTTAATGTACAGCTTGGCGTCGGGGTCGGCCTT
GCGGGCAGCTTCGAAGGCAATGCGGACGAAGTCCTCGCCTAGGACTTGGG
AGAAGACGGATTGACGCAAAGAGCCGTCTTCGTTGAAGATCTCGTTCACG
ACATCCCAAGCGCGGATCTTGCCCTTATACCGGGTCACCAGGGTGGTAACG
TGGTTCTCAAGGACCGAAGTCAGCTTCGTCTTGTCCCTGATGTTGGAGACC
CAACCGGGTAGTTGCGAGTGCCAGCAAAGAGTGTGCCCACGGATCGTCTT
GTTGTTCGCCGTGGCCCCAGTCAACGAGGTAATCACCTCCGGCGAAGTTGA
AGTTTCCCTCGGAGGGCTCGGTGCTGTCCCACTTCATGCTGTTCTCCGGAG
TTACCTGACCGAAGTCTGCCTTGATTACATCGGCGGACTTTCCAGTCGAAA
GACGGTTCTGGTCGGCGCAAGTTCCGTAGTATAGCTTTCCCTTGGCCTTGAT
CAAGGCGTCGATGCTGTCGGCGGCCTGTCGCTCGACGATCTCGGCGGGAG

CGGCCAATGCCGTCAAGGGGGCAAGTAGGTATAGGATAGCGGACTTCATGA
TTACCGATCTTACTGACACAACTAGGGATTAGGTTATGGGATGAAGCTTAGG
ATAGGAGGCGAGGATGGCGTGACAGCTGAAACGTCCAGATCTTATACTTTT
CCACCGAGGTATTACGGAGGACAAGGCCCGTCATAGGTAAGGCTAGACCTT
GGTTCCAAGTCCACATCTAATAAAGTTATACTTGGATACTTGGCAGCGGTAA
AATAGAATAGCATGATGATTAATTCCCCAAGGTGGAAATATAGCCTCTTTTG
AGCCCCCAAGTCTTCCTCTGTGTCATTAGGATATGATTGAGGGGCACTCGGT
CGCAGCGGGCCGCCAATATTAGCAAGGGCATGAGAGAAGTTTTCACTATTC
AAGGTGAGGACGTGCTATTGCCACGATGTGGGAGTCCAACCGGCTCCACG
AATTGATACATCGTGGAGTGCATGGGACCATAATAGCCTAAAAACCCATGCT
GGTATTGAGCATTATATGTGCCTGTTATATCGTATGGGGGTCAATCGACGTAT
TTATAGACGCTCGGCGCCAATACTCCGCAATAAAAGCTTGAAATAGGGCGT
ACAACATAATTGCATCCGCACCGCTCATACATAGCTCTCTCTTTCACTGGGA
GTTTCATAGGTCTAGAAATGGGTGAGGGATAGGGTTATATAGAAAAACAAC
TTGTCAAAATCGCCTCTAGCAGCTTGTTGCATCAGCGATGTTTTGCTCCCCA
TGGAACTAAACGTTTATATATATGTACTCACTAGTTATAACCTTAGATATACCT
CATTTTAAAGTTGAAACATTCAAGATGAGTGCATATAAGAAGGCATGTGAA
ACCCAGGTTTTCAACCTACACGTGGTATATAAGGCATTATATTAAAGAGAAA
TGAATGAGACAAAAAAGTGAACCATATACTATTTCACCCCTAATACAGGCTA
TGAAACACAAATAGTTATTGGTGGCTTTGGATTGACCGATGTCCGAGAGCG
TGGCACTCGCTTATCTGTCTCCTCGTTCTTATTTTCCATTTCCAATTCGGGGT
CGACTGAAAATGGTTATATGTTCACGTTTTCCTATATAACATGAGCAATCGA
GGAGTTGCTAATGTCTGTATATATATGAGTAAGCAAGTGATTCAAGTACCTT
ACCTTATATAGAGTATTAATAATTGAGCTTAACGAATGAAGATATCACCAAA
ATCTAGGTACCTGGTAATGACCCCTGCTCTATCAGCCTTGCCTTCGGTTCCC
CACAATGTCATGGGATACCGCTCGTGAACCACCAGATCATATAACGAGGGG
TCCTATACTAGATGGGAAGGGATTTCCAGGTCTAGCTCTAGCGTTTCTAGGC
ATGTAGGTTACGTAGGAGGGCACCTCTATCCTCTAAATTCCAAGTTAGTAAG
GTCGGATAGGCGTAGAGATGGATCCATTCTTTTTCTTTGTGTTAGAGATGGC
CATTTCCAATTTCTCCTCTTTCGTCATTCATCCAACTTTCATTCAATCGGCTT
CGATTTCGTCAATTTCATCTCGAGGGTTGCCGCAGAAGTTATCGAATTTCCA
AAGCGTCAGATATAAGAAAAGCATTGTTGCATTTTCGAAAACCGGGAGAAT
GTCCCTGTATCGATCATGAGAAGAACCAAATCAGCTCGTTGAATTGGTTCT
GCGCATTTCATAGGTTAGCCAGAGGGGAGACAAGAAAGGGATTCGCTAGG
AGGTGATCCAATAGTCCAGAGGCTTCATGCTGCAAGGGAAACGGGCGGAA
GACGAAAAAAACATTACGACCACGTTGGCCTGGCAAACACTTGAACCAAA
CATTAAACCTGACAATTTGGGAGACTAAATTAATTGATCCAAGTTCTCGTTT
ATGTTCTCGTCCTAAGGTTCAATTAGCTTCGAGTACGTATTTAAATATTCTAA

ACTTGCAGAGTTTAGATTATTAAGGTTCGCGCCGATTGGAGCTCTAAGGAC
ATTGCTAACTGCTAACTCCAAAATATAGGGTTTTTAGGTTGTAATTCAAGGG
CAAGTCAAGGCGTGAACCCAACTTACGACCGGGCTGACAGGCTCAAGGTG
ACACAGACACAGCCGCCATTTCGTCACCTGGCCCCTAACCGCTGAACCACC
TGAACCACTGAACCACTAACCTAATTCCCCCGTCTTTCCGTTTTGCTTTTTC
CGCGTTCCATCCAATAATGGTTCCTCGGAACGAGGAACAGCTACGTCGCCC
TGCCCCAAACACTCGCTTACTCTCAGTCCAGTCCATCGTATCTAAACATTCC
CTTCGTTCATATTCTTCTTCATTTGAAGCTGACGATGAACGATCAACGTCTA
CCGAATCAGGCAGAATGACACCGGCACCTAGCATCATCGAAGACGGCATAC
CTCCTACCCGCTATCATGGCGAAGATACTCGCCCGACCAGCCCCAAAGAGC
TTGCTGGTTGGTACATGTATGCCTTCGCTGCCGAGACCTACGTAGTATGCGG
TAAGTCATCCGTGCTTATGCTCTTTCCCCAACATCTGCTATGTACCCATGCAA
CATCAACAACAGTGCTACAATCAATAGCAATATGTAAGTAGATATATCTTCAT
CGTTTGACTCAAGGAGCTACCTATAAAGCTCTATAAACCCCATCACGTCCGA
TGCGAAATCAAGTAGATACCTATAATCCATCATGAATCAATACACACATATAA
TGCCAAGCTCGCCGTGCTTAAGCTTACCGATCCCGAATAGCTTCGTTTATTC
CGATTCTACTAGAAAGCTTGGCAAGAGAAAATGGAGTTCTCTTATCCGATC
GGAAAACACCTGCGGTGACAACGGAAATGGACAATGTGTTGTCTCCATCC
TCGGTGCCGAAATCAACACTGCTAGCTTCGCCATGTATACCTTCAGTATAAG
TGTCCTCCTACAAGCCTTACTAGTAGTCAGCATCAGCTGTGCCGCCGACCAT
GGAAATTATCGAAAGCGATTATTGTTGACATTTGCTTGGATCGGAAGCTTCT
GCGTCATGTTCTATATCTTCATCTCCAAGGAGATTTACATCCTCGGCGCATTG
CTAGCTATTATTTCAAACACCTCGTTTGGAGCATCCTTCGTCCTCCTAAACT
CCTTCCTTCCTCTGCTCGTCCGTCACCACCAAAAGTCCAAGAACAAGTAAA
CGGGATACCAGATGATCTTGACCTACAATCCGACCCAGCCACACTTGCCGA
AGATCCCCTAAGCGACTCCGTAACTGCCTTGCTACCCCGAATGACGCCGAC
TCCGACATTCATGACTTGCGACGGATACCGACCCACGAAGAACTAACTTCC
ATTGAACTCCAACTTTCTACCCAAATATCTGCGAAGGGGATCGGGATTGGC
TATGGTGCTGCCTTGTTCGCACAATGTGCTTCTATTCTAATATTAGTAGGCAT
GGGTAGCTCTACTTGGTCACAACGGGTCGTCCTCTTTTTCATCGGAGCTTG
GTGGGCTGTTTTTACAATACCAGCAGCTGTATGGCTGAGACCCAGACCTGG
GCCTCCTCTTGCAGAAAGCCCATATCGGGGAACCCGTGCTTGGATATCTTAC
ATCGCGTATTCCTGGAGAAATCTCTTCCGAACCGTGAAGCTTGCGCGGCGC
TTGGTAGATATTGTCCTGTTTCTAGCCAGCTGGTTTCTGATTTCCGATGCAAT
CGCAACGACGTCTTCGACCGCGATCTTATTTGCCAAGACACAGCTCGGTAT
GAAGCCTTGGGCTTTGGGTATGATCAACGTCATCTCTACGACTACTGGTGTT
TTTGGCGCGTTTAGTTGGTCATTTATATCGAGACATTTCAAGTTGAAGCCAC
ATCAGACTGTTTTAGGTTGCATTGCGCTATTTGAGATCATACCTATCTATGGT

CTTCTAGGTTACTTGCCTTTTGTTCAACGATGGGGAGTTCTTGGGCTTCAGC
GACCGTGGGAGATGTATCCATTGGCTGCCGTTTATGGATTCGTTCTTGGCGG
ACTGAGTTCATATTGCCGGTCATTATATGGAGAGCTAATTCCCCCGGGTTCT
GAGGCTGCGTTCTATGCCCTATACGCTATCACCGATAAGGGTTCGAGCATAT
TCGGTCCTGTTATAGTCGGTGCCATCATCGACGCGTCCGGCGAGATTCGAC
CCGCATTCTGGTTTCTCGCGGTGTTAGTTGGCCTCCCGGCGCCAATTATCTA
CTTTGTCAACGTCGAACGAGGCAAGTATGAGGGCGAGAAACTAGCCGAGG
TTATCGAAGGTTTTAAAAGCGCGGAGGAAAGCCAAAGAAGCACATCTCAC
GGACGGCGGAGAAAACCAACCGATACTTGCTGCTTATGACAGTGACGACG
AGGGAGGTAATGGCCACACGAGGTCCTAATTAATGTCGAATCCATTCCATA
AGAACGTATCTATCCATATTGCATAGCGACGGCGTTGACTATTGACTACAAA
AAGAGGGTTGTTCAATCACAACTCCTAGATACCTAGCTTTAAATCGATAATT
ATTTGATTAAATATTACCTATTTCACGCAAGTTGTTGCGGGTTTTCTCTATAC
GGCATGCAGCTCGCGGATATGACGCCAAACGTACTTGGCCTTATTACAACC
AATAAGGGAGCATATAATGAAAGATGACTCTGCTAGGTATTCATTCCAATGT
ACGTATGAAAATAACTCATAAAAATACGAGTCTCATATCCAACAATAGGCAT
AATTACCTAGTGACGATTCCTCGAGAGACAGACAATGCTTAATGTAGATAGT
TTCGAATACTCGAACAAAGAGATTCAAGTGCTTAAAATGAATACGTCACAT
GCTACAGGGGATAAGTGTTCCTTATGTAATATACAGGAGCTTATTGATCGAC
GGCATACATAAAAAAGCAATACCAGGCACTGAACTGGGGAGCTACGTTACC
CGGGCATAACCTTGAAGCCCATGGCAACCGCGCCGGGCCTATCAGTTACCG
ATTACGGTAGGTACCCTAGCTACTGCGGCAGATTATACCGAACAGAGTTTAG
GGAGTGTCTAAGGGCATCTGCAGCCTAGGATCTGGCATTTTCAACATGGAC
ATTTCCTCCACTTGAAATCCATTTTCTCCTCCCCATCCAATCCATCTCCTCAA
CCTAGGCAGCTAGGAAGCTCACTAGAAGCCCGCGGCGCATACACAACCTAT
ATCGCTGATGTGACCAGTGGTTACTAGAGGTGGCTAAATCGGAACGAATTA
GGACGCTCCCTCGTAATAATTCATTACTAATTCTTTTTTTCCCCCGATTCTTC
CACCCTTGATTAAGCAACCAATACCAACCTCAGAAAAATATCCCAGCATTG
AGGCCATCGCATCGTCCTAGGTCGGGTCTGCATCGCACTTTCCTCCTTCCTT
AGGTCTCCTTGCGCCTCAGAGCTCTAGCTCGCTATTGGATCTGATTGGAATA
GATTCTGGTGTAATTGTTTGGAAAGTCCTACGGCGCTGCCAGTTGCTACAG
GGTAGCTGTCGCTTCGTACAATTCAAGGTTTCGTAGGATTTTGACTTAATAA
CAGACGCGCCAAGCTACCAGATAATTCCCCCCTTCCCCGCCACAACCGGTC
CCGGCTTACAATCTTCCTGATCGCAACACCAACCGAAGCTCATCGAAGGTC
GCGAGCTCCTCACCTAGCAACCGCACAGCATTGGTTCCCCGCGCTACTGCC
TCCGCGCATACCCTCCTACTTTACATCTGTTTGGTGGCTTCCACTTCTGTTTA
GTCAGTGTCGATTGCGCGCGTGTCACCAAACTTTTTTTCTTCCTTTTTTTGG
TTCTATCGCATCGAACGCGAAGCAACTTCATACCTCTTGCAAGCCTACATTC

ACCATTGATCAACACAAGTTCGCGAATCCGTTCTTCACCAATCTACACTCCG
TTCGAATCTTACGATCATGGCAACAGCTCGTAAACGTCCTCGAGCGGAGAT
GGTAGAGGAGAGTCAGGCAGAATGTCCTTTCAGCGTTATTCACCCTGATCC
TAACGAGAAGGAAAAGAAGGAGAAGAAGACCAAGCGCAGACGTCAAGA
GTCCGAAGAACATTCCCACCCAAAGGTCTTGCTTCAAGCTTCGCCTTTCTC
ACCTATGGGCAAATTCAAGGATCCTAACAACACCATGACCGATACTATCAA
GTTCAACCTTACCAGAAGTGGATGGACATGACACGGTACAAACAGCTTCGT
CCGTATGTGCGCTTCAAATTCCTATTATGCCAATGGATCGTACTAACACGATA
ACATAGTTAATGGAGTCAAGTATTGGAGCGAAGGCTTCATATTCGTTGCGA
ACAATTCGACGATCGAGCGCCAGAAGAACCCGGGCGAACCGTACAACCTA
GAAAGAAGTCGGACGACGATTGGGTCGCAAGGATTCTCGAGATTCGTGCA
AGTGATGAACATCACGTCTACGCACGTGTCTACTGGATGTACTGGCCGGAC
GAGCTGCCTCCGGGTACGCAAGATGGGAAGAAAATGATTCGAGGAAGACA
GCCGTACCACGGCGCAAATGAGCTAGTCGCGTCGAATCATAGTATGTCTTA
ACGATATATCGTGTTTGTGGTGTTACTCTAACAAGGTATAGTGGATGTGGAT
CAATGTGGTCAGTGTCACAGCTCCGGCGACTGTTCAGCAGTGGGACGAAA
CCAACGAGGATGACGTCCAGCCCGCGCTTTACTGGCGTCAGGCCTTCGATG
TTATTAGCAACGAACTCTCGGTAAGTTTACGAAGCATTACCATACAACATAT
ACACTACTAACAAGATACAGTCTGCCGAGCTGCGCTGCAAGTGTAACCGAC
CCGAAAAACCCCGACAAAAGACTATTTGGCTGTTCGAACGATGATTGCAA
GAAGTGGCTCCACGATGACTGCCTGATCCACGAAGCTCTCCTAAAAACCTA
CCAACGTCTAGGAACCGATAAACCCCACAAGCCCGCGCCGATTAAAGAAG
AGGAAGGCGAAGGCGCGAAACGCCCGTTGAGTCCTAGTGAGACAGGAGC
GGCGCAAACTGCTGAGCAGTCGATCGATGTCAAGCCTGAAGAGCAGAAGG
AAAAAATGGACATTGATGGCGATAGTACCAAGGCGGAGGTGTCCGTTGCC
AGCTCTGACACGAAGCGAAGAGGACGACCGCGCAAGTCGGAACCGGTCG
AGAAGGCTACGGCCAGACCATATGAAGGATTGTTTGAGGCAGTTATTCACA
GCGAAGGCGATGGCCCGTCAGTCCTCGAGATAACGGACCTTCGAGAGAAT
GTCACAGGTGGAGACAAGTCATGGAAGGAGCCTCTCGAATGCCTCATCTG
CGGTCAAGAGATCGATTAACAATGATGCGAATACTTCCTAGGAGGTTACAA
CCCAAATAGGTTGTCCTAGTAGGAGTATATACATGTTCTTGTTTTGCCTGTG
AGCCTTATCATGTTCTGCTCGGCGTTCCTGGGGGCAAGTGATGATACCCTGC
AGTATTTTGCGGGGTGGCTGCTTTTTTAACCGTTTATCCTCTGGGCGATGAT
TTGGGTGTAGTTGGCGGAAGTAAATTGAAGTACCTAGTGAAGTGGGATGAA
GAAAAATGAAAATTGGGGGCAGCAGAGAACTCTGGTTTATGTGGACATGG
AATAGGAAATATACCAGAACGGTATCAGTTCGTTCTTCATGGATTGGTACTT
AATCACGTCCATCTTACTCGGTTTGGTGCAATCGTTGGTGGTTTATGCGGTT
GACTTCCTCAGTTGAAGGTGTACATAGCAGTCACGAAAGCTATCTATCGTAT

TACCAAAAGGCGTATACCGTTGTGTTTGTTGAAATGATTCCTTAGCACCTAA
ATAATGATCACTGCTATGAATAACCTTGACAGTCTTAATATGAATACCTACTT
ACCTTACAAAATACTTGAGTATAAGGCAGGGACGTTTAGAATACCTAAACC
GAGGCTGAGTTAAAGCTAGGACCTAGGTACAGTATCAAACTAAGAGCTAGA
GAAACGATGTCATATTCAAACTTAGAACACA

>Mosaic_sequence_22

CTCTCGCGCTCAGCTGATGGGTCCCGTACGTTAAAAAGAGGTGGTTGCGAA
AGGCCTTCGATATCAGGGCTTTGGAAGCGAGAAAGCTCATCAGGATGTTGC
AGGACGTTATCGATACGAACATCAGCATTAGGAGCATCGTATGGACTCTTCT
TTTTCGGGTGATCCATTTCTGGAGGTTGCACGATCTCGACAGCCGCCTTTTT
CCTCTCCGGCTTCCTCCATTTGTCCACAACTGAAGATTCGTCATCGCTGCGA
GCACGTTCATCTTCCTCTTTTATTCGACGTGCAAGTTCAGCCTCTCGGTAAT
AGCGATTGGCGTTTTCTTGAACAGCGTCCTTTTCGCGCCGCGGGGATTCAC
CTCGGCGCGATCTATTCCTAGAACTAGCATCACCACCTCGGCTATGGCTACG
TCCACGGCGCTCCTCAGCAAGTATCGCTGCGGTGGCGGCAGCAAACGCGG
CTCCGCTTACAGGGACAGTTGCATGCTCGGTAGCTTCATAGACATCATGCGC
ATCACGGAGCTCCCGTTCATACGAATCCTTTCGACTAAGCCTTTCTTGAGGG
GGAGAGTCATCGCCCTTATACTCAAATCGGGAGAAATCAGGTTCCCGCTCT
ACAGTAACGACATTCGGCGTTAAAGGTCGTTTTGGCGTCGTGTAAAGGGGG
GTTGGAAAAGCATCGTCAGCTACTTGAAATTGGAAGGGGTCAATGTGGCCT
TCACGAGACTGATACTCTAGTTTTTCCTGGCGTTTATAGCTTTCCGCGGTAG
AACGGCTAGTGCGGTCCTCATCACGGCGTCCATCACGTTTCTCATCTTTGCG
GACCTCTCTACGGTCAATCTCCCGATCCTCTTTTCGCTTATCCTCGCGTTTAT
CACGCACATTATCGTAATCATGATCAGAATCGTAATCATCGCGACGGCGCTT
CTCTTCAGACTCTCGTTTCAATTCGGGTACTATATGTTCCCGCCAATGTCTCT
CTCGTTCAGATTCCCGATCAGGTTCATAGCGTTTTTGCCGTCGGCGGTCGAC
GCGCTCTTCACGAATTTCGCGGGTTTCGCCAACATCCTCGCGACTCTTGCG
TTTGTCGGTTCTCTCATCCTGCACACCAGGATCTGAGCGCTTTGACCGACG
TTTACCATCGCGGTCGCGTTCGCGTTTACTCTCGCGCTTATCACGATCATCTC
TTCGATCAGAACTCAACGCCGCGCCTATTGCTGCACCAGCTATGCCTGCAA
TAGCAGTCTCCCCAATTGTACTTTTCGACACCGAGCGCTCCGAATATCTAGT
ATCAGTTTCCACAGAATCAAGAACTTTCCTAGAAACCGGCGCTATTGGCTT
CGGTTGTTGTAATGGGGGTGGCTGTATGGGTACTGGCGCAGGGCGGGCTCG
GGTGGAGAAATCTCGATTGCTAGACACAACCGATGCTCCAGCATCAAAATT
GCTCGGATCAGATGTTGGGACGGGATATAGACGTTGCAAGGGGCGGGTCTC
GGAGAAAACTTCGCTAGGTGGAATTGAAGGTTCGTATGGTCGGTGCGGAG
GACTAACCGTGGATCGGTCAACCTTCTCGAACCCACAAGGAGTCTCAACAT

AACCGTGAAGAGAGTTCACCGGTCCGAAGAGACTAGGGTCAACCACCGAT
TGTTTCTGGTGGATAGCCGGGCTTCGTCGTGGGGATATGAGAGACCGAGGG
GAGCTAAAGATGCTGCGCGGTGGTGACTTGGGTGACTTAGGCGATTGAGG
CAATTGGGGCGGTGGTGCAGCGGAAGCTTGTAGGCTGAGGCCCGAGCCGT
ATGCTAGCCCAGAGTCAAATTCGGAGCTAGACGAGTCATCTTCGTCCTCAG
AGGCAGACTCCCACTCAGAATCGTCTGGAGAAGAATCACGACGCGGTTTG
GAACTACCAATACCCCTGTTAGAATTCCCGCTATTCGTCCTGCGGAAGGCAT
TCAACGCCGAAGCTGCGCCAATCAGTGTCGAAGGCCGGCTTCTCCCAGCG
GCTCTAAGGTCTTCGGCATTTTGCTGTCTAGCGATCTCAGCAAATTTCCTTC
CTAATTCAAGAATCTCTTCGTCGGTCTGCGAGCGTCGCACCACCTGGGATC
TCCCATGGCTGCTGGTAGGACTGAAATCGCGACTCCTGCCCTTCTCGATATA
ACCTTTTCCGTAGGCAAGATCAGAACCCACGGACGACGAAGAGGAGTTAC
CAAATTTCAAAAAACGTCTCTTCTTCTTTACGCGATGTTGTTTTTGTCCACC
GCCAAAAAATCTACCAAGACCAAATCTCTGCGAAGGGCGGGAAATATTTAA
TAGGGAAGAAGAGGTAGAGGAAGAGGATGATAGGGGAGAGAAAGCACGC
GATCGTTCTTCTTGGTGTTCGTCTCGTTCTTCGTAACGCGTTAAGGGCAATG
GGACGGGCTGAATCTGAAGTTGCTCTCGAGAATCCCTTCGCTCTTTGCGCC
GGCGGCGAGAGTATTCGGGACTCCGCCGGCGATGATGCTCGCGGGTCATCA
TCTTAAAGGATTGGACTTCCTACGTATAATCGAGTCCATTCAGAAACACACT
CTCAAGAATTATTACGGAAAAGAGAGTATATTAAAAGAATATTAAGGATCGT
ATACCGTCGCGACGGCTCAAAAATTGAGTATAGGGTATAACGGTATAAACGT
TGTTTTGTTTAGATCCCGACTACGAACTGTGGGGAATTCAAGATGCCTAGA
GGTAAACGGCAAGCGGCAAGCGGCAAACGACCAGGTACAGGTATGGGAA
GGTTAAAACATGAAAACCGAGCTCTGCTGACAGCTGGTTGCTTCTTGCTCT
TGTTTCTCTTGGCTATTTTGATGGGCTGGGAGACTTTCGCCCCACATAAAAA
GGTCTGCTACAGGGGGTAGGTACGCTCTGTTCTCTTAATAAAAACAGAAAC
GCTCTGGGACTCTTGAGAAGAACGGGTGGCTGGGGGATTTTCGCCCGCTT
CTCACTCCTTGTCGCAAAAAAATAATCTAAAAAAGTTGGAAGGAGGCCTTC
AGCTCCCAAGACTTCAGGACTCAAGAGGGGATTCGGCGATAACCCATCAC
GTGCGATAAATTCAAAGCCATCTTATTACTGGTCAAATTCGATCCAATCGTC
ATCCTTCATCGTCGAAATCGATCTCAGGATTAATATGGGCTGCCACGTCAAT
GTGCCGCCAGTGTGCCGCGTGCTAGCCAGTCTTTGCCTCACACACCTTAGA
GGCTTAATGCACAGATCAAGCACGGGCAGTACAGGCGGATGACACCCGTC
TCTACGCAGTATCTGATGGAAGCTAGGGCTCAGATATCCTGATGCTAGCCGA
TGAGTTGACCTGTCGATAATGAAGCTCTGCTCGCCGGCTTCAGCTGCATATC
GTCTGTAGCACAAGATCAGCACCTAGGGCTGCCGTGTGTCCTGAGGGAATT
CGTCTGCTCATATTAGCGATGCCGCATTAACCGCATAAAAATGGATTGATCA
CACAGAGCCCTTAGCCTTGGCCTTAGATCAGAATCTTTTTTTTTCAGTCGG

TATGCTTCAATCGAGCGGTAAACTAGGAAGGAGTTCTAGTTACAATACGTAT
GTCTCGGACACCTAGTGTAGGTGTCGTGGTCGTTGATATTAAGGACAGTGG
TGGCAAAACGTAACATCGATACCTGTTTTGCCTTTCTTTGATCGACGGCTCG
GGCTTTCGGGTTCCCAATAGGAACTATTGATTCGTTCCTATCCGTTAAAGAT
GAGCTGAGAGACTACGAAGCTAGTCAACTTCGGAATACTTGCATGCATAGA
ATAATTTCTTAGCGGATTTACAAGATGATGCATTTGATACCTCGGTAAGTACC
TACGCAGTAGTAGGCATATAAGAGAGGGTAAAGCACGCCGATTCTTTGTCT
TTGTTTCGAGAACTCATTGCTTCTAACTGGTAGGCACAATTCAACAAGTTC
CCGATCTACTAACTGCCGTAATGAGGTGAACGAGTTCGGGCTCCCCTCTTA
CAGATCCATTGCTAAGAAATACGGAAATGAGCGAGCTGAATCATGGACACA
TGGCACAGCTGCCGAGACGGGCAAGCTGACTATATTGTAAGTGCCAACCTT
ACTAACCATCTAGGTAGGTACCTTCACAGCTACAGTACCCACGATAGTCGA
CACCTACGAATCGTGTAAGCGGCTGAAACACTTCTAAATACGTGGCTTCAT
TGAAGAATCAGGCTGTCAATTTGAATTTGCGATAGGGCAATACCTAGCTTGC
GGTCAAGTAACAGACCCTTCTGGATGTTATTTTCAATTGTTTGTTTCCTTACT
TACCTAGGTCCTAGGTAGATAGTTTCGTACCAACCCTCAATAAGGGCAGTAT
AGGTAATAGTAGCCCCGGCGGACTATATTGCTAAGTGATCATGATTGATAGG
CTAGTAATTGTTCGGTCCTATCGTATCTTACCATTCTCGTATACAAGTGCCCT
AGAAGAGATCTAGAACGAGCGAATGATACCTCATTAAGTTAGATGATTTAA
GCGCTTCTAGGTACATAGCAGTAAAATTTTGGTAGCTGCAGGTAGCCTAATT
CGATTCAGTACATCGTACCGGTTCACAGCATTAACGTTGGAAGTTGGCATCT
CAACCACATGGGAACAAACTCAATTCAACTCTGTGTCTGATCACAACGTTC
AACGTTCATCAGACTAACTTCAACATTTGAACTTTAGCCCTGAAGATCGAA
CCGATAGCTTCCATCAACAGGTGCATGTATGTAAGGGTCTAAAGTTAAAAA
AAGGTTAGGAGTCTCGAATTGAGTTCAAGGCACGTCATCTTGTATCACGAC
TGTACCGAAGCTCTGGAAACCCTTCAAAAATGCATTCCAGCCCGGAGCACC
TGGCAACTGACAACTTCCTATTATCTGTCAATCTTGTAATTACATAACCTGG
ATAGGCGATCCTTCTCATGTATCGTTGCGGCAAAAGACGGGGATTGATTGGT
CGGACTGTGCGAATGTGCTCAGACCAACAACATTCAACGAGTCAGATGCG
TCCATTTCCCTCACGTAAAATTGTACTAACATCTGTACTATGATACTTGGGGT
ATACAACATATGTGTGTTGAAAGGCGCCACCTTGATATATCTATATAGGTACA
TATATTGATAATTAGGGTAATATATGAGCATCTGGAATTGTCAATTGATATATT
TTCAGATGATGCACTTAACCACGAAATTGCATCAGAATATCCCTTAATACGC
ATGCAGCCAACCTGCAGCTTCCAAACGATTTGACATTCTTCAATTCAATTTT
GTCTGGCGACCCGCTTCATGTCTTCTCCCGCCCTCTTGCCGAGATCCTCCAC
CCCCTCGCTCCGCTGCATGACTCCTTGCTTCCAAGTATCGGTGATGGCTGAA
TCTACGGCCACAAGCGCTGCCGCCCCGAAGCCGCTGAACCGAATACCGCT
CGGAAGCTGGAGGGTCACCAGGGCGTGATGACTGGTTCTAGGCGGGGGAA

GCTTCCGGAACTCCTCTCTGATCATCAAGCCCGCGCTGTTCAGACTCAGCT
CGGGCGACTTGACGAGCTTGGGTGACATCGGTCGGGTGGTGAGGGGGTGC
ACGTCGGGCAGAGGCGCCGGAGGCTTCGGAGGGGAAGCCTTGAACCGGT
CGTATATCGAAGTCGACACGGGTGGCAGGTTTGGCGGCGCCTCTGCCCACA
GAGGCAGCGGGGGCGGCGGTCGGTCGAGTGTCGACATGGCCGCGAGGTA
GAGATTGCGCTCGGAGGGCGTGACGTAGAGCGTCGAGATACGGTTGGGCT
TGAAATCTATCGGTCTGAGACGGTCTTCCGAGATTGGGTGAAAGGAAGAC
GGCGGTAAGGGGCTGCTTGGCTTGGAGTGGTCGACGTGATCTCTGTTGGGT
GTGCGGCCCCTGGTGAGCGAGAACCAAGACTTGCGGTTTGATCTGGCAGC
CGCCTTCTTCTCAGACTTCTCTCGTTGACGTCCGATCGCCCTCTCCTTGGGC
GATGGGTGATGCTCGGCGGCGTGGTCTGGCATGGCGAGGTGCTGTTTTCTC
TTTGTAAGAGGGTGCGGGGTAGTGATCTGGTGAGATGGGATGGGCTACTTT
AGTGACGCAATCAATCTCGAGTGTCTAAAGTGATGAGTCGGATAGAAGAG
GAGAGACGAAAGAGAGGAATGGTTGTGGTTGTGGTGCATGGCTGGCACGT
TGACACAAGGGGAAAGCGGAAAGGGAGGAGAAGTGTGCAAGGGCGGGG
AAGAGACAAAGGGAGTAGGATCAGTTGGGATGATGGCTGATGGGATCAAG
GGCGTAGAGGGCGAGTGATGGTCAGTGTACCCATCAGCAATCGGTACCTAG
TCGTCGTCATCACCACCCACGTCATCCCCATCGACATCACCATCAGCACCAT
TAGCCGACTCGTTACCATTGTTCTGTCACTGTCAAACATCAAAGAGAGGAC
TGAGGACTGGTCTTGTCATGCCGCCTGCCGGGGCCCAAAAGCAACCTGATT
TACGTCATTCCTGCTCGCCTGACTCCGGTCCCGACCTCGGCATCGCGACTG
CCTTTCGCTTTCCACTGGATTTTCTGATCATTCCTCGACGACAGCACATATG
AGCAACATGTCACAGTCATCTCCACTACCGACAGGCTAGAGGACATACACG
CATCATAGCTTGATGTCAGACCTCCAAAATGGAGGCGGGCCACAAGTCGCG
GCACCGCCTTTACCACCTCGGCGGCAGCCCTCGCCACTGCCTGCAAGCAGT
GCAGAAGCCACTGCATCGACCAATACTCTACCGGCGAACGGGGTCATACCC
GCAAGAGGTCCCATGGATCTCATCGACTCATCAGCCTTGCCAGAATCTGCA
GTGGGCAGTAATCAGACTCAGGCCACTACTACAAAAGACTCGGATCCCGA
CTCGCTGCGCAGAGCCATGACACCCCCTCCTTTACCTAAACGTCCCGACGT
TTCCCAAACGACACCGAGCAGCAGACCCGGTGGGGGCCATAGGTCTGCCT
TGTCAGTGTCTGTCATCCCGGCTACTCCGCCCGATTCGCGTACCCCTGTAGA
CCAACCGCCCGTCACAGCACCATCGACACTTCCGACTCAGAACGATGTCG
CAGCAAGACCGGATACTGGTCAAGCTGCAAAATCGAAAGTCAGACAGGCA
GAAGAGACTGTAGATGTACAGCCTGGTGCCCAACCCACTAAGCCCAGTCTT
CGGCCCTTGTTTGCCCCGCCGAAAGACGGACCGGCTTCGGATTCCATGGGT
CCGACAACGAAGCTCTGGCTCATCGCGGCGAGTATGCTCGGCGTTTCATGT
CTCTTGCGACACTGGTGGTCCATTGTGCTTTCGGTCCTTCTCGTCGGCTACG
CAGTCTTGGCTTCAAGCAGGACGCCTAGGGTACCGGCGGACACTGGAGAG

AATGTGCGAGGGTCGGGCGGTACTGGTGGGGAAGCATCTGTGAAATGGAT
GTGAGTGAATGATCACATCGAGGGAAGCCAGGCCGACTAGCAGCAACACG
ACACTCGAGACGCTCTTTCCGCTCATATCGACAGACGTGCTTACGCCTGTG
ATCGACCTTCTAGAGGATGCGCTTATGGAGCAAGTACCGCCAATCATTGTAC
ATCTCCATGAAACCATATGGTGATTACTGGTTGACCTTCAGACTTCGGTACG
CCTCGTATCTGCATCCCTGGGTCGACAGCCGATGCGTCTCACCTCGTTACGC
ACAATGGACGACGACGAGTGGTTCTCACTTCTCAGTCAACACAAGACTGT
CAGCCACCGTCAGACAAATTCCGGTAGCAGTTTCGGTTCGATCGCCGCCCG
GTTCGTGCCGGGACACTCCCCAAATGACTCGCAGGATGCTTCGCTACGTCT
TTCGACTTCACGGCCACGATCCGATTCCGTTTCTGATCGGAGTGCGCAACT
CAGCCCTGGTGAGATCGAGCGGGAAGCGTCGCGACGGCGCAAGAGGGAC
GTCATTCTTCGCCGCCTGTCTCGTCGCAGAGCGCAGCAGTCCTCTGAGCCG
CCGTCCCCTGCCCGCCTCCAACGAGTGACATCGAGACTCGCTGGCCGAGAT
CCCGGACAACCAGATGGCAACAATCCACACAAAGAGGAGGAGACAGGCG
ACGCTGAGGCGCTGGCCGCAAGTGAGGAGGAGGCTGTCGGACAATACATC
AATTATCAGCTCGGCTTCGAATACCGGCGGACCGCCTCCACACTTCAGAAA
GGCTGGGGCTTGCACGTCCTTGCTTACTTCGGGATAGGTATTCGTGGACTC
AAGCAAACTGAAGCTCCCGTTTACATCGACCTTCTGTCGCTCAAGGGCACA
GTCAATCTGCGAATCATGCTATCAGCCACGCCACCGTTCGTCAGGACTGGA
ACATTCTTCTTTTCCAAAATACCTGAATTCGACATTTCCGCCAAACCACTAG
TCCGCAACGCGTTCAATGCCATGGATTTGCCGTTGATCAAGCCCTACGTCG
AGAAATCTGTCGCTGAGGTGGCCAAGGCCTTCGTCTCACCAAAATCGTACA
CACTGGATGTTGACAGACTGCTCCTTGGCAGGGAGGCGTCTCTTCGAACC
ATGTCAATCGGCGTTCTTCACATCATCATACACGGCGCCAGCGATCTAGCGA
GAACCGACGCGCTTGGCTCATGTGACCCATACGTGACAGTCTCGATGACCA
AATTCCCGAAGCCAATGTACTCGACCAGAACCATAATCAACACACAGAATC
CAACCTGGGAAGAGCCGGCGTTTATTCTCGTTGCCGGAGAAGCCGTTCAA
GCTGGCGAGAGAGTTCGTTTGCGAGTGTCCGATTCTGACCGCATGTCAGCA
GACGATGTCCTCGGTGTAGTGGAAGTCGAGCTGGCAGACCTCATTGATCAG
AGCTCGGCGAACGGCGCCGACGGTTTTGCAAGACGGATTGATAATCTCACT
GTGGACAAGCCAGGTATGCGAGTCCAAGGATCACTCAAGTGGAGTGCGCG
TTTCTGTCCGCTGTGGCAGATGCCCCCTGAGCAGGTCGCAGACAGACTGG
CGAAAATTCGACAGCGAAGAAAGGGCGAACCTGAGGGCCTCGCCCCGCC
ATGGTGGATGCAAGCGCTCCAGCGACTGGCTGGAGAGACACCTGACTGGG
AGGGAGAGAGGAAGGAGAGGCGAAAAGAGACATTGGAGTGGTTCACGGG
CGACAGAGAAAGGGACGAGCTCGAGGCGAATACCATGCCGTCCAATCAGC
TCCGGTCGGGTGTCCTGCAGTTCCACGTCCATCAGGCCAACGGTCAGTCTT
CGGAGGTTCCAGTATTGGTCGCCACTGACACATATAGAACTGGAAGTCGAG

TCGATCGAGGGGACTTATTCATCATCCACGCATCATCGTAAATCTGCTGCCG
GCGGAAAGCCTGCTCTCGAAGACATCACCGACCGCACCGCAACCGAGAAC
CCGGATCCGCCGTCAGCATATGTCGAAGTCCATCTGAACGATCATCTCGTAT
ATCGCACTCGAACAAAGCAAGTCACCCCTGAGCCATACTTCAATGCAGTAA
GCGAACGATTCGTTCGAGATTGGACGAGAGCAAAGATATGCTTCGTTGTCC
GAGACGAACGCGACAGGGAGCATGATCCGATCCTCGGTCTGGTTACTTTGC
CATTGTGGCAGGTTCTCCACGACAAGTGCCAGCTGACGCGGTGGTTCCCGT
TGACTGGAGGATTGGGTTGGGGATTACTCCGGGTATCTCTGCTCTGGAAGC
CCGTCGACATGGGTCTGCCGCCCAACATATCCGGGTACGAAGTAGCAACCT
TGCGCATCTCTGCCCTGTCATCTACGGACATCCGGCAATATACCGACAAAGC
GGTGTCGATGATGATCGAAACGGAGTCTGACTCTGTACAACTTGCGGGATC
GCCTAGTGTTCTTGGAACTCCTGTCACCGGGCGGTCCACTCTGTCCTTATCA
CAGGAACAGAGCCATGGAAGCGCCTGTGTACCGCTCGCCTCACCAAGCTC
GATTCGGACGACCGAAACCTTCAATTTTGACCCCAACGACTTGTCTGAGGT
AGATTGGAGCCTCGCAAAGCCTCTTCGACTCGCCGTGCAGTATCGGCATTC
CTGCTCGGTACTAGTGAGCTTTGCCTCGCGATCAAATGTGTTGAAGAAACG
CAAGATCATTGGAATGGGCTCGATCAGGCTCGACGGCCTCCCTGATGACGA
GGATATCACAAGGACTATTCCTATATTTGGGACGTCGTCCGTGGAACAGGCC
ATCAAGGCCAGCCAGGCCTATCAGGATTCATTGATGGCTGCTCCTGCTCCG
AGTGGGTCTATGGATGGCAGCAAAGGCGTTGGAAGCGCTCTGAGCGCCAC
TTCCCTTCCACCGCTCGACCGCAAGTTGTCGACGCGGTCAAGGCGCTCGA
CGAAGCAGCCGCGTGGTCATCGATAATGATGAGGACTTGCTAGCGCCGCAA
GTTGTTGGGCTTGTATCGGTCACCTTCAAGCTTCGCCCGGGCATCTCGCGT
GTGCACAAGAAAATTTGCAAACGGGATCTGAGGTTCAGGAAGACCTACGA
GGCCTGGGAGGCAGCAAGAGAAGTCGAGAAAGGCTTGGATCTCGTCTCGC
CCACAGCTGCCAGTCGTGCCGCTGTCGACGATCTGCTGGCAAAGGCTCGC
AAGGAAAAGGAGAAGCTGGAAGAGAGTGAAGATTCCGAAGACGTCGATG
ACGGTCAGAGCGAACAAAGTGGCCCGGGCTGTGAAGACGAGCAGATGGA
AGGAGGAGCGGGAGACAAACGCCTGCCAGGGAAATCGGATGTCACCGATA
GTGAAGACGAGTCGCAAGGATTCATGGGTGAGAGAAGGGCGCATTCTCAT
GCTCTACATAAGCGGGTGAGCGGCCAGTCGACTTCGGCCGAAGCGTACTTT
GCTGACTGCAAAAGCATAAGGGCATTTTCCAGCTCAAGGTCGCGCGGACA
GGCCGATTCGTCGCCGACAAACTGAATGCCAAGATCCGGCAAGGCGTCAA
TCGCATGGAGGGCAAGGACGACCGAGTTCGAGGGCCGGACCTGCACGTG
GAGCGCGAAGGGACGTCCAAGCTGTAGCTGGTCGACAGAGCGTCCAGCGC
GTCGATTGGCATTGTGTATCTGGGTTATCAGACTAAGCATATCAGGCGGGCG
ATACGGACCTTGGTGTATCATACTGTTGTACCGGTGTCCCAGAGCATATACA
CACACCCGCATCGCATGGACGGTTGGCCAAGCGGCAGATGTCAAACCGGT

ATCACAATACCCAGTTGTAGATGGTCGTCAAGAACGCCATGCCAGCGAGCG
AGAGTACCACCAGCGCGCTGACGGCCATCCACAGCAGCTCTCTCCGGTCA
GGCTTCTGGAACGGCCCGTCGTACTCGTCCTCCCCATCCATCATCAGCATCT
CCAGCTCCGAATCCCCCTCGTCGCGCCACTCCACCCCCACCTCGCCTCGAT
CACTGTCCCTCGATCCATCACGACTCCCGCCCTTGTACTGCACATCGTCCTC
GGGCCGCTCGACGACTTTATACAGCTGACTCGTGCGCCTCAGGGGCGGTG
GGACTGCGCCCAGCTCGTGCGCTTCGTGCGGCGCGCGAGGCTCCGTGC
AGGAAGAATGGGGAGGATGTGAGTGCTGTGGGGGATCGAGCGAGGGAGA
GAGAGCGCGAGCGAGCGTGAGCGCTCATGTCGAGAGGTGCGCTGGGGTTT
GTGAACATTGTATAGGGCGGAGATGATTGCAGATTGCAAGAGAAACAAAC
GCAGGGTATGACGGGCCAAAACCGCCACCACGAATGATCACTTTTTAACAC
CTATCTCCATCCATCGATTTCTCTTTATTCCTGTATCTCTGTCAGTTTTGAAGC
TACAGCTTCGAGACCGGCACCAATCGACTCAGCGCACCCAATTACACGTAG
AGAGACGCGTAAAAGTCATGCATGGCGCCTCTAGCAGTGGGTCGGGACAG
GCCCCTCTCGGGCCGGTGGGTTCATCGATGTTCTACTTTGCTCTTTCGCTCA
TTGCCAGACCCCAATGAACCAGGAGCTGGCCAAGGTCGTCGCATACCTTGC
CCAGACCAACCCCGAAGCCCTTCCAGCGGTTTTGAGCGCTCTGGGAACTC
CTGGAACCGTGGGAACGGGATCCGTCAGCAACTCGGCTCCTCTTACCTCGA
CTACGCCAACTGGACCCAATGCTCCGGCGGCTGCAGCTCATTCCACACCTA
TCCCAGCGGCCCCAAACGACGCTTTCTATACAGAGGGGACCCTCAGGCAG
CCTAACAGCCTTGCCCGAGCCGACACAGTCCCGCGCTTCGATCTCCAGTCC
GCCAGCAACATTCGAGCCGGTTGGGCGGCCTCGAGAGGCGTGAGGAAGTC
AACTGTCAGCCGTTCCTCGGTGTCTACTGCTGATGTGGGCTCCCAAAAGCA
CACGGAGCAGCCCAAGGGGCCTACCAAGGTAGCACAGGTCCCCCTGGCGA
AGGATCATGCGGTTCAGCTCAAGAATCAGCCCCCGGGGCCCGTGGGCGAT
CTCTCAGGCTGGCCCAAAGTCGCTCACCACAGCTTCTCTTTGTCCCCAACA
GCGGGATCCTATCCGAATATGCTCCCTGAACCCCAGAGAAGCATGACATCT
TGCGTGGGATCTGCCGAAGGCGCGAACTCTTGGGCTATCCAGTGTTGTCCT
ATGGGATGACCTCCGAGGAAGTCCGCCGTGAGGTCGCGAAGTGCCTTCCT
GAGGTCCCAGAGCTTGCCAACATCGACTATGTGTAGGTCTGAGGCTGCCTG
GGCTCTAGCTGATGCCTCAGGTGGATGTCGACCGACAGCAAGGGCGAGAA
CCTGTACAATGGCGTCCGCGCGCATTCGACCTCAGCTCGCAACCTCGATAA
GTTCTTCGACGATGACGGTTTGATGATCATCACCCCAATGGTGCCCATTGCA
GGCGCGCACAAACAGCTCGAAGACTTTGAGGAGGACTGGATCGTTTATGT
TGCCCCCTATGAGCTAAAGCAGGCCCGAAAGTCGACCGATGAGGAGTTCC
GGGCTCGAGTTTTCGGATCCCTAGCAGGCCCTAGGTTCAAGAGGAACGGT
CCAGGCCTCTCCAGCCAATGCGAGGACCGCCCTGGAACGACTTATCAGCG
ATAGCGGCAATGAGGCCACTCCTCGGGCCCAAGAGGTTCTCTCCATGACCG

GAGAGGGTGTGGCAGCACCTTCTTCGGTCACGGAGAGGACTTTGGGCCCC
GCCACTGACTCTATCAGACACATCAGACAAGACTACATTACCCATGTCATTG
GAGACAACCAGAGGCCGCTTTGGCCCGCCTATCGATTGGGACCATGGGTTT
GAGGCCCATGGGTCGCCCGGCAATGGTCAGGGCCTTCCTGTGGACCTTACC
ACGCCAAGCACTAGGGGCAAACGCACTCCCAGGCCAAAGAGAACAAGGA
TCAAGTTGTCACCTTTCGGTTCGACGAGGCAAGAATCGATGACCCGCCTTA
CCCGCAATTCTGCTCGCGCCGGCTTCATCGATAACGCCGCTAGCGCCGGGA
TGTGATCAAGGCAAGTCTGCTGTCTGTTCTGGTCCTTGGATCTCTACGATAC
CTGCTCTGGATGTCGAGATAAAGCTCAAAAGCAGATATCACCTTTCGCGCC
AGCTCTACATAGTCAAAGGATGGGTGGACGAGAGGTCAGGAAGGAGGTAT
CTCCGGGAGATAGGTATGAGGCCGAGGTATCTGCTTTTCCTTTTTCCTTCAA
CAGGAACTTGCTCGTATGCATTGAAATTGGTATGGGGCAATTGTCCAGGCT
CAGGACTATCGTCTGGCTGTCATTTCATGCATGTAGCGTCATTGTAAGCCAA
GAACGCCGGAAATTCGTGACGAAGGAACTCATCGACTGCTTGTCGACATT
GCCGATACCTTGCTTCACCCCAATCGTCCATTCCCAGCGCCCAGGGGCCCA
GTCTTCGCAGCAGAGCGTTCTGGAAGGCTGGTTCTTCTAGTGCTGAAGCCA
CTTCCGGCGGAACTTCACCTAAGACGGGCTGCACCTCGACCAGCGGGTCA
TCGTTCGCAATCAGCCCATCTGCTCTCCGGCCGTAATCGTCCAACCCGTATT
TGGCAAAGGCCAGAGGCCGGTGAACGTTGTTGTCCAGTCCCTGGTGGACC
TCCTCACCGACTATGGCCATTGGATCGGCCAGGATGTCGATGCCACGGTCTT
CGGCGTCGAGCACACCTCTGCATGAGCGGTCCTTTCCTGTCGGTCAAAGTC
AGGATCGACCACTCACTGTCCCACCACATCAAATCCGGGCTTTCACCACCC
AGGTCATCCATCTTATGACCGTTCCAGTCCCTCACGGTGTTCTCCAGGCTCA
GATTGATCATGGGAAGGTAGCACAGGTGAAGACACCAAAGGTCAAAGGGG
TTGTCGAGGCTCAGGATGCCTTGTTGCTTGTAGCTGTCGAACAACGTGCGA
TAGCGGAAGGTGCAACGCCACTGGAGGTCCACCCAGAAGCGCTCTATCCT
CTGATTGCGAGTTGAGGGCCCCTGGATGAATGCATGGTGAGATATACCTAA
AAAGCAGTCAGCCCGAAAACAAGAATTTTGGGAGGCCCGTGCGCGACAC
ACCTCTCTCTGCTTCCATGAACCTTTTGACGTCCAAATTCTCTTTCCCGTAG
TCCCCCCTGACTCTGCTAGGCACCCCAAAGCGTCCTACGGCCTCCATGAAG
CTCTCAAAGACGGTGGCGGCTCGGTTGTTCGTGCTAGCCTTGAGAAAGAC
GACTTTTCGTGACTTCCCGTCGATGCCGCCGTGTATGACGATTTTGACGTCA
ATGAGCTTGTGATGGCCATCTATATGCCACAGACTGTTGGCAAATGGGACTT
GATACACCCTGCGAGCTGTCGTCCTGGCAAATCGCCTTCTAGCCTCGTCCG
AGTCAAGATCGCGAATGGCCTCTCGCACCTGTGCTCTTGAGGCTTTGATCT
GCTTCCTCTTGAGCGCCGCCATGATCAGACGATAGCCCTCCATCCCTCCGCC
CTCTTTCCAGCAGCGATCGACTTCCTAAGAGCGGTCAGAGCCCAGGCTGCA
GCACAATTGAGACTCACCGCTTCCACTTGTAGGAGCGATAGGCCACCTTCG

GCTGCATTCCTGAGTCCCAGCTCCCGTTTCCACCTCTGGATGGTCCTGATGG
ACACATGTAGACGCTGTGCCAGCTTGTTCTCAGTCATTCCGAGCCCATGTA
GGTCTTTGAGCATGTTCTGAGGACATGAGAGTCGATAGCGGCCGTTGTCTC
CCTTGTACCATGACCTCTCACGCTGCGAGGGTCCTGGTATGGGGCCTAGGA
GGGTGTCTGCGGTCCTCTGAACGTCCTTGAGCATGTCCTGCACGAGCGTGA
GGTGTATATGCAGCGCAGAGAAGTCCTGGGCCGGCAGGTTCATCTCCATGG
CCCGAAGATCGAGGATAAGTTGGGTCACAGAGGCGATATGCCGGGGTAAA
CAGGTCTGATCGCGGTGTGGGGGGCTGGTATTGACCCAAGAGAGGGCCTG
GGAGCTGTAGTATGCGACATTACGCTTGACATTGGTGATCTCGACGGCTAAT
AAGAGCAGGGGGGTGCTCTAAAGGTCATTTTTACATCTACCCCGAGCCATC
ACTCAACGCTCCATGGTCACAGCCTTCTACACGGCCTCCAGACGCGATTGA
AGGGTCTGTCGACGCGTAATTTGATGCACTGATTCGATTGGTGGTGGTTTG
AAGGCTCTACGATGCATACAACGCGAGAAAGATGGAGAAGAAGATAAGTC
AAGGTGGAGAGGTAAAAATCGCCACCGGAATTTACATGGTGGCGGGATCG
GCTCGCCATAGCAGGGCGTCGCCACAATGTGGCACTTGAAAGTGGCAGAC
TCCGAGAGGCGCTGCGTGGGATAGAGTATACATCACAAAGTACGCATGCAG
CTCTGATGTCAGACCTGCAGGTGCGTAGGGAAAAAGTGATCAGGTAGTCC
GAGAATATCAATACCACAAGCGTCTCCGCCCAACCCTCGAGCACCCGGTCC
ATATGAGACGGACTACATTTGCCAGTTTTCTATCTACATCTTTGCTGACCATG
TGTCCTCGGACAGCGATCCCGGCGACGACGGCTACGTGTCGCACTGCGCC
GAGAGTTGAGTTGCCTCGAGTGGAGGGGAGGAGTCCGTCTCGTCCTCGTC
CGTCTCCGTACATTTCCATTTCCTCTCGACTCTCTCTATCTCAAAGCGCAAA
GATTCCGCTTCCGAATCCAGTTCAAATGTGCGGTTGCCCATCATAATACTGC
CTATCCTGGGGTACGGGAAAGACCGACCGCACGCCTTCAATCGGGTCTGTG
CCAAAGTCCGGATACGTGTCGTCCTCCATGAGCTTTTTGTGCACGGAGTCC
AAATATTTCCTGGTATCTTCCATCCGCTTGACTCGTTTCCTAGCGGCGCCCA
GAACAGAGTTGAACTCATCTTGCGTGGGTGACTGCTTCTGGTAGTACAGAT
CGTGAACCCTTTTCCAAAGGACTGACAGATTCTTGCCACTGACCTGGTCAC
ACTTTATCCAAGCCTGATGGAGGATTTCAGTGCTTGTTATCAGATGTCTAGA
TAACCAGTCATAGTGTGTGCCCCCCAAATCCTCTGAACTGTCAATCAGCTCT
GAGAGCTTGGTATGGGCCCTTTTCCACCGTTGATGCCACATCCTTTGCTCAT
CTTTGGCCTTCTTGACATTGCTCTGGAGGAGCTCGTCTGCAACGGCATTTTT
CTCTTTGTTGGATCTGACCTGCCATTGGGCATGAGAGCTGCGGAGGTTGGA
CAATTCAAAGTTGACTCTGGCCTTGAGGTGATTCAAGATGTTCCTTGCCTG
ATCATGAGAGTATAACAGACTCTTTGACTGGTCAGCCTCCCATTTCAGCAG
CTCATTCAGCTTTGACTCAAGGGCTTCCTTGCCTTTGATAAACTGAGGCAG
CAATTGTTTGGCATCGGCTGTTGATGCCAGGGCCATGGCACTGGCAACAAG
CCGACGAGCAGTCTCAATGGTGTGGTGAGTCCCTGTCATTGTTACCAATTG

CTGAATATCTGTGATCTCTTGGACCTCATGGACCCAGCTGTCAAGCTGATTC
GCTAAGTCTTTCCCTTGTTCTTGCTGCTCCTGAGCTTGTGAGGCCAACTCAT
CCTCAACATTGGGCTTCCCAAATGACGGGTCAGGTATAAAGGACTGTAGCC
ATGACTGGGCTTCTTCAAGCACTTTTGGCCAGTCCTTCCACTGTACCATCTG
CTTCAACTTCTCCCTTGCTGTTTCTGGATCAACATAAGATAGACGGATGAGA
CCCAATCTGGCTTGGCTCAAGGCAGTACTAAATCCACCAACTTCACTGGAC
TCTTGTGATCGCTTGTATGCTTTTGCTGTGTGATTCTTGATCTTGGCCTCTGC
CTGTGGAGCAATTCCCAGATAGGTGTCAGTCACACTTGTGTACACAGACAG
TGCCGCTTGAATCTGAACCTGGCTGTCCAGTTTTTAACAGCTTTCATGCGA
TCCCTGGTTACCCATCTGACCATACCTCTCCAATGAAGATCAGACATCCCAG
CAAGGAGTTCACCGTATGTTTTGACCTGGTCCTGCAGGATGTGTCCTGGTG
CTTTTTAGACTGAGAGTAGTCCAAGAGTCATATACTTTTTGGAAGGTTTCAC
TCTTCCTTAAATTGGCTTTCAACTGATTCCAGTGGGTCAGTGTCTCTGCAAG
AACAGAAATATTACAGGTTGTGACCTGATAGTATTCCTGTATGCTCTTTAGG
GGCAGCTCCTCTATGTTTCGGTTCTGCTTTCCAGATCTGTACTTGTCCTGCG
CTGAAATGCAGGCCAATTTCTGCACCCACTCCGTGTAGTCTTTGTCCAGCT
CAGTGAACAACTTAAGAGCGGTCTTGGCCTGCTCCGCAGTAGGTGTTCCAT
ATTGAGAGTTGCACTGTTCCATTTCTCAAGCAGCAGTTGTTGCTCTTGCAA
AGCAGAGAATCCCAAGGACACAGTTGTGCACTGATTGATTATGGACACTTC
TTTTTACCTGTTGGCAGTGTAAAAACAAGAGATAATTCAGCAGGATGTGGG
CTGCTAAGCCTCACTGCAATGAAAGGATGAAGCTCACCACAACAATGAAA
GGAGTGTCACCTTGGCGCACTAGCAAATTCTTCAACCCACTGAAGGATTGT
CGGAGGTATTTCAAGCATTGATGAAGTGATCGATACATGTGTCACGGGGCC
AGGTCAGTCGGCAACCGATGGGCGCTCTCACACGTGCTCTCTGTCAATGCA
TCTCTGTTCTCCACAACAAGAAGGTGTACCTACAGCCTCTTGTGCGTTAAC
AACACGGCTGCGCCGCTACTTTCTATCCACGGGATTCAATACCATGGTGCTA
AGGACGGTCTACGGGTGAGGACTTGTATATATATCATCTGTGCCCTCCTCTG

>Mosaic_sequence_23

ATAACTCCGACTTATTCTTGAAGACACTGGAACAACCTGGAGAACGACAG
AAGGATGCACCTCAGTCTTCCATAGGCCGAGTTAAGGCAGGAACGCACAC
AAGGAGAAGAGTCAGGTCCACAATGACGGAGGATAGAGTTATCTCACAGT
CAGGGCCACGGGACGGAGACTAAGAACGATAAGAACAACAACGGGCACG
CAAGGAGATTAGATGCGAGCCAGCACGGAGAGAAGACGATAGAAGAGAA
GAGAGACAACACAAGAATACTGGAGACTATTGTTAACAAGTAGAAACGAC
TACGAGAACGATGGAACAATCCACGAGAGACGATGCTAGAAGAACCAGAG
AGAGATTGGGCGAGAGCAGATGGATACTTATGGAGGAAGATTGCAGAGGA
AGTTCCTCAGCGATCTAGTTAACTAGTCCTTTAGATAGAACGTCTACATAGA

GAATCGACCGTACCAGTGACAAGAGATAGATAAGTGAAGTGAGTCTTGATC
GCTCCTTACCCAGCTTGGTTCCACTCTACTCTTGTTCATTACTATCTGTTACG
AATAGTCGTTGATAGTTAGCAGTTGAAACCGATAGACCGATTGTGGTGAGC
TTTAGTTGGATCTAAAACAGGTCGCTGAGTACAGGTCTGACACGACCTTTC
AACACCTGCCACTGCGGTAATCTCGGTCCCTGCAGCAGAATCACACCTTCA
GGCCCTGCAGTCTTTGCACCGGACTCTGGCAACCAACATCAAAATAGCACA
GTGTCACGATGCTGCGCTCTCCCGTTGGCAGAGGCCCTCTCGCACAATATC
AGAGTCAAGAATAAGGGATAAAGGGATAGAAACGAGGATGTCGAGGTAGG
ATCCTGGTCGAGGTTGGATCCTCGTCGAGGTTGCAAGGTCGTCGAGGTAAC
AAGGTCGTCGAGGATACAGCGTGAGTGCGACTCACCCACGCTGCGTGCCA
CGGAGAGAGTGAGATCGTCAATGATGATCCATGAGACTCAATCAGAGAGT
GCTGAAGAACTACGACGGGCGCCCGGGGCATCCTATATGACTCTGCGCCTG
CTTTCCGTCGTCGCGCGTTCATCCAACGTCTACTCCCTCGTCGAGGTTCTCC
AGCCCGAGGTAGGTCACGTGTCGGCCACGTGAGGGGTGTCGGTCACGTGC
GTAGGTCGTCGAGGGCGTGGCCGTCGTGACACACAGGAACATCATACCAA
ATTCTTCAACCAAAAGGTTAAGGAGTCTCCACAATTCAAGATAGGAGATTC
AGTATTCCTCAATCGAAGGAACATTCAGACCCTCCGACCCTCAGTAAAGCT
CAATCAATGGATGCTTGGCCCATTCAAAATCATTGGGACAACGTCTTCACCT
TTGGCTTTCAAATTGGATCTCCCACCTTTGATGAACATTCACCCAGTATTTC
ATGTCAACCTACTGGAACCAATGTGTAAGGGTCACCCTGATCAACCGCAAG
ACCCACCACCATGCATCAAGGTTGATGGTCAAGAAGAATATGTCATTAATAG
GATTCTCAACTCAAGAATCAGCTCAGACAACGGCTATGACTACCTTGTCAG
CTGGGAGGACTACTCAGAGGTCCACAATTCATGGGAGCCCTGGGAAAACA
TCTGCAATATGGCAGCATATTGAAAATTGCATAAGGACCATTTCCAGGACAA
CAACCATTTCTTCCCACCTGCTAGGGGTTACCAACAGACCAAGGCTCCATG
CAACAATAATCAAAACAAAAGATCTCAGTGTAGATCACCAAGGCTCAGAG
GACTTTGCCCTTAGAGGGGCAGCTCCTGTCATGACCAGCCAAGCCAGCAA
CCAGCCAAAACAACCTGTGCGCTCCGTGCGCTCCGTGCGCTCCGTGTGCG
CTCTTGTGCGCTCCCAGTGCAGTTGTGTGCGCCCTCTGTGCGGTCATGTGC
GGTCCTGTCACGTCGGACATGACACCGCCACCTTGCAAGCCTATATGTACC
ACAAACCACGAGACTCGGTCAGACTCGGGTTATCATGACCCTTTTGGTGCA
GTTGTATTAGCTGGACAGTGCAATAGCTATGTGGTAGGTTATTGGCATTGTT
ACAGGTCCTCTGTGCGCTCCTGTGCGCTCCCATCTGCAACTTGGATTTCTG
GTCAATTGACAAGATCGACAAATATTCTCTGGCCAACCGACACAATCGACA
ATGGATTGACATGATCGACAAAGTGTTCCCACTCAATCGACAGAGTCGACA
ATGTACCACTTGGCAGTCGACATGATCAACAATTCTTCCTAGACTTGTCACT
CAGGTCAACCACTTAGGTTTGGTAGTGCACACTTCACTTATTCTAGTTTCTC
ATATTCTCATATTCTTGTTGTCACGAGGCCTATACTTACCAAATAGCATAGGG

CGACGCACAGTAAACCACTGCACCATAAAAGGGTCAAGATGCCCCGAGTC
TCAGCTAGACTCGCGGTCTTTGGTACATATAGGGTACGGTGGCTGTCACCTC
TGTGACACTTGTATTCTCACATTCTTGAGTTACTGTGATCTAGGTACATCAG
AGTTAGGCTTCAAGCCATCTCAGATATACAAAGGTACTCAGATATATGAAGG
TACCAAACCAAGTATATTATCACGGGGCCAGGCCGGTCGTCGACTGATAGG
CCCTCTCACACGTGCTGTCTGTCGATCTATCTCTGTCCTCCACGAGAAGGTG
TGTCCTACAGCCTCTCGTGCGTCGACAACACGGCTGCGCCGTTACTTTCTAT
CCATGGGACTCAATACCACGGTGCTAAGGACGGTCTACGGGTGAGGGCTTG
TATATATGTATCACCCGTGCCCTCCTCTGCACCGCCTTGGGCTTTCTCCTTGA
AACACCATCGACGAGGCTTGAGCTCTAGAGTCGTCAAGGCTACTCGTGCGT
CGTCGATGCTACTCGAGGATCGACGATAGGCTCACGTGTCTGACAGGCGTG
CTGGGCTGGGACGAGGCTCGTAGGCGTGCTTTGGTGATCATGGAGGCGTG
GCCGACGTGACACTCTGCCCCCCTCTGAAATCGAGCTCCTGCGAGATGGCG
GGCGAGGCGTGGCAGCTTGTATGGCCTGGGCCTTTGCTCCCGCCTCGCGCT
AAGCCGTCTCTTTTGGTCATTCCTCGCAGAGCTGATCCCGAGGGTCTGTGG
GTGTGTAAGCAGGGCTTGGTCTGGTCGACGGTCCGTCCTTCCTGGTCGCTC
GAGGTCTCGCGCAGCTGGTGCTATCTTTCTAGGCGCTAGTCGGGGCGTGGG
CTGTGTTGCCGTCGTGGAGTGGCTCACGGGCACAGGTAGTGTGCAGCCCG
CATGTGTCCCGACGCGCGTGGTGTCGAGGGTGACCCCTGTGCTGGATAGCC
CGCCCGTTGCTGAGCTGTAGGCGTCTCATGCTGGAATTTGCGCGTATGTAA
GTCGACCTCGAGTCGACCTCGGCCACGAGGTCGGGGTCAAATCGCCCAAT
GCGGAAGTCGCCGCGAGATAACCGGTGATTGCCGCTCCGCGCTACGCCCTT
CTGTCGATCATGTTGCTCGTTGCTAGGCAACAAGTCGCCAAACTGTCGTGA
AAAGTCGTGTTGCCTGCTCTCGTCGTCAAGTGCCCTCCCGTCCCTCGTCCC
CGCCATGTCCTCTTCCAAAAGGTATGAACCCCCCTCGACGAGGCTACTCGT
CCCGTCCATTCCAAACTCTCCACTCCAACGCATCCTATGTTCTCCAACGCCA
TCGATCCCTCTCTCAAAATCGTCCTCCGAGGACGACCTCTGCGCCTGGTCA
CCATGACCAGGACCCCTTCCCCCCGCCCCACGACGCCGCCGTCCTCCCCCA
AGTCGGCTGTCGACGCTCCCCCTCCCCGACTGTTCCCTCGATGGGAAGTCC
GTCCTCGCACTCCGGAGCCCACACCCGTCGAGGAGATCGACGAGCTCGTT
GATGAGCTCGACTGGTCGGATGACGCCAGTGCTGCGCCAAGCGGTGAGTG
ATTGTTGAGCCACCCTACTGCTTGTATTGATGCCCAGGGCGTACAGCGATAC
TGCGCTCGCTGGCCGACATACACGCCGAGATCCGTCGGCACCAGGGGACC
GTCAGAGCCGCTAGGGAGAGTAGCTGTGCTACCTCCTCTACGTCCAGCGGC
TACTCAGACGCCTCGCCCTCGTCCTCCCAGGCGTCCGACGCGGCTTTTGCT
GGCGAAAGCCGCTATCGCGAAGCCCGCTCCCTGCCGTTCGAGCCGGTCTC
GCCTCGCCCTCTTCGCCCCTACGTTGGTGGTGGGACTGGATCCCCCATCATA
CCGCCACATCCCCTCGTCCCCCTCGGCTCGCCTATCCTTCTCCTTCCCCCGC

CTTCTATGCCTCCGCCATCCCGCACTCCGACTCCGGCAATCAACCCACGCC
GCCGGCCGAGTCGAGTCGCCCGGTTCCCCGGGTAACACTCCCCTGCCAAC
CATACCGCCTCCCCTCTCCCCAACCCCAAAGCGTCGTCGCGTGTCGCCTGT
TGGCGGCATGTACTCCTCTCGCCTGCGTTCTGCCTCAACCCGGTATCTCGGA
GTGTCTGAGGTAGCCGATGCTCTCCCGGCCGCCGTGGACAACTCAGGTATA
GGAGAAGACGTCGTGGAGGGAAGCATCTCGACGGGAGAAGGATCCGAAG
CGGGCAGCAAAGGATCCGCTGGGGAGGGAAGTGTGGCGTTGTCGAGGCA
ACTCGAGGCTGGCCCTCGAGTCCGATGTAGGAATGCGCTGGACCGCGCCTA
TTTCGGTACGTTCGTCATCTGCTGAGATCCCCGCTCATTCCCAGGGCCATTC
TACCCGTCGTTTGGCCGGGGGTCGAATAGGACCCTCCCATGGGCCTTGGTG
CGTTATCATCTTCTATCCTCCTGTCATCTCACGCTGATGTTGCAGGCTCAGG
CAAAAGGGCGACCTCTCCGCACGCTCAACGGCCGTCAGCGATGCCAGCGG
TGTCGTACGGACAACTTGCCGTGTAGGGTCCTAGACGGAGGAGGGCCGAG
GATGAAGTGCTACACCTGTGGCACGGCCACGGGCTGCTCGTTCAACTCCA
GCCCAACTCCTCGACGACCCGCCGGCTCGTCCTCGACACCCAGAGCTCGC
CAGGCTGTGAGTTTCGCGGATGCCGACCCGCCTTCGCCAACTCCCGCCCCC
ACGCCTGTTGTCGGCCCGGCCGCCTTCACCGACCGACTTCGCCGGTCGGTT
CGTCAACCCGTGCCGGCTCCCGTCCCGCCTCCGACGCCGCCTTCGGTGTCG
ACCAACGGAAATAGGTGGTACGCTCACATGAGCTTGACTCATCCTCGAGTA
GCCTCGTCGAGGCACACACACCCCTCCCTTCTCTTCCTCCTTCCTCCCATTG
CGACCACCGCTTCACTTTGCATTCCGCACCTGTCTGCTCATCTCTCGGGATG
GTAGGGGCCACAGGTTCGCGCCAATTTTTTGTTCTTATCAAGGCACTATGCC
GCCTGATTGGTTTCGGCCGTCGAAGCATATACCGAACTCTACAAGGTATGC
ATGTTGCCTGATGTCAAGACGCTTTTTAGTCTTAGACACGAATCATTGCCCT
TTTTCGTTGCTGTCTTCAATTCATTGGGGACGCAAGGAAGAAATCATAATTT
GCAGTTTCCAACCTGATTGTCGATCCTTTACCTTACCTTATATAAGGATATAT
GAAGTATCGGATTCACTCTCGCGACGAGGACATTCTTCGGAAATAACGCGG
GGTTCCATCCCCACCCATTCTCCCCTTTTCTAGCTTGGCAGCTGTGTTACTC
TAGCCTCTATCACAGATCTGTGCTACTTAGAGAACTTTAACATGATTGAACA
TATGCATGTCGTAATTGAGCGGCAGGAATCCCGATTATGTATGTGTCACGCC
GATGACCTCAGTCTTGGTATGCGGTTGACGTCGGACTGCGACACTCCGGAA
AGGAGAGGGGCTTCGAGGACCAATCCGATAGCGGGGCTGCTACACTTACC
GGATTTGGGTCATCCGACGGAGCGGAGGCGGACAATAAAAGGGCACGATC
TCGCAATAAATTCAAGGCTATGTCAAAGAAGCGCAGAAACTAAAGATTTCT
CATTTTCCTCTTGTCGTTTCTGAAAGAGATATCGCATTAGTAATCCACATATA
TCTTTTATTTCCATCTTGAGTTCGAGACCCCGGGAATAAAACATGGCGCCTG
TGGGAATAGCGATTATTGGTGGGGGAATCTTTGTTAAGGAGCAGCACATAG
TAAGTATTTTCGGGGTTATTTACGGCTCAATCCACTCCAATGGGTCGCTAGG

ACATCGGATTTAAGCAGTTTGAGATCTCAGTCGATCTCGACAGATACATTTG
GGGATCTAGATCATAGCCTAGCTAACATGTGACTAGCCCGCGGCTCTGGCTT
CTCCCCTGCTAACTATCAAAGCGATCTGGTCTCGGTCCCGGAAGACGGCAG
AGAATGCGGCAAAGGAAATTCCTGCTGACGCTGCTTCTTCGGTGGACCTGT
ACTCGACTGATTCTGGGGAGGGGAAGGCTTACGAGGACATCCTTAAGCGC
GACGACATATCTGGAGTCATCCTTGCCCTGCCAATCATGGACCAGCCCGCG
TATATCGAGAAGGCGTTGGCTGCGGGAAAGCACGTACTCGCAGAAAAGCC
GATCGCTAAGGATGTCGCTACCGCGGTCAAGCTGATCGAGTACTACAAGAA
GGTCTCGGCCGAGACGAACGCGTCGCTAGCTATCGCGGAAAACTTCCGTTT
CAATCAAGGTTGGGCTTACGGCGCCGAGCAGATCAAGAAGCTTGGTAAGG
TGACCGGCTTTGTTGTAAGGCTGAACTCGATGATGGTCAAGGAAAACAAG
TATTACAACACTCCCTGGCGCACAGTGCCCGAGTACCAAGGCGGGTTCCTT
CTTGATAGGGGCGTGCACTTTACTGCCGCGATTAGGCGATTGCTTGGACCC
GAGAATGCGGTTGAGAGCCTAATAGCCAACACGGCGCTAGTATCTACCCAT
CTGCCTCCAGTGGATTCTATCAACGCGGTGCTGAAGACAAAGTCTGGTGTG
GTGGGCTCCTACATCACGTCGGTGGGGACGACGATGGAGGCTTTCGAATTC
CACATCGCAACAGAGCAAGGTGTTGTGAAGGCGGAAGGGGCTAAAGTAGT
GACGACCCGAGGGATCGGTAAAGATGCGGTAGTGGAGGAAAAGGTTTTCG
AGAGGACCAGCGGAGTCAAAGAAGAAGTACACGCTTGGGCTGAGTCTATT
TTGTCGGGCACACCCAATGCGAACCAGACACCCGAGCTAGCACTTGGAGA
CCTGGAGCTAATGGAGAAGATGCTGACGAGTGGTGATCAGGATGGAGCGC
GCCAGAAGCTGGTGTACCAGTAAATCGCATAGTGGCCGCGGCTATAATTTA
CTAGAGAGCGGTTCCACAATTAGCTTCGCGAGGCCTTCTCGTAATTGATTAT
TCATCCCGTTAGCACTCGGCCGTTGTGTTGCGCGATCCACAAAGACGTGAA
CAAATTCGCCGACGGCCTTGACCTGCTCAACGTCTTTCTCGAACAATGCGG
CTTCGTATACCGCGCTTGATTTCCCTAACTTGTTGACCCGCAAGCAAAGTTC
GGCAACTCTAGGGTAGGAGATGGGCGCAAAGTAGTCACAATGTGAGTGGG
CCATGAGAGGGTGCTGAGCGGAGGTGGGCGGGTGAAGAGCACAGTAGTC
AATTAAATATGTATTAATCACAGAATCAAAGCTGAAGTATCTCGGACGTTAG
GTAAGTACGGATGGAGGAATGTTGGGTTTACAGACAGGAAATTATAAACTG
AGTTATTCATATGATCATACATGTCGTTATCGTTCCTATCTTGTATTAGACATG
GAATGATTGATGAGCCTCAGCTTTAGGGGCGCCATACCATCGTGTCCTATAT
TCGAGTAGGAAAGGGTAGTCCTTTCTGCTCCTCATCTTTAGTTCCTTGATCA
CAGTCATGGTAGAGGTTGATTAAGTGGGAATGAAAAATGGATGTGATGGAA
AGAATAAGGTTAAAGCGGCTCTCGCAGGCCGAGGTTCAATTGGGATTACAT
CGTTGATTATGTAACTAAGTGATTACATAACCGGATGTCAAGTTAGTCCGTT
CTCCGGGTCTAGCTCCAATTCGGAATACTGCCAGACTTCACCACCAACTTT
TTTAAGTGCCCACCTAGAAATCTGAATCCCCGCCAACCTGCAAGATCGCGA

TCAAGGGCGCCTGGATTTATACACGGCTAGCATTTCTTTCGGCATGATGTTG
TCATGAGCCTGGTTGTCTTGAGAGGTATTCGGAGACCGATACTTAAATACTA
TATGGCTCAAGTTGCAGAGATGCCGAGCTCTTCGGCAGCGACGCCTGTCGC
ACTTATAACACCACAAATTGAAGCATTAGCTGTCAACGAACTAGAAAATTG
CACAGACAAAAAGGCAGTCCCGTCGCCTGATCTAATTCCTTCAAGCGACA
ACAATCTGTCACAACCATATTATTTTGAAAATGGGTTGCGACGAGTTGCTCC
CTATCATTTCACTTATAAAACCTGGTGTAAACAACGATGGAGGGGCAGAAA
AATCTTAGACATATTTGAGAGCGAATTTAGAGACCGACCATTAGAATATTAC
AAGAACGCCATGGAAACTGGCCAAATACGCGTCAACGACAAACATATCAG
TCCAGACCATATCCTTGTGAATGGAGAGCTGGTGTCACATACGATACATCGC
CATGAGCCTCCTGTCACAGCAGACCCCATAGGAATTCTCCATGAGGATGAC
GATATGATTGTCATTAACAAGCCTTCCGGTATACCGGTTCATCCTGCTGGAC
GATATAACTTCAATTCGGTTATCGAAATCATGAAGGCTGAGAGGAGCCCGG
GTTGGATTCCGTATACTTGCAACAGGCTTGACCGGCTAACGAGCGGGATTA
TGTTCGTCGCGAAGAATCCGAAGGCTGCAGATCGCCTGGGTGAACAGATA
AAGCAAAGAACCGTGCGAAAGGAGTATGTTGCTAGGGTTATTGGCAATTTT
CCAGACGAGGAAATAGTGTGCAATCAGCCCCTACTTCAAATTTCTCCCAAA
CTTGGATTGAATCGAGTACGGGCAACGGGGAAAACGGCTCGGACAGTCTT
TAAGAAACTCGCATATTACCCCCACCCGAACCTCAACGAAAAGAAGATGA
TAAGCTTTCTCAGACAGCAGAAGATATTGCTAGAGATAAAAGCAGGCCGTG
GCTGCGAAAAGAGGGGTATTCTATCGTTCGATGCCTTCCCGTTACCGGCAG
AACTCACCAAATCCGCGTTCATCTCCAACACCTAGGATATCCTATCCAGAAT
GACCCGATTTACGCCAATCGAAAAGTTTGGGGTTTCAACCTTGGTTATAAC
GATCCCGACGCCCTCGAGAATACGGATGATGATATCATCGCGCGTCTCAACC
GTATGGGCAAAGAAGAGGTCGCAGATGCTGTCGCCTACCATGACGAGATG
GTGGACGAATATTACAAGAGGAAGGCAGAGAGGATGTCTGGAGAGCTCTG
TGAGGTTTGCGACACCGCCCTATACACGGATCCCGGATTGCAAGAACTTTC
GCTTTGGCTGCATAGTTTACGGTATGAGGATGCAGGCGGTGATTGGAGTTA
CGTTAGTCCTCTCCCTGCTTGGGCTCTCCCCCCGACTGGCATGTCGGGACC
TACCGAGGTGGGGGGCATGGAAGAGTTGGTATCAGCAGTAAAAGATGTCA
ACCCCGAAATAGCATGACCTCTTAGCCGATGCGTCAACTATATACCAAAGA
AATCGAAGTAGAAAACGATAAAATCGCTGTCTTTTCATCTTTCGATTCCTCT
AGTCTTAGCACTGGTTTGAATGATCAGCTGACTTTGACTACCTCAGCATAGC
ATCAGCTCCCCCTTTCATGTTCACTAGACTCGAAGAAGAATAGTGCCTAAG
TTCTCTAGCCATGGCTGAGAGATACGACAATGTGGCTCGTATGATGCGATCT
GCCATTGCGCTGCCTACCTCCCAACTTTAGGGATGCGCTGCCCTGCTCTGG
GCAAATCTCGGGAATTAGGTTTGGTACTGCAGTACAACACAGGTTTCCCTC
GGCTTCTCCCTTCATATGCATCTCTAGGTTATGGTACCTTTGGTATGAAGAAC

AGCGCTACATGGGATCGAGAATCAACCCGAGGTGGTTTTTCGTGGTCCTCA
ACCAAACGCGCTAGGTTTAGCCTTGATCAGGTTAATGTTGACAAAACATAG
TTGAACGTCCATTGGTAATCACCACCATCTACAACAAAAAACCCTTTAATTT
CTTCAATGGAAAAGTTTTCAAGTCTGGAATATGGATTGCACAATTGATCCGG
TGGTTGCCTATAGCGCTGGGACAGCATCCTTGGATTAGACCAGTCCGGGTT
GGCGGCTACCAGAGATGGAGGTCTAGACTCGAATCTAGACAAAAATGCGA
CAACCCTCTGATTTCTTCAACTCATCGGCCAGTCCTAATTTCAACGGTCCCT
GTACTTACGTGTGCTATTAGGCTTTACGGAAAACCACATCCTTTTGCCAGGA
GCATTAACAGCGCCCGCCTGGAGACCTGCTTAGGTAATTGGGATAATGGCC
AGTCACCCCCACCAAGATATGCCTTCAGTATGTAATCTTCGCTTAATGGCTT
GTAATGTGTTCTTGGCATAAAATATGGCAAAGTTACTTTTCTTACCGGCCCA
AAGCACCAAAGGGTTGTTCGTTGGGTTGGTAATACTTGGTATATTGTCCTAT
TCGTCGAGACATCATCTAAACTTCCATCAAACGTGCTCTCCTTACGTCCCTC
GAGGTCATCACGCGGAGTCTAGCTCCTAGCTCCATGTTCCCGAACATCGAC
GTATCTATTTCATCTCACACCTCATACTTACGTGCTGTATTGTTCGGATCTTC
CATGTCTATTCTCAGGAGTGAGCGATCAACGATCTGCAGCTAATAATGCAGC
GCAAAGTCTATTTACCATCCGCAACCTTGCTGTTAGTATTCTCTCCAGACAG
GGGACAGGGTTGAGCTGTCAACGTTTCCTGGGGCTAGTACCTAGCGGATCT
TAACGTTCGGGCCCGTCCCTACGATAGCAGAACGCTTGGTTTAGGTTGATG
TGTAGTATTCCTCAGGAGACCCTCGGAAAATTCTGACTGTGTGCCACAACG
CCGCCACACATATTCGTCCACTATGCTCTCGACTTTGACCGTAAGTATAGAT
GTAATAATTACTGTGCTGAAGACCTCAAAATGTACTAACAAGCTAGTTATAC
TAGTTAGCTCGAGTACTTGCATACTTGAGGTCTCAGGGAGTGCCCCGGATAT
CAATCCTCCCGCAAGAGAACTCTTCGAATCGTGCTAGATTAGCTTCATAGTA
TCCACCACCCATCTTCTCAGAGACTTCTTGCAGATTTGCGGCTACATGCAAT
AGCTACATCTTGGGAGAATTCCATTTGCCAGTATTTATTATGTGCCATCCCCA
TGAGTAATGTGTCCAGGGGCTGTCTTCTCGATGTTAAACATTAAACAATCTT
AAAGTTGAACTCGCCTTGACCCAAACAAACATATTCCACTATATCGGTATTC
AGAATACCGTGTGCTTCTAGGCGCGGTTACTACTACATATTTGCAAACCAAC
ATTAACCCTCGCCAAATATCAAGTGCGAGATGCCTGGCAACATGTCAGCAC
CAGAATCCTCCACCAAGAGTGGCAAGCGCAAAGGTATGATTTAACATATGT
TCTTATAGGATATGAAGTTAACGTATCAAACATAGGCACTAGAAGCGTATCA
AGTCTTACTCCTGCGCAACTCGCTAGAAAGCGCGCCAATGATCGGGAAGCT
CAAAGGGCAATTCGGGCACGGACGAAGGAACATATCGAAAACCTCGAAAG
AGAGATAGAGGACCTTCGAAACCATCAAAACCGAGATCAAACCATCCAGA
ACTTACTCCGGAAAAATGAGGCGTTAGAGGAAGAAGTTCGCCAACTACGG
GGCTCTATGGGAATACGGAACAGCGACGCCAACGATCCATACCAAGCTGGT
AAAGCCTTCCCTTGAGGTTAACCTATTTCTTGTAGCAGTTGACTAACAGCG

CCGTGTAGTCTTCCACAGCTCCAGCCCTACGCGACCATCAGCATTCCCCCAT
ACTGCTACAAGTTACCCTATCTTGCAGAACATGACTACTTATCACAACATAC
CTGATACCACGGAGCCATGGTCTTCTAACGTAGCATATTCCATCACATCTAC
AGCTTCAAGCCCTGCATCCTCTGGAGGTACAGACGATTTTGGAAGTAATTA
CATTTCCACCAGTGCTCCAACTACTTCTATTGAGAGGTCTAGTCTCCCATCG
AGCAGTCACTCTCCTGCTGCTGGTTCATTCGAACGTAGCGACAGCGGATTT
GAAAGTATGAAGCCAGGTATGTCCTAAATCTTCAATATTAAGGCAATACTAG
ATTCTAATCGGATACCCTTAGAATTTTCATGTTCACAGATTGGTGCTATGCCC
ATCAACCCAACATATCACTTCCAGCCCTGGAACACATATCCAATGCAGTACC
AGGCTTCCCCAACAACTTTAGATCATGTTCATCAGATTTCCCAAATCGGGAG
ATGCCAATTTTGATCTTAGAGCCTCGTATCGAGACGAGATGCACAACATATC
TCATGCCGAGCAACGCCGACGCTTGGTGGAGATGGGATGCGAATGACCAC
AATTGGTCGTCATCTTCGAGTCTACCCGCTCGGATGGAAGAACATCCCAGA
ATAATTACGCAGAAATTACATATACCCTTCATTTTCAATGATAAATGCAACA
ACGTCCTGAATTTATCCTACCATAGGGAACTGGTTGTCCCTTTCGTATCCAC
CTACTATCCTAACATTCACGCCGTTCAATTACGACCATGCTGCCGGATTCCC
AGCTTCGCGATTTAGCATACAGGGGAAAACTTTTCGCAATCAGGGCTTTTT
AACCCGCGATCAACGAAAGGACGGTTTGGATCGGCAATCGATCTAATTATT
GGCCCCACTGAGTGATGCTGTCATCAGTTAGCAACGCATCTACACGACTAC
AGACTTAACGCCGTACCTAGAGTACAAGCCCCACGCCACGGGCATAAAATA
TATCTGGCGGGATAATTAATCACTAGTTACGAACTACTCTACCGGAAATAAT
TTTCGTCAAAAAGAGAATTGACGATTACGCGTGAAATGGGGGACTAGTCAC
CCAACGAATTATTCACGAGACGCTATCCGAGCTGGATAGTAGATGAATCGG
TTATCAAAGGACAACCTCTCTATAGTATCTACAGCCACACCAAGTTTATCTG
GGCGGGTTACTGAATTTTGCCTTGCACAGCTCAAGCATTGGGAGGAGTGAT
GGAGCTCTTAGTGAGCATTTCGGAGGTTCATTTAAGTGGGAAGCCCATAGA
ATAGATCTGGATATATAAGATAGCCTCACCAAAATGTAGGTTCGATTCGATCT
TTAATCCAAATCATCTTTTGATTAACTTTGAATTCCACCAACAACTATTGAA
ATCCCATAATCAATCTAGTCGTACTCTCAGCCAGGTCCTAAGAGTTGTCTAC
CTGTGGCTCATGCGCTTCATCGCACTCATAATGATTACCAAGGTAGTTAGCA
ACCATGTTTAGCTTACGACGCCGTTTGATTAAGCTACGATACCCCACAGCAG
ATCTCGCAGATTGTTTCCCTCAAATTTCCACATAGAATATACTATGGGTGGGT
GGAATTCTTTAAGAAAATACCATCTACTTCTCAGATTCTACGCAACAAGTCA
TTCACCCTCAGAGATAGTTAACTTCAGATTCACCTAACCCGACCTAGCAATT
AGCAATTGTCCTATCTAGACTAGTAGACCTCTTTCTGAAGAAGTAGCTCAGC
GAAGACCGTTGACTATCAGAAGAACCAATTCAATCTATATAGCCTTTAGCAA
TTAGCACCCCCTTAAAGTCTTTATCGCAAAGGACACGACCAACTATGTTGTA
CTACGGGTATCCCGTAGGAGTCGTTGAGAATATAGCGTACGAAACCACCAC

CCTCACCCTTAGTCATTCGAAAACTGACTTGTGCAGGTAGATACCTAGGCA
GAGACCTTAAGATATGTCAATTCAGTCCATTTTGGACGATAGTTTGCATATTT
TAAACCCGTGAGTTACAGGTAAACACATCCATCTATATTCTTACACGATATTT
CATGCTGTGAAGAAAACCCCAACTAAAAAAAATGACAGAAGTTAATTGCT
TGCGCTTGATGTTTGGGTTCCTTTACTATTACGTCGAAGCACGTGTAAACGC
GAGATCTAGTCCAGACAACATTTAGCAGCTTCACTAAATGGGATTTATGATG
GAATGGGGGACGAGACTCACAGCGTTGAGGCTGGCGTGGCTCATGAGGTT
AGGGGGGATGTGTGTCAGCAGACTTTGTCCTTTTGTAATGGCCCCGTAGTC
CGGCTAAGGATGAGATCATGAGGATGTATGTTTCTACGTTCGTGATTGGTCG
AGGAACGTGAAAAAAGACACCACCAGAGACCGTTGAACTCTTTTCCTAGC
CCCTGTTCCTAAAGAGCCTCGAAAGGGAATCTGAGGACCTGATTGGAGCA
CGGCTTCCCCTACCCCGGACTGCCCGCCAACCTTACAGTAACCTTGCAGCC
TCATGCACAATACCCTGTTAGCTCCCTTGATCCGGGGACTTGTGGGATCACC
AACTAGAGGAGACGGCGCGGACCTGCGTACCTAAGGCAGGTCTAAAGAAC
CCGTGGGCTGAAAATTTCCCGGCTAGTCGGAATCTAGCCCGATCTCCTTTAG
ATGGATACTCTACCCATCTATCAGAGTCACAAGTATTTCTTATCCGGCATTCG
AGGACATATATAATGTCCGAGCTGTCGAATTTTTCTTCGATGTTCCAAAAGA
AGAACTGAATGGAAACCCATACGCGTCCTCCGTGTTCTTTATCTGAGAAGT
AACTATAGACATAGAACCTGACTCTTCATCTATCTCAAACTACTTCGCGGGG
CTAGCTGTTCAGGTGATACCCTATGACTTGGGTCACATCTAGTCAGTTATATT
CATAGAGAAACAAATCTGGCCCAACGGAGACATGATTACTCATGTTATGCT
ATATTTGAAAATCAGCCCATTTTGCTACATAAATATATGGTTCTCGCGTCTAA
ATAGACCACAAATTGGCGTCTATAATGCTCATCTTTACTTGATATCGACAGG
ATCGACAGCTATAAACTTAGGGACGTTCATTTGGCGGAAGGTAAGCACAAG
CCCTTCGCCCGGCAATCTGTAAGGTGTGGATGGGTATCCATATATTTGGCCA
AGCTACTGGGTCTAGGACAATGAGCTGGAAGAAAAGCTCCCTACACTAAC
GAGATCCAAGCCTGGAGTAGTGTGAATACTAGCAGGCCTAAGTAGCAGTAG
TGAATCGTTTGATTGGATGGATCTAAGCGAAGAATCCGAGTAAGAATTGGC
TAGTACTTCATAACTAGATTCCCATATAGGGAATATGGGAATCTAAAAACGC
ATATGTTCCGACACATTGGATTCTAGTAGTATCGTTTTAAGTCATGACAAGTA
CGACGACGACTCCTTTTGCCCGTAATCTGTAAACTACCTGAGAGAAATGAT
AAGACAGAACGAGTAGATTACATAATGTGTGAACTTCCAATGTTGAGGTTT
CCTACAAGGTTCTCAACTGCGAACCTATTGACATGATCTCATGATTTCATCA
TGAGTTAAGCTAGATAGATATTTGGGCTCGTTTTCAGCATATAATTGAGAGT
ACCAAGCCTGGGATTTACCTTTGGTATTTCTATAAGGGGTTAGGAGTGGCTT
GCCACTTTAAACCCAGGTCTGTTAAGGTTTGGTGGATTCCTTGACTGGAGC
GGGCCACCTTCTGGAGGTCACCCCTAAATCCCAACCCACACACCAAAAGG
TCAAGCCTCCTCTTCTTGGCATCCTTCACCAGAGAGAAGCTATCTATATAGA

TATTTCCTTAGCTATACGCGTCTAATCTTCAACCTTAATGCTATTGACGCTAG
AGCACCCAAGCTAATTTCCAGTTGTGCGGTGCCTCCCCTATTCCATAAAGG
GGTATGTAAACAGGTAAATATTGTCTAAGAAGCTTTTATCTTCTATAATTAAT
CAACAATTACAGGTAAAAGCATTATTGAGTGCTCTGGGACCCCTCAATTATA
CGAGGAGATAAGTCTGGCACAATTCTATCATACAAAAGTTAATTTTCTTGTT
CGACAAACAACACCTCGCTGTTTTCCAAGCCAAATGCGTCGACGAAATGA
GAATTGAATTAAAACAAGTTTCAAACCAATAGAAGGAATCACCGTCCAATG
GAAAATAGCTTTACCTAATTCACCTACCCAGCTGACCGTTGCGCATTCAAGC
TTGCGGAGTGTACCGAACCTAGTCCTTAAGCCCCTCCGTCAACCTAAATGG
TGAGCATGCCAAGATACTTAATCCTTTCGCTATGCTGTTAGACTACAGATGA
TCGCATCGGTAATCTTGAGATCGGGATTGCGGCATCATCTGAGCGCATGGG
AGCTAATTTGGCGTGCCAGATGTTGGGCAACTTAATGCGATCAGCTCAGGG
GCAGTATCGCCTTTTTGGGAGTGAACAACACCAGATAATTCACACAATCCT
TTTGGTACGCATACTTGACTCAGGAGTTTGATTAAAGTCGGAAAACCCGAG
CCACACTCAATACAGAGGCGGTGGGATGACCCCGAGAATACACGTGGAAT
ATTGGCAATTCAAGGTTTAAAACCACCCGGCCTTCGCAAACTGGTTCCGTG
CGGACTGACCCAGTTGCGCGTTCCATCAATTAAAGTAATTGATTAAATATAA
GGTCGAAGCCTCTCCACGGAGTTACAAGAGATGTGGTTGAATCCCGAGAAT
TGTGCGTGGTCCAGCTAGTGAATCACGATGACACCCGTCACGATTCAATGG
CGACGTCTCTCCCCTATATCAAAGACGTCAAGCCTCAAGTCAAGCCCCATC
CTCACTATCATCGTCTCCGAAGCACTCATCATTGCGGTCATTAACCTTCAAT
GCGCCCGCACCCGCTCCTGAAAGGGAAGAGACTGTATCTCCGAGGATATAT
CATCTGTTCAGTATAGCCTCCAAGCGACAGCGAAAACAACCCTTGGCATAC
TCATCATTTCCAACCAAGCTGGCGGATGACACCTCGAGTATTGGCACGAGT
AGCGAATGCTCCCTAAATCGACGATTGGTGAGGATAGTATGAATCGATTCAA
CACCACACTCGTTCGTGACATATTTCCGCACAGGCAATGTCATTAAATTTGT
AGCACAGTGCGTCTTTTCAAAGGTACATGTAGAATAATTCACTTGACGATG
CATCATGAAGATTTGACGGCTTTGATGACCATAAAATGATTGTGACACACCA
TGGAGAATATGAATAGGGGTAGTCATGATGAAATTCTGAAAGGGATCCGTA
CAAGATTGATGACGAAGCCGAACGTGTCGAACGCATTGAGAAATAACACA
AATATATAGAATAAATTCAACATCTAGTCTATTTAACTACATATTGCGATTGC
ATACTTATACATATTGCAAACACATGGCTGAAGCACTCATCACTTGCCGAGA
GTCATGTGTTGGGTTTGGTCTCTGAGCTATATCTTACCTACCCATAACAAAC
CAATGTAAAAGCGAAATTCCATTCGACGACCTAGGGGTCCTGTTCCATCTA
AGTATTCGATCGACATAAAAAGGGTAAGTATATGAATGACTAGGATACAGAG
AGATCTGTAGATGTAATAATTCATGGCAATATTCGATTACGTGTCCCATTGAG
CATCGCTTACAACATTTTATCACAAGTACTCATCGCAGCTAGGAATAGTATT
GTAGTTATAGTGGTAGTTTATATTAACGCTAAGGGAAATTTGTCTTGTAGTGA

ATGTCTCCCTGACTCTCCCAGTCATAAGTATCTAACTATCTAGGTGCGTAGG
TGACTTGACATCGTGAGATGAATCTTCATTGGCGTAGAGTACCTAAACATCG
CCACAACCTTCTCATCAACTTCGTCGCGGTAGGTATATCCTCTTATCGTCTCT
TGTATCCCTATCTCTGATATTCTTCGTAAAAGGGGAAGGCGTCGTTCCAATA
CTTGCCAGCATTTGAGAACAGCCAGAACGACCGACTTCGAACGGTCCCAT
GAATAGAGTGTTCTGGAGTTAAAGGTTTCGTTAGCAAAGCGATCAAATTGA
GTATGAGGACAAAGTAGACTTACCATAAGCTCCCAATCTGAGCTACCAATA
CCGGCTCCCAGAAGAAATGTGAGTTTCTGTACTTTGGGACAGACCTCGGGT
CTGTCCCGTCTGTCTCTTGTAATTTGTACCGAAGATAAGTGTACAATGGTCA
GTTGTTATACTGACCGCCGGAGAAACGGCGCCGTTGTCGCGCGCTGACGTT
GAGTGTCGACAGCCTCGCAGCTGCTCATTTAGGGAACGCCGCCTGCTTTTC
TCCTTTCGAAACAGTTCCATGAAGGGCGGATGATGGGATGGAGGTAACGTG
CACCCAGAGGAGGGAGCCAGCTCCCCCTCTGTTCCGGGCTATCGGCGCGC
AGGAGTGCGCACTACCTCGCACCCCATCATCACCCTCCATGGTGTTCTTTCT
AGAGCAGAAGTTGAAGTCTCCACCGTCATCGTATAAGCCCGGGTCGGCGG
GCTGGCTTGGTGTGAAAGTGAGAGTGGCAAGGGACCGATGGGTGCCTGCA
CCCAACGGCCCCGTCCCCAGCCCGAGGCATGGGTCCGCAGCGAGCTCGAG
CCCGCCAGCGCAACGCCATGTACATTTTGTATGTATGAAACGAGCTGGCTC
GTTTCATATAGCAATAGCTTGATGATAACTACCGCAGCTATCGTCGGCAGGT
ATGCCTATCCTCGCCATGCAGGCCGAGGATACACGACGCCAGGTCGTGTGC
TCTGGTGAGCCTGCTCCGAAGTCGACGTACTTCGGGGGATGCCTCTTTGGA
CCAGCCAAAGGGCGATCCCCATGAAATGTACGCATTAATGCGTCAGCACAT
CAGATGCCTGGGCGGTTCCGGCCCACGGTACCATGAAGGTGCGGTGCTGCT
AAACTGCATGCGCAGTGCCCTACCTAGGAGGTGGTCGGTGTGTTGTCCAGC
AACACCCCGACGGCACCATGGTTCCTAGGTTGGCTTAAAAGCGCATGAGTT
GACCAGCCTTTGCTGTTGTCTGGTATCTTCAATAGTAGTTCCCCCAGCATAT
TCCGGGATGCCAGCCCCCGGAACTCCTCAGAGGAATCTCTGCCTCCTAGAG
GCCTGCCCGCTAGGAGGCCGAGGTGTCATCTCCAGGACAGCGATTCGGGC
GGACCGACCATTTACCATCAGTCAACTCGATTCTCTCGTCTCCTAGAACGA
GACGAGTCCTCGGGAGATCTCGTAGATACTCCTACGGAAGGCACACCGGTG
GTCAGATCGACCGCGGCTCCGACCACCGGTAAGCTGTTAAGCCTTCCATGA
GACCCCGGGCGATCCATTGGGTTAAGAGGCGGCGATCGGCCCATGGTGAA
GTTCCTCCGTAAACCAGCCCTAGGCACTTGGCTAGACAAAGAGGGATTCCT
GGTTATACCGCGTCTCCTAGCACGTGGTAGTTTAGAGCTCTTCGCGCAACA
ATGGAGCTTTTTGCTTCCTCTTAGGTTTGCATCGTGACAACCACTGGTGAG
GTAGCCCAAGGGGACGCGACCGACCATGTGAGGTGGTGCATTGCTCAGCC
GCTCCCTGGAACATCAGGCAGTGCGGACTTGCCATGCGACGAAGGGGGAT
ACCGCGGACTAAGTAGTTTGGAGTTAGCGAGTTGAACTAATGGTGTCTTAT

GAGATGGGGGAGGGGATATATATTATCAGGGATTTCACTTACCCATAGGCTG
TATATATCTACAGCGATCCCGTCGCAGGAAATGGAACCATTGCCCATTGCCC
ATTTCCCACATATGATCGTACCGTCTTCCGACACCACTCTGGTGTATGGTGC
TGGCAGACGCACAGTTGTAGGGGTCCGCAGATGTTGACTGCATCTTCAATT
GACACCCCGCGCCAGCGCAGGGGTCTTCCCTAATACCGTACACCAGGTTCT
TCTGGTGAACTGTGAAAATATAGGTAAGTAAATGGTGGTGAGGATAGACGG
TTAGATGGCGGGACTTACGTGAAAGCACCTTGTGAATGCTGCGATAGAGGG
CTTGGAGTCCTCCCACACTATGATGATGGTGAGGTATCGCGGCGGCGAGCC
ACTCAGCCCTATAAGGCAATTTGATAATTGTATGTTAAGAGGCTATGCGATC
CCGTTGTCTCTGGCATTGGCATTTGCATTGAATATCCACTGACGTCGCCTGA
GGGGCGGCATCGGGAAAGGCCGCAATGGCCTGTGGGTAGAGCAACCACTG
CGATGTTTCTAGGAAGTAGAGGCAGGATTTATCCCTAACCATGTACAGTTAT
GGTCTGGCCAACCAGTGGCCAAACAAAAAACAAGACGATGAAACATCATC
AGAGATTTAGGTAGGATGAAAAGTGATGCAATAATGTAGAAGTTGGTTGAT
GTGAAGGAAAGAAAAAAGCTGGCAATTTAGGAAGTGGGGGAGTGATGAA
GGATGAGATTTTTTTCACTCCTAAGGTTGAAAAGTTAGTATTTTCTTGCAA
ACTTAGTTGCAAAGCTCGATGGGGGCTCATGAATCGTCGGTGAAGTTATCG
TCAGACAGAAGAATAAGTTATTCGCGCCTGAGTATGTAAATTTGAATATAGG
AAGGAAAACTGCTTTTTACTCCGAGCTCACTTGAGTACAGAGAGTGATAGT
TCAAGGGTTGATGGAGGTTGGAGTTGGCATAGGTGAAAGAAAGTGCCTGG
GCTGTTTATGGGCAGACTCATGGCTG

>Mosaic_sequence_24

AGACACGTGAGCCTATCGTCGATCCTCGAGTAGCCTCGACGACTCTAGAGC
TCGAGCCTTGTCGATGGTGTTTCAAGGAGAAAGCCCAAGGCGGTGCAGAG
GAGGGCACGGGTGATATATATACAAGTCCTCACCCGTAGACCGTCCTTAGC
ACCGTGGTATTGAGTCCCATGGATAGAGAGTAACGGCGCAGCCGTGTTGTT
GACGCACGAGAGGCTGTAGGACACACCTTCTTGTGGAGGACAGAGATAGA
TCGACAGAGAGCACAGGTGAGAGGGCATATCAGTCGACGACCGGCCTGGC
CCCGTGACAGTACCATCAAAGGCTGATGAGACTTTCAAGTCCCACTGCACG
CCGCCACACATCGTCCCAGAGCTTGATGAGCTGTTTGTCAAGGGGCCCAG
AGCAGCCAGACAAGTACGGTCCAGCCGTCCGCCATACAGGTTTTCGACATG
GGCGCGACCCCGATCCAGCAAACGCCCATTTACAGTATGACGCGTGACACG
TTCGCACTCGTCAAGCGGCGAATTGAAAATGTGAAAATTGGTGGAGTGGT
CATCGGCTGGAGGACCTTTCTTGGAGGTGATGAACGGTGCAATTCACAGCT
GGAGGACGTCAAGAGCTGCTAGGTGGCACTTCGCGGTGTTCGTTTGGCAC
CGATGCGACACAAAGGGAGCGTAGACCGCCGACTTCCACGGCTTAGTCTG
GGCCTTTCATTGCTCGTCCTCATTCCGATTAGCGTCCACGCCAAAGATCGGG

TTTCTCGACCCGTCCGGTCAGTCAGCTTGTCATCTATCGTGCTTCCCCACTG
ATGTCAGTACCGACAGCAAGCCCTGTCCGCTTTCGACGCCGAGCCTATCTC
CCAAGCGCCCGGCCGGTATGGCAGACACTTCTTTCGCACCGACCGTCCGGT
CGAACTCCAACCTCAATCAGGTAGCCTCAACGTCCACAAAGCCAGACAGC
TTTGCTCGACTATTTGCGACTTCGTTGTCCCCAAAATGCAGCTGGCTCATCC
AAGCTCTGGCCCTCGTGGCCCGGCTAGACCACACAGCGGGGCAACCCCTC
CGCGATCTGGGCTGCGCATCGACACCGGGCGTAGGGTCAACAAGCTCGCC
CCGACGGAATCTTAGCTTTGAGGCAATGGGATTCCGGTCACCTGTTGCCCG
CTCAGCACTTGCTATGAGTGATGCATTCAGACTCACCTGTTGCCCGCTCAG
CACTTGCTATGAGTGATGAGAAGGGCAACAGGCGGCGGCATTCTACAAGG
TCGTCGAGGGTGTCGGCGTTATGTGCGGAGTTATCGTTGGTGGCGGCTTCT
CTGCCTCGTCGAGGTTGAACCTCGTCGAGGCACAACCTCGTCGAGGATGT
GTGTGCCTCGACGAGGCTACTCGAGGATGAGTCAAGCTCATGTGAGCGTAC
CATCTATTTCCGTTGCCTCCCCTCGCGAGCATGTCTATTGACTCTGCTGCTCT
GCTTCTCCTTGTCTTCCCAAGCCCAGTAACTGCCGGAGCTGTCTCGTCTTCT
CGGCTGAAAGGTTCTTGGTCAGGATGTCGGCTCGATTGCTGTCCGTTGGTG
TGTATTTGAGCTCAAGCAGTCCACCGGCTTGATGCTCTCGTATCCACTGGGC
TCGCATGTCGAATGCTTTGTTCAACTTGAACCCGTGTTGATGTTTGGCCAGT
TGAATGGCTCCATTGTTGTCGTTGTAGATGGGCACTGCTCCATCTATGCCTA
GGTCGAGGTCGTTCAATAGTTGCCTGAGCCATATTGCCTCTTTGCCTGCTTC
TGTTGATGCCAGGAGTTCTGCCTGTGTCGTCGAGAGTGCGACTGTTGGTTG
TCGCCTGCTTTTCCATGACACGATGCCTCCGTACGTGTTGAAGACGTAGCC
GGTTGTGGATCGACGTGTGTCGAGACATCCACCCCAGTCTGCGTCCGCCCA
GCCTAGCAGTGCTCTCCTTCCTGCATGTGCGTCGAAGGTGAGCACGAGATC
GGATGTCCCTCGCAGATACCTTAACAGGTGCTTCGCAGCCCTCCAATGTGT
GAGTGACCATTTGCTGATGAAGCGACAGAGCAGTGATGCTGCGTAGGCGAT
GTCGGGTCGGGTGATCGTGGCAATATAGAGGATTGACCCGGCGAGTGCTGG
AAATGGTTTATCTCGAGCCTCGTCGACCTCCTTTGGTGTAGCCTCGACGAG
CTTTGCGTCAGGCAAGATTGTCTTGCAGGCTTTGCCGGCTTCACCGAAGCG
TTGAAGAACTGCCTCGATGTAGTGCTCTTGTGACAGGTACATCTTCTTGTTT
GGCCTATCTCGGTAGATGTTGATACCGAGGAAGTAAGAGACCGGTCCTTGG
TCCTTGCACTCGAGTTCCTTGTTGATCTTCCGTTTGATCTCGTCAATTGTTG
ATCGATCGTTGCCGGCAATGAGCATGTCGTCAACGTGGACTGAGATCATGC
ACCGCTTGTGTCCTTGTCGCAGCGAGTAGATACATGGATCCGCTAGGGTCG
GTTTGAATCCTAGCTCCCGTAGGAACTCGTCGAGGCGCTTGTTGAACAAGT
GCGGTGATTGTCGGAGACCGTATAGTGATTTCCGGAGGTGCAGAACGTGTC
CGGCGGCGATATCGCTGCCTTCTGGTGGCTCGATGAAAATGTCCTTGTCGAT
GTTGGCCTTGAGGAATGCTCCGATGATGTCTATTTGGTCGCATTCCCAGTCG

AGGTAGTTGACGATGGTGAGAAATAGGCGGAACGATGACTTGTGGGTGAC
CGAGGCAAAGAGTTCGTCGAAATCTCGTCCTGAGACCTGTTGGAATCCTCT
CACGACGAATCTGGCCTTGTGCTGGCTTGCTTTGCCCGTATCCCCGTCGATC
TTGCGGGTGAACACCCATTTGCCTGTAAGGGTGCGGTGATTCTCCCTTGGA
ACCATCTCCCACACTCCATACTTTTCCATCTTTGCCATCTCCTCGTCCATGGC
CAATTTCCAATCCTCCCAGTCCGCAGCTCGACGAGCTTCGGTGTATGACTG
AGGGTTGTTGAGTGACTGGGCTGATAACGCGTGCTCTATGGCTACTCGGTT
ATAGTCCATGTCGATGTCGTGCAGAGGGTGATGACTAGGTTCTTGGCCTGC
TAGTCTCCCCGACCGTCTTGGCTCATCTGCATCGGCCGCTGCGGGCTCGTTT
AGTCGACGATCGGGATCCTCTGGTCCGTCGTCGGGATTCGGTTCCGGGCTC
GGTGCATCCTCTCGGATGTACTCATTGACCCAGCCTCTTCCTTGCCTGGGTA
CGGCTTGGGGAGGTATGTCGTCCATCTCTTCCCCTTGTTCCTCTTCTTCTGG
TGCCTCCCCTGCCTCAACTATCTCGTTCAGCTCGATGGATGTATCATCATCCT
CGTCGAGACTCGTCGATGTTTGTCGAGGTTCAGGTCGAGGGGGCTCCAGG
ATCCGTTGCGGTTCATCTGAGCTGGGCATTGTCCTCCTGCCCGTGATGAATG
TGACGTCTCGGCTTGCGATGACCCTGTTGGATTGGGTCAGCACCTTATAGC
CCCAGCTCCCTTCTGTGTACCCCAGCAGTCGACCGCATTGTCCCCGCTCGT
CGAGCTTCGATGCAGGGTTCACGACTCGATAAATGCAGTGATGGCCAAATG
GCTGCATCCCTTGGTACTGCAGGTCGATGTCGTGGTTTGGTCGAAAGAGCG
TTATCGGGATTTTTGCATGGGAGCCTGGTAGCCTGTTCCGAGTGAAGACTA
CATATCGCAGTGCATCTACCCAGAACTGGGTTGGCAAGTTCGAGTCCGCAA
GACATGTCCGGATGTCATTGAGGATGGTGAGATGGACGCGCTCGACTCGAC
TGTTTTGGTTGTGTGCGTCGGTCGGCGTAAGGGTGTGCTCAATTCCCTTGCT
TTTGCACCAGTGGCTGAATTCCTTACTCGTGAATTCGCCTCCTTGGTCTGAG
CGGATGGTCTTGACTGTTCGATCAAGCTGATTCTCTGCCAGCTTGATATAGT
CTTGCAGTGCTTCGGCTGCATCCGACTTCTTCTTGAGTGGTATGGCCTGCAC
ATATTTGCTGTGGTCGTCATAGCAGGTGAGGAAAGTATTGGAAGCCTTCTCT
CGACTGGACGGTCGCTGGTCCCCATAGATCGACTGATACCTCGACGAGGGG
CTTGCTGGCTCGACGAGGGTTAGGTAGAGATGCCGAGCGCGTTGCCTTCCC
TTTCAGGCAATCCATGCATTCGACTCTCCGAAATGACCCTGTCCACTTTGCG
CCTAGCTTCCCCTCTTTCCCGAGCTCCCACACCTTCCTCATGTTCGAATGAC
CTAGTCGTTTGTGCCAGGTGAGGAGGCGAGTGTCTTCGGCTTCAGTATTTT
CTATTGCCTGAAGTGCTCGGTGAATGCGCGAGGTGGTGCGTACCGGTGTGT
CGTCTACAACCCATAGGTTGCCAAGTTTCTTGCCCCGGAGGATGACCTTCC
CTTTCTCGACCAGTGTAGCCGAGTCCTTCGAGAAGCGCCAGTGTGTCCCTG
TGTACGGACCGAGGCGCTGTGTTGAGAGGAGGTTGTGCGAGAATGATGGA
ACGTAGAGGGCGTTGTTGTACCATACGTTGTTGAAGGTGAGGCGGCCGGA
GTGTGTAGCTTTGACAATCGATCCGTCTCCAATTTGGATCTTGACGGCCCTG

TCGAGTTTGGTGAGATGTGAGAGAAGGTGCTTGTCCTTGATGAATGGACTG
GTGGCACCGCTGTCGATGATGATGGAGGTGGATGGGAGGTTGTCGTTGGCG
TGTAGTGCAATCTCCGTCGCATAATAGCCTTCCTCCTTTGTCTCCCTCGACG
AGCCTCTCTTGTTCTCACTCGATGAGTCATCTCCTGACTCCGTCTGCTCTCT
TGCCTTCTTCGCTTGTCCTCCCTGCTTCCTTCCCTTTGCTGCTTCCCTGGCC
GACCTTTGCTCCTGGATGTGCTTCGGCACTCCAATCACTTTCCAGCACTCAT
CTGTGGAGTGCCTCCCCTGATCCGGATGGTGGATGCATCTCTTGCTTGCCG
GTTTCTGCAGAGATTTGCTGAGCATCCCCATCTCGTTTACGCTCTCGCCCTT
TTGTTGACGTCTGCGATGTTCATCGGCAACCCTATCGATAACCTCGTCGAGG
TGGATCTTGTTCGACGAGGTCCCTGAGTAGAAGGTCGATGCGAGGAGGCC
GTATGAGGACGGTAGGCGACGGATGGCGGCGTATGCGCCAATCTGGTTGGC
GAATTGCTCAAGGGTGAGCTTGGAAGTGATGGCGGAGGAGGTGATTTCGG
CCAGTTGCGACCGGATAGCTGTGAGATGAGGTTCGGGGTCCTCATTCTCTG
GGATGGTCTCGCCAAATATCGATGACCATAATTCGGCTAGACGAGATCCGTT
GTCGGCCCCGTATGTGGTGACCAGGTGATCGTAAAGCCCCTTCGGTTGAGG
ATTCTCGAAGTCGATCAGGCTCGAGGGAAGCTTATTTTGGACAGTCTCTGT
CAGACTGCCGTACAAAGGTCTCCGCCTGACAAACCCGTCTTCGTTTCTTTA
ACAAGCTCACGCTTTTCGTGATCTGTCGCAGCCTTCACGCCCTGCCATGGA
AGAGAACCACGAGCAAAGTATACAAACATGTATCCTAGAGATTCCAAGTCA
TCTGCCCACGATTGCTCTTCGATTATGTAAGTGACAGTGTAGCTATAAGAAA
TTCTCTAGGATGCTTACCAAGACCCTTGTGGTTGTTGATACTGGCGTAGCGA
CAGGTGCCACCAAAAGGGAGACCTTGGCGGTGCTTGGGCTTCTCGTCGTT
AGAGAATTCCTTCGCTAGGCCAAAATCGATAGTATACAGAATATGCCCCTGC
CTGCCGATGCCTAGCAAGAAATTATCCGGCTTGATGTCGCGATGAAGAAAG
CGTCGGTGAATACGCTGGATTCGGGAAATGGCCTGGTCAGCGATGAGAAG
GATTGTCTTGAGTGAGAATCTTCTGTCGCAATAGTTGAAAAGGTCCTCGAG
TGATGGGCCTAAAAGTTCGAACACAAGGACCTTATAGCTGCCCTCTTCCCC
AAACCAGTATACCCGGGAAATTCCGATCTCTCCAGCCAGCTCTCTGTAGGT
CTCTGCTTCGTCCTCGAGCTCCCTGGGATCGTTCCGGTATTCCATGAGTTTG
ATGGCAACTTCATCACCGGTGTGCATGTCGGTTCCTTCATTAGTCAGTCTCT
GAATTGAGGGGTGGAAATCGGGGTGACAGACCGGAGTACACCAGGCCGA
AGCCTCCTTGGCCAATCTTGCGATCAACGCGGTATCGGTTATTAATAAGAAT
GTCCTAAACAGGTTCTAAGTAAGAATGTGTGGCAACAAGCCTGGGAAAGG
AATATCCACCTTCATTTCAATGTGCAGTCCGTCCGACAAGAAAAAGCTGAG
TTTGAGCGCGAAGCGCCTAAAGTCTGCTTGACATCACATCGGCTCCTGGAA
GTTTAGGGCGATCGTGGTAGCTTAGCTTAGCGCTAGTGCAACTAAGGCTTA
GCCTCTTAGGCGGAATGCTACTCCTTTCTATCCGCCAGCATGATAAATTTAG
CTTGGCGGGCAAGCTTGGCATTTCCCCGCTTTTGATGCGCGTAAATGAGTG

GTCGCGAGTGACTCAGAGCCAGCCCTCCGTATCGCATGCATTTGCATCAGG
CCAAATCACAGCTTCTTGACCAATCCTCAGTTGCCGCTCGACAATACCGAA
GACGCGACTCCAGACGAAGCGTGTCTAGGACTTCCAACCATTTAACCCCCC
CCCCATTCCATCTTCTCCATCTGACCTCCAAATATGGCCGGTTATGGCGAGA
CCCCAGAATCGAAAAAGACAGCGCGCAGACGACCTCTCGCAAGACCCTCA
CCCAAGCAAGAAGATCAAGTCGGGAGGTCATCACCATTCTTCAAATTTCTC
CCCAGAGTTCTGGGATAACCTGTCCAAGGTGTGGCTCACGCCTCGTGCCTT
GCGAGAACTCGACCGGCGAAACAACATTCGACGACCCCCAGAGCCTACGC
CACCGAAAGAGGTGTGCTCTAAGGACCTTGCCCGTTTTGCAAGGCACGGC
AATTAGCCTTTGTAGCCAATTTCCAAGGTACTTGCGAACTATGGTGACCCTC
TTTTTCTCCCAACCACAGCATGCTGTCTACTGTGGTAGGTAACAGTAGCAC
AATTTTAGATCTACTGTATACAGTGATTACCAGCAGGTAGGGGTGGAAGTTG
ATTTGTTTGTACGGTAGTGTTATACCATGCTTACTACATATTTTGAGATAAGG
TGGGAAACTTGTTGCTCTCGACTATAGCAACGGCGTCCAATAAGGTATCCG
TGTAGCCTCTTTGCCTTGGACTTCAATGGCTGCTCAACAAGGCCAGGGGCT
TACCGTTATATCGGTTGCTCCCATGAGACCGTCTAGTGTAATAGCTAAGATAT
CACGTAAATTAAAGTATTGTCACTTAGAGATACATACACGCGTTACAACTTC
ATACTCCAATATCTCAATACTTTTCTAGCTTTACAAGACGATACATATATTAAT
CAACTATCCAGAACAAGCTCTATCATCTTATATGGACTCTTATATTGAGTGAC
TATTGGTATTGCGATTAAGTTGTTTGAATACACCTTCCCTTCAAAGGTCAAG
CTCAGGGGACCACGTGCCATCCATGGATGGATGTACAGGGGTATTCACTATT
GAGCGCTGTAGCTTAGTGGATTAAAGGCGCTGTGAGCGCTAATTTACAGCG
CACCCCTGCTTAAGGGTGGGTCAAATCATCATGCGTAAAAAGATGTGCATC
CGCTCGCACTGACTTCACTTCAACGCCAGGTTCGAATCTAGCATTTTGCAA
CTTTTCTTTTTAACACGCATCGCTACTAGGTACGCACGAGCGCGCCTCGTAA
AATCGTTCTGCCGGCGCTTGCCAACTTAATAAAAATGATGCCGCGGCCATCT
ATTTTAAGATTAAAACTATTTTAAGCGCGCGCTTAATTATTATTAGCAATAAT
ATATCATAAAATTTCATTACTTGAAAACAGAATTAGGATAGCGGAATTTCGC
TTTTTCTTATTGTTAAGACTAAAAAAAAATAGAATGTTGTTTAAGGCATGTAA
GTATTAGCAACGCCCAGTCTCCTTACGCCCTTCGCAAAGGTTGGTTAGGCA
GGAAAAGGGGCATTATTAAAAAAAAAACTCATAAATTGTGCCAGCGTAGGC
TCCTGTTTTCGTAAAGAAAGACGGCGAAATATATCCATACCAAGCATACCGC
GACTTTCCTTCTTCGATACCTAGTCCTATCTTACACTCTAATAGAATGACGCC
GAACGACTTCATCACAGTGGCAGAAAGGTCCCGGCAGGACATGAACCAAA
ATCTGCCCAGTGCTGAGCAACCACCTCGAAATAGTCGACAGCGGAATCCG
GGAGGTCCCGGCACAGCTTCTGTTGATCGGAATGTAAGATCTGCTTTCTTTC
TCCCTTCTATGAGCCACTTGCTAGATTTGGCTAACAGGTTATAAGAATAGTG
GCTTATAAAGTACAACAATTTCAATCTCCCAGACCGGGTAAAAAATAATCGT

TGTATGCGGTGTCGTAAACCTACGGATAGCTATGTCAAAAATAGAGACATAG
CATTGGTCACCCTTCGCTGCGGCTGCCACTACCATCTTCGGTGCCTCTCCTT
GCCCAACAATCAAACCAATACAACCACATGTCATAATCTTACCGCCACATAC
TTTTACATCCAATATAACCTAGAATTCCGTTACGTTATGGTAGATATTAGCAA
CTATAGGCCTAGAACAGCGCTTCCTTGGGAAAGAAGTCAGTTGGTAGACCC
ATTCCGTACCCTAAAAGACGACGTGGAATGCGCTATTTGCCAGAGTCAGAT
TGATTCCGTGAATGTGAATACTCATTCTGTTGCGGTCGACTGCGGGTCTCTA
ACACACTATCATTATTTTCATGCGGCATGTTTGGCCACCGCCATTATTTATGA
GGAAAACTCGGTTAATCGATACAACCCCAGTCGGTCTGTCAGTTGTCCGTA
TTGTCGGCAGCCATTTGTTCGTGTATGGACTACGTCAAATTGGTTCTACCAG
GAGTCTTGGCACAGAACGTATACCGATGACCAAAGCGGAGTTGTGCAGCA
AATTCAGTTCAACGAACACTTCTGTCAGGACGATGCCCTGGAAGCATTTCC
ATTAGATAAATTGGGCAGCAGCAGTGGATAGTCTACGTGTAAAAAGCTCTAT
ACTATTCCTTATTGGATATCATGTTTGTGGCTTTATTTAATTTCGTATCTTCATT
AACTACATGATCAAAACGATCCCAATAACATAATCGTTACTTAGACTTGCAC
TTCCTCCTTAGCTCAACTAAACAAAACTGGGCCTTCCAGCTACGTGTAGTA
ATATGTTAACTCTAGATATCTCTGTGTAGAAGGGAATTGCCCTTAGTGTAGG
TAAAATACCCACTGTAGATGAGGAAAGGACGATACTAGAATTTAGGAACGG
TTTTGGTAAGATAGACTTATAAAAGGAGTTTGAGGCCTGTCACAGCCGAGC
TCGGATGCGGAACTGCGCCAGACATTGCTTGTTGGGGTCTTCTTTGACTATC
TAGTCGAAAAAAATGGAGGGTAGAAAGGGATTACCTCAAAAGTTCCACGC
AGGATCCTGTACGTACTGAACAGGCATTCCTCGCTAAACTATACGGAGAGA
GATGGTAGCATATGACTGTTACAAAGGTTCCAAAGCCACAATGGTTCGTCA
CCAATATTGGTTCACGCTAATACCACACAATTAACTTGTTTTAGGTAGCAAT
ATTAGTACAGCGCGGCCCAAAACGACGACGAGCATTCGACTACAGATATGT
ACAGCAAATTCAGTTGTTCGTCACACCCTTACTATCCTCCCTAGAGTCATGA
TGACCTTTGATGTCGGTCTGTAGAAGGGGAACTGGGGAGTCTCCGCTCTCG
TCCATTCGTCCTACGGGAGAGTTCCTTTTACGCGATTCGAACAGCGTTTCA
AACGTAACAAGCTAGAACCAATAGGGGTACCCTGAGCAGGCGCCAAGGGG
TAGGCGCCTTCGATTTGGCACTCTCACCTTCCGCGGATTGGCAATGGTATCC
CAGCAAAGCATTCTACGCTCTACCAACTTAAAATTAACTCTTTCTGGAACA
ATGGAGTGGTCAAGACGTGGCAAAGGATTCGAATGAAGGAGTGAAGCTCC
TCAGTGACCTTGGATTTGTGTGGGCGCCTCCGCGAACCGTCCAGGCCTGAA
AAGGTTTGTACGTATCCCAGCCATTCTGGCGGTATCTCAAGAAGTTGCCTG
ATGCAAAATATGAGCAAAATGACGATATACGTCCTAGAGAAACCTGCTACA
CGTGGTAAACGCCATGAAGTTACCCTCATTCCGGCCCTTGATCTTACCAATT
GTACCCTTGTGACAACTGGGCAAGTAGGAGAAGGGTTCGTGAGATCCGGA
TATGACTCATGACTAGCATGCGGCCCCTGACATGTGAATGCTGCTATCAGAG

GCTGCGGTACCGAGTACCTATATAGAGGAAGAAATTGAGAAATAGGGAGAT
CAGTATATGTTTGGCTAGACGCAACGCGGAAATGTGTCGTTCGTTTCCGGC
AAGCTACCCAAAGCCTCTAGAACATGGGGAAGGCCCAGATTCGGCTCACT
ATACTTCACTACATGTATCTGTACCCTGCCCTTGATTTGATTAGTGAGTCGGT
GAGTTCCTGCGGTGTCAGGTTTCGAAATGTAGCAGCAGATTTCCCTTTCCC
CTTTGACTCTACGACGTCCTCCTCTACAAGTAGTTTTCTTATCAAACCTAAT
CGTTCGCGAACCATTTTGCGCTTCGCGTAAGGCAACCACGAAAAGGATATG
AGAGGGTGCGCCATGGAAGGGGTTTTCTCTTCTCCTTGTAAATAGTACAGA
CACCGCCATTGGTAACCTTGCTCTCCTTTGCCTTCTACCTTCGATTTCACAA
GCCCTTTTCCCATCTCCCTTCGTAATTTAGTTGATATTTCTTGACTTCGAAAA
TGCCACGAAAAGACGAGAATCCCTCTAACGTCAATCACCCCCGAAGAAGC
CCAAGCCCAAGTGATAGGCGGAGAGGGAAACGTAAACACCAGTTCTCATG
CACACGGCCTAGGAAAAGGGCAGCCCATTCTCGACCTACTCCGGATGAAA
GTGAATCTATTCAGCTTATCAAACAGCCTGAAACTAGGCCGATCAGCCAGG
AACAGCTGATTGCTGAGGTTAAAGGTATTTACAATAGGCTTCAAATGGCCG
AATCGAAGTGTATCGAGACCGTTAAAGCCCAGTCAACGCAAAATGGTATAG
AGTTCAACAATGAACAATGGCAAGCATTGATCGAAATGCATAAACTTCTCC
TGAGGGAGCACTACGACTTTTTATTAGCCTCCCAACACCCACCCGCAAGCC
CAGCTCTTCGACAGATGGCCTCTAAACACGATATGCCAGGTAGGCTGTGGT
ATTATGGCATCTATTCACTTCTTGAACTATTACGCAAACGGTTGCCACACTC
TCGACAGCATATTATTATTTTCATTTATCTGGCATATACCATGATATCTCATCTC
TACGAAGACGTCGCGGTAGGCTTCAAAGATATCTGGGTAGAACACCTGGG
AGATTTAGCGAGATACAGGTCAGTGAGTAGCTTACACTACCCACTCGCCGT
TAATGCTCACACTTGATGATATAGAATGGGCATAGAGGTTTTCGATATCCGG
GTCAAAATACATTGGATGTCTATGTGTCGGAACTGGTATTCGAAAGCATCTA
ACGGGGAACCTAACACGGGACGACTTTATCACCGCCTTGCGACTATGGCGC
AAAAAAACCCCTCGCAGCAGCTTTACTACTATACGAAGTCCCTCTGTGTTG
CTAGCCCCTTTCCTTCCACACGGCAGTCAATTATGACCTTGTTTGACCAACA
TTCGCGACTCAGCCCAATTGATAGAGCCTTCGTCAAGGCGCACAGTATCCT
ATTTAGCAAAAAGTTCTCCGATGATTTCTCTTCCATGGTCGACGAGTTCTGT
GCCAATTTAGACAATCACATTGCCCGAACTGCCAGGAAGTGGATGGAATCT
GGGTATGTTCTATTCTTAATCCAATCCCGACTCCCTCTAGCCAAGCTTAACC
AAGATTGAGGCAGCTACTACATCGCTATCTCGAATTGTTGTGGAATCTTATT
CTATAGCCAAGAAGACAACGTTATCCTGAAGGAAATTTGCCCCCAACGATA
TACACCGAGCAGAATCGAAGAAGGTGCGCATAGGCTCTTCATTGGCACATG
CAGCGTCGTCTTCCGTTAGAATCAGCGACTTCAATGTAAGTCATCCGTTCA
ACCGACGTCTTACAGTTTCTTAGACCTCAAAACCTCATATTACGAAGCTAAT
TCCCGTAGATTCTCCCCTTTCTACACCTAGTGATGGTTTTCATATTTCATTTC

ACACACCACCTTAGCGCCAGGTCATTCATTGAGAATATTATACCTTGGAAGC
TACTTTCACTGCAGATGAATTCCCATCTTCGCTCTCGTAGAAGTTATTGCCA
AATCGAGGACGAGAAATTTCCAAGACAATCCCCTCGTCCGTTACCAGAAG
ACTTCGCGGTGAGAGGTCTGGGTTGGGATGAGAAATATTTCCCGGTTGATT
GGTTTCCTGATGATGTTGATGACGACGAGAAGTACTCTGAGGTGGCATCTA
TGACCAACCACCGTAAAGAGAGAATTCTATGGTTGGGAGTTCGCATTGCAA
TGCGTAGAAAGTGCCCGCTTACCTACAATGAGAAGTTGCACGAATTTGGTG
TTGTGCCTAAATATGAGGAGGCAGCTGACATTGTTACTGATGGAAATCAATT
GGCCAGGCCCATAGAGAACATAGCTCTTAAGATGGGTGATGGAAACACGGT
GAACGCGGATGTTGCCTTGACACTGAAACTTGTCATCCGATAATAGACTTC
TAAGAGACTCCCTATTGTCAACATTTATTGGCTTCTCTCCATCTGGTTAGTTT
CTATCACGCCTGCATTATACCTAAAATGGCTATTTGAATTGTGCTTGGTATCA
CCTAGGCTGTGCCTTTAGCCTTACTCGAAGCCTGGGTTCGAGAATGGGGCG
ATATCACACAGTAGACGATGGCTGCTACGGCGTGGGTGTAGAGGGGGGGA
GGGGGGGGGGGCCAAAAAAAAAAATCAAAACTACTAACATATCTGACCA
GGGAGCAAGGTTTACGCATATCCATAACCTTTCCGCCTGTTCCGTCCGGGA
GGCTCAGGGGTCGATTCTCTGAGAATGGCGGCCACATAATACAGGGTCGTA
AGCTAGGGTGATTCCTCGCTGTTAAGGGGGGTGGGCGGATATTTTGACTCT
CCGCCCTGGCAGCCTGGTTGGATCCTAGTTACAGTCTTGAATTCGTTGCATG
AACCCTTGGCTAGATGCCGGCTCGGTAAGGCAACTTCTCTGGTCGTTAGCG
TCTGGCCCTACCTCGGACGCATATCATCAGCTGGGCTGAAAAGGATTAGAT
AAGAACGCACAGCCAGATAACACTCAGCTACTCCGGCAGTGGTATCCGAG
CTTCCTATTGACCGACGCGCCTTCCTGATCCTACATACGGTGGTGGTGGAGG
AGGGCGGTAAAGCTACTGGTCCAACGGTCAACCACCTTCAAAGCCTCTATG
GCCTAACATACGAATTCGGGCTCGAAGGTACCCGATTGTTGCTTAAATCTAC
CTGAATACAAAGTTTACCTCTTTTGTATTATGCGTGCTCGCTAACCCAGACT
GGCAGGCGAAATGAGTTTTCGCCGAAACTTCGCACGCATAGACCCGTGATC
TATTGTCCTCAGCATTCCATGATCGATAACTATTTCACCGATCAACCATGGAC
TTTCATAAAAAGCCAATAGGCTAACAGTGGTCAAGATAGGTTCTCCCGAAA
TTATCAGAATTTTCGATATTACAATAGAACGTAAACTTCAGTACGTGTAGTG
GATGCTGTATCATAGCGTTGATTCTGACGCAGTATGCCTGAATAAAGAACAA
GGTGGCAACTCGATTTCTTCACTTTCTGGCCAAGGCAACCCAAGTATCCCC
ATCCTTTTCAACGTATAAGCCAATACGAACGTAGAAAGATCTTTGATATGAT
TTTTGTTCTTTGTGAAAAAGGGCCCGATCGTAGTCATGTCAAGGAACGATT
TTGGATCTCGCTTGTTCCGCATCTTCCCAGTGATGAAATCCACGTATGCTTT
GTCGACTTTTGCTAACGTCACCCATATCTGGTCCATGTTTTGTGAAATGAGA
ACGTAACTGGTTACATGATTAGCAATAAGTACTTATAGGTCGAGTTGATTGG
GATAGGTTATGCCATATACGGATATTTTTTGCCCGGTTTTAGATCCTTTGGTG

GATGACTGCTGACCACGGCAGCGAGTTCACCCGTTTCTTGGATCGCGTATC
CAACATCATTATTATCTTCAAATCGTCGTTCTGCCTTGGCTTCGAAAATCAA
CCAGATCTCCTCGCCAGACGAGTCCATACAAACGCCATCTGTCCGCGCTTC
AAACACTTTGACCTGGTCACCGTTTTCATCCAGAGTTCTGACTTGGAAACG
CTTCCTGTCATAGCTATACTTGAACATACCTCCTTGGTATGGTAGCGTTAGAA
GGTCAAAAAATTGGACAAGGACTAGATTAACAACTTGCTCGCCGTCAGCC
CTCTCCTGTTCTTTACCCCCACCGCCACCAGCATAATAAGAACCAGTATCGG
AATCCATCGTTAGGAACGATATATCGGGGAGACTGGAGGACTCGGAGAGGT
CGGCGGGCTCCGGGGTATGTGGTCTTGTCGTGGGCTGGGCTGGGTGCTGTG
ATTGGGATCTGGTCGTCATGGTCTTCGGCCGATCCTCGGGCTCTATTTCGTG
ATGGTACTTCCGGATCATCGTCCATGGGCCGAATTCAGTCACGGGAGACCG
CTTTGGTTGTTCCTGGAGATATTGGAGGTATCGCTGGAAGGAATCACGTTC
GTTGATAAGTATTCTGGCCCTGTTTATGGAATCGTCATCGACAAATTCGGGC
CTGCCGACTTCAAATAGCTTCGACAGACCACGGACCTCGGCGATAGGCCG
GCAAAGTATGGCTTGCATATGACCCATGGTAGACGCGGAATTAGTGAACTG
TCTATCATGAATGGTTGTATTTTGAAGTTTATATGTCGTGACTAAGCTCTTCC
ATCTCGCCTCAGATTCGGGCAGTGCAAGGTTAGACCGGGACATGGTCACAA
GCTTCTGAGGATAACCCAGCCGGAAGACATCAACTACCAAACCTAGATAAA
CATAGATGAAGAAATATATCATCCTGTTATATGGGAATTTTTGGTTGCCCCCG
AAATTTGGTGTCCAGTGAACAGCTCAACCATGATCATCTATCACTTGACGG
AACAAAACCTAATGTAACATTACCATGTAGAAAGACCTTTCCAGACAAGAA
GTGCAACAAAAGCGCGTCGTGCCTTTGATCCACTCTAGGATGGAATGAGCC
ATGTTACCACACCTTGCTGTCGGCACTTGGAAAGCAAGTATGCAGAATCCA
TCTCAGCAGCCCACGTAGTCCAGCGACTCCAGCCATCTCTCCTGACGCTCA
CGCAGATTCTTCTAAGTATGCTGATCGAGATCTAGACATCCTCAATATCCGC
TTTAATTCAACAATAATTGGCCGTGACCTCTTGTTGATTTCCAAGGCGTTCC
TAATCCCAAGCTGCCACGTCTAGCTGGTCTAATGGATCGCGACCGGGGGTG
TTGATATGCACGGACCATGTATGAAGTCATATGTAATTGAACTAGTGCAGTC
ACGCTTTATCAAGTATTTAGGTACCTCCATTCATACCTTTTCGTATATACAAA
CACCAACATATACCGAAATATCGCCTGGTCAAATAATACTGTTACTTAATAAT
CCACCATGGCTCAATCGAAACAAGTCCATGCTATCCTCAGATTCATCAAGG
AACGTTTGCCATATATACGAAACAGCGACGCGTCGTACGTGAACGAATACG
ATCCCGCGTTGAAGAACCTCGAAATCGAGATCGAGCCCCTATGCTTCGACG
ATGGTGACAACTCCGGAGATGACGGCGAAGTGTCTAGTGAGCCTCTGACAT
TTAGCAGAATGAGCGATCTAGTAAAGGAGAAGGAGCCCTGGGCTGCACCT
CATAACGTTATGACAATCTCAAGTATTCGCAAGGACAAGGAATTAGGGAAC
TCCTGGATAGCCAGGTACCTGGGGATTATGCTCGTCGATTACTTGGATCTGA
ATAAGGATGATGCCGCTCCGGGGATTTTGCAGGACCTCGAGAAAGAGAGTT

TGAAGTAA

>Mosaic_sequence_25

TTTGGTGGCAGGGGCGTACGTCCAGAGTCTGCAAGTCGGCAAGCGCCAGA
CCAATGACTCGACCACAGGTAATCAGACGCAGATCAGTATTGGGTCAGTCG
TAAGCGGCGTCGATTGTCGAAGTGCACAGAGCTGGGACTTGTACTACAACC
AAGCTGGGTTGATCACTCTGAGTGGCACGGACCTGGTGTTGGATGCCAATG
ACACGGACAACGGGGTCATCTTCGTAAGTCCTCCTTGTCTCGAGCGTACTA
TGAGTCTGACTCCCAGGTCAACAATGCGACCAATGCGACCACTCAGACGT
GAGTAGGGTTCACATTGACTTTGAAAGCTGACTGGGTACAGGTGGTACTGG
ACAAGGGACGACCACATCGCCATCTTCTCAGGCGAGAGGTGCCTCGACTC
GGGAGAAGACGGTCAAGTGTATCGCTGCTACGGCAACAATATTGCCCAAA
GTGCCCTTTCGAACTGACATGGACTGATTGACGCTGACAGCAAGCATGGAT
CGTCGGCGATCTCGATCACGTCCTCCAGGCGGCAGGCCTCGTCGGGCTGA
ACGGTAATCCCGCTTGTGCGGGTATTCCACTTGGACAAAATATAAACGACA
CGCTCGACGGCAACGTCAGGATCCACCGTACGTCTTGTCCACTACGCATTT
ACGCAATATAGAAGTGCTCGCTAGCTGAAGTGGAGCGTGCGGACGACAAG
ACCTCTGCGTCTCATCGCTAGACTGCACGCTGAGCAACGGCAACGCCACA
GAGTGTAGCAGTATCGGCGTGTAAGCGCTACACTGTACATGTACCAGCTGT
GGTCACTCATCGCAGGACATACTGCGTGGACGTGCATGATCCCAACGCGGG
CCTGCAATTCTGGTACATGACCCCGCAGCTGATCGGCACGCCGTGGTCTCT
CCTGAAACAACCTCAACACGTCCGCCGCGGAACTCGCTCAGTGCCTGACT
GCCGGGGAGGCCCCTGCCGATGGGGGCAACCTGACCTTGGGCAGCTGTGT
CAGCGCGACGAGCGGCTCTACGTGGGGATACGACACGAGGACCATGCAGC
TGAGCACGAGCAACGGTCAGCCTGCCCGAGGCTGATGGCCGTTTAGGCGA
CCACTGATCCCAGGTCAATGTCTGGATGTCAAGGCGGATTCGGGCGAGCGG
GACTCTCAGCCGTGGAAGAGCCTGATGGACCTGCAGACGTGGACGTGCAG
TGCCATCGACCCGCATCAGGTGAGCTTTGGATGTTGAGCTTTTCAAACGGT
TGAAGACTGACGTGAAGCAATTCTTTGTCCCGTGATGCCGCCTGTCTCGTT
TGTCAATGACGTGTGCATGTGCCGTAGTGTAGTACAACAAGTGTTTGCTAA
GTGGTGTGTTGTATGCGCGAGATGCAATAAATGTATGTGGTATGTCCTTTCC
GGACTGCCCTACCTGACCTGCTCGCACTTGATGAGCGCATTCGGCCACTGG
GCCGACTCGGTGATCCTGACTCGCACATCCTCTCCAGGCCCATACGACGAC
CCCTTGGGCGTGAACATCTGAGCCTTGACACCCAGAGCAGGGATCAATACC
GCCGTGAACGTCTGTGCGCCCTTGGTCAATACCGTGCTCATGATCGTCATTG
CTTCCAGATTGTTCAGGTCGGCCTGATGGCCTTCGGAGGCTGGCCAGCCAA
GATGCCGGGCAATGATGCCCGCTTGCCACCATGCTTCAGCACTCTTGCTCA
GCCTGCTGATACGCCTATTGCCGGCATCGGCGCGCCGTGTGACCCTCTCCA

CCTCTTCTGCGATCATTCGAGACCACGGAACAGCAACGCCTGTCTCCCGAG
CGAGATGCTCCTTGATCTGCCAATGGACGACGCAGTCTTCGTACCGCCGCA
GCGGGCTCGTGAGGCGCAAATAGCCGTTCGTGTCATCGAAGCCCATGATCC
AGTGCGGCTTGGGTCGAAGCGACTGTGTTCCGGCTTCCCAGTATAGACCTG
CTCTGACGATATCCCTCTGCGCTAAGGACCCGGTCGCAGAGCGTCGCGCAA
GTAGCTGCTCGATTGTCATACCCTCGTTGCTGGGGCCCGAAGCCACCATCG
GCCTGGCGGAACCTCGATACAACACGGGTATCGATCTGTCGTCACAGAACT
TGGCGCCGATCCGACCAGCAAGGATCATAAACTCGGCGATCATGCTTTGTG
AGGGGCTCATCCCGAGAGAGACCGAATAGTCTGTCTCCGGGACCGGTCCG
TGAAGAGCGGCTTGCGACGGGATTCCGGACATGGAGAATATGCTGGATGTC
GGAGCCAGGCCAGGTGATAGGAGCCGTATCGAAACCGCGGGATTCGCCCA
CTCAAAACCTGCGTTACGAAGTCGGCGGCCTCTCACGGCAGATGCGATGTC
TCGCAGCAATTTGAGGTCGTCGATATCGTCCGGCCTGACGGGGGAAACCTT
GCGGTAATTTCCTTCAGACGTCTCCACCGATGTACCGAAAGGCTTGAGCGG
CGCGGCAGGAGATGTTTGCAAAGCCTCGTCCACCAAGGCGTATGTTACCAC
TCGAGGCTTCTTGACCCAACCCAAGCGCACGTTGGTGTCTTGCATATCGCC
TGTTGGCGAGATGCGCGCCGAGAGGGTCAAGACGCCCTGAGCCCCGAAGC
CGTCATCGATGTCAGCGCCCAGCGAGAGATCCCTCATGATGATGTTCAGGT
CGAACAGCGGTATGCCGCCTTCGGGGAGGTAGAGCGAAGACCCCATGTGG
CTGGCCCGCGTCGCAACCGGATGACCTGGATGCAGGTATCGAGTCGGATCC
GCAATGTGCACATGGACCCAGACATCATCGGAGCCGTCTATCCTCTCCACC
GACACTCCATCGTCCAGCTCTTCCGCGTTAGGATCGTCAATGACAAAGACC
TTGTGACCGGTGAAGTCTGTACGGAGGTCGTCCAGCTCGGTGCCCTTGAG
AAGCTCGTTCGGGCCGCGAGCACTGGCCCCGGACAAGGCTGCGACCCTCA
CGGTCGATTCGATCGCCATGGAAGCTCGGGTGCTGTCCCATGGAGCGATCA
CCCCTACTGCGTACAAGATGTCGACCACAGCTTTGGAATCAATCTTTTCGG
GAGGAGAGACCAGAATAGTTTTAGCGATCATCGTTGACAGGTATCCTGATG
GTGGAGACTGTGTGGATCGGGTCTCATAGAGGCTCAACGAGAGGACGTAA
AGGATGTCTCGCTCGGCTTCCGTCCACGGCTCGATTTCAAGGCCGATGGGA
GCCGACCCTGCATTAAGAAGCCGGGCTCGAGAAGTCTCCGCGTACAGTGT
CATTGCTGAGCGCACTTTGTCGCAGAATTGCGACAGTATTTTCTGGTCCTGA
GGTGTCGCTGCCGCCACGATGCGGCTGACCCGTGCTGTCCGCTGCACTTCC
TCCTTGCTTCTGACAAGAAAGCGCCCGTCGTTCCACATATCGGAGGACTCG
GCGACGAAGAGGTCGGCACGCGCCATAAGCAGCTGGTGTGCTGCATACGC
GATAGCACTTGACGATTCAACCTCTCCTGCCTGTTGTTGCAGTATCAGAGC
GACCGCCTCGGGTGCGGTGACGCTCGTGCCGTCTTTCTCGCGAGCAAGTG
CGCGCCACACAGCTTCGGCGCCACCGACGGTCCCTTCGCGCACAGCGCCC
CCCGAGAGAAAGTTGACCTTGTTCTCGGTGTCAATTGTGATTCTCCGCATG

ACACACACGGCTTGACGCCGGGCCGCCAGCATGTCCGGCGTTTCTGCAGC
AGTAGGCTCTTCGCCTTTGGCCCAAGATGCAAGGAGGGAGCGCGACCAGC
AGCGAGTAGCGACATCGGGGGAGATGGTGTCGATCGGGAAGAGGAATTGA
ACGTCCTGTGCGGTCGCAGGCCAGATCTCGCCGGAGGGTCTGAGCAGGAG
AAGTCGACTTCGGCCATTGACGAGGATCGAGCCGAGGATGAGACCCAAGC
CAGCACTGCCAGATCTACCTCATGAGCTTGGGCCCACCTCTCTAAAGTGTC
CGAAAGCTCACCTCCGGACTTCGACAACTCTCCCTGCTGTGAGACCTTGCT
CTTCGCCATCGACATCTTCTACCTCAGACGGACTGGAAAGGACGCTGGTCT
GCGCGGCGGCGGACGACTCGGCCTGAGCACCGGCGGCATCGAACGCACG
CCGTTCTTGCGTCAGGTTCCCATCCTCGTATGAGAACCGAATCGTCGGCTTG
CTACTGGGAGCATGTCGGCGATCGACCGGAAGTCTGCCATCGCGATGCGAT
GATGCTCGTGGAGGCGGTCGATATTTGGCTTGTCGAGGTTTATAGGATGGTT
TCTGAGCAGATTCACGGTCGATGCTATGGCTGCTGAAGAGTCTCAGGTGAC
GTGCGCATCTCGTACAGCCCGGCACGCCCCTGGCGAGCGCAGGTATGCTCA
TGCTATGGTCGACTACAACGAATTCGGAAGGTAGAGCTGGAAATCTTGGTC
TGTCGGAAATATGGTTTCCCACGGTACTTCAAGTGACGACCGCGATGTCAG
ATCGGGTTACGACTATGCATGCATGACTGCTGAGCAGCTGGGCGGAACCCT
CAGCTGCGACCTGGAACTACGTCTGCAGAGCTGCAGAGGTCTCCGAACAC
AGAGCCGTTCAAAGCTGAGGTATCGACGTTCAGACGGGCGCGCGACGCAC
TATGGCAGCGACGGCCGTTAGTCGAGGATCGCCTCGACTTGCCGTAGTGGT
GTCCCAGGGCGGGGCAGAATCCTGGTAGATCCCAATGGGCCGCGGTCATCC
TGGGGGGCAGGCGAGATCAAGACGGGATACCCGGGATCTCAGGCAATATC
AACGGCGGGAACTCTGCGCCATCAGCCCAGCCCCTTCGCACCACGTCCGC
AACTCACCCTCACAAGCCAGCTCGATATCCTTGCGCATGACGACCGGCCCA
TAGCCATAACATCTGACCTTGCGCGTGATGCGCTCCGCAAGGGCCTCGGCG
TCCGTCTTGCTCATATCCACGAGACTGAGCACCGCTGCCCAGATATCGTTCG
CATCCAGCTCAAGATCGTAAATCGGATGCGGGGGCTCGAGGAAGGGCACC
GAGAGCGGGCCACAGACCTCGCCATGCGGCGGGATGAACGCCGTCGCGGG
GGTCAGGTTGGAGAAGTCCAGCAGAGCAGACATGTCCAGCCCACCCTCGT
ACCCTTGGCCTGGTACGGGCAAAGTTGCGCTGGCAGCGAGGGGCTGGCCC
AGCAGCGCTGCCCACGGCGTAGTATCTGGCGTTTCGCCCACCACGATCGGC
AGCCCCGTATTGAGCGGCCGGTTGACCGGCCGAGGGGGGTTGAGGTCCGG
TGCGCCGGACGTGTGACCCCTGCTCTGGCTGAACTTGGCGCACACCGCGT
GGATGGTGTTGAGGTGCGGGTCGCACACCGACGCGAGGACGGCGAGGAA
GGCCTGGCGGCACGCCTCGAGGTCGATCGACGCCACGCCCGGCGGGCCGA
TGCTCGGTGGAGGAGTGACCACCTGCGGGCCGGACGAGCGGGAGGAGCG
GAAGGTCGACGAGAGGCCTGGGGAGGGGAGGCTGAAGCGCCGCGACGCT
GTGCTGGGCGTGGACGGGCTGGGCCTGCATGATGAGCATCACCCACCACG

CTGGTCACTGCTGGCGGCAGCGAGATGGGGTGGACTCACCCGAGCTGGGT
CTGCGCCTTGAGACGCTCGTTCTCTTCTTGCAAGTTGCGAATCAGGGCGTC
CTTCGCCTCTCGCGATCGCTGCTGGCTCTTCCGATTCGTCTCGCGCCGTCGC
ATCAGCCGGTCCATCGTGGCGGCCTCTCCTCATGAGAGTTAGTACAATCCA
GGACGAATCCATCACACACCATGCATGACTACCCTCGTACTAGTGGCTATGG
CCACGGCGGCGCAGAGAAGGAGACCGAACCTCAAATGCCGCCAGGGCCA
TGCACTCCGAGCCCGAGACTTGCCTCATCGGGCAAGTCCAAATGGGGATG
CCTGCCCCTGAGGACAGGCAACATGACCATCCCGCTCCGTCCGACGCTGG
AATTTGTGAGAAGCCACCCCGTCTCTGCCAAAGCAACCGTCCAAAATGCA
AAGGGAGTAATCAGGAAATTCCACATCCCAAAAGGAAGGCGAAGACTACC
CCTGGCGATCGTGAGACGGCTAATCAACGGCACACATAAATTTAGCCTTCC
GAATTAACCGTCATCTTCAAACTGATAACTCGAGGCTTCATTTAGGTATACC
AGTTCTAATAGGTAGTTTATTCTTACATTTCAACTAGGTCAAAAAAAAGGAG
GAAGAATTGGTAGTATCGCGAAGAATTTATGTCAGGTCCCTAGTATGATATT
TACCACAACGCATCTCAAGCTGAGAGTAACTCGATGTCGCACACGCATAGC
CTATGAACAGGAAGCAAACGATTTACAATAATATGGTAGTTAGATCAGTAAC
ACCATCTTTCCCAAACAAGACATAGCTACTTATTAGAAGATAAAGCCAACA
GTAACGCAAGGAGTAGGTATTCATGGAATACGAAAATAACAGCCGAGAAG
CTTGATCAAGGTGCTTAGTCGTGTCAGTGAGTCTAGTTACCGCCTAGCCTCC
TTGGAAGTAACTCTTAGACCCATATGACTCGTATAATGATGACAACACGACC
AACCAAGATGTTGCGATCTATTTATAGGTAGACAACTTGTCGTATAATCCCG
TAATCTGACTTCATATTCAATTCATCTCAAATGCATAGAAACGTTTATGGAAT
CACTAATTGTGTATTGGGTTATTCGATTATTGGCCCATCTACCAATTATACGA
GCCAATATACAGATAGGAGATGACTGAAAGGCGAACATAAATATGTAATAA
AATAGGTAGTTTCAAATTCAAATACTACTAGGTAAACAAATGAAAGGAGCC
GTCTTAGTAACTCCTACAAGACTCCTTCAACTAACTCCCATCATGATATTAC
CTATTCCAGGGGTGCCAAGAACATATTTGAAAGGAATCATGGTTATATGTTG
ATAATGCTATTCACAAGTGAACTCTCTGTCGAGTCCATTGTTAACATTTACA
CTGGTAGCCCCTTTCACGTGACAACCCTCCGCTCTTCCCCCCCCCCCCCCA
ACTACAAACCCAAAGAACGGCCGGTCCTTCCTTGTCTGCTCCGAACCTCTG
CCGACAGAAGGGTTATAGGCAGCCCGAACACGGCGATCTTCCATCATCGAG
GGACACATTAGTCGCACTTCTGGTAGCAGGATCTCGTAGCTCAGGTGATCA
GAGCATCACATGAAAGTGTGAAGGTCGAGAGTTCAAGTCTCTCCGAGATC
ATTTCAACCCCCCTTCCCCCTCTTACTTTTTTTCTCGCTTCACTCTCAGAAA
CACCTCGCCTTGTGAGCTCATTATGATATAAAGCTGTGATTCCCTTTAACTTT
GATGACAGTATTATCTTGATCTTTAGTTCTCATCAACTCTTGTTGATGCTCTA
TGTACTTGCCAAGATGAGACCTATTTCAATGATTGGGGTACAACCGTCGCTC
GGATATCTTCCAGATCATGTTATCTAGTTGATACTTCAGACTTGTTATAGTTAT

CTATATCATATCAATCAAAGAGGGTTGCTCTAAAGGGCTTCGATGTTAGGTA
CTTGATAGGCACTTAACTACCAGGTAGGTCAGTGTATCTGCTATATATTCTCA
TCAACAGCATCTCCAGTCCAAACATATCATCTCTTACCGCACACCTATCATA
ACTCCACCTAACAGCTTACTCTGAAGTACTACTCACTCATCAAACACACAC
ATACACACTCACAAAATCACGATAGGGGAGAACCCATCAGCATAAGCTCGA
GGAGGAACCAAAAAAACGAACAAGTACAATGCCAGGATATTTGCCGAGTT
TCCTTTTGCTGCCTTTGCTGATGCCGAATCGATGTGCCAAACTCCCTGATGC
GGACTGTACGTGACTCAAACGCAAAGATTTCCCCCTCCCCCATCCCCAGCC
TGATGCTTTACACACACCCCGTGAAACCCGTGGATGACGAGAGAAAAAAA
AAAAACACCTTAGGACGCAAAAAAAAGGATAAACGAGCAACACCCATCCC
CTTTCCCCCTCTCTACGCGTGCGGAAAACACTACCTAGCCTTCTTCCATTAA
CCGGTCCTTTTTAATTTCAGAGAAAAATGAAAAACACCACTATGCCGCCCT
TCCCCTAAAAAACACCCCTTCTCTGAAACGTGATAGTATCGGTTGAATTAAT
TCTCTAATTTCGACGACATCCGGCCAAATCCTAGATGCGAATAATGTCGCAA
TGGGCGCCAAGAGTAAAGAGCGGTTATCGGTTTTTGTGTGTTCAACCTCGC
ATCAATGCCAACGGAGAGGACTTGCAACTTTCCCCATTGCCGAATC

>Mosaic_sequence_26

ACCGATTGCCAGTTGTTGAGAACTGGACTTGTGTCCTAAGGTTACATAGGC
GTCGCCGGTGATGCTTTGGTGAAACTTACACTGTCGTTGGAACGAACGGGC
CTAGATAAGAAAAGCTTTCAGATGGTGAGTACGTATAGCGATGATGACCTT
GATGGTTAGTCTGAGAATTCCTTATAGATCGCGGACGAGCCTGGCGCCGAC
CCAGGCGTATAGATAATTGCGTTGGTACCAGTTTACACTATCAGGGAATATT
AGCCATATAGACCATTAATATTTGAGAGGGGGTCAACAATAGAAAATGAGC
AAACGAACAAGGTTCTTCTCCCGGCAATACGGGGGTGTGTAGCCCAGGAG
CCGCCAAGGACAATGTTATGTTTCTCATCGAAGAAATCGGCTGTGACATCT
ATATTAGCGTATTCATTTTGAAAGGGGCAGAAACTGTCGCACCGGTGTATCC
TGGGTATAATGCTAGGGGCTTGAAGCCCTCGTGAGGCCGGAGTGTGGTGCT
AGTCCACTCCCACACGCCTCCCATCTCAGCCTGACCTGCTAGACGATTTCC
ATGAGCAGTACAGGCACGGGGTGCCAATGGCGAAAACCGACATTGGCGTT
AGCGAGGTCGATGAATAGTTCGTCTGTTTGAGACAAGCCACCATCGACCTG
AGAGGCGTTGGATGGTGGGGTTTCCTCCACACCGTCGTTAACTAAATGACT
GATTATTCCTTGAGATTAGTCTGACTGTCGCGTATAGCTGCCATGAAAAACT
TACGCATTAACAGCTGGTACAGTTTTCCCTAGCTTGCGCTCTGCTTCCGCCT
TTCTCTTCATGCTGTCAACGTAAGTGTATATACTTTGCGCTTCCTCGAACGTA
GGAAATTCTGCCTCCTAGCCATGTAGCACAACCTGTGAGCTCGTCATATGAT
GCAAATACCGGCCAGTCGAGTGCATATTTGAGTGGTACTAGTCCGTAAACA
GTACGCACTGCCTTTCCTTCTAGAAACGAGGAGGGGACAGATTGTTGACCA

TTGGCCCCATTGGCCCCATTGGCCCCATTGGTGTGGCCGTTTGTATGACCAT
TCGCTTTTCCGTTTACATGTTCGTTAGTACCATTGACGTGATTGGGGCTTTC
GGCCGCCCAAGAAGCAGGAATCTTTGTTGTTTTGGTGTTGTACATGTATAGC
GCATACTAAGAAGGATTAGAGCCGTCATATTTTAGGCTAACGATCAAGCTCA
CCTCCTCATTAGTGATGGGCCTCCCCTTGGCTTGGAATTTGTGAACTTGAAT
GCGTCTAGGTGGTTTCTCGTTATCCCTGTACACTGCTTAGTAAATATCAGCTA
CGATATCTAATTGTGGACTAACCATCCAAAGTGCTTGTTCGAGCTCAGTACA
TCATCTGGATCGTCAAGTCCAATTGTGATATCCTGCTCAGGTATGTCAAACC
AGTCATTGTTGACCCTTGAAGTATAGGCCTCCTGGGCTAGAGCCTCAAAGT
TTGGGCGCTTTGTAAGAGGCGGAGGGAGAGTTTTGTCACTTTGCAACATCA
TATAGAGCAATGTTTCCAAATGCATGATTTCATGCTCGAAACCAACCCAGAT
AGCTCTGCCGACATTTCTGGGGATATTATCCAATCCTACGGAGTAGAGGCTC
TTCAACCGGTCTCTTACCCTCTCCTGGTATTCTAGGATATCTTCGAGGGGTG
GCCATTCGTCCGGAATTTCCGAGTGTTGATGGCAGAGCTCGGGATTATCGA
CATCGGGGTCTATTCCTCTCTCAAAGATGGATGAGTAGAATGCTGGCTCCGT
AAGAGGATTCTTGGTTGTTTTGGTCAATTGTATGTCCAGAAACGTGGGATAT
GACCAAGGTAGAAAACGCAAGCGTTTCGAAGCTTAATGGGCTTTCTAGCA
GTTCCTCGTCAGGTAACATCCGCCTGGTGACAATGTCCCACACGGCCCATA
GACCCTCGAACTCGCTCAGGGACGGGACGTGAGAGCGAGCGTATTCCGAG
GGGACGGAGCTGAAAGACATTTTTGGCTTGGTTAAAAGATGAAGTCCTGC
AGAGTGTCAGTGTACCCGGTTTGGCAATCCACTCAAGAATCCACGGGTGGC
CTAGGGCCGCCCCGCATTTAAAAGCCACGGCTGATGGATGTAAGATATTAC
AGGCACGCCTCGACGACGTCAGGGTCTCCTGACAGGTCTCACGTGGGCCG
CGTGCTGCAGGGTCTGATAAACGTGGAGAAGACCCCTAATCAATTCGCGGG
ATGGGACGCTGCGGATGTAGGACAAACCCCATATCCTCGGGTTGGACAAAC
ATTCGTGGACCACTCACCGTAGTCGTCATCATCTGTCATCCAACGATCAGCT
TCGATCATGCCCGCCGACCTCCACAGATTACTGCAGCCTGCTTTCGAGTACT
TCAGGCTCTGTTCAACCTGAACACGTTCCTGAGCTCTGACGGTTTCTCCTA
GCACGGCAGTATCGCGGAGGGGCGAATAGAATGCTTGGTGGCGCCCTCCTT
CGTCGTCATATACGTATTCTCCGATGACCCGCCAGTCATTCAGGTCAAATGC
CTCGCGTCCAAGGATTTCATTTGCATGGACAAGCCCATTCAAGATGAACCT
GGACCAGTCAGCACAAGGGTCAGCGGCCGGGCGGACGTATGTATGGCGTC
ATTGACAACTGACCTATGTGTGATGCCGAGCTTGTCTTAGAGGAGCGGCAT
ACAGAGTTCAGCAAAGTTCAACTTGACACAAGTTTCGATAGTACTAACCGT
TGTATGCGTGACTACGGTTGGGCGTCAGCAGCCTGATAATCATTAAGGGAG
GACGCAACGGGGCGTTCCATCACTTACTAGACCTTGGAAGGATCGGTGCA
GGAGTCCAATCCAATCAGAAAGCTATCCGCAGGCTGGAGAACATCGGCGA
ACCCCTTCAAGAATGAGGCTGCTTCGTCCCGGTGGAAGTTACCTATTTAGG

CTGGTCAATTTCCAGAAATCTGATTTGGTATCAGAGCACGTTCGAGAAGTC
CGTACCGATGCTAGACCCGAGGAACAAGACACACTTGCGCTTGGAAGCGA
TCTGCGGCCTCTTTAACCATTCACGGCCATCATCGTACGTTCCCAGGAGCCC
GTGACATCGGACGTGACGAAAGGTCGGAAGCTGAGCTAGAGTCCTTGACA
GCTCATCTTCTGACAAATCAAGAGCATAGTAGTCGATCTTCTTGCCGGCATC
TTCAAGGGCTCGAAGCAACAGAGACACCTTCCTCAAGTTGCTAGCCGGGA
GATGGGAGAGTCAATCAACTACCCCTGAGCTTCGAAGGGTGGTGCTATCAC
TCACCCGCTACCCAGCTCGAGAACAAGGGCTCCGGATGGGATTGCCTTAGC
AATACGCGCAGCCGAACGGCGCAGTACCTGGATCTCGTGATTGGTCAAATA
ATACTCATCTAAATATGTGATCTAAAATATAGGTCAGCATAATGACATACAGG
CGGGAGTCGTATTTCCCTTACCTCCTCGAATAGCTGGAGGCCCTTCTTGTCA
TAGAGAAGCAGGGTCGGTAGTTTCCGAGGACCCTCCGAGGGACGAAGTAA
TCTGTGGATCTCTTCTTTGAGGTTTATCTCGACAGCAGCCTGCCGTATGTCG
ATAATATCGACATTAGATGGTCGTTCTGAAGTCTTACCAGCCCCCTTTTCCA
ACTTAGACCGTAAGGCGGGGAATTGAGGCTCATATGAGGATTCGACAGCAG
ATGGCATAGCTGTTGAAGAGTGAATTGCTTACTACAAACGGAGAAAAAAGT
ATTTGCTAAGCTTAAGAGAAGTAATCTTAACCACTTGGCAGGGGGGGTAAG
ATAACCGCACTTGATATATAACATAAGTTTTGACTGGGATGATCACGATAGC
TGATGGCGTGGAGAGTCACGCAGGTCACGGTACCCGGAATGTACCTACCTA
CCTACTTAGACCTAGTACAAAGTATACGATGGGATTGAGAAGCATTTGCAA
ACCTAGTTGCGAGGCCACGTTCCCATCCGTCAAAACAAAGACGACAGCAC
GCTAATGGTGATGTCAGTCCGGGCTAAGGGGCTATCCCAGTCCAGATGCGG
TCGGGCAAGTGACCAAGCTACCAGGTACGCCAGCCCTAAAATTGATCGGG
CGAGCTGCATTCACGGTGCCCATCCTGGAAATGGGGATCCGAGGCAGGGA
AGACGGGGAAAAAAGATGGCGGCAGAGGGCAGAGGGCACGGTGAAAAG
GCGAGAGGGCGCCTGTACGGCTTGCACGGCTCGTACGGTGTACCTTATGCA
TAAGGCGGGAGCATATTGCGGTAGTTCGATTCGCCAAGGGTTAGAATAAGC
AATTGATCGTAATCAATTGTGGTTTCCGGGACTTGAGAGAGCTCGGAGATT
TGAGAAAATCAGAAAAGTTTGTCATAAGTTGATGCAAGTTGGACTAGACTA
AGACTATGAGAGTGGGATGTAAAGTTGAGGAACAAATCAAGAATCAGAAA
AGAGCTTTTGGAGCCACGATGAACGTGAGCCACGGCTTCCCCGGGTCCTTT
GGTGCCCCCCTTTGGCCCCGCAACAGGGATGAGCTGGGATGGACATGGTTC
GGTGCTTAGGTGCGTGTGTACCGCCACGCTTTCTGCACGGTTTGCTGCATAT
AAGCAATACGGGTAGAGAGAGTATTGGCTTACGCCGCATTAACCTTGGTTG
GCTTTGGTTGACCTTGCTTCGGTTAATGCATCCCATCGCTTGCAATCGCCTG
TAATCGATTTTCCGCCTGTTGTGCGACAAGCCACGCTGCAAGCCCTTTTCG
CCCCTTACCCAGGTCCGATCATGTCCTGGTTCCTGGCAAATAGCCCTAGATG
CTCCCGAGGTTGTTCTAGACCCTTCGCCAATAGGGTACCGAGAATTTGTTAT

AGCATGCGTCAGGTAATTGTACCTATGAAAATGCTGCTGTGTGCTTTAATAT
GTTCAGACGGCTGTTAGCTCTGGCCTTTTATCAGAAAAAGAGCAAAAAAG
AAGAATTAGGTAAGAGCTTTCTTATCAATCGAAGTCTGGTAGTACCAATTAT
CTCCTAAACACTCCGACTCTAAAGAATTCCCGTTACGGTTTTAGGAAGTCG
ACAATAGAAATACACCCCCGGACCGAAACCCCCAAGTTACCCAGGTAAGC
TACGTCACTTGTATGTGACCATGACCTGACGATCTTTACCGAAAGCCCCCCA
CTTTACCCCTCAACTACAGGTAGTTAAAAATGTAAAGAGCCTCCCGCAGCC
AGTTGCAAACGAAAGCTATGCCTTGGTGAACTTGCCAATACAAGTATCGTA
CGTGTAGGGGTCTTGTATGGTCTATATTTGGTAATATTCTCATAACTCCCCTC
GCTGCCTCGCTATTACCTCAATGCTTCGATGGACTGATTTGCTAGAATGGCT
AGTGCTTGCTTATTGCTTGTAATTATTGCTTGTGGCGCTAATGGATACCTTGC
CGTTGATCATGGGATACGGCGTACATGGAACGTATATTGCTAACTTCAAAGT
GATTGCCTAACCCAGCGCCGTGCATTCGTTGCCGGACATCGTATTGATTATT
TGGAACATTCTATTGCATATTTATACACGCCTTTGATTGTGGGACAGTGCCT
GGATGCTACCCCCCGAACTGCACGTCAATTGTGGGGAAGCGACTCCCCGTG
ACACTTCCAAGGACTTGAGGTACTGGGTGACCTAGTACTAAAATACCCAAT
ATACCAAGATACATGGTGGATAACGTGGGAGAAGGAACGTCTCTCGAATTA
AGGGGTCCTGGATTGAAAAGGGGGGGTTCGCCTCGACTCCTTGCCACGAA
GGGTCTCTTCAGTATCATTTATCATACAACTTCATTTTCATGCTTACCTACAC
TTCCGACATTTTTTCTCTCATTTTTGATTTTTCTCGCGGCACCCTCGGTCATA
AACTTAAAAATGTCATCAGAATAGCAGCGGTGAACTCTCCATTCAGCCAAC
ACATCCTTGATCCGGTTCCAGAGCTTGGAAGTTTGTCAAGGTGCATGAACG
AATCACTTTCGATGCTGTACGACAGTGCCGTTATCCATTCAGCCACGGATCA
ATTTTCATTCAATTTTCATACAATTTTCATAAAAATAAATTGTACACAAGAGG
AAAGTGAGGGGATGATCATCTTTTCTGGCTAGAACATCTATTTTTTCCCTAA
CCCCCTAACCCCCTAACCCCCTAACCCCCTAACCCCCTAACCCCCTAACCCC
CTAACCCCCTAACCCCCTAACCCCCTAACCCCCTAACCCCCTAACCCCCTAA
CCCCCTAACCCCCTAACCCCCTAACCCCCTAACCCCCTAACCCCCTAACCCC
TTGACGACCGCTCGACGACTTGTCGCCAGAGTTTCGCTCCCGTTACAAGCG
ACGTCATACGACTGCTATCTCGACTCCTGGATCGGCAATGCGGGTGTGTTC
GACGAGGACGAGGGAGAAGGCTGGAGTGGATGGAGATAGCGCGTGTCTC
GCAGGAGCTCGACTTCAAAGGTGGGCAGAGTGTCACGACGGCCACGCCCT
CGACGACCTACGCACGTGACCGACATCTCTCACGTGGCCGACACGTGACC
TACCTCGGGCTGGAGAACCTCGACGAGGGAGTAGACGTTGAATGAACGCG
CGACGACGGGAAGCAGGCGCAGAGTCATATAGGATGCCCAGGGCGCCCGT
CGTAGTTCTTCAGCACTCTCTGATTGAGTCTCATGGATCACCATTGACGATC
TCACTCTCTCCGTGGCACGCAGCGTGGCTGAGTCGCACTCACGCTGTATCC
TCGACGACCTTGTTACCTCGACGACCTTGTAACCTCAACGAGGATCCAACC

TCGACGAGGATCCTACCTCGACATCCTCGTTCCTACCCCCTTTTACCCTTTAT
TCTTGACTCTGATATTGTGCGAGAGGGCCTCTGCCAACGGGAGAGCGCAGC
ATCGTGACATTCTCCTCCACTGAGAGCTCTGACATCCAGCCCTTGCCAACC
TGCTTGCCTGAAAAGATGACTGCGGGTGTGAGAGCAGTGCCATCTGCACA
GATGCAGGCCAGCAGGCTAACCCATTCTCGGCTGCCATCCATCCTAGCCTG
TGTGCCACCAGGCTCTCTGGCTTGGGTCGTGGGCACAATGCGCCGCTGCTT
GTTCGTCTTGCCCATCATGAAACCCTTCTCGTCCATATTGTAGATTGATTCAG
GACCTATGTTGAGGTCTCGGCATCTCTGCTCAAGCTGACCATCAGTGATCTT
CTCTTGGTGACAGTGAGGGTAACGTGATGACTCACAACGTCAAAGTACTCC
TCAAAGACGGGCCTGTACTCCGCTGAGTGACGGCTTGCCTCCATTCCTCCT
ACTACCATACACCGTATACGGCCATGATGCTTCTTCACAAACTGGCGGGCCC
AGCTATCTGAGGGTCGATGCTGGGTGATCTCGGCCACAATGTTTCGTAGTAT
GATTGGTGTGATAAAGAGATGTCTCTTCCCCATGCGCTCCAGGTGTTCAAG
AATAACATCCTCCTGCTCCTTGGTGAGCAGGGAGCGATGATCCTCCTGCTG
ATGCATTGGCTGGATTGCTCGGTCATAGTGACGTCGAAGGGTTTGGCGGTC
AACACAGTGTTGTCGGGCAATAGCACTGAAATTAGGGCGGGTCGGGCCTT
GATGAGTGTGACAGCTCTGAAGAGCTGTAAGTAGAGCAGCACTGATCATG
CTCTGAACGAGGCTTCTTTCAATGCAGAGTTGTACGATAGAAGAAGAGATG
TATGATCTTTGAGGAAGGTGACCGGTCAAAATGCCAAACCGACCATCACGT
GCGGGTACCCTTAGAAAATGGTCCACCAGACAAAATAAAAAGGCAAACCT
GGCGCCGCTGTCCTCCCTTGGCCTCGACGTCGGTAACGACCTTGACATTCA
CATCTACCGCATCGCGCACAATGTCATCAAGATGGCGATCGCATCCTCGTCC
CTCAATGAATTCTTCAGACGACAACGACAACAACGACCACCATCACCATCA
CCATCACCATCACGACGACGGGGTGCGTATGCGGTCGATACCCGACTGTCT
CTCGCTCGCGGCGACGCGTATGGCTCTGCAGCTCATGCGCCACAAGCTCAT
CGGTGCAAGGCCGGGCTATAATCACGAGAATGAGCTACGCAAGGCGTTGA
GTGACATACTTCAGACCCTCCTCGACGACCGCGCGCTCGATCATGTCCCCG
TGGTCAAGATGGACCAGGAATTCAACATGCGATCCCTCGACCAGGCGGGTT
GGGACGCCATGGATGAAGTGCGATCCCTGCGTGAAGTCTTTGACGCGGCG
GCTGCGGCGTCAGCAGCGGCAGCATCCGCTCCGATCGCAGATTCACTTTCG
GCCTTGGCCTCGGCCTCGGCATCGACATTGACAACATCGAGTGTGATGTCC
CGAACAAGCAGAGCAAGCAAAAAGTACGGTCCGATGACACCCGACCTAGC
CGCGACTTTGATCGAGGGACTGTCGCCTGGGCACCTTGTACAGGCCGGAG
CCCTCGGAGAGATCAAACTTGATCACTTCAATCCCGACGAGACGGATCTTA
AGGAGGCACAGCTGGTCGAGGGTGTCTGTCAGACCCTATGGTATCTACGCT
GCGGATCAAAAATGCTCGGGCTTAGGTTGGGGATGCTGATCATCAACCAAT
CCTACAAGCGCTTCTTCGTCATCGACTCGACTACCATTGCCATCGAAGTCG
AATCCGAACAGCAACTCGAGCCCGGGTCCACGACCGAGACCGAGAGGGA

CACTGAGACTGAGACGGCCGAGGAGCATCATGACCAGTATTGGGCAACCC
CGGAGACCCTGTTCGACATCCTGTCGGCCACCCCAAATCGCCGTTCCACTC
GGTCCCCTTGGACAGGCGCCCACCCGGTTGGAGCTACTCTTCCTCCTCCCT
CGCGTCGAGGCCATGTGCCCATCTCCGCTCTCAACGTTAGCGATGATGCTG
ATCGGGTTGTTCGTAGTCGATTGTGCGCCTTGCCGAACCATCTGTTCCAACC
CGACGACAGCCACGGATATGATGCCGCTACCCTCTCACCGCTTGCCGGATT
CAATGCCACTGCTCTTAATCCGATGATGGGATTCTGGGCAGTCGGCCTGGA
GAGGGCGTCTGTCGTCCCCCCGAACCGGTCACGCTTTGCTGAATCCCTTGA
GTCGGTGAGCGCGCTTTTCTCATCTCACCTCATCTTCAGCTTGCTAACAGTC
GTCCAGCTCAATCCCTGCATGACCCAGCCCTTGCCTCATTCCGCCTCGTCCC
AGCCCTCCCCGCCGCCCGGCATATCGTCCAGAACGCCATGTCTCACTGTCG
CCGTTGGTGTCGACAACACGTCCAGGATCGGCAAGCTCAAGCACGAGTGG
CATGCCGTGCATAGCAAGGGCAAGAAGCGAAAGCGAGCAAAGGTAGACG
CTCATTCGAGCACATCTCGCCGAGACCGCGGACCTGACGATGACGATGACG
ACATTGGTCCCAATGACGAGAGAAAACGTGAACGGAACGACAAAGAAGG
GGAGGAGAGTCAAGGGGACAGGAGCAAGGACGCGGGCTCGCGTGGGGG
AGCTCTGGAGGGACAGACGACCTATTCGGGCCATCAAAGACAGGCGCATG
GCGGCGGACAGGCGGACGAGCCCAGCAAGGCAGCACCGAGACGGATAGG
CGAGTTTGTGCTTAACCTTTCTTCTTCACGCCCGTCCGAAGACGTGAAAAG
GACGCGGGCTAATCCGTGCAGAAATTGTCAAACCCCAAAGGTCCGACCTC
CTGTCACGCGTCTATGGTTGGCGGCAGGGCGTCGAGACGCCAACTTCCAA
CGATGACGTGACCCTACCGCGTGTGACAGGGACCACAGCACCAGCACCCA
ACGACGACGATGCGGGGATCGGGACTGGCGCCGATGTCGAGTCTCTTATGA
CAGCTCTAGACATGCTCGACAACTCGCGCAATCTCGTCGAGGCGATGCAGG
TTACGGGCTTGACGCTCATCCCCGTCAGCTCTATGGAGATGGACTCGCTAGT
CCCGCGAGGTGACAGTGGGGAGGAAGAGATCAAAGCCTCCAACCCAGCTA
CCCACGAGACCCCTCTCACACGTCCACTGTCTCATCATTGCGGCGACGTCG
ATATGTCGATCTCGACGGTCCCCGATGCGTGTTCCTGTTCCTCTCGGACGGC
TTCCATCTCATCACACGGACCAGTCACCCCAGTTATCCCGGCATTTGCCGTC
TCATCAGTTCATGGCCCTGCCGATGGGGATGGGAAAGCGGTATCAAAGCGC
GAGCCGGGGTCTCATGATGTGATGGCCGCCAACGGATTGCCGCTCGTCCGT
CTCGAGGCATGATTTTCCCACTCACATGTCCAGCTTGTTGTACCTCGCGGCC
GACACATGAGCATGACTGCAATGGTATTTCAAGTATAGCTGTCTGTCAGACC
GTGCTCACTGACTGACTGATAACCACAGGCATCGTGTCAAACTCACTCACC
TCGGACAAAGAGTAGATTGGTTCAAATTGGGTCAGGCGATTCGATCCTCGA
CTGGGGGTACCCTCGACCCACTCTCTCGCCTTGACCGGGCCTTGATGGGTG
GTTGATGGCCGTGTGAAGGGGACGGGAGGGGTATTGTGAGGCCGGGCTTC
CCCTCCCTGCATCCACAGGCTCCTCTCGCTCCACGACTACCGTCTCCCCGG

GCCTGGTTCTTGTTATCGCATCACGTCCTTTCCTCCTTCTCGTTCTCGTACCT
CGATGGGCGTTCTTGCATCACGTCCTCGCATCATGTCCTCGCTGTCACGATA
CCTCTCTGTCGCTGTCCAAGTGCCCGTCACTGGGTGACCGACACTCTATGA
CTTCGGCGAGGCTGGGTGAGGCTAGGAGAAGTGGCAAGGGCAACGGCTC
GAAGGACTCGTAGAGTCTCAACGAGTGACATAAATGACTCGCTCACAAGC
TGGAGCAAAGTTTATTGATCAGAACCCAAGTCCTTATACCAGCTGAGCACG
CTGAACATGGTGACCAAGGCGCGTCCGACTGTTGTGTCGCATGCGACTGAT
AGGAAGGCGGGACGCTGGAAACCTCAGAATGCTGGCTCGGAGTGACAGC
TGCTATGGGAGGGGTCACCCGAGGAGGATCAAAGAGCTCATTGGGCCTCTT
TCTTGGCCCATTGACATAGGCTTTCTGGGGACACCCTGGGCCTTGACGAGG
TGCTGTGCTTGTGGCACAGCACTGCCTGTGATTGCCAGGGCTATAGGCTTC
TTGTTGTAGGTCATTATGCGCAAGAGGTGTGAGCGTGAACATAACACTGCA
TCTGTACTTTGTTTGTGTCTATTTCCGGGTTTGCCCTCAATCCTGCTCTGAGT
CAAAGTCAGGGTTTGGCGTTGAATGATAGATGGTGACTCAAGTTGGTGTGA
ACGGGGTTGAACTTTTTTTTGGGCCGGCGAACAGCCAGATGAGTGCTTCTG
GCATACTCTGAGAGGACTAGCTGCAGTGCAGAGACCAATCGCTGCCATATA
ACAAGAGTTGCGGGCAAGATGTAGCACGGACTTGAGGGGTAAATGAGGAG
CCTCATTCAAAGAGGAAATTGCCGGAAAAATTATACACGCGGTAATGAGCG
GGTCTTTACCGCTAAGATTACCGTAGACAATCGGTATCATTCACGTACGACC
ATTACCTGCTCGACTCGGCACTGGCCAATTCACCCAGCGACATGGAGGATG
AACTTCAACAAGCTCGCTGTTCACGACCGAAGCTTGCAATCAGCGGAAGG
GCCAACATTGATTTGACCTTAGGGTCGAAGGATGAAGGACTTGAAACACA
AGCTAGCTGAGTGGAAGCGCGACGTGCTTGTGTGCAGTCGAACAGCCTCC
CTATCACACGCGTACAACCGTCAATAGTTTACCGACTTAACCTGATCTACTC
TCTTACACCATGTCTGCCGTCCGAGACAGTCGCGGGTCGCGACGCCGTGTC
AAGCACCGGCAGGGTCCTTTTGGCACTCATCGTAAATCGGCGCGTGGGAG
AGCTTCGAAGGTACCACGGACAACGGCGGGATAGCGTTCCTCGGGGTGAT
TACGATGAGGAGGGCCAGGGTGCAGGCGGAGACGATTCGATGGAGCGGTT
GGTGCATGTGCTACAGTCCGGCATGGGAAACCCTTGTGAGGATTGGAGAG
GCGCATCGACGCCAAGCTGAGGGAGATGAGCGTGGACATGAAGGACTTAC
GCAACAACAAGCAGGCGAAGCGCTTGCCCTCGTCGGAGATTCAGAAGGA
GCATGAAGGGGGCGAGGACATACGGGTCCATCTTCTCATACCGTATCCCGT
CGCAAGTGTGCGAGCCTTTCGAAGTCGCGTTCCACTGGGTCAGCGTGAGC
ATCCGAGAATAGATCGCTTGGCGAGTCCTACTGGCCACCAACTTCCACGTC
CTCCTCCTGCCTAATGGCCCTCTGTTCCGCCGCAAACCCACAACGGCGGCT
CCATACATGGCATCTTGTTACGGTACTCCCCTTTCGCGGCTAGGCTCCGTCG
CTGCTAGGACCGAGAGAAGGAGAGGAAAGGAGGAAGGGGGCCGAGCCG
AGTCTCTATGAGACCATAGACACTCGGTGAGGTGTAACGAGCCTGGATATAT

AGGCAGGAGACTGATGAGTGCTACGAGCATCTAACGGACGCGACGGAGCA
AGAGGCCTGGTGATGATCTTAACGGATCCATGCAACAATGAGAATGCTATA
AAACTACAAAGAGTGTGAGCGAGATCTCCATGCGCTTGCAGCCCTTACGCC
TGTACGAGGATATGCACGCCGGTGTCGGATTCAATCCGTGAGATACTCACG
GACGAGTAGGTTAAAGCCGCGACCGGAATAGGACACTTGACCTGATTACG
AACAATACCTGTGTCGGATGGCTTGGACTGGGTCTTGGACGTTCGAGCGTT
TTGTGACAACATAGAGCCATCTCAACCCCTTGTATAGCCATCCTTTGTATATC
AACATAGCATTTCATGAGCTTTCACGAGGACTATCAGATTGAGAGTGACCC
ATCACCAGGCACTCATCTGGCATCGCCGGTCGCCATAAGTGACTGGGCCGA
CAAAGTCATGGAGGTTGACGTTCCTACCGCCTCTGGCGACCGTATGCTATC
GAGCCCCAAGTCAGCACCGAATCTTTTGTTGAAAGATTGGAGAGGCGACC
TGCTGGTCGGGTTTGTCAGTCAGTGATGCCGTCGAGGAGCAGTCAGCTGAT
GTGCCTTCAGGCGGAGCTCTTGGCCACCGTAAGCACCCACCCTGTCTCAAT
GACCCAGGAAGTTTGACCGGTCTATAGACCTTGTTCTTGTTTTCCCGCGATG
GGCGTGAGTCACAACTTCAGAAGGAAACAGCGCTCACGGCGCAGACAGT
CAAGGCAGCGGGACAGGACTTTAGCTCCGAAACAGCTAATGCCAACCCTT
TGCTGGTGAGACGCATTCCATTCTTCTTCAAGAAGATTGCCGCTCATCGCG
CAGATCAAGATCTACATCTCCGCTGGGTTTGGTTTATCACTTGTCGGCGTTG
GATTTGCTTTCTCCCGGGTCTCGGGATCGGTTTTGAATCCGTGTGTGGCTTT
GGGCTTGTACACCGCTGGGAGAATCGGTCTAACACGATTCATTGGTGAGTG
CTCAGTTTCGGGGCGGTCATTCCGTTGACGGTTTCGTTATCGCCACTGCCG
ATATGATTGGAGGTATTCTGGCACCTGCTCTCCTCGTATGGCTAACTCCCGG
GGACACCAAGGTCGGAGTAACCCTAGTCGAAGGACTGAGTGTCGCGCGAG
GTGTCTTCATCGAAGCTATTGCGACCTTCTCTTTGGTCCTTGCCATCTTGAG
CCTTGACCGAGGTGGGGTTACACATTATCACTGAGCTTGCTAAGCGAGCGA
TAGAAGACTCTCCGCAAACCGTCATTATGATAGGATCGCTTCTGACCGCTTT
GATGATGATGTCAATCGGGCTCACTGGCGCTGCTTTTAAGTGAGCCGATGAT
CTCGCTTTCGAGGCATGGCTCACACTCAGCACGGCCAGAGCATTCGGGCCC
GGCCTGCTTGGAAGGCTTCGAAGGCATTCATTGGGTGTACTGTCAGTATGC
TTTCCCCGTTTCGAAGAGTGCTTAACGCAGAGGGCTAGGTCCCTCGCTCGG
GGCATTCGCAGCGGCCCTACTGCACAGGCTTATCGATGCAATGAAGAAAAG
TGCAGCCCAACAGGCAATTACAGCCACGGTATAGAGCATCCTCGATGCAGT
GCCACTGTTATGTATGAGCACATACTTGGGAGACATTTCGCCTACGCGCGCC
AACAGTCTCAAAGTCTGTAGAGGGCGAACATAACGTAAAACGCGGGAAAT
GTCCTATGCTCCTTACTTGCACGTCATACATCCTCGTACTCTGATCCTACTTT
GCGGACTCCACACCAGACTCGTGACTACGGATTCTGCAAGCTGCCATCCTC
TTGCCACTTTACGCCACCACGCTATCTTTCTCTCGAGCTCACGAGCTTCTCC
TGTCCAAGGAACAACACCATCAATCCTCCTCGTCTCCCGGCAAAGCCGATC

TCCTCCCGCCTTGCTCTCAAACGCCAAACCCGCACACCCGTCCACTGCCCC
TCTTTCCCCCCGCCCCCCCTTTCTGACCGTTCCCCCTTAAGTTCAAGCCGAT
TAGAGATCCGCAGCGCGATCACCGTGGTCCAGGAGAGGAGGACATGGTCC
AACTTTCTTGAACATTACGAAGCGCTTCCAGAACCAAGGAAGGACGGTAC
GGAACTGGTACCCCCGGAAGTCGGAATTTGGGACGTTGAAGACCTGGACA
TCAGCGAGAATCCTACGCCTTGGAAGCATAGCTTGCCTGATCTAATTCTTCC
AAAGTGAGCCGCTTGGTCTCGGGGACAAAGACGAAAATGAACGCAAAGG
CAAGCATGTTCAATCCAGCGTAGAAACCGATGGCACGTGAGCAAGAATCA
GCCCGCGCGCGCATATCACCCGGTGCAAAAGACTTATCTTGCGGGGACATA
GCTCGAAGCATACGCGGAAAACTGGGCGTCAATAAGACCTGATTCTACACA
AAGCATTCAGCGAGAACTTACGGTAAGACCTAAAACTCCGGCCCAGGCAA
ATGTCCACATGACCGACATGGCTACGCCCTGCTCCCTTTGCTTCAGGGGGA
AGACCTCGGCTGCGTACATGAACGGGACCGGACCTTCACCCAGGGCGTAG
AAGATGCTGAAGATGTACATGAAAAGGGCGACCAAAGCCGTACGAGTGGT
CAGATCCTTGATGTAGAAGCTCATGCCAAGGGCGAGCAGTGACCAGAACA
TCTAAATCGTCAAATGGTAGATCATTGCACGGTCGCGGTTGACTCACGTTG
GGGAAGGTAGCCAGAAGCAGTGATCGGCGGCCAAAAGTGTCAATCATGAA
CACCGCAGGTATCGCAAAAACAAAGGACAGTGCTCCAAACCCTATCGAGG
CGTACAGGGCTTGGGTAGTGGTGAAGCCACCTTCGACAAAGATCGTTCCG
GAATAGAAAGCAATGATGTTGATGCCGCACATCTGCTGCGCCAGCATGACT
GTTCCGGCGGCAAGCGAGGCTCGACGACATCGAGGAATGGTGACAAGCTC
GATCAGGCGCGTGAAGTAGTTGACTCCTGAACGACCTGAGGTTCGTCAATT
TGCGGTCATCTCAAGGGAGAGGAGGAATGGATTGACCTTTTGTTCCTCCTC
ATACTGAACGTATGCATAGTACAGATCGCGAGCCGCTTGTAGGTCGGTATAC
CTCACACGCCGCATCGACCGCCATGCTTCAGGTATGCGACCCTTTTTCATAT
ACCATCGGGGTGATTCTGGACCTGCATGGCATTCAGCAAAGTAGTTGACGT
CAATCGCGACTGACAGAACCAAATCATTATTGCCAATGGAACTGCAGGGAT
GAAGGCAGAACCAATCTGGAGACGCCAAGTGATGCGACCGGCGTCTTTGA
CCACTGCGTTGGCGGCGAACCCAAGGAACAGACCTGTCCTGCTGAGCCAA
ATGTGACCGTGGCTCCCGCATCGACGTACCGAAAGCCGTCCAGAGCTGCC
ATCCCATAGTCAGTGCACCTCGAATGATGGCCGGTGCTACCTCGGCGGCGT
ACATAGGAACTGTGGCGGCTGCAAACATTAGGCAATCTTCGAATGAAATAC
AAGACTCACCTTTGGCCCCGATGCCGACCCCCATCACAATGCGGACAGCTG
CTAAAGCTTGCCATGAATGAGTGCATGCCGCTCCAATTGGTGTCGCGATAA
GGATCAGAGAGGTAATGAAGATCTCGCCTCGCCTTGGGAATCATTAGCTCA
CCATTTTGCTGGAGAACTGCCAGTAGTCAAAGTAGTCGAACACAAACTCA
CCTTCCGAACAGGTTATTGAGTGGGTCAGACAACCAGACGCCTCTTTTAGG
TCAAACTTGCGTCATTGAGGAATAGGTAGATAGACTTACGCAAAGCAAGCT

CCGATATAGGGTCCAGCATTGATGAACCCGAGGATCCACTACCAAAGTTAA
CCACGCGGGTCATCAACGTAGGACACTTACTTCGTCGCGACTTCCACCTGG
CTGACCAAAAGGTACGGCGATTCCAAACTCTTGGGGGAAAGACAAATCTG
AGATCAGCGCTTTCCCACCTTGGAAAACGCGCCTTACTGGCGGCATTCGAT
CCAGTCTGATCCCAGCCTTGACAAGCAGCGCCTATGGCGCAGACGGTCACT
AGATTGCGTCAGTTCAAGGTGCGTATATTCGCCACCTTGGGATCACCTACCA
ATATAATACAGTGAGAACGGCTGCCGCCACCTGTGTTCGTGCTCCAGCCGG
ATCGTTGTCTTCTCTTCCAAGCTTAACTCTTCAATGGAATCCCACTCTCGCG
GTCGTTGCGCGAGCAGCGCGCCTTTCCTAAAGGCGGCTGTGTGGTCTTCCA
GGCCGTGCTCCGTTGCGAATCGTTCCGCTTGAAAGAGCAGCTCCTGCTTTG
GAACGTAGGCGAGGACATTCTCAAAGTGCCGCGAGTTTGACAAGTAGCCA
TCGTTCGCGCTGTCGTGTCGAAGCTCCTGATGATTGATGACTTCCTTCTCGG
TGTCCATGGTATTGGTCATGGCTGACAGAGCTGTCGGCTGCTTGACGCGAT
TGATTCGAAATGAGGATACCCCACCACTGCTTGGGGGCGGGATTGATTCGC
TATTTGCCCTCAGCCGTCAAGATATGTAGTAGCGCCTCCTGTGGCAACGCAC
GGCCGTTGCCGGCCCGAGCATCTAGGTTACAGCCGTCTCATCATAGCAAAG
GGGCCATAGTCGCGACCCAGCGGTTCCGCTCGCGGCTTTGGCTGACTTAGG
GCCCCGTTGTAGCCGCCCGCGGGGCCGATCGGCAATGGTATGAGGGTGCA
AGAATTGTCGTCCTGTTGTGCACCAGCTCTGCCCCCTCGATCTGATGCATGG
TCAGAGATTGTCCTCGCGTGGCCATGCGTGAGCATCAGGTCAAGCATAGGT
CCGCAATTCGGACAATCCTGATGTTTAGTCTGCCGGCCCGTGTTTCGGGAC
TGTACTGTCTTTCAAAGGTCTAATTGCTCCCGTATGATACTCGCAGTGATGT
GGATTAGTTGCCAAGGTGTGAAATGCATTGAGATCAGCCGCAGCTCGTACT
ACAAGGTGCTACCTTCCAGCACGCCGCGGCACAGCTGTCATCAAAGTCCAT
CGTCTGCACGCCATGGACTGAGAATATCAACAATAGTCAAAGCCCGGGTCG
AATACTCACTTGTCCCATCGATGATCATATGGCTGTTCACGATCGGCAAAAC
GTAGATCTGCAGACAAGCGAATCTTCCCGAGGGGTCCACATTGCGCCCACT
GGCGTGGATCATATCTGCGACGAAGTAAGCGAAGGACAGTCCCAGTCTATC
CCTATCCTACGAGTATGATGGAAGACAACATCTCCGGCTTCGTAGTCGCCG
ATGAGCCACTTGTGGCCTCGGCCTTTGGTTCGACCGTACAATCCAGCATCT
CTTGAAAGCATGCCCTGAGCTTGCATCTATCGCACCGGTCAGGTCGCTCGC
ACTAAATATGGGCAGCGACTTACGTTGGAATTGAAGGCGTCAAGCCTCTCC
TCCTCGGTCATGCCTTTCTCGCGGCACATCTGGTCAAACTCTTCCTCCTGCT
GAACCGCAATTCCAATAGAGTCGGAAAGGTATATCAGACCTCCCTGCAGAG
GATCACAGTCTCCGATCGGTACCCAACCTGTGAGGAAGGTGGGCGGACCT
CCACGAAGGAAGATGTGGTCGTAGTGGACGCCTGTGGAGACTCCTTTAGC
AAACGGAAGGTTGGATCGGAACAGCTGCCGCCTCAGACAGAAGGGCTGC
GACCAGTCGGGGTGAATCTTTTGACGAATTGAAGCAGATCTGGCAAAATAT

CAGGTTGGTGAAACATGGCATGATTGCACTCACGATCGTTCTCTGCGAAAG
CATTGGCCCAATCTGACTGAATAGCCTCGCATGTTAAACGGAAATGAGCAT
TCTCCTCCGGCGGGCCTTTGATTTGGGGGGCGTTCTCGGGGCCTAGGAATA
GGTGGGTGTCTTCGTTCCCACAGTATATCCCATCGACGGGATCCGTGTTTTC
CTTCAGGATGCCGCTGGGAGCCACATGCTCGAACCACCTTTGATGATTAGT
CGCGACACGGTGTTCCGACTCCCAGAAGTACGGACTTTCTGCGCATCTCTA
ATACATACTCCCTCGGCATGACGCCTTTGACGTGAACCACGCCTTGGTTCTC
GAGCTCGTGCAAAATCTCCTCTCCGCTGGTCGAGGCCGGCACTGGACGCA
GCCATTTCATCTCGCTCTTCTGCATGCTTCCTAAATTCGACTCGAGCGGCAT
ATCTTCAAGCTTCAGAACTGCTCGTGGTCAGTCTGAGAAGTACGCATCATG
GCAGATGTGATTGGGCTTACCGGGATGCTCCCAATCGACGATTGGGGACAC
ACCCAATAGGTCTTTCTGCAGATGGGCAACGTCTGGCATGTTGATGGGCGA
TAGAGGTATGTGACAGCAAGTGGTCGATGACGAGTCGTAGGCTAGACTCAT
CTTATTGTGTCCTCCTTTACACCCGATCACCTGGATCCGCGAGCGGGATCAG
CCAACGGGACCAAAGTGTCAAATCCTTGCACGGATAGAGACCAACAGTTG
CAGCGCTAGTCTTCCCGCCGGCATCGTTGCCTCCGTGCGGATGGGCGTAAA
TCCGTCCGATACTTCCTTAGTCAGCATGGGATCCAAAACTATGCGTCTGCTC
TGCTGTGGCTCTTCGCTTCTCACCGAGCGGTGGTTTCGAAGGTCCGTGTTT
TCATATCCGGGTCAGACCCTGAGGTCCGCGCGCGGAGCGATGTGGTCTCTT
TCAAAGGCAGGGGCTCTCGACTAGCAGAGGAGTCTCTACCTCCCCATTTGG
AACGCTTGGTTGTCATGCGTGTACTGGCATTGAAGGCGGCCTGGGGCATCG
AAAGTGATGAGCTGGAGCAGTGGCTTGACAGGGTCAAGCAGAGCGGTTAT
GGTGCGCTGCCTCCAATTGGCGTGTGACTTGCAATGGTTGACGGACTTGCT
AGATGGCATAGAACTCAATCCCATCGGCCACTCTCATGATGCCCTGGAGCG
CCTTAAAGTGAAACTGCAGGCTTTGTCTCTGAAAGTCGTCATACAGTCAGT
GTCGAATTCGCCGATCCCGAAGAAAACTGTAGCTCAAACTCCTTCCCGCAG
GGCAATGTCCTCCTGGGACAATTACACGGGGCCGCGACCGGAAGGTCTTG
GGCCTGCAGATCATCTTAAACACTACAGAGAGAGGCTCATCTTGGCAAAGA
CCTTCTCGCCGATTCTTATCAATGTGCAGAGTGGAAGGTATGTCAAATGCTC
CCCTGTTTGACTAGCTCCTGTGCAGCCAATCCGAAAAGACTGTTATCTATGC
TTATATACTCAGCGACCTGTGGACAGCCGAGGAATCCATATCCTTTTACAAG
CAAGCCATGAAAATAGACGAAGACTTGGGCCTTGGAGGGGTTGTATGCCA
CGAAACACATCGCAACAGGAGTTGCTGGAACCTCGCCGTAACGACAAGAA
TCATCCGGGAGGTGCCACAGTAAGCATCGCTTCGCTACAATAGACCGCGCT
GACGCTGGAAAGGCTGCAACTGACTGGGGATATCTCGCATTGGGTCCTGGT
TGGCGAGCGACTCCTGAATGTCAATCCGACAGATGTGAAAATGCTGAGTG
AAGTTGTAACGAACGTTAGGCAACTAATCATCAATCCCAGCGACCCGACAC
TGACAACGTAGATAAGGTATTTGCATTGCCGTGTAGGCACCACACAATCTTC

GCAATGTCCGGAGCCAGATCACCCCAGCTTCCAGCTGGAAAAGGAGTTCT
TTTACGCATTTTGGAGAGAGGTCGTCGATAAACATCGTTCTCACGGTGCAC
TATCATGGTTCGCCTTCGTGCCGGAGTATGGGTCAGTCCACAATCGCTTGAT
CGTCAAATCCGAAATGTCATTTACTGATCGCGCAGTCCTTTTCCGTATCAGC
CTTTGTTCAGTCCGAGAACGAATTCCCAAGTCGCGGATACTGAAGCGGTGC
GATTGAGAGCGGTCCTAGCTCGTTGAGGGACCAGGATTTGTCAATACAGCC
AGTGATGTCGATTAGAGTCTTAGACTACGAGAGGGTTCTCATAGAGACCGC
CATATGTAGATCAGACAAGGGAAGTCAGGATGATCCAGCGTGAATCCAGCT
TGAAACCAAAACCTTTTCACATTGTAGAGTAAGCGATATGCCTTTCGTTGGC
ACACGTGCTGAGTGGGCCGAGATGCATCGCAGACAACAGTGTACACAATA
GGCTACACTTACTTCTCTGCGTAAGAGCGGCAGCAGACGCTTACAGCCATG
GATATTGCTCGAACAGACAGGAGCGTCCTGGCGGATGGTAACACATTCATT
CGGACAATCAAGTTATCTAGGCCAGGAAGCAACGTTCTCGATATTCTCCGA
CCTTCACACAATCCCTCAATCAATACAGGCTGAGTGTGAGAGGCCGATCGC
ATTCTACAGTCCATCGTTCGCTATTCAAGATTAGCGCGGAAAACAACACGA
AGGACGGAACAGACGTTTCCAATATTCCATCCAGCGCTCATCGTAAGGCGC
GTTGGGATCGACGAAGCGGATATCCGTGTGAGGAATAATCTTGTTCGACTG
ATTGACGCAAGACGCATGGATGACGAATGGATCATGAAAGGCAACATCGCC
AGGCTCGAAATCGGCCATCAACCATTTTCGCCCCTGGGTTGTGAGACAGCA
TCAAGCGAAAAGCAAAGTTGCCTTACTCGAAGGCTAGCAAACCCGCTCGC
ATCGGGGTGGATCGCACCTCCAGGCATCTGACCGTCAACCTACATGTTTTG
AAAGAGGTCGAATACTCGCCATGTTGATATTAAACGCGCTTTTCCGCTCCTC
GTTTGTGAGATGGGCGGCGTTCTTATTGAACTCCCTCTCCATCTCCTCTCCA
AGCTCTCGAGATCTATCGAGGTACATCAATGAACCCTGCCAGGTTGCGGCG
AAATCGAGAGGCGTCCAGCAAGTGAGAAAAGTAGGCGGACCTCCTCGGA
AGAAAAGCTGGTCGTCTGTCCTAATCAGCACAGGGACGGAACCGGTAGGA
TAACATACAGTGGACCAGAACACCATCCTTGCCAGGGACCGCCGCTCTCAA
CATTTGCCGTTTGAACCGGAGAGGATTATCGCAGTTCATCAATCCCTTAACG
AGATCCAACAACTGGGGCGTGTTGGAGAGTTGTTCAAACCATTCGGACTC
CTGTATAGATATGCTCTTTTGACTAAAAGTGTCAATTCCAAAGCCAAGTGCA
CAGTGTAGAGACCTACGTGAACGATGAGCACCTTGGATCGTCTTCGACTCT
GTTGTCTCGTATCGTGCCGGGAGCGATGTACTACAAGCCCATGAGCCAATC
CGAGCGCGTGATACAAGCAGACGACGACTCACGACGCGTTGCGCATCCTC
GCCACCGACGAACACGCCATCTTCTGTGGGATGATTGGGATCGAGTATGCC
CGTATCCTTGATGTCTTAGAGGATTAGCTCACGATGTCTTCTACTTCGCCAA
CACATTTACGCTCAAGGTAATGCTTCCGTGCCTCGAGGATGATGTCCGAGT
CGATGATGCCCTTCATGAAAACGTATCCATCACGCTTAAACCGATATTTGAT
CTCGTCTAATGGAGTATCACGCGGAGTCGGCAGAAGCTGACCGACCTGGG

CGTGCGCAAGAGGTCGGCTTGTGGGCCGCGTGTTTCCGATGATCAAGGGC
TCGTCACGGAGCGGTGAGGCGGGTGGATGAGCGAACATTCTGGGTCAGCA
GGAGTTTGCGTCGTTGCTCTGGAAGCCGAGCTCCCACTCTTAAGAGTATAT
AAAGTCGTCGGAAGCCGTAAGCGGGCTCCGCGAGCGGAACCGTACTGAAC
GTGTCTTATCGGAACAATCATCACAGACACGTCTGTGTGGATGAGGGGCGA
AATGTCTGCTAAACTCTTGCATTTCATGCATGAAAGCCGACAGCGGATGTCT
GATGTGCTGGGGGAATGGAACCGCGAAAGTCTGAGACTCGCATACACAAA
TGGCTTGAATGCTTTACGATCCCTTTGATTACATGCGTTTGATACAATAGATT
ACTGTAAGTCCTCCTTTGGACAGGGTCCCGGCCAGTCGAACCAGTCCAAG
AAAGCCCTCGAGTTAGCGCCGGGAAAATAATACCTGTGGGTCTCGCGCGCG
AGGGATCCGCAATTCGAAGGAGCTTAGAATCGATTCATGCGTAGTAAACGA
GACCCATAGAAAAGGAAGCGGTGCACCTTCTCATGCCTGAAACTTACTGAC
AAGCGTGCAACGACGTCTATCTTTACACTACAGACCGATGGTGCACACAAG
TAAACACGTCTTCGGCTCTTCGAAGGCAATGCGACTGTTCGTCGTGCATCC
ACATCAACCGCTCTGAAAGACTCGAGACCCATGTATCGCTCCCGCCCATGA
GTGAGCTTCCAAAGTTTGGCTGCGTACGGATT

>Mosaic_sequence_27

CTCCACTAACATTCTCCCCCTCCAACCTCTGTCTTCCAACCTCTATCTAACA
CTTGATCATCAGACGAAGATATCCCATCAGCTTTCTCTAAGAGGCAAACGG
GTGTATCATCTGCGAAAACACATCACCGACTTGCCCCACCTGCGGCGAAAA
TGAGTCGTGTCAATTCTCTATCCAGCTCTGTAACGAGTGTCCGAGAGCCTAT
TGCGCAGTCGACCCCACAACATCGACTTCGACGACCAAGTCTACCAGTAG
CCCCAACGTAGGTGCTGTCGTTGGTGGTGTAGTAGGTGGTCTAGCAGCCGT
CGCTATTATTACGTACTTGGTTTGGAGATTTTGCATCAAGAACAAGAGGGG
GCAGTATGAGCCGGAGCAATGGCGAGGGGACGTGGAACAGTCTAAGGAG
GGTATGGAAGCCGACTTTGCTTCAAGGAGATCCAAACGTACCTCGACGCAT
ACCGTACATTCTATCGCCTCGACTGTTCTTACTCGCGCTTCAAACATAATCC
AGATCGCCTACATCCCCGGCGTTACTACCGAGCTACGCCTACGTCACCACG
CTACTCGTTCCTCCCGTCCCCCAATTCCTATTGCGCACTCGGATAAAGGCAG
CAACTCGCCATTTGATGAGCAACACTTCTTCGTCCCAGGAGACCTCCGTGA
CTCGACATACTCAGGCATTTCCGGCTTTAGCGATCGCTCCTCCATGGCGCGA
ACTTCTTACGCTCCCCGATCTAGTGTTGCGTCCACCATATATGGCAAGAACG
CTGTTATCTCCCCTCTCCCAGCTCAAAAGGGCAACTTCCCTTAAGCCTTCCA
TTGTCAGCGTCAAATCTCGGGCCCCTAGCAGTACTAGCGGCAGTTCAACGC
CGCCCGTTCCAGCAGTCGACTACGAGAAATACGAGCAGCCGCCTCCGTCTC
CAGCATTTAGCATCGGAGCTACATTCTTCAAAAATGCTAGCGCATCGACAG
CAACCGCCGTGCGACCTCAAGTTGTCCGCGTTGGCAGTGGCCCTAAGGCC

ATTGATGTGAAATCGAAGTCCAGTTCCGCAACCGCTGTAGAATCTGACAAC
TCAGAGCCCCTCGTACAACTTCTCGACACGAGCAAGGAAAACAAGGAGAG
GCAGTCAGGTGCGGCGACAATCATCGAAGAATCGCCGAATGTGACCAGGG
CCCTTTCGCCGATCCACCTAAGCAAGGGCCACCAGAAAAATAGCTCCCTGA
GTGCGGTAATCGGGGAGTCCCAGCGGGCAGTAGATGGCAAACGCAAGTCT
TCGGCGGAACGGAATAGCAGTCCGTTCGGGGACGAAAATGCCTTACGAGA
ATAATCTAGCCGCAATCTAACGTGACGTCTAGTATCATGACAGGACATGGGA
AATACGCGGATATGGTGGGAATATAGCTCGTCTATCTTAAAAACTATTTCTAG
TATCTACTTCCTTAGTCTGGACTTTTTTTTTATCTTCTCACCTGTCTCCTTACT
GTCGGGTCAATCGAGAGACCTACCTACTGTTTGTCGAATTCTTATTCATACC
ACCCGTTTCGCAACGCGGCGGGAGAGGGCCTGGGCTGGATCTTTTCTTTAT
TTATAGGCACGGCGTCTCGGGCTTGGAGATACGAACGAATCTTGGGGGCAA
ACAAGTTTCTAAAAATGGCACCCGGTTGCCAGCGTTCGCGGTTTATTCGCG
GCTGGTGGAGCTGTCGTTTGCTTACGTATTGCTCCCCCTCTTTATCAAAGAT
GCCTTAGAGGCGTAGAGATGAAAAAATGCGATGTTATTTCAACGCACCGCA
CTATTGTGGTTAGATGATTGTCGTAGGCTGTGACCTGGATCTTGTGAATCGT
GAGATTAAGTACATAGGTAAGTAGTCCCGTATACTAGAGCTTTAGGAAACAT
GATACACTACCTACCTAGTAGACCTGCTTAGTTGTAGCAACGATCTTGTTCA
GGCCATTCGCTCTAGCAACTGCCGTCTAACCTCGTATATATTCCATTACGTAT
GAATGTCGTGGAGGTCTTCCTGCCTATGTCGACCAGCATAAGTCTCAATGTC
TGGCTTTGATGCGTGATGGCTACGAGGCAGCATATATCTTAAGTGGAAGTCC
TCAAGATGTCTCGAGAATATCGCATTATGGATCTTACAACAGCTCACGTTAC
ATGGCTCTAGAGAGGAGAGATCCTATCATTCTCCTTAGTTTAGGCGAATACG
TCTACCGAGGCTCGTATTATCAGTACTTGTATCACGGTACACATCGATATTTC
ACCGACAAGCTCCTTACAAGTCTAATTTATAGTTGTCACGTTCATCACCATG
GAAAAGCTAGGTACCTAGCCATGGGTAGATGCCCTAGGTACCGTAGGGAAG
CATTGAGCTCCAACTTAATGCTTCTGTCGTTTATTATCACCAAATACTTCGCT
TTAGCCCGGCCCCCTTTCCATGGGTTGTCATACCCAGATAGAATCGACATGA
CGCGCCTACTCCCGCTGTAGCTAGGTCACGCTACGTATATGCGAGGAGATAA
GCAAATGCTGTCAAACCTCCGCCGCGGTGCGTAGTTCGTAGGGCCAAACTC
CGGCCTCAAGGTGGTGACTCGCGCTGCAGGCTTTGCTGCACGTATCGGTGT
CCGATGGGGGCGGCATGCGAGCGTCTTCGCTTAGCAATGTTTTTCGGGAAT
GAGGGAGAATGTGGAAAATGAGGTGGAGAATAGATGTATACATAACTTATG
AATAGTAGCCTCGCCATCTATTAAAACGTCCGTTTATATAATATGTATAGCATC
TCATAAACATGACCGAATATCGTTTGTCACAATGACGAGTTATATCCCCCTTT
TCTCGCGGGCATGTACATACCTAGATAGCTTCGATAATTTCGATGAGTGGTA
AATATTCACATTAAACAACTAAGTGAGTTTGACTTACAAATGATATATCATAT
CGACAAACCTTCAAGTACACACAAACCGTATATGTAGTTCAATTTTGCCTTC

TCTCTTCACAACCACAATAACTTACCGACCTCCACCCTCCTCTCGCCCCTTC
CTAATTCCATCAGCAGCGTAACACGACCAAACACGGCGTCATACATCCATC
CAGACCGGCTTTCAAGATACCCACCCATCCACCTACCTGACGTTGTGCCAC
CCCCCTCATCCCCACCCACTCACCCATCCTCCCGTCTGCTAAAGTGACTGCC
TCTTCTACCCGCCCCTCTCCACCTTCTGTCCGTACCCGGCCTCCCGCCGTCC
ATGCCCGCACCCGTCCCGAGCCACGTGCCACATGCGGACCCCGCATCCCGC
ATCCCCTATCCCCTACACCAAGACTAGGCAGGTACCTAGTATGAAGTGCCGT
GTCTGCCGGTTATACGGGGGCATAGTTGCCTCTCGGACAGGTATACATAAGT
AGGTAGGTATATGCAGTTGTTATGTATAGCCCTGTGCTTAGGCGAGACGGAG
CTTGTTTATGCAAGGTACTAGACCTGCCTTGTACTTATGTATGATGTAAGTCC
GTAGTACACGTTAGGCGAACGTAGAAGGGGATAAAGAGGGAGAGAGGAA
GTCGGATTAGGACTCCGAGACCGGATAAGCGGGAACCATGGGAGTGCTTA
AGTTTAGCAGCGTAGATGGGTCCGTAATTACCTAAGGTAGGTAAGGTGAGG
TACAATACAGATTAGCTAGGTAGATCAGGGGTCGGGAGATATTAGATGGGA
ATCTGAACTATGGATGGCTTGATTCTTGTGGTGGATATATGCTGCCCGGGAG
GGAATAGTGAAATTTTGTGATATGTCGAGAAGTTACCTATCAGCTACGAGCA
TAAGTTCCAAGGTTCGGATGTATTTTGAATTTTCATATTCCTCTTATGTTGCA
AGTACTTCAGTCTGTGAGTTTGACCTGTCAGTCCCCCGAAAACGCCACACG
AGTACCTATTATGAACTCCTATCAATTTCTTGTTTCCCATCGAAATCATGTGT
GGAAGATTGAAATCTGACATATCCACTTGATCAACATGCATATAGTTCGGCG
ATATCCATACTAATCTTGATTTGCTTTCCCTGGCCGTGTTCCTTGGTACGGGG
TAAACTGGAATCCTATTGCCGTGAAAAATGCCTTCCCCATAACCCCGTAGAT
CTAAATTATATGGAATCACTAGAAATCAAAACATACCTCAAGACAGGTAAG
AGATCTAGAGGCTGGGTTAGAAAGTACGTGGTCGTGATATCTAGCGGTTATT
GCGTACTTTCTAAGTAGGTAGGTATTCCCATCGTTATTCAGTAGCTAGGTAA
ATGAATGACTAAATCGACTTAGAAAGGAAGCATGAAGTGTTATTGAATATAC
AGGGATACAATGATTTGATAGACGAGAATATTTGGACTTAGTCTCACATATG
AACAAATGTTAAGCAATGAAACTTATTCCGTACCATGTCCACCAAGGCCTC
ACAGATATCTCAACAAACTAAAACAGATATCGGTAAACATTGTACCTGTTCC
TGAGAATCTGCATGCCCTCTGCAATATAAAACCGCCACAAAATCCTCAATC
ACCGGCCAGCCTATCACCGCAAAGCACCAAGTAGCCACAGCAAGCCCAAC
GCTCCGTCCTCCGTCCAATATCCCCGGATACACCTACCTGTGCAGGCAAGTA
GCTCTACATCGGCACTAGAGACCATTTCCCCTTTACGTCTATCTTAAAGGGG
ATTGAACCCTAGATCTGCCTACCATACCCTAATCCATCTACGCCGATGCACT
CCGCAGTTCCAGAATACATCCATCCACCTGTCAGTAGTTCCTCCTGTGAACT
ACGTGTGCATGTACGTATAAGTATACGTATATACCTACCTACATATATGTAGG
AATGCCTGCTTACTCCGCATAGTTCGAGCTGCAGCGGCCAATTCCCCTATCT
AGATTTCAGGGGCATCCGTACCAGTTCGTGTCTACCCACGTAGAGGAATGT

AAGATTGGCTGACCCGTGGGGGTTAGGGTGTAGTGGATGAGACAAGCCAT
ATGCCGCCACGTCCTGCACTCTGACGCACGCGCATACCTCCCACATTTGCC
GCGTGAATCTTCCGATCAGATTCGGTGGAGGGGGATCGTGATGGGTGCTGA
TTCAGGAGACAGCTCAGCGCTCTTGGGTCCGACACTTCAAATTCCTCTTAC
CGCTCTAATAGACTAAGCTATACATACGTACATACCTTGCACAATGAGACTG
ATATCATCATGGGAATTCCTCATTGCCCTTGGTCAGCCTGATGATAAACACT
TATATCACGACGATAAGGAAGGAATTCTACTTAGTAACTATCAAGGCAGCAC
TAATCAAATCCCACTAGACCTACCGTATGAAGCAGAAGTGACCCCCGCAAG
AAGATCACGGTCCGTTAGTAATGCAGGATCCCACGTGTGGCTCAGGATAAG
CATCCCGTCAGCGGAGACTAGATGGAAGGAGTTCGCCGACGAGCTAGACC
CGAAATTGCACAATTGAGATCCGGCGTCTATAACGAGGACGTCGGAGTTCA
TTCCGTAGTACCGATATACGAAAGACCCTATGCAAGGAGTTACACACGCCC
GAATGCAAGTAGTAAGATCTCTAGACGGAGAAGCGTTAGTGCGTATCGGCC
GTTGTGAAGATGGACAACTCGCGGAATGGTTCCCTAGCATGAGCCGTTGCA
ATGCGTTGATTCCTATTCGCAGACAAGCCAAATCATAATTTCGATATAGGAC
AACCGCGCCTATCGCAAAATGCCGAGAACTCAAACCGCAAACCAGCAGAC
GCCCGCTGCCGGGAAGGATCAGAAAACCATGGTTAAAGCCGCCTTTTATAG
ACCGGTCAAGATAGTAGGACGGGACGGGCCGGCCAAGAAAGGAAGGAGA
AACACGGTGTAGAAGGGCGGATTTGTAAGATAGAAGAAAATTTTCTTCGAA
TAGCTTTAGAGAACTCGCAGCATATGCCAGATCGGACCATATGTTGATGGGA
CTTATGCAGCAAGACCGAAGTGGCAGAAACAGTTATTCCTTTCGGACGTCC
AGGTATCTGCACCGTAAGCATGCACCACGTGTCGATGTCGCAACGCTCCAC
TTGCTTGAATGCCATATCTCAGTAAACTTACGAGCAGAATCCCCTTTTAACT
CGTGACCACCTTCAAACTTTCCAGTTTTGATGACGCTAGTCGCTCTTGCGT
GCAGAACACGCGCTCGAGCCAGCGTGGCACGCGGGGATGAACCGAACAG
GGAGTCTTGACGTTGCAGAGGATCGTAATGCTTGACTTCTGCGTAGAGGCT
GAAAGAGCAGTGATTGACGAGGCGATGGCTTCGTTCGCCCTTTCAGATATT
CCGGGAATCAATGCATTGTTCCACGGCATACACTACAATTACGAGTCTCGA
AGGCAAACAATCACCGTCCGATAAAGTAGGATTGATGAATCAGAGCTCGCA
AACCGCGTCCGTGGCATTGGGAAGATTAAGCTGTGGGGTCGTGCACCCAC
ACAGGCCGGGGCTAATCGCCCAAAGCCATGGCTCTATTGTCGTGGAGGCAA
TCTTCATTTGCGGCCGTAAATGCCTTTACCGCTGCCTATACTAGTCGGCGAT
GGTAAATGGAGAAGAGAGACACCATCATTAAAGTCGGACCCGAAAACGAG
ATCGCACAAAGCAATATTAGGATTTTGTTGAATGATTCGCTGACACTAACTC
TGTAGGATATTAGGAATCGATGGAGTTGTGTTGAACCTTTCTTCTGGCGGAG
ATGGTTTGATGTCACCGAGCAAGGGGTTTAATCCCTTCACCTCTGAGTGGC
CCGTATTGGCATTTTATATCGGCGGTGAAAAAGTAAATTGGTTTATTTGAGTT
GAAACAACAGTTGGCAATTGTGTCGAGTAATGCCTAGAAAACATCCTGGCG

TCATCAGCAATGCCGGTTGAAGTTACCGATGCGCAGCATGTTGCCTGCCAG
GTCAAGGGAAAGGCGGAAAGGCATAAAATCCTCGTAGCGAGCCTCTTCCA
ACGATTGGGTAACAGGGTACGTAAGGTTGTCAAAATGCTTCGATTGCTTCT
ATTGCAGCAATACTCCTGGTAACAAAAATCCTCCTGGCCAAAAAAAAAGA
AAAAAAAAGGACATGATGCAAGGTTAATGATGAAAAGATATTCCTGGTAAG
GTAAAAGCACATTTTCATTACAATGGAAACACTCGACCTTCCATTTTGGCTC
GGGAACCTCCACAAGGCTACCAGAGAACGGTTGGCGGTTGCGAGGTTGGC
CTGGTTGGCAATTCATAATCCCATGGTGATACCAATGAAACGCGAAACTAA
GCGTGATAGCCGACATGAGGCACTGCAGGGTTCTCTCCACCATCTTGCCGT
AAGTGGTATTTAACTAACCCGAGGTCGAGGATAGGTACCGGGGTAAGTGTG
CGCAGAAGGCAGATGTAAATTGAGCCATGCCTTACGGCGAACATTGCCATG
CCGGCCCGTGAGAGTTGGCCAGAGGCTGGCCCGAAGTCGCCACAGTTCGT
TCCCACTCGAGACCGAAATATTGTCATTAGGAGAACAAAGTAGCCAAGACG
GTATTCAAGGTTGATTAGCGGAGTTGTAGCTCTGAATTCTATGGTTTCCTCG
AATATGCATTAGTCAAGCAATACTGGTACGAGACGGCTTGCTTGCCATGCA
GTTGAGCGCAAGTCCGGCACTTTCGGAAGAATCGCCACACTCGCCGCCGC
CACCTCCGATGCTCGGATACCGCAACACAGTCAGCAGTTGCCGTTGTAACA
CCACTTTTCTGCTTTTCCCGGGCGAGCATGGAATCCACAGAGCGACAGCCC
CACGCAAGAGGATACGGTCAAGCATGCGCGCAATGCCGCATGAAGAAGCG
GCGTTGCAAGCCCACCGGCGTTGAACGATGCGAGTGGTGTGACAGGACGG
GGAGCGGTGCATTTTCGTGGGGTGCGTGATTCCGGCGAGGGCCATCCTTTC
TTTCCGTGCAGCCGAAGCTGACGGGTCAGCCGTAAGAAGCGGAGCCGCCG
CCTCCCAGCTGTCCACAACGAACGGCGCGTCTCCTCTTGCCAAGCGGGTC
CGGGGAGACGAGTTCTCACTGGAGGCGTTCGGAGACGTCCCCTTGAACAG
CCAACATCAGCGCCAGCGCGATCTACCGGGCAATTTGCTACAGACGTTCGA
ACAGCACTCAAGCAATGACGACTCGATGTCTTTCACGCTGCTGGCTCTCGC
GGACGAACGGCTCGGCGCACCTCAGACGTCCTCCACGGCCTTGCAATTCC
CGTCAGCCCTTGCCTCGGGTTCCAACTTGATGCGGGACGCTGAATCACCCG
AGGCGAGCGTCGACCCCTACCTCGTCGATCCCAGTGTGACCCAGGTCGAA
CACAGTCACGGAAATCGCGAGAGCACGCTCTACACGACAGCAATGTCCAC
ATTCGTGGACACGGTCTGCGGAGACGTCAGGCATTTCGAGATAAACGAGG
AGTTGTTGTCTCTGTACTTCACTTGGTTTGCGAATCAGCACAATGCCGTCAT
CGAACGCCTCTTCAGACGTCCCGTCCTCTCCGTGCAACTAGCGCAGGAGTC
TTAGCTTATCAGCCAGGCGATATGGCTCGCTCACCACCTGGACCGTACTTTT
CCCTGCTCTTCTCTACGCCATCTACGCTCAGGCATCTCGTATAACCCAGCCT
CCAGATCGCGGGCAATCGTATACCGTGAGCTTTCTTCCGCCGCAATGCCTG
CCACTGACCCCTTCGTATAGGAGAAAGCTCAAGCCCTATGTATTGCCAGCAT
TTCGGAACCCCCATCAATCCCCATAATTCAGACTCTGCTGGAGTTGGCTGG

GAAAGAGCTCGCCACAGGGCAAGGTAGCAAGGGATGGCTGTTCGGGGGG
ATGGCCCTGCGCATGGTGACCGACATGGGCTTGCATAATTGCGTGAAGCAA
CGAATGGAGGATCGACGTACGACCGAGGAGCGCGAAGACCTCGAGGTCCG
CACGCGGTTGTTCTGGAGTGCTTACTGCTGGGACAAGTGAGTCAGGGATA
AATGACGTCCGCAAGGTCCTCATCATCAGATTCTATGCGCTCACTATGGGCC
GACAACCCAATGTTGGCCTTTCCCTACGGGGACGTGGACGGGCCGGTACC
GCTCACTCTCGCGCAATTATCCAATTGGGGCACCACACCGCTAAACAGCAG
TGGGGCGAGACCAAGCTCCAACTTCGGCCGCAAGCGCCGCGACGGCCCGT
TGGTTGCCTCTGTATCTCCCGGCACAGCAAAAATACCCCTATACGCCGCAG
CCCTTCCTCGCGACTGAGACATTGCACGCGTAGGTGGTTACGTCGCGACCT
GGGCGAGCGCTGAACGACAGGTCCTGCCTGCTCTACCGCGAGATCGAGGA
GATCATTCGTCGGCTTTACCAGACCATGTCGCTGGACACAGAATCGATCGA
ACATCGCGCATGGATGGACAAAATGACGGCCAGGCTCATCAAGTGGCGCA
GTCAGGTTGCACCAGGCGTCTTACTCGACACGAGCTATCTGCCGACCACCA
GCCCACCACCGCATGTCCTTTCGGTCAAGTGAGCATCTGTCCCCCACGAAA
ACGGGCGGGGATATTGCTGACTCGCAGCATCCTCTACCGTACCGCATGGAT
CCTACTCAAGCGGCCGAGGGGCGAATCAGGCCGGTCTCAAGAAGAGTTCA
ATAACCCTCAATCATTATGTGCGGAACGGTCGGGCGAGATACACGCCTTGTT
CAAGCTGCACGAGCGTAGCTTTGGACTACAGAACTTGACCTATCTCATGGC
TTACATGGCATACGTTTCCGCCACGGTCGACATCAACGAAGCACTAGATCC
AGACCTATATCGAGCTATGGCTGGTAACATGCGACTGACATTTACACTCCAG
ATCCTCACACAGGCCGCGATACATACGCCAGGTATACGTCGCAGCATTTCTC
ATCTTCGCCGGCGCATCGCTGGACAGACAGGCAGCACGCACAATCATGGC
CGCCGATTGGAGTCACGCACGACCGCACATCCCATGTTCCACGGCGGGAC
GATGGAGACACGCGCATCTAGCCCTGTGCGAGTCCTGGGACGCGACTTCA
GAGCAAAGTGCGTTCACCACGGCGGATCTGCCGAACACCCAGTCGGCCTT
GGAACTAGAGGAATTGTTCGCGACGCTGTTTCCTGAGCAAGGCGCATCGTC
ATGGCTCGGAGATCTGGGAGGTGTGACGCAAGGTCAAGACGGGAACGAC
GTGCGGGACTGGTTCGATACAACGTGGACGTGAGGGCAGTGAGCACTGAG
GCGATCCATAGTAGTATCACACATGCATCTACGTCTGTTCGACATGTCCGCC
GGGCTCGGCTTACGTCACGCCGCTGACACATGACTGTTTCGTGGACTTGCC
ACCGCAATTCATACTTTGCGCATTCGTTCTCTTTCTCTTGTCTGCAAGCACC
ACGATCCAAGAGCCATGGGAGCCGTGGAGATCCCGCCTGACCTCATCGGC
CAGTCGCGGCTGTACAACGACCCAGACCCGCTGCTGAGGCGGTAAGCTTT
CAAGTTCTCGGCTGTCCGCCGGAACTAAGACCAGACTCCGAATAGAGGATA
CAACAGGCGTGTCAATCCCCGACATTGCGCGGTACTTTCACGCCAAGGACC
TGGTCGTGCTGTACGCCGGCTCGGAGCAGGGCGCAAGTGAGGCCGTCCCA
CCTTTCCCTAGTCGAGGCCAGCTCGCTGACGACAGACAACCTGCGCGAGC

TGCACCGGGCAAGTCCAGCACCTGGAACCCATTTCGCATAGCTGACCAGC
AGGACCTCACCACTTTTGCTCTCAAGTATCTCAAGGTGGGTTAGACATCCG
GCCAAAGAGCAGAAAGCGCAAGAGAGGAGTCGCTGAGCCACAGAGCGCC
GCAGTGATCTACGTATCGGCAGACACGGACCCCAAGGCCGCCGCGCGGAT
CTTGGCCAACCAGCCTTGGCTCCGCCTGACATTCCACGACAACTCGGACTT
TGCACCCTTGGTGCCTATTCGTGAGGGCGCACGTATCACCGAGCTGGACGA
GGTGGCGGAGAGGGGAGGACTTTGTGCAGGCTGGCGTGGGTTGATCCATG
CGAACCGAGAGGGAAAGAATACCGTTGACGGGTAGGAGATCGAGGTCGGC
GCGGAGAAGGTCGACTTTGGCGCCGAGGAGAATGGTGAGTCTGCGGCTAT
GATGCAGAAGAGGGGGAGCTGACGGAGCAGAGAACGACTATGTGCGGCC
GCTGTCCCGCGCGGCGGTGACGGCGCTCATGAATGTCTTCTCCACCCCGGC
CATCTCGGTCTAGTGCGTGCTGTCCCACAGACAAGACGACCTCGTCTGATG
TCCAGTCATATCCCCTCTCACCGCTTCGTTGCCAGGAATGTCCGCCCCTCCG
CATTCAACTCGAAGAATCTCGAGACCAGTCACCGATTATGGAGTCAGGGAG
GAAGTCCGGCCCTCAAAGCCTCGGGTGAGTGTTGTTGTCACGGCCCCATC
GTCCACCAGCCTGACAAGTAGATATCTGGCTAGCCATGAAGTGGCCTCTGA
TTAGGCCTGTTCCTCGCTGCCCTCTACCAGCTCCTGGTCTATTATGGTGGAC
GTGAGTCGTTTCGTTGCTTGTTCCAGCAGTCTGACGACTGCAGCGAAATAC
GCATTTATACCGAAAGCAATGGACGCAATCAACTGGCGAGCGCAGCCGGG
AGCGGGCAAAGTGTTGTAAATACAGTACCTAGAAAGCATGTTCACATCTGC
ACCGCATACAGCTCCATCACAACGATCCGGTCGTAGGTCATTATCGTCTGCA
TCCAAGCCGACATGCACAAGAAGCCGTGTATACACACCATGCGAGTGAGG
AGATCACTGAGCGGTCAGCCCTGTTCAATCCAGTCGGGGAGACGCACAAG
AGACTCCATTTGAGCAACGACCTCGTCGAGCTCCCGTTTGGACTGCTGGTA
TTTTTCGACAGCTTCCTGACCTGTTAGCCCTGTGCTTCGTGGCATGATAGCA
GCTGACGCCGTGTTTGCGCTCCCACTCGTCCCGCTCCTGCTCGGCCCGTGA
ACCTGTCGCTCGTAGTGCTCGGCTTTGACGTCCACCTGGCGCAATCTACAC
AGGTCAGTCGATATGAAGCGAGCGAGAGCATGACGCACTTCTCAGTCGTGT
CTTTCAGGTCCTTCTCCGCCTTGTCCAGCTCTTCCTCGAGGAGCTGCACCTT
TCGCGCGAGGTTCTCGCCGGTCGTCCGGTGTTCTCGCCTTCATCTGATGCA
GCCTTGAGCTCCTTCACTTTGCTCTCTGCCTTTTCCAGCTCAGACTCGGCG
AGTGAGAGCTTGTGTTGCAGAGAGGAGATCTCCTGGTCGCGCTCCAGCAG
AGTCTGATTGAGCTGATCTTCAGCTGATGCACATTTGGCCGCTTCAGGGAT
GTACCTTCTTGTTCTCTGCCTCGGCAGCTTCTGCGCGGCTCTCGGCGGCCTC
GATCTTCTGGCCGAGAATGGTGAAGCGCTGCCAGTGAGCTGCACACTAGTA
CTGAGCAGCGAGGAGTCGAGCAATGTGACATGATGTGTGCAACGAGACAG
GTACCGAGCAGCCGTTCACTCACTTCCTTGATCTTGTCCTATGGATGTCAGC
ATCATTGTTCTCACAGCATTCCACCCTTTGCCACCGTGTGACCGGTATAACG

CAGTGGGCAGGATGCAGAAGACCGGCGCGACTCACCATGGTGTCTGTTCG
TGATTCGTCTAGGTCTAGGTCTGGTCGTGAGGCGTGAGAAGAGTGATCTAC
TTGTCAATGTCGATATGAGAAGAGCAGAGACACCCTCTTGTGACGTTCATC
ACCGCCGTTCGAGGGTCCGAGCGCGTGTCCAGGCCCAGCACGCGTCACCA
TTTCCCGCCCCTATTTTCTGTATTTTATCCATCCTGCCCAGCCCGAAACTCCC
TTTGCTTCTCGTCTCTTCTCCTCATCACGCCGCTCAACATCGTGGCGACCCG
CTCCACCTCCTCTGTGAGTGGGGACTCGGCCAGACGAGCTCATCAAGGCG
AGACTGAGGAGTCCTGAACCGCCCCGGACCCCATTAGTGGGCGAAGGGGA
GGAGGGAGGAGACGCCCCGCAGGACTGGTCCGGTCAGAGCAAAGGCCGG
GCGGTTGTATGGGGAATGTCGACATCTGCGAACGGTGCGTATACTCGTCGA
GAGACTCGATGCATCACTGCGTAAGGAAGCGTTGGCAGAAGGCGCGGCTG
ACTACTGATGGGCAATAACCTCATGTCGAAGCGCCTTGCCCTTCACTCCTG
CACACACCTCCACGCCACTTTTATCTTCCTGTCGGCTCGCATGACACGTCG
AGGGGATAAACTAGGTGGCTATAGCAAAGAATTTTGAGATTTCGCAGAGCG
TCCCTTGGATCTTGTCGGCGAGAGGGGCGTTTTGAAGTCTGCGACATGCCT
TGATCGTGGTGTACCGGGTGTGACCTGTAGGGGCCATCTTTAAAAGCCGAG
GCGGTATAGACTATACTGCTCGACAATGCAGGCGATCGTATCATTACTGGCA
CCCGTGTTGCATCTACTGCTCATCGTGTCCTGCCCGCATCTATCTGCTCACT
CTGCTACAACCGAAACTAATCCGATGTTGAGCGGATCTTCAACAACCACCT
CGACCTCCCTGCGCATCTCAAACCACCCGAACCGCCGATGTACGTATACGT
CTAGTAGAAGTGACGGAAGAGAGGGTTGCTGGGTCCCTCGGCGAGCGCCT
CGGTGGGCACGTCCTTGCTCTGCCAGTTGACACCCTGCTCCTTGTTCCTCC
TCTTGTTGTCGAGGTACATGTACATCCTGAGCCCAACAATGAGAGCGAGGC
CGAGCCCCTCAATGGCGGCAGTGATAGCGAGCGGGGTGACGTACTGTGGG
GACCACTTGTCGAGGAAGATGTTGGCCGAGACGATACCGCCGGTGTTGGC
GAGGAAGGTGAACGAGCCCCACGCGGAAGGCACGGCCGTCCTCGGTGGG
GTCGTTGCACTGGTGCCAGGTGTGGAAGAGCACCGAGGGGGTGAAGGCG
CCCATGGTGATGAGGAAAGTGGCGAAGTAGCCGACGCCCTTGTTGGTGAC
GGGGATGGTGGCGAGGATGACACAGCCGACGATGACGAGCACGAGGGCG
GACGCCAAGTGGAAAGCCACGCTCACGGTAGCGGTCAGAGGACCACGAG
GTGACGAGGAGGACGAGGGTGCCCAACACGTAGGGCGCCACGGTGTACA
GGTTGGTCTTGACGGTGCTGTAGTGGAACCTGCCGATGATCTGAGTAAGGA
AGTTGCCGGCGACGGAGGCGGCGACGCCGTAGCAGAGGGCTGGGCATCA
GCGAGGACTCGAGAACATGGCTTCGGGGCAAACGTACCGATGCCGGCGAA
CACGTAGAACTTCCAGTCGGAGAGGGGCTTGAAGAAGGTCTTGAAGGCAA
ACTTGGTGCTGGTGGCGGTGGAGCCGTCCTTGAGCAGGCGCATGACGGCG
ACCTCCTTCTGCCTCTCGGTGAGGAACCTGGCCTTGCTGGGGTGGATGGGG
AGGACGATGTAGACGATGACGGCAAAGGCCATGGTGGCGAGACCCTCGAT

GAGGAAGAGGATCTGCCAGCCGTACAGCTTGGAGTCGACCTGGAAAACGC
CGTAGGCGATCAGGCCGGAGAAAGCACCGGAGAGCGCGTTGCACGAGTA
GAAAGCCGCGATGCGCTTGCCGAGCTCGCCACGGGTGTAGAAGGTGGTGA
GGTAGAAGATGAGCGACGCGGCAAAGCCAGCCTCGAAGGCTCCCAGGACT
GGCCATCAGCCTTCTTGCTCGACAGGCTCGAAGCTGTTCAGTGACTCACGA
AGACGGATGATGAGACACCTGCAAAATTCTTGGCGGCGGCGTTGACGGTG
GCCATGAGACCCCAGCCGAACATGTAGCCGGGAATGGTGTACTGGGGTCC
GACTTTTCGCGTGAGCATGACGCCGGGCACGACGAGCACACAGAAGGGA
ACGTAGAAGACCGAGAGCATGGTGCTATAGCCGTTGGCGGGGAAGTGCAG
GTCGGCTGCAAGCGTCAGCGAATGTTGGCAGTGGAAGATGGGCAGATTGG
ACTGACTGGTCATGCCGTCACTCTTGGCGTTGGATACGTTGTTACGGTCAA
GCGAGTTGAAGAGGTAGCACCACCAGATGAATGGCAGGATCTGCAGGGTC
AGTACCAGTATGAGGCAAGTAGGGTCGTCAAGCATGACAGCATACAGCGG
TGGCGACAGGGGGTAGGGGACAGGAGGAAGGATAGAGGGGAAGACTCAC
ATGCCAGTCAAACTTGCGGCAAAGTGCCTGGGACTCTTCTTCGGTAAAGTC
ATAGATGGTCATGTCGACCTCTTCGACCTTGTGGATGCCCACATTGTCCTTG
TGGTGGTTTGCGGGGACGTCGCCCTTGGACTCGACGTCCTGGTATTGCTCG
ATGGCCGACATAATGCCACACAGTCAAGCGGGGTAGGCTCCGGTGAGCAG
AGAGGTAATGTGGGCGGATATGCGGAAGCGCGGTGGGAAATGGGACAAGC
CAAGGGCGGCTTCGACGCTGGGATGAGTAGGTGTCGTGGAAGAGAGGTGG
GTGGGATTGGGCTCTGTTGGGATCGATACGGCACCACCTTTATACGTCGAG
GAGCGTGAAGTGGTATCGCCCAAACCAGTCATCGTGTCGGAAGCCTCACG
GGCAGATACGAAAACAAGCACGCCTTATCTCCTCTTGGTCCACCTCGGCTC
GATGCACACCGTCATGCCGGCTCCCGCGAGGTCCTAAACGTATTCCCAGAC
CCTGCCAGTCAGCTGCCAGCCGTCGCCTCGGAGGTGGAGGCGGCTCAGGC
ATCATCTCTGTTCAGGGGTAACACCGACTGCGATCTTCCCCCACGCTATTCT
TCCGGCTGGGGAGACGGGGGGCTCTGCTCTGATGCCCAGCCTCCCGTCGTT
GCACAGTGAGGTCTGCCACCTAAAGACCGATTGCCAAGCCATCGAGCGCT
ATTGCGTCTTGGCCACACGGCTACGTCCGATCTGAGCCTTCGCAGATCACC
GGCGGACACTGTCCGCCTTCGCTCGACGCCGATACCTACTTCTGTGGAGGC
AGAGTGGGATCAGGCCGCCTCGACTGACGTGACTTTGAACACTTGCCTTG
AAATAGCACTTGAACATCCAATTTCGGGTTGGGCCGGAAAACGCTCTCGAC
ATTGATAGACTCGCCAGTCAAGGCCACGTAGACTCGTAGTATCCGGTACCA
CACAGCATGACAACACCTCAGAGCAAGGTCGCCATCGTCGGCGCCGGCGT
CTTCGGTCTGTCCACCGCCTTGCACCTCTCGAAAGCAGGTCAATCGTCTCT
GACAACAGTTGAGGCTCAGCTGAACACTCAGGTTACAAGGACGTGACTGT
CTTTGACTTCCAGCCGTACGACGAGAACGCCTACGATCCCTCGGCAGGTTG
CGACGGTGCGTCCGCCGACGTCAACAAAATCTATCGTTGTTCTTAGTGAGT

GGCAAGGGTCCTTTGACAGGGATCTTGATGGGATCTCGGCTTCGTCATCAT
CACAACGGGTCCTTTGGTGCCTTGCCTTGATCGCAGGATTCATCCGCTGAC
ACGATCAGCGGCCATGAGACCGAGTACCAAGATTTGGCGTTCTCCGGCCGC
CCCATCTGGCTCGACTGGAACGCCCAGATTGCCGCGTCCAAAGCCGAGGA
CCTGCCCAAAGGCCTGACACCAGAGACAAAGCTGTTCGAGCCATGCGGAT
TCCTGCGTCTCGCCAACGGACCCAAGCTGTCC

>Mosaic_sequence_28

AGGCGGCGGTGGTTGCATAGGCTGCAATGTTGCGATATTGGCGTTGGCGTT
GGCGGTGTTACTATTAAATTTCCAAGCTACGGAATCGGAGTGAGACGAGAA
ATTAGTAGAGGAAAAAAAGCGGATATGTGCAGCGGCAGCCACTCGTAAGC
CACTTTTACTCTAGCAGACGTCTGATGACGAATGCTTTAGCCAATTGGTGAC
GGGAATCCAGATAAGACTAAGACAAGCTTGTGTGGAGAAACAAAGATGGA
AAAAGAAAATCCTAACAATCCTACTGGGCACATGTGGCGGGAAACGTGCC
GAGGACGGTAGGTGGGCTGAAGCTACCCTTGCAAGTCCTACCAAACTGTG
GCTAGAGAATCAAATCGAGATTTTAGTCGAACTCTTCTTGCATGATTTGTAT
GTCCAATGTAGGACAAATCCATATTATATAATCATTTGGTATCAAATAGTATG
CATTTAGAATTCGGAATTCATACTTCTTATTCTTACCCTCTCAATCATACCATA
TAGCATTCATCCCAAACCCAGGCTCAGGAATCCTGCACCTAGCGACGCGAC
CCAGAAACTCGTCCTTCCACCGATATATCTCGATCCACCCGTCCTGATCATC
TGTGATCGCCAACCACTCCTCACTCCAATCGCACGGCGCTACCGCATTGCT
ATGTCCACCGCTCGTCGGTGTCGGATTTAAGCAGATCTGCTCCTCGATGGA
CCCACAATCCCGCAGCCGGAATGCGGCGATATAGCCCTGCAGATCGAAGCT
ATTGGCCCGACTCGTCGCGAACAGGTACCGGCCGGATTTCGTAAGACTACA
GACGTCGCTCCGATACATGGTGTCGTGCTTCGGGATCCCTGGCGGGATCAA
TGGGTACGAATGGTGCGTGTACACGGGCATGTGCGTCGCCGGGTCGATAAC
GTACTCGCAGAGTCGGTTGCCGGCTTCCATGAGCGCGTATAAGTAATTGCC
CGTGGGATGCATGGCTACCCATCGCGGATGGTCCTTTGGATCCGGCGCGTC
CACGCTTCCTATCAATTCGACCTCACCCGAGGGAGCTTTCTTGTGCACCCAT
AGCTTATTAGCCTTCAAGTCGGCTGAGTAAAGGTAAGTCTCGGTCGGATCG
AACACCATGTCATGGATTCCAGTGTTAGGTTGGTAGGGATAATGCTGTATAT
CTTTTGCAAGGGCTCCGGTAGATGATACCGAAAATACGGATCCATGACCGG
CATGTGTGTAGAATGGATTGCAGTAGACGGCGTAAGGGGGTTGTTTTGCGG
CAAGTAGGAAGATGGCGCGGGTGTTTGTCGTGGCCAAGCTTGCTTGTGCTA
CGTGCCGAACATCAGGGGTCAATTACCGGGTTGGCTAGAACGACTGGAAC
AGGTCCTTGCGAAACTTACGATCATGGTTCATCGGATGCGACGCTTCGTGG
ACGATCTCCGTCGGGCTCTTGACGGCAAAGCTCGACCATTTTTTCATGGCC
GCGCCGTATATGTTTTTCTTGGCATGCTGAGAAATGTGGTGAGTATTGAGTA

TTCGGGAAGTATGCCGGCTTTGGTTGGGTCTGGGTTTGGCCGATTCAACTC
ACATCGAAAGTCATCCATGAGATTGGCTCATCATGGGGGATCTCTGTCTCTT
TACCAGTTCTAACGTCAATTTCTCATCATCGAAAGCTACAGTGAATATGGCT
CCTGGTGGGGTCCATGTTCCTATCATCAAGTGATGAATTGGCATCCTTGCTT
CTTTGCGCAAAACGAACTTGAAGGTATTCCTCAGAAACTGCGGCCGATTCA
TGCAACGAAAAAGCAAATATGAAGACTCAAGGAACTGATCTCCACCGTCT
CGTCGACTTTAGTGCACAACTGATAATAACATCGCTCGACAATAATAGCCAA
CTCAATGGTCGAAACAATACAACTTCCTTCAACACGGATATGAATAGGGCT
AGAGTTATTCCCCGATTGCTTAAACTGCTATATTTTTTTAGACCTGTCATCC
AATGGATCACACTGACACTTCTAACACGGTACCTTGTTTAGCACAGTTAGC
ACAGTTAGCACAGTCTAGTAAGCGAAAACTCACTAATTCGTCCCCGCATCT
ACTTGGCGATATCTCGGTCCCCTCCAATGGATTATTGCACCATTGCTAGAGC
CACCGATGTGGGGAAGAATCGACGTTATCCTTCAAGCAATCGGCACCGCTA
GCGGGTAACGGTAAATGGCCACGGCACACGGGTTATTCTGCTATGTCAATT
CGTTGTTAGTTACCGAGCGAATGGCTACATTGCTGGATCTCCTTGGATCTCC
CTCGTTATTTGCCACGATGCAATGCCGGATAAACGCCTCATCGTCAGATTTT
CCCCGCCATACACCTCTCGGTTCCAAGGTCTACCGTATTACACTACATACTA
CATATCACGGTCCTGCATTACTGGGTCATCGATATCGAATTGGGTCCAATTG
AGTCGCCGGAATAGTTCAATGCTTTTTTCAGTGATCTTACCAACGTGTACGA
GACATCAGCCAGCACCAACCTGCTAATGAACTTATAGGGGTCCAACTTCAA
AAGTATAACGGTCATGACCAAGGTTTCATATGCTAAAGCAACCAATTACAA
GAGCTTAAGAAGCTATTTCCTTACCTGCCTAGGTCGGTACATAAATAGCATC
CTCTGGACCCGAAATAAACACGCGACCCCCCGAACTAGAAGCTTTAGTAG
AAACCCACCACTAAGCTTGTTGCCATCTTTAAGGGCGATTTATGACCCGAG
GGGCAATGGGGTTTAATATCGCTGCCGTGACAGCAATCCGATCCACCTACG
TACACATATAGGGTCCTTTTGATATGGCGGGGTTTTCTTGATCTTCAGGAAC
CAACCTATCAAAGATCATCCATTGCTCATACTGCTCGTACAGTAAAGAGGCG
AGTGGTAAATCTTAAATGAAGTCGGTGTTTCTATTCGATAGTTGTCTTTGTC
CGTGTACTAAAGTTCCTTCTCGCTGTTGAATATATCTAGCCGGTGTCCCAAC
GATGAGCTAGTCTGACCTTCGTATACACTCATCTTCCCCTCAAACAAACAGT
CATATCTGATTCATACATACACTCATCCCCCATTCGCACTTGTTTGCTCTGTA
CACATATAAGCTGCTTCATATTCCATCTGATCACATCCATCCGATCTCACTTG
TAGCTTTTCTTATATATTCAATTTCACAAGAGCCGTCGCACAAAATAACAAA
ATGGCTTCGGACGCAGCCAGCGCATCCAAACCCTCCAAATATGACCCCAAC
TTCACACAGCACGTCATCGATACGATGGGCCCCAACACCACGCCCCGAAAT
CGAGTCGTGTTGGGAGCTCTAATCCGCCACATTCACGACTTCGCGCGGGAA
GTCGACCTCACAATCGATGAATGGATGGCGGGCGTTAAGTACATCAACGCA
GTTGGACGAGTGTACTCCGAATCGAACCAAACGCGCAACGAGGCGCACCG

ACTTTCCGATATTCTCGGGTTAGAAACGCTCGTCGACGAGATCGCGCACAA
GATTATTTCAGACACCGACGTCGACCCCACTTCATCTGCTATTCTAGGTCCA
TTCTGGAGCCCTAACGCGCCCTTCCGCGAGAACGGGGGCACGATCATCCA
GGATCCGGCCAAGGACGGTCGCGTTGTCCTCATGCACGGCGTTATCTCCGA
CCTCGTCACTGGCAAGCCGATTCCGGTGCCGTCTTCGATATCTGGCAGGCT
TCCGCGAACGGAAAGTACGATTTCCAGGATCCCGACAACCAGACCCCGAA
CAATCTGCGCGGAAAATTTAAGGCAGACGAGAATGGGAAGTATTGGTTTTA
CTGCTATTATCCGACAGCGTACTCGCTGCCAAGGGATGGGCCCTCGTTTCA
GCTGTTGAATATGATGGATCGACATCCGATGAGGCCAGCGCATATCCACATC
ATGGTGAGTTTTTCTGCCATACTCCCCACTTCATTACATTTTTATGAATGCGA
ACCATGTTAGCTAACGCAAACCACATAGGTCACACACCCGGAGTACAAGG
GCTGCACCACGCAACTGTACCCGAAGGAGGATGAGTGGCTCTCATCTGATA
CCGTGTTCGCCGTCAAGGACGACTTGGTGGTCGACTTCAAGCCCCTCGAG
GGTGACGACAAGGCGAAGCTCGAACTGGACTACAATGTAATTCTAGCGCC
GAAAGATTATAAGGGAAAGCCTTTTTAAACAAGTTCGTCGATCACCAGCGA
TTCCGGGACCGGCGACCAGCCCCTTGGCCTTTGGGAGTCTAGGCAGTGGAT
AAACGTACATATTTGTGTCGAGATTTAGTTTGAGCGAGGAAATAGCAAAAA
GAAGCATAAATATCGTGGATGGAATTAGCGGTGCGATCGCGAGCCACGGCG
CTGAGTTGGAGTTTATGCCCCTGAAAAGTAGGGGTAGGGTGCTCTCCTATT
GGTGAGGACATGAGACATATGCATTTTAATAGCTTTGCGCCCATTTCTGTAT
CTATATCAGGGTGCATTGCGCTATCCTTGAGCTGCAGCTCGAGGTACGAGAT
AATGAATATATGCCTACGACGGCAATCTGAACATATACTGAACCTGAATATTA
TTAGTAGGACGACAGAGCTATTCAATCAGTCGCCTTAGGTTGGTACTGAAG
TAACGCATACTCAAACAATACTTATTAACAATATAATCGCGATCACAAAAAT
GCCTACCTACAATTCAAAGACCGTCACCCCGAATCCCCTTCAACTCCGGGT
CCAATCAGCCAACCCAATTCTCGACCAACCGGAGTTAGCGAGCGGCGTCG
TAACCCGCCATCGAACCAGAGAAAAAAACCAAACCAGATCAAATCTAACC
AAAAACCCCATCTACTCATCTACCATCTCCGATCCAGCTAGATCCCGAAGTT
TCGCACCTCGTTCTCCGCCGTCTTCTCGTCAAGGCAGAAAGCGATGCCCTC
CTGAATGCCGACACATCGATCTCCCGTATCCCCGCACGATTCCTCGCGAACC
CATTCCCCGTCAGAGGCACAAGCCTCTACGGTTGCGTTGCCTTGGGCGCAT
CGCTGCCAGCCCTGCTCACAGACAGTCTAAGCATGAGCGGAGTTAGCGGC
GGTGAACTCAGCGAGACGAGTGTTGAAGTCGTCGGCGTCCTTCTGGAAGT
CCCATGTGCAATTTACTCTGGTGTTGTAGAGTTGGGTGCAGTTGGGGCACT
ATGATGGTCATGAGTTAGCAAATACATACTAGGGACGCGTCTAGAGAAAGG
AGTCTGAATGACGGGTAGGGAGTAGGGGGTTCCTCAACATACCGGGCCGA
AATCCTTCCACACGTGCGCCTGCATGCACACTTCGACGCGATCGCCATTGC
CGTAACACTTGACCTCGCCGCCGAAACAAGACTCATCCTTCGCAACGCCG

ACCTCCGGGGGCTCCTCGATCTCAGTCTCGTTCTTAAGCAAAGCGCGTGCC
AGAACATCAGGCACATCGGGTCCCGGCGTCTGCACAGCAGCGTGTTCCAC
GGTACCAGTCGAGACCTCGGGAATCTGCACCGGCGCCGTAGGCGCGGCAT
CCGCGTCGCCATGGGGGAGTCCTGCCTGTCGAGGCGAGCACGTCACATCG
CCGTGCGCGTTCTGGAAGCACTGAGCGCAGTACTCGACATTCCTCCACCTG
CGCTCCGTAGTGCACATCTGCATCCACCTGTCCTCGCACCTCTGCTCGCCTG
TCCTACACATCGGGTCCGGGTTCGGGTGCGGGAGATCCTGGGGCACGCACT
CGACTTGCCCGTCCTCGACCTCGTTGCACGCCATGCAGGCCTCCATCTTGA
TCCAGCGGAAGCCGTCCGCGCAGAACTCCTCCCACCCGCCGTTGCAGCGG
AACGTGCTCTTCTGGCAGGGGGATGGGGAGGGGATCGCCGTCTCGATCGG
GATGCAGGTTACGCCGCCCGCGTCGTCCGTTATGCAGGCCGCGCACCGCGC
GATCGTGCGCCACCCGTCGATCGAGTCGTCGTACATCTCCTCGACGCTGCC
GTTGCACCGCAGGTCCCCCGTCACGTGTTCCGCGTGCGAGGGAGTCGCGT
ATGTCTTTGTCGCGCTGGTGCTGCCCCGACGGGCCCTTCTAGGGGGCGAGT
GCGTGCTCACGCGGGTTGGCGGGGGAGCGGGACGGGGGAAGGGCGGCTC
GCGACGCAGCTCCAGGCGCGAGTCCACGCACCCGGCGTCCTGGGTATGCG
CGTCTTCGACGCAGTGGGGTGGGCGGGTTGCACTGCTTGTTGTTGGACCA
CGTACCTAGTGTGCTGCAGCGGTAGACGGTGTCCTCGTGGCACATCTTGGT
GCCGGGTGCGCAGGGCATGGCTTCCAGGTTGTTGCGGGAGAGCATACGTC
CCAGGGCTGGGGCCGAGAGGCTGGCCGCGGCCAGGAGGAGGTGGTTAAG
ACGAGGCATGTTGGATTTTACGGATATCCAATGAGCTCGGCTATGTGTATATT
AGATGACTGGTTAGGTCGAAAGCGATCATGTATTGGTGATACACGTAGGTA
GGTAAGTATGTGGACGAAGAGCAGAATTGCCTATCCAAGAGGATGATTGCC
AAAGAACGTGTTCCGGTAAGAGTGGAATTCATGATGAACTTGGGTCTTCGG
GCCAATCTTATAGAGCGAACGGACAGTATGATGACCTTCATGATCCGGTAG
GTTTGTGAATAGTTCATAGCAAGAACATCCTATTGCCATTACGCTTTGAGGT
AGGTGCTTGGTGTATATCACTATTGAAGTTGATGGTGGTCACATGCCGCTAT
TTTGGTGCTTGCTCTCAATATTATATCAGATATTATCAAAGCAGTGGCGAGTT
GAGATTAATCGGAATATAGGAGAGTCTGTGTAACTACCTACCCCTATCTTGA
AAGTCAAATCTTAGCTTGAGCCTCTATTTAGCTTCTATCTTTTGGGCTTAGGT
CTTATAAGATAGGTAGGTATGCCCCATTTATGACCTCTAGATATGTCAGATGA
TAATAGCGCGCCATAGCAACACTTAAAGTCGAGTTCTTTATAGATAAAATAA
TCAGTATACCATGCGATATATTGCCACATGTTACCAGTACTATAACCAAAGTC
TAGGGCTTAACCTCGATCAGTATTTCCAAGTTATATGAATTATGTTTCAGAAT
TTTGAACGTGAGATTTAGGTAAGTGCGCCTGCGCTCCGTTAGTATATCGCAA
AGCTAGGGGGTATTTTAAGCTTCAGTCTTAGCACTATCAAAGTAACCTTAAA
AGCCTTTTCACCATCTCGGATCTATTCGGCGAAAATAGGTACTTTGACTCCA
TATATTATTGTGCGCACATCCATAGCTAAGTTGAAAGGCAGCTTATGGAATC

GCTACCCATCTAAGCACTTTCCTGTATTCAGAGTAGTTTTGTGATATGATTCT
CGAGATCTAGGCTTATTTTTTGAAGTCCAAGGTCAAGGTGCCCGGCGAACG
AATTGAAATATAGATGAGTTGCCCCCAACATTTTCTTGTGGCGTTCTTGAGC
TGAGATTCTCCGAATGTTGTCTCTCCCACCATCTTTCGACGCAGGGAGGAA
TATCAATATTCCACCCCTCTCCTTCTTGGATTGCAAGACCCCTTCCCCTTACA
TGACTAATGGGTTTATTAAGTGGAAAACATTTGTATTTGCTCAAATTCAAAA
GTGAAAAAATATTTCTTCAATTGTACCTCCTAAGGAAATTTTGAGTGTACAT
TTTTTAATAGACGAGTCGCAGATGACATTGGTGTCATTTTCGTGCTCCCAGT
CCGGCCCTGAGAGCTGATGGACGGCGATGGTGCCGTCGGGAAGGCAGTCT
GCGATGATAGAGATGATATCCGCCGCGATCTGCTCCCAAACGGTTGATTTCA
GAAACCGCCAATGCGAAGCAATGGCTTTGATGAATACCAGCCGATCGAGAT
TTCATTGTATCCGAACTGATGAGCGCGAGGTTGGCCGCAGGAAAGCATGAC
GCAGCCCGTGCACTCGGTCTTGCTGTCGGGATCAGTCTCCCAGGACCTCGC
TCTTCGGTACCGAGCCGTCATGAGCGCCAGAGCACGCCATGAAACTCGCTG
TCGCACCTCGTGCTAAGTCTGCAGCAGGCGACTTGACGGAGAAGGCATTT
GCGGCCCGTGCGCTGCATGATCGACTTTGCGCAGCCATGAACGTCTTCGCC
TGCTCGAGTACGCGATAGAGGCCGTTATGAGCCTCTTCCAGCTCTCTCCTG
GATGCATTATGCACGCCTTCGAGTCGGGATCCGAGGTCTGACCGGGTTGAG
AATGGTTGTTGGACAAGCAGCGACGCAGAGGGGTTGGAAGATCGTTGACC
GTGCTCTGTGTATACGCGATGGCAGGAACGTAGCGCTCGCCCCGGTCCGCC
CTCCCGCCCCCCGCCCGCCCAGCCGGGGCACATTTCGTCTTCGGAGCGAG
GAGGGCATCGTTAAATGCGTCTTCGGCGGCTGCATCGGCGATGGGGGCCAT
CCAGAAATCCCCATATGCGAACCGCTTGCCTTTCCATCCTTCCAAATCAACA
AAGCAGGGCTCTGCAGCGATTTTGATGATCTCGCGAAGCTGCTAGCGATGA
AAAAGTCGCTTGATCTGTGGTAGGATGATGTCGATGGCATTGGCGAGACCG
TCCTTGCCATCGGTGATGATGACTTTGACATCGGTCTTGTGAACGCCGATGA
GCTTGATGAAGGCAGCGATGGCGAGCTCGCAGCACTGCTTGGGCCCAGGT
CATAAAAAGCCGATCGCTGCGGTAGAGTTTGGTAAGTCGCTGAAAAGCCG
CATATAGGAATCATGGGGAGGCAATAGCGATTCGTGCGAAAAGTACAGTCG
ATGAAGAGTACTGTGCGGTCCATATGCGCCATCGTATGGGCTGCCGGATTGG
TAGAGTTTCCCGATATCCTTTCGCCAAGCCTTGAAGAGGTCGCCGCTGTCTT
TATGGCGCTCGGCGGCCGCCTCTGTAGCACTCCACCCAGCCCGAATTGAGG
CTTCATCCTGATTGCGGTTGTTCCAAAGGTCTGCTCTCGCAACGAGTGCTG
AGGAATGTTCGATCTTTCCAAGGGGGACGATCTGGGCCACCGCGAGATGA
GCGGTGAGTGGCGTCGGATGGCTGCGGGGGTTTCGGGGGGTTGCACCTCG
TGTGGCAGGGATAGGCGATATCGATCCCTTCCTGCCACTCAAGGTCGTGGG
TGTTGTCGATAGAGTGTGGGAGCCAGAGATGATCCTCCAACCTCCTCCCAG
CCGCTGGTGCGCGCCACATTGTCTCCGGCCGCCACAGGACAGGCCACTGA

CTGTCCCACATGAATGTGTGGCTGTCAGCCGCCGTCGAACCCAGGAGCTTT
GCGTGTTTGTTGCTTCCTCAACCTCCTCTCACGCATGTCATGAGACATCGCC
CTGTAGCCATCCAGCTCCATGCAGTGACTGCACAGAAACCCAAACTGAGA
GCGAGTTGCCAGGTGTGATTACGAGTGGCGAGTAGGGATGACTGAGGCTC
GAGGGCTGGGAAGGGTGTGAAGAGTCGAGTTGCGAGGGATGGGACGGCT
GGTCGAACAGATCATCCTCCAGATCACTCTAGTCGCGATCAGACTGTGAGT
CCTCATCCGAGCGTAAGATGTCGCTCGCAGACTAGTCTTCGATTGCGCTGT
CGATCGGCAGCTTGCGAGACAGGGCGCGGATGTCTCTGAGAGGCGAGCCG
GAGGTGTCGGGTCCGATTCGACAGCGAATGTCGAGTGGGGAGAGGGAAG
AGAGGTTTTTGACGAAGGACCTGTAGCAAACTCAAGGGCTGGGTGGGCGG
CTGGCTATCAGTCAGCCAATATGTCTCTTCTGAGCAACAGAGACGTACTGG
CCGATAGCATGGGCTTTGTGTATGGTCACAAGCCAGGGTAGAAAATGTTTG
GTTGTGAAATGCGGTGAATGGGGCGATGTGACGGGGACCGGTAGGGGGGA
GAGGACAGACGGGAACCAATGATGTCGATCTGAGTAAAATCGGGTTGCAG
GAGAACAAGATGGGTAAGAGAATGTAGCTGGAGACGTGTAGAGAGCGTGA
GGGCAAGACAAGCGTGATCAGAGACGATTTTTGTATGATGTCCTGAAAGAC
AACACGACGACGAGGACGGTCTCCCACGCTTTTAGGGCTGAGCGGCATTT
CGGCGTCGAAAGAAGGTGAAACGCGGCATCAAAGGCGTTACTCACAGTAG
AAGTCCATGAACACCACCCATGTCTGAGAGACACCCAAGCATTAGGCATTA
GGACACGCCCGTCCACCTGAAACAAATGGGCATTAACTCAAAGCCACGTG
GCGAAGATAGAGTCCGGACGCGTTTCACGCAACGCACCATTGGCCGACAA
GTACTTGGGAGACTGGGCGCGCAACACATTCGGTATTCTTCTCCCGATTGTT
GTTCTTTGCAGACACGTAGCCGCCCGCTGCGTTCCGTCGCTTCGCTTGCCA
GCGACACCGAGTTCCTTGCCAAGCTCCCCACCGCGCTTCCTCAACTCGCCT
GCTCGTTGTCAGGACTGTGGCACTGAGACTGTGGCGGAATGAGACAAGGC
CAAGTGCCACACGGCCTGACTCAGCCAACCGCCCACTTGCCTCATCCGGG
TGAAGTAGCCGAACTGCTAAATGCGGCACTCGGACTGTCTAGTATAGTTGA
ACGATTTTCGAATACATATAGTAGACATTCGAGTAGAGAAGATCTCTAGTCG
AATGTATCTCTTGTACTCTCAGTCTACTTCTAGTAACCCTACTCGATTGCATA
CTCCCTGTGAGGCAACCTCGACTTCCCAGCTCGACGCGGACTATCCTACGT
CCGGTCTTGACACTCGTCGAGTTCACCTAATTTATCGCCAAGGCTCAACGA
GTCCCTCGCCAACGGTGCGGAGTACGTGCTCATCAGGCCCCGGACAGAGA
AGGCAGACGTCGTGACAGTATTGGGAGAAGACGGGCGTCTGTATGTGGAA
TCAGGGCCGGATCGGGTGCAGATACTGAAGAGGATCCATGGTGTCACGAC
GGCCACGCCCTCGACGACCTACGCACGTGACCGACACCCCTCACGTGGCC
GACACGTGACCTACCTCGGGCTGGAGAACCTCGACGAGGGAGTAGACGTT
GGATGAACGCGCGACGACGGGAAGCAGGCGCAGAGTCATATAGGATGCCC
CGGGCGCCCGTCGTAGTTCTTCAGCACTCTCTGATTGAGTCTCATGGATCAC

CATTGACGATCTCACTCTCTCCGTGGCACGCAGCGTGGCTGAGTCGCACTC
ACGCTGTATCCTCGACGACCTTGTTACCTCGACGACCTTGCAACCTCGACG
AGGATCCAACCTCGACGAGGATCCTACCTCGACATCCTCGTTTCTACCCCTT
TATCCTTTATTCTTTACTCTGATATTGTGCGAGAGGGCCTCTGCCAACGGGA
GAGCGCAGCATCGTGACAGTATCAGAGAGTGCTACTACCTCTTCGCACAAC
CACGCACATACCCCGGGGCATATGCCCACCGAGACTCGACGTTCGACATCA
CTCAACCCGAACCACGAGCACCAAGCTTTACCACAGTCCCTTCGACTCCCC
CCTGCTAGCCGGTCTAGCACACCCAACAACATGGCGGATCAGGCTTCCCTC
GCTCAGATCCTGCAACAGCTCGTTGCCATCGAGGCTCGACGAGCCAACTCC
CCCGCTCCCCTCGACCAACGCATCATCGAGCGAGCCGAAGGCCAACCCGC
CCGACACTTTCGATGGCTCAAAGCCGGAGAAACTCGAAGGATTCCTCATGC
AATGTAACATCGTCTTTCGGCTACAACCTTCGAAGTTCACAGAGGAGTCCA
AGGTCATTTACGCCATCTCCTTTCTCAGAGGCTTCGTTCAAGACGCTATCCA
GCCTCTCGCGTCGGGAGAAGAACTCGACGACAAGATCAGTAGCTTCCAGA
GGCTATCGAGATACTTGAGGGACAACTTCGGGGATCCCGACATCAAGGGCA
CCGCCCAGAGGCGATACTTCGACTTGAAGCAGACCGGATCAGCGGCAGAG
TACTTCGCGAAACTTAGGGAGTACCTGGCGGTCTTGGGATGGGAAGCCAA
CGAGCAAGCTAGAGCTAGGGCGCTCGAGGGCCTCTCAGACAATCTTCGAG
ACGAGGTTGCTCGGACCACAGTGGAGCTGGAGACCTTCGGAGAGCTTCAG
TCCTATGTCATCAAGTTGGACAACCGGCTCAGAGCAAGGGAGGCCGAGAA
GAAGGCCGAGGTCAAGCGGTACGAAGAAGCTCGACGACGACCAGCTACG
ACTACCCAGGGACAAGTTCCCCTCCCTGCCTCGTCGACCTACCGCCCTCTC
CCGCCAATTCCGTCGGCCCGCCCATCACCAGCCCCTAGTGGCCCGCTTGGC
TACAGACCAACGCCTCCCGGAGGGCTCGACCCAGCGGAACGCCAGAGGA
GGAATGAGCAGCGTCTCTGTTACCGTTGCGGCGGGGTCGGACACTTCGCC
AATGCCTGTGTTCCCGGGCAACGACCTCCCGTTCCTCGAGGCAGCCCCGCA
CCCGGCCGACCTATCATCAAGCAGGAGGACCCAAAAGCTTGAGGGACGCT
GAGAACATACGAGAGGTCCAGCGGCCCCATCAAGACATCCTCTCGGCTACC
TCCCCTCCCCATTCACCTCCCACCCCCCTGTATCACAATCACCTTTAATCCC
GAACCTCGACGAAGAAGCGAAAGATGAGAAGGCACTGGTTGCCACGGAC
GGAATAGCTAGTGATGAGGAGGCATGCATTCGTGGTGAGGTTTGCTGTTTA
GGCAGAGAGCAGGCTGTCGTGTCAGGAAGCGTCAATCAAGTCGACGGGGT
GAAGGTGCTGCTCGACACCGGGGCCACTGGTAACTTCGTCGACTCTTTGTT
CGTGGAACGGCATCACATCCCCTCCTACCCACTCCCTCGACCCCGGCAACT
CCTCCTCTTTGACGGTAAACCAAGCTCAGCAGGACAAGTAGAAAAGGGAG
TGACGGGAACAGTAAAAGTCCTAGGCGGTGAAGGAAGGGAAGAGTTAGG
TGTGACAACATTGGCAGGAGCGGACGTGGTGTTGGGATATGACTGGATGA
AGGAGAACGGTGTGATATTGGATGCTGGAAGGAAGAAGGCGACAAGGAA

GAGGACAGGCGGGAAAGAGACGAACCAGGAGAACGGACCACAGAACCT
CGACGAGGTTTCTCGACTATCAGGAACATTGGAGACCAAGGCGGAAAGGC
GACAAAGTAAACGAAGGAGCAGGCGATCGAAGGCGAACAGCATTGAGCT
CCCATTCAATCGATGGGAGCAGAACCAGGGAAATACAGTCGAGCTATGCGG
CACAGTGACCGAGGAGTTGAGCGAGAAAGAGGCATATATCGACGTCCTAC
CCGACTATGACGAGTACGAGCTCAGCCCAGAGGAGGAAGCAGCTGTGCAA
CATCTTGTCCCGAAGCGGTATCACGAATACCTCGAGGTCTTCCATCCTCGAC
GAGGGGTAGAGACGCTTGCTCCAAATAGAGAATACGACCTACGCATTACGC
TTAAACCTGGAGCCGACCTACGAGTGGCCCCGCTGTATAGCTTGAACCCAG
ATCAGATGGATGCGCTCGACAGGCTGCTGGATCGAGAGAGAAAGGCTGGA
AGGATTCGACCATCGAACTCGCCCTATGGCTCACCAAGCTTCTTCGTACCA
AAGAAAGCCCAAGGAGAACTGAGGATGGTTGTCGACTATCGCAAGCTCAA
CGAGATGACGGTAGCGGATGCATACCCGCTACCTCTGATATCCCAAGTGAC
CGACGACCTAGCCAAGTCGAGATACTACACGAAGCTCGACTTGCCGGGGG
CATACCAGCTGCTCCGGATGGCTCCTGGACATGAGAAGTTCACAGCATTCC
GTACCCCTCGAGGAATGTTCGAGTCACTGGTGGTGCGAGACGGACTTCGG
AACGCGCCGAGCGTGTTCCAACACTTCTTGAACGAAGTCCTGCACGAATT
CCTGGGAAAAGGGGTCATCATCTACATCGACGACATTCTCATCCATGCGGA
GAACCTCGAGGAACTTAGGAGATTGACTAAGGCGGTGCTGGAGGCGTTGC
GGAAGGCTGGACTGTTTGTGAAGGCGTCTAAGTCGGAGTTCGAGCAGACT
AAGGTGAAGTTCTTGGGCTTCATCATCTCGCACGAGGGCGTCGAGGCTGA
CAGTGGGTATGTCGAGGGTGTTCGCAACTTCCCTACCCTCGAAACCTACAC
GAAACTCGACGGTTCCTCGGTCTTGCGAGCTATTACCGACGCTTCGTACCG
CAATTCTCCAAACTCGCTGGGCCTCTCTCGCACCTCACAAGGAAGGATATC
GACTTCCATTGGGGAGATGGACAGCAGTGGGCGTTCGAGGAGATCAAGAG
GCGAATGAGCGAAGCACCAGTGATCGCTCACTTCAACCCGACGTTCGAGA
CTATCATCCAGACAGACGCCTCGGTCTATGGATGGGGGTTCATCATCTCGCA
GGTGAACTCCTCGACAGGACAAGAACACCCTGTTGCTGTCGAGTCGGGAG
CCTTCTCAGGAGCGGAGCTCAACTACACCACCACGGAGAAAGAGTTCCTC
GCGATCGTTATGGCCTTTAAGCGGCGTCGACACCTCCTTCTGCAGGTTACCT
CGACGATCTTGACCGACCATCTCAACCTCACATACTGGATGGAGCCACAGA
AACTCAGCCCTCGAGTTGCGAGATGGGTGGATACCCTGGCTGGTTTCTCAT
ACAAGATCGTCTATCGACCAGGAACCAAGGCAGCCTACCCCGGATGCATTA
TCGAGGAGGGCCGACTACATCCCACAGGGCAAGGACGCAGAACCAGAGC
TAGTTCAGGCCCTGCCAAGCTTCGACGAGGGGAGTACGGTCACATCCAGT
CTGGGCTACATGTTGCGAGCGTTGGTGGAAGACGAAGAAGGAGGAGAAG
ACGAAGAAGAAGGAGAAGACGAAGAAGAAGAGGAAGGAGAATTGAGGG
GACTGTCTCGAGGAGAATTGAAGGAAGGGCAGGGCGGAGATGAGGACTT

GAAGGATACATTGGAGGAGCTTGGGGAACTAAGAGAGGAGAAGAGGATTA
CGAAGGAGGAGAACCCGAAGGCGTGGTCACTGGCAAAGAGACTGGGCCT
CGACGAGGCAAGCAAGATCAAGTTCGACGAGGATGGTCTTCTCAGGATAA
ATGGTCGACTCTACATCCCGGACATCAAGCAGGCAAGAGTTAAGGTCATGC
GGGATCACCACGAGGGGCAATTGGCTGGACATCCTGGGGTTGCGAAGATG
CTGGAGCTGGTTAGGAGGGAGCACTGTTGGTTAGGGCTAAGGCGGACGTG
GAGAAGTATGTGCGAGGATGCGCAGTGTGTCAGCGGTGCAAACCGGTGCG
TCAGCGTCCCCATGGGCTGTTACACAGCCTGGAGATAGCTGACAGGCCATG
GTCCTCGATTTCCATGGACTTCATCGAGGAGCTTCCCGCCAGCTCGGGCTT
CAACTCGATCCTTGTCGTCGTCGACCGGCTCACCAAATGGGCTATATTCATC
CCTACGACAACTCGGCTCAACACGGCAGGCCTGGTCGACCTTGTCCTCGA
CAATGTGGTAGCGCAGCATGGGCTACCCACGAGCATCATTTCGGATAGAGG
AAGCAAGTTCACCAGCAAGCTTTGGGGTGCAATGTGCAGCGCCCTCGGCA
TCAAGGTCAGCCTTTCGACGGCCTTCCACCCTCAGACGGATGGGCAGACA
GAGAGGACAAATCAAGCCCCTCGAGCAATACCTAAGGATCTTCTGCGACTA
CAAGCAGGACAACTGGGCTACTCTCCTCCAGCGAGCGGCGTTCGCGTACA
ACAACTCAATGCATTCGGCAATCATGATGACGCCGTTTGAAGCAAACTTCG
GCTATGCCCCAAGGTGGGCAGAATCCATCGTCGTCGAGGGAGAAGCTCGA
GAGATCCCATCAGTCGTCGAAAAGTTTGCTTCGCTCGAGGAGGTGCACGC
GTTGTGCAGGAAGAATATCGAGCTCGCCAATGAGGCATACGCAAGAGGGT
ACAACGCCAAGCATCAGGAGGCCCCCGAGATGAAGGTAGGGGACCAAGT
GATGCTGTCGATGGAGAACATACAAACTCGACGACCTTCGAAGAAGCTCG
ACTTGAAATGGTGTGGACCCTATAGGATCATCGCAAAGGCGGGCAGCGCGT
ACAAGCTTGAGCTGCCCGACACAATGAAGGTCAATCCAGTCTTTTCGGTGG
ACCGACTCAAGGTATTCGAGCCACCGACGCTCCCAGGACAAACATACGATC
CGCCAGGGCCTGTGGAAGTCGACGAGGAAGGGGAATCATTCGAGGTGAA
AAGGATCATCGACTCGAGGAAGAGGAGAGGACGATTGGAGTACAAGGTGG
AGTGGCTTGGATATGAGGGGACGGATGAGGCGACGACATGGGAACCAGCA
GGGAATATTGATGGAGCTTGGGCGAGCATTGAGAAGTTCCACAAGGAGAA
CCCGGAGAAGCCAGGAGGCCAAGCAACAACCAGGCCTAAGTGAAGTAGC
GACAGAGCATAGGCTGAGAAGCAGTGAGCGGTGAGAGACATACATGCGGC
AAGATACAAGAGTCAAGACGGCAAAAGCAAGAAGCAAGAAGTAAGAGAC
AAGACAAGCTACGGTCAAGATGTTCCCTCGAGAAACTCCCCAAGTGTCAA
CTCGTCGAAGTTAACACCCAAGTCACCTGCGATGTCTCGCGCCCGTGACGA
TAAAGTAGGTACGCATCGGTGCTTGAGTCTCTCGCCGAGCGAAGTGGCTCA
ATTGAAATAACTCTTGCCACCGGCGGTCTGGGGCAGAGGTCGAGGTCACT
GGAGCGGGTGGTGGTGGTGTTGGCGGCGGTAGCTGCATCATGGGCGGGGG
TGGAGACCAGTGAGAAGTCCTTGATGGTCCGGCTTGAGGTACAGGATCGA

GTCCGCCATCCCCTAGCCAACCCGTGCCAGCGTCATCCATCGGTTCCGGAG
CAGGGGGAGGGAGCGGGCTCAGCCTACTCGTCGAGATAGGATGCCCCGAG
CGGAGTCTCTTGTGTGCCAACGGTGACGAAATGGGCGAGGACGAAGAGCG
AGCCCGACGTTTCTCTCTTCCCTTTCCCTTCCCTTTCTTCCCAGAAGCCTTG
ACCGTGGCCGCTAGGTCGATGTTGGCGAGGCCGGCGGCCAAGGTCGTCGA
GGTGGAGCGACCTGCGGCGAGCCTGCCGGAACGCCGGATGCCTGGGGTGA
CGGGCGAGGGAGACGTGCCGCGAGCGAAGGACACCTGACGGCGCCGTGC
CGATGAAGAGGGGCCAGGAGTTACCGTGCCGAACGAGCAGGCCAGGGTG
CCGCACGTTAGGCACTTCACTTTCTTGCCTTCCTCGACGATGCGACACGGG
AGACCCGTCCGCGACACATGCCTCGCATCGCTCATCCTCCTCCAGGACCCG
GATTGGCCGTGAACCCGCTTGTTGCTGAATGTCAGAGGATACCAAGTAGCT
GACAATACTCACCCGGGCCCATGTCAGAGATCTGTTGTATCCCCGTTGTCCT
CGGTCGGGATAGTGGGAGCCTACTGATCAGTTTGGGCGAAACTTGCAGAC
CTTACTCACCAAAGAACGAACGGTCCAATGGCTCTGACTCTGGGACCGGC
AGTCCCCATCCTTCCTTCACATAGTATGCCTCCTCGGAGGCAGTACGCGGGT
CAAATCGCCGGCTAGGAGGAATCTCCTCCTCGGACAAGCTCGACGATGCTG
GAGTGTCGGGCGCTAGCTCGTCCACACCAGCCTCTACATCCAACTCCTCCT
CCCCCTCTTCCTGTGGGACATCCGCGGCAGGCGCATCGCTGGGTAGCTCCG
CAGCCTGTTCGTCGGGCGGTGGGATGGCTTCGACGGCGGGGAGTGGTGCA
ACCTCGACGACTGTAGGGATTTCGGCAGTCGAGGCGGGTTGCGTGGGGGA
TGGGAAGGGGCTACCTTCGGGTTGGGCGGTGCCGGCGCGAGACAGAGATG
GGTTGGACATCGATTCGACACTGACAGTCTCAAATCGAGAATCGGTTTCTG
TGCAGAGTGTTACTGACCCGTCTGAGTCAGATGATGCCTCGTCTATAGCTGC
CGTAGGGGTTGTAGAAGAAGGATAGGACAGCGCGTTGTCTAGAGAACTGC
TGTCGAAGAGGATGTAGGGAGACGGACGGGGCGAGCCTGTGGTGAAGGT
GGATGCGTCGAGTGTTCTTGGGCTCGAGAGGTGTAGCCTGGGCATAAGGC
GTGAGTTGAGGTTGATGGTGGGGATGAGAGCGCTGACGCCCTCTCCCCCCT
CTGTTTATGTCGTTTTCGAGCATCCCGCGACATGGCGACGCCCTCGAGCAA
CACGAGGCTACCTCGACGACGGATTGGCAGTCCTTTCACCGTTTCCTTGAC
GACCGCTCGACGACTTGTCGCCAGAGTTCCGCTTCCGTTACAAGCGACGTC
ATTCGACTGCTATCTCGACTCCTGGATCGGCAATGCGGGTGTGCTCGACGA
GGACGAGGACGAGGACGAGGACGAGGATGAAGGCTGGAGTGGATGGAGA
TAGCGCGTGTCTCGCAGGAGCTCGACTTCAAAGGTGGGCAGAGTGTCACG
ACGGCCACGCCCTCGACGACCTACGCACGTGACCGACACCCCTCACGTGG
CCGACACGTGACCTACCTCGGGCTGGAGAACCTCGACGAGGGAGTAGACG
TTGGATGAACGCGCGACGACGGGAAGCAGGCGCAGAGTCATATAGGATGC
CCCGGGCGCCCGTCGTAGTTCTTCAGCACTCTCTGATTGAGTCTCATGGATC
ACCATTGACGATCTCACTCTCTCCGTGGCACGCAGCGTGGCTGAGTCGCAC

TCACGCTGTATCCTCGACGACCCTTGTTACCTCGACGACCTTGCAACCTCG
ACGAGGATCCAACCTCGACGAGGATCCTACCTCGACATCCTCGTTTCTACC
CCTTTATCCTTTATTCTTTACTCTGATATTGTGCGAGAGGGCCTCTGCCAACG
GGAGAGCGCAGCATCGTGACACATGGGGAGGCATGAGGCCGTCCTCGCGG
GACACCCGGGGGCTGCCAAGACTCTGAAGCTGGTGAAGCGCGATTACACG
CGGGTCGGTCTTCGGCGTGACGTGAAGGCCTATGTGCGCGCATGTGCAGTA
TACCAGCGGATGAAGACCGACCGCCACCGTCCTCATGGACTCCTCCTGCCA
CTGGAGGTAGCGGGCAGGCCTGGGGCGTCCATCTCCATGTACTTCATCGAG
CCCTTGGCAGTCTGACCAATTTGACTCGATTCTGGTGGTTGTTGACAGACT
GACCAAGTGGGGCGGTCCTTCGGTCTGACGACGTCGACGACCACTCACGA
GCCTCGCCGAGTTGATCGTTGATCACGTTGACCGTCAACTCGGGATCCCTC
AGTCCATCGTCTCCGACCGAGCTTCCAAATTCACCAGTAAGCTGTGGGGGA
GCTTGTGCAAGGCCTTGGTCATTTGAGTGTCTCTTTCCACGGCAGTCCATC
CACAAACTGACGGCCAAACTGAGCAGATGAAGCAGGTACTCGAGCAGTAC
CTCCGGGGTTTTTCAACCTAACCAGCAAACAACTGGGCTAACCTCTTGCCA
TGCGCGTTGTTCTCCCACAACAACTGGAAG

>Mosaic_sequence_29

CAATCCTTTTTTGCCCTCTGGGAATGGGAGGATCAATGGGCGGCCTTATCAA
TGCATTCCTCGTCGACAGGCTGTATGGCGCCAGAGCAGTCCAATTCTGCGC
CATTCTTAGCGTTCTAGTGCTCGGGAGTTGCAATATTCTTTGTTACAATCTG
GACCTTGTTTTCGGCTGGTTCGGAGCTGCTAATCACTTCTGGTGGTGGCATT
GGCGTTACCTCTTAATTTACTTCGTTGGTTATGGTAACGGCAAACTCTTGTT
CACAGATGACGGCCAGGAGAGTCTTGCGCAGTGGGGGGCTTAAGCGTGTC
TTGCTTCATAATGAGGCCATGGATATGTTGAAGAGGGAAATATGGGGTTTGG
AAACAGCTAGCGGCATTCAGGGAAGTATTTTGAGTGTCGCGTACTCTAGTA
AGATATTGGCTCAGTCTTGAAGGAACGAAAGACAATGATGATATTTGATTG
CCCATTGCATTGAAACACAGTTACCTAAGCATATTTCGTCTTGCCTTATAGA
AGTTGAGCTATCAGACATGAAGGGAATGTTACTGATTGAGAATTAGAAGGC
TATGGCCTGTGCTCTAGTGTGCTGCCGGGCTCCTAATCCCTAGCTGCTAACA
CCCTAGGACCTAGGTCCTAGGTACCTACCTAGTATTTACCTAGACCTACGTT
ACACGTAGATAAAAGAATCAAATTGACCTTGAATCAAATGCCTCAATTCATT
ACCTAGAGCCTAAATAGGTAAGAAATAAGTTCAAGTTCCTGTGAGATATTAC
TAGACCTCAATTTAAAAAGAATAAGAGAAACCACCTGGGCACCGTTTAGGT
GATTACCTCAGTGCAAATGAGTAAGCAATTCTCATTATATAAGCAGTACTCA
GCGCACGGACCACTTTCTCTGGTACCCAATGTAGATCTCTCATCATTTCCCC
TGTCATTTCCCCACCGAGTTCCGAGGCTTCTCAAAGGCCTAATTAACCCGT
CCTATCCCCCAGAGTAAGATTTCACCCTCCTAAGGTGATAGTAGATAACGCT

GTCAATTTATTACATCTCTCCTAAGATGAACCTAGCATCAATCAATATTTATTT
CTATGCGCTACCAACATGAACTCACCAAGTCTAACCGGATATCGGCGACTC
ACATTTCAAGAGATTATGAGCCTAGCTCCTCTGCAAGATGAGATAGCAGAT
GGCAAGATCACAAAGCGATATATGAGCCAACGGAAAGCGTGGTGTCCAATA
GAAGATGTGCCGATTGCGAGCGAAAAGGCCGATCCCGAACAGGATCCTGT
TCGTAATTCACAACCGCTGAGAGCGGCATATGGTGGACATGTTTATAGCCA
AGCAGGAATGGCTGCAACTAGAGCCCTGGCAGAGATACAAGCCTCTCAAT
CAAGCTCTCCGAAAGAACTCGGCATTCACGTAAGTCATTTCTATTCACCTAG
TCTGCAATTCACCTACCATTGTGTCATTTTAACGAATCAATCGATGTAACAA
GACGAATGAAGGAAGACCTAGAGTAACGTACGGTTCGTAGAATCCTCATTC
TGCCATACTATATCTCTACATATCCATAGATTATCCAACGACCAACAGTCTCT
ACTAGTCTACCTGCCTAGCCCTACCTAGGTAGTTGTTTACAAGTTTTGCTGA
CAGATATAGACTATCCATGGATATTTTTCCGAGGCTGGGTTGATTGATAGGCC
CTTCATTTTCAGCGCGACTCCTATCGCAACAAATGTATCTTTTCCAAATCTTC
TTGTTACAGTGCGGCAGCCCCTATCACCAAGCACAAATGCTACTGGGGACC
ATTATCCACTCGCAGACGCCACCCTGCCTCTCGGTCCCGTCTGCTTCAGCG
CCATTGTGTCATTTAGACCGCCCGGGATCTCGCAACATATTGCCCAGGAACC
GCCTGTTCAAATGAGATTTATGGATATCCTGAATTCTCGACCACCCTCTGAC
TGGGATCCTGCGCCACATGTTGATATCGCCAAAAAGCTAGCTGAATTTCCA
ATGAAAAATCCAGGCTCCTTCCCAGCCGTTGACATGAAAAAGGTCGATCTT
ACTTCATTTAATAAAGGCAAATCCCTGTGCGACCGCCGAGAGCTGCTGTTAT
ACCGGCTTCTTGCGCCACTACCCATGGATCACCCGGACTCACATATCCTCAC
TCACGCCTACGAGGCAGACAGGAATGGGCTATTGATGGTAGGAAACCATGT
GGGTTTTGGCTATGATTTCGCCCGAGCGGCCAGCCTAAGCTATTCCTTCGTT
GTGCACGTTAATCCCGAACATGCGGTCATGGATTACGGTGAAAACGAGTGG
TGGGTTCAAGAAGTTTGCTTCCCCAGGATTGAGGCCGGTAGGGCTATCGTT
ATGAGCAAGATTTGGAGTCCGAAGGGTATTCACGTAGCCACGGAATATCAA
GACGGCGTAATACGACGTAATTGGAAGCCTGAGGAGAGGCAGGGTAAGTT
ATGAGGTATGCCGTCTTCATTAGATCAAGATTTAGCACCTGTAACGTTCTAG
GTACCTACCTACCTAGTAAGAACATTATTAGATAACTGAGGTACCTGTGTAG
TATCTAGGTGGGTGCAATTCTATTGCACGCAAAACAAGACTACCTTAAATCC
CAGTAGCTTTGCTGCTCTCATCAGAACTTGACTCTGCACGTATATCCTCTCC
AATCACGAACGGATCCTCAATTACGGCCTTTTCAACCTCGCCACAAGCGTC
ATAACCGAACAGCCAAGGTTGAAATTCGGGACAAAGTATGCGAATTGGAA
ATGGGCTCCGCTTGCTTTCTTCCTCGAGTTGCGACAAAAATGTTTCGTCTCG
TTCCTGTTGCTTCGGGCCATCAACAATCTGAAAAATGTCGCGCTAAGTTTTA
TCAGTAACCGCAAATAATTATCTTACGAGCACGGCGACATTCAGCTGCTTAC
CTGTATGAAAGTTTCCTTCACGATGGCATCTCCACGGGACTTCCGAAGCTT

CTCGTACATTTCCAAAGCCTTCCCAATTTGCTCTTTCCTCTGGACATATTTCA
GCAAGCGACCAAGCACGACGCCGTCTTCGATAGCAGAGTTAGCTCCCTGG
CCGAGATATGGTAGCATGGGGTGGCAGGAGTCGCCGCTGAGATATGATATTA
GTGAGACCAATGAACATTCAAATACACAAAAGCGACCTCGCAACTCACAT
GAACGCAAAGTTTCCCTCTGAAGAAGGATAGACCCATTTTCGCATTTCCCT
ACCTAGAAAAGAGATTTCGTATTAGAGCCTAATATTTCCTAACAACTACATG
AAATGAAGAGACTTACGATGCATAAGTTTCCACTTCTTGGCAGAATCGGCG
AGAGCGATGAACTTGGTCAAAATCGGATCCCAGCCCGTAAATAGAGCTCTC
ATTTCCTCAACTGAGCCTGCCTGTTTACTCACGCCTGCAGGGAGATCATCA
GGTACTAATAATACGATATTGAATTCAGTTGCCCCCCGGAGTGAATACCCAA
CAGCATGCGAGTGAGGCCCCAGCCATATATCAAGTGTCGGCTTGCTTAACC
AATCCCTCAATTCGGGATCTTGAATTTGATCTAGCGTTAATACTAGGCGATAT
GCCAAATCGCCGGTAGGTCTAGGAACCCCTTCAAAGTCTTTTGGGAGAAA
CTGGGCGCGGCAAAGAGACCACAGGCCATCAGCTGCGATAACGAGGTCCC
CTTTCGCACTCAAGCCAGTGTGGCTCGTAATTTCTCCTGTTTCGAAGTTTAC
CTTACTTACTCTCATCCCAAACTTGAACTCCACGCCAAGTTCCTTGGCACGT
GTGTAGAGGGCAGTTTGGAAGTCGACACGATGCAACTCAATGAAAGGGAC
ACCATATCGCGTCCGCATTTTTGTGTCAAAGTCTTTCTCCCGAACTAATAGC
TCGCCCGAATAACGCCAAACCGAAACTGAAGTTGGTTCAGTAGCTGAAGT
CCATAAGTCATCAGAAAGGCCCCATCGCCGTAATATTCGTGAGGCGTTTGG
AGTGATATTTATCCCAGCTCCTATCTATAGTTGTTCCATGTAAGTATTACATCG
CACTCATTACTTTCATGTAAATGGTATAAGTCAGGTTACCTCATGTAGTCCCT
CAGCTGCTTCGAAGACAACAACATTATGGCCAGACAGAACTGATTCTATGG
CAACTGCCAATCCGCTTATCCCAGCCCCTACAACAAGTATGTTCAGGCGTAT
CGCTGGCTGAAAGTTCTCCCCCTATTTCACATTAGCGAACTCAAATAATCAC
TTTATTACAAAACAAGGACAACATACCATATTTGATGCAGTAGTACTCCAAT
TTCTAGATCGTGCCTACTATCGAGGACCGTCTACGTAGGTAGATAAGTAACA
ACTATCAGACCTGATCCAGGCTTCAATAGGTGCTTCACCAAGACGAACAAC
GCAATCGCACTATCCAACGACAAGATAACAACAAGATAATATACAAATACTT
ACAGTCCTACAACTTATTTTTTTCTCTTGTCGGATGTGTCGGATAATATACA
GACTGGACTCCCCCGCTTCGCCGGGTAATTCTTATGTAATATCAAGATGCTG
ACCTGTGCCACAGCGGCACGCAAAGGTACATAACTTCGTTTTCTCAGTATC
GCCGGCGGTCCCGACAATAGACGCCGTCGTATAGCAAACCGATAAGAACAT
CGATCCGAAAATACTGAAGAAAGTGTTTATTTACCTGTGTCCTCAGCACTTT
CGAAAGTAAACAACAGAAACTGCACACCATGTTCCCGCTAGGTCGCTGTT
GCTCGATTGATCAACCCCCGATAAGTCTAGCCATGCACGCTCTCTATCGATC
GCTTTATACAGAGTAACAGGGAAAAGAAACTCCAGAGGACATTCCATGACT
AAATACCAGTCGACTTCACCTCGAGGTCCTTCCATGTCCATTGAGATTTCTT

CATGCTGCTTATCATTAATACCCATAGTAACTACCGTGTTGCCCCACAATACC
ACGCCACCGGATGCATGGACGCGAAAACAGACGTCTCTCTTCATGCTTTGG
TAGAGAGCTAGGAATCGAATACTCTTTGTCACGAAACCCGGAATGATCGGG
ACTTCTTCGACGATCTCGTAAACCTTCAAGTCTGTCAGATCTCAGCTGCAG
TCGAAAGAATGGTCCGCGTAGATAGATTTCAAATTCCGGTTTATCGTTGGCC
CTTCTTGGAACTTCGTGATGAGGGGATATTGACCCATTAACGGTACATATCT
AGTCTGAGCCGTGTTGATAATTGCCTGGGTCGGGACCCTCTCGATCGCGGG
ATTTTTGAGCGCATTGTCATGCCTTTGACCGAGTTGATATCGGGATAACCAT
CATGCCCACTCTATGTAAGCAAGAGTCCGCCACTTTCGCTGGCGATATAGG
AGAGCTTTCAGGGATTCGATCTTTACCCCATACGAAGAAGCGAGGATATCG
TAAGTAGCCATGGCATGACATCATTCAGCAGAGCTGTTTGGAGATACAGGC
CTTGCTATGTTTAGACACTAAGCCTTGAGTAGAAAAGAATTATTGACACTAT
CATAACTTATATCATCCATTATAGTAACAGAGATATTTAATTTTTTTCGGTTGT
GATGGGGCAACGAATTACGTGAATAGGTAATGTTAGGGATTCGTTGAAACT
TCAAAGTTTCAACAACTTAACGATCGCTTACCTACTATAACAACTGCACAAT
GTATATGATTACCTCAGGTCAAATTCTAGACTCATTGTAAAGTTGCTCTTATA
TTCTGCCATATAATGTACGATATCATATTGTTGAAGTTATTAACGTGTCCATCA
AGATGGAAATTCTATCATTTTTAGCTACTAGACGCCTGGATTGGTATACAGAT
AGGGGTATGTCATAACTTTATGGAAGAATAATTAAGGTCAATCTTTCCAAGG
TGAACCTATACAAGTGATTTTTATACTATAAATATGCTTAAAGGGGTGGATAC
TCAGCGACTGTACTTAACGTAGTTTTTCGCATCACATGCCTAATTCATATACC
ATGCATACTAACTAGGGACATTCCGCTTACCCCTATTCGTCTTGGCCCCTAC
CCTTGCGGCATGGTAGATGTAATACTACATTACCTGCCTCAAAGTAGAGCCG
TCAAAGTCAATCTTCTCAGAATTAAGCTTATATTATCACCGTGGCACATCCG
AGGAGGATCTGTGCCGCCGTCTTGCATACCTAGTAGTCATCCAGCTTATCTG
TATACTTTCTCAACCAAGCAAATTACATACCTAGGCATTCGATTATATGCTGG
ACCCGTTTATATCATTGCTCGTTTCTTGAATCACTTGCATCACCCTTATATCTA
TGTAGTTCCATTGCATGCTTACTATACACATCCCCGACTAAATCCTTCCGAG
ATGGGTGACAATATCAACAGCACCAGTAGTAGATTACTTATTGCAGTTTAAG
CATGACTGCTTCCATAATGCCACCAATTATTCTAGCACAGTGACAGAGCTGG
TGAGTTTTATTAGCATATAACTTCATAATGCAAACCCAACCACAGCTTTGAT
GAATCAGCTGACATGCGTGTGTTGACATCGACCTAGCACAAGCAGGAAAG
TTTTCCGGAGATCATGCTTCTTGCTAGATAAGGGGGGGCGGTTACGACGGA
CTAAGGTTCCGCTCGGATTCCTTCTTGGAGCTGGTACATTGTCGAGACGAG
GATTGGTAGTTGGATTGAATATGATTTGATCATCGGTAAATCCAACACAAGG
GTATCATTGGTTACCTAAGTAACTTACAAAACATTTATAACCCAAGTTGGAT
CGTAGGACTTTGCTCGGACACCGAGGTCGATGAAAGGATATACCTAATACC
TAAATCTCTTCATCCACATGAATTCTCTCAGACTGCATAGTCTCTCCAATGG

GTGAGCATGCTTGTGTACAGAACCCTCATTGGTCCTTAGTAAAGCAGATTCT
CCGTACATATGATCTTCGTGAACGGGGAGACCTAGTCTCGGATGCTTCCGAT
CGATCCTAAAGAATCTTAGCCGGATCCATACTCCTCGGAGTTACGGTAAGAC
ACATAGCGCAATTCGGCATGCATCTCTGACCCCTGAATAGGTCCCCGAATAG
GGCACAATGTGGCCTTATTCTAACGTCGAGCTGCAGTTTGACCTCCGCGGT
TTCTATAAGCATTTAATTGGTTCATCTCTAGGTAGGCTTTTTGTTTTCCTCTA
GGTAGTTTCATATTATCTTCTAGAATCATGTGAAGCAAATCGTAACCTTTCAA
GGCTAGGTACTAATTATCACAGGACAAAGAAGGACATAGAAAGTCGGCATG
TCCTAGATAACTGGGTCCTACCTATCCTACCAGTTTTACTAGTTTGTTGGGA
GTCCGAAGATAAACCCTTGTTTCATCTCGACGTTTGTTATCGAACGCCACA
ATGTAGGAAAGACATTACTTTTTAGCTAACGACATTCAGTTTGGGGCCTTTG
CTAGAAAGATTGAAGGCCTTCGGCGAAAATCCTCACATGGTTTCTTTCTTCT
ACACGCGCCGCTAATGTTTCTTTTGAGACAGGACAGGACCACGTAGTCGG
GTCATCAAAGACCACCAAGCTCGAGCTAGTAATGTCTGAGGTGTAAAACCT
CATTGGCCTGTATAGACTACGACGTTGCTACGTCTATGCTGCGGTCTGCAGA
GAGCAGTAAGCATACGTACCTTGGTGCGACCACAGGAGAGCCTCATGTCG
ATACTCAGATAGACTCATATGCAATCATTTTTAATGATTACGACCTATAGATG
TATCTAGACAATTTAGACGATAGGTCACTACCTTCCCTTCCTAGGTAATTATA
ACCTGGTCGATCTAACCCTCTTCCTGCCAACTTAAACCTACTACTCAGATTT
AGTGTGAGATCTTGAACCAGGCCATGCAGCGGATGTACGACGTCGCATGCG
GAGCATGTCTGCATCAAATACAAGCATCGCACCGGCCTAGACCCAGACGAG
AACCTTGGAAGTGTGATCGAGGGATCACTTAGCTTGTTCCCCTTTTCAATG
AGTCGGCGGTATTCTGACTCCAGTTTCAACGAGACGCGAGTGTACCTACCT
ACTGAAGCGAACATCGACTGATTACACGCAATCTCACAACAATATCTACAA
TCATGTTGCCGTAGGGATCTGCAAAGATTGTAGATAAGTGGAGTAAATGTG
GCGGCGCAGTATATACATAACAGCCCCACACCCGTTCGACGAGATGGAGCG
CTTCGTCCTGACCTCCGCAGGGCGGTACGGCATGGATCTGTACCGTTTCGG
AGGGGGCATGAAAGGTGCCCTGAACGCATTTTTGGGCTGCACCGGGGGGC
GGGGGGTGAAGGGGGTATTGATGGGCACACGGCGCGGGGATCCCAATGGC
GGCAAGTCCCCTGCCACCCACAACCGGGGAGCTGGCTGATCAGTTCTAGA
CGTCGCTGAGCTCGCGCCGACCGACCCGTCCTGGCCTGCATTCCTGCGAAT
TCACCCTCTCCTTCACTGGACGTACTCCGACATATGGGACTTTCTACGCGAG
CTCGATGTGCCGTATTGCACACTGTACGATGAGGGGTATACGTCTCTCGGGT
CGACCACGAATACAGTGCCGAATCCCACTCTCAAGAATGGAGATGGGTACG
ACCCAGCCTGGAAACGTCAGCTGCCTTCTCGACCTCCACGCAAGCTGACA
ACGCAGTGCAAGACGGCAGTAAAGAGCGCTGTGGGCGGCTGGACAACTC
GTCATGACATCTATCCTGACACTTTCAACGACCATGCATGCCGCGGCTGTAT
AACGCACGGCCACTCTCCCTCTACATTAATGCCAGACACGTTCCTGTTTGC

GACCGTTCTCTGTGTTCGCCTTTGCGCTTGCTTTTGCGTTTGTTTGCCCATG
TGATCGTCGTTCTCCGTGTTGCTGTAGACTGATGCTGATTTGAATACCCACC
AATACCCCAAACATATATACACTTGTTCCCAACATCCCGACATTCCACTTCC
AACATGTATCGCTCGCTCGCTCACTCCTGATCTATCCCTCCTAATCATGGCTG
CGACTGCTGCTGCTTTTCTCTATTCCCAGCTACCACATCCCCATAGCTCGCG
CGTCTCTCGCCTGCCCAAGCCACGTCCAGGGCGCTCGTGCTCGGCCGCAA
GTCGTCCAGGCTAATCCGACTAGACTTTCCCTCGTTTGGCCCCATAATCGCG
TGAGCACTGGCCGATACCGCCATGCCGCCGAAACCGTTCATCCTCGCCTTT
GTGGGTGTAGACGGAGAGTCTTGCCCTTCTCCAATATCGACCGACATGCCT
TGGGTCAACCGAGACAGCTCATCAACATCGGTCGTAGAAGAGGCCAGTCG
GTCCTGTGCATTGAGAGGAGACGCAGTCGACCCTGGGCGACTGGGCGTCG
ACTCAGCAGAGAGGGTTGAGAAGTAGTCACCCGCGCCTCCAAAGGACTGG
CTGCCGACTGCACCTGCCGCTCGCCCATGAATCGGGTTGTTCGGGAAAGCC
TGAGTGGTCGGCGTTCCGAGCTGGGCCGATCCGCCACTTGATCTCTGAACA
GGCGCACCCGGCGCCGGGTTGAGGAGAGGGTTGTAAGACATCGGATTTGG
CGAGGTGATTGGCAGACCAGGCGAGAAAGGCCCAACTGCACCTGGCGAC
AAGAAGCCTTGCGGAGCCATTGATGGGGTCTCAGGATACGCCTGAAAGAC
GAAACCAGGCATTGACGGTGTCATGGGCGGCAGGCCCCGTGTTTGCAATG
GACTGAGAGGGTTGAATGAATCCATAGGCTGGAAAGAAGGCATCGTGATC
GGTCCCGGATGGTGGTTGTGGCGGAAACCGCCAATCTGGGGAGTACTATCT
GCGTACTGGGCCGTCGATCGACCCGGCGTCTCTTGCTCTTGTTGCTGCTGC
ATTGACGTGTTGAGGGACCACGGCACTCTCTGCGAGACAGGAGATACACC
ATTCGCAGATTGCCCGTTGCGCGGCTTGGCCTGCGGTGTGCCCTGATCTTG
ACCAGGAGCTGGCTGCCAGGATCCTGTTGACTGAGATGCGCTGTTGTTTGC
CGATGCTGAATTCGCTTGGCTGTGCTCGGAGGAACGCCGACTGGCGTCTCC
TACTGATTGACGTTCAAGATGAACGCTGATCTTCCGGCCATCTATTTCTCGC
CTATGTGGTCAGAGATCATTTCAGATGAGTGGTGCAAGACATACCCGTCAA
ACATGAGCGCGGCATTGACAGCATCGCCCTCGCTAGCGAAAAGAGCCGTT
CCATAACCTCTGGATCTGCCATCGGGATCCGTAGCAACACTAACGGTCAGC
GCGTGTCACACATCAGTTGGATTGGTACTTACTCCCGCCCGGATGATGATAC
CAGCAGGGCGCAGAAAGTCTTTTAGATCCTGCCACTGCATCCCCACCGGAA
GCTGCGGATCAGCTGCATTGGTCACCGGTCTACTAGCTGCTCACATTGGTC
ACGAACACCACACGGTCGTGGAGTGTGGTGGGCGGGCCGAGCATAGCTCC
CTTGTTGACCAGTCCCTCCATGCCAAACGTGTGAGCGAGTGCAAGCGGCG
GTATCGGCAAATCTCCCAAGGACGAGGACGTCTTGGGACCATGACCAGAC
TCAGTGGGCGAGACACCAGGTCGCCCAGCATTGAACGCGGGCGGCATGCC
GCCAATATGAGATGGCGGATGGGCTCGAGAGGGTGCAGGCTGCTGAAGGG
CGGGATCCGGACGCGAGCCGGCATGGCTCTGCGCCGAAGCGTTGCGAGAC

ATGGGAAGGGCAGCTGGCTGCGCTGGGGACGCGCTGCGAGGCAAAGACG
TAGTGGACGCATTTGGCGAGATGGACTGGACAAAAGCCGCAAGAGACCCT
CTCCGAGGCTCGGGTCCCGCTTCTCCTGTCGGTCCAGCTGTGCCAGCCGAC
AGCGACCCGGCCAAGGGCGACGCACCGATGGGCTGGAAGGGATTCGACCC
GGTGCTCTGGCCGTTGAGGGGTAAGGGCAGGCCTCCCGGGCCGAATGCGC
CTGTGGGTGAATGCGGGCCGACTGGCGAGACGCCCGGGCCGACCATGTGA
GGCGGGTGAGGCGAGAGGTTCATGCCTTGCATCGAGTGGCCGCCAAACAT
GTGTTGTGACTGGTGACCCTGGCCAAACGCAGGCATGGGTCGCGGGGGCG
GTAGCTGGCCCGGTAGCCATGCGGGCCCGTGAGAAGGTGTGAGATGACCA
GGAAGGGAGAAAGGGGCGTGCCCGCCGCCGGACATGTTGTGGTAGGTGTA
CATGGCTGGCCGCATGGCGGGGTGGTGGTGATTGTGATGGGGCTGTGCTTC
GTATTCTGGCGGAAGGCGGTCCGGGCGAACCTCGAGGGTGCGAGTCTGCC
AAGTGAAGCCGTTGAAGCGATCTTGCGATGTGAGCTGCAGCGAGACCTGC
CAAGAAAGAAGCTCACCTATGGCCCTTGCCGCATCTTCCCTGCTCCCCATC
AAGACGGTCCCGTAGCCCCTGCTGCGATTGTCCGAATTGAGACTGACGTCG
GCGCGCAAGACGGTGCCAGCTTTCCGAAAGAGATCCTTGAGATCCTGCCA
ACGCACACGATACGGAAGCTGCAAGTCAGCTGCAGACAAGGGCATGGAAG
AGGAGCACATACATTGCCCACGAAAAGGTTGACCCGGCCGTCCGAGCCCA
CAGCGGCCGGAAGGAGGTTGGTCATGAGCGGAGTAATTGATCTGTTCATCA
CTGCTCGTCAGCGAGTGCCCAGATTACAGAAAGAAGCAGAAGCGTGCCAC
TCACTATCCATAGGAGGCTCCGGAACGGCGGGAGGGGCGGGGGAGCATTC
GCCTCGGCGTTCGCTATGACAGCGTCGATCTCGGCCGAGGTGAGGGGTCGC
GAGGGGGAGTGTGCGCGCGACTGGGAGTGGGACCGTGACTGGGAGTGAG
AGCGCGAGTGGACCATGGCGAGGGGGACAAAATCGGACGCAGGGAAGCC
GAAGGGGGGTGGAACGGCAAGTGCATTTTGCTGCTGCTGCTGCTGGGGCA
GGGGTATCTGGGGGTTCAGCAGCTCGCTCAGCACGGCGAGCTGGTACGGC
GTGAGCGACATGGCCATGGCCATAGAGAAGAGGCAAAGGCAAGGTGGGTG
GGGAGGGGTGGGTTGAAGAAGGAACGGGACTCGCCTGTGGACCGTCCGC
CTGGCCCGTCCCTGCGTGGTCGGGTTCGTGTTCGTGTTCATGCTCGTGTAG
AGGTTGAGGCTGGGGTTGGGCCGGTACCGCCGGCGGTGGGGTACCGTGGC
CGTCCTGCGCCTGCTGCGGCGGGGGCAGCGCCGGCTGGTCGTGCTCCTCG
CCCGGGTCTCCGAGCTCCGGGGGCGGCTCGTCCCCCTGCATACTGCGGCCG
GGGCCTCAGAGGAACAGGCAGGCAGGCAGTGGGCAGGCAGGAAGGGAG
GGGGAGGGGCGTGCGAGCGGTGCGCTCGTCGCGTTGCGATGTGTCCGGCG
GGTCGAGGCGAGGTTGATGGGGTGGCGGAGAGGAAGAGGGTCGGCAATG
GTCGCAAGGCAAGGTCGGAAAGGGAATTCTGGACAAAGGTAAGAGGAGA
GTGTCCCCGGTGAAGAACGTGGCGATGACGTGCGTGCTGGCTGTCGGGTG
GTGGTGATGGTGGAAATAGATGAAGGGGCCGCGAGTGAAATGCAGGTGAA

GAAGGCTATGCTGTACGACTGGAATGGCGTCGAGGGTGCGAAGTGGGTCG
GAGAGAAGGGGAATGAATGTGGGGAAGGGAAGAGGGAAGGGAAGGGTC
GAGCCCGAAAGCGACCACCTCGGGACGTGGTCGTCAATGCCAGTGCAGTG
ATGCTCAACGATCTGATGACAATCAAAGAAGACGAGCGGACACGTAGATG
CTATTGTGCCGTCTGCGTCTGTCCAGTAACGACTAGCCAGCGCCGCATCTTC
GGCGATCTCACCTTTGCCAGTTAGAGAGCCGCATGGTATGTAGATGGCAGA
TGGCTTCAGGAACGAACGGTGGCGCACATCTCAACACGCTAACCGCATCG
CTCTCTCTGCTCTGAACAGCCGGGCCTTTCCGGGGCCCGATACAAAGCCGA
AACCTTTCATGGCTACGTGATAGACCCGTTGCATAATAGGTACAAATGCTGC
ATGCCGCTGCCGCTGCTGTTGCCGCATTTCCGTCGCCGTCCCCCAAGCCAT
GCACATCGCTGGCTTAGGCAGTGTCGACGGTCTTGCCGGCAGTACCGCGCA
GGAGACCGGTACGGCGGGCAGCAATGAGACCGGCTTTTTGACCTGCGGGG
GCGTCGCGGGCCATGGTCGAGGCGTGACCAATGTGCTGGTGGTTACCACC
ACCGTGCTGTCCGTCAGCCTCATACAGGAAAAGACAATCGGGGTGGGACG
GACCGGGTGGTCGACGGGGTTCATGGCAACACCACGAGTACGGGGCCACG
AGTTCCTCTTGGCCTTCATCAAGTGGAACTTCCTGCCAGCCTTGAGGAAAG
GCTTGTCGATACGGCCGCCGCCGGCGACGATTCCGACGGTCGCACGGCAC
CGCGAGTTGATGACCGCCTTGGTACCGGAGGGCAGACGCACGCGGGTCTT
GCCGTCCTCGCCGTGGCCGATGACGGTGGCGTAGTTGCCCGACGTCCGGG
CGAGGGCTCCGCGGTCACCGATCTTCTCCTCGACGTTGCAGACGATGGTTC
CTTCGGGGCATTGGGAGATGGGGAGCACGTTGCCGACGTTGAGGGTGGCC
TTCTTGCCGCAGTAGATGAACGAGCCGGTCGAGATGCCCTCGGTCGCGATG
AAAGTCTCCTTGCGGAGCTTGTAGCGGTACGGGTCACGGAAGACGACGGT
CGCGAGGGGAGCACCACTGGTCGTGAGCACTGCGACCTCTGTGCCTCCCC
ATCCACCGATCCACCGGGCTCTCCATCGCGCACACATCTCCTCTCACTCTCC
CGCCCACTATCCTCCTTATTCCGCCGTGTCTTCTCTGCCTCCCAGACTGAAT
CGCAACTCACCGTCCAGCGTCGTGGATAATGTCCCGGACAACACCGCGGAT
GTACCCGTTCTTCTCCGCAAAGTCGAGGTTCCTCAACCGTGCCGGGTTCTT
GTTGTGGTGGGTGTGGGACTTGAAGATCCCCCCGGACTTTCTTTGGGCGCG
GATGACTCGCTAGGATGATCAGCACCGCCCTTCTGCGCTACTGCCGCCCCA
GCATCTCCCGCCGCTCGCAAGTTCTCCCTTCCTCGCCTGCTTGACATCCCTC
TCAGACGCCATGTCCAGCTCTCATCTCCCATCCCCACTTCCTTTTCCACAAC
TCCCCCCAGCGCGCGTAGGATAGGGAAGAGATGGCCAAGATCGAAGACTT
ACACCCATGGTCTGGACGTCAGTAAATACTACACTGAAGCAGCAGGTATGC
TCACGACTGATCGAGGTCTTGAAGGTTAAAGAAAGGCCTCTAGCTATGCAC
GACTCTGCACCCAAAAGGCAGGACCGAGAGCCAGGGACTGGAGGAAGGA
GCGGAGTTTCGGCGATATGACCCATGGCGTTATTTCCTAGTATCACTCTTAA
CTCCGATAAGGTCGGCGATTCCCCTTAGGCAGCTCCAGCTGAGCGTTTGCT

TCGCCGCCATGATGCATGCAGCATGCAGCGCCTCGTATCCCGTCCTCATGAC
CGGACCCTATGCGTACATATATTGATACAAGTGACGGCCTCATCCCCGGCCT
CCCAGCTTCCTGCCGAGCGAAAACCCAAGCCGTATCCGCTTCGACTCCTTC
TCTTTGACCGGTGTGCCTTCCCGTTTCTCTGTCCCGCCCTTGCCGGCAGGC
GTCGGTGTGGACGGCGCAGACTCTCTCGTCTTGCTCGGTATTGGTGTCGCT
GGTCGTTGTTGTTGGGCATCCCGCGCCTTCGCTCCGACCAACAACCCCGCA
AATCCCTCGTCTTCATCGTCCTCTTCCATCCTCTTGCGCCGCATGCGCATGG
CAACCTCCCCTAAATCCGTCTCTAGATCTGCAGTAGCCGCCGCGGACGTCG
GTGCAATGCGTGGTGATGCTGCGCCAGAGGACGAGTCGGAATCTGATGCG
TCGTCGTAATCTAGTCCCAGGGCTGCACTGACAGACGGGCCACCGGACGA
GGAAGTGGATGGCGAGAGCGCCGAGCCAGAGTTTGCGCTTCGGCGGCGGC
CCACCTTGTGCACGCGGTCGCCGTTTCGAGGGAAGCATAGTCGTCGTAGTG
GGTGGTTTCGGACCGGGCTCTTCGTCGTCTGAGGCATTGAAGTAGTCCTCC
TCGGCAGCCTCGGCTCGTCGGGCCGTGGACGATTCTGCCTCCGGCATCGTG
AGCAAAGGCGCCGGAGGTGGCTCGTTGTTCTGTTCCCAGCGAGAAGTGAG
ACGGACGATGAACCCTCGGAGATACGGCCGAGACACGAGTGCCTCTATAC
GGGGTCGATAAGAATCGAATAGGTAGTTGATGATCGGCTTGACATTCTCCTT
CCATATCAGCCTTGTTACCTCGAGCAACGCGCGGCTTACCTTGCGGATCAG
ATCAAGCACGTCCATACATGCCGAGCTTAGCATGTTATCGCGACCGCTTTCG
TGCTCGAGAATATGGAATAAGGGCGCCAACAGATCGTTCTTGACAAAGTAC
CGGTGAATGAAGTGGTTCTGGGATTTTAGACAGGATTTGACGAAACGCAA
AGCAGCTAAGCAGTCAGCAGGTATTCATTGGACTCAGAAGCAGGCTTACCG
TGCCGTAGGGGCTTGTGCTTGATGTAGAGGAGGGAGACCACTTTTCGTGAT
ATTGGGTTGGAGAGGATAAAGTAGGAGCCGCGATGCGAGTGCACCATCAG
GCAGTAGCTGAGGAGTTCCACTAGCTGCAAGAGCAGCGTCGCCTGTTCCC
TTGACAGCTGGTAGGGCGGATCCATGTCGGCCGTGATCTCCGGTAGGTCTA
TGATGGGCTTAAACAGCAGGTTGCACGAGGTGTCGTAGAAGAAGGTGATG
AAGCTCTCAACGATCGGTCCTTCCTTGCGACCCAAGAACGACTGACAGGT
TAGACAATATACCCGTCAGAAGAGATACAGCCCACCTCGGCCTCGGTAGCA
GTTTCTAACAAGGTCCGCATCGAATCGGAGATCTGGCTGACCAGTCCCATG
TTCTTGGTAGTCGACATGAGATTGATCATCTCCACAAGCAGTGTGCGGCGT
TTGGCTTCTTCCTCCTTGAGCGTGTGCATCCGTACCGCGTTGACATCGTGCT
CGACCGCAAGAGTGAGGCTTTCGGCCGCTGCGTGTAGCATCTGCGGCTCA
GGACGTCGAAACGCCCACTCGCAGACCGTGAACAGCCCTCGATCCATCAA
ATTCCGGTACAGGGCAAGGCGTGACTGGACCTGAATGCCTTTGCCCATGAG
CATCAGCTGGTGAAGGAACAGCATGATATCGCGTTTCTTCTCGTCCAGCGG
TTCAGTGACGCTCGAGTCGGAGTTCAAGTTGAGTGACTTGAAGTCGGCAA
AGAGCTCGGTCAAGAAAGCATCATTCGTCTGGATCCAGTTGATAATGTCGG

CCTGATTATAGAAGACAAAGCCGTTCAGGATACCGAATGTCGGATCGTCGA
GCACACGGGCCAGGACGACGTCTTTGAGGTAGAGGACACGGTATGTCTGG
TGGATCTTGTGGAGGATTGTCTCATCCTTGATCGGTACCACCTCTTTGAAGC
GGGCTATGTCTTGGAAGAACTGTCGATATGAAGCTTTCAGATTGGGGAATT
CCGGGTCGTCTGCCCATTAGCGTTCTCAAGAGAGGCACGAGGTTTGTCCCA
CATTCAAGCATCCCGATGACACCCATGAAGACCTCGTCTTGCAAGATGTAC
TCGAAAATGCCGTTGTCGTTGAACAGGACTGTAGTGAGTTTGACACACCTA
CTAAGCCGATGAAACATACGGATGGTCTGCATGAGAGCGCAAAGGTGATG
GAGGTCTTCCAGATCCTCCAGATCCTCAGCACTCTCAAACACAGAACAGA
GGGCTTTGATATAATGCTCCCGTGTCAACTCATGGTCGTCGCCTGTAGAAAG
ACAACTCACCTCATTCAGTATATGCTCCACCGCCTTCTCTCTGCCTTGTGCC
GACTTTGCCTGCATACGCAACCAGCCCTCCTGATCCCTACCAGATAAGCTC
AGCTCTTTCCCTGATCTGAAAGCTGACTTGATGTTCCTCAAGGTTGGAGGC
TCCCAAGGTATTCTCGCTGGCGCATCGCCCTGTACCGGCCCAATGACTGGG
GAGTCTGTCGGTGACGAAGTGCCAGGTACAGGCGGGTCATTGTCCACTGC
TATTGAGCCTGATCCAGCTACGAGAGTAACCATGCTCACGCG

>Mosaic_sequence_30

GCATGCTTAACCTTTTTGAACTGCCCTAATTGATGGTTTTCTGAAGCTGTTT
AGAAAGTCGCAACTGCGGGTCCGGGCATCACGTAAGAGCCACCGCCACCC
CAGAAGTTGAAAGTGAGGCCGGGGTCTGTCTTGCTGTAGATTCCGGGGATC
TTAACAAGCGGAGATGGCTTGGCTGTGCCAGTGCCTCCAGTAATGCGAATC
TGGGCACATTCCATGTCTACGTAAATCATCAGCATATGTGCGGTTGATCATA
AGAGACAGGGATAACTTACATAGCTGTGGCTTTCCGGCGTCTAGGTAATTG
TTTCTGGTTAGTAAGAGGTCGTGAAAGGAACAAAAGCGCAGACTTACGGT
TCTCGTGGATACCGATGTGCTCTCCTCGGAGAAGGTACTCGCCATCCTCAA
GAGTTGTTGGGAGGGTGAATTCCATGGTGTATTTGCCGTAGGTTGGCCAAC
CGCCGGTGCTGGTGGGGCTTCCGGTTGGGCCAGATTCCCAACTGAACTCTT
TAGTCTATTTTGTTCGTTCATGCATGAATGGTAATTCTACTGACACCTTGAAC
CAGTCACCAGATCCATCGTAGTCGCTCACGTTGCCGGGAGCCTTGGACATA
TAGAAGAATCCGGGACCCGGGTGCTCGATGAACTCGTTGTTGAACAACTTA
AGACCGATCTTGTCACCAGCCTTAACGTCCATGTAGGTCTGAGTTCCGGCA
CTGGTAGCACCCTTGTTGCACCTCAGATCATTGCTATATCTTGTCAATTCTCT
ATAAAAAGTTATTCTTGTTGTGGAGGAACACCAACGAGTCGATAATCTCCAT
AAACGACGGCATGTAGCTGTTGGAGTTCTTTCGGACATAGGTGTATTCGCC
TCCGACCTGCTTTCCATTGTGCATGACTATAAGGATGTTTAGTGATGGTTTAT
ACTTGAGCGAAATAGGAGGAGGCTTACGGATGTTGAGGATGTAGTGGGAG
CTGACTAGGGGAGCAGACAGCAGGGCTGCCGCCGCCGCGAGAGTGAAAG

ATCTAACCATGTTGACAAGTAAGCTAACCTGATACAGGACAAGAGCAAGG
AGTTTACGGTCTGTAATACATTCAACAGAGTTAGCGGATGCCAAAGCCTTTT
ATGCACATCGTTGCCTTGCATTCTACTCAGCAACCACAGCTTCGCAACTCA
GTTCCCGCAATCTAGCCGCGCATAGGTAGGTAGTCTACCTGAATATTCTCTC
GGCAATCGTGTCTATGCCGAGAAATCGCTAATGCCCCTTGTATTCATTACTT
GGAAGATGGTTGTAAATTTGCTAAGTCTCGGCAATCAAAGATCTAATCTAGC
GACTGGACGAGAGAATCTGGGGAAATGTCAGTTGCACTTGGCATACATGG
AAAATTACAAATAAGAGGCAACGGTTTTCCGCGTTAGGAATCCAGCCCACC
ATTCTCCGTGATGTGTGAGACCATCAAGACGGCATAGCCTCGCTTAATGTAT
AGGTAGATATTCTTCATCCCTTCGTCCAGTGGATCTTGTTCGCTTATAGGCAC
TCAACTCGACCACATCCAACCTCACAAGTTCAAATGGTTCTAGATGCTTCAT
GATCTATCACATGGTTTCAAGAATAGAATCTGAGATATAATTCGCGCGCATA
GTATCTGATCGGTTGCACAAGCAATATAATCTAACTCCGATCTCACTCAACT
TCGCTCTATATTGAAAGTTATGAAAATACACATCTACCCTACCGGTATAAGTC
TCTTGGGGCTTCTCAATGACTAATAACAAATGGTTTGAGTACCTTGAATTAG
AAATCTGGGGAAATCTTATTTTTCAAATGCTGGCGGACACTGTCAGAGAAT
AGTATGCCCCCCCTCCGACAATCTACATTCAGCCTAGGGAGAATAACCGGC
AGAAATGATGGGTCATATGATCGAAAATATACACATTTTGATATTCCTGTTTT
TTTAGATCTTCTAGTTAACTACTTTGATTACCAAGTTGTCTGAGGAATTACTA
AAATTTCAAGATAGCATACGACAACAGCTAAACTATGATTAGTCATCAACCC
CTGAGTATTGACCTTGTTTTCCTGCCCTATATCAGATGGTGTTACTTGGTTAT
ACGCCATTCCTACTGTTATATGTACCTACATATTCTGTCTGGCAGTCAGCTTC
GATTCAAGGGAGTCCAACGTGATGCCTTTGAATTTTCATGTTCTACTTAATT
TAGCAAAGGGCTATGCACCTATCAAAAGAAAATTTACCGACTTTCTAGGTC
GGTACTAAATCATGATCTGTACGAAGTAGATTCATGTGTAGACATAGCGCAT
TGTAGTTCAAATACGCTTCTTACGGAGAGATTTTAGCTCCGTTAACCAATAT
CTGCTTGGTGGTGTTACAGAAACACCAAAATGTAGGTCCGATGCTGACACA
TTGGTATCTTAAGTCTGCACGTCAGATTAACTGTCAGCGTAGTATTATCTCTC
CAGCCTTAATCATATCTTGGAAACCATTGAAGAGGCGGTCCACTCAAGTGT
TCAAGACCAATTTGTGTAATCCACGAGCTGTCGGACACGTCTGCGTGAGTC
TATATGAGGCAATCTGAATGGAAAATAATTTTCCTCCTATAGAATCAAGGCG
GCGATTTTTCCGATCCTTGGCCGAAAAAGGAGCGAAATTAACGATGGTGAC
ATGCGGGTCGTCAATCTGTTGGGCAATATAGGGAGATCCTACAGCTTGCATG
CCTGGAATGCCTATTCGCCTCATTATTGACGATATCTCTGTTGCATATGGGTC
ATGGGCTTCAATATTCGATTTTACAGTCGGTGGCGAGAGCGAAGGATCAGA
GAGGATATTCTAACAGAGATTTCCGATGTACTAGGTAAGTTATCCTATTCATC
CAAATTCAATGAGACAAAATCAGCATAAGATAGTATGGTTAAATGAAAGTT
TTAAGTCTATGGAGAAAAAAGATTGTCCGAGAAAGAAGCACGTATGGGTG

ACAGCTGACTACGACTGTGCCTACCTAACAAGTAGACATTTAGTAGTATAGC
TATTGATGTATCCTCACTAGACATGGCTGCCCTTCAGGAGTAGCATAGGTAA
GTACTTGTAGGAGATAGGATCATAGGCGTCCGTTCATAGGTCCGGGCTTCTG
TCAAGTATCGACTGAACAGTGTCCTTTATGGATTTCTCCAAGTCGATATAGT
CCATTCCCAGCATCCTCTTGGATTTGCTATTGTCGGACTGATATACATCGGCA
GGTAAATCATCCTGCGAATTCTCAGGTAGCCGAGACGCAAACTTCGGGAAC
GACTTTTGGATAACGTCTGCGATCCGTTTATTCGAGAAGTGTCCCGAAGCA
ATATAGAGACGATTACCACTAGCTTCAGGTACGGTGACAGCCCTGAGGTGC
GACTTTGCTACGTCACGAACATCGACAAAGGTGAACACAGGTAAAGTAGG
GGGCAAACCTCCTTTCATCTTCCCTTGTAGCATGTCCCGGATGCGATGGTTG
GATGTATTCATGCCATCCAGATTTCGAAGGCTACGTTGCAAGGGACCAAAG
GTGAAAGTGCAGTTGATTGTGGCAAGGTCAAAATTTGGCCGTTCGGTGTCC
ATAAAACTCCACGCAGCCTTCTCGGAGAGCACCTACAGCCAGGTCAGCAA
GGACGAATCATAATTCGCAAATAGTCGACAATACCTTGCTTGTCTCATAAGC
TGTCTTCGGATTGTGGATGTCCTCTAAGGTCCAAGGAGCCCAGATCGTCTC
ATTATAGACTTTGGCATGCTGAAGAGGGTTAAGAATAGCTGCAGACGATGA
TGTAATCACGATACGTTTTACACTCGGAGCGTGCGCCTTCACAGACATAAG
TATGCCTGTCGTCCCTTTGATTGCTGGATCTAAGAGATCTTTAACAGGGTCG
CGAACGACAAGAGTATAAGGTGAGGCAGTGTGGACAACATAGTCGAAAGC
TGACTCTGTTTTAAATACCTAGGTACTTATTATTAGCTCAACACTCGACCTTA
CATCTCCCAGAACGCAAAGGATGCGAACGTATCAAGATCCTTACAGAGTCG
AAAGCCCCCTCCGCTGCGATATCACCTATTATAACGTAGGAAATATTGTTTC
GTTGAGGAAGTGGTGTGGAATCGATGATGCCACGCCCTTTCTCGTCCGATC
TCACTGTTGCGACGACCTCGAATCTAGATAATGAGTCAGCATTAAGTTACTT
TGCTGGGAAGGGTGGTATACCCGTCGTCAAGGAATGCATCAATTATGTGAG
ATGCGATGAACCCGCTCCCTCCTAAATGACGTAGAATTTAGGAACCATTCA
AATGAGACATTTGCTTTTTCTAGGTGCCCGCGCTGTCTGCTTACCCGTAACA
AGGACTTTACCTTTCAGTAGTTCAACTTGGCTTCCCATCTCTCCTTCTTTTC
GTTGCGAATTATCAGTATTGTGAATAAGCTTAGTTATACTATTTCATCAACCA
GATTTGAATAATGTTTGGTTATAAATTAAAATCACACGACGATGCATGTTTC
AGTGTAGATTCTGTTGAAATCTTCGGTGTTCCGACGCATCTACCCCAACTGA
GGCCGAACGGGATCCTCCGATGTGATGGTGGCTGCAGACTCTGACAGCATG
GCTTATGAGCCTTTAAAAGTGACATTCATGTCTCTTCTAATTCGTGACGTCT
GGGAACTCAATCAGACAATTACCTAGGTAATATACGTTTTAATGGTAGGACA
TACATACACCAAAAACGCCATTGTCGAGACTGACTGAGAGCTGACTGTTAG
ATGACGAAGTAGGTCTTGGTTAATTACATAATGGCTCCCAAAGAATTCACTA
GCATCCACGTCGCGATCATCGGAGGAGGAATAACGGGCATCGCATTTGCGC
TGGGTCTTCAGGAGCGCCGCGTTGGTTTCACTTTGTACGAACGTAACCCGG

CATTCACGGAGACCGGGGCCGGCATCGGATTTAGTCCCAATGCTGAACGTG
CTTTGAAAATCATCAATCCCGAATTAGACCGCGTTCATAAGCAGTTAACCCC
TCGGACTAATCAGGAAGAGTATTTCTGCTGGGTTGATGGCTATAGAACGAA
CGAGGTTGCAGCCAGGACGTACGTAAGTAGATCTCGTACTCATGCGTGCAT
GGAAATTTCATTAACCTTATGTCATTCTCATCGTAGATTGGGGTGGATGCTTT
CAAGGGAGGGAGACGGTCCGACTTCGTAGACGAGTGGTCGAAGCTCATCC
CGAAGAATAACGTTCAGTTCAATAAGGAACTAAATTTCCTTGTCTACAAGG
ATGATGGCAAGGTGTCATTACAATTCAAAGATGGCTCCGTCGAGGAAGCGG
ACGTTGGTAACGTACCCTTACTTACTATTCCGAGGTTCCAACGATATATTACA
AAAAGCTAACTCATGAGGAAGTAATTGGCTGTGATGGAATACATTCACGTG
TCCGTCGATTGATACTAGGGGAAACCAACCCCGCCTCATACGCCACTTATAC
AAACAAATATTGCTTTAGGGCATTGGCTCCGATGGAAAAAGCTCACGCGGT
GTTAGGAACGGAGCGGACCACGGGGGATGGCAAAGTGTCGAACAGGTTC
ATGCTTACCGGGCCGAATGCACACCTCCATACCTATCCAGTAGCACAAGGC
CAATTTCTAAACATCCTGTTCGTCCTCTCTGACCCTAACCCATGGCATACCG
AGGATGGCAAGCACACGACTTCTGGAAGTAAGGGAGAGGCTCTACGGGCC
GTTAAGGACTGGCATCCGCAAGCCAGGGCGATAGCAGAGATGTTGCCCGA
CGAACTTCAGAAGTGGGCCCTGTTCGATATGTACGAGAACCCTGCGCCATA
CTATTTTCGTGATAGTGTGGCAATTGCAGGCGATGCGGCTCACGCTTCAGGT
CCTCACCTAGGCTCCGGAGCCGGCTTCGGTGTGGAAGATGCCCTGGTTCTT
GCGACAGCGCTTGAGGCTGTTAACACAAGACTCGGGGTTCAGGGTCAGAA
GGCAAGGGCCGAGCTCTGCCACACTGCACTAGCCGCATATAATAATGTGAG
ATACGATCGGACGCAGTGGCTTGTAGGGGCAACACGCGAGACTTGTAGATT
GTTCCACTGGCAGGACGAAAATGTCGGTAGCGATTTCGGAAAGTTTCTCCC
CGAGATTAACAAGCGATTTCATAAGATCTGGAACTACGATATTGGCGAGATG
GTCAAGGAAACCCTGAGAGAATTCGAGAGGTTGGGTAGCAAGTTGCCAAA
CTAAATTATGGCTAATATCGCCAATATGGTCAAGGCTGCCAGGGGTCGCGTT
TAGACGCTAACAGTTTGACTAGCAAACAACGATGTGTTCTGCGAACGACCG
GTTATGCGCCAAGAAGCTAGGAACCTTGGAATATTGAAGCATTGAAGGATT
CGAGAAAATTGAGCGACTCTGCCTTTGCACTTCCGTTGGTAGTTCGCACCG
TTGATTTCAAGTAGCCCAATCTCAAGATAATGTCTAGTGTAAATTTCCATATC
ATAGCTATTCATTAGTTTTTTTGACTATTATTCTCCTTTTTACCTCCTCCCATC
CACCTCCGGATCCTTTTCAGTCCTATACACTACCAAAAGCCCAGGGTATCTC
GTAAAAGCAAAGCCGCCGCCCCCAACCGGGCTATGGAGCCAAATGCCACC
TTCTATCGATACTATGTGCTGTATCCCATAGGGCGATGTAATAATTCGTTATGT
ATATATACTGTCGTGATAATTTATTATATATGGTAGTAGTTGTTTTCGCGAAAA
TTAGAATTTCTCTAAAATGTAACTGGTAGACATAGTTAGCATCCCTGGTACG
TCGAGGAGAATGCGAAGTTCGCCTACCAGATAAATCATTCGGACCCCTTTG

TAGGATGTTTCTGTCCATAATCCATTCAGCCTTGAACTATCCCTTTTTGAGA
CAGGGTTGGCTCGGATTTGAAGTCGAACCGGGTCTGACAAGAATAATCCA
GTCATAATGATCGACATTGTGTACAACCGGACTTCTCGGACCCTCCTCCTCG
ACAATGGGGTCGTTGTTTTTTAAACGAGTTGGTATTCCCTATAATATATCCA
ACTCTTGGGTGCGTTCTTATCTCGCGTCGAAAATGAGAGATGAAGAACGAG
AGCGCGACGCTGGAATGATAACGTCGTCATTAACGTCGTCGTTATGGGGA
CGACCGCGGGTGGGGGCGGAGATATCAGGCCATCTCGCACGCCAGGTGCT
GTCGATTTAGTTGCAAGCTCCTCGTCCATCTTTATCCAAGACTCCCTTTCGA
AACGCGACGGCCGACTCAACGGCTCTCCGCTGGCGTAATCCGGCGCGCTC
CGATGACCAGACCTTTTGCGCCTCGCAACCCGTAGAGATCCGTAAATCGTT
GGCGCATTTGCCACAAAGCAAGAGACGAACATCTGCACGGATGCCCATAA
GCTACGCGTAGTTTGCGAAGAATTCTCGTTGAAGATCAGGACGACGCGAAT
TATTGTGACGACGATGCAGAAGGTGCCCAGTGCAAATAATGAATATAGAGA
AATATATTCCGAGACAGTTCTCTTTCTCAGGCAGATCAGAGGATACGCGATT
AGGAGAACCAGGAAATCGATGACGATATTCGAGACACCCAGAAGAAGCAA
CTGAGCATAGGCGCGCACACATGATCCCGGATTAGGTTCCACTTGCCAGTA
AAGTCGAATCGGGCGGCATTCAAGGAAAGTTGCAAGCACGATGGCGATGA
ATGTGACTCCCAAGGTGATCCAGGTTATCTTGGTAAGTCGATTCGCCCAAG
CGATGCCAGAAGTAATGCGCGAATAAAATAAAAGCAGGAGGCAGACCTGC
AGCCAAAGACCAGCCGCGAGTAGAACCCTTGCCAGAAGAACCAGAATGCT
TCCAACTTTCAACCGATATTCATCGTCGCTGGAATGGATGCCATTGTGCAAG
GCATCGTTGGCGGTTCCGAATTGTAGATAGAAGTTGTTAGCGACGATCCGG
CCGATGATGACTAGCATCGACAGCACGACTAGGAAGAATGCGAGGTCGAA
TAACTCGCGGCGCCAGATCGAGATGAACATTCGGATGGCGAGGATGAGGA
CCGACAATGCCGCGCACGCATAACCGACGTGCTCCGCTTCGGATGACTGGT
ACGCCATATTGGCACGCGCAAGAGAAACGGTTTCTCAAGCGGTTCGTTACT
TAGTATACATGTTTCTTGTCGATATGAGCTACGACATCTTCAGCGCTCGTCAT
CAAACTAAAACTGCATCGGATCAGGCTAGCTAGAGGGAGAGGTGCTGGGG
GCAGATGGAAGAGGGTGATTGAGGAAATTGCTGGACATCATACGTATCATA
CGGCGGGCAGGAGGGGTGGAAATAATCAGTCGATGCAGCAGTAAGCGCAA
ACGGGTGACACCATCAGTCGTCAATAAGGGGCGGGTCGAAAGTGTCGTTG
ACGCTCAGTCCTCTTCCGAGCCCTCGTGATCGGGAAGGCATAGTCGTTCTT
GCTCCTGATCCGCCGTGAAGGCGGCGCTGTCGAGAATCGCCATAGAGGCTT
TTGAGCAATTGGGACTTATGCAAAGCGTACTCTATGTCGTCGATTGTAATAT
ATCCCGGCGAGTCGAACACTCCCTGATCGACCAATTCCATGATCTGCCTAG
CCTGCTCAGTAGGTTGACCGTCTTCTCGCAGTGGAGGAGCAAGGAAGCTC
TCAGCAGTCAGTGGTCCGATGTCTGACAATCCGCTTTCGGCGGCAGAGGC
GAGCGTCGACGGCCTAGTGCGGACGAAAGAGCGTGCTGAACTTTGTTCCC

GCGGGGAGCTAGCTGCGTGCAAGCCTTCATGCGTCCTCATGTGGGCGCGCG
TCGTGTTGGATCGCTGGGGTTCTGGCACCACCTCAACCACATCGCCCGCAG
ACGGGCGGAAGAGGAAGTGTGAACCATCCGACCTTCTTACGGTGACCTCT
GTCCCGTGGTGGGGAACAGAGTCGGGCACTCCAGTCTGCTGCCCTACAGA
TACCCAGGCGTTCGACTGGTTCAACAGCTTGTAGACGTCATTCTCTGGGAA
GCCCTCCACTAGATCTTCGCGCCAATCGGGAGAGCACGTCTGAAAGCGCTC
CCATCTGTGAGCGACATCTTGAGTGAATTGGCCGAGCAAGTTGCCTGTCCA
TTTGACGGACTCATCGACGCCTTTAGCGCTAACGGTAGGAGGCACCTGCAG
CTCCAGACCTATGGTTCGGGTGGTACCGTCGTTCCGTTCCTGAACCAAGAA
CATGCCGACTATGCAGCTCTGCCTGTCAGGATTCGAAGCTCCCACGGTATC
GGAGCCTTGCGCGGGAGCGTGGTTGACGGAAGAATAACGGGGATCCGCCT
CCCAGCTGGACGATCCCGCTCTCAGCCCGAGGGCCGAGGTTGGGAGGCCA
TTGGTGGCAGGTCGTCCGCTGTGGTGGAAACGGCTGGGGAGGCTGCGTTC
AGTGGGAGACCAACGGTAGGTGTCATCCACCGGGGGTTCGCGTGGTATGC
TGCTTTTCACGAGGGAGGAAGGCATCAAGTCTCGTTTGATGTTCTGACTCG
TAGGGTATGGCGGACCCAATGATTGTCGGGACATCCCAGATGCGAGTCTGT
CGGGGTCCATGCGAGCCATGGCTGATTGTGATAAGTCTCGGAAGTCTGTTG
AAAGCGCGAGAATTCGATGATGATGATGGGAGGGGGAGCGAGTCGAGGGG
GCGCATTGTTGACATCGGGGAAACGACCATTATATAGCCCTGCGTCCTTGTC
CATGCGCTCTTTCGGCGTTGTTGTGCAAAGACAAAGGATGGCTGGACAGAT
GCCCGTCTCCCAGAGCTGAATCATGGTCTGCCTCTGTGACACAGTCCCGTA
ATTATTCTCACGACTGTTTCCATTGTGATGCAGCACCGCTTCGCGGGGCAGG
CAGCTGTCGCAGCCCCAATTTGCGTAAGAGGAGGTGCAAGTGTATCAGCTA
AATGGCATCCGATACAGACGTCGATTCTTAGATGGCCCGAGAATTACTGTCA
TTCAAGCTCCTTTCACTCTAGCAGTCTGGGACATGTTTGTTCCGTGCGAAG
CGATGCACGTTGGTGTGCGTGCCCCGTGAGAAGCTGCTCTCAATTGCGCCC
CTAGTTCGTCCCATGGATAGTGCAGAATCGCCGTACCTTCGTCTACAATGCA
GATGGTTCGGAATGTACAGCTGGGGCGAAAGACATGGCGATCTTCGGTCAT
CCAGTCGAGCGTACCACTTTGGTCATTCCGCTATCTTCCGCTGTCGATAAGC
GGTCGTTTACCCCTCGAACAACTCGATGGCATCGCCCTCCTACGGTCACGT
CGGCCATGGGGGGTCTGAGACAAGTCAGAGGCGTTCCGGTGTGGACAAAC
GGAATCTCAGTAGACATTGAAGGAGCATCCCTCACTGCGCTTTGGCTGTAG
TTCAAGATGTGCAGTACGAGCGCGTGATCACGCTATATGAGCGGACAATCT
CAGAGTGATGCATTACATAGTAAGTTTGTGACTCGGTCTTTCAGTCAGAGA
CGAGGTAGCTGCCGACTTCATGCAGCGAGCGTCACGCCATGCCACCCTCCA
TTCCAGCTCTCATTCCAAAAGGGCTGGTCAAACGTGGAGTGACAGTACGC
AGAGGCTGACAGACATCGGCCTGGACCCAAATCAGGAAGGCACGAGGGG
CTCTGGAGCGAACCCCAATTGACAGCTTCCTGGCCCGAGTCAGGCTCATGG

CCGGCCTGATATACATCTCTGAAAAGTCCGGGGCTGAGATCCCACGCGTCT
CCTGGATACTCCGAGGGAGTTAAAGCAGACTCCCGCTCAGGACAGAGCCC
GTAATCTGAAATGAGGTCGAAGCTTTGTGTATCAGGCTCGAGGCTCGGCGG
CTCCCCATGAGGACCGTCGATGAATTCGTCGAGAGACTCCGCGAGATGCTT
CCCGTCATCGAGTGGTTCACGAGCGGGAACGGCGGGCCAGGAGGAATCAT
CCGTACCCGTTTCATTCACTGCCACGCCCCATGAGCCAGAGTCCGAGCTGG
AAGCTGCGTGCTGCGTCAGAGCTGTCTGTACTGTTCAAGATCAAGGGACCC
ACAACCAGTCGATGGCTCGTCCCGGCATCCGCTCGGTTCTTTGCGCTGGCG
CGTCACGAGTCCGTGCGAGTAGTTGTCGAAAATATAGCTGGGGGCCGAGAT
TGGATCGCTCAGTGGCCCAGCGCTCTGAATGACATCGGGCATGATGAAGCG
TCAAATGAGGTTTCCAAGTGTACCGGTCGTACTTCGTGTGAATGATCTCCTG
AGTATTCAGAGATCGTTGAATTGAATGATAAGCGAGTCACACGAGCACTGT
GATATATACGGGTTATATGGCTCTATGCAGTGGAGGGCAGGTCCCACGCGTG
CGACTGGCTGCACACCTGGCTGAATGGAGCATTGCGGGGGGTGAATCGGC
AGCGAGTAGCCCGTCGCAGGGGTGTGTCTCGGTCGCACCATCGATGAAGAT
GGGCGGCCTGTCCATCTAGAGGCTCCCACATACCCACCAGCGCTACATTTC
TTGTCGGAGTTGACAGCTGATGGATTGTCGCAGACAGCCATCCCGACTGCA
TCGGGGTTTGGAGCTCGAAAAACTCCCAATCATTGGCGGGACGCGATGGG
CGTGCACGTCAGCAGCGATACGACTGCTTTCAGCACGGAAATGCAGGGCC
GCTCGAGTGAAGCAAACAGCCAGAGATCTGGCAACTTCATCATAGCGTTGT
AACAAGGCGCCGCCGTGAGGAAGAGCAACAAGCCTACCAGATCCGTGCG
AACCCCTTGCTCTTAATCGCGAAAAAGAGTCATCTGTGATTCATACAAGTG
TCACTTTCAAGTCCTCTCTCTCTTCTATCACGTCCTCATGAGCCTTGCCCGG
ACGAATTGTCACAAAAAGGTGCCCGTGCGTTCTGTGGCATCATGTCTCGCC
GGCTGATCAGTTCTCGTAATGGTCACAGGCCATTGAAAAAATGACAGAACA
TGATGCAGCATACTAGCTGGACCGGGCGAAACAAGGGACATGGGTCTCGA
TGGTCACGGCCCGTCGCCCCATCTCCCACACGACGCTAATTGGAATGCCGG
ATGATGCCCCTTTCTATCTGGTACGCTTGGCCACGTGCGCATCGGCAGTCAC
CCTAAGGCGCCGTATCAAGGGATACACCAAGAAACAAGCCCGTTGAATACT
TGCTCTACTGTAGGCCTCTCCGCTTCCCTGATCTCTGATCATTACCGCTGTCC
CCGCGCAAGAAATGAAAAAAAGGCATATGTATGTGATGAATGACGGGTTGA
TGAGACGGGTGATAGACATGGATGCATGAGCAAAGACATACAAAGGCCTA
AAAACGGTAGGACCAAGATATGTAGGTGCGTGGCCACCGAGCGAGCGGAG
CTCATTCACCCCTGAGTTGCATCACCTTTGTCGCTCTTGGGTGTGTCATCGG
TCTTGATACGACTATTGGGCCCCTCAAATACGACCCTGCTTAGCTGCGTGGC
TTCCTGAAGCAGAGCCGCAAAGAACGCTGGAGGGCGTATCGTTTGTTCCA
AGTACTTCTTGATCGTGCTCCAGAGGTCAGACTCTAAGTTGTCGACCTGAG
TGCTATGATATTGCGACCATTTCTGCGCTGCGTCGCTTGGCTTCAGTCCAAC

AAAGACCTCCTGAAGGTCTCGAATCTCGTCAATACACTGGAGTGTGAGCCG
CACGTCTAAGTAATTCGTAATGTGCTGGGGTTGCGATTCCCTGATGCCCGGG
TCGAGCTAGCCGTCAGTACGCACAGTATGTCATTGGACGATCCCCATCCCTC
AAACAATGTACGCGAGCCTCCCTGTGTGCATCCTTCGACAATCTAGCAGAC
AAGATGATGACTCACTGCAGACATGTGAAGCCTCCGGATCAACGAAGCGA
GCTCAATACGCGAAGCCTCACTTGGCGTAACCATTGTAGGCCTGAACGTCA
GGCCCTCACGGCAGCAGCGGCCTGAGATCTCCAGGCGGGAAGGCTAGACT
TACTCAACATTTGACTCGCCCTCGGTCTTCACACGGACGGCATCGCTCTTAC
TGTCTTGGTCGGACATGCTGACGCTAGGTCTATGAGTCCTCAAAGCTGTAG
GTTGAGGGAAGAGACGCTAATCAAATAGAACCTGTTGAGGGATGGACGGT
GGCGAGTCGTCTCGTTCTTATACTCAAGCCAGTCGAGTGCCGGTCGTCCGT
CCTTTGCAGACTCCTGCGACAGTTGACAAACGAGGGGACCAGAGCTTTTT
CACACCCTGCGTTCGAGAAGGCTAGCGTCCTCTCCATGATTTGAAATGTTT
GATACAAACCTTCGTATCCAGCTCTGTTTGACAAGTGGCCAAACTCGGAAC
CAATATTTTTTTGGCAAGCTGGTCTACAAATGAGCGGTGGCGAGTCAGTTC
ATGGGACCCAGAGAGCCAGACGGCAATGAGAAAATCTTCTTGTACACGAC
TCGAGTCTTTGAACACTTTTATGCTGCTGCGTCACTGTGTCGTTCCCCTGCT
CTGCCTAGTTGTGACAGCAAACAGAGTACTCGTTCGTCCTTCCCAAAAGAT
GTGCCATCTCAGTCATGTCAAGCAGACCTCGACCCTCGCCCCCTCTGATTT
GCAATTTGGGGCTTCATGGCTCGAAGACCGCCTCCCCATTTGCTAAACGTG
ATGGAGGTATGCGAATGGCGAATGTGCTTCGTGTTGCGAGGCAGCTAGTTA
TGCGGCCCGCTCGAATGAGGGGCATGTACAACGCCAGCTTGCGCCTCACTT
CTTCGGAGCAACGCGCCGCGTTAGGACGAACACCAAAAAGCTACATGCCT
ACAAGATCGATGCCATGTCGTGTGAGCTTGAGGGGTGGCAATGTCATCGTT
GCTGCAAGCAGCACAGGCTTTGACTGCCCATCTCTCATGCCACATTCGCAT
CCGGGCGGTGGCAGAGGACAGGTGAAAAGTGTGTACCCTGTCATACTATTG
CGAGGATGAGGCCCAACCTTCAACGGGTTGAATCTCTGGGCGTAGGTCAA
CCCGCTGGTTCGAGCGGCCTTCAGAGGGAATGGGCATTGGCAGCTATCGGT
TCGTCCGTGTCGCGTCCGTGAGCCTCGTTTCGCTGAATAGAAGCGCCTCAG
TCCCTGAGCATCTTGCGTTTGGGGTTTCGTGTATCGGCCCTTATGTGAGCCA
CATTGGGGCTCTTATGATGTCTGAGACAGCCACGAAGAGGCATGTCAAACC
CAAGTATATTGGGACTTGGGGCCTTGGCACGGCTGTTGGGCTCGACCGTTC
GACTTGCAAGGCGACCCCTCGATTTCAAGCATTCACGCGTTCCTCAAGAGC
TTCTCTGGGACCGCTGAATGATGTCGCGTCATGAAGGGTGGATGTGCTGGT
GGGACTTTTGACCGGGTCTCGCCTACGACACATGCACCAACGACCACGATG
ATGATCCTTTGGATCTAGCAGCCTGAGATATGGTTGTTCCGTGCGCATCAGT
GACCCTAAGACAAGTTCACATTTTCTCCGACGCAATCTCCCCCTTCCCCCTC
TCAACCATCTGTCAATACAAGTGGAGGTTGCATAGGGAAGATTGGACCAAC

GTCTATGCTGCCGCAATGAGCGACAGTGTTCCCTAGACGACCAATGGAGCT
GAATGAATTAAATGCATCACGACAGCAATGAGAAGGCTGGGAAGATATAAA
CGCACGAAAGGTCCATGGCCGCACGAGGAGCGGTCAGCAGCTAATCGTGA
CTCGTTGAACCCTATCTGCTCATCCGCTATTTGTGGTTGTCGATTGCCGTCTT
CAAGTCGGACGCCCTTTTGTGAATATCGCAAAAGAAGTCCGGATCGCAGAC
TCCATCCGAATGCGCTTGCAGCGTAATGTTGTAATCAGACTCGATCTTTGCG
AGCTCCGTTCTCTGCGTCTGCAGCGTGTGCTCGTCCTCTGTACCGGTTTTTC
CACTGTCGTTTAAAGCCTTCTCTATTTCCACAATATACGCCAATGTACCCCCC
CTATACAAATCATACCTTGCATGCTGAAGCTCCAAGATCTTCAGTTCGACCT
GGTCGTCAGTCTGTCCGCCCTCCGGGCCAAGTCTCAAGACGAAGTAGGGC
TATCGCGCTTATCGCTTGGCCTGTGTCTCGACCGCCGGTCTCCCCACGATTT
ATGCTGGAAAGCATGATGACTCACTGCAGACACAGATTTACTAGGGCGGCT
ATTTATTGACGCGGCCTGCGTGGCCGCGGTGTCTGCGGGGGCTTCCACTTG
AGGTCTGAGGGTCAGCAGTCTCCGACCGCGGCCTGACCGCCAGGCGGAGA
AGCCACACCTACTCGCCGTGAGTTTCAGGCTGGTTCTCTGCGCGTGTCGTG
GAATCGCGGTGACTGTCTTTGTCTGACATGCTGAGTAAAGGTCTATGGATAT
TCCAAGCTTTACATGAGGTAAAGAGGCGCTTGCCAGATAGGAGCTGTTGAG
GGATGGACGGTGGCGACTTGTCTCCTTCTGCTCTAGCCAGTCCAAAGGAGG
GCTCCAGAGCTTTTTCACATCTTGCTGGTGATTCGGAAGGCGGGAAGGCAA
GCGTCGTCTCCTTGCTTTGAGAGGTTCGAGGCCTTGCTACTCTGTGGACTT
CGCATTCGTTTTGGTCTCTGAAAGGGCTGCAGCCGGTCAGTGTCCCGAGGC
GGGCCTGGACCCGGAGGATGAGTGGGCGATGGCGAATCTTAGCCCACCTC
ATACGAGTCTCTGGACTCTTGGACGATGCTTCGCAACAAAGGATCACTGCC
ACGCTGCTAGATAGGTTATCGAGCATGTAATAGCTCATCCTTCGCGAGAAGC
GGGGGGGGGGGGGGGGGGGGGAGAGGCAAATCAACCTTCCATCGAATG
CGTCCGAGCGTGAAGCAACACCCTTCAGTCGTCCCCTTCACTCTCTTGCGC
ATCACAGCACATGAAGGTCACCTCTGGATTTTCATCAGCCCGAGACAGCTA
CGAAGAGGCGCCCCAGGAGCAAGTACATTGGGGCTCGCGGCTTGTCATCT
GGATTGGTTTGGAACAATCGACTGGTTCGAGCGAGCACCTGTTTCAGCATG
CACGCGTCCTTCATGGGGTGCGTCATCTCGTGCATATCTGAGGGACGTTGCT
TGATGCATGATGGACGTGGTAGTGCATTTCGTCTGCCCTAGGCACAGAGGA
GGATTCAAGAAACATGGAACCATGAGTACAACGACGAAATGCGGCCCCGC
TCTTACCCGCAGTCCGGGACGTGGTTGTTCCGTGCGCACAAGTACTGCTGA
CCCCGTACGACACGGTCGACTTTTCTCGTAGCCCCGCCGCCTCCCTTCCAC
CTGACTAATGATGTTGGCACAGGAAAGCATGACCCACCAAATGAGCCCGTC
GCCGAGAGAGCCAATGGAGCTGTATGGAATGCATGGGGAAAGATGGACAG
GCTAGAAAATACTAGACACATGACAGCTCCACGGCCACACGAATAGCGGTC
GGCAGCCAGTCGCGAGTCATCGAGACCGTCTGCTCACTGGCTATTCGTGGC

TGTCGATATCCCTCTTCAGCTCCAAAGCCCTTTTGTGTACATTGTGAAAGGT
GTTCGGATGGTAGCTTCGATCCATGTTCCGTCTGATCTTGCTTCCGAGATCA
GACTGAATTGAGTTGAGCTCGGTGCTCTGCTTGTCCAACCATTCTTTGTCCT
TCGTGCCAGCTTTGCGGATCGTTTCCAAGGTCATTTCCATCTGCTCGATACA
CGCATGTGTGTTCGTCACATTGGAATCAGAGCTCACACGCTGATTCTGCATA
GTCTTCGGTTCGAGCTGGCTGTCAGTCTATGCTGTCGCCGCGCGAAGCACG
AGGATGATCGACCATCTCGCGACGATTTTTTTGTAAAACCTGATGACACAC
AGACACATACGTTCTACGGAGACCCCCGGTGATGGGTGTGGTCGATACGGC
TTGGGAGGCCGAAGTATCTGTCGGGACTCCCACTTGAGGTCTGAGAGTTAG
CAGCCTCTGAGAACGGCCCGACCGCCTGGCGGGAAAGCAACACCTACTTG
CCGGAAGTACCAGCGAGGGTCTTTGCCAGCGCGGATTTATGAGAGCTGTCT
TGGCGGGACATGCTGAATCAAGGTATGTGGGTAGTCAGAGCCGCAAGCCG
AGATGCAGAGTTGCTGTGGAGAAACGGCAGTAGTGCGGCAAATGAGTGGC
CTCGCTTTTGTGTTTGAGCGGAGCCAGATAGAGCACCGGTCGTCCTTTGTA
GCCCAAGATTCTCGCACGGGTTGACGACGGAGCGTTGCTAAGCGATATCAC
ATTCTGCGGCACCCTTTCACTGTTTGAGACGCCAAGGACTCGATGCCACGC
TTTACCTAGGGCACAGAAGTCGACGACCGGCGGGTCTTTTGACGCTTCGAC
AAGAGACATTGGCTCAGCGGGAGCCAAGGCACATACATGCTATGTGTGATA
CTGCCTCGTTGCCCTGACCGCAAATCGAGACGAATGCATGCGAAGAGATG
AGAGAGCCGAGAAAGTATAGGAACACTACAAGTTGTGGCTCATGCCGCCG
ACGCTGTCCTTGAGCATGTTACGATCAGATATCTGCTCCTGCAGAGCAAGG
ACCCTGGCCAACAGATCTTTCCTCGTAGCAGCCTTTTCATCGACATCAGAA
GCCAACTCCTCGACTGCCGTGTTCGTCGCTTGCAACCAGTCTCTCTCTTCC
GCTGTGTCGGGGTTGCCCATGGCCCGCAGATCCGCTTGCATCTTCTTGACG
TACGTGAGACAGTCCGAAGAGGGTGCCGAGCTGCCCGGTGGGGGCTAGCC
GTCAGTACAGTATATCGTCAGCGCCGCCCTAGGGCCTTCCTTGTGCAGCCTT
CGACGATCTGTCAGGCAAGTTTGAGGACTCACCGATGACATGCGCGCGAA
GTTTATAATATCCGTCATCGAGGGCCTCTCGGGCATCGAAGCGTTGCTTCGC
GCTATGGCCTGAGGGGCGCGCGTGAGCTTGCTGTGCTGAGGGGTCGCAGC
ACTCGACTCGTCCTTGACTCTGACAAGCGTGAGAGCGTCTTCGACGTTTTC
GCTGGACATGCTGATGTGAGGCCTGCGAGTACTCGGAGCTGAAGGTGGTAT
CAGTACTTCCAGCCTAGAGGACGATGTTGGGGAATGGGAAGAGGGCGGCA
GGCCGAGAGGTCTCGCTCTTATACTCTGAGTTCAGCCGCTGGTCGTCCTTT
GCGATCCACGGCGCTAGTGCCGCTTGACGAAGGAGCGCTTCTTAGGATTTT
ACGAATCTTGGCGGCTCGCAAAGGGAAGGAAATCTTCGTTGTCCTCTCACT
TTCCACTTGAGGTTGATTTGATCGCTACTGCAGCCCTTCATTGACCCAAGTC
ATCGGGACGATCACGAGACTCTGATAAGCTCGTCCCCCATGAAAGCGAGAT
ACTCTGGTGTCGGCGATGACTTATCTTTCCGTGTAGGGCTCGAGACTCTGTA

CACTTCATGCTGCTCTGTCACCGTCGATTGCTGCCACGCTGCCTCGTAAGG
CCCCATATGAATGTAGATATGCAATCTTGACCGTAGAGCTTCTCATACAACC
GTTCAGAAAGCAGAGCGGGATCAAATTTCCATCAAACGCGTCCGAGCTCA
AAGCATCCATCAAATCCATCCTCTCACTTATCTTCACTCTCAGCAATGTCAA
GATGACCACGACCCTCGCCCGCTCCGCCGCCCCTCCCCTGCCCTCGCAGCC
CTCCTCATCCGCCCAACGCCCCTCCCCCACGCCGGCGGGATCCAGCAGCGG
CGGTGGCGGCCAGCCCCGCTCGACCCAGACGCACTACTCGCCGTTCCCCG
CCGAACTGCACCAGGGCGCCAAGAACCGGCTCTTCCTCTGCCTGCGCTCT
GACATACCCGCCGAGGTCGACTGGGCGCTCCCGCGGCTCGTTGCGGGCAG
CCATGAGCTGCTGGAGCACTTTGACGTCTCGGTATGGGTGGACAGCGTGTT
GGTGCTGCAGCGGTGGCCGGAGCGCTGGGTCGAGCAGCTCGAGCGCGAG
GCGGCGTACCAGGCCCTCGTAGAGGGACGCCAAGCTGCATCCGGAGAAGG
AGAAGGAGAAGGGAGCAAGAAGCGCAAGCGCGACGCCTCTGCCCTCGGG
GCGCTGCCGAGCTGGACGAGCGACCCCCGCCACTCGGCGCAGGGCGTGCG
ACTGCCTTCAAATCCTGCGCAACGCCAGCCTGCGCGACTCCAACGCCAAG
CACATGGCGCGCGCGCACCTCGTCTCCTTTCTCGAGCGCTTCTTCGCCCTT
CCCCTCGCCTTCCTGTCCGACCTCGCCGTGCGCCACCCCGAGCCCGTCCTG
CACATCCTGTACATCTTCCAGTCCATCTTCCCGCTCTTACCGCCGAAGGAAA
TCCCCATGCGCGCCGTCGGCGCGGTGCTGCCCGCCCTGCTGCTCCACACGC
GCGATTCGGCGATGATTCTCGCCCTACTGCCGCTGCTCATCTCCGCCCAGGC
CCTGCGGGACGCCGCGCCGCCCATCGACGCGGCGGTTGTGGACCACCTCA
TGATGCTCGTCACGCTCTCGTCGACCAAGATCCTCGAGATGGCCCTGGATC
TGCTCATTTCGCTCACGCAGAACGCGCAGACGGCCCGCGCGATGCTGGCG
GACCCGGCGTTCCCGAGGCACCTGAAGGGCCTGACGATCCTGCTCGAGCA
CTCGGCGCGCCCGTTCCAGGCGGCCTTTGACCCGCCCAAGGAGCACCACG
CTGTGGCCCTGCGCAACCCGGCGGGCATGGGCGCGCAGGCCGACGACGCC
GTCAGGCGCAGAGCCATTGATCGCGAGAGGGCGCAGGCGGTCATGCGTGC
GCATAATGGCCAGGGATGGGTCGTGGAAGTGGGTGATAAGCCCCCGGTCCT
GGCGACCAATGTCAAGAAGCGGCTGTATCAGATGCGCGAGCCCCTCAGATC
GGTGCAGTGGTGAGTGTGCAGTCGTCCTTGTGAAACTATTCTGAAACGCAG
GATGCACGAGACGTTTGTCTACTCGTCTAATGCCCACCTCCCCCAGGTCAC
CTTCTGGCATGCGTACCGCGACTTTTTCGGCAATCCCAATGCGGTCGAGCA
GATGCTCAGTGCCTCCGAGGTCATCAAGAATGTCAACTTTGCTTTTCCCGG
GTCCAGTGCGAGGGTCTGGACCGCGCAGGACGGCACGCAGAAGTTTGTCA
TTGCTGGGATAGGATTCAGGAAGGGGTCCGGTGAGACATCTTCATGGGGTA
CGAAGGTCGCTGACATGACGCAGAAGAGGAAGACCGCTTTGTGTGCCTCT
GGCGGGGATGCCGATCCTCGACCGTCCCCACTTCTCCCACCAGCCTGTTCA
ACCACGTCCTCTCGGCCCATCTCACCCCCGTGCCCAAGACGTGCGGCTGGG

CACAGTGCCATCATTCCCCGTGCACCACCTCGCACGTCCTCACGCACACGC
CCGTCCTGCAACGGCCCGCCGTGCCCGAATACGTGACATCGCACCCTTCTC
TCTCAGCGCCAGAAGCCGTCGAATCGCTGCACGACGTCACCCACCGCCCG
GTCCCGCCGCTGCCCAAGTCGACCCGGCTCATGGTCAATGGCCACGTGAC
ACCGGTCGACGCGAAAAACTCTCCCACGGGCATCCCATTCCTTGCGGCTCT
AGTGATCCGCAACCTGGCGCGTGTCCTGCGTGTGGAGATGGCCCTCGCTGC
CCCTGAGCGACAAGAGGCCAAACGGCGTCACCTGATGGAAGAGCGGTTCG
GGCTGCCGATCCCTCCGAGCGTGATGAAGGAGGAAGAGGAGGAGGAAAA
GGCGGGTCGGGAGGAGGAGGGACTGACGTCTGATGAGCGAGAACGTGCG
CGACGTGGGTTTGAGAGTGTGGAGCGTGGGATCGGGCGTGTGGTCGAGGA
CAACATGTGCGGCGTGGGGCAGTATCTGAGCGAGGCGTGGGGGTGGTGAC
TCCTCTCCTGCTCTTCTAGCTACTATTAGTGCCACTTTGACATGGAAGTGAG
CCCCGGGATTTAGGCTGTTCTGGGTTGAAAGAGTAAATTGAAAAGTTGCCG
CATGGGGTAATTTACGATGGCGATCATCTCGGACCAATGACGCGAAATCAC
CACTACATGGTACATGGTAACAGTAACAGTATAGCAGCTGGTATATAGTAGG
GAAATATGGCGTGTAGCCGGTGTAGCAAGTAGGAGAGGGGTATGGCTAGG
AGGTAGTCATGCATGGGGTGGTGATGGTGATGCTGGTGATGATCTTCCAAG
GAGGGGTAATGCCGATACGCCGCGGCTGCAGACTGCCTCCGGGCATCAAC
CCTCCCCCGACATTTACTAATGAGATTAGGAACCGATTAGTATCGAATCGGA
CTAATGCTAAGACTGGTCTAATGCCACCTGACCCTGACCCTGAATTCCCGA
CGGTTGTCTCTCTCTTCGTCGCCTGTCAGGTCCCACCCACCTCCCACTCCTC
TGCATACCTCCTCGACAGCCACGGCGAGCGACGACGACGGCGCAAGGAGC
AGTGGTACGGCCCACCACACCACACGGAGAAGAGCCGCTGGAGGAAAGC
GCGCCGCGATTGCGCAGACTGGACTGAATCTTGCCGAGAGGGGTTGTTGG
TCTTCAACTACAAACGAGCGGATCGTCACGTTTCATCCCCCTTCTTTCGAGC
TCGACTGTCGACTGGCGAGCTTGACGGTGTCGCCTCGCCACTGCTGATCCG
ATCCGAGCACGCGCTGCTCTGCTCGACGCCGACTCGGAGTACCTGTTGCAT
TTTGCGGACGGACGGTCAACCCTTATCATCCATCGCTAGGAACGAATCACG
ACCCGACCCTCACCCTCACCACCTGTCTCAACCTCACTTGACTCGACCCCG
ACAATACCTCTCTGCGCCTCAACGCGCCTTCACCTCCGCCCAAATAACTAA
ACGCTTACAGGGTCGACGACGTCCGAGAGAAGTGGGAAGCGTAGCGTTTC
CGGACAATTCCGCCAATTGCACCTTGCTCGCCGGCTCGCTGCTCCTGCTTG
CTGCTTGTTGTGGCTCGCCCGCTCGCGCACGACGCCTGTGACCTTGCTCGT
TGGCGATCATCCTCTCCCCTCTCATCCATCTTGCTCGTCCCACCGCCGGATC
TCCCTCCAGAAGAGCTTGTCGCCTTCGCCGGCACCCATCGTTCGACGCGAC
TCGCCTCCAAAACGGAATCGACAACGGACCCAAGTCGCGCCGATCAGCTT
GTCCCGGCCATTCAATCGACAGGCAGGCGTTTGGAGACCCGACTCGGCCA
GACCGTCTCGTCTCCTGTCATCGCCGCCGACGACGACACCGCCGGGCGATA

CCCAGACACCCCCGTTCCTTTGACTGCCTTTCGAACGCAGACTCAAACGCC
CATCCTCCCGCACCCACATCGCCCTCCAGAACTGGGACCATCGCCATTCCT
ACCGGTGCGGGCTCTTCTGTGTATCTTGTGAACTCGTTGGTGTACATCGCTG
GACACGTGCCCCGCAGCGCGCACCCGCCTCTCCCCAGGACAGGAGGGCAC
ACCGGATATCGGATCGCGCAGGAGTGGAGGGATACTGCCGCCTGCCTGTCG
GTCGGATCAGTCGTGGAGGTCGAGCGTGGAGTGGCGAGGTCTTTGGCCTG
GTGAGTCGCGTCTCCCTCGTGCCCACACCACCAACACGTTTCCCCCGCTCT
GTCTCTGGATTTGTCTCTCCCCGGCCCTCCCTCTTCGGGGAGAGAACACGG
CGGATCCCGTGCGACGGGGCACAAAACTTGCCTCTTTTCGCGAAAATCCTC
CATTGCTGCCATCTATCATCCACCCCACACCACCATCCCCCTCCCCCCCCCC
CCCCCCCCCCCCCAACCCGCCCCCAATGCTCATCCCCAGATACCTGGCCAT
GGCCTTGCCCCATCCTCCGCCTCCTCTACCGCAGACCCATCCGGGGCCCTC
GCCGCACATCCAGCACCCCCAACATCCCCATCTAGGCATGCAACAACCCCA
GCCCGGCCCGTCCATGCCTGCCGGCGTTTCGGTAGGGCCCGTCGGCGGCGT
CAGCGTCATGGGCGGTCTTCCGCAAGGCTACGCGCACCCCTATGCCCAGGT
CAGGCCGCCTCAGCAGACCACTATACAGGGCATGGGCCCCGTCGCCATGTC
TTCCCAGCAGGCCGCGGCGCTGGCCCAGCAGCGTCAAATACAGCAGCAGC
AAATAGCCGCTCAGCAGCAGGCGGTCGCCGTGGCTGCTCAGTCTCAGGCG
CAAGCCCAGGCCCAGGCCCAGGCGCAAGCTCAGGCTCAAGCGCAAGCCC
AGGCCCAGGCTCAAGCACAAGCGCAGGCTCAAGCACAAGCGCAAGCACA
AGCTCAACGAGCCGCCATCCCTCCTTCGCCACATCACCCGTCTCCCTCGCC
ACATCTGGCACATCAGTTCCCCGTCAACGGACCAGGCGCCGTACATCCTCA
ACAGTACCCCCATGCACCCCAGCCGCCCGGTCAGCCGGGTATGCAGCAAC
CCCCTGGTGGCCCCTCGCCTGCCCAGATCCGTCAGCCCCCTCCGCACCCCC
AGGCAAGGCCCCCGCAGCATCCGTACCCGCACCCCGCCATGGCCCAGCAG
GCGGCGCAGCAGCAGCAGATGCAGCAGATGACCCCGCAGCAGGTCAATCA
GCCTCAGCCGGGTGTCAATGGCATGCCGAGCCAGCCTCAGGGCGAGTTTG
GCGATGCCTATGCCATGGCGAGAATGGGTAGTGGTGTCCAGCCTCAAATGA
CCGGTGGTCAAGTACGGCCAGACACAGATCTACTGTGAGTTCCCTTGTGAG
TGTCCGGCCTTCGGCTGATGAGATAGCCTCAAGTTGTATATTCACGACTTTT
TGGTCAAGAATAACATGCCTGCCTCTGCTGCGCAATTACACGCTGAAGCCG
GTCTTGGAGAAGAGACGGTCCCGATAAATGTCCGCCAGGGTCTACTGTCTG
AGTGAGTTGATGTCTTTGTATGTGCGATTGTGCTGATCAACAGATATTGGGC
CATGTTCACCGATGTTTTCAGTAATGGGGGCAAAGGACCCAGTCCTTGGAC
GTCATCAGCTGCCTACACTGATGTCTGTCTGACCCTCGAGTCCTGGGATAAT
GCTGATCTCAGACTATGAACCGGTTGAGAAACATACGGCAACAGATCCTCG
CTGGTCTTCACCCCCCTCAGCAGAATATGAACGCCCAGCAGCAGATGCTGC
AATTCCGACAACAGCAGGCAGCCGCTCAGGCTCAGCAGGCGGCTCAACAG

GCTCAGCAAGGACAACCGCAGCAGCAGAACGGTCTAACGCCCGCAATGCA
GGCTCAGCTCGCTCAGCAGCATCAGGCACAGGCGCAGCAGCAAGCGGCTC
AACAGGCTCTCCTGCAGCAGCACCAGCAACAACAGCAGCAGGCCGCAGC
CGCCGCGGCAGCGCAGCGATTCGGTCAGCAACCTGGTGGACCGAGTAGCA
TGGGTTCGCCCGCCCCGCCTCGGCCGCCTCAACCTGGCATGACCCCCGGTC
CCGGTGGGCATCCGATGTACCAGACTTCATCGCAAATGTCCTTGGCGGCCC
CTCCGAATGCTCAAGCTGGACCGAGCCACTCGCCCGTCGCACCCTCGCCCC
ACATGATGCATCAGCCTCACCCTAGCGTCATGCCTTCCCCGCACCAGAAGA
ACATGCGGCCTCCACCGCACGGCATGGTCAACGGTCCCACGCCTCCGCAA
CCTGGTCAACCTAACCCCCTCATGCGTTCCGTCACCCCGCAACAGCACTTC
CACCCCGGCCAGCAGGCCTCGCATAGTCCCGCCACCAACGGCCAGGGCCA
GCAGGTGAATGGTCTCGCCCCGGGCATGCAGGGTCAGCATGGTCAACCCG
GCGGACCGCCCATGACGCCCCAGCAGCAGCTTGCGCACCAACAGCAGCAG
CAGATGCTGGCTGCTCAAGCGCAGGCACAGGCCATGCAGCAACGCGCTCA
GCAGTCTCTGGCGGAGCATCAGCAGCATCATCAGGCTCAGCAGGTCGCCG
CTGCCCAGCAGCAGCAGCAACAGCAACACGCGGCCAACCTCGGCGGCCA
GCAGATGTCCCACGCTCAACTTCAAC

>Mosaic_sequence_31

TCACATCGCCGCCGTCATCTGGCCGTGGCGCTCCTCGCTCGACCGAGTTGA
CAATCTCGCGTCGAATCTCGGTGCTGATGCTTGGGTGTGGCCGGACGCGGA
TCACGTCGAGCAACGCGTCTTTCTGATCCGGTGTCAAGTCGGAGCTGTACC
TGGAAAATGAGCCCATATCGCGAGCAATACAATGTCACTTGCCTTTGTACA
AAGACGAGCAAGCTCTGGTGCCACAGCACGGGAAGCTTGTCCTCGCCCGA
TTTGCTGCGTGGCGAGTTGGCCAGGCGGATAAAGTGAAAGACGAGTGCAT
CGACGACTTTGTATGGCAGGGCGTACTTTTTGTCCAGCAGGATACGGATGA
AGAGGGAGTTGGGACCAGAGTAGTCCATTGAGGCGAGCCGCATCAGGGCC
GCGGCCGAGTGAAGCACGGGTACTGAGACTTTGGACAGAACCGAGGCGA
CGATTGCCGCTTCTTTGAGCGAACAAGCGGTCTGCGCATTAGCAGAACGCA
GTGGTGGTCAGTGTTGCCAAGCTCACCTGGCACAGAGGGAAGAGGATGCC
CTTGAAGAACGCTGCCGGCTTGTAGAGCGCCTTTTTCAATGCCTCATACAG
GTGCACGTTCAGCTTGCCACTGTTGGCTCGCATGTCATCGCGACACTTGTC
CAGCAGCACGCCCTCGTTGAACACCCGTACTTCGGATGGCTTGAGGTTGGA
GACGAAGATCTTGGTGCAGGCGAGGGTGGCGTGAGGTGTCCATTCGGGAG
GGTTCGTGAGGGCGAGCAGCTGAGCCCAGTGTGGGAGAGAGGGGAGGAT
TTTGAGGGCTTTGGGTAGGGGACCGGATTTGTAGCGCGACAAGAGGAAGC
CGACTCTGATCATAAGCTCGGTACGGGTCGAAGATTGTGATGAGCTGACTT
TGTGTACACTTCGACGACTTTCGGGTTGAGGCCTTTCTTCGGATCTGGTGG

GCCGTCGTCTACAGTCAGCAGGTTCGCAGGCGGAGTGCACCACGCACCCT
CCTCATCCAGCCCCTTTGAGACGGCGCCGCCCTCCATCTTGGAGAAAATGA
TATCTGCTAATGTCCGCCCGGCCCCTTCCGCCTCGTCAACTGCATGCTGCGG
TCCTAGAGCGTCCAGCGTCGCATGGTCTTCGGGGTCGATGTCCTGGATATAA
GTCCTAGGTCAGAGGAGAGACGGGAACATTCCAATTCCGCATATTCTTCTC
CGCCCGAGATGCTTCCCTCTTCTAGCTCGTCCTCGTCTTCATCCGAGTCCGG
TGGTGCCGCTCTTCTAGGCCGTGTAGATCTGCCATCAGCGCAAGCTCTGAA
CATAGCCATGAGACGCACGGCTCTCCCTCGCTCTCTTCATCCTCCCACTCGT
CTTCCCCTAGCTCCCGCGCCACCTCTTCTTGCTGATCACGAGCGAGGTCAA
GAATCTTCTTGGACATGCGCGCATCTTCTCCGACCTAGACGTGAGCTCAATT
GCCAGTGGAAGCAATACCTACCTGTTCTTCCTCTTCCTCGTCTCGCTGCTTC
CGCCGACCGGGCTTGGACACGCGGCCAAATTTTCGCAGATTGTCATCCTGC
TCGATGTCGACATGCAGTGGATCGTGTCGCACTTGTGACGACCTTTTGGGT
ACTCGAGGCATGCTTGGCAAGGTCTGTCGCTAGTACAGGCGAAGAGGAGT
GATAAAAGCCAATGCTGAAAAGGCCGTTTCAGAAATTCTCGAGCGGTGCC
GAATCCGATTGCCGGTCCTGAATTGCCCTGAACCCTCTCGTCAGAGCCGCC
CTCTCGCCAGTCATGATATCATGCATAGTACAATAGAGATCGACTGCTTCTG
TGACACTTGAGCATCGAGCAAAGCAGCAACATGCTCATACTTGTTCGAAAT
TGCGTCTGGTCCATAGAACCTATCCCGGCATGACGATACTCCTGTCTCACTG
AGATTCGCCCAGAGCGCTCTCGTCACCCAGCGCCATGTCCGAGCTCGACGC
CCATGAAGTGACCTCCCTCCTTGCGGTACGCGGCTACTTGACCAACGGCGT
GATCTGACCAGCAGGCCGAATACCCCCTGCTAGACTCTACGCTCGTCCTTG
CCATCGTGTACGATTATGACTCCTCTACACTCGGCGCTCGTCTGCCCGAGAT
CCGAGATCAGCTAGCCATATTCGAGGCGGCCCTAGTGCCCGATCCAGATGT
GCCCATTGCGCAAGAGCAAGCGGCTTCTTTACAGCAAGAAGAAGACGAGG
ACGACCATCTCCCTGCTATCGGCGCCCTCAGCCTGGTATCCGAAAGCTCGA
GACCCTCAACAAGCAAAGATGGGACATCCAAGTCGCACAGCAGCTCGAAC
AAGACCTCGAGTTCGTCGACACCGAGCGAACCGGGCCCGTCTTTGATTGA
CGAGGTGGAGCATCTGACATTGCTTTTTCCAGACACGTGAGCTAGCTGCAC
GGCCTCGCGTATGCTGATAGCAAGGCCGGTCTCTACCGTCAAAGAGGCATT
AGCTACGCAACCGGATCTCGAAGCTGCTACCGACTATCTCCTGTCGGTTGA
GGCAATTCGCGAAGCAGAACGCCACGGTTACTTTGCAGGAGAGGAGCCAT
CGTCCGACTCTGGGGAATGGACAGCTGTCGCCTCTCGCAGATCCCCGCCAT
CAGCATGGAAGGAGACAATTCCGTCTGGGTATCCTCTGATGCCTGTCAGAT
CGACCTCGTCCTCGCGCAAGAAGAAGAAAAAGAAAGGAAGGACGATACC
CGTCATCGACACGCTCCAACGCCAGTCTCCGATACCCAGTCGTCCCACGTC
GCGAAGCAGCTCACGGCCCGCGTCTGGCAAACCGTCGCGAGAGTCGTCCC
CTGCAAGGCAAAAGCCTCCGCTGGTCTCGACAGCTGGACACGGACCAGCC

GTGTGGAACACTCTAGCTTCGCTCGCGGGATACCTTTCCGAGTTACTACCAT
CCCGCCAGCCGTCCTATTTCCTGTCGTATCTGCACTCCCCTGATTACCGCTC
TGCATACAGCGCAATCCGTGCTGCTCTGTCCGCGATACCGGCACCACCACA
TTCGGCAGATGGAGCGTCTCAAGCGGTGTTCGAGGATATCTACGGAGTCAG
CTTGCTTGACAATGCTGGTGATCCACAGGCGCATCGCGACTTGGTCTTGTG
TACTAAAGTGGCCGGGATGGACGTGTCAAATGCGATGGACCTCATGGATCT
CCTGGGCGAGCTCAGTCAATGGCCGAGCGACACCGACGAGATTGAGAGGT
ACGATTGGGCGTCCAGTACAGACTCTCTCGTCATCCCACCAAAGGCCCCGA
CACCCATCGTCCCAAAGTCACCTGTCCCAACGCAGACCTCGTGGGACCGT
GGCAGGGTACAGAAACGTACGAAGCAGGAGCCAAAAGAACGGAAAGAGC
GTATCATTCCCGGCTCAGCGCAAGCTGCGGCCGTCTCCGCGACCCACACGG
CCACGGACAACTTTGGTCTTCCGTACGCATCTCAACTGGTCGTGCCGAAAC
ACCCTCACGGGGCCCACCCCGACAATTGGCGTTCGCCCAATTATCGCCCAC
CGCGTCGTTCAACGACTGCGCACCCCTATGCGTCTCATATCCCCGCTTATGG
CCGCGGACAGCTGCCGAACGATCCTACGCCCAACTCGCTCTTCTCCGGACC
GTCAGCTCCGCCGTCCGCGGCAGAATGTCTGCGTCGCGCACAAGCGGAGC
GGGTCAAGCGATCAGAAGCGGTCCGCGCTGCAACGCGGCATCTTGCGGGT
GGGAAAGCGGTCCAGGGCTCTGTTGCAGGCTACTACACGCTTCAGGCTAG
AGAAGCGGGCGATCGGGGCAAGCAATGGGAACTGCAGGCGGCGCAGATG
ATCGTCAACGATCAGCTCAAAGTCGGCAATACGATTGATCTGCATCACATG
ACCGTCGACGAGGCGGTATCAGTGTCATTGCAAGCGGCGGCGAGGTGGTA
CGCCCGTGAACGAGAGATCGGCTTTTCGGGACGAGAGAGCGCAAACAAG
GTGTTTGTGCCGAGCAGCGAGCTGCGCATTGTCACTGGTGTTGGCCGTCAC
TCGGCAGGCGGAGTCGGCGTGCTTGGCCCGGCTGTGAGCTCGGCGTTGGA
GCAAGCGGGCTGGAGGGTGAGCAGGGGCGATGCGTCGGGGTATCTCATAG
TGAGGGGGAAGAGGTAATCGAGGTTATAGTAGCATCGCATTTGCATTGTAAT
GCCAATATAATGCTTGTCTAGTTTTGTGAGTCTTTCTGCTAGGCATGAGGGT
ATCGAGCTAGTCAAACGCACGTCAGACCGATCTGTGATCCACTTGGGGCAG
TCGATACAGAGGGAGGATAGGATATGCATGCAGGGTGCGCCAGATGATTTC
GCTATGCTGCAAGCGCCGCCCTGGCCATAGCCAGTACTCCTTCGTATGTCTC
ATTCCCGCCGGTGAATCCGGAAGCGTGACAGAAGGTGCAGCCGTCAATAC
CAGACACCTTGCTGAGCTCGGCATCGCGAACTCCGCACCAAGCCTCCGGG
AGCGATTTGCGGTTTGACAAATGAGTCGGGCGACTCTGGGACGCACTGTAT
CCGCCATCTCGATCCAGGGGCGTCATGTTCGGGGTAGAGGGGCGTAGAGTA
TTTTCTGCGGAGGCGAGAGAGTCGGTTCTAGGGAGAAGAGGTGGTCTTTC
CATGGTGCAGCCTCGACATTAGCCAGGCAACATTGAACCGAATGCGGCTCA
CCTGCTTGAAAACGACAACGGAGCCGCTTGGATCGACTTCATGTCTACTGT
TGAGAGCCTCTTTGACGATATCCCGGGCTGGCAGCCAGGCTTTGGCAAAGT

AGTCGAGGTGCGCAATGAACTCTTCACCGGTTATTTTGGACGCCTCGGCGA
AACGAGCCTGACGTGAGATGGGTCATGTGCATATGTGCGCGTCACTCACAT
CGTATATAGAATCGTTGGTCGGCTCGTTCCAGTTTGGGTTGAGCCTCTTGAC
TCTGGAGCTAAGGTCTGTGCGTTGCGAGTATGCCAAAGGACCGGAGCGAC
GTTGACACCATTGTCAATGCCGTCCACGCTCTCGATCATCTCCTGAGAGATG
AGCGAAAGTCAGAGAATGAAAGTGCGCTCACAGAGTAGATTTGGAGCCAG
AGGATCTCCACCCTGGGGTCCGATTCGGGACAACCGAGATACTCTGCAATG
ATCCGCCTTCCAAAGTGCTTGTACACCAAGCCCGCGGACGAGAGTTTGATC
TTGTCGAAGCCGCCATATCCAAAGACCTCGGAGAAACCGCGTTGATGATGG
TCGTAACGGTGCTTCTCCGGGTCATACACCCCTCCGACATCCACGACGATG
TCTAGCGGTGCCACTGCTCCTCAGCACGGTTCAAACGACAAAGGAACGGC
TCACGCTTGGACATGTCTCTTGTCCGGACCACGTCCGCATCGCGGTACTCG
TCGGTCATGCGCAGCATGAATACCGCAAGAGCCTCTGCCTATCAGCTCCTG
TTTGCCTACAACAATGATTGGGCTGACCATCGCAGTGAAATGTCCCAGAAT
GAGTGCCGATAACCTGGGGTGAACCCTACATCTTTGAGTCCAGAGTAGACT
CACCTTTGTCGACTTGATACGTTTGGCGGGGGTTGGAGCGGACATGATGCT
TCGAGCGACTGGTGTCACCAAACGGCCGAGGCGATGAAGATGGACAAGA
GTCGATGCGATCTGTACACCTCGCATCCAATGAACGGGTCGCTTTTGCAGC
CGAAACAATTCAACGAGAGCCCCGCGCCGAAAATCCAGCCAAAGCCGGTG
GCAACTTTCCCACGATCTCCCTAAGAGATCCCGACGCGTTTGCCGGTGGGC
CAGACCGGTGTACCGAGTGAGCGGCGTTGTCCGCCTTCCGGCTCTTTGAGT
GATTGGTGCGCCGCCCGGCGTCATGCCCTCCCCCTTCTCCCCCCTCCTCGC
ATGCTTCCTACCCATCCAACAACGACCGCACAGTATCTTCATTCGGCGCCTA
CTAATGCCTTGTACATCTCGTCGCGTCCACCCGTCGATCCCCCCCACAGCAT
ATACACGCAGCATAGCTCCAAAGCCCACACCTCACCTGTCGCCCACACCGT
CTGCTCCCTCCCGAGGCTATCGGGCATGATGCTCGAGAGCCGTCGTAGACC
GGCGCCTCTGCCTGCAGGCAAGGATCAAGCTCCGGACGAGCTGCTGAAAC
TCAGCAGAGGATCTATGCGCAGTCAAAGCTCGCTGAGCACCACGCCCACC
ATGGATGACGATGATGATGGTGGGTCTTCTCTCCAATCAGCTGCTCAGCTG
GGGAGCGTGTCTTACGCGCCTCGGCAGCCCATGATATTGAGCTGACTCTCA
GACGTTCCACGACGCTCGACACGAGGTCGAAATCCTCTGCCGCCCTCCAG
CGGACCCCTCTTCCCACCCCTGCCACCTAAACAGCCCAAAAACTCTCCCAA
AAATTCGCCCATGCGGTCTCCAGCCGTCCGGCCTCCAGCATCAGCGACCCC
GGTGCCTACACCGACCACCGCCCAGGAGGCAGAGCCAGAGAGCAAACAG
GACGTAGCCGCCGTGCTCGAGATCGCGCCCCAGCCCGAGCCAGAGGCAAC
GAGCTTGCCTAGCGGCTCTGCTCCCAGCTTGACGCCCCCGCCCGCAAGCG
AGCCCGCCATTGATATGGACGAGGACATGCCTGTAGAGTTGGAGAAAGAA
GAGGGAATGGAAGAAGACGACAAATGGGAGTCTTATCGTCGAAGTCGGCA

GGTCAGGTCGTTTGGTACCGCCAAGCCTGCTGTCAAGCTGGAGCCAAACG
AGGAGGGACGAGGAGGCAACGCCGGGCGCGCCAAGCCTGTCAAGTCATC
CAGTGCTCTGTCCGTCCCAACAGCTTCAGGTGCAGACACTCCCGCCTCGG
GCATCTCGGACGAACGCGCCGATTCGGTCAGTCGGTCGCAGGGAAGGAAG
CGCAGAGGGGAGGAGCAGCTTTTGCTGGATGATCATCTCTTGCCTGAAGA
GCTGCGAAGGATTGGGTCGGGGCCTAGCAAGAAGGGCAAGACAGAGACG
GACGAGGGTGACGAGGAGCCGGCCGAGGAGGAGAATGAGGAAGAGGCG
GGAGTTCCCGACGTTACTCGATGCGTGTGCCAGAAAGAGGGTGAGTGAAC
ACGCTCCGCTCAGCTGACAGCAGATGGCGACCCCCTCATGATTCAGTGCGA
CAAATGCAATGTCTGGCAGCATGGTCCGTGTGTGGGCATATGGGCGGACGA
GGAGGCTCCAGATGGTGAGCTTTTGACGCGAATCTCGGGCTAACGTCAGA
ATATTATTGCGAGGAGTGTCGTCCAGATCTTCACGGCCCATTGAGGAAGTG
GCTGCGACGCTCGGGACGGAATCCGTGAGTCTAACGGTTGTGTAACGCTG
ACATGCAGAGCCAATTTCCTGCCTCCCACTCCAGACGACCTCAAACACCTC
GCACAAGGCAGCGATAAACACTTTCCCTCTCAGTCAAAGATGTGGGCGGAT
ATTGCTGCTAGGGAGAAAGCTAGAGCGGCACCCCGGTCCCACCATCGCAA
GGAAGCACCGAGCCCGGTTGATAGTCTCGACGCGCGGAGAAGCGGCCGAA
GAGCACCATCGTCAAATGGAAAACCGGTCTCATCTGGAAAAAAGGACGAC
AAGAACAAGCCGTCCACACACAAGCGAAGGGTGTCGATCGTGTCCGTCTC
TCCACAGAGATCGGTATCGCCCCCGCATCCCTCTCAACCAGCGAAACGGCG
CAGTACAATGAACTCGCGCGACGCAGCATACGAAGAGCAAGTCAAGGCGG
CCCTGGAGGCCAGTCGATTAGAAGTTATGGGTCCGCAGACTGAGGACGAG
GATCGGGAGAAGGAGGACTCGCCAGGAGCGGAGGGCAAAGAAAGGGCCA
AGGGGAAGCGAAAGCTCGAGGATGCAGGTGGGTAGTGTTAGAGGGATCAG
GCTGACTTTAGAAACCCCCGAACCTGGTGGCCCTGGTCCTTCGCGCAAGAT
CAAACATCCCAATCAGTATACATATCGCCCAAAGCCGATGCCTGGGCAGCC
AGCGGTGGCTGCATCCCCCTCGCGAAAGCAGAACGCTAGTACACCTGTACC
GACCGCCCCGCCGCCGCAGCACGAGCATGGCACCCGGCGACAAGGCGCG
ATCGCCAACGGCCTGATTGCTGTACCTGCGTTCACGGTGGATGTGAACAAC
CTCTCTTGGCACCTCCCGGATCACCTGTCCGCATTTGCCGACCTACTCCCTT
CCCCTCATCCAACCGCGCTCAATGTGCGAACTGCGAGCACCCTGCAGTCTA
TACCTCGGAATCACTTCAACAACCAGCACTACGGCCCATTCTCAGAAGAGA
GAGACGCGAGGAAACTACAAGGGAGAATCTGGGTGTATGTGGATGTATCA
GGGTACTTCGGCTAAGACGAAAAGGTACATCCACATTCTACTGCCTCACCC
GGCTGAACGGACGAATCACAACGAATCCGATTACCTTCAAGTCAATTCAGT
GACCTCACACTGCTTCTCTGGGATCAGCCACCACCCTACTGCGATAACGAA
AGCCACGCGATTAGGTGTTTGGTGTATTATGCAATCATTTCTATATATCAATAT
TTAATTGAATAATAAGGTCGTCATCATTACTCAAGTTACAAATAATTGTTTTA

CATGTTCGTCTTGATCCGTGAAGTCTTTAGAAACACGCGAGCACTTATTGG
CTGTGAAGCATCAGTGCCCAATGGAGATACCACGGCTACGCTAAATGTTTT
TAAAAACGTCCGAATTTTCTTGAATTGGGCGGAATGTGAACGGACGAAAG
CGGTTATGAGGATAACCAAACCACAGTTCAAGCTAGCAACACGTATTTCCG
GACTTAGATTTAGACGTTGTTTTAGTTGCCTGCTAATGAACGTTATTTTTTAG
GATGAATCTTAGTGCAAAAATAACATTTTAAAGAAATCTTCAAATATTATTAC
ATAATTCAGTTGATTTATTAACATTGGGCAAAACTCTCAAATGTCTTCATATG
CAAATATTCAATGCTCTATTGAAACTTACTCAGCGACCCGACTGATAGGTTG
TGAGACTTTTCCCACGAGTTCTTGGAGTCCAAGCCTCATTGGGAGCTGAAT
TATGGCCTATGACCTAGCTTCTCACTAATTCCTGCCCGCCTATGAAACCTTTA
GGGCAGTAAGCGCTGCATATCACCTCCATCAATCAGATGTCAGATTATTGGA
TAGTTAGATCTTCTCACGGCAACAATTTTTGGAAATATCGCTATAATTGTGA
ATGCAATTACAAACTCCCCTCAAACTATTGGTTAAGCTACCGGTAAGGGGC
AACAAAATAGGGGAGAGATATATCCTCAAAGTACCTCGTTCCTGAGCTGGA
TGTACCTTGGAAATAACCGCAGGTACCATTTCACCATCTCAAAAGTATCGCC
GCGATGCAGCGCTGAATCGCTTCAAAAATGCAAGTAGGTCCACAATAACAG
GTCTGCAGGTCAAATTCGACAGGCCGAGACAAATTTTGCTGAGTCTGGAG
ACCTCGTGTGTGGGCCTCAATTGGCCAATCGGCGCAAGCAATGGAGACAA
GGGAAAAGGGGAAATGCCCCCACGTTAAAACTTGCCTCGGTGTATGTTTGT
CACCGAATTGGCGATTATCCATCGCATTTGGACGCATCGGCACGAGGGTATT
GTTAATCGTAAGTTTCTCGTGACCAGACACTAGGCGAATATCAATATCAACT
AGAAAATTATATAATAAATACGTCTATGCTGTCAAGTAAATATAAGACCTGTT
CAACCTCCGCAACATACAGGAAATTCATACGATTGCGCTATAAACTTGTAAC
ATTGAGATGACGACGGCAATGCCGCTCGCCCGGTCCTGCAATGGCGGATGT
AGCCGGAAGATATTCGGGAGCGCGGAACATGTGAACCGTCGAGTTAAAAG
GACGCATCTGAAAAGCTTGGGCCACTAGTTGCCTTGTTGAAGTGGCAGAA
GTGGAAGCGTGTTTCTTACTAAGAAATAAGAAGCCTTAGATGACTAAAAAT
TGCTTGACCCCACCAAAATTTTCAGAATTGAGAACCTTGAAATCTCCAAAT
CAACTTATCTAATCGCAAAAAAAGATACCACCTGTGGATACTCCTGCGACG
ACTACCCATTCGCCGTCGAAGAGTCGGAACGAAAGTCCATACCAAATCAAT
TCGCATATTCCACTGTTGTCCACGTTCACACCGCCAACGATGGGAGTCACC
AACAAGCGCACATTATCCAAGACCCGCCGAAAGACGAGGGATCTCGACCA
AATCAAAGCCGATCTACGCTCGCCGAAGCACTTAGCCCAATACAAAGCAAC
GAAGGCATCAGAAGATTTACCGGACTTGGGCAAATGGTATTGTACTGAGTG
TGCCAAGTGGTTCGAGAGCGAGGTGAACCTTGTGGTCCATAGGAAGGGAA
AGCCGCACAAGAGAAGGTTCGTATTCTTGCGTATTGGCACATAACGTTATG
GTTATGGTATTGGGTATCTAGTCTAACATAGACAATATAGGGTGAAGCAACT
CCGCGAGGAACCATACACCCAGAAGGAGGCCGAGGCGGCTATTGGCCTGC

GTACAGACAACCAAGGTCCCGAGAGATCTGAGCACGCCGCCGACAACGAA
TTACGATGGCGACATGAGTGAACGAAACCATATCCCACGAACTCTTAGATA
GACACTCATTGTGTCACCCGACAAATCCACCATGGTGCTATCGATCAAGCA
CATAGGTAGCCAGACGCATTGCAATATGCGCAGATATACGATGATACCACTT
AAGGCATTCATATTAGACCATCGAGGCCATATCGAGTCCCCAGCTTCGCTCA
CTTCAAACCACGAACTCTCGGTTTGACGAGACCACTTGTGCGACCTCTTCC
GGTCCCACCTCTTCCCCTCGTTTGCGTGCCACCTCTTGTTGACGTGCTTGG
GAGGCGAGAATGACGAGTATTCGAAGCCGGAGTCTCGAACTTCTTGGGGG
TTGGTTTTGAGACGGTAGGCGGATTCTCTACGAAGGATGTGTCTGTAGTCC
TGGACAGATAATTAGTCGTAGCTCAGAGCTTTAGACCGAATCAACTACCTA
CATAAATGTTGTTTCACCATCAAAGTCGCCTCGTTCTGTGCTTCTAGATCGG
CCTGTAGGCTGTGGCTGTGGCTGCTGCTCCTCGTTCTCATTGAGTTTTGCTC
GACGAAATGATTCCGCAATCGGACCCTATAAATATGATTTATTAGAAATGTT
GAGTTTAATAGCAATGTGCCATCTTCCTACCTCGGGGAAATCAACACAGAA
AGCATTCATATTCAAGCCATATAAGAAACTCGCGAAGCTCTCGCTAAAACG
AGCTAGACTCTCGTGCATAAGTTGAAAATGCATCATATTTGCTTCGAGATCG
GCGACGGCATCCGAGAGCTCGGCAAAAGCGGGTTCGAAGGTGTTGAGCG
GAAACGGTTCGCTGCCCTCGACGCTACGCTGAGCGGATTCGCGGAACGAG
GAACGGGAGGAGGGCGGAGGGGTGTCGTCGGCCGCGATCGCGATGTGGA
ACGCGGTCTTCTCGCGCTATCGTGGCCTTCGTGTCCCGACATTATCTTATCTT
GAGATCTGTCGGTGATGAACTGATGTCGGTCGAAGTAGGATAGCCTGCTAC
CTTGGGCGATGGTCAACTGGCAGCCTATTAAACTTAGACTGAGCGTTGAGG
AAAACGATAGACTCGCGAGTTATTGCAATTGATGGATTTCCCAAATAAATCG
ATCCTTGGCAACAGATCCACTGTATAGGAGTAGTGGGGTATTTACTTTCCGG
GTTGCTCTAACTGCTTTGTATGGAGCACGTGCCACACTACTGTTTGAATATT
TCTTTCTGGAGCTAAGAAATCCGAAAGTGACGTAAACCCCTCTATTAAGAG
AGGATAATAGAGAAGGAACGGAAGCGTAAATGCGCGACTACTCGCTTGTG
GAAGTCACATAGATCCATGATAGGTCTTAGGTAATCGTCTCACTTTAGCTAT
CCCTAGTTTGTTACTAAGTAGCCGACAAGAAGAAGATTCATCTTTTTAATTC
TCAATCCAAGTATCGAATCCTATCTGCACGATCGTACCTAGGACATTATACCT
ATGGATATTCACTACTCATCCTAGAAACGTAGTCAATACCTAGTACCTAGGC
ATCAGGTTGCAAAGACTTCTACTCTTCTTAAAAACCAAGTTCGAGATCGAG
TCCGATTCCGAGTCCGGGTCCTAGAACATCACAAAATATTTATACACAAAAC
GTCCAGCACAACTTGGAACCTCGTTATTAATCTATAACAAATTCGGGGCCG
ACTTACCTAATTTAGTCTCGTATGCGCTTTTGGTTTGCGAGAACCGAATACG
ATTGGTTCGAGAAGCAAGGGGATCAAGGGGATCAAGGGGATGCAACAATT
ATTTCCCTATTACATCCCTACTACAACGTTCATAGTGTCAGCGCATCCGCATC
CGCATACGTCAACCCTGGCCGCCCAGAGATCCAACCTCTCTGAGAGCTCTC

CTCTAACATTTCACTGTCTGAGGTAGTGTTGACTCGCGTCCCGTCAGCAGG
TCGACATCTCTGCGACTGGCTTTTTTCACGTCTTAGCCCTAAGACAACCAG
GACAGTTAAGGCAGGCAACCAGCCTCTTTCGTAGGCTGGAAATCTGTGCTT
TGCGCTTACACGGCGATACAGCGGCTCGGGTCCTCCGGACATTAGTTGCGC
CCTCTTAGCATTGTCTTGGACCTTATGAAAGCTCCAGCGTCGCGGACATCG
CAGCCATTCAAATTCCCCACAGCTCCGAGACTTGGCTTTATTATTACTCGCC
TCGCTGGTCGTCCAAAATCTAATCTGCATTCATCTTCATTTCGTGCCTCAAC
CCTCTTGCGCGCATCCTCTAGCCCCGCCAGCCTTCTCACTCGACCGTTATCG
TCATCAAAGCTTGCACTGCCCCGCGGATCTCTACCACGCCGCAATATTTGCG
ATCGCAAAACTAATCTGCCATTGTTTACTTTCCAAGTAAGAAAAGCGCATTG
CGACAGAGGAAAATTGAGACAAGAATTTCGAAAATATAACTACAAGAACC
ATCGGATTCTAAAAAGTCAAATTTTATCTGCGCTATATATTATACCCCTAGTA
GTATTCGGCTCTTTCGTATTTTACGGTTGGGTTCCCTTTAATTCCGGACACCA
ATTAATTGCAGAAGACCCCGCGAGGAACTATTCCGGCAAGGAGGTTGAAG
TTGAAGTCGTCATGGAACAACTTCCAGGCAGACCGGGGAACCTGACCCCC
GAACAGGAAGAGAAGTTGCGCAAGTTCTGGGAAATGTTCTTGCAAGTATG
TGGCGTCCTTGGCGACGATGCCGCGGGTAACGATACGGAGAATGCTTCCGA
GACTGCGTCGCGAACAGGAACCAAGGAATCCGAGAAGCCCAAGAAAAGC
CGGATGTCCCTCTTCAAGAAGAAGAAGGATAAGGACAAGGGAAGTAGCAC
GCCTACCAAGATTGCCGCAATGGAAAGTCTTAAAGAAAACGACCCGGAAG
ACAAATGGGGAGAAAACAAGTTGTACCTCGAGACCCTAGCTACTCATCCTC
CAGAGCAGATAAGAGCTACAATCTGGTCGATGGTGAAGCATGATCACCCTG
ACGCCCTTCTATTACGATTTCTGAGAGCTAGAAAGTGGGATTTGCAGAGAG
CGCTCGTAATGTTAATGTCGACTATGAATTGGAGATGTTCGGATGCTCACGT
TGACGATATAATCATGGCAAAAGGCGAAGAATTCGCCGCCGTCAACGAGA
AGAGCGGGGATGCGAAAAGCAAAAAGCTGGCCGAAGACTTCTTGGCTCA
GTTGCGCATGGGGAAAAGCTACTGCTATGGCGCCGATAAGGCAGGAAGGC
CAATTTGCGTAGTCAGAGCTCGCTTGCACAAAGCAGGCGAACAGTCAGAG
GAAGGTCTCGAACTGTTTACGGTGTACTTGATCGAGACGACACGGTTTCAT
GTTAAGCCACCCTGTGGATACTGCTGTAAGTGTGCTTTGACATGCCTAAAAT
GCGAAAGCTAACGATAGCAGTGCGTTCTATTTGACTTAACCGGATTTTCATA
CGCCAACATGGTGAGTAGAAAAAAAGATATCATTAGGCTAATCCTAAGCAA
AGCTAATATTTCATAGGACTACACGCCAGTCAAGTTCATGATTAAATGTTTC
GAGGCGAACTATCCCGAAAGTCTCGGAGTGGTCCTCGTCCACAAGGCCCC
GTGGGTCTTCCAAGGCACGTCACCTCGCTAGCTCTTTGCATTATTCTCCACT
CTAACCGAAGTTTATAGGTATCTGGAAGATTATCAAGGGGTGGCTGGACCC
GGTGGTCGCAAGTAAAGTGCATTTTACAAACAATCTGTCAGACCTAGAAGA
ATTCATTGATTCGAAGAGGATACCTACCGATCTGGAAGGCACGTCAGGTTAT

TCGTACAAGTATAAGGAGCCAGTACCCGGAGAGAATGCGAAGATGCAGGA
TACGGAGACCAAGGAGAAGCTCTTGCAGGAACGCGAGCTGTTGTACGAGG
AGTATGAGAGCAAGACGCTTGAGTGGATCAACGAGCCGGACGCGGGGAA
GAGGGCCGTGCTTCACGCAGAGCGGGATGCCGTTGCTAAGAACCTGAGAG
AGCAATACTGGCGCCTAGACCCGTACCTCAGATCGAGGTCATACTACGACA
GGGTGGGAGTCATTAAACCTGATGGGGGCGTCAACTATTACCCCTCGGAGG
CTACCCCAGCTCCCACGAACGGAACGGCGGCGGCGATCGCCGCGGGAGAG
ACGGCTGCTAATGACGTGGACTGAATAGATAATGGGAAGTGCAGCGTAGAG
GGATAAAACAAAGACCGGGTGCATGGGAACGGGCAATTCGCATAGAATAG
GCATTGTATAAATACCATTGAGTTCGGTGGCTGGGATTTCACCATTATTATCT
CGCCCCTAAGCTGATCCAGTCATTGTGAATCTTGTCGTGCTTAGATTATTTTT
TATCATTTTGGGTAAAACTACATGAGAATATTCTTATAGCTATGAGGATACCG
TAGCTTTAGGTACGGGTTCTAGAGTTTAAAGCTGTCTTGATCATGTAGTTAC
ACCTTGGGCTTGATCTCCTCGAAGATGGCCTTCAGGGCTTCCATATCTGTCT
CCTTCTGAGGCCAGCCTACGCCGAGTCCTTCGGCCTCATTTGGCTTGGGGA
TCTGGTGGGCGTGTTAGTAGGGCTTGTGATAAATATACTAAGGGTTTGTGCT
TACCATGTGGAAGTGAACCTATAATTGTAGATAAGCATGGTCAGCCATTTTA
TTCACTGTAACTTGATCCTTGTTTATATGGGAATTGGGAAAGGGAGAATAAA
CTCACGTGGTCAACGACTTGGTGGGCGATGCGACCGTTGTTCTGGAGGATG
TTGAAGTTCACGGCTCCGGTGGCGGTGGCTAGCTTCTTAGCTACGGGAAGA
ACTTCACTCAATTGCTCGTCGGGGATGTCGGTTAGCTTCGCGCCGTGGAAT
TTGGGGATGACGAGCTGTTGTATGAGATGTAAGTATGTATTCGGTTAATCTG
TTGTGATATAAGGGGAGGTAGTGCTGTTGAGTCTTACAGCATGGCCCTTGCT
TAGGGGGTTGATGTCAAGGAAAGCGAGGGTTTTGTCACTTTCGAAGAGCT
TGAAGCAGGGGATTTCTCCTGTAGTTATTATATTAGTGATTAAGTTGTATAAG
TGTTCATGGAAGTAGTTATATAAATGAGTTGTTGAACCATTGCACGGAGAA
GCCATAGGATTAGGTGTAGCTCAGTCAATGCGAACACTATAGAATTAGGGA
GACTTGCCCTTAATGATCTTGCAGAAGATACAGGCGGCCATTTTAGATGACA
GTGATTTGCGGACACTCAATTAGGTTGATTAGGATGGATACGTGGACCTTGA
TGGCAGTTTGAATCTAGATGTCAAGACGTAAGAAATATATTCCGTGCTTGTT
ACTCTAGATCAATTGTTTGTTAAACAGCTCACTGTTTCGGCCTCATAACCAG
TTACAGCTTAAGACGTCACATATCGATGGAGGGGCTCGGAGCTACCCCGCT
ATCGTCCGGACTGGTCGGTAAATTGTCGGCAAGGCCATCGGACTTAAGAAT
TATGATAGATTCTATGATCGATTCTACGCAAAGGAAAAGAATCTCCTTGTATT
TGTGATATTTTGAAAAATGTTGAATATGCCGTTTGATATGAGTCTACAGAAA
TGTCATGTGGATGGTAGAAATATCAAATAGGTGCTGAAATGCAGAAGGGAA
ACGCGATAACTTCAGCTGCTTGTGTCAGTGACGTGCCAAAAATGTTAACGG
AACAAAGTCAAGCTTGGATGACATGTGCAAACAATCAAAAAGCAAATGAG

GATTGATAAAAGTGTAAAAAAGAAAAGCAAAAAATTTTGACTAGCAAAAT
TGAATCTGGAACTTCCAGAGTAGTGTTTCCAAAGATTAGTGTAAAGGAGCT
TTTTGGGTTGCGCAAGGTTGAGTCAGGTAAAAGGCATGAGTCAAGTACTTT
TATGCAATCCCGCTCCTGAAGAAATTTGTCTACAATTTGATCTTATAAACCC
AGAGGTGGGTTGGGACATTTGGGCCAATTAGAGCTGCCTAGGATTGGCCAA
TGCGACCAGCTCACCGCTTAATATTTGGACAGCTGCACAATGCTTCATTGCC
AGATATGAATGGGATTCTTCTTTCACAAGTGCATCTATGTTGCATAGCACAA
TTGCTTGCCATTACCGTGCCTCTCCACAGATCTTGCGATCTTGCTTAAGATT
ATCGTGGTGCCCTTCGTCTTTAAACTTGAAATACCCCGCGCATTTGGAGCA
AATCACTTACTGCTATATATGAGAAACAAAAAGAAGACATTTAAATGTCGA
ATATTGTATTGTTCTACACAATCTGTTAAGACATCAAAAATGAGGGACCACC
CGGGATCGAACCAGGGACCTAAGGATGATTGTGTAACTGCAATCCTTCGCT
GCGGAACTGTTAGAAACCAAATCTTGAATTTTGGAAAATGGGAGAATCACT
AACCTCTACCACTGAGCTATAGCCCCCAATTGATTGGTTGAAGTGGCGAATT
TTAAAGTGTATTATAGAGTAAGGACACCCTTAGCGGTCTTAGTACGACGTGT
CTAGTGGCTTGTCGGGATTGGAGGTTCTAGAAATAAGCCGGGCAAATAGCA
TTGCGGAGAGCGTATCCGGATACTTGTCCTTGTCTTTGGGGAAAATAACGG
ATATTGATAGGTGCATGTTAAGTGCTTTCGAGTAGGAAGAATCTGGCATTTA
GAATTTAAGAAGATAATGGATGAAAAATCACACACGGATCTAATTGAGCTA
CCTATGGAGCTAATTGTTGGCTGCTGGTTGCTAGCAATTTGCAAACGCGATG
ACGTGCGAAGGTCTAGGACCTAAACAATTCTAAAGCTGGGTTCGAATTAAG
GGCATGCATGAGAATAAAGTGAAGCCATTTAACGAACCACTAGATATCATAT
ATAAAAACAAAATTTCTATACAAAAGAAGAATTCCATTATAAATCATCAGTA
AGGTGTATCTGTATCTAGGTATATAACTAAACTTTTCGAAGGCCAGAGCTGA
ACCCAGTGTTGCTGCTCGGCTCATAAATCCTCCTGTACAATAAATCGCTAGG
TATTCACATATCATAACCCATTATATCATAGCAAACCATTATATCCATATATAA
ATAAACTTGATATCATCAAGGGGCTGCGAGACGACACGGGTCTGAGTATTT
TTAATCTCCAAATCACCGTCTGCTCGCAGTCCGTAAGCCCAAACTCCTCCC
AAAGAAATAACCATTCTATTGTTTGACCATAGGTATCCCCTGCGCCCTTAGA
AACTCCACTGCGTCGTCCATTCCGAACGGAGCATTGAACCAGTGTCCCCCG
GCCGGGTACTCGTGCCAACTGACGTCCATGCCAAAGTTCGTCAGTCCGTCC
CGTAACTGCTTCCCGTACTCGACGAACACGAGTGGGTCATCCGCGCAGTGT
CCGAGGAAAACCGGCGTATTGCGTACCAGCTCGTCGCTCTTGCTATCCAGG
AATTCCTCCAACCCCAGTATCTCCCTCGGTCTCGTCGAGAGTGCCCCCAGG
AAACGGTAACCAGGCCGAGAACCCCATGAGCCCGCATAACCGCGGCGGCC
TCGTCTCCTCTTCCTTCTCTTTCTCTTTTTCTTTTTCTTCCCCCTTTTCCTTTT
CTTCATCCGCGCTCTTCTCATTAACCACATCCCCCTCTTCCCCCAAATGCAG
CAAAACATGCGCCGCCGTCGCGCCCCCCTGACTAATCCCGGCCAACACAA

CCCTATCGTACCCTCCCACCTTCCTCGCCTCCCTCCTCACAATCCTCCTAAC
GCTCTCAACACTCTCCTTCAGCCCCAACGCCTGCAGCTCCTCCCGATCCGT
CATATCGAGGATATTCCAGACATCGAACCACTGGGGCCAGTGCTCGCCTATG
GGGCTGCTCTCGACGAGGTTCGGCTCCGCCTGCGGGAACACCCACCTGAC
GGACGGGAACTCGTCGATGAGGGTGCGGCCGCGCGAGTCGCGGGTCCAGC
GCAGCAGCGCGTCCGCCATGTTCTGCGACGTGTCGCCGCGCCCGTGCAGG
AAGATCACCGTGTGCGTGTGCGGGTGCGGCGACAGGGGCGCGACCACCGA
GCACCCGAGCTCCATTTCTTTTTGCCGTTTGCCTCTCTCCCTTATTTGCCCCT
TTGCAGATAGGTAGGTAGATAGGGAAGGGAGGCGATTGTCCTTATTTCCAG
GTCTATTCCCAGTTCTCAATTTCCGATGTTCCGTTTCCTATTAATTTGGTAATT
TGATAATTTGCCCTGATGTGGAATGATAGGATAGATGCGATGATGTAGTATGC
AGGTATCTGACAAAGGCGATCGGTGAAAGATACCGTACCTGAATCAATACC
TTTGTTACGCTTCCGATAAATGGTAACGGGCGCGGTGTGGTTCAACCTACC
CACCCTAATTAGGTGGTAGCGGCGCGCGACGGGCAAAGAAATGGAGAATC
GGGATAGGACGAAGAAATGGAACTTGGAAAAGAGGCGAGGGATAACAGC
TTCTAGATTAACATGACTCAGCCCCCTCTTCACAAGAACTTGGACCTTTTTT
TAATGTAAACTTTATTAAAAAAAAAAAAAAAGCCTCGGACGTGCAACCTCAA
AATGTTACGTGCGACGAGGTGTTTCTGCAAAAGATCGACAGATACGCCCGT
GCTGGTGTGGTAAGCTTCTGGCTGGTGTATGTAAAGTGGCATGTAAACTGC
GAGATAGATACAAAAGGTGCCGGCCGGACTGCGACCTTATAGTTTTGGGTC
CATCCTCCCCATAGGATTCGCTTATCCCGGCTCCTGTGCGCTTGCCAAGCCG
GAAC

>Mosaic_sequence_32

CGAATATAGGATATATATAGCATATGCTGGAATTAGTGGTTGGCTCAAATAGG
ATTTCTTATTGCTGTGTATGTGATGTGTCTACTGAGTTTTATTAGATTGTAATT
TTTAAATGGCCCCTTTTATTCCACATGGGGGACCCCTTTCCGCCCGCTGATC
CAATATTTTCACATCACCATACAGCTCCCCCAGTCAATACGACCGCCATCCT
GTTGGGTCCATTGAGAGTCAGACATGTTGATTGCAGGTGTGTTTGTTGCTG
GGTTGAATTTGTGTTGTTTAGAAAAGTTATGATAAGTTTTGTGGTGTGTTGG
AAATTGATATGACATATGCCGGGAGACAAGCGACAAGCAGCAGATTTACCT
CCTTAAATACTTAGTGCCGTAAGAATGCCTTTGTCAAGGTGTCTATGGATAA
CCCTTACATACCATGTAGGTGCAGACAAGCACAGTAAGAAATTAGGATAAA
GACTTCTTTTGTTATCTCAAACTTTCATTATGCTCAGCTCAAGTCACGTACA
ACATTGAAGATGCCTAGTGCTCCATCTGGTAAGTCAACTTTTATGGTTATCA
GATCAATGTTGTGCAACTTCTCACAGGTGCTTCCTGTCATGTCTTTATAGTT
TAGCTAATGTCATATCCTAGAATTGTGGTCTGTCATGTTTTACGCATATCTTT
CAAATTTTTGAACTTTAGCAGAATGATAATTTTATAAAATTTGGTGTAGCAC

CTTCGATCCTATGGCCGCTTGAATCGGATCTGTAAAGCTTCATGTCCTGTTG
TCAGTTAAAGTGGACTTGTTTCTCTCTGGCCAGTCTGGGGAGGCCAGTGTT
CGAAGATGTTTCTCCGGATACTTCTTATTTCTTTGGCTTTACTATCACATTTAT
GTTTAACTCAGGAACTTCCGGCACCCATAGTTGACCTCGGCTATGCACTGT
ATCAAGGGTACTACAACGCAACGTATGACTTGAATATTTACAAAGGGTATG
ACACCTTAAACATCTTATAAAATACGAAGTTATGATTCTGACTATTATTTAGA
ATCAGATATGCCGCACCCCCTATCGGCAAGTTGAGATGGCAGTTACCTCAG
CCACCTGCACAGGATCGTAGTCAGGTGTTTTCGGCTACTGAATACGCCTTA
AGATGTCCACAATCTGGGTGCGCTCCGAGGTACGCTTCTGATATATGTTTCC
AGTATAATGATATAGCTCTGACAAAAGAAGACCGAGGACGCCAACTCCGTC
GGGGTCTGAGGATTGTTTGTTCCTGAATGTCTTGGCTCCTGCTAACAAGAC
CATGTTACCTGTCCTGGTCTGGATTCGTAAGTCTATTCATTTCCTCGCTCAAG
ATCAATTCTAAGTGATTTATTTCATGCCTTAGACGGTGGTGGATTTGGGGTG
GGCAACGGCGCGTTTGAGCCATCACCACAAATGCACACAAACGAGAATAC
GTATATCATTGTGACAATCCAATACAGATTAGGCGCCTTTGGCTTTCTATCGT
CTGCGGAGATTGCAGAGTCGGGGGTACTGAACGCCGGAATCCACGACATG
AAATTTGCGCTTCAATGGGTTCAAGAGTACATCCATCTGTTTGGCGGAGATC
CGACGCAAGTGACTGTTGCCGGGCAATCCGCCGGAGGTGGTAGCGTCGCA
CTGCTCGCCATGGCCGATGGAGGTGTCGACAATACTACTTTGTTTAATGGCC
TTATTGCCTCAAGTCCCTATTTGCCTACTCGATGGTGAGGCCAACTTCCCTT
GTTCTGTTAATGTTATTACTGAGTATATATCATCTTAGGGAGTACAACAGCAC
CATGTCAACGAACTATTACTACCGTTTTGCGGAGGCTGTGGACTGCCTTGG
AGATGATGTCATAGCCAACGGGTCGGTATTCGAATGCTTGACTGAAGCAGA
TACTATCAAGCTTCAAAAGGCTAGCGACTATATTAGCACTATGGGAATTTAT
GGCCAATGGGCGTTCACTCCAGTTACTGACGATAAACTATTGCTAGGGCGG
CCAACTAGCCAATTACTAAACGGTGGAAAGCTCAACGGTATCCGTATGCTG
TCATCCAACAATCCGAACGAAGGCCCAGGGTTTATCCCCCAAGGAATAAAC
AACGAGGACAGGTTCAGGGGGTTTCTTCTTGGAACTTACCCCCTACTCAGC
GAAGAAAATATTACTAATATCCTTGAGCTCTATTCTGTTCCAGAAAACGCCT
CAAGTGCATTGGTTAACTCCGATGGTGAGAATCCACCATACTCTACGACTAC
GTCGGGTTGGGCGTTTGGCTGGCAACAGGCGGCTACTAATCTCTACGGAGA
GGCAACATTCATCTGTCCCTCATACTGGCTAGCTGACGGCTACGCGAGGAA
AGATGGCGGAAAAGCTTGGCATTACCAATTCTCCGTTCCACCTGGGCAACA
TGGTCTCGACCAGACTGCACTACGCTCTGACATAGATGTGATGGGTACAGG
AATGGACGAGGTGTTTCGTACCGCTATCCAGCAAATTTGGGGCAACTTCAT
AGTGAAGGGAGATCCAGCACTCAGCAAGTCACAGGCTCGTGCCTTGCATC
GCGGTAATATTTCAGCAGTTAGTGATGGGAAGTGGCCGGAATGGAAAGGG
GAACCAGGAGTGACTGGATGGTTAATTTGAACATGACTGGTGGCGTGCCA

ACCTCGACAACGTTTAACCTGGACGGGACCTTAGTCCACGTCGTTAGCTAC
GTTTCTGGTAATGGTACATCGCCACCTTTGGAGGCGAAATTCAATATTGTCG
AAGGTAGTTCTTGGGAGGGCGGAAGGGGCAAGAGATGTCAGTTGTGGGCA
GATTTAGGACCTTGGATCATGGCTTGATGAATTTTAATTCTATTTTGGCGGAC
ATGGTCGTATACTCTCGATTCCGGATCATTCGAATACTTTTTTAACTAATGAA
AAAAATAAGTATTTTACGAATTATCACAGAGAAAAAGGGGTTGTCCTCTAA
AGTACTAACTTCGATTGAATTATAATGCATAATTGATTATAGTCTTTAATATCA
TTGTTAGCAATCGTCTTTTCGGGAAGGGGAAGCCGCTCGACGCGTATGATT
TCTCTGAGATGATACCAATCAATCTTCTTTCGAATAAATGACACGATGATAC
ATGATATCAGATATGAAAGATATTATGTAAAATTGTAAAAATAGATAATGTTT
GGTGGGATAGAACAGGGAAACGCGTGTTTTCGTAACCTCGAGATAGGCATA
TATCCAGATTAGGCAGGTGAATTGGCCATCAATAAGTGTATTCATATCTTTAG
TTGCATGTTGTAGAGACGACAAATGACGAGAACGACGACAATCTCAACGC
TAGGGATCTCACCACCCGAAAATTTACCTGCTAGACATGAACATCGACAAG
ACTCACGTATGATGCTTCCGTGGTCAATTGAAGCTTTCTTGGCTTTATGTATG
TATATCAAGCATCGGTCTTTTCCGAATAGATTAGATAAGTACCTTGTACATAA
TATAGGGGCATAGAGGTGGCCAGACCGAACTTGCTTACCGAATTACCGAAT
TGTAAATGTAAATAAAATGCTGGGGGTTTTAGACGGTTTAGACCCAGCTTTG
ATAAGGAATGGGTGCGATGCAAGATATAATGTTCATGGCATAGCGTTCCGGT
CGCTGTCGTATTATGGTGATCTTCCCCGGACCCCACTTCAAGCATGAGATAC
GGGGGTATAATATGCAGTAGGGAGATAATATATCTCTTGGGTGCTGATCTGTA
GCCACGTGGGATGGCGACGCGCGAGATTACGGAAGAAAAAGAAAAGAAA
AAGAGGAAAAAGAGGAAGAAAATAGAGAAATAGAGAAATAGAGAAATAG
AGAAAAAAAGGGGAACGGACTATAAAAATATAACATTTAAAATTTGAGGTC
GTGTTTCGACGGAAAAATGAGATATTTCAAGTCTTATAACCGGGTCATCACA
TCCCGATCAGAAAAGAAACGAGTGAGAACTCACTCATCACGGTTGCCACT
CGACCCATTTTGGAGATAATGGATGCTTTTCCAGGATAGGAGCAAGCCCTA
GACGCCACCCACTTGTCTACCCTAGGTATATGTAGGCAGGCTGGCTTATCTG
GCCTATCTCCTCACCCATTAACGTCCAGGGTGACAATGATCTATCCTGCGGG
GTGAGAGGAGGTCCCGGAGCTCGGACATCTGTAGATGCATATAATCGAGAA
TGATCCCATGTATTTCGTGCATTCGTAGATCTTATCCTGTAGTTGTTCCGAAA
ATAATTCATCGAGGACCATCATTTGGTCGCAACTCGTGAACAATGACTCAA
ACACCAGAGATCGTGGCCGTCCCTATGGAGGCTAAGGGTCCCTTGGAGATT
CCTACCGACTCCGAAAAGGAGAAGGACGTAGAGAAGGATCAGGCGAAGG
TGATGGACGCCGAGATCAGCGAGAAGAGTAGCGTTGCGGCCGGCGACATT
CAGGAAGATGCAGGTTCGGCTTCACTCAATTAGTATATCGCACCATGATCAT
CGTACTTACTAGTAGACAGCTCCGAGTTACAGCGAAGATGAATATCATAAGC
TGAAGAGGAAAGCCGATAGGTACTTGTTGCCTCTTATGTGGCTCTGCTATG

GAATACAACAAACCGACAAGACATCTATCGGAACACAAGCCACCTTCGGT
CTCTCGAAAGACACGGGACTGCACGGCCAGCAGTACTCGTGGCTCACCAC
GATATTCTACATCACCTACATGTGCTTCGAATTTCCTTCCAACATTATCCTGC
AGCGATATATGATGGGCCGCACCCTGTCGTGCTATATGCTCTGCTGGGGTGT
TGTCGTCCTGTGCGTCGGCTTCGCGCAGAACTTCACCCAGCTCGTCACGCT
GCGCGCGCTACAGGGATTCTTTGAATGCTGCATTTCGCCAGGCTTCATCCTC
GTCGTCGGAAGCTGGTACACCACGCGCGAGCACGCATCGCGATCCCTGGTT
TTCCAGAGCGCCAACGCTGGCTTCGGTGTCATCGCCAATTTGATCCTGTATG
GAATTGGCTCCGTCCAGTATAAGCGCGGAACCGAATTTCAGGCTTGGCGAT
ATATGTCTTATGTATGCTAAGCCCCTCCTTTCCTCTGAGTATTGCAGCATTTAT
GCCTTAAATGTGTAAAATGGGATTACGCTAACGTTCTGGTGGTAATTCTATC
GGTATAGTTTCTAGGAGGTATGACGCTTCTTGTAGGCTGTCTGTGTCTTTTC
CTTCTAGGAACACCGTCTGAAGTACGGTGGCTATCGCCCGAGGAGAAGAA
GATTGCCAACGCGCGCATATTGTCGAATAACACGGGGCATGACAAGACTGG
CATCAAGAAGTGGAAGTGGAAGCAGGCGCGAGAATGCTTGATCGACCCTT
GCGTGAGTGTTCCCAATCTTCGTCCCCTGAATAGAGGGGGAAAAGTATCTA
CGTCGCGCAACTAACACCGAATTAAATTTCGCGATCCGTAGTTCTGGTTTGC
TGGCATCAATGCGTTTTTGAATTCCGTGCCAAACGGGGGTATAACTGCCTTC
GGCTCCATCATCAATACCAACGCAGGGTTTAGTAAGTTTCCGAATGAAACT
CTGAAGAGAGCAGAATTTAACAATTTGCTAGCCAACTTGCAAGTCATCCTC
CTTGATATCCCGAGGAATATCACGTCTGTATTGTGGTTCGTCTGTATCGGCCT
CATCACAAGCAGAAAGAAGGACTTGCGTTTGTAAGTTAGGACAAAGATTT
CCATGTCCAAGAATCAAAAGTCAGCTGACCCGAGATCGAAGGTATTTCATG
ATGGTTTCCGTCATCCCACCGGTTGCCGGTTTCGCTATAATGGCGGCGCTTC
CAAACGAACCTCAGTATAAGTGGACTAAGTGGGCAGGGTAAGTAGTCCTTT
AGATGAACGGGTTGTATTTTTGATGGATGTATTTCAACAATTGTGCTAACGA
AGGCTCAATTCCTAGATTCTTCATCACTGTTCCTTGCGTGGTAGCGCTCTTC
TTAGCTTGGACTCTGATTCCCTCGAATGTCGCCGGCCGTACTAAGCGTACAC
TGACCTCTTCTTTCACATTTGTCGGATATTGCGTGGGGAACATGGTCGGATC
TCAGATCTTCAAAGCGAAAGATGCACCGAGGTATATTCCCGGAACTGCTGC
TTGTTCGGTATGCTTTGGGTTAGAGTTCTTCTTGCTTGTCGCGTGGCGGGTC
CTCCTCGTCATGAGAAATCGACGACGGGAAAAGCAGCTGGCTGAGCAAGG
CATCAGTGAGGAGGAGAGGATAGAAAGAGGCAAGCAACTTGGAGAACAG
GATTGTACTGACTTTGAGAATCCTTATGTGAGTCAATATTCCTTTTCGCTCCC
CTACATCCTACTACTCCAAAGGCCCGTCTAATGCCGTGTTATGCAGTTCCGA
TACACGATGTAGCATTATCAAAGAACGAGTAATCTGCTTGAAAAAGAGAGC
TCTTTTGGCTATCCTGCTTCATTACCTAGTTTGCCTTGATATAATAAAAAATAT
GTTCTATGTTGTTGAATTGAGATATGGAAATTGCTTCTGTCTTAAGTGTACG

GTTCGGGAGCAGATCCAGCGCTGGGTTCTGAGGCCTTTGGCAGGCTTTAGC
CGTCCGGCGTACGGTTAAAACCTAGCTTAAAACTGGTACGAACAGGAAATC
GGATTGTCAAATGAAGATATGATATTGTAATGGTTAAAATGTAAGAACGAGG
CAATGCGATTTCTGCCGAGTGTGTTGAATGTAAGTTCTTGAAACACCTACCT
CATACATTACCCTGTAGAAGAGCTGTTAACGATATATTCAATAAGTCGGCGC
GAAGAAAATTCAACCAAGCGCAAGTAAAAAGCAGGAGTAGCCATGTTAGT
CATGACAACATAAGAATACCAAGCATAGTAAAACGACATTGGGGACCAAG
AGTCTGTAAAATTGCGAAGGGCATCTCGCACGTCGATATGCTAGATCGCGG
ACCCATTCCCTCGCATTGAATAGAAACAAAAAGGGGTATCTGCAGCGAAGG
GTCCTCCAAAGCAGATGAGGCGTCGAAGTGCGCCTTACCTCCCAGGACAA
AGATATAGCAAAAATCACATAACATAAATAGGTATCTCAAGGCTCATCCAAA
ATTCCGAACCCAGTCTTTGGTAAACGCCAAATTCCATTCCCCATATATACAC
CCAACTCTATTTTTTCGGTGTATCATATCACGTTACTAGAGCCCTCATCTAAG
ATGAGTAGATGGCGACCCATTGAACTTCCATCATGCTACCGTAGCCATCAAG
CGTGAGCTATACATAATAGGTTAGCTTCGGTGGACACCATAGTTAAACATAT
GTGAGAAATCTAGGTACTTACCAGGTTAGGAGCACCCGGCCAGTCACCACC
AACGGCAACATTCAGGATAATGTACCTAGATGGAGAATAATATTAGTAAATA
GTAAATAATCATTTGTTCGTGCGAGGCGGAAGCTTACAGAGGGCTGTGGGC
TAGAGCCGACCAAGTAGGGGCATCGTTGATATCAGCCCCAGTAACGGTAGA
GAAGGTGTTGTCATCCAACGTCCACGAGATGGTCTGCTTTGTCCAGTCGTC
ATTGGTACGGTCGATTGTGACACCCCATACATGGAAGCCATCGGCGGGCAT
GGCAGTAGCCTTACCACGTCCGATAGGCTCGTTGCAAGGGCCGCCGACAG
AACTTCCGCAGTGGAGAGTACCATAGCCAGTGAGGAGCCCATCGACTTGTT
CCATGATATCTATTTCTCCGGCGGCAGGCCACGAAGTACCGTGTCGCATCGC
GTCGCCAAGGGCCCAAAAGGCAGGCCAAAGTCCTTGTTTGTTTATTATAGG
ATTATCACCCAATAGAATGTTTGCTTGGATCTTGGTGACCTTTCCGGGGGCG
GGAGTCCAGGATCCTTTAGATTCGATACGACCTGAAGTCCAAGTGCCGGCG
GGAGACTTGCGTGGATACGAATTGGACAGTGCCACCGCCGCTGATCTGGAC
GTTTTGGTTGGAGGTAGTGTATGTTTGGACTTCGTTGTTGGTGTCGATAGCA
TCCATGATGTTCCACACATTACCCTTAGGCGAGGCACCTGCGGAACCCTCG
AAGTCCTCGGAGAAGACAACGCTCATGCCCTGGATACTTGGAGGGGTGAT
GGCCGAGACGAGAGCCGGAGCCACACAGGCGAATAAGCTTTGCTGGAATC
GCATTTTGAAAATATGTAATCGGAGTGACAGATATACTAATATCAGGTGCGT
AATGATAGTAAGGTGTAAGTGCCTGGTCCTATAGATCTGAAGATCACCTGGA
TAGACGAGGCCAAGCTAAGATGTAAGTCGAAGGGATAGCTGTCCTGGCTGT
GGTAGAAAGGAGCCTGTCTTGCAGGGAGGTTGACAGATATATAGTCAAAA
AAAGAGAGACGAAGTTAACAAGCCGATAGCTAACTGGCTATCGACACGGG
AGCAAGAAAAAAAGAGTGTGGTATATATAAACTCGGCTAGCAAGAGCGGA

AGAAAAGAGCGTGAAGGCACAATAGTTGGGCACGGAGGGGAAAAGAGAC
CAACGAGAGAGCTCCGAAGAGACTCACCTAGGAGATATATATTAAAAAGCC
CATAACAGACCAGTCGGTTAGTGTGTGGTGGTACCGGGCAAGGAGTCAGG
GTTAGACCTCACGATGTGCTCGAACAGATCCACAGGGATCTGAAGAAGATG
GTGGCACTGCCTAAAAAGTGGATGACAAAGGAACTTACACATATGCAGAC
AAGCAGACAAGGTCCTTGAAGCCTTAAAAGGATAAGCAAGCTGCCAGGGA
CATCGGACAAAGAGAACTCAAGACAGGAAGACACAGGAACCAAAGAAAT
GGAAACATTATGCACCGCTGGGACGGAAATTAATTGTGGGAGCTAAGGGTG
AAACTGGTAGGGGTGAGCTTTTCCTTTTGACTGACTTGAAGAGAGCAGCA
AGAGATATGCCTGATGTGGTGGTAGTATCTGCCGAAGCGCTCATACTCCGA
GCTCGTCAAGCATCCCGAGCTCCGTCATACAGGTCTACAGGGCGAGTGAGC
TGATTCACCTATCAATGCTCAGCGACGCTCACACGCAGTGGTTTTGCCGAC
CTGTATGTGACCTGCCAGCTTTGGACGGGTGACCATGAGGCCACGCTTCCT
TTCCGTACACCTTGGAAAGAATTCACAAGTCGTACACGTTAGTCTGCGCCA
CATGGAGCTCTTGAGGCTGACGTCCAGCTGGAATCATTGCATTGTCCTCCC
GATTACATATCCGTCACTGTTACTATCGTCTCGACTCGCCATCACAATATGGG
ACATGCAAGGGTCCGGGAACCCTATCCCAGTCGGAGGCTCCACCATGCCTA
TGTTTAGTGCAAATCGGCTCCTCAAGCGAGGTCAACAAAGGCTGCTCATTC
ATCGGGGTGTCCAGGCTGACCCGGCAACGGACTCGACTACTCCTGCAGATA
CGGATCAAGCCGAGGATGAGATGGGCCGGCTCGAAGAGCTGGTCAAGGAC
TATGAAAGAGGCGACATCCCCAAGATTGACTGGCTCGACCGGATGGTCTTT
CGGTCAATCGAAAAGGTTCACTCAGACGAGAGTGCCAATTCCGATAAGCTA
TACCTATACGTCGATATGCCCAGATTCGACTTCCCTGTGGTCTACTCCGAGC
AGGAAAGCCCATTACCTCAGGCACCACTGCCAGTTGCTCCCTCTCCCGCGC
CCGATCATGTCTCGGCCCTACCACCAGACTTTCTGTCATCAGATCCGCATCT
GTGGCGAGTGTTCGACCCAGACGCATGGCGGGAGAATCCGGTGGAAGTCA
AGCATCGCAAACTGCTGCGCAGCCAGAGACTCGGGATCAAGGCAGAGACC
TCAAGCCTGGACCGGTGGATAGAGATAGACTCAATGTCAGTAGAGATACTC
GTGTGACCGCTAATGAGCAGGAAATATTCCGAATGCCCCCGACCTCATCTTT
GAGTACGCTGGACAAGGATCTCTTGTGGAAGTTCCGCTTCTCTCTGTTCCG
TTCTCCACGTTCTCTGACAAAGTTCCTCAAATGCGTAACATGGTCAGATCA
AGTAGAAGCGAAACAAGCGGTCGAGAAATTACTGCCACTTTGGGGCCAGG
ACGTGGGCATGGACGACGCCCTTGAGCTGCTTGGCCCGGGTTTCACGGATC
CACGCGTGCGAGCCTTTGCGGTTCGCAGATTGGAGCGCGCAGAGGACGAG
GAACTTCTGCTATACCTCCTACAACTCGTCCAAGCTTTGAAGTTCGAGCCG
TCAGTCGCGGCATCTGCCCCCAGATCTGGCAAATATCAAGCTCGAGACGAC
GCTGGCAAGAGTGCGGATAGTGGGTTGTCGGACTTCCTCATCAGTCGCAGT
ATCGCCAATCCAGTACTTGGGACAGCCTTCCATTGGTATCTCATGATCGAAT

GCGGGCCTACTATGGCATTGGGCAAAATGTACGCTCGAGTGGCATTTAGGT
TCATGACAAAACTCTCAGAGGTGAGAGTTTGTTTCAGTCAGAACCCAACG
CTCATATCAGTCACCTGCGGGCGCTATGCAAAGGGATGTCCTGAAGCGCCA
GGCTGAGCTGGTCGAGACGCTTTCGACAAGGGCAAAAGAAATACGCTCCT
CTCGCGATTCGAGGTCCAAGAAGATTGACAAGCTACGAGCCTTCGTCGCG
GATCCAAAACATGGGTTATCACCATTGCCCTCGCCCCTGCCACTTCCCATTG
ACGCCCACGTATCAGTGACATCTATCATAGCCGAGAAATCGTCCGTGTTCAA
GTCCAATCTTGTGCCGTTACTCTTGTGGTTCGAAACGGCGGAGGGCCGGAA
CTCAGAGAGTGACGTTGACGGAGAGGCCGTCAATCTTTCGGCTGACTATGG
AATCATCTTCAAGAATGGAGACGATCTTCGTCAAGACCAACTGGTCATCCA
ATTGTTCACCCTCATGGATCGACTCCTCCGCAAGGAGAACCTGGATTTGCG
ACTCAGCCCTACAGCGTTCTTGCCACATCCATCAACGAGGGAATGGTTCAA
TTCGTTCCGAGTAAGAGTCTGGCAACCATCATGGCAGAGCATGGCAGCCTT
CAGGCATACTTGCGACTGGAGCACGCCGACGAAGGAGCGCTCGGATCATAT
GGCATCGAGGCGGGGGTCATGGACACGTTTGTGCGCAGTTGCGGTATGTCG
GATATCGATATATCATTCGATGAACTTATCAGCAGCGGGCTACTCTGTCCTGA
CGTACGTTCTTGGAGTAGGTGATAGACATCTAGACAATTTGATGCTCGCACC
AGACGGCCACTTCTTCCATGGTGGGCTGTCTTGTGCTGATTACGACAACGT
TGACGACCAGTTGACTTTGGCTATATCCTTGGTCGCGATCCGAAGCCATACC
CCCCACCGGTCAAAGTCTGCAAGGAAATGGTGGACGGAATGGGCGGCACG
ACATCGTCGCATTATGCGCGTTTCCAATCCCTGGCCTATACTGCTTTCATCAA
CCTGCGGAAGAACGCCAACCTGATCTTGAATCTCATCGCCTTGATGGTCGA
CGCCGGGATCCAAGACATTCAGCTCGAACCTGACAAGGCGGTGTGGAAGG
TTCAAGAGAAATTCATGTTGGATCTCAGCGA

>Mosaic_sequence_33

GAGGGTGCTGAGATGGGAAAAAGCTGATATGAGACCCGCGAGCAGATATA
CGAGCTTCAGGTGCTTGACGTCGCTGTTCTTGGTAGTGGTGGTTGAGGATG
TGAAGAAGGAAAAGAGCACGAGGAGCGCGCTGACGTATATTGGGGACGG
CTGCCAGAAGGCTGTTAGGTATTGCACGGTTTGGATGTCCCAGGTGCCGTA
AAATAGGGCTACGGTTGGGATGAGGTAGCCTAGCAAGACGGCCGGGAGGA
GAGCCCGCGCGTAGCTGATGGGTACTTCGCGACCCTGCTGGTAGTATTCTTT
CCCTCTGGAGGTGAATACGTACGCCGCATAGTAGACAGGCGCGATAACGGC
TGCGCCGATGATTTGGTAGAGGAGACCAAAGAGCGCAGCGCTATTATTCTC
TCGTCAGCTCAATCCAATTAAAAAACCACCTCCGGGATTTTGAAAGGCTAG
AGTACTCACAAACTAACAAGCCTTCCAGCATTCCTCGTCCTACTCGACTCA
ACAGCCCACATAACAACCACCGCAAACCAATTCATCAGAAAGTAAGCCTG
TTGGGCTTTCACACCGATATCCCACCCCGCAGTTCCGGCGAGAAAGGCCGT

GACCAACATCTTGGCTAGCTCATCTATGACCTTGATGCCCGTATAATGCGTT
ATGCGCGGCGTACCGTTTTCGAGCGTGGCGGTCTCGACCACGGTGCTGAA
GTTCAACGGCACCTCGTTGACGACCGTGTCCACGCGCATCAGGTACGTAAA
CAGCGCCGCGAGGGAGCTGAAGGCGAGGAAGAAGAGGGCATTGAGAGGG
CGCCCGGTGAAAGATAGGGTTGTGGCTTTTGGTTTGGCCATGTTTGCGGAT
GTTTTAGACTACGTTCTAGACGTTCTAGATAGGAAGATGAGAGGATGTTCC
GTTGAAGAATATCTCAGAGCGAAGATGTCTGAATATAAATGGTTTCCAACC
ATGGATATCCCCTTCACACTCAGCCTCACGGGTTTCGGGACCTAGCTAGGAT
CCGGGGTAGCTATGGATGTAAGCGCTAATACTCGACATATAGCAGTATATGT
CGTATGGCATGCGAGGACGGCGTTAAAGTAAGGTTCGTCTACGCAAAAGGT
ACGCTTTCTAGGATCCATCTTTGTGCATCATGCATCTCATGTGTGTTGGCAC
AGTTTAGTGGCCTTGGCTTCCGGGGCACCCGAAATTAAATGTGTGATATCTA
TTCAGACTTCTTAAGAGTGATATATAGAGGATTTATTATCTAAGGACGTTTAT
ACGAGCAACAATGGTTAGCCTCGTATGTAGGCCCGAACGTGATGAAGCTTT
CCAAGTTAGATGTTGAGATATAAGATTCAATTCTGAATGGGTAGTACAAATG
AGGGATATTCCTTATTATTTGCGCTATTGGAATATTCTGGTTATGAGATTTTCT
GATGTCATAGATGCTTATGTCATCCACCGGATAGTTTGATTTATAGCAAATTT
AGCGAGGTTTCCGATTTGGTGACTAAGCTGAAATTTTAATCATTCAAGATCT
TCGTGATATTCGCCGTCGCAACTTAGACATGGGATAGTGCACGATCGCCATC
CCTCAGTCTTTCACAGCATTTATGAGGGCGTTGACAAGAATTGCGGCGCCT
CGTAATGGAAGCCTCATAAAGTCTATCGCTTTAGCGGTAGCGAATCCAACTT
TTGGCATCTGCAGCCATATTGCGTCCATCGCATTAGGAGGTACACGCTGGTC
TTCATCCAGTTCAAGATGGAAGCAGCGGCAAGGGAGCGATTACCATGCAA
AGAAGGCCAAGCTACTTAACCTCAAGAGTCGGGCCGCATTCAAGTTACTTG
AGGTGAGTAGTTAGTTCATCGATAGGAATTAACACCAACTAAGCCTGTGGC
CAATATCCAGATAGACTCAAAGTACAGATTATTTAAGAGAGGGCAGACAGT
GATTGATTTGGTATGTATTCTAAATGTCAGTGGATTGCGACGCTAAGCAATG
TAGGGATATGCACCAGGGAGCTGGTCTCAGGTATGATGACAGCATGGCTCC
CCACTCCATCCGTTGCTCATTCACTACAGGTCGTCGTTGAGCGTACGAAGC
CAAATGGACGTGTGATCGGAATTGATATCATCCCGGCACAGCCGCCTAAAG
GTGTATCAACTATCCAAGGCAACTTCTTAAGTCCAGGTGTCCAGCAACTCG
TGAAGGATTATTTGGCTGAGTTTGCCAAACCCCGAGATCCGCCCGTTCCCA
TCTTAGAACCATCGAAAGAAGGGGAAATCATGGTTACCGAACGACAGAGT
TATATCGATGCCGAACGTGCGGAATCGGTTGAGTCAGAACATCTAGAGAAC
GACGGAAGACTAGTAGATGTGTGTATTTTATCACAGAAGCTTAGACCCGAA
GATGCTGACTATCTAGCAGGTGGTTCTAAGCGACATGTCAGACCCCTGGGA
ACGATTGAATGGACCTTATAGCAAGACATTGAGTAACCCGTATCGTAGGATG
ATGAACACCAGCGGCAACAACTTCCGAGACCACGCAGGTAGTATGGTAAG

ATTACATTCATTTTCGGATCTGAAATAGCAGCGTGTATGCTCACGGGAAAAT
TAAGGACCTATGCCTTGCGGCATTACAATTCGCGAGCGACACGTTGAAATC
CGGCGGACATTTCGTCTGTAAATTTTACCAAGGTGCCGAAGATAAGGACCT
CGAGTTAAAGCTTAGAAAGATGTTTGCGGCTGTCCATCGCGAGAAACCAG
AATCATCGAGAAAAGTATGTACTTGAATGTTATGTGTCTTTGATTTCGGTGC
TAACTTTTTACCTAGGAATCGAAGGAGTCGTATTTTGTGGCATTGCGACGGA
AAGGGGACGCTACGTTATCACTCTCATAAGAGAATTGAGACCACCCACCTC
CACTGTACACTATTTGTATTACATCTAGAAAACGGTCGGTTATTAAAATTTTA
TCATACAATGCGTCATACTAGGTATAATGGTATTTTCTTGTATGAATGTTGCT
AAATCTATTAATGTACAGTGGTATCGATGAAAGGCGCGTATAGCGCCACTTT
TTAACAAATTCACAGAACCAATGAACGCAATTCCAAACACCCACCCGATAT
GGATGCGTGTCCATGATAAGAATTTGGTCATGTTCGTGTCGTCGTTGGTGGT
AAGATGGCCGATGGTCCCTTAGGTCCGTTCTTGGCGCATCGTTCTTAGGTAT
GCTTCGATGAAACGCCTCTATTCGAGCTCTCCAAGGTATGAGTATATGGTCT
CCCAAGGAACATCGCCATATCATTCAGATTTAACAACACCATCCCGGAATAC
CGCTGACACCTTGCCCGAAGGTCACGTTACCAGGCGGTGCGCAAAAGAGA
TCGGTCCCATTGAGGCGTGTTGGGTTCACATTGAAGAAGGTCCCTACGATT
GAAAATTCATCAGTGTTCGATTCCATGAAGTTTCTAGCTGCTAGAGGTCATA
CCGTTCCGGCACTCGCAGACAGGGAAGTCAACACAGCAATGTGGATCTTC
GAATGGGATACACTGGGCGTTCGTATCGAATTTTTCAGTGAAATTACCCCAT
CTGGTGATATTGACGAACTCGGTAACGTTGGGAAACGGTGGCGCTGGTACT
GTGAACATTCGTCTAGAGAGCGCAACATTGTTGAAGGCTTGGGCGACGATG
AGACTTGAGAAAGTGATAGAAACCATCTTGACTTTGGAAATCTTGGGCTCA
ATGTCAGACGAGGTGGGCATGTGGGTGTGAAACGAGTGTTCTTCTGGGTGT
GTATTTTCCTGACTGACTATCCAAATCGCGAGTGGCTGATCGTTCCGGATAG
GTATTTCGGCTTGAGACAGTGGGAAGTTCTCTTTTTTTTTTTTTTTGCTTTCT
AGAAGAATTATCCTGTGGAGCGAAGTATATCTATACGTCGCACAGATACTTC
CCGCTCCTATCGGTCTTTCCGAACACTGAGGGACCAGATGTGCGGCTGTAA
CCATAGAAGCGAGCATTGCAGTTTCTCTGATATTTGAGGCATGTCCTTCCTC
ATGAAATGATTTCAAAAAGTAGGTGCTCCTCGAGAATGAGTGTTAAAGTTG
GAACCAGAGCCGAAGGTGCTGTCTTGTCAAACTCTCATTCTCCTTAACTTC
TATTCAATTCTTGGATGGGAGAACTTCCACCGGTCTCGTCTCGTTTTCCTTA
GAGATCGTCAGCTTCATTTCTCTCGTTGATAAGTTTCTTTCGCTACCGTACTC
TTTGGTTTATTTTCTTGCTTTCGTTGGTCTTTTGTTGGTTATACCCTCGCTTC
GTTTCAAAATGCCTATTGAAAAGGACATGTGGAGCTTTGGACCCGAATCGC
AGTCGACATCACCCGACGATCTCCATGAAATTTGTTTAGACGACGATGAGA
GGGACAAACGCGCCCGAGCCCCTCTAACCGCCGGGGGTCGGCCTGAATGC
TTGAAAGGACCCGTTAGGGAATGCATATTTGTTGCTCTAATTGCCTTTGCCG

CAGCTACTCCCGTTTTCCTTCAACGATCTATTGTAGTCGTGGCCGGGTCTAT
ATCTGAGGCATTGGAGATGACACCAGCTCAGATTGCATGGGCTACAGCAAG
TTCAGGGTATCATCACTATACCCTAAACTAAGTGGAGACTGACTCACCAATT
CGCAGCCTGACGACCGGCGCATTCTTACTTCCTTTTGGCCACATTGCTGATA
CATGCGCCTTTCTGCCCCGAAAGACCCTTCTCCTCACTAGCTTAGCAGCTTT
CAGTCTGCTTGCATCATCGACATCCCTTTCGCCGAACGGCATTGTTCTCGAT
GTTATATCTGGCCTGACTGGTATTGCTTGCGCAGCGAACGTTCCAATCGCTG
TCGGAATTTTGAGCCTCGTGTACCCTGAGCCATGCCTTCGCAAGAATCTGG
TCTTCTCTTCTTTCTTGATGGGCACACCAGCGGCGACAATAATCGGTGGCCT
CGGCTCAGGAGCCTTAGCTCTCCAATTCAGTTGGAAAGCTCCATTCATAGC
TTTGGCCATCTTATATGTGGTTATCTGTGCGCTTGCATGCTTCTTCGTTCCAA
GTATCCCGCAGCCCGAAGCAGTCCAGGAGACGGATGAAGTGCACACCATT
GCCGAGCCAGAGGTCTTCCCCATACTTTCGATCGAGCCCACTAGGAGAAAA
TCTCCTTTCCTGGAATTTGACTGGGTCGGATTGTTTTTCCTAGTCTCAGGTC
TTTTGCTCTTTGCTGTTGCTTTGACGATTGGACCACAAGGCCCAGAACCGT
GGAAGACACCGACAGTCATCCTCCTCTTGACTTTGGGAGTGTTGTCTCTTG
GATGCTTTCTTCTCTGGGAGAAGGTCACGCAGACGCCGATGATCCCACCAT
CCATCTGGGAAAACTTCACAGTAACTTTGGTAATATCTTGAAATCTGTTACT
TTACCAGCTCAAAGAATATGACTGACCTGGCATTTAGGTGATTATATCTACAT
TAGGTGCTTCCATGTCATTTTACTCTCAACTATTTTGGGTCTCGTTATTCATG
CAAGATCTCGAAGGTCTTCTACCATTCGAAGTTGCCGTTAGGTTGCTACCA
CAGGCTCTTGTGGGGTTATTACTCAGTCCTCTCGTTGGCTTAATTATGCACA
AAATCCCAGGTACAGTGCTATTGGGTGTAGCAGCTGGCGCTCTCGTTGCAA
GCAACGTGCTTCTCGTGTTTTTGCACGAGGGAAGCAATTACCTCAGTTGGA
TATTTCCTTCGGTCATGTTGAGTACTGTTGGAATGGATTGGACTATGAATGTT
GGGTCGGTGAGTAGTTTTATAAGCAATCTGAGAGAATAAAATGAAACAAAC
TTCTAATAAGATTATATATAGCTGTACATTTTATCATCTCTTCCCCTCAAACAT
CATTCTATTGGAGCTTCGGTCCTCCAGACGACAAGCCGTCTCGGGGTCCCG
CTAGGAATGGGAATTACCACTGCAATCTGGTCGTCATACGATGAAATGCAG
CTAAGGAGAAGTCCAGAGACACCATATACGAATACTTTCATAGCAACAGCT
GCATTCGCGGGGGTTTCGCTTTCTCTTGTGCCTTTCATTCACATTGGGAGAC
AGGGTTCTTCGAAGCAAGAGTTACCACACCAAAAAGAAGAGATCCGGCCA
AACTCGCCGACGCCTTCTCAAAATCATTTAGACCCTCGTCGAAGCATTCGA
AACGACAATGGAAATCGTCCGAGCAAACGTTGGTCGCCGGTCGATACGTT
GGCCACATCGGCTATAGGCGAAAGGCAACATACAATAGCTCCCTCTGTGTC
ATCACAGTCATCACGTAGGAGTGCGGACGCTACAAGCAGCGTTGGCACAG
CAAGGACAACTATTCGGCGTGGGCGCAGTCCATCCGAGCGGGTAGTCTGG
GTTGTCTGCGAAGAATGCGGCACGAGCAAGCGCCACAATCAACCCCGTAG

CCCCATGGGTGATCCGGCCCGCTACTTTAATGACGTCGCGTTAAGTGGAAT
GCCTGAGAGTCGCAATAACCAGGCAAACACTCCCACTACTGAGACAAGCA
GCAGCCAAAATAGCCAGCAGCATGCCTCTCCTCAACTTACTAGGCAACTCG
GTGGACGTCGGCGTCTACCCCTCGTGAACCGGCAGATTATGACTCATCAGA
TGCTCACGCAGGGTTTCCAGCCTTGATCGTCATGAAATTGGAAATTGTGTAT
TCAAGGCTTTGATAATAGTTTTGTGGAAATGGTTCCAAGTTAGGTATGGAAC
TTGATGATGGGAGATAAGCATGCCTCTATTAGTTCGACTTGGTCATTCAGTG
ACGCCATTCATAACCGGTTTTTGGTCAGGTCTAAGACCTCTTTGGAGAATTT
TGGAGGGGTAATACTAAGGCGTGGGTAACGATGTGACGCCTATTCCCAAGA
ACCCCTTTTGCTAAGTCAGATATACATGTTATAGAGATAGATAGCTCGCTGCC
TCGTCATATTGTCATAATACATTTAATATTTTGAGGCCATTGTTCAGTGCGAA
TATTCATGCTTCCTAGTAATTCTGGTTTGCAAATTCGAGTGTTTGCCCTTAGT
ACTCCGTGGCTTATGCTACGACGCTCCCCCAAGATACTCCGGTAACACAGC
ATGTGTACCTAGCCGAGACATACATTTAATATACAAGAGCTTCCTGCAGATT
CCCGCTTCCGCACCGATTGAAATTTCGAACAACTCAGGTACTGGGGAGGTA
CCCGATACTTAGATAATTCAACTCAATGAACTCATAGCTTGGCATTAGGCAT
CTGCCGCATAGCCTGACGCACACCCAAGTCAGCCAAAACTTCCTCAGACC
CGCCCAAGATCGCATCGAACTTATACGTCCGATGGAACATCTCGACAACAC
GGCCCATGCCGGACTGTGTCAATCCACGTCCGCCGAAGATCTGGACGGACT
CGTCCGCGATCTCGTGCGCGGCGCGCGTGATGCTCATTTTTAGGAGACCGA
TGGGGCCGCCTAGGTGCTGCGCTTGCTGCTTATACGGCATGTTGCACATTTG
GTATGTTATTGTTTCGAGCCAGGATTGGTTGGACTCGACGAGGGAGATCATT
TTTGCGAGTTTTTGGCGGATTACGGGTTGTTCGATTAGTCGCTTGTTAAAGA
CGATGCGTTGGTGTGACCACTTGAGACATTCTTCGACGATGGTTCGGCATT
GGCGGATTGTGGCGCCTGCGTGGGGGTTAGTATGAGGGCGAAGATGGGGA
AGAAATGTTAGAGGGGCTTACAGGCCATGGTCCATCGCTCGTGGTTGAAGT
TGCTGAGGATCACGTAGATGCCCTTGTTCTCCTCGCCGAGGAGATTCTCGG
CCGGAACTTTGACGTTGTCGAAGGTGATGTACGCGGTGCCGGCGGCAGGC
GAGTACGAAGTCTTGATAGGCTTGGTCTCGACGCCCTCTCCGCGTTCAATA
ATCAGCACGCTCAAGCCCTTATCCGTCTTCACGCCCGTAATAAAGTAATCGG
AGAACACGCCGTTCGTGATCCACTTCTTAGTGCCGTTGACAATGTAGTGCT
TGCCATCAGGCGTCTTCGTGGCCGTCGTCCGCAGGCCCGCGACGTCAGATC
CGGCGAAAGCCTCCGTGATAGCTAGACACATGTACTTCTTGCCGCTGAACA
CCTCGTCCATAATCGCCTTCTTGCGCTTTTGATCTTTCATGTAGTTCATCACA
CACGGCAGCCCAATAACCATACCAGCCAACTGCCCGTCTTGGAACCCTCGC
GCGTTCGCGCGCACGCCCTCCTGCGACATGATCAAGTCGTGGAAGTAATCA
AACTCCTCGCCCTTAATCGCGCCATCAAATAAATTCACACCGTGCAAATGCT
TTCCCGGTCCTAACCGCATGTGCAGCAAGCCAACGGCAGACATACGGTCCA

CAAGCTCCTGGCTAATCTTCTTGCCATCAAGCTCGCGCTGTTGCGCCTCTGG
CGTGACATACGTGTCGGTAAACACGCGCATAGCCTTCTGCAGCCTCCTATG
GCTCTCATTATAATACGGGCTCTTATACACAGGCGACAGCCACAGCGGCTCC
GCGTACGGGACCAAGCTGAGATCCCCCGGCTTCGGGGTGACGACTTGCGG
CGTCTCGCCCTCGAGCGTGCCTATGCATAGGTCTTCGTACTTAGCTAGGACT
TCGTGTCGGTGGAGGTTGTAAAAAGCAATGGTCGCGTCTTGCCCGGCTACC
TGTCGCAGCACGGATTCGCCGCCCGGGTGTGCGTCTACGAAGTCGGTTACG
TCGTAGACTCTGCTGTCGATGATGAACCAGAGCGAGTCTTCCGTATTGTGTT
TGCCGACCTCGGCGCGGGTGTACGTCTGAGACATCGTGTTCGTCGGTTGTG
TCGGTGTTACAGGGAGGATCTAAGAAGGGAGGAAGGTGTGGTTTCTTCAA
GATATAGGGTGCAATTGACACTATTGATACAATTTAGGAGATCCGGACGACA
CACGAAACTTAGAACGTCTTCTAGACTCTAGAACAAAGAAATAATACATTT
CAAGAGTAAGCCCCCGGATCTTATGACCTTTTTGACGTCAACTGGCTGACG
CAGTGGATCTAAGGGACGATGCCTCGGCTTGCGGTAGTAATCGAAGTTTAG
CTGCCTCTCATTGGTTGATTCGGTCTCTGCTTTGCCTGTTACGGGACACTA
CGCTGGGGAGTCCCGGATGTTCGTCCAATCCCTGACCGCCGACTCAGCTCC
TTAGCGCTGCCGCTCTCCTTTTTTCTGAGCATTGCATGCAGTCCACCACAGC
TCACTGATCGGGCCGTGATCCCGCGATGGGCGAGGGTTCTGAGAGTTCACT
TCGAAGGCAGGCGCCAACTCATCTACTGATATATGTTCACTTCGTCGAAAC
GCTCCTTGACAAGTGGGAAACTGATAAACGAGAGACAAGCTTGGCCTGAA
ACGCTCTTTGGGACGCAAGACTCCGCAAACGAGAAGATGCGCCTAGAGAC
CGATTACGAGTCTATAGGCAGCGGCTCGCCTACCGTTCCAGCTGCTGGTGC
GTCTGCTGCTCGACCACCTCTAACAAAGGGCATGTGCACCTCACACTGGCT
TCAATCGGTCCGAAGCAGTCCTCTGCTGGACGACCGCTCGACCCCAAACC
TGCCCGCGAGCGCCGAAGTGGTAATCATCGGTTCTGGGGTATGTACAGCCT
GGAGATGGGAAGCTCCTTGCCGACAAGCAGATCTCGGGGGCGCTGGTCGC
CATGTCCCTGCTCGACAGCCCGACCCCTCCAGCCTCCATCGTGATGCTTGA
AGCACGCGAGACGTGTTCGGGGGCAACGGGGCGGAATGCGGGCCACTGC
AAGCCCGATCTGTGGCGCGGCTTTGTCGACTACGAGCCAAAGCACGGTAC
AGAGCGCGCGCTGGAGGTATGTCTTCCTCCTCTCGCTTTTACGGGGGGACT
AAGCCGCAGATCTTGAAGAACGAATTCGAGACGTGGACCGCCCTCAGGCG
GTACGTAGTAGATAACAAGGTCGACTGCGATCTATGGGTCGGCAAGACTGT
GCGCCTCTTCGGCTCTGTGAATGTGCAATGCGGCTGATGAGCAGCTTGATG
TGATGATGACTCAAGAAGCGTACGACCAGGCCGAAGATGCGATGCGGCGC
TTTGTCGAGGCGGGAGGAGATCGCACCGCTGTCACTTCCGTCCTGGACGC
CTCTGAGGCGGAGAGAGTGTCCCGACTGAAGGGCGCCAAGGCTGTCTACG
CATGGGACGCGTCGACGCTGCATCCTTGGAAGCTGGGTATGTCCTCATAAA
GGCTGTAAACAGGAACAGAACGCGAGAGCTGAGGGCCAGTGGCCCACAT

CATCAAAAGATGCCTGGAGCGCGGCTTGAACCTCCAAACATGGACACCAG
TGACTGGAGTCACCGGCACAGCCAATGACTGGACCGTGCACACCGACCGT
GGTGACATACGGAGTCCAACCGTCGTACACGCCACCAACGCCTATGCGGCG
TACCTCCTCCCTCAGCTCAGCCCCGTGATCCGCCCCGCCCCGCACATGTGC
AATCGCGTCATCCCGCCAGCATCCCACTCGGGATCCAAGACACTGCAAAAT
TCGTACATGGTCTTCTATGACGATCACTACTTCACCATCAACCCGCGCTCCA
CTTCGGACGGGGCGATCTTGTTTGGCGGAAGTCCGACCGGCACCAAGCAG
CTGTATGAGCACATCGAACGACACCCTGCGCTCGCGACGGATGACAGTCTG
ATCGACTATGAACCGATATTGAAAAGTGTGCGGCGACTCGGGTCGGAGGGC
TTCCAATGGTATGTCCCCTAACCCTGGATCTGCCTGGATATTTTGCTGAGTG
CAGGGACGACTCGCCGGGAGCGCAAGTGAGGTATGACTACTCTTGGAGCG
GCATTACAGGCTACGTCCGTCGTCGCAAGTTATCCTTATGGCAATTGACAAT
TTAGACGCCCGATGATGTCCCGTTGGTCGGCGCGGTACCAGGGAAAGAGG
GCCAGTGGATGTCTTGCGCGCACAACGGACACGGTATGGTGGGTCTCCTGG
GATACACCTCCGAGAGCGGAGCTGACACTGCAGGCCAGGACCTTTACCTG
CTCTCCGGGGTTGGTCAAGTTGATGCAGGGCGCCAAGTGGGAAGAGGTCA
ATCTGCCCAAAGCGTTCGAGGTGACTCCGGAGCGTCTCTCCCGTTTGAGCA
AAGCGATGGGCACACTGTGAGCTGGCCTCGGCGAGTCGTGAGCAGATCCT
ATCATTCGTTGTGCACATGCGAGATGTATGCGAGATAGCGTCATGCGGCAAT
CTTCTTCCGTGCGCAGGGTCGCGTTCCAAAGGATGCCAGAGTCGTATGTAC
AATTCGATGCAGGGCTCGAGCTTCGTAACGTTCACATACATTGCAAAGTGT
ACATATATCCTGGACGCTGTTTGTGGTACAACCATCACTCTAGCACACAACG
TTACAGATCTCCTTCATAGTGAGTCCGGTCAGAACAACAATACGTCCCCGAT
GACCTTCCGCCACGACGGTTTCGCGTCGCCAGCATCGCGCAGTGCGTACAA
CTTGTTCGACATCAGCATCGGTCGGCTGGATCTAGAGCGTTTTCAGATGACT
TACCGTATTCAGACTCCTGAGCGCCTCTTCGCAATCATAGGCATAACTAGTT
TGGTACCGCATGTACATGACGACTTGTCGGGCTCGATTCCGACCTGCTTCGT
CCAGCATCATGCTGCCTGCGCACGTCAGCTTCTCTGACCAATAGTCGATCGT
GAAGCAGTGCTGGGACACTGACCGATAATGATCATCGGGACCAGGAGCGC
GATCTCGGGGAACCGGTTGGCCCAAGGAGTCGTCTCGCTCCTATTTGCTCT
AGCGCGTTCGAGGAGCGTCATCCCCGACATGGCAGCAGTCTGTACGACCG
GGTCCGCATGATCTTTGTCAAAGAGATCGACGTTGACGATCACCTGAAGGA
CTTCAGCGTCTAATTTACCCTTACGGCGCCTGGCCAACTGACACGCAAGGT
TTGCGCAATGATGCTGTTCCCCAAGTGCACCCGCGGGACAGTCAAGCTCGC
ACTGGTCCCGGCTTCCCATACGTCTAGCTCCAACATGAGGTCATGGGCTTTC
TGCCGATGTCGCAGATGCAGAGTCTGGTCGTTGAGCACCGAGAGCGCTTG
ATGCTCGTGCACGAGAATATTGACGCGGTTCATCTATCATCAGCACCAATGA
ACTGAGCTTGACTCGGCCTGTTGCCCAACCAGGGACATAGTAAAAGCAGA

CTGACCAGCTCGAGCATTCCTCTATCTGCTCCAAATGTGTAGTGAAAGGGC
TCCAGTTGCGCCCCGGGAGATGCTTCGTCCGCGAAATCATACCTGCACGTC
AACGACCACAGGTTGTTGGCAGCAGCAGTACGCACCACCAAGGCTGGAAC
GGCTCAGTCAACAAGCGCGGCGCTGAACCAGTCGACATGCACCGCCAAAC
ATCCACAATGGCAAGGTTTTCCAGCATGGTTCTCGTGCGCTTCAGAGTCGC
GGCGTCTACGCTCAATGCTTGCTGAAGTAGCCTTGCTGGCCCGCCCCAGCG
TGCTACCAAGTCGAGGGCGGTGTCGAAAGCCTCTTTCCACTGAACGGCAG
CAGACATGCCCGAAGCAACGATCGAGCATACCATGCTCGTCAACAGCGTAT
TGACTACCGAGAGTGGAGGAGGTTCGCTCGGATCCTTGAGTACGAGCGCG
GTGCTGGAGCTCTTGATGGCGGAAGCGACGAGATGGTTGCCCACCATCCG
CATCCTCGACGCGGACGCCGTAGTCGGGCTGCCGAGAGGGGCGGTCGGGG
TGCCCCAGTTCCCGCGCGGCATCGTAGAGAGGTAGCCGTTGGCACAGCGAT
GGTGTATGGCGGACCGGTGGACCGCCCCAATCGAGAGGAGAGACAAAAGC
AGCGCGTCCGACTCTAGCGAGGAGCCGCGCGGATGTTCCATGCAGTACGA
GATGTCGAAGAACTGCACGGGCTTATCGGTCTGGGGTATAGCACACAGGTT
GACAGTGCCTGCTTGTCAGCCGACAACGTCTCGAACGGGTGAAGAGCTAA
CCAAAGCAGATAAAGTGCCGCATCTATAGTCAGCAAGAACGACGGGGGTC
TCGTCATACTCACCAAGTCACGCTCTTCCGGCGCAGGCAGTATCGTGCGCA
AGATATCGATGGGCTCCAAGTTCCAAGCGGGGTCGAATGTCGGGAACGAC
GACCCGGCAGCAGCGACGCTGAAGAGCTCCAGCATTGTGTCGGTGGATAT
GAGCGGCATGTCGCTGGGTGAATCCTGTTTTGTATGCTCGTTAGACGGACT
GGCGAGGGACGGTGTTTGGGTGTTTCGGTCGTACGGTTTTCGAGAAGATG
ATGATGATTGCGAAGGATAGGTGCACTGGATGATCAGCATGCCCCTTCACT
GTCCATCCAGCCAGGTGCTTGCACTCTTGTGACGCTGCACAGCGGCGGTG
ACTGTGGGTGGTTGAGGTGACTGACCGCTCGGGATCCGCGTTGACAACGC
GTGCAGGCAGGTTTAGCCTCATCGCACCTGCAAGTATGTCAGTCCTGATCG
TATAAACCTGATCGTGTGACCCACTTGTGCTTCAAGACCCGGCAACTCAGA
CATCCTGATGAGACACATCAGCTATCGATAACGCGGCGGATGGTAGATCAC
ACCTGTACGACTCCGGCGATGCTTGGGGATGACAGTCGAGCTTGGCGACG
CCTCCAACGCTCGGATGGGAGACGAGGCGACAGGGGACATGATCGCAGTC
CGTGGTCACTGCACGTCACAGCATCCCGGATGAAGAAAGGCTCTCGAAGT
AACTGATTACGACCGACCGACCGGGAGCATCACCTCCGATGGCTATTTAAT
CCCACAATTGATAAGCTGCCTCCGATACACAGATCCTCCCCACGCGCACGC
ATCGATACCATGCATACATCCACTAACTAGATTGGTACAGCAGGTCTGAGCA
ATCATGCGTTGTCCCTACGCCCGCCCGTCCAACGTTGTATGGAAGCGTGGC
GCTGTCTAGATAGAGGAAACCTATCTGCTCCTCCGGCACGCGCATCAGGTC
CAGTATTAATAATAGGCAAACGCGGCTTGGGAAAGCTTGGCCAGCGGCCGG
CATGCACCTCGCAGACAAGTGTCCTACTTTGTAAAGATACTGACGCTGCGA

GTCATCAGCAAGACCATGGACGTACTTGGTTGGCCGCGCCATGACTTACAC
GTCTTCGTCTTGTACCAGATGCGTCAGGCCAACTTTTTGTGGCTGAGGGTT
GAATTTGCTGCTCTTTCCCCACACGAGAGCGTATTTGAAATGCGAGGCAAG
ACTCCGATGGATGCCGTGACACACCGTCTCGATTGTAGCGCCTTGGCGGAG
ACAGATGGGATCTGTTAGATCCGGGTTCTCCCCCCGTCTCTTCGTATATACC
CTGCGACGAGGTCAGGAGTCATTCGGGTGGAGAAAGGCATTCTCCAGACT
GACTTTACGAGTCCCAGCTCCTGAGCCATCAGCACATCGTTCTGGACACGC
TCGAGAATACTTGCCCGCCATATGCTGTCCAACAGCCAGTCGATACCGAGA
TCCATCTCACAACTGATCATGACAGTCTTGCCGTCTCCCTCGCGGGCCATCC
GATCCAAGGTCTCCATTGACACGCCATCGATCTTGTTGATGCACGTCAGTG
CGGGAATGTATTTCCTTGTCCCCAAAAGAACGTCGATAAATTCGTCGGTCG
TGATCTTCTCCATTAGACCGAACGGTGCCCATGATTCGGGCAACTCACATCT
TCACGGACCATAACGTCACAGTTGTGGACTGGTGTCTTCAGTGCTGTTCAT
GCGCTGCCAAGATGGAGGACCCACTTTTGTAGGTCTGCAAGATGGTCCGG
ATCGTTCTGTCGTCCGTCTTCGTCAACTTGACTGTGCAGTTGATCTGCTCAT
TCAGTTCGTTCTTTGTGCCGGAATTCTGGTATCTACAGTAATCTGCTGGTCA
GCAGGTGATCCCGGATCAAAGACACACTCACACCCCCCGCCGCCTTTTGCT
TGAAGACGACGTCCGGTTTGCGAGCGTTCAACCGGATTCCAACGGCTTCA
AGCTCAATCTCAAGCAACTTCTTCTGCTCCGCCGACTTGGTCGCATCGACT
GGCGCTGTAAGCGTCTCAAAATGGCACGACGAGACACACTGACTCATGAG
AATGATGAGATCCGCTGTCTTAGCCACTGACACGACCTGCCGACCTCGTCC
CCTGCCTGACCTTGAGCTTGGCCGAGGTCTCACGGTCCGACTGACCCTTGG
CTGCGTCCTGTACGATTCCTGGCAAATCGAGCAGCTGTATACGAGCGCCTT
CATACTCCAGCACCCCCGGTATAGCCTGT

>Mosaic_sequence_34

GCGAACAGCGCCTCCCTCGCCGCCACGATCAAGTCGCAATTAATAGCCATC
TCCATACCGCCCCCAAAGCAAATCCCATTTACCGCTCCAATAATCGGTTTGA
GGCCTCCCCGATTACTCATCCCACCGAACCCACCAGGCGGAAAGCGCAGAT
TCACACCTTGCTCGGGGAGCTCTTGTTGATCCTTGGTTTCCGGAGAGAGGT
TGAACGAGTCCCCGGCTTTGAGGTCCGCGCCGGCGCAGAAGGCGCGGCCG
GTGCCCGTGAGCACGGCTACACGCAGGGTGTGCTGCGTGTCGAACCAGTC
CCACAGGCGCTCGAGGGCGAAGTGTTGGAGCCGCGGGATGGCGTTTAGCT
GGTTGGGCCTGTTGAGGGTGACGAGGAGGATGTGCGGCTTGGGGAAGGA
GATTAGGGTGTTTGGGACGGGCGGGGGCGGCGTTGTGATGGAAGGGGGGA
GTTGGGTGTTGGGGGACATGATGACGCGGTGAGGAGAAAGGGGATGGTGG
TGTTTACTTGGTCATTCAAGGGATGAAGTCGGTGAGCAATGGATGAGTTTC
TGCAAGGAGGATGTTTCTTAAATTTGAGGTAAGACACAAGACGGACAAAG

GATATAAGATATGGATGAGAAATAGGCAAGAGAGTGGCGCGGTTAGGATTC
TAGTATTACTGATGTACCTAGGTAAATCTCACCTCCAGGGATTGAACGTTGG
GGATTGGGGTGGTTGTGGGGAATTGCCTCGGCATTCCGTGGGATCCACAGT
TCTCCGCATACCCCGGATATGGTAACTACCCCTGACCGTCGAGGACCGCGT
CCTATTTTACGCTTACCTATACGGACCACAGAAAGTTCATGTTGTAGAAGAT
GCGAGACGTAAAGATGGATTAATTTCATTAACATTGCATTGTAGCAATAGCC
CGTAACCATTTTTTGTTTCTGTGATTAGGGCGCAGCATCTGAGCGATTCCTT
ACAAGCTACTATGCACACTCGTCTCCGTAAATGTGTACAGAGGATTCCTATC
ATGCGCAATTTTGTATGAACACATCTCACTACTCTTATGGGAGTAATACCCTT
AGTTGCGAAGATCAACCCCATGTTCGCCTCAACTCCCCAGTATCTCCCCAG
TATCTCAAAGGCCTGTGCTATTTTGAAGCTACACGAAAATTAGAGGTTTTGT
TAGTAGAAGCATTGGCGACGGCTCTACATTATGCAGAAAGACCACGTTTCT
CACTCTCGGTGAAGAAAATAACCATTCGCCATAACTTCTTCACCAGGGCCT
CGGCCTCATCATCTAGAGCCTAGTGCACATAATTAGTAAACTGTTCCAAGTA
ATCATAATGGCGTTACATACCTCTGCTAGTTCTTCAACTAACTCGCCAGGCT
TGGCGTGCTTTCTAAGGTGTTCTTCAACAACCTCGATGAGCAACTGTTCTT
GAACACCAAGATACTCCACGAGTTTCTTTTCGACAAAAGGTCTGAGCTTCT
CTCGAATGACGCCTTCATCCAAGTAGTCCCACTTAACATCCCATTCCCATAG
CCCTTCCTTTTCGTTAGGAATTTCTTGAGCTAATGCACGGACGGCCTGGTCA
CGCTCTTCGTCAGACATAGCCGCAGCGGCAGCAGGCTCAAATTGAATAGGA
ATAAGCTGGCGTCTGGAAGTGACGTCTTCGTCTTCGTCCTCAAGTAGACCC
TCAACTTCGGCAATCGTCTTTCTTTGAGGAGCTGCACGTTGTGCCTGTGCC
TTCTGTGCGGCGGCGCCAAGAGATAGCTTGAAAGGCTGTGGCGCAGCAAC
ACCAGCTCGACTAGCCTGTCGTTGTTCCATCTCCTGAGCTTGTCTCTCGAG
AAATGAATCAGCCATGCCCCTAGCTTGCTCGCGCTCGGCCTCTTCCTTGATG
CGCTCCTGCTCTTCGGCGTGGCGATCGGCGTCGTCGGCTGCAATTTCTCTG
GCTCGGAAAGCGGTCCTGTTTCTTATCCACGCACTCCTATCCTTGTAATAAT
CTTCAGTTTTCCGGGATGCCTCTGCGTCATCATCGAAATGCTTGAGTCGTTG
TAAAACTTTGTCTCGGTTCTTAGCAAAGTCTTTAGCGTCAGACTCGTCGCG
GTCCCTCTCTCGCTCAAGCGCGGCTGTCCGGGACTTCTCTCGATTCAGCCA
GCGACGTTCGGCATCAAGGTAAAGCTTCTCCGTCTCGGCGTTCTGTTTCGC
AACCCGTCTCTTTTCTAACTCATCGTCACTGGCAGAGTCGTTTTCGTCTTCT
CTATTGTAGGCCGATCCCCCATTTGCTGTGCCGCCGTTTACGAAGTTGATTG
ACCTGTTATGATCTTTGCCCATGTCGGATTTCTGACCCTTTGGCCCGGAGGG
TGCGTTAGGCATACCGCGCGGTCCTAATGGAATGTTGTTTGCGCTGGAGGG
GGGCGGCGACGCTAGACGGGAGGGCCTTGACGCGCCATTGCGTTGACGTT
CCATTGCTTCGAGTTCTTCCTCCCTCTTCAACCTTTCCATATCACGTTTGATA
CTACGTTCTCTGAATGCGGCGATCTCGGCCGCGACGGTTTCGCGCATTTCA

GCCGGGATGTCGGCTAACTCGTCCTCCTGTGCCAAGGGGATATTAACCACC
TCAACGTTCTCTCCATTTTGCACCGAACTGTCACCCATGGTGACATCTCCAT
CCGCGTCTGCTCCAGATCGCGATGTAGGGTAAAAGAGATTTCTTATGATCTG
CTTCAACGCACCTCTGGCAGCCTCACATCTTGCCTCGGCACCTGAATCATC
AGGTCTGCCTTCCTTGTAAGCCTCCAAATATTTGGACGAGTTTTCATCGAAT
CGAACTTGAAGGGTTGCTTTCTCGAATTCGGCGAACGTTTCGTCATCATCG
TTGGCGGGCTGATCCGACGATACTTGCTTTTTGACGGGTATCTGAATGTCCT
TTAGAATCTTGGAGGCGATCCATAGACTCTCGGCGTCTTCGAATTGAGCGA
AACCAAAGGAAACCTCTTTTCCACTAGCGTCGGTAGCTCGGTCCCAACGG
CGCACCTTGCCTGCGGCACCCAGTATCTTCTCGACACCTTCGTCTCCGCTA
ATACCGTCTGTAATACCGCTTACGAAGACCGTCCGTACGATCTCCTCTTTAG
TGGGGGGGACCAATGCCTGCATGTTCTCGCGTAAAGCCTGTCGAGTCTGGT
CCAGGCCACGATCCATACCTATGCCCGCCCTTGGTCCAGCCGAAGCCAGGG
TCTCGTTATCTTTGCGACCTGGCATTCCGGAGGGTGTCGGTTTCGAAGAGC
TCGTACCGAGGCGGATGACTGGGGCGTTGAAGTTAATGTTGGGCATGTTCG
GGGGAGCTTGAAAGTTGGACGGTAGACCGCTAGGTCGATTAGCTTGGTTG
AGGTTGTGCTGCTGAACGCCAGGTGGTGGCGCCGTTCCTGGGGCAGACAT
GCCTGGAGGAGGTCCTGTGAGAAGTATATTAGTATGCGTTGCGCAAATATCT
ATGTATGTGCTTGACCATAGAGTTACTTACCTAGACCAGGAGCAGACGAGA
TTCCTGGAGCACCAGCGGCACCAGGATATGTATTGTAGGACGGAGGCGGGC
CGAAGCCGGGAACTTGACAGAGGTATTATTGTTAGCATATACCACAAGCCT
AGGTGGTTAGGTAGCTCAGTATTCATTCAAGGTAGCAACTTACCTCCGCCAT
ATGGATTGTACGCCATAATCGGAATGCGAAATAGAATACAAGGACGATTTG
GGAGAGGTGAAAAGGATGTTAGGGTCCGACAAAGATGTCGGTATTGAGAC
GTGTGTCTTAACAATTACACGTCTCTTGAGGTGGCCGATAAAGTGAAGATA
GTTAACAGTAAAAAGGCGGGTGATGGGTTAACTAGGAAATTACCTAGTGTT
GAAGATCGTCAAAAGCTCAAAAGTTGATGTTGAAAGGTTTGCTCTTGAGGT
AAAGCACCGCCACAAAATAGTGATGGCTGGAGACTTGGAGTACAGCTAAC
CTTCGAAGGTTGGAACAATGTGGGTCTTCAGGTACGGGCTATGGATGTACC
GATTTGTAAGACTCAATAGCCCCATAAATTAAGGTTAATCTTTCACTGTCCA
ACCGGCGACTGCCCAGGCTGCGAGGTGAGCCAGGTGTGCATCCGGTAATC
GTTTGTGGATTATAGATTATTCCGGCCGAGAAAAAGGTGAGTGGATCCGATT
GAATCGGACCAATGAAGTTTTCCAAGTTCTCCAAGTCGGAAATCGCAAACT
GACAAAGCTTCAAATCACATTTGTCTCAATTGTCGCCCATCATAACGCTGCA
AGAGCAGATCCGCGATCAGCAACCTGCGATCTGGCTGAGCCCATCCTGACT
ATGTATGGAAAGACCACATATCACGTAACACAAATATCATATCAACTTGCTA
ATTAAATTAGGGCTGAGCTTTACCGGGTTACGCAAGGCGCCTTGCGATCATC
GTCAATTCTGCGAAGCTATGCGTCAAGTGCGCCAAGACGGCCAGCATGTTC

AATACCCAGAATCATTCGCCCAATCGAACTCGGTAACGGCAGATTACCCATC
TCCTACGCGCAACATCAAAATTATTCGACAGATTATTCCACATCCCGTTTTC
CAGTGAAAGATTTATGGCCGGCTGCCAAGAAGGAGGAGATAAAAAAACCA
GACATGTCAGCATACGAGGACGATGATATTTTCGCACCCGAAATCGATTTCT
CAACCGGCGAACTCACAAAGAGCCCTAAAGAAGCTCGTCCAGAACTCAGC
CGACCCCCAATGCGCCTAATACCACGAACTGGTCGTACGATACGTGTAGGG
AAGACTGTTGATGTAGCAAGAGCCTTCACCCTCTTGCAAAATTCCGTCAAT
GCAAACAGGGTCAAGTCTGATTCCTTCCAACAAAAATACCACGAGAGACG
AGGTCTCAAACGAAAGAGGTTGGAACGTGAGCGATGGCAAAAGAAGTTC
AAGACCGGATTCAAGTCGTGCGTTAGGAGGGTCAAACAATTAACGAAACA
GGGCTGGTGAAATCCTATTTTACCGTGTACGACATCTATCTCGACCTGAACC
AAAGCTGCCCTAAGCTTGGTTGTATTAATGAAATCACGTCAAGTCATTCTCT
CATTCTTTCAAACGGCCTATCGTGGGCCAACCCGTGAAAAGTAGTCCCAAG
ATCCAAGTGGCAAGTTTATCGCAATCTATCAGGCTCAGCTTTCACGCGAAA
AAAAAGAATTAGGGAGGTGTCTTAGTTCATGGGCTTAATCTCATAATCTGTT
CATGAGAAATCACACTTGCCAAGCACCTAGCAACAAGACCAAGCTAGAAG
CCCTCCAATAGCGACTCTTTATAGTAAATTGAATGTTGAAAACAAATCACAT
AACCTTTGGATAGGTAAGTTTGCGATGAGCTAAAATTGAGCTAAATGGGCA
GATTGGAAGTAAAGACTCGGACAATCCAGACCGACCCAGGGCAAGAAAG
GAATCAAAGTCAGGCGGCATGGGAAATCCGGAACTGCGTGCACACCCGAA
GGATCCAGGGGATATATCAAGATATTTCGGGAACTCACAGCATAGGAAACA
ACTAGCACAAAGAAGCATAGAAGAAGATGCATCAAGATTTTGAAATGGCG
AAAAGCGGCAAAGATTGCAAGATAAAGGAGCCAATCCTCCAAACATGCGG
CAAGGAATATGATCTCAGCATCCAGCTTAATTCCACTTTTAAAGGTACATAC
AGTCTACTAGGGAACGAAATTGATAGGGTGAGAAAGAAGAGTTAGGGGGA
AGAATTCAAAGAATAGAAGGGTGAAGAAAATATCGAATGAAGCCCCAAGA
AATGCGTACGCAGCTCGAGATTAAAATCGCTAGGCGGCACTGGAGGCACG
AAGAGAGCAGATCAATCGATCAGTATAGGAGGCGACATTTCAAGAAGCTG
CTTATTGCAGCTCGCAAAGGCTTTGATGACGAGAATACACAGTTAAAGAAG
ATTCACGAAGAGGAGAAACAAAGCCAAGCCCAGATCTCGCGGGGTGCTTA
TGATACGTAAGTATATATGATGGCGGTCGGGGCTGCTACCGAGTGGAGTAAA
ATCTGGTTGTCAAGGGTTTACACAGATTTGACGCGGCAGTTGATGAAATGT
GGAAAACATTACAGGAGACGATTTTCAAGACTAGAAAACCGAATAGAGAT
AAGTTGACCAAGCATAACAGACTGAGAACTCAACACGAAGCTCTGCTCGC
CGAGTTAGATCAACGAAACAGAGCCCTTCCGGGGCCTGAGGCGAATGTTC
AGAACCTACATCGAGAAATCGATGCGTATGAATTGAGGCTCAACCAACAAA
CCCAATCCTATCGAGAAGCCCTGGAAGAATCACATCTTGATATAGAGTACCA
GGAAAAACAGCTCCAAGAGGCCAAGGCGCAGCTAGAACGCCAGACCACA

GAATTGGAATCTAAAAACGAACTTGTATCTAAGTTGAATCAGGAGATCCGC
TATTTGATAAGAGACTTGGGGGATTTAAAAAGGCATGAAACGGAATATCGG
GAGCTTCTAGAGGATGAAAAACGACATTCTCGATACCAGCAGAAGATATCC
AATAAGCTTACAGAAGAGCTGAAAGAGAAATTACGCATAGAGGAAGACAG
ACGACGAGAACTCTTCGAGCAGATCCAGGAACTTGGAGGGACTATTCGTG
TTATATGCCGCATTCGACCGGACAATCCCCGGGACTCCCTAGTGTATGAGGT
GAATAAAGGAGAATTCCACGGCGACCCAGCTAGATTGACGATAATCGAGGA
AAGGAAGACGCCACTGGGGCAATTAAAACCAACCAAATCGGACACGTACG
AGTTTGAGCGGATTTTCGGCCCTCATGAGACGAATAATGACATCTTCAAGG
AAATTAGTAGCCTCATACAATCGGTCATCGATGGGAAGAAGGCTTGCATCTT
CTGTTATGGAAAATCACGGGCCGGGAAGACGTACACCATGAGCAACCTAG
ACAACAGTCTAAGTCGACTCGGAGTCAACGGCAGCAATGACGGCATTATTC
ACAGGGTGAAGACAATGTTATTCGCGGAAAAAGAAAGGCTTGAGGATATC
GGGCTTTCTATGGATATCAGAGGATGCTGCTATGAGATATACAAGAATGAAT
TGTGGCTATTGAAGTCAGGCGGGAGCGAAAAGAAGTATATCTCCAGAAATA
CTCGCTCTGTGAGAGATCCTGGTCTCAAAATATTGAAGAACAGTCACGAAT
TCGAGGGCATGGTCGCAGTAGGGATGAAAACCCGGCATTTTGGAGAAACC
AATACGAACAGCAACTCATCCCGATCGCATTTTATTATCAGCATTGAAACCA
GAGTAATGTCAGAGAGTGCTCATGGCGCAGTACACGAAGGTGTACTGAATT
TAGTAGACCTCGCAGGCTCGGAGAGAACACGCCAGGCCGGTACTGCGGGC
GCAAAGCTACAAGAAGGGAACGATATTAACTCATCACTCACGCATCTGGGG
AGAGTCTTCGTTAGTCTTTCACAAGGCAGAACACCAACATACAACGGCAA
CATTCTCACGGAATTCCTCCAGCCTTCCCTTCAAAGAGGATGTATAACGCTT
ATGCTTCTCATGATCAGCCTCCTGAGAGAAGATTGGCCGGCAACAAAACAA
ACATTGCAGCTTGCCTTAGATGTAGGTTTCAAAAAAAGAAGGAACTTCGTT
GGGTTGATATTACTATACTAATCAGGTCTCGTATAGGCACAGTCTGCAAGGA
AAACAGTGCCGTCAAGCAGAGGCATGAAAGCACTAGCAAAAGGAGTCTG
GAAGTAGATTGAGCTTGAGTCATCACTGGCCTATCCAATTGAAAGGGTTTC
TAGGCTCTATTTCCGCGCTCTCATGGTTAGATGTTTATTTTGCCCCCTCGAAG
GATAGATTTCCTTCTTTTCTAGCTCATTTTACTAAAGCCACGAGGTCTTTTAC
TATTGCGAGAATCATTTCATAGCAACTATGAGACCCCTGAATCTATGATAAG
GCATGCATCACCTTTATCCTTGGCATGTGAATCCACCCATGGCCGCGATTTT
TTTTCATCATTAACGTCATATGTCATATTTCATAAGTCTCGATAATTGAAAAC
ATATTCTAGATGAAATAATCCGAATAACAAATAACCCCTATTTTCAATAACAG
AAAATAACGGTTTTTCGTGGGTATTTCTAGTCGTGTCAAATCCACGACCAAT
CCTGAAGCAAGTTCCATATCCCATGTCGTCCTGCGACCTCATAGGCGGCCG
TGTTCCCATAGAAGTTGACAAATTAAATACCCCGTCATTTTGATCCGATCAC
GTCGTATTCTGTGGTCAATGGTTCCCTAGACACCCATGGGATCTTCTCACCC

CCTTTCCGGTAACCCGGTTTGTGTCTCTTGACTAGTCTGCCGGGATTCGCTC
TCCAAACCATACACGCTTCAAGAACATGGGAAAGAGTTGATATCGACGCAG
ATTGGATTGTTCTAGCCTCTACTCCACAGTTTCATCACCCTGTGTGGAAACG
ATGGAAGGAACTTGAGTGGTCCAGCGGGTCGGGGCGACCTTGTGGGAGGC
CTTCTGGCAGGTCGTATGCATCCGAGGATGGTTGGTCGAAAAGCGTAGTCG
AATCAGGATTCTTTGAAGACATATCGTGACTGTGAGTGGTCGCTCGCGGCA
TGGTCAGCGGGATCCACCTGTCAATAGAGTCCGGGCTCTTCGGCCAATGCG
TCTCGAAGCCAGAGCCCCCTTGGGGGCTACGCACTCCTTCGCAATCATTAC
ACCGGCTCCTTGCAGGACTATAAAACGTGATGTATGGGGTGGCAAGCATCC
CGCTGTCGTTGGTGACGAAACGGGAAGGTCGATACTTCGGCAGCTCTCGCT
TTTTCTTTCCTTGGCCAAGGAAGATCATAGACCTCTACGGACACCCTAGGT
GACTGCATAGCCTGACCATGTTCCCTAGAGGTATTCACATCGTGATATCTTG
CAGTCAAGCCACTGTCGCGCGAATCAAGTCGGTATTCTAATATGGTAAGCA
AATGGCCATTTCCAATAGGATGATTAATCTATTTCTTGAACTTTTAGTTGGAT
AGCTGGGTTGGTCTGAGTTATTCCTGCATCTAGGGATTACTTTTAAGTCCGC
CTATACATTTATCAACATGGGTATTACCTAAATATTGAACGCCCATGATCCCA
TCCAAAGGGTCTTCAAGACTGGGTAATAGTTAGCTATCGATTGCCCTTACAT
ATGATGTGATATAGAGATGGGCGTCCTAGGAGGAAGTTTTCTTTGGCGGCA
ATCTCTGATTTGGTGAACTACCTAGGACAACATGTGTTCCTATTTCTGGTCT
GTCGAGTTTACTCGTCGGGGATTCTCCAGCGTGACGCGGCCAAACAACGA
GTTTGTAGGTGGAACAGGGACTACGTAGGTAGATATGTGATATTGAAACAA
AAACAAAAGTTCAGTTGTTATACTTCCACCAAATGCCCCTGATTAGCATGA
AGCAAGGAACTATGGTGCAGTACGTAGAAATTGTTCCCGGAAAATTCTAGG
TATGGGACAGTGATACAGGACTTTCTTCTGTAATCAAGTTTTGCAGCTGAGC
ATAGGCGTCTGGCTGGTATCTAAGAGGTATACTAGGAACTTATATGACAATG
TAGCAATTCGAAATCGATAGCGGGTCGACATAGTACCGTGAGAGCACAGAG
GCCAGAAACGGCGTTCCTCGAAGTCTCCCCTTCAGACGATCGAGCAGCGG
AGATCAATGTCGCGTAGAGAGACCGAAGACTGCGAAGCTCATGAGCAGGG
GGCATATCGGCGTGATCCCGGGTGAAGACGCGCTGCTGGAGCAGCTGTATA
GCCTCGAGCCAATATCCAGTCCTGCTGAAGATTTCGCAAACATTGAGGATG
CCCGAGACGAATTCCTTGCTCTGCACTATGCTCGAATAGGCCTTGCTGGAC
GAAACTTTCTCGACCCACCGCACGACTTCGTCGACTCGACCAGCTCGGGC
TAGGGACGCAACGAAGGTCCTGAATGTCGGGGCATCCGCCGGCGGCGGCA
GGACAGGCGTCGAGGCGGGAGCGGACAGCGAGTCGAGCATGGACTGGAA
GATCTCGTTGGCACGGGCCACTTCGGACACACTGACCAAGGATTCAATGGC
GGATGCGTAGGCGAGGCGGATGTTGCCTGATAGACGTGCCTGCGCCCGTCT
ATCCAGAGCCACGGAATCGTCGCGAACTTCGGCGGTGTCTTCCGAAGAGG
CCAGCACTTCGTCTTCCGCGTCTTCGATGACATCGGCGGCGTTGACTTCAG

CTGGCGGGATGGCTGCAAGGGCATGTTGGAAAGCGGTCCAGGTCGCCTCG
ACACCTTTTTTGTCGCCCACTCGGCCGTAAAGACTGAAGACGAAACGATAA
AGCGATTCGACTCCGGCGATTGATCGACTGCGAGCGAGAGCGTTGCGCATG
ACATCGCTCGCACCTTGGACGTCGGGCGGGTTCTGCGCCGCACAGGCTCC
GAGGACATGCGAATACGTGGTAGCGGCAAACGCCCAGCCGCGGCCGTGCT
TGAGGATGGCAGCTTTGAACAGACGAAGGGCAGCGGCAGCATTCTCCTCG
GACTTGTAGCTTTCGAACAGTGCATCTTGCTCGGCAGCTTGCGACTCGTCC
CATAGCAGTGCCGAGATTCGCCGCGCGCCATTGACCCCAGAACTTGGCCTT
CTCCCATCGAGCAGCACTGGACTGTATATCCGCCTCTCGCGCACACAGGGC
GTGGATTACATGGGCATATGTGCGTCCGTTGGGGATGACGTCCCGCGCGAG
CATCTCGTTGTATATGTCAAGTATGGGCGCAATGGACTCGCCGGTCCGGCGT
AGTGTGAGGAGGGCCGAGAGACAGGCGTTATAGGTGCGCACATCGTATCC
GGTGGGCAGAGGGAGATGTCGAATGCCCGTTGATTGAGGCTGGACTTGTG
AGTCGATCTCGGAGATGAAGGGTGAAGATGTGCGCGGGGATCGGTAGTGC
GCAATGAGGGCCTCGGCGTAAGATCGATCGTTCTGCTTTCGAGCTTGAATG
AGAGCGTCACAGATCTTCCGGTCCTTGTCGTCTTCAGCCGATTTGTGAGAG
GTCTGCTGTCGCACTCGAGGAGTGGAGGTGGACGTCTGAACACGGTTCGG
CGGGTCGTCAAGCGAAGGGCGTTGTACGGCTGCGACGGAGTATCGACGTG
GTCGACCGATGGAAGAGACGGTACTGAATACGCCTGCGAGATCTTGCTCTA
TGCGTCCTCCGTTCTTGGCCAGCGTGGGCTTGGTGACGGCCACAACAAGC
GCGTCCGTCTCTGCCTCTCGGGCAAGGTCCAAGGTTTGTTCGTCTGTGGCA
GGAACAAGGACCTCTGTTGCGCGGGAAGAGTACGTCCGTACAATACTGTG
CGACGCAGGCGTCGGAGGACCCACTTGCCACAGCTCTACGGTGCTCCTCG
ACCTTCCCAGCGCACTCTCAGGTCGACGACGGCGGGAAACCGAGCTTGGG
CTGCTGGCACGGGAAGGAGCTTTTGACCTGGGCGGTGTCCTGGGGCGGCT
CGGACCAGCGATCTGCTCCTGCTCCAGCTCCTCCACTGCACGCGTTTCGCT
CGCGGCAAACGAGCGCCACTCGATGGCACGCCGCAGGTCTTCCTGGCTGC
CCTCCGGTACGTCATCGAGTTGCAGTACCTTCCACTCTTTATAGGTCGGGGC
GCGCAAGTCGACGATGCGCACTTCGCGTCCGGGTCGAGTCGTAGCAGCCG
AGGCATCCTTCTGCTGCCGGACCAAGAGCCGCCTCTCGCGGCGTTTCTGCT
GGCTGTCGTCATTGCCCGTCAAGCTCTGAGACGGATCGACGCTGCTGGTCT
GGGGCAGGGACAGGAATGCCCGCGCATGACCGTGGTAGCCACCACCGGCT
GACGAGTTTGAAGATGACCCATACGCGCCTCTAGCGCCGTTGGTAGTCTGC
GATGCCGGGGACGATTTGGCGCCGACTAGCGAGGTGCCGGTCGAGCCGAG
GCCGTTGGGAAGAGTGTGGAGGAGGGAGGGGTTGGAGGTGAAGTGTTCG
GGGGACGGGACGTGCAGGCGTAGGTACGGCCGGATATGGCCTCTGAGGGG
TCAGCCAGCTCATACCCTCCATGGATTGAGACGCACGCTGCCTTGCCAAAC
ATCATGGTTGCTGCAAGTATTTCCAGATGGCCGATGTGAAAGGGGCGAGTG

GACCGATGATGGATCGGGCAAAGGATCTATGCGCTCATGCTGCGCCAGGTG
TCGTGATGATACGATGGGGAGTCACCCGTGTCCGGGTCGAGTCGGACGGG
ATGATGACCTTGCCCAGAGACAGGTGAAGAGATGGAATAGGCTAGAGAGA
CCAAAGTCGACTCGGCTCGAGCCGCGCAAGTCTTTCAACATTCTCGGCGG
AGCAAATTTCGGATTACCTCGCACAGAACACCGGCTAATGGGTGATTAGGT
TCCGTCGTATTCTCCGATGCAAGCCGAAGTCCGCTCATTAGTAATTGGAATA
GCCAAAATTTGGTATTCCCCTCGGGCACTCTCGGCCAACGTCGGCGACGGA
CGACCCGCAACCGCAACCGCAACACGACATGAACGACATGATACGATATGC
CATGCACCGCCATGAGGGACACGACTCGTGTCCGAACTATACACACAAGA
GGAGAACTACTATACCTAACGCTACAATGCTGGAGATCCAGGGTCGGTTCC
TGCTCACCGCCGTGTCGAGCGAGCAGACGGCTGATGCAGTTTGACGTGAA
TCGGAATGACGCCCCCGACGCCGCTATTGGGCGGTCCAAAGCCGCCAGGA
ATGGGCTTGCGACATACCTGACATCAGCCTTGGTCACGAGCACCGCCATAT
GGAGTCTCACCGGACATTCGCCCTCGACAACCCAGCCACTGTCGAGCTTCC
ACAAGCCTCGAAGGACCTCCTCCACCTTGATCTCGTCCTTCTCGCCGAGCA
GATCGATCGTGACGAGCGGATCGTCCATCATCCCATCTAATTCTTTTTCCGC
CAGCCTTCTCTTGTGCTCGTACAGCGCGTCTTGCAGCAATTCCTTTGTCCAT
TGTGCAGGCTGAGGGAGAGCGCCATGAGTTACCGTTCCTCTGGCGCCCCTT
GCTCTGGCGCTGCCGCGACCACGGGAAGCGATACTGCGACCTCGTCCGCG
TCCTCGACCTCGGGGCTGGAACGCAGGGAAAGGATTGACTTGACGAGCGA
CGGCAGCTCGCATGGAGCCGTGGAGACATTTTGCACACAGGATATGGCCAC
TGAAAGTCAGTATAGAGGCCCTGGTCCGCAAGGAGACATACCAAGGGGTG
ATCATTGGGCGAGTGGGTTCGGAGAAACACACAGGACAAGCTGGGACAAT
TTAACACCATTCTCAGCCGGCAAAACAAAACTCCTCACTGTATGCACTTATC
AAGTTCTCCTCTGGGACAGCTTCCTCGCCGTCTGCATCCTCTTCTTCCAAG
ACCATGTCCTCCTCGTCATCCTTGTCCTCGTCCGGGACGCTATGGGTGGCAA
TCGGTCGTTTCTGCACTCCTACCACCTGGAAGCTCTCATCGCCCTCATCATC
CTCAATCTCGATCGGCTCCACCACAGCCCTCCCTTTACCCTTCTCCGCGGCC
GTATACCGCCGCTCGCCCGAGATGCTATGCTGCGAAGATGAGGCAGACGCG
ATAGACTGCCGATCGAGAGGAAGAGACGGAGCTCGTGCGAGCGGCGAAA
ACATGGGCAGATATTCGTCTGCAACGCTATCGAACGAGTCTTCGAGGAGTG
GCGAAGGAGAGCCTTCAAAGTATTCTTCGACAGCGGACGAGCGGCGGGTT
CTGGATGCTGCGGGTGGGGCGGGGGCAGGAGCAGGGGCCATGGCGGAAG
ATTGAGAGCGGGTTGATGGGCGGCCTGATGTGCCGCGTGACATTGATGCTG
AGCCACGGCGAGCGCCACGACCACGAGAGGAGGACGTGGAGGCGGTAGC
GGAGGTAGATGTGGAGCCTGTACGCTTGCGTCGACGGGGTATGAGGGGTA
CATCAGCCACGGTAGGTCGCGGACCGGGGGAAGGAGGTCGAAGGGACAT
GTCTGTCCGAGCTTGATAGGCTTCTCGCCGTCGTCAACGTGTCCACTAGAG

CAGGCAGATGAAGCGATCGTGCTTGGCCATCTGCCGGGTCGGGAGAGAGG
GTGATGGAGGGGAGAAGAGATGATGGCAAGAGGCAGAGGGAGAAGAAAA
GGTTCAATAGGAGACAACCCCCTGCTTGCGTCACAAATCCGAGGTCCACGC
GCGAGCCTACTCCTCCCCTGATCGGGTTGGCAGAAATAGGCTCGTTCCAGT
TTTGTGACAGGGGACGTTTTGCAACTCACACACTCCAACTCGCCACCACA
GCTTTTGAGATTGAACTACAGATAGAACCACAATCCCCTCAATACCTCGCA
GTTAGAACGTCCCCGAACAGAGCAGTCGATACAGCAGTATGCCCAAGGTG
ATTCAAACGTGACTCCGGTGTGGCGGAGCTGACGATGAGGCCAAAGGAAA
GGGTGGCAAGAACAGGCGTAGAGGGAAGAACGACGATGATCTCGCCAAG
CGCGAGTTGATCTTCAAGGAGGACGGGCAGGGTGAGTGTCTTGTTCTCCCT
ATGGACGTGTCTGGCAGGCATCGGAGGGCGGAGAAGGGGTCACACGAACT
CAGGACCGCGAGCGCGTCTCTGTCGCGTGCCATGCTTCTGGGGGGAATGG
CAGCGTCAAAGAGCCGTAGGCTGACCCGAAGAATACGCACAGGTCAACAA
GATGCTGGGTAACGGCCGTATCGAGGCAAAATGCCAAGACGGCGAGACAC
GTCTATGCCAGATCCGAGGTCAAATGCGGAAAAAGGCGAGTCTCTTCCCTC
CCTCAGACGTGTTGCAAAGCTCACACGGGGACAGGTATGGATCGTAGTAG
GCGACATCATCCTCATCTCCCTCCGTGACTTCCAAGACGACCGCGCCGATG
TGATCCACAAATACACCGCGGACGAGGCTCGGAATCTCAAAACGTACGGA
GAGCTCAAGAGCGATTTCCAGATCCACGAGAACGCACAAGAGGGTGGTGA
GGGCAGTGATGTCGAGGAGGGCGGTGTAGACTTTGAGGAGGCGGAGATTG
ACGATATTTGTGGGTTGCGTTCCTCCTGCTGCGATACTCGCCTTCGGGTCAG
AAGAGGTGTCATTCGAAGGCCAGGGTCGCTGTGCCGCATCCTCATCAATCT
TCTCCTGTTTCTCCAGTTCTCGTGCACTACCAAGGGCGAATCGCTGACGTT
CAGAATGGCAAAATGTACAACCCGCATGTGCCCGTATCTCTTCTTCTCAGC
ATCCGGTCTGCACTGTAGCACACATGCACCCGTGGCTTCCCCAGTACAGTG
TGGCGTCTGATCAGGGAAAGCTCTCGCCGTAGCGTACGTCTTTGTCTGAGC
TCACAGCTTTGTCCAGCCAAACGGGTCCAGCCATCGGTTGCACGGCTCATT
GTGTATTGTTGGCGGTGTTAACTCGCCCGTCGTATCTTCACCCCGGCGTAGC
TGGGACGGGGTTTGTCAGCTGTGCAACTGGCCATGGCCGAGTTGTCATAAT
GCTTTCGACTAGCAGCTTCGAGCTGACAAAGGCGAGACATATAGACGAGG
CTGTTACATATCCACATTTTAGCCGCCCACTTGCCCCGCGCCAAGTGTCCGC
TTCCCCACCTCCACTCTGTCTGGCCACTCCCCTTGTAGACGCGCGATACGC
GCTGCTACAACGTATATAAGGGAGCCACTGTCGACGAGCGGCTAGTCCGCA
TTCTGTCTGGGATACGTGCGCATCGCAAGATGCCCTCCGAGTGGACATCCG
TATCCTTCGCCGACCCCCGATGGTCGCTCACCAATTCGAGCCAGAAGCCCC
AGGTGTCGAGTGACGGTACAGAGCTCACTCTGCTCACTGAGCCGGGCACC
GACTGGTGGCGGGTCCCAGAGCGAGATTCGTCGGATGGGGTCCTGTACTCT
CTCGCTCGGAAAATCGGGGAAGGGTTCGAGATCAGCGTCGAGCTGAGCAT

CGCCCACCAGGCCCAAGTGCGTTCTCAACCTGATCTCGCGGAACATAGAGT
CGTCCAATGCTGACGGGCAGTTGTAGGTTGATTCGGAGTACTCTCGAGGGA
TAAGCTGAGACGCAGTGACCAAGCGTGCATCATCCTGCAGCTCGGAGACC
GGTGGGTCAAAGCCGGCCAAGAGTTCGAGAGCGGCAAGCTGTACGACTCG
GTCGTCTCCTCCAACACGTGGTCGGATTGGTCCATCCAGCCGCACGTCGGG
GAGACACGGTTCACCATCTCGTTCGCTCCCGGCGTGCTGCGCGTCTTCTTA
GGGGACAGGATGATGCGCGAAGTGAGGGCCTTTGGGCTGGGTGCGAAGG
GAAACGAGGTGGTTACGGTGGGCGTTATGGGCTGCTCGCCGAAAGGCGGG
GGTGTGGCGGCTGTCTTTAAGGGGTTCCAGGTGAGGGAAGGCGCGAGGTC
GGTGCACGAGTGACAGAGACAAGTCTGGAGATGATGCATCTTAACAGGTG
TCAAGGAGCGATGCTGGAGCACAAGGCTCTGAATCTACGAGGTTGTACTAA
GGATGCTCGGTCGAGGCAGCGCTTGCGCTGCGCTGAGTCTTCTCACAGGG
AGAGTCCCGCTGGCTTGAAGCAACCGGAGTACTGAACGTCTGAGGGGGAT
CCTCAAAGATACTAGCGGGGAGGAGAGCAGTGAGTGTCCGGGCGTGCCCA
CGAGATGGCTTCCTTGGGCTTGGCGGGGACTCAACAGGGAGCGACTTTTC
GCTAGCACCCGTCTCGCTGTGAGTCGAGACGGGGGATGAGTCCGCAGTGG
ACTGTGCCACTGACGAGACCTGGGGAGGTGATCGGCCGATGTGGTCGAGC
AGAGTCCGCATTCCAGAGGGAGTCTTGGGGCGCTTGGTCTCGGGAGACGA
CGTGTCGGTCCTGGTCTTTCTCCCTGACTTCTGGATGTATGTGATTTGCCCA
CGCATCAGCAGATCCATGACATCAAATGTCTCCCCGGGCTCCAGAGGGCGA
GGGGCAGGGCAGCTTCTGTCGAGTCAGCTCGAACAGCGGTGCAAGGGACT
TGCCCTACACCCTCGGCCTCGTGGCGGTAGAGGAGTCCCGTGTCCAGTGG
GTCGCTGGATCCAGAGCCTTGTCGTAAGTAGCCATGATTTCGATGGGGAGG
ATTACAAGCGGTCAGGAGCTAGCTATATCCACCGGCTCCAGATACGCTGTTG
AGAGGAGCCTGGTGGCCTTCGGATGATGCGGTAGTCTTTGACGGTCCAGA
ACTGCCTTGCGGTTCGCTCCTTTATGTCGGGGTTCATACTTTCTCCCGAGGC
TTTTCGAATATCTTAGACGGCGGAAGGCCTTCCTCCAGCACGGCCGTGCGG
TAGGGGGTCGCGAGCAAGGGTTCAGAGACCGGGACAGGAGTAGCAGAGC
ATGATGCATAGTTACGGAAGTCTTTTCGGGGTGTTTAGTCATCGAAATTGTA
GATCTTGTCGCGCCGCCCGTTGGACAGTTTGCCTTCCAGAGCATCGACAAT
CTCGGCGAAATGGTGTGCGGTCTCAGCTTCCCACGCTCTCCTCGTTGTTGG
GGTTCTCGTGCCTTTGTTACGAGACAGCGAGCCCGAAGGGGAGGTCGACG
AAGCAAGACGCGTGGAGCTTGTGCTTGGCCGAGTCGATGACGATGGCTGA
GTTGTGCCGATCCTTCGAACGGGGCCGGTTACTGAGCTCTGGCGTGGAGG
GAACTCGCCCCTCTGAAGCTGTCGGGCCTCTGTTACTGAGAGCAGAGTGG
GTGTGTGGTCAGAAGTAGCCTCTGAGTCTTCTGGAATTGTCCCCTCGGTGG
GGGAGTAGCGGGAGAGTGGAACAGATGGTCGCCTGGGCGCAGCCGTCGTA
TCGGGCCCCGACACCTTGGGGTACAGATCAACGGGCAGAACCGTACTACG

GGGTCCGCGAGCTGGTGGGAAGTTCCATAAGTTGTTGTCTGATTTCTTCCT
ACTCGAGGAGGGCGCAGCACGAGGAGTCTCGCTCAACAGAAGCGGCATG
TGGTTTCGCTGCTCTGTGGACCTCCGCGACTTCCCACTTGGCACAGACGAA
GACCTGCTGGGCGTTTCATCATCGGATGAACTCGGTCTGTCCGAGCTGTTA
GCTGAAGTCCGGCTCTCATCCGACCTCTGCGAATCACGCTGGGAGGGAGA
ACGGCTCATCACGGGGAGGCCTTGATCATGGAGTGGACACTAGTAAAAATG
AACTGAGGATAGGTGATGGGTGCAGGAGCGGTACAGAGTGTGGGCTGTGG
AAGGGTCAGCTCGCCAGGTCAGGATTATAAAGGCTGCGAAGCGGGCGCAG
TCTGCCAGAACGACCTTCGCGCTCTGATCACCTTTAATGTGATGTCCTTTGT
GGCGACCTGCGCTATCTACCGAAACGCGCCATGCTTCGAACAACCTGTCCG
CTTTCGGCGGGGGCAGGTAAAGTGTGCGGGCTACTGGGAATCGCTGAGAA
AGAGCATCAGAAACAGTAAGTCTAGCATAAAGGAGTGTCTAGCGACGAAT
ATGCGAGAAGCGTTGGAGAAGTCATCAGGCTTCTCCCCTGACTACATATCC
CCCCGAAATTCCCTTGGCTGTGCGCAACTCCGCCGGCTGATTCGTCGTCAG
CGCGGCTCCTGGAAGATGGCCGTGGAGTCCGTGATCAACGGCGCCAGTCG
GAGAAGGCATCAAGAATAGGCCGCTATCCGGTCTCGCGAGCGGGTCAGCC
TTCTGGCTTCGTCCGTGCGGACCGCAGTCCCGAGCGGAGGCGTGGTGATC
CGCGCGGGACGACCTCCTTGTGCTCCCTCTTTCATCTTCATCTTCCTGGGAA
GAGTCCTCGCGCAGGAACGATGACTCTTCGCGGACGAGGGTGCTAGCGGA
GCGCTGCACCTCCTGCGGGGGCATTAGC

>Mosaic_sequence_35

CCACCACCAATCGAATCAGTGCATCAAATTACGCGTCGACAGACCCTTCAA
TCGCGTCTGGAGGCCGTGTAGAAGGCTGTGACCATGGAGCGTTGAGTGAT
GGCTCGGGGTAGATGTAAAAATGACCTTTAGAGCACCCCCCTGCTCTTATC
AGCCGTCGAGATCACCAATGTCAAGCGTAATGTCGCATACTACAGCTCCCA
GGCCCTCTCTTGGGTCAATACCAGCCCCCCACACCGCGATCAGACCTGTTT
ACCCCGGCATATCGCCTCTGTGACCCAACTTATCCTCGATCTTCGGGCCATG
GAGATGAACCTGCCGGCCCAGGACTTCTCTGCGCTCCATATACACCTCACG
CTCGTGCAGGACATGCTCAAGGACGTTCAGAGGACCGCAGACACCCTCCT
AGGCCCCATACCAGGACCCTCGCAGCGTGAGAGGTCATGGTACAAGGGAG
ACAACGGCCGCTATCGACTCTCATGTCCTCAGAACATGCTCAAAGACCTAC
ATGGGCTCGGAATGACCGAGAACATGCTGGCACAGCGTCTACATGTGTCCA
TCAGGACCATCCAGAGGTGGAAACGGGAGCTGGGACTCAGGAATGCAGCC
GAAGGTGGCCTATCGCTCCTACAAGTGGAAGCGGTGAGTCTCAATTGTGCT
GCAGCCTGGGCTCTGACTGCTCTTAGGAAGTCGATCGCTGCTGGAAAGAG
GGCGGAGGGATGGAGGGCTATCGTCTGATCATGGCGGCGCTCAAGAGGAA
GCAGATCAAAGCCTCAAGAGCACAGGTGCGAGAGGCCATTCGCGATCTTG

ACTCGGACGAGGCTAGAAGGCGATTTGCCAGGACGACAGCTCGCAGGGTG
TATCAAGTCCCATTTGCCAACAGTCTGTGGCATATAGATGGCCATCACAAGC
TCATTGACGTCAAAATCGTCATACACGGCGGCATCGACGGGAAGTCACGAA
AAGTCGTCTTTCTCAAGGCTAGCACGAATGTCACGATACCTCTCTGTCTCTG
TCCAAGGGCCCGTCACTGGGTGACCGAGACTCTTCGACTTCGGCGAGGCT
GGGCGAGACTAGGAGAGAAGGGCGAGGGCAAGGGCTCGGAGGGCTCGTA
GAGTCTCAACGAGTGTCGACGAGGGTAGCCGAGGTTGACTAGGCAAGGCG
AGATACGACAAGGCGGACCGACAAGTGCTAGACCTTAGCAGCCGTCGGCG
GGACGGAGCGAGAGAGAGTAAGTGAGGGAGTAGTCCCATGCAACTCAATT
GCGAGAGTACTAGAGTTCTACGAGGACTCTGCGAGCCTTAAGTATCCTACA
GCCCGTCCGTCGATTCTGCGTCTCTGTCTCTGCGAGCTCGACAAGGTTTCG
CCCAATGTACGTCGAGCTCATTGTTCTCTCGTTTCCCTTAGGCGAGTTCGCC
GCATCCGGCGAGCTCGCTGAGTAAGCATCGAGGAGGCCTATTGTTCTTCGG
CGGCGAGCATGGCGAGCATGGCGAGCACGGCGAGCCTCGAAGAGTTCCTT
GCTGTCGACGAGAGCTGTGGTGACGACGGGACTTAGTTGTGCGAGAGATG
CATCCTTTTGTCCGACGAATGCGGTAATTTCACGTGGGCCGGTCTTGACGA
GTCGCGGCGAGCTTCGGCGAGTGCCGACGAGGGCTCGACTCACGTTCCTT
TGGCTCGGTCTTGCTCGGTCTTGGTGCTGCTTTGGTGTCATTCGTGACAAC
GAACAACCGAGCCGCCACCGTCTTTGAGAGCTTCATGGAGGCCGTAGGAC
GCTTTGGGGTGCCTAGCAGAGTCAGGGGGGACTACGGGAAAGAGAATTTG
GACGTCAAAAGGTTCATGGAAGCAGAGAGAGGTGTGTCGCGCACGGGCCT
CCCAAAATTCTTGTTTTCGGGCTGACTGCTTTTTAGGTATATCTCACCATGC
ATTCATCCAGGGGCCCTCAACTCGCAATCAGAGGATAGAGCGCTTCTGGGT
GGACCTCCAGTGGCATTGCACCTTCCGCTATCGCACGTTGTTCGACAGCTA
CAAGCAACAAGGCATCCTGAGCCTCGACAACCCCTTTGACCTTTGGTGTCT
TCACCTGTGCTACCTTCCCATGATCAATCTGAGCCTGGAGAACACCGTGAG
GGACTGGAACGGTCATAAGATGAATGACCTGGGTGGTGAAAGCCCGGATT
TGATGTGGTGGGACAGTGAGTGGTCGATCCTGACTTTGACCGACAGGAAA
GGACCGCTCATGCAGAGGTGTGCTCGACGCCGAAGACCGTGGCATCGACA
TCCTGGCCGATCCAATGGCCATAGTCGGTGAGGAGGTCCACCAGGGACTG
GACAACAACGTTCACCGGCCTCTGGCCTTTGCCGAATACGGGTTGGACGAT
TACGGCCGGAGAGCAGATGGGCTGATTGCGAACGATGACCCGCTGGTCGA
GGTGCAGCCCGTCTTAGGTGAAGTTCCGCCGGAAGTGGCTTCAGCACTAG
AAGAACCAGCCTTCCAGAACGCTCTGCTGCGAAGACTGGGCCCCTGGGCG
CTGGGAATGGACGATTGGGGTGAAGCAAGGTATCGGCAATGTCGACAAGC
AGTCGATGAGTTCCTTCGTCACGAATTTCCGGCCGTTCTTGGCTTACAATGA
CGCTACATGCATGAAATGACAGCCAGACGATAGTCCTGAGCCTGGACAATT
GCCCCATACCAATTTCAATGCATACGAGCAAGTTTCTGTTGAAGGAAAAAG

GAAAAGCAGATACCTCGGCCTCATACCTATCTCCCCGAGATACCTCCTTCCT
GACCTCTCGTCCACCCATCCTTTGACTATGTAGAGCTGGCGCGAAAGGTGA
TATCTGCTTTTGAGCTTTATCTCGACATCCAGAGCAGGTATCGTAGAGATCC
AAGGACCAGAACAGACAGCAGACTTGCCTTGATCACATCCCGGCGCTAGC
GGCGTTATCGATGAAGCCGGCGCGAGCAGAATTGCGGGTAAGGCGGGTCA
TCGATTCTTGCCTCGTCGAACCGAAAGGTGACAACTCGATCCTTGTTCTCT
TTGGCCTGGGAGTGCGTTTGCCCCTAGTGCTTGGCTTGGTAAGGTCCACAG
GAAGGCCCTGACCATTGCCGGGCGACCCATGGGCCTCAAACCCATGGTCC
CGATCGATAGGCGGGCCAAAGCGGCCTCTGGTTGTCTCCAATGACATGGGT
AATGTAGTCTCGTCTGATGTGTCTGATAGAGTCAGTGGCGGGCCCAAAGTC
CTCTCCGTGACCGAAGAAGGTGCTGCCACACCCTCTCCGGTCATGGAGAG
AACCTCTTGGGCCCGAGGAGTGGCCTCATTGCCGCTATCCCTGATAAGTCG
TTCCAGGGCGGTCCTCGCATTGGCTGGAGAGGCCTGGACGTTCCTCTTGAA
CCTAGGGCCTGCTAGGGATCCGAAAACTCGAGCCCGGAACTCCTCATCGGT
CGACTTTCGGGCCTGCTTTAGCTCATAGGGGGCAACATAAACGATCCAGTC
CTCCTCAAAGTCTTCGAGCTGTTTGTGCGCGCCTGCAATGGGCACCATTGG
GGTGATGATCATCAAACCGTCATCGTCGAAGAACTTATCGAGGTTGCGAGC
TGAGGTCGAATGCGCGCGGACGCCATTGTACAGGTTCTCGCCCTTGCTGTC
GGTCGACATCCACCTGAGGCATCAGCTAGAGCCCCAGGCAGCCTCAGACC
TACACATAGTCGATGTTGGCAAGCTCTGGGACCTCAGGAAGGCACTTCGCG
ACCTCACGGCGGACTTCCTCGGAGGTCATCCCATAGGACAACACTGGATAG
CCCAAGAGTTCGCGCCTTCGGCAGATCCCACGCAAGATGTCATGCTTCTCT
GGGGTCCAGGGAGCATATTCGGATAGGATCCCGCTGTTGGGGACAAAGAG
AAGCTGTGGTGAGCGACTTTGGGCCAGCCTGAGAGATCGCCCACGGGCCC
CGGGGGCTGATTCTTGAGCTGAACCGCATGATCCTTCGCCAGGGGGACCTG
TGCTACCTTGGTAGGCCCCTTGGGCTGCTCCGTGTGCTTTTGGGAGCCCAC
ATCAGCAGTAGACACCGAGGAACGGCTGACAGTTGACTTCCTCACGCCTC
TCGAGGCCGCCCAACCGGCTCGAATGTTGCTGGCGGACTGGAGATCGAAG
CGCGGGACTGTGTCGGCTCGGGCAAGGCTGTTAGGCTGCCTGAGGGTCCC
CTCTGTATAGAAAGCGTCGTTTGGGGCCGCTGGGATAGGTGTGGAATGAGC
TGCAGCCGCCGGAGCATTGGGTCCAGTTGGCATAGTCGAGGTAAGAGGAG
CCGAGTTGCTGACGGATCCCGTTCCCACGGTTCCAGGAGTTCCCAGAGCG
CTCAAAACCGCTGGAAGGGCTTCGGGGTTGGTCTGGGCAAGGTATGCGAC
GACCTTGGCCAGCTCCTGGTTCATTGGGGTCTGGCAATGAGCGAAAGAGC
AAAGTAGAACATCGATGAACTGTCACGCTCGAAGGGGTAGCCCAAGGTAA
AGGGCACGAGGGGGTCTAGTCTCTGGCTCGTAAAGCAAGGGGACTGAGGG
ATCGAGGACAAGAGTGCTGGGTTCAGACCAGCTGTGTGACTTTTGTGACG
GGACACAAGAGATCCATTGCTGAAGAAACTATCTTGGCCGGAAAGTGGCC

TCCTTATATAGACAAGAGGGCTATGTACGCCGAAGGGATTTGCCACCCGTTT
TGCCACCCGTTCCATGAGAAAAGTTTTCCTACTGCCGATATTTCTCGAGTGC
AAGCGATTCTCCTCGCTGCAGATCTGTCTATAATTACTGGGAGCGGAAAGG
TGGCTGAAGGCCGCCGAGTAGGATCACCGGAAAAAATACTGACTTCAGGA
AAGATCACCGATAAAAGTATTGACTTCAGGGAAGATCACCGATAAAAGTAC
TGACTTGTATCGGCGATGTACCAGAAGAGGTAATGTTTGAGCAGTTCGGTG
GAAAAAGTACTGTGTTTTGTAGGCGAATGGCGTAAAAAGTGGTGACTTGTG
TTTGAGATGAGGTCGGTCATGACAGGAGCTCCCCCTCTAAGGGCGAAGTCC
CCTGAGCCGGGTTGATCTGGACTTAGATCTTTTGTTGGTTGCTGTAGTGCGC
TTTGATTTTGTTGAACTGGAAGGGAAATGGTGGTTTGGGTCTCGAGCATGT
TGTCGATGGAATTTTTGATAGGCTGTAGTGTCGTGGACATTTTCCCATGGTT
CCCATGAGTTGTGGGCGCTTGAGTAGTCTTTCCAGCTGACAAGGTAGTCAT
AACCATTGTCTGAGCTGATTCGAGAGTCGAGGATTCTGTCAATGATGTATTC
TTCTTGTCCATTGACTTCAATGCGTGGTGGTGGATCTTGTGGTTGCTGAGGG
TGTCCGTGACGCATTGGTTCGAGAAGGTTGACGTGGAAGACTGGGTGTATG
TTCATGGATGGCGGGAGGTCCAATTTGAATGCGAGCGGAGAGGAAGTAGC
CTCAATGATCTTGAACGGTCCGAGCATCCTTTGGTCGAGCTTGAGCGAGGG
TCGAAGTGTTTGTATGTTCCTTCGATTCAGGAAGACTTGATCTCCTACTCTG
AAGAGTGGTTGATCTTTGGCGTTGATCTCCTTGACTTTTTGGTTGTAGAACT
TGGTGTAATGCTCTTGGGCGGTCTTGATATTGTTGGAGAGCTGTTCATGCAA
GGAATGTAATTTTTCGAGGTGTGTCTGTGCTGCAGGGACAGTTGATGTTGG
CGGAGTTGTTGGATCGTTTGGATGATACCCATAGTTGGCTTCAAAAGGAGT
GAACTTGGTTGACGAGTGGACCGAGTTGTTGTAGCTGTATTCTGCCAAGTC
CAAGAGTGATGACCAGTTGACTTGGTCGTAGTCGCAAAATGATCGGAGGTA
ATGTTCCAGAACTTGATTGACTCGTTCTGTTTGTCCATCGGATTGTGGGTGA
AAGGCTGTCGAGAAGTTTGGTGTGATACCAAGGCCTTTGACAAATGATCTC
CAGAATTGCGAGTTGAAGATCGATCCACGGTCTGAGACAATGTCGTGTGGG
AGTCCATGAAGTCTCCAGATGTTTTGTCGGAACATTGTTGCTAAGTTGGGT
GCGTCAAAGCCGTCTTCCTTGAATGGTACAAAGTGGGCTTGTTTGGTGAAG
CGATCAACAAAGACCATGATTGAGTCGTAGCCGTTTGATAATGGGAGCTTG
ACAATAGCGTCCATAGAGATTGAGTGCCAAGGTTTGCTGGGTACTGGAAGC
GGTTGAAGAGGTCCGTAGGGTTTGTGTCGAGGTATTTTATTCCGTGCGCATG
TGTGACATCCGTCCACGTAGGTGTTGGTGAACTGTCGCATGCCAGGAAAGT
AGTAGTGTCGAGCTAGAAGTTGGTATGTCTTCTGTTGTCCAAAGTGTCCTG
CGGTGGGGGTGTTGTGACACTGGTGAAGTAGGTCAATTTTGAGATCCTGAT
TGTTTGGGACATAGACCTTTTGTTGATATCGGATAATGTTCTTGTCGAGGGT
GAAATCTTTCAATTTGTCTTGTAATTCCATGGGTATTGGTCGATTTGGATTGC
GCAGATTAGGCAGTAGCTCTTGTAGGATCGGATCCTCAGGCTGGAGTTGCT

CTATTCTGGCCATGATGTCGGAAGTTGTCGAGTTATCAGAGTTGCTGTGTAA
AGCACCAATGATGAATTGTCCAGGTTTGAGGAGTGTGTGTGGAGGTGCTTC
TGCAGCTCTACTCCCTCCTTGAAGGTCTGACCTCCTGGTCAGGGCGTCTGG
TTTGCCCATCTTTTCTCCAGGTCGGTAGTGTATCTTGAAATCGAAGTTGGCT
AAGAGTTCTGCCCAACGAGCTTGTCGTCTGTTGAGCACCTTGGAGGTTGTG
AAATATTCGAGGTTCTTGTGATCTGTGTAGACTGTGATTTCTTTTGCTCCTTC
TAGGTATGGCCGCCAGTGTTTGAATGCTTCCACAATTGCAAGCATTTCCTTG
TCGTAGATCTCATAGTTGAGTTCGGCCGGTTGGAGTTTTCGAGAGTAGTAG
GCAATTGGGTGAAGTTGCTTCCCATCTATGTCGGAATGTGAGAGGACACCT
GCGATAGCAAAGTCTGAGGCATCTGTCTCCATGATGATTGGCTTGCCAGGC
TGAAAGTGTTGAAGTATCGGAGCACTGGTGAAAGAGTGCTTGAGTGTATC
AAAGGCGGATTGTTGCTTGTCTCCCCACACAAATTCGACTCCCTTGCGTAG
GAGGTGTGTGAGCGGTGATGTGACCTTGGAGTATGCTTTGATGAAGCGTCG
ATAGAAATTTGCAAAGCCAAGGAAGACTTGAACATCGTGTACCGATTTTGG
TGGTGGCCAGTCTGTGACAGCTTCAACTTTGTGAGGGTCCATTCGGATTCC
TTTGTCGGAGACAAGGTAACCAAGGAAATCAACCTCGTGCTTGAAGAAGG
TGCACTTTTCTGCTTTTGCCCAAAGGTGGTGTTCTTGTAGTCGTTCCAAGAC
TTGCTTGACATGCGTGATGTGGTCGTCCTTTGATTTGCTGAAGATGAGGATG
TCGTCCAGGTAGACAATGACAAAGACGTCCAAAAAGTCTCTGAGGACATC
GTTGATCAGGTTCTGGAATGATGCGGGCGCATTTGTAAGCCCAAAAGGCAT
CACAAGATATTCGTAGTGTCCATAGCGGGTGCGGAATGCCGTTTTCCATTCA
TCACCTTTGGCAATGCGGACAAGGTTGTATGCGGCACGTAAGTCGATCTTG
CTGAACCATGAGGCTCCTTGAAGGCGATCGAGAGATTCTTGAATGAGCGG
GAGCGGATATCGGTTTTTCCGAGTAATGCTGTTGAGTTGTCGGTAGTCGAC
ACATAATCTTAGGGATCCGTCTGGTTTCTTGACAAACAGGATGGGTGAACC
AGCGGGGCTTGAACTTGGTCGTATAAATCCTTTCTTGAGGTTCTCTTTGAGG
TATTCGTCCAAGGCTTTGAGCTCGACCTCTGACAGACTGTAAATTGGGCCA
AACTTGGGTTGGGCATTGCCCTCAAGTGTTATGGCATGATCGTAAGGTCCG
TGGTCTGGTAGCTTGTCGGAGTTGGATTTGTTGAATACTTCAAGGTGGTCAT
GGAATTCTGAGGGAATGATCTCCTTGAGTTGCTGGAAGTTGAGATCCGAGG
TGTCGTCATCGTCCTGTGCTGGTGATGGATCGATGAGGCCTGAAACTTGAG
TTGTCGTGGTGATGCCATAGATTTGTGCGTTTCTCATGGCAATTCTGAAGGC
TGCCGCGCTGACCAAGGCTATGTGTGGCGGAGCGTATGTCGAGGTTTGTTG
ATCTTGTGATGGCGTACCTGAAGCTTTCTTGCTAGGCAGGTTACCGGCGCG
CTTTCTTGTAATTCGATGTGACTGAGGAGCAGAATCTGCCTCTACAGGTTTG
TCATACCTGCTGCACTGCTCCAGGCAGAGCGGAGAGGAGAACTTGATGGT
CTGACGGGACCAATCAATGCTTGGGTTGTGTCGAGAGAGCCATGGAAGGC
CAAGGATGACTGGATAGGTGCCGAGTGATGTGATGTCAAGAGAGATTGTCT

CTTCATGACCATGAAGGCACAGTGACATAGGTGCTGTGTGGTGTGTGATGG
CGCCAGAAGCAATAGGTCGCCCATCAATGACTGAAACCTCCAGGGGCGTA
ACCTTGGAGATGCGAGGGATGTTGGTTGTATCGAGGAAACTTGAGTTGATG
AAACTGGCTGTGGCACCAGAGTCAATCATTGCAAGAGTGTCGTAGCTTGA
GTTCCCATGTTTGATGACGATGGGAAGGCAGAGGTGGTCCTTAGGTGTTGA
TGGTAGTATTGTTGACGATAGCGATGTTGCCGTGATATGTAGATGTTTTTGCT
GTACCGTGTTGTGCCCTGTTGAGAGCCCCAGAGGTGGGGACACGCCTAGG
CCTGGGGCTGCGTGTTTTTTGCCTTATTGTCGGAGGCAATGACACTGTTGG
ATGCAGTCGTGGTGGCGCTGACCTGGGCTTTCTGCTGCTGGTTGCGCTGGC
CTGTACGGCATTCCCTAGCCTTGTGACCAGGCTTGCCGCAGTACATGCAGA
GGTTTTCCTTGATGCGTCGTTCACGCTCAGCTGGAGTGAGCGGTCCAAAGG
TTCTGGCTTGAGTTGCATCGATTTCCATTGGGGTTGGAGTGAAAGTTGTCG
AGGTAGTTGTCCGAGGTGCAAAGGTGTTGGTAGGCCGGATGGAAGGGCTA
AAACGAGTTGCCGTAGTGCCGCTGGGAGTGAAGCGTGTTGATGGTCGAGG
ATTGTTGTTGGTGCGACGTCGTTGGTAGAGAAGGTCATCGTACTCAATTGC
TGCAGTGACCAAAGCTTCATAATCCTTGAATTTACCAGGAGCAAGAAGTCG
ATCCTTGACGTCATCCTTGAGTCCATCAAAGAAGTTTTGCTTCTTGGTCTCG
TTGTTCCAGTGGACATATGCTGCGTGGCGCTGGAACTCTGTGGCATAGAGA
CTTGCTGAACCGGTTTGTCGGAGGTTGCGGAGCTTGAAGCGGGATGTTTCT
GCCATGTTGGCATCGCCAAAATACTTCTTGAATTCAATTTGGAAGTCGTCCC
AGACATGGAGCCAGGTTGGTTGATCATCTGCGTCCATGCCAAGGTAAGGTA
AGACCCATCGATAGGCTTCTTCCTTGAGTAGATTGGTTGCAAAAGCGATGC
GAGTGACGTCCGTAGAGTAGGTGCGAGGTTGAACAGCAAAGACCATATTG
CACTTAGCCAAGAATTCCAGAGTCTGATCCTTACGGCCACTGAAAGGCGGT
GGATGAGAGATCTTTGGTTCCTTGGTATGCTCAGGCCTGCCGGTAGCGGAG
TTGACTTGAAGAGCATTGATCTGAGCTTGAAGTTGAAGAATCATGTCGTGT
TGGGCTTGAAGAGCTTGCTCGGGAGTGAGCTGAGTGGTCGTGTTCTCGGA
TTCCATGGTGCAAGAGGTGACCTGTAAGTCAGCAAAGTTCTGAGGACACC
CCTATCACCCCAATGCACTTAAGGATACGCATTCTACAAGTCTGCATCGCCT
TAATCATAACTGTCACAGCATGAGGTCAACGAATGGCGTGGGTTCCAGAAT
ACCACGTATATCGATCGATATAGCTTGTGTATCTGTTGTGAGGTTCAGCAGTT
TGACGAAAGGTTCTTCTTGAATGTCACGCAAGAGCTAGACACCGAAAACG
CGCAGTGGACCACACGAATTGAGGGGAAATTGATTGCTTCATTCTCGGCTT
CTGTGTGTTATTAGTTCCCATAGAGGAAAAAAAATGAAAAGAAAGAAAAA
AAAATTGGTTGAACCGAGATGAATACAGAGATAAATCCCGTAACTAGAGGC
AAGCAATTTGGCAGTGAACGATTACAGTGTTGAAGCTTGACGCATTGGTGC
CGTGCTGACTTCCCCTGTCCAGGTAGTGACATTGATTGATAGGCGTTATCTA
GATTGGGATAAGTGAGCCTTATTCCCATCAGCATTCACAGTCAGACATTGTA

ATGATTATGATGGTTTGGTGGCGGAATAGTAAGGTAAGGATGCAAACTTGT
GGGTTCGGGATTAAGAGAGACATATTGGTGTCCTCGCCTAGATACGTTAGA
GTGATGTGTGTGAGTCAGGATCCCAGGCTCCTACGTGGCGTGATGATCTTT
GAGGCCGCAAATCGCAAAATAGACAACGATAGAAGGGGAAAGAGGGCAG
ATAGATGCAGTGGGCAAGGCGGAAAGTGGGGAGGAGCCGGCCGAGTGTAT
AAACGGGATGCGCAAGGCTTCACCCGAGAAAAAAAGATGAATCCGAGAG
AACATGTGATGAGACGATGATTGATGATTGAATCAGGCGGATGACTTTTTG
ATGGTTATTGTTCTCCTTAAGCGCGGTTGACACGACACATAGTGAATTCAGG
GGGAATGTACATATTTCAGGGGAAGCTAAGCACAGTAGAAGTGGAATCGAT
AAAACTTTCTCTAGATACAAATTGTTGACGAGAAAGACTCTACTAGGTAGT
ACGTGGTTGAAAGTATAAAACAATGCATTATCGCCTCGAGAAAAATTAACA
GCACTAGGTTCCATAGGTAACTTATTCAGCGATCGAGAAATACCTAGAAGA
CCTAAAAGGCAGGAACAATAGAGAGAAAAAAAGAGACAATAAGCATTAGA
AGATAGAGCCTGGACCTTTGGTTAGGGGGGGAAGGTACATTGTTCAGTAGC
GTGTGTCTTGCCCCTGGTCATGGCGGAAGGCCGTCACCTCCGAATTTTTGG
CGCAACTGGAATCTTGTAATCCATACCCATACACCCTTAATACCCTCCCTTCC
CTACTAGAAACCGCGTAGGACAAACACCATCGCCGAAAAAAGCGCGACGA
GAAAGCCTCGCCGGCAATTTCGACTCTGAATTAAAGTATGTGGAAATTTATC
AAATTAATAAGAATAAATGCTAATTTTTCTATATAGGCAAACAATTGATTTGA
TTAGGAGCCGCGCGCGCAGTGAAAGAAGGTCGAAGGCAAAAGGGGTGCC
GGGACCCCCCTCCCCTAAAAAATTCAGACAGAACGAAAAAAGTTGTCAAG
TGCTCTTGCCTCTGCAATAGCACTGGCACCTTCCGAACGATGTCTTCTACCC
ACCATAAATCGACGCGATCGCGTTATTCGAGTCCTCTCCAACCCAACACCC
CTTCAGGTCACAGTCGAGGTTTAATTATGGAAGGACAACGGACCTTCATGC
AGCGGTGGCTGGAACCCCCCGTACAAAATAAAGCCAGTTTTCAAGAAGCA
GGATTAATTAGAGGAGGCGTTGTCGAGAACATGGCGCCTTTAGGGACTCTA
CCTAAGTCGGCTATGTATAAGAAATCCCCCGTGAATGGCGATGGATTACCCG
CGCCAGCACCTGCACCTGCACCGGCACCGGCACCTGCCCCTGTCAAGACG
CGTATCGTACTGAAGAAGCCTTACGTGACGACAACGACGACGACACCAAC
CCCGACACCGACGCATCGTGAGAGTGCTCCTCGCATTCGGGAACAATCAAT
GGATGAGGTTGAAGCTGAGGTGGGTGCGGCGCCATTGTCGCCATTATCGCA
GCCTTTAGCGGCACCTACAACAATGGATGATGGCGAGGATGAGGACTACAT
ACCAAAGAAGTCAAAGATACGCACCTCCCAGCAACAGTCCGAACACACAA
CGCCTAGCAACAATCGTCGTCACTCTGCCAGGCGTCGGAGTGCTCGACCA
AGTCCCACACCTGTTGTCATTACTGTACCAGCCATATCCACACCAGCCCAAT
CCTCCCCTGTTCAAGCACCAGTACCAG

>Mosaic_sequence_36

GCGTCTGTATGTCTTGAATGCACGTATGTATGTGCGTATGTAGGTATGTGTGT
ATGTATGCACTATAACAATTACTATCGTGATGTGGTATCCAAACGCGCATGC
GAGATCCGCCGTCCAAAGTCCTTCGCCTAACCAGGTCCTCTGTCGTATTTTA
ACATGAGGCCGACCTTGGCACTGCGTGCTGCCTAAATAGGCAGCGCTGGC
AAAGTTGCCGGGGGTTGAGATCAGGGAAACGGGAAGGTCATTGCGGCGGT
GAGATCTGGCTTCTAGAAGAGACGTCCAACTTGCAATATAATTAGTACACA
GATGTGTGTGACTGAATACGTTGACATGTATAGGAGCAACGCTTAGAGGAC
GCTTCCTATTTCCGAATGTCTTAACGATCACTTCTCCTTTTTACATTGGTTCG
GCTGTTAAAGGCTATCAGATTTTAGACAAAGAGAGGATGCGGTGTGAATGT
TTCTCAACAAGACCGAGATCCACTTACGCATATTGAGGCAACGAGACAACT
ATAAGTCCTAGGCAATTCTAGAAAAACCAAAAATCGATCTAAGACTCATATT
CATGTCGCATCCCGTGTCCATGTACCTAGCAAGTTAGATTCGCCTAGGTGCG
GTTATCGTATGTAGGGCTTTGACATCATCTCGCCGTCTAATTACTAAAAGCC
GGATTCTGATTTAGATATTCCTATATCCAAAGACTATTATAGAATGGTCCGAG
CTAACTTGAAAGTTAAAAGTAGTCATGCTGGACCGAAGAACCCGGAGCAT
CGAATGCGGCAAGGCGACGCTTTGCAAATCGCAATATAATAGTGCTCGTTT
CGTAGTGGGACAAAACCGTCCACCTGCATTAGTTCGCTGCTGATAGGGGTC
CAGTTGGGTGGGTTTTCTGCAAAGACATGCAGTGGACAATAAAACCAGAG
ACTCAATTTAGACGCAATAAGATTAAGCTAGACGTACTTGTTCAAAAGCGG
GGAAATTGAAGGTATTCACAAGCAAGAATCGAAGTAGCATTATGTGAGTTT
GCAGAGATTTTTACTCTTTTCCATGTTTATTTACGAGTGCTGTAGGCATACTA
TCATGCGACATAAAGTCTAGTATGGGAATGAGAAAGCCATTTAAGGTATCGT
CCCATATGCCTCCGAAGGTATTCCACAGCAAAAGTCCTCCACAAGAATTAA
GATCCTCCCGACTGGGGAGTCGATCGATGTATCGTATCGTGTGGCACGCCG
TGATGCTGCTTTCATAATTCCTCGGCCTCGCGAGGCGAGAGTCAAGCGCCT
CTCGACATTCCGAAAGCAATCGGCGAACCCACTCGCCAGCAAACGTGCCA
GTGCGCACACAAGCGCCCACCCCATTTTTTTCATCATCCTCCGCCGAAAATA
ATTGTTCACAAATTGCCAGTCTTTTTCTCCAAATACAGCCTCGCCCATTTTTT
TATGTATTGTTCCTCTCCTTGCGCACCGTAGCTCTTGTGCCGAAATTTGGGC
GCCATCTTCTGGTCCTCGGATCGATGGGTGGAAAACGCCCGAACGCACAG
AAAGGAGAAATCAGATTTTTTTGGTTGACAATCGTTCGTTTTGGTGGTTGAT
GCAATGGCAGCACGCTCAATGGGTATGACTGAGAAAGGAAAATAAAACGA
AGATGAAATCGAAATCAAGCAAGTCGCTGATAATGCCATGGAGATGAAGCC
TAAACCGTCAATTATGCAACAATCTTTTTTTCTTCCAATGATACACATCCATG
CGATAGTCTCAAAGATAGTTGGTGTCGTTCATATGTTAAAAGAGTTTATTGA
GTGAATCTACTCGAGTTTGTAGAAGTATTGTGTCGGGTCATCATGATCATTA
CGAGGACCATCGGGCCACTGTATTCGTCATACGAGTAACCCGAGTACTGCT
GTGCGTGTTGTTGCGGTTGTGGTTGTTGTTGTTGTTGGGGATACCCTGTCAT

GTTGGGAGGCTGTGGGGTGGGAGTGACGGGGCATCTAGGCGGGACATACT
TGTTCATGGGGTCCGCGGCGCCGAAGTTGACTCCGTCGGGCGTGATAGGCG
GTGTAAGATATCCATCCGCGACACCCATAGCAGCGCCGTGTCCTTTCTGGG
GAGTGCTGTAGATGCTTGAGTGTGCTGACAGGGAGGACTCGGCTGGGGGA
GACGGCGGTCCTTGCGCTCGACGGGCGATAGCAGCCTGCTGGGCAAGAAA
CTCCGCAAGCTTTCCAGAAACACGGGACAGGTGCTGGGACGAGTACTTCC
TTGAAAGAGTAACCGACGGCTGGTGTAGGTGCTGAGAAAGGGCGAGGAC
AGTAACGTTTTCTAAGTGGTCCCACTCTCCATCATTGACCTCTGGTCGTCCC
AATATCGCACGCGCGAGAGCAAGCGAGGCGCGCGCCATGATCGAGGGCTT
GGTGGAAACGAAGTCTCGGTGGTACAAGGCGATCTCACAGAGGTAGGCAG
CCATATGCTCAACCTCTCGGTCGTCAGCCTCCTCGGCAACGATCAGTTGAG
TAAAGAAGTCGACGGTAGGGTGTCCGATAGTCCACTCAAGAGTGTTGAGA
ACGTGCATCTCCATCTGAGTGAACATTCCAGAGTCGTACAATCCACAGCAC
ATGTTGTTCAGTTCGTTGATCTGGGGCACGCGATCCTTCTTGTCACCGTACT
TGGCAGCAATAAGGAGGGCCGCACATCCTACTAGCTGGTAGTGTTGCTTGT
AAACCACTCGCTTAGAGCAGTATCGGTCTAGCAGATTCACGGTTAGGAATA
GGGTCTCGGGGAGAAGAGCGAAGGCAGCATGGGCCTCGATCAAGAAGTC
GATCAGGTAAGGTCTCATGAACCACTGAATCTCCCGTTGCATATCAATTAGA
TTAGCGTCCGGCAACGTCTCGTCCTGTTCAATCGTTAGCCACTAAGTAGGTA
TATCAACCGCCAACCCAATTGAGTCCGGGAATGGATAAGCCAAGACCTGGA
AAAGGGGCTGGGCAGGGGAGCTTGAGGACACTAACCTCCATGTGCCTCAT
ATGTTGCATTATATCCTCCAGATATTCATCACCGGCTAGACGGGACAGCTCG
TTGGCAATCATCTCTTGTCTGTTGCGTGCCAACATCTTTGCCGCATCCTTCA
TGCTGATCCGGGGTTGCTGACGAGGCTCATCGGACTCGACGAAGAAGCCA
CAATCTCCTGAGGTTGAGTATGCCATATTGACGGTAAGATAATACAAAAAGT
TGGGGGTTTCGGTAAGGGTGATTATCGCAGGATTCGTTCAGAATCAGGATG
TGTTCTTAAAAGAGTGACGATTAGGTTCTCCAGGAATCTTGATACTGAGAG
AGCGTATTAGGTAGTCGTCGCAGGTAGAAATTCGAAAGAGCGTAATTATTC
CGGCTGGACGAACAACACAATTATTACTATATTAAAGAGCGTGATGAACAA
TATGCAGTAAAAATAAAAATTAATGAATGACTGTGGCCGAATGGCTACGGA
AAGACGGCGGAATGGTGAGATGAAAGAAGGCCGGAGAGACGGCCGTTAA
AAAGAAGACGTCGAGTGAGAGGGCTCTGGAGTGGAATAATATTAGCGTCA
GGAACCAGAGACCCAATTGAGAAGACTAGGAGAGACAATGATGGACCAA
AGGCAGACTAGGCGGATCAGGGCACGGCCTTCTGGAACCACACGATCAGG
GGCAGAATACCAGGCATTCTGATTCGACCGAGTATCTGTGGGGATCTGTTC
CTGGCACGCGGCGAGAGACGTCTGGCAGGCAATTGATTCTCGATGGAGCA
TTTAATGCAGCATACGTGCATACAAAATGACGTTGGTCTGTGTTATAATTTC
GCTGAAGAAGGGTCTCCCTTACCGCCACGATTAATCACAGGAAATTAGCTT

GATTCCTGAATGAATTGGTGGGAACAGCGAGTTAACGAGTCCCACGAAAG
GCAAGCGTCTTGCTCGACGGTCGGATTTGAAGCGTGCGTGAGTGATCGAG
TACACCGTTGGAACACTGTACCTAGGTAAGGTGGACCGTCGGTATGTACCT
GCGAAGGGAGGAAAAAATCCAATATCAGAATACTTTGTTCGTCTATACAAC
CTCTCAACCCCCTTCTGAGCCCCTTCTAACCCCCCCTCTTTCGCATCTATAG
GGATATCGGCAATTATTGGTGACAGAAACGTACCTCTTACACCTGCACGCTT
CTAATGGATTATAGTCGAGCGACTGGGTTCATGTAGGGCTCGTTCGCGAGAT
CCTCGAATCGGTATAAGACTCGTGTGGAGTCTAGTACCGATAAGTCTTCTGG
AGTAATTGTGATGAGCCAGAGTCAGATTCCTGGTACCGTTGGCACGGAGGA
GAGAATTAGGAGATTCGAATTGCCTTGTGTCTGCCCAGCAAAGACAGACA
AGACTACTCTCCTAGGGGAATGGATTGATGGACGGATGGATCTTGTCGAAT
GAAGGGAAAAGGGAAAGGATTGGTTCGGATGGCAGGAATGGACGAAGGG
TAAATCGACCGCCTCGAGGGACGAAAGATTTAACTCAAGCCTTTGTAAGCC
TTTGAAGGCTGAAAGGTTTAAGAGGGTGGCGCGACGAAGGGCGATGAGGT
GGTGGAGGGTGGTGGGAGGGAGAGGTGCGAGTGCGATGTGTTAAATTTGG
GAGATTCAAATTTCCTAGTCCCCAAAATAAAAGGCCGAGGAATTCGAATTC
CCAAAGAGAAAAATCCTGAAATATAGGTAGGTTTATTACCTGGAAAATATTT
GAAGAGGACAATGACAATTATCGAAGCTGTGCTAAAGTCAAAGGAAACTA
TAATAGTATTATAATTCCTTTACTTCTCTCTTCATTTATCTGGTCTTTGGACTT
GGACTTGCGTCTTGCGTCTCTCTCTCCCTTCTCTCTTTCTACAATTATCTCTC
GGCCCTAGCCCGTTTTTTTCTTCGTCTAGTTTCTGGTCGTAGAGGCCCGGGA
TTCTCTCTCCTCCTTTCCTCCTTGATAAAAAAGAACAAGGAGAATGATGAG
TAAGGATGAGTGTCGAGGGAAAGAGGAAATGAATGGATCGGATTGAAGAG
GATGAGGAAAGGAAGGAGAGAGTAGAGATGGCCTCAAGTTGGAGGAGGA
TGGAGGAGATGAAAAAGGATTGGAAGAGATGGTTTGGTTCGATAGGTACC
TAGGGTACTTTTAAGACAACCGGTCCTAGCAGGTCCCAGCACGGGTCGTAG
CTTACCTAGAAAACGGAGTGGTGAGAACGAGAGAGGAAAAAAAAGCACC
AGTAAAACCCACAGGTACCGAAGAAAGTAGGTAGGCTTTGCCTAGGAACG
CTAAGAAACATTTGAGGTATGGATGTACCAGGTACTAGGTACGGGACACTG
GGGCCACAGATTAAGAAAGAAAAAAAGAGCAAGAAGCCAGGGGTGACCC
AGGAAGAGCCTAGCAAGGCACCGCGCTCGCGACAGCCTATTATATTCGAGC
ATTCAGCTAAGGTTCCAACTGGCTCTAGGTTGGCTCTAGGTTGGCTGCGGT
CGCCTTGCAGAGCGTCCTATCCTACTGGGTATTCCAGCAATGTACTTTCCCA
ATTCCTAATTCCCAGCTTACTTTGCGCGCCCTCTTCCGTCATTTTTTCCCATT
TTTTTCTCACTTGCCTCCTAATTTTCATAAGGAGGTACATTCCCTCACCTTTC
CCTGACACAAGAGTAGCAGCTGCACCTTGCAACCAAGACCTTGTAAGTCG
CAAGCAAAGCAACAAGCAATTTTTAGCCTGGTCTCTTTTTTTTTTATTCCTT
ACATCTGCATACATGCACACTCCATACACCATACACCATACACACATACAAT

GTACGCACGCTTTATTACTACTAAATATGTATGTACGGTTCCTCTTGAACCTG
GCCCTGGTCTCCGCGTGATGGAGACTGGAGACTGGAAACCAGACGATTTG
TGTACGCAGTACATATTAGTACTATTAGTACTATGTAGGTATGTACGTGTGTG
CGTATACTGGTGGAGCAACACACGATTTTAGATGGTTTAGATGTTTCCAAAC
TGTAGTATCTTAGCTACCGAAAGGTTTTCAAAACGGCTGGCTTCTTCGGCTT
CTTTTGATTGATAGACTCGGGGGTGTCTCTCACACATACACATGGTACATGG
GTTAAACGGCAAAGAAAACAGCCACTAGCGATTGCTATGACTATGCTATGG
AGGCAAATATCAGACCGCTCAAATCGGTACTGCCCAACGAACCCGATGCTG
ACAATACAATATCGAAGGTGGAAGGCTGCCGCGGGTGGTGTACGGAAAAC
ACTATCAGACTACACTATGTATTCTACGCTACTTATGGATGTATTGTACAATA
CGTCCAGTAAGTAGTCCTGGGAGAGAGGCAAACGGTACGTTAAATTGTTG
GGGGCCACAGGGCCACAGTATCTCAGACCGTCGGAATAAATCGAATTCGAT
AATGAATCCAACTATGCAGATGCAAATTTCAATATTAGAGGTATAACTGAGC
ATCTAAGGATCTAAGAACGGCACAAGCCTCCTTGGTCCTTTGGTGGTTGGG
AGACCGCGGTTGGGTTAGCCGGGTGTCTCGCCTCTCACGATTCGGCCTAGG
CCAAGATGGAAAATGTCGCTATTCGATTTCTGTTTGAGCCTCTTCTTGTTTAT
TGTCATTGTATCATACGCACATATCATACATGACTCGCCACGATTTGACAAA
GATCTATTTGTTTGTTTGGTAAATGGAAGGTCTCCTTACTATAGACCTTCGAT
GCATAATGCATTGTACCCTCAGTCGAGATTTGATAGGCGTATGCACGTAATG
TCATTCTTTAGGGCAGTTTAGCCCATTTTGTAGTCCGCCTCTCGTTGATGGG
ATTGATCATTGATGGGATGCTAGTAATACGCCCTGTATGCCTGTATGCCAGCT
TGCCCAGGTACATCACTAGATAGGGTCGCCAAGGCCATCGTCGAATGAACA
TCATGGAATTATGCCACAACGACCGGGGCACGACAATTCGGATATGGGGTG
TATGTGATACCTCCAGGGCTAGCTTTGCTTAGCTACTCCACTGTCAATCCTG
AGCAGTTCCTACGTGCATAGTACCTAACAGTAAAGCAGTCCTTAAAGCCTC
TACTACGCAGGTGAGTACGCTGCACTCCTTCTACAGTGTTTCGAACTGTGA
CGAGGCCTCAGCACAGTAAGAGGGGATTCGGAGGCACTGAGCGTGATGCA
AACGTTTCGATTGAAAATAGAATGATAAAATAGCGGAGGCTCCTGCAATTC
GGGTAAGTGTCGCACCGTGTTTGTGCGTGCTCCAGTCTGAGTCTTTTTACTA
CACAGTATTTACAGTATCATACACACTGCTGTATCCATTAACGTGACAACCC
CTGAAGACTTACATACGGTACATTACTGACATACATTCGCAAAAAAAGAGC
GATAGAGAGACTCGATACCTGAAAAAAGATTATCGCCTGTCAGAAGCTGCA
AAATCATCCTCGTCGCCCTATTGGACGTAGCTACGGAAGAAGCGTAAGATG
TCACTCGCCAGTTGACACGTCCAGGACACAGCATTTGGTAGTGCGCAGTGC
GTACAGCATACGACAGGCTACAGTACTTTAGATATGATTACTGCAGATCTGC
CTACAAGGAAGCAGCCAGTTGAGTCGATCCGACCGGCTTGGAGGGAAACG
CTGCTAATGCATACCAAAAACGTGCGAGAGAAAAAAAGTCTACGCCTTCAT
CATCAAATCGTCCGACGATACTTCCTAGGAAGGATAATAATCCCTAGTAAGG

AGAATTATTACTGAAAATATTATGTACGTATCTCCATGGATGACACACGGTAT
GGTCCTGTCGCTCCTTAAGTCTCTGCAAATCTCAAGCAAAAATCCTACGCC
CAGTTCGCATGGAGCTATGAACGGAGCTTAGACAGGGGCTATTTCATTCGG
GTTAGTGTCGCCCATTCAGCCCATGAAAATCTGCAGGAATACAAGGCAGAT
GTCGCGCCAGTAAACCAAATCATCGATCCCTCTTAGCCAATCAACATATCCT
GACGCGGTCTTACACGACTTGAAAATCAATTTATCATGCGTGTCATGTGTAA
TGGGATCCATCCCCTATTACGTCCGACTCCCTAGCTATCATCACATCAACTTA
CCACTGCTAAGGCTGGTTTATGTACGCTATGTAGCTACATACGCTTCACCTA
ACCCTTGGGGTTTTGTTACGCAAAACACACCTTCTTGTTCCATCGATTCGTC
TGTTTTGAAGACCAGGTTCAATAGGCCTTACCTCTATTAGCACGCGATGACT
ATTATAGTACTCTAGTATCATGGTGTCTACTAAGTAGGTAGGTATTGTACATG
CACGTTTGAAGGTCTAACCATGTCAGATCATCGCATACCTGGTACAGGCTCC
CCGTTACCAATAGGACATGGCACCGAGCAGGCTATCTGCGGACGCCGGCAT
ACAATTGAAAGCCACCCACACAGCCCGATCGAAAGACCAAGAAAACACA
ACACGACAACAACAAAACAGTAGGGTAAGATGGCGACGATCACGTCTAAG
TGCTTGCTTACACTACCTACCTCCCTAGTGCCTCCTATACAGAGGGGAGACG
CATCAATGGCTAGCATGCAACGAGAGGCGGCGACCCAAAGAGCCGGCCCA
GTAAGGAAAAAGACCGTGAAACCGATATGGTGGGATGCTGGCTGGCTAAG
GGTTTGCCATGATTGGATTGATTGATTGCCTCTACAGATGCTGTGCTGCCGC
ACCCCGTGGGACAGCTCATCTTCATCCCACCGACTCTAACTCTATCTAACCA
ATCGTGTCCGACTTCATCTTGCTTCATCTTGATGGTGTGCGAGCTGAGATTT
TTCTATATCGTTCAATCATGACAATCGCAGCGTCTCCCTTCACGTCCCCGCT
CTTGCTAGTAATATGGTAATAGGGTACTAAGTAATTAGGGGGATTAGGGAAG
AAAGCAGGGTGCTGGGTTTGTCGGAGTCTTGATAGCATCATGACGATATGT
ACAAACTTATTGTCTGTATCGAGCCTAACACGTGTGTGCCTACCCGAACAA
GGCAGGACATCTTGGATTTTCGACGATTAGGGGGTTCGTCCGTTCAGTCGG
CGACTGGATTTTAGTTGCCCTGGCTCCCCACGGACGCCGATAGGTCCCATTT
AGTTTCGGTAATCTCCCGTATTAAGACCAGTATATGGCCAGTATAGTCCTATA
GCACCAGCTCCTAAAAGGAGTGGTACACCTTTATCCCTACCAGGTACAGAC
AAAGACGAACAGGGAACGAATAGATCCCTATTCTTCCGGGTCGCAGTCCCT
ATATTCTTACCAGCCAAGCGATCGCCTTACCATCGCCTTAGGATTGCGTTGC
GTCGCATTGAATACAATTTCTCGCTTCACGTCTCAGAAGGCGAATCTTAGGT
GCGCAGTAAAGCCGCATCTTTCTGATGGACGTAAATAGCACACATGGCGTT
TGATCCCACAGAAGTTCCGAGTTGTTGGAACAAAGCCATCGTGTGTTACCA
GAGTTGCGTTTGCAGGTTATTATCGGCTGCCGCGTTGTGGCACAACCGAAG
CCGGGAGACAACACTGAACTGATATTCGCCTTAGCTACCTCTGTATCCGATA
TTCAAGTGTGGTGGCGCGGTCCGGGTTGCAATGGTTTGTCGCTCTGTCACT
AGACTACGTTACAAATGTTACAACTATTGCCTCTTGGACAGAAACATAGGT

AACTTTGTCACTCGGATAAACATCTATTCATTCTTGTGCCTCAGGACAATTA
AGCATGATGATGTCCTGTCTAGTTGGCAATGCCAACTAATATTCGTTACTTC
GCCTGTTGCGCCCTGAATATTAATTTTCTCCCGTCGGCAAGGAACCGTCATC
TGCCCCTCACTTTAGAGAGGATAGCGGGAACAAACTTGCCATCGATCGTCT
AACCAATCACCACTCACGTCACCTTGCCTAAACAGCCTACAGTAACTACAA
GAATTACGGAATATTCATGTAGGACCTAGGTATTCTATACGGGTCATACATAC
GTAGTAAGTACAAGTCCAGTACTATAAAATCTTGGCCAATCCAGATACTGGC
CACATGTAAAAAAGATGAAATGGGGGCCAATGAAGCCATTCTCGAACTAGT
TTCCTGAAACCCCTGGGTTGCTTTCTCCCTCCATAACGGCTGCGCACGAGT
TCAAATCTTTCTGCAAGGGCTTTTCCTGATAATGTAACCGAATAGATTCAGC
GGATGAGCTCTGGCTTGCGACAAAGCCAATTTGGACCTGATTTAATGATTA
GTCTAGTGGTCTAAATGCACCAGGCAGCTAGGAAGGCCGCTGTTAGGTCTG
TTAGGTCGCTGTAGGTCTCTCAGATCAGATCTTATCCGAATGGTTACGCATT
CAATTTAAAATTAAGTGCCAAGTACAGCTACGAATATTGACAGTGGTCAGT
CCATCGCCCTGCTTCTGCTCGCAATTGATCCTGCGGATGGCCAAGCCATCTC
TCAACACAAATTCGCATTCGGCTACATCCTGCAACATGCAATCAACCGCCA
GTTCGCCATCTCGATGTGTTGTTTGATCCTCGTGGTCCTCGTGTTCCAGATT
ATGGCACAGATGCAGGCGTCATCGCGCTTCGTCTTCGCGCTCGCTCGGGAC
AATGCCACCCCATTTGCGGAGAGCATCAGATATACCAACTCTGCCAAACAG
CCGGTGGTTGCCAATTGGGTCGTAGTGGGACTCGCGGCGCCGTTCGCCTGC
TTGCTGATCGGCGGCAAAAATGTGCTGTGGAGTGTCCTCGCGGTGACGGC
GAGCACGCTGAGTTATGTCGGCTACGTGAGTCTGCGCGTACACTAACACAC
AAGCTGTATGACTGACGTGAAGGGTGTGCCGGTGGCATTGTATCTCTTCTC
CCGTATCAATTTGCAACACGAAGGCCGGACGTCCTGGTCCCTTCGCTCTTT
CTCTCGGCCCGTCGCAGCTATCGGCATCCTGTACGCTCTGGCGGTCATCATC
GTGCAGATGTTTCCGGGCAGCGTGCCCGTGACAGCCGAGACCATCTCATGG
TCACCGGTCGTCCTAGTCGGCGCTGGCGTCATCTCCTTCGGCACATGGTAC
TTTTACGGCCGAAAGAACTATTCTGGCCCCATTCGCGCTCTGACAAAATGG
GAGACGGGCGTCGAGATCGACCTCGCTTCGACACTCAACAGTCGCGTCCG
AAGCTCAAGGCCAGAACACATGTCGTACGGCGACGAAGCGGACGGTACGC
CGCGCCACCCGCGAGTCTCGCCCGGTACAGGCGGCCTGTTCACACCTCTTA
CACCGCTGGGAGAAGAGGGCGGCGCTCGCGGTGGCGAGGCAGGCTTCAA
GCTGCCTGTCGCCGCGTATTTCCCCGAAGTCACGCATACGGTGACCATCGA
AAGTGCGCACACGTTGGAGAGTGGGCTGCACACGGGCTGGACGTCGGGC
ACGGATAGCGGCTGGACATCCGGGACGGGCGGCGGCAGCTCTGGTGGGAG
CGCCAGCACGCCCACGCCCACGCCGACCACGACGCGGCGGACGATGTGAT
CGCAGAAAAGAAGATTTGGCAGAAAGAAAAGAAAAATATCGGGTTGTATC
GCTCGTCTTGGTATTGGGTTTCTTCGAGGGGTTATGCTCGGTAGGAACGGT

GTATAAGTATCCCCATCTATCTCTTGGGACCTTGTAGATAGACATGTCACGC
AGCAATGCATGAATCAAGCCGTATGCGCCATCTCACTTACTTTGACTGCCAG
GGAGTGGTGGTTGAAATGTTACACATTATGCATGATGGTCATAGATTATACTT
ACAAGAGGGTCGTGGGCTCACTGCCGCCGCGCCACGGCCAAAACAAGATC
CCGTCTCCAGCCAATCTTCCACTGTGCCAGTCGCTGCAGTTCTCGCCAGTT
CTCTCCCTCTGGCGCAGGCACGTTGGAGGGGAAGTAAATGACGAAATCGG
CCTCGGAAAGCAGGCTCCGCAAGAGGGAACCAAGCAAGAAGCACACGAT
GCAGAGGACGACGAGCGACGGGAGAGACAGTGTCACTCTTCGGCTGAAC
GCCTGTGCTGCCGTTGTCGAAGGAGGATTTGTGCTGGCGGAATCAAGGGA
GATCTCTGTGGGAGCAGGTAGAGTGGCTGAGGCCGCTTTGACGGGCGAGG
CGTTGACAGATGTCGAGGCGCTCGGCGTGAGCCGTCGTAAGCGCGGGTAA
GGATATTTCCAGAACTGGTACTTTGTGCTCGTACCCGGTGGCTCGAGGGTG
GATACGCTTAGAGCGGGCTTAGACACAGTACCGTGCGTACCATTTGCCAGC
TCTGCAGGCTCAGCGGGCTCGCCTGGAGGAGTTGGCGTCTCTTTGATCGAG
TCGGCCCCGGTCGATTTGGTGGCAGTGGCGTACTCCGTTGCGACGACAAG
GGGCTTTTCAAGCGGGATGGAGTTGGGCGTCTCCCGATTAACCAATTGAGG
GATCTTGGTAGTCTCCCTTGCTACCTTTGTCGTGCGCCTCTCTTCTTCCACC
TGCAGCATCTGATTGCCGAAGAAATACTTGCCGGTATTGGGCTTCGGAACG
ATCGAAAGCTGTACCGCCGCTCCATCATTCAGCAGATCCAGCAGCCATCGT
GGCCGAGGCAAAGGTGCCGTCTTGCCAGTGAGCGGATCTGCTATTGGCGTC
TCATACCCAGTTGTTGGCAGAGTCACTCTGAGCAAATGTCTGGACGCAGAC
AGTTCTAGCGAAGCTGACTGCATGACGGAGGGCGAGAGGGCGATGACGGC
GCATTTGAAGGGCAGTTCCACCGAGTTATCGGCAGGTAGATTGAATGGGAG
GGTATCATCGCTCGATATTGCAATAGGAAGCGGGACTCCCATCAGTGAGATG
TCGCAGCCTTTCTGAAGGGCCGAAGCAAGCTCGATCTCGAAGACCACAAG
GTCGCGCTTGCCACGGCGGATCTCTTCTGCCAAATCGATGACGGTAGAGCG
CCCTGATTGCATGCTAGACCGGGAGTCGTGGTGGGCCAATTTCGGCGAGCT
GGGCGAAAGTGAATGATCTCCGTTACGTTGTGGTGAGATAGGCCGAAGCA
CAGCTGTCATGGCGAATTCGGTCTCCGAATTGCGCACGGAAACACCCGTGC
GCTCGGACTCGACACCCTCTAAGGACCAAGGAGCGAGTGATCGGGCATCT
GGCGAGAGGATGCACATCGATGGCAGGCGCGGTCGACTTGGAGCCCCATT
GGCCTTCAATGCCCCTTCGATCGTGGCGACTACCCGGGGAAGAGCGGCGTT
GAGCATGTTGTTCACGGACAGAGGCATGCTGCCACTGTAATCTGCCGATGC
GACATACATACACCTTGTGGAGGGGATGGCGTAATTTTCATGCGAGTAAGG
ATCGATAGTTTCGAGAACCCAACCTTCCAAGATCAAGTTTCCCGTAGGTAG
GTTGAAGGGATTATACTTTGCGGGGTCGAGTGAGCATACAGTCTGGTCAAA
TGAGGAGGTAGATGCGTGAAAGATGGTTGACAGCGCAGAATGCGCGTGAA
CAGATGGGGAGGGAGGCGGGCCCTCAGGCAATCTAGCAATGACCGTCGCA

GCGAGCATGCTTCGGCCACGGAAAGGGAATGAGGTGTGGGCGGTGAGATG
AGAGACCGTGATGCCGTGACCGAAAGAGAGCAGCTGCACTGGGGCGTCA
AACCGCTCGTCCTTTCGAAGACTCGATACCGCGGCGGATACCTCCTCAGCA
GTGAAGCCCTCGATGATTCTACCAGCTCGATAGACTGGAAACGTCTCGGAA
ACATGGCTGACCGTACGGCGCTCGATTTTCAGCCCATCGCGATCCGAAACG
GGCTGCCAGCTATTGGGGTCCGTGGACTCCCCATCTCTGTCCGCATGCATCC
TCGCCAATTGCGACAACGCCCGTATGGCGGCATCGACAGGAGTTGATCCTA
TCTCAGCTGCAGGAGTGGCAGAAGTCATGGGCACGATGGCAGCTTTGGTT
TGCTGAGCCGCAGCATTATACCATCTTCCAAGCGGTGCCGCCAGCTTCGCA
TAAGTGGACGGCGTAACGGTTTTGAAGGGATCGATGGCTAGTTCAAGAGC
GCTCTGTTTCTTTCGGATGGTGCCGAGGGCCGGAGGCTGGTCCAATGGTGC
CCGTGTGGGTCGAGATCGTTTTTGCCGTTTGAGAAG

>Mosaic_sequence_37

CTAATTGGCCAAGGTTAAGAAGCCCCCTTGCCCGAACTTGGTGCCACGGCA
TGCTTTCCCCAAACTCCGACGAAAACTCCGATGGCTCGAATTGAACCACCT
GCTATACAGTGTTGAAAGTCTTCTCATCCTTCAAGGCTCCATATCGACAAAA
TGTCCAAGCTTCAAGCATCAAGCGTGCGATCTTCCATCAAGACTGTCCTCG
CGCAGTCCGGCGAGACCACCTACAAGGAGGCCGGTGGCAAAAAGAGGAA
CTTCGTGGAGACCATCGAGCTCCAGGTCGGCCTCAAGAACTACGACCCCC
AAAGGGACAAGCGTTTCGTTAGTCTCATCACAGCATGGGTCGGAAGGCAA
TGCTGATGAGCAGTCTGGTACCGTCAAGCTTCCCCATGTCCCTCGCCCTCG
TATGTCTCTCTGCATCTTGGCCGATGCCTTGGACGTCGACCGGTGAGCTTGT
ACTCAACTCAACATGAGACCAGCTGACATCCAGTGCCAAGCAGCTCGACG
AGGAGCTCCCCTTCATGACCGTCGAGGACTTGAAGAAGCTCAACAAGAAC
AAGAAGCTCGTCAAGAAGCTCGCCCAGAAGTACGATGCTTTCCTCGCATCC
GAGGCTTTGATCAAGCAGATCCCTCGGTGAGTCTCGCGAGGCACAGGAGA
AACAGGGCAGCAAGTCTGACCGCCAGTTTGCTCGGCCCCGGTCTGTCCAA
GGCTGGCAAGTTCCCCACCCCGGTTTCGCACGCCGAAGACCTCGCCCGCA
AGGTCAACGAGGTCCGCTCGACCATCAAGTTCCAGCTTAAGAAGGTTCTGT
GCCTCGGTGTCGCCATTGGCCACGTCAACATGGAGGAGGACCAGGTCATG
TCCAACGCCATGATGTCGATCAACTTCCTCATCTCTCTGTTGAAGAAGCAG
GCGAGTCGTGTTTCCGGGACTTATTCGAACGAGCTGACTTGGGCAGTGGCA
GAACGTCGCTTCGCTCCACATTAAGTCCACCATGGGCAAGCCTCAGCGCTT
GTTCTAAGTGGTCACTCTTGTGAACGCGAGCCGAGGGGAACGGATTGGGA
GGAGTGGGAGGAGAAGATACAACGAGTCGACACGGATCGTCATAACATGG
AGCCGTCGCACGCTTACACCCAGGCTCCTGCCCTTGATTGGGTGCTGGAGC
TGAGGGGAGCCGTTTTGTTTGACTTGGATGCATCTATTCTTCCTCCTTCTGT

GCACTCTATGTACTTTCGGATCCGCTTGTAAGGGATCGACGGAGATGGATGT
GTCGGGACCTGAAAGTGACTGGATTCAAGGCACGGGCCGAGCCGGGGCTC
GTCGCCAAGTCGATCTCAGCACCACCACCAAAAGAAAGACGGGCTCGGGA
CTTTTGAACGGACAGGCTGCGGTGCAGTCGAGTCAACTTTACGACTCGTTC
GAGTCGACCTTGTTGGTAAGCCTGGCGTCCTGCTGGCTAAGCAGAAGACA
GCAGGAGCTCAGAGGCGAGACAGGGATCGCTCATCGCATCGCATGGGACG
TCATACGACCGTACGAGAGTCGCATGGCCGGATGCCCACTCGAGTCGACTC
TAGCTCGCCACGTCAACTCGCCAACAAGCGCCAATTAGACATATACGCTGC
TTGTTAGCTAAGACGTCCCTTCGTCGAGATGTCATTGCGCACTCGCCGATCA
CCGTCGGCGTGACGTGACCATGTCGGTTCCGACGTCCACCGGTGGGAGAG
GCCGCTCTGCCACGTCGCCATCCTCGTCCCCTTCCTCGTCCCCCGAGACATT
GCTCTCCCCTCCACTCTCGCAGTACTCGCCACGGGCGTCGAGTCCCCTGTC
CGGCTCCGCAGGGCCAGTTCACCCCGTGTCGCTCTTGAGCCAGAGTCAGG
GCCGAGCTCATGGTCGACCTGGTACGCCCCCCAGACCCCCGCTCGGCAATC
GCCAGGCATCGGACAATGGCCATCTGTCCCCGCCTTTGCCGGGCGCTCAAC
GCCGAGCTTCGTCCCCGGCTCCGTCCTTCGACTCGCGCATCGAGCCCAGGC
GCGCGTCATCGCCTGCCCCTCCCAGCTACGCCCGCAGCTCGCTCGATAGGT
CGCCAAATGTCAACCCGCTCGTCCTGGACGATAGGCCCCGTGTGCCCCCGC
CAATCTACCTGCGAAGCTCGAGCCCACAACCATTGTCCAGCAAGGGCAGC
CTCAAGATCCATGTGCCTGCCTGGTACGAATCAGCCTCTTGCACACGTCCG
TTTCTGAGCTGACTTGAAGGGGCGTCGTACTTGTCCGTCCTCCCCGGCAAC
TTGAGCTGCACCCGCTTGAAAGTGGCGCTCGCGAGCCTCCTTCGGATGAC
ACGGTCCTCAGTGGTGCCCTTGAGGTGATCATGAAAGAGCGGCGCCGCTG
CCAAGCCATCTCCGTCGGCGTCCAGGGGGTATGTCGACTCCATCTCGGTGC
TCGCGGCTGGGAAGAGGACGGTATCTTCGAGCGAGGGGTAGAGGTTCTCG
GAGGCGACTCGGAGGGCATATGGCTCGAGAAGGGCAGCCAGTCCTTCTCG
TTCTCCATCCTCCTGCCTGCCACATTGGCTACGCACGACTATCACCAATTCT
GTCGGTTGTCGTACATCCTGACTGCGCGCGTAGAAGGCATGGCCTCCACGT
CTGTCCTCGCCTCATTCGGCAAAGGTAAAGACTCTTCTGGTATCAGCGACG
ACATCGAGTTCAAGCGCGATTTCGAGACGGTCATCGCACGATCCGACAAGC
TCGCCCAGGACCTCGCGCTGGCGCACACCCGTTCGCGAGACTCGTTTGGG
TCGCCCCGCGGTGTCCATTCGCCTCTCGCAGATGTGACATCCCTCGGCTCA
GGAGGTCCAGACGGTCCGGACGACTCGGCCATTGCCATCGGCGAAGGATC
TCCGTCTCTCATGGGACTGTACCACCGACGGCAGTCCTCGGACATACAGAG
TATACCTCACCTTTCCCTCGGACCCAAGGCGGATGACGCGCGCAGCATCAA
GTCGTACAGGAGCACAGCGAGCGATCTCAAAGCTGAGAAGGCCGGCTGGC
TCAAGGGTGACCTGGTTGCTTCACGAAGTCTGATTGTTCATGCGAATCCTT
CTCCCGGAGGTGGTGTTTATCAGCTGGACATCCGCAAAGAGGGATTCGTAG

ATGGCATTGGTATGTGGAAATTCACTGCCACGGCGGAACAGGTGTGTTTCC
TTTTCAACTCGATGCTCACGCGGCCTTCAAACGCTAACACGGGCAGTTCTG
CATCGGTGCTGTCTGCCTCGTATCGTTACACATCCCATGTCCTTCCCCTAAA
ACCACAATCTTCCTCCTTCGCCTCATGATACAACAGACATACAGCGTCATAT
CCCCGCGCACGCCAAACGACAAGGCGATCGTGCCAGAAGCCCCCAAGTCG
CACATTCTGTATCAAGTCGGCCGCGCCCACAAATCTGGGGAACGATTCCCT
GCTCACAACGTGGAAAGCTTGTGGCGGGGAGGCGACGCTCATGGGCCTCG
CGGACCTTCCAAGTCCAGGTCTGGATCCAGCAGCAAAGGCCCGAGCACGG
GAGGATTTCCCGGTACAGGGACGAGTACAAACAGTGTAGACGAAACAGGC
TGGAAGACTCGTGCTGTGGCTCGCATTCCCAATCACGACAAGATTCGACCC
TCCACTAATGACGGGTGAGTCGCCGTTGTTCCGCCAAGGTGAGATGTGGGT
AGCAAGCTGACTTCACAGAACCATCACGCCTATCCGTGTCAAGCACGATGT
CCTGATTCAAGTCTTCTACTCAGTCGACGGCGAGCTGGTCACCGGCAAGCC
TGTCCAAGGTCCCGGTGAACTGCGCATGATGCAGGTCAGAGTCAACATCA
CAATCCCTTCTTGCACCTGTACGACCGCGGCGCTCAACCTTCCCGACTACC
AGACCGCCGATGCGGAGTCTGGGCGCAACATCGACGAGGTCCTCAACTCG
CCCCCGGGCAAACACTCTTGTATGTGCGGCTACACATTTGCCGAGTTGGGC
GAAGCGGCCATGCGGCGAATGCAGAGCGAAGTGAGGGAGGACCAGGAGG
CGGGCACCAGGGACGCATCAAGCAGCAGCGCCAGTCAGTCAGTCAAGGA
GGCCGAGGCGAGGGCATGAGTGGCACAAAGCCAAGACCTTGCTGGTGGA
GTAGAGTGAGTCGTTTGTTGTCCTGTAACATCAGTCCCCCTAGAATCCGTAG
AAGCTTGGTTGTCACTCCAGCCAACATCCTTATCTATCTTCTCGGTCCCACA
GAATCTAGAGGTCATTCAGAATATAGTCACAATGCATGTCGTGTTCATTATG
CTTTCGGCTGCGGTCCCGTCCGTGCCTTCGCGACTGGCTCCCATGCTTGGA
TTTTCGGTGTCACAGTCGAGTACGTCTTGAAGGCTCCCCTTGTCCCTGTCA
AGTTCTCCTTGAAGGGCTAGCGGTCAGTGACCGCCTTCCACCTCCACGATG
TCAGGAACGCTCACCGCAAGCCAGGGCGGCCGACTGGCATTGACGACAGG
GTCCTCGGTGGGCGGAAGATGTCTGATGCCGTGCAGCCACGAATGCCATTC
CGGTGTTGCCTGCGAGGCGTTGAAGTCGTCCTGCGCATACTCGATCCATCG
TCCCCGGCCTGCGTCTCAGCCAACATGCCCCTCAAGCTTTCTGAATACCCA
CCAGGTACTTCCTCCTCTGGCACCAGGTTCTCGTAGTACTTGTTGCCCATGG
CGTCTGTCCCCACCAGCCGGCCCTTCTTTGCGTCTCCGATATATCTGCAAGT
CAGAATCCGTTCGAGACCTGCCGAGCGATGCAATACTCACGTCATATCCCG
GAACCACTGGCGCAGGCCGACCGCCCGCACGTTCTTGATTGTTCTTATGAG
GGACATGGCGATCGCTACCAGATGACGGATGGCGACGAAAGCAGAGCAAA
AGGAAACCCGCGATTGGCCAAGGTGCTCTCACGGGCCTAAACAAAGCACA
AAGGGGATAGGCTCGCTGACCGCCACCGGTCTAGCCCCGACCCGGGACTC
GGCAACTCGCTATCTCACCGCCTCGCCTCGCCTCTCTGCTCCGTCCCTCTCC

ATCCGCCTTCTGCCTGCTGACAGACGACACGCGTTCAATCATCCCATCTCG
ACATTGTCTTCTCAACATCAATACCTCTGCAACCCATCCCCACGTCTCAGCA
CATAGCATCCCTGGCACCTTGCCCCCCGCCATAGTGTTCTGTAGTCGCAGCC
ACCGAGGGTGGTAAACCGATCGCGCGTTTGCCACCGTTCCCACCCGCCGC
AGACCCAGACCGGCCTTTCGCCATGGTCTTCGTCATCAAGGCCACGCTTAA
GGATGAGACGCGGAGGGTCACATTCGAGAGCAGCAAGTTCCCGCCCTTCT
CCGAAGTGCAGAATCGTGTGCGTGCACCTACCTGTCTCATACTCTGTTTCCC
CTTTCCGCCTGACTTCGGTCACGCGGCCGACCGTGATAGCGGCGCTCCGTC
TGTCCACCCGCTGACTAGCAGCTGAGAGCGAGCTTCAATCTTCCCTCGACG
GCGCACACGTTCTGGGTCAACGTGCTGTTTTACCCCGACGACGCCCAGGAT
GCCCGTATCATGTTCAAGCGACATGTGTGTGACGCCGTGGAGTGAGTGAGA
CATCTCTCCATTTGCCGCTTGACTCCACCTTACCCTACGACCGTAAGGACAA
TGCCCGTCGCCCCAAAAGTCATCGAGGCCGATCTAGACCCATCTTTTAGTC
TCTCTCCTACCTTCTTTCCATTCTGGAACTAGGCTTTCTTTCCGATTGCTCCC
CCTCACCTCCTTTGTTCCAACTCTGATCCACAGGTATGAGACTGCGCAGTC
CCCATTCCTTCACGGTAACTTCGCTGCACCAGCTCTTGTATTCACCATTCTC
CTCTCCTCTGATCCGCGTCTCGACAACATCCACGGATTCCATCGTGCAAAC
CACCTCATCCAGTCGGCGGGCGACCTTGCCATCCACATAAGCGGACTAGAG
GAGGGCCTTGCCAAGAGCGTCTCCCTCCTAACCGCCCTTGAGGCCAAGCT
GCAATCGAGCGAAGACACCCACGACCGCATGGCAGTGAGTTTCTGGGGTA
ACAGGGTCGAAGAGAAGCGCGAACACACGCGTAAACTGCAAGACCAACT
GGAGCGCTGCACCAAGGAGTTCGACCACATGAACCACGAACTTGACGGCG
GGATGTTCTTCGGCTCTGAATCTCTTCGCCGCTATGCCGAAGACGAGAGCA
AAGAAGAGCAGTCTCGCACTCATCAGACGCTCGATGAGCTCGCCGCCTGG
ACCGCAGGCAACGACGATGATGTCGAGAATCTCTTCCCCGATCTCGAGACA
CTTCTGCGGGCCCCACCTCCCCCAGTGCAGCCTCCAATGTCGTGGAACAGT
GCCCACACCCCACGAAATGACAATCACGGCTTCCGCACCTTGATCGACCGC
GTCAGCGACGTGATGCAGCATCCCGAGAACGCTCGATCGCTGGTCCCCACT
CAGGAAATCAAGACAATCCTGGACAATTTCCTCAAGAACCTGTCCGAGCA
GCTCGCCAACACCTTTGACGGGGCAGCTGCGCCTCATGCGCGTGATGGTCC
CGTTCCTGGAGCTTATCAAACCTCGACTCAGGTGCAAGCCGATAGCCCAGA
ATTGTCGCAGGCCCAGGTCCAGACCGATGAGACGGCCAAGCCTGCCGGCA
AGCTTGGAAAGGGTGGCTTCCGTCACAAGCACATCTCGTGCGATGGCTGC
CTGACTGGCATCCGTGGCATGCGCTACAAGTGCGAGCAGTGTCCCGACTAT
GATCTCTGTGGCTCATGCCTACCGCTGCTCAGCACCTCGGAGTTGCATCCTG
TCACCCATACTTTCCGAGCGATGCTACACCACGGTCTGGAACAGAGGATCA
AGCTCCCTTCCGACGCACAAGCTTCGGACGTGCCAGTTCGTCATCCCGCAA
GTTGCGATTTGTGCTCGCAGCACGTCCTTGGCGTCAGGTGGAAATGCTTGA

ACTGCCCTGATTGGGACTGCTGCGCGTCATGTGCTCCTACCATCGCTGAGA
CCCATCCGAACCATTCGTTTGTCAAGATTCACGCCACCAACGACTATGTTG
ATCGGTCGGACGATCCTGCCAAACAGGCAGTCCACTCAGGAACTGTATGCG
ATGGGTGTGACAAGACGATCAGAGGCACCCGTTACAAGTGCATGCACCCG
ACGTGTCCCGACTACGACCTGTGCGAGTTGTGCGAATCGGCTCCGTTCCCG
GTTCACCCCGCCGATCACCCGATGCTCAAGACCAAGACGCCTCTGCGTGTC
GAGTTCGAGTCGACCCTCGAACTGCCCGGCCGAGGAATCCGATCATCCATT
GCGAATCCGCTCGCTGCTGGTCGTCACGCTTGCCATCGGGGCCCAAGGACT
TTCCCGAGGCTGAGCCGCGCTCCATGGCGCCAGATGCACGCCAGGGAGCA
CGTAACATATGCCCCGGCTGTCAAGCCACAGGAAGATGATCAGCCCGTGTT
CCCGGGCAGCTACGTCCAGCACGTCAGATCCGAGATCAAGCCTGTCGCCA
ATCCATTCGAGGATCTCCTTACGCCTCTCATCCCGGACAGCGCCTCCGTGAA
GACAGAGCCTCCGGTGTACGCCGACGCCAGATTGGCCGAGGAAGTTACTC
CTATCGACGACGTCAGGCAAAAGGAAGCTGTGGAGCAGGAGCAGGTCAA
AGAGCCTGTTTCCCCGCTCGACATTTTCTCCTGGGCCCGACACACGACCAT
GCCTCCTGGTACCATTTTGCCTCCTGGTGCCGTGTTCACCAAGTCTTGGAA
GGTCAAGCACTTTGCTTCGGGCGCAGACTTTGACTTCGACTCTGTCCGCCT
TGTTCACCAGACCAAGCCTTTGCTCGGCCCCGCCGTCGAGAAGCTGATTGT
CGTCGCAAAGGACGATATCAAAGAAGGCGAGGAGGTCCTCTTCCACATCG
AGGGGCTCAAGGTCCCCGACGAGCCTGGTAGGGAGATCGTCGAAGAGTGG
CGCTTCGAGGACGCGGATGGCAACACCTATGGGCAGCCTCTTCGTGTCAGG
TCAGCCGTGTCTCATATTCTCGCGAACGATGCTGACCGATAGGATCACTGTC
GAACAGGACAAGTCTTGCCCACCCTCTATGAACTCGTCTTCGGTCATCATG
CCCTCCGGTGTCTCGACTCCCGCCCTCCGAACACCGCAGCTTGCCCCGATG
CTCCCGTGTCAGATGCCATCTCTGGCTGATCTGCGCCGTGTCGCCGAGGAG
CAACGCCAGATCCGTGACTCGCCTGCACCAGTGGACCCTGCTGTGTTCGAT
CCCGACCTTCACGTCGAACTGAACCCGGCCGCGGTCGAGGATGCCGAAGA
GGAGGAGTATCACATGGCCGACGACGATGATGACGCCTCAGTCATTTCCGC
TGGTGCTCACTCGCTCCGATCGTCAGTTTCATCATTCTTTGACATCGAGGAC
GCTCCCACGATCACCACTCCCAGCGTCTCCGCGAGCTTGAACGAGGACGA
GTTTGACTTTGTGGACGAGACCGACGACGAGACGGCTGACGAGCTGTGAG
TCAATGGTCTATGGGATGTGGACTCTGCTCTCTGTAACAAATCCGCTTCTTC
CTCAATCCGTTGTGCCTTTCCATTCCATATGACTGACTGCCCGTTGTTTTCAC
TCTACATATATCGTTTTCTGCCCCTCCGGTGTTCGTTGTAGATTCAGCCTGCA
CATCTCATGTATTCCGTGAGCCGAGACCATGCATACCCAGTGCCCATCAATA
TCTATGTCTGAGCTCACAGAAGTGGTGGTGCCTGAAAGCTCAACCGACCG
CGAGCTCGACGCGACCAAAGTCAAAAGAGCTTGCCCAAGTTTCCCGTTTC
ATCAACCAATTCTACTACATTTGGCGGTCTTCAACGGTGGCGCCGGATGCTC

GTCGCCCATCTCTTGGGGCCTGCCACTACTCCGGGGCAGTTACACTGCGCG
TACGATGCCCCTCAGGCGGGGCCGAGCTCCCTGCCGTATACGCCCACCGTT
TCTTCCTCCCGGCAGTCTGGACGAAAGGGCAAAGTCGGCCGCTCTGTCATT
CCGGCCGCCACAAGTGACCATGGGTCGGATCACTCTGGGACCGTGGAGGA
GCTCGTATGGTACGAGCAGACTCTCATTTGGAGCCGAGGCGCACAGACAG
TGCGCAAATATACCTACGAGCACGAAGGAGAGGATGTCGCCTGCGCCTTGT
TCGCCCGCTTTTCGCCGCCTGATCCGCCGGACAGCAGGGCTTCTCATCAGG
CGCGAACGAGAAGAGATGCAGCGTCGGTCAATGGAGGACTGACAGGCGT
CGATTATCTCCTGAGCAGCAAGGGCAAGCACGCCGAGACGTTTGGGCCGT
TCCATCATTCTCAAACGGCGAAATGGGGTCAGCCTAAAGCCGTTTCCTCCT
CTGCCACCTCGCCGAGCCCCAGCGAGTCTCAACGGGCGCTGGTGGTCTTC
TTGCAGACGAGGGCTTGGATCTACTGGCCAAATGGCGAGAGTGTAGTGGT
GCATCTGCCCTTTCCCGTCGATGGGGCCTGGGCAATACCAACGACGGCCAA
GAGTAACGCTGGCGGGAGCGCGGCTGGGCCTGGATCACTGGCAGGAGGCA
TCATCGTCCAGCGCGCACTGGATCGGAGGGAGCGGCAGCCGATTCGGTCA
GTCCTAGGCGGCATACACGATGTCTCGACCTCATCGGCGCTGGACGAGCTC
GACTCTTCCTTGGACGATGCACTTCCGCTTCCTCGCCTTTACACCCTCGGCC
GCCCATTCGAGGAGCTAAGACCGGTTGTTTTGGGAAGTGTCCAGGACGGT
GCGCCCTACTCCCGCCGCAGTGGACGTCTTTTGGGCCACGGTGAAGCTGC
AAGCAGCGACCTGGCCATAATACAAGTACTCGAAGAGTACGGATTGGTCGT
GCTGCACAACAAAGCGCGAGCGGAGATCATCCTCTGTCGCTGGGTGTCCG
CGCCAGCCGACGCGACCTCTCGACCTACCGATGTGTCGTCACCAGAGTTCC
CTCTGCGGCCTATCGACGGTCTGCCAGCGGCTTCGACTCCAGCTGCCGGCG
TGCGGACCATGCATCCCGCCGAGCTCCTCGCCGAGGCACCCATGCCGCAGC
CCGTCCCGCCGAGAAGGCCACCTCGACCGTCTTTGGCCCGAAATCCATCTT
CCATGACAGCCTTTCCTTCTGGCGATAGGCGTGTGTCTTCAGCAGCCGACC
CCCTCGACCGCACACAGCGACGCGCACCTCGGATGTCTCGAGGGACTGCT
CCGCCTGCTCTCTCCGCACAGGCTGAAACCGAGAACAAGTCAAAGCAGTC
TGCGCAAGTCGACGAGCTGCATGCAGCACTGGATCCGCCCCCCTTCACCGC
ACTAGCGACATCGTCAGCATTCATCAACCAGCCGGCTTCAGCATTCGGTCA
GCCGAGTGGCGCCGGATCGAACGCTCCACGGGCGTCTCGAAGTAGGGCCT
CCATCGGTCCTGGTGTCGTGGCGGCCAACGCATCCGATGACCGACGCTTGT
CTGGCGCGTCTGCCTTGGCTGGTCGAGAAGCAGAGTCCGCTCGTATAGGCA
AAGCACCCCTTGCAACGATTCATGGGGATCTCAGGGAGACGACGATGATG
ATGGGGCTCGAAAGGGACGATGATGTACAGCGGAGCGAGTGGGTTCTGGA
CAGAATCTGGATCTGGAGACCACCCCAGTATAACGGAAACTTCTTGAGGTT
ACTTACGAAGCGTTCGATAGTGCCTGCGTACCACTGGAAAAGGTCTCCGGG
TCGCGTACAGGTGTTTTTGTAGGTAATTTCAACAATGATCACCAGTTAATGC

AGCTTCGAGATCCCAATCTCACGCTACCATATGCTGTCACGGGAGGTGGCC
CAACCATCTTAAGTAACAGGATCAACTATGTTTTCAATCTTCAGGGGCCCAG
GTAAGCATGCTCTCCCGCTCACTTAATAGCTAGGTAATGATGAGTTACTGAC
TTGTGTGGCTATAGTATGGTGGTAGATACAGCCTGCTCCGCATCAATGTATG
CTCTACATCTCGCAGTCCTATCTCTTCGGAACGGCGACTGCGACGGAGCTAT
TGTTGCAGGAGGAAACTTGATTCTAGGCCCGGATCACCAGATTATAACCAG
TAAATTAGGTGCCGCGTCACCTACATCTCGCTGTCACACTTTCGATGCTCGA
GCGAATGGATATTCTAGGGCTGAGGGATTTGGCGCCATCTACCTGAAGAGG
TTATCAAGCGCACTTGATGATGGTAACCCAATTCGCGCTGTTGGTATGTCGA
TTCTGGATTTATTCGTGGCCTTCGAGTATCTTGACATTCCCTATAATAGAGAC
CCTGGACCCTTTTTAAGATTCAATCTTGGTCATGTAGTCATTTGTCGAAAAA
TTAACTAATTCGAAAATGCAGTACGCGGTACATCCTTCAATGCGAACGGGA
AGACAGGAGGCATCACACACCCAAGCCCAGAAGGCCAAGAGGCTGTCATT
AGACAAGCATACAAGGCTGCCGGAAATCTAGATCCGAACCTAACAGGATAC
TTCGAGTGCCATGGCACTGGGACACCGGTTGGTGATCCGATCGAAGTTGCT
GCTTTGGGAAATGTGTTCGCGTCAGGTCGCCAAGACGAACCATTACTGATA
GGATCGGTAAGTTAGGTTCTTACTTACCTATCTACCTAGTAGATAGAGGCTC
CTCTAACTGTGTTCATGTAGATCAAGCCGAATCTTGGTCATAGCGAAGCTGC
AAGTGCTATGGCACAGCTCATGAAAGGGATACTGTCCTTGGAACACAATGA
AATCCCAGCAACAATCGGCATCAAGGCTTTCAACCCTGAAATTGACTTTCA
GAAGGCAAGAGTACGGGTTGTTACTGATATGACTCCATGGCCCTGTGATAA
GTTACGTCGAATCAGTATTAACAGGTATGGTGCTTTGCTTATCGTCGTCCTTT
ACATTTGATAGCCACGCTGACCTTCTACCGGTAATAGTTTTGGATATGGTGG
TGCCAACGCACATTGTATATTAGATCATCCGAGCTTGGTCATCCCCGGGTTC
CGGTTACATGGTATTCCTTTGTCGCATCTCAAACCTCCTACTAATGGCTACCT
AATCGACGAGACATACGTCAATGGCCATGTCAAGAGCAATTCATCGAACTT
GGCAAAGTGGCCCCAGCCCGCCAAGCCCATCCAAACAGAACGAGCTGGA
AGTCGACCTTTCATTATATTACCATTTTCTGCGCATGATGAACAAGCCCTCA
AAGCAAATGTTGCCGCAGTATCAAAACGTATCCAGGACTTTGACATCGGAG
ACCTTCTTTACACTCTTGGATCCGGGAAATCACGGCTCTCACGTCGAGCCT
TTACCGTCACAGACCCTGTGGCTCTCAAGGGCGGACTAGTACTTGACTCTA
TGTCTTTTGGGAAGTCGTCTGGGTCGCCAGTGAGACGTATTGGTTTCGTATT
CACTGGGCAGGGTGCCCAATGGCCAGAAATGGGAGCAACTTTGATACATG
AGTATGCCGTTTTCAGACAGACAATCCAATATTTGGATGATGTCCTTACTAA
TTTGCAACACAACCCCTCATGGACGATTGAAGAGGTGCTCCTTGAACCGGC
TACTACCAGTCGAATTCACGAGCCCGAGTTCTCGCAGACTATATGTACTGCT
TTACAGATTGCGCTGGTAACTCTATTGGGCCAATGGGGAATTCAGCCAGCA
GCGGCAGTTGGCCATTCCTCAGGTAAAGATATGACTTCTCATGATGGCGTAT

GGCTAGCTAATGAAGATTCTCCAGGGGAAATCGCCGCCGCATATGCCACAG
GACATCTTCGGGCTAGCGAAGCCATTGTTCTCGCATACGCCCGCGGTCGGA
CTGTGGCAAACAACGAACGAGAGGGCTTGATGATAGCGGTTGGGCTTAGT
CCAACCCAGGTAATGACCTATTTATATGATATTGAGACCGAAGTAGCAATTG
CTGCCGTCAATTCACCGGAGAACACCACAGTATCTGGTGATCCGGTCGCAA
TCAAAGAATTGGCCAAAAGGTTAAGCGCGAATAGTATCTTCAACCGTATTT
TGAAGACAGGCGACAATGCATATCATTCTCACCATATGCTTCGCTTGGGAG
AGTCGTACGAGGAACTGGCCAAACAGTGTCTTCATGATGTTGAACCCATAA
CATTAGCAGAACCGTCCATGCCTCACATTAAATGGATATCATCAGTAACTCC
CAACGACGAAGTTCTCTCCGCTTCGTCAAAATACTGGCGGCGTAATCTCGA
ATCACCCGTGCTCTTCTCCGAAGCAGTCGAAAGACTCGCGGGGAATAATCT
GGCAGATCTTCTAATTGAAATTGGCCCTCACCCTGCTTTGAGCGGACCCAT
GAAGCAAATTCGTTCGGGTTTGGCAAAAAGCGCGATAACGCTTCCAACTTG
CTTGTCATCTCTTCGGCGGGGTGAACATGACATCTCAAGTATGCTCAACCTC
GCTGGATCTCTATTCCTTTGCGATGCTCCTGTCGACATCGTAGCTGTAAATG
CGACGGAGATGGTAATCAAAGGTAAAATGCATCTCCGACATGGATACCCTT
GCGTCGATATGCCCCAGTACAACTTCACGTACCCAGAGACACCACTTTACT
TCGAAAATCGATACAGCAAGGAATTCAGGCTACGAAAGCATCCTCGCCACG
ACCTACTAGGAGCTAGACAACCTGGTTGTTCGGCGACTCATCCATCTTGGA
GAAATGTGATTCGGAAGAAAGACATACCCTGGCTAGACGATCATAAGCTCA
CACCTCATACTGTTCTACCGGCGGCGGCCTATATAACGATGGCCGTCGAAGC
CGCTAGTCAGATCCATTTCGAGGAGGAGAGCCCTCTTCCCATAAAGTCTTT
CAAGCTTCGAAATGTCACTATAAGCTCTACGCTTCGTGTGTACGACGATGA
GTTTGGTGTCGAAGTTGTCACAGACATGCATAAACCAGCTCTAGGAAATTC
AAACACTGCGTCAGTATGGCATAAATTCAGCATTGGCTCAATTTCCCCCAAC
ACTGATACTTGGTCTAACCACTGTTCGGGTTCTATCATGGTAGAGACTAGAG
ACACTACAATCGATGTTGGTCAAAGACTGCAATTAGACCCCCGCTCGAGAT
CGCTTGACATGAAGCGGTGGTATGAGAAATTCGCAGAGGTCGGTCTTCTTT
ATGGCCCTACTTTTCAAGGACTCTCCAAACTGAAGGCTTCTCCCTGTATCA
ACGCCGCTGCTGCCGATGTGGCTCTTAACCCAACATCTGGCGTAATGGACG
GCGGGGAATCGCGGTACTCAATCCATCCCGCGACTCTGGACACATGTATCC
AACTTGCTCTCATAGCATGTCACGCAGGTCAAGTAGAATATGTCCAAAAAG
CATTCGTCCCCATAATGGCCGATAGCATCGACATATGGATCCCAGAAACACA
CGAAGAACAAGGTTGTGGTGTGGCCAGCGGCCAACAACTCGGCCTCCGCA
GCCTTTATGCGCGGGCACAGCTCTGTTCCCGGTCCGGGCCCCCACTCCTAG
ATATAGGAGAGCTTAAATGTGTAACCTATGATGGGAGTTCCGATGCGACAAC
TCGAAATATTCCTCGTGAGCCATATTGGAGGCCTGTCTCACGGGTCGACATT
GATACACTGACCAATGATTCCGCTAAGTCTATGTTTCCTCTTCGGAAAATTC

CGCCCGAAGAATTTGGAAGCTATACCCAACTCCAGCATCTTGTCGATCTTCT
AGCCCACAAAAATCCCAAAATGCGCATACTAGAGATCAACGCCGGGACAG
GCGGCGCAACGGTGTCTATCTTAAAGACTCTAAAGTCCAATACGCCATTTA
AGAGATACCAAAATTTTGTGTTGGGAGGTCCCGATCTGTCGCAACTTGAAT
ATGCACGAACCGACTTGAAAGGTCACTATGGTATTTCGTGCCAGCTTTTGG
ATATCCGAAAAGACCCTATCGCCCAAGGCTTTGAGCCACATTCATTCGACCT
AATCATTGCTTCTAG

>Mosaic_sequence_38

AAAAGAAAAGTCCTGATGCTGGGCGTGCGGGCGTGCGGGTGTGCGGCGGC
ACGCCACCATGCAAGTGCTCGATTCTAGTTGGCGCTCTCCCGTCCAGTAGA
ATCTCGACGACCCACCAAATCAAGATCTTCTTGGCAATGGAGCAGATAGCG
AGTAGAATCTCTCATCGTTTGATTGAACGCCGCAGTCTGTTCGCGGATGGC
GAAAACGAATTTTCTAATTTTTCTAATATCTTTTTAAGCTTCGGCTCTCGCGT
ACCGAGGATTGGGTCATTGACAAGCAAGGGGTTCTTCTCACGTTGTTTCTC
AAACGAACCTATCAGCCCTTAGGGGACTCAGGGGAAATGTCTCATATCCTT
TTTAGTATTCGGTGTCAGCTTCCTAAACGAGCCTCGACTCCTGAAAGAATCT
TATGCCCACATATGCGCATCGAGAACGTCGGTGCGTTCGCTGGCAGATGAG
AAACTCGGCCAGACTTTCTAACTATCTTTTATTTTTTCCCTTCCTTGCAAATC
AGCGCAGAGCTTTAGATGACTACACAGTCGAGACTGCTTACTAGGTACCAA
TTGTCCACGTTGCGATGCATCCCTTGCTACCCTATGAAACAAAACTCATTTT
TGGTAGCAGTGTCCTCATCAGCCTAGAATAGATCAAATGTTTCTACTCTGTC
ATCTGCACACTGTTAAAATCCCTCACCTCAAACCACTAAAAGGAAAAAAAT
ACATCGTACGTACGAACGTGAGTGAGTGTAGGCGCACGGAAATAGTCCCG
AGCCCTGCTGGGGTTTGAAGATCACTGCCGTCGTGATACGCGATCGGACGG
AAGATGATACCCCTGAAAGCTGACCCGAGTTAGCTTGCTCGGGCTGGTCTT
CCGCTGTGCTGGACAACGCCTTGTTGATCACCACCTAAGGCCGTATCTGGG
TCTCGACAGATCTTCAGCAGCCTGCCTTGATCTTTGTCCCAGCTTTCTTGAT
TGAAAATTAGATTCCATGCGATCCACGCTAATATTCATTCGCCATAACCATGC
TAGAATGGAGATCATCTTGACGTTGGAAACGTGGTCAACGTGATCAATGTG
GGCAAGTCGCTGAACAGCTTCTAAATACATACCTACCTAAGTACGCTTCTGT
CCCGATTTCATGGAACTAGCCCCGTTATTGGGCAAGAATGAGAAGATCTTG
CGATCTACGGTCGCGGTCGCGGTCACTTGAAGTGAGACTAAAATTCAGGAT
AAGTCAATCATGATCTATGAGCATGAGGAGCTCGCCTTCGTCGAATATCACT
TAATAGACAGCAGCATCCCTCTGAATTATAACCGAAACCCAAGATAACCAA
ACATTTGCTTTTTTATGTGTTTGAAACGTGCATGCCTAGGTAGCTACCAATAT
CCAAGATGGTAATTAATCGCAACCCTTGTTCTAACAATATATAGGCTGTTCG
ATATGTTCAACCATGTGCCCTAACTATTGTAAGGACAGCTAACTATTTATTTC

GCATACTGGGACTACGGGCAAATACCGAATCCCATACCAGAAACTCGTGGG
ATAAATTGCTTTTAATCTACTATTAATATCCCTTCACTCGATGGATACTCAATG
GAGGAGATTTTGCCACTCACAGTAGACCCTCCCAAGCTGGTTTTCTCAGCA
ACATCCATTAACTAGACTACCACTGTAATCACTAAACTTACAGTTTGATCGT
GATCGATTGGAAACAACCAATTTCATCAATGTTGGTAAAACACACCAAATC
GCCATGGCCATAACCAAGCACGGAAAAATAACGTTAAACTAATACCTATCTA
CAAAGTGATACTTGATTTTATGATGCATATTATAGTAGGTACATAGGTATAAA
TAAACTTCACTTACCCCCGAATCATTTACTTACTTACCTACATAAGCTACCTT
GATCAACTATCGTCTATCTAGATACCCAATATATATCTAGGTAACCGCAGGTG
ATGTTTAGGAACAATTAGGTATGCTTGAATAATATATTACAGATAAACGTCGT
ATACGTATCCATTGTATGTGAAAAATCCGTATGCTTACCTACCTAGATATGAC
CGTGGAATAATTGAGTTGTCATGAGCGATGGATTCACGAGAAAGAGCGTCG
CATGCCTTTGAGCTTCAGACCTTCCAAGGTTCTTAGTGATGTAGATACCTAG
GTAGGTAGGTAGAGCCTCGCACCTTCATCGCACATTCGATTGAACCAGCAG
CTGCTATCGAGAGTATGGTCACTTGCTAGAAAGATATCGAACGAGTACAGC
TCCGTGAAGGTTAACTTGAGCTATCTAGACATTCAATCAAGACTACCAGGT
ATCCGTTTCTCTCAACAGATTATACGACGTTAGTCTAGTAGCCGAATTTATTC
CTCTTCATTTTATAGCGCTCCTGATGTCCAAGTATACACAGATTTCGGATAGT
CGGTATGTTTGTAGTCTTCACGGGCTAGTTATCTGCTTTGAATGCGAGCTAG
ACTTCTCCGTCTACAATTATTTGGTCAGATTGCCAGCAGATGAGCTAGAATC
GGAATTGCATGCTCTAGAACCAGACTTCAGATACTCCTTAGCAGACACACT
TGGATTTCTTGAACGCAGCAAATCACCCGACCCCCCGCCAAGCGATATACC
TCCGCCATCAGACTTACCTGCTCTATCGATGGAACGGATGACCATAGACCCT
AGCGATCCTAGTACAGAATGGTTAATACCACAGTTGTTTGTTTCTCCATTTT
GGAGTGCCCTTCCGTCGGACGTTTTCCGCGAGGTTTCTAGCCCTTGGTCGA
AAGGAACCACCTGCTTTGTCCACCGATCTGACTCGAGGCAGCTATTAATCT
TCGCTGGCGCCATCCAGGTGTGGAAGAAGTCGCGTAATCCTCAAGCCGGCT
GGGCACTTTAGTTTAGGCACACGGGCAACGGCTCTGGATTCGCCATAGAAT
GCGGTCGTTTAGAGGACGCGATGGTCGGATCACGCGTCGTTCCGAAAACG
CAACAGCGTGTCGACATCCGAGTCCTGATTGCAGCCCTCCAACACCGGCG
GTGGGCAGCCGACGGGTTTAATTCCTTGGTCATTGCCACAAGTTCGGGTTA
TGTCGTTAACGCAGTTGTATCGCAAATAAGAAGATGGCTATCTTCTGAGTGG
CAACTTCCGATGCCAGAAGAAATTCATGACAACCGCGACTTATGGCAGATA
TTCCTCTCCGAAGTTGACAAATGCAACGCAGAGGGCTTGGATGTGAGAATT
TGGCATATCGCACCAGGAATGATACAGCACGTATATCTGGAGGCCGAGCGG
GCAGCAATGGGGCAGCGCTGAAGGATTGCGTAACGATATGGCATGGTTAGA
TTTCAACCTGACGGCTTATAGATAGGCATGACAGTTCCATAAGAAGTCATTA
ATCATTACTCCTTAGGGTCTGCGCCTCTCACCTCTCCGAATAATTACGCAGA

GCTGATGCAGCGCAAGTACCGCGTATAGTCTCTGATAGCTCTATTGGCCGGT
ACTTCTATGCGTATTATAACCAGCTGAATACAGCCAGCCGAGGATGTAACAC
TGGTGCCTCGGAAGGATGCGACGTTGCTGACGGTGTTGTCTTTCTACCCCA
TAAAATAAGCCATAAGCTGACTTGCGGCTACGAGCTAGGGGCTTTGTGTAA
TATTAATGAACTGCTTCGCCAATTTTGTTTAGGTATTCGAGCGTTTGTCGCA
CTCTACCGGCTTTATAGTTGTTGTGCAGCTCGATATTGGTTTCACGATGTCTC
TCTACTCGTATTGGACGCAGTTCTTTCCTCCGCGCGATGGAGCACCGATCAC
CGAGGCCAACGTGCCTAGATTAGATGGGAAGGTGTTCATAGTTACGGGCGG
GTCGTCTGGTATAGGATATGAGCTCGCGCGAGTCTTATACGGCGCCGGTGG
GAAAGTGTACATGCTCACACGAACGAAGAAGAACGCGGAGGAGGCTATCG
AACGCATAAAATCCCACTATGCGGACAAAGATGCGAAAACGGGGAGTTTG
ACATTCGTACCGATGGATCTATCAGACTTCGAGAGCGTCAAGGCGGCAGCG
CAGCAATTCTTAGAGAGCGAAGGTACGAACGGGAGACTTGACGTCCTATTT
AACAATGCTGGTACTGGTGGTTGGAAAGACGCCCCGCGCACCCCTCAAGG
GCATGAGCATCACATCACAGCCAATTCGGTTGGGCATTTCTTGTTAGCCCGG
CTATTAACACCCATACTCAACCAGACGGCTAAGAAATCGCCTAAGGATACT
GTCAGGGTCGTTTGGGCTGCATCTATACTCGTTGAGATGACCAGCCCGATA
GGCGGAATAACGAAGGAATTTTTGGAAGACCCTTTCGGCGAGCAGCATGC
AGGTATACTATACGCGCAATCGAAGACGGGGAACTGGTTCCTGGCTACTGA
ATTTGCGCGGCACCAATCTGAATCCGGGCCCTTTGGATCTGGAATCGTTCAT
ATCGGCGGTAACCCGGGCAGTTACAATACTAATATCTGGCGTCACGTCAAA
GACCTAATCAAGTATTATCTTGTTTACCCCACGCTGCGCGACCCGGTGTACG
GCGCATATACGTATATGTGGATGGGCTTCTCAAACGAGGTGACCATGGAAG
AAGCTAAAGCCGGGAATTATGCGATATGCGATGGGAGGTGGCACCCGGGAC
AGAGAGCGGATCTTCTACTAGCAATAAAGGGCGAAGACGAAGGCGGCTTG
GGACGGTCCAAGGAGTTTTTCGCCTGGTGTGAGGAGAAAGTGAAAGACT
TTGTATGAATTAAGGGTTAATCGGACCTGTTTATCATTTTCTTTCCTATTTGTA
AGATTAGATAGAGACGCTAGAGAATTATCCATAACTTCCCCAAGTACTCGTA
AATTGGGAGATATCTGCGACTGGAAAATCGGACGGTATTCAATATTTACGTC
CCGTCTCGTCTGAATTTCTAATTAGCTAAGAGGACCTGAAGACGGTAGGGA
GCCACGGACGCCACGGAGGGTAGATGAGGGGTTACCTAATATGAATACCCA
ATAGCTTATTAATTATCAAGGGATGGAACTGGCTTAAGTAATCATGGGGGGT
CGCAGAGGGCAAAGAGCTGCCTCTCTTTACAAAAGCGCCGATGCGTCGGG
GTAGATTTCTAGTAACCGAGCTAGATACTGTGCCGTAAATATCGGTCCATATA
ATAGCCCCCCCCAACAAGGACAGCTGTCAACTTATCTTAGGTTGTAGTCA
ACAAGTTTCGAGCCCTGTGTGAAATATATCGAGATTGAGGGGTTAGCATATT
ATTGCAATTGCAATTATGGCGCAAGAAATTGATAACATATACGAAGAGGCCG
TTGCGTCGGGACTCCTTCCGGGGGCTTCTCTTATTGCGGGGGATAAGGATG

GTAAAGTCTTCCGCCTCGATGGCAGCTTCAATACTGTACTGACATAGAAAC
AAGGAAACATCCTGTATTCAAAGTCATTCGGAAAGGCCAGTCTTAAAGAG
GGTGACAACCGGCCTTTTACCGAATCCACCATTGCCGCCGTCGCTTCGATG
AGTAAACTCATGACTTCGATAGCCGTCTTACAATGCGTTGAAGGTGATATTT
TGAACCTGGACAAAGACGTCAAGTCTTTGCTACCAGAGATTGGCAAGTATG
GCTTAATCGTCGGCTTCGACGATGTCAAGAACAGTGCCATATTCGCACATAA
CTCTACACAATTACGCTTCGCCTACTTCTCACGCATACCTCTGGCCACGAAT
ACGACTGGTTCAACCCCTTACTCCTGAAGTGGCGCGCTTCACGCAATGAGC
CACCATTAGGTGGTTTGACCCTTGAGGAACGATCAGCTTTGCCTCTTGCGT
TCGAACCGGGAAAGGGCTTCGCATACGGTGCCGGACACGATTGGGCGGGT
AGAGCGATTGAACGAGCTACTAACTCCAGCCTAGACGAATTCATGCGCAAA
CGTATCTGGACGCCCCTGGGGATTGAGAATGACGCTAGTTTCTATCCAAAG
ACGATGGATAACATGAAGGATAGGTTGGCTGATGTCAGCACCTTAGATGCG
GATGGAAAACCGCCAGCTGTGCATTTTCCAGGCTTTGATATTTCAGGCGGA
TCACCTGATTGCTTCGGTGGTGCTGGTGTCTACACATCGGCAAAGGCATATT
ATACCTTTCTGACCGCGGTGCTGAGGCGCGACCCAAAGCTCCTGAAGCCTT
CGTCGTATGAAGAGTTGTTCCGGCCGCAGCTCAACGAACAATGTGAAAATG
CCCTCAATGATGACATAATCTCCTCCGAAATCAAGACGCAGTATCTAGGCAT
GCAGGTGCCGGCATCGGTACGGAAAACGTGGAATCTTGCGGGTCTGGTAG
CAAAGGAGGCCCAAGAAGGACGTTTTGCGAAAGGGACCACGCTTTGGGG
GGGATACCCGACCTGCGAATGGTTCATCGACAATGAGAGCGGGATATGCGG
TGTAATGGTCTGTCAGCTCGTGCCGCCGATGCAGCCGGATGTCATGAGATT
GCATGAGCAGTTTCAGAAGGCTATGTTTAGTAAGATCAAGGTAGGCGCAAG
ATTATGAGTACTCTTGAGCTAGGTACTATGAAAGGCAATAAGAGTTTCGGCC
CACTCGTTTAAGGAGACTATTTCATTGTCGATATATTTGTCGGAAGGATTCAT
GAAGTGCTCGAGGTAGTGAAGTGTTGGGGATTCTATCAACATCCGAAGCAC
TCTCGTGACTCATTAATGCGGCCTTGGCTCGACCGGATGCTGAAATCTTAGG
CTACAATACACGAAATATTAAGACCGTAGCATGACACCAAATTGGGAAGTG
CTTTTGACATGAAAGAGGGCTTGAATTGATGATGTGTTCCAGTAAGTAAAA
TGTTTGTTGAGTGGTTTGAGAAAGTGAATGCATATAATATGAGAACGATCCA
TGGGTATTTGCGATTGGTTAGTAAATACAGCAGACCCATAAAACAAGGGCT
GAGAAGTAAAAGTCTAATTGGTTCAAGTCTTTAAGATTTCGGCCCCCAACC
GCGCGACCCCGCTTTTCGAAGATTTGGGCGAATAGACAGCATCGAGTTCGA
TCTTTAGCTTCACCGGTTTCCTCCAGGGCTCTGACCTACATATGTACCTAGG
TACATGCCGTATATTGATGGAGGGGTTCAAGTCCCTCGTCTCGTCTAATGGA
TTTTTCTTTTCAGCTCCTCACATGCCTCTAACCACCTAGTATCGTCGATCGTC
GCATGAATACAACAGGTTACCAAGAAACGTCAAGGTTTAAAGTGACCTGAT
CGGACTGTCAGCACGGCGCAATATATTTCCTTGACTATTCCCCTATAGCCTC

ATTCGGCTCACGGCTCACGGCTTACGGAATGAACAAACAAACAAACAAAT
AATAGTAGCTGGTAGGACTGTAGGACTACCAGGTAAGGAGCTACCCATGAT
GTAGGTAGTATTCATGGACCATGATAGATGGATCTTCCTTCCTCTTGCATTTT
CAAACGTTGTTACTCTTCCTTTCCCCTCTCTTGTGAGATGTTAGTAGCTCCT
TAAAACTAAGGACATTTCCTTCCATTTCCTTAGCTCTTACTCTCCTCTTCTTG
CCAACTATCAATTGCCTACTTCTGAGCGTCGTTTCCTCTCTTATTTTTACGGG
CCTGTCTATATGACAGCCCATCTTCGTCTTACCTCTTCACCTCTTACCCTTAA
GCCTCTCCCTTACCTTACCGGACTCTTCCTCCTAGCACCCACATAGTTGTCA
TATAGTTGCTATATAGTTGGTCCTTCCCCTTTAATAATATTGATCTAAACCGTA
TATTTACTTTGAATCTCGGATATCATGTCGGTCATACTCCCATCACCGCCTTC
GGAGGCTGGATACCTTAACCAGGACGTCGAAGGACGTACCGTAATGGCGG
AGGTACCTGTTACCATTCCTCAAACTCCTAAGAAACCTAAGCAACAAACTC
CTCAGCAAGCGATCGATGAATTCTGGGCTAAGTTTACTACTAAAGCACCTG
GCAAAGGTATGTGGCCTAATATACATCTACCTAGGACCTAGGTATATAACTTC
TGGGAAGTTTGACGCTTACTAACCCTGCCAACTTGCCTCTTGTTAGCAACC
ACTGTTATCCCGCACAACGAGTACGCCGAAAAAGCAGCGAAACGATCAAC
TAAGGCTGTTGGTACAAACACCCAGACTTCATACGAAGAAGCCGCTGCCGT
CTGCAAGGCAAAGGTTGCCAAGATCGTGAAAGAGTGTCGCCGAGTGAACC
AAAAATACCGTGATCCCCACTTCGATCTCGAGTCTGACTTGAAATGGGGAG
GCCGTGATACGCTTGAGTCGCTCTGCAACACGAAGGAGAATACAGAATCC
GATTTTAGGCCGAAAAGCGTCAAGCGCGTGGTTGACATTTTCGATGATCCT
AAGTTCTACATCGAGGGTCCCACTGCCAACGATGTTAGGCAAGGCCGTGAC
GGTGACTGTTGGCTGCTTGCGGCTTTATGCACCCTTAGCAACAAACCTAGT
CTGATTGAGAGGGTGTGTGTAGCTCGCGATGAGCAGGTTGGCGTCTACGGC
TTCGTTTTCCACCGCGACGGCGAGTGGATCTCCGAGATTATTGACGATAAG
CTATTCCTCACCAAGCCTGACTTTGATGAGAGTTTCCTCGAACGCATGCTTT
GGGAAGATCGCGAGAGGATCAATTCCGAGAGCAGTATCGAAAAGCCTACC
AGAGTGGTTCTGGCGCTCTGTACTTTGCTCAATGTGAGCACGAAAATGAGA
CCTGGCTGCCGCTCCTTGAGAAGGCTTATGCCAAGGCACACGGCGACTAC
GCTGCCATCGAGGGTGGATACACCGGCGAGGGTATCGAGGACCTCACTGG
CGGAGTAACCTCGGAACTTTACGCCTCAGACATACTGGACAAAGAATACTT
CTGGAAGGAAGAGCTCATGAAAGTCAATGACCAATTTCTCTTCGGATGCTC
TACTGGCATGTGGGGTGTCGGATGGGGTGAACGCAAGGGAATTGTCGAAC
TTCATGCTTATTCGATCATGAAGGCTGTTGATATCGACGGCCAACGCCTTGT
TCTGCTGAAGAACCCTTGGGGGAAAGGGGAATGGAAAGGGGCTTGGGCT
GACGGCAGCAAGGAATGGACCGCTGAGTGGTTGCTCAAGCTTAATCATCG
CTTCGGCGACGATGGAAATTTTTGGATCTCTTATGATGATCTGCTGCGTAAA
TACAACTCGTTCGACCGAACTCGCCTCTTCGGACCGGAGTGGAAAGTTACC

TCCCTTTGGTCTACACTTTCAATTCCTTGGACGTTGGATTATCATGAAACTTA
TTTCGATTTCTCCCTAGCAAAGACTGGCCCGGTAGTCATTGTGCTTTCGCAG
CTAGACGATCGATATTACCGCGGCCTTGAAGGCCAATACAGATTTGAGCTG
AATTTTCGCTTACACAAGAACGGACAGGAAGACTATGTTGTACGCAGCATT
AGCCCGTACTGTCAAAATCGTTCTGTCAACGTGGAGCTTGAGTTAGAAGAA
GGCGAATACGTTGTCTTGTTGAAGATCGACGCCACGCGTTACATGGATAAA
CTACCAATAGAAGAGGTTGTCCGGAACAATGCTCGTGATCGCAGGGACAA
ATTGGTCGCTGTCGGAATGTCTTACGATCTAGCTCACAGTAAGGGAAAGGT
CTTAGAGACCCCGGAGGAAAAGAAAGCTCGAAAGGAAGCAGAGAAGCGC
AAGAAGGACAAAGCACGCCAGGCACTCAAGAAGAAGATTATGGAAGCTC
GAAACCGCCAACATTACCTCCGCGTCAAGGATCATAATAAACGCCAGGAAA
AGAAAGCTAAGGCCAAAGCAAAGGCACAGGCTAAGAAAGCTGCCAAAGC
GGAGAAGCTCAAGTTGGCTGCCGAAAAAGTCAACAACTCTAAAGAACAG
CAACCAGAATCCGAGAAGGAAAAACACGTTCAAGTGCTGTCTCCGAACCA
GGCCCCCGAGCCTGGTTCTGAATCAAGTACCGATCCTAAAATTGAGACTCC
CTCTACAGACCCCAGCGAAGCTTCTGTAGATACGTCGTCAAAGCCTGAAGA
GCAATTGGCCGAAGGCTCCAAGGAACCTGAGGCACCGAAATTGGATGAGA
CTTCTGATTCTACCAAGCCTGAAGCGAAAGTTGACACAGCTATCAAGCTAG
AGGAATCGAAAGATGAGAGTTCCAAACCAGACGAAAGCGACAAGTCTCA
GGAAAAAGAAGGGAGCCAAGAAGACGAAGAAGAGTTCTCCGACGAGAGT
GACATTTCATCTGTATCCGAGATTAGCGATCGAGAGCTAGATATCGAACTCG
AGCGCCCGACCGCACCTCCCATGCCACCCGCTCCTCCGCCAATGGGAGTGT
CAGAGGAAGAGCCGGATGAATTTGAAAGCAACCCGTGGAATGCTGTCACA
ACTGTCGGCCTCCGCGTTTACTACAAGGTGTCGGATGAGGATAAGGACAAG
GAAATCGTAAAGTTGCGCGTAGTTCGTCCAAATCTATATGCCAAAGACAGG
AAAGACAAGGACGATGGGGACAGCGACAACGGAGACGACGAAGACGAC
GAAACCGAGGATAAGCAGAACGAGCCCAAGAGCTCCAAGGGTCTCGACG
TCGACGACAGCTCTAAAGACGCCACTCTAGAAGGAGATAATGAGCAAAGG
AGGAAGAGCATCTTAGCTTTGCTACCTAAGCAACGGTAGATTCTCATGAAT
GACAATGTTCCAATGGAATTGGTTATTGCGAATAAGATTGGCTAATCATCGG
GGGTTGGGGAAAAACCAAAATCGAGTATAGGTATCTTAAAAGTAGCATAAA
AGAAAAGGAAAAGATTAAATTTTCATTCTTGGAAAAGTTGTTCGTTTACTG
AGTCAAGGACACAGAATATTACCTACCAATTCGTTCAATGAGAAGTCGCAC
CTATTATTCCCTGTTCTCATACCTTTCTATAATTATTCATCATATCTAGTTCCGT
CATAACCTCATAATTTCATTTTGAGTTAAATATCATTCATTCGTAAACTTTAG
GGGTATCATTATTAGTAAACAGCCAATAAAGGACTTGACAATTCTAGACTCC
TCATCTGGAAGATTTCTTCCCCATGTTTCTAAAACTCATCCAGCAACCTCTC
AACCTTTCCCCGTTCCACAACTTCTAGATCCTCCATCGCCGCATCCTCACCA

GACCCTGGTTCCGCATCGGGTAGCTGCGCGACGCGGGCCGCGACGCGCAC
GAGCAGGACGCGCATGCGCTCGAGCTCCTTCTCTAGCTCGCCTTCGCGGGT
GACTAAGTTAGGCTGCACCTTATCTATCCCGTCCATCTCGTCTAACGCTCCC
GTTGCGCCGGACGCAGATGTGGATGTGGGAGCGGAGGCCGAGACGGAAG
CGGAGAAGGAGGAGGAAGGAGGGGGGTAGGAGGTGGAAGTAGAAGAGG
GGAACGATGTTAGAGGTGCGAGGAGTGAGCTCATTCGGCGTAGGCGTTCG
AGACGTGAGGAGACTTCTTTACGGTGGGCTGAGAGAGAGGTCAGTGTTGA
GGCGAGGTCTGCGTACCGGTTCGCTTCGGCGGGGTGGGCGGCAGGACTGG
GTCGGTGTGCTGGAGGTAGGTGATGAACACGAGCCAGTGGTTGACCCAAA
GGTACGGCAGGAAGTAAACGAGGGCGACTTCGGCGACAGATCGCTTGTAC
GCCCAGTAGCCAAGGGCTGAAAGGGTGATCACGATGCCAATGTCCGAGGC
GATGATCTGCCAGTACTCGCGAGCTTTGAAGATGACGGCCTTGGGGTTGAA
GTGGTTCGTGCCGGCAGGGTAGTGCAGCTGACCCGAGGCATTGCGGATGA
GGTACAGCGGCCAGCCGAAGACCTGGCGAAGGAAGGCATTCCACAGAGTC
ATGATGGGCGACTCCTCGAGAGCCTCGCGCAGCTCGATCTGACGGAAGGC
GGACACACTGAGCGGTCAGCTGGGGTACCATGGAACTTCAGCGGTGCGCA
CTTGATACCAAGGACCTCGCCGTCCTCTTTAATCTCTGAGATGCCAAGCTCC
TTACGGGTGCGGGGGACGAACACCTCGTCTCGGGTCAAGTGACCGGTGGC
AGCGTGGTGGCGGCCGTGCTGCAAGTGAGCTCAATTTGGAGACTCGGTCA
TGCTCCCGAGCACTCACCGAGATGCGCCAAGAGTGGTATGGGACGAGCAG
AGCCGAGTGAAGAACCCATCCGACCGAGTTGTTGATGCGCTTGGAGGGCG
AGAAGCCCTGGTGGCCGCACTCGTGACTGGATGTCAGCGTGGAGATGATA
GTCATGGGGTGGTTGCTGACCCGATGATCCAAACGCCGGTGCCGAACAGA
CCCGTAGCGAGCATGTAGGCGATGTAAAGCGAGACTCGGGCGGCCCACCA
CGCAGCAGTGGGGAGCGAGCTGTTCTCAGCGATTTTCGAGACTATCGGCG
ATAGCGACGCGGAAATGGACACTAACAACCTGGCCAAGAACGAGTCGATG
TGGTAGAATCCATACACGAACGCGCCAATGGCCGCAAAGTCCTGCACGATG
TACAGGGACGATCGGAGGGGCGAGCGGTGGAAGCAGTGAGCGGGGATCG
CGTCGAGCAGCTGCTTGACGGTAAAGTTGGGCACGATGAACCTGCGCGTC
AGCTCTGGCTATCCGCACTGCGGGGCGAGCGACAGATGAGGGGGAACGCA
CTTCTGGCCCTCTGTGAGCTCCTTTTCTTCTGCCTCGCGCAGCAGCTCTGCC
TTTTCGACCGAGCCCTTGGCGACCTGGACGCCAGCGGCGGTGGGGCGGAC
GGACATGGCGATGGGAGTGAGGTAGTGTAGTACGAGAGTGCGGGTGAGGT
GAGACGCAGAGGCCTGCTCTATGTATCTACTACGGCGTGGTGGGGGTCGAT
CGAGCCAGTGCGAAAGCGAAAACGAGAACGAAACCGAAACGTGTCCCGG
GTCTTCCCGCCAACTGTTGAGACTGGGGCGTGTTACCTCGCCTGCCTTTTT
GGGTGTCGTAGACTAAGTACGATGTCGAGATGGGATGACAGAAGAAGAAG
ACAGTGCGAGTGAGAGGGGGTGAGGGGGGGTTGCCAAGTAGCTTGGAAG

GGTGTCCAAGTGGGAGCGAGGGAATATGGAATTGGGGGCAGCAGCGAGGG
GAGGCTCGGATCGCGCGTTGATTGTCCCTGAGTTTTCAGTGAAATGTTCAC
CCATGGACCCAGGCGGGGGTTGGCGTTATCGGACCTCTGTCAAGGAAAAC
CGACGATCCGTCCCTGACCCAAAGGAACCCCATCGGAGCCGATCCCGACTT
CCGATTAGGCGAATATCCTTCAGTGGCATGTCAAATGAACGATTATACACGC
TCAGGGTCGGCAATGTCCGGACGGGTCCAAAGTGGCGGTCCAGGGCTCAC
CAGGTATAACCTGGAAATGCCAGATCATGCCCGTAGATCGTGCCAGGTTCG
TCATAGGCCATACAGTGCATGAAGCTAGAATACAACTCGATCATGTCACTTG
CGCCTCTTGGGCGTGGACGGCCCAGCTACCCTGTCTACCTCGGCTACGGCG
GGTGTGTGCTCGTTGACGATCTGCCGCAACTTGGGCAGCAGCTCCCGCAA
CGGGTCCACAAGCATCGAAGCCGGGACGGACTCCCCTCTGCTGGCTGACG
ATCAGCTGCCCGTACTGGTATGATTCAGTGCTCACCAGGCCCTCGCAGCAT
GATCTCCAGCGTGCCAACCGAACCAATGGCTTCGCGGAGCTGACCACCAA
GTAGGGAGCTGAGCTCGCTCACGACAGCGGGCCTGGAGAAGTCACAACG
GGAAGAGGTCCCGTTGGCGACACACTCGGCGCATGCCGTCTCAGGAGCTG
CTCCTCGCACGATGCACCCCGTAAGACACACAGGTGAGCGTAACTGTCTCG
ATCTGCACCCTTCGCATTGGAATGGCCCCATTCGGACATAAGACTGGTTCGC
GTTACACAGGTTCATCGCTTCCCTCAGCTGTTTCTAACAAGATATTAGCTAA
GAGCTATGAGTTTGACACGCAAGTTGAGCCAAGTACCTCTTTGCCGGTCTC
CAGAATACGTTGCCCATCCTCTCCTAGTTCGTTCGGTGGCAAGACCTTTTTC
CCTTTAACCGTATTCGGGAGGGTAAGGTTGCCGGACTCGGCGTAGGCGGGC
CAAAAGCGAGGGGTAACGGCTTTGCCAGGTGTGTACTCGTATTTCGCGTCG
TGGGCTGTGCGAAGTCAGCATAATGCGTTCGATATTCACAGCAGACTGACT
GAGTGGTTGTGGTTCTGCGGATGTCGTTGTGGTTTCGCTCATGTTGGCTTAG
GTGAGTGGAGATTCGAGGGATGTTGCGGGTCTCGGGCGGCATAAGCATGC
ATATACATGGTTTCTACGAGTTGAATGAAAAACCACACAACAATACGTCGC
TAAGCTTAACTCGGTGAGGTATGACCGAGTACCTTTGATACAGGAACGTCA
AAGGAAGGGGAAGCGCAGACCGGTCATTGTCGACATGTATGTGCCAACGT
TGGCAAAAGTTGGCCAAAGGACGCACAGCTCGATATGTAATCACAACAATA
CACTCGTGGACGCGGATGTACGTTTGATCCAGTCGGACAAAGCATTGAGCG
ACTTGCTTGGAAGCGCCAGGCGTTGTCTCTCCACGAGAACAACAGCCTCG
TCGGACGTGAGTTGGCGTGTCTCGATGATTGTTTGTATGAGCCGCCATATGG
GTGCACGACGAGAATAGAACTTGCGCTCGGCGTCGTTGGTCTTTTTCCAGG
ACTGATCGTCGCCCTCGTACTGACTGCGGATGGACGCACGCCCATTGATGC
CTATCGTGTACTCGGTCCAAAGGTCCTTTACCGTCGCCACGTTTCGGTCCAT
TTGATAGGGCGTAAGCAAGACCAAAGGAACGTCGGGCTGATGATGCTGAT
CCGAGGGCGCTCCTGTGGCCGCGGGCGCAAGCGTCCTTTGATCAAAGGAC
CGGAATGAAGCGGACTAGATGACGTTGTGGTGCGCCCTAGCGAGTCTAAA

GGAGGGGATATCAACAAAGGCCGAGATAACGCTTGTTGTATGGTGCCAACC
ATGCAGAGCACCGCCTTTAACGTCTCACCGCTGCTTGTATTTGCCAATGTCA
ACTTGTTGATGGCCGTATCAACATGATCCAGCTGTTGTTTGCTTGCTTGATC
ACGTTTTCGCAATTCGTCAACGAGAACAGGCAACGCAGTCTCAAGCCGTG
TGTCGTACGCGGAATGATTGGTTGCTGTGGTCGCTAAAGCCATCCTTTCAA
AGTCCAGGTATTCGGGATCTTGAAACAGCAAGTGCCTGAAAACAAAAAGC
GTCGGATGCGAGCGCCTCCAATGTGGTGCATCCTGAAGAAAGGACGTGCG
CAACTCGTCCAAACACCGCAAAAAGGCTCGAGCAGCGAGATCTTCTTCCT
TGTTTTCGCCCAAGTTGAAGAAAGCCATCCTTTGTCGGTCAGCTGCGCTTT
GATTGAACTAAAATAGCTTGCCATCGATCTAATTGTGGAAATACCTTTCGCA
CTAAGGTAGGGGGTACCTTTACTGCACGAGGCAAGTGGAAGGAACCCCCA
TTTGGCTTGAACCCAGCGGACGCTCTGAGCGCAGGCAAAGCAATGTGCAT
CAAGTAATGCTTCTCCAAAACCGTGTCATGCCAATTGCCGGCGCGCGCAAC
GTCGCGACTTGGAGCTCCAGCCAGCTCAGCTAAGCGCGCTCCAGCTTTTCT
GTTGATGTGTGTGGCTTGAGTGGTGCGGATGCCCGAATGTTTGAGTGCTGC
TTTGATCGCGACCCTTTGTGTTTCATATGGCACTGGCTCTGACGCGTTTCGA
CTGTTTTCGAGCAACCTGATAGGGTACCAGCTGGCACGAGTGTGAAAGGA
AGGGAAACGACTAGCGCTGACATCATAATCAAAACTGACGTCAGGATGAG
CTCGTTTCAGTACAAAGGACCCTCACCGAGCAAATAGGTACAGGCTTAGGA
ACCACACCGGACACATCTCTAGCTTGGCGTTCCTCATGAATGCTGCATACTG
TGTCTTGCCGGAATGATTGGTCTTGCCTTCGTTGGTGGCGAAGACAATGAC
TGGACACAGTGTTGGACCCTCTTGCGTCAAAACATCACAGCTCATGTCTGA
GAGACACATTTTTCGTTGATTGTCGCTCCGAAGGAGGGCAAACATACCAAG
CGCGTGCGCACAGGCTGTCACGACGGACGTAGGGAGAGCACATGGAAGCC
ACGTGGGGAGACGGAGCTCGGACTCGTTGATTCAACGCATCACTCGCCGA
GCTCACCGAGGGACGACGGATACTTAGGAAGACTGGGCGCGCGATACGTT
CCGTAGTCTTCTCCCAATTGTTGTTCTCTGCAGACACGTAGACTGCCTGCTC
CGTCCCGTCGCTTGGCTTGCCAGTGACACCGAGTCCCCCGCCGAGTTCCCC
GTCTACCTCCCTCAACTCGCTCGCGCTCGTCGAGTTCACCTTCCCGCTCGC
CGAGTTCAGCGAGTCCCTCGCCGCACCCCTCGCTCAACCCGTTCACCTCCT
TGAGCTCAGCGAGTCCCTCGCCGAGCTCCTCGCTCACCTCCCCGAGCTCAG
CGAGTCCCCCGCCGAGCTCCTCGTTCACCTCCTCGAGCTCAGCGAGTCCCT
CGCCGAGCTCCTCGCCCAACTCGCTCATATCTATCGACGTCAACGACCCTC
ACGCCGACCGTTCGGAGTACGTGCCCGCCGGGCCCTGGACAGAGAAGGCG
GTCGTCGTGACACAGGCGTCCCTAAGCGAAGTCTCGCTGTTTCGTGTAAGA
TAGTAGTCGTACGTTTTGATGATATCCTGAATGTCTTTGTATCCGTTGTAGTA
TTGTCCTTTGGGTTTAACCGAGCATCCTTTCATCAAAGGAGGGCTGCGCAC
CAAGGCCTTTGTCCACGAAAGTTTTGACCTCTCTTTCATGCTTGAATCTGT

CAAGGCGCTCGCTATCGCTGCAGTCCAAGGCGATCTTGGCGTTTCATAGCT
GTTCACTCCTCGCGAGACCTGGCTTTTCCAAATGTCTACCAAAGCTGCCAT
CCACTGATTGAAGCTGCTCGGCGACAGATTGGTGTTTCGTACCAAGGCTAG
ACTGACTCGCTTAGCTTGAAACTGATTGAAGTAGATCTGTGCGCACACTCT
TCGGATGTGATTAGCGGAGCCTCCTTTGACCATGTGGTGCTCAACGTTGGC
TGGTTGATTGCCGCCGCTACGTTCACAAAGTCTTGCTCTATGCCTTCTACCG
CCAGGACTTCTTCGGTACTCTCTTGTGAACACTTGTTAATGTCTGGGCTTGC
GGGTATCTGATGTTTTGCAAGTACGTGTTCATAAAGGAACATAAGTGCGGTT
GTCGGTGTTACTGTATCCCTGTATGGTCATCCTTTCGCTTGCTCCCTCCTTTG
CCGGCCAGTCCCTGCCAACGTTGGCAAAAGTTGGCAGATTTGAGTTCCTTT
GAGCATAATAACGTACCGCAACGGCCAGGCCTTTCCCTCGCACCACTTGTA
CCAGTGCTTTGCCCTTGATTCGTATTGACGTGTGGTGTTGGCCGCATGTGTC
TCGGATCGCAGGTGTGTTGACGCATCTACAGCCGCTTGCCCTTCGGCGAGC
ATCATTGGCTGAATTCTAACGGTCATTGGGTTGCTTTTGATTGCTCGTCCCAT
CTTGTACAAAGATCAAAGCTGATGAATACGCTCAATGATCGGTGAGCCCAA
TTAAGATTTCCTGTGTGATAACCTTCTGGGTAATACGTGGCTCAACGCGTTT
GTAGTATAACCAGCGTTTACGCCTGATGTAACTGCACTTATATGCATGTGGA
ATTGTAATCCGATTACAACGTATTAGGATAAGGGTGCTTCCCGAGAGGGAG
ACTTACCCGATTGGGTAATTTTGCGGTCGGACATTTGTCCCCGAGCGGGAG
CATTTTTGGCTGGACTGTCCAGGTCTGTCCGGACAGGACCTGGGTATAATG
CAAAGTATGTCAAATGCAAAGTGTAATGCATGCAAGTCTATTGTACGTGAA
AGAGACCCAAGATTCCGACCACCCTCCGTGTCAACTAGACGCCTTTGCCCT
TGGGCGGCAGCTTGAAGTTGAAAGCCCCCCTGTCTGTCTCGTGTAGCCGGC
GCAGCTCGAGCAGCAGGCGGTACATCTGCCGGGTCAGTTTAGTCGTGCTGT
GATGACGCTGGACGCACGTAAGATTGCATCTCGGCCCATGACCAGTACAGC
TCGGCGTACTTTTGGCCAGCCTCGCCAATCTTTCTCGCTAGGTACTGTTCAT
GAGCTGCCGTTCCAAGTCTCAGGTGAATCGCAACTGACGTCGTGGCCTTTG
CTCTCGTCAATGTTGCCCTGGTCGTCGACCGGACCGACAAAGAACGCCATG
ATGTCGTACAGGTCAGAGTAGTCGGGTTTGAGCGGGATATAGTGGTACCAT
GGGACTAGAATGTCAGCTGAAGCGCTATATCCACAACTGTCGTGGCGACTC
ACTCAGACGCTCCATGTGCCATTCTGGAAACATGGTGAATTTGAGCACGGG
GCTACCCTGGGCGAGCAGACGGTGGAAACGCTGTGACCATCCGTTGCCAT
CGACATCGAGCGCGTACTTGAACGCAGCAGCCTGCTGAGGCCAGACCTGC
GAGCCCCATTCGATCGTGCGCATGATCTCCTCACCGAGATGTGGGTGCTCG
TGGGGCATCTTACCGTCAGCTTTTTTAGCTCGACAGAAAGCACTCACGCC
GATCAGCTTGCCAGCGAGCTTCACGTCCACATATGCCTTGCTCAGGACACG
ATCCCACCGCCGAACAGTCTCATAGCCTCCGTTGCCTTTAGGCATAATGACA
TCGCGCGACTGCTGGGCCCACCATGCGTCGCCGCCCTTGGGACCATTGACC

ATGAGATGGAGGCGGAAGCGGTGGCTCTCTTTCCAGTCTCGTTGAACGTTG
TATCCACCCGTGCTGCTTCCTCGCCAGAAGAGGCGGTTGTCTGTCTTCTCCT
CCCAATCGCGCAGGGCATACCACGAGTCTGTATCGCTGCCCGAAAGATTGC
GGTAACCCTCCATGGGAGACGGCTGGAAGGACAAGTCGTTCGGGAATTTG
GAGTTGACCAAAGCTGGCTTGAGAATGGATTGGGAGCGGCCGTTGAAGTC
AATGCTACTCGTTAGCGTTAGTTCCTGTGCGCTGAGGGTCGCCCCAACTGA
CGACATGGCCCCATGCAGGCGCTTGAGCTCGGGAAAGTCGCAGAAATCCA
TGGTTGGCAGATGGTCGTGGATGAAAGACTTGGCTGATGATGAGCTATACT
GGAGCGCAGTCGACGATCAGCTCACGCAAAATCTCGTTGGTCGCGTTCTCA
GCCCCGGGCCGAATATTGGCCGGAGAGTCGAGCGGGCATGCGCTCTGGTA
CTCAGCAAGTAGCAGGGGGGGAAACGAGGAGAGACTGACAAACCAGCCC
CATGCCGGTGTCCTGTTAGGATCCTCGAGCGCCTTGAGCTCTGCCTCGTCA
AAGTCTGGGGTCAGCAAAGGCCTCCTCCTGGCTGCCGTGCCACTCACACT
TGCCCTGCTTGACGAGCTGTAGAGCCCGGACCTGCTGATCCTTCCCAATGA
CCTGACTGCCGGTATCGTGATCGCTGCCGCTGACCTTGAGGCGGAAGCCCT
CAGGCAAGAAGCGCCTGAACGTCTCGATCATCTGCGCATACTGCTTGGGGC
GAATATGCGGCGCCCGCGGGCCATGCACCTCGGTCTCCGCCTCGCGGTCCA
CCTCTAGGGTATACGTGAAGTCGCCACCTTCAAGGGCGGCACTGCGGCGCA
GGAACTCGGCGGGCTGGAGCGCGTGGTGGGGCAGGATGGTCTTCATCATC
TCGTCGTACTCGTCCATGATGACCACGCCGCGCGACCGGGCGTACTCGAAC
CACCGGTCGAAGCCATGCGGGGGGTCGATACCATATCGGTGCTTGTACTCG
CCGACGGCCCGCCTCCAGAGTCTGCGACTGGCTCGCGAGCAGCGCGTTCC
AGCGCTCCTCTGCGCGCTCCATAAGCTCGTAGAACGGGGTGCTCGGCCGCC
TGGCCCGGGCGGCTCGGCAGGAGCGTCGCCATGCCCGTCTTGCCCCAGAA
GAGGCTCGACCGCCTGCCGCGCCGCCCGCCGGGCGCGGCGAAGCCCAGC
GGCGGGGGCGGGATGCGCTCGATGCCCAGGCCGTGCCGGATGCCGAACCA
GTAGAGCAGGAAGAAGATGCCGATGAGGTACAGGATCTTCTTGGGCCGCC
GGATGGGCCGCGGCACGCCCATGGAGCCCGTGGAGAGCGGCATGAGGCCC
GCCTTGGCGCCGCCGGACCGCCGCCCGCCCGTGGAGAAAAAGCTGCGTGG
AAGCGCCATCTTGGGTGAAATGTTGCGCGGCGACAGGTTGCGAAGAGGCA
TGGTAATTGGGCTGAGGAGCCCGCTTACCAGCCTAGGCGTCTCGAGGGGTA
TGGCGGGCGGTGATCACTTGGGGTGGACAGAGCAAGAGAGAGAAGGGAG
ATCAAAGTCGACGTGGGTCGCCGTCGTGGTGGGTGAGTGGGAGGGTACGA
GACGGACTTTGCATGTGTGGGGTGGCCGCTTCGTCGCGACCTTTGCCCTGA
ACCTTATTTCAGGTCCCAGCCAAGCCCAGCCCGGTACGAGTAGCCTGTCTC
GACGTTACATATCGTGCATTGCAACAAAGCTTCATGTACAACAGGAGTCGT
TATGTTTGTGCGTTGTGACGCAGATGCGATGCAATCTCTCCTCGTCCAGGTC
GTGAATGTCATGCTGCAGGAGGTCCAGGCTGTCCAGCCGAAGGTGGCGCA

CCACGAGGAGCGCGAGTTCCCTGTCGTGGACCTTTCTTCCATCTCTGGCAC
GTGAGCCACAGGTTCATTGTGCTGAGGATATGCTCACAGAGTGGTCTACCG
TCGTGAAATCGTCAACATTCATGGCCAGATGCGTGAGCTGTCTGTCAGCTC
CAATGTGTGACAGACGAGTCAATCCGACTGACATAGATGCCAAGTCTAGCT
AGCTCTCTGTCTTGGACTCCTACCAAAGTCCCGGCCTCATTGAAGGCTCGG
ATCTGCGAGTCAGCTTGACAACATGAGTCAGGTGCAGGCATACCCTCAATC
CTTCACCGGGATTCAAGGCAAAGTTTCGCGAAAGATCGTCGATGTGGATGG
TCTATTTCTCAGCGTATAGCTCATACACAGCCTGGCGGAACGTCACAAGTTC
TGGGCTGGAGGCTTCTTGAGACTCACATTCTTCGGCGACCACTGCGGGAA
CGACGCCCAGAAGTATGCCAGCGGCTTTACCCTTCGCATCAACTCCGGGCA
AGGCATGACGGCTTAGGGTTGGACCTACTCGTGCTTGTACGGCTGCCCATT
CTTGAGCGAGAAGATCTAAAAATGAGCCGCAACGTCAGCTATCAGGAGGG
CTTCTCACGGGGAACATCGAAGTTCGATCCGCGACGAAGCAGACCTGGTT
GTCAGCCCCGGAGATCTGCGGAGTCTGGGGCCATCTACTTTGTAGTTGCGG
GTGCCGCCAATGATGCCCAGCTCGACAAGCTTGCTCTCGACCCAGCGCCA
ATGCGTCTGGCTCCAGATGACGATGTCGTAATGCTCATACGCTCTACTCTGT
CAGTACGAGAAACTTGTGGTTTCTCTGAGAGCTGACAGTTCCAGGAAGTC
ATGCAGACCGGGCCGTGCACATTCGTCAGCCGGTAGAGACCCAGCGAGGA
GAGGTTTCGTGTCGACCATAGCTGTTCATGAGCGGCGTCCAAGGAATGATC
AACATGTTACTCACTATAGTCAAGATCCAGCACCAGCAACCGCTTGCCGGG
TCTGGGCTCGTTCATCACCTGTCTCTCAGCCGTCTGCATCGTACGCGCATTG
GCTGGCACTCACGGTGATGGGGCACTCTCTGACCTTCTGCTGGACCCTCCT
GATGTTCCTCGGGTCTTCTGCGGGCGCTACCTGGCCTTTGCCTTCCAAATGA
CTGGGGTACTGGATATCAAAGTCGTCCCTGACCTCGGGCAGGTTCAGCTGG
TGCGGATCCTTGAAGCTGAGGTCTTCTGGGGTCCCGATCATCGTGAACTTG
GTGCCGTCTTTCACGCCGATTGAACCGAAGCGCGTGGGGTCCAGCTCTGC
GGCCAGCTTTCCCTTTGTGAGACCGATGAGCTTTTGCCGGACGGGCGGCAC
GTTGGTGAGCGATTGAATCAT

>Mosaic_sequence_39

CAGAAATTCTTCAAGTCTCGATTTGGCAAGCCATGTGATGTGCTTGTGCTG
CCGGACACTTGTCAGTATTGTCACTCGGCGCGAAGGGCGCGCTGACTTCAG
TCGGATATGTTGCACAGTTACCCCAACTCGCCCGACTCGCTGGATGTACCA
GCTTCTTCACCCAAAAGCTGTCTTGGAACGCAACGTTAGTCTGAACATCCA
TCCGCGCGTCCAATCTAACTTTGAAGGAACACCTTCCCTCTTACGACTTTC
AACTGGGTCGGCCTCGACGGGTCTGAAGTCCTCACGCATATGACACCCGCT
GTCAACTATAATTCGCAGTGTGGCATCGACGATATCAGGAAATGCTACACG
AATCACCGCAATCTAGACGCCGCCAAAGAGAGCATGCTCTTGTTCGGCAAC

GGCGACGGTGGCGGTGGCCCAACCCCACCTATGCTCGAGAAACTTCGCCG
CGCAAGGGCGGTAGGCGAGTCAAGTGATGCATCAGGCGCAGAGCTGCCCA
AGATCCGGATGGGTGGACGATGTCTGGGTTCTTCGACCACGTCAGGGAA
GAGACCAACAATGGGCAAGAGCTCCCGGACTGGAATGGGGAGCTGTATCT
TGAATATCACCGCGGGGTAAGTTGGAGCAACACCGCGTGGTATGGATGCTG
ATGGCAGACATACACGTCCCAAACCTCGACGAAGCGTGGGAACCGCAAGG
GTGAGAATCTACTGCGCGACGTCGAATACTTTGCAACACTCGCCTCCATCT
CGACGGACTACAAGTATCCTGCCGAGACTATAGAGAGCCTCTGGGAGGATC
TGTGTCGCAACCAGTTCCACGACACCCTGCCGGGAAGCGTGATCGACATCT
GCGTCAAAGACTCGGACGAGAAGTTTGCTGGTATCCAGGCCACTGGAAAG
CGCTTGGTTGAGGAAGCGCTTGCGGCCCTTGGGTGTTCAGCATCCGCCGAT
AGCGGCGAGGTGGTGGTCATCAACCCCGTTCATGCTATCTCTCGGCAGGAG
ATCGTTCCCAGCCCGGAGCCGGGAGAAGGACTTGTGATTGCCCAGTCCGC
CGGCGACTCTGTTGCCCTCACCAATGCTCTTCCTTCCTCGCCTGTGCAGGT
GCAACTCTCCAAGGATGGGTTCACCCTCTCCAGTGCCACTCTTTCCGTTAC
GGTCCGAGAAGGACGCATCGCCGAGATGATCGACCTCCAACTCGGTCGCT
CACTCATACCTTCCGGCAGAGACGGCGGCTTCAACTTGTACGAAGACCACC
CGGATGAGTGGGAGTGAGCATCTTCTACCAGGTCTTACATTGCCCGTAGCT
GATATCAGTGCGTGGGACGTAGAGTACTACCATCTAGAAAAGGGAAAGCCC
CTATTCTTCGACTCCTACAAAGTGGTGCACGAGAGCCCATTGCGGGTCACC
GTCGCATGTCAGATCGCTTTCGGCGCCAGCACCGCCACGGTCAACATCACC
TTGGACGGAATAGGTGCCTCGCCCAGACATGACTCTCGTAGTCTTGTCCGT
TTCGATCTCGACGTTGACTGGCACGAAGACCACACGTTCTTCAAGTTCGAG
CTGCCCACTACAGTATGGGCACCTGAAGCGACGTATGATGCCGCCTTTGGG
GTGGTCCGCCGCCCCACGCATAGGAATACGACGTGGGACAGCGCCAAGTT
TGAGGTGTGCGGGCACAAGTTTGCCGATTGTAAGCCGGCGCCACTCCCTTC
ATCAGTTGAATGCTCATTCTTGTAGTGTCTGAGTATGGCTACGGCGTCGCCC
TTGTCACCGAGGCCAAGTACGGCTACGCGGTCCAGGGCAACGTCATGCGC
CTGTCACTGCTGAGATCGCCCACTGCGCCGCATGCGACCTGTGACCGAGG
GAAGCAGAACTTCAGCTTCGCCGTCTACCCTCATGTTGGAACGTTCGGCGA
GTCGGACGTGCCAGAGGTAGCGCATGCGTTCAACAACCCCCTCATGCGTG
AGTCAGCGCATCTAACGTCAGAACGAGCATGCTGATGAGACAGTCCGGTAT
GGCACCCCTTCTCCTCTTCTCAAGAAGACTCCTTTCAGCTTGACTGGCGCG
ACCAATGTCATGTTGGAGACCATCAAGCGCGGCGAGGCGGACTTTGATGC
ATTGTCACACAGGGAGAACGGAGGGAAGACTGTCATCTTGCGTCTGTTCG
AGCACAAGGGCGGTGCGGCGACCGTGCACCTGAACATGTGAGTCACCGAA
ACATGATTCATGCGGCGACTTGCTGATGGTCGGTCACGGTCTCGCGTTTGC
CGCTGCTCATATCGTCAACATCGTGGAGGATGAGCTCAAGGAGGTTCCTTT

GGAGCGCACGGCGCAAGGGTCCGTGCTGAAACTCGCGTTTACGGCGTTCC
AAATCCAGACTGTCAAGTTGACGATGGGCTGAGTCGGCGTATGTACATAAA
ATGAAGCAAATACAATGCGTTTTGAAGCTCTGCCTCATCCTAGAATGGATGC
GCATGCTTTACGAATTACTGTCACTGCGAGTCATTAGGAATGTCTGCCAAGA
ATCGTGACCTGCCGGGGCCCAGGCAGCGCGCGGCGGGGGCGCGTTGGGGC
AGGGCAGCTCGACGGAGAAAAGGCGAGAGAACGGCCAACGACCGGTTCA
TATCCCGAGTCTAGCCGTTGTGATTGTTGTCTTTCGTCTCCTCGCCACCTTA
ACGACCAGTGCAGCCGAGCTGGTCTCTCTGGTCATCTCTGCAACATGTACA
ACCCACAAGGACACGCTAATGCCATCGCCGGGCCGTCAACAATTCCGGAC
ATCATCTCCGCGCCTTTCGACCTGGATGATTTCGCTGGGTCGCCCCTGGGG
GCTCCTTCGACATGTCCCTCGGCACTATAGACCCTCGCCCCAAGCGAAAAG
CGTTCAGATCATCCAGAGCCTGCCTGCCCGTGAGACGAGCCTCTGTCCTCC
GACATGCTACGGCTGACAACTAGTGCCGTGCTCGTAGATCAAAGTGCGCGT
CGGTACCGCCTGGCCCATGTCCCAGCTGCCTGGAGAACGAAACGCGTTGT
GAATGGCCACTCAAAGATGGCCGCTCCAGCAGAGCATCGGCGCGAAAGAG
GTCCACTCCTCGAGTCTCGACCAGCAGTGCCCTACCATTCGCAGATCCTCA
TATGCAGGTCGCAGGTGAGCTCATTCCTCCCCGGCCGGGTCAACCGTGTTC
ATCTCAGATCCCAACCTTCCAGGGATCCTCGATCCTCCTCTGCCTGTCCCCA
CATCCTCCCAGCCGCCCTTTATGTGGTTCGACACCTCTCCATCGCAGCAGCT
TCCCTTTCCAGATCCATCACTCGCTTGGGCCGCCGCATCGAGGCTACCCAC
CATACAAGAGGGCAGCGTGGGGGACCAGTCAACCCCATCCGACACGTCGC
CTACCGACAGACCCGCCGGTTCCGCCGCCAGGATCGTCAAGATCACCTGGT
GGCGCCCTCATGGCCAGACCGCCATCGCACCCGGACTCAAGCGCACCACA
CTTCGAGTCAAAGTGGACGTTCCAGAACAGCGATGGCGGAGTCTGTCGTC
GGATTCCAGATTGGGTATCACCTCTCCCGGGGGCGGTGTCAAGGTGCTGGA
TGATCAAGGGTGTCCGAACTCGGCGATCATGAAGCATCTTCTAGACATCTT
CTTGCTGCACTTCGGTTCCCAGTACCCGTTCGTCGAGCGGTCGGTTATGGA
AGAGATGGTGGAAAAGGGCACGGGATCGGTGTTCCTCCTCAACGCCATTG
CTTCTGTCGCAGCTAGGTCAGCACCGCCCAATTGTCGCCGCCGCAATTGAC
TTCAGGTTCTCGACCCATCCGGTCATCGCGCAAGCCCATCTGCAGCCTTAC
CAGTATGGCAATGTCTTTAGCGACCAAGCCAAGTCGATGCTGGGGATGATG
CTGGGCATTCCGTCCCGCGAGACAGTGTTAGCGTTGATCTTGCTCGCTCTC
GCCTCCGCGGGCAATGGTGAGTTATCATAGCGACTCCTGGAGCTCACGTCA
GATTCCGAGTCTGAGGTCTGGATTCTGGTTGGCATTGCTGTGAGGATGTCC
ATGGACCTTGGACTGCATCTTGTCAATCCATTCTGCTTCAAACTCTGGCAGA
ATACTGAAGACTCAGGAGCCCGAGCCAGGGTCCCACATCTCTGCCAGGGA
TCGTATTCTGAATCGACTGCTCTTCTGGTCTGTCCTATGGCAAGATCTCGTG
TTGTGCTTTGGCGAAGGGAGACGGACTACCTTCCGGGTGGATGAGATAAC

GCAGTCACTCCCGACCGAGGAAGACATCCGATTGTTGAATCCCGTCACTGG
CTCAGCCACGCTCTCCGCCTTCCCGTATGTCGCTCAGCAGATGCTTCACTAC
GGGCCCATCATCAATCTCTTGAATGCGCCTCGTGAGAGAGACGATGTCGCG
ATACGGGAAGCAGCCCGAGCAGATGCGGTCAAGCACTACAATGAGCTGCC
GGTCGAGCTGCAATGGGGTGTCCGCAAGTCAGTGCCGCTCGTGATCAGGA
GCTGGGCGTTGCGAGACTGACTACAGTCTGGAGCTGCACTGCAGAGCCAA
CCAGGGACAAATATTCATCGTGCTGCATCTGTGGATGCACACCATTCTCGTG
AGTCGATCGCCTAGTCCGCTCAATTGGTCCACCCGTACTCACGTCAAAGAC
TGCCAACGTGTCCCGTGCCGAGCCCTCAGCGGTGCTACACTCGGCTCGCAC
CATCGGGGACATCCTGGTGCTCAGCGACATCATCCAACCCTTTGCCTATCTG
GCTGTCCCATTTGTGAACCAGCCCTTCTACGTTGCCGCCTGCTTGTACATCA
ATGGTGAGATCCACAGTCAAGGCGACCGATCTGATATCAGAGCTGGAACGA
GCACAGAATCCACACGGCTCGACAGAGCACAACTTGCTGCTTACCAACAT
CACGCGCGAGAGCATTGCGACCCTCCGGCAGGGGCTCGCAAAACAGGCA
AAGTACTGGTCCTGCGTCACGCGCATCACGCATGCTCTCGATCAGCACATG
CACGGCTCGCACGATGTGGATCTGGCCGATATCACGGAGCGCATTAGTACT
GTCTTGTCACACCCCGATCGGACACGCGACGAGCCCCTCGATGGACAGGA
CGCTGTCCCTAGTGATTGTCTTCAATAAGTCAGCGGATCCTCTACTGATGAC
AGGTTCGGATGAGCTATTGAACTGGGTACTGGGCAGGGATGCCGAGGATAG
TCAGGCTGGATTGTTGGAGAAGGCAGTGCCATTCTTGACCAATCTTGACTG
GATCACAAGTGGTCATTCCTAGGTCTGCTGTGACGACTTCAGGGTCGAATC
GGCACACGATTGTTATGACAACCTCGTGCTACACTCCACGGCGCAGGATTA
ACCTTGGGTCCAATGGGCTGTACTGCATCCGCCGACTCGCGGATTCCCCAA
CCGTCCCTACGAGACTGCTGTACACCGTTCATGTGTTCCGCCGTCAAAGGA
TGGAGATACACCGCCTTGCGCGATTTTTGTCGCCTCTCACGCTTGCTTCTCA
GGCGGACACACATATGTACTGTATGTAGGGAGGAAAGAAGGGCTGCAATC
ACGGGTGAACTTCTTGCCTCTGCACACGCTATTGGGGTTGCTGCAACAACT
CTTACAGGAAGTTCAGTGAGACAGACCATCTCGCAGTGAGCTCCAGGTCC
TGGCCAGCCAAGATATTGCTCGGGACTGACGAAGCAGGCTGCAGTATGAA
GGTGTGTGTTACCATTGCCATGGGTCTACTCCTCTTTGGAGCCAGCCCCGG
CACCGCAGCATCGAGCAAGGAGCGGTGAGACATTGGCATCCGCATCTCTTC
TTGCCAATTGACAACTGCAGAATCGAAGAGATTGAAGCTGCTTCTGCTCAA
CGTCACGGCAAGATTGGACCACTTCGACTGAAGCCTTCATAGTCCAGCTTG
ATCCCCGGTAAGCTTTGTGCGGCCCGCTCTGAGCCCTGGCTCAGTTGCAGC
CATAGCAACCAGAACGGCGACAATGCTCCATCCTGCAAAAAGAGTTCGGA
TTGCCCTACGGGCTATCACTGCAAGAAGGTGGCAGGGATCAAGTCTTGTGT
CCCCAAGAAAACGCCCTCACAGAGTGGCGATCCGCCGTACAACGGGGCTG
AGCGGTCCGGAGTGGACGTGCAGTAGCCTCGGAGGCGATCCGGCGCATAG

GGAGATCGTCTGCGACATACCACAGACAGCTCCAGAGACTCGAGGCGGGA
AGCAAGTTCGCGATGCATCATAGCCCGGCAAATGTGCGGTATTTATACTGAC
GACTTGGACCACGCCGCCAGACGAGGAAACAGTGCTCTGGCATTCTTCTC
CCATACCCTTTGTGTTTCCTCTGCGCCCAGCCGCTCATCAACGGCTTGGCTG
AATGCCACCATGCCCTCTCGGACAGCGTATGGGTGGTCACTGCCGATGACG
AGCTTGTCTTCGGGGACGAATGCTTTGGTGCAGTCGAAGGCGGGCGACGT
CCCAACGAGCGCGGTGTCGTAGTGGAATGACTTGAAGCCTTTGAGGACCT
GATCGGGGGAGGTAGGATTCTTGAGGGCTGGGGCAGAAGTCGTGAATGCG
ATCCTGTCGGCAAGGAAGGGTATAGTCCCTCTTAGCCGAGTTAGATGCGAG
TTGACGGAAACGCCTGACAAGACTTACCCTCCGTGACACAGGACAATATTC
ACCTTCGAGTATTGCGCCCGGCGCTCAGACATGATGAGGTCCGAGACCTGT
ACAGTCAGAGTAGATCTATAGGACTGAATACTCACTGTCTTGGTTGTCTCGT
GGGGAAAGTCAAAGGCGGGGCACGGGCAGTACTTCTGTTCGGGCATGGGC
TTGTAGCCTTGAGGCGGTGTGGGATGGATGATGACTGTCGTTGCGAGTCGG
TCAAGCTCTGCCCAAAGGGGTTCAAACCTGCAGCGGCATCAGCATATGTCC
ACTGTCGTTCGGTCCACTCACGTCGGATGACCGAGGTACAGTCCATTGTAT
TGAGTAAAAAGGCAGACACCGTCGGCGTTCAGAGTGTCCAAGGCGTAACG
CATCTCGGCCAGACAGCCATCCATGTCTTCGGCGGGGTTGGACAGTGTCGC
GTAGAACGCAAAGCGGTCTGGGTGGGCTTCTCGGACACTGGAAGCATAGA
GGTTGCACTCTCGCGCAAGCTTCCGGTTTCCTTCTTGGTCAAGGAAGAGGC
AGCCTGTCCGCGTCAGTCGTCTCTGACTCGTCTAGCTGGATGAGTGCGAAC
CCGGCGATGTGACGCTCAAGACCGACGTGGTTATCTTGAGCTCATCCTGGA
CGTCGATGGCAGCTTCAGGAGTCCATTCACTGGGACGACGGTCAGAATGC
GCGCCGGGTGACGGGTGGCCAGAGCACTTACGGAATGTGCCAGCCTGCCG
GATCACCTCCAAAAGCCAGTAGAGCCTCTCTGAACTTGGGAGGCACGATG
TGCGCGTGCACGTCTATCTTGATTGGACGACTGGAAGACATGGAGGGGTAA
AATGTGCGGAATCGTGGTTACACTACTGTGGACTGACATGGATATCGCATAA
ATAGCTGAGGGCATCTTATCAAGGCCGACTGTGCCCGGGTAACGGGCGGAA
CCAGCGGATATGCACAAGGCGGGGCTCTGCGGGAAGGAGCCCTTCTCAGA
ACATGTATGGCGAAACTGTTTGTATGAACTGAACGATCCGCACCGCCTATCA
ACGTCTCGGTTCCAGCATGGCTCCCCTCTCCTCAGTCTTGGCCTTGTCCCCT
CGCTCTCGCCAGCCATTGAGTTTGTCTGGGAACTCGGGTATGACCCCTTCC
AAAGCAGGATCATGCGCTATAGCTACCAAAACATTGTCCATCTTGTGCGCC
AACGCAACTTTGGAAATACTGTCCCGAGCGTCCGAGGGCTTTGCATGCATC
GAAGTGGTCGGTCCAGGGCCGTATGTGGTGCCAATGTCCCACCCAGAACC
GCAGCACGGCGAGAAGAAGTGCCGTGCGTGGCATACATCGCCGCCCATGA
ACACGTACCTCTCATTCAGCCACATATCCCGGCCTAGCGGAATGGCCTGGA
CTCACTCGTCTTCGCCTGTCCGTGCCAGAGCTCCAAGGTGACCGGGCATAT

GACCCGGTGTATCGACCAGCAGAAAGCTGCCATCGCCAAAGTAGTCGTGC
ACCCGCTCGAATGGGCCCCAAGACGTCCAGATGTCATCGTCGTAGGACATC
TCCCTGACCTTGCCTGACGGAGGGTGGAAGCAGTAGGTCATGAATGGGCC
ATCCCGCTCAGGCCAGCCGGGGTATGCGGCAGCTTTAGCCCCAGGTCCGAC
GACGACCTCGGCACGGGGGAAGAGCCTAGGATCTCCCTTGACCGTCAGCT
CCAACATATGACTATCAGGGCGAAGACACACAATGTGATCCCAGTGTAGAT
GAGAGTAGATGATCGCGTCGATATCGGACGTCTCGATCTCCCCAGTACGCA
GGATGTCTGCGGCGTCTTGAGGTACCTGTTATCAGGCTACGCTCTCCCTCGA
AATGGTCTATCTCACTTCCGGTGTCACAATCGAGACGTCCTCCTGGAGAGC
CTGCGAGTAGTCGCTCAGCCTCTAGCCTTGTTAGCCTGAGCCCAGAATCGC
TCTGGTGAGAAGAGACGGACCTTGTTGAACCCCATGTCGAACATGAGCTT
GGCTCCCGAGGGATGCTGAATGTGAAAAGCATAGTCAGGACACCTGTCCA
GCTTTTCCGAATGCTCTTCTCCTGGAGACGGTCAGCTCATGCACATCCCGA
GGTGACTCACCAGCAAACAGCCATTTCCGCGGGAGGCTGAGCCATCCGCA
AGGCATCGCGAACACGTCCACTATGACTGCTCCCATGTTGAGCTACCTGAT
CTGTTATGCGAACTCGATCTGTGAAGGATGGTAGCGTTGCGAGATTGAGCT
GGCATGATACCGGTACATGTTGCAGATGAGCAGGAACAAGGCAGTCGTCA
CCGGGGAAGAGGCGGGAAGATGAGCTCCCCGCATCGGAGCTCTCGCCGAG
CCACCGGGCCCCAGCCGGCATCTCCTCTTGCCCTGATGCATTCGTCTATGGT
GTATCTCGCTCATATGTTGCGAATCCATGAAACAGCACATGATGTACGGTCC
AAAGAGCTGTAGAGCACGGAAGACAACATGTCGACAATCAACGGAACGA
GCCTAGATCTGACCATTCCCAGTCAGCATATTGCCTGTGTTTCGGAGAAGGT
GGTGAGTGCGGGTGGACCCGTTCTGAGAAGCTTCGTCAAGCAGGCTGAGG
CATTCAGGGCGACTATCCCAGCATTCGCAATGTGCCTACTCCCAATGCCGA
GTCGATCCCCGCCGGACACGCTCTTGTTCGGATCGCCTTTGCTGGGGTGTG
TCACTCGGACGTCGCCCTTGCTCTCGGGCAAGGTACGCACAGACCGGTCG
AGCCCGTCATCTCCGGCCACGAAGGCGTAGGATACGTCGTGGCACTTGGCC
CGCACATCAGGCCGGGACGTCCGACCGCTGTTGGAAGCCGGGTCGGCATC
AAGTTCATAGCGGACACATGCGGGACGTGCGGCGCATGTCTGGCGGGCGA
CGAGGAGATGTGCGATACGGTGAGTTCTATGCTTAAGCCGCCTTCGTATGG
ACTTGAGTTGAGGTTCAGCCGGAAGTCGCAGGTATCAGCAGGCAGGGCGT
CTGTGAGTAAACAACGCACTTCGTGTCTGGAGTGAGCTAATCTCTCTAGTC
CAGCAGTACGTCATCGCTTCGACCATGCACTTGGTCCCGATCCCGGACGAT
CTCAGTATGGAGGTCGCCGCTCCGGTACTCTGCGCTGGATTAACGAGTGAG
CCGCTCTTTTGTCCCTGATTGAGCTCATGCTGATGATGAACAGCCTACAAAG
CTGTTCTGAGCTGCAATCTCAGACCCGGAGACTGGATCGTTGTACCGGGTG
CGGGCGGGGGTTTGGGGCATCTCGCAGTCCAGGTTGGCAGGGTTCAGCTG
CAACTGAGAGTTTCACTGAACGAGGTAGTACGCGCTGGCGCTCAATTTGA

GAGTGATTGCCATAGACACCGGTCGAGAGAAACAAGAATTGATGGAGTCG
TATGGCGTACACTGCTGGATCGACTTTGCCACTGAAACAGTCAGTTCCCAG
TTCAGCGGAGAGACTGCACGCTGATAGGTGGCAGGACATCGTCGGAGCCA
TCGCAAAAGTCACGTCTGGACGGATGGCCCGTGCGGCCATCGTCACAGCG
GGATCCATCAAGCCATACCAGATGGCGATGAGCTACCTCTCGAACCGGTCC
ACTTTGGTTTGTGTGGGATTCCCAAAGGGCCAGACAATGCAGGTCGACCCC
ACGCAGATGGTGCTGAAGTCTATGCGGATCATCGGGAGCTCGGTCGGGAAT
AGGCGGGAGCTCAAGGAGGCACTGGAGCTCGTTGCGATGGGCAAGGTCA
AGGTCGAGCTCACGCAGCGACCGCTGGAGGAGCTGGCGGAAGTGTACCG
GGATCTCGAGGGCGCCAAAGTCCGGGGCAGAGTAGTCGTCAAGATGTCTT
AGGGCAATTACGAGTAGTTTGCACTCATGCACTTGCAATAGTAACCGAGTT
TTACACACACCCCTCGTAAGGCAGGCTTTCATTGGTAGCTTCAGCTCCTAA
AGTTGGTAGATCGGTACATAGACATGGGCGCCGGTAAATGTGACAATAGTAT
ATCTAGCAAGACGCCAGCGAATCCTGCGCAAGGAGACCAGCGGCCACCAT
CTCCCTCAGGTTGCCTTCCTCTGAGACGTCCGCTTGCTTTTGTGCCTTGGCA
TTAGGGAAGCGCTCGTTCAGAAAGCGATCCGCAACCGCGTCGACATCATAG
CCGTACAGCCACACTTGGGTGGACGGGTCCGACCACTGGTTGGCCGAGCG
GACATAGTGCTCGTCCGACAGAGTAGCCGCCATACCTTCATCTCTCAATCGC
TGAGCGGGGCCATCTGCGACTGTTGATCAGCTTGGCCACATCGAAACACAC
CATTGGAGAGATGAGGACTGACAGTGCCAGATACGCCCATTCAACGCGCTG
CGGTATTGCACCTGGTACGCGCGCTCTATTCTTATTTCTTGGAACGCACTCG
TCAGGTGAACGAGGTCCCCGAATTTGGATTTGCCATATCGCGCCAAGCAGC
AGCCGAGCGCTGCGGCATCTTCGATCGCCTGCGCGGCGCCTTGAGACATAT
ACGGGACCTGCAGACTCAGCACGAACCGGGAGGTTGGAGTGTAAGACCA
ACCATTGCATGGACCGCATCACCCATCATGACAAACTTGCCTGATGGGTGC
TTCCAGCTGTCCAGTCGCGGGTATGCCATGAGCTGCCACTTGGCTGTGCTC
TGAATCTGGTTGATGATCTTGACCAGCCTGGCGAGTGTCAGCTCGTGAATA
GACCTGCAAGAGTGTATCACTTTGGGTCCCACCCGGCATAATTGTCTTTCAT
CTCCTGCAACACTGCGTCGGCGTCTTTGGGCCAGCCATCCGTCTGGCCGTT
GTATGGGTGAGACAGGACCATGTTGAAAGACTTGCCGCCGCTGATTGGGTA
GGTCATGACGTGTCGGTCGGGGCCTATCCAGAGACTGATGTCGGGCCGTTC
GACGATATCTGCCAGCTCAGGATCGGCGAGCAAGATGTCCTTTGCGCTGCG
TCTCTTGCGCGACTCACGAAGACTGAAGAACAAATCAAAAACCTCCCTTTT
GAAACGACCGCTTTTTCTCCGACGACATTGCTTTCATTTACTCGAGCGACTT
CCAAGCCTACTTCTCAAAAGTGGTGCATCGATTAAGGCATATAAGAAGTCG
TTGGGCTCTTTGACCTAGAGAGGCGATTGAGATGATTTCGTATTTTCTTAGT
GGATTTGTCCGTAGGTAACCGAGTACCCAAGATTTCAGGATATCCCCAACA
CGCCGGGGGTAGACAATGGCCTGCCGTTCGAGGTTAAAGTGACCGTAGTTT

TAAGGTAGGTTAGCAAACACGAAAGGAATTATCGAGTAAATAATCGAAAGA
TATTGGTAGCCATTATTATCTCACGGATTATATTATAAAACAAGCACCAGTTC
GAGCTGCATAATCTTGGTAGCAATCGCGTGTGACCTTAAAATCGACAGCTAT
TCAACGACCCAGCTTTAGGTAATGTTTCTGATCAACATGTTCAATTCAGCTC
TGAAAAGTGTAATTGATTGCCATTTATTAGCTAAAACAACCCAATCCCCATA
CAATACACTTTACAGTATCAAGTCTGCCGCTCTCTCAGCCACGGCATAAAG
CGTCGCCGTCAAGTGACCACTTATCTGAGTTGGCAGGATAGAAGCATCAAC
AATACGGACGTTTGAGGCGCCGTAGAGTTGCAAGCTGGGGTTCACGACAC
CACCAAGCTCGCGAGACATCATCGAAGCCGTGCCTAGCGCATGGTGATTTG
ATGTTAGCAGAGATCCGAGAGCTGTTGCCCACTGCGCGTCGCTGGCGTTGC
GAGGTAGGTCTGCATCACCAGGGACCAAATAGCCGGCGACCAATTCACTG
ATCGGCTCCGTATACCAGAACTCTTGCGATATGCGGCCTATCCCGGTTTGAA
CGGTGAGATCAAAGTCGATATCGAAGTATTGTGGATCAATAGAAATAGCCG
GACTGTCGGTGTCTTCTGCTGAGTTAATGTGAACACTGCCCCGGGAAAAAG
GCAGCAGTGTCCAGAACGCTGATATTAATATATTGCCGGAACCAGACGTCA
AGCTTTCCGCGATGGTTACATGTTTATTGAATATGAGATCGTGTTGGATTTGA
AACATCTTTTCTAACTGACTCGGGGTAATGGCTCCTCCGTTGGCGTCCGAG
ACTTTCTGAGCATATTCGGGGATTTTGGAGTAGATTTCATCCGCCACGGTGG
AAGTCTGGTTGCCAAATATGTCTTGGGCCGTAGCGAATGTTGCGTAAGGCA
TGGTTCCTGTCAAGTTGACGCTTGAAGAATACTGCATTGACGTGTTTGGCT
GATCAATTAGATTCTCACCCACGCCATTGAGCTCTATCTTCGTCTCAATGCC
ATATTTCTCCAATATCTTCGGATTCCCAACTCCCGATCTTTCCAAGATTGGCG
GGGTTCTCAAGGACCCAGCGGCCAGCAAGACCTCTCTCGTTGCATCAATG
GTAATCGTCTTGTTGGCAGAGTCGCGATACTCGACCCCATCAGCTGTAAGT
AGGCTATTCGTAGATGAATTCGCCCACGCAATCTTGGTTACCGTACCCTGAA
TGACCTTGAGATTGGGCCTTTGCTCAATGGGATAATAAAATGCGCGTGCGG
CGTCTTCACGTATGTCCGCGTCTCTATCCAGAGTCTGCGGCCAGATGCTGA
AACCAGCAGTGCTCCCGCCGTTAACATCAGAATTCAGAGGATACCCCAACG
TCTGCCAGGCCTGTTGGGCCTTTTCGAAGAAGGTCCCATTGAGCAATTCGA
ACGGGAACCCGGTCGTTAAAGCGCCATCTTCGCCGTGGTATTCTGGATTGA
AACTCGCGCCTGCGGCTGTTTGCGCGGCAGTCGGGTAGGTGAAGCGTTCG
ACACCTTTGAAATACGGATATAAAGATTCCCAATTCCAACCATCATTTCCCA
AGGTTTCCCAAGCGTCGAATTCGGCCTTGTCACCTCGGATATAGGTCATCCC
ATTGATTGTGCTGGTCCTATTTGGAATGTAAGTTCACAGATTCTCAAATGCT
CTTCCTAACTCACCCGCCTATTGCTTTTCCTGCATGGTAAGCAAGGGTCCGG
TTCTTTGCGCCTGGTTGCAATGTCGTAGTATACTGCCAATCTATCGCTGTGC
CAAAGGCACGGAGGAACCGTGTCACATCTGTCACATTGATGTTATCGCGAA
CGTCACCGCCCGGCTCAATAACGGCCACCCTGACAGCGGGGTCTTCGCTCA

ATCGGTTTGCGAGGACCAAGCCGCAGGTTCCGCCACCTGCTATGATATAATC
AAACGACTCGCATCTCACCTTTGCGGCAGACGCGAACAAGATCAAAGAGA
GAGTTGCCTTCATCGTCAGCTGTGTTTCGTGTGACGAAAGCTTGCCTGTCC
ACAGAATTACCTGTGATGCTCAACTGTGTTGAAAGATCAACATCAAATAAC
ACGAGAAAAAAGCAATTGACTTCACGATCCATAGAAATCCTAGCACGGTCA
CGTTCGCTTACATCGTGCTGTCTTACGGACCGATTATTCCTACCTAGGTATAT
AGAGCTGTGTTTCTTTCAGCCATTGCTCATCAAATTGTTGCCTTGTGTTACC
CCAATTTTCGAGCTGGCTTCTGAAATGGCTAGAACGGCTGACATTGCGGGG
AAGATATACGGCTAGAAGTGCGAGGATCAGATTCCTACGGAAATTGATCCG
TTTGCTGAAGGGGTTGGAGAGTGACGACGAGGGCATCGATTGATGCAGGT
TTCGTATGAATTAGAAATGTGAAGACCACAGCGCGTTAACAAAGGTTACTA
GGTATTATTCCCGTCCCGCTTATTTTGATTAGGTAAGTTGGCGGCATCCGCCA
CGGTGGATCTATTTTCCGAGACTCTCTTACCTAATCGTCTTACTGGACAATTT
CGCATCACGGTTTGATGATTATCGGAGTTTGGCGGTTCGATGCATCGGAGTT
CAAATGTTTTCCATCCGTTTGCCGCCCCGGAAATACCCAGATGAGCCACGG
AATTCCATCCCTCAAAGAGACTAGTGGTTTCGGAGAGATATCGTATCCAAG
AGTGACGCTTAAACTCGCGTGTTCTAGCGGCGCAAGAGCAGGATAGCTTCT
TGTTCTCGCGAAATTAAGGACAGACGATAGAGGGGAGTCAAAAGACAATC
GACCTAGGAAATCTGATTGGGCAGCGCCTCGGTTCGCTGCCGTCCAAAATG
AGCGCACGGACTTGGATTATTGGCAAACAAGAGGAATGGCAATTGCTCAG
GTGGTCGTTTGGTCTTTATGATTTTTCATCGGCTCATTCTGGGTTTGGAGGA
GGAGCATAAGTATATATAGCAGAGAAGAGAATGAAACTTGGAAAACCAAC
ATTCTTAGGTATTTTACCTGGTACACGTCCTTGATGCTTTTGCAAATGGATGT
GGTGCATGTGGTACCTGAGATCCTCTGAATGGGTCTAAGTCGCAGCAGATC
TTCCGGGAATAGGAAGGTGGATAGCCACACACACTGCCGGTTGCCGCCCG
ACGAAGCTGAGCCGTCTGCCAATCACGTCATCAGACTCGCTGTCGTGCAG
AAGACGTACTCCGGACTGCCCCTTTCATTGACCGCGCCCAGACGGCAGCTT
CGAAGGAGAACATGGGTTGAGAGGCCAGCCGGCCAGACCGTACTTCACCC
ATCCCATAAATAGTGTCCCATAGATGTTATTGCCCGCATAATATCGATCGCGC
ACCGTATCTCGTCTCCACGAGCGGACCCGCGTGCTGCCGAAAGTGGAAGG
CGGGCGGCGAACCCCTGAAGGATCTCAAGTTGAGTTACGAGCTCCTTTGTA
ACGTTAGTAATGTCAGGTAGCCTCTCCGAGGGTTCCTGGCCAGTAGTTTGT
CCCATGAATCTATTCAATGACCCGGATTGGATATTTCACATGCTGATCTGATC
TTACTTGAGCTTATATTGACTTTTGTCCTGATCCCCTCTCCTGTAGGAGTGGT
AGGCCGGATGCATTTAAAGGGCACCGATCTCTCCGTTCTTGGCACATCGAA
ATCACCCTCGTTGCCATCCTCAGTAAACCGGCTCAATCCCTCTTTAGTCCTA
ATTTGCCCATAATGTTCGGCTCTAAGGTAATCCAGACCTTGCCAAGACAGAT
CTATCTGAAAGGATTCTCTGACATCGATTATCTAGATCCTCTCCACCCTCTTC

TTGGCGGCTGTTGCGTCCGCTGTCGCCATCAGCGAGAGACAGAGCCAGGA
CTGTGTCGCCACTTGTGGGTCCGTTTGCTATTGGCAATCTGATATCGATGCC
GCCGTGAAGCAAGGATACGGCTACTACCAGAACGGCCAAACCGTGGGCAG
CGACAAGTACCCGCACGTCTACAACGACTACGAAGGCTGTATGTAATGCCG
ATAGTTCTGCTTGCTGTTCATCTGAAGAAGGGATCATTGACTTTTGGGTAAT
GCAGTCGATTTCCCCGACAGTGGTACCTACTACGAGTTCCCCATCCTATCAA
GCTTCGATGTCTACACTGGAGGATCGCCCGGGTAAGTAATGCCAAAGGGAA
ATGATAGGAGTTTCTATTAACATGGATTTATAGCGCCGACCGAGTTGTGTTC
TTAGGAAGCGATGGAAACTACGAGGGTTCCATCACTCACACCGGCGCGTCT
GGAAACAACTTTGTTCAATGCACTTAAGTTGTGGAGATGCTGATGTGTTGG
TCCTAATGGGTATCACGTGACACCCAGCACGTCTCAATACCTATAAGACACA
CGATTATCAAGACAATTCTGTAATATATTTATGGATATTACGTACAGTATATAT
ATTCAAACTTTCTTCATCGGTTGTTCAAGCTGTAGAATCTCCTTCATCGCAA
TGCTAGATGCAGTCGTAAGATGTGTTGATTAATCCTTTAACACACTATTCTAT
CTATCCTTCGGTGGGCTCGAAAGAGAGGAAATGATGAAATCAGAATGAAA
GGGGAAATTGGATATTAGTGAAGAGATAAGATGATAAATAATGAACGGAAG
TTGGTAATTTAAGATATGGTAGATACTGCTTAGGTATATATGTATTTATCATCG
TCCATCATCTGGCATCGGAACGAGAAAGCGGGACGACCAGTCCGTACGAA
AATGGTTGCCTGGTCATTTGCGGAAGGAGCCCCTCAAACTTAGACGATGAA
AAGAGGCAATCAGACAGTGGGGTTAATGCGTGTACAGTACGGACAAGAAA
TAATAAAAAAGTTGAGTCACCGTCTCCTAGCGCGTCGCCATTCGGTCATCG
CCTGGCAGAACTTATATAACCACCCAACCCCGCGTCCCATTTTCTTTCCTTC
TTTTTCTTTCTTTCTTCCCATAAGTAGCCTAACTTTTAACGTGAGTTTAACGC
ATACAGGTTCATATTCACAGACCATCTAAAGAAGTTCAGTTTATATTAACTG
ATCATCTAAAGAAGTTCACTGGTTCACTGTCTTTTTGACTCGACTATTGAAT
TTTGATCTTCTTGATCTTTATCTCCTAAGCTGAAGCTGAAGCTTTGCGACAT
CCCAGATGACTTCCCACCACTTCGCGGCACAGCGCGCCGTAAAGGACTGTT
ATCTTCTGAAGTCATCGGTCGGGCCAGTGGTTCGATCGGATGTTTTCACAAT
TAACCCGATTGTCACTGGATCTAGTAACGGGAGCTCTTAGTTCGGGTTTTCA
CTTAGATTCAGCGACAATCCTATGAAAAATGAAGAGATGGGCTTGGGGGTT
CACTACACTTGTAAGTTTCCTATCATCTTTCAGTAGTCGAAACGACAACATT
TACTAATTCATCAATCTATCAGACGATGAAGACAGTAAGGGTCTTCGTATTA
CCAATCAACATCTGATCACTATCAATTTCCCACGGGAACAAACAACCATGT
CAATCAAGACAGCCGATGAGCAGCTGATGAAGTTACTCCCTGGAACTCCG
GCAAACCTCAGTTTGATTACCTTTGAAGTCGAGCAAGATGCTTCGATATTTG
TTAAAGGATTTGGCGTCCCTTTCGGTGATTACCAACAATCAGAAACAAGCA
AGGATACGACTATCACGGATGACGTGATCATAGCCGAGCTTTTGCAGAAGA
AAGAATTCTTTTTCTTGGTTACATCACCAAGCGATATGTTGTCGAAGATCCT

AGACATGGGCAAATGCATGGCTAATCCTCGAGCCTCTAACCCATACGGCAA
CAACCATGGCTGGAACATGGATAAATACAAACTGCAATACTCACAAATAAA
GAGTATGCGGCCCTTACCAGCTTCTCATAGGTAAGATACCCTGGTTCCCTTG
TATCTGATTTTTATTTTCTGCTTCTCGTATCGGATGCCTCCGTGCACTCCCTT
ATTGTTTTCAACATGAACACGCTACTAACATACACCAAATTAGTTTCCATGA
CGATGACGAGCACATTCGATCCCTCGAGCAGGCAGTTGTGCAGGACGTATT
CTGGTTAGAGATGGCTGCCGACCAGATCGCGCAGCGCAGGTTGGTCGGCT
ACTTCGTTCCCCTGAACGGGATACCTCTTTCCCAATCTGGGTCCGACACCC
GCTATATGATCGTTAAACACGATGGAATTCCCAGTGGCTTGTATAAAGCGGC
ATGGAGACGCCTCACCAAGGACGGCTCCTTAAAAATAGTAATCTATGAGTC
GTGGGAAGACGAGGATCCTGTAGCCAAATGGTATGTCTACCTACACTCCCC
GCTTTCCATGTGTCATAACTAACTTAGGGTATTAGGAATGGAACCATCATCG
ACCATCCGGATACTATGGTAGAGCTACAGGGTCACGCCGTCGATGCAAAGA
ACGAGCTGGTGTTTGCCCTTCAAATTCCTCGGCTTCCCCCTCAAGGGGCCA
AAATGCCTAGTAGATCTTGCATGAAGGTCTACACGAGTCGCTCAAATGCAC
AGAGGGCCTTACTGAAGGGAGAGAGCCATTGGAACTGTCTAGCATTACGG
TTCGCTTCCCAGCTCACCGATCCCGAACGAAGAGTGAGCGCCATCTGCGAC
TTTCGGCCAGAATCATTACCGTCAAACCGACGTACTCTTACAGATCTTTCAG
CCGAAATTAAAGAGGTCAAGCTTGCAGAGATCAATGACCGCATGGACTTGC
ACCGCGCGCTCTTGCGTGGTAATGGTTTTTACGACTGGATGACCAGCCCTG
CCCCAAGTGTAGGCTTAGTCTCCTCGATAGATACCATGAGTATCCGAAACAA
GCCATCCTTACGTCCTCTTCCCACCGAAAATTTTTTAGAAACGAGTAATCCG
ACATTCATTAATGCTTTGCTTGATGAACTTCTACCAGAGGACCAGCAAAGAT
TCAAACGCTACCTGCAGTGCCGACCTCTAGGATTCGGATTGATCACGGCTG
CGCCGGGCTTCGGAAAGACGAGCATCATTGCTGTCGTCGCTGCTTTGATGA
AAGTTCGTTTTGGTGTCATCCTCTGCTCTGCCCCGTCTCACGTAGCAGTATC
GAACCTTGCGGAACGTATTTACAAGGCCGTTGCTTCGGCCTGTGAGCGCCT
GAACCGAGACAAACCCGAAGCAGACATTCCAAGTCGTCGTTTGCTAGTAG
TCCGAGGTTACAAGGACGTCGAGGAGGTAACCGCAACTATTTGCATTCTTC
GAAACCCCGAGGCTGACCCTACAGTCACGGCAAGGGAGGCCCGTGTGACG
TGTCATTGGAAGTTAAACCTGTCTTTGGCATATTGGGTTCTTTGCTGCCTAC
GCTCTACGGGCGTTAAGGGATTGCACGAGGACGATAGCGCAGCTTTGCACG
ACCTACGAGCCAAGATCGACGACTCTCAGGAACTCAGTAAACTTCGAGAT
GTCGCGACTGGTCGAATGTCGTGGAAAGAGTACAGCAACTCTGGAGACGA
CATGAAGGGACATATAGTCCGCATCATGCAAGAAATTTTCCCCATAATCGAT
ATTCTTGCAATTACACCTGCCGCAACAGTCAACATTAGGTCGTATGCAGTAT
GGAAGTCTAACAGGGCAATGGGTGTTGTGGTCGATGAAGCTGCCGCAATG
CACCGCGCTGATCTATACACGGTGTGGGGAAACACATTGCTACCCTGCTTC

CTTGCTGGAGATCCTTACCAGCTCCCGCCTGCAGTTCTGACTGAGAGGGAG
ACCGATGGAGAGGGAAACCTCTTCAACCGTTTCGCTGCCACGGGTAGGGT
CTCTGCACTCGAGGGATTCTTGGCTTCGGGCATGCCTGTGTTCCGCATGCG
AGTGCAACTACGGATGGCCCGCGGGCTTTTCGACGTGGTATCACAGACCAT
CTACAACGTGCCTCCTAGGTACCACTCATCATGCGGTGTAGAGAACGAGAA
GTTCAAAGCTGGACGCGAACTCGAGGCTTTCATCACAAGTCGATACCCGG
ACGTTTCACCCTCGCTCCCCGACAGACTGAGCCCGGTGTTCATACAATGCG
AAGGTACGAAGGTGGAGACGAACCCCACTACCGGTTCGAAGAAAAGTCA
CGATCAGGTCGTTGTGGCGCTGAACTTGATCTACGACTTGGTCAAAGAAAC
TAGCGTGAACGCAAGCCAAGTCACAGTTCTGTCCCCGTACTCGGCCAACG
TGGAGTTGATCCACACCCTGCTCCTGCGGGAGAAGTTCGCGAGGTCTCTCG
CCGAAATAGGCCAGCCCTCAACCATCGACGGCTATCAAGGCAAGGAAAAT
GACATAGTCGTCGTGGTGATGGGAAGCTCGGGCAGAACAGGCCCGGGCTT
TACCGCCAACAGGCATCGCCTCAACGTTCTGTTAACGAGGCAGAGGGCTG
GCCTCGTCGTGGTGGGAGACCTCCGAGCTGCCTACCTGGACGGACAGCCA
AACCAGCCGGCTCGCATCGCGAGGTACGCGCCAAACCT

>Mosaic_sequence_40

GTATTCTCCAAGGAGATCGCAAGCCGAATGTAGTAGTTGTGTGACGAGACT
GCGAGACTTGACAACCTCGCTGCATGTTGTACCTGGCCAAAAGGGAACTAT
CTACATGCAGCCATAGTCGTTCCCCACCCTGCCACCGCGACCCTGGCAAAG
TAACCGATATATACTTATCTACCTTGTACTCCCGTGCCCATCTCATCGTCGGT
CTGGATCCTTCTACTCCCTTTTCGTTTGTCGTCGCCAGATCGTTGTGATCTA
ATTCAATGGCCGCTCTTACTGCCTTTCAGTATCATCAAGTCCCGTGAACTGG
GGAGTCTGGTATTCCTCTTCGCGCGACGAACATTCATTCGTTCAAATTATTT
CCAATGGTATACGCCCAACATAAAAATCGACACGTAAATGCTTCCATAACCA
AGTTTCGACTCATGTAGCTAGTGAAAGATACCTAAGACGGGGCCAAGAGA
GACGAGAATTATTGATTCTAAATCGCTCTCGCGCTTCCGCGCCCGAGATCCC
AATCTGGTCGGCGCATTAAACGTCGAGCGCTCGACGTTAGCGAGTCCTGTC
GATATCGCCTAGGTATCTTCTAACCCCAACATGGCCGAAGAACCAGTATCAG
CCATGGCGGGTGCGTCAAGGACACTTTCAGAGCTTGCAAAAAGGATATATA
TCCCCTTGACAGCAACGAGCCCGCCGGGTTTGGTGGAAGCGAACACTGTC
GATCCATGGAGTGCATCTGGGAAGTACGGATTGGGATGGACTTACTTCAGC
GTAGCCTTAATGGGGTTGGTGGTGTTGGTACGAATATGGCATTGGTGGCAA
GACAAGATCCGACAGGCGATATACAAGCAAGAGGTGGAGCAACATTACCG
AGATTTATATAGTCTCGACCCGGAATATGCATTGGCGACCGGCATGCACACG
GACCAAACTGGAAGACAGTTCTTCCCGGAGGAGGGCGGAGGAGAGAAGA
GGGACTTCAAGCCGAAATCAGACTTCTCCGCCGTCGGTCCTGTTTTGGACA

CATTCGCTCTCTTTAGATGGGTATTCTACCGACCCATACCGGATATCGTGTGG
CGGAAACATCGATTCACCTTTTCGTCCCTCGCAGTTTTTATTTGTCGGCTTC
ATCGCACTGGCATTCGTGTCTTTATATGTATTCCTCAAGCAACCCCTGTATTG
GGACAGCATCCAATACGGCTCACCTCCGGTAGCGATCCGAGCAGGCATGAT
TGCCGTCGCCATGACCCCTTGGATTATTGCGACCAGCATGAAGGCTAACCT
CCTCACACTCATCACGGGAATCGGACCGGAGCGACTTAATGTGTTTCACCG
ATGGGCCGGTTATCTTTGCCTGTTCCTGGTCCTCGTACACACCATTCCGTTT
TATGTCCAGCCCGTGTGGGACGATGGCGGTATGGCCATATTTCAGACGCTAT
TCCCTCAAGGGAGCGGTGTTATCTACGGTACTGGTATCGCATGTCTAGTACC
ACTGCTCTGGCTCTGTGTCGCGTCGCTTCCGTTTATCCGTAGGATAGCTTAC
GAGCTTTTCGTTACGCTCCACGTGCCAGTCGGCTTCGTCTACCTAGGTTGCT
TATTCTGGCATACTAAAAACTACTTAGGGTCCTGGGATTACTTATATGCTACG
ATAGCTATTTGGGTTATATGCTATTTTCTACGCATCTTTAATCTCAACTGGGTC
AAGCCGTGGCGAATGTCGTTCTTGGTTGGGGGACGAGGCAGCTATCACCCT
TATGACCGAGAATGCTATTAAGATCACGATCCCCACGCAGATGAAGTGGAA
GCCCGGACAATATGTATACCTACGTATGCCTGGCATCTCATTTTTCGACAAC
CATCCTTTCACCATTTCGTCACTATGCAGCGAGGATTTCCCGTCGGAATATG
GCGAACAGTATAGAGACTGCATTCTTGTGTTCAAGCCGTACGGCGGGTTCA
CTCGACGGGTCTTGGAGACGGCTATTGAGAAAGGGCCATTTCACACATACC
GGGCATATCTCGATGGACCCTACGGTGGCATGCGCCGAGAATTAGCTGCATT
CGACACATGCATTCTAATCGCTGGTGGAAGCGGTGTAACTGCACTGATGTC
TCAGTTATTAAACTTAATCAAACGTATGCGAGATGGAAAAGCCATCACAAA
GAAGGTGGTGGTTGTCTGGGCCCTCAAGCGACTCGAGGCGATGGATTGGT
TCCGAGAGGAGTTACGTATCTGCCGAGACTCCGCGCCACCGGAGAGCGTA
ACCTGTAAATTTTTCGTTACTGCAGCCAAGCGACAATCTCGAGTCCCAGCC
CCTCGAGAGCGGGGACCTCGACCCCTCAGCAATATGTTCCATGATAGATTA
GACGGGTTCGTAGCCGGAATCGCCTCAAAGCGTAATTCCCAGTTAATCCGA
GACGAAGCGCAAGGCAACCAGGACTACGAGCGCGAGCTGCGGGCGGAGG
ACGAGGATAGAATTACTGCCCTCCCCCAGCAGAAGTATCTACAACCACACC
ATATCCCACCGCAAGGTCCACCGCCCTCGAATCGCCTGTCACCTCAAGAAG
AGTCACTTCGGAGGCTAGAAGGTCGAGATCACAGCGATACTGAAGATCAA
AGCCACGCAGCACCATCCCTAACGGAGGGCTTCCCCGACGACAAGCCCAA
GCGGGAATTCCACTTCCCGCCGCTGCAGACCCGTCCCGACCAACAGCCCC
ACTTCAACTACGCACCGCCGTCCCCACGACGGATACCGCCCGGTTCCAGCG
AGGACGAAGCTTCAGGCGGTCCCTCGGAGCCCGTAATCCGGCCGCCGGAA
TTTGCACATCTGCGCACGACAAATCTGCCTCCACAACAGCCACGACCGAC
ATCAACGTTCGGACCGCCGTCCGGTTTCGACTTCGGATTCCCTGAGACGCC
GACCGAGTTTCAAAAGAGCCTAATGCGGTTCGCATTCCCGGTACCACATCA

AATCGACGGTGGTTGGAGCGTCGAGTATGGACGTCCGGACTTGGGGTACAT
GCTGCGTGAGTGGACGATGGGAGGCTCGGACGGAAGAGGCATCTTAGGGA
GACGAACGGCGGTGTTCGTTTGCGGGCCGCCAAGCATGCGAGTCGGCGTA
GCGAATACGGTGGCACGGTTGCAGGCGGAGATCTGGGGCGACCCGATGTT
GGAAGAGATTTTCTTGCATACGGAGAACTACGCGCTGTGATCGATATATGGC
ATAACTTAAGCAAAACATTACGACACAAATAAGGGAAATATATAAGATATAG
GGAAAAGGGGGAGAAAAAGAGGAGGGAGGGTTGTACATATCTACATACAT
CATACACCTAATATCCCATGCCGGGACGAGCGATACGATACGACGTACACA
AAACTTAACGACTCTACTTCCGGCGTTTGGGATCGGATACCTCTGGTAGGAT
AGGAAAGGGATAGGGATTAGGGGATGGGGGTGGGAGATACCACGCACAGA
CGGCCAACTTGGGCTTTATAAATCCATATACCAGCCGGGACGGTGCTCCCG
GCGTGTATTTGTATCAAACGGGTTGGTTGGGGACGGGTATGTTAAGGCTATA
AGGGGTCATGTATACCTTACCTATGTGCTTTGGTAGTTGAACCTGGGGCCTC
GAAGCTTGAGCCCGTGAAATGTAGTTTAGGGTGAGGGCTTGGATGGATGG
AATTGGACTACGTGCTTTGTGAGCGCGTCGAGCGTATACCTACTAGGTATCT
ACTTGTGTGCCGATTTACTTGGGTACTAACTTAATCTACTCCGATGACTGTT
ATTCCTTTCCTTGCGCGTGTGATTATCTAGATATTTGCGAATGATTGCTCATA
GGTTTAATGGGTAGCCCTGAGAAATGCCCCTTGTTATCATAGCTGCTATTCT
CTAGCCAACCAAGTTTCCAAGATTAGGTACACTGTACGCAAAATCCCTTATG
ACACATCCATGATCCATTACCTCCCAAGCAAATTCCCAATAGCACGTTCCCA
AACATCGGACGAAAGTTCAATTGCCTCAGGGAAGGCACATACCTAGGCCC
ACCTGATTGGCTTTATCTACTACCTAACCTTGGACATTTACTAGTCAATTCCT
GGTTCCAATAGGAGAGGGCGCAGTGGCAGAGTGGTTATTGCGTATGACTAG
AATAGTGTACTATCCTATCATATTCCCTTGGGAGCGCCTGTTCGAATCAGGTC
TGCGTCGATTTTTTTTTAATTTTCTTTTTGTGGTTCTTGTTTTGTTTTTGGTGA
TTGCCCATTTGTTGAGTGCTTTTGTGTTGTTGTATAGTCTATGTGTAGTGATG
AAGGATGATGCTTTTTGTCGCGGGTTGTACCTTACTTGTATACGTCCACTCT
GTACTTCGAGTACCTCATTAAGAGATTAAAGGCTATTATGTTGTCATCTATGG
TGAGTGATCTAGATATAAGCGAAAGTATGGATCAATCGCATCAGAGTAAGG
GGCAGTAGTTAGGGAGAGGGGGTAAGGGGTGAGGGGTTGAATGAGAACAT
GGGTGTTGTGGTTGATGATAACCTCAATAACACCACGTTTAAAGTAGAAAT
GCGTTCATGTTAGATTGTCTTTCGATTTTAAAATGGGAGGTCGGTATGTAAC
AATTCGACTTAGTTACGAAAATATGTGCTCAATCATGGTGATGTGGCGGCGA
AATCGTGTGGTGTGATTGAGAGGTCGGTGGCGATGACGATGACGATGTATC
TAGATGCGAACATGAATTTGAAAGAAAAATGAACGAATGATAACATTTCCA
TGAATAAAAATGCGAATAGGCATGAGCGTGGTTGAGAAAAGAAAATTGTC
AAAAAAAAAGATTTGACCGTTGAGGGAATCGAACCCCCAAGCCCGCGACC
GGTACCAGGCTTGGTACCTCTATACCCTCTATAACCCAACCTCTATATCCAAC

CTCTACCCTCTATACCTACCGTCTATACCACTGAGCCATCCAGTCTATGCGCG
CCATCCCAACCCGGCCTTTTTTCTTGTTGGAATCTACTGCCGTAACTTAGCT
ACATATGGTTGCTTATCCGGAGGTACGCCGGCAAAGGGAAGGTCATAGCTT
CGTGACTGCACTTATAGTACTGTGTGAGTCATGGGTTCCTTCCGTCTATGTC
TGTCAGAATATTCCATTGGTGGATAGTTACTGACACTAGACCTCACAAAGG
GTACACCTTCAGTCCTATATCGTCTTATCATATATTCCATTCTACCTACACTTT
GGACAGTGTCCGTGCCAATTCAATCTAAGTCGGGTATAGGTAAATGGTCAA
CAAGCTTCATCTGCAGTCCCAATTGCGCCAATGCCTTCACGGCGTTCTTATC
TCTTAGCTTCTCCGTCTCCTCCTTTGTCGTCAGCACAAAGTCATGCTTCAGC
AACAAGCCTCCTTCTGGAAGTCGTATTCCTTAGCTGTCGCAGGGTCGACCT
TGTCAATGTCAATTATGAGGCTCTGCTTTACGCCGAACACAGCATCTGATGT
TTCATACGGGTCCCCGCGAACATAAAGCGCGCTATTGACGTCAGTTAGTTTA
TGCTGGTCTCTTACGCGACAGACAACCCCTTTTCCCCGCCCCCTTGCCCGG
TCAAGTTCCGAGTACGAAATACGAAACAGATGACCTTCACCAATTGGACAA
TATGCCGGAAGAAAAACACAGACGCAAGAAAACATCCGACTCACGTAATC
AGGTGATCCCAACCCTCCTTCTCAAACATAAAATGCAAATGCGCCGGTCTC
CAGCAGTGCCGCCCCAGCCGCTCTAGCAGCTTGCCCACGGGCCCGTCGTG
CGGGATCGGGTAGCTCACGGGCTTGATCCCCTTGAACCAGAAGCGTCCCTC
GCTATCACTCACCATGACGCACCGCTCGCTCGGCTCCGAGCGGTCGGAGTG
CTGCACGTCGTAGTGCCCGCTGCTGTCCGTCTCCCAGATGTCGATTTTCACG
CCGGAGACCGGGCGTCCGGAGGTATCGCGCACCGTGCAGATGCAGAGGAG
GGGCTCGCCGGCTGGGTCCGAGGCCATGTTTGAGCCGTTGGGGAGAGTGG
GGGCGTCGTGCGTGTGGAAGGGACCCAGGACGGAACCTTCGGTTGAGGA
GGGGGGTTTGGGGTGGTTGATGGAGTCGACGAGGAGCGAGAGGCCGAGG
ATGTCGGAGAGGAGGATGAATTCCTGGGGTATGTTAGTTCTACTTCTCTATG
CTCATTCTACTGCTTTCCACCCTTGATTTGCACTGGGTTGGGGGAGGTGAC
ACATACATGCCGCACGTCGCTGCTGATCTGCCCGCAACCCACGAGGAAGTT
GAGGGCTGCCATCCACTCGTCGGTGCTAAGTCGCGTCTCGCGAGCGAAGT
CGTGAAGATGCGTCACGAGCCGCGACATTAGGTATTTCAACCGCGGGTTCT
GGCATTGTGAGTTAATCCGATTCGTGTTGGGCGTGATGTTGTCGATGGTCAG
GTCTAGGAGAGCTGGGATCCCGGCTGACGCTGGCATTTTGTCGATATTTCTA
TGTTTATGGTTATGGATATGGTATTGCTTTTTTCTTAACTACCAAGTATTTCTC
AATTACTGAGATGTATACGGAGAGCATATGAGAGGTCAATTGGGAGTCCAA
TGGGATCAATGGGGGGAACTGGTATAGGAGAAGTAATGAATTATAATTACTG
CATGGTAAACGCCAAACTGGGGACAGACGGCCGTACGAGGTCGTTGTGAT
CCCTTATGTAATTCTCTGTCATCTATTCTGTTCACATCACTTGAGGGTCTCGG
GTTCCGGCAGTTGAAAGGCGGGATCTCCAATGTCTCCAATCCAGGAAGAG
CTGTGGAGGTGGAAGAAGGTTAACCCTGACGCCAGACAATGCAAAACGTA

CGACGTTGCTACTGCGAGGGTCCGGGGGAATGGTTCAGATGGTGACACTTT
AGGAGTTTGCCAATCGCATGGCGTGTTATTTACCTAGTTTTTTTATGTCACGT
CGTCGTTGCATCCTCGAAGACGAACCCTTTGACATGTGCATGCATAGGTAC
CTATATACGTGCCGATAGTATGTGAAGACCATCGTCAGAGCTAACGGACGTG
GTGTGGGGTAAACAAAGGTACTCGGGAAGCTTTCAAGTGAGGGGCAATAA
AAAAGGGGGGACTGAGGATGGCCGGGAAGACGATGCGGAGGCATTATGGG
GAACGATCGTCGCCACTATCGCGATAAGTACGTAAGAAACGGAAATGACGC
CAATCAGTTGTTGCATATTTTCAATTGAGGGTGCTCCATTGACTCCCCGGGT
CTCGCTAAAGATCCAGACCTTGTCTCATCTTTGTAAGCTCCTACGCTCTTTT
CTCAATTTTCTCCTCACCTATCTATTCTAACCCCAATCACATGACTGACCATG
ACGATGTTCAACTTCCGATGAACAAAACGAAAGAAGAGATTCATCGAGATA
GTATGAATAAGAAAAGAGCCATGGGCTCCCTCTCTCGAATTCAAAAGCCTC
GGAATTGTGGTGAAACGTCACGACCTCATTCACCGCTCTCCCCGTTAGTAC
GAAGCCCCTTCGCCTAATCGCCAGTACGCCTTGAATCACTCATGGATCACTC
ATACTCGCACCTACGATATCCATACCTGTGACGTCCACCGGCGATGTCAAGC
GCGTGCCGTCAAAAATGGATTATCTTGTCAACTTCATCTTTCATATTACTTGG
AGGCTCATCACTTTCTGCCTGAACAATAACTGTCATCTAGTCAGATTTCATA
CAGGACACATGGGGGCGTTGATGCTCACCGCATCAACAAGTCTCTAAAAA
AACCCCAAATCTTGTTTTTCATGTCAGTATTTGGGAATAAAGTGAGAAACC
GCAAGAGAGGAGACGAAAGCAATATTAATTGTGGCGCATTATCCTGCCATA
GTGATCAGATGCTCTCGCTAGCTGCATATTCAGAGTCATGCTTCCTCTCGAG
GGTTGCCTTGGGATTTCGGTATCAATGGGTGGGTAAATTGCGACAATTCGCT
GCATAAAGGAGCAGGTGGCAGTGTTGTCCATTACTGAGGACAATCAGATTT
TCCATTCCTAATTGACAATGTCTCAAGCAACACTTCCTAAGTCCAATGACGA
AGAAATCGGATCTAGCGAGCCGCAACGGAACGACAAGACCTTCGAACATC
CTGCCATGAAAATTAACCATCGAGAAGTAAGGCATTTAAGTCACTTACTGC
TGAAGGCGTGGCGAAGCCCAAAAGCCGGCCCTATCGATGCCCAACGGATA
GGTACCCTACCCTAGTCTCAAACCGTTACCTTCGAGAGCCTTGCGATGGGT
CATTGGATATCGGCGAGTCCCTAGAGCAGCTCAACCTGTCGGCTACGTCTAT
GAAAATAGGCATCCCCAACAACCTGGAAATATATCTATTTTTCATCACGTTT
GATCGTACATACGCAAGTACTCACGGGACTGCGAATTCATGTACAATAATAT
GAGGTGCAATCTGCATTCAGCCTCAATTTAAAAAGTGTTTCACTGGCCCTA
AATCCCCGCGTTGCTCGTCGAATCTGACTGGTTGATGCCTCTGAAACTGCA
CATTCTAGAACAATCCTTCACACAAGTCTTACCACACCTTAACCGTATCAGG
TCCAAGTGCCATATTCTTCGTATACGAAACATACCTATTCACAAATTGTCTAC
CTCGGCATACGACCGTGATATATCAGTTTTTGACCAGGATACAGGCAGACAT
CTCAGCCTCTACTCCATAATACACTTACTTCATGACTGTACAGTATCAAGATA
TTATGGCCTGTGGCGCTTTGCCACCGCGGCTGCTCATCCTTCCCGGACTCTA

TGACCGATAGGTAGTGAGACGCCAGCACGAGGCACGGCGTTCCTTCGGAA
GGACTTCCGTCAGACTTCAATCTTGACGTCTACCGAGAAGTCGATCTCAAA
CATGGTAATTATCTACATTTAAAACTAATATGTTGCTACATCATAGCCGCAAT
GGTTCTTTCTTAGATAGGAAATCGGGTGGCAAACGGACATGGAGCTAATGG
CAACCTACAACTTGCCCCCTATAAGGTACCTAGCTACACGGCATATAGGACA
ATACCTTGGCAGTGGATTCCGATTTTTCACATTTCCAGGCGGCCTACTACCT
AGTCTAAAGGACAATGACAGAAATGTTTAATGGGTAGATCAACATGACTGT
TTTATAGGAAAGTATGTTTACAGAGTCAAGGAATGTAAATTGCAAAGTACC
GCCGGAGGTACCTGTGTCTAAAGAATCATACAGGAAATCCGCAAATCTAAA
TAACCGCCAGCTCATTTACCCGTCTATAGAAATGAGAAAATCTTTAAGAAAT
AACACTTTTTTTTCTCTTTTAATTTATTTTCCCCAATTGCCTTGGACTCCTTTT
CCCTTCCAAGTAATACACTGTCTATATCAAAATGAGAGATCCGCCGCCCAGT
TTCCAATTGCTGGATCTATTCTTTAGTCGTAACGCCGGTCCCAGCCGAGCCA
TGATATCAATAAACGCCGATGGACATCTGAGAGAAAGACCCATAAGGAAAA
TAGTAGCCGATCGTGAACCATAAACAGTCTTCTTATCGTGTTTCAACACCGT
TCTGTCGTTGTATTTGAAGACTCATAGAGTGTAGAGTCGAGGAGTCTCCAT
GCTTTTGCTAGGGATAACAATAGCACAGGGTGTAGCGTCGGTCCGTCTGGG
AGCCCAATGTAATTGAATTGGCTTCTTTGAAGGTTCCGGGTGAGCTGTCGG
CTTCCTAACTTTGGAAGCGACAGGCACAGCACATTTGATGATCGCCGCAAA
AGACGCACGACGGTGTTAGCTTGACTGTCATTGGTCCCATCTTGCACTGAC
AGCAGAACCACATAGGTGCGCTGGTTGGTTCAGGCATTTTGAATATCTTAG
ATTGTGGGTTAGACTTGGTTTGGTTGAAGAGTGGTGTGTTAGGAGTAGAGT
GGTACAATGGTGGTGGATGATGATAGATAAATTCGTTTTCGTTCAACGGAAT
CCGAGTCGAATATATAATCGACTCCGTGGTTCGCCCTCCTTAGGTCGCCTGC
TACATCGTCGACGTGATCATCCGTAGGGCAACGCTTGTGGTTGTAGACACA
GCCATGAAGATGGAGTCATCAGGGTCTCAATTCATATTGTTCTCTAGGGCTA
ATTGTATCGGGCTTTTTCCAGGACAGCCAGTAAGGGGTTTTGGAAGTTGAG
TGGATCACTCATTCTCAATTGGTCTCCCTTTTCTCGGTGTATTATCTGTTTTA
CTCGCAGGACAGATTCTTCTCGTGACAGTCGCAACTTCGTCTCTCCGCCCG
TCAAGATCCACTGAGGACATTCGGGAGCACAAAGCTAAGCCCTCCATTGTG
TCATATATCTAGGTTTTACTAGTATCGTAAGCTTCTATTGATTTGAGACAACT
TACATACCAAGTCACTTGAGTGATGCTTCTATCGTCCGCCTCGGGCAGATCG
ACTGTCCAGGCGCGGGCGTCGGTCCCGGGCGAGTGAGATTCGAGCTAGCA
AATTAATTGAAATATGAGTAAGATCTTTTAAAAAGCGGATCGTTTAATATTCA
AAGTAAATACAACATTTAATGCTCACATGGGAATGACCTGCCTCGCTGAAC
AAGTAGTAAGGTTGAGGGAGGCTCACATAAATTCTCCGAAACTAAGCATTT
TCTCGACACTCGTAATTCATGCTATAGTAATTTCTCAGTTTTGAGATACGGCT
TAGCCCCCAGGGCGAAACATGTTAACATGTAACAAGTCTGCCCTATAAGGC

TCGGCTGTGTGTTCATCGAACGACATGATTTGGACACATAACCGAAACTTC
GTCTCACCGAACCCGTTCGTATCCTGCGTGCGACTATCCATCATGTTTTCCC
TTCCAATCCTTAAAGTCACCAACTATAGTTGATGTCACCTGCGTTTGCGTAG
GGCAGAGTTAAGGTTTGCAGCACGGACTATCGTTCCAAAGAAGAGGTATTT
CTTGGTGGATTCGGACCACTAATCGTGAGGCAAGATGAACGATTGAAGATG
GTTGGCAAATATTTGTGGGCGTAAGAAAGGTTATAGCGCGAGTAATAGGGG
GGGCAGTGCCCGTGGTCATTTGGTAAGGTATCCATTATCCATTTGCCCGGGG
CAGTTTCGGTGAGCGACAAATGTAAAAATGCCTATGCTTGGCATCAATTTAA
TGACCACGGTCGCAGTCTTACCGATTGTGTTAGAAATCAGTAATAAAGGCG
ACGGAGACCTTATCAACTTCGATTGTTAAAGGAGACTTGCAATTTCCGATC
GCTTGAATGCAAAGGCTCCCCTCAATTGCGCACAAATGGTACTAGCAATGG
TGCCAGTGCATCGAAAGCTATAAACATTCAACAAAGTCAGTCAATAGTTCA
ACAATCGTAAGAAAACTTAAGAGATGCTTCTAAAATTTTCAAATTTCAAAA
AAAAACCAAGATTTCGAAGTTAACCCACTCAACTAAGTAAGATAGATAGGT
ATGGTTTGATCGAGGATGGATGCATCGACGAAGATATAGACAATTTAAGCAC
GAACAAGATATTTGGTCTGATAAATTATAATTTAACCCTCTGCTGAGTGAAC
AAGATATATCAAACTAATCGTGGGGGGAGACTGATGCTTTATACCTGATAGC
ACTTTGTATAGTGGCCTCAGTCTGATATGGAAGAAGGGAGCCCATGCGGCT
CAAAGTCTACGTGTCTGCCGCCGGCTCTTTAACGACGCAAATGATGCTGGA
TCGCCATTCTCAGGGTGTCCCTCGCATGCCTGTATCCGAAGGCAATACACC
ATTCTTGGGCAACACGCCTCAGATGAACCAGACAGAATACTTCCAGCCATA
TCAGCCGCCACTGGGCCCAGCTCCACAGGGTCCACCTCCTGTCGAACGGA
CATCAAGCGATTCCCGTTCAGATGAGCGCTCGGCAGGAGGACATATTATGG
ACGCGTGGAGACCGATGCCCCAGGGATACATGCCACCCGGGACATATGTTC
CGACAGCACAATCCCCTGATGCAATCCGGTCTCACGGCATGTATCAGTCTA
CGCAAGGATACGACAACCCCTATTTTCCTCCAGCGTCGCCACCACTCTCTC
CTGGCCCTCACGTTCGCCATTCCTTGCCAGTGGGCAGAAGCTCGCCAGGGC
CGTCTATGCCCTCACATACTCGCACGTGGTCATCAAGTGCCTTGGGAGCGT
ATGAGTCACCGCCTCTCACGTCTCCCGCCATCCTCCCGACTACTCTGTATCA
TCAGCAAGGTCGCGCTGGAGCGCCACGTAATGCCGAGGGGCTTCATCCTCC
GCAGCTTGCGATACCGAAGCGGCGCTCGCTCAACAACCTCCGCGACGAGT
CACCCACATCTCAGTACTCCGACTCAGACAAAGAGGCCGCCAGACGCGGG
GACCGACGATCGCGGGATACACGTAAGGCCAAGCGAAAGTCCAAGCAAAG
TTGGTTTGGCCGCGCCCAAGTGACCGACTCGTCAGAAGGAGAGAATGATG
CCATTTCCGATGACGAACCAGGTCCATCACGTCGAAGGACAAGACAATTG
ATGATGACCAACGAGATGAGAGAGCCGGCCCCGTTCGAGCCGGTCCCACT
TGTCACAGGACATGCGGGGACACCGAGGGGCTGGAAGGCTGCACTGGGTC
TGGGGAAGAGGAAGAAGTTGGACGAGGTTGCTGAAGCTGCGCGCGACGA

GAACAAGGCTCGAAAAGCTGCACTGGCTGCCGAGTCTGGGGCCTTGTTCG
CCGGCGTCGAGGCGCCAAAGCCCAAGCCGAAGCCTAAGGCCACAGCCAC
AGCTGCCACACCAGAAGCTGCCGTCAAAGGCTTCAAAGTCAAGCGAGCA
AGCCAATTGCCGGCGATCTCTCCTGTCGCGCAGGTCGGATCCAATGATGTA
GCGTCTAGCAGTGCGCGGCCAATGAGAGGGACCGTACCGGCCATCTCGCT
GGAACAGCCCAGTCCGCCCCGCTCGTTCGTCGTCAAGCGACCAGGGGAGT
CGCCATCATCCTCCCCTGCTTCTTCCAATCCGCCTCGAGGATCGTTCAACAC
CGCACCGTCTGTGGCGCCAGCGTCAAGGGCTTCCGTTACCCCTTCACCAGT
CTCATCCACGTTGCTTGCTGCTACCGAAGAGCCAAAACGCTCTTTCAAAGT
CAACAGACCGAACCGAGCGCCTCCGAATGCTCAGACTTTCTCGGTCCACC
GGCCGTCCTCGCCCTTGATGGCGATCTCGCCCACATCGCCAACCAGTCCCA
TTGCTTCCACTGCGGGCTATCCCTTGTCGGCCATCGTGCCCGTCTCTGAGGT
CAGGCGCCAGAGTGTGGCGGCCTCCATCACCCCTGAGAGGCCTCCGAGAG
ATTCCAGAAGAACAAGTCAAGAGATCGCTGCGCAGAGGGCAAGCACGCAT
CTCGACCTTTGAAGGACATTACTGTATCGGATTATCTATCTAGCATATCTCCG
CACAAGACACGGCTTATTTACGCTTCTAACGCTCGGAATCACGATACAATG
ACATGAATCGATAACAGAAGTATGATGTTCTGCTATGGATGCATGATCTGGC
CATGCTGCGAAGTAGCAGGAGGAATCGCTCGGTCGATGGAGTGTTCCGTGC
GTTTTTTCCGGAAGAACAGCAATGAAGGCTAGCTCACGAAATTGAAGGTG
TGCTCCACACGCGCTACATACGTCCCAAGTGCCTCCACTTCTCCTGACAGA
GGGTGCGGTAGGAAACATACTGAGCCCGTGACAGTACATACAATGTCGCTC
TCGAGGCTATCACCGAGGCGCCTGGCTGGCGCCCGGTCGACACACACAAT
GTTCTGGAAAACGACAAGGTTACCTAGCCCTTTTGACGGTCCGCTCCAGCG
AAGGCTTGCTTCTGAGATGTTTGACATATTCAACGGAACGGAAATGCCTCA
GCCGATGCGCAAACTGCTCCTGACCGAGCTCACGAGTTGCTTCTGACCGA
GGACTGGCAGGCTCCTTCGTGCTCGCCGGGTTTCATGTCGCCTGGACCCCC
ATCTTCTCGTTTTCGAAGATACTGGCAGAATCGTCCTTTATGGACTCATCAG
GAAGGCCCCAGACCTTTGTATTGGTCATCCATTCAAGACCTGTACACAACG
TAAGACGTGAAGTTCTCGCCATGATATAAAATGCGCATCGAATCGGTCAAC
GATCACTACCTGTGCTTTTGCTTTACCCCTCTACTCTCGCTGGTCCGATTTTC
CGTACTTGTCATCACTCAAGAGGACGTACCGCAACATCATCTGATAAGGTAT
GTATGTTCAGATCTTGAGCTGCAGTCACTGAGCTCCTGCCGCAGGCTATAC
AGCAACGAAGCTAGAGCAACGAGAATGAGTAACCCGGCAAGATCTGCTCA
GGGAGACGGCAACACGTCCCGACTCAAAGCGCAGACAACTGTGAGCACA
GACTGGCTTGGACCGACCCACGTAAGCTCCACAAAGGATGCTTCTGGGAT
GCACGTCGGCGGCGTCATAGACCTGGAATCCAGGCTGGCAGACCACGTATC
TCTCGCGCCTGATGCCCGCGCCTACCTGCCAGCAGGCTTGAGCGACTACGA
GGTAGCTGTCTGCACAGGCTTCCCAAACTTCGAATACAGGAGTCGGCACG

AGCTCCTTGAGCAAAGCTACGCCGCATTGCGTACCGGGAACCCGGGAGAG
GGCGTACAGGCCGGCCCTACCGAGAACTATATCGCTCAATCTACTCGCACG
ATCGGCGACTATGAGTATCAGTTCACGCACGATACCGAGGTCTATCCATTCC
AATTCAGGGGCGTCTCTGTTGGACGGAGTGGGCGACCGGAGCGTGCCTTG
GAAGCCTATGTAGAGCGGCCTGAAGAGCTCGGAGTCAGCTGCCACAGGCG
TCTGACCGTCAAATTGCAAGGTTTTCGGCGAATATGAGTTTGTGAGGACCC
ACACGATTCATGAGGTTACCATCAAATCGAAATGGGGTCGACAAGTGTTCA
GATACATACACTGGGTTGGCCACGACGGGTCGCGAGACGTCTGGGAGGGT
GCGATACCCCCATGGCAAGGTTCAAATGGTCAAGCTCGTTGAGGGCCCCGG
TAGACAGTACATATAGCCCGTTGATGGACTATGCAGCTACTTAGCTTCTCTT
CCTGACCAGTGAGCCTTGTACATCAGCTGGATCAGTTACCAGCGGCCGAAC
GACGGCTTCTCATGAGTGTGAATACCTGCCAGACGCATGCCATCCCGCGCA
CGGTACACAACATGATGACGTATTCTTCTGCTTGGAAGGGTCCCCAAGGGA
TTGCTGCGCCTGCCATGCAGGATCTCAGCGCCCAGTCGAAACACTCTCATG
CAGAAGACTTCTCGTCCTTTCATGTGGGGCGATGTCAAGACGATGCAGGGA
AGGGAAAGCAAGATTCGTTCGTTCCAGCCCTCCAGTCATATAAGAAGGTCG
CCGCGAAAAGGCTGTTCTTCCCTGCTAGACCATTTCTTGCTGATCCGGACG
ACTCCAACTTTCTTCAATCTGAAGGTCCTGTAAGTCAAGCGGTTCACTCGC
AACACCAACAACATGGACCCCCACCACACAGTGAGTGATCATCCGTGCGA
TATCCGGCCTGTGGCTGAGGCAGCAGGCCAACTCGCCTTCCGGAGAAGGC
AACATCCAAGCTTCCCTGCAGACTGTTGTTCAGCAGGTTTCGGCTGCACAC
AACCGGCAACGCGTTGCGGCGCCCAACACCAGAGATCTTTACGGTGATTAT
CAAAGCGCCACTCCCCCGAAGGTGGAGAGTGACAACGGATGGCTCATTGA
CCCTGACGCCAGAGTTGACATTCCGACTCCGGCTAGTGTCGACAAGGGCA
CTATGTCCGACACTGATACTGCCCTGCACACTGAACGAGCAGAACCAGTCT
GGGAGCGGGTTGAAGACGAAGATGAATCTCAAGAGGCTCTGCCCCCCTTT
GGGGACACGGACGCCGACGGCAACAGTGCTAGACTCGCATTCAGGTTGAG
TCAGCATCGCTCTTTTGCCCCTGGCTCGGATGCATATCTGCCTCCCGTATCG
AGCGAGTACGAGCGGGCGGTCTTGACAGGATTCCCCAATTTCGAATGGGG
AAGCTATAACAAACTATGCAAGCAAGCCTATCGTGCCATCCACACCGGTAG
ACATGGATCCGGTTACAGGACCATCGCGGACGGAGTCACCATCGTGGAAC
GCGTACTTGACGGGGAGAAGTACAAATACAAACAATACACCTCGAGGTCC
GTGCCCTGGAGCAGAGTTGGAATAGACGACACGAATGGCTCTAACTACTTG
TTCGGAACTGTCCAATGATCCAGGCGATTTGAGCACAGGGTTTGTGCGCGG
TCTGTGCGTCAGTCTTCCCGGACACGGGATGTACGTCTTCTACGCTAGTGA
GTCATACCACAGGGTCGCTATCGAGTCAGACCAGGGTGACACGGTTTTCGA
GTTTGAGCACGGTCACGGTCTCCCTGATGTGCACCGTGGCAACATACCAGT
TTGGCAAGACCTGTCTACGCCAAGCGCCTCGGAGTAAGAGGGATCGCCTG

CTTGTCCAATCCGAGCCGGAGAAGGCAAGGCATGACCTTACCTCGTGCAC
ACTTTTCATATCGCAGTTTCTCATTGAGGAACCCGTTGACAGTATTGTAGAC
ACAGCCCATTGATC

>Mosaic_sequence_41

GGTTCGTTGGTACCACGAAACTCATGTCTCTTACGCGTACCTGAGCATGTA
AGACTTAACTAAGTAGCAACATCTTTTAACTGTGGCTTTGTTCCAAGACTTC
GGGATTTTCGATCCCAAGCTTCGGCCGGTTATTAAAGTTAGCTAGATGCGGC
TCTTATCTTAGCCGTGAAGATGTCGATCTCACCCTAGAAAAAGGTTGTCAG
AGGTCAACCACAAGAGTTCCGAGATTGATGACAAGCCATGCTTATACATGA
TTGACTGACTTGCGCTGTCACCCCCACGTGGAAACCTCACGTCTATCTATTT
GCTACGGATCGACGTGGCCGATTCCGTCATCACATCCTTAAGAAATCTAGTC
ATCATGAACCGTAATTTTATATCTACCGCAAAACAGGTTCCCACATTTTATCG
GTATGTGATTTGGCAGGAGTCCTCAGTCGATGTCTCCTTTCTACCCTTTCTA
CCGACACTTCAAAGTCCTACACAACTTCGATAATGGAAGTTACTTATTGTAC
CTACCTAGTTAATATAGTAACCCCGACTGATATGAAAGTTTCATTAATCATAC
ATCAAGTGGATTGCTCCAGAACTAACTCCCATCTAGTTCCCGCTAGATGATA
TTGAAGATAATGTATTGGGTGAGCTAGTTGTCCACGGTGGAGTGAGAAAAC
TGCACTAGCTCTTGCGAGATCGAATATGGTGAATATAGCGATTTGCCCAACG
GAAATGTACTGCTCGTAAGGCCTCGCTCGTTTAGTTGAAACGGAAGATTGG
CTTGACGAAGGCGCTTGCATAGAAGTGTCTGGATTGCATGGAAATGGCAAA
ATGACCACCGTGGTGGCTTGCAATGTCAACATGGCGCCGGAAGTGGTTGG
AAAATACTACCCACCCACTTGAAGTCCGGTCAGGCTAGATTTCACGACGGA
TACAGGCCTAAACCGTCGTCCCACACACCATGGGTAGTGAATAAAATGTGA
TAGGTCTTTTTGAAACGTAGCAGCTTTTTCAGCCTTGAAGCGCTTGAATCG
CCCAGGCAAAAGAACTTGGAATAGTCAAGCTCTAGATAGGTATCCGATATC
GTGGGCTAGGATGGCGCATCAATCGTGGTGGAGGGCTTAACCGCTTAGCTC
GACAGGGTTAGCCGATGCCAACAAATGCCGTAAGAGTACTAGCCCATCCTT
GTTTTAAGGGCATTTTTTAAAAAGTGATCCGTTTATGAGTGGAATTCCGCGG
TCAAGATCAGGTACAAGGAGAATACTTATACACCGCAAATCGAGAAAATAC
CTACTAAGCAGTAAAATACCTCCGCTTGATCGTGAAATGGGGCGACCGATA
ATTTCCTGGAGCAACCACCAACTTTCAACTTTTTCAGGGGTGCAAGGAGTG
GCCAGGACTGCCAGCACGGTTAGGGAGGTTAGAAACGAAACTAAATAAAA
GGGTAGAAGATGCTCGCTTGAGATATTTGCTTAGTCCTTCCTTTTCCTCTCC
GTAGACTCCTGATTCGACCAATTTGGAGATTGCAGATTAGTTGGGTGTAAG
ACTATCCCCAGGTACGTTCAAGATCTTCTCTCTTCTCTTCATCTAATTTGAGC
TGTTATGCATCGCATGTTTTTGCTTGTTCCCCGCCCTTGTCTTTGCATGTGAT
TGAACGCCTGCACGAGAAAAAGAGGAACAAGCCGTGGTGGGTTGATGAC

AAGACGGCATGACGAGAAAAAAAAAAGAGAGAGAAAGGAGAGAATGCA
GAAAGTTCTCGTGGCGTTTGAGCCACGTCTAGATATTGTGATATTGTTAAAT
GTTATATTGGATTATAACATAAGCAATCACCTTTTAACTTCAATCGACTCGAA
GGCTATACAATATCGGTGCTAGCAAGTGCAACCGTTTGTCATTAGTTTTATTT
GAGCAACTGATGAGGCAAATGGAGATGAAGGAAGTTTGTTTCATTGGCCG
GGGAAGGGCTGCACATAGTCGAAGGCAAAATGCAGCTGGCAAGCACAAG
CAAGGAAGAGACTCGCTCTAAATTAGTGTGCATTACGTCAATGGTGCATTC
GACTATACATCAACTATACAAAGTGAACTAACCAGCTAACATTACCACCCCC
CCTTTTTTAGGTACCTATAGAAAATTACCGTAGCTGCCAAAGCTCCGACGCC
AACCAAATTTTACCCCGCCATTCTACCCAAAAGTACAAGTCCCAAAAGCCA
GAGGCTTGAAAGTTTAGAAGGAACAATCGTCATCCCAACCTCTACCTTCTA
CCTTCTACCTCCTGTCGTTCTACAATACAAAATTTAAATCCTAACATCTTTTT
CCCTCTTCCTTTATAAACCTCGTTTCGACGAAGTTGTGAATCAACTTTGGGA
GAATTCGTCGTCGTCTTCTAATTCCGTACCCTGATATCTGGACTTACCGAAG
CTTTCACATCTACGCATACGATGGCCCAAGCAATTAAAAGTGCGCTGCCTTC
CCATCTCAAGCCCGGCGGGAACGCCGAAGCTGAGTTTGAGGGCAGACACC
ATGGCAAAACTCGATCACACATGGTATGTATAGCTCTATGCCTTCTCACAGC
TTCTCTACTCCCTACAAGCATATAAAACATTCGAGACGAGTTGATAGCTACT
GGATTTTCGAGCCTCGAATTCCTGACCCCTCAGAAAACAATGGAGTTGCAG
ACTATTCACAGCTTTGTTTCTTTGTATTCCCCCTATCTCGATGACGAAGCAA
CCTAATACCCCACGTGTATCTGCCTCAGAGCTTCACATCTCACGATCACACC
TATCCCTGAGATAAAGTTTCTACTTCTATGGCTACCTGAAGCTTGAATTCTTC
AGATGTCAGGCAAACCATGTACTACCAAACATGACTGCGGAACACCCAATT
TCCTGAATCACTTCCTCATCCCTATACCATCACGTACGAGATCTAAAATCTAT
AAAAGCCAAGGAAGCTTCACGGTCATCTTATATATATTTGTATTTTTCATCTG
CTTGATGTTTTCCCTAAATCATCTGAGATCTTTACCCTTCGCCCTTCTTTGCC
CTTCCAAATTGAGGTCATTTTGAGATGGTAGAACAAGCAAAATGTCTACTT
TGCTAACTGACCGTAACAAGGCCTTCGAGAATGCCTCGACTAACGTCGCCG
CGGCGCAAATGCGTAATTCCCTAACTGAACTCGCGGAGACCGTCAAGGAC
CCCGAGCAGAAGAAACTATTCGAAACCGAAATGGACAACTTCTTCGCCCT
CTTCCGTCGCTACCTGAACGACAAAGCTAAGGGCAACGCTGTGTATGTGTA
CCTCATATTGTAGCAGTACTTGTAGTACGAAAGAAAAGTATACTGATATTGG
GTTGACAGCGATTGGGATCGTATCGCTCCTCCCGCGCAAGGCCAGGTCGTC
GACTACGAAGACTTAGCTAACTCCGAGTCCGTCGGTTTCCTCAACAAGCTT
GCCGTTCTGAAGTTAAACGGTGGTTTGGGTACGTCCATGGGTTGTGTTGGT
CCTAAGTCCGTTATCGAAGTTCGCGATGGCATGTCTTTCCTTGATATGTCCG
TACGACAAGTCGAGTACTTGAACCGAACGTACAACACCAACGTCCCCTTC
ATCCTAATGAACTCTTTCAACACCCACGACGACACTGCCGCCATCATTAAG

AAGTATGAGGGTCACAATGTCGACATTCTCACCTTCAACCAATCAAGATTC
CCCAGAATCTACAAAGACTCGCTATTGCCAGTTCCCAAGAGCATCGACTCC
CCGATCAATGAGTGGTACCCTCCCGGTCACGGTGACGTATTCGAGTCTCTG
TACAACTCTGGTCTCCTTGATAAGCTCATCGACCGTGGTATCGAGATTATCT
TCTTGTCAAACGTCGATAACCTGGGAGCCGTGGTGGACCTGCGCATCCTGC
AACACATGGTTGAGACGAATTCCGAGTACATCATGGAATTGACAAACAAGA
CCAAGGCTGACGTCAAGGGTGGTACCATCATCGACTACGAGGGATCTGTGC
GCCTGCTAGAAATTGCTCAAGTTCCTAAGGAGCACACAAACGAGTTCAAG
TCGATTAAGAAGTTCAAGTACTTCAACACCAACAACATCTGGATGAATACC
AAGGCCATCAAGCGCGTCGTCGAGAACAACGAGCTTGAGATGGAGATCAT
CCCCAACGGTAAGACTATCGCCGGCGACAAGAAGGGCGAGTCGGATATTTC
CATTCTGCAGCTCGAGACTGCGGTTGGTGCTGCCATTCGCCACTTCAAGAC
CGCGCACGGTGTTAACGTCCCCCGTCGCCGATTCTTGCCGGTCAAGACCTG
CTCAGATTTGATGCTAGTCAAGTCTGATCTGTACACTGTAAGCACGGTCAAT
TGCAACTGAACCCGAGCCGATTCGGTGGCGCGCCGCTTATCAAGCTCGGCA
GCGACTTCAAGAAGGTATCGGACTTCCAGAAGCGCATCCCCTCGATTCCCA
AGATCATCGAACTGGACCACCTAACGATCACTGGTAATGTCAATCTGCATC
GTGGAGTGGAACTAAAGGGAACCGTTATCATCGTCGCGACAGAAGGTTCG
ACAATCGACATCCTCCCGGATCGATTTTGGAGAACGTTGTAGTCGAGGGTA
GCTTGCGTCTGCTGGAGCATTAATCTGTGGGATTCCCAACCTTGTTGTTATA
GTACTCCCGTACTTCTTATCGAGCGTTTGTCGCGACTCAACCCTTCGTCCGC
AAACATCTACCGCTTTCCTATTCCTCTATAGGGATTAGGTATTTTATAGAGAT
AGCTAGCCGTAGTAAAGGTGCTGCGGGACGGATGTGGGGGATAATGATTGA
TGTTATGAAAATGGGCGTGTTGGAGCAAAAACGCCAGGAAGATAATGCGAT
GCAATAAATAAAAAAGGTACTAGGTACACCTTGTGGTATAACCCTGCCGTG
TCTTTGCCCACCAAGTGACGAGATGTACAGCATTGTGACATTGTGACTTAC
TGGTAACATGGTTGGTAACTTGCCTAGCACTCATTCACACATAATTAGTGGC
TAAATTTCATAGTTCTATTTTGCTTTACGAAGATATTTTGAGCAACAATGGGT
GTGGTCTCATTGATTCTTCAGACCCCAAAGCCCCTAGTACTAAGGCATAATG
CGACTAGCTACCTAACCACGATGAGAAAAAGTGTCTGACATTGTCCTGATC
CCTTTCGGGCTTCGGGATACGTGAAGGTTGAGCACGTCTTCCTATGCTACCT
GGAAAACCTGCAACTACCCGTCATTGTCCTGCTGACCGCATACGCCACCAC
ATTGTCCGTCAGTATTCCCCCAAATCTCTTCCTCAAGTAACTCCGTCCCCTT
TTCTCTCTTCCAAATTTGCACCCACTGTCAGTCAAGACAACATTTCCCAGAT
AACGACCATGATAGGTTTGACCGATTTCGATTCTGATGATTATCGATATTGTC
GCGTAAGGGTGATCGGAAATTAGTCGAATATGCGATTAGTAATGCTTAACAA
TGCGTAATACGCGTGAGCGACGTGGTGATTCGTTTTGTTGCGTTAGGTAATG
CAATAGACTCCCTCCAATGATTGGGTATCGATGTATGATTAATGGATATTAAA

AGAGTCAGGAGAGAAAAAACTGAAAAATAAGAAAACTTCACTTCCCCTTC
TTCTTCATGGTCTCGGTGCAAGAGGGACAGTACCAGCGGCCCTCGGGTTCT
GATTTGAGGCCCACGCACGTCCAGTGGAACCACTCGTAGGGACACGCGTT
GTTGTCGCAGGCTACCATGTCGCCATATGACACGTGCTGGCAGAGACAATA
CTTCTTCCCTTCTTCATCTTCGTCGTCGTCGGATCCCCCTGAGCCAGCAGAG
GCAGACCCTCGATCCGGCATTTCCTCATCTCCTTCTGCGTTAGGGTGGTTAC
GACGGTGTTTATGTGTGTGGGGACGAGGAACAGGCGTTCCGCTGCCGGCT
CGAGACCCAGCACGCGACGATCGAACGGATTCTGCGTCGGAAAGCTCACT
ATCTGCCGTCGATGCCGGGGATCCTTTCGATACGCCTGCGCGTCTCACGCG
CGATAATGTCGATTTCGAGTTCGGGGGTTTCTTACGACTCACTCCTGCTGTT
GGTGTGCCTCTTCTCCCAGCTCCTACACTTCCTGCTTTAGATGTAGCCCGGG
GCCTGCACTCCCAGCCCTGACAGTTGCTCCCGGCGTCCCTGCCTTGGGTGT
TCCTGGTCCGAGAGAAGAATGCCGAGCCAGCCCACTTGATGCCGTGGGCA
GACTACCCAGACCAGAACTCACCCGTGGCCCTCTCTTCGGAACCCCACTTC
CACTAGATGGTATGTTAGTACTTGTGACTTATGTGTGGCTGCGAATGATCGG
TGAGTATTGATGACGATTGTGCCTTGGTGATATATGCAGACTACTGCGGCGA
TGGGACGGGGGCTCCCAGGAAGAAATTTAATGTTGAAAAGATGCAGTAGC
TGGGGACACAAAATGATCGCTAGGTTACTCACGGTCCAGCAGAGGACTCG
CGCTGGTTACGGTTGCTCATGATCATACTCGCGGCAGGAGACGTAGGTGCT
GAGTTAGAGATATGCGTCTGCGACTGCGTTGTTCGGATCTGAGGGTTGCTA
GCTCGGGCAGTCGTCGGGGTCGCATTTGCCGAGACGGGGTTGGCAGGTGT
GATGTTGGACGTCGTCACAGTCAGGCCTCGGAGGCTAGGGTTGTTGTTGTT
GTTGGCGGCGCTGGGTCGGACGGTGGAGGGAAGTTCGTCGGGGTCAGTGA
AACCGGGCTCGCCTCGATCGCCCAGCTGCTTGATCTGCATGTCGAGATGTC
GAAGATGCTTATCCATAAGAACCTTAGCCTTATGCGCCAAAGCGATCTTCTC
CTCAGCAAGCTTGTCAGCCTTCGCATAGTTGTCACGGATGGTGTCTCGCAA
GGCCTGCTCGCGAGGGAAGGGTGCATGCTGCCATTTAATCGAATCCAGCGT
TGGATCTTAGCATCGCAGTCTTCGATAGTCTTGACATGGTCGTTGAACAATC
GGTCTTTGTAAGCGATCTCATCTTGGTAGAATCGTAGCTCATCGGGTAAATT
GGCAGTTCGGTATATGAAGTCTTCGAGGATGGCGGCAGGGTCTTGGGCCTC
GGTGGAGGGCGCCATCTTCTTTACGAAGTCTATGGAGAGATCGTCTCTAGG
CATGGTTGTTAACCTGGCCCTCTAAGCGGGGGGCGCGCGTTAAGTGGTAGT
GAGGGATGTTCAATGATGTGAACGATGCATGCGCATGGTTGTAAAGTTGAG
AAGGAATATCATAAAGGGAGGGACTAGATGACGAGACGAATTTGCTCTGAT
AGAGCTCGCAAACCAGTCTCGTTTTGTTGATTTTTTTCCGTGCTTGAGTTTG
TTCTAAATTTTCAATTGACTCTCATGTTTGTTTTCTTGTTGCGCAATTGTTGC
TTTGTTACCTCGACCCAGAACGGTGCGCAATGTCTGCTGGGGATCATAGGT
GGCAAGACAATATTGACTTGAGGGTTGGAGGTTGAAAAAAGTTGAGAGAT

GGTTGGACGATGGAAATCACATGCATATAAAATTATAACGGTTGTGAATTCG
AGCTGTGGCTCACCTTCCTAACCAGTCCCCAAGACTTGTTCGCAGAAATAT
GATCTCCCAAGTCAAGAGCGACGCAGTATGAATTATGAGTACGCCACTATG
AATGCGTAAAGAAGGAACGAATTACCTAGTATCGCTTCTTTGGGCAATCGA
TAGGAGCGAAATGGCTTGCTCGGAAGGCGCAAACACAAGTAGTGGTCCAG
TCCCCGGTGCCAATGTGGGGTGTCGTCTTGGAGTGGATGAAATTATGAAAG
TGAATGTTAGAAGGAAGAAAATTGCCGAGCCAAAAGTTGCGATGTAAATC
AACAAATTACGTAATTGAGTCTCGTCACTCGATATCGGTTTACCAGTGCTTG
ATTACTAAACCGTTAAACATTATAACCGGTATAAAACAATTATTGAAGTATCA
ATGCAACATCTTACTAAGTCAATCACCGTGCTTTTCTGAATAATATTCATAAC
ATAGACCCAAACCATACATAATTAATACTTATACACCGTGATCCGTTGTTCAT
CTGAAGTCATTCTGAGTTTTAGGGGGTTTTCAACAAGACGTCTCTTTCATCC
ATTTTCAGCCGTCTTTTCTTATTTCATATTTAGGGGCCGGATGACTTGCACAG
TCTAGGCTAGGTGCCTACGTATTCCTCCGAATGCCGTCTACAGTGTCCGTAC
CTACCAGTATGAACAACAATCAAGCCTCATTCACGGTGGCGTTACCTTATTC
ATATCTAGCAGTCCTCTTACTAGGTAGAAATACGACGTCTAGCCTACTATCAT
GTGCTACTGACGTCGGAGAACCTGGGCATATTCCGGAGTTCCTGAAGCTTA
GCCAGGCTTTTTCAATGCTGATTAGTTATTGGTGTAACTATCTCGGGCGTATA
AGAGTAAGGCGTCTCCTAAATGGAAAAGCAATGCATGTCTCACCTTGATTC
CCTTATTCCTGTTGTGGATTGGGGGCTATTTCGAGTTGCCAAGCCTTGCAAA
AGGCATATATTCTAATAACTGAAGTTAAAAAGAAAGCATCGTCGATCCAAGT
TCAAGTATGACTAGTGGATCCCCTATTTTGAGTTCGGGGAGACAAGTCAGA
CATCCTGAGGCTCTAAGCCACTCAAGCTACATAGTAAACTAAACAGCCAAG
ACATCCCAATAGGTACTATTATCTGGCATCTTTATATCGTCAGGGTGTCTCGC
AATACTAGGTAGGCTTCCTTCATACTTATTAAGTCTTGGGTAGTAAAGGAAA
CACGTACCCTCGATGATTCTTCGGGATTCACCAAAACGAATGACGGTCATAT
GTAACGATCATACTAGATAATGTACGATACCACAGCCGTCCCGCCAATTATG
ATTTAAATGATCGAGTTGCACATGCACATTCGATTTGTCTTAGATCTAGGCG
TGATGGTATTTAGTCTAACGTTCTGATACCTACCTAGCTACTGGGTATGCATA
AGGTCTCACAGATTCATGATTCCTGAGTGCCACAGGACAACATCAGCATAT
ATACAGTAGAACTTGACTAGGTACTCACTTAGTCAAGTTTATTCTTATTTGG
CACCAAGTTTGCGTGCTGAGTTGTCTCATACAGCGATCGACGACGCCCGGT
TTTGGTGGTGATGATGATTAGGAGCTTGAGTCCTGCAAAAAGTCAGATCAG
AAACATTGTGACTCGGACCGGATCAACTTATTATCTCCCGTTAGAAGCCGTA
CACGACATTCCGGGATCCTTCCGCGTAATCTTACTCAATTGACCTAGCTTCA
ACTGATCCTAAAATGTTCGGGTAGCCTCTCGGTGTCGTAGTTGGAAGGTTC
CCCACCATACAGAGCTACGGCCCCTATCTACTACATGTGTCATTCCTCGTGG
TCCTGTCGGGCTAAACTAGCTTCTAATCTAGCTGTTTGGGATGACGAGGTAC

CTACCTACCTAGCTAGGTACGCTATCGATCCTACTGTAGGTCTGTTCAAATG
TTGTAAAGTGTCTGGTTAATCATGCAACCGGTATTCCCCTTTAGTTCTTGAC
TGGATATGCGAACTTCAAGCAACGGAATACCACTTTCGACAGCGTATTCGA
GGTGTAAAAAACGAACATAGAACATGCCGGATCAAGTTGAATATACATTTC
CCCACAGGAATGCCTTAAGCTAGAATTAGCTCAATTAGCACGAGCGTTCTG
GCACGGACACCGGATGAAATACCCAACGCCCCCAAGGGGGTCCTTGATGT
TATTGGCTGTAGAACCAAACTGCGCCCCGATCCAACATTCAGGTGTGAACT
CCTCCTGAAATCCACCAATCAGTTGCTGGTAGTGATACACGCAGATAATCTA
CATGTGCTTAGCTACCTACTAGGTAAGACTCAGGTATCAATCATGCCAACAT
ACTTTCACTTAATATATCATTTCTCGCCAAAATATAGCAATATGGTCCACGTT
CGGTGATGGGGGCTGAAGCAAACGACTAGTGACATAAGGGTAAGGAGTCC
CAGCTTCAGCCCTACCTGAATCCAAACATAGCTCACGGCTTTCGAGGTAAA
TTAGCAAGCAAAATGCGTAAGTTTGTTGTTTCTTTATGACGCAAGGCCCAA
AAAATGCCTTGATTGCCCCCTTTCTTACCTACATATTCTGTCTCCCGATGTAG
GTGTGTATTTGTTGTCTTATTTTTTTCATTCATGCATACAACTCCAGGTTGTG
TAACACGTCCTGCATAAGGGTTTCCATCAAGCTCTTAGGACATCAATAATCA
CCAGATGAAACGTGGAATCCTGCGGCTAGCCAAGTTCATTGAAGAGCTTCA
TATGTTTCTCGAGAACTGGTTTTCGTCCTTCTCGTGGCCACGATAACCTATC
CAGGCTCGCTCTCGTTTTCGTATGTTGCTCGTTGACTCGACGTCGTCCGGCG
CGGAGGGAGTTCCTGACGCGCCGAGCATTGCGGTCCGAAACCCTGAAGGA
GAAGGAGATGAACGTCCAAAATGGAAAGGAAAGGGAAAGGCTATTGTCCT
AAAGATAGACGCAGAGTCCCCACCGGAATTGCCTGGACGGTGGCGAGAAA
CCCGTCGATTGGACCCAATATGATCCCCCGGAAGGCCTGAGAGATCCGGCT
GGATGCACTACAGACATTGTCATTCAAGTCATCCAGGAATCAATCGATAAG
GTCAAGGCTCGCATAATTGAGGAGGCAGAGAGGCGCGAGGCAGAGGAGG
AAGTCAAGAAATTAAGACAGGAAGAAAGACTTAGACAGGAAAACGAGGA
CGCAAAATACGCCGAAACTTTGAATTCGTCATTAGATGAGACACGACTCGA
CGCCTCAACGGAGGCTTTGTTACCAGATCGGCCAGCTAAATCAAAGAAAC
GCATGTTGATGAACCTTCTCCGCCGCCTGAAAAATGTCGAAGAAAAGGGC
GAGTCCAGTGCAGCTGGGGCGGCTCGCCATAAACGTGACGTTTCTTGGAC
CAGCATTGAACAGAGTACTCAAGCCGCGGGGAGACGCATTGTAACAGAGG
TTTTGAAGAGAACAACAACAAACAGTAGTGGCAGCCCTTCGGCCCATGAC
ACAGAAGTGTAAGACCTTCAATGATAAAGATATCGATCAAATTTCAAGTAG
CACTGACACCAATCTAGCGAATGCGTCTCTTGTTTAGACGACTTCAATCCC
AAGAGATGGTTAAGGCTCCGGGTCACAGCTATTGCAAGCCCTGCTTTCTAC
GCCTCATATCCACAGCCTGCCAAAATGAACAACAATGGCCACCAAATGCTG
CTTAAATCCTATCCCTACCAAAACCATAACCCCGAATATCAATGACGAACTA
AAGAAAACATATCGTGATAGAGCAGCTGAATGGGAACACCAGTTAGCGAG

CGCATGTACTGTAACTACGCTAATTGTAACGTATGGATCCGACCAGATCACA
TCAATCGCGACCGAAATACCGCGAGATGCTCAGCAGGCCATTGGTCTTGTA
TTATCTGCCGTGGCCCTGAACACCAAGGGAATGAATGCCCACAGGACAGG
GATCTGGCGAGGACTAACGAGCTCGCAGAAGCAGAGGGATGGAAACGGT
GCTACGGCTGTCACGCATATGTCGAGCATAGAAAGCTTGTCAGCATATGAC
CTGTCGATGTGGCGCCGAGTTTTGCTACGTGTGTGGAGCCCGTTGGCGAAC
TTGTAGTTGCAGCATGGAACAGTTAGGTGCTGTGAAAAGAAGGGCGGAAG
AACGACGCAAGCTCGTCGGATATAGAGGCTCAGGAAGATGCCGAGCTACA
AGAAGCTCTACGTATGATAGCGGAGTTTGAGCGCGGAAGAAGTCCCTTTCA
CCGGCGCTCGACCAGCTCGTGTGGAGCATATTGGGGATAGTTTGGAGCTGA
CGGCGCGCTAGATGTCCCGGAAACATTGCTGCTGCCGAGAGAAGAATGTCT
TCCTCGTCGACCGGGTCGTCCACAACGGCGAGAGGCCTGACAATCGGCGA
TGGCGGCGGAGCAGCGCGCATGCGTGTCACCTCGCTATACAGCTGCCCTTG
GTCGTCTTCCCAGATTCTGAGACAGCTTCAACAGCGGGAATGTCTTCGGCC
TCGAGGAGCGGCGCAGTCATTTCGCCGTTGGTGGCACTGGGAGTGCTGCA
GCGTCTGGTCGGCGAGGCGCGAATAGGGCAGGGGTTGAGACCTGAAGAG
GCCCGCGATGACCAGAGTCGGAAGACCGATGGCCTCCATGGAGAGAGGTG
TCTGATCCTCTCCTTTCAGAAGGACGATCTTGTCACAGCGCTGTTCTGTTTC
AAGACTAGGCGATAAGCTTCCGTCTTCGCTGGAAGAAGAGGACGAAGCGT
CCGTATACAAGTCTGTCACGTAGCCGCTATCGTGCGTTATGCCTGCATAGAT
CGTCTTGCAACCTTCTTGATCGGCGTGCAGTTCTAGTTGAGCTGATGGTCA
GTATGGAACCCGACAAGGGCACACGAGACGCGCCGCAAAGCTTTCGATCC
GCCACTTCTTTCAGACGACCGGCGTCAATCACTGATTCTCAGTATCTTCAGT
ATCGACCCTGCCAGGCCATGACATACCGGATATAAGCTTGTGCGCGTGACT
CAGGCCGTCCCAGAAACCGTCGAAATCTTTGCCGCTCACACCATGTTCCTC
GGCGGTCAGTTGGGACTTTCGAGTAAAGATGCTCTTACCAGCCATGCCCTT
CGCAGCCCTTTCAGTCTTGTGTAGACATTGATCACGATAGGATAGGGTGGA
GATCCTCTCGTAGAATTCCGCTCAATGATCCGTTCCTCGCCTTGGAGCGAGG
CCTGGATACAGGTCGAATCCAACCGCACTGGGCCCTCGGCGGGTTCAGGA
GTGTTCCCCAGGAAGGCGTGATGATCGCGAATGGCGCTCCATAGTCCGACG
TCCAGCTTCCCTGCCGCCTGCAAGACCCTGTCTCATGAGATCGTCATCGAA
CTGTCCTTCAGCTCGACGGTGACGAAGGGAACCAGGCGAACCAGCAGACC
GTCTCCATCGATGAGGACGAGAATGAATGGCCGAGAAGCCCATGTTGTATT
TGCCGCTTGCGTTGGAGCCACCGGGGCTATGGGGTGCATCTTGACTGCTCG
CGGAGTTGCACTAGCACTGCGGTCCGTTGTAGTAAACGGAAGTTGGGGGG
AGATACTTAGCTCGTCTGGTTGCGGATTCAAGGTGGATACTCCAGGTTGTTG
CTCGCTCATAGCGCTTTGTGCAGCGAGGAAGATAAACCGAATGGGAAGGT
GGTTGAGAAGGTTGAGATGCATCGTAAGCGTCAGCGGAAAAAGCACGCGA

TAAGCGATCCTTGCGCGTCCGCGAGGATCCTTTGCTGCCGGTACTTGAGCG
TTTGCCACGACTACACAACGCCGTGAGCGTGTTCAGGATACGCCGTGGGA
CGACATTGGGTGAATTTCTAGCATGTGGAGCACTGTTCAGTGGCATTTATTA
TCCTCTCGCGCGGTAGTAAATCCGTGCGTCCAGAGCGCGATCTTAAGAATG
CGCGCCAGGAAGATCGTTGAACCAGCACCACTTACTACGAAGTCAGCAAT
ATTCATGATTATCTGGCTTACTTTTGCTTCTTGCAAGTTGGACCATCCGGGC
ATTTATGCCTGACGTGAGCTTCCTCCGTTCTCTAAGCGATCACGCACGAGTA
GATGCAATCCTGTTCATCAAGATGGCCTGACGGTCAGTATTTGGCCTTGGG
GGATATCGTAGATGCGGGATAAGCCCACTCACACCCGCCATATTTGTGTGCG
GACAAGCAATCGTTTAACAAGGTTTTCTTAGTTCTTTACGTTGGTCGGGAG
TGAGATCTACTATTAGCGGTCCACCAACTTGCGAGCTTGGTCTCACCATAGT
CGTGACCGTACTCTTTGAGGTGTGAACGGTCGTCTGTCCGAGGACAAGCA
GCAGCTGCCGGGATCAGTACAGTGGAGACCTCTCACGGCCCTCCATCAAA
AGACGGACTTAAGTAGTGTCTGTTGGTTAGCAGGGATAAGCCATACAACGA
GGGCGAGGACTGACGCATGGCAAGGTCGGGGCTTGAGTGTCCTCACAGAT
CCCGAGACCCGCTTTGACTCGGCATGCGTGCTCGCCGCCTCTGGCATTTGA
GTTTGAGGAATGCCGTTCCCGATAAGGGTGATATCCTGGTCCGCTTCTACCT
CTGGTCCTGGTCCTGGGAGGGTTGCGCCACCCTCTTCGTCGAAGATCTTGC
GTAGAAGCTGCTCGGCGCGGTCAAGCGAGTGCGTCGAGGAGTTGTCGCTG
GCGACACTTTCTGCCGTGGTAGGCTCTTCCCCATCCGTCATCAGATCCGGTG
CCACAACTGTCGAAGAAGCGATCGGTCCTTTCGGGACGATCAACGCCTCT
GATCGGAATAGATCCTGGACAGCCACTGTTGGGAGATGTAGAGCTTTGATT
CCTGGAGCTATCTTATGGAAGCCTTTCAAGAGCACAATCTTGTCCCGTCTTC
CTTCAGTGGATAGGCTAGAGTCAGTGGGTTCTGACGGGAGGCATGGCTGG
CTCACGAATTGAGGGTCGTCATGTAGCTGTGATCGTGGGCGACGCCTGCAA
AAATCGTCTGCAGTCTGGCACACTTGCGAAAAGATCCATCTGTGCTGCCCA
ATAAGCGTATCTTACTGGTCATTGCAAGCTGTATACCTTTAATCTTGGCATCT
GCGCCCTCTTTCATATGTCCGACGTCAATCACTGAAGCTCAGCCAGACGGT
ACGATCTCGATCCAGCTCACTCACCAGACAAGAGCTCGTGAGCACCTGATA
GACCATCCCAAAACGCCTCGATCGCCGCCACATCCAAGCCTGACTTCTGAA
CGGGCTCAGCATGTCCTCCGCCTCCCGGCAGATACTCACCGCTAAGACGCC
GTGCAGACCTTTCTTATTCAGAAACACGTTGATGAGGATAGTCAACTTGTT
CTGCCCATGTGCTTGGCCCCGCTGCGTCTTGCTCTTGGTCGAAGGCGTGCT
GATGACTCGCGGTCCAACCAGTATCTCGCTGGTTGACCTGCTGATATCCATC
GCGGCCACATCGCGGATGGCCAAGTACAGCGTTCGGGCCGCTGTGAAGCC
TCCCGCGTAGCCTTTTCGCATGTGATGATCTGCAAACTGGCCTGTATCAGCT
GAGCCCAATGCCGCCAACGGGATAGTCACGATCAAGCCATCGCCATCAATG
AGTACGAGCACGAAGGCAGAAGGTGATGGAGGCGGGGGTGTTGCCCCTTT

GCGAGAGATCGGTTCGCCGCTGAGATGAACATAATCGCTCGCCTCCTCGTC
GTCCGGCAGCCTCAGCCTGCCCTTTCTTCGACTGAACGAGTTACCCATCAA
GTCGGAAGCGACAGCTCAGGCCGTGAGTTCAAGCGATCTTTCGGGAAGTC
GCAGAGCGCCAGCGACAGGCTACTAGTCAGTGGTGAGTCTCTGTACAAGT
CGGAGCGAACAGTTTCTCCTGCATGCCAAGCTGACCCAGGCAAAGGCGAG
GGTGGTTGGATGACGATTCCAGAGAGGTAGACGAGACGATCATCGTATAGG
CCCGCATCGGGTGAAACACTTGAGAGTGACGTGTCATTTTGGCCTTCTTGC
CTCCTCGGCCGGGCTGAGTTTTTGTGAGACGAAAGCGCCTCATCGGGTCG
ACGACACCTTCTAGACACCTTCTCTAGAGCTGCTTCTTGCATCTCGCTCAG
AGCTCACGTAGTCACTCTCAGACACTCTTCGACTACCCATCCGTATCGCAC
AGAAACAACCCAAGCAAAATGCGTGAGTGTCATGATCCATTTGCGTTGATC
GAAGTCCACCTTGGGCGTCCACACGAGGAAATTGAATGTCAGGCGAAGAG
GTGCTGACTCTTCAGCGCCCAAGAAAGACAAAGCCAAGAAGATGGCCTTG
GCCGACTTCTTCGCCGACACCAGTGAGTTCTCTCGTTCGCCACTGCCTCCA
ATGCATCAAGCAGAGGTGGAATGACGTTGGTCGGGCCATACTAAAGGATAG
CCTCCGGGTCGTGGGCGGACGACATGGTCGACGTTCCTACAGCACGTAAG
CATTGGGCCTGCTCCGATACTCTCCGCATCGCTCATAATCAGCTGCTGCTCG
GGACCCGATGCCGGTCCCGTCAAAGGCGAAGCAGGATATCTGGATTCAATG
CCTGACCGCGCCGCACGCGCCGATCGACCAGGTAGGTGTCTCAGGTTGAA
CTCCGACTTTTCTGACAGATAGCCTTCCCCGGTGCGCCTCTTCGTGACGAG
GTCCCTGTCCCCACGGTCCCCCCTTCACCGCGCACGTCGGCAACCTCACCT
ACGAGGCCGATGAGGAGGCGCTGCGGCACTTCTTCGGACCGGCCGAGCCG
TCAGCGTCCGGATCGCCAAAGACATTCAGAGCGGCCAGAGCAAGGGCTAT
GCCCATGTGGACTTCCCCTCTGCCGACAAATTGCGAGAAGCTCTCAAGCTG
AATGGCGAGCAGATGCAAGGGCGCGGAATCCGTATCAGCGTCGCTGAGCC
TCGTGCGTCTCACAAGCCTTGTGGAGAAGGTCTGGCTGACATGCAGCTGC
CAACAGGAAAGACTACATGCCTTCGGCCGCTGAGGAATCAAACCAGTGGC
GTCGGGCTGGGCCTCTGCCCACCCGTGAGGCGCCGGCGCCAGCACCCCGC
CGCGGGTCATCGTTCGCTGAGCCGGGACCGGATCGCGATTGGACAGCGGC
CAGGGGAGCAAAATTTGTGCCCGCACCCCTGCAATGGGTCTGGGTATCAGA
CGCGAATCATCGGGAGCAGGACGCATGCGCGACCTTCCTCCAGCGTGGCA
GATGAGGCAGGGACAGTGGAGGAGCGCTCGACCATTGGTCGAGGCACGC
GGACCGGCCAATGGCGGTGGGTCGGGC

>Mosaic_sequence_42

TGGTACTCGCGAATCGCGATTCGAGACGGTTTAACAAACGAAACTGAATCC
GAAGCTGCACAAAGGTCTAATGCTATTTCCGAATCTTGCAGGATTTATCGAA
CACCGCGGGATCCAGTGGCTTGGAACCTTATGTCCGTCCTGAATCTGATTTG

ACTCCGTAAGCCGCGGAATGACACAGGTACAGTCTTTGTCGAATAGCCGAT
GATACTAAAAATGCAGTTTGTCAGCTATACTGACGTCTTATATAAGACACCA
CGTGTGTCTGGCAAGGGAAATTCCATGTGCCACATTTTATAATAAAAATAAA
AAAAATAAAAAAACAAGACACCGCACAAACTTTGGTGTCTCGGTGTCTAG
TAGCACTTGGGTAAAGATCCTATGCAGTGATGGCACAAATGACACGGATGC
CGTCTCTGTTTGGCAAATCATCGTCGAGGAAACTTACACACAAGAACGACC
CCTCAAACTACACACTAGTCCTGTGATGTTGTTCGATGGGACGTGATTATAA
ATTTTATCACGACAACGGCGGTATTCCGGAAAATCGAGTTGTTGGAGACTT
GGTTCGGGAAGGTCTTGACGCAATGTCTATTGTCTTAAACTTGCGTCTTGC
GTCGTGCAGGAAAATAAAGTCCGGACGATGTGAGAACCCGCTCGATTAGA
CGTTGTGAACAGACTATGAGGTGATTGTGGTCTGGTTCTATTATCGAACTAG
TGCCGGGGAAGGTCGACAGAGATCCGTGGGGTTTGATTTTAATATTAATGAT
GGTCGTCTCGAGGTTGACAGAATGAAATTCGACGGTTCTGGACAAGCCTAT
TAAAAAATAATGATAATCAGGTATAATAACTCGAGCTCAACTCAATATATTCG
ACAATGTGTATATGGAATATGGTCAGAAACGAAAGTGGCAAAAACGGGTAA
AAAGCAGCCAGGCCGAAGATAGTTGAAAAAAAGACAGAGACTCAGGTTG
AGTTGCAAGAACTTCCAGAGCTCTGCTCTTGCTGTATTCACAAACAGCTTG
AAGAGAAAGGCTTGGGTCTAGGTGACTGAAGATCCAGATAGACGAGACGC
CTCGAGAACCGGTCCGCGTCCTACTCAACTTATTAAGATCAGTTCGATCTCC
TGAAGATAAGAGATGGAGGACCTAGTGTGGAGAACTATCTTGATGCTTTAG
ATGGCTCAAGTGCTTAAGCGGGTGCTCCAACAAGCTTCTACTCCGTGCTTG
GACTGTGGAGTGGCATCAAGGGTGGATCAGAGGGTTAAACGGGTGCAACG
AGTGGAACAAGGTAAGGGTACAGGGTACCGTAGGTATCGGGAAAATAGTG
GGGGTCCCGAACTATGTACTACTAAGTACGTACTTCGCTGAACTCTGAAGG
AAGCCGGAAGGAAAGAAGCTTGTTAGAAAATAAGCCCTGAAGAAAATGG
AGTGGCGCCTACATGCGAAAAATACTAGACCTTTTACTTCGAGTTAATACCG
TGTACGGTAATAAACCCGGTACGTGTGTACCATCACCCCGTCAGCCCGTATA
GAAATGCTGCATTTGAGCAACTTAAAAGATCGTGATAACAAAAAATGTAGG
GAATGTAGGGTACGTGGCGGTGGTTTGACCCACCATTACTAGTACTTCTCCT
GTTTTCCCTAACAGATTCTGTGAAGCGTTTTCGCGCCAGGTCTCAAATTGC
AACCCTGAGCCGGGGGCCAATGTAAGCGAGCAACGCATAAGGGGATTAGA
CTCGTCTCATCTTTCCAGCTCCCACCGCAGGGCTTCCAGGTGCTCGCAGGC
AGTGGCAGGCTATCTGCAACAGCTTCCGATCAATCAAATCGTCAACCAACC
GGCGATTGGCATGGGTTCTTGAGCCTCCTTGAGCCTCCGTGATCTCCTGCG
AGCATAGTTGCATAATCCCGAATTGTTTTGACCTCTCACTTGAGGACGAATC
CTGCGTCTGGAACATCTTTGACCACAATCACCTTCCCTGCGAAGATCACAC
GTCCAGAAACATCTTATTAGCCGCCAATCTACCGATACGATGTCCATTGGTT
CACAGAATATTTCGAATTTCGAGAAGCAACCAATCTTTTCGGTATCTCGAAG

GATTTCTAACTGACAAGATGTAGCTAGAAACTATATGCGTGGTGGTGGCCAT
CAATAATAGACACCACCAAAAGTGCCTTGTTGTCGCCATTACTATTGGTTAG
CCGTCAGCGCGCGGGATGAAACACAGTTGTCGTTTTTCGTGATGTCCCAAA
CGTGACCCTGTGGCAGTTAACGAGTCTAACAGACCAGCCAAACTCTTGGC
AAAGCATGGGCAGGGCATACCTGATGATTAGCTTACTGCAACAGTTGCCGC
TCGTCCCGTTCGTCCACCAAATGCCCATCCGATCAACATTTTGCCATGTAAT
GTAAACAGAGCGGTCCTATCAACTCAACCCGATGATCCACCATTCCTGGCA
GGAGATCCTCATTGTCTTCTTAGAAGCAATGTATCCGTTCGGGAGCATCAAA
CATGTCAACACGGCCCCAACGCCCAACCTGGCATATTCCGAACCGGCGGTC
TGGTGCATTTTACTGTGCAAGCTCCAGCCAGTCCACATTCCTAGAATACACC
CCGTGACACGTGTTGGTCTACGACGTAGTGTTTAAGACTAGAAATGGTGTC
GCTCATTTCCAATGCAGATCCGAAGCTATTTTCTCTTCCATCGTAGATCGTGT
GAACTGCTTCAAGTTTGCGCCGGTACGGTTGCCATCGTCCATTAGAGTATCA
CCGTCGTTCGTGGTATCCGATGAGCAACATTGTTGCTGCGCAGCGACAAAG
GCATCATCCCATTCTTTTACTGTGAAGCTTCCTTCGCTCTCCACGAGGATGA
GTTCGTCATGTGACGTAAACGAGAAAACGTGGAACGAGTCTGAAGTTTGG
ATCTCTCGCGGCGACGGGTTTATGACGACCTTCTTCGATTGGCCATCTGCGA
TGATAGCCAAGGAGGTCGAGGTCAACGTCGCTGTCAATGGAAGGGCAGCG
GAGAGGAGAGCTAGTACTGCTGTCTGTAGTAGAAGCAGGTAGAATAGGCA
GGTTCTGAGATTGCATGACATAATCAGTACACCCTACATCACGCCAAAATTA
TATGAAGCATATGAAAGTATGACTTACCGAACTAGCCTGCACAACTTTGGC
GTTTACATATTCGTTCTCCGGGGCGGTGGTGACCTGTAATATAACTTGGATG
AGGGTTCTGGGGGAAGTTGTTGACGAGGATGATCTGGCGCAACGTCGACT
GAAGTAATGATTCCAGGTGTCTTTCTCTTGTTCCTGGAATAACACGGAGTC
AGATAACATTTAAATAAAATTTCATGACGGGTTTCAATGTCGAACCTCCAAC
CCCTGCAGCTGGTCGTACGATCACATCGACGGCAGCTTCCTCTGGGAGCTC
ATCTCGTCTCTGCACCTCGATCGGCCCATTTACTGCCCCAACCACAGTATAC
CCGGCATATGAAAAAGTAGCAGAGCCGTCGGCGTTTTGCAGATGAGACAAT
AGAGCTTCTGGCTCAGTGTTGACCGTCATTTTTCTAGGAATTATCGTGGTTT
TGCTGGCGATGAATGGCGCATGTGCTGGCTCTTTAGAGATCAAAAAAGTGA
GATGACGATAGTTCGAACTTTTTCTCAATCGGGTATCAACCTGGGGTAAGGT
GGGTTTGGTGGGACGATGTGTCGTACAAGACGGGTATCTAAGCTCCACCTT
TTTGTATCGGTGCCTTGCCTGGTGCTCCTTCCGCTAGAAGGTACCTAGAAAT
CTAAGACATTGGATGTTGTTTGTACATGTGAATTCACTAACTGAATTATTCAT
TAATGCATGATAGAGGGGTAGTTGTGGCGTTACTTCAATGTTGCATACCTCT
GATATCCATCACGGATCTTTATAATCAACCCGTCTCAATCATCTTGTGACATC
TTTTCACGATTCTCATGTATAACACCTAATCTTAAGCAACATCTTACAGAACT
ATATTTGTGACACTCATAGTGACAAAATGGCAACTCAACAGTCTATGCAGTC

TACAGCAGCTCTTCCAAATCTGAGAGACAAGCTTCCCAAGCTAGAACCCC
GCCGTCGCCGACCTGCACCTTCAAACCCTCTTCCATTACCAGAAACGCCAG
CCTTACCGCCACCACCGGATCTTTCATCTCATACATTCCGGGTCCCTAGCCG
ACGAATCTTATCCACCAAAGACCATGAACTATTTCTCTCATCACCATCTTATA
ATCTAATCGTCGCATTTGTCTTCGGTCTCTCAGATGCCGTGATAGATACCCC
AATATCTGCCGTCAAGGATGACGACTGCAGTCCCTCCGTAAAGTCTTTTCT
GAATATTCTTGACAAAGCAGAAGCTCTGGTTCTTGAAACGCCTCCGGAGGA
TGCAGAAGGGTCGAGGTTCGGGAACAAGGTGTTCCGCAACTTTCTCGACA
AGGCCAAAGTCGCCAGCGCCTCTTGGCATGAGGAGCTTGGGCTGACATCT
AAACCTGCCATCGCAGAGTTAGAAACGTATCTCAACAGCTCATTCGGCAAC
CGGACACGGATAGATTATGGTTCAGGCCACGAGCTAAATTTCATAATTTGGC
TCTTATGCTTATATCAACTGCAGATTGTGAAGACGTCGGACTTTAAGGCGCT
AGTACTGAAGGTTTTCATCCGATACCTGCAGCTCATGCGAACGATTCAATCG
ACGTACTACCTCGAACCAGCTGGTTCTCACGGTGTCTGGGGCTTGGATGAC
TACCAGTTCTTGCCGTTTCTCTTCGGAGCCAGCCAGCTCCTACACCACCCC
TTCATCACTCCCTTGGCAATCCATCAAGAATTGACTCTTGAAGAGTTTGGTC
ACGACTATATGTATCTTGGACAGGTCAACTTTGTCAACAGCACCAAGACGG
TCAAGGGTCTACGCTGGCATAGTCCAATGCTGGACGACATATCCTCGGCAA
AGAGCTGGACCAAGATAGAAGGTGGCATGAAAAGGATGTTTGTGGCAGAG
GTCCTACGAAAGTTACCAGTCATGCAACATTTCCTTTTTGGCTCACTGGTGC
CAGCAGTTGAAGGCATGAGTACCGAACTCCAAGGAGATCTAGTAGGCGAA
GAGGATGAAATCGAGGGCGAAGGCGTTGTTGTTGAGCATGATGGATTTAAG
CACGTACACACTGCTAACAGCTGGGGCGATTGCTGTGGTATCAAGGTACCC
AGTGCAATTGCAGCAGCCCAGGCGATGAGAAAGAATGGCGCTGGAGAGCT
ACGCCGTATACCGTTCGACTAAGTGTTCGATTAGGAGGCTATTGGTTTTAGA
GGTTCCTATACAAGAGAACATTAATAAAACAGTAAACGCAATATCCCGACA
CAATATATATAGGTACGATGCACCACGAGATATAGTGATAGAAATTGTATTTA
TACATTCTGACCGTAAGTGTCTATGAACGGATCTCAGAACGGAATAAGCCA
ACGCCTTGTAATTTAACCACCTAGCAAAACCAAAAGGGGTATCCTTAATCTT
CCGGAGCTCACTAACTACCTTATTCACATTACCGATTTTTAACCTCTTAATCA
GCCGTCTCAGGTGTTAGCCACAGCCAACCGCCCCAGGACAACCAATCTGA
ATCATGTACATTTCACATGTACATAAACCACCTCATCCTACTCTATCCGTAGA
GCTCCTAGCTCGCACGATACAAGTTCATAAGATCTGTCCGCATTTTCTGCCG
CGCATCGGCATCCAACCTCTTCTGGTTCTCCAGATCCACAATCTTGCCCCTG
AGCTCCGCGATCTGCGCCTTAGACGTCTTTATCGCCTCGCGCGCGGCATCA
CGGCCCTTTTCGAGGCCCCGGGAGCGAGCCGCGTGTCTGTCTAACGCGGA
GCGAATGGCGTGCTCGGTGTTCTTGTCGAGCTGGACGCCGGTTAGGAAAC
CCATGACCTCATCCGTGATAGTAGGGTTGTTCTTTCCCTGGGACGAAGACG

GGGTTGATGACGGTGTTGTCGATGTGTGCTTAGGTGTAGAGGCAGACGCAG
AGGTAAACGAGGTAGAAGCAGCAGGAGTAGAAAAGGATCCGTTGTTATCG
GTGCGTGGCCGCTTTGCAGCACTCTCTTGGTCGGCGAAGAGAACCCGGCG
AACTGGTTTATCGGTGAGTGGGGTCGGCAGTCCGTCTTCCACCGTGACCGT
GCGACTCGCGGAAGGCGTCTCAAATGCATTGCGATGCCGGGTCTGGGTCTT
GGCGGAGTTGTCCGTGATAGTCGCGAGCTCCGCCTCGTCACCGGAAGAGA
AGTCGCTGAACTCATCGACCTCCGGTCGCTTTCTCTTAGATCCTGCCGACG
GCGTGCCATGTATCGCGCCTGACTGCGGCTTGTCGCCTAATTCACGGAAGA
TTTCGTCTATGTCATCCGAATCCTCGTCCGAAGTGAAAAGGCTGCCCAGAT
CATTGTTGGTCGACTCTTTGTTCGCACTCGAGCTGCCGCCCCTCAAGGTCT
CGCTCTGCAGAGTTGCAGACGAGGCTGTGCTCTGGCCATTTTGATCGCGGC
CGAGCAGACGATTCAGTTCGGAAATGCTAGTAACCGGAGTCTTCTCTCCAT
CCGGCCTCCTCTCCTTAGCTGGAGTAATGCTCTCGTGCAACGTCCTCTGCCT
CTTCGGCTTACGGCTCGGTGTGTTGTCAGCCTCGCTGCGCGAATTAGTCAA
GACAGCGCCAACTTCACGCGCATGCGCATCCTCGGACCAAAGGAAAAAAT
TGCATTTGTTCTGCTTATTTTTGTCCTTTTGGCATGTGTAAAATGAGCGACCC
TTGTTGGCAGTTTCTCTTGTGACCGTTAACTGGACCGCTGGGAGTCGAGGA
GTACAATCGCCTATGAACAACCGAAGCAAAAGTTGATTAGCTTTCTATCCG
ATCGCGGTCCGTGCAGTAGGATGTTCGGGATGATTTTAACTTCAGGGCGTA
ACTTACAGTGCCAAACGCCGTTTCTAAAGAGGCCCTTGAGTGGAGTTTTGC
TCGCCGGCGTGCTAGGTGTAGTAGCTTGTGACCGTTGCATGGTGGCGAGAA
TATTGAATCTCACTTGGTCTTGTACGTATCAAGACGTTTGCAGATATGTTACG
ATTCGCACAACGGAAACTCGAAATACTTAATATCCCGTAGAGTTGCAACTC
GGGATATTGATGTTTGATGCTTGTTGTCGTAGCAATGACCATGAGTTTAGGT
AACAGCGGGGCAGACTAGAACTACCAGAATTAATGTACTTGTTTGAGGTGG
GATACATGGACCCATCCCGATGTACAAAAACGGTGGCTGGGACGACCGAG
CTGAAACTAAGACACGTGGGGCACGGGTTACTCCGCACTAATGTTACCAGA
TCTCAAAATTCTTGGCATGTTACCTGCATAACTTGCATAACGTCGCAGGGGC
TTCCCATCTTTCTCAACCAGCTTCGAAAATCAAAGCGGGCTACCTCATCGA
GTAGGTAACACGACAGTAGTAATTCTAGTGAAATCCGGTCAAACCCAGAAA
AAGAAACAGGAAACACAAAGGACCAGGTTTGAAAAGAATGAAATCGAGT
TTCTGGATGTGGTTACGATATCAATAATCAAAAGGAGATAGACATTCATTAC
ATACATTACAATAACGACTCTCAATTCATTCATGGGTATATCATCTATGCGTAT
CAAAACAAAATGTGGTATCCTCAGACGATGCGAACCTCCCTAGCTTCCCTT
GCGGGCGTCTCTGAATGTCGATTAGTTCGGAAACCAGTGCAATAGTACAAC
CCCTCCAAGTCCTAGATCGCAAGCTCAAATGAACAGCAAACGATCCTGTAC
TTCTCTGTCAGAATGATAACGTCCAATAATGCCAACCCAATCACTTCATACA
CCCTCGCGTGTAAAATCATATCCTCTTCAGATCTCATACCAATAGTCGTCATT

AAGCCCCTCTCTGCTCGGTACCACACCGGCACCTTCCGGAGGTCCGGCGTA
GGTGGCTTCCATCCGAACGTTGAGCAGTCTGATCTTCATGCCGTAGTCTTG
AACCAGCTTGATGAGCTTATTGGTCTCCGATGTTATCGGCGCTTGACCTAAT
AACATCAACAATCCGTCTAGCCATTCCGAAGCCACACTATGGTTCAGAGGA
TGTAGGGTAAGTAACGGCGTTTCCTTGACTTCGCTCAGGTCCTTAGCCTTG
CTAACTTCGTCAGGATTAACGTAACCGAATATCGTAATCTTTGTGCTGTTAC
CTGCCCCGTTTATGTTCTCAGTAGACGGAGCAGAGACATTAGAGGCAACTG
AGCTAACTTGTCGTAGGTCCACCTTTTCGGGTAGAGAATCCAAATTCGGGT
CTGGACCAGTCCGTAACCCAAAGTCGGCGTAATGAAGATAGCGGCGGTTGT
GCGACAATTTGGCATATCGCCAAAGTGTACCATCAGTTCCGCGGCGATTTTG
ATAGCTGGTTCCCTGACTATCCTTTCGTTGCGTCGGTTTCGAAAACCAAGA
GCCGTCTAATAAGCATCTTATACGCTGTTCCGTAATGAACGTAAGAGCTTCT
TGGTTTAGGCCGTCCCGCACTTTGTAAAGATGTTGGCCCCATTGATCATCAA
ACGAGAGTTCCAGAAGCTCCATCTGCAGCTCGCGCAATCGTCCAAAGTCAT
ATTCCACTAGGTCTTCCTCAACCTCCAAAATGTCTTTCGATCGGCCAGCTTG
TCCTATAACTTGATCTATGAGTATACGAACCAATTCTGACACTTTTTCGAAAT
CGTCCTGGTCTGCCCCGGTAGATCTCCAAAGCTTGAAGAAAGCATTAAGTC
CACCTGTATGCAACCTGGACCACTGGAGAAGTAATGGCCGGAAGAGCTTG
TCTTGGTGCTTTCCATCGGATAGTTGGTACCCCCTAAGATCTTCCATTTCAG
ATCTATCGCAATCGAAATGCTCGTACAAGATAAGCGTAACCGCCAAGCTCG
ACCTCGCAATAGGACAGCGCTCGTGCAGTGGTTTGGTTGACTGCTCCAAGA
GCAACTTCTGGAAGCCGTCTTCGTTCTTTCGCACAAAATCGGCAAGATCCA
TTAGGCCCAGGAATCCAGTTTTTTCGAACTCCGCAGCCGGACTCTCCGTCT
CGAAGCCAAGACGCCTCCATTTCTCCGGATTGTGTCTTCTGCTTCCCTTCTT
TCCAGACTCTTGGAGCTCTTCGACTCTCCCGATTCCGTTCGCATGGTCTCTA
TTCGGTGCGCTCGCAAGATGGAGTCCTTTCAATGCCCTTCTATGTTCCGGTC
TTTCGACGTCAACCTGGACCTGTCGCCATTTTTGAAGCAGTACCTTAGTAA
GGGACTGAAACTCCAATAAAGGGTGCGCGAGGTCTTGCAGTGCTGAGCTC
TGCATCAGGTTGTATACCGCCTTGATCAAGCCCAAATCTTGCAATCGCCTTA
TAAATTTGGGCCATTCTTCTCCACTGCCTATCCTGCTGTTTTCGTTGCTGGAT
TCGCTCGAAACGGCATCCCTTATGAGAGCATTGATCAACATAAGGGCGTTC
GCGCAGAGGGCGTGATCTGCAGACTGGAGCTGCTGAATGACGAGTTCGAA
GAACTGCGGGTAAACGGCTACAGCTGGCTTTAATGCGCGAAATCCGAACGT
CACAGGCGCACGGCCCCCAGAGGATGTAGATTGCGAATGGCTAACAAGCG
CAACGAGAATGGACATAGCACCTCGAAGGATGTTGACTAGCGGATTCGTGA
CAATCAGCTCGACAACGCGCTCGACAAGATCGCCCGCCGTAGGTCCCTCG
AAAATATCCCATCCCATATCGACCTCGAGCAATCTGCTTAGACTTTGTAGCG
AGTAGGCGAGTGTGTTTCCGCCCGTACTCATGATCAAGCGTCGTAGTATGAT

CAGACCGCCAGAAGATATGAAGACATCTGCAAAAGATGGATCGTTTATGGA
GGATTGTAGCTTAAAAACTGCCATTTTTCGGGCAGCATCTTCGTCGCTGGC
AAGTCGCGACAGTAACGCAGGTATGTCTGCTTGATCCATGGTGATGGTAGG
TAGATTGAATCGTGAGGGTTTGCGACTCAAAGGGCGGGGAGCGAAGGGGC
CGGGTGTGAGTCGGCATGAACCAACGCGAAGTGGTTACTAAAGGGCTAAA
GAACCTCAAGGAGGCACTATAAATGCATAATCGTCAGTATAGTATGCAATGA
CATGCGCGCGAGCTGCGACGTTTTGCTAAGAGCTGTGATAAGCTAAAAGGG
AGAAACTAGCAACGCGGTTCGAGCGTGGAAGATTAAAAAATAGGTGGATA
AGGAGAAATCGCACCTACCGTGTATAGCGCATATGGTGAATTGAGACGGTG
CGGTGGTATTGGACAGGGAGAACCTTCCAAAGGTTCCGACACGGGTGTGG
GGGCAGGTTCCTATTCGGTTCTTAGCTTAGGTGCTCCTGGTAGCACATGCAA
GGAACAGAGGTTTCCCAAGAAACGGTAAGATGCTGTAGTGTAAAGTTTAA
ATTGCATCAGTGCGCAAAGACAGCAAACCTAGATCCGAGTGTTTAAGAGTC
AAGACTCAATCTTGGAAAGAGAAATCAAATGGATTTAACATATTTAATCTCC
GTATGAAGTCTCGATTGATGAAATATCATATCATACCATATCTTACTTCATTTC
CGTACGGCAGATATCAAGCTTCATAATTCCACGAATCAATAGTTGAAGATAT
AGTTTCATTCGCCTCAGGTGGTATACTTAATCTACTAGCTATCTATCCCCAAT
TTGATTATGGCGCAAACCAAAAAGGCTTTAATTTTCAACTATTTCATCGACA
CTTACTAAAGCTCGAATATTAAACAATCTTGCTATGAATCACTTTACGCTGTC
TGAACCGGCACCTCCTTTGCCGATGCAGTCTTCTCCAGCTCTTCGTGAACG
ACGGCGTGAACATCTTCTCCGTGTTCCTTGCGGTTCAACACAGCCTCGATT
TCGGCTTCAAGACGAGAGCCGCCCTTCTTAGTTTTCCATGCGGGCAGTGCG
CCCTTGGCAAACAGCACTTCGATTTCTTCGATCGTCCTTACCGCATGTTTCG
GGGTAAGTGAACCAGGCCTGGATAGCAGCACCAATGCAAAGTACTCCAAA
GATGATGAAGACCTTGTATTTGATGTTCAGGAAGGCTGGAGGAGTGAAGA
GACCAAGGGCGAAGTTGAAGAGCCAGTTAGCTACGGCAGCCAGTGACATA
CCTGTCGCACGTGTTCCGAGTGACCAGACCTCAGCAGCGTAAACCCAGGC
AACAGGGGCTAACGTGAGCGCATAGACGATGATCAGAAGGTAGCTGAATG
CGATGACGCCGTGTGCGGGGGAACCTGTAACTCTAATAACAACGTTATGGT
TGCCACCGACACCCTCGGGAACGGGAACGCTGTAAGTGCCAAGCAAGCCA
CCAACAACAAAGTGGCAGACGGCCATACCGATGGCGCCATAGACCAGGAG
TGATCGGCGACCAGTTTTGTCGATGAAGAAGAACATGATAGTTGTGAAACT
AAATATAACTAATTAGAATCGACAATACAGTGTCCACGGGTATAACCTAATC
AACTTACATGATGAACAGGGCATATTGAATACCAGAAGAGATCAGGTTGAT
ATCGCCTTGGAGATCGGCCATCAGGAAAACGTATCCGATGTAATAAACGAT
AACGTTGGCCCCCGACATTTGCTTTTTACACGGTCAGTTTGGCACAGTTTC
ATGCAAGTATTTACTGTAAGAAACTTACCTGCCACGCCTGTACACTCATTCC
AGCAAGCGTCCTCTTCCACATTCCATTTATAAAGAATTTTCTCCATCCTCCG

ACCGTCTCGCGTTCAGCGTTAAGGACAGTGAATATTTCCTCCCATTCGGCG
ATAACAACGGGGTCGTCCCTGTTGCCACCGGCTTGGATATCGGCGAGGGTT
TGGATTGCTTCCTCCTCGCGACCCGACCTTTGCAAGCCATCTTGGAGATCT
GGGGAGGAACGGAAGACCTAGCATAAGCCCGACACAGGGAATGAATTGTG
TTCCCCAGGCAGTTCGGAATGATGCCGTACCCTCGATCTTGGAGCACTACA
GCATCGATAAACGTCAGTCCCAATTTTCAATTTCAATTTGGAAACCGTGTAC
ATGAAACACAATAAAATAGAAAGAATCACTTACGCCGTAGCCAATGAAGTA
CATAATCAAGATACCCCATTCAATAGCAAGTTGTTGGATGACGATAATGCGA
CCCCGAGTGTCCTACGAATCATTAGATACTGCTGCTTATTGAGACACAGATA
CACGTATAGAATATGACTTACGCGTGGGGCGATCTCGGCGAGATAGACTGG
GACTTGAGAGGAAGTAATACCGACACAGACACCGTTCAAGACACGGCTCG
GCGATCTGCAAGACGGCAGTGGGCAAGGGTTACTCGGTGTCATCGGTGTC
GTGAGGTGTCTCCGCTGGCTCTCAGTGAGTCGACTGACATAGAGATCATCT
GGAAGTCCATATCGCACCCCAGCGGGTCATACCCCTGCATGGGCCAACGAA
GTGACATGGCACAAAGGCGACGCCTTTACTCCATCGACATACCGAGACCTC
GTCGCTGCTTCCGGAGCGGTCGTACATACTCTCGGTATTTTGCTGGAGGATA
CGGGCTACAAGGGGCATCTGAGAGACGGCAATGTACTGGGTATACTGGGC
AGCGTTGCGAGGGGCCTGATGGGGGATGCAGCGGGCAGGCTGAAAGGTG
AGAAGGAGCAGAGGAGGGGATACGAGGGCATGAACCGGGACTCGGGTGG
GTCCCATTCCTGCAGATCGAGCTGCTGGAAGGGTTACTGATGCCAGCTCTC
GCTGTGCTCGACACGATGCTCCACACTCCTATAGCCGAGGCAGGCTCCTCT
ACATCCCGCCGACCGTTCGTCTACATCTCCGCCGCCGACGCCTTCCGGCCC
CTCATCCCGCGCGGATACCTAGACAGCAAGCGCCAGGCCGAAGTGGCCAT
CGCCCAGCGGTGCAGAGCCGCGGACCCGCCGACTGTGCGGCCAGTGTTCA
TACGCCCTGGGCTGATGTACCACCCGCACATCCGGCCCATCTCGACCTTCC
CCGCGTTCGGCCTCTCCCTCACTGCCGCGGCGCACGATGCGCTGCCGCTCT
CGCTGCCGTTTGCGCGTGCGTCGGCGCTGGGCGGCGTGGCGGAGGCGCTG
CGCACGCATCCGATGCATGTGGACCATGTGGCCGAGGCTGTCCTGCGCTGC
GTGGAGGATGGTGAGCGGGAGGGCGCGGTCGAGGTGGATGAGATGCGGG
AGTGGGCTGGGTTTAGGCGGAGACGGGCTGGGCAAGGGCAGCAGTCCCG
ACAGCAGCAGCAGCAGCCACAGACGCAGACGCAGACATAGCGTCGGGTA
CAGGTCGTAGCGTAGGTCATGATGCATAATGAGCGTACTATACAATGGATCT
TCCTTCCTTCCTTCCTTCCTTCATTCTCGTAGATCTAGTCGAGACAGTGTCTA
GGCATCCCCTCTGACCTGGAAGAACCGCCGCCACCAGCTCCTGCCCTCCCC
GCGAGCCGATCGATGTACGCCATCCAAGATCCGCTTGTAGTACAGCGCATC
GTGCGCCGGCCGCAGCGTCTTGGTAGAGAGAGGATACTGCCCTCAGCCCC
GTCCACAATCTCAACCGTTAGGTGTCAAAAGTCAGGCTCACCTGAGCCAGT
GCAGAGCCGGCGGTGAGACGCTCGAGCGGCCCGAGCATGCGTGCTACACG

CTGTTCGTCCACTGCGCCCAGTGCGCCAGTCGCCGATGCCGATGTCGATGC
CGAGGAAGAGGCAGAGGCAGAAGGGAACTGCCGTGAGATTATACTCTCCA
GGGCGTCTGAGAGTTGAGGTGAGCCCTGTGGGCGCAGAGGTGTAGGAGG
GAGAGATCGCACGACCTCGACGAGCCGGGCGAGGCGGGACATTCTGCTTT
GCGCTGCTCTTCGATAGTAATGGACGGATGAAGGTGAAGAAGATGCAGAA
GGAGATCGAAAATCCCGTCGGCAACTTCTGACGAGCCGATCGGCCAACGA
CTTTGATTTTGCCGCTAGCCCATTGACCGCTTCTTCACTTTCAACAGACATG
CCCCTCTCGGTGGGAGACATCATTCAGCGCGGCACTGTCCTCGCCAGCTTG
GGCGTAGCTGTGAGCTAGCCCTTCCTCTGACGATGGCTGGAGGAGCTGAC
GCCCTGCAGGCAGTAGCGGGTGTGGCTATGGTGCAGGGCTTCAACGACCG
GCTTGCGAGGCGGAAACAGGCTGTAAGTTTCCCCGTATATGTCGACGAGTC
ATGACAATGGGCTGATGAGCAGTTCCGGGACGAACAGCTGGCTTTGGTATG
ATCCCTCTCTTTCACTATGGGTGGCGCTGACGTGCAGGCGGAGCAGCACGT
AGCTGAACAAAGCCCAAGTCTCTCGAAACCGTAAATCACCAATTAAACTG
CCAGGACACGCGATGCCATGACGACCTCACGACTCCCTATATCACTCCGCT
GCTGTGCTTTCAGGCCTAGGCCCATTCATACTGATAGACGGGCCCCTTTCTG
ACGCTTCCTCGCAAGACACTGGGGGAGATTCAGGAGGTGGCATGCTTTAG
GATCGATGGCCGTGAATGGAATAAAGACGAAAGGCCCCTTCAGGCTGCTTT
GGACTATACCACCGGCAGTGCTGAGCAGCGAGAGGCGAAGACCGGTCGCT
CCAGGTCCTTTCAGCGCCCACAAATGGGCTGATCTCCTGATATCAAAGGAA
CGTCCCCCCATCCCTCGGTCCCTCGATCCCATCCCGCCGAGGGCAGAGGCG
AAGACGGTGGACTCTGGGTCTGACTGACGCGCGACAAGGTCGTCTCTCAC
TCTACTCATCTCGTGATTCAACCCAATCGCGCCCGTCACTCACCCTCGTCGC
TCGTTCTCACCACCAGGCTTACTTATAACTCATCTTAGCCTCCCAGATAAGG
GACGTGGTGCGACCGCTCCCCGCCTCCCTTGTTCTTCCCTCTTGATTACGAG
AAGGGTCGACGGTGATGCCCCATGGCGACGGTGATTGACGATACCAACGC
GTACTTCCAATGGGTGGGGACGTCATGGTGAGTCGACCGTCTCGTGAGAG
GTATCACACTGAGTGCAGGCTGTACAAGTATCCCGGACACACGGGTGAGTG
GTCCGTGGGCGGGAAGAGAAAGGGCGTGCTGAGATAAGATGACGACAAG
ACGACCGACTACTTCAACTCGACCTTGTGAGCTTCATATTGCAACTGATGC
CAAGAGAAGCTGACGCTTAGCCACGGGACCACCAACAACGTGAGTTGGCT
GACGGCTCCTTAGTGCGAAGGAAGGAGTGCTGACGGAAGGGCGACTACTG
CTTGATACACTGGCAAGGGAAGGACATCTCCATCTTCGGCGCACGGCGGG
GCAAGTGAGTCATAGTCCAGCTTTGCTTGATTACATTGGCGGGCGGCTCGG
GCGGCTGAGCTGAGCGCAGTCACGGCGTGTACGGCGTCTCGGTCGACGGG
GGCGACGTGTCCTATTTCTCAGGCTACGCCTCGCCCGACCAGTTCCAGGCC
GTGCTCTACGCCACGTCGGGCCTCGCGGAAGGCGACCACCAGCTCAAGAT
CTCCAACGAGAATGCCAAGTCGTCAGGCGGGAGCTACATCTGGCTGGACA

TCGACGAGGTCGCGCTGACGGGCACCATGTAAGTCATCTCGTCAAAGTAGT
GCACTGGCACTCACGGCAGTCTGCCCCCGACCCCGTCGGCATCCTCGACTA
CTACCACTGCCATCGCGACAGCAGCCACGACCAGTACGACCTCCACTTCCC
CCTCGTCCTCGTCCACCTCGTCCACCACATCCACAGCGCCTTCCTCTTCCTC
GTCGTCCTCGGCCAGCTCCGCGGCCAGCACTAGTGCTTCCATCAGCGTCGC
AGCGTTCTCAGACAGCACCACCGTCATACCCACCACGACCATCGGCGTCTC
CCATGCGTCCTCCAGCGCTGGCGGCGGCGCGCCAGTGACGGGCGCATCGG
CGTCGTCCTCGGCAAGCAGTGCCAGTAGCGAGGAGAGCAATTCCAGTTCG
ACCACATCAAAGTGAGTCGATCTTCCCCACATTCTTTGCCTTTACCATGCAG
TGATGCTGACTGAGTGCAGCAACACCAAGCTCGCCATTTGCGTACCGGTCA
TCGTCATTTCCGCCGCCATCATCCTCGCCGTCGGGCTCATATGGTACGTCCG
TCGGCGGCGGAGACTGCGAGAAGAGCGCGGCGAGGAAGAGAAGCGTAGG
TCCCTCTCAATACCACCTCGCACTCATGCTGAGTGAAAGAACACTTCAACC
GCAGCGAGGCGCGCAATACGTGGAATCAACTCTAGTTTACCCCCGTGTGTA
TCCGAGCTGGTCTCATCTCCCCGTCGACTTGTACTCGATGCAAGCATTCATC
TCGTCTGGCGAGCATGAGCATACCTATACCTGCTTGTACCTATAGCGATATCC
AGCGCATACCTAAACACGCCCGCGTATAATATAACACAAACCCTCGGATAG
CAACGAGCGTGATGCGGCCGCTGCGACTGGGGCAGGGCCGCGGATGATGC
GGGCAAACGTGAGACGAAGTGTCCATGTACAGATCTCCCCCCTCCTCTCCC
TTGCTCTGCTTCCCTCCTACTTCCCTCTACCGCGCCACTACTCCGCACGGCC
CACGTCGTCAGAGTAGGCCGAGGCGTCCGACTGGCGTGCCGTGTTGCTCC
TTGTCCGTGCCTCGGCCGACTGAGGGGCGAGGCCGCCAGAGGGCGGACCG
GGATGGGGCATGGCGTCGACGACGCTCGAGCTGACCGTGCCGTTGTTGAG
GTGAACCATCGACTTCATGTCCTGCGGCGTGGGCGGAGGAGGCATGAGCG
AGGCGTCGGTGTTCTTCTTCCCGCCGAGCGAGGGGAAGAGTCCCCGCATG
GCAGAGTTCTGCCTCACAGGCTGCGGCTTCTCGATGACAGCGGGAGCAGT
GGCGGGTCCGGCGGCTTGCGACTTTGCCCTGGCAGCGTACACGGCGAGCA
TCTCGTCGGGTCCAAAGGTCTTCTTGC

>Mosaic_sequence_43

TTGTCACGCCCTTAGTCGTTCTCACACGCAAATTTTTCCACGGCCCCGGCA
CCGCAGGAGCTCGCCACCGCAACGGGGCAAGCGCGTGCCGTCCTCGTCAT
GCTGTGCCGGTACCTTGCGAGCGGTGCATTTTCGCACGTGCTGAGTGCCTG
GATCCCCGCGACCGCCACATCCCTGTCAAACGAGTACGAGCCAGCGGGAC
CAGCGTGGTCGTCAAAGCCGGCACGGACCGGGCACTGTTCGAGCACGAG
GCCGCGGTCTATGCCGAGCTGCCATCCACCGATGATTCGGGGACACGGCAC
CCCTGGCGGCGGTTCCTGCCCAGGATTGTTGGCCTCTATGTTGGCGAGACT
GATGAAAACCGAGCGCGTTCTCCTGAGGTACGGAGCACACTCTACCTGATT

GTCATGGAGCATTCTGGGTGGCCTGCGGCGCCAGGTGGTGATGTGTCGGGT
CTGACAATGCAAGATCGGTACGTGCGAGTTGTCCCTCACCGCGTCCGTCAT
CCCGTCTCAGGCCATGCCTTGAAGCCACATGCGACAACATGCCGAGCATGC
TGACTTATTACCAGGTCCGACTGTGTCAATGCCCTCGCCGGTATACACGACA
GTGGCTGGCTGCACGGCGACGTCGAGCTGCGGCACTTAAACCGTTCGCCG
GCAGACGGGCGGATCAGGTTGCTGGACCTCGGTCGCGCGGAGCGTATCGA
CAATACTTCCAAAAACCAAACGATCGCAGCAGCCGATGCGGATGCAGCCC
GAGCCCGAAGTCACGAGGTCGGTGTTACCAGGATGCGGGCCGAGTCGGCC
CGGCTGATCGGCCTGCTGGACGGGACTTGCTGGTGAAGGGGGATTGGGAG
GGACCCTCGCAACTGTTTGATCAGCGCAGCGTATGGACATGTGGCTGAGAC
AGGTTGGACACTTTGACGTTTTGACCGACCATTCAGCTGTCTCGAAGGCCT
GTTCGCTTTGACAATGATCGAGCGTTAAATGACATACTGATTCGGCGCTGTG
TTCCCCCTGATCTGTTACACAATGATGAGTGCAATGCATTCGCCCACTTGGG
GCCGAATCTACATATGTATTACAGTCGGAAGGTCTGTGGACAGGGCAGCCG
TCATGGTTTTATAGTTCCTACCTCATCCCGCCGGAAGAGGCAATTATCACCC
TACAAGGCTTCGTCGGGGCTTATCAACAGTGCCCCTGGAAGCTTATGGGCT
GCAGGTGACCGCGACGGGTCCCGTCATGACAAGCATCAGCCCCTGACCGT
TCCCAAGCCCATAGATCTTCGCTCCATAACGATGTAGGCCCGGGGGCGACG
GTCGCACGGTCCAGAGACCTGCCTCCTTCAGGCTGATGAGAAGGGATCGA
CCCGATCTCCGCCAATATGGACCCAATTTCGGTGATCTTGGTGTCTGATGAT
AGCTCCGATCCTCCTTGGTGTTCTGCAGCTCTCCCTGCACTCCTATGCTCTC
TGGTTCTTCGTGCGCATGGTGATAAAGGTGGAGGTGGATGAAACGAGACC
AGATCTTCGTGTTGAACTTTAGGTCAGCCGCCACGGAGTTACAGGAATTAC
CGTAGACAGACGAAACTACATATAGCCACCACTCAGAGAGTTTGTACAATG
AATTCTCTCTTCCCAGCTCGTCCGGGATTCAACAGATTTGGAGTCAAATATA
TCTAAAGAGGCAACTCTGAGCACAACTACCACCATCATGTTGACCACCAAA
ACCACTGTCAACAGCACTGCCATCAGCATCCCGATTCTGAAGGGACTGGGC
AACTACTTTGTGTCACAAATGACACCAAAGCAGCAGCAAGATCGAGCCAA
AGACACGTGAGTCAATCACTCGTCGGCGCTTGCCAAACCTGGCCGCGACT
TGTCGAAGATCAACCACGCGGAATTACCGCATCTGTCGAACCAAACATTGG
TCTCACTTGTTGGTGCTTGCCGAGGCTCGCCACCACTCGCCAAGACCGACC
TACCGCATCCGTCGCACTAAGGGATCCACCTCGTCTCACAAGTCACCACAG
CTCTCGCCAGCAACAAAGAGCTCGTCGATGCTCGCCGTGCTCGCCCTCCTC
GCCGCCGAACGACAATAGGGATCCAAGACACTTCCTCAGCAGACTGGCCG
CATCTGGCAAAGTCGACCATAGGAGACGGACGGTGTCACAATGCTACGCC
CTCCCGTTGGTAGAGGCCCTCTCGCACAATATCAGAGTCAGGAATAAAGGG
TAAAAGGTATAATCGAGGATGTTGAGGATGGATCCTCGTCAAGGTTGCAAG
GTCGTCGAGGTAACAAGGTCGTTGAGGGTACAGCGTGAGTGCAACTCAGC

CACGCTTGCGTGCCAACGGAGAGAGTGAGATCGTCAATGGTGATCCATGA
GACTCAATCAGAGAGTGCTGAAGAACTACGACGGGCGCCCGGAGCATCCT
ATATGACTCTGCGCCTGCTTTCCGTCGTCCCGCGTTCATCCAACCTCTACAC
CCTCGTCGAGGTTCTCCAGCCCAAGGTAGGTCACGTGTTGATCCGTGTCGG
TCACGTGAGAGGTGTCGGTCACGTGTGTAGGTCGTTGAGGGCGAGGCCGT
CGTGACACTCTGCCCACCTTTGAAGTGGAGCTCCTGCAAGACACACGCTAT
CTCCATCCGCCTAGCCTTTTCCTTCGTCGTGGTTGTCGAACACGCTCGCATT
GCCGATCCCGGAGTTGAGATAGCAGTCTTATGACGTTGCTTATAACGGGAG
CGAAACTCTGGCGACATGTCGTCAAGGAAACGGCAAAAGGACTGTGGATC
CGTCGTCGAGGTAGCCTCGTGTTGCTCGAGGGCGTGCCCATGTTGCAAGGT
GCTTGAAAAACGACATAAACAGTGGGGAGGGACGGCGTCAGCGCTCTCTT
CCCCACCACCTCTGACTTACGTCTCATGCCCTGGGTTCACCTGTCAAGCCC
GAGAACACTCGACGCATTCCAGTTTGCCAACCGCACCTCCTCCCACGTTGT
CTACGACTCCAGCTCCATACACAAGGCTCTCAACTATCCTCCTGCCTCCCGT
TATTCTACAACCCCTATAGACGACGCATCCTCTGACTCGAGCGAGTCAGTAA
CACTGTCGTGGTGTCGGAGAGGTCGGAGGTCGGATCGTCGGACGGACTGG
ACGAAGGCTAGGCTGGACTGGAAGCGCACTACTGTGAGTGGCAAACTGGA
CTGGACTGGGATGACTAGTCTCTGGCTTGTCTGCTGTCTTGCCTCTCTGTGT
AACAGAATGATGGCGCAGGGTCGCACTGGACGTGGTCTGGGGGACTGGGG
AACTCGGACTAGGAAGAGAGACAAGGGAATGACTTTTGATCCATAACTGG
GGAAAAGGTCTTCAGCGGCAAACAGGCCTGCTTATATCCCTTTCACCAGAT
TTGCTAAGAACTATGAATTTGCCACCCAGAGCGCTGGGCAGATTCTACGAA
TGGTGCTCAGCAGGTGCTACGCAGGTGCTGAGCAAGTGCTAAGCAGGTTC
TAAGCAAGTGCTAAGTAAGTTCTAAGTAAGTGCTACGCAGGTTCTAAGCAG
GTGCTGTGCAGGTGCTGAGCAACTTCTGTGCAGCTTCTACAAATGTTACCC
TTAAGTTCCGGACTTTACTCTATTTCTTGGATCCTAACAGTATAAAGGTTCG
ACCGGGGGGGCCGGGTCGAGGTCTTGACCGGGGGTTTCGCCTCTCGACCG
GGGGGTACCACCCTCCCTCGCACACCTCGACCCACCCCTCTCGCCCAGCTC
TCGCCTCGACCCGGCCCCCTTGACCCACTGCTTCGCCCTCGACCGGGGGTC
AGGCGATTCGACCCTCGACTGGGGGTGCCCCCGACCTACTCCCTCGCCTCG
ACCGGGCCCCGCCCTATGGGTGATCCAAGCCGACAGGGCGCTTCTCTCTCC
TCTCATTCTGCGGCCCCCATTTTCCGTGCGAGCTGGCACCTTTCTTCCCGCG
TTCCCGTGCCATTCTTTGTGCTGGTTGGCACCCTTCTTCCCGCGTTCACGGT
CTCCCGTGCCATTCTTTGTGCTGGTTGGCACCCTTCTTCCCGCGTTCCCGGT
CTCCCGTGTCATTTTTGTCCGAGTTGGCACCCTTCTTCCCGCCTTCACGGTC
TCCCGTGTCATTTTTGTCCGAGTTGGCACCCTTCTTCCCGCCTTCACGGTCT
CCCGTGCCATTTGCCCTGCCGGTTCCCACCCTCCTGCCCTCATGCGTGCACC
CGTACAAGAGGGTTAGGGGGTTAGGGGGTTATATGGGGCTCTGGGGGGCG

GGGGTTTTGCCCCACTGCACCTACAGAGGTCTGGGCTATACCATTAAATAG
CTCTGATCGAGCCGAGTTCAGATCTCTCTTCAGATTCTGTGGGGGTGCAGT
ATTTGCGGAGCAATTCCCCAAATAGTGCGTCCACTCTGCATCAATGATGTCC
CATTGCCCCTCAGCAAATGTCCCAGTGGTAGCGGGACATTGACACTCTCAA
ACAACTTAGAGAATTTTTTTCATTGCTGTGGGACATTGTCCCATTGACTGGG
ACTTGACTATTTATCCTATAATCTGTCTATTGGTCACTAAGTTCTCATAGTGG
TTGTCTTTGAGCCAAAGCCAGAAGGCTGTCCCAGGCATGTCCCTGTCCCCC
AACTCCATCCATGTCCCAGTGTCCCTGACCTGGACACCTTCAAGACCATGA
CATGAGCATGATAGGCTTCACACAAGGCCTGAAGAGTGAGATGATCCCCCC
AGGTGTGAGACAGTGCCATGTTGTGCAGGTAGAGGTCCCACTCATCCTGCC
CCCCAAGGATGAATGGGACATACCTGTCCCAGTTGGCTCTGATGTGGGACA
CAGCTGTCTGCCTCACTTCCTGGTGGGACTGCCTCATCCTCATACCTTGTGC
AAAAGCTCTGAACATGCAGCTGCCATCTGCCTTTGTCCCAGTGGTGTGGTA
GAGTGATGGGACAAATGGGACTCTTTTGACTTGAGTTCTGGAGAGACTGGT
GATTGTGTTGAGAGGAGCCTGCAGTCAGTGAGGGCTCTGTCTCAGTGGGA
CATACCAGTCCCAGTCCTGGATGGGACGTGAGTGAGGGTGGTGTCCCAGT
GGTGGTAGGAGCCTGTTGTCAGTGGAGTTCAAGTCAAAGTTCAACTCACT
GTCTTCACAGCTGCAGCTGTCTGTGCCCCAGTCTCCATGGTCCCATTCTCCC
TTGTCCCATTCCCCCCTGTCCCATTCCCTCCTGTCCCACTGTCCCTTGTCCC
ATTGCCCCCTGTCCCATTGTCCCTTGCACCTCCTAGAGGGTTGAGTCCCACA
GCCTCCAATGGGACATGTCCCTCATCCACCAACTTTGCAAACCACTGCCAA
GCAGCAGGCTCCTGCATGGAGTGACCACAGACCTTGTTTGCACACCTGAA
CCTGACTGGCTTTGTCCCACTGGCATGATACCTGTGGGACCTGTCCCTCTG
GCATGGGACATAGCCAAGACTGCTCTTGTCTCTGAGGATCTTGATGAGAGT
CTTGAGTCCACTGGGACCACTGTTGAGATGGGACAGTATAGAGTTGAAGTT
CATAACTGTCCCCACCCAAGTCATATGTCCCAGTGTCTGTCCCAGTCCCAGT
CAGCCACCAAGCAGTGGGACTGTTGGACTTTGTTGGTGCTTTCCTACCTGC
ATTCCAGCCCAGTGAAGACAAGACATCAGCCACCACTTGCTTCTGCAGTG
GTGATGGCTGTCACGGGGCCAGGCCGGTCGTCGACTGATAGGCCCTCTCAC
ACGTGCTGTCTGTCGATCTGTCTCTGTCCTCCACGAGAAGGTGTGTCCTAC
AGCCTCTCGTGCGTCGACAACACGGCTGCGCCGTTACTTTCTATCCATGGG
ACTCAATACCACGGTGCTAAGGACGGTCTACGGGTGAGGACTTGTATATATA
TCACCCGTGCCCTCCTCTGCACCGCCTTGGGCTTTCTCCTTGAAACACCAT
CGACGAGGCTCGAGCTCTAGAGTCGTCGAGGCTACTCGAGCGTCGTCGAG
GCTACTCGAGGATCGATGATAGGCTCACGTGTCTGACAGGCGTGCTGGGCT
GGAACGAGGCTCGTAGGCGTGCTTTGGTGATCATGGAGGCGTGGCCGACG
TGACACTCTGCCCCCCTCTGAAATCGAGCTCCTGCGAGATGGCGGGCGAG
GCGGGGCAGCTTGTATGGCCTGAGCCTTTGCTCCCGCCTCGCGCTAAGCCG

TATCTTTTGGTCATTCCTCGCAGGGCTGATCCCGAGGGTCTGTGGGTGTGTA
AGCAGGGCCTGGTCTCGTCGACGGTCCGTCCTTCTTGGTCGCTCGAGGTCT
CGCGCAGCTGGTGCTATCTTTCTAGGCGCTAGTCGGGGCGTAGGCTGTGTT
GCCGTCGTGGAGTGACTCACGGGCACAGGTAGTGTGCAGCCCGCATGTGT
CCCGACGCGCGTGGTGTCGAGGGTGATCCCTATGCTGGATAGCCCGCCCCT
TGCTGAGCTGTAGGCGTCTCATGCTGGAATTTGCGCGTATGTAAGTCGACCT
CGAGTCGACCTCGGCCACGACGAGGCCGGGTCAGATTGCCCAATGCGGCA
GTCGCCGCGAGATAACCGGTGATTGCCGCTCCGCGCTACGCCCTTCTGTCG
ATCATGTTGCTCGTTGCTAGGCAACAAGTCGCCAAACTGTCGTGAAAAGTC
GTGTTGCCTGCTCTCGTCGTCAAGTGCCCTCTTGTCCTCGTCCCCGCCATGT
CCCCTCCAAAAGGTATAAACCCCCCCTCGACGAGGCTACTCGTCCCGTCCA
TTCCAAACTCTCCACTCCAACACATCATATGTTCTCCAACGCCACCGATCCC
TCCCTCAAAATCGTCCTCCGAGGACGACCTCTACGCCTGGTCACGATGACC
AAAACCCCTTCCCCCAGCCCCACGACGCCGCCATCCTCCCCCGAGTCGGCT
GTCGACGCTCCCCCTCCTCGACTGTTCCCTCGATGGGCGGTCCGTCCTCGA
ACTCCGGAGCCCACACCGGTCGAGGAGATCGACGAACTCGTCGATGAGCT
CGACTGGTCGGATGCGGCCAGTGCTGCGCCTAGCGGTGAGTGATTGTCGA
GCCACCCTACTGCTTGTATTGATGCCCAGGGCGTACGGCGATTCTGCGCTCG
CTAGCAGACATACACGCCGAGATCCGTCGGCACCAGGGGACCGTCAGAGC
CGCTAGGGAGAGTAGCTGTGCTACCTCCTCTACGTCCAGCGGCTACTCAGA
CGCCTCGCCCTCGTCCTCCCAGGCTTCCGACGCGGCTTTTGCTGGCGAAAG
CCGCTATTGAGAAGCCCGCTCTCTGCCGTTCGAGCCAGTCTCGCCTCGGCC
TCTTCGCCCCTACATTGGTGGTGGGACTGATCCCCCATCATACCGCCATATC
CCCTTGTCCCCCTCGGCTCGCCTATCCTTCTCCTTCCCCCGCCTTCTTTGCCT
CCGCCATCCCGCTCTCCGACTCCGGCAATCAACCCACGCCGCCGGCGTCGA
GTCGAGTCGCCCGGTTCCCCGGGTAACACACCCCTGCCGACGATACCGCCT
CCCCTCTCCCCAACCCCAAAGCGTCGTCGCGTGTCGCCTGTTGGCGGCATG
TACTCCTCTCGCCTGCGTTCAGCCTCAACCCGGTATCTTGGGGTGTCTGAG
GTAGCCGATGCTCTCCCGGCCGCCGTGGACAACGCAGGTGTAGGAGAAGA
TGTCATGGAGGGGAGCATCTCGACGGGAGAAGGATCCGAGGCGGGCAGCG
AAGGATCCGATGGGGAGGGAAGTGTGGCGTCGTCGAGGCGACTCGAGGCT
GGCCCTCGAGTCCGGTGTAGGAACGCGCTGGACCGCGCCTATTTCGGTACG
TTCGTCATCTGCTGAGATCCTCGCTCATTCCCAGGGCCGTTCTACCCGTCGT
TCGGCCGGGGATCGAACAGGACGCTCCCATGGGCCTTGGTGCGTTATCATC
TTCTATCCTCCCGTCATTTCTCGCTGATGTTACAGGCTCAGGCAAAAGGGCG
ACCTCTCCGCACGCTCAACGGCCGCCAGCGATGCCAGCGGTGTCGTACGG
ACAATTTGCCGTGTAGGGTCCTAGACGGAGGAGGGCCGAAGATGAAGTGC
TACACCTGTGGCACTGCCACGGGCTGCTCGTTCAACTCCAGCCCTACTCCT

CGACGACCCGCCGGCTCGTCCTCGACACCCAGAGCTCGCCGGGCTGTGAG
TTTCGCGGATGTCGACCCGCCTTCGCCGACTCCCGCCCCCACGCCTGTTGT
CGGCCCAGCCGCCTTCACCGACCGACTTCGCCGGTCGGTTCGACAACCCG
TGCCGCCTCCGACGCCGCCTCCGGTGTCGACTCCTGCCCGCCCGTCCGCGC
CAGCTGCCTTGTCGTGGACACCCTCGACGAGGCTCGACATTGGTGTTCTCC
AGCACCACGTCACGCTGGCCCAGTCCAACCCGGCGGATAGGGCCTACCAC
GTCGAGGAGGCGAGGAGGCACGCGAGGCTCCTGGGACTGGACCTCAGGG
TGATGGTCGGGCCCATGTTCGACGACGTGGCGGAGGCGGGTGGGTCGAGG
TCGTGAATGTAGATAGTGTACATAGTTCTTTCTTAGCTCATGTTCATACGTCG
TGTCGTTCATGTTGTACAGGGAATGCAGGAATAAGACTCAGCTTGGGTGAC
GAATCTCGATCCAGATCATCGTCACCGCCAGTAGTCCATGCGTTATATTCCT
CTTGGTTTCCCTGGGTTCCTCTTGTGGAAGCTCTCGACGCTTTCAAGAGCA
CCAGTAATGTTCTCCACTGGTTCCCAGGTCGTCTCCTCATCTGTTCCTTCGT
ACCCCACCCACTCGACGAGGTACTCGACCTTTCTGCCTCTCTTCCTCGAGT
CGACGACGTTCTTCACCTCATAGCTAGGGCCCTCGTCGTCGACCTCCACTG
GTGGCGGAGGGTCGTAGGTCTGCCCAGGGATTCTTGGCGGGTGGTATGGCT
TAAGGCGAAGGACATTGAAGACCGGGCTCACTCTCATCGTCGCCGGTAACT
CCAGCTTGTACGTTGATGGACCCGTCTTAGCGAGGACTTTGTATGGTCCCCC
CCATTTGATGTCGAGCTTCTTCGAGGGTCGACGAGTCTTGATGTTCTCCATC
GACAGCATGACCATGTCATCCACCTTCAACACTGGCGCATCCCTTCTCTTTG
CATCGTAATACTTGGCGTACCACTCATTGGCTTGCTGTATGGCTTGTTGGCA
AGATCGGTGCACCTCGTCGAGGGACCCAAGCTTTTCCTTGACCGACGGTAT
CTCCGCCCGCTTGGCGCTCTCTCTCAAGTCATCGGTCCATCACGGCGAGTA
CCCAAAGTTCGCCTCGAACGGACTCATCTTGATCGCCGAGTGCTGGGTATT
GTTATAGGCGAAGGCTGCTCTCTGCAGCAGACTCGACCAATTGTCTTGTCT
GTGATCGCAGAATACCCGTAGGTACTGCTCGAGGATCTGATTGACTCGCTCT
GTCTGCCCGTCGGTTTGAGGATGGAAGGCTGTCGACAGGCTGATCTTGATC
CCCAGGGCCCCACACAGTGCTCCCCATAGCCGACTAGTGAACTTGCTCCCT
CGATCAGATACGATGGATCTTGGCAACCCATGCTGCGCCACGACATTGTCG
ATGATGAGGTCGACGAGTCCCGCCGATGTGAGGCGAGTCGTTGTCGGCAC
GAAGATAGCCCACTTCGTCAGGCGGTCTACCACGACGAGGATGGAGTTGA
AGCCCTGGCTGCCGGGGAGCTCCTCGATGAAGTCCATGGAAATTGCTTGCC
ACGGGCCCTCCGGGACTTCGAGACTCTGGAGGAGACCGTGAGGGCGCTGC
CTGCTTGTTTTTGTCCTCTGGCAAATGGCACAACCCTTTACGTAGGCCTCAA
CATCGCGCCGCAACCCCAGCCAACAGACGTCCCTCCTGAGCAACTCCAAC
ATCTTGGTAATTCCCGGATGTCCCGCTAGGATCCCCTCATGTCGATCCCGCA
TGGCCCGGAGCCGCCCATCGCCCTTGCCAGTGAGGTATAGCCGTCCATTGA
GTCGCAGGGCCCCGCCAGCCTCTATCTGGGCTCGCGTGGGCTCGTCGAGGC

CCATCCTTTGAACCAACCCAAACAGTTTGGGAAATCCTGCCGCATCAATCA
CCACCCCGTTCTCCTCCTTCTTGATTCTCTCCAACTCTTCTCCAGCATGCTC
GAACTCCTCATCCTCCTCCCAGATGCTCCGAGTATCCGTCTGCGGGTCCTCT
CTTGTCTCTGGATCTGGACTCGACGAGTGCCCGAAGCATGTACTCCAGCGA
CGTCTCGACCTTGCTTCCTTGATCAAACCCGGGTAAGGCCTGCACTCGTTG
CTCCTCCAGGTCCAATCCATCAGGCTTGTAGTCAGGCCTCCTTGACAGAGC
GTCTGGGTAAGCCGCTTTGAGTCCGGGACGGTACACGATTCGGAAGGAGA
ATCCTGCCAGAGCGTCGACCCATCGCGCAACCCGCCCACTCAGTTTCCTCG
GCTCCATCCAGTAGCTAAGGTTGTGGTGATCCGTGAGGACCGTACAGCTTA
CTTGCAGGAGGAGGTGCCGCTTCCGCTTGAATGCCATCACTATCGCCAGGA
ACTCCTTCTCCGTAGTCGTCCAATTTAGCTCTGCCCCCGAGAACGACCCAG
ATTCGATGGCCACCGGGTGCTCGAGGCTCGTCGAGGGGTTAATCTGTGATA
TCACAAATCCCCACTCGTAGAGCGAAGCGTCGGTCTGCACGATGGTCTCGA
ATGCAGGGTCGAAGTGGGCCAATACAGGGGGCTCGCTCATTCGCCTTTTGA
TCTCGTCAAAGGCTCTTGCCTGCGCGTCTCCCCAACCAAACGGAACTCCCT
TCTTTGTCAGCAACACTAGGGGCAGCGCTACCTTGGCAAAGTGTGGTACGA
AGCGGCGGTAATAGTTTGCCAGACCCAAGAACCGGCGTACTTCGAGCAGG
TTGCTTGGTGCTGGGAAGTCGACGACGCCTTTCTCGTACTCCGGATTTGCT
TCCACGCCTTCTCGGGAGATGACAAAGCCCAGAAAGTCGACCTTCCTCTTC
TCGAACTCGGATTTTGACGCTTTCACGTACAGTCCCGCCTTCCGCAACGCC
TCGAGTACGGCCTTTGTGAGGGAACGAAGCTCGTCGAGGCTCTTGGCATG
GACCAGGATGTCGTCGATGTAGATCACCACTCCTCGCCCAACGAACTCTCT
AAGCACCTCGTTGAGGAAGTGCTGGAAGACTGCTGGCGCGTTCTTCAAGC
CATCTCGCACTACCAGGGACTCGAACATCCCCAGAGGCGTCCTAAAGGCG
GTATAGGCTTCGTGTCCTGGCGCCATGCGAAGAAGCTGGTAGGCTCCCGGT
AGGTCGAACTTGGTGTACCACTCTGACTCTGCCAGATCGGCTGTGACTTGG
GAGATCAACGGCAAGGGGTACGCGTCCGACACGGTTGCTTCATTGAGCTTT
CGGTAATCCACCACCAATTGCCATTCTCCAGGAATCTTCTTAGGAACAAAG
AAACTCGGCGACCCATACGCCGCATTCGATGGCCTGATCTTGCCCGCTGCC
TGCTCCTTGGCGAGGAGCTCCCTGAGTGCTCTCATCTGATCCGGCGACAGT
TGGTAGAGCGGTGTGACCCGTAGTTCTGCGCCTTCCTTCAGTCTGATCCGC
AGGTCGTATTCTCTACTCGGTGGTAGCGTGTCGGCGGGTCGACGAGGATGG
AACACATCGAGGAACTCGTGGTAGTACTCCGGGACGATCCTTCGCACCGCC
TGGGACTCCTCCTCTGACAGAGTGTACTCGTCAAACCCAAGATGCCCGTCC
AAACGCCCCACCACTTCCTCGAGCTCGTCGGTGACAGTCCCCCGTAACCGC
TCTGCGGCAAGCCTGTTCTGCTCCCATCGATTGATCCGCAAGGGGATATGGT
TCGGGGAGCTCCTACGAGACTGCCCCGTACGCTTTCCTCTCTCTCTTCTTGC
TCTGGTAGCCAAAATGGCCAGTTCACTTGTAGCAGCCTCGACGACCGTCGA

CGAGGCTACCTTTCCTTCTCCACGGGTGACTTCCATGCCCCCATGTCCATG
ACTACCTTCTTTCCTTGCATCCAATCGCACCCTAGCACGACGTCTGCACCTG
CGAGCCGCGTAACCGCTAGCCGTTCTTCACTTGTTTCCCCAAGCACCTCCA
CCGGAGCCACCACTTCGTGGGTGATTTCCCCCGCAGATGACGGTTCACCGT
CGAACAGGAGAACCTTCCGGGGCCGCTCCAACCTCTGTTGGGTCATGCCG
TGGCCTTGCGCGTAGTCCTGATCAATAAAGGTCCCGCCAGCGCCAGTATCT
AGCAGTATGCAGGCGGCCGTTTCGTCCACCTTTCCTGCTACGACAGCTTGA
TGCCTTCCAAGCCTGCAAGCCTCACCGCTCATGCATTCCTCGGCATCACTA
GCACTCTGTACAGACTCCTTCTCGGACTCCAGGTCCTCTGCTTCTTCTTCAT
GTTGATCTTCCTCGACGAGGGATATTGAACATCTCATCTATGGGATACAGGT
GGATCTTGGATACTTAAGCGAGGTCGTACAAGGTCTCGAAACAATCTGCAA
CAACCTTAAAAACTTGGAATAAGAGAGCCATTCGATTTCTGTTACTTCCAT
TCCTCGCTTGGCGTACACACATGCTCGATTTCGCATGCATCAACCTTTCAGC
CCGCATGTGACCGCTATCTCTCTCTCATCACTTAGTAGTCATATCATTTCTCT
TACCCAAATATCTATTATACCTGTATTCGGTAACTTTATTCCTGCTGGTAATCA
CCCCAACACCAATCTCGAAGTGGTTCATGTTGGGGCGGACTGCTAGTGCGA
ACCATTTTGACGCTACCGTGACATACACCCATGGATGGATTTCCGGTAGCTT
ACCTAGTGTAGTAGGTACCCAACTTACAAGGGTCAGGTGTCCCCGGTTTCG
TCACACACCAACACTCATTTGCAATTTAGTGATTCGCTAGATTTTTGAATTT
TCTTATCCAATACATTATTCTTGGGACGATCGGATCTGACATTCATAACGAGC
GTGGTGGTCTTCTAGATTCCGGTGCATCTTGGTAGCGGATTTATCTTTTGAA
AAAGTGCCACTTGCAGGTAGATAGATAACTATTTGCGCAGGTAACATTAAA
AATACAAACAATAAGTCCCGTCAAAGGCACGAATCCAGTATATTGTCGACT
TAGTTTATAGTTTACATATTTCATTACTAAAGCTTATTATCTCGGAGCATGAA
CAGATGGATGATTACGTGAATATCGATTTCATCTGGGACTTATTATCATTACC
TTAAATGGCGTTATAGGGCGGACCCAAATATTTCCATTAATATCCAATTTATA
TTCGAAACTTTGCTCAAGTGCAAAACACAAGACTTACTAAATGAATAACGA
CAGATAATTGTCAAATCAACCTACTAAAGTCCAATGCATGCGGTACTGTACG
CCTGTGTAATGCGGGACTTGGAATGCAACCACATTAATCATCGGCCATCTTG
GGGAACCACAACATGATCGGACTTTTCTACCAATCAATATCAGTGAAGAGG
TTGCACGGGACACCGAATGAGGTTCAATGAGTCGGGACATTTCAGCTCTTC
AAGGTGTAAGGCACGCATAAAGCGCAACAAGCAATTAGTTGCGACTAGAC
GAGCCACGCGGCGGCCAATGACAGAGCCTCACGCAACACATGAAAACTCC
GTCAGCTCGTTGGATACCTAGATATGTATTCTATTTTAATCAGTTGATTATATA
TCGTACACTTCTATTTAAGTAGGTACTTAGTAGTAATTAGGAATACTTTAGGT
AAGATCGTTATTACCGAAATTCATATCACCAGACGTTTGACACATACTTCCA
TGAGGTCTTCACACCTAACCTCACATTTTGCGTCACACAAAACCTTGGCTC
TGGAGCGCCCCTGCTGCGCGCCAACGGCGGCCGTCCTGTGCACCATACCTG

GGCAGATAGCTGTTGTATTTTCTAGGTGTCAGCAACTTCGTCATCATACCTA
CTAATTTCGCGCAAATATAGGTACTAGGTACCTAGGTAGGTAGGAAATAGCC
AGAATCAACTAGAGCCCTCAACTTCAATGGCATCCTATTTGGACTAACTTCT
TTTTGATGCTTCAACTAGAACCCTCGACAAAGAGTCCAGTAGTGTTAACTC
ACGAAGTAATATGTATCAGGTGGTTTTAAGTTGAATACTTTCCACCTGTTAC
TTAGTAGGTATCCATAGATCTAATCACGCACTAGGTAAGGGAACTAGGTATG
ACCGACATAACGTCGGCTATTAGTGGAACGAACGGCTACGCGAAGCTCCAC
TCCGGGGTACCTGCGGGGTATGCTTCCCCTGGATGACTAACGCTACTAATGC
TAGGTAATTTCTCCTTGCGGTCTCGGACTTTAATTGGACTTGAACTGTTACA
CAAAAAGCAATAAGTTACGCTGGCTGGTTGTGTAAGTTATAGTCAATCATTG
CAACAATTCTGACCAAAATCTTATACTCGCTGTTCATAAGATCATCATAGTA
GATCATGAGGACTTTGGGAGCCAGGCTACCCACAGCCATGTTTAGTAGAAA
GCTGATGCGGGTCCCTCATCCTGGTCCGTCTACTGAGGCTCGATCTCCTACC
ATATATGTGAGATCAATGGCAAGTCATGCCAAGCCGTCAATGGCTATTCTCG
ACGATTACCTAGATATCGCAAAGCCCCACTTTAGTCATATTCCCCCATCCCA
ACTCTCCGTCACCGCCTTCCACGACAGCCTCCCAGCCTTTAACCACCCACA
CACCAGCGCCGCCGAAAAGCAAGCCATCATCGACCGGCTAAAGCCCTTCA
CCATCATCTCATCTATGCGGGAGCGCACACCTTTTCCCGGTGACCTGCTCCG
CGCTCTCCCCAATCTTAAGCTCTTACTAGCGACAGGAACACAGTTTGAAAC
TTTCGACTTGACCGCTGCCTCCGCACTCGGGATCCGCGTCGCAGCCGCGCC
CGGCCGGGGTCGCACGGACGGAAAACCGTCGTCCCGGCCACCACGTCCAA
AGCTCGACATCAAGAAAGGCGGCAGCCACCCGACGACTCAGCACGCATGG
GCGCTAATCCTCGCCCTCGCGCGTAACGTCGCCGCCGACGACGCAGCGATG
AAGAGTCCGGCGAAAAAATGGCAATCGCAACTCGCGCTCGGACTAACAGG
CACGACCCTCGGCGTAGTGGGGCTCGGGCGGCTCGGCGCCGCGGTGGCAC
GTGTCGGGTGCTTGGCGTGGGGAATGCGGGTCCTGTGCTGGAGCGAGAAC
CTGACGCAGGAAAGAGCGGACCGGAAGGCGGAGGAGATGGGGTTGCCTG
TTTCGGGCGGGTACGCGCTGGATCCCGGCGAGCCGACTCTTAAGGCGGTTG
GGAAGGAGGAGTTGTTCCGGTCGGCGGATGTGGTTAGTTTGCATTACGTGC
TTAGTGAGAGGTCGCGCGGGATTGTGGGGGAGAGGGAGTTGGATTGGATG
AAGAGCTCGGCGTTGTTGGTGAATACGTCGAGGGGACCTTTGATTGATGAT
GAAGCCCTATATGGTGCGTTGGAAAACGGGAGGATACGGGGCGCCGCGCT
AGATGTATTTGACGTCGAGCCCTTGCCGGCGGAGAGTCAGTGGAGGTCGA
CGAAGTGGGGCACTCAGGGGAGGAGTAACTTGATTATTACACCCCATATGG
GATATGTTGAGGATGGAATCATGCATACGTGGTATGAGGAGACGGCTGAGA
ATTTGGAGCGGTGGCTTCGGGGTGACACGCTTCTTCATGGTCTCAATTAGA
CAGCGCCTAGATTGTATCGTCACTGAGAGCATCTAGACTTGTGATCGGATAT
ACTAGCTAGGTATGTATAATGTAATAAGCAAGTAAACCACCTTCATAACGAT

AATTATTGATGGTGTGTACTCGGTCATGGTCACTAGAGTCACACATCCTTCG
GAATTCCGCGAGGGTCCAATTGTCAGGTAGGAGGTAGGTTTCATGACTTAG
GATACACAACGGATGGCTTACACTAGGTCTGTAATGAGAACTAATCAGCTG
CGTAACAAATACAAGTTAGTCATGTTTACAGATTCTTGACGCACAATGTGAC
TATCTAGGTGTCTAGGTACCTATGTAACTAATTTACGAGTTTCTCGAATACCT
AATCTAAGGTTAGGTCTATCAACAAAACCAGCTCCATACCTACCTAGGTACA
TATCTACCTATGTACTAAGTAGATACAGAGCACCCGGGCAGGTTACTCTGCC
CAACAAAATGCGATCTCTTACTTTATGAATCTTCATAATATAGCAATATGGAT
GAACATTATTAGGAATGTGGCAATACAAGGGGTTCTTTCCCGCTTTCTAGTA
TGTTTGATGATGTATAGCACAGGGCACATAGAAGGCTATTGGCCAAAAGGC
TAATCGAGTTATCTTTACTTCTTCCTGTAACATTAATGTAGGTACTTAGTGGC
TAAGAGCCTATCTATTATATTTAATCCACGACTGTCAAACTGTCAGTACATAA
TCATCAGGTTTTTGAAGCGTTAGACAACACTGATTCACCATAGGGTCATATC
ATAATTCGCCGTGACATTCCAACGGGTGAATGACAGCGTGTTGCCACTCAC
AGTTTATTGATATTACTCATAGGAATATCTTTCTGCATGTAGTCGTTGGGAAT
CTTAAGCTACCATTTTCCTACCTAACAACAACTTCATACATTATCTTCCATAA
TAGTACCTAGGTATCTACATTTCAATATAGCAATATTTCATCATGTCTAACCA
CCGAGACTACAATCACCAGGGTTTAGTCAGCTTTGATGGAAACCCGAGATC
TCACCACGCTTCCTCGAGTGGAAACGTGTTGCTGGATACTTTCCTCGAAAT
CGTGTTCAGATTTTTAGCAATGCCAGAGAACAGTCAGATCAACCAGATGGT
CACGGCATACAATACTGTCAGTATGGACTTGGAGCGAGCAGCTAATTTATCA
TTCGAACGCCAAAAACTTCTCTGGCGCATTGATCTTTTCCAATCTATCTCGA
GGGTGGAGAAGATACTATACCTACAACAACTGAGGGAATTATTACCACCTC
GAGGTGGACCGATTTTCGACCGGATAGTGATTGTTATGCCTTACATGGAACC
ATTTGTACGTTCTATTCGGAGACGCTCGTATGATCCGAGATTCGATGGTAAC
TGGTTCTGTTGGTGGCGCCGATCTTCTCAGAGTGATCCTGCTGAGAGAGCT
CAACTGGAACAGGGAAATTATTTCGCACGCCATTTGTTAGAGTACGAAGTT
ATCGAACAACTAGTTCCTGCTCTGTCTAATCAGCTTCCGCCCATTTCGGATT
CCCGACGAACAGCTGAAGGAGAATCTCTCCCATCAGTTATTCACTCACCAG
TGTTTAGCAGCAATATAGGTTACGTTAGTTTAAATCAAGCTTACGCTGGGAG
TAGCGTCCAGTATCATAACAGTCGGGACAACAGCGGACGGGGTCGCCGTTC
CTAAGAATGCTTGGCGTCCATCTTAGATAGGTTTTCAACATTATTATATAGGG
CTTTTGAGGAATATGTATGTGTCATGGCGTTTAATATACCTAAGATATTAACT
CCAATTTACCAAATAATCCATACCTACTTGTGCTCATATGTTTCAAGCACCTA
CTAAAGGTGATCTCCAGGAAACACCTAATTACCTAGGTAGGTACTGTTAAA
AGATAGAAGCCACTGAGAAACTTTAATACACATGATGATTCACTTCCTCCTT
CTAATAGGTAGAACCTTTTTCTGACAGTGAAGAGGACTTTAGACGAAAGTC
TACTCTGTATCATGATGTGGCTTGACAAGACGTAGATTTCAATTACTCTTCA

GTAACTGATTCAATTATTGATAAGTGACACCAATATGTCCCTGATGCATTTCA
AAAGAGAGCAACCTACGACCAAGTGCTAATAGCCATCTCGGCTACATATCA
TAATTCAGAATTGTACAGATCACGTCTCATTTTATTATACAGACAACTGTAA
GTAGTAATAAAGAATACGATGTACGAATCATTACTACTACTTACCACAATAC
GAATACATCGCATACCTACATTAGGTACGAAAGCCTCCAGAAAATCTTAGGC
TAATCGAGTCACCTCTAATCTTTTCTCTGCCTGTATAGAGATAATAAATAGAT
AGGCGATGATTCACATAGAAATTATCGATGTAATGATTAGGCGTCTTGATTC
AATTCTTTGAAAGCCGACTATTAGGTATAGGAGGCATTAAATGATACTTATTC
ACTGATGGTTTCTATCGTAGTAAGACACTATGAACAACACATTACCTACCAT
GTACCTACTACCCATAGAATATTTTTCTATATCCAGTAGTTGAACATCCTCTC
TAATCTCTCTACACCACTCTTTTGAACACCCTAAGCCGACAATCCAAAGTCC
TTGGTCATCTCATCATATATTTCGCCGAATTCTTCGTTAAAATAGTAGAAAAT
TTTCACGATGTCTCAACAGCACCACCACCATGCCTCTATCAGAAACACAGC
CGGCATGCCCAGATCCGGCATGGTTATACGTCATGGTTCCGCAAAGGCTGA
TGAAGTGGAGCGGACCGTTATCCAGAATTTGCGTTATATCTTTACAAGACCA
CAAGCAGAGCAGCGCGAGACTTTGATTCTGTCTTGTCGAAGTATCAACCTT
GATCTCAGGTCGTTCCCGCAAGATCAGATGAATTTAATACACAACAATTGG
ATTCGTCGGTGCCGTCTATTCCTCTCTACCCCAAAGGAAGACAGGTTGCTG
TACCTGCACCATCTACAAATGAAATTTCAGGGGCGCCCTGACGCTGATGCG
AGGTTTCAAATGGCCACAAAAATCTTTCCTAGCTACGAGAACTTTGTCCGC
AGCATCCGGGTGGGTATGTACAAGGAGTCGCTTGATGGCAATTGGTTTTCT
TGGTGGAATAAGGCACTCACCGCTATGGTGAGCAGCAACAATGTAACTCGG
CCAGATCTACTGAGATGGGACCAGGATCTGATACGCCACTTGATGGAATAC
GAGATTATCAGAAACTTGGACAGCAACGTCGGATCCAATCAACTCCCACCC
ATTTCGAGTGCCCGTGAAGGAACTCAGGGTACTCTACCACCTGTTAGCCAG
GTAATGAGAGGAAGTACCAGTTATGGTTCCAGCTATCAGAATTACGGAAGC
TCAAGCTACAGCAATGCGGGGAGCAGCCATCAACAATCTAGCAGCCGAAG
CCACAGCAGCCGGCATGGTCGCAGGAACTGATTCTTAACATTTCTCTCGGA
TAATCTTGCATATGATATCGGGTAGGGGATGATTCCTTTGGTGTACCGGTTTA
GATTGTTCCTAGGTTTGGATGGGATTGTTAGTGTAAAACAAGGCACACTGG
ATGTCCTATTGTCTTTTGGGTGAAAAAGTTATCAGTTTTGGGACATAGGCAG
CATGACCTAAACGAAACTGGGAAATTGATAGATACTAGGTATCACAGAAAA
AGTATAAGACTGTTTATCTGAATTACAGAGAGTTGCGTACTCTTCTTTGCCT
ACTTACCTAGATCGCCCCAGGGAGTTCCTTGCCATATTAGTACGGATATTCTA
ATTGAATCCAAAGGCCTACCTACGTAGTACCTAGTGTCGATTTCTCCGTGAA
GCTAACACGTTGCATATTTGTCACACATATAAGCCTTGGTTGCGCTCCCGCA
ACTAGGCGTCGCCTTTTGCGACAATATTGTAATCTACCTAGTAGCTATCATAC
TCGCGCATGTATAGTCTGTGCCCCATACCCAACCTCACGATAGTTACAGTCT

ATCTAGGAAGTTATCTCCATACATGGACTGCCAAATATCCAACAACCGAGCT
TTACGATTTAAAAGGTATGTCATGTAGCAACACAATAAAATCCCCGCGTGTT
GCTGCTTTTAGCCCCTTATTTACACCGGGAATCTATTGTCTGGTAAAGGGAG
CAACTATTCTATAATTGAACCTCCATTTTTCTTGGAGCCCATTAGTACCTATT
CATATTATTCACTAGTTGTTGCTTATATCCAGGAAATTACTTTTATTACACTAT
ATGAAATTCTTAATAAATGCTTTGCGGACGCCCTATTGCTAGCAGAACTCGG
ACGCCCCGCAGTCATCTACTAGTAAAAGTACTAGGCTATATTTTCGTAGGTA
GGAACTTACACGCTTCTACCTAAGCGGATCTTCGTTCTTTCTAGAAGCATCC
GGTCCTCGTCCAAAATTCTTTGTTCTTTATTGCAGTCTACTCGCAAAAAGCC
GTGTCGTCTGGAGGTATACCACCAGACGAACCAGTTCTCGCCGGGGCATTC
AGGGGCTTCACCCATGGAAGTAAGAAGGTGGAAGATATGAATCCCTGGAA
GAAAGTAAGCTAGGTACAGGCCATCAGTTTTGTCACCTATGCTACGCCATTC
GATGAGATTTTTTAAGTACCGTCTTTGTTCAGGCAAAGGCAAAGTCTTGAA
AAGCTTGTACCTCTTAAGAACCCACCACACCTGAGCCCCCAAAGCATCGTA
ATTGTAAAGGTTGCCAAGGATATGGGATAAATCGATGAATAGCCCACTTCTA
CCTCCTATATCGAGTTTCTCGATTCGTCGAATTTCGTTACAGATAAGGTGGG
TGAATGAAAGCGATTGGTCCTCAGTTAGGGGAGCCTTTTTGTTCCTTTCGG
TACTCGGGGCCACTCTTCGAATAAGTAGACGATGCCGCTGCATAAGTTCATT
GAGAAACATTTTGCGTACCTCCTCGGAGGATTTCTCTTTGATGTGATGCATT
GTGTGTCTAGGAGCTTAATATAGATATGTTTAGATTACTTTTGATAATAACCA
TCAAAGGTATCTACTTCTACTTTACTGTTTACTGAAATTTACCTAGTGATATA
CCAGATAATCCTGAGAGTAAGGAAGTAGTACTCTACGCTCAAGACTCGGAG
AGAAATTTATACCTACCTATGTTAGATAGATTAAGATAACTATCTACAGTCAG
TCTTAAATTTCAGCTAATTAAGGAATGATTCCAATAGAGGTGGTATCTAGAA
ATAGCCCTGTCCCTCCTCTTCTTATTGATGTTGAGAATTCTGAGTAGACGAG
TGCTTTGCTGCTAGGTATGTAGAGAATGCTTATAAATCAGGGAAACTGGATA
CGTGCGATCATATATTAGGGTTCAATAAAGAAACGTGTTCAACGTAGTGGA
AGGACCTGTCATAAATCAGATCGAAATACGAAGCTACATCGAATGACAATT
CATTGTTAATGAATGTTCGGAATGAATGCTGCTAAATTTCCCTTTTTCTAGCC
CTTTATCTAGGATCCAACAGCCCTTTTTTACTCTAATGGATATAGATGAAAAC
CATAATGCTTCCAGAAAAACTAAATAAGATCAAGCAAGGTTTATATTTTACC
AATTCATATCTCAATTCAATCGAACCATCTTGAAAAATGATTTAGATTTGAA
GTATATGTAGGTAGGTATCGGTTCTAGAATGGGATCGTGACAAAGTCGTAGC
CTATGCTGCATTACGTGTAGAAAAAGGGGGTGGGGAGTGGAGTAGATTCCA
AGCGAGACGAAATTCCTTCAACTCTTTGCACGCATTAAACGTGCAATACGT
AATTTTTACTCATAGATAAAGACTATAACACCCAGTTGTTTGCAATCAACTAT
CTAAAACTACCAAATGAACGTTTCGTGGTCCTTTCCAAGCCTTTAAAAGGC
ATTACGAAACCAGTGGATTCAGTAGGAAGAGAGGTGACTTCCGCATGAGA

CTATGAAGCAAGCATCCTTCTTGCTCCAGCTTAAAAAAACGCGATAAATCAT
TTGTCACGGATAAGCTAGTTCGTTACTCCCTCGAGCTGATACTACGACTAAC
CGACAGTAAATTCAAGGTGATGATGTCGCTTCAGCCACGAAGCATGCCGGA
TCCGCGCCTGAAGGAAGCCCCACGTCAACTTCCCCTCCTCGAACTCCTCCT
TAAGCTCATCGTGCCAGGTTGGAAACCTTTCCTTTAGGCCTTTGATCATAAA
AATGACTTTCACCTTATCTGGCAAGACTATACCTTTCTCGGCAAGGCGCATG
CAAGTCCTATGGAAGCCGACATTGAACTCCCACAAGGTCTCATTCTCTCCC
ATACAAGCATTTGCTATGTCGTCGAGGAGTTGCGTAGCGGAGCGGAGCGGC
AGTTTCTTAGTTATAGAATGGAGCAAGATCCAAAGCACCTTGGGGTCTTTCT
CTGTCTCAGGGTCCCAGCCTGTAACTACCACATCGTTCCAAGCTTTTCCGAT
CGACTTAGTGATAAGAGACATCGCCCGCGAACGCTGGCGAGTGAATTGGA
CCTTACTCTCCTTGTCGAATGGCTCTTCGGCGGTACCCTCAATAAAGGCCTC
AAGTCCATAGTATCTTGCTTGTTCCAAGGTGTAGTGTTTCCATTCGACGAAG
TTAGATTCTCCTTGAAGAAAAGGAATGGTAGTTAGGTGGGCCGGCTCATGA
GGCTTAGTTGCAACCGTCATTGTAGAAGTAGCCGAAACTGACCACTGTTTA
GACATGATAGCACTAGTAGAATGTTAAGAGTTGTCCTTCGGACTTTGTGATT
TGTGAAGGTCGACGAAGCGCACCTGCCTAGAGGGTATTCGGGTAAAATCTA
GTAGCGAAAATTAATAAATTTATCTTCTCTTCCATAGATAAAACAAAGATTA
GTATGACTTCAACTCACATACCTATAGTATATATAGATAGTACGCGAAACTCG
TGAAGGGCCTTAGTAGTGTGATGTAATCTTTCGGAGCTGATAGAGCTGAGC
GTACGAGTTCACTGATTTGATGTTGCGTAAATAGAGAAGGATAAATTTTCTT
CTTGGGCCTAGACATTGGGAGAAATATTTATATAGACATTTACGACTCATTG
GGCACCAGTGCCAATAAATAATTAAAAGCATACGGTGCGAAAGACGGAATA
GAAGGTACCGCCGCCAGGCTTGAAGATAGTGTGAATTTTATCTTTACAAGA
ATGGACCCTCAGGCATATAAAAGACACTAAGTCAACTCATAGGTCATTTCCC
TATTATTAATCACTAATAGCTAGATCTTCAATCACAATAAACACTAATTTTTAT
TGTACAACTCTACACACACTGTAGTACCCATATAGTACGCCTCTTTCTACTAT
GCTCAAGACATCGTACACTTAACGGCCCGTGTGTCCCCCGCTCTCCTCCCC
GAGCTTTTGCCTCTTACTCGAGGCCTCAACGCTCGAGTTCGCCGACCTTTT
ATGGCCCCTCTCGCCATCCGATAGCTCTCGCCCCTCGCTCGTAGCCGGGGTA
ACCGAAGAACGATCCGGCGTCGATCTTACGAACAATTCCTCAGACGAAGC
CGTCGACTCCGACATATTTCCCAAATCTAAACGATTTATACCTTCCTGGATAC
GTCCCTCCTTCTCTCTCTCACTCTCGCTCTCCCCACTCCCTGCGCTATCCGC
GCACAACACAACGCCTCTCTCCACGCTCCCCCCTTTAACCCCCATCCCCTT
AAACCTCTCGAGTCCCCACTGCGGCACATTCGCCTCATCAAACAGGGTCCT
AAACCAAGCCTCTCTAACAGCGAGCCCAAGGGCCACAGAAACCTGTCTCG
CAACACTGTTCCCAATGATCCTCCACTGGTTCGCCGGCGACGCGACCAGCA
CCTCATCATCGCGAACCCCCTGCGCGCGGCGGATCTCCATGATCGTCAGGG

GCCGTTGCTGGGCCCAATGATTCGTATACCCGACCCTCGCGTCCGTAGGCA
GACACTTCGTCGAGATCGTGCTGAACAGCTTATCCGGGTGTACACGGCCCC
ACCCCTTCGCCCCTTTCTGGGCGCGGCCGCGCCCGTCCGCGGGATACATGG
CGCGTTCGGCGGGTGTCATGACGCGAGGGAGTCCGGGGCGGCCGTGCCAC
GCCTTCGAGAAGTTCATGCCCCAGGGCTGGGTCGGGATAGCCAGGAGCTG
GTTGCGCACGGGCGGCGTGACGCCGATGGATAAGCGGTGGTCGGGGAAGC
CCGGGCAGAAGTCCGGCTTCGCGTCTTGGATGTCCGGTAGGTCGCCGATGG
CGTCGCGGGCGCTGACGTATTTGAACGGGGTGGAGACGAGGACGCGGCGG
TCGAAGGGCCGGCCGCAAGACATTTCGCCGAGTTTTGAAAGCATTGTGCC
GGGTGGGTGAGAATGAGATGGTCTGGGCACTTTCGGCATGCGGAGACCGG
GGGCGCTGAAGCATAGGAAAACTCGGCTTCGAGATTGAGGTGCGCCGAAG
GACCATGCGTCTAAGAACATCACTTGGACTTGATAGCCTAGGCCAACGAGG
GCGCAACATAATTGGGAAAAGACGCATGCGTCACGGAAGTTTTTCGTATTC
ACCACCTGTGGCACGTTCTCGAGTATTCCGTAATAAGGACGGTATAGGTCG
ATGAAAGAGGCGAAGGATGCGATGAGGGACTGGTTTTTCCGTTGGTCGTCT
GTGGTTCTGTCGGGAGTAAGCATAGAAAATCCAGGACACGGGGATCCTGC
GGATATGAAGTGCACATCTCCAGGTCGAGGTATTTTCTCGCCGTCACCAAG
CAATGCCCGACGAAGTAGATCAGCAACCGAGCCGAGAAAGGGGGTACAG
ACGCCTGGCTCACTGTTCGCCATGTATGTGTGGACAGCCTCTGTCCAGATG
TCGTTTGCCCAGCGCATTTCAATGGCGCCGCCGTCTTCAAGTCCACGTCCA
AAATTTCCACCACCACAGAATAAATCAAGACCTTGCAACTTTTTCCCATGAT
GTATATTCAGGGGGTCGAACCCTTGTCGTAGCTGACGTACCAAGGCCATGT
CGAGAGGAACATAACTCATCTTACCCTCCGTTTCAATCTCCTGATGTGTCAT
AAAAAACATGTCGCCGGTGCCGTCGTGGTCATAGGGGCTCGGTATTTCTTC
TTCGAGATAGAATGCCCTTACTAAACAATGGCGAAAAATTCTACGAATGGA
AATTTCGACAATTTGGTCCGTATAAACAACTTCATTTGGACGTGAATTAGGT
GAATTCTGATCCACGTCTTTCCTTCGCAAAAGTTTTCGTAAACGAACGTATT
TCTTCTTTCCCTCATCGAAGAACGCCTCTACAACGAAAACCTCCAATTGGA
GGGACTTTGGGTTCGTTTCAGTGAGTACGGCATCGCCGATCTTGTAGGATA
GCTTCGCCTGAAATTTGTCGTCCTCGCATGTCAAGTGTTCTTTCTTGAGAGT
AGTCCATCGACAGTCATCGGAAAGATAAGTTTGGCGCACAAAAAACTCGG
CAGACGTCGAAGGATTCCCAAACCACTCAACAGTATGCGTCGCGAGTATTT
CATCCGCCTTAACTTTGGCTGAGTTACAATGACAGGTGCAGTTGTCGGACA
GAAAGATCTCGTTGTCGTAGGGATACTTCATAATGCCACACGGAGTATCGAT
TGGTTGATACAACCAGATGACATCGAAAGAACGTTTCCCCTTTGGCGACAA
ATGAATCTTTTGCACGAGACCAAACCATAGGTGCTCTCCTTGGTAGTCTTTG
GACTTCTGTTGTTTCCATTCCGTGTCTGTTCCTTCGTCATCCGGGTGCGTCG
AGATTACATCTCCTACTTCGATTGAAGCAGTGAAAGCTGAACGATCCTGGC

CAATTCGTTTCACAGTACGCAGACTTCCCAGTTCTATAGGCATGTTGTTATG
TATAAAAGTATCTCGTTTCTTCCTGATGTTAGCGGCCGCATCTACTCGTTTGA
GGACATTTCCAAACACCATATGGGAGAATAACTCGTGTATGTATGGGGTTAC
GATAGTTGGTGGTACCCGATTATCTCTTTGATTCTTTCTGATCGGATCTGTCA
TCTTGCTATACTCGCTCAGTGATATCTGCCCAAGCGAGAGGTTAGGCTGATA
ATAATCCAACGTCTTGACTTCCTTCCAGAATTCATGCCATGTAACTATTGTCG
GATCTAAGCCATGAGCTTCTTTCCATATGAAGTTGATATTTGCAATAACAGC
GCTTCTAAAGTCGTTCCCTCGGTTGGCTGAATGCCACCGTCTGAAAGTATC
TGATTCGGCATGTTGTTGCTCTAGCCAAATACTGAACTGCGATTTGAAATCG
CACAGAAGAGCGCGCTGCTGTCGACTCTTCAGGTAGTCACAATAGTCAAG
AACGTGCTTCAAGTCCGCAATCCAAATAAAAGGTTCGTAATAACGTAAGTA
TTCGGTTGAGGGACTCTTTAGTTTGTAATAAACTTCATCGCCTCGTCTCTCG
TTAAGATCCGACCGTATCCAAATTTGATCGCCAACAGTGTGTTCATCTCTCC
CATAATTTCCAATGGGAAGCTAACGGAAATGAACCTTTCTAAGAAAAAAAT
GTTTATCGCCGCGCCGAACCACTCCATCGAAGTAAAAGTTCTCGCCTACTC
GGGTAGCGAACTGTTGAAGAGGTCTAAGTGCATTGGGATGAACAGTACC

>Mosaic_sequence_44

ATCACCACGAAACGCATGCTGAAGCAGCAGAAACCGCAGAGCTGTTCAAG
GAACACGAGCTCGGCTAGGTTGTGGACCGTCGAGTTGATCCAACGACGCC
GTTGCGACAGCAACACCTTGGCCGAGTCCGGGGCGACGGTCTCGGCATGC
GCATCTCGAACAAACTTTGTCTTGTATTTTGGGGAAATGCTTCAAGACCAA
GGTCGTCAGATATCGATCTTCTCCCAAGTGAAGGAGATTCTTCAAGTGAAG
CGTATCGACACGGTTCTCCGAGTAGTCTTGGATTATTTGGTTCGAGATGAAC
AGCGGTTTGTGTGTGTCCGGAGTCCGAAGGCGGTACATGAGGAAGCAACC
TGGCAGACAGGTGATACTGCCAAACAGCGACTCGAACGACTTGGCAAGAT
GATGTGAGATGAAGTACTCGTAGACCTGGCGCAGCCTTAGCCAAAGAAAG
GACATACTGTACACATGGGACACGCACCTGCGACATAGTCCACGATGGACT
GCTTGGAGTTGGCCAATGAGGTCTCGCCGCAGACACCAATAATCTTCTTGT
CATGCATCATGCTGTATGTTAGCCAAATCCGCCACAGGAGCGATCGTGACT
GACGCAGCGACGAGTCGGTTGAGCGAAAACTTCTCGACAGTCGTATCGGC
GTCGACCATGAAGAGGTATTCGTAGAAAGACGGATTGACGCCAATGACATT
CTTGATCTGATGGAACATCTCAAGTTCGAGAGGGTTCATGGGGGCATTGAA
GTGTACCTGGAATGAGCAAACACTTGTCGGCATCTGTCAAAGCTCACCTTG
TTCAAGAAGTGCATCAATATCATCTGCGAGTCCCTCTTGCCGCGGTTTCCCG
GCTTGGGCCTCTCGGTCGGCTTGCCCACCTTGACAATCACCATATAAGGGA
CGATATGGCCATTTGTCTCGTACAGCCCAGCGTAAACCTTGCCCATATTATG
CTGTTTGCACCCTCGCCGAGAGACTGGAATGACAGTGGCTCAGGATCCTG

GTGTCCTTCGACTCCAAGAATGTCCAGCACGATAGCAGGCGTAGACTTGTC
ATTTCCGTAACCCTTGATGTTTCCGTCACAGATGATGACGAGCAACTTTCGC
TTGTCATCGTACTCGAGTCTGACGACGGAGTCTATCGTCTTCCGTAACGACT
CCTCGCCTTCCCGTGTAACAAGGCACTTGGCAAATGACAAACTTGTCCGGA
CGCTCGGGCGCGCGGGATGAGCCGAAATGCAGCGCTGCGATGAACTTGAA
GCCGATGATGGCTACCATCATGACGGAGAGGGCGAGTAGGATGTATGTTGA
AAACTGGCATTGTGCTGAGTCTCGCGTGTCGAGCTTGCCAATGAGGAACA
GGTTTCTCAAGCAAGTCTTCTGCCAATCGACTACATTTGCCCGATCGTCGA
GGACAGCCCATCGAGCAGGTCGGTCACGTCTTTTCCAGAATTGTAGGTGAA
AACGCTGAGCACCTGGTCCGACATGGAATTGCCGATCAGCTTGATCTTGAC
TGGTGAAAGTGTAGCCATCAGGAGCCTTGAGACCACCGCCATTCTGTTGTA
TATAAGTCGTGAGCTCGTAGACCATGTCCTCGTATATGCCGACGGCTCGACC
GGCGCTGGCCATGTTCCTGAGGTCTTTTCTCGTATACCCCATGAAACCCTTT
CGGTATTTGTGGCGCATCATGATCATTTGCTCTTCGTACCAATCTGGGCGAG
AGTCGTTCGTGTAGGCTCGGAAGTCGTGGTATCTCGAATAAGTGTCTGATG
TGTTGGTCGTATCGAGAGTGACGTATTGGCTGAGAGAGCCTGTGGTTCCTG
AACACAAAGCGGAAACCTGTACAGGAAAGAGATCCGTCGCGTCCAAGCCG
CCATAATTGAGAACATTCTTGACAGGCACGATCGATACGGCTGTCAAGTGT
GTTGGAGCAAAGGTCGTGAGGTCGAAGACCTCCCCTCTGATGGCCACGAA
TGCGTTGTTGGGATCGTTCTTGTAGCTGTGACTCGCAAGCTCGGAAGCCGA
ATAGACATGTTGCGTGGGGCATATGAGTGGACCCAGTGCGGCAATGACGAA
GACGGTGCAGGCGCAGATGAACCAAATGATCAGATTGATGGTCAGCTTCTC
CCGCCAAGCCTGCCGGATATCCTGTCGTTTCATCCCGCCAACTTTGCTGAGC
ACAAAGTTGGGTATCCAGAAGGTGAGGATGGAGCACAGCCAAACCCAGCG
ACGACGCGCAGAGCTCTCACGATATTCCTCGGTGGTCTCGCCATCCTGTATC
TCCTCGTCGAGGATATCCTTCTCGCCCTTGTGGTCCATATCTTGAAACATATT
CCTTGATGGAGCGTAGGTTTCGGTCCCGATGTTGGAAGCGGTATCTCCCAA
ATTGGCTGGCCAGCTGGCTAGGCGCAAGAGACCGTGACTGATCGAGATCTT
CGAACGACTCTTGCGGCAGAGGCTGTGCGTATTGCACGAGAGGAAGACCG
GCAGCGGAGTCTTGACGTACAAAAGGCTGAGATTCGATTGCAGGCGAATG
TATGGGGGAAACATCGCGGGCATAAGGCGAGAACGGGTCGATTGGGATCT
GCTTCTTGAACGGCGGACCATCGTCCATTTCCATTTGTGTGTCCGCGGTCCA
TCTCCCTCCTCCGCCCGCAATCGGTCTTCAAGCTGATGAAAGAGCCCGTGT
GATAGGAAGATCTGTCCATGAGTTCGACCTTGGCATCATCAGGAGAACCAA
CTGTTGTGGTTCCGATGCCCATCTGAGTTTCCTTGAGGTCCTTCAATCTCCT
GAGGTTGTGCAGCTTGTCCAAGTCGGCACCGCCGTTGATGCCTCTGGCACC
AAATTCGCTGGCATAGCGTTCGCAAAATTCGCCGTGCGACAGCTTGATCTC
GTAACACACCTGAAGTCTTTGTGCCATCTCGGTGAGGCCGTAACTTCGGAC

CTGGGACTTGACACTCCGCGTCTCGACCTGTGACGGCAATTGGGAATCATT
TGGTCGGATGCAGAATACGAACCACGGCTGAGCCTGATCAAGTGTTGACA
AGAGGGTCTCTAGGGCGGCGCGATGCTGGCCGGCGACGCACTGCATGTCT
GTACTGCCCATGGTCGGTTCGTCATTCCCTCCTCCCACAGTTTGATCATCTT
CCTCGTCCTCCTCGGTAACCGAAGCGAGCCGTCGGCCTTTTCTGCGACGGG
TAGACGGGGCTCGCATCGGGCGGACGGGTTGCTGAGCGGCGACGATGGTC
TCGTCATTCCTGGGATGCGCCTGCGTGGTGATCGCTTTGGAAGAGAACAGA
CTTTTGATAAAGGGGTTGTTGGAACCCTGATCCTCGACACCAGCCCCAGGA
GTCCCTCTGAGCAGGCGGAGGATGTCGGCGCTCGTCTCGTTCGCATTACGC
TCAAGGAAGTTTTCGGTTGTGTAGGTGACGGCGCCAGAAAAATGGTTGAT
GGTAAAAGCAGAGCCTCCCTGCCGAGATGCATTACTGATTGAAAAGGAGG
CGTGACCGCTGTGTCGCTTGCCGAGAGCTTCGATGAAGGACGTCTCTGTCT
TCTTCTTCCTCGCCTGATCGTCCATGATGTGAATGACGCCTCCCTTAGGCTC
AGCAAGCATCTTGAGGCACTCGGCATTGTCGTAGTACGGCACTTTGGCAAT
CGACAGCTTCTCTTTCGACGCTTCTTCGTAAGGAACATCGTGTATCCGCCTC
AGAACCCAATGCTGAAGCTTCTCGTTGGCAAAGTTGAAGCAGAATTGATC
GACCGAGTTTGATGCTGTGGTTGATCCCGCATTGTTCTGCGGTCCCGGCAG
ATCGAGCAGGGCAATGAACGATCCAAAAGACTCCTTGCAGAGCTTTTGATT
GACGTGCTCGTTCAGCCAGGAGAACAGGAGGGAGTAGAGCGTGCGCGCTA
ATTCGTCTCGTACTTGTTCCGCTTGCTCGGGGTCGAGAAATGTTTGTGCAG
ACCTCTTTACGGACGAGGACCGTCTTGAAAGAGAACAGCTCGGCGAGTAG
ATCAGGATGCACACCGAGGAATTCCGCTGCCGTATCCAGAACGTCGAGATT
ATCAACGATAGCTCCTTCATGGTTTGAGTCGCTGCTGAATTGTAGATTGCCG
ATATGAAGGATAACCGCTAAGAGCTGGCACACTTGGGCCACAAGCCGAGTT
GACATCCCGACTGTCTTGAACGCCTGCTTGAGCTGGTCGAAACGCTGCTCG
TCGGTGGCTCCAGTCCTCCGGACGCGACATTGCAAATACCGGTAGTCTTGC
ACATGTCCGAGCTTGAGAAAAGTCTTCTCCTCGCCGCTGACTCCGGAGAC
GAGATAGTAGAAGACGTGAAAGTTGCGCTCGCCCGTCACAGGAGGTTGGG
ACACTCGCGACCTCTCAAAGTAGTACTCGATGGTCTTCAATCCGTCTAGAC
GTCCGCGCTCGTTGAACTGAAGCTCGGTGTAGTTGCCAAAGCGCGAGGCA
TTATCGTTGGCAAGGGTGTGGGCATTGCCGAAAGACTCGAGGATGAACTTT
ATGTCAACATTGATCTCCGACACACATGACGCGACGGCTGACCTCTGCGTT
CGCTATCTGCGGTCCGACTTTGGCTGCCTTCTTCCCCGGGAGCGGCCACGC
TAAGCTCTGAAATAGCCTTGATTGCGAGTCGGCGCATCTCGGACTTGCCTG
AGCCAGTCGCTCCGCTGCTCTGTTAGCGAAGCGGCCGGAGTGGGTCCTCC
GGGCTCACCTGAGCACAACGACTTGATCCTGGCTGGTGCGGCGCATATTGT
AGTAGGCCGACAGGGCCTGCCTGAAGATGTGCGGCCCGAGCCTCTCCTTT
GCACCTGCCGCTTCGTCCGAAGCGTTCTTGTGGTACTCTGCCACATAGTCC

TGCAGAGGCGCATCGCCGTTGATGGGGAGGTAGGTGAGAGGGTTGACCGA
GACGAGCGACGACGATGAGAGGGCGGTGTATGGTTGCGACGCCAGGTAGC
GCTCACGCAGGGTGGCAAGGACTGTGTCCTCTGCGATGGACGACAGGGAG
CTGGCAGTCAGCCTCAAGCGGATGGGGGGCAGCGGCCGAGCCATATCGCA
TCCCTCACCATAGATCGGTCACACCCTCGAAGCGCGATGCCGCTCTGCGAG
ACGACTCTGCCATGATGAGACGAGCTGCCGGTCCTCGGCTTCGCGATGGCC
GATCGTCAGATCCTCAGCTCTTTCAAGCGGGGCTGCCTCCCCGATCCTCCG
CCAGACAAGCAGGTGACGATGTAAGAGTAGTGAAACGTCAGAAGAAGAG
TGGATACGAGTGAAATTGTAGAGTATAGCGTACAAAGGGATCTTGTCTCTG
GTGATGACTGTACCCCCTTCTCTTTGGCGGGGCGGTGTCAGTGCGGAAACG
CCCAAAGGGAACCAAAATAAAATGACGGTGTACTCACCTTGGCGAAAGTG
TCACAATACGGGACACTCACCTATGCTTCTCTCTCCGCTCCTTGCCATAGAG
GAGGGCGCCAGTGTTATGCATGCATACTGGTTGTATGCTGCTCATGCTGCTG
CTTCTCAAACACATGCCAAGATGACGCGCCTGTCTTTCGATGGTCATGTTTC
AGAATAGACCTCCACTCTTCACAACACGTGGCTTTAAAGCGAATGCGTTAT
CAGATGACGCCCCTTGCCTCGCCCCGGCGGTCAATGCGCTTTCGAGACTTT
TCCATTCGTCTCTCATCCTCTGGTCCGTATAGACTTGCCTGCTCTTGTCAAG
ACTTGGCAGCAAACATGGTGAGTCGAGGCATGTGGCCGTGTAGAACCGCT
ACTGACGAATCAGAACAGGACGTGAGCTGGACAGCGGCAGACCACGTCCT
CTAAACTGATCACATAGCGTTCTCTACGTCTCCGGGTTCTCCTCCCAGCTTC
GAGCCAAGGATCTCGCATACGAGTTCGAACGCTACGGTCGTCTCGTCCGAT
GCGACATCCCAGCTCTCAAGACTCCTCAATCGGCTCGTGAGTGTCATGACC
AGGATGACGATTTTCCCATCTCCATTCCACCGAGCGTCGTGTAAACGTCCCC
TCCCGAGCAATATCCGTGAGCGTTCGTCCCGAATCTGTCCGAGATCTGCGT
ACGAGTGCCGTAGCCTCAGTGGCTCACGGACGAACACGATCGATACGGTC
GATGTGGTACATTGGCGATCGGTCCACCGACGCCCGCTCTTCCGTTCTTGAT
GCGAGGTCCGGTGGCGAGCGAAGACGGGTCGAGACGAGACGAAGCTAGG
CGCGTGCTGGCTCGATATCTTTGATCTGGCCAAGGCATCGAAAGATGAACG
AGCGATATCAAGGCCGAGGATAGCAGCTCACCCCCGACTAGCGTTCGCTTT
TGTCGAATTCCGCCGCGAGGATGATGCCGAAGAAGCCTACTACGATATGTC
GGTCGCCTCCTCATTTCGCGAGGAGTATTGCTAAATGTTAGGCATGGACGAT
CAATCGACGGCCGCCGTATCGCCGTTCAGTGGGCCAAGCGACCCCCCAGC
GCGGCGTGGAGGCACGATGGGTGAGTCCAGGCCGGCAGAGTCGAGCGATA
GAGCTGAGCATACAGAGCGCCCGAACCGCCCAGGCGCGACTCTGGCCGGG
ATTACGGACGCGACTCTGGTCGTGACCGAGACTACGACCGCCGCGGGCCG
CCTCCGCCTCGTCGCCGATCCCCGTCGCCCAGGAGGGACCGCGACCCGCG
GACGGGCGTGACGCCCCAAGGGATGACCGCGACCGCCGCGAGCGCGACTA
TGATCACCGTCGCTCGGTCTCTCCCCGCCGTGACCGCGATGAGCGTGACGC

TGCCCCGCGAGGCTCCGCGTGACGAGCGCGATGAGAAAGCCAGCCTGAGC
CCAGGCCGGCGGAGAGGAACGGTCACGCCGAGGACAAAGACGATGAGAG
GGAGAGGCGCGATGACTAGAGTGGCCCGGGTTTCGCTTGATATCAAGCAA
GGAATGCTATACATAGGCTGGCGGGGCGCAAGTCGTCAATGCGGACGGAA
AAGAGTACATAGCCATGCACAAGCATCTCGTCCCCTCACTGGAATCGCCGG
ACATCACCGGTCCGCGGTCCATCGACGGCCGCGTCAGATGTGCAACGTGA
GCGCTTTCATGTCGTTCTGATTTGGACATTGTCTTTCTTCGCAATTGCCACC
ACCGCCAACATGAGTTCAGAACCTGCTACGACCATCCTAGTCCTACATCCG
CCAGAAGTCGCCCCCCGTCCATTCCTCGATCGTGCGTTGCTCAGCTGTGTC
CGCATGCCGGGCGCTGAGCCACAGGTCTCGTCTCTTCAGGCGACGCGGGC
CCAAGCACTCCCAAACTCGTGCCCACTTCGGCCACAGCACACGATGAGGA
GGAGCTCTTGCCATGGACCATCGACAACAAGTACTACACCGCCAGTGTACA
CCTTCGCCCCGTCGCCCTGGACTCTCCTGACGCACTGACGGAATGCAAGG
AAGATGTAGTGGTCTACCTCTTTGCGGAGCCGGTCAGCCCGACGACTCTTC
GCTACAGAAGGGGTGCTTATGCGCAGCCATCGAGCCTGCCGCCCGAGCTC
GGCAAGATCATGTCCACTCCTCGCGAGATCGCCCTCGCTGTTCACCTCGCT
CGATCACCTGGCACGGGTGTGGACGGCTCGACAAGCCGGACATCACTGGA
GGGCGTGGAAGAGATGTTTGGGGAGTTGGGCATGGAGCTCTTCGATGAGG
ATGCGCCCTCAGACGACGACGATGAGCGGCGTGCGTCGCCACTGGCTCTG
CTCCTTCGGCTGGCTAACGGCCAGCTACGTCCGTTTTGGACCGTATTCGCG
AGACGCTCATGACGCACATGTGGCCCAACATGTCCATGAAGCCCCGAGCTG
GCCCGTCGGTCTCCGCTCCATCATTCGACGAGCCGCTCGAGGACGATCGCG
AAGAGGGTGAAGACAACCACGCCACGTTTCCCGTGACGTTCAATGCTGCA
CAGCCTGCGGCACGATCACCTGGCGCACAAGCGGACAGCGACGAGGCGTT
CCCGGACCTCGAGACGATCAAAGCTGCTCTTCGGGCCGTACGGCTAGCCG
AAGAGGCTGAGGAGGAGGACGACAATGACTGGGGAGAGGATTTCGGACC
GGGGAAGAAGAGTATGCCAGGCTGGACGAGTGGCTCGACGAGGACGAGC
CGGACTTTGAGAGCTTTGTCCGCGTTCCAGGTGGCGCAGAGACGTCGGAG
CAGCAAGAAGAACCGGAAGCGGGACCGTCTCGGCCCCGACCCGCTGTAAC
TGAAGTCGAGGAGAAGGATGAAGGCTTCGAAGACGACTTTTCGGCTTTCC
ACTCTGCTCCGCGGGCCGACATCCCGCTCGACCCCACGCCTATGCTCCTGC
ACCTCCAGTCGATCCGGTCGGAGATCTCGGCGCTCCCAGAGGACGAGCGC
CGCGTGCGCGCTGGCAAAGAGGTCATTGGTGTCATGCGACAGCTGGGGCT
GGACATGGACGACCTGGGGGATCTGGTGGACATGGACGAGCTGGACGCGC
TGGCCCAGGCCGAAGAGGAGTTTGATGATCTGCAAGACTTCGAGCTGGAG
CTGGCTGACGCGGCTGGTGAGGCAAGACTGGCCGGCCGGCCAAGCCGACT
GGAAGACCTGGACAGATTCTTATGAGGCATGGTGTGACAAGGCGTCCTATG
CATGTGTGTGCAGCGCTCCCTGTGCAAGCTGGTCAAGGTCAAGCGTTCGCC

CCGGCCCCCGTCATGTCCCTCACGCCTGTCTCGCTGAAGCTCAACATACCC
CCTTCTTGCATCTGCTCCACCACCTCTTCCGCCTCTGCGACCTTCATCGCCC
CCAACACCTTATTGACCACATCCAGGCTCTCTGTCCGCAGTGCCACCTTGA
GCTTGTCTGGGAACGCCTCGAAGATCTCCCATTTGCGCTGCAGGAACGCGC
GCACTTCCTCCATATCGAGCGTCTCGGCGCCCTCGCCCTCGATCTTCAGCTC
GGCGGGGGGCGGGCCGTCAGGCAGGTTGAAGGAGATGACGAGCGACGGG
TCTTCAGCTACGAGCTGGATCTGTTCCCGTTCTTGTGGGTTGGCCGCTTCGT
CCGCGAGGATCTCGGCGGTGCGCTGCGCCACGCGTGTGTAGGTCCGTTGGT
AGTCTTGAACGAACATGTCGATGGATTTCGGGTTGCGTGCGATCATCCTGA
GTGATGTCAGCAACTCTCGGGAGCGACGTTTCGGACGGTCGGACGCACCG
CTGGAAGAACAAGCCGACGCCGTCTTTGCCGAGCTGCCGGCAGTACTGAA
TCAACAGGCTCTGGTGCACGCAGCGCTGCGCCAAGGCCTTTTCGCCTCGC
CGCTCGGCGTCAAACGCCTCGGCAAGCAGCGAGTCCTGCGTGCGCTCTGT
CAAGACCGACGAGTCTTTCTGGATGAACGCGTAGGCTCGCTCGTAGTCGCC
CACAGGTATCGACGCGAAGGCCTTCGCGGCCGGTGTCAGTGGGCCGAGGT
CGGGCATGTCCTCGTCCGTCTCCACGTCTTCGGCAGCGGCCGCCTTGGACG
ACTGCCATCAGCAGAACTCCATGTGAGTATATGACGCTCACACTCGCCACT
GCGCCCGGATTGAGCACCTCAATCGTCTCGGTCGTCTCGTTCTGCTTGGCT
TTCCCCTTGCCTTTCGCCTTGGGCATGTCGTCCAGCGGGCTCGGCTGCGCC
TTGGCGACACTCGAGCTGCTCCAGCCTTCGTGAATATCCTCGCTCGTGATCC
TCTTCTTCGACTCCTTCTCCTCCTCGTCGATCTCCTTCTGCGCCTCGACGTT
CCGCTTGTCCAGGTCCTTCAGGTGCCACCCTAGCCGGTCCACAAGCGCCTC
TCTCAGCTTCGCCTCCTTGCTGTCGGGCACAGCGCCGCTCAGCCAGTCCGG
CTCGTCGCTCGTATGGTCGACCTTCTTGCCATCCTTCACCACCTCCCCTTTG
GCGTTCACACTGGCCCCCTCGGTCATCCACGCTCCTTCGCGCAGCACGTCC
TCCAGCAGCTGCGCCAACATCTGGTCGTAGGTAGGCTGGTTCGGCGCACCC
GTGGCGGGCTTCTCGTCTGACGGCTGCTCTTTAATGCGCCGCTGGACAGCG
CGATAATGGTCTACGCCTTTCTCTGACGTGCCGGTGAGCACGGCCTGTATGC
GTGGGCGCAGGACGGCGTTGAGCTCGAGCTCGGTGTGCAGCTTGGCTATC
CGCAGCCGGCGAGCTTCGCGCTTCTCATGAATGTCGCGCTGCTTCCATCTAT
CAGTCAGCCTGGGCTCTTGGAGGTGCTGAGCGGGTTGCTCTCACCTGATCA
TCGACTTCTTGTCCACATTCGGGTGCTCTTCAATGTCCGAATCATCGGACAG
CTGTGCGTGAGCTGTGACCCTTCAAAGGCTTTCCAGCTCACTTCGAGCATG
TCCCATTTCGAGTAGTTGAGCGGCATGTTCGTCGCCTCCTTCCTCGTCGATC
TGCCTCAAAGAGTCAAAGTCATCAACCTTTGCAGGTACAACTCGGACCGC
GTCGAAGAACAGACCAGAATTCCGGTCATGAAACACGTGGTAGCCACGTG
GGTGCTATGACCTCACTGCCTACGCCAGCCGAGCAACACCGCATAGCTTGC
TCTCACCCATGTCCATTCCTCACTCTGATCTTGATATAAGGCAGCAGCCATG

TCGATCAGCTTCTCCGAGCCCAACTTGCAGCCGACGTACCAGGCCATCATC
GATGGCTCGGCGTCGTACGACTGGGCGCTCTTCAACCAGACTGCCAACGA
GCTCAAGGTGCAGTCTACTGGGAATGGCCTGGATGAGTTGGAAGAGGAAT
TCATGGACGGCAGGCGAGTCGTCAGTCCCCATGTAAATCGCGACTCATGCT
TAGGATCCAATATGCCTTTGCTCGCGTCAAAGATCCGGCGGTGCGTATGTCG
CTCGGCGCGAGGGACTGCGTTGATCTTCCCGCAGAGCTCGCTCGACAAGT
TTGTCCTCATCGCCTGGTGCGGCGAGGGTGTTCCAGAGTCGCGCAAGGGG
CTATTCCGTGAGTGATTGGACATCGGTCTTGCAGGATTTCCGGCTTATGGCA
GACACGCACATGTCCCAAGTGTCCAGCAAGTTCCTCAAAGGCGCTCACCT
GGTCATACAAGCCAGATCTGAGGTGGGCCAGCCTTGCGATCAACGTTTGAA
GCTCACGCTCAGCTTGATGTAGCCCCTGCCTTCATCCACAAACGGATCCAG
GAGTCTTCTGGGTCCAAGTACTCCTCCTCAGCCGCAGCGCCGAGCCCAGTC
TCGGCACCCAAGCCGACCACGAGCTACAGGCCATCCCAAGGCCTGGGCGG
CTCCCAAGGCAAGGTTCGTGCAGATGTTCGGATTACACATCGGCATATCGC
TGACGACTAGCCCATATCTGTAGCTGCCCCAACAGCGACCTTCAACAGGCC
ATCGCCAGCCGCAGCTGCTCCGCCTATCAAGCCTGTGCAACCGACTCCGCC
GCCCAGGCCTGTCACACCGCCACCTGAGCCAGCTCCGGCCCCTCCACCTG
CCGCCCCCTCGATGCCAGTCAGGCCAGCAGCGACGACAAGGATTGCATCT
GCTCACACGCCCGCCCCATCAGAACCAGCCAAACCAGCATACGAAGACCG
TATCGCTCCAGTAGGAACGGCTTATGAGCCGGTCAAGCTTGCTACACCGGG
CAAGCTGGCGAACCGTTGGAATCCAGGAGGCGCATCTGCTGCCGCCTCAG
ACGATGCACCAATTGGCGGCAGCATCAAAGATCGCATGGCCGCGTTCTCCG
GAGGTGGTGCAGCCACGTCATTTGCCCCTCCTCAACCGACTGGGAAGAAG
CTCACTTGGTCTGAGAGGCAAGCCGAGGCGAAGAAGCAACGCGAGGAGG
AAGACCGGAAGAGTGCAGCGGCATCGACAGCTGTAGGCGTCGGCGCTGTA
GCTGGCCTGGGCGCCGCAGGTGTAGCAGCAGTGGCGAAGGCGACTCCGGC
TGCTCCCGAGCCTGAGCCCGAAGCGCCTGCCCCTCCTCCCCCACCTCCGGC
TCCTCCTGCTGCTGCACCCACCGCTGGCAAGTCTATTCCTCCTCGTGCCCCC
GACTCGGACGAGGAAGACGAGAACAACGACGATGAGTGGGATGCGCCCC
CACCGCCGCCCCCGCCAGCTCCTTTCCGTCCAACACCGGTCACAGAGGAC
GAACCCGAGGCAGCGGCAGCTCCGCCTCCTGCCCCACCCCGCCTCCGCCT
CCGCCCATGCCCACTGCGGATCCTGTGAGACGATCCGCATGCCACAGCGTG
CCGAACTGACCCGTAACAGCCAAGTCGAAGAGCTCGAGCGACTCAAGACC
GATCTGACGCTGGAGGAGGCGCCAGGTGCCGGTGCGCCACCGCCTGTACC
TTTGGGTTCAAGGCCTGGCGCTTCCGAACCAGCGCCCCCAGCGCCTCTGG
GCAAACAAGCCCGGGTCGCATTCGAGTACGAGGTGAGCTTCGACTCCGCC
GCTGACGTGTGAAGCTGACTGGTAGGCTGGCGAAGATGTGGGTTCCGCCG
CGACCAAGAGCGCGCGCTGACGAGCAGAACGAGATCTCGCTTCGAGAGG

GCGAGCTCATCACCCAGATCGAAGAGATTGACGAGGGCTGGTGGAGCGGC
ACGAATGCCGCCGGCCAGTCTGGTCTCTTCCCCTCCAACTACGTGGAGCTG
GTGGAGGGCGAGCCCGAGCCGCCGGCTGAAGCGGCCATCGTCGCACCGCC
TGCCCCGCCACCGATGCCTGCTCGGGTGCAAGCTGCAGAGGCGGCAGAAG
AAGCGGAAGAAGTGGGCGAGATCTACATTATTCAGATTGCGTACTGAACAG
GGTCGGTAGTGGTTGACCCCTGGCTGTAGCCATTTGGACGGATAGAAGTC
CGACTAGCGATGTTATTCGTCAGTACCTTCGCCCATCCTCTCTCATTATTTAG
TTGAGAGGGAACATATACTGACTTTTGTAAACCCCTTACCGAGGCCGTATAT
GACCTTAATGGCATTGGGATTTCCAAATGATAGTGCATTTGGTCCTAGCCGC
ACAACATCGCCATACTTTTCATGCAATTTGATATGCCACTGTTCAGGGTGTC
GCAGCCAGACCTTGTAGAATCGGAGCCAGTCTGTAAAATGAGCCAATGCTG
GCCCGGGATATTTGTTTAACCCTCTATTGAAGTAATTTCGAGCTAGATAGAC
GATGGCCGCTATCAACAAGGCTAATGGCCATTGCTGAGAAAGCCACTGGAT
AAAAGAACCTGCGATCATAGCGACAGCAATACTTCACAGAGAGATCGTTTC
ACTAGAAAGAGAGTATGAATGAGGCTGTCGTTTTCGTTCAATGCTTACTAG
GTAGGTAGCTAGTCTACATTCTTCCAAACACACCTACATATACCTTTTCAGA
GACGTAGGACAAGCTCTAGAGAGCAAGGATTCGCGAACATGCATAGAGCC
CTGAGGAACCCATTCCGAGTGACATAGCTCTGATAGACATGCAGAATAAAA
GTCTGCCGGTCGAGGATTCGAATGTCGGCAGTTGTAAGAGGCGTTTATAAT
GACGCTCGTAGACGGGGTCCCATTTTTAAATCTTAAACTAGCTAGAAAGGA
TCCAGATGATCTAGCTAGGGTTGATATTACCTTCTATGCTTTCTGCGGGGGG
CAGAGGTTCGGCTTGCTTCCTACAGAGAGATTAGATGGTGATTATGTGAAG
GAACCCCGGACGTATGGTTACATGGCAGACAGGTGGAGGGAGGTGAGGTA
AGGTGAAGTAAAGAAAGGAGGGGATGGTGTAATAGAGAAATAGGGAGCA
AATCTGGGAATCCGGCAGGGCTTAGAGCTAGCCGGGCCCGGAGATGCTGTT
CCAAGCCTGAATCGGATAAATCGTGTAACCCTGATGTTCGAGGCGCATCGG
AGGACCCCCCGGCCGATACCCGCCCGCCACCGAAACTGAATAAGATCTCCA
CGTAATCTTTGTCGATGCCTGAGTACCTGCCCAGGTATCACATCCTGCATAT
CGTATCGACTTGTGGGCGCCCCAATGAGAAATCCTCTTTTCCCACGGACCA
ACGTTGACCCTATCATGTCATGTCATCCCTACGAGCTACTGGCTACCATGGT
ATAGAGGCGTAAGGGCAGGGGGGCATCGTAAGAGGTAAGAATAAGATATAT
TGCCCACATTTAATAACGGCAAGCTCCAATGAGTTTTAAGGATTTCTTTAAG
ACCTACATGATTTAACGCCGCTCGCTGGCTTAAAGAAAACTCCGATGTCCC
TGTTTAGCTTACAGAGCATTCGGATTCTAGTGAGATAAGCAATTCAAATCGT
CTAGGAACCTTTGAATTCTCGCCCTCGGCAGTTCCAATTTCGTAAAGCCGA
GAGTATTCCCGCTTGAAGATATACCTATTGTCGGCGTTACCTGGGTATTTGA
ATTAATTCCGAGGTTTCCTTGGGTATTTTAACCCCGGACAATGCTCTCGACG
ATGGGGGCATTTTGGATGTCAGTGGAGAAAGTGCTGATAATTATCTCGGCA

CTTGACGCTTACCTAGATTTGTAAGGCTTTCAGAATCAAGGCTTCGCCACC
ACAATGTTTAGTGTTATAAATTCTCCTAGCACGGGACGGTCCGCCGTCCGCT
GCGCCAAGTGATGAAGGATTTGACCTGCAGTTCACAGGGGATTGGTATCAC
GAGATAATTACTTCTTCGTCAACCCCATTGGGTAAATGTTACGATCTGTGGG
AATTATGCTCCCGATTGATGCTCTACCTACAAAGGTCCGGGCCAAAAGTCTT
GCACATGTTGCTGATGGGTAAAGGTGGATCAGGCCGTACAAAGTCAAGTTG
CGCGTACTGTGCAGCGTACTGTTTTAGTGGCTCAAATGCCTAAGCCAACTA
ATTACCTATTAGAATATAGCGGCTTCATCCACTAGCATAGTGAGATTATAACG
AGCACATCACTAAAAGACCTGGCCTCAAAAATTAATACAGTATTTCTTGGC
AATTACCTAATCACGCACGGGCCAAGACCATGTGCAAGACTCTTGGCCCGG
ACCTTTGTAGTTATTCTTTCAAGGATTAGGATCGGGATTAAGTAGGTAGGTT
AAGCTAGCGGGTCTAGATTGTATCGTCTATTCGCATTGATCATCTTAGACTCA
AAATCAGTTGGATCGGAAGTGGCTTGTCGTTTTTAATGTACGCGAACTCGC
ATTCGATGAATAGCAGTTCCTGTGATGCTAATAGAAAACTAAGGAATTGCG
AATGCCTTCAGATGAAGACAGACGATAAAAATGACGAAAGTGGCGATGGT
CAAGGGGGTGATCGTAACCAGAAAAGGTCCTCTGGAGACATGGGTGCGTC
TGGAACATGCATGGGTTCCTTGCCCTAGATTGGGGTTGAAGGGATTCGAGC
ATACTAGTAATCATGTGCTTTTGCTGAAAATCTCTTATGGGCACGTTCTTGAT
TTGATAGAACCTCGACCTTCTCGTATATTGGGAAAGTAAGGCGCACGATCTA
GAAGTAATGTGCGAGATCAATGTGGCCATTCCCATCCAGTAGAGGAAATAG
GTTGAGGGCACCGAAAAACTTGATCTGCCTGGTCTTGCTGGTCTTTATTCG
AGAAAGAATTTCCAGAGTTAGCATCGAGCGAACATCCCAATGATAGCAATA
AAGGATCGATGTCGTGAAAAGGTGAAGTAGGTGAATCATGCGGAGACAGA
TTTTGCGGCAAACTGGTGTCTTGGGGATTGGAGGTAGTTGTCGATCCAGCG
GTATTGGAGATAAGTACATGATTTTGATCTGGGGATTCCTTCAGCATGAGCC
CGAATAGCTGAGAAGAGCTTTCCAAAATCTGAAGTCGTTCATCGATATCTTT
GCTTCGGAGATGTTCTAACGCACTAGACAACAAAGACTTTGCGATGAGTGC
ATACCTGCATTGCATGCGTGTCGTTCTTCGCAAAATATTCTAGCGCGGCAAT
AGACATGCAAGCATATTTCTCGAGAATGCGACCAAATCCTCCAAGGAGACC
AACCCCGAGAACCAAAGACGATGCGAATAACCACGATCTTTCCGACCAATT
AGCAGGGGCTAGAAATTTCAAAAGAGGCGAGGTTGAACTCACACTAGTAA
AGGCATGCGTCCGTCTATAAGTTGTTGATCTATCAGACCTAACACTGACTCG
GCCATTAAACTTGCTGCGTCAATGCATGCATTTGCGAGCCTCGTCCGTCCTG
AATTATCGCTACTGCTACTTCGATGGCCGGTGGTATCAATGTGTTCTGGAAG
ACGTTTACATAATTCGTACATGAGAAACGGTCTAGCAACTAGCATGACGGC
ATAGTAATAGCATGTCATAACTTGGCAAGCCCCGATGACAATTGGTATATCTT
TTGACATTTTGTCGACGGCTTGCTTCAGCCGAGACAGCCACTTACGCGACC
AATCTCGGAGCTGACGTGAGATTCCTTCAGTCAGTTGAAGTGAGATCTTTC

TTCTGGAATATACCTGGAGTACGATGTCTTCCGTAATGGATAATATTTGGACC
GACGCATTTAAGAGGTCAAATTTCTCCTGTCCGTCGGCGTCTAACTCTCCG
CAGGGCACCGTACAATCTATATCCGACATAGATGGAGGCCTACCCATGGATA
TGCTGAGGAACAAGTCGAGTACTCGCAGACTCTTCCAGAGACGATCCCTA
ATTATCATTAGTACTAACATATTGGTATTTTTGCTTGTACCTTACCTTCGGATG
TGTAACTCGGATCCGAACCGAGAGTTGACTTCTGTTCGATGCAAGCCAATA
GAGTATGCCGCTCGCGCAGCAATTCCTTGTCGGTATTAGTTGTAGTGATCGC
CCACAGTACGATGCATAGCTTACCAAAGAAGAGGAAGGCGCCGTTAATTTG
ACACGCTCGGAGCATATATAAAGTGATGAGGGCAAAAGCTTGGACGGTCTC
GGTTCCAGTGTCTCCACCAAGAGATGACAAAGCCAAGGCCTTCGCATACTG
GAAGTATTGGGCAGAAGATCCTTCATCGACAGACTGGGAGCCAACAGCTA
AAACAAGGTAATTGACAGCGGATGTCGCGTCCTTGGGACGTCCTCTTCGAA
TGGAAGCCCACGCTAAGATGTTTTTCCGCTAGGTGAGTTCTCTCGAACAGA
TCAACGAGCCCGGATGTCTGCAGCAAGAGTCAGACAAGGAATTATGGGAA
ATCGGAATAAAATATGATGCAATAGAATTACCACTGAGATAAATATAGATACT
AATTGTTGGACATCGGTGGTATCCACATGTGGCTCCTCGTTATTTTCGGAAA
ATAATGGGTCGGAAGAAGCATTTTCTAGCATTGAGACGCGGCCAGTTTCGA
GGCGTAAATGCATTCGGATCTACTCGAGATACTACAATTTGGCGTATTGTTT
GGTAAAGGGAAAGCGGCGCGCAGTCGCCGACAAAGGCTCAATAAGAAATA
TCAATCAGAACATGGGCCTCGTGACGAGTAACGGTAAAGTCTCTCATACTT
AACTTCCCCTGGCATCACACAACAAACGTGCCTCCCGAGGGACGTCTGCTT
CTTCCTCGGCCGAAAGATCGCCATGGTTAGTGAGGCTATTGTCACCACTACT
GTCATCGGCTGTACGAGGATTCTGAACCAAGGGTGCGACAGGGGGATGGG
AGGAGGCGTCTAGGCTGACGGATGACGGAACGGAAGACTGAGGTGTGATG
GATCCCGGAGAAGGTAAATGCGGCCTCCTTGCTCGGCGACGACCGGCAGG
AGAAAAGCGACAAACTACACTACCCTGCCGACTACTACAGTAGCGACAGG
GTAAGTTCCCATCACATTTTACTACAAGAAGACTTGTCAATCGACTGTCATA
CCAAGATTATGTGCATGGATAGGTACTTTTCTTTCTTACCTACCTTTCCGTTG
TTTGCACGTATCGCAAGCATTAGCAACCCTGATACGAGTCATCGGAGAAGA
CATCCCGATTTCTAAACCTGTAATAATGATATTGAAAAATCCATAACATTCCT
CCAAACTACAGCTAGCCCTGGAGGTACTTAGTAGGTAAGTAGGTTATGTGA
CTAAAAAAATAATAGACGATATTAGGAGGTATGAAGGTGAAAAAAGATCCG
TTGCGGATGTCGGAAGATCGGAAGATTGGGGGTGTTGGGGAGGTTGAAAG
GTCGGAAGTTATTAAGAAAGAACTAGGCTAGGTATCTATAGGTGGAACTAC
TAGGTACCTGGTACAAGTACGATCTGACCTCCAGGGCTAAGCGTCACTGGG
TAACAGTGACAAGCTGCTGTGCTATCCGAACTTCCTTGATCAACTCCGAGG
CCATATCCGACGCCCCTTAATATATACCTAGGTACCTACACATGCATATGGAT
ATGATCAAATCTATAACGGGGTATAGGTACCATATATTACCTAATCTAATCCA

AGTCTAATTCTAATTTCAATACTTCTCTTTACATCAATTGCAGTTGATCTATG
CTCCACATCCACCATCTCAATCCCCATCAATGATGCACCCAACCTCTTAACT
CTCAAGATTAGAGCTTACCTAACTTAATGCTACCCCCCCAACGCCTTAATTC
CCGCTACGTACGTCTCATCTCGAGTCTGTTTTAAAAATGGTATCAACGCCGT
CCCTCCTGTTCCTGTCAGCTCTCTTTCTGATTGATGATTAGCTCGCGATGAC
GCAGCAGCAAGATTCATTGCCAGAAATTGGCTTTGCTTCCTTGAGGGGACG
ATGATATATCTTGCCACAAGTTGCATGTGCTTGTTTCGAAAGTCCCCAAGAG
CCTTGGTCGCATATTGGAACGATAGCCATAATTGTCTATGCTCATCAGAATC
ACGGAGCTCCTTCGCCAAGTCCTGCAGATTCTCCATAGACGAGATATGTTCT
AAAAACCTTTTATGTGGGCCGGGCATATATGAACGGACCTCTTCGTGAAAG
TCTGGGTTGGCTCACCCGCCTCTGAGGATACGGTTGATTTTGTACGCGCGT
CATTGCCGGATGCTTCATGTTCAATACCAAGAACAGCATCGAAGAATTGGA
TAAGCGAGCTTTGCCCGTTACTTCCACCCCTCAGCTGTTTCCAGCTGAGCG
CGCCGTCTTCGCCCTGATATAATACTCCATTAGGAAGTCCTGCTGCACCCAT
ATTCTTACTCCCGGCTAAGTATGGGCGGATTTGGTGATAAAAAACCATGGG
ATCGCATTTCTCATCCATCCGATCAAGAAGGTTGCCAAGCCTCCCGATGCA
GATCGCCAATTCGTCCAGAGCCCTTATTATCGTCATGTAGTCTCGTTCCTGA
ATAGCGCCAAGCGCCTGCAACATGACCGGAATTATATAAGCCCCCTGTGCTT
CCATTGCTACGGATACCGCGAAGAACCACGATTCATCCTGTGTTCCACTGA
ACGTGTGCAATGCTTGTAGTTTGTCAAGATCTCGGAAGTCGGTTCCACTGC
ACGCGAAGTTCCAAAGGTTCAGAGCGGCGTATGTTGCCACCGGGGGAAGG
CTAAGGTAGTTCGAAACTCGAAGAAAGGGAGCTGATATTGCTTGAGGGAG
TACCTAATTATTTCATCTGTCAGCTATGCACCTATCTAACACATGTTGATGAA
TTCATACTTCCGACGGCTTCTCGCCCCCCCAGATGTATGCGTGTGCTAAGAA
TGATAGCACCACATAGGCACGACGCCACTCTTCTTGAGTGTGGAGTTTGCT
CGTCGAAAGCACTTCTATACGGCTGACGGACTGTCGTACTGTCCTATCCTTG
ATTAGTGATGGAAGGTCGTCCAGCAGGGTTTCCCATTGGGCATAATATGGAT
TTGGTAGACGCCTTAATGGTTCTCGTCCAGGAAGAAACCCATTTTCGGATAT
AGAATAATTCAGGAGGTAGTCATATTGATTGAAGCCATCTTCACGTTCTAAC
AGAAGTTGAGTACGGCTCGGCATTATAAAATGATACCTAATTTAAATACTCG
ATCTAACAGTTTCGTTTGTGGATTGTACGTAACAGCGATGCAATGAGCTTTT
TAAGGTAAATTCAAATTACCTCACAATTGGATTGAAGCTGATTTGAGAAAG
GGGCTTGGATATATACCTACCTGCCTAGGTAGTAGGTTTATCACCCGCAGGT
GCCTTTCGGAACTCGGATAACGGATCGCGGAAGTCGTCACGGATAGCCTCC
ACTTGTTGCCATAAATCCATGTTCTCCATCAGTTGATATTCGTAAGATAGAG
GAACAATAAGCGATACTACGGCTTCACATCACGTCATCGTCTCGGTGAATAT
TGTAATCCTCAAATCACATGTTATTCATACTACGGCGAGAATAGGACAATGA
TGTCGTTCCACGAAGAACTTCTGAATCTCTTTCGCTAAAGATTGTAGTACAA

TCATGAGAAATAATCGCTGCCTACCTGTCAAGCCTTCTACGAAGATTCCTAT
GAGCTAGGATCGACCCGCAGAGGAGTTCCCTAGCTGCATACCTCGCTGCTC
AGGTAGTTCTTAGATAGACAAAGAGATCCTTCCGTATATAGTAGGGTGCAAC
AATAGAGTCTTTTCCATGCTTCTTGGTTTGCATCGTGACACTACCAAAGGTG
GACGAGTAACAGACCCTCCTCTTGACTGATCGGGCGTATTTCTTAGAAATTA
TATTCCCTCGACGATGCTAAGGGTATGATGACATTCCAACGACTCAGCACG
GCGCAACCTATTGTCGTAATTACATAGGTATATTGTCTAGCTAGGCAGACAG
CACTGACATGATATAATAAATACCAGGATCTCCTCGCTAATGATTAATAACAC
AATTGTCCAGAGGCTAATTGGAAGCTCGTAATGTCGAGTACCTACTATGATA
GGTTAAAAAATCTCCTCAATACCTAAATTACATAGACAGAGGAGGCCAAAT
AAATTTCGGCGGTATTCAATAAATATAGGCCTCCTTTTCCAATCGTTCCCGTC
TACCCCTCGTGAAAATACACCATTAAACACCTAGGGAGCCATCCACAAACG
CCCATAACCAGAAGTCGCAGGTGTCTCGGCTATCTAGGTAATCTAGTGGAC
CATCGTGTCTGCGAACAGACTTGCTTGACTCCCAACAGCCTACCTAGGTAC
AGGTAGTGGACCTCTAGCTCAAGCCGCTAGCGCTGTGCCAGAATTTATTTC
CAGTAAAGTTTTGAGAGGACTACAAAATCGACAATCAACATCAACTAATAA
CTGCATCTTAACATAACGAGTCTAGTTTAAAACAAGCCGTTGATGCCATGAT
CGTTTTAAAGAGATTCGCACTTCATGTTGGACCGGGTCAGCTAAGATATAGC
AACGAACGAACAAATTTACGCCTATCGCGCATAAGTGATGGCTACATGCAG
AATACTACATAAATGACAACTCACTTTGTGTGAAATGAAAGCCTTACAGCA
AACGCGCAGACCCTTACAACCTTGTATAAATATCTCTGGCTGACGAAAGAG
ATATGCTCGCTATAATCCTTTACATCTTACAAGATTTACTTAGGAAGAGAAG
AAGTTAGGGATCGAAGGAATGCAAGGTAGGTACCTACTTACCTATAACCTC
AATTCATCCCGTATCTTTGATGTGCCGCTACCTACCTTCTAACTTGCGAACTT
CACAGTTATAACAAAGTTATTACAAAAGCTCTCGAAGCCTAAGAGGGTCAC
CTCGGGGGCGGAGGTTTCTTATATGTACCCACAGCTACACCATGAGGTTTC
GAAGGCCGTGTCTCCCAAGATCAAGAAAATTTTGTTTCACGAAGAAGAAG
GAAGCAACGCCCTTCAAGAACACAAGACAAATGTGATGGGCAAATTCAAG
TGCGGTAACGATGGCTGCTCTAATCGTGGATGGACAAGCAAGAGGGTGGC
AATCGTCATTAGGGGGTATGGTAAGAACGGGTACAATGCGGTGGTATTCAA
CCAACGCTGCCAATCCTGCAAAACGCTCGGCGTCTTCACGTTGGATGAGA
ATTCATACGTAGAACGGATCGCATATCGACTCAAAAAGTGGGCAGGTGTTC
GGGTAAAGAAGCCTAAGTATGATGAGAATCAAGGTCCACCACATAAACATG
AGCTCTGCGAGGGCTGCAAGAGAGGCGTATGCCAGGAGAGCAAAAATGG
ATGGATAGAGAAGACGATTGCGTCGAGATTCGTCGCTCTAGGCTAGGTACT
TAGGCTACATATAAGTACCTCCTATGAATGAGATCGATCAAATTGAAGAAAT
CCGATTTGGTGCTTAAGGTGGGGTTGGGGTTGGGGTTAGGCTACCCCCGCT
GCCGCTGCGTTATCCTTCAAACTTGTACCAGAGTTCCACTCCCAGCCGTCA

CAGTCAAAAGACGATCGATTTTTCAAGTACTTTTTACCTTACTTGAACAAGT
CACAAACAATCAAGATCCTCTTCCCAATCTACAGCCTACCTGTTCCGCGAAT
TTTGGTGTCAGCTTACCTTCCCATAAGTATCCATTTCTTATAATCTCCTCAAT
GAGGATAAGGCCCGCCTTCCAATACCAAAATCCCGACTATTGTGTGCCGTG
AGGTAACATCATTGCTACTAGGGTTAAGATAAGATCTTGTTGATCCAGTAGA
AGTATAGTCGCTATTGAGTATCCAGTTGTAGTTTTGGTGAACTATAGCGTTCT
ATTGCCCTAAAATATACGCGCCCTTATGGATTGGATAAAGTGGGTTGAAACA
TCGCGAAAACTTGAAGTCATTGTTGGGTTGGTGGATTATCTTTACTTGAACT
GGGATGCTGGGGGCGGGGTGGGTAGGTATCGATCTAGTTGCTGAATGGTGT
GTTTTATTTACCCCGGACTTCTGAAGTAGCATATGTTGCATCTCGGGATCGTT
AGCAATGTTACACTTTCAGATATAGGTACGAATACAGATGGGCGACTTCGTT
AGCATATATCTTGCCGAGGTACGGCAATGGGTCAAAGACCTTATAATGGGA
GTGGTAACTCGGAGGTATGCACACTAGTTTCATATGACTATAGCTAACATAG
GTAAATTGTCGCTATGCAATCCTTTTCTGCGCTGGACATTTCTGCAAGTTGC
ATGTCTAGTGTCGAGTCGAGCTGTTGAGTATTCCAGTTCTCGTCATCTCTGG
TACAACACACCAGGAACCGGTTTCACCTCAGGTCTTGCTATTGGAAATGGA
CGGATCGGTGCCTTGGTACTTGGATCTTTTCCTGAGAGAATTATATTGAATG
AAGCCTCCGTCTGGAGTGGCTCATATCAGGATCGAGTAAATCCAAGTGCTC
AGAATGCCTATCCACAGGTTCTCGATCTACTCAGTTCAGGTCAAATCACTGC
TGGTTCTGACCTCGCTTTGCGGGAAATGACTGCCATTCCGACTACTAATAGA
GCTTTCAGTGTCACCAACGACATGAATCTCGACTTCGGCCATTCTATAAATC
AGACTTCAAATTACGAAAGGTGGCTTGATACTCTCACTGGATATTCGGGAC
TTACTTATGATTATGACAATGTCACCTACAGGTATATTATCATCACACTGATAT
GGTTTACCCCGACTCTACTAACTAGAGCTTAAGACGCGAAGTCGCCGCGGC
ATTTCCATCAGGTGTCATTGCGATAAGACTGAACGCCAGCGTGAAAGCTAG
CATTAACGTGACGATTAGTTTATATCGTTCTTATGGTGTGCTTCAGACTGTAG
CCTCGCTGGCGAATGGTACGATTTTTCAAGATATCGGGGGACCAGATGCTC
GCTCTATCGCGTTTTCGTCTGGCTTAAAAGTGATAGTAGACACTGGTAAGG
GCCCTATCATTTTTGTGCTGCTTGATCTTTCTGAGATAATATCGTAGTAGAGG
CATAGGAACTAATATAAATAGGCGATATAACCTCAAGGGATGGTGAATCCCT
TTCTATAACAGGAGCAACGACTGTTGAAATATTTTATGATGCCGAAACGGA
ATTTCGGTGGGAAACCGAGGATCAATATAAAGCCTCAGTTAGCAATAGGCT
GAATGAAGCAGCAGCAACCGGCTTCCAGAGGCTGAAAGCGGAGTCCACC
GCAAGGCATCAATCTATGATGAGCCGTGTGGATATCGACCTTGGTATAAGTA
ATTCATCAACAGCTCTGCTGCCTACTGATCAACGCATCACTAGCTATGAAGC
AGACCCGGAATCAGATAAGGAGTTAATGGTCCTCTTGTACAATTTTGGTCGT
CACCTGCTCATCTCATCATCAAGCGACGCCGGCAATGCTGGGTTTGGGGTG
CCGGCTAACCTGCAAGGTATTTGGAATGAAAACTATAGTCCTCCCTGGTGC

GTTCGACCCTATACCCATCAACGCGCGCATGCAAGTGCTGATATAGAGCCA
CATCGTGTACAGGGGGAGCAAATACACTGTAAACATTAATCTTCAAATGAA
CTATTGGCTCGCCGAAACAACTAACCTAGCAGATACTCTTCCTCCACTCTGG
GATCTGATGCATCGTAGCCATGAAAGGGGCCAGGACGTTGCAAAACGCATG
TATGGATGCCCCGGATACGTATCGCATCACAACTTAGATCTCTGGGGAGATT
CAGCACCCCACGATAATGGAACCGAGTATACCACCTGGGCGATGAGCGCGC
CCTGGTTGCTCAACCATATGATTGAGCATTTTCGCTTCACTGGCGACAAAG
ATTTCCTAGAACCCAAGTCTGGCCTCTTCTCGTTGACTCTGTAGCGTTTTAT
GAATGTTACACCTTTGAGTTTGAAGGCCATCTTTCCAGCGGGCCCCTCCCC
TTCTCCAGAGAATGACTACATCATCCCAGAGAGCATGTCAAGAAATGGATC
AAAAGGGTCGATCGATATATCGCCAACCATGGACAACTCCCTGTTATATGCT
CTCTTCTCGAGCTACATCGAAGTAGCATCAGTACTCAATATTTCCATCGACG
AGAATGTCGAATCAATGCGCTCCAATTTACGTGACTTCCAGATAGGACAGT
ACGGTCAAATTCAGGAATGGCGGAATGATTATACCGAAGCAGAGCAAGGA
CATCGCCACCTCTCAAACCTTTGGGATCTATTTCCAGGCTATCGCATTAGTC
CTCTTGCCAACCAGACTCTCGCTAATGCCGCCCGCGTTTCCATAGATCGTCG
TAATAACGGTACGAGTACTAGGACAGGGTGGTCGCGTGCCTGGGATGACAG
CCTGCCTCGCCAGACTACTTGACGGTGACGCTGCTATCAATGAAGCGCAAG
CTCTTCTTCAAAACCATCTTCTACCAAACTTGTTCACACGTATATCGGATGA
GACTTTTCAGATTGATAGTAATTTCGGATACGTTGCGGCTATCACGGAAGTT
TTATTGCAGAGTCACCTTGGGTTTGTTCATCTATTGCCAGCGCCCGCTTCGC
AAATTCCGACCGGTACCGTTAGCGGATTGGTTGCCCGCGGGGGTTTTGAAG
TCTCTCTATCTTGGAAAGATGGGAAGTTTGTTGAAGCCAAGATCAAGTCCA
AACTAGGTGGGAAGTTAACAGTACGAATCGCTGATGGCATCGCCTTCAATA
TCGATGGAGAAGCTGTTGAAGCGGTCGAGACATTGGCTGGTCAGACCTATA
GCATAACGGTATAAAGGAAAATCTTTCGTCGCAAGATCTCATAACTTAAATC
CCAGCGCCTAGTGGTGATTATCAAGAGTATCTTGGAGCCAATATCTCTACAC
GACAGGTTATTATTAGTTCTGTAACCTCTCCCTACGAATAATACTTTACCCTG
AATCCACGTCTTGGATGCAAAGTTCCCTTGCGAGCAAGATCTAGTTGGCTC
ACTTACTCCGTACTGTTTCTCGGGAGTATAGTCTATGGAACAATTTGAATTG
GTAATCGTAGGAAATACCATTCTAGAAACCTATTAAACATTTAGGGCTTCGA
CTAGAATTTTCATAGAGGGATGAGTATGGTAAGAGTCAGACCGGTCAAACG
AATCTTAGTAGCTGTCATTTCCCTGGGATATCCACCGGTCGCTTATGCTCAG
GACTGCGAACAATGGGTAGATACTCGACCAAGAATATTAGAACAGAATCAG
TGATTTTACTCCCAACGCCCATCCCTTTACCTACCTACCTACGCTATGGGCC
AAATCCAAACCTACCAATGGTATCCACCGTATTGCTGTATCTTGAAATCGCC
ATCAAGTTACCCCAAATGATTTTCACTACCAACTACCATAAACTGCTATATCC
CTCGAACTTCTACCGACCAAGAATTTATATTCGCCATCGACAACTATAGCCC

AATTTTGCGCTGTGGTGTCCCAATAACTCAAATCGCGCCCTTGTCAAATTGA
ATGTTATTGTCTTACTCTCGCCTTTTTCTAGCGATAACTTGTCAAAGCCGCGT
AGCACCTTTACAGGAAAATCGACTGTAGATACGCCTTGTGGGTATTCCAGA
TATAATTGTGGAACAGTGCTGCCTACTCTGTCGCCAGTATTCGTGACGTTAA
CGGAAACTGTGACGTAAGTGTCCCATAGGTCAGGATTGCCACCTTCTTCGC
CTCCTGCGCCAGAGGGTGGTTGTTTAATGTCGTATCCGTCAGGATATGGGTA
TGGGTTAGATACTATATCATCAACAGCATCGAGGTATGGATAGACATATTTTT
CGAGCTTTCGGAAGCCTTCTGGAAAAAGCGCCTCCTTTTCATCCGGGATTG
TAGTATCATATTCAGGAGGGACTGCAAAATCCTTGGGTCGTGGCGCTGGGA
AGGCGCTTTTCTTTTTGAGTTCCTTGACAACGCAGTCGCTGAAGGTGAAAG
TTGTATAGCTTAAGCCAAATCCGAACTCGAAGCGGGGCTCTATATTGTACTT
ATCGAAGTGTCGGTAGTCGAAGTAGAGACCCTCCTTGAAATCCTGTTGTGG
CACGACGCCGTTGGGTATGTACATGATCTTGGCGCCATCTCCGTAGTCGTCT
AACGTCTTGCCGATGGTCCAAGGCAATTTTCCGCTCGGGTTAACATCGCCA
AAGATGACGTCTGAGAGTGCGTTTCCGCTTTCTTGGCCAGGGAGATTCGCA
AGAAGCACTGCCTTAACATTAGGTAGATCGATCCACTTCTCCATCACGACG
GGGCCGACTGTATGAATGACAACAAGCACATCTCCGTCCCCACCGTGACAT
CCTTGTGCAACTTGAATAATGAGATCATCTCCTCCTTTCTGTAGAAACAGGT
CCGGTCGATCGCCCTTCACGTTCTCCCAAGCTTTGTACCCTTCGCCAGAGT
CAGCGTTCGCGAATACGATACACAGCTGAGCGTCTTGCAAAATCTCGGGCT
GCTTTGCGAACGGCGGCTTATTCGTGAGGTAAGTCGACAACTTCACATTCT
CGTTGCCCTCCCAACTCCTAGATAGAGCGTCGACTGGCGTGACGAGGTAGG
GAAACTCAACAGAGCCAGAGCCCCAGCCGGAAGCCAACGTGCCTTGATTA
CATCCGCGATCTGGACATCGGTTCGGTCCTTCACCGGGACCCGCGTCCTCT
CCAAATATGGCGACCTGCAGCGGGGCCGTCGCGTCGCCGTGTCTGATTGGA
GAGGGCGTTGGTATCACGAGTGAGAGGCAGCAAGCCGTCGTTTTTAAGAA
GGACCGTTCCCTCAGCTGCAATCTGCCGTGCTAATGACGCGTGATCGCCCA
TCACATTAATGTATTTGTTAACTGGAACTGTCTCCTGGTCGGTAGGACTACC
TGGTGCAGAGACACCTTCAGGATCGTCCGTCCATGACGAGAAGTTAGGAC
CTTTGCGATCGTATTTTGTTTCGTCATCCTGACCCAGCTGATACCACGCCGC
GACGATTCGGGTTACCATATCATTCAACCTATCGAGGGGCACCGACCCGTTG
AGGATAGACCTCGTTAGCTCGGGACCCCAAAAAGATTTACCATTCTCCCAA
AATTCCCCGTCGCCAGGCATTGTCATATCTAGACCAGCCAAGGCAGTGGAG
ACACCACTTCTTTGCGCCAGCCAGTCACTCATTACGAAGCCTTGAAAGCCC
ATCTCATCACGCAAGAGACCGTTGAGTAGTTTTGAGTTGCCGCATGCGTAA
GAGTTGTTGATCATGTTGTAGCTGCACATTATACTCGCCACGCCGGCCTTGA
TGGCGTCTCCGAACGCCCACATGTACTCGTGAATCGTTCGGTCGTCGATATT
GGAGCTGATCGCATTCGGAAGACCCCACTCCCATGGTTGACGGAAGTGTTC

CTGCTCGTTTGCTACGAGATGTTTGACAGTAGCCATAACACCCTCATCCTGA
ATTCCTTTTATAGATTCAGCTGCTGCGATACCTTGCAGATAAGGGTCCGCAC
CAAAGCCTTCCCAGTTTCTCCCGCCAGCAGGCATGCGACCCAGAGGACCG
ATACAGGGACCTAGGATCGCGTTGATACCTTTCTTCCTCGCCTCGATCGCGT
GGGCATTTCCCCATTTATACATTAACCTCCTTGTTCCACGTCGCGCCAACCG
TCACACCAGCAGGAAATGCGGTAGAATTGTCAGCAAACCGAATACCTAGC
GGGCCATCCTGAAGTGCGAGAGATGGGAACCCTACGTGGATGGCAGGACC
GGTGTTTCCTACGGCTGGACCCATCTTCCACCCTGTCCCTGTCGTCACATTC
ACCTTCTCGGGTAATGTCCATCTTTGCTACCATCTCGCTCGCTTTCCGGTAG
CTATCAGCCCAGCTTCTCGCCGACCCTCCTTTAGGGGTCGGGTACCAAGGC
GGTGATAGGCCATCTGGTGGAGATTGTTTGTATTTCTTAAACGCAAAAAAG
CCACCCGCGCCGAGGGCCAGCCATACTAGGGCGAACACGCCCAAGCAGCC
AATAATACATCTGGTTGAGCGGCCAGCGAAACAGCTGCAAGGTTTTCGTAG
CCAACGATTACGAGGCTGGGTTGAGAAGGCACCAGTGGATGGTCTTGAGG
ACGTAAGAGGATCGGATTGGAGAAATTCGGTGGCGTCGAGATCTGAGTCA
AGGTCGAACGACTCGCGAGCATCATAGGATCGTCTAAAATGAGTCTGCTCG
TAGTCCGAGCCAGGACTCAGCAATTGCTCTTTGCTGGAGCCCATCGTTGTC
GGAGGCCCTTGGGCCTTGCTTCGTGCAGTGATTCGACAGACTCTCGGTTCG
ATGAGGATATCTTTGGTATGGAAGCTGATCGTGAAAGGGCGTGTAATAAGG
TAGGTATGGAGATAGCCTTTAGTGGAATTTCTCCCGGTGCGTTGGATTTTCA
AATAGCCAATGTTCGAATACGATAAACTTAAGAATTGCTTGTTATAGTGACA
CGACTGCAACACATGGCAGATTATGAATTTCTCTCCCTTGTATTACAGCCAC
AGAAGCACCGGCCAGACAGCAAGCTTTGAAATTGTAACGCTTGCCTCGAC
ACGTTGATATATATAACAAAGTTTTAATAGAACATTGAAACTCGGTCAACG
TCGCCTCCGACATTGGAGTTGTTACTTGAAACCTGGAGATCAGCTTGGCGT
TCAACTACCTGACTACACTAAGTGCTGAGCTGGTAGAGTGGATATGTTTAA
GGTGTGCTAGTGTGCTGTTGCACCCCCCAACAATACCTAGCTCCCTGTGCA
GTGACCCTGTAGTGTTTATCTTAGCTAAAATATGAATTCAGGGTTCCCTCTA
ATAGGTACTACTAGCCAGGGGGGACTCCACTAATCGAGTCACGATATTCGTC
AGATAAGCTTAGGTACATTGGCACACTGGGACTGGGTCAAGAATGCGGGG
GTTCTTCTAGTGGGTTAATCACTACCTAGGCCCTAGGGTCCGTTTCAACCTT
GCAACGCTGTTGAATGTCCAGAATGCCCATCTTCAGCTTTCCCATTTGCTAA
AAGAGGAGTTCCTAGACGGGAAGCTTCTCTGTGATGATACTGTAACATCAC
ATTCGAATCTACGGAGCTCTGATTGGAAAGCTTAAGAGCGCTTTCGGTCCC
GGCAACGGGAAGACGATATTCGAAGTTCGTATTGTTCGCGGCGGCTTCTTC
TCTGATAGGATATGGGAACGGTAGGTAGTCGGGAAGCAGGCCTAACTGCTT
CAGTAACGTTGCCTGATCCCAACTTATATGTTCGTGGTAGAGACGATCGCCA
CGGATGTTAACTACGCTCGTAAAGGGGATCTTGATTGATCGCCCAGTTGCC

GGAATACCAGGAGCACTAGAAATATCAGTACTAAGTAAGTTCTCCCCCACC
ACCTTCATGTTATGAGTATACATACAGCCAATCGATCATACGGTCATGAGTA
GCCTTGAAGAGGAATTCATCCACTACACGATCTATGCCCACGGTCCGACTC
ACAAGTTCAAGCTCTGTGTCGGGCGGGTTGCTGAAGATAAAATGGTCCCTG
TAAAAATTAGTCAAGGCCTGTCGTCCGACGCCCCCGGTCAGCGTTGGGATG
TGGTTCACGTAGGGCTCTTCGACCATAGTCGCCATCGTTTTCTCGACCGAG
CGCGCTTCAAACTCCCAGTACGTGTGTTCATCCCAAATTTTTTCAAGGTCA
AAGTGAGGTCCGCCTACGAGCGGCTTTAAGAAAGAGAGCGTCCGCGTATG
GGACACAGAGGCCGAAGATCGTTGTAATCTTCATGAGCTGGGATGGCGAA
GAAAGATCCCGTACGAGCATAGTGGTGGCTCTTCACATTTCCATCACTGCTT
GCGGTATTTCCTTCCGCCTTGGTAGCTCCAATTACGTGCATCAGCAGCGGTA
GCTTGCTTTGAACGGTACTCGTCGAGGGCACACCGTACCAGATCGCGGCAC
ATATTGCTGGATATGCCTTGATTATTTCCAGTACGATGGATTCTACAACAGGC
ATATAGACTTTTCGTTATCATACCATCTTGTCAGCTCAATCATTATAACTTTAT
GTACACCTAGAATGTTCCCTCTCGCTCACTAATGATTCCAATTCTCGTAGTTT
GGTCACAAGACGACAACTTCGATAGCCCCGAAAGTGCTGAACAGAGTCCA
TCGCGTATAAGACCCCCGTTGGATGTCGTGGCCCTCAATTGAGCAACAGCG
AAACCCTCCTCTGCCCATTTTATCAACGGTGGCGGGTCTAAGGTTTTCTCGT
TCTTCCCAAGATTAAGTTTTTCGTCGACAAGAAGGAGTAGTCCTGGGCCGT
GGCCTCGTCGTGAAAGAGGAGGCATAATTGTCAACCTTGAGTCCATGTTGA
CTACCTAGGTCCTAGGTAGCTAAGTAGCTAGGTACCTAATAGGTAGTGATTT
TTAGGGGGATAGATAGCTGTCTACAAGTCCAGCATTATTCCCAAATTTGACC
ATGGCAGATATTAGTGTCAGTAAAAAAGAAGTCGTACATTCACCAGCTGAG
AGATATAAAGCTGCACGTGTACCTATGTTACCTACTCGTGTCACTGTTTCAA
GCGGGGTTGTGGAAAATAAATCTAGAGCCGTAAGGGAAGAATACGCGGGT
ATCTTGGTCAACGTCACCCAGAGATCATAGGTGTACTACACTGACCCACAG
TGAAAATAGCATACATGTATTAAATTTTGACCTCCGCGGTATTGATCGATTCT
TACTTTAGGTGGTAGATAATTAGCCCGGCAAGCATTCGGGTATCGCCAGCTA
AATACATAACTTTGACAGTCAGTGAGAGCTCAGACAGACAGCGCTCATCAC
AGCTATTTCGGATCGGGTTTTCCCTATTTATCCCGTAGGTACAGGCATAAGA
ATCTCACATAATCCAGGTAGTCACGAGACTTT

>Mosaic_sequence_45

CGCCTTCAAAGCATAGTATGTAATAATATCGACCATGCGATAGCCGGATAAT
GCCACGCGCCACATCTGCTCACGATATGTACTATGCGTATGTACTATCCGTCT
GGGTCTTGCGTACGGATCAGAAATGCCGAGCATAGTTTTCACCGAATTTAC
GCCAGACTTGATTTTTTCCTCTATTCTTCTTATTTCTTTCATCGTGCTATAGA
AAGAGATCGAAGATAGATCGAATACATCATATTCAGAACCTGTTACATCTTC

CAGCTGCCAAGTCTGAGGGGTGGCCGAGACCTGTTGATGACTGGTTAGCG
AGGTTGGTAACTGTGACGGATAGGCGCAGAGGAGCCTGAGAAGGAGGAA
GAGATGGTGGGGTCAAAACCAGTGGGGAAGACGCTGACGCAATAATGCAG
TCCAATCCTAGCATTTTTACCTTGTCTAGCTTTTTCGTAAGGCCTCCATCGTC
AACTAAGCTTAGACCACCTTATAAAACTCGTGATGATCTTCGGGAAATGTAA
CTTCGTACCAATATCATCGATATTAATGAGATTAGGTAATGTCCAAATCTTCA
TAAAAACATGGAAATCACGAGATCCTAGTTTGACTACTGTGGAGGACGTGA
ACTGTGCTATTCCAAATTTTGCAAGGATTCCCGGACTGTCGGGCGCTCATTA
CATTCTCCATATATAAATAGGAACTATATCTGATTCACGACCTAGTTGTCGGG
CATCAAATATAGGTATCTAGGGATGTTCGCGACTAATCATTGATGAACAAGA
TGCTCACAAACCCCTGATCAGAATGCCCCTGAATCTAGCAGAGAACCCATC
GCGACTCTGGATATTATTGGATATGAGATGAGTTCGATTTGTTGTCCCTTCCA
TCGTCGAAGCCAATTGCCCAATTATGCCCTGATCCACACTTCAGAGTTCCCG
GGCCGAGCCGGAGGCTGATATATCTTGGGCGGTTCGAACTCTGTCGTTATTA
GTCGGTGATTTCCCAAACGCCTCTTAACTGCAGCAGCTGCAGTCGAGTCCA
CCGGGAATCAGGGACACGTTTGAAGGTGTAAATGATACACGAAGACCCGA
TTTATATTAGGCTACCATCCCTCTTTACCAGAGTTATCGTGATGTTATAAAAT
CTCACAGCATCTCATGTGCCTCTCGATTCTCAGCGGATAGGTATTGGGTAAC
CATATTTATAGTGCCAATTATCTAGTATTACGGTGGCGTTAATGGTAGGCACT
CTTGTTCCGCGTCTAGGTAACCTTAAATTACGGCTTTCCAGGATTTCCCAGT
GATAATTCTTCTGAATTATTTACCTAATGGAAATCCTATTCAATTAACATCCC
AGTTATCTCTGAAAACATTGTCCACATCTAGTGCTCTGGGAATTCTAACTCA
TGTCGAACGGGCTGAAAATGGGTTAATCTGAACATCACGAGAAAAACAAC
CTAACACTGAACGAACAATTTGGATGCCTATTATACACCGTTCTATTATTTAT
AGGTACTATGTATGCAATGCTATATGCAACATCATGCCGAGAGGCATTCAGG
TTTCTAGGTACTTTAAGGTATATATATAGTCTCTCCCTTCCGCTGCCTTTACTT
TGAGTTGAAACTCATACCCGCATTCCACCCCAATGCTCGCTATGAGATGATA
CCATGGATATCTTATAATAAACAGAAAGAAAATACCTAGTCGGCTTTTCATC
TTGAAGAAGCGCTTCATATTATCTCCTACCACACGTCATCCCTTTTTTCTTCT
TCGGAGTAAGTAAAGACCTCGGCAGGTATCATGGTACTTCCTTATAATGCCC
CTCACCAATCAACTCCGCGGGCCTCTCCGGCTCCGCAAGTTCCTGTGGGTA
TTGTTGTTGCTGTTGTTGATGTTGGTAATAATACTGCTGCTGCTGTTGTGCGT
TATAATCGCCAGCCGGCGCACCATACGCCCACGCGCCTTCCTGCCCAACAA
CCCCTTGCTGCGGGGGGTATCCGTAGCCCTCCGCTCCTGCCGCACCGGCAT
ATCCCGGCTGCGCCGCATACACGCCTTGCACCTGCTGCCCGTACGCATTCA
CATTTTGCACGGGATACCCCTGCGGCACAGCCGCATACCCATCGCGACTCC
CCGCCGGTTTATCCGCAGCCCCGACGTACGCGCTCCCGGGGCTCACAGCAC
CGCTCACGCTCGTCCCCTCTCCTGCAGCGGAGGCAGCCTTAGCATCGAGCT

TCCGTTGCCGGTACCTGAGGAACCAAATCGTAACGCCCATCCCCGCAACCG
CGATAACGCCTAACGTAACCCCCACCGCAATCCCCACCTTCGCGCCATCGC
TCAACCCATCCCCATCATCCCCAGCCCCGGTATCCGTCGAATCCGTTCCAGA
GGAAGTAGCAACCTGACTCGCGTTATGCGTCGCTAGAACACTGGCCTGGAC
CCAGGTCCCGCCGTAGCCGTTCGCTTCGACGACCTTGCCGCCGCTGACATA
GTACAGACGCATGACGTCGGAGCCGAACGCGTTCGCGATGCCGATTGCGC
CGCCCGCGGTATCGGCGAGCGGCCACGCGTTCGAGACGGTTTGGTTGAAG
ACGCTCCAGACGTAGTTCTTGTTGCCGACTTGGTAGATGTTGCGGTCGCTG
CCGATGTAAAAGACGCTGCGCGTGTACGCGTTGTCGATGCCCACGGCGAG
AGCTGATGCGTTGCCATTCCAGGCTGGTAGGCTGAAGTTGGCGGTACTAGT
CGTTAGGGTCAGGTTTGATGCGGAGGTTATGTTGGTGAGGTTGTTTCCCGC
TAGGGGTTCTGGGAAGGTAGCTGGGAATGGTGTTAGTACTAGCTATAGCTG
AAGGGCAATGGAGTAGACTTACAGAGATGCCATAGGTTGTCGCTGTTTAGA
CGCGACACTCCTATGTTGCTATCGTCTCTAGGCCTCATCACCGTGATGTTCT
TCTTAGAGAAGGTGGACGCAATGGCTTGAGTCGAAACCTTGTCGTTCGAA
ACGATGCCATAGTATGTCCACCCTGAGGAGCTAGTGTACCCAATCGAGTGC
AAAGTCCCTTCAAGGTCGTTGTAGTAAACGCGATACCCATCCATAGCTCCC
AGCAGCACGACTGATAAAGCCGAGGTCGGCGACACTTTAGGCGAACCGCC
CGAAGTGACATATTCGCCCGTGTTTATCCAATGCCCCGTGTTCCAATCGCAT
TTCAGGAGTGCATTGACAATTTCGTCGTTGTTATCCAAATAGAAGATAGACG
CCTGTTCGCTGGCGTCAGCAAGTCTCGTACAATCTGACCGGCTCCACTTAG
CTCATCTCGCGATGATGAGAGCGTCTTAGCTTCAACTCACCCAGGCGGTGT
CGCTGTCAGTCCAACCGGCCGCGGCCAAACTCGTCTTGTTCTTGGGAATGT
AATTGTAGAAAGGGGTCGCGAGAGTTTTGTCGTCTGGGAATATTGGCGTGT
GATTGCCGTTGCAGAGACTATATAGTATATTGCCTGTATCGTCGTCCTGCATT
ATGAAACTGGGTCCTCTTGTATTCCACCATGCTGCGATTGAGCCGCAAGAC
ACATGTCCAGCTAGCAGCCATATTGAAATGTGAAATAGCGACGCGAGTCTC
GTCATTTTGTGAGAGGGCGCGCAGGCTTTTCGGAGATCCGTCTAGGTAGTG
AGGTGGGACGCTAGCACAGGATAACGGCGCTCATGATCGTGTCAAGCCCC
AAATATGCCAAACTGGTCCAAATTAAGACAAAAAATGGGAAAGAAAGAAG
AAAAGATTGTGGAAATAGTAAAAAAGAGGTGAAGATTGACAGCTACAAAA
ATAAGAGGTCAAGAAAATAGAAGTTCATGTGAAGCGGGACTGATGAGAGA
TCTTATTAACCCGAATGACATTGGGATCTCTCGTCAGCAGCCAAATGGCTGT
CAGAGCTGCAGGGGTCCATACCTCCTAGGGAAATATGTCCTAATTTGAGCG
GATGAGGTTTGGTTCTAAACGGGGTCTCGAACGGGTCTTTCTGGGCAACCC
TAAGCTTGAGATCGGACAGGCATTCTTATTATTGATGTTTCCATGTCTACGTC
CTAGGTTAAAGGAATCAAGGCCATATGTCCTCGGGTCCGGGTCTGTAGGTA
GGCGATGTTGCATCGGGAAGGATTTGGGGCTACATGCACGGTTCCTGGTAT

AAACACACATGGGCTTTCAGGTAGTATAACTTGTGAAAACCATAAGCGAGA
TGGTGGAAATCGGTGGAAATTGTATAAAAAAGCCTATTGTTTTGCAATGTG
GCGGCCAAATGCAACTTTTAGTAACGGTGCGTCATATATAATGAGCGAAGA
GGCGTTTAGCACGTCTTGCAACTCAAAATGTGTGAAGAGTGAAGTGACCG
ATTGCAACGGTAGCGTTGATATTAAATAGACGATACACTGTCACTGTAAGAG
CCCTGGAGACTCTAGTCCCTTCGGCACTGCGCTTTAATCAGGAAAATGGGC
AGTAATTCTTATAATACCTCATTGTATATAGCATCTTATATAATAAGAAGTATG
TCCTTAATAAACTTATTCAAAGTGATAGTAATTATATTAAAATTAGTATATCGG
CTGACTTTTGGAAATATAGCAGTTGTTACAACTAAAGCGCGGTGCCGCAGG
CATGAGGAGGCGTCACAACCTGGAAGCCAGTACTAGTAGCCAAGTCTAGG
TGTATGGTAAGACGGGAAACGCTTTAATCGTCTCAAATATAAATCAAAGTGC
AAAGTAGCATTATAGCAAGTCAGGAGTGGGGGATATACAGAATCTAGCAT
GAGAAATCACCTGGCTGCATGATATAGCTTCAAAAATTTTTGTCAATTTTGA
TCAAATGATTCGTTATCATGAACATGGGTTGTAAGCTGAGAAGGTCCCAAG
AATAATTGAGGCTGGCATCAGACTATACCAAACGGGACGAACCTCAAACGG
GCGTCCCGAACATCTACTGGGGTCTGGGGATGACATCAATCCTTACCATTTT
GAGACTGTGGCTTGTCTGAGAAACGCACGGATGAAATATTGTGGCGCAAA
GAAGCTCTCAACTGCCGTCTCAACAATTTTGTAGTCCTGGAGCTTCCCATC
CAACGTGTAATGGTATATTACTCATCATCTTCGTTCACTATCTCTATATTCCTA
GATAGGAGGTACACAATATGTATTAAAGAGTACCCATGTTGGCTACTACTTG
AAGAACTCATGTCTATATGTTTCAACTGAGCAGCATGAGAAACGTCGTTGA
AGAATGAAAATCCTCTTCCCAATCGCGTGAGCACTACCCCGCCGTCTGTGA
TCGAGGTCAATCAAGATGCGGTCCGTGCTCCTCCTTGTGTTACCAATTCTAT
TCAGACCTAGATGATGAACACGGTCCCGAAGCTCTGGAAATCCGATGCAGC
TCGTCCCAAGATCGTCATCTGATACTTCAGCTCCCGTTGAAACATTATGCGT
AGAAGCATTCGATCTATCTAGTTGACCCCGATTGGCTTCCCGGAGTATCCAA
ACCTTGACGCACATACGGGTGTAGTTGGGATGTGTCTGAGATGAGCCGGGT
TGGCGATCCTAATAATCTGATCTTTTGCACCTAGTCCCTGTTCGGGGTATATA
TATCAGCTGTGGATGAACCACCGTCATGGAGAAGCCGGATAGCACGGTTAC
ATGCGCGTTCCATTTTACGTCGATCCTCGCGTAAACCACGTGCCAAGATTCC
ATCGTGCCTCATTCTAGAAGTAGGGGGATTATACCTACATATCAAGGAAGGT
AACCAATCAGAAATCACCACTCAGGGGGAATGCCGTGCAGTTGTCCGGAA
GCCTTACTAGGCGCGCAGCGGGATACACTAGCCGTGACCCTTAAGCTATCT
CTGGTGACATATTTCCATACGAGGTAAGTAACTGAACTTGAATATGAAATTC
TCTAGCATAATATATCTAAGTAATCTCGTGTAAGTCATTTCTATGATGAGAGT
AAGATTTTAAGGCTGTTTGATCCGTTTGATCTCTTTACCCCGGCCGAGACAT
CCTGGCCGGGATAATAGCGCTTCCACCAGTTGAAATCATGAAAATGGACGT
GCCTGCTATTTTTGTGAATAAAGCAGCACTCAAAGCGAATGCTTATTTACCT

AGTATTCAGTTAGATTTTCAAGAGGTTAGCCACACATACGACGCCAGTACG
ACTATTAATATGTAATAATGATAGAATATTTGTATTTCCTTAACCCTAAAGGA
GAATCATAACCATGCGCGTATAACCGACCCAATCCATCTACCTAGGTATCAA
CCGGATTTCTAGTCCCTGAACCCTACTTCCGTCCGTCTTCCATCAGTCTAAT
CGACCGATTTACTCCTTAATAAGGCCGCGCTTCGCGTCCTTGGCCATGAGCC
CAACTAGGCTCTTCTCCGGCAACGGATACTGAGGGCGTCCACCGAGAATAT
CTACAACTTCCTTGTTATCCAGCCAGTGTGCTACCGTGGGTCTTCGGTTCTT
CCAATCGGTTTCATTCTCCTCTAATGCCTCCATGGGCGAGAAAACCACGTCC
GCGCCTTTGATACCGATGACGGAGGCGCTAAGGGATCTCTGTTGATTTTCTC
GGGGTTCTTCTGGGGCTTGGGCCCGTATTTCTCGAGATGTTGTATGCACTTG
ATAGCTAGCCTGACGGCACGGCATCTGTCCATTGGTGATGGGACGCCACCT
TGCTGAACGTGGCCGGGAATGCTGTCTCGTGATTCGAATCGATCATGAGCT
TCCTCACGAATGATATCCGCAATGAGCTTGGCTGAGAATACTTTGCTCGCCT
TTTCGTTAACGAGAATCAATCGTCCCGCTCGTGACTGACCTCTATCCTTTTG
GAAAACTTCTTTCAAATGCTTGACATCGGCGGTAAGCATTTCTAAACTGATA
CCTTCTTCTGGGATGTAGACTGCGCTTGCGCCGACACCTAGACCTCCTAGA
ATTGCGGCATATCCGCAGCGACCTCCTTGAGTCTCAATGACGAAAACTCGC
CTCCGCGTAGCAGAGGCAGATTGTTTAATCTTGTCACAGTAGGCGACAAGT
TCATTCAGGCAGGTATCCGATCCTAGAGAATATTCTGTACCGGGAACGTTGT
TTGAGATGGTTGCTGGCAATAGGGTCATAGGGATACAGAGCGAGGGGAATC
TGGTCCTGGCTTTGCGTAGCTGCGAGACGGAGTGGAAGGCTTCGAATCCG
CCGATTAAAAATAGCGCATCAAACTCGTACTTTTCGATTAGTTTGGCAATTA
GTTCTATGCCAGACTCTTCGGGAAGTTCGCGGTTTGTGCCTATTTCTGAGCC
ACCCTTGCTTGCCCATCCATCGACCTCCAGCCAATCAAAATGCCGTACAGC
ACCGAGAGGTGTGTCGCCGTGATGGCGCGCGAACCCAGCAAAACCATTGT
GGATCGCAAGAGGTTCATGCCCCCCTATTATTCCTTTGTCAGTCTTTCTTGTA
CACACATAAATAAATAATGAGGCAAATTTTGAGCTTGTATCTAACAAATCGA
CGAAGAAACTATTAGAAAATGGTGTCTTACGGGCTCTGCAATAAGCTACGC
CTGCACGAATTGCAGCGTTCATACCACCAGCAGGTGCTCCTACGTTAATAA
ACCCGATTTTCATTCGCTACAAATAGTTAGCATTCGTTGAAACTCGCGTTGT
ATTTTCAAACGGACATACTTTGCTGTTTTCTAGGTGGATATCCTGATCAAGG
CCCATGGTCGTAGTCATCATATAGGATTTATATTGGTCGGAGAATTCGGTATC
ACGTAGGGAACATAGCTCTATCGAAGTCTTGCGCTTCGATAGCCTTAGCGA
CTTCTTTGGTTTCTTGTACGGCCTTCATCAAGGGTTTTCGGACGATCTTGTT
CTCGGTAATAGCGATGACACAAGTCTCAGTTTCCGGTGTTGCCTCGAGGAC
GGCCTTGACAGCTTCTACTCCCTGTAAGGTGGCGAGCATCCTATCGTATGCA
ACTGCGGTACCACCTCGTTGGACATGGCCGAGGGTGGTGATGCGGGTGTC
GAGAGCGAGGCCTGATTTGTCGGCTAACAGGTCTTTTACCATCTCGGGTGT

GATCTTGGTTCCATGTCTATCGAGGGCACCTTCGGCGATAATGACGATAGTT
TTACGCTTTCCCCAATCACGGTGCTTTAGAGGTTAGCTTTCATTCGTCCTAG
GTCTCGGTTTTCGATTAAGATACATACCTTCCGAATTACCTGGTTCATCTCTT
CTTTCCAGTTGTCCTCCCTAGGTTTCTCTGGGATAAAGATAAAATCGGCACC
GGTAGCAACACCGGCCATTAAGGCAAGCCAACCACAGTGTCTCCCCATCA
CCTCTATAACGAAGGCTCGCGAGTGAGAGGAAGCAGTGGCCTCAATATAAT
CGACCATCTCGCAAATACGTGCTAAGGCGGAAAAGGCACCAATGGTGGCG
TCGGTTCCAGAAAGATCGTTGTCGATTGAGCCGACAAGACCAACGATGTTG
AGATGTCGGTAAGGTTCGATCTGTTTCGCAGATAGTTGACCGGTAGACACA
AGTTCTTCGAGTAGAGAAGGCCATTCAGCTCGAAACCTGTCAGCACCAGT
AAGAGAGCCATCACCACCGCAGATGATGAGAGCATCAATGCCGTTGAGGA
TTAGGTTCTTGGCAGCAGTTAAACGCCCTGGGCGTTCATAGAATGCCATAC
ACCTTGCAGTGCCTATCAATGTACCACCTTCAGAGAGGTATCCCCTGACGTC
GTTCCATTCCATTTTTCTAATGAAGTTACCGCCCTGAACCAGACCCTCGTAA
CCCTCGTAAACGCAATAGGGATCGCAATCGACGTGGATTGCCATACGTACG
ACGGCTCGAACAACGGCGTTCATTCCAGGCGAATCGCCGCCGGAGGTCAT
GACGGCGATCTTCTTCTTTGGCGTCGCCATGTTGGATATATTGACTGGGCTA
GGTATATTGGTTTAAGTAGAGCTTCGAGCTCGAAGGTCGAGGTAATAGCGG
TATAGAATGAAATATTCCAATTGCGTTTGACGAATATTGACTAGGGTCTATTT
GAAGTAGGTACAATGTAAAGGTAGGTGTATTCATCTAAGGTTGAGCAAAGA
CGAGCTAATAGTAGGTTGGACGTTGGACGTGTGGGCGCGAATGTGGGCGA
CCAGAAGAAATTATATTAACTAGGACCTGTAGTGCGTTCTTTTCATACTATAC
TAAAATCAGCCCACAGGAATAACGCTGGAACCAAGATGTCAGGAGATACCT
GTAAATTGTCCTCAAATTGAGGGACAGCAATCGGACCGTCCACTGGTTTGT
GGTCCGGTACGTTGTAGTACGGAAATGGGTGGTGAATTGATGTGACGAGGG
AAAAAAAGACTGCTTACCCGCACTGGATCCTCCCCTGTTAGTCCTATCATTT
AATGCTGTATGTCATCGTTTTGGAGAAGAAAAAAAAACTACGCCAATTCGC
GCGAACAAAAAGAGCTCTTATCAATTATCAATGAGGGGCAGCTACTTCCAA
GTTCCCAGTTACGTAGTTGCTAGTATAAAGGCAAGTCCATCCAGGGTTATTT
TACCTCCTACCCACTAGAAGCCCACTATATGAAAACATGTATAGTACCCCGC
TTACGTAGCTCTGTGGGCGACGGAAGTAACCATAAACCCCTCACTACTCCG
TACTCCGTACCTATCAGGTACCCCTAAGATCCATGGGGAAATCATTAATTCG
CAGCTGAGTGGTGAGGCTCCTACTTCTGAGTTATTTTTATTTTTACCTTTGG
AGTTTGTTGAGTCTTTTATTTCTTATTTATCTCATAAGCTGCATTTGAATGTTG
AAGTTCTAGCTTTAAATACCCCCTAATATTTGTTGAAATCGAATGAACGAAT
AATGAAGGTCGATACCTGTCTATTATGCACCTACTCAACTAACCATGACATA
GCCACTGAATTGGCGTCTTGGTTAAAATCCCCACTTGACGTATTCTTGCTTA
AGTTGAGCTATAAAATTTGAGATATACATGTATAGATTCGAATACGTGTATAT

AATACATATTCCTCATCCTATAGACGTCAAATATCCAATACACATGCCGAAAT
TCCCGCAGGAATAGGCGAGCCTCCGTAAGGATTGTGCTAGATGATGGTCGG
TGGTCGTTGCTCCCATCCTCTATGCAACCTGGTGTTGTACCAAACCTTCATG
CATTTCCTAGCCGTTGTCCGATAGGTCGATTAATATCTCGAAGCATGCTGTT
GTCGCAGTACTTCTCGAGACAATCTTCAACAACCTGATCCGGCAATGATATA
AACCCACAAATGTCTCATCGGATTTAAGTAATAGTATGAGCTAGTATGATGG
GGTCGGCGTGTCGCGTATTCTCGAATGACCTTTCACTAGATGTTATAACAAG
ACAATGCTTTGCGCAACCTCACCAGATGTTCCTGTGGTTATGCTGTATATTC
GGGGAACACTTGGGCAGCCCGAATCATACCACAAATATGGTGAGAGTTGA
AGCCTGTAATCGCTTTCCAGCTTTGCCAACATGAGGAAATCATGATCAGTA
AACCGGTTCAACCTTGGGCGTACCGCAGACATATCGAGATATCGAGACCTT
AAGAGGTGATGAAGGGTTACCCTTTTTAGGGGAATAATATCTCGTCTTGCAT
GGCCTTTCAGGGCAGTTAAATGGCTCGACGCGAGAATAGGGCCTTGCTTGT
AAGTGACGAGAGTCAGTAGCTGGTTGAAAAAAGGTAGGTAGACAAGATGA
CAATGTACATATTGTGTACAACCTGTGACCCGATTGATCGATCTGTTCAAGG
TTACAGGGAACAGTTACAGGTGGGTCTGATCCGCTAAACTTTAAAAAGTCC
GGAAGCCTGGATCAACCCTTGTTGAAAGCCGGCTTAAGGTTCATTATCCAC
TAAATCATCAGCCACAGACACGCAGATCCTTGCTAACTTCGAAAAACATAG
CACTTTATTCTGAACATCCTCAACAATCCGCACCGTCGTAGGTACATGCAAA
CTACGAACACGCGAATATCTGCATTGTTTGAGCTCGAGTGCCTTCTAGCACT
AGATTGCGACAAAAGCAATAATTTAGGAATCATTCTTCGAGCGATTGGCCC
ATCAAAATGGCATCCTCTAACTCGCCGTTCTCGTTGCGCCCGTGGCCGACC
GGCGACAAAAAGCCGAAGAACCTAAGCGAATTCATCACGCGCGTCAACGC
CGAACGCGGCGGTTTCCGCAACATGACCGAGGAAAAACTGCGAGAAGAA
ATTGCCGCCGAAAAAGATGGGCAAATAGAAGTTGACCAGTCTTCGGATGG
GGAAGAAGAGGAGGAGACCGATGAGGACAAGTTGAAGAACGTCATGGCT
GCGCGAGAAGAATTCTTGAGGAACCTCGAGTAAGCGATCTCGCCATGAAA
CTAGGTGGAAATGTTCATACTAATTAGGCCTACCTAGGTCTGCACACCAATC
GGCTATGCTGGGTCTCGATTTCGTCTCTCTCCTAGCTTCCAAAAACAAGCTG
AACGATACAGAGAAAGAAAAAGAAGCCAAGGCTTCCCTCTCCCCTGCCCT
ACGAGAGCTAGTACCGATCGGAACGCTAGGCGGTTCAAAGGTGAACGCGT
CGAACGTATCAGAACCACGAAGACAGGATGAGATCGCAATCGCCACCGGA
TGGAGACTTAAAGGTATTAACAATATGGTAGATTCTATCGTGGCAGCCGCGG
AGAAGCTAGAGAAAGAGATGGAATTGGAGGCGAAATACTGGGCCGACATA
TTGGCTGTCAGCGAGAATGGATGGGCTGTTTGTGCTTTACCCAACGAGTCT
CATACGTTAGGCGTTCGATTTGGATTTGCCGAGTCTGCGCCCGAATTCCGGA
ACAGCAGCATTGCTCCCCTGAGGCGTAATGACGATGGAACTGTCCGACTAG
ATCTTGGTCGCGTTGGCGGTGGTTCACAAAGGGTCAGGATTACGCTGAAGA

AGAACGGCGAAATCGTGGATCAGTCACCTTTACCTGGACGAACACCGGAC
GATGCGCCTCTTCAAGACCGAGTGCTTGAGGCTAGGAATACCGCTTTTCAC
CAGGAGCTATGGTATGAAATTAACCGGGAAGCTCGCGGCCTTTTATCATCA
GACGTCTACTCCAATTCATCAAGCATAACTTGGAAGCAGAACTCGGAAACG
CAGATGATATTTACCCTCGAAGACCTCGGCGAACCGGATAATACCTACGAG
AATATTTCTCATATGCAGTGTTCTTGCACAGCATATTACGTCTACATGCAATT
CCTTCTATTCCAGGGCCACAGAATAAACTACAACCGACGCACTACAATGAC
ACAGCTCCCGCCCAACCGCGGGGCCTTGAGCCAGGGATATGTTATTATACG
TGCTGTAATCGCGAATCACGAGTACTTTAAGGACGGGAAGGTCTTTTCTAG
CTTCCTAGAGGATTTGGTCTTCACTTTAAAACGCGCCGGCATTTCCACGGCT
ACGTGCAAATCAGCATCCCAACCTCTCGTCCCAAGCCTTCTACAAGCTAGC
TCGACCCGCCGAAACGCAAAGTCGGAACTCACCTTTATCAATCACCTTATG
GGCCGCCTCGATTCTCAGTTCGAGCTGACCATTACACCAGAAGCCCGGTTG
TTCGCTCGTGGCCTTATCGTACTTATGCCGCAATGCGGGATGGTCTTCCGCG
TCTCCCTAACTCCGTTCCCGCCCGTCGGCGAGAACTCCGCATCTGCAAACG
GTGATAATAAGGACAAGGGGCCGCCGAATCCCCTAGAGCACACCTACCCAC
CGCACGACCAGTATCCTAATGCGAAGGAAGCTATATTCTATATCCGACAAGT
CGCGATCCGTGCTTTGGCCCAAAAATTGGCCGAAGAAGCGAAGGAGAGGT
TACACAAGGATGATATCGAGTTTACCGAGACTATACGAGGCCCAAGCGTTAT
ATAAGCTGATTTCGGCGGCAGCGTATGTGAGCGATGTAGCAGATTGCATGT
GAGATTTCTGACTGAGGCGACAGACTGGTATGCCGTTCTTTGTTCAGCCGC
TCTTCATTGCCGGGTAAGCCCATGGCAACTTCTACTTGCCGATATTGATCAA
TCAGCATGTCGACTATCTTCGAGATCCAATCTCTTCAAGCATCTTTGCCACA
GCTGCCCGCCAACAGCCAACATTTCTTGTCGATCTCCAAGCGCAATCTTGC
CACAGTCATGGTCGCTCTGTCCGAGATCGAAAAGTACTGGTCTGGAGCTCG
GTCCGCCTTGGGAGTTTTGGAGAAACGTGTGTCAGGTTTCGGTTGTAGTTG
GTCGGGCAAAGATGCTCACGAGACTCTGCAGGTTCCGGACAAAGGGCGGG
ATGCCACATCATCACCTCCCAGACCCCTCATCAATCTTCCTGATCGTGGACT
GCTCAAACGATTCACCCTCGACCCGAGGCATCCCAAACATCTCGCACAACC
AACCGACACGTCTCTTAGAGATTACCAAGCCAGGATGGATAACCCCCCGCA
AGGCATGTAGATCTTGAGCAGGAGGCATAGGGAGCTGATGACGGGCAGAT
ACGAGCTTCCTGGCGGATCTGCTGTCGGACTACAACTTTGAGAGCTTCAGC
TTCTTGCCGACCGACATGACAGATATCGGATTGCGTGGCTCTACAGCAAAC
CCCGAGCTTTAGATTGTACACGCCAGTGACGCCTGCGCACGCGTCTTGATT
ACATCGCGCAACAAGAAGCTTGCAATTGCCACGAAAGCCTCTCCATGACG
CCCAAATGGCTCCACGGAGCATGACTGAGGTCATGAACACTCCTCGGACA
GACACCAGCGGGTCTATGGTACATCATGTAGATGCGCACGGGGTCACACGG
TTTCAAAGGGAGCGATAGCCATACCATATAGGCAAAGAGTGACGGCTACAC

AGTACTCTGCGCCTTGTGTTCGGCTCGTCAGTGCTCTACATCGAAGGCATG
CATCTCATGACGAAGCAAGTCGGCGGAGGGACGTGCGCGACCGCCACTGA
CAGTCTTTCGGAACTAGGCCGCCGAAATCTCACCCGGAGGCTGATAGCGTC
GTGGGTCAAGCGCGGAACACCTTCCGAAGCCTTAACTAGTTACCGATATGA
CTGGCCATAGTGTCGCACTGCCCGAGATGCTTACACTCGGTCCTCGCACAA
GCACACGAAATGCAAGCAGCCACACCAGAAGACGTGCTCGGTGCCGCCG
AGAAGCTGGGCTTCATCGTGCCTTCTGGCCACGAGCACGACTATCTCGAGC
TGCTGAGGAAGACCGATGCGGCCTGCAAGACCATCTTGGCCGAGCCTGGT
GAACCGCGAGCGTACAGCAGCTGCATCGGGCTTACGAGACAGACTACAAG
CCCGTGGTAGGACTACGAGAGATGGCCACGAACCAACATACATCGGCCGG
TTGCCGAAGCGAATCCTTCGAAAGCGTGGGCATGGCGGGCTGACGCGGGC
GACCCGCTGGAAAGCAGCGGCAAGATCCTCAGTGGGAAGACTGTCGTCTT
CAAGGACACCATCTGTATGGCTGGGAGTGCCTCTGCTCTTCGGAACAGACG
CCTTTGAGGGCTACATTCGTGAGCCTTTCTGTGTCTGAGGGTATGACACGG
TTGACACTGCAGCCGATGTGGACGCTACGGTGGTGACCAGGGTCCTCGAA
AACGGAGGGCATATCATGGGCAAAGCAACATGCGAAGTGAGTCACAGCAG
CTTAGATACTTGGGCAACTCTGACGCTCAGAACTTCTCCCATGGCGCGACA
TCCTCGTCCTCTGCCTATGGGCCGGTCGAGAACCCCTACGCCGTAGGGTTC
AGTACGGGCGGTTCCAGCTCCGGCTGCGGAGGCCTCATTGGCTCAGGCGA
GATCGACATGGGCGTAGGTGGGGACCAAGGCGGCTCTATCCGCATTGTATG
TCCGAGATGAGCCAGCTGTGATGAGAGAGCTCCAGCTGACGCTTAGCCTG
CATCGTGCTGCGGCATTGTTGGTCTCAAGCCCACCTTCGGACTCGTCCCATA
CACTGGCGTATTGAGCTCGGCCGCAAGCGTAGATCATGTCGGACCCATGGC
CCGCACTGTTCTCGACACCGCTGCTCTGCTGCAGGCGACTGCTGGCTACGA
CAACCTGGACGACCGACAGCTTGGCGCTCCTCTGCCTGCTCAACTCCCGG
ACTACATCGCAGCTGCAAAGACCACCGACATCAAGGGCTTGCGAGTTGGT
GTTCTGAAAGAGGCTTTCGCCATGAAACTGGTGACTCCGACTGTTGCTGCC
CTCACAAAGGCAGCAGCAGCACAGTTGGCCGCGCTTGGAGCCATCGTAGA
GGAGGTCTCGGTGCCGACGTATGACTTCATTACCAGTGCCGTGACGCAGCT
GACGTCCAGTTTCAACCTGACGAACCCGATCAACCACATCGTGAACAAGT
TTGAGTCAGGCGGCACCCGCCAGGGGAATCGATTGGACGACGAGGACTCT
TCATCAACGGCTACTGGGACGCCCTGCTACCATGGGAGCCGGCAAAGTATG
ACAAGGCCAAGTACTTCGTGACCGGTACGGCCATGTCAAGGTACGACTTTG
CACTCGACGTTCTGCTGATGCTGACTCTGCCAGCGAGTATGGATGGCTCAA
ATTCCCCACGGCATATGGTCGAGCCATTAACTTGGCCCGTCGCATGACCGA
CGAGTTCGACGCCATCTTCGAGAAGTACGGTGTGGTTATCATGCCCACTTG
TCCCGAGCCGCCCAGAAGGCACATCCCGGCGCATGCGGGTCCACTGGGAT
GGGCGGAGCATGCTCGTCAGTCAGCACTATATGCCAAGCAACGTCAGGCTG

ACGATTTAGCTGGCGCAGCATCCATCACAGCTGCCACGAATCTGACTGGTC
ATCCTTCCATGACCATCCCCGTGGGCTTCTCGGCGCCAATGCCGGACGACA
TCACATGCGAGGCGGACAAAGCAGTCAAACTCCCTGTGGGACTTATGATCG
CTGGGAAGAAATTCGACGAGGCGACGATTCTTAGGATCGGCGCGGCTTATG
AAGCCTCGGTCGACTGGAAGACTTTGGGTGTCTAAAGGGAATCACAGGGC
TCTGTGCGGTAGATGGGATTGCATGCATGCTTCTCGCTATTTAAATGACACC
GTCGACGTGCTGTCCGATCTGCCCACAGGGCGCCACGGGAGTCTGCCCGA
ATGCCTTCTTGGCATCGTACGGGTGAAGCTTCTTCCAGTGCTCGGCAATGTC
TTGACGACGTGCAAACCAGACGCCCTTGGACTGGCCGTATTTGATGAACTC
CTCGAGCCAGTAGGCGCGGTTCGCACGGCCGCAGATGTGCGGATGCAGCA
GTACGGTCATCATCTTTGCTTCGCCCTCTTCGCCCTCCTCGAGCAGGCAGTC
GAGGGTGTCGCACTGGCGTTCAGTCTTGGACGATATGCAAGACAGGAAGA
CTGACCATGTAGATGAACCAGTCTTTTGGGCTGGCCCAACCGGAGCCGCGC
ATGTTGAAGCGGAGATCAGTGGTGTCGTAGGTGTAGGGCACCATCAGCAGT
CCCTCGCCGTCTTTGGCCGGCGATGGGACCCAGTATGGGAGATCGTCTTGG
AGAGAGTCGGACGAGTACTCGAGCGGCAGCTCGCGAGCTTTGTGTGCGAG
GGTGTAAAGCTTGGTCGATAGGGCTGATCGCCTCTCAACAAGCCAGCCTGA
TTATCAGTCCTAGTCGGAGCAGGCGTGTCTTTGGCGTACCTTTGGGCACCG
CCTTGTCCCCTGTAACGTTCTGAAGGATATCGATTGACTTCTCGATGAGGGC
GTCTTCCTCGACGGGGTCGATGTAGAGGTTCTCCCTATACTTGTACCCGCCG
AGCGAGAGCTCTGCACCGGAATCGACGATCGGCTTGACCCAGTACGGCTC
CTTCTCAATAGCCTTTGCGACTGCACGTCAATCTCTATCCCGGCTGGAAGA
AGGTTGCACCCTACACATATTCCATGTCACGGGAACCTTGTGTCTGTTCGTC
AGTCATGCTGTGCGCCGGACGAGTCTCTGAACGTACTTGTTGAACATGTTC
AGCAGTCTCGGAACGCCCTCCCGAGCGCCAAACTCGTACATCTGCTCGGTG
CTCTCGTTGATCTCGGCAGTGCTGCCACCTTTGGGGATCTCGAGCCACTCC
CACTCTGCCACAGGATCGCCATCCTGGATGTTGAGTTCCTATCAAGTCGTCA
GCTCAAATCCTTTACATCGAAAGCAGTATCGCTCACCGCCCCCATGTAGTAC
TGCACGACCAGCGACACGCAGGCTTTGGCGCCCCCGGGCCATTGCGGGTC
GGGCATGCGATCCATGCCGTACCCGATGAACTCGCGCTCGAGCCATTCCTC
ATCGTACTTGCCCATCTTGCGCGGTGTGTGTCAAAGCGATGAGAGTGTGTA
GAGACCAAGTGAGGGAGGGCGTCTTTCGTATGCGTGGGTCTTGGTCAGAC
GGGACGGGGAAGGGGCGGGCATAGGGGGTCAGCAACCACTGCGATGCCTC
GGGTATCTCGTCGGGATGCCATCTTCGGAATGTCGGACTGTCGGGCAGTCG
GCGTAGATGCTTATCTGCGGAATGTCTTCCGAAACGTCTGCTCCCTGCTCTG
GCTTCCGTCAGCCTCGTAAGCAGCCAGCTGCCCGTCTCCACATGCATGACC
CTCGATAGAAGACCAAGAAGACCCCACGAGCCCGTTCGTGCAGTGAGAGC
AGCCCCGAACTCACTTCTAGCACTCGACATCGCACGCCGACGACAGCACA

ATGATCGACAAATACTGGCTGCCCCCGCGCGACTTCGTGGGCTATGGTGTT
CAGCCCAAGCGTGAGCTCTGGCCTGGAGGCAAGAAGATCGCGGTGTCTTT
CGTGCTCAACTACGAAGAGTGGGTACCCATGGAACTGCGCGACGACTGCG
GCTGAGATGCGCAGAGGAGCAGAGTCGACGCCTTGGAACGGCGATGCCAA
GTCGTGCCCTCACCTGCACGAGCTGCACTATGCCCGTCCGGCGACGACCGA
GGGCAAGAGAGACCCGCTGGTCGAGGACATGGTGAATAGACTGCACACTC
GACCAGCGATGTGGCTGAATGACAGTTCGAGTGGGGCATCCGACAGGGCT
TGCCGCGCCTGCTCAAGCTGTTCAGGAAGCATGGGTGGCAGTGGACGACC
TGGGCCTGCGCCCGGTCATTCGAGGTGACGGGGGAGTACCCTAAAGTCCTT
GTTGCCGATGGGCACGAGGTGGGTCTCGGTCAAGCGCATCAGCTGGGGTA
AAGAAGCTGAGTGCAGATTGCATGCCACGGCAATCGATGGCTCATGCACG
GCGACAACTTTGACGATGTCAAAGCCCACATCGAAAAGTCTTTCGACCGC
CTGCAGGCCGTTACGGGGCTCAAGAACTGCCCCACAGGCTACTTCTGCGG
CACCGGTCCGCCTCAGATGAAGCTCGCGCGAGCTATCGTGCACAAAGAGC
GCGGTGTGCCGCTCGTCTACTGCTCGGACACGTACAAGGGCGACGAGCCG
TACTGGGTGGAGGATCCGGTCGCCGCCGTCGACGGCAGCAAGGACGAGGG
CATGCTCATGGTGCCCTACAGTCTGTGCACCAACGATCACCGATGTGAGCC
ACCTCGGCACCCTCCTTTCACTGCAGGGCCGAAGCTGAAACGCTAGTCTTC
GTTGCTGCCGGTGCCGGTGTCAGCAAGCCTGACGACTGGTTTGAGCTCCT
GAAGGGCGAGTTTGACCAGTTGTACGCGGAGGGCGAGGCCGGTCACCCCA
AGATGATGTGAGTGTCCAGACTGTGCTTTCCTTCCCTGTCCCGTTTGACTCT
TCAGGACCGTCGCCATGCACAACCGATTCGTGGGCAAGCCTGGGCGTCTG
ATGGCCCTGAAGCGGTTCATGGACTACATCGCCAAGAAGGAGGACGTGTG
GGTGTGCACGCGAGAAGAGATCGCCGCTGTCTGGCGAGAGAGGTACCCGT
ACGAGAAGGTCGGGGCGACTGGACCTCTTTCCCACTAGATGATGATGAAG
AAAGATGCATCGTTGTGTGCTTTGCCAAGAGCCTCTCGGCCCGAACTCACT
CCACGTGACTGACTCATCTCATCTATTGTATCTCGGGTTCCATCTGACGTTG
TATTTCGTCTCGCATCTCGATGGAAGCGACTCATGGGCGCTTCGCCGACTCT
TTCACCTCTGTCTACGACGCCCAGTCGCCGGATGATACGCCCTCGTCCAGT
GGTCGTCCGGCGAAACGACCTAGATCTGCGAAACCCAAGCGCGAGAGGTG
CGCCAATGCCTGCCAGAGATGCAAGAGCAAGAAGATCAAATGCTTTGCTC
AACAGGACACATCGTCAAGGTCAATCTGAATTGGCGAGCACTGGGATGTC
AGGCTGATCAGAAGCACATGTATGGCTTGTGCCAAGGCGGGAGTAGCAGA
ATGCGTGTTTGACGAAATTCGACCGTCAGGCAATCCAAAGTGGGTGACTCG
GTAACAGCCGATGAGAGGAATGCTGACTCGCTCGCAGGTATATCGCTGCCC
TTGAGACCCGAGTAGCAAAGCTCGAGCATGCGCTGGCAGAACTTGATCCG
CTGCACCCAGAGATCAATGATCATTATGCCAATGGCCGAGGTGCAGAGGAA
GGGCCATCGGCGACTCGGTCCGGATTTCTTAGCAGGTCCGTAAGGAATACT

GGTCAACAAGACAGAGGCTCATGGTCAGTCTCTACCGGAGCCGTCCTTATG
CGGCACAATCTGCAGACCCCACGCTGGTATCGCAGCCAGGAGCTCTCGCTC
CTCCCTCCATCAATCCAGATCCGGTGATCCCCTCAGATGCCGTTGCCCAAGC
GCTCCTCCGGGGTGTGCATGGTCATATTCAGGCCCGGTACCCTTTCATGGAC
TGGCCACGAATATCGGAGTGGCTACGAGACCGAGAAAGGCTACGTTTGGT
CGATCCTCGAACCGCGCCACCTCATGAGCAGCTTAAGGCGTTCTTCATTTG
GGCTCTCTACGCAGTGGGGGCGCAACTGGACCGGTCTCTGGGCCTCGACT
TGCCTGCTGCGTACTTCGAGCAAGCTTGGAAGTATGTCGACGCTATCATGG
CGGCTCCCCCGAGCATTGTCTCGCTGCAAGCTATCTTGTTTGCTGTATTCTA
CAGCTTTCGCTCTCCTTCGGGTCCTTCACTTTGGCTGCTTGGTGGCTTGAGC
ATGAGACTGTGCATCGAGATGGGACTGCATCGCAAGACTTCTCGCGTGAAT
CCTCTGGAGGACCAAATACGGAAAAGGGCCTTCTGGTCTGCCTACGCTTTT
GACAGGCTCATCTCAATCGCCTCAGGTCGACCCGTCAGTATAGCAGACACC
GCCATCGACGTAGATATGCCCCTCGATATCGACACTTCGGTCACCGATCCTC
AAATCATAGCATCGTACACGGGTCTGGTCCGACAGGCTAACGACATGACCT
CTGCTGTACATTCCATAGAGCTACATCGCATTCGCTCGCGGATACATGCGGC
GTTCTACTCTCTCAACGCCCCTCCTCCCCCACCAGAAACGACGAAACAGTT
CCTCACCGAATTGGAAGATTGGCGAAAACGGATACCTCAGAAAGTTGAGG
GCTTCCCGATGCAGCTGGAGATACGGTTTACCATGCGGTATTTCCACACTGT
CCTCTACTGCCTACGTCCAGTCATCGTTCGCGCGACAGCGTTCGATCCTCTA
CTCAGCTTGGGTGCCTCGGCGGCAGCCGAGGCATGCGAGGTAAGAACTAC
GCCGTCAGCGAAGGCTGTAAGATGCTGACTCTTCTGTTTGATCGCAAAGTA
CACCAGGACCATTCTCACAACAAATTGACACCTGTCAGCATCTGCCACACG
TTTCTGTGTGGTGTCACACTGCTTTATTGGTGTGGTCGAATGTCTCTACCCA
GCATCAGACACTGACGGGACAGCCTGGCGATTCAGCCAAATGTCATCACTC
AGCGGCTATCGGGCAAGGCGTTGCGCATGTGCTCGTCGACCCTGGCCGTGT
ATACGCAGACTTTCCCTGCTGCATCCCCATTCTCGGATCAATTCGAGTCTCT
CGTGGATGAAGTTCTGACCGGTGACCCGAACGCCGTCATTCCTGCCATGCA
GCAGATGCTGCAGTTCAATTATGCGCCGCTCACAGCGTATGTCCAACATCA
ACAATGTTTGTATCTGACATTCAGCATGTACGACAACTTGCTGGGAAGCGG
TAGACTCGGTCCACCTCGGCATCCGGACCCACAGACCACATCGGCGCCAC
AACAAGACCAGCAGCAACAGCAACAGCAACCTCAACTGGTATCTCAACTA
GGTCAACCGCAGCAGGCGCAACCCGTACCGCAGCAGTCATTGGGATTCGA
TATATCGACTTTGTTCACTGATGCGCCGTCCACAGAAATCGACTGGAACCT
GCTTCCTTTCGTGGATATGTCGGCATTCATGGACCTCGGGCTCGATATGCCC
GACGCGACCGGGAATAACGATCTCTGGTGGGACCACTAGGAACACCGAAG
CTATAGCTACAATTGCATAATGTATGATGTAGATGTTGTACAGGATGTGATTT
TAGTCTCATGATATGGTCTTTCGTGTCCCGTCATGTCCTGTCGTTTCTGCGCC

TCCGTCTCCTAATGGCTCGGTCTTCTCATTCGAATCGACCACCGTCGTCGAC
GATGCCGAGCTGTTCAGACGATCCTCCCTCGTACTCGAGAGAATGTCAACT
TCGTCCCCCTGCCCCGGGCACCATCGCCTCGGCTCTGTCCGCATCGGAAAT
GTCCTTTGCCCGGCGCTTCTCCGCCTTGAGCTCTGCCGGATCTTCTCTGAA
GCGTATTTCCTGGCGGGTCAGCTGGTCCCATCTATCTCTGAAGATAGCTCAC
CTTAATTTGTATACTGAGTAGGATGCACACCGCGACAAAGGCAATGAACGA
TGCAAAGACCATTTTGATGCTGTCCGAGTACGCCTGGCGTACTGCGCGCAG
CGTGTCGGGCGCTAGGGCGGAAAGATCGGCCAGCGCAGCGTCTAATAGGG
ACGCTTGTTGCTCCGCCGAGAGGCCGGGCACTGATGTGAGGCGCTTAGCG
ACGGCCGTCTGGTAGATCGCGTTGGAGACTGCTTCGTCAGTCTTCTTCTCA
ATGAGGGGAAATGAGAGTCGACGTACCTGCGAGACCCATGGCGCCGCCGA
GGGTGCGGAAGTAGTTCCGACTTGCCGTCACTACGCTGCGGTCTTTGTTCT
CCGTTGCCGAGAGAGCTGCGACCAGTGCTGCCGCGCGTAAGCGGACGTGG
GTCTTCATTGTAGTGAGGACTTACTGGTCTGGAGGTTGAAGCCAATCCCAA
TCGAGTTGAGCTCGAGGAACCCGATCATCTCGCCGAGCGAAGTCGACGGC
CCCCACAATAGCTGCAGAGCCACCCCGATGGTCCAGAAGATCGTGCCAGC
CACCTGTTCCAGTTAGCTGAGGTCCAGGCTTCTTGCTGGTGCAACTTACGA
TGACCCGCTTGTAGTGGTTGGTCTTCGAGATGTAGAACCCCGATACGACGC
CCCAGAAACTCTGCGGGAACGTCCATGCCAGGACGAGCGAGCCGGACTTG
AAAGCCGAATAGCCCTTGACATACTGCATGAAGATGGGGACATAGTAGACA
TTCTGCCTGGTCAGCTCTGCTGCCGAATGGTGCTTGCTGCAGGGCAAGTCT
CACCCCGTACATCGCTGCGCCGATGAAGAATGATTGAGCCATGACGATTGC
CGACGTCCTCGTCTGGAAGAGGCGTACTGACCGTTAGTCTACGAACGTCAT
TGAGCGAAGAGAGAAGAGCTGACGAGGAAGAATGGGCAACTTGGCCAGT
CGCCACTGGCTCAGGACGAACACGATGGCGAGCAGGAACCCCAGGATGA
GCAGCACAATCACCACGGCACTGTTCCATGCAAATGTCGAGCCGCCGCCA
GAGATGGGCACGAGCAGGAAGACGGTCGCAGCGGCCGAGAGGAACACCC
CAAGGTAGTCGATCTGCTTGAGCTTGGTCTTGACTGATCCAGTGACCGGCT
TGAGCGGTACGATGACCTGGATGATACCCATGACCAGGGCGCCGAGGAAG
GCGGTGATCCTGTTCGGCTGTCAGCGTCACTCGATCTCACTTGAAAGAGTT
GTTGGCTCACCGGAAGAGCCAGCGCCATTTCCCGCCGTTGGCCATGCCGCC
GCCGAGGAACGGCCCCACTGCATTGCCCGTAGCTGCCGCCGCACCAATCA
AGCCTTGGTACTTTCCGCGATCTTTGAGCGGCACGATATCCGATATCTCGCA
CATCAGCCGCGGTCACATGTGATAAACGAAGGACGTACAATGGTCATACTG
AGATTCTGCATTCCGCCACAGCCGGCCCCGCTGATCGCACGGAAGATGTAC
AGCTGTATCGGTGTCTGCGCAAACGAGCACGCGATATTGCCGACGATCAGG
AAGCTGATCGCGGACAGCATGGTAATCTTGCGGCCGAATATGTCGGACAGG
CGCCCGTA

>Mosaic_sequence_46

CGAGCTCTCCTTCACTGTCGTCCACCAAGACCTTCTGGGACAAGCACCGTC
ACGTGCTCGAGCCGCACCTCCCCGCCTGGTTCGTCGAGGGGACCGATTGG
TCCAAACGCAAGGCCTCGCCGCTGGCTGGCATACGTACGGGCCCGCCGGC
CTCTTGAGGCTGATACAGGTTGTGCCCACGCCCGCTGTGGTGGACCCGAAC
GGTCCCGGGTGTTCCGACTATCTCCGACAGGCTCAAGTCTCTCACGAGTCG
CGAGGGGAACGCGGGACATCGTGGGAAGGCCCGGGGACGGCCGGATTTAC
GGTCGACGAAGAGGACGTGGCGGCGGGCGAGGCGACGGCGACGGCGGCG
GCGGCGGCGGATAGGGCCATGAACGAGCTGGCAAAATGGCACTCGGCGTG
GATGGGGCAACGCGAAGGCGAGTGAATATGCCGAGTCAGGCAACCTGCTG
CTGTTCGTTCTCGCGTTCGTTCGTCACTGATGGCCAGCACCAGAGCTCTGC
TATCTCATCCTCTCTCGCTTCGTCCGCGTAATCTCTCGCCACGCCATCCCTCC
ACCCGCGTCACTCGCCCATATCCATGCCGACCCTGATCCAACACAGCCGAC
ACGGTCACGGTCAAGCTCGATCTTTCTCAGATTGCGCGACGGCGTGGTGAT
AACCGTGATGGCAGACTACGCGGGAAACAACGCTCCGGCGGGTGCGACAG
GCGTGCTGCTCCTGATGCGTTCTGCGGGGGTGTGTCGACGACGAAACTGC
GATGATAGGGGGAGAACGTCAATGGGAAGGAGGGTGTCTGTGGAGCGGGA
GCAGCGGACCGTCAGTCGCTCCGGATATAGGTGATTGCTTGGGCGCCATGT
GGACGGTGTGTTCTCTCCTTCTTCGCTCTGTCCCTGCGCTGCAAAAGCCGA
GGCAAGCCCACTCCGGCCCGGTTCCGCCCACGGTCCCGAGCCATTGATGAT
ATGGTTCCTCGCTGAGTATCAGACGTGGAGTGGTATCGCCGCTGGCTATGCC
AGCTGCGCCGACACGTCAGAACGGCCGTCGACTTTGGTCAATCGGTTCGC
TGCACCACGCATCGAGTCGCTCGGATCCTTGTCGCTGCTAGAGGTGCTGCG
ACTCGTCGTCGGCGGCAAGACACTGGAGCGGGCGTTGGCATTGGACGCTG
CAGGCACCCGGTCGCAGAAGATGGCGTGCAGGGAGGTGCTGGACACGCT
GCTCACCGAGGTCTGAGAGTTTTCTACTCGAAGGAACGGGTAGAGTACGA
ACTCATTGACGATGCATTGGCATTACTCGAGTACTGCATATACACATGGAAT
CCAGCTTCTAGATCGCTTTCAGGGATCATCTAGATTTCTTTGTAGAAGGACC
CCGCCGCTAAATATCAGTTCCAAAGATCGAGGAGCTTCTGCCGTTGCTGCT
CGATGGACAGCACTTGCCCAGCAAGTTGCCCAGCAAGAGGGGCGTTGATC
GATACAGAGTCCTCGTCAGACTCAGACTCATCGAAGGAGTCGTCCTCGATA
CGCTGCTGTTGCTGCTGCTGCTGCTGCTGCTGCTGTCGCTGTTCGTAGTGCT
GGGGGGTGAATGCAGCGGTCGAGGTGTGGCTGCTGCCAGTGTGGCCGGGA
CGGTGGGGCGGTGTCACCTGTCCTTCAAGAAAGAAGCTAGCATCACTGCC
TCGGGCGTCGTGGCGCGGAGTGGCTATGTGGGATTCAGACATGACCGGAG
ATGAGAGGGAGAAACTCACAAGTCGAGAACCCTGCGCTCCCACCGGTCAT
CTCCAAGCGCGCCAACGCGGCCTTGAGCTCGGCATTGTCCCTCTGCAAGTC
CTCGACGACGCCGCCCAGCGCTTGCAGCCCCGCCGTGGCCGCCTGAATTA

GTGCGTCCTGGCTGTGCGTGCACGCTCCAGCACGGCGAGACGCTGGACAA
GCTCGAGCTGCTGCGGGTCGTCTGCTTTGTTTACCGAGTCCTGAGCAGTGG
GAGGACCAATCGGGGTCGAGGGCGGCGGGATACCATTAGATCGCGCGGGG
TAGGTGGGTGCGAGGTTCGAACGATAACCGATACCGCCAGCCTTGACCGG
GGTGGGGATCATGGAACGGCGGGGGATCCTCTGGCGGATGCGGCTAGGAC
GGGCTTTCTCCCCGCGGGGCGAGAGTACGCTGCGAGGTGGTTCCATGCGG
CGGCGGGGCATTTGGCGCCTTGCGGTCCCATGGCTGGGATGTACCAGATGT
TTCGGACCTGGGCGCCGGTGTATGGCGCAAGGACAAGCGCGCTATGGAGG
ATGAGCCCGCGTCGCTTCGAAATAGAAAGCGAAGTCCGCTCACAGCTGTC
CTACTTTGTCCGTGCCAGACTTGTTGAGCCCGCGCATGTGCTCGGTCAGAG
CCAGCTTGGTCCTGTCGAGGAACGACTGCGACGTGCCGCTCTCCGAGCCG
CCGAGATCTACCAGAGTCAGGGCGGGAATAATGATCCGGAATGCGTCGTTA
CTCACTCGTGAGCACGACAGTAGTCTGTCTGTCGCCGTTCGTAGGATCCTC
CTTGGCCTGCAGAGTCATCCTCGTGCTCCCTCGCGGAGGGAGGAGCGAAG
GAGGAGGACCCACCACGAGCTTGATCACAATATGAGCGTTTTCCCCCGCAT
CATTAGCAATCTTATTCACATGCTCCCGCCAAGCACGATCACTGCAAGCGA
ACTCGAGCGACGAGACGCTGGCGATGTCGATGTCGATCGAGCTGGACCCC
TCTGCAGCTGCTGCCCGGTTGAACAAAGCCTTGGTGTTTTGCGAGCAGCA
CACCCCTTCGGCCATGTGACCAAGGGTCAACAGGTCATGAACGACATCCG
CATCTTCCCTGCCACCTAGCGAGATGGCGCTGAGCGTCCAAGTTGGCCTTG
TTGTTCCGGGATCGCCCGAGGTCAGCTGTCCTCCTGCCGTGTCAGCTAGTC
CCCGGCTCGTCGGCTTGGACTTACTCATGCGGGAGAGGATGGCGCTGAAC
AGCTGGGCCTTGTCGGGCGACGATGGGCCGTGCAAGTGGATAACCACTTG
ATCGTTGCCTGCTCGGTCAGCCGGACAGCCACGTATCCCGCTCAACTGCGA
CCAAGCACCAACTCACCCGGCGTGAGCAGTGGAGCAATCTCGAGTCTAAG
AAGCGCATCCACGGAGGCTTCATCGCGCACCTGGAAACCCTCGTATATTCC
GGCTGTAGCTGCCGTCAGCATGTCCCAAGCTAAGAGTTGAAGGGCAAGGG
AAAGGGGGGCGGGCGGGCGAGAGGCAGACTTGCTCCACTTCCAGCTCGTC
TTGTCTTTGGACAAGGCCAGACCAGGCTGACCACAAAGCTTTGATGCATCG
CCCGGCAGGGGCGACACGGTGAAGTCGTGTCGAGGAGAAGCGAGACTGA
ACGAGGCAGGAACAAGCGTCAGCCCACTTGTCTCTCTCTCGCCTTGTCACT
GGACCATCGACTCACACCAACGTTGCGCCAGTCTCGATGATGGGTGCTCGA
CTCCCTGTTGGCGTGACCGTTACGCCAAATGACGAGCGACGACGTCGTGC
ACTTTCAACGTACCGCGCCATGGCTCCTGCGCCTTGCCCTTGCCAAGGGAT
GGATAGAGTACGACTAGGAGTAGAGTCGTCTTTCAACATCCAACAAGAAAT
CCAATCCTATCCTCGGATGACCACGGCAAATTGTCAAAACTAACAACTTGT
CGATGTGGCACCGGACCACTCCGGTGGGTCATGTGATCTCTTTGGAAAGCC
TATCGGTTTCCCTAGAGCAATACTAACCCGCACGCCTAATAGATGAGACCCC

GAAACTAACAATTTGTCGATGTGGCACCGGAAAATTGCGGTGGGTCATGTG
ATCTTCTTGATGGGCCTATCGTTTTCCCTAGAGTGACACCAAGCTGCACGCC
TAATAAATGACGCCCCGAAAAACCAACAACTTGTCGGATGTGGCACCGGA
CATTTCCGGTGGGTCATGTGATCTTCTTGGTGGGCCTTTCTTTTTTCTACAGT
GACGCCAAGCTGCACGCCTAATAGATGACCCCCCGAAAAACCAACAACTT
GTCCGATGTGGCACCGGACCATTCCGGTGGGTCATGTGATCTTTTGCAACA
GGGGCCTATTGTGTTTCTTCAGCTCGCGCACTCCTAAGACATAAGTCGAAG
AGGCTTCACGGCTACTGTATTCCCGTGCCGTCGCCTACTCGTTTGGCAGTCT
CTTCCCGGGTGTTATGTTCATTCCTGTTCTGACGCTCAACAAAGTCCCGGCG
CCGCCCTGCCTCCGGGCAACGCAATCGTCGATCATGACCAAAGTAACTCGC
ACCAAGCCAACATCCCACTCCGCTGATCAGAGTCAGGAGGAAGGAGAGGA
AGATTCTACATATCTCGCCTGAACACTGTTAGAAGAAGGCGATTGGTGACT
TGACACTCGACTGCGTGGTCACTTTTGCTCAACAGATTAAAAGTCCCAAGA
CTCAGCCCAGCTATCGAGAGCTCGCTGGACATCACCGCTCGAGCCGTCGCC
GTCAGCCGGGACGCTCTGGATGCCGCTAGACCAACAGGATCGTTGCAGCA
CCCCGCACAGATCGCCGTAGACAGTCTTGGATACCTTTATCGCAAGATGTC
GAGGAGAATCAACATTGACGCCATCCCAAAGCTGTTGCACGACCCGTCGT
CATACGTGCCGTGGCGAGCAGCTGTCGAGATTGCTCTGCGACTAGGAGATT
GCTGGAATGCCGCCTTGGGGCTCAATCCTGAGCCAGGGCGTGTAGAGACC
GCCGAGCGCCAAGTACGTGCTGGATCTATCATGCCGACAGGATCTGAGGCT
GGAAGCGCTAGGGTCACCGAGGAGAAGAGGGACTGGGAACGGTGGCAAG
CTCGAGAGAGCAGGGCGCAGGCGTTGCTCAAGTGGACGGTTTCATAGGCT
GTTCGGCTCGACATTGAAGACCTGCTCGCAGCCGTCGACATGTAGCGTTAT
TGCGATAGGGATGCACAAGGTCGATCTCCTGGAGAATCAGAGGATTGTGCA
AGCACGCCTGTGGACGCTCGACCTCCGAGGCGACGCCTTTGCCGATGAGA
TGAGCGCGCATGTCGAGATGTTCTCAAAGGCTATCGCAGACGGCAAGCGT
GTCGGGATCGTCCTGAGCGACGGCGCGCGTGCGCAGAGGTTCCTGTCAAT
CATCCTAGATCCCGCCTTCATCATCGTGAGGGCTGAATGGCCCTCTATCGCC
AAGCACCAGCAGTCCTGGGCGGTCGTACTCTCGAAATACACCGCCGAGAT
CGCGCGACGGCGGGCTAGGCCGCCGCACGGAGCATCGCTATCCCACGGAC
GAGACGGCCGTTTTGACAGATACGGGTTGACCGAAAAGATCCATGGAGAC
GCTGTCCTGATAGGGGATCGTCGTCGCAAGGCTAGAAGACTGACGAGTGT
CGACGATGCGGTGAGATGGGTCGTTGGGAACGGGACTGCAAGGCTGCACC
TCCGCGCAAGACCCATAAAGACCGCAGGGACCGACAGAGGCGGTATCGCG
ACCAGCAGAGCGATACCGAGGATGACGACGGTGGCGACGATAGCTCTGAT
GATGAGCGCTCGGTCGCAGACAAGGACAAACGCATCACCAAGGACAGCCT
ACTCACGGTCGGCGAACTGGAATCCTGGATCGGTCTCACCGTCGGCACTAC
CGAATGGGCCGATCTGCTCAGCAACCCATGGGCTGCGACCAAGGCCGTCAT

CACCGACAAGATCGCCGAGGTCTCCGAGACCGTCGAGATCGCAGAGACCG
GCAACACCGCCTGTGAGGCGGCGACCGCCGAGGACATAAGGAACGGTGTC
GAGACCGTCGAGATCGTCGAGAAGGCCTGGGATATGGATAGCGGGACCAC
GCATCAATTGACCCCGCACCGCTCGATCCTCAAGAGCGTGCGAGCGCTCGA
GACGCCCGTCGCCTTCGGGCTCGCTAGCGGAGGCACCGTCGAGGCCGTCG
AGGTGAGCGAGGTGGACGTAACGCTTGTCTCGGGACGACGGCTTATGTTG
CAAGAGGTGTATCTTGTGCCGACTGCCAGAGTCAGCGTGTGCTCGTTGTCG
AGGCTGCTTGCCCGGGGCTGGCAAGTGGAGATCAAGGACGACGGCGGCAT
GCTGAAGAGAGAGGACGAGAAGCTCACCATGAGGAGTAGAGAGTCCTTGT
GGACCGTCGCTCTAAGCAACCACCAGCCCGAGTGCTTTGAGCCCCACCGA
CGTGGGCAACATCATCGCCGCGTGAGACGTCGAGGCCGGCCCCGCCGTCA
GAGATTCGCAGGGCGATGAGGAGGACAAGACGACATCGTTTGGTGATCAA
GAGGGAGCAGATAGAGCCTGCGGTGAGAGTGAGGCCGCGGACACGGGCC
AGAGAGAGGCGAGACGTCAGAAAATCCTCGACACGGGCACCGCAGAGCT
GGGGATCAGAATGAGATCCCGCTCACCAAGTCGCCCGCCACCGCCATTTCT
GCACACCATCTTGAAGCGGTACGTCAAGAGGATCAGTACGCGACGCAGGA
CCATGGCTACATGCGCATGTCGGCCGTCAACGATCGAGAGACAAGCGGGC
GATACAGAGACGATGTCGAGTCGTCGTCCTGCAGACGGGGACGACATCGA
GACACATCACGTGATCGAGCCAAAACGCCCGAAACGCCGATGGTCAGCGC
GCACGGGATATGAGAGACTTGTTCAGGGGGAGCGAGGGGACACCGTCGGA
GCCATGGGCTAGTATCGATCACCAGATTGACCGAGATTGGTGAGGAAGTGA
GGGAGAACACCAAGATCGCTTTTGGGCAGCCTGAAGGCGGATCAGTGAGC
CGTGCGACGTTCGCGTCAGTCGTTCCCAGGTCCTATTGTGATGAAGCAACG
ATTGACAGGCTCCAAGATGCTCCAGAGGTGCTCGACGCTATCACAGAGGCC
TTCGCAATTGGCCTGCAGCCTGCCGACTTTGAGGGGGAGTGTTGAACTATA
GGCCTGAGACGCCTCATAGGGTGAACTTGCCTCTTGCGGACTGTCACGGG
GCCAGGCCGGTCGTCGACTGATAGGCCCTCTCACATGTGTCCTCCGTCAAT
CTGTCTCTGTCCTCCACGAGAAGGTGTGTCCTACAGCCTCTCGTGCGTCAA
CAACATGGCTGCGCCGTTACTTTCTATCCATGGGACTCAATACCACGATGCA
AAGGACGGTCTACGGGTGAGGACTTGTATATATATATCACCCGTGCCCTCCT
CTGCACCACCTCGGGCTTTCTCCTTGAAACACCATTGACGAGGCTTAAGCT
CTAGAGTCGTCGAGGCTACTCAAGGCTCGTCAAAGCTACTCGAGGGTCGA
CAATAGGCTCACATGTCTGACAGGCGTGCTGGGCTGGGACGAGGGTCGTA
GGCATGCACTGGTGATCATGGAGGCGTGGTCTACCTCTCTCTTTGATGGTGT
GGATTGTTCCAGTGTCTTCAAGTATGAAGTCGTCGTTATTTGTAACTAATAG
TCGTGGCTCCCTTTTGCTCATATCTGTTGGTCCGTCGGATCTTTTGGTTATTT
CCGTCGGAAGTAGCCTCATGGTAGTCTCGTGGTCAGGATGCCGTGTCGGTT
TCCTGACAGTAAGCGGAACAAGCAAACAACTGAGACACTTTCGTGTAGAG

CGGCTGTGTCACGCGCTGTGGACCGGGTGGCTGAAACCGACCGGAGGACT
GAGGGCAGTGGTACTATGAAGTAGTACTGATAGGGCTAGTCTCTGGCTCGT
AAAGCAAGATGACCACTCAGGAGGGAGCTGCGGTTGCTGGGTTAGAACCA
ACTGTGTGACTGTCGATGGAAAGACAGAATGAGATCCATTGCTGGAGAAA
AGAGAACTACGGGAGAAAGTACAGGGATTATATCATTTTCACCCATCCATGT
TTTTCCTGTTCATCCAACTGTTTTCCATGTCGAATCGAACCCACATCGGAAC
TGCTCGTATCGCAATTACTTCTTACAGTAACAGTTGGAGTGTCAGGAAACC
GACACGGCATCCTGACCACGAGACTACCACGAGGCAATTTCCGACGGAAA
CATCCAGAGGATCCTACGAACCGAAGAACGTGAGTACGGTAGAACCGTCA
CTATTAGTCACGAATAACTACGACTTGCTCTTGAAAGCGCTGGAACAACCT
GGAGAACGATAGAAGGATACGTCTCAGTCTTCCATCGGCCGAGTTGAGAC
AGGAACGCACGCAAGGACAAGAGTCAGGTCCACGATAACGGAGGATAGA
GTTATCTCACAGTCAGGGCCACGGGGCGGAGACCGAGGACGATAAGGACG
ACGACGGGCACGCAAGGAGATTAGATGCAAGCCAGCGCGGAGAGAAGAC
AATAGAAGAGAAGAGAGACGACGAGAGAATACTAGAGAGTATTGGGAACG
AGTAGAAGCGACTAGGAGAACGATGGAACGATCCACGAAGGACGATGCTA
GAAGGATCAGAGTGAGAGCGGGCGAGAGTCAGCAGATACTTAAGAAGGA
AGATCGCAGAGGAAGTTCCTCAGCGATCTAGTTAACTAGTCCTCTAGATAG
AACGTCTACTTAGAGAATCGACCGTACCAGTGACGAGAGATAGATAAGTGA
AGTGAGTCTTGATCGCTCCTCACCCCGCTTGGTTCCACTCTACTCTTGTTCG
TTACTATCTGTTACGAATAGTCGTTGATAGTTAGCAGCTTGAACCGATAGATT
GATTTAGGTGAGCTTTAGTTGGATCTAAAACAGGTCACCGGGTACAGGTCT
GACATGGAGCTTAATTTCATTGATAGCAAGAAAGTGACAACATCGGTCGAT
GTCTGACATCATAGGTCAAAGAAGTGATGTTGTCGAGTTGGTGACCCGAGG
TCATAGGTTGGGTCAGGATCTGATCAGGTCATGACATCATGCAGCTGTTCTG
CCTGAGTGTCAGAACAGACTTATGTAAGCGGTCATAAGAGAGCCTGCAGTG
CATTCACACTGTACCAAAAGGTATGTTAATGGATCCTTACCCTAAGGAATCC
GCACCGCTTATATTAGACTCGTGTCAGTTTCCTGACACTGAGGTGCGGTCAT
GACAGGCTGTTTAGGCCCGCCGCACTCCCTTCAGGCTTACTAGTATGTTCC
AGCTGCTCTGACTCCTCCCGAGCGAGCAGGGGCAAATGAGTGAGCGAAGC
AAGATGGGTCGAGCGCAAAGGCTAGGTTGACGATCGAACGGGACTCAGTG
ATCTGGGTCGGTTGGACGGTCGAGTTACTGCTTTCTGGATGTAGTAGGGTC
ACTCTAGATCCATGTAAAGCAGCTCAGGCTCATAACCTGTTGAGCGAACGA
TATGTTTGCATAGAGAACTATGAACGAGCGCGCAGTTTCGAGTACTCTCTAG
TCCAGTCCTTATTCAAGCCTCCCTGTAACAGGAACCTGGTGAGAGCCTCAA
AGATGGACAAGGGACAAGGGCTCGTGATAGACGATTGCCTCGTCACTCTA
AAGTCTGGTCAGTGGAGAGGAGATAGATACGTCGAGGATCAGAGCTCTCT
CACCTCGCTCCAAGGCGCAGCCGGACGACGATAATGAATGGGCATGGTGTA

TAGGAGTGCACCAGATGGTATCAACAAGTGTCACGTGTATGTATCAGTAAA
GTACGAGTATTTGTATGTGATGACACCGAGACTGTAGTGTATGGCGTCTGGA
GCGGAACGCCTCCTTCGAGGTCGGCGCTGGCCATGGGGCTGTCCGAGGCC
GCTGAGAAATGGTTCATGGTGGAAGAAAGGCTGATCGTCGTCGCTTTGGA
GGACGCCGGGCTGTTCGAGCGGGCAGGAGCGGTGATACGCCAGGCCAGG
GGTGAAGCTCATCGATTCTGGGCGGACAAGAGCGTATGCCAGGATATGACA
CCAGATGTAGAGTTCCGTCAGGTAATGGGCTACTATGCATGTGCCCTTTCAC
CAGCGCCTAGCTGCTCTGAAGCTCGACATGCAATCCCACCTCTTCAGTATC
CAGGACTCATCACCTCGGTGACACGTCCCGACACTTTCAGCAATCAGCAAT
GGGGGATGGGATGGATCTTTGCAGCAGAGGGAAAGGGCAGAATCGGCATG
TCAAAGTACAGTACAGTGGCCTACCGGAATTCATTGAACAGCGGAGGTGCC
TCATTCGCGTCGAATCGCGCTACTCTTTCTGTGCCCGTTCGTTCTTGTTGTC
GCGTTTCTACGGGCTATGCATGTTTGTGGTTGATCAAACTTGGTTTACAACT
GCATTGTCATCTCAAGATGCCAGGAGAGTGCTTTACACCTCGAAAGCGCTC
AAGGATAGCCAAGCAGGGCTCACCACCCCCTACAGACAAGTTGCAGAAGA
CAATGACTGCTCTGTGGGGTCGGTCTCTCGCCTGGTTCGAGAACTCCGCCA
AACCGGCACCAATCGACAGCCTCTGAGGCCAGGAAGGCCTTCACGCTTCT
CTCTACGCGCCCTTGCCAGCATACGCCGCATTATTGTCAAGAATCGCAGAGT
ATCACCCTCTCAGCTGGTACCCATCATCTGTGAAAGCGGTTTCACCCTTTCC
CTCACCACTCTGCGACGCATCCTAGCCATTCTAGGCTTCAAGCGACGGTAG
TAGCTCGCATCAAACCATTCTTAAACAAGAAATCACGCATGAAGCGCCGCC
TCTATGCTTCCAGACACCGGTCTGATAGCTTGGATGACTGGAGAAGGACGA
TTTATGTGGATGAGGCGTCACTGAAGAACAATGGCACCCCTTGTGAGCTGG
GTGACGCGCAGGGAGGGGGAGGAATGGCTAGAAGAGTGCCTGGCACCCA
AGCTGCTTGGTGGGCGAGCCAGCATCATGATTTGGGGGGCTATATGGCATG
GAGGGCGGTCAAAACTGGTGCGATTTGACACCAGCAGCAGTGACGGGAG
AAGGAAGGGTGTTACAGCCGCCATCTATCGCGACCAGATCACCAAAGTCG
AGCTCAAACGATGCTGGACAAACCTCAATAACAGCTGGAGAGGCTACCGA
GGAGAATCAAGGATCTTGGAGGACAATGCAAAGATACATACCGCCAATACC
AAACAGAGAAGTTGGCCATCAGCTGCGATTCAACTATATCGACCACCCTCC
CTACTCCCCAGACCTCAATCCCATTGAGAACTGCTGGTCTTGGATCAAACG
AAAGCTTGCACAGCTTCCCAGGCGTCCGACAAACATGGAGGATCTGTTCTT
AGCAGCTGAGGCTATATGGAATGCAATTCCACAGGCCTTTATCGACAGGAT
GATAGACAGCATGCCTAGGAGGCTGGAGGATGTCAGGAGGCGAAGGGGAT
TGGGAACTAGGTATTGAATATTGTGAGTTTTCGACCCCGCCGTTGGATGCGC
ACGGATCACTTGTGCTAGAACGCTGATTTCCAGGTTGCTAGATGGATGCAT
GCATGAATGACAAGAGGGAGCAGAGAAAGCAATCAAAAAGCGCACGGAA
TTCCGGTGTTCAATGAATTCCGGTAGGCCACTGTATATCGACTGGACGTGGT

TTGCTGTCTTGGAAAGAGGTACTCGAGTAGGAGCTCTGCATCCGGCGACTA
GACACCCATCATAGAGATGCATCGCAGATATACGGGAACTGGGAACTTTGG
CCTTGACCCGCACCCGGTCATTGCAAGCGTGACTGCAAGGCTGCACGTCA
GCGTCAGCGCCAGACTCATAGGGATCGCAGGGACCGACAGAGGCAGAGGC
GTCATCGCGACGGGCAGAGCGACACCCAGGATGACGATGATGACGACAAC
ACACAATGATAGCGACGAGGAGAGCTCTGACGATGAGCACTCGCACTCGG
TCGCAGAAAAGGACAAGCGCATTGCAAACCCGGCCAAGTGAAGCCTGCTC
GCAGTCGGCGGGATGGGAAAAGAACGCATGGATTGGTCTATGGGCCAATCT
GCTCAGCAACTCGTGGACGGAAAGCAAGGCCGTCGTTCCCGCCGACAAGT
TTGTTGAGATCGCAGAGGGCGAGGCGAGGCGGCCGCCAAGATCATTGAGA
CTGTCGCCGAGAGGGACGCCGTCAAGATCGTCGAGAAGGACTGGGGTGAT
CCACAGCGGAGCCACTCGTCATTTGACCCGGCACCGCTCGATCCTCAAGA
GCGAGCGAGTGCTCGAGACGCCCGTCGCCTCTGGGCTCGGCTCGCCAGCG
GAGGGACCGTCGAGGCCATCGAGGTAGGCGACATGGACGGCATGCTCGTC
TCCGGACGACGCCTCACGCTGCAAGACGTGGTTGTCTAGGCTGCTTGTCGA
GGCTGGCGGGCAGAGATCAAGGACGACGGCGGGACGCTGCAAAGGGAGG
ACGCCGCGAGAGACGTCGAGGCCGACACCGCCGCCACAGACTTGCAGGA
CGATGGTGATGAGGCCGGAGCGGCGTTGTCTGGCGATCAGAAGGGATCAG
GGGGAGCCTGTGATGACAAGGAGGCCGCGGACACTGGCAAGAGAGAGAC
GAGACGTCATAGAACCGTCGACACCGTTGACACCGGCACTGCAGAGTGGG
GATCAGCCCGCCCGACCTATCAGAGTGAGGTTCAGCTCACCAGGTCGTCCG
CCACCGCCACCGCCGCTGCCGCACACCATCTTGAAGCCCGAGGTCAGGAG
GATCAGTACGCAGCGCGAGAGCACGGCACGGCTGCATGCGCATAGCGGCG
GTCAATGACGATCAAGAGACGAGCAGCGATGCAGAGTACTGGAAAAAGGT
GGAAGGAGGCGGTGTTCTCAATCTCTCAGATGGATAAAGTCGAAAGACGC
GCAGAATGCTGGATCATCAGCATCGACATGATCACTGGAGGCAAGCACTCT
GGACCGACTCTTTGTCATCTCGTCGACTTGTGCCCTGTCAAATCTCCATCCC
TTGGCATGTTCTGCTCTCCCGTCGATGCTCCTTCGAAGGTGCTCAACGACC
TGCTGGATCGGGGCCTGGAGTATCCCGGCCGGATCGTCTGCGCAACGGGCG
GTCAGTAGGGTATACTGTTGCAAGTTGATATACTCCGTTCCATACCCGGTCT
CTACTATCCGCACACGAATGCGACAAGAATTCGAACGCCACAGATTCGTAA
ACAAGCTACCAGTAGTCGACGTACTTCTTATGCAAAACAATGCAGAGTACC
AGGTTAGTGGACTAGCGATCCAGCGCACAGCAACTTGAAATGTCGAGGAC
TTCGTGAATGCGATTGGAGGGATATCAGGTTTTTGCAAGGGCAGGTTGCTA
ATAGTTTGAACAATAGGAAACAATGAATTTCTGGAAGCAGTCTAATCACGT
CATGTCGTACTTCCCCGAGGAGAACTTCCGAGGAGCTGACAGATTACCACA
CAACTTTATGAAGGGTTTCCTAGAGGCAAGCTTCTACTACAGTCACCTTGAT
GCGGAACTTTATCAGCTAACAGCGCCTCAGGGTCGCAATTAGATATGGATG

GGTATCGTCCCTTGTATTGTACATAGAAAGAGACCCCAGCAAAGCAAAATT
TACCACCTAAGAACGTCGTCTTTTATACTCCAAACGCCGAGAATGCCATGC
CACCTGTATTGAGCCGAGATGTTTCCCGGCAGCCTATGCCTCTATCTCCAAA
CTTCTTTGCTCCGCTTCTTCCTGTCTTTTGCGAGCCTGTTCCGGTGTTGCTT
CGAATTTCTCTCGCTCGGTCCAGAATTCCCTCAGAAACGACATCGCATAGAT
CCTCTAGATCCTGGAGACCCTTGGCCAGAGCGGCGGCGGTCGTTGTGT
CTACGTGCAAGAAAATTAGTACCACAAGAATGAGGCTCGATATTTATGACTC
TTACCATAAGTCTGGATTCTCAAGTTCATCTTATCCTCGGAAGGATGGGGA
TGGCGTATGCGCAAAACTCAACATCGGGGCTAAGATAATAGTCAGCCATAC
GTTCTCAAGACAAAAGGTATAAACCGTACTTTTTCATGATGATATACCGCAG
GGCGTTGCCAAGTGTGTGGCCCTCTTTCAAGAACTCAAAGGAAGCTGCCG
TCTCGGAAGATCCAGGAAGCTATGTCCGATTGCCATTAGCGTTGAGTCTATC
ACAATTGTTCGCGACTTTCCACACGTACAATTCGAACTCGTTGAGGCTCCT
CTTCTTCCTCTTCAGGTTCACCACTTTCCGACCCGTCCTCCTCCATAGCATC
TCCATTCTCCTGCTCTTCGGTAGGCTTCTGTGCTGAGGTAGGAGCGTCGTCC
ATAGAGGTATCTACCTCCTCAGACGCATCATCGCGAGTCTTTGTTCGTCCAG
GCATTTTCTGTAAATTGACCTAAAAATGAATTATAATGTCGCAAGAGAGGTG
AAATTGGCGACTAGTTTTGGTCGTTTATACGTAATGTTCACTGGCGAAACTA
CACTGTTCAAGAATTTTGGTGGGGTCTGATAGCGATCAAGCTCTGAACGAT
AAAATTCCAGGTTCAAAGCCCAAAGCCAATCTCTGATTGGGTGGCTTCATG
ACTAAGAATTCTTGGTGGGCCGATAATTTTCCGTTCCTTCTTGTGGATAAAA
TTCTTGGGTAGGTAATCATGCATTTCGAAGTCTAAGTGGATTTCAGATGCAA
GTTAACCCTCACTGAGACACTCGGATTTCATAGATCAACAATGTAACACATC
CATGATATATTATCATTCGTATGCAATTATTATTCTTGCCACGGTCAATTTAAA
CTCTTATTACATTATATGGACTTATTATGCATCTAGTCCGAAGCAATGAGCCA
GCTCATCCGGATGAACATACTACGCAATAGTACTATTTTTCCCACAAGACTC
GCGGATATTCCCCAACTGCTGTCTCAAGTCACCCGTGTTCTTGGGAAGACA
AATTCAATATTCAGTCCGCACAAATCTAGGCGCACGTGGCAGACTGTATATG
AGCCATGTCCCTTTGGGTCTGTGGGAGCTGAAATTCTCTGAAAAACGCGAC
CCGCTTTCCGTTGTATCCTAAGATGGACTGATCGTACGTAGGGCGGTTAGGT
TCGGCAGCAATTACTCGGACACTGCATGTTGGTAATTTGAAGTGCGGCTGT
TGCCTCTCGACGGAGTCAATGGCTTCTTGGATTCGTCGCTCAAGGATCCGA
TTTCTTACTTTCGGCTCGACGAAGACCGCATCCGAGCTGTAGACAGGATTC
CTATCGTCGCTGACGAAGGTGAGATGTTCTATGTTATTAAAATACATATGCG
CTTGGCATAACGTTTGTACAAACGTTAGCTTATTGGCTCTTCCTCTCCGATTA
ATGGAGCCACTATAGGTAATGAGCCCCTCGCTGATAGCGATGCGGTGGACT
TGGCCTAGATCTTCTTGCCGGACTAGGGAGACGAAGGTATGAAACAGCGTC
TCTGCAGCCGCGATTCCCCGCCTTGGACCAAAATAAAGCGTGTCTTTCAAA

GGGTCGAAGAAATACGACCAGGTTGTGGGGAGGACCCAAATAACAGCCGG
TACCGTTTCAGCGCTTCGCGTCGGGACTCCCTACAAACATGTAGATTTGTCG
GAATAGGCGATGTGGACGTACAAACGACAGGGCTGCTACCGCTCTGTCGC
GGCTGAGATGCCTGAGGAGATTCGGTATTGCATTTTATTTCCACGATACGCG
GGCCCGGAAGGTTGTAAGCCCATATCTTGAATCGAAGTTCAACGGGGAGCT
TGGCAAACGGATGCTTGGGTTTCTGTTAGCGCTCGTCCGTCCCTTCATTCCA
TTAAAACTTACGAAGACCTGGGTGCATTCTGTCGTGGCTGCCGAGCTCTTA
TCATGGACAGCCATGAGTGTCAGGTTCTCAGAGCAAAGGAGGTGTGCTAA
AAATGATGATAGGGTCCCAGGAGCGTGGTCGAGTGTGGCGCCGAAACCCA
AGCTTCTGGTAAGTGGGAAGTGTGCATCGGCTGAAAAATGTGTTCGGGAG
GTAGGAATGGACAAAGAAGTGACTGCCACGTACATATAGGTGCTTCAGCTA
TTCTCTCCCGATCCACAAAGACGGATATCGCCAAGGATACAAAGAGATTATT
ATGGAAAACAAGCGGCATACCAACGATATCGGTCAGAAGAGTAACACGGA
TCGGTTAGGCGTTGTGATACTTGCGCTTCGTCCTTCCTAGGGACACGTCCG
ACCCACCATATTCGTGTATGTACTCGTAGCTCGTCGAGTTCCCCATCACAAA
CACTCCTTACCTCCATATGACCAGGGCACTCTGTCCAGCGGCGAAAGGAAT
GGGTGAATCAATCCCCAGAGCCGTCAATAAGAATCTGCCGATAAATCGACC
GTCTAGAATTCCAGATATAGACAAAGTTTAGCGTGGCCGAAATAGATCCAC
CTTGACCCGACAGTCCGGAACCAGCACATCAAACTTGTCTCAGATAGGAA
ATCCAAGAACCTACATACATGCTTACTGCATATAATGTACATATATGTAGCTAT
GGTTTGTTGCGTACCCTCAGTATGACCCCCTCATCATGATGGACACTTGAAT
CTTGTCGGTGGAGGACAGCTCCCAAAATGGAAACCTTTACGAGGCAGTCG
TGTATCCCGAGGCGTGATGACTCGAAGCAGATACAACCATGTCCTCCCCTC
AGGAGCACATGTGGAGTGACAACTTGCCTAAACTACCTGGATACGCTTTAT
CACGCGTGAGAAAGTCCGGTTCCGGTGAAAATAAAACACGTGAGAGCGGT
GTCTACAAAAGTCCCCAGGTGATCGCCCCCAATGGACTCGAGTGGTTACAG
AGGTGACAATCTACCATACGGATGCAAACCCGTATCCCTGTCGTCATCATCT
ATGTACTCAAGATAGAGATTTTGCTCTTGCAATACTTACCGATGTATGGTACC
GAGTTTCCATGCGTCCCAACGAAGAGCTCGGCATATGGATCCATGGAGTTG
GTAGACCCAATGGTCAGAAGAATTCTGCAAGCTAGTCGATCAGTTCCGACC
TGTTGGTACCTAGTCATCACTAACCCTCGGCTTGCCTGCTTGATACGTACGT
ACACCTAATGTTGGAAGCTCTGACTTGACGGCTGTACCATACCGCCTCTCG
CTGTCACATATGTTAGGAGTACTGTACATTATTGATTGACAAACTGATTGTCT
CTGGGCAATAAAGCAGGCACATTGAAATTTATACGCATAAAGTAAGTGTTA
ATTATATAAGGAGCAAACTACTCTAAATCTCGAGGCAAACGTTTGGGCTGTC
TATGATGTGGAGACTCATTAGCTATAGTACCCACAACTAAACAGACGTCAA
GGCAATCCGCTCTTATACCTCTGACTGCATAATCTAGATTTCCCGGGTCTTG
ACGTCTGCAAAGCATTAAGTATGGCGACCCTTGATGATCATCAGCCAGACT

ATCTTCAAGGTAAACTTTTTACAAATACCGACATGCCAAACTTACCTCCACC
AACTCGCCGGAAATCTTCGAGAATGGCCCCCTTGCAGCACACCGAGATCA
CTTGACCTATCATATTGAAACAAAAAGCAAACTCTAAACTAGAGACTGCTT
GAGTGCATATACTAGAATTGATTTACAGTCACTATAATAAATATCAAAAGGC
CTACAAACCAGTCCGCGTTGATCAATGAGATCATTCATCAACTGCACTCCA
CAAAGCCGTTTCGTAATTAATGTACTGATAGCTACTTGACATATGCATCAGA
GGTCCCTTACATCGTCACCTACTAGGCATGTAAGTACGTTGTTTATAGAACG
GATAATCGGAGTGTGGATGGTTCGAGGCTGGAGGTCTCGGACCCAAGATAT
CTCCTAGTTGCGAAATGTCTACTATATGTATTCATATGGAAGCATCATCCACC
ACACCTTTTCATCTAGGTGATATAAACAGACACAAACTATTCGCTATTAATAC
ATTCTAACGTCTGTTCTCTGTCGTACAAGGGTTTCTTTGAGAATCCCTTGAG
TGTTCGCGATGTAATGCCAAGCATCAACGTGGACGAGCCCTAAAGCCCTGC
GATTCGGATCGCAGATCCCTTAAACATCTCAGGTAAAAGGACAAGAGACTA
ACGAAAAATCTAGTGGTTCCCCTTTAACTTGCAGAGCGCCCCCAAATGTGC
CACAAAACAACAACCTGCCACGAGCATTTTCCCAGAGGAAACCGGTGTAA
ATCCTCCCGGAGACTCGAGCCCTTTCTCCCCACACCAATATCTCCGAATGTC
GTGCATTCGACTTGGCCTTCGACCCAGGCCAGTCAGCCAACCCATCTGGCC
CTGAAGCTTCGCATCTAGATGCACTTATAGCTTATCTCGCGCTCGAACTCCA
CGAAATTAGCATTCTTCATCGCATCCAGCCAATCTGAATGCCGGCATAACTT
GCCATCGCAGGGAAGGCATCTCAGGCGAGACAGGGCAAGGTAACATCCGT
TAGCGCCACGGACTTTCATTGGGCGTTCGCGCCCCCACTTCCCCGTGTCTG
TAAGCGCCCGCGATGGTTCTAAATTAAGGCCAGAACCTGGGTCGGCGTGTC
GATGGGCCAATCCGCGACGGCCACCTCCCGATTCTCTGCCATGAAATCTTG
CTGGGCACCCTGGCATCGGGACCCCGTCACACGCGTCTGGCGGTTCGCCCT
TGGACGGGCTAATATCGCCGGCAGGGTGTCGAGTGTGCAGTGTTATGTACG
AAGGTATCACCCGCACTGCTATCCTTAGGACTGTGCATGCCGGAAGCGCTC
TGCTACCTCTAGTTCAGGGAAAGGTCCGCGCAGACTAAGTTCTAGACGGG
ATCGCTTCGTCGATTATAGTCGACGTATATTCATTACATATTTGTCGTTCTCGC
CAGGTTTGTAGATTGTCTCTTGTTTCGACATCTCGCTATACGATAGCTACTCA
CACCTAGGCTATCTGTTTTCCTTTCTTCGTCGTTTCGTTTCGTTTGATTATAG
TTCTCGTTGCTGAATCGTCGTCCTTCTGTGAGGGTTGAAACGGATTGCCATA
TACGCATCGCACAGTATACTATATTGTATCACATTCCCTATCTGTCTATCTCTA
CGTCGCTATGTCTTCCGAAAACAAAGGCCCGGCTCTTCCGACTTACTCCGA
GTTCGACCCTCATGATCTTGAGTACCGGAGATTTATGAAAAAGATATACCTG
GTGGACAGCGCCCGCGTCGGTCTCACCGGTTTCGCTCTGCTCTGTGGCTTG
ACTATCCTTGGTACCTCTGCCGATACTTTGGCTGTATACAACGCCACTCATCT
GCCAGGTGATTTCCACCTTCCGTTATGGCCCGACCAATTCGATATCCGACCC
ACGATAGCGCTCGTTATCGGAAGTAGCATCGTGGTGCTCTCTCACATCGTAT

CCTTGATATTTAGCAAGGTCAAGGTGGTAAGTGCAACATCGTTAGGAGACC
CTTAAAAATTTTCATTTTATACTAAGAACCTCAAAGCTTCGCAATCGGGCCG
TAGTACACACGTCCTTATCTATGACTGCACCGTTTGTTGGTTTCACGGCTGT
TATGATTAGCATGATCTTCTTCTACGCCGTCAATACCTCAATTACGAATGACA
CGCTTCAGAGCTGGAGCTGCCAGTGGAAGGTAGCGAGCATGGGCACGAAG
CCACACTTCGGCACCTTGTGCGATGAAAGCCGTACAGCCTTATACCTATTCC
GTCATCTTGGTACCAATTGAGTTGATCGTCTTTACTGTTGCTGGCTATCAATC
GATCCTAGAGAAGAAATCGATTAGCGTGATGTCGTCCCGCAAGGCCGGCAG
CCCCACGCCATCAGCGTAGGGCACTATCAGATCTCAGGCAGCTCTAACCCG
GTTCTCAATCTGCAAAAGGGTGGCATGTCAAGCTCTATAAATGAAAATGAC
CCCCATGCTCATCTGTCGGAGCCGGGATAAGCTCCTAAGGATAGATAAGGT
GTGCAGGATAGGATGCGAAACGACAAGTGCAGCCGAGTTTTGGTTGTGAT
GTGTATAACAATTGTAACAGGATATTAGGGACATTAGGGAGGTGTGGCGAC
GCCAATTCACGTTATCTGAGTCGGTTCTGCGCGTTGACGTTCTAGGTCACCT
CGCTATGTATATCAGGGGCAGACTGATTGGTGCAATATCTCGGATCTCGGTA
GCCCATAATAAGAATTAATTCGGATTTCACCGTATTCTTTGAACTAAGAAAT
AAGGTGATGAAAATGGTCTCGGATATGCGACAAAATAAATTAACACAGCCA
TCCCGTGGGCGGAAAGCTGCGGCTCTAGCTTGAAAGGTGCTTATTTGCGA
TAAGAGATTCTCCAACTATAGATGACCTAGGTGACCAAGATGTCATTTTTG
GACACGAGCATCGTCATCTCAATTTATTGAAGACGAACGGCGGAATCATTC
AGCGCCTTCTCCGACCGCTTATCTGGGGTTTCGACACGGTGAATGGCGCTG
TCTTCCATTGCTTTTCTGCTTCCACCAAGGAATGCCCTCATCCCGCGGCAGG
AGATGTCGGTTTCCTGATTGCTATCAAATCGATGGTGGATGCAGGCGGCCTG
AACGGGTAGCGGCGTGGTAACAGCGTGGTAGGACGGGTGGTGTTGCACAC
CCTTAGATGGCTATCTTGGCTACCTTGGTACCATGTTTTACTTAGATCCCGCC
TGTCGTGTGCTCCGTGTCACAATCAACTCATAAAAGACTGTTGGTAGGTGA
ATCCGAACTGAAAATTTTCGATTTTGAGGTTGAAACATTAAAAATAGAGAC
TCGATCACATGCGAATTAGAAGCAGGGCAATTGCACTGGGATGATACTATG
CAAGAGAGTAAGTGCAAGACACAAAACGGAGAAAGTGCTGCTACCAGCT
TGGAACCCCGGCGATCGGTACCTGCCTCATGCAGCTTGACGACAACATGCT
TAACCTGAATTAAGTTGAGTTCTAAGGAGTTCTAAGAGGTTCAACGTTGCC
CAGTGAATGTTGTGCAAACGGACAAAGTACTTACCTTTTCAACTTGTTCAA
CCTATTTGAACCAATTCAACTTCGACATTCCCAAGGTTCCCCTTTTATTACTA
CATCCCGTTCGGAGTCCCCAATTGGAATTGGAAGTCCATTTTGACCGGTCTA
AAACCAGTTTACTTCATTTGGATTCAATTCGCCCTGCTGAAATTTCAGCTGT
TTATACTACCAACGACACCATCTTACGATAAAATAAGAGCTAGGCTAGCTTG
CCAACGCCCACTTTGGTTTTATCCTATAGATCAAAAGACTCCACGCCCTCAA
TCCAGTTTACAAGGCGAGAAAAGGGTCTGGATCGATTATCTACTTACCCAA

AACAGACAATCCCACCTCCACGTCTTCTACAGCCCAACACCAATACGAGTT
ACTACATGCACTAAGCCAATGCAAAGTGTATCCGCCTGAACGGTTTTCAAG
CAATCATTGTAACCAAGAGACACGGATTCGCTTGCGAACGACTAAATCTGG
AAGGACATAGGTCGATAATACAAGTGAATTTGATCTATCATACACCCTAATG
CGTGCGTAACCATGGCTCGCCCGGACTCCTTGCCAGCGAGGCTCCCTCGTA
TAAACCCTCCACCTACAGGTAACAACAACCCCTCCGCTGCTCACAGTGCCT
CGTTCGCGCAACGAAAACATCAGCCTTCGCGATCACCAAAGGTTCATATTG
TCGAGGGCAACAAGATGATGCGCGATATTGACCGCCCTCCTCCGGGAGACC
CCACGGGCTCCCGGAAGTCCCAATTGCATCCGGAGCTCAGTAAGAAGAAG
AGTGCTTACTGGGAAAATGAGTTTGCGGCAGCCAACCGGGATCCGGACCC
GGTCCGGGCTAGAATTCAAAATGAAGCTATCGTCATGGCTGATTTGAGAAC
GAACGTCATCGTGAGTTTTTTGTCGCCTACCTCTTCGTTTGCAAGTACTGAC
ACAAAATCGCAGATCAAGGACGAGTTTTCTTTCATAACAGATCTTTCATACC
ACTTATCCAACAGATATCAGCGGCCAATATCCTCCATCGTCATCAACTTGAA
GCACGGTTGCTGTATGATGTTTGGTGGTTCCTTCGATCCGGCGTATACCCTC
ACTATTCACGCACTGCCAAGCCTAGTTCAGCCTACGACAAATAAGAGGAAT
GCGGCACTCATTCAAAAGCACATTCAGGATACCTTGGGGGTCATAACATCA
AGGGGCTATGTGCGTTTCGTCGCCACTTCCGAAGACAATGTTGCCGTTGGG
GGCAAGACGATTGCTGCCGAAATAAATGAATCGGGCAATGCCACGCCCGA
CGACAAGCCCGCCGAGATCAGAAAAGGTGCCAAGTCAACCCGAAGGATC
GGTGTTAAGGTAAATACGATTACCCTTTCCTTGGCCGCAAGATATGAATCGG
GTAAATGGCCGGATTTGCTAATATCGAAGCAGTCTTTTGGAAGCTTTAGAAC
AGCTTCAATGTCGGATCTTAGAAAAGTCCCTACGCCACCCCCAAGCACTTC
AGACGAGACCACGCCTATTGCGACTATTCCGGAGGTACCTCCTACGCCCCC
CGAGGAAGACAAGTTGGGCGAGCTCATTGAGCAGAAACCGATCAAGACA
GCGCCACGCCGAAAAAGCCTACGGTTCAGTCTGTTTAGCGGAAGAGTGCG
GCGTAGACGGAGACAGCCCACAGTCACCATGAATTTTGTGTGGGCTGCAC
CTTACGAACCATACAGCCCTGCCGCCCTTCATCTACGATTGAAAATATTATAT
TCTGATGCACAAACTAATTAACGGAGCGACGGAACATAGGTCAGCATATATT
GCATCGCATAGCTAAGAGAGGAGCCTTGCATCGGAGTTAGGGATTGGTACC
TTGGGAAATAATTGTCTTATTGCTTTGAACAATATATCCTTGTCATTTCCTTC
ATGCTGGTTTGACGAATCATGGATAATGTAGGAGTTACCTATCAGCTATGCT
GCATAGTTACTCGTCTAATGAGAGTGTGTCGCCATAGAAGATATTAATTCAA
CTAGACTCCGTTGAGGCCTTGGTATATTTCCCCGTCACTATGGGGTTCGAAG
ATACTGAGACGTGGTCCGTGTGAGTGAGTTTATTGTAAGGATAAATCTGTAA
GTTTAGCATGAGACGGAATTAATACACCCTGGATATAGCCCCGGATCCGGTG
AGATCCCGTAAGCATCGGAGAAGGGCTTGAGCCCGGAGTTTATGACCGGG
TACGACATGACCCGAGACAGCTCTGGAGATGGTTCTTACTTGGGCAACGTT

TAACATTTTCATCCTTCTAGCTACTAACGATTTTCTAAAATAAGATGTTATTAT
TTCCAGTGTCTCATCGAGTCGGGTGGTGAGTGACTCAAGCTTACTATTCGG
ACTTTCCAATCTTATTGTTGATCCTGCCAGGTTTGGGTTGGACGATTCAAAC
TTGATCCATGATACGAAGATGGTGATTGAGAAGGAGTGAGTGAGACTTATA
ACCACTAGGATTTGAAATTGACAGGAGTTAGAAATTAAGTACTTTTTAGGTC
GCGACGCTACTCAAACAAGAGTTAAAGATGAAAATAAAAGAGTGAGGGCA
TGGATGAGCTTATTAATATATCTCATGTCTCATATTCGCACTAGCCAGCTGTA
ATCGTAGCAATAAACTCTGATGACAGCTGAGAAAAGTGGGGTTACCAGCTG
GCCCTAACAGTATACTTGTGCTCCGCCAACTCCACCTTGAACTTCTCCCGCC
TTTCACCTTACTCACTATTATTTCTAACGAACGTCGAAGGGAGCAAAAGCC
CTCCTTACTTTCTGCGTCTTATATGTGGCAGGGCGCTTCATCTCCCCCTACTC
TGCCAAGGACCTCTATGACATTTCGGGATTTGCTCATATTAGTCTGGCGCGA
CATGGTGTTCAATTAATTCAATTGATTTCGGGATCAACTATCGAAAGTGTCA
CATCAGAGCCCGTCGACGTTGAACATTCGACATTGAGTGTGAAGCAAGAA
GGCAGAGCTGAACGACCTACCTAGGTCAAGGGGGCCGAGGAAGTTAAGG
GATTCTACCAACTAGTTACCTAGCTGGGCGACTTGAATTCGCCCTCTTTCTA
CCTACCGAAATACCCTACCGACGTTGTGTACCTCCGCCTGTTGTTTCTTTGC
CGCTAATTATTGCTACCGCCGGATTGACGAACACAGCAGAGAATCATCATTG
GTGCGACGGGTCAAGGTTGAGTCAGCCCGCGCCAGCCTGTTGGTTCAAAA
CCAGCAAATTCATTCGCCGCCGCATACAGAGCTGACTCAGAATTCCTCATC
CCACAGGTCAAAGTACGACTTCAAGCGGACACAACGTTCGCTCTAGTCGA
TACGGGAACGGAACACGGGTATTCCCATCGAGTTCCAAAGTCCCATTTACC
CCATCTGTCTTCCAATGTCGCGGAGGAGGGGATTATAGGGCCGCCGCGCGT
TTGATTACCCTCTAGATCACCACCCTCTACCTAGGTAGTTATACCTACTCGCA
ACATGGAGGTGAGACATTGACTTTTTGGTTTCCTTTTTCTTCTGCTCTTTTC
CAAAGTTCCTTACACATTTCCTTGATTATGAATCTCCCAAACAAAAATGTCG
TTCGAGCTGGCGTTGCCCAGTGGCACAAAAAACACCGCCGCGTATCATCAC
CGACTCTTTTCGGGAAGGTCATCCATCATCAGTCGAAGGCTACGAATACTTT
GTGCCAGTCAGGTACATCGATCGCCGTTATAAAAAAATTTTCCCTCTTACCA
TGCTGCGTCTGACACGCAGCTATGGTTTGCAGTGGTCGCATCGGGTTTAAA
AGCACATTATCGCCATTGTTATTTTTATTATCATCGCATCATCTCCTGGGCGCT
GGGCATAATTAGGCAGACACCAGGCCAAGTACGGGGTTGAGTTTGCAAAC
GGAGGGATTATTATATCCTAATTACCAATCACCATATACCCGAAAAAATTACC
TGGATGAATTGGCCGAGCTGTTTGATTTGTACGACGGTGAGATCAGCAATG
AAATCATCGCGCCCCTTCTTACAGATTATCAGGAGCAGCATGCCCTGTTTTG
GTCCAGCTGAACGCCCCGGACCCGTCGCCTCATCCCGTTACCTAGTATGAG
ATTTGGTATATTCCTGGTCGATTTCGCAATACATGCAGCCACCCAGACGTCG
CAATCTCTGAGCGTTGTGATCTTGCAGAGTTCCATTCCCCTACCCGGTCCCT

TGGATTCGGCAACGTCGTTTGCACATTGGCTCATTAGCTAGCTCAATAGCTC
TTAGCCTCTTAAAACTCGCCGCCAAGCCACAAACCACGATCCTACTCAATT
GGAAGGTCGTCTGAGATTCATTGGGAATATTGTGACCCTCTCGTATCACTCA
GCAGATATGAACCAAGGAAGAATTTTATATTCATACAAGGAGCACTTCCGCT
CTCATCGGCTCGACTCTTGATGTACTCGGACCTGGTGTTTTTTCGTTGATCG
GCGTTTCTTCGTGTCTTTTTGTCTCATTCAACTCAGTTCATGTTGTCGTTATG
TCTTCTAAGCTTCGTGGTGTGGACGCCTTCGACCCTGAGGATACTCAGGAA
CAGTTAGGCGGGACGCCATCCAGTCCTTCTAGTCTCGCGCAGCTAGCTCTA
ACACGTTTATATGAGCAGGATCAAAGACAACGTAAAAATGGCCCGAGGGC
GCCTGAACAACCTCCGGATCTGCCAACCAAAGGGAAACCTAGGTTGCTCC
TGATGGGACAGAGAAGGTTCGCCGTCACACGTCGCGCCCATCGAACATAC
GTGCTGATCATACCATAGGAGTGGCAAGTCCTCGATATCAAGCGTAGTCTTT
CATAAGCTACCGCCGAGCGAGACCCTGTTTCTTGAGTCAACAGCGCGAATC
CAGAAGGACACTATGCCGTAAGGGCTCAATGACACCAATGAGTATGGTTTC
TGTTCCTCTGACATATTTAGGTCATTCATGGATTTCCAAGTTTGGGACTTTCC
GGGTCAGATCGACATATTTGACAACCCAACCTTCGACATAGATGCAATGTTC
GGTGAGATCGGTGCCCTTATTTGGGTCATCGATGCTCAAGATGATTACCTTG
AGGCCGTAGCCCGTCTAAATGTGACGATATTAAACCTCCAGCGAACATATCC
GAATATCAAAATCGAAGTTTTCATTCATAAGGTAGATGGGTTATCGGATGAT
TACAAGTTGGATATCCAACGAGATATTACTATACGAATTCAGGACGAACTCT
CAGACCACGGTTTTGAGAATGCGCCCGTCACTTTCCACTTAACTAGTATTTA
CAATCACAGCATATTCGAAGCGTTCAGCAAGGTTATTCAGAAGCTCATACC
GCGTCTCGGAATACTTGAGGCGATGTTGACTAACCTTTGCCGAACGTGTCG
CTTCGAAAAAGCTTACCTGTTCGATGTCTTGTCTAAGATTTATATTGCGACA
GACAGTGAGCCGGCGGATATGGCGAGTTATGAGATCTGTTCCGATTACATTG
ACGTCATAATAGATGTCACGGAAGTCTACGGGAGCTGGCCTCGTACTCAAA
GATATCGCGAAGCTCTGGAAGGGCCGCCGTGGAATCAAAAGATAGAGGAC
CAAGTGGCGTCGGGTTGTGCAGAGAGCTGCATGGTGTTAAGCGATGGAAA
TAAACCGATAATACTCCGCGAGGTCGACAAATACCTTGCGCTGGTTGCGAT
CATGAAGGAAGATAGTTATGATAAGATGCCGCTTGTTAACATGAATGTCGAG
GTGGTGGTGCAAGGGTTGAAGGAGTTCTTTGAGATTACGAAGCCGAAATA
AGCATCGGCTTAGCTTAAGTCGAGTTCTAGCTTGGAGCGAAGGAATCAGGT
ACAGGAACGGTGTGCATGAAAACACCTCTAGTGGACAAGGCTCTGTCTAG
AGCTCAAGGAACCGTCCACGTTTCTTCAGGATCAATGCGGTGGTCTGCGGC
TTATGTCGCTCGCCTTGGCACACGTGGCTGAGTGAATTTCCAGCGACAACA
TGAACCCTGACCCTGTATTGAAATGTTAACTTGAATATAAAAATTGATTCTG
CTGAAAATGTATGCACGACTTGTGCTAAATAACCTGTCTTGAGGATTAGCTA
ATATACTAGTTGCCGTGATCCGAAATGTTACACCGTGGTATGCAGCCAATCA

AATGAGGAAATTGGAACGCGTGCTTACGAACGGCACAGGGCTCTTCTCAAT
TTACCCTGCCATCATCGCGCGGTATGGCGGATATAAATATGATCGTCACTGA
GAAACAATAATTTCTCTCTTTTTAGACATGTTGGCATATATCATGTCATCATA
ACAAAGGGGAAACTTTGAGTTTAATTGACAGGTGTCATCCTGCGCCGCCCA
AAGGCCTCCGAGAGACCAGGTCATTTCACTTAGCGAAGCCGGAATATTCGG
GGAGACCGAGCTTCAAGAACGCCGCAACTGCGCCGCACTGATCGGCGCTC
GCGAGATACATTTAAACAAGGCGGCTTGTTGCCTCTTTGCTTTTCATCTTGC
CACAGGCTTCGCCTAGCATAATACCTGTACTCAAGGTTCAAGATGCGCAGC
TCATCCATGCTCAGTTGCTGCTTAGCGGCATCCGCGTCCCTCGCCGCGGCC
ACAGCCAGCTTCGGGAACACCAACTCGCTGGCCCAATTCGCGCTGCAGAA
GGTGCTGCAAGATGGGAGGGAGGTGTTCGGGGACTACGCCACCACGCCCA
GTGCTTCGAACAATAAGTTCGCAAACTGGATGTCCAAACTCCCGGACTCGA
CGCTGCTCTCCCATATCAATATTCCCGGGACCCACGACGCGGCGACGTGGA
ATTATACGCAGGCGACTCAGGACTCGCTTGTTAACGCGACGAAGTGCGATG
GCACGACGGCTAGTCCGGCCGTCGTGTATAGATGTCAACGAGAGAGCATTG
CTGAGGCCTTGGATAAGGGGATTAGGTTCTTTGATTTGAGGTTCGCGGCGG
ATCCCCTAGGGCAGAGACTCGTGTTTTGGCACTCGGCGGCGCTGGTTAGCG
CGACGGCGGGGGTGGGCGATGTGCTGTTTGGCGTGTGGGAGTGGTTGAGC
AGACACCAGGGGGAGACGGTGTTGCTGAGCTTTCAGCATGAGAGCGGGAC
TGTCGCCGGTGCGAGTTTCGATGCGCAGGTCCAGGGGTTGATGAGGGAGG
CGTTGACTGGGGAGGTTGCGGATAGGTATGTCTGGCAGGGAAAGGGGTAC
GTGCCTAAGCTCGGAGAGGTCAGGGGCAAGGTCGTGCTGGTTAGGAGGTT
CGATATGGATAACGACAACGACGCCGACAATGGGGTGGGAGAGCTGCCCG
GCTTGCATATGAGCCCGAATAAGTGGATCGATAATAACCCGACCGGGTTCG
GACTCGTGTTCAACGAGACGACGAACGCGACCGCGTATATCGAGGATTACT
ACGAGCCTGACGTCCTAGGGGAGAATTCGACGGTCGTTAGTAACATCGACG
CGAAAATGGTCGCCGTGAAGGCGAACTTGCAGAAGGCGGCGAGTGGACA
GGATAAGGATAGCTTGTTCATTACGTTCGCGTCTGCTGAGCACGATTCGAAT
ACCCCACCTGTCGTTCCTCAAACTATGGCGCTGGGTAACGGGACGCAGGTT
ACGCCGGACGGAGGTGTAAATCATCAGCTCGTAGATGTGCTGAAGGGGTT
GCAGGGGAAGAAGTTGGGAGTTGTGGTGCTGGATTTCTTCGAGGAGCCCG
AGGACTTGGTTTCGCTTGTCCTGGGAAATCAGATATGATGGGAGAGATCGC
TTTCTCTAGAGTTGGGATATTCATCGCCAATAATGAAGATGGGGACTTGATA
AGTTATGATGCATTAAACATAATTAAGATAATATATTTGGATATGATCCGTGAT
GCGATGGTTGCGATGATGCTTCGAAATGAGGTTGAATGCGAAAACTATATAT
TTATCTATGTATAGATAGATCAAATAACATCGTAGATGACGACGGCACAATTT
GCATTAAGGTAGTACGTCGGGGTGGACGAGGCGTCACAGTGCGCGCATAC
CCACTCCTCGCCCGTGCTTGCTCAGTTTAACATATATACTGTTCTTAGACCA

CTGTCAAACAACCAGGCACAGCCGTTGAAGGTTGCTCCGAGGAAATTTCT
TGATGTGGCTCAGGTAGTTGAAGATTTCTTGGATGTTAGCAAATATTCGTGA
TTGGACGAAAGTGCCAGTTTGCCTCTTGGTGGGTCAAGATACAAATGGGTT
GCACATGATAGAATGCTCCGACGAAGGTCGCTGGTTCAATTCCAGCGCCTG
GTGTTTTTTTTTTATTTTTTTAGAAATATTTTATTCTTATTTTATACGGTGCTGA
TTCACGACGCCTCTCTCTACCTTTGAACCATGACATATTATTCATCAACACAT
AATAGGTCCATTAGTATCATCTAGAGACACTCATGTCGCTGCCGCGCATCGA
TAAGCTTCACTAAAATTTGACATATGAAAACTACATGCTTAAATACGACAAC
CCAAGTTCACAATCACAGGAGTCAAGTACGAGCAATGGTTCATAACATACG
CCATCCAAGGGACGTTGGCGACTGGAAAATTTGAGATGGACATTCTATTCC
CCAGAATGAACTTAACCATATAATCGTCAGTATCCACGGCGGATCACTCCAG
TGGGAAGCTCCAGCTGTACTCTCATTTGGATATAGCGATGGCGGCACGAATT
CAAACATTTTAGGCATCTTTTCGTAGTGCCGCGATTTCATTGGGAAATATTC
AATTCCCCTTAGCCTTGCAGCTCTCATCCTAGACGATGCTTCTCAATGCGGG
TGCCCACCGGATTGCGAAATGACGATGTTTCGAATCCTCTATGCCTATGCCT
ATCTGTTCACTTCGATTTGAAAGACCCCTATCTTCACATCTACTATAGCACTT
GGATAAGAATGAATTGTCATCAGAACCACCATATTTCTAATCATATGGTCAG
AGGCTGTGGCGAAGATTCGAGAGATTGTGTTGCATAGTACAGTCTTCGTGA
AACCAGCTCGGTCCGTTTAATTGACGTCTTTCTAGAAGGGTATCCGATCTTT
ATATTAAACTTGCGATAACATTCTAGTATTTGGAATTCTTCCAGCCGAGAAC
GATACGTCCGGAGACAACCGTGGCGGCCCGGCTCACCGTTTCCAGTGGATA
GAGCCGGTAAAAGGGGTTCGCTGCAAGCACCGTTAAATCCACGGCATTGAT
GCGAGCGTCTCCCAGCCGTGGATATAATAATGTCGATTCGATCCCGATTTTC
AAACAAGCCTCGAACGCCAAGGATATCTATATTTGCCTTTATTACCATGCAT
CTCGCATTCCCTTAATTACTACGCCAAGTCCGAAATGATGATATCACCAACA
AGGGGAACGTATTAAGAGCACGACATGCATTATAACCCCCACGGAAGAATG
CATATTTTCATTCTAACATAAACGAGAGTGGCTTACCCACCTGGAAATATTG
CGGATAGCACACGTTGACAACTGACAACTGGCTCCGATATGTCTTCCCTGA
TGACATCATTATATTTATATTTAGGCAAATAGTGGCGAGGACTTGTAGACAG
CTTTTCTGGCGATAAGATATCGCTTACAATACCTTCGCTATAACGGATATCCA
TGTGAAACATCACCAAGCCCTATAGTGAGGTTGTACAATGAAGCTAGAGAG
CAGAATGCGGGAATTGATTAGAAGATGACTCAATCCCAAACAGAATTTTCA
GCAGCTGAATGTGAGTCAAAATATCTCCGTTATAGGACGCCAACGATGATA
GATACTCCGGTGATAGCTTCCACTTAGTGCTTGACGTAGATGGCGGGACCC
ACGATCGATGTTGAAACAGACAGCCCGGAAACCGCGGGCAGCCGTCGGCG
ATTTTCACCTTAGAGGTCTAGAACAAACATTAGGCTCGGAACATGTTATGG
AAACGTAGAGCTCGAGGAACTAGCTCTACGATAGTGTGATAGTCATACGTT
CAGTATAACCTCGTCGGATTTAAGAAATAGTAAGACCTAGCGACGCCGGCT

AAAAATTCATCTGAGATGGCCGAGTAGAAATTGGATGCAATCGAAGCTGGG
CTAAATGAACTAGCAGGGAACCAGGGATGAGGAAAGGGAACGACATTGAA
AAACAATGAGCATTTCCAAGACGTAATCAAGAGCTGTAATTCGACAACTTG
GAAACTTGTTGAGCATCCCTTAATTTGAATAGGGTCGAGGGATCAATTAAA
GAGAATGGATTGGCAATAGGGCAGATACGGATGTTTCTAGTGCGTGTTCTA
GTAGATGCGTCCCCGCATTTGCGCCCTGATGCCCTGATCCTCCCGCCGAGG
GTATTCATCCTTCCGTTACACAAAATCTCATTGCTTGCGGAGGAAATAGGGA
ATGAATGTTCCAAGGTTCAA

>Mosaic_sequence_47

AGATCAAGGTCCGCGTTCTTTCAGCTCCAGCAAGCGCAACTTCGTTTCAAA
GTGCAGTTGCAGCGACAAAAGCCAACGGTGGTGGTCTGCCAGCGCCTACA
TCCTCAGGCGCAAGCGTCATCATTGGCACGGCGTGAGTCTTGCTACCTGAC
CTGCTTGCGAATGAGTCGCTAATTCCTCCCAGCGTACATCCAAATGGCAAC
ATCGGAGAGTTCCTGTGTGCTGCAGGGCTAGCAAAGGTGCTAGATGTGAGT
TGCAGATAGTCCAAGAGTTGTCGCACTGAGACATAGTGGCACGCAGGCATT
CTCGCCCCATATGGCGGTCTGGAGAAGTTGCGTGCGGCTGAACGCAGTGC
GAAGGAAAAACGCCTCGCTCTATGGGAAGGTCTTGGAGCTTCGGCGGCGA
AAGGCAGCAATGGGGCTAGCCATGCTGAGGCTCCATCGACAAGAGGCTCG
ACCTTCGAGGCGACAGTGGTGCGTGTCTGGGGCTCGGATCAGCTTAGTGTT
GTTGCAAGAGGCGACGCCGCCGGCACGGAGAGGAGGGTTCAGTTGGCCT
CTGTCGCGGACCTCGGTAATGCTGTCTCTCCGTGGAGTCATATCGCAGCTG
AACAAAAGGGGAACTGGAGCCAAAGAAGCGTATTGGGCAAACGAGGCAA
AGGAGTTTTTGCGGAAGAGGCTCGTCGGCAAACACGTCAACGTTCTGGTG
GACTACGTCAAGCCGAAAGATGGTGAATACGAGGAGCGCGAATGCGTCAC
CGTCACTTATGGAGGACAAAACGCGTTGGTATTCATGTCGATCTGAAGCTC
TGAGTCAGGCTGACTCACAGGAATGTCTCTGAGCAACTCATTGAGCGAGG
CTTGGCTACTGTGCTCAGACATCGTCGAGATGATGAAGATCGCTCGGCAGA
GCTCGACAAGCTGATCGCGGCCGAGCAAAGGCAGGTCCCAGTCCTGATCG
TTGAATTCTGACTCACGTGGCAGAGCGACAGAAGAGACCAAAGGCTTGCA
CTCCACGAAAGATGTCTCTCTGCCCCGTATTGTCGATGCTTCCGAGAGTGG
CAGCAGGGCATCTCAATATCTGCCCTCCTGGAAGCGCGCTGGTCGGCATGC
TGCGGTTGTAAGTCTGTGTAATTCGCATCAGGTAGAGTACTCATCGACAGG
TGGACTTCGTTTCGGCAGGATCGAGGTTCAAGCTGTTCTTGCCAAAGGAG
AACGCGAAAATCACATTTGTCCTTGCCGGTGCGTATTCGGTTCGCCTAAGA
CTTGTCCAAGCGAGGCTCATGGCAGGTATTCGCGCGCCACGAACAGCACG
AGCGGGCACAAACGAGAAGTCGGAGCCATGCGGCCCAGAAGCCTATCGAT
ACGCCATGAAATTCATGCAGAGAGACGTTGAGATTGGTCATTGATCCGATT

TCTTCATTGATGAGGCTAACGAATACAGCCCTTCGACAGTACCGATAAGCA
GGGCGGTTTCATCGGAACGATGTATGTAGGAGGAAACAACGTTGCGGTGG
AGTTGGTACGCGAGGGGTCAGTCTACGCTCAGCACGCATCGGAGTGACTC
ATTTGTTAGACTGGCCTCAGTCCATACGTATTCAGCAGAACAGTTGCCGTAC
GGCCGAGAACTCGCCGCCGCCGAAGAGGCTGCAAAGAAGGAGCACAAAA
ATGTGAGTGACTCCGTTTTGACTCCTCTTCCTCAACTCACTATTCAGCTATG
GGCAGACTACACCGGGGAAGAGGCAGCGACCGTCAAAGCCGATGATGCG
GCCGGTGCGCTCGCCCCGGATTACTTGGACGTATATGTATCATCGACGAGAG
ACTCTGATCCGTTTGGGTTTTCAGTTCAGATCTTGGAGCCGTCCAGTACGTC
TGGGCTCCAGCGAAAAGTAAGCTCATGCTGACTAACAGGCGTTGCTTCGCT
TTGAAAAGCTCATGTCCGACTTCTCGTTGCATCATCGACAGTCGACCTCGA
ATGCCCCGTCGAGCTTTTCGCCCAAGACTGGCGATCTGGTGTCAGCCAAGT
TTACGGACGACAACCAATGGTGGGTTTTGAGAAAAGTCGCGTAGTCTTTGG
CTGATTCCAGGTATCGAGCGAAGGTCAACGTGCCAGTGCCTTGAAGAAGG
AAGCCGTCTTGACATTCATCGATTACGGGAATGAGGAGACGCTTCCGTTCA
GCCGCATCAGGCCTTTGGATGCCAAGTTCAGGACTTTGCCCGGAGCGGCTC
GCGAAGCAAGACTAAGGTATGCTCTTGCTGAACGATGGAAGATACAGGGC
TGATGTTGTAGCTTTGTGAAGCTTGTGCCCAAGAGCAGCGAGTACGGACCG
GAAGCTTGGCGCCGCTTTGGCAGATTGACTGACGTCTGCTATCTCCTTCCT
GTTTAGGTTCAGAGCTGACCACCAAGGGCAAGAAGCTTGTAGCAAACGTC
GACCAGAGAGAGGGCAATCTGCTCCACCTTCGTCTGATCGATCCGTCAGAC
CCCAACGCCGCTGAGGACCCGCTTGCATGCCTGAACGCCGATCTGGTCCG
AGAAGGTCGGCTCTTGATGTCATACTGCTGCAATAGGAGACACGCTGATGA
GCAGGCTTGGCAACGATCGACAAGTCGTGTCGCTACCTCAACGCGTACCC
GCAAATCGTCAAAAAGTTGCAAGATGGTGAGCTCCGCGTTGGCACTGCCT
GATAGAGGGGGCTGACAAGCAGCGACTGAGGGAGCCAAGCAGGATCGTCT
TGGTATCTTCGAGTTTGGAGATGTGAGCGAGGAACTAGGGAGGATGAAATG
TGTCTTCATGTCGTGTCTTCATCATTGCACTACCAACACATAGGATCTACAA
CCAAAGCAAAGCAAACAAGCAGCACAAGCATGCAAATGTCCCAGATGCCC
AACAGCGACGAAGATCAGGTCTCGGCCTCCTGCGGCAAAGGCGCGTAATG
CGTTCCAAGTATTCGTTTCGTGAACTGGCCAAATGTCTCGGTGGAGGAGAT
GAAGTCATCAACATCGGGTCTAGTGCTCTCAGTTCGTGTCCTGGCACTCTC
TACGAGGCAAGTGAAAGATGAGGCTCACTGGAGGAAGAACGCAAATGTCT
CTCGTGAGATACCCCCGATCCCAATTGAATCGACCTCGCCTCGCTCGCTCAT
CACCCCAGAGACGTAATGAGGAGTAGCGGCGAGCCGGCCGCCAGTCAGGA
CCTCCATCGCCTCGCCTATCTGGAACGCCAGGCAGTCGGCCGGTATGTTCA
CCTTGACCGCTCCGCCCTGTCCGCCAGACGGCGAGGTTCCGCGTGGAAAG
ATCCATAGGCCGGCTTCTTTCGATGGCGAGGGTACGACTTCAGCCGGCGAG

AACGAGGCGTCAAGGTAGAGGGCAGAGGTGAGGCCTGTCAGGAGGGAAT
GGTCGAGGTGCGTCCCGCACAGAGCGTCGGCCGAGTCGGGGATGGACGAT
TGGGGGAAGTAATGCAGCAATCGGGCCTGTGTATCAGACATACCGTCGCGA
TTCGGCAATTCTCCAAGAGACATACCTTGTGACATCTTGAACCGGTCAATA
AGCACTCGACGCCTTTGCTTTCCGGGACATCTGATAGGTTGGGAGCCACTG
GCGCTCAGCAAGGTTTGAGAGCCATCGCCTGCGCTATCCCCTGACTTACCG
AATGCCTCGCATGCTCTCGCTAATGCGAGCCCGACCTCGAAAATGAACTCG
CCAAGTCTCTAAATCAGCCTCAACAGCATGATCAGAATCTTGATAATGTAGG
ACACGACGTACGCCTTGAAGGCCAGTTTGAAGTCCTCAAGCCCGTCCTCCT
CTGGCCACACATTTCCGTGATAGTACCTGGATCATCAGCTACCCCAGTCCCT
TCAGCAACTCACTCGGGATGCGCAGCACGCTTTTCATCTGATACGTCTGGC
GAGTCCGACAGGGGATTTGCATAATACGACCCTTTGAGGGTATCAGGCCTC
TACGTATGAGCTTCGCATAGAATGAATAAGCATAGCAAGCTCACCCCGTTCA
TGATCTCCTTCCCTGACTCCATCCGAACATGTACTGCACATTCTCCCGCGCG
TACTTCTCTCGTACGGCTTCGGGCAGCGATGCGAACTTTTGGCCTAGGCGA
AAGAGCTTCTCTCGCAGCGGTGGAAAGTCTGGTGGCAAGTCTAGTGGTGA
GCCGCGGCCGAGAGGACGGAGAAGGCGTTCACCAGATACAATTATGACGC
CAAGGCATCCGGGCTCTGAACCGAATGCTCGAGAGACCTCATCGCGAAGC
TCGTGGGCTTTGACCGTAATCTCGCCAAAGAGATGGTGACAGGGGCAGAA
GCCATGAGGTCCCGCTGGAAGCTTGCTGCCTTGATACGTGAATCGGAGCGT
CCAGTGCCAGAACAGAAGGCCAGTCTGCCTTCCGCAGGCGCCCACTCGAG
TCGAACCAGATACGAGAATGCGAGAGCGTGCCCGTCTGGAGAGCGAAAGA
TAACGTCATGAGTTGGGTCGAATTTCCCGTTTGGGCATTTTGACGAGGTCCT
TCGCGACTGCTTTTCTTGTGAGATACGCGGAACCAATAAACGTTTCAATTTC
GCCGACTAATTGTCATTGAATAGGATATCCGACAGTCACAGCCGGTGTGCC
ACCCCAGCGTGGTAGTTATGGTCTGGTAAGCCTTTCGGGCCCGAGGTGCTT
GTACCGTTCCAAAACCGAGCGAGACTTGGGAACACGCGGGATGGGGGGTT
CGCGCATCCCGTTGACGGCAGTTCCAACGGCGCCGCATCTCGCTCTTACTC
GTGCGGTACGGCCGAGAGACACGTCTTTCGCAATGACTGCTACAGTGGTG
ACTAACCGCCCCCCTTCAAGCCGGAAAGCGCAATTATGCATGCTGGGCCCC
GTTTGACTGGGCCCAGCCTGTGTCAGTCGTTCGATGGGGTGCCTGAACGAT
GCTGAATCGCCCCCACCTTCACCCGGGGCAGGGCATAGCATGACCTCTCCA
AGGGGCGAAGCAAAGTGTGGGTGGACGGGATCAGACGATGTATGATGGAA
CGCTGGAGCGCTTTCAGACCAAAACGGAATGAGCGAGGGGTATGTCAGCC
CCCGAACAGACCCCGCCGTGTCCCGCGACGGGCCTTCCAGTACGTGATGC
GTCCAGAGGCCCACTTCGGATGACTTTGTCGAGGCGGAGCGTGGCGAGGT
CGTGGGAATGTCGCTGGTTTTGTGGTTGGCTGACTGGAGAAAGATCTTGGG
CCCGTTCGTCATCCCGTCAGCGGAGGGGGATCGGGTCACACGTATACGGAG

AGACGGACGATGTCGCAGGATGACAGATTGAACTGTGTCTCAGAAGTGTC
AAAACGTATAAGAAAGTGGAGTGAGTGGAATCCTTCTGCATCGACACCCA
GGTAGTCTTCTCGACAGCCAAGTCCTTCTTGACATCTTCCAACCCCACGAT
CCTAGTCCAGCTCGCAGCATCTCGCAGATCATCTTCTCAGCGCATCCCGCCA
GCAATCACCAACGAGTGGCTCCTCATTGCAATTAAGCTTAACCCGAATTGG
CACTGGCTCACCCGAAAACGTGACGGCGGATCCCAGTCACACCGCAGTGC
TCGTTGCTTCCTCGCCGACATTCCACCTCTGTAAGACGCTCCTCATGCCTCG
GTCACCGAATTCTCGCAACTCCATTCCCCGTCAATCCACCTCCTCGTCTTCT
CGCCCAAGAACCTCTTTCGCCCTACGTATCGACACCGAAAGCTGAAGATTG
ACTTCTTGCCGATGGGCGGTGAAGCCGAGCCTTCGCCAGCCGCACAACGA
CCCTCCCCGTACCCGCCGCGGCGACCAGACGCGACCGGTGCTGGTGCCCG
CTCCCCTCCTCGAGAATGCCAAGCTGTGAGTATTGAGGCGGATCACGGCCG
AATTGGGACGCTCATCCGTTGGATGAAGGTCAACAGGCTTCTAGACATGCC
TAACAATGAGCACGTCGCTTGGCCCGGCCCCCGTGCGGGGAGTCGCTCCA
AAGAACAAAGCCTGTGACGAGACAGAACGAATCGCTTCACCTGGTCATAC
AGCGTCCTTCCCATACCCCCAAGCGATCGATCCCGCCGAGGCGGCAATGAA
CGAGTACATGACGTTCAATGCTGAGTCTCCGTTGCCAGAGACAAGTTGGAA
TGAGCCAAAGCAATACGAGTCCTTAGCAGGTGAGTGCTCATTGCGTCAGTC
TCGTCTGCTCGTCCGCGCGTATATCTCAAGCTGATCATAAGGGCAACGGGA
CAAGCGAGACTACCCAAGGACTGCAGATGCGAGCACAGAGTGCGCCGCGC
TCGGGGCGCTGGAGCTGCCTTCACAACCGCAAGACTTCGTGCCGCCTGCC
TCGCACAACTTTCGTTCTCTCTCGTTGTCGACTCCGAAGGCTTCATCGATTA
GCTCACCCTCCGCACGCAAGGGCCGCGACATGTATTTGAAACAGGACCCG
ACACCGGACCTAGACGGCTCCCGTCCTTCACGCACCACGCAGGAACGCAC
TACCGACTCCGACTGTACACCTGAGTTGCTCAGCTCGTCCATACGACAGGC
AAGGCGGGTACGAGTCGCTCCAAATCGCATCCAGGATCTTTCGCCAGAACA
AGAGATGATTATGACCCCGCCGGCTGTTGGGCATACTGGCAGGCCCGACGT
CGTGCGACCTCTCCAGACCCCTTCTAGCTCCCGGCCTCGCCAGGCACTCGA
GTCTCTACGACGTCAATCAGACCCCGCGGTGCCCTCCTCCAAGGGCAAGA
GAAGCATCGCGCGGAATAACAGCTTAGCTCCTCAGGACCTGAGCACCCCG
GATGCGCGTCGCAGGCTACAGGTGTCGCCGAATTCCCCAACAAGTCCATTT
GGAATCAATGCCCCTTCGCCACGGCCCATACCGTCTCGGCAGATATCGTGG
CGGGAGGCGGAACGGAGGGGCAGTCTGCCATCGACCTTGGCAGCGCCTTT
TACTTCGCCACCTCCTCCGGAAGGTTTCACTTCGCAGGTTCCCCGTTCCAG
CTATACCAAGCATCTTCCGCACCACTCCTCTGCCTTCGCCGACGGAAGAAT
CCGGTCCCTCGACGGTACATCCTTTCCTGTCAACTGGATACCAATTTCCGGC
TCCTTCCTCACAGACGTTGTGTGCTCAAGGCTCCCCCAGAACACCCGAGG
GAACACTTCCTCTTCGCAATGCTCGGCTGTGCGCCGAGCATGAGCCCAAAA

ATGTCCTCCAGCCGCCTGGTCTGAATACCGGGAAAATTCCCGTCTCCCCCG
CCGATAGCCTTTTGGACGACTATTACATGGACTGCCTGGATGCTCGCTCAGA
TTCGGACCCGGACAAAGATCTCAACGGCAGCCCCGTGATCTCAACCGAAA
CGGATGTGACCATGACCCCGCTGTCGACCAGTCACACATTGCCGCGGGAG
CTCTCAAAGACCATCGCCTTGTGAAAGCCTTCGAAGAGAAGTACGAGCTT
GGGGACGAGCTGGGGTACGGTGGCTTCGGTTTCGTCTGCGCTGCCAGGCC
TCGGCTCGGTAACGGACTACTTGGTCCCGAAGTGGCCGTCAAATTCGTCTA
CAAAGACAAAATGGCACCCGAAACCGCTGAATACTATGACGGCTGGCCGG
GAGAGCCTTATATCCTCAGCCAATGCGAGCACCCAAACATCGTTCGATTCTT
CGATTATTTCGAGGACGATCGGTTCTTCTACTATGTGAGTGACGAAGAGTCC
GGGGAGAAATGGAGAGCAAAATGCTCATGAAGAGGTAACCGAGCTGCACG
GAGACCCCTGGCAGCCTGGACACACTCTGGACGAGGTCGTCCGGCCATCG
CTTGTTGCCACCACAAGGGGCCTAAATAGGCCACCGGAGCTTGTTGCCACC
CCAGCTTCTTCGATCTCTGGCATGCTGTCTCGACTCGAACAGACGCCGACT
ACTCCGGGAAAATCGAGCAAGAGCCGGCCGAGGATGGGACGGCGCGCAA
GTCATGACTTGTTCGAGTGCGTGGAGAACCATCGCTTCACCGAAGAGCAA
GCACGTCACGTCTTCCTACAGATCGGTGAGTCACTCGGAAGAGTCTCGTGT
AGGGAGCGACGTACACTGACGTCAGTGTCCGCCGTCGACTACATGCACAA
CAAAGGCTTTTACCACCGGGATCTGAAAGACGAGAACGTGGTGGTGGACC
GAGATTTAAATGTTCGTCAAACCACTGTCCTTTCTTGACGTGCCCCTCCTTA
CCATACAGGTCAAATTGATCGACTTTGGCAGCGCCGTAGCAGAAGATGCGG
ACGGGGAACAACAGTATTATGATCAGTTCCGGGGTACAATGTCGTACGCTG
CACCAGGTAAGTTGGCTATGATGGTCTGCTCTGCTCCTTCTTGCACAGGGC
TGATAAATAGAAATTATCAATGGGAGCAGCTATCGTGCTGGCCAAGCCGAC
ATCTGGGCCCTTGGTATCATACTCGCCATACTGCTCACTGGAGCCTCTCCTT
TTGTCAGCCAGGAGGATGCTCGCATGGCTATATCCGGCTGAAGACGCGGGT
CGAGGGGGACGCGCGTCACCTGCTTGGCTGGTGCCTCTCGGCAAACGCTG
AAAGTCGCCCGACCATTGCTCAAGTGGTCCGCCACCCCTGGTGCCAGGAG
ATGGGCCTCGCCCTCTGACTGGCATCTGACCGGCTTTCCTCCCGCACGTTC
GATCCCGTTGGAAGACCCTGAAACGATGAGCTCGTTGTAGAACCGTGCGCT
CGGGCGATATTTGGGCAAGGTGTTTCTTGGTTGGTGTTTGTTTGCGCAGAG
CATAGATCAAGTGGTTTCGTTTACTCGCTTGTTCCCGTACACTGTTGTTGTT
TTTGTTCTGGGATGTCATCATGTCTGGTCATTGTTTTCCAGGGTGCCACTTT
GCCACACTCAGCTTGCCACTTTCTCGCAAGTGGAGGATGTCCACCTTCTAT
GCGCGCTCTATGCTTACTCGTAACGTCTTCGTCGCTTCCTCTCTCCAGCATG
AATCCCGCGAGGCAACTACGCCTTTTCATTCCATCATCAAGTAGCACGATAC
GTCTCGAGGCGCGCATATACTCTGCTGCCCCGTCACTGCCGCCCTCGAGCT
CTCGTCAATCGGCTCCTTTGTCCGCAGAGGAGGGCGAGCGAGTCATCGCA

CAATGGCTCGAGGCAGCGAGCGATGATGCCCCAAAGCGGCTGGTAGTCGC
CACGCATCCATATGCACCGCTTGGGGGAAACATGCTGGACCCGTAGGTCCA
CCACGCCGGCCCCCGCCGCTGTTGGCGCAAGGGACAGACGCTGACTCCAA
GGGTCTTGACGGATCACCTGGTGCCTGCAGCTCTGCGACATCCCCGAACAG
CCGTATTGACCTTCAACTACCGCGGTGTAGGACTATCTGGAGGATCTCGAG
CCTGGGTAGGAGCGGAACCTGCCGTGGCGGATCTTGAGGCGGTCGAGCGA
TGGGCGGTGGATCAGCTCAAAGTCGAGGAGATATGGCGACTGGTGCGCGC
GACCTTCGCATCCCCTACGAATAAAGCTGAGGACTTTAGGGGTTCTCATGG
GGCACCATGGCTGCTGTCTGTGCAGCACCGACTCGGCTCGCCGGGATCATG
CTCGTCTCGCCGCCCATCTCGGTCTTCGGGTGGACAGCCTACCTTTCGACG
TCTTTCGCGCAGGGCCTGAAAGAGACAATCGACTCCTTGTGTGATGGCCAG
ACCGGCGAAGCGGTGCGACAGACTTCATCGAAACATCCCGCTGTACTCATT
TATGGCACCCGCGATGAGTTTACTGGCGCTCAGGCCTATCGAAAGCTTCGG
GAGAAGTTGCCGAGCTTGCATATGGTCGAAGTCGAGGATGGAGGGCACTT
CTATCGGTCCGAGCAGGAGGGTGCTTCTCTGGATGAAGCTCTGCTAGACTG
GCTAGGAGCTTGATGCTTGATGCTCTCCCCTGGCTCCTGGACTGGGAGGTG
ACATCCGGCGACAATCAGATTAGCCCAGTCCCCCACCCCCGAGTCTCGGGA
AATTAGCCGAGCCCGCTTTCGCCCGTTGTCATCCCCGTTTTGTTCCCCACCT
GCTGCTACCTAGCAACTGCAATTATACCACTCGACAGCTCGAAGAGCATGG
AGCGCGCTATGAGCCTGCTCACGAAACGTAAAGCGAAACTCGCAGGCATA
CTCCAAGATGAAATCGATCAAGATACAATGCGGAAAGAGCGACATAAGGA
CGGCATGCGGAGGAGGCGGGTGCAGGAGCAACTAGGCTTGAGATTTCGGA
CCGATGAGGACTGGAAGTTGTGGAAAGAACAAGAGGAGGCAAAATTGCG
ACTCTGGGCCGAAGCGCCCTACTCGATATACCGGCCCGGGCAGAGACGATG
CTTCAGCGACCTGCCCGAGGAGGTTCTGGCGTTGATCGTGGAGATTTCAGA
GTGGCCCGCGATGCTGATGCGTGTGAACCGGGTGAGCACGGCTGGGCAGG
CACATCGTCACATATAGCCAAGCTCACCCCCAAGACAATGCTCAGAGTAGC
GGGTAGAGTAGTCTACACGTCCGGCGCGATCCCGACGTCCAATCCTCCTCT
GAAGGAGTGGGCAGGGGCGGACCTTGCGGCCCCAACGCATATCAAGCCGT
TGGGGGAGACCTCAAGCGGTCTTTGCTGGGGTATGTCGAGCATCTCAAGCT
GAGAGTTGACATGTGGTGCGTGTCTTAGTGCCCGGATGTTGAATTAGTGTC
GGACGAAGGTGGGCTGATGCATCAGGACGACCTATCAGGGCATATTTCTCT
CGAGGGTACCGCAACAATACGACCTGTTCCCTAACCTGAAGACACTCGTCA
TGTTCGGACCGCCTCACGGGCGTCATGCGACGCCGATTCGGACTTGCAGTG
TCGCCCAGTTCTGCAGCCGCGTGTCCACACAGCATGCCTGCGTCACCGATC
GACTTGCCCGGGGATTGACAACGCAAGGTTACGACATCACGGAGCTGTGG
TTCTCCACCCCGCCACAAACATTCACCAGTCATGATGTGCGTTGCAGCGTG
ATTTCCGATTGGGAGGAAGGTAAGCAGGGGACCGGTACTGCCGTGTTATGG

GACTCTCAACGTCATCGAGCTATCTCACCACGCCGGCTACAAACGGCGTGC
CGGCGATGATGACACAGCCATGAGCAGCGCCGATGACGAGCACTACGATG
TGGCGAGTCATCCTGTACCCTCCAGTTTTCGATTCAAGGCCGAAACGACAC
AAAAAGCTCGTCAAATGGCTGTGCGATATCCTTGTTGGAGCTCTACCCGAG
CTAGACGAGGACGAGGAACCTGAACCTGTCTCGCCCGAAGAGCAGGCGCT
GCGGGACAAGACGACATGGCGGTTCGTCTTGCCGCCATGTGGAACAGCGT
CCGACCAAGGGGCCTCAAAGAGTCCGAGTACTTCGAAGGGGAGATCTTGA
ACAGGTTGCCGAGCATGCGGGGTCGTGTGACGTGCAGTCAACTGGCGCCC
GGGCAGACTACCGAGTGCCTGGCGTGTCTGATCCAGAAAGCTGAGGGTCC
AAGTCGAAATGAAGCGGTGGAACGTCAGGCATGGCTCAGATAGTCTGTCC
TTGCGAGTGCTCTGCCAGTACGAATACACTGCATGCTCTGTACGTAACAGA
ATACACTGCTATCGCTTGTCTGGAGGCGGAGGGTCCCGCCTTGAAAAACGC
GTCCGATCATCTGGAGCGCCTCGGTAGCGTCGACCATTCAATCATACTCTGG
TTGCACTCTGGTCCTCGACCTCGGCCCAGTTCGTCATGCCTGCCTTGATGA
CTATCGAGGAGGCGTCGGACACGTGAGCTCTCTGATCTGGCTCGAGCTTTC
GACGAGGAAAAGGAGCAGTATGAGAATAAAATACGGGACGATGAGCGGAG
GGAGCGGGTGCAAAAGAATTTCAATCTGCTATTCACCAGCATTGAGGCATG
GGAGGCGTACAAGGCGCGCGAGGACGACCTTCAGCAACGCTATGCGAGAC
AGAGCTTCTCCACACATAAGGCCGGTCAGCGACGACATCTCATGGACCTGC
CTCTGGAGGTTTTGAGCAGGATCGCAGCCTTGGTCGATTGGCCGGCTACGC
TGATGCGCGTCAACAAGGTATGATCTGCGCAAAGATTTTGTGGGGAGAACT
GGACGACAGACCCTACTTCACGTTGCCGGACCAAAGCTCTACTCGTCGAG
CTGGAATAGATTATCGCAAAACGATCGGCTTGCATACACTGGCATCGAGTTG
ACTTCGACATCGCCGGAGAAGCCGTTCGGCGGGGATCTCAAAAGCAAACT
GCTTGGATACGTTGAGCATTTTCACCCGCAAAGAATTGGTGAGTCTGCGAT
AACTACGCTCAGAAGGCAGTGTATCGTATTGAGCGGATTCAGGTCGAGATC
TCTCAACGAGATCCTATCCCAATTGTCGCCCTCTCGACTGCTTCTCCCGAAT
TTAACGACGCTGGTCATGCCGCTAAACGTCACCCCTTCAGTGATCGCAACC
TCGAGTTCAACCATCCGCTTCTGTGCTCGACTTCGAGTGCAGCATGTCTGT
ATGCCCTTCTTCCCCCTCTCACCCCTACGAGCTCGAGAAGTGTGGTTCGCC
CGTCCCCCAAAAACGTTCTTGGTTCACGTGAGAATCTTTCGTGATTGCCCT
ATGCCGGCTTTTGAGTTGGCCGCACCACTCTCGGTGTGCTACGGGGCTCTC
AACATCATTCAGCTCGCGGAGGACGATGTCCTAGGCCGCGACTACAGCGA
ACCGGGTTTGTTTTCTGGTAGCTGGGACTTCGCAGAGGGCAGCAGTCAGA
AGGACAGCCAGTTAAAGGCCACCCAGGGATTGATAGACTGGTTCTGCGAC
ACGATCCGCACCGCCCTACCGGAGCTGGATCCCGACCACGAGAGCGACCT
CTTTCTGCCCGGCGAGAAGGAGATGCACGACGAAACCAAGTGGCGATTTG
TTTTACCGGAATGGTGAAGAGCGGGCAAGACGAGAGTGTGACGGAGCAA

GAGTACTTTGAGGCGGAGGTTCTGCGCAGACTGCCGAGTATGACGTTAACG
TGCTTGACGACGTCGGGGCCGGATACCTGGGAGTAGTCCTCTAGCAACTTG
CGGATAGGGTCGTTAACTTCAATCAGGTCATCTACATACCATCCCTTGTTTC
TCTCGGGAAAGGAAGTCTCGTTTGTGGCTGCCATCGTAGGAGCTTCGTAAC
GTGTATAACGAGGTTTACAAGTGTGAGAAATGTTCGTAGACGAGTGATGAA
ATTAGAAGAAAATCGGCGAGTGCTCGTCGCCGACTCAAAGAAACCACATA
GTTGAAGCCCTTGCATAAACACCACATAATTCTTTGTCATAAAAAACATTGT
AAAAAAATATCCCTTCGTAAACTCGGGGTACATGATTCCTCTAAGAATATAA
CATCCCTTCGTAGGTGATGAGGCTGTGAAACCATAAGGCGTCGTTACCCGA
GAACGGCACAGTAGTACCGGAGTAACTTGAGTTGTTATATATGACGATCGA
AGGTTAGTAGTTTACCTACCTAGGTACCTATACTTGCTATCGTATTGCTTTCG
GTTCCGTAACTGTAGCTTTTAGTCCACGTATAGGAGCCGTAAGATACGTGAA
GTTTAACCAATCTGGCCACTAAGGATCGTCTGATCGCATCAATAAATGAGCC
AATCTGCATTTACGAGTCTTTAGCGACCCGAGACATAAACGGAGAGGCCAA
CAGAAAATGAATGGAACGGACCGAGCCATTCTTAGTCATAAATTCGATCCG
GCCATCGAGGCAGTCTCTTCGTTCTTTGTTGTGAGAAGGAACTCTTGAGGT
AACCTACGAACTGGTTCAGTACAGATGCCTATCAGATCCGTTTCGATATTTC
AAAGTTGCCTGTCAATGTCTTAGATCAGCAGCTGCTAAATGTGGCAATTCC
CTTTGTGCAGTTATATACTTAGTCCTTCTAAGTTGTTTAAGCTTAAAATGCC
ATGCTTACATCCAATCGACATCAGTTAGTTAGTAAAACACTAGGTACGATTC
TATGGTCAGATTTGATACCTAGAGAGTCAAAATTCGACAAAGTGAATTGTCT
TTCTTGAGTAATTCTCCATGTGATAAAATGCCGATGGTGGTTCCGTGCGCAA
ACGCTAGAGAGATACTGCTGAAGGGAAATACGCAAAGGGATAGCTGTGGC
TAAGTGGCTTGCAGGAGGCGTTGGCATATGATACTTTCGACATAAAACACT
GTTCACAGCATAAATTATGCATCGTGATAATTATGATGTGCATTGTAAATATT
TGAACTGCTTCAAATCTTTCAAGGCAGTAGCAGCTTATGAGTTAATAGTAAG
TAGGTAGTTACCTACATATTGATATCCATGGATGTTATCCATCGGGTCCGAGA
AGTTGAAGCTGCCTGATCACGCGACGTGAGGGGTTAGATTTATGGTTATTG
CAACTTCTAATGAATTTTATTTGGTGCCAAAGACATGTACGTATGATTGAAC
TGATTGAACTTGCCATGTGTTAATTTTAGACAGGTGATGTTGGAAGGCTCG
ATCGGTAAGTATTGAAAGTTACCCGTGGACGAATGTCTCAGATATATAGGCA
CATAGATGCAAGCGATGTCACCTCCCAACTTTATTGATTATCACTTTCTTCAT
CACTTTCAATGAGGCTCATAATCTCCATAATTTCCTTTCGTCACGTTGATTTC
TCCTGTGACTCCTGGGAAAAATATATCATTTTGCTTTTTTTGGGTGGGTTAC
ACTGGTCTAACCTGGATCAACCATACAGCCTACCTCAATGTCTGCCCACCGT
ACTCTGCCTACCTATCTGAGGTCCGGCGCCTACCTGGGTACCAAAAGGTGG
CTGCCAGCCCTCTATATTGCAATGAGAGCCTCGACCGAGGAACTTTACAAA
AGACACTACATATTAGGTAGCTAGATGTTTCTTCTATGATAACTGAGCCAGG

GGTCCTATATTACCCTACCTGTCGCCGAATCCACTTCGGGTTCTTCCAAATG
CATATGCATGTGTAGGTAGCCTATGAAAGTGCCGGTAATGTCTTCCAGCTTT
TCCCAGACCGCGCTGGTGATCGCCAGGGCCCTTAGTCCTCTTCCAAGGTAC
CCGTTCAATCCAAAGAATGACGATACTTCCAAAGAAACCGGCATTCTTAGG
GATCTAAAGTGTTTGAGCATTACAGACTTGAAGACGTTGAAAGATGTCCTG
GTTCTTCGATAACTGGGCAACAAGATGACGATACTTTCTTACAAGAACGTC
TCGTCAATCTACTAGCCAAACTTCCACCTCATTCCCATTTATGTAAACAGTC
TACCGACGTATTTATCAACTCATTATGGGGTGTACTCAAACATCCTCCCGCT
CAGATCACAAACGGGGGAGCTCCCTATCGCGAACCAGATGGCTCAAACAA
CAACCTTGATCTCCCTACCATGGGAGCTGCCAACACAGCCTACGCAAGAAC
GACAACCCCTCTATACTTCCAAGACCCAAATTTACCAGATCCCGGGGTTCTA
TTCGACAGCCTTTTGAACAGAGGAAGTGGAGAGTCATACCGGACACACCC
CAACAAGATATCTAGCGTGCTGTTCTATCTTGCTACAATCATTACCCATGAC
ATATTCCAGACTGTAAGTTATCCAAGTTGGAAGTTCCACCATCTCATTCATC
ATCTAAGCATTTCTAAAGGATTCTCAAGATCAAGAGCTTAACCTAACGTCCT
CTTATCTTGACCTGTCGCCCTTGTATGGTCGGAATGCCGAAGAACAAAACC
TGATGCGAACATTCGAGAACGGCTTGCTCAAGAAAGACTGTTTCTCGTCGA
CTAATCCATATGGATTTCCCCCTGGGATAGGGGTGTTTTTAATAGTGGGTAC
GTCTGATGATATGATTTGAGTTTTCGCTGACTACAATTCGTTTAGATGTTCAA
TAGATTCCACAACCATGTTGTAACTCAGCTCGCAGCGTAAGTACTATGATGG
GGATAGGGGCAGGACTTTAAAGTATATATGTATCTTGGTATGTGCCGATGCT
AACACGTATAACAAGGATTAATGAGGGAGGCCGTTTCACAAAGCCGATCCC
TATTAAGGATGGCGGGAAAGAGTGTGATTGGAAGAAATATGACAATGATCT
GTTCCAAACTGGACGACTCATCACCTGTGGACTCTACGTCAACATCATTCT
GAAGGATTACGTTCGGACGATCCTGAACCTGAACCGCACGAGCTCAACTT
GGAGCCTTGATCCCCGGTCACACGAGAAGAAGAATGCATTCAACAGCAAA
CCGGCTGCTGAGGCTACAGGAAACCAAGTCTCAGTCGAATTTAACCTTCTC
TATAGATGGCACAGTGCATTATCGAAACGGGATGAGAAATGGGTGAATGGC
GAGTTCCGCAAGATACTTGGGGGTACTGATCCCGCTAAGGGCTGCCTATGA
GGAAGTGTTGTATGCTCTGGCTAAGTTTCGGGACAGCATGCCCAATGATCC
AGTTGAACGAAAGTTTGCCAATCTAATACGAGGCTGTGATGGCACATACTC
CGATGACGATCTCGTTGAGATCCTGTCGTCCTCCATCGAGGATGTCGCTGG
CGCATTTGGCGCCAACCAGGTCCCTAGCGCATTCCGAGTAATTGAGATCCT
TGGGATCAAACAGGCAAGAAATTGGCGTGTGGCCACATTAAACGAATTTCG
GCAGCACTTTGGCATCAAGAAACACACGACCTTCGAGGAAATCAACCCGG
ATCCGATTGTTGCTGCTAAGCTGAAGGCTTTCTATGACTCGCCTGACTCGGT
TGAATTATACCCGGGCCTTGTTGCCGAGAAACCTAAGCCGGCGATGGATGG
TAGCGGATTATGCGTGAACGTAACTACGAGTCGGGCTATTCTATCCGATGCT

GTCGCACTTGTCCGTGGAGATCGATTCTATACCACAGATTACGTCCCTTCAA
ATTTGACGAACTGGGGTTTTAATGAAGTCCAGTTTGATTTAGAAGTTGATCA
AGGACAGGTGATACACAAGTTGATACTTCGTGCGTTTCCCAACCACTTCGA
GGGTGATAGTATTCAAGCACATTTCCCCTTCGTTATACCTTCGGAGAATAAG
ATTATCCATGATAAACTTGGGACATCCAAGCTGTATTCGGTAAGAATACTTT
ATATATTTTGATTCTTCCTCACCCTGCCCCCTTTTTCCGCAAAGCTTCCTTCC
GGGTTACTATTCAGAGTGCTGATTCATACTATGACAGTGGGAGAGACCAAC
GAGGAAGTTGGATCCCGTGATAATCAAATCCCACACGGCTGTCATTGAAGT
CTTGAACAACAAGACTGACTTCCTCGTTCCCTGGGGTGGAAATATCCCCCC
CACCTGCTGGTTCAACAGAAAACCTTCCACTGATCTCTCCAACGGCAGCCC
GACCCACGATCACTTACTGTACCATCCAAAGAACTGGGAGTCAGAGATCCG
ATACTTCTTCGACCAGACAACCAAAAACCTCCTCCAGAAAGCTAGCCAGA
GGCTATCTCCAAGCCCTCTTAGAGCTAAGAGCGTAGAAGCAGAGTTCGAA
GCCGACATCATCAGGGACGTCATAGTCCCGCTCAACACACGATTCATCGCG
GAGTTCTTTGCGCTACCGATCAAGACAGCAGAGACACATCCCCACGGGATC
TACACGGAGCATAAGTTGTTCGAGTTGCTGATGGCCATGTTCTCAGCTATAT
ACTTTCACAATGACCCCGTAAACTCGCTCAAGCTGCAGAAACGGACTCGC
GAATTTGCGAGGGAATTGGAGAAACTTGTCAAGGTTGAGGCGAACCCGAA
GGTCAGCTGGATTGGTAGTATCATCCATCACCTAGGATTCGGAGGCGCTGG
AAGAAGGGGAGCTCACGTCGTGGGCGGGCAACGTTGGCCCGGGTTACCGA
GTTATGGTGTGCAACTAATTTCCCGGGTGATGAAGGATAAGGAGAAAGCGA
CCGATAAATATGTTACCGGGACAGTTATGCCTATCAGGTAAGCCGCGGTTTT
ACTGAGTGATATACCATACTAACGGACATTTGCAGCGCTGCCGGAGCTACG
ATAATGTCGGGTATGCTTTCACAGTGTATGGACTACTTTCTCGGGAAAGGGT
GCCCGCATGTACCTGAAATCCAGCGTCTAATTCGTGAAAATACGGCCAATTC
CGATGAGATTTTGTTGCATTAGTAAGTCCTATCACCATCGCTCACAAATATG
CTTACGTTCTAATCACTAGCAATCACCAGCATGCTAGAAGGCATCCGCCTAC
ACTCCACGATCACCCTCTCGCGTCTCGTTTCCCCAACAAGCGCTGCCCAAA
ACATTACCGACGACGAACCTTGCGTCGCCGACCCATACGATCCATCAAGCC
CATACCCTATTCCGAACGACGAATCCACTCGTGCCTCCTCGACACGCACATA
CACCCTCGAACCGACCCAACGCGCCATAATCGACCTAACAACCGCATCCCA
CGACGACCTCGCCTTCCCCTCCCCCTCATCCCTGCGTCTCGGACCGCCCCC
TCTCCTCCTACCCCTCCTACCTAATCTGGGACGACCACCCCAGCCAAGACC
TAACCCGGATCGGTCTCCTCTCCGCCTTCAAAGTCCTACTCTCCCTCCCCGA
CATCCGTCGGGCCCCGGGACCCCGCGGCGAGATAAAGAGCGTGGTTTATAA
GGAATGGCGGGGCCAGGTAGACCGTGAGGGCGTGCCGGGGGGAGGCGAG
GAGTGGACTGGGTTGAGGAGGTACATGACCACCGATCAAAGCGCGTTTAC
GGTTGTCCCGAGTAGTATGCGTGTTCGTTGGGGCGGGACGGGGGCTGGGG

ATGATGTTAATGGGATGGATTAAAGCGGGAGTAATAATACGGATGTGTACGA
GTGGTGTAGGATATAAACTTAGGACTTGGAAACTGCGGTGTTAGGTAAATT
GTAAAGGATAGGTATTGTATGGTACTGGGTTGGGTAATAGGAAGAATACTTA
TGGAGCTATGGAAATTGTAAGATGGTTAGGAAGGTTATTAATCCGCTGGGTG
TTCTGGAAGGCGTATTGTTTTTGTTAGCTAATGGTTTAATACACTCACTTATC
CACATCATATCAATCCACGTTAACCTTGACAAGCTAGACACCATAATGGTTT
GGCGATGTAATGTATGTTCTTTTAAGGTATGTAAGTAGGTAACTATACTATAC
ACATTCTTTCCATGCCAAAAACTCTTCTCTAGACAATAAGTTCTAGGACTTG
CGAGTCTTTCAATCGGTAGGACTATCGTTTCTTTTCCTTTGCACAAACGCCA
ATCTAAACCGTGCGCCCAATCTATGTATATACACTACTACGCCACGCCTGAG
AACCCAGGAATTGTTTGTTATGAGACGGTTTTTACTCAAGACCAGAAAAC
CTCCGACCCTCGTCGCTACTACTCCCCGTGGTCGGTTTCGCGGCACCGACC
CGGATACCCCGACCGCCTCTCATAGAATCCAGGCTAAGGTTGCTAGCAGCG
CTGTTACGCCTGCGATAGTTCTTTTTCTGCGCCGTTGGGCTCGGGGTCATAG
GGTCGGGCGAGTCGTTGGCACTATCTTGACCTACTATTGTCAATGTTTCGGA
CGAGGGCGATGCGACGTTTTCTACGATGGTATTCATGGGGTGGTCCTGGGT
ATTACTTTTTGCCGTCTCCGGAGATTGGATTGGTTCCGTGGTGAAATCCGAA
TGATCCGCGGGGACTTCACGCGCTGTGTGGGTTTCGCCGGTGCCCTCCCGA
GCTTGCCCATTAGGTCCGTTAGATGCCATGGAAGTCGAGTCTGATTCGGGC
GTGTCGCTATTAAAACGAGCGCTGCCATGATTTAGCTCTCTATTTCTACTGG
AATATTAACGATGAGGTAAACTCACTGTTTTATCTCCTTCGAAGCTGATAAA
CTAACACTTCGCGCCCGCTGATAACTCTCAAATTGCTCTACGGAATGCTCCG
CACCTGTAAAATTCTCTAAGGCATCAAATACGTGCTGGAAAACCTGAGCAT
CGACGTCGCGAGCCCATAAAAAGTCGAGGTGCTCATAGTGAGGGATCTCA
GTAGCGACCGTCCGATTAGGAAGTTCTCTCATCATAACCCTGATGTCGACTA
GGGAGTCACTGCCTCCGTATACCAGAACAATAGGGGTCTTGATATTGCGGG
TCGGATACTTGGCGACTTTCGTGTACTTGTTTAAGCCATTCACAGACAGGG
GCGTCTGTACATCGTCGTCATACATCTGGAACGATTGATTGCGAATAATCTG
AAACCAATGAACGACCGACTTGGTGCTGGTGAACGAATATAGGTGCGGGT
AAGCGGCAAGCTTCTGGCTGACGGACAAATTCTTAGTCTGCCATCCGAAGA
GAAAAGACAATCCCATATCTATAACTTTGATGAAGATGGGTGGGTATAGTAT
AGACTGCCACATCGTAGCAGAGCTCAGTATGGAACGCCTGCCAAAGAGGA
GAAATAGGACCTGAGGTGAAGCCTTAATCAGAGCATCGACGATTCCATTGT
TTAAGCCGGCCGGCGCCATGGCCGGAGCCAATGCGATGAAAACATTGACCT
GTTCATTAAGCTTGGGGTGTATGGCCAAACTAGCGAATGCTTGCGCAGTTC
CCTGAGAGAAACCAATATATGACAAGGATGTTTGTTTTGTGGTTTCCAAAA
CGTAATTAATGGAGTCCGGGATATCATAGAATGCAAATTCGTCAATCGAAAA
ATTCCAAAATTCGGTAGAAGTCGGCGAATATTTTGCTGATTTCTTGGAGTAT

TTGTTCCCACGATTGTTGCCCAGCTAAGTTCTGTTGTCAGTATACCCATCTT
AACTGTTGCGCGAATCTACAATATTTTCAACATACCCAAACATCGAAGCCCT
TTTCTACCAACACAAAAGGTAGGGACCTTTGCGCATCGGTAAGACAAACCC
AAACTTCGCTGTTCATCAAAAGCCCGTGGTGGAGGTAGACGACGCGCTTCT
TAAGACTAGCAGGTCCGTCGTTGACCTTCATATCTTCTTCTCCCTTTCTCCAT
GCTAGGCGGTGTATACCGAGCAGGTACCCATCCTTAGTTTGTAGTACATGTT
CCTCGGCTGTATAGCCAAACATGGCGCAGAGGTCGACGAAGTCAGAGGCA
TTTCGAATCGAGGTCGAGATAGCTAGGCGCAACGAGTTAGTGTGGTTGGTA
GATTGGGCTTGAGGACAGGCGAATCGTACATTTCCTTCTCTGCTCCGCTCTT
TTCTCCGCTTCGGTGGTTAATCTATTAAATACCCGTCGCGAGGCTCGGTAGC
AGAGATTTAAAAGCGAAGTTGCTGGATATAGGATGTTAGCACGTGGGAGCG
GCGCGCATAGTGGCATTGACATACGTAAAGCTAGTGTTAATACTCGGACGA
AGGCCTCGAGACCGACGAGTAGAAACGAACCCACAAGGGCAAAATACTC
GGCTCTGTGCATTGCGTTAGAATTCTTGAAGCGACAACTAGTTTCTTATTGG
TATAGAGCTACGAACATGTCTAATCGACCAAGAAATGGCAGGGGCATTTTG
GCCTTATTCAATGTTATGATTCGCACAACACTTAGCTCAAGAAGGGTAGAA
GCGGCTCGATGATAGGTTAAGCGCTGCGCTATCCTAGCGAAGCGTTCATTTT
CTGCTTATTTGATCCTTGGTCGAGAAGATATATACGAAGGCGAGAATAGTTA
TAGTTTCTAGAGAGGGTAGGCGTTCCGATTGAAAAGAATCATCCGTCGAGG
TGTATGGTGTAGCTATTGGTGATGTCACATGTGAATACGACTGTAGGGAGCA
CACGGTGAACTAGCTACTGAGCTATAGGGTGTTTCAATATACTGTGACAAA
AATGCGGCCTCAAATTAGTAGGAAAAAGAGAAGAGAATTGGAACTAGAAG
TTGTAAAAAGTGTAAAAAATCGAGAATAGTCTCAGTAAAAGACTTTGAATA
TAGTTTAAGAAAACATAATGCTAAAATCATTAAGTTGAATAATAATATCCAG
ATTCCAAGGTCCTTTCCTCATTTCATTTCAAACCGCTGGCGAGAAGCTCCTT
TCTTTACATACCGCCCTTGTGCTCCCCACTTATGATATCCAGGAAGGTGGAA
AGCGAAATAGAACTATGATTTATGACATCGGCTAGCTACCCCTGCCTTAAAA
TGCACACTTTATACAGGCTTATGTCCGCGAGCTAAAAGATTGAACCAATCG
GCATATATTTGATTGTGAGCTTGGACCAATTCACATTTCGCAACTTTTGCGA
CGGCAACATCTGATTCATCCCGCCTGACCGAATTGGTGGAATCGTGATTCGT
GACTCTCAGTCTTTTGGTTCACAAATCCTCCAGCCCAACTCAAGATGCCTA
GCTCGTGTAAAGAGATAAGTATGTATCGATATAAATTTACCTAAGCGCGGGT
TCCCTCGTACGCATATTCAAATCCTATACTGATAGCCCAACTCGTATAGGAGC
CGCGCTGGCGGATTGCCTGCAACAGTCCGAGTGCGTGATGGTACAACGAC
ATACAGCAGCCGAATGCTTAAGGTCGCCTCTATCCGAGGACTCTCCCGACG
AAATGCCAACAGCTCAAGAGGGGCTTCGGCGAGTGTAGGAGGGGTATGAT
CGACATGCGCAAACGGTTCCGTGGCAACGAGCCCATAACATTTCAAAAGCT
CGAGTCCACCGAAAAGTCGGGCGACGGATACCAATTATACGCTGGTCGATC

AGCGTTTGCTGGCGGCGTAAGGAGAACCGATGGGAACGAACCAGAACCCA
AGGACTGGCGAGAAGTGGAGAATGAGAAATTCCGGAGAGAGCAGGAAGC
CCAAAAGAAATGATCCAAGTTGGTACTATGCATGACAATTATGACAATAGTA
TTCTGGATGGGTGGTCGAATATAAAAAGCATTGGGTGGCGTTGGAGTTGGA
TGGGTATTTCTCGATTCCTCTTCATGGGATTGACGGCTTATCACCTTCAGGC
ATGGCTTACGATCAACTCTTCTCAAACGGTAAATTATTTATCCTGTGATGTCG
ATCAACCGCTTCATCCTCGATTATTCTCGACGATATGGATTCTCAGACGCTG
TCTTGACTTCGAGATCCTGAATGTCTCAAACTGACTAAAAGCTCCAGTTTC
GTGCTATGCGGTGCGACGATCTCCTGTCTTTATCAAGAGAGACGCCTAATCT
CCATGTTTCTCTGAATTCTCGAATAGAAACTGGAAAGACAAGATTTATAAAC
CCGAAAGAAATTAGCTTCAATACATGATCAATCCAGGTTTTCTCCATGTCAC
GAGCATCAAGCAATGAAGTATCAATTCGAGCCTCGAGTTATTCATTAAATTC
AGTATACAATTAATTATTCAACTAACCACATGTCATACGTCCGTGTTACGTTG
GATAAAGAACTAGTTCGGACTTGGCGAAAACTCAAGATTCGCATATGACGA
AACCTTCCAGGTTTATTCGGGCTTTATTCCCGTCAATTTCTTAATTTACCTCT
CCATCGCTCCATCAAGCTCGGATGCGGTGTCAAGTATGACGATGTGTGCAA
CTATATAACCCCGGCCGGATTGATATCCAGTCTATTAGCAGTCGCGTGTCGA
GGATTCACAATATCAACAATTTGACCCCTAGCTCTATCTGCTCCGACTCAAT
CATAACCTCGTGAATACACTCAAGGTCAAGTCTTCAAGTGCATTGGATAGT
CCAGGACGAAAAGTAGCGGTATCATCAACATACCTACCAAACCAACTCTAA
TCATCAAGCCCGGCTCTCCTTCAATGGGCCGTCTCGACCCTCCGCCGTCCG
TAACTCAGCGGCGGAAACACCAGCGACGCCAGCTCGTAGGCAGCAGTATT
ATTCTCGCGATGCTAGCCCTTGTCCTCCTCTCCTACACACGCCCCTCTATAA
CGCTGCCGACAATACTCAAACCAATGGCACACCCCGACTCCGTCCTTGCCT
CCCTTCCTGACCTCACCCACCCACCTCTCCTCGCCAATCTCCCCTCCTCTGC
GATCCCGAAACCCGGCAGTCCGGGTCGCCTAATCGTTATCGGAGACGTCCA
TGGACAACTCTCGGCCCTCGATGCCCTCCTCGCGAAAGTCGAGTTCGACGC
CTCGCGAGGCGACCATGTCGTGTTCACGGGTGACATGGTGAACAAGGGCC
CGGACAGCGCCGGCGTGATCGACCGCGCGATAGAGATCGGCGCCTTTTCCG
TGCGGGGGAACCACGAGGACCGCGTATTGAGGGCGTGGAAGAAGATGCAT
AAGAAGGGGAAGAAGGGTGGGAAAAATCACGCGAAGGGGGTTGACGAA
GAAGGAGAGGTGGAGGTGGAGGGAAGTAGTTCAGGAGAAGGAGGAGAA
ACAGCACTGGAAGAAGGAGATGGAGAGATCTCGAAATCCTCGGCCCTCGA
CCGCTCCACCGCCGCGACTTTAACCCCGGCACAAGTCTCCTGGCTCGCGAA
ACTACCCGTGATCCTGCGCGTCGGCACCGTATCCCCATACTACGGCGACGT
CATCGTCGTGCACGCGGGTCTAGTCCCAGGAATCTCACTCGAAGAGCAAG
ACCCGTGGGCCGTAATGAACATGCGAACCCTAATCTCGCGCTCCCACTCGC
ACTCCCTCTCCACCTCCGACCCCGACTCCTTATCCCTCTCGCTCGACCCTCT

CTCCCTCTCTACCGCCAAACGCCCAACCTTCAAACCAAGCGAAGGCCGCA
AAGGCCCGCCCCTGGGCCAAAGTCTGGAACGAAGCGCAAAAGCAAAACG
CCAAGACCGAAAAGAGCGGGAAAGGCCACCATAAGACGCACAAACAAAC
CGTAGTCTACGGCCACGACGCGAAAGTCGGGCTCAGCGTGCGGCGGTACG
CGTTTGGGATCGATAGTCGCTGTGTCAAAGGCGATAAATTGACGGCGTTGG
TTTTCGAGCCTTCTTCCGTTTCGAAGTCTACTGCTGGTTTGACTTCCGCGTT
TGAAGATGGATCGGAATCTGGATCTATGTCTGGTTCGGTCTATACGAGCGAA
GAAGAAAAAGAAGAAAGGTACGATAGCAGCAATGGGGGTGATGAGGCGG
AAGACCTAGCGGAGATGGCGAAACACGGTATAGCGCATCGTATCGTCAGTG
TGCGCTGCAAGGAGTGGTCGGATGATGATAAAGGGGGTAAGAAGAAGCAT
AAGCATCATAAGGATAAGGGGAAAGAGGGGAAGAAGCATAAAGACCAAG
ACTAAAGGGATGAGGAGATTTATACATCCTTTACTACGAGCAATCGGGGAA
GATTGGGGTTGGGGTTAGAACGGGCTTGGATAGAGGATGAGATGCACGATT
CTTGTTTATATGATGTTTAAGTATTTGCGATACCCAAGTCTATATGGAGATTT
GCATAGCGGTTACAATTTACATTTCTATACCTTATCCATTTATACCACGAATGT
TATACGCATGAGAACTTGACAGCACCTCATAGGAGCAAAGTGATATCGCAT
GATAAATCAACCGTGACATTTGTCAACTCTAAATTTTATTTATTTTCACTGGG
GACACTCGGTAGAACATGCCAACCTTTATGTTCTACAGAGATCCATTACCAA
AGACGTCCCGACGACATCCCAGAAACGAGAGGCTCAGAACTCATGCATTC
TAGACTGACACAAGCAGACAGCCCAGTTACGTTACTCAATGCACAACGAC
ATCCCTCTCCCTATTACCGACATGAATAATTTATGCAGAGATCTTGCTCTTGA
TGATGTCCTTTAGGGTGACGGCGTCGGCGGCGAATTTGCTGATACCCTCGC
GGAGCTTCTCGACGGCCATTTGCTCCTCGTTGAAGTCGAATCGGAAGAGCG
ACTCGTCGCTAATATATGTCTTCTTGACGATGTCTAGGGAAGAGGCGGCAG
AGGCGTCGAGCTTCTTGGGGACAGCTGTGCTCGAGTTCAATAGCTCCTCGA
GTAGGTTAGGCTATTAAGTCTAGTTAGTAACATTCTACCGAAAGAACGTCAG
TTTTGATAGACAGGGACATACGGAGATGGTTAGGTAATCGCAGCCAGCGAG
TTCAGTAATCTCGCCAGTGTTTCGGAAAGAAGCACCCATGACGATGGTCTT
GTAGCCGTACTTCTTGTAGTAGTTGAAGATAGCCTGGACGGACTTGACACC
GGGGTCTTCCTCCTTGGCGTAGTCCTTCTTCGTGGCGGCCTTGTACCAGTC
AAGGATACGGCCGACGAAGGGGGAGATCAGGAAAGCACCGGCCTCGGCG
GCGGCGATGGCCTGGACGAGAGAGAACATGAGGGTCAGGTTACAGTTAAT
GCCGTGGTCGCGCTGTAGGATCTCGGCGGCCTTGATACCCTCCCATGTTGA
GGCGATCTTGATTAGGATACGGTCCTTGGAGATACCTTGCTCCTTGTAGAGC
TATAATTGTTGCATTAGCAGGCCGTAGTTCAGGATTATTGGAAATCAAGTTT
AATTCTCAACAAGAACATACGTCGATAATGTGAAGGGCCTTGTCAACCGAA
GCCTTGGTGTCGAAGGAGAAGCGGGCATCGACCTCAGTCGAGACCTTGCC
GGGAATGATCTTGAGGATTTCCTTGCCAAACTCAACCAGGAGTCTGTCAAG

AGCAGCATCGACTTGGCTGTCGACATCACCACCCTTTCCCTTAGCATATTCG
ACGGCAGAGTCGATTAGCTTGGCGTATTCGGGCTTCTTGGACGCAGCAAGG
ATGAGAGAAGGGTTGGTGGTGGCATCTTGAGGCTTGTACTTTCCGATGGCT
GGTAATCTCGTTAGTATTCATTATTCTATATTCAAAACGGGACGAAGCAATG
AAGTTATAGACGAGACGAATTGGCCTCATTGCTGTCGGACCGAGGTTGCCA
ATGTGGCTTTGAAATGCGGGATATACTGACTTGCGAAGTCACCTAAAGGAA
TATTTGGTCAGCGCCACTTTCGTTTATGAGACAATGGATTCAGTCATAGCTT
ACCAGAGTCGCAAACAACGACCTAGATTACCAAAATACATGTTAGTAATTG
TTAAGAATGCTTGAATGTTGATATGTGATATGTTATTCGTGCAGTTCACGATG
TTTTGTTCGGTATATTCCTTAGCGACACAGGAGACTGAAGTCTCCCGACAA
AGGTCCAAAAGCTCACGATGGAGGGGTAAAAAAGGAACTCACCGTGCCA
GAGGCCTTGAGCTGGTCGAGAGAGCTAGACATCTTGTATTCGTGATATTAG
GTTATTCTGTGATAATTATGTAAATATTCAATTTCAATAAGGGACAGCTAGTT
TTCAAATGTAGTTGTAAAGTCTCAGGTGTTTGAGGTTGAAAGTAGCAGGTT
GTTGGGGAAGAACGGTCTATTATTTAGTATTGTACCTACATGGATACATGGG
TGTATTGTATCGGCTTTGCCTACGATGGCGATGGCGGGGCAATTTGCTTGGG
ATAGAGCGTCATATGCCCCACAGTACTATGGGGTACTCAGCAAATAGCCCAC
TTACACACTCGCATACGCTCGCTTGTACGCACTTGCATGTACATCCCCGTAC
CCGCACACTAACAAATAGTGGGAAGCACCTGATTTTCAGTCGTCGACTTTT
CTTTCCTCTTCGACCTGAAGCTCTTACATTTTAGCAACGACTTAGTGATTTAT
TAGTACCCCTCTATGGCTTGACCTGACTGGCCCCCGATTCATCCAATTCGCT
CTTCGCCGTCCCGTTGTTAGCCACCAAGCAACGGCAACTTACACCCGGAAC
AAAAAGACCGGAGATATCAAAGAGTCTTATTCCAGTGGCGTTGTATCATATC
CA

>Mosaic_sequence_48

TTACCTAGGTAGGTGATAAAGTAAGGATAAGTAAGGTACCATCGGGGCCAT
CGGAGTGATTTCTTGTCTCATTCGTCATTCGCTACATAACTATTGGACATATT
GGACCCTCCCACCCTAAGGACCCTCTGGAAGACGACGAGTCCTGTTTCTGC
ATAACGCGCCAGGACGCAAGAGCCAACATGGTCAAGCACTATGTCCGAAA
AAAGAAAAGCCGATGAACATTCGAGCGTTGTGTGTCGGCAGACGAAGCCC
CCCCAAGCCCACTTCGAAGCCCCCTATGCCATACTCAAATCGAGCTAAGCT
AAAGCTACCCAAGTACGGAGTCAATCTCTCAAGCTTTGGGGATCGAGCTCT
CTGATAGGAATGTGAACGCGAGCGGTGAAGCAAGAGTTTTATTATGTGAAG
AGGGAGATCAAACTAACTGCATGTGTTCTGAACACGTATATGTTGTGAAGG
ATAGAGACAGGTCTAACAGCTCAACAGTCAACAGTCAACAGTCCAACTGA
TCAGATACCCAAGTCCCAAGACCCCAGATGGGTATACCGACAGCGTCTTCG
CATAATGATAGCCCGCAAGACTTGGAGAATCGAAACATTCGAAACAACTCA

CACTTACCTAAGCCATGCAGATTAAACAGACTAAACAGAACTAAACTAGCA
CCGCTACCTGTTAGGGTCCTATGTAAGTGCTAGGAATCCATCGCCCACATTC
TTTTTAAAGCTGGACAGGCTAGTAGGCTAGGGCAAGCCTTGTCTACGGAGG
CTTGGCGGTCGAATCTCGACCTAGACCGTCCGTGGACCCGGCCGATTTACT
AATTGATGAGAGGACGCTCTAGGACACCCTGGTCGCGATGGGCGTGATGG
GGTTTCTAGAAGGATGAGAAAGCGTGGGAAAGGATGAATAGAGTATGTGC
AAACGATGGAGCCGATGCAGGCGCACGACAGGATAAGAACTCAAAACAGC
CTATGACGGCAGACAACATATGACACCCATGGATCAGTATCCATGGATGCCA
TGAACGTAGTATGCAGTGACAGGCCATGGCAATCAATGACAAGCAGCGAC
AAGCAGCGACAAGAATGATGATCATTGATGGTTGAGTATATTTCGTACTTTT
TTCGTACTTGCGCAGTGTCACCCCCTTCTGCCCCCTTCAAGACCCTTTTCTT
TTCCTAATTCAGTACCATACCAGCCTGCGAGCCCGCTACTCGTAATGCTATAT
TATATATATACTCCGGCTGTGTTAGTTACGTAGTAATAAAGCTGGCGCAGCA
ATACGAGTTCCAAAGTTCCATAGTTTTTTAACCCATCCACAATCCAAGCTC
CAAGCCCATCTTCCCGTCCTGCCCGTCGCAAGGACGCAAGGTTTCAAGATC
CGCCGGCTCCGCACGCAGCCCTAGACGCCGTCCCGACGCCTTGACGCCCT
GGCACGCCCTGTACACGCCCCTAGACGTACTCACATAAATCAGTGTCTACA
ACGATGAAGATTGCGGGAGCGACGGATGCGCAGGTGGTGGCGTTTAAATC
TTAATGAAGAGGCGGACGAACCGAGCAATTGGGTCTTGCCCTGCATATAAC
CGCACGGCGTAATTATCTAACCGACGGGTCCCTTAATTTATCTGGAAAGCAA
GACAAAGCATGGGATCCCGTACTTTGGCGGCGATCGATAGCCTTTGATTTCT
GCAGGTTGTACAGGTTCTGTAGGAATCTTTAGACTCCCAATTAGTATGATCT
CCAGGGCTAAGGTTACGCTGAATAAGACCCGTGAACCCCTGAAATTCAAG
GGGTCTCTCTGTTGGGGTCTTCTTGGGGGTGTGGTCCCACTATTATAAGGAA
TGATGAAATTATGACTAACGCTAAATAACAGCTGATAGCCGCCGATAGCCAG
CTGCTCCTAGCCTCCTACAGCCCTTTAGCCCTGGAGGGCTGTTGGTGCCCC
TCTATACCCCACTAGATCTTGAAATCTCACCATCTCCAGCATTTCCAGCATTC
AATCCTATACTCCTTGGCAATTTGACACTCGAAGGATCAACGGTTTGTCTCT
CAACCTCTTAACCCGTCACAGGACACAATACAACCAACTCGGAGCCCGCAT
ACTACCAGATCACACCATTAGGCTATTCGGATAAGCCGGATTAATCAGCAAT
GGCGTCTGCGGAAGAGGGTGAGGCGATACAGGCGCCAGGTATACTGGAGA
CGATCCACTTGGAACACGTTCCCGTCGACACACACAGTTCATGTCGGTCTC
TTCAAAGATGTAACCAACGCAGACTTCTTGCAGAAGCAATTACTAAGTCGG
AATTCCGAGTTTGAATATGCCTTCATCGATGCCACTTCTGTATGTTTCTATGA
ACTTCTGTCTTTTTACCCTTGGCTTCACCCATTACCCCTGTGACTCATTGATC
TTCTTGATATATCTCAACTGTATAGGGGCTTAATATTTATAGAAAACATTAGA
GTTAAGCAAGTTTCAATTTGTATCATTGAAACGAGAATCATATTGTAGACTA
ATGATTCTTATGAAATGAATAGGTAGTATCGCGCTTCCAAATCTTAACCGCC

GTCTATAAGGCTATAACAATACAGATGAGCGGTAACATGAAAACACCAAAC
GTCCACTCAGAGATCGTATGCTCCTTAAGTCCGAATAACAACGTATGTGTCC
TCCATACTCCATACACGGCTCCATTCCCTTACCTGTTTACCCTCATACTCTCA
TATACCACCCTTCACCTATACTAACGACCCCCAGATCTCCGAAGCCTACCGC
CGCTTCGGCATAACCGGCACAACCACCTCCCTCATCATCATCAAAGTCCTC
ATCACGCCCCCTTCCACCCTCACCCCCGCAGCCATAGAACAACACCTCCTC
ACCCACGTACAAGGCACGCCCACCCCCCTCACCGACGAGGTCTTGCAGTC
GTCTCTTACGGACTGGGCCAAGGTGCGCAAGTACTATAGCTGAACGGCGTG
AACTGGCTTGACTCGGTCAAGGACGAGGCGATGAAGCGGGGAGAGATGG
AGGTTTTGGTGCTGGAGGGTATGGCGTTGAGGGGATTATGATTGTTCTTTGG
GGGGTTTGTGGTCGTAGGTGCCTGCTAGGTACATGATATAAGGAATAGGAG
AAGATGAGAACTCTAGAAAGAATTTGAGAGATATCTATGAAAACCTCAGAC
GTATCTGAAAAGGGGGATCCATAGGATATGGCGGAGTTGCCATAGTGAAGA
AAGCGAAGACAGCTTCACAAGTAGCAGAGCACCAGGATCAATTACCAAAA
AAAAAAAATTCTCGAATTGAAGTAATTTTTTTCTTCAATTCCTAATGAAAAA
TCGTGGTTTTCTCATACTACTACTCGAAGCACCTCGATCTTCCTAGGTGGCA
GCAGCAGAAGGTATCCGCGTAATTACAAATAGCTCATATCGTAATTCATATCT
CGCGCCGTGCTCGACTCTACATGAGTCTTCAATAGCACGCGCTTTCCTCAG
GGATTAATCCACAGCACATCTTTTCATTCTCCCACTTCCTTCATCCTCCCCCT
TCTAAACTCCTCACCACTTACGCAGCAAACCCCAACCCCTCCCACCCTCCT
CTTAACCTCCTCCCACATGGCATCGCGTCCACTCCTCGTCCTCGCCACCGCA
TCCCTATTCTCATGCGCACGTCTCAACAAATACTTCATGCACTCTCCCGTGG
TCCCCCAGACGATATACTTATACGCCTTCGGCACATCCCCCTCCTTCCCCTTC
TCCTGCCCAACAAACCCCTCACCCCTTCGCCGCACACACGAGCTCACAAC
TAACCTCATCCGCCATCCCCTGCAGCTGCGCGATAGCGAGTTCGGTCCCGC
GGTCCGCGCGCGGACTATCCCTGATCGCGCGGGCCTTGCGCACGGAATCTT
GGTTATTGCGATCCAAGTAGGATACTCACGCGGGGGAAGCTGGTCTCCGGC
GCTGCGACAGGAGCGGGGAGAACAGAGGACCATTGCCGACATATAAGCGC
CTCCGCGATCCCGTTATACGCATTATCCGTATCGGCCTTAGTATCATGGATAA
TATGCCGGGGATCGGCGCCGAGGTACGCGCCGCGCACGAGCTTGATGCCG
AGGGTGAAGCCCTCCTTGGCCGCGGTGGCGAGGTGCGCGGATAGCGTTGC
GAGGGTCGATTTCAGGTATGCCTGGTACGTGCCGTAGATGATGGCGTGGCC
GGCGTCGTGTTGTACCTGCGCTGGTACTCCAGCGTCCAGTCGTCGATGCCG
GCCTGTACGGCCGTCTGCTCCGCGTCGAAGAGCAGCGGGACGTCTCTTTCG
GCGGCTAGCTCGCAGATCTCGTCGATGGCTTTGCGGAGTTCTTTTGGTGGC
GGGAGGGCGGTTCTTAAGCGCGTATAGGGCTGCCGTCCGGCGCCGCTGAA
CTTCAAGGCGACGAAGTCGGTCGGCCGCGCGAGGCGTACTGTTTCGAGTG
TTCCGGCGGCCCAGGGCCCGATCTCGGTTGCGTACGCATTCTTCGGCTACG

GGCCCGAGTCGCACGCGGAGAGGTTCTTGGTTTGTGCCTCGGTGAGGACG
ATCTCGCGGCGTAACCCAGGATAACACCGGTAAAGCCGATCTTTGTTAGGC
GTCGACGGTGCGACGGACTTCGAGAGCGTTCTCGCCGGCGCAGAATGTGG
GCGTAGAATGTCTTCTTGAGAAACCATCGCAGTAGAGGGTATTTGTCTGGG
TTGAATATCGCTCGGTTGGAATGGGCGAGGACGTTCATGATGGCTAGGGAG
GGAGGGAGCAGGACTGGCGAAGATGATATGCTAGTGGTGGCTAAATACGTA
GCACCGTCGACAAAGGAAGAACTGATAGGGGCGCTGGCTTAGGCATCGGG
GCGATTCTGTGTGATCTGTGAGGGATATGATTTACTGATGTCACAGCAGCGG
ATAGTGTAGATTGATCTGCAGGAGGCGATGACCTTGTTGTGGATGAGTGTAT
TTGTCTCCTCCTTGTTGACATAGCTACCATTGTTGTAGCTATCCTTAGATTCT
GGTTGGTGCCATAGCGGCCTATAGCATAAAGGCCGCATTGTGAATGCCTTAC
TGTAGCCAGAGAAACTGGTAGCAGGGACGACCTGCTTAATGCCAAAGCTT
GTACCATTGTTAGCGTTTTGCGGTACCGCAACGGCACTGACGACACCGATA
AGTTGTTTTATGATAGGCTGATATGTTAATAGGTAGGTATGACAAAGGTATTA
TGTGATAGGGATAGGATAGGAGTAGATGTAGGGAAGGTAAGGTGCGGGATG
TGAGGTAGGTATGTCAAGTATAGTAGCTGATAGGAAAGTGGCTTAGGTAGA
TGAGAGATATAGTTCATAATAAAGAACAATGGAAGGTCATAGCATAGTATGT
ACGGAGTGGAAGGCTATACTTTTAACTATCGAAGGCTTCTACTGCTGAGTAT
TCTAAGTGACATCTTTACTCCGAATCGCAACGGCTTCAGCTGCATCTTTATG
ATGAAAGCCGACAGGGGAGCATCGTACTGCATCAAGGAAGGGACCGAATA
TGGCGAATAGTCCACCACCAATGCCCGATATTCCAATCACGACTCGATGTTC
ATGGCGATACTGCAGGACCAATCCACCGGGAGCTCTTATCGTAGATGATCA
CGTGGACCCATCGAGTTCGAGTATGCCGCAAGTAGCCGTTGCGCAAAGAC
GTGCAAAGAGGTAATAAAATGGTGCCATTGTCCAATCAGAGACAAAGCCG
TAGCATAGCGGCAGGGAGAGACCCGAAAGATTGGGCGATAGCTTGAGCCT
AGCGCGCTCGGTCGACGAGCAGGTATTCCTTGGTATTCCTTGCACCGTATTG
GCTGTATTGGTTCTCTACCTAAGGTAGACTGAGTAGGGTCTAGTTCAAAGTC
CGGTCCGGCTTCCAAAGGATAACCAAATATAGCAAGAATAAGCCGCGCGTA
AATTTCTACTACGCAATACTAAGCGGTACTATGCAGTAAAATTCATAGCACT
CCTAGTGTTCCCAATGTTTCCATTATTCTGCGCCGTTCGAGGGCCTCTGGCC
AAAACGTGGGTTATCCAGAGCCAAGCCGAATGTTCGGTCGTGCCGCATTGT
TTAGAGACCCCTTGCAAACTTGATGCGATGATGGCCGAAAGCGCTAATTGA
AAATAATGGTTATTGAGTAATCTATAACGTAACTTTTCTTGACTAAGCCACGT
GCCCCGACGGCTTAATTTCCGACTATATCTTGTGATTGTATCTTGTGAATGTC
AATTTTCGTTAGATTTGAGGGAACTTGTTGTAGCGACTTTAGCCTTTCGTGA
GGCTCTCGAGGTTTCTGTCACGGCATAAAACCCCGCAATTTTTTCAGGGTT
CCAAGGTTTTCCTGTGCATCAAATTGACCGGCGGCGGTTAATGCGGAAGTT
ATATGCGGAGTACAGTAGTTGCAGCACGCATCAAGGTGTCCGAGTGAGCCG

GTTGAAGATAAGGCCGCATGCAAATTGAGAACTCCTTAACACCATCCGGCT
TTAAGCGATGTATGAAGACTTCTGTCTTCGGTGTCCCCTTTAGTTCGTAAGG
ACGATGTTATATCATCAATGTCGTTTTCTCCTCAATTGAGTGGCTTTCCCAAC
TATGTCTTCGGATAACTCGACATACCCGACGGGTCGGCCTTTAGCAAAGGG
ATAGCAAGTCTTGAACAGCGCCATATGCAAGCCCGAATTTCAGGCCTTTCT
TGCAGTTCTTGCTGTCTCAGCGGCCGAAAACGATCTGCACCAGAATGTGAT
TAGAGACATTATATGCATAGGATGCCAACAAGTGATTCAAACAGAGACTGA
GAACATACTCCAAAACTGAAGCCACCAGCCACAGCTAGACTAGCCATAAC
AGTGCTGAAGAGATCTTTGGTTCCTCGATAGACGTCAAACAGTTTCGTGCA
AAACATTGCGGTACTCAGAACCCGAGTCTTGCTCCGACTTTTATTCCTTCCT
TTACACCACCTAATGCTAAGTGATAATTCTTGCTCTTGTGATAAAGGTACCA
TCCTGTCGTAGATGTTGGAAGTCGATGAGCATTTTCGGCTCTGAAACGTAA
ACCTGCCGTCTTACTACCATGCACAAGCCCTAAAGACATCCCTATACTAAAA
GATGTCAACGCAGCAGCTGGTATGCGTAAAAAGGGCGGGATAGATAGGCG
CGACTCGTTCCATATCTGTGCGTATTCTTCTCTGGTGGGAGCCCATGTCTGT
CTAGCTTCTCTGTCAGAGTTGCCATCTTTGTTGCGCTATAACCTTTGCGAAC
TGTCGCAAAGGCGAATTTCCCTATGATCTCAAAATAATTGATATTGAGTGAG
AACTGATATATTAAAACGTTTTCTCGTGTGTTTTGGTAAACTTTGAATGGTTT
ATGCAAATTGTAGAAATTTGTATAATATTATATTTGATGTCTTGCTCCTGCCA
ACTCTGTAAAAATGAATGCTTCAAACTGAGAGCTTAGGCCACCGCTTACTT
AATATGGGGTTACCTACAGGAAAAAGCACTTTACGCGAACACGTGCCGCGC
GTCTACTTGGATACAAAATACATATGCACAATTATTTAGCAAGCTCCATCCTT
GCATCTCTCCAACCGGGTACAATCGCGTTTCAACAAAACTCACTTTCACTT
TCGAGATCTCACTACAAAATTGATTGACCCTAAGAAAACAATTATTTGGTAT
TTGGAGGGAGGGCGCTATCGCTCATCGGTTGTCAACATGGTAAGCAATTGA
ATGCATCCTTTGCATCCTATACTTAGTATGACCTCATTGCGGGCTTGGGTCTC
TGGTTTGAACGGACGAGTGCATCTCTCATGCCAACATCCGTCTCGGCAGCG
GATATAAGATGTCATTGCGGATTACCCAACACACGTATCATCCGACATCGCT
GATTTACATGTAGGTCAAACGGGACGAGCTGAAAGATACGGATCGGTCGAT
CAGCTCAATATGGCGTCCACACTATCTTCCTATTCGTCGACCCGATGAAACC
TCTTATCGACGTAGCAGAAGAGCTGCTGGAAATTTTAACAGAGCGCTATCC
TAAGGGATTGACCTCCTTCTCACCGGAGAAGGGTCAATCGAAGATCCAGCT
ACCCGACGATCCTCAGCAGATCGAGTTCGCTCTACCTAAGGTGCCGACCGA
CCTTAGCCAAGGCTGGGACGCCATTGATTCCGCCAAAACGCAAACTCCCGT
CGATATTGGTCTTCAAAACAACAATGTAGGCATTCACTTTCAGACCCTCTGA
TTCGGACTCGGCTTACGAGCTGGATTTCCAAAGTCGAATATCCCTGAACTC
GATGATGATGCTGCCGCTGAAGAGTAGCGGAAGTATTCTATCGCAGACTTC
TATGAGATACGATTTGACGCGTGTGGATTTAATGAATGACGAAACCTATTCT

GAGCAATGATTTCGGAATAGAGTGATGTTTCTTCTCATCTTGGAGAAGGCTT
GAGGGGTGTATTTTGAGCCCCACGTTAGGAAAGAGGGTTATTTCTAGGTCT
GAGATTAGATGACGATAAATTCTAAATTTCATCAATCGCCCTCAAGATACTA
ACCTACTAAGGCGCCTATTAATGGTGTTGTAGGTACTCATACGACTAGTCAA
TTTAAGTTACAACGACACTAGTAACTCATGAACCAAATCAATAAATGAAGC
AAAAATAAAGCCACGATCACAAATAATATCAACTGTGAGATATCAATTCGTA
TCGCTAAATTTAGCTAAATTTAGTGATATTTAATGCTTGCATGTGATACGATAT
AGGGACCCAGGCAGCTACCAGATCGCTATAAAGTCCGAGAGCCGACGCTT
CTATTTAGTGGGACACCCCTATCGAGCCCCTATTTTCCTTCTAGCTTCCTTCC
AGTAAGACACAAGGGATTCCTAAAAGGATTCCTAAAACTTGTTTCTTATTCC
GTCTTCCGTCTTCCATATTCTGTACCTAGTCTATATTCTTTACACATAACACA
AAACGACGATGCTCCAGATGTTTTCTTTTTTCTTTTTTCAAGTACCAATTTTA
GGTGCCATTCGTTATTGCTTAAACGGCTTCGGCCACATGCCGGCGTCTACAT
GTCCCCTGCGACTATTCTTCGCAATGTTTGCGAGTCCTCATCCCGATCTGCA
TAGCTTCAACTATTTATCTATATCTATATCCAGTCTTTTTAGGATGTGCATTTC
CGCTTCCACCCCAAGAAGCCAACAAGACCGAACCCGAGGACTCGGCTACG
GCATTCAGCATAGCTACGACAGCATGTAATTCCTTCGAGTAACGGAGCGCC
GTTCCGACTACTAGCTCTCGGCGATCCACAGCTTGAAGGCGACACGTCGAT
CAGGAACTACACCGCGAATCCTTCCTACATTTGACCTGGGCTATTCGTCACC
TTACCTACAAGACCGAGCATCCGTCGCTACGTAGCCGTTTACGACAGGTAG
TGCACGATCTAGTCGACGTCTTCTTTGACGATATCCCCGACACGGCTGAGT
CGATTCGCAAGCGTATAGACTTATTCGGGAACGACTTCTACCTCGCCCATAT
ATATCGAACGCTACGATGGTGGACTAAACCTACCCATGTTACAGTTCTTGGA
GATTTACTCGGAAGTCAATGGATTAGGGATCCTGAATTCAACCGACGAAGC
TCGAGATTCTGGAATCGTTCGTTCAAAGGAGGTGAACGTGTCCCCGACGA
GGTTGCAGCATACCCTGCGGACGAATATGAATTGGCTGGTTACTCGGCGTC
GAGGACGAAAATGCAGCGGCTTGGCGACGCAGGATTATTAACGTCGCAGG
TAACCACGATATCGGATATGCAGGTGACCTGACGGTTGAACGACTAGAACG
ATTCGAACGTGTGTTTGGGAAAGCTGCCTACGAGCTACGATGTGAGCTCCC
CGTTACCGACCCTGCAACCAATGCGACTATCTACGATGCTGAGAAGAACCC
CGAATCCGACCGACTGCCTCCCGAGCTGCGAAGTGTCAACGTCAATAACAT
GAACCTTGATACTCCGCTGCAGACCAGAGCCTTCAGGACAAGACATACACT
TTCGTGAACGATGTGATCAATACAGCCTCTGCCGTCGAGTTCGCGGACAC
TTCACGGTAGTTCTCACGCATATACCATTGTACAAACTAGAGGGCATCTGCG
TTGACGCTCCATTCTTCAGCTTCCATTCTGGTGGCGGCGTTAAGGAACAAA
ACCAACTGTCGGCTGATGCTAGCCGTGGATTTCTGGAAGGTATTTTCGGAC
TGAGCGGTGACCCCAACGCACGGGTCAGGGTAAAGGCGGAGGGGCGTCG
TATTGAACGGACACGATCACGAGGGCTGCGACACGTATCATTACATCAACC

AGACTGAGGGCGAGGAGCGTCAATGGAAGACTACTTTGTGGGAGACTGCA
AAGACCGGACAATACCCAGGAAAGCAAGGTCTTCCGGGTCTAAGGTAAAT
CACTGTTCGAAGTATGATGGGTGGATTTGGAGGCAATGCTGGATTACTCAG
TGTTTGTTCGACGAGAACAATTGGGAATGAGATTTGAATTTGCGAAATTGC
GCGTTGGCACTCAGTACCCTTGGTGGTTCGTCCACATTATCGATCTCATCGC
CGTTATCGGTCTCGTGCTTTACGGCATTTTGCAGGCGTTGGCTGCTGTGGGG
ATCGATTTCGAGAATCGTCCGAAGATTCCTGTGCCTAAGATCGAAATCAAG
CGATCACTCCGACGACCGAGCAACCCGACGAAAAACCCGTCTCGATTAGC
CACGGCGTTATTAAGAAGGGATAGGTTCTTAAACGACCAGCGGCGGACCTA
TAGCACGGAGGTGTAATGAATCACGAATACTTGTACTAATATGAAGTATAGA
CTAGGATCCCAACGGATTGTTAATTAGAGGTTAAAATATATATTATACACGAG
GGTTTTCTCTCGACATAGGCATATATATCAACTGGATATATAAAACGTTTACT
GATTTACAACTTGAAATCGAGCACTACAACATCGACCTGTGCATGAGAGTC
TAATCCGACCCGTTGCTAGATCGCGTGAAGATAGACAGCACATTATCGTACT
GGAGCCTCGTGGCATCGACCAACGGCTCCGTCGGCCGGGAACCCGTCCTC
CTGCCACACGAGCTTGTAGCAGAGTAATCGCTCTTCTTCGTCGCCTTCAGG
ATCCTTAGATGGGCGCCGCGTGGTCCGGTAGAATAAGGACAGATCATCGTC
CTTGCTCACGGCGAGCCGTCCCCGCTTACCCCCGTATACGCCGTCGACGTG
GGGAAGGGCGCGCTGAGTCCAAGTGCCTATTTCTATCAGCACGTAATTCAC
TAACTTGTGTGTGAAACGGGGACGAGGACTTACCATTAGGCGCTCGATAGT
AATGCTTCCACCTCTGCTCACCCCCACGGCGTCGCGGTTCAACACATGCAC
GCCCCCTCCCATCCACGGCCTGCGCCTCCTGGTTCGCCAGCCCGGATCCCT
TCGGGATCTCAAACGCCACTATACCCGGCGCGTCCGGGCGCACGCCTCATC
CCCTTTTTCCAAATCGGCTACCACGTCGCCCGCCCCGTTCCGCCATACCAAC
CCGTCGTTATCCGAGTACGCGTAGCAGATATCGTGGTTATTAGCTCCGGAGT
TCGGGCCGGCTTGCTGCTTGTGCTTGGTATCCAGCGGGTCGTCCCAGCCCG
GTAGTGGACGAAGGCGCGGTATACCCAGGTTGCGTGCAAGCACCCGGAGC
GGTGGTCGAGGCCGTGGATGTACGGGTTGTTGGCTACGCTTTGAGGTTCGT
GCCTAGGACCCGATAGCGTAGACCGTGGATGAGGTAGGGGAAGGGGTGGC
GGTGGCGGAGTACACGGCGAGGTGGTCGTCCCCGAGACCGGCTTTGCCGG
TTCGCCAGGTGAAGAGGAGGGAGGTGGGGGTTTGCACGAAGCGCGGGTA
CGTTATGTAGCTGAATAAGGCGTCTTGCGATGAATCGAGGCGGGGAGGGCG
AGATAGGTGAGGGCTGAAGAGGGATTTGGTCCAGGTGTGGGATTCGGGCT
TTGACGCGACGCCGGGGTGGGAGTGGCGGTAGCGGAGTCTGGAGGTGGTG
AGTATGGTTGAGGGTGGTTAAGCTTGAATGAGTAGGACTTACATGTCGCAG
TGGTGGTCGTATGAAAGATGGATCGTCCCGTCGCCGGGACAGATACCGAGC
TTGACTTCACTCAGCTTCTACTTTCACTATTCGAAAGAACTTGAGTCCTGTG
TTACCTGGACTGTATTATGCCCGTCATCCGTCGTCTGCGGGTAGGTCGTCAA

AGACAAACGTTTCCCACTTTCCCTCCGGCAGCTTCCTGCGTGATAGGTGGA
TATACAGCGGTTCCTTGGCGCTGTCCGCCGAGAGCTGGCTGTAGAAACAGG
CGTACTGCCATCCGTTATACGATTGGATGGCGTCCTGCTGGAACGCGGCCCC
GTTCAACACGCAGTTCCGGTGCGTCGGGTCCAGGCCAAGAACCGATACCG
AGGGTTCTGGCAGCGTCATCTCGACTCTATTATATAAAATGATACTTTTTTCA
ATTAATTGAGGTAAGATAGAGTCTCGGAAGAACGAGTAACTGTAGGATGAA
AGGATATGAAACAAAATGAGATGCGCCGGTAGAAGCGTGTGGAGGGTTCG
GGAGGCTAAATGCAGCTACGAGGATCCGGCGACGGGCGATGTGACGGCGC
TCGCCTTCCAGAACGGAATCGAACGTGGCGCTATAGATTGCGTGCAGCTCA
ATTGCTAACAGATATTAGATTCAATTGTCGCTATAGCCTTCCTATCTCTATTCC
GAATCAATTGTATAGCGGCGCTCGTACGATGGAGTTTTACCTTCGACTATGT
ATTGTATGAGTGTTGAAGTGCCGTGTAGTATCGCCCGAGTAGAGCTTCGAG
TCGCAGTGAAGGTCGAGAAGACCCATAACATACGAGATGACCCACAACTG
ATGGATTTGTGACGAAATATTCTATTCAATCGACAAGTTGCAATTATGTTATA
TCGTTAAGTCTTCCCACCTTAAAGATTAATATACATTTATGACTATAGTATGC
TAATTGCGCCGTGTAACATAGTTGTGGCAACTCTTGTTACCCCCTGAATGTT
ACCCATCTTGCTTTTGTACGACACGATTTAGCAATGCAGCCTGTCGATGAGG
ATGATGATATGAGCCCAGTCGAGAGCTATCGAGACGACGACGACAGCGAC
CTCGATTACAGCCCGACGACGCACGACGTCAATCATGTCACCACCTGTTCA
GAGTCGTCGGATCAAGTTACCCGTTCTCGTCAACGCCGCTGGCTCGCGGTC
CGTCTTTACAGGTTTACCCCGTATCCGCCTCTGCCCCCGATCGACGAGGATC
TCGTAAACGATCTGCCGGAGCAGAAAAGGACATTGAAGGCCCGTCTGAAT
CACCTCCGCGACCTTCGACCTCCTCCCACGTTCCGGGTCGAATTCGCGTCG
ACATCCAGAGGCAGCGTCGTTACCGACGACGCTGCTGGTGCTGGTACAAA
GGCGACCAGGAACAGCACCCTCATCTCATTTAGCAACATCGCCCATGATCC
CTCGCTCGTCCCCACACGATTCAAGACGGTTAACATGACTCAAATGGCGAA
CCCCTCCGCAACTCGCTCGTGCATTTCGTACGACGACTTCCATCCGGCGTC
GATCTCTTCCCCGACGCGTTCTTCCCCCCGTCCCCGCCCCGCCGCCACCAA
GGCGCGTCGACAACCGAATTCACCGCCGGCAGCGGCGGCAGCGGCAGTAC
ACGTCATGACGGACCTGTATGCTCGACGCGCGGTGGAAGCTCGAACGATCC
GCCCGTCGGGGGAGTCGGGCCTCAGCCACTGGTTCAAGACGGCTCTGTTC
GATCACCTCAACGCCCTCGCTAGGGCCAGATATGGGTCCAGCTGCACCCGC
TATTGGAAGGTCATCGGGATCGGTTCCACGGGCAAGACCGACTGGGGTCTC
TTTGTCGATGGTCATCTCGTCGCCCTTGCCGAACTCAAGCCTTCCGTCGTG
AGTTTGTCTTGTCCTTCCCCTTCCTCTCCCCCTTCCTCTTGCCGTCCCGGCG
GTATGTGGTATGCTGTATGTGGTATGTGGCGCCGACACCTCATAGGTCCTCT
CCTCGATAGACATGGTCCAGGTATGCGGCCCTATCGGGGCACACCCCCAGG
GAGTGGGCGAGTACAAGGACTCGAACGACAAGATGGCACGGATACGACTG

CAGGTGCGCGCATTCATTTGCTTGCTCTCCTTGTAGTGTGACAGACTGCTG
ATGAGCTCGCATAGATGTATGCGCAGTTCTGCAATACCGCTGCCAAGGCCC
GGGTCCGCTTCGCTCTCTGTATCAATGAGACGAACCGCGATGCCAGCGGGA
ACAGGACGCACGAATCCTCCGCAGTCCTCTTTGAACGCACCCCGCATTTCA
TCCTCGTCGGGTCGAACACACTGACCGAGGCACTCAACGAAGACCCCACA
TCAGCCACTCCCGTCCTCTTCCCCCACCATGGCCCTTGTCGCCCTCACCTTC
ATGCCCACTTCGCAGCTCGTCTACTCGACAACGACAACTGGCGAACCCCAT
GACCCCACTTCGGTCGATATCGGCATGACAATGGCCACCCCGCCTCCTTCG
CATGACTTTGCTCCTCTTCCCAATGCCAACGCCAGCGCCCACGCCTACGCG
CACGTCCACGCCAACGCCCACGCCAACGCCCACACCAACGCCAACGCCAA
CGCCAAAACCGCAGCTGGGACTGATAGCGGGCCAACATCAGGCGGATACG
CGATACGCACCCGGTCTAAACGCCTCGGCACTCGGCGGGTCGGTCAGGCA
GATGCCACCACCACCACTTCCAACCCCACCCCCACCACCACACCACCGGC
CGCGGAAGGGGGAGTAGTCGTCGTGCAGCCGGTGTTCAGCGCCGCCGTGC
CCGCAGTGATCCTCATCGAGCCAACATGACGGCCGCAGCTGGAGCGATGG
ATGCGGGAGGAGAAGAACAAGAAGAATGACAATTACTGCACGACAAGGA
CCGCTCGCGCCGACCCCTTGCGGTCCTTCAAGTCGATCATACAAGGTTGTC
TCTCAGGCCCGGCGATTGCGCGGTGGAGGATGACAGTGGGCGTGATGGTG
GGAGCTTGAGTCCGACCGAAGATTCGTCGTTTGAGAGTGAATTCGGTGAAT
TCGATTTGGGCTCGGTGCGTGCGCTTTCCTTGGCTCAGACGACTAGCGATA
GGAAGGCGGGTCGACTGATGATCAAAGCAAAACGAGAGCGACAGTACTGT
ACCGACCTCTCCCGTTCCAGGAGAGATTAGCCTCGACGAGGTATGTCCTCT
TGTATTTTCTTTCTTTCTTCACAGCAGTCAGTGTTACGCTGACGCATTACTTC
AGCTGCCGCTCCTCCGCCTAGTCACTGCTTCCCGCCCCGAGTCGCCCGGCG
ACCTGACCCTTTACGGTCCCATTGACTGGTCCAAGCATGTTCTACCCGGTCT
CACGCCGGTGGATACGCTTCCACATCTGAATCCTCCTCATCCCGATCCCGAC
CCTACCCGTCTGGGCTACGGCGCCAGGCCGACGACGCGCACCGTTTGGATA
GGTAGACAAGTGGGTCAAGGTGCCGTATGGACCGTGTACGAAATCTCGGC
CGACGAGGTCATCAAGATATGCCGACCCGGCTCGTTCCCCATCGCAGCGGA
TGACGATCCCGTCTTTGGGGATCGGGGGTACGATCGAGTCGAGGTCAGAGC
CGCGATCAGAAACGAGCTCGACCTTTTGTTCGGTCCGTTGAGCGCGTTGCA
GGGCAAGGTCGTTCCCCGGGTCAATGGCGTCTACGTCGGCGGTTCCGCCGC
CGCCGCCGCCGGCGGGCAAGAGGTGTGGGCGGTCATTATGCAAAGGGGCA
ACCCGGTGCTGGAGACTGTGGACGGAGAGGCGGCCATCGCGTTGCAGCAC
CATGAAAGGTCGTCCCGGATGTGCAAATGGGCACGTGCAGTCTGACACGG
CAGGCATGATGTCTTGAACGCCTATCGCGAGCTTCACAAGGCCGGAGTGCT
CCATCGCGATGTCCGTCTCGACAACCTCCTGTTCATGCCTCTGTCTATCTC

>Mosaic_sequence_49

ACTTCTTGTTATCATAAAATTTCGAAAAGGGGTATCTACCTGCCTGCTACGT
ATAGATGCGTCGCACCGCCACACCTTGAGGCTTCAAGCTATTGGGTTTCGT
TACGACGAGATTAGCCACAGTAAGGGGTTCGAAGGCAAGGATAACGGTATA
GTAATGCTAAGATTGCATAAATGCCTGATACAAGTCCGATATACCGCGGTATT
TGTTAGAGAATCTAAAGGGATTGAGATCCCACTCACCCTTCAGACGCCAAG
AGACCGTTGATTGGGCTAGGGGATGGTATGATATGATATCTGCTCGACAAAC
CCTAGGCTTAAACCCGTCTGAAAAAACACCTGCATATTCCGAATTTCTAAA
ACTTACCTGAAGTTTCTACAATACCTATCTTTCACGATAAGTAATCTACTGTA
ATTCGTCCGCATGTCTTTTAATGAGATCAGATAGTCGGGCGGCCCTCGTAAC
ACACGAAGGAATTTATAAGTGGGTAGCCAAGAAAGCGTTATCCCCCTTATT
ATTAATTAAGCATTGTCCGAACGGGTACGCGGCGAACGAAACGCTTTGCCT
GGCTCGGGCGTCGTGGATCGCTAAGGGCCTTGGCAAACGCATCGAGCAAT
CTTATGTAGAGCTGGGCAGGCGGCCGATCGCAAGACTTGCCCGAATCAGGC
TTTCCCGCTCTTTCTCTCCCTCCCCCTTTCTGCTTACGCTCTGGTTCTGAATA
GCCGGGAAAGGCTGTCTTGGCTGTTGGATAACCGCGGGAGGGCAAAAGCA
AAATTTTGAGGGAGTCGTGGATGGGATGGGATGGAAGGCATTGAAGAACT
CGGATGGTGCTGGACTTGACCGGTCTACAGAGTAATAATTAGTATGGGGAA
TGTCTCGCGCTTCTGATCCATAGCCGTTCTCCAACATGCAATATCCGTATATG
GTCGGAAGTCCCCATTGTGCTTTGATAGTCTATAATATCAAGACGGCTGTTG
ATAGCCTGCTTCTACGACGGGCTTAAGCGCGGGTACCGGCTTTCTGTCTAC
ACTTATTTGGTATGAACTTGTCGATCTGTTCGGTGAGGTAAGGCAAGGGAT
GCCGCCGTGGAACGTTGTAATCGCAGAAACAAAAACTGCGCTGGCATTAG
TATGCTTACGGTTGCATCGGTAGCATTAGGGCACCTTTTCTGCTGACATGAC
TGATAATAAGGATGCCTGGATGGATTGCGAGGCTGCGAGGTTAACTGGCCA
AGGTTAGGGAGGCTCAGCTATCGCCTTGCAACTTGGCTTAGCTGAAACTCA
CTTTTTTGGCGGCTACCTTTGTTCGTCATGCGACAAGCTTATCAATAATTAGT
AGATACCTAATTACCGATTTGATGTAGCCGTGGGGTTGAATGTGGTCATGAG
ATATCCGGAGGGTGATATTGCATAGTCATAATGATATTACTCATCTGGAATCG
GATTGCACAAGAGATTAGATTTACAATTTCCACGTGTTTAGAAACACTCCGC
ACTTTCCATTGTACAAAGCAGGTTAGACATTCTATATAATCCTGGTTCTGTTA
TAATGAAATCTCTTTAGACTCGTCTTGGCTTACCATGGGTATAGGGAGTCAA
GCAAGTGTAGGTGAAGGGTTGAGGCTGAGAACCTGATTGTGAGCAGGGCC
TTATTGCCATTACGTCCGAATCCCGAACGTCGTGTAGCCTTAAGCATTATTTT
AGCTGTCTAATTAGTACTTTTGTGGCTAGATGAATTCCATGACAGAGAAACT
AATCTCCTGACAGCGTGACCCGAGTCATTGATCGTATAATAGTACTCCCCCC
CAAAATCGGGATCAACGTGCATCGATCTGGTTGGACGAACGAAACGATGG
AACCATAATGAGTTGACAGCGTTGGAATATACGATGGTGTATTGTAGGCACA
TAGTAGGATATCTTAAGCTTGGCGTGGAGACGGCTTATACCCCTCAAGGATA

CGAAATGCAGTTTTGAAAATACGGGCTAGAAGACGCAGAGCCGAAAAAGC
TTGTCTCGTAGCCGAGCGATGCTGACCATTGGAATAGCTCGGATCGTAAGA
AATTGTCGGCTAGACCCCGAAGAATAAATGTGTCATGCTGCATGGTGTCGA
AGGAATACCACATACCGATGGAATGGCCACCTGCGGAGTAAACGATCCAGC
ACGGGCGGATACGTGATTCGTCGGTTGCGGCGAGGTTGTCGCAATGTTTGC
ACTGACTAAAGCGCATTGGGTCTAATGTGCGAGAGACTGAATCCTTGTTTG
GCAGTAATCAAAGACATTGTTGGCGCTCAGGAGTGGTTGACCTGGGTCTTT
GCTGAGGTTCTCGCCGTAATAGGGACCGGCCGCGTCATCTTGGCAAGGCAA
TGTTACGTCCTCCTGACTGGTTATAATTAGGCCTGTTAACAATAAGAGCGGG
GCTAGAGATCGGATTTCGATGGCAGAATACGTCAAAGCAACCGTGCAGCTG
AACGTTAACCGACATTTGAGTAATGAGAAATGGGTTTGGTAACGTATAGGT
ACGTAGAGCTGGTAGAGGCATCTGAAGTTGCTGGAGATACTAACTCACGAT
TGCCTGTGTAGCATGTACCCTTTTTGTTTGGATCCAACAATTCTTTTGTCCTG
TAAATATGTAATAGATCCGTACTGGATTCTTAGAAAAGCAGTTAATTACCGA
CAATAGTCGTGGACCGGCCGATGTTAGCTCACATCTAAATCCGTTCGTTGTT
GTTTCAAGGTCAAGGGCACAACGTGTGGGTTTTGGAGCTGGGGGAGGATC
TCCAAGTTTCGGGAACGCTCCCGCTTACGTAGATGCTGTCTGATCTCCAAG
ATCACCGCACTGGTCAAGTCTCGACTCCCTGGATTTCAGGATGCTTGGAGG
ACTGGAGGAGGAGCTCTAGAAAGTTACCATAATGGTGGACTCAATAGCCCT
TCGATGTGGGAAATCTATGGCAATCTAGTTAAAATTAGAACATAACATGAGC
CGCAATCGCGTATTAGCCGATGACGCCGTGTTCCTAGTACTGCCAATGATTC
TGGAAGATGTAAGGCTGTAGGAGTAGTAAAAGTGATGCATGCCAGGCGCCT
CTGCATTTCTTGGCCCTTGGCATTTGCTTCTTTATATGGATCCATTTGTGGCC
TCTAAATATTAATCTGTGCCGTCTTGCCCGTGCCGGTTTGCCGTTCTTTCAG
CACTTTCATCAGTACCTATGTCTATGATACTGACCTGTAATTCTGTTGCTGTC
GCTGCTATTCTTTGTTTCATCCGATGTTAGTGACGATTTATGACAGTCCACA
ATCATGTGGATATCATCCATTCTTATTTACATAGGGTTTCCAGGGTTTCTAAG
GGGTTCTCGAGATGGGATCAATCTGAATCAAACGAGTACTTGGTATTTTGG
CTCTCGGACACCTGGAGTTCCCGGGGTAGACAAATCCGAGCTCCAGGCGT
CTCAGCTTCTGTGCACCTAGCCGTCAACCCGCCTAGACCGGCGAAGCTTTC
ACGTGGGGGGAAGTTATTTTTCCCGCGCACAGTCGGCCTCGGCGGGGTCA
CCTTGGCGTGATGTGGCTTTGGATTTGTATGGATTTTGACGGATTTGTAGGG
CTGGGTGGTTTTAGTGCTGATCCAGAAATAGGGGGCTTGCTCTGAGAGGTT
GAGCGAGATTAAGCCGCGTAAGCCTTTTATCATTTTATCGAGCTAGCAATTT
GACAATTCCGTTTCTGGTATGTGGCTCGCGCTCTACTTGTCACAACTAACAG
CGCCGGTACCTACGCCAGCTTGAAAACAGTTAGTTATCGAAAGAACCATAT
TTAGCAATTTCTAGTCATTTTTAGTATTTTTATTAGTTAGCTGTCGCGTGCCTT
GCTTTGCCAATATGGGAGACCGTTCCCGGCGTTCAGGGACGTTTCCGCGTA

TATTGGCGTGCAATATTTCGCGAGTGGGCGCACAACTGTGGGTGCGCTATTA
CTATCGACGGTGGCGAGTCGGCAAGCTGGCAGATAACTGTGCTTAGCGTAG
CGTGTCTGTGGACATGTGGATGTGAAAAATGTCCAGGTGGTGAGAAATGA
CTGCATCGATGAACTCCGTGAGTTCGGCATTGGCCCGCATTGTTGCATCGG
CGGGCATCGCCAAGACTGTCGGCAGGGTCGGTGAAAGGTTATGTAGCTAGT
AGCTAAGCTTTCTTAGTAGGTTATATGTATTATGTAGCTGAACTTCCATCTCG
GTACTAAAGCATGATGCAATTGTATGCCCTTCAACTAACAGAGCATGTGGG
CTGGTGGGCTTGAAGCCTTGAAGCCTTGAACAACTGAAACATCCCGTCTCC
AGAGTTTATAGAAGATCTTGTGGAGATAAGCTTAACTGGCACTGACTGGCT
TAATCAATCGCAGCTCGGTGTCGAAGGTGTAGTAGGAGAGAAATGGTAATA
TACAGCAGACATGCCTATCCAGGTTTAGCAAAGCAGGCATATGGTCATTCGT
TATGTCGATCATTAGTTGAGCCCTGCTAAAGAAACAGAACAGATAGCAAAA
TTGACGACATTTGAAGCCTCATTGGTCGCAAAGTGGCTTTAGCGATCTATG
GCAGGTCGCAGAAACAAAATCGCGATCTTCTTTGCCGCATCTAACCAGTCA
GCCCTGCGTCAAACCAGTGCATCATGGCTGGGAGTTAGGACCGAAGCCTG
ATACCGGATGCGCTATTGTCCATTGCAGCTTTACATGACGGGGAGATGCGGG
AAACAATAAACAAGGAATGTGAAAGTCTTGGCCGTAGCACGATCCGTTCG
GACTCGGTGTTCAATGTTCAATGTTGGATTTTAGGGTCGTAGGGTAGCGCC
GGAGGCGTCTAGGGCGTCAAGGCGTTTTGATACAAGTCATGAATTAGGATG
AAGATCAATTGGCGATTGCTGCAATTGGTAGATGAAAAGGTGAAAAAAAA
GAGGAGCAGATGAGTTGAAAATTACCTACTCCTATGAACCTTGGAGGCCGC
ATCTGAGCTACCTAACCTAGCAAATAACAAGTACATTCATAGAAGCAAGTA
GCACTTGCCAGCACTAAAGCAATTAAGCAACATTTTTATAGGCGTGGCGAT
ACCGAATGACACAATACATCCACTTGTATACTTGATTTATGTATAAAATGATG
TTGCGGAAAGTGAAGCCAAGCATTTGTCGGTTACACTGCCAAGTCTCTTGG
AAGGAGGCGCTGGACAAATAAAATGCGCCAAAGACTTGATCGAATGCCGA
TTATCCGGGGAACGGGGAAAAATAAGATACATGAATTGTTTCGTGATAGAC
ACAACGTTTTGTCAAAGACGGAATTGAAGTTATCGCATGTGCTCGATATCG
ACGATGATAGATTCAAATTCGCGACAATAATTTCTATAAGGGTCAAGTAATT
AGATAGCGGACCGATAGCTTATCCAATTGCCAATCTTGCAAGTCGGTCCAGT
TTCTTGCTCTATAACATTGAACCAATGCATGTGCATAGTGTAGTTCCAATCTG
CGGACCTACTCAGAAAAGAAAAACCGGGACTTCAAGGTGTTGATCTTATAG
CCCTGGGGGTTAGTGTTTTGGTGGATATGGCCTATCATATCGTGCTATGGAG
TTTTGGTAGTGCATTTGGCGTTGTTATAGCCCCACAATATTGACGATTAATCT
AGGTTAAACGCTACCGAATTCAGTTTAGGCAAGATGTGTTCGATTTCCGTTT
TTTCCATTGGCCCTTTAGGAAGGCGAGTTTTACGAAGTACACGATATACCTC
GCTTTTATGTAACGTGCCTACACACGGCATGCAAGGAGGTACCAAGATGTC
AAGTACCCCAGGAGAGGCGTTCGGTACAACTGGAACCAGACCAGACTCAA

TCACATCATTCACAACCTTGAAGGTTTTGATACATATGTATATATGTACATTC
AATGTTGTAATTTCCAGCAGACGCAGGAGAAGAAGAAAGAGTATCAAGGA
GTTGTACGCGTAGGCAAAGCGCAGGTAAGATTCACCCAAAATCCAAACATA
GCACAAGCTTGAGCTGTTTATTTGGTTTTATTTGGTTTATTCGTGTCAGATTG
GAAATAGCTACTGTAGACTTGGGTAAGATTGTCAGATTGAATACGCAATTCG
CATAATCGCCTATCTTTAAACCTAGTCCATTTACGAACTGGACCTGCCTTGA
CGTTCCTTGACGTGCCCAGCTAGAGTGCCCTGACATGCTGTAAACAGGACG
CTAGGCGGTACATCGAAGAGGCTGAATTACGGTTGTGGAGACTGACCTAGC
AGTGGGCAGTGGCAGCGCAGGTAAAGTTTGTAACTTGGTCAATCCCGTCCC
GTTTCGCTTCGCTTAACAGCATGTGCAGTCGCTGGTCAGGTACATCTTTCTT
TCTACCAGAGTTTTGACCCAATCTTTGGTCCAATCTTTGTTCACCTTTTCCA
CATTTTCCCAGTCCACCCCGTGCTGCCCGTGTCTTCCATCTGGCTCTAAATT
TTCGCCAAGCCGCCGTCAACCTGGACCAACCTGGACAATCCTGAACCTTC
ATGGGGGTTTACGGATCCATAATACATAGCTACATTCATATCCCCCCATATCA
AATCAATCACATTTGATCCTCTTTGCCAAGTCCATCTGGTCCATCTTGCCCAT
CTTTCCCATGTACTAATCGTCGGCCCCTTATTCCCTTATTATTACATCCTCTTA
AAAATCCTCCCACGTCCATTCTTTTTAACCCTCTTCTTTTCTTCTACTGCGCA
TTCCGCCTTTATCAGTTGCGTGTGTAGGAACTGACCTCTAATCCTGTCCTTC
CTGTTCTCTTCGTACTTCTTTTTTTAATCGACCGTATCCTCAACCCTGAAAAC
GTTTAAAAGCTTCCTAGGAATCTGGGAATTGGGAATCTGAGTGACGTATGT
GTATGCGTGTGTTGCGTGTAGCGTGCGAGCGAACGAGCAGAAAATAGTACC
AATTACCAAATTTACATAATATCGCCTCCTACCCATTGAGACGGGTATTCGA
ATACCGCTTGTCCTATCATCCGACGCCTCTTTTTTATTCTTAGCCGCGCCGTT
GTCTCTTAATATCGCCCTAGGGACAACCTTGGCACCCTGTTCCCGCATCTCC
CTTGTGTTGCGCGCGTCCAACATTTCCAGACCAGAACCGGACCCGTAACAT
TCCAAATCTATTCGCTCCAAGTAGCTACCTATTCAGCCACCTGGTTAGCCGC
CACTTACACCAACAAGTCGGTGCTACATCTCAACGATTGGATTCCATCTGAT
AAGGTGTTCTCCGTTTCCGACTTGGACTTTTGTGCGAGCCTTTCCCATCGA
ACCATCAACTTTCATACATCAAGGAATGATATAGCGAAATTTGAGCAACACC
ACCTTCCGATTTACCTCGACAGCCCTTCACCATGGCTATTGGCAAGGAAGG
AAATGTCAAATGGGTCGATGTAAGTGCACAAATTATTCTTTTCAGTCATCTC
CGGACATTTGGGTGGCGGAGGATGAATAGGGATCTAGTGTTTGGGGATCAA
AACATGAGAATTTACCGATAGGGATGTGGGCTGTTACGGTTTTGCCAAAAA
CAACTATTGCTTCAAGAGAACTGGTGAATAGTCGGAGTGGCTTCGGAGTTT
CTCATTATCGACAATTAGCGACGGGATGGCACGAGGAAAACCCGCTTTGAC
TCTCAGCAGTCAGACCAATATAAGTAACTAACAGATTTTTATATAGGGTCTT
CGAGGTCTCGCATCTACCCTCGTAGTATTGACTCATATCGCCAGAGCTTTCG
ATGGCGAACTCTTCCTCCCCAGCCTCTCATGAGGGCGCGCAGCCCCGACCT

ATGCAATTACCGTTTTTGCGAATCCTCGTCCAGGGTCGCATCGGCGTTACTA
TTTTCTCTTTTGTTACCGGATACGTCTGCGCCCTGAAGCCCCTTAAGCTTTT
CCGACAGAATAACCAGGAGTCCGCCTTCACATCCATCGCTAAATCAGCTCT
TCGCCGCGTTCCTCGACTCGTCATCCCCTGTGCTTTCGCTACCTTTTTGATC
TGGATTATGGCAAACTTGGGCATGTTCCTAGTTGCTAAGCAGAGCGACTGT
TGGTGGTGTGGCAAAACCGCACCTGATCGTCAGGCCACCGCCTTTCTCTCA
GTGAAGCACTTGATTCAGAATATTGTTTCAACATGGACGAGAGGTCAAAAA
CGCTTATGATGGAAACCAGTGGACATTGTTACCTCTGCTGAAAGGAAGTAT
GGAGGTTTACATATTCGTCATCGCTACTGCGTACATTCGGCCGCAAATTCGA
ATGATTTTGAGCTTGATGATGTTTGCCTACTTTTGGATTGGTTCGGACTGTA
AGTTGTCAATACCACTATATCTTTGGACTCTATCTAACTATAGCAGCGGCTTT
CGGGATGCAATTCTTCTGGGGTGTTTTCCTCTGTGACCTGCAGAATCATCCC
CCCGCCACCGAATGGCTTACTCGTCGAGCAAGATTATCCCAGGTCTTGGCC
GTGGTTTTCTTGTTCCTAGGTCTTTATGTTGCCTCATACCCCGAAGGACATC
CTGAATGGGCAACTTGGTCCAACTACCAGTTCAAGGTTCTCAAGCAAATTC
TACCTAAAGACCCAGACTTCCCTCGATTCGCTTCTGGAATTGGTTTAGAGTT
GATTACTCTAGGTCTGCATTTCAGCCCTGCTCTTCGAGATATTCTATCGAGC
AAGGTCCTCCTGTGGTTTGGAAAACAGTCTTTTGCCGTCTACCTGCTTCAT
GGACCTCTTCTCCGTAGTGTTCTCTGTTGGATGGTTTATGGAATTCGCGTCC
CGCCGCCAATCACTAATGACAAAGGCGAGACGATCCACGGTACACTTATGT
TCCCTAGCGGATGGCGATTGATGATCTCGCTTCCGTTCTGGATTCCTCTCAA
CTATGGCGCAGCAATGCTTTGGACGGGTTACGTTGACCCGTGGTGCGCGAC
CTTGACGGAGAAGATCGTGGGCAAGGTGATGTTGGCACGTGACGAGAAGG
GCGCGCTGTTGCCACAATAGGGGAGTGGCACTGATCGTATCGAGAAGCGC
AACGACAACATAGAATTGGAAATGATAGAGAGAAGTGAACCTCAAGAAGG
GGTTCTAAAGGACTGATGCTATAATCAGCATATCCTATCTTGCCGATTAGGAT
ATCAGGGGCAGCATTGGTTTACTAGAGATGGAGGATAACATCAATCGTTAC
CGACAACATACGACCCTATTTGCGACACCCTGGACGATAAAATCGCTCCTG
TACATATGGTACTATTCCTTTCTTTGATTTGCGATATGGGTGTTTGGGTTTTTC
TGGCGTACATAAGGGTCAAAGGCCATGGGATTGCATTGGTCAAACAGGCAT
TGGGAGGCATACAACGGCCGAAGAAAAAGTGCATCGTACATAGCGCCTGG
GGCGTCACTTGCTTAATTTCTACAGAGACGGCCGCGCAGCATTAAGACGCA
TGGAGAGGTGGAAATTCTTGCGACGAAGTTTTTATTTTTTTCGCCGGCTCG
CAACGGTTGATACCCAATGATTATGAAAGAAAGCCAAGCAAGCATTAGAGC
AAGATGCGCAAGACGATGCCACATCGCATCGCAACCCATACAGTCAATAAT
TGATAAAATTCTGACTACCTGAACATTACTATTTTCATGGTGAATTGTGCTTC
TCGTGATATAGGCTATTGGGATGGGGTGAAATGGTGATGAAGCCGTTTGGG
GACTAATTTAGTGAATACGATTTGAAGGAGCCGAACCAATTTCCCTCCAATA

AAAGTCTTATTTTCAATCACAAGTACAGTCGCGAAAAACAATCGGTTCAAA
CTTCATCCTACTAATTCGGTAGTATAAGACGGTTTATCGTGTCGATTTGTATT
CTCCAATATTACGAGTACCAAGACTCGGCCAAGAAGGCCAGCTCCAATGTC
TCGAATATTGCTCGTGGCTCACTATAGGCGCACCTACTGTCTTAGACATAGG
TAAGCTCACGATGGATGATCTTTTCTAATCGCAGGTTGAGACGGCGACGAA
AAGGCCGACAGCTATTACAAATATAAATATTGTGATTATTCATTATCACCCAT
GTTTCTGCATGGATACGATACAATTCAATTGTTCTAGACTCGAAGTCTTCAA
TATGTCCGTTCGTCGGGTATGACAAAAATATGTACAGGGCAATTAGGCAGTA
TACACGGATCGTATGACGTACCTGCGTAGACGCGTCGAAGGCAATAAAAAC
AAATTAATTCTTCCATAGCCGCAAAAGTCGCAGATCAAATATAGATCAAATA
GATAGGCGATATAGATGGATAGATTGGTCTAGGTAGGGTGAGATACGTACCG
TTGTGTATTAGATACTATGTAGCAAGTAGTTCTTGTACATGGATGTCATATGT
CAATATGCGGCCGAATAGGCTATCTCACTGGTTATCCCACCACACGCTGTTC
CGCGCATTGTCAGCACGATCGACCCCCCGCGCGCTACCGAAATTCTTCT
CACGTTCCCTTTCTTGACTCGCATGTAACCCACCCAACGGTCCGCTGGTCG
TATCCGGACTGTCGGGTCGTCCAGTGAGAGCTTAGCGAGCTTACCGGAATA
GGTAGTATGTCGCGCAGAGATGTAACCTTGCAATTGTATATTCAACCGTCTG
TTTAGACTTAAATTCATGGTTGCGATAGCCTATTTACTTATAGGCTTAACATG
GCTGAGGCTCGCATGCAGTTGAATTTTGGGATTATAATCAAAAAGGTATACA
GTAATGAATATCATGTTGGCCAAGAAGCCGTGGTGTAACTTGAAGTTATAGC
AGTATTCAGGGGCTTTTGAGTTTGCTCGACTTAGGTACCTTGTAGGTACAAT
AAAAGCCACCAATTTCTCAACCTCCCACCCCCTATCTCAGATCCCTAGCCAT
GGTAAATGAAGAATAGGACATAACTGCATTTTTTTCTTCCCTTTTGTTTTGG
AATTGCATGTCATTATACCAAGGTTGTTCCGATATACCTAACACTTCAATTAA
GAATATGACGTATGACGGAAGGATTCCCAGCGTAGCATTCTGCCAGGTGCA
CGAAAAGGGAAGCTTCTCCAAACAGTGACCTGTGAGTACTGCATAAGTGG
GTATGAGGGCGTTTCTTCAAAGAACTAGGTAGTCCCACGATACCTGCCATC
TCGTGGTCAAAGTGCACATGTATTATGACGCACGAGATCTGACTGTATTCTC
ACATGTTTCGGGTACGTTCACACAGCTTCGTGGTGACAGTACCGTCGTGTT
GGAAGGCTCACGAGACGTAAGCCGTATGAGTTGCGGGGAGGCATCATTTC
GAATTGGTTATGTGACGGGGAAATTACCTACATAGCATTATGTCAGAGATAT
CTTCAAGCTATCTAAGAAGAAAGGTTCAGATTAGTATTTGCTAACGACAGC
ATACCCCCATGAGGCACTAGGAGGCATGAAACCTGGCGTAGAAAAAGCCA
ACAGGTCCCTAGAAGTACCTATCCGATTGCTTTTCCGAATAACTTCTTCAGT
AATCGGAAGGTTACACAGTGGCTATCCAGCGGTAGAGACAGGTTTGATTTA
CAACTCAATTACGTTTGTTGGAAATGTTTCTTAAGGCACGTGTGAGTCTATT
CGCTTTCTTTAACATAACTAATAGGTTCTATGCTAGAAGAGAAGAAGACGA
AAAAGAATTCCGTCTAAAGTCACAGCTTAATAGTGTCCCAAGACCGTGATC

TGCGGGGTCTTGTAGATACTCCAGTCTTCCGACACAGTCGGGTCCCCGAAA
GGCCTTAGCGTAGCGAAACGCCACCTGCGAGATAATCGTCAGTATTGTGGC
ATTGTGGCTTGGTAGATCTTATATCAATGGAGTTCGGAGTTACTTACGTGTA
AACGCCGTTCGCGATCTGGCTTCCGTTGCCCAGCCGCCCGAACCACGAGT
GGTCCCAACCCCCGCGCAACTGGTCGGTTAGCGGGTACGTGAATGTGTCGT
TCGGGTTGTTGACGCTGGGGTCGAAGTAGCTTTGGCTACCGTTCAGGATGC
GGCAGGCCCGATGTAGCCGTTTTCGCCTTCTACGGGAGGGTATTTCCACTG
GCTCTCGTTCCAGTTGGCTTCGTAGATCTAAGAGAAAGGGTTAGGTCGGAG
CTGGCTTAAGCGTGAGTGTCAAAGCTACTTACATCCCACCGGATTTGCTTG
GTGCCCCAGCTGATCATGGTGTAGACTTTTGGGAAATCCTGCGAAGACAAG
CTCAGGTTGAACGTGTAGCTATTTTATGTTAACATCATGAAGCTGTTTGGGA
GAGCGTGGTCACGTACTTTGACTTTACGTTGATGGACTGGTAATTGACACC
TGAATTCGAAAAGGGTAGCCGGGGTAGTAAATGGGATCTAACTCCGCTTTC
AAGTCGGCGCCGAGTCCTAATTGGAAAGGTCAGTTTGAAAACCCCTATATA
GATATAGATGTAAAAGAGAACTAATTACCCAGGTATGGGAAAGAAAGTTCT
TCTCCGAGGCTCGATTGAAGGATAACTTTTCCGCTGTATAAGGGGAAGTTA
GAGGCGTTGTAACCCGCCGATCCCTGGGGGTTTACGAAGTTGACGCTAGAT
GCACGCCTTATTAGACTCGACGGCATTATGGTGTGTGGGTTAGATGTACTCA
CGAAACAGTTTTGCTCTCGCCAGCCTTCAAAGTGAAGGTCTCAGGTAGCTT
CACTTCGACAGGAAGAGCAATCGGCACCAAGTCGTCAAACGCTTTGACCT
CCTTGGTACCGCTATTGTTCTCGAGGATCTCAGTGCCACCAGCAGCTTCCA
CGGAGAACTTGTACTCGACATCCTTAGCACCATTGTTTGTAACGGTGATAG
AATGCGTGTGCTGGAAGTGAGCAGTGTCGTTGAGGTTGAACTTCTCATAAT
CAATCTCGGTGGTGTAGTTCACAACCTTGAACCCGTTGACCAACCCGCCAC
CAACCTGTGCAACGCTGGCCGTGTACTCGGTGCTCTTCTCCGAGCCATCAA
ACCACGGCAGCGACGCGCCACTTGCAATAATACGCTTGCGCAAAGTATGCG
CGAAACCCTTCCCATGCACACTTTTCCCGCCGAAAGCACTGACATATAGCG
CCGCAATACCGCGACGTACGGCGTGGCCATCGACGTACCGCTCATAAATGC
AGTCACAACGATTGAGGAGGCAGTGGGCTTGTAGGACGGCGAGCTTGGCG
AACTCGTCTCGTGCGAGGACCCAAAGCAGAGAAAAGCTATGCAGAAAATC
GCAGGGCCACCGTGTAGACCTGCTGCCGCGACCGGCTGACAGCGATGGAC
CGTGCGACAGGGTACGGTCGGGACATGAGACATAAATGCATGTGGCTCGA
GACGATGCTGAGAGGTGTCATGTACAACATACGTCGGAATGATGCGCTATA
GTAGAGTCGAAGAAACAGGACGTGTCGCTCGGTAGGTCACTCGGTCCTGC
GGGAGACCTTGGGTGTCAGTTGCGCATCATATTGCACGACTTCACACTTAC
GCCGTCATCCCATCACACTGTGTTCAGGATCTGCTTTGCTTGTTCAAACAG
ACACATCTTGGTCTCGTGCATCTTCTCCCGTTGATGGAGTGCCGTTGACTGG
TCGGCCCACAGTTTGCTCCTTTCCATGGTGAGCTTGTTGAATGCCTTCTCGC

GCTCGGAAAGCTTCCTGTCGTGCTTCTCGGCGACCTTTTCCCTGCTGGCGA
CCACCTGCTCCCTGAGCTGCACGTTCCGCTCCTTCTCGTCGACCGCCTTTTC
TTTCTCCGCGACCTTCTTCTCCTTGACTCCAAGCTGCTTGATCTTCTCCTCG
TACTCTTTCTGCGCAGCATCGAGCTTGTCCTTGTCATCCTCGAGCCGTGCGC
GTTTGTAGCGCTCGTCTTCGAGGTCTTCGCCGCATCGCTTCAGGTCTTCGCT
CATGTCGTCCCTGATTCCGTCAAGATCTCCGAGCTCAGCTTCGAGATTCTCG
TTGGCTTGGGTGAGGTCGGCCACCTCACGCTGCAACTCTTCGAGCTTGTCT
GTGAGGGAAGCCTTGTCCGCCACAAGCTCCTGGTTTCGAGCAGTGAGCTC
CTTCTTGTTCGCCGAGAGGCGCTCCGAGGTATCGGTGAGTTCTCGAGTCTT
GCTGCGCAGCTCGTCATATGCAGTGATTGCGGCGCGGAAGATAGCCATCGG
ATCGGGATCGCTCTCAGTGGTGGTGGCTGACGGGATGACAGTGGAAGCGG
GAGGAGGAAGCGCCTCCGACTGAGGGGCAAGGGCGGTGGCAGTAGCGCC
CGTCACGGGTGTTATCGCGGCCGCGTTCTCTTCGACTTGCATAAGGTCCTTC
TCGGTCTCCTTGGGGGAGTTTTCGGGCGCGTCCTCGCCCTGAGGCGCATGC
TTGATGTGAGACTCCGCGACGGACTTCTCGTCGTCCTCGCGTTCGGCCTCT
GAGCCCTCTTCATCGTCCTCATCGGCCTCGTCATCGTCCTCTTCATCCTCATC
TTCGTTCCAGTCTTCATCATCCTCGTCCTCTTCGTCCTCGTCCATGCTCTGAT
CGGCATCAGAGAGGTCGTTGTCCGAACCGAGACCGGCGGTCGGCGTACCG
GACGGGGTCGGGGATCCTTGACGAGTAGACTTGGGGCTCGACTGGGTGGG
CTTGTCGAGCGGGGATAAGTAGAATTGCCGACCAAGAGACATCTTCGCTTC
CTTCCCCTCCTTCGGGTCGGAGGCCTGGAATGAGGTCTTTCGCTCCACGAG
CTTACCGCTCAACACTCACGCTCATTGCGACAAGCATTCCGGGTCTGGATT
CAGAGGTATTTGGCGGTATGTCGAATGGTCACTGATAGAAACGTTCAGATG
TCGGGTGAAGGAAGCAGACGAGAGAAGTGATGTGATGGGAAAGAAAGCA
AACGCGGGTGGCGAGGGCGTGATCTCCCACGACGGGGTCCTTTATATTCAT
TCCACGTATCTCGTACACGTCACCTAGGGGGTCTGTGCGCACACATGAACA
CATGTACACTATTTGCTGATCTCGGAGTGCTGAAGTCGTGTAATAGAAGCTG
TACGTACCGCTCGCCCACGCTCATGTCTCAGACACAGCTTGGGACCATTAG
TGATGGTGGTCAGTGAAGGTGATTAGCAAAAGGCCTCGAGTATCGCTTACA
TGCGTCGCATACTGATGATACAACTTGTCAACTCCTCGCCTACCGCAGGCCC
GCCATCGGCACGGCGAAGCAGAGGGTAACCCAAATGCACGCACTCACACC
GCCGACGATGAGGATGTCCCTGATGACACTCTCGAACAGCGCTCGAACTCT
GGGGTCTTCAACGACACGCTCAGGCCCAAAGATTTAGTGGGCTTGTCCAG
GTCTTCCGGCGCGCCCTCGCCGCTGACGCGACTGACACCAAGAGTGTACC
GGCTGAGAAGAGCGGCGAGATCTTCTTGCTGAATCTGGGAGGCGCCCATT
GATACCTCTGCTCCTGATGGACGGACCAGTGGATCTCCGGGGCCATTGTCG
GGTACATCGAAAGGTGAGATCTCCGACCGTTTCCTAGGATCCGAATGAGCC
GCTGAGCCCAGCGATGCTGTCCGAGCGGTCGAGTGGCGTGCGGTCGAGGT

ACCGCCTATAAGTGGCGAAGTCACCACCAGAGGCGAGGTCATCGAGTCGA
AAACTGGGAATGCTTCTTCGGGGGAAGGCGCCTTGACCGTCGCTCGCCGT
ATCTCCAGCTGCCTTTTCTCGACCGCGGTCATCAAGTCGCCTCCTTCGCCG
ATTTCAACCTCTGGACCGAGGTAGATAGGAGCGACCACCTCGCTACGCTCG
AGCAGGACTGGCATGGGAGCACTTTCCCATTCCCCCTTGTCGGCGGAGGAT
GACACTCGCATCATGACCCCGCTCATATCGCTGAGTGGATGGACTTCTTCCC
CCAGATCGCTCGCCGTGCTGAGCTTTTCGCTGGGCTCACCAGGCGGCGCG
GCCACCGAGCTGCCTCGCGGACCAGTAGCCGAGGAATCTGCGCTGCCTCC
GTGATGACTCGAATGTGCTCCGCAAAGTATCGCCTCTTCGTCGATATCGTCC
ATTTCCCAAGGTCGAACCTGCCTGCTGCTGCGGCCCCAGACTTGGGTACAG
AATCCTCGGGCAGCCGAGTAGAATTGCATGCTTCCGAATGCAACGAAGATC
ACAGAGGGTAGGCGGGTAGCGCGAAGTCCCCACGGGTGAGATTTGAGACC
GAGCGTGAGACCAAGAAAGAGGGCCAGGCCGATGAGGAAATCGATTGTCC
CGACAATATACCTATCACTTAGTATTTCGATTGGGATACTGCCTGCATTACCT
ACCAGAATAGCTGCTTCGGTCGATTGATATTTGTCTTGGCATATGAGAGGAA
GTGAGGCAAGCTCTGAGTTTCGACTATCTGGTAGATGTCCTCGTATACGGG
CAGAAACTGGAATCAGTGTAATCCTGGGATAAACTGCTCGTGAACGACGC
ACCACTTCAGGCGCACAGCTCCGCTTCAGACACTCTTTGGCGAATTGCCTC
AGATCATCGGCGACACCAAGCTCCCTGCTGCCGCCACCTTTGAGAAAGGT
GGCAAATGCGCGGCGAGCCTCATCCCTCATGGGTTGCTCTCTCGTTTCCAG
CAGTACTGTGCCGGGCGGCGGCAAGGGCGGATTCAACGACGTGTGATGTA
GGATGGGTCTCGGTGGTTCCTCATTCTTCCCCTTGGCCTTATCCTTCTTGCC
AAAGATGCTCGACCTCCGTGGCGCTGAGTGGGCGTGGGAGAGATCGTCAC
AGCGGCAGTTGCCGTCAACCGCCCATTGGCATGGCTTGAACCGGGCGTCC
CCTTCCCAGCCCACGCCCGAGCTGATGGTGTTCTTATGAGGCGAGGGAGGA
TTTGAGGAGCCGGACGCGTCTACGCTTGCCTGTCGTGACTGGACTCGGCCT
TCCTCGATCGCCTTGTCGAGATACCCAAATGGCCGATATCGATCTCCCAGCT
TGGTGCTCGGGATGGGAACCGCTCGCCTGACCTCGTCTGGAAGCGCCTCC
CACCGTGACCGATACGATCGGAACCAGATGATGAATAAAAGATTCTCGGAC
GAGAACTCTTTCTGAGAGATGAAGGCCGCAAAGTCGGGAAAGGAGATTGG
GGGACACGTCTCGCCATTCAAGACGTGACTCAGCTTGAGCTCGTCCAGAG
TCGACACCGCCGACATGGACCGCCGCAGTCGCTGCTTTCCGCCCACCGATA
CCGACTCTGATGTGGTCGTGAGAGTGGTCGTCATGGAGAGGCGTGAAGAC
TTGCGGACCGAGGCAGCAGGCTGTTGGGCGGTCGAGGATGGTGTGGCGTC
GGACATGGTATACAGACAGGTGTGAGTCGACCCCGAGGAACGGGGCTAGA
AGGCGGTTGTGCTAGTCTGGAACGCGCGGATCGACGTCCGTCCTCGGTGTG
CGGGTAAGTCGCTAAAGATCTGTCTGCCGACTTTCTCTCAAAGGACAAGGT
CGAGTGGGAAACATGATAGCCGTTCGAGGTGTACGTGTATGTAGACAAGAC

GATCCTGCCCGGTCAAGATTGATGCCGGAGTCGCGCTTGACTAAGGGGATC
AAAGTACGACGAACTGATAGGCAAGGCTTGACTTTGAGCGCGTGAGGGGC
TGGGCGTAGAGGATTCCCGATTGAAGTACGAAGTGAGTAGAAAGAGGCGC
AATGGTAAATTACGACCCCCCGCCAAAAGTCGTCCCGTCCATTCGGCATGT
CACAGCACTGTGTGCCTGGCTCTGTGCCTGTACCTGTGCATCCTCCGAAAT
ACGCTAGCACCCTGAGCCGAAGGGCGCCGACACGTGAGGCTGGGGCGTAC
ATTCAAGAGCGGCGCCCGCCGTTAGCACGCTGTTCTGCATCATCGAGACGC
CAGTCGCTTCACGATGCATGCCAGGAGGCTGGGAGGGGCGCTGACAATTT
GTTCTCCGGCTTGCTTGTATATTCTCTGCCTGAAATACCGCCTTTCCATCCCA
GTGACCGCCGCCCATGCTCCTTTGGCGGCGAGGAGCGGAGATGAATTAAAT
CTCCGCTCTGACGTTCTTGGCTTTTGCCGCCAGAGAGACGTGAGCTTCGAG
AAACACTTTCTTTCTGTCAGCGCTTCTCAGTATCTCGTCAGGAGCGGCTTTT
CGACGATGTCCATGCAAGCTGTCAGACCGCTACGGCAAGCCGTGACATGTC
GATGCTCTCCGCACGCCTCGTCCTCTCGCGTGCGCCTCCCAGCTGCCCGCT
CGATGTCTTTAATGGCAAACCGACCTCAAGAAGCTGTCCCGTTCTTCACGC
CGCCATCTACCTCCCCTGTCTGGTCATCATTCAGAAATGCGAATGGGGCGCT
CGATCTCAAAGGCAAGGGGAAAGCCAAGCCACCTCCGAGCGGGGAAGAG
GAGGTGGACGACGGGGAATGGGAAATGAGAATAGGTTGGTAGTCCTGTTG
GCACCTCTCCAAAAAGGCGCAAGTGAGCTGAGATCCAGCTCGAGTCATGC
TTCATCTGCGAGAATCACTGCCTCAGCTGTTTGACAGCCGACTGGCATCAG
TCGAGCTCTTTCCTGCGGACGTCTTCAGCAAAAATGTTGTCCTCAAGCTGC
CGGCACCTCTCCCCCTCAAGGTAAGCTACCATTCGGTCTAGTTGAGATCCG
CGCGTGGCTCACATACAGCTCCACTCACTTGCTCGATATTCGATGGCGTTCT
CCATTGCTCGCAATGGTATGCAAGGTCCGTCGAGCCCTATTCATGGCTGCCC
TGTAGCTCACTGTTGCAGCACTGCACACTGATCTGCGTACCACTCTGGAAC
GCATGACTTTTTCGCCGTGTCCATCGTCTTCATCGAGTGCTTCGGCAACATT
ACCTCTCAAGCCGATCCCTTCACACCGACACAAGCAGATTCGTGTACTGTT
GCAGGTCCATGCGATTCCGAGACTGCCCCCTCATCATGTCGGCTACCTTCGT
CTTGCTTCTCACACGAGCTGATCCTCCAGGAGCATACATGGCATACATCGTC
CCTATACACCTTCTCGCCCTACTCTGGCTTGATAATGTCGCATGAGGTTGAG
ACCATCCGGCCACTGCCTGGCGAGGGAGTTGGGGAGTTCCTCATGACACG
GCTGCTTGGGTGGACTTCCAGAACCGGGCAGGGGGAAGGTGCGATACCCG
CTCCCGGGCATGCCGCTGCGAGTGAGGTCGAGGTAGTACCTGTCAGATGTA
CGAGCAGCTCGAGGGATCAGAGTTGAGAGACGAAAAACGCATCTCCACGT
CGTATCTTATGCATAATCACTCTGCTCATAAGCGATGCTCTTCCAGCTTAAGT
GGGAGATTCTCGGCATGGCGTTCGTTCCTGAAACGAGATGGTGCAGCTCGA
ACCGGCAAAAGATTTCGACACCCTCTACTGTCATTCTTCTATCAAGTAGCAG
CTCGCAGCGGTCAAACTGCGCCTCACATCTCTCGTGCGGGCGTCAAAGATC

GGCATCATGCCCAAAGCATCGCCGAACCCGCCTTTGCCCCTCAACGGGCTG
TTTCCCATCGCCAAACCCTCTGGGCCATCATCGTTGGCTGATGCTTCCTCCT
ACATGGACGAGCACGCTGACAGGCAGCATGCGTGTCATCGATTCGATCACG
CCCTTGTTGGTGCATTCCAAGCTGTTTGATGATCCAGAGAGGCGCCGGCAT
GCACAGGGCAAGATTCCCTGGAAGAAGAAGAATACAACGCATCTTGG
GTTGAAGATTGGGCAAGGGGGCACGCTTGACCCGCTCGCAGATGGAGTCC
TTGGTGGGTCCCTAATCATTCTGAACAGCGCAGCTTCTAGATATGCCTGGGA
CGGATCGAGCTGACTGCTAGTAATCGGGGTGAACAGGGGCACCAAGCACC
TCAACAGGTTTCTCGAGTGCACCAAGGCGAGCAACTTAGTGCGACTTCGG
TCGGACGATGGCTGACGAGCAGACGTATACGTCCATTGGTATGCTGGGCGC
CTCGACGACAACGTACGATGCTGAAGGAGAAATTCTCACGACCGCGAAAT
GGGACCATATCACTCGGGAGGACATTGAGGGCGTGCTCGACAGGTTTCGA
GGGGAGATCATCCAGACCCCACCGATGTGAGCTGGACCCGCGTACTGTGG
CAGCAGCAACGAAACTCATGTCAGCTTTTCTGCGCTCAAGATGGACGGCA
AGCCGCTTTATGAGTACGCCCGCGCCTCACAACCCCTGCCACGAGCGATAC
CCACTCGAAAGTGCCAAGTCTCGATCGAGCTGCTAAACTTCACCCCTGCAT
CAAAGACCACCTCTCCCCCACCGCGCCCAGATACCGAAGCTGGACCATCA
ATGCCAGTCTCCGAGCCTGGTGTCGGCACAGACGGGGGACACTTGTATCGT
TGGCCAACAAAGCATCTCTCAGCAGATGAAAAGGCCGTCTTTGACAAATT
GCACCAGCTCGTACACGACGCTTCTGCCGCAGAGCATGTTACCGAGCCACT
CGTACCCAACCTGAAGCAGGAACAGGTCGCCGAGGTTTTACTGGACGGCA
CGCGCCCACCGACATTCGAAGTCCGCATGACCGTCAGCAGCGGCACATAC
GTCCGGAGCATCGTCAACGATATCGGTCTCGCACTTGGCTGTGGCGCGCAT
GTCGTCAAGCTTACTCGGACCCGACAGGGCGAGTTTACGCTGTACGACGA
GGTTCCACCTGCCTCTGCGATGCAGGAGGGACAGGAAGCAGAAGCTGGGC
CGAGCTCGGGGGCCACGACGGCCTGCATCCCTTGGGAGGTGTGGGAGCGG
GCCATTGCCAATCGGGAGAGCAAGCTCAAGCAGAAGGAAACGGACAGGG
AGGAAGCGCTTGCTACAGGGGCGTCAGAGGAAGAGGCGCAGAACAATAC
GGCAGACAAGGAGGAGGGTGGCAAGGAGCAGACGGTGGAGAGTCTTGGG
CAGCTCGAAGAGTGGGAGCAAGAGGTGCTGAGGAGATTCGTGCCCGTCCC
AGTGCCTATCAGTGGGTCCAACTCGCGGGGAAGTCCAGTCATGAGGTACCC
ATGAGTCTTGCTACGCGAGATACAGGCCTGGATCCCATGTGTTGGGCCAGG
CATATGCATCGGCTCGCTAGCATATCGGCACATCCATAACTTATTGGCTATTC
GCAGGGAGATACTGTCCACCTCCCAACCTCCTTTCGGCTGCGCCTCGAGCA
GCTGCGATGTAAAATGCTCGCTCACTTCTTTGAGCTTCTCGCCGGTCAAGG
GATAAGGCGGCAGCGGTGGATACTGTCCCTCAAGCAGTGGCTCCTGGCCAT
CCGGGTCGGTGGTATCATCAATGTCGTCGAGCAGTGCAGGCAACGCAGTGC
CGTCATCCGCGGTTGCAGACGGTGCATGTGCCGACGCCGTATCCGTCGGCC

GTGCAGCGCCACTGGGAGCTGACACGCCGTGGCGTCGACGGATCCTTCGC
GAGCAAACACCACGCGAATGAAGTATGGAACTCTGGATCGTCGCCGTGGT
ATACGGGCAGGTGCATTTTGCTCAACAGCGGCTCAAGGGCACGCTTGACCC
GGTTCAGCTGTCATTGGTGATCGCTCCAATTACACGCCCCTTCAAGACTCA
CCTCTGGCTGACCTGCACCTAGGCGAAGGGCGAAGAACGCACGGCCACCC
TGGCCCACCCCGCCCGTCCGCGTCCCGTTATGATACACCCTCACGTCGCCC
GCCAGGCTGACCGCCACGCCCTTGCCCTCGGGCAGCGCACTCCTACATGCT
CGAAAGACGTCATTGCGAAAGTCGTCGATCTGCGAGCGACGCAGGGCAAG
TGGGTGCGTGAGCGAGACGTGTAGGGCCGGGTTGGACGGGAGGTAGTTGC
GCACGACGCGGTAGCTGGGCACGAGGGGACTGTATGTGATCCGCGAGGGC
GTTGAGCGTAGCCACGAGCGATCTCGACGGGGTCACTGGGGTCAGGGGTA
GCCTCGAGATGGCGAGGACTTAGCTCACGAGACACAGAGACGTGTGTGAT
GTAGTCGCCCTCGACGTATGGCCTCGTACGCTTGCGCCCCTGATGCTGCGC
CGGGTCATCTTTCGGGTTGACCCCCGTCTGATATGCGCCCGGCAAGCCCGG
CAGGGGTTTCCTCTTCTTTGACAGGGGCGTATCGAACGGTCCAGCTGCTCG
AGCAGAGGTGCTAACGCTTCGACGGGTACTAGCCCTTGATCTGGCCGAACC
TGAGGAGGAGGCGCTCGAATCGGATGTGTAGTCGACCAGCGCCATCTGCTT
CATGCAGCGCGAGCATGCTGCGGCTTGCCTGAGTGGTGCGCGGGGCTGCC
AGACTCTACAGAGAGATAGCATGAACGTGCAGAGCAAAAGTGCTCTTGCG
ACCGTTTTGCGCGACGGGCTTGGGTGGCGGATTGGTGGCGGCGACAATGT
GGCAGGTGGCGGCGATCAAGGGTTCTTCATCGTTACTGCATCTATTCTTCTG
CGCTCTATGTACTACTCTACTGCTCGCATCTACTTCGACTTCTTTCACTTCAT
CAACGTCTTCCGTTCCGTGTACGCCTAGCCGAAGTAAGGCTCGTCATCATA
GTCGGTCAGCTCATTGGCGCCGCTCCAGAAATGCCCGTTTGCTTGAACATC
CTGACAGTGAGCACAACCCTTGACCAGCAAGAAGGGCGACTTACCTCCAC
GAACAGAGGAGGCGCGAGTTGAGCAGCCCTCTTGACGATGCTGTGATGCC
GTGCGACGTGGCGCAAATACGGCTCGGCCTCCATCCGCCTCTTCTCCCTGC
CCCTGCTTTCCCCCCTCCTAAGCGTCTTGCCTTGGCCAGTGCGCAAGAGGT
CCATGCTCGGCTGCAGCAGCGAGCTCAACTCTCCAGCAATATCGGACGATG
AGGTAAATTCTGTCCTGCCGAGGAGACGCCCGGTGTCGAGATTAAACTTGT
CCGGGTGGATCTCTGTTATTGCAACTACGTCGGCAATTGTTCATCGGGCTGC
GCGCGAGAAGGTGGTCGAGGTCAAGGTCGAACTTGTCCGGATGAATCTCT
GCGAGCGAGTCGAGGTCGGCTAGGTCCCTTCTTTGCAACGTTGATCGTCGT
CGGAGTAGGTCACCGAGGTCGAGGTCGAGCTTGTCTGGGTTGATCTCCGC
GAGTGAAATAATGTCTGCAAGGTCCCTTGTCTGAGGAGTACCACGGCGGA
GAAGGTGACCGAGGTCGAGGTCGAGCTTGTCTGGGTTGATCTGCGCGAGG
GATACAATGTCGGCGAGGTCTCTAGTTTCTGACGGAGCGCGATTGAAAAGC
TGGATCATGTCTAGTGCGGGCTCGTCCGTGTCGACCTGGACCTCTGTCGGA

CTCGTCGTATCGAGCGAATATCGTGCCTCTGGCCTAACACTACTGACTCGCT
TCAGAAGATCCCCAAGGCCAAGACCGAGCTTGTCAGGGTGAACTTCGGCA
AGCTCTACCCCCTGGACCAAGTCGCCGACTTCCTCCTCCAGCTGTGTCCCG
CGACGAAGGTCATCCGTCCTGAGCCGATTCACATCGATCTGCACGGCCGTG
GTCGCGTCCAAGAGTGTGTCCAGGCTATTGATATCCCCATCCCGTTTCAGCA
AACCGCCCAGGTCGAGTTTATCCGGGTTGACAGCCACAAGCCCCGTAGCG
TCCAGCAGATCATCAAGATCGTCTTGATGCTCATGTCCGCTTGAGAGTCGG
AGAGCACATTCTTGCCGACGACAGCCTCGATGTTCGCCAGGTTCAAGAGAT
CATCGCCGTCATCAGGCTTCGGTACCGTCGTCCCGACCATCGTCTCTGGCTG
CTGCTGCTGTTGTTGCTGGTCCAAGTCATGCGCGGCGCTCTCGGCCGGGAT
GACGATGCCCAGTCCTGCGAGACTCGAGCCAAGCACACCTTTCCGCCTGA
GATGCGACAGGTCCAGTGGGGCAGCGGCTGCTACCCGGCCCAGAGTGTCG
GTGGGCCACGACGCCTCCGACACCAGGGAGGTCATGTGCGGTGCTTTCGA
GCTGCGTGACGGAGGACAGCGCCGGCGATACGGTACCCATTAGCGGACTC
AGCGATTGAGGAAAGGAAATTGTGGGGCTACGCTGCAATATCAGCTGTGTT
CCGCAGGAGTATGATCTGGCTCACCGCCTCAGGGAGGGGAGAGCCAATGG
CGAGCGAGGTGAACGCCGCAATGGTCGCGAGCATAGGAGAGCGCATTTTG
CCGGTTTTTTGGCTCCAACGCGCTTGAAGGAAAATCTGGGGACGATCGTCA
GTATGTCTTTCCCCAAGGTGCAGGAGGCGCTGTAGGGGGAGACTCCGCATA
GCGACATCTTATATCGCCAAGCCTTGCGCAATGCTCATGCATAGCGGCGACA
ACCTTTGCTTTCGGAGATCGCAATCGGCTGTCTCAGCTGCAGATCTCGCAC
ATGGCTGATACGTCCATGTCTCAGCAACACGCCGAAACAACCAGTCAACTC
TGAAACAACCCAAACGGGTATCGCAAATGCGTCCAAGCTCGTCTGTGCATA
AACACATCGTCAATAACATTATCATAACTCTTCATCGGAGTCCTCCGCTACA
CGTGAGTTGTTGGACACACTGCGAACACGTAGCTCACCACCGACACTTGC
GAGGAAATTGACGAAATCCGCTTCCTTCTTTTGCTCGACAGTCGGGACAGG
CATTGCCTTGGCGGCTGGCATCGGTCGGACTTTGTCCTTTCTGGCCTTCTTG
TGCGAGGTACGAGTTTGGATCTCCTCGATCTCCGCCGTTGGCTCTTCCACGT
CCATTCCCATGAAACCCAGTGTGAGCTCGTCTTCGGTTCTTTCAGCCGCAG
CGCGGCGCTTTTTGGCTTTCTTCGCGGCCTGCTTCGCTTTCTGCCTGTCGAG
CATTCCATTGGGCAGCACAGACAGTTCGTCGGGAGTGAAAAGCTTCGATG
CTGCGCCGGAGGTAGGAAGGACAGGCTTCACAAACGTCGACTGGATCGGC
GTCGGAGCAGTTGCGAGTGAGGGAGGGCGGTATGCAGGTCGAGCAACTTC
GTCCTCATCTTCCGACTCCGAGTCGATTCGCGCGAAAGCCGGGGCCGTAGG
GGCCGGGACTGACGTAGGTCCGGAAGGCTGGGGCGGGATGTCGAAGCTTG
GGGTCAGCAAACATTGGCGTCGCAAACGGGACTGACTCTTCTTCCATTCCA
CCAGCTTGGTCCTGGACGTTCTCCTCCCTGTCCCATGCTGCATCATCACCGG
TCACGTCGAGAAGCCCGTCCAGCGTGAAAGCTTCTGAAAGCTGACTGAGG

ATCTTGGCACCGCTTGGTATGCCATTCTGGTCGTTCATCTCGGAATCGCCAT
TTCCAAGTGGAGCAGTGGTAGGCACAGATGCGGGATGATTGGCAGGAGGT
AGTGTGAAGTAAGGAATCTTGCCGATGTTCCAGTCCCGCAGTACATTCACT
GCCGCTGCATCTAGATCGGGTACACCTCCCTAGTCGGTCAGTTGCGCGCTC
TCAACCGATTCGTTGGGTGCCAGCTGACCTTCCCCAGTCGCCCCTTGGTCA
GTGCTATGCGGATCAGAAATTCTCGGACATCCTGGTAGGGCGGGATATTGTA
GAGGTTTTGCAGGATTTCGGGCTGTACTTTGGACAAGATGACCTCGACTTT
TGATCAGCAAAGAGCCCTGCTCATCCCTAAAGCACCTACCAGGGGTGATTG
GATCATCCACTAATTCGGCCTTGATACAGTTACGCAACATGATCTCAGCCCT
CTTCTTCTCGCTCTCCTCTCGGCTTAGATCCTCAAGCACGACACCTGGACA
GTCGAGAATCTTGACTCCTTTATCCAGTACCACCTCTTGGACGACCCTAGTC
TTACCGGGCATGGCAGCCACGGCGCATGCTCGGCTTCGTTTGAGCGAGTTG
ATCAGGGACGATTTTCCGACATTCGGATACCCTACGACGCCGACAGTCAAA
GACGCATGGGTGTTCAGAGCGTACTGCTTGAGCAGCTGGAGTAATGCCGGT
GCGCCGAGCGAGGAGGATGTAGAAGGGAGTTCGGGAAGGGTGACTTTCTG
GCCCGGGACCGAAGGTGCTTGGACGATCGGGACGGAGTTCTGAGAGAGGT
TTTGGCGCTGATTCTGCGTGGAGGACTTGAATGGCATGCAGGGGAAGGAAT
GGCGCAGGTGTTTCAACCATGCCTCGAGATTCGCTCGTGGAACCAGATCTG
CCGTCAGCTACAAGGAAAAAGCCCAGAAATCCAACGTACCAATCTTGTTC
ACGACCGCCAACAGCTTCTTCCCATCAATGTCCCTCTTCCTCACTTCCTCCT
CGACCCATCTGCTACGAGTGCCATCGGGATCGCGAGCGTCGAGGACCTGG
ATGATGACGTCGCTTCTCTCAATAACCTTTCTCAGCTCGCGCATGAAGGCTT
TGGAAGAGGAATCGCGAGTTAGCGAGGAGTCGATGGGCTGATGCCATGTC
GAAGAGGACGGGTCCGACGGGAAAGCCAATTCGGATGCTACAGGCAAAAT
CGACGTGGGCGGGGCAGGCTGCCTCTCAGCCTTGAGCAGCACTTGAGTCC
GGAAGACTCACTCGAGCTTTGGCTACATTGACTGCCTGCTTCCCCGCTAGC
TTTGAAGACTGTAGTTTAGGCACACCCAAATCTCGCTTCTCTGCGAGAATAT
CAGCGGGGTGACCATGGCGCGGAAGAAGTGCTCACTTTGTGCAGACGGGA
CATTGTAGACTTTGCCTTTGCCGCCATTGTTGGACTTGTCTGCACGTAAGCT
AGTCCTTGTCAGACTGTCGCGGGCCTCTCACTTGCTTTCGGCATGCTTCCTT
CTGGCTGATTGTGATCTTTTGAGACGAACAGGCATCTACTCGTCATTCTGAA
CATTTTGGCCGGACGGTGATCGCCGCCAGACCTCGGATCCGAAGCAGCCTT
CCAGATGATCAAGCGAGCGCGAATTCGGATCCCATCCTCGATCTAACACTA
CCAAGCCGCAACATGAGTTTGCTAAGCCGATCCGTACGGGCGGCCGCATCG
TCTTCTAAGCCCTGCCTCTCCAGGCGAGCTTTGCACGCCTCTCGACCCGTT
CAGGAGCACTTCCTCGATGCCACACCGGAGGTGGGTTCTCTGACTTCTCCC
AGATGCTCGGGGCTGACTTTACGAGGTGTTTGAGAAACGGGTGATGGACT
CTGCAGCCGCCAAACCCATTCTTGTCGACTTCTATGCCTCTTGGTGCCAGCC

ATGTCGAGTCCTGACACCGCTGCTCAAGAGCGCCACCGGACCTGAGAGCG
GGGCGGACCTGCTTACTCTGGATGTCGATGCCCACCCGGAGCTGGCGGCA
AAGTACCAAGTGAGCGTTCTTGGCCTTGCCTTTCTCTTGACTAATGACAGA
TATCATCACTGCCTACTGTGATAGCCTTCAAGAATGGCCAAAAGGTCAACC
GATTCAGTCAGCTACGTTATGCACCCACATTCTAAGCTGACGAGCAGTCGG
CTTCCGACCCGAGGCAGATGTCAAAAAGTTCATCGAGATGGTCAAATAGGC
ATAGCATATGCAGCGACTTCCAGCATTCACGCGCGCTCTGCGTTCCGGTCAT
ATCCAAAGCAGCTAGCATGGTTGATCGGTTTTTGTCATCTGCCTCTCTCCCC
ATTCCGGCTTCGTGTGCTACTCGACCTCCCCTCCAGATGATCCTCCCGCCTT
CTCGGCGTCCTTCTCCCTCCCATCCCGTGTGGTGTGACGATCCCTGTACTTC
TGCCGCAGTCTGCCGATTGTGCCCCCGACAAGACTGTCTGCGCGGCCATAC
CACTTGATCTTCTTTAACTCCTCTTTCGAGCGGGGCACCGGCACTTTGCCGC
ATTCCCGCCCGATACAATACGTCTTATCGCAAAGACAGTGCAGCTCCTTGTT
GGCGCCCTCCTTGAGGTGGCGGTAGTTGACATGCTGATGGTCGAAAATCTC
ATCTTTGACCGAGGTCGGGAGACACTTGTACTCGAGGCGGTTGAAATCTCT
CTTGATCTGCTCGTGCAGGTCCGGATCTTCGACCTTGGCAAGCAGATCTTG
CAGCCTCTTCGCGCGCGAATGCTTGCTCTCGTTTCCCTCGTATTTGACGTGG
TGCTTGTCGTAGGTGCGCCGTCCTTCGTGGTACGTCCATACCCAGAAACAC
TCGCAGCATTCGAAAAGGTGGTAGGAGCATTTGCCCCAGCATATCGCGCAT
TTGTAGCAACATTTGCACCATTGCGTGCACATCGAGAAGAGGGACCACACT
ACTGAGAAGGCGAGCAGGGCGATAACTGGGAGTTAGAATGCCAGGTCAAT
TCATCTCAGAAGGGCTTGAAGACACGCACTGACGTAGACCACATATTGGTA
TTTCTCCCAAAGGTCGTACAGCTGTTCGTCAGCCGAAAGGCTCATGCAAGC
TCGAACTGGTGATGATTTACGTGTTGAATGGTGTTGACCGCGTCCTGCGCTT
CGCCGGCAATTGTCGCAATGTTCTGCGTTGTCAGCGAGATTACTGAGTTGT
ATCCAAACACATACTCCTGCAGCATTAGCCGCCGTCTTGCCGGCGTCCGTC
ACCGTGCCCGCGACATTGGTGGCGGTAGTGGCAATGGTCTTACCTACGTCC
GTGGCGCCAGTAGCGACGGTCTTACCCGCATCTGTGATCCCTCCGATGACG
TCCATCCTACGCGATCCGGCTGCCCTGCAGACAGCTGCGCTCTTGGAGACC
TCTCTTCGTAGCGCCAGACGAGGGGCAGGAGAGCTCTACACAACCACGCT
CTGACAGGCGTACAAGTGTAACATCCCTCTCCGCCTCAGTAGTTGAGGGTC
AGCCAGAGGTATCAGACGTCGAGGTGTTTCGAGCGGACGGCCGGAGGAGA
AGGGTCAAAGGACTGTCTTGGTGTTTGGAGGAGAACAGAGGGTGAGCGA
GAAAAGAGGTCGAGCAACTCACATGTACAGAGGTGAAGGGACGGGGCAG
ACTACTTCCTTCTCGTCAGAAGGGATCAGTGGTCAGTCGTTCAGTGCCAAG
CGTCCACCCCGAGGGAAGGTGAGTATGTACATACATGCTTATGAGGTGCGG
CTGCTCGCCGGGCGCCGCCTGCTCTCGACGTTTGGGCTCCTTTGAGGCAGG
ACGGGTGACTACCGCTGGCTACCACGGCACCTTGTCCCCGCGCCAGTCAA

AGAACCCTCCTCCCCACTCCTGCTTGTCGTCCCGCCTCGCCTGACGCATGA
CGCCCGTCATCTTCTCGATGGCCTGATCTACACTAAAGACCCCTTGGTCGG
GTCGCGGCGTCGCCTTGGACCCGACGACAGGGCGGGAAAGATCGGTCAGT
ACCGTGCCGGGGTGATAGGCCATCGCCAGTCCCGACGAGCTCTTATGCACC
AGCTGCGCTCAGCGACTGCGGTCCATTTCCTGTCTCCGGCCGAGCTTTAAA
ACTCACCTCGTGGTCGAGGGACCGTATGACCTGGTTCAGCGCTGCTTTCGA
CCTGCTCTCAGACGGATCCAAAGGTAAAGTGAGGACTCACGCTCGGTAGC
TGTACCATCCTCCACGCTCATTGTCCGCAATACTCCCCACTCTCGCACTGAG
AGAAAAGCACAGGCTCCCTTCGGGATCGACGAGTCCCTCGGCCGGGTCGC
TCTCAGCCTTGAACCGGTCCGAAAGGGCATTGAAGTCTTTCTTGCTCGGGA
GGAGAGGCACGAAGTGCTTATAGGTGAACAGATGCCCAAGGGCATTGATC
TGGAACGTCCGTAGTGCTTCAGCGGGGTCGATGGCGCCGAGCGACTTCTC
AGGCCGGAGCTATCCTTCAACCTTCACTTGCCGTGAGCGCCGAAAATACTC
ACTATGCCCGCTAGACACGCGATGAGCCGCACGTCGTGCCCGCCCGTCTCG
GCTCGCACCTGCTCTGCCGCCCTAGCGAGCGAGTCATCTTTCGTGACGTCC
ACATCGCCGATGACTTTGAGCCTGTCCTTGGCTTCCCCCGCACTTCCGGAC
AGCTTGGCCTCCAGGTCGGACGCCGAGCGCGAGGTGAGCGCGTACACTCG
GAGCGAAGTGGTTTTGAGGAGGTGCGCCGCGAGGGCGGAGCCGAGGCCG
CCCGAGGCGCCTTGCAAGATGGCCGTGGACATGCCGATTCTGCTCCAGATG
CGGACCGCGGTGCTGATGAGTGAACACGGATTGCATGGTCTGGCATGCATG
CATCGCGACGAGTTGTAAGAGGAGAGACGTCATTCCAAGGTGGGTAGAGG
GCGATCGCGATCGAGACAAGCTCCGTCCGGTCCGCGTCCGAAACAGCCGG
TCAGTCGTTTTCAGGCGAGCGACTTGTATCAGGATGCCGTGGAATGCATGA
CTACCGACCGCTGCGGCTCAAGATATCTCGTCGCCGTCGTCGTGTCTCCTTC
CTACGACCCAAGTCATGTATCCAAACGGCCAACTATGCAAGCTTCTGCCGC
TACTCCATATCCATTCTGGGAATGGCGAATCATCCCCCTCGGCC

>Mosaic_sequence_50

CCCACCTGACTTTGCTCTTCCATGTATGTGGTTCGGAACGCAAGGGAAACG
CCGCCTCTATGTCGCAGAGAAGGCCACGAGCAGTTGACACCGCCTTTGCG
AATAACGCTGCTGGCGCCGGGTCGCTATCACCTTGTCCTTTGGGGTTCGTA
GATATCGGTGAGTATGAGGGGCAATTTATCGATTCTAGTCCCCTGATTTCCT
GTCGTAATAGCAGGACATGGTTCATGGCGACGTTTAAGTCCTCGTCTGCGAT
CTTGTAGTCATGCTTGAAGCGAGATATCGCCATGCTCAATCTTGAGATGACA
TCTAGCATGCCCACCACTCCGGTCGTAATCGATATAGGATCCATGCTCGGCT
TTTGGTAGGGATGTCAGAAGTGGAATAGTGATAGCCAAAAGCGTAACAGC
ACGCAGGTTTAGTTCATTGATGTGTCTCTAGAAACTTCAGGCCTCCTGATAT
AAGCCAGGAGAATCACAAAGAAAGATTTGCTTCAAGACACTATTGTTGTAT

GAACTATAGACAGATAGGTATACATTCTCTCAACTCGCTTTTAACTAGTCAG
TAGACGACAGCAGCAGCAGCACCACCAGATATGAACCGGAGCCATGGAAG
ATGGGATGGACCAGACTTGATGGGGGCCAAAAATCGAGGCCGGGATCGTG
GATAAGAAACCACCAACAAGGCAATAGAAGCGCCCTGCCCCGCGCGAATC
AACAGGATTGCAGCAGCCATCAGCAAACCTAGCTCGGTCTTGCGCTCCTGA
TTGGCGACGTGGAGTCTGCTGCCTCATTCTGCTCGCCTGGATCTCGCCCAA
AGGCGTTTCAATTGATGCTGTTTATTATATCTCTTGAACTGACGAAAGTCCC
CGACCGGGGATTAGGAGGTTTTTTGTATGTTATGTCCGACCCATAGTATAGG
CCATAACTGGCTTGAAGCCATATTACCTGGTTGGTAGGTACTAAGGGCCTTG
GTAATATGTCTCAGTCCCGCACTGGGACTCTCAGTCATCAGGAATGCACTTT
TGTAGATAATCTACACCTTAATTTCTGGTCGATGACTTGCGATAGTTAAGTAA
GTAGCTGTTTAGCTTGGAGACCTAGTATAGGTAAGTTATGAGAAGAAGAAT
TTGTAGAAGATAACGGGATGACGCGTAGCTATAGTACTGGAGGAGCAGCCT
TTACGCCACTCAGCTGATTTTGCGGGGGGGCGGGATGACAGGGGATCGAA
GCCGAAGCATCTCGTCGTTCCAAGTTTCAACATTATCTAAGATGGCTTTGGA
CGAGCTAGAGCGTGAAAATGGGCGATAAAGTATTGCTTACCTTGAGTTCAT
GAATTAAAAACGTCTTATCTGTTAGTAACTCATTGCTAATTTTATGATCGTTA
GGTTTTCCATCGCAGTCTTCGAGATGCGTTTCCCGCGTAGCGAAATCCCCG
ATCCGATAATAACGAGTGTCGTCACGATGATTTCTAGGAAGAAAAGGTCGG
GCTAGGAACAGACCTTATATACAAGCCTTGACTCACGTGCTCGTATAAGCA
CAGACTGCCATCGTCCCACGAGAATGCGACTGGATATTAGTTTCACGCAAC
GAACATCTTTTATCTTTGCAAAAGTTTTCTCGGAGAGTCCGAAGAATGCCTT
ACCTTCCTACCGAGCGTCTAAAAAGGGACTATTTCAGGGGCTTCTGAGTAT
ACTGTCCGCCTTGGGAGACAACCCGCGCTTTTCGCTCGTTTTCGTGTCTTG
CAATTGGCTAGAATATTCAGCTGTTGCATACATAGTCGCAGGTAGTACTTCT
TGGTATCCACTAGGAATTCTGCCAACAGGAACACTTAAATGCGAAGCTTTG
GCCGGCATATCCTGGAAAACAGCTTTATCTCGAACGCGAATTCGATGTAAAT
ACTCGACGCAATTGAGAAAATGCCACAGGTGCAGTCCAGTTTGGGGCCCC
CTTCCGACATGTTTGCGTGGTAGGAGCCGGTGTTAGCGGGCTTCGGGCTGC
TGGACTGCTTGCCGCGGCGGGCTTTGAAGTCACAATTCTTGAGGCGCGAG
ATCGTATCGGCGGCCGTATTCAGCAAAGTTCCCAATTGGGTCTTCCAATTGA
CCTTGGGGCGAGCTGGATCCATGGAACACAGGGCAATCCGTTGGTAGCTCT
GGCAGAGAAGGCGGGAGCAACAACCGTCGCTTGTGGTGCTGTCTACTCAA
TTTGCGATTCAAAAGCAGACTGGCTGAACCGTGATATAGCATGTAGTCGCT
ATGAAGAAGTGTGGGAGATACTTGAGATGGCAATCGAAAAGAGCCGAAAC
GAAACCGCTTCGCTTTCTGACACGGTGAAGATGATGGACTTCTTCCGTGGA
GAGGTCGGAAGAAGACGGCCACAGGATAAACATCCAGAGGCTTATGAGAT
CCTGATGATGAAAATTGTCGAGATGTGGGGGGCTTTCATGGGCGATGATTG

CGAAAGGCAGAGTCTTAAGAATCTGTGGCTAGACGCCGGTTTGGAAGGCG
GTATGTTAGGTGTCGGTATCTCTCCTTTGATGAGTGGCGGTTGACCAACAGG
CGCGCAGACAATTTGTTCGTAGCGTCCACTTACAAAAGCATTCTGGACGGC
CTTTCAAAGCAAATTGGGAGCCAAGCTAGTCTGCGGCTTAACTGCGAGGTA
ATACGGATATCAAACATCCAACCTGGAGTTGTTACCATTGAGGCTGCTGATG
GATATCGAGGTTCCTTCGATGACGTAATCGTCACAGCACCGTTGGGTTGGC
TTAAACGCAATACAAGCGTCTTCCTGCCGCCTCTAACACCTCCTATATCAAA
CGCCATCTGTAGTCTTGGTTATGGGAATCTTGATAGGGTTTTCATCAAATTTC
CCAGCGCGTTTTGGGATCCTCGCAGCTCTGGAAAGAATAGTCATCGAAGAC
AACAGCCCGAAACTACAGAAAACCCATCGTTTCCCGTTGAATCGCTTTTCT
TAAGTCCCGAATATGCAACTAATACAAACCCAGCTAAATGGCGAGTGGAGA
TCATATCGTTCTCTGGTTTGCCAGAGCCGTTTTCACAACCCATCGTTATGTTT
TTCATATATGGTCAGTGGGGTAGGCATATCACCGGGCTTATAAGGGGTATGA
AGAGCGATTCTGCCGAATATTACCGAATTCTCAATGAGAACTTCCGGCCGTA
CTACTCCAAGCTCCCTAATTATGACCCGGACTCTCCTAACTGTAAACCGTCT
GAATTCTATCAACAGACTGGCAAAATGACAAGTTCGCGGGCTTCGGGTCGT
TCACCAACATGCCCATTGGGTCTGGGGATTGCGCCCAGCACTTTGAGACTC
TTAGAAAGGGCATGGGAAAAGATCGCGGTATCTGGTTTGCCGGTGAACAC
ACATCGCCACCGGGTGGGCTTGGTACTGTCACAGGTGCTTATTGGAGCGGA
GAGGAGGTTGTAAAGAGGATCGCCCAACGGTATGAGACCAAGTTAGAAAT
GTAATATTCCGTAGATAAATCACTTGATGTAATGGCTTAGATGTTTTGAAAG
ACTTCTACCAGGTAATACAGAAGTGTATACGTAGATAAGGATCGCATGAAGT
TTCAAGCCTCAATGAAATGAAGGAAATATCTACGAGATGGATGTTTTGTAA
GATCCTTATGAAGCTTGTTTTTAGGGGGTAAGGATAGTGTATGTTACTGGTA
AGATTAATAGCGCCGATGCCCTTCGCTGTAAAACGCCCTATTTTTAAATTTA
CAGGCGGGCACCATCGATCAGGGAAGCCAAGATTAGGTCAGCATTTCCAGT
CTCTTACGGGGCACCCCTCCCCACAGCGTTAGTTAATACTGAGCTAATGCA
AAGTCTACTAGGTGCTCTTTTCGCCAGGGCTAGTAGCACGGCACACCGGCT
GACCGAGCACAGGATGGTGCAGGTTTCAAGCCGGGGGATTAAAGATTTCG
CCTAGCTTGCTAGCTCCGCATCACCCCACAATCTATCTTTCTATGGAAAACA
CCAAAAGGACATGACAGCGAGCACATCGCTATGACTTGATTTTCTCAAGTA
GATCATGACACAGATTGTGTATTTAACTAAGCTCTCGATATTTACCTCAATAC
CTTAGTACCAAAGTCCCGAAGGTAAGGCTTAGCCGGAATTTAGCCGCAGAT
GATTTTAATCGGCGTTAAGGGGTAAGGAGTTAAAACAGTCGACGATGTTAC
ATTTCCGTTATCCTATCAGATCATGGCGATTTACTCACGAATTATTCTCTATCT
ATTTAAATGATATTAGGTATCTAAAAAGACGTCAGAATGTGTGCCACCATTC
AACTAATCTCGGTAGCAATAAAGTTACAGGTAGGTAGGTAAGAAATAAATC
GTAGGTAGGTAGGTAGGTATCAACATATATGTCATAGATGCCTATGTTATGAG

GCTTTCGTAGCCAAATACATCGAGCTCGTAAAAAAATTTGATTCGTTGCCTT
GCCTGTCTTCCTACTACTATTCAGATGATTGACATTTTACCACCCTTCTTGCC
TTTCTCCCTTTACTATCACCCCCATCGCACACTCTTTGGAACACGATGGCTT
TTAGAGAGTTCTGGAAGAGCCTTCTGAGTCATAATAAAAGTCCTTCCCAGA
AGTCAACCCAGATCATAAACGACATGAATAACCAACACGGCGAAGAAAAC
ATCTCATCCAGCCAAAGGGCAATCGCAAGCCGACGACAATGTCATCAACTC
CAACAGGCATGTCAATAGTGCCGAACCCACCAGGCTATTTCCTCCTCCTGC
GTATTAAATGTGCCTTCTGCCGAACACTTTTCGCAAATGACGGACATTCCAA
CTGTCGACACCGCCAATGCCACCAACCTTCGTGCTCCGGATGGTTTAAAGG
TGACATTGGATTTCAGTATACCGAGACAGTTCCATCCTGTCTTACGGTGGGG
CGAGTACATTTACTGGCCCCTATCCTATATCGATAATAGAGTTCGATGGCGAT
AGTTATAACAGACTCGGCAGATAGAGTTGTCAAGATCCTTGAAGCAGAGGG
GGCCCGATATCTCAAAGAAATTCAAGTCCGCGAAGAAACCCTAGAAGTAC
AGTTCATCGGCCAAGCCAACCAGGTTGCCACACTTCAGTGGCAGCATCTCT
TCGTAACTCCAACCGTCAACCCACAATCTCCGACAAATCTACACTCCAACG
AGCCACGACCTACTGGCATCCCGTTAGTAACGAATGAACAGAACTTGGATA
AGTACTTTGTGCCTTCCAAGACGACATATACGGACGCAGATTTCGATAGTAT
TGCAATGTTACTTAGCAAAATGGGATCGCGAGAATATAGCAAGTCACCTCG
ACTATACACACTCTTGCGTTATCTAGACTGCCCCTTAGGGGATCTGGATCGT
ATTCTTGCAAGAGGCCTGTCGGATAGCTCATTGCCTCTTTCTCAGGGACAG
CTGCCGTCCGATTTCAGGGAGGGTTGGAAGATTCGATTTCGGGAGGCTCAG
GGCCTTGTATGCGATCATTCCGACGTTGTCCAGCTGATAAACATGGGCAAG
CATATGCCTTTCTCGAAACCACCGGATTACTTCATAACCCAAAAACTCTTTG
GGAAAGGAGGCCGCGGAGAAGTTGATGAGGTGTTTTGCACGATTGGGCAA
TCCAAGCATTATGCAAGAAAGAGGATGCTGCGGCAAGATTTTTCAGCTCGC
GATATTGCAACCATGACCGCGTTTCGTAACGAGGTCGAATGTATGAAAACG
TATCGTCCATCGACATTGCGTTCGGATTGTAAGTTCGCATGCTTGTCTTTCGT
CCGTAGGAACAAGTTGTAGCTAACTTTCATTTAGGTGGCAAGCTATACGGAT
CCAAGTTCCTTCGCCATATTGATGCTGCCGGTTGCAGACTCCAACTTAGCAC
ATTATCTCCAAAAAGCAGTTTCGTCAGACGGCGAACGAAGTTTCCTTCCCG
ACTTCTTCGGTTGCCTTGCAAGAGGCCTCTGGCACATACACCGCCAGCGAC
TCCGACATCGTGATATCAAGCCCGAGAATATCCTAATCCACAACCATAGAGT
CTTGTTCACTGACTTTGATTGCTCTCTCGATTGGTCTCACACGGTACATAGC
ACGACTGACCAAGTCCCACCGAGACGAAGGAATACGCTTCTCCAGAGGTG
CTCACTCAGGATTCGTCCAGAATATCAACATCAATAGTTCGTCAGATATTTG
GTCTCTAGGTTGCGTATTTCTTGAGATTATCACTGTATTGAAAGGGCGACCA
CTACAGGACTTGAGTAATCTCAGGGGTGCATGTTACTACAACAACATTGATA
AAATTCTTCAATTGATAGAGGATCTCCAAAGTGTTGATACACCTCCGACGTC

AATGAACCAATCACTAGGATGGATACGAGACATGCTTCAGGAAGATCCAAA
TAGTCGTCCGACTAGCCATAGGTTGGTTGAGATGACGTCGGAATTCTGCTG
TTTTGATGTAAGGGGGAAGACGGTGGTTCGCGTTACGCGTGATACCGTCGC
ACGACACCTGAGAATGATCCTGAAGATCTGCAGCATTTTAATGATCACATCG
ATCTTATGGAGAACTTTATCTACCTACTTATCAGCATATTTTTATGTTGAAGTC
ACACGACGTCATCACATAGCGAAGCAACATATGCCTAATCATGAGCTCATCT
ATATTCAAACTCATGTACCTAGGTAGATAGGGTAGGACAAGTTTCAGACTGT
CTAGCATCGTCATGGAGATAGGATGTTTTTAGATTGTCATGTTCCTTTTTGAA
TAAACTATCAATACGTAACCATCTATACTAAAAGCTGTGTCAACCGCTCTGG
ATTATGTATATTTCTATAGAACTACCACTCCGCGTATATCTAATATTCGAAGGG
CACTTTTGGGTAACGGCGGATCTCTTAACTTAGCATTCCCCTCGCTCATTCC
GAAAGACCAATAGCCTCCCTCGGATGGCAGACAATGCTGCATAGAGAAAC
CATAAAGTAGACAGGCCTACCCTGTTCACTTAGTTCATGTAGGTATTCTCTC
GAGATCTTCTCTATTCGAGCTTCTATTGTTCCATTCCTCAGGCTGGAATAAA
ATGAGCCTAACTCCCTGCCGAAACCCGTTGATGCCCTAACACACCACTTCC
ACTATTTGGTTCCCCACTCGAACTAGGAGACTGCATCTGACAATACATTTAT
TATTTATACGAAATACGAAGCCATCCACTGAGTGCTATTTGGAGTGAAGATG
ATGTTACCCGTGATGTGTGGAGGGTCACACTAAAACTCGTGGGGCTGGCTC
ACTGGGAAATGGCTGTGTACCGCACCACGAATTCATTTCTCTGCTTGGTCTA
TCAAGCCAGTACTTGACGCCCATAGCCCCATTAGCTCCGGGAATTTTATTAC
AGGTAAAATACCTACTCTCCAGAGCGAAGATCGACCTCTTAAAGAACCTTT
GAGCTTTTGGAATAAGCCCCTCGTTAGCGAATGATTCGTTTCACTAATGTGA
ATGTGCGTTACACCCCGCATTGGTTCCGGACGCAATCCTATTGAATGCGCAT
TGTCTAAGAGAATCATACATATTATTGGTATGGGATGATCTGATTGGGTTAAT
CTGGATTGATCCTCATTGATCATCATTCAAGTCATCGATAAGTGTTTTCTGCG
CAACATTTGCCACAATATAACAATCCCGAGGCCTCGTGTTGCGAACAAGTG
TATTTAACCAGCTGTGAAGCTGTCGGTCGCTTCTCCTTCACAGGCTCTAGCA
TCTTATCTACCCAAGCGATAGGTTCATTATTCTCATCGCTAATAGAGATAAGT
CGCTGAGTCCAAGTTTTGATTGCTTGTTGCTTGATATGGTACGGCGGTGCAG
AGGCACCCGACCGCCTCTACCAAGGAAGTCGAGCAATCCATTGACTGTCTG
TCCTTTCAGCACAGTAATAACCTCGAGGAATATACATCCTAAAGACCAGATA
TCCGAAGAGGAATTCCGTGGTTGGAATTGGGCGACTTCTGGCGCGCAGTAT
CTTTTAGACCAGCCAGGACAAGTGCCTTTTGTTGTCGTAGAATGTCCACCG
GCCCAGTCTAGACTAAGTCCAAAATCGGCAATCTTGACGGACCCTTCATGG
ATAAGTATGTTTGCTGGCTTGATATCACGATGACGTATCTTGATCTCTTCATG
AAGGTATTTGATCCCACTCGATAAGCAGCCGAACAAGCTGCGGAGGAGGG
AGTACCTCTCAGGTAAGGAAGCCCGCCTTTCAGAAAGGAGTTCTTCGAGAT
TCAGTTCAGCAACTGGCGATATCAGAAGACCTAGGAACGTAGGAGTCGTGT

AACTGCCTATCTGTGAAACCAATACAGATTAGCTTATATCTTACCTTGGTAAT
AAACCCGACTTACAAACTCCACGCAATGATGGTGCCTTATCTTCATCATAGA
ATTTCGTTCCCTTTCGAACACTTCCATGCCGGCTCTTGATATCGGTAGAATC
CATGCTCTTCGGGTCCTTTTCCTTGCATAAACCCTGTGGCTTATCCTACTCTC
TACTTTGTCAACTTCTCCAAATCCGCCCTTTCCCAATATGGCCAATCTCTTG
AAGGCCGAATCTTCGTCTGTCTCGAAGTGTACATGTTTCGATTCCTTGCCCC
CTTCAACATCTTTTTCTAGAAAAAGATCGTCGGTTATAATAGCTTTTTGAGC
CTCAAGGAACTTTTCTTTTTGCTGAGGAGATAAACACCACGGAGTCGTGCG
AGCGTTGAACGGGAACCAGAAGTCTGAGTTGATGCCCCCATCAATGATTCT
AGTCATTTCAGATTCCAGACCGATCAGGCGTAGTACACAGTAAAGACGAGG
AACACGGCTCCAAGAGCCCGCCATTGAGACGATGGACATGACAGCAATTC
CGCGATTCGGTTTAGGTCGGAGATGGAAAACCTGGGTACTTGGTGAGGACT
AGGAGGAAAGTACTGTGACATTTCCATGACAGCTGGCTCGAAACTTGGGA
ACATGGTCAACTAGATGCCGTATCCCTTGTGATTCAACTGTTAGGGATCGCT
GTCAGCTTAGTAGAATGTCCACTCCACACAATTTAAGGCCAAGCAAGATAA
CACAGAACGGATAGTAGAAATGAACCTAAGTAAGTCGAAGGAGGTACTGA
TGAAAATTCTAGGCGATAAGTTCTTACTGTGGCGACATGGGCATACGCCCA
CAGTTACTTCGTACCTCCGCACCAATATCGAGCTTGCCTTCTATTTGCTCTAG
GCCCAAGCCTCGGACACTGGTCTTTTCCCTAGCCGCCAAAAGAAGAAAAG
TGATGTTCGGTAAATCGAAATATTCACATTCGTGCTCTAAGCCCCCCAGTAT
GACCATGGATGTCAGCTAAGCATCCATTATGTACGCCTAGAAACTATTTTATA
GGTACTAGTACCACCGTAAGTACCACACTTTCCCGAGATAGGGTCGAAGAA
AGCAAAGCATTATTGCCTAGGTAGGTACGTTAGGATTGAGTCCTAACATCTC
ATGATTGCCCCACTAACCTAGGTAGGTACCTATTTTTTCAGGCATACCTAAA
CTGCTGATCTTACCCGTTTCCAAGCTTGCCCAACTAATCAGCCTCATGTGGG
TAGATATTTGTATACTAGGTACCTATATGCACAACGCCTCGCTTCCATGCCGT
TAAGCCGTGAATACTTACATTTATGTGAAAGAGCCCGATCATCATGTTAGGG
CTTTGGTTCCAACTTTTGAATTTTCATCGTACCAGAAAAAAATATCGTAAAA
TAAAATGGATATCTTTAATACAACCATAACGTCTTTGCAACTTCTGTACGGTT
TCATCGATGCCTGCGCTGGCTTCCCCGGCGACGCGAAATCTCTCTCTATAAG
GCTGCGCCGTGATATACGAGTTCTTCAAAACGTTCAAGCCTTCTTCCAGGC
CCGCAAAACATCGAGCAGCTTGACAGACTCTGGGCTCGCTCCAGAAGATG
AGGAGTTACTGGGAAATCTCTCAAAGCATCTGGATGGATTGATCAGTAAGG
CTCTTGCTATGGAGGGGATCTTGAAGGTCCATGGAGGTTGGAGAAGGCATG
CTAAACAACTATCATGGTGGCATTATGGCCCAAAGGTGCGAAAGCTCCAAC
AAGAACTCCATGAATGGACAGAGAATTTCGATGCCCGTCTCATATCGTTACC
GCGGGGTACAGAAACCGTCATCAACTTCTCGGATGATGGAAAACCAGCTAT
TGTGGCAACGAAAGAACGGATCGAAGGCGTCCTAGCTGAGATATATTCTCA

AACTAAAACATCGGTCAAGATGAGCAAATTACGCATTGATGAGTACGAAAA
CCGCATCGGCTTTGGCGTTTTTTCTACTCTTCTCCGATGTAGTGCAACGTTT
GACGGAGTTCCGGTACTTCTCGAATTTCGACCTTACGATCCATCTCTTCTAG
AGCCAGGCAACGAAGACCTCCTTGAACAACTCTCTCTTGACCAGATGAAA
CTAGCATATATTCTGAGCAACGTCCAAAGCAGTGGTTTCGGCCTTCCTCGAT
GCATGGGTTTTTTGAGGTCCCCGACAACATTTGGCCCTCGTTTGTGCATGTT
TACGAAATACCGCGCCAACCGCTAGAACGGGGAAATATTACAAGTCTTCTA
GACATTATAGACGAAGCTAGATTTGCATCTAGCAAAGGCCAACCAAGCCCT
CATCATTCCCTCTCAGAGCGATTTTCTTTTGCTCGAAAATTGTCCACTGCCT
TGCTGTTGATTCATTCCGCAGGATGGGTTCATGAATCAATCTCTTGCGAGAA
TGTATTGGTCCTTGAACAATTTTCTGGTCCTGCGAGCAAGCCATCTCCATCT
ACTCTGGGCAATCCTCTATTTGTCGGCTTTCATGCATCCCGACCCGATAGTG
CAGACACAACACCGTCCTATTCCAAGACCAATCCATTGATCTACTACCATCC
ACACCGACTTCTAAGTCAAAATCATCCCCCCAAATTCGTTCGCGCCTACGAT
GTGTATAGTCTCGGCGTCGTATTGTTGGAACTTGGACTCTTACGCTCTCTAA
GCAGCGAGGTGGATACACTTCCAATGGAGCAAATACCTTTCTATCTGCAAG
AACTTTCCAAATCTGTGAGCGTCATTATGGGCGACACATATACATCTGTTGT
GCAATGGTGTTTAACCCGGAGCGAGGAGGATTTGCCTGTTAATAAGTTTTT
CAACGCAGTGGTGGAGAAATTAGAGGGTCTCTCAAGTATTGTGTAGTGAAA
GCACGTGCCCTATAGTGGGTCATTAGTGGTGGCCATTAGCGGTTCCAATGCC
AGGTGCCTAGCCCGTCCTAAAGTCTCCAACTGCAACTACAAACACTACCTA
AATCATCACGAGAAAATAGGCAACCGTTTACCAAAATTCGACCGAATAACT
CGCCAACAAATGATCGTGCCAGATCCACGAACTATTTCTTTTCTTCCACATC
GTCTAACCAATGAGCAACTAGAAATAATTATAGAGGCGATGATAATTCTTCG
TCAGCGGGTCGCATAATAGATGATGTATCAAGGGGTTGTGCAATCTACACA
AAAGCCCATGGATGCTATTCATGATTCCAGGACTCACTTTCACCACTATCAG
TACATAATAATATTTACGAAACTAAACGTTGCAGACAGGTGACATTCCAAGG
TTCAAAAACGAGGTCACCTTCGGAATATATAAAAGTTTTCGTATCTCTTATT
CACAGCAAGGCGAAACCTGAATCGGTATAAGCACTCTACAGCGATTCATTG
GGCACGCTGGAAATTGATCTAGGGGGTTTCAGATTTGGAATAAGCAGTAAA
ATAGGTAGGTCCCTCAGCACTGCCCCAGCCCCTATTCCAACCAGCTCGCCC
CATTACCACCCACTTCGTACCATCTTACAGGCAGATTAGCAGGTATCCGCTT
ACCTAACTTCGCTAAGATCTGAGCCAACCATGCCCCTGGGTCCGCCCCAGA
TTCTATTCTCCTTTTGTGGATATGTGTGTCTCTACCGATCCACATGCAAAGAG
ACCCTGACTTTCTGCTGAGATAGAAAAAACAAGACATGAGGCTGACTTAG
GCTAAGTTAATTTCGTTTGGGAAAGTCGTCCCTGAAATAAGTCTCGCTTCTC
TCACTCCTTCTCCAAACGCGATAGGTATGGCTTGGTGAAACGAAACTCGTC
GTTATGCCTGAAACCCCAACTCCAACGAGGATTGTCCCGACAATGCACGCG

CCTGGCGTTAACCTTCCAGACATCTTCCTTTTAAGGGATGACGATCATGGCG
GTGTCGTGCAGGATCCAGGGGGAAGCACGGATCGAACCGAGTACAAGGTT
GAAGTTTACCACTTTACCTAAGATCTGGATGCTAAAACCGCATCATCTAACC
AATATTATCCATGCCAAGAGATTTTGATACGAGAATATACAGTTGTTCCATGG
GTCCTCTTATTATCCAATTTTCGGCCGACCAAGGCGATCCTCGCATTACGTG
GGTGGGTTATAGAAACATAATATCTTTAGTTTCTTACAATGGTGTTGTTGTTA
CTGACCGATATTCTTCAATGCTTCTCTGTTTCTCTCGTTAACTAATCTTGTTC
AACTCAGTCATTCCAAGAAAAGGCCAAGCAGCTACAACATTACCAAGTCTA
TCATTTATACACAATTTCATTATGAAGGTTTCATTACTCATCACCCTGGGCTT
CCTAGCCCCTAGCATTACCGCTTCTACCCGCCACGCCGCCACATCACAACC
CATATTCGTCCTCAAGACAACCCAGACCGCCACATCTTCACTCAGCAAAGC
ACTGAGTAAACTGGGATTCATACACCAAGATACTCGCCAAGCAAACTCGAG
CGTTATATCCAATGCAATTGCAAACACAAACACATACGTAGAAGTCACATC
AGACACCCAGCTTCTAGAAATCACGGAGTCACACCCGGAGGCCAAATTCA
TAATACCAAGCGGGAGCCGGTTCCCTACTGTGGGAGAGGGCAATTGGCTCG
CGCAAGCATCTCTTTCAGACAAGAACTTGAACGGGATCCTGGAGCTAGAC
GTGTTGGCGTTGGAGAAGGGGACGCAGGCGGAGAATTGGGTGCGATTGTG
CGATTTCTTAGGCATGGGGTACAGCATCGTCGAGAGGTTGGGTTTGTGGCA
CTTCCCTCAATGAGAGGGGATGGTGGAGGATGATGGATTGAGCATTAGAGT
TGGAAATGCATGGCGGACACTCTTCGCCTGCTCGTCACGAAGTCGACCCCA
AACCCCGCTCGACTGGCTGTGTTGATCGGTCACACCGCTGGAGTCATTCTG
CGACTGAACGATTCCCGGAAGCAAGGAGGGTCAAATTACCAAACGGAACC
GATCAAATCGACATTCAGAAGGTCAGTGGCCATCCGAGATCTCTGTGTGGT
CGTACTCATGCCATAGGACGCCGTTTTGTCCTTCTACTCCTCCCACATTCTG
GGAGCGAAGGCTGCTCTGCCAGGCCACGTCCCGGTCAGTCCTCGTTCGTC
GGCGTCGCATGTCTGCATATTGACATTTAGGCGGCTTTCGATGAGTTCATTC
AACATTTCGTCACTTTCGAGGATTTAAAATCAAAGCTTCTACCTGTCGCAG
AGCGAATGTTGCTGCGTTCTCCTGAAGTCGCCCTGCCTCGTGAGTGACACC
CTCGAGAAGAATCCAAATTTTTAATTTCCCAGTGACGGGACGTCTGCTTTC
CTCATGTACTTTTGACATCACATCGCTTTTGCCCGGCAAGCTCGTTGCCGCC
GGTCTGTCGTCGTCCAAATCCACGAATGGCGAGACTCGGCAGAAGGCGGT
ACACTTCGTGGACGTCCTCACCACGCGCTGCACAGATACTGCAGCTCTCGG
TAAGATCGCCATCGAGATATTGGCGCTGCCCAAGACCGGCAAATCTGCCTC
AGCAGAGCATCGGATCTGTCTCTTCGATATGGCAGCATCCCTGCCTCCCAGC
GATGAAGTGTCGAGTATCGTGGTCGAAACTCTCCCGCCTCTTATCAGTAAA
GAAGGGAACGAACTGGCCCTCAACGCCTTGTGCTCAGCCTTGACTGTCCA
TTTGTCCTATCTCGTGACGTCGACGACTTCCGTGTCACCAAACTCAGTAAAT
GCTCTGGGAAAAGAGCTCGCTTCGGCCAAATTGCCAGCAAGAAGGGGCGC

GGCGAACAGCATCGGTTCTGCCTTTTGGGCTGGTGCATGAGAAAGGGTCG
CAGATGTCGAATGAGGGTGCGAAGCTGCTCCAGACCTTGGCCACGCCACT
CGCGGCGTGCTTGACTTCGGCGGCCGGAAACGCTCCGTCAATGCTGGCGG
TTTTCTGGAGGGATATGTTGCCATCGCATTGGCCATCGGACCGTTGGGTCAA
GCCGCTGGAGCGTCTCTCGCCTCGAGCCCCGAGCTATCCACGATCCTTACT
GCTGGTGCCAAGCCCTCTTTCCTCTTGAATGAGAAAGTCCACGCTCGCCTA
CCTGCAATGGAGGACGAAATCTGGTTGTTGCGATCGTTGGAGATGCTCGTC
ACGCATCTGAGCGACAAGCTCACAACACAGGGCGTAGGTATGTGACCAAA
CCCCCACGCGAGAGTCCGCTCATCGGAACAGGACGGCCGTTGGGTTAGCG
CTCATTCACCTGGCCTTTGAATCGAAACATGCTCGCGTCAGACGAGAAGCA
TACGCAGCGGTTGCTCGTGTATCTCAGGCAAATCCAAAACCCATCAGTCGG
ATCATACGGGAAGCCTTAACTGCATGGCTCAAGATTCAAGATGCCAAAGTG
GCCAAGGCGTCCGTCCAAGATGAGGATGAGGGTACCAAATCCAAGTCTCG
GGAGATCGGCCGCCTCTTGCAAGCAACTACCCACGCTTCTCATATCGCCGA
CCAAACGATCCGCGAAGACATTGCTGTCGATCTCCTCATTCTTGCTCATCAT
CCTGATATCGGCGATGAGGCACAGGTGTCTTGGTCGGCTTGGTCCAGTCAC
TCAAACTCGATCCGGCGGCGGTCGCGTTCGACTTGCGAGAGAAGATTTTCA
AGCGCCTATGGGATGCTGCTGCGGCACCTCCTGCCGATTCTCGTCTGGCCG
AGGCAGCTTTCCGAGCCATCACAACTTTGTGCCTGGTCTGCCCGGACATCT
ACATTGAAGCCATTCTCTCTCAGCTTAAGGAGGACCTCGATTCCAGGACTT
TGGATTTCATCGGTCCCGAGGAGAAAGGGATTTGGGATACACCAGAGGGT
GAGCTGTATGTGGACGGTGAGTCACTACTGCTCGTTTTTGGTCAGCGATAG
CTCAGCGCCAGTGCTATCGAACAAGAAAACTGCCGTCGAGGACAAGAACT
CGAAAGACTACGCCACGAAGAAGTGGGAGCAGGAGGTTCGAGACTCGCT
GAGCAAGAAGAAGGCTGCCTCTAATGGCATCACCTTGACGAAAGAGCAGA
AAGCCCTTGTCGCTGCCCAGCAGGCAAAGGAGACTGAGATTCGCGCACGG
ATCCGCCAATTGCAGCCCGCCTAGCGCGTGGCGTTCAGCTGGTGAGAAGCT
TGGTTGTCTCGAGCGCCGAGAGGGTCACGCGACACGTCGGAATTCTCGCG
AGCTCGATGATTGAGAGTGTCTTCGGCCCGGCGGCCTTTTTGGCGGATGAG
CGAGCATTCACAGTGTTCATTCAACTCTCGACATTGATGTCCGACCGACTT
GGTGAATATCGGCGATTGTCGCTCGCGGCTACTCTACGAAGTCTGGACTCG
CCCCTTGTGCCGTCTGACTACCTCGACGAGTCTCTCGGCGAGCTAGTCACT
CGTCTGCTGCACCAACTACAATTCGTCGCTGCGCAGAGTCCGTTCGATGGC
TCAACTTATACGCTGCTCAGCTTCCTCTTGGCTCGCGTCGTGAAGCTTGGTG
GTGTCGGAGTGGGCCTCCTTCGTCCAGCGAGGAGGCACAGGAGCAGCTTA
CTCTGGTGGTCAACATCATCGCCTCGTGTTGTGGCGAATTCGCCGATGAGA
GCTATCCCCGCTCAGAAACGGTCAAGTTGTTGATCGATCTCATCAGCACATA
CCCACGTCTAGGCAAAGAAGGCTGCGTCTGCCCTGATCGACCTCGGCGAA

CGCCATCAAGGATGTCGCCTCGCCTACTGAGAGTCGACAACTGATCCTGGG
GACTCTCTCGAAAGACTCTGTCGTCCGAAATGCTGCGTTGCAAGCACTGCA
GCCCGTCGACTTGACGACTTCGAGTACTCGGAAGAGTTATGGATCGCCGTC
CACGACGATGACGAACAAAATGCCAGTCTCGCTCTCACCTTCTGGGAGGA
CAACGGTTTAGATGTTCCCGAGACATACCTAGCTTCGCTCTTGACATATCTC
ACGCACGACTCTCTTGCAGTCCGGACTTCCACGGCGTCAGCGCTCGCAGC
AGCTGCTCAGCAGTATCCAAGCCAGGTTGTGCCACCCTCGAAGGCTTGCA
GGCTATGTATGTCGACAAGGCACGGCTTCTGCAGCCCGAGTACGATCAATT
CGGCATGATCATCCTCGAGACGGTCGATCGACAAGATCCCTTTGAGGCTCG
AGTCGCCATCGGCTTGTCCGTCGAAAAGCTTGCGCCGTTGCTGCCTCAAGA
GTCTGTCATGACCGTCTTCGATTTCTTCGTCTCGCAGGAAGCTCTTGGCGAT
CGGCATGGACAGGTGCGGAAGCCATGCTTGCAGCTGGCATCTACTCTAGTC
GATCTGCACGGCGGAAGCCATGTTCCGGAACTGATGAAGATGTTCGAGACT
ACTTTGGCGTCTGGGGCCAAGTCTGAGACTGCAGACTATATTCAAGAAGCG
GTTGTCATTGTGAGGAGTTTCATAATCAGCACTGGCAGACTGGGCTGACTA
GGATAGCTCTTTGGCCGCTTGGCTAAGCATCTCGAGGCGAGTGACTCTCGC
ATTCCCAAGTTGTCGACCGGCTCGTGGAGGCTCTTAATACCCCATCCGAGC
TTGTCCAGTCTGCTGTTGCGGACTGCCTACCGCCACTTGTGGCTGGTATGG
GGGAAGAGGTGGAATATCTGGTAGACAAGTTATTCTCAACCCTCACGACTG
GGTCCAAGTACGCTGCCAGACGGGGTGCTGCCTACGGCTTGGCGGGCGTG
GTCAAAGGCCGTGGCCTCGCGTCGCTGAAGGAGTACGAGGTGATGGATAA
GCTTAAAGAAGCTGCGGAAGACAAGTCTGCGTATCAGACCAGACAGGGCG
CGCTCTTTGCATTCGAGTTTGTCTTCCCATCTTGCGAGGACCTTATGACGCT
AATAAGACAGGACACTCTCCTCTACCCTTGGTCGAGCCTTCGGTAAGTTGA
TGTCTAGTTCTTTGCGGAGTCTAGCTGACCGTCAGAACCATACATCATCTCG
TATGTCTTGTTGGACATCTGGACGTCATCTGACTCAGTAGAATCATCCCGCT
CATGCTTGCCCTATTTGGCGACGCAAATGCCGATGTTCGCGAGGCGACATC
GGACGCTGCCAAGGTCATCATGTCGCGAGTCTCTGGCCACTGCGTCAAGCT
CATGCTTCCAACCCTTTTGAGCGGACTCGAAGAGAAGCAGTGGCGGACGA
AGGAAGGGGTCCATCGAGCTGCTAGGGTAAATCTTCTTATGCCTTTTGTCTG
ATTGCGCTCAACCTCTCAGTTCCATGGCTTTCTGTGCTCCACGACAGCTTTC
CCTCTCACTTCCAACCATCATTCCGCAACTGACTGGCGTCATCAACGACTC
GCACTCCCAGGTCAAATCAGCTGCCAACACCAGCCTCAAACAATTCGGCG
AGGTGCTGAACAACCCTGAGGTCAAGGCGATTCAAAGCACTTTGATGAAG
GCATTGGCCGACCCTACGGCAAACATTGGCAAGGCTCTCACTGCACTGCTT
AAAACCACCTTCGAGCACTACCTTGATGCACCCTCTCTTGCGCTAGTCATG
CCCATCATCGATAGGGGACTGAGGCAGAGGAGTTCGGAGACCAAGAGGCG
ATCTGTGCAGATCGTGGGGAACATGTGAGTCACCCAAAGAAGCCCATGATA

TGATGCTGATCGTCCAGGGCGTCTCTGACGGAGTCACGGGACTTGATTCCG
TACTTGCATGAGCTCATGCCTCTCGTTCACGAGGTCCTCGTCGACCCAGTG
CCCGAAGCCAGAGCTACTGCGGCCAAATCTCTTGGAACGCTGGTAGAGCG
TCTGGGAGAGAGTAATTTCCCCAATCTTGTCAACGACCTGCTGCAAACGCT
CAAATCGGACACCTCTGGAGTGGATCGCCAAGGTGCAGCACAAGGTCTCT
CCGAAGTCCTCTCAGGCCTTGGAATGGAGCGTATGGAGGGTCTCTTGCCCG
ACGTCATCAGCAGCACAAGCTCTCCGCGCGCGTATGTTCGAGAAGGATTCA
TCTCTTTGCTGGTCTACCTCCCTGCGACATTCGGACATCGCTTCTCCCCCCA
TCCTGGGCAGGGTCATTCCTCCTATTTTGAACGGCCTGGCAGATGATTCCGA
ATATGGTGAGCGGTCTAGGATGGGCACAGGCCTTGGCTAATAAGCAGTCCG
GGAAGCCTCCCATGCGAGCGGGCAAGATGATCATCGCCAACTATTCAAGCA
AGGCCATCGACCTGCTCTTGCCGGAGCTCGAGAAAGGAATGTTGGACGGC
TCGTGGCGCATCAGGGCATGTCATGATCTTGCTATTGAATCCGACTTCGCTG
ACTCCCACAGCAATCTTCCATCTCACTCACAGGCGAGCTTCTGTACAGAGT
CACCGGTATCAGTGGCAAGGTCGAGCTGGAAGAGGAGGAGGTCCCCGCTC
ACGGCGCCGACAGCGCAAGGAAGGCCCTGCTGGAAGCGCTGGGGCCAGA
GCGCCGGGACAGAGTGCTGGCTACCCTCTACATTGTGCGCCAGGACAACG
TCAGCACGGTCAGACAGGCTTCCATTCATATCTGGAAAGCTTTGGTCCAAA
GTGAGTAGTGCCAGAGAGGCTTGTTGTATCAAACTGACAGCAGACACCCC
GAGGACTACTCGCGAAATCCTGCCAGTGCTCATGCAGCTCATCATGGGTCT
TCTTGGAGACCCACATGTCGAACAGCAGGAGGTGAGATACTCGATCAGCTA
TCCTCTCCGGTCGCTGACATTAGACCGCCTCCCGCACACTTGGAGAGCTTT
GCAGGAAGAACGGAGAGCGAATCTTCGCGGAAATTGTGCCCATCCTGCAA
AAAGCTATCTCTGCGCCCGATGTGGCCACTCGAGAAGGGGCGTGTCTGGCC
TTTGCAGACGTCATGTCAGTACCCAACGTGAAAAGGGCTCAGAAACGCTG
ACATGGCAGGGCTGCTTCCAACAAAGACGCCATGGCCGATCACGAGGGGG
CTATCATATCTGCCATCCGCTCAGCGCTCGTCGACGAGGAGGCCTCAGTCC
GATCGGCCGCGGCCAAGACATTCGACGCGATGCAGCACTTCATCGGCAAC
AAGGCTGTCGACCAGACTATCCCTACATTGCTGGAGGCTATGCGCAACCCA
GGTTCGGGTTCCGAGACTGCTCTACAGGCGCTCAAGGAGGTCATGAGCGT
GAGTCGAGCTCATTCTGAGGCTTTGGCTGGGCTGATTGCCAGGTGCGCGCC
AATAGCGTCTTCCCCGTCCTTATTCCGACCCTCATCGCACAACCCATCTCCG
CCTTCAATGCTCGCGCTTTGGGATCTCTGGTCAAGGTTGCTGGCAGCGCGC
TCAACCGACGACTGGACACTCTGCTCACTGCTCTTGTCAAATCTCTCGAGA
CCGAGAAGTCCGAGGATATCCTCATCGAGCTCAAGGGTGCCATTGAGTCTC
TCCTCGCTGCAGTCACCGATTCAGACGGCGTACACCTGCTTGAGATGCTGC
TCATGGAGTGGTCAGTCCATCTTGGGATTTTTTAGACGATAGAAGCTGAGC
CAATAGGGCGAAAGATCGCAATGCCACTCGTCGCGCGACAGCGTGCAACA

TATTCGGCACCTTCTGCCAAGTCTCATCGGCAGATACTTCCGACTACCGGGT
TGACTGGATCCGCATCCTGATAACCCTCTTTGCCGATCCGAACGAGGTTGT
GGTAGATGCCGCATGGAAATCCCTCGAAAAGTTTGTCAAGACCGTTCCGCA
AGACGATCTCAAGGGATCTTGTCGTGCCCCTACGTCGGACCATGGAGTCCA
CTACATCGGCTGGAACCAACGTACCAGGCTTCTGCCGTCCCAAAGGCGTGC
AATCCATCGTTCCGATCCTCCTTGCGGGTGTCCTCTCCGGCACGCAGGAAC
AACGAGAACAGGCTGCTCTTGGTGTTGGGGATGTTGTGCAGCGCACCTCG
GAGGCGGCAATCAAGCCGTATATCATCCAGCTCACGGGTCCTCTGATTCGT
GTCATTTCGGGCGCAGGCATCGCCCCGCAGATCAAGACCGCCATGTCAGTC
GCTCTACACGATGCAAGATCGTTGCTGACTGCAGTCTTTCCGCCTTGACAG
TCTTACTGGAAGAGGTTCCGCAGTTGGTCAGACCCTTCCACCCTCAATTGA
CAAGGACGTTCGTCAAGAGCGCGTCGGATCCCGCTGCTCTATCAATTCGGA
ACAGAGCTGCAGCTGGTCTGGGAGAGTTGATGAAACACCAAGTGGGTCTT
GTCCGCAGTGGAATTTCCGGCACGTGCTGACAATCACAGCCTCGCGTCGAC
CCCCTGATCACCGAGCTCATCGGTGGTGTCAAGGCCGGCGATAAAGACATC
GCTCCATCTATGGCACAAGCGCTTGCTGCTGTGTGCAACAGTGCAGGAAAG
AATATCGGCACTGCGGCCAAGGCGGCAATTATCGATCTTGTCGAAGACGCT
TTCCTGGCTCAGGCAAATGGTCGGCTCGCTCATTACGATGCTTGGGCTGCC
GGAGCTGATATGGCAGAAAACTACAACGTGGCCATCGCAAAGTCGTTGCA
GGTCTAGCAAAGCACGATGCCACAAACATACAGTCCATAGTCGAGTGAGTC
CGTCTCGAGCAAAGCGATCCGACTCTGACTCTTCAGCACATTCTTGGCCGC
CCCTACCCACCTTTGGCTCTCGTCAGCGTCACGATCTTGACGTGTCTGGAG
GATTGCCCAGATGTGTTCTTTGACTTAGACGTGGCCCAGGATATAGCCGCA
AAGATATCGTCGAGTGTTGCGTCGGACTCGTCTAGCATAGCGCGTCCAGCA
AGGGAGGCTCGAGATCTGTTAAGGAAGGGAAAGTATGGGGAAGACCCTGC
AGTGCAGGCTGCTCTCAGATGACGGAGTAGATGGAAGAGACGGTTGTCTT
GCATTGCATATAGCGCATGCATAGCGCTTGAAGCTGACAGGGGTATGGCCC
GGCGCTATGATAAGGTGGCTTGCGGCATCTGAATTCGTTCAGTCTCATTCGT
ACCGACAACCGGATCCAAGGAAGACGACAGACCAACCAAGCTGCTTCATC
TCTGCGACGACGGGTCGTTGGATGCGGATACATGCAACCCATGCTGTGCGA
AGTGATCGGCTCTCACTGATCATATGACCGAAAAGTCGCCAGGGAGGATAC
AATGACACCATTCGACACGGGATTTCTCCTCAATGCCGTGCTCCAGGCGGA
TCCCGGCGCATCGCACTGCGAGCGATCAATGTGGGTGAACGAAGCTTGCG
AGCAAGCAAAAAGACCATGGGACAGCGTCGAGGAGGTGGGTGGACGGTA
CTGACGCCGTTCTGAGGCCGAGGCAGCTGTCGGGACGCGCTGATCAGGTC
GAAAGAGGTCTTTGCAACCGGTTGATTGGGAAAGAAGGGGAGGGGCAAG
GGGAAGCGAGTGACGTGATTCCTGAACCTCAGTGAGACTGAAAGGAGCAC
CAACTCACTGGGTTATGCACTGAATGGGCTCTCCAGCTTCATCGGCGCGAG

TGGAGCTGTACGAGGTTGATTACCAGAGAGGAGGGGGTGGCACGAGTCCG
ACCGCGCCAAGGACGATGTCGTCACGGCAATTCGCACTTGATCTGAGGCAT
CATCGATCTAGAGAGACGATGGGAATGCCTGCTGAGTTGTTTGGATGCTCC
ATGTCTTTCATTCTCACTGTCGACGGTTTCGTCTTTGGGAACTGTCTGACGG
TGCGTCACATCGGTGCCGGTCTCCTCGAGCGAAGCAGCTCTGGGTTTCTTA
GCGTCAACAGCCCGGGTGGCCGAGGAGGAGCCCGTAGCAGGTGAGGCGC
TGCTCGAGATGGGGGTTTTGGTTGTAGTTGATGAGCGTGTGAAGGAACTGC
GCGCTGTGTCGATGCGAGCGGCTCAATACTCTTTCCTCTTCGGCCGCGAGA
GAGTTCGAGCAAGTCGCTTTCGCGATCTGCCGCAGATGATACATGTCCGCC
AGAAAGTCGAGATTGAGCTCGTGACCAAAGCTATTTTGAAAGGCAAAGCG
GCATGGCACTCGCCAGTTGTGTCGGTTCAATGGGTACAGGACGAATCCCTC
GTCGGCGGCATCGCAGCGATCGCATCGGATCCCGGGCTCCATGCTGCATAG
CCGACAAGGTAAGAGCGCGAAAGGACCGTAGTAGGGAGTCTCCGATTAGA
CCTCGCCAGAGACAGGATTGGCCCAGCGACCGACAGCGAAGGCAGGTGA
ATATGGGATCTTTATGCCTGCTGGATGAGCTGTTTCTATTGTTGGACTACAG
GATTGGAGAGGCTGAGAGCGAAGACGGATTCTTCAGGCTTGACTAAAACC
GAGCAAAGACTCACTTATACCAAGCGCCATTGCCTTTTCCCCTAATCTTCTC
AAACCCTAGAGATTCCATCATAGATGGCATTGCTAAGCATGTACAGTTCTCG
CAACTCGGACATGAGCAGAAAGACGGCGAAGTACGACAACAAGTCAGCA
GCGCCCAGCCAGAGATCCGTGCACCCAGATGAATGTGGCGGTTTTTATTAG
CACTTGGTCTCCTGGGAATTGCCATCCTCGACCACCCAAACAACGCACCGG
CTTCCGCCAAGCCTTGTCTCTTGTCTCCATTGTGTGTTCTCAGCTGCTCGCC
CACCATGACCCCCGGCTCGCTCGGCGGTGCTCCGCCGATCGTGCCGACACT
GTCAATGGGGCTTTCCTCGATGTGGCATTAAGCGATGTGTGGCAGTCTGGC
CAGGGTCGGCCGAGCTCGGTCGGACCACTACCTTCGGGGTCTTTCACTAGA
TCTGCGTGACGGGTCGGGGCAAGGAGTGCATTTCGATGTCGCGGATCTGCC
GAGTATAGTACGTCGGCCGGTGGGGAAGGGCAGAGCCTCAGGGTCCAGGC
CCTCCAGAGAGATCGCAGTCGAGCGTTTCGAGCGCCTTGAAAGATCTCTGC
ACTCATGGGTGCAGGCAACTTTCGGATCGACTTTGTCACTCGCTCGTGAAA
GTCCAGACTATCACTCGCCCCTCCTATCTGAAAGGACAATTCTTTCGGGTCC
GCCGTCGGCACCGTCACTACTGCCGTCCCTCCCGCCTTCAATCTCTGCAATC
GGAGCCTTTCCAACGGTCCCTCTTGCTGTGCGTGGGAGGGAATCTGCCAGT
GAAAAGGGCCGTTTCCAGGAAGACGAGCGGGATACTCTGCGGTCTATAGC
GCCCGACTCGCGGTGTCCCGCCGATGGTACCTTGAAGACCAGCGATACGG
GGCGCAGATCGTGGTTTCAGTGATGGATCATCGCGCGAGAGATCCAAGTGC
TGCCTGCCGAGATCGGTTAGTGTCATGCACGCGCCTAGCTCGACAGCTGGG
TTTGGGGAATTCCTTTTTGTATCTTGTCTCACACCCTGACCGCCGCCTCCTT
TCTGCTGGACGACGCGCATCGATATGACTCAAATAAGGGCAGTGGGCGAGC

CGACACCGCAACGGCACCCCCGCACCGACTTCACAAACTGCATGTGCTGC
AAATGGAGGCTCTTGGGTTCAGGCGGGCGGCGCGGAGCGACGGTGTGCTG
TACTGGTAAGTGATCAAATGAGCGGTAGACATCGGTGGCTGAGAGCTGCAG
GCATCCCTCCGACCCTTTCTTCACCTGTCAAAGATGTGGCGACCTGAGCCG
AGCAGCGGATGAGGCGTGTTTGTGGCCGTCGCTCATCGCGCTCAAAGGCG
ATAAACACCACTATCGTACGAGAAAGTGCTTCGGCTGCGCTCAAGCGGGTG
TGCCATGCTCTTGTGCGGTCGACCCGAAAGACTACGATTTTGTCATCGAGC
CAGGCCAAAACACGGTCATCAAGGACTGGGCAGGCCGAACGATCTCGATG
GATGTGTTGAGGGAGTCTCATCAGAGAATCATCGACGCAAAACGCCTTGA
AGGGAGAGAGCGAGAGCGAACAGGACGCAAGAGAAAGCGCGCAAATGTA
CAAGACGAGGAGGCTGCACAGCCACTCCCGACTACAGCCTTTAATCCCGC
CGCAGCCTGGCAAGGACCCGGATCCTCGATCAGAGGAGCTGGCAGGCAGG
GGCAAAACGAATTCCAACGCGCTGAACATCGTATCACCAAAGGTCGACTTC
GAGTACCAGCGAGGTCAACTCTAGCAAGTTTTCACCGCTCCAAAGGCCGA
GTCACAATTCGACCATAGAAGCAAGTACTCGCCCGCCGTCTACGTCCCCCT
TGCCGTCTCTATGGCGTCATCGACATTCAGCACGTACTCCTGACGTACTCCG
AGCGAGTCCTCCTGCACGACCATCCGGCATGATCCTTCCCCCTATACTCGCA
CCTGGGACCGGACCCTCCCGAGATGCCCACCTTGCCGAGACTGGTGGCAG
CACCACTCGAGGTACGCCATGCCACAGAAGCCCTGTATCTGTCCGATTCCA
CTTCTCCCGGCGGAAAAGGAGATGGAGAATGGCAAGCCCGCGCGAAGTTC
GTTCGTGATGCCGAGCAGCTTTCCGGAGTACCTCTTACACAGAGCTTCCTA
CACGCTTCTAGGACTCAGAAAGCCGATGGTCGCGCCGAGCATCACCTACTT
GCGGCTGATAATCTCATGCGGAGGTCACGACTTGACCTAGAAGCCGCTCAT
CGCAAATTGACGGGATCCGGACCTATCTTCCGCGGAGGCTCGGGAGAACG
ACCAGTACTACTGCCTTCTGGTGTCGAGCAGGACGAAGAGGGGCGATACAT
GGGAGCATGGCGAGAGCGAGCGAATGGAGAATTGGACCTGCAGACGCGA
CGCCTCCATTCTCTAAGAGAGCGGTTCGAATTCTATGGCAAATCCTGAAGAT
GCTGGAGCCCGCGAGGCTGATTCTCACTTGCGGGATGGCAATCACAGCGC
GAAAAGCGATGTCAACAGGGAACGTCTTGAAAGCCAGGGTACCCAATATG
CCGACCCCAGATCGTACCACCGATAGGTCATCTGCGGACTGGATTTAGGAA
GAGAAAAGGCGGCCATTGCTGAGTCGGGCAGGAGTGGCATGATCAGTAGG
GAACGGAACGATGTCTGCGCTATACATAGAGAATAGATATGCAAGCTTCAA
CGCAGACCGAAATGCGCTCGGCTGCCCGAAGAGCAGGAAAGGAAAAAGG
GGTGACCAGTACCGATGGGTGAGTGCATCTCGAGTGAAGGATGGTCTGCG
CATCTTGAGGGAGGTCCGATGTTTGCAGTGCATTTCTGGGTGCTATTTCGTC
GGGCCTCACCTTTCGTTATGACGAGCATCCTTGATGTGAGCCTCAAACCCTT
GATCCAAGGTGGCGTTGTGGCAATCCGAGAAGAGGTGCCACATTGGGAAA
ATGAATGCTCCGTGCTCCAGCATTGATGCCGTGGCGAAACCCTGAGCGGCG

TTGACTCGTGCGATGCTGAGCGGAAACATAAACGGCACATGTCTACTTGTC
GCAGGAGCGATGCTTGAGGCACCTGATCTTTGTTTACCGTCCGGGGTGGCT
GACCGAGGTCGTTAGCGACACCAAAGGCTTCTCCGTTAGTGGAAAGGATG
CGGACGCAAGCAAGTTCCTAAGCTATCCGCTCCTGCCCGGTAGGCCGATAA
TTGCTGCACTTGACTCACCAGCTCCGCCTCCGACGTGCCACATTCATGCGA
TGCCATGATGATCAGGACGGACATGTCGACACGGCGAGCCAAGCGATGTTT
GCCTGCCCCTCGTCCACCCCTACTTGAGCGCCTGAATTGCACATGTCCTGG
AAGCAAAAACGGGTTGATCCACTGACGTTCGGTTACGCAGGTACTGTCATC
AGGGGGCTCACCTCTGGATTCGATGCTTGATTCTCCCTCTCTGTCCAGTATA
AGCAAAGGGGGCATTGAGAGGTATGTCATTCAAGGTGTTGGCCCTCAGATT
TTGCAATCTTGTCGGCCTATTGTATCTGCTTCCCCTACCTGGCGCTTGGTCC
GTGGTTCTCATCGCGTTTCGTACACACTCGAGGAACGTGCGGGTGCAGAG
AGGAGGAATGGTGGACCAAGGGCCTACATTGTACCTCTCGGTGTTGTCCCT
GAAGAGTGAGTTCGGTCAACGAGAGCAGAATGGTTTGCTTTGATCGGATG
TGTGAGGTCGCCTCTTGCGACACAAGTCCCGTGAGCTCAACCTGTCGCTAC
ACCTCGCGAGGACATGCCACCCCGAGGGTGTGAAATGGGTCCTGATATGAA
CACTGCTACCGCCCCGTTCGGAAATGACTCACGCCGTTGACTGAACGGCTG
AAATTTCAGCCTCTTGAACAGCAGAACAGCCCTCTTGGAAGGACCTCTACC
GCGTCGCGCGCGGATCTCACTCAAGCGAAACCTGGTGCTGCTTTGCTTGGA
TGTCAGGCGCGTGCTCTCGGTGGACGGAGACGGAGACGGGCATCAATTCC
TTTTCCTTTGAGTAAGCCCGTCGCGGAGAGCTTGTCGCGGTTTGTTACTGC
TCGGCACCCACGCCGGCATCCATCGGATCCAGGATGTATGCGATGCTTCAA
CGCGTTCGGGGGATCGTCAGCAGCATTGTCGCCATTTGATTGCCTTTTGAG
AGGGGTTATAGATGATGACGAGTAAGATTCAAGGTTGAGGGAGAAGTTTGA
CGACAGTCAATTTGATGAAGATCCGTGAACCCTTCCTTGAATTCCTTTCTCG
ATCCGCTTGATGCCGCACTTACAACCGTCAGTCTCGCTGTCAGCCTTCATCC
CCTCACATCTCCACCGCCCCGCCTTGATTCATGGCTCGCAAGCAAGACTAT
GGAGAAGTTTGGATTTCTAAAAGCAGACACCGGAGATGACAAGGATTTATA
CTGGTGGGCTTTCCTCCCTCTCAACGCAAGCTCACTGTAACGTCAGGCATC
CCACCGATTCATTCTTCACCTGCCAACGCTGCAAAGAGCTCTTCCCTCATAC
CAAAGACGCCTGCCTCTGGCCTCCGCTTGTCCCGCTACGCTGGTCAACCGA
CCGGTACAGGAGGAGAAAAATGTTGGCAATGTGCTCAAGCGGGCGTCTTA
TGTTCGTGCACGTCAAAGGGCTCGACTTCAGTCTGTGGCTACGCCCGGGGC
GAAACGCCAGTCTTATGAACGCACACGGGGAGGAGATCAAGCGGGAGGTG
GTGGAGGAGGCTCACAGGAACCGCCTCGCGGTGCGCAGACTGGACGGCG
GCGACTGGGACGACGGGGCATACTTGGACGAGGCACCGAGCACGGCTTCA
ACGGCGAGAGGCTTGGTCATGGACGGCGCGAGGACCGCTCCTCTCAGCGC
CCAGCATTCACTCGCATTGGGCGAAAGAAGTCGCGGGACCCGACAGCACG

ATCTCGAGCACACCGTTCCTCAGATGGGCAACCGGGGTGCGCTCCTGGTTC
TATCACATTCGACACCTTCACGGCCAATCTTCCTATTCCCCACGCATGTTAC
CGCCTGTCCCCCTCTCCGCACCGGTCCCAGATATCCTCGGAGACTCTCGCC
CGACTCGCCGCCTCTCAGAGATTTCGCCCCCTGCCAATGGCATCCGCTCGC
CCTCGCCTCAGCGTCGTTCAGTGAGTTGGATGTCCCCTCAAGGCTATTACCT
CCGATTCGTGACGCTGGATCTGCCCGGCCACCCGGCGCTCAGGCTTCAGCA
TCTCCTCCTGCTCAGACCGAGCTGACATCCGTTGTGCTCGAAACGTCTCAA
GAGGAGCCTCGGCCCGAGCGAACCGCTTTCATCCGCGAGGCTCAAGCTTT
GAGCGGCGTTCCTCTTACACGCTCCTACCAACTCGCAATGGCGGCCCGCAC
CCCCTCGCTGGAGCGAGAAATTGCGAGGCACCTCAACAACGCGGAGATGC
TGTTGCGAGAAGCTCGCAGAGAGCTGGAGGTGGCGGACCAATGCCTGGCA
GAATCTACAAGGGCAGTCCCCCAGCATAAAAGACGACGGCGAAGCGGGGG
AGGGATCGCCGGACAGCTCGGTTACACCAGCATCGGCCAGGACTGCGCAA
CATGACTCATGTACTTGGCATCCATGTTGTGCCGGGCGGTGCCCGACCGCG
AAAGACGCATACAGAATGGTGCGTCCTCACATCATAGATTCAGCCAGGAAC
TGAAGTTCCTCCAGTTGCCGACCGCCAGCGCCGTTATACCGGGCATCCGTA
AGAGGATCGACTTCGTGCTGCATGATTTGAAGCTCTCTGTGCTGCGTCTCC
TGTCAGCACTGACCTTTCTACTCACCCTTGGTCTTGCTGGGGCTAACACCG
CGCACCATTTCCACCTCTCGCCAATGCGTCTGGGATCCCTCGTCCATGCCAC
TCACCCTGATTTCACCGAGACCGGCCCGCAAGAAGCTGACCGTCGCCATC
AGACGACCGGGACAGTGTCATTGCTGCGCGGTCATTCAGCCATAGAGCATG
GTTGCCGAGCTCGCACGTTCAGCATGTCCCAAGGTCGACCAGAGTCTTCGC
CAAAGCCCAACGATACACATCACGTGAAACCGCGACAGGATGTGGCGGCT
CGTTTGTGGCACTTTCAGAGATGCTGATTTTTATACTCCACGTCGTTTTGTC
TGACATGAGACATTCACCTCCTTACACTGACGACGAAGAAGAGACTCCCA
ACTTGATTGGTGCTGAAGAGCAATGTCATCGCCAGCATTCATCAAGACTCC
ACCCCACGCTGCCATGCCCGCGAACACAAGGACAATCACCATGCGGACAA
AAGGCCCAAAGGACGCACTGCCCTGTTTGACCGAGCGGCCGACTCTCGGT
CGCCGGATGTCCGACAGTTCATTGTAGGACCCGTCTCGGTAACAAATCCAA
GCTTACAAGATCGACAATAACCAAGCGCAGCCATGCGCCACATCGCGGCCC
T

>Mosaic_sequence_51

GTTTGGGCGGGGGATGGAGGATGTCGCCGGAACCCGGAGTTCGGGGGTCA
GTTTGGGGTTTGTGGCACTTCGGCCGAGTGGCTATCCGGCCGTTCGTTTCG
ATAATGTTTCACGACACTGACAACGGGTATATATTGAACTCGTCGAGAAGA
GGACGGCGCTATGCCGACTGATCCCGTATAATCGCTGGAGTGCATGCTTTGC
TTGTTGCACAAGGGGATCTCAATGCGACTCCGTCAAATCCCGTGCGCTCGA

CGAGGGTATCTAGCTCGCCGCCAGTCGATGCGGTCACTTGCAGGCGTCCTT
GTCGTTTTTCAGAACGCTCCCCTGGGTCTCAGTCCGACTTTGTAGTCGTTC
AAGCAATCTGAGATTCGAAGGACGAAAGCCATACCACGGAAGTGCCCTTT
CGCACTTGAAGCGATTATATTACGTAAAGTCTTGCTTGTCCTACGTCATTTC
GATCGCTCCCCGGTGGTGACTGACCAAGTTTCCACCGTGTTTTCAGACGCG
AGCAAGGTATACTCAAAGATGTTTTGCATCCAGCACAGAAAGCTTTATCTA
GTAGACGGACGGGGCACTTGATGCCCTCCCGCTTTGTCGCCATATCAGCAC
TCTCGCGTATCCTTCGATTCTCAAGGCCTCATCCTCCCATTTCCGCCACCTTC
GGATCGAGCATCGACCGAATCTCGGCGTGATGCCCCGTCGACCGAGGGCAT
CCAACCCAGCTCTGGAGACAGCAGCGTCCATAACTACAGCAGTGGACACC
TCACCAGGAGCCTCTTCGGGCCGACTCTGGTACATGCGCTTGATCACATAC
CTCCCAAGAACGCCCCGCTGAGCGACGACGATTTGACGCCCCCACCCCCA
GTCGAGATAGCAGTGGACGCAGCGGTCGAACAGGTGCTCGAGCAATCGAA
CAAACGTCGACGTATCAGCAAAAAAGCTGCAGAGGACATAGCCGAAGCTA
TTGGAGAATCTCCAGCTAAACAGCGGGCACCTCGCAGGGCGGCGCAGAAA
GTCAAGCAGGCGGCCTCAGCAGGAGTAGGGGAGGAATCGGAAGAGGTCG
AGGAGCATGAAGAGCCGCCGAGCAAACTCAAGCGCACTCCAAAGAAAGC
CAAGGCGGAAGATGAAGCTTTCGAGGACGATGAGCCCATGACGACAATTC
CCTCCAAGAGAAGACACCCAAGAAGAAGACAGCGCCAATCGACGGGGAT
GAGGGGAATGGTGAAGATCTGTGAAGAAGAAAACGCCCAAGAAGTCTCG
GATAGCCAAAGACGAGCCTGAATATGACTCCGAAGGCAATGAGATCGTCA
AGAAGAAGCGGAAACCTAAGGTCTATCCAAAGATAGAGTACGATATCCCGC
CAGTAGAGACGAAGGAAACGACTTTCCGAGGTAAGCGTGGCAACGATATG
CTGAGCTCTGCTCTGATCGACAAGGTCGCCTGGGATATGCGTGCCTCAACA
CTGTTTTAAGAAAGACGAAACCTGACAGCATCTTCTGCTCCCGAACTTGCA
GGATCGCCAGCATAGAGGAAGAGGGCATGGAGTTGCCTAAAGGCCTGGCT
TTGATGAATTTGCGTGATCTGAAGACGATGGTTCAATGGAACGAGGATAAC
AAGTGAGTTGCCATCGAGGAGATCCATTCTCCACTAATTGCCTAGGATCCGT
TTCATGCGCCTCTCTCAGAGATGTTTCCTTTCGCTTCACACGAGAAGTATGG
ATACGATCTATCCTTTGCGGCGACAGAATTGAAGGAGATTGGCGACTTGGC
GAAGAGCTATGGTCACCGCTTGACCATGCATCCAGGACAGGTGAGGTGTTG
CTGACGGGAGGATGCTCTCGACACTAACCTGAATAGTTTACGCAGATCGGT
TCACCAAAGGAGAAAGTGGTGGAAGCCAGTGTACGGGAACTCAACTATCA
GTGCGAGATCATGGACCGCATGGGCCTGGACCAGGACGGTGTTATGATGTG
AGTCGATTCGCTTGCGAGACATATGATCTCACCTCCAGTATCCATATGGGCG
GGATCTATGACGACAAACCGGGGACTTTGGAGCGCTTCAAACACAACTAC
ACGACCAAACTGTCAGACACCGTCAAACGTCGACTCGTGCTCGAAAACGA
CGAGTTGTGTTATAATGTCGATGACCTCCTGCCTCTCTGCAATGAGCTGGAA

ATACCGATCATCTTCGACTACGTAGGCGTTCAACATGACTGTCCAACCATCA
GCTGACGCTCAGCACATGACTGGATTATCCCTCATCGAAGCCGCCCTCAGA
GCTCATTCCTCTGATCGGCGAAACGTGGAAGAGAAGGGTATCAGGATGAA
GCAACATCTCTCCGAGCCGAGGCCGGGTGCCGAAACTGTCATGGAGAAGC
GGGCTCATGCCGATCGATGCAAAGCGCTTCCACCCGACTTGCCGGATGATG
TCGACCTGATGATGAGGTGAGCTGTCGCGATGTATATTGGAACTTGCTCACT
CGACAGGCAAAGGACAAGGAGCAAGCAGTCTTTGAGCTGTATCGTATCTA
GTCAGTGCTCAGGAGGCCTGCGAGGCATGCTGACCTCTCAGCGGTCTTGA
GGATGTTGTATGGGACAACCTACGTCCTCCCGATCCGGAGCCCGGGCTTCG
TACAAACGGCCGCAAATCCAGCGTCAAGAAGAAGCAGAAGGACACCGGT
GCGGTCGATGAAGACGGCAATCCCATCATGCTGGAAGAGTTCGAGAAGGA
GGGCGATGGCGATGTCGGAGACGAGGGCCTGATGGAACCTCGAATCGACG
AGCCGACCGAGGAGATCAAAGCGGAGGTAAGGAGACCAAGACACCGGCG
AAGAGAGGCAGAAAGCGTGCGTCTGGCACGGCAGCAAACCTTGATGGGG
AGATCAAAGTGGAGGTCGTGCCGTCTGCGTCTGCGGAAGAGACTTTAGTC
GTGCAGATGGCTCCCGAAGTGGAGGAGACGTTGATCAATCGCACGGAACA
TGACGTGGACAAGCCATTAGAGGAGCCTAAGAAGAAAAGGGGAAGGGCG
AAAAATAACATCGCCGCTTGACCATGATCATGCAACGCTCCCGGCACTACG
CGTCTTGCGTCCTCTGCGCTTGCTAACGAGGTTTCCCAAATCTAGTGCGCC
ACTTCTCTGATCTTCGGCAGTAACCTTCCCCCAATCCTGATCAGTGGAGCA
GACCTACAGTGGGGTCCATGAATCACTGAATGCTTGCCACAGGTCCCGGAT
CTCCCACCGGCTAGCAGGAGAGACACCGGCCCTTTGGAAACCGAGATCCT
TAAGGGCAGGAAGTACGTACTGGTACAGCATCGAGAAGCGGAATGCGGGG
CTGGAGATGACCTCAAGCAAAAGTCAATAGCGAGCAGAGACGGTTTGAAC
TAGGCTTGGCACAGAAGGGTGAAGCGGTCAAGCGAAAAGCGTGCATGTAC
ATATAAGTTGCTGAGAAGAGTGAAACTGGGATTGATGTGCGAATGCTGAAG
TCACTGCAAGATTTGAGACACTGGGCGCTCTGTGAAGCTAGCCGTAAGCCT
CTATCTGCTACGTCTTTCCACGTCCTTCGTGCTCCTTGCAGATGTTCTGGAA
CGTGATGCTCCTCTCCATCGCCGTCAAGGGGGCAACAGTAAGCGGATGCCG
TCCTCGCTCAGCGTACAAGATGTTCCAAGACGCTTAGCCTCCCACTCTACAT
CATCCCTCAGCATGACTTCCCACTCCTCGTCTGTCACGTCCCACATTCCGCT
TGGGTCCTCGCACGGGTCCGTCCCATTGTGGTATTCTCTGTGTGCGTCCCGG
AGTCGGGCTTCAGTCACGACTTTCAGCAACTCGTTCCTGGTCCACCCTTCG
GTCATCTTGCACTGACACATGAATTCTAAGAAGGGCTTATGGGGGGCCCCT
TGAAGCAAAGACTCGTCAATGAAACGAACGAGGTACGCGTCGTGTTCTAC
CTTCGTTATAGGGACCTCTCTGCCGAGGGAACTGCCTTCGGAAGCTTTAGC
TAGAGCCGTCAATCTTGTGTTTGTCGTTTCGTCGCTCGAGATATACTTTTGG
AATTGATGTACTCCACGCTCGTCTTCGAGGGACTTGCCTGAATCGTCCTTGT

TTCGGTCTGCCATCATGGAAGCAGCATCAAGGGAGGCTGGACTGGAAGAG
CCTCTCTTAAACACCGTGAGCATTTAGAAACACCGGTAGCCGAAGCGCTCA
CGGAAAAAGCAGCAGCCCTATTGTGGCGAGCATCACATCCGGCTCCAGTAC
AATCACTGGTGGTATTCCTGAAGCGATGAGAAAGTGCAGTCATATCTAGTT
GAGCGGCTTCGGATGAGAGATTGTCGAGAACGGGATGTCCAGGCTTATGA
GGGTTACCCTGACGCTCCAAACCGGTCTTGTCTCTGAAAGGAGGCTCTCGA
ACAGTGCAATGCTGTGTCATGTTGCAAAGGCTCACAGAAACGCGCTCACC
CTTGCTAGCTCGCCTGACGTTCGAAGTGGTCTTCGGAGCGCTTTCTCTTCG
TTTGACCTTTGCAATATGTGTCATTGTTAATATTTCAAATGCCTCCCCGAATT
TGAGTTCCGCATTGCGGCCAGCCCGGGGAGGTAAGATCTAGGGCGTCTATC
ACAGGCTTCATCGTGGACCTTCAGTAGCGCTCAGAGAGGATTGCATGCACA
GCGATATACTAGTCCACATCACCCGGGCCCAAATCGGACCCCGGATAGTAA
CCCGTGAAATCCAGCTTCGCCCGCTGCTTTCGACTCGTCTGCATCTTGCTG
GTATCCGCGAGCCGCCATGCGACACCAGCTGGCATTCTCGCGAGCTTATCG
TATGGCCCTTCGTCCAGCTGACGAACGGCTTCAGTCTGGTTACTGAGCTGA
TCAGAAAGAACAGCCCCAACGAGCCAGGGATCAGTTCCGTAAGCTCCCAC
TTTGTAAAGGGCTGTCGACTCGGCCAGAAGGTCATCTATTTCTACTTCCGTC
AAGCCCTGGGTTTCCCTGCACAACTCGGCGTATTCGTCTTCGCCGACAGTT
GGAGGGTCCTCTGTGTATAGGTCCCAATCCGCAGAGGGGTCGTGCGCTTTG
AACTTCTCCCTCTGGTCGAGCCGGTATGCCAGTTGCTCATCTTTGTCCAGAA
ATCGCTTGTGAGATAGCGAGGCCTGGTCTCCTCTGGCGTCAGTAATTGTCG
CCGGACTCTGGTGTTGTTTGAAGGACAGGTTGCAGTCAGTGGCCTGGACG
GTCCTCGACTGAGTTGAAACGACGCGCGAGGTGGCCTGTGACAGGCTCGG
CCTCACCTGTTGTGGTGTAGTGACATGAGAGGTCAGATTAGCGGTCTGTCG
AAGACTCTTCGACAGTCGCGACATGCTGTTTTTGAAGCAATATCTGTGACC
GGGCAGGGAGAGCGAGTTGTCTTTGGCAGATGGGAAATTTTTCTGGAAGC
GCTATGTGTGGATCAGGTCAGGCGGGGATCCGTCGTCTCGTTTATAGCTGCA
CGGGCAAACTTGCATGCAAGGGCCTAATCCGGTTTCAGAGCCCGTCCAGC
ATAAAGGACTGACCGGGCTTTTGAGGGACCACTTCCATCTGCGGAGATAAT
ATGTCGCAATCACAGCTCGTGAGATGCGACGGATTCGTGAAGGATGCGATG
TTTCACGATCATGACGTGCGCATGACGTGACTGATGCATGTCACTTCAGGT
CTTGTCGGAACGGTCTATTGTTCCTGCTCTGTCTCCAGTTTGGTCGATCAGG
GATCGTCCGTGCGCGAAGGAAAGGGCATCCCTGTCTTCATTTTGGCCCCCT
CTGCGGCCATACGCCACGGCACGACCAAATGAGATCCTTGTGAGTATTCCC
GCATATAATTGGGGCAGCAGGTGATCAAGCACGCTCTACGTGTCCGTACGG
TGGCAGAGCGGGCTTAATCGCATATGCGTCGTAAGAAATGCACGTAAAAGT
CTGTCGGGAGCCCGCATCCGAGCCCGAGGAAAAGATCGCTGACTGCACAT
CATACTTCTTCTGACAAATTGGTGAAATGCATAATCCACAGTGAATCAGATT

GGTGTTCACAATACGAAGTAAGCAGAAATGTGTCCGGCCCCGAATGCGGG
CGCTGACGCACGGAAACAACCCGTTATCCCGTTATCACGAAAGGCTGTACA
GTAAGACTGGCACACTTCACCTGGAATCAATCAGTCTGATATCCCTCTCAA
ACCTTGCCTCCAGACGGAAGTTTTACTCCATGCGTTGTCTTCATAATGAACA
CGATGCCCGCCGGAGTCAGAGGTCTCGATCTCAGCTCTGTCGCCTTGTCCT
GGATGCAAGACTTGAGATCTTTCTCGAATCCATCACTTCTTGGCGCTGCATG
AGCTTTACACTCCGCAAGCATCATTATCAAGTCATCGAGGGTCCACCCGGG
CGCCGTGTATTTTGTTGAGCTGGCTGTCGGGGGGCCTCCATAACTACCGTAT
CTTCAAGCCGGTCCGCGAGCGCGGTGTCAGCATCTGTTTCAGGAGCTCCGC
CATCAGCGATGGTGGCCTGCGGAGCTGGACAACTATCTGTCTCGTGGGTAG
AGTTGCTGATAGGGTTAATAGACACTCCGCTCATGTTGACTGCCGATAGAG
CTGGATCGATGGTCCGGGCGCAATGGTAGGGAATTCAGGTGGGTGGCGATA
AGTATAGCTGCGCGGCGACGTTTGCAGAGACTACAGACCTGAACCTTTGTT
CCCTGGACGCTGCTAGGTTCTTCGCTTGCTTGCTATCTGGTCGACGAGCCG
TGTATCCATTCTTCAAGCGATAAGTGAAAGATTCTTGAATGTGATATGCTGT
CTCTATTGTCTCACGCCCCCTCTCCCCGAGACGGGCAGATGACGGCGGGAG
ACCGAGCGCGCCGCTCTGTGACTCTCAGAAGCCAAGCGCCGGCCATGATC
CAATCGTATGTTCTCGGGCGGTCAAAATAGGTATCATCGTAAATTGTATGTA
ACGCAGTATTGACATGAAACAAGGTAGAGAATGGAGTGTAGGCCCACTCTC
GATACTTCTAGCAACGGTCGACCTGACTTTCCTGCCCACCTGAGCCCGCCT
GCTCATGCCTTTGGCTGATGCTCTTAAAGGCATCACTTCTCGTGTATGCAAA
GCCGGGCGGCATGAAGTATCTGATGCCATCTTCAGAGAGTAGCGTCCCGCG
GAACCATTCCCGCTGGTCTTCCAATACCCCTCGAAGATAGTACGCGTACTCC
CCAGCTGAAAGGTCCTGGTCCTCGAAAGTCAGCCGAGCTCCTCGAGGTCG
TGGATGGCCTTGCCTTCGACGATGAGATCGCCTAATTCGTCCCTTGTCCAGC
CTTCAGTTGCCTTACACAGCTCGGCAAATCTCTCGGGGGAGCAGAATTCAA
CGCCCCATGTCGACTTCAGGTTGTCGTACCGTTCCTTGTATGGCTCGGGCTT
CTGACTCGTCAGTGCATTCCACTTGAGCGAACCTTGTTCGAGGTTGAGCAC
CATATAAGCAGCCTGCTGGCTTTCGGTGAGAGTCGTGTTGGCGGTCCTGGG
TTCTCTGTCGCCCACTGTGAGTGATGTACGGTCGTCTTGGAATGAGATGGG
GTCGTCTTCCGCATCGTTTTCGACGTAGAGGTTGCTCTCTTCGAGAGCCTCT
TCTTCGACTTCGTCTTCCCTTTCCGACAGGCGCTCTCCCACAGCTCTCTGG
ACCCCCATGCGGTAGGAGCAGCAGCGTCGCGCTCGAAGATTGGTCCAGCT
TTGACGGAGCGGTGGGTCAAGTGAGAAGAGTTACACGACATTGCGGGAAA
AAGCACTGAGAACGTAGAACTGCTTGTAGCTATTTGTATCGGACCTAGTCT
ACGGGCTGTAGTGAAGAGTTGTTCTGACCACGGGGCCCAGGGTGGCTGAA
TATAACCCGACTGCGAAAACATATGGTGTGCTCTGTGGCAATCTAGACGAG
CCGTTATTGCGCGAAAGCTGACTGCTTGCGCAACGTCTCGACTGTTCCTTT

GCGCACGCTTGGAGGACTCATCGAGGGCTGTGTTAGGAGGACTGCTGAGC
TGAAACAGGGGATTTGGGCAGACGCAAGGATGGTTAATACAAAGGAGTGC
AAAAGGGCAATGTGCGATGTCTACGTTGCAGCGAGCCAAGAGTCAACACA
GTCATATGAGCTGGCATCATCCGCGCCAATCTCATTGTGACATGTTCCGGGT
CGTACCCCTAATAATCACTGAATCACTCGAAGCCCAGACCGTTCCGCATTGC
CGGTGCTCCGTGCGGACCCCCACCTTTGAGCTCGATCCACCCCATTATCATC
TCGAGATCCTCGAAACCCATGCTGACGTACATGCCCTGGGGGCCAAGGTCG
ACGTGATCCCCCCTCAATCGGTGTATTGGCGTTCGCACCCATAGGTCTCGCC
TCTTGCCGGTCACTGGGGTGAAGCTGAGCCACCAGGCTGTGCGTCAGCGA
TAGCGCGTTCCGATCAAGCCTACCCACATCATGGGGCCAACCCATCTTCTG
CGGACCCTCTCTACATCATCCCACTCAGCCATCTTTGTGCTCTCGCCCCAGC
TGCTCATACTCGGTGACCATGGCGTGCCGTCGTATACGTCCTCGACTTCGTT
GTAAAGAGCCCTTCTATCAACCAGACGAGGGACCATCTTGCCTTCCTCTGC
TTCTGGATCGAGATCTCTAGCGGGCCGAGTCGTTCCTCGTTCAAGTGACGT
GAAATTAGGATAACACGAAAAAGCTCGAGCAGGCTGAGGTTTACGAAGGG
AGGGGTACAGTAGTTGATGGGGCGTGATCAAAGGGGGCGTCTTGGGAGGA
GTCGGAGGAATTAAGGAGGCTGATAGATGGGGGTCGAAGCGTTCGGGCGC
GAACTCCTGCAATGTAGGCATTGTCGGAGTCAAGGAGTCATGAGCCCTGGC
CTGGGGTGAGGGGATCAGAGGTTGCTGCACATCTTGTCGCAGCATTATTTG
ACCTGTGGCAGAAGCCGACCTGGCCCGCCCAAACACCCATGATCGTCGAC
GGCCTTGTCCGTCTTCCGTATCACTGTCTCGACTGCGAACGAGAGTGAGAG
GGTTATAATCGGTGCTTGAACTTCCCGAGCGCCTGACACGTGGCATGACTC
GAGAGATACTCGATCGAAATGAACTGGCACCTTCAGGTACGCCCAGAGAG
GAGGATGGCATGGGCCCTTCCGTCAAACCATAGCTCGTATTTCTCGGACTT
GTCGGGGACAAGGACCTGGCCAGAGGATAGTATTCTTCGTTCGAATTTGCG
GAAGGACTGACACCAGCCACGGGTTTCTTGATAGGAATGGCAGCAGAAGC
CTTCACACTCGTAGCCGTCGGTCCACCATCCCAAGCTTTGCTTTCGAGATTG
CGGAGTAGTGGCGGATGTAGCAAGAGATGGGCGTTCGGATTCACGAATAA
GGAAGGCTTTGACAGCAGTACTCCAAGGGCGATGATCCGGATGTCTTGCAA
ATGTAGAATCACGCGCACTTGGGTCGGCTCGTCAGCAAAAATTGTTCTGAT
GTCTGAGCATCAGCACAGTTCGCACTCTTGAGCCGTGGTCCCACTTGAGCT
CGTTGTATATTTCGAAAAGGATCGAAAGTGCGAGAGTAATATCGGTTGATGC
TGCTTGCTCCATTCCTGTGAAGCGTGACCCAGCGCTGCTGCGTGAGCTACT
TTCTCCTCAACACTCAGCTGCTCACCGTCCAAGATTCTTCGTGGGTGCACC
CAAGATTGCGGTGCTCAAGCCGACCTAGTACTCTGCATGTCAGCCCGAGCA
CCATGCTTGGGAGTCTTCGCACCTCAAGAAGACGGCTCTCCATGAATCTCC
CTCGCGCTTGTACCCTCTCCAACTAGGCAAAAATCGGCGCTCATAAGCCTC
CTGCCAAAGGGTGTCCTTCCACCTTCCCGTGCTCCAATCGTTCCAATATCCA

CTTTGGTCGAGCTCGCACCAGGACATCGAGGAACATGTAGCCGCCATCATC
CCGGCAAGAAAAGTCGCAGTGATGGGAGAACAGCAAGTAAAGGAGGATAT
GGCGAGCCAGCATCCAGCAATGACCTAACGACACCAGGCAGTGGCGAATG
ATGACTTTTCGTTAGGGCGAGCAAGATGGGATGCAGCGCGAGAAAGGGTA
AGACATCTTCAGCCGGGAGATACCTGATCCGTGAGCACAGCGCCCGGTTCT
TCCACTCACGAGGCTATATGACTTAGAACTTCGTCGGGAAGGTCGAGGAAC
AAGCTCGGAGCCCGAGCCTTTCCGCGAGCTGGCATAACGGCCGTCCTGGT
GACCGCAATAGTTTCCAAGCTCACTCGCCCAGCCTCCCAGGTCGTCTTCCG
ATTTTTGTTCCTCAGCTGTGCAAGGCCCTTGAGGTAGGGAGAGATGTCGAT
GAGCGGGAAGGAGACGTCTGCTTCGGCCGCGAGGAATCGTCGTTGCATGG
AGCCCCGCAATGCTTGGCCTGGGTGTGTGGAGGTGCCACATTGACAGCTG
CTCCAGAACGTCGCATGACATCAATTGTTCCGTGAATGCTCTCCCTTTCTCC
TCTTTGTCTTCCTATTTCGCAACGAAGACTGTCAAGCACCATCGGAATCAG
CTCGCTCATCCACCAGACCGTGTCCAAACGCTGCACATGGCGTCCGTGATT
ATTGCGGGATCCGCGCGATCCGCAACATCGCGGACTCTCCTTCGTCCTCCC
GTCCGACCTATCTCAAGGGCATTCTTCTCCACATCTCCATCCTCGCGAGCTC
CGTCATCGAGTAGTGCGGTTGATGTGCCTCCATCAAGTGCCGCGACTGCGG
GAGCATTGCCACTCTCATGGCCCGAGTATCTTTCCTTGCGGCGACAGAGGA
GGTTGTGGTCGACATTGACCACTGTGCCAACTACTTTTGCGGGACTCACTT
TGGGCGGAGGCTACTTTGCGAGCCTGGAAGCCGATCCGGCGCAGCTCATC
ATGGGGATAGAGCCGATGTGAGTCATATCTTGCCCTGCGCAAAGCCTTGCT
GACCCCAAGGTATGTTTATATCGGGGCAACGTGAGCACTGCAGATATCTTAT
TGGTGCGCCGGGAGCTGATGCTTCTTCCCCATTTAGCTGTGGCTGCATGCGT
GCGTGAAGATATCCTCCACAGTATGCGCGGCTGACGTGAGCAGTTCTTGGC
TACCTCCTTGGTCCGACTGTCGGGTCATCGTTGTTCTCCCTAACGCATCCCG
CTCTTTCTCGTGGCAAGCCCGCTCCTCTTGAGGTCATGGATCGCGAGTTCTA
CCATCGCATACAGAGGAAAAGGGTCGACCCCAGCTTTCAAAGCGTGAACA
ATCCCGCTCAGACTTTTACGGGGAGAAGGTGTGTCAGGAACGCTTGACTCT
TATTGTATTGCTGAAACGATAGATCGTCTCCCTGTCAACGTACCGCCGGTGG
CTACGAGACCAAGCCGCCTACCAGCGGAAAGCTCTGCACGGAGTTCCAGG
AGAAAACTGGCGTAAATCATCTCGATATTCTGTTCTCATCTCCTGCTGGGTC
TATCGTCATGCATCATTCATCGTGTGCTCGTAATGAGTTGTTTCGTCACGTG
AGGGTTCTGGAATGGGCGGCGATCTCCGCTGTTGTTGCTCTCAGTGTACCT
CAGCTCAGAAATCCGTCGACGTGATTGGGTCCCGAGGGTTCCATTCCCGGC
TTATTCCGGCGTCCCCCCACTTTAGACCCCCTGTTCCCGATCACACCCGCCG
CCCTCTCACATCCATCCTGTTCGTCACCATCGGGCTCCATTGAGATCTATCTT
ATACTCTGTACTCCTGGACACCAGCTATCTTCTCGAATCGACCTGTGACGAC
CTCTTGAACGATGATTCGCACTACCGCTTTCAGATCTCTCGCGTCGACCTCA

TCGGCAGCTCGACCCACGGCCTCGTCTGCCATCGCGGGAGCGAGGACTATC
CATGCGAGCGCCAGCCAGCGCTTCGCAGAGCCGGTAGATGGACCCAGCGG
CAAGCAAGGACCGCTGAAGGAGATCAAGATCTATCGATGGGTGCGTCTCA
CCTCTTCATCCCAAGTTGCATTGTACAGACTACCGCTATCGATGGCAATGGG
AGAAGTTCATTCGCTGACGATCTAGAACCCGGATACCCCGCCGAGAAAGCC
CAAACTGCAAAGCTACCAGATCGACTTGTCGCAATGCGGTCCTATGATGCT
CGATGCTCTGGTGAGTGACTGTGGAGACTATTACAGACAACGTCATGGCCC
GATCTTCAGCCGTACGCATGCTCACGGACGCAGATCAAAATCAAAAACGA
GGTCGACCCCCACACTCACATTCCGCCGATCCTGCCGAGAGGGCATTTGTG
GTTCGTGTGCGATGAACATTGACGGTGTCAACACACTCGCATGCTTGTGCC
GAATCGACAAAGACACATCGAAGCAAAGCAAGATCTACCCCCTACCCCAC
AGTAAGTCACTGCATTGAATGCCGGAAACTCGTCTGACATGCCAGTGTATAT
CGTCAAAGACCTCGTCCCCGATCTTACCCTCTTCTAGTGAACGGCTTCCTC
GTTGCCCAGATCGAGCTGATCGTCCAGCAAACAGTACAAATCCATTGAGCC
ATTCCTGAAGAATGACACGGTGCCGGAAAAGGGAGAATATCTGCAATCGCC
TGAGGACCGAAAGAAGCTTGACGGCATGTACGAGTGTATCTTGTGTGCTTG
CTGCTCCACGTCTTGCCCATCAGTGGGTTATTCTTCCTGGCCTATATCATCGC
TGACATCGCAGTATTGGTGGAACCAGGACCAGTACCTGGGTCCAGCAGTAT
TGATGCAGGCTTACCGATGGATGGCGGACTCGAGAGTCAGCCTGACGCAG
CATTATTAGTCAGAAATCACTGACATCCAGGATTCATACGGCGCTCAGCGAA
AGGAGAAGATGCAGAACTCGATGTCCCTCTACCGTTGCCACACAATTTTCA
ACGTGAGACTGGGTCATGAGCACCAGCAGTCGGGTCGCTAATCATGCAGT
GCGCCCGAACATGCCCGAAAGGTCTCAACCCCGCAATGGCGATTGCCAAG
ATGAAGCTCGAAATGGCGACGGAGCAATAGTCCTCGTACAACGTTCACGTT
CGGCCACTGTCAATCCAGGAGTCTGCGCTTTCACAGGAGGATGTTTGTATG
CATATGTCGCAAGCAATCCGAATGGATCGATAGGCTCTTCAAGTTGACCTG
AGAAGGCGTAGAGAACGAGCGGACGGGCCGCCGTCGACGCTCGTCAATG
ATCGTCATCATCGTACAGATGACAGGCGTTGCATTTATGGGTATGCTCGTTC
AAATTCCGTTAATTTGGGTCACTCGGGTATTTGAGAGAATGCAGGGACCTA
ATGGTAGACTCATCGGAAACTGCATCTTCTGGGTCTCCTTCACGGTCCTTGG
TCAACCCTTCGCCGCTTTGGTATATTTCTATGCCTGGCAGGCAAAATACGGT
TCTGTCGCCAAAAAGATGGCGTCGAACCAACGCCCGTAGCAATGGGTTAA
GTTATACCCAAATTAAACGGGATACGAATTATGCAAGTTGGGAGTTGAAAA
AGGGAAGGTTTTCTGAATTTCATCTTTAATTAATCCTCCCGGATATCCGAAAT
GGTGTTTCGGGAGATCCGGTCTATAATATCGATAAAGTATAAACGAGCAATC
TTGAGAGTAAACGGTTAATCGAGTGCTCTAATAATAATTATATCTCTCCTACC
GATCAAGTAATAACTTCTCGCCCGAGACATGTGTGGCATAAAAGGGGTTGT
TTTCATCTTGTTTTTATTTCGCTGTCCCATGCACTATATGTACAAACCGTATTA

TTACACGTAATTTAGCCATGCTACCTCCCCTTTCGATGCTCCTATGCTTTCCT
ATCCCCCTTGCCATACCCCTATACCGTTCTTGTACAGTCTACTATACGCAGCT
CCCTATGATCACATGCTAGATGCCCGACTCCTACTCCTATACTCACCACGCC
TGCTACTCGTGAGACCAATAGCCAGACTAGCAACGAGCAGGGCGCCGTTG
AACCCGGCCATCATGTAGAACGGGGCGCCGGGAGACACGACATCGAACAT
GTATCCGCCAAGCTTCGTTAGAAGCAATATAGCGGCGCCACCGCACCACGA
GTACACACCCGCAATAGAGCCCTTAAGCTGTACGCGGGATGCCGGTTCCTG
CTTCCTTCTAATAATTTCGCTACCTGCGTCCGACCCCAGCAGGGCATCCCTC
TCGGACCCCTCTGCGTCGAGGGTCCTATCCTCCTCGTCGACATCGACGGCA
GGCGCATCAACAGCCAGGACACCACGTCCAAGGGACCCAAGACTACACAC
AATAGCCCGATCTGGCTAACGCCAATAAGCACCGCGACCAGGAAGACGCC
CGGCCCGCCGCCCCGTCCGTCCACATCCCTATACTCGGGACTCTGCAGCTT
CGGGAACGCGATGTACCCCGCCATACCCAACAACGCCGCAATGACAAGCG
CGTAATCGACGCCGACGCCCCTTCGGCTAGCGTACCCGAACAGCGGGGCG
CATAGCAAGGCGGCGAGCTGTGCTACGCCGGTTAGGATAGAGGCTAGCAC
GTAGGCGGCACGGCACTCTTTCTTGAGCTCGGGGGAGGGGTCGTTGGGGG
AGCCGCGGCAGAAGCCGTTCCCGATGAAGAAGGCGTTTACGTAGAGGGGG
ACGAAGAGCGATATGGCGACTGTGCTTGCGCGCGCGACGAACCCGCCTAG
GTATCCTAGTGCGATGTGCGGGTCTGTGGCGCCGAGGCGGAGGGAGGAGA
GGAGAAGGCGGTGGTAGGGAAGGGGACCTTTGGTTAGAGTGAGGGCCGA
CGATGAGGATGAGGGCCCGGCGGAGTCGCTATTAGAGTTGGAGGTGGAAG
CGTTTTGCGGCTTGCGCAAGTTGAGAAGCATGCGCCCCACCCCTTGCCTTC
CTCGCCCTTCAGTCCGCGGAGGCCGAGAATACGAAGATGGCTATAGCAAGC
GCAACGGTGCCTACGACGTAGAAGCTGTCCGTGATAGCCTCCGCAGGCGTA
ACACCTCTTTCTTAGAGAACTGAGCCGGTAGCGGTAGGAAAAGGATCAAA
GCAACTAAAGCTCCGCAGCCTGTGAACAAGCCGACGTATCCGGCTAGGGC
AGACGGTCTGCGGCCGCTTCCCTCAACACTACCGTATGCATCCGAAGAGTC
GTCTGAAACGTAACTAGTACTCCCCGTGCTGCTTCGAGGCGCGTTGCGTGA
GTCGTTCCGGAGTAATCGTAGCATCGCTCTCCAAGCTCAGTGCCACACTATC
CCTAGCATTATGGCCCGACTTACGGCTCGAAGCAGCGCTCGCCACACTCCT
GCTCTCCCCATTCCCATTTCCATTCCCCAACACCTCGTCCTCCTCATCGCTC
ACTTCCCTATTATCATCCGTAAGTACTGGAAGTATCGCCGTAACCATCGTCG
CCGCCGCCGTAGCCCCAAGCGCGAACGCGATCCGCGCAAACACCAAGCTA
GGGTACACATCCCTCGCCTGCACGAAGCAGAACAAGCTGATGCCGATAATC
GCGTATCCCAGCACCGCGACCCATCTTACACCCAAGCGATCGCTGACTAGA
CCCCATGCTGGACACGCAACTAATGCGACGACCTCGTCCACGAACCCCAGT
GTTCCGACGATATCTCCGACGCCCGTCTTGACGCCCAGCAAGTCCGTGATC
ACGAACGAGACGGCCGAGTTGAGGAATACCAGGAACGAGATGCTGAAGA

GGCGATGCCTAATAGGTACGTCGCAGCTTGCGTCGCCGTCGCCGTCGGGCT
GAAGGGCAGGATGCGCGTGAGAAATCGGGAGCGCGCCATCGTTGTTACTT
GGGTTTTTGGGGTGATTCGTAACGGGCGCACCGGTCTTGCGGAGGGGTCG
GACACTTCGGACTTTTGGTCGTACGATGGCGAAATCCGTTCGTTTCGTAGA
CCCGTATACTATAAAATTTACACAATTAAAAAAAGGAATTAAGGTCACATCT
TTCGTCGTCGTAGACAATTTTGAAAGATGTCGGCTGGTCAAGATTCCAGCT
TCGGGTTGGTGTTGAAGTTGGATGGAGTGGGTTCAATTATCCCCCAACTTA
GAACTTGGAAACTTGGGGGAGCTTGGGAGATTGATAAGATGTAGTTGGCTC
AAAATGGAAATGGTAACACCCAACCCATCGATGGTATGGATCCTATTTTAGC
CCCGGAAGCCGGTCCTTCTTTAGACTTTGGCTCGGTTGCCGGCCGCAGGGA
TAGGTCACGCTTAACTCGACCTTGACGGTCTCACTTGTATCACATTTATCAG
GTACTGTTCCTATTTAGGTATATTCCATGGCATTAAGACTCATGAGTGGAAGT
TAATCGACCCTGTTATGCATTGGAACGTATCTCAACTTATGCATAAGAAATC
GTACAAGCAATATCACATTATTGGATGAACCAACCAATCTAGACAATACAAA
GACAAAGGGTACATACATACACTCGAGAAAAGGGAAAACAGATGAGTTGT
TGATATATCGCCCAAATGAAGCGAATAGGTTTCATACATCGTTGAGCTGTCT
AAAAATAATTAGTACTCTTTTGAGAGCACATATCCCTTGCTCATGTCCCTTAT
ATGGTCGTTATGTCGCCATTCTCCAAGGCAGACAGACCTCCACGCAAATGT
CTTACAATGATCCTAACGCCGGGTGATGATCTCTCTATCCCCAAATTCCGTTT
CTTCACAGGATCCCATTCGTGATGCCTGAAGACCTTGCAAAAACAGCGCAC
ATGTACAACACCAGGAGTGTCGTAGCTAACTCGACCGGAAGAAATACGAA
CGAGCTAGCTATCGCCATTAAGGACCACCGGAAATGTGCAATCCTTTTCTCC
AAATTTGCATCACGCGAAGACCTCCGCGCGAATTGATCCCAAGTCGGTCGC
TCATCTTCAGAATTGACGTCTTGATCATAGTCATCGCCGCTGAAAACTTCCA
ATTCAGACTGGGATTGTCAACCCGAGAAGTGGAAGCCACCGGGGAATCCC
TGGGAGCGCGTGCGACCGCCGAATCCGCCTCCCCCGAAACCTTGATGACC
GCCAAAAGACGCGCCACCGTTCATCATGCTGAAAAGGATCTCAGGGTCGA
TGCCACCGCCCATTCCACCTCCCATGCCGCCGCCAAACATGTCAGAAGGAT
CTATCAGATCCTCGCCGCTGTCGTACCGGGCGCGCTTTCTGTAGTTTATTCG
TTAGCACAAGTAATATCAGAAATTTTTACATCAATACATACTGAGGGTCGCT
CAATGTTTCGTAGGCTTCACCGATGTCCTTGAACCGTTCAGCAGCAGCCTC
ATCGCCGGGGTTCTTGTCCGGGTGGTGAATGATGGCAAGCTTGCGGTAAGC
TTTCTTGATGCCGTTCTCATCGGCATTCTTGTCGACATTGAGAATTTTGTAGT
AATCTTTACGTTGGCTCTTCTTAAGCTCCAATTCCGCTCGGCGGATCTCCTT
GGGAATAGAGCGATCCTCTGGGTCTAACTCTTGGATTGACTTCCACTCACG
AACGCAATCCTCCCATCTATCCGCTTGACCTAGTGCATTAGCCTTGGTCTTC
CTAGCTTTGATGTAAGTGGAATCAAGAGCAATAGCTCTTTCGCAATCAGCA
ATTGCTTCAGTGAATTGCTTAAGCTTCAGTCGGCATAGAGCACGGTTCTGG

TACAGCTTCGAATTGGTGCCTCTGTTTGTGGGATCCACTTCCAAGGCAGCA
GAATATTTGTTAAATGCAGCATCCCCATCTGCCAGCTTTGTAATCAGCATTG
CCTTCTTCCTTCATTCGGTCAAGTTTCTGCACAGTCCGAAGCCATTTGACGG
CATCCTTGAAATCAGGATCACAGCTCAATGCTTTTCGGAAATGCTGGATAGC
CTTGTCATTCTCGCCTTGAGCATATAAAGCACGACCGCGCAGCACCAACGC
TTCGGGGTCCTGAGAATTATATCGTAGAAGGCCCATAGCCACATTCATCGCT
TCTCCGAAGGAATTTGCGCCTCCCATCTTAAGATAAGCGTTACCTCGCATTA
GCTGCCATTTTCGGGGTTTTAGACCGCCGGGTCCGAGAAGCTTTTCAGCCT
GATCTAACGCATGTAAAACCATGGAACCACTAGTGCCACGGTCGAGAGCGT
CCTGGGCAGCCCTGATGTGATGCATCATTTCTTTAGCAGCAGCCATATCCTT
AGCAGAAGCTGCCGGACGAATGCGTTCGAATGTTGTCAGAGCTTCCTCCG
GACGTCCAAGACTTGTGTAGATGCGAGCCAGACGCAAAAGATACTTGGCA
TTGTTAGGGTCCAAGTCCACAGCGCGAGTGCAATCATCCAAAGCCGACTCG
TATCGACAATCAGACATGTAAGCTGCGGCACGGTTACTAAGATAGGTCGGC
TCGTTGGGCATAAGTTGAATAGCTATAATAAAATTAGTTCCGGATACTTTTGC
GGTTTGCTCAATTCTGCAGTGACTTACCACTTGTGTATTCGGCAATTGCTCG
TGAATAATCCCTCTGCTTAAAGAATCTATTTCCGTCACTCTTGTGATTTTCAG
CTAGCTCTGCGTCATTAGGCACTGGGCTGGAAGGGTTAGACCTGTGAGGCG
GAGGCACTGGACCCTGTGGCTCTTCGGTTGTACCATTCTTCACCCCATTAGT
CTTGGGGGTAGCGGTGACTTTAGGTGACTTGACAGGAACGGTGAAGGACG
ACTGTGGCTGTACAGGAGGAGGTGAAGACGGTGTTGCGGTGGGGGTATCC
TTGTCGACATCCATCTTGTCGAGAGACGCCCGTTGGTTGTTCATGTTGAACT
GGGCAGATAAGCGTTTAATCTCGTCAGGCGGCAAATTGAGGGGATTGAATG
TTAGTGTCAACTTTGCTACGTCTCGAAGACGTGCTCTGATCGGTCGAATGT
CTAGTGGTGTAAGACCGAGAGCTTCGAGACTTCAGCTGCTCCCGCTGAGC
AGGTGATGCGGGGCGTGAAGGTTTCTTAGTTGGAGATTTGGAAGAAGACG
AACTAGCAGAGGGCTTAACAGGTGAGGTCTGCTCTGAACGAGGGATTTGC
GGCTGTGGCTTTTCCTGGAGATGGTGGGGGCCGGATGTAGAGTCTGTGTTG
TCGCCTGCTGTCGTCTTCTTTGGAGATTTTTTATTAACGAATAGGCCCTTCAT
TTGGAAAGTGGAATGCGAGTTTTCGGCTTTAGTAAAGCGTGGTGTTAAGTG
GTACGTAGTGGTCTCCGAGTTCCCACACCCCTATCGCTATTATGCTCGGTTT
ATTACCTAAGACAGGGCAATTAATCGATCCTTCCCCGAAAGGCCGCCTTCG
ACAGCCTTCTCTACTTGATGCTGGATCCCGGTCGGCTCTAGTGGGACAGGA
AAAAAATTATGTTAGTCGAGTTACCCAAAAAAACGAGTAAGAAACCAAAA
TATTTAGTGCTTTGTTAAATTTCATCAAAATGATGAAGACCGACTCGAGGTA
GGACAATAGACGTTATAAGTTTATATGCAGAAAAAAAGAATAGGGATAATA
GGAATAAGAATAAAAAGAATTGGTCGGTTATTTGAGTGGCGTATTTCTTTTC
CCCTTCCCCTAGCATCCAGGCATCCGGGAACCTAGTAAAGCGCTGATTAGG

CGATATCACTTACACTGCAATCCTGACTTGACCGCCGTACCGGTCTTGCCTT
TGGATAACCAACAATTCCTAAGCGGGTCGCGGGTCGTGAGCTGCAAAGAA
GAATTAAGGGTACTGTCGTTTCCCCTCTTGTTTCCAATATTTATTTTTTCCTC
AATCGTATAGGTGGAGAAAGAAAAATTCGTCCTAGAAAGCGTAGGTCGAC
GGCGAGTGCTAGGACGACCCCCGGATAGCGATAGCTTTTTTCAAGGTAGGA
CTAGGAATGCCCAAGGCTGTCAAGTGCGACGAGAGGAAAAAAGAAGAGC
TAAGGGAATTGTGAGGGAACGAGAGGAATTGGAGGGTCTAAATGTCTAAA
TGGCTTTCTTTGGTCAAGAATGAATTTGTGAAAAAATGACAACAAGGAAC
AACGATTTATCAGCTTGTCGAGAAAGATCAACGTTTACGGGACAGCAACTG
CAAGCTGCGCACAGCCAACATGCACTAAAGCAGATAGTGATGTGCTTCAG
GCTGCTCCAGTCCTGTTGAGAGTTCAAGGTTGAGAATAAATCGTTAGGCTG
GGATTTGAAATTTGTGAAATTTTGGGGAAGCCCCAGAAAAGAAAAAAAGG
ACGGAAGTCCCGAGTACTTGGTCAGCTAGCGGGTGCCGTGCACTGACGGT
AAGTTGCTCATAGTACCTAATACATGAATACATATCTAGGCACGTAAATGCC
ACTTCCTGGACTAAGCCCATCAACCAGCAATTGCCAATTCAATCCAGACTG
TCCTGACTGTCCAGCCACCCCACTATCGCATACTGGGTATGTACCTACGCAT
ACACTACTACCACATACTGCAAGTAGTAACTTTTACCACTTATCACCACATC
ACCACAATCAAAAATCCATACTATTACTATAATTCTACCCAATATCCATTCAT
GTTGATTAAACTTAGGGAGGTTGTATGCTCGGTTCACCAATAGCCAGGTCA
AATACATGATTTTGTTTACAATTCTTTAGCGTCTGTAACCTAAGTATGGGGTT
TTGAATGGCTATATATCTCCGGTACGCCTGCTCAGGTAGTATTCTATCAAGCC
AATCCCGTCATCACACTCACAATAGGAGAGGCTGAACCACACCAACTGTCG
TACTTGCGACAGCGCTCACTGCGGCAACACGTCGACACACCCTCTACAAC
CCTATTCCGAACTTCTATCCTGCCTTGCCTGCCTCCCCTCCCCTTCTCTGACC
CTTCTTTTGTGATATGAATCTCATCAAACCTCAAATTGATCATCATATGTATAT
CCCGAGGTCGCTAGTACCTTACGCGGAGTACTGAATCCTGGCAAGCCGAGA
TGAAAAGATGAACAGGGGTCACAAGGATCGTTCCGAGGAGCGGCAGGAG
GACCCTTGTCATCTACTGCGTAATAGACATTGTTACCCACTTCGCCCACTCT
CCCACCTTCCTCCGACCAATGAGGCTACTTCGTCTTCCACCGATTCTCGAAC
CTTCTATGGGGAAGGTACAAATCTGATGTAAGAGACTGAGGTATACAGGCG
CTTGGGCCAAGCGGGAAGATGCGTTTAGATGATGTAAGTACTCTGTCGAGA
GTCTCTGATTTTCAATGTTCCCTTTGCTTTTGCCAAGCCGCCAAAGCTGTGA
TTGTAACCGCGTATAATTCCTATTGTTCCCACAGCGGGCTGAGGTGGGCGA
AGTGGATGATGGTTGTGGATTTGTTTCAACTTCAGCTCTTTGACTTGTAATA
ATTATCAGATTATGGTTATCAACCAGTGTCAACCGTGTCAACCGTGTCAACC
TGCAAGCACATGGTCTCACATGGCTCACCGAGTTCCTAGTCACCTATATTGG
ATTATCTGTTATTATTGAAGATCGTTTAACTCGGCGGCTAGTCCCGATATGCT
CACATGTTTGAAAGTGGAATAGGAAGAAAACGGTTCACTCTATTCTGAAGG

CCAATCAGAGTTTAAGAATTAGACTCAGGACTGGTTCGGAGTTTGATTGAA
AACGCTTAACGTTGTTTGTACTACAACCGTATAAGCAGCAAACCACATCAA
GTAATCGAAGGGTGGAGTTTGTGGCAGCGTCGGTCCTCTATATTGATGAGA
AGTGACGATTTTCATAGGCAGCTACGTTGTTTGCTTGATAGCCTATGTATCA
CCGCGTTTATGAATATTTGCATATAAATATGTTAGTATTATTGTTTACATAAAT
GTTTGAAGATGGAGCCTTAAACATTGGTCTATCACTTTCAAGCCTATCAACA
ACAATTTAGTCTTGATAGTCTAGCCTCAATGAAAATCTTGGGTAACAATTCG
ATGATTGGACAGTATTGTAACATGGGATGCGATCTGTTTAATAAGTAGCAAT
CACGGACCAAACAGTTAATTTCGTGTTTCACATGACTTTTCAATAGACCGTA
TAGTCGGTCACTACTTTATTAAGTACTTCAACAACAATGTTGAGACAGTTGC
TCGGAGTTCTAAGCGAACCTATTAGCAGTGTAAATATCTAAATTCCTGCAAT
ACGAATTCTTCGTATCGATCCGAGTTGCAGGCTCATAGCAATAACCCTCACC
ATATTTGCCCACTCATCGAACTCCTCAGCACCAAGGCATACCTATCCGAGGA
ACTTATTGATAAACCTCCGAGTGAAACCTTAATATCGACAATTCCACATGTT
ATACAAGTTCCTATCACCAATATGGGCTCATCCTTCGGAGCTTTCTTGAGAT
GTGGGTTCTGATGATCCGTTTCCGCCTACGATGGAATTCACGAACTCCTGAT
ACTTGAAAACCTCGCCGCGCTTACTCAGACCGCCAGACATGCTGTTTTTGC
TGAAAGCCCTTCTGCCGCTGAACCCCACCATGATTTTGTCAACCTCTAATG
GTGTCAGGGCCCTCTCTCCTGGCTCCGGCGCTGTCTGTAACAGCGCGTCTC
GTAGCTCATTGAGATCTACTTGACCACTGTCGTCGTCGTCGAAGGCAGAAA
AAGCTGACAGGAGCTCTGCGCTTGGAGACATGGCGGTCAGTGTCGAAGCT
ATCGAATTGAGGAATACCGCGAGTGTCGTGGTCTGCGGACCTCCCGGTGGG
AAGAACTGTGATATGTCGGATGGGCTAGAGGGTAATCCTAGTAATTCATATA
TGTAAACATTGGTGTCCAATGGCTTGATTGAGAGGTGTGATATACCTAGCTG
GTTGAGCATGTCTGCGATGTCTTCCTATTTACGACCCCATCACTGTCTCTATC
TAAGATCTGGAATCCGTCTCTTAGAACGCGGACCTGCGCTGGCTGTAGTTG
AGAAAGTGCATTTCCCTGTATTGCTGCTTGTGGCCGTGACGACGATATATGC
CACGGGGGAAGCGAGAGGACGGGGAGACGGCATCTCTTTCGTCTGCGCGT
ATCCGCGAGGAGTGTAGCTATCTGTACTACTCGTGGGCGACTCTACTGTGG
CAGCGAATCTAGAGGGTGTATGCACGATGCCAACTTTCGTCGGTGTTTTGG
TCGGCGTTTGTGTCGGCGTCGTGAATCTAGGTGCCGAGGGAGACGCAGGG
GATTCCGGTCTACGGAAAGGAGAACTTCGAGGAGAGCCGTAACCTAAGGG
CGATGGCTTATAACCCGCTGGCATGGCCTAGATCATGTAAGCATTTGGTTCG
GTCAATCGTATTTCGAAGGGAGAGATGATCGATGCAGGGAGCTCCGCCGGT
AAGTGGATTCATTACTAACCATTGTCGCGTGTTAATTCCCAGGACCGTGTTA
CAGATTTTATCGATAACGAGTAAACAAGATGGAGAGAAAGTGCTTCTTCAG
TGGTGTGTGTAATGTTGATGATAGTCGGCAAGAGACGCCACCTTCGTAATT
GAGATTGAATTTGAGGTTTAGTGTGTCGCGAAAGGGAATGTCAGATTCGTA

TCCTGTCGAATTCCAGGATGGTCTAGGCGCGACCTTCTGTATCGCACAGTC
GATGCATCGCTGGATTCGTTCGCACAAGCCCGTTGCGGAGCAACCGTAAAC
CCACTTACCAGATTCGGTATAATGATGTCACTGAAGATTCTCACGTGCACAA
ACCCATGGATTTCAGGGAAGTCGAACACAAATTTGATATTTCGAATAAAATT
ATAAAAACCTTCAATTTCTGATAACTATTAGGCACCTACCTATTTGTAAGAAT
TAACACTTCTTATCATAGTGCAGTGAGAAGCATTGATTTTTGGAAATGTTTG
CAAGAATGGAGGTTGCAGTCTTCCCCTAAAATTCGTACTGTAAGGCTGGTT
TATGTCTCAGTTCGGCCATCGCGATTGAATTTCTGGTTGCTGTCTCTGGTCC
TCTCAGTCTATATATAGCATGGATTGCTTCGATTTGTCTAGCATCTTAGTTGG
GTAGTATTTGGAGAAATTGAGAGAACTTGAAAAGTGTCAGCTTTAAACAGC
CATCTAGACAGTCAGACAGTGTGCCCCTTCGACGAAATTCGATCTAAAGCC
ACTCGGATAGCGCTTCGACAAAGTCCATAGGTTTCGCCGAAGTCAGCCATA
AGGGTGGCTACCATTTTGAATCAATACCCACACTAACAGGAGCTGAGGCGT
AAATTAGGCTGACGTCCGTATTGCAGTATAAATCCTAGGTGTTTCCTCTTCG
AACGAGATGGCGTTGATGATGACGATGATGATCTTGAAATACTGAAGTAGG
TGTCATTTTCCATTTTCCTCCGTCCTAAAAAGGGAATTCGATAGATCGAGCC
GAAGAAATAAAGTTATTTAGAGAACTAATTGCACGGATAATCCTTGACTTCC
ATAGCTTCAGATAAGGTTCACGACCAGCTTGTGGCTTGCGCCATGTGTTGA
ACAGGAAGCGGTTTATCATACCATTGATGTCGTACAGACTGGTCTTGGAAG
TGCGGATGAACTGTCGGTAAACTACGACCCGCCTCCCCGCTTCCTTTGGTC
GATCGGGCTGCGATAACTGGATTTGATTTGGTAGCTGCTGTAGCAGAACTA
GTCGCAACAGGGAGGTGGGGGTTAAAAAGGAAAGGGCGTTAGAAGCCCT
AGAGGACTTTTAGAGCCTGAGAGGCGCTAAGAGAACACTAAAAGGGTTAA
CCCCTAGCATCCTAGACCCCTAAATAGCGCCTTAAAGCCTGTAGCCGAGCT
AAATCATTGGCCTGTACGACGAGAAACAGGGGTCTTCTGACCAAGGGTCT
AAATTTGATACGAAACTCTCACTCTTTCGCTTCGCTGCTCTCGGTACCTTTT
TTCTCCCCCAATCTTGAACTCAGAGGACCCCCCCTTGCTCAGGGTGAAGTA
CCGCCTTTTGAGTTCAAACAACACCGAAGAGATGAATGTGTAGCAGATCAT
GTCTCGGCCATTGACTTCTCTTACTCCAACTTTTCACATCCGATTGCCGTCG
GCGTTGGCCTTGTGCGTCTTCTGGTTCGACCTTTGATCTTGCTCGGTGGTAC
TTCGTACGCTTTATCGGTCTATCGCGACATCGCAAAATAGTGTGGACATTGG
ACAGTGACTGCGCAACCCCTGAGTTGCGCATGGCACAGCAGTGCTGATCG
AATCCCCCGCCACGACCGGACCACCAGTCGCAATCCATCCTAATTCAAGTA
CCATATTCTTGAATTTCGGCGCAGTAAGAGGGTATACATATCTCCGTGATAC
ATACTTCCATTGCCAGCCAAGGAGCTCCGGCTTTGAGGATCTGCGCTTGAT
CACTGCCCCGAGCAGTCCCACCACGGCGGTCCAGTCAACCGCGCGACGAT
CATACCAGACCCGAGTTGTTGGTTGCTAATGGCATCGGCTCAAATGGCGTT
CCCCTATCTGTCTCGAACAAATACTAGCCAATCCCTTTCTAATGGCGCAAGC

GGAAATCACCGCGCGTCTCTCACGTCCTTCTTATCTTCGCGATCCCCCTCGC
AGATGTCTCATACCTCGCGACCTCCACGATCTCCACAACCGCCCATCAT

>Mosaic_sequence_52

GGGTCGGCTAACCTTATGATGTATAGTGCGTTTATCATTATCACTGTGTGTGT
AGATTTGTTCCGGCTACGAAACCTCTCCCGAGATAAAAAGAAGGACAACTA
CTTTGTACAACTCGGCCAATATAGCGAGGATGGCATGCCAAGGCATATGGG
AGCACCTCCTCCGTACGAGGCAGTAGCAGGACCTCGGTAGCAGGCAAAAT
CAATCTTGAATAGAATATGAAGCAAGTATATGAACCATACCGACTAATATGAT
TATGCCGTGAAATTCCTATGGTAGAGACAGCAATGCGTAGCCACTATGCTGG
CAGAGAGGGATGTGGTAGGAGAGGAAGAATCCAACTGTTCGGACCTATAA
ATATCGACTCCATAAACTTCTGAGTCTTAACATGAGTATGAGTTCATTCTCA
ACAGAAATATCAATTAGGTACTACCTAAGCTCCTAATATCATCCTAGGTATGT
ATAGGGGCGGACATCTATGATCAAAAGACGAAGCCACCTCTACGTCATAGG
GATATGGTATTAGCAGCCACACCGAGGTGAGCCTCCATCAACCAGCCACAC
ATATCGATAAGAACGCCAAGATTTTGAACCAGTTCTGAAATCCACGAAAG
CTTCGTCTTTTCAAAGCTCACACCTAGACTTCATTAACCCCGCCGCTGCCG
ACGTCCAGTTTCAAAGGAAATACAATTGATCAAAAAAGAGTATCGCAATGG
ACAGCGAAGCATTCTTGGAAGAAGCCAAGAAGCAGATCTCAACCCAGCAC
GCCATAGAAAATGCGAAAGAACTAGTAGACGTACGCATCATAACTCCGTGC
CCCTCCGGGCTCAAGCCTGCAATTGCGAGAGATCTAACCCTGTCTTCCCAT
ACAGAAAATCAACAAGCACTGCTTCGAGAAATGCGTGCCTAAACCAGGCA
CCTCGTTATCGAGTACCGAACAGAACTGCGCCACCCAATGTATAGAGAAAT
ACTCGCACGCGTGGGGCCAGGTCAGCCTCACATACGTCAACCGCTTGCAA
CGAGAGAGGTGAGGTCTCCCGTTTCGCAATCTCTCACGCACGTATCTGGTC
GTCCCTGATCGTGGTAGACTTTTTGCCCTTGGCCACAGCATGGGCAAGGAT
ACTATTATTGTTGCGAAAGATTAACGAAAATGGGCTGAAAATATTGTGGCGT
AATCGAAACAACGAAGACATTTGGCTTCGAAACAGGTATCTTATCTAGATA
AAGGCCTGAATGGATATATGAATATGAATTCTGCTCATGACTTCTCTGCGGC
AGCTTTTTTCTTGGCCTCCTCGGCTTCTCTCTTGCGTGCAAGGTATCTCTCT
TTGGCGCTCGAGACGTCTGATTCGGTTTTGCGACTCTTCGATGCGCGCTCG
ATCTTTTGCTTTTCCTCTTCCTCTTCCTCCATAGATCTCTTGAGGGATTGTTC
CAGCTGCGCTTCAAGCATCCGAGTTTGCCTTTCGCGCATAGCCTGCTTCCC
ACCGGCACCGACAAAGCCACGCGACAGGCCTCGGTCGTCTCTGCGGTCTG
GCCCTCTGCTCGCCTCTTTTCGGGGTTCCTGTTTTTTACTCGCTCCTATGTTA
AGACCACCCTTTAGTAGCATACGCTTGTCTACTACTTGGCCTTCTTCGTTGA
TAGCAATAGAACCGCCCTTCTCATTGATCTCCCTCGCCACGTCTGCGTCTGT
CTTGACTTTCTCTTCTTGAACTTCATCCTCCTTGGGGCCTGCTTTTGCGCGC

TCTTCCGCCGCTTTAACAACTTCCGCGTGTCGTTGATCGCCTCTCTCCAATA
GGTCCTTATAAAAAGCAGTCATACCGCCACCCTTATTCTTTTTCGCTTCTTCT
TCCTCGCGCTTCCGTTCCTCCTCTTCTATCCGCCGGTTCTCCTCTTGCTGTTT
CTTGTATGCTTCTGTGACGAACTTCTCCTTATCGGCGTATTCTTCGCCTTCCT
CTGCCCGTTCCCGCGCAATCTTCTTCTCTTCCGCTATTAAGGTGTCACGTTT
TCGAACGGCGGCAGACGCGATCAGGTTCTTCATATATTTTGGTTTCCTCTCG
ATGTCTTCCTGTGTAACCTTCTTCTCCGGCTTCAGCGATTCGTAGACCGCGT
CGTAGTCGTATATCGAAGGATCTAGTTCCTCCGCAGCTTCTTGGTGTTTGCG
CGATTCTAACGAGCTCGATAAATCGCCGTACATTAATATTTGTGGTTTTTTGC
CTTTGCCGTTCGGGGGTTGAGACGGGGGGCCATCATTCAACTTTGGCAGAG
GTTTTTTGCTGGGTTTGCGACCCGGAGAGTTTGTCGCGAAGTCATCTATTTC
GGATATCGTAGTTGATTGGGGCCCGTCGGCCGAACCGTTCGCCTCGTTGTC
TGAATCGTCATCGCCGCCAAACATCGGCTTCCTTTTAGGGGGAGCCGGTTT
TGAGGCGCCGGGTTTCTTGGCAACTTTTAAGCCAAACGAAAAGCCTGGTTT
ACTCATATTTGCGATTCCCAACTTTGTTCTTGGAATATCTTGCGTGAAAGAG
CAATTATCTGATTCAATTCGTCAAGTCGTGTCTCGTTCCTCGTCGGGAGGTG
GCGCGAAAGATCCACTATGATTAAAGCAGCCACATTTGAATGCATTCAAAA
AGTTCCCAATTTCAAGGTTCCTTGATTAGATAAGATTAAGCTTAAGCTTATC
GATACGAGATAACCAGCATCCATATATGACCGGAACCCGTACCTTGGCCCAG
AGACTCGGGAACCTCTGCAACTTTCTGCATAGTCTAATTTAACAACTAAAAT
ATTTTAGGAATATTAAGATAATATTGCCATAAATTGAAATATGGAAGGTGTGT
TACATATTTGTGTCCAAGTAACAATTTGTGAGTCGGATTCACGACAGTTGTA
ACCCCCAGTACTACCTATATGACAAAGTTTACAACGTCGTACAACAGCTGA
GGAAAGTTCCAGCTATGAACCGCAACCCCACCTACCTGTCCTAGATATTAG
GCACCTGTAGCGACCGTCGCAAACACCGGCCGAGGTTTCCTCCAGGACTG
TTCACGTAACTCAACATCTCCCTATAATACTTAGCCCCTTTTTAGCACGGGTT
TGCTCCTATTCACGATTTATAGTTATACCAAGTAATTTCCATTTTGTTAGTTTT
CACCTATTTTGACGTCGTGAATATGGTATCGTCCAAGACCTACCAGGCCATC
CAGAATTGGATCGGCCGACAACAAGAGTACGAGAGAAAACACCTAGAGCC
TGCTCCGTTGACCGACTCCCAACGGAAGCTATTAGATAAACTCGAGCTTGC
ATTACCGTCAGAAACACCACTCTCAGCCGAGCCAGAAGTTGGGGAAGTCA
ACTGGGTCGGTCTCCTACAAGGTAAATATCCCTCGCTTCCATATCACCGTTA
TATCAAGAAGCTCTAGTGTCTAATGTCTCCTTAGAATATCAAGCTGCTCGTC
AGCTAGTACCTTCAGGCACTCCCGTCAGTAACTTCATAGAGGAGCAAGGCC
CGACTATAGGCAATGAAATAAAATGGTACTGCCGGGTCAAGATCGATGAGA
ATGTGAACCCGTTCCCCGGTCCAGAGGGTGGTTCAGTGGACGGAAGGCAG
CCATATTTCGACGAAAGAAAGACGCCAAGAAGTACGCAGCCAAATGTGCA
TACGAGTGGCTTAAGGCAAACGGATATCTCTATCGATCCGAAGCCGATGCT

GCGAAATTCCCTCAAGCCCCTCCAACCCCCCAAGCCACTCAGCAACAAGC
GACGCCACTATCTTCTCCCGCAAAGAAGAAACCGAAACTCTTGCAATCCTC
GCCTAAGAGTCCCCCGGGTGTCACCGTCAAAACCGAACCCAACACAGCTT
CACCGCTTCCCATCTCTGCCTTCAACAGCGACGAGGTATCAGCCGTGGTCG
AAATCGAGCGCCTCTGTGCTCGTCTCGGCCTCCCAAACTCCATTAGATACAT
AGTCCAAGAGAGCACGGAGTTCGTCGGCTTTTACAACGGCCACGCCGATC
TAGGCATCCTGGCTACGAGATTCCCCATAGGCGCCGGCCGCGTTAAGAACG
TCATCGGCAGAAAGGCGGCCAAGGAGAAGATCGCAGAGGAATTGCTAGCG
CATATGCGCAAATTGGGCACAGAGCATGATGTGGCTGATAAGCAGTTTCTG
GATGCCCTCGGAGCTAAGAATGAAGAGGTACCAGTTCCCGCTTGATCGGAT
CACAGCTAAGTGGTGTTGCGAAGGTGACTCGGAGTGCAACTTATGTTGCGC
TTGGTAGGTCTTCATGATTGTATGAATGAATGGGCTATGAACATAGGAATAA
ATGGGCTTCAACTCATGATATGATGTTGGACGGATAGTCATTAGGTGAAGGG
CCATTCGTTATAAATACTACGGGTTCGTCCATCTGATCTTCGAGATTAATCAC
GGAGAGGATAAGGCATAAGGTAATTGATTTATCTACAGTCACACCCTAACG
AAGAATAGGATGGTACTTTACGTTCGTCACAGCTTTCCACAAAACCAGTAG
CTCTTATTTCACCTTGTCGCTGTTGCTAAATCGCCTTTAGTATACAGACCTAC
GTGTCGGAAGCCTTATGAGGTCAACAATTCAGTGGCCACTCATTCAATAAT
GTTGGTGAGCTAGAACCATAAATGAATGACTTACAGACGTAAACATCAAGG
CCAAATATTTACTTATAGAACTCTTCTTAATTAAAATTCGTCTCCCTTTATCA
ATTATATACTCGTTTCTTCTTATATTTGCAAATACTGTTGCGCAGCATCGAAG
CCTTCCAGTTTCCTCTCTCCCATCATCTATCTATCTGGTGAGAAGATAAACTC
TCCCCGACTTCATTTAGTAGGCAGTGTACCACGAAGGCGACAATGTTCGGT
TTCACTATTCTCGTCCCCACTTTGCTAGCAGTGTCATCCTCACTAGCAAGCC
CTTTCAGTAACGACCTATGTCCCTCTTGGTATCTCCGCAAGGGCATCTACGA
TGTAAGACAAGGTGCTGTCTGGGAACTTGTCGCCACCTGCTCCATCGGCGA
CGGCAATTCACTCGAGTGGGTTACAAGCAGCTTGAACATCGACCCGTAAGT
TTTCCACCGAACATTGCATATTCTCAAGGTAGCTAACGACAAATCTCATGTA
GGTGCTTTACCAACCACAATGGAGTCCTTGAACCAGCCGTTTCGGGATCAT
CCGATGAACCAGGAAGATTCACCCGGACATGCGACGGTTGCGGCCTGGAT
CTTGAAGGACCTGATGGTGACGACAAGAAGGACTGGCATGTAGCTATGCAT
TGCGCCTGTGTGAAAAATCCTATCAACTTCACAACTATCAAACTAGGTAAG
CCTACATCCTATCCTTCTATCATAAAGAGGCATCTTTTAACAGTCTCTCAAAA
TTAGAATATGGAGTCAATGTTACCAATGGCATTTTAAGTTGCATGAAATACA
TGGGGACCAAAGTTGATTACTCTCCTACTCTCAATCCGCCCATGCTTCCTCC
TCCAGGCGTAACGACGTCGATCAGGACACTGACCACAACGGCAACCGTTA
ACAATAATGCTACAAGTATCGCCACGATTACAGCTACTGCCAATAATACCAT
TGTCAATACCGCTACTGATACAGCTCTCACCACGGTTGTCAGCTCCTGCCCG

TCGCCCAGCCAGGTCACAATTACGAAAACTCATAAAGGAAAGGCTCATAA
GGTAACCAAAACTACAACGGTGGTACAGACACATCCGGTCACAGTCCTGG
TTAGCATTATGGTGACGAAAACTCCCACTCCTACGCCTACGATTGCAGCCC
AACTTCACTCGACGGTCGTAGCAGACTTTACGACCATTAATGGATAGCCTG
ATTCTTTGGCCTATTCTAAGAGAGGCATGGAGCTAGGTAGGATAAGAATTAT
GAGATTGGCTAGTCATCACATGGGAGAAAGATGGTATTAGCCTTGGGTGGT
TTCCTAAAATTTGGTTTTTCTCAATCAGTTGCAGGTTTACGATTTGTTTATCC
TTAGATTGCTTGTCATTTCGATTCGATACCCCTATTTTATCTACCTCGTCATAT
AAGTCACTTATGGTAAATGGACATCTCTTACAAGCATGTATGGTTTTTGTGG
CCTCAAAACATATGTTATGGTGTGGTGTGTGTCTTGACAATGAACGGGAAG
AATTGTTTGACACATCGTGATCATGAGTACTTGCCGTGACAAGCAAAAATG
AAAACCAAGTGATTTGCGTCGATATTGTGAAAATTAAAGTACAAAAAGGAA
TAGGGAAAAAATATTCGTCTATAGGTGTAATATTAACTCATTCAGAGTTAGA
AAATTGTGTGGTGTCTAACCATGAGTGTTCTTCGACTCATGAAACCATGGA
CGTACACACGGTAAGTTCACGTTTGGTCTCGGTCTCTGACGAGGTGGACAA
CTTGTATATATACAAAATCATGAGTCACTATTTGGCAGTTGCCATAGAAAATA
CATCCAGCGTGCCTTCCAAATCTCGAAGTGGGTTTAAATTTATTATTATTAAC
GATATTTTCTTTTGCATTTTTGTTTACTTCTTAAAAAATCACACTTGCTGTCA
TCACCACACCAGATTATTCCCAACACCATCAACATCCGAACGATCGATGTG
GTAGACAGTTAACGATGAAGATTTTCACCTCTTGCTCCATTTTTGCTCTCGC
TTGCTTCCAGACTGCAGTTCTTGCAGTGGCACAGGTTCCCATTAGAGACCC
GGACTACAATCCGGCTTGGGGACCAGCTTTCGATAGCAACCTATGTCCCGA
GTATCATCTACAAGAAACGGGTACCAATTCATCATCCATAATCAATCTCGTC
GCCACCTGCTCCGATGGCAGTAATGGCACACAGTACCACACCAGCAGCCT
GGATCTAGACCAGTAAGTGGGCGGCTTGATCCTCAAGCCAGCCCAATTCCA
TGCCCATTTGCCGAGTAATAACATAGCCGTTCTTACTCGGCTATATTATTACG
CTCTCTCTATATCCCATGAATGTATCTTGGCTAACAAGCTTCTTTTTCTAATA
GCTGTCTGTATAATGAAGAGGGATATCTTTTCGCTTCGGAGCCCCAGGACTT
TTACACAGGTCCTTTCTCGAAGACATGCAGCAACTGCGGCCTCTCGTACTT
GTCACTCGACGCAGAAAACGAAGGAGTCTACATCAAGTGTACCTGTCAGG
TAGGCTTAGACGATGATCGTACGCCAAGGCAATCGATGTACCTTCTTGGTAG
GTTTGCGTGCTTCGTAGCTCATTCTCTCACAGCTTCTATCTATCCCAATGAA
ACCAGAATAATGACGATAATTCTAACCTGTTGATATAGGAAGGGTAATCCAT
GTTCAGAACTATACCCTTGGCTGCTTCGAGTTCGTTGGCGAGCTAACCGAC
AACTCCATAATCTACCCGAACCAGGGTCTTCCGGCAAGCAGCGTAGCGACG
GTCACGGCCACCGCTACCGTGTACACGAACATTACTGTCAGCCCTCCGATC
AGCCCTGCAAGCAATACCGTCCCTTCCACTATGGTGCCCTCTACAGTCACC
ATCACGGAGACAAAGTGGAAGGCGAGGAAGGCGGTGACGCTTATCAAGA

CGCAGACCGCGTTCTCTCCCGTCACCATCCTAGTCAGTGTACCGGTCACGG
AGACGCCCACGCCTACTCCCCAGATAAGTGCCATACTGTTCACGACTGTCC
ACGCTTCGTTTGTCACATTGAACTCAAGCACGAATGGAGTTTAGGTCTAGG
AGCTGCGTGTGTGATTCCTGAGGATTCCTACTTTGGTCCGAGAAAGGTGAA
CGCTGATTCACTTGGTTTAATTTCGTATTACATGGGAGGTCTGACATCCAAA
TCGTAAAATGTTGGATTTGATATTGTGTTTGAATGGGTTTGGCCCTATTCTGT
GTATTCAGATTGTTTAATGTCGATAGTGGTTGGGGGTTTAGTCCCACGTAAT
GATATTCTCTTCTCTTCTCTTGACGATATAGAATATCTACTGCATAGTCTATAA
GAACCTCAACTCTCCTGTCCTCTCCCTCTCCCCCCTCTCCGTCCCCTACCAC
CTCTCGTCTTCCCCATGGGAACCTTCGCAACAATGCCCTCCGGCAAATCCG
CAACCTTCTCACCATCCCTCCACAGCACGCCAACCCTCTGCTTCCTCCCCC
TTACCTCCTTAAAATACTTCAACGGCACGACGCCCTGTTCATCGCCATCCGT
CAATACGACATCATTAGCACTAATCCACCACTTAATCCCCCATCCCTCAAAC
TCCTCTCCACGTCCACGAAAACGAGGAGCTCGGCATCACTCCTCATGCCAC
TAACACCTTCTCCCCCCCTTCCTGCTCCGGACCGCGGTCTTGGTCCAGCAA
TCCCGTCCCAAAATGCACATGTCTCCGCCCCATCTTCTTCAAACCCCCGGC
CGCTACGATAGCAGGCCAGAAGGCGATGTACGTGCCGTGGACGACGGTGG
CGGGTATATTGCCCGTCTCGAGGGTAACCGGGGTAAGCAGCCCCTCCTCCG
CGAGCGCAATGCTGTGGCCTTGGTTAGCGCGGATAAGCCAGTCCGCCGGGT
TATCCTGCACCGTAGCAGGATCGGTGACAGATGGGTCCTTGGGTTTCATAG
CGAACCGCTGTTTCGCGTTGTCGGCGACAGACGCGCGGATCTCGTCGAAA
GTTGGGTTTAGCGAGCGCAGGCGTGGCCATTGCATCTGTGGTGGGTTAGCA
ATTTATGTTTTCGGTACTCTTCCGGACCTTTTACCAGATTTTGTCTGAAACA
GGGGATGATCGTGTAATAATCAAAGACAATAAAAGGGGTGAAGGGACGAA
CTCACTACTCTGTCTAGCGGCGCAAACCCCTCAGCGTCGAGCCTGATCCCC
GCATTCTCGGCCTGGTGTCTCAGAAGCTGTGACAGTGCCTTCGAAATCTGC
ATCTCACGGTCTTGGGGACGAGATCCGCCTCCCCTTCCACCGCGCCTATCA
CGGCCTCCTCTGCCTTTGCCACGGAGAGCCGTACTCTCCATTCTCTCTGCTG
CCTCCTCGTACTCGTCCTCCATGCTTAGATTCTGTCCCCTTTGACTGTAATGT
GCTCTATAGCCACTTCCGCTGTAGTATATACTCCGGCTATGAAAGTAGAACG
GTTTAGTTTGAGGCGGGGATACCACCGGCCAAATTCTCGAGATATGTCTTGC
TGAACCGTGAGAAGGTAGCGACTTATTTAAGATAGCCCGAGACAAAGCTAG
CGCCACGATTTGCGATATAATGGAGCCGAGTAACTCCGCTTGTGTCAACAAT
AAAAACGTAGAATCTTATATGTCAGTGAAAGAGTAATGTTGGTGGTTAGGG
TTCTTGTGATCACAAGGTAAGGGTGTGTGTAGTGGTTGATGAATTGATTCTA
TTATGTACAAGGGAGGGTTTACTACGACGTGGGTGTAGCCATATAAGGTTAC
ACCAATAAGTGGCTCTGTAGGTAGCCTTCGCCCTGGTCGCCCTAGGCCACC
TTGTCAGGCAACACACTCGGTCATGTGACCTGGCTCTAGTGGTATGCCAAC

AAGTAATGTTTTGAGATTATTCTTTGAATTGGCAACTTGAACCCGAAGTCGA
GGTAGCACTTGCTGTTAAAATCGATTGTGGAACGAGATTATTTGAAAGCTG
CACGAGATGGGGTCTACTTCGAAGGAAAGTGAAAATAGCACTGTGAGAGG
TATTTAGGTCTAAATCTTAGACGGACTCTTCGAGCCTTTATTCTTAAAAATAC
GACAGCTAAAGCTCGCTAAAAAGCTCCACCTATTCGGTCTGAGTCAGTCGT
ACCTGAATCACAAACGTCATCCCGGGCGATCATTAGGCAGCTTCCATCTCTA
TACGGTATATAAAATTGCAAATCGCAAGGGAGCTGAATTAATATTTAAAGGC
ACTAGGTAGTAAAAGACTTATGTTCATTGCAAAACTTAATATTCATACATTAT
CCGCCATACTCTGGCGACCTATTTACATAGTACAACTGGTGATCGATTTACC
ACTCGAGCGCCTGTAAGGTTTGGTATAAAGGCTTTCCTTCCTGTCCCCTAAA
GCCGCCCGCCCCCCTATTCGGTATCGAGACCTCACAAAGGAACCCTTAAGC
GCGGCCGCGCCAGACGCGATATTTTCATTCCTGTCAAGATATGATCAATATC
TAAAGACTCCTCGCCGGAGTTCGCCACCCTTCCGCGCCACACCCCTCCGTG
GCTTTTTTTCGCCTTTTGTACCTTGTACCTAGTCGATATACAGGAAATTGCAC
TTATTAATCAGTGCTCGTCTTCAGACGGAGAGAGGCGATAGAAGAAATTCG
TAACTTTTTAGTAGACGCGAGGGGTATTATAAAAAAAGTAAGGAGGTGCAA
AAAACAGACAAATAAAAAACCGTCTAACTATGCAAAGTTCGTGTTGATGGG
GTTTGCCGAGGCCCAAATGACTTTCTATTGTTACATTGTCATGAGCATATCAT
AGTCGTTATAGCATTATCGGTACCCGCCTGATGCGGGAGAGCTCGTGGCAAT
CATACTTGCACGATTGGCTCGTTTGTCTAGACTTCCGGCGCGGGATATGGCG
GAATATTGGCGTTGCGGCCGATCTTCCACAATTCTGTCTCCGATGTTGTACA
TCGTTTCACGCTCGGGTGTTGATCGGTCAAAATACCGCCGCTGCGGTGTAG
CGATGTTTGGGGACGATGTCGCCGGAAGCACTAGTCTAGTGTTGAGCGCCG
AAGTCCCAAGCCGTCTTCGAGGAAGAGCTGATGAGGCCAAGGATTCGGAG
TCTTTTGAAGGTGGTGGGGGTAGTTGAGAGGTATATTCGCGGGTTCCGGAT
CTAACGCCCTGAACCTCAGTAGTAGCGCGTGAAGGGGCGCGGAAGCGACT
TTCATCATCATCGTTGTTTACCGTTCCTCGACGGGTTCGGATCAGCAGAGGC
TTGCGGTCGGATAAATCCTCAGGCTCTTCTGTAGTAGCGCGACGAGTTCTC
AGCAGCGAAGATCTGCGCCCTTCTTCCGGATCCTCGGTCCCGGCTCTACGA
GAACGAGATATCAACATCGCGGTCTTCCGGCCGGCTGCAGGCACCATGTCA
TCGGTAGGAGTGGCGGGGACAGCGTTGCTGTATCGCGGGGTGGGGAGTGC
ACTGGTCACTATCGAGTTAAGGCGTCGATCTTCGAAACGGGACAAAGCTCT
CGGGCTGCTATGCATTGTTCCAATGTTGCGATCAATAGCGCTCGGTCGTCTC
TGATTAGCCACTCCAGGGAATGTGGTCGTTGTCGTTGTCTCGGGGAATTTT
GCCATGGTAGGGCTTGTAATTGTGATATCTTCCGGCACTGTTGCCGGAACTT
GTTGAGGTTTGATTTGTGCTGCTCCTTCACTTAGGGCAAGGCACAACTCAG
TGAGACTTCGACAGATACTATCTGCTTTTCGTCTGAGTTGACGATCAGTGAT
GGTGGCACCATTACCGCCATTGCCAATGCTACTTGCACCGCTCGAGATAGG

TCCCGGCTGACCAGCGGTGCCCATCATCGTGGAAAGGGCTAGCGCATCATT
CGCGGCCGTCTCCAACGCCTCAAAAACTTCCACACTAAGGAATTGTTTGGA
CTTGGTAAGGGCTGAATGCAGTAGCGGGTGACCGTCTTTGGACGGAGTGTT
TAGATCTGGTGCTTGTACGGCACCCCCAGAACCGCGCTTTGGTGAAGCGGA
GAGGGTGGTCGCATTGGTATGGGCGGTCGGTGGTCGCTCTTCGGATGCTCG
TGATATGGCTGCGCCGGAGGTTGGAGGAAGCTTTCCAGTTAGCTCAAGGCG
ATGTATGCGAGATTTAAGGTCATCGAGTTCATCCCATACAGTTGAAGGTGCG
GCGGTGGAAGCCGTCGAATTTGTTCCCTCGACGCCGTGATTTGCGTCGTCC
ACTTGGGTTTCATGTCTTTGATAATCCGAAGGCTTAGGCACTAACGGCGAA
GAGTTGTAGGTGCGAGGGTGGCTGTAATTAACACTGCCCTGTTTAGGGAT
GACATGCGAGAGGGGAGGGGAGTGCCATTGTCAACTGAGCCCTGTCTTCT
TCGGTTAGAAGCAGATGCTTGGTCAGAAGAGAGGCCATCTTGAAAGACCA
ATGATCGAGGGGTCAATGGAGAAGCGCGGAGGGGAGTTGTGCTGGAGCTC
TTGAGTCTCATCTCGTCTGTCTGCTTTTCTCTAGCTATGCCTCTGTAAGTTAG
ACTGCGGGCGATTCTATCTCCTGACTGATCTTCACTATAACCTCGAGACCTG
GAACTCTCCTGATCTGACATACGACGAACTACTTGGGGAGGTGAAGAAGG
TTGATATGAAGAGACGCCCACGGAGAGAGGTCGTCGACTAGATCGTGTTAC
TCGAGACTATATCACGCGTTAGTATAGATCCATTCAACAATAGAGGTATTTTT
AAGCAGATATATCTGCGACGTTATCTAAATGTTGGTAGTACCAGTTCAGGTT
TGCCAGTCCGATGCAAACTCCAAAACTAGGAGATGTGTATAGACGCTTTGG
CAATTGTGTGCCTTTTGAAACAATTGAGGGGGCAAACAATCGGTTGACTTA
CGATGGCGCTTTGGCTGTCTCCATTATTGTCAGCATTGCGGCGTAACGTGTC
CTCTCGCGCAATCTTTAGAAATAGATCGCCGCTGTCATCGCCGTCTGGACG
ATCTCCCGCCGAATCGGGTGGTGTTATCTGTGAAGCATCGTCGAAACGTCT
CCCCTCTAATGCGCGCAATGTCCTGTCGCGGTGATTTGAATCACCATAATCC
TCACCCCCTTCGTAACCACGCATCGTGCGGACGTTCATGTCTAATACTAGAC
TCCGGCGCCGCTCGCCGGAGGACCGAAACGATCGAATTGGCGGGATAGAT
GCAGTGCCGCCCGAATTGTTGGAGCTTACGGGCGGTTTGTATGATACCGGT
GGTCGTCTCGCCACGGAATTCCTCCTGTCATCGGTCGCCGTTCTCTGAATG
GTCTTTGGCAATAGGAGTTCCCGCTGCCCGTGCGCCAACTCCTTAGTTATCT
CTTCTCCTGATGACGCGCCTCGACTATCGTCAGATGCGTGCATTCGATGGCC
AGATGATGTCTCGCTAGCCGAATTACCCGACAAAGGCGCACGTCTGTGTCG
GGGCCGTGCTGTGACAATAGTGTCAGTTGGTACTGTAGTTGATACTGTGTG
AGTCGCGACGCTAGTTCTGATAGGCGTTGGTTTATTAGTATTTCATGAACAG
GGAAAAGAAGCGGGACTATCCTCTCAGCCAGCTTTAAACGAACAGAATGT
GATGCTGAGAACTTGGGGCTGTGCGCGAGTACGCAAAACCATTTCAGTCCC
CCCTTCCCACTCATTCCCCGCGGCCAACCTACTGCGTAGAGAAGGGAGAA
AGGTGGAGGGCCCACCTCGGCTAGCGTTGACACAACAAGGCTCACCCTAT

CAGACTGATCAGACTGAAACGACTGGCTACAGAGACAGGCAAACAAAGG
ATCTTTTTGAGGCTTTAACGAGAGTCTCAAACAAAATATTGCTACTTCCTGG
GCGGCGTGGACTCGGCCGCGAGGTCGGTGGCGGTGCAAGCACTTGCATTC
GATAAGCAAGAAGGCTTCACCTGATGGCCACTGGGCTTCCCGCCTTGTCCC
TCTCCCCGACTAAAATCGACAGCACGGTGGACACAACTGGATGCCGATTGG
TACCACACCTCTAGGGTAAAATGGAAAGCATGAAGCATGAAATGTGCGCCG
GCCGCGGGTGCAATCAGCTTTTATGACACGGACGATTGGTCTTCAGGCTTA
GCTGGTGTGTCAAAAGTAGGGCTGGAAGAATGACGTGGATCACCTAGCAC
TCTTCCCTCTCGGGACCTGCTTCAACCCCACAGCGGTTGAAGGCGAGTGC
GGGAGATCACGGTGTTTCAACACCCGTAATGTTTTTGAGTGATAGATGGC
GGACGGATACGTAACCAGAGAGGAGAGACCGGGGGCCGTGTCGCACAGC
TTCTGGATCTTATCTGCCTTGTAGAGGTGTGAATGTGAGGCGGCATAGCACA
TGGAGTCGAATGTCCACAGAAACGGTATTTAAACAAAAGCAGGGAAGGGG
GGGAAGAGTCGCGAAAGGCGCACAGACTCACAGCTCAAAGCAGGACATT
TGGGCCAGACTGGCTGATCAGGGTCGTTCCGAGAGCTGCAGCCATGGGGG
TACGCAGGCTGCGTCACTGCCAGCCACAAGGGCATCAAGCAGCATCTGCAT
CGCGTAGCAGCTCTGTTGAAAAGACGCCAAGTCTGCGAGCTCCTTGTAGG
ACCGTTCACTTGCTCCATGCTCCGCCTCTAGGAGTTCGCTTTGCTTCAGCGG
GTCTTGATCGAAATTCCCTTCAAGCCGAGCGGCCTTGGAAGCCGACGTAAT
GTCCGAATGTCCTCCGGACGGATCATCTATCCAAGTCCTTCTCTTTCGTCGG
TGCTCCCAACCATTGTTGATTTGCAAGGCGTATCGTAGCGTCAAAATGGTG
CGGATTCTCTCCGGGCCAACTGCAGGGTCCGTAAACCGGATGTCTTTAGCA
ATCCTCCCGAGATCCAGCGAGTTATTCTCGACGTCTTCGATGGTCCCGGAC
ATCTTGTTGTCAGCCACAGCCAGCAAAAAATGCTAAGGGTTGCTGACGCAC
CTCCCAGGGACCCTTTCGTATCGACGGCTGACCGGGTTCAACTGATCTATG
AGTACTGGAGCTGTCACGCCATGGCTTACGTACCATTTGAATGAACAGCGA
AAATAGACTTTGCGGCCGAATATAAAGGGCTTTTTGTGAGATCCTTCACGTG
GCTGTCTACTCGGAGAACTGTGTGTTCTACGCGCGCTCCAGCTTGGCCAGA
GCGAGCTTGCGAACATCGCCCCACGTTGGATTATCCCAAACCCCCTCGAAG
GATTTCAGGCGCGCATCTGCATCTTTCGCGTCAGACTTTAGACGGTGCGTG
TTCAGGCCGGTTTCCAACCGTCTACACTGCTCTGCGGTCAGCGTTGTTGTG
GGCTCCTCAACATCGGCCTGAGTGGAAGTGGACATGATAGGGAAGAGATC
GAGAGCAATTGCTGGCAGTCCAAAGTCGAAGAGCGAATGCGGGGTATATA
CAGTAAGAGATACGAGACCTAAAGTCTTCTTGTCTAGGTTGACCAGACTTC
TGCGGCAAGTATGAAACGTGAACCGCTTTTCTCTCCTTTGTGCACTCTTCA
ATGGTGGACTCCGCCGGTGTAGGACGTTCAACCTTCTCGAGGCCTGGACCC
CGAAGGCGACTTTTGTGCATCGCCATGATGTCGCAGACCGCACAGTGGGC
AGAAACACCAAGCCAGGTTAGCGGGTCACGAGAACAAAGCGAGACGGCC

GGACGCGAGGGAAAGACTGTCAGAGGAGGGGAGGCCACCCCCTCGGTCA
TTGGGCACGGTGGCTTACAAGCTCTCGACACGAATTAGTTGTGGGACGTCA
GCCAGTTCTCGGATGCCTAAACGCGTCGAGGGGAGGCCCATGATGAGTACT
TATTGAGTACAGCGACCTTTCCCTAGTTGGGCCGCAATCAATTTGGGTATCC
CACGTAGGGCATCTGCATACAACGGCGGTGGCTTGCGGCATGAATGGCTCT
GTTCTCATCATTGAAAGAAACATCACATGTAGGTGAGGAAACGCAAGTGG
GACCGAAGTTGTGAGTCGCTTGCAGCCCGCTGAAGGGACTGATGAACGTG
AGGACTCGGGATCCCTCCAGTAGCTCCTTGTCATCCATTCGCAGAGGGTCC
CAGCTCTCTCGCCGCTGGGTAGTCCCACTGCGTCACATCATCGGTGACTCT
CAGAAAGCGCCTCCAGAGCCCCGTTCTCTGATCCACCGGACGGGGCGGTC
ATCCGTATATCTCCAAGTGGTTTTGTGCGAGTGTCACTATCTCGCGTGCTAC
CCGTGCGAGACCGCCCACCGTCTGCGCCCAGTTGCGCCATCATGGTCAAAT
GGGCGCCTTCGATGGATCCTCACGTCGTGGATCTCGAGATCAAAGCCCCCA
ATTTGAGCTGGACGGCCTGCAGGACTCGTAGAACTCGTTGTCCCGGTGCCC
TTATGTATCCAGGCGTCTGTCGCTGCCGACACCACGCAGTGGAGATTGCAA
GCCTCGCGTCAACCATGACGTCGGCCTCGCTGTCTGGCGCCAGGTTTGAGC
AATCATGTCGGCGACTGGAAGGGATCGTGCGCCTTCGAGCAATAAGTCGTC
TTCTGAAACACACTGCACAGCCTGCCTTGTCAGTCAGCACGACGTAGTACG
ACTGGTGTGGTGGCTACGAGCCTAGGGATCCATCTCTGTACTACATCTTCCA
GCTAGGACGGAAAGCGTCACTTAGAAGGGGTACAACATCGGGTCCGTGCG
ACGAGCCGTGCACCTCCGCGAGCGAACCGCTTGGTTGATGGGATCAACAG
GGAGAGACCTCGGTACCAAATGCTCATGATCCTGAAGGGAGGCTGAAGAT
GGCCTAGCATTATCGGTACATGAGAGGCGCTTTGATGCTGCATCTCTTGCAT
AGGGGTGCTGCACAAAGTGAACGCAAGTCTTCAAGTTGAAACGGCTGAA
ACGGCACGTAATCGTGGTGGCCTGCAGGCGGTCGCGACCGTGTAGCTGGG
AGATCAACTGTACGAATGCATATGATGGTCGTCAGATGGCTCACATGGAGC
AACTACGAGGTCTGTACTTGACTGTGACGCCGTATACTGACATCTTCAACAT
ACGGCATGGCGGTAGGCATGGCGGTAGGCATGAGTTATGCTACAGTATTTA
GGTAGGCGTGTCCCCAGCCTCTCCTTCTGTCGATTGTGCAGACGAGGAGAC
GTGCTCAGTCCCGAGCGGTTGAGAGCCTTCGCATTGGTCCAGCAGAATCTG
CATCTCGTAGCAGGTTTGTTGCAAGGCCGCCAATGTTTCGAGTGCCAGGTA
CGAGTTTTCAATGGCTTTTGTTCAGCCCTTCGGAGATCATCCTCCGGGCCTT
GGGCGTCGTGGATATCCCACATAGCGTGGTGCGCTGCCCACTTGACGTCTT
CCCAGCCCGAGGACGGGTCATATACCCTAGGATCAGATTCTTTTCTTCGCAT
ACTCCATCCATTCGCAAGCTGGAGCGCGTAACGTAACGTTACAATGGTGCG
AATCCTCTGTGCTCTGATCCCCGGGTCGGTACACTGAATTGTGGCAGAGAC
TGCACTGAGGTCTAAATCCATATGCTTGACGTCCTCAATGGTCCCGGACATC
TTGTTGTCAGCCACATCTGAATGCCGATACGGGTTCTCTGTCTTACCTCTAG

AGTGCCGTTCCGTATAGATGGTTGACCAGCTTCGACTGAGGTGTAAGCTCC
ATGCTCTCTCTGCAGTCAGGCTGAGGCTTACCGGTTGAGTGAGCCTCAAAG
ATTGACTTTGCCGCCAAATACAGAGGCCCGCTGATGAGGTCCTGCACGTGA
TCATCTATCTGTTGAGCTGTGTAGGGGATGGCTAAACTCAGCTGCGCAAGC
ACGGTCTCGCGTAATCTGGGGTGTGGAGGACGGTCTGGATTCTCTGACGTG
GCCAAGCCTGTCTGGGACGTTCTCAGGTAGGAACTTGCCTCTTCATAGTCC
AACTCCACTTTGTCCACATTCAATCCCGTCTCCATTTGGTTACACTGCTCGC
CGGTCAACCTCATTACCTGGCGCTCATTATTGAGCCGAGTTGGGCTGGACA
TGAGAGAGATGCTACTAGTGTTAATTGAGCCAGAGGAATTGACGTAAGACA
CAAATGTGACCAGACGCGGGGGGAGAAGGGTTGCCTTTTGTATTCTACGGT
CGAATGTTTGCTGCTGCGGTCATAGGTGAAACGCGTCAGCTTCCTTTTTGC
GCGGGAGAATGGTGGGCCTACAGTGGTGCAGGTGGCTCAACCTTCGCAAG
GCCTGGATCTCGAAGGCCACTCCTCTTACCATTGTACCGCAGACCGCGCTG
GCAAGAGGAAGGTCATTCCTTTGCTGCACAGGGTGAAACGCGACGGCTGC
GCTCGGACGCGACGGACAAACAGATGTGGCAGAATCACGTTCATCGAGAA
TGGGCAAGACACCATGCCGGGTGCTCCAGTAAATTCTGGAGCTGTGATATG
AGGGATCGTGAGGGCCTTTGGCATCCATGAATATTCCAATCGTGGCGGTGG
TCAAGGCGAGAGGGGCGCGCGCGCTCGTCTCAAAGGATATGTACAACA
CCAAGCTTCCACCTCAGGTACAGTACGACGAGGACCACACTGATTGCTCA
GAAGATATCATATATTCATGCAAAACCAATAATCTCATATGTCCATGAAAGTG
ATTTCGTATGTTTATAATCATGAAGGTGGGACCAAAGAACGATCAAGAGCG
TTCAGTACGCTGACGAGTAAGATGAAGCTGAGGCCTCAGGTAGCCCTTCTA
AAGCCTCCTTGCAGCCCCTCAACATATAGAGCTTGGTCATCAGATCCCCTAC
TGTTTTCAAGTCCGATTGCGTCTTATCATTAGTGAATCTAAGTGTCTCCAAG
GGGTCCGCATCTCGGTCCACCGGAGGCGGCGGAATAGCAACGTCTCTCTCC
ATGTCCCTAAGAGCGAAAGTCACCATCTGACCTAGCATTGTCGCAGGAGGT
CGTGTGTCCTCGTCCAGCTTCATGATTGAGGTTAGCTGGGCGCCAAACCGC
ACCAACGCACGTTGACGAGTCATCTCGTCCTGGACTGTTGAGGTATTGGAG
AGGTCGCTAGGCAAGCTCTCATTTCGAGATGGGCAGCCCGCATCACTTGCA
GAACGCGTTGCCCCTGTACCAGTCCCTCCTAAGTCAAGATATCCCAGCTGC
GCCTCCACTGTGACGTCGGTTGTCTCGTTTGCCCTTAGGCCTGAGCGGTCC
AGTCGGTGACGTGAACGGATCGCGGGCATTCGGGCGTCGGCATCCCTCTCA
CCTTGTAACATCGATGAAGGCTCAGTAACGATGGGCCGTCCCGTGAGTGAT
TCGTTGTCAGGGTCGATCGTTTGAAGTCGTCTCTGAGTCAGTCGCGTGAGT
TTGCACATTGGGGGCGGTCGTCTGGGCCCACTTGATGCGTCACTTACTGTT
GAGTTCAGCGATTGATAACCTTGCAGGAACCCTCTCTGCGAGGCTGCACCT
TTAGCCCGCTATACTGGCGATGGCCAGTCCGATAAGACTTACTTGAGAGAC
GCTGCACTTCCGCGTTGATCGCACCATCTGTCTTCAAGTCTTCTACGGCGG

AGTAGGCGGTCCCTTCTAAGCCCTCGAGGCATCAGCGGCGCTTGCCAGTAT
GCGTTCGCAGGCCGCATTCGCTTGTTCAAGTCGTGTGGTCAGAGACTCGAT
GTCCATAGTCATTTGTATGTGGTTTGTACGTGGGTTGAAAGGCGACGGTCC
GGATGGGGGGTGTCGCCGCTGAGAGCGAGTTTGGGAAGGAAGCAGGAGG
AGGCGTTCTTACGAGAATACTTATAGGCTTCACCAAAGTAGGCCTGGAACC
CGAGAACGACTGTTTTGGAAGACAGCTTCATGCGCCGCACAGTGCATACTG
AAAGGTGTGGCAAAGCAGAGAGTTCGCTGGACCTCCCTCGTCATCGCCTT
GTAAAGCCTAGACCTGGAAGGCCACCGTCTGTTGTGGGCCTTTATCTCGAT
TTCATTTCGTAGGAAATGATTGTGTTTTAAGATACTCTGAGGTCCGACATGT
GGGGTACAAGCGAGGAAGCCGAGCAGTAAACGCCTCGTCCAAGCCCAAG
CAAGCTCAAGGCCGTCAGAGACTGTAATTTCCAAATTTACCATAGGGTATAT
GCATATGATCGATGAGGCATGCTATTGTAATGTGCAAATTGCGCATAACGAC
GTATGTACTGTACAGTATATCAGGTGATTATACATTAGGGCGGTGGGAGCAA
GCTCGACGAGACAGTCTTCAGGTCTCAGACGTTGCCGAAAGGCTCTCAGT
TGTGCGCATCTGGCACGAGTAGTACTCGCGCATGACCTGCGTGACTGCAGT
CAGCTGTGCGTGGATTCTGTCATTCGCTTTTCGCAGTTGAGACACCGGGTC
AGCATTTCCAGCGGCACCATCTCCGACCGGACCGCAATCGTGCATCTCCTT
GTCCGTGTGTGTGAGCAAGGCCAGCACATCTTCGGGTGTCATGACCGAGG
AGGGGGCGTTCCAGAAGTCTGTCAGGTAAGCCGAAACTGTGCACATTCCC
ACCAGGCGCCACTCTTCTTCGGCCGCGGGCGTATATCGCGACCTCGCGGAT
GCCGATGCAGAGCCTGTATAAAGGCATGGAGTCCCAGAACGTGAACACGC
TCCACAACCGTCACTCGTGGAGAGCGAAGTGGTTCGTTGACTAGCCGCTT
GTAGCCTCTGTGTTCTGGCTGATCGAGCCTCTGCAAGGAGACATGGGACTT
GCCGATGAGCTTTGTGCCTTCCCAGGGACGGTATGAACATTTCTGTACTCA
CTACACAAGCGCTTCATTGTTTTTCCAGCCTGAAACCACTGACCTTGCTGC
AATCTCGTGAAGATGGTATCCAAGTCCCCCTTCAGGGTCCCCACTGATCCTA
CAATTGTCTTGAGGGTCTGATGTTCCTCGTCCCACACGTCGTCCAGACCAG
TGTTGGCGCGCATGAGAAGCTTATCAACTACCGACATAACGCCGTAGTTCT
CGATGAGCTTGCGGGGTTGCCTAGGAAGTGAAGTCCGCGGGAGGAGTTGA
AAGACGTGACTGACGAGAAATCGACAAGCTGACGTCGCTGAGTTACTGCA
AGCACTCACAGAGGGCGAGAGCAAGAACCCTAGACCGCTCGCAAGCCTG
GATTCTCAAGTTGCCACCGAAAGGAGTGGTCGATTTGCGACCCCAGGCTTC
TGCTCTCTCTGCTCCAGGCCTTGAACAACCTCACGAGGCTCCTTCCAAATC
CGAAGTGGAGTGACCGTCAAAATCGAGCCAGCTGCGCACGGATGGAAAGC
TGGGGTGTGCAACGCAACCTGCGATCTGAGCTTGCTCCCATTGCCGCACCA
TACGCTAAGGAAGGTGAAAACACCGTGTATGACCTAGAGGGGGATCCAGT
TGACTCCGTGAGTGCCGTGAGCTACCGTTCGAACACCGCTCCGGCCAAAC
GTCGGTCTGGACCAGCTTTCCGACCAACAATTTCTCTAAAGAGAGGTGAA

GGATGATGCGCTTCTCATCGATGCCGCTGTACGCATCATCACCCACGGTCAG
ACGAAGTACCATGGCAATGGAGCCATGGGCCTTGCCGGAATAAATGACATT
GTCGAGAACGCCTTCAGCCACGATTGAATCGATGTGGGCCCATATCTGCGG
AATTGCCCAGAGACAACTCGCCCCAGACTGTTCGCGGACGACATAGCGGC
CGACTTTGTCACGCGTCTGCGAGCTAAGGGACTCTCCAATAAAGGCATCAT
CGCTTCTATCACCCCTCAGACATGCGAAGCCATCACTCAAGGGTTAGAGCT
GTTCGTCATCCCCCAAAACGGCTATTTACACGAGGCTTTCGTCTGTGGGGG
CGGGGCGAACCACCCGCCTTTTATCATGAGCGTCTTGATAAACTGCTCTCA
ACGTCTCGAGTGAATTTTACAGGATGGGTCAGGCGTTCCTGCCGAGGCCAT
GACTTATGCTGTTTGGGATTTCTTGTGTGGGACATCGATCGGGGCCCATAGC
CACGGGCGAGAAGAACAAGGAATGTGCGATAGTGGGGAAGATGATTCCAA
GCTCGAATTTTGGTGTTCTCCTGAGAGCGCTCTGTGCGGAAGAGAGAGGTA
GCTTCGTGCGCAGAATCCACGTATCCGATCTAGTTCCTTGGGCGGTAAAATG
CCACTCTCTTGGCTGGCCAGTATGCCGCGTCAATCGTGCTAGCAGACCCAG
ATTTCTCCCATGAGTCCACCATGGAGATGCTCTAGCGCCGTGAGCCCGTCC
CGCATCTAGGATCCTGATGTGCCGCGCCCTGGCTCGCCTCCTGGATTCGGCC
CCATGGACTGACGGGCTGAAGAGTCTGGAAATGCCCACTGCCACTGACAG
CCTGGACAGACGAAATCATCCGCTGCATACTCCATCGACTCGTTCTCTGTCG
TTGCAGATGGGTATACTGTCACTGACACCATTTGTCCGCATCAACAGACAC
ACAGTCCCCCTTCCAAAGACGGGTATGACCACAGGTAGCGATCACTCCCAC
CCGCAAGCGTGACCGGGTTCAAGCGACATGTCTGCCATGACACCAAGTCT
TCGCAATGAATGGCCTTGGACTGTTCTGCTCAAACCAGCAGCCAAACTTAC
ATTTCATGGGGACGCCTAAGTGCAGCGTGGGCCCCGCTCACCCCTCCTTGA
CACGCACATTGTCAAGCCGAAACACACAACTGCATTCAACCTTGCCCGTTA
ACGTACGGCCACACAGAATTGAAAAGTTACAACGGCAGCGCTGTGACAGC
AAGCTCACGACGATGGCGCCACCTTACTTTGAGTCTTTCAGACACCTCCCT
CAGTTTCCCTTACTGTCATGACATAAAACATTAACGGTGTTCAGTCTTCCAT
ACGATGAAAAGAGGTCAGCGCGTGAAGGGAAGATGAGACTTTGCAGATTC
ACCGTCCTAAAATTCGCCACTTCATGGGGGCGGCGGGGCTAGGTCTTCTCG
ATCACCCTCTTTTCCTCTGCTTCACCTTGAGACCCATTGAAAGAACACAAC
CCTCTCGGCAAGACCCTTGACCAACTTTGGCAAGTTAGGTGGCTTTCAGCA
CCTTCTTCAAGCAGTATCAGTCGTTGTCCCCGCTCAGCAGTCCTCCCTGCC
GCTGTCGGATCCCAGACAGACCAGAATGTCAGATTCCTTCAGCAAATGGAC
CGTTCGAGAGCCCTGGACCGATCTTGAGCCATACATGACCGCCAAGCACTC
TTTTCTGCATGAAGATGACAGAAAGACGCTCAAAAAGCTCTTCAAGTCATT
ATTGGAACCTGGATTCATCAAAGTCAAAGGCAAACATCACATAGATGATAT
GCAGAAACTACGCGACTTGCTGATCTGCCCCACCGGAACCACCAAACGGA
CCAATGCAGATAACACTCATTACCCATTTACAGTTGATGAGTTTATCCAGAC

ACATCCAACAGTCTGGGCAAGAATAGGTCTAAATGATTCTCACTTTGAAAT
GCTGAAATCCCTGGAGACTGGGCTCAACGCAACTTTTGCAACACTGACAG
CATCAGAGAGAAGCAGAGTCATAGGGTGGCTGGTACGACTAAGCCAAAAC
TTGAGCATGGAGCCCTTGAATAGGCACTACTACGATGCCAGTGCCGCAAGT
TATGCCCAAAATCCTAGCAAGCCCCCAATATTTGACTGGAACATAAATGACT
CATTGTGGTATGAGCCAGAGAATTTTGCTCCTGCAGAGAAGCCAGCCGCCG
GCACAAAGAGCCTCGATGGTGGCTCGAACAAAACTGCCTCCACCGCAGCT
CTGACAGATGACTCAATGATGCGAGAGGCGCCAACCGATGAGTTCCTATGT
TTAGACACAATGGCCTTGTTTCACAGCACTTTGGGCCGCCTGGAGGAGTAT
GCTCAGGAACTAGACCAGTTCAAATTCAAACAGGTAAAAAGTCTCCTCTCT
TGCGCATGGAGGGCAGCCACGTACACGGATCACGGGGGCCAGAAACTCCC
TTATACGGTGAGCAAGTATTTTGGCAAGTACCCATGGGATTGGCTTGAGTG
GGGTCTTGAGCTTGATGATTTGAAGACTTCGGAAACTTTGGAGAAGCTGCT
CAATGAACAGTTTCCCCACCACACCTCAGAGAGAGCGAGGACGTGGCGTG
GATGACACAGGAGACCGACAAAGTGCAGGCGAAATATCATGACCACATGT
TGTCAAAGACCAAGAAATTGCATGAAGCAAACAGAAGGGAGGATGAGGC
CCTAGCCAATTGGCGACTTGAAGATTCCTTGAAAACCACAAAACCCAAGC
GCGACCTTCATGATGTACAGCAAGCGCCCACCCCATCTGGTTCGCAGGTGG
AAGCCAATGATGTTCAGACACAACCAGCTGCAGAGACCGACATTATTGCAA
ACAAATTTCCAGGCGCCTCTCCTGGCCAAGCAACAACGCAAGATACAGTC
GGTTGCAGTCAATGATTTAACGGTCCAACCATAGGAAGAGTCTGTTGAATT
GTCATAACCGTCAAGAAGCAAAATCTCAAGCCTCAACCCAAGTCCGATTCA
TGGATATCGAGAGGACCGTCCATTGTGGACTCGGCAGAGACCCACTGGAG
TAATTATAGCACATTGTTGAACAGACGTAGAGCTGTGCATCCCCAAGGTATG
AAAGCTACCTCTGAATCACGTATCTTCTCTATGCAGAAACATATTCTACGAG
ATCGGCGGCACCAGGCTGGCGGGAGAACGAACCGTCCAGGGGAGCTCCTC
GCGCTTAAGATTCTCATCCGTATAAATGCTGGCGCCTCCCATCTTTGGCGTT
ACAGGCCAAAGAAGATCCGCGAGGCCAGTACTCTTGAACATGTCGGTAAA
CTCCTTCTGCATCTCGGACGCTGATGGTGATTCCGTCTTCTTGCCCGTCCAG
TCCGTGCAGACCTGGTAGCCGCCATCCCCCGCCAGGCTCCAGGCCTGTCGT
GTCTGCCACAGGGCCTCCTTGCCCCGCGTCACATTGCTTGTGACTCTCAGC
AGCCTCCCGTTGGAAACACAAGTCGTCGTGCACCCATAGTCGCCCTTGCGC
GCAGCAAAAGTCTTTGGTGCGCCGGTCCAAGCGCTTGACAAATCCTTTTGA
GTTTCAGGCGATACTGTTCCTCCACATTGTGCGACGGCCTCCTTGAACTGCT
CGCGGAACCGGTTGACCCATTGGGCAGTGGTGTGTATCCCGGTCAGATGGG
CTCGTTCTGCCTGTTGCTTTTCCTCTGGCAAGTCTGTCTCTTCGGTCGTCTC
GTCCAGTATCGGAAGGTCGGGCTCGAGAATAGATTTCCAGAATGGGGCCGG
AGTTGAATGGAATCCTCCAAAGAAGGGTTCCTTGATCCTGAAATCCTCGCG

GTCCTTGGCTTGTTTCTCGGCGAGTTGTGATTTCACTGTTTTGAGGGATTCC
TCGACCCATTTCTTTGTCCTAGTCCTGGCCTGGCTTGGGTGTGGAAATGTGC
TGTCAAAAGCTCCTCCGGAGTGCCTCGAGTTTGCCCATGACTTGGTCGGTG
AGGCCGCACTCGAAATGAGAAGGCCATCTCGTGAACTCCTGTTGTGGGGT
GATGGCGTCTTTGCCGATGAGGACTTTGCCGCAGGAGCAGCCGACCAGCT
TTTCCCATTTGTCAAAGGCCTTCGGGGAGACTTCACAGCTCGCCTTCAGCG
AGTCGAAGACATCTGCATAAGTGTCCCGATCATGCTTGCTGAGAAATGCGT
CTGTGGCGGTGAGCCGTGCTTCCGCAGCATGTGCAGCGGTTCCGGTGGTG
GTTTCGCGCGGGGCCGCGTCGGACTTGTCTGCCATCTCTGAATTTGGTTTT
GCGGTAGTTGTGGAGTTTGTGTGACTGGTCTCCGATGGGATGGTCCGCTCT
GGCTCATGCAATGCTTTTACGCTGTGACGCCCATATTCCCAATCTGACTTGA
AATCTTCGGGATGCTTCTTGATGAGGAACGCATTTCTCTCGTTGCAGTACCT
GATCCACATCTCGTCCAATACGCTGTCTTTTAGAGAGTCCGTCCACCCGGTC
ACCCGCTTCCTGTCACCCTCGCTGAGATTGGCGAGAGTCTGCTGGACCCCT
CGGAGGGACGCGAGGTGGGCATAATCGGACTCTTGCAGGCCCATCTCATCA
AAGACCGCCTTGTGCACACGGCTGTACGCCTCAAAGCTGTCAGTCGGCTC
GTGCGCGCCGTTCCGGGGCGTCTCGAGGCTGTCAGATGGCTCATGCGCGTC
GCTCCCAGTGGTAGGGGCTGGAAAGATCAATTCCGTGAGCCGCTGCAGGT
GTTTCAGCTTCCTGATTGCCTCGGGCGGCTCCTCGCCCGTGACTTGGATCTT
TCCCGGGGTCAGCCAGGACTTGATCAGCCTCGTAAAGGTCTCCTTGTCCTT
GTCCGTCAGGAACGACGTCTCGGCCGACATGTACGGCGATGGATCCGTCCA
AAACTCCAGGCGCGGCTCTGTCGGCGCACCCGGCAGGGGTGGCGAGTTGA
TCCGGGACGTCGTCTCACCGCTCGTCTCCGTCTGAGCCTCGGTCGAGCTGT
CGACATAGCTAGGCTCATCGTAGGCGTACTTTCGGGGATGGTCATACGGTAC
CCTATCCTGTTTAATCCAATGAGGATGCTCCGCCCATTGCTCGTGCGTAGCC
TCGTTGTGTGCCCTCGTGTGCAGCTCCACCTTCGTCTGCGAAACGAGGGCC
TCCATCCACTCTGTCACCGCCTGCCTCTCCGTCTCACCAAGTCTGGGGAGA
GTGCTCTGCAAACTACGCTCGAGCGCCCTCACGCCTTCCAGGTCTTCCTCC
TCGACTCCCATAGGCGCCCAGACGTCCTCATGTTTCTTTATGTACTCGTCGA
CGCTGACGCCGCTCGTCTCTTCATCGAAAGTGGACCCAATGGCACAGTTTG
TGAGCTCCTGCAGGCGTTGTAGTCGATATGAGGCTAGTTGGCGGCCCCCGT
AGCGCGTACTTGGCAATTGGATCTTGCCGGGTACAAGCCATGTCTTGAGCA
CTTTTGAAAGGGCCGTCTTGTCTTGTGCGGTCAACAAAGACCTGCTTGCGT
CCATGAACTGATCAAGATGTGTCCAGTACACGGGAGGAGGCTGGAGAGTC
GGCTCGGATGATGTCTCGGTCATGCTTGAGAGATGCGGCGCTGTGCCGCTG
GATGCTGAGGATGTTGTGAGAGTGACTTGGCGGGTGCGATCTCAATATCTG
AGAAGGAGGAATGAATGACGAAAACACGAGAAAGAGGGAGCTGGGAAG
GCGTTGCCACTGACTGGCCGCTCAGCGAGAGGCGGTTAGATCGAACATGT

CGCACAAGTTCCCTCCTTTGTGCGCCCACCTCGCTTTCCTCTCCCCTAGGA
GGAAAGGCGGTGTGAGGCTCGGGTGGGGGCATGTTGTGAGCTACCACTGA
GAGCGACATGTGGGGAGGGAATGCAGTTGGGGTCAGTGACGTCGAGCACC
CCCTTTGGGACTGCCCCGGCCAGAACTACTGAATTTTGGCTGGTGAGGTGT
CGACGTCATACATCTACCTTTCTGCACTCATGACACCCACTTTTGACCTTGC
AGACTTAAGACCGGCTTCCCATACGACCAGCAGCATGTTCAGACGAAATAG
AGTGCCATCATTTGGTCATACCGCTCTTACCAAGCAATGTCGAGCGCATCTC
AGAGCACATGCATGCTCATTATCAAGTGACTGGATGTGGTACGTATGGGATG
AGCGGTCGACAAAGATGCGATCCCGTATGAGGGAGTGTCGAGAAAGGGGG
TGATTGAGGTTAGGAATGTTGATCTCCATAAGCTAGGCCTGGAGACGCCTG
TCCCCTCAGGTGAAGGAGATGGGTCACGTCTGCACAAAGGTAGTGTAAAC
CTTCTCCGGACCCCAAACACCCGGGCTCGCGATGAGCCGTGGAATCCCAG
CTGAAGGCGTCCGAATCCAATTCGCCCGTGCATGTGAAGTCCGTGCAGATG
AGGCCCGTGCGCTCCGCCATATCTTCCAAATCGCCACATCGCAGCGCTGCC
ACATTGCCACGTTGCTTCATACTGTAACCCGGACTAATATGCCATTGCATATG
CAAACTAATGATACAGCAGTGATGATGCCCCCACAACCACACAGAAACAC
GGCGCAGTCCCTGCGCGCGCCAATGCCCAACAAAGACAAAATGATATAAAT
GCGATACGTGTCGCTCCCCTCCACTCTTACACCTTGGCCCCACCAGCCAAG
ACAACATTCGGCGCCGACATCCTCCTCCCGTCCAAAACAGCTCCCTTGCCC
AGCCCTGGGATAGGGAGACCCTGACTCCAGCTTCCAAACTTGTGTCTCGAA
TCGAGAAGATTGGCGTGATACACAGCGCGCATCGCCTTGGCCGAAGGCGAT
TCCGGTTCGTACAGCGACAAGCCGTTGGCTGAAGAACGCTGCGACGGCGT
GGACGGGCGGTCCTGTGAGCCCAAGTTCGATCCAGAAGGGAAAGAACGA
GGGACTGCGGGTGACCGCGGACCCGGCTCGAGTGCTTCGGGCCCAACCAT
CGAGCATGGCAGCGTGAGCGGGAAGAGCGTGCGGAATCGCTCGGTCTCCT
GCGATGCGCCGTCAATGACGGGCTGGATGATGCGCGAACCGGACGCCTTG
GCTTCTCCGGCGAGTCGCTCCAAGCGCTGCGACCAGAGACTGCGGTTGGC
GGCGCACGAATCGGCATATGGCTGAAGTTCTGGCAGGATATTCGACACCGA
CTGGAAGAGCGGGTGGGTGAAGAGGTCAATGAAGCCGACCTGACCCTTGG
CCTGCAGGGCCGCATCGGCAGAAGCGATAACTGAGACTGGGATCTCGAGA
TCTTGCTCCAGGGAGGCTTGAATAGCCCACTCTTCTAGCAAGACCGAAGAC
CAGTGTTCCGACACATCCATGGGCCGAGTCTGCGCATCAGCGGGAGACCAT
CCCAGTGAAAGAGGGACTTACGGGATTCGATATGTCTGCACACTTGATGAG
TGCTTGGCAGAACATGACCCGATCCTCGTCCGCATCGCCGGGCTGGTCGAG
CGCTTCTCCAAACTCCTTCAAACGCTGTATCCAGGCAAAATGGAGAGACAT
GTCGGTGGCGAGAACTGAAGAATACAAGACCTTGCGGAACCCCTTGCGAT
CCCAAGCTACTCGAGCAGGGTATGCCTTGTCCTTGACAGACATGGGACCGA
GGAGGAAGCCAAAGTTGTGTTTCCGCAAAAGTTGTACGACCAACATGCAG

TGCATGTTCTCGAGGGAGGATTTGTCGTCGTACACCTGCGAAAGTGGTGTC
TTGGCATTTTTCTGCCCGTCAGCTGAGTCGCAGTCCAGGCTGAAAGTAACT
GACCATGAAAGCATTCGACAGTCCCGGGTGACCCACATCGTGGCCCATCGC
CCCGATCAGCAAGGCAAACACGTCTTGCGGCCTCAGAACCTGTCGAGCGC
GATAGGTCCCAAGGCCTAGCGAAGCGAGTTTCTTCTCGTTTGGTGGCGTCC
ACAGCGGGGTCTCCTGGGTGAGGGTACGAAGGTATGAGAAGGGCGGTACG
ACCCCAATGACGATGAGGAAAGAGTACGTCGCTTGAAGGACGTCGATGGC
GTGGATGTAATTGTGGTATGGGTTGGGAGCATGATAGATGGCACGGATAGC
GAAGAGAAGACGACGGACGTCCTCCCAGTCGAGCTCAAGATCGGCGATGC
CTTTGGTGCAGAGGATACCCTCAAACATGACACAGGCGGCGCGATAAAGAT
CTCCTTCTTCAAGCTGATGCGGCTTGAAGTTCCACGTGGCCAAAGCGTCGA
CCAGTTTGGCGCGATGAGCGGGCGACATGGTCGCAGAGAGTCTTTGGTCA
GCAAGAGAATATACAAAGCTAAGGGCACCCACCTGGCATACTCGCCCATCT
GAATATCAATCGCCCGACTAGTCTGGTAGAACATGGCCGACAAGAGCTCCG
CTAGTCTTGTGACACCTTCCCCATCGACATCCGTTCTATCCGCACCATCGTC
CTCACCGTCTGAGGATTTGAGTCCTTCCCGTGTCGGGGAAGAGGGGAAAG
TGAACCCGTTTTGTAGCCGTGAGGGCTTTTTGGGCGTCACAGCAGTCGCTT
CAAAGACTCGCTGTGCTCGTTTTAGGGCGAAGTTGAGACCGCTGATGTCGA
TGCTCCGCCTTCGCGCACTGAGTTTTGGCGGAATCAGATATAGGGATTCGA
GGAAAGTATGATAGTTGTCACTCTCCTCTTCGGGCAGTTCGGCGGTCATGC
CAAATGTACCAGCAGAAACGGAGTGCTTGCGGGACGCTGGCCCATTTGTC
GAGTTGTGGCGCGACTTTTCTGGTCGCGAAGGCCACTCACGGCTCATGGA
CCGCTTGTGATTGTTCAACGCGGCACTCAAGACCTTCTCACCCTCATGCTC
TCCGCCAAAGCTAAGTGCAGTAGGGGGCAGGACCACCAGTCGCTCGTCAT
CATCGAGTGAGCGCAGCTCGTAGGCGGGTGAAAGTGGCCCAGCGGCGCTC
GAGAGGCCGACGACAGAAGAGATGCGGCCTTCTCTGGCGGCGCGGACCAT
GCGCCACAGCAGAGCAGCAGTCTCGAGGTCATAGGGGGGTCGGAGCACGC
CTGCGGCTCCGGCGGCGACAATCGCGTTGATGGCGGCCGGGGTGATGCCT
GGATTCGCAGAATAGGCAACGATCACTGGAGCGGCTGATGTCGACGCTCCC
GACGCGGCATGATCCCGCATCACCGCGACGGAGCGAATCTGGTCGACTGC
CGAGTTGAGCCACTCTTCTTCTGTGCGCTTGGCACGGTCGAGCAGGTCTTG
CTGGAAGCAGACGGCCGAAGGCGTGGTGCCCTGTGATAGAAGGAGGGAG
AGCGATGGGAGTGATGGCGAGTCGCACAGCGGCACGAGGATGACGGGTAT
GAGCTGGGCTGGGGTGGAAGGCCTGGCGATGAGAGGCGACTGATTGATCG
AGGCGAAAGCTGGTGAGTGGCTTGATGATGGCGAGAGAACGGAGGGGAG
GTGAGAGGGGTTTCGAATGACGGTGGCTGTGAATCGGCGTCAGCAAGTGG
CCGTCAAAGGTGGCGAGTGTAGCAAGATGAAAGGATAAGTGGGAGCGGG
GGAGGTGCAGAGGGGAGAGAAAGGCATGATTTGAAGATGAGGGATATAAG

AAGGAGGAGGGCGCCTCTTGAGAGAGAGACAAGGATGAGCACGGTCACG
GGGAAGATCGACGTTTGCGTTTGACAAACTCAAAGGCACCTCCTTTGCCAT
CACTCTTCACCATCACCATCATCAGACCAAAACTGTGCCTGGAGACAACAG
ACTAGAGAGAGAAAGCAGCGACTCACCTTGTAGTCCACTGCCACGGAGCA
CGGCCACCACCTCGTCACCCTCCCAACCAGGTCGATTCCTGCCCACACCGC
CCCCTCCTATGCCCATCCCACTGGGCAAAGCCACACTGCCTCGGCTCACAC
CCGCAGCAGCCAACAAAGCGGCCGGACCTGGCTGGGCGGAAGACGTTTG
GGGTCGCCTCGTCCGTTTACCGGTGTTCGGGTTGAAGCGTTTGGCGCCAAA
GACAGGGGCCTCTGAGGAGGGGTAGGCCGCTGCGCCCTGCCGACTGGTCT
CGCCAAAGCCGTTGGTGTCGTGCGGGCCTGTCGACACGGGCGACATCCTAT
CTGGGGATTGCATTCGGACCTGGGTGCCTGCACCTATGCCGGGCGCAAGCG
ATAGCCTCCTTCCAGCGGATACGACCGCGTCGATGGCTTCCTCCTCGAATGT
GTCCTTCTTTGACCGCGTGAGCCTCGAGCTCATGCCGAAGGACTTGAGAG
ACGGGACCGTGCTGCCGGACTGTCGGGTTGTGAGGGGTGGCGGACGAGA
CGGAGGGACGATGGGCGCAGGGCCATCCGCGGTCGGCGGCAGCAGCACG
AGCAGGGAGAGGTGGGCCTGCTGGCCCTCCT

>Mosaic_sequence_53

TTTGCCTCCTCTTCGATACGCTTGCGCTCCTCCATAGCCGCTTGCATGGCAG
CAATGTGTGCTGGCACCTTTTTGCCTTTGGCTACTGGCGCGGCGGCTGGTT
CAGGTTTGGCGGCAGCCTTCTTCTTCTTCTTTTTCTTCTTGTCTGCACCGCC
AGCTCCATCCTCGTCGCCCTCGTCTTCTTCCGCTTCGGCTGCAGCCTCGGGT
GCCGGCGTCGATTCTGCGGGAGCGGGGGCGCTGTCCGCTGCGCCGCCCTT
TTTCGCCGCCGCTTGAGCCTTCTTCTTGGCCTACACGTCAGCCGAATATCGG
AGGATTCGACGAGAGCTGACCTTTTCCTTTTCCTTTTTCAATTTCTCCTTCT
GCGCTTTTGTCAAGATTTTGGGTCCATCATCGTCGTCTGCCTCCTCTTGGGG
CTCTGCCTCCTTGACCTCCGGTTCCGGAGCCTTTGTCTCCTTCACAGGCTCC
TCAGCAGCTCTAGGCTCGTCTTTGGGCTCGGCACCCCAGTCGTCCTCGTCC
TCGTCGGCTGCCTTCTGCTGCTTGCCCTTCTTCCCCTTCTTGCCCTTCTTCG
GCTTCACATCCTCCTCGGGCCACTCTTCGTCCATGTCGACAGCAGCTGGCG
CCTTGTCATCTTCGACGGGCGCAGGTGCCTCTCCGTCCATCTCGACGTCCT
CGTCATCCTGTTTGGCTTGTTTGCCCTTCTTGCCTTTCTTGCCCTTCTTAGGC
TTGACATCGTCCTCGGGCCACTCATTGTCCATGTTGACCCTGCTCTCCTCGA
TCCCGGCGGCTGGCGTTGCCGCTCCTGACTCTGCCTCTTCGTCATCGTCAA
ATGCGAACGCGTTCTTCTTCTTCTTGTCCTTCTTCTTGTCCTTCTTCTTGTCC
TTTTTGGAAGCGGCAGCTTTGACAGCGGCCATTAGGCCTCCGCCGCCAATG
TCGTCGACTTCCTCCTCGTCGAGCATGTCGAATGCGCCAGAGATTCCGCCA
TTCTCGGCCGGCCCTGAGGCATCTTGCTGCGCCTGTTGCTCGAGCGCGGCG

GCCTTCTGCTCCCTGCCATGTGAGCAGTGGTTCTGCCGATTGAAACGCTTT
GATAGCCTCGCGTCATGCGAGCATTGACAGGAGCCAGGGGAAGCAGTGAA
GGTGGCTCACCAGTAGTCATCGTCTTCGGCCTTCTTGCCCTTCTTGCCCTGT
TCTTGGGCGGCATCGCGACAAGAGTTGGTCGCGAAAGAAGCTGAGTGGCC
AAAAATGCTGGTCCAGAGTCTTTTGTTCGGTCGGCAAGAGAACCGGAGGC
TCAACGTGAGATTTGATCCCGTTGTGGCAACCCCCAGATTGAGCCTAGTCC
ATGGTCCATCTCTCATCGTGTCGGATCTCCTTTTGGCATTGACAATGGTTAC
GAGACAAAAGAGAGACCATACACCATCTAGACTCTCATAATATCGCTATAGT
ATCGATCGGTGACAGTTCTTCACAACAATCTACGAGACTGCCTACCCATTTC
TCTTCTCTTCAATACTTGAAAGCGACTCTCGAGGCAACATGCCTGGCAAGA
TCCCAGATATCCCTGGCAGGCTAAACGAAGACCCGAATATCCAGCACTTCC
TATCCGAGAGGGTGTCTTCGCTATGCGGTCTCACCAGCAACAGGTTCCCGG
GCTCCCAGCCGGTTTCGTTCACCTCTTCCTCGTTGGACCTTCTAGAGAACAT
GGACTTTTGGGTGTGTGAGAAGAGCGACGGTGTGCGCGTGCTGGTGTTTG
TGGTGTTCACCAGGCGACACAACAACAGGAAGTCTGGCTGGTATGTCGTG
GCTGGTTCAAGAGGCTGTGAGCAGCCATCCTGACCTACAGATTGACCGCA
AGCAGCGCTTCTTCGCCATTGAGGACCTGCACTTCCCACACTGGGAAAAG
CCTGACCAGCCTTTACACGAAACCCTGCTTGACGGCGAGATGATCATCGAT
GAGGATCCGAAGACTGGCGCAGAGACTCTCCGGCTGTACGCATTCGACTG
TTTGGTTTTGAACGGGGAGAACATCATGCAGCGACCCATGGTCAAGCGATA
CGGGGTGAGCTATCGTTCGTTCACTCTGGCACCTAGCTCATGTGTAGCGTTT
GAAGCAGTGGGTGATCGAGCCATGGCAAAAGGCCCTGAAACAGTACCCTG
AATGGAGAGAGGTCTTGCCATTCGAGTAAGTCCTCATGCTCGACATTGAAT
CCGATCCTAACGGCAGTGTTGTGGCCAAGACTCAAGAGCTATCTTACCACA
TGAAAACGGTTCTTGAAAGCCATATCCCGCGACTTCAGCACGGCCATGACG
GCCTCATCTTCCACGTGTGCAGAGTCTGAATATACTCCTGGGACAGACGAG
AAGATGTTGGCAGGCTGTTCCGTTTCCCAGGGCGTAGGCTTATCTCAGTCT
CAAATGGAAACCGCCATCGGAAAAACTCAATCGACTTCAAGCTGGAATTG
CGCTTTCCCCTCCATCACCTCATTCGTCCGAACCCGACTTTTACGCGAAACC
CGATTTCCTTCTTCATACCTGGCTTGGTGGGCACAATTACGAGTACTATGAT
GTGATGGAGGTGGAAGATGAGGAGTGGAATCGTCAGCTGCATGTCTTCCTC
CATAAAGTCTAGCTGATGAGCAGAATGAAGGAGTCGGGCGAACATACGATG
ACCGTATAGTCGAGGTACATTGGGACAATACGCGCAACGGCTGGCGATTCA
TGCGCATCCGAAATGACAAGCACGACGCGAATCACAAAATCATCATGGAG
AAGATCCTCATCAGTATCGAAGATGGCGTAGAGGTCGAGACGGTTCGTTTC
ATCTCCCATGCTTGAACCCTTTCTGACTGGCTAGATTCTCGCTCGGACCGAT
TCGATCCGCCAGGCTTGGAAGGCCCGCGAAGCAAGCCGACAACAAGGTG
GTGCAGGAGGCCCGCCACGCCCGCCTCCGGGGCAGCAGCAACAACCTCA

GCGGAAGAGCACATCCTACGCCCCGCAACAACAGAGACCGGCGGGGTATC
CCGCCACTCCAGGAGGCCTCCCTCAAACACCTGGATCTGCCTATCCTCATA
CACCCGGCATGGCGCAGACTCCAGGCGCTCCTCCCGGCGTCCTCGCTGGCT
TGAAGAGATGAGACGAATGTGGGAGAGTCGGGTTTGTTCACCTCATATCAT
TGTACTGCGTTACCACATCAATTTTGTATGCATCTACTCGCAGAGATTGTTC
GGACCTTTTTCGCACGTGATTTGCCGAGTCGTCGACCAAGAAAAGTCCGG
AGACGGCGAGACGTTGTTTCACGAAGTTCTCCTCCGAAGCATTCGAGGTAC
CCAGGCAGTCTTGCCGGTTGTCAAGCAGCGAGTACAATGGCGACCGAAGA
GATGAGATATTCTCGACACGGGGAGGGAAAGAAACGCTCTCTTTCGAAGA
GGTATGTATCAAGTTCACCTTGGGATTCAACTAACATTTAGGCGGTCCTCAC
TGGTCTCGCACCGAATGGAGGGTGAGCTTTGCACTTCACGCGTTGCGCAA
CCCAGGGCGGATCATCTGACCTTTCGTACGAGTATGAGAGGAGCCGTAGCT
GACAAGCAGCCTCTACATCCCCACACACATCCCGTCGCTCCCAACCGACTG
GGAGACCCGGTGGTCCACCCTTTCCTTCCCCGAACTATCATTCGAGATCCTC
TCCCTCTTCATTCCCCGCGATGTCATTCCCGGAGTCCGATCTAAGAGGGATC
ATCGAGCAGTCCTACGCTACCTTCCGCTCCAACCTCACCACGCCTCTGCGC
CAGACGGGCGAAAAGGAGAGGGTGCTGGAGCTGTGCACGGCCCGACATG
GGCGTTCAAAGATGTCGCCTTGCAGTTCCTGGGCAATCTCTTTGCGTACTTT
CTGGAGAGACGGAATGCGAAGGGGGCTGAGGAGACACTGACAGTGGTAG
GAGCTACAAGTGGAGACACGGGATCGTGCGTCCCCCCCCCCCCACCTCCG
CTGGGGACAAGGCTCATGTCCAGTGCTGCCATCTACGGCCTGCGGTCCAAG
CCCTCCATCACCATCTTCATACTCTACCCCGACGGCCGCATCTCTCCCATCC
AGCTCGCCCAAATGGCCACTGTGCCCGATGCGAACGTGTTCTGCGTCGCCG
TCGAGGACTCGGACTTTGACACGTGCCAGTCGATAGTCAAGACGCTCTTCT
CTGACGCCGAGTTCAACGCGGCCCACCGACTGGGCGCCATCAACTCGATC
AACTGGGCGCGCATCCTCGCGCAGATCGTCTACTACTTTTCGGCGTACTTCC
AGCTGCCCGCGACAAGCGCAAAGACGTGCGTTTCGTCGTGCCCACCGGCA
ACTTTGGCGATATCCTCGCGGGGTGGTATGCGAAGAAGCTGGGTCTGCCGA
TGGGCCCGCTGGTCGTGGCGACCAACGAGAACGATATCCTCGAGCGATTCT
TCCGCACCGGGCGGTACGAGCGGGAAGACCACGCGCCTAGCAAGCCGCA
GGCGGGCGAGACGGCCGTTGTGGACGGCTCGAGCGATGGGCAGCAGGCTT
CTGGCGCAGCGAGCGGAGTCAAGGCAACGTACTCGCCTGCGATGGACATT
TTGCTCTCGAGCAACTTTGAGAGGCTGTTGTTTTACTTGACGCAGAGCCAG
GCCCAGCGGAGAGGGCGAGGAGAGGCGCTCGGGTGGCCAGGCGATTTTG
AAGGGATGGATGGACGCGCTGAAGAAAGATGGCAGGGTGGACCTGGGCC
AGAAGGCACAGCAAGAGGCCGGATCGGACTTTTGGGCAGAACGGGTGTC
CGACGAGGAGGTGAGTATCCTCCTTTGAGTTTGTCGCAGAGCTGATGTGGC
AGACACTGGAGGAGATCCGAAAGTACTACGAGCGGAAGACTTTGGCTCGT

ATGTTATTGACCCGCACACCGCCGTTGGTCTCGCAGCACAAGCCCGGTCGC
AAGCCAAGGCGTGAGTCGTTGCGATCGCAGAGAGGTCCGTGCTTATAGCA
GTCCCTCGGCGACATGGGTCACGCTGTCCACCGCTCACCCGGCCAAGTTCT
CCGAGGCCGTCGACCTCGCACTCCCCTCCTCGCGCTACTCTGCCTTCAACT
TCCGAGAACAGGTATTGCCAGAACAGCTGAGAGAGCTGGAGGTATTGCAG
AAGCGGATCTACAAGGTCAAGGGGGAGCAAGGGGTGCGCGAGCTGATCG
AGCGGGTGAAGAAGGGCGAGGTGCCGCCGCATGATGGTCGGGCGTCCATC
TAGCTCTGTTGCATTATGCATATCTAGACCTAAGACTCGAGACGTAAAGGTG
GAGGTGGACCACCGGGTGTCACCGTCGCCGTCCCATCGGCCGATCCGACTA
TGTGTCTGTGTCTGCGTATTCTCTTCTCCATCACTTCTTTCAATCTCTCAGAG
CTAGCAAGATGCCTCTACCAGACAGCGCCCTCGCCGCAGAAGGCAGCTTT
GTCGACGGTGCGTACCCAGGTCTTCACTGTACAGTAGACTCATACCCCATTA
CCGAAGATGGGGGTCCGATGGTCTGTGTCTGATGATGTGCAGCCGGTCTAG
CAGAGACAATCGTCGACCCTTCCCCTTCCAGCAGCACAGATCCACATGGCA
CCGCATCCAGCCAAGACGATAGAGCGACCCCGGCAGAGAGAGCAGGCAG
AGGCGGAGCGAGAAGAAGCACCCGCTCGACGACGGGCGATTCGGCGCGC
CGGGCACGAGCATATACGACTACAGGCGGCTCAATATGCGCAGTACAGAGG
TGGGCATGTGGACCGGCGTCCTCACGGGCGGCGCGGCGAGTGAGTTCCCC
CAGTAGGAGTCGATGACCTTGAGCTGACTGAGTGTAGCGTGGGGACTGAG
GAGGTGGTCGCCTGTGCAGGTGCCGAAGAACGGGCTGTTCTTGACGTTCA
TCCGTACGTCCCCTCCTCTCCTTCCCCTCTCCTTACCCTCTCCTTCCCCTCTC
CTTTCCCCGCTTCCCGTCTCCTCGCTAAATCTCTCATCACTACTGCTTGCTCT
TCTTGCTTCATGTGTTGGGCTGACCTCAGTCACCGCCACGTCCGTCTCGTAC
TACACCTCCCGCCGCATGCTGTATCGCGGCATGGCCGAGCTGAACGCGCGA
TACACGCCGCCTCCATCCCCGGAAGAGCGGTTTACTCTGATTGACACGACG
GCATTTGCGGACGAAGATGTCCATCCCGAACAGGGGGTGCCGGACGTCAG
ATTCGACACCCTCAAAGAGATGCGAGATCCGTTCTTCCGTCCGGGGATTCC
TCGGCGGCCAGCATGAGTCCCCCTTGGCTCATCCAAGACATAACCGAAAAG
ACACTACACATCACTAGATTAGACTACAAGCATTATCGTCATCTTCATGGCG
ACGCCATCATACTCCCCACTCGACCCAAAGCGGCCTCTCGTCACCTTTTCAT
CCTCTTCTTCCTCGGCCTCGGCCGCTTCGGGGCTCATCGCTCGCCCCAAAC
TGCCTCGTATCGCTCTTCCTCGCTGGATTCGCCGGCCAAGCGGACTAGGCG
TGTTTGATCCCTCTCGATGAGCCGGGGCTCGCCAAGCCGCCTGCTCGACCG
GCCTCAGATGTACCCGGTGACCCAGGTGAGGGTAGGCATTTGCGAGAATG
ACCAAGCTCTGGACGTCTGTCGTGATGAAGTGGTCGAGCGGCCCAGAGTT
GGTGGAGGCGGTGGTGAAGGGGATGACAATGTCAGCAAGAGGGCTTTGA
GGTTGGGCAGTGTATGCCGTAGATCCAGCATGCGGACACTAGCTTCTTGCA
TGAGCTGGTGATTAGCCTTGTAACCCTCATGGCAGTATACATACCAGCTCGA

TGTTCTTATTGAGCAGGAACACGGCATACTCGAAACGGTATCGCTCGACCC
CACGCGCGTAAAGGGGAAACCTGACATCAAATCAAATCCAAGCGACAAGC
TCGACCAACTCACGACCTTGGACCTTGCATTACGCTCACAACATCTTTGAC
AAGACTCCTGCTGCCGGCGCACGTTACAGGATACGGCAGTCCTCCAGATAC
CCCGTTCAGCCCCCCGAGTAGCTGCAAGACCATCGCAACGTATCCGAGGGC
TGCACTAGTACTTCTCTCATCGACCTTGATCCCTTCTGGCAGTTCGGCAGCT
GGCAGTGTCAGAGGCGGCGCCGGTTCTTTCGGATTGGTAGGTATAGGTAAC
GGGACGTCAAGGATGGTGTAGAGCAAGCGAGCGGGATCGAGCGGGACGAT
GGGGAAGAGTGCGTCGAGCTTCTGGACAATGCTGCGATCTGAGAAGGTGG
ATCGCTGGGCGTAGAGCCGATGTCTCTTCGAGGACGAGCTCGATGGCGGCT
TCCTTCTCTCCTGCGATGCCGCTGAGCAGATGATCCTGTACCTCGGCCCCAC
GTAGATTCTCTCGCCGACGATCATTCTCGTTGTAGGCTAGAGTCGTGCGGCG
CTCGGACTTCCGCCGAGGTCTTTCTCACTTCAGATATGCGATCTCGAAGCC
ATTCGACCCTCGATTCCCGCTCATCAATGTCACGCCGCTGTCCTCAGCACCG
CCAAAGGTCCAAGGCATAGCTTACAAGAGCTTGGTGGCCCACATCAGACA
AGAGGAGCGTATCGATCTGCTTTTGCACTGCTTGTATACCTCGCTGTGTATC
TGTGATGACGGCTTGGATATTGACGAGTCTATGTCTCACTCCGGGTTCCCCT
GGCAGGAAGGTCAAGCTGACTGCTGCAGGGCACTCAATGTCGAACCCTTC
TTCATTCGCGTTTCTCGCTTGCTTCTCTCCAGCACTCCCCTTGCGGTGTGTC
GCTTCATCGCAGAGGTACTTCTCGTCCGCTGTTCCTTTGGCGGCAGGTAAA
ACGCATGCTTGTCGTCGCTACTCAGTGTGAACTGAAGAGTGTTGGGCGGG
AGTTGTATCTGAGCTGAAACAGGTCGCAAATCAGAAAGATCCACCGATCCA
CCCAAACCTTCAAGCTTTCGCCATTTTACAGGGGTGATCGCATCGTCCTCTA
CCCAGACTTCGACTCGTAGTGTTGTCGCCGCGGCGCTTTCAATCGTAAGCC
ACGAGGCAAAGTCGGTCTCTGGTGCGAGCGAGAGGAAACGCGGATGCGT
GGAGGGAATTGTGTACAGCAGAGACGAAAAAAGGGACCTCCAACGGATC
GTCTTTCCGTTTGGCCACTGCGACAGTGTCGGCAGAACGAGTGACTACCTG
AAGCGATGGTCTATGCGGTTGGAGTGATCTGGTCGAATTCCGCGGCGACGG
GATGGATGGCCCTCGCTGCACGCGCTGAGCCTTCTCCAAGGCGGGACGGT
ACACGAGCGAGCTGGCTTTTCCGCTCTTCAGCGGCATGCGACAACGTCCTT
CTGATAACATGGGGAGATGTTTTGGATGCTGATGTGGTCAAGGAAGTCGTC
CGTGTTCTGACAGGGGTGTTGGGAGGGGAAGCGACGGGTGATCTGGAAGG
CGGTGATGCTCTAGATTTGCCGTCAGGGTTGATGGCGTTGAAAGTGATCTG
GATGAAGAAGCTCTATTGAGATCGGGAACTGTCCCATACCGAACCTTGCTA
GGTGGAAGATGGTCGCTGTTGCTCCTCTTTGGCTGCGGAAACGGTACGCTT
CCTTGATTCTCCGCGGGTGCGGGTGCGAGCAGCTTGAACGCCACAAAGCA
TCGTGCTAAGCGCGATGCTGGACCGCTTGACTCCTCATCCTCCACAATGGC
TGCTTCTCTTCTCGTTGATAATGGGTCATGAGCATGCTCCAAAGCCTCTCCC

GCAAGGTCGGCGCTCGTGGCCTAGTGATGCGACTGCGGGTTGTCGCCGGT
GAAGGTGGCCCAAGAGAGGAGACAGAGCTAGAGCGCTTGTGTCTAGTAGC
CGAGAGGCGGCGGTCCTTCCTGCTGGGATCAAGAGATGCGGTGCTGGGTC
GGCGCACGGATCTGCTGGATTCCCCCAGCTCGTACCCACCGAATTCTGTAC
CGGGAGACGGGGAGGGTATCAACTTGGAGGCGAGAGCTAAGGATTGCGGG
AGGGTGAGCTGGTGGATGCGGACACTGGACGTCAGCTATAGTGACGCAGT
AGGTGACTCAGCAGACCCCGTGATGTCTCGGATACGCGGCTGCTGGTCAGC
CAGAAAAGATGAATGGGGAGAATCGCCCACTTGATCCAACAGAGAGAAGT
GGCTGTCCTTTGTGTCCGACGCAGCTTCAGCGGGCGGTTCCGCATCGTCCA
TCCTGTTTGATTCGAGGATGTGCCCGATGTCGGTCTCGCGGCCCCGGCTAG
GTCATGTCTGCTGCTTGGTATTGCGCGGTTACAACTGGCGTTTGCGACATTG
CGAGTTGAAATGGATGCTGGCTCTGGCGTCAGTCCACGTGGGTACCCTGAG
CCTGATCACGTGAGCTGGAATGAGCTCGGCGATGAGCGATGCTTCTGCCTG
GCGGTCAACGAGGCCGAGATGACATCTTTCGAGCATATTCCATCTTCGCATC
ACTTCGTCGCCCACCCGCCCACCTCTGCGGCGACGACAGCGTTTCCGATAG
TGAAGGACAGTGGCATCGCTGCCTGTCCGCACCCCGCCATAGCCAGCGAG
CTGTGCCCGAGCTTTTGGCTCTCCCCGACGGTCATCGCTCGTCTGATCTGAT
AATCAACAGCATCTAGACAACATTCTCCATCTCACTTCTTGTGCATAGACCG
TAGAGGCTCCTCTTCCACACCAAACATGTCCAACGCAGCCGGTCCATCTGC
GCCGCCGCGAGGCATACCAATACGTCAGCTCTCATGGAGATACCTATAAGAT
GAAGCTGATCAATCAGTATCTGGCCCGCCACCAGTTACTGAATCCCCCAGT
CAGCTTGCCAATACCTGCAAGGAGAGTCGAGCTGACCAGCAGACTGCCGT
TCATGCGGGAAGCAGTTCAACCCTCTCTGGCGGCGGTCGCACGCATGCGG
TCACTGCGGCTACGAATACTGCTCGTCCTGTCTGTCCGACGGCCAGGCGCT
CATGCCCCGCCGTCCCGGCGAGCAAAAGCCCAAGGGTATCCTTGATGAGAT
CAAGGCGGCTGTGACGGATGAGCCGAGAGTGCAGAACGGCTATGAGCTGG
AGAGCGTGTGTGTGCCCTGCTTGGGGATGGTTGCAGGGTGGGGACAGCTC
CGTCCGGACCGGGAGAGTGACTGAGATCCAGTCACATCAGCCTCGGTGGA
GAGGTTGCGGGCTTTACCCGTAAAGAGATTGAAGGAGTACCTTGCGGCGTA
CGACATTACACTTGTCGGTCCCAAGGAGAAAGAGGACTTTGTTCAGGCGG
TGGTGCGGGCGAGAGATCCGCCACTGGCTGTCTGTCGCCCGAGGCAGAGG
TGCGCCACTAGTCGCAGCGATGTCGTCGTGCTGATCAGTAGAGCTTCTATC
GCAGAAAGAGTGTACCCAAGTTCAAGCCGCCTCAGCAACAGCCGCCACAG
CAGCCGGCTAGAACCCTCCGCGGCACGGCCCAATGGCTACCCTCAACAGC
AGTACAATCCGCACGCATTCCGACCACCGCCTCCCACCAACGCGTATGCTC
CACGTCAGAACACCCAAGCCCGTCCTCCGCAGCCATATGCTCGACCACCAC
CTGCACGTCCACCAGCTTCGACTGCCGCTCGCCCAGCAACTGCTGCCCCG
GCTCGCCAGCCATCGCCTCCAATTCCGACGGTGCTCAACCTCGTGGGCCTG

CAAAATCGTATCTCTCCACCTTGACTGTCGGACAACTCAAGGGTATTTGTT
CGACAATCATGTCAAGGTGGACTTTAAGCAGGTGCTGGAAAAGTCAGCTG
GATCACCAAGGCAAATGGGATGAGCTGACAACAGGAACGAGCTCATCAAC
CGCGTCTCTGAATGGTGACCGAAGAGCGCAAGAGACTCGAACGTCAGGCC
CGTGCCGAAGAGGCCGAGGCTGCGGGCACGACACCGCCAGAGCCTGCTG
GGTCTGAAGCTGAAGAAGCCGAGGCGGTGAAGAAGCCTGTTCCCAACTG
GGCCGGTAGCCGATTCTGATAGAGGTCTCTGTATCGTCTGTCAGGACGAGG
AGGCGACATTGGCTGTCGTGGACTGCGGTCATCTCTGCATGTGCCAGCGTG
AGTTACACCACTTCAAGATGCATTGCGCTGATCGTCAGACTGCTCAGACAT
CATCATGGCGACGAGTCAAGAATGCCCTCTGTGTCGGACCAGGATAGTCAC
CCCGCAGAGGTTGATACGCATATATCGGCCATGAGAAGCCGCATCGCGCAT
AGTTGTAGCAACAGTGAAGTTTGTACAGGCTAGGTGCTACAACAATACGTT
TGAATCCTTCGCAGTGGCCGTCGTCGCCGGCACTAGCTGGTAGGACTCTCA
GCTGACCTGTCCTCGCCGACCAGCGCGAAGGGCTTCAAACCCCCACTCGC
TGTCTGATCCACGGAGCTATGTGCACGGGCAATCTTGGGATTCGAGGCGCT
TGTCGGCACACCTGGCTGAGCTGCATTGGACGGGTGTCGTAGAGGTCGAG
GATCCGGCTGAGGAAAGGCCAGACGGGGTGTGCCCAGTTGATGATACGTC
CTCAGCGCTTCCGACATGATGGATAGCTTGTCGTCAGCAAGATGGCCGCGG
CTGTGGGAAAGCCTGACCCTGTTCATGATATCGTCTACGCCCTTTTCTCTCC
GCTTCAGCCCCTCAAGCCCCTCCATACGCTCGTCGTCCTCCCGCCACACCT
TGCGCCCCGTCTTGCCCTGCATATCGGGGCTCTGCAGGTTCTCCAAAGCCA
GATGCATCCTCCCAATCTCCTCTTGAACCTTGAGAACCGCATCGAGGTGAT
CCTTGAGCGCGCGTACCATCGAGTCGCCCTCTGGCGAGAGGGCCGCGTTG
AATATCTCGTAGATACTCTTGTCACCGCGGTCCTTTGGCTGGCCGTCCGAGT
GCCCCGGGTCCATCGGCGCGGCGCCCAGCGGAGAAGGAAGGAGTTTGCCG
GGCGTGGTGGATCCCTCAGCTGTCTGCGGCTGCGACTGAGGCTGGGCCTG
CGAAGGCCGCAAGCGGGACGGGCATGGGCGCCGAGCTGGACCGCGCCAT
GGCTGGACGGAAAAAGTGAGAGAGGTAGGTATCATGCGGCGCAGGGGGA
GGGAGCAGACTCGGGTCCAGCTGCCGCGGCATATCTTTGGGTAATACGGGC
GAGGACGGCTCGGTATCGCTGTCAAAGGTGTACCGCTCGAACGCGTCGTC
CAGCCTCAACTCGGGTGGGCGGGTAGTGGGATCCGTACGGTCGGTGTCGA
GCGTCGTGATACATACGGGCTCTGGCGTGCGACTGCTTGTAGCCAGGTAAT
CCCGACTCGCGGACTCAGGCCCATCACGCGCATCCATCAAGGGAGGCAGG
GGCGGCTGGGCGCTAAGAAGTGGGACACGGCTTCCGTCGGTCAAATGGGT
CTGCGGTGTATCTTCGTCCACTTTTCCAAGAGACATCTCCTCTGCGGGGTCT
GCGGCAGAGGCACGATAGGTGGAGAAGGGGCATGGTTGCGGCTGCCCAGC
TGCAGGGACGGCGATCGGTCTAAGAGCGGGTGTGAGGCTGAGACGGGCG
ACTCGACTAGGGCCGAGGAGTACTGGACGGACGGGACGGCTACTGGCTGT

GGGTGGATTGAAGAGGCTGTCGACGACTTGCGCCGCACAGACGAGACCGA
GTAGGTTGGCCACATCGCAACGCGATGCGCAAGACAAAGCAAGCGATGAC
AGAGGAAGCACCCTACAGTATGTACCCTCTGTCACGCACAATATATTACCAG
AAAAAGCGGCGCAATGACGTATTCCTGTTCGTATAACGGCTAGTATGCTCGC
TTGTCATCAACTCTGATGTCGCGGGAGATCGGAGTTCGATTCTCCGACAGG
AAGAATTGCTTTTTGCAGTGCAGCATGTGGTCGACTGCTCCACAGCGCAAG
GCTGGGAATCATGCCTTCAGCGGGCCCGGGAGTTCGCGATTACCTGCCAAC
GAGTGCTGGATGATCGATCTAAAGTCAACTCTTTGCGAGGAAAATCTCTTT
TGACCCCATGCTTCACTCCTTGTCAATGTGCGCGCTTCTATCGTCGGTAATG
AAACGATTCTTCACAACATCACTGTCACTTAGGCCACGTCACCGTTCCAGG
TTCTGCATTTAGCACCCCAGCGACATGCCCCCCCCCCCCCCTCTCCCCCCC
GCGCGCGGCCCTGCCACAAGCAAGCAAATAGTTTGATTCTCTGACAGGAA
GGATTCGAGGTCCGAACCGTGCGTAAAATGGCTCGTCACGACCCGTGCGG
CAGATTGGGCCGTGGGCTGTTGAGAGTCGCCTGAGGGTCATTAGGGTCCAC
GACAAGCACTAGAGAAGGATCGGAGCCCAGAAGCCTTCGTGATCATTCTC
CGAGAGTGATAACACGGGACGCTGCGGGACCACTGGAGACACGTGCCTCG
CCTGATCTTTAAGGTGTGGGTGGAATCGTTGTCGGTGGTCACTTCCAGTCC
CTATAACAGATTGCCAAGCGGGGGATTACACCGCTAAGACGTACACCCCGA
CGAGGGGAGGGCAGGGAAAGATGGGAGGGGGCCGCTGGGATCAAGACGT
ATTAAAGTCTCGTCTTTCCGCGCTATGGATTTCCGACACCTCGTCCATGCGT
CAACACTTACCCTTCCGAATTTCTCCTGCCCTGACGCACAAAGGTAGTCAC
CCCGCTGTCACTCAGAAGCTGGATTCATAGGGACATGAGTGATCACAATGT
CGCCGCCGCTTACGCCAGGCGCCCAATGGTCCAGGCGTCATAGTACGAGGA
GTCGTACGGCACTAGGGTGTAGTTGAAGGCATAGTCGTCTAGATTCCGGGC
ATACCTTTCTAGGAAATCCGGGTCAATGGAGGAGTTAGGGATAGGGTGAAT
GTCCGTAGACGGGTTAGGGAACGTCCAAGCTAATGAATTCTGTAGGCATCT
TGATGATCACTTCCGGATAATTATACGTATGTGAGTGTTATATCAAGAATTAT
ATCACGCACGTCGGTTTTATGACTTGGGTGTGGTTATTTATAAGGGTACGTG
ATATAGCGAATCGTATCGCGACGCCGATGGCGACAGTTGCGGGAGAATCAC
GCAATACCAACTAGGCCGGTGCCGGCCCGTAACACTCCTCATCTCAACCCC
GACCTGATATATAACGGGTATAGTCGACATTCCATCGTCAGGTTTTTGATTTA
TAATCGTATGGCTTACCTGATGAATTTGGTCAAACGCGCGTTATTTTGGTGA
TCATACGTGAGGGGGTAATTTCAAGTGATGATCTAGGAGAAATTCTCATCCT
TCCGATATTTCTCAATAGGAGCAAGTCTATGAAATTCTCTCCAATATACCTAA
TGAGTTTGATATAGTGTGAAATTTACATTCATTGCCTTATTAATCGTGTATAG
GTACTAGTCCGTATTATAGATATAGGTGCTAAGGGGGTGACGTCCTAGCGCA
ACCAACCTCAAGAGATATAACCCGACTAATACTTCCGATAAGCTTAGTCCCA
CTATAACCAAGATGAAACAGCCCTCATTACTCAGGCATATAAAGGGTCTTAC

TTGAATGTATAGGTAGTATTTTTCTTCAACCGAGACTTTGTCAGGGCAAGCT
TCTAGATACTGTACCCCCACCCTCAGGCACGGATAGGAGTGCCCCCAACAA
ACTAACCAAAGGCAGACCAGAACTCCCTTCCTTTATATCAATATTTTACGGG
TTCTTTCTGACATAACTTAATGTCGCTGTTTATATTATTTCTAGTACAGATACG
CGATACTCTTCAATCAATATGCTGGAGCTTCAAACTTCTTAATGCTAAGAAA
CGGCGATATCGCTCGTCAGACAAGACCACGGTATCCATGTTATACATATAAA
CACCGTGTTCCAATCTGCCTGTTATTAAAGAGACTATATAGTATACTTTTACA
TTGAATCCTGAGTAAAAGTTAGGCAGACAGTTTGTGTGTTATCGCTTCCCA
ATTTCATGACAGGGACCTTGGCACCAACAACATTGTCAGAAGCCTACTCCG
CAGGATAAGAGAGTGGGAATTTTATCAGAGTATAGATGGATCGAAGATCTAT
CTGCCTGTTAGAAGATCGCAATAGCCTCATGTGACCCTGGGGTATCTGTTAG
CAGCTAGCCGTGGGGTAGATTAGAAAAGCAAGATTCATGAATCTATATCATG
CTTCTTGGCAGTTCATAGTTCAGAACCCCTTTCATAAAACCATCGTTATTCA
ATGTCTCCTGAACACTCCTCGTGACAGACACTGAGTCTCATAAAGCTACTC
CAAACGCGTATGATAGACGAGGACGCGACTTCAGTAATATAACGGGAAGGG
TTCTAGAACATCGACCTAATAGCTTTCTGTGCCTGAATGCCATGCGGCTGAG
ACAGACTAAGCGTGTTAGAGTCCGTCTAGCTACGGCACCTGGTGAAAACA
ATTTTAACAGATCTTCCCTGTCCCCAATTAGGAAATTCAGAGATCTAGCCGA
GTCATATGGTTGGGTATACGTAAGTCACGGTAAGTTCTTAAGTCCTAAATCG
TGGATAGTGCCGCTTGCTTAACCCAACTAATAAATATCTTGCTCATGTCGAG
CTAGTGACCACTATGTTAGCGTGAGGCAGTCCGTAAAACAGATCCACGGGT
TCGGGTTGACCGAGACAGAATTTGATGGAATAATAGAGCGGAATAAACTCC
GTTGGATTTGACTGCGATACAAGTAAATAACCTCCTATATTTTGTTTCATACA
AATTCGAATATATTGATCATATTGTCCGCAAATATATCAGGTGACAGTTATGT
TTCATATAAAGGGGCAGCGATTATGTAATTTATTAGTAAATATAGATGATATC
AGTGAAGTATATATTGACCTTAACTAGACGTCATCAAATGAACTTCTGCGCA
TTTATTCTTCAATGAAAGATTCAGAGTCCCATTTCTATCTGCTATGTTGGAGA
TTTCCCCGGACATCAGATGGACGGAATTACAGGTATACCTGGATAACCTCG
GGTCTAGCAAATTCGAACTAGGAGTAACGGATGTGAATGAATTTTTTCTATC
TTGATAGATATAAACATATTTTCCCTATAATTCTGAAAAGTCTTACGTCGTCT
AGGGAACGTGATCACAGGCACTGATAGTTAGTTCGTAGAATAGACTACTAG
CAAGGGTCTGTATACTATATTTTATTGCCTGGAGACAATATCCAGAAAATCTA
GACTTAGTTGACGACATCAAGAATTGGGATTAAGGTTTCCCCGTCCTAGTA
CTCAAGGACTTCAGTCAATACAGAGTTTGCCAAGAATTATCGTGACTTAGG
ATGACACGCGTATGAGATACCGGCGACTCGGGATTTGAAAGAAAAATCCAT
CTTGGAGTATTAACTAGTTAGTAGGAAATTCCATCTAGCCTTGATCTAGCTAT
TTCTGATGTCTGGGACTATTAAGTGCCTAATACAAAATTTTCCGCTTCCGTG
CTATTATAGGTGCCGGGTACTAAACAAGTCCTTACTGTATGGATGAGTACCA

TCTTCATGTTAGCGTCGATCGTCAGAATGACACTTGCCCCCGGAGACTAGA
TCCAATGCGGGAGTAGGTAAGCAAAGACTGCAGACGAAGTTAGCATCGTG
AGCTCCAGTGACTATAATAGACTCCGTGGTCTAAGAGAAACAAACACCAA
GAAAAACACCAAACTGCAATTCTTCTCCAACTACTACTACATCGTTAGCTTC
ATAAGCTTCATCGGCCTCCGCCGAGAACCTTTAAGACAAGCAGAAAAAAA
TGCAAACCTCTAGCATTTGAATCTTGCTCACGATGTTATTAGTTTTAGTATCG
TTAGTTGTACCGAGGATTGCGATGCAGTCGTAAACTCACGATCGTCAAGAA
CAAGTCTAACACCTACCTAGAGGTAGATAACTTAGACCAACATGAGCATATT
TGCTCACATCTAAGGTCTAGAGCGATATAGTAATCTTGCGTTGAAGGATTGC
CAACATGAACACAAAATCTCAAGTTGAGTAAATAGTCAGGTCATAACGATA
TACGCAGTTTGGTGATCAAGACGTTATAAAGGCACATGTAATCGGTTATATA
GCCCGGTCTGGCAGGTAACAACTTGTCGTTGTGATAACCATCAACCTGACG
TAACGTTGTTAAGCCCTACATATATGATCGGAATCAAGGTTACGGTTGTGAC
CGGAAGTCTGGGAGATCGGACTGGGGTGGAGCAATCAGACCTTGAGCGGC
GCAGGGGTGGGGGGTCGATAGGACGCCTCGGTTCTAGGATTGTAAGCCGG
GGAAGGGAAGAAGGATTTTGATTACCGCGTTTCGAAACACCATCTACGCGT
GTCACCAATAAACTCATTTATCGAAATCCTAAAAGATGATTTCTCGCTTGCT
TGTTAATATGAAAATATGTGTATTACCAACATAACCCCTGAATTGCAAAAAG
CTTCGCTATCTACCTAATGGATACCTACCTAGGTAGGTATCCAAGGAAGCAT
GGTTGGTGAGGATCCATGCCCTGCGATTCTGCAAGAATCAGCAAGATCGAT
TACCCTTCCGCGTTGAAATCTTTGAGATGCGATGAATTCAAAATCCATCCGA
CGCGTCCCTCCCTCTAGACCGATAGATCTAGCGCACTTCCGTAAGCCACGA
CGCTTTAGTACCGGCGGCTGCCGGTGACTGACTCGCGATGCTGCATCATCC
ATATCCACAGATGGGGCACCTATGTCATGGAGAGCACACCGAGCCACACCT
TCTGGCGAAGAAAAGAAAATTCGACCCAACTACCTCCCAGACGTAGGCAG
CTCCGATTAGGACTAGGGTGGTATGTGTTTGTTTCGCCTAAGGTCTCGGTCT
AATTCTTGAGGGTGTCCAGCATAGCGGCTCAGCGAAGCAAACGTCATAAG
GGACGAAAGATCGCGTGCCTGAGAGGAATGTTATATAAAAACCTCGAGGA
ATCCGTTCCTGCATCTAAAAGTAAAAGATCGACATCGACATCCCAGGACTC
ACTTACTCATCCCTTCTTTTACGCTTTTATATCATATATATATCAAAATACCCC
TACCCTCGTTCCCCACCTACCCCTGACGAGCCTCACATTCCAAAGACCTAC
CTTGTTACCTTGTCTTAAAAGTTTTACTTCACAATGGTTTCTGCATCCGGCC
TCCTGGCTGCCGTCACCGGAGCCCTCAGCGCGCAGACCGCCCATGCCGCC
ACCGGGGTCGACCCTCCCAGTGCCAGTGCTACCGGACGAACGGCACGTCT
CCGGCCTACTTCGCGCACTACAAGTTCTTCGACTTCCGGAACATCGCGAAC
CCGTACGTGCCGGGCGCCATCGACAACCGGGCGCAAGCCCTGGCCGCCCC
GGTGACGCACTCGTACTTCAACACGAGCAACTTCACCAACACGTGGTCGA
TCCAGGCCTGGGTCGACAGCGCAACGTGTACAACGAGTACAGCAAGAACG

ACGTCTACATCGAGTCCAACACGGACTCGAAGCCCAACTCCAAGACCTGG
CTGACGCTGCGCACGTACCGACACCCGGCCAGCAACGGCAACTTCCAGTC
CTCGGCCGAGATCCAGAGCATCTCGTCCACGTACCAATACGTATCCATGCG
GATGTACGCGCGCACGAAGGGAGCCTCCGGCGCCGTGACGGCCATGTTCA
CCTACAAGGACGCGGCGTCCGAGGCCGACGTGCAGGAGGCGGACACCGA
GTTCCTGACCTCCGACTCCACCAACGAAGTGCACTACACGAACCAGCCCG
GTACAGTAAACGGCGTGTCGCGGCCGAACGCCACGAAGGAAGTCAAGATC
TCGGGCTCGTGGTCGAACTGGCGCGTGCACCGCTACGACTGGACGCCGGG
CAAGAGCGACTGGTACGTGGACGGCGAGCACACGGCGCAGATCACGTACC
AAGCACCGGTTGACCCCGACCACGCTCCTCTTCAACGCCTGGTCCGACGG
CGGCAGCTGGAGCGGCGCCATGGCGAGCGGCAGAGCGCGAACCTGCAGA
TCCAGTGGATCGAGCTGATCTACAACAACACCGCCGAGCCCTCCAACTACA
GCTACTGCAACGCCCGCAACATCTGCAGTCTCGACCAGGGCAACACCGTC
GGCGTCCCTGCCCTAAGCAGTGCCCACTGAACCTCGCTAGAGGGGTGGGT
TCTGCATCTGCTCCTCGCCCTGTTAGTCACCCTAGCGCTCTGGTACTGAAAG
CAGCGCGAACAACAATTTCTCTCGGGCGGTCTCCTCAGGTACGAAAGCTG
GATGGATATGGTAATGCGGACCATGACCACTGATATCGAATGGTCTCTTCAA
AAGTAAACAAAATGGGTCTTATGGTATAGAGGTGGAGTAAGCGTGTAAATT
CTGTGCTATGGTTAGCACACATCTGGGATTTTTTATTACCTTCTATTTTTTGTA
TATTATATTTCACTATACCGGTTTAAATAAGAACGAACGATTTTTATATATCCG
TCTTTGCTTGGATGTGCCGAGCTTGCTTCCGCGTAGTACAAGTGACCAGAA
GGGTGTTTCCATATGAAGCCATCCCTTGGTGTGGTATGTTGCCACAAAAGAT
ACCTACCTTGATGGCACGTGGGACTTCAATAAACAGCTTTGAGGGGCTGCG
TAGACGCGGATGGCCAAAGGGTTTTCTAGATCGTCCCTATATAAAAGGCCTA
GGTATCTCGAAAAAGTGTCAGCAAAACACAGATAGGTAGGTAGGCATGATG
CGGTTAGGATGGCACATGGATGCGATGAAAGAGAAAGAAGTAGGGAAAGA
AATATTCTCAGAAAGAAGCAATCTTCCAGGAACTCGTATCGAACCGCCTCA
GCTCGGTTGTACATGTTTCTAACCGGGATGTCCCTCCGAGTATAACAACAG
GTGGTCCAAGTGAGCCAATTGATCCATTTCTTTAAAGACTGTTAGAAGGAG
CACGATGGTTTTGGTATATAACCTCGCTGAAGGTAACTCATACAGTATAGAT
GGGTACATAAGAGGTAGGGTAGCAGTTGACGAGGGCTGACAAACTCAATA
GGTTCAATTGATTGCTCGTGGCTACATATCAGTCTTCCCAAAGCTATGCATC
CGTCCATCCATATGACCCGACAATGCCTTTTTGTTTTTTTGCCCTTCCCGAC
GAACGTATCCTCGGACCCGGTCGACGAATACCATTCGCGATGCATGAGACC
CAGTACCGACACCATCAGACAGCATCAGACAGCCATTAAGCGCAAAGCAG
ATGACCTCTAGATAGGGGTATTCGAGATGACAACAAATGGAAAGGGTAATA
AAAGAAACAGTGAAATAAATGCCAAACCCTTCGCCAGGACATATTGTCCGT
ACCCAAGGATATATATACCTAATGGATGTTTTCATGAACCTGATTAGTCGTCA

GATTTCGTGCATATGGCGTGACCTTGCCTCATCCCAATCCTTTTCTATCCCTA
CTTCATTCGCAAACCTCCGTTCTAACAGTGCACCAGACAACAAAAAAAGG
GGAAAGGAATCTCGCCTACAACTTTCCTCAATCGCCCATAGAGGCCATCCC
AGCCATTGGAGCGAATGTGTCATAGTCTTCATCGCTACTATCTATCGGGCTC
TCGGGGCTCTGAATGTTGCTGATGTCTGCGCTGCCCATTCCGCCATATAGGT
CTCCGACAGAAGTGCCCATACCCATGGATAGGATGCTCTGTCGTCTTCCGG
GATCGAATTCAATAGCGCTTCCGCTGCGCTCAATTCCGGTTTCTCCGGGAGT
TTCTTCAATAGACCGGTTGATATATCAAGCATACCCATGGCAAATTCAGTCA
GACCTTTAGATTCGCCAGGTTTGCTGCTGACACTCAACGTCCATCGGTATTC
TCCGAGCCTACGCCTATACCAATCAGCCTCCTCACGACCTGGTGAAGTCGC
CCATAATAGCGACCCAAAAGTGCCAATTAATGCGAAGTTGGTCTTCGAAGC
AAAGAACCAGAAAGATCGGAGATGTTTGAGGTGTCAGACGATTCACAAAG
TCCATGGCAGATATAAGACGAGCCTTTGCAGCACTCCGGCATATGTGTTGA
ACATAGGGGTCAACAGCCTGCGAATCCGTCTCGAGAGACCGTATAATTCGA
CGATGGAGTTGTTATCTCCGTCGCGAAGTAAGCCAAGTGCAGATATCCGAT
ACTCGATAAACGAGTGGATGTGACGCTACCGGATTGGAAAGAAGAGTCCA
TGCGTATCGCGGTCGGAAGCGCGGCGTACCATTCCTTGAGCTTCAGCTGGA
TGGGCTTTGCCATTCCAAAACAAGGTGGGTACCTTGTGGACCAGCATTGCT
AACGTTCTGCATGGCTTCGAGCGTGTAGAACGTTTCGAGTATCTCGGCCAG
GACTTGCGTAAGATAGACCATCTGAACAAATAACGTCCGGCCTCTCTCGAT
GTCAATCTTCCCCTCCGGATCTTGGTCATCTAATTCAGCTTCCGGGAAGTCG
TTTGGCGTTAGAGGCTGCACAACCCAGTTGGAGCTAAGATATGTGATGGTC
GTCCGTGAACAAGGGCTCCCCACTTATCTTGCATATACAGAGCCCAGGCGA
GTCTTTTCCTGAGTCCCTTCTCCAAGGTGGAATCTTCCAATGTGTACAATCC
AGGTGGAGGCCGAGCTCTTGAGCGATTGCCACGATCTGGGCGGTCGGAGC
CCATTGGTCACCCTCGGGTCGCTGCGAAAGCAAGAGTCCGGCCTGCACAG
TAGAAAGCTTTGGGCGCCACATGGCATCAGCCAGAGAAGATCGTATTAATC
TCTCAAGTTCGCGCACATTGGGACGCTGATTTTTAGAAAGTTCCTCTGAGT
GGTCCCACCAATTGATAGCCAGGATGTAAACGGCGGCCAACAGCGCTGGC
GAAAATTCCCGGTAGTTTCGATCGTATTTCTCGTAGAAGATGGTCTTCTGAA
TAATGGGGAAGGCCGGGTGTACGACTTCGAAATAAAGGTCGATTAACTTGC
GGCCATAAGGTGCCACCAGCCGTTGAATCTCGTCGGTGTCATCATACTGAT
GCTCGTGGCCGGGCGTGGCATTGTCAGGCAGCATTAAAAACGTATCATTAT
CGCTCACTTTCCTCAAAGTACCTCGAGCAAGTAAGCTCTCATCTTGCGGGT
CAAACGGAGACAGATTGATTAAAGAGGGCTCAAAATCGGTGGTTGGGCCG
ATGTATGGCTATAACGGTCATCTTGGAGACCAAGAGAGCGCTTGAGCAAAG
TCGGACCACAAAATTAGCCATCTCTTCGATTAAGGAAGTGCTAGCAGTTGT
ACTTGACAAGCTGGCCTGGCGTTGCCGCCTTCTCCTGTTATCAGACCTGCC

GGGCGAGCTGCATTGGCCAATAATATGTCAGCGATTTTTTTTCTGTCCAAG
AGTCTGTCGACTGTGGGTTGCAAGTGATAACGTAAGGATGGGGGTCAACG
AATGAAAAAGTATCGGTGAGCACTCACCCTCTCTTCCCGTTACTTTCTTCGG
CAGAGTCACGCGCTAGCTTTCTTTTACGAGGCTGAGGGCTCTGATTTAAAG
AGCATGGGATTCCGTGGACTTGGCAAGAGATGCAGCTCTCGTCCTCGTCCC
CGATGACACACTTAAATCGCCTTCTCAGACAAGATTCACAAGGGGAATCCG
CCTCCACTTACAGCAGAATCAGAGTCGGTTGAATCATTACTGTTCATACTAT
TGAAAGATGCTTCCTTGCTATGCGAGCGTTTAGAAATAGCTGTAAGGGTTTA
GGAGCAATAGCAATAGGAGCTGTCCGAGGAAGTGTGCTAAGACTGGACAT
GGGCTGAAATTATTAAGCGGATAGCGCCCAGGACCGCTAGCTGTGTCCTCG
CTGAGGTCGGCTTCGCCGTATCACTGTCGGGACCCGCTTAGGTAGACGCCA
AATGACGGCTCGTCTGGTAGGACCGGGCTACAAGGTGACAAGGTGATGTA
ATGAACCTATATCCGGACAGAGGGGGTATGATGGGTTACCGAATGGGGGTG
AATTCTGGAAATGCCTTCTGAAGGAGCAGGGACCGGCTACCGCAATACAA
CGTCGAATCGTAGCAGAGAACCGCAGATCTCTGCACTCTCCACTGCTTCGA
GGAACTCCTTATGGGACCTTGTTGAGACCTTATTGGGAGGTTTATTGGGATG
CTTGATAAGGAAGTTGGATGATAAGGCTGTTAAGTGGCACGGGAGGGCGTC
AGGGGGACTGTCAAGGGATTGTCAATGGATTCGGGAGACGCAGGTAAAAA
TGACCACGCGAGTTACGCCGCGTGTTTCAACACCACCCAGACCAGTCTGG
TGGGATCTGTCTTATCGAAATTGAGGCCGGGGAAATCAAACAGGT

>Mosaic_sequence_54

GTTTACTTTGACGAGCGAGGACGGAGATGAGGGGTTTCCCGGAGAGGTCC
TCGTTAAGGTCTTCTATACAACTGGAAAAGTTATTGAGAATGGAAAGGAGG
TAATAGTTCTCGGTCAAGAATACGACGCGGAGCTCGTAGGAGGTGCCGAG
GAGACAGTAGTAAACATAACTAACCACTCGTATGCTCTTAAGACTAAGAAT
ACTATACCTGGTAGAAAGTTGCTAACCCAAACAGGTACTTCAACCTTTCGG
GTGGCCCAACCATCGAGGGAACTGTAGTCCAACTTGCGACCAATTCATATC
TCCCACTGAGCGAATGGGGAATTCCAACGGCCGGACCGACGGTATACTCCA
AGGTTGAGACGACTACTCCATTTACGTTAGGCCCCAATGGCCCAGACTTCG
ATGATTGCTTCATCGTCGACCCCTCAAAATCTCCCAGCTCCGTATCTATTGA
CACCCGAGCTCTTCCCCTAACCAAACTTGTCTCGGCACAACACCCGGAGA
GCAAAATTCACCTAGACGTTCTAAGCACCGAGCCTGCATTCCAATTTACA
CAGGCAAGTGGATTGAAGTGCCAGCTGTGGGTGGCGCTCCTGCTCGTGGA
CCACGAAGTGGCTTCTGTGTTGAGCCGAGTCGGTTCGTAAATGCTATCAAC
GTTGAGGACTGGCGGAGCCAGATGCTGCTTAAGAAGGGTGAGAAATATGG
TGCTCGCATCGTGTACAAGGCGTGGAAAGAGTAATTCATCACGATATATAGT
GATGGGGTATATTAATCGTCGGAAAAGGTATAACGCATATGCGCTGAATTTC

TGTAATTACTAGGTAACCATCTCAATGAGAACGAGCTATTACGGCTGATCAT
CGTCATCTCCAATATCCTGGTCAACTGGTGCGGTCGGCGCTTTAGCCTCTTG
TTTCCTATACAGGCCTGCCAGAAATTCACTACTACTAGCTTCCTCGATTCCC
TTATCCTCACGTTTCTTTAGAAAGCGAGGGAATCGCAAACTCAACCCCCGC
TCTTGACTAACTAGTCCGATTGACGCCGTGTATGTCGGCGAAAGCGTAATAT
CGGCAAACGCCATCTCCCATACCTCTTGAGGTTCGAACCAGACATCTGGCT
CCGGGCCGTAATACTCTATAAAACTCGGCTTCGTTCGACGAATATTCTTGTA
TTTTTGAGCATCGGCCTCTTCCGTGTCGTCATCTCCATCCTCATTTGGTTCCG
TAGCATTCTCTTCTGGTGCTTCTTCACCGGAACCGTCGTCGTAAAATTTCTT
GTTCGCCTTGTAAAAAACGTCCGTAAAACCGGAAATGCATTTACATACGGC
TTCTAGAGATCCCGTCGCGTCGTTGCGGACGGCCATGAGGATCGGAGACCA
CCACTTAGCTTTTCGGCCTTGGCCATGCCATGCGGCAATAGGCACAAGATC
CAGTGTGTCGAATGAGCTGCTGTAATCCTTCTTTACCTTGAGCCACGAGTC
GAGTCGCTTGTCGGGCTCATAAGTGGCGAGTAATGCTTTCCGCCGTGAAGA
TGCTTTCTTCTCTTCACCAGTCCCGTCATTTGAGGAGCCTTTGGACTTTCCT
TTACCCTTTTTAGCTTTAGATTTTGAGGGGAGGGTAAGGGTCTCGCTTTCGT
CGACTTCCCCGGCCAATTGGTTCATATCTCCAGCAGCTTGTGTTGGAGCGA
GCAGATCCGGAAGATTGTCTAGTATCTTGACCATAATGCCCTCACATTTCGT
ATCTGTAGCTTGCTTGAAGAACTCCAATACAATTTCAGAATCTTGAGATGTA
GCATCCAGACTCTTGACCCAGGTGAAGCGATTCGGTATTTCGATAAATAAG
GATCGTAGTAATGATCTTCGCTCACGAAACGGCCGATCAAGTAGTGGCTGG
CCATTCAGATACATGAGGTCGAATGCGAATAAGCATACCTCAATGGTTATAT
TACCAATCTCCACGTCTTTTCGCGCCCGGTTCGTGAGAGTCTGGAAGTTTTT
CAATTCTCCGGACTGCTGGTCCACCGCGACGACTTCGCCCTCCATGATAAA
ACTGCTAACACCCTCGCCGCGGATTTTCGGAACGAGCTCCACGAGGTCCG
GATACTTATCAGTCATAACTTCCAGATGCCTTGAGAAAATTGAAACTTTGCC
TTTATCGTCGCAGTGGACTTGGGCTCGTTGTCCATCGTATTTGAATTCACAC
GCGAAATCCCTGCCTTGTAATTTCGTGAGCATCTCAGACAAATCCCTTGTAA
TACTCCCCAACATTGGCCTTAAAGGAATATGCAAAGCTAGGTCGCATCTCA
GTAATAACTCGTCGCATACACCTATCTCGAGCATAGTAGGTATGAGATCGTT
ATAGTTAGGGCGCCTTGCGAAACAAGCTTTGACGATCTCCTCGGCTCGACT
CCAAATCTCTGTCAGATCCTCTTTTTTCAAACGCCTTAAGGTCATTTACGGA
TTTGAGGGAGAACTCGGCATCAGGCGGGCGCGAGAGTAGAAAGGCGCGC
GATAAAGCTATGAGCATAGTAGTCTTGACCGCGCCAATCCGAAGGTGTTGA
CACAACGTACGGACGACATAGCGACTTTCCTCTCCACCCCGAGCATCCTGA
AGTAGTCGATCCACGATTCGCTGTTTGACTTCCGCGCTTCCTTGCCCTTGGG
CGTTGGCTACCTTAACGAGAGATTGGTAAACGCCCTTGATCGTTAAGGGCC
GTGGCTTCCTTAGGGTAAAGCTCTGCTTCTTCTTTGCCTCAAACGCGACATC

ACCCGCATCGCCATGTTTATCGTAGATGGCCTTTAATGATCGATTGTCCAAG
CCGCAGACATTCCTAAGTGCTTTGGAGATAGCTGAACCACCTATCCCGAGT
TCAAGCGAGATATATGGGGGCGAGATTGAGTTCGTCGCCAGCCAGACCTAT
ATTATCTCATTAGCACAAGTGGCAAGGCCTCAAGATAGGATGATAGCTTGCC
GCCGGAAGTAGGCTGTCGGGATCTGCTTCTATGAGAACACGGAGACAGTT
GACTAGAGTATCCACTATTTTTATCCGACTAGATGTGCCATTGACAAGCACA
AAGCAGCGCGTTAAAAGCGCATACGATGCATCGCCCCCCTCATTCAACCAG
TGTTCCCGAAGTTTCGGCACGTAGTCCGTAGGGTCGAATGTTAGAGGGCTC
TCATCAAAAGGTATGGTTTCTGAAATGCTGTCTACGGAAGCTGAAACGGAT
TGCAGCGACAAGGTGCCTTTGCCTTTATTTCCAATCTTCGTTACCTCTGGAA
CAGGATCCGGAGACTCCCGAAGCTTTTGGGAAGTTTCCGCAACGGCTGGT
GTGGTGACGGATGTGGATATTGCGGAAGGCGGTGGAGATCCGGAGGCGCG
TCCTGTAGTTTCGGTTTCCTTGTTCCACTCAGCTTGGAGCTTTCGGGCCAGT
TCCTCATCCGTCAAACCATGACCTTCATCTTGTATCAAAGAGAGCTCATTTT
CTCGCTTATCGTTCGTTTCATTGCTTCCAGGGTTATTGTTCTGATTCTGCTTC
GAGAAAAAGTATTCCAATCCCCTAGAAGGCAGACCCGACGATTTAGAGCTT
CCTGTATTCAGCTTACGCTTTTTGGTCGGGCTAACCATCGCGAATATAGATG
TCTTTCAAAATCAACAGCGTCAAGGGGTCATTGCTTTAAAATATCCATCTAA
GTAATTGTATCCTTCGCTCTCAGATTTTGGTGAGTGCCTTCATTGTTGATGA
AGGAATTGAATTGATGTTGTCGTCATTCAACGAACCATATCTGTTTTCTGCA
TCGGACATTCAAGTAACCGATAGTTACATATCCGAAAGAGAAATTGATGAC
ATAAGCAAGACAAAAGAATTGTGGGTATCAATTTTCAATTCCGACTTCTCTC
TTATGTAATCAAATCAACCCCTTTTTCTCATTTTCCACTTTTGGCAAGTCTTA
TTTATCCGGGAACGTCTAGCCGACGACGTCAACATTGACGATTGTAGAAAA
TGTCGTCTAAATTCGACATATAGTAAAGGCATCGGTTGTAACACAGTTCACT
TGTCACGGCCGGCAATACGAGATGATCTGCAAGTGAATGTAGTTGATTTCA
ATCTTAACAATACTTGGAACGAACGACGCTGACTCTATATCCTAGGTACCTA
GATATCTGACTGCATTGAACCTGCCATCTTGACCAGACTATCCTGAGAACTC
ATCTTATGCTAGGCCGAACCAAGCCATGTCAAGCTGGGGGTGGACGGGCA
CAGTATGCCCAACATTAGCTGCAGAGTACCCCACCAATTTGGTGCCATTTCC
ATACACTATCTGAGTGTAGCCTGACTGGGGGGTATTGGTTACATTCTTGGTG
AACTCAACGCCTAGAAGGGCAGACCATTCCTTCAATTGCTCAGCTAGGTTC
TTGTAGTCCACGAAATAGTCGATGGTGCCGTGTATGTCTGCATGCGAGGATA
AGTCCCGTTGAAGCTGGGGTACATTGCTTTGACTTGCTGTGCCCACTCTTC
GCCAGTCTTGACGTTTTGCCCACTGGCACACAGAGGATCAGCGCTTTGCGG
GCTAGATCCGGGAGAACCTGCGAGACAGCCTGCAGCGAAGCCCGAGTAGC
ATGATCCAGCGGCGAATACGTCGGGATAGACCGCGAGGAGCACGTTCGTC
ATCATGCATCCCGATGAGGATCCGGTAGAATACACCTTGGTCGGGTCCGCG

TTATACTTCTCAATTAAGTATTTGACCATGTTTACCAAGCCCGTGCTATCGCC
ACCTCCATTGTTGGTAAGAGTCTTTTCGGACGCAACATCCTACGATATCATT
AGTTCAGCTAAGATCCTGAGGGCTGTTTCAAGTTTTATAAGAGGACTAACC
CAACAATTACTATCCTTCTTGGTGGATGGATAGACAACTATGAACCCTTGCG
CATCGGCATAAGTATCATAGTTTGCCATCTGATAGTATGCTGGCCCAGTGCC
ACCGCAGTAGTGCAGCTATATCCCCCTTTAGTCTCGAAACATCTATGATGGA
ATCGTCGCTATCTTACAGCAAGAACAATCGCAGGTTTCTCGGCTAACTTAGC
AGGAAGGTAGACCTGCATCTCGAGGTTCGTCGGGTTCTCACCCCAGTCAGC
CACTTGTGTCAATGCTGCGTCTGTCGTGGAAGAAGATAGCAGCGCTGCTAG
AAGACTGATCACTGTGATTCCCCGCATGGTAAATGTAGCTGAAAGGTCTGG
AGGATTGAGAATGTTTCTTAGAGAACATGAGATAGTTGTCGAACCAGTCCG
GATGGGTTTCTGGCCTCGTAGATATTTATAAGAATTATTCTAGTATAAGCGCG
AAGCCGAAGCTGAATACCGACTAAGGTACTGTAAATGTATGCCTGCCAGTT
TAGGTGGGCCGGAGTTCAGAGTCGGCTAATTGGGTATCCATCCAGAGCAGC
CAAGCGTCTGATGTAACCGGGCAATGGAGATGGGTCGAATCGTCTAAATTC
AATCCAATCTCGGATCCCCACCTACGGTTCCATCCCATCATGACCAAATGCC
GTTACGATGAGCCGCCTACATATCTATCTCTATACAAGAGGACGACAGCTTA
TGAGAAGATCCAGCTCCAATCATTCTTGACTGCAGCTTCACATGGTAAACC
TTGAAATCTGCTAAGTACTAAATGATCAGTGATCCTACAACTGAAATCCATA
TCTCTACCTACACACGGTATGTTTCGTGGATAGGGAGTTCGGTTAAATGAAT
GGCCACTTGGAAGCAGACTATTAATTCCAGACTACCTATCTCACATTTATTC
AAGATATATGATACATTTTCTTGGTTATTATCTTGGTTATTATTAATCAGATAA
ACTGATTTACCCCATAGCTAGTAGTTAAGTACCTAGGTAAGCACCAGCCTGC
TATTTCATATAGTAATAGACGATTATATACTACCTTGGCCTCCAGTAGCATTCT
AATCTTGTTTTTTTTCACGTAAAAATCAACCCTAATTCCAATTTATCATAAGC
CAATTGTCATGAAAAGAGCCTACTACCCCAGACTAATGATGACCCTGGATA
GCCTCTCGCCGTTATCATTATCCCAACAACTCTTGTATTACTATTCAACCAAC
CGATCCTTACCTCTAGAAAGGTAAGCATCATGATGTGTGAAATGTGTTGTGC
TGTGTGGACTCAGAACCATTTGTAACTCCATTGTTGTTACCATTATGAACTC
CAACTGTATGTCCAGTATTGCGAGATTGGTTGATGATAGTGGATACGTCAGT
GGTCTCGAGGCCCTTTATCAAAGACCCTTCAAGCCATGGGTCACAACCATC
AATAATATCCTTCAGTTCTTGTAGATACGCCCTGTCAATCCCTCTACATTCCT
CGTCGCTAAGCTGGCGGAGCTTCTTTTGGTCGTCTTCTGGGATGAGTTTGT
CAAGGCCATCAAGTAATTCTGATATGGTTTTGACAAATGCGTTGAATTGGTC
TTGCTTATAGAAGACCCACTTGACCGCTTCGATTGTCGTCGATATTTTAACT
CTCCGCCTGCGTAGGAAGTCCATAAACCGGCTTCGAATGCTCTTAAGATCA
AATGGCATATGCAATTCCGGGTCAAGAACCTGTGCCTCCTCGGAGTTGTCT
TGTTGAAGCTTTCTTTGCATCTTTCCCACCCTACGTTGCGTTGTGCGCATGG

TGGCGCTAATACTCTCTAGCAGTGCTACGATAGCGTTGCGATTGGACCCATC
TTCTCCATTATTTTGCTGAGCCTTATCATTTTCACCGCTATTTTTGCTTGTGCC
AAAGCCGGCAATCTCTCCCCAGCGGGAAAGACGAAGTCGTAGGATATCGA
GTTTGAGCTGAAATGTTTCGAAGTCATCTTTAAATTGGGTTGCGAGTTGAAT
GTTTCCTAGAGTATCAATGGCAAATTGGAAGAGACTAAGAGCAGTAGTTTG
TAGCGTCCATGGTGCTTGGAGATGTTGATTTGATCTTTTTACGCTTGGGATG
GCACGCAATAATTCGTAAAAAGGAGTTCCCGGATGCCTTCAGCATGGCGTC
TTACGTCGTACAGTACTGCACAGGAACCTGATATATATAGGTACTGGTAAAT
GAATATATCTACTGTACTCAACCAGTCAAGGAGGGTAGGTCAAGTAGTATTG
TGATGAGCCGAGGCAGACTAGTAGCTAGGTAAGTAATTAATAGCCACAAAG
TAGGATGGGGGCTCACAAACTTTGACTGGTGAGGCGCCGTACCTCGCCTTC
GTAGATAGTATGTACCTAGTAAATAGAGGCAGTGAATAATATACAGATTCAG
TCAAAGTCCTGGTAGTGGGGAAACTTACCTACCTACTAAACAATGTTATAAT
CTAGTTGGCTGACGGCATGTGGATCTTGGTTCTGGCAGACTTGTATTTGGA
GACCTTGGCATTAGGCATAATTTTGACGCCTCTTTCGAAGATGCCTAGTGGG
TATCGTTAACAGGAATGGTGATGACATGGCGACAGTCTATATATTTAAAGAT
TAGTATTGGATGGGTGCTAAATAGGTACGTATGATGAGTTATAGTAGAGGCG
CCGCTATTTGGCGGAAATCCAAATAATTTAACATGAAAAGTACTCAGTAATA
GGCGATTAATTTGCCACGAATTTCACCAAAGTGCTTCCGTAAAGCGCATCAT
TCAGCTGATGAGGAAACCTTGGTTGAGGCAAAGGGACAGATCCCCTATCCC
CTCACTCGGCGGGCGATTTTTCTAGATCCGCTACCCTAGGTACCATTCTCAT
TACGACGTTCAAAGGTATACGGTAGTGACCCAATAAAAGAATTACGGGACA
TCTTATATTCTATACTACGCCACCTATTTAACAGCGCATATTTCGGTAAAAAC
TCCTATGGCCTATGGCTAGCCATAACGCGTGTTTCTTTATCTGGTCAATGGA
CGAGAATGGTGATTCTCACAATCATACGTTTGAGTGCCAAGTTTCTCGCGAT
CGCCACAATCAATTTGAGACCGCCATCGAAAGTTACAGCTTTGATATATGTA
CCTACCTACGTTCGATGCACGTCGTCATACGTTGATTGGCAATTGAAGATTT
GCCCGACAGGTCAACATGTCGGCGATCTAAAAATGTATAAATTACTAGGTA
GGTACTAAGTATCTAGGTATGTATCTAGGTACCTAATATCCCTTAAAGTGGCA
GTTGTCCTCAGCCAGGGATTAGGTCCATTTATACCTATTCACCTGACGAGTG
GACCAGACCCAGTATATTAAATAGCCAATTGCATTAAAGGTACAAGCTTTCT
CACTTATTTTGCCTTGTGCCACTTGTACCAACAATGATCAGACCTACAAAGT
GGTACATTACACGTAGCACAACGAGATCTGGTCTTCTTTTTGAAGCTATTCG
CAGTACTCTCAGGCAGGTCGGCTAGCCCTTTACGAGAACGCCCCCAAATCG
TATTCCCTCTACAAGCTTGGCAAGGAGATACATCCTTACCGTTGCCCCTTAC
CTGGTAGCGTTGTGGTACGTGTAAATTACCTCCTTTTGAAGCAGTCCTCGA
GTCATGCATACGAGAGTTAGATACTGAACCGTAGTATCCCAGCCCTTCTTCG
AGCAGTAGATCTAGCCAACCGGGTAACGATTCAGCTGCTTTCCAAAAGGGC

TGTCCCCTTTTCTGAAGATAATACGTATTAGAACAAATTAATTGTGGCAAAA
AGCTCAACTGCAACGCTTCAGCCGCCTGCCTTCGGTATCGATGCTCGAACT
TAAACGAGCCTCGCAATTGGTCGTTTAGGTCCACGGCGTTCATATGATGATT
ATATTCGTCTATAAGCAACGGTATATCTAATACCTTTTGGTAGTTGTAGTCAA
AAGCCTCGCGTACAGACTTATTGGCATGCCTTCGGCATCGTACGTTCTTTCG
CCACTCGCTGACTAATCGTATAAACTGTTGAAAGAAAGTTGACTGGACCGT
TATCCTTCCAGATAACTTGGTTGACGAGATTATCTGGTGTTGGCCACTCTTT
CGCTTCGTTATATTTGAGTTCTTTGTGTGACTGTATTGCAATAGCCGCAATAC
CGCAGTCCTTACGGCACGTGCCTGTAGCACCTACACCTTGAGCCCGTAGCC
TTGCAAACAACTGCGGGCTGGAGAACAGGTTATCGACGTAGACATGCAGT
GGCAAATAGCCGTTCCTCTCGAATAATTGGTCCACAAGTGCCAGGACCACT
CGTTGCGTAGGATTGAGGGCATCGTCACCCTTGGCGTCGGATGGCTTCAAA
TTTGCAGGTCTCCTCGGCACACCAACAGGTCCTGAGCCAGGTCCTGTGCC
AGGAACGTGCCAAAAGAAGGCCAATATATACCCAGATTCACTGTAGGCCCA
AACCTTAAATCCCGTAGGGTCTGGCTTAGTAGGTATCGTAGTAATCTCTTTG
GATCTGCCAGTAAACTTGACGATAACCTCATCTACAGCACCTTTACTGCCGT
AGGTCCACAATTCTTGTGATACCCTCTGTATCCACTGAGACCACTCCTCCAC
GTGGTCGAAAGCACGTGCGTGGTGTATTGTAGGATCAGCTGTTCTGAATCG
CCTCTTCAGTGCCTGAAAGCGTCGAAGAGTCATATGCCTTGTAAACGGGTG
AAAAGGGGCTTCGTTGAGATCATTCCTCCAATAAGTTGCAAGGTGCTTTTC
TGGGTGGCGAGCCATATATATAACCACAAATAAGAAGTAATATAACTCGTTT
GCCGTTGTATTGTACCAATCGTTTGATCTCGTATTAGGGTGCTCCCAGGTGT
CCAGGTCTGGCTGCCACCTATTGGTAAATTCAGCCCACTTTTCGACGAGAT
CTTCAGGTACGAAAAGCTTAAATAGACCTAAATGTGTATCAGGTAACGGGG
ATATACGAGGCTCACGATCGGGTATCTCTAACGGCTCGAAGTGGGTGCCTA
CGGTTGGATTAGGTCTCGCTTTAGGTCTACGTGTGGCGGGATCGGTCTCGTC
TGGTTGGTTATTCGCAGGATCTAGTTGGGGGGCAACGACGATACACTCTTC
TGCCGGCGGGGAATCCATATTAGAGGGTATTCACAACTTAGGGTTTATTGCA
GTACTGAAAGGTGGTAAATAAGGTGGTATAATACCTAAGTTTTGAGCCTCA
AAACAATGTACTTAACCGTATAGTGATCTTCAGGGGGCTGTGGGTGTTTTCG
ACCAATGAGAATCTAGCTTTTGTGGGTGTTTTAGCGCTTGGTCAGTGCTATA
ATGTACTACCCCTCCTAATCCCTGGCTGAGGACAACTGCCACTTTAAGGGAT
AATGGTTGGAGACTTGCGCCAGTTTGGGGGATTGGTGATTGGCACTCCCCA
CTAGAGGGCGGGCCATGGTACCAAACATGTCAACTGGTCCACAAAAGTTAT
TGAATAGTAAGTGACATGGTTATATACTAATACAAGTATGCAACGGAAATGA
GGCTCAGCGTTGAGGCTGATACGTGATATGTCTTGTTAGATTCTAACACATG
TAAAGTTCGCGGAGACATCTCATGATTAGTATACTCAGCTAGGTTATATTCA
AGTGAATTAGGTGTCGAATTGACAACGTTGGTTCATAATGTGTGTGATTATG

TTAGGCACATCTACAAAATACTCTACAAGGAACAAAATACTTACTTCCACCT
ATATGAAGCTCCCTTTTTACCCTTTAGTGCAGACAGATGGAAGTAGATCCCT
TTTGAGTAATTCAAGGTTGATGTGGTATTGAGACACAGTCAATTAGCATCTA
GTTGTCATGACAGTGGCTTCAAGCAAAACATGGAGCTTTCGTATTACTTGAT
ACTCTTTTAGGCCATGGTGTCATTTAGTAGCAAAAGGGACAACCCACAAAG
CAAATTTGAATAATAGGCAATGACTGATGATTTGTGTCCTAAATGATGTACT
GCCTACCTAGTAGGTATCTACCAGGGTTTGCACGAGCTGGGCAGCGGTGGC
CTATAGGGACGCCCAGAGCACCGTCATGGGCACAGCCTGGGGTTCCGCTTG
GCAACAATATAGTTGCACAAAGCTCGTAGTGAGTCTAAATCTACTTGCTTCA
TGTCGTAGCTGGTTCGGCGGCTGTATAATGCTTCAACATGATTATTACTCGA
CTACCTAGTGTCGAGTCGGTAAAGGTATTGTTACATAGACACATTAGATTTG
TGTCAAGCTTTCCATAGCTGTGGTTGGAGCTGTGATTAAACTGAAACTCCG
GTGGCTGGCGGCTTTGAGAGAACGTGGAGGGGATGTGCCTCAAAGAAAA
GGGGTTAGCCGCCTGCTTTTCCACTTTCTTACAAAGCCGCCAGCCACGGAG
TTTCAGTTTAATCACAGCTCCAACCACAGCTATGGAAAGCTTGACAATGTG
TACTGTTTAAATAAGACAGTAATTAAACTGTGCTTATCAGAATCGTTCTTTG
TGGCAGCTTGTAAAATTATTAACTAGATATAAATCCGAGTTTTATACCACAG
GGTGCTTTGAGAAACCTTCTAAGATGATTAGATAGGTAGCCATTGGGGGCT
AGGTAGTTACGGAACTTGATCTGGCTAATAAGAGATTAGGTTAGCTTTGGCC
CTTTCGTGAGAAAGAAGCCCTTGGTAGCTAGCCCTGAATGTGTAGACAAAG
GGAGTTCTCGTATGTATACTGGGTTAGTTAGCATAAGGAGGTTTAGAGCTCC
TTGCGCTGCAACACAGTTATTTTCCATGTACTTGGGTTCCAGTCGTCACAAG
GTAGATAAATAATGTACTCACTATTATTAATTATAAATACCGGAAAGGCGCTT
TTAATAACTCTATGTTACGAACATATACCTAGGTACTCAGGTATACGGGGTGC
TTCGTGGGTTTCCTTGTTTGTTAGCAGTACTCGTAGAGATCAGATCAAAGTT
AAGTCTGTTCGTAAGAGAGGCTTGGAGTCCTCTCCCTATTAGTTCGATGCG
GTTGAAGTAATAGACTTAGATTGATAGCTAAAGAGCTGAATGAATGACTAG
ATACATAGGAGCCTGCCATGTTTATATAGATATCCTTCTCTTGTGGGTTGGTC
CCCTTCCTTATGCTGGATCGTTGAATCGCTATTTGAAATGGCGATTTGAGCG
ATCCTTAAATCGCTTGAATCGTTCTTTGTGACCGCGATTCGAGCGATTTAGT
CACGTGATATTCGGGTGATGATTTTATCCGTGTGGTTTCTTAGGTGGGTTGT
GACACTCTACTGTCTAATTTGCATTGATGATACCTAAAATCTACGTTTGTTTT
ACTTTCCCATTAAAGATGCTTGCTTATATGAGTAAACACGTACGAAATCTAC
AGTAGAAATGGGGTATGCATTAACTATTAACTTACGTAAACATTGAGCGTAC
TCGAGATTTAAACGAGTCAAGTACTAGTTATTTCCCATACGGACTTAATGAT
TTTTAAGGTTTGGTTGTAATGACGTCGGTGGATCTTATGTAACCCCTTTTATT
ACGTGAGATATGATGAAATACGTTATATTTTAATCTGGCGCAGAGAATCGGC
GATACGATTACTCTCTTCGGCGTTATATACACCTAAAGTAAATTAATAGGTTC

CTACGTATGCATCTAGGTCGTAATATTGGATGGAGCTGAGATAGCATGTAAC
ATGCTAATCATGTAAATATATGTAGCTGATATTATATATACCAGGACAACTACC
CCTAGGAAATAACATAACGTATATGATTGACAGTAGGTACGCTACCTAGGTT
TCTCATTAATTATTTAAATTCGAGCAAAGTTCAATTTTCAACCACTATGTCCG
GTCATAGCTATACCATTAATCAAAACAATAATATTGGAACAACACAAGGGGT
GCATTATGGTGACAATGTCTCTTGTTAGTAACCAGATCCCATTATATATCTAG
GTACCTACCTCTGCTATTATTGACATACTCAGATAATATCACTAGAGGAGGTC
TCACTCGGAAACACATTGCACTCGAGACGCAAAACAAAAGGAAACAGAC
TTGGATGGAATCTGAAACGTGCGACTGGTTGTTGATTGAGAAAGAATTCAA
ATCTTGGGAGGAGACTAAGACAGAAAGTTCTTTCTTTGGCTTCATGGCCG
TCGTAAGTATTTCCATCTATCCATGTACCTATATACCATGTTATTAACTTAAGT
CAAGGTGGATGTGGCAAATCAACTCTATTGTATGCTCTCCTCTTATGATCATA
CTCACTTGAAGATTAACAATGTTGTTATATTAGGTCTCGGATAATTAGCAAA
ATATCGCAAGACATAGCTTGGAAATCTTCCGATTCTTCACGTCTATTATATTT
CTATGTTGACAACGATAGCGATCAAGACACGGATGATATTTATCAAAACATA
CTGATGACCTTTTGGGAACAGGCGGTAAAATCGGCCAATGGACAATCTGCC
GATCTATTTGGGAAATTCCCGACACATCAGTCGCTGGAAAAGGAGCTACAC
AAACTCCTAACTTCATCACTAGGAGAGATATATATCATTATTGATGCGCTAGA
CCAACTTCCTCCTAAGTCTAGATCTGATCTGATCCTCTGGCTTCACAGGTTA
ACACAGAAGATCGATGATAATATATGCGGTAGTCACCTTAGTGTGGCTATCA
GCTCTCGTGACTGCAAAGATATCGACATGGAGCGAATGAAATCTCTATTTCC
AGTTGAAGTCACTCCAGAAAGCAATAGATGTGATATTGAAAAATATCTAAA
AAAGAATCTTGATTCAGATCTCCTGAGCACAGAAACTGGACTTAAAGACCG
AGTATTTGAGGAATTGAGAAAGAATGCAGATGGAATGTGAGAAGGAGAAG
CAGAACTCGCACCGAAAATCTCGCGCTCAACTGGCCGCTGGCCCTCCGGC
AGGCTGCGCATATAAGCAAGGGAGAAGCGATATTGGAATTGGCGATCGTCT
GTTGGCGGAGGCTGTTTGGGTGCACCATTCGCCGATCCGTTCATAGCCTCA
GCAGGCAGCATACGCTGGATACTCTCTCGAGAGAGTGAGTACACGTCAACC
TGCCCTAGGGCGGGCAGGCCAGACAAGCCGGGAGATGATGTTGGAGAAGT
TGGTGAACGGATTCGAGGTGTGCGTCGGGTCTCTGCCCTCCGTTAGCAGTG
TCGCTGTTCCCGGGTGAGAGAGACGCACTTGGCTTCCTGGTCCGCTGCCAT
CATGGCTTTCCTCTCCTTCTGCTTTTCTGCCCACGTCTTCTTCTTCTGCTGTC
CCATATCCTCTTTTTTCTTCTTGACTTCTACTTCTTTGCCCAAACGTTGTAAT
GCTTCCAAGACCGTTTCTCCTCTCTCCATCAACGCGACAGCATCTCGCATG
AAGCTCTCTTCTCGTTTCCTTCTCTGATCCCGGATCTTCGTCTACCCCCATCT
CCTTGTCTTCTTTTTCTCTCTCTTGCCGTTCGCGCTCTTCGTGCGCCTTCCTC
GCTTTCAAGATTTCCTCCTGGTCGAGGGCCATCAGCCAAGCGTCGTGCTTT
TCATTCGGATCCTTTGTGTTCTCGACATAGCTCTCCCCATCGGCAGTGAACT

TGCCCTCATCCATCTCGTTCTTCATGTTGAATGGCGTGATTTCGAAACCCAT
ATCTCCGTCGAGCCCTTCTTTCCGTTTCTGCACTCTGCCCTCCTCGTCATCC
TCCTCGTACTCGTCTCCTCGAGAGCCGAACTCTTGTCCTTCGACGTCTTTCA
GATCCATGAATTCCTTTTCTTTGCTCTTGCCTTTCCCCTTGTCTTCCTCAACC
ACGTCCTGGGCGAACATGTCCATATCTTCGTCGTCTCCGCCGCCCTTGCCGG
CCCGGCGGGAGGGCACAACGCTCTCTTCGTCATTGGAGGAGTCGGACCCA
TAGCCCTCTTGATCTTTGAGTTGTCGACGGGCCCGCGTGCGCTCTCGTGCT
GCGCCTTGCGGGATGTCTGCCTCAAGCAGCGACTCGTCTGCCTCTAGGATG
TGGAGGACTCGGCAGCGGGTGACGGTGATGCAAATCGAGTTCGCTTCTGC
TGGGTCGATGGGCCCGGTCCAGCCCGTTTGGCGGAGGACATCTCGGGCGG
GAGGTCGAGGTATGCTGTTGAATATAGTGAGAGTATGAGTCAGAGGAAGCA
GACCGGCGGACGTGACGGCGAAACGTGGCCCCGGGTGGCGGAAACCTGA
AACTCGTGGTGACGATCCCTCGAATATAGGAGCAGCGATATGCATGGAGGT
GCTTGAACTTTGAAGATCTCACGTAGCAGTGCAGATCTAAAGTGCAGCATA
TCACTTTCTCAAACGGCCCACAAGTCCAAACTGAGCGCATCGTCATCTCTG
ACGCTGCTCGACTGGAACACCAAAGTCTTTTTGTTCAGTCCGATCTTTTGT
TTCGTCTTTACGCCTTCCCTGACATTTCTTGGTTTACTTTGATAGACACTCCG
CTGTCAACGTAGGACAGCGGGGGAAACCCCCTCATGTTGCCTTTGATACGT
GCTGGACGCCTTCACTTGAAAACAAACTCTTTGTCGCCCACATTACGAGCA
AGACAAGTGTCCACCTCCACCTCTCGAGCTCTGTCCAGTTACAGACACTAT
CGAGCATCAGCTGCCCAGCCGACAGCGTCAGAGCCACGCGGACCAGCGAC
ATCGCGTCTCCTTCCCACAAGGTCTACTCAGGATCAACCACACTCCTCACA
CTTCGCCTCGAGACGCACGCTCGCCAGCCTCGTTGACGCTTCGTAAGTACA
GCAATCTTCTGCAACCCAAATCCCAAGCCTTCTTCCTCTCCGCCACAGCCA
CAAACGCCATACAGAGATCTGCAGAGGATGCTGTCTGAGCTCGCTCAGATG
CTCGCTGCCTTTATCTTCATTCCGCTGATCTCGCGTTTCCTATACAGCTCGAC
CTCGAGACCGCCACCTGTCGTACTTCCTCCCGTCCCCATGACCGACTCACC
CTTCCCACCATGCCCTCCGATACCCAAAGCTGCGGGTCCGGTCGATCTCATC
CTGCCTCCTACCGACGATCGCCCGCACAAATACTTCACCTTGCCGAACGGG
ATGGAGGTGGTCGTGGTGTCAGATCCAAAGGCAGACAAGGCGGCAGCAA
GCATGGATGTGGGAGTGGGACACTTGTCAGATCCAGACGATCTGCCAGGAT
GCGCGCATTTTTGGTAAGAATATGCTCGCATCTGAAGTACATCGCCTGACCC
TCAGTGAACATCTGCTCTTCATGGGAACAAAGACGGTGAGCCACCCCTCA
GTCAGACACATGTTCAGAGCTTAGATCAGTACCCCGCCGAGAACGAGTATT
CCAGCTACCTGTCGAAGAACAATGGTGGCTCTAACGCTTGGACAGCCATGA
CCTCGACCAACTACTACTTTGATGTAGCACCAGAAGCTTTGGAAGGAGCTC
TTGAGCGATTCTCCGGATTCTTCATCGAGCCTCTGTTCAACGACTCTTGCAC
CGAGCGCGAGATCAAGGCCGTCGACTCTGAGCACAAGAAGAACATCCAAA

ACGACATGTGGCGGTTCTTCCAGTTGGAGAAGTCCATTTCGCGTCCTGGCC
ACCCCTACGGAAAGTTCGGAACAGGCGACTATAAGTCGTTGTGGGACATTC
CCAAAGCCCGCAGGCAGGGATCCGCGGCAGCAACTGATCTCCTGGTGGGA
GAAGGAGTACTGCGCGAGACGAATGAAGCTGGCCATTGTTGGCAAACACG
ATACCGATACGCTCGAGAAGTGGGTCCGGGAGAAGTTCGAGAAGGTGCCC
GTACGAACAGAAGGGCTACCTGCTGTGGGCACCAACGGTGTCCGAATCGT
TTTTGAGGACACTCCTTATGGTCCCGAGCAGCAGGGTGTAGGTCTGCTTCT
CTGGAGATGCTCTGACTGACAATCAGCTTGTCACCTTTGCAAAGCCTGTCC
GAGAGGACCGTGGCCTCGAGATCACCTTTCCCTTCCCCGATCTTGACCACA
TGTACCAGTCGAAGGTGGGAATGTACCATTGTTTCCCATGAGCCGGGACTA
ACCCTGCAGCCAGGCAATTTCTTGAGCCACTTCCTCGGTCATGAGGGCCGC
GGATCGATCCTCTCGTATTTGAAGAAACAGGGATGGGTCAATACGCTGCGG
GCAGGAATGTCTCACGGTGCCGCTGGATTCGACTTCTTCAGGGTCACCGTT
GATCTCACACCGGAGGGTCTTGGTACGTGTCCCAGACGGGAGGAACTAAG
GCTGACGTTGTAGAGCACTGGTCGGATGTCGCAATGAGCGTATTCCAGTAT
ATTTCCCTTCTTCGCTCTCAACCGCCTTCCAAGTTGGCATTTGACGAGATCA
AAGCCATTGCCGACATCTCTTTCAAATTCGCTGAGCGGGGCTCGACAAGCC
GGTATTGCACTGGGCTGTCAGGCTGGATGCAAACTCCGGTACCCCGGAGA
AGATCATCAGTTCCAACTGGTTGGTGGAAGATTACCGTGAAGACGAGCTCC
GGGCTGCGCTCTCTTTGCTTGACCCGAGGAAAGCCTTGCTCGGAGTGACG
TGCCGAGACCTACCGAAGAACGTCGAAGGGACGTTCGACAAGAAGGAGC
CCATTTATGGCACCGAGTACAAGCAGCAGAGGTTGCCAGAGGAATTCATCC
AGAAAGTGAGTACTCGGCCTCATACATGCAGTGAAACGGCTGACATCAGG
CAATGAGCGGTGTACGGATAGACAGTCTACATTTGCCTGGGCCCAACTTGT
TCATTCCGGAGAAGCTGGACGTCACGAAAGCCGACGTTGAACATGTAAAT
ATTGAGCAATCTTAATGATGCTTCAGCTGATTGTCAGCCCGCCCTTCGCCCA
GCGCTCCTTCGCGACTCTGCAGTATCTCGCCTATGGCATAAGCAAGATGACC
GATTCTGGCTTCCGAAAACACAGTGTGACATATCCTTGCATTCTCCCCTTCT
CGACGTCACCCCACGGAATCACGTCTTGTCCAAACTTTTCGCCGATCTCTAT
AACGACCATATCACCGAGGATGTATACGACGCATCTCTTGCAGAGTTGAGC
TTCACGATGTGGCACAGTGGCGATTCGATCAGCATCTTCAGCGGCGGTTTC
AGCGACAAACTGGCAGTGTTGACGGAGACCATGTTGCAGAAGATGGTTGA
ATTCAAAGTCGATCCGGAACGCTTCGACAAGATCACCGACACTATCAGAAA
TGAACTGGAAGAACTTCAACCTGAGCGAGCCGTACATGATGAGCCACTGG
TGGAGCACATATGCTGGAAGCGTGCCGATGTGGTCGCATGCTGAACGTCTA
GCTGAGCTTGAATGTGAGTGGGCGACTAGAATGCTCGATCGCAGGGCTGA
CGACCTAGACATCAAGCCAGCCGACGTGGAGCAGTTCGCTCGGGATGTGC
TTGCGCGCTTGCACATCGAGACGCTCATTCACGGCAACACGGACGCCAAA

GGTGCGCAGGAGATTCAAGATATGATCGAGCGAGTTCTGCACCCGCGCCCC
CTGTCAGAGGCCGAGAAGGCCGCACGGCGGTCACTGTTGCTCCCGAGTGT
GAGTTCATGCCTGCACGGCACAGCAACAGAGCGTTGATCCCCAGCATCCG
AGCATGTCCTATCAGTCCCGGTGCCGAACGAAGCCGAAGTCAACTCCGCG
GTGTGCTATGCTGTTCATGTCGGCGATCCTGCCGACCAGAACCTTCGCGCG
ACTCTTGAGCTTCTCGAGCAGGTTTTGAGGGAGCCCGCATTCGATCAGCTC
CGCACGAAAGAGCAACTGGGCTACATTGTATTCACCTCGAGCGTCGACGG
ACCGGGAGTCATGGGATACCGGGTCATCATCCAATCCGAGCGAGATCCGGT
GTTCGTCGAGACGCGCATCGAGGCGTTCTTCGATTGGGTCAAGGACCACCT
GGAAAAGATGAGCGCGGAAGACTTTGAGGAGCACAAGCGGAGCTTGATC
GCGAAGAAGGAGGAAAAGCCGAAGAATCTCGCGCAGGAGAGTAAGCGCT
TCTGGGCCCGCATCTCCGATCGGTACTACGAGTTTGGCAAGCGTGCGTCCC
ACTATTGTCAAGCGGATGAGAAGTGGCTGATTACTGCAGGCGAGACCGAC
GTGGCCAACTTGCGCAACACGACCAAGGCAGACCTCATCGACCTGTTGAC
AAGTACATCCACGCCTCGTCGCCCGTGCGACGCAAGCTTTCGACGCACCTC
AAGTCACAATACAAGGGCGTCAAATTCGACCAGACGGCTCTCGCGCCTCT
GCTTGCCGCATTCACCAAGCACGCTATCACAGTGGACCAGTCAGCCCTGCA
GGGCGTGTTGGTGTCCAAGCCGTCGCTTGACACCATCAAGGCGTTCGCCA
CTGCCGCCGTTGAGAAGACCGCCGATCTGGCGGCTGAAGCCAAGGTCGAG
CTGGAGAGCATGATCGCCGAGCTCAAGGGGTCGTCGAGTGAAGAAGACGC
CAATGTCAAATTGCGAGACGGCAACGTGTGGATCGACAATCTACAGCAGTT
CAAAGACGGCCTGATCCCGTCCAAAGCGCCCTTGCCGATCGAGCCGCTCA
AGCTGGCCGCCAAGCTGTAGAATCACGATACAGAGTCTTTGACTTCTTGTA
CATTGCATACCATGCATCACCACTATGCCAATGCTGGGAGATGAGCCGATGA
TCTGCGGGATCACCTGTAGAACTGGCCGTCAGCTGAGACGACAACAAGGA
AGCCGTGGGCATACGTGGTCGGCCTGGAGGTTGGCGGCGATCTCTGCTTCT
GCGCGGTCGCCATTGCTGAGGAGCTTCTCCTTGTTGTACAACAACTCCTCG
CGCGTCTCGGTCGAGTACTCGGCGTTTTGGGCTGGTCATCAGAGACAGGA
ACATGTGGAGACAGCCACAGGTGAGGTGACGCACTCTCCACGATGGCGTC
CACGGGGCAGGCCTCCTGGCAGAATCCACAGTAGATGCACTTGGTCATGTC
GATATCGTACCGCGTCGTCCGCCTCGATCCGTCCTCCCGGGC

>Mosaic_sequence_55

ATTCATTTCATTTCTAAATTCCTTTAGAGTCATATAGATTGAATCAACAGGAA
CTTCTCAGCAAGATTTGGGGAAATCTTATATATGCTATGTACCCACCAGAGT
ACCTACACAATACCTCATCGCTTTGCGACTTATTAAATGCCAATATGGGTTTC
ACCACTAAGAGTGATTACCTGGGTACTTATATATACGACTACCTAAAAATCA
TTAATATCTCTCATATGCCTATTTACCTAGTTTCATGTGCCATTTGTCTAAATG

AGGACCTCACGACGCTTCAGACCAGATATGAAGCATACCGTTACAAGGTGG
TCCCGTTTGGACTGACAAATGGACCTTCTACCTTCCAGAGGTACATAAATA
ACACGTTCCAAGGATATCTGGACGAGTTTTGTACGGCGTATATTGGTGACAT
CCTGATTTTCTCCAAGAACAAGAAGGAACATGAGGAGCACGTCCGTAAGG
TACAGACCAGACTGCGGGAAGCCGGGCTACAAGCCGAAGTGACAAAGTG
CGAATTCGATGGGTAACAACCTGAAGCATACGTTCTTCCGTGAGTTGAACA
GAGCACAATACTATGGCTCCCTTGTGTAGCCGTTACCGTCAACGAATCTCCA
TGGAGATAGGAGCCGACCTCAGACAGAATTGCATTGTGTTTGTCTATCCAG
ATGAGATAGACAATACGTATGTAGGATACCTATACCTATAACCTACATCTCAT
ATCGTCGAGCGCCTATTCATGGACTCACAAGGTATGAGAAGTTAGTCTGCC
TCGATACAAATTTCACCATTCGAATGCCGAGCGTCCTCTCACGGATTCTCTG
GGGACATCAAAAATTTATCGAATGGAGATCCCAGACCCGAGCAACGATGTA
ACCACGCTGTGGGTTCTCGCTCGCAAAGAAATATACCACCCGCGTATATTCC
AGATCTTTGCCATATGGACCCAACAAGCCGCGGACGTCTCGGACGACTCAT
GATTCCTCGCGAAATTTCATGGATAATTGTTGGCGGACAGCTGCCGGCGTG
GAAGAAAGCCAAATGCAAGATCGGCAACAACACCCCCGGAGCATGATCGG
GGAGATATCAACGATAGATTATCACGATGGGAAGAAATCCCAGTCGAAGTA
ATTCGGAGAGTCTGTTTATCGCGATATCCTCAAACCTAAGAGGGTAGACGG
ATAGGATCCGTTGATGAACGTCATCGCGAGCTAGACCGCGGTTTTTGCTTG
AACTAGGTAGCATGCCGCCCTCCTAAACAACGGTAGTGGCTAGCCTGAACT
TTTTTGGATCCCTTCCTTCTGGAATGTGAGGGTTTTGCCTGTTGGTATGTGC
TGTGAATACTTAGAGACCTTCCCAAACGTCTAGCTTCGGCTAATACATGTTC
AGCAACTACTTCCAGCTCTACCGCGTGACGGAGATCCAGAGTACCTCGTAC
TCTCACATCCAATACCACCCGTTGTTTCTCGCCAAAGGACACTTGGTGGATT
GGAATCGGAGGCATACTGTGTGCCACGAGGGCCGCATAAAAAACGCTTTTA
CACCGGCAGGACTAGAGTCAGTCTTATGCCCCCACGCATGTTCTGGGGGAT
CAACCTAGGGTCATCACATCTACCAAGTCCCTGAAGTTGTAGATTGTCAGA
ACAACCAAACCAATAATTAGAAAGCAATCCATATCATTGAACGTATTCGTAT
TCCATGCATCTAGACAGTCCGCGCCCGCCCTATCCATCACCCATGGCCCATC
GACGCCGTCTAATGCACAAGTCTATCTATCGAGGTATCTTACGACCGTCTCT
TCGTCACACGCAAGAAAAACAAAACGCATCACCCACATCTATGGCCGCCG
GTACCTCGTCCACTCCACTCCAGTCCATTTCCATGATTATTTTCCATATGTAT
GTATGCATGTATTATCCATAATTACTACGTAGACACCCCGATGATAGCTCAGG
TAGGCAGACAAGTCCTTACAACGCTACTGCGCCGCCATAGCTCATCGTGTT
GACCCCAACCATCCTCTTCACGCTGAGCCAGAGCTCGAACGCCTCGAGCTT
AAGCTCGACACGTAGGTACCCCACCCACGCGGCGATAAACCGCAGCGCGT
TGCCCGGCCACAGCAAGCTGCCAGGTCGCTCTCGTGCGTCCTCGACCTGTT
CGCGGAAAACAGTGCGCGCGCAGTACCTAGCCAAGATCATGAAACTGCGC

ATGGGGCCCTGGCGGGTATGCAGGTTTTCGTCGAGGCTGCGTGTGAGATCG
TTCGTCTTGAGAATGAGCAGGATGATGCGGGGGACCTGGCCGAGCATGGT
CACGAGATTCGAGAGGAGCCCTTCCTGCAACGCGGTGCCCATTGATTTCTT
TTCCTCCTCTGTGCGAGTCTCCAGGATTGACGACCTAACGACGGTGTAGTC
GCGGCCGGTGATGGCGCTGGCGAATAAGGGGAACTGCTCGTCTGTGATTCC
GGCGACCTCGTGCGCGTATCGCTTCATGGCCGGCATATCTCCGTCAATGATT
GCTAGCCACATCTTAGCGTACGAGCGTTGCAAGCTCTCGGGAATATCGCGA
TACAATCCGTGATCGTATATGATAATCTCGAAATTGGCGCCGCCCTTTCTTCG
GCGGGTTGCTTTTCGGATGGCAAGGTTGCCGCCGTGTGGATCACAGTGCAA
AGAGGCGTTCTTTCCGAAGATCATCTCGTTGAAGATTCGGGCGAGTGTGGC
GGAGACTTCGTCTCGATCGATGTGGTTCGAATCCAGATATTCAAGATCGTCC
AGACGGTGACCCGACTCGTTCGCCATGACCAAGATGCGCTTCTTTGCCCAA
AGGACGTCAGGAACCACAAGGGGCAATTCGGGAATGCGAGCAAAATATTC
CTGTGTGCGGGTCGCATTTGCAGCTTCTAGTTGAAAATCGAGCTCTTGCGG
TAAAGATACATCCATCTCAGAAGATAACCACTCTAGATCATACTCGGGGAAA
AACTTCTTCAAAGTCGAGAAGGTAAACCTCGTCAGGGTGAGATCAAGAGG
TGCCCATTGAGCCAGACCGGGGTGCTGAACCTTCACAGCTACCTTTTGGCC
ACTCTCCTTTACGGTTGCCAGATGGACTTGGGCAAGGGATGCAGCCCCTAT
TGGCTTTTCCGAGAACTCCGAGAAGTAGTCCTTAAGCTCTTCACCCGTGTC
AGAACGGAACATCTCCTCGATCGATTCATATGAGGACACCGGACATTGGTC
CTGGAGCGGTATAAACGTATCAGTCCATTCGCGTGGTAGAAGGTAAGTCAT
GGCGCTCTGTCGAGTGGGGTTAGTAGGTATTCAGTAGGCTGTGTCGTCATA
GTAAGGTTGTCGTATTACCAAGTGTTGTCCTAACTTAATGAAAATGCCTCCA
TTCTTCTCGAGGACCTTGAGTGTCCTTTCTGCACACCGCTTGTGGCATGATC
TCAAGATAGCATCTTGTTCCTTTGGGTCTTCACTCTCGTCTCGTTTATTCAG
GGTGGTTCGATAACTAAAGACTTAATTTAGTCATGATCTTTGACCCCTTTCT
ACTTGTTATACAAGCATAATAGGTAGGTAGGTAATACATACTCATTGATGCAT
ATTGCCAATGCTGCAGCAACCCTGCCCGTCCTCTCCGTAGCATCGTATCCAT
ATTTTATGTCGTCCGTGAAAGCCAGCACACCAGCACCGGTTGCTGCAGTTG
CGGCAGCAGTTAACACGGTCAGTCTCTTTCTTCGAGGTCTTTGTCGTTTGG
AGTAACCCGAAGAACCGGTCGCATATTGTCTCGCAAGAGGATGTTGTGTGG
TCTTAGCATTGGTTATGAGCTGGGTGCGACAATTCACGCAAGTCCATGATCG
GTTAGTGGCGAGACGGCCAAGCGAGTTGATCGAGAGGCATTTCATGATAAC
CCGGAGCTCACGTAGCTATCGTAGTTCTCGTCATTTGAGGCGGTAAATTGAT
TTTGACAGACCAATTACAAAATACTTTTTTATGAGCAGAACAATAAAGACAT
GTTGTCCTATTTCCTAGAATCTCCTTGCTGGTGGAGAATTTATTCAATGATTG
TTGAATCCTGAATTATGTTGGGATTGCACTTTGCACGAAATGAACTAAGTAA
TAAAATTTCACAATCTTTTTTTGGTCTATCGATCGGGATATGCAAGTCCCGGT

TCGTAGAATACCTCGGCGTCATAGGCGCAGGGGTAAGAGAACATTCTATTC
TGGATCGTGTGATGCACTGATCAATTGATGTACAAGCCTACAAGGTTGCTGT
GGCTGGGAACTTATCCTAATTTGGGACAATAAATCGGTTTACAGGTTAAATG
GTATTAGTCTGCTTTTATGTATTTAGAAAGACAGTAACTTCGGGGCTTCTTTC
GGTTTGAGGAATATACTTCTAGAGCAGTTGACAATGATTGGCTCGAATCAC
GAACAATCACATTATCCGTACAACCCAGTATGCCCCGCAAACCCCTTAACG
CGTTGTTACCCCTCTTACGTGCATTGCAGGGATCGCGTTGCTTTCCTACCCG
CGTTCCTCTTTATTAAACTCGACAGTAGAAATAGGCGGCATCTTTCTCGTCT
ATCTGACTCAATTAAACAATTAAATCATCATTACAAAGAACGACAAAACTA
ATAAACCTCATTCATCAATTCCTCCCATCAACTATCTAATATACCGCCATCAC
ATTCGAGATGTTAGCAACCATGCCGATATTCAATCTCGACTTCTCATCGCCG
CATAGGCCGGCGCATTCTTCGCCATTGTCATCATCACCTATCAGACCTAGTT
CCCCGCCGCCTTTAGGACATCGCGATCCAAACACTCTTCCCCGGTTCGACA
CTCAGTCTTCACCCACACAAGAATCATCCACCAAATTCAAGTTCGCTCGCA
ATGTGAAGCCGAATCCGTTGAGGCAAAACCGAGAGAATGCGCAAGAGAGC
AGACGCAACCTCTTCCTTCGGAATGTGCGGAAGCGGGCTGATGACAGAGC
GTGGGACAGAAGAGGAGGTGACCAAGAGGTATGAACAACTGGTCCAATTA
TTTCGTTTATATTACAATGCTAACATGTTCTGTCAGACCCTAAAGCTCGAATG
GTCAATATTGGATCGGCAACGAAGAGAACAAAAGAACGCTGATATCGATGG
TATCGTGTTCGAAGATGAGCTCGATGATATCCTAGAGCTCCAACGCCCACA
GATGGAGCAGCAGCAGGACCCCGATGAGATGATGGTCGATGCCATGGTGC
AGGAAGAAGAGGAAGAGCTAAATGCGATGCTATCCATGCTCGAGACCCAC
TCATCTCAAGCGCGTGGGGGAAAAGCGCCATCCACACTGTCCGATGATGAC
GACTATGACGATCTCTTCATAGATTTCTTATCGCAACAAGGCCAAACTAGCC
AGAACGCAAACCAACAATTCGCGTCGTCCAGACAGATGGACATGTCGTGA
GCTTCAAAGGACTAATTACATTGATTCCACAACAGCAAGGAGTTCTGCGGC
ACAGGTTATGATTTGGCGTTGCGGCTATATTGAAATCGGGGGTCTATGGTAC
GAGGCAGGGGGATAGGATAAGTTATGTAGCATCGCCTATTATATGGCAATGT
TAGGATTGGGACTTCACACAGCCCGGATGTATAAATGGCGAAAATGAGGGT
TCATATGTCCCCCTGTATATTACTGCGGCGCGCATGACTCATCAGGCGCATC
AAGCACTTCTTTGGCAGAAGGCATATCTCCAACTAGGTCATGAGGTGGCGA
ACCTGCTGCTTGCATCTGTTACTCTAGTTAGCGATGTAATAAACCAACGAGT
CTCATAAGAGTCCTATGAGACGATAAAACAACTCACTTTTTGCATTCGATCC
ACAATGTACTCTCGGTCCGCCACATTGGAATCGGCATATGTTGACTCCTCAA
ACTTGGCAACCATCTCTGCAACCAAAACTTGTTGGTCCTGATACCGCTTCA
AATCGTCTTGTGGCAGAGTAGCTTTATTCTTCTCCATCCATCCAGGGAACTT
GTCATTCAATTCTTTCATAGGTTCGTAAAGAATTTCCTTATTGGTTAGTTGTT
CCATCATCCCCATTAACATCTTGGAGAAGTCTTCTTCGTTTCCTCCATCGCTA

GGGAGGTCCTTCATAGCCTTTAGCATCTCAGCAATGAAATCGTCCTCGGAG
CCTTCGGCGGCCGCTGCAGCTGTGGCCTGGTCGCCAGATTCCTGCATTCTT
TCCATCGTTCGCCGGATCGTCTCCTGGAATGAAGCATCTTGGCTCAGATCCT
CGGCTGCTTTCTTGGATGACTGCGGTGTTGACCCTGATGCGTTCCCTGAGC
CTTCTCCGGTAGCTTCTCCAAGCCCTTTGAATATGTTCTCGAACTGGGCTTG
CATCTCGGGCTATATTGTATTAAATTCAACGTACTGGCATGCGTGGTGATGGT
CTCTTACCGAGTTATCCAGACCACTCATTAGCTCCTCCATACCAGCTTGTAG
CTGCTTCTGAAACTCATCAAGATCATCTAGTGAATCATCTTTGTCTGACACC
GTGGCTGCTGTTGCTGCAACTGGTGCGGGTGCATTTGTGCTAGGCCTTCCA
GGACCAGATGCTGTGGCGGGCTTCTTCGCATCCAGGTTTGCGCTAGAGAAT
TCACCTAAAAGTTCATCTAGGTCATCAAGGTCGTCTTCATCTGGATCTGGCG
CCTCATCTAATGAGGCCTGTAGATTGACAGCGGTCTCCTCCGTCTTGCTGAC
TTCATTATGCGGAGTTGAATCGACCTTAGGAAGCGTCGCTTTATCCTCGTCC
ATTTCGCTATATCAAGGGCGTGGTCTTTTCTGCCCAGATTGTAAACCGTGGA
ATATCGCCAAACCCAACTACTTAAGTAGGTACTCAGTACTTGAAAGTTGTG
AGCCCCGCGAACCAGGCGTACGGATTGGCCAAAACAGGTTTAGTCGTCAA
GCATGTGAATAGTGCGAGTTAACCAAGAATAGCAATACAATAGGGACCCGA
ATGATGATGTTTGCGAAGAATGTGGAGAGAAAGCAAGTAAGTTTACCATGG
AATGATATAGAATAACGGGTTTGAAAAGGCCGCTAATAGAATAATGAGATGC
AAGTTGCGATTCCAACTCTCGAATATGGACGAGTTGCCGAGGCCTGCAAGT
GCCTTCCAGGAAATGCTTGGCAGGAATGTCAAGTACCTAACAAGTAGCCAG
ATTTTACGTTATAATGCTGATTTTTACTGCAAACCCATTCTGAAATACAGTGG
CATTCATGAGACGGTTTGACGTAGGTACTGTATGAGAGCTCGGAATTCATGC
GATAAGATACCAATACCATAGATAAGGTAACCCCACCATTCTAAAGTTCCAG
TTGTGGCTGACTAGTGAACCCTGGCAGAAAATGTCTGGGTCAGGAAGTCA
CCGCCTTCTGTTTTCATTAAAAGTCCTGACAGGGTTCTTTTTGCCCTGTCCG
TTGCCCGGACTTAACGTACCTGCCTAGCAGTGATAAAGAGAAAGAGAAAC
TGCTAAGGGCGTGCTGGAAAAGCGCGGGAAGTCCCCTGCAAGAAGAAGC
TTCCGGTTAACATCAATTCACGTTCATGCATTTCTTGCCTTTCGAGCCTCAC
CTACGGAGGCTTAACCTTCTTTTCTTTCCTTCTTATCCTTGACACACAATTTC
CATACCATCATTTCAGATTCGTTAATCAACTTTTTCTCTACCTTTTTACTCGTT
GTGCGGCCTTGAAGCCATCAAAGTCTATGACCTGATCGCGCATATCCTAATA
GATTCAAGGTCAAGATCGTATTCTCTACTTTTCATAACACTTTGCGCGAAGT
CTCACCGCACGCTCACCATTTGATTCTATCACAAGAATAGCGGAAAACCCA
ACCACCATAACAAGTACAAATCCTACACTATCGTTGCCAAGCAGTTTGTTCT
GTAGCGATGTCTCTCTGTCAATTGCGCCTCGGAGAGGAAAGGTAGGCTCTA
CCAGCTATCGCTGGATCTTCACGCGAGGCATTTGTCATCATCTAGTCTCCGT
ATTCACTAGCTGACATCTATGTCATAATAGAAAGCAGTGGCGAAAGGACCA

CCCTTTCGGATTCTGGGCTAGACCCGTCAAAAATGCTCAGGGCGTCCTAGA
CCTGAAGGTTTGGGAATGCGGTATTCCTGGCAAGGAGAAGACGATCTGGG
AGGGGGGTCTGTATAAACTAACCATGACCTTCCCTGATGGTACGTTATAGCT
ACGATACGAGTTATTTCAGAAGCGAGCAATACTAATCATTGCCTCACAGAG
TATCCTACGAAGCCTCCGAAGTGTATGTCTACCTATACCTATAACTTCGCACC
CTAAAGTTTCATCGGCTAGGTACTAACCTTCTCTTCCAGGCAAGTTCACACC
GCCACTATTCCACCCTAACGTCTACCCTTCCGGCACCGTCTGCCTCTCAATT
TTGAACGAAGACGAAGGATGGAAACCAGCCATCACAGTCAAGCAAATCGC
GCTTGGCATTCAGGACCTCCTAGACAACCCCAACCCCGAATCGCCTGCGCA
AGCCGAGGCCTATAACCTGTTCAAACGTGACAAGGTTGAATACGAGAAAC
GTGTCAGACGAACGGTTCGGGAGAACCCAATCTCGTAAGCTTAAAAGTTC
AAAAGCGTTTCGGGTGCTAGGCAGATGGTCTCTCGGGGCTGGGACTATTAT
ACTTGGCGTGCTTTCGGGATTGGAGTTTAGGCTACGGATACCACACGGATG
GCTGCTTTCTGCATTTGAGCCCTCGGAGTTTGACACAGGCGATTACGCGAA
AGATGTCAGACATGATTTTTAACGTTGTGGCCACGGGAGTCCACGGTTTTC
TTTCACCGGCGAGAATGATCTAGCTTGTGTTTTAGGAAATCGTGGCATGAG
CTCAATGCAGATACATTCTCCGAATATTTATAGAGTCTACATTCAGTATTCCC
GTTTATGACCATGACGGTGCTTGTCCCTTAAATATGAACGTCTACTCACCAC
CTCGTTCTCTTTCGATGTGAATACTAATATATCCATGCTTTTCTTCGTAAACC
AAAAGGGTCGGAGCCACTTATCCAAGATAAATAGGGGTATCTTCTACCCAC
TTTGCTTTCAATATTGTTTAAGCTGACCTATAAAGAATAAAAGTGATATCCTG
CTGCAGATATCTGTACCACACGGGACGATCGAGCCCTGTTTAAAGGCTATTT
ATTGGATGGCACATGGTTATCTAGATATGATGCAAATATTACACAAGACACT
TACATTCCTAGGAGCTTCATTTATTTCTATTTTCAAGGTATCACGTTTTGTGT
AAAGTTTATGTAGATTTAGAAGTTTGAGAGGTGAGCATTCCGACATAGGAC
GAGTTAGTCTATTTAGAACTACGTGAACTAAGTGAAGTATCTAGAGAATGAT
ACATATGGACAGAATATACAAATCAACATTAAATACCAACAACAAACATCCA
TGAACTTAATACTGCTTAATATCTTACATAGTGTATATATGAGATTAGTCCTGG
AATAGATACGCATGTGTACATATGCACCAATAGATAATGGGTAATTGGTAAG
TGACTTTATTTTCTTTCTTGTCCGAAATCGGCTACCTTCCGACTCGACGGTC
TGAGCTAGCGAGAAAAAAGATTATAGGTACCATCGAGCTAGGAAACCGCCC
CTTACCTGTGCTATCTGGCCCATCTGTCAGAGCAAACCCAGTCTAGTATCAG
AAGGCAAAAATAAGTAATAAAACGTACTAATTGACCCTGATAGCAATAGAA
ACTGCCAGTGTGACTCAAAAAGTCTCCTGTTCCGGTATTGGCGTCTAGAAT
TCTAGAGTCACCTTCGACGCTCCACCGACCTAGCACCTTTAGACATCTACCT
TTTCTACCCACGCTTTTCTAGATCAGGTCGCGTGGCATACCAGTCTTCTCTT
CCCAACACCATCACACCTTTTTTCTCTCGTTGCATTCGACGAATCCGTTTCG
TTCATACTTTGACCCTACCTACCTGCACTACCACACTTCCAGACGAGCAAG

ATGTGTGGAATATTTGCTTGTCATAGGTAAGCTCACATTTCTCCCCTGCATG
AGAGGTCGAAAAGTTATGGAGGGGCAATTCGCGAGAATAAAGAAACCCCA
CCAAGAATAATTATTATCGATTGGATTTGCTTGTGCTGCCATTGGGAGAAGA
CGCCGTATACTGACAAGAGACTTGCGCCCAGCCATCCCGACGTCTGTAAAT
TCAAGCCGTTGGCTTTGAAGTTGGCGAAACTGTATGTATAATCCGTATTCGA
GCTTTAGAATTCCAATTGACCTGTTTTGCGCAGTATTCGTCACAGAGGACC
GGATTGGAGTAAGCCATAGCACGGTGAACTGACTGCAATTTGTATGCGAAT
GCTAATTACTCTTACGCATTCTAGGTGGTAGCTGTATCTGCAACAACACCAG
TAAGGGTGACCTGTCTGAATGAAACGTATCGAGCGTAAATTAGTCTAACGA
GTGTGCCTTTAGTTCTTTGCCATGAACGACTAAGCATTGTCGGCGTTGGTAT
GTGGTCATCCTTTACACCGAGGTGCTAAGCACAGCACCGTATTCATACTCAC
GATGTCATAGAGTCCGGCGCACAGCCCTTGACCAACGCAGACGAGTCTATC
ATCCTTGCCGTCAACGGCGAAATCTACAACCATCGGTTGATTCGAAAGAAC
TTGAAGCACCCATATCACTTCAAGACTGCCTCAGACTGCGAAGTCATCATT
CCTCTGGTAAGGACAACCGAATAAGTGTGTGAACACGGGCGCAAGACACT
CGGCCTAACGTGAAATTAGTATATGGAATATGGACTCGACGCACCTAAGTAC
TTAGATGGCATGTTCTCGTTCGTGCTATACGATAAGAAGCTCGATCGAACAA
TCGCTGCCCGCGACCCTATCGGTATCACCACTCTATACCAGGGTTGGTCCTC
TAAGCAACCCGGTACTACATACTTCGCCTCTGAGCTGAAGTGCTTACACTC
GGTTTGCGATGAGATCCGCTCATTCCCTCCTGGACATATCTATGACACCAAG
AGTGGTGAGACTATTCGATATTTCCAGCCAAGCTGGTGGGATGAGTCTAAG
GTCCCCGAAACCCCCTTAGATCTTAAGGCTGTTCGAGAATCACTAGAGAAG
TCTGTGAGGAAACGGCTTATGGCTGAAGTTCCCTATGGTGTGCTATTGTCGG
GTGGTCTTGATTCGAGTTTGGTGGCGTCTATCGCGCAAAGAGAAACATTAC
GGCTGAAGGCGGCCGCTTTAGCTGCTGCTAACAGTGCATCCGATGAAGCA
AAGGAAGACCCTGACAAGGGTGAGGGCCTGGTCGGTATTGACGACGATAA
CAAGCTCTCTACCGTAACATACCTCCCACAACTTAACTCTTTCTCCATCGGT
CTACCTGGTTCTCCCGACAACGAGGCCGCTATTAAGGTCGCCAAGTTCCTC
GGCACGAAACACCACGTTATGACATTCACTATCGAGGATGGTCTCAACGCT
TTATCCGATGTCATCTACCATCTAGAGACGTACGACGTAACAACGATCCGTG
CCTCAACACCTATGTACTTGCTGTCGCGAAAAATTAAGGCTATGGGTGTCA
AGATGGTTCTTAGTGGCGAGGGAAGCGATGAGATTTTCGGAGGATACCTGT
ACTTCCACGCTGCCCCGGACAAGAAGGCTTTCCACGAGGAGACAGTTCGC
CGAGTCAAGAACTTGCATCTCGCCGATTGCCTACGAGCCAACAAATCTACT
TCGGCATGGGGTCTTGAGGCCCGAGTGCCCTTCCTCGACAAGGAGGTATGT
TTGTCCATAATTCGAAATCGATGCTCTTGAGATGCCATATACTGATACCGTCT
AGTTCCTTGAAACCTGCATGAACATTGACCCTCAAGAGAAGATGATCACCA
AGGAGCGAATTGAGAAGTGGCTGTTGCGAAAAGCGTTCGACACATCGGAC

GACCCCACCGCGGAGCCGTATCTACCGGACGAGATCCTCTGGAGACAGAA
GGAACAATTCTCCGACGGTGTCGGCTACGGCTGGATTGACGCCCTCAAGG
ACAACGCCGAGCTCCACATCACCGATGAGATGATGAAGAACCCCAAGCCC
GAGTGGGGTACCGACATTCCCGATACCAAGGAGGCGTAAGCTCCACCATTC
CCGTACCCCAAAAATGCTTTTGCTAATGTTCGAAATAGTTACTGGTACCGCA
TGATGTTTGACGAGCACTTCCCTCCCCACTGCGCCTCAACCGTAATGCGAT
GGACGCCGACTTGGTCGAAACAGACAGATCCTAGCGGCCGGTAAGTTGCC
ATAACGAATGAACCATTAATTTAATATTGTAACAGTCGCTAACATGCAAACT
GCAGAGCTATCTCGACGCACGTTGCCAAGTACGACGAGACTGCATAATTCA
ACATCCTGTGATGTTGGGTAATTATGGTGAAACCAAACAAAAGCGCAGGAG
TCCAGGGTTTGAAATAGTGAGCTGAATAGAAGGGTCATTTGCTGAATAAGG
CAACTCGGATTGGTGAAAGGGAATTACACCATTATGGGAAACTTCGAGCAA
GTATGGAGAGATCTGATTGGCCTCTTGAGCTTTCGGTTAGTAGCCCTGGAG
AACAAACTTAGAGGCTTGTACCTGTAGCATAGACCTGTAGCTGCTGTAAGT
TCCCGTAGATGCCTTTAGATGGGGAACTGCCATAATACCAAGAGATTTGTCT
CGGAAGACGTTTGCACTTCGAATGTTCCTTCCCGACTATGACTAAGAAGTT
CCACGTTTGAATCGGTAGAATGGGTACCATTGCGTGCATAAGGTAGCCCGT
ATTTGAGGTCCTCTAGGTCGTTCCGAATAGAAACATTGAAACTGATCATGG
ATGTCTCCAGTTGGTCGTTCTGTTTTAAACCACTCCTGGTGTAGACCATCCC
CACGTGGACAGGGGTAAATCGAAAGGTGAGGTCGTACAAGGAACATCGAA
TTATCATGTACCCATGTACCTCAGTATGTACATGAAGTTAATTGCAGGTTGCT
AGGTGCTACACGCCGTAGTTGCCCGGTTGTCGACGATTAGTGCGTTTTTAG
TTCATCGGAACCAATTGGAGGCTGGTAAGGTGCGCTGTAGAATACCGACAA
GAACCCGAACGGGCAGAGGAAAGAGCCAAAGAAAACAGTAACAGTTTCC
ATCAGACCGTCTTGTCGTTTGGTACCAAATCTAGCATCACCCAATCCGATTC
ATTGAAAATCCACACACAATACCCCAACACAATTACTTTTTTTCTCATCTG
CATCTGCATTTGCTCTACATTTTTCTGTCAAAACAACAACATTCCGTATTGC
GAAATAACCATAACCACTGATCAAGATGTCTATTCCCGCCTTCGCTGATATC
TCCAAGTCGGCAAATGACGTGCGTAAATCCAGATCTTCAATGCTTCTTCGT
GCATCTGGAGCTACCCCCCTCCTCGGCCATCAATTTTCCAAGCCTCAAAATA
AAATTTCCCGCAGCTCGAACTTTTTGCTCCTGTCGCGCAAACGAGGCAAAC
AGGTCGAATAAGGCTAACCAAGGCGCGTACAGTTGCTGAACAAGGACTTC
TACCACCTCTCTGCCGGCACCATTGAGGTCAAGAGCAACACGCCCAACAAT
GTCGCTTTCAAGGTCACCGGCAAGAGCAGCCACGAGAAGGCTACTAGCGG
TGCTGTAAGTACTCCTCCCGTTTGACTCATTCGTCGTTATACTAGCTACGCC
AGGCATCAACTGCTATGCGCAAGCTATGCGGACAATTCTTCTTCCATAGTCG
AGTAAATTGATGGCTAACGATTATATGTATCGAACTATAGATCGAGGGCAAA
TACGCGGACAAATCACTCGGTAATGATGCCACCACCCCCAATGTTCAGAAA

TCCAAAACAGTCACCCGCAAGCGCCGGCCAACTGCAGTTTCGTTCTCATTA
GGTCGAATAAAGGTGCCGGGTCCAACGGCCTTGCCCCTCGAATTCGCTGCC
AACTTGAATCAGCAAGGATCAGGATTGCTAATGCTCTACCGACACGCAGGC
CTTACCCTGACCCAAACTTGGAACACTGCCAACGCCCTTGACACCAAGGT
CGAGCTCGCCGACTCTCTGGCCAAGGGTCTGAAGGCTGAGACTATCTTCTC
CTTCCTCCCCGCCACCCAGAACAAGGGCGTCAAGCTCAACCTTACCTTCAA
GCAATCCGCTTTCCATGGCCGTGCTTTCTTCGACCTTCTCAAGGGTCCCACC
GCAAACGTCGATGCCGTTATCGGCCACGAGGGTTTCCTCGCCGGTGCTTCT
GCTGGCTACGATGTCCAGAAGGCTGCTGTCACCGGTTACAACGCCGCCGTC
GGTTACCTCTCTCCTCAGTACAGCGCCGCCGTCACCGCGACCGATAACCTG
AGCGTTTTCGCCGCTAGCTACTACCACAAGGTGAACAGCCAAGTCGAGGC
TGGTGCTAAGGCTACCTGGAACTCCAAGAGTGGCAACACCGTCGGTCTTG
AGGTCGCCAGCAAGTACCCCACTATGCATCCGCCGCCTCCGCCACCGCCCT
CTCTCATCTCAGTCCCAGCTCAACCGCCTCCCGTTGCTCAGCCCCATGGTCT
CGGTCTCTCTGCGCCCCTGAGCCGGAAACAGCTCGAGGATACGCAGAAGC
ATGCTCGATGGGCCATCTCAGCTCTCGACTACGACGATATTGAGACTGCGA
AGAAGGAGCTCCGCCTCGCCCTCGCCAATCTGGGCGGATAGAATGATTCGC
AGAATCAGGTCATTCGAGAGCCTCAGTCATCCGCCTGTTATCGGCACTGAC
TACAATGGACGATATGCTGCAACCGCAAATTCGCCCAGCCCTCCTGCATCG
TCTGCTGCCATCGATCATGTAAGCACGCCTACATAGTCCCGCTCTACCCAGT
ACACAAAAGTTCAAGCAAAGCAGTCTGTCTCGCGCCTATCGTTCATCATTC
GTGCTGTGCATTATGTTGTCATTAAGAGTAGTGTCCAGTGTTTATCATGCCC
ATAGCCATTCATGCATTGTGCATAGCTGTACACCGGACTAGGAGGGATGGTG
GAGTGTCGCCTGCGAGATGAAACAGTGCGACCGAGCAGGGGTCCTTTGAG
CGGAGTTGTCATCGCTCCTGTCGCTGCAAGTGACAGATGCCTTACGACATT
TGCGATGTCCAATATCGTTGTTCATTGAGCACTTCCTGCACTCTGGCACTTC
ATCTGCCTCACAGCACTCGCTATCAGATACACAAAGTTAAGGTCCCGGACA
TAGCCAACCTTCGGCCGAGGAGGGTTCAATTGAGCCCGACGCATTCCGTCC
CAAACGGGTAATTCTGCGCCACCCGTCGCCGTGGATTTGGATTTTGGGCTG
CGTCGCCAAGCTGTTTTCTGAATAAGCACCCAGTGATCCAGTGAACCAGTG
ACGACACAACTCTCTTACACGACGACGGCGACGACGACGGACGGTGGGGT
CGTGCAGTGAGGTGTACAGCTGCGGAGCGAGGGGATCGTCGCTTCACAAC
ATCAACACCGACACCAAACACCAACACTCGAAGAGAGGACTCGTCTCTCT
TGTTGTTGGCCCTGAGCGACGCCGCCTCGGCCGACACCTTGCGCCTCTCAT
CGCATCCATCGCACGGCATAAAAGTCTGCTACTCGTAGCCATGAGCAGCTC
GCACACCCGCTCGGCGTCGGCCAACTTGGCTTCGCCCTCTCGGTCCATCCC
CAATCCCAACCACACCTCGCAACACTCGAGGACAGCATCGAGCTCGGGGC
TACTGTCGACCATCATGGCCTCGCCACTCGCGCTCTTCTCGCCCAAGGCGA

CGTACGGCCAGGTCCTGCTGGAAGATGACGAGGTCAAGCTCGGCGAGTCG
AGCAAGTCTGCCTTGCTCGACCCGGCCGGCTGCAAGCGCGTCGAGCTCCG
CATAGGCGGCATGACGGTGAGTCCCTCTCCCTCCTACGGCTTGGGGCTGCG
GGGCTGTGTGCTGAGCTCAGTGTGGGGCCTGCGTCGCCAGCATCGAGGGA
CAGATGAAGCAGCCCGGTATACGCAGCGTCCAGGTGTCTCTCCTGGCGGA
GCGCGGGGTCGTCGAGTATGACGAGCACTTTGTCGATCCCAAAGGCCTGC
ACTGGACCGACGCGCGCATCGCAGAGGAGATAGAGGACATCGGCTTTGAC
GCCGAGGTCGTGGAGCGCAGCGAGGTCGACGAGGTCGAGTTGCGCATCTA
CGGTCTCGAGAACGCCGACCTGGTCGACTCGATTCTAGATATCACCACCGC
GCTGGCCGGCGTGCACACTGCCGCCTGCCCCTTCCCCCATACACATCTCCT
CCTCTCGCACTCGCCCCTGCTGGTGTCATTACGGACGATTGTCGATACACTC
GCCGCGGCCTTCCCCCAGCTCTCCTTCCTCCCCACCTCGACGCAGAACGAC
TCGCAGCTCGCCTCCTTGCAGAAGCACAGGGAGACGGCGAAATGGCGCCG
CACGTTTGTGCGCGCGGCGTGCTTCGCCATCCCCAATTTCATCGTGGGCAT
GCTCAGCATGTACCTCCCCAGCTGGCTCATGGGCTGGACAATGTGGAGGCT
GTGTCGCGGCATATACATCGGCGATCTGGCCTGTCTCGCCTTGACCATCCCT
GTCCAGGTCTGGCTCGGCCGCGGCTTCTACGAGAACGCGTGGAAGTCGCT
CAAGCACAAATCCGCCACCATGTGAGCCACCCGCCCCGTCCTCCTTCACTG
TCCTAAGCTGACGTGAAGGGACGTGCTCGTAGTGCTTGGCACTTCCGCCGC
CTTTGGATACTCTGTCTGCGCCATGTTCTTCGCCATCTTCAACCCGGATCCC
GACTACCGTCCCAAGCTGTTCTTCGACACATCCACCATGCTCATCACCTTTG
TCTCGCTTGGCCGGTACATCGAGAATCTGGCCAAAGGCAAGACGTCCGCTG
CTCTCACCGACCTCATGGCCCTCACGCCGTCATCGGCGACCATCTTCGTCG
ACCCGCCCGCAGAAGGGGAGAAGCTCGACGCCTCTGCAGCCACGCGCAA
GATCCCCACCGAGCTCGTGCAGCTCGGCGACGTCGTCCTGCTCGTTCCAGG
GGAGAAGATTCCCGCGGACGGTGTGGTCCTCAGCGGCTCGACATCCGTCG
ACGAGTCCATGGTCACAGGAGAAGCTCTGCCCGTGCCCAAGACGGTCGAC
TCTCCAGTCATCGGAGGCACAGTAAACGGTCTTGGGACCGTTACATTCCGT
GTGACCCGCGCAGGATCCGACACTGCCCTCTCGCAGATCGTCAAGCTCGTC
GAGGACGCGCAAACATCCAAAGCCCCCATCCAGGCGTTTGCCGACCGCGT
CGCGGGAATCTTCGTCCCGATTGTCATCGCCCTGTCCCTCGCTACTTTCGTC
ATCTGGATGCTCATCTGCATGCTTCGCCCTCTGGATAGTCTACCAGGCGTCT
TCCACTCGCCAGGTGAGAGCCGTTTTGGCGTATGCCTCAAGCTCTGCATCT
CGGTCGTCGTCGTCGCTTGTCCCTGCGCCCTGGGTCTCAGCACGCCTACGG
CCGTCATGGTCGGTACCGGAGTCGGCGCCAGGAATGGGATTTTGATCAAGG
GCGGGAAGGCGCTCGAGGCCTGCCGCGATGTCCGCCGAGTGGTTCTGGAC
AAGACCGGCACGGTCACCGAAGGCAAGATGCGAGTGACAGATATGGCTTG
GGCGCCTAGCCCTAATCTCGCCACCCCGGGCTCAAATGTGCCGAACGAGCT

TGACGCCGCTGCAAGCTTGTCGCTCACGACGGCTGCGCCACCTTTACAGA
GGCACACGGTCTTGGCGCTGCTGGCACTGGCCGAAGCGCGTTCCGAGCAC
CCCCTCGCGGTCGCCATCGCCTCGGACAGCCGCCAGGTTTTGGCGAACGC
GCGACTTCTCTCGCCCAATGGAGAAGTCGTCGAATTCGAGTCGGTACCTGG
CGAAGGCATCGAGGCGCTTGTCAAGCTGGCCAACGGTCGAGGGCAAGAA
AAGATCCGTGTGGGAAAGGCCGATTTCGTGCTGTCAAGTGACGACAAGTC
GGCCGAGGCCTACGGGGCCAACTCGCTCCCTGGGGCGTTGCGACACTTTG
AACAATCACAAAGCGCCCAGGCGCGTACGGTCATCTTCGTCTCGGTCATCC
GACCGTCCTCCCCACCCATCCCCGTCCTCGCCGTCGCGCTGTCCGACGCTC
CCAAAGCCTCTTCTCTGCAGGCCATCACCTCGCTCCGCCGCATGGGCATCG
CCGTTACGCTCCTCACCGGCGACGCGGCGCCCACCGCCCGCGCCATCGCCC
GAGAGGTCGGCATTTCCGAAGACGACGTCTTCGCCGGCGTGTCCCCCAAG
GGCAAGGCCAAAATCGTGCGCGACCTGCTCGCGCAGGAGGGCGGCAAGG
GCGTGGCGATGGTCGGAGACGGCATCAACGACTCGCCCGCGCTCGTCGCC
GCCTCCCTCGGTATCGCCCTGTCGTCCGGCACCTCGGTCGCAATCGAAGCC
GCCGACGTCGTGCTCATGCGCTCGGACCTGCTCGACGTCGTCGCCGCGCTC
GACCTGGGCCAGACGATATTCAGCAAGATCAAGGCCAACCTCCTCTGGGC
GTGCTGCTACAACGTCCTCATGATCCCGCTCGCAATGGGCATCCTCCTCCCG
TGGAACATCCACCTCCACCCTATGATGGCCGCCGCGGCCATGGCCTTCTCC
AGCGTCTCGGTCGTCTGCTCCTCCCTCACACTGCGGTGGTGGCGCCGCCCG
GCGTCGAGTCTCATGCCTGGCCAAGCGGCCGAGGTCAGCGGAATCCGCGC
CGGGTTCAGAGAGGTCTATGCCGATGCCGGGGACCTCCTTGCCGATGCGCT
GCGGTCGCTACTGAGCATCTCGTCCGCCATTGAGCGACGAGCGTCCACTGT
CCCGCTTCTCAGGCGCATCAGTCTTCGCAGACCCGATCGCTCGGCGTACGA
GGCGATCCCTCTCGGTGCGGGAGACCGCAGTGCCGCTCCATCCCCGCTGCC
TCGGCTGGCGGTATGATCTATCTATCCACTCACGCCTTACGAGCTATGTACC
GGTACAACAGGATATGCATGTTGATTGTGCTATGTCTATACACCTCCGTTGG
CTGGCTAGCGATGATGCACCGTCCCGCCGCGGCCCCATTTTTCTTCCAAGA
ACTCGGTCATCCACTCGTGTAGCTCGCGATACGCCTCGCGCTTGTCCATCGA
GTGCGCTCTGCAAATCAGCATGAAGATCGTCCGCGAGGGAGGGTGGGATA
CTGACGAGTCGGTGAACATCCTGAACCTCCACCCTCTCGTCTTGCTCTGCG
TGAGCTTGTCGACCAGGCTGGCGCTGTTCATGAAGTGGACATTGTCGTCGC
CCGTCCCGTGCGCCAGCAAAAAGTCGGCGTTTCCAAAGCCCGTCATGTTGT
TGACCGCGCTGCGCTCGTACCCCTCGGGGTTCTCTTCTGGCGTGCGCATGT
ACCGCTCGGTGTAGATCGAGTCGTAGTACCGCCAGTCCGTGACGGGCGCA
ACGGCCATGCCGAGCGTGAAGATGCCCGAGTCGGCCTCGATCGTCTTGCAC
GTCATGTAGCCGCCGTACGACCAGCCCCAGATGCCTATGCGAGTGCGGTCG
ACGTATACCTTTTTGACCATCTCGCGGGCGGCGGCGATCTGGTCGACGACT

TCCCAGTGGCCCAGATCGTCCGTCACGGGGTTCCGCAGCTTGCGGCCCTTG
AAGCCCGTGCCGCGTCCATCGACGAGAACGATGATGTACTTCTTCTCGCAA
GCCAGATATGTGTGCCAGTCCCGTGACCAGACGTTGCTGACCATCTGCGAT
CCGGGCCCGCCGTACACCCTGATCAGGACGGGGTACTTTTTGCGACCGGA
AGCATCGAAGTTTGGCGGGGTGATCTGGATGAGATTCAGCTCGTTGTCGTC
CGACACAATCGTCGTGCGCGTGACAATCGGCCGCAGAAACTCGGCAAGTG
TGTTGTTCAGCTCCGCGTTGCCTTCGAGCAACTCGGAGAAGCCGTCTTGCC
CAGTTTGCACGAGGCGTTGCCACGGCACTTCCGGTCCGCGATACTGTAAGG
AGTAATATGCTCCTCCGGGGGACATGCGTGTCTCGTAGTATCCAGCGTGAG
CGGAGTCGGTGAGGGCCGTCAAGTCCGGGGTAAAATCGGCATTGGCCGAG
GAAGGGATAGCCGCCGCATACAGGTGACGGTCAATCGAGGGATTCGCAGC
CGTGAAGTACCTGTATATCAGATGAAGTCGCAAGTATAGAGCAGACGCTCA
CACTGTGCTCCTCGCCGCGTCGATACCATTGATTTCCGTCACCTCCCATTCA
CCCTCGGTGAGCCAAATAGGCTCCGTCGCTGAGAGAGGGGAGAACAGGGC
GATGTGCTCGTAACCGTCCTTGTTGGGAATGATGTCGAGATACGCGTTCTG
GCCGCCAGAAAGGGGAAGGACGCTTTGCCCCTACTTGATCAGCACGTGAG
GACGTTCAAAAGAGTGTTCTCACCTGCTTAATCCATCCGCCTTCACCCTCTT
CCCCATCTTTGCCAAGGGTGCGGACGGTCGTCCCTTCACTTTCGCCATACT
GGAACAGAACAGCTTTGCCGACCCGGCCAGATCGGTCAATCTCCTTTACCA
ATAAGGCATCTTGGGCAAGCCACGCCACCTCTTGGATAATGCGGCTTTCAA
GCGGGAGCTGCCCCTCCCAGTGCAAGATCTTCCGAGCCTGTTCGAGAGAG
CTAGAGGACCGGTAAGCTGACAAGGAGAACGTGTGCACGGTGACGAGAG
GGTTTGGAGTACCGGGCTTGGGATACCTAAAGTTATCTTCAATCACAGAGG
TGCAATCAAACTCACTTCATCTCCAGGTCCGTAAGGTAAGGATGCGACGTG
AAAGCATCGCTGGTTGGGTTGTAGTATTGTAGGGTGTATTCACGGACATTCG
TTTCGTCGCTGCGAAGATAGGCGATGGACTGGCCGTCTGGGCTCCACCATA
GGGCAAAGTCGGTGCTGAATACCTCTTCCTCGTACACCCAGTCCGGTACGC
CGTTGAAGACGCTTGCGCTCCCATCGTCGGTCACTCGGATTGCATTCATGC
CGGCTCCCACCTCTTGCGCAGTCACGATGTAGAGATCGTTACCGCTCACGT
AGGCAAGCGAATGCGAAACGGGCGACCAAGCGGCATAAGCGATCTTCGGC
GGCGAAGTTGGAGCAAGCACTGGCCATGTCGACGAGTCAGACACGCGGTG
CACCCAATAATTGCCGTGAGAAGAGTGGCGCCATTGTTTCTGGTAGTTGGA
ACGGAAGAGAACATACTCCATGTCGGGTGAGATCTTGTAGCCGTCCCACTG
GAGCCTGTTGCCCTTGTCGTCTAGGACCTTGGACGTGTCCACAAACAGGCT
ACTGGTGTTCCATAATTCGGCAGTCCCGAGGACGATATTTCCCTGGGCAAC
ATGCACGAATGTCCCGTCCGCCGCGCCCTTGACCCACTCGATTGAGCGCGA
GCGATAGCCAAACGTGCCGTTGAAGATGTGATCCATGGTGATATGCCGGTT
GCCCGACTTGGCCGAGTATGACGGTGCAGAGTATGTCCATGCTGACAGGGC

GCCGATGACTCCTGCGAACGCAACGATGGCAATGAGGACCGCCAGTATCTT
CTTTGATTTTTTGCGCGGACGAAGCTGCGTGTCAGCCCACGCTGGCTGTAT
ACATCACGGGGAGCATGTGAAGCGATGACCCACCCTCGACGGCTCCATCG
AGTACCCCTGTCCATCGGCCTCGTCGCCATCTTCCATTCGGGGCTCATCCTG
GAACCGCTCGTCCTTCTCGTGCAGGTCATCTAGGTGATCCCGGTACACGAT
ATCCGAATCGATATCGCTGGACGTGTCGACCGAGCGCCGTTGGGATTCGCG
TGACGGCCGAGCAGCGGCATAGCTCGTGTCGGCAAGAGGATCGTAGTCTC
CCGCCATGCTGCCGCCTGCTCTGATACCAGTTGGCGGACTTGGGGACGAGC
TGCAGCTGAGAGAAGGTGAAATGAAAGGCAGACTCTCACCAGAGAGAGG
GGCAAGGCCTCATGTATTTCCGTCGGAAGCGGCCGAACCACGCGCTCGGG
CCCGCTCATTGTCCGTCCTTGTGAATGCCTAGTGTTGCTTACTTACCATTGA
CACTACCAGCAGCGACGCGAGGCCTCGAGTAGGTGTCGCAAGATGATCAA
GTTCATCTTGGTGCAAGTGAGGCTACCGACTGCCCAGCGACACGAGAGCT
TACCACGCAGAATCGCCAAGGGAAGACACGGTTGTCGAAGTGGTATGCGC
CTTACGATGACGACGAGAAGGTGCGCAGCAGTATGCGGCATGTGCAGAGG
GCTGACCGCCAGGTGCGACTGAGAGGAGAGGTTCATCGTCTCATTGCACC
ACGAGATCAGAAATACCAATCCAACTTCGTGGAGGTCAGCTGCGTCTAGAC
AGAGTCGTCATGTATAGCTGACCGGCAGTTCCGAAGTGACAAGATTGTCTA
CCGCCGCTATGCCGGGCTGTTCTTCTGCGTCTGCGTCGACTCGAACGACAA
TGAGCTGGCCTACCTAGAGGCGATCCACCTCTTCGTGGAAGTGTTAGGTAA
GTGAACCCTATCCTCACGGCATGATCTGTGTTTGGAAGCACCCAACTTGGC
CGCGATGCTTGTTGAGCTCTGATGCTGACACGCAGACGCATTCTTCCAGAA
CGTCTGCGAGCTGGACCTCGTCTTTCTTTCTACAAGGTATATTCGTCGGCG
TAATCGAGGCATATTTGCTGAAAGC

>Mosaic_sequence_56

AAGTATGAGTTGGCGCCCTTGCATCACCAACCGCGATAGCTGTCCCTGCAA
TAATGAGAGATAGATTGTTTGGGGACAACTAGGGAGTTTTTAACGAAAATA
CAAGAATCGTATCCAACATAGGTATAATGATCTCTGATCCTGGTCGATGTTAT
CATTCTACTTACTTCCAGGAGGAGGATCTTGAGGATATGGGACATGGCGAC
GTCGTTGCACAAGGAGCTCTAAATGACTCCTTGCTATCTACCTGGGTAGCG
TATTCCAAAGTAGGTAAAAAACCCTCCTTCATCTAATCTACGAAAGTTAGCT
GCCCCAACAGGTCTCTCCCTAGGGATGCCAAGGCTATCACGGTTTTCTTCT
GGACCGATCCCCAATTCTCCGACCAGCTCAGATGAAAACTTAGGTTGCTAA
TTACCTCTCACGAGAAAGGCTTCCAATGCCCACGATGGAGCAGTTCTCACA
AGTCTGGATGCTTTCTATGTCATTAGTGAGCAGGAAAAATATGCCGGCCTCG
AGGAAGGAAGACGACTGCTCGTCGTCACTAACAAAAAGCTTCTCATCCCT
TCTCGCTTTCAAAATCTTGGGTTTGGCTCCTAAGTTCAGCGATCTTGACCTT

CTCCATTACAACAGCTCTGTGAAATGGATTAGCGACACTCCTATTGGCCTTT
ATCATCATGTTGGGACATATGTGAACTGGGATATCCGACGAGAACGATAAA
AATGCTTTCGTCGCTAGTTGGGATGCGTACGCTACCAATGCCTAGGGTTGGT
CAATGAGTGAAATGCGACAGCACAGTACCTACTAATTTTTCATTAAACTAGG
TCACGACATATACCATCCCATCTCAGAAAGAGGTTCCTAGATGAGCCCTACT
GGGCTTGGATGGATACTCATTGATAGCATAGACACTCTCATGCTCATGAACC
TCACTGCCCTTTGGCTGAGTGTCGCAAATGGTTGCATCGCAGCCTAAGCTA
TGATCAAGATCAAGATAATGAACACGTTTGAGACTACTATCCGCATGCTTGG
AGGACTGTTGTCCGCACACTACCTAACTACAAAGCTCCCGAACGTCGCGTC
TCGTAGAGATTCGGTATATTTAGCTAAAGCAGTTGAACTCGCTAACAGACTT
CTGCCGGCTTATGAGTCGCGCTCTGGTATTCCTTATGCTAGTGTGCATCTATC
TCGAGCCAAAGGCCTGCTGTCTCATACAGATGGTGGGTCTTCGTCTACCGC
TGAGGCTGCGACGCTTCAACTCGAAATGAAGTATCTATCTTATCTGACAGG
AAATGAAACTTACTGGAGGAAAGCTGAGAAGGTGATGAGGGTACTAAGCA
GTAACCACGCTCAAGATGGCCTTGTCCCTATCTTTGTACATCCAGAATCGGG
AAAGTTTACTACGAGAGAAATTCGACTTAGAAGTCGTGGAGACTCATATTA
TGGTTTGAACTCATACTATGGCGCAATCAAGTGAGAATTCGCTATATCTATTA
GAATACTTAATCAAACAATTCCTACAAACCGGCGGAGAAGAATCTGTTTAC
CTAGAAATGTGGGAGGAGGCTTTGGAAGGAATCCGAAAACACCTTATCAC
CACTACTGAACATTCAGGCCTTAGATTCGTCGCCGAACTTCCGCAAGGAAT
CGGTGGAAAACTGTCACCTAAGATGGATCATCTCGTCTGCTTCTTGCCTGG
GCTGATCGCCCTCGGTGCCACACGGGGCCATACCGTGTCTGAGGCACGTAC
ATGGTCTACCTGGGATGACGAAATGGAGACCCAGATCAAGCTCGCGCGTG
ACTTAATGAAGACTTGCTGGGGGATGTATGCTGTGACCAAGACGGGACTTG
CGCCGGAAATCGCGTGGTTTAATGTAACGAAGATGACCTACGTCCGGTGCC
CGGCAATCGACCCGCGGCGCCTAACAAAAATTCATTAGCAGCATGGAAGA
GAGACTACATAACCAAGCCAGCAGATGCGCATAACTTGCAACGGCGGAGA
CTGTGGAGAGCTTGTTCATGATGTGGCGGATTACCGAAGACCCCCTCTATC
GTGAATGGGGCTGGAAGATATTCGTCGCATTCGAGAAGCATACCAAACTAA
GCCAAGAGAAAGGTTATGATTCCGTCAATGATGTGAACACAGTTCCATCGC
CCACGAGGGACAATATGGGAGCTTCTGGCTGGTAGGTCATGGCAACAGCC
AACATAATCCCCGTCTCGTATAATCGCTTATATTGCATGGCTTCTTAGGCAGA
AACTCTCAAGTACCTCTACCTCTTATTTAGTCTTAATGATTTTATTCCTCTCA
CCGAAGTTGTTTTCAACACAGAGCACATATTTTCCCAGTCCTTGACAAGGT
TGAATGGCAGACGGGATGGAGCAGAAGTTGATAGTTGGGTGGTAATGAAC
CTAACCTTAGGTGTGGTACCTAGTGGCTATTTTTAGGTACCTCCATTGCTAC
GTGGTCGCTACTAGCGCGCAATTATAGCGACCGGAACAACCTCCCAGGACT
GGATTGACTGCCGTGTAGCAGGGGCCTTAGCTACGATTCTCCCTGTCTTCTT

AGAGCGGTATATATATTTAAGATTTCCGACTGTATGGATATGTTGGAATTTAT
CATATGGTTAAGACTGAACGGACTTGATGGTTCATTTTGTATATTCCGATACC
TTAAATTGTGATAATGACTGTTTGGCGAATTTCGTACCGTCCCCCCGCTGTT
GTTTGATAAGGTTCCTGCCCTTGCATCTTTCTGGCGATACTCTCCCTTTGTGT
AATACCCTCCCTTGGTAGGACATCACCAGACTGTTTAAATATAAAACCCTCC
TGTATTGCTTTCAGGTATAATTTCGTATCTCATGTGAGAAATCTTACATTAAC
TCTCTTGAATGTTGTCTTCTTCCTCGTGTCACGATGTTAGAGCTACCCACAA
AAAATGTTATTTTTACAGAATGGGCAACGTTGGTGTTTCTACTCGGCTCCTT
GTGGTACGTAATAAATAGCACCTAGACCCTTATCCCCCGACTAACGAGGATA
AAAGCTTGTCATAAATCCAATCTATAACTTGTACTTCCACCCCCTAGCCAAC
GTCCCGGACCGTTTCTAGCTAGGGTGAGTGCCATACCTAACTGGTACTATGC
CTACCGTGGAGATCGTCACATCTGGCTATGGCAGCAATTTCAAATTTACGGA
GATAAAATCCGCCCGGAGCCTAACACGGTTTTGTTCTGTGCCCCCGAAGCA
TTTACTGATATATATGGAATGAAGTCAAATGTGCAACGGAGCAAGTTCTACA
GTGCATGGAGCCGGAACGATGCGGACAAGAGTACACTTAACTGCGTTGGT
GTGGCAGAGCATGGAAGAAAGCGTAAGCTTCTCAACCTCTGTTTTACAGA
AAAGTCTATACGTGCAGCGTCCGATTTTATCATCAAACACGTAGACCGCTG
GAATCATCTCCTAGTCGAGAAGAACGCTCGAATTGGTCCGATCCGATGGAC
TTTTCGGCAAAGGTTGATACTTTATCATTCGACATCATGGGTGACTTAAGCT
TCGGGAGGTCATTCGATGTCAAAGAACCCGGAGACAATCCCTTGAAAGTC
GTACCACACAATATTGCACAATATCTACATTTCATTTATCCGGTAGGAGTTCG
ATGCAAGATCCAAATTCTCGATTACCAAAACTCATGATTTGACAGATTGCTC
GCTCTCCTCTTCTCGGTCTTATTGTCTGGCTTAAACCCAGGGGGCCTTGATG
CGCTACTCGAACTAATAACCCCACCAGCTGTACAAAATTACTACCAATTCAT
ATACGACAGCGTTACGAACAGGGATCTCTCTACAGCAAGAGCAAACTAAG
AAGCCGGAAAGCGAAAGGAGGAAAGATATGTTCTACTTCCTGTGCGAAGC
TCGAGACCCTGATACCGGGCTCCCCGCTTATGACGAAACCACCCTCCGCGC
GGAAGCCAACCTGCTCATAATCGCGGGCTCCGACGCTACCGCCGTTTCTCT
CACAAGCATATTCTTCTACCTCACTGACAACCCTTACCAATACGAAAAACTA
GTGAAAGAAATCCGAACTACGTTCGAGACAGTGGATGACATCGTTTATGGA
CCTAAACTTTCGTCCTGCACGTACTTGAGAGCGTGCATCGACGAGGGCATA
CGGCTTTCGCCGGCGGGACCTAGCGAGATGTCGCGGGAGGTGCTACCCGG
AGGTATCACTATCAACGGGGAACATTTTCCAGAAGAACGATCGTAGGTACG
GCGCAGTGGGTCAATTCACGTAGCGACATTTACGGGGACCCTAACGTGTTT
CGGCCGGAGCGATGGATCCCGGACGAGGGTTCGGTAACGCAGGAGCAAAT
CACGCGAATGCGCTCCATGTTCCATCCATTCAGAGCCGGCCCAGGCAACTG
CGTAGGCAAAAGTTTAGCCATGGTGGAAATGATGATTACCGTAGCTCGCAC
GCTGCACCGTCTAGATGTTCGGAGGGTCCCCGGGTCGACGATGGGGGGCG

GTGCGCGGAACTAATGTGGGGTGCGCGCGATAAGAGGCAGCTTCAGCTGG
TTGACGTGTATATCTCACTTCGCCACGGTCCTGAAGTGCAGTTCAGAAAGA
GGGTGGATTAATTATGTATTCGCGACTAACATACTATCCTAGACCACGGCCG
TCCAAATAATTATGCATAAAATTAACTACCTAGTACCTAGTAGGTACTCAGG
GGAGGCCATTTATTATCCATACTTCAGGACCCAAAGTTTAGTGGCCGCGATA
GCGGTCAACGCCCCGTAGAGGCTATTTCCGCGTTGAGGGAGGGAAACCA
TTTAGGACCATCCTTGTTTCGGCGACTTAGACTTTCCTTTTTTGATTAAGCG
ATCAGTTAATGCCACCGTACTTTCAGATACAACTCGGTCAAAGGAGTCTCG
ATCTTGTGGCTTAGATGCCGGCTCTGCTGGCTGCTGAGCACTCTGCTGCAAT
TGCTTTTGCACCGCTCTCGCAGAGTATCTTGCAGACAATGCTTTAATTTCGA
GAAGTTGTCTCTGTTATAGAATTAGCTTGGAACTAAAAGTAATCTGCCTGTG
CTATAACTTACATCAGTAAGCTCCACTCGGGTTTTGATTGTATATCCGTCAAG
TTGAGACTATTGTAAGATCAGGATGCGAAATTATTAAAGTTTTGGTAGATAA
TTTGTACCGATATGTAATACATGTCTTCCTCTAGTATATTCTGCATGTATCTTA
CCAATCTCGACGCCTTGAGGTATCTATCGGTTATTCGGTACTCAAACCATTT
GGTTTTGGTCTCTGCCATTTTATTTCGTGCATCAAGCCCCGCATTTTTCTTCT
TCATTGTGAATGAGATTCGGTAGAACTGTTCTAAATGAAGACATCCTGGAG
ACGATACTGCATCTTGCTAGATTTAAATAGAGATTTATGAGCGAAAATGCCT
ACCGTCATGACACAATATCTAGTTTGTAAGATCAACCTGACCTTATATCTTAG
AGTATTAATACCTTTTTAATGGTGGTATTTGTCTACATTCAGGGATTAGGAGT
GGTACTACACTCAAGCGCTGACAACCAGACCGTAGAATCTTTTCTCATTCA
ATCAGATGGTTTTGTAACGTCCATGAGTAGTCTCTTTCTATCAGTGGTGGTT
TATATGTTCGAGAATGCCTTAATCTCGCTTGAGGAGTTTGGTAATTGGATATT
TAGGCCAGCGCAGGAAATCGGGCAGGTCGGACTGACTGGGTAAGGTTAGT
TCTTATTTTTAACCTGACTGATGGCTTGTCAGGCTAAGTTTCAAATCTATACC
TATTTTTATATGTATTCTGATTGATTTATTAAAGAAGTACCTAGTTTTATCTAAT
GAATATCCACTTTCGGATTTAAGCTTATTAAACAACCCACTTCTCCCATTTGC
CCACCTAACCAATCTAACTGCCTGACTAACCCAAGTCAGGTTGCTCTAGCT
CAGCGAGCCAATCAGGTCAGTTATAAGGTCTGGCCTGGCAGCCTGGTCTGA
CTGCCTGATTTCCCGCGCCGACTTAGGCATGGATTGGGTTCTAGTAGGCAG
GCAGTGGGCTGACTAATTAGTACCTAACGGTCCTAACCGATCAACAACCCC
TCCAAGTCGGGATATCTTTTATCCGGCTTAGTCTCCATGATCAGCTGGAAAC
ACGCAGTATGCCAAGAGGCAACCAAAGCAACAGAGTAGGTATCTTTTGAAT
CATTCCATAAAACCTACAAAAGAGTTCTCAAATGTAATTATCTCTTCTCCAG
ATCTTCCCATAGCGTAGATAATTAGTTGCTAGTTCATCGGCCTATCTCGTCGT
CTTCGCATGGGGAAGCTAGCCCTCCTACTAAATACCACACTATCCAGGACA
AGTTACGGCACGAAACGCGTCGCCAATGGAATCGACTTCTTTGTCGCTAGA
GATAGTCCGCACCCTCAGAGGAGGCTTCATGCCTGCGAGATTATCAATGAG

GCTGGAAAATCATTTATTCCCGATGGGATACTAGAACATCTCTTAGATGAGA
ACACTGTCCGTTCACTTTTAAAACAGCTTGTCTCCCAGGGCGAACCTTTGA
TAAACGACGAAGAATATACACCAAAGTTATGCAATCAGAATCGTCCTTTTC
GTAAGATACTGACGATACTTCTTTGATCGAACTACCATGCGCGATCAAAGCG
TTTATGGACCTGGGTATTGATGATTCAGCTCTTCCGATGCAAGACCCAGAGC
GATTTCTCTCGTGCGAGCATTTAGAACCTTCCAGTGACGCAGATTCCAAGA
GCAATTGGAAACTTCTAGCTGAATCAGGGTTAAATCATGCAAACTTACATG
AAATCTATAGGAAGCAGTGGTGCGTATTAGCACCTCATTTTGGCGAACGAG
GCAGTATCCCTCATTATAAGTTGACGCAAAACGATATCCTACCCTTTCTACA
GATACCCTTGCAATCGCCTAGTCACTCTGAGGTACTTCAATCTTCAAATCAG
GCGGTCATATACGGATCATTCAGCCAGGTTCGAAAGGTAGAAATTCACCCA
AATCATCACGATTTTGGTGACTATGGGGCAAGTTACAGGATTATTGTTATCTT
GGAACTACTTAGCTAACGAAAAACGTAGATCAGTAACCCGGACAACATTTC
GCCATAAAATCCCTACAGACTAACAACTACGATGATTTTGCTCAGGAGATC
GAGATATTCAAACGCTATTGGAAATCTAAATTACATATTGTATCGCTTCTATC
AACCTTTGAAATTACAGAAACTGTCTTTGGCAACCACTGCCATACCTATTGT
TTAATTTTTCCCTGGGCAAAGGGCAATTTGATGACCTTCTGGGAAATGAAC
GGATCTCTCGTTCGGAATCAAAAAGTCCTCCCATGGATTACCCGAGAATGC
TACGAGATTGTTCGAGCACTTTCATACATCCACGGGGATATTGACGATCAAA
TAGCTTCGGCAGATTCTTTTGGAAGGCACGGGGATATTCAGCCCTCTAATAT
TCTCTGGTACCCAAATCCGCCTGAAACAAATCTCGATGATTTCGGCAAATTG
GTCCTTGCAGATTTTGGACTGGCTGATTTCCATCGTGCTTCTTCAAAGAGCA
ATTCAAGCATGTCAAGTCTTCCGGTATCTGTGACATATCGGGCCCCAGAATT
TGATACTTCTGAGGTAATTTCACAAAAAATCGATATCTGGGCTCTAGGGTGC
ACATTTTTAGAGTACATCACATGGTTTCTTAAAGGATATAATGCAGTTCAAG
GCGAATTTCGAGGATACCGCTCTGAATTTGACAAGTACAAAATATTTGCCG
ATATATTCTTCAGAGTTCAGACAAACAATGGGCTTCAGGATGTTTTTCTCAA
ACCTCAAGTTATGAAGTGGATTATACTCTTGCGCCAGGAAAGGTCTTGCAG
TAAATACCTAAAGGAGTTTTTGAATATCATCCAGGAAGATATGCTTGTTGTG
GACCGTTTGAAGAGAGGGTCGGCCGAAAAGATATGCCAGAAACTTGAAAA
GCTATACCGTCAATACTCAGTCGATATGGACTACTGTCAGCATACGAAACCT
GCATCGCCAATAACAAATGAGAAAGCTATGGCTCGGAAACAGACCCGCAA
GTTTTCTGAGGAAGGTCTTCCGTCGACATTTTCGTTACAAGAATGATGTTAG
GCAGTGAATTGTGAGAAATTATGACTTAACACGAAGAATATTTCATCGTAGG
TAATGTTAAGGGGAAACCGGGGCTAGGGCCTTCCGGTGGACCCATACGAG
GTAGCGGAGCGTAAGAAGCAGGCTTCGCCCACTTGAACCGGACCCACCTA
CCTACCTACCTAGGTAGTAGGTACTCTCATGACATACCTACTAGGTAGCTAC
CAATATTTACAGACACAACTTGCGAAGCTCAGTTCATGCGAGACGAAGGG

GGTGGAAATTAAAAAAAGTATAAATGCGTACCGCCCCAATAATTAGCATAA
ATACCAGCTAAGATACCCAAGAGCAGAATGAGACGGTCTCATTACGCTAAA
ACATATCTTCCAATCCGCTGCAGTGCCCAGGTGGGAGGTAGGTAGGTATATT
TTACGTCGCAGAGCAACGTGTATTTATCTAGGTAGGTAAATACACATCTGTT
GGGCCATATTCCCGCTTTAGGACGTCTAGAGTACCTAGGTAGGTAGGTAGGT
AGGCAGAGCTAGAGTATCGTCTGACATAAGAAAGTTCTTATTCCACCACTC
CTGCTCTCCGCGCATTACTCTAACCAGGTGCGTCGGCGACCTCGGCTAGCC
GATCTATACCAGGGAAAAACCCTTTCTCCGAGCCCATTTTCTTTCAACATTT
CCCTCGAGCGGAGTGAAAAACTTGAGATCGGACGGTTGGTTACCACAGCA
GGATTCGAGCCTACTTGCATTCGCGTAGTCGTACGCTAATGCACTGCCAGGT
GTTTAATCACTACACCATGTTGGCCTGTCACGTGCCATATGGACGACTGCGG
CTGACAAATTCAGAAGCAGGACCCTGGCCTTTACTCACATCAGTCAGGAGC
CATCCCGCGTCAAAGATGGTGTCGTTTCCTTGATTTCTGGATTTCATTTTCAT
ATGTGATCGTGTTCTTGTCATTTGCCCTCCTGCTAAAGTTGCAGTTTAGTGC
TGCGGTTAAGGACCATTCAAAGCCTCGCGAGACGCCAAGACAACAAACCG
TCAGGGTTTCCAATATACCTTCTCAAATTGACCGGGAAGAGTTCTACAATGC
CATCACAGGCCTATCTCTGTCCGATTCGGAATTATCCAGCGAAGCCAATCTC
GTAGGATGGTCGTATGCGTCGGCTGCCACGGGGCCGTACTTTGTAGCGACT
GCCACCTTCTTGAAGGCTGTACCGCTGCACGAGATTGAGACGTCTCTGAAG
ATTGACTTCGGTAGAGAAGCAGCAAGACTTAGAGTCGATACGGAATTCTTG
GGGCTTACCGTGCTGCACGAACTGCGGGAGCAAACAACTGTTGAGTAAGT
AAAGATGGTATCGTTAATATATGGGCGATGTCATTGAAAACTGTTTATCAAC
AGCATCATCGCCGTTACGGGGCTGGCTGGACATGCATTTAGCTCGTGGAGA
AAGCGAGAGGCGGAACCGCCATGTGGCTTCGCGATTTCTTGCCCGAGAAA
GTACCCGGATGCGCGGATCTTGATTTACGGCTATGATACCACACTCCCTGGT
AGCCAGTCTGAAGCTTCTGTAACCGATCTCTCACGCAAGCTTCTCGAGTCT
ATTAAGAGCAGCTGAAGTGACAAGCGAGCAGTAAGAAAGTTTCTTGGAAT
TGCCCAGAGCAAAAGATAAACTGACTGATTTGTCCCAGAAAAGCAGACCA
CTTATCTTCATTGGACATAGCCTTGGCGGGCTGGTCATAAAACAGGTATGCA
AGTTGTCTATTTCGTTCGTCGCATCAAAGCGACACGCCCAATAAATTAACCA
GACAGCGAACTTTTGTAACAGGCCTTGCTTGATGCATTTGAAGGCAGCGAA
GACGACAAAGATGTGTTCTTCGCTAGTACTGGTCTCCTACTATTCGGTGTCC
CTCACAGGGGGCTGGAGATATCGAGTCTGATGACGATGGTGAAGGGTCAG
CCAAACGAGGCACTCATTCATGCCTTATCCGGCTCATCTCGCTTCTTGAGTC
GCCTTCATCAGCAGTTTACAACTCGCTTCGCGGATGATGATTTAACCATAAT
CAGCATATACGAAACCAAAACTACAGCGACAGTTCAGGTAAGATGATTATT
TTATGCACGAGATTGAACCCAGCTCTAATACTTTCCTTGTTGTAGTGGTCAC
AAGACGCTGGAGCTTGACAGAGAACAGGCCCCGAAACCATGATGGTACCT

CATATGTCTGCAATCCAATCCAGCCCTGGTGAGAAGATGTATGACCAAATTC
CTATCAATTCAGACCACTCTGGGATTGTCAAGTTTAGCGATGAAGGTAATTC
AGACTACGTTGTTATAGCTTCGAGGATCGAGAAGCTAGTTAGGAGAGCGCC
AACAGTCGCTACAGAACGTTATGCAAGGCATCGTGAAGGTACATACATGAT
AATATATACCTAGTCTTTACATTGGCGCTGACGTAGATAGGAATTTCTCGACA
TAAGCAGCAGTTTATTCGATCCATCCATGCCCCAAATTACCAGGGTTTTCGG
ACTGACTTAGTCGACGATCCAACGCCTGGCACACTCACATGGTTCCCTGCA
GTTGCTGAATATGACTCTTGGTACTTGTCGAACGAGTCGTCCATGCTATGGA
TCAAAGGCCCGGCTGGTCAAGGAAAGACCATTCTCACCAAATTTCTCTTGG
GACATCTTGAATCTTCCACATTCGCACATTCAAACACAGCAGTGATATACTT
CTTCTTTCATGGCCAAGATAATTCTCTTTGTACCATCGGGGCAGCCCTCCGA
GCACTAGTCAAACAACTTCTCTCTTTCCAGCACGTCAATGGCTTCGATGTC
ATTTCGGTCCTGGTCCATCCCGAGCTTTCAACACTTGAAGAAGATATACTGT
GGGAAATGCTCAAAAGACTACTTGAGTCGGCCCTTTTCGACCGAGTTTACT
GCCTTGTGGATGGATTAGATGAGTGCCGAGACCCCGCATCACGATTAGCAT
TCCTGCGTTACTTCCAGAGGCTACATCAACCAAGACGTACAGGTTCTAGAC
AACACCGCGCTTCAGTCTTGAAAACTATTTTTACCGGCCGCCCAACAGTAG
AACTGGATAGGCAGCTTTCATCTACCCCAACTATTCACCTCAAAGCAGATCC
TCAGGACTTGGAGACGTTCATTTGCAGCGAGATTGACTTTCTTGGCTTTGA
TGGGGAGATGCGAACAAATGCGGTCCAATTGCTGGTCAGACGCGCCGAGC
AGACCTTCTTGTGGATATCCATACTTATCAAGAAACTTAGGCACGAAGCCCT
TCTCTCAGTCGCAGACTTGGAACGAATCGTGGATAGCAGTCCGACAAGTCT
CACCGAGCTCTACAACAGCATCGTCACAGATATCATGGATCATAGTAGCCTA
GCCCAACAAAAGCTTCTGGTATGGGCTGTGTACGGCCAGCAAACCTTGGA
CTCTTTTGGAGCTACAACACGCCATAGCCGTTCATGACGATTCTGACTGTAT
AGAGTCAACCAGAAAAACACATGGTCCATCTTACCAAACAGTCGGTTACTA
GCGCCGTTGGTATCATCCTCGATATAGGAGAGGATGATAGGCTCCATCTGAT
ACACCAGTCAGTGAAAGACTTTTTACTCGGAAGCAGCCATTTATCTGACGC
AGAGTTTTGTCAGGGACTGAAGCCGAGTGCGTACCTAGCAAAGACGTGCT
TAATATACCTTGGCTTCACAGACTTTCGAGTCATGACGAATCACAAATGCCT
ACCAGGTTCAAGCCAACCGGGTGGTTTTCTCAAGTACGCCGCCAAGTACT
GGTTCAGACATATTAGAGATGATCAGGACATGCGAAGCTTGGCGGGGCTCA
TCTTTCGGATAACGGAACCTCGCTCTGCCAACCTATCAACTTGGGCCCAAG
CAACAGGCCTCTTAAATCTGGAAGAGGCTGAATCTCGATGGGAGGTTGCCA
CCCTAGCCAACATTCTGTGGCTGGCCGAGTTCGATCTTGCATGTTAGACTCG
ATATTGGATGCGAGGAAAGTCCAGAATGCCATTTCCCGCGGAAAAAGAGG
ATACGAATTTTGGAGCAACTCGTACAGCGGCCAAATGTCCAGATAAACCAA
GCGGCTATACACGCCATAATTCGGTCTTTTGATCGGAGGATGGCATATCTCT

TGATCGTACATCAAGGTGCCGTACTAGATTCGGACGTGGCTGGGGCCATCG
CTGAGAACATCATCCATGGGGAATCTATTCTACGACTATTTTAGACCACAA
GGACGCGAGATCAGTCACAGCTACTCTGGAACTAGTCCACGGGCTCGCTTC
AAAACCGGAAAGATACGTTTCTCATCCAGGCCTTGATGTGTATACTATCCCG
GCTGACGGGTCCGATGACTACTCCTACAGCTATGCTCCGCGGCTCCGACGG
ATTATTTCCCTCCTAATCCGGAGTGAAAATGTTGAGTTGACCGATGCTGCGA
CTGCTACGATTTTCAAAATGTTCCGTCGCCATCCACCTGATATTGAGCACTG
GATCGAAATTCGCAAATCTGGCATAGTTACGGACCATGTCGTGCAAATCGT
CCTAGACGGGCTTGCGGACATCCATCTCAGTGTGCTGTGTACTGTGTTTACA
CACTTCAGAGGCCAGATTGAGATCTCTTCTAATCAAGTGAGGGAGATGCGA
AGACATGCACACTGTGAAAGTCTTTTAGGACCTTGGTTCAGGACTACGAGG
CCAAATTGGTCTTGAACGAAGACTCGTCGGTTCGATATTTTCTACGCTTGGG
GAACAGCAAGCAGCTTTCTTACTCGAGGCACAAGGCGAAAAGTTCAAGAT
CACAGAGCAGATAGCCCAAAGAGTTGCGGAGTTCGGAGAGCATGAAACGA
TGGAATTGCTTCTCGATAGTCGCGGAACCGAGTTCTCGGTCACGGAGAAAG
TGTTTGAATGTGCAGTGAGAAACGGAATGGAGATGGCAAGTCTCTTACTCC
GTAGGCGGGGGGCCGAGTTCAAGATCACCAATACGATATTACAGGTCGTGG
CAGCAAGCGGGAATTGTGAGATTATGGACCTACTCCTTACTGCTAGGGGAC
AGGAGGTCCGAATCACAGAGAATATAATAAAGCATGTGGCAGCCGCGCATG
ATGGCCTGAGTATGCTGCAACTCCTATGCAAACGACGCAGACAGGAGGTTG
TTGTTACTGAAGCTATAATGGTGGAGGTTATACGAAAAAGAATAGACATCTA
CTATATTACGTATTGGATACATTCGGCAGAAGCATATCCGTCACAGATAACAT
GTTGTTGGCGGCCGCAACGGAAGGGGAGGATACCCTCGAGACTGTGTTGG
ACGCGATATTTTGGAGATTTCCAGAGAGTCTGAGGTTAGTCATCACTGTCG
ACTTTCTCCTCCGCCCGGAGTTTGGTATTTGCCCAGAGCTTGAAGAACAGC
TTATGAAAGAATGGGTGCTACTAACAGGCTTGCGCACCGCGGTTGATGCTG
GAGATCGGAGAACACTTGTGCATCTGCTCTCTGAAGGGGTGATTCCCAATA
CAAGAGACTTCCATGGCGACACCTTAATCTCGCACGCGGCCAAGTGCGGTC
TTGCAGATATGGTGGATGTCTTGGCCCAGAGTGGGCATTTTGACATCGATCC
CCCAAATTCCTATAACTGGACACCACTCTATAAAGCCTATGACAAGGGCGTC
ACAAATGCAATCGAGCCACCGCAAGTAAGTTGCCTTCTAAGAGACTCTGTA
CAAGGAGTGCTGGAAGAATCACGGGGATTAATTGGCGGCCATGACGAGCG
GTCAGAGATAGGAGAAGATGGGGGTGATCCCAAGAGTAAAGATGAAGGTA
GCAACAACCCATCCTCGGCGGCAGAGATCAGATGGCATAATAAAAGTGGCC
AACTTTCAAGCCCCTCCACTGAAGGATACGACTTTTATGATCTTTGATGATA
TGAATTCGATTTCGGCTGCAATTGACGTACCGCGCTGTACGACTCCCACAA
GGGATCTATCCCCTTAACCCTTGAAAACCCCAAGCCCCCCCCCCCAAAATA
GTGTTCTAGCTAATGAGCGTTCTCAAAACACTGACTAGTCATAAAATTCTAC

AATGAAATCGATATTTTAAGACCTCGGTTATAGTAAACTTTTATTGAAGGTTT
AAGTGTGGTTGTGTTTGTGGTTGAGGTTGTGACGGGCTATGCCCGGTTGAC
TGGCCTTGTGGAGTCTCGGCTGCCCCGACCGGAGCCGAGCGAGGCCACTT
TAAACGATAGTAAAAGGACCCGTTAACTGGCCAGATCTGGAAAATATACAT
AATAATCAATCATAATTGAATGAACCTGTTCACCGGTGTTGCGAGTACCGCC
ACCGCGTCCCGCCGGAATGCAACATACGTACAGCTTCAAACTTGAATACCT
CAGTAAAAATAGCGTCACAGCGGATGCAACCTGGGTGAATGAATTAAGTCC
TGAATGCGCTTTTATATGAACCAAGTTGCGACCGATTTGAGATAGTTTTGAC
TATTCTATAATTGAGTGTAGTGAAGCGCGGCAGACAAAGGTGGCGAGCGAG
AGCGATGAGATGAAAGGACACGTTGATTAGGATAGCACGTTCGAGCGGTAC
TCACTTCCCAAGCAGCGAGCGATGGGCTATTCACGGTCTAGGCTCACGCCT
CCTGGTGCCTGGCCCGAATGAGATGCGGTGTGAGTCTGTACGAGTAGCGGT
GAATGCTGTGTCTGAAGTCTGCATTGTCATTGTGAAAGAGGTGGTCAATGT
CGACGATGATAAAGGCATGAGCGGGATTCAATTCGGCCGCAATTCGAGGTT
TAGATGCTGGAATCGTCGACGCATCCTTTGGCAGCACGGACAATCCACCAG
GCCACATGGGGTCCCAGCCGTAATCCACCTTTTTCGATCTGGGACACATGA
GGTGGATGCAAGATACAACACAATTGCTCGATAGCGATACGGAAGGCAGTC
GCGTCGGGGCCAGCTTATATCTTCGTGCTCCTGGTCAAACCCAGTCCCGGT
TGCCGACGGTCGGATCGTGTTGCGCAGCGATGCGTGGTCGGTTTCGAGGGT
CATCGACTGCGGTACGGCCCGGGATGCGTTGACCCTATGCCGTCCTAGGGC
AACCGGCACGTGTTAATTGACCCTGATGCTGCCGCAGCCTACAACCTCCAG
CTGCTCTGACGAAGCCTCCGTTACAGGTTTAGAGTTGGCCGGATGACACCT
GACTAGCGATCGAGGGGGATTCCCAGGCGAGCAGGTGAAACATGCTTGAC
GGCAAAGCCCCTTCGTCATCTTTCAAAGGGCCGCCAAGATGAACGAATGA
GAAATGCCGAGATACGATCCCAGACGCCCAGGAAATCCATTCCTCCGCAGC
TTGGCACTATCATGAGGGTATGGACAATCGACAGGGAGAACCAGGATGGC
AGGACAATACCGATGCCGCCTCGCCTGTGCCGGTGCCGATGGCACGCCGCC
GCCGCCGCTGCGGTATTACTGCAGCTGACTGCGGGAAGCGGGGACGGCTG
ATTAAAACGCTGGATACTCATCCTTAGGCAGTTTCTGCGGTTCTATTTCTCG
CTTACGCCACCTCTGGTCACACGACTGAACCTGCGATGCCATGCTGGCCAC
GCTCGGCCCCCGGTCATACTACCCCGTCTGATCCTGGTGTCCCTTTGATCTT
TGGACCGTGGACCCCTTACTCTGTCTGGGCAGCGTGACCACGTCGCCACTA
GGCCACTAGGCCCCACGTCCACGGCAGCCAGACGCCGAGGCCGATAGTCC
GCAGTGCGTCGCAAGTCTGATTTTGGCCCGCTCGGCCGTCATCAATCTGCA
GCATCACTGTATCACAATGTACAGCCATGGACGCAGTCTCATCCAACAACG
CTGACCTACAACACCGAGGATCTAATCGTTTCATCATCGACATCGGCCATTC
TACTGCTAGGTACTCGACCTCGTACCTCCCTTTAAGGCCAAGGCCGTCCCTC
TACACAGCTCGGGCCTCCCTTTGACCCCTCAATCCTTCTTCCCCGGTCAACC

TCAACGCGGGTCGCCTCGGCCTCGCGACCTCTTCCCTCGGCAACACCTTTG
ATCACAACACCTACCCTCTGATCTCTCATCGCTTCCTCTCGATACCTACTTGC
ACCGATACCACACCACAATGTCCAAGGCAGAGGAGATCCCGGCGCTCAGC
TTCATTCTCGAGCCAGCCCGTAAGTACACCACCGCCCTCCTACAATTTGCCA
CCGCCATCAACGCTAGAGGAGAGCCGCGTCAGCTCACATCGCAGTGGTCG
CGGGACTGCTCACGGCCGGATGCCTGCTCAACCGGCGCTCCCCCGCTGAC
ATCCGGCTCGTCCACTCGCTCATGACCCCGCCAGCGACGCCCGCCGTCCCA
GGCCAGGCGAGAGCCGATAGGGAGATGCGCTTCCTGTTCTGGAATGTCAA
GGTGCCGGACAACAGCCCGTTCAGGATGAACTTTATCTCGCGGCTTCTCGG
CATGTTTCCCTTTTTGTTGGAGGTCTGGTACTGGCTCTTGACGTGAGGACCT
CGAGACATGTACAGCGAGGAAGGGATGTGCTGATGGGCAGGTACTGGACG
TACCAGATCGCCAGGGCGATGCAGGCGCTCACCATGGGCGAGGGGACTAC
CAAATTGGCCGAGAGGCACTCGAAGCAGATCCTCGCGCTGGAGAAATGGC
TTCATATCGACGTTGAGCTCGCCCTGCAGCACGTCGTCATGAAGAACAAGC
TCGTGCTGCTCCTTGCCAACAAGTGAGCTCCCCCAACCCCTTTCCCCCGTC
GATGGGCTGGAAGCTGATGCCAAAGGACCTACGCCATGGTGCACATCCCCG
CCACCATCCTCTTCATGGCCTATGCGTACCGACACTTCTCAGCCCCGACGTT
CCAGTCTACCCGGCGTACACTGGTGCTGTCAAACTGCTTGGCATTCATCATC
TTCTCCACTTGGCCGTGCATGCCCCCGCGCCTCCTGCCCGCTCAGTATGGCT
ACATCGACACGCTCCACGTCGGCAAGGCCGCGAGTAGTACGCCTCATCCCC
TTTCAGTATCGATTGAGCCTGACCAGCAGTCTGGACGACCAACAAGTTTCA
GAATCAGCTTGCGGCCTTCCCTTCGCTCCATTCGGCTACTCTTTCGTCATGT
AGGTTTCGATCGTCTGTCTTCGAGATCTAGTTATTAACGACCAGCGGCGTAT
CGCTATTTGTCTACTCGCCTCACCGCCTCGTCAAGTTCCTCGCCCCGCTCTA
CCCGGCTCTCATCCTCACCGTCATCATGGTAAGACTCCTCTCTTCTCGCACC
CGTGAAAGCTGACGAGCAGGCAACGGCAAACCACTACCTGCTCGACGCGT
TCGGCGGTTTCCTCGTTACCATCTGCGCCTACCGATTGAACCGCCTGCTACT
CAACTTGCGTCCGATCGAGGAATGGTTCTTCTGGGCGCTGAGGACAGAGA
AGCCGATGGACAAGGCGGACTTTGACACCTGCGTCAACAGGGAGAAGCTC
ATGGCTCCGGCGAGCCCGGGACGGGAGAGGGACATGGCCCAGCGCCCGCT
GCTGTCGAGCATACGCGAGTAGGCCGAAGCTCGTTGGTACAGCATACGGAC
CGTTCTTCCCGATCCTGCCTGATTGCATCATGCACACGTTCCATCACGCACC
CGTGTCTGACCTGCATTGCGTTGCTTCGCAGCGCTGCGCTGGTTGACCTCG
ACATCCGGCTGACACGGAGGACCGCGAGATTTCGAATTGCATAGCAGACG
CTTCCGTTCCAACGCATGGACGTGCTGCGAGGCAATAGGGAAAAGGGCTC
ATCTCTTGCGACTTTATGACACACCGGCGACCCGCGACTCGGGATCCTTTG
GCCTATCGCCTTTGAGGAGATGGGGAATGGGGTGAGCACTGAGTTCTGACG
AGTGTGGCTGCGAGTGTGGCGGAATACACCGTCCATAGGACACGGCAGAT

CATCTCCACGGCGGTGCAGTACGACCCGATCGTCGCCGCCATCAACACGTA
GATGTCGCGCACGATCGACGAGCAGGTATGCGCAGTCGAGAAGCATCAGC
ATCAGCATCAGCCTCGGTACACGGAAAGCGGAGAAGCGACGACAGAGCCG
GCCGCGTGCTGTCTCGCACGCATCTACTTGAAACGCCTCGGCTCCACAGCA
CGTCCAGGGGCTGCCGGCGGCATCCCAGAGTCCAGAATCGCAACTGGTCC
ACGACTTCGGTAATTAAAACGAGGTGCTGCGTCTGGTTAATCGAAAGGAGC
TCTCAGCAACCTGTTGCTCGCTTCATGGCGACATCAGCTGACGACTGGCTG
GGTTTGACGTTTGCACATGCCTGGCCCCTGCCCGCGGCTCTGGTCCTTGTC
TTCTGGGCATTCGGGACTGCGCTTTGGCTGAGTTTTGGGGTGGCAGCTGAT
CATCCGTACAGCCGGAGCTGAAGGGAGGGACGGATGGGCGCGTATGCAAC
TTCTGACCAGAGTGGCGGGTCGTCGGGGCATACCACTGGCCGTCCACGCC
GGTCCAAACCAAAACCACTGACTGGACGAGGCGATCGGCAGTCACCACAC
ATACACCGCGTCCTCTGTCCTCTGGTGAGCGACAGACAAGACCAGCCTAGC
CGCGCCGCTCGTGCCGACGTGCGACGCCCGAAGATCGAGTCCGAACGAGC
GGACGACGGACGAGCTAAAAGGAAGCGAGCACACAAACGAGGCAGGCCA
AGACTTGATCAGACTTGATCAGACACGCCGCCGATCCCTCCCGGGATCCCG
GGGAGAGGGGTCACAACGAGGCAGCGCAAGCGTGCCAAAGGGAGGGTGA
CTCCCCTCCCGCCGTCCTTTCGCTTCTCTCTGACTCTAGTCGCATGATCTCC
TCCCTTCCCCTTGGCCCGCGGACCCTACAAAAGACGAGTGGGAGGCACGG
TGATGCGTGACTACTCATCACCCATCACTGAGTCGCTCGGCCGGGACGCAC
CGAGCACCGAGCCGCGGATTGGTCTCGTCCTGTCGTGCGTTTACGTTTACG
CTTGCCCTGCCTTCCTTTGGGTATGTCTCGAGCCTTGGACCATGCCACGGGC
AGAGGAGAGGATAAGGGGTACAGCCTGCTGCCTCGCAGACAGTGCGAGTC
CGACAGCTTCTCCGTGGTCGGGTGTGCGCCGTGTAGACAAGAGCTAGCTTC
TTTGCTCGACTGGGTCCCGCCGATCCCCTCCTCCCTCGTCTCGTTGGCTGTC
CTTTCGGACGCACAGAAAAAGGGCAGGCAGGCAGGCAGGCAGGCAGAGA
TCGCACAACGCACAACCTCACTCTTCTTCTTGTTCTTCTCGCGAAATCCGTC
GTGCAGGTCGGTCTCACCGCCTGACAACCGCCCGAAGCGACCCGCGACAC
CGGGGCCGAGACTAAACTGTACGGAGCACGGACCTCTTCTTCTCGGGAATA
AAAAAGAGGCGAGTCTTGCGTATATAATACCTTGTGTCTGTTGTCCCTGGTG
CTCCACCCACACCCACATCCATACAGACAGAGTAGATCGCCAGTGTCCTTT
GCCCTTGTCCAGACTCACCTCAGATCACCATCACCATCGGCGCCGACCGAC
CCAGCCACCCACCCACCCACCTTGCGGCACCCATCTATCTTCGCATTCGCTC
CTCTTTGCTTCGCAGGACGTCAGCTTCTCACGCGGCCCTCGCTCGCTGCGT
CATCTCTTCCAAATCTCACGTAGTCTCCCCAAAATCGTACAGAGAAGAAAA
AATAAAAAGCCCACAACCCCCGTCCTCAGAGCACCCCGCTCGCTCGCCAA
GAGACATGCTCCCTTCCCACACCTCTGGACCATCTACATCCACCACCCAAT
GTCTGCCGCCCGACGGCCACGAACCTGATCATGATCTCCAGGTCCCGGACG

ACGCCCCCGCCCCGCCCACAGACATGTTCGACCCGGGATCTGCTTCTGCCA
CGGTCCCAGTCGCACCTGCCGCCGAAGCACAGCTGCAGCCGCAGCAGCCG
CCGAGACAGCTGCAGGTAGGTAGCTGGTGGGATACGGCAGTCGAGCCGCC
CCAGCCAGCCCAGACGGCGCTACTCGCCATGCCACAACCGCCTCAACCCC
AGCCCCAGCCGTACATCTCGACGTTCGACCCGCCTCCCCAATCCATCGCGC
TCGCATCGACGTTCGACACCAGTCACACGCCGTCAGCGTGCACGACCGCC
TCAATCGGCTCAAACACCAACACCTGGGACATCTCCCACTCTCCCTCGCAC
AGACCCATCAGGCGACGAGCAGCTGGGAGACATACCCCTTTCCACCCCTG
CCTTTCTTCCCGGACACCCCGGCAGGATACGCCAACGCGGCAACGACAGC
TCCCGCAACGGCATCGGTGCCCAGCGCCGCAGTGGCGCCCGACTGTGCTC
CCGCCGCAGACGACCTCGCTGGCGTGACCACCCCTGTGCCCGTACTCCCTT
TCCGGACCACCATAGACGGCCCCCCAAGTCATCCGTCGACAATCTCATTGT
CACTCGCATCCTTTGCCGGCTCCCTGGGGGAGGATGACTCTGACATGCCCC
TCTCGGCGGTCGGCTCAAGTTGGAGCTCCGAGCCTTCTTCTTCTGCCCTCG
ACTCGGCCGTCGCCATGATGGCAGCGATGAACGGTACCGGCTCAGCTCCTG
ATGCTGGCTCGGAAGGTCTCGTCAATACGGTCAGCAACACGGGCAGCCTT
GCCGCCAATGGGGCGACCGGCGACGCCCAGCCTCTACAGTGGCAGGGCAA
CCTGCTGGACATGCGAGGCGGCTCGCTCCTCCCTGCCGCCGGCTTCACCTC
GGACCTCTTGCCCCAGCCACCACCGTCCCAGTTCTTGGACGAAGAGCCCG
CTCCGTCGTTCCCACTCGCACAATGGGACTGCCCTCTCCCTGGCGGCCATT
CCTTCTTCCCGCGCGTCGACCCCAGTCGCCCGACCAGCCGCGGCCGAGGC
AAGGGCAAAGGCAAGTCAGACGAGCCTCATATCTCCCCACCAACCCACGA
CATGGCCCCATCTGCAGGAATATACAGCGGCG

>Mosaic_sequence_57

ACGTCGATCGCGTCGAGCTGCAAAAGCTGCTTGAACATCATCTGGAAGTCA
GCTGGCGTGCCCAGAGGGCGACATAGAGGAGAACGAGGACTCACTCGATT
ATGCGCGTGCTCCCCGGTAGCTACCCCTATACGAAGAGGCTTGAGAGCTTT
GCGGATGGCTGCGTGGCCGACAGCGTCGTCTGGTGCAGTGGGCTCCTCGA
TGAACCACGGTCGGAGCGGTTCTAGCTTGTGCATGTACTCAATAGCCTGCG
GGACGTCCCATACCTCTTCTATGTCAGCGAGGAGAAGAACGATCTGATGCG
GGGACGAACGGTTGGCGTCCACCATAAGGACACATCCTGTCGGTCCGGCG
TTCTTTCCCTCAATTGTCTTCGGGTCGATAGGCGGGCGATCAGAAGGGATG
TTGGCGGGGTTGTCGATGATACTTCGCAGCAGTGTCCCGCGGCGAAGGTCG
TCTTCTGGGTCCGGCACCCACTTTGAGCTGAGATCAGCTCCGGGAAAGAC
CAGCACAAAGCTCGGTTCGGCTTGCCTTGAAGTGGTTGAAGCCTTGGGCC
AGAGCTGATTTGGTCAAGCGCTCGACCTTCTCGTCGGAATATCCTATAGCGT
CAGCTTCGTCGGTGATACAAGTCGGACTGACCCAGCCATCCCACACTGGTC

GTGTATGCTGGATAGCCTCTTCGCATCACTTCCTCTTCTCGCGCCTTCTTACC
CGCCTCGTTCTTCTTGAGGAGGGCGAGCGCCTCATCAGGTGTGAGTGCGTC
CGTGATGTATCGCCACGACACGGCTTTAACGAATCTGCAATTAGTGATGCCC
AGATTGAGGGGAGCAGGAAGCTCACTCTTCCGGTGTGAAATCGCAGATGA
GCTTCCACAGTGGTTTCCCACGAGCTTTGGCATACATATCCCACAAGGCATT
TGACACGGCTGCTGTGGCGAGGTGGATGACGCCCTTCTCTGGTCCGAGCC
AGCGGTGCTGCGGATCTCCAACCACTGACTGTCAGCGGGCATGCTTGGGG
GACGTGTACTGTAAGCCATACGAAAGTTCCAAAACGCCCCCATATTGCGAA
GATCTCCTCGATGTCCTTGCCCACTATGCGCTTGGCGACCTCTTGTATTGCG
AAGCAGACCTGTGGTCAGCAACGACCGATGACTTGGTCATGGTAGTCACTA
TCTCGTTTCCTCTTCCGATAGCTGATCGTCAGCGTTGCCCCTTGAGCTTCAG
GAGAACTCACTGAACGTCATGCCCTGCCCCTTCAGCGCCGACGTGGTGGT
CACCTCGATATACGCCGCCGAATAGTCGCACTCTTTGTTCCTGCCGTGAGAC
TACTTGAGCCAAGTGCATTGGAGGATCTCACATGGCATCTGTGCCGTCTCC
GCTGACGTTCTGTCTCGTCAGTCTCGGTCTCGCCCTCTTCTCCTCGAGGCC
CGCTCACAGTCGGGAAGCGGACATCTGCCGCGTCAGCTGGTCCACCTATTC
CGGCTAAGAGCATGTCAGCTCACCGTGAACCGTGAAGCCAGAAATCTTTG
CGCCTTGTGACATTCTGCGACTGTTCTTCTCCTTGACAGGCCGGTCTCACA
GGAAAGAAGCACGATACATATGATTCCCCGCACGAACAGCCTGTACCCGTC
ACTCGGTAGATCCGGGCCGACCCATCATGCGGGGGTGGGGGAACTGATCCC
GTCTCTGATCGGCGACCGCCGCTTGGATGATGGTGCAGGACCCTGAATTCG
TGTCAACTGCTTATTGTTCGTCGACCCTCAGCTGTTCATTGTACTCTGTGAC
ACCCTCGTCTCGCCCCTTTCGGAGACTCTGCAAGAGAACAATGAGACGAG
CTGCCAGGGGAGGCAGGAGTCATCGCTGGGGCGGCAGAAAAGAAGAAGT
GAAGCAGCTGGGTCCAAAGCGCCGCGAGGCGCTTCGGCATGAGATCATGA
GACGCTGGCAGAATTGTCGAACAGCTTCTGCTGTTCAATCACTACTGACAA
GTCGTCATTCACCTCTTGACGTTCCTCACCACCATTCATCAGCTCGACGCTC
AACATAGCAGGCACAAGAGCTCGGACTGGACCGTCGTTGGGCGAGGATCT
ACACAACGCTAAACGGGGGACCTTGATCGATTGTCAAAGGAAGCACTCAT
AGTTCAACATGGAGGCATCTTCGTCTACCCAGCATCGTCCTTCTCCATCATC
AATCGCCACGCCGTCCACCCCGCCACCTAGGCATGCGGCTGCCTCACATAC
TTCCCCTGCCCGACCCCAATCCAGCGCCCGCCCTCCCGAAGCTCACTCTAC
CCGCCAGTCGCCTCGATAAGAAGTACGCATACGGACAAGACAGCCAGTCC
CAAAACGCCGCCTCGTGTCCCGGCCTCGTCCACGCGCTCCCTCACCACCCC
ACCCACGCTCCAACGGGCAATCAACACCTCTCTTCCAGTTTCCCCGCCCAC
ATCCCACTCTGCCTATCGCACACCGCCGTCCTCCTATTCTGCCCGACTTGAG
ACACTGGAGCTGACACCGCTCTCGAGGAGGAGTCACCTGAGAGAAGAGG
ATTCGCCATTTCACGATCGGCATTCGATCGTTGCGGAAGAGCCCAACCAGC

TGGACGACCCCTTCGAATACTCTCATGCCAGGAGTATGAGCGGGGGTAGTG
GCGGGCGGCAGAGGACCATGAACGGCTACAAGTTCGCCTCCCTGTCCCCA
CCAGCCCGCAGGGAGAAGGAAGAGGTGCTAGGATATTCCGCTGGAGAGCT
GCACGAGGTCTTGGAGCGACCAATCTCAGAGTACAGGCGCTCGCTTTATCC
CTCTCAACCTCCCATACCCATAACGACCATTGAGCAGCACCCGACCTGCTA
CCGTCTCGGAGCACTCGGAAACCGTCCAAGCCGCCTGCAAAACACGATCA
AGCCCTCTTTCCGCACCCTCTTCTCCCTCGCAAGACGAAAAGACTACCTCC
TCAACTTCATTCCAGGCCTCATCCTCGCTTTCGCATCCGGCCTGGTTCCCCG
TACCATGTCTTCGGTTGTCGGTGACGCCTTTCAGGTCTTCGAGTCATACCCG
CTTGACGACATTGCCACGGACGCACAGCGAGCCGCTTTCACGTCCGGCATT
CAGTCCACCTGCATTAAGCTGACAGTGGCTGGTGTACTCGGAGTCGTCTTC
AATTACTGAAGACTGTTGCCTGGATCCGACATGGCGAGACGCACGTTGGCA
GGCTACGGCAGGCGGTCTACGAGGGGATGCAGCGCAAGGGAATGGAGTGG
TTTGATCTGGGGAATGGGGATGAATGACAAGTCGGGCGTGTCCGAACAGA
ACGGCGAGACAGTTGGAGCAGCAGGTGTCATGTCCAAGTTCACCAGGTCA
GCTACCAGCGGACTAGGGTGAGAGAGCTGACGATCCAGAGAGACGGACG
AAGTCCGCATTGCCACGTCTCAGGCGATGGGATTGGCCTTTACCAATGCTG
CTATCTTTATCTTCGGCTTCATCCTTGCCATGATCACGTCTCCCAAACTCACT
CTTGTGACTTTGTCCACCATACCTTTGCTCGTCGTTGTACAGATCATCACAC
AGTTCGTCGTTGGTCCCCTATTTGCAAATGAGCGTCGAGCCATGGCAGAGG
CGTCGACCAACATCGAGCGCGCGACCACTGCCATCGCGACCGTCAAAGCC
GCACAACGCGCAAGCAAGCGAGACGACACGATTCGATAAGATCGTCTCGC
GAGGCAACACCGCCATGGTCAAGCAAGGCCTCGTATGGGGCTTCATGTTCG
CCTCGACTGACTTTCTTTCCCTCGGCACATTCGTCATCGGCTTTTGGTACGG
CGCAAAGCTGGTACAGAAGGGCGAGCTGTCCAACGGAGACGTCATGACGG
CTTCTGGGCATGCCTCATCGCGGCCGGGTACTTGCAGAACATCGTCCCTCA
GCTCACCCTCATCACCAAAGGCAAGAACAGCATGGCATCCCTCCTCACCAT
GATCCAGGATGTCCCGCAGCGTCCTCTGTCCGGCTCGTCCTTCTCCACCTC
GCCGACTTCGCCTACCTTTCCCGGCAAACGAGCTTCACGGCGACCCACTAG
CTTGCAGAAGATCCGCCCGCCTAGGTGTCGAGGCGAGTTCAACTTTCGCAA
CGTTCAGTTCTCTTACCCATCTCGGCCCGATAATCCCGTCTTGCGAGATGTC
ACCTTGTTTCTCCCTCCAGGCGAGACGACTTTTGTAGTCGGCGGATCAGGT
TCCGGCAAGTCGACCATCGCCCAGCTCCTTCTGCGTCTGTACGAGCCCCTC
AGCGGTGAGATCACCTTGGACAATCAATCGTTCGCGTACCTCGACGAAGCC
TTCACCAAAGAGCACATCGCCGCGGTACAGCAGGGCTGCATTCTCTTTGAT
CTGAGCGTGCATGACAACGTCGCAATGGGTCGAGCTGGCTCTGGCCTCAA
CGCGGAGGACGCAAATGCCGGGGAGTCCATTGGACGTCACGCGGAGGAA
GTCATTGAGGCGTGCAAGATGGCCATGATTCACGACTTCATCGAGAGCCTT

CCAGACGGGTATGAGACCAAGTTGGGGACCGGTGGATCGCAGCTTAGCGG
CGGGCAGAAACAACGGTTGGCGATAGCTCGAGCGAGGATCCGCGATCCTA
CCATCCTCATTCTTGGTGCGTCTGTTGACCGTCAAGGAGGTCGTGGCTGAC
CGTTCAGACGAGGCGACTTCTGCCCTCGACGCGACCTCTCGGGTAGTTGTC
TTCGAGAACGTCAAACAATGGCGAATTAACAAGACGACCATTGTCATCACT
CATGACCTGTCCCAGATCCTGCCTGACGATTTCGTTTACGTTATGAAAGACG
GCATTGTCGCCGAACAGGGCTTCCGCTCGGACCTCATGCAGAGACACCGAT
ACATGGACCTGATTCTCGGCGTCTTCGCCTCCATGGCGGCGGAGCAGGCTG
TTGAGCCTGTGCCGCAGAAGTTTGACGACTATGCGGATGGTTCGATGATCG
AGGGAGATACTGGAGGGACGATGCGGACATCTCGCCTGAATCGAATAGGC
GAGACTCGCACTCCCGACCACACACCCCGAGCTTTGCGGCTGGGATTCGA
CCGGGAAGCTCGATGTACCTGGATCTCATCGACGAGTATGTCAAAGGCAGC
CGCAACTCGATGACGCCCGCGACAGAGAGGAGAAACTCGCGGAGGATGA
GCAATGCGCAGAAACGTCTCAGCTGGTCGCCTCAAGAGCTCGACCGTCGG
GCTAGTCGATCTAGTTTGGTTCACAGGCCCCCTTCCATGGCCCGCCCGGCG
TCCGCGACGAGCTTGCGAGTCTCAAGGCAACTGTTTCCTCCTGCCTCGTAC
GACCTTCACCGTACCAACTCGTCCTTAGAGTCTGGCCTCAGCCCCTCCCCC
TTCATCGAGAAGGATGGACGTCTCTCTGTTGTTCCATACGACTTTCGCACCC
GCACGCTGTCCCAAAACCTTGACGACGAGCTCAAAGCGACCGACGTCGAG
ATCGTTCCTCATGCTCCGCCGACCGAGGTCGAACCGGTTCCATCTATCTTCA
AGCTGTTGTATCGGTACTACCCGACACTCGTCCGGCGATACCTGCTGGTCTT
TGGCGTCGTAGCCTCCATCTGCCATGGGGTCGCCACGCCCTTCTGGGCATC
CTACCTGTCCAAGCTCATTGCAGTGTGCAGGGACGGGCACGACTGGATCCG
AATTGACGAAATATGCCCTCATTGTGCTTGCGCTCTGCGGTGCACAGGCTG
CATCCGACTTTTTCCAGCAGTACTGTCTCTACGCCGCATCCGCCCACTGGTC
GACATCCCTTCGCACTACTGCGTACACCGGCATGATGCGTCAAGACAAGAG
CTGGTTCGACAAGTCCGAGAACGCGCCTTCGAACCTGGTGCAGATGCTGG
TGAAAGATACAGACGATATCAGGGCTCTGGCCAGTACGGTCGTGGGCAAAT
TTGTGGTCTTTGTCGTCATGGTTTCTCTTGGCATTATCTGGCGATCGTAGTTG
ACTGGCGGCTAACCTTGTGTGGAGTCGCTCTGGCACCAGTTTTTGCGGGCG
TCATGATGACAAACGAGTCGTTGATCGGGAAGGCGGAAGTGCGTAACAAG
GCCAAGCGCGAGGCCCTCGCCAAGGTCTTCTACGAGGTCAGCTTGAGATG
AGCCTCGGATCCCAGCTGACGTTGTAGAGCATTGCAAATATCCGTGGAATA
CGTGCTATGGGCACTTGACGGTATTTTCCGAGAGAGGTTTGACCGAGATGC
GGCTTTCGCCAGGCGGGGCGGTCGGCGAGATGCCTGGAGTGTCGCTGTTG
GCGCTGGCATGTCGGGTGGAATGATGATGTTCTGCCAGGGTGGGTCGTCTT
TGCGCAGGGCGGGATCGTGCTGAACGACAGCATTGCTGAATTGGGCGGGG
TATCGGTTTATTCTGAATGGGTGGCTGAACTACGTGCGGATGCTGCAGGTGT

ACACCTCGTCCTGTTCTCTTTGGACGTTTGCGACTCAGATGCTCGACTTTAG
TGAGTTCTGTCTCGCGTTGGAGTGAGACTAGTACTGACGATAGTCCCAACG
ATGAGCAAGGCCAAAGTCGCCGCACGCGACCTTTTATCGCCTCGTCACCCT
GCCCACCACCACGTTCGAGACAAGCGGAGACCTGCGCTTTCCCATCACCG
GCAATGTCAAATTCGACCACGTCTCGTTCTCTTACCCAACCCGACCTGACG
TGCCTATCCTGGCCGACCTTTCGTTCTCCCTCACGCCAGGCGAGTGCGTGG
CCATTGTGGGCCCGTCGGGGTCGGGCAAGTCGACCATCGCCGCCCTGCTCC
AGCGACTCTACGAGCCGACTACCGGCAAGATACACATGGACCGCTTCGATC
TCAGCCGGTCAGACGTCAAGTGGCTCCGGAATCACATCGCCGTCGTCTCGC
AGAGCGCCAACCTCTTCGACGCCAGTGTAGCGGATAACATTGCCTACGGTA
GCGATGTGCCGATGGGCGAGATACACCGTGCGGCGCAGGCCGCCAACATA
CACGACTTTATTCAGTCCTTGCCACAAGGCTACGATACGAACCTCGGCGAG
AATGCCAGCTTGATCTCTGGTGGTCAGGCACAGCGGCTGCAAATTGCCCGA
GCGCTGGTCAGGCGGTCAAACATTCTCATCCTGGACGATGCACCAGTGCTC
TGGACGTAGACAATCAGCGCGCTGTACTGGATACGATAGTCAGAATCAAAG
ATGTGAGCGGCTTCACTCTGGCAGCTGATAAAATGCTAATGGCAGACCCGC
ACGACCGTCTTCATCACACACAGTGTCGAGGCCATGAAGCGGTGCGACAG
GATCATCTGCCTAGGCGATGGCAGAGTCGCGGAGGAAGGCTCGTTTGACG
AGTTGACCAGGAGAGGCGGCGTGTTCGCCCAGCTCATGCGGACGGGTGAG
TGGGAGTAGGCATATAGACAAAGTGTCGTTGTTGTACGAAAAGTATATTACC
GCTCTACATGTTAAGACCTACGAAGTTGGAAAGGCGCTCCGCGTGCACCTG
AATCGATTAAGAGTTCTCCTGCGGCTGTGGGTGCTTAGTCGAGTAGCGCAC
CCACTGCTTGGCATCAGCGTCGGAAAGTCCAGTGGCTGCGGGTGGACTCA
CTGTTGGATTCGATTCGCCGGCGATGCGGTCGACCTCCCAGCGGGCGAAGA
GCTGTGCCAGAATGCCGGAGACATGTGGTCGGGTCCAAATTCGCTGCAGG
ACGTATGACAGTCTTGCTCGCCTCTGGCACACGTCGGCATGGGGTGGAACT
CAGTTGCTAGATCGATAAACAGCGGAATGACATCAAATCTGACGAGCGCGA
GCAGACGCTGGAAGGTGTCGTAGGTTGCGTAGACCGTCAGGCGGATAATG
CTCGAGTGGACAATCACCATGGCCATTGTCAGCCGGAACGGGACGGCGAC
GTTTCCGGGCTGGCTCCTCGTCACCGTCACCTGAAGCCCGCCTGACGGCGT
CTCGCGCATCTCGATGCTCGCCGGCACCTTGACTTTCGCCATCGGGGCGAC
ACAACATGTGCCGGCCCATACGCAGCGAGAGAGAACGGCTTGTTAGACGG
GTCGGCCACCGTGGACGACTGCTCGCTGCCGATCTCAAAGTCGAAGCGGG
TCTGACGGATGCTTTTGGTCATGGCTTCGGTGATCTCGATAAGAAGGAACG
AGGCCAGTTGGGACTCGTACGTTCCCTGCTTGAGGGAGATGAGTTCTACCT
GCATCTCGTTGCGCTGTCCGAAACGAGCGTGACACGACGACATGAGCACA
GATCCCGAGTCAGGCGAGTGATCTGGCACCCATATGGTCCTTGTGAACAAT
GGCGAGTGCTGTATCTTCCTCCACAACATCTCGAGTCGTCTCATGAGGGGT

GCTTCGGGACTGCTTGTTGCTGAGCATCTGAGCTGTGACAAACAAAGTGG
TAGCCTCATGATCAAAGGAGACACCGCTTGCACCTGACGGGTCGACCAGAT
CAGCGCTCGTCTACACACCGAGTGGTCAGGCAGCAACGAGGCGGTGAGGG
GCGAGGCTGGGCCTCGCCAGAAACACGTTGCCAGCCTTGTTTCGTGGCGC
CTTGGGCGCTGCGTAAGCGGCGGCGACATTGTGATCACTCATGTCCCTATG
AATCCAGCTTCTGAGTGACAGCGGGGTGACTACCTTTGTGCGTCAGGGCA
GGAGAAATTCGGAAGGGTAAGTGTTGACGCATGGACGAGGTGTCGGAAAT
CCATAGCGCGGAAAGACGAGACTTTAATACGTCTTGATCCCAGCGGCCCCC
TCCCATCTTTCCCTGCCCTCCCCCTCGTCGGGGTGTACGTCTTAGCGGTGTA
ATCCCCGCTTGGCAATCTGTTATAGGGACTGGAAGTGACCACCGACAACGA
TTCCACCCACACCTTAAAGATCAGGCGAGGCACGTGTCTCCAGTGGTCCCG
CAGCGTCCCGTGTTATCACTCTCGGAGAATGATCACGAAGGCTTCTGGGCT
CCGATCCTTCTCTAGTGCTTGTCGTGGACCCTAATGACCCTCAGGCGACTCT
CAACAGCCCACGGCCCAATCTGCCGCACGGGTCGTGACGAGCCATTTTACG
CACGGTTCGGACCTCGAATCCTTCCTGTCAGAGAATCAAACTATTTGCTTG
CTTGTGGCAGGGCCGCGCGCGGGGGGGAGAGGGGGGGGGGGGGGCAT
GTCGCTGGGGTGCTAAATGCAGAACTGGAACGGTGACGTGGCCTAAGTGA
CAGTGATGTTGTGAAGAATCGTTCATTACCGACGATAGAAGCGCGCACATT
GACAAGGAGTGAAGCATGGGGTCAAAAGAGATTTTCCTCGCAAAGAGTTG
ACTTTAGATCGATCATCCCAGCACTCGTTGGCAGGTAATCGCGAACTCCCC
GGGCCCGCTGAAGGCATGATTCCCAGCCTTGCGCTGTGGAGCAGTCGACC
ACATGCTGCACTGCAAAAAGCAATTCTTCCTGTCGGAGAATCGAACTCCGA
TCTCCCGCGACATCAGAGTTGATGACAAGCGAGCATACTAGCCGTTATACG
AACAGGAATACGTCATTGCGCGCTTTTTCTGGTAATATATTGTGCGTGACAG
AGGGTACATACTGTAGGGTGCTTCCTCTGTCATCGCTTGCTTTGTCTTGCGC
ATCGCGTTGCGATGTGGCCAACCTACTCGGTCTCGTCTGTGCGGCGCAAGT
CGTCGACAGCCTCTTCAATCCACCCACAGCCAGTAGCCGTCCCGTCCGTCC
AGTACTCCTCGGCCCTAGTCGAGTCGCCCGTCTCAGCCTCACACCCGCTCT
TAGACCGATCGCCGTCCCTGCAGCTGGGCAGCCGCAACCATGCCCCTTCTC
CACCTATCGTGCCTCTGCCGCAGACCCCGCAGAGGGAGATGTCTCTTGGAA
AAGTGGACGAAGATACACCGCAGACCCATTTGACCGACGGAAGCCGTGTC
CCACTTCTTAGCGCCCAGCCGCCCCTGCCTCCCTTGATGGATGCGCGTGAT
GGGCCTGAGTCCGCGAGTCGGGATTACCTGGCTACAAGCAGTCGCACGCC
AGAGCCGTATGTATCACGGACGCTCGACACCGACCGTACGGATCCCACTAC
CCGCCCACCCGAGTTGAGGCTGGACGACGCGTTCGAGCGGTACACCTTTG
ACAGCGATACCGAGCCGTCCTCGCCCGTATTACCCAAAGATATGCCGCGGC
AGCTGGACCCGAGTCTGCTCCCTCCCCCCTGCGCCGCATGATACCTACCTC
TCTCACTTTTTCCGTCCAGCCATGGCGCGGTCCAGCTCGGCGCCCATGCCC

GTCCCGCTTGCGCCTTCGCAGGCCCAGCCTCAGTCGCAGCCGCAGACAGC
TGAGGGATCCACCACGCCCGGCAAACTCCTTCCTTCTCCGCTGGGCGCCGC
GCCGATGGACCCGGGGCACTCGGACGGCCAGCCAAAGGACCGCGGTGAC
AAGAGTATCTACGAGATATTCAACGCGGCCCTCTCGCCAGAGGGCGACTCG
ATGGTACGCGCGCTCAAGGATCACCTCGATGCGGTTCTCAAGGTTCAAGAG
AGATTTGGGAGGATGCATCTGGCTTTGGAGAACCTGCAGAGCCCCGATATG
CAGGGCAAGACGGGGCGCAAGGTGTGGCGGGAGGACGACGAGCGTATGG
AGGGGCTTGAGGGGCTGAAGCGGAGAGAAAAGGGCGTAGACGATATCATG
AACAGGGTCAGGCTTTCCCACAGCCCGCGGCCATCTTGCTGACGACAAGC
TATCCATCATGTCGGAAGCGCTGAGGACGTATCATCAACTGGGCACACCCC
GTCTGGCCTTTCCTCAGCCGGATCCTCGACCTCTACGACACCCGTCCAATG
CAGCTCAGCCAGGTGTGCCGACAAGCGCCTCGAATCCCAAGATTGCCCCG
TGCACATAGCTCCGTGGATCAGACAGCGAGTGGGGGTTTGAAGCCTTCGC
GCTGGTCGGCGAGGACAGGTCAGCTGAGAGTCCTACCAGCTAGTGCCGGC
GACGACGGCCACTGCGAAGGATTCAAACGTATGTTGTAGCACCTAGCCTGT
ACAAACTTCACTGTTGCTACAACTATGCGCGATGCGGGCTTCTCATGGCCG
ATATATGCGTATCAACCTCTGCGGGGTGACTATCCTGGTCCGACACAGAGG
GCATTCTTGACTCGTCGCCATGATGATGTCTGAGCAGTCTGACGATCAGCG
CAATGCATCTTGAAGTGGTGTAACTCACGCTGGCACATGCAGAGATGACCG
CAGTCCACGACAGCCAATGTCGCCTCCTCGTCCTGACAGACGATACAGAG
ACCTCTATCAGAATCGGCTACCGGCCCAGTTGGAACAGGCTTCTTCACCGC
CTCGGCTTCTTCAGCTTCAGACCCAGCAGGCTCTGGCGGTGTCGTGCCCGC
AGCCTCGGCCTCTTCGGCACGGGCCTGACGTTCGAGTCTCTTGCGCTCTTC
GGTCACCAATTCAGAGACGCGGTTGATGAGCTCGTTCCTGTTTGTCAGCTC
ATCCCATTTGCCTTGGTGATCCAGCTGACTTTTCCAGCACCTGCTTAAAGTC
CACCTTGACATGATTGTCGAACAAAATACCCTTGAGTTGTCCGACAGTCAA
GGTGGAGAGATACGATTTTGGCAGGCCCACGAGGTTGAGCACCGTCGGAA
TTGGAGGCGATGGCTGGCGAGCCGGGGCAGCAGTTGCTGGGCGAGCGGC
AGTCGAAGCTGGTGGACGTGCAGGTGGTGGTCGAGCATATGGCTGCGGAG
GACGGGCTTGGGTGTTCTGACGTGGAGCATACGCGTTGGTGGGAGGCGGT
GGTCGGAATGCGTGCGGATTGTACTGCTGTTGAGGGTAGCCATTGGGCCGT
GCCGCGGAGGGTCTAGCCGGCTGCTGTGGCGGCTGTTGCTGAGGCGGCTT
GAACTTGGGTACACTCTTTCTGCGATAGAAGCTCTACTGATCAGCACGACG
ACATCGCTGCGACTAGTGGCGCACCTCTGCCTCGGGCGACAGACAGCCAG
TGGCGGGATCTCTCGCCCGCACCACCGCCTGAACAAGTCCTCTTTCTCCTT
GGGACCGACAAGTGTAATGTCGTACGCCGCAAGTACTCCTTCAATCTCTTT
ACGGGTAAAGCCCGCAACCTCTCCACCGAGGCTGATGTGACTGGATCTCA
GTCACTCTCCCGGTCCGGACGGAGCTGTCCCACCCTGCAACATCCCCAAGC

AGGGCACACACACGCTCTCCAGCTCATAGCCGTTCTGCACTCTCGGCTCAT
CCGTCACAGCCGCCTTGATCTCATCAAGGATACCCTTGGGCTTTTGCTCGCC
GGGACGGCGGGGCATGAGCGCCTGGCCGTCGGACAGACAGGACGAGCAG
TATTCGTAGCCGCAGTGACCGCATGCGTGCGACCGCCGCCAGAGAGGGTT
GAACTGCTTCCCGCATGAACGGCAGTCTGCTGGTCAGCTCGACTCTCCTTG
CAGGTATTGGCAAGCTGACTGGGGGATTCAGTAACTGGTGGCGGGCCAGA
TACTGATTGATCAGCTTCATCTTATAGGTATCTCCATGAGAGCTGACGTATTG
GTATGCCTCGCGGCGGCGCAGATGGACCGGCTGCGTTGGACATGTTTGGTG
TGGGAAGAGGAGCTCTACGGTCTATGCACAAGAAGTGAGATGGAGAATGT
TGTCTAGATGCTGTTGATTATCAGATCAGACGAGCGATGACCGTCGGGGAG
AGCCAAAAGCTCGGGCACAGCTCGCTGGCTATGGCGGGGTGCGGACAGGC
AGCGATGCCACTGTCTTCACTATCGGAAACGCTGTCGTCGCCGCAGAGGTG
GGCGGGTGGGCGACGAAGTGATGCGAAGATGAATATGCTCGAAAGATGTC
ATCTCGGCCTCGTTGACCGCCAGGCAGAAGCATCGCTCATCGCCGAGCTCA
TTCCAGCTCACGTGATCAGGCTCAGGGTACCCACGTGGACTGACGCCAGA
GCCAGCATCCATTTCAACTCGCAATGTCGCAAACGCCAGTTGTAACCGCGC
AATACCAAGCAGCAGACATGACCTAGCCGGGGCCGCGAGACCGACATCGG
GGCACATCCTCGAATCAAACAGGATGGACGATGCGGAACCGCCCGCTGAA
GCTGCGTCGGACACAAAGGACAGCCTTCTCTCTGTGGATCAAGTGGGCGA
TTCTCCCCATTCATCTTTTCTGGCTGGACCAGCAGCCGCGTATCCGAGACAT
CACGGGGTCTGCTGAGTCACCTACTGCGTCACTATAGCTGACGTCCAGTGT
CCGCATCCACCAGCTCACCCTCCCGCAATCCTTAGCTCTCGCCTCCAAGTT
GATACCCTCCCCGTCTCCCGGTACAGAATTCGGTGGGTACGAGCTGGGGAA
TCCAGCAGATCCGTGCGCCGACCCAGCACGCATCTCTTGATCCCAGCAGGA
AGGACCGCCGCCTCTCGGCTACTAGACACAAGCGCTCTAGCTCTGTCTCCT
CTCTTGGGCCACCTTCACCGGCGACAACCCGCAGTCGCATCACTAGGCCAC
GAGCGCCGACCTTGGCGGGAGAGGCTTTGGAGCATGCTCATGACCCATTAT
CAACGAGAAGAGAAGCAGCCATTGTGGAGGATGAGGAGTCAAGCGGTCC
AGCATCGCGCTTAGCACGATGCTTTGTGGCGTTCAAGCTGCTCGCACCCGC
ACCCGCGGAGAATCAAGGAAGCGTACCGTTTCCGCAGCCAAAGAGGAGCA
ACAGCGACCATCTTCCACCTAGCAAGGTTCGGTATGGGACAGTTCCCGATC
TCAATAGAGCTTCTTCATCCAGATCACTTCAACGCCATCAACCCCTGACGG
CAAATCTAGAGCATCACCGCCTTCCAGATCACCCGTCGCTTCCCCTCCCAA
CACCCCTGTCAGAACACGGACGACTTCCTTGACCACATCAGCATCCAAAA
CATCTCCCCATGTTATCAGAAGGACGTTGTCGCATGCCGCTGAAGAGCGGA
AAAGCCAGCTCGCTCGTGTACCGTCCCGCCTTGGAGAAGGCTCAGCGCGT
GCAGCGAGGCCATCCATCCCGTCGCCGCGGAATTCGACCAGATCACTCCAA
CCGCATAGACCATCGCTTCAGGTAGTCACTCGTTCTGCCGACACTGTCGCA

GTGGCCAAACGGAAAGACGATCCGTTGGAGGTCCCTTTTTTCGTCTCTGCT
GTACACAATCCCTCCACGCATCCGCGTTTCCTCTCGCTCGCACCAGAGACC
GACTTTGCCTCGTGGCTTACGATTGAAAGCGCCGCGGCGACAACACTACG
AGTCGAAGTCTGGGTAGAGGACGATGCGATCACCCCTGTAAAATGGCGAA
AGCTTGAAGGTTTGGGTGGATCGGTGGATCTTTCTGATTGCGACCTGTTTC
AGCTCAGATTACAACTCCCGCCCAACACTCTTCAGTTCACACTGAGTAGCG
ACGACAAGCATGCGTTTTACCTGCCGCCAAAGGAACAGCGGACGAGAAGT
ACCTCTGCGATGAAGCGACACACCGCAAGGGGAGTGCTGGAGAGAAGCA
AGCGAGAAACGCGAATGAAGAAGGGTTCGACATTGAGTGCCCTGCATCAG
TCAGCTTGACCTTTCCTGCCAGGGGAACCCGGAGCTGAGACATAGACTCGT
CAATATCCAAGCCGTCATCACAGATACACAGCGAGGTATACAAGCAGTGCA
AAAGCAGATCGATACGCTCCTCTTGTCTGATGTGGGCCACCAAGCTCTTGT
AAGCTATGCCTTGGACCTTTGGCGGTGCTGAGGACAGCGGCGTGACATTGA
TGAGCGGGAATCGAGGGTCGAATGGCTTCGAGATCGCATATCTGAAGTGAG
AAAGACCTCGGCGGAAGTCCGAGCGCGCACGACTCTAGCCTACAACGAGA
ATGATCGTCGGCGAGAGAATCTACGTGGGGCCGAGGTACAGGATCATCTGC
TCAGCGGCATCGCAGGAGAGAAGGAAGCCGCCATCGAGCTCGTCCTCGAA
GAGACATCGGCTCTACGTCCCAGATTCTCGAAAAGATAGAGAAGTTGAAA
GCTGAGAAAGCCAAGAGGGCCCAGGATGGAAAACCAGAGCTCGAGCCGA
TCAATGGACCTATTAAGGGCGTGTTTGAGTTGAAGAGCTCTGGTAAGGATG
CCGATAGAAACAAATCCACAAACCAGGGTTATGTCGGGGAGCGAAAGTTT
AGGCAGTGGACAGACGATAATCTCGATCGTCAGTGCGAGGTCATGGAAAA
ATACAATAACGGAGACAAGCTCTGGACCAAAGAAGATCTTCGCCGTGTCG
AGAACGACATCAAGGGAGAGGCTCCCTATGTCAAGTTCTATGCTTATGCTG
GAAAGGATAAGCTCATTCCAAAGCAGAAACTGTATGTTATCTCATCGTTCTG
GCCTTCAACCAGGATTTGCTAACATACATGAACAGCTTGAAGGATCAAAAG
ATCATGGTAGAGTTCCGCGGTTTCCAGGACCTATCATGTCCTCCCGCCTCTT
CACCTGACAATATGTTGTACAGCTCCTTGTCGCTCAAGACTGTCAAGTACG
ACAATGAGGACTACCTGGCCCCTAGCGACCACTGGACCTGGACAAACTGC
TACAAGAAGTCGTGGAAGAGCTTGCCTATCTCCCACGAGCTGTACGCGATG
ATCATGCGTGTGCCTCTGCCGCCTATTGACAAGATCGTGTGCATCGGCCTCG
GCCCGATTTCTTTCAGACCCCACATTGAGTCTACCGAGATGTCGAGGAACG
TCTTCTGCCACCTTGGGGCCCTGCTGATTGCCGAGGCTCTACGTAGACGCT
TCGGCGCTACTCCACGGCTGCTGGCGCAAGATCCCGAGTACAATCCCGATT
GCAAGCGGATGCTGGAAGGTAAGGGATTCGAGATTGTCGGTGAACATGGT
GCTAGTGGCCTTGCCGAGATTAATGACCGAACCTTGGTCTACGCCCCCAAC
CCGGGCTTTTGTTTGAAGGAGGTCGTGGCCGACATCGCGCAACCCGCGGC
CATGTTCTGGAAGCCGGTGATCACACCCGAGCAGGAGGATGCCGAAAACT

AAGTCCAAGAACCCGGAGGACATGAACGTTATGCGATTGAGTTGCTTCACT
CAGTATGTATCCCTTCTTCATTCTCATTCGGCACTCTCCTTCCCCACGATAAT
GTCCACTCCTTGAATATTCAAACCCCAGATTGAGACCCCAAAGCTAACTCA
CTCACCAGCAAACGCAGCGTCGACCCCGACACCCCCCGCGTCCGTGCTCT
CGTACAGAAATACGACAAGCACTTCTTCCCCGACAACAACCTCTTCGGGA
AAGTCGTCCTATACACTCGTCGCGGGTCCTACGTCCCACGCCCCGAGCGCA
CACCTCTGCTCTCCATGCCAAAGGTCAGCCGCATATCCGACGACTACGCGC
ACCTCGACGAAAGCACGCGGGACGGCGCACCCTACAGCTTCGAAGCCGCC
GCCGCGGGCGAGTCCTCCAAAGGCAAAGCCAACGAGTCCCCCCAAGACC
CGATCCCGGACTCCTCCCCCTCAACCTCCTGCGCCACCGCCACCGCCACCA
CCTCCGCCACCACCTCCGCCGCTCCTCCCCCTCCTCCCCCTCCTGCCCCTTC
CGCGCCCGGTCCCCAAAGCGGTCTCACCCGCAAAACCGGCATCGCGCATC
CCCCCTCTAAACAACGCCGTAGAAGACTCATAGTCGAGGTCAACGACGCG
CTCGTCGGACAGATGCAGAATCTGCCACGGAGAGACGAGATGAATTATACG
TGGAGATACGTGCGCGCGCCTGGTGTTGCGTCGAGGGTGCGCACGGATTAT
GCCTTGGGCCAGGCGGAGAGGGAGAGGTATACTTTTATCGAGTACCCGAG
GAGTCGGAGGCGGGAACAGCCGCAGCCACAGGCACAGGCACAGGCACAG
CAAGGACAGGGGCAGGGGCAGCAAGGGCAGCAGGCTCCTAGGGATGGGG
ATGAGAGTGTGGATGAGGATGTGGCGAGTTTGTATCGCGGGATGGGGTATA
GCTTTTAAGCTAAGTAATAAACTGGGGTAAGCTGGGGTAGGAAGTTTGTGG
ATTCGATTGGTCCTGTTGTTAACGCAGTCGTGATTTTGGGGGTGGGAAACA
ACCAGTTTCGTGTTGAGTGGTTCATGCTTGAGTGTTGTGGTTATCAAGTTAA
ATTCCCATAAGGACTGGGTACTTTTTGGGGTCTGTCTAGGAGTAAGGTTCA
AGATTGAACTACTTTTTTTAAACCGCAGATGGGCACAACTACTAGGTACGT
GGACAATAGGTGTGGATGTTTTTACAGTCGTTTGAAGTTGGGGTACGCGAA
TAGCAGCTAGGAAAGTGGTTTTTACAATTATAAAATGTTCCCACCCCCTAAC
CCCTAATTCCTTAAGCGGCGTATCCTAAGCCTTTTTTTTCATAGGCTAGGTAG
CGCGGGTATATTCTCTAATAAGATGAATATCAGCGAAAACCTAGTACCGTTG
ATATGCGAATCGGGAGTAGGTAAAGATGGCGTAATCAAGTTCTGATTGGATT
TGGTAGGTAGATTTGTATGCCGTACATCCGTGTAATTATGTTGTACTTCGGGG
GATATCCATATATCTGATGTAACTATAGCGATCTACCGCTAGGCACCTAATAA
CCTTGGAATACACAAGTATATCCCACCCAGGGAAGTAGATCACCAAGTACC
AATTACTAGGTACACAAGCTACATCAAATTACTCAACATCGCCACAAAGATC
TTCTCAACTTCAGAATCATCTGTACTGTATTAAACCACGACTCCGACCCGAC
CTACCGTAGCAGGTTTCGGGGCAACGATGAGCACATCTGGCATTAATTGGT
AGTAATAGTAGTAAGGCACCTTAATCCGACAACACACGCGTACGTACGTAA
AACAAGAACACAGGCAGCGCGAAGACTATATCAACAACCATAAACCGACA
CACTGCATGCAAGAACACACACACACACATAAACACCCGTTAGATGTAGTA

ACACAGAGCCCGTCGATTGAACTAAGCCAACTAACTGTCACTGTCACTGCT
ACTACCCCTGACTGATTAGCGCCGCGTTTCCTCGATCACTACCTAGGTAGGC
AATGACCTTAGCTAACGGACCACCTATCTTCGTCATAAATATCAACGCACCC
CTTCACTTTCGACCTCCTCTTGACTAGTAATTGACAGCTTGTATAATGCGCC
CTTTTATACACACCCCTTATTCCGCATCCGCCACGCGCGCCATAACCAGTAA
CCAGCCTCGTTCAGCATGGCTCGGGTCGGCCCCAATCCCATTGTGAATCTT
GGCGAATGAATAAAAAGGTGTAAAACGGTAAAGAAGAGGAACTTTTCTAT
TCCCAAGCTGCAAAACTCGGAATGAAGAAGAAAAAGGGAAATGAATTTTG
CGAGGCTAAAAGCCGAAAAGAAAAAAAAAGAGTAGTGGTAGTGATAACC
ATCTACAATTCCCGCCCTATCCCGTTGCCATTAGGGAATAATCCTCCGTGATA
TGTCCGTTGCACAAAAACTCCGATTCTCCAGTATCGTTGAACGCCCCTCTA
AGGTAGAACGACGGATAAAAGAAATGCGTGCCCGGGTTTCTCAAGTCGAG
TAGAACTTTGCGCCCGTCCTAATCGCTCGGTTTTGGAAGAAGCTTTCTCTTT
CGTCTTCCTTTGGATTTAGGTGGTTCTTTCTTGTTGCTAGAATCCTGAGGTT
CAGGTTTTATCTTGTCACTAGATTTCTGAGATTTAGGTTCTTCGTCTTGAGCC
TTCTTTGCTGCCGTGGCCAGTTTTTCTGCTGCAGCCAGTTTTTCTGCCTCAG
CCAATTTTTCTGCCGCGGCCCATTTGGCTACCGCGGCCAATTTTCGGGCATC
CCGTTCCGCATCAAGTGCCAGTAAGGCCGGGTCTATGCGTTGGCTGGGCGT
CGGAGAGACGGCCAATTCAGGCTTATCAGATGGTGAATCTTTAACTGATCC
CGTGGGCGAACTTTTATCATCAGAACTGTGCAATCCAAAAAACAAAGAAG
GACTCTAGGCTTCCCTGTTGGGGGCTAATGAACCGCCGTTAGATGTCGGTA
CCGCCGTGGGGGTTCCGCCGTTGACCGCAGGCGAAGCGGCCGTAACCCTG
GGTGAGTTAAGCTGTTGCTTTGGACTCTGGCTGGTTGCAGCATAGTTTGGC
ATGCCGGCTCGGGCTGCCGCCAGCCTCGTTGGGTCACCTCCTAGACCGATT
CCACCGAGGCCAACCGGCGCGCCGACTTGCCCTTGCCCCTGACCAGACAT
AGAGTCCTAATCCCCTCGGTTGTCCTCTGTGCGCTCTTGGTTGCGTCGAAG
CGAGCTTTAGCTTGATCCATAGCTGTTGAACCTGATGCGGCTGTAGAGATGT
ATGAAACGAAGAAGTGCCAGGAGTCTGCGCGTTCTGGCCGTGCTGACTGT
GCTGATTAATTTGGTTATTAGGCATTGAAGGCGAATTAGTAGTGTTGTAAAA
CCGTCGCGAAGGATCATTGTTAATAGTTGGCTGGCCGCTATACGATGCTTGC
CTCACGGGTGTTCCGCTCGGGTTGTATCCAGGGTGGTGAGGAATCGGCTGG
TGATGAGCTTGTGGCGTTGGGTGGGTGACGTGAGGAGATGCCGTCCTTTGC
GGAATAGGTGTGGCGCCATTTGTGTATGGTTAAATGGCTGGTGCGATGGTGT
ACCCTGAATCTGGTGAGGCTGGTGTTGCGCTTGAAGGGTTGGGTGTGATAA
TTAGGAGGCGGCTGGGTGCTCGGTGGATAAGATGGAAAACGCGCTTGTGG
CGTTTGGGCGCCCATATTAGGATAGCCCGGCATTGGTTGGTACGGAGGCCG
GACTTGGGTGGGGGTGTTCGCATACGAACCCGGTTGCGAATATGGGGCGG
GCGGTTTATTGAATTGCGTGGAATAGCCTGTGCCGGGATACTGGTTCGGTG

GTCGATAACCTGCGTTAGACGGCGGTCGCGGGGGATAAGCCACAGGGGGC
ATCGTCTGCGGGGGACCTCGCATAGTTTGGTTGCTGAGCGCGAACCGGGGT
AGACGTATTGTAATACTGCGCATTAGGCATCGGCGCACGATTGGGATGATAC
TGCTGTTGTTGGCCGTACTGCATACCACTCGATGAACTCGACGCCCGGTGC
GTGACTGGCCGGGGGTTAGCCGTGGCAGCCTGCGCGGCTCGGGCGCGAGC
AGCTGCTTCATCCTCTTCCATTAAACCTTGATATTCGTCGGTTCTAACTTCAA
TCTTGGTCGATATGTTCAATTCGTCCAACTTGTCCGAGTTGAGGCGTGCGAC
TGCGTAAGGTGGCAGCGATGCAATGACAAGAGCCAACTGCGCCTTGAGAG
CCTGGTAGGTGGCTTGCTCTTCCTCACGGGCTGCACTGGAGTTCCATTGGT
AAGCATATCGCTATGGGCCGGATCTGCCGCATATCGACTCTGACTGGATGCG
GACGGAAGGGTCAAATTGCGATTCTTTTGGTAAGACACTAGCGTCTGGATC
ATGTCGGATACCTCCTGAAGGATATCATCCGCGTCCATCTCCTCTCTGGGTT
TCTTCGGACAACAGACCTGTTCTAGGCTAGGATCCACCATAGTCTCGTCCC
AGTTATCTAGCGCCTCCTTGATCAGTGCTTCGTCTATCTCTTCAACAGGAGC
AGCATCATTCTTGTCTGCGTCCTCGGGCGTATCTCCTGGCATATCGGCCTCG
ATTAAACGATCGAAGCTGCGCTGTCCTACTTTCTGCCACCACATGGTTTGAC
TAATCATACCCGACGAAATCATGGCAGCAGAATCATCCTTGGATGGAGCAA
AGCTGCTGAACGCGCCACGAAAGAGCGATTCCATTTCGGACATCTCAAGG
TCGGATGAAGAAGGCTTATTTCCGGCAAGGCTTAGAGCCCGTTCGCGTTGT
TTGGTCATGATTTGCGACGGGGGCGCGGCGTTGCTATAGTCTAACCAGCGA
CCAGTGGTGATGTTCTGGCTGAAATATGACCCGGCGCGGTACTTGGACTTC
TCGGTGAGCTCGGGGTGGTGCCACCCGACTTGTACACCCTTTACGGTGCTC
TTAGGCGCCTTGGGGGGCTGCAGAGACGGTAAGTTACGCGGAGAAGGGAA
TAGTTCACCTAACGTCTTGGCCTTCTTGTCCTTCTCAACAGGCGAAGAGAA
GGCATACGGTATGGCCCTGCCGGTCCTGACACCATTAGGCAATCCGCTTTCT
CTAAGAGGTCGGATGGCGATTTCGGTTTCGGTACCGTCGCGGCCAGGGATA
GACTGTTGTAAACTTGAATATAAGGACTTTCCTCTGATCTGTTCTCCATTCA
CTACCAGGAACATGTTGCCACTGGCATTGGACTGTAGATCGCTAATAGAGT
TAAGTTGTTCCAACACGCCAGAAACGTGGGTGTTGTCAGGATACGCGAGC
TCCCTCTTGAATATGCCATAAGCTTGTCGCTTGAAATCAATGATCTTAGCGAT
AGCTTCGTCGTTCGTCTTGCTGTCCTTTCCAGGCTCTATGTGTCGCAATTCA
TTGAGGTTGTCCGTGATGGATGCGGTGATATCGACGATAATGCTATCAAGGT
CGCCATATTGCTCCTCGCCACCTTGTCTGCGATGGAAGGAAATGGGGTGCC
ATCGTCGGACTTCTTCCGTTTCGCTTGGGGCTCGTTGGAAGTGTCTTCGGG
GTCGGGAAGAGGGCGTTTAAGAACGGAAGGCATAGGGTCGATGCTATGTA
ATCGCCAATAGTAAGCAAACGAATTGATCAATGAATTGTATGATGAAGCAA
ATGAGGTCACGGAATATAAAAGTACGAAATGAAATAGAAAGAATGCGCAA
ATATGTAAGTCGAGGGGAAAATATCAAAAGTGATGCTAGAGGGACTCGGGA

ATAGGATTGGTAATAGAAGAGGATTAATAAATGGGATCAAGTTACCTTTGAA
GAACATCATAATAGTTACGTATCGTAGGTTTATCAACCTTAGTCGCGTTGGA
GGTGGAGGGGGAAGGGACGGGGACGGTATTTGTAGAAATAGGACTTTGTT
GGCTAGGATTGAGGCCATTCGGCGGTTTCTCACTAGCATCGGTGGATTCTC
GTTTGCGTTTAATTGAGGTGCCGGCCGTGTTTTGCGCAGCATCAGCATCGG
CGTCGATCATGATGTTAGCGTTAGGAGAATTAGATGTGGTAGCGGAAGCGA
CAGCGGTGGTCGTGTCGTTGGTGGGATTGGTGGTGTTATTGGTGTTATTAGT
AGTAGCAGCTGCGGCGGCATTGGGATTGACGTTATTGTCGCTGCTATTGTTG
TTAAGCTGCATAGGTGTGGAAACACCGTTCGGGAGTGCTATGGCTGGCGAG
GCCATCTTTTCCCGTTAGTCGAGGTAACTTTTTTTTTATTGTTGTTGTCCTCT
CTAAAGCGGGATTCGGGTAGTTGGGGTGGACGGTTGATTGACTGTGATTAA
AATGCTAAGAATTTCGCGGCTCCGTCGCGCTGCACGTTTAGTAGGTCGCGA
TTGAAAAAAAGGGAATTTAGGACTTGGGATTTTTCGTCGTTGCTTGTTGGT
GCTTAGGTGCTTGGTGCGACCGCGACACTCTGACTTTCCCCTAGAATAGAA
AGAGCCTCCTAGGCAAAAAAAAGACCGGGTCGCTACTGCGTACTGCGTCC
TGCCGTAGAAACTGCCACCTTCCACTGTGCTAAAATGCCAGGTCTAGGGAC
TAAGGAACATTGAAGGAAGGTACAGGTTCGGGACATGGACTAAACCGCAA
AAAAACTTCAATGTTGCAAGGGCGATGGATAAAGAAAGTGGACGAGATTC
ACCCATCCAAATTCAAATGATGGACGAATCGTGTTAATGCGGTAAGAAAAT
TAGAAGTAGAAGTGATGTATCGAATAAGGCCAACTACGGCTAATCGTGAGA
AGATCTTAAACCATCCGCGAACAATTTCGTTGTCGGGATGCACGCGATATTT
TCACTGATAGTCTTTTGGTTACGAAAACCTCATTCAATACAAGAAGTCTTGT
ACCTAGTATTATGAGAGGGAGGACGAAGAAGTGCAACGAAATGAATCTGT
CTGTCCTTCGACTCACCTGCAAATGATGGCTGACGCTAAGCTCGCTACTAA
CCTAATTACGGACTAGCCGCACGCGCACGATCCACCATCCGCCCTCGACAC
GTGATCTCAACCAAGTCAGCGCGGTCCCGTCTTCCAGTCTGCCAACACCGA
TCATGTACTTCTGTACTTCGGTATTCCGTACCTTGTCGCCAACAAATTGCGA
GGAAAGTGCAACTATGATGGACTCCATCAATTTAGCTGTCATACGATTTTAG
AAATTGCTGCTAACGAGTAATGGAATTTCATATGACTTTTATAATAGATTCTG
AAGACGGCGTAAAATAAAGGTTTTGTACGGTTGTCACCGATGTTACCAGTC
AGTTTATTGTACCTACTACCTAGGCACATTTATAGCAACAGTTGCATATTTTA
TCAACGTAAAATCGGGTTGGAATGTTATTGATCTTCACGACTTCCAATTAAC
CTAAATGGGCAATCGGCAATATTGAAGATGTCGGATTTTTAAATGTCATAAA
TAACACGATCTTTACTGGTGGTAATTCACCACAGGCGTCACCAAGGAATAC
ACCTTATGATACCCTACGGCGTGTCGCAATTAAATGCTAGCGAAGTACCTGG
GTGGGTAGATACGTGGCGAATTACAAGCACCAAAGAGATCCGTACACTTCA
TAGTGTAAAGCAGAATAAAATCGAATTGATGATTATGCGCTTCACATTATGA
TACTTGGGTAGGTAGTTTCAGCACTCCATAGGTCAATAGAGATTCCTTGAGA

AATTTGATTGTTGTACCAAGCTAAACGAATACGTGACGTTGAGTAATTTACA
ATGGAGACTAAAGTCTCGTCATGCTTCGAGACCAGCACGTTTCGAGGGGA
ATACCCTGAAGGAAAAAAAAACACCACCCCAGATGAGGGTTCAATTGGAA
ATGCAATCAAACGCGTTGTCGCCCTCATATTATGATCAAACCAGTACAATAC
TCATGTTGCGTCGCAATCTCGTCACAAACCATACATGATAGTTCATAGATCC
TCCCCAAAGGTTCCGCCGACTTTCACCTATTTGGCCTTAGCCAGACCGACG
GTGCCCTTGCCGAGGTCATAGATGGAGTACCATCTTCGGAGGAATGCGTCA
CCAAGGATAGCTAGAGGACCGACGGGCGCGGGGAAGTCCATACCCATAAA
GGTAGAGATGCAGCTGCCCTGAACCTCGAGGATGTAGTCGTACGGTCCGAT
GGCGAAGTCATATCCGGCAAGCTTGAAGCTGATATCAGGAAGCGAGTCGC
GGACAGAGCAGTCGACAGTGTACTGGCCGTTGAAGCTCTTCTTGGCGCCG
ATCTCCTTGTTGAGAAGCTCAGCAAGGTCGGTGGGGAGAGCAATGAGAGA
AGTACCAGTGTCAAGAATGACACCAGTGTTCTCGAGCTCGGCAGTCTCGTC
ACCGAAGGAGATGGAGTCAAAGTCCACCTCCCAGTAAGCCTTACGGCGCA
GAGGAATTTCGGTGATCTTGCCGGTGTAGTGGTCCTTGTCGACACCACCGA
AGACGACCTCGGAGTCATCGCCTCCATTCTCGCCGCTAAGGTAGAAAGCGA
AGACAGCATCGTCGATTAACTTCTGGTTGACCATCTGGTAAAAGGGAGGAA
CGATGCCGTTGACGGAGAGACGGTCAAAACCCATTCCAAGAATACCATCG
AATCGGCCGAAAGCGAAAGCGAGACCAGGCTCGCTGGTAGCTTCGGCAAA
GTCCTGGTTCTTAACAGTGAGATCACCAATAGTTACGGTATCCTGAGACAC
GAAACCGCTCAGGCTACCAGATCCATAACGGATCTCGAACTCGGTGCCGTT
TTTCTTATACGAGGACGAGGACGAAGAGTCGTATTTAGAGTGGAGGTAGCA
GGCGATGCTGCCGCAAGATGACGAAGGAACCCAAAGGTTAGAGCTTCCTG
TGTCGAGGATGACCTTGAAGTCTTGAGCTGGTGTGCCGATGCTGATCTCAG
AGAAGTCTAATAATTAAGTCATCAGCAAACATGCTTCTCAAGCATCCTAGGC
TTTTACAAGAGGTTTCGGATTCTCGGATTACATACACTGAGCGTTCATGAAG
TTGGAGATGGGAACGGGATGATCTGTGTCGGCGTGACGGAGCTGCCTTGG
AAGACCGCCTGGGTGTGAGTCTCGGGTCGAGGTCCCATATACTTCTGGCCG
AGGCCTCGCATCTGGGTCTCGATCGGGATGCTCTCCTATCACAAAGTATCAA
TATCGAGTCGCATTACCAGTAAAGAGCAAACACGAACCAGTTGCTCCGAA
AGAGGTATCTTCTTAAGCTTCATCTTGTGGACTCCGGCCTGGGCGGAGCCA
AGCAGCACAGCTGCGGTAAAACAGAGCTCCCTTCATGATGGGCGACTCCG
AACAATGACAAATTAATTAAAATGAAACAACTCTTGATAAAACAGAACTAT
GGAGTGAAAAATGATGTCCCGATGACTTTCAATTCTCTGATCCTCGGGATG
GGGGGAAGAGAGATGATTGATGGCAGATGATGGATGAAGAGTGAGATGTA
ATTAAGTAGGAGATATTAGGAAGAAGAGATGGGTCGATGATGTTGTTGAGA
GACTTGGGAGATAAGACCGAGCTCCCTTTGATCAATCAGCGCCTAGAGTTG
CTAGGGGCAGGTGGGAGACGGAGCATGCAGGCTATTGCGGTAGTAGTAAA

GTGGGTAACTTTTGGTCTCGTGCGCCAGATACAAGTAATGAAGTATAGTAC
ATTCCCGCTGCGTTCAGCGTCGCTTCAGTGTACTCTCAGCGTGTGTTGTTTC
CTGTCTGCTGCCTGTTACGACTTAGCCTTTCCTAAAAGGGCATTAGCGTTTC
CAGTGACGCAGGTGTACATGTTTATGTGGGTCTCTATTGTTCCGGGCAACCA
GAATCTGGAGGCAACAACCGCTGATGACACAAGCAGGTACACACTCAACG
CCACCTATGCGGGTAAGTTTTACCCAATAGAGTTTCATGTCGTCTGGTTCAT
CCGATCCGGGGCATTTTGCAGGGTGTCCGGGTACCTGAGTTTTAGGAGTCA
TGCTTGTACTGTACATACATTGGCGTAGATTAATAAGCTCTCTGCATTCATGT
TTATGCGTTGTTACAGAAACATCCATGGATAACATCGAGCTTGGAAATATTA
ATTCTCAGCCATCTAGAACCTGGATACTCACATGTGACCAGGCTGCCAGGT
ACAGGTGTATTATCCTAATTTGACGACGACGTCTATGCGTTCCAGTTGACCA
AAATATCTCGCAGCTTCAGAATTATTTCGGTAGGCCGCTCCAGGGCTTCGGT
AGGTTTTGGTCATAAACTTAACAGTGAAAGTTTGGATAATTACTACCAGAA
GCTTGGCTGCATAATTGTCGCATAATACAGCGCATAAGTATTTGCCTCTTCGC
CTCTCAGGAGAAGCTAAAGAAATGAAAAGACTCTTGGATTATCTAGACTCC
TTAAATGCCTTCGACAATAGTCGGTGTTCCAATCCGAAATTACTGAAAAGG
TTCCTGTCCGATGAATTCGGAAAGTATGAATCCGTATCAGGTTAGCCTCTAA
ACTATTCAGAACATCCCATTCCAAGCCATTCCTAAGCCTCACAATAGTGACG
AGAAAATGCGCAGGTTGGTTTTGCGTAGACCGCTTTCCAGGTGGGTAGGTT
GAATCCAAATGGTTGTCTAATAAGTCACGTCCTGAATAGACCTTGTTTTCTT
TCAAGCCATCAAAGACATTACCCCTGAATTTAAGTGTCTTGAATGTCAAATG
CTTCCTGGTCTAAATACTCGGGTCCTGTGCTTAGTCAGGAATTAATGCTCTA
GGTGAGCTTTAGATCCAGATAATTCAGCTTATCATTGACTACGTATGAGCTAT
ATCTTGCAACTAAGTTAAAGATAGCCAATTAGCATTTGATGTAGCTATGGGA
ATATACTCTTTATAGAAGTTCTCAACTTAATTTAACCAATGAGGTTCAAACG
ATTCGGGGTATTGTCGAAGACGTCTCCAGCAAGTCGCCAAAAAGCAAACA
GTAACATCTAGAAAGAATGGGTTAATACCGGCGTTGAGCACAATTTCGCCC
ATATTGAGAACTAAACATACATCGAACTTCAGGATTTTGTATATGATATAATT
GGTGGTCGCAGTTGTCAAATTTGCTTGTGAAGTCTGCTCCAAGTTCACTTT
TTAGCCCCGTGTCACAGTTGTCAGTTTCAACTCAAGACACGTTTGAGGTGC
AGGCATTGTTCAACCTCAGAATTAGGAAGAGTTCTTGGTCTGGAATATACG
ACAATCTTTATACTGCTAGCATCTATCATCTATCATCAATAAACTCATTGTTTG
CGGCGATTTGATAATAGAAAGCTCCAAGTTTGCCCGCAGCGAGCAACCGCG
ACCACAATTCTATACACTCGGAACCTTGATGGAAGGTTTGATTCGCCGACTT
CGGTATGGGTCTACCAATGGAAATACAGAACCCCAAGCTACTCCGCTAGTT
ACTCCGGATCCTAGTGTAAGCAGGACCAGGAAGGGACCAACAGCTCGTCA
TCACCCCGCCGTTTAGCATCTGGACCCTTTCTTTACTTAATACGAACCCCAC
CAATCAATTGGAGCCGAAGAAAAATTATCCTTCTCGCCTTCTACTCTTTGCC

CGTCCTCCGTCTTCCATCCTCCGCCTTTTCTCTTTTTTTCACTAAAAAGCTA
AAAAGGCTCTGTAGTCCGCTGGCAAATTTATCTTGCTAGGAAGATTCTCTTC
TGCAATCGTTTCTATCTTGTAATTTGAATCATACCATTCTCCTATTCACACAA
TGGCCGCCAACAACACCTACGCTCTCTCCAAACAGCACAAGGACGTCAGT
AGTGCCTATGCAATGACACCATGGTGAAGAGTTTTACTAATCTCACGATAGT
TGCTCGAGAAATCTCTTCTCGACTCTGATCCGGAGGTTGCGGAGATTATGG
TAAGTCTCCTAATAAGTCAATTGGTTCAAATTGATCATACGACTAATTGAATT
GGTTACAGAAAAAGGAAATTCAGCGACAGCGAGAGTCCATCATCCTAATC
GCTTCCGAAAATGTCACTTCCCGTGCCGTCTTCGATGCCCTAGGCTCTCCCA
TGTCCAACAAGTACTCTGAGGGTTACCCCGGCGCTCGATACTACGGAGGAA
ACCAGCACATCGATGAGATCGAACTGCTTTGCCAGAAGCGTGCTCTCCAGA
CCTTCCACCTCGACCCCGAACAATGGGGTGTCAACGTTCAGTGCCTCAGCG
GTAGCCCGCCAACTTACAAGTCTACCAGGCTATCATGGCCCCTGGTGGTCG
ATTGATGGGTTTGGACCTCCCCCACGGTGGCCATTTGAGCCACGGATACCA
GACTCCCTCCAGAAAGTAAGTTTGCTTCGGATAATTCGGAAAATTTTGTTAC
TGACTCGGTCACTTTTAGGATCTCGGCTGTCTCGACTTACTTCGAGACCATG
CCTTACCGAGTCGACCTTACCACTGGCATCATCGACTACGACAGACTCGAG
GAGAACGCCCAATTGTTCCGTCCTAAGGTCCTTGTCGCTGGTACCTCCGCC
TACTGCCGTGAGATCGACTATGCGCGCATGAGGAAGATTGCCGACTCTGTC
GGAGCTTACCTTGTCGTTGACATGGCTCACATCTCTGGTCTGATCGCCGCCG
AGGTCATTAAGTCTCCCTTCCCCCACGCTGACATCGTCACCACCACAACCC
ACAAGTCTCTCCGTGGCCCCCGCGGTGCGATGATCTTCTTCCGAAAAGGTG
TCCGTAGCACCGATGCCAAGACTGGCAAGCAAACTTTCTACGATCTCGAGT
CTGCTATTAACTTCTCCGTCTTCCCTGGCCACCAGGGTGGTCCCCACAACC
ACACCATCACCGCCCTTACTGTTGCTTTGAAGCAGGCTCAGACGCCCGAAT
TCAAGGCTTACCAACAGAAGGTTGTGTCCAACGCCAAGACTTTGGAGAAC
AAATTCAAGGCTCTCGGCTACAAGCTCGTCTCTGACGGTACTGACTCTCAC
ATGGTTCTCCTTGACTTGAGACCCCAGTCCGTCGACGGCGCACGTATCGAG
GCTGTCCTTGAGCAGATCAACATTGCCTGCAACAAGAACGCCACCCCCGG
TGACAAGTCTGCTCTCACTCCTTTCGGTATTCGTATCGGAACTCCTGCCATG
ACTTCGCGTGGTTTTGGAGAGCAAGATTTCGAGCGTGTCGCCAGTTACATT
GACGAGAGTATCAAGATTGCCAAGGAGCTCCAAGCCAGCCTCCCCAAGGA
AGCTAACAAGCTCAAGGATTTCAGGGCTAGACTTGCTGTTGGTGACATTCC
TAGAATCAACGAGCTCAAGAAGGAAATCTCCGGTTGGTCTCAGACCTTCCC
TCTTCCCGTTGAGGGCTGGAGAGTGGATGCCGGCATCTAAATTTTGTCACG
TGTATGATTGAATGACGAGGACGAGGGGGATTTATTGTTCAACATATAGACT
TGAAGGCGAAAGAGGGGCAACTCAACTTATCTCTGGTTTGCGGGACAATAT
GGCGGGATTCATTCTGGAAAATGGGATGACAAAAAGTGGACAATTGCATGC

ATTCATCAGGCGTTGCAAATATCATGAGCTCTGGAGTTTGGTATAGCTCTAG
GGTCTGGGTCATCCGCGGCACTGCTTCAACGCGGTAGATTATAGGGAATTG
ATATCCATTACAATGATTTAGTGTAATTAGGGAATTCGAGAGCACCCATAAG
GATTCTCAATTGAATACGAGCTGCGCAACGTAGCTTCAGACCAAAAGTCTT
GCGAGCAGAACACGACGTGACGAACATGATACGCCATTCTCCAATTACCTA
TCACGTCATCTCTAAAACGCTACACGAGGGTATTCATTACTCATTATAAATC
GGTATTTCCCCAAGACAAGAGTTGTCTCTTTTACACAGCATCTACCTATGTT
CACATAGATTTCCCAAGCGGTCAATTGATTTTCTTTCAACCTAGGTACTTTTT
ACTTTTTATTCAAACTAACCATCAAGATGCCTCTTCCTGATGCCGACCTACA
TCCCGTCGCAACAAGTCTCGCCAAGGAGACGGTCGATGTACGTTCCAAATC
ATTTCCCTTCATTTTTTTAACATAGCAAATATAACAAAAAGAGCACCAGAGC
CACAGATATACACCTATGCACATACCATTAACTAACTCTACTTCATAGAAAC
ACGCAGCAGAGCGGCCTCTGAAATTGTTCGCGGGCTGGTTCTGCCCGTTCG
TGCAGCGCGTCTGGATCACGCTCGAGGAAAAACGCATCCCGTACCAGTAC
ATCGAGATCAACCCGTACCACAAAGGTCCGGAGCTACTGCGCGCTAACCCG
CGCGGCCTCGTGCCGACGCTCGAGGTCGCGCCTGGAAGGCGTTGTATGAG
AGTACTGTGTTGTGCGAGTACCTTGAGGAACATTATCCGGAGCATGAACCA
AATAGTAAGTACTCCTCTCTGGACATGTTTGGTGGTGGTTTATGAGATGGGA
GTGGAATGAAAGGGGACTGATTGAGGTGGCGGGGGTAGTATTGCCAGAGG
TAGGCAAGGACGATTACGCGAGAGCGAAGGCGCGTATTTGGGTTGACTTC
GTCACGTCCCGCGTTATACCGGCGTACCACCGACTCCTCCAATTCCAGCCCT
CGGCACACGCAGACGGGGCCAAGAAACTCGACGACCTTCGGCGCGAGTT
CAGATCCTACCTACTGGAGTTCGCGCGCGAAGCCGATCCCGAGGGCCCCCT
GTTCTTCGGAGCGGACTTGAGTCTGGTGGATATCGCGCTTATCCAGTGGGC
GGTGAGATTGTGGGTGTTCGAGGAGTTCAAGGGCGGGTTGGGGATCCCGG
AGCCCGGGAAAGGGGGCGAGGATGAGGAGGCGTGGGGACGCTGGAGGAA
GTGGGTGGGTGCGGTGGAGGGCAGGGAGAGCGTGGCGAATACGACGAGT
GATTATGTGCGGTATGTGCCGATTTATAGGCGCTATGCTGATGATACGGCGC
AGTCGGAGCTGGCGAAGGCTACGAGGGAGGGGAGGGTGTGCCGTGATGTT
GGGGTTGGGGAGTTGAAGGGGTTCTAAGTGGGCTACTTCGAATAGAGGCA
TACTTCAGTCACGAATTATGTTCAAGTTATTTTGGTTTTGAAAAAAATATATA
TATATATATGGTTTATTCAGAGTAGTTTCTAACTACCAAGGTATCCTAACGCC
AACATGCTCAAAGGCGAATTAAAGTCAATCAAAAATCGATCAATTGACGAT
ACTGCACCACTTGTCATTGCCGAAGAGATTGGCAAGAAGCTCGACGTATGC
CTTATCGACAGTGCAAAAATAATCATCGTATGTGCTTCTTACGAGCCCCACA
CGTTCTAATTCAGGGAGTAAAGGAGGCATACGCATAAAGTAAGAGCTGATA
TGCCTCGCAGTATCATCGGTAGAACACTCCTCAAAGCGGGCTCTCGCTTCT
CTATCCCGACAATCGAGCAAGATGGTAAGATCAGGGATCGGCTTACCCAAG

ATCGAGTGAGAATCCCAGAATTCATCCACGTTCCGTGGGAAGCCGTCTAGG
AGGATAGCCCGATACCTACCTGTCTTGGTCGCCCACTGCATCTTGAGGTACA
GAGCTGGACAGAGGATACCCAGGAGAGTAAGTCGGCCACGGTTTTGCAGA
TGGCGATTGTGTCCTCAACGGTATCATCGTAGTAATTTCTGGCTTTTTCTGTT
GCGTCCATGGGGAGAACGCCACCACCTGCAACGAAATCAAACATGTACCG
CTTTACAGTCTTCTTAACTAAGGGGGATCTTTCCAACTCCTCGAGCAAGTCC
GTGACCTTCAGGTGGTATAAGCTAAATTGGTCGGCGAGCTTAGCACCTATTT
CGTCTTTTCCCTTTGGGAAAATTCCGAAGATGCTGATAATTGGAAGTACTTT
CGCAGGCATTATGATTGTGTTGATTGGGTATGAAGGGTTTGGGGTTTGCGCC
CCGGGAGGGCGGATTGTTTTAAATATACTTGAAGGCATTCGCGTATTCTTTT
CACGCAGCGTGACGATGATACAACAATGTCGACAACAAAGCTTCTGTCATA
ATCATATTATTTTAGAATTCTATGCTTTGTGATGGGATACCGTTAAAAATGTG
ACTAAGAAAAATATCGATGCTTCTAATTTTAGACACTGTTTGATGGAAATTG
CACAATGACACATTGTTGAGAAATAATTGATGAATAGAACAAAAAGAGAAC
TATCGACCCCTCCTCGAAAGCCCTCCTCAGGCCCAGCCCAGGATGTATTCG
GCTCCTGGCTACTGCGCCCCCGCTGTTGGGTACTTATTTGCCTCACATTTTT
CAAGGTCCTTCGCGGAATCGAACCCTAGGCCACCCCATCTCAGAAGTGCCG
TTACGTATGCAGGAACGATTGAGGTGTCTGAGGCGTCAACCAACTTGACCA
CTTGGCCATGTTGATCTGATACGCCGCCGATGGAAACTTCACACCGTATTGT
GCTATTTAACCTTCACTTCGGGGCAGGTGCCTGGCGAATTGACCACTCGTT
AATAAGTGTTGCTACTGTGGTTGATGACGAGCGACGCGTGACGCGTAACGC
GACGGACCTGATGCGTAGGTACGTACTTACCTAAATCTCTAGTACCTAGTAT
AGAGTAAGTTAAAGGGCACTTGAACAATCTTATCATGACTTTTCCATCCATG
TGAGTGAGACAAAGGAACGTGGATGAAACTTCCATATATAAAGTCTTTGTT
CTTGACTGGACCTATGAGTCTGCAGCAATGATCTCGACAATAAAACAATCC
TTGCCGCGTGGTACACCTCCTTTAAGCTTATCTCCAGATCTACGTACGAGGT
GGTACATGAGCAACCGCGCAATCAAGCAACATCGCAGGGCGTCCATGTTGT
ACGCATGTATCGCTGACACAGCAGGGGGCATGCGAGGGACACGACGAGAA
CGTTATTCGGGTACGCGCGTTGACATTCATGCTTTTTCCAACGGGAGCAGC
CGTCAATTGAAATATTAAAGATACCTAACTTCTTCCCTCTGCAAGGACCTGC
CGGATAGATGAAGTTTCATTTATATGATACAATCTCAGCGGCGGCATGCAAT
GTAGGTAGGTATTAATTATACCAAACGTCTGCCCTCACCTCAGTCGGTCGAC
GATGAGGAAATTATATCAGGCGAGTTTATTACGTTTGTATTGTTTAGCACATA
TGAACCTTGCTTTTCGCAAACTATTGATTCTGCGATGCGAGAAGATGAAAG
GTCGGGACTGGGCGGTTGACGTTGGCCCGTATTTACGATATAGCGTGTATTC
CGAATCTCCCTAACTGGGTGCAAATTTAGTATAAAACCACGGTATCCTGGCG
GATAAGATTTATCACTTATCTCATCGAGCCCTTCGAACAGCATATCTGATCGT
ACTGTCGGACCGTCATCCCCGGATTTTGCCACGCCAAAATGCGCTTCCTAC

TCAGCTTCGCTGTCACATTAGGGGTGGTCGGTGCCACGCCCTTCGAACTCA
TTTCTAAACGCGCACTCGGAAACAAGAGAGCCGGGGATGATGCTTGTCCA
GGTTATCTCCAGCCTGGACAATTCGAGTTCCCCCACTACATTACGCAGATCT
CGGCGAAGCAGCCGGATCGAGCGTTTGGTCAGCAATACAACGCCAGTGTC
ACGCCAGGAGACATCTCGGCTCTCTTTGCACTCGATGTCCCAAAAGACCGC
GCCGATGCTAACTGCACTCTTGAGTTTCTCTTCCCTACCCAGGACCAGTTG
AAAACTTCCTATTATAACTATAGCGGACCTGGCAATTTCGTGTTCACGGGAT
ACTTGGCTGGTAGTTGTCCGGATTCACAGACGACCTACAATCATCAGCCGC
AGCCTGATATCTTCCCTTCCCACCCCATCCATATGGAGCCGGGCTTTGCATA
CACCATCGATGTTGGACCTTGCACTTTTTCGGCCGGGGGATGTATATCTGGA
AGTAAGTTCAACTCTTTTTTTTTCACGTAATTTTAGGAGTACTGACACGAAG
ACAGAGATATCGTCGAATGATACGAGCCTCGAGTTCTTTCAGGACCAAGAT
GATTGCCCGATTGGACTTTACAATACGTACAGCTATGGGCTGCCTTGTCCTA
AGGAGTACTGCGGGTAAAGCTGACAATATTTATGTATAATGGAAAATGGACT
TAGATCTCTGTATGTTAGCTAAGTGCTGAATTTGGTATATGCAGATAACTCGG
ATCGAAGAGACAACTGCAAAACTTTAGTCAATATAGTGACTTCCTGAGCGA
CCTAAGTTCCTAAGCGCCTCGATTTCTCAATCTATACCTGATTTGGATAATGT
CTAACGAATGAGAGATTAGCAATATCGGTGTAGATGTCATTGTGTGACCTTT
CTATGAGGAACCCCGAGAAGATGTGATGTGATAGGGCTGCCAGATTCCCCC
ATCGGCGTTCAGTGGCCTGATGGCCCCCAAATGGCTTGTTGCGTTCTCTAAT
ATGACTGGCTAGCCGTATCTATCTTATAGTCAACTCTGAAGTCCATTATAAGT
CGATTTCGGTGTGTGTCTGCACAATAAATTTTCTGTAACCAACACCATACTG
GCCAAGCCTTGGTGGGGAAACTAGACGAAGTAAGGGAGACATACAACCAC
ACTCAAACCGTGGCAATGTATGTAGGTAATTCATACAAACGTGTGCAGATA
AAATGCAATTTATGCCCATAGGTCTAGATAATCATTAGCTTTTCCTGATTTTC
TCGATTTTGCTTTTCATCCTGACTTGACAATGTGTATCGTTCCAACTCGCATA
CAAAAAAGAACTCTTATGCTAAACGTACCTACCTACCTAGCCGACATTTCTT
AAGCTATCAACCCGAAAATATCACGAGGCTACGGGTGTATCCGAGTTGCCG
CCCTTTTGGCGACAGAGATGATGCGCTAAATCAATTCTTGGAGACTGCGAT
GTTGATCAGTTCATACAACCAACGGGTGATCGCCAAAAGCATGAAGACGC
ACCTCTACTTCTTAGCCCTAATTGTTAATGGTGAGTATTCCGCGGCTATAGTC
TACCTACCTAAACCATTGGATAAGCTAGGTACTAAATCGTAAACCCAATAGG
CGGGTTTGCCGTCGTAGACTCTTGTACTACGTCAACCGTTTCCCTCGAGGA
CGCAAGCTGCCGCCGCCACAGATGCCCCAACCAACGAGAACGACGCAAG
CCAGATCAGTCTCGAGGTCACGGGCTGCGACAATTTTACCGGTTTTCCAGA
TCTGAAGACCGTGGCTCGCGTCAATTTATATAAGAACAATTTCACGGAACT
CAATCTGACTAGCTTCCGCATCACGGACCAAATCCGCATCGCCGGAAACCC
GAATCTCGAGACGCTGATACTCCCGGACCCAAAACCAGGCCAGACGATATC

TCTTCCGCCGACGGGTACGGATGCGCCGCAGTGGTCGGTTGTCGAGATTGT
GGATAATCCCTTGCTGGATACCTACAGGATCAAGTATCAGGGCGGATCCAG
CTTTTGGGATTGGGGATTGCGCAATCTAACACGGTTTACATTCGCGGATGGA
AACTTCCACTCCGATTTCTTGTGAGTGATTGTCGCTTGTTGAAAACTGTGTA
GCTCCTTACCCCTATTATCGTAAATGATCCACTAATCTCAGGCGCAGCTACC
CCTTATCGTCGAATGACCCAGAACAAGGTCATGTGTATGTAACTGAAAGTT
TCTTTCTAACCTCGACGGATTCGACCTATGATTGCGCCTACCTAGACTCGCT
AAGGTATCACGGTTCTTTCAAAGGAAATTACACTTGTCAGGGTAGGGGGGT
TGTTCCAGCCCTCGCAGAGAGGACGTCGAGTACTCACACAAGCCTACTGG
GTGGATTGGCAGTGTCATTTTGGTTTTTGGACTTGCTATAGTGTCTTCAGGG
GGGCTCATATGGGAGAAAACCGAGCTGAAACTTACAAATTCTCGTTATTGG
GGTTCTTCAGCAGGAACCACCGAAACATACCTATATCTTGAAAAGCTTGAT
AGGATAAATAACGTTTGACCTATGACTCTATGTTACCCAACACTGCCAGAAG
TCGGGACGGTCGATATTACCTAACGATGCATACATTCATGTACCTAGAAAAG
CTCATCCCTGACTGCTTCTACAAAATCCCCAGACGTCCTTTCGCCCAATTCC
TCTTTTGAATACCTGCTTGTAGATCTTTGTCATTAAGATGATGCGATCCATCG
AATTAAGTTCTGCAATTGGGCGATGTTTCCCAAATTGCACACTGGCTCGAA
GAAGGCGCCCCGTGGATAGGCAATGCCAGAGTGACAGCTAAAACTGGACA
TTATCTCGCTTCTCTTTTTCTTCCTCTTCTCTTATTCTTCTCCAACTTCAACAT
AGAGAAGCTCTTCGAAACAATTCCATTTCTGACTGACGTTTCAAATTTCTAC
AAAATGTCGCCCCTAGCAAGATTGTTATACATGGCTGCTCATCTTTGGGCTC
TCATTTACCCGCCCTTCGCTCATTTCTCTCTCGTTCTTGTCTGCCTTCGTTTT
ATTGTATGGTATTTCTCCAACGAAACCTCTCGCGAGTGGTTTCGGCGACGG
GGTTGGAATATTCCCTCAACCCCCGCGTCCTCTGATCACCCGGAAATTCTGG
CGGCTCTGAGACAAATCGAGGAGACTTTGGAGAGATCGAATGATCTCTCCG
AAGAGCGATGCGAATTGTTGAGGATGATCGTCGATTCCCTGGCTGAATTGA
AGAGATTGACTGCGGAAAATCACGTAGAGATCATGGGTGCCCAGTCAGAA
CTACTGATGATCTATACTGATTCCCTTGATACCGCCCGTGCTTTGCTCAGCA
GCCTCGACACGCTCGGTCATGACGTACACTTGCTGACGTTTGAGGTGGATA
GAATCTTATGGGTTTTTCTACAGTTGGTTTAAGGGGAAGAATCTGAGCATCC
TATGGTCGGTAGCGACTCAGAGGACTCGGCGTGAAGGGCTGACGAACAGC
CTTACACCCCATGGGGACGGGGTGGGCATTCGGCTTCGGCTGAGTGTCTCC
CCAGCGAAGGGATTGCATCTTGACGCGATGCTGGGGCTTAACACCTTCGGG
TGAACTAAGACAATCCCATTTGTGAATGTTGCTCACTTGGATACGGTTCGGT
GGGTGTTTGATTATTGAGACGGCTGTTGGTTGGTTCTGTAGTAGAGTGGATG
AAGTGGCCGGTCTACTCCTTCCAACACGGAAGTAGTTCAGGCTCATTGCCT
ACATCATTGTTTCCCCCCAAGCCATGTCCTTCATTATGTTAGTTGTGACCATG
AAGTGGGTTCATCAACTTAGAGACTATCGCAGATCAAGCTTTACCCCTCTA

GCCATCTGCTCTTCACCGCACCACTTGGCTTATCTCTGTTTTTGTGTCTTG
GAGTTCTGTGGTCATTGCCTGCCATTTCCAATGGGACAGTGAACGACAATT
TCCAAAGTCATGATAAGTCGTAACCGTGACCATCCATCCATGAGTTGGACTA
ATCCACTCTATAGAAGCATCCCAGTCTGTCAGACAAAACTTACACGACCCC
GTACAATGATAGTCCGCTTCATCGGGTGATTTTCTGGCTATAGGGGGTAGGT
TGACGCTGCTCAAACTTGCTGAGCTAACAGTTGCATGTTTACAGACCTTGA
GGTATTTGCACGCGAGGTAATCCTGCAAGGCTTGATTATCCGCATCTGGTTG
CATCCATCTGTACGTGGCGGATAAGTAAAGCTCAGGGCCTACCAGTTTAGC
TTCCCACGACTCAAATCGGAGAACACAATGCCCAACAAGTGAACTTTCAA
GGCAAAAGAACGTGTAGTCGTTGTCTCGAAAGTGGAGTTGGAGATACTGA
TTGCCGTAGTATTTCAATTGGGTTGGAAATATATATGATAGATATGATGGAGG
AAGCCCATGTCGAGGGCCAAGAATTCCGTAGTTTGTCACAAGTCTTGCGGA
ATTATAACGCAATGTGTACATCGAGCAATCGAATGTTACGGGCCTCGAAGTT
TCAATGATTGTCCCGGGAAGCCCGACTTTCTTTGGCTCATACATCCACCTGT
CGACATGTCTCCAATGAAGGCCATGCTGGTCAAATGGGATAAGCTTATTGC
AGGAACAGCAATAACATACCCCGGGTACGTCTTTTTCTAAGCGTTCAAGGA
GTGCTCCTTTGTTGATCGGATTGAGTTGCGTCTTGCGACTCTTGATTCCTAG
AACGGAGAATAGGCCCTTGCAGGTCAGAGCAAGGCAAATTCTGTCTTCCG
CGGGGAGATGATCCGAGATCGTTAGGATTATGTCGACAGGGAATTCTAATA
GGGGAGATGTCATCTTTGGAGGCTCAGGTTTCGCGTCTGTCTTTGAGCGCA
GCGCAAAGTGCACCAAATTCAGCAAGAATGACAAGAATGACATCGTTTAG
AATGGGAAATGGGCCTTTCTTGAAGTAGTGAGGAAGAAGGATGGAGGAAA
AGTTAGGAGTTGTCTTGGGAAATGGGACATGAGTAGGAATTTTATTAAATTC
TAGGGTTAGCTTTCAATAAGATTTAATAGAACTTACACTTCACGCGCGTAAG
AATCATTTGAGGATAGTGTACGCCTGTGGAAAGCATATACCCGCAGAAAGG
TTAACTCATCACGACTAAAAGGAGCAGCCTGGCGCGAATTGAAGCCATCCA
TGGATGCTTGTTTAGGAGCTAGAATGGCAGACACAGAGCGCAATATACTCG
CTTCACTTGAAGCCAATCAAGCTAACTACTTGGTGTTTCGTTATATTCGTATC
TTATGATTTGTCCTGGGTAACTGGCCTTTCTTTTCCCTGAACTCCTTTACATG
TTGCGAGGCGAATAGGGTATAAGTAATCCAAAACCGTGTAATCTGAGACGC
CTGTGAGATGGGTGGCGCCAATGGCTGAGCTCCTTAAACTATAGCGCTTAG
CCACTAAAGTTTAGCGGGGAGAAAATTACTAGACCTGAACTTTAGCTTCAC
TGCTTGACATAAAGACGAAGGCTTACTTAATCAGACAGGAGAAAGATGGA
ACTGGCGAGCGATATCTTCGTATCCTTGAGAGGATAATGGTAAGATTAAATG
CCCGTATCGAATTTCGTGGCTTGGCGTCTATGCTCTTCCGGCCGTGCCGGAC
AAGACACCGGCACTTCTATTGACCCACATCGGCGGTTGGTTAAGATGACTG
GTATATCGAGTGCTACCAGTAATCTGGATATCTACCATGTTGTCCTAAGACCT
GGGCTTCTTTTTAATCTCATACCTTCCTATTCCGCTGTTAGATCTACCATGATT

GGTCACGATTCTGGCTTTGATATCATTAGCGTGTGCAAAGCAACCTATGAAC
AAGAGACATCTAGAACCCCCCCCCCCCCCCCAATCGTACTAGGCAAGGAT
AGGGCCGGCAATCGTTGATTTTAACGTCCATTCTTAATACCAAACGGGTTGT
TTTTGGACCTTTGACGTGAACTAGTTAGGAAAGCAAGGAAATATCTTGATT
TGGATCTTGGATGTGTTGGCTTGGCTTTTCTAAAGCAATATCATGCCAATGC
TGACCAATACCGAGGACTTTGCCGTAGCGGCATCTTGGTTCGCAACGATGG
TGTATAAATCATCGACGGTCTCCGTGATGGGTTGAAACTTTCCGATACTAGA
TGTAAGGTACTCTCCCCGGATCGCCCGTCTGAAAAATGCGTATCTCTGCTTT
ATTTGCCGTTGCTTTGGCGCCCCTCATGGCGCTGTGCGCTCCGAAATGCGC
CAAACAGGGGGCCTCGAAATTCTGCACGCGGATCGTACCAGCCCCTCCTTC
GGAAGTGACAGAGGCTAGATTCAACGAGTTCGCATACCTGTTCCTGGTCAA
GAAGGATATCAATTCGGCGTTCCACTTTATCCTTCCCGAATATATCGTACGTC
CACGAATTCCAATTGCCGAATCCCCCCCTGTTCCGGACTTAGCTGATAGTCC
TCTGTAGAACCACAATCCCGCGGTCCAGAATGGCGCTCAGAATGCTCTTGA
CTTGCTTGGCCCGATTTGGGCAAACCAGTCCATCACTGTACTAGGCACGAA
ATTCGAGGGCAACATGTCGTGGCTTAATTACCAAAGCCCGTTCGGTACAAT
CGTGGATCGATTTCGCTGGGAGGACGGATGCATTGCCGAACATGTACGTGA
CCAACTTGCTTCGTGATAAGACCGCCAATGACTGACATCTCTCAATAGTGG
GACCAAGGAGAAAAATTCCCTGACTCATAGGCGATTTAGGTACGCTTGAGT
TTGCGAATTTCTTCGGACAAGCCGCATGGTTATCACCAGCATATCATAGAGT
GCGATTCTGAGAATATTTAGAAGAAATGAAAAGAAGTGATGAACATCATAT
TACTTAATTATGTGTGGGTCGTTATATTAACCTTGCATTTTGTGCTTGCTGTG
CCGCAGCTTTGGGCTTCTTTATTGCGGTTCTAGGAGATCCTCGATCTCCACC
ATGGATTCAATTGCCTACCTACTACCATAAGATTATCTTAATGGTCGGCATAA
CGATGATGAATTCCGCTTTTATTACACTCTGTGCGAAACATGACTGAAAATC
GCGCAGGTACAGCAGCAAGACAGTGAATAGAAATCTCAGATGATGCTCAG
GGTTGAGGTGGTATTAAATCGTCCACTTAGGGCTGAGTGTCCGGGTTGGCG
CGAGTTCAGTATTTATTTTGGGGGGTTGTTAACCGAAGGATCGGAACCGAG
CCACCTAAAAATACTCGCTGAAGGCTTTCAGTTTGGCGAGTTGCTTAATGTT
GCCTAGGTACCCGTTGAGGCTGAGTTTCAGGTACGGTCCACTGCATGGCGG
GATCGTTAGCGGTGAGGCTTGAAACTGGTTGATTCAGTCGACGAAGCAGTT
TCATTGGCGAATCGACCCGAAGGATTCAAACCATTTACTACCTCATTTAGGT
CGGACCACACGGTAGGTGCTTCTATTGCAAAAACATGGGTCTACTGCCAAC
AACACAGCTGTAATAGGCCTAGCTGTGCAGGTTACAGGGAGCCGTGGGAA
CAATTCCGCGCTGAAAGGAATGGAATAAGGGTTTACAGTGGGCTGAAGAA
GCCTTACAGCGTTTATCATGATATCGAAAGGCGAAATTCGGCTAATCATGGA
ATGAATTGTGGGAACACAAGAGCCGTATGCAAGTTTCGTATAGATAAATTG
GGCGTACCAGCAATTGTCAGCTAGGACTTGTTGAAATCCTCAGGCCCTTCA

TTAACTTCTTGCCAGCCCTGAGACGGGAGGGGTTTTACTTCTTCCTTTCCT
CTCTTTTGTCAACACACGGCGGCGACAGACTAACCCAAAATTTCAACGCTG
GCCACGATGACGCGGTCGCTGAGATCCGCAGTCGCAGCGTTATGTCTTTTG
GGGATAAGGGTGGTTAACGGGGACGATGTAAACAAATTCACATATCCGACA
GAGGAGGGCCTGAAATGGAACTACCGCGACACGATCATCACGGCATATGA
AGGCAACATCTCGGCACCGGTAATGTACACATTCTGTGGAGATTCCAAAGG
AAATGTCGAACGTGGGTTGCAGCAAACACCGAGCTATGGGTTGGCAACGT
CGCTGATGAGAATATAGAGGAACGGTACGACCATGTCAATGGGCCTAATAC
GACCGCAGCAATAATTCTCGATTTCGTCGTTGACAAGCCCATAACATCATGC
TGGTTCAACATCAGATTCGACAAGCCTGACAGTGCCGTTGGTGCTAACAGC
GTAAACTTCCAATATAACTCCACGGAAGGAGAGCGGGTACGTCAAGTTCAA
ATACTTATTTAGACCTAGGAACCGTGATCCGACGGCGCAGACGAAGGCATC
GGGATCTCTTACCCGACCCGGACCTCTAGCGCGATTCGGCGTAGCATTTAG
CCGAGATGAGTCTCGGGGATAGCCGATGGACCTCTTCCCTATTCCTCGTTCG
GGTCCCTCGACGGAAGGATGGACGGCTAATGGAAGAATTTACCAAACAGA
AAACGTTTAGTCTCGCGCCGACCTCCACCTCCACATCTAGTTCCCCAACTT
CAACTTCAACACAATCCGGGGTCAGCACCACGAACCCCGCCATATCGGCA
ACAAGTACGACGAACCCGTCCGCTAGCGCCGCACCTAGCCATACCCCCGG
GTTATCGACGGGGGCTTCAGCAGGCGTTGGTGTTGGGGTAGGGCTCGTCG
GGGTAGCAATTGGAGCAGGGTTGGTCGCTTTCATCTACCGACGGCGCAAG
AAGCGGGGAGTAGCAGACGACGGCAGCGAGAAAGGAGGAGCAGGAGGC
GGTCCAGGTGTCGCATCGACACCCCCCGAGTTAATGGGCTCGTCGCAGTAC
CCACCGCCATCGGCTGGCGCCCCGCCAAGCACGATGGCTTCGACTGATCCC
ACCAAGGCGGGTTACTACGCACAAGTTCCTAACAGCGAGATACACGAGGC
GCCCGCGTTCCAAGACGGTGTTGTGAAGGCCGAGTTGGGTGCACCGTACG
TCCCGTACCATGATGCGGCAGCCGGAGCGCAACAGCAGAAGTATCGCGAC
ACGCCGTCGATCGCGCCGACGCAGGAGATGGAGGCGATATCTGTAGTGCAC
GAGATGCCATAGAGCGTATCCATTAGCGCATTTGCGGCTATTATTCGCCGATT
CTTTAGGGAACTTATAATGTATTAATAAGAAAGTGAAAAAAAAATCCTGTCT
TCAGGCAAGTTACCTAACCCAGCCGGTTTTGCTATGTACTAGGTAGTAGGC
AGGTTTGTATATACAATTATTGAATATTGAATAGGGCTTGCTTTAATTTGCCG
GCGGTGGAAGAAGAAGCAAAAAGACTGGGCAGTTAGTCGGTAGGCTAGTA
CATATATTCGATACCTCCGTGTTAATTATTCATCGTTGACGCACGTCGGCAAG
GTAGCACGCAGCCGATGTCGTATAGATGACTGGTCGGGATCTTGCCGAGAC
CGGGTTTACGTATGCGCGTTGGCATTTTTGGCGCTCAACGCAAGGTAGCAT
CAACTCACCAAATCTTAGCCATGTCTCTTAACCGACCTATAGTTCGATGGGG
AGTTCGCGGAGGGCGGGTACGTGCACATGGTAACAAGGGAACAAGTTACC
CTCGTGGATCCTTTCCCCATTCCCCAACTTCCAAGGTTTCCCTGAATACTTT

GCAGTTATGTACTGTGATCTTCAGCGCGTACATACTAGGTAAGCACCGAGAT
GCACGGCTGTTCTTTGGGATGTGGACGTGTGTCTCCGAGATTTCAGGCTAT
GCGCGTCATCTGGGCAGGCAAGCCATGAAAACCCTACCGAGGCTAGAAGC
CGAATTTGGGGGAGACAGAAGAATATATGTAATAGAATAAAAGCATTGCTC
TTCCCCATTCTTTACGATCGATTCTATTCCCCGATCTCCAACTTCGATACCCC
CGCGGACCTATCCACGTTACGTATATGCTGCGGAGTTAAGCCCTGGGAAAG
AGAGTCGGAAAAGCAAAAGCCAAAGCCATGGCGGGCTGGTATTCTCTTGG
GGTCGCGCTGTTCGCCGCCATCGGCACATTTCTCTTCGTACGTTCATTCAGC
TCGTTTTAGATTCAAGTAAGAGCTTGTACAATGGATTTCTGTTCTAACAATT
GCTACCATAGGGCTTTGACACGGGCATTGCTACTACCAGTAAGTTTCTCACG
TAAAGACGGAGATAAAAGTCTAAATGCCACACGAACAGCTCGCTGACTTA
ATTTCTATTAGCTATCGCCCACGAGAGTTGGATCGATTACATGAACCACCCC
AACAATGGCCTCACCGGTGCGGTAGTAGCAGTGTACATCGCCGGCGAAGC
AGTCGGCGCTCTCACCCAGACTTTCATTGGAGATAGGTTAGGTCGCATCCG
GTTCATGCAGCTACTATGCATTATCGTGACGATCGGCACCACGATCCAGACG
GCCTCCGTCAACATCGGCATGTTCCTAGCGGGTCGCGCCATCGCCGGCATG
GCTGTCGGAGGCATGGTCGCAACTGTGCCCATTTATCTTAGTGAGATATCGG
CACCCGACCAGCGCGGTCTCATCGGTGGGATTTCTGGTTGCGGCATCGCAT
TCGGCACCATGATGTCCAATTGGGTTGGCTTCGCGTGCTCGTATGCGCCGTA
CGGCCAAACGCAATGGCGATTGCCCCTCGGCATCCAGATACCCTGGGGCGT
GATCATGTTCACCGGCTTGTCGACGTTTATGCCAGACTCACCGCGACAACT
ACTACGCAACGGCAAGGTCGAAGCTGCGAAGCGCGAGTTCGCTAAGGTGC
GAAGAGATTTGAAGTCGCATGAAGTGCATGAGGAGTTCGCGCTCATGCGC
GCGCAGATCGAGTTCGAGATGTCGCGTGAGATCAAATCGTACCGCGAGGTA
TTCACCCTATTTGGACACCGAGCTTTCGTCTCAATTATGGTACAAACTTTCA
CCTCCTTAACAGGTGTGAATGTAGTGCAGTATTATCAAGTTACGTTGTATAA
GTCGCTGGGCATACAGCAACAGATTATTCTTCTATTAGCCGGCGTATATGGC
ACCATTGCCTTTGTCTCGAATACTATAACGACGCGATTCTTGACCGACCAGT
GGGGTAGACGGAAGATGATTATAGCGGGCACGGCCGGTCTGATTTTGATTA
TGATATACACTGCGGTGATGCAGCGCGAGTTCCAGAACACGGATAACCGCG
TTGGCAAAGGTTTCGCAGTCCTGGGAATTTACCTATACGTGGCATGGTACTA
CGCTATGATTAACAGCACGACGTGGCTCTACGGCGCCGAGGTCCTACCCAT
TGCGCTGAGAAGCAAGGTAATGGGGCTCGCGGCGGCTGGCCACTTTATCGT
CAACGTTGGTATAACGGAAGCCGGACCCAGTGCGTTTGCTAATATTGGGGA
GAACTATTATTACGTGTTTGTCGGGTGCACGCTGGTCTTCTTAATCATTGCG
TACTTGTACTTCCCGGAAACACGCCGGAAGACGCTTGAGGAGATTGCGAG
GGCGTTTGGAGATAAGGTCGTCCTTGTTGGTGATGGCCCGATTGATGGGGA
ACATGTAGGGGGCCAGGACGAGGAGACGGCAAGCGTTGCGAAGGAGGAT

AACAAGGCTACGGCGTCCACCACTTCTACAACTCCCCCAAGCCACGAGTC
TGCGTCACAGTCATAACAGGTACATAGACAAATTCTTATCTGAGGTAATATC
TATCTAGTTGGGTAATACAATGTATCAAGCCATCACCAACCCGCCTGCATGC
ACAGATCTCGCATCTCATCGTGCATCTACAAACACACAGTTCCACATATACT
AAACTTAATGCAGGTTAGTTTCAGGGGTAGCTGCTTATGA

>Mosaic_sequence_58

CAAGGCCGAGGCGTCCCGGGCCAACGTGTCTTGGGCCGAGTACCACAATC
TCGACGACGTGATTGCCAAGTCGGACGTTCTCTATGCCACCCGGGTGCAGA
AAGAGCGCTTCGACTCGCTCGCCGAATACGAAGCGGTCAAGGACATGTAC
GTCATCAACAACGACGTGCTCGCCAAGGCCAAGGAGAGCGCCATTGTCAT
GCACCCCTTGCCGCGGGTCAACGAGATTGACCCAGAAGTCGACTTTGACT
CGAAGCGCGCCGCCTACTTCCGGCAGATGCGCTACGGTCTCTTTGTGAGTC
ACAACTGAGCGCGATTCAGGATGGGCGCTGATGAACAGGTTCGCATGGCG
TTGTTGACGCTTGTCCTTGGGGCGTAAGTCAAGGCGAAAGAGAAGGAGAG
GGAGCCACGAAAAGCCGAAAAGTCCATACTCGCATCGCTGTCACATACCTA
ATCATGCTCAACTATTTACATTCTCAAAAGTGCATCCCATCATCTGGTCAGA
CTAGATTAGCTACATATCCACAATGCATCGTTCCGTGACTCGTATCCCAGCA
GAGACCAAGACCAGCTCGCATACATCCGCATCTGTCTACATTAGGTGGGTAT
CGAGGGGGCAGACTAAGCGCACCCCCAGCGCGCATTTCGACGATCCAGCA
ACTCCTCCTTGTCCCGCGTGCAGACAGCCTAGAACAGGGCGTCGCAGCAC
ATCTCCTCGGCACAGCAGCAGCAGAGCACTGTCGTCAGTCCCGCTCACATC
TCCATCTCGTCTCGGTCGAGCACGACAACGTACTTCCAGCACAGCAGGCG
AGACACGAGTTGCCGCCGCCCCCGCCGCCTTTCTGCGGCTGTTGTTGCACG
TACACCGGCTGAGGCTGGGGCTGGGGGTAGCTGCAAGTCAGCGTAGCTGG
CGGCGCCTGCGTGCGCAACGCAACGCACTATTCTGCACCTGTCAGCTGCGC
TCATGTCCAGCACGCTGGACTCACGGCCTGGCTGTTGGTACGACTGTTGCG
GTGGCGGGGGGTAGTAGCCCTGCTGCTGGCCGTACTGCGGCTGCCCGCCGT
ACTGCGGTCCGCCGTATCCCTGCTGCTGGCCGTACCCGGGCCTGAGGTCAG
TCTCCGTTTCATTTTGACGGCCCTTGCGCTTGAGCCCTCGGGGCGGCAATG
GCAACGCCGCCCTGACGATGACGATCGTCCAACACGAATATGAGGCGGGA
GCTCGACTGACTGGTTGTTGTAGTACTGCGTGTCAGGAGGGCCGGGGGGC
GGAAGGAGGATCACGGACGTCTTTGGCCGACATGGTGCCCTTCCCTTTGGC
CTTTGGTTGCGGCTCCTGGCTCGCTCGATCGGGGTGAGGTAGCTAGCCTTG
GCTTTGGCTGATATCGGCGATCGGCTGTCCGACGGTAACGCTGTTGACACT
CCAGGGAGATTAGGCGACGAAGGACCAATGTAACAGACAGTGCCGCTGCA
GTCGATCTTTTCGGTGTTGCAGGTTATAGGTTATCATCGGCGTCACCCCCAC
CAGCCTCTTCCCGAGTGACTCGTACCGCCTCAGGGTAAGTCGGGGACGAAT

GTGGCAAAACCCAAAAGTGGCACTTTCCACCTTCCTCGCCACGGGCTGGC
GGACTACGGTTTCGCGCGGCGCATATTCTCTTGTCGCGCATACTGCCCGCCA
CCGCTCGATGCGGCCGTCTTGGCCTGGCCCTTCTCCGCGGCATCGCTATCGT
CGGACTTGCAGCAGCTGCCACAGCCGCACCCGTCGCATCCGCAGCCCGCA
AAGCACGCGCGCACACTGCGCAGGAGATCGTACTTCATCACGAAGGCTTT
GACGCGGCCGGTGGGCGAGGTGTAGTCGATGATCCAGCAGAGCATCAGGA
GTGCCCACGCGGCCCAGAGCGCTAGAGTGAGCGAGCGTCCGCGCTCGCGG
GACCTGGAGACTCACCCGCGACGCCCTGCCCGACGACCGACCAGCCCTGG
CCGCTCGACGCGCCCTTGGCGGCGTTCGCCACTACGGCCGCCTTGATCCCC
AGGCCGAACACCAGCAGGCCCAGCACGGCGAGGTAGAGCCGCACGACGG
CGAAAGCGAATCGCTTGAGCCTGTTCTTGAGCGCGCCAGCGCCGGGGCCG
GTCGAGGATGCCGGCGGCGGCGGGCGGAAGAAGCGGCCAAAGGTCAGCA
GGAAAAGGATGAGCGCGAGGTAGAACGCCGAGAGCGCCCCGAGGCCCAG
GGTGAGGTAGAGCGAGGCCGTGTCGACGCCCGAGATCGTCCCAGACGTGC
CATCGCGGCGCGTTGCCCCCGCCGAGGACGACGCAGCGCTGGTCGAGGCC
GAGTCGGTAGACGATGCCGCTGTCAGCCATGTGCACGTGCTGCCCGTACAC
GTCTCTATCTGCTCAGCGCGACCGCCTTGAAGCGGGCGTTGCCGCGGACTC
ACGCAGCGCGCCGACGTACACGCCCGAGCCTGTGTCGCGCACGATGGCCC
AGCTCTCGGCGGGGCCAAAACACACGAGGCCCAGGAGGACGAGGACGAC
GAGCTCGAGCAGCCACAGCCACAGTGGGATCGAGTAGCCGGCGTACTTGT
CCGCCCGCCTTGCCTGTATCTCCATCGTCGGCCCCGGTCCCGTCTTCCCCGC
CGCGGCCTTGGGAAGTGGTGGTCTTGGTGTCCGCAGGGGGCTGGGTGCGA
TCGGTCCGTTTGCGCGTATCGGTCGTCCTATCTTGATTTGACGACATCACTC
CTTGACCGCCGCCCAGAGCTTTGCTCTTGACAATCCACTATGTTCCCCTTGG
GCTCGTTGCCATGTTGACGCTTGTTACTTGTCGACTCGGTACCCCGTGACA
GACCTCAATAAGGATCTCCTCTCAGACTAGCAGAATAAAGACATCAGACAC
TTTGAAATAGACCCGCTCCACCACACATCCCGCATCACATCGTCGGGAGAC
CTCCAACACCGCCGGGCAGCTCACACACTCAGCGTCAAAGGACAGCAAG
AGGGATCAGCAAAGGATCGAGACCACCCGGGTGACTGATCCCAAAGGACT
TGTCACCGCGCTCAAGTGTCTCATCAGTTTCGCTGACCGAAAGCAGAGTG
GCAAGCGGATGCATAAGCAGTGACGCGTGTCATTGCAGTTCAAGTGCAGCT
AAAAGCATCCGCCGGCCATGCTCAATGACCGCTCCCGTTGCTGTCGAATGA
CGGCGACTGGCCTAAATGAGGAGCCTGGGAATTCGGTACGAGATGCTGGC
CCGGTGGCAAGAATTGAGGTGGGGGCTGGCCAGGGAATGGGAGCTGGGAT
TGGACGGATGCTGCGGGTCAACATCAGCATCGTCAGCCTAAGGCATCGTCA
CTCACATTGTGGGGTTTGAAGGGGCGATGCCATGCTCATTTGGGAATGACT
ATGACTTGGAGGTGTGGCGAAGTACATTCCGGACTGGTTTGGAGGCTGGTG
TGGGATGTGACCGGGACGCTGAGGGGTGTTGGGGTTATATTGCAGGTGAG

GCATATACTGTGGGTGTTCTGCGATATCAACTCCATTGAAGCACAAACGAGT
GGCAGCACTCACGAGGAACACCGTTTTGATAGTAGATTGGCAGCCCGCTCG
GTCCGCGGGGCGGTCCACCCATATGCTGCTGCATGGTTTGCCCAGGGTGTC
CAGACAAGGCTTGGAAGTGCTGAGGTGGAGAGAACATCGGATTTGCCCCC
ATCTGGACAGGCATCCCTTGCGGCATGCCCTACAGCGTCAGTCATACACGT
GTCAGGCAATACGATGGGCTGACCTGATTGTGCGAATAATACTGAGGGAAA
CTCGGCACCATTGAGCCCATCATAGGCGGCACACCCGATGCGGTTCCGACC
ATTACGTGAGGAGGAGGGGAACCGCTTTCTTCGTATCCAGGACCGCCCGGT
GCACCAGCTGGACAGAGAAAGACGCGTGTGATCGCCGGCCAGTGTATGGC
CATGCGGGCTCGACAGTGGAAGCGCTCGGGACCTGTTGATGCCGCCATGG
GTTGAAGTCAGATCTTGGGTCGACGCCACCTATCCTGCGCGGGGCTTGATC
CCGGAAGAAAGGATTTTTAGGTGCGGTTGTGGCAGACGACGGAGCAGCGA
CGGCCTGCGCGGGACCGAGTGGACGCTCGGAAATCGTGGGTGACGGGGA
AAGTACCTTCGTCTGCGGACTGGTGGAGGACTGACCGGGCTTGAAAGCGG
TGGCATTGGGATTTGGTACGAATGCGTTTGCTTTGACGTTGAGCTTGGCAG
CGCCAACCGCCTGACTGGTCTGAGAAGTGATGCTGGGAGTTGGTGATGTG
GCGCGAGGGACGTCTTTCTGCGCTGTTGGCGGAACGGCTAGCGGAGCGGG
CTTCCGTTTGGCAGGGTTGAAGGGCGGAATCTCGGCAATCTTCATGGACAA
TCTTCTTCGGGGGCGCAGACTTCGGCGTGAGCTCCTTAGTTGAAGGTCCTG
AATCTTTCGATATCTTCCGTGCTTCTGCAGCTGCTTTGGCCGTCGCCAGTGC
TTTCGCAGCCTGCTCCTCAATCGCTTTCTGCTTCTCCTCGTCCTTCGCCAGG
ATGGCAAGATATCCTTCGGCATGGGCAACGGCACCTTGAAGTTTGCCTGAA
ACTTCTTCAACTCTGCGAGCTGGCGATCTCGTTCTGTCTTCGCCATGGACTG
CTTCTTGGCTTCGACTCGCTCCAGCTCCGTGCCGACAAACTGTCGGAACGA
GTCAACCACTCCAGGCCGGGGAGTTTCCTGCGTATCAGCTGGCACGATATA
CAATCTCCGGTCGTATCTACCTTGACGTCAGAGGCAGCGGCCAGCGCACCT
GGCGGGGCGATGGCCGATGCATCAGCGCCTGCAGACGATGGTTTGACTTCC
TTCGTTGGCGGGGGAGGAGCAAGCCCGTTCGCCTCTGCAGAAAACGGCTT
GAGTACATGCATCTCGCTGGTCGTCTTAGAAGGCACCGAAGGCGGCACAG
CAGAGGTGGTCGCGGGGGTCGTAGCAGGAGATGGGACGATGGCAGGACC
GGCGGGAAGCGGGGTTTGAGATGGCGTTGGAGAAGACAATCCATTGAGAC
TGCCGTTGGTGCGAGTCCCGCCACCATTGGCAATCGTGGGGTTTGCGGGCA
ATGAGGCTCTCGGCGCTGATGCTGGCAATCCTCCCGCCCCGCCTCGCCTGA
GTGACGGCGGCACGTAAGCATTGGGGTTCCGGGCAGCCGCAGGCGTGGTT
GCGGCCATAGCATCCGCGTGTCCGCGCTGCACCCCAGAGTATCTGGGATCA
GCGCCACTCGTCCCCAGCGAGGGCCTACAACACACTTCTCCTCCTCGTCCT
TGGCCCAATCATCCACAATCTGATTTCGTTCCTCCTGCACATGCGCGTTGAC
GCTGGTTTGCTGATCGTGAGCTGGACACTGGCGCGCCGGGAGCAACTCGC

CCCCATGATTTCGCTCGCCAGCCTGTCTGCTTCTTTCTCCCTCTTCCGGTAG
TCTGCACCATTCCTGTTGAGCTTGGTGGTGTAAATCTCCTCCTGGTAGCTGG
TCTTGGTCCCGAATAATCTCGCGTTGGTCTCAAATTGATCCCAACGAGCTCC
GCCGGCGTCACCAAACGTTTCTGAGTCTTTGCCTCCCCCTACTCCGTTCGG
ACGCGGGCCGTTTGGCAGGATTTCTGGCCCATCACCCGCCCAAGGCTGCA
GCTCACGCTGTGAGCCCGGTGTAGAGTTGGAAATATCGGTGTCTGTCTGGA
ATCCTTTCCGTCAGTACAGCTTACGAAGCGCGATCTCGGCCCACCCTTTCCT
GGAGCGGGCGAAGGTCGGTCGCTGCCGATTCTGCTCATGCTGATTGTCAAC
TTGATCCCAAATGAGCACAGCTTGACTGACTGGTCGTTTATGGACGGGATA
GCAGGTCTCGGACTGGGAGAGGCCAGCGGGGTCCCGCCTCGTGAGGGGTT
CCCTGCATATTTGGGGGTCCTCCTCGCCACGAACCTCTTCTGCAGACAAGT
CAGGCTCCGGTACCCATCATATGCGAGCTCGTGACCAACATACCTAGAGTC
GGGACCGCCCCCTTGACTCCCTCCGCCGCGAGGTCCGCCTTGACCCGGCC
CACCGCCTCGTCCTCCTCCCCTCCCACCACGCCCGCCGGTCGTCACCATGT
TGCAAGGTCGTAAGTCGTCGTGAATGCGTCAATGATATGCTTGTGTGAATG
GGTCTTCGAATGGAAGGAATGATTGCGTTGTGGCTGAGGTGTCTGTCGAGT
CAGGCTTGGTTCTGAGCTTGTGTTCTATGGGAGGGGTGCTGGAGAGGCAA
GGGAGACGGCAGCTCAGGATGAGGGAGAATTGACAGTCCGAGGTGTACGT
TCTGAGCACATGTTGGTGGGTAGCGATACTACGGAGGAGAAGCATCCGCGA
ACCAAGGGGGAAAGGGCGCGTGCTCACCCCGATCTGACTGACACACTTTG
GTCCTGCCGTCCTTCTGCTAGCTAGGAAATCTCCACCGAACGTTGCTGCCC
ACGCGTAGTGTCGAGTCTGGTCCCGCCCAGACGGATCGTGTCGTGGTGAC
AAGTGCTTTTGAAGCTGTAGTTAGAAGCCCAGAGCGAGAACGAGACTGTG
AATGGGATGATGAATGGCTCGGTTGCGTTGTGAAACCCGGAAGGTTCCCCG
CACGCTGGTGACGCCAAACGAGCACCGCCAAGGGGGGTCCTATTATCAGG
GAAAGAGTATTTTGAGTTAATCCCGTATCTCACCGCTCATTGTGGCACACCT
CCACTTCTAAGGTCACTCACTCTCCTTTCTTCGACCTCTTTCAACCCACCAC
CCCTTCATATCCTTGGTATCGCTTGTCTACTCGCGTCATCTGCCATACATTGT
TCGGTCATCGAGCACACATCCCATCACCTCTCGAGATGATGCCCTGGCGAT
GACTCTGGTGATTCCCCTCGACCCGCCCACTGAGGCAGGGCCTTCCCGACA
ATGTCCCGCCACCTTCGGCACTCCGGCTGCCGACCCACTTGAAAGGCTGGC
AGATGTGGTCAGGGGTTTGAGGAGAGCAAAGCGCATCATCGTTGTCAACG
GTGAATACCTTGGGATCCAGATCGTATCCGATCTCAGGCTGATGGTACAGGT
GCAGGCGTGTCCACCGCAGCTGATATTCCCGACTTTCGGTCTGCGTCCGGG
CTGTTCAGGCGAGGCAAGAGTGCCAAGGATCTTTTCCACATCAAAGCTCTG
ACAGCAAGTCGCCTATTTTTCCCGCAGACGTAACTGATGGTCACAGGATGA
ACATCTGCTGCCGGCTCATCATGCCTTACTCAACGAACTATGCTCCATGTCT
GTCTCGGCCGATCCCACCCCATTTCACCATTATCTTCGCTCTTTGGACACAT

CTGGGTGCCTGCTTCGATGTTACACACAGAACATCGATGGTCTCGAAGCGA
AAGCTGGTCTCGAGGTTGGCATACCGGCGAAGCGGCACCACGTCAACAAA
AAGCATCTACTTACGGTCACCGCGGATGTGGCCCATGAGATGCCGCAAAAG
GCTACGCCCAAGTGCATTCCTCTGCACGGCCAGTTGACGCACCTATACTGC
TCACTTTGCTCTACTACACTGCCGATAGCCGATCATCTTCCTCTACCTGCAG
AACCGCTTCTCTGCCCGACTTGTCACCTCTCTGCGATAGTGAGGGACGCAC
TGTCCGAACGGCGCCGGCGAGTCGGTCAGCTTCGGGCTAGCGTCGTACTCT
ATGGCGAAGACCATCCTCAGGGAGAGGCTATCGGTTCCATCGTCGAGCGGG
ACCTGAGAGGCTCCAGGCGGAAAGGCGATGAGCAGGGCAGGGTCGACTT
TCTGCTGGTGGCCGGGACTAGTCTTGCGATCCCTGGAGTCAAGCGAATCGT
CAAGGAGATGGCGCGGGCGCTACGTCCAAGGACTTTATATTCCGCGGACGG
CGCTTTCGAGACTACTGGAGCCGTCCGAACGGTCTACGTCAACGAGGAGC
CGCCCAAGGGAGCCGAGTGGGACGGGATATTTGATGTCTGGGTTCAGGGC
GACGTGCAACGATTCATCGCTCGAGTCGAGGATACAAACTTTGCCTTGGCA
CCACCCGTCACACCGCGAAAACGCAAGGCCATCATAGGTCCTAATGGTCTT
CCAACGCCGCCGAAATCGAGATCGCGGACTCCGGCAGCCAAGCGACACAA
GACGTCGAGCGATCTACCGAGCCCGACCCCGAGTCCAGCAGCATGCGGCC
CTTACCTGCCGACGCCGCGGGCGACACCAGTAAAGTTCCCGCCATTAGCCC
CTGTACGACAAAGAGATGAAAGCCTGACGCCTTTATCAGAGCTAGAATGTG
AGCTGGACAATCCGTTCGCGCCATTATCGCTGGGCATCAAGATGTAGCATGT
AAGGTCATTTGTAGAGATTTCCATCCTCATCGTTGTTCGTACCCACGCTGTG
ATATGCATATTCTCGCATCAAGCCACAGCGTCAAAGCTCGAGCGTCGGGGC
GCCTGTTTCAGATAGTGAGCACGGACGTTTCGGTGAACGCAAGGACCTCCT
GTGACAAACCATCAAAGGACCCGCGGGTGGGAGTCTGAGCAAACAATGG
ATCCTCCTCTCTCAGTCTGAGATGTCGGGGGTTATGCATACTGCCATCGTTG
CTCTTCACAGAGGAGAGCAGTACGTCCGGTGACTTCCGCAGAAGCCCTAG
AGACAATGAGACTCCCGATGGGACGCTCGGCATCAGTCCCAGCAGGCAGA
CAGAAGGGCAGGTCGAGAGACGCCCCGGCAGACCGAGTCAGAGAAGCCA
GAGCAGAGGGGCCCGCTCCAGCGACCAGCTCGTCCAAAGACCAAGCAAC
AACGACCCAGGCCGCGCATACGTCGACGTCCACCGATAGCGGGCCTGTGA
AAGTAGGCACGACGCTCGCTGCGGCCTCATCTTCGTCGGCATCGTCTTCCA
CATCCGCATCAGCTGCGGCCTCGCACTCGTCCTGGTCGTCCGCCGCACGCA
CTTCCAGTAGCTCCCTCGGCACCCATTCAAGCCTCAGTAGCTCAGTGACGC
ATGCGACCTCTTCAGATCTCGCAACCTTAACAAGTACCAGCGCGAGTCTCA
TATGTGAGTCTGGTAACGTGGCGCTGCCGCGCGCTGTGCGCTGAGAGCTGC
AGTCCTCACCAGCTTCGCGCCAGACTTCCCAGTCGAGACTGCGAGCGCCG
CGGCCAAGACGTCCGGCCCTGACCATGTGTTTGTGACGAACCAGCTGGGT
GACAGCGGTGAGTGTCCGGTGTCCGCAGAGGCAGCGCTGAGGTTAGCCAT

CATCTTCGCCCTGTTCTGGGTGGCCGGGCTGCTGGGCACGATCGCGATGAT
CCGATGTACGAGATGGACGGACGGGCGGCGGGGGCGGCAGGTGGATCAGA
TGATATCGGACGATATGAGGTGGGCGGAGGGCAGGGAGGTCTTTGGCGAG
CGCAAGGTCGTCTGGGAGGGCGGGACCGCTGGTGGGGCGGACGAGGCGG
CGGGGCTGCTGGACGGTCCCGTATCCATGCCAGCTCAAGACGCTGGGAGA
GGTCAGCGTGGGAGGGCGCCCCTGGGGCCGAGCAAGCTTCGTCGCTAGGC
CTCTCTAGGTCGTCTGGTTCAAATGCGCTGGATATCAGCATTTTCATGGTCT
GGCTCTCACGATTGAACGATGATTCCAGCCCCGTTGCATTCCGGCTGCTCG
TGACCATGCGATGAGACTGGAACTGGAGTAGCCGCACAGGCGGCCTTAGG
CGGCTAGGACTATCACCTGGATCATCCGTGTTCCGACGCCGAAAAGCCAAA
AGTCCACTTCGAAACGTCCCTTTCCTTCTCAACCCACTTCTCATTCTCGCCT
CATATTACTCACGACATACATTCAAGATGGCCACACGCAAGTTCTTCGTCGG
GTACGTCGTCTCAATGCACATACACTTGCCCCGTTGGACCACGTCCAGCTC
TCCTGCGGGCTGCCTCCCCGTCGTCCTTCGAGGGTTCCGCCCTAACGTCCA
GAGGCAACTTCAAAATGAACGGGTCGATCAAGTTGATCACCCAGCTCATCG
AGCAGCTCAACGCCGCCAAGCTCGACGGCACCACTGGTACGTCTTCCGGC
TCCCCGCTTCCCTCCTTCACCTCCTGGCGCTGAGATGAGCGGATACACCCT
CACGGTGGACCCAAACGGCAGCTCCTTTGGCCCACCGACCACCCATCCCG
CCGTCCCTGGATTAGAAGCACATCACTGACTCTCGCAGAAGTTGTCGTCGC
CCCGCCCGCTCTCTACCTCCTGCAGGTGCAGTCCACCCTGCAGCCTCCGGC
ACAGGTCTCGGCGCAGAACTGCTTCACCGAGTCGAGCGGTGCCTTCACCG
GCGAGATCTCGCCAAACCAGCTCAAAGACGCCAACGTGCACTGGGTCATC
CTCGGTCACTCGGAGCGCCGCTCCCTGTTCGGCGACACGGACAAGCTCGT
CGCGGACAAGGTGCGCGCCGCTATCGGTGCCGGTCTCGGGGTGATTGCGT
GCATTGGCGAGACGCTCGAGGAGCGCGAGGGGGGCAAGACCAAGGATGT
TGTCGAGCGCCAGCTGGAGGCTATCGCCAAGGAGATCGACACGGCCCACT
GGGAGTGAGCCTCAAATGTTTTTGGGTTACTTCAGTGCGGTCGCTCATCTC
AGCTCGGTCGTCATTGCCTATGAGCCCGTTTGGGCCATTGGTACCGGCAAG
GTCGCCACTGCTGAGCAAGCCCAGGAGGTGCACAAGGACCTCCGTGGCTG
GCTCGCTAGCCGTGTGTCCAAAGAGGTCGCGGAGAAGACCAGGATCATCT
ACGGCGGCAGCGTCAACGGCAAGAATTGCCAGGAGCTGGGTGAGTCTTGC
ATATGATATCCCATGCAGCTGCTCATGCTCAGCCGCCGCTGCCGACATTGAC
GGTTTCCTTGTTGGTGGAGCTTCGCTCAAGCCCGAGTTCATCGACATCTGC
CGCTGCAAGAACTAGAAGACGTCACGTAGCTATGCGAGGGCAGGAAGAAT
GTCCAGATCACGCGAGGCCGAAGCAAAGATGCATAAAGCAACGAAGCAAA
CAAAGTCGATCACCATGCCCACCCGCTGACCGTCTTGCGTCGTAGCACTCC
ATCTTCCCTCACGATCCCCGCGCTGCGCACCTTGTTGAGCTCTTTCTCCCAT
CTCTTCTCCCGCTTCTCGGCCACCGACTGTCCCTCTTTCAGCAAGACGTGT

AAGCGCGCGGTGGGATGGTGCTTGATGCCCGCCCGACCTCTGCCCTTGACG
TCCAGCCGCATATGCTTTGGGCCCTTCGATACCCACGTCTCAGCTGTCCGTA
AGCCGAGATTCAAGAATACGTGTAGGTCCACGCACCGACAACCAGCCGGC
CTCTACGCAGACCCTTGTCGATCGCATGATCCCTCGCCAGACAAAGCGTGC
TCTTAATCCACCCACTTGCACGCTTCTCGCTGAACTCCATCTGAAGAATCGC
CTCGTCGATTGGCAGACCGGCAATCTGCCTCGAGAGGTCGTTCAGCTTTCG
CGGCGACATCTTGCGCTGCGCCGTCTTGTACCGGTGCTCCGTGAACATTTT
GCCTTTCCGCTCTTCCGCCTCTGCCTCGGCTTGCAGGTCGTCGAAGAGATT
CCCACTTATGGGCGTGGATGCCTCCACGGTGGTTGGTGTGTCGATGTGGAT
GCTGATGGTTCTGCTTGATCTTTCGGTCGCAGGGAAATCGGGCAAGCGCAG
ATTGCTCAGGCCAAAGATACTGCCAGTCAGCCAAGACTATACCAGATGCGC
GGAATCATGCCTCACCTTCGTCTTTGTTGTGAGGAGGATGCAGTAGGCGGA
CGGAGGAACGGTCTGAGAGGCGCTGAGGACGGGCCAGCAATGGCTGATTG
AGCCGCTATGAGTGAGCTGACTTCACTTCAAATATGCGATGCTCTCACAAG
CAAAAGCTGTGCGGGCTGCGCGACTCATGATGGCCTTATGAGCAAGCTTGC
AGGAGTATCGTCAGGTCTCGAGAGCAACTTTCGTCCAAAGACCAGGAAAG
TCAGGAAGTCCGTGTTACCCCCACTTAGCCACGTGTTGACTCGTGCACCAA
GCAAGATCAGTGAGTGTCCATTCTGCTGTTTGCCAGTCTCCTGCACGCCAA
ATAGCGGGATCTGGCATATCGACAGACCGCAGGACGCCCCTAACTACCCTC
CAGTAGTTCTGGTCAGCCGGTGGTGGAGACGAGACGAGCAATATGGCCAA
TCTCACCGCGCTTGACTCCGGCAACCCCGACGGAGACGATGTCTCCAAGG
CGAGCAAGCTCGCAGCGCGCGCCGCCCGCTTCAGCAGTAAGCTCCCGGGT
AATCGATACAAAGAGGTCAGCCCGACTTGCTGCAATATCCCCGCGCTGACG
CTCAGCTCGAGGACATGCGAGCTCGGCAGCGCAAGGCCTTTGAGCAAGAC
GGGCTCATCAAAGTCGGCAAGACCGAGCTGGGGGATGCCATCGACATGCG
GGGCACCTGCGAGACGATGTGCTCGGAGTATGAGCGCGAGTTTCGCGAGT
ACACCCGTGAAGTGCACCCATTCGAAGCTGTCAGTCTGCAAGCGCTCGTG
CATTCATTCGCGCAGCTGATGACGAGGGACCGAGTAAACGGATCGACCCTG
CGAAAGCGGTCGCTGCGTATTCACGATCAGATGCCGGAGCAGGCCATGGTG
ACTCTGCCATCTTGCCTTCTGACTTGCGCACACCGCAGACCTTAATGGTCC
GTGCTACCAATGCTCTCGTGGGAAGTGTCTGACGAGCAGCGCACCCTCGA
CTACCTCTTCTCGGAGGTCATGCCCCTCGAACCCCCATCAGCGTCGACATC
GTCCCCCTACTCCCGGGCGCGCAAGGCCCTAGGGTACTCGGCTGGATTTAT
CCGCGATCGTACACGGGCCATCAGGAAAGAATTCGCCATGCAGAGCAGCT
GGGGCCATCAGGAGGCCATCAACTCTTTTGAGCGCATTGCGAGATGGCACA
TTCTCTGCTTGCGAGAGCTGCAAGAGGAGACTGGTACGAACACTGATATGC
ACATCGACGCTGCAGAGCTGGGAAGATGTCAGCTCCTGGACGGATACTGC
GGATCTAGCTGACATGCTAGGCTTCACAAGTCTGCGGCAGCAATACAACGA

TCGTCGCGAGGAGTCCGGTGTCGACATGCCTTGTCCGAACGAGCCTGAATT
CCGCGCCTATATGCTCATTTTCGACCTCGCGAGTAAATCCGTCTCGATTCCT
ACAGCCGAGCTGCCGTCTGCCATCGTCGACCATCCTTTGGTGAAATTGGCC
TGGGAGATCCGCAGAGCCGCTCAGAGAAACTTCGACAGCCAGAAAGAGG
GTAGCAAAGCCAATGCCGAGCTGGGAGCGAACCTTATCACCCGCTTTGTGA
GGCTGATGAAGGCCCGACGGGTACCGTTCCTCATGTCCTGCCTAGTCGAGA
TCAGATTGAGAGAAATACGGCGGAGTGCACTGAGAGCGCTCAACCGGACA
TATCCCCGACTGCGTGGAGAGCCAGTCAGGCTTAACGAGGCAGGGGAAGT
GGTGGAGAGGAGAATGGTGCTACTCAGAACACTGGACCGGATGCTGGGAT
GCGAGCAACAAGAAGCGGAGGAGTCCGCGTGGGACGACGTGTTACCTGC
GGATCGCTCATCCGATGCCGAGGCCGTAGACGTGGTAAGAAAGCTCGAAC
TCGAGGTCTACTCGGATCAGAGCGGTCCCGTCGGAGCCCTGATCAACGCTG
GGGCATGGTTCAATGGTCGGTCAGACCTCGGCCTCCGGGTACACGCTGATC
GTGGTAGACAATCGTGACGCCCCGTATACTCGGAGATGGCTCTCAATCACC
GCCAAAAAGGGCGATGTCTCGTATGTGGACATTGTTAACGGAGATGCTGGC
GTTCGAGTCTCTGGTACCCCGATATCCTCAGAGACCAAGCCTCCCAGAAAA
GCTTTCGACAATCGTCCTCTCTCCGTCCCCTCAGCCGTCGGTGCGCCAAGG
CTTGATTCTAGCTTTGTCACGACTCCTACTGTGTCGACCACGCCAAAGAGA
AGCCCGCCAAGATCAGCATTCGAATTCCCCTTTGCGCCGGCGACCACCGCG
CCTTCAGAAATTCCGCCAGCTGAAGAGAATCCGGTCATAGCGGCTGCTACC
AACCACTTTGCCCCTGTCGCGTTGCCCCCGACGCATAGCTCGCAGCCCGCA
CCTGCAACCCCACCTCTGCCAGCGTTCTCCTTCACGCCTCCGACGCCGCCG
GTGCCGGTCAAGCCACCACAGCCTGCCCCACTTTTTCCAAGCCTGGGATC
TTTCAAAGCACGGCCCAGCCAAGCAAGCCGACGAAGCCGCATTCTTCATC
TCCGTTGGCCACGGTGGCTCCAATTTTGCCTTCATCTCCATCCCCGACGCCA
CCGCCTGTCGCTGTTACTCCAGTGTTATCGACGCATCATTCGCCCCCAAAGA
CGCCCTCAATACAGCCGCGAAGAAGAGTATCGCCCGAAGAGCGCGCTAGG
CGCGTCGCTACAGCCCCGACCCTATGCTACGATTTGATTCGAGAAGTCGTT
GATCGCTTGATCGACGAGAATCGACCGATGTTGCGGTCCGTCATCAAACAG
GAAGCTGCTGCGACGCAGTACACAGCAATCAAGTGTCAACGCTTGTCAAA
GATAGAAGAATGGGCGCGGGGACTTTTCGGCATGTTGTACGCCGATAACGT
CAAGTCCATTGCGAAGAAAGCAATCCTTGGGGAGGTTGCCCGTCGCGGCA
TCATCAGGAATGCTATCAAGCATTGGCGCCGATGGGCAAGACACCGACGCT
ACGCTCGAGAGGGTTCTGAACGAGACCACTCCGAAGCCTTCGAACGCCTG
GGACGGATGGGTATTGGTCGCGGCCAAATACTGGGCACTGCCCATAATGAA
GTGCCAGACGGCCCTGACGACGGCTTTGACGCTACAAGACCCGAAGTGCG
AGACCGACAAGACGAAATGGACGTTGACAACGCGCTTCACCAAGTGGAA
AGTAAGAAAGACCGATTTTACGCGGCCTCTACTTTCTTCCACACTATCGCCA

GATACGTGGCCAGTCTGCTTAACCCGGTAATTATCAGAGTCTGCATTTGCCT
TGTTTGTGTTGATGGGTTAGGAGGTTAGGGGCATATCTCAATACAAGATGAG
GTAGAAATTGAGTTTACAGTGGAACTTGACTCCTGCTGTGTCTAGCATAAG
TGGTTGTAATACAACATCCCTAGATGAGCATTGGGTGGTTTTAGTGAAGACT
CCTTTTGCCCCTTTGTGGGTAGCCGTTACACGGTCAAATAATCCCTCAAGGA
CATGACTGTTAGACATAGATTACTCTGAATGATATGGATGTGATTCGTGAATA
TATTCGTAGTCTCTTAATGGCATTAGTCAGATGTACAATTTTTAGTACATGAT
GGTTGGGAACCATCGCCAGCATTCAGCTGAGACCTAGCATCCTCACAGGTT
GACCTAGAACGCCGACTCATGCTTCTGCTGTTCAAAGGAGTTACGTCTTGC
TATCGTAATCTCTTCCCCATTATCGTACAAGGCGGCGACCATTCCTGTCACA
AGTTCGGTCGTAATGCGCCGAAACTCCGCAATACGTAGTGACTCTTATGCC
ATGGTTGATGAACAGTCTTTCCCGTCCTATACCATATCCTCCCATTTTGGACG
CATCATCTCTTCTTCTTCTCCGGGAACGTGAGTCTCCCCTTAAACCGCGAAA
GGTGGTAGTTGATCTCGCTGGCTTTCGGCCTCGCAGGCGGAGGTGCGTCCA
TGCAATTCCGTAAAGCCTGCTCAATCGTGGCACATCCTGCCTTGTTGTGTCC
CGCAGATGCCCAGCATGCTATACAGGTTAGATAGTTAGCCGAGGTTCGATAT
AATGCGACGGCACTGCCAAATGCTGTAGGGTATAGCATATGGTATATAGCAG
AGAGTATATTAGATATGGGGTTGATGGGGGTTGATGGCGCATACCTAAGACG
GAGGACATGAGGGCCACGCATGGCTTTTCCCTCGTACCGTTCGGGTTGTGA
ACTCGGAGAGACCTCAAGGGAGGAAGACGCATTGGCTTCTGAGTGGCCGA
CATGTTGGAAGGTCGAGCTAGGTATCCAAGTCGTAGACTGATGTGCCGTCA
ATGGTCTTGTCAATCGTCAATCGAAAATCGCGTGGGGCGACATAAAGCTTT
TCCGATTTTTGATTTTCCAGTTATCCCACTATTCCCAAAGTAACCAAAAAAT
CAAGAAATTGGAGCTAGCTAGAAAAGATTTGTCACGTGTCTTAGTCTGTTC
TTAAAGGCGGGTCTGGGAACATAGCGCTCGTTTACGGAGTCTAACCCGTAC
TAGGGCTAGTGTGATTCAGAGGCTGGTCCTTGCTTAGCTCGTTCCGCCCGTT
GTAACCATTATAAGGCTTATTTTTTATTTCCACCACAATTTCAACCTGCCCAC
ACCACCCATAACACCCATAACCATCCAACCGAACCCGATAATCAGATCGCAT
AGCATCTGATAATCTTATACGCTCCACTCCCACGAGTCCCACGCCCGCACCA
TTGGAGATGAGGACGGGGGATTTTTCTGGAATAGATGAGAACCTTAGTCTG
CATATGCATGGCATACCCGTCCTCAGTCCTGTTATTCGAACCTCATGATTGGT
ATACGACGTCGTACACGTAATCCTCGTATGTAGCTCTTTCAGAATGGCACGA
ATCACAAACCGGGCTGTTACCTTGTTGCGGCTTGCTACCAGTCTGCACCAC
ATCTTGATTGCCCCGAATTCTGGCCGCGATATCGGCGCTTCCTGTTGGTGTG
GCTAACGCCGTATAACCGCTGCTGTTTGACAGAAGTGGTAACGAGCTGTCA
GATCTGGCAGGACCGCGCGACCTGTCGCACTACACTAATTCACAACTACTA
CACAACCTTCGGCATATAGATGATCTCGAAGAAATCGGGTGATTTATTCGCC
CAACTGCCAACTTATTTGCAAGAACGTGAAAATAAGCTCGGGCTAGATGCT

AAATTCAGCTGAAGCAGGTTGTCGTCAGTCAGATGATCTATCGGAGTTCTA
CTGCGAGTTTACGCCATTGGTATACGACAATTATGGAGGCTCTCCATAGACA
AAGGCGTTAGCGATCGGTGATCGCCAGAGAGCGTCTCTTCGACGCCTCTAT
TCTAGAACAATAGAACTCTAGATCTGAAAAATTTATGTGCTGATTCGCCTCT
GGGCTAGTCTCGCGCTTCAGCCAGTCGCAATCCGTCTTACATACAGCTCTA
ACGTTTATGGAGCAGATTCCATCTCTCGGCCGTGCGAAATCGCACAAGTCT
GGGCAGGATGGACTTTCGCATTATGCCTACCGTGGTCTCGACGTAATGCTGC
CCGGACTATTGGGATACGCCAACGAGATCGAGAATGTGATCCCGTTATTAGC
TCTGCTTAGTGTTGGTCCTTGGTCACGCAGGTCGCTGCTGATGGGTACAGA
TGGATACAGATGGGTACTGATTGATAAGATAAATTTTTCGCAGGGATCCGCT
TTTCCGAATATCTGATCGGCTAGTGCACCGATTTCAGCTTAGCGGAACAAG
GAATGGGATGGTTTGTTGTGGACGTTACACACAATGGTCCACTGAAATGCC
TGGATTGCCCGAACAACCTTCCCCGTTTAGTATTGATTCGATGTACCACTAG
CCCATCTCTAGACCAACTCACGCAACCACACCTGGTTAAGGATTCCCTCAT
CTCAGCTCAGGTACAAGGCATGGGGTAATGAGAGGGATGGTTTTACGTAAA
TGTCTCTATTTCAATAGAGGAAGAGAGAGGGTGGAACGAGATGGTTTTTTA
GGCATGGGCTTTTGTTAGCTCAGAGACACCATCGCGGGACAGTCAACCTTT
TCAGGTATGGGTTAGAATTGGTTTATTGCTTAATCATGCCGAGATTTTTCAA
ATGTTCTTTCTTTCTATTCGCGATCAAAGCTATGCACCATGTACAATTTGTAC
CGACATACAGCTACATGCTACATACTACCAGGTAGGTACATACATAGCGTGC
CAAGGTCCTAGGTCCTAGGTATACGCAGCATGCAAAATTTCTTGACCTTTAC
CAAAGGAAGGAACCTCCACTTCGGTCGCCAACAACCCCCACTATTTTAGCT
ACGTGAACGGCCAGGTCCAGAGATCAAGGTTCGGGTTCGCATCTTTTCCCA
CCCTACGCTTTCCGTCAATGCTTGGAATATAAGGATACAGAGATCTCTCTCC
CGCCTCAAGGTTACTCTCGCTTCTCCTTCATCCCTCTCTTAAGCCAACATCT
CAACCCTTCTCGGCAGAGAAGCTTTGCTTCGTCTGATACATTCTTTTATATA
CATTGTACATTTGTATATAATAGGAGTATATAGAGGTCTCGCGTACTCCGAAG
TGGCTTGCAATTGTCACCCAATAGCAAGTTCTAAATCGCGCCACAGACTCA
GGTCTTCATTACAAGAATCTAAGAGTCAGCTCTGAATTGAAATTTTCAAATT
CTGCTTGAGCTTCATATTCAGCTTCAAATTTAATATTGGCAAAATGTTGAGA
GGGCCGCTCGCCCGGTTCGCCCGGCCCACCGAGGTGCCGGTCGAAGAGAA
ACCTGCCCCGGACAGTCACGACAAGGAGGCCGGCGTCATCGACAGCCAAC
CTCACCACAGCGATGCCGAGTCCGATCCCGAGGCTATCGACACCACCGCTC
AGGCCGGTGTCCAGAAGATGGAAGCCACCACCAAAGTCTGGTCGAGATCT
AACTTGATTCTAGCCTACGTCTTGTAAGTAGCGACCATACAGCAATCATTGA
GAAATCCGATAGATGATTTCTCCGTCCTCTTCACATATGCGTGAATTAACAA
AATTGTAGCATCTGGATTATCTACTTCGTCGACTCCATGCAATCGGGAATGA
GCTCTCTCTTGACTCCATACGTCACTAGCGCGTTTGCCCTGCACTCATTGAC

CGCCACAACGAACGTTATGTCTGGTATTATTGGAGGTGTTTCTAAGCTCACT
CTAGCTAAGATCCTAGATATCTGGGGTCGTCCTCAAGGTTTCTTAATTGTCG
TCCTCCTGATGACTCTAGGTCTTGTCATGATGGCGGCTTGCAACACCGTCG
AGACCTATGCCGCTGCGCAGGTCTTCTACTGGGTCGGGTTAGTAATAACTTC
CTCTAGCCGTTCAACTAGATCGCCTAATAATTTGTCAATAGATACAACGGTC
TCAGCTACTCCCTGTCCGTTTTCATCGCCGACACCTCTCACCTGAAGAACC
GAGCTTTGATGTTGGCCTTTGTCTCATCTCCCTACATCATTACCGTCTGGATC
ACCAGCCCTATCGCCCAGAGCGTTCTATATGGCATTGGCTGGAGATGGGGTT
TCGGTATCTTCTCCATCGTCACTCCGATCATGTGCTTACCTCTATTCTTCCTC
TTCACCTGGAACTACAACAAGGCCAGGAAGGCTGATTGATCGTCAGCACC
CCAAGCGGCCGAACGACCTGGGAGAGCGTCAAGTACTACTTCTGGGAATT
CGACGTCATCTGCCTTATCCTCATTTCTGGTGGTTTCGCGCTGTTCCTGCTC
CCTTTCAACATCTACTCGTACCAGTACTATGGATGGAGGGACCCGTTGACTA
TCTGCCTGATCATCTTCGGTGGCCTGCTATTGATTGGCTCCGTCGTTTGGGA
GAAGTGGTTCGCCCCGGTCAAGTTCATGCCTTGGGAGCTTCTCAAGGACC
GAACCGTTCTCGGTGCCTGCATTCTCGCCGCTGTTCTGTTCGTCGAATACTA
CATCTGGACCGCTTACTTCTCCTCGTTCTTGCAGGTTGTTCTTAACCTCAAC
GTTACCCAGACTGGTTATATCAGCAACATCTACAGTTTGGGATCGTGCTTCT
TCTCGTTCATCGCAGGTTTCGCGATCCGATACACTGGTCGCTTCAAGTGGA
CGGTGCTGTACTTCGGTGTGCCTGTCACTATTTTGAGCATTGGTCTTCTTATC
CACTTCCGACAGCCGGGCACTTACCTGGGCTGGATTATCATGTGCGAGATC
TTGTACGCCTTCGCTGGTGGTGCCTCCGTTATTGGTGAGCAGATGGCCGTC
ATGGCTGCTGCTGCTCATCAACACGTTGCTGTCGTTCTCGCTATGGAAGGTA
TGTTCAGTAGCGTGGGTGGTGCTATTGGTAGTACCGTCGCTGCCGCCATCTG
GACTGGCCTGTTCCCCAAAGCGTTGGCTAAGTACCTGCCCGAGGATGCGCT
GCCAAACCTGACTCTTATCTATGGCGATCTCGCAACACAGCTCTCTTACCCC
GTCGGCTCTGCGGCAAGAGACGGCATCGTACTCGCCTATGGTGACGCGCTC
CGATGGATGTTTGTCGCTGCTACAGCCATTACCGGTATCGGTCTAGTGGCCA
CGGCCATGTGGAGAGACATCAAGGTGAAGGATTTCAAGCAGGTGAAGGGA
AACGTCATCTAAAGCATCTAAAATTATATACGGAAGTTGGAGAATATGCGTT
GCCGATCTGCGCCAAAGCGGTTAGGTGCAAATTTAGGTAATGGAAGGTGT
TAATAGGGGATACCAGGTTAAGAGGGAAGGTGCATTTGATATCGAGGTGGA
CGAGGTCTGACGTGTCTGACGCTGTAAGATGATACGGTGATATAGATTTACG
CTGCCGGCTGGTTCATTGAATCGCGCTCATTAGAATCTAATAATGTCCAATT
AAAAAGTTCTATATCTGTTTCTATTTTCACGACGCCTCTAGGGCTGGCACTA
GCGCGGTTAATGCACGCGGTGGGCGGGTCACGGCGGTGCTTGTCTGGCAG
AGTTGGTCTTGGTACTGTAGCTGGGTATTACATATAAATGATACCTGGGCAC
AGATCCAAGTTGAAACAGTAGCGCAGGTATCTCCCGTAATACTTGGCCACG

GCTATCATTCAGCCGTTAATGTTACAATACCCGCCGTTCTCTCCGGACTTCC
TAAATTGAATGGTAGCGGACTGCCGGGCCGGCACATTGGTGGCCCACTACT
CTACGCCTAAATAGGCGAAAACGAACGATGGAGGACGAAGGAATGACGAA
GGAATTAGGAGGGCCGACGAAGATTTAGAGACATGCGTTAGCTGGTAGGG
AGGAGGTCAAAGAGTTCGAAATGAGAGACGGTAGATCGGCAGGTAGGTTA
GGTAGGTAATTGATTAAATAAAAAGTACGTCTTCTTCCACCCGTCCTTGTGC
GCCATTTTAGTTGCGATCACAGCATCACTATTGTTCATATTGTATATATCACG
ATGTCTTCCAAACTCCTCTACGCAAGCCTCTTCGTCTCGCTCCTGACGCGC
GCGGCCGTCGCGCAGGAGGCGTACCCGCCGCTGACGACGTATAAATGCAC
GACGTCCGGCGGATGCGTGCAGCAGAACACGTCCGTGGTGCTAGATAAGG
ACGCGAAGTTTGCCGCCGGCGCGGCGGGATCCAGGTCCGCGGCAGACTAC
GCGGCGATGGGGGTCTCGACCGACGGCGACGCCGTCACCCTGTACCATTAC
GTGCAGTCGGGCGGCGCGCTCAACGCGGCGTCCCCGCGCATCTACCTCCTC
GACGAGAACAAGGAGTACGTCATGATGCAGCTGCAGGGGCAGGAGCTCCG
CGTCGATGTAGACTACTCGCAGGTCCCGTGCGGCGAGAACGGCGCTTTCTA
CCTCTCCGAGATGGCGGCTGATGGGGGTGGCTCGTCTGGTGGTGCTGGGG
CTGGACAGGGGTACTGCGATGCGCAGTGCCAGGGCTACTGCTGTAATGAA
ATGGATATATTGGAGGCCAACGCGAAGGCCAACGCAATGACGCCGCATCCG
TGCCAGGGAGATAACTGCGATAAGTCGGGCTGCGGGTACAACCCGTACGC
GAGCGGGCAGCGCAACTTGTGGGCGACGGGCGGCACTGTCGACACGAGC
AAGCCGTTCACTGTTGTCACGCAGTTCCAGGGAAGCACCAGCAGCTTGAC
CTCCATCAACCGCATCTACATCCAGAACGGCAAGCAGACACCCGGCGGTAC
CATATCTGCTTGCAACGACGGACAAGGCGGTCTTGCTGGTATGGGCCAGGC
TTTGGGACGAGGAAGTAAGTGATCACCATCCCTATAATCTTTTAGCCTTCTC
GGATTTGGAACATTTGCTGACTTGAGATTTAACAGTGGTCTTGGCCATGAG
CATCTGGAACGACCCGACGCAACAGATGTCGTGGCTCGACTCCGGCAACA
ACGGACCTTGCAGCATCGCGGAGAGCACCCCGGCCAACATTCAGGCTCAA
TACCCTAACACTCACGTCATCTTCTCCAACATTAGATGGGGAGACATCGGGT
CTACCACCCAGTCCTAGATACAATTAGTTATTACCTAGTATACTGTATTTAGT
ATTCTCTTCACAATAGGGTCTTGTCTTGGTCGTCCCTGGTTAGGGGTACGCA
AGAAAGGAGGCTGAGTTAAAGTTAATAATTAAGATTTAGGTTGATCTTTAAT
TTTGAGTTCTCGTTTAACTTTATTTCAACCGTTATATGCCGCTTGAAGTCCTA
GCCCCCATTGCGGAAGCTGTGAAGTATAACGGACAGGTAGGATTCCTAACC
TTGATCCATCTGATGCCTCTTACTGGTGCGACCATTTGCCACAGATCGTAGA
AATGTAACTGAAAACTACCTAGTAGGCAGTGACTGTGTGTTGAGTCGGATC
AGTCAGATCTTTAAATTGACGGCGGCGGCTATACCCATGGCAATCCTTATGT
TAAAGATATGAACATGATATTGTCCAAAGCATTGATACCAAACTCTAGAAAA
CGATTAGTGCTAGCGATAAAACCTCTTATTTGAGCATGGTTTCTCCTTATTCC

TCATAAGAATGCCAAGTTCTTTCTAATTAGTAATTCGGTGGCTTTGATTGTCC
GATCGTTGCCTTGGCTCTAAACCTAGAAGGTTTTGGATAGGAAAAATGTTC
TGACGATATAGGCTATCACTGTCATGTCCGTATTACGCTGCACGTCAGTATTA
TTCAGATTCTGCGTGAGTAAGATCATCGTTGACAAATGTGGCGTGAACACT
GGCAATGCAGTCATATCTACAGGTGGAATTAATCTTATGTCCGATCAGAAAT
CTTGCTGGCAAGACAGGACGGGCATATGGATGTCTCCTGAGAGGTTTCATG
GTCCAACGGTCGGGTAGTATTCTTATCATATTTCATTGTAACGGACGGCAAT
GAACGGGTATAACTCGAGGTAGATCACTCGAAGAATATATAGGTAGGTATCT
ACCTACGGACGAGAACAGATGTCAGTTTACGTTCGATTCTGCAACAAATAG
TAACAAAATGACCGCGACGTTTCTTATCACTGGCACCAACGGCCTCGTTGG
TTTCCGCATCCTCACTACTGCTCTTGAGGCAGGTCACAATATGCGATATGCG
GTACGATCAGAAGAAAAGGCCCTGGCAATTTCTCGAATCCCGCAGTTAAGA
AGCTAGCACCTGGTGACCGGCTCTCCGCTGTCGTGATCCCGGACTTTACTAT
TGAAGGGGCATTCGACTCGGCTCTGAAAGGTGTTACGCATGTTATTCATACC
GCCTCTCCCGTCCCTCTGCCGTGGATTGATCCAAATAAAGATATGTTTGAGC
CTATACGCAAGATGAGCTCCAATATACTCACCTCTGCGCTCAAGACGCCTAC
CATTCAACGCGTCGTCATCACCTCGTCTATTGTGGCGAACATAGGAATAGTT
CCACCAAATCATGTCGTCTCGGCCGCTACTCGCTTGCCACTACCTGATCCTC
TCCCACAGACCTTCAGCACCGGGTACGAGGCATACGTGCTAGGGAAAATA
GCGGAAATGCATGACACGGATAAGTTTATCCGCGAGAAAAACCCGCCGTTC
GGCGTCTCGCACGTGGTTCCCGGCTGGACGTATGGCCGCAACGAATTGGCG
CTCGACGTTAAGGGGATTAGCGAGTATAGCAGCTCGATTAATTCCATGATCA
AGGCTATCAATGGCGGCACAACGACGATGCGAGTCCATAATGGTTTCGTAC
ACATAGATGATCTGGCGGAGATACACCTACGGGTCGCGCTCCTAAATCCGA
AACTCAACCCAGACGCACCGAGGGATTTTGGAGTGGCAATGAAAGTCGAC
CACGAGAAGATATTCGGCTATATTGAGAAAGCATTCCCGAAAGCTGTGCAG
GACGGTGTGCTTAAGAAAGGAAATATGCCCATGTTGGATTTTGAATATGATT
CAAGCGACGCGGTGAAGCTGATGGGTGGGAAGATGAAGAGCTTTGAGACT
GCCGTGGTGGATGTGGCAGGTCAGTATCTTGAAATACTGGGAAGAGAGAA
GGCTTGATTTGCTACCATCATCCGACCTAGCTCTTAACTCTTTGATAGTTCTG
ACTTTGTATGAGATTCTAGCATACTATCTTGATGAACTAAATAGCATCTCTTT
CGCTCGTTTGAGTGTCCGTCTTTTGTTGAAGCAACTTCCTGCGTAATTTTAT
GGCGCGCACAAACACTGCGTGAACTATAAGCAAGTCAGCAGCCTTTCTCC
GAAGTGGTTCAACCACATCTAGCCAGGTGGAAACGGCTTCCTATAAATATT
GAAATAATCATTGAGAGTGTATAGAAGGTATTCTGGAATGAGTATTTAGACA
AGTTGCTCCATTCAAAAGGTTGGGAATATTGATACTGAAGTTGTTTCTTGAA
TCTGAGTTGAATGGGAGGGAGTGAATAAATACCCCCCATAGCACTTTAGAA
TTTAGAAGGTGTACAGCGCTAAACATATCGATAAGTACCCCGGAATGCGAT

GTGGGGTTGCTTTCTCAGCCCTATTGCGCGCCGCATGCGGATCTAGAGGAA
CATTTAACCTTACAGCTTCATCATTTTCTACACCAATTCATCAGCCACCCATT
TACTTCTATTATAAACGTCTTCTCTTCTGTATTCCGGTAATTCATGTGGGATAT
AAATCTTAAATCGAATTCTGATCAATTTCATTACCTACCTAAATTTCCTGGAA
TAAACATAACAGCGACGCTAAAGACACACTAAGATACGCGCCATCCCGGCA
ACCGTCTACGGAAGAGGCTACGGCAGCTATCTGCTCATCCATTCAGAATGC
GTGAGCTCTTCTTAACGGCATCCGTCAAGGATGCTGACTTCTCTATGACATG
CGCGGTTTCTGCAAGGCTTGACGTGGATGACTGCGCGTCAATCCCTCCATC
GTATTCTATTCTTTCACGGCCAGCCGCAACCGAGAGCTCTTAAGAACGCGC
GAGCTTTCCAGCAGTCACGTTTCCCGTCGCTCTGGAGGGAGCTGAATAGTC
AGTTAGCGCGATCATCTTATGTTCTTCAAGTAGCATATGAGGTCGTTTTGGA
CAAGGATTTGGGACAAGGCACTACCATGGAATTCAACAACCTACCCGGTAC
TTGAGATGGACAGACCTACCAGATCCTCTCAAAGATACGCCGATCACGCAA
AGAAAGAAGATAGAGATACCCGACCAACTTAATCTTCCAGTAGCTATGACT
GACAACGGCTTTCAGTAAGATTATACTTGCACTAGCTATTTGTAAGCCAGGT
TTGATACTAACAGGCTTCATTAGGTACAAGAATGAGATGATACAGGAGAGC
TACTCCTTCATCAGAGAGAACATCGAGTTTGTGTTCAGCAGATACTATCACA
TACCAGACTCACCATCGCCAGGACAGCCAGTGAAGTCTCTCCCGGCATTTG
ATAATCTGAGACCGGTTGACCCGGCGCAGAAGTGGGTATTAAACATAAAGC
TGAACGTACTTGAGGACAACCAGCCAGATAAGGTCAGGAAGGCAAGCGA
AGAACTCCTACGTGTGAGGATAGAGCTTGAGAAGCTGTTCGACTTCAAAAT
TCTAGACCGACGGATCTTCGACACTAGGATTGCGCCTCCACCAGTAATACC
AGGTCGTGGATAAGACGCTTCTGTGAAGAAATTTGACAGTATTATATTCAAT
ATACCTAATTTAGGCTTGTGTTAGAATATATACTAATATCATGCTAGTCGATAC
CGTGAGGAAATGAATGGAAAATCAATGGGGTAAAGTGCTATTCTCAGCTTC
ATATCAACCACAAAGGACAATTGGAATCTACTTGAACAGTGTGTATTACCA
ATGTGAGGTGTGAGTATTAACATACCACTATCGTTAAATTCACACAGTCGCA
AATGCCTTAATAACATTGAAGAGTGGCTAGAATCGCAATTTTGGAAGGGAC
GACCCCACAAGAGATACCTACTAGGTGTGCTAGGTGCCTTCCTCGGTAAGT
GAGGAAGGTAGGAGGGTAGGAAGGTACGCCGAGTTTTGTTGATGCCATGT
AATATTGAATGCCGTTCCTTCCTTACATATAAAGCACGTCGACTTAGCGTCC
TCGTTCACATCCACGCCCACTTAATGTCCGTCAAGTCATAAACCTTCTTCTT
CCTTCTTCCTTCCTGACCTGATAATTCTACTCCAAAAAAGCCTTCATCTCAG
AGCATAAAGTTCAAGCCTAAGGGATATTCTAAAGGTTATCGTGAGAAGGAT
TTCGAAGATGTGCGAGTACTGCGGGCACAGTGAACCTGACCGATTCTTCAC
CCTGCGAGAACTACGCGTCATTTTCAGAGCCGCAGTACTCTTTTTTTATCGT
GAAGCGCGCCGCTACTTCAGGCGTTTACAAATAGGCGTAATTGATATGATGC
GCGAACAACGACAGATCAGACAAAGACGGTACAATCGCGCAGCGGGAGA

AGACGTCTCAGACGACGAGGACGACGAGGAATAATCTACAACACGATCCT
ACATATGATCGCCAATCGTCGCCAATCGTCGCGAGTTCCATTTTTTGCGGCA
ATCTAGATACCATAAACACTATCAACAAACTAGCCCACATCTCTCATCTAGA
GACTCCCCATTACTTTATCATATTCAGCCATCTCCTCCTACTTCAACAATCAC
TATTCCCATCTCTCAACACCCAAGAAAGCACTACCACTATGGCTACCATCTA
CGACTATGATATCCCTAAAGAGAAGCGACCTGAGGGCTCTCGCAACTTTCC
ACTTGTTTTCCCATCTCCCGTCGACGTACATCCCGATTCTGACAAGACCGCC
CTTGGAGGAGGCTCTGCCAAAGAGTGTTCAGGCCATGGTCGTACAACATCC
AACCACAATATTCCAGGTAAGCAATACATCTCAATTTCATTGGAACAATTAT
CCAAGACCAAATTCATTAACAATGATGGACAGCAACTCGAGGCTTCCCTGC
CTATGGCAAAGCCATACCATGGTTGGATGCAGACTACCCTTTCGACAAGAC
CGCGAACACTGTTCAATGTCCCGAGCCAGACTCAGACGGTCAGGATTCGA
CCACGTCAAACACTCCTAAGGTAGTGCTTCCGCCGCTTACGAAGGAGCAA
CTTGGCCCTAGTACGCGCAACCCCGTTCACGATCATCACCGACAGATGACT
CGCATGGTTGCCGGAGATCTATCCCAGCATGATCTCTATTCACGAGCTACTC
GCGTACCTCCCGGATTCAATCAGCAGTCCCCAAGTTCGCAGCATCCCTGGA
GCTCCCATCGCTCAAGTTCCCACCCGACCTTTCCGCTCCAACGATCCCCGC
GATCCCAACTTTAAGCCTGCTAGCCAACAGATCATCAGTGACCCTCGCCAA
ATTCGAGTCAGTAGCCCTACGTTCAGGGCCTATTATTCTCCGCTGCAATCTC
GTGGCGACCATGGTCAAGAGGATTTCTACTTGAAAGATCTACAGCGCGCGC
AGATGGCGGGCACTCCTGACGCACCTGGAATGCAACCCCTTCGCCAATCGC
AACGCCTTCAAAAGCTGCAAATCAGACGTCGGAACCTTGAGATTCGCATG
GCTCGGGAAGAAGCTGCTAGAGGTTTCGATTCGGCAGATGACGACGAGTT
CTACCCTCATAAAGATTAGATGGGTTAGGACAGAGGACTTCTACCGCCTTTT
TAGGGAACAAAGGCGAGGGAGATAGTATCTAGCACGCATCATGGCTGCGGT
ACGCTCCTTTTCTTCACGAGGTCATGGATTGGTGGGCAACGGATGCCCTTT
TTCCCAGTGGAGGATCTCATGATGATCCTCTTCGCATTTGAGAAGAGCATTT
CCACATTACGGATGCTTGATTTCAGGCAGTTTATTGCGTTACTGCCGTTTGC
CTTTCTAATACGATTGATTTCATAACTTTTCTTGTGTTGCCCCAAACTTTGTG
ATCGTAAAATGATAATAAATTGGCCTCAGGTCTAAATAGGGATAAGGATGTA
TTTATCACGTAAAAAATGCTGAGACACTATACCTGTTCCTTTGTTGATTTAG
ACAAAAACAGCTACATAAAGTAGCGAAATAGAATAGAACGGCATTTGGTAA
AATTTGACTTGTCCAATCTAGCCACTTCTCCCATACTAAGTCTCCCTCAACG
AGCTGAAATCTACTCATAACCTTCCACTTCCTATCAAATTCCCAATTCTCGTC
ACTTCATGTCACACCTCCCCAGTGATAACCTCTCCATTAACCACGTAATCGA
TCCCGTCACTCACTCTCCTCCTTAACCTCATCCACATTCCTAAACAACACCC
ACAGCAAAACTGTAGCGCCGCCCAAAAGCCCCGACAGAACAGCGTACATA
GTAACCAAATGCGGATCCCTCGTCAACGGCGTAAACGCAAGAGCTAAACA

AGACCCAATCCCCGCCATCGCGAGCCAAATAGCCTGGACCAGTGTCTTCAT
CCTCTCGGGCGCGTTGTTATACGCGTACTCGGTGCCCGTGGTCAGACAAAA
TACCTCTGCTAACGCGCCCCCGAAGTAGATTGGGATTTGCAGGAAGACGGA
GATTTGGTTGGGGATGGTGTGGTGGTTAGAGGCGTCGCAGGAGAGGGGAG
TGGAGTAGCAGGGCCCGGCGTTGTAGATTAGTTTTTGCACGAGGGTTGTGT
AGACCATGGAGAGGGTTAGGACGGCGAAGCCGACAGTCATGCGGGAAATG
GGGGTGAAGGGGATTTGGTGACGCGTGAGGAGGGGGTAGAGACCTTGTTG
CACGATGGGCGCGACTAGGATGCCGGATATGGCACCGGCGATCTTCATCAT
GTCGTTTGGGATGCCGTAGGTGACCATTTGGCCCGCTTGAGAGATCAGGTT
GTTGAATGTCTGGTTGTAGCAGAGCCAATGGATGGGAAAGAGGAGCAAGA
CACGACAGGTGAGAAGACTACGCTTGATATCCTCAATGAAGGAGTCATTCC
AGGTGACTTGACGACCGTGGTTTTGGAGTTGAGCATCTGGCTTAGCTGCGT
CCATTTTAAAACCACCGGCTATGCCATATCGAAGGGCGGTTGCTACATCAGG
CATGATATTGCTTGTTGGCGATGTTCGGACTAAAGACATATCAGCTAACGGG
TCTTGACGATGTTTAAAGCAAAAGAATAAACTCACCGAAGTGATTTCGGCC
AAAAAGAATTGGAATTAAAGCTATCCACATGCAACAGCATGGGACCAAGTA
AGCCAGCCAGAAGCCGACATATTTCTCTAATAGGGGCGTAGAATCCGCGAA
GAAACAAATTATGTTAACAGCCCTATAATAATCGTCAGAACCTATATATTTCG
GTAGGTGTGAAGTATATACGGACCAATAAAACGCATTGTAAATATACGTGAT
AGTCAACTCTCGACTGCTGACGACCCTCTCGCCTGTCTTCTGGGTGCGGAT
TCGAAATTCGGTTTGGTCATATTGATCAGCTGGCAGAAATTAGTCCAACGTA
CGCATCCGATATGAGAAATAACTTTCGAAGCTCAAAAATTACGAAAGAAAA
ATAGAGGCAATTCCACTCACCAATGAATGGTACTACAGTGGTCTTGAAACC
CCCTGAGCCCACAGCCAACAGGACGCACGCCACTATCACCCCGCCTAAAG
CAGCGCCTCCTTCGATAGCAGCTGGTAGTGAGGTCGCAAATAGGACACTCG
CGCCAATACATTCAATTCTGCGCAACTGTTAGCTTTTTGTCTAAAGGAAAAA
AGATACATTATGAGAAAAGGATTGAAGAAAGAATTCCACTTTACATGGCAG
CATATAGCATCGTCCTTGTAGCGGCCCAGCCAACTATCCGCAATGACAGCA
GCCGGTACGGGTGTGATATAAGACCCGATCATTAGCGCATTCACAATATTAG
TTGCAGCGGCCTGACCCAAGCCCAGAGCACCGGGGACTTCGCCTCCGGGC
GCATTTTGCAGGTAGTTCTCTATAATACAGTTAGCGTTTCCTCTACTAGAAA
AACAAGGTCAGGATTCAACTCACGCAAAGGAGACGTAGCCCCATACCAAA
CAAAGCGCTCTAGAATGCCGGCGACACAAGCGATCCAAAGCCTGACGGGG
ATCCGATCGACTACGTGTAAAAGATTGCGCACCTCGTCTTCGGTAGCCACG
CGACCATCATCGCTATCGTCGACAGCTGGTGCATCCGAAAGCGATTGATCG
CTCTCACGACAAAATTCGGAATGCTCCGGGGCTATACAAGGAGAAAACAA
GCCGTGATTAGCAACAGTACTCTCATGATTAGTTTCGGCTAATACCAACCGT
ATCTGTGAATTTGACTCTGCGATCACCGGCGCAGAGTCATTTACGTCGGGC

GCTGAGCCCGACATGTTTGAATTTTCCGGATGAGTGCTTTGGCTGACGGAG
GGCATTTTGGAGGGTGTCTTGTCTCGTGGGCTTGAGAACTATCACAGGGGG
GCATGTGTGGAAAACAATGCTTTTGGCTGCGGCGGGAAAACGGAATTTGG
AAAATGAGAAGTGAGAAATATATTCAGCGCCCTAAATACTAGGCGTCACAT
TTTAAGTAAAAGCTGTGCCTTCTGACTGCTGATTGTAGAATTAGGGATTGAT
TGAAGCAGGCCAATCATCGAGGTCTAATGTGACTTCGTCATTTCAGAAGAT
GACGAGAATTGCTGCGCCCCTGTTCTGCTGCTAAGGCTACGTTAGCTACCT
ACCATTACCTATAATAGACAACTATTAGTATAAACGGCTGATGAATAACTAGT
AGGTTTATTGAAACTTACAAATTGTTTTGCAATTCACTTTCATCTAGAGCGC
GAGCTTCATGCATATTCTTTTTTTCCAATCATTATTCGGGTATGCGAGTAGAG
ACTTCTCTCTCTGAGGCATCGTGATTGCAGCGAGCTGCCCTAATTTGTTGGC
TAGAATTTTAATATTTTAGTCTACTTCTGTCAGATCAATCACTGTAATATCGG
TGAGAGAAGCAGCATCGGTTGAGTGTTTCTGATGAGTCGTAGGAACTGAG
GGTTCCATAGGAGATCCATTTCTAATGCCATCATCAACATCATCAATCAGCAT
CCGGTGCCTTTCCGAAAGACCCTTTTTGTAGCTGTTCGCGTTAAGAAAGTC
GGTTTGGTTCGTTTGTCCTTCTAGTCTAGGGGGTTTCTTGAGCGGTTGATTG
TCGAGATCCAGTGGATTGTAGGCGCTATCTTCCCCTAATGAATTAGGACCCC
TCTTCTTAGACAGCTGACGCTTATTAAGTGACTCCCTTTCTCGAGAGTTGTG
TGACGATGCACGATTACTCATATTAGTCATGGTTTGTCCAAAGGGCACTTTC
CGCGATCTGCCTCTTGATTTCTTTTCAATAATTTTCTCGGTGAGATTTGGTAA
AGCCCGTCGGACCACACAATCTAAAAGTTTAGCTTCGACTCTTTCAAACTC
GTACCCGAAAGGAAGAACGTCAAGAAATTCGGGTATAACGAGCAGCGGGT
CAACCATCACAGTTGCTTCTTTCCTAGATTCGGTTACAATCTTTTCTGCGAT
GCCATAGTCGAAATCATTGGCTTTTTCTCGAAGACGATGGTTGAGTTCGATT
GGCACTAGATACTGTACGGGCCAGTAACGACTTTTACGAGAATGAAAATGC
TGTAGAAGGAATATGTACCGCGCGTACAAATTTGGTCCAAGCGGACGAAAC
CAATTGCTGACTAACCCGAGAAGCTGATGGTCGGAAGAAACGTTTGGACA
AGAACAGTTCTCCAGGACTTTGGGATTTGAGAAACTGTTCGGAGGCAGGT
TGAAGTTTTTGCTCGGAAAAGTATCTTTCATCATGCGGAAGAGCATTGCCC
GCCATTTGCTTAGCTCCCGATGGTTATTTGAAACGGAGGCAGCCAATACTTT
CGCAGTTTCTTTGAATTTATGGTGCTTCGCAAGGCCCAGAAGAGTGCTTGA
TCCGAAACCAAACAAGCCATCAGCATAGTCACATCCTACGAGGATTGCATA
CATCAAGACGCCTTGCTGTGAAAGATGACTGCGATTTTTAATACTTTGAGCT
GTGTATACGAGTACATTATCCTCGCTCTTGAACTCGGAGCCTTCTGGTCGGT
AAAATTGAACAACAGTATCGCATCCGAACATGAATGTATCACTATCATCGGA
CCAAACAGCGTCTACAACCCCTAATCTTTGGAGTTTAGCGCACTCCGCTTC
GGCCTCTCCAGGGGCTTCATGGCGGGGAACGCCCAGCTGGTCTAAAAGTT
CCTTCAGTACAGCAACGTTCTGATCGCTGTAGTCCTTCACTGTCGAGTGCC

GTTTTACGGGTTTATTCGGTCCGTCGAACACAAAAACAAGTTGAACATTCA
TCCGTAACAAATAGCATATGCGCTCCATTATTCGTAGTTCTCTGGGATGCGA
GCCGGGAGAGTCTAGTTCATTGTTTAGCAAATTAGCTGGCGATCATTTACGG
TCCTCCTAGTGGAGCGGCGCGCTATAGCACGCTAGAACGCGCAAGGAAGG
CACCTAGACACTTACTTCTTTGGATCTCGGCTTCCTTTTGTGGCGTCACATT
CTTAAACCACCAGTTCGCTTGGTCGACAGCAATTCGAAGGGGCCGATGATT
ACGCTCAAAGTAGTCGGAGGACCATTGAGATATTGATCTAGTTTCGCTGATC
GCCCTCTCTTTCAAAGAGAATCTAGCACGATCTGTTAGAGATCGGGCGTAA
TAGGATGGAAAAGGCACACAAACTGTTGGAGACCCATGTGCCTGTGAGAT
GAGGTTATGTCAAATAGCTTCCTTGGGTACAATTCATCAGCTGAGAATTTCT
TAATCCACGGAAATAGACGTCGCGAGTGTTGAACAAATCGATGACATTTCG
GTACTTTTTATGCGTTTTAATTTTGTGTGCATGTGTCATAGATAATGCGCACT
CAATTCATCAACGCTGACAATAACGTTCCAGTGCTTGAATAGACGTCAAGA
TTTAGTTGATGTTGCGTTTGGATGGGGTTGTCCTTGAATGAAGTTTGGAGGC
AAATTTAGTTCCATTAATGGATATATACACCCTGGACGATAGCAAGAGAACC
CGCCAACCGGTCACATCCTGTGTCTCAACCTACTTTTAAACAGGTGCATATA
TTCATATTCCTCCCGTAACACTTCACATACACATCGCACCTTCATCCTAACCT
TAAAAGCAACTTCTATCTTTAAGCTTCATTTCGCGTAGGAAAGTGCGACCG
ACAAAACAGTGCTGGGGATATAGGTAATTCCATAGCTCCAGTTTGGATTACA
TAATCCCAACTCCAAATAAAATGCACGTCTTTTCTCATGCATAATCTATAAAT
TGTGCCTGGATCTCACCGTGATCTGTGCAGGGTAGATGACGATGTGGCTGA
GAGTTCTGCAGGCGAACGAGGGGTAACAGGAGAAGAAATTTGATATATTG
AATGAATGGTCTAAAATACATCAATCGCTATCATGAGACACTAAACAGTGTT
GGAATCCATCTGATCATTCGAGCGCAATATCGAGGTTAATCTCTGGCTGTCT
CGATCGGAATTGGATCTTCGATGCCTGGTCGACACCATTGCCAGCCTCGAA
CCTTCGAGGAGCCGGTCTTCCCTCACCTGGAACGAATCTCCACTCGTATTT
GAGTAGTAGATGGCACAGCAAAATCTTAACCTCGTTTGCCGCGAAGAATCG
TCCTGGACAAGCATGCTGGCCGTGGCCGAACAGAGTATGGGCTGGGTTGG
TCGTAACAAGTTGCCAACCATTTTCTTGGCCTGGCTGTTGTCTCATCTTCAA
GAATCGGGCTGCATCAAACTTCTCGGGCTCAGGGTAGATAGTTGGGTCCAT
GAAGTTTGTACCCACTATCACGCGTGAACCACGCGGCAGCACGGTGCCATC
GGAAATCTTGACGTCCTTCTCCACGTATCGGTGCATAGTAGCTGGTTTCCTG
TTAGCTGGATCAAACGTGATGGGGTCATGTCAAACATACAGTCTCCCATCG
GGCGAACTCGCTGTCCTTCCTTCAAAAAGCTGTCCATAAGCTTGAGTTTATA
CAACGAAGTCTTAGCCCAGCCATTCTCGCCAATAACCTGGATGACCTCCTC
TCGAAGTTGCTCCGCAACTTCAGGGTATTCGCAGATATCAAACAATGCCGA
ACAACTCGTTTCCGTGGTTGTGTGCATAGCAGCCATTGTCAACGAAAGCTG
GCCGGCAACGAAGTCCTTTTCTTTGCCCTTAGCAATCTCGTACATCCAACC

AATCGTATCCGCAACCTTGGGTGGTTTCAAACCCGCAGCTTTAGCTTGGTC
AACAGCAGCCTTACGGCGTGCTACCTCGGGATCGATGAGATCATGCGCATC
CTTGACGGCCTGCCTTAATCTTTTGCTTTGCGGCAGGAACATATAAACGATA
GGGCGGAACAGTGCTGGGGCAGCT

>Mosaic_sequence_59

GGGCCCGGTGCACATCCGCCGCTGCACCTGTCGCAGGTGCTATTGCTGGCA
CGCAGTGTCCTGTCTCACCCCCGTTTGCGTGGATGTACGTCTTCCACGCTC
ACCCTCTCACTCTACCCTGTGAATGAGCAGACATTGACCATCAGTGCCAGC
TGGGCCGGCATTACCCGCCCAACGCACCCCGACCATGAACTCGCCGGCGA
GTCAACGAACAATCCAGACCGCGCTTTGTCCCGGATAATGGGTTAAACACA
TAGACTGGGCATGGATGCCCAATGGACAATGCATAGAGTACGACTGTCAAC
ATCGCACCTGAGAAACAAGGTCCGTGAGACTGACGAGGTTTCGTGAGTGT
CCGCTCAGAGACTCTCACAATGGAAAAAACGGAGTCCGGGAGTCGGGCGC
TGCATCAAGATGCGCTCCACTGATCACTGATTGATGTACTCGTGAGACCAG
CTCATCGCTATATATTTCATCAATCAGCCAGTTAGCAAGTAATCGATCATCCG
TCCGAGATCCCCAATTTTTATCCGGGCGACGGACGCGCGCCATGCACGGAC
CCACGAGGCGCGCGTCAGCGGGCCTCGCCGCTCAGAGTAGCCCAATGATT
GATCGAACAGGGGGTGCCTCATGTAAGTCCGTCCGTCCTGATTGCACACAT
GCGGACTGGCAAGATCCGGACCGTGTGTTTGTCCGCACACATATCTTGCTT
CTCGCCATGAAAGTGTCCAGATACATAAGACCTTCTGTACCCCGTCAGACG
CCCTCTTCCTCTTTCCCAGTACCTCCTTTCCTCCACCCGATCTCCATACTCCG
TGCGAACGGCGACACCCACAACTGTTACGTCAAGCACGTCGAAGACGACC
CACGACGCCTTCTATCTCCGCTACAATGTCCCCGCTCGATGTCATGGACCCC
GCGAACGGCCTTCGAACCCTCCCTGAACGAGTCACGGCGTGCCAGTGTCA
AGTCTGCAATTGCCCGAGGGCCGCCGTAGTGAACGAGACCATATGCACGG
CTGTCAGGCGTTCACGCTGTCGCAGAACACCAAAAGCGCTGACATCAGTG
CACTGCCGGATTCCATGAGCCCAATGTGTATGCGCTGTCAAAATGCCAAGA
TAGCTCCGGGAGCAACCAAGAGGAGGACAAGGCAGGCTCGGCGTCGATAT
CTGCGTGCGCTTCGGGAGATTGGTGAAGGCGGTGCAGTATCTGCCGGAAC
AGAAAGAGTCGGGGGCTGTACGGCTACGTACTACTATAGCAAGTCCGATGC
ACACAGTAGCGTCCGTCGGACCAAGCTACACTTCGGCCGGCAGGAATGTG
CGCTGCAAATCGCCCTCCTCGGAGTCGCAGTACGAACACGCGGAGTGGGG
CAGAGAGCGCCCCATCCCGGCAGCGACTGCGTCCGCGTCCTAGACGCGCA
GCGGATCTCCCATACGTCTTGCCAAAGATAGCCAAAGAGCTCATACTTTCA
AAGGTTTTGCTCGATCGCACGACCGGACATCAGACAGGAACATCATGAGC
CCTCAGCCGATCCCTAAATGCGCTTGTACGAATTGCGAATGTCCGTGCGAG
ACGTATCTCGGCCTCTGCCTGCTTGTGCGCGGTCTCTCCCTTCCTACTCGCG

TCCAACTGACTGTGCAGTGTCAGGAAGGCAATCAATCACCATCGACCCGA
GTCTACTTAGCACGACACACAAGTCGGCTTACTCTATCCTGACGAAAGTAT
CGTTACCCCTTCATCTGCACGCCGACGTTTCAGCTTGTTATGCATGCTCTCT
AAAGTCACCTCATTAATGACGATCGCCCTACGTGTCCATCTGTGACTGTCTG
AGCTTTGAGCATGTCTGGCGGTAAGAGCGCACCGATGATCGCTCCTTGACC
TCCGTTCCGGATGGATACCGCTGTTCCTCTCAACGCGCGCCACCTCTGCTCT
GCCGAGCGCCTGTCACGTCTTATACTGCATGCATGAGGTACCATGCAAAAC
GACTCCATGCAAAGCATTGTCCCCTTTCACACCATTCTCAAGCTGCCGCTG
AAACGTGCGCAAAGCTGCCATAGAGCGCTTTACGTACATCAAAGTACACCT
TGCCCATAGCTCCCTCCAGCACAATCTCCCCCGCTGCAAGCTTCCTGACAG
CTACCAAGCTGCCGCCCTTCGCATCTTCCGGCTTGAGCACTCCAGGACCGC
AGATGAGCACGCCCTCTCCCGCAAACTCAGCAGGGATGTTGAGCCCGGCT
AGTTTCGCCTTGAGAATGGCCAGTCTTAGATCGCCAACAAAAAGGCTGGA
CGGAAGGGCGAGAGGGGGCACTGGCCGCGACTGTGCGCTTGGCGGCCAC
TGCGGCAATCTCTGGAGGTGATGGCGAGGCCGGAGCTTCAATCTTTACTTC
GACTGGCTCTGTCTCAGCGGGCATATCCACATCTGCATCCGCGGATGCCGC
AACGGCCGGTGATGTGGTCTCTTGTGGACCAGCAGGGGCGGCCTCGATTTC
AATCTTCGCTGGGAGTGACTCCAATACCGCCGAAGCGGCTTCCAGGTGAG
GTATGGTAGAGCCGGCCGAAAAAGCGATCTTGCCATCGACCATCGTAATTT
CAAAGCCTTCGAACTGGATATCAGCGAGACTTTGGATCATGTAAGATTTATT
TCACCTTGGACCACTTGCCGGCCAGGCTGGCTGAGATGCTGTTGCCCAGCA
TGAGCGAGTAGGACTGGACGTGTTCTCCTATCCGGACAACTTCGTTGACGG
CGGGCTGGTAAATGTCATGCGTCACACCATGTGACGATTGTAGGAATGTGA
CGAGGTGCTGGTCGCGTCGGGTGTTGAATGCACAACAATCTGACTCATCAG
CCATCGCGGATTGACAGCATATGAACTCACGATTTTCCGCGGCTGCAGATC
GGTGATGACAGTCTTGATCGCCTGGCCGTCCAGCTGACCTTCCATGTCCAC
GAAGAAGACGGAAGCCTTCATTTCGACAGTGACGATTTCCGTCACGTATTT
GCTTGGCGGCTCGGGAGGTTCCTTCTGACAACGTCAGACCTATTCTGATGT
GGGATCAATGACCAACCTGTCTTTCCTCTTCCATTTCTTTCCGCCGTTTGGC
TTCGCGCACCTCCTCGGGTCTCCCCCTCCTCCTCTATTTCCTTGCCTTTCCG
GATCCATTGTCCGATGTCGAGACCTTCACCATAGTCGTCGACCTTCCTACCT
CGCCTCTCAATGAATGGGAACATCCGGAATCTGGCCATCTCCGCGCCTCTG
CCTGTCCGCATCTGTTGGCCTTTTACGAAAATGTCGAAAGACATGGTCATG
CCTGCGTTATCCGCATCCCCCGCATACGCATTGGCACCCTCTGCTCCGCCAC
GACCTGGCCGGATCCCAATCCCATCGGCTGCGTCTCCATCCGCTGATTCGG
ACTCGGATTCGGATTCTGACTCGAGATCGTCTGCCTCGAGCATACGCTTGG
AGCGGTCAAGAGCAGCTTGGTGCGCAGCTTCACGTTCCTTTTCCAGGCGCT
CGGCCTCAGGTGTGCCTCTAGCTCGACACCAATGAGGGGCACTTTGGAGTT

CATCTTTACCGTTCAGCTTTGGGCTTGGATGTCTGACGAACGAGACGTACC
TCCAGAGTCGTAGTTCCAGAGAGGCGGGCAATGTGACCAATCTTTCCTTGG
GCCCATTTTGCATCCGGGTCTTGCGTCAACGCCCACTGATCGTACACGTCTC
GGGCAACTGAGTCCACTCCACTCATAGATGTGAGCAGGATGACGTTGCCCT
CGATGCCGCCCATAGCCGTGAAGAGCCATCTGGAAGGGCCGTAGCTCATGG
ACGGTGGGATTGCCAGAACGAGTTTCGGTCGGCTCAGGCCTTGCTTCTGCA
GCAATTCCATGGGCGATGCGAAGAAGCGAATATGCCTTGAGCGAGTCAGCA
CCGTCATGACTCAGCCAAAAGCAACTCACCTGAAGTCGAGTGCTCCGTATT
CAGAATTCATGCCAGCGACTATCCGTTTCTTCCCCTCCTCGAATTATCCGGT
AGACCCTGGACGACGTCCTCGCCCTCTGCTTCTCGGACTACACCTCCCATC
CACTCGATCAGACTGCGGGCGAACGCCATCATGTCCTGCGCTGTCCGGCTG
ACAAGACACAGTGTATAAGGCCAGGCATTCTCTGCGTCTTTGGGCGAGACG
TAAAGGTGATGCTTTCTATCTGGTGGAATTTGCTTGAATGTCCAATGTTGAT
CCAAGAGTACGAGCAACTCAAGCAATCGAGGAGAAGGATCGACCGGCATG
AGAACCGAACGCTGTGATTCGAGCGTGGATGTGACAAGATCTACCTGCTCA
GTTGACGTTTTCAAGGCTCAGGCAAAGGACGGACTCAGCAGCGCAGTTTC
CCTCTCTCTTCGCTTCGGATTAACAACCGTAGCTCTGCTCCCCTCCACTATG
AGAAGGTCTGGCCTGAGCAACCCCTCTTCGTAGCCAGCTGGGCCTCCTGA
GCCTGCTGGTCAGACGAAACTCAGGGACTTCGCATGTCCTCAACTCACCG
CTGACCATGCCATCGAGGTGTCTTTCGCCCGTGTGATTCATTCCAACGGCAT
ATAGAACAGTTCCGCTCGTTGGCGAACGAATCTTGAACAGAGTTCCTCCCA
GTGTATACCCGGATCTGAATGGCGTCAAGAGCAGATGTGATAGATCGCCTAT
TGAATGAGTGGGAAGGTTGCCTCACAAAATTTGCCCACCTTCGAGATGCA
GAGGCTGGTTGTATCTGACCGCCCTGATAGCATCAAACGCTTCATGAAACC
TCTTCCACGGTTGGCACGAATGGCCCTCTCATGGGCCGCTTGCCCTTGTCG
CTGGGATCGTTCGCTTCCGGACTATCATCGACCCCATGCTCCGAACGCCAC
GACTCGACGTCTGCGAGGCAGACAACTCGTCCCATCTCCACCGTGGGTTG
GGTCGCGTATACCGGGCATTGCAAGCCCCATCTCGCCCTCGCGTATGGATAC
AAGGACAGGTACCGAGCGGGCGAATGTGAGAGGAGGACGAGGGACAAGG
TCGGGGCGAGTCTGACTTGTCAGCTGCTGTGTCGATAGACGGCTCTGATCC
GAGATTCACTGTCTTACTGCGTTCTCGTACTCCCAAGATGACTGCGATGCTG
ACGCCCTGTAGTCCCCCTGCCCCAGGTCGAGAAGGATCCGAGCCTGGTCG
ATCTCGAGGAGGTAGCAGAGGGGCTCAGTCGTAGATGACGAGCTCGCCGC
GGACGAAAGGGGCGTTAGGGTGATCATTCTTCGTGTCTCTTGGCGATCCCG
CCCTCGCGATTCGTCGTCTCGGCGAGACCTTGGCGAGGCCTAGAACTCCGG
CCACTATCTCGAAAGTCGACGTTCAAACTAACGAGTCCCAGGATATGAGGT
CCGTCCCGACACATGGGCAGGCGTGGGGATTAGCTTTTCGCCTGATCAGCT
TTCTAATGTACTTTCCTAATCCGAATAGGGGCCGATTATGGGGTCTTCGGTA

AAAGGGTAATGTAGATCACTTTGAAACCTTGTCGCGGCAGGGGCACACAC
ACCCACCCACCCGGGACAACATCTAAAACGCCTTTCAAGCACCCCTTCACG
CAACCCACGACCCTACCACCAGTATCCAGATCACCCACAGGCAGCTGTCCT
CTCGCTGTCATACTCATACATCACTGCCCAGCAGGACCACGACCATGCCAC
ACACCCGAGCCAGCTAGCTACCCAGCACCCGACTCCGCGTCATTCGATCTA
GCAGAACCCTTGCACGCTCCGACGCAAGAACGGGGATTCATTGACATGTC
CTCGCCTACCCGCTACTACGCTTCGACCCCCGCGGGCTGGTTCTCGACCAT
CTTCACGCCCAACTTCGCCGTCCAATCCCGCCGAGGTCCGCTCTCTGGCAA
TCGCCAAGCTCAAGCGTGCCGCATCGATCCCTCGTCAAGCGGATGGCAGA
CGACAAGGAGGTGCAGGGACCCCCGAGCAGCAGCAGCAACAACAGCAAC
AGCAGCAGCAGCATGTGGCGGTGCAGTATGCCTCAGTCAACGATCCTGGA
CCTTCAAGCTACGTCCAACCCGTCGAATATCAAGACTATGATCAGACGCCA
ACCAAGTTCAGCGGACATGGCGATGTCGAGGTGCTCTCGCCATCGCCCACC
GCACCGACCTTCCCGCCGTCAGCTTTTTCAACCACCCTCGCACAGTCATC
TCATGCAGCGCTCGGCGTCTGCTTCTTCCTCCTACCATATACCTTCACCCTC
GCACTCGGCCTATGGTCAGCCCATGAGCGCTCCTTTTGCAGCTTCGCCAGA
CTGGGCAGCCATGCAACTGGCTCAATCGATGCTACCCTCGCTCTCGCCATC
GGCTGCCGCTTTTCCTGTCGGTCGGAATACTCCCAGTCCCCTGCCAACACT
TGGCGAATTGGCAAGTCTTCAACGATCAAACTCGGCAGCGGCCCGAGCTC
AAGCTATGTCCAAGCTCACCGGTGGTCGAAATACACCTTCGGACGAGGAC
GTCATACTTGGACAGGGGCGGCCAGAACTGCATAGAGCAGATACACTTGCA
GCTCCAGGGATGCTCGGAGCGATTCTCGCACGGAGAGTGAGAACACCCCA
ACCCGAGGATCAGGCCAGGACTGCTTCCGCACCAGCAATCGATGAAGATC
AAACAGTGTTTCAACGACCTGTAGGAGAAGCTCGGCCGCGACTACAGAGG
AGCTATACAGTCTCTAGTTCCAACCTTGGCGAGGAGCGACGCAGTGTGGTC
GGGCGACGCATGATGGAGCGTTTGGGCAAGAGGCCCGGTACAAGGGAGA
AGGAGGAAGAGGAAGTGCGACAATTGTGGCGACAGCGCCGAGCAGAAGC
GGAACGTCAGTCGAACCGGGTCTCCACTCCTGGGTCCGACACGGACGGAG
GGGGCGGCATCACTCAAGAGGAGGCCGACGACGAGCGATCCTCCACTGCT
CAAGGATACCACGACACGACAGATTCTGAGCGGGAAGAGCACGTGGTAGT
CCAAGACCGACCGCCGTCGAGGGCAACAACGACCTCGGTCGGGGAAGCA
TTTGAATACTCTGGCCATCTGAGAAGGAGCATGTCAAGTCGCACAGCGAGG
AATGCCCCGGGTCTCATCACCGAAGCTGTGCCCAATATGCAGACGCAATCT
CCTGAGAACGCTTTGGAGCAGTTGAAGTCCCCAATTCCTGTCGACAATGGA
CCGCCCGCGCTCGCCCCTGCCATACATACCCCGGTACGACCGAGTACTCAG
GCCCCGTCCTCTGAGCCAGCAGACCATGGCAGCTCACCAGGAGCAGCTTC
CACCTCAACCAGGGATCCCTTAAACTCCATGATGTTTGTCGTTGGCGGTCA
CAGAAGCGGAGAAAGCTTCCCTGTCGAGGTATCGAATAGTAGCGAGTGGG

GTACGCCTGCCAAAGACCTCAGTCGTAAGCCCTACTTTTCGTCGCATAGGC
ATGACTCACCCTTCAGATCCTACTTTCTCCGATACACCTGGGCATAGTCCTC
TTGTGGTCCAAACTCCCCGTCAACCGTCAACTATCGAGTCCGATACCGAAG
GCACTAGGTCGTCTCATCGAGGCAGCATCATGTCGTGGGAGGAGGTTGGA
GGCGCCGAGGATCAGGAGGTCCCTTCGGACGCGCGTTATCACAAGAAGTC
AGGCAGCATATCCGCTAAAATCGGAAGATCTATGCGTCAGGCCATTCGAAA
ACGATCCAATTCCCGAGGCTCGACAAGCTCAAATACCGGAAAGAGCCCAC
CTACCAGTCCGCGAATCCTTGCCGACGGTCTGATCAAGACTTTTAGTCGGC
GAGGATCATCTTCGTCAATCACGCATTCCGCAGCAAGCACTTCTCAGCCAA
TCTCAAGGGCATCCCCAATGCCCTTGAACGGGCATGACCGGTCTCCTTCTC
ATCAAGCTTCGATCAGCTCGATGTCAGGAGCCAGTCACGAGCCACTCGACT
CGGCAAACAGCGCATTGATCCAGCATCAGCTCTCAGCGGAACGAGTCCATC
AGGTCTTCATGCCACGAGCGGACATCAGTGATCACCGCATCAACCACAAAC
TATCTCCGTTTCCAGGCTTTGCTCAGCTAGAGGAGAAAGCGCGTCGGGGAT
CCGACGACAAGCCCGACGCGCCGAAGTTCCTGCATCAAGCTTCTGATTCTG
TTGTGCCCAGTCAGAATAGAGTCACAGTACAGGGGGAATCAATCTACTCTC
TTCCACTGCCAGTGACGGAGGACGGCAAGCGTGGCTCGGCGGATAGCTCT
CTCCGCCGAGGATGGCTCGCGAAGGCCTTTGGTCAGACGACTTCACCAAG
GTCGTCTGGGAGTGTCTCGAGAAAGTCGAGCAATCCTGACATCGTCATCGG
TACTGGTCCTCCGAAATCTCCCGGCCTGAAAGGCAGCGCCGTAGGCAGCTC
ACCGCCGGCACTTGATGTCGATCCCTTCGCACCTCCACCCCCACCGCCCAT
CGCAACGCCGCGCGTCAAAAAGAAGACATCGGCTTCGCCTTGCGTGTCTG
TGGTGCCCGAGGGGAGCGAAGAAGGGAGCCGGTTGACGAGATTTACTGCC
CGTCCTGACTTGGCAACGCGGGAAGAGGTCAGCGAGACTGAGGAGAAGA
CGCCTAATGGTTTGACTCAACGATCCATGGAAGTCTTGAACCGGATGGACG
ACTTGTTGGCGTTGGGTCCGGATGACCCTGCGCGTCCGGACATACTTGACG
ACCCGCCTCGAAAGCTTCTGCTCTCGGCGCAGATTTTGCAGGTTGTCAATG
TTCATGTGAGTGGCCCTGCCCGTCGACCGGCATGTCTGACGACCAGACCGC
CAAGGACAGATTTGTCTTTCTCTTCAATGATATCCTGGTCATCACCAAACCT
CTGATCACCCATGGCATCCACGCCAACCTGGACATGCAGTTTCTCGTTAAAT
CGGTCGTGCCTCTTGATGAGTTGTCCATATCCGGATTCAAGGAGGACACAT
CGCACGAATCAGATCGACGCTCAGTTGTGCAGAAGTTCGTCGGACACTTC
GCCCAAGACCCCGAAGCCGCCTGCCAATACCTTGTCCAACGCTCGAACCC
GAAGGTGGACGCAATGACTCTAGCGTCCCTCATCTTCAAGACATCCGAGTT
GGACAAGACTCAAGTCGGGGCTCTACTGGCCGGGAATGATGTGCTCATGA
AGGCATTTATCGACCGGTTTCATTTCACCGGGGTCCGCATCGATGACGCCTT
GCGCATGTTTCTCCTCGCTCTACGCCTGCCAACCGAACCGCTCGCGTGCGA
AGGGCTACTTAGGGGATTCGCGTATCGCTACTTTGAGGCAAACAAGGACAC

CATCCCATTCAACAAGGATCTTGCGAACGAGCTGGTGCTAGCCATCATGCA
GCTCAATGACGCCTTGTATGGCACATTCGGCTTTGCCCTTCCCAACCATGCG
ATTACAAAGGACATCTTCATTGCAGCGTTCAATTCGAAGGACACCCATCATT
TGGTACCGGATACTCTGCTTGCCGATGTCTACACCTCAATTCGGCAGGCCA
AGTTAGCGGAAGCATTACCCGCGGAAGATTCAGCACAATTGCGCGAGGTTT
TGGTTACCCCAGCTCGCCTTCCTAGCAGATTGACCTACAATACGTGGTCGG
ATCCAATTTACATCAGCATCCCGAGGGCGGACTCCCTATTCAAGATCAAGCT
TCACGGTGATGGCTTGGAGTTCGAGCCGCCTGTCCTTGACTTCTCTGCAAG
TACGGAAGAGGCGTTTCGAGTCAAGGGAGTTGCCTTGGGCGCGAAGAGCA
TACTCTTCGACAGGCAAGGGGTGAATGCGCCCCTGTATGCCGGACTCGGCA
ACACACGCACATTCACTATTGAGCGAGCCTTCATGCGTCACACTTTTCAAG
TTGGATTCACCTCCCATCTGGGAGTCAAGCGCAAATATTGCTTCTCGCTTCC
CGATGCTGCAACCAAGGAAAAATGGGGCATATTGCTCCAGCGCCAGATTGC
GGCCACGAGACTGGCCAAATCAATTACACCGGCAACCCACCAGCAGCGGG
TGCGGCGTACTGCTGAAGCGGTCGCTTTGCAAGTCTTGCGCGATGCCTTGA
TTCCCCCTGAAGACGCGCCGTCTGCAGTAGAGATGACCGAAACTCCCAAC
GGGCTGGCGCGCACCAAAGCGTCGGACAAGCCTAAAATGAGAAGTGGTTC
CGTGTCGATAGCCTACGGGCTTTGGACGGGGCGGGTGAGGAGGCTCTTGG
ACCTCTGGTAGCACCGAACGGAAAAGTGAACGGTCATGCGGAAGACCTCG
GCAACGGCGGCGGTGTTGGCATGGCGGGCGTGCAGACTGGGAAAGAGAT
GGTCTTGCTGTGTAGGCAGAACAGCCTCCTGTCAGGTCTCTTGGAGCTATT
GCAAGCGGGAAAAGAGCGTCCTCTGACGCCTCTGACGCCGACCACAGCG
GACGGATACGAAAACGGTACAGGAGGCGTGCACAATCGCGCTCGATCGAC
GGGCGTTAGAATATGAGGCGGAGCGGAGGGGACGGGGGAGACCGAGAAC
AGCATGTAGCGCTGGCGGGAAGTTGTACACAAAACTCATGATCCCACGAC
ACGACCACACGACACCATGACCCTCACGATGATTCCCAGCAAAAGGATATG
GCATGCTTCTCATTCGATGCAACGTTTGCTCATGTCGCCACGAAGCTGTGTA
TGGGTCTGCTAGCTAGTAAAAGGCCACAGTGTGCAGCAGGGTGTGGGGGT
GCTTTTCCGAGTGCAGCTGTAGGATCTCGTCAATCTCTTCATCTAGATCATC
GGCCGCTTCGATCTACCACTGTCAGCCGACCGCCTTGACCCATGGAGCGCA
CACGGGGTGTACTTACCGATCTGGCCAATTCGAGAATGCTCTGGCCGGCAA
TCAATGCTTCTTCTGGGTTATCCTTGATCTCAAAGTTGATGACGTGAATAGG
GCGGTTGAACTCTCCTCCTCGCGCCAGGAGATCTGCGATCAGTCAGGCAG
AAGTAATGAGCAACGAAGGGCAGCTCACCATCGCACACAAGCGTCAAAGC
AGCGCTCCTCACATGTGATGACCACGTCTGCCAGCACGACCTTTTGCTCCT
GCCATTTCTCAGGCGCTATCTTGACTTTGCGATTCCGGTCCAACATGGGCAG
GAGGCCATTGCGCGTATATCTGATCTTAGCACGATGATGGATGGGCTGCGCG
ACGACTCACAGAGCCTGGTCTTTGCTCGCGAGGTCGCGATAGATGGTATCG

TATGGCGTGCCGAATCGATACACGTTCGGTTTGTCTATTGCTGGTCCGGGCA
GCCGCACGGCTGAGCCTGTGCCCGCTGATATGACGCGAAAGGCGGCTTTG
CTACCAATCAGCTGGGACATCGATGCAATCGTCTAGCTGACCTAAGAACCA
TATGTGCCTCCATAGAACGATTCTTACAGTCAGTCTAGCTATGTGAGAGAAG
AATGTCACGCTCACATTGTTGCTGGCGCATACGACGCAGAACAACGGCCTG
TTCTTCCCCTTCAACACCTCAGCGCCGGTCGGTGCTCCCACTGTATCTGCTA
CCGCTCCATTAGGCTTCACGTCCATAGGGCCTGGTCCAGCAACCGGCACTA
CAGTCCCAGCTGGTGGTCTGGGCGGGTACATGGCAGCAGGCGGCGGAGTC
GGAACCGCTGGCGGGGGCGTAGGAGTGGCAAAGTCATTCTGTGGCCGTCT
TCTGGGATCAAACCCTCCCGATGACCCGCTTTGGCGCGGATCTGCTCCCCT
TGGCGGTCCCGGCTGACCTGCAGGAGGAAACGACGGCGGTCCATTCGACT
GAACCGGCGCCGGGGGTCGTTGACGCGGATCAGAGCTGCCGGTCGCAGGA
TATGACCCAGATTGAGGTTGTTGTGGCGGATAGTGCTGCTGCTGAGATGATT
GTGCAGGATAAGGGGGGTAGGCTGCGCCTGATGGGGGTATGATTGTTGGTA
CCCTTGGGGCGGACCTTGCTGTCGAGGAGGGTAGTTTGGGGGTGGAGGAG
GCCCACCGTTCTGACTCGGTGGGCGAAAGCTGCGTCTGCGGGGATCCATC
GTGTCGAATATGTGACGAGCTTGTGCTGGGATGTCTGCTACCTGGACATGA
ATGCTAGATGGCTGAGGTGTGATGTCAAAATCAATGACAGCTTCGGCTGGA
GCGTTGACGAGTGAAATTCCGTCACGTCGGCGTTCATTCTTCTCTCCTCATC
AACTTCTTTCGTTTCGGCTCCGTTGTTTTCTTTCGAGGGGCTCGTTTCTAGA
GGCTGTACCGGGGAGACTGACGGATGGAAGCCTTGGCCAGCCCAAAACTT
GCCTGCCATGTCGACCTCCTTCGTCCCCTACGGCTTCGCTCAAGCCCACCTT
CCTCCTCCCCCTTCACCACCGAACTCCATCCCAGAAGTTCGCGTGCCGACG
GTCCGACCTTACCCAGCGACTTCTCAAGCATTTTCGACCCCTCTGCACCCC
GAGCATGCTACATCGGCCATCCGAGAACCGCTAACAGGAATCCTCGCAAGG
GCGGTACACAACGGGTACGTGCTGGAGCTTCGCACACTCGACCTCTTTATC
AACAAAGACCGGCCAAAATCGGCCGAGGGGACTGAGATCCTGCGAATCTT
CTTCCCGGACCCGCTGAAGCCTCTCGCTGAGGGGTGCATCATACCCAGACT
AGAGGAAGGGAAGGATATTCTTGCTGCTGGTTACCCAGGCAAATGTCATCT
ATCGTTTGATTTTCGGCACAGGGGACTTTCAAGCGGGCTCCGGGGATCGCG
TTGTGTTCAATCTACTCGCCAAAGACGAGTGGTGCGAGGAGTGGTCGGTTC
CGGACGAGACTACCACAGCGAGTGGAGGCGTGGGAGCTTGGACCTTGGTG
GACGAGGATGAGGTCGTTCTTGGGTGTAGGGATGGAGGCATCATTGTCTGT
TCACGATCTCACCGTTGGGGCAAAGGTCGGTGTCTTATTGGAGCATATGTA
GTGTCTGACAGGCATTTTAGACGAATCGTGGTCCGCTGCTCACTATCGTGC
CTCCTCTCGATTACGGCTACCGTCCCTCTTCTCCCGGTCGACCTCAGAAGA
CGAAGTCGTCTCCTTCGCCCGATTTGACGAAGAAGCTCAGACCAGACTCTA
TGCCTTGTCAAGAGACAGCAGACTACGAGTATGGAATACAGAAAACGGTC

ATCTGGACAAGACCCTCGACTCCGTTCGACAGTCAGTGCGTCCCGGGATCT
GGTTCTTCGGAGATCCCCAAACACCGACGCCGACTTCTGCAGGCATCACCT
ACGACACTTCCGACCACGCCCAGTATGTGCAGGTCGTCCGACATCCGGTAC
AAGCCTCGAAATATACTCATTTGGTCATCGCTTTCGTAGCGTCACCTACGGC
CTCTCCGTCGCCAGGCACATTCGTCGTCTACCGGGTTAGCGCATCAGGAGA
GCTGTCTCTAGCCGGAGACAGGTCTTGTTCTACCCGCTTCGCCAATGCCGC
GTTGCGAGGCTTCCAAATCTTGTCGCCTACCGATGAGGGATTGGCGGGCTG
GCGTCTTTTCGCCGCATGGGACGTCGATGGCGGGTTGGCCGGGGAGGTCAT
TCTGATGGACGACTTATTTCAATTCACTACCTTCGAGGATCGGGTACAGACT
TCACGCCGGTACGGGATTGGCGAATGGTACACTCTGGCTTGCAAAAACAC
GCCTCGACGCTACTTTCTTCGATAACAGCCTCTCACTGGAACCGCCAAATG
CTCAAGAGCCCTTCGATAACGATGACATACCCTCCGCATTCCTCAATCAGCT
ATTCTTCCGGGCAGATTCTCGTCCTTGACGTTGCAGACAGCGCTTGACGAC
TACGTTGGAACATTGGCAGGTAGAAATACCCTACCGCAAATCAGCGCTGCA
TACCCGACACTTTCCGCCAAATTCGCAGCTCTGGTCGGATGCGAGCTCGCG
ATAAGGTACTGCTCCCAAACTGGCGGACCGGAGGTCTCTGAATTCCGGCGG
GACCTCAAATTGCATTGGCTGGGCATTTGGGCTCGAGTCACCGATCTGGAC
CGTCAGGCCCGCTGGCCTGTCAACACGTCCACAATCGACGACCCAAGTGT
TGATTCTCTCGCGCGAATGTCTCTCAAGCGTTGTGGCTCAGGATACTGCGA
CCTTCGTTGGCCAGCTCGCAACTGCGCCGGATTCTGTCTCTGATGTGTTATC
CGCACCGGACAGTGTCTTCGAACTCGCTCTCCGAGGATTGGTGGAACGCA
AGACTCGCCAGAACGTACTCTCGATTCTGGCTTGCGGGCGGTCTATTCGCG
GCGCCTTGGGAGAACTCGGAGCCCGAAGCGAATTCCCCGGTCCTCGAGTC
TTTGACAGACGACTTGGACGCAGTGCTTGCAGCTGGAATCCAAGAGCCCA
TCGAAAACATAGCAGTTCGACTCTTCGACGAGTATCTCGACGAAAACCTCT
CGGAAGACGACAAAGCTTCTCTGCGGGACGAGTTTCCCGATCTGGCAACC
ACAATCGAAGGCTTATCCACTTGCCTAAATCTATTGTCGGGCAGTCCTGTAG
CAAAACCAGCCCCAGAGATTGATAATCTGTCGTTCTCGGGTGCTGGCAATG
CTTTGATTGCCTCGGCATTACAACACAACATCACTGTTCGACATTACATGAC
CATCAATCTCCTGCTCGTCGCCCTGTTGTGCCTGTCTGACCTTTCGAACCCA
AACGAGGAAGGACGATGAGACTGTAGCGTTCACCTCCCTCATCGCCAGGG
TATTCTCAACTTATCAACGATACCACATCCTAAATTGGGTTCAACGAGCAGA
CTGCGGAAGAGGCACGCAATGTTGCCTGGTCACGGCGCTCGAAGCAACGG
AAGACACGGAACGATATGCTGTCGGACGGTCTTGGGGGGTTGCCGCATGC
AGGAAGAAGCCGAGGCTGAACTCGAAACAGATGGCTTTGACACAGCGTAC
TCCCTCTTACATGCGTTACTGGGCGACATCCTGGTGATCCCGCCACGAGAC
GATACTTCGAAGGCGCTGGAATTGGCTACCTCGGGGTTTCTCGATTTCTACT
CCTCGCCGGATGACGATGATGATGTTAGCCTGGAGGCTAGCAGAAGTTCTG

TTCGGCTGGCATACTCCATATTTTCGAGCGACATGCCTGCCCTTTCGTTATAC
TCATAGTCAGATCATCCTCCTCGTTGTTGTGGATCTGACTATAGTATCCCAGG
CTGCCAACCTGTGTGCTCTGAGACAAGAGGCGTGTGTGGCCTGTAAATTGG
TAGAACTGGCTGCAAGAAGTTGCAGCTGAAAGAATGAGAATGGTTCAACT
CATCACGATACCACATTTCATACTGAGTTCGGATCATTGCCAAAATAATGTTT
GGCCATTGTGATCTCTATTGTGATAGCTAGGGATGACATATGGAAGAAGGAA
AGGAGTGAGTGTGATTCGAGGTTAAATCACATAGTAGCACGCTTCTCTCCG
TCGCAGCCTATCCGGATGGAGTTGATTCTTACGTGCGACGTGGCGCAATTG
GACCCAAAAAAAGAAAGAACTTGTATCTCGACTAGGTACTTAGCAATATAG
ACCCAGCTAAGAAATACCAGTAAATGAAGAAAATAACTATTGCTAGCGAAC
GTATTGCTTGGTGGATTTAATCCCAAATATTGGATGTTTGTAACAGGTGATG
ATGTCACTTGATCATCCTTACTACCAATGTGAGAACAGACGTTTGTTTGACT
TGGTGTGGTCATGGGCATGATTATCAACAATAACTTTATGGCTCACGATGTT
ACACTACTCAATCGGTCCTTATGGAGTTACTTGGAATATATGTACGTGACATT
CTCGCATTTAAGTGACAATTAGTAGGAATGGTTATTACGGGTTCATTTTCTTA
TGTCTTTGCTCGCCTTCATCGGAGCTGATGTCAACGCGATATTACAACAGTA
CATTACAGTACACAGAGCATAAGCTCATACACACAGGAATATTTTTCTAACC
CAGCGAAAAACAAGTACCCGTTATTGCTGGTGAAATGGTATCTCTCCTAAC
TCCCGCCAGACTAGCTTGGTTCATGATTCCTTCCACAAACGCCGTAAAGAC
AACTCGTGAGATTAGAGTCCACGGTAGGTGTGAGGCTTACTTCTCTTCCTT
ATCACCTTCGCGCTTCTTCTTCTCGGCTTCCTATGCCTCTTGTGCCTTGGCA
ACAGCGTCGATGTCCTTATCACGACAGCTCCAACCATCATACTGCGCTTGC
AACACCTTGTTTCCCTCGTGCATGGTGTGAATCCAGGCTAGGAACACATCG
CTGGCGGTGTCGATCATTTTCTTTTCTTCCTCCTCAGCAGCAAGTCTTTCCA
TTTCCTGGCGCTTCTTCTCTTCCTCCTTTCGCAGCTCATCTCTCGCCTTGCG
CTTTGCTCGGCGATCCTCGATCTCGCGCGCGCGATCGGCTAGGTAACGGAC
ACTATGGCCAAGAGTGGCGCTTTCAGCCGAGAGTCCGCGGTGCGGGCGAT
CTAGAGGTGGCTGAGTAAAGAACAGCACGCGCCCAGCCTCATCACGCTGG
AATTGTTCCCGAATATCGGCGGGGATCTTGTCGTTCAGAGCGTCATCCAAAT
GGTAAACCTCGACCGGACGAGGCGGGTTGTAAGCGTTTGCTTGAGCATGC
GCAACTGGACCAGGAGTGGCTGCCATGGGAGGACGAGCAGGCGATGGCG
CAAGACGATGCGACGGGTCATAGGTGGGCATGGGAGAGTTCATTTGATGAG
GAGGGGGGCGTGGTTGAGGCTGATGAGGTACAGGAATCGGCGTGGTGCTG
TACTGAGGCGGGAATCCGTTATAAGTAGGAGGAGGCTGGTGCGGTTGATGA
GCCTGGAAATGCGGAGTCTGGGGCATCTGGGGCGTGTGATGACCGTGGGC
GGTATGATGAGTAGGCTGGACTCCTTGAACGGGAAAGTTGTGATGAGCGG
AGGCGGGAGCGTGATTACCATATTGCGGAACAGGCACCGGAGTAGGGCTG
GGACGCGCTACGAAGGGATGCGGTGATGCATACGCGCCGGCATGTCCAGG

TGTAGGCGTAGGTGTCGCCGGAATGGTGGAAAAATGGGGAGTGTTACCCAT
CATGATGTCGCCCTGGCTATCAGTAGGGCGAGCTGACTGCATCAATGAAGG
ACGTCGAACTGGGTCCATGATTGGAAGAGCGGGAGGAGGCGTTGGTGTAG
GCTCCGGGGGTGGAGATTCCTATAGGAAGAATTTATGTCAGCGCAAATGAG
AAATTAAATTAATATTAGGTGACGCGCTTTAGTGAGACAATAGAAAAAGGA
AAAAAGAGGATTAACATCCATCCAATATAACATACGTATCTTTTTTTTCCTT
TTTGTAAAGGGAAATCTTACAACGCAATCGATAAGAATGAGAAGGGATGGG
ATCGGGAAGAGAGATACATTAGAGGAGGGGCAGATTAAATTCTCTTCTCCT
CTCCTCTCCTCTTCTTTTTTTTTCACTTTCCTTCTTCGTATGCCTTTTTTTCCC
TTTTTTCTCTCTCACTTTGTGCGTATCAAGGGGGTAAGGAGTGTGTATGAAG
ATGTGGAGTGGCAAGTGACGAGGCTTAGGCAGGCAATCAAATAAAAAAAC
AGGGAGAACTCACATTCGGCTCTCTGTCCCGACAATGTACGGCTCCAATGA
GAGGGGGCGCATTTGTAGCTCCCCAGGTCGGCTTAGGTAGAGCATCCGTTG
GCTTCGCATCATCGCGTAGCAGGTGCTTGATAGGGCTGGGTACCTTTCTCAG
TTTGCGAGGAACCTCAAACAAATCCATCTCATAGTCCTTCTCGCGAACCTC
GTCGGGCACACAACTCGCCCACGTCTTGATCTTGTTGATTTTGTGCTTCTCT
TCGTTGTATCGAGCCTCGCAGACATATACTTCCTTGTCCTTGGGGAATCCTC
GAGGTCTACCCTTGTTGTATCGAGTTACGAACATGACGAAACATCGATCCA
CGACCTCGTCGATGCGGTGGTCTCGGTATTGACCAGTCTTAACTACCTCGTT
CTCTAGGAAATGCTTTTCAAACCGATGGACAGTCTGCTCTGGACGGTAGTA
CCAGCAAGCGTTCACCCACTTTTGTCCCGACTGTCTTCCCAAGTTCGGTAG
ATCTGCGCGACGATAGGCTTTGTTAAATCATTGGGGTTGGTGATATGAACCC
AATCGCCTAAACAAGCAGAGTTAGTGTTCCAATACAAAAACACGGGACTA
CAGGCAACTTACCAATTCTCCAGACCTCTCCTCTGTGGATAATCTCAGGAA
GAGGTCGTCTGCCATCTTCGTCTTCGAAGTCATTGTCAGGCTTGGCCTTCTC
TTGCTCACCTAGTATGTGAGCTTGACGTTGCAACTCAACCGCATCGTTATAG
AGTTGGCTATCCTCCTCGTTGTACTCCTTGGCATTTTCGAACATCAAGTCAA
GATCTCTCAATACTTGGTCCACATTCTGATACTTCTTTCTCTTGGCCTTTCGC
TTGATGAGATCCAAGGCGATAGGATTCTTAATGGCAGCAAAGTAGTCTGGC
GTCGCCTGCTTGTCCGGGAGCTTCTCAAATGGCACGATCAATAGCTCTCCG
TTGGGGTGCTTGGGCTTTCGAAGACCCTTCAACAAGGCGTGAATACGGGC
CTCGAGTGGAGTAAAGACCTTAGGGGGTCGGCCTCGCTTCTTGTGTGAATC
ATCATCTCTCATATCAGATTTTCGGCCTGACGTGGAGGAGCGACGGCCACC
TCGTCTACCTCTTCGACGGATCCCATCGTCGTCCGAATCGTCGTCTTCATCA
TCATCTTCGTCATCCTCGTCCTCTTCTTCCTCGTCCTCAGGGTCCGGAGGTG
GAGAATCTTCTGTTTCCGGGATCTCGCCTAGATCAGGAAGCACGGCCTCTT
CGGGCTTGATGGTTCCGTCGTCGACTAACTTCTGGAGTTCTACCTTAAAGAT
CTCTCGTAGACGAACGGCATCTTTGAAGAGCATGGATGATGGGCGGTTGTA

AACCATGGCATTATGGGAAATCTGAGCGACATCCCTAACGAATTCGGAGAA
AGCAGTATACTGCTTTTTAAGCTTCTTGGTCTATTGACGAAACATTAGAAGT
TAGTATGACAATTTATTAAACATAAAAGTATCGCTGCGTGTCTAGGGATTGT
AAGGACTGTAAGGATTATACTCACGCGAATGGTACTGAAGGCGATAGGCTC
TTTTATCACTTCGTGGTACTCGGGCATGTGTCGTCGGTTGGGAATACGTTGG
AAATGGGCGGCAACTTGGTGGTCACTGTAGCGTTCATGTTAGTTAAAAGCG
TATGACATGTGGAAAAATAATAAAAAAAGAAAATAGTAAATAAGAGGGATA
AGGAAAAAGGAAAATTTACCCATCCTCTTTATAGGTAGAGAGGTAGTTCGC
CGTGTTCTCAATAACGGTCATCATATTGTGATCAAGTCGACCAGGGACTCGT
TCTTCCTCGGGTTCACCATCGGCATCTACTTCACCTTCCGCATCTACATCTTC
ATCCGCGTTGGCGCTGGGATTGGGATTGGCGTTGACATTGGGTACTGAGAC
GGGGCGCGACACGTCTGCGCTAGTAGTAGCATCACCCGTATCTATATCCATT
TTTGAAGCGTCATCATCGTGCGAGTGATCCTTATTGTCGCCTGCATTGTCGT
CGCCGGTGGGTTTGGCCTCTATGGACTCGCGAACCTCGGCCGCGCTTTCGG
TTGGCTTGTCGGCTTCGTCAGCCATGACGAGAGGATGAAAATTGAATAATA
GAAATCAGGATAGATGGAATTATTCGTTGTCGAGCTTCGAAAGTGTGTATTG
GAGTGTACGATTTGATGAGGGGTTGGCAATTGTGTGAAAGCTGAACGTTCT
CGATGCCGATGCAGCAGTTTGAGAGTTCTTGATCGTTGGATCGTTAATAGTT
GGTTGGTTGGTGTTAGTGGTTATGGTTAGCGTCCGTGGACGCTTAGCAGGT
GCTAGTGCCTAGATGCTAATGTGCACAGTGCAGCACGCTAAAATTAAAGAT
GGAATTTTACCCATCATTGTTCCGATACCGACGATAAGGAAGTACAAATGTA
CATGGGCAGCTTGTCCCAAAAGTGGCTCGATTTGTGTGGCTGTGGCTGTGT
ATATTGTAGCTAAGTAGCTATTTTCAATCCCTATGAATTTCTAAGATTTGCTA
ACAAGACCAATTGAATCAAGCCCTATTACTAGGTATATATCCTTCATTAACCA
TCGATTTCTGGTGTCTTTTCCAATCTAGAACTCTCTATATCTGCCTCCAGCCC
CGGCGATATGACTGTATCCCGAGTTTGACCTTGTCGTCGATAGTCCCTGTAG
CTCAAGAATAGGATGGCAAGGATCGTGCCCAGTTGTCCCACCGTCGTAACA
AGCACACTGATGAATCCGCCACGAAAATTAGGAGCGCGGCTGGCAGGAAC
TGGACAACCTGTGTGCCTGCCGTGATTGCTTGACCGATGGCATTCATACTCG
CTGTTACCACCGCCCGCTCCTCTGCATCTCCCGCCATGACCACCGAGGCCA
GCCCATTGTCAATGGCGAACAGGCTAAGTATGCATTAGTATTTCATTTTCAC
TCAGGCTGAGGGTGAAGAAGGGAATGGCAGGGGGGAAGAGAGAGATGCC
AACTCACCAGCTTCAAATCCCGTCAGGATGAACCCAGCTAAGCGACCGCTT
TCACCGACGTCCCAAGCTACGAGCAGTATCATGGCCACAAGCACTGGGAA
AGTAACTATAATCGCTGGAAGCCAAAATCGGCCTGTTCTGTCTGCGATGATA
CCACAGACCAAGGCAACTACAATACTCACTGCCGTAACGATAGTAGGTATC
GTGTTGACCTGGACAACCGAGTATATGTTGCTCTTCGCTTTCAAATACAGCG
AGAATGGCGTACTACCAGGTAAGAAGTTTTGGTCCATCAGTGTCCATTGAA

GCACAAATAAGTACCAATGCCACCGAGAAAATACGCGCCTAAATATGTCAC
GGGAAAGCTGCAATTTCGGCGGTGCAACAAGCCCTCGGAGCCGCTCACTC
GCCAGAGCATGCTCCTCCTCTGTGATGAAACGCGGCTTTTTCCTAGACGGC
ACATCGGGAAAGACGACGAAACCGCTGCAGCGAATCAGTGCCGTAATTTT
TGGTTTACTGCGGGTGGTTGAGATAGGGCAGGCAACTTACAAGGCTGCTAT
GGCAAGAGTTATGATAGCATCAACAATGAAGAGCCATCTATCCGAATGGTT
AGCACATTATCCTAAAAATCTAAACTAGATTATCTCGTCGTGAGAACCATAC
CTCCAACCCGCCAAGCCATGTACTTGATTCAAATTAGTGTAGGCTGCGGCC
TGAAGATATCCGGCAAACATAGTTCCAAGGGGACTGGCGATGAAGAAAAG
AGCAACGCGACGACCGATTTCCCCTGGTTTGTACCAGGATCCGATGACATA
AATCGTTCCCGTGAAAGAGCAGCACTCGAATAGGCCAGTGAAGAACCGCA
TCGCATACACCGTCTCGACATTCCTAGCCCAGGCCAAGCCAAAGGTAGAAA
GTCCCATCCAATCTCCAGAGCGGGAAGGACTCGGTTAGCAGGGTAATGGGT
CAAGAAGTAGCAGGCCGGGATCATGAAGACGCAGTACGATGCCCAAAAGG
TCGCAGTGATGTAATTTAATTGGTCTCCGTGCAGTCCCAACTCTTCTTTCAT
ACCACTACCAGCAGTGATACGCTCTTAGTAATCTGAATGTACCGAAAAGTAT
GTAAAACACGTACCTGACATATGCATTGGTGATGGATTGCTGGTCAAGATAT
TTGTAAAAAATGACAGGCAGCCAAAAATCAGGATACTCAGGTCAAGTTTTA
AGATCAGCCGTCTCTCCTTTTCTGGCATATCTTCTGGGTACCATCCGATAGAT
GCCATAAATTTGATCCAGGCGTGCCCGATACCAGGGGTCCTGACATTGTGG
TGTTCATTCTGACGAGGTTTATGTTGTACTAATGAAGGTGTTATCGTTGTCC
GGGTCTCCTCGTCTGCTGCCATTTTTAGGATCACGCCCAGGCGAGTGACAT
AACATGAGTCTATACTTGGATATAGGATATACCAATCGTATCTTTCAGTTGAT
GGGAACTGGGCAGTACCTAGTACATATATATTAAAGTGTAAGTCTAAAATGA
TTAGAATTATATATAATCAGTGTCGTCATCAGCCTCTACGATGATGGAACAGC
CCTGGTCTCGTTCAGCATGGGGACTATCGCAGGGTTCAAGATGGAGTTCCA
TATGTCTTAGCCATCTTAGCTATCTGAGTATTTGAGCGCACCATATCACATAT
CCCTATTTAGACATTGATTATACCTATTTGTACCTGGCAGATAACCGACTTTC
TTGGGCGTGCTAAAGCACCGATTCCCCTCTTCTGGGACTACTCTGTAGGTT
GCGTCAGTGGCACTCGCTATTGCGTGACGAGGACAGCCATCAAATTTACAA
CGTTTCCAGGTATTCCTAGTGATATAACAGGAATGGCCACAATACCTGCCTA
GTATAAAGTTGTCGCGCCTTTTGTCAAGTAACCATTGCTATCAGTAGTGCGT
ACCTATATATAATACAATGCATTCAGATATGATGACCAATATCAGGGGGTTAG
ACTAGCTAGCCCAGGACAGACTGTTGAGCTGATCAATCATATGAATACAATA
TTATAATATCTTCGCTATGTGATAAGAGGCTTCAATGATTTATGAGAAAAGCT
TCATATATATGTGATTTCGATGAGATAAGTCAGCTTTGTGCTGCTGACACTTA
CGTAGCTTGCCCACGTGTTGAGCTTCTGCTATTTGATGCGACATAGCAATAC
GTACATGCTATAACTCATCGCGATTGTTATAAGCTGCCAAGTCAAAACCGAT

GTTGGTTTGTTACTCTTAACAATGCTTACATAGTACTACCAGATATGTTTGTG
AATGTATTTCCTTGATTCCTTGACTAAATTGTTAATAGTTTGATAAAATTTAC
ACTTTACATTTTCGTAATTGAGTTCAATTTTTCTTCTTCTTTCATATACTCTTG
AATCGCATGCTATTCTTAAAGTTAAGCTTTTATTGCAATTAGCCCCTGGTTAT
AGCTATAAAAGGAATCGGGTCTAAAAGTGTTCCGTCAAGTTCAAGTCCGGA
ACGTGGGGATTTTGATCAGCTCCCCTCTTCCACATAACGCTACGGAGTAGTA
CTACATGACTACCTAACTACCTGACCTCACATTTTCTTTTATCTACCTAAGTA
TATATCGGCAGCGCCTGCTGTTGGCTTGATGATCTTGCATTTTCGTGTATATA
GAAGCGGGTCTTTGGGTCTTAAGGGGTTTTGCATATGAAGGGAGCGTCTAC
GATCTAAGGGTTCAAGGGTTCAAAGGTCCAAAGGTCTAAAAGCCTCACGA
TCCGACGGTCCTACAGTCTCACGGTCTCACGGTCCTGAGGGTCTAAAGACT
GGCTTGCTTCTGAGTTGGTTTTGGCCCTTTAAGCTTACCACTAACCACTAAT
ACCACTGATCGTCTTTAGGGGAATCAATACATACCTAGGTAATCTTGAAGAA
AAACCACAGATTCATCTTTTTGTCTTCTGCGCCAACGTGTGAATCGACTTCA
ATACCGAGTCTCACCCTCAATCTGCCTTGTCCTGACTTGGTCCATCTTCCAC
CTTCCATTTGCCTTACTTCCCATCTTGTCATCTTCTAATCTAACCATTCACGC
TCATACGCGTATAAATACATCTCTCCAAATAACACAACGCCTTTAGGGCAAC
AATCCTCCTTTCATCCCCCCGACACCACACCTAGCACCTCGTTTCACTCGTC
CAAGCCATGAAGATCCGATACCGAGGACCCTCTGGGATGGGTAACCTAGAG
CTTGCTGATGACGCTACTGTCAAGCAGCTTCTCGAGGCCCTCAAGCCTGCT
ACCGGATTGGACAACCTAACAGTCATGTATGGCTGGCCCCCCAAGACACTT
GGTACGACACTTGGTACTGACGAGGGCGCCGCCTCTCTGCAATCTCTGAGC
CTCGAGCGCGAAGCACTCACCATCGTACCCCTAGAGACGGCCCCCGCAGT
AGCTGAAGAGGAGCCTCAAGCTGCTGTCCCCGACTCAGAATCATCTGAGT
CCGAAGCTAAGAGTAAGGGCAAAGGTGTTAGGGACCAGCAGATCACCGTC
TTCATGCCTGAGACTAACTCCACCCTCGGTAAGCTGCCCAACCTGTTTAATT
ATCTGCGTATTTTAGTGAAGCCCCCTAGACTAACGTCGCTTGCCTGTCGCAG
TTCTCCGAGTCATGCCAGATGATAACAGCTGCCTCTTCAACGCTGTCGGTG
GCGCCTTGCGCGGTCACGGCACCGACCCCGACCGTACCTTCGATGCCAATA
CGCTTCGTCACATTGTGGTAGGCCACATTCTCGCCAACCCCGAGAAGTACA
ACGCCGTCATCCTGGGTAAGACCCCGGAGGCATACTGTCGCACTATGCTGG
ATCCCGAGACCTGGGGTGGCGACATCGAGCTCTCCGTCCTTTCCGAAGTTT
TCCGCATCGAGATCTCCGTTGTCGACGTCAAGACCGGAACCCTCTATCGAA
TCGGAGAAGGCAACGGATACGATATGCGCTGCGTCGTAGTCTATTCCAACG
TCCACTACGATCGCATCGTCGAGATCTTCGTCGAGGCCAGGCCTCCTTCGA
GTTCGACGTCGCGCGCTGGGTCGTAGACGAGAGTGACCACATCATCCAGC
ACGCCCTGGAGATGTGCGAGAACTTGCGCGAGCAGCATCACTACTACACC
GACACCTCCGACTTCGTCGTTATGTGCAACGAGTGCGGTTGGCTGGGTCAA

GGGCAAAAAGCTGTTGTCGATCATGCACTCCAGACTGGCCATAAGGACGTC
AGCGAGATTAAGGATAACTCGTAAACAGCCTGCTATGTGTACCTTTAGCATG
CCTTGATCGTCGGTTGCCCATAGGCGTTGGTCGGCGCAGTTTTTGCAAAGT
CGCGTGTGTTCATTTTCGCGGAGATCGCTGGCGGGGCAGCTTTGCGTAATC
AGATCCATACGACTCGTGCAACAACAGACGATACCCATTTGCGAGACAAGT
GGTGGAATAAAGTAATGGAGAGAAGTGGCGAAGAGAAGTGGTGGAGGTG
TTGCTTTGTGTTCATAGCCTCCGGGGAGATTTTGACGGTTGGCCGTACGTAC
ATATCAGTCTCTGGTCTCAAGATTTTCGTCTTGGCCGTGTATTTACGAACAG
GTGATGGTTTAGTCTACCCGTCCACCATTGCCAATATATAAAGTGCTCACTG
AAAACACGACTTATATCGTAGACCACATAATAATCTAGTGAGAAGTTCCCAG
TAATATCTTATGCTTCTCGCATACATAAGATTAGATAGGGAATCGACGGGGA
AAAGAAGCCTCGGATGTAGACTCCCTTAATGTTATACACTACACTTACGCGT
GTAGGTCTATTATTATATTTTATACGCCGTTGGGCTCTATTCTACGTAGAGAG
GAGTGGCTTTCCCGATATAATACCAACATCAATACCATTAGGTTAGTTGATTA
GAACTAGAAGACAAGATGCTGCCTTCTAAGCAAGATATCCTCATTTGTCCA
ATTCCAACTCGAACAGGACATGAACAATGTCATCAATGGTGGTGATTTCGC
CGTAACCCTAACTCCAGCAACCCCCTTTCCATGCAATAGACCATGCCAATCC
ATATCATTAGACCGACATTATTATAATACAAATTTCATGCTACCTAAGCTGTC
GTTAATCAACTTCCCAGCTACGATTATCGCGCCGGCTGCTGTTGGCTGTCTG
CGGTACGCTACTGCTGCTACTCTGCTTGCTCTTAGCCATGGCATAGTCTTCG
TAATCTTCGTCATCACTCCAATCGCTAAGCTCCCCATCAGAATAATCCCACT
GGACTGGGATTCTCTCACCGCCTTCACCGAAGCCGAAGACGAGACCGCGC
CACTCGCCCGACGCGATCTTGGCGGCCACCTTCAGCATCAGGTTCACGAGG
TGGGTTGGCGGCGCCAATATCATCTCAACCGTCTTCTGTATGATGGCGCCCG
GTAGTCTACGGACAACATCCATACGACTGCGAGATGCATGCCTCTGAGGAG
GCGAGAAGAAGTTGACAGCGGACGGCAGCGGCGAGAGAGCCGGTCGGCT
AAAAGACAAACTATTAATTCGTGGCCGCGGCCGTAGCTGAACTTCTTGGGT
TTGAATGTCCTCTAAAGCCGATGTATGCGACGAGTCGGAAGACTCATCGAC
AATTGGATTTGTAAGGTTAGAACTTTCCGGAGGGCAACGCGATAGAGGAGA
AGATATCTCGTCTGTGTGGTCGGTAAGACCTGCGGAACTAGGGACAAATGT
CCGATCCGTATCCGGCGTACGCATGAATACCTTGTCCTTTCCCTGCGACGGC
GGACGAGGGACAACAATATTCTCATGAGGGTGAGGAAATTGTGATCTAGAC
GGTGTGACCGGTCGTGACGATGGAGGAGACGAGGTAGTGGGGCGATTGGC
GAATGCCAAGACTGTGTACTATGGAATGAATCCGTGGATCCAAGAGGGGAA
GTCTCATCTGATGCTTGACGCTGAGGTGCCGGAGGAGGAGGGGGCGGCGG
TGGGAGGCGGGAGGTGGTTGCTTTCGACGACGGTGCCGACTTTGAGGGGG
GCGATGTCACCACAGTTAACTGCGGAGGAGCAGTGTAGGCACGACCAGCT
AACATCCGGGTCATTCGCTTCCTAGTAAGATTCATAGGGGCTACGCGACCG

GACCCTTCGAAAGAAGAAGGCTCATCGACAGCTTCTTCATCTTTACTAGAA
TCAAGAATCACCTCTTCCTTGCCTACATTGGCGTTACTAGGTGACTCTTCTC
GAATACGTCGCTGTTTCGGCGTGGTAGAAATATTCGAGGAAGTACGTAAAT
CCTTAAAGGTATTGGCGGATGCTGCCTCGGCTTTCCGCCATTCATTTAGGTT
GTGATAATACCGAGCGCTGTAGTCATTTCCTTCCGCAACTTCTGGCTCTTCA
AGGTTCTCTCTCTGCCAGCGCTTAAATCGTGATTCCTCTAACTCATTGAGAG
GCTCAATGCTGATCCTCAGAGATGCCATTTCGTCGGCTATGGAGTTGGCAC
GTTGATCGGCAAAGCCCTTAGGCGAGGGCGAGAGGATTGGTAAAAATGGA
TCGGATATTAGATTCTTGCCGTCAGGTGTCCAAGCTTTCTTTTTGACTTCTA
GCTCCTCTGGTAGCTCTACTGTAAAATTCCTCTGGAAGGGACATGCTGTCTT
CTCAAACAGTAAAGCCTTATCGAGGATATCTCTCAGTAATAGAGCGCGCTCT
TGATCGTCAGGGTCGGAAACGGGGAGCTCGATGCGCCAGTATTGTGGCCG
CCGTATCTGCAGGACAAATCTGCTGTTATCCTCGTCGAGACACCAGCACTG
ACTTTTCGGTAGGATGGGTTGTAGGGCCGAGCCGCAACTAAGAAAGGCAA
CGGATCCTGGCGCACGATAAATGCGGAACGGGCCTTTTGAACAGAAAAAA
AAAATAGCGTTAGTCCGAAGTTAAACAGAAGGTTTCTTGGGTCAACAGGCT
CAACGTACCCACGGCTATCGTTCTCTCGAGACGCGATGAAGCTGGTAGAGT
ACCTGGTCTATCCACATCGTTGGAGGATTTGCGAGGACCAAAGGAGGCAC
GCTGAGTAGATGTAAAGGCGATGATCTTCACGGAAGGATGACTATAAAGAG
TCTCGACTAGGTCTTCGGAACGCGCCGATGGATTGGATGCGGATGTAGATG
TAGATGTAGATGCAGAAGAGGGTGGAGGTAAAGTCGAATTGGGAGCGGGA
AGTGAGCACTCAACAGGTGCTGTCACAACCCGACGAGGTCGCGACACATC
TGATCTGCCGGATGATATTTTGGAATTGTCATCAGCCATAGTTAGCACATTCC
TGACTAGAAGCAAGCCTTTTTATTTTTGTCTGCTCGTTTGATAGAATTAAAC
TCCCAGGAAGGGTTGTCGGCTTGCCCAAGAAGCTTTAGGTCATGGCTAAA
AGTTGCCAAGTGCTAGAAAAGAGAGAAAAGCAGCTAATCAGGGCAGAGA
AGATCCGACACGTATTCCTTGAGAAACCAAATAAAATAATCGCGTGCTCGT
TGAACCAACTCCAGATGTTCTCTCAAAAGACTCGAATGCTCAAAATACACC
AGAGAAAACAAGTATGTAAATACAGAGCATCGAAGTAGAGAAGGTTAACT
ATAAACCAGACTAAGGTATGTCAGAGCTGCATTCGGGGAACTAGGATTGAC
GAAGGTTTGCGAATGCACGAAAGGGATCGTACCTAGGAAACCACCTGGGC
AGCTACTGGATCACCTAAACCACCTTAGAAACCACCTAACACACCTAAACC
AACTAGACCTACTAGACTTATCCCTGAAAAGGCGCCACAATTATAAACGGT
ATGTGCCGATTAGAGGCTGGTGTGGTGGACCAAGTGGGGTAAACCTAAGT
GTCCAACAGTCCAAGCTCTTTCCCGATTACCCCACCTAGGTAAACCTGATT
GATCCATAACACATTTGATTGGCTGCTATCTCCATATTACGTAGAGCATCCGC
GGTATCCATGGATACCATCAGATATGTACCCAGATGCGCAGACCCAGGTTTA
CAGGTACCTTAGGTACATCCAACGCTGGTGATTGGGTACATACATAGGTATA

TTTTAACAACCTTTCCAATATTCTCATCCTTCCAAACATCCATATTTGACTTC
CTCTAAAACTCAAAACTCCTGAGTATCTCGTGGTGAACTGTCGCGTTTGGT
CCCTTCTAACCTCAACGCGCCCCACGCGGTTTGTGCGCGTGCATGCTATCTA
GCAGCTACGTGCATACATAATACATACTTGCATATGTACCCAGAAGGATCCA
TCCATGATGGATGTGACGGTCGCTGTTTAGCGCTATCTCTCCCCGACCGCGG
CGCGATATTCCTTGTCCGGATTGGCTGACAGAATCGAATTCTGCGACATAGA
CCGCATTCCCCCAGGTGGAGAATGCATGATTTGGAGACCTCCTAGCATTCG
ATTTATATACCATTTTATTCGATAATCATACCATCAACCTACGCACCTGTTAAA
GTGCAGCTCAAGTGGTCAGAGTAGTGAAACAACTCTCTCCGCTATCTTCTC
ACTTGTCTGATTCTCTAACGCGAGCCTCACATATTATAACATGGAACTTGAG
AGACGACTTGTTAGGAACTGACTTATTAGGTACATTGCAGGGCTTTCGAAA
GGAAAGTAAATCTACCATAAGCAAAGCCGGAACATTATTATAACAATTATCC
TCGCATGATATATGTTCACCTATGGTAGTTAGGCGACATCTAACGTAGCTATT
ATCATCTTGTTCTCCCCTACAACAACAACGTGAAAATATCAGACGACGGCA
CAAGGAGTCGGACAAACCCAACATACCCGGAATCTCGCTTTTGTCTCCTCA
ACTCTCTCGGTTGCTCAGGATTTATATGTCAGGCCTCCGCCGAAGCTATACT
CACTATTCTTACCCCCAAATGTAACTTCGCCTTTAGCTACACCGGTTTCGAA
AGCACAGGTCCCCATGTCCAATGCTTTCGCTGGTCCCGATGGCAATATGTCG
AAATCGAAAATATCTGCGGGTAAACCAAGAGTCGTGCACGCGTTCGGTATG
TCAACAATACCTGCCACATGCCCCTGAACAGGTGCCGATGACAACAGAAG
ATAAATCTGGTAGTCGTTGTACCCGAAGCGGCGAAGGTACTCGATACACCC
TTTTCCCGTTGATCAGTTAAATGCCGGTTACGGTCAGGAATCACCCGAAGT
TTATAGCACTTACGCAAGATGGTCTGTCTGTAAGCAACTGTCACATCCATAT
AATGTTGCTTTCCATGTTCGTCCACGGAGAAGCCTTCGAAGTAGATACAGC
GGCCCGGTCCGAACTGAGGCTCGACTGGGCCAGGTAGGTAAATGGGCGAT
TTGAGACCTAAATCCGCCACACCGTTCTTCATCACCTTGAAGTTGATTGTGA
TGATGCCCGCCATCTCGATTGCTCCACAAAATGAGATCTCACCATCGCCCTC
TGTTTCGGGTCAATATTCTTCAATTTCTTTCCCTCAGAGGATAGGACTATTAG
AAGGGGGAGCTCACGCGAGAAGTGCAGATCTCCGACGCTGAATTTAGCAC
CTGGAACATGGACTGGCAAGTAAACCTTGGAACCTCTGCTCAGGTTCTTGA
TGTCGCAATTGCCTCCGTGTTCTGGCCGGCCTGGAACAGTCCTAGCCCCTT
CTCCGCCAACCTTCTCTTTCAGAGCCGCATCAGAAGCGCCGGCGTGGATAC
CTTTCGGTTCTGGTGGCAACGCAACTTGCCTCTCAAGCTTATTAGCTGAAAT
CAGCTCGGCCTCGCGCTTGTTCCAGGTAGCCAGGACCTCGGCTGATGGTGC
ACATCCCAGAATGCCAGGGTGGATCAAACCTGGAAATTTTACATGTGGGAT
ATGTCGCGAGGTGGCATAGATGCCCTCGAAATCCCAAATAGCTTTGGCAGC
AGATGGGTAGATCTCGTCGAGGAACCCTCCTCCATTTTCCTTGTGGAAAAC
GCCCGTGAACCCCATGGTTGGTCTTGGAATGGCTGCACGTCCATTATCTCA

ACTAACAGGACATCCCCTGGTTGGGCACCTTCGACCTCGAAAGGGCCCGT
GAGGTAATGAATTCTGGTTAAATCGACATTGCGGATATCGTCCGCGCTATCA
TTATTCCCTATTTGGCCTCCGGTCCTATGGAACACGTTAGATTTACAAAAGT
CACTATCAGCTGAGGCATATCTAGGTGTTTACCAATCGAGACACTCAATCTT
AACTGTCTCTCCATCCTTGATGGTACCAGCAAATGGAACTATATAATAGTCA
ATACGTAATTCTAACTACCTTAAACATTAGGGCACGTCTAGGGTTCATACTG
TCGGGATGCCATCTGTTCTACTGTATATTAGCTTGCGAAGTGGGCAGTGATT
TCACCGGTATCACCTACGTGGAGATTTGACTGTTCGTACGCCGGCTTGTCG
AAGCTGACCTTTCCGACAGTGCGGATTTCGGTTTTGCCCATTGTGATTTCGG
ATATGGATAGTGATATCTACCTTTAGGTATAGTACAGAACCTACCGGATAATT
CTCGCACAATGATGTGTTAAACAACTAGCGTCGGTATCCCTTAGTTGGTGA
GTGTTATACGATTTAGTTAGGGATGTTGTACGTCCAGCTTGGTATGGACAGT
TGAAACAAGCAACGAGATTATCATGTTGAGGCTCCTAGTCATCACCCGGCA
GGTCTTATTTATATACACACATGTATACAGATATACATCCAATATCTGGCAGAT
ACCCCGCATCGTAGGAGCGGAGAGGCGCCAATCGCAATGCCTCAGACGTC
GTGATATCTCTCGAACACACCTACGTGCATAGCGGCCTGCACCATGTCCGTG
ACGTCGGAAACATGCGAGTTACTTAGTAGGATCCTACACATACTAAGTTCAT
AGATATACATAGCGTGTGTTATTATACCAATCAGCTGCCCTCGCCCCCACCAT
GGATTCTTCTGCTTATCACATCTATTGACACCGAGGAAATACAAACGTCGAA
GCTGATAAGGAGAGCCCCAATTCAGGGTCAAGGGCAGTGCATACCACCTAC
CGTTATACCTAGGATCTACCAAAGTAGATGTTGTAGCTTATCTCGGTAATTCG
GTGTTCCTCTCATCCATGAATAGCTTACCTATACCCACTTAAGATAGTACTAG
GTGCGTGTGTATAAAGACCCGAGATAACGATGAACATCGCAGAATTCTATAT
ACTAATTCTTATTGCTATTTTGTGGCACGAGT

>Mosaic_sequence_60

AGCCCTTTGGCAGAGCCGAGATCTCAAGTAGGTAATTGATCAGATTGACTT
GAGCTTGCTCCTTAGTCTCCTCGACGGACTTGAACAGGCCGTGGTGTATAT
GCTGTCCCCTACAATTACCAGAATTTAGTCGCGATCATTGTGGGCAGTATTC
ATAACGGTGAAAAATATTACCATAGGGAGTAGTAGTAATCCGAAGCTATCTC
ATAATGCTTCTTGATCCTGCCCTTCAGTGAGTCTGAGGATTCATGAGCCTCA
AAGCTCGGAATCTCATGCTGATCACCAGGGTGGAGTTGATCGGTTTCGACT
TGGGCCATGTTTGCAATGCACCTATAGTCAATCTCATTAGCACTATCCGAGC
AAACATACAGCACAGGGACGGGTCAATTAGAACTACGTACCTGTATGGCAA
CTAGGAATCAATAACGATGACGAGGAAGTCTCGGTATGATACGAGGTTAAG
TTGGATTTGCGGCTTACTGGTTTAAGGCAAGCTTGACGATCTTGGCCGAGG
GTTCCTGGATACGACATCAACCTTGGAACATCCCCTCTTTTCGATTAGATCA
ACGCTTAGACGGGCGACGATGCATGTCATTTAGCGATTGTGTTACGATTCCT

GAAACACTGATCTACTCTTTATCGTAATCCCCTAATCCAGAACGAGGCACGC
CACTCTAAAGCCAACGGCAACTTCCAGCGGAGCAATTGAGCTATTATCACC
ACATTTGTAATTGACCTCAGGAGTAGTCGTCAATCCAAAAACTCGGACATAT
GCTAGACAACGAAATGAATTTATAGACATATCAGCATCACCTAAATATACCT
ACTATATATGTAGTAGGTAAGAAGACATAGTAATCAAGTTTATGCTATACACA
GATCTCTCAGCTTTCTAAGTAAGTGGTGAATTGCCACATGAACATCCATCCC
TCATATCCCACCTCATTATACAATACCCAACCTGTATATAGAGCAAGACAAA
ACAAAACTAGAAAACCAAAGTCGAAACTCCTCATGAGCTAAGTAATAGATA
TGCCGGCCGTCACAGTAACGTTCGTCGCGTTCGCGTGAAGAACCAGACCA
AAACCAAAAACGTGCCAAAGCAAAGCAAAAAAACTCCTTTCAAAGCAAT
TTTTGGCAAAAAATTGAAGAACCAGTTCGCACGCGCGCCGCATCCCATTAC
CATTAAATGCTCATGCTCATTGACCAAAAATGCCCCGTTCTCCATTCCGAAG
ATGCCAGTCTCGTTTCCGCGCAATCCGTCGTCGAAACCCGCGAACAGAAC
GGCCGATTTTGGGGGCGCTGAAGCTATGCTAGGGGCCCGCCGTAGGTTGTA
TATAGCACACGTACTTTTTCTTTCCCACAATAGGAAGAGGGGATAGAGGGG
GATGTGGAAAATTAAGGGGAGAGAGAGGGGGTTAAGCTAGAATGGCTTAA
CTTATCCCGTCATCCGTCTTCCCGTTCTCAAAACTCGGATACGCGCCCCCCG
TGAACTCGGCCCAGTCCCAGTCCAAACCGCCGGACATCTGCCATAGCTCCT
GCGGCAGGAGCGGTTGGTACCCGTTCTGTCCTGGTAAGGGGGGAGATGCG
ATCGGTTGCCCGAAGCCGGTGAATTCGGAGAAGTTGCCGAAGTCCATTGC
GTTCGGATTCCACTCCTGAGAGCCGTTATTGTTGTTTGAAGTCTGCGCAGG
TGTCGATGCGGCGCTGGGCCAGGCGTTGGGTCCGTCGCGGGGTGCTGCTC
CGTTCGTTGAGGAGGCTGTGTTCATGGGGCTATATCCTCCGTTGTTATTATTG
TGGTTGTTGTTAGCACGGTTCTGCTCCCCATTAAACTTCGGCGACAATTGCG
TAGCGTAACTCGGTGTCGGCGTCGCGGCCGATGACGACGGGCGCGGCGTG
TGATGTGTGTGCGCGGACTGTGATAGCGCGGAAGGAGAGGTCACGGAGGC
TCGGTGGTCGTGGCTCTTGCGCTTGCGCTTCGAGGTGTCGCGCTCGGCTTT
GTCGATCACGATTTTGGCTATCCGCTCAAATTCCGAACACACACCTAACATG
CGGTGGACACCGCCTGTTTCGGCTTCTTGCCCGAGTGTTGAGAGGAAGGT
GACAACGAGATTCATGAGCTTTGTGTCGGATTTGGCGCGTGGGTCGAGGG
GATTTTGGAGGATGTTGCCGAAGAGGGTTACGAGGGCTGAGACGGGGAAG
TAGAGGATGAGCCTGGATTTTGTTAGCTTGATGAGAGGGGAAGGAGGAGG
GAAAGGGGTTGGTGCTTACCACACGCAGGAGAAATCGCCTTGCGGGATGT
ACTTGAGTAGCTGGATACTCGCTCGGGCGGCAGATGCGCATAGAGCTGCCG
AGGCAAACACTCGCGGGTTTAGGGGTCTGGCGTTTAGACCTTGGATTGCGT
AGTTGGAGAGTCGACTCGTCCAGTAGCCGTGATGTACGGACATGCGGTGG
ATGGTAGTGAGAGCGTTGTAATATGAGAAATGTAACATGACGACGTGTAGT
ATGAGAGGGGTGTGAGATGCCTTTATTTCATGTTCTGGACGGAAATCTAGT

GGTATGTTGTCCTTCCATGTCTCTAATTGTTTGTCGAGTTCGCCAATTGTTTG
TAATAGCTCACCCTCTGATAACTTGGTGGCTTTCGTCGAATAGAGCCTCTTG
TAAACTTGGCTTTCGATGACCGTTAATTCGCACATAGCTCGGAACAAATTCA
TTTTCCCCTTTCCATCGGCAAGAGGGATATTGCCAATATTGTCAATGGGGTC
GGCATCCGGGAGCTCGACATTCATATCGTCGTCGTCCTGTGCTGGAGGACG
CCCAGATCGAAGACATAAATCCTTATCCAACATATACGCTATCCAGAACACT
CGTTTCTTTGCTCAATCTCAGCCGGGTTAAGATTGAATCCACTTCCACGCTT
ATGCAACCCAATGCTGTGTGACAATCGGATAGCTGCCGCGACGAGTAAGAA
TGAAGGTTGTGGGTTTGGAGTACCTTGCATGAAAGAGGCCATCCCAAGTAA
AGCTTGTACACTTAGTAAGTCAGTGTTGCGCATCGTTAATTCGGAGAAGAC
ACCCATAGCGTTCTTCATGTACGCCCACGACTTATCATCATCTTCTTGGGGT
ACAAGATTACTCATAACTCGAAGACGATGTGCAATGGCCAAGGCGACGTTG
AGACTCGCCCACCATCCGGAACCTTGGTAGGGATCATTAGAGTACTGCCTC
TCAACGAGGTGCATAAACGTCGGTTGGTGGAATAACGGGAACATGCAGTT
GAAGTTGTCGAAATAGTCCTTAAGCAGGGATAGCGCTTCAGGCTTGGGCGG
CAGTGGCCGGTAGACGGGACGATGAAAAGAGATCGCTGAAGATCTCAGGT
TTCCAGTGATTCCATTTATGATCGTCAATGGAAACTTCGGAGATCATCTGCT
GGAATGATGTGTCCCCTGTCTTCTCGTTCACCCACTGTATACCCTTTGGTGA
GAATATAGAGAATCCGGAAGAGGATCCTGGCGCGAAGTTAGCGGATATGCA
CCAGGACATGCAGCCAGCCGACTAGCTTGTGTGGTAGCTTGCAGGCACTTA
CCTATATATCTTGTTTCGCCTGATTGGTTCGTCACCAATGAACACATCATTTC
GGATAGCGCCTCTACTTCTTCCTCATCTTTTGGCTTTTCTGGTTGTGGAGAG
TGGTCTTTCCTGAAGTCCTTTTCTCTACTCGGTTCTGGTGATGTTATTGAGC
TTCGTGGGGTCGATACTGTGCCATCGTGGCCGGAGGTGGCTTGCGACGGTG
ACGTAGGATTCGAGGTGAGTGCTTGTGAAGCTTGTCTCGATTGTTGATGCT
TTTCTGAGAGTCGCTTCTCGAGCGTAGCGAGGTCGGTCGATTCGTCATCGT
CTCCAAGCAGGCCTTCGGTCGCGTTAGTGATACAGTTGTTGTAGAATATTTG
AAATACGGCGACAACTCGAGACTTACCCGACAACCTCAGCAAACTTTCCAT
ACGTCCTAAGCGGTTTTCTAATCCCTCGATATACTTCGCACTACAAGTTCAA
ATCAGCCTCATATGAAGCTTGTTAGTTGGTGGTACCGTCTGTGCTCATACCC
TTTCGGAGGATTCCTCTTCTTCTCAACTTGTGTAAAGACACAATCTGTTTTG
TAATTGATGCAATGCGTGCAAGCTGGAAGCTTTCCATCGCACTTTATTTTCT
TCTTTCGGCACATATCGCAAGCCTTCAACAATCGAGTCAGTCCATGTATAGC
AGTATTATACTACCTAAGGTGAATGTATAAAAGCTCACCCGTGCGATTCTCC
GTCTCTTCGCCTCGTTCGCATCGTCGAGCAACGCTGATGGGTCGAATGCCA
TCTGAGCCGCGAGCTCCGGTGGTAGCACAGACAAAGCATCATGACCATAA
GCTCCGAATATCTGTGGCGGTTGGTTCATCAGTTGTGGACGGGGCATTCCT
GGGATCTGGAAGCCCCCAGGGTCGACTCTCATGTGATCCATCATCTGTCTCT

CGTCGTGTTGGTCTGTGGTCGTTCACTCTAGGGATGATTAAAAAAGCTGTAT
GCGCGGCTATGAAGGTACCACAAGGATGGTCTCGTAGGGCTCTTAGGGGAT
GGATTAGGTTAATTAAAGTAGATGTAAGTTGACGTTTTCCTGTAGAAACAAT
TCGATCTAGGTAACTTTTAGGTAGGTACTTAGTTCAGTACCTGGAATCCGCG
GGTTTAAGAGTCCCCACGAACAAGCCCCGGGTTCTCTTGGTGGCTCTGGTG
GGTGGTATGGGTGTACCGTGTGAGCGATCGATCCCCTTGTCTGTCTCCCTTT
ATGAACTAGGGCGAGATGACCAATCGACTTGTCCAGCTAAGACCTGAAGC
GAGCTTGTAATTCGCCCGGTAACTTATCTAAGGGGTCCGGAGCGGGTTTAA
ACGCCGAGCAATCCAGTACGGGTGTACGGGGGCTTTCAGATTCGCGGATTC
GTACTCGTTTTTTTCTAAACTTGGTAGAACCGCCCGGTGGAGGCACTGCTT
GCTTTGATAGGTAAGAATTCAGAGATATTTTAAGATCTTCTATGTATCGATAT
GTAAGTTGGGTTACTCGACTCGCGTGGCTTAGAGGCTTAGTGGAATAATTA
AAGCGCTGTTAACTATCAGAAAAATAGAATTCAAAGAATAATCTAGATTCG
GTTTTCTTTTCTTTTTCCTCCGACTGAAGAGGAAGAAAATAAAAAACAAGA
AATGCAAGGTTTGAGACATTGGATAGAAATCTTCGTCTGAGACTAATATTTG
ACATATACGTTAGTCTAACTACTTGCACAGCCAGGACAGCCAACTAGCCCG
ATAGTACGCAAGGTACGGACGCTCGGGGATGTGCAATGACTTTCTTCACTC
CTAGTCCTGACAATGATCCAGTGGTCCTCGAATATATTGACAAATCAAAGGC
CACCCTGCTTTACCTGTTACTACCTGAAGGTCTGTGATACTCAGTAAATACC
ACCTTGTTTATAAGGATGCCCTAGCATTATTAGGTGCATCAAGTACCGTTGC
ATTTTGAGGGGATCTTTCTGCCTGAGTGCCGGCAGGGTCCTGCCACACGCG
CACATTAGAGCCCTGGAGTATTTCATATTCGCAATCCCTCTAAATAATCATCT
CAAGTTTCTTACTATCCTTACTATCACACGCTTGCCACGCCGCTGAGAACCT
GTTCACCCCAAGTCTACTCGAGACAGAATATTGCGCCCAGGAGGTAGAAA
ATTTTGCTGGGGTGTTTCCGCCAACACCTCGTCAAGCTTCCCGAGTTAAGA
CTGAATATCCATTAGGAGACTTAAGGACTCAAGCACCTAACGCTAAAGACT
CCAGGGCTGCTACCTATGTAACCCCTCCTACCCTTTTTGGCAACTAAAGCTC
CGTGCATTGGTTAGTTCGTCATGTCAGCGAAGTTAGTCTCTTAAGTCTCTTA
TAGAAAACCAGCTAGAAAGACGGATTTCGGGTTTGTTACGATTACGAAAGA
ACGACTCCGGGAGCTAATAGCAGCGCAAGAAGTATCTGCCCATTTTGATCT
CATGAACAACTTCAGTAGCATATGGTTCCAATATGTTATCAGCATACATTGGC
CGGCTTAACCGGCAATTCCTTCAAGTTAGTTCTGGTCCAATAAGCTGACGC
ATTCTCATCAAGTTGCGACATAATGTAGACATCATCATATCATGATACATACT
ACTTTACCGACTTACCAGGACTTAGGTGGCGGATGGCTGAATACTTGACGT
AGGAGGCCGCCGACCGCATGCATATGGGGGTAGTGAATAAGCTTGAATCCT
TCTAAGGGTTTCCCCATGGAGCCAGGTTACGTGCGTCCCAGTTGTCTGATG
TGATCACCCTAAAGAATTGGCTTTCGTTTCATCCGCCGTGCATGGGGTGGG
AAGTCCTAATCCACACAGTCTAGGCTCGACAGACTCACCATCTGAGCGGGA

GCCGGAAGCTCGTCTTATCATAAGTACGTTAGCTTCGTCCGGGGGAAAGGC
ATACGGAGAATAAACTAAAGATGCTAAGCTTTTTTCTTTCTCGTTTTCACCG
ACTGAACATGCTTTGTCACTACAAACCATCGGCGCAATAATTTGTCATACGG
CCAAAGCGTTTGTGGGTAGGGGGATGTGAGATCCAGTCGTGTTTCATAAAC
GTCTCAACCTCCCATCTCGACGGGGAATTTTTCTGGTTTGTAGATCAAGCA
GCGAACTAGCGGTGCGCGACTGGCGCCCAGATATATGTCCACCAAGAACCA
TTATCAAATACTGTATGGAAATATCGCCTTAACTCCTCGGCTCCGTCCGTTAA
TGTGGGCTAAAATAACTTGATACCAATGCCTTGGATATGCTTAATACGCGTAT
CCTGCCCACCGGCATTATAACACAGCTCGTTAGGCTCGGGCGCCTCAAAAC
AGTCCTCAGACAGGTCCCGAGATAAGCATGGCTGAAAAAGAGTTTGATGAT
AATTATGCTTGATTAGGAGCAGGCCCGGAGGAGACCTGCTGCTGCGATCTC
GACTCCGCGATAGCCGTGCCCTTGGTGTTCGACCCTGGCAGCCCGAAGGCT
CGGGACGACACATCTGGCTATTTACAAGAAATGGTGTGGTTTTTGCATCTG
ACGTAGGTGCGAAAAATAAAAAGTTGTTTGCGCGGCTGAAAAGCTAGCCT
TTCCCTTATATTCCCATCCCTTTCCCTCTCCCCCTATGAGACCCTCTTTCGAG
ATATTTTTATTTCAAAATGTCAACGCCAACCCGATGCTGTGACGAAGCATGC
ATGTATGGACGCCCGACACATAGATAACAAACAGGTATCATCAAGAAAACG
AAATGTATACAAGCACAAAGGTAGGGTATCAATCCAAGGGTCATAACACCT
CGGTGAACCGGGAGACCGAAACATGGGACCGGTTGGAACCGTCCTGGGA
AGGACGAGATGGTCCAAGCGGATCTCTGGGAGGAGGCTCAAGGGGCGAC
GTGTCTGGGGAAGTTAAGGGAGCTTGCTCCGTGATGGGTGTCCGGGCCGG
CCCCGGGTTGTAAACAGGGATACCGCTATCCGGCCGCATCGGGGCACCAAC
GGCTAGCCTAGTCGGTCCCGCGGGGGCTAGTCCGGGGAAAGCTTGTGAGT
CCCGGTAGTCTATCGACTGGTCGCTCAAGATGGTATCTCGAGGATCCGAGT
AGCCATCACCTCCGAATTGTATAACCGGAGGCAACTTCCTACCGGATACCTT
AGAAAACCCTCTCTCGCCACCATCCGACGAGGCACCGCTACGCCCGGAATT
TCGCTTGTGACCGGTCAAGCTGGCGAGTGCAGCGGGAATGGCGAAGGGGA
TCGAACGTCGCGTTTCCGACATGGCACCGCTGTTTCCTCCACCGGCGGGGC
CGGAATTTCCAACCAACGCACCGCCGGGACCCCGTTCGCTACCAGACCCTC
GCATGAGTTGTTTGGTGTTCTGACGCTTCTTCCATCGCAGGAGGAAGAGTG
CTAAGACTAGGAGAGCTGCGAGTGCAGCCACACCTCCTAGCACGCTGCCC
GCGACCGTGGCTGCACCAGGCCCGCTAGAAGACGAGCCCGAGTCGCCGTC
AGCGGTTGACGTGCTCGAAGAAGAGGGTTGCACATTGCCACCTAGACCTA
CGCCGTCACCACCCGAACCCGTAGAAGATGCAAATGCGGAGGATGAATCG
GAGGTAATCGATGCGGAGCTGGAACCGGAGCTTGTCGTAGTCGTTACCGAA
GTCGAGGACGAAAAATCAGAAGAAAGCGAACTACTACTAGACAATACTAG
TGATGATGTAGAAGAGCTATTGATACTTTCTGCGGAGAAGGGAAGAATTGA
AGTTGTTGAAGTTGAAGAAGTACTGTTGGAAGAAATCGAGGTCGATGATG

ACGATGATGCTTCGGGTAAGTTAGATATTGAGAGCGACGTAGTGGGAATAG
CAAATGAAGACGAGGATTGGGACTGGGATTGGGAAATTGAAGATGAAGAT
GAGGATGAGGATGAGAACGAAGATGAAGAGGACGGACCTTGGGTTGTATT
AGTCACGCCAGCCAAGGAAGGAAAAACGGAAGAGCCTGATAAAGGAGTG
GACGTCAAAGGCGAATAAGATAATGTGGGAAACGAAGTAGGCATCGAAGA
CTCCGACGGTGTCACGGTCGGGTCTATCGTTGAAGTGGAAGTTGTCGACGA
ACTATCGGCGCCTGCATCGTCGCTAGTTGCTGTTGAAATAGAAGATAAACCT
TGGGCAGATACGGACTCAGTGGCCGGGTCGACGGTGGTTGTGTCGGATCC
AGGGATTGATCGTAGTCGCTGGCGTAAAGCCTCCTGAGGGCGTAAGGGTCT
GAAGAGTAACGGTCGCACCAGACGAATCGATTATCTGAAGAACGGAAACT
GTCTCGACGATAATAGTGGGTTCGTCAGAATCAGTGCGTTTGTGGCCCCTTT
TACCAGAATCAGGATTGGTATTGGCGTTGACGTTGACGTTGACATTAACGTT
ATTATCGCTATTAACGTTGCTATTGCTATTTCTGTTTTGAGAACGAGCGTGGT
GGTTATGGTGGGTGTGTTGATAATGACGGCTGTGGCTGTCTCTGGAGTTGT
GAAAATGATTGTGTTGATGTACGTCTCGGGCCTGAGGGAGTGAAGACCCCG
AGACTGGGGTTGTCATCGCGCGTGGCGGTGAAAATGGAGAAATACGAGCT
TTCCAAGTTGCGTTGCGTTGCGTGTAAGCTTGTACTAGAATACAACTAGGTA
GTCTTAGTTTTTTTTAGGTCAAGAAGGTCGAGTCGGATTCGCTGACGGGTT
CGGGGGCGCGCGGTGCGTTTCTTGACTCGTCCGGCTTCTTTCCTTCTTTCTG
CTTGCTTGCTTGTTGGATTCTGGTTTCTGGCTAGAGACTGGCTGACTGCTGA
CTGGCAGGTCGAAAATGGTAAATCTGGTAAACGATAGAATGAATGTATACG
TTAAGTTTGGTTGAAGATCGTCTAGTGTAGTATTCAGTATCCCGTCATTATCG
TATTATTATCGTCCCCTTGTATCTTTTCTTTTCCTCTTTTTTCGAATCCTTCTTA
CTACTACTGCGGCTGCTTCTTCTTCAATCTCTTCTGTAAAAAACCCTTCTGA
TGGTTACCGGGTCCTCATCGTTGGCGTGATAGGCTGGAACCGAATCATCGA
CACCAGAGCGACGAACGGTCGACTCGACGGTTAGGGAGGAAGTAGGAGG
ATGAGGTAAAGGAGTAAAAAGAGAGGAAAAAAAGAAATTAATTGAGACCT
GGAGGCGGCTGCGTTTCGATAATATGTTTGCAGACCAAAATTAAAATGAAA
TGCTTCTTAACAGTGGACACCACCCCCAAGCATCGTTAGCTTCACCGGGCA
ACTCGATTATGGACTTATTATGGCGTACGGTCCTAAACCTGGAGTGCTTGGG
GGTGCCTTGGGGGGTTCTTTGTCTCGCTGTAAATGGACGATGGCTTGCTAG
ATGGGTGTTTCTGCTCATTTCGCTTCGGTGAGCGTTGTCCCTCTGACAAGG
GGGCCATAAACATACGAGCCAATCATACGAAAAAGCTACTACCAGGGACAT
GACAGGACCACCTATAATTGGCCAGGGACACGGGGAATTTCTTTGCAAACA
TTCGGCCATCTCTTGCTATCCTCGTTATCCTTCATCATTATCATCATCAATGTT
GCACCACTCCTGATATTTCTTTCTGTCCCCTTTTCCCTATCCCCTAAAAAGTT
GTCCAGGTTTATGAAACCCCTATTATTTTGATAAGTGACAATTCTCTTTTCAT
ATTTCATTTTATGCCGTCTGTGCTGTGTATGGATATATGTATATGGTATGTATG

TCTTATATTTTTATTGCCTATTAGCATTGCTATTGTATTAGGAGACTTAAGAGA
TGGGAGACAGTTGTCAAAGCTGCTGACGCGAAACAAAATCTAAGACACAA
CTACTAAAAAAACCCGCCAAAAACACTCCCTAATCCAAGTCTACCTAGCCT
CTAACCAACATATGCAGGAACACGGAACACGAAATCGGGACTGCAATAAG
ATATCAACTAAAATCCCACACAAGCCCACTCGAGGTTAGGCACGGGTAGCT
AAGGCAGGTAAGATATCTCTTGCATACCACTTGTCCCCGTTGTGTCTTGGAT
AGCCTATGGGTGGCTGTAGAGCAGCTCGTATTCCCTCGGTGCATGTGCCTAT
TGCAGATATTGCAGATCTAATGGAGAGCCACGCGATTCAGATTGATTGATTT
CTCTATGTGGGTAGATAATCTGGGTTGGGTTTGTCACACCAATTTGGGATCA
AGTTTACGGTTGCAATAAGAAAGTCTTCTATCAATAATTCTCGCCGACTACG
GTCGAGATTTCAAGTTCGTCTGCGTCTACCGCCCGGTTTTGTTGTTGATGAA
AGATCAAGGTCTTGATCTCCTAACTCTAGCTAGATCGGTCTAGCCTCCCGAC
CTAATAATAATTAGAAATCAGGACCCCGGGACGATGGGTGGACCATCCTGG
CCGCCTCATTTATATTGCGGATGCTTACGCGCAGTCCCTGGGTACTGAACCA
TCTGAACCAATTCTAAAATCACCCATCCTCCACGAGAGATTGCGTCATAATC
GGTAAGAAATTATCGTCTATGAAACGAAATTAGTTACTTTGACCTTCAAAGA
TCTAGTTATTACGATCCGAAATCAAAAAAAAAAACATAAATCCAGATTACGGC
CAACATGTAAACTTGATTTTCGTCTGTACTCTACGAAGTCGAAGCTCTAGAT
ATTTTTTTTCTACTAATGCATGTAACACTTCAAAGCTCCAGGCCAAGATCTC
ACTAGTGGTTAGCTTCGGCCTACATCCTAGTTTTTCACCAAGCCGCATGCGA
TACACGTACCATCGTGGACCATCGTGGTGTTCTAGCTTCATAGCAACGCGTG
AGCCTCTTCTGAAAGAAGCCTTACCCATGTATTCCTGGCCAAGGAAACAGA
ACCCGCATCAGAGACAAACAAGCTCATTGCCCTGGTTTACAACATTCGGAA
GCATAAGCGACGACAGACATGGCTAGAAAACAGAATAGTAAGGTAAAGTA
TTAGTGATGACCACAGAGTTGAAGAAGGATCACTAGTAGCACTATAAATAT
GTAAGCAATGGATCTTATAATTTTGTCGTACTAGCTGTATCCTTGCTAGATTC
CTCGTAAATCTCTTTTGAGTTGAAAGGCCAATTGAAAAAGGAAGTTCCCTG
ACTTGAACCCGAGAATCTGGTATCACCATGAAATAAAAATGAGCCCTTTGG
GAATATGGATGCTCATCAACTACTAAGAAGCTTGCATTCCCAATAAGCCATC
TCTCCCCTTTGCCGATTTATAGGTAACCTTTCTTATGTATTGCTAGCTCTAGA
CGGGTTCAATCTCATTTATGAGATTCCTTTTTCAAGATAACACAGCGATTCA
CTGAATCTTAGGTAATCTATGTTGATCTCTTTTGTAGCATAAGCCGGGCTTCA
GGTTATGCCCTTGATCATCTACGAAGTGTATTTTCTTGTCTCTTATTTTTCAAT
TCATCATTTCTTCCCAAATCTATAGCGGTTCGCTCAATTCGTCCTGTATATTC
CCTTAGTAGCCATCTCATCTGCGAGGTAGGCCAGCGTCCTATTTTTATTGTC
GTCATGGAATATAAGAAGCATGGATGTAGGCAAATCTTTCTCTGGTGGCTGT
TGCTCTGCCTCCCTTCGCTTCAATCCATATAGGAACCTCATGAGTCCGATTAT
TCGATCGGAGCTGTATCTGTGTCGGGCTGCCTCTTCGAAGTGCCCTTCGAA

CCGTCGTTCAACCGGCGTCGGGAACAGTTTCATGTAGATGCGTTCCTCGAG
TTTCCCATCTAGGTATGAGATGGTGTATGGTACACCTCAATTCTCTCTTCGTG
CATAATGTATTTGTTGCATAATTTTTCGGATCTTCTTGGCCTAAATTTAGGAC
GCTGGGTTCGAAGTCATCGCTAACAGACATCGTTAACGAGGCAATGGGGTT
TGTATAAACCTCGATATTGTCAACACACCCTCATACATGCTGGCAGTCGGCT
GACCTATTTCCCGTCGCCTGATGCCGTGCCTTACTTAAGTAAGAATCTAACA
AATATTTCACATGAGTTGGGTCAAGAATACCTGTGCCTACCCAGTCTTCGTC
GGTCTCCGAGAGGGCGGGGACTACTGTGGAGGATTCGAACGCCTCTATATG
ATCGATAATTATTACAACAGAAATAAAAGGAACATTTTCTTCCCATAAAAAG
AGATTTAGGAGTGGGAGGGGATGATCGATGCGGCATTTGATTCATCTAGAA
AGAGACGCAATACTATTTCGCAGTTCTACCGAAAGGAAGCACCAACGTGTA
AATACGAGGCCTGGATTGCTCTCATACACATCTCATGTTGTTTTGGTAGAGT
TCCATCTCTCTCTGCTCTGGCTTGCATCAAAACCGTTTCTAAAAAATAAGAG
TTTGATACGTTGTTGAACTCCATAGCTCGGTATTCTCAAAATGTTGTTTAGC
ATAACGGGTGCTTACACGAAATGTATCATATTGTTAAGCAGATAAGTAAAAA
GAAAGGCGTTTCATACGATGGCTACCTGATAACAAACATATATATATGAAAT
CTCCAGGGAAAAAGATGACTGAAGTGATCTGGAGAGAAATATACGATTTGT
AGGGAAAAGGCACGTTTAAATAGAATATGTTAAGAAGCGTTGGATTGTAGA
ATATTTTAACGTACGGCGTGAGTATGTGTGATTCCACAGATGACATAGCAAT
CGTGGAACTCTGTAATGAACAAAAAAATCAATCAATTTCTAAACATACAGA
GCGGTTTACCCCTTAGAGTCATACACTATAACACACCATCGGGCAGAACAA
CATCTAGTATGATGTCAGGTTATGACGCATAGGAGTCGTCTTGCACATGGAT
TCATGCTGCGGAGAAAAGTATTAGGTATGCTCAACTTTGACTTCGGGGTATC
ACGAAAATGCCTTGATGCTAATCTCATCTATGAGAGGTCGAATCTTCTCGGG
AGTAACCCGAACCAACGCCCGAACGCCGCCCTTTGATGTTAAAACTCATAC
GAATCTGACTCTCCTCGACCTTAGGCGAACGATACCCTCTTCTTTTTCGATT
TCGTAGCCTTCGTCCCGGAGGTGGTGGCATCCTTGACCACAACATCGTCGG
CATCTTGGTGGGGTTGCTTAGTTCGCTTCCTCTTTCTCCTCAGCGATTCCTT
GGTCTGGTCGAGCTTCGCCTTCTTCTCCTCTTCCTTCTTTTCCGCCTCTACTC
TTCGCGCTTCTCGGATCTCCTCCATTTGTCGGGCTTTGATCATCTTGGATTCG
ATTTCGCCTCCTTATCCGCTTGAACCAAGGCGAGGATGGTGCTCATGCTCTC
CTGTTACCGCAGCGACACACTTTAGAACTCCGAATTGCGCAAAATGCTCGT
ATGAGGTTGGTAGCCTTACCTTATCGTCTACAAAGACCTTGCCCTTCTTCTT
TCCCTTGGGTTTCGTAACCCCGACGGGTGTGACCATGTTCAATTCTGGGATA
CCAAGTTCTTTCTCTGTGTAGACCTTCTTCTTTTTCTTCTTGATCAAATCGGC
GATGGATTTGCCTTTCACTTGCTTCGAAAGGACCAGTCCTTTCCGCTTCGAT
TTGCTCACGCCATCTGACGGGCCACCCTTTGACGATTTGCTGGAGCTGATG
CCAGACTTGTTCGTCGCATGCTTGTTCTTGACGGTTTTGTTCGCGACGGCG

CCATTGTGAGTTGTGCGGAGTTGCGACGAGAAGCAACTTGAAGAAATCGG
TATTACTTGGAATTAGCTGTTGTAAAGTTGTAGAGCAGAAATATTTTTGGGA
ACCTTTCCTTATCGATGTTATCGATAGAGGGGGCTGGTCCAACGGTGGGGC
ACTGTGAGCCTGTTACACCAAATCTAGTAATTTACTTTGTCCTAGTTGGTCA
ATCGTCTGCACTAGCTTTTTTTCCTGCGTCTAAGCCGATGCTCGGCCTATCA
GAAAATGTTGATTAAGGTTTATTACGTCCCGTTCCCGTTCAGCTTAACAGTC
ATATTTTAGCAAATCTCCAAAATTCATTTGGATACAAGTCAATTTGGCTACTT
CAGTCTTTTCGAAAATGGGTTTAGAAAAACCTAGCCAGCGGTGGGCGGCG
CTCGCTCGCGACACAAATGAGACCAAGATCCAACTAGCCCTAAACCTCGAT
GGCGGGGCCTTCCCCCCAGACACCGACTCCCGCCTGCTAGACTCCGTGGC
CGAGGGGCACGCCAGCCAGTCGAGCAAATCGCAAACCATCAGCGTGAACA
CAGGCATCGGCTTCCTCGACCACATGCTGCACGCCCTCGCTAAGCATGCCG
GCTGGAGTCTGGCTCTCGCCTGCAAGGGCGACCTCCACAGTACGAAACTA
CACCACATATTCCACCCGTCGTACCCTGTCCTACATTTCGTAATTTGAAAAA
CGGATAACCCACTGACGAAACACCCAAACTAAAAGTCGACGACCACCACA
CCGCTGAAGACTGCTGCATAGCGCTCGGCTATACCTTCGCCTCCGCGCTGG
CAGCGCCACGGGCCTCGCGCGCTTCGGCTCCGCCTACGCGCCCCTCGACG
AAGCCCTCTCGCGCGCCGTCGTCGACCTCTCGAACCGGCCCTACAGCGTCA
TCGACCTCGGTCTCAAGCGCGAGAAGATCGGCGACCTGAGCTGCGAGATG
ATACCGCATTGTCTGCAGAGCTTCGCGCAGGCGGCGCGCGTGACGATGCAT
GTAGACTGTATAAGGGGCGAGAACGACCACCATCGGGCTGAGAGCGCGTT
CAAGGCGCTTGCGGTTGCGGTCCGGCAGGCGACGAGTAGGGTGCCTGGGA
AGGAGGGCGAGGTGCCTAGTACGAAGGGACTTTGAGCGTGTAGATGGGTT
GGGTTAGGTAATACTCATAGTCAGATCACAACGGAGGAGGAGGAAAAGAT
CTGACTATGAGTATTAATGTAGCAAGATTATAACACATCGATGCCGATACCCC
CTCGACCCTCACTACAGTCCATACCCGACTCGACGTCGTCCAATCTATTCAC
CCATACCGCTGTACCCCACCGTCTCTACCCCCTCTTCGCCGGACTCACATTT
CACGTTCTTCCCGCCAAGATCCCCGATATCGCTCGAGTGTATGACAGCATAG
ACGAGCTAGGAGGAAAATGTGTCGCTCTGGAGCACGCAATAATCATCATCA
CTGCTCTTCGTGGTCGACCGCGCCTCGTGCGAGCTCTCGGCGCTGTTGTCC
TCGACAAACGGGCGGAGTCTATACTCGGTGAGGAATATGTGAATGATACAT
ACGAAGCATGCCTACGTGCCTCGGTGGACGATCCCGATGGGGAGGAAGTA
CCCCATCTACCTAGTCGGAGGAAGTATCGCGTGATCTCGCCGATCCCACCTT
CGCCGGAATCCCAAGAGCCGTCCTTGACCGACGGGAGAAAGAGGAAGAG
GGAGGCTTCGGAAGAAGGAGACCTCGACATCCTGCCTGAGGATGTGAAGC
TTGTGGACATACCCAAGTGGTGTGTTCTTCGAGCGTGCCCGCTAGTGTGTG
TGAACCAAGACATCGTAAGTCGCCCTTTACTCCTAGTGGAGAACGTATCGC
TGAGTTTCAGATCGACGCGATCAAACCTATCTACCTGTCTCGCGAGCTTGA

GGAGAGAGGGCAGCTCAATTCGAATGTCCTGAGCTACCCGACGAAGCATG
AGCGTGTGTGGCCCATATCCCTTGGAATCGTTCTGACCCTCAGATGCTCAA
ATGCCAGCTAAGCCACCCTAGGTTATGGTCAAGCTGATCCAGTAGCCGTAC
CCCGGCGCATACGCTCTGGCAAGGAGGCTATGAAGCTGCTGGATGTGGGA
GAGAAAGTCGCAGCACGTGCAAGTCACTTCATAACCATGAGGACCCAGCT
AACATCCGTCAGATAGACGAGTTTCTCCAGACGGGCACTGTAGAGGAGTC
CGAGGAGATCTACGGTCCCCGCGGTATCAAGCGATGAAGGAATTCGCCTCG
GTCTACACTGTCGGCAGTGTCACCGCGAAAGAGCTGTACGACAAGTGGAA
CTGCCGGACTCTTGAGGATGTCAGGAAGCACTACGAGTACATAGCGGAAG
AGTCGCCCGAAGTGAGATTGAAGGAGAAACTGAGGCGGCGGAAGCAGGG
GGGCATGCTGCATGTCGACATTGTCGAGGAGTGGATGGCCTTGAAGGAGG
ATCTAGACACGCCGTGAGTGGTCACGGCAGCCGCACTGCTTCAGCTCCGA
CGGCTGCCTCAGCTCACGTCAGGATACCCCGGCAAGAGGTCGAGGAGATC
GCGTCCTTGCATCATGGAACATCTTGGCGCCTTTATAGAAGGGTGTGAATAT
ACGATCTGCGGAGGGTACGTCTCTACTTCTAGGTTCAGTCAGAGCTCATAG
CAGGTACCGACGAGGCAAGACACATCGAATGACGTAGACGTAGTGTTCTG
CCCTCCCGAGGACGGGCAGGACATCGGTCTGTTACGCCAGCTTACCTCGTC
TGGGCGCTCTCGACCTGGTGCCCCATGTGCTCCGTGGGTCAGGCTTGGCCA
ACGATGTGAGAAGCTGATGTCAGATACCACGCAGCGCGAGGAAGGCACGC
CGATACATGCGACCCCGAACAATTTCGATAATCTCGACAAAGCCTTCACGA
TCTTCAAGCTCCCCGGGAAGGGGCGCCTCCATCGGCGGGTCGACCTGATTT
CTGCGCCCAAAGAGCGGTACGCGGCGGCTGTTCTGAGCTGGTCAGGCAGC
ATGATGTTTGAGCGGGATCTCAGGTGAGCTTCGCAATGCAAAGCAGGCATC
AGCGAGGCTGAGCTTCAGACGATACATCGAGCATCGCGGATTGAAGTTCCG
TGCCGGGCTCATCGAGCCGTCGACAACGACCGAGATCAACCTCCCGACCG
AGCGGGCTATTTTTGACTATATCGGGCTGCAGTATGTCCCTCCTCACCTGCG
CAACGCAGATGGGTAGTGTATCATGTTCAGCTGTAGCATCTCTGTAGCATCG
TAGCATCTATTGTCGTCGTGAGTTGTATAGCCATGCAAGGTGACAACCATTT
CTCGCTAGATGACGTCAGTCCACGTGCCTGTCGATCATATCCAAAGACCAC
GAGTCCAGTCTCGAAGAGACGAACACGAGCTCTGCTTTACATCTTAGACGC
ATAGAGATACCTCTAGACAGCGCCATGCCGCGCTCGAGACGTTCATCTCGC
CCTCTGGCTCATCGGCCTACCCCCCATCCCGGCGCGTCCTGCCCAACACGC
CCGAACCGTACCCGATACATACCGACTCCTTCGAGGCAGTATAAGCCTGTG
GATCAAGTCGAGCATGAGCAAGACTGCCGGGAACGGCGACGAGTACTTTA
CGCACCCAAAGAGGTCGCATACGACCTACGGTCTGGTCATCCGTGGTGAG
ACCCGTCCTTCTTTCATCAGGCTAAGTCACGCAGCTGAGCACAGGGCACTT
TACGCAAGACGTCACTGCCGACGAAAGTATTCTGGGGCAACACGTTCGAG
CGGCCTATACGGGGATGTCTTGCCGTGGGGCACGAGCGTCATGACGCGGTG

CATGCAGTGAGTCTGCACCGACCTCCAGAATAGGGAAGGCATGGGAAGGA
AGGGAAGGTGGATCGGGGCTGAGGGCAGATGGCTCGATCCCACCGTCACC
ATGGACATCTACGCCGACAAGCCTTGGGCAATGGCGTACATGCTGGCAAGC
ATGAACTGGATCGCGCTCGGCCCCAGTGGCTCGGCGCCCTCGCCGGGCCT
GATCGTGCAGGAAGACTGCGCGTACTTGGAAGAGCTGGGCGCGCCAAAGT
GGGAAGGGGACGAGATGGCGCGTAAGCAGGCAAGGAAGGCGTGGTTTGG
GACCAAGGAGATCGGGAGAAGGTGCGGTTGAAGGGTTTGGAGCTGGCGTT
GGAGTTTTGCAATGGCTACCTCGGTGAGCTTTCCTCCCTCTGTCCCTCCTTA
TCTGCTTTGGGCTCTCGTCGCCTGCGTACCCCGCTGATTGCAGACCTCGAC
TCGCTCTCCGTGACTCTGCCCCGCCCGTTCACCCTCTCCATCCCCCTCCTGC
GATACTGGGACGGTCAGCCCGTGACGTACGTCTGCCAGACTGCCAATACCG
TCGGCGAGAACGTGCATTTCTCAGCGGCCTTTGAGATCGTGGGCGAGGCG
CCGGCCGTAGGGACCAGAAGAGGGCAAAGGTGAAAGAACCGACGGACGG
CCCCACCCCTTCCGAGGCAGAGACCTCAAAGGAATCAGAGAGCAATACCA
AGGAGACCAGCGGACGATGATGTGGACTAGACCCGACTCCTCTCGGCAGA
GAGTATGTTGTACAAGTGCTTGTTGAACCCGAGATGGTAGACATGCCTACA
ATGACATAACGAAATTACAACAGGCCAAATGGCGACCATGATTCTAAAGAG
ATACCCACAGCCCGAGTCCATGCTCCCAAGCCCCATGCGATTGCAGCTTCA
TCGTTTCTGACCGGCTTCGACAGCCGGTGCGCCCTGTAGCGCCTGCGCTTC
TTTCCTCTTTGCGTTCTCTTCTTTGATCTTGGCATTCTGAGCCGGTCAGCAA
TCGTACTTTTTTCCGAGCATGTATACCCACCCGCGCCTTGTACCGCTCGATG
CGCGCCCGCTGCTTCTCGTTCTCCACACGGACAGTCTGCAGCTCGTTCTCG
AGCTCGCGGACCTTTTTCCGTAGGGCTACCATCAGCTTTCCGCAATCAGATC
TGGACGGTACCTACGGGCAGTGGCTGCTGCGACCATCGGTGACTCGCCCTC
TCCGCCCAGGCCCATACCTGCCACGCCCATGCTCAGTCCTGTGCCTAGAGG
AAGGGCGAACGGCGGTCGAGGGAAGCCCGCCATGCCGCCACGACTGGGT
GTGGGTATTGACGGGGCCGAAGGTGATGCTGCAGGAGGGGTCGAAGGAAA
GGATGCGGTCGAGGCGAGGAGCTGGATCGCATGAGGTCCTGGCCGTGCTT
GGCCTTCTGCGCCTACAGGGTCAGCTACCAGTCCTCCGGACGATGAACTAC
GCACCTCTCGCCGGACCCCATTCACCACTGTCCTCATGGTCTTCTCCCTCTC
CTCAGCCATCTCCCTCAGCGCCTTGTTGGCCTTTTCGAGGGCATGTGCTTGT
GATGCCAGAGTATCGAGCGATGCCCGCAAGCCCTCGTTCTCCAGCGCAAGC
TCTTCCGGGGTCTTGCCGCAATCAACAGCCTGGACTTCAGCTCTTGAACAA
ATGCCGCCTGGCCAGCCCCAGCGCCAGGCGCCCAGACGTACGGCTTGGCA
CCTAGGTCGGAAGGGGGTTCGACTAATACCCACGGCTTGTCCTTGTCCTTAT
CCTGCTCAACCGAGGAGGCCCGCTCGTCATCCACCACATCCCAACTCTCCT
CGTCCAGTCCACGTTCCGCTTTCTTCTTCGAGCTCCGCCTCCGCGGGTTCTT
ATGCCGCGTCTCCTTCACCTCGTACACATTCTCCTTGATGGGCGGAAGGTCC

AGCGGCAAGGTCGTGAAAGCCACGGGGTTGCTCACTTCGTCGAGCATATTC
TCGAGCATGCCCCAGAAGCGCGAGAACGGGTCGTTGGTATCATGCACGCC
GAAGTGAATGTAGCTGCTCTCAGGGTCGGAGGAGGACGATGACGATCCCA
GTGGCGAGAGGGTGTGGTCGAACGGCGTGGGCGGGACGGCAAGTGCGGT
CGCAGGGACGCCATGCGGGTCACCAGGTGCGCGGATGGCAAAAGGTGGC
GCGTCGCCAGGGGACGGGTCTGCACACTCGTCAGCCGCGCGTGCTGCGCA
CACGGGGATCCGGTACTCACTCAGTCCGCCAGGTGCGCCGTGAGACCGAT
GAGCGCCGGCAGGTCGCCGGGTGGGTGTGCTCGGCTCTGGCGCATTCTGA
GCGGCGGCGGCGCTGTTGATGCGCCTCTGGAGGTCCTTGACGAGGTTCCC
GGTGCTGCGTGACGAGCATCCCCAGTGTCTGCCTGGCGCCGGGATCCCGC
ACATCGTCGTTGGACTGCGCGAACAGGTCCGCGGCCTCTTGGTACAGCTTC
AAAGCCTCATGTAGGGCTTGCGGGGTCTGGTTGGGCGGCTTGAGCAGCAC
CGACGCCTGAGCCGAGAGGACGTGGGCCTGTCGGGCAGCTGGGGCAGAG
GCAGCTGCTGTGTGATGTGATCGGTGCGTGGGGTAACTCACCCGGCTCAGC
GGATTCTGATGCCCGTGGTCCTCCATGACGCAGAGCAGGGGGTGGCATCTG
CTACAAATCCACACCAAGGACAAGGACCAATTACTACGACATGGGTCTTCG
ACTACCAATCGGATCACGAAGAAGAGACAGCTGCTCAAAGCAGATGTCCC
ATTCAGCGCCCGCTTACGGCAGAACACGTGATCTGCCACGTGGCGGGTCTC
GGAGATGCCGTCAGACATCGCCGAGCCAGGATTCCAGCCAAAAGTCCGTT
GATGCTATTGTTCAACGCCCTCATGTCATTTCCACCTCTTCACTCCTGATCAC
GGTGTCGAGGGGTCCAACTGCGACCGGATATCATGGGCCCCGTCCACCCAT
CGCTGTCCCTGCCTATCCCGCTGGCGGTGGGCATCACGCCCACCGCTGCCC
ACGTCCTCTCGCTTCTCTTCGCCTCGTCGTATGTCGGTTCTATATACCTCTCT
GCGCACCTCCCGCCTCTGCGCCCGCGCAAGGCGGCCAGCACTGCCGGTAT
ACCACCTGTCATAGTGACAGACTCGGATGGCACGCTTTCGCCTATTCCGGC
CCGCATCGGCCGTGCCGTCCAGGTTGGGATCGCCTGCGCCCGGCGAGCGA
GATCATCCCGAGACGATCAAGAGCCGGATACGCGCTGTGAGCGTCGCGAC
GGCGCTCAGTCTCTTTGGGGTCGGGTATACTGTGTCTACCCTTGCGGACTTT
AGGTCCTGGACCAGCGTGGTGGGTGTGTCGTCATGCTCCTTGAGTGTGCTG
ATGGCAGATACGCCCGACCCTTCGGCTGCTGGGCATCCCAACCCGCCTGTC
TATCCCGACAGATATAACTAGTGCTATCTTGCCTTACGCTCTTGCGCCCACTC
TTCTCCTCGGCCCGATCTACGCGACGTATATTGACGCTGCATCCCACCCGCC
GTCCAGGCTGAGCTGGTGGACCCGCATCAAGCTGGCATGGCAAGATTTTG
GCCTCATCCCTCTGCGCAATTACATCGTGGTGCGCCCGTTGTGCTCCTGAGA
TGATACGAAGGCTGATGTCAGGGGCCCGTGACTGAAGAGCTTGTCTTTCGT
AGCTCCGTCCTCGCCGTCTCCGTCCTCGGCCACTCTCAACCCGATGGATGG
TATTCGGTACCCCTATGTGGTTTGGCCTAGCACATGCGCATCACGCAAGGGA
GTCGTTCAGACGAGGCGGCTCGACGCGCGCAGCGGCCGTCTCTGCCGCAA

TGGCATGTTGTGAGTCCAGCGTGGCGTACTAGATCGGCGTAGGGATGCTGA
TAGTAGTGTTCCAGATTGGCTATACGACGCTCTTTGGCTGGTTTGCGAGCTA
CCTCTTCCTCCGAACAGGTGTGTATGACTTGGTTTGAACAACTCGAGACTG
ACTGCAGCTCGGTCTTGCCGCCCCTTGCCTCGCACGTCTTCTGCAACATCA
TGGGCATCTACCTGCCTACTACTGCGGTGCAGCGGCATCCCTCCCATAAGCT
CTGTGCGTGGTTCCATCTCTCACGTAGAGCAGAAGGGAACTGATCTCAGCC
ATCTGGACAGCATACCTCGCCGGCATCGCCGGCTTCATCTACGGCATTCGCA
GGTTGTAGTATCAGCCTGCCCATCATACCCCCATCTGTGCCAATCGTCGTAA
TGACCAAACGTAACCCATACCACCGTAGACACTAGACCGATGCCACCCTGC
CGAGCGAGTCATCGCCATGCATGATCATACACAAGTAGTCCGCGCCAGAGA
GTCCCGATCGTGAGAACCGCCTAGACGACCGTCCCGGCGTTCGAGGCTATA
CGGGGCCACAGGTCCGCGCACTCCTTTGCGACGCTGCCCATCAGCTGCTG
ATTCTCGTAGAGATGGCAGCAACTCACGGTCCGTTGATCGCACTGCCCTTC
ATCTCCCCTTGGCGTTGACAATGACACCGGCGTTGTCCTCAAAGTAGAGGA
AGACACCGTCCCTGCGCCTCCAGGGCTTGCGCTGGCGGCAGATGACGGCG
GGCATGACCTTTTTCCGGAGCTCGGGCTTGCCCTTCTTGACAGAGGCCATG
ACCATGTCGCCGGCGGCGGCGGCGGGGAGTCGGTTGAGGCGGGCACCGA
AACCGACGACGGAGATGACGTAGAGGTCTGCGATTAGTCTCTGTCCTCCAA
TCCTCCATCTCCATGTCCTTCTTGTGGGTTTGGGCTGTCCCCCCTTCTCCTT
GAAGAAACCATGCCTCGCTTCGCTCGGAGCAGAGAAACAGCACCCACTCT
TTGCGCCAGAGTTGTCGGCGCAGTTCATCACGGCGCCCACGGGCAGACCG
AGGGTCATGCGGAATTTGGTACCGGTAGCAGCGGCTGCACGTCAGCGAGC
GTCCCCTATCTCCATTTCTCTTCTCGCTTCTCTCCACACATCCACCATCCTCC
TATCCAACGCTCATCCCAGCTCGCATGACCTCGACTTCTACCCTCTTCTGCG
TCTCCCTCTGCGAGACGACCTCCAATATCCCTCCGGTCCTCGCCCCTGACG
CGCTTCTTTCGTTCTGCGCAGCCTTGCCCTTGCTCCCCCTTCACACTCTCGA
CCCCTCTGAGATCGCCGACTCACTCTTGGCGGACATCTTGACGGTGTCGAA
CAATGTTAAAGGCCAATCTCCCTCAGTGCTGCTTTTCGCACCCCGCCAAAC
CTCGAATTCGTCCAGGCCAAGACGGAGTTTCGCAAGGAGGAGATATCTCCG
ATTCCGTTGGGTGAATGTGGCGATTGCTTTCGATCTCGGGTGGCGATCGTCG
GCGATCCCCGCCGAGATGACTGCACGCGTAAGGCTCTCCTTTGTCCCCGCT
GCTCGACGAGAAAAAAGTGAGCGCGTCTCTTGGTGACGACATTGGATATCC
TACACCTCTCTCTTCACTATCCTAGAGACACGATACCCCACAGCCCCTTGCC
CCTCGTGTCCACTCTGTATATTGTCCAGTATCTTTTTGCCTGCACAGCGCTCC
CGAACAACCGTCACCCATGACAGCCGACGTGTCGCGCGCCTCGTCCGAAC
TCGCCTCCCTCCCCTCCTGTCCCATACCGCCGGCCCCTCGACCGTCTCGTCC
ATGCCCGCCAGCCCCCGATCTAGCTCGCCAGGACGCGAAGACAAAGTCAA
GATTGTCAATGGTCACGGAGCGGCAGAGGAGAACAGCGTGGACGGCGCC

GAGGTGGTGGATGAGGTGAAAGACGCCGGCATGGGTGACCCAGACGTGA
GTAGTGATTTCGTGCCCCTCTATCACCTTGGAAGGACTAGCAGCGCGTGTG
TCGTCGACAGTGAGAAGGATGGCAGACGTGCTCTCTTCAGCGCGTCAAAC
CTGCTCTTCCGATCATCATTCGTGCTCGGGGCGTCGAGAACGTGCCAGACG
CGCAATGGACATGGCTGACGCGACTCCTTTGTCCTCCGACTGCCCTTGTCT
CTCCTCTTGCTTATTCGGTCTCGTGTTCCCCCGAGTAGGCCTCTGCACGCGC
AGCCCGCCTCACCTTCCTGCTCGACAAATCGACCATCTACGCCAAGATCAT
CGGGGATCGTATGGCGAGACAGCAAATCGAGAAACGCAGAGCAGAACTCC
GCGCCGAAGTCCGGAAAGCGGAACAAGGAGAAGAAAGACGCCTTGCCCC
AGGCTCAGGCGCACAAAGAGGGCCTGCGGACAAAAGACGAGAAGAAGCC
TCATATCGTAGACGAGGAGAACGGCAAGAAGAGGAAGCGCAAATCCGACG
TCAACGGCGCGGTAGAGAAAAAGGCAAAAGTCGAAGTGAAGAAGGAGCT
TGAGGCGGACGCCGAGGTCAAGGCTGAGGCTGGGGCCGATGAGCTCAAC
AAGGATGAAGAGGACGTCGACGATAATGTGGAGTACTCGTTTGTTCAGCCC
GAGCTGGTCACTGGGGCCAAGCTGCGCGACTACCAGCTGGCCGGTGTACA
GTGGATGATCAGCCTGTATGAGAATGGTCTGAACGGCATTTTGGCAGATGA
GATGGGTCTCGGCAAGGTCAGCTGTCTGTTTCGCCTTCTTACGACTGAAGC
TGACAATTAGACGCTGCAAACCATCTCCTTCCTGGCACACTTGCGCTCAAA
GGGTACATGGGGACCCTTCCTCGTTGTCTGCCCACTCTCCGTCCTCAACAA
CTGGATTATGGAATTCGAAAAGTTCACCCCTACCATCCCCGTCCTCATGTAC
CACGGCACGCCCGAACACCGGCAAGAGCTCCGATCCACACGCCTGCAGCC
TCCTTCATCTGCCGGCGCCGGCAGCGTCAAGGGCAAACGAGGCCGCAAGT
CTACCAGCAGCGTCTTTCATGGCAACACCACCGACACATTTCCGGTCGTCA
TCACCACCTACGAGATGTGCATGAAAGATCGCCAGTTCCTCTCGGGCATCA
TGTGGAAGTTCATCGTCGTCGACGAAGGCCATCGGCTCAAGAACCTCGAC
TGCAGCTCATCCGAGAGCTCAAGTCGTATACCTCCGCCAACCGCATGATCC
TCACCGGGACGCCCCTCCATAACAACCTGGCCGAGCTGTGGTCGTTGTTGA
ACTTCATCCTCCCGGACATCTTTGACGATCTCGACTCTTTCCAACAGTGGTT
CAATCTCGACGACATGAATGACTCGGGCGCTGACACCGACAAGTCTTTGTT
GAACAAGTCGTCCATCGTGTCCTCCCTACATGCTATTCTCAAGCCGTTCTTG
CTGCGTCGACTGAAAGTAGATGTCGAGAAAGAGCTGCCGCCGAAAAAGAG
TACCTGCTCTATGCTCCGTTGACACAGCAGCAGAAAGACATCTACCAGGCC
ATTGTCGCCGGGCAGATCAGGCAGTATCTCGTCGACAAAAAGACGAAGGA
CGCCAACAATGCGGAAGACGAGGTTGCCGAGCTTGAAAAGCCCATGTCAC
TGGAAAGCGGAAGACGGAGAGGGTCAACTACAAGATCGAAGAAAACGAC
TCGAAGTATATCCGCGACCTGGAGCGAGGTACCGTTAACGAGCCCAATGGC
GTCAAGGAGAAACCGGCTGCCGTAGTGGGCAAGGAATGGGCAATGAAGC
AGCCCGCAAGCATGTCAACAACATGCGCCTCCAAAACCTCGTCATGCAGCT

CCGAAAGATCTCTTCGCACCCTTTCCTCTTCGACTGGCCTGTCACCGATGC
CAACGAGCTTGTGGTCAACGATGATCTCGTCAATGCTTCTGGCAAAATGCT
GCTCCTGAACCGATTGCTCCAGGAGCTGTTCGCGCGCGGTCACAAAGTGTT
GCTCTTCTCGCAATTCACCACTATGCTCGACGTCATCGAGGATTGGGCGACT
GTCTACAAGAAATGGGAGGTGTGCCGCATAGACGGCAGCACCAGCCAGGA
AAGCCGGCGCGAGCAGATGGAGTACTTCAACAATGGCGGGAAAGACGGGT
GCAAGCTGTTCCTCCTGAGTACTCGTGCGGGCGGTCTTGGTGTAAATCTTG
TGTCGGCCGACACTGTCATCTTTTTTGATCAGGACTGGAGTGAGTCTCGTAT
TGGAGGACTGATAGGTTCAGTGCTGAGAAACAGATCCCCAGATGGACCTG
CAGGCGCAAGATCGTGCCCACCGTATTGGTCAGACCAAGCCGGTCTTGATT
TTCCGACTGGTGTCCGCGCACACTATCGAGACCAAAATCTGCAAAAAGCA
GGCAACAAGCGGAAACTGGAGGCGCCTGTCATTTCCCAAGGCAAGTTCGG
ACGGATGGTTGACGAGAAAGGTCGTGTCCTGCTCGGTCGATCCAAGGGCG
GCAAGATGGAGTCTGTTGCAGACATGGCAAAGGCTCTCTTGGCGCTGGAG
GGCGAGGAGATCAACGTCGCTTCGGCCGACGACACCATCATCTCGGACGC
CGACCTTGATATCCTGCTCGACCGCTCGCCTGCCGCCTTTGCCCGCGAGAA
AGGCTGGAGCGCTGGTCTTGTGGGCGGAGCGGGTGCCAAGCGAGCCGAG
CAGATCAGTAAGGGCGAGCGCACGGCGTTCGAGGTCTTCGTCCCGGCCAA
GGACGACGCTGCGGACGGCCTGGCGCAGATGTTTGGAGGAGACGGTGAG
GCCGAGGAGCAGTGATGAGGGGGTGTATGCGTGTGCTCGAGTGCTCGGTT
GTTCACAGCTATGGCAATGGCTATGCCTATCGCGGTCCTATGGACCATGTTG
TATCTCTCGTCATAAGCATATCTATGCATGTAGACATTAGACTCTACGTGTCT
TCCACGCCGCCACGCCTCCACCACGACGTGTCCGCAGCAGACCATTGCATT
CCGACTTTTTTTTCTCCTTTGCGCTTCCTGCTTCTCCCAGCTGTGCTGCTGT
CCAACGATGCCGTCCCCGTTGGTCCGAGTGGCCCTTGGGCTGGTCTGCCTC
CCCCTCCTCGTCCTACTGCATCTCGCCTTTGTGCTCTCTTCACTGGCTCTAC
GGCTCTCCGAGCTGTTCTTCAAATCCCCTCCGCCCATCTCGCGCAGCGTCG
ACCCCCCTCGGCACGTCGCGTTCGTCTTCAAACCTTCTGCGCGGCTTAGCG
AGGGTGAGAATGCGCGGCGGCTGGCGGAATGCAGCAAGAGGGCAGTGAG
GTGGGCTGCCCAAACGGGCGTGCGAGATCTGAGCATCTACACAGGGCAAG
GTGCGCTTCTCTCTGGTCTCCGGTCTCCCCTTCAAGTGGGGAGCCCACAGG
TACGGAAGGCGGGCTGTATTGATTGATAGATATGAACTCTCTGGCCAGTGTG
CTCAGAGCCGAGATGGGCGTCTTGCCGCCTTCTCCGCCGTCGTCGCCTCCC
TCTTCTCCACGAACCGCGAAATCGTCTCTGGCGCTACCAGGGATAAAGGAG
GGCTCCACGGCGCCAGTCATCAGCCTCGCACTGTACCCTCTTCCTTCCCCG
CCAGGTCAGTTGTGCAAAGTGGCCCGCCGTGTGAGAGAGCTGAGGGGCA
GGTTCCGACGACGAGGACGTCTCGCCTGATCGAGCCAAGACGCGGCAATT
GGGCGTGCACCTCTTGCAGCCTTCTGCTGGCCAGGTGTATGGCCGCATTGC

CTCGGAGTACGCGGCGCGGTTCGTCCCGCCAGGAGAGATCACTCAGGATC
GTGTAGACCGCGATATACGCTGTGAGTCTCCTCGTCCCTTACCTGACCCCAC
TTCACCACCGCTGCTGCCTCCTTCACCTAGTGTTCAAACCACCTGTCCCCC
GACCTCCCGCGCCTCATCTACCGTACGTTCGCCCTGCTGACACCTGTAGCC
CACCTCCCCTTCCCCGACCCAGACCTGCTCATCCTCCATCCCCTCGCGCCCC
TCCCGATATGGCGCGCATTCCTACCCCGCCCTGCGCCAGAAATCTCAGGATA
CCCATGGTGGCTCCTCCGCGTGACCGAGATCTACCAGCACAACACGCCCGA
CCTCCCCCTCGGTCTTCCGCGCTTCCTTGCGCCTGTGCTCTTACAGGCCAG
AGCGTCGAGGCTGCCGGCGGTCAGGAAGGCTGCGCGGGCTGTACCGCTTC
CTCTTCCACGGCAGGAGGAAGGGCTGTTCTCTCGCGCAGAGTGGGAAGGG
GCCTTGCGTGCTCATGCGGGGGTGGAGCAGCGCCTCGGGCGTTGACGACG
CGAGGATACATGCCACGAAGCTACGATCTGATGATCTGATGACCTGCATGC
ATGCATATGTCTTGTCTTCTGAACTGACAGATACGTATTCGGATCCGGACCA
CGTGGGCCACGTTCTCAGTGACGAAAGTAAACTCGGGCAGCCGACGGTCA
CATCTCTACGTCGTCTTTAACTTCTCAAAGCAAGATGGCCCTTCCCGGCACC
GAGACCGTCCGGCTGCGCCGGCCCATACCGCAGGGCACGGCCGTCGCGCC
CACCGCGCGCATCGTCGACGAGAAAGACGAGCACGGGCGGCCCCTGCTGG
CAGGCTGGAAACGGCGACACCAAGGTCTCCTGCAGGACCAGACCCGCCG
CGCGGCCCAAGGCCCCTTTGTCCCCTCCCTCAGCTTCGCCGTGCGCCTCCT
CCTGCTCGTCCGGACGTGCGGCGCGATGTACGGCCTCATCGCCGACTGCGA
CGAGGTGTTCAACTTTTACGAGCCGCTGCACTATTTCAGCCACAATGCCGG
TTTCCAGACGTGGGAGCTCTCGCCCCAGTTTGCCATACGCTCGTGGACGTA
CGTCCTGCTGCACTGGCCGCTGGCGCACGTCGTCCCGCGGCTGCTGAGGC
TCGGCAAGCGCC

>Mosaic_sequence_61

GAAACTGGAGGAGCGAGCGTCGCCTCTCCGTGTCCGAGGGATTGTATTCTC
TGCAATGAAACGACTTTGAAGAGGACAGAAGTCATGAAACCCTTGGGTCC
GTCTGCGATAAAGTATGTCCGTGTCCGGGGAAGGTCTCATACCGGTCACCA
TGCCTTCGTGGCTCTATTGGGCAGTGTGACCATCGGTCATACGGCCCCCGT
GCCTTTCGGTCATCGCTTAGTCCAGTCCGTGCGCCATCAGACGTGAGCGGG
CCATCGCCCCTGAAACCACGCAACTAGTGGCACATGCTATCCTTTCTACAAT
CATCTGACACACCATTCCGCGTATGCGAATCATATACACCCTGGCCGAGAAA
GGACCTCTCGGCCACGCACCTGTGATGATTGCACGGGGGTCAACAAAGGA
GGTCCACCTGCCGCCTTACGCGTTCATACTGTTTACATATCAGGCGCCGCGT
GAACGCCACCCATCACCCTGCCCCAAGACTCGCCTCAGCACAGCACAGCA
GGGATGAAGACCGCCGTTGCCCTCGGCGCAGCCGCCGCGCTCCTCTCCGG
GTCCGCACTGAGCACGCTCGCTGCCCCGGCTCTCAAAAGCGCCCACCGTA

CCCGGGACAGCGACACCGAGTTCGACAAGACCGTCACGCTCGTCTGGCCC
AACTTCACCCAGGTCATCCCGAGTACGCAGCACCAGCAGTGGGCAGGCGG
GTACGGCGAATGGTCCTATGCAAACTTTGTGACGGAGGCCAGCGGATTCCA
AGCGGAGGAGTTTGAGCTGGTCATCTGGGCGGGAGAAGGGCCGTGGGAC
CCGCCTCTGAACGCGGCACCGGTCGACAAGGCCAAGGTGAGTTGACGGCG
CAGACGCTGGGCGGCAGGCTGATAGCAGTGTACTATTTCCGCGAGCGGTCT
GGCTAACAACCTGGAGGAGTATGTGCTCCTCCAGCAGGCGCCGTATGTCGA
GAGTGTCTGGCCGACTCCCGACCGGTGGGCAAGGTCACCTGCGCGAAGGA
GTGACCGCGGCTTTCTGCGGCGACAGACGGGGCCTGTCTCAGGCTGGTGA
CTACAGCTCTTATGTAGCCCCGTATAGGCTATCGCACTAGTTGCGGACATATA
TGTGCATGACACACCTACTTGCACTTCGTCTACCTCCACAGCCCAGAACAG
TCCGTGCGCAACTTGTCCAGTCCCTGACATCTGCTGCACTCGCTGTATTACT
CTGACCCCTGCGTGTCTCCCTTCAGAGAGACTTGGCGACACGCTTCATCCG
CGAAACTCGTCCAAACTCCTCCGGGGCAGAAAGGAAATGCCGCACCATTC
AATCACCCCGTCTGATGCATGTACAGTGAGTATGAGGGATGGGGATTGAGA
GCGGAAAGGAGCATCGTGCGCTTGGAGGCGCCCACCTGCCATCTGAAAGC
ATAAAGCCTCTGTCTGACCCGTCCATCCTCCCCCTAAGAGCTTCGCTGGGG
ACTGCAGAACCGAACACACAGGGCCAACCCAAGCACCATGAGCACCATGT
TCACCGCTCTAGCCCGCAGGTCGGGATCCCTCGCGCTCATTCTCGCCATCTT
CTCCCTCCTGGCGCGCAACACCCGCGGCGCGGCCGTCTCCGTACCGGTCTC
CAGCCTCGCCTTCGACCCCGCCCTCACCTCGGGGCCATCCGGCTCCCTGCC
GGCCGTGACGACCACCGCCTCCCACGCTCAAGGGGCAGCGAGCTCTGGCG
GTAGCCCGACGCTGACGGCGGGGCCGAGCGCCACAAGCAAGGCCGCCATC
GCCGAGGCGATCGTGCTCATGTGGCCCAACTGGACCGTCGTCAATCCCATC
GGGCCCACCGTGCCGTTCGCGGGCGGCAACGGGCAGTCGGGCGTGGCGCT
CACCACTGCCGACGACGGGTTCGGGGTCCAGGTCTTCGCGCTGAATATCTA
CCCGGTGACTGGCGCGTGGGACCAGTCGCGGAACATGACGAGCGTCCCCG
GGAATGACTCGGCTTTGGTGCGTGCCCTACCACTGCATGCACGACGCAATG
GGAGGGAGCTGACGGGCAGTGCACCATATCGGCCAGCGCCAAGCCCGCTT
ACATGGAGGGGTATGTCATGCTCACGTATCCGCCGTACTTTGAGAGCGTGT
ACGACACTCCCGAGCCGGTCGCCAATCTGCAGTGCAGACAGCTGTTCGGT
GATCTGATACAGAGTAGTCCGACGTCATCTTTGTAAAGTCCTGCAGCATGTA
CTGTTTGTACTGTTTATACTGTACGAGAGTAGACGAGATACCCCAGACGCA
GATGTATGTTCTGTAAGTAGCCCAGTGTCGCTGTCCCAAATTCGTGATCATA
TACATGATGGCATGATGAGCGCTCGGCCCAATGGCCGTGACCGGCCACAAC
GAGTAATCTAGACCCTTCAGATGACCCATTGCACCTCAAGATCCCTGCGAC
CAAACGCCGCAATCCCCTCTATGACCACTTGTCCATGAACTCTTTGTGGAA
CGTCTCGATCTTGCCCACATAGTCCTCAACACCGGGACAGAGGGCAGTTAC

ACGGAGATGATATGTCCCTGGCACCTGACCGAATCCAGATCCTGCGACAGC
GCAAATACCAGTGGCGTCTGCGACCGTCAGCTTGTGGCCCGAATGCAAAG
CTTAGCCCACCTAGGAGGTCCAACGCGTACATCACATCCGCCTCCTTGCCC
TGCTTCTTCGCCTCATCGATCGCCTTTTGGGGCATGTCGATCTTGGGCATGA
GGTACATGGCGCCATCTGCCGGCTGACATGACATGCCGGGGAGCTGGTTGA
ACTTTTCGACCATGTACTCTGAGCGGGACTTCAGGTTCGAGAGGATAGTCT
GGGTCTCTTCTTCCCATTGGGCAAAAGAGGGATCTCCGGGTTTCGGCGGCG
AGACGAGGAGGTCAACGCCAACCTATACATTAGCCAAAGACCAGGGCTAG
GAAAACTGGGGATTACCTGGCCGGTGACGGGTGGGCACAGGTTGACACTG
GCCATCTTGTACACCTGCTCCATGACGTCGTCCTGCACATTCACCACCTCAA
AGTAACCTCCGCGCCTGCCGCACTCGCCGCTCATGCCCTTGGATATACTGTG
GAAGCTGATCAGCTCGACGCTGTTGGCGACTTCGGCCGGCATGTCCCTGAG
GACCTTCTTGAACGATACGAACGGCTTCTGCTTCGGGTGGAAGATGTTGGC
CTGATACACCTCGTCGGCGAGCAACAGGATGCCCTCATCGTAGCAGAGCTG
GACGACATCGCGCATTGCCTTGACATCGAGACATGCGCCAGTAGGGTTCCC
AGGGTTGATGATGACCAACGCCTTCACCGGGGTCTCGGACTGTTTAGCCGA
CTCGATGGCGTCGAGTAGGACTTGGTGGTTCATCGACCAGTTCTCCTCCTC
TTGCAGGTAGTAAGGCAGGGGTGTCGCCTGCAAGTTGGCAAGAGTGGCGG
TATAAAGGGGATATTGTGGGATCGGGATGAGACAACCGTCGCCCTTGCGCA
GCGCGACGTGCATGATGGACCCAACACCGCCCGAGGCGCCGGCAGTGAGG
AAGATGTGTTCTGGGTCGGCAGGATGACCGTCGCGTTCTATCATCAGCACC
ACTGCGTACGCAATAAATCGGCTCCACTCACCCTCGATAAACTTGGCGACT
CGCTTGCGGATCGCGAGGACCCCCTTGGAATGGGTGTACGCGCCGACACT
GCCTATCTCGTGATACAGGCTTTTCGCCCTGGCGATGGCATCGCTGGGGTAG
ATCTGCTTGGCGATCTCGATATGCTCCTCCATCAACGCCGGGTACTCGAGCA
GACTGATGACCTGGCGCCAGAACGTGATCGGGCGCTGGTCAAGCCCCGCC
TGCTGGGGATTGCCAATGTTCGCGGTGACGACCTTGTCGAACGGCAGCTTG
GCCGCGGCATGGCCGTTCCCGTTGGCCTGCGCAGAGCCGGTGCCGTTGCC
CGCTGGCGCGGCGGCATCGGCGAGCTGGTGTGCGTACTTGTCGGCACGGA
GGGCGAGTTCGCCTCGCACGGCATAGGCACATCGAGGACGGAAGGATTGA
TGGTGTCGAGTCGGAGGGCAGGCTGGCGAGCGTGCCCGTTCCCGTTTGCG
TTTCCGTTTGGCTGTGCGGCGGGCATGGTACTGTGTACGAGGCGCCTCTTG
ATGCTGGGACTGGGCCGAAGCGTGAGGCGAGGAGAGACGTGGATAGACG
CAGAGGGGCCGGAGAGGCCGGGGCATCGAAATGTGTGTTGGATCGGTGGG
GATCGGTCCGGGGGCCGGGTGTGTCGCGGGGCAAGAGGGAGGGATGAGA
GAAGCGATAATGAAAGGGGGGATGCAAGGGCAGGAGTATCAGCGTGACCT
ACGGCGACGCAACGATGCGAGAGAGAGGAGGGAGAGTGGTAGCAAGGGA
TAGAGGAATATCGAGTAGTGAGGGAGAGAGGGCCGAGTTGCCGGAGCAAG

GGGGACGGAATAGGCCCGACGATGAAACGGACGGCCGGCGGACACGACT
ACTGTACTCACGGCTCGCAATGTATTGTATGTGGGTAGTCTACGGTCTTTCT
GCAGACGAAGAGAGCGTCGAGGAGATGGGGATGAAGCGGATCAAGATAGT
CCACACGATGCTATATAGCCCGACGAGTAGAGTGCTTCGCGATGACGGCTC
TTATCACCCACCTTTGATGAGCTTATCACGACTGTCGTCGGGATGAGCAGTC
ATCACATCGACTCGGAGGCTGAAACAGCCGCCGCGCACACGAAGCGACCC
ATACAGGTAGAGAAGCAAGTCCTTTCAAGGCCAGCCCAGAGAGAAAGACC
GAGAGAGATGGGGTGTGATGCATCGCTGCGGGCTGATGGACCCCAAGCCA
CCACCCCATCCACCCGACCGGCCGGACCAAAGCGCATCGCTTCCAAGGCA
AGATGGGCCTAGCGGGTATCCGTAGTGATGATGTACAACATGGCGAGCTCG
GGCGCTCCTTTCTACTGGCTGACAACGGCAGTGTTGACGGCGAACCGGGG
GCTCGCTTGCTTATCGCCACCCTCAAGGCGTTGATGGGCTAGTTCCACTGG
CCCGCATCCTGGCCATCAGCCGGCGTCAGCCTAGCCCAGAGAGACGAGTG
AGCCCACCATCATGCGCTCGTTGGTCGCTGGTGACGGGCTCTTCCGTTCCG
CCTCGAGCTTGGCGCTGAGTTGTCCAGCACGGGGATTGGACGAGTTGACA
CCCTTGCTCCGAGCCTGCGTGTCAGCTCGCCAGCCTCGGTGTCATCCTCGG
CCTCCTCCTGCGGCTCTGAGGCTTCGCGTGGCTAGAGCGGACAGCTCGAC
GCACGCTCTTCTGCCGTTCTTCGGCGGCCGCTAGTGCTGCTGCTCTCGGAT
CCCCACCGCCGTTCACGGCACCCTCCCCGCCGAGCGCCTGGGGCGGCGGT
GCCGGTCGTCCATTTGATCGGCTGTTGTTTGGTGCTGATGGAGGCGGTTGA
GATGGGCGAGGGCCCGAGCCGAGTTTCTGTCCACCGCTCGTCTTCTCTTCG
GGTGAAGAGAGGCAGGCGCCCATTGCTGTTCACAGAGATGGGGGGAGCTA
GCGTTGATTCTGCTCGAGGCGAGGGGTCTTTATCTTCGAGGTGCCGCACAA
ACAAACCCGGTCTGATATGCAGGCCGACGTGCTTGGATAATGGCGCTTTGG
CCAGAAGGGCGATGAGATGTCGAGGCTATGAGTAATGATCAAGGACATAGG
AGTGTCGTGCCAAGACGAGCCGAGTGTCCGGTAATCCCGCCTACGCCACC
GGGTGAGCATGCAATCACACTGCAGTATCGACTGCGGACCGAACGCTGCCT
CTGCCCTCTCTCTGGAATCACCCGAATGAGAAGCAAGTTACGGTGCATAGG
TGATACATTGTGCTATAGTGAGAGGTGCCAGATACTTCGTCTGCATGTTGGG
GTGGTGCTTTATCCTTCATCGTCCCCCGCATCAACCACCTCCGCTCAGCGGC
CTCGGTAGGTTCCTCTCCATTCCATCCTTCCTCCCCAGGTCCCCCCGCCAGA
CCACACTTACTATCCGAACTCGCGGCACTTTCGAACGCCACCGCCGCTCAG
GCCGTCGGGACCCCATCGCTCAGCCGCTCCCACTCGGTCCCACTGCCCATC
CCGCTGCTCGCGCCATTCGGCGAGCCCATTTCGCCGCCCGAGGCGCCCGCC
GAGCCCGAATCGGGCGACGCATCATCCACGTCGGTCACGCTGGCCTCGGG
CCTGGGAGGCGGCGCGGCAGAAGCGGCCGCACTGGCTGTCGCGCCATCGG
TCGGCGCTTGGAGGGTCGTGAGCTCGATGACGAGGGCGAAGACGGGCGG
AGGAACGACCTTCTGGTAGACTGCCGGTCAGCACATGTAGGCGTGCGAGA

CATGCGGGCGTACTCATGAGCAGCTCAAGGGGCTGGGGGTACTCGCGCATC
GCGCGGTAGAAGTGGGCGGCGGCGTCAATGTGCATCTCGGGACCTGTGTG
TCAGCCACTCGGAGCACTGAATGCCCACTGCGCCTACCTAGCGCAGAGAG
TTTCTCGCCCTCGGCGACGTTCTCCTGGAAGTACATCTCCTTCTGCTCTGGC
GACTCGGGCCGCGGCTCGGCCATGATCTTGGCCAGCACCGCGCGCAAAGC
CTGCTCGTCAGCCTCTGATGATCTCCCTGCGGCAGAGTGGCTGACCTTGGC
GTTGCGCTCCTTCTGCCTCGTCAGTTCCGCCTCGACCGCCTGCACGGCCTT
CTTGTGCTGTTTGCCTGGCTGTCAGCATGGTCGAGTGGGCGCAGAAGGCA
CAGACGGATGCCGTCCCGGAAGGTCTTATTGTTCCTGCGCATATAGTCAAA
GTAGACGCAGTACCCCAAGACGCCGGTGGCGACTACCGCAGCGGTGGCGG
TCGCTATTTGCGCGGCAGATCGGGTGGACATGTTGACGAGTATGTCGGTGG
GTGTGTCGATGAGCAGCAGGTAGTCCACGATGAGACTCTCGATGAAGCGG
GACAGACTGGAGAGAGTGGAATTTGTCCCGTGGGATTAATTGCCAACTATC
GTCACGTGACTCGAGGTGTCACTTTCGGGCGGTGACTAGCTGGCTTCAGA
AGGAGCAACTCTGTCTCATTTGCACAAAACACTGAACTATCACTCGTCTGG
CAGCTCATAGAACAAGCGCAGTAAGCGCCAGGAGCGATAATGAGCGCTCC
TTACCGCGCTCGCGAGGTGCAGTCAAGCCCCAACACCTACCGACCTCCCG
GGCCAAGCCATGCGACTCCCTCACAACCACGGCAAGCATGGTCGTCTGCG
CCCTCCCGATTCGCCCCTACTCCCTCTCCCTCCTCCCTCACGCCCGGTCGAA
GCTCCAACAGGGGCCACAGTCGGGCAACACCCGCACTGGCAATATACGGC
GACTCGTTCGTCGGTCCGTTCACTCTGTACCGCGATTCCGACAGGAGGATA
TGCAAGTTCAAGGGCGCGTCGGCCAAGGCATGTCACCCAATTTTGGGCCAT
GCGATGTCGCTGAATGGCAGGGACTGAACAACCCTCACTCGACCCTACGTA
TAGCAGAGGACCTCTTGCCTGATCTCGAGAGACACTTTGAGCTCGACGCGC
TGAGACGAGGCCCCAAATTCGCCCTGCTCATCTTTGGCAACGCGAGCTCGC
TGTCTAGAGTCTGTGTGGGGCTGAGACACAGGTCGACCTCCAGGTGAACT
ACCTCTTCAACCTCAGCACCAAACCCCTCACCACCGCCTCCGTCCCACCAG
CCCACCCGCTCTCGTCTGTGCTCCCTCCTCCCGAACCAGGCCAGCCCAGTG
CCCTCGGGCCCAAACTGCACGTCGAAGCCGTCGCCAAGGCGTACATCTCCT
TCATCGAGCGCGAGATCGTCCATGGGCCGGTGGGCAAGTCGATCAAGCTG
GCGGCTGCCGAAGCGGCATCTGCAGGCGATAGTTCGACCGGGCCCCGAGG
GCCGAGCAGAGTGCTGGTGTGCGGAGCCCTGCCCCCCGTCGTGCCGGACG
ATGTGCAGCCTCGTATACCCGAAAAGTATGTCGTACGCCTCGGCAAGCGCC
CCGACGACGACACGGAGCGCGTCGACGATGCCCGAGGGAGGACGGACCT
CTCATCAGCCCTGTCCAGGATGGATATCTCAACTACTGACCCCGTCCCGCTC
GACACATCCCCGCCGCCTTCCACTTCCAGCCTCCCAGACTCTGCCTCCCAA
CACGGCACGCCCATCGACGTACTGCTCGAGACCCAGCCGGGACTGCTGAA
CCTCTCCGTCCGCACACGCATGGTCAAACACTTCAACTCGCTCCTGGCAGA

TTTCTGCGCGGCGCACCCGGACGTACTGGGCTTTGTCGATATCGGCCCCCA
GATGCTTGCTGGGTCCAGCACGCCGGGCTGGCAGTCCCTCGACGGCGAGG
TGGACCAGGGGACGTGGCGCGACGTTGCCGACCCGAGCAATATCCACCCC
CTGTGGGAGCCCGCACTCCCTTTCTGGCTCGAAGCCCTCCGGGCGCACGG
GGTCGCTGTGGACGAGTGGAAGACCAAGGTCGATCCGGAGCGCACGATGC
GGTTCTTCGAGCAGGAGAAGGGCATGCGCGTGGCGCGAAGAACCTATGGC
AGCTCTTCGCCCGCGTCGGCTCCAATTCCCCCGCCGACTCCTATGGCGGAC
GGGGCGGATGTGGCGCTTGTACCCCGCGCAGTCCCGCCTAGCCCAGTGTCG
ATCGTCGGTCCCTCGACAACGCGCGGCAATGACGGCAAGTACGTCCCGCC
GCATCGGACCGCGTCGGATAGGGGACAAGCCCGGCTCAGTAGTGCATCTG
GAGCCGACAAGGCGTACCTACCTTCACCGCCCCCGACCCCGACGAAAGGC
GATGCGGGGTCGGGTGTTACTCGGTCAGAGGCGGAAGTGCCCAAGTGGCG
CCCGTCCGGCCGGTGGACGCAGAGTCAATCGCAGCGGGCGTGACCGCGAT
GCCTTTACTGTGGTACCTCCTATATCGCACGTTGTACATGCATCAGCTCCTGC
ACGCCGAACAAACCACCATGCGTATCCATGATTGAATGATACGTTGCATGCC
TCGGTAGTATCGATACATAATGTGTTTGCTTCTCATTGACTTCTCTTCACACC
AATCAATCAGTTAATCGGTCCGCTGTCCGCAAAGGCGATTGCTGCAAGTAC
GAATCGATACAAGTGGACCACGTTACAGATGGACCACGAGACACCTAGGT
CGTCTTGAACTTGGGAGTCATGCCCTCCTTGAGCTGCCCGTCAGTCGCGTA
TTCCATGCGATGGACGAGCTGTAGCACTGACTTGACGGATGTCCTCGTTGA
GCGGGGGGAGCGAGTCTTCCATGTGTCCCTCACGCGTCTTGTGGCGGTACT
TGAACAGGGCAGTTTCGCTCGAGATCAGCACAAGCAACCACGAACACGCT
CGTAGGGCATGTCAGGAGAAAGCGGCTCACTTTGGGTTGGTGCCCATCTTG
TCCTGCCCATCCCACGGGTTGACCATACCGCCGGCCAGCAGGGCCCGGTGC
GGCTCGGTCTCGGTCGACTTGGACATGCTGCGAGTCAGCCCGACGACTTGT
GCAAGCAGAGGGGACGTGCAAGAAGTAACCGGCACAGGCGAGACCGGCG
ACGACGATGAAGCTCAGCGGGTAGAGCGCGGGATCGCGTTTGGCGAATCG
GTTCACGCGCTGGGTGGCCTGCTGGAAGTGCGCCGTGTGCGAGAGGTTGT
GGGGGTCTTTCTGCTTGCGCGAGTGGCGAGAAGAGGGGCGCAACGTAGCG
TGGTTTGGGCGAGCGAGGGTGGTGTGGGAGGGGTAGGAGTAAGGGAGAG
GAAGAGAGAGCGTGAGCCGCGCTCTACTCCTGGGCGACAAGACTCACCAT
CTCGGTGCGGGGAGGAAGGGACGACATGGTGGTGTTGGTTGCTTGTTGCT
GTTGCCGATGCCGATGCTGATTGTGAGTCGGTTGAAGAGAGAGAAGAGAG
AAAGCGGCCGCCGATCTTATACTTGTACTCTGCGGCAACTCGGGGGGCGGG
GTGGGGGAAGGAGGGGAGGTGACGAGTAGGGGGCCGGCCTGGATCACGA
TGTCGCCGATTCAGGATCGACCGAGGTCTGACCGAGCTAATACACGGCCTA
CTCGAGCAACCAAAACATTAGACTCACCTTGCTCAGACTACAGACTGCTTC
TCTGATGGAAAGAGAAGGAAGGTAAAGCTAAAGAACCTCCCTCATCGAGG

TAACTATGCAGTGGAGCACGAAGTCTAGTGGTGACGTCACACGGTTAAGA
GCAAGAGATCGGCGACCAAAGATACCGGTCCACTCGACGGCGATCACCGC
TCGGGTGGGCCCCGCACTCATCCCTCAGCATAGTGTTGGTGTTCCTCAGAA
CACAGGCGTGGAGGAGGGAGATGCGGCCGACCGATGAGCAACAGCAGGC
CTTCGTGGCCGCGACCCGTGGTCTCTTCGGGGGCGACAGTGCTGCCCGAC
TGTCCATTCGTGACGTCGGTGTCCTGCCCGAGGCAGACGACCACGGTCAC
CGAAGACGAGTCGAAGGGTTCGGGGTGTCCTTCCAGGGGGTCGTCGAGCC
AGGTCAGTCACTCACCCCCTTGGTATCACAATCAAAGACACTGAGATCAGG
ATGGAGCAATCACTTGGGGATGATGCATGGCGCTGCGTACTGCTGGCTAGT
CGACACGTGTGTCCTTCTGCCTTGCTCCGCGGGCGGCGCTGGCGCTCGGTC
AGGGCGCTGAGCGTATTGTAGCTGCACCTCGGCCACACTCGTCGCACTGAG
CACCGACACATTCTGGGGCGCGCCCAACCTCTCGGGCGTCAGTGTCAGTAT
GGACGTCAAGTACCTCCATCCGGCGCCCGTGTGAGTATCGTCAGGTCTGCT
GACGCGAGCAAGGAGCTGACAAAAGGGGTTCAACGGTCATTGTGAGGTGT
ACGGTGCTCAAGATCAATCTGACTCTTGCTTGTGTGCAGTGTGATGTGAGG
GCATGCTCCCACGTCTGCAAGTGGATGAGGCTGAAACCAGATCATGGATGA
GACGGAGCGCGTGCTCGCCACAGGTTTGCACGTCAAGACGTGGCGGGCGA
GCAGAAGCAAATTATGACTCGTCTCCGTCCGAGGAGATCATCTGCCATGAG
ACCCACCATGCACCCTGGGGCCATTACAAGGAGGTGGTGTAACTTCGCAAC
CGCTACTCGCTTTCGTTCCCATCGCCCACCAATCCCGCCCGCCGCCGCTCCT
CTTCCTCTTCTTCTCTTCTGCGCTCATTCTCCCTCTCTTCCCTCGTGTACTTC
GAGAGGTCTACCTCCTCCGCATCATCCTCATACAGCTTGTCGTCTCCCGCCA
GGTTGCCGGCTTCAAAGAGCGCTTTGCCTGCCCGTCAGCCTGACCGACCC
CCTTGCCTCACTCCAGCTGCTCTCACCGGACAGGCGCTCTTTCCGCTTGCG
CACCTCTTCCCTCTCCTTGGGCGCCAGCGCCTTTATCCGCTCCTCTTCCTCC
TTCTCCCGCTTACGCTTGAGCTCGGCCATGAACGACTTCTTCCACGCTTCG
AACGTTTCTCGCGTCAGGGGTGTTCCGCGCGTCCGCGCCGCCTCCGCCTCC
TCGTAGGCCTTGGCTCGGCGGTCGTCTTCCTCCTGCTCCCGGATCGCACGT
TGCTCCATGATATTCGTCAGGGATTCGCGCGCGGATGCGGCGAGCGTGAAT
GTCATCGCTATGCCGAGATTCTCCTCGCCCTGGGGTCAATCTGCACTTTCTG
CCCCGAGCTTGTGTGTCAGGGGACTCACATATTGCCTGAGGGTCTCCAGCA
CCTGCTGTTGCTCGTCCCCTTCTAGCCCTCCTACCTCTTCCTCGTCCTCGTC
GGCATCCTCTCTTGGTGCTGCAGGCTCGATGGAGATGAGAGGCAGGACATC
GGGATACGTCTCGGGATACTCGAACGCGAGCACAAAAGCCACTGATGCTC
AGATGATCGCCTGGCATCTGCGTTGCGCGCCCAGCCTACCTGGTTGGGGGA
GGAATCCTTCCTCCGGCTCTATCCGAATGGCCACGCTACTCTCGCCCCGCTC
TGTTCCTCAGAGAGCAGCTCGTCACACCTCGGGGCAAAACTGTTGGCCCA
CTCTCATATTCGTCAGGAGGGAAGATGCTCTCCAGCACCTCGAACTCCTCT

TCTAGCATCGCTTTGTAGTCTGGTTGCTATCAGTCCTTGTGTCCGGCGTATC
AGAGTAGAGCGTACCCGTCATCGTGCTGTTGGTGTCCAGTCGGGTTTCCTA
GGACTGACTTCCCTAGACATTGTCGCTGCGAATGTCAAGACAGAAGCGTTC
AACGCCACGTGGTAGGTCTTCCGTACGATTTGAAGTGGAGGAAGGCTGAA
ACGTCCTTTCTTAAGTGATCACGTCGTGCCCCAAGTGCTGTGGCTAACACC
CGTGCCCGGCTCCATGTTCGTCTCCACTCTACTATCTGGCGCATAGGCCGTA
TCGGCTCTTCTTTACTCACTCTTGATGACCGACTCGATGTACTCGACGTCGT
CCTCGGAGGATCTTTCAGCCGCAACGGGCAGCTTGACCCGGGAGATGCAG
CTGTGGGCCGCGTCAATAAGGCAACAGGGTATCGTGGACGTGACTTATGAT
GATGCGCTGCTCGCGACGTTGCGACCTTGGCCCGGAGCTGTCGGATCGCCG
AGTGAGATCTTCAATGTCCCGTCCGAGCTGTATGACGCAGTCCAAACTAGC
ATAGGTGAGGTGAGTAGTGGGTTTGTTTCACTCGTGTGCATTCGCCGGACG
GTGGATGAGAAGCAGCAAGTGAAGATGCGTATGGATGGCAGACGGCGCCT
GACAGAGGTGATGACTGATCGGCAGGATCCGGGCAAAGCTCCCTTGGAAG
ACGCCATGGCCGGATTCACCTCGCTCCCGTCATCATGTGTATCGCGTTTTCC
GCCTCTTTCCCGCCAGCTGCTCGTGCTCTCGGTCTTCCGATACACGGTCGC
GAACATCCGTCACATGCGACGGAACTACTGCGAGGAGTCGCTCTGGCAGA
GCAACATGGAGAGCATGTTCAACGTTCTACTATCGTCGTCCCAGTGGCTCC
ATGCGGCGCAGTGAGTGTGCCTTCCCCCCCCCTCCTCCTCCTCCCCCTCCTC
TGATGGGAATGCATTGGCCAGGTCGGCGTTTGTGTAACCGCGGCGAGACCA
TGACCATTGAATGCTAATGTTAGCACCGATACGCAAGTTGATCGTGACGTG
GCCGACCAATTCGGGGACTTCGTCGAGGTCAAGCCAGATCTCACCATCGGT
ATGCTCAAGGCAGAATCGACTTTCGACAGGGAGATGAGTCGATACCGTCAG
GCGAGGAGATCGAGATGTATGCAGCGAGTCGGGGTGTGCTGTACAACCTTA
ATCAGCGTCAGTGATCCACATTACCTGCGCGTGTATACAAAACAACGCTCG
AGCTGACAACCAGTCTCGTCCAGCCCGTGGTTCCCTCACCTCATCTGCGAG
ATCAAAAGTCTTGAATGCTCCCCGGCCGAGGTGGAGAGACAGATCATTATC
GGCATGTACTACGCCCTCAACATCCACCTCGAGCTCGCAACGCTCGCTGAA
CAAGCCGACCCCGTCACCTACGCCCGGAACCCAATCGACCATGTTTTGAGT
AGGGTGTACGGCTTGATCTTTGTCGGAGAGTACTGGACATTGTGGATGCTG
GCCGGGCGTGCGGATGGGGCTGAGCCGAAATACGTAAGTCGCGACGTCGT
ATGTGGTGCGGCGTGACTTGTGGTTGTGGTATCGACCAGTTGGGCATCGTG
GGGGAAAGGGCGGCAAAATGTAGTCGAGTCGCGCCGGCCGGGAGAGGCA
AGCTCTCGCACAGAGTTTGAGCTGATGATCAGCCAATGCTCCAGCTCGGCC
ACGGACTTGGGCGGGATGTTGGCGACATGAAACGTCTCTTTGTCCTACTGG
AGCGGATCGAGGCAGAAGCACAGAAACGCGTGGAGGCGATCGGATTGTG
GTTCTACTGTTTGCGCAACACATCTCCAAAACTCAAGCGGGCGCGGGCCCA
GGTCGAGGTGGCAGCCCGCGATGAACAAGAAGAGGTCACGCCCAAACAG

ACCCGACGAGCGCGACAATCCAGCTCGGCTCGTCACGCTGAGGCTGGGGC
TGGCGGCACCGCTGAGGAGAGAAAGCAAGTCGGCGCGAGAAAGACAAGA
CAGAGACCGCTTCTGAAAGAGGCCGAGTCCATGCTGGAGGCAGATCGCAT
GAGGAGTCGCCGCATGAAGCAGGCAACTCCCAAATGTTCGAGCTAGCCAG
GGTGCTTGCGCGGGATTGCCGAAAGTCAAGGCTCAGGATCAAGTCATCGA
GCGTCGGCAGAAGTGCTGCGTCGGTCTCAAGATCAAAGGCCAAGGTTCTC
GGTTGGCTCTCAGGCGTGTCGGCTTCGGCCAGCAGCGACGATAGTGGCAG
TGGGAATCAGCCGTTTGAAACGATGAGGCCAAGGTCGTCAGGGCGTTATGT
CGTTGTCCTTGGACAGGGAACGAGAGCCTGAGGACGTGAGATGAGCGCGC
ATTGCGCCGATCTTGCAGTCTACGCTGACGCTGGGGGACGTTGGAATTGGG
CAATACGCTCTCTTCGCCGAGTCAACCTCTACGACTTCCAGATAGAACGAC
CTTGTAGAATGGTGCCTCCTGTTGTTCCTCATCACTCATAGCAGCCGCTGAG
CGAGCGACAGATGATCGGGACTACACAGCCAGAGAGTACGAGACTCCGTC
GGGTGGTACAGTTTCGTGTCACACTCCGAGAGATCCCAGGAGAGCACTCT
CTGACATTGCAATACTCCATGCAAGAGGATGTCTTGCAAAAGAAGAGAAA
AGTGTATCAAGACAGATGGCGAGTGACAGTCGTCATTTTTCGTGTGACCGG
TCAGTGTGCGCCCGATACAGTACATCGACCCTTTGCATTGACTGAACTAAC
ATCCCTTGAGCGCCGGCATGCCGACTGTCCAGTGTGATCCTTTGACTGGTC
TGAGCTACCTGACGAGGACCTCGACGAGTCTGGGCGCCGCGCTATGTTAGA
CTAAAGCACTGTCCAGCTCAAAAGCGAGGCCCGATTCCCGCAAAGGTCAG
CGCTGCACCCTCGATACCGATCGAGGGTCAGGGACGCCGGCTGCAGGCCC
TTGAGCACATATAGGAGCCACTCTCATCGCTGATGCGGCTCATCTGGGTGAT
CTTACTGCTCCCGCTTGCAAGATGACAAGCAAGAAGCCTGCGCACTTCGTG
GCCATTCCGCTGGGCGCCTGGTACTTGGTATATATCTCATCTGCCACGGCAG
AGACTGATGCGCAGGGGCCACCTGCGCCCTCATCTGCACCTCCTCCTCAAC
CTCCTACGGGTGCACACCGAGTTACACCTCACGCTCCTCATTCAACCTTCTT
CGCTGGCCAGCGTCGAGAAGGAGCTCAAGTCGGTCGCACTGCAGCATTCA
CAGCCGGCGTCCCTCTCCGCGAGGGCCGATGCGCCCGAGCTCGCTGCGTC
GGCGGGCGCGCCCGCGGTGGACGACCGCATGCAGCTGATTGTCGTTTCGT
CCCCTGGTGCGAGCAACAAAGCTGGCTTCGTCCCCGACGACATGGTCGCC
GAAGCCATGGACTTTGCCAAGCTTTTGCCCGACGTTCTCAAGGGCGCCTTT
GCGGGAGAGAGGAAGCTCGGGGTTGGCCATATGGCCGAGAACAGGTTCGC
AGATGTGCCCGTGTCCTAGCTCATCTGATCGTTCGGAGCCGTGGCACCTTC
GTTCCTGAGCAACGACTTATATCTCTTGTTCTGGTAGTCTGGTTTCGCCAGT
TGGTTGCATTCTATTAGCATTCGCTTGCGCGTATCAGCTGCATTGGGTAGCTA
TCGCTTTTGGTTTTGCTATGGGTAAGTTATTCTTCAGACTCGACACCCACCC
TCCATATCACAAGTCTAATAGACCTGCGTGGCTTCAGTTTCCTTTGGCACAG
TGTACGCCCCAAATATCGCCTTAACTTATCTCATCGATTGCTATACTCCGTTC

GCACAGGAGATCCTAGTTGTCGTCAATGTCTTCAAGAATCTCGTTGCGTTCT
TGTTCGCTTACGTTGCAGTTGACTGGGTCAATTCTCAGGGTTGGATTCAGG
TCTATATGGTTATGTTCATGGTGGTCACTCTGTCAATGGTTTTGGCTATACCT
GTCTACTTCTTTGGGGGAAAAGCACGCGTGGCGTTCGAGAAGATCCTTGTG
ACGGTCTCATTGGTTCCGTAGATGTGATATGGTATGCTTCTAGTTCACCCTGT
TCCTTAACCCGGAATTCGAAAGTAGGCTGGATCGAATAGGTACATCTAGCCT
GCCAGTCCTAGGTCCTTAATATACTTGAGTACTGTTGTGCTGTACTTTGAAA
TTAACCCAGCCGTAATAAACTAACCCGCAACTCCGACCAGCCAAACACTAA
TACTCCGTGACAAGCCTTTGCATGGTCCTCCATAACGATCCGCATCCGCTCA
CATATGCGCCACATGCCCACTTCCCACGTATCCTGGTTATCCGATATGCCGA
AGACGCAGATTAGGACCCAGCACGTCATCGCAACTAAGTGCGCCACGAGG
AGCGTCTCCATCCGGGCGTCTGTGTTGAATTGGTAGTTCCACACCCCGGGC
GTCGAGATGGCATAGAGAACAGGATCCGTTCTCCGAAAGAGCGCGGATAA
GCGGCCGTGGCTGAGACAAGCCTCCGGGGAGCTTCCCGTTGGATATTTGGG
AGATTGCAGTGGCGTGATGGCTGGCGCCAAGTCTCGGGAATAGGAGCAAG
AGAATTAAAATGTTCTAAACGCTTTTCATGTTTTCATCATTTACCGTTTCAAT
GGTGACGGACCGTTTACGTCGAATAAGCCAAACCGTTCATATTCTTGCTGT
GTAACAACCCGATCTCGTAGGCAATCATATCAATATAGTGACCTTGTGGCGT
GCGGAGGCAAAGCCTTATTCGAAAGCCGGGCACACCAACTATGAGACCGC
TTCAGTAAGTCAAGTCAATGATCTAGCTATCATGTCCCGTTTCTAAGAGAAA
ATCTTTGGAGGGTTGGGAATTGCTATTTCCTATTAACCGCAGAGGCTTCGAT
AGTCAGCACATCCTTCGTCACACTCAGGTCTTCAAAAAGTTACCAGGTAGG
TACTAGGTACCTAGTAGATAGTCCAAAGAGTCCGAGGTCCGGGAATTATCT
GCACAGGACCGCGGTCGGACTCATGGTTTTATGGGTTAGGCAGCCTGTTCT
TCCAATGTGACACGAGGTAAGTTGAACGGTGATAACGATTATATTATCCTTC
ACGCACCTTGCGCGAGCCTTAAGTCTACGTCTAGTTGTATTTCGGAGCACAT
AGGAAAAAGCCTATCGAATAACCCTACACACAGCAATACCGCCCTAAGGCA
ATATTTGCATCTCCTCTTTCCTGTCAGGGGAAAACTCGTCCTTGTATCGATC
CCATAGCTTCTCCAGCTCGGTCACGTACAAATCATGTAGTCGGTTGATTTCT
TCCTGATCCACGAGTTCGATTGCTTGACCTTGATAGGGGCTCCGACCACGA
TATTCAACGGGCGTCGGTAAGGCATGAGGCCTACGTCGTAGTTGAAGATCC
CTCGTCCGTGTAGGAAAGGAAGGGTAAACTTCCACACTTTTAGGATGAACA
TCTGGATCTTGTGCACCATGGGATGTTCATTAGGGTGTAGCTGGTCGTAGAG
GTTGTTTTCGCCAAATGCTAGCACGGGTACCAAATCGGCGCCAGTCCTGAC
CGCAATCTTAACGAAACCCTTGCGTTCCTTCAGAATCAATCGCAACTGGCC
TGGTTGCGCTTCAAGGGACTCTCGAGCACCTCCGATGACAATAGTCACGCC
GCGACCCATACCCTCTCCGTTTGGTCCACCCTTACTCAGAATATTGACGATG
GATTCTTTCGAGACCGATCGTACACCAGCAGCAAGAATATATTCGCGATATA

GTGGAATACGGAAGTTCGAGTCTAAGGTCAGTAGACTGTTTGTGATACCTG
GGAATTTCTCCGAAAATCCCAAAGCATCGGTAGCAAAAGCCGCCCAAGCA
CCGTGAGAAATGATGCCATGGGGATGATAGCCAAAGATATATTTCCGAGTG
GGTGGCAGCTCGTGGGTCTTGTAGAGCTTGGCTGGGAAGTAGCCCGCAAA
GAGATGCCAGAGATAGCTTTTGCGGAACCACTCTGATCGGTATTTAAGTGTT
CCGTCTGTAGCAGCTTTAGAGCTAAGGCAGTGCAAAAGATATATAAGTAGT
AACGGCCAGAAAAAGGGGTTAGCAGAAAGAGCGAAGAAGACGGAGACTG
TCAAAGCTATACACATGCAGTGCATAAGCACAACGAGGGTCTGTAGCCTGC
GCTTCAAAGGTACATTCCATGGGGCCCATCGGATTCCACCATTTGCGCTTGG
CTTTGGAGGTAGGTGAATTTCGTCCAGCGTCATATCATCTAACTCGGGGTAC
GAGTCATCGTCGTCTTGGGCACGTCTTAGAGGTGAGTCCACTGTTTCTTCA
ACCTCGCCGTTCAGACCAGATTTAAGAGCGGATGCATAGCTCGCCTCATTT
GTCTCATCAACGTTTGCTGTGTTTTCTAGGCCGACGAGTGTGGTAGCATCA
CTGCCTTCCATAGAAATGATACTTCTCTCATGATCCGGGAAATCATGGGTTC
CCTTCACAGGCTCGTCGCCATTCTGAGTCGCTTCCTTCAAGTTAGACGGCA
CGTTTGCGATCTTGACACTTTCTTGGATGTTGTCCATCTTGGAAGCGTCTTT
CGGTCTGGGGTCTTTCCGTTTAAAAACCTTGTACGATGGACTATTCTTAGAA
ACCAGTACTCGTGGTTTATGGGACGGAGTAGTTGGATTTGGGTCCGCTGCT
CTCGCTAGGCTTATTGAAAGGTGAGCGACGAGTTTCGGTTGCTTGTGGAAG
GCGAGCGGGAGTGAGAGTGGGGGGAATGATCACTGGTTGTATGAGGAATA
CGTGCTTTGTTTGCTGCAGATCTAGGTGTATGGGTTGAAGCACGAGGATTG
GACAGGTCGCTGCTATCTTGACTTGCCTAGCGAATTTCAATTAGCATCGTCG
CGTTCTTGCGACAAGTAATGGACAAGCAGTGATTGCAGAGATGCGCAAGA
CATACTATGCTTGATGAAGCAGAGACCTCCCGCCTGTGCCCTCTAGGAGTT
GTAGGCTTAATATCGCGAGTTGGGGTATTCGACGGGCTACTCTTTCGAGAAC
CTAAGTTAGAGTCAGAATCGGCCGTTTGTGCTGCTTCACACGGGTCATGAT
GGCATCTTCGTCCCAGGCGCGGTAGCTTGGAGCTCTGAGAACTGTGAGAG
CCGTCTGACGGCTCCTGGCGCTGACGCTGATGCTGGCGCTGGGGGCTGTG
ACTCTCGTCGTCCGCCATAATTGATCAATCTAGGATTCGGGTGCTATTCCCG
TCATTGAATAAATTAAAGACAGAACATACCTTAGGGCAAAAAAAAAAGAA
AGAAATGTACGCAATGTACGAAATGTGAGACGTATCGTCAATCACCCGTTT
ATGGATTAAACTTTAAACGTTGTCTTAGAATGCCGGCACTGGCTCTGGCCTT
ATTTAAGGTTCCCTCTAAGGGTTTTAAGGGTCCTTAGGTATGCGCCGCGTTG
CGATGACCCCCAAGACTAATTATTATGTGAGGCAATGTTAGGCAATGCCGA
AAACAAACTTTACAAAATTCAGAAATTTCGCAGCCTCGGTAACCTCGGTAT
GTATGCGATTTGCGACAGCGACGAATGAGAAAAAACGGGCCAAGCTAGAA
TTCAATGACCTGCATCGCAAAAAAATGTGCCGGAAGGAGCGATTTTTAGAT
CTTCCCAATCTCTTCACATAAATGGCAAGGCTCGGATCCGAAATAAAAAGA

AGGCGTGAGCCAACTGGTTACACAATATCATTGGGAAAAGCCATCTTACAA
AGCGACAAGCGTTCCAAGTTAAGAAGGTTGCAAATTGTAGGGAGAGAGCT
GAGTGTTTGTAGCTGTAATTTCTAGTTACAGTGGGGCTTACAGCTCATCGCT
AAGGCAAGGGCATAAGTGGGCATAAAGGGTGAATGACGTATGAAACCGCA
CGAACGCTAGCATGTGCAATTCATTGGCTCACCTGCACAGTTATGTACTACG
ACGCTATAACATTCCTGTATGTAATTAAGGAAGTTATGAGTATATCGCAGCTT
TCTTCCACTATCTGTGATACTCCTTTCCATTACTGGATTTCTTCATTAAACTT
GTACGGGAATGGTATTGTGTTCTTTTGGTTTTTTATCTCAAGATACATCTCAT
ATAATTCTCATAAAATGAGAAGAAGGTCATCAGTTAAGTTGGTACAGTAATG
TTACTAAACTGTTGCGGTAGCCTTCTAGGTGATGGATTCAAACGTGCTGAG
TCCTATTGATTCAGTATACTATACACATATAGGTACCAATGAACTCAAGTTTC
CAAGATAAACCAGAAACTACTGCTTCAGTTGCAGTCTTGCGTTCCAACCCA
CTATACATCTGATAAGATCCCAATCGCCATCTTGGCCAACCCAATCCGTAAC
CACTATACCAGTGCTTCCGTCTCCTACCTTTAAACCCGCTGGCCCTTTACCA
TCTTCACCGTGTCGTAAACAGAGATACTCGATGATAGCCGGGTTTGCCTTG
GCTGCAATCTTCTCAGGCCAGCAGTTGGCATTGAAGAAATTGCTAGCACTT
AAGAAATTCACGAAGAATGGCTGTGGTGGCACGCCCTCGTTGTAACCTTCA
ACTCCGGGGATGAGGCATGTAGGCTCGCCTGCTCGTTCTAGGTGGCCCCGT
GCTAAATCAAGCTTCTTCTGAATATTGTGGCTTTGGGTCAATATCGTAAAAG
TCTTGTACACGACCAATCCCACTTCCAATCAGGCCATCCTCGCAGTTGTCCG
GCCAGGCGCTGGCGTCAAGCCCCCATCCTCGACCGCCCCATTCGCCGTCCA
GGCTCGGGTCATTGGCGTGTCGACGGAAGAAGCACAATCTTTCCTCGCACC
TCACCGATCTTAGGAATACGTGGTTCCGTATACCATCGTCTTCCGTCTTCGTA
AAAGCGGTCCTTCAAGATCTTGGATAACTGATGGTCAGTACCCCTGCCGGT
GCCCTCGCGCTTCACGCTCATGATGAGTGTCTCGGACGGATTAGCCTCCAA
GAACGAATAAACAACATCAACCATGTCTTTGAAGTACTTGGTCCCGGTGAG
TGATATGGGGAAGGCGCTGTGCACGAGCGCGAGGTCCGAGGAGTCGGAGC
CCACATTGACGCGGATGTCGAAGAACCGTACGCCGTTGTCCAATTGCTCGC
GCACGTTGACTGCTTGGCATCGCACTGAAGGAAGCGCGACGTGGCATGTT
GGGCTGTTGTGCGTGCCTGGTATGGATAAGGCTGAGAGGGGATATTCATCG
TGTAGCTCACGCATCCAACTTGAGAGATTGGCGGAGCTAAAGATGGCTAGA
CAACTACCAGATTGGACATAGATGGCAGTGTATTCGTTAGGAGCACCGTCT
AGCTTTGTCATAACCGTGCTGCGCTGCGAGGCACTCGGCACATCTACCTGG
TACCGGTGACCATCTGACTCGAAGGTCAGGCGGATAACTTCCCTGCCGGGA
TCAGCTGTGCGAACGTCCGTCGTGCGGGTCTCGAAGCGCCCGATGGGAAT
AGAAACATCACGACGGTCTTTAGCCTCGCCCTGGATGCGGACGTTGGGCG
AAAGAGAGGTCGAGTTGAAAATGCCGGTAATCCTGCCGAGAGGAGTCGAG
CGGCCGGAGACAGACTGTCCTTCGAACCGCTCGACGAGCTTCAGCTCCAG

CGGGGTGGCTGTGAGGTTACGGATTATGAGGTCCGCCATTGGTAAGGATTT
TAAGTTGGGGAGCTCCGGTGAAGTGAGAAAAGGAAAGCCATTCTAAGCGG
AATTACATTATTACTATGTATGTAGAGCCACAGGGCCACGGAGCCACGGCTC
GGGCTAAGCGCCGCCAGTCCAAGAGCATTGTCTTAGGCGGAGGTAAATGA
ACAAGGCGATTGGACGAGATGCTGTGGGGAGCTTCGTCAAATGGCAGCTG
AAGTTTATTTTGAATTGCTTTTTTTCTTGGTCAAAATAGTGCCACCACCA
ACGAAATCTGGGAACTGGAGGCCCTCTTCATTATGTCGAGATTATCTACAAG
GTGTGTGTCACAAAGTTAATAATTAATTTGTAACATCTGAGCGCAAGATGTG
TGAAGAAACATTAATATTCACATAAAGGAGCAATTCAAATAAGGTGTGAAC
CACAGCTTATCGATATCGACATAATGAGATCAATAATGTGTTTAGTGTGAAAT
CGTTTAGAACAATACTCTCCAGAACTCTTCTCATCATCATCTATGTCCAATGT
GAATCCCTCACTTGAGGTTAGAAGTCATGCGTCCCCGTTTATCCACAAGCC
TTCGTCTGGCTAGATATAATGGGTTATATGTGAGAAAATTTAGTAGCCCGCCT
ACGCCTGCTCAAAAAAATTAGCCTCACATTGTCGACGAAGACTTCTAAGGT
AGGTCCATTCCACGTCGGAATCCTTTAGTGGGACTAATATTCATGTAGCAAA
CAGTCATAGAAGCATTGAGTCAGCATTTCGACCACTCGTCTAAGAAATCAT
GCAATGTTTCGGATTTTGGAGCTGCTATCTTCGCTTCGCCCGATTTCGCAAC
TTGGCTAAGAGATGAAAATTTCATGGGAGGTTTGCTTGAAACCCTCTCCAA
ATCAAATCACAATGAGAACTCCAAACTTAAAGGCCTCGATGCACTATGCGT
GGTAACAGACGGGCTTACGCCGGAAAAGGTATTCGGCAAGCCTCAAGCAG
GGTTTTCCATTTTATATGGCTCTACGAAGAACATCTTTCCTGACTTGCGGGA
TGTTGGCAGTCATCAATCAGGTGACAACGACCAGGATATAGAATCATCGGT
TTCATTTTTTATCAAATCCTCTACCTAAAGATACACGGCCACTGGAGATCAC
ATTGCCATTGGCAAACACCATTTTCCAAACTGGAAGACGTACTACCTTATTC
ACAAGCCGTTGGCAACGTGATATTCACGGGAACTTAGGGCTTCCCGAGACT
TTCATAAAACCAACACAGCGGATATATCCTGGAGTTAATGTGGCCGACCAC
ACCTGTCCGCTTATTCCTTTATTGCCACTCATTCCTCCTCGCAAAATTGTCGC
TGGCTTGGGCAATATTGTCCGCCAAGTTGAAGTCAATGGTTCTCCAACACC
TGCTTCAAAGGAGCTAGAAGATATTGTTCCAAAAGTCTTTGACGTTAGGTC
TAAGAGAGATGGATCATATTCGCCCGGGCCTATCGGAGTATGGTGTTGGGTT
ATACCTCCTCATATTGTTGAGAAGCACGACCTTCTTAACTTAAAGGTCTTCC
AAACTGAAACACTCAAATCGGAAGCGGAGCTAACTCTTGAGAACATGGAT
GTGTTTTCTGAAATGATATCTTCTGGTTGTCGGTTACATAAAATACGTATGTG
TCATACAACTTCTCAGGGGTTAGTATAAGCTAATGTGTGTCTAGTCAGCGGT
GGTGGAGGCTGGGGGCCTCAAGCAGGGACTCCTTTCCCTTGATCCCGAGA
CGAAGGCCTCTCTACCAGATCAAGATGATGAGCATGACATTGAAATGTTTAT
TAAAGCATTCAATGATATCGACAACCCCAATCCATCTCAGGGCCTCGTAACT
CCAGGTTCGTACATTATGTTCTGTACTGAGCCACACTGGACCGAGAAAGAA

TAGGGGAAAGTCGACAAATGACACCAACGACAGTGCTCGGTGTAGCCCCA
AGTCATGACCCAGAATCTAATTCTGCAAGTCTATCAAATGATGTAAAAATCA
TCGATGATCATTTCGGTGTGGTATCGAAGGCTGGTCTCTTCCTGAAAGCCAC
GTCATTATCACATATGGACAACTCAACCGAACCACCCCAGGTATTCACAAC
GAAGATTGACTCTCCACAAGCTTCGTTCTTTATGAAATGATTGCCCCGAAGT
ATCTTCTGGAATAGATATATAAATTCAAATATAAAGAGATGTAAAACATTCTT
ACAAATACTCCTTATGTAGCATGACGTTTGTACGTGCAATTAATAGTAGTATA
GCATGTCGTACTAAATTAACATGGTACTTTGTGCTGTATGCAATTATGCAGCT
TTCCGATGAAGGGTCCACGCTAAATCCCCGCTAGCCATTAAAATAGCGCCA
AAGCTCTGTGGAACCGTAGTAGCCAGGTACATCTATACCTGGACCCGTTAC
ACGTACAACTAAGGTTCCTTTGGACAAAGCCGCGTCTTTGTCGCTGACATC
TACCAAACTTCCATTTCTTCACCATACCTCAAAAATGGATCGTAGCCTTGAT
GAGATCGTTGCTGAGTCTCAGGTACGTAATTGGACTTCTATTCACCCAAATT
AACGGTTTTTGATCATTCCATTGCCGTGCATGCTAACTGAAGCTGCAGCATA
AGAAGCGAGGCTCTCAGCCTCGACGTAGAGGAGGCGGCGGTGGTGGTGGT
GGTAACGGTGCTGGCCGATATCGCGAGCGTGACAACTATCCCCGCGACGGT
GTAAGAAAGGTAAATTCTATAAATCATGTTCATGGTTTTGGCTTGCAACATC
TAATCACTCTCCATCTCAGGTGCATTTTCCCTTTCCCTCCTCGACTACGAATA
CCTTTGCCTCGAACGACCGGTTAAGTTATCGACTTGCGCTCGATGCCTCGG
ATTTCGATCGGTGATGGTTTATTCGTGTAGTGACAATGGCTAACAAGCAATC
TCGTTCTGCGAGACAGTCAACCAGAGACGATGCGAGAAACATGGATTCGT
GAGTAGAATTGCCAATATCTACATATCTTTAACTTATGTACTGACTAGAATCT
AGTGAGTGGGTTCACGACAAGTTCGACGATGCTGGCTGTAAGTTGATCAGT
TTAACTACGGACGGGCCACAACGACTAATTGCACTCTAGCTCGTAACCGCC
AGCGAAACCCGCGACGACGAGCCTCTCCCGACCGATTCCAGTTCGTACAAT
TCCAGGTCCTTGCTTAGAAGAAGCAGCCATCTAATTACAAGAATAGGGAGA
CTCGTGGTGTCAAAGTCAAGGTAGACAACTTGCACTACGAGCTTACTGAG
GATGACCTCGATGTAAGAGTACAGCTTCACTCTGCTCTAGAGACATGTATTG
ACCATATGTTAGGGTCTATTCAACAAGATTGGCCCCGTTCTCCGACTCGAGC
TGCTGTATGACCGCGCTGGACGCTCGGAGGGCACGGCCTTCGTCACTTACG
AGAATCGTGAAGACGCAATGGAGGCCATCAAGCAGTACGATGGCGCCAAT
GCCAAGGGTAAGCACACTACTCCTGATATAAAATCTAGCTGGATGTCACAG
ACTAACAAATTCCCCATAGGTCAGCCCATCCGTCTATCGACCGTGTCAGCA
GCAGCACCCCGACGCAACCCCTTCGACACCGCATACATGCCGGGCCGTCCC
TTAGCCGAGCGCATAACTCGCCCTCGTTCTATGTCGCCTCGACGCAATTCAG
ATCGTGACCGGGGTGTCGACCGCTTACGTACCCGGCCGCGGCAGCCGATCG
CGCAGTCCTCCCCGCCGACGCGGTGGACGACGACCCGGAGCTCGCCGCGA
AGGAGGCACAAGCACTCCCAACGGCCGGGAGCGCACCGGTCGGGATGGC

CGACCCAAGAAGACGCAAGAGGAGCTCGATGCCGAGATGGCCGATTACTT
CAACCCGAGCGGCGGTAACGACGCAGCTCCCGCAGCACAGGCTAGCGGCG
ATGCCCCGGCCGGTGACGTGGACATGATTGAGTAAATTGGACCGGTGGTTG
GTGGTCATTCTATTGGTCATTGCATGCATGGTAAACACATGATTGCTTGTAC
GGAGTATTAAGACGGGTCAACGAGAGTGCTTTTTCGGTCCGAACTTTTCAT
GGATTTTTTCGGTGGTTATGCTAGGCAAATAAAAGATACTGGCATCTATATG
CTGTTTTGTTGGTCCGTGATGCAATTGATGTCTCTTAGGCAAGTTGTGTCAG
TAGATAACTTCGACTCCTGCCGTCCTGTATGTCTCGGGTCTGTCTATGCTAG
TCTTGAATGGTTGTGATATTGGGAACAACTTCATATGTCTAGCAAGAGAGCA
TTCAAGGCAACGCTACGGCTTCTATGGTAAGGCCGAATATCGTCTAAATCTA
GATCTATAGTCGGCATGAAAACGCCATCATACATCTTGACCCCAACTTGAAC
AACATATATGCGTCTTCCAATCACCTATTCATTCTTCTTCCTCTTCGTCTGCA
ACCCCTCCGCCACAGCAAGCGCCACATCCTCCACCGTGGTCCCCCTCTTCC
TCTTCCCGATCTTCTCCTGGCCCAGCACCGTCTCATCCGCCACAATCCTGTA
TACCTTCGCAGCACCGATAAACGAGTCCGGGTCGAACCCTCCTTCCTCCGA
AAGCGTGCGTGCGATCTCCTCCATCTCGCCGACCCACCGGTACGCTTTAGG
CGCCATGCCTGTGACCCCTTTTTCTGCTACCTTCAGGTGCGTGGGTAACAC
AGTTGCGAGCTCCTCTTTCATTACGTCGAGGACGCCGAGCCGGTGCGCGGT
TGTGAAGGCTTGGATGGCGAGGGAGGTGAAGCCTTTGCTGAAGCTTGCGA
AGCACATTTTTAATCCGCTGGCCGCGCCGATATCAGGGGATATGTGCTTCAT
GTTCAGCGTGGAGAAGAGCTTCGCTCCATACTCTGGGATCGAGGAGAGTG
GGTGGGGACCCGAGGTCGGGATGCTGGGTAAGTACCATTCTGCCGTGTCG
GGCTTGGGGGAGGGGGGCCCGCCAAGATGGAGCCGTCGACGAAGCGGAC
GGTGGAGGTGAGGAGGGGGCTGCTGCTGAAGAGGGAGGCGATGGATTTTA
CGCTTGAGGGCGCGACGGCGTTCAGGTCTGCGTAGTAGAGCGGGGTTGAG
GGGTCCCGGCGCACGGGGCCGGTGAGGGCGTCTACGATGCGCTGCGCGGT
GGCGACGGCGTCGCGGGGAGGGACCACGGAGAGGATCACCGGACAGGAT
TGAGTCAAGGCTTCGTCGGAGTCGAGGAGCTCGACTTGCGCGGCCTTGGC
GCGGGCGAGGGTGTCGTTGCTGTGAGTAGGTTAAGCATGGTATTGGTTTAA
GGATGTTGCATTGAGGACCAGGTATTGGGTGAGATAGAAACGGAAGCAAG
GAAACAACCCGGAGACGAATAATAAGCTCGAAGAAAGTAAGTAGTGCTGA
TTAGCTGACTGACCTTCGACCTTTGATATTCGTCGCAACTGAGAAGCCGTT
GGCGATCAATAGCTTGGCAATCCCGACCCCCATATCACCAATAGAGATTATG
CCGATTTTAGCCAATGAAGACGCCATTATCGGTTCTCACTTAGTTACCTAGC
TATTTCGGAGGATAGAATTTTCTTGTAAGACAACCTAAATGTCACATGCGTT
GATTATCACAAAGTCTTTCGATGAGACTTCTTCTCTCATCACATACGCGGAA
AACAAGGCCCCTCAAAGCTATGCGGTGCCATGATGAGCTTCGTACATGGGC
ATATGTCGGTCGCTTTGACCATCGATATCCCGCGGGCCAATAGGGTTTAAGA

TTGAGATTCTTCTATACTAGCAGAAATCGCGTGAGAGGGTTGTAGTTGGAG
ATAAACCGTCCGATATACGGAAGAATTGAACAGCGATCATTGTTTGTAACAT
GAAGCTTTTCATGATGACTTTAGGATTTTAGGAGTCGATACCTAGTAGCATA
AAAAGTTTAGGTGCACTTTCCGAAACTCATAATAATGTCGCAATTGTGTTTC
CTTAAGTGCGCCAGGCCGTTTGTAAAATCATATCATCAAGTATGGATACTTG
TCAAAGTAAACTCCAAAGAGAAATATAGCGTGCTATAGAAGTACATGTACA
CCTTGAATGTTCATAATCGCTTATATCTGATAGGCTGGAAAGAAGGTCCTTC
AAATCAATAAATGTGCTACATGTATTCAGCTGTAGTTGGAAGTTGCGAAGG
CGACTAGGGTTTATCCCACTAATCTTTTGTATTGACCTTCAATGCAAGTGCT
CGCAGTTCATAACTCAACCAACTCAACAACAAAATATCAAGAATAGGAAAT
ACCACTGATTACCTTGATCTACAAATCCGCAGCTACCCAATCGTATTCATTC
CCATCTTTCGGTCACTTTGCCAATACGGAAACCCCTTCGGCTGCCCGTCGG
GGGCGCAGGGCTAGGGGAACAGGCTAGCCACATGGTAATTTGGGATACCA
ACTTCGAATCCATCCGAATCGAGCATGGACATCGTCTTTAAGTCTTGCCGTC
TCCGCGTTACACCACTTTCATTCGCCGCGCCAACAAGGAAAACTCTCCGCA
CAGAAGTTGTCCTAAAGACCTAAGGACCCAAAGACCCAATGTCCTAAGCAT
TCTAATCGCCCAAAGAATGAAGAATTCTGCTCGCAGTCCTAGCCTGATGGA
GTATTCAGATCATGGTCATATTACGCTACGTATCAGGCTTCGCAAGTGCCGT
TCAGCCTCTTATCATCCGAATCTGTCGCAAATAGAAACTTTGGAGCCTTGGA
ACCTTGGAAATCTATCCGGCTGTGCGTTTGCGGGGCTTTTCATCGGTTCGCC
CATCATTGATGGACGTCTGCATGTGGCATGTGGGTGAGCGACTTTTATGTGC
CTCTGTGCCGTGCCTTTGTGCTTCTGTAATCTGCTTCCTTGGATAATGCCTTA
AGCGAGAACGAGAGGAATATTGAAACTAAGCTAAGTAAAGCTGGAAAAAA
TTCGTGACGCGTCCGTTTGAACACTTTTAAGCTGACCATCATCTGACATCCT
ATTGCCTTCTTTTGTCTATTCGTCGCTGTAAAAGGTCAAGTTGAGGTTGAGG
ATCCCTGCAACGTTCACCCGCGCTCATATGTATCAGGGACCAATCCTGTGGG
TCGGCCAGTATCGGATTCGCTTCATCCCGTTTCCATTAACCAGATCTTCTCTT
ACAGTAATCAATTCACAATACACGATTCGCCCGTAGTGTATATAAATCGGCC
ACGCTAGCTTTTAATCGTCCCGGCGGCCTTTTAAATACATTCCATTCCATCG
GTATTAGTTCCACTCGACCCAATGAAGTCCATGACAACTAGACTGGAACGG
CCTTTTCTATAAACTCCGTCCCCCTGTTAAACGAAACACTTCAAACTACTCA
ACTAGGATTAATCTGTTATTTAATTGGCTGGACACTCTGTAACGATTATTGGA
CTCGTCATGCCAGCGCCCTATTAACTTGAGAATATTCGCATTTTTTTATCAAC
AAATTTCACTTTGGGTCAACCATCTGATTTCAAGACATAAAACGGGCGCTG
TTTAAGAACATTGTCAACTCTAACTATTTCTGCTCTGGTTGCGGTTTGGACC
TTCTGAGATCGGCCTCTCAACGCATGACCTTTCGGCTAGCCCCCGTTCCGTT
CATCTAATAAGGCGCATCTTCTAGGATTCGATTTGCCTTAATGTGGCAATAGC
CATCTTTGGATTTGAGAAAACCTCTCAGTAACCAATTTAGCATCTACATTAA

TTCTAATCATACACAATATCAACGATGCTGTCTACTCTGCGAAATTTCTCTAT
CGTATCTCTCCTTTCATCCTACGCCGTCGTCTCCTTCCAGCCAGGATGGACT
AAAATCACAGCTATTTGGCACATTTGCTGCCATCTGGTTTCTCCAATTTTCC
TCTACTACGTTGTTTGGAGGGTAATTCTATATCCAAAATTATTTCTCCTCTC
CGTGGTCTTCCCCAACCCCAGGATGGGTCCTTCTTCATGGGACAGTTCAAG
GATATAAGTAGTCAACCGACAGGCATACCGATGAGGAAGTGGTATGTTTTCT
CCCTATTCATCATGCACATTGGTTTTGCGCTGACATAATTTGAAAGGGTTGC
TACTATCCCAAACGATGGTCTCATTCGATATTTGGGCCCTTTCAACCAAGAG
CGTTTGCTTGTGACTGGACCCAAGGCCCTTGCTGAAGTCCTAGTCACTCGT
AATTATGACTTTCAGAAGCCAGGTACAATTCGATTCGGTCTCAGTCGCCTGC
TTGGTGTTGGGATACTTCTTGCCGAGGGCGATGAGCACAAGTTCCAACGTA
AAAATCTTCTGCCCGCATTCGCATTTCGACACATCAAGGAACTCTATCCGAC
CTTCTATTCGAAAGGTCGCGAGGTTGTGCAGGCTATGACAAAAGCTGTCAT
CGCGGATGCACAAAACGAGGCTTCCGTCCAAGATCCTGAGAAAGCAAAGA
AAACGGGTGTGATCGAAGTCGGTGACTGGGGGTCCCGGGTAACGCTGGAC
ATCATTGGTCTCGCCGGGTTTGGTCAAGATTTTGGGGCTATTCAGAACCCAT
CGGGGCGAATTAAGCCAAACATATCGAAGTGTATTCAAGCCATCGAGACAG
GCTCAAAGATTGGCTTTCCTTATCTGCTTTTCCCCAATTGGCTTCTTGCTCG
ACTTCCTATCCAACGTAATAACGACATCAATGTAGCATCCGAAGTTATTCGA
AGTGTATGCCGAAGGCTAATCCGAGAAAAGAAGGAAAAATTGGCACGCAA
GGAGCTCAAGGATCGCGATATTCTCTCCGTAGCTTTAGAGTCTGGTGGCTTT
TCAGATGAGAACCTTGTCGATCAATTGATGACTTTCCTAGCAGCGGGGCAT
GAGACGACGGCTACTGCGATGACATGGGCTATATATATGCTCTGCAGACATC
CTGAAGTACAGACACGACTACGTGCTGAGATCCGAGAAAACCTACCCCCT
ATCGACTCGGATCAGGAAGTCAATAGCCAAGATATCGACCACCTGCCTTAC
TTGAACGCCGTCGCAAACGAGGTCTTACGCTACTATGCCCCTGTTCCGCTT
ACACTTCGCGAGGCTTCCGTAGACACTACGATACAAGGACAAAAGTGCC
CAAGGGAACGGCGGTCATGTTAAGCCCGTGGGCTACCAACTTTGATAAGAC
TCTCTGGGGCCCCGACGCGCACGAGTTTAACCCGGACCGTTGGGTGCCCA
AGGGCCCTGAAGACAAGAAAGCCGCTAGCGGAGGCGCGTCGAGTAATTAC
GCATTCATGACCTTCCTTCACGGCCCAAGGAGCTGTATCGGATTGGCCTTTG
CCAAGGCCGAGTTTCTTTGCTTATTGGCGACGTGGGTTGGCCGATTCGAGT
TCGCTCTTAAGGACGAGGCATACAGGGACGAGAAGAAGATACTGATTAAG
GGGGGCATCACTGCTAGGCCGGACAAGGCATGCATGTCTACGCGACGATCG
TGGACGGATGGTGATATGTGTATGTGTATGATGAAGTTGTGGATATGGATCT
TACTTGTATATTGATGCTGGCTGCGTTTAATGATCTAAGAATTTGTTCATACC
CATTGGTCTTAGGTATAGGTAGCGATTATAGATTCTAATGAATTAGTGCTTAT
ATTCTCGCACTCTTGCGATTTAAGTATCCTGAGGGAGTGAAAAGTGCTATCT

AATCCTTAAATTGTACATTCTAACCGATCTATAGTATTTTCATAACCACCTGG
GCATGTACGACGATATTATTATTCTGCGTACACCATTTTTACAAGCCCTTAAT
ACCAACCTTGAAGTACCACGGGTCTTCATCGATGCGCTTCCTCAGCTCCCC
GTTCTTCAACTCCTCCACGTCCCAGCTCGCCCACACGAAACGGCCGTGTAG
GAACTTGGCCTCGGGGCTAGCAGCCCAGACGGCGAAGTCGCCCGGTAGTT
CGGCTATATTTGCGATCGGCGTTAGTTAGCCCCCAAAGAGCAATTCAACAA
ATTAAACACGATACTAAAAGGAACCGAGAACGAGAACGAGAACGAAAGC
GAGAGAGAAAGAGAAAGAGAAAGAGAAAAGGGGGTGGGGGCGACGTAC
GTTCATCCCAAGGCAGGGTCTGGTCGGTGTATCCGGCGTCCTTGGCGGCAT
CGGTGAATATGGCTCCCGGGTGGAAGTTAATGACTTGCATCTGATCGGGCG
ATACGTCTAGCGCGATCGTCTGGATCGCCGCGGCGCCCGCGTTCTTGGTTA
GGCCGTACGCGCGCGCTGGGTTGATGGCCCATTGGTGGATCGCCATTGTTG
AGACGTGGATTAGGTACTATGGAATATATTAGCCTTGCGGTATTGGATCGAA
AATTGCATTTTATATATTGGGTAGCGCACCTTCGTCTCCCCCACCCCTTTTCC
TTGTTGAGAGTAGAACCGTCCCGCCCAGTCAATCTGCGCGCGCACATTCAC
ATCATAATGCTTCGTCCAAAGATCCGCGCCGACCTCGAGCAACGGGAACAC
GGGGGCATGGCTGTATCGCTTGTCGCCGGTTTCATGGGGGGTATGGACGCC
GCGTTTAGAACGAGAACGTCGAGTACCGTGCCTGAGCCCGCGAACTTGCC
CCAGAGAAGCGCCGTGTCCACCGGGCTACCCATGTCGCACTCGAGGCCGA
TGACGGTCGTCGGGCTACCGAGGGAGGTGGCGAGGGTGCTAAGGGAGGA
GACCGCTTTGGAGACGGCGGTTGGACGGCGCCCGAGGATTATGAGTTTACT
GGTGTGGGCTTGCACGAAGGAACGTGCGATCGCGTAGCCGATGCCGGCGG
AACCGCCGGTTACGAGCACGGTCTTGCCGGCTTGGGAGAGCTCGGGGCGT
GAGGGGGAGATGGATGGGTAGCTGGTGCGGTGGGAGGTTTTGGTTGGGGT
GTCCCACTTGAAGGCCGACATCGTGAATAGGGGGCAAAAGTAGATGCTCA
ATTGGTTAGGGAGGTAGGTAGTGTTGGAATTGGGGATGATGTCTTAATGTAT
TCTGTGAGTGCTATGGGTTTCTTTTCTCTTGAACTTTGAGGATAGATCAATG
AGTCTCATTTATATACATATTCTCGAACTCACCGACGGTCTATAATTTCCATT
GTCTCCGTCTTAGCCAAGCTAAGATTATACTATGATATCGTCATGGCGTGGTT
TCTCATGCCATATTAGAAATCCGTTAACTACGGCAGTCCAAGCACAGCAATG
ACTTAAAACCATTAACGCCGGCGGTATGTAGCATTTCCCCATACCACATCGT
GAAAAACACCTACTAGACGGTCGCGGAGAGGGTGAACGACACATAATTTC
CCGCAGCTCTAATTGGTTCGGGGTCGTGATGGTTTCCATCTTACCGATTATA
GTGAGAGATCGTCTCCACATTTCTCCGTGCTGCGGGCTGACAAATATAGCAT
GCCCCCGACTTATCCACATGAATCGTATTTATTCCGCACCCATAGGGCTGTT
CTAGATCTTAACTTCTTTTAGCTCATTTCCGCAGATTGCGGAAGAATTCTAC
AGTGTGCGTTACGTCGAACGGATCGGCACTTTGAGCACCCTATCACGACTG
CGGATCTGGCTTCACGATCTAAGCGTATAGATCACGGCAAGCATACCTGAA

ACATACTTTTACCTGGACTTACCCGTTGAAGCATGGCTTCGGTTTGGAGAA
GGGCTTGCACGAACTGCACCAAGGCCAAGAGACAATGTACGAAAGGTTTG
CCCATGTGCCGACGGTGCGCGGATAAACGCATCCCCTGCGTATACCCACCA
GCGCGGCGAATAGTTCGTGCGGAAACAGAGGCAGCGATGGCGTCAGCATT
ATCAGCATTACCATCAGATGGTAACGCGTCCACGGACGCGCCCCCTATGAG
TCAGAGCGAGGGACACAACGCGGTCTCAGGTGATCTTGGCTTCCCCCTCTC
ACGCTGACGCCAGCGGAAATAGCCGGGAGTGCTGTGGGATGTCGCGCAGC
AACACCGCCGCCAATGAGAATAGCGGAAGAGCCCCAGTCACACCCGCAAC
CGCTCCCCGATGCTACTCCATCCTCGGACGACTGGTTCTTAACGCCGGAGT
CTTGGTCCGCGGATTTTACGCCACCTCCGGACCAGCCAGCGGCCGTGATGG
ACGGCGTAATCCGGCGGTTTCGTAGAGAGCGTGCAAGACTGGCTACGGCG
ATGGGTAACGGAGGGCAGCTCCCCACTACACCACCGGCATCTCTACCGGGA
CAAGATGCCCCGGCACGTCCAAGACGCCTACACCGCAATCGCCATGTACCA
CACCCTCCCCCCCAACACGCCGAACCCCAACTTCCAACGAAAACCCAGCA
CAACGAACGACGCGCGCGTAACCGCCCACCGCATCCTCGACGACCGCGTA
ACCCAGCTGCTCGAAGACCAAGCGCTGAGCTCCTCTCTCGGCCAAACGCC
TGATATATTCGATCACCTGAGTCGCGTGCAAGCGCTGCTCGCGTACCAGAC
GATCCGCCTCTTCGACGGCGACGTGAGGATGCGGGCCGCGGGCGAAGCGT
TGATTCCTACTTTGGCGCAGTGGTCGCGGCAGCTGTTGGACTGTGCCAGGG
AGACGCTGACGCGGAACGCGAGGTACCTGCATGAGCTCGTGGACGCGGGG
TATTTTACGCGGAACGAGTTTGGGGGTGGGGGGGGGAGGGTGAGGTGTGG
TTGGGATTGGGATAGTCCCGGATCCGATTCCAGCACTGGGAGTACGGAAAG
TGAAGGAGGAAGCTGTTCCGAGGAAGGACTATGGCGGGCGTGGATCTTGG
TCGAGAGTGTGAGGAGGTCGTGGTCTATCGCTACGTACATACAGGAGATCT
ATCTGTACATGAAGCAGGGCTGGTCGGAGTGTCCTGGGCGCGTCATGATTA
CGATGAGGGGCGGGTTGTGGGATGCGGCGTCCCCGTATGCATGGTACCGTC
GCGCGTGTAGGGAGGGGGACGCGCTGTTCTTGCCGACGACGCGGACGGAG
AGTTTGTTTCGTGAGAGGTGTCCGGAGGATATAGATGAGTTTAGTTTCCTCA
TTGTAGAACTGGGATTTGGACAGGCTAGGGTGGAAAGGTGGATTGGGGAA
AAGGGTAGGGACAGTAGGAAGGCGTTGATGATGGAAGGATGGGATGAAGC
GGAGATGGCGATGTGACGAGTTCAAAAAGCAGAGGTATGTACGATACATGT
ACAGTGATAGTGGCGTCAATCAAGAGAGCGTATGGACAAATTACGGACATT
AGAAACGAATTAATAGAAGGTCAATTGACATGGTTAGGCAATCAAGGCACT
AGACTAGGTGTCTTATTAAGTGGCTGAATAACTTCACTCCGACTGGAGGCA
AAATGTCTTCCTCATGGCGATGCAACTAGGCATCGCTCACAACGAAACGAA
AGTTTGAGGACATCAAAAATTGCGCTCACTACGATCCACGAGGGATAATAG
AAAAGGGTCGACTCCGCGCTGTGCAATGCAGGTCTCCCCCTTTGGCAGCC
CTCATGCTTGAGCGAGGGTTGCCTGAACAAGGTAAGTATGAAGAAAAAAG

GAAGAAAAGAAAGTTGAAAAGTTGGGAGAGAGGACTAGTATTTATAACTT
TCGGTCGGGCGAGCTAGGGCGGTGAAGACCCGCTAAATCAGTCAATGAGT
GGCAGATTATTAGTGGGTTTGATTCAAAAAGCCCGACCAATCAACGGTACA
AGCTCATTATTGGGTTTAGACTAGCAAGACTGACCTTTGTGACCTGTGCGG
CAAGCGAAACCCATCTGAATCAATCAGCGTCTAGAACATGATATGCAGCTAT
GCATTTACTGGGGAATGATCTGATCTGTGATTGTAGACAGCTTCAAATATCC
AGCAATAGGTTGCAATTTTTAGTAGGCGGATTAATTCTTTATCGTGGCATGA
CCAGATCATATTTCATGTCATGTATTTCTAAAGCTCCAGGTCTGTCACCAAA
GATTACGCGTATTATACAAACTCAAACAAGACACCCATTTTACCATGTTATA
ATTGAAAAGCTGCTATTCTGGTGTACGGACGGTTCGACTGAACCAAAAACA
TGTGACCACCCTTTTTCATAGTCTCATAGTTGAGTATCCTGATCACCAACTC
GGTGGATGATGAATGCTACATCCATTCAATTGTTCTACTTCCATTATAACGCC
AAGCCTGCTCTTTACAGTCTTAATAGGTATCTTCTTGATCCATTAACATCACC
ATGTAGAATGGGAACCATTGAGCAAAAACAAGTAGATTGGTACCGGAGCG
GATTGTAATATCATGGTTTATGACCATGGAAATAGTCGAATAAATTGTTTAAA
TTTACTCAACCTTCTTGCTGTGGCTCAAGATGAGGAACAACTCGGGCACAA
GAATGGCAACCCAGAGTAGACCAAGAGCAAGGCCAGCCGCGAAGATCTTG
ACGTAAAGCTTAACAAGACGGACCCACGAGGCGCGGGAGAAAGATTGCC
AAGCGTCCTTGATAGTGACGAAACCCTTGCCACCCACGATGGCCGGCTCAA
CTTTACCACCGAACGATCGGTCGAAGGGAATGATAGTGTCGTCCTCGTCGG
GAAGGAGAGAGGCCTGAACACGAGTGAGGACGACCTGGGTGGGGATGAC
GAGGAAGATATGTAGGGTGAGCGACACAAGTAACAAGATAACAGCCTTCC
AGACGTCGTTGACGTCGCCCTGGGGGATAACACCAGGCTTCTTAGGGTCG
GGCAGGTTCATAGACATGGCGTACGCGAGCATCCTAGGGACATATGTGGAG
AGCTCGGCAGCGAGGAAGTACATGCAGGTAGGGAGGGCGGTAGCGTGGA
ATGCCTTCTTGAACGGAGGGAGACGCTGCCAGAAGGTCTTGGAGCTTGGG
GCCG

>Mosaic_sequence_62

ACATGTTGCAGCCCCTAACCCCCGGACTTTTTTTCTCCAAAGTGCGCTGAC
AAATTCGTCCGCCTTCGTCCGTCCGTAATTTCGGCCGAATTCTCTTAGCATC
AACGTCAAGGTGAGGAAAGGGCCTCGTTCAACCAGACGAGCAGAATATCG
ATACTTTTATGGTAATATTTTTCAATTTTATTACTTTGATTCTTATATACACTTT
TGGGTGTTTTGTGTGCGAATAGCTACCAATCATTCCTAATTGACGACTCTGA
GTGTATACTAACTCTACGACTATTGCTCCTACACACGAAAGGAGAACGTAC
CAATAAGGCGTGAGTGTAGTAATTAACTCTATCATATCTCCCAACTATACCTA
GCAAAAATGCCCCAGCTTCCTGTGAGAAGTGCAAGTAGTGGGGGAGACCC
AGAGGCAAAGAGAAGAAAGATCCGTAAAGGAACTCATAGTTGTTGGGAAT

GTGAGTTCTCATGCATCCAACCCTTTATATACTTGTATAGAAACTAAACATAC
CTACTCTTGCCCATAAGGTCGGAGGAGAAAAGACCCGTTGTCAATATTCTT
CTGCCACGGCCTCCATATGCATTGGCTGCGAAGCTCGAGGGACAACCTGCG
TAAGCCAGGAGTTTCCTGACGAGCAGCCGCCATCAGCAGAACGAGGTCTT
ACGCAGCGGATGACGAGGGTAGAGAGCATGCTCGAGAGGATAATTGAGAG
GATAACGCCCGGATCTTATAGTTCCAGTCGATCAGGTGGCCAAGCGTCTAC
CTCCTCCGCCTCGGCCGGAGATGAGCTAGAAGACTCGTCACCAGATATCCT
CCGTCCGTCGGCGGCAGACCACCCCCACGTAATCGGCATCTTCGATGCCTT
ACGCGAGGAGGGAGGATCTTGCCCTTCGGCTAACACTTCGTTTGTACCGAC
CCCAGCATCGACACAGGCGACGCGGCACCCGGTCTCCCTGTCAAGTTCTC
GCAGATCTCGAAGACACTCCACGCGGACATTCCCATGCCAACATGACATCA
ACGCCATCATGGAGGCCGTGGGGTCTGGCCCATATTTCATCGTGACTTACTT
CAGTGGTGTCGATGGCCCCTCGGAGGACCCATCTACCGTCTTAGAGGTTCC
GCCAGTGACCAGTCATCCTGCCTTGCTTGCGAAGAGACTAATGCAATTGAC
CAACTGCATGCAGAAGATATCCGTAGATGCAATACCGCAGGACCTCGTATC
AAAAGAGCCAATACGCTTCCAGATGAATAGGATTGTGACTGTAATCTCGGA
GTTAGTGGCCTTTAACGATGACCTTGTCGGTTTCTGTCGAGGGCCTCGAGA
CCTTGAACTTTATCGCCTTATACCAGTATGTAATATCATAACCCCCCAAGTCC
TAAATCAGTACCTAAAGTTCAAATCGTACTAACCGACCCCAGTGCAAACGC
AGGCAATCTCCGCAAATCCTGGCTCGCGCTCCGGCGCGCTCTCGCTGTTGC
GCAGCTGATGGGCGTAGACCGATGGCCCGACGACAAGCCCCTAAAATCTG
TGGACCCACGTTCGGACCCGGGCACGCGCACCCGTGCCAGCGCGCTCTGG
TACCGCGGCAACTTCACCGACCGGTATCTATCTCTCCTCCTCGGCCTTTCCG
CGGCCTCCGACGACGACTCCTTCGCAGACCCCCTGCGCATCGCGCGGAAC
ACCCCCGCCGAGCGATTGGAGAAGCTACACACCGTCATCTCGGGGGCGATT
ATAAAGCGGAACCTGAACACGAGCCGCACGGTTCCGGACGCCGCCTTCGG
CACGACGCAGGCCATCGACTGCGACCTCGAGATGGCGGCTAAGTCCATGG
GCGAGGAGTATTGGCGGCCTTTATGCCCTATCGACGCATCCACGGCGACAC
CACGGGAGCTTGTGAAGGCGCACACCCATGTCATGATGCAAATGAACCATC
ACCATCTCCTAATTCTACTACACCTGCCGTATATGATGCGAGACCCTAAGGA
AAGGCGTTGGGACTACAGCAAGACGACATGTCTGCACGCCTCGCGCGAGC
TACTGCGGGCCTACCTCATCTTTCGAGATCTTACCGATGCGGCCTCCGCCTG
TCGACATACGGACTACGGCGCGCTGACTGCGGCCATGACGCTGCTACTTGG
TTACCTCGATCCCAAGCTACGGGTGCGGGAACTGGAAATAGCGAGCCAGC
GTGACGCGGACCGTGATGTAGTGACGAAGGTAAGGAATGTCATGAAAGAA
ATGGCCGAACGAGACGGCGATAAACTAGCGCGAGAGGCTTCCGATATCATA
ACCAGACTACTGCCTCTGCGGACATTGACTTGACGGTGTCAGGAGGCAGC
GAGAATGATGCCCCTGTGCGTCTCGAGATCCCGTACCTCGGTACCATCAAC

ATCAACCCAGTGCGCAGGCGACCGCAACATAAGTTCCCGACAAGATAACA
ATAATATCATGCCGGCTCCGACCAGCAATAATAACGATGGGGGAGAATGCG
CTGTACTTGAACGTCTTGGGGTTTGTCCTACGTCCAACACCTACCAGGCTC
CTGATAATCCCGACAATATGGCGATGGACATGAGCTTACAACAATTTCCTCA
AACATCCCAAAGTTTCCTGTCCACGCTCGGAGAAGATGCCAATTTCTCATT
CATGCAATTTGAGCCGGACATCTCTTCTCCGTACCAATTCCCTGAGCTTGCG
GCTGATGTGGATCAATGGACTTTCCAGGGTTTCGATGCAACGTTCTTCGAG
AACCTGCTTTCGTAACAGAACTCGATGCCTTTTAGGTCTTATAAGGCCAATT
ATTGACATTTCAGATTCATATGTATATAGTTTAGTAAGATATTCGCACTTATAT
ATGTATATATAATTTTAGGAATGCCTTGTAGACGTCCCAGGAAATAGAAACA
TATGTACAAGAATTATTTTTCAATCGGACCGTTGTTAGGTTTCATGATTGATA
CTTGACTAGCAACCTATAGTATTAAGATCTGTCCACCTGTGTTTTTCGTGAA
CTTCGCTGCAACCTTCGGTTCTAGATTTAAGATCTTCTCGGCAGCGAATTCT
GCCGCGGTCACGATCATGGCCGAAGGGTTTCCGGTAACCATACCGGGGAA
AATCGACCCGTCGACGACGAAGAGGTTGTCGGTACCGTAGACTTTGGTGTC
GAGATCAACGACAGACGTGCCGTTCTCTTTGCGGCCATCGTCTGGACCCAA
CTTGGCCGTTCCTATGAGGAGTTAGTGGGATCATTATGATTATTATTAGTGAA
TACTAACCCATCCAGTGATTTGAGCGACGCTTGGCAGACGTCACCTCGATG
GCATCAACATAAGAATCTGTGGTCTGGTTTGTCTTTGGCAATTGAAACTCA
AGGTCCTTCACTGGTGCTCAAGGCTTTTCTTAAATTCTCAATACCTTGGATA
ACAGCTGCCTTGTCGTTGGGGTCTCGGAGGTAAGGTGGTGTTGAGACAAC
AGTGCTCAATCCGGGGTTGATCGTCATTCGGCCTCGGGATACCTGGCCTCTA
CCAAGGTATTGGCTCATAATCATGGTGTCTATTCAATTTAGCATTCATTTCTC
CACGTATTAGTAAGTGCCCTGATGACTTACTAGTGCTATTTGTCTGATCAGC
ACCTTCGACACGTCTGTCCATTGTATTTGACGGACTACACCGTCTGCGCCTT
CGATTTCATCCCAGAACTATCTTTCATTAGAAGTGCTTTCAAAGATGGCATA
GTCAATTAAACGTACCACTGGACCGATGTTGGGAGCAGACTGTGCAAGAAT
ACCGGTACGGTTTGCTGCAAGAGGTCAATTAGGCATTCAACTAATATAGCA
CCCATTTCTTACCAAGATATTTCTGCTTGTCGGAGGGTATTGGCGTCGTCCA
TGCCTCATAGAAGTCGTAGAATACGACATCGGGATGCGTGAAGTAGGTATC
GGTCTGTAAATCATCATGTTAGACAAGCCCGATTACTTTCTATTGATAACTTA
CGTTAACATGGTCCATCAAGTTATAACCGACGGGCAGATCAATCCAAGATTC
TTTTTCAATGAAGGCCTTTCCGTCGGAAGCTGAGGCTGCAACAATCTCGAG
TTGGTCGGCCGGACCAATACCACCTAATATAATCAGCATATCATTCAAGTATA
GATGTCGTATCAAGACATACTCCTTAAGAGGAACTTAGCCGAGCCGAATGT
CCCTGCGGAAACAATAACTCGTCCAGTGCTGGCAGTAACGTCGACGGTTCC
ATATCCGCCTCCTTCCACTGTACAGTCCAGCTCAACTCCGGTGATATGACCG
CCGGTTCTAAGAGCTCTCCTGACCGGAGTGTTCATCCAGAGGGCGAAGTTG

TCACGCTTGGCAGCTGTAGCTAGGTAGGTGGTCATGGGCCCACCTCTCTCG
CCTCCAGAAAACATATATGTCGTAGCACCAAAAGTATGGTTCTTCTGAGTCG
GGGCGTCATTTGGAATTGGTACATTCTTCCATCCTGCCTTCGACAATCCGTT
CGAAATAACGTCAAATCCTTGATGCATGTAAACTTCCCCATCTTGAGACGG
CGCCGTGGTTCCTGGGATACGTGTAAATGTGCGGTCGGTGGCTTCCCGGAG
ATCGGAGTTGTGCCACCCGCTGGGGAAGTTTTCATCCCAATCCTTCGGGTT
CGCCTAAAGCATATTAATATACAACCTCTGAAACTGCCTTGAAGACGTCTTA
CCTTCCACCATAAACCGGCATTGACAGCTGTGCCTCCGCCGAGGACACATC
CGGCCATTTGGTCAGTATCAAGACAAGCTACACCATCTGAGTCGACCCAAA
TCTGGTTGCACAGACCCGGAACATCGAATCGCGTTAGATCAGTCTTCTCAA
GCCAGAGTGGCTTCATATTTCCACCCCACATTCCCGCGGACGGCGGCCCCT
TCTCGATCAATAGAACGCTGTGGCCCTTCTCGCTCAGGCGGTCTGCAACGG
GTATACCACCGGCTCCGGCACCGACGATGATGTAGTCGAATGTTTGATGGG
CCATCTCTGCAGCACAAACAGGAGTTGTTGGCGGTGGTGCGTTATCGCCGC
ACATTCACCAGGAACGACCTTGGTTGCTAACGCAGCCCAGTCGGGGTACT
GAGCCTTCGTAATACCTGCTAAGGGAGCGCCGAATTGCCCAAAGCTTTCAT
GTTGTCTGAAAGAGATTTCAGCAGGACATTCTGGGTTAGAGGGAACAGTC
GAAGCCGAAGCTCGTCCCAAGATAAGCGTTCCAGTGCTAGTTTCGACGTG
GCCGCTAGTACCCTCTTGATCCCAGGAGAAACAATTCTGGCATCGGTATATG
ACCTCGAAGCCAGTGTCGTTAACCTTCGACGAGATCTGCGTCAGAGTTGCG
TTTCCAGTGTAACACCTGGCAAATTGTAACCCGTCGCGAAGCGGAAAGAC
GTTAAAACTTCGCCATTGTAGGGCCAAGCAACAAGCAACAAGGCTTGTGT
CATCTGCCCTGACTCGCCATGCGATACACCACACCATCCACTGGAGCATTG
CTAAAGGGATTCTTAGCAGAGCTTCGAGTACGTTACTTATAATTACATACGA
GTATGCCAATGTATTCCGATGCGTCTGTAGTCAAGGCATCCTCAGGAAGAGT
AAGACCAATTGTCTGTCCACCGGAAGACCATGTTCCAAACTCTATCCCCGT
GTCCGGATCGGTGTATGGGACAGCTTCTTGGGCAAAACAAATTCGGAAAG
CTAAATGAAATTTAGTGAATGCTATCATGCGTTAAGGATGCTAAAAAACATA
CAGGTAGCAGCTGCTATGACAGCAGTCGCAATTCGTGAGAAGGACGGCAT
CTTGAGGTCACGGGCCAAGCATCCAAGACCTAGGGAGACGTGGGTAGGAA
GCTATAGAGAAAGGGGATCTGAATGGATTAATAAATGGCATCGCGTCGGTA
GGATCGATGAAGCCAAGAAATCTCAGTTCTCGCCCGGTATTCCGGGAAGAG
AATGTGATAGGACATGATCTGCAGCACAGACATAGTATAGCTACTGTTAAAG
TCGAAGGTCTTGGCTAAAGCACCAACAGGATTACGGAGGACGATGCCGGA
GGAACCTGTATGGAAAGGGCTTAGCACGGATGATGCCAGTCTCCCTATAAT
ACAGCCTGGTATATTGTTGCAAATGCGGAAAGAGTATCGCCTCGCGAGCCG
ACTGGGTGGAATAGGCGATAAACGCCCCATGGTTGCCAACCAAAAGCGCG
AAATAATAATACGAGTCGCATTGATTCGCGGAGATATTGTAACGAGGAAGAT

ACAGGACTCAGGAGCTCATTGAGCCTGATGGTAAGCTAGCTCTTCTGACAT
CAACATTGCCACAAGTAAACAAGGTGACTAGGTAGGTAGGTAGGCTAAGAT
CCATATAGATCCATATGGTATAATTAAGCTAAGGTAAGGTAAGATCGAACATT
TGCAGATATTCCCATATGTTCCAGCATATGAAAATATCGGCTCGACCAAGCC
AAGTCGGGTGTGTAAGGGGTAGAGCAGGCGATCGACGGAAACTAAACGAG
CCCGGGGATTACATTGCTGCCAGGTACAGTTGAACTTCTGGAACCTGAAGC
CGTCTGCCAATGATAAGAAGCGGAATGGTCAGGCATTGGGGTTTGCGCATC
CGTTAAAGTTGGAGATAGGGAAGATTTTAGTTGACGGGTTGGACGATTTGG
GACGATTGCACCCCAATTGACCCCGTCTTGGCGGCGCTACGGAGTAATTAG
GTACGAGAGGGCTGAATTTTCCCCACATCTTCAGAGCTCTTCAGGGCCGTC
ATTCCACTTTCCCCCACAATCTTAAGGTTCTCCCCGCTTCCACTTCCATGCC
CCTGCCTTCCTATACTTCCTACCTTCTGTCTATGGATGGTCTATGATTTTTAA
GCATTTGAGCACATAACTTACTTCTTTACTAAACCTTGAAATCATGAGTTGG
CCGGGGATCCCATGGAATGTCTCTATCGTGACTATACAGGGGGTCGATTAGC
GAGCCATCCCCAGCCAAGTATAAGGTTAATTGATTATTGATTTGGATAAATTT
AATTCGATTGGATACTTTCCCGGTTTGTCGTCGGCGTTTCCACCTGGCAATG
GTTACCCAACCTCGCTTCTCAGCCTATATTCTCTTTGAAAAGAATCCTTCTC
ATACTCATGTAAGCGTCGAAGTCTGCCCATCCCACCTAGACCTGCTAGATCT
AGCCAACCCTTCCGAAACTAACCCGCTCAACAGACCAGTCGGTTGTGAGG
GCCGATCAAAGTTGCTATTGCGATCACAAGATCAAATCTTCATGCTATAAGT
GTGAGAATTCCTTGAATTCTTTGCTTAAGCCCTGGCTCTTTTCTCGCTACAT
ATTCCGCACCGTGAGACGAGAAGAGGGCCATCGACGGCTCGCTCTTCCAC
TCGCGCAAGATAGACTCGGAGTTCCGTCTCCATTCACGTTAGCCGTCGCCC
TCTGGAAGTTTCTAGAAGGAAAAACGGCAATCCCTTTGACATGATTGGGCG
ACAAGAGAGGTTGTGTAAAGGCTGGGAGGTACATATCGAAACATTTACCAA
ACCCACCCACGCATATAAGTGTACGTTGGTTTCCCGATACACCTAGCAAGGT
CTCGATGCTTCTATGAGATGTAGCATCTCTGTATCACAGAACCAAGATTCCA
CATCATTCGAGTGAAGATGGTTGGTGACACGTTCATTTTCATCAATTATCGA
GGTATCGTGAGTACTTTCCACGTACTAATATATATACGAGTATGCCAAGTGG
AAGACTACTAACAGCAATAGCTCGTTTATTTAATGAGTGTATATGAATGGTC
TGGGGACTTCATTATGTGTAGATGAGTCGGAGATATATGACAACAAAATCTA
CCTATGTATTATCCCAATGCTACCTACTTAGAGATATAGTTTACCTCTTAGAG
ATTCGTATAAAGGGATGTCGTGTCGGTGAATCAACAAGAATCCCCTGGTAG
ATATACTAGAAGACCTAACAGGAGAGTCTCATGCCCATTCAGATTCAATAGA
CTTTACGGAGAGATCTAGACTGCCTCATAATATCCAGTATGAAAAACACCTC
CTAACTCAACTCTCCTGGCACTTGTTCACGATCCTTACCTCAGCTTTGGCAC
CTAGTAGGAAAAGGTCAAATTGAATCTTTCAGATCACAGGACTATTCAGTG
GCGAGAAAAGCTTACTTGATCCTGATCTAAGGCGATCCTCGCTAGCTAGAT

CTCGCATCCATTTTTCCGGGGGTCCCAATGCCGAACCACTACTCGCTGTGA
GAACATCGTGCATATAATATCTATTTAGAATGTTAATTGCCCGGCACCACAAT
ACGACGCGGAATAAGCCACAATAGCCAACCACAGCAGATTTCTAACCAAA
CTGTTTAATCTAAATCATCAACAACTTCTCCTATGATGTTCAGTTCGCAGATT
TAATCCCCGTCTCCTCGACTTGGCTAACTAGCCCTCAAGTCTTAGACCCAAC
CAATCTTCTGCATTTGATGACTGGAGCCGCGGCCCGCTTCATATACAGAATC
TGGGGTTCAACGTACACAAGAACCAGCCAGAGGGGACGACAACTGCCCG
AGGAGAGAGGTTCTGGCTGTTTGTTTTTAAGGATGGCTGAGAGCACCTACG
TAGGTAGCTGGCGAAGCCTGCTACTTGCCAATTCCTAGAAACAGATTCGTA
CCACTCTTCCATAGATCATCATGAAGAACGTCGTCGCTCTCGTTGCCACAGC
GGCTCTCGCCGCTCAAGGCGTATCCGGCCACTACATCTTCCAGTCGCTATCA
AAAGGCGCCGCTAAGGGTACCGCATACGAGTTTGTCCGACGAAACACCAA
CTACAACTCGCCCGTCACCGTTTGTCGCCCTATTCCAACCTTAGAAGAGATA
ACATACCCGTACTAAGATATGACCAGACCTATCTTCCCCGACCTCCGATGC
AACGTCGGCGGTGCCACCGGAGGCAACACAAGCACTCTTGAGCTAAACGC
CGGCGACCAGTTCACCTTCAGCCTTGACACGCCCGTGTACCACCAGGGAC
CCGTCTCCTTGTACATGTCGAAGGCCCCGGCCCTTGGCTGAGGAGTACGAC
GGCAGCGGGGACTGGTTCAAGATCAACGACTGGGCGCCGATCTTCAACGG
CGGCTCCGCTTCCTGGACCATGTCGGGTAGCTACTCCTTCACGATCCCCAA
GTGCATCGCTAGCGGCGAGTACCTCTTAAGAATCCAGTCGCTCGCCATCCA
CAACCCTGGCGGCGTCCGCAGTTCTACATCGGCTGCGGCCAGGTTAGCGTC
AGCGGTGGCGGAAGCGCTTCGCCCACGCCTACGGCTAAGATCCCCGGTGC
CTTCAAAGCGACCGACCCGGGCTGTAAGTTACCCTACCCTTAGTAAGACAA
AGTACCATATTAACTATGATCTTAGACACTGCCAACATCTACAGCAACTTCC
ACAGTTATATCGTCCCGGGTCCGTCCGTATTCGCCTGCTAGACGCCCTCTTA
GCCACGGGCGCCGCCATGACGTTGTGGCTGGCTGAACAATAGGTGGGGAT
GTCCAGATGTGGCTTCAAATGATGTATTTCCCTGCGATAGATCGTGACGAGA
CACACTCGTTTTTGCCAGTATTGTACACATTATTGATTAATTGAATATATTTTG
AAACACGCCCGCATATAAGAACATGAAAAGTCTTCATAGCGTACCCCCACT
AGATCAAGAGCTGTTCCCAAAGAACTTGGTTATCTTCTGTGTCTCTGGTTG
CTTCGTCTTATTCGGGCTCTTGGTCCGATTGCTAGTAGCGCTGATCTTCTGC
CTCCCACCCTTCTGCGGCGAAGGCGCTTTATCCTGCACACTAGAAAATTTC
GTCGTCGTAGGTAGCTTCTTGGGGGGCTTCTCATCAGCCGACATATCCTCAG
CTTCTCTCTTGACCCCACTTCAACTTTGCTTTCTTTCTTAACTCCGATCGAAT
CGTGTGTGACGAAACTTTCCTCCTTCTTGACTTCGAATATCTGGCTGCTCTT
TCTTCACATCTTTCTTCGAGGCTCCTTTAGCGAAGAAATTTGCTATGTTAGA
CTTGTTTTCCTTGCTATCGATGGGGACGATGAAAGTAGGCGAGTTTGTTGCC
CACTTTGCCGACGTCCTTACTAACCGGGTACACGTTTAATTCCCCTTGGAAG

GGGATCAACAGAGCCTGTAGCTCTTTTGACCATTCGTGGCGTTTGGGGTCC
AGCCACGTGCGTAAGTCCTCAGACCCGTTGTCCAATATGACCGGCATGCGA
TCGTGGAGAAAATTAAGCTGTTTGTTTGAGCTGGTAGTGATGATCGTGTAG
GTGTAAAGCTTCTGCTCCTCGTCTATGTGAATTAATTAGCACTGTAATTGGT
CACCGGAGTTCGCTGAACTCCAGGGCTACTTACTCTCATATTGCACCACATC
CCATAATCCAGCGAAACACATCAATTTGCCATCTTTTCGCTTGACATAATGT
GGTATCTTCTCTTTGCCTCCGTCCTTCTTCAGCCACTCGTAGAACCCCTGTG
CAATGACGATACATCGTTTTCTCGCCTTCATAGAGCTCCACATGCCGCCACT
TTGAGCGAGGGAATCGTCCCTGCAATTAATCGTTTTCATCATCGAACCGTAG
TCCGGATTGCGTTTTGTCCAAAAAGGAACCAATCCCCATTTCATGCTTTGCA
ATTTGTAACTAGTAGGTTGAGAGGTGGAATCATCATGTCCCACCTTATCCTG
ACCGTCACTGTCAGTCGGGGCCTCATTAGAAGCATTGGCGTGCTGCCTTCG
AGGACCGGCTCCCAATCTGGCGCGTCTGCACGGTAGGACAATGCCGTGGT
AACCGGGAGCAAAATTATAGCTTTGCCTAGGTGCCCCGTCACCTTCGTCAT
CTGGTGCTTCGTCCACGGCCATATCATTATCCTGTAACATTTGGCGAACTTG
CGCCGGCCGCTATGATTTGATTGCTATTAGTCCTCTAGATCGTTGATAAAATG
ACCAGTGACATACCAAGGCTAGTGAATAGCGTCCACACATTATGTGATGTG
AAAGCCGCCAAAACAGATGGGTAAAAAGAACATATTAGACGTTAGAAAAC
CTGATGAACTAGGTGTCTAGTAATTGGGCCTTTTGAAAGATTTAGTTTTGTC
AAACGCTAAAATCTAGAAAAAGCGAGTCGAGGTTAATTAAAAGGAGCTAT
GACGAAATGATTCTACCATCCAACCACTTAATCGCCAAGTCAGGTGATGATT
TCTTGAATTCCCAGTTCCACGGGAATCGGGCATATCATCGAACATTCAATCA
TCTCAAGTCATTAAGACGCTTCTCTTGTATCTACAATTGACAATATAGATCAG
CGAGACAATTTGGTATGCGAAATTTATGCTTTGGCATTCGTGTTTTATCTTTG
GGACCCTACGACATACTCGGCTATTCCGTTCTGGCAACATAGAAATTCGAC
GGATTGTCGGCCATAGTTTGAGCGGGAAAACGGTCACAAGTAGTATAAAGA
CGTGGTAATTCATCATGTTCTGTATGATTCATTTTGCAATCCCCGTACCACGC
CAAGCCATGCAGAAACCATTCCCGAAGCAGCTGTCTCCCAGTTGAAGAGT
GCTTCCATGGTATATCCGAAGAGACGTAAACGCAAGACCCAAACCAACCCG
ATTTTTCCCTTAGACCTTGAAACCACGAGACCGTCTGCGACCACGGGCACG
CTCGAACTTGCGGCCCTTGGACTCAACGTAGGGTTTCTATGAAGTATTAGC
ATGACGCAGAGTAAGATAACTTCCAAATAACGTACCTTGTGCTTGTGAGGG
CCCATGCCGAAGTGCTTGACGGCTTCACGTGCGTTCTTGGGCCACGGAGC
AACAGAGTGTTGCTACCAGTAGGGGATCGGAGGGCGAGCTGGTCGAGGGT
CAAAGCCTCACCGCCGGCAGCGACAATGCGAGCACGGGCGGTGGCAGTG
AATCGGAGAGCGGCAATAGAGACCTTGGGGACTTCGAGAAGGCGTTGTCG
TCGGTGACGGTGGCCAATGACAACGATGATCTTACCCTCCTGGTCCTTGGA
GAGCTGGCTCTTGACACGGGAGAGGGAGACGGGGGGTCGGTTGGTGCGC

GACATAAAGAGGCGGCGGAGGACAACCTTGTTGAAGCTGGAGTCCGTTCG
GCCTAGATATTAGATGCTAAGTTAGCATCGTGTCTCAAGTATACGGAAGAAC
GCAAGTGAATTGTGAATGTGACTTACGGGCCAAGAAACGGTAGAGCTTCA
CCAAGAGCTTGAGGTAGACATTGTCGCTCTTGGGTGCCTTGCGGTGGGTGC
CGCGGACGTGGTGGCGGTCGAGATCAATACCTATTCAGAGAAAAAGCCAT
GGTCAGCTTTTTGTTCAAATATGCTCTTTCAGGCGCATCTCAAAAAGATGGT
TCCCGATTGTTCGCAGACAGCGAGAGAGACAAGTTGCATTGTGACAGCTT
GCCTCTCACACTTCCACTCGCATAGACATCTCTAAATAAACATTAAACGTAC
CCATCTTGACGGTGACTCTTTTCTTTGAGATAGAAAGTAAGAGAGTCGATG
GGAGGAAGTAAAGCTGTTGTGCTGTGGGTTAGTACTGACTGAAGATGGAA
GAAAATCTCGAGTGGACCGAAAAAAAGTTCTAAATCAAAAAGACAGACGA
GTTTGTGCGGGTGGGCGGTGGCTGCCGGCAGTTAGGGTTGGTCAGTGGGG
ATCCACTTCCGTTTGAACAGTTTTAACCCGCTTGGCCCGAGCGCCAGATAA
CTTGATTATGTAATATTGCTGAATAGAGTCTCTCAAGGTTCTAAGTTGATACG
ACGGGAGCTCTTAAGACTTGTAGGTTTCGAAGTTTACAGCTATTTCACAGA
TTCTAATAGGCATATAAGGCAGTCTCAAAGCTTTCATTTTTCACTTCATTTGA
TTCTTCTATATAAAACGTTTCCTTGAAGCTATATCTCTAATAGGATGATGATCT
CGCGCTGCTCGTATCCGTCAACTCTAGCCGTACCTATGCGACGACGTGTGA
CATAAATACAGCTGCGATTACTCTGCGCAACCCATATTATCATCTCTGCTAGA
TAGCCAGGATCTTCGTGTTCCTCTATGTGTTTATATCTACCCTTGCCAGTTTT
AGTGTCTGATAAGTTATTCCGCAATGTAGCATCTGTTAGCACCGACGCCATT
CCACTTCCTACTAGGTAGGTATCGTAGGTAAGAGCCCTGCCACAAGCAAAG
TGCATGTCTAAAACGTAGTCCGAATCACAGCCCGAATGTAACCCAGAAAAT
AAAGGTATCCAGCTAGAATTCAGCCCCTTTCCCTCTATAAGACCACCGGTTC
CGGCTTCAAACCGACCAAGAAATCTCTGGTGACTTCGAACTAAGCCAGTT
GGCAGCCATGCTAGGAGCCACCTTGAGTAAATTCGGAATAAGACCCTTGTA
CAATCCACGCGGGCCTTCTTGGGTTATTATCACCTTGACAGCGTCTCCTATC
GACTTGTACTTATAGCCCATGCCAAGACATGGTATTAATCTGGAATCTTCGT
CGCAATACGTCGCTACTTATATGTTAGTGAATTGTGAACTAGGATACGAATT
GATCAGAACACCCACAATGGATATGTGCAAGTCTGGGCAACAGCCCCAGAT
ATGGCACCAGCAAGGAGCTTTCGTGCCGCGCTGGGGTTTTGATCACCCTCG
GGAGTTAGAACTTTGCGGACGAATTCGTACGTCATGAAGTTCAATCCTACC
TAGGACATCGTCAATAACCCTTGTCTATATCGAAATAGCGTTGTACCTGGAA
AACAACTCACATATGAGCCACTCCAGCAACAGTAGGTATTATGCCACGATAT
AATGCAGGCACACCGCCCTCTTCTTTATACATCACTGCCATCGTAGTCCACA
TTCCGGGCAGTTTGTCTTTCGATGTGTGACCGAGCGCAGCAAATGACGCCG
ACTGAATAGAACAGACGGGTTCGCACAATGTCGAGAGGGTACGTTAAAAA
AAACCGAGGTGATTCCCGCGATACCACCACAGATCAATCTTTCAAAGGGAG

ACAGAGGCGCTCCTGGGTACTTCTCGAAGAAGTGCTACAATCGTCGTCAGT
TAGCTTGGAATTACCTCTCTTCAAAATTGCCAAATATTACAAATGCCCCAAA
TTCAACCGTGCCAGATATAGAATTGTACGTGACAAACTTACCCGTTTGTAGA
GATTGTAACTACCGAATTGTACTGCAGAGTAGGGGACGATGCGAATACAAT
TGGTTCCATTACCGGCCATAAAACCCCTCCAACCCTCTTCCCTCCACATCTT
GGCAAGTGCCTTGCCCACTGATAACTTATATTCATTGCGACCAGCACTTTGC
ACCTGGAAAAGGTTTTGAGACGTTCGAGTGGGGATACGACTGTTCGCGAA
ACGGCACCAGCGACGCCGCCAGCGCAGAAAGCAGCGACTACTGGTTGCG
AGATGAAATCGTTGAATTGGCTGCCTTTGCTTCCATCGGCATATTTAGGGCT
TGACGTCTTCCGAGCTCTAGAAGATTTGAGTAAGCTACTGCTATTGCTGCTA
GAGCACGCCCTATTCGGCTTTCCCAATTCAAAAGGCGCGCTATGGTGGAAA
CGAAGGAGACATACAGCAGCCATGTTGCAACCTCAGCCGTGCGAAAACGG
TAAAGTCAGCCGGCGCAATGGAAGGAGACGGACGGCGTGTAACTCGCACG
AGGCTGAAGTAGGGGGTCGGGAATTTTTGCGCGCGGCTGAATACTTGTGCA
TGTATATCGGAAAAGTAAAAGATATTTTCGGGTACGACGATTATGGCGATAA
TGTAATTTCTATCGAGAATAACTTGACGATGCGGGCGACATAGTCGAGCTTG
TAAGACGGTTTATCGAGGGCAAACATAGTGACGGCGGTAACTGCGCAGGA
ATCCCAAAATAACCTCCAGAGGGTGGTTTGCTCAGGCGCGTCGCTCTACCA
TCCGTTTGGTCAGATCATCGAATTGACCAAGGTACATAGAGCGCTCATGATC
GTTCCGCCTCTGAAATATATGCTACAAATCAAGACACTTTATCGCGGAAGAA
GAAAAGCGAGCGTATACGATATGAATATGCTACTTAAAAAGGCGATACGAAT
TAAGAACTTGGAAGACCAAAGGGTGAGAAGAAGTAAAATAAAAAGCTTTA
GAGCTAGAAATTTTAAGCTACGAAAGACGGGATGGCAGACCGCAGTTGCT
GAAGTTTAAGTGGTTGAACTCGGGTAGGAGGTCGGCCAGTCGGAACTTGG
AGAAAAATCTGGTTCTAATCTTGGGGGTCTTAAGGGGGCAGTCACTCGCTG
AACTCACCTGAGACTTTTGTACTGACAAATTGATAATTAACTGCAAGTGAC
CCGTAACACCTTGCTATTTATGCATTCTTAATATTAAAATGTCAGTGGCCTTG
GAAGGAACTGAGAAGACCTAACAGATATTTGGCAAGGACCCATATACATAT
TACGTATTAATATTTCACGGGCATTTAAAATTTACGAAGGCGTTTAACTAAAT
AATACTAACAATAGAACAACCATTAATAGTTAGCAGTTTGAAGCTTCGAGC
GAAACGCCAATAGAAAAAATCTAGAATGTCTACCTATAGGGGTATAAGTTA
AGTGATAAGTATAGGTACCCTCCAACACGGTAGGGGTAGCATAAAATCCAG
TGGTTCAAAAATACGTTGAGCATTGTAGAACCTCACTAATAAAACGTCCCT
CGACTTGATCCTCGGCATTGCGGCCAGCCCCGTAGAGCTTAGACAAAGCGG
AACATTGAAGCATCGAACAGACCAATACAGGACTCGAGTCTTGCGGGATC
AACTCTAGGTTTGGAGGATTATCTGCATCAAGTACGACAGTGCACTGTAAG
AGGACGTATTCCTCCTTACATCCGTTTGGATCCTTGTACTGTTATGACGTGA
CTAGACATAAGGGAGGTTTGGTAAGATTGGGTACAGCTGTTGCACGCTCTA

GTCTGTGTTTCCGTGACAAGGCTGCAGGCGTTTGAGGCGCGCGAATAAAC
GAGTCTTTTCCGCCGCTGGGCGGGACCAAAGGAGCCAGCCTGGCCCTGGA
TGCTGACCGCAAGGCGAAAACCCTCCTCGCTAAGCTGTACAGTATGCTGGA
TCGAGCACAACTTCCGTCTGCGTGACCCCTCTGTACTAGATCATCGACGAG
CTCCTCGCGCCATCCATCTTCGTACCAACCAAAAGCCACTGCAGGTCATATT
TCGACGAGCCCTTGATCATCATCGGTCGAGCGCAGCGCGATGGGGTGGCGT
CTGGGGATGACGATTCCAGTCTCGCGCTTCGTTACCACAACGAGCAATACT
GTGATAGGTAGGGACACCCTCCAATACCCCTTCAGTCAGCACTATATAATCA
TTTGTTAGAGTGATCGCATTTGTTAGAGTGGTTTTTGCATTATGCGCCATAGT
TTAGTGGTCAGAATTCTCGCCTTCCAGCTTCGTGCGAAAAGCGTGAGACCC
GCGTTCGAGTCGCGGTTGGCGCACTCTTTTGTCTTTTTTGACATACCTCTCA
AGCCCCTTTAGACGTTGTTTGAACCTACCAGGGTGATACGCGCATCTTGCTC
GATTTTTACACCCCGCGAGAAGAGGGATACGATTAATCGATAGCGAGATAG
CGCCTTCGAAGCTGTACGAAGCAAAAAGAGCGGAATCGAATCACGTGAGA
CAGCGCTGACGCGTGTATTGGATTCGAAAGGGGAGAGGAGTGCATTCTCTT
GTCATCTTATCGACGACACACGTCTTTGCCCGCCAGCATCTCCATAAGGCTA
TCGGGGACTCACTTGTGCCGCTCAGATAGACCTGGACTCTACGGCGGTGCG
CCATGGTGGGCTTGACGACCGCCCAGGTGCGCTAGAGCTGTGAGACGGGA
GCGATCAATGCTGATCTCAGGTCAACGAGCTGTCGGGGACGAATGTGTGA
GATCAGCTGGGTCACTGGAAGGAGACGGGGCTGATGAGGGCAGTGTGATC
CACCCTTTGGGGCTGCTCTCGGTGGTAGATCATGCGTCGAGAGTACATCTG
CCGAATAGAAGAGAGTACTGGGCGTATTGCTGGGGCAAGATAATGGAAGC
ACCATCAATGTTGCCAACTCGCGCGCTCCCTTTCTGTGCCTCCAAGGTCAC
GAGCTGAGATCAGGTTCGCCGTGCCGTTCGAGGAGGATGATCGGGATCCCC
GGACGTTCTTCCTGGATTTGGATTTCGTCCGAGGAGATGTGGCGCATGTTC
AGGAAGGTCAACGGTGGGTTTGGTCTTCTGGTTATATGGGGCACACGCGGG
GGCAAGGAGCAAGGGCTCGCAGCAACAAGAGCACAGGGAGGTGGAAATG
GCGCAGAGCTGACATAGTAGCAAAAGAACGACCTGTTGGCTTTTATCATAC
CGGACCTCGTCTAAGGTCGTCCGACCTGGAAATCACAGAGTTGTTCAAGC
GGTTCTGCCCAAGGCCGGTAATGGTCATTGTGGATGTGAGGGCGTCGGGAG
GGAGGGGAGACACGGGTATCCCAACGGATGCGTACTTTGCAGTGGAGGAG
ATTCGCGATGTGAGTACCTTGTTTGAATGCGCCCTTCGCCCTTCTTGGCCAC
ATTGTCCCCGTCTTTCCCCTCGCGCGAGGCCAATCGCACACCCCCTGCAGC
CGATCATGGGGTACCCCGTGAGAAAGACAGAACCTGACGGATGTAGGACG
GCACGCCACCCAGCGCACATTCACCCATATTCCGACCATGATCGAAGCAGA
AGAGGCCGAGGAAATGGCGTAGAGCACCTGTTGCGAGACATCTCTTCCGC
CTCCGCATCGTCGATCGGCGCACCGGCTGGCAACGCCGATGGCCCGACGA
CCACATCGCTCCTGACGACCGAATCGCTCGGCTCGCGCGTCACCGCCCAGC

TGAGCGCGCTGCGCGGGCTGCACGCACGCCTGGGCGAGATCACCGAGTAC
CTCGGCGCGGTCAGGGCGGGCCACTTGCCGGTCAACCACCAGATCGCGTA
TCAGCTGCAGGAGGTCGTGGGCCCTCTTGCCGCAGCTGGGGGCAGACGTG
GAGCTCGGCAAGGCGTTCAGGACGCAGGGAAACGACAGCGGGATGGTCG
TGTACCTGTCGGCGCTCATTCGGACTGTTCTCGCCCTGCATGATCTGAGTGG
GTCTCCCTCCCCCCAGCTGCCCCGCTCCTCGGTAGGCTCGTAAGGCGCGAG
CAGTGTGCTCACGAGCAGTCGAAAACCGTATCAGCAATGCCCGACAGGAG
CTGGAAGACGCCAAGTCGCCGGAGGAACGGGCAAACGAGGCGAAAGCTC
TCGCCGCCGGTGTCAAGGCGGAAGATGTGGAAAAGGCCAAGAAGGAACA
GGAAAAGGGAAAGGACAAGGATCCCAAGGGCCCGTGAGGGACGGCTGAG
GGAGCATGCGCATATGCATACGAGTCATGTTTTACACTTTTGGATTTGGTCC
GGTACAATCGCCCCTCACGTTCCGAACTTCACATAGCCAAAGGTCATTGTA
GAGGCTCACGACTGCTTGTCAGCGCTTCGCAGCCAATTTCCTGAAGACCCA
CCTCGACGACAGCTTGTTGAGGTGGATATACACGTCCGTTCCGTACCCCTC
CATCGAGATCAAGCGAAGGTCCTCCGCCGAAGAACCGGGCGTACAGGCGG
GAGAGTGGCAGTCCATATCCGAATCCAGCCATGGGCGCTTTGAAATCCCGA
ACTTTCGATGTTCGCCTCGAGGCCCTCGTCAGACATGGTGGTGTATAGGTAC
GTCCAGATCATCGGGATGGCCGAACGAGGAATGCCGCCCCCTTCGTCGGA
GATCTTGATGGTGATGTCTTCTTTGCCCTCGACAACGACGACTTTGATTGGA
GGGAATTGGTCCTCGTTTTCGACTCCGTACCGCTCCACCACAGCTCGCAGA
GAATTCTTCAGCAATTCAAAGCAGATGTGCGATCTGGGCTCAGCTCAGGTC
GCACAGTGGAAGTGGGAGGAGGTAGCTTACAGATGGCCCGGGACATAGGG
AAAGGTGAGGTTCGGGTCGCAGAGCAGCTGTATTGGCGGTCCCTTGAACA
GACCGTAGTGCTCTTCGCAAACGAAACGGGCGTTCTCAATTGCCTCGTGGC
AGATATCGTGAACATTCTGTCATATCAGCTGGGCCAACGTGCCAGATTTGAC
TGACGGGCTCGTTTGCAAATGATACCGACGTAGTCTGGATGCGGTTGAAGC
GTGTTCAGAGCGATATCTGGATATCAGTCAACGTCGCCCATTTGGCTACCAC
ATCACAGCTCACGTTGTCCGATCAGAAATCGTATACCGATTCGCGTCATGTA
AATCGATCCAGCCATTCTTGCACACCTTGCCCTATCCGTCCCGTCTTCTGTT
TCCTCTTCCACTCCAGCACTCCCTGAGCAACGGTAGTCACGGTAGGATCGT
GCCTCTTTTTGATGTTCTGAAGGAGATTCGTGAAACGATCATTATAATCGTG
CACCTCGGGTGGGTAGAGCGTGTTGGGAGGCGGTGGGGAGAAGTATCTGC
GCTCGATGGGTATTCGCATACGTCCCGCCGAGCCTTGTGGTCCGTAGGTACC
GTTCGTTTCTGGCGGTTGCAGAAACGAGGAGGAGAGGGACGACGAGGGG
GAAGGGAGTTTGGAGCCAGATGGTCCCTCGTTCATGAGTGGATCGAGAGA
TGGATTTCGGGGTGGCGGAGGGAACGGAGCAGCCTCTGAGTGTCAGCCGT
GCGGCAAAACAGCGTAAGCGCTGACGGACCTGGAAGGCGGCAATCTGAGT
CGTTCCGCGAGATCAGGGGGCAAGACGGGTTTGGGAAAGGTGACGAGTTC

CTGTTGGTCAGTCGTCGTGCGGGCTTGCAGTCCTGACCGACCCACCTCGAA
GCTCTGCGCGTACCATTCTTTGACCTTTTCTATACTGGGCATCTTGTCCAGC
CCATCCGGCAGGCTCTCGAGCTCGACCACGCGATGACTGAGACGGATGGG
CAACTCTTCCGAGAGGAATTGGACGCTTTGAGGAGCGTGCCTTGTGAGGG
ATTTCTGGCCAAAGAGGATCATCTGCTGCAGGGATACTGCCAATCAATATGT
ATCCCGTAGAAAGGGCGCCAAGCCAGACTCACCGCCTGTTTGGGGGAAAG
AAGCAAAGTGGTGTATGCGATCCCATAGCGCTGGCGAGATCTTGAAGCGGG
ACATTGTGTCAGGGGTGCGTTGGGGTAGCGGGAAGAGGAGGGGGTGAAG
AGGCCCTCTGTTGCGGATTGTGCTATACTTGCTCGTTTTACGCTGCGAAGCG
AGAGCCAGTCGCGTCGAGTCGGGCCGAGGCGGGACAAGCAGCAAAGAGG
TGACGGTCGATCGATGAGGTCTCACAGCTTGTTGTGGCTGCCTATCGGTCT
GTCTGTCCGTTTGACGACCCTCTGATGTTGTCTTGGAGTAGGTGTATGAAC
GGATGGAAGCAAAGACAAAGGTGAGATGGGTTTGTACGTGGTGTCGAGTC
TGAAAAATGGTAGCCCAGTCCTATTGGGTTCCACTGACCACTCGCTACTGA
CCCGGTGGCGCTATTCCAATTACCCGGTGGGACCATCGGGACATGGGCCCG
ACTAATCCCGATTAGCATAATAGTATTCCCCTTCCTAATTGAATCGCGGTTGC
CTTTTTTGCCTCCGCCACCTCGCCCACACACCGGCGGCGAGCGGGCAAGA
GATGATGACGACAACACCACTTACTCCGACGCCGACGCCGCCTTCAATGTC
GACAGCGACACTGCGGGCACGGATCGCGCCTCATGTGCTATGTCTCGCTGC
GGTGATCGTGGCCTCTCGCCGGTTGCAGGTCAAATGGCACGCCACCCGAG
CGTTGACGCCCCCCGCAATCTCTGGCTCCAATGACTCTATAACTGAGGCGA
TCAATGAACCGACCGCCAAGCTTCCACTATGGAGAAGGATCAAGAAGAGC
AGGTGGTTTGGCGCGCTCTTGGCAGGGTTGGCGTCGGCATCGGCGCAGTTT
GTGGTGATCTACCTGCTCCCAAAGTACGGTGCGGCTTGGATGCTCGATCTG
TTGTGGGGCAATGTCGAGGCGACGCTCAACGTCTTCAATTTCAAAAAGGC
GTGTGATTACTTGGACCAGTAGCGCTGGCTGAAGGCTGATGCGTAGGCTCT
CGAAGTGGTACTGCCCAGCCCGACAGAGATATCCTACGTCCCCATGCCTCG
CTCGGGCACGTACGCGCTTGCCTCACGCGATCCAGACGGTCCGCATGATCT
ATTGCATCGCCCGAACACCCGGTATATCATGTCGGCCCTGCTCACTGGCTTG
TGCTTCTTCGCGGCGATGCATATGACTCGGAGTGGTCAGCAGTGGCGCTGG
CCTTGCCCGGGGCCGCGTTGTGCTCATGCTGTCTCAATATCACTTCCAGAG
GATCAGCACTCGGCGATTCGTCCTGTACCTTGGTGGAGCTTGCGTATGTATA
TCTAACACTGTTCGGGTGAAGCTAATGCTTAGACGGTGTTTATTGCAGTATT
CGGCGGTGTGTGTGGGGTCTTTTGGCTGCTTGGCAAACCGATGAACTCGCC
CCCGCCAGCGGACAGGGCTGTCCCGAGCAAGTGGGTGCAGAAGCTCATGG
AGGACGTCAACAATGTCTGGGGGTACATCCCTCCCCTCTATCTGGTACGTC
ACCCCTGTCACACATCCTTTTACGAGCAGAGCCACACACTGAATGCAGGTT
GCCATGAACCTGCGCTACGATCAGCGCCGGGTGCCCACTGCATCCAGGACC

GAGATTGCCACGCTTCCCACGGTCGGCCCATCCCTGTCGTCCACATTCACC
TCGCCGACGTTCATGGCCTCCCTGCTCGCTCTCGCAGGATCAATACTCGTGT
ACGGATTGGTGCCCAAGGAGCAGGCAGACCAGGTGGGGCCATTCGGCACG
GTGCTGATCACCGTGCCGATTGTATGTGCTGCCTCGGCATGCCTACCTTGGA
TCAGGGGCGAATGGGCCGATTGGTGGCGGTACGAAGAGATCTGGGAGCCG
GGGATCCTGAACGAGAAGACGTCAGCAACGGATATGGTTCCGCTCGCGGG
TCAGGATGAGGAGAAGGTCGCAGGGCGTGAGGGCAACGGTGTAGAGAAG
ATGTACTCGTAGCGCTTCAGGCTTGTCTGTGACGAGCAGTCATGCATGCATC
CCCCTCGAGACACTACGAGTATTCGAGTAGAGTAGCAAATCGGGCGAGTG
ACTGAACTTTAAATTGTCCGTGTTTCTGAACCCTGAACTCCGTCTCCCCCTT
TCACAGCCCCACTCTCTTTCTTCGATCTGTCTATCTCTCTCTGTCTATCTTTC
TCTTCCATCCATCCCTCATCGATCACATCGACATTATGCCCACCGTTCTCGCC
GACGTACCCCTCGACACGGTCGTGTCTCGCTTCCTCACCGTGGCGGGAGTG
ACTCTCACCTCTCGCCGCCTCCTCACCCGCTGGAGAGCCGTGCAGACACTG
GTCCCACCCGTTCACTTGGCCGAAGCACCGCCCGCTCCGCCTGTCAAGAA
GGGCGAGGAGAAGCCTTCGGTATGGCAGCGCGTGAAGAAGAACAAATGTG
CAACGTTTACATTGTTCTTCAGTATCATCATGTCGGTGGAATTCGCGGTGGC
GTATTTGTTGCCAAAGTACGGGACCAGGCTGGCAATGGACCTGTTCTGGGG
CTCGCTTGAGGCGCTGCTCTTCACGCTGGTCTTTTACAATGTGAGTCTCTTC
ATCCGAAGACCTACCTGCGACTGCGACGTGTCGATCGATGCGCCCGAGACC
GAGGATGGTGTAGCTGACTCACAGGCCCTCGAAGTCGCCTTCCCACGTCCC
GTCGAATTGTCTTACACACGCCCCGATCCCAACGGTCCATACCTCACTTCG
AAACGCCAGATCCACACCCCAGAAGACGGGCTTGTCTCGCGCAACCCCGT
CCGTATTGCTCTGGCTGCCGCAATGACCGGCGCTTGCTATGCGGCCGCGAT
GACAGGTCACTACTGGGTTCTCGGCACGATGTCCGCTGCGTGGGTCATGAT
GATGCTTCACAACATGACCTACCCCAATCTGCACACCAAGTGGGTCGTTTG
TGGCATCATGGCCTATGTGGTGAGTCGCGCCATCAGAGCAAGTGTAGTTTG
ACTAACCGCAGGGTCTGCTGGTTGGCGTGTGCGCCATTGTCATTGCTATCGT
CGAGATCGTCAACAAGGTCAAATCCAAGTCGCCAGAGACCCCCTCCGAGC
CTGCAACCAATAAGGATGACAAGTCGGACATGGCCACCCGAGCGACTGGC
TGTTGCGCATCATGTTGCTCATGAATGATGTTTGGGGGTTCATGATGCCCTC
TCTCTTGGTGAGCTTCATCCGTCTCTCACCGAGAATGGAGCTGACTCACAG
ATTGCCCTCAACCTGCGCTTTGACCATCGCCATGAGCCTGGTCTTGGCGGC
TCGCGCACCTCTGTTGAAGTGCCGACCTCGGAACCTGTGGGCTCGCGCGC
TCCGCTATTCACCACCTCGCTCATTTTGCTCGCGACGTGCGAGCTTGCCGTG
TCGCTGCTCCCCAAATTCCTTGCCGAGACCTTGGGCGTGATGAACGGTATC
GTCATCACGGTGCCCGTTGTATGTGCAGGAACCGCGCTGGCGGCGTGGCG
GCGTGGCGAGCTGGCCGAGTGGTGGGCGTACAAGGAGACTTGGGTGGCCT

GCTGCGCCCAAGGTCGAGGATGTCATTCCGACCGACGACTCAGAGAAGGC
GCCGAATGCGGGTGAAGAGGAGCTGGGGATGACGATGCAGCTGGAGCGG
AGAAGAAGGTGACCATGGTGTCGAGCGGGACGCTTCACCTTTTTATGAACC
GGCCTTCGCGGCTTGTCCCCTTGTCCTACCTCTCACCCGCTCACTTGAGCTG
TGTATAATGAGAATGAGAAGGTAGACACCAGAATTCCTTAGTCCATGCGTG
TACATGTATTTGTGTCTCAATACAATCAACGATCACTCGAGAGACAACGATA
TGCTGGTACAGCCCTAACCGATCCAACGATCTCCCCAGCGCACCTCGCCCC
AACACATGGCGCCCAGTCCAGCAGCAACGTTGGCAGCTGTCTCTTCGCAC
GACCGACCTCTTCTCACCTCCAACACGACCTCTCGCCCTCGTCTCTCCCCT
GGATGGGCCCCGCCGTGGCGGTCAAAGGCAGGGGGGCGATGTATAGCTGA
CACCACCCCGGCGTAAGAACAACGCCGGCGTGGTCCCACGGGAGAGGCA
GCCCATGTCTGCTCACGGGGCCCATCGACCGCTGCACATTGACGAGTTCAG
GCGGGGAGGGCGCAGAGGAGATCTTCGACCCGGGCGGCCGGCTGGACGC
GCGTGAACAAGTCGCGGAGCGAGTCGGGCGAGTCGTCCTCTGTCAGGGCC
GACTCTGCCAGCGCGCAGCTCGAGACCGAGGCGGATGGCGATGAACCGGT
CGAGGACGCGTGTCTCGCGGCGGTCCCCGTCCGTCTGCGCCTGCGTCTCCG
CCCTCAATAGAGACGGCCCTTTCTCGGTCGCCCCGCCGTTCGAGGAGCCAG
GATCAGTTGAGTGCGCGGCGAGCGGGAGAGGCGACATCGGGTCCGCGGTG
CACCCGATGCTCAAGCCCCGGGTGTATGCTGGCAGGTAGTGCTGCCGCAGG
ATGGCCATCGCCACCCGCCTCAGCGGCCGGCACACGGGGCGCACCCCGCG
GAACAGCGCGTAGCGCCTGCGAGGGAGCTCCTCCTCGATGGAGGTGCACC
ATCTGCTGGGCGTGGCCAGGGGGTCGAGAACGTACGAGAGGACGAGGTGC
AGGATGTGTAGCGGGAGAGTGGGGAGCGTCGGTTGTCGCTGGGGCGTGTG
AGCAGTCCCCGGGATGATCGGCGAGTACGGCGGCGGGGCGTCCGTGTCTG
CGCGTCAGCGTGGCTGGCACGGACGGTACGCGCGCGGAGAGCAGCTCACT
TATATCCATTGCTACCAAGTCGTCAAGGTGGCCATGCATCGCTTCCTCGGCA
TGCTCGTGGACCGGTGTGGATTCGCGTCCACGTGGCGTGGTGATGGTCATG
ACGTCAAGAGGCCGCTCGGCGACGTTCAACTCGAGATCGCCATCTGCGCG
AGTACTCTTACCACTTGTTCTCGTATCCTAGCATTAGCCGCAGCAACAGCAA
GGAAAGATGTATCGCCTCGCGCGCCTGACCCCCGCGCGCCCATACCGCCTC
TCCCGAGGACTCTGCGCCTCCCTGCCGACCCTCACGACGCCATCCTCGCAA
GGACACGCCACGCGCAAGTCGTCCGACCCGTCTCACAAGAACAAAGATGT
CCAGTCGGCCTCGGTCACAGCTGCAGCCGAGTCCAAGGCCTCCTCTCGTTC
TGGCAGCGGCAAGGGCACGGGGCGGACCAGCCGTTTGATGCCGCCAGGC
AGGGCGGGTCGGGGGGAGAGAGCAAGCGCGGGTCCGAGGGGCGCGGCCC
GGGCAAGGGGTGGGAGGGGAGCATGAAGGACCAGGTTGGCGGACAGCGC
GGCGGTGCCTCGCGCAAAGGTGGCAAGGAGGGCGCGGCGGGGGAGAGCA
TCCCGGAGACGATCGCCAACAAGGTGGTGGGTCTTCTTCCGCGGTCAAAC

GCGATACAGCGTGTGTCGCTCATGCCAGCGCGGCCTGCACACATCTGCAAG
CGTGATGTGGCCCACCTCGCCCGGCAGCATTCCCGACGGGAGAGTCGAGG
GGAATGCTCAGCCGACGGAGAATCTGCGTGGAGAGCAGAACGAGCACCTC
AAACACAAAGGCGCGGACGAGGCGGACAACGGGGCCGGGAACGCCGCG
AGCGACCCGCACCTCCCCTCGCAGCAGAAGAAAGAGGGCAACGCAGTCA
AGCGCTCGACGGCTCCAAGAGACGGCTGCATAGCTCTGCTAGGTCGGCCAT
GCCTACCAAGCAGTCTGGCGGGTACGCGCAGGCCATGGACAGCGAGCCCA
AGACGACCTCGGGCTATGTGGGTGGATCCCAAGAGGTACCGAGAGGTGCT
GATCTCAGAACGTTCCTCAGGAGGCCTTGCCGCCGCAGCTTGACTCGGCGT
ACAGCAAAGAGGCGGCGCCGCCTGCTGAAGAGGACATGATCCCTTCCTCT
GACATGCCGCATTCGAGCACCGCCGTGGACCCGCCTAATGAGACCCTCAGG
CAGCAGGCCCTGTCGGGCGGGTTGAAGGAGAGGAATCCACAGCCAAAATC
AGAGTTTGGGTGAGTCTGGTCAATCTCAGATTGTGGTCTGATCAGCAGACG
TAAGGGCATTGATGAGGCGTGGAAGAACAGGGGCATTTGAAGCCGTTGAG
ACAAGACGACCCCGTTTCCAAGTCACGTGTTTGAAACATGTACCGATATAA
TCACTCTGGTATCCGAATGCACTGATATCAATCCCATGCGGCAAATGATCCA
CTCCCTTCATTGAGATTCAGCTCGGAAGCTGCTCCGTCTCTGTCTCTGTGAG
AGCAGCCGCAGCGCCCTCGATCCAGTTGTCCGTCACCGTATCCCCGGAGGG
TCGATTCACTTTATCCACCAGCGACGGCATCCGACTGGCCAGCCCATCTCG
CATAGCCGCAGAGAATCCCTCCATCTACCCATCAGCTCAATTCAGACGAGA
TGCGTGATGCGTGCGTTCAGCTCACATCTCTAACCCCCAGCGCCCACGCCC
ACGCCCCATGCGCCGAGAGCGGCCATACTCCCCACAAGGGTGGTGGCTATT
CCGAGAGCCTGAAATCCGAGCAAAGGGCTGACCCCCCTCGGTCTCTCCGT
CTCTGGAAGGAGGTGCTGTACCCGCAGACAGTGGCGAGCCGAAGAGAGC
CGTTTCTTCTCGTGCATTAAACTGCTTGGAGAGTAGATCTGTAGAGATTGCT
CTGCTACCAAGATGATGTTCACGGTCGGACGAAGGGTGTCGCCGTTCAATT
AGGTATCGGTTGGTGTGGAATGTCTCCTTGGGTGGCCTGGCCAGCTTGGAC
GCCTGCCTCGCATACGGAAGCACGAGCAGCGTCAGTCCGACCGTGATTGC
AAACTTGTCTCAACGATACAAGCCAAACTTGTCGAACAAGGAAGACTCAC
AGCCGCGCCAGAAACGTACACGGCTACTTTGGGTATCGGCCGGCGTTTGGT
CTGGTTGGAGTGATCGAGGTGAGCAACTGCTACACGTTCTGGTAGCATAGG
AAGCTCTGCTTGTGAGATGAACCGAGAAGGTCCCGCGTCGGCATTGAGGA
AGCGCATCGTCGCTGTATTGTGTATGCGAGGGAAGAGGAGAGAGAAGATG
GACGAGGGATGAACGAGAATGAGATTCGGACACTTTGCCATGGCCGCGGG
GCTCCGCCTTTTGGCAAATTTCCAAGGATCCTCTCTGATCTTCGAGTGTTCT
CAACGATGACCTTTCTTGAGACGGCAGGAAGCATGTCCGTAACCAAGCGA
AAGGTGTCTATAGGGCGAGACGTCGATAAGAAGGTCAGACAAGCCTGACT
ACGATGGGGCATCAGCTGAGCTGCAGATCAAGGCGGCTCGACTATCCCCTC

CCCTTGCTGCTGACGACCTCGATGGGTTCATTGATTCCGAAACGGATCAAG
GAGACTTGGACGGTTCAGACGAATATGGGGAAACGTTGGATCCGATGATAG
ACCCCCAAGACTCTCCAGCCTCATCAGATATGGAAAACGAGGTCCCAGAC
GACGACCAAATTGCGCAGAGGGAAAATGAAGGAGTGAAAGAGAATCAGC
CGCAGGCTGGGCCGTCCAGACCCAAGACGGGCCTGTACGCGCCGCTCAAG
CCGCCCCCATCGCAGCTCACCATGATTCTGCCTTCCTCCTCATCCTCATCCT
CGACCTCGTTCCAACTCAGCTTGTCAGCACTGCTGTCATCCACCATCGTCC
CACAGCAGCCCGCCCTAAAGTCTCTTCTGTCTGGTGTACACGACATGATCG
CCCATCTGCCCAGCCTACCGCCCGTATCGCCCAAGGAAGCACTAAAACGAC
TCGGCAAAATGCGCGCCCCCCTCGTGGCGCCCGACGAGTTTGGATTGGGC
GACAACGTGAAGTGGAAATTAGGGTGGGAGAAGCCAGCTGAGATAATGGT
CGGAGGTACGTGGAGCGTGGCTGGGAGTTATAAGCGTGCAAAGGGGAGAG
TGGGAGTGTGGATCTGGTGTTGATCATGCCCGAGGTACGTCCTCTTCACGG
ACGACGACAGGGAGGGTCTAAGCAGCAGTCACTGTTCACGCCTAAAGACC
GTCTGGATTATCGGTACTTCCACAAGAGGGCGCATTACCTCGCTGTCGTCGC
CAAGGCCTTGTCTGAGCAGACTGCAAACCTCCAGGGCGCAGAAGTCGCTT
GGGCATATCCGCATGGCGATCGACGACGGCCAGTCGTGCAACTCTCGGTGG
GCAAGGGTACGTCTTGGATCTTAGCACAGCTGGAGCTTACTCCTAGCCCAA
GGATTGAGACATGCGACCGAAATCATCATACATGTAGGCATCCCTGCAGGC
CTCTTTCTCCCATCAGGATTGTCCCCCGCAAGATCCCTGCTGCGACATGCGA
GCCTGGACGACTCTCCGACACCACTTTATGCCTCTTCGCTCGCTCACGACT
CGTCACACAAGGCCAACTTGTTACATCTACACCGCTTAACGCAGTCTCTGC
GGGACAACCTCGTAGAGCGTTTCCTAGCTTCTTGGAGAATCTGGGCAAGAC
GGCGCGGCATCAGAAGAGAGCGCGGTGGGAGCGGGTGGTTTGCCCAAATG
ATTCTCGGTTGGATTGTCAACGGGGAGAGATCGGTGGTAACAGTGGCTCGA
GGGGGAGAATGCGCAAGAGCCGAGGATTGGGAAAGGGTTTGAACGTTTG
GGAAGCCCTCAGGGCGTCGTGGGAGTTTTTGGGTGCGTCGATCTGCGTCG
GGAAGCCATGCTGATGGCAAGCTGAGACCGACTTTCGAAAGTCGCCGGTG
TTGATGGCAAATACGGAAGAACCGCAAATACCCCCTCGCGACTTTTTGAAC
TCTTTCGGTCACGTCCTGGTAGACTTCAGCGGTACGGTCAACGTTCTCGCG
GGATGGGAAGAGGGCGACGTTGAGCTTCTGCGGCACCACGCTCGGGAGA
GTCTTGCGATGCTCGAAGAGGGCCGCGACCTCTTCCCAGAGGTCTTCCTTC
GGGACCGCACATTAGGCCTGGCAGCCTGGGACGAGCTTGTCCGGTAAGCC
GACAGCCCCGCTCGAAGTCTTAGCTGAAGTGGCAGCGTCGACATTTCTCTG
TCTGCCTTCTCCGGGTCCCTCCTCCAGCGCATTGCCTTGCCTGGACGAGCA
GATCTTCTCGCCCATGGTGCCGCCGACGTCCTTCGTCGGGGCTTGTCAGAC
CGCGCGCACCTTGTGCACGTGTCGCCCATCAACGAGGCAGTGCTAGAAGT
CGGTGTCTTGCTCAACGCCTCGCATGCCACTCGACTCATTGACATTGGTCC

AGCCTCGTCAGACGAGGCTGGTTGCAAGATCTTTCGCGAATTGTGGGGCG
AGAAGGCTGAGTTGAGACGATTCAAGGACGGATCAATCTCCGAGTCGGTT
GTATGGGAGATCACTCGTCCAGAAGATGCCACCCGCATACCGGGTCAAGCT
ATCGCCTACCTGCTGGAACGCCATCTTCATGTGACGGACGTCTCCATTATTC
ATCTCTCATCCGAGCCTGAGTGGCTGAAAATCGTTCAAGTTCCCGATTCGG
CGCGCAACGCAATCGCCTTGGGCGGTTCGGAAAAGTTGGGGTTCAGGCCG
ATGATGGACGGCTACGACTATCTATACAAGCTGCTCAAATCTGCCGACAAC
GAGCTTCCCCTAAGTATTCTGAATGTTTCCCCCGCTTCCGAATTGCTGCGAT
ATTCCTCAACCTTTGTGCCTCATCCCATCGACGCCACGCGCTTCGCATCGGC
TCCTGATTGCCTCAAGGACGCGACTATAGCCGAGGTGGTAGTGCAATTCGA
ATCTTCACCCAAGTGGCCGGACGACCTCGTTGCGATACAGAAGGTCAAGCT
TGCACTATTTGAGAAGCTCGCTCGGATCGTACTGGCTCGTCAGCCGAGCGC
CTTGGCATCCGTTGCGCTCGACCATGTGGACAGCGAGATCGAGGATCAAGC
TGCTCTTGAGATCCTCCTTCCTTCCGGTGTAGCTTTTCGCTTGAGAATTCAC
CTTGAGCGGGAAAGGACCATTCTGGAGCGAGCCTTGAGTGACAAACCAAA
GACAACCCGCGATCCTTGGTACGGCAACGCCGCAGCCATCTCGCGGCTCCG
CCATCCCCGCGCTGGCAACTCTTACAAGGCGATTTTTACACCTGCCGTCAC
ATCATTCTGCCCTAGCGCCAACGCACCATCGCTACCCTTCTATTTCGTCGGC
GACGAGACTGCTTAAACGGTGGTTGGCGGCGCACATGCTTTCGATGCACGT
CTCATCCGAGGCGTCGGAACTGCTGATGGCTTCCGTCTACCTCGATCCGGG
GTGCCTTGGGGTGCCAGCTAGCGCAGTTGCCGGATTTGTGCGAGCCTTGGA
ACGACTAGCCGAGTGGAGTTGGAGGGAAGAGCCGATTTTTGTTCCCGTCG
TTACTGCTGCGCGAGAGCCCTCGGGCAACGGACGGGTCACGTTTCCAGTC
GAGAGCCGGGCAAATAGCCTGGAGGGCTTCGCCAAGATGCGCAAGCGTGA
CGCCGAGGTGGCCCGTGGTGCATGGTGTCTGGTCACGGAGCACGACGAGA
CGGGGTTGCGCTGGACGAGGGAGCGACCGGGAAGAGTCGTCGCGGCTCG
TATTGGTGCCTTGGCGAAGGCAACATTGGCTGCTCTGCCTGCAGCAGCGTC
GCCGGTCGGTGTCAATGTAAGAGTGAGTAAGATGTTGTGATACGGAGTGGT
AAGCTGATCTTAGAATATCTTCACATCTCCTACTGACGCGTACGACCTCCTC
ATTGATCTGAAACCCTCGCTGGTCTCTCGCTTGGCGCAGGGTGTCTCGGCC
AACGCGGACGAATGGGAGTCCAAAATCCGCTCGGTAGTGCGTAACGCACC
AACGCAGTCTGTCTTCGGCTCGGAAGTACGACCCGGATTCGACCTCATCGA
GTCGTTTGTCGTCGACCTGGAGAGATTGTATGGCGAAGCCTTGCTCTTCTTC
CACGACAGGCATGGTGGAACAACAATAGGCGGAATCTGGAATCCGGCGAA
AACGCAGCCCCGAAATTTGAAGGCACTGCTAGGATACTCAACCAGACCCG
TAGCATCGGAAGTCAGTCATCGTGAAATTCCAAGGGAACGTGCTGAGAGA
CAGTCTGGGCTTGTGACGTTCAATAAGGCGGCGGTGATAGCTGAGATTGCT
CGACTGGGTGATGGATTGATCGAACGCATGCAAGTGCACGAGACGAGCTA

GGAGTGGGTTGCGCAGACGGAGCAGACATGACCC

>Mosaic_sequence_63

AAGTTGCTCGTGCATTGCCACCGTCCTCTCAAACAGAACCACTTGCCCACT
CGCACTACGCTCCCGACTGCAAACAACAACCATCTGGTGAGCACCCGTTTG
CATCTTCGACCCCACGGCTGACCCCCAGCCACCATGGGTAACATCATCTCC
GCCATTGCGGGCGCCATCAACGCCGTCTTCAGCGCCATTGCCTCATTCTTCA
TGGCCATTGTCAGCGGTATCGCCAGCGTACTGATCGCCATTTGGAACTGTAA
GCGCCTCTCCTCCAAGTCGTGCACATTCGCTGACCTGGCAGTCATCACTTG
CGGATGCTGCTCCGGCGGTCGTCGCCGAGGAGGAAGAGGCACAATATAGT
GGGTGGTGGGTCTAAGGGGTGAGCTCTCAGCTCTCCGCTATAGGTTATGGG
CTGCGCTGACGGATCAGGATCTCATGATCGCCTCTCACCACCCATTATGCCG
GGCCTTGTCCACGATACCCCGTACAGTATAATATCACCCGCATTCAGGACAC
CTGTCTGCAAGTCCGCCCGTGTGTAGCCATAGTCATACACGACATGCAACG
ACCATGATAGTTATCCTCGCAATCCCCCGCAGCAGAGTCTTTCAAGCTTGGC
GCCCCCTCCTTCCTGCCAGTTCAGCACACACACCAACGAGCTTGCCGACA
CGCTCACCATATGCAGATTCGCCTCGATCTTATACGTCCTTCCTACCTCCATC
CCAGCCATCTTCGGCGTCTGGTCCTTTCCCAGCTTGTCGGCGGCGTAAAGG
AAGACATACGTGTCGTTCTCGTCAGCACGATCAAGACATTTGCACTGAACC
AAACTCACTAGGCTCAACACCCTTGATCTTTTCATACGCCGAGAAGACCTT
CCCCAACAGTGTCGTTGGCTTGACTACAAAGTTCGTCTCAACTTCCTTGTC
CTGGACAAACTATGATCAGGAAGCGAGCTCCAGCTGGCGACTAGCTCACA
GTAACCACGAGCTGCACCTGGATGCATTAGCTTGGTTGTCGAGGCATCCAC
GCTCACTTTTCCACTCGACCTGAGAACCTCGTCTCTTTCTCAGAGTCGGAC
ATGGCAATGAATCGTACTTAGGAACTTCTGCAGCGAGAAGAAGCAGAGAT
GAAGAGGAAGAGGACAGATGTGCATGGCATGGAAGTGATGAGATTGATGG
TGATCAGGGTCGGGCATGGACCGTCCGGGTGGCCCGTGCTCATTCTGAGGC
TTCTATTGACATTCGACACTAGACACGCAACACGAAACAGCAGATACAGCA
AAGACAATACGTGACGATTATTGTGAGGCCCTTCATCCGCCTGTCTGCGCG
ATCAGTGCTGCACTCGCGGGCTAGTCAGGGCGTACCGGTTGGATCATCGCG
TCAATACGATATGTCCCGCCAGCTCTCATGTTGCAATCGGCAGGCGTCCTGA
CGGTCTGCCACCCGGCGACCACGGTGCAGAAACCGGTAGACTGCAGAGTC
AGCCGTGAACAAAGTCAGGCAGCCCACCTCCGCGAACACGCAACCCGCG
CAGGAAGCTCTCGAACACCTGTCCAAGAGGATGCGTACGATGAACCCGGT
ACTCGATCATCTGATGTGCTTGGTCCAGGGTTGCCTGCGCGTCAATCGCAG
CGCAAGGATGATGCAGCCCAGTGCACATAATCGAAAATCACGATCTTGACC
TGCAAGTCAGCATTTGACCGAATGACACCGAAGCTCTTGCCCTCTGACTGC
TTCTGAGTTCCGGGCATTCCGCCTCGGAGTCCGGATCATCTGGGTTTGGGA

CTTCCCGCGCGCTCAGATATTCAGCTTTGGGCAGATTCGAGGCTGTTGGGG
AAACATACATCGGCGTGATCACTGCGGTGGGCGTGCGCGAGGGTGCGAAC
AGCGGGCTCTCTGGGACCAGAACATCCCGTGTAGCTGCCGCACGATGCTCT
GACCGAATGGGCGGGACGGGCGGCCGTGGGGGCGGGACTGTGCGGACTG
TCGCTCCTCCGACTCGGAGCTCGTACGTCCTACCTATCTTCATGCCGAGCTT
TTCGGGCCAATCGCTGGGCATCACCCTTGCTTCGCGCCAGTGTAGGTCGCA
GGGGCCTGCTATGAGCGTCCCTGTCAGTGGGCTGCGGTCTCGTACCATGCG
GCAGACCCGTCTGATGTGTACAGCTTTGCAGCATTCGTACGAAGAGCACGG
TTGGGCGGACTTGGACCACCAAGTCGTTCGAGGGGTGAGACAGCTGACCC
AGTCAGACGGTCGCACTGTGCGTTGCGCTGCGCGTACGCGGAAAACGATT
TGGACCTGTTCGGTAAGGTATCATTCGGGTAGCACCAAGGTCCACAACGTA
CCAGGCCTTCCTCTTTCATCAACGCGTCTTCGTCATCATCGTAGTCCGACAT
GGTTTGCTGCTCGACGAGTGGCATCAATGTAGTGCGGCAGGAGATAAGCG
GAACATGAGAAAGAATGAGAAGAGGTGAAGATCAAGTTAGAGGTCTGTGC
GCTGTGTTTGAATGGTCGAATTGCCCAGATCAGATGCCTCCCCTGTGCCTTG
CAAGACGAGAATGTCGACGCACGCACCCATGCATGCTATCTTACAAGAGGT
TCGGGGAGCGTGATAGCTCCTTGTGCTGTGGCTGCAATGCCCGAGGAGATG
TAGCCAACGTAGGGCATAAGCGAATACGACAATGTCAGGTTGCAGACTCGA
CACCCCTTCATACGGCGTCAGCAATCGACGTTCTCGTCACACGAAGACGGC
AAATCACCTGAAGCTGCCGGACGGATGTCTCCACGGGTACTTGTTCTTGCC
TTCCTCGACCCGGACCTTGCCCCTGCCCTTCTTCCCCTTGCCCTGCGCGAC
CTTCTTGCCCACAGCCCGGAAATCGCGCGTATCTGCGCGCGTGATGATCCT
GTCCCACAGCACGACCTCATGGCTAGCACCTGCCGAAGGCTCGTCGTACGT
CGCTGTCAGGTAGAGAAAGAGCTGCTTGGTATTGTACGAGTTGAGTAGCGT
TGTGAGGTCGGTCCGCATATCGAATCGGAGCGAGGCGAGCTCTTCATTCCT
GGCGCCATAGCGCCTCAGCCGCCCATGCTGACTGTTGCCTCAGCTTTCAAT
TGACTTGTGGCTTAGGTAGTCTGACACGATAAGATCCTTGACGTCTATCTTG
CCCATCTCCACCTGGGGCAGGGCGAGGAACGATGCCACCGAGATGAGCCC
GAGAAGGATCATTATGTAGGTCGTTGCCAGTGACGTGAGGTTGTTGAGGCG
CTGAAAAGTCGAGTACATCTTGCTCTGAGCAAGCGCCGCCTGCTCAGCCGT
CTGCCCTCTCTGTCACTCTCCTCTGCTTCGTTGAGCGTCGTGGGTGGACTG
CTGAGTGCAGCTGACAGGCGGTCTTCCACGCGCCTGCCTTTGTGGTCGGG
AGAAAATGCGATAAATGTGGTTCTGGAATATTCGCCGGTCTGCCAGCGTGT
CAGTCTCATCGTCAGGCGACGTGGCAGACGCGCTGGAATTTCCATCGACTT
TATATACTGTACAAGGCGAGGAGATATTCGGCACGACTCGCACCGTTTGTCT
CACTTCTCCAATCACGATGGCCTATCCGCGAAGCAGCATACGGCCACGGCG
GCGTTCAGCCTCGTCGTCCATCCTCTCGAAGCTCACTCTGGTGGCCTTTGT
CCTCCTCGCCGTCATCTGCTTCCTCCCAGTGGGTAACCAGGTCAAGGCGCA

GGATGCAAAGGAGAACGACTATGGAACTGTTATTGGTATCGATCTGGGAAC
GACTACTCTTGCGTGGCGTAAGTCTGCTCACAAGGGGAACTCGAGGTCGA
GCTGACTCTACCAGCGTTCAACGAGGAGGCAAGGTAGAAATTATCGCCAA
CGACCAGGGCAACCGTATCACACCGTCATGGGTCGCATTCACTGAGGAAG
AACGTCTGATCGGCGACGCAGCGAAGAATCAAGCGCCTCAAAACCCTGTC
AACACTGTCTTTGACGCCAAGCGGTTGATTGGCCGCACGTACGATGACCAA
GATATTCAACGGGACATGAAGCACTGGCCGTTCAAGATCGTCAACAAGGAT
GGGAAGCCCATGATTCAGGTCAACCACCGTGGGGATCTGAAGAGCTTCGT
TAGTCAAAGTGTTGTTCTCGTATCGGACAATGCTGATCACCTAGACACCTG
AGGAAATTTCCGCCATGGTCCTCACGAAAATGAAGGAGACCGCCGAGGCC
TACCTAGGCCACAAGGTCACCCATGCCGTTGTCACGGTCCCAGCATGTGAG
TCGCTTGAACTCAGGCAACAACTTCATTTGCTGATCCGACAGACTTCAATG
ATGCTCAGCGTTCGGCTACGAAAGACGCCGGCACCATCGCTGGTCTCACTG
TCCTCCGTATCGTCAACGAGCCGACAGCCGCTGCTATCGGTGAGCCAACAG
GCATGACTCTTCGTATATGCAAGCTGATATACAGCCTACGGTCTTGACCGCA
CTGGCAAACAAGAGTCCCAGATCATCGTCTACGACCTTGGAGGCGGTACCT
TCGATGTCTCCCTTCTTTCTATTGAGGACGGCGTCTTCGAGGTCTTGGCTAC
CGCCGGCGACACCCACCTCGGTGGTGAAGACTTCGACAATCGAGTGATCG
ATTACCTGGCCAAGGAGTACAAGCGCAAAACAGACGTGGATGTCACCAAG
AACAACCGAGCCATGGGGAAGCTCAAGCGTGAGGTGGAGAAAGCCAAGC
GTACCCTGTCCAGCCAAATGAGCACCCGCATCGAGATTGAGGCATTCGAGA
ATGGCAATGACTTCTCCGAAACCTTGACCCGCGCCAAATTCGAGGAACTCA
ACATGGACCTCTTCAGGAAGACGATGAAACCTGTCGAGCAGGTCTTGAAG
GATGCCGGAGTGAAGAAGGACGAGATTGATGACGTGAGTAGTTGCATATAT
ACTGGCAATTCCGTCACTGACACCATAGATCGTCCTTGTTGGAGGTTCAAC
CCGCATCCCCAAGGTCCAGCAGCTTCTGAAGGAATACTTCAACGGCAAGG
AGCCATCGAAAGGCATCAACCCAGACGAGGCGGTCGCGTACGGTGCCGCT
GTTCAGGGCGGTATTCTATCCGGTGAGGAGGGAAGCAGCGGCGTTCTTCTC
ATTGACGTCTGCCCTCTCACCCTCGGTATCGAGACGACCGGCGGAGTCATG
ACCAAGCTCATCGCCCGAAACTCCGTTGTCCCCACCAAGAAGTCCCAGATC
TTCTCCACCGCCGTCGACAACCAGCCGACCGTCCGTATTCAGGTCTTTGAG
GGCGAGCGGTCCATGACCAAGGACAACAACCTGCTCGGCGAGTTCGACCT
GAACAATATTCCCCAGCTCCTCGTGGCGTGCCCCAAATTGAGGTCACATTT
GAGATTGACGGTGAGCTTCTCTAAAGCCCGACTTGCAGTATACTAACATGC
ACAGCAAACGGTATCTTGAAAGTTGCTGCTCTCGACAAGGGTACCGGGAA
GTCGAACTCGATCACTATCACCAATGACAAGCGCCGATTGTCGGCCGAGGA
GATCGACCGCATGGTTCGAGAAGCCGAGGAGTTTGCCGATGAGGATGCTG
CAACGAGGAAGAGGATTGAGGCTCAGAACGCCCTTCAAAGTGAGTGCTGT

TGTCACAGCTCGAGGACACGAGCTCACAAATACCAGACTTCGTCTACTCGA
TGAAGGCGCAGGTCAACGACGCCGAGGGCCTGGGAGGAAAGCTTTCGGA
GGAGGACAAGGAGACTATTCTGGAGGCGATCAAGGAGAAGACGGAGTGG
CTCGACGACCACCCCAATGCCGACGCCGAAGACTTTGAAGATCAGCTCTC
CGAGCTGCAGGCAAAAGTCGGGCCGATCACCGCCAAACTGTACGCTGGTG
GCATGGGTGGTGACGATGACCAGGTTCCTTTCAGCCACGATGAGCTGTAAA
CGTGTCCTTGATCCTTGCATAGCATATCAGTTTCAGACAAAGAGAGAGAGG
CCCAGAAGAGATGCATGCATCAACGACACCATGACTCACTGATTGATGTGC
CACTTTGATGCCACGTGACATTGGATCCTGACTGTTTGCGAGGTCGTGCCT
TTGCTTCCTGGTGATTTCACCCAAAAAGCACCCTGGCCATCCAAAACACCT
TGTACTCTCTTTCATCTTACCTGGTCGGCCTCTTCATCCTCGTCTGGAGGGG
CGATGAGCATCCTCACACCCTCCAGAGGTAACCTTCTCCCCATACACAACC
CCTCTCATGGTCCCCTCCAACCCTCGGCTATGCCCGCCCCTCCTCCCTCGGC
ATCCCCTCGTCCACCCGCGCCGTTCCCCGTCTCTGACAAGATAGCGCAGAG
TCGAAAGGCTCAGCAGGTCGAAGCCGATCCGATACAGGTCTGGAGAGACG
CCATGATGGCTGCGCGAGGACCCGACGGCGCTCGGAACGCGCTGGCACCT
ATCGCGCCCAACAACGCGCATGTCAACTCTGTACAGCGTCCGAGCGCAAG
CGCGAGTGGCAGTAAACCTGCTCTCAGCGCTTTCGCCGCCAAACAATCTTC
CCTTTCCGCCTCTATACCACGTCCAGCCCCTTACCCAGACGTATTCGCCCCG
CGGGAGAGTACGGCTTCTGCTACTCGTCGACTCTTCGCGACTTATCGGTCA
ACACCCCTCAACATGTTGGCAAGAGGCAGAGGGAGTATGGCTCGGCATCG
ACGACAAAGAGGTCTCGAGTAGCGGAAGGGAACGCGACTGAGAGGTCGA
GAGACGCGACGCAGCACGAGTGGGCTATCAAATGGCGCCAAGGCGTTTCC
CACTCTCGTGTTCCACTTTGAGATTGGCCTGGAGGATGGGTCCGCCCGAAC
CTCAAGCCGCGCTTGATGAAGATGGGCAGTGTGAGTCAAGGCTGTTTGTTA
CTATTAGCAGTCACTGACCCAGCCAGCGTATTGACCAATTCTTTTCGCAGAA
GATCACGCATCTCATTGTCAAGAACGCCGGATCTCCTCAGAAGCCCAAAAT
CATCGCCCCAATTCGGCAGCTTGATCCGCGGAATTCCCCTCGTAACCCTTTC
AACGATAATATGGGCGTCACGGATCTGGCTCAGAAGGCAAAGGCGATGGG
TATCAAAGTCTGGTCTGTCAAGAGTGCGTCTTCCAGCTCCGATTTTGTGAA
AGTGCAGACTGAGACATGCAGAACTGGCAGAGATGCTCGATCTCCTTGCTC
CCGTGGAGGCTTCAGCTCGAGACTCGCTCTCGACCCTGCTCGAGGATGAG
AGGCTTCACGGCACTCGCGAACGCGACGTGAACGCTCCGCGGCCTGACTA
TTACTACTTCAAACCAGGCAGCAAGCATTTGCTCGTCGAAGATGCGACTGG
AAGACACCGTACCATAATGGTCAAAGAGTACAACTCGAAGCCGGGCGAGG
CTCCAGAATGGCCTGTCCTCTTTGAGACTTTCCTCCGACCGTCTTCGTCCA
GCGTAGCACCGGTTCCGATCGATGAGCCTCAAGGCACGCGCAGAGGCTTT
GTACGTCGATTGCCGCCCTTATAAAGGCGAGCAGCCGCCAACAATGCTGCG

CCGATCAACATCCATGCAGCAATTCCCGGGCACTCCGAGACTCCCTATCGC
CCGACCGTATCAGAATGCATCGGGCAATTCAGTCGTCCTCACCTCGAACAT
CGCCTCGACTTCCCACTGCAAATCAGTCACCCATGATGCAAGACGGTGTCC
CCGCATTGGGTACCGGAAAGGATCGAGCGATCTTGCAGATGAGTAAACGA
GTCCAGGTCCTCAAAGGCAATGCCCGACTGGCTGCTGCGAAGCGCATGTC
CGATGAACAGCCTCTGGCCATCGCTCGACGTGCGTCCACGAGCTCGACTTC
GAGTCAATCAGCCCACAAGACCTTCATGACCCAGGATCAGGTTGTCAAGAT
GCTGCGGCAGGCGCGGGGTCCAATTCCTGACGACCCGAGCGTGACCGCAG
CTATGCGGATTCAGAATCGAAAGAACGTTGATGCTGGTCTCAAAGCTCGGT
CGCAGGACACGGGTGCTGGTTATTGCGAGAATTGCCGCATCAAGTCACGG
ACTTCTCATCGGTGAGCTCGTTATCCGATGTCCACGGGCAGGGGCTGACAC
AACCAGCATGTGGTGTCAAAGAAACACCGTAAATACGCAACTAATCCTGCC
AACTTTCGTGAACTGGACGCCCTTTTGGCCATGCTACAGCGACCTCTAAGG
TCCTGGAGTGCTCCCGACCTGCTGCTGCCTTGCACAGCCAAACACGGCAA
AGTCTCCGACTGCGAATCGTGCAACTTTGACTACGACGAGCATGTGGCAAA
ATACGAGGGTGAGATTGAAGAGGAGGACGAAGAGGGAGAGTCGAGCGAA
GAGGATGAGACTGGTACCGGCAGCCACGAGGGCGGCTCCGACGATCTCTC
TGCGGACGAAGATGCGTCTGAGGAAGATGACGAACGTATGAAGACGAGTG
AGGATGAGAGGAGCTCAGGCGGTCCCGACGCATAGAATTTGCATACGGCC
AACTACAACAGGTCAGCATCACCATGTTCCACCAGCATCGCATTGTGCCAT
GTACGTACATATATGTTTTTAATCTTTGTGAGCATCATGCAGCATGTGCAGAA
GGCCATGTCCAATGTCAAGATGCATGAGATCAAGAGAGGCACAGGGCACC
GGTGTTGCTAGGTGTATGTATATTCGTACGTATCAGTCTGAAGATGGAGGAA
CGTCGCCCTCGCATACTCACTTGTCTTCCGTCAAGAGGAAGCGAACGACGG
CCGACACCGTGTCGCCAATCTTGCTCATCCCCTCCAAGACCTTCACCCTAC
GCTCTATATCGAGCCTCTTTTTCTCCTGCAGCTCCTTGCCACGGACGAGATA
CTCCATCATCCTGCCGCCGGCATTACAGCCTCGCGCGTCTGCGCGGTGTAC
CGAGCCAACTCCGCCCCCCAGCTCGGCTCGAGCGACGCGAGCTCTTGCTC
GCTCTCGGCCGACTCGTCAATCTCCTCTTCTTCCGCCATTGCAATGCCATCG
AGCACTTTTAGCACCTTCGTGATGAATGTGTCGACGCGCGCTGGGGTGAAC
CCGCGAGCTGAGAGAACCTTCTCGAATTCGCGACCAGAGAGGAAAGCCTC
GTCGGTGGCGTACAGGTCTTGTTGCAGACCCCGGCGAGTCGAGTCGATCTC
AGTGGCAATGAGACATGAATGGGCGATCCAATTGGCAGCGTCATGTTTCGG
CAAGGACTCCCTCACTAATTGCAACAATTTCCGGAGCGTTTTAGACCCAAT
TTTGATCGGGTGCTCGATCGGTTCTGCGGCGGAGTTGGTCAAGGGGGGTTT
GTTCTGCCCGTTGAGCACCTGAGGCTGAGCCGTGAAGGTGGGTGTAGGTA
CCGTGCCGTAAGCAGGTGATTGGCTTTTCATATGGTTAGGAGGTCGTTCGG
CTGTGTTGGGTGTTGGAGGAAGTGGTGACGGGCGACTTGAGCATGAGGAT

CGACAGGAGCCAACGTGCTAGCTGGCGGTGTAGATCGCGATTCGACCTTCA
CCATCGGTTGGTAAGGAGGCATCGACTCAGACGCTAGTGCCTGCATTTTGC
TCTCAGCCTCAGATGTGACACTGTCACGGCGCGGGTCCATCAACTGAGTGG
GGACATCGGGTTGGACAGTCGCCTCTGATGCCTCGGCGGGCAGTGATTCGC
GACGAGGACGCTTGTGCGGAGATGGCGAGTTGACTGATGAGACGGGAGAT
CTGGATGTGAGCTGGTCATCATCGGCCACGTGAAGAATCCCGCACGAAGCT
GGTATTTTCGCCGGAGTGGCCTCCCTGACCTCGGGCTCCCTGCTACGCTTC
CGCGCGACCTCCTCGACCGTGGCGGGGGCAGCTGACGGTGCCGAAACCTC
TTGCTGATCAGCTTTACCTCCCCGTGCCCAGTCAGGTAGATTAGGGAAGTC
CGTCGCCACGCTTGAGGGAACCAGTCGAGGTGGCTCCGCTTTCACAACAG
TCAATTTGCCTCGCCAGTTCCGCCATATTTCCAGATCGGGATCATTGGCCCG
TCCAAATTCTGTGTCCAGGTCGATTGGCTTTTGATTCAACGCTTGTAATGCC
GCGTCGCCGGCACTGAGTTCGAATAGGCAACATAGGCAAACCTGGGCAAG
GTCGCAGAGACGTGGTAGATGTCGACAGCCACACTGGCATGTCAGCTGCTT
TGCATCTGATATGAGATAGGTCGCACATATTCCCGAATTGGGAGAAGAGAAT
GCCCAGATCCTTCGCCGATGCATCGTTGGGAAAGCTGCGGCGTTAACTACA
CTCAGGATACCAATACTGGACGCACCTGGTTACGAAACAGACCCCTCTTTG
ACTTGCATAGCATCCTCGTCTACCTCGGGTTGCTGTATTGCGTCGTCGTGCG
CCACACTCATCGGTTTGGGCGGCAGAGACGGATGTCGTGCGGATAAGTCA
AGCTTGAGGTCATTGCCGAGGGGCACGTTGAGAGGTTTGGCAGGTGATAA
GACCGGAGGTTTTGGTCTGTCGAAGGGTAGCTTGATACGAGATGGATCCGA
GTCTCCTCGTTGCCAAGGCCCAGCTTCGCAGCTTCCAGCAGGAGCCGGATC
CCCGCCAAGTCGACGATACGCCTCTCCGAGAGTCCCCTCCTTTTTGCGTTT
GAGGTACGAGTTGCTGGCGGCGACAAAGGCGATTTTACCGGCGAGTACTT
GACCTTCAAGTTGAGGGTCTTTGGCAGCCAGACGAGAGTCATCTTGGCTTG
CAAGAATTCTGTGCAGACTTCACGCCACACGCGATCGAACCGGAGAGCCT
CGGAACAAGAAGACGTATCGATACCGACGATGGCGGCTTGAGAACTGCTG
AAGACGACGAGCTCGTGATTGATGGGGGTTGCATCCTTGATGGACTGATGC
AGCCGCTCTTGGAATCCTTCTGCCGCCTTGCAGATCCCTCCAATTTCAGCAT
TGAGAGCATCTTCACCGTCAAGAGCGCCGCGCATCTTGCTTCCTGACCGCC
CAGACTCGGATGATCCACTCAACGGCAGCAGACCAGCCTTCGCCAGCTCTT
GCAGCGCCATCGGCGTACCAGACTGCCTCGTTGCGCATGCACAGTTGGCGG
TGAACGTCAAGGGCGTCATCTCGACTGAGTGTCACGTACTTGACAATCGGC
AGATGACTTGGGGGGAGGGCATGCATGGCCGTGGTGTATGTGCCAACGGTC
ATCATCTGGGCTGGGTGGATGAGTGACAGCTCTTGAGAGTCGACATTGTCT
CTTGAAGGTATTGGGCGACGATCGGGGAGAGGTCCAGACCGTTGTGGCGG
GGAAACGATGCGTGTTGCGGACGATGGAGCGGAGTAAGTGTCAGTCCTGA
TAGGCGGTATCGCCGATGGTGGTTGCGGAGGCAGCCGCAGGGCAGGCGTG

CCGGGGTCATAGGAAGCTTGAGCAGGCGAGTACGGGTCGTAATGCTGTGC
ACTTCAAAGGACCTCCGAGGTTGTTCATAAGCATAAGCCGATCTTGGTTGT
CCATCATAGCTCTCGTAGGGTCGGTATTGGGCTGGTGGGGGAACGGCGGGT
GCGGGAGGGTAAGCATAGGCGGGGAGTTCGCGTCCAGGTGGTGAGGTGCC
GTAAGGTGAGGTTTCGGAATAGGGTTGACTGGGAGAATACCGTCCCGCCGC
TCGTGTGGGCGGGAATGGCGGGAAGTTGCTCATCTTGCAGGCGTGTTCGTC
GTCGATGGTGTGATCAGCAGTCGTCAAGAGATAGCAATTGCTGCAAGATGC
GTGAGGCGCGCGTGGTAGGATGAAACCCTGATCGTCAGAACGGAGTCAGA
CTGTGTCAACTCATAGACGGGGCCAGCAGAGTCAGCCAGAACTCACTGCT
GGTCAGACCATTAGGTCATCAGACGTCTAGACTTGTGGTCGGGATCTTGAT
GTGACAGGTCTTGTAGGAAGGACTGCCCAAATTGATCGCCACTGGTAAAG
AGTACGGGAAGGGGGGTTGGGGAACAAGAGAGTCAGACTGAAAAATCAA
AGATCGTATGAGCGGATTCTGAGTGGCCGGTATGATCGTATATCTTGGAAGT
CAATGCCGGCGCAGCAAGAAGACAGTGCGTCAAGAAGCTATTGGATATCG
ATTGGTGAACGAGGTGACAAGGTAGAAGAGAGAAAGTGACTGTTCCACCC
CGGCCAACGGAACTCTGGGCGGAAGTATTTTAAATGGTCCTCCCCCTTTCA
GATGTCCGAAGAAGATGTATCTGAGAGGTCGGTGTCTTGCGGTCACCACCA
GTCTGAGATGGCTCCGCTGAGCCCTGATGCAAAGCGCTTGCGCGTGAGTCC
CGGTCGCTCACATCCGGAGGATCAAGCTTGCTCGTGATACTGATGAGGCAG
AACATCATCATCTCTTTGCGGTGCTCGTGGTCACTTCGGCCATCCTCTACCG
TCGGGGTATGCTGGTAGCGGAAACTGGCTGGCACAACTGCTAACATGATCC
AGCTTTTCTTGGTGAGGAGCAGCGTCGGATACCACAAATCCCATCGGATGG
ACATGTCAAGGCGCACGACAAGATACTCGGTGCTGGGGCGAGCGAAATAG
GGGGGGCTCCATGGCAAGCACAAGACCACAAGACCGGCAGATGACGACA
ACACTGCCCAGGGTCGAGAACACACTACAAGGGCACCCATGTCCCCCAGA
CAGGCTGTTATCGAGCAGATCACGCATCCGCGACAAAGGCGGGGGGCATG
GCTTGATGGGGCCCGCAGAACCACTTCGTAGTGCGCAGAACGAGATCGTA
GTGCGAGGCAGTCGCTGTTCGTATGTCAATCAGGTGCATGCCGTGATGTCA
TCTATCCTCGTCTTCCATCTACCGTCGCTCGGCCGGCAGGACTTTGGTGCCG
GGTAGAACATAGGGGTTGAGAGAGTAGTTGCACTGGGAGTCAGGCGATCT
TTCGAGTGAACTACATCTGACGAACATTCGAGTACGCGACACCGGCTCCAA
AGCTGCAGATGATCAGCCTAGCAGTCTGACTTTCAAAAAGAAACTCACCCT
GTTGAGAGAGCCACGGGCCACCCTCGACCTGTAATGGTCAGTATTCACGCT
CATTCAGATATCCTGACGGCAGCTGGAGCTCATCCATGCGGCTCGGTCTCG
CGAGCCTGGGGAGGAGGTGGCCTTGGATTGCCATGAAGTGAGATTGTAGG
ACTGGTCGAGACTCACGCCTGAAAAGTAAAACCGATGCCACAACGCCGAC
ACCGAACCCGATGCCGGTGTTCACAACCATATCGGCAAGCTGGTAAATCAG
TCGCCTTTCCTGGTTACATCCATCACGATGAGGATCTCATGGCGACACATCC

AGAAGTGGTCGAGCTTACACAGTTGTCAAACTGTTCATGAGCTTGCTTGAC
CACGATTACTCGTTTGGTAACAAGGCCCGGGAGAGAAGAGGGACATTGGC
GCACCTTGCGAGCCACGATCTCTTCGCTTGGGATGGGGGCCGCGATAGCCG
CGGTGGCAGCAGGTTTCGACGCCATGTCTGTCGGTCTGAATCAGGCTCCCG
GGAGGTAGAGTAGGCTGAGTAGCAAATGCGAGGCGAGAATGACGGAAAG
CGAAACGAGTAGAGCCGCGACAGGCGCGCGTCGGGTTTGGCAGATGATGA
ATAGGCACGCGAGTCACGACCCTCGTCCTGTCTGCTCTGACAAGGACTTGT
GCAGTGATATATGAGGATCACAGAAGTGAGCTTTGGTTGCTGTTTTTCCATG
TCTTCACACAACACTGCGATGCTCGTGATCGTGTCTGCCTTGATCATATACA
TGTAAGTCCAGTCCAGACTACAAAAGTACGAGGAGATAGTGCTGTCCGATC
TGATCCCTCCGGATTTCCCGAAAGGTAAAAAGGACCCGGGAGAGGGGTTT
CAGGGGTCCGGAGTTCAGGGTTTGACAGTTAAGAGACGGAGTAGCACTGT
ACAGTAATGAGGCTCGATGGCAACATTGACCGTTTCAACGGTGCCGAGCA
GAACAGCCAAGCAGCGAACAGGATGATTCGTGCATCTTTCTCTCTTTGGCA
GTTGGAAACCGATAGACAGATACCTTTTATATACCCTCGGTGGTGACTGGCG
CCGTGTAGTCGATTCCATCTCCATTGCATCACTCGTCCCATTCGTCTCAAAA
ATCAACCTCGGCCGAAGTAATCGGTCATCGGTCCTCTACTCTATATACCAGA
TCAATCACCTAGATCGACGCGTATTCGTCTCAATCCACCTCCCTTCCTGTCT
CTCTGCCTCTCTCTTTCCTTCTCACCCTCCCTCCCCCTTCTTCTCCTCATCTC
CTCCCAGTCGCCGACATGGGCGATCTGGCGAAAATCTCCACCTCCGATCTC
GAACGTGCAATCTCGCTGGTCGCCGTCTCGTCCACATCGCCACTGGCAAAG
TTCACCAACTGGGCAAACACGTTCGAATGTAGACCACAGAGGGTCTTTGC
ACCAACAACCGAGCTGGAATGTAGACAAATCGTCGAGCTTGCACGTAGGG
AAAAGCTCGACCTGCGACCAGTCGGTGTGGGACATTCCCATCCGATCTTGC
ATGCACATCGGGTGGATGATTCGCATGGACCACTACGAGGGACCGATCAAG
GTGGGCCATCTGCGACCTTCTCTGCTTCGCGATCAGCCAGCTGATTCAACA
GGTCGATCACGATACCCGGACCGGCACCTTCAAGGCCCGGGACCACCCTTC
ACTCTGTTCATCGCATTCTCAGCGCTGCTTCACCACCACTTGCCATCCCCAA
TATCGGTTCAATCTCTGATCAGACGATTGCTGGTCTCATTTCGACCGCCTCA
CACGGCAGTGGCAGACAGTTCCCAGTCCTTTCCAGCATGTCAAGTCTCTCA
AAATCATCCTACCCCTGCCTGGAGCACCAGTGGTACGCGTTTCGAGAAAGG
AGGACCCAGAACTGTTCCAAAGCCAGCCTGTGCGGCCTGGGCGCGACCGG
ATTGATGCTGGAAGTGGAGATCGAAGTTGAAGAGGCCTTTCGTCTGCGCG
AGACCAAGACAACCATGAAGGTTGACGATGCCTTTGAGCAGATCGACGAG
ATCATGAGCAGCGCAGAGCACGCACGCATGTGGTGGTATCCAGACGGTGAT
GGCGTTGTGGTTGGCCGCGCTGACCGGACGTACGAGGTGAGTCCAGTCCT
TTGAACATGCCACGAATGAGCATATGCTGAATGGCAGCCCGCCAATCCACA
ATCCTCCTTCTGGGGCCCGATCCTCCACTACCACGTCACCCAATTCTTTCTA

TTCTGGGCACGTATGTTCCCCTCGCTCACTCCCCTCGTGGGCAAATGGGGT
TGGTGGCTTGCCAAGACACATACCGAGGTGGTCAACGACGGATTCAAGGT
TCTGAACTTCGACTGTCTGGTAAGATGGTCCACCTCGTTTGAGCTTGCCGT
TTCTGACCTCAGTTTCCCCAATACGCCCTTGAGTGGGCTATCCCGTCTGCCA
ATGCAAAGGCGTGTTTGAAGGAGCTTCGTGCATGGCTGGCGGAAGAAGCG
GCAGACCCCAATGGGACTGAAGACCCACTTCCCCCTCGAGATCCGCTGGT
CTTGCGCCGACGACATCTACCTCAGCCCGAGCTATGGAGTCGAGACGGTCT
GGATCGGTGTCGTCACTTATAGGTGGGTTCGAATCTGTTCAGGCAACCGTT
TTGCAGGCGCTGACCTGCAGACCATACGGACTCGCAGTACCATATCGAAAG
TTGAACGAAAGATTCGCCACGATCCTCGCCAGTCACGGCGGACGCCCACA
CTGGGCCAAAGAACACCATCTCAGCCGAAAGCGATCGAGGCTCTTTACCC
GGAGTTCGACAAGTTCCGCGAAGTGTGCGAGCGCGTCGACCCAGACCATA
TCATGCGGAGTGAATATCTTCGTCGACATTTGGATGGCGATGATATCGGAGA
GAGGGTTTTCAAAAGGCGAACGCTAGCAGGGTCCTCACTGACGACCCGTC
CGTCGCCGAAGCGCAGGAGGACAAGATCGTCTCCAGGCGCGCTTAGACAA
TACTAGACTAGACATAGAACAATTATATGCATTGACCGCGGCGGGCATCATG
TTAAAGTTGGAAAGTTACGGGGCAAGTGCACAAACGCAATAGGGCTATAGT
GATACAGGCATCAAGAAGTCATGAAAGAGCTTTTTGAGCTTTCCCCAGATT
TACCCCCCATAATCTCGATTGGAACATATCGATGTAGATGAACTGATACAAT
GATATATTCTTCTATCCAATTTCTATACTCACTACACGCAACTTCCGGTCCTC
AGGGCAAATCTCAACTCTCAACTCCTTCCTCAAATGACAATATACGATTATT
TCGCAACAATTTGAGAAGCTACATTTCAAATTCATATTGATATAATCTAGGA
AGGTTTAATTTGAAGTTATGCTCAGAGATTACATAAATTCAAAATGGACTTC
TCGATTCTTTCAATTACCACATCGTCAATATCCTCATCATGAGCACTGAACC
GTCTGTGAAGGATGGCAAAAAGCCAGCTCAGGACACGTGCGACCATTCAC
CTGGGATCGATGGCCACACAACTCTCCTTCAACACCAAACAGCTATCGCCG
CGCTTGCGAGCCTACCATTGAAGACCGTCAATCTCCGTCTTTCGCCATCAAT
GGCATAAGGACAAGTCCCGGCTCACCTTCGCCCACGCCGAGCAGTATGTAC
ATGAACATGACCTACCAAGTCCCAAGATGGAATGGCCAAACTTGAATAAAT
ACCGCCCGCATCATTGGGAGGTTCATAGATTTCAGCTGAAGCTACTGAGGG
AAGATCTCGTCTAGCACCGCGAGAAGTTCATCAGACTGTACTTAGATGCTAT
TATCGAATCAACTCGCCTCCCGAAGAAGGAACCGATTACCCTATCAGAAAT
TCGTAGCATTCGGAAAACGTTGTATGTTATTTCTCTGGGCGTTTTTGATGAG
TCGACTGATGTGGAAGTATAGTAGTAACGAGTTACGCATCAGTCTTCCGAG
AGACCTCGCCAAAATCTACTACGGCAAGGTTCCCGCTTGCATGGACGCCGC
AATCGAGCCTGGAGAGTTTAAGGATTTAGTTGATCACTTCGGGGCAGAGAG
GCACCCCATGATCCTGGCTGTTCCTATGTCTTTGCATCTGTGGAGCATACAC
CAAGACTACCCAGATCTGCTCCCCAAGAATCCGGCGCCGTGGGACTGGAC

TATGCCCGCAGATGTTATCGAGGCTAACCATTGGAAGACCAAAGAGGATCT
GTTCTAAACCCTATGTTTGTCGCAACGCAACGACGAAAAGGTGTATTGAAG
CGGTATCGCTAGCACTGATAAGTCCCGCGCATCATCAATCAATAATAGTAAT
AATAACATCCTCAAGTTAACTCATTCCGAGTCCGCGCTCTAAAATGCACCCA
CCTATGTAAATGGTATAAATACGGCAAATCATCTTTGTCCACTAAGGTAATAT
TTGAAGTACTATTCTAATGGTAACAATATCACTATGATGGCTTAGTGGCTTGA
CCTCCTTTCCACCTATCATTGACATCCCGCCTTCGTCCGAGTACGTCACTCT
CGGCCGCCACCCTTCATACTTATAACTCCGGTCTCATTTAAGACTTGGGAGG
TGCATGGATCCTTCAAATATTCCGAGAATGGATAGCGACTGTTCACAGCCTA
CGCATTTCGTAGCAGCCGATAGGCGTGATCTTTATTATTGGGGTTGCCATTTT
GTACCTCAAAGTTAGCCATCGCGGATGTCTTAGAGATGGCCCATTTCCATGT
ATACCAAACCCAAAATCGAACGCCTAAGCTACCAATCTCCTAAGACACCCA
TAGGGTCTTTGATGTTATCTAGAACGAACTCGCGAGTCAGATCTTCTTGTCT
CTCATACCCAATTTATATACTCCTGTTCTACTATATATTTAGTAAGCAACAATC
CCAAACAACTAGAACCACCAACGCCATGTTTTATTTCTTAAGTGTTCCTGTC
TTTCCCTTTTCGTTCCTCAATACGATTTAACAGCCCCGAGAGAGTCCACCTC
TTATCCGAGTCCGTGCCAGGCCCGGAGCCTGAGAAGAATGAAGTAATCGAT
CGCGACGTTTTTGTTGAAGTCCCTATAACGTCTGTAGGTCGACCTATTAGGG
CATTAGCAGGTACGTAGCGACGATACGATACCGATTAAGGCAGAGCCGCAA
TTCTCACGTATATTCCCCCTCCCTCCGATCCCATACCCCCTTAATTGGGTATC
CCGCTCCCCAACCTCGACTTCCTCTTCGCTCAGCTCGCCCCCTCCATCTCTT
ACACCACCATCATCCTCACTCCGGCGCCTCCTCGGCAACGCGCCGCCCACT
GCGTTATCTAATCCCCGATTCGTCGTGTCCCGCGGGGAAGGCGACCTGAGA
TTCGCCACGGACACGCTTCGTACAGGTGCGAGCCGACCTTGTATGCGCACG
CGATGCTGCCTCCGCTTCTCGGGTAGCAGGCGACTCGTGTCGGGGTCCAAA
TCTGCCAAGCCTACCTCGCTACCACCCACACCATCTCTGCGGTCGCTATTAG
GTAGATACGTGGAGAGGTTGTTGAGTAGCGGGGGGAGTGGGGGCGGCGGC
GTGTAGGGGTGTGAGCGCATGGTGTCGGAGTAGGTGAAGCGGATGGATTT
GGATGGGTGGATTGGTGGGTAATTGGTGGTAGGAACTTGCAAGCTCAGCTC
TGTCCAGTTCACTTAGGGGATGGGATTTTTCCCCTAGGCTTGCTAGCCGCTA
CACTAGTGCCATCTAGCCCTCTGAAGGGAGGGCCGCTTAAGCTAAACTAGG
GATAGGATAGACGTTATTTTATTAGTCAACGACGACTTCAACTTAAATTCGA
CTGCAATTCAATTATGCCATGATGGAACATTCAATGCAAGAATAATAATAATT
ATTATACACGTAGCACGATTACCAGCTAAATGAATACTCTCGGGTACCTGGG
TTAATACTTGATAGACCTTAAATCTCGTATTTCTCGTATCAACCCCTCGAGGA
ATTTGATGCCGTTATAGCACACAGCCTACTTCGAGCCATGTGAGCACATAAA
CATTGACGATTATTACTGGAAGGGGATGATTCGGTATTTGTGATGTCGGCTC
ACTAAAAGCAAATAAAGATGCGAGGTAAGTAACACGACAGGAGTTCAACG

GTCGAATGGTCTGTTATAAAGGTGATAGCCTGCCTACTACGCGCAGGATTTC
TCTGTAAGGCTGTCTGAAAATTCCTACTTTGCCCGCCGTGGCGTTTCCCATG
TCCTACAGTAGATGATTTGATGTCAGTGGTGTGGTAGATATCAAGTGATGGC
ATGCTAAAATGACAAGTTACCTACCTACCTCTAGGTAGGTAGGTATCTTTGT
AGTCAATATTTAAGTGCACACCCACCTGCTATGCCAATTCTTGCGTAACCTG
CCAGAAGCTGTGTTACTAGCACCAGTCGGTATTTGGATGGTCCTATGTGAG
ATCAGAAGATAAGGCTAGCAAAATGGATTGCCGATGACCGAGAAATCATAT
GAGTTTCGGACTCATATATGTGGTTGGGTTTATCCAATACTACGAACAGCAG
GCAATTGTGCCGTCTCATTAGTGTATCAGATTTGTGAGAACCAAGTTCGTTA
TAATATAAAAGCTTGATCTCTAATGTTATATCAGTAGATAATGAGTCTAATAC
AAGGTAGATTTATTTGAGCTATGGAAGTCACCTGCACATATATACCTCATTCA
TTTATGAATTTCATAATAATCTCATTAGAAAGGTGAATACTCTCTAAACGATA
GGTAAGTATATTGTCTGTGGTGCCAAGCTTCGGTACCTCTTTGTATGCATTG
CAAACCCTCTTGCCTTTATACCATGTTAAGTCAATGACAATGATGAAACAAC
TTGGACCTACCTGGGTGCCTCCATTTACGACACTACAACATCATGACGAAC
ATAAATTCCAGCGGGCTGGATGGGTTCAAGACTCTCATCAGTGCCAGCATA
TACGGCGGTTGTTCTCATATGTGAAATATATCCATTCGTATTGTTGAAGTTTC
TCGGGAGCCTCGGGGTAGATTGTGTAGACCGTGATATTAGCTCATCGCCATC
CTCGTCGGAGCTTCGGTTCCAATTGCCGATGAACCTGGCCTTAGAGATGCA
AGAGCAGGTGTTGAATAAGCTTTGGAAACCTTCACGGTAAATGTGTTGGAA
TAGGAAGAATATACTTGGGAGGCTAATGCTGATGATAGACCACCCTATGTCA
CCTACCATCCAGGAAACTGGAGTAGCGATTTTATCTGAATATACAAGCTACC
TAATAAGCTCGTTGGTCTTGAAGGATAGAAATCTCACACGTTGGATCTTGAT
TAAGACTGTCACCAGCAACGATAATGGCAATGAGGCGACCAATAGATGATA
CTATGACCCTATGAGTTTGAGTTAGCTGAGGAAATAGGAAGAATGTGATACT
TTCAAGAATATTGTAGGATGATATCACGTACAAATATGCGCAAAAAATACCA
GGGAGACTCGAAGTTTGCGCGTTGCTTCATTCGCAACCCCCATAGCATTGG
CAGGGGCATTATTATGACGACAAAGTCGATAATAAGACTAGATATAACGCTG
CCTTGCCAGAGTTTGCTAAGGTCCATACATGTCCCCGGGGTTTAAAGGTTTC
CAAGCCTTTTCAACGGGTTCACACATGAATCCAATGACGAAAAAATAACCG
ACAATCCACATGACATTTATAATGTGTCCAAACCATAGGGCATATTTAAATCG
CACGTTTCTCACGCCGAAGATACGAGCATAGAAAATAAGGCTGAAAGCTTT
AGGAATGCCGATTCCCGCGTTGAAAATGAACGAGACGATCCAGAGCAGCT
TTAAAAATAGCACGGTGTTTTGTTGCGGTACTGTCTCGACATGAAGTCCCA
AGCCAAGTTGAGTCATCTTTATACCCTATTGCACACACGGCAATGTTCAACC
CCTAAAGTCATATTAATCCCGGAGGCTTTGGAACGGGATGGACATGTAACAT
ACGGCAGCCACAAGTACTGTCCAATCGTCCCACCAAAATCTAGGCATTTGA
GCCCCTGACACAGAGGACTCTGGTATGAGAACGCGACTCCATACTCTGATG

ATAATTATGACTGTTGGAATGATCATCATGGAGATAAAGAGTTTCCGTAAGA
CATCCTGTGTATCATGTGTGATGCCCGCCGGCTCCATCTTGATAGGATGGTG
AGACTAAAACTTGAATATGACTGAGCAAAAATTGATAATTGCCAACTCAGG
AGCGACAAAAATCGTAAATAAGTAGTAGCAGGTACCTACATAGTGTTTAAA
TATGCACACGTAAAACAGACAAGATCTTTTAGGATAGAGCCGCGCCTTAGA
CAAGGCTACAAGGTCCTCGGTTAAGTTTAGAAGAGGGAACATCACAAAGC
CTTATTCTCACTGCGATATCAGTTGCCTATAGACAGGTCTGCACTATATTGTA
AATCGTCGTTGTTTTGGGAGCTCTCTCTTTACTAGTGACCAACGATATTAGA
GTATGTGACTGCGGCAATGCACGTAATTATCTGACCCCTACTTTATAGCCCA
AATGAAGTGATCAATATGGCACAAGTGTAATAAATTAGCGGTAAATGGATCG
AATCGGCACATTCTGTGTTGATAAGTATGTCTATACCCCAACAATACTACCC
AAGCACATCATAACTAGAGCCATGCCGTGAAACATATTGGAAGTGGGCGTT
TCTAATCAACAAGCATTTGGGGTTATATTGTCTAACTGATATGCACCTTAGGC
ATCGTAACGTGACAGCCCTGGGGCGGCAAGTCCATACTTTGAATGGTACCC
AAGGTAGGTAGGTAGTCACTAGAATCCTCCATCATAAGGTAGTCTCAACTTT
CTTTTCTATCTCCTTCATCAGACCAATCTATTCTCTTAGCTCTCGTGGGAGAA
TATATCCCAAGTACACCCTATCCTGCAAACTCCTGAGAAGATCCCTTGGGAT
AACCCAAAGCGAACCTCATTGCTCTTGAGACTTCGCACAATGGGAAGCTA
AGTCCGTACCTTGGTCATTTTATAGCTTTCCCGCTGCTTGTGGCCGAGCCCC
TTTCGCTCTCGATAACGCTATTGATAAAATTCTGGAGAATATCATACACGAC
CAGGAGGTCTAGAAAAGCTGTCCTAGATCTCAGTTATATTTGCCTTAAACAC
CGTAACGACGAACCTACCTACCTACCGACCTACCTATGCTGACAAGCAAAG
CGAGAGCGGTATTAGATTCATTGAATTGACTCAGTAAAAGAAAATAATGTAC
GTGCCCCGTCGTTTACCCCTGAGAAAGTCTCGATCCTATAATTGGCGAAGC
ATTTACTGATAGTTTTACAATGTAGGCAGTAATTACCTACTTCTTATATGGCG
ATTTGGCGAGTCTGGACAGATGCCCCCTTAAAGCATGGATGGAATGACGTT
GTCGAGTTTCAGCTAACCCCCTTTTTAGGATAGGCGTATCGTAGTAGTTATA
ACGACATCGATGAGCAAGCTAGAATCACCGATGTAGCTCGCAGCTATGCTA
CATAGGGGTGAAATGGTATCAGATATATCCTCACATTCGAACGCGCGTTTAA
TAGGAACAATTGCAGAGATCTTGCCCTGGACCTCAGGGGGTTTCCTCGGTT
CCTCATCCAGCAACGGGGTACGCACTCAGGTCGGACGTATCACCTTGATCG
AATGCCTTCTCTAGAGTGATCAATGGCTATTAATTGTCAGCTATCAGTTCTAA
TAAGCGTCAGAGGGTACTATACGCCTACCTGCCTACCACTTTAGTGATTGCC
ATGGACGCATGCTTAGGAACCTTTTCCGGGGAGGCATATGGATGGATTCGC
CGATAAAATATTGATTATAACTCCACGTTTAATCTCACGAGTGTCATTTCTGC
TAGTCCAAGCCTTGGGTTCCTGTTTACGCATGATTATCACTTTCTTACACATG
CAAATTAGATTGACACGGGCGTGTATTCCTATAAAGGTAAATTGGGAATGTA
TAAAAAAGAGCTTCATCAATTTACTCTGAAGGTATGTGATTTCTGATGGTCT

AGAAGATCTTAAGCAGTGGATACCGTTGTAGATGGTCAGCTGTGGCATTCG
GCATCTGACGCTCCTTTCACATTCCGGCCCGCGGCGAGGTAGCCACAAC
AACGCTTCGAGAACTAGAAGATCTAGATGCTAGGACTCATATGCCATTATGA
GATGTCGAAGATAATCTATCTAGACCTTGGTCTTCTTTCTAGCGGTGGACTC
TTATCTCAAAACGAAGTGTTGGCCGAGAGACTTGCCACGGATGGCGTCTAC
ACGCTACTTTCTGAAAAAGAGATACCTATTAGCTACTAGGTAGGTACCTACC
TTTAGGCATTTCTGCAATAGCATCTCCATGTCAATCAGATCCCACATAAATCG
TCTGGGGTATCATTAATCCCTCCTTGTAAGATAGGGAGGATCTAGTCTTGCC
ATACCATCTTCCTATCCCTTCTGGAGTCCCACATTCACCTACCTAAACTATCA
GTAACATTGACACAGAAAGAGATAGGGAGAAACTATTGAATTTGGTTGCTT
ACTGACTGAAGCGGGCTATGTCGCAATAATGAGCGAAATTGTGGCTAGGTA
ACGCACTTGCAGATCGAGTTTGCAGGCTATTCCCAGCCCCAAGGGCAAGTA
TTGACACAGGAAAGCCACTATGCGCCACCGGTACTGATTCTTCGTGTGTGG
GAAGTCTTCAAAATTGAATATGGAGAAATTTGCGGAAGTGGCTGTCTGTGA
CATCCTGACCTAGGTATCGTGGTGCCTGGGAATCTCATTGCTAATGAATCGT
GGGCGGGTCGAGTTGCTGCTTAGGGCAATTTCGTGTCCTATTCGGTGTTCA
GATGATGGAATGGCTTTTGTATAAATTCAACACATGTCTCATTTCAGCCTGC
GTCTTTTTGTTGATAAGCACCCGTTCATGGAAAGCAAATGCAGCATATCTGC
ATTTTACTTACTAGATTCAAATCTTTAGCTGCTAGAAACGTGCCTTCTCAAAT
ATACCTACCTACCTATTAGGTAGCTGCCTACTTACGTAGGTACCTACCAGGTA
CTCGTGATATGCACAACACCGGCAACGCTGTCCCGTCCCAGGATTTTCCTA
GATGTTCCCATCATTGAGTGGCTTATGCAGCCTCTTTAGTGTCAGCGCCAAG
GAATAGATGGCGGACTCCAGGCAGTCAATATCGACGGGAAATTTGTCACTT
GTCACATCTGCAAAAAGGACAATTCGAAGCTCTCTCTCCTAGAATGCAAGA
CATCGCTGGTGCACGATTCTGGCGCAAGGACAAACCATGATTGTGAGATAA
CGGTGTACCTAGCAAGTTGTAACGAAGGGTAGATGCATAAGGAGAGGGGT
ATGGGTGGCTGCATACCGTATCCTATCAAGCCACCATCGTGCTTTTGGACGA
GAGGGCTTGGACATAAGATCAGGAGTGATATTAGCCTGACCGCCCTTGAGT
CCTATTGGGCTCTTCTGGCATTAGGGAAAGGATTCATACTGGTACCCACCTA
GGTTTACTCATTCAAGGGCCGTATCCAAATTATTTTATGGAATCTATCTACCT
ACTAGGCATCATCAAAGTAATGTTGTGGTATAAGGTACCTACTATCATTGATG
CCACCACTATAAGCCATATATCAACTCCCCGTTCGCGAAATTGAACTAGTAG
GTACCTACTTATTCACAGCCCTATCCAGATTCACGGCTTAGTTTCCGGACTG
ATGTACCGGCTAGTGTTTCACTGTCTCGGGTCGCATCTTGGAGCGGCTGCC
GACCGAGACACAGCCCTGAAGGTCTCTCATATGGCGGGGGCATGTCTCGGT
CTTGGTCTCGGTTATGACATGTAGAAGATTTTGTGGCCTTTCGATGGCGGCT
CCGAGACATCATTCGGCATTCAAGCCGCTTGGTCAATAGGGCAAAGCCCGC
CCTTCCTACGATCAATGGAGGCATGTCGTAGCTCTGTCTTCTTTCTCAAGAC

CAATTTCGATAGGCGAGCTAGTGAGTACGTGCCGCATCTGGTTGGATTAGTT
CCACTGGCGGCCGAGAATGTAGAGTAAGCAGTTTATTTTCCTAGCGGATCG
AAGATATTCGGAGTATGTAGCGTGGCGCGGTATCATGCCGAAAGACCAAGT
ACGCGGAATGTGTTTTAGACAAATATACCATGTTCGATGGCGAAAGATGAA
GTGAATCCCGCTTCCAAATGAATGATCGGGGATTGAGACACCTTAACGAAG
ATCCCTCGTGTGGTTGATTACATGGTGGCCACGCGGTGAACACGCGCACGA
TTGCACGGTCCATGTGCTGATCTCTAATCCACCGAATCTCCATCGTGGAGTT
GAGTTGCTCCGAGCTCTGATTAAAATTCGCGATGACAGGAGGTACTCACGG
CAACATGGCTAAAACATCCCCGGGAAAGGAGGGTCTATCGAGCCAGATAG
ACGAGCCCTAAACTTCTGCTCGTCAACTCGTATACCATTTTGACCAGTGTTC
CTCGCCCCTCAAAGAGATTGCGGTGGTCGTCAATAGCATGCGCCACCATAC
CAGAGTAGAGCCTGGAGAATTCCGGGCTGAGGCGTGGAAAAGCAAAAAG
CAAAAAAGCGCACGTCGAGGCGCCTTTCGCACCACTGTAGGTACTTAGAG
ATGATCGAGATAATCGAGTTGTCTCTTGTATCTCATCACGGGTGGCTGCTCC
TCGCGTGCCGCTTTTGATGGGGGAAATTGCTAATGATCAGCAACGCGTCGA
CCTTGCTACGCCCCCCGTACTGCTTTCTCCACGAACTAGGCCTCGTGCCATT
CTCTGCTCATTGAGACATTGGCAGCGGGGGAACTGACACTTAGGCAATGCT
AGGTCACTATGGCGGTTACTTGTATCATAAATGGAGTCTGCCGTTTGGAGAG
TGTTTTGAACGCCCGCTGTATGTGTTTCTGGGCGACTTCTGCGCTGGTGAC
AGTGAGGGCTTGGATGGGATGGTTGCCGGCGCAGGCAATCTGAGTGAATAT
GGATCAAACCACATACGATCTAGCAATTCGCGAACACTTACATACCTCTTGC
CTATCTCTCTCTACCTCTTTGAAGAGAGCATGTAGGCTGGATTCCTTCGTTA
CACGGCATTTATTGCTTTCCTTAGCGACCGAATATCTGACGACAGTGACCAT
GTACTCAAGTTCAGCGGATAGGCACTGATTCTGGCTCTACTACAGAGCTAC
TCTAGGCGTCTATGCGCCATTTCTACGCGTAGATGGCAGCCATCACATGCAC
CTAGAAACACCAGGACACTTTAGAATCTAGATACCTAGTTACCGCCCCTTTT
GACGTTAACGAACTGAGGTCACTATCATCAATACCAACCATGCACGAGGCG
AAAAATGTGATGTATGCACAGCTTTTTCGGCCTACATCCCAAATACCTATCT
GCATAACGACTTCAAATTTCGCGATATGAAAATGGAGGGTTATCGTCGCCAT
CACATCGCCCCTTATTTTGGCGATTCGATTTCCCGAAATGGTCGATCTAACG
CCGATAACCCCGATTTCGTGGAAATACCGGCTTACTATGATCGGAAGCGTTT
AATAGGGGCTACCGCACATCGCGCATCATACATACATCAATACCGACATACA
CACGCATACATCTGAGTACATTACATATACATGCCATTACTCACATGAAGATT
CGATAATGGTCAACCAGTCATGGCGCGAGGAAAGCAGTAAGCAAGATAATA
AGCTCCGCCCTTCGTATTTTCGTGTTCGACGGCAGGCGTTCGGAAATGAAC
GGCACGCAGGCAAAAATCCCCGATAGGCGGGATGCGGAGCGGGCAGAAG
GTCGCGAAATTAAGGAGGAGCTCTTTTCTTGCACGGGGTTTATGTGATTGT
GACGAAAGTTGGACCGAAGCCCACCGCCCGGTCGGCATGTATGTATGTTGA

TGGTGGTTGATTACTAGGTACACAATCACATTACATGGAGAAATGCAGAAT
GCGAAATGCAACGAAATGCAAAATGCAAATAATTTATGTCGCGCGGGAAGC
AAATCGCGAAGATGGGCTTGGGGGCGGGAGGCATCTCGATCTCGAAATGT
GCAAAACAATGCATTAATAGATTGGATTCTGACCTGTCATGTTGACCAGCCT
CTCTTATTGTCTTCGAATGCGTCAATTCTATGTAATTGTATGTATTGTATGTGC
AATTAAGATTATCAAAGTGACAGAATTGACGATAACCCCTGATAGCGGCTC
ACCGGTGGAAAAGGGGGTCAGCAATTATTGGATGATGGGTTGGATTTGGTA
ATTAATTGTTAGTAAGGTTAGTAAGATATAGGGAAAATAGAGCTTGGAAAG
GGAAATAGAGGAAATGCATCTATTTCCAACGTTGCTGTTGGTAGGTCAATAT
TAGATAAGATCCATTTCTAAGTTCTCCAACTGCAACTGCATCGCCCCGGTCA
TGCTGGTCACGCCGGTCATGTGTTTGAAAAGGGATTTGCAAGAGTTGGAA
GTTTCATAACTCATACGTCAGATTTGAGGCTCTTTCGTCTAATGTCCTATCAA
GATCGACGAGATTGGGATCCGTAGGTGATAGAACCATGGCTTATCTATGAAG
GGTTCAACGTATGCCGCAATGTGGAGCCGTAGATCCATTCTCTCATGGGCAT
CGTGGGTGTTGTTAGGGTGTGTTGGGTGTGTAGTGTGCTGAAATAGGCAAC
TTGACCTAGTCTGCTAGCTCTGACTGAGACGATTTCAGTCAATCAATCAATG
ATGTGTGGCACAGCTGTCCAAATTCTGTTCAGTCCTGCTCCGCTATTGTTAT
TTGATGCCATTGATGTTATTGATGTCTCCATCTTCATCATATCCATCTGTTAGC
CTACTTGCGACATTTACGACCAACGGCCAACGACGTGCTAGAACCAACATT
CTTCAGTGGCTCTTGTCCACTACCGTTAGATTGATTGAATTTTGACTCGTTC
GTAGGGCTTAACCTGGAAATAGTGTAGTGTAGTGGCAACTAAGTAAAACAG
CCAACGATGTGGGTAAGGAGGGTCGGACGCTCCCGCGCAACTTGCTCGGC
TTCTTCGGGTTTAAATTAACGACTTCAACATGATGTTAGCGACGTTCACTAG
ATTCACTAGTAAGTAGAGTAAGAAGAATCAATTAGATGAGAGGGTTGGAAG
TCGATGAGGCATATTATTTCCCCTCGGAAGCGCGATTGACGCCAATTGGCAT
GATCAAAATGGCCTCAAGGAAAATGCCAACAAGGCTGGACCTTGTTTAGA
CCGGCTTCAATAGATTTGCTGGCTAATGCAGACTTGCAATGCGCGCGCAGC
TTTCGAAGGAAGATAGTCCTCAACATCATGCACCTCTTTGGTCTCACAGAA
AGGCTAGAATGGTCAGCATGACTAGACCTCTCCGCTCAAGCAAAACGAAA
ACGAAAAGACAAAAAGAAGTGGTGATGAAGAAAGGATGCACTTTTGGCG
GTGTCAGACGAACATTGCCGGCAGATGCGATGCGATGCGACACGATCAAG
CCTCCAATTGCGTCAATATGAAGCCTCGGCTCCCAATGCAAATTGATTTTCT
CGCATGTGGCGAATACGCTGTAATACTCTGTAATATCGGCGCAGATACGGCA
GATGCTTCAATTGGCCGCACATCACGTATCGAGTTTCAAATGTGCCAATTGA
TATATGTTGTCGTCGATCGTTTGTGAAACAATGGATTGGACGAACATGTGAT
GGCTTCATCTTCAATGCTCCCCTAATCGGCTGTCTTATCCTAATTGTTCGTTA
GCCACATGCACATCGACTAGGTTGGAGAGTGGAGATTTCTTCTCTACCTTG
GGCGAATTTTTGCTGGGTTCAATAGTACATTATCTACTTGTATGTACGTATGC

ATGAGGTGTTTTCGGTTCGTTTTTAGTTAGGTAAATTCCTAATTCTTCAATAC
TTCCTAGGTTGTCTTCTGACCCGGTCTGTTGGAAGCGACTTTGTTCATCGTG
GCTACGTGTCATGCTCACCTGGGGTGTTCTATAGTCACAAGGTCGAACTCC
TTCCAGAATGTCGCAGTTCGGGGAAAAAAACATTCCATAGAAGAAAAGAT
CGTAGCCAAACACCAATACCGGTGTTCTGGAAAGGTGTGGTAGTATTGGTA
GCATTAGCATCCTTAGCATCCTTAGCATCACATTCTCAGCTCATCGTCAGCA
CTTGGGCACCGTATTTGGTATTCGGCCGAGCAACCCTTGAGACTCAATCTAT
CTGTAACCTTGCGCAACTCGAGTTAGAAGAGTAGCAGATACCGGGCTGAA
AAAGATCAACGAGGGTCAAAATCGAGATTTAATCCATGAATTATAATACGCA
ATCCGTAACAAGGCATGGGTTCGCATGGGTTCTTTCGTCAGTCGACGATCA
ACAGGATCTAGAACTTCAAAAATCTCTGCTGGAAAAGACCCTGAAGAACC
CATGCGAACTTGCCAAAACTGACCCTTACCCTCGTCTCCCCAGTCGCGTCA
ACTGGCAGACAGACTAGGTCGAAGGGTCGAACATGCCAACTTTAGGCTCG
TGTAAACGTAATCTGCGTAATTAAGGTAAGAAAAAAAGGGGGCGCAGCATA
GACGGCAAGCTCCATCACGATGCGGTGTGGAGTTTGGGTAATTGACCCCGG
CTCCGCCGCCCCTGTCTCCCGGGGACACCCAAGGATCCCCTGAGGCCCCCC
ACGGCATGGGCGGAAGCACAGGCGGAGGGACTACATTGGCCGACCTTAGA
CAACTCGAAGCGTTCAACGTTCAATGATCTTCATTGCAATAACAATACGTAG
TAAGGCCGGCAACGCCGCCAATGCCGTCGTGAGACAACGCGTTTCGACGG
CGTAGGAATTCTTCATTCCGTTTCATTCATGGCATTTCCCGCTACTGACTTTA
GTTTAATTAATAACGGTCCGTTAGTCCCCGTCAGCTAGTCGACCACTGGGTA
ACTATTTGCTTTGCCTAGACTGCATGGAGAATTATCAGAATTAAGACTCCTG
CTGCATAGGGAATGGATCGCTTTTACGACGGACCCATCACCCGCATGCAGC
TCGACTAATCCCAGCTAGCCTGAATAATTCGCCGGCGTTGGTCGTTGTTGTC
GTTCGACTAACTGGCGGAACGGTTCGCTGGCTGGCATGGTACTGTCAGGA
AATAGTTCTTAGTAGTCTTCACTAGTAGACTGTCAGTTAGACTGTTGGTTAG
TGTATCGCGTCGACTACCTTGTAGGTGTCAACGACTAAACTAGTGAGCGAA
CTGCCGCCAAAGATCCCTCGCAGAGGTCCACGCCGTCGGTATTGCCTTAGT
ATATGCCAGGACATGGCCAGTACGCTGACATGGTTCGAGTTAATAGCATGAT
TTCGAGGCGAGCAGGGCGAGAAACCTGAATGGTATAGAAACGAGCGAAG
GAATAGGACTAAGCTAGGGTAAAGGAAATATTTTTCCACTAGAAGCTGTAT
GTATGCACTATGCTGGTCTCTGGTCTGTATTCTGGCTGGTGCGATAACCTTG
GTAACGCAGCTAAGCTGACTTGGGAGTTCTGCTTTGACTTCTTGGTAGACG
ACGAGCACGTGTTTTCGGTCTGCAGAAGGGTCTGCAGAAAGTACAGTGCG
TATTTGTCCCAGCCCCTATTTTCTACGTCTCCCTCGCTTCGGCATGTATGCAC
TGTAAGAGTAGGTACATAGCAGCAAAAATCATGCACGTGTGTAAATTCTCAT
GTAGGTGATTATGTAGCTTGTATGTATGAGATTATGCTAAACACAACTATGTA
CATGTATGTAGTATATACTGCATATTGCACCGAGGAAATAAGTCCCTCCAGG

CATTATGCATACCCAGGGGTCTCATTATGTGCTAGTCGCTTAGACATTAATGA
TGGGTATCTCTTCTGGCTTCTACTCAAGTAAAAAAAAAGCAAGTCGTGCAA
GTGACAGTACAAGTAGAACTTTTAACAGAGAAAATAGACCAAATGCTGAC
GCTCCAGCTCAACCTGTACAGTAGCCGAACAAAAAAAAGCAAAGGGAGG
GTTTTTAGCACAATCATCGCGAAATGAACTCCCAAGCCCTGCCGTCCCAAA
AATAGATGTACAAGCCTTTTTGTAGAACCCATTCGCGCGTGCGCGCCCGTA
CAAAACAAAGATCTAGGTAGAAATGTTTCTAGAAATCTAGTTTAGTTTTGGT
TCTTCCGACGGCAGAGTTTTTAAGCTGTCATCCAGAGACTCCAGCTTCTTC
ATGTCTCCGTACTTGGCCGCCCACTCGTTGTTGACGTTGCGGTAATCCTCGG
GACCGTAAGAGTCGTAGAGCTCACCGAAGAATCCCTTCTTTCGGTAGATGT
TTAGACCCAAGTCGCGCTGCAACAAATCAAGATATGGGACAGCGTCGAAG
ATGAAGTTAGGGCTCTTGGAACCCCAATCGGGTCGGGTAACGCCACTTTCC
GAAGCGGTTGTATAGAACGGTTTCGTAACGCAATTGCTCCATCGCCTTCTTA
TCACCCAAAGATGCTGACGGAATCCTTTTCTAGCTTACCAGCGAAATAAGC
GCTGATCCAGAGACCTTGCAGGTGCGCGGTGATCATGTTGCTAAAGTTAGA
GACGATACCGATGAATGCAGTGTCGCGGGCACGCAGGAATCGCTCGGACG
AGGGCACGATGAAATGGTGAAGCATGTACGGGGTGAGTGGCTTATAGGGG
GTCACTTCGTCTGTGCTGGTAATACCTTCTGTTGTGTCATGGGTATATAATTC
TTGTTCCACACGGGTTGATCCTTCAATCGGGGGAATTTCTCAAGGATTTCCT
TATCGGCTCGTTCAATTAATGCTTGGTTGTTGGCGAGATCATTTTCGCCTCC
ATCCTGGTTAGGGGTGTGCGGGACACCCAATTCGGCCTCGATTCCTTCGGG
TAGGAGCTTGATTGGCGGAACGTGCTTCCAGCCAGTGTGGGCGAGCAGGG
CCTCAGACTCAAATTCGGTTCCGTCGGAAAGGTGGACCTTTCCTGAGCTCA
AGTGGTCAATCTCGCTCACATAGACGTTAACAAGATCGCTCTTCACGATATC
CATGAAGTTGGTGTCGTAATTTCAAGATACTGAAACTGGACCCTGTAAACA
TCGCATCCGTCCAGGGCTTCAGCTTAGCTGTCTTCGGGTGAGAATCGTAAC
CCATGAGATTGATAACTCTCCGCCAAGAACTTTCCAAAAAGAGTTGACGAT
CGTCCTTCCGACAGCTGTGCCATGGTAAAAGCCACGGACGCCGCCATAGCC
GTCAACATCTCCCCAGACGCAAGGGCTGAACCAGGTAAGGAATCGGATATC
TGTTCGATTTGTTAGAATTTCAGATTATAAGGCAAAATGTGGCTGCGACAAA
CTTACTAGCAAGCTTCTCAATCCACTTCTTTAGTGGTGTCACATATGATGGC
GCGATCCAGCACGGGCCGTGACCAGTGGCTGTCAATTATGGTTAGCCAGGT
AAGCCAACATCGTCACTGACCAAGATGGTCGGACGAAAAGGGAAAACAAT
GGCAAAATAGAGGAAAAACATACATCGAATAACCCAATCGACCTTGACGCC
CGCAGTCGCATAGGCGTAAGACAGCATCCCAAGCAAACTTAGTGCCGCCA
AAGATCGTCACTCTCTTTGCCGTCTTTAGCGTATCGGCGTTCTCTTGGAATT
GCTTTCCGTGAAATATTCTGCCCCATATTCCTCTTGGCCTTCAAAATGTGGC
AAGAACGGTTCCGAGGTTAAGCCTGTGGCGAAGACGACACGGCGCGCATA

CACCTTAGTCTCGCTCTGGTTCGGTGGTGCGACTGTCAAGACCCAACCGCC
TTCTACAGTATCTTGGTGTTCGGCAACGAGCACTTTAGTGTTTAGACGAAC
AAGGCTGGCGATTCCAAACCTAGTCGCATATGCTTTCAGGTAGCTGTTGATA
ACTTTACCGGGCATGTGCTCGCCGGGCTTGACGCCGAAGGTCGCCGTGTCC
ATAGGAAAGTCGGGGTACTCGAAGGTTCCTAGCAAGTTGTTGCTCTTCAAG
CCAGGATATAGACGGTGGTCGGCCCATGTACCACCCAAGCTAGACTGAGAG
TCGAAGACGGCTAAAGAGCATTCTGGTTGAGTGCAGTGAAACTGCTTCGC
GGCGGCCAATCATACCAGCCTATGATGCCGAAGCAAAGTCAACTAACAATC
CTTTCTCGCTACACTAGGAGGTAGGTATGTATCGAGTGATAATGATATACGTA
TAGTATAGTAGTAAACTCATGCAGGTATGACGGAGGTGGAAATGAGGACGA
GACTAAGAGAAGGGTATGGCTTACCAGCACCGACGACGACGCAGTCCAGC
TTTTCCATGGTGATATTCTCGTTCTCGCCCATGGCGATAGTTTCGGTTTTCTC
GTTTGTTGGTGTTGGTGTTTGTGATAGTGATGTTGGTAGTGGTGTTGTATCT
GCCATGACGAATATGTGTGCAACTCACACTCTCTCGCTGGGTTTTGTCCGA
CAGCCCTACTGCTATGAATGCAACTAGATATTACTTCTTTCTTGGTCTAGAA
AAGAACCGGGAAAGCGGATAATGGTCATGCGTAGGATAAAC

>Mosaic_sequence_64

CACTTGACAAGTTGGTACGAAGATGTCAATTCCCGGATTAGGAGAGATATT
ATCGGTGGTGCGTGTATTTCTATTAACCCACTCTGACTGATTACTAGGTAGC
TAATCTTGGAATAGGCGGATCCGACTACCTCATCTACAGCCGCCGCATCGCC
AACTACCACGGAAAACCTCCAACCTTTCTGGGAATACCGATTTGAAGTTCC
TCACGGATCCACCCTCGATATAAAACTGCTATCAGGGACTGCTGAGAAAGA
TGGCACCGAACTTGCTCCAAATACACCTTACACTTTTACCGCGACGAAGTC
TAAGATAAATACCTGGCATGGATGTACTCTCGAGATAATATCTACATCTGGG
ACTTACGATGCATACACTTCAGAGCCGACGGCGGATGAGACACCCATGGTT
AGCTATCTGAACTTGCATTTTAAACTCGAAGCCATTCGCGCCGCCGCAGAA
AAAGCAGGACAAACTGGGCCACGAGTATTGGTCGTCGGACCGCCGAATGC
CGGTAAGACGAGCTTAGTAAAGATGCTGACGGGATGGGCTACACGTATGGG
CCGTCAACCCCTTGTCGTGAACACAGATTGTCGTGAGGGTATGCTTGCATT
ACCGGGTTCGCTAAGCGCTGCCGTATTCGCGACTATCATCGACATTACAACG
GATTGGGGCAGCACGCCAAGCAGTGGACCAAGCGCCATACCAGTCAAGCT
TCCGATTGCGTACAATTACGGTTTAGGACAACCCGAAGAGAATATAAGACT
TTTCAAGAGAGTAATAAGCAGACTAGCTGTGGCAGTAACATCCCGGCTCAC
AGAGGACCCCGACGTAAAGACATCCGGTCTGCTAATCGATACGGGCGGTGT
CAGCGCTTCTAAGGGCGGCTACGACCACATAGCCCATATAGTCTCCGAGTT
CTCAGTCAACATAGTAATCGTGATAGGTTCGGAACGTATGGGCAGCGAGAT
ACAGAAGAAGTTCGCGGGTCAGAAAACCTCGCTGGATGAGCCCATCACCG

TGGTTAGTCTGGATAAGTCTGGCGGTGTGGTAGAAAGAGACTCGGGATTTC
TGCAACAAGTTCGCGAGGCAGCTATTAGGGAATACTTCTTCGGCAATGCTA
ATACCACATTGAGCCCATTTACGCAACAGGTAGACTTCTCCGCCTTATCGGT
CTGGCAAGTTAATGAATGTACGTGTGCTATTAACTACTATGTAGGGCAACGA
TACTAACAAATATCTAGCACCAACCTCGGGGTCTCTTGAGGATGAAATTTAT
AATGATGCAGTCTTAGAGAAGGTTGAGCCGTCGCTTATGATGCAGAACTGC
CTTCTTTCAGTTGTCTATGCGTCTGTGCACGACCCAGCAGACACGATCCGC
GATTCTAATGTCATGGGATACGTGTACGTCGCTGACGTCGACGAGAAACGG
CGAAAGCTTAAGGTTCTAGCACCAGTGAATGCCCGCTTAGGGGATAGACCT
CTCCTCTGGGGAAGTTGGCCTGAGCCCATGGTCAGCTTACTGGGCTAGACG
TCGAACGAAATTCTTGTTTTTTTCGAAGATACACAGAGCACTATACCCACAT
GAAGTCTGCTATTTTTGCACATAGATACTCAGCCAGGGTCATTCTGAATTAT
AGGAACGGAAAATATCACGAACTGCAAAAAAAAGGCATCCACTTGACGAG
AAAACGAAATAACGATATAACTCGAAGTCTAAGAAGGGAGACGGAATCGA
CTTGTGCACGACGTCGCACCTGGATCCTAAACATAGTTGGACAACAGGAA
GAGGGACGACTCTAGGAGCAACATGAGAAAATTAAAAAAGCGCATAAGAA
TATAATCACGACGCAGATACCAAGGCCGGCATTCCTATGCCATTTCGGCCAC
TCTTACCATTACAATTCAAGAGGTTCTTACGCGCACAGAGCTGATTTGTTTA
CGCTGGGGCGACTTGGATATTATCCTAAACACTGGTTACTTCACGCGGCTAA
ACCTATGATTCCAGATCTGCTACCATAAACTACACCCATTCAAGGAAAGGC
GTTTGATGATCGGGTCACCTATTAATCGACTCTGAGAAAGTGTCGGGCTGG
ATTGGCTAGCAAAATACCACGGATATTCGGCATAGGAATTGCGTTCTGGACC
TAGTTTGGTCATTGATGTCGATTATATCTGGACTGCTTGAGGTGAACTGTGG
TAATACTTCTCTAATGGACAACATTACACGATATAAGAATTGCTTCTAGATGA
CGAATGGAACTTATATTCAGAACAAATAATGACTGATATGGATCACGAATAC
TTGCATCAGTCGCCTTCAACTTATCAAATAATTATATTTTAGCAATAACTTAT
GGCTACCTCCAGTCTGATGACCTTTAAGTTCTTTTGCTCTAGCTGCTAACGA
TAATATCAGTTACAAGCTGCAGAGTCGGTTCCAAATCAACATCCGAAGCGA
CATTACTTAAGTAGTTGTCATTGTTTCAAGACAGCATGAGGTTCCTCTGACC
GCCGCGTTTGGCCCCTTTCTTCTGTCTTCAGCCACGAGGTGAGGTAACACT
GTCAAACTCCCTATTACGGGAATTGATATATTTAAGGTTTCATCCTTGCTATG
TCTGTTTCCGGAGCAGTGCAGGTAATTTTGAATACGCGAGGCACAGTGATT
ATCTTATGAGTAATGGAAAATGAAGTTGACGTGGACAGCATCTCCTGCAGG
CTGCAGAGCCCTTAAGGTGTGAACGTTACACGGTCACAGCGCGTTAGATTA
TCTGGCGATCAGAAGAGATTGTCACTCAGGAATAGCTGGTAGCCATGTGGA
CTATCTCTTGGCTACATGCTTCCGAATCTCCAACACTCATTTATTCCCTACCT
ACCTGGAAGGTAATCGTGTTCTAGCAGTACGCGTGTACGTAAACATAGGCA
ATTTCACAACACGTCTCAGACATGGCTCTAACATGGCGTTGCTAAAGGCTT

TTCACGTCGGTTACTTGGCGGAATCGCTATAACTCTTGGCGTCGACAGAAG
CGTAATAGGTGTCCCCATAGAAATCTGGTCATATGAAAGCTAATGTTTTTGT
ACTAGCATCGTTATCCATGCTGTCGGCGGACTCACTATGTTTTCAGCAGACA
GTGGTAGCAGCGAAACTCGGGTATCGGATCGTTTAGGCCTTTGAACCGTAA
TGGCTGCTCTGAGAAGCCAGATGCTAGTCTACATAGGAAGATTTCTCCGTC
GACGCAAAGCCCGACACCCACGACGCCCACGGAGAATAACACATCTAGCA
TAGATAGAAGTAGGGTATGTATTACTCTCTTGATTGGTGCTCTTGTCCCTTTT
CTGCAAGTAACGGAGACGATTTTGACCCTATTTGGACTTGGTCATAACTCTT
CTTGTCCTTCGATTACCAGGTTGGGGCAGTGATTACAAGCAAGGCTTCTTC
CCGTCTTTTACTACTACGTCAGGGTTGCAATTGTGACTGCGCTGACTTGCTT
GCAGCAGGTAGCCCTTCTGCAACTCTGCAACTCTGCCAGTATCTCAGTGAG
CAACGCAGGGGTATCATGTAGGATGGTAGTCTATGATTTGGACACGGCATAG
CAAGACACCTAACTACATACCTGACTACTAAGAAATAATAAAAAACTAATG
TCTACCTAACCAATTGCGGCGGATACCTAACACGGCGTAAGCGCTGGTTG
AATGTAGTAGCATACGACCTGACTGAGCGTTATAGTCACATCATGAACAGCT
CAAGTGATCTCCGCGTTCGGGACTTAGCGATACGACACGATTAATTGACTTT
CAGGGCGATGCCACCCTCCGAGGCCCCCTAGTCTTCGGTTGCTCCCTACTC
CGGCCACGACTAATATACTAATTCTCCTAGAGCAATTTACGTGTTGCAAGGA
GAAATTTCTAACCGCCGAGAGGAATAATCTTTTGCTGCACCTAAATAGGTCC
GAATTATAATCGAATCTTTATAAGTCATAAGTGACGCTTGAGGATCTTGAAG
ATCCTCCACTCACCCCCGGAAAGAGTCAAAAGTTGAGACGAAATGGCATA
AAGATCGACCCCAGCAAGAGAGTGAGAGGGAAAAAAAAAACCATACCCT
TACCCCTGTCTGGGTCTACCGGAAATCTTCAAAGTCTAGTTCAGGTCAACT
CAGGTCACTAATGATTTGCCCCGTCTCTTCTCCACCATTTTTCCTCCGCACT
GCAACGCTATCTCCCGTGTGTTGCTGCTAGCCCAGATTTTACTCCAACCTCT
TCCACTTTGCTAATTGGAGCCTGACGTGAAAGTCTCCTCGCTATACCCTCTT
CATACTCGTCTGCTCCAACTTCTTGCCCCTTCTTGCCCCATACCCCTGCAGC
GGACTAAAAAAGTTGTCATCTTGCAACCAAAGGTCCTAAACAAGAGGATC
TATGTCGAGATGTCCCCCCATCCATATCCCCATACCCCCTTCTCGCATCATCG
GTCTCCCGACCCCCTTGCTTTTAGACTCGGCCGGCTTACACAATTTTTCCGG
ATTTCATGCAGTACCGGACTGGGATTCGGTGCAAGGACCCTCCGGTTAATC
CCAAGACAGCAGGAGAGGCGGGGGATGCTGAACGATGGTGGAATGCTAGT
CGAGGATAGCGAATATAGCGTGTCTCCGGAGGGGGAGACGGACCTAATTTC
AAGATGACGAAGGCTGGGCATGTTGTCATCGCTAGAGCCCGTAGACTCAGC
TTAAGAGTGTCCGTCGGGGTGACTTTTCTACTCGCCTTCCTTATCCTCATCC
GTCAGTTGACTCTGTTCAGTGTCAGTGTCAGTGACAGTCAGTCGATCGACA
ACTCAAAGCATCCAACCATGTGGAAAGGTATCTTCCCCGCAGGGCTGCTGT
CCCTTGGATTAGCTGGTTCGGCATCCGCTACCCTCCTTTATGTGGCCTCATAT

GCTGGTACCGTCACGACGCTGCGTCTAACCTTGCCTAACGATGACTGCGTC
GACGGCACTGCTGCTACCCTCGCGACCATCTCCAATTCGACGGGCTGTGGA
CCGAATCCGTCGTGGCTAACGCTCGACTATTCCAAAAACATCCTCTTCTGTC
TAGACGAGGGACTGAACGATCCCTATGGAGGCGTGGCATCCTTCTACGTCA
ACGATGATGGCACACTCACCACACTGAACAGGCTTGAGGTGATACAAGGC
CCTGTAAGCATAGCTGAGTTCGGCGCTGGAGGGCACGGACTCGCCCTCGC
ACACTAGTAAGCCATCCTTCTCCCATCTCCTCCGCGTGCGGCGAACACGTT
CGGTGGTTCGCAGTCCACTCCTATGTCTTGGCATCACCTACAATATCAAATC
ATGGTCACCGTCTAGATAAAAGGGTTTCTGCCATTGCTGTCATCACTGACCA
AGCTATCCAGTTCCGGTTCTAGTGTCAGTGTCGTCGGGTTCGATCCCGAAG
GCAATCTCACTCTGGTGCAGAATGAGACATATACGCTCGCGGCACCTGGGC
CTAACGCTGACCGTCAGGAGGCGCCGCACCCGCATGAAGCGATATTGGATC
CTACCGCTTCGTACGTGTTGGTCCCAGACCTAGGGGCCGATGAGGTGCGCA
TTTACCAGGCCGATGAGACCACCCTCGGGCTGACCCCTATCGCACCCCTTA
CTGTGCGGCCCGGATCTGGTCCGAGACACGGTACATGGTTAGTCACTCCGG
AGAAGACGTATTTCTACCTCATATCCGAGCTAGCGAACGCCATTACCGGATA
TGAGACTATTTACAATGAGGATAAGACCCTGAGCTTTAATGAGATATTCTAC
ATCCCCACACACGGTGAGAATATAACCCTCCCGGAAGGGACAGGCGCAGC
TGAGATCGTCGTATCGGTGAGTGTCACTCGTGATACGCTTTTTTTCTAAAA
GGAAGCACCTCGGCTAACATGATATGAAGCCTGACGGAAATTTCCTAATTG
CGTCATCTCGCTGGGAGAATACGTTTAATATCACGAACTTCGATCCTAACAA
CAGCACGTTGATCCCCTCAGATCCACTCATAACTGTGAGTACTTACCTATCT
GTATTTCGAGATTGCTTACTCCCTTTTCGTCCACGTGATTTCTTATTGTTTCT
TGGCCGAGGTCTCGTGAACTCACAAGATTCATATAACTCCGCACAAGTACA
TAGATTACTAACTCACGAGCGTTACAGTTCTCCATTGACCACGCCACTGGC
AAATTAACCAAAGTCCAAGAGTTTCCCGCCGGCGGGTAGCGGGCCTCGGC
ACTTCTCCATTAGTGCGGATGGCACCTTGGTCGCCTCTGCGCTGCAAGCGG
ACGGCCGCGTCGTGCTCATTAAACGGGACGTCGAGACGGGCAAGCTCGAG
GACTACGTCGCGTATGCGGATATCGAAGGCGAGCTCACTGCGGCCATCTTC
CGCGAGGAATATCCTACCTTCTACTAAGTAGTAGGTAGTCTAGACTGCAACT
TTAGGTGCCTACCTAGTGGGTTGCGTGTTGAAATAACAGAAACGTATATACG
TCGTACGACGCGCAGAAGCAGCTAGCCCCTTTCTGTGGACCGGTTTCATCC
TCGTAAAACTTTACTTCGTGAAGCAGATGTGAGTGGCCAATGGCCGGCTGA
TACGGATGGTGACAATACACACGTATATAGGTAAGATTAGTTATGTATAATAG
AATCGTGCATAATAGGTAAGATATAGATATTATATCTGGGTGCTTTTAAGGTC
ATGTGGCATTTAGTATCTTCACCGAGCGACACCCCTGGAAAAACGCTCCAA
AAACCTCCCAGAGCCTCATTCACGAGACCGAGGGGGGCGATGCGAACATG
TGTTAATTGGAGCCTCCTGCGGGGTTCGAGCCAGTAGCTCACACAATATAG

ATTGAGAGGAGAGATATTACGGTGCGGGGGAAGGGGTCATTTTTTTAATATA
GGTAGACACGTAAGTAAGCCCGTCGAAGTTTGGTAAGCTAGGATGGGGAG
GATAAATGAAGTCGCCACTGGTGAGATTACTTTTTGTGAGGTTCCTCAGGG
ACGAAAATAGAAACCCAGCGCGAGTCTTCCGGGATACAAGGCCTGGTCTG
GCTAGGAACGGGATGGCCGCTGCGGAAGAGCGTCACCTTGGGATGTGTGT
ATGTGGTACCTAACTTGGTGCTCGAATCAAGCTAATGTGCCCTGCGTATGGA
AGATTGTTGATCAGGGGCGAAAGCTTTGGACATAGCATTCGTCATCCGCGC
CGTCGACCTCATCATAGATAATCTCCTTACGGTAACAAGACGACGGCGGAG
AGGCTTGGCAAGCCCGTTTCTGGAACTGGATACGGGAGATCGACAGCGCT
GGCAGTCTTCAGGGTCACGATTACGTAGCAGTCCTCCGTTCGAAAGAATCG
TCTTTTCTTGTTCGTACGATGGAGCTGAACCAGGAAATCGTTTTGGGATTCG
CGTAATTATATTAGCATCGCATGAGCACATCCTGTATGACTTACAGCTTGGTC
CACACCGGACTAAGGTGACCTGGTCACACGTTCGAGACGGCAGACTGCGG
CCCTCGGTAACGGTACCAGAAAAGCTTGACCATGTAGGTCTGTAGCGGGTT
GGTACGACGTAGCGAATTTCCTTTTTTTCTTCTACAGGGTAGGAAATGTAAG
AAATAGCACACACTTGCAGGTATGTCTCAGGTCTCAGGTCGGTAGAAAGGG
GGGTAATTGCTCGATCTGGATGTGCGGAATGCCGTTTTGGGTCCGATGATTA
ATCCAACACGGCTGAGGATGTCAAGGTGTTGACGTCTAGACTTCGATCGTG
TGCTTTGCTTTTCCCCTTCTTTTTCCTTTTTCTTGGAGAAGCAGAAGAATAA
AAATTTTGTTGAATGTCAGGCATGATACCGACGTTGCACTCGTTTAGTAGTC
TCTGCCCAAAAAGCGCACGGCCTCTCTCCTTTACCATCTTGCGCGTCAGGT
ATGTAGGTATTCTTTGCATATGTCTGGGACGGCAGGTATCACGATCGTGCCT
CTCTTATTCTTTCTTATTTTTGTGTTTTACTAGGTTTGTATCAGGTCTGGTCAA
AGCTGGTTGGCGGAGCCAAGATAGACGTGCTCGTGGTACTTAGCCTCATAG
GTAGGCTGGTGACGAGAGAGGGTCTATCCCTATTCTAAAATCTAAGAAATA
CTACCCCGAACCGAGCGAGCCCATGTCTTACAGCCCGTAACTTTGCGCATG
CGGGGTCTGCGGCGAGGCAAGTGAGCTAGCGAGAGTCTGAGCAGGCTGG
GTAGATGCACAGGAGATCTGGTAGGTCGATCATCAAAGAAGGGATGTGAC
ATTTTTGCGATGTCATACATAGTACATAGGAGTTTTTGTCGAATGCCATATTG
CAAGGTTAGTTCAAGGTTTGTTCAGGGTTACTAGGTAGTTGTCCCCTGTCG
GAAAGTTGGCCGGGAACGTAGGAGCCGACGTCGTTGGAGTCTATCGCAAA
GAGTCTGCCGGCAGCGGAAAGACTCCGAGCGGCTGGGCAGATTTAGTAAA
CTCACGAGCACGAGCATGGGGAAGCATTAAAGTTATCGTAAGCTCTAACAG
ATATTGGAGCAGCTCGTACGAGTTTCGCCATAGGTCGGCCACGTTATGAGT
GCAACCAAATGCCCACTTAGTAGATTAGAATGGTGGACGGTGTCTTGGCGT
CGAGGACAAGAAGTCGAGTTAGTACGAAGCAGTGAGTAAGAAATACACGA
TACTACGTGCGTACATTTAGAGGTGGTAACGGTGGCTACCGGCTGACCGGG
GCTGTGGGAATAATATCAAAACTTCTCGTAATATAACTCTGTGCTGTTATTAG

GTAGCCTCTCTGAACTGAAATTGTTGTCGGCTCTGGGCGCGTGACTAAGGT
AAAAGTTCCACTGCGGGAGCGATAGAATAGGTCCTAGGTACTGATACAACC
CCCATATGATCTACTTCGTTGACAATATGGAGATACCCGGGACGTTACCCTG
ATGAGGTTCAAGGCTCATATAGCATTTCTCATTGTAACGAGATGGTGCATGG
TGCGACTGTGTACGTACGCATTAAGTATGACCGTCTTGGGCTATTCTCTTAC
ACCACGAGACCCTCTTCGTCGTCATATGACTAAAGATGACAAGAGAAAGG
AAAGGCCGAAGGATGCGCTGGCTGTGGATAGTCGAAGCTGAATCTTAGTAT
GTAATAATAATTCATGGTGATAATGGTAATATCTGATTAATGGCAGGTAGGTA
ATCTTAATCTTAAATCTCAAACCGGACTTCTCAGCCTACGGGAAAAGCCGT
CGTGTTTGAGAATTTCTCTTTCCCCACAGAAGTGATCTACTCTGTTCGGACG
ACTTTTAGTAGATGCTAGGCCTAGTCGGAGCTTATAAGTGAGAATTAAGTGT
AAGGATAATGCCCTCTAGGAATTGAACTCCGCGCCGTGGCTTCGGCGCAAG
GTTGCCACGTTTCATCACTCCCATACTCCCACTATCACTCACTATTCGGCCC
GTTGGAAGGCTTTGGTCATGCAAGTCACCGTCAACACTCAACAGCCGGCG
AGACAGTTATCCCACGCACCAAGCCTTGGGTTATAGCTGGCTAAGTATGTC
GAGCGTGGGTGAGAAAATAATTCCTAGGTTTCGGTTTGAAAAAAGATGGAT
CGCTGGCGCTGGGCATGAAAACCGGGCTCATTTCTTGAGTCTTCCAGAAGA
TTGCTAGATCATAGTAGATCTTCATGCTCGCTGTAGTAATCCGCGGTCTGCCT
GAAAACTCCGGTCCACTATTTAGATGAACTCGTCCCCGTGTGGAGCAAAGC
TTCCAAGGTCAGCCTCGCAGGTTTTCCCGTCCAGAGGGTTATAGGTTGGGT
ATAGGGTGAAGCGTTTGCCTGGTAGCGAGAAGTGATCGTCTAGTTCCTCCT
GCGCTAGCGGAGCGACAGCGAACAAGTACGGCACCCCCAGGCTGTCGGCC
GTAAAAGAACAGAAGGGCTTTCCAGAAAGGAAGGTCAGCTACCTTCACTA
ATAGAGATAGTCATCCGTTCTTATATTGATGTCGCAAAATAGCGATGGTAAA
CCGGCTCTCGTGACTTAAGCGAGACGATGTAAGCAAATCGCTAAATGAAGA
CCAAGGCCTCTGGTCAACTCATTATATATAAGTTGGAGCATCTTTCACGTGT
GGCCAGGTTAGATTGATAAGTTGGCGAACGGCGAAGCTTAACTGATGGTTT
GGCCACACTGCCACAGCGTCGACATCCAATGATGGGACACCCATTACATGA
GGAGGTAGGTATGGTCATTTCGGAGGTTGACTTGTCTTCAGAGGATTCTGG
GGCTTATAGTCGAATTTGGTATAGTATGTTATTGACCAGTATCATATAGTATAG
CATATGAGATGCGTCGTATCTCCTTTAGGAATACTCATCTGAAGTTCTGAAG
AGTCTTGAAGGTCACTACCCTTTGGGGTAAGTGGAGATCTGCTAGGTAGGT
GAGTTTACTTGCCCAGTGATGGCTCGGCCGAGTACTTTGAAGATCTTGACT
TGTTGTGTGTACCGTAGACTAATGAGCGGCTTTCGGTAGAGGCGTTCTAGG
CTTGGTCATCATATATCTGCTTAAATCGTGAGAATCGAGGTTATACAGAGAA
ATACAAATCACTAAATAGCATTCCGATGAGAAGTTTTTTCAAACTGTCAAAT
AAGCTACTTGGTATGCAATGTAGTGCATGTATCTCGCATGCTCGTGTGCCGG
ATCCTGGGCAGTTGGTAGATGGGGATGACAAGGGTCACCTACCGCACCAG

CCCGCACCTATAAGTACCTGTTAGCTAGTACATGTAATAATATGACTTGTAAC
ACTTTATCTAAAGTATCTCCGCAGTGGCGTACGGCATTCGGCTGCTCCAAAT
CAGACGATTAAGGGTCCGAGCCGAATTCATGTACCCCATGCATATAATTACA
GCACATAATGTCTGCTGTTTGGGGTATCCGCGAGTGGCATCTAGGCTTTATA
CGGGTAGCTAAGATATCATAAAATGAAATATACGAAGACAGAAGATGCGTC
CAGACACCAGACACCGAATTGCTTTCCCAGCTTGATTCTTGGAGGATTACT
GCGTACTGTATGTGGTTTATGTTGAGGTTAGCTTCTATTGGCTTTCGTTAGCT
TGTAGCTTTAAAAAGTAAGCGCCTATACGAATTTTTGCATAACCAGTATTTC
CCTACGTAACTCGAGGAGTATGAGATAAAACGGTTAGTGTCTAACGATTGC
TTCCGTTCCATGGCAACTTCTGCGACTTCTGCAACTTCTTCCTCTGGTAGGG
CTAGTAGCGCTAAAGCTAACCGAAATTTTAGTTTGAGAGTTAGAGTTAAGC
AACCGCTATGGATTGTATGGAGTAGCAGCTGGAGCCCTGGAGTACCTGGCT
TGTGTAGCCTCTAATTGTGGCAATTGTGGATCTTGCTAGCAGTAGAAGAGC
CTGTGGGCTACAGCTAATAGCAGCTGTGCCCACCTTTTCAGATTCTTCCCCC
TGTCAAACAACCCACAGCACACCCTACACCCACCACCACTCCATCCACCA
AATCCACAACATCCATGATCTTCCAACTTCACCAAAAATCTTCGTGCCGTCC
CATCTTCAATTGCTATCGTGGATCCCACCTCAACTACGGAAATTTTCATATTC
CCTTTCATCCGGGTCCTTGTCCTGCGCAGGACCCCAGATCCCATCTCCAAA
ATTGCATCCTACGTACTTTTGTACCTTGTCTTCTAACAATTCATTGTCTTCTT
ATTCGTCAAATTTGCGAGTCTAGGTCTACCGCAGTATAGAGGTACAGTATAC
TCTCAGAGGTATACTCGTCCGCCTTCATCTGAAGATCTCTGTTCTAGAAATG
ACATTTCAAAGTTCAGACCGATCGGTACTATATCTCAAAATGTGTATACCGA
TTTTCAAATCGTCCCATGTTTTATACTATTCATGGAAAGATCCTGGCCGTAGC
CAATGCGCTGGGCTGAATTATACTATGTCGAGACCGTATAGTCCTGCAAGCG
AGGATTTGAGGATCTGCTTATTTGTAGTTCCATGCTTCTCAAAAGAAACCCC
GTTGATATCAGCGCTTTGGCCGGTATCGAGTCACAGGTGTTAAGTATCACCC
GTGGCAGAAGCCCCATCTTCCCCATCCTCTTTCGGTCCCTCTGCATAGTGTA
GGGCGTGAAGGCATGGCAGAGAGTGGATGTGCGATGCATGATTCCCGCCTC
CAGGGCTTGCCTGTTCGCACGAGGCATTACGAACTTGTATCACCATAAGGA
CCTAACTACCTAAGAACCTGTTCATACGGAAGATGGTTATCCTGAAAACCG
GTTGGTCAGTCTGGTTAGTGAGTTCTGAACGGGCGAACGATCAAAATTGCG
TACAATACTGTACCGCAAGCTTCCAGAAGATGGGCTCCAATCATGGGTGGG
CGTGACCGGGTAGGGATCGAACCCTTGTCTCCTTGCAACGTCCCGCTCTGC
TGCTGTACTGGGTACCTTTTGACGACACTGCGCCTAAACCGCTATAGGTAG
GAATTTTCTGGGGTTTTACGGCTGGCTGTCTCGCTGGTCCCCCTCTATTCTT
TAGACGGGCTGGGATTAAATACTAGGGGCTCAACGATTCTGCGCCGCTTCT
GCAGGTGTGTACCTACCATGTGGAGGAAGACATAACCGTCCAAATCTTCCC
ATTTCTCCTACGGTTTCCCGTCCTTAATACATGATAAGCTCGTTCCCGTGCCT

TGCAGTTCGGATACCCTCTCATCTTTTTTTGTCCGAATCTTTGTCCCTGTAC
GGCATCCTGGGTAAATACTTCCTCACCGAAGTCGTATGCTTCAACTCTTGGT
TCCGGTTACACGGCCCTCATACGAAAACGTAGCCAGCATATCGTCTAAGAC
ATAAGCGAGACTGCCCGTGGTCGTTTCCGCCCGTTGAGGTGCTCGGTGATC
TGTTGGATTGGAAGATGTCGCCGTCGTCTACGGGAGACTCACCTCTTTCCG
CCGGCGCAGCGAGTAATTCGGGACGGCCGTTTCCCTTAGCCTCAAGAGGA
GACGTTCTCGATTGAGGTCCCTTCGCCCCAGCTTAACCCGGTCACTAACGG
CTCAACAATAAAATCTCCGACCTCCGTCAAGGCCCAGCGCACTGCAAGTTT
CAGCCGTGAGGGAATATTAGGCTCGGCACAAAAGGCCCGAAATCTTTCAC
AGACGACTGAAATTCGTACCGAGCCAACTACCAATGGCATGTCGAAAGCA
CCGAGCGACGAAGGGGCGAATCCGCTAAAGAGGCGGAACACAGATGCGG
CTGTTGACTATCCCCGGAGACGGGCCACTATTGCGGTAGGTTGAGAAGCAA
TGCAAGTAATTTGAATGAAATTCTAATAGAAGTTTTCGTAGTGCGAAGTCTG
TCGCTCCCGGAAATCAAGATGTGACGGTACTAAACCGAAGTGTAAGCTCTG
CACGGAGCTGGGGGCTGAGTGCATATATCGAGAGCCCGGAATCAAGCTTG
ACGCTGGCGACAAGCTCATTCTTGAGCGATTGAACCGAATTGAAAGCCTGC
TTCAAATCAACTTGGCAAACGGTCATCCAAATGCCATACAAATCGCACACG
ATTCTCCGCCCATAAGTAATGGGACTGCTCTCAGCGGTGAAAATGTTATGAT
TCAGAATTCCTCCGGAAATTTCGTCTCTATAATACCGAGCGGCGGCCTAGGA
ACATGGACTAGCCATGCCCAAGGAACGAACATCTCCACGATGCCAAAGGTT
CACACTAATGCGGCACTGCATCTACTCCAATGGCCACTGATTCGAGATTTAA
TCGGACGCCCATACGATCCCCAAATTATTCTGCAACTCGAAATGGCACGTG
AGCCGCTTCATTCACTGGCCAAGACACCTTGCGTCGATTTATCGAATACTAA
CGCTTATATCGAGGCCTACTTTGAGAGGGTGAATGTGTGGTATGCTTGCGTC
AACCCTTTCACTTGGAGAAGCCACTATCGGACGGCCTTATCAACTGGATTT
CGTGAAGGTCCAGAGAGTTGCATCGTGCTGCTAGTACTTGCTTTGGGCCAA
GCAAGCTTACGCGGCAGTATATCGAGGATCGTACCACAGGAGGACCCCCCG
GGTTTACAATATTTTACGGCTGCTTGGTCACTATTACCTGGCATGATGACTGC
TAACAGTGTGCTAGCTGCCCAGTGTCATCTACTAGCCGCGGCATACCTGTTC
TATCTAGTGAGGCCTCTGGAAGCCTGGAATTTGTTATGCACCGTAAGCAGC
AAGCTACAACTTTTACTTATGACGCCAACTCGTGTACCGGATGACCAGAGG
GAGCTACTAGAACGGATTTACTGGAATGCTCTTCTTTTTGAGAGTGATCTTC
TTGCGGAACTCGATCTGCCCCATTCCGGAGTGGTTCAATTTGAAGAAGGCG
TCGGACTTCCGGGTGGATTTCAAGGGGAGGAGTCGGAAGCGGTTGGACGA
GATGATCTCTGGTACTTCTTAGCAGAGATCGCTTTAAGGAGACTTCTCAATC
GAGTTAGTCAACTTATTTACTCGAAAGACTCTATGGCTTCAACTACTAGCCT
AGAGCCTGTGGTGGCGGAGCTGGACTTTCAGCTCACGCAATGGTATGAAA
GCCTCCCGTTAGCCCTCCAGTTCCCCGTTCACCCGTACAACTCTGCCGGAC

CCGGTGCAAACGGTGCTACGATTACGTTTCTTTGCTTGCCGAACCATTATCT
ACCGTCCGTATATCTTGGCGGTGCTGGATAACGAGCAGGCCGTATTGGACC
CCTCAGTGCGGGAGAGCTGTCACAAATGCCTCGAAGCCTCAATCAGGCAA
TTGGAGCACATATCTGCACAGTAAGTAAAACCCCCCTTTAATGATTGGTCAA
TTTCTCTAGCCTCATGCCTTGTTACCGGCTCTTCAAATCATGACCCAATCTA
ACGATTTGGCCGCAGTCACGCCGGCCATATGCCCTACCTCTGGCAAGGCGC
GCTCTCTATCGTCTCGCAAACGTTATTGGTAATGGGTGCTACCATGTCACCT
TCTCTATCCAACATCCTGTTGACGCTCGTCCCTCACCGTGAAGTCATTGATC
AAATCATCAATGACGTAATTATGGACATCGAGAGGATGGCGATACTTGCCCC
AAGCCTCACCCTAGCTGCCGAAATTATCAAGGAGGCTGAGGTACGGAGGC
GAACATTCTTGAATGGCTGACAGGTTTGATCGGGAATGACGCATTAGTATTA
AATTATTGACGACATGTCCGGTCCGAATGCTCTGGGGACCCCACGACGACG
ACGATCTCTCGCTCCGTCTTTGACTCGGCAGTCCGCCACCTCTACGCTCCC
ATTAAAGTGTTAGTTTTTGCGATGTCATATCTACCCGTTGTCTGGGCCGGCC
TGTTCGCGTATCTGGGAATAGGTGCAGGCACTTGCAGAAAGGTGCTGTCCA
ACGTTCTCGGCTATGCTTGAGATGCGCCGTCTCTATTGCACGAGGGACTTCT
CCCGGGGAATGCACGCTGCGGTACGACGCTCAATCGGGGAGGTTATGAGA
ACGCGCTTGGCGGCGACTAACCATCGGATCCTTTCAGCCTTGTCGGGTCTC
TAGGGATCAAGTCCTTCCGTGGCTGATTTCTTAGGTGATTGACAAGCTCCC
AGGAACAAGGCCGACAGAGAGACCTCGGACATGTAAAGTGGCACACTCAT
GCGCTTTGGCAGCACTGCAATGGTCTGGGGTTGCATTCTGGGGTTGAACCA
GCGAAGCTGGTTGGCGGGGGAGACTCTTGGTCTGGGGATTGCTAGGCTCT
ACGGTCCGGATTCCCGTCAACCCTGCTAACAACATCATTCCCTATTCCTGGA
TCCGGATGGGTCGGGCAACACGACAACCACCAAATATTTTCATGTTTAGCA
CAGTTGGAGAAATACCTGTTGATATTCCCTATACGTTGCACCGCTATTCAGG
ACAATGCGCGGTTATTCTCATCTTCCTACCTACCCCTTTTATCACTACCACTC
ACGACAACATCCTAAGGGGCCGATGAGGTCACAGACAAGAAGAATCTTGG
ATCAAGGGAAATGAGCTAACTACCTAGTTCATCTAACCCCCCAAAATGTTTA
CATCCATGCATCATTGTATTCTTTGCGCGAACATGGTTGGGCGGAACGTGTC
TTGGTTAAGCGTCATTTCACAATGTATTATTCTTCGCATTCATCGCAATTTCT
AGAGGATGAGGTAGTCTTTGGTTCTTGTTCGGCGCCACGGTCTCTCTTGCC
TCCCTTTCCCCCTGGACACGCCGACCTGCTTTGCCAGGCCGAGGTGCACGC
ACTGCGCGAGCTGCTTGGCCGTCGAGCGGAACGAGTACGGCGAGAAGGG
CATGTGGATCTGGTACAGGTCCACCTGGGGCACGCCGAGTCGCGCGAGCG
AGTTTCGGCAGCCCTTGATCACGCCCGGCGTGAAGAGGAACCAGTTGCCC
GGCCGCGGGATAGGCAGCGTGTCTGCGTGTCAGCGTGGCCTCGCTCCTTCG
GACCATAGCTTCGACTCACACTTGGTTGCAATGTAGATCTTCCTCCGCGTCT
CCTCGTCCGTCTTGGCCAACAGCCGCCCGATAATGCGCTCCGACTCGCCCC

ACCCGTATATCTCGCTGCCCGTCAGCTCCCAGTGCTGTCACGCCGAGGGTG
GCTCACGCAGAATCGTACCCGGTCAAACCTACGGTGTTGCAGGAGTCCCA
GGCCTCTTGGATACCCGCCAGGTCGTCCGCTTTGTAGCCCCACGTCTGCGT
GTCGCCCCATGACCACGTCCCGATGAGCATTGGGAATGCGATCTGCTCAGC
GCCGAGCACGGTCTTGGACATCTTGGCTGCACTGGCGAGGCGAGTTACGG
GGCTCATTCAGATAGTTGCGATCGTGCCCGGTCCCCCAAGACGCCCCCGGG
GCATCATATATACTGCGACTTACAGTGGCCAGATGAGCTCGTTCGTCTCAGG
CACGACCGTGTGTCACTGGTGGTGACTGATCGCCGCTGTCCGCTAAACTCG
GCCACGGACAATCAATCCTCTCCGCCGCGTCGTCATCTCTTACAACCCATCT
CATGCGCAGCGTCCGAGGCTTCACCCCTCAGAGAACCACTGCTCAGCTCAT
ATTTTGCTACTCACAATAGCGACCGGAGCTCGGGGACGGAGACGGCGCCC
GGCGCCGACTCCATGGATCCAAACGTTTATCTCACGAGTATTGCGTCTCGA
AGGATGACCGTCGGCCACCTCCTTGCACATCTCATAGGATTGACATGACGG
GTCTTCGGGGTCGCATGATTTTATACAGATCATCGCCTAGTTTCTACGCTGG
TCTAGTGCGCTCGTTTCTCCATGAGCTTCTTCTGGGTGTCCCTGACAAAGTC
CGCCGCTTCTTCGCGTGTAAATCTGCCTGTCAGCGATGGCGGACCTGATAC
AGGTAGACTCACCCCGGCATGGTGCCGCGCACTTCGGGACTCGCGTCGCC
CAGCGCGAAAGGGTTGAAATCGCGCTTCTGGTCCAAATTGGCCAGCGAGT
GATTGACGAACCGATGTGTCGCTTCGTCCGCCCTGACCGCGCGGATCACAT
CGATGAGTTTGGCCGAAGGGGCCAGGCGCCAATAGTCGATGGCGATCTGC
GGCGCCGGGACGTCTTCCCACTCCGGCACGAGGCCCTTGCGCATGTCCTCG
ATACAGTGGGTGCTGCGGATCAGCTTGGGTACTATGTGTTGAGAGAAGCAC
GTGCTATGTCTTGACGGCCTCTTCTTCGAGTGCTGCGACGAAGCGGTGGGC
GTCTTCGGGCTGATGAGGTAGGTCGCGAAGAGGAGGTTGTACATCAGGCC
TTGCGCAGCGAGGATGGTAGCGCGCAGGAACCAGCCGGGCTGGGCGATGG
TCATGAATGTCAAGAGGTGCATCCGCTCGTTCTCCGCTTCTTCGAGGAGGG
TGTGGGATCCATCCGCCATCACGGCGCTGTCTGTCAGCTACGCGACTTCTG
ACATGATGTGTGAGGGCACTTACAAGCCGTCTTAGACTCCGCAAGTGTCTC
AGAGTGCCCGCTACCATCCCAGGCACGCCAGCGATCGATTCGAGGACGATG
ATCCTCGACATCCACATCTTGTCCGACAACAACATGCCGTCTTTGCGCAGC
TGTTCTATCGGTATTGGGCGCATGTCGAGCGTCTCTTGAGGTATGGGAAGGC
TTTGTAGCCCGTCACCCAGTCGAAGATCCGTCTTGGTAAGCTTGACCGAGC
CGTGGGCGAGCTTGTCGCTGAGTGTGACCGTTGGCCGATAGACGATCTGGA
CCCCGTCGAGTTCCTGCTGTGTCAGTATGCACTTACTTGTTCCGGAACACG
ACGCACCTTCTCCGTGTAGATGGGGTTGAAGAGGGTCCATGATCCTGCGCC
ATTCAGGTCGGTGAGAATCTTGTCGGTGGTCGCGCCGCCCCTGGTATCTTT
GCATCAGCATCGCATGATGAAGAACATGAGCAAGCCTCACCATGGAAGTGC
AGGCCTTCAGAGAGCTTGTCCTCCACGCCCACCACCCTCTGGGTGATGCCG

GACTCTTCCTCGCTCTGCCTCACTTGAGGCTGCAGAGCAGTACTGGGACCG
CCCCGAGACGACGCCTCCGACTTGGCGGCGTACTCTCCGCTTGTTGACGCC
TGCGCTTGCGCTTGTGGTGTGAGACTGGCCGAGGCATATGTCCTAGAGCAG
CTCCCTACGCCCAAGCCGCCGTAGGTGGCCAGGACGCGGGCTGCACCAGT
CCGTGAAAGAGGTGAGACGGGGCCGACCGGAAGAGTGGCCGGGCCCGGA
CGAGTACTTGTGTGGTCATTGCTTGGAGTGTCAAGTGGGTTGCGTCGTGTT
GCGCAGGGCGAGCCTTGGACGCGAGTAGGAGATGTTATATCCTCTGCGCCC
ACCTTTGTGAGTCGGGTCGGCTCGCCGCCGTCTGGAAGTCAAAACGAGCC
GGACGACCCGTCGTCTCCCGGTCAATCAGAGCGCTCGGAATGCTACGCCAC
TGGATAGCTGCGCCGGGACCGGGAAAGAAAGGGGAAAGGCATGGCCGGC
GCCGCGTCGCCGAGGTAGCCGTGCCTGTCAACGATCGCTTTCACCCAAGCT
CACAATCACTTTTTCAGCGTTTCAGTACGTATCTCAGGTCGGGGCACAGCC
TAGTACACCTCGCTGTCACAAACTGCTCGCTTCTACCTAGATTAGTGCCCAA
ATGAGAGGTCGACGACGAAAGGCAGCAAAGGGTCTCGCAGACGATCAGA
GACTGCACGTGATCTGAGCGCTCCGCTATGTCCCGAGTCGCGTGGTACGAC
GTCGGAGCGATTCGCCTCTCTTCTGGTTGGTCGAAATGAGCGACATGCAGT
GTGATCTGACTCGTCCGAGGGCTGTGTCGAATGCGCGCGCAGGGATGAGG
CCTTTTCCGTCTACACTTCGAGAACATCATGTAATTCAGTTATGAGATCGTC
AGGAGGGTGGGTCGGCATTCACTTCGGTGATGACCGCGGCCGAGATAGCC
GGTCCTGACCAGACCAACTTACCACTTGGCCTTTCACCTCTCGCAGAGCGA
CACGACGCTATGCGTACAGGTCGGATCGGACGACGAGGACTCGCAGGATG
GAGGAACCTCGTCGATCGGGTCAGATCTACCTTACAGATTGAAGCTGGGCA
CGACATGGCAGGTGCATGTCGCGTTTGAGCGAGTGCTTCCGGACTCACGAT
CAGGCGCTTGGTCCTTTTCCTAGGTAGATGGCCGCTGCTTTAAGCTGAGTA
CTGACAGAGAAGTGGTATGCGGGAGCATCCCTTGATGTCGTCACCACTGGC
GGCTTACCTCTGCCAACATCCTGGAGACGATGAGGCTATCGCTTTTGCCCG
ACTTCCTTACGACGAGACGCACAGCTGGCCTAGTGAGCTCGGTACGGGAG
GGCGGGTGGGCTGGAAGACATTCGAGGCGCACGAAGATGGATGGCTCAAT
ATCTCGTACCCGGATATCAAGTACGCATGTCTTTGTTTCCTCTGCCGCACGC
TCAGATTCTAGCTGGAAGCAGCTTCGGGCCGACCACGGCTGGGCTGCGCT
GCAATATCAAACGATCCTGCGCAGCACCTACACGTGACCGGAAGCTCGTGG
CGCTCAAACGTCCTGGTCGACATGGTGCAAGGTGCCGAGTATGCATTCATC
TCTCACTCCGAGCCGACCTCGCCCACAGTTTGGTATAACGGAGATCTGTAC
GCCTACGCCGACACACCCGCAGGTCGACAGGAGCTCGAACCCGGTGGTCC
TTCCCGGCTCGCCAGACAGGCTCTGCCTTCGTCCCGGTGACTATACCGTGC
TGATCAGAGGCACTGTACGAGATCCGCATGTTTGGCGATTCGCACGACGGC
CCGCCCACCATCCGTATCCGACCTGAAATATGGGCAGACGGGGCTTCGTCT
TTCCACGTCGTTGACGGTGTTGGGATCATCCCACACGTTGTGGACGGCTGG

ATCATGGGCGATTGGCTCAGTATGACTGCTGAATCCTCAGACGGAGATACT
CAAAGCATGAGGATCGTCAAAGTCCAAAGTAGATCAAGTTCAGTGCGAAT
CAAGCTGCTTCACCCTGTCACGTTAGTTCCCGGACAGAGGCGCCCTGTACC
ACTGCGTATACGTCAGACCGCCCCGATAGGACCGCTCGAGTTCAACTTTGA
ACTTGAAGTGTATCATCAGGCATCAGTTGGCGGATACACGTCCGAGAGCTT
TTGGTATATCCCATTGAACATCACTCATCTCGGAACGCGACTCCGTTTCAGA
TGACGCATGCGATCTCGCGGCCCGGCACTCTCCCGCCTCAAGTATCATATGC
CACGGTCGTCCCACCGCATGCGTCCGCTTATCCGTCGACCTCATCCGCCAA
GCCGCCAGTTGTGCTGTCGCTACATGGCGCCGGTGTCGATGTTGCCTGGCC
GCAGGTTGCCGCTTCTATGCCACGCCGAAAAGACATGTGGGCGGTCTGTCC
CACCGGCAAGAACGAGTGGGGAGAAGACTGGCACGGTGCAAGTATGGCC
GATGCGTGGCATGCGCGAGAGGCTCTTCCGACATGCCTACGTAAGATAGGC
GTCGAGGTGTCGGACAAAACACTGTGAGTCGAGAACTTTAGACACTGATA
AATCTGACCGGGAAGACTGATAGGGCATTCGAATGGCGGTCAAGGGGCCT
GGCATCTTGCTGCGCGATATCCCGACAAAGTGGTCGGAGGTGTGTCGCAGC
CTTTCGCTTGCCATGGCTGAGTGACAGTTATCGCTGCTGCCGGGTATGCCA
AGATACAAGACTATGTCCCGTATCTACACCTGTTGGTGCAAGTCTCGCTTAG
AAACGTCTGACTGATATTCGGAACGTCTCAACACTTCGCCGATCCAGCTTT
CATGGGGATCATGATGGCTTCTTTGACCCCCTACAACAACGATCTGCATCTC
TCGAATCTAGCTCACGTCCCCGTGTTGGCCATACACGGAGGGGCAGATAAC
AATGTCCCGCCACGGCATAGTCGGTCACAGGTCGCCCTGCTCAGCGCATGG
CAAGGGAAGGACTCGGTCAAACTGGTGGAAGTGCCAGGCAAGCCGCACT
TCTGGGGACGAGATATTCCACGCTTTGCACGTCGATGATTTCATCGACAGC
ATCCCAGAAAGATCGTGGGAAGACGACAGGAAGAACGGATTCACATTGAC
CACTGGCCAATCCAGAAGAGTGCGGTGGCAGAGCTGGAATCCGCATCGTC
GAGCTGGACATTCCTGGCAGACTGGGTCGACTGGACGTCAACGCCAGACA
GTGGAGCCGAGAGCGCGGGCCTGGCTTGGATCTGCGAGGTACGAATGTCA
AGCGGATAGCGGTCACTACAGCTGGCAGAGAACAAATCTTGACGAAGACT
GGGGAGGACTGGCTTGTGAGCTGTCATCGTCACAGATACGTGCAGCTCTGA
TCCATAGGAGCGCGTACCAGCATACCAGCCGAGAGTCTACGGTCCTATGAT
CCGCATGTTAGCGGGAGAAGCTCCTTTGGCCATAGTCGTTGGCTCGAGCCC
CCAGCACATGAGCATTGCCAAACGCCTGGCCCACGATCTCTACGTGTATCA
TAGAAATGATGCGGAAATCCTCTCGGATCAAGAGGGGCTCGAAGCAATCGC
ACGGACTGATCTACCGGGCAATGTGGTCATCATCGGCAGACCCGAGGAGA
ACAGACTGGCCGACTACGTTCTTCGTCAGGACACAACACCCCGTAAGGCG
TCGATGATGCTGGCTCAACTTTCATTCAGCTGACGCACAGTGACTTTCCCC
ACAAGAGGGGTCATGTTGCTCGCCGATAGGCTAATTTACGAACAAGGTGCA
GGGATAATCAGCCTTCTTCCGCACCCTGTCGCAAGCGTCCGCGGCCTTGCT

TGCATCATGGCGGGGAACGATCCACTCGGTCTTGAGCTCGCAGCGAGACTA
TTCCCGGTTCGTACGGGCGTACCGGTGAGTGTGTCAAGCCAGCTGTGTACA
GAACTCATAGTCAGATTCCCGACTGGGCGATCGTCGGTGCTCAGAGTCAGT
GGAAAGCTGCTGGCGGTCTCATCGGAGCAGGGTGAGTGGTCCTTCTCCCT
CAAACCCTGGACCATGTTGCCCCAGAATTCTTGAAGAAAGACCGAGATCG
ATCTTAGTAATCGATTGCTCGTGACGCAGGACAGGGGACACCTGGCACGGG
GCACGAGGCGCAATTGCTTTTTCGCAGAACTGAGGCGCAGATTCTGGAAC
GGCGAGTGGGAAATGGGGCGAGGCGATGTCGTGGATAGATAGATAGACAA
GTGCTCTTAGGAGGAGAGGTGACTGTCCAGAGAGAGGGTCCCTCGAAGGG
GCCAGTGAGAAGGATGCAAAGGAGATGGAGATGAGCTTTGACCTCAACTT
TGGCCGCCCGTCCCAACGCCTCAAAATTGCCCAAACTAGTAAATTACGTCG
CTTCACCGTCTCCGGACCAATTCCGAGCTACAGTCTCGACATCCCTCTTCGC
ATCTAATACTTCTCAATCTCCTCATACCGCGCCCGTTGCCGTTTCGTAGTGTC
TCAAAAGTCTGCCAGCCGTCCCCCTAGACGCCGTCCTCTCCGACGCCGTCC
CTGTCGAAACGTCACGTCTTGCGCGACGGCTCGACAGTCTGTCCCCCTGA
AAGACGAACGCGACCGAGCTTTCGATCGTCCTCCGTCTTTCCGTCACGACG
TCTCGGCTGTTGAGCCTCGCCACCACCATCCCTCCCCTCCGTTGTCATGTCC
AAGGCAGGTCGCATCCCATTCGTG

>Mosaic_sequence_65

AAGCGAGAATTATTCGAGTTGCATTGGACTGGATTGTTCCAATGATGCGCAT
AGAGACAGAAGAAAAGTTCGACATTCTTTTGAATGGTTAATTAAGAGAATA
GTTTGGTGGTCTATGAGTGAGCTACTTACTGAAGTGGCGTATGTAAGGGAG
CAATACATATCTCCAAGAAAGAAGTCCCGAAACTCAACGGGGTATAATCCT
GCGAAAAACAATCGATACTATTTGCTATTAGATTGTATCAAGTATCAGTTTAG
GGGTTAGTACTTACGTGGGAATACATAAGCCATTGACGACTCCTATGATATA
GGATCGGCGCCGGTAGCAAGAGAATAATCAATGTGATAGCAATAAGGATCA
CTGGGTAATACAGGTACAACTCATCCGACCCGTACCTGGTAAAGTTTATCCA
GAAAAATATGCCAAAGAGCAACAGAAAGAAACTTGGAAAGCTTGAAAGTT
GTCTCCAATCTAAGTTATTTCTTGTATCAAACTCAAATATGAAAGGATAGTT
GATCTTATTGCGCGTCCACATATAGCAGTCGAGGCAGAATGCGAACATAAG
AAATAACATCAGAAAGTAACCGGCGTATAATTGCATAAGGTAGCTGGTCTG
CTGTTGCACCTCAGGGTCTGGATCAAATAAAAGTTGAGATCCATATACAAG
GCCTTGGAGGGTGAACACGGCTCCGGTTCCGATAAAAAGCCCGTTGCGGA
ACGCGACGGCGGATTCGTCTCCCGGTCTTCGGTTCAAACGATGCAATTTCC
CCGAAGCGATCTTATGATTCCCCTTTTCGAAGTATCGTGCATAAAGGTCCTC
AACAGAATGAATATATCCGTCCACGGTAGTGCTATTTACGAACCATGACTTG
TTGACTTTCTCGTTCATGTACCGGTAAGGTGGTCGAGCATTAACCGCCTTGT

CGTATTTCTTATTCAATTTTCGAAATGCAGTCCGGTTGAGTTGCGCATATGAT
TGGAGAAGTTCCAGTCCTCTGTAGAATTCCTGTAATGCTAATTTCAACTTTC
CGCTTCGCTGATCTATAAGATACTTCGTGGCTTGCTGGTCGTTTGATATAATC
TCTCCGCGAATCTGGTGCCCTTGGCTTCATTACGGGAGTGAAGTGTGGTTT
GGAAAAAGCTTTGGAATTAGCTCCGGGCTTAAATATCCTAGACTTGATCGG
CTCAACCCAACCATTAGCACCAGAGGAAATTCCCTTGGCATCATGACTGCT
ACCAACTTCGCCTCTCTCTTTTCGTTGTCTTAACTCGGCCAGTTCTTCGTTT
CGCCTATTCCGCATCTCGTGCAGTTGCTCGCGTAGAGCTGCCAGGCGTTGG
CCAGCTTGTTCTTCTTTCTGCCGGTAAAAAGTCTCCACTTTGTCCAATTCGC
TATCCAGGAACTCGAAGAATTCCTTTTCACGATCTCTAACCGTGTCGATAGC
CTGCAAGCCATAGCCGAATTTTAGACTATCTTTCCTCGTAAGCTGCTGGCTT
AAGTGATGGCCTGCTGAAAATATTCGTCGGAGTCTACCAGGTGATGGAGTT
TGCTCAAAGGTAGGCCTAGAGCGGAGAGGAGTAGCTGGTGGAGGGAGTAT
TCCTGATGGAGCCACGCCCATAGACATACTACGTTGTGCGGAGTGGCGGCT
GACAGACTTCCGGAGTCCATCGGAAGGCCGCGATAGAGGTACATCATTTGG
TATTGCAGGCTCGCCCAATGCAGGGTCAGGCAATTCGAATACATTCCAGCT
CTCAGCAACCGAAGGCGGCGTAGGAACGAAGCTACCATATTGCCCTTCGTT
GCCTGGCGACCGCGTCAGTGATTGGCGCTCTGTCCTAGCGAGAATCGGCGT
TGGCTGAGACTTTGGCGCAATATGAGCTCCCCAATTCGTTCGCCCTATGGGT
GCGGGGCTTCCAAGCTTCGGCCGAGATTCTATCTCATCTACTTGGGGGCTG
GCAATTTGCTGACGGCCGAACTGGCTTGTACGAAAGTTTCCTCACGCGGAA
GATCACCTTTTCTTCCTATACCTCTCGGGGTCCCGTTTGCTTTATTGATTGAC
CTTGAGACAGCCTTGATATATTTCTTTCCAGCTTTATAATTTAAATACTTAAC
TCGCCATTCTAGATTTCAGATCAGCTTTCATGTCCACACCCAGACCGCCAAG
AGAAGACCGCCCAGATTTCAAAAGTCCGGTGGAAGTTTCATAGGTGGTAC
AGTATGTCTCGGCACTCACCTGGGACGAGATCTTGCTCGAGTTCTTTAGCG
AACTTCATGACGTCAAGGAGATAAGCAAAGGTCGCAACCTTTCGAATGCTA
GATCTACGTTGTTATGCATAGGAGGTGAATTGAGTTTGAGATTAAAGGACG
AGTGAATTGAAGATTTAAGAAATCTAAGGAGGTTTCAATAACGAATATACA
GAACAGACAGAGCAAGTCATAGAAACACTAGACGCTCTAGACGCTAGTTG
TATTGTAGGAATTCGCGCACCAATGGACAAAGACGTGATTAGCAAACGAAG
AATTGAGTTCGTTAGTCCATTTTTGTTTGGTAAAAAAAAAAAAAAAATCAAA
GTCTAGAAAATCTCATCTTACCTGTTTGGTACTATAGCTGATTACAAGGCGC
GGTTACCAAGGCTTTTTTTCTTCTTCAGTGGGTCGTCCCCCATGTCTGTAAA
CAACGTTCCAGACCCATCTTGCCATCCATGATGGATCTCTGCCAAGGTTTAA
ACCTATTTTGGGTTACAAGTGCAATCTTCCGATGGATAATCGGCACTAATCT
GTTGGTTGAACTTGTCTTATTAGTTCTACATTAATCCCTACTGCAACTCACAC
CTTCTCTATGAATTGTTCATCCAATATGTTGCTTTTTACCACTACACACTCAT

GTGCTTTCGCTTATGGCTGGTATGATGCATATAATACGCCTATGACGTTGTTC
TTCGGTCAAGTTGGAGACCGATGCCCTCCCCCGACTTCGGCTATCTCCGTC
GCATATCCGGTTCAGCTTAAGGAGAGTATCGATATTACGCCTCGTTCACTGA
ATTGACATGTGAGTGTGTAGAAGGATGAGATGGAGTTTTTTTATACTCAAA
TAATACTTCACGTACAAGAAATACAGGTTCGTCATTGGTGACCACGTCGTAT
CAAATTTCGTTTATATGAGTTATATGAGACATTTTGAAGTGGCTCGGGTGCC
TATCATCTTACATCTGGAGCCTGACGTTGTGGCTCCCTGCCCCTATTGGTCTT
TCGTACGACATAGACGTTAGCTAACTTAGTAAGCTATATTCCGCGATGAGAT
CCGACCAGTACAAATCAGTTGTATGAGTTCTCAACTCCGACTGGAGCAAAA
ATAGCACAGCTTCGTATCACATATATGAGTGCCATTCTGGCCATTTCAATTCC
TGAATCCTAGAGTCGCACGAACGATTGATAATAGATATTACGTTCTTGGAAA
GACAGAACGTACTTTGTAAGTAGTAATCAAGAATCATTACATAACGCTGGG
GTTCATGTAAGGGAGAGACCCCGGAACTCAGGGTGTCTTCATGAATCACTT
TAAGTTACCAACCTACCTACCTAAATAGGAAATCGATATTGATAACATTTAA
ATGTTGGTTCGAGGCCACGTGCGATATGTCAAAAAGTCTTATTTATGAATGG
CAACGTGCACGCTATATTGATTGACAAAGCAATTGTCACTCATGAAGACTT
CTATGGATGTCTCATTCACCCCTATTAAAATAGCTAAGCCCGAGGATATGGC
AGATAATAAAACATCATAATTGGTCAAATTAAGCACAATGTCCACATCAGAT
TTAACAGCTTTCCAGTCTAAGGCATGAACATTAAAACAAGTTGCTCCATAA
CATCAAAATTGAAACATGTCAAAATATGAGTTTAGGCGTTTATATAATGGATT
TTACCCCAGGAGGTAAGAATGTGTGAAGTGGTAAGTTTTATAGGGGTTGCA
GCGACAGTTATAGCGGCAGTCAGCAACTACCCTGCCACAATCCTTCAAGCT
TATTTTAACCGTCACTCCAAATTTCCCCACAGCCTCAGGGTATTCACTCTCT
ACAATTCACCACCTCATTGACAAGGTCAATGAGGCTGAATTCACCAAGCTC
GGTGTGAATCATTTTTTTCAGAGTACTTAGTAGATTATAGAAATACATTTTTA
TTTTGAAACCATCTTGATTTGCGAGATCTCATCTCTCTTTCGCCCCGAATCT
CGGGCTATCTCACAATGGCTTCCTCATCCCCCTCAGCGAGGCCAACCCTCG
CATTATCATGTACTACCAAACGACCCACGACCCCGACGGCAACCTAATCTC
AACCCTACCCCTGATAACCCAACCCAACATCTCACTAACGCACATAATCGT
GGCCGCGATCCACATAAACGAGGACCCCAACGGCCTCACCCTCAACGACC
ACGCGCCCTCGGACCCGCGCTACACAACCATGTGGGCGGAACTCCGCATC
GCGCAAGCGAGCGACATCAAGGTCCTGGGCATGCTCGGCGGCGCCGCGAA
GGGGTCCTACACGCGCCTGGACCAGGACCAAGCCACCTTCGAACGCTACT
ACGCGCCGCTGTACGATCTAATCCGGGAGCGCGACCTAAACGGCCTCGACC
TCGACGTCGAGGAGCCGATGTCCCTCGCCGGCATCCTGCGGCTCATCGACC
GGCTGCGCGCGGACTTCGGGCCGGAGTTTCTGATCACGCTGGCTCCTGTTG
CCGCCGCGCTGCTGGATCCGAATCGCAATTTAAGCGGCTTCGATTATGAGG
CGCTGGAGGTTATGAGAGGGGGGCAGATCGCGTGGTATAATGCGCAGTTCT

ACTGCGGATGGGGCGATGCGAGTAATCCCGCCATGTACCAGATGTGTATGG
CGAGGGGATGGCTGCCGGAGAAGGTGGTTGTGGGACTTGTGACGTCGCCG
GAGAATGGGCCGGGCTGGGTGCCGTTCGATGCGCTGGCGCAGACGCTGGC
GTTGCTCCAGAGTCAGTTCCCGGGGTTTGGGGGAGTCATGGGGTGGGAGT
ACTTTAATGGGTTGCCTGGAGGACAGGCGAAGCCATGGAGTGGGCGCAGA
ACATGACGAAGTTTTTGGGCGCTGGGAAGAGTGATGCCGCGCAAGCTGCT
GGTGCTGCTAGTGTTAAGGATGTGAAGGCTGCAGAGGTGGATCCGGATGTT
GCGACTGGCCCGAGTCCAGGCGTTCCGCAGAGTTTCGAGTATTACTCTGAT
GGCGTGGACGAGGATCGTGATTAATGGGAATGAGTCTACAGGTAGGCCATA
TTCACAAATTTGGGGACGTGGGAATGTCTCTTGTTGGGACAGATGGGAGG
ATGGTAGTAGTTACATGCTCACCTAATTGAGATCATTCCCACATTCTAGACC
ATAACCTAAGACTGCAAATTTGCAAACTGTCTCGTGATGTTCAAATATTGGT
GAATAGAGATGGGATGGAAACTCGATTATTAAGCCAAAATACCACCTGAAT
CACGTAGCCAGTCATAGTTGAATATGAGGTGGCTAAGGGCATATTCTACACT
GTGATGGATTTAAAAGGAGTTCACAAGCAAGTGAAATCTTCGAGCTACGGC
TCTGATTGAGTGCGTTACACAAACTGGCGCGACTTGAAGCTACTCACGTCG
AAACGTCACACGTCTACCCATGTGGTCCTGACGGAGATCTTAGCAAACATC
ACTCCGAGGAAGAGTACATATTCTAGCGTTTTATCTTTAGTTAGGACTTCAG
GACTTGAAAATGCATGCCTTTGATCAAAGCATCAACCTTGAACTCTTACAA
AGTTATCGATTTCCTGATACGTACTGTGAAGAAAATAATTGATATAATTTCCT
CGGAACCTCCCCTCCTCTTAGTTAGATTGCCCATTACTATATTTCTTGCACAG
GAGAGGCTGAAGAATCTCGAAGGCCAATAATCTATCGCTATGGCTGAAGAA
TCCAAGAAGACCGACGGGAGCCGCACCCCTGAGAAACATGAAGATGTTAA
GGACTCCCAAGCCTCTCGGATGTCAAGTATGACATCCGACAGTCAGCAATT
CATGGACCAGCTTAGGGAAAAATTTCCGAGCCTTGACTCCACCAAGGGGG
TAAGCTTTTTTTATAGCTTTATAGCCGTTTTTTTCGCTAAGTATTAACAAGTG
TTTTAAGTCTATCATCGAGGATAGCCGGGGCCGACGAACCAGTATATCGCAA
AGACTATCCGGAACCTTAAGCGGAGCTAGTAGTCGGGCGTCAAGTCTCTCG
GCAAAGAGCAAGGAGCTATGGAAGAAGCATGCGCATATCTCCATACCTGAA
AATTCAAGTACTAAGCCGAAGCAGAAGGATCCCCTTCACCAGTGGATGGTC
GATCATTCTGGCGGCACTGTGAGCGATAAGCCTTGATCCTTGAAAGCGTTA
ATTAAGTGGGAGAAACTTTGCTTTTCCGATGACTTCGGCGACTAGCATATCC
GGAGTTTGGCTTGCAGCATATCGTTGGGGTTTATTGTTGAACGCCACAAGG
AGTTATGGCGTTGCGATACCGGATGGATTGGGAACTGCCAGGTGGCTGAAG
AGATATCCTGATGGATTTTTTTTTCTTTCTTTAGTTTGATACCCAGGAGTTT
TCATCAATGAATGTCACGATTTCGGTATTTTTGAAAATCTATGTCAATTCAAT
AGGGATATTACTACCTATAAAGTTGCCGCTCATGGAAACTATTCGTGGATGT
CTAGGCGCTTACCATTTTGGTGCACTTAGTGCTCTATGCCGAAAAGAGAAG

GAACGTGATAATAAATAGCCATTTATAGTTACTCGATGTATGAATTTTGATAT
ATATATGTATATGTGTGTGTGTTTTTTTCTACATATTCTGACGAGGTGTGGTT
GTAAAATGAGGCTGCCGTTCGTCATTACCACCAACGGGGATCGATCGCGGA
CTTCCAGAAGACCCCCGTTTTACCAGGATCAGCCCTGCAAAGAACCCCTGA
ATGGCCTGCATCATTCTTCAGCACGCGTTTGCCATCTTATGCGCGTTTACCT
CCAAACCTGGTAGACTCATTAAATTTTTTTGTCCGAGGGTCGAGCTCAAG
ATCGTACTATCTGTGCCTGGTAATCTTCATTTTCCCACTCATATATTGAACTA
AGTCAGATTTAGTTGAACATGGCCGGAAGATATGAATCCCCGATGGAAGTG
AGCGATGCCGACGACCAATCTGAGGTTGAGTCTGAGGACTTCGAGAAAAC
GTTTGCTGAGGTCCGAGGTTGTCTGTTAAAATGCCTGAATGGAATCAAAAC
CACTGGCAATGTTGCGACTTCCAAGCACTACGAATCATTCATCAACCCGGC
TTGACAGTCGCGGATACCTTAATCCCCTTACCTCTATCTCCTCGAGATGCCG
AGACGATTAAGAATGCATCGAGACAAGCTCCCTTTGGTAGGGGCGACGAG
GTATGTAAATTCGAATGCAATTCATAATCAATACAATATTGGTCTTTATTCAA
ATATGCCCTATTAACTCTTGATACTTTAGACTGTGGTAGACACGTCCGTGCG
TCATACCTGGGAACTCGATCACACCCAGTTCAAGCTCGCAAATCCTCGCTG
GAGTGCCTATGTCGACTCGTTATTAGCCGAAGCTGTACGGTGTCTGGGTATT
CAAAAGATGAGAGCTGAGCCTTATAAATTGCTGTTATACGAAGAGGACTCC
TTTTTTAAACGCCACAAAGACTCCGAAAAGGTTCCTGGGATGATTACGTAT
AACATTCAGACTTCATTCCTTCCCAACTCCCCTTCAGCCCACCTTAGGAGC
AACAACTACCCGCTCGATCTACTCCAAGACATGCTTTGAGATTTCCTACGTA
ATGTATTATCTAGGTACTAACTTGCTGTAGTGGCAACAATGGTCATTTGTCTT
CCCTCGAAGCACGAGGGAGGTAGTGTCCATCTATCACACGGTGGTAGGGA
CTACGTCTTCGAGACCGATGAAAAATCTGCGTTCGGCTTGACAACTCTCTC
GTGGTTCTCAGATGTCACACACGAGGTCAAACCGCTAAAGTCTGGATATCG
CTTGGTATTGACTTACAATATCATTCACACGGGTGGTCCCCGCATGTCTGCT
AATGCTATTGGAGGGCAGTCGGATGAGCTGCACTCGATACTTTCCAAATGG
CAGGCGATGATATCCCAGGAATTCGATGGCGAGGTGCACAAACTCATATAC
AAACTTGACCACAAATACACAGAGTCAAGCTTGGCGTTGAAAAACCTCAA
AGGCCGGGACTATTTCGTGTGTCAGAGCCTCTATGAAGTCGGTTTAGAATG
CGGCTTTTTTATTTTCCTAGCTCAACTGAACCGATCGCATAAGGAGGAAGG
GTATGATGATTATTTTGATGATGATTGTGATAACGATCAACAGACTGACCTTG
AATGCATCAAGACCTGGGATGGGCATACTATCTGTCGTAGCATAGAAGTTCA
GGAAGAAGATATACTTGGGAAAATATGGAATCGAAGAGCTGACAGCGAAG
TAGAGGGCGGTTTTACTGGCAACGCAAGTATGCCCTCGACTCTTCGCTATC
ACGATACAGTGAGTTCCCATTATGCCTCGCTTCTATTGCGTGTCCTTCGTTG
ACGTGAATTCTTAGGTTATCATTGTATACACCGTTGATAACATATGCTACATC
CCTATCTCAGACCTCGCGCCCCGTGTTTTTGTGGATTTGGTCCATCAGATTC

TTGATCATCGACCGAAGAAACGTTTCCCAACGTCCGCTGTATTTAAGAGTT
TGGAGTCGCTCGTTCAATCTAACGTTTCCGACAGCACACTCACGTCCGACA
TCCTAGACATGGCGATAAAATATGAAAATCAGTCATTATATCGAGAGACCGT
CTCCTGCTCGCTAAGTATTCCCGATACCTGGCTGAAAGTATTGGGGAAAATC
TTCCAGTATATGAAGGACGAACAGTCTAAAGATGGCCACAAAACTCTAGAT
TGGGATTTCTGGTATGCTCACCTACTATCCATTTACTTCTGGTCTTTCTTTAA
CAATTTATTGGGTTTGGTGGCGCCTTGGAAGCAACCTTTAAACCGACCTTG
CCAAAATTCCAGGAAGCCATGAATCAAGCTGAGTCGATGCTCCAAAAAGA
CGAGGATCTGAATGATATGGTTCCATCTTTCCAGGATTGGAAGTTGTTAATC
CCAGATAAGATGATAAAGTCCAAGCATCAATTGGACGTGGATGACCTCGAC
TTCTTGAAATCACGAATGTTCTCACAACCGGATGATCACGAATGGTTGACT
TGGTATGTGTTGCCGATACCTACTCCCATATTTCTATCTGAGACTTATTTAAC
GTACCAACTAGGTTCATGGGGATCTTGCCGAAGCGTGGTACTAGGAAACTC
ATTTGCGAGATACTGCAAGCTCTCTACGAGAAACGTGATGACGATATTTTCA
CGATAGCTCAGGGCCTATTCAAATTTCTCGTGACCAAAAGCGCAAACAAAT
TGGCCTTGCAATCCAAAGACTTTCCCGCGTCTAGCTATGTACCAGGTTCTCG
TAGACCTCCTAAAGATCCAAGCCAACCAGTTTCGATTACCAGATGCATTATC
GAGGTTATCGATCAAGCATTCGACATGGGACTCGATAAGCAGGCGACTGGT
CTTCTCGACAAAAGTTGTATTAACATTTGTCAATGTTGTCCGAAATCAGGAA
GCAATTCAGCCCCTGATCCTTTGATAGTTCGAGACTTCTTGTTATGCCTCTT
CACAATGCTGCAGAAACACCAGTTCACGCCTCCCACATAGTTGAAGGTGAT
GTTCGTGGCTCTTCTGCGGAATATTCTCGTTTGCAACCCGCCAGAGTGTCC
AAAGCCTCCTCGAGGCTGGGCTCACAAACCTAGACCTAAATGCCACCAGT
ATTCTTACCCTTGCGCAGATTGCAATACTCTCAATCGCTTCCTTGAGGATCC
GGACATGGAGGCGGGCGGGTTTCGCATGACCCTAAACAGACAACAGCATT
TGATATATCAGCTTCCAGATTCATTATACTATTACCAAACTGAAACAAACCC
ACCGAAGCTCATCGTATACAAGTTAGGGAACGAGTATAAACAAGAGATGCA
TGTCTATAAGGCCAGGCTCACCCTTTTTGAGGATCACGTTCGAGCTTTACGG
TGCGATTATGTGAAGTCGCTGCTAAGTGAAGAACTCTATAACGAGCTTGTC
CTTCTGAAAGACATACCGGACTCGGATGGTTCTATGCAGACTAAACGCTGA
GAGAAGAGAAAACCTGAAGAGGAGGGAGATGGTTCGAGCGCTTCGAGAC
CACAGCTCATAGAATAGGCAATCTAGGCGGTTGGGATTGTACGAGGAGAAA
TGAAGAGATCAATTCATGTGCAGTATGGTTAAATGGTGAAGGTTTTCTTATT
TATTGTAGGTTGGCATAAAAATGTAATATGAATTAATATAGGGAGGACGGAT
AGAATTGGCGGAGAGTGAGTGTATCATGCAATGCACATCGCGATGTCAGGA
ATCAAAATATTCAAAGCAACAAAAAGTTAATAGGCCCAACCTAGGATCTGT
TGATCGGCTCCTAGCTATTGCGACCCCGCTATTGGGTATTTCTTTGCCAACC
CTTTTTCAACGGCCCATGGTGCGAATGCGCATGCACTCTATGGGACTCGAA

CCCTAAACCACCCGTTTCAGAGCAGTTGTCAGACAGGAACGAGAAGGTGC
CTGTGGAAACGTACCACTTGACCATCCGACTGGTACGGTCAAGCAGTTAGG
ATCCGCCGATGGAAAACTGACACAGTATTCTATTATTTAAGCCTTGGTACTT
GGGTAGGTGACGAGTGCTGCTTGGTGGTTAGATCACCTGATTGGCCAATTA
TCTTACTAATGGACTGACAACACTGTGTAGGATAACCTTTGGCAGGGAAGG
CGCGGACGGCGTTTGTAGTAGTTTGATACCTCAGGTACACGAACAAACAAG
GCCTCCCAGATGCTAGAATATATGGATATATTCCACCTAATCGTAACCACGCA
CCACGGCTTTTATGCATATCAGGTAGATAGCTATGGATTCAGATATAATCTTA
TATCGCCACGTTAGTCAAAGAGATGCAAGGGAATTTTTGAGTCAAAACGTA
CGAAAGAAGCTTCAATCCAATGTGATGACACCATAAGTCGTCAATTGCCCT
CCCAAACGCCCCAACCATATGCTAAACCCTTACGCAAGCAAACACAGGTAA
CAACGAAATGGATATATCAACGCTATCGTCGTCCTAAACGCCCATCCCCCTT
CTTCTCTTATCACCTTCCACCAAACGCGCGATCCAACTCCTCTCCCTGATCA
ACCGCCATCTCACTAATCACCCCTCCTCCTCCTCCTGAATACTCATCTCCGC
AACCAACCCCGCAACTTCCGCCTCCGCCTCCCCATCCCCCATCTCACCAAG
CCCAGACCCAGGTCTCCGTTCCGGCTGCAGCAGCCTCGCCTCACTATCCAA
CCACCCCGGCGCGACCCGTCTCTTCTCCGCCAACTCCCTCGCGCGTATCCG
CTCCTCCATCTCCTTCAACGCCCTCTCGCTCTTCTCCTGCGTCGCGGCGATG
TGGGACCGCAGCGCCTCGCACGAAGCCACGATGTCGCTTGGGTGCGCGGC
GTGGCCCGACGGCGCGACGACGTATTTTGGCTTCTGGTCGGCGTTGTTGGC
GCCTGTGACGCCGGAGATAGGTGGAGGGAGGGTGGATGGCATGGTCTTGT
ATGTGTAGGGCAGAGTGGCGGCGGTGGGAGGGAGACGAAGGGGGAGACG
GTTAGGGGGACCTGCGCGAGGAGGGGGTGCGTGCCTAGGTGCGTGTTAGC
GATGGGATACTTGGATTTGAAAGTATATTTTAAGGGGTTGAGACTTACGGTC
GTCGCGCATTGTGACCATTGTTTTGTCAGCGGCTTTAGGGCTGGCACGATG
GATGAAAGCGAAATATCCTATTTAACTTAGTACCTATGTTGAAGATGACGAA
GTTGCTGACGGCCCTTGGCAGCTGTTCAAGCTTGTCATGTGGGGAGCAATT
CCGTTATCGATAAGAGCTAAGGTCATTGTGGGGAATCCCGACGGTTTGGGA
ACGCTCACGTCATTCAAACTAAGTCCTGCGCAACAACCCATAGGATTTTAG
TTTAGAGTTAAATCGTTATAGTTAAATCATCAGCTATCTTACTATCTCCTTCAT
TTTCCTTTACTTCCAAGGTTCTTTTTCATATAAATTTACCTATATGGATCACTA
AGTGAGTTGTACCTGGCACTGGTTTAGAAGGTAGGTAGGTAATGTTGAGGG
ATTATTCATTCCTATCAGTCAACAGTCACATTCTTAACCCGACATACGAAGT
TAATTTCTTCCAATGGGATAAGCGTCATTCCCCAATCTGATTAAAGATCCGG
CAGGACGCAAGAAGATGGGGCTGCGAGGAGAAGAGCCTTTAGAGACGGC
GATAGGCCCCTTGCTGCTCGAACTGCTGCGGGGTCTCTTGCTTGTTCAAGT
ATACGCCGTCTTCCACCGAATCCAGACGAATATCAGTCATTTTACTGCTTGA
TGACGAGATCAGGCGCGTGAGCGAATTTCGCAGTTTAGTGAAGGAGATGT

AACGCGCGAGACGTTCTTGGATAGGACGGAAGGTAGGGAGGCACGCGCTC
AAGACCCCGACGTTGGTTTCGATTATGGTCCAGTAGTACACTTGCGAATAC
ATATCTATGGTTCCCATGTCAGTATTACATGATCGCGAAACCCTTGGGTTATT
GATGTCAATCAACGCACAAGTGGTATCTGCTTCGGGGCTCGACTCGAGCAC
CCCGTACACCTGAGTCAAATAGTGGATTCGAATGAGGCCGACGACGCTGAC
TAGGCCACCAAGAAGGAAAATGCCTACTATCATGAGTTTTCGTTCCATAGG
CATCTGCAACTGCCAAACTTTGTAGATAGGTAAGAGGAGGACTAGGACTGG
TATACAAGTAAGTTGTCATCTGCTAAAGATACGATTTGATGTCCCCGATATGC
TTACAATCTGTAGTCACATCCGTCGCTAAGGCAGTAGTAAAGAGTGCCACT
AGGTCGACACAAGTATATGGGATGTCGAAGTCCCACGATGAGACAATTGGC
ATGCACGAGACTAAAGTGTCAAGTGAGTGTTAGAGAGATTGGAGACACTA
CAGTGTTATAAAGATAAGACATACACATGAGCGCAAATGTGAAGGCTATCG
TCCAAGCAATATTAAAACCAATCAGAATATTGGCAATTATCTTGAACTTTGG
AGTCGTAAAAATCCGCTTATACATGAGAACGACGCTGACCTTAGTGAAGCC
CACTGCCGGCCACGTTAACAGCTCGAGCGCGTATACCGTCTGCGTCATATAT
ATTCAGTCATCATCGAATGACTTCGCTGTATCAATTGCAGAACTTACCTTGC
CATAGACGACAATGGCTTCGGGATTAGCAGGATTGCCGTTATCATCGTATTG
CGCATGGGTTCCAAGACCACCGAGTTTGGTCACTGGCAATTTGCAAGTTGT
CAGGATACTTATTGATGGAGAAGATCACGTCGTAGCTCACTAATAAGGATGT
CTACCGTGACTGCAATCCCTAGAATCTTTAACCACCACCCATTAGCAAAAAT
AACTTCACTAGTGTCAGATGGGGGTGGATTTCGTACCAAGGCTATGACGAT
AGTCCAGTCATCGGATCCCAGCTTCTGGCCCTTGATACGCCGAACCTGAAA
TCGGAGTACGACGGCGATAATGGCGATAATGGGGAAGGCAATGCCTAAGCC
CCAAATCGTCGGCTGCTGGGTGTCAGCCATCTTAGTGTACCTAAAAAGCGT
CACAAGAGAGTCTCTTTGAAGTGAATAAGAAGTTGCACAGACTAGACTGG
TCTCCATACCGAGTCGACTGACGCGGCAGCTTGGCCCTTTAAAGGGACGCC
CCTACATCAACTCATGATCCTTTTCTCACCTCTTCGACCAACGGGCTGAAAT
GAATGGACACTTACATTGGGATTGGTGGCACTCGTGGCACAGCATTGGTGT
GTCACATAGGTACATAGGTACTTTCTATGGATGCAAGAATCAATATTTCAGG
GGGGTGTGCTTACCCATGTCATAGGTACACCCCCTGAGTTGATAGATCACTA
CGAGAGCCTTACCATCTGAGTGCGGTGTACTATGTATTGTGTAACCCGTCGA
TATTAATATGTACCCAGGTATGTAGTACCTGAGACTAACACAACTTTGGAAG
CCTCTTTTTAACCCTTGCACTAGCTTCGAGTACTTTACAACGCTTTACAGGA
GCTTCTTCACTATTTCAATAGCACACATTGTAGAGTCTTTGCAGAGCATTTAT
CCACCCTGACCATGTTTATTGAGACGCTTTTGCTCTTAGGAGCTTCCTGGAT
AATCGCAAGTATCTTCGATAAGTTTTCACAGTCTAAGCGGCTTGCCAAGAG
TTGGAGCAAGTCCCGGACCTTTCTCCCTTTATATGTGGTGGGCTCGATGGA
AGTGGTATAAGCATGGTCACGATGATGTGATCAAGAATTATGAACAGGTAC

GAAAGTGCGATAGAGCTAAATCGTTGGCTTCGATACTGATTATGATCTGACC
GGAGTATAGTCCAAGAACACGAATTATGTCACTCAGGCTTTGACGGGCGAT
ATCATTGTGCTCGCACCCAAGTTCCTCGGTGAGCTCAATATGCTTCCAGAA
ACTAAACTGAGCTCGACTGCAGCCTTGGTGGACAGTGTGATGGGTAAATAT
ACTGGCGTGGATCTTCTCTTACGCGACCACTTAACATCAGATATCTGCAGAG
GCCCCTTAACGAGAAACCTACGTAAGACTGCCAAAGGATCAGTGGCAAGA
CTTTTTGAACACTAACTAAGGAATAGCCATATTTCTCCCACATATGGCGGAA
GAGCTCCGCGCGGCTATGATGGAGAAACTAGATCAACAGACACCTGAGAG
TGAGTTGAACGATACGATGCCTATTTGAGCATAGAAATGACCTGGTTAACC
ATAGTAACTTGTGTCGCTTACGACCTCTTGTTATCCTTCATTCATCGCATTAC
ATCTATGGTCTTCATTGGCAAGACATCCTGCCACAACCCAATTTGGTATGCA
ACTATCGATAATTTCCCCCATCCATTCAGGTTTTGCGATGAGTGGAAATCAA
AGATAGGATTCATTTAGCACATTAACCTGTATACTTTTAACTCTTCATATCCG
TGCCTCTCGCAATAGCTAATCCGTAAAATTGCAAAGGACAAATGCTGTAAC
GGACTTACCTTTCGATGTTGAAATCACAAAAATTATCTTGCTGCCATTTCCA
GAATTTCTTCGACGCTTCATCGCACCACTGATTCCGCAACGGAACAAGATC
TTCCGGAATCGTGTTGCCGTAAGAGATATTCTCTTTCCCCCATCGGAACCGT
TAGAAGCCAAACAGGAGCCTAGCGTGATGAAGCTGTTTATCGAAAGTGGA
AAAGACAAAGATCGGGACAGTATAACGGCCAGGCTTCTGCTCCTAACAGC
AGCCGCAGTAAGATTACCTACCTCACGAGCTGAGACACAGAACTTGTGGCT
GATTGTGGAACAGCTTCATACATCCTCAATGGCAATCACACACGCGATCTTC
GATCTCTGTGTCATGCCAGAATACATAGAGCCTCTACGATCTGAAGCGAAA
ACGGCGCTTGCTGAAGAAAATGGAGAATGGAAATTCTCTACCATTAAGAGG
CTTCGTCGGCTAGACAGCTTCCTAAAGGAGTCGCAACGTGTGAACCAATCT
ACGTTCCGTAAGCAACATACGTCCATGGTCTAAGTGAAAGTGTTTGTTACTA
AGTAACTATATAGTCGGGTTCGATCGCAAGGTTATGTCTCCCATTGAGTTGT
CTGACGGAAAGACAGTGCTCCCACAAGGAGCTTCAATCGTCATTCCTGCG
GGACTCATGGCGCGCGACCCAACATTCTACACGAATGCTCAGCAATTCGAT
GGTTTCCGATTCTATCGTGAAAATGAGGATGAGAACGTAAGTTCTAGGGTT
AGTATGCAACAGGATTACACAGGTATCGAAACAGGTAATCTCTCTTGGGGA
AATGGCAGGTTCACGTGCCCGGGACGATGGTATGGAGCAGCGATGATCAA
GCTGATCGTAGCGAACTTGCTACTGGAGTACGACATTTCGTTTCCTGCAGG
GCAAACGGAACGGCCGCCGAATTCGAAGTATAACACGGAGGTGCACCCAA
GTTTTGAGCAGAAGATAGTATGTCATGATGTATAATACCGATCCCTGCTGGT
CAGCATCTACCCGCCGCACACGGCATTCCGCTTGCCCGATATGGCCCATTCC
TGCCCGTTAGCTGACCGTCTACCCAGTCCCGGCGGCTTACCTGGCGAGACG
ACCCAGGCGCGTTTCAAGGCAGGAGGCAGCGATTCTCGCCGTTTCTTGCG
CGAAAAGCGCAGCTCATCCGTAACGCGCGACACCTCCAATTTGAAGCCTTC

CGGCTCCGCACCCTCAAGAACATCCCCGTCACCCGCGATGGTCTCCTCATC
TTCGGTGTCTGAGTGCGGTTCGGCATCTACATACGTGACCGACAGGTCCGC
CTCCCCCGGCAGCTTCGCCCATCGTAGGCGAAGAGAGCGGGACTTGGGCT
TCGTGCAGCCCAGAGTCGATGGCTGCTGTGACTATTCGGACTGAGGGGAA
CACGCGATAGAGCGCATGCAACGCTTGTCTGGAAACCAAAAAGGCAAGGA
AGATGATATTCTCTTCTGACCTGTTAGCTTATGTTTGGTCCGAATGCTCCGGC
AAACCTGGTATACCGTGATCTAGCAAGACCCGGATGGCCATCACTGGCCGT
CAGCTGCGCACGGAAGTAAAGAATTCGCCAGTCTGACTCGCTGCTGCGCC
GGTGCCCATCTCATGATCAGCAAGCTCGATATATAATAGCCGGTAGGAGCCT
ACCTGCGAATCAAGCAGGAGAACCCGCACACTCCCACTAGTCTTGCTCTGC
TTGATGCACTGCGGCAGATCACTGCTCAGCAACAATGGCTCCCCAGTCTTT
GGGTCCGACTGTATGAGCATGGCGCCGATCTGCACATCTCGTATAACCCGA
CGCAGGCCATGCGAGAAAGGACCACCGGACCGCAAAATGGATATACCTAC
CAGTGGCTGCTCATGAGCTTGCCCTCAGTTCTACTTGTCATGGCTTACATCA
CTTGTCGTGGCCATCCCGTCGTACTCCACTCCAAGCGGGGTTTTGCACGGC
ATGCTTCTCGTATGGAATGAGTGTCAAAGCTCTCTCCACGATGAGTGTGGA
CAAGCGATCCGCGTAAAAGATAAAGTCGCCTCGTTCGGTCGTCTCGTCCCG
CAAGATCGTCAATATACCCTACGAATGAGCGAGGAGCCGCAAAACCGATGT
ACTAACCTGTAATTGATTCGTCTGCTCCAGGACTGTGACCGCGCGAAGGCC
GGATCCCGTGGGGGAATGCGCAGGTGAGGTGGGGGACCCCGCCCGCCCTT
TTGTTCTCCTCTTCGCCCATGCGAGCCAGCATATTCCGAAACCGTAGTGATC
GCGAGTCGAGCTGCCGTTTGATATGTGTGACGAGTAGCTCGATGGCGACCG
AGTTGGACGATCCGGGGACGATCTATAATCAGGTAACGATGGTGTACATCG
GCACCGGCTTACCACGTCGGCATATTTCGACGAAGGCTGCACAAAGTTGTC
GTAACTGGGCTTGACGAAGCGTAGATACTTGCTGTGAGTCGCCGTCGACTT
GCGAGGGAGGCGAGTCTCACCTGATCTAAGATCCCTCCACATCTCGTCCTC
TCTCTTTCACGTCCCGCTTAATCCGCCCGAGCCAGCATCAAGTCGGTATCGC
AGTTCTGCACGTCAAAGGAAGCTGATAGCGCAAGCTAAGACTCTCACGAC
GAAGACCTTCAGATCGTACAGCTTCCGCATTTCGGGACTTTCCAGTGCCAT
GATGCCTTCCCTTCTATGTCAGCTCAACAATTGGTGATATGCTTCGCCTTAC
ACAATGATGACGCTGGCGCCGTACAGATACTTCTTCTCTGGCATCCGCTCTG
GCATTCAGCAATGCGCCGCATCACTCTGAGCTCGGGCAGAGCGCACGATG
GTGTACAAATGAATAGATTGGGATCTGCAACACTCAGCCCAAGACCCAGTG
ACCCCCGGGGGGGACAAACCTCTGTCGCCTTCCCCTGCTTCAACTCTGCTA
AGCACTGACCATCAGCGCATGGGCTATTCGCCGAGAAAGATGCGCACTTTG
GCAAAGAGGGCGGTGTCTATGGAATCGGGATGATCTGCCCGTCAGACAGTC
CGCCATGCGCCGGTAAAGATAGATACTGGCCTACGTCGAGCTCATTTCGAA
ATGCCAGCTCTAGCTCCTCCGGGGTATGCTCGTTGTAAAGGCGTCCTGCG

AGAGGATCAAGACAGTTGGAATGTAGTTGAGCGCCGACAGTATCGACCTC
GGCGACGGATGTCTGGCTGTTCATCTTATGTCTCACCAATAACTGAGTGTAC
CTCACCTTGCCCGACGCACTGCCACCCGCAATGCCAATGACATACGCCTCT
ACGTTCTTGCCGTCCGGGCCATACCACGGGGCGCGCCCCGTGTGAGGCCAT
GACAACATTCTTGTCCTTGCTAGCCGACCTTTGGGTCTTGCGGCCGAGCAA
TTTGGGCCTCGACGGCAGCTGTGGCGTCGCCATTGTGCTGTTGAATGACCC
CCTAGTAAGTCAAAGAGTTAAGCATGACACTTATCGGGAGACATTGCTGTC
ACTGGACCGAGAAGTGATCGACGCGGACACGTGGTTCGAGCGGTGATCAC
CGCTTGCGCTCGGACCTCGCTCTGGTCCGAAGTTGGTCCAACGCGTCAAC
AAAGTTGGCCTGCTCGCTGTTGCCACTCGTCCTGTCGTCATATACCCCTACA
TAGAATATTCTGCGGTTCGAGAGGCGACACAACGGAGAGGGACGGTCTTT
CTCATTGAATCGATTCCCGTCGCGAGCAACGTCCTTTGACATCCTCTTTGGC
AATGGACGGAGAGCCGCCTCAGGAAGAGGACTTTTCCCAAATTCCAATAAT
TGAACGTAGCCAGCACAAGGTGAGCAAGATATTAGCAGACGCTGTGAAAC
CCATCACTGCGAACTTGGGCTGATGTAGCAGAATTGGAAAGCCCGTGTATC
AGCATATACCGAAGTCATCGCAAGATCAGCAAAGACAGCTTCAGATTCCGA
CCCTTTCTTCCGACCATACGTGTCTGACGGACCATTGCTGTGGGCGCTCTTG
CCGGCGCCACAGAGCTATGCTAACGCATCAGAAAGAGGTGGTGCCTAGATT
CGAATGTTGTAGCCCAGGAGAAGGGCGTGGAGGCTATTCTAGCCATTGTGC
AATACAGCGGGGAGACGAGCGTCAGGTTTGTGTTTGACCATTTGACTTGCT
TGTCTGACACGCAGGACACGAAGCGATGTCGTTCCCGCACTGGTGGACAA
GGCCTCGGGTCGACGAGAGCCGGGACGAAACGGAAAGCAGGGGACCTCT
GCGCGATGTACGTGGAGATAGAGAACACGGGCGACGGTGTCGTGGTATGT
GCGCTTGGTGAGGCTGGAAGAATCTGCTGACTCGCCGAGACGGACGTCCT
TGTCGGACTTGACTCGAAACAGCCGAAAGCAGTAGCCGGAGCGGTCCTCA
CATTGAAAGAGCTGGTCGAGTGAGTTTCCGTCCAGGCGTTAGCAGGGCTC
ATAATGCAGGTCGTTCGGTATCCCCGCTCTGGGCAATGTCAAGCCAATCCTC
AAATCTATACCCAAGATTTTTGCGCATTCGGACAAGACAGTGCGGGCCGAA
GGCACGTCGCTCGCTATCACTCTACATTCCTACCTCGGAGACGCCCTCCTAC
CCTCCTTATCCGACCTGAAGCCTGTCCAAATGGCCGAACTGCAGAAAGGTT
TCGATACTATCCCGGGCGGAAGCGCGAAACCCGCCCGCTTCACTCGCAAG
GCGCAAAGGGAGAGGGAGGAAGCAGGACCTGGTGGTGCCGAGGAGGTCG
CGGCAGCAGCAGAGCCGGCAGCGGTAGATCCGCGGAGTTTCCTTGACCCA
GTCAATGTGCTGAAGAGTTTCCCGCCAGACTTGATGGATCTACTCGCCTCAT
CGAAATGGAAAGAGCGTCTGGAGGTTCTCGAAGCTTGCAACTCGGTGCTG
GCAGATCCGCAAAACGGGCGGATATCAGACTCCAATGTCGACGCATACGGA
CCACTAGCGTCTACTCTGGGCGCGAAATGCAAGAGCGACATGAACGTCAA
CGTGGTCATGGAGGCCGCAAAACTTCTCGAAGGTTTATGCAAGGGAATGG

GACAACCGTTCGGTCGCTATCGGAGTGTGACCATGCCTGGACAACTGGAG
CGATTGAAGGAGCGCAAGGCGAATGTCGTGGAGGCTCTGGGGAAATCGTT
GGACGCCATGTTCGCCAGTGTAAGTCTTCACAATTCCTTCGAGCATAGTCTC
ATTGTTAGACGAGCTTGCAAGACATCGTCGACGATGTGCTTTCGGCACTCA
AGTCGAAGAATCCTCAAGTCAAGGAGGGCACGCTCAAATTCCTCCATCGG
TCTCTGTGTACGACAACGGATGCACCCGGCAAAGATCAGATCAAGCCCGTT
GCAGAGTCCTTGGTGGCCCTCCTGAGTGACAGCGCTGAGCCTGTCCGGAC
ATCCGCGGCGGACTGTTTGGGCACGATGATGAAGATTTTGGGAGAGCGGCT
TTTCAATCCGTTTCTCGATGGCGTGGGAGATCTGCAAATCGCCAAGGTCAA
AGATGCCTTCGAAAAGGCGGAAATCCGCTATCGTGCCGGCGGGTCCAAGC
CTGCTGCCGCACCTGTCCAGAAGGCTGCGCCTCCTGGTAAAGTGGCGACT
CGTCCAAGCCCATCAAGTTCCTCTTCCGGGCCATCATCATTTCCTATTCGAC
CTGCCAAGACCATGCCTTCGCCTGTAGCCAAACCGTCAGCAAAGTTTGATT
CTGACCAACTCGTGAGCGATTTTGCGCCGCCCGCCAGACCTCCCCCGGCGC
GATTTGGAGGTGCCAAAGTCGCCAACCCAAAACCCTCTGCTCCCAATTCAG
GTACGACGTCTCCTACTCGACCTGCCCCAATTGCTCGTAAAGCGCCCATCA
CCGCTGCCGGCCCGTCAAAGCCCGCAGCCACCTCGGCAAAATCAGCAGTA
CCATCCACATCGAAGGCTGGATCCTCCAAGACGCTGGCCGTGTCTCCATCG
GAGCCGGTCAAGTATCGCTTCACGCCAGAAGACGCGGCTGCCCAAGCCGA
GCAGCTTATTCCGGCCGACTACCACGCCAAGCTCGCCGATTCTGCCTGGAA
GGTCAGACTCGAAGCGGCAGAGGAGCTTCTCACCTGGATTTCCGATGGTG
GGGCTCAAAACGTTGACGCCGAGGTCATGATGCGATTTCTCGCGAAGACA
CCGGGCTGGGGCGAGAAGAACTTTCAGGTTGGTGCGAACGCCCGCATCTT
TGCACACACTCACTTTGAAGGTGTCCGCCAAAGTGTACGAGGTTATGAGAT
TGACGGCCGAGAGAAGCCCCACTTTTGGTCGAGCGGCCGCCGCTCTGGCC
ATTGGCCCCCTGACGGACAAATTGGGTGACATTAAACTGAAGAAGCCATCC
GGTGAGGCGCTGACCGCCTTTGCGGAGAAAATATCCTTGGCTTTCGTTCTC
AGTCAATGTAAGACTCTTCACCTGGTCGTTATGACAAGCCTGACGATGAGC
ATACGAGCCCATGACCAAGCAAAAGGCGCCCAAAGCTCAGGCGGACGCAC
TAGGTTGGATCAAGCAGCAGCTCGTTGACTTTGGCATCGCTGGCATTCCTC
TGCGCGAGATGATCGGTTTTCTCAAAGTTGGTCTTGGCTCGCCAAAATGCG
GCTGTCCGCTCCAGTGCAACGCAAGCCTTGGTCACAGTCAAGATTTTCGTC
GGAGCAGATATCTCGGGTTTCCTGGAAGATTTGAATCCTCAATTGCTTTCGA
CAATCAATTCCGAGTTCGACAAGGTTGCTGGCCAAACCCCACCCGAACCG
ATTCGTCAACAAGCAGACCTACGCGAAACCCCCGCCGGGCCCGGCAAAGC
CAGCAAGGGAGGTGCGGACCCGCTAGATGATCTCATCCCCCGCGTGGACCT
CGACAAGCTCGTAGCCTCGACCTCATTGATCGCAGACTCCAAGAGCGAGG
CCTGGAAGGTGCGCAAGGAGGCTTTCGAAGCATTGTCGGGGTTGCTCGAG

GTAAAGTCCAACTCTCGCCTCAAGCCAAACATGGGCGAGGTGGGCGCTGT
CTTGAAAAAGGCCATGGCGGATACCAATTTGGCGGTGAAGATTTTGGCATT
GAGCGTTATCTCGAAAATTGCATTGGGCATGGGTCAGCCGTTCGACAAGCA
CCTCCGACTGCTGACCGCTCCGGTCTGTAGTGTCTGTGCGGATCAAAAGTC
GACTACAAGGACCGCGGCGGTCAACACGCTTACAGCAATGGCTGACGCCG
CCGGAGCCCTGGATCACATGTACGCTGGCCTGGCGACGAGTCTGGAGAATC
CGAACCCAGCTCTCCGATCTTCAATCCTCGGTTGGCTTGCGGAACGGCTAC
AAACGGACCCCCCTTCCTCGCACGCCGATCTGACACCGCTGGCTGCGCCA
CTTCTGTCTTGCCTTGAGGATCGTAATGGCGACGTCCGCAAAGCTGGCGGA
GCGGTTCTGCCGTTCGTTGTGGCGAGCGCAGGCTTTGAATACGTCATGGAA
CAGACTTCGGGTTTGAAACCGGCCTCAAAGGCCACCATCGTGCCACTGATC
AACAACGCAAGAGGTGCGGCCACGGGCGCCGGTCCATCGAAGCCGTCAGT
ACCGGCGCCATCGAATGTCTCGGCGGCAGCGTCTGCGACACCTCGAGCCA
AGCTCCCCTCTGCAAAAATCGTGGCGAACAGCAGTCCAGTTGCCCGACTC
AAGAGCCCTGCGCCTACCACTCGTCCAGTGGGCGTCGCTGGCAGATCATTA
GCTATGAAAGCGCTGTCCTCTGCGCCGGTGACGCGACCGCCTTCCAGTCTG
AGCTCCGGTGACGATCGTCCAACTGGCCTTCCTAAAGCTCGCGTTCTCCCT
CGACCGGCAACGTCTGCGACATACAACACTTCCGCAGTGCCTACGGCAAG
CTCGAGCCGATCTGTGCCGTTCTCATCTGCCAATACCGACGCGCGGCTCTT
GCGATTGAAGAAGGATACTTCTCGTTGGGTATTGGATCCCTCGCCTCATCCG
AAGGCCGACTTGTCCGACTATCTCGCGCAGCAGATGGAGCTAAATGTCCCG
TCAGAGACCTACGCGCAACTTTTCAGCAAAGACCATCGTGCAGAGGAAGA
CTTCATGGCGGCTCTGAGCTCGATCGCGGATTTCTACTCGGATCAAGCGGC
TACCACGTACGAGCTCGATGAAGCGGAAGTCGCTGGAAGACAGGTCGCGA
ATGTTGATCTGGCTCTCAAGTATGCGGCAATGCGGGTACTTGGCAACAACA
GTCAGCTTAACAATCGGTGTTTGGAGGTCATTAGTCGGGTCGTCGAGACCA
TGACTCGGTTGAATGAGCGATTAGGAGACGCCGAGGTCAAGTTATTCGTCC
CGGCTCTCGTCTTCAAGGTGAGTCCGCGACAGTCGCGTGGACGATCACTG
ACACCCAGCTCGGTGATGCCAAGTTCGTTCCAAAGCTACAGCCGATTTTCG
ATGCACTCGGTACGGTCACGGCCACCTCACAGATTCTCGGTCTTTTGGTGC
AACATGGCTTGGATGACAAATCTGCCGGGAAGACGTGCAAGAATGAGGCA
CTGCAGTTGATCGAGAAAGCGTACAAGCGCCGTGGATCGATCCTCCGGATC
AAGGACGAGCGCGGGCTCTACGAAACAGTCGCCAAGTGTATCTCGGACAG
CGGTACAAGACAGGCAGCTCTCAGCTTGATGGCGTGAGTGATCGCAGCTT
GATGTCGGCCGATGCTAATGGCAGATTGCTTCAAATACAAGGTTCGTCTCC
AGCATTACAGCAAGTTGTGGACGGCATGCCCGCGGCGGCCAAAGACATGT
TAGCCAACCGCCGAGCCACTATGGCGGCAAGCAAGCCGACGAGCTCGTCG
ACTGTCTGCCCATCGTGGCAAAGGTATCCGCATCTGCCATTCCCACCGAGG

GGTCACCTTCCAGATTACAGCGCCCATTGGGCTCAGCCAGTCCCGGGTTGT
CTCGATTGCCAAAGACAAGCGTCATGCGCGCCGAGTCTCCAGAACCTCGG
AATCTGCCAGTCGCGAGTGGTTCGAAGCCTCATTCAGCAATCCCTTCACCC
GCGCCGAGAAAGACCGCAATGTCTTCACTGCCGCGAACGACACAGACAGG
ACCTCCCGATGCATTCGGCACGTCGAAACCCGCCTTGCAACCGCTTCGCAA
GACGTCTGGGCCCACTGCGCCTCTTCCTCTTCCTCCGCAATTGCCTCCTCTG
CTTCCTACGAACGGTCATGCGCCGCCACAGAATGATGTACTCATCGCCATC
GATGCGATACGGCAGGACGACATCGATTCTTCTGTGGACGCACTGAAGACC
CTGCAAGATCATCTGCTAAACAACTCGGAAGCTTTCCGATCGTGCACTTCT
ACTCTGGTCGACACGCTGTGCGACGAGCTGGATCGGGTCTTCAATCCTCCA
ACCAATCTCCTCGATATGGCCAAATTCCGTCTTGTCAAGCATCTACTGCAAG
CCACAAACAACTTGTCCGCCAACCAAGACCTGATGCGCCGTCTCAAGTAC
GCCGACATCTACACGCTGATCTACTGCCTCTCGTTACGACTTGTACAGGCCG
ACCGCATGGGTGGTTCGCCGAAAGAGCTCGCGGGCTTTATGAACCAAATCA
TGATTCAAATCATGTCCATGGCGGAGCGGGATCTGGTCTTCAAGGTCATGTT
CCGGCTACTGATCGACCTGTCGGGTGACTTTAGTCAAACGAGACCTGAGCA
CGACAGCGAGGTGGTCGCGCATGCGGACTTGGTCTTGAAATGCCTCTGGA
AGCGGTGTCGGGTCATCGACGATGACCTTCGTCATGGTCGCGTCCAGCCTG
GCACCATGCTAAACATCCTGGAGGACTTTACGCAAGCCGTCAATCCGATGG
AGTATAGAAAGCGAGAGGCAGAGGGTGTGGCGCTTGGGGAACTGCCGCTT
CGCACGATCAAGACGATCATGCAGCGCATCATCAGTGAGCAAAACTTGTCA
GATTTGCCGGGCTGACAAATAGTCTATGCGAAGGAGACCGACCAGGAAGT
GTTTGACATTCTCCGGGCAGAGTTTGGGGACAGAGCGGCGGACACTATCGT
CTACTCGTACATCTTCCGTCTTCGTGGGTTGAACTTCGTCAGTAGGGTGTAG
CTGATCCTCCAAGTCGGCAAGGATGAAGCCGCCGCGGCTCAGAACAGGCA
TCGAAACCGGAGCCCTTTGCTGCCTGTTTTGAATGGCAATGGCAACACCGA
GATCTCTCCGCCGCAAGACTACTTGTCAGCTGGATTCGCTCCCCGTGGGCT
GAGTCCAGTGACCCCTGTCGAGGAACCGACCTCTGAAGCAGAGGCGCTCA
TAAAGGGTCTCGGATCGCACAATCAAAAGTCTCAGCTGGACAAAGTGCAC
GCGTACATCACGGCAAACCCGGACAAGGAGGTGGAAGTGCAGAAAGCCAT
TTCCGCTCACCTGTCAGATGCCGTCCAGATCTACGTCCGCCGTGCACTCGA
GCAACGTAAAGCGGCCGCCTCGCCTCCCGCGGCAAGAGAGAAGGTCTCTC
CCGTTCGTTCCCCCGGCCGACCAGTGACATTGCCCGCCTCAAAGCCCGCAC
CTCGACGATCATTGGCAGCCGGACCTCGGCCAAGCAGCGTGGCCGTGGAC
AAGAGCGCACCGATAGACGATCAGCTCTCGGCGTACAAGGCTTTGTTCAAC
CGAGGGTTGGGCGCCAGCCAGACGGGCAGTGCGGGCGGCAGTCCGGGTG
AACCCCTCCGCGAGATCGACCAGCCGCAAGCTCTGGACTCGTCGATCGAC
TAGTGAATGCATCTATTCTCGTGGGATCTCTCAGCATGGGCACCAGTCATAT

TCTGTAATCGACCATGCAATGCATTGATTTGGGCACGTTGACAATTGTGTGA
GTGGAGCTACAACAGCGTCTCGCCTAGCGCTGCACGGGTCAACGAGCAGC
CGACCGCGATAGTTTGGGAGGCAGATGGCGCGTCGACCAGAGACGGGTCA
CCTCCTGGCTGTCAGCTGAGGCATGATGAGGTCACTCACCATGATGTCGAG
GGCGACCAGCAGTCCCGTCTCTGCGACCGCCTCGGTGATATAGTGTCCTTC
ACGGAAACTCAGTCCACCACGGACCGGTGTGCCCGTACCTGCCGTCAGCA
CTCACTCTGGCCGCACGACTCACTCGGCGCAACGGTCGGGTCGAGCGCGT
CGACGTCAAGGCTGAGGTGGATGGGTTTTGTGCGGCTCGGGTTGACGTGA
TCCAGGGCCATCTCCACGACACGGCCTATCCCGTACTTGTCCACCTCGTGC
ATTGAGAACGCCTTGATCCCATGTTCCCGCAAGATCTTCTTCTCTCCGGGGT
CAATGTCGCGCAGGCCGATGTACACGCTGTGCTCAGCCTCGTCCGGTCATA
CCAGCTGAATGCTTACAGCTCATCGGGCTTGAGGCATGGCTTCAGCCACCG
GTTGAACGGCTCGACATCGGTGCCGTCAGGCCGAGCAGAAAAGACACCGG
GCACCCGTGCAGATTCCCAGAGTCGGTCGTATCGGGCGTGTTGATATCGGC
ATGCGCGTCGATCTGGAATGAGCCGGCCTCGCCTCGAGCGCCAATGGGGA
CCGACCCATATCACACACGCGTCCGGATACTTGGACTTGCTACCCGCTATCG
TCCCCATCGCCTGGTATCAGCTCGGTTGCACGTCAGACCAGACGACGGCGA
CGCACCAAGGAGTGATCTCCGCCGAGAGTCAGGGGCAGCCAGCCCTTCTT
TGCGATGCCGCCCACCTCCCTGCCGACCATCTCGCACACCGCACTGACCAG
ACGGGGCTTCTTCATATTCCCAATGTCGGGATCCGGCAGCCTCTGCCCGAC
CGCCGTCTTCGAGCCGGGCCGGACAGGGTTATATGGAATCTCGGCAAACTT
GAGAGATGACTCGTAGTACACCTCCCAGCCGAGGTTGGCGAGCTGGTCCA
CCAGGCCGGCATCGATGAGCGCGTTCGGCCCGAGGTCGACCCCGGCTCTC
CCCTGTCCGCCGCTGAAAGGACACCCGACGATCTGTGGTCAGCGAGAGAG
CAGTGATATGCACGTGAGGACCCCACAGCCACGGTCGCGGGCTTTGTGAG
ATACTTGTAGTCGTGCGTCGGCCCGGTGTTGCCGTCGGGCGTCCCGTGTAC
GGTGAATTGTCCCGGGGAAGTTCCATGAGTGGTCGACGTACAGCGAGATG
GTAGGACAATGGGGCGCTTAGGTACCACATGTCTTGAGCATGGTACAACGG
TCGGCCTCGCAGCCCGAGAGCCTCGAAATGCGTGCCCGAGCATCTCCCGG
CCTCTCAGGTTTGAGACCTTGAACCTGCGACAATGTATGGACGAGCAGAA
GGAGCGACGATATAAATGGGCGCTTGCCTCCGAAGCGCGCTTAACAGAGTT
CGAGCTTTAGTCTTGTTCTCGGCAGTGCGTTCTCGTTCGTTGCACCCATTGT
CACGCCTCTGCAAGTAACGAGTCAAAATGGCGACAGCTCCGGACCACCTC
TGTCACGACGGCCACGCCTCGACGACCTACGCACGTGACCGACATCTCTCA
CGTGGCCGACACGTGACCTACCTCGGGCTGGAGAACCTCGACGAGGGAGT
AGACGTTGAATGAACGCGCGACGACGGGAAGCAGGCGCAGAGTCATATAG
GATGCCCAGGGCGCCCGTCGTAGTTCTTCAGCACTCTCTGATTGAGTCTCAT
GGATCATCATTGACGATCTCACTCTCTCCGTGGCACGCAGCGTGGCTGAGT

CGCACTCACGCTGTATCCTCGACGACCTTGTTACCTCGACGACCTTGCAAC
CTCGACGAGGATCCAACCTCGACGGGGATCCTACCTCGATATCCTCGTTTCT
ATCCCTTTATCCCTTATTCTTGACTCTGATATTGTGCGAGAGGGCCTCTGCCA
ACGGGAGAGCGCAGCATCGTGACAGTATCAGAGAGTGCTACTACCTCTTCG
CACAACCACGCATACCCGGGGCATATGCCCACCGAGACTCGACGTTCGACA
TCACTCAACCCGAACCACGAGCACCAAGCTTTACCACAGTCCCTTCGACTT
CCCCCTGCTAGCCGGTCTAGCACACCCAACAACATGGCGGATCAGGCTTCC
CTCGCTCAGATCCTGCAACAACTCGTTGCCATCGAGGCTCGTCGAGCCAAT
TCACCCACTCCCTCGACCAACGCATCATCGAGCGAGCCCAAGGCCAACCC
GCCCGACACTTTCGATGGCTCAAAGCCGGAGAAACTCGAAGGATTTCTCAT
GCAATGCAACATCGTCTTTCGCCTACAGCCTTCGAAGTTTACAGAGGAGTC
CAAAGTCATCTACGCCATCTCCTTCCTTAGGGGCTTCGTTCAAGACGCCATC
CAGCCTCTCGCGTCGGGAGAAGAACTCGATGAGAAGATCAGTAGCTTTCA
GAGGCTATCGAGATACTTGAGGGACAACTTCGGGGATCCCGACATCAAGG
GCACCGCTCAGAGGCGATACTTCGACTTGAAGCAGACCGGATCAGCGGCA
GAGTACTTCGCGAAGCTTAGGGAGTACCTGGCGGTCTTAGGATGGGAGGC
CAACGAGCAAGCTAGAGCTAGGGCGCTCGAAGGCCTCTCGGACAATCTTC
GAGATGAGGTTGCTCGGACCACAGTGGAGCTGGAGACCTTCGGAGAGCTT
CAGTCCTATGTCATCAAGTTGGACAACCGGCTCAGAGCAAGGGAGGCCGA
GAAGAAGGCCGAGGTCAAGCGGTACGAAGAAGCTCGACGACGGCCAGCT
ACGACTACCCAGGGACAAGTTCCCCTCCCTGCCTCGTCGACCTACCGCCCT
CTCCCGCCAATCCCGTCGGCCCGCCCATCACCAGCCCCTAGTGGGCCCGCT
TGGCTACAGACCAACGCCTCCCGGAGGACTCGACCCAGCGGAACGCCAGA
GGAGGAATGAGCAGCGGCTCTGCTACCGTTGCGGCGGGGTCGGACACTTC
GCCAATGCCTGTGTTCCCGGGCAACGACCTCCCGTTCCTCGAGGCAGCCCC
GCACCCGGCCGACCTATCGTCAAGCAGGAGGACCCAAAAGCTTGAGGGAC
GCTGAGAACATACGAGAGGTCCAGCGGCCCCATCAAGACATCCTCTCGGCT
ACCTCCCTCCCCATTCACCTCCCAACCCCCCTGTATCACAATCACCTTTAAT
CCCGAACCTCGACGAAGAAGCGAAAGATGAGAAGGCACTGGTTGCCACG
GACGGAATAGCTAGTGATGAGGAGGCATGCATTCGTGGTGAGGTTTGCTGT
TTAGGCAGAGAGCAGGCTGTCGTGTCAGGAAGCGTCAATCAAGTCGACGG
GGTGAAGGTGCTGCTCGACACCGGGGCCACTGGTAACTTCGTCGACTCTG
TGTTCGTTGAACGGCATCACATCCCCTGTTACCCACTCCCTCGACCCCGGC
AACTCCTCCTCTTTGACGGTAAACCAAGCTCAGCAGGACAAGTAGAAAAG
GGAGTGACGGGAACAGTAAGAGTCCTAGGCGGTGAAGGAAGGGAGGAGT
TAGGTGTGACAACATTGGCAGGAGCGGACGTGGTGTTGGGATATGACTGG
ATGAAAGAGAACGGTGTGATATTGGATGCTGGAAGGAAGAAGGCGACAAG
GAAGAGGACAGGCGGGAAAGAGACGAACCAGGAGAACGGACCACAGAA

CCTCGACGAGGTTCTCGACTATCAGGAACATTGGAGACCAAGGCGGAAAG
GCGACAAAGTAAACGAAGGAGCAGGCGATCGAAGGCGAACAGCATTGAG
CTCCCATTCATCGATGGGAGCAGAACCAGGGAAATACAGTCGAGCTATGCG
GCACAGTGACCGAGGAGTTGAGCGAGAAAGAGGCATATATCGACGTCTTA
CCCGACTATGACGAGTACGAACTCAGCCCGGAAGAGGAGGCAGCCGTGCA
ACATCTTGTCCCGGAGCGATATCACGAATACCTCGAGGTCTTTCATCCTCGA
CGAGGGGTAGAGACGCTTGCCCCAAGTAGGGAATACGACCTACGCATCAC
GCTTAAACCTGGAGCCGACCTACGAGTGGCCCCGCTGTATAGCTTGAACCC
AGATCAGATGGAGGCGCTCGACAAGTTGCTGGATCGAGAGAGAAAGGCTG
GAAGGATTCGACCATCGAACTCGCCCTATGGCTCACCAAGCTTCTTCGTAC
CAAAGAAAGCCCAAGGAGAACTGAGAATGGTTGTCGACTATCGCAAGCTC
AACGAGATGACGGTACCGGACGCATACCCGCTGCCCCTGATATCCCAAGTG
ACCGACGACCTAGCCAAGTCGAGATACTACACGAAGCTCGACTTGCCGGG
AGCATACCAGCTGCTCCGGATGGCTCCTGGACATGAGAAGTTCACAGCATT
CCGTACCCCTCGAGGAATGTTCGAGTCACTGGTGGTGCGGGACGGACTTC
GGAACGCGCCGAGCGTGTTCCAACACTTCCTGAACGAAGTCCTGCACGAA
TTCCTGGGAAAAGGGGTCATCATCTACATCGACGACATTCTCATCCATGCGG
AGAACCTCGAGGAACTTAGGAGATTGACTAAGGCGGTGCTGGAGGCGTTG
CGGAAGGCTGGACTGTTTGTGAAGGCCTCTAAGTCGGAATTCGAGCAGAC
TAAGGTGAAGTTTTTGGGCTTCATCATCTCGCACGAGGGCGTCGAGGCTGA
CAGTGGGTATGTCGAGGGTGTCCGCAACTTCCCTACCCCTCGAAACCTACA
CGAAACTCGACGATTCCTCGGCCTCGCGAGCTATTACCGACGCTTCGTACC
GCAGTTCTCCAAGCTTGCTGGACCTCTCTCGCACCTTACAAGGAAGGACAT
CGACTTCCATTGGGGAGATGGGACAGCAGTGGGCGTTCGAGGAGATCAAG
AGACGAATGAGCGAGGCACCAGTGATCGCTCACTTCAATCCGACGTTCGA
GACTATCATCCAGACAGACGCCTCGGTCTATGGATGGGGGTTCATCATCTCG
CAGGTGAACTCCTCGACAGGACAAGAACACCCTGTTGCAGTCGAGTCGGG
TGCTTTCTCAGGAGCTGAGCTCAACTACACCACCACGGAGAAAGAGTTCC
TCGCAATTGTTATGGCCTTTAAGCGACGCCGGCACCTTCTTCTGCAGGTTAC
CTCGACGATCTTGACCGACCATCTCAACCTCACATACTGGATGGAGCCACA
GAAGCTCAGCCCTCGAGTCGCGAGATGGGTGGATACCCTGGCTGGCTTCTC
ATACAAGATCGTCTATCGACCAGGAACCAAAGCAGCCTACCCGGATGCATT
ATCGAGGAGAGCCGACTACATCCCACAGGGCAAGGACGCAGAACCAGAG
CTAGTCCAAGCCCTGCCAAGCTTCGACGAGGAGAGTACGGTCACGTCTAG
TCTGGGCTACATGTTGCGAGCGTTGGTGGAAGACGAAGAAGGAGGAGAAG
ACGAAGAAGGAGGAGAAGACGAGGAAAGAGAATTGAGGGGATTGTCTCG
AGGGGAATTGAAAGAAGGGCAGCGCGGAGATGAGGACTTGAAGGATACAT
TGGAGGAGATTGGAGAACTAAGAGGGGAGAAAAACATCACGAAAGAGGA

GAACCCGAAGGCGTGGTCACTGGCGAAGAGACTGGGGCCTCGACGAGGC
AAGCAAGATCAAGTTCGACGAGGATGGTCTTCTCAGGATAAATGGTCGACT
CTACATCCCGGACATCAAGCAGGCAAGGAGTTAAGGTCATGCGGGATCACC
ACGAGGGGCAATTGGCTGGACATCCTGGGGTTGCGAAGATGCTGGGAGCT
GGGTTAGGAGGGAGCACTGTTGGTTAGGGCTAAGGCGGGACGTGGAGAAG
TATGTGCGAGGATGCGCAGTGTGTCAGCGGTGCAAACCGGTGCGTCAGCG
TCCCCATGGGCTGTTACACAGCCTGGAGATAGATCTGACAGGCCATGGTCC
TCATTTCATGATTCATCTTCCCGCCAGCTCGGGCTTCAACTCGATCCTTGTC
GTCGTCGACCGGCTCACCAAATGGGCTATATTCATCCTACGACAACTCGGCT
CAACACGGCAGGCCTGGTCGACCTTGTCCTCGACAATGTGGTAGCGCAGC
ATGGGCTACCCACGAGCATCATCTCGGATAGAGGAAGCAAATTCACCAGCA
AGCTGTGGGGGGCAATGTGCAACACCCTCGGCATCAAGGTCAGCCTCTCG
ACAGCCTTCCACCCTCAGACAGATGGGCAAACTGAAAGGACAAATCAAGC
CCTCGAGCAATACCTGAGGATCTTCTGCGACTACAAGCAAGACAACTGGG
CTACCCTTCTCCAGCGAGCGGCGTTCGCGTACAACAATTCAATGCATTCGG
CAATCATGATGACGCCGTTTGAAGCAAACTTCGGCTACGCCCCAAGGTGGG
CAGAATCCATCGTCGTCGAGGAGAAGCTCGAGAGATCCCATCAGTCGTCG
AGAAATTTGCCTCGCTCGAGGAGGTGCACGCCTTGTGCAAGAAGAACATC
GAGCTCGCCAATGAGGCATATGCAAGAGGGTACAACGCCAAGCATCAGGA
GGCCCCCGAGATGAAGGTAGGGACCAAGTGATGCTGTCGATGGAGAACAT
ACAAACTCGACGACCTTCGAAGAAGCTCGACTTGAAATGGTGTGGACCCT
ATAGGATCATCGCAAAGGCGGGCAGCGCGTACAAGCTTGAGCTGCCCGAC
ACAATGAAGGTCAATCAGTCTTTTCGGTGGACCGACTGAAGGTATTCGAGC
CACCGACGCTTCCAGGACAAACATACGACCCACCAGGGCCTGTGGAAGTC
GACGAGGAAGGGGAATCATTCGAGGTGAAGAGGATCATCGACTCGAGGAA
AAGGAGAGGACGATTGGAGTACAAGGTGGAGTGGCTCGGGTATGAGGGA
ACGGATGAGGCGACGACATGGGAACCGGCAGGGAACATTGATGGAGCCTG
GGCGAGCATTGAGAAGTTCCACAAGGAGAACCCGGAGAAGCCAGGAGGC
CAAGCAGCAACCAGGGCCTAAGTGAAGCAGCGACAGAGCATAGGCTGAG
AAGCAGTGAGCGGTGAGAGACATACATGCGGCAAGATACAAGAGTCAAGA
CGGCAAAAGCAAGAAGCAAGAAGCAAGAAGTAAGAGACAAGACAAGCTA
CGGTCAAGATGTTCCCTCGAGAAACTCCCCAAGTGTCAACTCGTCGAAGTT
AACACCCAAGTCACCTGCGATGTCTCGCGCCCGTGAGATAAAGTAGGTACG
CATCGGTGCTTGAGTCTCCCGGCGAGCGAAATGACTCAATTGAAATAACTC
TTGCCACCGGCGGTCTGGGGCAGAGGTCGAGGTCATTGGAGCGGGTGGTG
GTGGTGTTGGCGGCGGTAGCTGCCTCATGGGCGGGGGTGGAGACCAGTGA
GAAGTCCTTGATGGTCCGGCTTGAGGTACAGGATCGAGCCCGCCATCCCCT
AGCCAACCCGTGCCAGCGTCATCCATCGGTTCCGGAGCAGGGGGAGGGAG

CGGGCTCAGCCTACTCGTCGAGGTAGGATGCCCCGAGCGGAGTCTCTTGTG
TCCCAACGGTGACGAGATGGGCGAGGACGAAGAGCGAGCCCGACGTCTCT
CCCTTCCCTTTCCCTTCCCTTTCTTCCCAGAAGCCTTGACCGTGCCCGCTAG
GTCGATGTTGGCGAGGCCGGTAGCCAAGGTCGTCGAGGTGGAGCGACCTG
CGGCGAGCCTGCCGGAACGTCGGATGCCTGGGGTGACGGGCGAGGGAGA
CGTGCCGCGAGCGAAGGACACCTGACGGCGCCGTGCCGATGACGAGGGG
CCAGGAGTGACCGTGCCGAACGAGCAGGATAGGGTGCCCGCACGTTAGGC
ACTTCACTTTCTTGCCTTCCTCGACGATGCGACACGGGAGGCCGTCCGCGA
CACATGCCTCGCATCGCTCATCTCCCTCCAGGACCCGGATTGGCCGTGAAC
CCGCTTGTTGCTGAATGTCAGAGGATACTAAACAGCTGACGATACTCACCC
GGGCCCATGTCAGAGATCTGTTATATCCCCGTTGTCCTCGGTCGGGGTAGTG
GGAGCCTACTGATCAGTTTGGGCGAAACTTGCAGACTTTACTTACCAAAGA
ACGAACGGTCCAATGGCTCTGACTCTGGGACCGGCAGTCCCCATCCCTTCT
CCACATAGTACGCCTCCTCGGAAGCACTACGCGGGTCAAATCGCCGGCTAG
GAGGAATCTCCTCCTCGGACAACTTCGACGATGCTGGAGTGTCGGGCGCTA
GCTCGTCCACATCAGCCCCTACATCCAACTCCTCCTCCCCCTCCTCCTGTGG
GACATCCGCGGCAGGCGCATCGCTGGGTAGCTCCGCAGTCTGTTCGTCGGG
CGGTGGGATGACTCCGACGGCGGGGAGTGGAACAGCCTCGTCGACTGTTG
GGATGTCGGCAGTCGAGGCAGGTTGCGTGGGGGATGGGAAGGGGCTACCT
TCGGGTTGGGCGGTGCCGGCGCGAGACAGAGATGGGTTGGACATCGAGTC
GACACTGACAGTCTCAAATCGAGAATCGGTCTCTGTGCAGAGTGTTACTGA
CCCGTCTGAGTCAGATGATGCTTCGTCTATAGTTGTCGTAGGGGTTGTAGAA
GAACGATAGGACAGCGCGTTGTCTAGAGAACTGGTGTCGTAGAGGATGTA
GGGGGATGGACGGGGCGAGGTGGTGGTGAAGGTTGATGCGTCGAGTGCCC
TCGGTTTGGAGAGATGAATCCGGGGCATAGACGTGAGTTATGAAAGGTGGT
GGGGAAGAGAGCGCTGACGCCCTCTCCCCCCCCCTCTGTTTATGTCGTTTT
CGAGCATCCCGCGACATGGCGACGCCCTCGAGTAACACGAGGCTACCTCG
ACGACGGATTGGCAGTCCTTTCTCCGTTTCCTTGACGACCGCTCGACGACT
TGTCGCCAGAGTTTCGCTCCCGTTACAAGCGACGTCATACGACTGCTATCTC
GACTCCTGGATCGGCAATGCGGGTGTTGTCAGGAAACCGACACGGCATCC
TGACCACGAGACTACCACGAGGCAATTTCCAACGGAAACATCCAAAGGAT
CCTACGAACCGAAGAACGTGAATACGGTAGAACCGTCACTATTAGTCACGA
ATAACTACGACTTGCTCTTGAAAGCGCTGGAACAACCTGGAGAACGATAG
AAGGATACGTCTCAGTCTTCCATCGGCCGAGTTGAGATAGGAACGCACGCA
AGGACAAGAATCAGGTCCACGATAACGGAGGATAGAGTTATCTCACAGTCA
GGGCCACGGGACGGAGACCGAGGACGATAAGGACGACGACGGGCACGCA
AAGAGATTAGATGCAAGCCAGCGCGGAGAGAAGACGATAGAAGAGAAGA
GAGACGACGAGAGAATACTAGAGAGTATTGGGAACGAGTAGAAACGACTA

GGAGAACGATGGAACGATCCACGAAGGACGATGCTAGAAGGATCAGAGAG
AGAGCGAGCGAGAGTAAACGGATACTTAAGAAGGAAGATCGCAGAGGAA
GTTCCTCAGCGATCTAGTTAACTAGTCTCTAGATAGAACGTCTACTTAGAGA
ATCGACCGTACCAGTGACGAGAGATAGATAAGTGAAGTGAGTCTTGATCGC
TCCTCACCCAGCTTGGTTCCACTCTACTCTTGTTCGTTACTATCTGTTACGA
ATAGTCGTTAATAGTTAGCAGCTTGAACCGATAGATTGATTTAGGTGAGCTT
TAGTTGGATCTAAAACAGGTCACCGGGTACAGGTCTGACAGATAACTAGAT
CGCTCATATCCTTCGACAAGCCACCAAATCAAGACGACATGTCCTCTGCAC
CTACGACCGGGTACGGCCTTAGGAACCGAGGCGCGACAGGCAATCCCGAC
GCAGAGCATGAGCTGATCACTCCTCCCACTGGGGACTACCACGGAGGAAG
GAGAAGGAGCCCAAGGTACGCCAGGCGTGTACGAGCCAGGCGCTCAAGA
AGACACGATGGAGTCTACCCAGGATGATGGGCTCTCTTCGATCAGGACCCA
ACTCGATAAGATTGGTGAGGCACTATTGCTTTTGAGTCAGAATCAAAGGAT
GATGACTGAGCATCGAGTGAACCAATTCGAGACTAATACGAACAAATCGAA
CAACAAGGAACCAAAGGTCAACTCTCCGGACGAGTATCAAGGAGAAAGG
AACAAGCTCACCGGATTCCTGACGCAGTGTCAGCTGGTCTTCGACTTACAA
CCAGCCCGATACGCCACCGACTATGCCAAGATCCGATATATGATATCGTTCTT
TAGGAAGGCGCCATTGCACTCTATTGAGGCGCACTTAGCTAAGGATATCGA
CGATCAGCCTAGATGGCTTCACGATTACGGGTTGTTCATCAAACACATCAA
ACGAACCTACGGAGATCCAGATGAAGTTCATACAGCCGAACGGAAACTATT
AGCGATCCAACAAAAGTCTTCTGTTACGAATTACTTTGCAGAGTTCGAACA
GTTATCATCATTGCTGGATTGGCCCGATTCGGTTCTCATCGCCAAGGCTAGG
GAAGGCTTGAAACCCTCTCTCAAGGACGCCATCGCTACCAAGGTGGAGGA
GATCCCTAAGACCTGGAACGACTTTGTCACGGTGATCACGAGACTTGATGA
ACGCATGTTCGAGAGGGAACAAGAGAAGAAGA

>Mosaic_sequence_66

CATTGAGCGTGTGTTACGTCATTCCGGATTGGGACGAATCCACTCCGTCCC
ATTAACCGACCAACTGGGGCACCTCTTGCATTCGTGTCTCTCCTTCTCTCTA
GTTCTCATATCCGCATATCTCTGGAAAGGAGACAGCAGGAAGCCCGTAATAT
CCTATTGAGCCAGAGACGGCCCCACTCGCACATCACTTCTTGGACCCATCA
CTCAACATGACAGCTGAAGTTCCCAAGGCCATACTCTACTCGTGAGCATCC
GGCGAGCTCGCGTGACGGGGCATGGTACATACGGTCTATGAGCCCCGCAGA
TCAGACGCAGGAGCTGATGGTCAGATGGCATACCTCGGTGTGGGGTACGGT
GCCACTGCTCTGCTTGCACGAGAAGGGATACTCAGAGGACGAGTATGTCA
CGCGGCAGGTGGACATCAGTGAGTAATGGCTTCGATGTCAAGGAGATGCG
CTGATTCTGCAGCTAAGGGAGAGGTATGCCATGTCCGATCGAACACTCAAA
CGGATCATGCTGACATCATATAGAATTTCTCTCCCGCCTACTTGAAGTCAGG

TCCAAATGAGCTCCCAGATCCAAGCTGACAGGCAGAATCAATATGAATGGC
ACGATACCCACATTGGTAGTGCCGATGCTGGAGACCACCACATCAGATGTG
GACAGTCGGTACAGGTCTCTACGGGACACGATTGTGTGTCCCTCTCGCGGA
TACATGGGCATCACTGATTCGCAGACCATCTGTGACTTTCTCGATTCCGCAC
GAAACCCCAACACCCACAACACGACATCACATCGGCCAGCTCCGACTCTG
GCACCAGCTACCATCGAGGGTAAGATCCTGTTTCAAGTAGAAGGCCTTCTC
GCTGAATGAGCTTAGGCAAAGCGACCAGCGATCAGCTCATCGCGCTTTGCC
ACGAGCCTTCCGTTGATCCAAACTTCCTGGCCATGGCTTGCCGAAGTAAGG
AGGAGCTCAAGGAGAAAGCCAAGGGCGCTCAGGGGACTATTGTGAGACA
GCGCAGGGCAGCACTGGAGAAGTATATCAAAGAAGCGGAAAAGATCGCGC
AGGAGGCGAGCGGCGGGGGCTCGAGCACAAACGGCAAGGCCTTGACATA
CGAGCAGAAGGTTGTCGCGTTCCTCGAGGAGAAGTACTCGTCAAACCAGA
TCGTCTACGAGGTTTACAGCGAAAATGTTGGACCCGAAGAGGAGAAGGCG
TTCTTTGACGCGAGCATCCGGGGGTGGACTCAGGCCTTGCCAGAAGTCTTG
GGCAAGATGGAGGCCATCATCAAAGGCCCCTTCGCTCTAGGCGACCAGGT
CGTGCGTATCCTTGGGATCGAAGGCTTGATCTCGTCTGATGGCAGTGCCTG
GCCGACCTGCACATGATCGCCTGGCTCGCACGTCTGATCGGTATTGCAGGT
GGCAAGGTGGAGCCGAACGACTACGAAAAGTTGGAGGCTGCTATCAATGG
GAAGATTGGCGAGAAGCTGCGGACGTTCTGGAAGACATGGCTGGAGCGAG
AGAGCTTCAAGAAGACGTACGTCTTCTGGTAGGATAAGCAGGTCTATGTTG
ACGTCCAGCATCTCGGCATGGCCTGACCTCGTGTCCGACCTTCCTGCCAGC
GAGGTAAACATTCTCTGAATCACTATCGCTCCCGCCGTCTGATCGTCCAGG
AATGAAGTCGAGCAAGGCGAGAAGGTGTATACATCCAAAACTATGCATAGT
GCTTCCTCATCACTTCGGGTCGTCAGAGGAAGGATCTCGACCCTGGCCCTG
GAATGCTGAAGCGCCTTTGCCCCATCTACCCGCCTTGGGTCCCCAGGTGTC
CTCGACGCCCCATACATGCTGTGGGCGAATGACGGGCGCGTCTTCCCGAGG
ACCTCCTTCGCCCATATTGGCCGGCCGCGAGTATCCCGCCTCGACCGCGAG
ATCGTCGTGGCTTTGCTGCAAGCCGCCTTCGCCTGCTGACCGGCCTGCTAG
TCTGCTCGAAGGAGATGTCTTCGTGCCAGCTGCGGGAGAGCGTGAGGGTA
CGGACCCTTCCCACGTCCTGCCCTCCGGAGTCAGTGGCAGCCAGGCTTCCT
TGCGATAGGCAGGACTCCGCGTCATCTCCACCAAACGAGACACAGCGCGT
TTCTGGCATGAGCTCGACACGCCCCAAAACATAAGAGACACACATATGCGA
ATCGCTCGTCCTCGCCTGTAAAGATGCTCAGAACGGAGAAGCTCGTGGCGC
GCTTCTCGGCATCTTTGACCATTTTCTCCCGCTGAGTTGAAGAGAGTGGATT
ATAGACCGAGATGTTGGGCCGGTAGGGCGCGTGCAGAGTCTCCGAGAGGG
ACTCGGCAGTAAGTGGGTCAGAGGTGTCGGGTGCCCCTGACATAGAGGCA
GGAGAGGGGTCGAGCGCATCGGGAAAGAAGAGGTCTTCGGGTGGGGCTG
CAGCACGCACGAACACTTGACATCGCGCCTCATCTGTATTTAGCAGGTATCC

CAAGTAGCCAAGTGGACTCACACAGGGCATCGACGAGGTTGATGAATCGT
CTCGCTTCATTCTTCTTTTTCAGAGGAAGCGACGGGACTTCGTCGATGAAG
ATGGTGGGGTATCTCGCAGAAAGAGTGATGTAGTCGGCAGGACCGAGCGT
CTGTACTCCATGAGCGACCTGAATTTGGTGCGGAGACTGACTGGCTCACCG
CCTCGCAGAGGTCCTCAAAGGTAAAGCGGCACGCAGTCGGAGTCGACAGG
GGGACCCGGAGTCGTCTAGAGTAGACCGACAGCTCTGCTGGCTCCGCATG
GCCCTCGCCAACAATTGCTTCCCAAGCATCATCGAAAGAACGCTCGGTCAG
GTTGGACCAGCTCGGCGAAACACCGGCTTCGCCTAGCGTACGCCAATCGC
GTCCGCCATCGAGCTCGTATACCTCGCAACGGGTCTTGAGGGCGTCCAGGA
AGCCTGCCATGCGTTCTTTCTGCACGCCATGATGGTACAGGTCATCGGGGA
CTCGGTTCTGCTCTCAGCATATGTCTGGAGCCGGTGAGCACTCACCGAGCA
AGAGACGACCACGCCGCCAAGACGCCAATACCATGAGAGAACATCCCTCA
GCAGCGCAGCAGCCGAAGCATCCAAAAGCTGCAGCTCATCAAAGCTGATC
GTGAGCTGTGACGCCCCTCAATGGGGATATGAGACGTACTAGAGGATGTGA
TACTCCATGATCATACTCCTCGCAACTTCTCATCAGCCCTTATCCGAAGTCA
ATCGGGAGGCTGGGACTCACTCGCAAACGGGATCGTCTCCTCCTGCACCTG
CACTTCTCCCTCGCCCTCATTCTCCCATCTTCCTCCCGCAAAGACCGCCTTC
CACCCTCTGCGCGCGGCAAGGTCCATCCGCTTCCCGTGCACCTCAGCATTC
ATACCCTCTCGCCGGCGCTGCATCTCGACAAACACCTGGCGGTACAGCCAC
AGGGTGAACGCGTGGTAATGCCACCGTTTCTTGTGTGGCGTGGGAAGCGC
GTCGAACAGCATCGACAGGAGGAGCGATTTGCCCGTACCAGGGGGACCGG
TGAGGAGGATTCCCTGCGCTCAGCTTGTACCACACTGCGAAGCGATACGCA
CCTTCGGAGTGGTGAGCGCCGCGAGCTCTTCTTCTCCGCTGAGGATCCGCA
CGATTGCTCGCTCTTCCTCTGTCTGGCCGGCGGCGCTTGATATGTCGGCGGA
CTTGCCTTTCCACCATGCTTTCGACCGCGTCAGGGGAAAGTCGGGCAGCCG
CGCGAGCAGCTCTATCGGGGGAGCGTAGTCGGCAAGCTCTTCCAGGAGAT
GCCGCAGCTGGTCATTCAGCGAGGATCTTTCTACTGCAAAACCTGGCCAGA
TTCACACGCATCACACAGCGCACTTGCTCTTCGTCCCATTTGAGTCGACCC
GTGGCGACATAGCCTCGGTACGTGTCGAGCAGGTCATTACTGACAGGCAGG
CCTGCCGAGTCAATGTGGGAGGTCTCCCGCGTTGAGGAGCTCTCAGCCGC
AGACTTGGCCAACTGTCGGGAATTGCTCGCGATCGCGCGAACCTGGTGGC
CCTGGGCCTGTGGCTGCGATTGCCTCGCTGCGTGAGGCGGTAGGAGTGGG
GCAGCTCGAGGAGGCCGGATGTGCGACGCGCACCTCGAGCATCCCTGCCG
GGGTAGTATCCGCGAGGTCGACATCCTGCCGAGTCGCTGAGCGATGAGGA
GAAGGCGAGTAGCTGGTGGACGAGCTATCAGATAAAACACAACGTGTGGG
TCGATACAGCAAGTGACGATCGGCCGGTGAAGTGCACAAGTGGAGGCGGG
GTCCCGGTTACTTTCGATCCGTGCAAGCTGGGATTACCGATGTAGCGATCGA
CCATGACGCTATACCAAAGAGCCAGAGCCAGAGCCGCAATGCCCAGCCGG

CTAGTAATGACGCTATATATTCCAGCCAGCTCCCCCATCCTCGCTCTTCCTCC
TGACTGAACCAAACAATCAAAATCAGCATGTCTCTCCTCCGGCTTCGAACC
TCCCTGCGCCTCACACCTGCCACCCGCGGGCTGAGCAGCACCGCGCTCCG
GCGCACGACGGCGGGGTACGGCGACCCGCAAGACGAGAAGATCGAGAAC
AAGACACCTACGCCTCAGGCCGCCAAACCTTCTTCGGCATCGAAGAGCGG
CGCACAGAGCGCCGACAAGGGCACGACGGATCCCGAGACGGCGCGCGCG
GCCAAGGCGAGCGGCAATGCGCCTCGGACCAGAGGCGGCGCTGTGGGCA
ACGCCGGTGGCGAGGAAGGGAGCGGGGGGAGTCAGGTGGATGCCCAGGA
GTTGAGGGAGACGAAGAAGGTCGGAGAAGAGCCGAAGCAGACGACCGA
AGGTGGCGCCGGACCCAAGGGCGGGTGATCACCGTCCCACTCGATCAGTG
AGCTCCCTCACAGAAACCTGTCCCGGCTGATGCGTAGCTATGCATGTTGAC
ATGAGAGTCATGAGTGTAATACGCGAGAGGGTTGATGCAGTATATGTGACA
AATGCAGGTTGTGATGGTGCTGATTATGATGTGTACGATGACGGATGCGCAC
AGTGTGTCTCTCTGTCCATGACGAGCATTCGGAAATCTGTTTGTGGGATCA
CGACTTCATCGCCTTGATGAAGTCTGCAAAGGTGCGACGGAAGTCTTTGGC
CTGCTGTGTCCGCAGGTTGTCGATCAACTGGGTGGCGATATGCGGCGGGCA
CTGCAACTTTGGCAGCAAGTCGTTCAGAAGAAAATCGATCGCTTCTTGGCC
GCGAGCTTGGAGGGTATTGCGAAGGAGCATGGCAATGTCGTGCAGGAGCT
GCATGTCAATTCTCATCCTTGCATATCGCTTGTGGAGACTCACCGGCTGACC
CGACTTTGCCACAAACTTGTCGTTCGCGGGCACCTCGAAGCACGCAGGCA
GCAAACGGCCATAGATGAATGTCTGGTAGCCAGGCAACGCTTGAGATGCTC
GTTGCGTCTTTGGAATCGCATGCTGATTCGTGGCCGCCTGCTGGCCATTGGC
GACCGACCTGCTGAGCTGACTGAGAGCACTCTCGGCAAAGACCGAAGGG
GCAGCAGCAGCTTCTTCAGAAGTACCCCAAGCAATGACACTCTTGGCAAA
GAAGGCGAACGCCAGTCGTTGAGTTTGCTGGTCCGAAAAGTCCGAGGTCA
TGCTCAATAACGCCTGTAGGACGTTCTCGAATTCGGGCTTGTTGCGCTCGG
TGATGAACACTCCATCCAAGTTTGCGTTCATGAGAGAGTTTGAGAAGCTGA
TGAACGTCTCGGACAGCCGTCGGTGGATGACAACTTCGTCTGTCCCAGTG
GCGGGTTTGCTCAGGACTGCGAAGATACGCGAAAGCAAGGGCAGCAGGA
GCATGTCCATCGTCTCGAATGCCTTGTCCTACCGAGTCAGCTTTTCCTCCAA
ATAACATCGCATCCTCACCTTGAGTCGATGCATGAGGAGATTGAGGAAGAT
CATGAAGTCGACCAATTCAGAGGGTTCGAACTCGGTTGCTACCTCGGAGAC
GAACTGAGGAACAAGCTCAGCAACGCTCTGTCCGATGGCAGAGACGAATT
GGGAGAAGGAGAAGCGAGCCTGACTGTCAGCCTCGCGCCATGCGTAGGTA
AGGCGACCTACCGAATCCCTGACAATGCGTTCCGTTTTCATGGCCCGCAGC
GCTTGGAGGAGCACCTGGGTCATCTGCTTGAAAGGCTCCTGATAGGGCTGC
GGTGTCGGGATTGACGCCATCGTCATTGGGAACCCTTTGGCAAAGTGACCG
AGAGCCATCAGGTGATGGTGGACCGAAAGAACCGCTTGCTTGTCCTGCGG

GTTCGCGCCGTACGTCTGGACGCTAGTTGTGAGACCGTTCATGAGGGACCC
GGCGATCGCCTCAAGCAATGGGATTTGCTTGACCGGATCCTCGCGGCAAAT
GGCAACGAGATGTCCGGCAGTCTCGAACAGGTAGCATTGGTCCGAGAAAT
AGCTCCTGCTGGCCGTTGCTTTCTGCAGCAGATCCTCGTCGGTGTCGGTCG
GCTCGGGCAACTCGGCCTTGACAATCAAGAGATCCTGCATGTCAGATCGCG
CATAAGACGATTTTTTTGTTTTTTTTTTTGCTCACCTGCATGCCCTGGAGG
ATAGGCTCGACGAGTTCAAGTATTAGATCACGACGACATTCCCGTATGAACT
TGCTGAGCAGATAGAAGCATCGCTTTCTGACGCCTTCGATCGGGTGATGGA
TGCCTCGAGCGTCAAGCATAGCTTCAAGCATCGGTGCAAGCGTTTCCGGCT
TGGACTTCCAGAATTCGACGTAGCGGACGGCGATCTCGAAGTACTGCGTTG
TCACACTCGGATGAGGGAAAGTTGCCACGCCGGATGTCATGCAGAGCGCG
AGAAGTTGTCCGAGAGGAGTGAGAGGGTACTGGGTATAGTCGATGCGCTC
TCCAAAGTGACCTCCATTGATCGAGGAGACATCTACAGCAGACCCGTTGGC
GTGCGTGGGCGTGCTCGAGCCGCTTGATACAGACATGGCGTCGGCGGCGTT
AGTGAAGCTGAGCTTGCGGGAAGGTCGGGCAGATTTGGCAGCTACATCGG
GCGGCAGGTCGTAGAATGCAGCGCGTGTGTTAGCTGTTCTGTAAGCCGCGT
TCAGGTGAGAGTTCCTCGACTCACTCTTCGTCAACTCGCCGAAAGAAAAG
ACGAGATGAAGGGCTAGCTCGGCGTTCTGCCACTTTACTGCCGAGGGACC
GTTGGTTCGGATTTGCTCAAGGGTGCTGAATGCGATATTGGCCACGATCTCC
GTATGCAAGGTCCGGTCGATGGCGGCAATGGACTCGATGTGGCTACGGCAG
GTCTGAATTCAGCGCCACTGTGGTCGACGGCAGGTCAAACTCACTGTGCG
CAACAAGCGGAAGTTAGCCAGCTCATCATCAGGATCCGCCTCGCAGTCTGG
TGTCTCCCATTCTGCGTCATCGGGCCAGGCCAGCTGGCGAATGAGAATGTC
GAGAACGGACGCAAGGAAGTCCCGTTTGTCCTGTGGTAGTTGCGGTACAG
GGGGAGGAGGGGGTGCCATCGTGCCTTTCGGCGGTTGAGGCGGATTCTTC
GGCACATATGTCTTCTTGTTGATTCTCAAGTATTCAGAGATGAAAGGCGAG
ACGGAGAGAGGTACATCGTGTTGTCGATCGCTTAGGAAGCGCAGGACAAT
TGGCATCCCGGCTTTCATCATCGAGTCTGCCTCTCCGCGCACTGCGTCCGGT
ACTTCGGGCTGTCCGTGAGCTCGACATTCTTTTGGCAGCAGATACTAACGT
CTTCAACAAGATCCAGTAGATCAGACTCATACGCCGAGAGTATCGCGCCAA
GAGCGGCCCTGAAAGCGGCAGTCTCCTCGTCGTTGGCAGCTCGGGTGGTA
GATTCAAGGGGATCGACAATGGCCAGCACATCAAGCACTTTCAAGACCTG
CAACTTGTCCTGCGGATTCTGCATGCCCTTTGCTGCCAATGTGCGAAGGAT
GTTTGCCGAAGATATACGGACAGGGTTGATCGAGGTTTGTAGGGTGCGGCG
GAAGAACTGCAGGGAGCTGGGTGTGATGGAGACAGAAATGTCCACCCAA
GCTACAGGTGAGCGCAGTCTCCCATCAATGATAAGACCTACGAGTCCATGC
AGCCAGCGTCTTGAGCGCTAGCTCCGTAGTCTCCGCCCAGCGCTGCTGATC
CTGCCCGCGCTCGATAATGTCCAAACCGTGTTCAGCCACAGCGAGAAGGG

CGTTGACCGCTAGCTTTTCATCTCCGGATGATCGGATCGTATCTCGTATCTGA
CCATCATGCTTGATACGAGCCTCAGAGTGGGGACGAGCACTCCTGATTGCA
ATGTCATGCACCTCGAGGGCGATCTCACTGAGCAGACGGATGGCGAGAAG
GCGGGGATGAGGGTTGGAGCTGGAGGATGGATCCGAAGGGGAGAGAAAG
TTGATGAAGGGGTGAAGGAACTGGGGGACCGGGGAAGGGTAAGAGGCGA
GGAACAGGTAGGCCAGGGTGCAGGCCAGCTTGTTGCGAAAGACTGAAATC
AGCTGCAACTCGACTAGATCAACGACACATACAGGATTGACCCTGCTCACA
CGACCCGTTGACCCATTCTGTCTGGACGAATTCCATGAGAGCGTTGTACAT
GGCAAGCTGAGCTTCTGGACCAATGACCTCTGCTCTGAAAGGGTCAGCTAT
CGCATGACGGACACGCGGCGCGACCCACTTTGAGATGAGGACCGTGTCCA
CGACCTGCTCGCAGTACATCCGCAATTGTTGGTCCAGCTTTTGCTTGCCATC
TTTACCCGCACTCGATGACTCGGCGGCCCCGGCACCCTGAAGGTAAAGCG
ACAGGCAATCCTAGTCCATCAGCTTCTTGCATCGCCTATTTCGTTGACCCTC
ATCACGGTTGCCGCCAGCTCACGCGAGCAGCGGCAGCGTTGCAAAGGAGA
ATGCACGGACCTGCCATGTCGACTCGCAGAGTTCCTTGACCTTGCTGAGGT
AGCTCAAGGCCTGCTCCTTGAGGGCAGGATCAAGCGATGGATCGACACTA
GCGGCAATGCGAACTGCTTCTGGGATCTGAGCAAGGTGAGGGGAAGAGCT
CATGCTGAGTGTGCGGTGGCGTTGAAGTACTTGTCTTCACGCCACCGCTAG
GCCGATGCCAGGACGAAAGGGTATGCCGGGAGACGACTTGTTATGACAGG
CGACTTGCAATGCAATGCTTGATGATGATGAGAGAAGGTGCAGACAATGAG
ACAGAGGTTTGAATGGTTCTTTCTGAATAGAGAGACAGAGACGATCCGTCC
GAAAGTTCGTGCAGCGAGGCGAAATCTCAGGGCGAAGCGGAGAGCTCCG
AAAGCCTACCACCAAAGACTGGCTGATGAAACGTGGACCTGCAATGTCGT
TGCAGGCTATGCATGGGAGATGTACGGTCGCACAAGTCAGACCGCTACGAG
TATAGGTGAAAGGTATGCATGGCAGGAAGGTTGGAAAGTGCAGATTGCCCT
CCCTTACTTCCGGTACCACCGGACACCAGCGCGGTTGCGGCGCATCTTCCT
CATCAATTGCAGCATTCCGTAAAGTAGTGCCTCGGCGGTTGGCGGGCTGAT
CGTTAGTAAAGATATAAAAGCTCAACAGTCAATCTACTGACCATCCTGGCA
CGTAAATATCGACGGGGACGATACGGTCGCATCCTCGAACCACCGAGTACG
AGTAATGGTAGTAACCACCACTGAGACGTCAGCGGCGATCCTGGCAGGAA
GAGTAGCTGAAAGAAGTGTCACTAACCCGTTCGCACATGACCCCATCGAA
ATGACCCATCTCGGTTCGGGCATTTGGTCGTACACTTCTTGTCAGCCCAGG
ACCCAACAAGCCGACCAACGACGAACCTTTACGCAGAGCAGGTGCCATCT
TGTTGGTCAGAGTTCCAGCAACGATCATGATATCACTCTGCCTCGGACTCG
CTCGGAAGACAACACCCAGTCGATCTTGGTCGTATCGCGCCGCGGCCATGT
GCATCATCTCGACGGCACAGCAGGCCAAGCCGAACGTCATGGGCCACATC
GAACCCTGCCTCGCCCAGTTGACGATCTTGTCCATGGTCGACAAGACATAT
TCTGCGCCGTTCCGCGGTGTCTCGAGTGACAATTGGTTGCTTCCGCGCTGA

GCGAGGGCGGTTACGGGAGTTGTGAGGGAAGGTGAGGCATCTTCCTGGTG
TATCGATGCCGAAGGGAGCTGACGGGGAACGATGGCGGTGTTGTGGAGGG
GTCGTGCTTCTCATCAGTCAGGTTGACCAAAGTAAAAGTGGCGAGATGATA
TACCGATGGCCAGAGAGGGTCGGAGGGAGGAGCTGGAAGCTTTGGCGAA
CGCGCCTCTACCTCAGCTGACGCACGAATCAACATGAAGTACAGCTTACCA
GAACGCAAGCCGGTGCTGATCATGCTCGCCATCTTGCTCCTTATTCACAAGT
CGAAAAATGAGCGGAATGAAACAAAAGAGGTCGTTGACGATATCGATCTT
GGCCTCGTCGATGCCGTGGTCGGGACCCTCAGCCAATCGATGCTCTGCCGT
TTAAACCCAGCCGATGACTTGGGGCGGCATTCGCCGAATTGCCGCTCAGTG
GCACTCCTACCCAGCACCCTTCGGCTCCGAGGACACACCAACTGTCCGTCT
CGACAGTCTCGCAGAAAAGTTCGAAATCAAGCATTGCATTCAGCATCGTGT
CTTTTCGAACAAGTCATGTTGGTCCGGCAAGTGGCCAGAGGCGCATACCGC
CCCATAGTACGGTAGGCCGACTGCTCGAAGGCCCAGTGCTCTGCTGACCTG
GCAGGGCCTCGCCTAGCCCCTTGGCTCGTCTACCTGCGAACGGTCCTTCGG
TCATTGCGCTCGGCAAGCGACCAGCACTATATGGGCCGCACCTCTCAGCAG
TGTCGCCGGTCCTCAGCCGGGGCTTCTCCTTTACTCCTTGGAGGACAAATA
TGCCGGCAGAACCTTCGACTGTCAATACACTCTCGACTGAAACAGGGAGC
GCGACAGGCCACGAACCGATCCTCACTCAGCCCCACCCGGATCTGCAGCA
GGCGAGCACGGCGCCAAGTCCAATTACGGTCGAGAGTACTACGCTATCAG
AACCAGTCGCCCAAGTGTCCGAAGCAGTCTCCCAATTGCCCGCAGCTGGC
ATCACCGATCCTTCACTCTTCCCTCAGTCCCCCGCTTTGCCCAATGTACCGT
CCCTCGAAGAACTAGTGACAAACGCCACAGTTCCTCTGTCCGACATCTTGA
ACTCGCCGGAAGCCGTGCATGCTGCTGTCAAGCTCTCAGACCTCAAACTC
ATCGGCCTCGAGCATGGCTTCTTCGGCCTGTCCGGCTGGGCACGAGACGCT
CTCGTTTCGATGCATACGCTCACTGGGCTGCCATGGTGAGCGTCCTCCCATT
TCTACATCATGTGTGCTTATTTGAGGTGGGGCTCCATCGCCGCATTGACCAT
CTGCCTTCGACTGTGTCTGTTCCCTCTGATCGTGCGGAATCAGAAACACAA
CGTCCGCATGCAAGCGGTCCAACCACAAATGACGGCCTTGTTCAAGCGTTT
GGCCGAGGCGAAAAAGGCCAACGACGTGGCGGCTACGCAGACAGTCGGC
ATGTCCCTCGCGGGCCTCATGAAGGAGCACAACGTCTCGCCCTTCCGTTCC
CTTGCCCTGCCGCTCATCCAGGCGCCTTTCTTCTTGTCGATGTTCTGGGGCC
TCGAGAAGCTCGCGACGACTCCTTTGCCGCAGTTGAAGGAGGGAGGGTTT
GGCTGGGTGACGGACCTCACGATGTCTGACCCATACTACATTCTTCCAGCA
ACGAGCATGCTCCTCACCAATCTGGTCTTCCGCGTGAGTGACTCAGCTCCT
GAGGTCGTGATTAGCTGACAAGTAGTTCGGCGCAGATGGTACCGGCGCCAC
TTCCTCGAGTCCGAACGGGATGGACATGCGCCATGTGCGCAATTTTATCGG
CATGGCGTCGATTGTTGCTATACCTCTGCTAGGCCGCATGCCAGCGGTGAGT
ACCAATACAGCAGAGGGATCAGTAGTTGACCCGCAGGCCGTGCTGTTCTAC

TGGACATTCACAAATGCTTTCACGCTCCTGCAGGCTCTCACGCTTCGATCC
AGCACAGTCAAGTCGTTGCTGCGGATACCTGCACCGCCCAAAGTGGCAGC
ATCCCCAGACGTCAAGCCGGCCGATCCATCCTGGCTTGACACGATACTCGC
GATCAAGAAGCACTTCACCGACACTTGGGCCAAGACGAGGGAGAACGCC
GCCCAGATGCAGCTCGAGCAGATGGAGAGACAGAGGAAGTTGAAGAAGG
GACAGGCACTGTTTGTGGATCGGGTCAAAGAGGGCGGCGCCGGCGAGAG
CAAGGGCTCCGACGCGGTTGTGAAGAGCGAGAGTTTCATGGAGAGCGAGC
AGCCGAGCCCGGCGCAGAGCGCGACATTGACGAGGGAGCAGGCGAAGCG
GCAGAGGGTAGAGGCAGCCAGGGAGAGGCGAGCACGGCATTGATGCACT
CATCACTCATCGATCGGCGAGCGCGTGGCTCATTGGCTGTAGACTGATACAT
GCGCCGCTGCGACGTCTATCTAGCAGTAGGACCGAGGAACTTGCTCTGTTC
AAAGCCGAGCACGTCGGCCTCGTGGTGATCCCTCAATCTGTCAAACTCTTC
CTGCGATCCTCTTCTCCTCAGCACTGGCGTCCTCGCTCCTTCACCGCCGTCA
TCCTCATCGTGCAGCAGTCTGGCGTGATGGTGCGGCGGCGAGTGCAGCCG
GCGCGGAGTGGCCGACTCGTCCACGCTGCCTCGCGGGCTGTGATGCGCCC
AGGCGTAATGCTTGAAGCCGTCCAGATTCTCTAACGAGGTGTTCAGCGTGG
CTGTCGGACTCTCTTCCTTTTCTTCTGCCGAACGTGCCAAGACTGAGCTGC
CAGTAGAGGACAGAGAAGAGCCAAGCTCGGATGGGGCTGTTGAGGCCGT
GTCGGACGCCGAGGCAGACGCGGACGAATCGGCGCTGTCTTGTCTTGGCG
GTGGCGAGCCTGCGCGGGGGGAGATACTGAACCCGCCCATGGCAGCGGTC
GGCTCTTCCTCTACAACGTCGAGGCCTGGCGAGCTGGGCGACGGCTCGGA
CGTCTCGGATATGACCTCGGACGAGGGCTCAGAGGCCCTGCGGCGGAAGA
TGCGCGGCGAGAATGTCGACTGGGAGGCGCCATTCGCACTGGCCGTCGCG
GCGGACGCGGACGAGGCTGTAGACGCAGCAGAGGAGGAGGAGCGAAAGC
GGTTCCAAATCTTGGACGCCGAGTCTTTGGCCAGCTGGCCTAAGTCGACGA
CATCGCTGCCCTCAGCACTGCCCGCATTGTTTGCCGGGGAAAGACTCACTT
GTGCTTGGCCTCGTACTGCTTCTTGTACGTGTCCCACTCCGAGTCGGTCATG
TACAGCACGCCGCCGCCTTTGGTCCCGCCCGTGCCGTTGCTCTGCTCGCTG
AGGAGGTTCTTGCGCGCCAGCACCCCGAGCGTGATGGACGAGCGCCGCTG
CAGCTCGGGCGGGCGTTCGGGCAGCGGGGCAAACTTGATCTTGCTCCCCG
CTGCGACCGACGAGGACCGGCGGATGCTCCCGGGCTCGGTATAGGATGTG
GGTGGTATGGAGGGGCGACGGCCTGGGCGTGCTGGGGATGCGGGGAGGG
GATTGAGGAGGATGGAGCCCGATGCGCCTTGCGAGAGCGAGGTGTCGGAC
GGGCTGCGTAGGGAGGACGGGCCGGCCTCGGCCATTTTGAGTCTCTGGCG
TAAGGTGTAGGTGTCACGGGGATGGCTGGGAGGTGTCTCTGTGCTTGATGC
ACGCGATGGCGATGAGTGGGAGCTGTGGATCTCTTGCAATGGCGAAGAGG
GGATAGAAGCGGGGTTGGCAGACGGGGATCTCTATTCGTTTACCTTCTCGG
GGCTCGGTGTGTGGAGGTCCCACGGGGACAGCAGTACCGAGCCGAGAAG

ACGGCATACGGCATCGTACTGACATCTCGCATCTCTCCATTCGTCTATGACA
GGTCTGCAGCTGGGCAAAGGACAGTCACCGCCTCTCCATGACCAAGTCGA
ACGGGTGAACAGGCAGCAAAGTCAACTCTTCCTTGGCCTCCAAGAACCGC
TGCTTCACCTCATCAAATGTCTCCCCGGGCAACATCTTGGGCTTGAACCTG
AACGTCTTGCACAGGTGGCTGAGCACGCACACCATCTCCACCTCCGCGAA
GCGCTTGCCGATACACTGCCTCGAGCCCCCGCTGAACCCCGTGAACTGCTC
CCTGTTGTCCGCCCCTTCGTCGCCGAGAAAGCGCGCCGGGTTGTAGGTGA
GCACGTCGGGCCAGTGGAAGGGGTTGCGCGAGACGGCGGGGTTGTCGATG
ATGACATGCGAGCCTTTCTTGATGGGACACACGTGCTGCGTGACGTTGCCC
TCGTCGTCCCATGTCGAGTAGGGGATGGTCGTGTCCTCCATCGCGAGCTTG
GGGAGGGTCATGACGAGGTCCCTCAGGCGCAGTGCCTCCAGAGCGCACGC
GAGCGTCAGGCGGAGAGCGTTTACGTGCTGGTAAGCTGAATGTCAGCGGT
GCTCATGGTCTGACGGCAACTGACTAGGGTATGCGGTGTCTCCGCACGCGT
CGGTGATCTCCTTGTGGATGGCCTCTTGCCACTCGGGGTGCAGCGCGAGGT
AAGCCAGGGTAAAGGTGAGCGTATGGCCAGTGGTCTCGTGACCCGCCAAG
ATGAAGATCCCTCTTCATCAGCTGACAACCCCTTCCAAGGATCGTTACGATA
CTTACAGACGCTCCCGACCGTCTCTTCGGCAGTCAAGCCCGCAACTTTGTT
ACCCGTTTGCGCCTTTGCCTCTTCCTCAACCTTCATCTGACTCGCGACGATG
GCACCCAGAAGGTCTGTTGGGGCCTTCATTTCTCCCCGGCAGGAAGACC
AGCTTCGATCGCTTCCAGCTCTTCACGCTTATTGGCGATCATCGTCTTCAAG
TGATGCACCAAAGACCGCTGTGCCTTGCCGAGCCGGCGCACGCCCGCCGC
TGGAAAATGGTCCAGCGCCCAGAGCGGGATCATCAACTGGGTGATCATGG
ACAACTCGACAGTATGAAGAGCCTCAGGGAAGGGCACTGTCGTCAGCCCC
ACTCCTCTTCTCTCGTCAGGACTTGCGTATCTCTCCGTGCGTCTTCGGGATG
TGCCAGGGGAACTGGACGCCAAAACCGCTCATGCCAATAATATTCAGCGTC
AACTGCGCATCAGCCACGCCCCCGTGTACCTTGGTGCACACCTTGATCAAC
ACATCGCGTACGTCGCGCATTTCGCCTCCGTGCTCGAAGCCCTCTTCCCGG
CACATGGCCCAGAACTCATTGGCCGTGTTCTCCCAGCCCTTCATCATGACC
GCCTCGCCAAAACAGCCCTTCACCACGCCCTTGTGGCGCCGGTGGTCTGC
ACCGTTCTGCTGTGACGTGATCTGCCCGCCGAAAATGTTGATCACCTTGTA
CCGCGTCATCTCGGTCGGCTTGCCGAACACGGCGCCGCGCGAAAAGATGG
ACGCGGCTGTCACGGGCGACGAGACGATGTAGACCGCATACGAGGGGGTC
GTCAGCTGTGTGGCCGCGATCAGGTCGCTCTTCGCCTTGGCGTATTCTGCC
ATGAGCCCCAGGCGGCAGCAGGAGGCGCCGACTCACTCTTCCACGGGTCG
TCCAACGTAAAGTCATTTCTGGGCAGGATGACCGGCACATGCGGCAATTTG
GTCCTGGCGCCTGATTCGAAGAATTCGAAGACCGAATGGATGGTCTTGTGC
CCGCGGACTTGCTTACTAGTGAGTTACGAATACGATATAGTCGGGGAGGTC
TTACCAAGCGGACACGCGTGTAGCTCCAGAACCAGTGCAGGAGGTAGGTA

GTGAGCGGGATGATGAGACCGGCAATCCACGCATCGCGAGTCTCGAGGTT
GCGCACGCGCCTCGGCAAATCCAGCAAAAATCTGGACACAACGCACATTG
CGGGCTCGATGATCCCAGGTGGAAGACCCCCATCTGGCTTTAATAGCTCCA
GGGCAGGCTGCTGCACCTCGTCGCCGAGGTCGCTGCAATCTGCAATCCATC
AGACCAATCGGTCCAGCGACATTTCTCACTATTTCTGCATCACGACCAGCT
GTCTGTGTCCTTTTGCGCTAACGGCGCTCCCCAAAGGACCGCAGTCCTGGC
GCTTGATGCTTACGTCGGCATCGCATCGCATCGCATTGCTCCACGCCGGTCC
CAAACCCCTGAAGCAATCCCAGAAGCGGGCTGATCGATTCCAAGCTCACG
TATTATAGCTGACAACTTCAAAACGTCGATAGAAAAAGCTGCCATAGTCCA
ATATCCTCTTGCGCCCGCCACAAGAATTCCTCAGTAATATCTCCCTTCTAAAT
TGTAGCTATATCCATCCTTAGAAAGCTCGATCCTATTATTTTAGTACGGCTAA
GCATATTCCGACACTGCTACAGCTGTACTACTCCTTCTTAAGGGGCAAGATT
ACCTAGTCTTCCCTGAACACTTCAAGTCATTCCTTATAATGGCATCCCCATG
GTGCATTATATGGCGGGGAAGCATAAGTATTTGCTGGAGGTTGCTGTCAGTA
GAATATGACGCGAGCCAAAATTAGCTGGACCTGCTGCTCTCTAGAGATAGA
GCCCATTATATGCTTGTATTATAAATATCAGGCCTAGGCGCTCAGTGCATATA
GGGAGAAATAGCACTGAGAAGAGTCACGATAGAGCAATTGCTAAGGTAGG
TACCTAGGTAAGTATGGATTTGTGTCATGAGGAAAACAACTTAGGGCTATTT
AATTTTGATTGTAAATTATTAACACAACCTACCCAGGTCTCCTTTCTATCGTC
TATATATATGATGATGAACATGCAGTTGAGAAAATAGCAACAGCGCCAACAA
CGAGATTATCTAAACCCTATGCTCTCCCTAGCCTCCCAGATTACCCCTATTAT
CGTAACTCCCTATAGTGTTACATCTTCTCCCTGATTAATAACTAACGCAAAC
GACTCCCCAGCTTATGTGATTTTTTTCAAATCAACCCACCCGATGTAATCCA
TTGCTCCAGCCAGTCTCTCGAGGTTCTACCTATGTCTCCTGACATCCACCAC
TGTTTCTTTTCGCTCACCCATTTGCATGATCCATAGCCGAAGTAGAGAAACC
ACCAGGCACCGACAACCACCTGAGCCTATGCGAGCCGCTCCGCCTCTGTG
CTATCACATAAGCCGCCAGATCGTACAAGGTCTGCCCGGAAGAAGTCAATC
TGTGGGTTCTATAGCTCGACAAGGCCTCCGCTACTTCTTCCCTCCACTCGGT
GCGTGTCTGGGCCGGGGCGTCTGCCCTAAGAATTATGATGATGAAGGACCG
GCGGCGGTAGACTAGGAATACGCACGAGTGGAAGCCGGATATGTTGGCGC
TTAGATGGTCACGGATTTGGGATGCTCTGCTACGGAAGTAGTGCCTGACATT
AGCGGAATCGCCTCCGGGTAGATCGATGAAGGGCATATGACTATGCTGCTT
ACTTTAGATACTGTGCCGTCTATTGAAGGATTTTTCGAGATTGTTGGCCGAT
AGATTGGGGGTTGGTTTCTTCCCGTATAGGGAGATAGAGGTAGAGGAGAAC
TATATGGCTCGAATTCTGCTCGGTCATATAGATTTTGCCCATTGTCCAGTAGT
CAGAGTCTATGGTTATTTATAGAGAAAGCAATCAGATTTGATGGCATTTTGA
AATACGATACATATTAGTACGTGACACTGATCTATAGTCATTCTCTTGTTAGG
AATATCATAAGTAGATAATTGTAGGTAGTGTCACGACGAAAACATAGACTCC

TCTACAGGCCGATCCTAGCCCTGTGGCTATCACGGGATCTTCGTAGAAGAC
TTGACAGGTTGGTCTCATGATTGAACCACTAACACTTATACCAAAGTAATAG
TAATATATATATGTTGATAATAGGTGATAGAATAGATACTCGGGGGTAGAATA
AGTACTAGGTAGGTAAAGATGGCTCCGGAGTAACTCTGGTCAACTCAGGCA
GAACTCAGCATCTCCCATTAAAGTCCATTCTGGCCGTGATATCCATTCGTATC
CCCACCAAGTACTGTTGTTCTTCTTGACAATCAAATACGAAACCAAGTCTG
ACAAAGTCGTGTCGGGATCTTCTGGGAAAGGTTCGACCATCGATAAAGCCA
ATTCTACTTCCATATCAAAGTCCTCTGGGTCACTAGTCACACAGTCTTCGCT
GAGGATAACCACGATGTATGCTTTATTAGTGCTCAAGAAAACTTTAAACTCT
TCGAAGTTTGTGACCAGAGTTTCAAGGTAACCATTGATTTTATCTCTGTTGT
CATCAATGTACGCTTTAACTGCGGCAGTTCCATCTGAAATAGGGGCCATCTT
CGACGAAGGTAGGTAGTAGGTAGCTGTGTAGCACGGTATTTCTGATACAAC
TGAGAGAT

>Mosaic_sequence_67

GACCCTAACACCGTCGCGGATACCTGGCCATCGCCCCCGCCGCCGGCGG
GTACGTCGCAATGGTCGACGTGAGTCCAGCTGCCCGCTGCCGCGCATCGGC
GACGCCCTCGCTGATTCTGTAGATATCCTCTGCCGACCCGTACTATGCCCCA
GCCAATGTCCCAGCCGACAGCGCCGCCCTGTACACCTACTGCACCTACCTT
GCGCGCTACTACTACGTACGCAGCAGCAGTGGCGACAGCAGCACCACGTG
AGTCCCATTCGAGGCAGGCCTTTTCTGACATCCAGAGTCACCGACTTCACC
GGGATCGCGTCGTCGACCGCCGCGGGCATCAACTCTTGGGACTTCACCGA
CTTCGATTCCGACGACGAAATCGGTCTCACCTACGTAGGCGCGGGGACCTC
GGCCGCCAACCTCTACGTGGATCCCAACAACGCTCGAAAGCTGTATACAGG
CCCTTCGGCCGCCGCGATCAACTCGGCTTACTCACTGTCTATCGCTGAGAC
CGATCAGGTGACGATCACCCTCGTGATGCAGCAGGTCTAGTTCGGTTACAG
GATGATGGGAGCGGTGCAAGAGGAGACGCGCCTTCTCCGATGAACAGTCA
GACCGACATTGAGGGTGGGACAGGTAAGACTTTGCGGTCGGGTAGACAGA
TTCATGGACATTCGACTCGCGTCGCATACAGTTTTGAAAAATGCGACGTGT
GACCTTCGTGGATACGCACGGATCGAGTACGTTCACTCGACCCTCATTCTA
AACATTCGCCGAACCGTTTGTATTCACTACCGGTTTGCTAACTATGGTGCTC
ATTGTATCATCGATGTTGACGCCTCTCATAGCCAGTGCCAAGCCTGTGCCCA
CATTCGCAGCTAATGAGGGCTGGTTGACATACCTCATCGGACGGAGGCTCG
AGTTGACAGGCACCCGGATCATGTTCTATCATATTCGGTTCAACCTGCGCCA
CCGCGCCGACCCCTTGAAAACAACAACGCAAGGCTGAGCCATGCCGTCTC
AGCAAAGAATGCGTGCGAATGCCCAATCTGCATCCGACTACGCCTTCCTTT
CCTCCGTCATTGTGGCGGCTCGACTTGTCTGTCCGACGGCGAGCACAGCTG
TCCCACCCGCTTCCGATCTCGTCCAGCCCGAAGGGTCTTGACAATGCCTAT

CTCCTTGTCTCTCTGCATTCGTGTCGGCTGAGTCTTGTATGCTACGCGCAGT
CGCACTCGCAGCTCACTGTCCATCCTGCTGCGAGCTACCAACAGCATCAAC
AACATGATCCTATGCCTTGGTCACCGGCACATAAGAGGCGTCCATTCTAACG
ATCTGCACTAACAAGCCTCTCACCGCTGAAGAGCACCCTCACGTCATGTCC
CGCTTCGCGACCCTTCTCCTCCTGCTTACAGTCGCCCTCCTCAGCATGAGC
GGGGTTGGCGCCCGCGTCCTCCCCGAGGACTCCTCGACCTTTGTCGGCGA
CCCCCACACTGCCAGGCTATCCAATGGAGAGCGATTCAAGCGTGGTCTTCC
CCCAAGAGCCCCGCAACAGCTATACACCCCCACGCGAGTCCTCGCCCCGC
GAACAAGTAACACCGCCACCACCCCCTTCTATGCCGAGGTAGCATATGCCA
CCGAGCCAGCCACGGTCATTGGCTACCTGGCGCTGACAAAAGCCTCTGGA
GGCCATCTGGCCGTGACAGTGAGTCAAATCATCTCGGTTCCGATCTCTGAG
CTGGGAATGCTGATGCTAGACACTCGCCGCTGCCGACACGTACTACGCGCC
GACCAGCGTGCCGGCCGACAACGCCCTCTTGAAGAGCTACGCAGAGGCGT
ACACCGTCAATTACTACATCAACTCGAAAGAGTCCACGACAGTCAGCGGG
TTGGTTGAGACGGCCGTCGACGAGCGGTGCTGATGTCCAGGGTGTACAGG
TTCAGCGGGACCGTCACTGTGAGCGGCACTACCTTCAACATCTGGGACTTT
TCAGATTTCGACGCTTCAGACGATATTGATCTCACGTTCGTGGAGACGGGC
TCGACGTCCATCAATCTGTATATGGCTGGAAGCGCCAATACCGTCTATGGTG
CGGCGTCGGCTGCGGACGTCAACTCGGCGTATTCGACAAGTATCACGGAG
AACAGCCAGATCGTCATCACGCTCGTGATGCAGGCGTAGCTTCAGGGGGA
CATGGATGGTAGATATCGATATTGATGAGCTGGGGGACGAACGGACAATATC
AGTAGAGTGCTTCTTGGTATCCTGCTACTATGGACACTGATACATTTGATGG
GCATCACGTCGCATCACAATCTTCGCATGACATCGTTACTTGATCTCGTACA
TAACTTAAGGCTTCCTACCAGTACTCGTCATCACGCCAGATCGAAGCGGTC
GAGGTTCATGACCTTCTCCCAGGCCTTGATGAAGTCCTTGACAAATTTCTTC
TCCCCATCGGCGCTGGCATACACCTCGGCGATAGCCCGCAGCTCGGGGTGA
GAGCCAAAGAACAAGTCTACACGGGTCGCTGTCCACTTCTTCTCGCCCGA
CTTGCGGTCGCTGCCCTCGAACTTGTCGAACGAGGTGGGCTTCCACACCGT
GTTCATGTCGAGCAGGTTGACGAAGAAGTCGTTGGTCAGCTGACCCGGTC
GCTTGGTGAGAACGCCGAGGGACGAGCCGTCGTAGTTGGCGTTGAGGACG
CGCAGTCCGCCAATGAGGGCGGTCATCTCGGGCGGGCTGAGCGACAGGAG
GTTGGCCCGGTCGACCAAGAACTGCTCGAGCTTGACGCGGCCGGTCGACT
TGCCGTAGTTCCTGAACCCGTCGGCGTGGGGCTCGAGGTAAGAGAACGAT
GTGCGCGTCGGTCTGTTCCTGCGATGCGTCACCTCGACCGGCCTTGAAGTT
GACAGGGACACCGGCGGCCTTCTCGACGGCGGCTGTACCTGCCAGCACGA
TAAGGTCTGCGATGGAAGCCTTGGTCCCGGTTTTCTCCTTGACTTTCTCGAT
AGCCGACAGGACCTTCTGCAGGACCTTGGGGTTGTTGGCTTCCCAGTTCCT
CTGCGGCTCGAGGCGGATGCGCGCACCGTTGGCGCCGCCTCGCCAGTCGC

TGTTCCTGAACGACGCAGCGGACGCCCATGCGGTGAGCACCAGCTGTGCC
GGCTCGATACCGCTGTCCAAGAGCTCCTTCTTGAGCGAGGCGATCTCGTCC
GAGGATAGCTTGTGGTCGGGCTGAGGGACAGGGTCCTCCCAGATGAGCAC
TTCCTTGGGGATCTCGGGGCCGATCCATCGGGTCCTCGGACCCATGTCGCG
GTGCAGGAGCTTGAACCAGGCTCGGATGAAGGCATCGGCGAGCTGGTCCG
GGTTCTCCAAGAAGCGTCGAGAGATCTTCTCGTACTCGGGATCGTGCCTCA
GCGCCAAGTCGGACGTGAGCATCCTCGGCTTGTGCTTCTTGGACGAGTCG
AACGCGTCCGGGATGATCTCGTCGGTGGTCTTGGCGGTCCACTGGTTGGCG
CCCGCAGGGCTCTTGGTCAACTCCCAGTCGTACTTGAACATGTACTCGAGA
TAGTTGTTGCTCCACTTGGTCGGTGTGTGTGTCCAGATGACCTCGAGACCG
CTAGTGATAGTGTCTGGCCCTTTGCCAGAGCCGTGCTTGTTCTGCCAGCCG
AGGCCTTGCTGCTCCAGCCCAGCACCCTCCGGCTCGGGGCCGACGTTGTC
GGTGGGTGCGGCGCCGTGTGTCTTGCCGAACGAGTGGCCGCCTGCGATGA
GCGCCACCGTCTCCTCGTCGTTCATGCCCATGCGGCCGAAGGTCGTGCGGA
TGTCCCGCGCAGAGGCGACCGTGTCGGGCACGCCGTCCGGTCCCTCTGGG
TTGACGTAGATCAGGCCCATGTGAGCCGCCGCGAGCGGCTGCTCGAGGTC
GCGCGTGTGAATGTCCGAGTGGCCCTTCTTGTGCTGGGTCGCCATCGACGA
CGCCCGACTCGGCCAGACCCTCATTGCCGTCCGAGTACCGGACATCGTTGC
CGAGCCATGTGGTCTCGCCGCCGTAGTAGGTCGACTGGTCGGCCTCGTACG
TGTCGACACGACCGCCGGCAAAGCCGAATGTAGGCAGGCCCATCGACTCG
ATGGCGACATTGCCGGTCAAGAGGTAGAGATCGGCCCACGAGATCTTCTCG
CCGTACTTCTGCTTGATGGGCCACAGCAGCCGACGCGCCTTGTCGAGGCTG
ACGTTGTCGGGCCAGCTGTTGAGCGGCGCGAAGCGCTGCTGCCCCCGCCC
GCCGCCGCCGCGTCCGTCGTACATGCGGTACGTGCCCGCGCTGTGCCACGC
CATGCGGATGAACAGCCCGCCGTAGTGGCCAAAGTCGGCGGGCCACCACT
CCTGCGAGTCGGTCATCAGGGCATGCAGGTCCTTCTTGAGCCCCTCATAGT
CGAGGCTGTTGAAAGCCGCCTTGTAGTCGAGGTCCGGCACGGGCGTGCCC
AGATCAGAGAACGCCCGGAGGCTGTCGAGCCGTACGGTGTTGGGCCACCA
GTCCTTGGCCTGCTGGCCGCCGCCGCCGCCAATGTTGGCGTGCTTGACGGG
ACACTTGCTCATGACTGATCGTGCAGCGATGCTTGTCGTCAAGAGGTGTGA
ACGTGGGAGTACTGCCGAGTGGTATGGGAGGGATGGGATTGGCGTGCCCA
GCAAAGGCCGGCCTGCTCTATATAGGCGAGTCGTCATCGAATGAGAGGAGA
GGTATTGTCCATCACTGCGCTCTCTGCCGGCACTCGTCGTCTCTGGCGTCTC
TCTCGTCTCCCCCCTTGTACAACCTGCCGACTAGTCGGGTAATCCAAGAGA
CCGATTGCGCGGGTCTCGGTCGTACGGCGGCTGCACGGGTATCCGGGGGTG
TTAATGCTTCTCGGCGGACTCGAGGCGGAGGCGGACGGGCCCCGCGCGGC
ATCTGCACTTTGGTCTACAGGAATTTACCAGTCTAGCCGCCCCCACAGCATG
CGACCCCTTCTATCCTTCTCCGATTCCTCCGAGGCGCAGCCGTGACAGCATT

GCACGTGAATCACGGCGGACATGGCGCGGCTTGGCGGCTAGTAGCCGACG
AGTAACGATGCTGCTGGAACGTGTTGTGCTGGACAACGAGCGGCTTGGGC
GCACAATCAGCGTTGGTGCTACAGATTCACGGCACCGTGTGCGAAGGACA
CAGCCATAGTGGATGCATGAACGAGCCAGAGTCTACCCTGTTGTAACGCTA
GCTGCCCCCTGTAACTGCTACTCCATTGGGCTCCTGGGCGGACTCTCGTCAT
GGGACAATTGAAGGACCACCCAACATGGAAGAAGACATCTAGCATCGGTA
GCCGCCATCGACAACTCTGAATAGCCTTCAGCTCTGGTCAAAATGTCGGTG
CGTAAGCAGACTCACATGTCAGTCCCCGTGCAAAACGTGCTCGCGTCTGA
ACCCAAGAAGATGTAAGCACCAGCAAGCTCGCGATACTCTCCTGTCCTATT
GAGAACCTGTCTCGACAAGGCGTTGGTGATCTCGGACTCCGGTGCTCCCTG
AGCATCGCCCATGTAGCCTGCTATCGAAACGTCAGCTCAACTCGCCATCGG
CTCTCACAAGGGATGACTCACGCGAGACAGAGTTGACTCTTCCAAATTCCT
TAAACTCCTGTGCCAGGGATCGTGCGAGCACGATCACCGCCGCTTTGCTGG
AATTGTATATTCCTTGGTTGGTAGGTCGGATGTTGGCCAGGCCCGCCGCAGA
GGCCGTGATGATGAATGAGCCCCCGGGTGCGTTTTGCCAGACTTGACCCAG
GATCTGGCTCTGGTAGGCCACTGCAGTGTAGTTGACCTGGATGATGTCTTC
CCAGTGCTTCCGATCGCCGTCCAGGATTGTGTCGTCTTGAGGAATGCCCGC
GTTGGCGACCTAGTGATCAGCCGATCTTCCCGTCTACATGTGACGAGAGCA
ACGTCACCCAGATGTCCAGCCGATTGAAATCAGACAATACAGTCCTGACGA
GTGCTTCAGTCTGCTCGTAGTCTCTCACTGCTCTTGTGAGCTACAGACGAC
CGAGATGTGATCCTGAGCAGGAAGCCTCACCATCAAGCTGGTAGGACTTG
ATTTTCCTCCCTGTCCGGTGTCCCAGTTCGGCGGCAAGACTCTCTGTCGTCT
CCTTGCTGCGCGCATACAACATGGCTACATCTGCTCCGGCTTCTGCGTACGC
CGCGGCAACTGCCAGTCCTATACCGATAGAAGCTCCCGTTACGACCGCAAC
CTTGCTGATGGGGTCAGTCTGGAAATCTTGAGACACGCGTGTATGCTCACC
CCCTCAGCGAGAAAAGCCTGGGTTTCCGTCAGTGCTGCCTATGCGATCGGA
CGCGTGAGGGGCTCACTCGAGAGCGTTTTCCGCAAGCCTGGGAGCTGGTT
TGGGGTCAGGCGGCATTTCGATGGTTCAACGAGATAAACGAGGATTTGCGG
GACGTGGCCCTCAGGGGCTATGTATGAGCACCGTCCTCCTTACGTCCAGTC
CTATGCATGACTGCCTCACCAGAGCATTGTCAGCGTCAAAGCGTACCGCCA
GTCAATGGCTGACAACTACCCGCCAACGCGCCAGCCGACTTCGGCTCGTCT
TCGCATCGATTTCGCCGCTGCTCAGGTCGTCTCGCACATCTTCCTCATACAC
AAAGGAAGTGGAACTTCGCCCACTGTACCTCCAAAGAGCTGCCCTGTTCC
GTCTACACTGTAGCAACATGTCCGCTCCGCATCTCAGCAACCCTCTGATCCC
TCCGCCGGAGGGGGACGAAGATCCCACGCAGCTGTCTCTCATGATCCAAG
GCAAGCCGTACTTGGCGTTGGACAGCTACCTTGATCGTCTCCGCAATACCG
GCGCTATCAAACTTGACAAGATCGTCAGCGAGCCCGACGGCGAGAAGCGC
ATGGATCTTTGGCGGGAACACGCCGTCATCGGCCCCAATTGCATGATCGTC

CAGCGCTTCTTTTGCGAATATGTAAGTTGCCCTTCGCAGCGGAGCAGCGGC
AAAGTCTGAGCATTCTAGGGATTCAACCTCACCCTTGCTGGGGAGAACTTC
ATCGGAGCCAACTGCACATTCGTTGATGTCTGTCCTAGTAAGTGCGACGTT
CGATGGCATATCGATCTCGAAGATGACCCCAGTCTACATTGGGGCGCGAAC
GATGATCGGACCCGATGTCAAGATATACACCCCGGATCATCCTATCTCGCCA
GAGGCCCGTGCTGGCCTGAACGGCGAAGAGTGGGGTAAGCCAGTGACCGT
CGAAGAAGACTGCTGGATCTGCGGTAGCGTTGTCATCCTCCCTGGAGTGAC
CATTGGGCGCGGGAGTACGATTGCGGCGGGCTCGGTGGTGACTAGGAGCG
TACCCCCGCGAAGCCTCGTCATGGGTACCCCAGGTCGGGTCGTGAAGACG
ATCCTTCCGGATGGCTCGTTGGTGAAGGTATAGAAAACTTTGTGGAGTATG
CTACACATGCCTTAGTCCACCGTCAAGCTCGGACTTCAGATTGATGACCTG
CATCCCGCGAGCCGCCTGCGCTCTTATGACGTATTGAGACTGCATGCCCCG
AGCTTCGTATTCCCCATCCATAACGCTACTGTCCGGGGTTTGAGGAATGTAG
CGGCTGGTCAGACTGCTTCTTTTGGCCGCATCACTCTTTACAGGCCCTTGC
ATATGACAGCTATGGCCCTATCCTCAAGGTACGATCGCCTTTACATGCACCT
CATATAATTGACCATCATGGAACAGCTTCTCCACCGCAGCCGAGGTCCATGT
CGGATGAGCACCCTTGAGGTACTTGTCGACAGCCTGCACAAGCAGCATCA
GCCGAGCTCAGTGATAAAGAATCCTCCCACCTCAAGCTCGATTGGCAGATG
TTCAGGCAAGGATCCGACGTGGTAGGCGGTGACTTCGAACACGTTGCGCC
AAGCCTCCTGGGCAGTCCCGTACTCTGGGTTCGCCTTGAGCAGGATGTTCG
CGATTTTCTGAGGTGTTAGTCCTGCTCGCCAGGGGAAAATCTCACCTCGAA
GACCAAGAACACTTGTGTTTTGAAGTCACCGAAGGTGCCGTCGGCGTTGT
CGCCTACGACTCCGGAGATGTAGAGGGTCTTGGCGGAGGATGGGATGATG
ACGGCCGCGGCTGGAGCGAAGTCACTGCTCCTATCAGCTCTCGGTCGCGA
CCGCCGAAGAGAGGGACTTGCCTGCCCAGCTCTTTGCCCTCGGTGTCGAC
AGCGCGGAAGGAGAGAGACATCTTGGGATTCTTATTTCTTTTTGGAACGAT
GTGAGCAGTCGCAAGTTTAGTCAAGGCCAGATAACGGGATCCTCTTGTGTA
CACGAATTCAGAACGATGACTCTCGAAGCTACTCCAAATGTGCATGTTCGG
CGACAACCTCCCAGGCGCCCCGGCGGAAAGGCTTTACGCGCAAAATGCGC
CGCGCGGCAAAGCGCGTGCGTGAGGTCAGGCTCCCTCAGACCCTTATCTCT
TTCGTCGATACATGCATGATCGCCAATATGGTCCAAGCGTCCCAGCATACGC
TCCAACTATGACTTCAAAGCAAGCAAGCACATTCGAAGAACTCATTTTCCG
CGAAGAGCCGCCCTCGACGTCAATCAAGAACGGGCGACTGACCATCACCG
CTCCACCGCTCGTGGACTGGTGGCGGAATCCTGAGCCGGTACTTGTGAACC
GCAAAGACGGGCCACTGAAATATCGCCTCATCGACGGCTCCCGACCATTCG
AATGCGAAGTATGGCTGCGATCAGGTCTGGTCGGCCTGTACGACCAGGCAT
GTATGGTCCTGCACGGAGGCGACCTCAGTGATCCCAAAGCGCACTGGCTG
AAAACGGGTACAGAAACATTTAGCGAGCGTCAGTGGATCAAGTCAGTCCA

GCGCGGTCGCTGCGGATGAGCACTGACAGTCAAGCGCGGTGGTAGCCACA
CCATTCTGTGATGTCTCAACTGTACCGGCGCCGAACGAACTGTCAATGGGC
GCTGAGGGCGACTGGGTCTATATCCGCCTGACGAAAGAGTTGACCATTACC
GGACACACCATCCTCATTCGATGGGCCAAAAGCCGGACGCCCATACTGGAC
AGAAGGCTGTCCGACAGCGAGATCTGGAATCTGAGGGAGGTTCAGGCATT
TGGAGTAGACGATAGTGGCAAGCGGTCGACAGACTGCAAGTGGGCGATTG
GATTGATGGTGTCTGGACTCATGAATGATGCCGGCGCAGTGGGGGAGTTTG
AGGGGTTCTCATTTCGGTACTTGGACGAGGAGTAGCGATGACATGTCACAT
GTTCCCTTGGCGTTGTATACCCAAATCGTGTAGGAGCGGGCCGTAGACATG
CACGGTACCCGCCATCGTCCACCGGGCGTCGGCGAAGGAAAACGAGGGGC
GGAGAGCAGAAGGCGCCTTCGCGCTGCCGCTCAGCCGCGCCCACCTGCAG
AGAGCATATAGCCCCGTGTCTGGCTTGCAGCCATACCTCAATCAGTCTCACT
GCTTTCGAAATGGCCCCAATCGGTGTCGCTCTACTGGGAGGCGGGATCTTT
GCCACAGAAGGTAAGACTCTGGTACAAGCACCCGAGGGAGCGACAGCTC
ATATACCAGCGCACTTGCCTGCACTTCTGAAGTCCACCGCGGGGCAGAGAG
TCGCACTCAAAGCTATCTGGTCCAGGTCGAGCTCGTCAGTGCAGAAAGCC
CTACAGGCATGGAAAAAGCTTGACAATACCTCGCCACTGCGAGAACAGAC
TCTTGCCTACAGTGGGGACGGCGAAGAAGGGAATACGCTCCAAGCCTTGT
TGTCCAGACCCGACATCGAGGCAGTCATCATTGCTCTGCCTATCGACTCTCA
GCCGGACATCGTTCTCCAAGCTCTACGTGCTGGAAAACATGTGTTGTCAGA
GAAACCTGTGGCCGCGACGGTCCAAGCAGGGCAACAGATGCTGGACGAG
TACAACAAGGTGTACAAGCCACGGGGACAGGTAAGTAGCATTTCTGCGGC
TGGGAAGAGAGAGCCGCAACTGCTTAGGGCTAGATATTGAGGATCGCCGA
GAATTGGGAAGTCGAGCCAGCACATCTATTCGCCGCTGAAGCGGTGAGAA
GTGGCCGCATAGGCGCGGTACGAACCTTCTTCATCAAGTCCGTGCTGTTCG
TGGACGACGAAGGCAACAAGTATTTCGAAACGGCCTGGCGTCGAAAGCCA
CAGGTGAGCTCACACTCCTGGAGCCGAGAAGGCGCTGAACCCGGACAGTT
CCAAGGCGGCTACCTACTTGATGCGGGCGTAGTGAGCCATCGACTCGAGTC
TTGGAGACATCAACTGATCTTGCAAGCATAATGCTGCGGTCCTCCGCACGG
TACTGCCCAAACACATCACGCATCTGTCCGCCTACACCACTCTCCAGAACG
AGCACCTGGCCCCTAGCGACTACCTAGCCGCTGTTGCGCGATGCGGTGCGG
ACATACATGGCCAGATCCTGATTGACTATGGCGCACGCGGTTGTCAGCCCG
CTTGGGAGCTCCGGATCACGGGCTCAGACGGCGAGATCGTCTCGCAAGAT
GTCGCGGACAACGGTGTTGACTCTATCTCAGTGGAGATTCGGACGGGAAA
ATTTGGGGCTGAGTCGACTGAGCGCAAAACCTTCAGGAAGACGGGCGTTG
CGGAAGAGCAACGGCGATTCTTTGCCGCAATCCACGGGGACGAAGATGGT
GAGTCCATTGGAAGTAGGGGTCGGGTCAACCACTGACCTCAGGCTTTGGG
TCTGTTGAGGGCGCCCTGCACGACGTTAGTGTGCTACAAGCAGCGTTGAC

GTCGAAAGGCACGGAAGTCCAGCTGTCGGCTCTGCTTTAATGCAGCCTTGA
GTGATGTATGTCACCATGAAGATGCATCGCTCGCTCAGAAGAATACAACCG
AGATGTCGAGCCGCTTGCACTCCTCGAACCAGTCTACAACTTGCCCTTCAC
GATCAGTCGCCGCCCACGTCGCGTCCAGCGCCATAACCATATCTTCCAGGT
TCTTCTCTGCGCCAAGCCTTTGCAGACTCTGCTTGCACCATGCACGGTCCT
CCGTATTCACCAATAACGCCCCAACAAGGAATATCGGCATCGTATAGTTTAA
TGTCCAAAAGTCGATAACGTTGTTCTCAGCTTTGACTCGAGGGACAGTCAG
GTGCTGCAGGGACCTGAGAAGGCTGAGGAGCAGGGCGATGCAGTCTCTCA
CTGTGGGGGACAGCACAGAAAGGTTGTTGACGTGTCGCCAAAGGAGCAA
GAGGCCCAGGTTGCGCCAGATCTCGGTCTCGACAGCCATCTCCCCGGCCCA
TTCTCGCGGATGTCGGTCTGCATTCCGGTCGTTGGCAGCGAAGGGTGAGCG
GAAGTTTAGAAGGTCGGCTTCGATAGCGAGAGACGAGGCAAAAGCTGCCC
CTGACACTCTAGTATCGGGTGACTCGCCGAGCTGCGCGATCAAGTTGATCA
AGGTGGACAGACACAAGACGAGACCGGCAGGTAGGCCAAACCACTGGTA
AAGGCTCAGGACAAACTTCGTTGCTCAGGATGAGAAGCTCACCTGATCGT
GAGACCCGTCATTCAGGTAGAGACCGTTGTTCAGCTTTGCTGAATCTGTGA
TTTTGAAGAAGGTCCGACGTCGTTGAGAGATACTGCGATTCAAATCGATAG
TCACAAATGCTCTCATGTAGAACGGCAGATAGCCATTGAGCGAAAGATAGT
CGACTTCAGGTTGTGGACCGTACGTAACCGACAGCAGACGCTCGGCTATTT
CCATGATGGCGTACGATCTCGTCGATTCACAGAAGGATCCGACGGCCATCT
GGCCCGGCGTCAGCTACAGTGGGCGGAACGGATCAGAACGTACATGAAGG
TCGGATAGAGCCCAAAAGTGGGCTTCCATGGTGATGTGGTCACTGGGATCA
GCGCAGACGCACGACCGTTGTGCACGGTCCTCTCATGAGGCTCACTCCTTT
GGGGTGCGGACCAACAGGGTGGCATCACGCAGAATGGTCTCAGGATCGAC
TTGCAGTGCCATGTACTTTTGCGGTAGGTAATCCAGAGGCGAACCCCAGTG
TCCCACCCATATGACTCCATGCGAGTATCTGCGACGGAGAAGGATCAGATC
TGCACCATCAGTGTCAACGCTGCGCACGCTTGCCGTGCATCACTCACACAC
TGCTGCCGCGGCTTGCCGACACAGGCGCGTCACATGGTGCGCGTTGGTGAT
TTCTCGATATCGAGCAATGACAGCCTTACGAGCTCGCTCGGGATAGGTGATC
GCCCAGGCGGGTAGTAAATACTGATCATCTTTCGAACTGTCAGCGGCGCGC
ATGCTGCGTGATAGGGGGACTCACAGAAGTCAGACAGGACCGCTTGCTCG
TTCGTCATCTCCACCTGTGTAGCAATAGGTGGATTCTGAATGCTGCCGGTCC
GCGAGCTGGACGCTTGGGGTGTCCTGAGGCTTATCAATGAGCATGGCCGTG
TCGTCTAAGGGGAACTGGAAAGCAGGGGAGAATCGGAACGGGTCGTTGG
GGTCGACTTGGCCGAATGACTCATATAGAGATAGGTCGAAGTTGGGTAGAC
TTGCCCCAAGGAGCGCATCGAGCGACTGTGTGGTCCAGAAATTATCGTCCA
CTGGTATCGTGGTGGTCGGGTCGAGCAGACCTTCCTGCTCCGTGGTCATGT
CCATCGTAGCGGTCGGCATTGCTGGGGCTTGGATATGATGGGACCCCAGAT

CGAGCAACGATCCCACCGCCTGCCCTAGTCCTTGATGTTGATGTGGGTTTG
GTGGCAGCTGCTCTGGAGAAGCAGAAGCGGCGCCGCTCCCTCCTGTCCCG
TCTGGCGACCTGCCCAGCCCGTCTATACTCCGTCGCATGGTCGATTCAAAG
GGCAAGTCCTCTTCCCGGATACACTTCCAGCCGCCAGACGAGCACCTCAA
GCAAGCGCCGTTGTCATACTATCATGTGAGCTCATGGCTACAGATTCACAGA
TAAGGGGGATGAGGTGTTGGGGAAGAGCGGCAAGCGCACCTGTTCTGGGC
ATTTCTTTTCTGGTGGTCCCGCGGGACATCGGTTAGCCCGCGGCTAGCAAC
AACAGGCAGGTGGGAGGGTGTGATTGTATGTATGGACTCACTACGCTTGCG
ACTGCAGGCGCGAGGCATCAGCGCAACGTACAGCGCAGTACCGAGGACCA
CTCACCAGGTCAGACAGCCCGTTCGCGAACGGCGAAGAGGCTTATCTCCA
GCTGGTCGGAGCTCCAATTTGTCCAGAATGGGCTTCCGTCTCGGCTTTGGC
TTGGTGCTGGCCTGTCCCACTTCGATGGAGGTCATGTCTGAAGTCGTGGGT
GGGGCTCGACAGCCGCGAGATGATGATCCTACTGTACAAGCCCTATGACCA
CCCTGCTTGGCAGACGTAGAAGAGAGGGAGTTATCGGTTAGAGCGGGAGA
TTCCCATCGGGCCGGAACTACAGTACCGTCTCATCGCTATTTGCATGGGTGG
CGATTGTTGCTCTGATACAACTTCTCTTCAACTATGATATGTCACTCAGAAC
GACCCTCAGCCCTCTCGTCGCTGGCAGTCTCTGTCCTAAAGCAGCCTCGTT
GAGTCTCGTGATGTTCGGACATCCTCACCGGGCAGCCATCGCTTCAAGAGG
ATCCGACGAGCTCACCGGACCGCCCAAGCGCTTGGGCGGCTGAGTAACGA
GCATCGCGATGTCGACATTTGAGCAGCACGGTGTGATACATCTCCTTACGCT
TTGCGATGCGCACCGCCTGATGCCACCCACGCTATGAAGACCTTCGTATAC
AAGACTGTAGAGCAGCCCAGCCCGGCAGGGTTGCTGCAGCTTCGAGTCGA
CGTGCATTCGCCGTCTAAAGCACTCCGCTCAGACTCTCCGGTGCTCGTCTA
CTTCCACTCGGGTGGACTGACGGCGGGGTCCCGTCACCTCAACGAGTGGT
TCCCTCACTGGCTCCTGGGTGAGCCCCGGATGCTTCTTTACCTTGTCCGCTG
ACACGAGCGGACGAAGCGCTGCAGTTGGGCTTTACTGTCCTGATTCCCGAC
TACTCCTTGCTTTGCCCAGGATCCGCACGCGATATGCTTGATGACGTGGTCT
CTCTCGGAAAATGGATACGAGCGAGCCTGAACATGGCTCTGGAAGGGGTC
GGTCTCCCCCGTGCTCGTACCGAGGATCTGGCAGTCGTTGGACAATCCGCG
GGCGGGTACCTCGCATACCTCATGGTGGGCATAAACTCGAACTTCGCGAGC
TGCTGGAAGCTGAGGAACTAGGCCGCCCACGCAAAACCTTCTCCAAAAGC
TGTCGTGGTGTTCTATGGCGTGGGAGGAAATTTCTTGTCTGATCATCACGTC
ACCAAGAAGAGCGTTGGTTCGCCTTTTTCTGCGCACAGAGCACTTCGTAAC
AGAAGCAGGGGCAGTTCCAACACTGGATGCCATTTCACGAAGATCCGGCA
CCTTTCCGACCTCTGGAAACCGCGACACGGACGGATGGAATCGTGGCTGA
AACGGATACGAGCGACGAGGCGAGAGTCGGCTTGTTCTCATGGTTGCTGC
AGGACGCAACTTTCCTCGATGTGCTGCTTGAGACACCAGGACTCAGTGCG
AGACTGCAAGGAGTGGACGAGGAACATCGACCCGAGATGCTACCGCCAAC

ACTGCGGCCATTGATACCATCTCTCTGCATCTCCAGAGACTTTCCTCCGACT
TATCTGGTAAGGAATCTTCAAACGTCTCGTGTCGCTCACTGACGAGTCAGA
TTCATGGTGCTCGTGACAGTGCTGTGCTCGTCGAGGAAAGTCGCAACATCG
CAGCGCTCCTCCACAAATCAGGTGTCGACCACGTCTATCGAGAATTCGCAG
AATTGGAGCACGGCTTCGACTGGGGAGATAAGGCGTGCAGGCGTGACCTG
GAAGACCTCTTTCCTTTCGTGCTGCGGCACCTGGGCCGCTGAGGCAGCGG
GCTACAGTCCTATCACTGCGTCATGCATCTGTCATTCTGGGGTCTTTCCCAG
CTTACCCTCTGTACTTACCAAAATGAGCATTAGCCGCCGAAGCGCCAGAAC
GAAGCGCGGAAGCGCGACCCCGAAGGATGCCATCCCGCTCCGGTCACGGC
TTATCATTTGGTCTGTTCACACCACCTATCGTGCAAGTCTGGCGGGGTCATA
TATCAGTCAAGGCATCATGAGAGAGGAGTACGCTGCGCCCCAATCCCATTC
TGAAGTACAACTACGAGATGTCTTCGCCTTTGGACATTCAGCCAGCGACAC
CCCTCCATCACCGAGACTCATCGGACACCGACTCAGCCAAGAAGGCTGCC
GCCGCGTATATCGCAGTGGAGGACGTCAATGTTCGAGATGCCGATAACGGC
CTCGCCGATCTGGGTTATGTGCCCCAGCTCAAGCGGGTAGGTCTCCCGCCC
ACGCTGACAAGTTTCTTGATTGACGTCGTGCCAGAACCGGGGTCAGCTTG
AGATCCAAACCATGTTGCTCTCCCGTCAGCATCGTTATCTATGTTGCTCGCG
GAAAGCTGACTTTCAAGTCTTCGCTGTTCCCTTTGGCGTGTCGTCCGGGTT
CTACACTGCCATGATCGGCGGAGGTCCCGCTTCCTTGCTTTGGGGCTTCATC
CTGGTCGGTCTGCTGCAGGAAGCTGTCGCCATTTCACTTGGCGAGATCAGT
TCCCGCTTCCCCACGTCTGGGTCAGTCGCTCCCCCCTGTCGAACATCGGTG
ATCGGTTAACTGATCCCTTTCAGTGGCCCTTACTACTGGTCCTTCGCTCTCG
CTCCCCCTAGCTTCCGCACTATGCTTAGCTATGTCACCGGATGGCTATACATG
CTCGCTATCTGGACGCTGGATCTTGGCACCCACTATGGCACAGCTCTTCTGA
TCATTGGTGGCGTCAACATCTTCCATCCTGACTGTTCGTCTACAGCGCACGG
CCACCTTCCAAGGGCTGACATATCGCAGGGACCCCTAAAGTCTGGGTGATT
CTTGTCATCACCTACGGTTTGTACGCCGCCTCGACATTGACGACCTGGAAA
GGGCATCGCTGGGTGCCGACTCTCGATGTGAGTATCCCAGCAGCCTCGAAT
CATCCACATTTGATACTCTTAGACCATCAACGCAGTCTTCACCGGGATCTGC
CTGATCGTGATCACCGTCAGTCTGCTCGCTGTTGCGGCAGAAGGCCGTCGT
CCCGCCTCGTTCGTCTTTACCCACTACGACTGGAGCTACTCTGGGTGCGTC
ATTCATCTCGCCTTTACTTTGGCATCGAGCTGACGTTGCTCTCGCAGCTGGG
GCAAGGGGTTCACGTTCTTCATTGGCCTGCTTCCAGGCTGCTTCGTCATGT
GTGGGATCGGTTTCATCACTGCCATGTCCGAGGAGGTCGCCGTTCCGACTC
AGCAAGTAAGACACCTCTTCTCGACTCGTCGTAATCTTCACTGATCTCAAA
GATTCCCAGGGCCATGGTCATCGGCGTCCCCTTGGCCGTTGTGTCCGGCCT
TGTGTTCATCCTTGGCTGTCTCTTCACTCTTCCAGACATCGAGGTTCTCCTC
GCTGCTCGTGAGTTCTCCGAGCTTGAGTGGTCGCATGAGTTCTGACGAGGA

AAGCTGAGGGCGCTCCTATGCCTGTCATCCTGGCAACCGCGACCGGCAGTA
GAGCGGGGCGTATCATTCCGGGAGGTCCATAACCCCCACGCGGACCGTAAC
CTCCTCGTCCATTTGGTGGTGGTGGCCCGTATCCACCACGGCCAGGTGGGC
CATACCCGCCTCTCCCCGGTGGTGGGCCATATCCACCTCTTCCACCTCGTCC
ACCCGGGGGACCGTACGGACCACCATAACCGCCCCTGCCGTAACCTCCTCG
CCCTCTATAACCACTTGGAGGTCCACCACGACCTCTCGAGTTCATACTTTCG
AAAGATGGCCCTCGTCGCATACTATAATTATCCTGAGGTTGATCTATCGGATT
GCCGTACTGATCCCTGGCCACTGAGGTGTTCGAACCGGATCGCTGAGGTGC
GTTGTACGTAGTGCCGGACATGCTGGTGTCAGCAGGTGCGCCATTCGGAGA
TCTATCGGAGGGGGATCTAGCAGTTAAAGGTATGCGCTCGTCGCTGCTACG
ATCGTCTTTCTTCTCATACGAAGCAAATTGGGCTATCTTGTCTGCAGGACCG
TTTGCTCCACTGACAACGGGAACGGTCGGTTGTCGTGAAATATTTGTGGTA
GAGGACGGGACTTCTTGCTGGGCTTGGCGGTTGTAGTAATTCTCCCCACTC
ATCTCGGCATTCTCTGCAATTCGCCTCTTCCTTGCTTTTCTACTCACCAGAG
TACGGCGCATAGCACACGAAACGATGAAGCTTAGGAATACAAGGATGGTAG
CAGCCAAGACAATGTAGGAGCCCCACGCCATATGAGGAACGAAGAGTAAG
ACGTCGATTAAAAATGCGAGAAGGCATACTAAAAATGTGATAATGCCAAAG
ATGAAAAGAAGTAACAGATAACGAGAAGAATGAGAGGGCGAATGAAGGT
GAGCGACGATGGCAAGAACCAACATAATTAGGGTGAGCAGACATGCAACT
GGATGGACTACGAGGATGGCGGATAGCGTCGTCCGTGTTCCAGCGGGCAA
GTCGAAGGTTGCCGAGTCTGCCTGAGAAAAGAGTTTCGCTACAATTCGTTA
GTTAACGATCTAAGATAGTCTTCCTCACAAGCAAGAAGACAAAGATAGACC
CAGACATACATGTATCATATCCAATTTCTATTGGACTACACTTGTTGCTGCTG
TCGCAGTAACCGAAGACTCCAAAGCTAACGCCTTTGTAGGTACCTAGAGGA
ATTGCCTTAATAATAGGAGTCGACAAAACTGCGAGCAACAACATGGCGAAT
GCTGCAAAGAGCAGCACCGTCAGAGGAGTGGCCGGTCGCAACATGATGGC
GGCTTAGGAGCGTGCGCCGACTCGGACGAGTCGCGACGCCGTAGAAGTTG
TCGTCGCCTTCGTTGGATCGGATTTCGGGACCGGTCGACCTAAAACTTAAA
GGTGGTAATATGTTTCCGCATGCGAGGGTTGCCAATTGAAGTAAACGGTTC
TCGGCGGCGACGACATGTAGAACGATCGGGTCTCTTAGATTTAGGCGCCT
TGATGGTTTGACGTCGTTCCGTTATACAAATGCTGCTCGCCGTTCGTCGTTC
ATGAACACCCGATAGCCAAATACGCGAACTCGAACTCGAAATCGAAATCGT
TCCACTATTTCCTAATTTCCTCCTTCTTAAACCTCTTAAATCTCTTTTAACCTC
CTAGACCCTAATAATACTGATATTGTTGAATAACCAAAATATCGTCAACCTAA
CACGCTTTCCAGAAACGAGTGACTAGAAGTAATTCAGGACAATATCGATCG
AAGTGGCGGTACTGGATTGCCTAGTTAGACTGGCTCGGTGGCAGTGCCAGT
GGTGACGGAGACGGAGAGTTGAAGATCGGGAAATCGACTTGAGAAGTCG
AGTTGATTGGGTTGATTGGAAGGAAGAGCGTCTGGTAAGATTGTTTGGGTG

GGTGGGACGGGACCGTGATTGCAGATTGCAGAGTGGGTGGAAGCACTGGA
GAGCGCAATTGGTAAAGGAAGGGTACAAATTACTTACATGCAAGTGGGTCA
AGTTGGAAGGACTGGGAGGACAAAGGGGGGAATTTGCTAGGGACCGTAC
GCAATTGATTTGGTGATCTCGCAGGTCTGGATTGAGCAACCCTTGTATTCTC
CTTGTACACCCCCTGCAAATGTGATTAAAAGAGTGGCTTAGCGCACTATTAG
CCCTGCTAACCCACCTGCCCACGCACTCCCCCTACGTTTAGCGTCAGAAGC
GTTAGAAGGCGTCTTGACGGTGGATACCGTACCTGCCCAGGAGACTAGCA
GAGCTTATTACTGCGTATCTGCGTATATGCGCGTGCGTAGCCCTAGAGCCTG
AAGCCTAGAGGTATTGGGGAAGTGGCCTTCAAGTCCTCGACAGTTAGAGCT
GTTAGGGCTAGCGGCCGGGGCTAATCGGAACAGGCTAGGATAGGACCGGG
TGAGAGCCCTGAAAAGTGACAGGACTGAACTAAAAGATCTAGCGGGAAG
AGCGCTAGAGTTAGCGGGTTACTAGTTGATTGACTTGACGCTTCTTCGTCTG
AGCAATCTTGAATCATTCTTGATTTGTTCATCCTTCGCATTTACATACTACAT
GCTACGTGCTACAAATACAAAGCATTACCTTGATGATCCAGATGAACCAAAT
CGGTTCTAGAATTCGTCATCATGAATACACGTACGACGCAGATAAGGACCTT
CGTAGACTACCGAGTTCTACAGTAGCGTATGCGAGAAGCCCACCATTACGC
CGTGGCTCGGTGATAAAATCCTGAGCGACTCAACTGCAAACATAGCCACCG
TCTGATATTAACTAGCAAAAAGCAACATGGCAACCAATACGCCATACGCCAT
CCACCATACGTCCCACCTTAACAGGGCTTGTCATTACAGCCTAACAACCTT
GGAAGACAGCAAGCTTCAAGCTAGTCAGGCGGCGTTTTTACCCAACCTAC
CAAGTCTGTTCATATCCGAGTTTGAATTAATCAGGTCAAATGACGTTGAAAT
CAAGCAGCCGATTATCATGACGATAATTATATGTGATGCTGTGATGCCGTGC
TTCGACAAGCCGGAAAACGGTGCCGCCTTGGTACCTGCTGCAACCAGTGA
CAGGTAGCATTTCTAGTTAGGTACACAGGCCAGCACCGCCTGAGTTCCGTC
TGAAGCGAACGTCGGAATAAGGCTACCCCCTACCTGGGCGGCTCCCGTTAC
CGCACGTCGTATCGATGCGATGAGCTGGCAGCCAGTAGCTTATCCGTAACTT
AGAAAGCCGAAGTGCTGCAGCCCTTCAGATAGGCTAAAATTGAATATCACG
GGATTTGCCGTTGATGTTGACGTGCTTTGGTGAACCCATCGCCTACGTCTGA
TGCTTTGACTGTCGGAGAATACTCAACCGGGCATATACGCTAACCTACACG
ATGTTATGTTGCATCCCACGAGATTCAGGGTCCCTAAAGCAAGCGGTATAGT
ATGAACCGAATATTTTGTCTACTTGGTTACTGACTGCATTATCCACCCAACA
CAAACTTGTCCGATACACACCGTAAGATACAGGACCTCGATGCAAGGTCTT
GGGGAACAAAGCTTAAGTTGACAATTGGTAGACTACAAGGAGTAATTAATA
GCCTTATCATCGGCTCTTGAATCAGCTACGTAGGCCAACACTCCAAATACAC
AAAGATGGCTCAAAGAAGAAGGTCGGTGGAAAAAAAGAACAAATCGATG
TACAATGAGATGAGATTATGTCCTCCGTGAATCAGTCCACCAATCATAGCAA
TTGGATAGTTTTAAGCCACGGATGTGATTCGACTTATCATATACCTCATACAG
ACCTAGTACAAACAAGCAAACATCTTCTATTCTTGTAGCGTCGGTAATTCGA

GGGAGATTGTCGTTCCTAAACTTGGAAAGGGAATGATTTACTTCGACTGGT
CTTAGTCATCTGAAGTCTCGCGAACTGTTCCCATCTTCGAAACAACTTGATA
CTGGGTCACTCACAGGCTCCAGCGGATTCATAGGTTGATCACGAACCACAA
CTTATGATATGTGATCGAGATATGCATGCAGCATTCCAATATCGGTATAGTGT
ATGGTTCGCTATCGAATCCAAAGCATTGCTCGGTCGAAACTTCAAGGTTTAT
GGTTGAGTTGATTCTCATAAAAATCACCGATCATGAAGAACCTAAATCTTAG
AAAATAGGCTTTTATGTGGGATGAACCAACCAGAATAGCGAAACATATGGG
TCAATACACGATCAACGATGAGAACAAATCCTAGAAGTTTGACCATCGAAC
AAAATGTGGTCTTGGATCCTGGTAAATGATCGTGGAGAAGGGCTCTGGCGC
TCTCTACTCCATCGATGGTACGGTAGTTCTCAATCTAGGCTTCTGATTTGCG
CTTGAATTTGAGGAATAACCCAGCAAATAACCCAGCGTGGCGCGTTTCGTA
ATGCGTAAGCTTCCTCAAACTTCCAGGGATCCCAGACCAAACTTGTGATCT
ACGCTGCTACAGCATATCGGTTGGTTTCTGATGCAGAGCCTCAGATTACAG
GGGACGACCGTTAAGCGGCTGAAGTTTTAGGTGTCATGAAACTCGATTTT
GGGTTCTAAGGTCGTTCTTATTTTTCTTATTTAAGACCAAGTTTGTACGCCGT
TTGCGAATGGTAAGAATTGTTCGAATATTCTCTCCACCTTCCGGACATAGAA
TTACTTGCGGGCGTATTAAGTTTCAATTTCCCCGATATAGTGGGGAACAGGA
CCCCAGGTCTACAGATTTCTACTGCGCATACGCGTGTTTAAATCAGTCGTGA
TCGCTTATTATTCACAATAATTGACTGAAATAGCTCATAAAGTACCAAATTGT
GGACAATATTGAAATAATACTGCTACTAAAATCGATTGCTATGATTACAAATG
TCAGCTTCAAGTGGGTGTAAATCAGAACTCAATTGATGATTACGTAATGAA
CAATTCCCCAGCTGAGAATCGGCTCCGACGGTCAAACGGGTCTAAAGGAC
TCCGACTCTTGGCAAGTTGAATGACGGACTCGGCGTCAACGGAGATAAAG
AACTAATAAAGATAAGAAAAAAAGCATCTTTCGTCATCGATCGCAACCTGC
AGCGTCCCTGCGTTACTACATGTAAATTCATCGCACATCGTAGCATTATCCCT
ACAGTGTAGAATATCTTGTCATACGACTAGTTTGTAGGTGTTTAAGGGGCAG
AATTACCTTCATGTACCACAAGAGGCCGGAAGCTCGTCTTCCGCCTCCTCC
AGAACCTGTCACGTGTGGCAGCTAACCAGCCGGTCTTCCCTCCGCGAGAC
AATGTTCCGTCACGGGGCATCGCGGCTGCACGTCCGCAATTTCTCGGCGCA
CATACCACGAGCTCTCATCCGGCGCCCCGGCCTCACCCAACAACTCCGCCA
TGCGTCCTTCGAGCCTCTCCGCCCACCTTCCCCCACATCCCTCGGCAAGCC
CCAGGCCGCCCGCGACTACAAGCGTACGCGCAAATGGGGCCGGCGACTTC
TCGTCGTCTCGCTGATCACCGGCGCCGTCTACGAAATCGACGACCTCGTCT
TCGCGAAGGGTGTCGCGCGCTCGCTCCTCACCTTCGGCACCGGCCTGCTCG
TCGCCCTCGACTACAAGCTGAATTTCCGCGCCGAGCCGCTCTTCGGCGGGT
CCATCGAGGACCTGCACCGCCGCAGCGCGGAGCGCTTGTTCGAGCTCTTG
CGTCGAATGGGGGATTGTACTTGAAGATCGGCCAGGCTATTGCGATGCAGA
GCGCGGTTTTACCTCCCGAGTTCCAGAAGATGTTTGCGAGGATGTTCGATG

ATGCGCCGCAGGACGAGTGGAGGGATGTTGAGAAGGTTATTAGGAAGGAC
TTTGGGGGGAGGGGCGTGGAGGAGGTTTTTGGGGTTAGCTTTACGGGGGA
GGAGGGGAAGGGGTTGAGTATGTATATCCCGAATTCCTGTTCTCTTTTTTTT
TCTGGTGGTGTTTCTGCGCGTTTTGGAGTAGTAGCGTACTTTGAGGCGTGC
ATGATATGAGCCAATCCCCTCACTGAAAGTGCAATTACTGATTCATGACGTT
ATAGTGGAACGAAGGGCTAGGGCGAGCGCGAGTGTGGCGCAGGTTCATTG
GGCGAGGTTGCCAGATGGACGGGAAGTCGCAATTAAGTTGCAGAAACCGG
AGATCGCGAAGCAAGTCGGTTGGGATCTGTGGGCGTTTAAGTAAGTCTGG
GGAATAGGGGGCGGCGAATGTGGTGTGTTGCTGGTGAGACGCTAATCAGG
ACGATAGAGTTGTAATGCGGATTTACACATGGTGGTTCGATTTACCGTTATA
CAGTTTGGTGCCTTTCATCACGGAACGGCTACTGCTAGAGACGGACTTTGA
ATCAGAAGCTAGGAATTCGGAGATGATGAGGCAGCTTGTGAACTCAGAGC
CCAGTCTCAGGGGCAGAGTGTATATACCCAAGTCTATCCTGAATTCACGAC
AAAACGAGTCTTAGTCACGGAATGGATCGAAGGTGTAAGGCTCTGGGATA
AGAATGCAACGGGAGGTCGTTGGTTAGGAGGACCTGGGTCCTCCTGGTGT
CCATGGTGCCCAGCTGCAGTTTAGCCAGTCAGATTGGCAAGCTGCTCGCCG
AGAGCTGCGAGAGAATCCTTTCAAGGAAAAGCTGAAGCCTGATCGGGAAT
CCTGGAAAGGACGGAATGGCAAAGGCGGGTTAGGATTATCTAGTAAGGAA
GTTATGACTACTATGGTGGATCTCTTTTCTGCTCGGTACGTATGAGAAGGAT
TTAAGCTTAGATCCTCCCCCTTTTTTTCGCCACGATGATCCATACGCTAACAT
GTCGGCAGTGAGTAACTAACACATACTGGCATATTTTTTTTATGGAAGACTA
ACATACTGCAGAGATCTTCAAATGGGGAGTTGTGCACTGCGATCCCCATCC
TGGTAACATCTTCATCAGACGGTTACCGTCAGGGAAGGCCGAATTGGTCCT
CATCGATCACGGGCTTTACGTTTACATGTCACCTAAGTTTAAACACCAATAT
GCTCTGTTCTGGAAATCTCTCATGACATTTGACAACGAAACCATCTCCGAG
ATCACAAAGGAGTGGGGTGTTAACGCCCCTGACCTATTCGCAAGTGCAAC
ATTAATGCGCCCTTATGAGGGCGGCGATGGAACCACGAGAGGTCACATCAT
GAAAGAACTTAAGGGCAAGACGCCTTCTGAACGACATACGAGGCCCAGCA
GCGAATGAAGCAAGGAATTCGCGAGATGTTGTCGGATGAAGAGAAGTGGC
CTAAGGAGCTCATCTTCATTGGCCGCAATATGCGTATCGTGCAGGTAATTTAT
GATCCATATACACCCAAAACTAATTCCTTTAGATTAGCTCTACTTACACGTTA
TTCCAGGGCAATAACCAGTTTATGGGGTCTCCTGTCAACAGAGTCAAGATG
ATGGGTAATTGGGCAAGTCGAAGTCTATTCCAAGACACGAACCTTCCGTGG
AGAGAGCGCATGGTGAATATGTGGCAACATTTCCTGTTCAAAACGGTTTTA
TTCGCTACCGATGTGGCGTTCTACACCTTCAAGCTAAGACAGTGGCTTGGT
TGGGGAGGGGGCATGGAAGACGAGGTTGAGGCTCGGATGAAAGACATGG
CGAAAGACTTTGGCGTCGAATTGCAGCATGAAGTTTTCGAGGGCTAAGAC
CTAGATCTAGAGGATATGAAATCTCCAGCTAAATACCTTGTAGAATCGTAAA

AAGCATAGATGGGTTGGAGTAGACGGCTGCATATAGAGCCTACGGCCGGCT
TGTACTATACATGTAGAGTACCTGGAACCCCCTCAAAAATATTATAATTCCAC
TTCGCAAGCTGTAAATTGTTTTAGCGAATTTTCAAGAATGATGCCCTTTTAA
TATTTTTATATTGTGGCATTTTACCATGTACTATTTTTATCGAATATATCTACGA
GGAACAAACAGTGACGTAGATCAATATAAGACCCGTACTAGCCACACGGTT
TCCTTAAAACGGTGCCGTTTTATTGGTTCTTGAGGCGGTTACCCAACCTATC
CTAGCCATGTCTTGCTCGGATTGAGGCATATACCTGTATCGAGATGGTTGGC
AACTCATATATGATAGGGTCTCGAAGCCTACGGTATGGATGTACTGTAAGGT
CACTAGACGTTGGTAGCCGAGACAAATTAGGTGTACCCAAATTCTGCAAGG
AATTAGCTAGTAGGAACGGCACGTGGAAGAGTGCCCGTTCGATCCCACGC
GGATACTGTGCTGGTCGGGCAAGTCACTAGTAATATATTAATGCAGGTAACC
GCGTGGCTGTTCGTGTAGGTAACTCTTTCCGTAGGCTGCATAGGCTGCATCA
TAATTGATAGGGAAAACAAGTTTGGTGAGCTTATGAACTTGTTGGATTCGTT
GGTGCTACAACGTTGAGGCAATCGTCGATAACGAAATTGTTTCCGAAGGTA
ACTTTCGAGATTTTAATACATTTTAAATTCCAGAAACAAGAAAGAATAGTTC
CGTAGGGAATATGTGCATATAATCAGGAGATACTTACCAGATATGAAAATAC
CCCGAAGTATTGGATATTACAGTGGCTATTACATGCATCAAACCTAAGCAAC
CTAACTGGCAACCTAAGCCCTTTTTTATGACGCTTTACCCCATCAGGTAGCT
AAGTACCTCCAATCTCCACCATTCAGCCAGGTCGTCGGGATATTTCTGTGTC
ACTTCCTCCGGAACCAACTCTGACTAGCTTCAGCGCTAGTCCTGCTTGGTT
GCAGCACCTTGCCCAATCACGAATCAAATTTCACATGAAACCACCAACTTC
AGGCGTGGCATCAGCGTGGATACGCGTGGGATACTTGGGTACCTGACACTA
ACTGCCCAGGTAGACGCTAGCAAGTCCCCTAGCACTTTCCCTAGCACGAAT
GCTCCCCCCTTTTTTGAATCAGATTGGCAGAGGGAAACATTTTAGGTTAGG
AGCTCATCTCGTGTAATCGTCGCGATGTTTGAAGCTTGACAGATTCATCTTC
CGATTTTTTTTTCTGCTTTCTGCATCGATTTTTCTTCGATGATGTGTGCTGT
GTACACCCTTGCCAGCAAATCTTGTTGTTGAACAAGCTGTGACAGTTCTTC
GTTCTAAACATATCAATGCATTGACTGTGGAATATTCCTTCAGCAATCCTCA
ATTCTGCACGAAATCGCACTGTCGTCTGATCTTCAACGGCATCCGGGGCGC
GGCACCAGAGACCCTTTTTTTCTAATCTGCATCGACCCAATTTTGCGAACA
GCCCGTTACTCACAGTAAACGCACGCCCACCTGCGCACCTCGCACGTCTTT
TAACCAAGATCTATTTCAGCACGACAATCGTGAATTGACCACTTTTTTGCGA
TTCGGCATTCATTCACTATTCGCATTTACGTTCTCTCATTCGATAGGTAAGTT
GACTTGTTTCATTCGTTCAAAGCGCTTTCTATCCCGTCGCATGTACACAAGG
TGTTTCAATCCCTCTTTTACGAGATAGTTCTAGGTGGGTTGTGTGGGTTCTA
TGTAATCGCACGACCTCAAGCTGTTGCGCTCTGCTGCAGTCCTGCAGGACG
GTAGATCACGGAGGGAAAAACTCTCGATTGGACCATCCAGCCCATCCTACC
ATTTCATCTCAGACGTCCACATTGGTTTACGTAGTACGTCGGAGGGGATGCC

AGTTGAGGTGCAATACTCTCCAATCTAATTTGCGCCCACTACCACCGACGC
GCCATACAAGATCGAGTAGGAAGCGAGAAAAAAGCAGAGCTCGAAGCTAG
TTGGTTACAATTGCTGCATCTTCCTTGGTTCACCTTCTACCCCTACCTAGGTA
GCATCTTTTATTCTAAAGGGTGTTTTCTCATCCCCCTTAATGCGCCAGACATT
CCTAGGGAAGAAAAAGAGGGACGAGAAACATCTGCGATCTATTTTTTATTT
CCATCAGTTCTACGCCAATCGTGACTTTCTTGTCCGCATTGGCAAGAACTTC
ACCTTTTTTTATCGTAAGACGATTCTATCTTCACAGCTGGCTGACCTTTAATA
TCTGCGATTTCTAGACAGTTTCGAATTACTTTGCGTTGATCGTCTGTCATTTG
GTTTTGTCCATTTGCCCTCGTTCTTTATCCGGCAGTAGAATCTGTCCAAGTT
GAGATACCCAAGCATAAACCGTAAAAACCATCCATTCTATTTCAGTCTGGG
AATAAGAGACGAGACGCACGATTCTCAAGATTCGCATGCGCAGGTCACTAA
ACCTTCAATCGAAACTCGGGTCTACCTACCACGCACCTTTAGGTACAAGCT
ACCTGGGATTTCCAATACGATAGGACAGATCGAACACGAGATAGGAGGATA
CTCTTTCCCCAAATTAGACACAAGCAACAACCTAGAACCTTTTTGCAGTAG
CCCGCCCTCCTTTATCATCCCGGGGTACCAAAGATAATTGCGAATACACCTT
CTTCGCACCCCCTCACCGGGAAAGTCATTTGGCAAATTGGACAGAATAAGG
AAATATCGGTCTTGTAGTCTGAACTTTATTTTCGAACTATACACCGTCCAGTT
TGCGGTACCTTCTTCACCTATTTTCGTCTCGAGGATACTTTTCCCCTGACAG
AATTCCAAGGCATTCGTCATCGTTATAGCACCCGACATCCTAGTCTGGATAG
CTATTTGAGTTTCTAGCATATGCTAGCTAGTTTTGGAAGAATTAAACTATGAG
CCAACCGCCTCGTCGATCTAGCTTCGGGATGTTGCTTCGACGTAGTAAATCG
GGTGACCTTGGCAAGGGGAAGAAGCACCAGAAGGAGCAACACAGAGAAA
CCGTGCCCAAGTCACCTCCACGTCTTCCGGCCCTCTACAATGGTGCTCAAC
CCCCTCCACCTCTGCAAAGTTTTGGAGGCGATAATCGCCCAGATTCTTTAGC
AATCGTTTCTGGAACCGCAGGACCACTCTTTTCACCACTATCCTCCCAGAG
CATCTATTGATCCTGGCCGACCTTCGATGTCTTCGACCTTTGCCAGTCCTCC
AGTGCCGCCTGTTCCCAACGGAGCTTGGGTCGATCCCTACGCCCGCACCGA
AAGTATGACGCACCGTGGCCGGTATAGCTATGCGAGTAGTGCCGTTAGCAC
CATCAACAGCCCTAGAAGAGTTCGTCGAAGGAAGGATCCTACTCCTTTCAA
GTGAGTATTTTCTTCCCCTCTTCACTTCTGACAATTTGTTTCTCTCTTTGCCG
AACCTAGACTTACACATAATTATAGTGTTTTGATCGTGGGTACCCGTGGTTC
TGGAAAGACCTCGTTCCTCGAATTCCTCAAGACGTCTCTGGCTCTGCCACC
TAAGAAACGATCTCAGCGATCAACCGAGATTGAGCAGGAAGTCACCGCCA
ATGCCACCCCTTCGGGCAACTTCATACCACACTATCTCGAGAGTGAGATTG
ATGGGGAGCGCATTGGATTAACCCTATGGGACTCGGAGGGGCTCGAGAAG
AACGTAGTGGATTTACAATTGAGAGAGATGTCGACCTTTTTAGAGAGCAAA
TTCGAGGAGACGTTCGCTGAAGAGATGAAGGTGGTCCGATCACCTGGCGT
CCAGGACACGCACATCCATGCAGTGTTCCTGATCCTAGATCCCGCTCGTCTA

GACCGAAACATTGCGGCTGAGAAGGCTGCTTCTGCCAACGGTTATGGCCC
AAAGTACGACCAGAATGCAGGTCGCAGCGCGGGCGTCCTGGATGAAGATC
TCGATTTGCAAGTCATGCGAACTCTTCAAGGCAAGACAACTGTAATCCCTG
TGATTTCAAAGGCCGACACTATCACTACCGCCCATATGGCAGTCCTCAAGA
AGACAGTATGGGCTGGTCTCAAGAAGGCGAACTTTGATCCCCTCGAACCA
CTGGGACTTGATGATGACGATGAGGCTACGCCAGACTCTAGCAGAATCGAC
GAGGCCGAGGAAGAAGATGAGGAGGACGAGAAGCCAGCCGAGAAAGCG
GAGACTAAGGAAGACAAGGAAGAATCTGCTGTGGTGGAGGATGATGACAT
CGATGCAGACGATGACGACGATCTCCCCATCCAAGGCAGGGTTTCTCCAGC
CTCAAAGCGCCGATCGAATAACTCTACACGTCGATCCAAGTCATCTGAAGA
GGAGACAAACGACGAATCTCCTTTCCTCCCAATGTCTATCATCAGCCCCGAT
CTCTACGAGCCCGGTGTTGTCGGTCGTAAGTTTCCTTGGGGTTTCGCGGAT
CCGTACAATGAGGAGCACTGCGATTTCACTCGCCTGAAGGATGCTGTGTTC
AGCGAGTGGCGTGGCGAGTTGCGTGAGGCCAGCAGAGAGCAATGGTATGA
GGGATGGAGAACAAGCCGTCTTAAGCACCGCCAACCTGCGACCCGACGTC
GATGAGGCTGAGAGCTGAATGGCCAGCCGCTAGTAGATGAGACATTTTTCT
CCACGCTTAGCACGTTTTCTTCAA

>Mosaic_sequence_68

CTTCTCGCTCGTCGCATTCAGGTAGAACCCCGCGGCCGTGCCGAAATCCCA
GTCGTCGTCCTCGCCGTCGATGCCCGCGCCGCGGGGGGACGTGTCGGGGA
AGACGACCGCGAGGCCATGCTTGCCCGCGGCGTTGAGGAGGCCGCCCTTC
TGCGGGCCGGTGTCCTCGGTGCAGGTGAGCCCGGCGAGGTAGTAGAGGAC
GGGACGGGCGCCGAGGGGGACGCGGCGGCGGGGACGAAGATGTTGAAGG
TGGTTTCGAGCCCGCCGAGCGAGGCGGAGGGGAAGGTGTACTTTGTGAGG
GTGCCGCTTGAGACGCGGTTCGAGGACTTTTAGTGAGTGCAGGGACCATC
TTGCTGCTGTTCTATCTTGATTGGTCCTGCTATGCAATGGAAGGAGATGTAA
TGTAGATGAAAGGAGAGCTCGCAGTGCGAGATCCTCTAATCTCGCCGGACG
TCGATACGTCACCGGCCAACTGATTCTGGCCGATCAAGACTCCGGCCAACC
TCGCATCACTGCATGGACCTAGAACGACAACATAGAATATCTTGACAACCA
AGCTAGTAGAGACAGAATATTAAGATAAAACTACACTTCAGGTCTCCACCG
CTAACGGATACCCCTGCTCTGACTGCCGCATATGCAGTCGTGGCCGTCCCCT
TGGCCGCTTAACCACTGCTGAAGGGGTCTTCTCCCTAGTCTGGACCTCGAG
TGTTGCACGACTATAGGCTGCCGTGTCTCGTGGCCGTTTCCGATGGGATGG
CAAAGGTGTCATGACCGGAGCAGCGTCGTGCTTTGCGACAGTCGAACCAC
TGACTTCCGTGACACTGCTCGATGAAGTTGCGCTCTGTCGCCGTGTGGCCA
GCATCGCTTCGACTGGAGCATCGTCCAGCTCGTCGATCAACTTCTCCAGCA
CAGCCGAGGGTTTTTGAGGTGGTGCAAAGCTGCTCGACCCGACCGCGCGG

GATGTCGTGATGGGCTGGTAGGCGAGCAAGTACGTCTGCGGTCATTCCGGC
AAACGACTAGCCCAATCAGTGCGGATCCTTTCGTCATAGGCTGAATCCTCA
CCGTTTCTCCGAGATCTGTGAGCGGCGAGGCGATTGAGCGCGGAGCCTCC
ACACGACGATCCACAACCGAGCCGTCTTCGGTCAATCGTCCAGCGGCGAC
CAGCTCCGCTCTCTGCTGTTTGGCAAAGGTCGAATTGTCTTTGGCGTGCGG
TACCGCCAACCGGTCTTCAATGTCCGCAGGGCCAGTCTCGCCGTCTCCTTT
CCGTCGCACAGACTTGTAATGCACACTCTTGGAGAAGAGAGCTAGCCACA
ACATCGTCCTCCCTGGTTTGAGTGGTGGTACATATTGAATGAGAGCATCGGC
GTAGCCAGGAGAAGCGAGAGGGAGCGTTGAAGGGTTGGAGAGTAGGTGT
ATAGTATCGGATCTTGTCCGACGACGAGGCGTGTTCAAGTCTTGGGAAGAC
AGTGGTTCGGAAGCATCGCCAGCATAGGCGATGGTCTGTGACGACTGAGAT
CAGATCAGGCGCTTCACCACACAACGACGATGGCTCACGTTCGGCAATATG
ATCCGGATGCTACGACGGAACACATGTGTCGCAGCGATGACTTCGAACTCC
GTACCATACGTCACTGCTCGTCAGCGTCCAGGTCCGAGGTATAGGCTGATC
GACCCACTGCTCTGGCGCATGTTCCTGACATAATCTGCAAACGTCTTGATG
ACCTTTGAGCCTATCGGCGGGGTCCACCAGAATGCGAAATCTTCCTCGTGC
ACTTCGAGATAGTCGCAGACGAGCTTTCGCACGGCGGCGTGTCGGCTCTG
ATCACCCCACAGCTGATCCGACAATGCACGAAAGAGACAGTTGCCATCCCC
TTTGATCTCCCTCAACGAAAGGTTGAGACGGTTGATTCTTCTCGTCAGCCC
AGGTCGCGTGACCTTTTCCTCCACACACCATCAGTCAGATCCTTCTTCTCCG
CCTCCAACGTCATCCAGTGTGATAGTGAGCGAGTCATCGGACCGGGAACG
GGTCGTCCGTGGCGGCTGTGAGGGAGCCGGTCGGCGGTGGCGGGTACGAG
AGGAGGAGCGCGTGACAGCTTTGCTGCTACCGGGGACCGGTGTGCGGAAG
GGCGAGCCGAGGGCGGATTGCGAGGTGTACGGAGTCGTCGACTGATGTCA
GGTTATGCATCGCAGATCTGTCTGCGGGTGAGACTCACGGCGGTGGTCATA
GGTCGATCTTGGAGATCGTCGAACAGGAGGTGTGTCATATCGTTCGTTGAA
TGTCTCGGCGCTCGGACTCGGGTGTCCGTGGGCCGTGCCGAGCGATGAAA
ACCGCTGAAGCGGTAGGAGAAAGCGGGAAGAGAATCTAGGTAGGAGGAG
TCGATGCGTGTTGGCGCATGGTCATAGCTGCCGAGCTGTCCAAGACGGGGT
AGAGGTGTAAAGTGCTGCTTTGTCCCTGCCCACTTTGAGACTACGATAACC
GGAGGTCGGCTGACCGGTAGCGGCGGTGCTTTGGGCGAGGTGGGCTTTGG
TCTATCGGCAAGACGGGTTATCCGCCTGATGGCAAGCAAGATAGTGTATCTC
CTGAGGTGGTCGTCGCCGTGTCGGAGCTAGGTAGCGCGTCTGTATGTACAA
TTGGCCTGGTCGCTGTAGGTTGCTCTTTGAGAGGTGGTGAAGAGGTGAGAT
ACGAGGTGACTCTTTTACCGGGGAGTCGCCGAGGCTAGGGAAGAAAAAAG
AGTACGTCATTTTGATACCATTTCGGGTAAGTCCAAGTCAGCCTGCCTCCAC
CCATCTCCCCACGCCGCAGAAGTAGCCGTCGTCAACGATGGGGAGATCATG
ATCCTCTTCTTGACCAGTGTCCAATAGTATAAGTGGGTCGAGCCCGGTAGA

CAAGGCGAGAGGTGGCAGCTGATAGTCTCCCAGTGTCCCGTCCAAGCCAA
ATTCGAAGCCGCAGTTGCCATCGTCGGCGAGCTGCCAAAGGATGGACCGG
TCCAGCCTACCTCCGACGACAGGCTGGCGGTGAGTCACGCCGAGGCAGAG
GCAAATGCGAGAAGAGCATTGCTAACCTTTGAAGTTCTACTCTTACTACAA
GCAGGCCAAAGAGGGGTGAGATCAAATCTGTGCCCCGACCCAAAGAACCA
GCTGACGCGATCAGAGACTGCAATGCCCCTGCTCCCGGTACGTGATGCTCG
CGTCCACTCCTCTCCGACCTGCTGACGAAGCAGGCATGTTCGACTTTGTCC
GCCAAGGCCAAGTACAAGGCCTGGAAGGCACTCGAGGGGCTGAGCAAGG
AGGACGCCATGAAGAAATACGTCGAGCTGCTCGAAGCGGTACGTCCACGT
GTCCGCCCCCCGCACAGACCCCAGTGCTGACGTGGAAGATGCTCCAGAAG
GCCGACGACGAGCAGTCCAAGAGTCCCTCAAGAAGCTCCAGGAGGCAGG
TGCAGGGGAGGCAGCTCCAGCTGCGACTGCATAGACGACGGTCGTGGCGT
GACCATCAGCTTGAATGACGAGATATGCATGACAGGCGACCTATTGTGATG
GATTCCCGTGTCGCCAATCTTGGGCTATGGCTACATGGTGCAAATGGTACAA
CATCTCATGCAGAGTAGGAGCGATTACATGGTCTGAGAGGGTCAATAACGC
ATGTGACGCCGTCACTGCAAGCGTTGAAGATGCCCTCGACCGGGATGAAC
TCTTGTCCCGGTAGCGGGCGCTCCGATTCATCCGTCCCTTGTGGCCATATCT
CACCTGCAGGAGGCAGGTCCGGAATCTGCAAATCAGCTTCCCCCCCGACT
CGGACCTGAAGACACACTTTGAGGACGTCGAAGTGGACGTAACGGTGTAC
AGTGCTTGCGTCTTTGCCTGCACCATCCGTTACGGGCGAGGCTTTTTGGCT
GACGCGATGAGGCGTTTGACCAATTTGCGATGACTCGGTCGGACAATCGAA
TAGAACGGCTTGTCCGTCACCTGCTGAGTCAGACTCTACCCTAACATATATA
AGTCGGAGGTCACCTGGTGCGTATTGACAATCACATCGTTGCTCGCCCACA
TGATCTTGGCCGTCTCGGTGAACCTGTCCAAGAGGAAGAACGTGCGTTTG
CGGCGTTGCGATACAGGTGCGAGGTTGTAATAGTCGGCCGGGTCGAAATCT
TGCAAAGCGGGCGGCACGGACAGATCGGTGACTGTGGTGGTGGATGTGGG
AGGGGACGAGGTGTAAGAGGGCGAGGCCGAGGAGGAACGAGAAGAGCC
CGCGCCGAAGGACGAGGCGGGAGAGGCGGTCGACGAAGGGGCGACGGC
GGTCTTCTTTGCGGGTGGTTTCCATTTCTGCGAGTCAGTCACATTCGCGACC
GCTTGGGCGATTGGGGCGCACGCGTAGTTTGACGTCGCCTTTCGTGACCTT
TGTCACTTCAAGCACATCGCGTGCGCCAAAGGGATTGCGCAGGGGAGAGC
GGTCGGTGACTCTGGAACAGGTGGCAACTACCGAGTCGCTGACGTAGGAG
CTGCGCGGGTTGTCAGCGCGAGTGAACCGTACCCAGAGTGAGGGACTCAC
AACGCTGTACATATATCGAGATATTCTCCAGACTTGTGTCGTATACGGTGATA
CGCGACGATTGCCATGCTCTCCTCCTTGCCGTACATGCTAGCCAGTGAGCG
AGAACTTGAGCAGCAGCAGCGGTGGCAGCTCTCTCACCTCTGAATACCGA
CTAGCTGAGGCGTCTCTTCCGGGTGCATGAGATCGAAGAGTCGCGTGCCCA
TGAGCTCGTGGGGCTCGTAGCCGAGGATATCATAGATGCTGGGCGAACAGT

AGACCCATTTCGGCTCGAACATCAGCACTTGCGTCTCCTGTACAGTCGCGG
TCGCGTAAGCAACGGGGCACTCACGTCCGAGTCCAGTTTGGCCGCCCACA
CAAAGCTGACGCCGAGATTGGTGTCCTCGCCCAGGGGCTCTTCGTCAACC
CGCCCATCCGAGCTGCCCGATGCGGACACCGAGGGGATCGCCGACGAGTC
CCGGGCACGCTTGACGTGCTGCTTGTTCATCTTGGTGTGGTGGTCATGTGT
GGGATGGGTAAAGGGGAAGATAAGCAGTGGGGTGGTGTGGATGTGGATGT
GGGTGGTGGCGGATAGTCGGGGTCCGTGCTCGGGCATGCTGGTGCGTTCTG
CGTGTTCTGTATGCTCTGCCCGCGCTCGCGTGTTCCACGCCGGGCTAGTCG
CACATCAGTCGAGATCGGTGGGACGGCATGGTGACCCACTAGGCGCTCAG
CGGTGCAGTGGACAGAGTGCGGCGCAACAGAGAATACGAAACGACACCA
GCAAGTATGACTCTCGCTGGTCTCCGCCTCTCCGCGACGCTCCCTGCTTCTC
ACCTGAGCGGGTGCGGAGCCTAATTGCGAATCGAGGCAGGTAGGACACGG
GGGCAGGAGGGGAGGGCGAGCGGACCGAATGTCCGGCTCGGAGAGTACA
TACCGTAGGTAAGAGAGATCTGGCTCTCGTACAATCGGCCCCCCTAAGATC
CTATGCTCCTATCCAACTAGATCCTACGAACACGGGCACGGGGAGGGTCAG
CGCCAGCGTCAGGAATCCCGATGGAGAAGCGGCAATCCGCCCCCCGCCCG
AGGGAAGGGGACTGATGCCGGCACCTCAAAAGCACCGACGACAAGACAA
GCTTTTACCCATAATGCGCCCTGAGGGCCGAGGGGGTCGAGGGGTTAGTCT
TTGCTGGAACGAGAACTGAGAAAAACCTGGAACATTGAGAGTCTGTTTCC
AAAGACGACAGTCGATCTCCGTGCGCCTCGTCGCGCCCCTGTCTAACCCTC
CTCTGTGACTTGGCCCAGCACGCAATATATCCCTGCGCCACCCGGCAGCGC
CTGCCTGCCGAGGCTGGGCGGGCCTCGACCAATCGCACTCGCGCCAAAGT
CAAGCGACGACGGCAGCTCGGCATCGAGCGCTCAGGCGGTCGCGTACGAT
CATGCCATGCGAACAGTACCTGCACAACGGTTGGTTTCCTGGGCCGATGAC
GGCTGATCGCCTTCCGGTCAAGCAGGACAATTGAGGACTATAGCGTGCGCA
TCTGCGGCGGCCAGCGGCCAACGGCCTAGACTGGACCGCCGGAGAACATG
GAGAAGCGGACGCGGAGGCGGAAGGGGGACGGGCTGCGCGCGCTCCACA
ATCCCCCAACTCTCATCTGACCTTCCCCGATACAGCCAAACATTCTAGAACC
CGCAGACGCCATGTGCTTGTTCCGGAGGCGGGCTTGGCTCGGCACGGCGC
GCTAAACGAAATCAAACCGAAGCGAGCCGTACTGTGCTGGGAAAATGGTT
GATGCGCCAGGTTGCCCGACTCGGCGTGACGCCGAATTAAGCATAAAGTCG
TGACATTCCCTCTCTCCCAATCCCAGCGAACAGGGTGTGGCGTGCACGATC
AAGTGATGCATGCTGGACATTATAAGCGACAGATGCCGGTTGTTGGGGCTA
CATGCGATGTGCAGAGACCGATACATAATACAGTCAGCCGGGCAGATCAGC
AGATCTGGGTGATTATGAGACAAAGTTGGAACTATGATGTGGGAACGTGAT
GGGTGGCTCCTGGTGGAGATAAGTCGGAGATGGCTTCGTGCTTGACCGAGT
ACGATTCGAGTCCAATGCGATGCGGGCGTGGCGACGTGGTGTCAGCGACC
CGTGAGTGGGTCTATGACGCACGTCACGCCATCGCTGCACGCGTTGAAGAT

GCCTTCTACAGGGATGAACTCTTGCCCGGGCAGCGGCCGCTCAGACTCGTC
CGTCCCCTGGGGCCAGATCTCGCCCTCTGGAGGCAAGTCGGGTATCTGTGA
AGTGTCAACCACTGCTCAAGCTCGCCTTGAGGTTCAGCGGACTCACCCGC
AGCACGTCGAAGCGCACGAAACGATATATGCTGTTGCCCTCGCGGCTGTCG
TGTTTCTGCAGCACGGGTGACGCGCGTTTGGCGGCGTCGATAAACTCCTTG
ACCAACTTTCTATGGCTTGGACGGACGATCGAGTAGAAAGTGTTGTTGGCC
AACTTTGAATCAGCCCCCAACTCTACTTGACTGGTGACAGGGAACAAACTC
GCCGAATGCGTGTTGACAATGACGTCGTTGCTCGCCCACATGACCTTTGCC
GTGGTCGTGAATCGGTCTAGGAGGAAAAATGTGCGCTTGTGACTCAGCGG
CATGGCCGGAAGGTCAACCACCTCCGTCGGGTCGAAATTGTCGCCCAGAG
AGACGTCCGTGACCGTTGGCTGCGGCGAAGCCGACGAGGAGTGTTCTCTG
CTCATCGGCTGCGAGACCGGGGCGGAGCCAGCAGTGATGGCTGCTTTCTT
GGGCAGCGTCCATTTCTGCAAGGAGTGAGTTCTTGGGTCTCGCATGGCAGG
AAAGAAGCATACTCTCAAGCCCCATTCGCCTTGGGCGACGCGCGTAACTTC
GAGCACTTCGCGCGCGCCCCACGGCTGGACGGTACGGCTCGCGCGACTCG
GACGCGCGCGAGTACGTGGCGACGCTGCGGTCGTATACGACAGAGCTACG
AGATGAGAAGTGCGCCAAAAAATCGCAGTGACGAAAACACACTATGCGGC
GCAGACGTTGACCCACTGGCCCTCCTTGTGTCCGCATGCGAAGGTATGCGA
CGCCGGCCGTGCTTTCAGACGGTACCTGTGTGCTGTCTCGTAAGCGAGGCT
CGGGGTGCTCGTTTCCGACCAACGCATGTATGCCTGCCACTTGCACGAGCT
CCTCGGGGTGGATGATACTGTAGGCTGTTTTACCTACGAGCTCGTGTGGTAG
GTAGCCGAGGACGTCGGTTATGCTTTCGGAGCAGTAGACGTATCTGAGCTG
GGGACATCAGATTCATCGCAGCACTTGATTTCATTCACCGACCTCCTTGTCC
AAAGTGGAGCACATGATGGCACTGACACCGAGATTGGTGTCTCTGCCATCA
AGTTGGCCGGACTCCGAGGTGGTCGCGGTGGTCGCGCGGGACTTGCCGCT
CGACGAGGCCCTGGTGTGGACATTGTGCGTACGTTACCTGGCGACGACGG
GAGGCGAAGAAGGAATCGAATCAATTACAGAGACAACTCAAAACGAGGC
AACGACCAGCGGCTCGATGCGACTAGTACTGGTGCTGGTCACTAGCCGCG
AGATCCCAGGCGCGGGCAACATGAAACGAGACGGGGACCGCCGCCTAGCG
TGGCGTGCCGGGACACGGGGATGGCGATGTCATGGGCTGACGGAGAAGCA
AGGGGCGAGCCAAGTGCCGCTGTGGTGGAGGGTGGAGGGTGCGCGAGAG
ATCTGGGGACGGGGGGTCACAAGCGGCGTGCACGAGCGCGAGTTGCAAG
TGTGAAAGGAATGGGGCGTTGTGTTGGTCCTTTTCGATGGTGATAGTGTTG
GTAGCGCTGCGTTGGTGCGCCATGCCTCCCTTGGGTTCCGGGGCTGGGGGG
TCCGAGCGCGCCCCTTCTCACCTCTGAATCGCAAAAGCCCCCGATGACCGG
GCAGGGCTTTGATTTTGAGACTGGGTCGGATCACCGAGCGGCGGCCAAGC
GAGCGCGCGAGCGAGGGAGCCAATTGCAGGAGCAGTCAGTCAGCAACTG
TTCCAGGGTGGGTGGGTGCTTGAATCGGGGGGCCATCGACCACATGGGAC

GGCTGAACGGAGGTCAGACGACGAGGGCGACGCCGCTCGGGTTATTTCTT
CCCCTTGGTGTGTGTTTGTTGCTTCGAGTACTTGCAAGACGAAAGAACAAT
GGGACCAAGGCCAAGGCGGAGCATCGGAGTAATCGAGACCCCGGCACAG
CTGCGGGACCGCGCGGAGCAAGGTGGTCGTCATCTCTAGATCCCTTGCTCT
CACAAGGTTTCCCCTCCGCTTTTCCCTGTGCCGTCCGCGCACCCCTATCTCT
TTCTCGGGTATTCTTTTTGTCATCTCAGAGCCAACAGCAGTGAGAGCGGAC
GCTAAGCCGCGCTTTCAGTTTCCTTAAGCTTGACTTCCAGAAGCTTATCGAC
CATCTGTCACACTCATCGTGGACAATGATGATGACGATATGACGGATCTGTA
TATTCGACGCCAACGCCAGTCCCCAGCGCCATCCCTCGCGAAACAACGCTA
GAAAGAGACCAGGGGCGACCACGGCCGCCACCCGACCTACGGACTGCCC
CTGGGCTGGACTGGCAAGTGCAATCGCAATCGCCATGCCCCACGGCTGCA
ATCGGATTCCCGTGACGCATACGCGCAATGGAGCAGGGCACGAGCGGACT
CTGACGCCGCAAAACATCCGAACATCCATCAATTAGCCAATTTTTTCTTCCA
TTCCAGATCCCATCCGATCCCGGTCGTCCGAGTGAGGGTAGAGGTGTATAG
CAGCCAGGAGCTGCGCAGACGGTAGGCGACACGCGAGGCTATGCGGTTCC
GACAGAGCGGGACAGGCGAGACGTCCATATGCATCGTTCTTTTCCCACCCC
ACCCCACCGAACAAGGCCATAGAGATCCAGGGGCAGGGTCGTGTCCCCGT
TTCCCGGGTACGGGGCCATGGGGTTTGTGGTTTGGCCGTGTCGAACACCCG
ACCGCTGCATGTTGCAGCAGCCTTCAGACGAGGGGATGCTGCGCTCGTGC
GCATGGCGTTGGCGTTGACACTGGCGCATGCACGCGCACGCTAAGACAAG
ACGCCGATAGATACCTCTTGCTCCTGCCATCTCTCTGTTCCTCCTCTGGACG
ATCGGTCCAACTTCCGACGCTCAGTCGACGACACCCTGATGCAGATGAACG
CTAGGCGAGGGACGAGCGGCATGGCGTTGTCGAGCCAACTCTCGTCGCGG
TCGTTCCATCCGCTGCAGCAGGAAGAGAAGAGGGAGCATTTGTCGGAGCC
TCCGACGCGCCACTCTGACCCCCATAGACGCCGAACACTCGACTACGGAC
CCACGGACCGACCCGGTCTAGATCTCTCATTGCGCCACTTGGCCCTTCTGG
AATGAATCAGGTGCTCTCGATTGCATCTCACCTGTTTAGATCTGTTCTTTGG
CTGTCGGTCCTAGGTACGCGGCACCAATTTCCTTATCGACCTGACTCGACC
GCTTTTCGGCGTTTACTCGGAGGAGCACGTCCACGTCCAGCAGGCAATTGC
ACGTCGTCGTCAGTGACGCCTGCACTTGCGACGACACGTCGCACAGTTGA
TGGGGCTGCGCAGTCGGACTAAAGCCATTGGACGAGGAGCCTGCGACTCG
AGCAACAAGATGGAAGTTGACTCTGACCGAAATAGCACCACCTCCCACCG
ATGGAGCTTCCGTCCAACAAGATCCTGCTACGTTATAACTTCGGACTCGGAT
GCACTTTTGTCCAGTCTGCCGTCACCGAGATCTAGATCTAACAAGTCACAT
GGCCAATGTACAGGACCTCCACTGGCTATGAACAGGTCATCCGACAGACCC
CCACGCACGTTTATCGCTCGACCAACCGGACTTCGACGAGGTTTCTGTGCC
TGACGGATCCGCCTTCTCTGCGGTGTGTCGCCGTTGCTGCAGTACACTGTA
CAGAAAGAGTATGTAATTTACCCAAAGCAAGCAGCTAAATGCCTCTCCTCG

TGCGGCATGCACCGAATCGGGCTGTCGGGCTGTGGGCTGTGGGTTGGCGG
AGGCAGGGGGCAAGAAAAGCAAAGAGGGACAGGGAGATCCATCCAATGG
GAGGACCAAGCCGCAGAGGGGTCCTAACCTGACTGACGAACGTTTACGGC
TATGCTCATGGTTTCAATGGATATGGGTCTACCACAGGGCAGACCCGCCGCT
GGGCCGTAGCGCTACCACGGTACCATCTGTCTGATTTACGCTTGCGTCTGCC
CGACCCGCTCTAGCGTCGATCTGCCTCTGGCCTCTGAGCACTGATTGATCTC
CTCGTCTCGACGCGTCGAGCTCGCCAGATAGTCGACCTCAGTACCGCTCGC
GTCGGTCCCGAGTCGTCGCAGAGCGCATAGACACCATGGCGACATTTATGA
AAGGGGACACATTTCTCGAGGTGAGCTCTTTGCCGCCAGTACAGCACAGG
CAGCTCATAGTCAGGTCGCATCGGTTCGTACTGCCTCGCTGGCAAGCTCAT
GAGGTGAAGCTGAGCGCAGTGCCTTGCCAGGACAGATATCCACGATGCTG
CGTTGTGCAACTTTGCCCTCGAGGTCGTCAAGCGGTACGTCCGCTCCAAGA
TCAAGAAGGGGAAAGGCAAAGAGGTCGGCGGGCCAAAAGGCAAGGTGA
GTCCTACTTCTCTTCTCGCCCTCGTCGGGATGAGAAGCTGAAGCCCAGCTG
GAAGAGCTCGAGCCGCAAGTACGCCCACCTGGGATACCAAGACATCTTCTA
CTTGACCCGCCTGCAGCTCGGCCTGCCACCATCCACCCTCGACCTGGGCCG
GCCGACCTCGCCATGCCGCAAAGTCCTGGTTCGCGCAAAATTCCCCCTCGA
CCCAAAGTACCAGCTTCCCTTGGAGGACACAGCGAGCTTGCGCCTGTCAG
CGGCCATGTCCCGCTCGGCCGGCCCATCGGGCTCAGGCTCCACCTCTCAAC
TGGGCGCCGGCTACGCAGAGAGCTCCCGGTCCGTCGCTTCAGGCTCTCGG
ACCGCGCGTGGCGGTGCCGGTCCAGAGGTCCTTCGACGGCCTCTGATAGA
CCGCGAAGACTCCAGGGGCAGGTCGCGCGAGCGGCGCGATACCAATATGC
GGTCTCCGGCTCTGGGACCGACACCGTTTATCGTCGCGCCTACCCCGCCGG
AGGGGCGGAGGGAGAGCGAGACTCTGCCGAAGACACCGGAGGCCCTGCC
TATACCTGGCCGATCGCCGCTGCATCAGGCGGAGACGAGAGACCTGTTTGA
CCACTCGGTCGATCTGGGCACGCCGCTCCCGCCGCCTTTTCTGGGTGCTCA
TGGTCGGACACAGGTAGACACGCCGCCAGTACCTGTGCCCAAGAGGGGAG
GCAGGGCCGAGCCGGTACAGGAGGACGATGTGGACGTGCGGCGCATGCTC
GAAGCGCGCATACAGGCTTTAGTGGGTCTCGACCTGGTCGTGGTAACACGA
CGCTGATAGACAGACCACCGCGCTCAAGTCGCTCGATGCTTCCAAGTCATC
AGCCGACCTTTCAGTCCCTCCCCCTGCGGCAGCTCCGGTTCCACTAACAGA
TGCGCACCCTCCACGAGTCCCGCTACTGCGCATGCCGTCCGGCTCACGACG
GTACATCCCCCCGGCCTCTCCCCCACCCGACGTCCACTCCCTCCTCTGCCC
ACCACTATCCCATCCACTCGTCCGGCACCATCGTGTTCATCTCATCGCCACG
CCTCCGACTGAACGGCACACCGCCCCTCACACCCGCCACGCCCGTCTCGC
CAGGCGCGGCGGTCATCCCGTCCTCCGTCCTGGACATGCACGTGCGTGGCG
CGGTCAGCCCGACGAGTCACACAAGCGCACCCACGAGTCCTGGCAACGA
GGGCATATTCGACCTCGACCCCGAGATTGCCAAAGACCCATTTGGCAGTCC

GACGCGGCAGCTTCCTCCTGCGCATATCCCGAGGCTCGTCAAGCCCACTCC
GCTGTACGGCGCCGATCCGGCCCTGGCGACGAACAAGACCTCGCATGCTC
GGGCAGGGCGGGAGACGGCTGCGTTCGCGCGGAGTCAGGATGATCTGCGC
CGCGTCAGGGCGATGAATGGGTACGATAATGGACGGCAGACGCCGCCGCC
GCAGTATGAGCGGTGAAGTCTTAGCATTGGTTGCCCGCAAGACGCGATCCT
TGTTGGGGGTTTGACAACGATTGTGCGGAAAGCCAAGAGTCTCACGACCC
TGTATGCAGTATAGAAGGTACGACCCGTTGGTCCCGAGAGGCCGAGCAGG
CCAGACGCGAATGTCTAAGCATCACATAGATGTGTGAGCTATCCTTTGAGCT
GGACTTGGTCCATTGTGCTCAAGGGAGCGCACTGTACAGTTCATACGCTCG
AGAGGTCCGCACTGTGGCTGGGCGTGAGTGCATGTATACTCTTTGTTGCAT
GACATATCTACCGCACAACGAAACGGCGATGGCAAGCACGACGGTGAACT
CGCACCTGCAGTCTACCGCGTCCACGTCAGCCTCAAACCCAACTCACACGT
TGTACTGGAATGCCAAGGTTCGGGTCGGAGACCGGCACGCCACCTTCCTG
GCAAGGTTCATCCTCGAGGGTCCCCGAGCGTCAGGGGACGACGCCTGAAT
TGCACGTGAAATCGTGTGTGAGAGACCTGGAATCCGGCTGCCGCAAGGTG
ATGGACTGGTTCCCTGAGCAGCCGTGGTTCACGGCGGCAGAGTGTGAGGC
GAATACATTTCACTGGGAGGCGACTCTTGCGGAGCAGGCCCAGGTCTTCAA
GTCCAAAATGAGCTCAGTGGTAGATCTCGCAGTCGAGGTGGATCCGCACGT
TTGGCATCGTCGTGACAGAGCGGTTCCGGAGATCGATTATTGCAGCATGGC
GGGCTTTGCTTCGAAGAACTGGGGCCGCTTCATGCCAGTATATCTTATACTC
GCAGTCTCGAATCAGACTCGGCGCACGACACATCGTAGTCTCCGTCCTTAG
TGTGCCAGTGAAGCCCCGCTGGGCTCTGTGCATGCATGAATATATTGATGTA
CAATCAATTGTGATTTTCTGCCCATCGCCACCGAGATAGAAGTTTTCCAAGC
AAAAGAGCAAGCGACCACTAGCTGCCGCATCTTGCAGATCAGCCGTGACT
CGCACACCATGTATTGGGACGCTACCGCCAACGTCGCCACCTCCGCCACTA
CCACTACGGACCAGGTGACGTTCAAGGCAAAGTTTATTGTTGTGGGGGCCC
GGGATGTTGACGGCCCCTACGGCGTTATTGAACTCGTGCACATCACATCTTC
AGACCATGTGCTCTAACTTGGTCGAGTGGCACACGAAGCCGCGGTGGTTC
ACCACGTCCAAATTTGGCGGTCGCGAATATCACTGGATCAAAACTATCGAG
TACCAAACCCAGGTCATCGCGGACGAAATGAGCCAAGTGGCGAACTTTGC
AGTCGAGCGGGATGAGGGCATCTGATCTCGTGAGCGCAAAAAGCGGCACT
CAGTACTATATACAGTCACCCGGGTGCTGCCCGCTGATCCTGCCGTGGCCG
AGGCCTGTGATCTAGACGAAGACGACCCCTCCGATATTGTTTTGGAACTAC
GAATGGGGCGAGGATTCGAGTGAAGCGGCGTGCGATGCAGGGTGGGGCA
AAGTTGACGTCCATTGTGCTACAGTGAAACATGGCGCAGGGAGCGGGAAG
GCCACGCTGGGCTATTGAGTACTTTAGGTGCGCGCTTGCGTCTTGATGTACG
CTTCAGCTCCTGCAACTCCGCTCTGCGCTGTCAAGCATGAGCTTGATCTCG
CGGCTGCGCTCTGCATTCTCCAGCTCTCCTGCTCAACACACCTTGCACTGG

GATGCGAAAGTCGACATTGACACCTCGGCGTACCCGGCCCTCCCGGGCAA
GGTAACATTCAAGGCATTGTTCGTCCTCGAAGGCCCGCGCTTGTCCAAAGA
GGCGACCGAGCAATTTGAAAGGTGCATTGCATCCCTCAGGTGCGACTGCC
GTGATACCGCCAAATGGTACGAGGAGAGGTCGGGGTTCAATGCGGAGGAT
CTTGAAGCGCGTACATACCACTGGAAAGCTACCCTCGAGATACAGACCGCT
CTTTTTGCGTCTGACATGCGCCAATGGATCGACTTGTCAGTCGAGTCAGAC
GTCTCGGTCTGGTCTCGTGAAGTTGCGCGGCGGCCGTCGCTCAGGTATGAA
AGCACTCGCGAGCTGCGCTATCTACTCGCCGCCGACCTTGGTATGAACGTG
GATTGGCGGTGATGACCGCTTATAGGCACATTCATACCAATGTTGCCGATGA
GTAGTAGGGGTTGTCAATTTTGGTATCCTTTCAGGGACCGATAGTTTGATCT
GTCAACAAGTTACCCAAAGCGGTAATTTTTGATACATTCAACATGTACATCT
ATCAACGACTTTGAGCTTGAGTTACCTCTGTCAATTTCTCACCTCAGACGC
GTCGGAAGCGGCCTCAACACCATCCCCGCCGCTTCTCCTGGTTCTTACGCT
TCTTCTCATGCCTGAATTGGCGCCTCCCGTCCCGGATTTGGCCCTGTCAATG
GATGGCCCTTCCGCTGACCCCGCCAGGACGAAAATCACCATTCGTCTACCT
ATGGCAGTCCCAGTGCCCAACGCGCTTCCCCCGCCCCAAATCCCTATTGCT
CCTGCACCAGAGGCGTCCCAGGAGGAAGCTGGGCCTCCTGGGAAGCGGA
AGAAGGCAAGAACCGTCGATTGGTCCCGCGATGGAGTGGGCGGCCGGCCG
TCCAGCCCTTGACCTTTTGCTAGATTGGCTAGCAGCCCCAAATAACATCGAC
AGGTGGCGCAACGGCTATGGGGGGATCAAGCAGAAGGCTGCATGTCAGCT
ATGTTCGGACTGGCTCGCTGCGAACGATTGTCCGACGCCGAGAGACAGTG
ACGCCTGTAACTTCAAGGTATGTCACCCGGCGGTTCAGATGTCCTTTATGTC
GAGAGACATTACTCGGTCAGTTCGCATGCTAACTGAAAGATTCGCAGCTTG
TTCGAGAGCTGGAAGAAAGCGCAAGACTACAAGCACACCACTGGCCACG
GGAAGGCCTCACAATCTTGTGGCAGGCTGAACAGACGGGAATGCAGGATA
AGATGGATGCAGCGGTCAGGATGGTCGAACGTATGTCATTCCTATTGGCCCT
AGGACGCGTCTGGGACTGCTGACACGCCCTGTAGGCTCAGTCAAGTCCCT
CTGCCCCCTCTGGGACCAGCTAGATCCCATCTTTGCCGAGCGTGACGGTGG
CTGTGCTACTCTACGAGCGGACACCACCTCTACCTTCAACCCGCCCGTTGA
GACCCTTGCAGCTGCTCTGAGGGAGTCCCAGGGGACACAGGCGGCCGACG
ATCAACATTCAGACCTGGGATGGGGTCCCAGTGACCGTGAGGACGCAGGA
GAAGGTCAAGATCCGTCCGTGGCGGGCGGGGACGCAGTCACAGTCTCAAG
TCGTCGAGGTTCGGTGCGATCGGTGGCCACAGTCTCAAGCGCGACGTCTAT
CTCGGATCGCAACCAGAGTAGGCCTGCGAGATGCACAGACACAGGCTACT
GACCTATAGGAAAGCGCTCTCGCTGGGCCGGTGATCTTGACATAGGTTCCG
ACCTATTCCGTCAAGATACAGAGTACCGCAGCTCAAAGCTAGCCAAGCTCG
ATGAAGCTAACGCCATTCAAGCCCGCAAGGTCCGCGCGGACGTTGGTAAA
GCGGTCATTGAGGCAGCCAAGATCCTGAAAGACTTATCACCATCTCGCAGC

TGGGACGACTGCAAGACCGAGGCCAGAAAGGAGCTCTGCCTCGATGAATG
ATGTGTTCGCGGGCGGAGATGACCGCTAGGGCTATAGACTGCATGTCGTAG
ATCATCCTGAGAGAAGCGATGGTTCATAGGACAACATGTCATCCACTAGCT
GTTCGCGGCGGACGTGATTCTCTCTCATACTCTCATATGAGTTCCTATTGGTC
TCAAAGTCATTGGGGACGACCGCCTCGATGCCCCAAGAGGCCCGTAGATCT
TCAGCGGTGAGAAACTGGTCGTAGGTCGAGATGGTGAGGTTATGTAGGGA
CGAATGCGGCCTGGATCATCGCATAGGCCTGGGCTTCGTCTCTCGGTGTCTT
GAGCCTCATCCGGGCGTTCCGCAGCATCTGCCAGCGCCCCTTGAGGATGCC
GAAGGCGTGCTCAATGTTCACCCGCGCGGGAGAGGCCTTCGTATTGAAGA
ATTCCTGATAGATCAGCCTTCCTTCGATGTCATTTCGGGAACCGCATGCATA
CCCTTCTCCCCGTCAGCTCGGATTGTCCCGGCGCGCGTTTGTACATTGGTAT
GATGGTTACCCCTACCCTATACATATTTCGGAAAGTCGACTACTATAGGGTA
GCTTCTATAGGGTAGCTCCCACCAGACACATACAATAAATCTCATAAATCCC
AGAGGATCCTACCCTATAACCACCCTGTAGCCACTCTATAGACCGCCCCGAC
TACCCTGTAGTTACCCTGCAGACTTGCCCGGACTATAGGGTAGTTCAGGGTA
GCTCTTCTACAGGGTAACCCCACGGCCTTACAGCGCCTTACAGCACCCTCC
GTCCACTAAATGCCCTGTAGCCCCCTATAAGACCCCCCTAAAACCGCTGCC
CCCTAAAAGGCCTAAAGCCAACCCGCCCGATTTCCCCGCGTCTCGCCCAAC
CTACCCTAAAGCGAGGAGCTGGCTCCTTCCAGGCGGCCTCGGCCGGGTAC
AGCCCCCACCCCCCCACAGCGGGGAGCTAGCACCCCCCTATAAGTACCTAC
CAGGCCCGGCTGGGCTGCTATCGTCCCTTGGAGGGTTCTACGCCACGGGGA
CATTTCCCGAAAAACACCTCCCTAAACACGTATAGCCCCCTACCTGTATTTT
TCACCTCGCTGACGCCTATAGCGGATTATAGCGGCCTCGGCGCCGTGTCGTC
GCGGGCGGGCGGACCTACCCTGAAGAGCGTGCTCGCCAGGAATTTACAGG
GTAGGTTCGCCAGGCGGTGGTTACCCTATATTCCGGGACGTGTCGCCTTCTG
TAGGGTAGCTCCGGCCGGTGTTGGCTCGCGGAGATGACCGAAAGAGGCCT
GCCCTGAAGGGCAGTTCGCATGCGGGCGTGTACAGGGTAACTCCGCCGGG
GCTCGGATGCGTTTACGACAGTCTCCGAGTGCACCTACCCTAAACTTACCC
TATAATATAACACTTGAAGACAAGACGCAAGTTGAAGAGATGGGACGGGTT
ACAAGTCCGAAGAGCCCTACCCTGTACTACAACATATTAAAAAAAAAAGG
ACGGCAAGGATGTTTTATTGAAGTTTAAACACCAAACCCACTAAGCCCTCT
TTGATAGGCCCCCCTAACGCTTCACCTTTACAGGTACTTACGTCCACTAATC
TCTTATAAGCCCAACTATAAGTTAGGCGGTATATGAGAAATCTAGGCAAAAT
AGGGCTTACCCTCAGCAGATCGTAACAACAAGGCTACTCTACTGCTTACAG
GAACCCTTTTGCACAGCTAAGTCGTCTGCAAAGGATTTAAATCCCACGCGT
GTTAAAATTACAATACGCGAAATACTACCGAACCACTTCCGGCTCGATAGA
AAATTCGCCAGCTAAGATGCGAGGCCGAAGCCTATTCGTATCCGTCTACAA
CGTGTGGGAGTATCACCGCGTTCTGGCATGGATTCTGACTTAGAGGCGTTC

AGCCATTATCCAGCAGATGGTAGCGTCGCGGCACTGCCCGGTCGGACAGCC
GCAAAAACCAATTATCTGAATCAGCGGTTCCTCTCGTACTAAGCTGAATTAC
CATTGCGGTGAGGTCATCAGTAGGGTAAAACTAACCTGTCTCACGACGGTC
TAAACCCAGCTCACGTTTCCCTATTAGTGGGTGAACAATCCAACGCTTACC
GAATTCTGCTTCGGTATGATAGGAAGAGCCGACATCGAAGGATCAAAAAGC
GACGTCGCTATGAACGCTTGGCCGCCACAAGCCAGTTCCTGTATACCCACG
ACGCGCTGAAGCGTGCCGCTCCGACAGCTGGGGGTATAACGCCATACCCTT
AACCGCCGGCGCCTTCGCAGGCGGGAGTAGCCCGTACCTTAAGCGGCCGC
CCCCGTATTTTAGGGGCTGCCACCAACACCTTAGCGAGCGTTGAACCGCTG
AGCCCTTGCACCCGTCCGTACGGTGAGTTTGGCCCAGTCGAGCTGCGGATT
ACCGCTTACCTTGCCGGATTGTTACCCGCAGTAGCAAACTGCTTAGCTTAG
GGGGGGTTATCCCCTAAGGTTGGTACCGACAGGCTTTACGGCTTCCCCGCA
TCCAGGTGTTTCGCCCATCTCAGGCCCTAATCCGGGGGATTTAGGGGGCCT
AAGCGCCGTTACCTAGCGCACTACCCGCGTTATCACAGTGGGTGGTAGTGG
TGGACTAGGGGCGGATTGCTGCCGCCGCCCCTGGGACTGATTAATCCCTGT
GGTAACTTTTCTGGCACCTCTAGCCTCAAATTTCGAGGGACTAAAGGATCG
ATAGGCCACACTTTCATGGTTTGTATTCACACTGAAAATCAAAATCAAGGG
GACTTTTACCCTTTTGTTCTACTGGAGATTTCTGTTCTCCATGAGCCCCCTT
AGGACACCTGCGTTATGGTTTAACAGATGTGCCGCCCCAGCCAAACTCCCC
CACCTGACATGTCTTCAACCCGGGTCGGCCCGCGAAGGACCTTAAAACCA
GAAGTTGGACGAAAGTCCAGCCCCGCTTCATTGAATAAGTAAAAAACAATA
GGAGTAGTGGTATTTCACCGGCGCCGAAGCTCCCACCTATTCTACACCTCCT
ATGTCTTTTCACAATGTCAAACTAGAGTCAAGCTCAACCATTTTAACCGCTA
GCCACTGAAAGGGAGTTGTTGTTTTTGGTTTTTAATGTATAAATAATCAAAT
AAACCCCCGAATGGCAGCAGATGACCAGTCTTTTAGACCCCGCTATGCTTT
GTGCTATAGGCACTCCACACAACAGCAGGCTTTCGCTGGGATTAGCACGTA
CCTTAAGCCAGCGCTGTAAATACAGCGCTAACCGACACCTTTCCGCGCATT
ACGACGGCCGCTACAGCAGCTCGCCACGGGATTGCCGTAGGGGGTTACAG
CGCTGTAAGCACTTATAAGCCCCCCCGTAGGGTTCCCCCGTACCAGGTTGT
CTCGCCCGCGGCCACCTATAGATATTGGCCGCTGGACTAACAGCGCTATTAG
GCTGCTATCGACCCCGTTGAGGTTGCTCAGGGTCTTCTTTCCCCGCTGATTC
TGCCAAGCCCGTTCCCTTGGCTGTGGTTTCGCTAGATAGTAGATAGGGACA
GTGGGAATCTCGTTAATCCATTCATGCGCGTCACTAATTAGATGACGAGGCA
TTTGGCTACCTTAAGAGCATTGCTCGCCCCCGAAGCTTTCGCAGGGGGATC
AGACTATATCTTAAGCCCTGCTATACAGCAAGGCCCACCGCCGTCTAGTCGT
TGAACTGCATCCTCCCTACAAGGTTGTTACAGGGTAATACCCCCCGTAACTC
CCCTACAGGGTTAGCGGACTTGGCTGCGGATTTTCCATTGTAGTATCCTGCT
GCAGTGTTACCGTACCCGGAGCTATTAACTCGGCCACAGGTGGCTTTCGCC

ACCTGCTTGGTCGCAGCAGGCTTTAGGAGGTCCCCGCAATTTGACGATGTT
ACAAGGCGTCAGGCGCTAAACCTGAACCCCATTAGCGCTTCTTTCAGCACT
TGACCCTCTGTGCAGCCCCTAGAACAGCGCTGTATTTACAGCGGCCGTCCC
CTGAGACTGCAGGTAGTTTGAGTCATAGTTACTCCCGCCGTTTACCCGCGC
TTGGTTGAATTTCTTCACTTTGACATTCAGAGCACTGGGCAGAAATCACATT
GCGTCAACATCACTTTCTGACCATCGCAATGCTATGTTTTAATTAGACAGTC
AGATTCCCCTGTCCGTACCAGTTCTAAGTTGGTTGTTAACCGTACGCCGGA
CGGCCGAAGCCTGCCAAAGGCGTCAGGACCCAGGTGCTGAAAGCCGCCG
GCCGGTTGCCCGGCTAGTGGCGATCTGCTCCCGAGCCGTCCACCAAAGGC
CCAACGTACCCAACCCTTAGAGCCAATCCTTTTCCCGAAGTTACGGATCTAT
TTTGCCGACTTCCCTTATCTACATTGTTCTATCAACCAGAGGCTGTTCACCTT
GGAGACCTGCTGCGGTTATGAGTACGACCTGGCCTGAGAATTATTCCCTCC
AGCGGATTTTCAAGGGACGTCGAGGGCGCACCAGACCCAACAGAGGTGTT
GGGCTCTTCCGGCCGTTAAACCCTAGCTTCCGGACAAACCGATTTCAGGGT
GATAGACCGTTAAGAAGAAAAGAGAACTCTGCCCAGGGCCCCCGCCGTCG
TCTCCGCTTTCAGTTGCGTTGCCGCGTAAAACCCAGATCCAGGCGCCGGAA
TATTGACCGGCTTCCCTTTCGCCACACGGGGCACAAGTGCCCACTTTGAAA
CGGAACTTCCCTATGGCTTAGGATCGGCTAACCCACGTCCAACCGCTGTTC
ACATGGAACCTTTCCCCACTTCGGTCCTTCAAGTTCTCATTGAAGTATTTGC
TACTACCACCAAGATCTGCACTAGAGGCTGTTCTACCCGGGCTCACGCCCT
AGGCTTCGTCACTAACCTCCACGGCCGCCTACTCCTTAGGGCATCGTTTCTA
CCCTAAGGGCGAGGTATGGGTGAGACGCTTGAGCGCCATCCATTTTCAGGG
CTAGTACATTCGGCAGGTGAGTTGTTACACAGTCCTTAGCGGATTCCGACTT
CCATGGCCACCGTCCTGCTGTCAAGATGTACTAACACCTTTTGTGGTGTCTG
ATGAGCGTCCACTCTGGCACCTTAACCCCGCGTTCGGTTCATCCCGCATCG
CCAGTTCTGCTTACCAAAAATGGCCCACTAGTGTTGGTACATTCGAATGCCC
ACGTTCAACTAAGTAACAAGGGCTTCTTACATATTTAAAGTTTGAGAATGG
ATGAAGGCAATATAGCGCCCCCGAGTCCCTAATCATTCGCTTTACCTCATAA
AACTGAAGACAACACTGCTATCCTGAGGGAAACTTCGGCGGTAACCAGCT
ACTAGATGGTTCGATAAGTCTTTCGCCCCCATGCGCAGATTTGACGATCGAT
TTGCACGTCAGAACCGCTGCGAGCCTCCACCAGAGTTTCCTCTGGCTTCAC
CCTATCCACGCATAGTTCACCATCTTTCGGGTCCGAACAGTTATGCTCTTAC
TCAAATCCATCCGAAGACATCAGGATCGGTCGATGATGCACCCTAAGGCTC
TCACCTCCGTTCACTTTCATTACGCGTGAGGGTTTAACACCCAAACACTCG
CACAAATGTTCGACTCCTTGGTCCGTGTTTTCAAGACGGGTCGTTGACGAC
CATTACGCCAGCATCCTTGCCGAAGCGCGGTCCTCGGTCCCTGAAGGGTAT
TACGCTAGGGGCTATAACACTCCCTAAAGAGCTACGTTCCCAGGCCTTTATC
CCCCTAAGAGAACCGATGCTGGCCTAAACCAGGGGGAGTGCACCGCTGAG

AACAGCGGTTGATCCCCCTGGAGAAGGTCTGGTCGCAAACGCTTCCCTTTC
AACAATTTCACGTGCTATTTAACCCTCTTTTCAAAGTGCTTTTCATCTTTCG
ATCACTCTACTTGTGCGCTATCGGTCTCTGGCCGGTATTTAGCTTTAGAAGA
AATTTACCTCCCATTTAGAGCAGCATTCCCAAACTACTCGACTCGTCGAAG
GAGCTATATAGAGGCTAGGTATCCAACCGTACGGGGCTCTCACCCTCTATGG
CGTCCCGTTCCAGGGAACTCGGAAGGCACCGCACCAAAAGCATCCTCTGC
AAATTACAACTCGGACCGCTAGGGCCAGATTTCAAATTTGAGCTTTGCCGC
TTCACTCGCCGTTACTAGGGCAATCCCTGTTGGTTTCTTTTCCTCCGCTTATT
GATATGCTTAAGTTCAGCGGGTATTCCTACCTAATCCGAGGTCAACCACTAT
AAGTTATAGGGGGTTTTACGGCAAGCAGCTACAGCCTCCCTGAAAGCGAGT
TAAACTACTACGCTTAGAGTGTGCTATAACCCCGCCACTAAATTTAAGGAAC
TACCCCTCAGTGAGGGGTAGATTCCCAACGCTAAGCTACAAGGCTTAAGGG
TTGTAATGACGCTCGAATAGGCATGCCCACTAGAATACTAATGGGCGCAATG
TGCGTTCAAAGATTCGATGATTCACTGAATTCTGCAATTCACATTACTTATC
GCATTTCGCTGCGTTCTTCATCGATGCCAGAACCAAGAGATCCGTTGTTGA
AAGTTTTAACGTATTTGGTTTTTAAAACCTTGGTTTGATCACCCCCTAAGAA
CCGTAGTTTATAGGTGGCTTTTGCCAATTTTATGAATTCATTCAGACCCCTCA
GAGCTTTGAACAATTTTCGAGCAGATATTGTAGCTTGAAAACAAACGACTG
CAGGGTAAAGAAATTATTATTTCGAGCAATAATTCCAACCGGGAAAGACGT
TTTAGCGTAAAGAAAAAAAGCAAAAGCAGATTTTCGATAGTTGTTCAACGC
GGGGTTGGATGCACTAGGTGCTTAAGTTGGAGCGGTCCTACTCGGCGCCGC
CGAGGCCTTGGTCAGCACCTTCACTGCACTAACCGTACCCCGAAGGATAGG
TAGCACTCAGGCACCTCCCCAGCAGAACCCCCCCTTAGGGAGCCCTGTGG
GTAGGCTAAAGCGGTACGCCGGAGGAAACATAGGTAGGTTCACAAAGGGT
TGGAGTTTTTGATAACTCAGTAATGATCCCTCCGCTGGTTCACCAACGGAG
ACCTTGTTACGACTTTTACTTCCTCTAAATGACCGAGTTTGGATAACTTTCC
GGCCCCGGGGTGTCGTTGCCGACTCCCCTAGGCCAGTCCGAAAGCCTCAC
TGAGCCATTCAATCGGTAGTAGCGACGGGCGGTGTGTACAAAGGGCAGGG
ACGTAATCAACGCAAGTTGATGACTTGCGCTTACTAGGGATTCCTCGTTGA
AGAGCAATAATTGCAATGCTCTATCCCCAGCACGACAGAGTTTAACAAGAT
TACCCGGGTATTTCTACCAAGGAAGTACTCGCTGGCTCTGTCAGTGTAAAC
GCGCGTGCGGCCCAGAACATCTAAGGGCATCACAGACCTGTTATTGCCTCA
AACTTCCATCCGCTTGAGCGGATAGTCCCTCTAAGAAGCCAGCGTACTGCC
AAAGCAATACGGGCTATTTAGCAGGTTAAGGTCTCGTTCGTTATCGCAATTA
AGCAGACAAATCACTCCACCAACTAAGAACGGCCATGCACCACCACCCAC
AAAATCAAGAAAGAGCTCTCAATCTGTCAATCCTCATTGTGTCTGGACCTG
GTGAGTTTCCCCGTGTTGAGTCAAATTAAGCCGCAGGCTCCCTATAGAGCG
TTGCTAGGCGGTACTTTCGTACGCCGATGGACTTTATCTTAGGCTGCGAGGA

GCACTGCCGTTATAGCGGTACTCCCCCGTAGCCCACCCCTATCAAGTCTCTG
AACCTTCTCCGGCACGTGGCTACGGAGCTTGGCTGCGGATCGTCCCAGGGC
CTGCGTTCTTCCCCTACCGAAGCTGGTTAGGCTTCGCCATACGCTCTATTTC
TAGGCAGTACTTGGGAGCAGACCCTTTTAACAGGAGGTCCCCGCAATTTAA
GGGTGTCGCCGGGCCCTATCACAGGGTAACCGACTAGCGAGCTATAGGGCA
CCACCGCGTCGACTGCAGAGAGTCGACTCACTATTCAGGGCAGAGACTGC
GTGTAGTAACAGCTGCGTTAACCCCTGGGTGGTGCCCTTCCGTCAATTTCTT
TAAGTTTCAGCCTTGCGACCATACTCCCCCCAGAACCCAAAAACTTTGATT
TCTCGTAAGGTGCCGAACGAGTCAAAAAATAACATCGTCCGATCCCTAGTC
GGCATAGTTTATGGTTAAGACTACGACGGTATCTGATCGTCTTCGATCCCCT
AACTTTCGTTCCTGATTAATGAAAACATCCTTGGCAAATGCTTTCGCAGTAG
TTAGTCTTCAATAAATCCAAGAATTTCACCTCTGACAATTGAATACTGATGC
CCCCGACTGTCCCTATTAATCATTACGGCGGTCCTAGAAACCAACAAAATAG
AACCGCACGTCCTATTCTATTATTCCATGCTGATGTATTCGAGCATAGGCCTG
CTTTGAACACTCTAATTTTTTCACAGTAAAAGTCCTGGTTCCCCAGCACAC
CCAGTGAAGGGCATGAGGTTCCCCAGAGGGAAAGGCCCGGCCGAACCAG
TACACGCGGTGAGGCGGACCAGCCAGCCAGGCCCAAGGTTCAACTACGAG
CTTTTTAACTGCAACAACTTTAATATACGCTATTGGAGCTGGAATTACCGCG
GCTGCTGGCACCAGACTTGCCCTCCAATTGTTCCTCGTTAAGGATTTAAATT
GTACTCATTCCAATTACAAGACCCAAAAGAGCCCTGTATCAGTATTTATCGT
CACTACCTCTCCGTATCGGAATTGGGTAATTTGCGCGCCTGCTGCCTTCCTT
GGATGTAGTAGCCGTTTCTCAGGCTCCTTCTCCGGGGTCGAGCCCTAACCC
TCCGTTACCCGTTGTAACCATGGCAGGCCAAGACCCTGCCATCGAAAGTTG
ATAGGGCAGAAATTTGAATGAACCATCGCCAGCTCAAGGCCATGCGATTCG
AGAAGTTATTATGAATCACCAGAGAGCCCCGAAGGGCATTGGTTTTAATCT
AATAAATACATCCCTCCGTAAGTCGGGATTTTTAGCATGTATTAGCTCTAGAA
TTACCACGGTTATCCAAGTAGTAAGGTACTATCAAATAAACGATAACTGATT
TAAATGAGCCATTCGCAGTTTCGCAGTATAAATTGCTTATACTTAGACATGC
ATGGCTTAATCTTTGAGACAAGCATATGACTACTGGCAGAATCAACCAGGT
AACTATCGTACGCCGGGGTTGGCCAACCGCGCCTTGCGGCGCGGAAGAAT
TAGTACCGGTTTACACCACTGAAGGTGTGGCGCTCCAGCGGTCAAGGCTAG
GCCTATCACGCCGGGGGACGCTTCCGCTTTTCCCCTCTAAATTCCCCAGGG
CACTGGTCCGCCACGCCGTAGAGGCGAGGCTTACAGGCCCCAAGAGGAGA
CCTAGGCCCCGCGGGCGCCTTAAGGCTGCCGCGAGGTAGGTATCTCCCGAT
GCTTAGTGGTATGCTTAGCCAAAGGCCAGCAACCCACAGAGCCCAACCAG
CAGCGCTAAGGCGAAACGAGACGCCTAAGCACCGCTGTGCATCCTGGATA
GATGCGCCGAAGCCGCTAAAGCTTCAGCGCCCCAGGGGGGTCGATAACTG
CTATGAATCCCCCAGTACCGAGCGGAGCTTCTAGGTTGCACAAAGGACCAC

GCAACAGTTATCATGCTAATAAGAGACAATCGTTTTGGGGTAGGCACCGTC
TAGCGGTGACTCCCCCTAACCCAAGACCTACTGGCGCAGCTCTCAGCGGTC
GAGATAGCGGCAACCTCAGCCCGTCAGCCCGCTGAACGAGCCACTATAACT
ACGCTACCGTACCAGCGGCAGGCAGACGCTACAGGTGGTAGCACCCCTAC
ACTTAGCGCTCATTACTGCAAACCGGCCGAATGCAACCGCGCCGTCTACTG
GGCAAATACATGCCTCCCGCAAGACAGCTTAGTTATAACGCACACAGCTCC
AGCTACAGCCAGCCGGCCGAATACACGGCCCCGACTGCTAACGCCCGTAG
AGATGTTACCTAGCAGGCCATGAGAACTTCCTTCCGCGGCCAGCATTACCT
GTCGCGTAGCGAAAGCCTCAGCCACCTTACCCCCCGCTGCCAGCACTACCT
GTAGCAGGTGGGTAAGGCGGCCATCCCCCCCCGCTGCCAGCATTACCTGTA
GCAGGTAGAGGATGAGACCTACCTTCAAATAGCGCCCACTAAACCCACTGA
GCACGATTAGCGCAATTGCAGCAGCCCGCCAGCGGCCATCAGGGGACTTA
CAGGCTCCGAAGCTGCGCGCCCGGTGAAGAGGGAGCTTCATAGAGCCTGT
AAGCTGCTTTGAGTGAGCGCTAAGCCGAACCGTCAATCGCTAAAACCAATC
GGACCTTGGAGGCCGGGGCGTACTTTTCAGCAAACGCCTCAGCCTACGCA
GGCGATTAGGCATACCTTTCGGCAAATGCCATAACCATAGGCCTAATATAGG
GGCGACCTCAGCATACTTTGCAGCAAATGCCAAGGTCTTGCGGCCTATTGC
TAGGTCGCCTACCTAATTGTGTTTTGAAGGCAATTAGGCAGAGGAAGCGGA
GCAGCCAACACTTTTCCTTTCAGATCTGTGCTGGCCGCCCACTTCCTCATGT
ACCCACCGGACCCACTTGGGTCCCAATCTAGGTCTAGAGCGTTCTGTGCCC
CAAAACATTTTCCGAAAAACTAATTTGTATGCGAAATTTTAGAAATCGACTC
ACAACCATACAAACCAATAACCGTCCTGCACTTTTTTTTTGGGACTTGGCG
AAAAAAAGTTCAACTCGGCCCAGCCAAAATTGAAGCCCCCCCACCCACT
AAACCACCTACGCCGCGACCACCTACAGGGCATTACAGGGCACTTAGCATA
CCACCACCCACTAACCCAAACCCCCTAAATCCACTAAACTCCCTATAAACC
CCCCAAAGCAAAAATTTTTAGGGGTGCCCGGCACACCCATTCGAGCACTGT
TAGCGGGGCCTAAGGGTCAACTCCCCTACAACCCCCCGTATCTCGAGCGCT
ATTGCGTGCACCGAGACGAGACCTATACCGTTCGATAGCGCTCGTCAAGGC
GGTTCGATTGGTATAGGTTTCGTTTGGGTGCATTGCCTACTCGAGTTTTCGG
GCGGGTGCGAAGTTATGCTGCAGGGTGAGGTCACGTGACCCGGGCTTGTA
CTGGGCATTTTTTTCTCTGGCCCGTACCCCGCGCGGGGTGATATCTTTAGGG
TAGGTTCAGGGTAGGCCCCGCGGCACTTGATTTCCTAAGGGTATTTATTTAT
TTTACCTCTTGTATCTAAGCCGTTATTTGACGTATCTTCTTATATTACATTACA
TTTATATATTATGTTTATTTAATCGTATATTTTATTTTCATATATCATATTTATATA
TTATACTTATATGTCATATTTATTTAATCATATATTTTATTTTCACATATATATTTT
TTAAAGATATCATGTTTTCATATTATACCATTTTATTTTAATTAATTAGTTATAA
CCTGTTTAGTTCTTTGTTTTTTTTTGTTTTTTTTTTGTCGTCTTCTTTTTTTAA
TAAAAAAATAAATTATAACCATTCATTTATGTATTATAATATTTTATTACACATA

CTTGCACAAATATTACATTTGATATGTTATGTTATTTTTAAGTTTTTATAGTTA
AAAATAATATATTTATTATTTCATACATATATACTTGTTATTTAATCCCTGTAAA
GTCGTCTTGGGCAATGGTGAAATTTTTCTTTTTAATTCATCTGTTGATTTAAA
ATTGGATAAGTACTTATATAATATAACTTCAGGAACGTGGCGTGAGAAGATG
ATTTATTACGATTTTTTCTTTTGAAAACGTCAATCATTCCATTATTTCTCACCA
ATTTTATATACTTATATTTTCTTTTTATTTTATTATCTATCTACAACTATAACCCT
ACTATCATCTCCGTACTATACTAATTATATCCTTAACCTATACCCCTTAAGCCC
TTAAAACACATAATACCACCCATTATAATATAACCTACCCTACCCTATACATAT
TTCGGAAAGTCGACTACTATAGGGTAGCTTCTATAGGGTAGCTCCCACCAG
ACACATACAATAAATCTCATAAATCCCAGAGGATCCTACCCTATAACCACCC
TGTAGCCACTCTATAGACGCCCCGACTACCCTGTAGTTACCCTGCAGACTTG
CCCGGACTATAGGGTAGTTCAGGGTAGCTCTTCTACAGGGTAACCCCACGG
CCTTACAGCGCCTTACAGCACCCTCCGTCCACTAAATGCCCTGTAGCCCCCT
ATAAGACCCCCCTAAAACCGCTGCCCCCTAAAAGGCCTAAAGCCAACCCG
CCGATTTCCCCGCGTCTCGCCCAACCTACCCTAAAGCGAGGAGCTGGCTCC
TTCCAGGCGGCTCGGCCGGGTACAGCCCCCACCCCCCCACAGCGGGGAGC
TAGCACCCCCCTATAAGTACCTACCAGGCCCGGCTGGGCTGCTATCGTCCCT
TGGAGGGTTCTACGCCACGGGGACATTTCCCCGAAAAACACCTCCCTAAA
CCACGTATAGCCCCCTACCTGTATTTTTCACCTCGCTGACGCCTATAGCGGA
TTATAGCGGCCTCGGCGCCGTGTCGTCGCGGGCGGGCGGACCTACCCTGAA
AGAGCGTGCTCGCCAGGAATTTACAGGGTAGGTTCGCCAGGCGGTGGTTA
CCCTATATTCCGGGACGTGTCGCCTTCTGTAGGGTAGCTCCGGCCGGTGTTG
GCTCGCGGAGATGACCGAAAGAGGCCTGCCCTGAAGGGCAGTTCGCATGC
GGGCGTGTACAGGGTAACTCCGCCGGGGCTCGGGATGCGTTACGACAGTC
TCCGAGTGCACCTACCCTAAACTTACCCTATAAATATAACACTTGAAGACAA
GACGCAAGTTGAAGAGATGGGACGGGTTACAAGTCCGAAGAGCCCTACCC
TGTACTACAACATATTAAAAAAAAAAGGACGGCAAGGATGTTTTATTGAAG
TTTAAACACCAAACCCACTAAGCCCTCTTTGATAGGCCCCCCTAACGCTTC
ACCTTTACAGGTACTTACGTCCACTAATCTCTTATAAGCCCAACTATAAGTTA
GGCGGTATATGAGAAATCTAGGCAAAATAGGGCTTACCCTCAGCAGATCGT
AACAACAAGGCTACTCTACTGCTTACAGGACCCTTTTGCACAGCTAAGTCG
TCTGCAAAGGATTTAATCCCACGCGTGTTTAAAATTACAATACGCGAAATAC
TACCGAACCACTTCCGGCTCGATAGAAAATTCGCCAGCTAAGATGCGAGGC
CGAAGCCTATTCGTATCCGTCTACAACGTGTGGGAGGTATCACCGCGTTCTG
GCATGGATTCTGACTTAGAGGCGTTCAGCCATTATCCAGCAGATGGTAGCGT
CGCGGCACTGCCCGGTCGGACAGCCGCAAAAACCAATTATCTGAATCAGC
GGTTCCTCTCGTACTAAGCTGAATTACCATTGCGGTGAGGTCATCAGTAGG
GTAAAACTAACCTGTCTCACGACGGTCTAAACCCAGCTCACGTTCCCTATT

AGTGGGTGAACAATCCAACGCTTACCGAATTCTGCTTCGGTATGATAGGAA
GAGCCGACATCGAAGGATCAAAAAGCGACGTCGCTATGAACGCTTGGCCG
CCACAAGCCAGTTCCTGTATACCCACGACGCGCTGAAGCGTGCCGCTCCGA
CAGCTGGGGGTATAACGCCATACCCTTAACCGCCGGCGCCTTCGCAGGCGG
GAGTAGCCCGTACCTTAAGCGGCCGCCCCCGTATTTTTAGGGGCTGCCACC
AACACCTTAGCGAGCGTTGAACCGCTGAGCCCTTGCACCCGTCCGTACGGT
GAGTTTGGCCCAGTCGAGCTGCGGATTACCGCTTACCTTGCCGGATTGTTA
CCCGCAGTAGCAAACTGCTTAGCTTAGGGGGGTTATCCCCTAAGGTTGGTA
CCGACAGGCTTTACGGCTTCCCCGCATCCAGGTGTTTCGCCCATCTCAGGC
CCCTAATCCGGGGGATTTAGGGGGCCTAAGCGCCGTTACCTAGCGCACTAC
CCGCGTTATCACAGTGGGTGGTAGTGGTGGACTAGGGGCGGATTGCTGCCG
CCGCCCCTGGGACTGATTAATCCCTGTGGTAACTTTTCTGGCACCTCTAGCC
TCAAATTTCGAGGGACTAAAGGATCGATAGGCCACACTTTCATGGTTTGTAT
TCACACTGAAAATCAAAAATCAAGGGGACTTTTACCCTTTTGTTCTACTGG
AGATTTCTGTTCTCCATGAGCCCCCCTTAGGACACCTGCGTTATGGTTTAAC
AGATGTGCCGCCCCAGCCAAACTCCCCACCTGACAATGTCTTCAACCCGGG
TCGGCCCGCGAAGGACCTTAAAACCAGAAGTTGGACGAAAGTCCAGCCCC
GCTTCATTGAATAAGTAAAAAAACAATAGGAGTAGTGGTATTTCACCGGCG
CCGAAGCTCCCACCTATTCTACACCTCCTATGTCTTTTCACAATGTCAAACT
AGAGTCAAGCTCAACCATTTTAACCGCTAGCCACTGAAAGGGAGTTGTTGT
TTTTGGTTTTTAATGTATAATAAATCAAATAAACCCCCGAATGGCAGCAGAT
GACCAGTCTTTTAGACCCCGCTATGCTTTGTGCTATAGGCACTCCACACAAC
AGCAGGCTTTCGCCTGGGATTAGCACGTACCTTAAGCCAGCGCTGTAAATA
CAGCGCTAACCGACACCTTTCCGCGCATTACGACGGCCGCTACAGCAGCTC
GCCACGGGATTGCCGTAGGGGGTTACAGCGCTGTAAGCACTTATAAGCCCC
CCCGTAGGGTTCCCCCGTACCAGGTTGTCTCGCCCGCGGCCACCTATAGATA
TTGGCCGCTGGACTAACAGCGCTATTAGGCTGCTATCGACCCCGTTGAGGT
TGCTCAGGGTCTTCTTTCCCCGCTGATTCTGCCAAGCCCGTTCCCTTGGCTG
TGGTTTCGCTAGATAGTAGATAGGGACAGTGGGAATCTCGTTAATCCATTCA
TGCGCGTCACTAATTAGATGACGAGGCATTTGGCTACCTTAAGAGCATTGCT
TCGCCCCCGAAGCTTTCGCAGGGGGATCAGACTATATCTTAAGCCCTGCTAT
ACAGCAAGGCCACCGCCGTCTAGTCGTTGAACTGCATCCTCCCTACAAGGT
TGTTACAGGGTAATACCCCCCGTAACTCCCCTACAGGGTTAGCGGACTTGG
CTGCGGATTTTCCATTGTAGTATCCTGCTGCCAGTGTTACCGTACCCGGAGC
TATTAACTCGGCCACAGGTGGCTTTCGCCACCTGCTTGGTCGCAGCAGGCT
TTAGGAGGTCCCCGCAATTTGACGATGTTACAAGGCGTCAGGCGCTAACCT
GAACCCCATTAGCGCTTCTTTCAGCACTTGACCCTCTGTGCAGCCCTAGAA
CAGCGCTGTATTTACAGCGGCCGTCCCCTGAGACTGCAGGTAGTTTGAGTC

ATAGTTACTCCCGCCGTTTACCCGCGCTTGGTTGAATTTCTTCACTTTGACA
TTCAGAGCACTGGGCAGAAATCACATTGCGTCAACATCACTTTCTGACCAT
CGCAATGCTATGTTTTAATTAGACAGTCAGATTCCCCCTGTCCGTACCAGTT
CTAAGTTGGTTGTTAACCGTACGCCGGACGGCCGAAGCCTGCCAAAGGCG
TCAGGACCCAGGTGCTGAAAGCCGCCGGCCGGTTGCCCGGCTAGTGGCGA
TCTGCTCCCGAGCCGTCCACCAAAGGCCCAACGTACCCAACCCTTAGAGC
CAATCCTTTTCCCGAAGTTACGGATCTATTTTGCCGACTTCCCTTATCTACAT
TGTTCTATCAACCAGAGGCTGTTCACCTTGGAGACCTGCTGCGGTTATGAG
TACGACCTGGCCTGAGAATTATTCCCTCCAGCGGATTTTCAAGGGACGTCG
AGGGCGCACCAGACCCAACAGAGGTGTTGGGCTCTTCCGGCCGTTAAACC
CTAGCTCCGGACAAACCGATTTCAGGGTGATAGACCGTTAAGAAGAAAAG
AGAACTCTGCCCAGGGCCCCCGCCGTCGTCTCCGCTTTCAGTTGCGTTGCC
GCGTAAAACCCAGATCCAGGCGCCGGAATATTGACCGGCTTCCCCTTCGCC
ACACGGGGCACAAGTGCCCACTTTGAAACGGAACTTCCTATGGCTTAGGAT
CGGCTAACCCACGTCCAACCGCTGTTCACATGGAACCTTTCCCCACTTCGG
TCCTTCAAGTTCTCATTGAAGTATTTGCTACTACCACCAAGATCTGCACTAG
AGGCTGTTCTACCCGGGCTCACGCCCTAGGCTTCGTCACTAACCTCCACGG
CCGCCTACTCCTTAGGGCATCGTTTCTACCCTAAGGGCGAGGTATGGGTGA
GACGCTTGAGCGCCATCCATTTTCAGGGCTAGTACATTCGGCAGGTGAGTT
GTTACACAGTCCTTAGCGGATTCCGACTTCCATGGCCACCGTCCTGCTGTC
AAGATGTACTAACACCTTTTGTGGTGTCTGATGAGCGTCCACTCTGGCACC
TTAACCCCGCGTTCGGTTCATCCCGCATCGCCAGTTCTGCTTACCAAAATGG
CCCACTAGTGTTGGTACATTCGAATGCCCACGTTCAACTAAGTAACAAGGG
CTTCTTACATATTTAAAGTTTGAGAATGGATGAAGGCAATATAGCGCCCCCG
AGTCCCTAATCATTCGCTTTACCTCATAAAACTGAAGACAACACTGCTATCC
TGAGGGAAACTTCGGCGGTAACCAGCTACTAGATGGTTTCGATAAGTCTTT
CGCCCCCATGCGCAGATTTGACGATCGATTTGCACGTCAGAACCGCTGCGA
GCCTCCACCAGAGTTTCCTCTGGCTTCACCCTATCCACGCATAGTTCACCAT
CTTTCGGGTCCGAACAGTTATGCTCTTACTCAAATCCATCCGAAGACATCAG
GATCGGTCGATGATGCACCCTAAGGCTCTCACCTCCGTTCACTTTCATTACG
CGTGAGGGTTTAACACCCAAACACTCGCACAAATGTTCGACTCCTTGGTCC
GTGTTTCAAGACGGGTCGTTGACGACCATTACGCCAGCATCCTTGCCGAAG
CGCGGTCCTCGGTCCCCTGAAGGGTATTACGCTAGGGGCTATAACACTCCC
TAAAGAGCTACGTTCCCCAGGCCTTTATCCCCCTAAGAGAACCGATGCTGG
CCTAAACCAGGGGGAGTGCACCGCTGAGAACAGCGGTTGATCCCCCTGGA
GAAGGTCTGGTCGCAAACGCCTTCCCTTTCAACAATTTCACGTGCTATTTA
ACCCTCTTTTCAAAGTGCTTTTCATCTTTCGATCACTCTACTTGTGCGCTATC
GGTCTCTGGCCGGTATTTAGCTTTAGAAGAAATTACCTCCCATTTAGAGCA

GCATTCCCAAACTACTCGACTCGTCGAAGGAGCTATATAGAGGCTAGGTATC
CAACCGTACGGGGCTCTCACCCTCTATGGCGTCCCGTTCCAGGGAACTCGG
AAGGCACCGCACCAAAAGCATCCTCTGCAAATTACAACTCGGACCGCTAG
GGCCAGATTTCAAATTTGAGCTTTTGCCGCTTCACTCGCCGTTACTAGGGC
AATCCCTGTTGGTTTCTTTTCCTCCGCTTATTGATATGCTTAAGTTCAGCGGG
TATTCCTACCTAATCCGAGGTCAACCACTATAAGTTATAGGGGGTTTTACGG
CAAGCAGCTACAGCCTCCCTGAAAGCGAGTTAAACTACTACGCTTAGAGTG
TGCTATAACCCCGCCACTAAATTTAAGGAACTACCCCTCAGTGAGGGGTAG
ATTCCCAACGCTAAGCTACAAGGCTTAAGGGTTGTAATGACGCTCGAATAG
GCATGCCCACTAGAATACTAATGGGCGCAATGTGCGTCAAAGATTCGATGAT
TCACTGAATTCTGCAATTCACATTACTTATCGCATTTCGCTGCGTTCTTCATC
GATGCCAGAACCAAGAGATCCGTTGTTGAAAGTTTTAACGTATTTGGTTTTT
AAAACCTTGGTTTGATCACCCCCTAAGAACCGTAGTTTATAGGTGGCTTTTG
CCAATTTTTATGAATTCATTCAGACCCCTCAGAGCTTTGAACAATTTTCGAG
CAGATATTGTAGCTTGAAAACAAACGACTGCAGGGTAAAGAAATTATTATTT
CGAGCAATAATTCCAACCGGGAAAGACGTTTTAGCGTAAAGAAAAAAAGC
AAAAGCAGATTTTCGATAGTTGTTCAACGCGGGGTTGGATGCACTAGGTGC
TTAAGTTTGGAGCGGTCCTACTCGGCGCCGCCGAGGCCTTGGTCAGCACCT
TCACTGCACTAACCGTACCCCGAAGGATAGGTAGCACTCAGGCACCTCCCC
AGCAGAACCCCCCCTTAGGGGAGCCCTGTGGGTAGGCTAAAGCGGTACGC
CGGAGGAAACATAGGTAGGTTCACAAAGGGTTGGAGTTTTTGATAACTCAG
TAATGATCCCTCCGCTGGTTCACCAACGGAGACCTTGTTACGACTTTTACTT
CCTCTAAATGACCGAGTTTGGATAACTTTCCGGCCCCGGGGTGTCGTTGCC
GACTCCCCTAGGCCAGTCCGAAAGCCTCACTGAGCCATTCAATCGGTAGTA
GCGACGGGCGGTGTGTACAAAGGGCAGGGACGTAATCAACGCAAGTTGAT
GACTTGCGCTTACTAGGGATTCCTCGTTGAAGAGCAATAATTGCAATGCTCT
ATCCCCAGCACGACAGAGTTTAACAAGATTACCCGGGTATTTCTACCAAGG
AAGTACTCGCTGGCTCTGTCAGTGTAACGCGCGTGCGGCCCAGAACATCTA
AGGGCATCACAGACCTGTTATTGCCTCAAACTTCCATCCCGCTTGAGCGGA
TAGTCCCTCTAAGAAGCCAGCGTACTGCCAAAGCAATACGGGCTATTTAGC
AGGTTAAGGTCTCGTTCGTTATCGCAATTAAGCAGACAAATCACTCCACCA
ACTAAGAACGGCCATGCACCACCACCCACAAAATCAAGAAAGAGCTCTCA
ATCTGTCAATCCTCATTGTGTCTGGACCTGGTGAGTTTCCCCGTGTTGAGTC
AAATTAAGCCGCAGGCTCCCTATAGAGCGTTGCTAGGCGGTACTTTCGTAC
GCCGATGGACTTTATCTTAGGCTGCGAGGAGCACTGCCGTTATAGCGGTACT
CCCCCGTAGCCCACCCCTATCAAGTCTCTGAACCTTCTCCGGCACGTGGCT
ACGGAGCTTGGCTGCGGATCGTCCCAGGGCCTGCGTTCTTCCCCTACCGAA
GCTGGTTAGGCTTCGCCATACGCTCTATTTCTAGGCAGTACTTGGGAGCAGA

CCCTTTTAACAGGAGGTCCCCGCAATTTAAGGGTGTCGCCGGGCCCTATCA
CAGGGTAACCGACTAGCGAGCTATAGGGCACCACCGCGTCGACTGCAGAG
AGTCGACTCACTATTCAGGGCAGAGACTGCGTGTAGTAACAGCTGCGTTAA
CCCCTGGTGGTGCCCTTCCGTCAATTTCTTTAAGTTTCAGCCTTGCGACCAT
ACTCCCCCCAGAACCCAAAAACTTTGATTTCTCGTAAGGTGCCGAACGAGT
CAAAAAATAACATCGTCCGATCCCTAGTCGGCATAGTTTATGGTTAAGACTA
CGACGGTATCTGATCGTCTTCGATCCCCTAACTTTCGTTCCTGATTAATGAA
AACATCCTTGGCAAATGCTTTCGCAGTAGTTAGTCTTCAATAAATCCAAGAA
TTTCACCTCTGACAATTGAATACTGATGCCCCCGACTGTCCCTATTAATCATT
ACGGCGGTCCTAGAAACCAACAAAATAGAACCGCACGTCCTATTCTATTATT
CCATGCTGATGTATTCGAGCATAGGCCTGCTTTGAACACTCTAATTTTTTCA
CAGTAAAAGTCCTGGTTCCCCAGCACACCCAGTGAAGGGCATGAGGTTCC
CCAGAGGGAAAGGCCCGGCCGAACCAGTACACGCGGTGAGGCGGACCAG
CCAGCCAGGCCCAAGGTTCAACTACGAGCTTTTTAACTGCAACAACTTTAA
TATACGCTATTGGAGCTGGAATTACCGCGGCTGCTGGCACCAGACTTGCCC
TCCAATTGTTCCTCGTTAAGGGATTTAAATTGTACTCATTCCAATTACAAGA
CCCAAAAGAGCCCTGTATCAGTATTTATCGTCACTACCTCTCCGTATCGGAA
TTGGGTAATTTGCGCGCCTGCTGCCTTCCTTGGATGTAGTAGCCGTTTCTCA
GGCTCCTTCTCCGGGGTCGAGCCCTAACCCTCCGTTACCCGTTGTAACCAT
GGCAGGCCAAGACCCTGCCATCGAAAGTTGATAGGGCAGAAATTTGAATG
AACCATCGCCAGCTCAAGGCCATGCGATTCGAGAAGTTATTATGAATCACC
AGAGAGCCCCGAAGGGCATTGGTTTTTAATCTAATAAATACATCCCTCCGTA
AGTCGGGATTTTTAGCATGTATTAGCTCTAGAATTACCACGGTTATCCAAGT
AGTAAGGTACTATCAAATAAACGATAACTGATTTAATGAGCCATTCGCAGTT
TCGCAGTATAAATTGCTTATACTTAGACATGCATGGCTTAATCTTTGAGACA
AGCATATGACTACTGGCAGAATCAACCAGGTAACTATCGTACGCCGGGGTT
GGCCAACCGCGCCTTGCGGCGCGGAAGAATTAGTACCGGTTTACCACCACT
GAAGGTGTGGCGCTCCAGCGGTCAAGGCTAGGCCTATCACGCCGGGGGAC
GCCTTCCGCTTTTCCCCTCTAAATTCCCCAGGGCACTGGTCCGCCACGCCG
TAGAGGCGAGGCTTACAGGCCCCAAGAGGAGACCTAGGCCCCGCGGGCGC
CTTAAGGCTGCCGCGAGGTAGGTATCTCCCGATGCTTAGTGGTATGCTTAGC
CAAAGGCCAGCAACCCCACAGAGCCCAACCAGCAGCGCTAAGGCGAAAC
GAGACGCCTAAGCACCGCTGTGCATCCTGGATAGATGCGCCGAAGCCGCTA
AAGCTTCAGCGCCCCAGGGGGGTCGATAACTGCTATGAATCCCCCAGTACC
GAGCGGAGCTTCTAGGTTGCACAAAGGACCACGCAACAGTTATCATGCTAA
TAAGAGACAATCGTTTTGGGGTAGGCACCGTCTAGCGGTGACTCCCCCTAA
CCCAAGACCTACTGGCGCAGCTCTCAGCGGTCGAGATAGCGGCAACCTCA
GCCCGTCAGCCCGCTGAACGAGCCACTATAACTACGCTACCGTACCCAGCG

GCAGGCAGACGCTACAGGTGGTAGCACCCCTACACTTAGCGCTCATTACTG
CAAACCGGCCGAATGCAACCGCGCCGTCTACTGGGCAAATACATGCCTCCC
GCAAGACAGCTTAGTTATAACGCACACAGCTCCAGCTACAGCCAGCCGGC
CGAATACACGGCCCCGACTGCTAACGCCCGTAGAGATGTTACCTAGCAGGC
CATGAGAACTTCCTTCCGCGGCCAGCATTACCTGTCGCGTAGCGAAAGCCT
CAGCCACCTTACCCCCCGCTGCCAGCACTACCTGTAGCAGGTGGTAAGGCG
GCCATCCCCCCCCGCTGCCAGCATTACCTGTAGCAGGTAGAGATGAGACCT
ACCTTCAAATAGCGCCCACTAAACCCACTGAGCACGATTAGCGCAATTGCA
GCAGCCCGCCAGCGGCCATCAGGGGACTTACAGGCTCCGAAGCTGCGCGC
CCGGTGAAGAGGGAGCTTCATAGAGCCTGTAAGCTGCTTTGAGTGAGCGC
TAAGCCGAACCGTCAATCGCTAAAACCAATCGGACCTTGGAGGCCGGGGC
GTACTTTTCAGCAAACGCCTCAGCCTACGCAGGCGATTAGGCATACCTTTC
GGCAAATGCCATAACCATAGGCCTAATATAGGGGCGACCTCAGCATACTTTG
CAGCAAATGCCAAGGTCTTGCGGCCTATTGCTAGGTCGCCTACCTAATTGTG
TTTTGAAGGCAATTAGGCAGAGGAAGCGGAGCAGCCAACACTTTTCCTTTC
AGATCTGTGCTGGCCGCCCACTTCCTCATGTACCCACCGGACCCACTTGGG
TCCCAATCTAGGTCTAGAGCGTTCTGTGCCCCCAAAACATTTTCCGAAAAA
CTAATTTGTATGCGAAATTTTAGAAATCGACTCACAACCATACAAACCAATA
ACCGTCCTGCACTTTTTTTTGGGACTTGGCGAAA

>Mosaic_sequence_69

GATGATTTGCTCCGAGTCAGCTCCTCCACACACTGACGCTCAAGAATATCT
GGGATATGGCTCACCAGGAAATCATGCTCGGCATATTCGAACACCATGTAG
ATGGCCTTGTCCTCCAGTATAACCTCGCGCAGCGCGGTGAGGTTCCGATGG
TGCAGCTCGCGATTCAGCTGCGCGTCAGCCCCCCTCCCCTCGCCAACCACC
AAGTGGCCCAGTGGACCCGCCATGATCTCGCGCGCGCCGCTCTGGCTGATC
CCGGCGTACGTCTGCACGTCGCCTCCTTGTCCGGCTTGAACTTCTTGATGG
CAAACACGTCGCCCTCCTTGGCTTTGCGGTCCCCGGGCCGCATCCTCAGCT
CGGGGTTGTTGAGCGGGTCGTCCGTCCGGCCCCTACTGCGCCCGCTGCCCG
AGCTCGTCGTGCTGTCCGGGGCGAGTGCGGGGCCTTGGCGGCGGTGAGTG
AGAGCGCTGCGCGGGTGGAGGACGGGAGGGTCGCGGCGTTCTTGCCGGCT
GATATCTTTGGCGGGGCGAGCAGGACGGCCTTGTACACCCTGCCGTAGGTC
CCTGCGCGTCAGCTCACCGAGCCCCTTGGACGAGGCGAGAAGGCAGGCA
GACAGACATACCCGACGAGATGAACCCGAGGATCTTGTACGCTTTGAGCAC
CCTGCTCGTCAGCTGACCCCTCGCGGACAGTGAAGGCCCCACGTCCGCCG
CTCGCGGTCCCGCTTGGCGCGGTACATGTGCATCGGGTCGGGCGTCGTGCT
GGGCACCATGACGGCGGCCATGGTGGCTCTGTGCATGGCCTCGACACTGCT
AGGTTGCAGCAGTGAGCAATCGTCAGAAACGACATGTGCGGGGCACGTGA

CACGGAGATGTCCGACTGTCCGCCTTTAGAAAGAGGAGACAAGATGCATC
CGACTATCGATCATCAGAGAGGGTGAGGGCTATGGCGGCAGTGACAGTCA
CTGTCAACGTCCTAGCAGAGTCACAACGAGTTGCAGCCTGACCGACTGGC
CGCTTCCCCGGGAGGGTTCGGCTACGGCGTGATGCCAGGCGTGATGATGGT
GGCTGGTGAGCAACATGAAACGGGGTGCAGCAATGCCCTCTATCCAGGAC
AGCATCAACATAGCGCAGCGCAGCGGGAAGGGTGTTCATTGCCGCAAGCG
AGTCCATGTGACCGGTGACAGGCAATACAGCCAGAAGCAGTGCGAGGCAC
TCCGTGACTCGGCCAACCGAACTCCTATGGTGCTATTATCATCCTGCAGGAG
CTCGTCCCGCTATAGACTATTCACATCCATTCCTGGCCTTGTTGGTATCCTGG
TGTTGCTATCCTGGTGTGCTGTCCTGGCTGAGTCCTGGCCGTTGCTTCCGGT
TCCAAGAGCGCAGGGTGGGGAATGTCACGTGGGGTACGGCAAGCGAGCG
GCTTGCAGCGAGATGTCGCACGACTATTCCATCAGACATAGGACGAGACTG
GGGGAGTAACGGTGGGCTTTTCGATGCCAGCAGTGCCAGCAATGCCAGCA
GCGACTGGACGACCATCGTCGTCGTCTCCTCCTCCTCGACCCCGAATCTCC
CCACGCCGACCCCGCACACCCGCTCAGCCGCAGACATCACCGAGGGGAGT
GAAAGAGGGAAGAGGTCACACGGCATGTGAGGGAGAATACAGTTCCCGCC
CCGCGCATCCGTAGCCGTATCCGCCGTCCTAGCTATGCTATGCCGTATGCAC
CCCCGATCTTATTCAGACGGCCGACATCACTTCTTTCCCACCGACCAGCGA
GCTGTCAGTCGAGCATGAAGCCGTTCCGTGACTTGGCTGCCCGCGACGGC
ATAGCATGCGAGCTGTAGGGCAGGCGGTCCATAAGGATAACTTAGGATGTG
ACCGTGGCTAGGCGATGGCGCCCGCCGCTTGCGTCAGCAGCAGCCCCATG
CACAGCCAAACCCGAAGAAAATCAGAAATCACCCACGGGTAAATTTTCGT
TGGAAGAAAACTAACAATCCGGAAATCTGGAAACGAGAAAATCTACTCGA
GTAATCTAGGGTAAACCAAGAGGAGGCGTCGCAATTGTTCTCTGCTTCTGG
CGCATCACTTCAGGGTTCAGGCACCACCTTGCGTCATGGAATGATCGACAT
TGACTAGGAGGTGGTCAGCCGAACAACCACCCACTTGCCAAGAGGGCGCA
GCGTTTTCATCGTGTCTCGCACTAAAGCCTACTTCTTCGACGACGGGCGAC
GGGTGGCAAGGTTATACCTGTGACACCGCGCTGGTCGCATCATCGGACATG
CGGTCAGGTCAACAAACACGAGGAGAGGACAGGTTGGGTTGGCAGAGGA
CAGGACAGGCTGAAACATCCTCCACCCTACGAGCGAGCAAAGAGGAGGC
ACCATCAGCAATTGACGTGAAACGTCCTCTTTTCCAACATTCAGTATGCTTC
GGTCCGCGCTTCGACGGCGAGCGAGGATCCGAAGGAGCGGAAGCACCGG
GCGGCTTGGAATGTCGGACGTGCTGGTCTGACGACCAACGAGCGACACAC
CGACACCGATACACCGGCACGCCGACCGACGCCCTTGCTCGTGCTCGTGTA
GGTGTGGTGTGCTCGTGACTGTGACTGGTAACGTGACATGGAGACCCGTAC
CCGTGACCCTCCTCGTGGCCACCCGGGGCGGTTGATGAAGGGCTGATGGT
GATGGTGATGGCGAGACTCACACTGATGGCCAACCCACCATACCCCAAACA
CCTGCAACCATTCAAGACTATTGACATCTTGACGCTGACATACCGTGGGGC

TTAGTGTCTCTGGGACGGTGTATGCCATCGCCGCTTTGCTCAGAGTCAGGT
CGGGCGTAGCTTGGGCGCTCCTCCTCCTCGTCTAGACCTCTCGGTGTTCCT
GATTCGTTGAGGAGGACGTACCCTTTTGATGGGATCATTGATGGCTGATGGC
TGATGGCTGAGATGAGATACCAGGATAAAAAAGACGCTAAAGTCGATCACG
ATCAGAACACACAAGGGGACGACCCTTCATCAATCATCATCATCATCATCG
ACCACCATCACCACCATCACCATCACCATCATCCTCCCCGACACCGTCTCCC
GTCACGCCTCGACGACCATAAAGACGTGCCACCCAGGACTGCCCGCCGGG
TGTACATGCAGTCACATGATCTGCAGAGCAGCCTGCAGCCCATACAGCCAC
CCCTGGGTAAACCGTCGTCCAACCTGCCCGCTGAAACGTCCTCCCCGTCAT
GACCGATGCACAGACGCTTCGTCTGCCAGGACATCTAGAGGAGGATAGGAT
AGGGCCATCATCACGACCTGACCTGACGGATCGTTACTGTAACTTGTATCCA
CACCACCCTTCCACCCTCCCTCATCCTCGCCCCGTAGTCTCACCCCTCCGCA
CCCTGTCCATAGTCATAGTCACAGTTCTTCTGTGCTGCTTGCTCTTGCATCC
CATCCCATCTTCGCCTCGTCACCCAACCATCGCTGCTCTGCTGTGCCTCGCG
TCTCCCTTGGCCTCTGACCGACATCACCGGAGCATCCCGAGCTAGCAGAAC
CCGAGCGCAAGCGAGAGAAGCATCCCACCCGCCCGACCCTTCTCCTCCAA
CCCTCTCCTCGCAAGTCAGGCCTAGCAAGAACGGCTTGTACCTCTCCCCCG
GTCCCCTCGCTCCCGCTGCTCATCGGCACCGCGAGCCAAGGCGAGGCCATA
ATATCAAAAGCTCATAGAAAAGACAAAACTTCCCCGTCCTCTTGGACACAC
ACCGACCGCCCTGACTCCCTCCGCCCATCGACAACAACCACAACCCACAA
CCATTGTACGTCCTCGCTATGCGTTTCGCCTGCCCGCCATCACACCCTAGCC
CCCTGCCCCCCCCCAGCGACAGCGACGTGTTGTCCCTTACCTATTGCTGAA
GCCATGCATAGGTACTCGCACCCCTCCCCCCTCCCTCCCCTTCCTCACAAGA
CCCGCCGCCGCCGCCGCCTTGCCGAAGATCGACCCGCCCAGCATCACCCG
GCGCCCGCTCCCACTCCCAACCATACGCCACCGACGATCGCCGCTCGCTCG
CCGTACTGCACCACGCTTATAGCTGCGTCGACGCCATCTTCATCTTCCCCTC
ATTTCCACCAGTCGGAAAGGAGGCTGCGTCCACTCTCCCTCGAGCTGCCTT
CGATCTGATCGACACGAGGCCGACGTTCCCAGTCATCCACCCATCCATCAG
CATAGAGCGGACGAGAAAGAAGCAACACCCAGGAGGGGCCCTCATCCTTG
CCTTGTCCGGCACCAGTCCGGCCAGCGAGTCAGGACTCGGTCCTCAGGAG
TGAGGATCGAGGAGACGGACGCACAAACGTGCCGTCCCTCCCACCCTTCT
CCCTGCTTGTTTAGCGCGTGACGAGCGCCGCGCGGGTCAGTGTGAGCAGA
AGAGCGGAGCACATCACATTGATATAACACCCCTCACCCTCGCCCCCGCCT
CCCTCTTTCCCATCCGTCTTATTGTTCACCACCCCCGCCGGCTCATTACAAT
GAGCACGGCTTAGGCTTATTGTTGGCCACGCCACACCCCACCCCGTGTGGC
CTCCCTCCCCTCGACGCCGGCACACGCACGACCCTGCACCTACCTAGCCGG
CTTCACCAGCTCGTCCATCAGCAAGTTAGTATTGCCGCAACGTGCCTAGCG
GTCCATCGCTGTCTTGTCTCGAGTAAGTTAATAAGAGGGGTCATCGGGAGT

CGATCTCTGCCTCACACCGCCGCCACATCGTTCTCGGCAGATCAGCATTCC
GAGTCTTATCTGACACGCAGCGCCCCCTCAGTCCGTCCGCAGCGATTGCAT
CCACGGGAATCGACGAGCGATCGACAGTCGATCAGATTCCAGCTCGCCATA
CAGCAAGCAGGCTTCAGCTTGTCCGTGAGTGGTCCGTTAGTTTCGTCATGA
AGGAGTGGTTCTCGGGATACGGTTTCGCCCCACCCCTTTCATCTCCATTTAT
CAACTCCACTTGTCGATCTTGGATAGATGGGCAACCATCGCTAGTCCATGCG
CTACCACCTCCATCGCCTGTTGCTTTTCGCCAGCTTATTACGGTGCCTCGAC
GGGACCCGGGTGATCGACAATTGCGCATGCTCTGCCATCCGGCATATCCGG
CCAGGACTCTTTTGCAGCGGCATATGGCATGGGAAAGGCGGGGTCTCGCCA
TGCATATGGGCCGTCGGCATGAGGCAGGGTTAAGCTGGAGAGGCGAGGGC
GGCATGTCGTGCCTCTGTCGAGCTTAAACTGTGGCATTTGAGGGAGGGAGG
GAGCAACGAGCATGAGGCATGAGCCAGAGCCAGAGTTATCTTTCTCTGGCC
GGCTCGGTCGTCGTACATCGCCGGACAAGCCGGCGGTGCGACGTCGACGA
GCGCGATCAGCGGCGCCGGGCTCAGCGGGCGCACTTGAAGAGCAAGGCG
AGGCGAGGCGCGATGAGAGATACCCAGCGAGATGGGCTCCTCGGGTCCTG
ACGATCCTGCTAGCCCTGATCCTGATGAGGCTCGCAAAGACGTCAGAGCGC
GCGCGCGCTTGGCAGAGGCGTTCCAGCCTGCTTGCTAGCGCCAGTGACTG
TCTCGCAGAATTCAGAGATGTGACCGTGGGCGCTGAGCGGTGGGCGGTTC
TGGGCCCCGACGAGTCCTAATCTCCGGGCTAACAAAACGCGCTGGCTCTCG
AAAGGCAATTAGGATTATGGCTGACCCGCTCAATAGAATCCACGACCCCTG
AAGCCCGCGCCCGCTCAAGAGCCAACCTTCGAGCCGCCGAACCGCCCGAC
ACGTTCACCGCCTGCCCGGTCGAGGCCCCGCGTCCCGTCCCGTCCTGTAGC
CGCTGCGCTGCGCTGCGAGATAACGCGAGACCGAGAAATTATTGCATTGCC
AGCGGGTAATCAGCCGAAGGGTGAGTTTGGCACACCGAGTTTTCTGGTCTT
TCTTTGCTCGTTCGTGGTGAACTGGACGGTGGTCGTCTGCAAGCAATGGGC
GATGCGAGCCATGCGCGTGGGATCATCCTCCCACACGTCAGTGACAGTGGC
GGTCACCTCACTGTCCAAGGACAAGGATGAGCAAGCCCGGCTACGGCACC
GGGAACGCAGCCCTGCTCTCGGAAGAGGTCGTGCGCTGCGCGGGCACGCA
GATTTCCATCTCGCAGCGAGTTGCAGGAGGAAGAGAATTTCGCGTGACAC
GCCCTGCCTCCCTGCTACGCACCCAACCTCCGTTCCTCAGAGCACCGGACT
GGTCGAGCCGTCGCCATCCCATCGGATTGAGCCGGTCACGTCTCATCTGGC
TCTAATCTTTCAGTCTGCACGCGCGAGATTTCTTGCTTGTGCGTGCTTGTGC
AGCTTGCGCTTGTGCAGCAGCCCTGGGCCCCCGTTTTTCCTTACCGAAGGG
CCGAAGCATTTTTTGCGGGTCTTTCTTTGCCTGGCGTGACGGGTTCAGGCG
CTCTCTCGATCGGAACTTGCTCTCTCTTCACCACAACCCTGACCGCTCCTAT
CACCTTGTTACCCAATTTCCCCCTTGCCTCCGGCTCGCTGGCTGGCGTTGGA
ACCCACCTAGCACCTAGCGCTATGCAAGAGGCTAAAGGCTGCCCTGGCTGG
ACCTTGTGGGCGGCGCCGTGACGCACGTCCTAGACCCGCTTCCTCCGTCAT

GTGCCAATCGCACCCAATCCCACCCACGAAAAGGACCGACAGCAAAGACT
GCCAGCCAGCACTGGGTAGGCAGGCAGGTCTGTCAGGTGTGCTCCTATCCT
ATTCCTCGCCCATCGCCTTTGGCGTTGCACCTTCCTCCCTATGCTCGCCTGT
GGGTTGGGATCACCGCAACACGAACACGGCCACCCCGCTCGCTGCGTTAA
AGATCCACCACGCTCGACACCACCCCGCTCTTCTCTTCTCTCCTCCTCTTGG
CCGTGGTCGTCTGTGTCTCGCTCCTCTCTCATGATTTGACATCTATCTGCGCT
CGCAACGCAACGACCGCTCGACGACCTCTTGTTGCCGTGTCTCGGGTCTTG
ACGACTCTCTCACTCTTGTTGGACGACCCGCACGAAAGAACCTAGAGCTC
ACGCCGTCTATCGCTTCCCACTTCCACTCCCACTCCCACTCTCACCCGACGC
CCGACGCACCCCTCTCGCAATCATATATACCGTCGGCCGCCCTCGCCCTCGT
CCCGTCAACACCCCGTCCCGCCCACTCCCACTCTCGGCCTCCTCTCGGCAA
CGTGTCTTGGGTGTTCGGTCACTGGTGACTCAACCCCGTCTCCGACCCCCG
CGCCGCATCGGTATCCGTATTGGTACGCCTGACAACCGCCAATTGATAATTG
GGTCTGTCTCACACCGCGTTTCATCGCTGGTCGCCTGTATCTGGTCTGCGCG
TCACCGTCACCATCACCACACGTACTCTACCGTACCTGCTCGCCTGCCCGC
CTTCTTTCTCTATTCGACTGGTCTCGATCAAACAACCCCGCGCGCGCTATAT
ATCGCCCCTCGCTCCACCGAAACCTTGCCTCACATTCCCACTCGCCCCTCG
ACCTGACTGACGCAGAGCAGCGCTCGCACCTTCCCGTCGACAGACTTGCC
CGATTCGCCCGACGAGCTCCATTTCCAAAAGAGTAGACGACCCGCTGCTCT
CTCACGTTCATTCCTCCTCGTTCTCTGTCGCCTGTCACTTGTCGACCTCCCC
ACTTCTGCTTCAGCACCACCTCACCCTTCAACCACCACACCCACGACACCC
ACCACACACCCCAACATGCCCGCCGCACCCGTCGCAGGCGCCTCCAAGGC
CGCGCGCCACAACTCGATCTACGGCTGGAGAAAGAGGTCGCAGTCGGTCT
CGCCCAATCCCACCGCCGGCCCCTCGGCCGGTCCCCACGTCCCCCAGGCCC
CTCCCCCGCGCATCATCCGCGCGGTCGAGCCGACGTACTATGAGGAGCAGT
ACCAGTCCGAGATCATCGAGTACATGATGAAGATGGACGTGAGTGGCCTGC
TTGCCCGCCTGAAACGCGTAGTTGACGCGTCAGGAACTCACGCTCGCCTC
GGCCGACCTCATGGACATGCAGCCCGAATTGCAGTGGTTCATGCGCCCGTA
CCTCATCGACTTTCTCATCGAGGTCCACACCCAGTTCCGCCTCCGCCCCGA
AGTCCTCTACCTCACCATGAACATTGTCGATCGCTACGTCTCGAAACGCGT
CGTCTACAAGAAGCACTACCAGCTCGTGGGCTGCGCCCGCGCTGTGGATC
GCCGCCAAGTTTGAGGACGGCAAGGACAAGGTGCCCCTCGTCCGCGAGCT
CGCCGAGATGTGCTGCAAGGCGTACGACGAGTCGGCGTTCATCCAGATGG
AGGGCCACGTCCTGTCGACCATCGGCTGGACCATCGGCGCGCCCACCGCC
GAGGCGTGGCTGCGCGTCCAGGCAAACAGCATCTTCAGCGAGGACATCAA
GATCCAGCACATGGCGCGCTTCGTCATGGAGATCACCCTCTTCCACCGCGA
GTTTATCGGCCTCCACGCCGAGCTCATCGCCGCCGGCGCCCTCATGCTCGC
GCGCTACATCTGCGGCCGGCCCCAGCGCGTCAGTCCTTCTTTGTGTGTGTC

GCGTCGAGCGCTGCTGACCTCCTCGCAGTCGATCAAGCGCATCGAGAGCG
TCGCCGTGCGCGTGGCCTATGCCATCGACCGCCACTTTGCAGAGAAGATGG
ACACGGTCTCGGACATTGTCTGCCGCAAGTACGCCCCGACCTACTACCACC
GCGTCTCGGTCGTCGCGCGCGAGTGGTACCTCTCCGGCCGCCGCTATGCCT
TCCACCCCGAGGTGCCCGTCACCCCCACCGGCCTCCCCGCCTGCGGCCTCG
CGCCGTCCGGCTCGTGGCTCTCGTCAGGCGGCGGCGGCGGCAAGCGCGAA
TCGTGGGTCTCGGGATCACCAGGGACCATGTCGACGTCGTGCTCGTCGTCC
GAGGCTGGCGACGAGGTCCCCACCACCCCCATCACCCCCGTCCACGCCAA
CGTCGTCAACCCCTTTGACGCCTCGGCCAAGGAGAACGTCGCCCCCTCGT
CTGCCCAGGTCTTCCGCGCCCCATTACCGAAAGACCTCAAGGGCGTCACGC
CCGTACACCACCACCCCCACGCCGTACCCGTCGGCGTCACCCAGCCGAGA
CCGCCGCTACATGCCATCACGCCCTGCGGCGTCGCGAGCTTTTACGCCTCG
AGACGGTTGTCAAATTAGCCTTCTCTTTCATACCAAAACCGACACCCATTG
CATTGCGCACGAGCAAGAAAAGTACCATCAACATCCAGCATTATATCAATAC
CACCGCCCGGCCTCCCTGTTATATGATTCCCAACCAGCACACAGAACACAA
CACATTGCATCCATTTCAACTACGCTCGACCGCCTCGCGCCCCGCTCGCAC
CCACGCACCCACGCACGATTTAATGACACTTGACCTGTGCCTACACGCCCA
CGACCACGACTAGACCTATCACGTACGTGCGTGTCCGACAGCACCGGCGC
GTCGGCCGGCGACCAGCGACAGCCACAGGGGTGGGACGAGGCAGAAGCG
CAAACGTTGCATGTATCATGCCCCTCATAGTTTTACGTATTGTGTCTCTACAT
GCATAGGATGATCTGCTACGTCTGCTTGGCTACGTGACTTAATGGCAATGGC
GCGAGCTGAGACAAGGGTTCTGCGGAATGGCCTGGTGGAATTGGGAGTAG
GACAGGGACGTGAACGCCTCTGGCAAGCGGGGATGGTGGACTTGGGCTTG
GGCTTGGTATTCCAGCCCAGGACGGAGCGGAGCGGAGAGGGGAAAAGAG
GCGTCTCGTCCCTCTCACAGCCAGCCAGCCAGCCAGCCCTGGATCTCGTAT
CTAATCGCTCTCTTCCAAGTCTAAGTGAGCTAGTGAGGATACCATGTCTCGA
GTTAATGTCGTGTGAGCAGTCACCCGCTACCTCAAAGCCGAGAGAAAACT
GGGAATTGAGAATGGACGGGATTCATGCGGGTTTTCAACACGTTCACAGTC
TGCTAGTCAGCGACGCGTCTCTCTTGTTGAGCTCGCCAGCCGCTGGCTCGC
TCGCCTGTCGGCTCGCACTCACTCCAACTTCTCCAGACCCTCTGTGAATGA
CTGTACGGTCGTCTGGAGGCTCTCGGTGTTGGCCCCGTCTACGCCTTGCAC
AAGGAGGATGTAGGTCGCCGTGCGCGCGGCGATGAATGGCTGGGGTCAGT
CGACGCTCCTCCCCAGCCTCCACTCCCAAGTAGCTCGCTTCTGTAGGAGCT
AGCAGGAGTGGGAGAAGAGCTCGCAGGTTCGAGAGTGGTGGGGGAGCTC
GCTCCTTCCGGAGCTCGCTGCGCTCGCTGCTCGCTCACCTTGCCCCCCAAC
TCGGCCAAGACCCAATCCGCCGTCCAAGCCTCACACTTCGCCTTGCCCTCT
CCCCCCAACGCCAAATTGGCCGGAACCGCCCCATCCCCGCCGGCAGCAGT
GGGGTCGCCCTGCCTCGCAGAAGTAAGCAGCCCGTCGTACACTTTCCGCA

CGTCGTCTGGCCCCACATCGAAAGCCGGCGTGCTGGACGCCGCGAGGGAG
AATCGTCAGAGGCGTACAGTGCGGCGCGGGAGACTTGGGCGTTGGACAGG
AGGCGGTTGGTCAGGAGCGCGGAGGGGTTGCCGCTTTCGCCCTGCGACAC
CTGTCAGCCCCTCTTCCCAACCTCCTCTTCGTACCCGCTCATTCGCGCCCAC
CTCCGGCGGGCTCGGCAGTGGAGACCCACCTGCGACTCGAGCTTCGCTGC
TGTTGCGTGCTCCTCGGTGGCCTCTTTGTGGCCTGACGACCGCGCGGGCCC
GATGTTCTTCCGTCGGCCCCTACCTACCTCTCACTTCTGACTCACCAAGTTT
TGCGTGGACATTCTCTCCTTGCTTAGCTGCTGCTGGTACAGTACTACTCACC
GGCATGCCTCTGTGGGTGGTCATGCATATATACCGCCCCACCTGGTGATCAC
ACCGACCGGATGACATCACCCGCCAAGGTTTACGATCATCGGCATCGACAC
TTGACACTCGATGCCCTCTCGTACATTTTATAACTCACTGTGCAACTCTCAT
CTATGTCTGACAATCTACTGTGCGATGTACTATGCAACCAGCCTCACGGCAT
GCCCGCCACGTCCACCTCGGGTCCTCGCTTCGATCGCGCAGCCAAGCGCAT
TGACCCACACGCTGGCTTCGCGGACCGCCTCTGAGCTCCCCTCTTTCATCA
TCATGTCCAAGGCCATTCGGACCTTCTCCACATCGCCCTTGACAGCATTGA
CCACACGCAGCGCCCACTCATCGGCGAGGCTGAAGCTAGCTAGCACCTTG
ACCTCAACTCTTGTCGCGTCATGCCCCATACTTCGGCAGGGTGACGGACGT
GCAAGTCCAGGCTGGACGAGCGAGCTTCGACAGCGAAGCGTTGGCCGTCT
CGCGAAGCACCTTGAAGAGCACAACAGCCGGCATGTCCTCCAGTGCATCG
TCCAGCTTTTCCTCTTCGGGATTCCCGGGCTTGTCCTCGCGGACGCGGATC
GCCGACCGCAGGATGAGGGACGAGCCGTGTGGGCCCGACATGACCGATAG
GACGGTGCGGAACGAGACCATGGCTTCCACTGCCTCTCGCAAAGCTTCGC
GAGCGACTGTCCTCTCGGCAGTCGGCACCGTGTCGACCGGGTCCGGGTCG
GGGAGGACAGGGACCAGAGTTCCAAAGAGCCTCCTGACCAGTCCCTCGA
ATGACATGATGGCTTGCACTCGAGGGTCGGCCTTGCTTCGAGGGGGTGAA
GGTGGTCGGGAAGCAGCGCGAGAAGGCAAGATGTTGAGCGAGTTCTGAA
GCTTCGACGCACTCCCACCGGAGCCGAGGGCGTCGGAGTCGCAGCGCCTT
CTTTGGACATGATAGCATGGCCGTTGACATTGTTGAGGATCGCCTTGGTCAC
TTGACCAGGTGAGGGTGGTCCGCCTGAGGTGTGATGGTCGAGACCCGCAA
CAACGGTCATGCCCTTCCATACGACAGGCACGATGATGCCAGTGGCCCAGC
CTTCACCGTCCTGCTTGCAGGCCCGCGCGATGAGGTCGAGCGCTTTGTGCG
CCCCATCAACTCGGTTGGCAAATTGCTGCAAGCATGTCGGGATCTGCACCT
GCCTCGGCTGCCCGGGCTGCGAGACCTCCTTGGTGAGGCGGGACCGGCCG
GACGAGCTGACACTAGCCTCCTTGCTGATCTTGACATCAGGCAGGGGTCCA
GCAGGCGGGGCAGTCACTGATGCCAGCCCTACAACTTTGCCGCCGGGAGG
AGACGATCCCTCGGTCCTGGCAAGCGCGCCAAGGAGGTCTTTCTTGAGGA
GCAAAAGCAGTGGCTGGATGACCTTGCCCCACGTGTTGTCGAGCTTGTCC
ATGAGAGGGCGCAGGGTGTCGCTGACAAACTTGGGCCATCGCCCCAGTAG

CGAGGGTGTCCTCGAGCGCTTCGCATGTCTCCCACGCAGCATGAGCGACG
GAAAGAGCGAAGAGTTGAGTGGGATTGAGGGGGTGCGTAGCGCCCACAC
GAAGATTCATGGCCTGCCCGAGAGCCGAGTCGGTAATTGCGGGAAGCAGG
AGCATCTCGAGGCGATTGATGTAGATGGAGAGGGCACGAGTGGCTACGCG
AAGTATGGCGCGCAAGACGTATGGGTCGGAAGATGGGCTGGGGAGCTCCC
TGCCCTGTCAGTTGTGCCGCATGCAGATAAGTGTCGCGTGACTCACTTGAC
AACGCCATTGCCCACGGCCCATCCTGCCTCGCGCTGGATGGCTCGCTTGGA
CTTGGAGTCTGCGCCTGCGAGGGCCTTGTTGACCGTCTCATTGAGGCGAAG
GAGGATGGCATCCATGTGCCCGCCACCGCCGGCAGCTGCGTCCGCCGCAA
GGCCCGGTGTGACGGGTCGGCTGTTGGGCGGGGAGATCTTCTTGCCGAGA
GGCTGAGACTGGATCAAGACCGGGGTGCCGTTCACCTCGTCGGCGTGGTT
GAGGTGCATGGCGGCGACCTTCTCGGTCACCTGCTTGCTGAGAGAGCGGG
AGCGCGGCTTGGCGGCGTCGATGCCCGGGGCGGCATTAAGGCCCATTGCAT
TGGCATTGCTGAGCTGTGCGGCGGCCGGAGTGGGGGAAGAGGGGCGAGA
GGGACGGTTGAGGATGGGGAGGGTGCGGAGTGCCTGCATCTTGGCGCAGG
AGGTGTGGTCGACTCTATGGGAAGGGGCGAGTCGGACTGGGCGATAGGGG
GCTCTGGCGCTTTATCCCATCCGGCTCAGATATGGGGGAGGGCGCGGCGAG
AGAGGAGTAGTTGTATATTTTATGCCAGGACCTCCTGGCTTTTGAATGGTGT
AGGCGGGGTGTCGAGCCAAATTACGGCGGCTTCTCCCCAGAGTCGAGGAG
GATGGCGATGAGGGGCGGGAGGCGGAGCAGTCGGCGTGCGTTGGAGATGC
TGTTGTGGCGAGGCAGCATTGCGGTAGGTAGACAACAGAGGGGAGGCGGG
GGGATGTCGGTGTTGATATATGAGACAAGACAATTGGTTCGGTGAGGGTGG
AAAAAAGTAAAGGAAGGGGCGAGGCGACCGTGCGTGTGACCAAGGAAAA
AAATCTGTCAGCCCACGTTCTTCTTCGCCGTGCCCCCAACAGCCCTCGCTG
GTCTGAACATGCACTCTGGACTCACCCGACTCCGCCCTTGTTGAGGTGTGC
GTTCAAGCCACTATTAGGGAGTTTGGACTTCTTCATTGAAACCAGGCGGTC
GGTCCGGGGATGGCGGTAGGTGGTGGCGATGAAAGCACAGACGCTGTCTG
GCAGTTACTGCGATGTGTCTGGTGTGTTTGTATCTGGTGGCGAGGGCGAGT
GTTTATGCTACTTTTCGAAGAGGATGGTGTTATAATGCTTCTAAGGTATGAG
AGATGTCTGAGAAGTGAGAGCGATGGAGATGAACAGAACAGAACCAATCG
AGGGAAAAGGATTTCGACACCCAAAGGAGGGGTTATTCAGGGTAAACATA
CCATTCAGGGTCAGTCAGGGCGAGGCGAGATCCGGCCGTGGCGGGGCAGT
CGGCAACAACGCGTCATGACATCTAGTTTCTAGTGTCTATCACCTGTCTACA
GTCATAATGTGTACTGGCAAAACTCGTCGTCCCCCTGCAAAGCACTCCAAG
TGCTCTCGCTCAGCGTCAAACTCACCCACAAGCCCTCGTCTCCCCGGGGCG
GCCGGATATAGTACACCAACCCATCCACGACCATGCGTATCGGGACTCGGA
GGCAGCCACACCATATCCGGCTTCCCAGCCCCAGGACCGAAGTCCACCTCG
TAAAAGTGGTCGAGCTTGTTCCAAGCGGTCACATAGAGGTTCCCGTTCCCA

TCCACCAACTTGCTCTTCACGCGCGCCTGGTCAGCTGAGTCGAGCGAGCC
GAACCACTGTAGCGTCTTGCCCAGCTCGGGCTGGCAGAATGCCGCGTCGTT
GATCGAGGCGTGGATCCAGCTTGCGACGTCTGCCAGGCTGCTCTCTACAAT
CTGCGCGAGGTGATGGTTGTCAATAAGGGGTTGAGCACCGCGTTTCCAAA
GTATGCAGGGGGCAGGCCGTCCGCTTTGCCCCGCAAGTTGTACGCGTTGAG
CAGGTGCGGCAGTGATAGTGGCCTGAGCTTCGGGTCCAGTTCCCGTGCGC
GCTGGCGAGCGAGGTGCGTGCTGCGATAGAGGTAGGCGGTAAGCGCCGAG
TACGTCGAGACCCAGCCTGTGCTGTTTGCAGGGGCTGCGCGGCTCTTCAGC
TCGGCGAGCTGGGCCGGGGAGAATCGCAGCAGCTTGCACTGGATCGGATC
GGGTGTGTACTCCTTTTGTGTCCCACTCGGCTCTTCAGCTGCCTGCACCCC
AGGCGGCAGGTCGACCGGGGCCGGCTTGACGGGTGGGAGATGGAGTATCT
CTGACCGGTACGACCGCCACGCTTCCAGGTCGGCAGGCGAGGCTTCGCTC
CTGAGATTCGGCAGGAAAGGCCTGACGACCGGAATGGTATCAAGCTCAGC
AGGGCTGTCGGTCGAAGAGGCGGAATACAAGGCGCCGATATCCCTCACGA
GACGAGCAAAGCCCTCGCCGTCCGTAATAACATGCGGCATCCGCACGCCCA
GCGCGACGGAGCCGCAGGTAAAAGTCGTGTGTTGGATATGGAGGAACGGC
TCGTCGACCCATGCGCCAGGCCGCAGCTCGAAGGGAGGCACGAGACCATT
GCCCCCTCCGCCAGGGAGGTCTTCTGGGCGGGAGCGGTGGTTGAAGGCGG
AGAGGGGACGGTCGCAGGTAGCTTCGACGAAGCTGATGCCTTGGCCGAGC
TGCCGGACGTGTGGGATGCCTGGTTCGGACGTGAGGTCCAGTCGGCCCGT
CAGGTGGGGGTAGTAGTTGAGCACATTGGAGATGGCCCGGCGTAGTCGGT
CGGGCCGGATGACAGGAGGGGCAGGTGCGTGCGAGTTGGAGGGGGAAGG
GGCGGAAGGGGAGGGGAAGATGTAGACGACCGAGAGGGGCACCCAGAAA
TACCGCGAGCGAGTCGAGTGGGGAGAGACGGAACTTGCCGTCGGTATCGA
GGGGCAGGGTGGACAGGCGGGATGTGTCGGAGAGATGGACGGTGTGTCG
GGCGGTGATGTCCAACTTGTATGTCGGGGCCGTAGGGCCTGGATCGGTAGT
CATTGTGGATGTGGCCCGGTATGCAGCTGGGCAGGCCGTCTCATAGTAGCA
CACCTTTCATATAAGGCAGAGGTGACTGACTGCAGTCGAGATCCGGTATTTT
CGGATCCGAAACCCCGTGCACCGGAGGATGTCTACGACAGATGCATGCCTA
CGAGCCGGCGAAACGCTTCGTCCTTCTGCAGCACGCCCCACGTTGCTTCA
GTCAAAGTCAAATTGCCCCACAGCCCCTGACCCCCTGGCGGTCGGAGCCA
GTACAACAAGCCGTCCGCCGCCATCCAAGCATGGTTGAACGGCGGCCACA
CCATCTCGGGCGCCTCACCGCCAAACGTCGTCTCGCCAAACAAGTCAAAC
TTGTTCCATGCACTGACCATCATCTGCGCTGGCCCATTCACGAACCCTCTCG
CTCAATCTTGCTCTTGTCCGGCTGGCTGGCAATCCAGCGGAGTGTACGGTC
TATCTCGGGCTGCTGAAAGGCTGGGTCGCGCGCTGAGGCGTGTATCATCGC
CGCCAGCTCCGCGAGGGGCATGTTGGATAGCTGGGTAGTGTCCAAGCTCGT
AATGGTCGGTACGGAGACGGCGTTGGGGAAGTATTTCGGACCGACTCCGA

GCTGAGCGCGGAGGTTGATCGAGTGGAGGTAGTGAGGCGGTGGGAGAGG
AGGGAGGGTGTGATCGAGTAATCGGGCTGTCAGGCGGGCTGAGTGGATCT
TCTGGAAGAGAAACGCCGTGAGAGCAGAGTAGGTCGAGATGCGGCCGTTG
CCATCCCGGGGCGACGCCAGACTCTTGATCTCGGCGATCTGGTCTGGGGAG
AAAGTCAGAACGCGCCCGACTATGCGTTCGGGTAGGACCGTCGTCCGTGG
ACTTCGGAAGACACGTCGGGCGCCGCAGGCTGTGCAGGTTGCCCTCGTGA
AAGAGCCAAGCCGGCAGGCTTGTACGATAGGAAAGCGTCCTTCTCATCCTC
GGACGCAGTTTCAGCCAGTTCGGAGAGGTAAGGGCGGATATTCGGCGAAA
CAGCCAGACGCCAGTCTCGTAGATCTCGCTGAGGTCCTTGACCATCTGCGT
GAACCCCGCAGCGTCCGTCAAGGTGTGCCGGACCGACACGCCCAGAACA
GCCGAGCCACAGGAGAAAGTCGTCAGCTGAAACGCGAGCAGCGGGGCAG
TCGTCGCCGCCTCCATGGACCAGTCGATGGTGGGCAGAAGGTCCATGCCAT
TGCCCGGGAGCTCCTTTGGGTCGTCGCGCCATCTCGTCGAGTCGAGAGAG
ACCCCGGAAGAAGCTTCGGAAAAGTCGACACCTGTGCCAAGGCGGTCTAT
GTGGATTGCGCCAGGCTCGTATTTGTCGTTGAAGCGCCCGCTCAGCTGGGG
ATAGAACTCAAGGAGCTTCTCTATGGAGTGTCGTAGTCGCGTAGGGGATAT
CGCTGCAGACTTCGGGTAGACGTAGAGGACTGAGATGGGTATGGTGGGCG
GCAGCAGGTCAAGCGGACCCAGTCGGAAGATGCCATCGGCATTGGTGAGG
TTGTCGACTTGGCTGAGGTCGGACGGGTTGAGAATATGCCTTGCGACTATT
TGGACCTGTCCAGCCATGTTGGCGGGGGAGTCGGAACAGCGATCCGGGTC
CCATCTCGCCAATATATCGCACACTCGACTGGGGCGCCGCCATTCGGCCCAT
CCTTTTCGGATCCGAGCGATGCTGAGGTTACGTAACGTCTCCTTCGCTCGG
GCCCGTTTACCGGGATCTGTCTCGCGTTGAACCCATCACCACCCCCCACCA
TTTCCTTCCCTTAACTTTCCCATGCCCGTCCCAGTCATCAAGCATCGTCCAT
CACCTGACAACTAGTGATTTATCGCTGCGCAGCAGGAGGAGGTCCAGGGG
GAGGAGCGTAGTCGCCAGTAACGCCAGTCTGCATGGGGTCGACCTTGGTG
CTTCCCATAGGCGGAGCGCCGCCGCCCTCGTACATGGGCGGACGGTTGTTC
GGGTCGTAGACTGGGGGCGGCATGGAGTGCATGCCGTAAGCACCGTTAGG
CGGGGGTCCGTAGCCCGGAGCCGGGTAGGTGGTGTAGGAAGCCTGGGGCC
AGGCGTAGCGCGGGTCGACGCCGGCCTTTTCTTGGCGGGAAAGAAGCACT
ACGACGGGTTAGCGTTTAGTCGATCAATGGAATTGTGGACTTGGGGCGGGA
GAAAAGAAAAAAGGGGAAGAGGGAAGGGGTACGTACGTTGTGGTAGGCC
ATCGGCTTCAGTCCTTTGTTCACGCGTCGCTTGGCGTGCATACGTCCAAGA
ACGAGGTATAGCGCTAGCAGAACGGTGATGCCGAGGAATAGCGACCACTT
GACGATGACGCCTGTCTGTAATGTGGCGTTAGCAATGTATTCTTTATCAATT
GATCTAATGAAATAGTGGGCTAATAGCGCTTGACGTACCCGAGTATACCAGA
ACCAGACAAGGTGGCCATTCTCCTCGACATATTGACGGCGCACTAACTTGT
CCGCCGACGGGGCATACACCGGGATGCGAGTCTTGCCGAACATGATGACTT

CTTTTTCTGTCTTCAGGTCTCCGATCCGAAGAATAATAGGAAAAGAATTGC
AAAAACCAATAGAATCGAATGAAAAAAGAGGTTGATAAGAAGCGAAGAA
AAATACAGCGGTCGTTAATTGAAAAATGCACTGCACTGCGATCGCCGGTTC
TGGTCCAGCCGACTCTCAAATAATACGAACGAGGAAGGGAAAGAAAAGAA
TGAGAAGAGGAAAAGAGGAATTGAATGCCAAGCTTTTCCCCAGCGGTGAA
AGGGTCGGGAGATCACTATTTCAATGGATTTCCTTAGGCTAGGGGTTAATTG
TCAATGGTAGGGACAATGGACGAGGGAGCCCAATTCTTTTTTGGTGATTGT
GAAGAATGGGTTTGTATGTGTTTTGGTGGCGGTCCTCACAATCTGCGGCAG
ACTTGTGACAAAATTTGGTGTTTCAAAGAGGTCTCGAGCGACGGGGGTCG
GTTGGCTGGCCGATGTTAGCTCTTAAGGATCGCTGAGGGTCCGGTCAGGGG
ATGGCACAGATCGGGGAAAGAAAGAGGAAGACGCGTGTTACATGCGAGCC
GACAAAGCAAGCTTTAAACTTTTAATCGTCGTCAGGGTTGTATCGTCAAGT
GCGAATTACGTACTGTTTAGAGTTCACAATTCTTAAAACAGAACGCCAAGG
GGGGAAAGACGATGATCAGATCGCCGTGCCTGCCCGTGCTCAGATCTTGGG
ATGTTTGTTTGGGAATTTTTGGGAAACGGCGAATTGCAATAATTGTTAAGGT
CGTCTCTATTTCAACTTCCTCAGCGCATGCTTTTTGTAGTTGCCTACAAATA
AATGTTTCTACAAGGTAACACCTACCTAATTGCTAAATATATTAAGTGCAATG
CGAAGCGACAGAAAGGACAGCAAGTCGGCTCGTACCATGATTCATGATAA
CCCCTGACTTGAACGATGGCTCAGCCATACCAAGCTGACCAGGTAAGTAAT
CGATATGACCACTTCGGACGACTTATATCCTGTTGGTCAAGGGTGACGAGT
CCATATTTGCCATTATATTCGATCTTGTCATCAATAATTTATGCCTTATTTTACT
GGTGCTACGTCTCTCCATTACTCAAATGCACAGGTATATAATATAGTAGATAG
GATATCATCGCGAAACTTATTGAATAAGATTTGCCATCACCACTAAACCTGT
TTTTAACTCACCAGTTCTAGACAATGGTTGAGCTACACAAAAAGACTTTATT
TTCCTCCCTAACCTGAGGGGCATCTATAAATCATTCGTGTCACTATTTATGGC
CTCATTCCCCACGCTATTCTAAGTCAGGATCAAGCGAATCGAAAGATTTGA
ATGAATTGTCAGCAAGCCCTCAATGCCACATGGGCTATGTGTGTGCTTGATG
GTCAAGGGAAGAGATTAGAAGGTATGGATTACCGAGGTTCCACAAACAAA
TGGCAAGATTTTGAGTATTAAAGCCTAGGAAGACCCTGGAGGCTAAAGTCA
CAGAGTGATTCGTAAACCCGTATGCCATCTGCGTAATGCAATGCATGTCGTG
TCAATTACAAGACTAACTAATATGTCGAACCTTCCCCAAAAAGTCGTGTTAG
AGTCCAACACATAAAGTTGGCGCGAATGCGCAGGTTCTCATTAATACTTCG
TGGGCCGTATAAGCTAAAAAGTTTCCTTCAATATGCCTAGGGCACATGGCAC
ATGGCACATAACACATAGTCTTGATGTCGTAGTTCCGTATAACGCTCTTCCG
ATCGATGTGGTGATCTGCAGTCCTGTGTTCCTGATTAACATTCTTCGTGGTC
TTCCAGTCGGTGATACAAGTCCACCGCTAGTCGTCGTGGCCGTCCCAATAA
TTCCGAGATGACCCAACAATGCAACTTGTTGCAAATGCGATAGCCTAAGCC
CGTATCGAATAGATCCGAGGCTCCCTTAACGCCATCAATACGCCGGGCTATT

CCCCTCTGTTGCCCCTGTCGCGGGTTCAGCGTTATATTCTGCAGCAATTATT
CGTCGTCTCTCCTCGCCAGCCTCGACGATCTTTCGTAATGTGACCATTCGCC
TCTCAAATTTGTCCACTATATTGGCGTAGTCTTCGGTCTCCCCTCTTTCACGA
TCATGGTAGCCGCTGCCACCGCCACTTGCCATCCCGGAGCCTTGTCTGCTA
GCCTCCCCAGACGTCCCAGCCTCTGAGCCTAGAGCGCTGGTTTCTATACCG
TCTATCCGCAGCATCAGACGGTCAGCACGAGCACTGCTGTCCCCGCCTTCG
TTCCCATCGTTGCTTCCGTCCGATGAAGGTGGTGGTATATGACTTAGTTGTG
TATCCAGCACCGCCCATGACGCTGATAAACTCGATGCCCAAGCAACACTAT
ATCCGCCGTTAGAGTCGGGCGCTAGGTCTAATATCATGGCTCTGCTGGGCTG
TAGGCTTGGGTCGGAGGAAGATTCGTCGACACCAGTATCAAGTTCGGCTAA
TGCTCGAGTTAGGACGTCTGGGTCATCGTCGCTGAAGATGTAGCGCACATG
GGGATATGAGTTGTACGCGTCGACGTGTTATTTATGAGGGTGAAAATAGGC
GCAAACGGAGGCGGATCGAGGTCTTGATCGTGGATGTCGTCGCGGTCGTG
GTCGTGGGAAATATCCGCTATTTGCGGTTGTTCCGGTATAGGCTCCGTCATC
TCGAAGATTGGCAGCCGGAAGTCATGGGGATGGAGAAATGGTAAAGAAGT
TACGATGGAAATATTGAAAAGGCTGGCGCATCGTATATCTGGGACTCCATGT
GTTGGGAAGTTATTGATGGATAAGAATAAGAAATGGCTCCAAACGGTTTAA
ATGGTTTGCTCCGGAGTAGGTACCTTTAGGTGATCAATGGAAGACTGCAGG
TGCAAGTGGTGTCATGCACGCTGCAAAGCAGTGACATGGAAGTCAAGTGG
CAGAGTGGTACCTCCCCAGGTACCCACCTAAGAGAAAGCCTAGCTCGCCTA
ACTTTAGCTGTATGTCCAGGGTTGCCGTAGCCTTTGCAGCTTAGAGTGGTG
CTCTTTTGGAGCTTCTATCTTATCTGCATGTGGGGAAATAGGGAGCTCGGGG
TCTTGGCGTTTTTTCGGTAAGAAATGCCATATGCAGGGACAAGCTTCGTCG
TGGAGATATGGAGCATTTGATTTGGAGGCGAATAGGCAAAAGATGCATTGG
CAAACTCCGAATTTAAAGCACGACCTAGTGAGTTGGTACATGTATATTCTAT
GTCGTTGATTGGAATTAGGTAAGCCTGTAATTCAAGGGGTGAGAGCGTGGA
GATCACTTACTTTCACCGACTAGACTATTGACTATGGATATAAGGGTATAGC
AGGTAAGCATAGAGAAGTCAGAAATCGTATAGAAAGTAGGTAGACAGGCA
AGAAGAGCCATTTAGCTCTAAAAATGCATAGAATCATCTAGAGCTTGTCATC
ATTGGTCTTCAACTATTGTGTCTTGCTGCTCTTCGACGGATTGCCGAGGCAC
CTCAGCCTCTCCCCATTCAAATTCTGTATAGTACATCAGTAATAATGTCGTTT
GTTTAGGGAATTTCAACATACTCTGCCGACACATTGGACATTGTCCTTTGGC
TGAGTCCTGCTTTATCCATTCCATGATACAGTGCTTCTCACAACGTTAGCTT
ATTTAGTTTAAAAAGTTACGTTAATCAATACTTACCATATGAAAGTTATGACC
ACACTTTCCAGATACTAAAAGAGTCAATTGGTTAACGTCTGTAGATATGCAG
AGAGAATATAATAAGGCTCAATGGTTTCTTACAGAGACTACAATCATCTCCT
GGATATTTGCAAGTCGGACACGTCCCGTCAAAATGGACTTGACAAATTCCA
CACACGTCGTCTTCTGGAAGATCCCATCTCCATGTTGCGACAGCATCCCAC

TGCTTGATCTTGACCTTCATTGTCTAATATTATCTTTGCAGAATAGTAAGGGG
ATAATGCAAAGACTAGTGAATCAAGAGTGTGTTCTTCTGGCTTATTTTCGTG
TTGTTGGCGTGTTATTATTGTTAGGATTTGTAGCGTAATGAACAGTCACAGT
AATCAACCCGAACCAACCCCACTAGATCAGACTATACCTAAACATATGCAA
CAAACCACGACACGTGCACGCGATATATGCATTAGCCCGGCACCGCAACTG
TTTCTAGTAAAATGTACCCCACCATTGAAATCTGGTGTATGGGCAGGCGCCT
TTTTCGGCCAGGTAAACCTTGCTTATCGGCGGCTATTGGTAATTGGCGATTG
TAGAATTTACACTTCACGCCATCGCAAACTCTATCTGCAGCTCGTTCTCTCA
CCACCCGTCGATTTGTTTACTTGTAAGGCCCTACTGGTCATAGGATTGGATG
CGACAAAGGAAGTGACGACTACAGATTCCGACAAGAACCCCGCATTTTAC
AATTTCAAAAGCTACCAGGCAAATTGGGAATTATTCGTTCATCCGGACATCA
CTTTGTTTCACTCAATCAGAACTGCTCAACGGCCTTTCCGGACGAGGTGCT
GATTTGGTTTCTCGTTTTCTATACGGCACCCGAACAGAACGCTTGTCTCAGA
GTGTCACCACACCTAAATACAGATATATACGATCGACGTCTAAACAGTTACG
TGCATAAACAGCATAAGGGAGCTCCTCATTGAGCGGTCTAGCTATGGCTAC
GCTCGGTATATTCCGCTCCACAAGAGCCTTCCGACCGCGACAAAGTTTCCA
AAAGTGTCAAATTGGATTCGTAGAGCTGCGACGAAATCTCTCTTCCTATTTA
GTCACACCCAACGAGCTAGCAGAAGCTCTCAAGAAAAGCCCACCATCATC
CATCAGTCTCGAACCTCGAGTTATCCCCCTTTGCGCCGCTTGGTTTTTACCA
AATGATGAACGAAAAGGTATTGAGGTCTTCCGAGAGACGCGAATACCGAA
GGCCCGCTTCTTTGACCTTGACAAAGTTATCGACAAGCGAAGCCATATCCT
CATATGCTTCCAAGTCCCAAGACATTTGCCGAAGCGATGAGTTCCCTTGGT
ATCCGGAAGGAAGATACTGTTGTTGTATATGATTCGAAAGAGCTGGGAATC
TTTAGTGCGCCAAGAGTGGGTTGGACTCTAAAAGTATTCGGCCACCCCAAA
GTACATATCTTGAACAACTTCAAGATATGGGTGGAGGAAGGACATCCTACA
GAATCTGGCGAGCTCTACAATGTGGAGTGCAACCCGTACCCGATCCCGAAA
CCCGACGAAGTTAAGGTCGCAAGCTTCGAAGAAGTACGAGAGGTTGCCCT
AGACTCAAACAAAGAAGGTGCCGAGGGCGTTCAGATTATTGATGCAAGAC
CTAATGGTAGATTCACCGGCAGGGATCCCGAGCCCAGAGAGGGTCTCTCTT
CTGGTCACATGCCAGGTTCCATCAACATCCCCTTCAGTGCCGTTCTCGATCC
TAAGACGAAAGCATTCTTGCCGAAGGAGCAGCTGCAAAAGTTATTCAAGG
AGAAAGGAGTGGATCCATCGAAACCTATCATCTCGACTTGTGGTACCGGAG
TGACAGCGGTTGTCATCGACACTGCTCTTGAAGAGGCTGGCTTTGGTACTC
CTGAGACCCGTAGAGTCTACGATGGTAGCTGGACGTAAGTTGCGCCTTATT
TCTTTTAAAATTTCTTGCTCTTTACTAATAAATGAACAGTGAGTGGGCACAG
CGAGTTAATACTTCAGATTCATTAATTCGTAAGGATGAATAGGGGGTGGTGG
TGTGAATCGGAGACAGGATGACGATGAGATACCCTCCTTTTTCAGGACTTC
GTTGTGCTTTTGTCGATTTAAATACCTACACCCTTCGCGCATACCTCGCTACC

CTACCTCCGCAACGTGCATCTTTTGCATCAAACATCCATGGTTCTTACATGG
TTTAACTCCACTTAGTGTTGGGACAGTCAATTTGGTCCAGGATTATTTTCAG
GTACCAAAATTCTAATT

>Mosaic_sequence_70

GGTTATTATGCAAATGATTCTTCCATATTTTTATGCGTAATGCATAGTGTATTG
GATGTGTGTCTTATGTAATCCACGGGGAGCCTGAACAAACACTTCAAGGTT
ACCTAATCGAATGTTTGCACATTACCTAATGGCCCCAAGCCACCACAGCCA
CATGGACTAATTCTTTAAGCTGAATGTTGACGGAGAGTTTATCTTTCAATTG
TCCTCTTTTGGCCAAGCCGGTCAAACTCGAAACTGGAATTTGTTCTCCAAT
CATTCTAAACAGACCGCGACGAGGCGGAAACGCGCTTTTTTTTCGACGACT
ATCCAAATCTTGGACCCAGATAACTTGGGCCAAGGGCCAACTTCCCCCATT
CCCACATTCCCAATCGCGATCGGAGCTGGCGTCGCACGTTTTTTTTCTCGGT
GGAGCTCCAGCACCCTGAACACCTGGGCACTTAGCACCTAGCACCTAGCAT
CGTACCCAGTTCTACAACTTCTACTATTCCGTATTCCGTCCAAGTCTCACAA
GCTGGAGGATTGTGGACGATTGAATTAGGTCAAGTCAAGTCATATTTAAAA
CGAGTCAGAAAAATACTACGGAGGACGAAGACGGTTCCTTCACCGAATCA
GTCAAGATGCAGCCTGGCTACCGTCCTTACCCGCAGCAGCAGGTCGCGCC
GCAGCGTACCCCCCATTCGGCCGGACAGCGACGAGGAGGCATTGGTATGTA
ACTTTGGACCGACCATACGATCTGCTTTTTCATCCTGTATTATTTTTATTTAA
ATTGTCGACGATCCAGCGCCAGACAAACCCCACTACGTCCACTACGCCCAC
GCATCCACATACGTCCACTATATAGGACCACTACGATGACCCCCTTTGGCTC
CACCGCTTCATTAAGCCCTCCATTGCCAACCTACCACATACTACCACAATAT
GCACATAACTCACATACATACATACATGCCTACATACACGTCGTGTACCTATA
ACTAACAACTTGCTTCCTCCTGTTTATCCCCGTCTTCGGACCTGTAAGGGGT
CTTGTTTCACCCCTCTCTCGTCGTATCTCAATTTCTAACCATTTCTGCCGTCA
CCTAGGTCCCATGATGTCGTCCGGTCCTCATCACCAAGTGCCCTTGACTCA
GGCGCAAATCGCACAGCAGCAACAAGCTGCCGCCATCCAGGTTGAGATGG
CAAAGCGTCGCAGTCGCAAACCAACCGATAAGAACCTGCCAGACAGCCTT
GACGATTGTACTGTCGGTGACATGGCCGTGAGGTACCGGGATCTAAGAGAT
TTTGAGAGAAGACTCGATGCAACAATGACGCGAAAGCGATTGGATATTGTA
GACTCGGTCAACCGAAGCGTTAAGGTATGTCTCGCGTGTGATATATATAACA
TAAGTAAGAACCGTATCGGCTAATTGCAGCTCAGCGATGGAAGACTCTCCG
AATTTGGATTACCAACACGGCAGAAGACCAAGTTTGGCAAGGGAATGGTC
TCAATATCGACACATTTGACTTCAGCTCCAATCTTGAGCCTACATATCAGGT
CAAGATCGAGGGACGACTTTAGATGACGATGACGATCTTGATAAAGACGAC
GATAGTGAAGATGCGAAGGAGAAATCAGACGATCCCGACCGTATGGAAGA
AGATCCGAAACCGAAGGCGGCCAGTGCGCCAAAACACAGATTCTCGCACT

TCTTCAAGGCATTAAGCGTGACTATCCCGCCAACAGGAAGGGTATCGATCG
AGAGGGTCGAGTGGAAGAAGCCGGAACGTACTGGTCCTTCATCGAGTCTT
CCTGCCGCTGCCGACTTTGACGAAATGACATTCAAGCGCAGCGGTGACGA
GAACCTCAATATCACGATCAACCTCTACAGACACGAAGAACCCGAGCGATA
TGCCATCAGCCAGGATCTAGAAGACATTGTTGATTTGTCCGAAGCCACACG
CCAGGAGATTGTTATGGGTGTCTGGGAATACATTAAGCTGATGGGCTTGCA
AGAGGATGAGGAGAAGAGGAATTTCCGCTGTGATGAACTCTTGAAAAAGG
TGCGTTTCTTCTGTGTTGGTTTATCCATTCAATTGACTAACCTACCGGATCAC
TAGGTTTTTGGTATCGAAACCGGCCAGATTCCCAAGTTACACGAATACATAT
CCCCGCACTTGAAACCATTACGCCGGTCAAACTTCCTTACACCATCCGACT
TGACGAAGAGTTTCATAAGGATCCTCAGCCTACCGTATATGATATTCGCGTT
GCTGTCGATGACCCATTACGTGAAAAGATGCTCTCATTTTTGAGCAATGCCG
GTTATGCCAACATGTTGAAGGATGCAGCAAATCTTGATGAGCAGCTTGCTA
CGATTATCCAGGCAATTCACGTGTCTAAAGCCAAGCATACATTCTTGTCTTC
CCTCAGCGAAGATCCTGCCACGTTTGTGAGGGACTGGCTTAGCTCGCAGA
AACGCGACCTAGACGTGATCATGGGAGAGTCGATGAGAGGTGTGGGCGAA
GACGCAACCGGAGACGAGTGGAGAAAGGGTGGAAAAGATAGTATCTGGTC
TACCGTCAACGCTCGCGAAAGCGTCACGGTCATGTTGTCGAAGCAGCCTAT
TCAGGTGCAGCGATAGGATTGTCGCGCTAATAGTCCAGAATCGACAAGCAA
GGCAAAACCAGAAGTGGCTCAACCGGAACTTGGTCAAGGCGTTAGGTGTT
CGAGTGGTTTCCCCTGGACGTATCCCAAATATCTCCCGTCCAGAGTTGCGTC
TATTAAAAGTGATAAGTGATATGGGGATCGCTTGAGGAAAAGGTTGTATATC
CCTGTATTAGTATACCAGCTTTCTCTATAAAGGATGCTTCTACGCATGTGTGC
TTAGGTACCTACCTAGGTAGATGGCTGGCTTGGAGCTCATAATAAATACATT
TTGGAAAAAATAGCTCTGACTATTGCGTGGTGTTGTGAGATATTCGCAGGCT
TTCATAAGATACATCCGGATGCTTCAATTTCCGTCGATAAACCCACACGAAC
CGTAGCATATCAATGGCTGTTGAGGACTATGCGATGCACGCTACGTCATTTT
AGTGAAACGTACATGTATGTCGAGAGTTAGCATCGGGCTTTAATGTCTTATC
CCCGAATACGGAGGGGGTTTGGTAGGATATAAACTTTCAAAGACTCCTCGA
GATATTGCTTGGGACGTTCGAAGGAAGACAATCCGGGTACGTGCGAGCTTT
TGAAATTCCAGATATAATATTCAGAATGTCGGCGATCTTCAAGAAGGCCAAC
ACGGCCGTCATTACGGGCGGAGCATCGGGGATCGGACTCGCGCTCGCGCA
GAAGTGTATTAGCTATGGTATGAAGGTGCCTTGTGCCGATTGGGATGCTTCT
GGTCTGGATGCTGCAAAGGGTAGTTTAGGAGAGGCGGCGACGACGCTGCG
TGTTGATGTTAGCAAGACGGAGGACTGGGCGACGATTAAGAGCGCGGTTG
CTAAGGACTTTGGTGGTATGTATCGTCTGTCTTTGTTTATTCAATCAATATCT
ATTTCTATCGTTATGAATCTTGTATGCAATCTTAGTTTCTAATGATGTGTACAG
GCCAAATCAACCTCCTAGCCCTAAACGCCGGTGTTGGCGCCCCGCACTACC

TTCGATGGCCCGGACGCAGATCCTGCCACGTTCCGGAAGGTCCTCGACGTC
AACTTCTTCGGAATCGTAAACGGGATCACTACTTTACTCCCCTTGATTAAGG
AAAAAACCGCATCTGCATCCTCCCTCGAGTCGGCGATCGTGATCACGGGGT
CTAAGCAGGGTATCACGAACCCTCCCGGTAACCCGGCATATAATGCTAGTAA
GGCGGCCGTCAAGTCTCTTGCTGAGCAGTTGAGCTACGATTTAAGAGCGCA
GAAGGGTATCGCGGTTCATTTGTTAGTGCCTGGGTGGACGTGGACGGGTCT
TGCCGGTGCGCGAGTGGGAGAGAGAAGCCGGATGCACCGTGGACGCCGG
CGCAGGTTGGTATGTGATGTTTAATGTTCTTCCCTTTTTAAGCCTATCTCGGG
TGTGACAGATCCTTGCTTGGTCGAGCTAACCGGGGAGTTTGTTGCTCCTCG
AAGTGGATTTGACTCTCGCGCCTTCCTACTCCCGAAAACCCTTTTTCAAATG
AAGAAATTACTGACTCCTATCGTTTAGTGGATTACCTAGAGACGAAGATGG
GAGAGGGCAAGTTCTGGGTCTTGTGTCCGGATAATGAGGTCACGGAGGAG
ATGGATCGGAAGAGGATGCTGTGGAGTTCGCAGGATGCGGCTACTGGACG
ACCGCCGTTGAGCCGGTGGAGAGAGGAGTATAAGGACGAGTTCGCGAAGT
TTATGGCGAGTGATGCGTGAGGCGACGGCTTACGATGACCAGGGTATTATG
GCAGTGTCGCCTATCCAACATATAAAGTTTAGCCATAAGGCATCACAGTTCA
ACGCTATATTCCAAAGATGGAGAGTGGTGTATATGATGTGTTTCATTGCAAA
GTTAGAGCTCTTCGTGGCTCCGGTGACAGTGATGACCCTAAACGCCTAACT
ACATGCAATAATGTCTGCCAATGACGGCTGCTTCGTACGTCTACCGTAATCT
GTTCGTCGTCGATATCTAGACTCCCCTTTGCAAAGCTCCAAAAAGCAGTAA
TTTCAGACTGCCGTGCCGTCAACAAGTAGGTAAAGCAATAGAGTAGTAAAT
CGACTATATGTATACGCCCCTCTCGGCACCACCGCCAGTTCCCCCAGACCTC
CTCTAGACCGCCTGGGTCATCTGGCGGGCAGGAGGCACGGGGTTCGGGGG
CATGTTCCGGATCTGCTTAATAAGGAACTCACGCTCGTCGATGAACTGCTC
GTCGTCGGTGCGGTCCTCGAGGAAGTGGGAGAGGAAGTTGAGCAGCTTGT
CGCGGTTCATGAGGAGGATCTTCTGCACGGCGATGGACTTGTGGGGGTTGG
CCACGAAGACCTTGAAGACGTGGAAACCCTCGTACTGGACCATCTTCCGG
TCGCTGCGCAGGAGGTTCATGCAGATCTTCAGGTGCTCGCCTCGGTCCACG
TACGCGGTCATGACATTGTAGTTCGAACGGTCGAGTAATATCTCGCCGAGC
AACTTGATCGACTGACGCTTCGTTACGTAGCTGTTGGACTGTACTAGGATGT
GGTTGTACTTGTCGAAGAAGAGCTCAAAGTTGATCGTGAGGTAGCGAGGT
ACGAGCTCTTTGTGCTTGGTTAATAATTCCTATGGCAGTATTTGTTAGAGGAT
ATTGTATGACTAGTCTAGGCCGGATAGCCGGATCATTGGATCGCTAGACCTA
CTCGGAATGTGGTGAAGGCGTCGGCGGCAACTTCGAATGAACTCTTGTCG
ATCCAATCGAAAAAGCGCCAGAATACTCCGTTGCCACTTTGCGGTCGTTCC
TATCGATCGCCGTGACACCCTTCAGACTGGATCCTGGCTCTTCCCCGTCATC
GTAGAGTATAATGGCGGCGGCTGCCTCGCTCTTGAGAACCTCACGTAGTAC
AGTACCGGCTGGTGTCGCACTCTCTTTGTGATCATAACCTCGGCATAATTCC

AACAATACTTGTGGCTTATTGTTAACCACATAGCTCAATGCGACTGGATCGG
TCTTGGGCGACGCAGTTGGGGGTCGGAAACGGAAGACGTATGAGAATATG
ACTTGAGTATCTTTGCGGGATTCGAAAGGGAGGCGGTGCAGGTTCTGGGC
GAGTAGAAGGAGGAGGTCTTCTTCTATGAGACCTGTGACGAGTTGGTAGA
CTTGTTCAGGGGTACTATCTACTTCTGTTGTCGAGACAATGTCAGCATTAGC
GTTGGGCGGGTACATGTTTGCGCAGGAGATAGGGGGATGCGCCACCGCATG
CGACAGTGCTGAGTTGAGGTAATACCTGGTGTTCCTTGGAGGATGAGCTTC
ATCTGGCTGAGCACCTTGGCCAGTTCTTCGGCCTAAAGGATACTAATTAGC
ATATGAATCAATGTTATTAGGGGTCAAGGGGTGCTTCTTCACCTTTCCGAGG
CCGTCGAGCTTTAGGACATGCTCCCTAGCTTGCTTGGGGAGATCTAGAGTG
TTAGTTCTAGTTCTCGCTCTCGAGAATAGAAGGACATGATTGCGATTAGCCA
GATTGGTAGACCATCTGAGTTTGAAAAGGTCTCAGATGATCAGATGAGGTC
GCAAAGTCGAGTTAGAACTAAGCATAGAATTGGTTCGGTAAGGAGTGGCC
GAGTACTACCCGGCGATAGTTACAATGGATTCTTTTGAGGCCCCCAAAAAT
CGGATTGTGTTGTTGTGTTCAAGGTTTTGAGTCAACCAGGTACGTCATTGTT
GTTTGGATTGTTTGGATGGCTGTTGGATAAGTTTCCTTTATCGCTATCCAGA
AATTGGGGTTACCTTGGAGGTTTTGGTTAGGCTTGAATGTTGCCATGTGCCT
CCTTTGCTGGTTGCCGCAGTGTTTATCGTAAATCCGGGGATCGGAATGTTTA
TTTACGGGCACAAAGTTATACTTCACTTTCAGCTGAGACCATGGGTCCTATC
ACACTGTGGTATCACAGTACAGATATCCTATCGGGGTCTCGGTTTCTTCGTA
AAAGCAGGTAGCACAAACCAACAAACTCTTTTTGCGGTTAACTTCCTTTAG
GTTCATATTTAGTCATCATTATATTCCGCAAACCGTTTTATGTAATAATTACCT
GCTGTCACAAAGCCAACAACCAAGGGCTCATCATTTATCAAGGTAGGTATT
CAGCGAGAATGAGACATTGAGGAAACTCAGAAACCTCTATTTTCATGCCTA
ATCGCATTTTTAAAGCATGGTATGATTTCACATCACCATTCCCCAAAATTTAT
CTCAGTGTCGATGGGGGGATGCTGATAATATGCCATTGTCATCGAGCGTTTT
TTGATGCTTTCTGTTGCTTTATGAGCTCACCATGGTCTCAACAATATTCGAG
GTATATAGACACCATAACTCACAAGGAAACAGTCTTTTAATCTAAGGAACTC
ATTGACACATTTGTCTTTATGAATTGAGTTGTAATCGGCAACGATACATTTCC
CATACACGCTTGCCTACACGATAATTAATATCCATCATGTCCCGCAGTTTTGA
ATTATGTCACCAGGGAATATTACCTCGGCGGAGCACTGCGAAACGGCTGCG
GCAAATTTCTGAATTGGTCGTTGACGTGGTGGCATTGTATCAGAACCTCATA
GATCTTGGAATATGAATATGAAAATCGGACAAATTTGACAAATTTGAAGCTT
GATAAAGTTTATAAAGTTAACCTAGTATTGAAGTCCTACTCCCGAACTGTTA
AATTGAGCTCTGATAGAATGTTACGTCATAATATCATATGCAAGTAAATAAGG
AAACTCTATGCATTTATAAGCACATATTTGAGTAACGTTAAATAGCGTATACG
GTGGTATGTACCTAACATGTTGTTGTTATGTACATAACTGTAGTCCCACTAAA
TTGTAGCTCCAACCTTCAATTCATTGCATACAGGTCGCGACGCGACTATTCT

AAGTTTTTGTCTGAACTCAATTCCAGGCCTATAGTCAAAGTCAATTCTACCA
AGTTAGAGCTGCCTATCTTCTGTGCCTTCTGTTTTCTCTGAGACATGAATATC
TTCTCTTCTTCAATCATGTTGCCTAATCGTTGACTGATGAATGACTATCGGCT
TGCACCGACCTTTCAACCCACTAGAAAACTACACACACCTAGGTACTTACA
TCCATAGTACCTTCTAAAAGAGCTGTAAGCTAGACGATGCGTACACCCACG
ATTGCCGCTACTTAATTACGACTCTACTATGGCCCTCTTATACAATCTTCGGC
CATATCTCACTGATTTGTCTGATACATCATCCGATGAAACTGAACCTCATGA
AACCATGGCTTCATCTGCTGTGCCCTCCCATGAGAGTCTAATCATGGATAGG
GACAAGTACTCGGATGAGGGTATGCTTCCTTTTTCCCTAATTCTCTATCCTG
CTAATTCCTGCTAGACATCCAAATTCTCTACCATATCGTGACACGAGCCGAA
GAAATATTGAACGACTACACGCCTACTTCCCGATTACCGACCCATGCTTTAT
ACAATGCCTACGACGAAATCCTGCCTCAGTATGGAATCACCCAGACGATGA
CCGCCTTATCAGCGAGCTTGTGTTTAAAGTTGGGGGTGTCAGAAATACCGA
TTCACTTAGGGAGAAGTTCAAGACGGTTATGTCCAAGCTAGGAATTACAGT
TCAATGGGACTTCCCCCGGCGCATGGAAACGAGTTTGACGATGCACATTCC
AACTCGGCCTACACCTCCGATGAGATGTCTATTGTCGACAGTGAGGATCCC
CATGCCGTCATTGGTAGAATTGTACAAAAAGAACACCCTGTTGTAAACGGT
AATCATGACGGTTATGACCACAACTTTCCCGGGAACCGGTATGATCATCATA
TGGATGATGTCAATGACAACGTTTCTACTAAGGAGGATCCCGCCAATCTCTC
AAAGATCGAACAGCAACTCGAATGCAGCGCCATTGCTTTTCAAAAAAAAA
CATCACAACAAATTCACTGCGATGACAGCCGTTCGTCAATGGCAGAAGAA
GTCTAACTATATTGGCACACTATGCGAGCAATTCGACGCTGCTCGGCAGGC
CGACTTAGAAGAGGATCTCGAAGCGAAATTTGAAGAATGAGAGCCATATCT
GCCGATGTGGATCGTGTACCACCACACTGTCTTCCGCCAAATGCATACTCG
AAGCGCCTCGAGGGGCTTGCCATACGAGCTCGCGAGATTCATAATGCTAAA
ATTGCCTTGAAGCGATGGCGACAAAATGCAAGAGAGCAAAGTCGTAGAGT
CCAGAAGATCGAGGAGTCATCAGATCCTATCGAACGGGTTGCGGCAAAAG
CACATAAAAACTTGATGTTATCACGTGCATTTACAAATTGGTCCAACCGGCT
GGAAGAAGAATCGGACAGGGCACAGATGGCTGCTAAAGTTTACGAAATGA
GATTAAAGTCCAAAGCATTTGGCATTCACCGTCGACCAGAGCAAGACGCCC
AAATGAACGAGACTCGTCATAGTCCTGTTGAACCTTCGCCTCCTGCTCCTAT
TGTCAATTCGGAAGACGCCCCCAAACCTCCGCCACCAAGCGAAAACGTGA
TACCAAACATTGCCATCAACAACGCTAGTCCACCTCAAGCTATACCTGATAC
TCGTGAGAAAATCTCCCCTGAGATACATACGAAGAACGTCGATTCCAATGA
TGATCCGAATGATTCTGATGACGAGATGGATGAAATAACACTGCTTGCACG
ACGACATATTCTCCGGATGCGTTATTATGATATCTGGGAGAAGTACACATCG
GATAAACGGGAAAAGGCTAGAGAGTTCCAAATCGAGCAACAGAATGAACG
AGCTGCTCAAGCAATTCCTGTGTGGCGTTTCCAGGCAGAGCAAGCTTCTCA

GCAGCAAGAAACTCTACGATATAATGCAAAAAGAGCCAGCTACTACAACA
ATGCTATTAAAGCGATAGATGTATGGAGACAAGAGAACGCAGAGAGAACA
CAAGGACATGATCAGCTTCTAGATCATTATGCTGAGCGAGCCAACTTTTACT
ATAAAACCACCAAGGCGATGCCTATCTGGCGGAGCGAAACTGAACGTGCT
GTCAAGCAGCAAGGAGTCCTTGAGCTTTACGCCGATCGAGCCGAATATTAT
AGCGGGGTTACAAAGTCACTTCCTATTTGGAGAGCACAAACCGAACGGTC
GACCGAGCATGAGGAAAAAACACTCGCGCACTATGCTCAGCGAGCCGACT
ATTATTATAGGACTAGAGATACTCTGTTATCATGGCAGGAGTTAGCTAAGAA
GAAGCGCAAGCAGAAGTTGAAAGAAGCACATCTGGAGACTCGTCGTATTG
TAAAGAAATGCATGGGAGAGCGATGCATCGCGCAATGGCGTGAGAAGCTG
CAGCCAAGCTTTGAACGATACGAAGCGATGAATGCAGTTTTGGAGAATATT
GTTGCAGACCGAGAATATCGACAAAGCATGGAATCATTCGACATATGGCGT
GAGAGAGCACTAGAGCGGAGTAACATGGGCATTACAAGCGACGTAATGGC
CAAAGAAAAGATCCTACAACAGTGGAGAGATCGGTCGGAACGCCATGAAG
ACCTACGCATCGAAGCTGATCAACATTGGGAAGAGACGGCTAAATCGAAA
GCGTTGAAGAAATGGAATCTTACTTCCCTTCAGATACCCAATCGACCGCTC
ATAGTAGCAAATGCCTTGGAGAAAAAAGAAGAAGACAGCTACGAATACAC
TTCGAGAGTTGGTATGGTCGAACAGCAGACAAGCTTGTTCCCGTTGAGCTC
CCTAATGGAGACTATAGAAGCGTCGATCAAGTAGTAGAAGACGCCCAACAT
CGGGCTTCACTCAACAAGGCGCGAGGGTTGTTTGATAGGTGGCAAGCTGC
GGCGAAGGCCAGGAGTGAAAGTGCGCAGGAGGAGACTTATATACCTACTC
CAGCGAGACCACGCCTTTTCCTAGGAAGCTTAGGCACGACTACGCCTTTGG
CTCCTCCTCCTAATCGACTTAATTGGCGAGCTAGTGAGATGGCGCCGAGAG
GATCAGCTCTTAGAGGCAGGACTAATCGTTCTGGTCGCCCGGAAAGGAATC
TTCGTGTGTCGTGGGCATAATAATCAAGTAATTGAAAGCATTGAGAGCATTG
ACGGCGTTCTTGTTTGTCCCATCTTTGTACGATGGAAATATGCATGGAACAG
GCAGGCTAGGCTTACAGGTCTATATAGTGATACCCCCTATTCTCTTGATTGGG
AGATCTGGAATATTCAAAGATTCGAAGATACCCATAATGAAAGACATCCACT
GTTGAAGTGCACCTCGATTCTTACATGAGCGTGTTTCCTGAATAAAAGTTCC
CGATTTGAAGGTATTTTTTGCACTTAAATGATCTTTATGCAGATATCGCATTT
GTTCCCACTGTAGTCCACCGTAGTCCACTGTAGTTTGGTTCTAATTATGCAG
TAACGAGCTAATCGCGGGTCCCTATTAATTGATTTTGGCAGGGATTCGCGGC
CCGTCCCACGTCATTTTGCAGGCGCAAGCCGACGCGAATGGCCGCTGTAGC
AGCGACATCTGACAACCTGGAGAGCACCTGACATCTCAACACCCTCGTTAG
AGCTACATAGCTACGACGCTACGTACATCGGCCTAAAATATTTCTAAGGTTT
ACATCTTCGAATTACGATCCTTGAAAGTTCTACAAACGGGTTATATGTAGAC
CAGATTGACTATAATACTAAGACCATTTTCATTGACCAAATCCTTCTGCCGA
ACGCCGAAAGCTTACTTCTCGTAAATTGCAAGGGGTAATTTTGGGATTTCG

AACCAGCAAAGCCGAGCGCTCTTTTCCTTCCATTCAACCCGTCGCACCGTC
GCATCACCCTCCCTGAAGCTTTAGACATGTTACTACTGAGAGAATTGCGAC
GATAGTATGAAGGCAGTCCTTCCAGGCCAGCCCGAAGCTAGCCTTCAAGCT
GTGTCGACCGGTTGTTGGGACAAAAAGCGCATTATCGTTTACATCACAGGC
AACGCCCTAGCCATCCTCTCCGACCCCCAAACGATCCTCCAAACTATATACG
ACGATGACGAGCGGGCGCTTGAGGCTGTTTCCTTTGACGAGTCGTCAGGG
AAGATAGCTGCTTGCACAGATGCGACTGTGAGGATATATCGGCCCTTGAGT
CACGGTGACGACGGTGGTTCGCCAAAGGTTACTACTTGCCCCGACATAGAA
TACCATGGTGTTTTATTAACAAATAACTTGTATAGTGGGTATTACAAACATCC
TTCGAGGTCGATCCTGGTCCAACCCCCTTAACGCTATCATGGGAAGCTCCG
AAGAGCTTCTCATCGGACGATCCTCTCTTTGGCTTTACGACACGACTACAA
CACCCGCTACTTGTCATTGGCAACAGGGGCTTGCTAGTCCCGCGAAAGCCG
CGAACCTATCCTATGACTCATCTTATATTGCTTCCGTTGGATATCACGACCGG
CTAGTTAAAGTGTGGCGCAGATTGAATTTCGGGTCGGAGGAAGTGCATTTC
GACTTTGGCTATCTTCCACACCCGAAAGCTATCACTAGCATGCAATGGCGG
AAACCCTCATACGTCGGCCAAACGATCGAGAATGTGCTCTATACATTTTGCG
CCGATAACGTCATGCGCGTCTGGACAGGTGCGGATGGCTCAAGTTGTCAGT
CGCTACGTCATGGGGAAAGTTGGATCTGGCGGCAGCAATACGGAATGAGC
ATGAGACGCGCAAACACTACTTGAGATGGGCTTTTATCCTCAGCCAGCGGG
ACCTTGCAAATGCTGCAGAAAGAGCTGTCCAGGAAGAAGGGGATGTCGAA
GCGAAAGAAGACGTTGCTCTTCAGTACTTGCTATCTATCGCGAACAGAGAC
ACGGAAATCTGCGTCGCCTTTGACGGACATGGGAGTATGTCGTCCTGGGCC
TTAGAGAATATAAGTAGCAAGTCCAGGGGTGCTGAAAACGTGGACAACGT
CGCCTATGTCAAATCCAAGGACTTCGATTTTCTCGGAGATTCGACAGGGGA
CTGTCATGTTGAAATCCTCAGTTACTGCAACAAGAACGCAGGAAATTTAAA
TATGCTGTTTCATCACTTCGATGGGAAGGTCGAAGTGTACCAGAGCAATATT
GCAGATTTCCTTGACCCAACTCCCCATCCCAAGAATCGCTTGGTTCCTTGC
GCAACTTGGTCTGGACACTCTGCCTCAATTAAGAAAATTGTGCGAAACTTC
AGCGGTCGTGCGATCGTATCGAGAACTGAAAAGGGAGAAAGTATTGTGTG
GCGACATACCCCTGGTCCGCTGCGGACGAGATTATTGCGACATAACGTAAT
CCCAGAGACACGCCATATTCATCGAATATGTTTAATTAGAGGCGGCAGATTT
GTCGTTTTTCTACACCATGATAGCATCTCAATCTGGGATTGCACTTCACCAA
CGCACACGCTGCTTGCTGATCGCAAATTCGATATTCCTGGTAAACCTCTTTG
TATCCTCGTGCTTCCGAGAGAGAGAGCCAATGATGATTCTGTCGCACATCTT
GCAACCGTTACGTCGGAACAGCGAGGCATAGTCTGGGAACTACACCTCCC
CTTAAAGAACGGTGATACTGATAAACCATATACTAATGGTGTTGAACCATGC
CTTCGAGAATTCTGCACATTCAAGTTAGATGATGCCGGAGACCTCGCTTATG
TTTTGCCGGTTGATCCCGCAGGTTCCTCGCCTGTGGTATCTAGTTCCCTAGA

CGTCTTTGCTCGCGATGTTGCTATATCATATACTCACAGTGGACGCGTTGAA
TTTTGGACTGCTAGAGTGTCTCGAGACCGAAATCAAGTCGAATGGCTTTCG
ACGTCTTCGATGGAGACGGGAGTGTCCGAGCCCACGTTAGTCAGTGGTAG
CACCATGAAAAAGGCCGCTCTCGTAAACGGGGATCGCTCAGAATTAACTAT
ATGGGACATTCGAGGAGCCCGACTAGAGTACAAGCAAGATTTCGAGAATA
ACAATACCGTGCAGGATCTTGATTGGACATCTACACCAGACTCACAGTCAA
TTCTTGCTGTTGGATTTCCGTATCGCATCATTCTACTTTCTCAGATGCGTTTT
GATTACTTAAACAAGGGTCCTGCATGGGCGCCAATCAGGGAAATCAGTATC
CGTGATTTTACGCCACATCCAATTGGAGACTCAACTTGGCTCAGCGATGGC
AATTTAGTCATAGGTGCGGGCAATCAGCTATTCTTATATGACAGGACTTTTG
GGACAACCGACCGATTAATATCGCATGCACGAGTGCCACATCGCAAAGATG
GAACAAGAGACTTATTCGACATCATTCAAAGATTGAATGGCCCACTACCAG
TCTTTCACCCTCAATTCCTCAGCCAGTACATATTGGCTGGAAAACACACCTT
GGTTAAGCACATACTCCTAGCTTTAAACCATACACTGAAGTATCATGTGGAA
GGGGATGTAATTGACGACTACCTAAGTCTTGACCTGACGGAATTCTACATAG
CAAAGTGGTAAGTAACGCGAATTACTCGGAGCTTCATTTCACTAACTCCCT
AGGCTTCTATTTCTACTAATGGGAATGGTTTCATCCTACCTCGTAACCCAGA
CTTCATTTGATGACAACGACGAGGCCTTCACGGAGGATATCGCCATGAATAT
ATGCGAGAAACTCCAACAAATAACTATCCCTCAACTTTCTGGCCACGAACA
AATTCAGCTACTCGACATTGTTGAATGTCTAGGGGTGGTAGAAAAGCAGCG
TCGCTCGATGGACGAAAACGGCTCGAGATTCATGTTATTCTTCAGGCAGCA
TGCGCTGAGGAAGAGACGGACTAACGAAATCCACATGTCATGGAGGGAAA
TTAATTGGGCTTATCATTCTACTAGTCAGGACATACTGACAGACTTCGTATCT
CGTCAATATCATGGCACCCTACTTTGGGAAAATGCAAGAGAAAGCGGCATG
TTCATGTGGCTTACAGATGAAGCTGCCGTGGTAAGCAAAACTGGCCAAAAT
GTGTTTCTCGTCTTACTAATCGGTCAACAGAAAGCACAGTTTGAAATTGTG
GCTCGTAATGAGTACACGAAGAGTGAGATGAAGAACCCTGTAGACTGCAG
TCTCTTTTACCTTGCCCTCAAGAAGAAAACTGTGCTTCAAGGTTTGTGGCG
CATGGCAAGTTGGAACAAGGAGCAAGGCGCAACACAACGACTCTTGGCG
AATAATTTCGATGATCCCAAGTGGAAAACGACGGCATTAAAGAACGCGTAT
GCTCTGCTTAGCAAGCGCCGTTTCGGTATGCATTTCGAAGTATCGCTAAATA
CATATTTACTAATATAACCTCTAGAATATGCCGCTGCATTCTTCCTTCTTGCG
AATCACTTGCAAGATGCTGTAAATGTCTGTCTAAACCAGATCAAGGATCTTC
AGCTCGCCATAGCTATTACAAGAGTATATGAGGGCGATAATGGGCCGATACT
TAGGAAGTTGCTTGAGAATGAAGTTCTCACTGTCGCAGCTCAGGAGGGTA
ATAGATGGCTCGCATCGTGGGCCTTTTGGATGCTGAAGAGGAGAGATATGG
CCGTTCCGCGCTCTGATTGTTAGTTCCCACCCAAATTCACTGCGCGAATGGC
AAGCTAATTTCATCAAGACTCCCGTTTACACCTTACTAGAGTCGCCAGTCTC

GTCAAGCAACTTGGAGGCCAAACTCTTCTTAACAGATGATCCAGCCCTAGT
CGTATTATACTCTCAACTACGACAGAAGACTTTGCAAACGCTCCGCGGGGC
GTCAAAGGTTATGCCAAGGACAGAGTGGGAATTTGTGTTACACAACGCAA
GGCTTTACGATCGCATGGGGTGTGATCTATTAGGCCTTGATCTTGGTGAGTC
GTAAAGTACGTATTATACTGATAATTACCTAGCTGACCGTTTAGTTCGCAACT
GGGAGTTCCTCCGACAACCGCCATCAACGGAACCAAGTTTGGCGGGGAGA
TCGATCCGAGAAAATTACTCCGACGTCGCAGTTCGTTGGTCGTACAAGACA
TGCCTATGTCACCCCTTGCTCCAGGAGAGATGAAGACGGGAACAGGAAGC
CATGGTGTCAAACCGCCACCAAGCGTATTCGAAGAACTGGATGCCAACTCG
TTACTAGACAGTTTTGGATTCTAAAGGTTGTCTGCAGTGTGTAGGCTAGATT
GCAGCGGACGCTTTCAAAAGCGATATCCAGGTGCCGGTATTGCATTAGCGT
CGGTGGGCTGGTTTCCAACCGTGTATCTAATTGTCTTGGCTGGGTATGGATA
CGGTGTGCGAAATGGCGTTTGAAGATGCGTTTAGTGCGGACTACATCCTAC
AACTGCGTGTTTGTTAAAGCCTGGGTTATAGATAGACAAGAACTCGGGATT
TAGAGATGTATTATTAGTGTATCGGAGGCATAGTTGCGGGGTTTGAGTAGAA
AAGGAGGGTGTGTTATGAGTCAGCTACCCCAGCTTATATTAATAAGCACCCT
ACGTTACGACTTGAGTATAATTGGCTACCCACCGGCATTCCACACCTATAAT
GCAGTAGTGGCAGTGACACAGCGCCGGTATCCCTTTTCAGTGCAATTTTGG
ACAGATCTAGGACGCGGGGCTTGAAAGATGACCCGCCGTAACCCATTTATA
TACGTCATCTTTGCTTGTCTCCGATCAGGGTCGGAGGTTATGGAATGAGGC
GTTAGGTGTGACCTATGAGGAAGAGCCATGGCAACCCGGTCTACACCATAA
CACGGAGCTCCGGTAAGAGTCAACGCTCACGGCTTCAACACCCATCATAGC
CCCTATGGGGTTTATGGAAATAGGAAAACGTGAGATGCTTGGCTTCATAAA
GCCTAGAAGTCCTAGACTATGTATCAATTCTTGTACATGATGGCCGGCTATAT
ATTCAATTCTCTTTCCGCTTCGTTTCTATCCTCCTTGGCTCATCGGATCAAAA
GCTTCAGTCTCCTGTATTGCCGGCGCTAGTTTTCATTTGAGAAACTTCCAAA
TTCCAAATCAAGATGTCTACTTCCCAAACCCCTTTTCCAGATCTCCCCAATA
TCGGTATCAAATTCCCACACTCCCCCATCACGCTCGCCGCTCTTGAGTACAC
AAAACAGCACACCACGGCAGCGACATATAATCACTGCATTCGTAGCGCATA
CTGGGCTCTTATCCTCGCCAAGAAATTACCTAATTTCTCCTCTCTCTTATCTT
CATCCTCGTCCACGTCATCCGGACAATCACATTCCAGCTCAGGTCCTCGAC
AGTTGAATATAGAGACCGTGGTCCTGGCAACCATTTTACATGATATGGGATG
GGCCACGACCAAGGAGCTTATATCCAAGGACAAGCGATTCGAAGTCGACG
GCGCGAACCTGGCTTATGAATTCGTGGGACGGTATGTTGCCGAAGGGGGTG
AAGGAGCAGAGGACTGGGCTGGTCATCAAAATGAGCATGGCCACGGGCTC
AGAAAGAGTGTTGTCTGGGACGCCATAGCCCTTCACTGCACCGGCTCCATC
GCCTTACACCACCCCAGTCCCGAAGTCGCGCTCGCGCACCTAGCCATCATA
ACGGATTTCCTAGGCCCCAAGTTCCCTCCCGGAGACGGCAAAGTGATTACC

GTCGAGGAGTACAAGACAGTCGTTCGCCACTTCCGATGAGTGGATTTGGA
AGCGACAGGTTAAAGGAGATTATGTGCGGGCTTTGCCGCGAGAAACCCAC
GGACTGCGTACGATAACTTCGTGGGCGTATTCGGAGCAAGGTATGGGCTCG
ATACGAAGGGTGGGGGAAAAGAGGAGTTCAAGAAGGAAATGGAGAAGGA
AGATACGGTTGGGATGTTGCTAGATGGCTTGGATTATCTGGAGGGGATTGTT
CAATCCCATGAGGACGACATAGATTTCTCCGAGCAGGATCTCCCGGAGCTC
GACATCATTGACCAGGACTCGGTCCTGCACCCCATCTACGGCGGACCGCAC
TTGGGTGAATGGAAGACCGTCTTCAGGAAGAAGAAGAAGGAGGAGAAAG
TGGATCCGAGCGAGTTGCTTGACAACCATCTCAAAGTCGAGGTCAACCGC
CTTTACGCCTCTCGCAAAATCAAGATCGTCACCTCCGTCTCGCGCGCCGAG
AGGGGTGTCTTTCACGGCGACAAGGCGCATTTCATCGTACTCAAGGTTGAG
CGGAAGGGCAGCGAACCTCTGGAGCTCGAGAGCCTGTACATCAACGTGAT
GGTCGAGAAAGTTCAGCCTGCGCCCTCTGCTTCCGCCCCGGCTCTACTTCG
CGACTCGACCAAAGCGCTGAGTGGTCTCTCCCTCGACTCTGGCGCCCAGA
CGGGCCCCCTCCCGACGAACACTAGCCCCGACGCACCAGACTCCCTGGAT
CCGCTTCAGATCATCCGCTTCGCGCCGGAGAAGGTCGTCGGACTGCCCTCC
ACCATCAAGTACACCGACGACCTTGAGATCGAAGGCGATCTCGGATTCGAC
GCTTACGGGGCGAAGCTCGGCGCCAAGGGCACTCGTAAATGGGGCAAGGA
GTACGAGCGCGAGGAGCGCTTTCAGTGTCTGGGCGAACGTTGATGACGAG
GCCACGTACGAGTCCGACCGGGCGGCAGCGGCTGGGAAGACCAAGAAGG
AGGATTCTGGGATGAACGTGCTCCACGTCTGCGCGTCAGCAAACATCCTCC
ATAATGAGGGTATTCCTAACCTCACCATCGGTCTCGTTGTCATATCGTACGGT
CAACCTTTCATTCTCACGGTCGTCGGGGAGGCCAAGCCCTTCATTGGGGAT
AGTATCTTCTTCCGCCCCTTCTCCCGCGTCTCGCCCGCCAAGTTCTCGTATC
AGAGCAAGGCCCACTTGCCTCACGGCCAGGCCCCGATTACGGACGACTTT
GGATCCAAGAACATGAACAAGCACTGGAAGAGGCTGGTGGACTGCCGCTT
GAATTCAGGAACGTGAGTCAGACTCGGCCTGTTGAGCTTTGTGCGCTTACT
AGAAGAATCTGCTGGGCGGTGCGTGACGGAGGGCAAAAACAGGTCGCATC
AGCGGACTCGTCTCTATTTTCGCTTGGTGGTGATGACAAGTTCTCAAGTGTT
GTATATACAGGGTCGTTTCCGCAATCAACAGTCGTCCCCTCACGTAGCTTGG
CGGGGCCATGACCCGCTCCAGAGTGCTATGAAGGTTCTTCGCCCGACGAGC
TGGTCGGCTGCCTTGCCTATTTCGGCCCAGTCTTTGTTGCAAGGTCGTGGC
AGTCACTTATGCATGTATTTACACCTCCGCCAAATGTACACAGTCATCTTCG
AACCAGACTTGATGGCCCTGCTTCCTGACTTGCCCGCATATCCTGCATGACC
GGGCTGCCATGGAGGACATAACCCGCCCAATCTGCGACCTTGCGATACCGC
TGGCGGACGTCGAGAGTCGCTTGCTGTACAGCACGGGCGACGTCCACCAT
TCCGTGAGTGGTCTTCATGTCGGCATGACCCAACCACGCGAGGAATCTCTC
GATGAAGAGCGCCGCCGGCTCGACTCTGACGGGCCACATGGTGCCGACCA

CGGTGGCGGCGCCTGCAATCAAGGCGGCAGTCGGCAGTCCGAGTGGCTCG
TCGGCGGGTGAGATGGAGTGCGCTGCAGAACCACAAGCGATCAGGATCAA
CAGCGAGTTGGGCAGCTTCAGCTCCAACAGATCCTGGAGGCCCAGCTCCC
CTGACTGGTCAGCTGTGTACGCACAAAGTCGTAGGCGTACCATCGGAGAG
GAGCAGTCTCTCGTCCAGGAGTCCAGATCTCGGAAGGCAGTGGCCACAGA
AGAGGACCGCTCGGCGAGTCTGCAGAGAATCGGCGAACGACTTCACGGTC
ACTTGCTCGTCCCAGAAAAAGTCGATGCCAGCTGCGACCGCCGCGTCGCG
CAGACTCGCCTCCAATCCCTGCCTGTACGGCATTCAAATTTAGCTGTGTTCG
CCCGCCAGGAATAGGCCGATAGGTGGCTACAATCACCGCCTCCTCTCGAAG
TGAAGTGTGGGAAGTGTTGGTCGGTGCCATACTGCGATCAACTGATGTAAC
GTAGCTGGCAAAGTTGTACGCGTATGACACGGGAAAGCGCTCGAGAAGCG
GTACGCCAGTTTCGATGTGTAGGGCATGTATGGGCACTGCGTGTAGTCCTCC
TGTCGGGCAGATCACGAAAACATCGTCTGCACTCGCCAGGGGAAGAAGTG
GCTCCAACAGTCGGTCCAGCGCACGAAGCGCGGCAAGACTTGTCCTCGTG
CTTCAAAGTTGATAATATGACTGTCCAATAACGCTCGAGCCAAATTTGAAG
AGACGACGGAGTGACCCCGTGACGAATTCATGTATTGGTGTCTCCCCAAAA
CGGGCAAATGCGAGGTGAAGAGTATCGTATTCTCCATCTTCGGTCCAATCC
AACAGTACCACTCTTCGCCCGGCAGTTCCGCATTGAATAGGTTTGGCGCTC
TAGAACCGACTGCTCGGACATGTCCCGGAGGGTATGCAGGTCTAGATCCCT
TTGCAAATTGGCGTTCGCGGGAGCCCTGTGCTGTATCGAGTATGGGTCGTG
GCGAGGCATGAGTGACAGGCTGCTCAACTGTTTGAGGCGTTCCAACTGCT
GTATAAATAAATAGGGCAGTGCACGTTGGATCCGACGATCCCCTTTCGACC
ACATGACGAGGTCGGCGCGCGTTCGTCGATGAGCGATGAAAGCCGCAGTC
ACCTCTGCTTTCCCCTTTTGCTGCCAGAAAAGAGCTTGAACAGCATCGCCA
GCCTCTAAAGCTGCTTCACGCCCCGTTATGTAAATAGTTGAGGACACACCG
GGGAACGGCCGTGATTTTTGAAGCGCCTGAAGCATCCAGTAGATCGATCGG
AACTCTTCCATTCTCTGGCGTAGCGCCGCGCGTGAAACCTTCTCCACTTCC
AAGCAAGCCTGCCTAAGTTTGAAAGAGCCGCCAATTAGAGATCCGGTCCG
GAGCAACCTCCTCTGAACGCCCAAGGCTCTTGTCCGTGCATATATGTACCC
GGGCATGGGCCTTCCATCGAGGGCAGTTTCAGGCACTGACAGATATTCGTG
ATGAGCCGTTGTGAGCTCGAAGATGAACTCTTCTCCGCGCAGAGAATCAG
AGCGTTGAGTACGGTTGTCAGCCATGAACGATCGGACCACAGTGCGCAAG
GCGTCCATGCCCACAGCCGGGAGCTGTTGACATTTTGGCTTGGCCGAGTTT
ATTCGAAGGGTCGTCTCAATCTCCTTAGCAAGGAGTTCAAGATGCTGCCAC
TCCAGTGATAGTCGTCGAGCTTGATCCAAAGGGTCTCCCTTTTCACAATTTT
GCGGAGCAAAAGATGCTCGCATATCTTGCCAGGTCCGCTGTTCGTCGGGAT
CATCGAAGTAAATCGGCGTTTCTGTATTCGCGTCTTCGAGGCGCAACAGGT
GCTGAAATAAGGAGAAGAGGTAGTGGTGCTCGAACACGTGCATGCACAGC

AGCGTTTGCTCGCGCCAGACTTGAGGTCCATATTCCCAATGCCAGATAGAC
CACAACCACCAGAATCGACGGACTTGTGATTGGGTCCATTGCACGGACTC
CCAGAGAAAACTAAGTGAGTGTCGAGCAGAGTCGACAGACTGGAGCCCTT
TGGTACACCATTGTGGTCACTGTGTCTTTCAGCACCACTTTCTGACCTGCTC
CGATGGTGATCCGGATCAGTTCGATTTAGTCTGCTCCCAGCAAAGACTTCA
AGGACGAGCGGGTGACGGGCGATGTAATGCGAAATTCCGTGGGGCTTCCA
GAACGTCCAATGGTTCGAAGCCCAAAGAGCTTGCCGAAGGTGATGGTCCA
AGCTTTTGGTGGCAGACGAATCGTAGAGCAGGACAGCGAAGATCAACGAC
TCGGCTTCGTGTGCATTTTCCGTCGATTGTACAGAGTTCAAGAAAGCGGCG
AACATATTCATCAACGCGGCCGAGGACCTGGTGTCTTTAACTTGCTGAAGA
AGGAAGATCGCGTCGAGTGCTTGTAGGTGACGTGCGTGGGAGTTCCCGCT
CAAGTTGACTAGATCCGACCGAAGTTGCTCGAGGGTTTCTTGTTCCTGATA
CAAAAGCTGGACAGGATCAAGGTTGAGGTCGAAGCATGCCAACAGTTTAT
GGAATCCTATCGAACAACGCGGGTTGCAGCGCTGGAAAGACAACATCACC
CGGCGGCGAAGGAAGAACTTCAACTCCGGTTCGAGTCGGGCGCTGTTTTG
CCAGATCCAGTGACAGGCAATAGGGCTGGAACATCTTGCGATATGAAGGGC
ATGATCCATGACTGGATACCCAGACCACCTCTCAACCGATGCGATGAGCAG
CAGATGATCGATGACAAGCAGCTGAGGTTAGCAGTGATCTTGTCTAAGCGT
TCAAGCGCAAACCCAGTTGAGACACGACCTGAAAAGGGAGCCTGGGGGC
TCGATTGCAGGACGGTAGTCGAGAACACCATCCAGCTGCATTTTGGACAAT
TTACGATGATCGGCCACATCAATGACGCCAAGCGCGAATGACTCATCTCGT
CAAGGCAGCGGCCGAACTTTTGCGACCAACGAAAGTACAGCCAGTGGGA
AACTCGGCGTGTATTTCCCCACCATCCTTCAGAGACAGCGTCCATCTCCTCG
ATGAATGTGTCGACGGTGTACCCAAAAGTCGTGTGCAGCTTTCGATAGCAT
CTGAGAGATTGACACAGCCTTCCCTGGGCACGTAGATCACGCGCAGCGAG
GATCGAGACGTCACGTTGCCGTCGGGTGACAGGGTCGCCTGTCCAGCGCT
TCAGGAGTTTGTCAGGCCCCATGTCGTCCGGGAACGGGTTAGGGAGAGCC
ATCAGGCGGTCTATAGCACTGCACAATGATTCAGCTGGGCATGTGCGCTGG
GGATGTGAAGAGTGAAGACGAACTCGAAGACAGCTTGTCGGGCCTTGCAC
CATTTGGGATCCTGCTCCAGATGCGGCTCTGCGGCGTATGGCGGGGGACAT
GTCACAGCCTCAAAAGGCTTGAGCAGTGGTGCCAGATGTTCCCTGATGCGT
TCCTCCAGCCGCAGATCCCCCGGAGTCGGCTCATGGACAAGCTGCGTGAAT
CGATGTCCTGGTCGATGAGTGCTTGCCACGCCAACCGGGTCGGGGTCTGCG
CCCATATCTCCAGCTGTCGTCCAGGTCTGGCCACTCCGAGCTACCAGCTGC
AGCTGTGGAATGTTGGGCAAGAGTCAGGCCGTCTAGTCGTAGTCTTGCCCA
GGAGAGAGGAGAGGGAGGAAACCAGGTGCAGAGCTGCCAGACCCAGTCG
AGCCAGGGTGGCAATACGAGCGGGCCCATCTGGAGGTGCGATATGCGACA
GTCGGATACGATCGAAGAAGCAATGTGATAAGGCGATTCTGAACGAAGGC

GAGTGATGTTGGAATGCTTCCATGCACACAGGTATGCTTCTTTCTGATGGAG
ACTCGTACTCTAGCTGTCTTGTGTATGGACAGCACACACACACACACTATG
TGCCGCCAATCAAGCTCGACGGATCTTCCAGCTGACCCAGTCCCATGTCTC
GCATGACCGGGCTTCCATGGAGAACATATCCAGCCCAGTCGGCTGGCCGCG
GGAACCTCTCGCGTACATCGAGACTCGCGTGTTGTACCGCCCGCGCGATGT
TGATCATCCCGTTGTCCATGTTCGTCTCTGAGCGTCCCAGCCAATACAGGAA
CCGCTGGATGAAGCGTACAGCGGGAACCAGCCTGACCGACCACATGGTGC
CCACGACCGTCGCCGCACCCGCCACGAGGGCAGCAGTCGGGAGTCCCATT
GGCTCATCTAAGACACCCATGTTCTGCGCGGACGACCCACAGGCGATAAGG
ACCAATAGCGAGCTCGGAAGAATGCAGTCGAACAGGTCGGAAGTCGTCAG
GTCGCCTACCAAACAAGCTCTCAGCAGGCCGCACAAATCGATGTACTTCAC
CGGTATGAAGGACGAGCCTCGGGTCCGTCGCAACCCAGTCAATCGGCGGA
AGCTGAAGAGCAGTGCCCACAGAATAAGACTACCCGGTGAGTCTGCAGGA
GATCTGTGAAGGACATTGGGGTGACCTCCGCGTCCCAGAGGAAGCGCGTG
CCGGTCTGAGTGGAAAGACCCTGCAATATATTGTCTACTTCCGCCTGAATAT
GGTCGAACTCTGGATCCACAACCCGATCAGGTTGTGGACGATAGATCGCGG
CAATTGCGGCATCGCTTCTGATTTTCGAAGGGCTCTCGCTGCGGCTCACGC
ACGCCATGTAAGTAGCCAAGCTGGTGGCGTACAGCACAGGGTACCGCTGG
ACAAGGGGTACGCCGGCAGCGATATGCAGTGCATGTAGAGGAATCCGGTG
AAGCAGTCCTGTCGGGCAGATGACCAGCTCGTCGTCAGGCAGCAGCAGCG
TTTGCAGAGGCTCAATCAGGTCGTCCAGATCACGGAGGGGCGATACGGCCT
CGGGAGGGGACCGAGTGCTTGCTCTGCGTGTCCAAGAGTGTCCAGTCCAG
TGTCTGACGTATGCAGGGCTGATCGCGGGGATTGGCAGATGAATTGGGGTC
TCCCCAAATCTCGCAAAAGTGAGGTGATACTGCCGAGTTTCTGTTTCTTCA
ACCCAATCTAGCCAGACAACGCGGCGCCCAGTGGCTCCGCGAGGGGCGGT
GGGAAGGAGATGGTCCGACGGTGAAAATATGGCTCGCAGACTGCTGCTGT
CCAAACGCCTTCGAGAGATAATGTCTGATATCCCTCGATTCCGAATTTGCGC
TCCAGGCTGATTCGGTAATATGGCCTGAATACGTCGATCGAGTCGACGAAG
CCGTTCGACGCGTTCAACTGCAGAGGCCCACGGCAGTGATCGACGGGAAT
CGCTCTCCCAAAAGTTCAGTTCAGCGTGGACCCAACGATCGACGACGGAA
GCAGAAGTCAGTTCTGCTTTGCCGCTTTGCTGCCACCAAAGAGCTTCTACA
GGTCGCCAGCCATCAATGCGGACTCTCGACCGGCCGCGTATACCGCAGAAA
GATCCTCATATTGCAGCGGGTTCTGCAAGTGATAGAGCCGAAAGTAGAGAG
TGGGCGACATGATGGATTTTTGCAGATGTGAATACAGCTCTTCGCGCAGCG
TTCTTTCCACATGAAGGCAAGTGATCCGAAACACAGGCGGTCCGCCGATCA
AGGAGCCCGTTCGGAGCAATTCTCGCTCGATCCGCAAAGGCCTAACTCGCC
GTGTTTTCGAGCCGTCGACGCAGCTACCTGCACGGGATACGGGACACACAT
TTGTATGTTCTGAAGGCTGTAAGACGGAGGGTTTTGACCAGCCAACGCGA

GGGCCGAGACATATTCGTGGCGAGCAGTCGCGAGAGCCAGCATTTTCGCTT
CCCCGCTCATCACGCCGGCCAGCTCGGCTTGAGCATCTGCCGCAAAAAAG
TGCACGTCGACGTTCATGGCCTCGATGCCACATGATGAGAGACGTCGTGCC
TTCGGCAAGGCAGCGTTGCTTTGCAAGATGGCGCTGATCTCACCCAGTAGC
TGACCGATCAGTTGCTTCTCGGCCTGCAGTGCCCGGGTCCTGAGGGCAGG
AT

>Mosaic_sequence_71

CTGACCGTCCGCTGACACGCGGTGATATTTGCGGCGGCGGAATACTAGCCA
TGCGACCAGAGATAGGATGAGGAGGCCGCCGACTGCCCCGCCTACTGCGC
CGCCTATCACCCCTGCGTTGCTCTTCCCACTGGAACTGGTTCCAGTGCCAC
TCCCACTGCCGGCGGCTGCGCCGCTGGAGGAAGCCGAGGGGCTTGCAGAA
GTAGTGGCTGCCGTCTGGCTCTGCGCGGCGCTGTCCGACGTCCCGCTCGAC
CCGACCGTAGGCACGGACGCTGTCAGAGCGCCCACGGCGGTGGTGTATAC
TGCCGAGGGCCATGTGCCCGTAGGCTGAAAGTCGAGGCCGGTGATGCATG
AGTTGCTGGAGCCGCTTGGCGCGGAGTCGACCGAGTAGATGGAGGCGATG
CCGCCGACGCCGTGGCCGTTGTAGTTGCTAAGTGTCAGCCCCAGCCGACC
GCAGGGAGGCTACCCCTCATCCAACAGTACAGAAGGAGACTCGCGAGTAG
AGATGGGAGGAGTGGGTAAGAGGTAGGTAGTGGAAGCTCGCCCTCGCCGC
TCGCCCTCGCTGCGCTCGGGCTGGGGCTCGGTCTCGCTCACCTCATCATCA
TCGTATACCTCGTCCCCTCCTCCAACTGCAACCCCCAGTAAAACGTCGAGC
TGCTCGTATTCCCAATCGCCACGTCGTACGGGTGAAAGCTCCCGTCGAGCG
GGATGATCGTGAGCGAGTACGGCCCCCGGCCCACCGCGTCGTCGTAGTAGA
TGCCCATCTGCCCGCACGGCACAGAGTCGCCCCCTACTGTAAAGTAGAACT
CGCGCTCGACGACTTTGACGCCCGACATGCACGAGTCGTCGCCTGATGATT
GCACCGTCTGGACCTCTTTGGGGTTGCCTGCCGTCAGCCCCTCCCTCATCA
CGCTCCGCTATGCCTCCTGGCATTGCCTCGCTGCGCTGCACTCGCTGCGCTA
CGCTTTGCTTGCGCTCGCTACTACGGTTCGCTGCGCTGCGCTTGCGGACCC
ACCCTGCACATTATACTCGTTGCCCGTCTCAGAATCCTCAATGTACACGACG
TAATCCGTGCTCGCGTTCAGTGCCAACGGCAGCTGCTCGTCAGCCACCCTC
CTCTCCTCCAGCCTATTACACTGACGGTGAAAGACGCCGGGTCGCTCGCTG
TGGCCGTGTCGGTCGTCGCGCCCAGGTTCCACAGTATCGGCATGCCGTCCA
CGGGGCTACCCGTCAGCCTTACACTACATTGATGGAGTCGCCTGCGGCGAC
TAGCGACTACACACATGATGAACAGCGTCCACGGCGCGACGCCACCGTAG
ATGTACATGCTGATGGTGTCGCATTGCGCGGTCGACGTCGGCCACGTCCAG
GTAAAGTTGGACGCGAGAGCCGGGGGCAGCAAGAGCAGGGCCGCTGCTG
CCAGAGCGAGCTGGAGGGCGCGCATGGTGTTGGTCGAGTGAGGGAGTGA
CTGACAGAGAGGCAATGATGACAATAATTCGAAAGGCTGCCGCTGTCTGTG

TCTGTCTGCGGGACGGTGATCACTGTTAGTCTCCCTCTGCTCTGCCCTTCTT
CCCGGCCGACGGAGCAGTCACCTTTGCTATTGCTAGTCAGAAGAAGGAGA
GCCCTGATCTGTCTTCTGATCTGATGCATCCGGCCACTCGGTCAGTCATTGC
CTCGTGTCTTGACTGCTGCACTGCTGCACTGCATAAGACAAAGTCGAGGAA
GAAGAGAAAGAGGGGAGACACGGCGTCGGGTGCCTCGTCACCCTGATTTC
AATCCACTAGTCGTCTCCTCTCTTACCGTCCCGAACAAAGATACCCTTCCGC
CCGTACACGACTCTTCTGAGTCTTCGTCAGCAGTCGCCAAGAAGACTGCCG
CTCTCACTTCGGCGTCGGACAGCGATGTGGCGTAGAGCATGAAGAGGCGT
CGCTGCGATATCGTACAGCGGAACAGAGTGACATGACTTGCACGTTGCCAA
GACACGGCAGACACGGCGACCCCTCCTTTGGGACCCTGAATTTATACACAC
ACCCAGGCACCGGGCGCATCACACACGTAACTACGTCGCCGTCTTTTATCC
GCAGGCAACCCTACGAAAGTGGCAGTGGCAGTGTGGCACAGCATTCATGG
GCGATGCAGATCAAAAGTAGCTAGGCAGTACAAAGAGCTCGGTGACTGCT
CGACTACGCCGGATGGACTACGATGCGATCTTCAACCACAGCTACGCCCGA
GTGCGACCCAAAGTGATACTCTTCTTGTCCGTGCGCTGAGTCTATGACTCG
CCACCGTCTTACGCTCAGCAACTCTCAACTCCGACGGTGCTCTGTCCATTT
AGGGTTCCGAGCCCAGCCAGCAATGCGTGATGGAGTCGGTATCAAGCCTG
CCGCCCATCTACAGCGTGAGTCTCGCTCTCGGACTAAGGCTCGACGTTCGG
TTCGGGCCCATCGGCAACATCGTCACCAACGATGCCGGCTTCGCCGTCATG
AATCGTGATCATGCCCGCCGTTCCCCCGCTCGCGCGAGCCGGCTGTAAGAG
ACCGTCAGAGGTCTCCGCAGCCGCTGATTGCGAGAACATGAGGATCCTAG
AGATCCACGACGCGGGGACCCATGGAGGCTTCGAGAGATCGGGGTAAGGC
CGCTCCTCCGCTGCGCGCGTGAGAGTAGCCGTGTCTCCCAGGGCTTGTTCT
TCTTCAAAGGCGAGATCATGGGGTTGACTGGGTTGGCGGCGCCAATACCTC
TCCTTGTCCCGGAAAACAACAGTGATACACTTGCGGAGCACAGTTACGCG
CTCTTCGGTGGTCAGGCCGGCCCATCGCTTGTAGGGGTCTCGTCCCGTTAT
GAGCTGGAAGAAGGCCATGCCGTTCCCATGCTGGAGGCGTCCGTCAGCGT
GGATTGAACCGCGTTCCGGTGTTCAACGCTGGGGAAAGACTCACGTGGGT
GCCGCACCAGTTCAGACTTTCCGGGAGGTTGTCATATGTAGCACTCCTTCT
GCTGCAATGGCGGTCTGGTCCCGTCCAATACGTACTCACCAGACTCTTCAC
AAAGTAGGGGTCGTATAGGACCTCGCACAGTGCGCTCTTGCGCCTTCGCCC
GGACACGCTGCCACGATCGCGCGCCATCGTGCGGCCTGCATCTTCCCGGCT
TCTCACCGGTTGGGTTGCGAGCGGCTGAGGAGCAATCTGATGTGCGAGCG
AGAGAGGTGGCAGAGCATTAGGCGGCCAAGCGGGCAGTGTTCCAACCGG
AACGGCGCTCCTTAAAGGCGGTAGCATCTGTGTGCTGTGGTGTGGAACAG
AGGGAGCGGGAGCCCGATGCCCATACGCCCCAGCACTGGGAGACACAGGA
GCCCACTGAGTAGGCTCATTGAGACGAGGGAAGGTGGTCTGCTGGTTGTC
AGGGATGCGGACTGGGGGTTGCGGCCAAGAGCTGGTGGTGTACACTGTGG

TGGTGCCGGCGACCTGGGATGCGGTTGCCGGCGCGCGAAGCGTAACACCA
GTCGTAGGAGTGGGCGATTCGATATCGCCATGAGACTTGTCTTGAGAAAGA
TCCGAGGCGGACATGACTCAGTGCCGTCTTGTCGACGACTCGGTCAGGTG
TTTCTGACAAGGCGAGGTCCTAAAACGAGCTCAAGCTACACGGGAGCATG
GAATTAGAGCAAGAAGACAGTGCAGTCAAAGTCCGCTCTCCCCCTTTCATG
CAACCCACGGCCAAATCAAGGTATTTCAGATTACAGATTTCACTCGCCAGC
GTGACAGCCGGCGCGGGTAATGCCGGTCCTTTCGACGAAGCCAGACTTCA
CGGTGGCAGTGCCAGTGGCCAAAGCAGAGTTGCACAGCCTTCATGTTGGC
ATTGCCGCAGGCAATAGAAAAGCTTGGGTGAGTTGCTCGCCTAAGACGGC
TGGGTCCAAGTCACTATACAATCTTCAACTACAGCGATGTCCCAGTCCTAAC
TCTCAGGACTCGTCTGGTCCGTGTGCTGAGTATGTGAAGCAAATCCAGTCG
AAGAATCAACCGTCGTCCTGTTCCTCATCTTCGCTCTCATCTCCCAAGTCCA
CCTCGGTCCCAGATGTCTGTCCCCTCCTCCGCGCCATGTGGTCCCTCTCCCG
CTGTATCAAATCTTCGAGGCGCGCCCTCACCTGCCCCTGCTCCTCGGCGTTC
AGCTGGTCCAACCGCTTCTCCTGATCTTCACGCGCCAGGAGCTGCCTATAA
GCCAGCGCGCTGTGGCCCTAAGACGCTCAGCTCCCGCCGCACACGTTCGC
CGCAGCAATAGTGGGACTCACATGCGGCTCACACCAGCTGAGGATATGCGC
CGGAAACTTGCTCGTAAAATACACAGCCGGCCTCCCGCACGTCCCGCTCGT
CCATCCGGGGCGGCTCTTGCAGACTCTGGGGTCGAAGACCACCTCGCAGA
AGCGTCGCGGGAGTGGCAGACCGGGGTTGGTGGCGACCCATGGAGCATAT
TCTTCGCCCGGCGGGATTGATACCACCAACGGATATTTGGAAGGGTCAGA
GGTTGGCCGTCTGTGCCCGATGGCTAGCGCAGCGAAGGAAGGGTCAGCGA
CTGATCAGAGGTACAGAGCTGGGTGGACTGGCTTTGATCCTGTGCCCCGGT
TGTAGTTTGAACCGAGGCTGACGGCGCGTCGAGTTCTGTGGTATCCGCGTA
CGAGACGCCCAGGTAGGAGAGGGTCGGCTCTGGGTAGGAGGGAGGAGTG
GGGCCAGCGAGGCGGGAATCATTGGCAGTACGAGCTGTTGAGGCCGCCTC
GCCTTTCGCGGCTGGGCCATCGGCGCCGGTAGTGGCATGCCGGGACGAGT
CCTGAGGAGACCTGAGGAGAGACATCAAGATTCACGGTCCAGTATCAGAC
GGTCTTTCAAATAAGGGTAGGGATCAGCGAGTCTAGGCGGGACATAAACA
AGTAAGGGGATGGGGCACAGGCCGGCCATTTGGCGTGAATGTAGCACTAG
AGTCGTCTTTCCCAAACAGATTACATCCTTTGTATTCATATTCATTTTAGCCT
TACATCTCCGCTTTGGTCAAGGAGGTCGGGAACCCAATTTGAAGCTGAGAG
GGAAGGCTTGAAAGAGGAGCCCTCGACCCCAAGCAGCCCGAGGAGCCCG
AGTGCCACAAACATAGTGAGATAGCTGGGTGAATGAGTATATACATGCATAT
AGGAAGGATCCCACTGCATATGACAGTATGGATAGTCAAGTCCGGCGGTGC
CGGGCCTACCTGTCGGTGCCCATTTGCACGACGATCGTGTCTTGTATGATCC
CAACCGAGTCCGTCGACCCGGCGGATCCCTCGATGCCCTGAGTTGCCTCGT
CAGAGTCGGACAGAGACTCTTCATCATTGTTCACCGCCCCGTCATGATCAG

ACAACGGGGTGTCATCAGGGGTGCCATCAGAATCGACCCGTCTGGTCTTCC
CCGACGAGCGGACAGCATTATTCTTTCCTTTGGGCCGACCCGACTTTGCGC
TTCTCCAGCACGCCGGGAGCCAGGAGTGGATGGAGAGGTCGGGGTACGGT
TCCTGCTCAGCCGCGCGGGTGAGCGCGCCGGAGTACTCACCGGAGTCCGA
TAGGACGCGCTCTAGGGTAGGATCCCTGGTTGAGGCCAGGCATGAATCGAT
GAGCACCTTGAACACCTTCACCCGCTCGTCGGTGGTCAGCTGCGGCCACTT
GCGATGAGGATCTTCGCCCGTCATGAGCTGCTTGTAGGGATACCCAGCCCT
GGTCTGGGAGACGCCAGTCAGCTCTGACCGTCTAAGGAAAGCAAGACCGA
TTGACTCACATGTTGTTGGCACCACCTCAGACTTTTCGGAAGGCGCCTATG
ATCAACAGCGTCTCGAACGCACCGGCCATCAGTCCAATGTCCGGGATGACT
CGTCACGAATAGGGGGTCTCGCTTTACCTCACATCGGTCTCTGACTTTCTTC
TTCGCTCCAGCCTTCCTTCGAACAATCGGGGGGCGGGGGGCGGACGGGTC
GACAGCCTCATCATCGTGGCTGGGCCCCCAAGTATCATTGCTGTCCATGGTA
CCGTCATCAAGCAGCTCGGTGCCCTTGGACAGGCGAGCATTGTTCGGGTTC
GCTTCAATTTCTTGGTCCCCAATCTTGTGGCTCCAATCTTCGGGGACCCAGG
GCAGGCGCGATAGGTCGGGATAAGTGGACTCCTCAACCACGAGGGTGATC
GCGGGACGATTGGCCTGGGCGCGGCGTAGAAGAGAACTGGCGTCTTTGCT
GCGGGACCGGCCCACGCGGTCGAGAAGGCCGATCACGACATCCACCTTCT
CATCCGTGGTCAGTTCGGTCCACAGTTGCCGCGGGTCTTTCCCCGTCATCA
GCTGCAAGTACGGATAGCCACGATGGGTCTAGAAGGCGCCAATCAGCTCTG
CTCGTCCACCGATGTGTCACACAGGGGCTCACATGCCCATTGCACCACCTG
AGACTCTTCGGAAGGCCTTCATGCTCAATAGCCTGTTTTGTGCACCGATTGT
TGGACCACTGTCCCTGTCCGTCTCTGACGAATCGCGAGTCTTTATTCACCTC
GCATGGGGATATTGTTGTTTTCTTTTGGCGACTCGAACGACGCGGGGGCGA
CGCCGGCGTAACGTGCGACTGGGATGTTGATGGTTTCCTTACGCGACTCGA
CTGACGCGGAGCCGATGCTCCCGTAACGTGCGACTGTGATGTTACTTTTTT
CTTTAGACGACTCGAACGACGCGGGGCCGATGCCTGGGTGAGGTCTGAAT
CTTCGCCAGCGGCCGCCTCCGCCGTTGATGGGGACAACTCGTTTTGCGATG
ACGGCAAGATGCTTGGTGGGCTGACGGCACGCACCTGGTCGGGCTGATGT
GCCCCAAGCATGGGATCGATTGCCATACGGGCCCAGGGCTGCGGCGACGG
GCTGACGGCGCGCACCAGATCGTGCTGTTCTGCCCCGAGCATGTGATCCAG
TGGCAAAGAGGCCCAGGGCTGCAGCGACAGGCCGTCAGCCCGCATGCCGT
GCACGTCGTCACTTTCAGGCATCGGAGGCCACTGCATATACATGTCAGCATA
CTCATCTTCATGCTGCGCATTAGTGTCGAAGTCGGTGGGCTGGGGGTAGCT
GAAGGGGACACCCCATGAGTCGTCGACGAACTCGGAATTGGAGTTGTCAT
GTTGGCTGGCCTCCCAAGCGACGTCAAATCGCTCATTGGTCGTCTCGTACT
CGATCGGCCATGCAGGCATTGCCCTCCTCATCCTCCTTATGGTTGCTGTGGT
GAGGGTGCGCGGAAGACGACATCAGATACCGCTATAGCCAATACAAGTACG

CCCGAGAGTAATCTTAGGTTGGGAGGTCGATGCTCAAGAAAGAGTGCAGG
CAGGCAGCCCACTAAAGTACAGCATCGGCAGGAAGGCAGTCAAGACGAA
CCATTGATTCTCCTTTTGCTGGATCCCTTGATCACCTTGGGCGTTGCAGCTT
GCGGACTTGTCGCTACTTTTTTGCGAAATCCTCGAGCCATGCCAGGCAAAT
TCCAATCGACCCTCCTCGGTGGCCCCCATGAATCCTACAACCTGCCTGAAA
CGTACTCTGAGGAGAAGGGATGGATACCCTCTTGGGCGCTTATGAACTTAG
AGTCGTTTTCCAAACGAATGCCGACCTTTGTAATCATGTTCGGTTTAGCTCT
CCTTCCGCTTTGGTCAAGAAGGTCGACAATTCAGTTCCAAGCTGTGGTAGA
AGGCTTGTTAGAGGCGCTTCAGCAGTCCGGACACCCCGGGTAGCGCAGTC
ATAGCGCAATGGCTGCATGTACCAATATGCACACGTGTCGGAAGGAGCGGC
AATCTTAGTCCGGCGGCGCCGTGCCTACAGTACCCGGCCAAAATTCTTGGG
ACACCGGAAATTCCGTGCGCAAATCGCTTCTTCATGCGCCAACCGTCATTA
CTCATGCATGCATGATGATAGGAAGCTAAGACTCTGGAATAATCTGCGGATG
AGCCTTTGCTACATGCATACCGAAGGGCTTTCTCACCACTTCAATAGTCAAT
TGACAGCCCCTTCGCTTTGATGACTGCCTCCAACCTCCTTGGCATGCTCATT
ATGGCATTGTCTATGACTGCTTGTGGGATCGCAGCCCAAGCTTTGTTCGCCG
CGACAACCATGTGTTCTTTTCGAGTGGGTCGAGGTTGAACATTTGCCAGGT
CGTGCTTCAGAACGGCGAAGACGTTCTCGATGGGATTGAGATCTGGCGATG
CAGGGGGGTGATCGATATAGATAAAGTGCAGTGATTCTCCCATCGCTTTGGC
ATCGGCTGTGTGCACTGGAGCGTTGTCTTCCAGTAGCCTAGCTCCTCCATAA
CCCCTCCAGTTGCTGTTGACTTGCTTCCAGACGTCCCTCATGTGTCCCTGCA
CGATTTGCTGGCTGTAGATCTCGCCGGTCACCCCTCCCTTTGCTCCCTTGCT
TTCAGAGCAATCAAAGGTGAACAATTCCGACCTCCCGCCATACCAAATCGC
TCCCCAAAGCATGACAGACCCTGGCCCCTTCTTGAACTTTGGTGCCAAGCA
GTCCTTGTTCATCGCGTCGCCATCTACGCGCCATACCCAGACTCGAGGGGC
TCGATCAAAGTGCATTGAGGATTCATCAGTGAAGATGGTTCGCTGCCAGTC
CTGCAGAGTGTCTGAGAGGTGGGATCTGGCGTAGGCAAGTCGCTTTTGCTG
GTGGGTCTTGGTAAGAAAGGGTTTGGTTTGAGCAATGCGTCGATGGAAGC
CTAGGGAGGCTGCAAGGCGGCGGAGATGTCGCTCTGAGATTGTGAAGCCA
AAGGAGGAGAGGTGAGGGGCGAGTTCTTTGGGGGACATCTTGCGATTCTC
TTCAATCAAGCGCTTCAGATGGGCAAGGGCTCGTTCATCGAAGCGTGGAC
GGCGTCCTGGGTGCTTTTTTGGGTCGGTAGAGCCTGATTCTCGATGCTGAG
CAGCAATCCTTGCAGTGGTAGACTTGCCACATCGAAATTCGGAGGCCAGAT
CGCGACATGTGGTATACTCGGCTGCTCTGGCAATAACGGCGCGCCTGCGGG
GAGATAAGGTCTTCCCAGGCATGTCTGGGAAGAGAGGCAGAGTCTCAGAT
ACTTTTGGGGGCTTGGACCGCCCTCATGGGTGAGAAACGCGTCAAAAGCG
CGCCTTGGTCAGGAGTACGCGACGTGTCAAGTTACCATCAAGGTACCATCA
ATTTGTCCCAAGAATTTTGGCCGGGTACTGTACCTGTGGGTGCGAATTTGC

ACAACAATCGTGTCTTGTATGATCTCAGCTGAGTCCGTCGACCCCGCGGAT
CCATCCACACTCTCAATACGATCGTCATTGGCAGATCCAGACTCCTCATCGC
TGTCCACAATCTCATTATAGTCAGACAACCGTGAGCAGACAACATTCGACG
TCCCTTCGTCGAGGAGTCTCAACCGACTGTCTTTGGGTGCCGCAGACATAA
TGCGATGGCTGAATGCATATGTACAGGCGGGGACAAGGATTACCCTCGACC
GTGGTCGTTTGCATCCGAAGATTCTCCTTCTACGTACGGACAATGATAGTGT
CGCGGATCATTTCAGCCTCTTCCACTGATGAACTATCATCTACAGTCTCAAG
CTCATCATCGAGATCCGACGACAGGTCCTCCGTGTCGTTTGCAACATCATC
GTCTAAATCAGAGTCAGAGTTGTCAGTGACACTCTGACTGGTTTGTTGAAA
ATGTCCGCGGTCGCGTGTGCGGTAAACCTCAGCGGTCCAAGATGGAGGAA
CCCATGAGAGTTTGGTAAGGTCAGGATATGGTTTCTCCTCTGCAGCAAGGT
TCAGAGCTTCAAGGTCCTCCCTGGCCTGCTCAAAAGGGTCATCAGTGCTTG
TTTTTCCAGCACGTGACAGACGTCTTCTCTCATATGCCCACAGTACAGTGAT
ACACCTCTGCATTACTGTGACTCGCTCTGCAGTGCTCAGCATGAGCCAACG
CTTATGGGGATCTTCACCCTTCATAAGCTGCTTGAAAACAAATGCACGGCTT
TTCTTAGAGCGCGAGTCAGTTTTGTAACGGTGTCTGCTCTGCATGAGGACT
CAACTCACATGGGCGCGGCACCAATTCAGACTTGATGGGAGAAATCTGTTT
GCAAGACTCATTTGGAGGCAGCGGTTGTCCTCCCCCCATATCTTACCTCTAC
TGCCATGCACAGAGGCGGGGTCATACTTTGCCTCGCACGGCCGTATGTCGC
CCAATTCCCCTTGAGAATCTGCTTTAGGTTTGCGCGGGCGTGGGTGGTCAG
CCTCAGCGGTCCAAGATAGAGGAACCCATGAGAGTTTGGTGAGGTCAGGA
TATGGTTCCTCCTCAGCAGCAAGGTTTAGAGCTTCAAGGTCCTCCCTGGCG
CTCTTGAGATTGTCATCAGTGCTTGTTTTTCCAGCACGTGACAGATATCTTC
TCTCATATGCCCACAGTACAGTGATACACCTCTGCATTACTGTCACTCGCTC
TGCAGTGCTCAGCATGGACCAACGCTTATAGGGATCTTCTCCCGTCATAAGC
TGCTTGTAAACAAATGCACGGCCTTTCTTAGAGCCCAAGTCAGTTTTGTAA
CGGTGTCTGCTCTGCATGAGGACTCAACTCACATGGGTGGGGCACCAATAC
AGACTTGATGGGAAGGCTCTGTTTGGAGAACTCCTTTTGAGGCAGCGGTC
ATCCTTGTCCCATAGCTGACCCCTACCGCCGCGCACAAAGGCGGGGTCGTA
CTTTGCCTCGCACGGCGGTCCGTCGCCCAATTCCCCAGGTTCCTTCCCTGG
CGCGGAAGGGGGCTTCGTGACAATTGAGGCTTCACTCGTTGCAGTTTCAG
CTGCTGGCTGCAGCAGCTCCCCTGTCCGCGATGACTGCGACACACTCGGC
GGAGCCCCAAGAGGCCATTCAGGTCGAATCCCCAGAGGCAGAGTGGGCCC
GAGAGGTTGCACCGGATGTGTTGTGTTGTTTGGGCAGTAGGATAGGTGGGT
GCCTCTCACCTGCCCGCTGACTGCTTGCGGGCTCGGGACCCACTGAGAAG
GATTGTCATTTCCGGGGCGGCCCTCCAATTGGGTGTCGCATGGCGCGACAG
TGTATGGGTCGTTGCAGCCGCCGAACGGGGTGGGATTGGACGCGAAGAGA
TGCGGGTCAATAGCCGCTATATCGTCATTGGGCAATTCGACCGTGGCCTCTT

CATCGTCAGGATAGCCGTACCAAGCAGGATTGTATGCCCTCCGCATGTCGC
AAGAGTCCTGACCAACAACTGGCAATTGGAGTCGGTATCGACTAAACTCA
AGCGTGCGAGATCAAGAGACCAAGTGAAACGAGTTATATTTTCGCCCTTTC
ATGGAGCTTCCAATCACCTACAGCTCCGGCCTCCATGCAAACATCACTCTG
CTGTGACAGGAAGACCCCACCAGCATGTGGCGTTTGCGGATCTTGCTGTTC
CGCATGCTGACAACGGGCCACAAGAGTATACAAATGGCGATCACTGTCCTG
CACTTGATATCCTCGATGCCGCCGAGATACGCATGAACGTATGCACAGGCCT
GATCTTGCTGCTCACAGCTACGCGCAGTTAGTCTGTACTCAAGGTCTAACC
GAATGGGCTTAGCCTTCGGCAGCCCATTACAGCCGAATGACGGCTGAACAT
TCCTCTCCTTGTCATCCGTGGGAGGGACGGCGAGGGGGAGGGGGGAGGAG
GACTGGGTGGAGGATGTTCCATGACGCACCGCCCGAGCGCCATTTTCAGTA
TAGGGCATGTGTGCACTTTAGGGGGCAACGCTTAAGAGACACACTCGATTC
GAAATCTCAAAACGTCTGTGAGCAAGACAGGTGTGCCGCGCGGGATGATA
CTGGATCAGGAGCATCCGCCTTGGGGCGCCTTTGATCAGATTCCCAGCGCA
GGGGCGGCAGGCAGACGTTAGGGGTGGAGAACATGGGGTAGCTTGCGGG
CGAGCTACGGGACGTTGGGCATGGCGTGACTGAGGGCACATCTCAAGGAG
ATAGAGCATTGGCAGAACGACGCCGAAGCCAGTCAGGTCGTCATCAATCG
CTAAGGAATAAAAATATTGTCCGTCCAAGATTCCGAGCCGTGTGTATTGACC
GAGCAGTCCGGATCCTTTTCAACCTTGGTACTGGTCTCGTCCGTACAATAG
GTCCGTCTAGCCATTCTTCCTCGGGCCTTTGGACCATCTCAAATGCGCGCCC
CTAGACGTTGTAGCGCAGTCCGCAGGTCGACAGAGAGTACATTGTACAGAT
TGGGCCAAAGGGCGGTGTCCGCACTCTTCAAATGACCTTCGAGACACGGA
GCCACCACTAATACATCCTTGAGTGCACACTGATTCGAGGCGGATGCAGTC
AACCGAATCAGGACTTCTCAGCCTACAGATACGTCGACGGCCGTACAATAA
CTGTATTCTCTATGCCTTCAGCCGGGTCCTTCCACACCGCGCCAGTGTCCGC
GGACTCGGGCCCATTCGTGGAGTGCGGCTGACAACTGCCACTTTTCTGACC
ACCGCAACCATCTGCGCAACAGTCTTTGGGGCGACTCCTCCGTCTGGTGCC
CGCATGACGAAAACAACGGTCCGAATCTCCCGAAGTGGGTTCGCTACAAC
GCTTGCCGCTGCGATACGTACAGCATGTATCCCAGTCTATGACCTTCGAATT
AATCTATTTCGGTCAGTCAGGCTCAGAAAATTTCTGCGCAGATTTGGCTCAC
ATGTACGTGGCAGCGATCCTCATTCGGAAGAGCGAAGTTGCCACAAATACT
GTTCTCCAGTCGATACGAGCACCTAGTCTTTCCGTGCGAGAATTTGGCTCC
GGGATTCTGTGCAAATTAGACACAGTAGGTTTGAGCACGGATACGACTCAC
ATGTACCCAGCAAGCACCTGGGCCCGATTTGACACAGGTACATTGACGCCG
ACGGCGGGTAGGTCTCCATTCTACGGTGACCGGCGGTACCTGCTGGGTCTT
TGGTGCGATCGGCCGCAGACTGACTCCCAAATGGGTGGCTGAGGTCCTCTT
GCCCCGGCCGCGTTGCGAGGCAAGCCTGTCGGCTTGTTGGGTCAAAAAGT
GGTCATATCGGGAGATTTGTGACAGTTGAGCCGATGTGTAAACATGTCCGC

CTACAGACAGACTCTGGCTCGGAGAGGGCCAGCGGTGAGGGTTAGATCCA
GGAGGGGCGGAAATCTGAGCGCCAGAAGCGGCAAAATCAGCTCTCGATTC
CCTCGATGTACTGAAGAATTCCGGCCACATATCATAAGTGTGAGGGAGGGA
TTGTAGCATGGCCCCTGCCGGTTGCGGTTCCGTGAGTAGCGCCTCCGGTGG
GCCATAGATGGCATGAGCACCGGTGGTCGTCCCCTAATCGAATTTAGTGGTA
GGTCGCAAAGGCACAGGCCATGGGAACGCGCGGACGCACCTTCTCACCCG
CAATACTGGAAGGACGCGTCTCTTCAGACATGTAGTCTGACTTCTTCAAGT
AGACTGAGCCGAACGGCGATGCAGGCCCGTTTGGATCGAGCGTGGACTAA
GGTCGATGGAGTGGGAAGAGGAGAGTGTTGTTCGGTAAGACGATCGTTCA
TAGCGTGGAAGGCTCTTCCTCTTCGGTGCTCTTCCAACACTCCCTTGACTG
GCTGGAACGCCCCGCAGCAGAGCTACATATCTGCCTTCATTCACGACGTCG
AAGCCACGTCGCCCTCGAATCATTTACCTTGTTTTTCTATTCGTCCTTCGAG
ATGAAAATTGGCATGCCCTTCTCCCAGCTATTTGAATTTCACACAATTCTGA
GCGCTTCCTGACGAGCCAGCCCCGTGCCAAGTCCTCCGGAATACAGTGTCA
AGAGCTCTACTTTTCTCAACCAGGATAGAGTAATCCAGAAGTTTCTGGCTC
GCTGGAGATCTTTTGACTGAAAATGCAGACCAGGGACAGCGGAGGGGACG
GAGACGGGTGGCCCAGCTTGAGTCCTTCGGGCTACAAACTCTACGTGGCA
CAAAATATGCACACAAAGCAGGAAGGCTGCATGCAAGAGCATTATGGTATA
TTGAACAGCAGTTCAGGAGCAGAGACTCCTGCACAATCCATGCGATCATAG
CTTAAGCTGCCTGAGCGACTGAAGGCTACCGGATATGACTGAGTATTGAGA
ATCGACCAGGTCAGATATGCATTAGCCCCGCTGACTCCCATGCTGGATGCA
AAACTCCGTGTTGCCCTCCCTCGGATTGGTACATGGTCGACCCTTGTAGTAT
CGATATGAGCAACATTTAGCCCAGTCAGGTTGAGATGTGTCAATCTAAACTT
TATCAGTCGTCGTCCAGATAGACCACTGAAGGTAGACACTGACATGTCGGT
GGCAACGACCGTTCGAAGAGGCCTGATTGCGGCATTGGCGGGTTTCGAAA
AAATACGAACATTGCGCTGACTGAGGCCCAGTAATGGGACGCTGCCATCAG
TTCGCCACTGCAGCGATCACGTCAACCATAACTCACATGTACTACGCAAGC
AGTCAAGGAAAATGAAGCACATGTGCAGTCGCCCAGAGGGTCATTGAAGA
ACGTCACGGTCGACTGATTGGGGCGTGGCTCATTTTTACGACCGCCCGACC
GCTTGACTTTTGGGTGGGTACGTGAGGTTCCGAGGCTTGGGGGACTGGCG
GGACCAGCGGCATGATACGATACGTCAGGGGGAGGAGCGGCACCAGAGGC
ATTGTCTTGCGGCTGAGCCGAGCCCTGCGACAATGAAGAGACTGAGAGCA
GCTGTGAGGCTCCCGAAGGATCGCTGGGATCTGCTGGGGCTGGAGTCCGT
GCAGCTACTGGCCGAAGCACCACGTCGTCAGCGGGGTCATTCAGCATGCTA
TGAACACTGAAGATTGATGAGGCTGGTCGTTCCGGGGGTGGCGATCCCGTT
GGTTCACTTGACTGGTAGTTGGCGCGAGGAAGACCCCTTATGGTGGGCTAA
TTGTTTCGAGCGTAAGCTATTCGATCGAAGGCTACGCGGCACTGCAGACTC
ACCTCCTCAGCTGCAGAGCCGGTAGGGTACGAGTAGCTTCGGTAAGACATA

TGTGTTGCTTGGCTTGGCAAAGGTATACAGTTGCAGGCGATTTGGGTGAAA
ATGAACCAAATGCTGTGGGGAGTGTCTTCTTGAGCGACTTTCCCTCTTATAG
ACTGAGCGCGATCTGCACTTGTGTCGGTTGTCTCATCCTCCTTTTATACAGC
GAAACACGTAACACCGAAGACGCCTATCTTCAGAGCTTCGAAGCGAGGTC
ACTTCCTTTGCCCTTATTTCTCGCCCCGCCCTCCGACACAAAGCATAGCACC
GCACCTTGAGAGATTTTGGTGTTTCTACAGAGGTGGATAGCTGATCATCGA
ATAACAAGACCTCTATAGACACCTTTCAAATTGCTCTCCCCTTGTATTTCAG
CCGTGACCGTGAAAGAAACGTCACTCCTCCAAACCACGATGCGCGTAAGT
GGCGCGCCGGGACAGCCGTGTGGCACCATTGCAGGCATCCGCTCGTAAAG
CACGACCTGCAGTACGCCCAGGTGCTCCCGTCAGCAAGAAGCCGACGTTC
GCGCTCCGCCCCCCTCCTCGTCTCTTGCCGTTGTCTCTGTCGGCTACATTTG
GGCATGACTGCATTCCGGCCTGCCGGGAGGGCAGGGAAATACCACAAGAG
TCCATATATCCTCCTCCTGTTTCTCCTTCTCCTCCTCCTGCTATCCTCTCCCAC
ATATCCGTCGTCATGAGCCAGTTCCTCCCTGCCTCATTCCTCCCCTTCCCTTC
TTCCTCCTGTGCCGATCGACGGGCGGGCGGGTGGGACGATCGGCGACAGC
GACACGGCAATGGCGGGCGTGGTGGCCGTGGGCGAGGAAGTGACGGTTG
AGAGACGGTCTCAACCTCGCTCGGGGCCTTCAAGTCACTTCCCTGATTCAC
GTGACCGTCCAAGCTTGACATCCGCGTGCAGATACTCCGCGTTTCCTTTGG
CCGCGGGCGACAACCATCGGGCAGAGAAAGAACCCGGCCCTACACTAAAC
CCCTCTGACGCCCTAGGACATCACGTCTATTTTCCAAAAGGACTGTGGAAA
GGAGACTGTAGGCTGCCGGAGCACGAGGAATCAGTTTCGGAGTCACGCTT
GAGGCCGGCCAGTAAAGGGGCAGCATCTTTCAAACCCCAACCACGTTCAA
ACACGTCAGAATGACTGCTCGTCGCCTGACAGCGACGGCATGCAGCAGCG
AATTCAATATTACTAATCCCTCCGTGGTTCGGTAGAACGCTTTCTTACAAAG
CGAGAGAAGTCTTCAAGAGTAGCTTTGGGGATCACGGCGAATGTGCGATG
GTAGAGATGAGACGGTCTTGGCAAGTATTTGCAGCTGCTTGCGAAGGATGA
TTCGGGAACACACGATTCACCCCTACGCCCAGGGCTCTTAAAGAGCCTCGA
AGTCACGCAAAGACTTTGGCTTGAGAATATGGAAGATATGTCGTCCACTGC
TCGGTCTCACAGACTGTCCTTTGACCTCTTTGTTGAGTGCGACCACCCTGC
CATTGAGCATGAGGTCAGGGGGGACTGCCAGCCCGCGTGAGTGGTCGGAT
GAGATGCCGGACGGAGACAGACTCTCATGAAATCAAAAATGTCTGTACAG
AACGAAATAACACAGATAGGAGAGCAGATCATGTTACAAGTGCGACGAAT
GATGGTCAGCCTCAACAGCTTTGGGTGTTTACCTACTGCCAACCATCGCAC
ACGAGATCAATGACCGTGACCGGATGCGTTTTGCACATCGTACGCGGACAG
CCCCTCCTGCACAGGCTGTCGGGGCTTCCGTTTTGGGCGGCCACTGACAGT
CGTGCCCGCTCTTTGAGACTTTCCACCCCGTGACGGCGATCTGGAGCCGCT
CCGCATGTATGGAGGAAGTCTCCCTTTGAGAGCCCGGAGGGCTAGGCCTG
GATAGAAACGGCCCGATTCGTCCTCGCGACCTTCCACAGCTTCACTGGGCC

CAGAGGTCACTTGTGCGGGTGAATCGGAGGTGGGAGTGTTTCACTCCACT
GCCGGTAAGGACGGTGAAACCTGCAAAGCGTTGTTGTCGTCCAATCCCCCT
GAGAGGTGATCTGAAGGCGATGATGCTCTGTCTGACCGCCCCTCATCTCTG
TTCTCCTGGCTTGGGTCCTGATCAGGGGTCCATACACCGCTATTGGCGTCGT
TCGTCTCCTTGGCCCGGCTCGATTCCGGTATAACTGGTCCATTGCTTCCGCC
TCATCCGAATTGTTATTTGAGCCGCCAGGGGACTATATGTACGTCCACTGAC
TGCGGACAGCGCTGGAAATCGATAAGTGGATTCACGAACTCGCTATCGGGT
ACATTATTTGTGCGCCAGGCTTGATGACCATCGGTTCGTCGCGACGTTTCAC
CTGTCTGTCCGTCAGCAGGCATTTGCAATCTAGGCTTTCCAGCTAGCAAAA
GGCTGCGGAAAGACTGGCGGTAGACATTGCAGACCCCCCGACTGTATACTT
ACCTCGAAGTCCCGGGAAAGTGCTACAAAGCTTCGATCTACCTCTCTCGCT
GTTGGTCAGCATTTTCACTCATGCACCGTACCCACCGACGCAATCGCGTCT
CGAACGGGGTCAAATCTCCAGATTCTTGCGTGCACTACTGAGATGGAGCTT
TTGAAGATAGGGAAACTAGTTTCGATGGCCCATCTATCATGGGCTTGCGGTG
GACTGGAGAAGAGGCAAAGGCACGTGGAAGATGACCTCGGATCCCACCTA
ACTTCCGAGATCCGGATTATCTCCATCGATATAGCTCCACTGGCTCCAATATG
GCAATCATAGCAAATAGGATTAGTCATGTGTTTAACCTCCATGGGCCAAGGT
AAGTCTCCAACCAAGTGCTTAGAATTTCTTTGTAATGACTGATAAACCTCCT
AGTTTATCGTTAAACACAGCATGTTCTTCATCCCTTTATTGTTTACATACGGC
TGTCAGAGCAATCGCAGCCGGTGACTGTGAGGACGCAATTGTTGCGGGTG
CAAACCTCATCATGAGCCCTGAGCAGCACTTAGAAACCATGAAAGGTGGT
GTGCTTTCAGGTACCTCTAGTTGTCATACGTTTGATGAATCAGCCGACGGCT
ATGGCCGAGCAGAAGGAGTCAATGCTGTTTATCTCAAGCGTCTATCATCTG
CTTTAAGGGATGGCAATAAGATATGGGGCGTAATCCGTGCAACAGCTGTGA
ACTCGTTAGTGATCCCCATCTCTATTATTCTTGGAAATGTTGACCTAATAATT
TGTTACAGAAATGGAAGAACCCCTGGAATCGCACAACCAAGTGCTGACCT
ACAAGAAGCAGTCATAAGGAAAGCGTATACCCATGCGGGTCTAGACTTTCC
TGATACGGATTATATAGAGTGCCATGGGACAGGAACTTCAATAGGAGACAA
AATTGAAATTGATGCTCTTGGAGGATGTTTTGCTTCTAGGCAAGGGCTTCCC
TTGATGATTGGTTCGGTGAGTATGTCCCAAATCATAGATCTAAATAAGTATG
ATGGAGTTTTGCTAACAACTTGTAGATCAAGTCCTCAATGGGCCACTCCGA
AGCGGCAAGCGGTTTGACGTCGCTCATTAAAGTGTTATTGGCATTCGAACA
TGCTAGTATCCCACCAACCTTTGGTGTAAAGAAATTGAACCCCAAATGTAAT
GAAAGGTTCTTTGAATTTTCCTGAAACCCCCACACAGATATCGGAAGCATC
TGGCTGATCCCTTTACTTTCTTTCTAGTGCGTCTCGACTCCTTCAACATGAA
AGTCGTGACTGAGGCGGAAGAGTGGCCTCGATCGATGCGTAGAGCTAGCA
TCAACTCGTTTGGCTACGGAGGCTCTAACTCGCATATAATCGTCGAGTCCAT
TGACAGTTATCTTGGAAGGGAAGCTTTGGATCAAAAGTCAATCGCGCCTGT

CAATGATCAGTTACTTCTCTTACCTATCTCTGCTAAGTCGGAGAAATCACTT
CAACTACGTCTAAAGCAGATCTCATTGATGGCACAAACTCCCACAATACCT
CTTAACAACCTCGCTTTCACATTAACCCGGAGAGGATCCCATTTTAGAAAC
AGGCAATTTCTGTTAGCTAGACAGAGTTCTTATGACGCATTAGACTCAATGC
AGACTTTGGACGCTGGGTCCAGGGATCATCTACCCCTGGCCTATGTATTTAC
GGGTCAGGGGGCTCAATATGAAGGGAGTATGTATTCCAAACAATTTCTTCG
CCTCTTTATTGTTTAATAATCTCACTGCTAATCTATGGTACTATAGTGGCGAG
AGAGCTTTTCTGGAAAAATCGGACTTTTCGTGAAACCATCAAAAGGCTTGA
TGCAGATTTGCAGTCTCTCCCACCTAAGATATCTCCAAGTTGGTCTTTAGAG
CAAGCAATTACCGAATCACCCAGCAGTATAATCAATCAGGCTACACACAGC
CAGCCTCTCTGTACAGCTATACAGATTGGACTTGTCGACTTGATGCGAAACT
GGGGAGTTTTACCAGCTGCCGTCGTGGGCCATTCCTCCGGCGAGATCGCTG
CAGCGTATGCAGCGGGTTTGCTCACCGCATCACAATCTATAATAGTAGCGTT
TCTCCGTGGTTATGCTGTTTCAAACATACTATCGCCTGGAGCTATGATAGCT
GCGGGTCTAGGACCAGAAGCTGCGAAACAGCTAATTGCCGACAAGAACTT
CTCTAAAGAACTTTGCGTTGCTTGCGTTAACGACTCGGATAACGTTACGATA
AGTGGTTCTCAAGAAGCCATTGGCATAATTTTTCCGAGATCCAAGGGGAA
CGCAAGTTCGTCCGTAGGTTGGAAACGGGAAAGCAAGCCTACCACTCACA
CTTGATGCAAGATGCAGGAGCTTACTACGAGGAACTAATCGCCCC

>Mosaic_sequence_72

GTAGGTCTGTTATGCAATGAGGCGCGCTTGTTGCCGTGTGGCAATCTCCGT
CTGCACATCATCGTCGAGTCGCCCGTCTCAGAATGGGCCCACCTTCGAGCG
CTGAGTACTTTTCGCCCAGTCGGCCATCAGGTCGGTAACATTGCCTTGGTC
GTGCATGTATAGAGACACCCTGTGTTGATGCTGTACACCCGTTAGACTGTGT
GTATAAGCCTCACTCAGCTCCTCCCGCCAAGGCATTGACGACTTACGCAGG
AGCGGTGTTGTGTATGTCCCCGGCAATGAGGACATATGATTTCAACCCCTCC
AAGCCGAGCTCTTCTGAGGCTTCTTTGGTGGCCGTTTGAGCGGCAGAGTG
ACCCTTGTCTACGAAACCAGCTGAAGGCTCTGCACGATCAGTGTTTGGAGA
GTGAATGTCAAAGACATCTTTCCACGCCGAGTGGCTCACCCAAGCTTTTGC
AGGCAGAGTAGATTCCGGTTTTCTTGGTGTAGGTAAAGCAGCCGGCCCGAT
GCTGCTCTACGGCAGCGAGATATAGCTCTGGAACATTGGCCTCCGTGCGAG
ACAGAACCAGGACCATATTGCTAGCACCTTAACGCTTAACGTAAAACTTCA
AGGTCGAGCCTGACTAACCGTCTGCCACCAGACCGTTCATCCGTGCTTCAG
CAGCTTCGTCCGTCTTCGGCTGCACAATGAGGTTCCGTCCCTGTCCGTCTC
AGCTTTGCCCAGAGTACGACACTTTTACGAGAACCGCGGGCTCCAGATCTT
CCATCGAGAAATTCGACGGTTCGGCACCGTCCTTCAACTTGTGAAGGGTTC
TTCCCACCCACGAGGCTTCTGGACGATTTGGGTATGGCTCCGGCCGGTCGA

CCCAGAAGTAATGTTCGTGCTTTTCGTTGTTTGAAGACCCGTCGGATCGGT
CGAGGCCGAGCTTGGGAGGTAGGCCCCGTTGCGTTTCCGACTTTGGTACTA
CTGATTCGACATCAGAAGCACTGGCTGCCGTGATCTGCGGAGCGGTGAAAT
CGGTCTTGGACATCGAGCAAAACGGATGAGAGGTACTGTATACGGGGAAG
TCTGTGTACTATGTTAGCTATGAAAGTCAAGTTGGAAAGTGGGGCATGCAG
TCTGGGGCGCTCTTGCAGTTTATACCCCTTCCTGCCCGTACTGCGACATGTC
TGATCATGAACGAGGCGGAGCAGCGTCAAAGGGGGACTGCGATTAGCAGG
ATCCCCTTTGTCCGTTGACCGAGTTAGGTGTGACATCTTTCCTTCGCGTATG
TGCAATGCTTCGATGGAAGCCATGCAAGGCTGTGCTCCGTGTAGGGAGACG
AGACTCGAGGGTCTCAGACGAGAAGGGATAGAATGACCTGGTCGGTCCAA
TCGGCAGCGACAGGGTGAGATTGAGAGCTGATGGGGAGCGAAGGAGAGC
TTACTGTACATGTACAGTACATGATTAATCTATGATACGATGACATGCCACGA
CCGAGAGATTGTAAATTGCCCTCACACGTTGAGCTTTGCGTTACACTCCCA
CGCGATCGTCCGCGTCGACAGAAGTCAGAATCGAAGGCAGCTCATTGGCG
ATCTTGGCTTTCTCCGCGTCCGGTAGACTGTCTAACGCATTCTCCGTAGCAG
GCCGTCTGTACTGCGTTTCGTAATTTATCCTGAAATACAAGCCCAACAGCTC
AGCAGCGTGTGACACGCCAGGCGTCATCGCGATGGTCGGTCGAGGCTAAA
GCGCCATTTCTACCGAGTCAGCAGAGTGCGAGCACGGTGAAATGCCGACT
CGCCTCGTACAAGAGTGACAAATGTGGGGACGGTCATAAAGTGAGCCTGT
TGCAATTGCCTCTTTTCACTCTACGTCGTCTCAGCTGTGGATAGCTGAGGAA
GCGAAGCGATCCCTCACCTTCGATCTCTGCAATCTTGTGTCACATTGAGCC
ATTAGGCCACCGATCCAACCGTCGTAATCGCGCAGTGTGGTGCTGCGCTGA
TGATAGGCGCTGAACATCCTGCGATGATCAGCCTCCCTACAGCCAAGCATC
GGGAGCTACCATCTCACGTCGTGTCGTTGATTTCCGGAGCGGTTCTCGAGA
GCACCTGGACAGTCAGACCGTCCGCAAACTGGACCGCAGCTGAATCCGCG
TTCGCCCGGGAGTCCTTCGATGACTGAGCAGGCAAGCGTCCGGGTAGCCC
GATCTCTTCACGTGCCTCCTTGAGCCGAGCTGCGTCGGGAGTATCTCCCTT
GTCCACGATACCGCCCACAAGCTCTGTTTGTCAGCCTGAGCATCCGACGAC
CAGATATCCCTGTCTCGCCGTGCGTCACGACAAACGACGACTGACCCCAGC
TGACTATCGCGCTGTAAACCTCTCGTTGAACTGGAGGCTCTGTTGCCGTTC
CGTCTTTGTCGGTGATCGATAGCGTGAATTTCTTGCCTATCTGCGGTGTACA
CGCGGGTCGGGCTTGGCTCAACGTAGCAATGTACAGCTCTTCGCAGCCGTC
ACCATTCTTGGCGCGAATAAGGGGAATTTCATTTACACCTAAAAGCTGTCA
GGGTGCGCACGGATCACAGACGCGTGCAGTAATCCAGTGTGCTCTAACGA
GGAGACTGACTGTCTATCTGGATCTTACCGGTCGTGTGCAGCTTCACAGTT
TCCTCACTGACCCGTGTACCTTCAGGCCCTGTGTACACCATGACGTTTCGG
CCCTGCAACGCTCAGCTCTAGCCCCTTCCGTACCCTAAGACTCCTCGCGTT
GTAGCACGTAAAACTCACCACTCCCAGGTCAACGGCATCGACCGTTTCTGA

CGGAGAAGCCTCAGAAAGATCTGTCAGGAATTCGCAGAGGCGTCTTTGCT
CTCGCCTTACGTAGCTGGTGTCAGGCCGACAGTCAGTCGAGACCCAGCCA
AGGGACTTCATTCGCTTCGCAGACTCGAGAAATTCCTCGCGGCCGAAAGT
GGCAGGAAGCGGGTCCCTGTCCTCAGGCTGCAAAGAACTGGCACCAGGA
CTAGCGGAGGCAGACGTGCGCTGCGTCTTGATATAATAAGGTAGAGCGGGG
TGGGATGACATCCTGAGGTTAGTCCGGGCGAGACTGTACGCAGGTCTAGTC
AAGCGTTCGCAGAGAAGCAAGCAAGACACCTTGCAAGGCTTTACGGTGGC
GAACATATGTGTATCTTGTACGAGAATCACCCCTTCGCATGAGTCGTTGCTT
CTGTAAAGGGGTCGACGTCGGTGAAAGGGCATATACCAAAGTTACAGCAA
AGCCGTTTTGGGTCACTCCCTTTATCCCCAAGCGAGGCGTCTCGATGACTC
ATGTCTCGAGGTAGTGGTCTTCTATTACGTCTTCTGGAGAGCGCCTCTGTGC
GGATTGGCGTGCGTCATTCGGCCAGCAAGCATGCCCTTGTTCGAGGATGGC
AGTGGCAAGCAAAGCGTTTGTCTTTTTGACGCTTGCTTATCAAGCCCGTCA
CTTTCGCCCAGGGTGGCGCATGAGGTGTTTTGCGCTCTCGCGAGCTTCCTT
ATCCTTCGACCTGTAAGCTGTAGATTGACCCACATACGCGAGAGCACGGTC
GACGAGGCTTCGATACCAGAGGGCATCTCTTGGTCATCCATAGAAAGACGT
GCCCTCCCACCGGACCACAGACCAATGAAGGGCAGAGCGGAGAGAAGCC
AGATAGTGACTGTTACCGCTGGCTTCCCAAGGACAGTGTTCGCTCGCAATT
CCAGTCGAGTTGTGTAGCTTCTGAAGTCACCATCATGCGCATACGTAAAATA
CTCGAATAGATGTACTGACATGTCTACATCGCTTGACTCACAGAGAGTCGA
AGGTGTACAGTCATTGGCCATATTGGCCATAGTTAATGACACGGCCGAAAAT
CGCTTTTGGCCGTGATTTGGGCGGAGGCGATTTTTGCCCAACTTCTGACAA
CTATACAAACATGCATATTCTTCTAAGGAGCATGAGATACCTATGAGAGTAG
CGATGGGGAGATGTGTGCTTACTAAGCCGCTCCTATGCATGCATTGTCAAGA
CAGTGAAGCCAGGATTGACCGCAGAAGAGGGAGGACTTTGATCAAAAGCC
GCCAAAATCGCCATTACACCCTTACATTAGAAAGCTGTGCATCACTTGTTCT
CTGACGCACCTCAGTGTTTGCAGGCTGTACTAAGTCTTGAGAGGCAGGGAT
TTTTCAGGAATCAGGTCAAGAAGGCTGTATGCTTAAAAATGACAGAGTTGT
AGCGTCTGAAAGTACGGTCAAAATCGCCTCAGCCAAAATCACGGCCAAAA
ACGATCTCGGCTGTATCAACTATGGCCAATTCCAATGTCTGTACCTACCAAA
GTCTGCCGAGGACAGTCTATGGGGCTTGAGCGGTTGACGTTGATTCCCTCT
TGTAACCGCAATCCTCGGGGAAATCACGGACGAAGGCTTGCGTCAGATCGT
CCTCTGAGGCCGCTGGGCAATGTCTGCGGAAGCCTGCAGCGAACATATCAG
CCCTGTCTGCAAGGCTGAACACGCCGTTGGTTCCGAGCAAGGTCGGCCTC
GGATGGGTCAAAAGTTCTGAAACATTAGAAAGCCCCTGCGCGCGAGTCAA
ACGCCCGATCTTACCAGTGTTGATCTCGCAAAAGTCGGTAATTGTTAGAAA
ATGCGCCTGTTGCAGCTGGCGGATCTCATCCTGTCACGTCAACATCAAGAA
TAATAGGGCATAGGAAGGGGACTCGCCCGGTCACGCTTGGTCTGCTGGTTG

GAAAGCGCCACAAGGTCTTCTATTGGGCCCTTGTAATTGAAGAGAACGGTC
TTGCGCTGGTGCAGCACGGTGATGGTTCTCGAATTTGAGCTTCGCGCCAAG
GCGAAACCACTTCAACAAATGTACAGTACTCACGTGGCATCGTTGTTGTGG
GCAGGCGTTTGGCACAGCACTTTGAACTCTGGATCCCTAATACCGACCTCT
TCACCCAGCTCCCGTAGGCCGTCTGCGTCAGCTTCCTTCAGGTTCTCTATAT
GGCCACCGGCGGCCTCTAGGACAGGTCAGCTACAGACTTTATTGGTACTTG
GATCCAAAGTTGCAGCGTCGACGGAGAAGCCTTACCCATATTCAATGACAT
GCCAAAAACATCTTGCTCTTCAGGCAAATACGTCTCCGCTTCGTCGGCATTT
GACTCTCTGAAGCGCATGGCGATGTTCGTGGAGATCGCGCAACCAGGGCG
GCATTGCGTCACGACAGCAGTGTAGAGAGTATCTTTACCTTGTCCGTCTTTG
GCGATGATTAACGGCAAGACATTCACGCCTGGTGTCTGAGCTTTCATCCGA
GGAGAATTACTGCGAATGCAAGTGTTCAATCTCCTGCAAAGAAGCCGAGG
AGAGAGTGATCCACTTACTTTTCGCCACCCAGCCAGAGCCGGGTTTGAAGT
TCGGGCCCGTAGAGCCACGGCGATCGGACGTGACATTCCTGGTCTTCTTCG
GTTAGCGATTACCCCAAAGCTTCGCGCTGTAGCAGGATTTGCATGCGGAGA
AACCTACCAGCCCGGCCGCGCCAGTATCAAAGTCGCAGACATATTGTTCAC
CTTCTCTCCTCCATCGCGGATTCGACACTTCGTAGAAGGACCGCGAGACGA
GCTGATTGAAGGGCGTTTCCGATGCGCTACCCTGCGCAATGATCCCGCCTT
GCACTCTGTCTAAGGAAGCGTAGAATGCGTCCTTACCCAAGGTGGCGCCAA
GGAATCTACCCTTGACTTCAAGGTCGACGGCAGACTCAGTCTCGCCCGTAT
TGTCTGACTCTGTCCGGTCGATTTGTTTAGTCAGCCAGGGAAGAACGGTTG
GCAATTCGTCCCAGAGCTCATCCTTGCGGACGACGGCGCCAGATGCATCTG
TACAAGCCGGGGCTGCCTTTGTGAACAAGGTGGCTTTCGACCCAGTAGTTT
CCTGTTTGCCGCCAGTCTCTCTGGTCGAGAGGAGCTCGACGGTGGGCATTG
GTGTCTTACCTGAAGCTGAAGACCCTCTTGGATGTATTCAATCGATCTGACG
GTCAAGGTTGCGGTTGAGGGGAGTCAGGTTCGCTCAGACGACAAGGACA
AAAGGTATGATATACGCGGCAAATTGTAAGGGAGGACCCTTCGGTCTTGCA
TACTGCAGTCTTCCTCGAGGAGGCGATGACGCATTGGTAAAACCCCTCGGT
CAGAAGCCGGAACTGTGACGCATACAGCGTGCACAGCCTCTCAGATACCC
ATCGTCCTTTGTTAGGAGCGACTAGATAGTCAGCACAGCATTGCGTCATAAT
CCAATGCATGAAAGTCTTCTCGACTGACCGTTTCACTCATTATTCCCTCCAA
CCAACGATCCGTGCGCAGCTCCGATATTCGATATTTGACTTTTTGGGTCGAA
AGTGGCATTCTATCCGAGGAAGACTCACTTTGTTTGTTTCATCTTTCTGCAG
CAGTACGACCTCCCACGATATATTCAAATCTCTCTGGTCAGACTCTCTTCTA
GCCTATTTCGTGCTACAAAGGTGAAACGTGCTTGTTGCTCAAGTGAACGAC
CCTGAACCGCCCAGTCCGCTGTCGAAGGGGAAACAGTCGCAGGTGTATGC
TGCAGAAGCCATATAGCTCTCGCTCTCCCGAGCCGGGCCGAATAATGCATTC
ATCTACCTCTGTCATGTCCCGACAGGTGTGTAGCCGTGTCTCGTGAGGTGT

GACTGTTGCAACTCAAGCTCTACGCGGCCAGATAGAGAGCCTTCTGGCGCC
TAACATCAGACCCTAAAGTGCTCGGAAGTCAAAGTCAGAGTCGAAGACGT
AAGCAATAATAACGACAAAGAGATCGAGTGCAGCTATAGGCTTATAGTCGG
CACGTACAGTATGTATGAGCGATATCCAGACACAAAGCCACAGTCTGCTCC
GAACACATGCGACTATCATGGTGTCATATGTGAAGTGCGATAGCATCGAAG
GGGAACATTGCCAGTGTACTCTGACCATGTCACCCGTGGCGCTCTGATAAT
TTACACGCCACCGTGTGGTGCTTCATCGATGTGACAGGATCTCATCATAAAG
TGCTTCATTGCCGAGTTCCCGTGCGTCAAATCACCAAATCACCAATGAGGC
CTTTTGTCCAAAAAGTTTGATACATGAAGTGTGGTATGAAGTGTGGAACGA
TATGTACGTACATTCAAGTCAATTTGAAGCAAGTGCCAGCGGCGAGTGCGC
GAGTGCGCTGCAATCCCCAGTAGTGAATGAGCTTTCTATCACTTGTGCGGC
GAGACTCAGCTTGTTGTCTCAATCTCTTCACTGGGCGAGGGGAGCACCAGT
TGTAGCGACCTCACTCTTTGCCTCTGTCGGAGAATCTACAGTCTCCGTTTCA
GCCTGAGACTCTCCACTCTCCGTCTTGGAATGTGACATGACTTCCGACCGA
GACTTAACGTTGAAATCAACGCTCCCCGAAGGAGTTGCCCAGACTTTCCCA
AAGTACTTGACGGAGTGATTCTTTCGGTAACTGTCCTGCTCTTCCTCGGAG
ATTCCCATCTCCCTCATCATTGAGGAGATAGTGTTGACATGGACGGTGTACG
AAGCGGCAAGACTTTGAACGAGAGGACCAATCACGATGCGCCTGTCTGGA
TTGAGAGATTGGCCTGCTCACTCAGCCACGTACCAACAGCGGTATTTCCGG
ATGACACGCACCCTGTACATGACCTGGAAGATCCTCGAGGGGCACATAATG
CACCATTTGCATCAAGGCTTTCTCCCCCTGAGCGAATCAGCCATGCAACGA
CCAGGTGCCTCTTGCCCCGGAGAGGGGTCCGAGGGGCAATGAACGAGCAC
TGACCATATCCCGATCGACACGCGAGTCCGACTCTTTCATCACTTCCTCGAT
CGAGGTCGGGAAGTCGTAGTAGCTCATGACATGATAATGAGTACCGTCTGT
CCGGCTACAGTGGCGTCAGCCGCTATACATACACGGTAATTGCACGTAGTA
GCAACTCACACAGTGTCGGATTTGAAAGGCGCAGAGATTGACAACTCTGT
ACGGTACTCGTGCGTAAACCCAATCTCTTCCTGTAGTTCGGCCTTGATGGCA
TCCCGAGCAGTCTGATATGGCTTCACATGGCCTCCGCAAGGCTCTGCTCATC
AGCGAAGCAGGTTCTCTTTCACTTGACCACCACCTCTCGCCCTCCCGTACC
AACTCCACACCTTGGCCTTCTTCGCACCGGAGAGCCGGAACTGGCCTCAA
CTCACCGAAGCTGAAAAGAGCTTCGTATAATGTGCCCTCGACCATCTCTGT
TTTGCCATCCACTGTCTCCGGATACCATACGATCTGCGAGGTTTCTATGTTG
GCGCCCGGACGCAACTGCGAAATGTAGACCGTCCATAGTTCCTGCTTTTGC
ACTCTCTTGTCATCGACGTATTCGACCTTTGTGGTTCTGAGCACGGGAAGC
TCGCTGACACCTTTGCGCATAGTCAGCTCCTGAGCTCCTGAGCGACAGTAA
CGGGCAAAGAGAACATGAGTGAATCGGGGCCTTGCGTAGTCGAGGTGCAA
ATGACATGTGCACACCATACTCACTGTATGCCACGAACGGTTTGCCTCGCTC
AAGTATTGCCTCCTGCCTCCTCCCCTCGTGCCGTGCATGGTATATATGGCGT

GTCTCGGTCTGTCAGCATCGCTGAAGACATACACCCAGCAATCCCAACTCA
CGGCCAGTGGATCCGACGTCACGAACAGCGCATTTGCTCCCTCCTGCTCTT
TCAGTGAAGCCTTATCGTATAAAGACATTGTCATCCTGCTGGCAATGGGCGC
TTTACGTCCTATCCAGACTTTGCCGTCTATGCTCTCGTTTCTGAGCCTAACA
CTCTCCATCATCTCTTCAACTCCCATCGTGGATAAGCCTGTCTCATCAGTCG
ATAAGGTCAGGTAGGGCGAGCAAAGGTGCGTGCAACACGGAGGTACAAA
AGCGAGCCGCAGCTCGACATCGACTCACGGGTCATGGTAGCGAAAGGCAG
GAAGAGAAGAACGGTTAATCGAATTGAGCACTAAGCAGAATGTAACGAAA
CCCTTCGAAGGTCAGCGTTCACCAGGCTTGACGATTTGCCTTTTTATAGCG
AGCTTCTCGCTCAAAGGGTGGGGTAGTATGGATGGGGATTGCCAAAACAGT
ATCCGTAGGCGTCAGCACTCACAGTAGGTCAATGGACCTCACCTCAGTGGG
CCTAACCGACTCGAAAAGGTGTTATGTATCTTTGCACATTTCGTCAAACATT
TGTAGAAAAGACTGCCCCATCCATTGACACCCAAGTTCGATTGCATTTGCG
GATTCGACAATCGACGATCCGATTTTGAGATGATCTCTTACTGCTCCTGCTT
CAGATACTGCCTTCCTTACATGTCTATTCCAAAACCCAAAGGGCACTTTCCC
CTTTGACGACCCTAGCAAGATTCTCGGCCGTATGCTCCCACATCTCGGTCA
GAGTGTCCTCGAACGAATAGCCGGTATGTGGCGTCATGAGCACCTCGGATC
TCCCCCCTTCGCCCCATGGTACGGTGCGCCACCTGGAGTCGACCGGCAGGG
GCTCGAGGTCATAGACGTCGATCGCTGCGCCTCTTATGCAGCCGCTCTCCA
GCACCTCCAGCAGCGCGTCCTCCTGGACGATCGGGCCCCTGCTGGTGTTGA
TAAAGAAGCTTGTTGGCTTCATTCGCTTGAGATCTTGTCGTCCTACGAGAC
CGCGTGTCCGATCCGATAGCACCAGCTGTATCGACAGGATATCTGACCGGG
CGAAGAGCTCCTCCCTGCTTACAGCCTTGTATGTTCCGGCGGGCAGTCCAG
CCGAGGCCGCCTGCGCATCCGCTTTAGCCTGTGTGAGGTTTTCCGACCACG
CGATGACCTGCATGCCATATGCGAGATGGGCTATGCGGGCTGTGCCCGCAC
CTAGCTTGCCCAGACCAAGCAGGCCGATCGTCTTCCCCCCGATGAATGTGT
TTAGCGGTAGCGCGTTTGTCCACGTGCCAGCCGGAATGAGGGAGTGGTCTC
GGACAAGGTTATTGGTGATGCCGAGAATGAGCGCCCAGGTGTGCTGAACA
GTCGCATTGAAGTCGGGGGGTATCGGAACCCTGGGCAAGTGTTCAGCCGG
TGACCTTGGAGGAAAGAGAGAGGTGTCGCAAACTCACGCTGTGGGAGCAT
CGCGTGCCCTGGGAGTCCCGGCGACAATGATTCCCTTTTCTTTGCAGTGCG
CTACATCGATACCCAGATTACGCATGCCCGTCGTGAGGATGACCTTCAGATT
CGGAAGTGCATCGATTAGGTCCCTGGGAAACGGAGTGCGTTCCCGCATGGT
GCACAGTACGTCGTATGGTTCTAAACGCTTGATCAGACCTTGAATGTCCTGC
GATGGGAGAATGGTGTCGCGATAGATGGTGACATCGGCTTGGATGCCGGAC
AAGGCGTCGGCGGCAAGACCGTTGTAGTCGTCCAGCACCGCAACGCGCAT
CGACTTCTTCGTCATCATGAGATGCGGTGAGTCAAACACGAAGGAGGGAA
TGTGGGGATAGCACAAGTTACAGGAAGGGGGTCGAGAGGAGATGACGCA

ATCGGCCCAACCTCATTCGGCCGGTAATTAACCGAACCCGGTATTTAGCAG
AGTCCGTGCACAATGCAAAGGTCATTGAGGATTTTGTAGTTATGGATTGCAT
GGGGATGGCTATCGATGTGTGGCATAGAATGTACGAGATTAAGCTGCGGATA
CAAATGAGTGTGTGTGTGAGAGACGCAAAAGACGGCTGTAGAACGGATTG
TTGAAACAAGAGGCCAGTAAGATTGGGTTAGTAATCCAGGATGAGGTCAC
ATGAATGAATATCGACAAAAGGTATATATACTTAACAAGCGGTCAAGAGGA
GTGTCAGAGGACTCTCACTGAGGCTGAACATGATAGTGTCTGACCTATAGC
GAGGGAGGACGAGATCAATCAAACCTCGAAAGACAAGCGGCATCGATCAG
TACTTCTTCTTCTTCTTGAAGCTGTCCTGCGTGAATTTGTCTCGGACCGCTC
TGAGATTCCGAGGGCCCGGCCAGCCTTTGACTTCAGCCTTAAGGTGATCGG
TGAGCACGGTAGCTGATTGGTCAGTCATTGTCTTTGAAGTGCGAATCACAA
GCATCCCGCCGTCTCGCCAATTTCGGTTTTCCCTCCCTGCGCGTTATAGGCC
TTCCACACGGATTTCGCATCATGGGTTTGCCTAGCGAGATTGCTGTCCCTCA
CATAGTCCCTGTCATCAATGGCTACGGAATCAGCTGATGCTGCTGTCTGCAG
TTTGCCGAGTACCCGCCTGCATCGAGCTTCATAAACTGTTCGAATGGGAGG
ACTATGGGGGCGTGCCCAAAAGACTGTTGGTGCTTGTCTTGTCAGCACTTT
CCCCGGCGCATTCACTTGTGTTTTAGTCACCTCGTCTAGCATAGGCCTTTCC
ATGACCTCCCTCATGCTCTCCTCTGTCTCAAAAGTGAGCAGCACAGTCATG
TATCGGCTATTCTCGTCAATCGTCCTGCAAGTCAGCTCGGAAGATGGCGTC
GCGGAGGATCACACACTGCGCGACGGGGTCTTGCTGCAGAATGTCGACCA
GGGGCCAATCTCCTTCGAAATCCTGCTCGGATGCACCCAAGCCCACGTTCT
TACGGAGATGAGTGAACACCGACCCAATGGCCAGTAGGACAGGATCCATG
GTGTTGAGGACATCGTTTGTGAACAATCTGCCCCCCGGGGTGGCTGCGATT
AGCGTCATCCGAGCGCGGCCAGAGCGCGAGCCATGTTTGTTTGTCGGTGGT
TGATCGTGAGCTCACTGATGAAAGGTTTGCCTTGAACCACGCTGCCTTCAA
TCAAATACTCATGCTCGCCCCAGACGACGGGCATGGTGCTTGTCTTCACCG
ACTTGCCAAGGGCTCTTGAGGACCATTGAGACATGACGACCGTCCAGAGC
TTGGATGGCTTCTCTGCGATTGTCGCATAGGTGGCGATTCCGAAATCTGGGA
TTCAGCGCCGTTGCTTGCATAATCCTCGTCGTGGCGGGAACTGGAGTGTGA
CCCTTGTCGAGTGAAGCTGCTAGTCGATGCAGCAAACAATGTCAGACTTAC
CGCCAGGAGTTTTGCTCAGCACTTCCTTGTGCTCATGAGAGAGTCCCCAAT
CCTCCCTGATTCGGATCTCTCGGGCGGGCACAAGACGTTTCTGATCTGGGT
CAGTGTTTATGGATTCCTTGCAAAGGAGAGTCGACTGACCAGTCGGTGATG
TGCTAAAACTTTACCGTCCGCACTGCATGGGAGCCACGACCCAAGGGAGA
TGAAACCGTCACCGATGGTCTTGGTCATTTCATAATTTGTCCAGTCCTGGTC
GTCTTTCTCAGGGGACCAACCCTCTTTCTTGAGGTTGGCTGTCGAATCCAC
CGTTTTGATGCCGAGCAATCTGGTCACATTCACGAAATGCATGGGCTGATC
GGCCCGGGTCTTTAGGAACGACAGGGCGTAGTCCAATGTCCAGGGCGAGC

CGCTCGGGATTGGCCCGCAAGAACACTTCAAACCATTAAGCAGGCGCACG
TACGGGATATCGTTATTGGTCTCCTCTGCTCCTTTTGGGAGTTGGGCCACCC
ACTTAGTACCGCCGACCGACGGAGGTTTGCCAGCCCAGCGAGTTTTGCCA
GTCCACCGAACAGTGCTGTCGTTCGAGGCAGTAGACGCAGGAGTGTTGTC
GACCCTCTCTTCATCTTTGGTCGACTTGGTGCCCGGATGCGACATGAGCAA
AGTGAGGGTGTGACGGACTGTTGCGTATAAGGAGTGATATCGAACGATAAT
AGACGAGATGTCTGAAGAGGCATAGCGCTTGGACGTGATCGACCTTATCTA
CCTGCACTTCGACGCCGATTGTACCATAACTGAAGGGAACGTCCATCCCCA
ATCGAGAGATTTTCGAATGCCACAATAGAGTGCCGAGCTTATCGCCAAAGA
GCTGGGTAGATTCTCACGCTTGATCCAGGGGTCTCGTGCATGAAAGGGAGC
AATGAGTCTCCAGACTTGCTTATCAAATCCTGTTGCCGACTCAGAGTGCTC
CTTCCACCGCACGAAGACAGCCACAAGCGGCCAGGAGCCGTGTTGCGCCA
AAAACATCAAAGGTCGGATATCCACAGCACGAAGCAATCGATACAGCTCGC
TTCTGTTTCACAACGATGAGAGCTATTCATTCGTCCTATCTACAGCTCTGTAT
ATATGAGCATCGCTCTGGCGGCCTGTGCGTGGCCGCTCGCTCATGGTCTTAT
TGTCCTACTCCGCATCCTACCTATATAAAGAGGCAATTCTGACCCTATGAGG
CATCCCTCGGGTCCCATTGTTCAACACGCCTCCGAGTCGAATGTCCCGAAC
ACGTACTTGTTTGACACTACCTTGAAATGTGATGGGTCGGTACTACGCTTGC
CTAGATGTTCCTTGATTTTGTCGTCAACCAGCAGTTAGTTGAGAATCATCGA
AGTTCACACTCTTGAGCAATCCCTCACCTGTCAGGTCATTTACCGTCCAGG
GAATCTCGGTTGTCTGGCGGTCGTCGAAAACGATAGCAGCCACGATGAGC
AGTTAGGTATTCGGAGAAACTATTGAGTATGTTTGGCATGATTGCTTGACCA
AAGCGAGTGATCGAGTGAGTGAAAAGAAAGATGAAGACGAGTCTGGTGT
GCATCATTGCAAAGGTCATTGAGGGTACAGTATAATTGCATCAAGGTAGCA
GTGGTGGGTGTGGGATGTGGAATGTACAAGATGGATCCGTGTATGGTTGCA
AGTGCGGGTCATAGACGTGAGGGATTGCTGCAGTAGTGGCGAAATTGATTA
GCAAGCAATGATGCGGACACATGGATTCGTCTCAACAGTAGGAAATTATCA
TTGACAAGGGCTCGAACAAGGCGGATGAGGAATCTGACCGAAGTAGACCG
AAGCTGTACGTGGCAGACTCTGTCCTTTAGTGAGCGGGGACGAACCAAGC
CAGGGACCAGGACGTCAAGCCTTAGAAGACAGGTGGTTTGGACTAGTAGT
CCGTCTGCTTGAAGCTCTTCTGCGTAAAAACGCCACTGATCTCTTTCAGAG
TCTTGGGTCTCGGCGTCCAGCCCTTGATGGGTTCGTGAAGATGTCCAATAA
GGGCGGTGTCTGATCGATCAGTCATTCCGTTTTAGACTTCAATTCGAGCGCA
TCAAGCCGTCTCACCGAGAGATGTCCCTTGCTCCTGATGGGTCTTTAATATT
CGGTTGATATCATGTAGCTGCCTGGAGACACCGCTGTCGATAACGTAGCCCT
TTTCCTCCACGACTGTCAAGTTAGCTGATGTCATCGCTTAGTCTCATGGTAG
GACTTACTTTCAGCGAATGCAGGAACATCTTTGATGTCCATGACATGAATCG
AGTGTGCGAAACTTGTTTGATGCTATTTGTGTCAGCGCATTCCCTTGGGCGA

GCACTTCTCTTATCTAGTCACTCTATCTGGCATGCGCGCGTTTAAGGTTGCC
TTCATATCATCCTCCGTTGCGAATGTTAAGAGCACAGACATGAATTGGTCGT
TCTGGTCAATCAATCTGCATATCAGCCTTGAAAACATGGCCGTCACAGGCG
GAGAGGTCACGAACCTCACGGCCATGTCTTTGTGCAACATGTTGAACTTGG
TTCGCGCTTCGTGCTCTGCTACAGACACTGTATCATCCTTGACCTGAACTGA
AGCACCCAATCCCAGATTCTTGCGTAAATTGGTGAGTACCGACCCCAAGCC
TAGTATCACATGACTTTTAACGTTTTCGTCTTCGACGTCCGAAGCGAATAGT
CGGCCCCCGGGGGTACCTGAAATCAGCATCAATCAGGTGCGACTTGATTGC
AATCGTTGAATCTAGGTCGGTCTTCGATCGTGACCTCACTGATGGTTGGAAT
CCCTTCTACGGTCGCCCCTTCCTCATAGTTCTCCAAAGTCCCCTTGATTGCG
GGTTTGTCCACCCTGTTACCGAGGGCTCTATTGTCCCAATGAGAGGTGAAG
ATCGTGTAAACCTTGGATGGTTCTTTTTCGAAGGTCACGTAAGTGGCCACG
TGGAAGTCTAGCTTTCAGCTTCCCAAGGTCGGAATACCCTCTCTGATGGGC
AGGCTCATGCTAATCTTGAGAACAAAGAACACCAGACTTACGGCCGGGGG
TCTTCTCCAGCACCTGCTTCTGCTGACCAGTGAGCGAATTCTTCAGCTCTTT
GCTGAGTCGAGTGAGCCTGGCGGTCACAACACGTGCCTGACAGGCGTAAG
CGATTGAGGCAATCCTTGCGAGAAAACATGTCTCACCAATCGGTGATATGC
CTCCGTGGCACCCTCTTTATCGGTCGGGAGCCACTTTCCAAGGGCCAGGTG
ACCATCATTGATCGCTCCGGTAGCTGAATGATTCGTCCAGTCGTCGGCTACC
GTGGGGTTCCAACCCTGTTTCAGAAGGTTTGCTTTGGGATCCACCGTGAAG
ATGCCGAGCGTCTTGGCCCAATTCGTGAAGAACATGGGATCCTTGGCGCGA
GCGTCCAGGAGCGTCAAGGCTCCGTCCTTCGTCATGGTCAAGCTTTCCATG
TCGTTCCACGAACACTTGATCCCATTGATCAGGTCCACCGGCTCGACATCAT
TCGTGGTCATTTTCTCGACCTCTGGGCGTGTTGTCAGCCATGCGGGAGTGG
AGGTAGTCCGGAGTCTGGACGGAAAAGACTTTAGTACGGTCGAAGCGGAC
ATGGTTACGAATGCTCCTTCTAGAGTGAGGCCGTGAAGCAAGTGTGAGTGC
GACGAGTAAAGTCAAACGAGTAATATTAGACAGTGATGTCTGAGAAGGCAT
CATCCTGGGCCGGGATCGTCCTTATATACCTAATCATGGACGCAGACCGCAC
CAGTGGGCAACCGGAGGGAACACCCATTCGCAATCAAGAGATTTTCGAAT
GCAACAATGGAGTGCCACGTTGATTGCCAACATTGATGCGCTCCACAGATC
CACAGGGAGAGCTCAAGCGAGAGAAGTTTGGATGGATTTCCACGCTTGGT
CTGGCCCTCGCGTGCAAAAAGGTGGGAATGGATTTCCATACTGGCTTATCA
AGTCCTGTCGCCGATTGCAAACCCCTCCTTTTGCCGCGCGAACAAGACACA
AGCGATCAGAATAAGGATCAGCTCCAATAAGCTGTGTTGCGCCAAGGACAT
CAAAGGACAGAATACCAGAGCACGAAGCAATCGATACAGCTCGCTTCCAG
GATCGACCCGCTCCCTTGTGGGGTTAGAAGCCGTGTCAACCCGAGTTGCAG
AAGCAACGCCCCCTGCCCGAGGACAAAAAGGAGGTAAAATCACATTTGGA
GACCCCGTATCAGATACGAGTCTGCGTTCTAGCGAGAGAAGCACGCAATCG

GTCCGAACCTGATCAATAAAACTTCTCTCGTTGAGAAGACTCGCAAAAGCT
GTGTCGACGTGAAAACAGATGAGACGGGAACTCACGCACTGATGGCAGGA
AGCATCTTTCAGGTCCTGTGACGATTTGCCTTTGTGGCGCCGGACGACTGC
GGGCGCCTTTCGCAATCAATCGTGCGTGCCATGAGCCCGCTATGCCCTCCC
AGTCGATATTCATTGGGCGAGATTTCCCTAGCCGCTCATAAACAACCAGAC
AATGAGCTCGTTGTCATCGTCAGTCTCGGCCAATCCAGGTTAGCAGTCGCG
TGATACAATGCTTGCGTCATGCATGGACCGCGGGCGGGATCACCGAGTTGC
AGTCTTGCGAGCAGTCTCGACGATACCCTCACTTCCGCCCCACGCAATCCC
CGCAAAGGTCGCCTCTTCTTCACTAGATGTGACGATCCCATCCCGTATCGGC
CGTGTGGCATGTACCGAGCTCGGCAGTGGTGATGCTGCTGATTGGGCCGAT
GAGATACTTTGGCCCAGCAGACCGGGCAAGCGGTGGCCCTGGTCTCCCCG
TACGAGGAAGAGCGAATGCAAAGCATTGACGGCCGCTCGGTGCTCAGCCA
TGGCAAGAGAGGATCCGTGGCCTTCTGCGAGCTTCAGCCCGGAGGGAAGA
AAAATGCCGATGTTGAACAGAGGCGATTGCGAATTGACACCTGTGGATGCG
ATTACCCTGAGGGTAAGTATATACCGCCCACGAGACTATGAGACGTATCGTA
CCTGGACTCGAGACGACCAGCGCCGGAAGACAGGGGTACTCCGGCCTCTG
CCAGATGCTTCCCTACGACACTCGAGAGCACATGCCTCGGATTCCGGAAAT
GGAAGAGGGTGGTCATGTCCAGATGTCTGCTCAGGAAGAAAGCCTCGACA
AACTCTCGAGCATTGTGCACTCCCTGTTCCTCAGCATATCATAAGCGAGAG
AGTGGGGACTAGACTGACCTTCTCTTGGTATATCAGCCCCACGAAGGCCCG
GACTGCGCTGGCCACAATATCTTCGAACGTTTCTTGTCTCTCTGGGCGCCG
GCGGCCCGGATCGTCCGGCCGCGGAATGTACTTTTTGAATCGACCAGCTAT
CGGCAGTGACTCAATTTCGGCCTCTCTCTCGTCCCGCGCCGACGTGTCCGC
GCGGTGAAAGCGTACTGAGACACCGCCCGTCGGATGTTCTGGGCCGTTCA
ACCCCAGACCAAGCTCGCGTGCGACGGAATAACACGCTTTCGGCCCCACG
AAGCTCGTGACTGCCAGCTTGAGCGCTTCGGTAGGCAAAAGCGGGTACTG
CTGCGCAAGGTGCTCGGACGCAAAGAGACCAAGGAGGGAGTTGCCAAGC
GAGGCTAGGAGCTCGTTGGTCCTTTGGCTGAGTCGTTGCGCACTTCAATTT
CTCAATTAGACCGAAAAGTAATTCAGCTGTCAATATGGGTTCCTTAGCACA
GCACGAGGAACACCAGTACCTCGATCTCGTACGTGAAATCTTGGACCAGG
GAGAGCGTAGGCCGGATCGGTAAGTTGACGACAATTGCAACAATCCTATGC
GCAATACCAAATACTGATCCGACCCGTTCAAACTACAGAACAGGAACGGG
CACATATTCCATCTTTGCTCCTCGGCCCCTTAAATTCAGTCTCAACAATGAT
GGCAAACCAATTCTCCCCCTCCTCACAACCAAGCGTGTCTTTCTCCGCGCC
GTCATAGCCGAGCTTCTCTGGTTCATTGAGGGCAACACTTCTTCCGCCTCCC
TCAGTGCACAGGACGTCAAGATTTGGGACGGCAATGGATCGCGCGAGTTC
CTCGACAACCTAGGTTTAACTCACCGCGAAGTCGGTGACTTAGGGCCAGTG
TACGGCTTTCAATGGCGTCACTTTGGCGCCGAGTACGTTGATGCTAAGACA

GATTATACGGGCCAAGGATACGACCAGCTCGCAGATGTTATCCATAAGCTAA
AGAATAATCCGTATGATCGACGCATGATCTTATCTGCGTGGAACCCGGCGGA
CCTAAAGAAAATGGTGCTACCGCCCTGTCACATGTTCGCACAATTCTATGTC
TCATTCCCCAGGCAAGGCCGGGAGGAGAGCGATTTAGAGAAGGAGAAACC
GAAAGGACATCTTCACTGCCAGCTGTACCAACGATCATGCGATATGGGTCT
CGGTGTCCCATTCAACATCGCAAGCTACGCGTTGTTAACGCATATGCTTGCA
CATGTTTGCGATTTAGTCCCTGGCAGCCTAACACATGTCATGGGTGATGCTC
ACGTATATGTTGACCACGTCGACGCGTTGAAGGTTCAGCTAGAACGGGAAC
CACGGCCTTTCCCTGAGTTGAAACTATCGCCTCAAAATGGCGACTCTATCG
ACGGCTGGAAGGTCGAAGATATCGCCGTCCTGAATTACGAACCCCACAAG
ACGATTCCGATGAAGATGTCTGTTTAATATGGAAGCGACGTCTGGGGATCTC
TAGACTTTATATACCCCATGAAACATCAAATAGAAGCAGGGCCTAGACGCG
AATACAGGAACACGACGCAGTATGATAACGAGTGTACTATTCGTGATGTCAT
GAGGTGGGTTATACTACCCGCTGGATTCAATCGTATGTTGGATATGGTGTCA
GGTCCACAGCATAGAGATTCATGAGTTCCATAGAGCCTTTTAAGCGAAAGC
GCTAGATGTTAATCGTGAATAATGGTTCGGAATAGAGGTGCTGGAAATACAC
TCACAAGGAAGTGTATTGAAGCCGGCAGTACAGGCATAATTAGCTTGCTTA
TCTAGGTACGAGACCCTCATATATGGATTTACAATACATTGGTCAATAATTTA
AACAAACCCCCTTTTACGCGAGCCTGCGACTAGGTAGGTAGTCCACCAGG
GCCATAGGAGTTGCTCCTTCATCTGAATCTCATTCTGGCATGTGAATGTTATT
ATTAATATAAACCTTGTAGCACAACGACATGGGAAACTGCCGCCCAGACTC
CTCAGGGGCTAGATAGAATATGTGCCTAGTATCTAAGATCTATGTATTGGTTT
CGTCCTCCAGAGCATCACATCGATAATTGAGTGCTCTCCATTTTTCTACTCG
ATTGATTTTCCAAGCTACTATCTAACCATCATGGCTAACCATAATAATACCAC
GCTGAGAAAACCCACAACATTCCGAGAATTCGTAAAGCTGGTGGCGAGCC
AACCGTCAAGCTTGCAACGGTATCTCAACGACATAAATCTCCTCGTGCTTAT
GCCCGAGGACTGCGAACATCACGGAGCCTTGAAAGGGTTGAAACTCGAGA
ATTTAAAAAGTCTCTCGCTGGCTAAATCAAGCAACAACACCGACGACGCCT
TGCTTCCATATCTCCAACCCCGACTGGAATCGCTATTTTTCTATGGCGGTCCC
ATCTCAGATTTGTTTTTTGAGAAGCTACAGGTTTGTGACATACCACACCCAA
AATATAGGGGTATGGCGCTTACATGCCGGTCAAATCATCAGTCTCTCTGCCC
GTCGCTCAAAGAACTATTGATCTTCAACCCTCGCCATACCCAATGCACATCG
GAAGGACTACGACGATACGTCGAGGGCGCGACATCTTTGAGGACCCTACA
GATATTATGTGGCTTAGATTATCCTGTTGCCAGCGACATTTTCTGTGCTCTTG
CTAAGACGTCCACTCTTAGAGTCCTCGACTTCCGGCTCCCGCTCACTACAC
AACTCATCCAAACAGCTGCCATTGCGCGACAACACTACGCCAACAACAGC
CAATTATTTAGCCGCCTTCAAAGGCTAGTATGCATTGCAGATTATGAAGCCC
TCCTAAGACTACTCCCTCATTTACACAACCTTGTCGACCTCGAAGTGAGCAT

CGGGGGCGAAGTGCAAGACCTGTTCGCAGAGTTTTGTAAACTCGATTTGC
GATTGCGCTTTATCCAATTAGAATACCTAAGCAGAAGCCATATTCATATCAAT
CCCGTAACGATGCATAATTTTGTAAGAAGAAGAGCCTGGGATATAGAAAAG
CTATCGATATCAGGTCACAGCCTCTGGGGGAGAGGCTTCACGATCCCCGTC
CTTGATATAATAGAGCGTGCGACTTCACTCAAGATCTTTCGCCTATGGATGG
ACAACGATCTTAACGAAACTGCACTGCTGACCGCTGCCACGTGTGGGGCT
CAAAGGAGATTGACTGAACTTGATCTCGGCGGATGTCATGATTTTGATTTAC
TCAGAAGCCAATGGAATGTGAAGAGATTCAAATTCGGTGATCAGCTCACAG
CTCTCAATCTTGGGATGGTAAAATGGCCTGATGGCAGATGGCCTAGTAACG
GATCCTACACTGTGGGAATGAAGGCCGCCGAGTTCGCCCGCTTTCTGATGA
CGATTGCTCCCAAGTTAGTAGACTTCAATATTCGGGCCAGCTGGGACCCTTT
TTCTTCTACATTTAAGAGTGCCGTGTTAGTC

>Mosaic_sequence_73

ATACGACCGTCTTCGCCTGAAGACCATAGGACCAAGTCTGAAGTTATGACG
AGTTCCGCCATTGTCACGCATCTCCGAACTGCGGAGTACGGTCTGAATCAA
TCCACAGGAGGAAGCAGCCCTCTACCCGGTATCTTTTCAGGAGATTTCAGG
GTTTGTACGGTCCTGCACAGAGCAGTTTTCAAGTGCAAAAGCTTTGGATTG
GAAGTTCGCCACGGAGCTCTTCAAGTCCTTGCCCATTGAGTGGGTCGAAC
ACTCGACCTCCCCCTGCAGTTTGGAACCTTCCTGGCACTGTACGAAGGGGG
CGCATCTACCACTGTGCTAAACAGGCAGCAGCACAACCTCAACTTATGTGT
GCCTTATGATGTTGTGACAATTCAAGTTCTTGGGCCAAGATTGTCCTTTTTC
CGCGTTTACAGGCCACCCGGTTTGCCCATGAGTGGTGCTCATCATTTAAAA
GCTTTAGAATCGCCGAAGTATCGCGTTGGGCAAACAATAGGGTTGAGGGAT
GGCGCAGAGGCAGCTTGACCTACGGACGCCTGTGCCGCAAGTTTCACTTG
ACCTATGCATCCCAAACCCCCCTCATCCTTCGCGCCGCCCTCTTTTACCCCT
CTCCTACAAATTTCAAACACGCAGCTGTCCTGTGGGACAACTAGCCTCGAG
GCAAACCGGCCAAGACCCCAGGGGAAGAAAGAGGAAATTCAACCAGAGC
CGGGGTGCCGGGCCGGCCGGTGTAAAAAGTCTCAGGGACGGTGTGTCCCT
GAGTCGTGTACCTGATCGGGGTGTTGACACGGATTGAAGCGCGTGTTTACG
CTGGGGCCTCTTGTACTGTAGATGGTGTTGTTGTGCAGAACAGCGACGACA
GCGAGCGACAAAAAGCGTTGTGTCGACAAGAAGTGGATTGTACAACGACG
ACGCAGCTGTTTACTTGGGCCCGAACGTTAGCCCACCCCTTGGCGGCTAGG
TGAAACCCCTTGGGCAATCGCAATCCCCCCTCTTGCCCCTCCTTTCACCCC
AGTGACCCCTTGGTGAGCCGAACAAGCGACGACGATGTTGATGCTGATAC
GATCCTCTCACCATGACGCGGCAACTCGACTCGCTGCTGGACTCTCTCCTC
CCTGGTCATGAAACGCAGACGCAGACGCAGCCGAGCACGACGATGGCCGA
GTCGGTGACCACCTCGGCGGCGAGCACGGTGGAGACGAGCGCGGTGGTG

AGCAGCACGACGACGTCGACAGTGGAGACGAGCAGTGAGTCGAGTCGCG
TAGGGAGGCGCTGAGAGCAGGCTCGACAATGGTGAGCAGCACGACGTCGT
CGACTACCTCGACCACGTCGACGAGCACCATCCCCACCTCGACATCCACCA
CTCCCACGTCCACGTCCACCAGCACCAGCTCGACGACGCCCACGTCGACG
TCGACGTCGACTACAACCACATCCACGACAAGCAGCACCAGCACGACCTT
TTCCAGCCAGAGCGTGCCGGGGCTCGTGACCAACGTGACCGCCTCGTCGC
GCACATCCACCAGCGCGTCGCACACCGCCAGCAGCGCCGCGGCGAAAGCG
AGCAAGGGCGGGGTGGGCGGGTCGGGCCTGTCGCTCGGTGCGCTGGTGG
GCATCATCATTGGCGGCGTGGCCGTGCTGTGCATCATCGCGCTGCTGGTGA
CGCGGTCGATGCGGAAGCGCGCGCGGAGCAAACGCACGCGGAGCCGGTC
GAGCATGTTCGAGCCGTGGCCGGCGGCGGCCGCCTGCGCCGGTGGCGCTC
GAGGAGCCCGAGTACGAAAAGGAGACGACGTACGAGGCGGGGGGTCGT
ATGCGATGCAGCCGACGCAGGGCGAGTACGCGTACGGCGAGTACCCGCAG
TATGCGTACCCGCCCCAGCCGCAAGAGTACGCGCAGCAGCCTCAGTATCAG
CACCAACTGGGGTACGACTATGGGTACCAGGCGCCGCTGCAGCCGGTGTA
CGAGGGGACGGCGACGGGGCCCGAGGGCCTGAGAGACGGCACCATGGCG
CGGGTCACGGTTGGCTTTGTGCGCAGCTTGGAGGATGAGCTGGGTAGGTCT
GCGACGCGTCGTTGGACTAGGCTCATCCCAGCGATCACCCCCGGCCAGCA
GCTCTACCTGCACACAATGTACGACGACGGGTGGTCCCTGTGCGAAGACC
AGAACGGCAACCGGGGCGTAGTCCCCGTATCCTGCCTCGAGCCGTGGACA
GAGGGCAACATTCCCCGCAGCAACACGACCGAATCCATCGCAAGGTCTGA
ACGACGATCGAGCCTGTATGCAAACGCGCAGGCGGGCCAGGCATATCTCCA
AGCGCCGCAGTGATATAATGTACAGTAGATGTATGTATGGCTAGCTCGGTGC
GTCGAGGACAATCTCCATCTGGGGTCAGCGCCATTTGGCTTGGAGACGTAC
AAATTCGTTGTAATCCATCGTGAGCCGGCCCTCGCCGCGGACGTCCATTCT
GAGATTAGCGCGGTCACGCGACCACACTCACCGTCTGAACGCCTCGGTGT
AGTGCTTGACCGTGACGCAGGCCATGAGGAAGCGGTCAAAGCTGATGCCC
TTCATCGGGGGCTGGCCGGGTTTGGGCGGCGGCGCGTAGCGCTTCTCCAGT
TTCCGCACCATCTCGCTGGGGAGGGAAAAGCCGAATCCAAGCAGCGCCGA
GTGGAGCTCCTTGCGGTCGATCAGGCCAGAGTTGTCCCGATCGAAGCGAA
GGAAGATCGCGTGCCAGTCCTACCCTCAGCCCGATCACCTACGCCAGACTT
ACTTTGATGTAGCGGTACAGACCCTCAAACTCTTGAAAGTTGATGCTGCCG
CTGCGGTCCGTGTCGAAAATGTTCATCAACATCTTGACGCAGTCTTCGCGG
GCCTCCATCGTTGCGTCTTTTGCGAGAAGCTTTTGCAGATCGTACGCGTTG
AGCTGGCCCGTGCGGCTCCCGTCAAACTGCTCGAACCTGCGGTCAGCGTC
GTCGAGGTCGTGGACTCACAGAATCTCGAGCTCCTGCTTTGGAGGCTGCA
CCGCAGGCGGGGGCGACGCGGCGGGCTGCTGCCACTGTTGCGCAGGAGG
GGGAACGGCGTTTTGCGACCACTGCGGCGCGTGCCCTTGAGACGAGGGCG

GGCCGGGGTACCCCGCCGGCGGATTCGAGTACCGCGGGTTCTGCGGATTCT
GCTGCATCGGGGGCGAGCCGTACTGCTGTTGAGGCTGATACGGCTGTTGCT
GGTAGTTCTGAGGTGGGAAGTTTTGCGGGGGTCTCGGGAGCGGACCCGAT
CCACGATGCTGGGGCGGGGCGGGCTGAGCGGGATAGCCCCCTGGCGGCTG
GCTGAAATGCTGCTGGGGCTGATTCGGCGAGAAAGGAGGTCGGGGCGGCG
GTCCGCCATACTGCCCGCCGTACTGCGGTTGTTGCTGGGGAGGAGGGTTCG
CGGCTTCTTGCTTCTTCCGTTCCTCCTCGGCGGCCTTCTGCTCGGCCTCCTG
CTTCTTCTTGGCGTACCGCTCCTCGTACCTCCTCCTTGCCTCGTCGCTTACG
CTCCCGTACGGCGAGGGCGTCGCCATCAGTTGGCCCCCCTGCGACGGCATG
CCCGGCTGTGGTGATCGTCTCGGTGTGGGATTCCGCTGCGACGGCGGCTGC
GGGGAGGACGTGTCGTGCAGGGCTCTGTCCCCAATGGCAAAAGGTGAATC
TCCAGCCGAGGGTCTTCCCAGATATGACATGATGAAGGGAAGCTCTAGCTT
GTGGTTCAGTACAGTACAATGGAGTAACGGAGAGCGGTGGTCTGTGCGGT
GTTGGTTCGTGTGTTACAGGCACAGCCACTCGGCCCCGCACCAGCCACCG
CACATGCCACGCGACCTGACTAATGATCTCAGGGCATTAGTATTTTTTGTAG
TGGGGGGATTTCTAATCGTTTATCCCACCACACGCCGAGCACTGACGTCGA
GGCGATCCTGCAGCACCAACTCTTGACTCTCACCACGGCGACTGACAGGC
GACATGGCTGTGGGTCCCCCTTGTAAGACTAGAAACGCGCTCACGGCCAG
TCCAAAGACCCCCTCTCCGGCCACCCCGGGCACCTGTGAGCTCCCCCAGT
CGCCAAGCGGCCCACTGACGCGGTAGAGACGAGGCGCAGACCGCCGCCCT
TGCACAGTTCCGCGAGCTGCTGCTGCAGTCGGGGCTCATACCCGAGGACA
CGGCCGCGTACGCGCAAAAGATCGGATACGACAGATACGACGACACGACG
CTGCTGCGGTTTTTGCGTGCGAGAAAGTTTGACCTGGCAAAGAGCAAGAT
CATGTGGGAGAACAACGAAAAGTGGAGAAAAGAATTTGGAGCGGATGAG
ATTGCGGCGTAGGTTGTCAGAGCAGCAGGCGGGCGAGCTGACGCTTCTCT
TCTGATGTATGATCATCTCTGTCCCGATGTTCACCAATGCCTCGATCGACCC
CTTGTCCACCACGCGATCTGTCTTTACGCAATCTCCCATCGACTGCTGAACA
CCTCGGACCTGCACTCGCTTCTCCCACTCGTCTCATCCCAAACAACTCCCA
CTCGCACTCGCTTCGCTCGTGCTCGCTCGCGCAGAAACGGCTTCGACTACC
CCGAACAACCCGAAGTGGTCAAGTACTATCCCCAATTCTACCACAAAATCG
ACCGTGAAGGCCGACCAGTGTACATCGAGCAGCTGGGCAAACTGGACATT
GCCAAGCTCTACGCGCTCACATCGCAAGAACGCCAGCTGAAACACCTCGT
GCACGAGTACGAAAAGTTCCTCAAAGACCGCCTGCCCGCATGCTCGACAC
AAACCGGCACGCTCGTCGAGACGTCCTGCACCATCCTCGACCTGCACAAC
GCGGGCATCTCCACGTTCTACAAGGGCACGTCCCCTCTCCTCTTTCGGTTTA
TCTGACCCCCAGTGAAAGACTACGTGTCGGCCGCCTCGACCATCGGCCAG
AACTACTACCCCGAAACGATGGGCCACATGTTCATCGTCAACGCAAGTCCC
CCCCCCCCCCCCCAACCCATACCCCAGCTCACACTGACAGGCACCCTACCT

CTTCTCCACCGTCTGGTCGCTCATCAAGCCTTGGCTCGACGAGGCGACCGT
CCGCAAGATCCACATCCTCGGCAGCAAGTACAAGACCGAGCTCGTCCAGT
ACATCCCCGAAGAAAACCTCCCCGCCGACCTCGGCGGCACGTGCAAGTGC
GCCCAGGGGTGTAGTCTGAGCGACGCGGGCCCGTGGAACCCCTCGGCGGC
GTGAGCAGCGGCAGCGGCAGTAGACGTAGACAGTAGTGTGGTGTAATGCA
TAGTCCCACTGCGGCGCCCGCGCAGCTACTCCTTTGCAGAAGGCAGGGGC
GGCCAGAGGTGTCCTTTGCCTTTCCACTCCTCCCGCGCGCGCAGCAGACCC
GACACCATCGCCGCAGTGTCCACCTCCCCCGTCCACCGAGCATCTTCCCAT
GTCCTCTTCTCCGGACTCACGCCTAGGCGGTCCAGCAGCTCGCCCGCCTTG
GCAGGGATGACGCTCTGCAGGAGGATGCCCGCGATGCGCAATGTCGACCA
GGCCAGCACCACCACCGCCGTGGGGTCCCGCCCGTCCGCCTTCCACGGCG
CGGCAGCAGTAAAGGCTCGATTGGCCTCTGCCACAGCATCCATCACGCCCT
CGCACGCGCGCGTCACGGCGTACACCTCCATGCGCTTCTCGAACACGTCCC
GCATCTCCTCCAGCCCCTCCAGCCCTCCCTGTTCCGCCTCGTCCCACTTCGT
GACCCTCTCCAGGATGCGCGGGCTCGCCAGACGCGCCAAGAGGTTGCCCA
TCTGATCCGCCAGGAGCCGATAGTGCACCTCGAGGTTCTCCGCCGAGTAAT
CCGCGTCGGTCGCCAGACTACCGCCGCACCGCATGAGGTACCACCGCATCC
CGTCCACACCCACCGCCGCCATCGCCTGGAGTGGATCGACGACATTGCCGA
GGGACTTGCTCATTTTAGTCCGGCCCATCGTCCAGTGCGCATGCGACAGAA
CCGTGCGAGGCGGCTCGAGCCCTGCCGCGCGTAGGAGGGCCGGCCAGTGC
AGCGCGTGAAACTTGACAATGTCTTTCCCGACCACATGCACGTCTACCGGC
CATCCTGCCATTTCGGGAGGGTCGCCCTCTGGCCAGGGGTAGCCGAGGAC
AGTGAGGTAGTTGACGAGCGCGTCGACCCATACGTAGATGGTCTGTTCGGG
GTCGTTTGGTACCGGCACGCCCCACTTGACTCGGGTGGTCGGACGGGATAC
GGACAGGTCTTCGAGCGAGTCGAGCGAGCGCAGCAGCGCCAGGCGCTGG
CTCTCGGGCTCGACGGCTGCCGTCAGTCTCCTCCTTGCCTAATAAGAGTAG
TATACGCACACTCAGAGCGTGAGAGCCATTCCCGTAAGAACGGGACATGCC
GCGTTAGGCGAAACTTCCAGTTGGTTTCTTCCTCCCAGACCACCTCGTTGC
CCGTCTCTGCAGCTACCATCGTGCCGTCGTCCCGCTTGGTCACTTGGGACG
GCGCAAAGAAACTCTCGTCCGAGACCGAGTACCACCCCGCGTGCGTCCCC
TTGTATATGTCGCCCGTAGCCACCAGGCGGTCCTGCCATCAACACCTCTCCT
CAAACTCATCATCATACACACCCAGAAATGCTGCACGGCACGCAGGTGCCT
CTCCTCCGTGGTCCGTATGAAATCGGTATAGCCCACTCCCGCCCGCCCCGCA
AGGTCGCGAAAGCGCAGGGACACTTCATCGCAGAACGCCTGTTCTCCCTT
GCCCGCCCGCTTGGCCGCTTGTTGGATCTTGAGCCCGTGCTCGTCTGTCCC
GGTGGTGAAGACCACTTCTCTGTCCGGATATCGCAGCTTGGCAAAGCGCGC
GATGATATCGCTCAGAAGGAGAGAGTGCAGGTGCCCAACGTGCGGTGCTG
CGCGTTAGGCCTGTCAGTGAACAGGGGAACCGCTTACAGGCGTTGACGTA

GAAGATGGGGGTGGTGATGAAGAACGGCTTCGCTTTGCCTTTGCCCTTGGG
GTCGGAGATGATCTTCGGGGCGAAGAGCGACGAGAAGGATGGGCCGGATG
AGGTGTGTCGCGCAGCTGGGACCTTGGGTGAGCAAGACGGACAGGTTGG
ACGGGGGATGGCAATGGGCCGAAGTGCGGTGTTGAGCATGGGCGAGCGGA
GCCCGCGCGGGAGATGACGCCTCATCGTGCACCTCCAGAGACTAGACCAG
AGCCAGCTTTCAGACAGCGAAAATCTCAGGAGCCGCTCTCGAGCCTGTAG
GATGAACGGTGGAAGATGCAGAAGAGAAGTCGTCGCCGCCCAATTCTACA
TCTCATCCACCTCTTCTCTTCTCTCCGCCATACATACATACAGCGCCTCTCAA
TCCAGCCCCACGACCACAAGGGCGAACGGGGTGCTCGACGCGTCGCTGAT
CCCTAAGGCTCGTGCGGACATCGAACCACCGGGGCTCATCGTCCCACATCG
CGCCATGGCCAGCACCATCCTCCCTCTCGAGCTGGTCGACCGGTGCATCGG
CTCACCCATCCACGTCATCATGAAGAACGAGCGCGAGTTCACAGGGACCTT
GATGGGCTTCGACGACTACGTCAGTGAGTACCCGCCGCTGCATCGGTGAGT
TGAGCTGATGGGAGACATGGTGCTGCAGAACGTAAAGGAGTAGTGGGTTT
CTTGATGTGCTGAGTTGGGAGAGTCGCTGATGGACAGCGAGGTGACGGCA
AAGGGGATCACAGAGACGGAGCTGGGGCAGACATTACTGAACGGCAACA
ACATTGCGATGGTGGGTCTCCCAGTTCTCCTTACGATGCACACAGGAGAGG
TTTGACTCCCAGTTGATACCCGGCGGCAAGGGCCCGAAAGCATGACGAGC
GACGTTGTAGCATATGCACCGCGGGATGTGGCCGCAAGTATTGATCTACGTC
GATAGATTGGCACAGAATGATGGGTTGCATGGAGCTGACCAGACTGACCCG
GAATACCTCGACTTAGATTATACAACTAGTGGACCCAAACTACCAGACTATC
AAGCTACATGACCAACTCCAGCCCCCCTCGCTCCAGAGAGGCCTCATCGAC
ATCCTCGCCAATGACGACCAATCCCCTCCGCACCGCCTTTCTCGCCCTAATA
CTATCCCTCTCCCTCTGCGCGTCTCCCATCCACCCTCCAGCCATATCATTCAC
ATCCTCCAAACTCCGCGCATGGACCGGGGGCGGCGGCGAGCCCGGCTCGT
AGTGTATAAACTCGAGCTCGACTCCGGCCGCACGGCACACATCCGCGATGG
GCGCATGATCCCTCGTCGTGCTCATGCGGTGGGTGTACGACACCCTCAGCG
GCGAGTCAAAGACGAATTCGGCGCCAATCTGTAGGAAGTGCCCGGGGGAG
TGCAGGAGGGGCATCTGCGTCGCCCGCTTGAGCGTCTGTCTGGTCGACTCC
ATGGCAGATTGCACAAGGTACGATCCTGCCTCGTCGTCGGGCCCGCTGTCC
CCCGTCTGCCGCGTCAGGCCGAGCGCGCGGTACAGCGCGAGCGAGGGGTC
GGTGTACATCCGATACGGCGAGCGAAAATGTTTGTCTGGAGTCAGCCTCTG
CGGCTCGTCTCACTGCCAGACGCACTCCTGTACCCGTTGAGCATCTTGTGT
GACCCGTTGCCAATGATGATGAGATCAACGTTGGCCTCTTCCAGCGCGTAC
GGCGAGACCTCGGCCAGGATCGAGTTGATGTACTGCGCACATAACGGGCA
GAACCCTTCCTATCAGCCCCCACCCCGCACACCTTGACCAACTCACAATGC
CGGATGAAGATGACAATGGTCCTTCTATCCCGCACCAAGTCGCCAAACCTC
CACGACGCCCCCTGCTCATCCAACACCTCCAGCATGCTCGCCTCAAACAAC

GCCTTGAGGCTCGGCGGCTCGTCCGCCGAGAACTCGTCTGCCTCCCTCTTC
TTGCGCTCTTTCTTCTCCTTGCGCGTCTCTTTGGGTCGCGAGAGCCGGTCG
AGCCGTTCGGCCGACTCGATCTCTTCCAACGTCGACCACGTCCCGCACGTC
GACGCCGAGCCGGACGAACAGCCGAGCGAGCTCGACTCGGAGACCGAGG
CCGAGACGGAGGGAGAAGCACGGCGATACGACAGGATCTTGTATGGCATC
TTGTGCGCCTCGTTACCGCTCTTGACTTGATAGATTGCGCCGAGGTCGAGT
GGGGGTCCTACTTCCAGCCGATAGGTGATGTGTCCTTCTGGGGATGAGGCC
TCGAGAGGACAACAGCTGCTGGAGGTTGACGAGCCGGAGAGGCTGCTTC
GTGGAGTGGAGAGAGACAGCAGTTCGAGCGATTCAGTGAGCGCAGGGGG
CAGCTTGCGGCCGAGCCGATGGCCAGGCGAGCGCTGTGGGGATGTCGAGG
AGCGTCGTGCTGCCGGTGGTGTTTGGGGTGTTGACGGGTGAAGAGGTGCA
GGCGATTCGTCTGTCAAGTCGACAGTTGATGGAAGATATGTCAAAGTCAAA
GTGTTCTTCTGCGTAGAGACCTCGATGTCTTCAGAGGGCAGGCCCATAGGG
AAGCTCGTGTAGCCGGGCATGTCGGGCTCGATAGGATGGTGAGAGGGTCG
CTAAGGGTGCGCAGATGAACAGTGGTGTGGACAGACAGGGCCGGAGCGA
AGCTGTCTGCGCGTTTGATACGCTGGAGGAGCACCGGTAGGGTCTGTTGCC
TGCGTGGGTGAACGGGACAGCCTATCGGTAGTCACACTATAGCTGCAGGAC
GAAAAGACGTTGTCGATATGAGCTAGGTGAGACAAGCGGGAAGGACGGG
AAGGGTCGGTCGTCCCGTTATTCAGAGCAGTCCAGTTGGCCAATGGGTCAT
TCTCGAAGAATGGTCAGACGTATAAGTATACATACCGCTGCTATTAGCGACG
AGACACTCGCCACTACCTGCATGCATGACGTCGAGCGTGTTGGATGGATGT
ATGAGACGACAAATGTAGCGCTGTTCAAAGAGTCGTCCCACTTCATCTATG
TTCGATCGAAAAGTGATCGACCGGGATGACGCTCCAATTACGTTCCCGCCG
AACCGAATTCCCCTAATCCCCGACCCAGATCCAACGAATCATGCAATGATG
CTACAGTCTCACTCGCCATACGCGTACTCGACCTCGTCCACAGCGCCAAAC
TGTCTCTCGCGCTCGTCGGCAGTCTCTCGCTCGATGGGCATCGGACCCCGC
AGCGGTTCATCGTCGTCGTCGTCGAAGGGATCCCCTTGTAAAGGTCCACCG
ACGAGAACAGCTCGGAGGGGCGAGTCCCTTCCGCCCGAAGCGCCAGAAG
ACGACGACCGCCGACGCTCAAACATGGCTTGCGGCGAGCGGAGGACCGA
AAGAGTGTCCGACGACATGTGCTGCGTGTCAGCTAGAGCGCTACATCAAG
ACAGAGGCTCACCTCCCATGTATTCGCATTCTCCTCCTTTCGCGAGCCACCG
TTCATGCCGTCTTCGCCCAACCTCGCGTCGTCTCCAAGCTCGCCGATGCTG
ACGACCGACTCGCTCTCACTGATCTCATCCTCTTCCAAGCCTTTGGCCCTAA
ATATCTCGTTGAGCCTCCTCGCTCCTTCCCCATTATCGTCCCTGATTTTGGCT
ACACCCGCACCCCACTCATCATCCCAAGTGTTGCCAGAGACGTCGTCCCCA
AACACCTCACCCAGGACATCCTCGTACTCGTGCAACCAAAAGTCAATCGCG
CTGTCGGGATTGTACGGCAGATGGCCACCATCCTGTACCGACTTGCGAGGT
GGGAATACATCCCCTTCGGAGCTTGACCCCGAGTATGTCCGGGATCGCGAC

TGATCGTGTTCCATATGCAGAGCCGGGTCCTCGAGAATGACAGCAGAGGTC
GAATCTGATCGATGATGCTTGTTGTCTTGAGAAGGATTCGGAAGGGGCGGT
GGCAGAGCCGCGATGCTCGCTGGGTATCGCGTCTTGTTGTAAAAGTGAATC
AGCCATCGCAGACCATACTCTTGGGGCTGCCGGTCAGCTCATCTCGTCTCG
AGCACTCACCAAAGCCCCTTCCAGCTCCGTCTCGTGGTCCTGACCAGCAA
GCCACTCATCTCTCAGATCGGGACGACAGTTGAGATAGATGGCGGTGATGA
GCTTCATATTATCTTCTCAGTCAGTCACATTCCTCGTCCGATTCCAACCCACT
CAGTCGCCACTTTCTGCCACACCAGGGCATCTGAGTCTTGATGAGCTTCAG
AGTCTGCAACTGCATCATGGGTTGCTGGATCTTCAAGATCCGCTTGAAGAC
TGCCGACGCCTTGTACTGGTTGAGCATAAAGGTCCGGTGACTCCGATGCTT
GGTCATCTTTTGCAGCACCTTGAGAAAATTGATGGCGCTGAAAAAGTTTCG
CCACGAGTACACGGTGATGATCTCGACATCAGTCGGCTCGGTGGGCATCGG
TGAGACGCTGGGCGTTATATGGGGCTCTTCCGCAGGCTTTCGGGCTTTCGA
AAAGTTGAGCTGGCAGTATCGGAAAAAGCTATCATTAGCTCACACTCCCGC
CTAGATTCGACCCTACTTCTTCTCCTCAATCTCATGCCGATAGGCAACCTGT
TGTGCCAGATCGCCCATGCCGAACATCTTGAGCACCAGCAGCATGCAGTTG
CTGTCAAAGAGGAGTTGCGCAAAGTAGTGAAACTTGAGCACGTGCGAGGC
CTTGAACCACTTGAGCAGTAGGATGAGAATACCAGAGATCGCTTTCGAGAC
AATCTCCGAGGTACGGGCATCGTCGATCTCCTTTGGTGTAGGGTTGTATGGC
GGCAAGGGGGCTGAGGGTCAGCTGCTGGCGGACTCGCCGCACTCACGAG
GTTGCTCTGTCGTCGGTGATGTTGGAGGCGGCTGAGTCTTCTGATGCATTC
CGGGACCCGTCACGTTCGCCAAAAGTATCTTCAGTATGGCGATCGTGCAGG
GCTGTAACAGGGGCAGAACGCTTCTCTGAGGATGAGCACAGTCAGTGTCG
AAAATTCACCCACATAGAACACCTCTACCCGATGAAGCCTCCTCGAGGCCA
GCTTCTTCTCCTGAGCTGCCTTTGCCCCGTCCCTGTCTCCCTTCTCTCTACAT
CGCATCTCCTCTTCTTCATACCGCCACTCCCTCCGCATCGCCTCTTCGCCCT
CCCTGTCCTCCTCGTCTTCCAGAGAGAGACTCGAAAGGCCGATCAGGCCG
CTTCGTCCCAAGCCCCGCTCTTCCCGAATACACTCCTCTCTGATCTCCCATA
ATTGGTACATGCTCAAGCTGACATATGCGTGTTTGTGGTAGAGCTTGTCCGC
TTCGTCGATGGCATACGGTACCATACTTGCTGGCATTCCAGATGAACGAGA
GAACGGGAAGACAAAAGGTCGGTTGGGGTCCCGTCTGAAACTGCTGTTTC
TTTGGTCTCGGTGCAGGTGATCCGGGTGGTGTCGGGGCTGGCGTGCCAGG
AAGAGGCGCAGGAGGCGGAGGTGCAGGAGTGGGTATGGGAGGAGGTGGA
GGTTCTTTTGGCACAGGCTTTGGTATTTCGGTAGAGTGAAAGTTGGGTCGT
GGAGGGATTGGCCGGATCGATTCGGCGAGCTTGTCGTTGGGGATGGTCGTG
GTGAAGGACGGAGAGGGGGAAAAGGTAGGATATTTGACTGAGATGTCTCG
ACGAAAGGTAGCGATATCGACGGGTGTAGCTTTGGTGAAATCTGGGAGTGA
GCGGGGGAGCTAGGGTCTGGATCAACTCACTTTTCATCTCGGGACCAAGGC

CGGCAAGTTCGCGACTCAGAGCATGGGCTTTGCCAACCTCGTTCATACCGC
CAAGACAGGCCAGGATCGTCTTCCACAGGAGAAGGAGCAACTGAGATAAG
CCAGAGTCAAGAATGATGGACTAACCCTTTTGAACGGATAGCCATGTGGCG
GGCGATCTTTGAGCCCGCCCAGCATCTGGAACAGCACAAGAGGCAGAGAT
GGGCTCATCAGCACTGCGAGTTCAGCTTGTCACCCCAAGCTCGCAACTGG
ACGCACTGAGCTCGTCGCCGAACGACTCGTCCGATCGAGACACCTCCACG
ATGAAATACAGCAGCCCAAGCGACTCTCGCAATTCCTCGGCCGCGTTCATC
AATGCCGCTTGTGCAGACGAAGAGGAAATCCCGTTACGGCTCACAGCAGC
GGCCCTGCATGTCAGTTTGCTCACTCAATACCCGTCGGCCTCACTGAGCAA
TATGATACCTCTTCGAGGCATCACGAAGACACGAGACGAGAATGACCACTC
CGTCCAGCTCCCGTATCAGGCGAGCGTTCTGCATGACCCAGTGCAGCTGCA
TTTCTGCGGACGTTGTCTCGGCAAAGACCCCTGAGCGTGAGCACAGATCT
GAGTCAAACTAAAACGCACCTTGCAAGAGATACATGAGCCGTAGTTGAGC
TTCACTCCGCACGTCGAGTGATGGAGATTCGATGAGTTCGAGCTGCTTCTC
CATAAACTCGCGTCGCTTGGCGCCGTTCTCAGGAGCTGTCCACGCTGCCTG
TCAGCTGAGGCTGGATAAGAGGTCGGAGCGACTTGCTAAAGGAGCAGACC
CGCTCAAAGGCTGCGGGATAAAAGTACACAAGGTCGCACTCGACGTATGT
GTAAAACTCTTGTAGCTCGTTCATCATCGTATCAGAGTCGTCATATCGGTAG
TCGAAGAGTTGCGGCTAGTCCATCAGCTCATTCAGACTGGCCCGCTCCCGC
TTCCAGTAGCGGAGAGCGGTACTGCGCCATGACCCACCTTTGGTTTCGGCG
CCATGGCGGCAGTATGAGCCTTCAGCTGGCCAAGGGTGATGCTGTCCGGGT
TGCCCCACGACTCGTCTCCACCGCCGGCCGGGCCCATTGGACTCATGTCAG
ACATCTCCGGTGCCGCCGCGCGTACTGCCAGCCCAGGTCGCGGTCGCGAAT
TCGGCAGCGGATTGCGCATACGGACGGTGAGAACGGGAGGAGCCGGGGCC
GCCGGCCCAGGCGTGGACAGCGCCGGCCGATCATCCTCGTCACGAGAAGG
AAAGCGGCGGAACATCCTGTCCGGCCTGACCTATGCGTCCCCTCCCGCTGA
GCTTGGTGGCAAGTACCTGGGAAGGAGTAAGAAAGTCCAAATGCGAAGCA
AGAGGTGGACGTGAAAGGCGACGCTCGGGCCGGTTCCTTCCACGTGGTTT
TGATTCCGCTCTCGATTGACGTTGCAAAAGTCGGCAGGACAGTGCATCTCA
ATCTTGGCTCAGTACAGACAACATGTGGCCATTCACCTCCTCGCCCGCGTC
TCCGGCAGCTACTCAGGCTGCCCCGCACCCTTTACCCGCGGAGGGAGCTG
CCGACAAGTGTCCAGTGGACCACACGACTCGCCAGGCATGGCTGCAATCC
AACTCCTCGGCACCGCACCCATTCCATCCCGAGACTGCTGCACAATCATCT
TCCAAAGGCTTATCTCACGACCGCGAGATCTCTTCGATTCCCCGGTCTAGC
AACTCCACCCCGAATGTCGAGTCTCAACCTGTTCCAGGCCCGTCAAAGCA
CACTACGCCACCGCCTGCCACCCAAGGCGATCACGGCAGCGGGCGTGCCG
CTGAGAAGGATGCCGATGGAAACTGGGTGTACCCCTCCGAGCAGCAATTCT
TCAACGCCATGATGAGGAAGAACCATAATCCTCGCGCAGAAGACATGCGG

ACGATTGTGCCGATACATAACGCGGTGAATGAGAAGGCTTGGGAGCAGATC
TTGTGGTGGGAAGCAGGCATGGGGAGTGAGCGGTGTGGTGGGCCGAGACT
CAGTAGCTTCACAGGGCGGCCGAGAGACCGGAGTCCAAAGGCGTATGCTA
TGGGCTTGCTGGGGTCAGTAACGATCCCGTAGGAAGTGTTTTGGCAAATGC
TGACTTTAGCTACGCCGCCCCGTTCGACCGACACGACTGGATAGTCGACCG
CTGTGGGACCCAGGTACGATACGTGATCGACTTTTACACTGGCAAGTCGGA
CCCAAACAATCCGAGGGCAATGGCTTTCCACCTTGACGCCCGACCGGCGC
TGGACAATTGGGACGGCGTCAAGACGAGGATAGCGGGCTGGTGGAGATGA
CGATGATCACGCATGCACTGCTCATTGCTTGTCACCTGTCTCAAACAAGCG
CCCTGGATACCTTTCAGGCTACACTGGACATCCCCAATCTGACCTTACAACA
GTTCCACAGATAGACACCTACAACACCTTTGACACACGCTACCCCAATCCC
TCGATCCTCCAAGACCCATCCCGAGTCTTCTCCCAATCGGCCCTGCGCCATC
CACCCGCAAACTCCACTTCCTCCACTTGAGCCTGGCCTTTCTTCCCATGCTC
GTCGATAGGGGACGGACTGAGCTGTCGTGTCACCTGAGAAGGCACTTGCA
CTATCGGTATGAGTGTGGTCGTCCCCACCACAAGATCTGGAAGATCCTGCC
AGTCCTCGTCCTCGATCGAGCGGCGAATCCGCTCTGGGTCCCCGCTACCCT
GTGAGGGCATGAGTAGCAGCACTGAGAGGGTGACGTCCGGCTCAGGATGC
GCTTCGGCCTTTTGCGAAGTGTCTTTGGGCGGGACAGACGTCGACATGCAC
AGTGGCTGCTATCAGCTTTGCCTCGTTTCGCCACCTATACTTACCATATACTC
CTCTACGCTGTCCACTCCGTCCACCCCACGCATATCTGCTCCGGATGCAGGT
AGCCATACCTCGTACATGTCCGGCGCTTTGACCTCCTTGCCATCGTCCTCCA
ACATGGCCAGAATATCTCTGAGTCGCCTTAATCGGGTGACGTCTTCTCCGA
ACATGCCGGCTCCGATGCCGACGCGATCCTGTCCAGACCCGGGACCGGCAT
GTAAGAAACCCCACCAGCCTCCTTCTCGCTCCCACACATCGTGAAAGGTCG
TGTTGTCACCTTGGCGCCGCTGGGTTCGAGCAGACCGCCTGATGCGGCGG
ACACGGAACAACAGCGAGGTCAGAAGTAGCAGCAGGGCCGAGCAGACGA
AGGCGACGCCGATCGCGACGACGGTCGAAGTGGGCACATTGATCTGCCCG
TTGGCGCTACTACTACCGCCGGATGATGAAGCCGATGTTGAGGTAGAAGAG
GTAGTGGCGGACATGATCGATGTGCGTGTCAGGGCAAGGCGAAGCTGTATC
GTGACATGCTACGCAGGTGAAGCCGTCTCTCGTCGGAACCTGTCTGATGCA
CTCCAGTGGCAAGACGATAGGAAACTATTGGAACATGTCAGAGCGATAGAT
GTACGATGCCGACGAGAATAGCCTCGAGCTCTCTGTGTGTCTCGGGAGTAG
GGAGATGACGACGGCTGATCGTCGGACCACTGTTAATCTGAATGTGGCACC
GAGGAATGTGGCACTCGATACCTCGAGGCATGTAGAACAAAGAATGAATC
GGAACAAACCACTGCATATCGATAGGCGAGTATACAGAAGGACTGCAGATT
TCGATGAAGAGCGCGAATCATAAGTCGTCTTCCTCTCGATCCTTGGCGCTTC
CGTCCTCATATCTGGGGTTCCGGTGTGGTCGGCAATGACCGAAATCCTGCA
GACTCCGCAACCACCACGGGGACAGTCTCCCGTCTACCCGAAGCGTACAC

TGTAAACTCTGGCCTTGAGCCCGTCTGAGACGCTGTAGCCCTCGACTCCAG
CGTAAAGCTCGCCGGCGGGAAGTCCGGATGGGTCGCGACAGGATCATACA
GTGTTGTGTATCGCGATTGCACCAGATCTGTCTCGGTACAGATCCCTTGTGC
GATGAGGTCGCTGGCCGTTGCGTATATGACGATGGTCTTCAACAGGTCCAG
GATCCTCATCGGGACCTCCTCGCTGGATAGAGCTGACAGTGCCTCGTCCAA
ATGCTTGTTATACAAGTCGAGAGCGCTTTCGCCTGTTGTGCTAGGTTTGCGA
TCGAAAATACTCCCTGCACAATGTCAGCAGCCGCAGGGATCCGAGCATGGA
TCTGATGTGGTCTGGACAGGTCGAGTGTCAAGATCGATACGGTCTGAACGC
AAGCCAGTTGAACTATGTACTCACGGTCGCAGCCATACGTACTGGTGATGG
CATCAAAGACGTCTTGGTCACTCTCCATTTTCATTGCGTTGGAGTGATACAA
CGTAGTCAGATGATGTTCAGTGACGCTTCTGGGTAAGCAGAGACGAGAATC
TGAAGCGCGGGTCATGGGTGGTCGACTTTCCCCCTCGATGTGTTACTCTTTC
GGCAGCAGCAACGGTGCATGACTCACCTTTGAGCGGGGCTCTGGAGGATT
TTCAAAAAAAAAAAAAAAGTCCCAAGTTATAAGGGAGAGTGGATGGTTAC
CACTCCGACGATGCGATACAGGAAGGCCGTGCAGTGGCTGCATCCATTGGG
ATATGGAGCCCCATGTTGGTGGCTGTGCGACCGCCAGAGCAAGGGGCCGC
GGCAGTCGGTGGACAAGTGGTTCTGCCACTGCTGGATGTCGCATTGACCCT
TCGCAGCAGATCTTTCCAGGCATGGCCAGGGTATACCCAAGCAGTATACGC
ACATTATGAACTCCTGGTTCCCATCACTAGGACCGATATCAAATGCAACATC
AAAGGTTGCAAGTAGAGGATGGGACAAGGCAAAGGAAAGATATGCATCTA
TATACAATTACAGATGAGAGAAGGTGAAACTACTGCACAAGAAGCCTGCCT
ATGTGACAGGAGTCGATATGAGTGTACGAATTGAACGATCCCGTCGCGTCT
TCAAGTTAGAACGGCCTGGCAGACCGTTGATAGGTGACGGGAATGGTGCC
ATTCGGGGGTTTATTTGATTATTATACATTAAAAACCAAAAACAACAACTCC
CTTTCAGTGGCTAGCGGTTAAAATGGTTGAGCTTGACTCTAGTTTGACATTG
TGAAAAGACATAGGAGGTGTAGAATAGGTGGGAGCTTCGGCGCCGGTGAA
ATACCACTACTCCTATTGTTTTTTTACTTATTCAATGAAGCGGGGCTGGACTT
TCGTCCAACTTCTGGTTTTAAGGTCCTTCGCGGGCCGACCCGGGTTGAAGA
CATTGTCAGGTGGGGAGTTTGGCTGGGGCGGCACATCTGTTAAACCATAAC
GCAGGTGTCCTAAGGGGGGCTCATGGAGAACAGAAATCTCCAGTAGAACA
AAAGGGTAAAAGTCCCCTTGATTTTGATTTTCAGTGTGAATACAAACCATG
AAAGTGTGGCCTATCGATCCTTTAGTCCCTCGAAATTTGAGGCTAGAGGTG
CCAGAAAGTTACCACAGGGATTAATCAGTCCCAGGGGCGGCGGCAGCAAT
CCGCCCCTAGTCCACCACTACCACCCACTGTGATAACGCGGGTAGTGCGCT
AGGTAACGGCGCTTAGGCCCCCTAAATCCCCCGGATTAGGGCCTGAGATGG
GCGAAACACCTGGATGCGGGGAAGCCGTAAAGCCTGTCGGTACCAACCTT
AGGGGATAACCCCCCCTAAGCTAAGCAGTTTGCTACTGCGGGTAACAATCC
GGCAAGGTAAGCGGTAATCCGCAGCTCGACTGGGCCAAACTCACCGTACG

GACGGGTGCAAGGGCTCAGCGGTTTCAACGCTCGCTAAGGTGTTGGTGGC
AGCCCCTAAAATACGGGGGCGGCCGCTTAAGGTACGGGCTACTCCCGCCTG
CGAAGGCGCCGGCGGTTAAGGGTATGGCGTTATACCCCCAGCTGTCGGAGC
GGCACGCTTCAGCGCGTCGTGGGTATACAGGAACTGGCTTGTGGCGGCCA
AGCGTTCATAGCGACGTCGCTTTTTGATCCTTCGATGTCGGCTCTTCCTATC
ATACCGAAGCAGAATTCGGTAAGCGTTGGATTGTTCACCCACTAATAGGGA
ACGTGAGCTGGGTTTAGACCGTCGTGAGACAGGTTAGTTTTACCCTACTGA
TGACCTCACCGCAATGGTAATTCAGCTTAGTACGAGAGGAACCGCTGATTC
AGATAATTGGTTTTTGCGGCTGTCCGACCGGGCAGTGCCGCGACGCTACCA
TCTGCTGGATAATGGCTGAACGCCTCTAAGTCAGAATCCATGCCAGAACGC
GGTGATACCTCCCACACGTTGTAGACGGATACGAATAGGCTTCGGCCTCGC
ATCTTAGCTGGCGAATTTTCTATCGAGCCGGAAGTGGTTCGGTAGTATTTCG
CGTATTGTAATTTTAACACGCGTGGGATTAAATCCTTTGCAGACGACTTAGC
TGTGCAAAAGGGTCCTGTAAGCAGTAGAGTAGCCTTGTTGTTACGATCTGC
TGAGGGTAAGCCCTATTTTGCCTAGATTTCTCATATACCGCCTAACTTATAGT
TGGGCTTATAAGAGATTAGTGGACGTAAGTACCTGTAAAGGTGAAGCGTTA
GGGGGGCCTATCAAAGAGGGCTTAGTGGGTTTGGTGTTTAAACTTCAATAA
AACATCCTTGCCGTCCTTTTTTTTTAATATGTTGTAGTACAGGGTAGGGCTC
TTCGGACTTGTAACCCGTCCCATCTCTTCAACTTGCGTCTTGTCTTCAAGTG
TTATATTATAGGGTAAGTTTAGGGTAGGTGCACTCGGAGACTGTCGTAACGC
ATCCGAGCCCCGGCGGAGTTACCCTGTACACGCCCGCATGCGAACTGCCCT
TCAGGGCAGGCCTCTTTCGGTCATCTCCGCGAGCCAACACCGGCCGGAGC
TACCCTACAGAAGGCGACACGTCCCGGAATATAGGGTAACCACCGCCTGGC
GAACCTACCCTGTAAATTCCTGGCGAGCACGCTCTTCAGGGTAGGTCCGCC
CGCCCGCGACGACACGGCGCCGAGGCCGCTATAATCCGCTATAGGCGTCAG
CGAGGTGAAAAATACAGGTAGGGGGCTATACGTGGTTTAGGGAGGTGTTTT
TCGGGAAATGTCCCCGTGGCGTAGAACCCTCCAAGGGACGATAGCAGCCC
AGCCGGGCCTGGTAGGTACTTATAGGGGGGTGCTAGCTCCCCGCTGTGGGG
GGGTGGGGGCTGTACCCGGCCGAGCCGCCTGGAAGGAGCCAGCTCCTCGC
TTTAGGGTAGGTTGGGCGAGACGCGGGGAAATCGGCGGGTTGGCTTTAGG
CCTTTTAGGGGGCAGCGGTTTTAGGGGGGTCTTATAGGGGGGCTACAGGGC
ATTTAGTGGACGGAGGGTGCTGTAAGGCGCTGTAAGGCCGTGGGGTTACCC
TGTAGAAGAGCTACCCTGAACTACCCTATAGTCCGGGCAAGTCTGCAGGGT
AACTACAGGGTAGTCGGGGCGTCTATAGAGTGGCTACAGGGTGGTTATAGG
GTAGGATCCTCTGGGATTTATGAGATTTATTGTATGTGTCTGGTGGGAGCTA
CCCTATAGAAGCTACCCTA

>Mosaic_sequence_74

CCGCAAACTCTTTGGATGTGTGAGTCACAGCTATGTTAGTGGATCGAAGCT
GACGCGTCTAGCTGGCCTCATTGCTGCTTCGGTTGCCCTTTCCCTCTTTTCC
CCACCCAATTCTTACCGTGCTACAAACCATAGCCTCGGCAGGTGCGAGCTC
GATGCCTCTACCTCCTTCCGCGCCAGGATCAGGCTACAATACTCCCCATGGA
CCGCTTCATGGGACTGCATAGCAGGCAGCAGCCGGGAAGGGGGCATGATC
GGCCACAAGCTGAAGCTTGGGGAGTGGAGTGGTGCGGGATCTGTGTAGAG
GAGATCAGCCGAGCTGGGTTAAGCGACATCAAACGGTCGGTCTCTCTCTGT
GGGTCTGCAGAAGGAACGCTAAGGTTGCAGGAATGCCCTTCGTCGACATC
TCGAGTCCGACATGACCGCCGTCTTGGCGACGATCACCGAGTCCATGGCAC
CTCCGAGCGGCTTTAACGATTCCGAGGTGGTCGGGGCGAAGCTGAAGGAA
GCGGAAGCTGCGTGTCGCTGCGCAGAGGAGCTGGGTCTCCTATGGCCTGG
GCGGGGAGTAGGTGTTTCCTGAGAGAGGATTGCAGCTGGAGCTGACGCAC
AAAGCGAGCTCACGGCCTTGCTACCACATCTTTACGGCCTTTTGCCTTTGC
CTGCTGCATCCGCCGCGGTCGTCGAGGTCCTATCCGAGAGCCTTTTCAAAT
ATGGCAAAGGCGCCAAAATTCTCACTGAGCCGCTGCTTGCTTGGATTGTCG
GACCTTCTGGGCAATCCCTGGTTGGTACTTGCGAAGGCGGTGTGGTCATTC
ACAAGTAGAGGAGCATCATGCTGACTCAAGAAGATCCTTCGGAAGAGGTG
ATCGGATTCGCAAAACTCCTTTGCGCGCTCGTCGAGCACTCTTCGGACTGG
TTTATGGCTCGTATCGAGCAGCAGGATGTGCAGAGCCTGCTCGCCACAATC
CTCCGTCTCACTTCGTGGCAAGGAACGGGCGGCGTTGACGAGAGTGTCAG
CGAGGTGAAGGCTGATTTAACATGGTGATCACCTGCTGACATGCAGCTGAC
TCTCCCGATCTACCCTCTTCTCCAGGAGGCGATCATGGACTGCGAAGCGTT
TGCGCAGCCATACGAGACGGCTCCAATTTGGGGTATTGCCAAGCACTTCTT
CCGCGAACTTGTCTCGATCACCCGTACCAAATCTCGGTGGCCAGGCTTCGG
CGAGGACCCAGATGGATTAGGGGGCTTGGACAAGGAGGACCGAGAAACCT
TCGAAACCTGGCGGAGAGATGCCGGCGAAGTGATCGTCTGCGCGTGCGTT
CTTCACAAAGCTTGGAGGGTCAAGACTCATTCGAAGATACTATATTCTTCGC
GAACACATGTTGACGGGCTTGGTAGAAGAAGCGAGACAGCGAGCTTCCAC
TGATTCGGCTGGCAGGTCGGTCATCTCGTTGCAATTGCTAACAAGCTGACG
ATGAGGACATCGAAGCGACATTGCATTGCGTGCGATACTCCAGCGAGGCGG
TCCCACTTGGCGAAGAGAGAATTTGCCAATCCTGTTCAGCGACCAGATCCT
AGGCAGATTAGCACAGCGACCTCTTTCTGGGCTAGGCGAGGAAAGATTGC
GTCTGACAGCAGTCTGCCTGATTCGTGAGCAGAATCTCTGATGTACGCTCC
TGTATTGATTGCAGAATCGTACGAGGAGTGGTTCAAGTTCCACCCCGATCAT
CTCTTGCCCGTCCTATCTTACCTTGTCCCATCCCTTACATCGACTAAAGCCAT
ATCAAGGTCTGCGGCCGATTCGCTTAAAGCGCTCTGCGATATTTGCAGGGC
AAAGCTTGTCCAACACATAGATGCATTCGCCGAGCTTCACGGTAAACTTGG
CCAGTTGGGGGTGAGCTAATACATCTTCGCAAAACGTCGGGCTTACTCCTC

AGCCTGAAGAGCAAACAAAGGTCATACAGGCAATAACAAGTGTGATACAA
GCTCTTTCCCCCGCCGATGCCGTTGGCCCGGTAGAGGTGAGCAGGACCCAT
GACATCGTTACAAGGCTAAGGGATAGGGGATCTTGTCACCGATCTGCGAGC
AGCTCGAAAGAGCCATTCACAGTGCCCGCACCGGGCTCAGCCGAAGCTCA
ACCAGCGCTTGTCGCGGCAATCGATGCGTTGACCGCCTGTTTCCGAGGCCT
CTCGCCCTCCGATGACGACATATTCGATGTCGAGGATGAAGCGGATCTCGC
AACAAAGGCAGATGCTGTCAAAGCTGTTTAGCTTGATCAGCGCATGTTGGC
ATTACGAAGCCGCATCGAACAAGCGGTCACAGGGATCGTGGAGGTGTGGC
AGGGTGACGGCGAAGTCGCGGACGTGAGTTGGTTCTCCACAACTCCCGGA
CTAGCCTGACCTGCTGCAGTCAATATCATCACTTGTCAAGCACTCGACATTG
TCAATCCTCAGAGCTTTGATCTCGCTCTCGCCACTACCGCTGCTATCGCTTG
TCACTACCGCCTGCGAACGAGCACCCTCTGCTCTGTGGATGTCCTAGGTTC
GACTCTGGTCCTCCGAATCAACTCGCCACCCTCTCCGTTGACGCAGAAGCG
GGACCGAAGTGCTGCTGAAGAGGCCATGCATCAACAAGAGGAGCAAGAG
CGCTGGAATGTCATTGCGGACTCGGCATCGCGTCTTTTGCCCATCGCAAGC
AGCCTATTGGCCGGACCGAGCGGCATACACGATGTGAGATCAGAGCTCAA
GACTGGTGATGAGCTGATTTCAGAACCCGGACGTCTCCGAGGCGTTCTTCA
AGTACGCCTCCGCTGTCGCCTCCCGCTTCCCTGGGGTCCTCCTGCGTCTTTC
GGCTTCGATCGTGGAAGCATATGTCCTTCTGGGCATCGCAGGCTTGGGAGC
GCAAGAACGGTTCAGCGTCAAGAGTGCCACTGAGTTTTTTGTTGCTCTTTT
CGCCACGACTAGGTTTCCGTCCCCACTTGAGCCCCAAGCAGATTCGCTCGT
CAAGCACTTTGGACCTCAGATGCTCCGCGCTATCCTGCTCAGCGCCGGTGC
AGACGGCCCGCGGTCCGTGATCCCAAATCTGGCAGAGCTGCTAGCAACCTT
CGTCACGCGCGTGAAAGGCGAGGACATGGCGGCATGGCTCGATGCGATTC
TGGGACAGGCGAGCCGGCTGTCTGCCGGGAAAGACTTCAGCTTATGTATCA
GGAGGGCTTCCCCGACCCACGAGCCACGTATGACTCGAAGAAGAAACTGA
AAGAAGCAATATTACGGTGAGTGCGGCGGGTTCATCACAAGTGCATGTGGA
CCAATGAGACAGATCACGAACGACGAGAAAGATGCGCGAGGCTTTACATG
AGTTCGCCCTTGTCGCCCGGGGCTTAGACAACACGACCTACGGCAATGCCA
CGGCGGTGTAGAGGGACAATCTTGCAACAATATCAATCATTGTCGATATTCA
TCATCTACGATCATCCTGCATCATGCCATACCCTGTTTCTACACCCATAGCCC
TCTGCGATGTCTGAGATGTCTGATTCTGCTCATGCACTGTTGTCAGGCCGCT
GTACAGACGCCGGCGCCCTTGTCCGTGTCTGTAGACGTGTTGCCGATAACG
GCGATATAGGGACCACTGCTGCTCCTGTCCATTTTGCATCGCTGGTGGACAT
TTCGTCTCGCTTTGGTCCACTGCCGGCCGGGCGGGCTTGCCGGGGAGAGA
GACCACTAGTTGAGTGAGCACGTTGAGCTGCGAAGCAGATAGAACTTTGA
CGCCTTCTCGCCTGCACACCTTGATTTCGGCCTGAGAGAGCTTACGCGGAG
CACAAGCTTTATCCTTCCCCTCGCCCACACCTGTAAGCGAAAGAAGAGGCA

TTATTACTGCGGGTGGACGTTCAGAAAGTGGGAGTGGCGACTGTATCGTGC
GGGAGACACATAGTGCTGTATACTGGATTGTGTCGTCGAGGTTGGCAAACC
AGAGGGAGCAGGCGAGTCCCTCACCTTTAAAAGAGCTGCGAGCGAATTGG
CTGGTTTTGCAAGTGGACAAGAAAGGAGTAAGCTGTCCTTTGACAGCATG
GCGTCTCGCTCGAACATTCCATCTGGGACGTGTGCGTCAGTGCCGCGTCAG
AACAGCCCTTCGCGAGTAGGAAGATTGAGTGAAACTCTTCAGCGTCAGCG
ATGTCTCGATGGGCGGCGCAAACGCCCGGTGCAGCGGTACGAGTACAATAC
ATGTATGCCGTTAACCGCGCTAGAACACGGCAATATAGTCGTTCACTCTATC
ATTGGCGTCCTGCGCGATGCACTCTGCGATCCACGTGAGATTCCGCACCTC
TTGCTCCTCAGCTTGACGAGGCTGTAGAACACGCCGTACTGAAATTGCTGC
AGGAAGGTCTGTTTGTTGAGCTCGACGTCGTACTTGAAGAACTCGTCCTCG
AGGCTGCTGCCGCCCTCGTCGTCGCCGGGCCGTATGACTCGACGCGCCCGC
CTTGTCGAAAAACGCGCGGTATTCGGAGATTATGGTCGACCGCGGCGACGA
CGGCCTCGATGTCGTCTGCACGTGCGAGGGCGTTGTTGCCTTCCGGGTACA
GGCGGCCGATGGTGGGGAAGAGGCGGGCGCGCTGATCCTTGGACAGCTCG
GTGCCGAACGAGTTGATGGTGATGTTGAGCGTCCGCCGGTCCGCCTCAAA
CGCCAGGATGCGGGACATGATCTCGTCCGTCGGGCTCGGGAGCGTCTGGC
AGAAACGGTGAAAGTCCTCGAGGTACGCCTTGTAGAGCGAGTTGCGGATG
ATCTCGATGTTCAGGTCGTCCAGATCCGAGGCGCTCAGGCAGTCCTGGAAG
TACGGCGCGAGCGGTGTCTCGACGAGCACCGAGTGATACAGCTCCTCGAC
GTTGGTCGCGACGCACAGCGCGGGCATCGTGTCGAATACGCCGAGCGGGT
GGCATCGCTCGAGCAGCTCGTGTGTGTCGCGCTCGTGCAGTGTCCCGAGG
GTGAGCAGGATGACGTTGTCAATCATGTAGGCGTAGGTGATGTAGTCCATG
AATGTCGCCAGGGGCTCGTTGGCGTTGGTCCGGATGAAGTTGAACTCGGC
CACGAGCTTCTCGGTTGCCTTGTCGGCGATCGTTGCTGTCGATAGCGGCAA
AGGCTCGTTGGCGAGAAAGTTGCCATAATCTGTCGATGATAGCTGCATGCG
AAAGTCTGGAATCAGCATCGATGTTCGCCGACAGATAGGCACAAGTCCGAT
GCTCACCTTCCAGGTTCTCGCATTGTGTCAAGTTATGGTAGTTGTTCTGGCT
CAACAAAGCTCCTTTGTATCCTCTGACCATTCCCTCGAGAAGCCCGCCGAC
ATCTGCATGATCAGCAGGTGCCAAATCGCAGTGATACAGCTGCTCACGTTG
AAGCTGAGGGCCTCCATGTTGCGAGCAGCCGAGAGGCTTCCCTGTCCACG
ACCTCAAGGTAAGGCCAGACCGGGCCGAAGTGCCTCGATAAGCAGTGTCG
GTTGCGGTCGAGCCAGTTTTTCGCTACTCAATTTATGGACAGGATTTCCTTG
GTCCACTAGTGAACCTCCTCGGCCTCGACTCCGGTTGGGTTTCGGGGAGAA
CAGCAGCAAGTTGTAAGTATGATTCGTGCAGGTGGCCAAAGACACAGCGA
CGACCGTGGGCAGCGAACCGGTGACGCACTTGAGTGGGGGGATGATATAA
GACTGTGGCATTCTCGGCGTGACGCCCCCGGGAATCAGAAGCGCCCAGGT
GTGTGGGATAGCGCGTTCAAAAGCATATTGCTTTTGTCATTCGTCTCATCCT

AAGGGGCCAGCGAGCACGATGGAGGCCGCCCCTTCTCTCTGACCACTGCG
ACGGGTACCCAGTCGCCGCTCCCCTCACCGAATCCCAACGCGCCCGCCGA
GAGCACTGCTGAGCCCGTTGCCGCCGCCGGCGAGGGCGAGTCCTCGACGC
CCCGGCCATCCTCATCGAGTACCGATGCAACCGCCACCCCTTCGCCTGGCA
AGGATGATAAGGAGGCTCCAGCAGGGACTTCGGCAACGGCCTCTACCCAG
ACAACGGCAGAGCGAGCATTTCAGCATGGGTTGAACCGTGAGAACCTTGA
ACAGGAGGTGGCCAGGTGATGGGAACGCTGAAGAGTTGGTGGGTGAGTAC
AGGGTCCCAAAGGTAGTCGTCGGGGCCTGCTGGCTGACGACTCAGAAAGC
CTCTCCGTTTCAGGTCTCAGAGAAGAGGTTGACAAGACGCTCGCTCAGGC
CAAGTCGGACCTCGACTACCTGCGCACGGCCAAGGTGGAGGTGGTCCGCA
AGGATCCCGCCGAGTTCCGCGCAGAGCAGGAGCGAGACAAGGCGGAGAA
GAAGGCAAAGGCGGTGTCTCTCGACAAAGGCAAGGGCAAAGAGGTGGAT
CTGACCGACGATGCTGAGCCTCGAGAAGCAGGCTCAGCTGGAACCTTTAC
CGGCTCGGCAAGTGCCCTGTTGAAGTCCCTCTCCTCGTCCACGACGCAGCT
TCAGCATTCGCTGCAGCATACCCTGCAGACGACGCTCGCGTCCGCTCAGAA
CAATCCTGCATTGTCGAACCCATCTCAGCTGCGCGCGCAGCTTGCCGAGAA
CCTGCGTCTCTCCTCGGCCCGGCAGACTTGCAAATGTCAATGAAGCAGGCC
GAGAAGCTCGCCGAGGACTACATGAAGAAGGCGATCAGTGGGTCAAAGAC
GCGGAAAAGTGGATGGAGGACGCGGTCAAGATTGTCCCACCGGGAGACG
GAGACCGATTGGTCGGCATGAGCTGGGACGGCAGCGACTTTTACTCGTTCT
CCACTTCCGCCGCCCCTCAAGCTCAACAAAGGAGGGGGATGTCATCTTCG
ACGCCAAGAACAAGACTCCCCGTCCGTCAATGTCAGGCGTGGCGCTCGCG
GGCTCGAGGAAAGAGGCACTACTTCGACGCTTGCGCGAAGACAAAGATCT
GCGTTGGTGGACCCGGAAGGCGAGGCAGAGTCAGCAGAGCGAAGAGACG
AGTTCAAGACCTGGGTCGAGAAGCAATGGCCGAGCGAAGGACCGAAAGG
CAGGAAGGAGGAGGAAGGCAATGTGGGAGGCATCCGGATGGCCTTAGGTC
AGCGCAGATCCCCACTCTATTGCGTGACTGACGATCAGTCCCCGAGCAATG
ACGGACGAGCAGTTCTGGCAACGCTACCTCTTCCACAAACACATGATTGAG
GCCGAGGACGAGAAGCGGAAAAAGGTTTTTACAGGGTGAGCGATGTCTGC
GTTGGGGACTTGTCGGGCTTCTAACCCGCTAGCATCGCAACAGGCTGCCCA
AGACGATTTCAGCTGGGACGATGAGGCGAAGAGCCCTCTCCCGTTGCGCC
TGCGGCCCAATCGACCACAACTCTTCGGCCAGAATCTGAAACACCCGAGG
CTCAGGCAGCTGCTGGTTCCGAATCTGACAGTAAGGCCGGGCCACCCACT
CTAAGGCGGACACCACTGTTAAAGTGCCTCCTGCCCAGGCGCCAGCTGCTA
AAGTGGGCGGGTCGACCGCGACCAGTCCTCGAGACTCTGAAGAAAGCTAC
GATCTCGTCAGTGACCAGGGTCAGAGAGGGAGCAAGGTGGCGACCCCTGC
TGTTGGAACTGAAGACGAGGATTCGGACTGGGAGTGAGTGGAGATGAACA
TTGGCGTTGGATTCTGGATGACTTCGATCCTTGATACAATTTGCTTGTAGTT

GTAATCCGATGTATATGATAATCTTGTGTGTGTCAACCTACGAGTAGTCCAA
GTCAATCTGATCCCGCGTGACTAGACCCATTTGATACGCGCTCGGACCCAG
TTACGTCGCACTTCCCGCACTTTTCAGTACTTCATCCTTGTTTCTCCTCTCTC
TTCTTGTTCTTGTCCTTCTGAAACGCAACTCGCAACATCTGGCCCCCGCCAT
CCCCCCGAGCCGTCCTCCCGTCAATCTGTCTGGGACAGGACGCTCACTCTC
TTCATCGCGTCTCCTTTCCCTCCCCAACCGCTGCCCTCACAATTCAACTGCG
TCTCCTGCTACTTGACTACCGTTCCCGGCTGTCGGCCTTTGGCCCAAACCTC
GATAGAGGCGAAGCGGCACAATGGCCAACGCGTTCCCCGAGTCCCTGCCT
CTCGAGTTCGGCCCCCCCTCACAACGCAATGAGTACGCCTTCGAGCCCACC
CAGCTCTCACAGCCCATGTTCTCCCAGACCCAGGGGACGCAGTTCAACTC
GCAAGTCGAGCCACGGCGCAAATACTGCAAGTCTTCCATCGCCATTGGACC
GCACCGCTGACGCCCAGGGGCCGTGCTCATTCCCACCAATGCCGCACACTC
TATCCTGAAGATCCCCTGGAGCAGACCTGTCTTGCAGATGGGCGCGGCCAC
AGCCAATGGCAAAGAACCACGTCGTTCTGCAGGAGAAGCGCGTCTCGAAT
GTGCATTGTAGGATCACGCTGGGTCTACAGGGAGTGAACGGGAGCGGGAC
GTCCTATGCCAGTGTCCAGAGCTGGAAAGACGGCGAGGGCGAGCCAGAGG
TCTGGATCGAGGATCTCAACAGTAGCAACGGGACCTTTGTGGGTTTTTGTT
CTGGTGATGGTTTAGAGGTATCGGCTCATCTCCTAGGTGAACGGGGAACGG
GTCAAATCAAGGCGTCTGCTTCAGCATGGCGATGAAGTATCTCTTGGTCAC
GCTTCGACACTCGAAAACCATGACGTGCGGTACATCTACCGGTCTGTCGGG
CACAAGGGGTCGCGTTACGGGGGTAGCAAAGCGGGTGTCGAGATGGTCGG
CCAAGTGTACGAGCGATATCAGCTGCTGGACAGGTCCGTCTGCTCCTCTCA
TACAGCTGGATGCGTGTGACTGACTCGATAAGACTAGGTAAAGGCACGTTT
GCCGAAGTACGCAAGGCGGTCGACATTGAGACGGGTGACTTGCGGGCCGT
CAAGCAGATTGTGAAGCACCGGTTCGCTGGGGACCAGAAGACACTCAACC
TCTTCCACCGAGAGATCGAGATCACACATTCCCTGGAGCATGTCAGCTGTA
TTCGTTTCGGCCTGGGAGCGATAAGCTGACGGTCAGGATAACATCTGCCGA
CTCATCGAGTGGTTCGAGGATCCGCAACATATATGCCTTGTGCTGGAATACA
ATGATGGCGGGCTTGCTTGATTACATCATTATGTTCCTGCCAAGGACGGCCG
TGGCCTGCGTGAGTCCTTTCATTCATGCAGCCAAAGGCAACACATGCTGAT
CTCATGTGCAGCCGAGGAACATGCGGCATCCTTTGCCCTGCAGATATGCCG
CGCCATGGCCTACACGCACTCCATGGGAATCACTCATCGAGATCTCAAACC
TGAGAATATCCTCCTTACCCGCGAGACCCCGGACACACCGGCGACGATCAA
GATTGCCGATTACGGTCTGGCAAAGATGGTCGACGACCGCACGATGCTCAA
GTCACTGGTCGGCACACCGCAGTATCTGGCACCCGAGGTGGTGATGCAGA
GCAGACAGAAGCCCGGCTATGAGAATGTGGTCGATTCATGGTCTGTTGGTA
TCATCGTTTTCAGGTGGGCCTTGAATATCGCACGGCCCTCTACCAGCTGATC
ATGCCAGCATGTTGACCAAAGCCTTGCCGTTTGACGAAGATGATCAGATGG

CCGTTGCGGTGAGCGACGTCGGTTCTTGCATCGGCCAGAAACTAACCCCTA
GGAACGAGTCAGGTCTCGCTTCACACAAGAGCCTGATCTTGGTCTGCTTGA
AAGCATTGATATCAGCGCTGCGTGTATCGACTTCATCGCACGGCTACTTGCC
AAGAAACCCTCGCAGCGGATGACGATGCAGCAAGCTCTTCAGCACGAGTG
GCTGGCAGCTCCGATATCGTCACAAAACTCGGTATGACATGCCTGCTGCGG
CGCTTGGCGGTGACTCCATGTGGTCTATCGAATCATTCGATGATGTCAACTC
ACTTGCCGAGCCCCTCAGATGACGGCATGCAGCCCGACGATACCTGGGAG
CGCCCTATGACTGCCTCGGGAACGAACTTGGAGGGTCTGTCCGGGAACCC
TTCTGCTCTATACGGCGGATCCGATGAGAGCTTCTCCCAGCCACTACAAAA
CCTGCATCTTGCCACTCCTGCCACTCCGAGCCGCGCGGGCAACTTTCTGCG
CAGCAGTAGTTCGCCCATTTTCGTTGTGCCCGAGCCTGTGCCCGTGCAGAC
AAATGGTAACCGACACACATCGTCGCCGCCTTCGCCGCCTTTGACCGAGGA
GAGGATGAACGAGGACACCACTCCAGTCGCCTCGGCACCGCTCGCAGCTC
CGGCCATGAACGGTCATACTGGAGAGGACAATCTACGGACTCCTTGGACG
GCGCCAACCCTCACAAGAGGAAGTTGCGGTCTACAGGCGCCCCGAATGGC
ACAAACGCCTTCTTTAGCTCGGGCTCGCTATCGCCGCCGCCCGACGTGTCT
GACCGATCTACCCCTGGTCGGGTCGAAGCTCCTGCTATCAATACGCCGACC
AGCGCACGTACACCCGATACGCCGCCTTCAGCTGCTCGGAGCAAAGGCAA
AACCCGCTCGGCAACGAAACGTGCTGTACCCACTGTGTCTCCCTCAGCCGC
GCCCACTGCACGACGCACTCGGAGTCAAGCTCGCCCTCGGAAATCGCTCC
GCGTGGTCTGACGATCTGTCCATGAATATATCCGGAAATGACTATATACGAC
TCGGACAAAGCTCTTGATCTGTTCATCGAGTAACGACCCCTGATACGCCCG
GCTGGCTCTGAATGATTAGCCCTTATTCCCTACGGCATGTTCACATTGGATC
CCGTTCCATGTGCATTCATATATCGGCAACAGAAAACGAAAACCCGCCCGG
TCCGCCATTGGTGGCCTGCGGACTGAGAATATCTAGAGGGAGTTAGAGGGG
AGTGGATAAGTATGTGTGTAGAGAGCTTCAGGTCGTACGAGTGAGGTTGTT
GAGTATATCCATATCGCAACGGTCGAAGGTGAATGCATGATCGATAGGCATA
TGATGTTTTATGCGGGCTTGGTGAAGCGCAAGACGTACAGCAGTTCTCGAC
CAGCTTTACGTCTGATCACTTGCTCGGCGATGAAGCCTCTCTTGCGCATGTT
GTCGCATATCTCCATAGGGCGATTCTGCTCGACCGCGACGAGGTAGAAGCG
GCCACCGGGAGCAAGGATCTCCTGCCGACATCAGCTCAGCATCAACGATAC
ACTCTGAGATCCACATGCAGTGAGTCGAGAACGATATCCGTGACACGCATC
CCGTCTGCACCGCCCGCCCAAGCCCCCACCGATATCCCTCCCAGCTTGGGT
CGCTGCCAATCTGCCGTCAGGCCTACCCATCCCCATCGGGGAAGAAACGCT
CACTCGGCCTCGTCGGTCGGCACGTACGGCGGGGTTGAACACGAGGAGAT
CGACCTGCTGCGCTATGCGCTGCTGGAGAGGAGAGAGGAGGTCACATCGC
ACCACGTCGCCCGGCACCTATTGTCAGTTCCGCAACACTCGAATATAGAGA
ACTCACATCGTTTGCTTGGGCCGTGCGGAGTGTAGCGCAACATGCGTGATC

GTTGATATCGAGCGAAAGCACCACTGTCCTCAGCCATCGGGCTCCGGCATC
AGGGAGAGGGAGGAGGGAACGTACCGCACGTCGGACCGAGCAGCTTGTG
CACAAAAGCAGAAACGACTCCCGAGCAGGAGCTGCCTTCATCAGCTTATC
TTTGCAGCTAGAGGAGAGGCGGACTCGCCCTATTTCCACACACAGGGCCG
GTCGAGCATCTCTGAGCGCCTCGGCGTCATTCTCGAGCGCGTCGAGCAGG
ACAAACGTATCCTCTGTCCGTCAGCCAGTTCTTGCAGGTGCCAGGAGGGAT
AGGAAGGAGACGTACCCGCCGGCTCGTAGATGTGGTCGTAGTCGTCTGCTG
TGAAGTGGGCTATGGACGGTGTGGGGATATGCGTGGACATGGTCTCGTTGA
CCGCATGTCCCCTCCTGTTCACTCCACCTGGTCACTGGAATAGACGATGTA
GATGCAGATGCAATGCCCAAAGATTGTTGGCGGCGAATTTCATCAGCCGAC
AGCGGCGCGACTGCTTTCTATTCCTTCTGAGATTGCGTGCGGGTTCGAGCA
AGACAAATGCACCCTCGCTACTCTAGACTACTAGCCTACCACTCCTCGGGC
TCGTCTATCCACTCGCCCTTACGCCTCAGCTCCCGCTCCATGCCCCTCGTCT
CCCTGTACCCTTTCCATCGACCCTCCACACCGAGCTTTTCATTCCTGCTCAC
TCCCAGCTCCCTCCTCAGCTCGCCGACACCCTGCTCCCACCGCTTCTCCCA
ATATACGCCTACAAGGCTCTTTCCCTGTCTTCCCGTCCGAAGCGCCCACGG
GACCCAGTCTTCCAGCCACACCTGCCGCCGTGCCGCCGAGAGGCGCAGTG
GACCAAATGTCGAGGACAAGCCAGCGACCGGCAGCGACATGTTCGCCAGC
TCTACCACTTTGAGCGTGAGCTCATGTGGCAGGGAGGTAGGTAGGAGAAG
AGCGTATGGTACAGGTCGTGGGTCTGCCGATAGCGAGTCATGGTGTAGGCT
AGAGTTGGCGAGTCGATATATCCGCACAAGCTCCCTTGTGTCGGGAGTCAC
CTGATTCCGCTCGAGCCACTCAACGTATTCTCGACCCAGCGTGCCGCGCTG
CAATTGCTTCAGAGCGGGAATATCGACTGTTGCTGAGGACACCAGCGGGC
GATCCTTCAATATCTGCCGGCCTTCTGGATGAGCTGTCATTTTCTCGTGCAA
CCGAGGGAGGAATGTTGATGCCGTTGATTCGGACAGAGTGGCAATGAGGT
CTGAAGATCAGATCTGGCGTACTGAATGAAACTCATTCATCACTCACCCCCT
CGCGAGGTGTCAAGGACGCCCATCACCCCACTGCCCACTGCCAATAACGC
ATTTTGCGACCAGGACAGCGGGACGTGGCCGGGGTAGCGGGGCGGTGCAT
CTCGAGAGCCGGTGAAAATCTGACGTCGACAGGCGACGCCGAAGCCGAC
GGCCAAGGCAGGAAGCGTTGTGGCGACGCCCGGGAGTGAGAGAGGCAGA
CGACTTGGAAGACAAGTGCGGTTCGCCAATCGACCCAGAGCCAACAGGTA
AGCCATAGCCGGCGCTGGTTCTAGAGATACACCCGAAACATTTCGGCGTCC
CTGGTCGTCAGGTGACATCGGGTTGACGATATTGACGAAGGCCGAGGGGT
CGTTTGTCGGCTGCCAATCCGATAAAGACCCGACGTTCTGGGACTGTCTGC
CGAACCGTAATGAGTCGGCGGACTGAGAGGACCATTGTGCGATGCTGAAA
GAGTCGCGGTGGGCGGAGTACCTGGGGCCGAGCGTGGGCCAATGATGGGG
CTTGAACCTCTTCTGAAACGCTCGACGCCGTCAGCGATAGATGAGCCGCTT
GTTCTGCGTGCTGCGTTGCTCAAAAGTAAAGGTCTGGGCAGAGGGGTGGA

GGAGCCGCTGACTGGTCTTTCCATGGCAGATCTCGGGGCAAAGGCTTGAC
CCAGTGATGCCAATCGGTCTTGTAAGCTGCCGACCGAAACAGCTCGATGAG
ACAGTATCGGATCGTTCACGTTGAATGCCATTTGCTGCAGATGAAGGCTGA
GATGTCATAGCCTCCGCATCCTGCACTGAATGACCGTTGCATTGCTGTTGCG
ATCGGCGCTGTCGACTTCGCGGACTAGAAGGGAGAGAGGTGTAAGGGCTC
TTTCGGCTTGAGGCGGCTTGGAAGTGGCACGTTGGAAGAGGCGGGTAGCG
GCACCTCCTAGGCTGTTGAGATTGGCCGACTGGCCAGCCAGTGGGTTGGTG
GGAAGAAAAGAAGCAAGTGACCTTTTGTGCTGCAATTGTGCGTCGCTCGA
CGTCTCAGGCCTCGATGCCGTATATCGTGCAAGCTGGGCAGAGGCAACCTT
GTTGGCCGCATTCGCCTTTTCGGCCAACCATGTCCAGCTGGCCGAAGGAGC
CAGACGACCCGTATCGACAGTCGCATCCGGATCATCTCCCGTGAGCTCGGC
TACCATCCTGGCATCTTTCAACTGCCGAGCAATAGGCAGCACGGCGTCGAC
ACCGCCGACCGCGCGTAAAGGGTATTCCGTGAACATTCTTGCGACCTCTTT
CTGTACCTCATCATGGACTGGCTGGCTGTCCCACTCAGGTTCCGGGTCTTG
CGCCCCGTATTCGTCCCACATGCTCCTTTGCTTGGGTCGAGCGATCGACGG
AGGAGGAATTGAGGTGACAGTAGCGGCAGCCTGAGCTATGACGTCTTTCG
AGGCGAGGACGAGACCCGCTGCCAGGTTGATAAGCGCGTCTAACCGAGGC
TGCTTCGCATTGTCTGCAGGTGGTTCGGCCAGAATGACATCCCAGAAAGCG
GGTATCGCGAAGACTGGTAACGCTGCGGCGAAGGCGGTCGTCAGCCAATG
CGAGGCATATGAGGACAAGGTGACGCCGAGGCTATTCTGCTCGAGGAAGC
GTAACAGTTGACCGTCACCCCAGCGTATCCGGCTAGCAAGTTTGCGCAAAG
GTAACTCAGCGTCAACCGAAGAGTCGGGAGAGGGCGATGTCCAGCTGGCT
CTCTCCTCCATCAATGCGGTGAATGCCCAAAAGGTCTGCTCCTCGGCATATC
GGAGGGTGTTCTGCGGACGTGTGTACGTGTTGCGCTTCTGTTTGCCGATCA
ATAGCCCTCGAGGTAGATCGATGCCGCAATATATCATGTACAAGGGCAAAGC
ATGTACAGCAGGCTATCGTCGTATGACCAATGTGGATGCGCACGACAGCCG
GTGTAGAGGATGCGGGTGAGGATCTCGGCATGGGTGTCTGGTTGGAGTAGT
GAGCCCTGAGAATAATGAACTCGCCGCCTGGCCAGGTGAGGGGATGACTT
TTGGTGAGACAAGCGTGATAGCTGCGGATTGTGAGGAGGAAGGCTCTTCG
GCGGGGACATTCAGTGACAGCTTTGCAGGTCGCAAGGAATCGAGTCTCGA
TGCTATGGCAGGAGAGGGCTGGAACGGAGTCGAAGCGAAATGGTCTGACG
TGTCGGGCGACAGCGTGATACGAGGAGAGAGGTGATCTCGGGGTCTGAAG
TATCTATAGCTCCGGATATGCCCATCTGGCCATTGGTTTCGCCCACGCTCTCG
ACAGCTGCAGGGTTTGTGCTCGCCTGCGTGGCCGGCTGAGGAGGAGGTAT
CGATAGCGACAGCGTTGTAGAGGTAGATGATGCGGCCGCGCGACGCCGATG
TACTGCTTCTGCGACTATCTCCGCTCGGATGAATGCAGCTCGTCGGCAGGT
CAGCTTCTCCACTTGCTTCCGCTTCATCTTCGCCTTTGACATGGGGTCATCT
TTGGCCCTGATCGTTGTCTCGTCCTCGCTGGACCCTGTCTGCGATGACGAG

CTGCCTCCCGTCCGAGTCGTCTTCGACAGGCTGGATGATGCTGTTCTCGGG
TCTGGGCGGCCCCAGAGGGGACGAGCGACCGACTTGACTCCGCGTGCGCA
GGAATGGGTTTGCCGACTGGAAGCGGTCGACATCGTCGCGAATGTTTGCCA
GGAGGGCATCTTCCAAGCTGAGAGGGTGGGTAGGCCAATGATCAGCACAT
GAACCCATGCCCAATGGTGTACCTACCGGCGAGGCCGACTCAAGTTCCTCC
ATGTAACGCTGCACCATAGCCTGGTCGTTAGAGCGTCGTTGAATGGCTTCC
GAGGCCGCTCACATAGTATCCTCGCGCTGACGGCGGGCCGCCGCATGCCAT
GCTCGTTTCGCCGGAGGCAACGTCTCTAGCAGCAAAGACCATGCGAGAGG
ACGAAGGTGCTTCGGCTGATCTGGTATGCCTGGTCCTCGTGAACTGACATC
CTTTACGCTTCTCTAGCCCAAAAGGGGGCGACTGACCTGTGGCGCATAATC
GCCGGAGCGTGGTCATCTCTACAATCTGCTCCGAAGCCTCCCTTGAGCCGT
CCAGCAAGGTCTCGAATGCCAAGCGCCTGAACAACGAGCGCGTAAGCTTG
ATAAAGTGTGTATTCACTACACGGCACTCACCGAACATGGTCATCCTGATCG
TCCATGGCGCTGGTGACGAGCAGGCCTGGCCGTCTCGAACTCGACAAGAG
ATGACTGCAGACAGCAAAGAGACGATCACAGGACGCCAGCTCAGGCGAC
GAGATGATGGGGCAAGTGATCGAGCTGCATCACACCGTGAGCTGCTCAGC
TGTTCGAGGTTTCGGAAACAGACGTCAGTAGCGTGTCAGGATGCCGCGTG
CCTGACAAACCACGTGGGAATCACGTGTCGTCCGTCGGCTTCTTCTAACCG
AGTTGTGCAGAAGAATCTCTGCAGTCGACTCGTCTTCTCATCTTTGCAACA
GCTGACGCAGATGAGAAGGGCACGGGGAGTGGCAGAGGCTGTTGGACAG
TCATGCCTGATCTCAAGGCCATCATTTGCCAGCTTTCCGAGAGCTGGAACC
ATCCCCGCCCGCTCACTGTCACCAGTCACCCCTGCCCTTGCGAGCCTCTCG
CGAGCACTTTCGACTGCTCGCCTTGACGTGAAACCGACATACCTGGTCTAT
CATCATCATGCAGCCAGTCCTCCAGCGTCGAGTACCGCCATCACTTATGCGC
GGCTTCGTGATGCTTGTGAATGTCCGCAATGCAAACACCCGCATACTCGCC
AAAGACCCATACACCTGGCCATCACCTGTCCACGTTCCCCAGCGGGCCTTC
TCGTCTTGGACATGGACCCCAGATTAAAGAGATAGACGAGACCGAGGGAA
GGCCTGAGCATCGTATGGCCCGATGACCATCAGTCGACATATACACCTGAA
CACCTGCAAGCCCTCGGCTTCACCCGAGAGCGAACGCCAGAGAGCCAAAT
ATCTCGAGAAGACCGCGCATCGAGTGGCGTGGGATCGCCAGACTCTTCTAG
ATAGAAGTCACTCCCTCCGTACCTCCTTCACCGAGCTCAGCGACGATGGAC
GGACCGCGAAGCACAACACCTTGTTGAAGGTGCTGGAGCAGCTCCATGTC
TTTGGTCTGGCCGTGGTCACTGGCGTTCCGACCGACAAGACCAGCAACAC
CGATTGTGCCCTAAGAACAGTCGCCAATATGGTTGGCGAGATTCGAAATAC
ATTCTATGGCGAGACGTGGGACGTCAAGAATCTCGCTGCGGAAAGCCGGA
ACGTGGCATATACCGATCTGAATCTCGGTTTGCATATGGACCTTTTGTGAGC
TGACCAGCTCCACTCGACTCAGCTCACGAGACAGGTATTTTTCCTCTCCTC
CCCGCTTTCAGCTTCTCCATTGCCTGCGTAATCGCGTGGAGGGTGGTCAGT

CTTATTTCGTTGACGCCTTCGCCGCTGTGGAGCGATATCATGGACCGCGGCT
TTAAGGGATATTCACCTTGAATACGAATACGACAACGATTCGCACTACCTGA
CCCACACCCATCCAGTCGTGACGGCTGAGAATCTGTCGCAGCTGCATACCG
CCGTCAATTGGAGTCCGCCGTTCTTGGGCCGACCCCTTCCTCGCCACCCTG
CACCACAAGATGCCGCCGAGGAGAAGCGGCAAATGCAGCTCGAGTTGGA
ACAGTACGAAAGTCTGGCAGAGTTCGAGAGGATCCTGGACGCAGAGGATC
TACGGTACGAATTCACGATGCAGGAGGGGGACGTGGCCTTATTTGACAATC
GCCGGGTGTTGCACGCCCGAAGAGCATTCAGAGACCTGACCGAGGACGAA
CGAGTGCAGAGAGGGTGCAGGTGCAGGCGGGCGAACCGAGCAGGTGGCT
TAAGGGGGTGTTACCTGGACGGCGAAGTGGTGTGGGACAAGCTAGTCACG
TTGAATAGGTTGCGAGTCTCGGGCAACTTGTGAGTAAAGTGGTAGAAGTTA
GATGTACTTTGATGCCAGCTCTATGAGCGATGTATTAGTTACTGGTTCTGTAA
TGAACTCACCGGATTGGTTGCCAACTTGTGCCTGCTCTACTGTGCCTTGTTG
GCTCGGAGTCGGGTCATCTTGAGGTTCGGGTTGACCGAGATATCGCGCAAT
CATCCGGCGGATGCCTGGATTCATGGAGTATTGGGGATCCATATTTCGCTCT
TTGATCGAATATGATTGGGGAAAGGCTGTTTAAGTTGAAGTTTGTGAACTG
TGATTTGATTGATGATACAATTCCTTCCAAGTGAAGGGAAATGATGGTTGAA
GAAATCCCGATCCCCAGGAAAGAAATTTGATGGATGGGCTGTTATGGGGAA
AGGGAAGAGGAGAATATCTGGGAAGGATGATGGATAGAGAAGAAGTCCGC
AAGGAACAAGTAGGTAGGTGGGCAGACAGAAAGCTGGCCAATGATAAATG
ATCGAAATCGACAAACTGCTGTATTTATCTATCGTGGCTATTATTGCGGCTGG
TATTAAACTACGAAGGTCCGGGATTCGATCTCGACTCATGAAAGTGAGATC
GAAGAAATGTGGTGGAATAGTCGTGAGAACGACGTTAACGATAGGGGGTT
CTAAGGAGGCATGTGCCTTCTTAGCTTGCGTGTGGTTATTCCTAGTTAATGG
ACTAATGGTCATGAACTGTCGTTTCGTCTCGCCGTGCTAAGGACACCAGCT
GCCCTTACTCTTAATTCCTCTGCTCAACTATACCTACTAGTTGATATCTGGTA
GGCAGACCGCTAAGTAGCTCGTTCAATAACTAAACTTTTATCAGAACATAA
GGCTGGACATAGAGGATGTGAAGTGGCATCTTCAATGTTGATTGAAATGAT
ATATGATCACATCTATAACGAATCTCGTGACATCGAAGGCCGTCATGCCATG
TGATATAGTACTCTTTGTTATTGATGCTGTTGTTTTTGAGAACCTTTACGCCG
TTATATGCTATGCCCAGCTTGACTCTGACAAAACTCTGTGCCAAGTGCAAAT
ATGTTTGCAAGTGAAAGGTCGCGGGCCTACCCCAGAATTTCTTCACATTGC
CCCCAACATTTTTCAAATCCAATAAAAATGTATCGTGACCAAACATACTCTG
CTCTTCGCTCAGCTCGACATGCGTCACACTCTTATTCCCCGTCAGGCGGATT
GCGTCGGCAACCTCTCGCTGTTGCCAAGCAGGGAATAGGATATCGCTTGCA
ACACCCATGACAAGAGTAGGAATATTCTTCAGAGATGATAATCCTGCAATTA
AATCTTCGGGAGGGCCAAGGTGTCCGGAGCTCGAATTATCAGCATTAGCTC
CGGGTTGTTCAGAGTACGGAGATTCCGGCAACGTGAGACTACACGAAGCG

TCGAATGATTTTGGAGAGGAGCCAGATTGGATAAGCTTTTCCCTCTCAATCC
GCCTCTGCGCAGCTGCTTGTTGAGTTTCGAGACCTAGGTCAAAAAGATCCA
TAGCCTTGCTAACATATAAGAGACTGTTTGGATCATATTTTAAGCAAAACTT
TTCGCCCGGCGTGGTCTAGATAAGTTTCGATAAGAAAGTCGGGACATGTGC
CGGGGGCTTCGTCGGATCCGCTCTCCTACGGCCAAATCGTTGTTCCCACTC
GGGTCCGGATCGATACGTCACCGTAGCGATCTCTCTCGCAAGCTTCATACCG
GCATGCGGAGGGACTCTGCCATAGTAAAACCCGCGGTTCCAATTGGGATCC
ATCATGAGCACTTGCCGTTGCGTGTGTCGCATTGCGATACTATAGGGGTGGC
TGCGAGCGCAGCCGCTAATAGACACTATACGACTAACCCTCGAGGGAAATA
GCACACTGGCTGCTAGAGATTGCATTCCTCCCATACTAGACCCAACACTTG
CGTAAAGCTTTTGGACACCTAGATGGTCTAAAGGCGGAACTGCGCTCGCAC
CTATCTTCCATTGTGAGGATCGGAAACCGGGTGGCATATCTCTGACCATCGG
CTGGAGCAATGCTGCTGGGTCCAGTCGACCCAAAACAACCCCCGATAACAT
TTGTACATATCACAAAATATTTGTCAGTGTCGAGACAGGCTCCTAGGACCG
ATGAAATTTTCCCACCAACCGGGTTGTGGATTGCTCTCCGTCGAATGGGCG
TGTGACGAAGCAGAGAGGCCGGTATACAATAGTATCACGTTGGACTTGTCC
GAGTTCATTTTGCCCCATGTTTCGTACGCGATATCGAATTCGGGAAGTACGC
CACCCCAATCTAGAGAGAGAGGATCTCTACAATGAAAGACCTCCGTGGCG
CCGGTAGTGTATGCGGGCTCGGGTCCTGTCGATAATGACTTTGCTTCAAGCT
TCGCTGATCGAGGTTTCTATCGCGTCGAGGCATGGAAATGCCATAGCGGGG
TTTGATGCCTCTCCTGGGCTCGCGCCGCTCCGCCTAGCAGGCTGGGAATGT
ATTAATCGCACTCCATGCTGTATGGTAGGAGTTTTCGAGGACGAAGTTAAA
GTCCGAGTTCGGAGTTGTGAAATCGGCGACGGCGCATTGCGAGTCGGCCG
TAGAGAAGTAGATACGCGTCGCAGAGCGGATGTGCCTATATCCTAACCTTC
ATCGTGACACTTCTTTTAGGCGAGAGGCGTGTCAAGTGTGTCAAGTGTACG
AGCACTCAACGGACGCGGACGGCTGTACCAGTCTCTATATACCGGATTTGT
AATACCCATGATGGAAATTTAGAAAATTAGTAAATTTATACCGAGACTAAAC
CTTGAAGGGTTCTAACTCAGCTAGGTAGCACCGTAGCACAGAGGCGAACT
TGCGCGAATTGTACCTATGGTGCGACCTCACGTGCTTTTTCTCCAGCTCCTT
ACTGATCACATCTTAAATGTACTATCACCCCACTTGCCATTTTTGCTCTAGGG
GCATGATCTACATTTCAAGCCTGGACCGGGGTCTCGGGGCATAAATGCAAT
CGATAAGTGTGTACGCTGTTTGCTGCCAGGTACTGAGTTATGAAGCAACAA
ATGATGTCACAATTCGCTGCTATACTCATTCACGCGTGCGGCATATGAAAGA
GTCTTCATACCTTATTTATACCCTTATACAAACCAAAGTCCACGCGGTAGTTA
AGAAGCAGCAACAATTAGTCTAGCCACTGGCGACCACTCTTCTCTCTGCAC
AGATGTTAGTTGCGCCACGGCACGATTCTACGCAGGCAGAAACGAAATGCT
GACGTTGATAGGTGACACAACCATGTTTAACCGCGTTTATTGGCCTTTTAAA
ACGTCCCATCGAGAATATCCTGACGTAGCCCTATCGGCTTAATTAGGACAAG

GCAGGCACTCTCCAGTCATTGTGTGACTTCCAAGTTTACGCTGTCAATGAA
CTTAATAGTCGCCCCCCGCGTCCGGATCTAACACCTATCTTGGTTCTGGAGA
CCCCGGAAAGTTTTCACGTTTGCCTGTTCCGTGACCTACCCGTCTAGTATTT
AAAGTCCACTGGTTAATTGTTCTTAGCTCCACTTACCTTTTACAAAAAACAT
GTTTGATCTTGTCGACATCGCCCTAATCTCCCTTATTGCAATAATAGCTATCC
CCCGACTTTTCAGCATCAGCGAAAGAAATACAGACACATCTACCGTCCCCA
TCATGATCAACCGTAGAGGAGGGGCAAGGATCCCGATGAGACGGATACCTA
TACACCCGCCTCCGGATAGACGCGGCGAACAGCCACCGTCGACTCCCGTTA
GCTTCTACGACCTAGACCAGAAGATATCGTCGAGGTGAGGACATCTTGTCC
AAAGCTAGGAACATACCTCCCGAGTTGGTCGATATGATCTCGATCGTGCAG
AGTACTGGGCTTGCTCTGCTACCGTTATCGATTATAGCAACCATCCTCAACA
TAAGATTACAATTCTTGGCGCACGTCCAGCCCAGAGCCAGTTCTTGGTTAG
TAAAATTGTTCAATGACACTAATTCCATTCCGCCAATTATTCACTAACGCCG
ATTGACAGCTTCGTACTGAGCCGTTAGACTTACAAATGGTCGCGGTCTAGC
CAAGATCTCTGGAGAATTGAATCCGCAGCCAAAAATCTCAAGAAGGAATAT
ACAGAATCTGATTTTCAAGGCCTTGTAGATGGTCCCTTTTCTACATTAGAGC
ACCCATTCCGCAAAATTGTTTTCGAAATTACGAGCAACGATCAGGGTTGGA
GTAGCGATAGCGAGCATCACGGTCAGTACGAAGGTTCATGGACTTGGTTTG
AAGTAGGATTGGAACGTTTCGACAAGGACAATACGTGTAAGAATCCCGATG
TAAGTGTAGTTTGATCGATGCGCTAATCATAATCTAGGTGTAACGGAATGTC
CAGATCACTTAGGTTCGGAGGAACTAAAGGGGACCCCAACCATTCCAACTT
GCGCAATTCGTCCAATCTGGCCCAAGATAAGATATACCCAAGATAGTGGCG
ATACCATGGGATACGATCATGATTTATTACCCAACGAAGACCACCTCATACA
AAAGAACAAACACGCCGTGGGAACTTTTCAGACCTATCATGTTGAATGTCT
TGGGACGATGATATAGATCCAGAATCGCCGCTCGCGACGCAATCCTTGAAA
GAGGCTGGCCGAGGTACAGGCACTGGAAATGGCGAGTTTCTCAGGACCCT
GAAGCTTGGCGACATGATTACTGTCTGGGGTCATGCCAGATTCCCAGGATG
GACGAACAATATCCAAAGAGTTGAAGTTAAGGTTTACTGGGCGTTTTAATC
ACATCGACCGCACATGGTGGGGGACATGAACGTACGGCTTTTCAAAGCTAC
TCGTCGCACTCATACAGTTCTTGCAGTTCACGACTTACCTAATGCTTACTAT
GTAACGAACTCATCCTTTACATGCTCATTCATAATTAGGCTGGCGTCAGGAT
TCTATTTGAGAGTTGAGGCGTTGTGGGTTCGGATAGTAGTGATGCTTGGGG
CTCCTATCTTAAAGTACTACTTCCCCTATTCCTCATCTTACATAATATCTAATA
TATATTGATATTATCTCCTACTAACTGCTTCTTGGTAGTAGCCATTCTGTGAC
AGTACTAGTTCGGATAAATGTGTAAACATGCGATGATTGTAGAGCAATGAG
AGTCCAAACATGCTTAGGATTCCTCAATTTACGAACGGGCAACAAGCAATG
TGACAATGCTGCTCGAAGAAAGTCTACGGATTCATTACAACGTAATAGGTA
GATAATTACTATATGTTCGTATGCATATGTACTGTATTATTATCAGAGACGAAC

TCTCCCCCCTTGAGCTCTTGCAGATCCTTCTTCGTGAAAGGAACATATCTCT
TTTCACCGGGAGGTAACCAGTATATTGCGTCCGGATCTTCTCCTTTTCTTTA
GCGGCCTTCTCGATAGCGTTCAGGTCAATTCCTTCGGCTCTTAATTGCCCCT
TCTGTAATTTCATCGTCCCCGTGAACTCGAGCTGCTTCGATACTCTGACGAA
TATAGGTACTGCATATCGCGGCAGCTCTGCGAGACAATGCTCTGCTAATCCC
TTTAGGTCCAGGCCTCGTCCACTTGCTATGTCTTCCGGTGTACTCAGAACCC
CTTCCGTGGCAACCACGGACACATCCGGCTCGACCCTCGGGAATTCGGCA
CCGCGACACCGTATACGTTCGCCACGGCAATCTGCGGGTGCAAGCCCACCA
CATCGCCAACCTCCGTTGTGGACACGTTTTCGGAGCGCCAGCGAAACGTAT
CACCCAACCGGTCCACAAAGAACAACCGTCCCATCTTATCCAACTTCATCA
CATCTCCAATCCTCAACCACAAATCCCCCTTCACAAACACGTCTCTTACCAT
CCTCTTCTCCGACGCGGCATCATTCCCATAATACCTCGGCACGCCCGCCGGA
TTCGTCGGATCAAGCCTATACACCAACTCCCGGGCTCATCCTTCCCACACCT
AACCGCAACCCATCCTTCCCCCGTACCATCTCCTCTGTGTCTGGATCAACCC
TCACGCGCGCCTCCCCCGCGCCATTCACCCCAATGCCAAATCGGCCCCCTC
ACCGCAATAGCGCATCGCGAGAAGTCCCCCTTTTCACATTCGCAATGATGC
CGATGCCATCAGTGCTGCTATAAAACTCGTAAATCCGCTCAATCCCAAAGC
GCTGCCGAAACGGCTCCCACACATCCGCGCGCATGCCGTTGCCCCACGCC
ATGCGGATGCTATGGCCGGTGTCGAGGGGGATGGGGGCGCGTTGAGCACG
TACCGGCATAGCTCGCCGACGTATTGCAGGATTGTGGCGTGCGAGGCGTGG
ATCTCCGGCCAGAAGGTCTGGTGCGAGAATTTGCGCCCGAGGACCATGGT
CGCGCCGGTCGCGAGGATGGGGAGACAGCACACGCCTTGCGCGGAGGCG
TGGTAGAGGGGGAGGCAGGTGTACCTCTTGTCGGAGGGGAGGAGGCCGAT
TTTGTTGCTGGAGGCGCGCAGGAGCATTGTGCGCGCGCGGCTTACTATTGT
GCCTTTCGGCTTGCCTGTTGTGCCGGAGGTGTAGATTAGGGAGGCGACGTC
TGTGGGTTTGGCGCCGGCGCGGCGTTCGTCTGGGAGTGGCTGTTTGTCTGT
GTGGGCGCTGAGAGAGGTTGGGTCGTAATACACCGTTTGCGTGCCGCCGG
AGGTCAGTTCTTCCTCCACTGGGGAGACGAGGTGGCGTACGTCGGAGTCG
GCGATGAGGTATCGTGCGCCAGCAACCTTGACGGAGTGGACGAGAGGGTG
GCTGTTAGGTTGCAGTTATGAAGGCGACAGATGCACCGATGGCTTCGAGGG
CGAACCAGACCATAACGTACTCGGGACTGTTACCGCCATCTAGGGCGACCA
TTTCTCCCTTTTTTATGCCCAGATCTTTCATGAGCCAGTTTCCAATAGGCTGT
ATTGCCTTAAAAATTTCCGCGTAGGTGTATTCTCGGCCCTCGAATATGATGA
ACACGAGGCTAGGAATCCCTTTTGGCGTGTTCCTCCAGGATATGGTAAATTG
TGTCTTGCCATCGCGAATCTGTCTGATAATATCAGCTTGCCACTTCGCAGCT
CGTTTCTTGTTACTACCCGCGGCGATATCATTGCGGATATGAAAACGAGCGT
CGAGGTACGCAGCAAAGGCAGCGCTGCCAGCAGCGGCTCCCGCTATAGCT
ACTAGGGTTCTTGGTCAGCTCTCATCGGGATGACGGACGAATTAGATGAGA

AAAAACGCCACTTACATGCCATATCGTGGTATGGGACGGGATGGAATGTATT
CCTCCTATCAGGTACTGGTTAAGTTTCAAGCCTCAGTGAGTCTACAAAATC
AAAACATGGTTCATCTAGCGCTAAACACGCGATTTATCATTGACACTTTCTC
TCATAGCTTTGTTGGTGTTAAGAAGGTGCTAGATACTAAATATTTTTGGCAG
CTTCTAAGGCAGCAAGGTTGCTTTATAAAGTAGCTGAAGTACGGTACTTAA
GGTTTACAAATAGTTCGGAGCTGAAGGGATTTAGCGATTCAATGCCGAGGC
AGGTCCTTACGAGGTGCGTTTAGGGGATCCACAGGAGCTTTGCTAGAGGCC
GGGTTTCGGGATAATTTCCTAGTATAGACTCCGGCGGCATTTGCACCTATCC
TCGTACCTATCAGGTACCTACGCAGAGACGCAATATTCATGAGGTATAAGGT
CGCAGTAAAAAGCCCTCAGCGTGCATGAACGCCACCGTCACCACTATCATA
TATTAATTACTGATCTCGGGAGATATCGAATTCCGTCGTCGTCGCACAAAAG
CACGATTGATGTTGTGCTTCCTTACACGGACCAATCTTGTCATTGTTGAGAC
TCCACGTTTTGCGGATCGCGGCCGATGAAATAGTGGGTTAGGTGATCAAAA
GGGTCCGTGTTTGCTTGGATGCCCCCTACGTGAGCCCACTAGGGAAAACAT
GCATCTTTCAAGTCTCATCATCTTGCAGTCTTTTTCATGCCAATTTAATCATA
CATATATATCTCAATGGAAGGAACCTGCCCTGCTTGTTTAACCTTCTCTAAA
GACATTTGTTCACCATCAGTTCGAATCTCGTTTAAACCTGGCTTATTGACTT
GCGACTGAAGCGATATCTAAACGAAGTGTAGCAGTAACCGTGAGCTGAAC
ACATTTGAACCCGTGTACCTGGCTAAGTCAATTTACCTACGTACTTTCCATT
CATGTGTATAATACGCGTATTATATATTGTCTCGGTATTATGAGAAAACTTTTT
TTTAATTAAAACTCCTAGTCTTCTCAATAAACCCCATCAAAAAGCATCAAGC
TAGCCCGATGAGAAAACGATCAAAGAGTGAATGTTTATCTACGAAGTTTCC
GAATCTTCGGTAGATAAAGCCACTAATCTACTAGCTGTATTCCTTCCGCTAG
CATAAGTGAGACAAACCAAGACGCATCCTGAAAGCCTGCCATAGGGCTTC
ATCTTTACCGATAGCAGTACCAAACTCGATGGCCAACAAATTTCCTAGAGTA
AGGGATATAAGCCACTTCCTGCGCTACAAACTTCGGATGTTGCTAATCAGA
AGGAGGGATAGATGCACAGATCTAGAAACAGCATCATTCGGAATCTGACAG
AACTTGGAAGCGCTCCAGGAACCGCCCTTCAATATCCGTGTCTGAGCTTGA
TACTGAAGAGCGGGATGTGTAGCTAGCGCAATAGCAAGCAAGAGAGCCAG
CACGATGATAACAAAATGCTCGATATGAAAGCCGTTTCGTAGAAGGCTTTA
AATTGGCCCCATTTTTAGGGGCCAGTGGTCCAACCATCTTCTTCACTGGCTT
GATGGCGTAGAGCGCCCGGCTTTGAGACCCCGCTGCATGCCCGGTAGTCCA
TCCATGCTCCAGGCGTTTTCAGGGCCCAATAGCGGTCCATACTCTCTTTCTC
GTTTCCGGAGAGGAACCTTTCCTCCTTGCGTTTGAAGAAGTCGTAGAGCGT
CGTGCGAAACTCTTTGAAATCAAATAGAGGCACGCTATATCCGCAGGAGGT
ACCAACCTGGTGGATGTCGACCACGATCACCGATCGGATGCCCGGGATTAT
CTCAATGTTCTCTTGCTGCACGATATCGTTGAATTCGGAGACCCCCTTCTAG
CACCCGCCCCTTACCCCAGAGTCTCAAGATGCGCGGCGGCCCCTCAAAGG

CGTTGAAGAGGATAGTGACGCGGCCATTGCCTGGCTCGTGAAGATGCGAA
ATGGTCTCGTTACCGGAACCGGTCATGTCTAGATACCAAAACGTCTTCTCAT
CGAGAACGCCGAAGGCTTGTCCACCTTTCGGCGAGACATTGACGTGACCT
TGGCCTGATAACGGGGCCGTCGACACCCAGAAGACCTTCTGATCAAGAATC
CACTGGATTAGAGAGCTAACAGTCGGGGTTAGTATTCCTTCAGCAAGAATC
CTCAGGATGATGATTGCTCAATTAGCCCAATTGAGAAGGGAATAAAAGAAC
GTGCGGTATGGTACATACGTTGGGATCGTCTCGAAGAAGGCCCCCATAGCG
AATAGAACTAACGACCCGATTTATTACAATGTAAAAGTAAAAAGAGTTACT
AGAATGTCTAGCCCTGTTCGCTAAGAAGAAGGATGATATCAATTTGTAAGTT
GTCCTAATCTGCTTGGGTCTATTAACAACATTGAATGACCACAGTACCGAAT
CCGAAGTTACATTCAATCAGACATGACGTACAGTATGAAATATCATTGATGG
GTGTTTGTATTGGATGTACTAAGCAACTTCTGGACTTCGGCCCCCCGATAGA
GGCGAAGGTGCACCGCTTCTTGTCTGAGTCAGTTGTGGTAGGTACGCTGCT
ACGTATCGGTGATAGATGTGGGTGTGGGGTCGAATCGCGAGCCTTCCCATT
ATCATATGCACTCAAAGTTCCGAGTTCTTTGTGTAATCGCACAGCAGGATCT
CCAATTCCAAGGTCTTGGGAGCTCTTGCCGATACCGGATTGACGCTAACGC
AATTCACATAGACTTTGACTTTATTCTACCAGAGCCAATCGATGTTCAAGAT
ATCTCGGTTATTCTAGGTTTAAACGGCTTAAACTAATTGGAAATCCTCCAAT
GAGCTGAGAAACTCAGCATCTTCGAGTACGACTTTCCAAAGAGAAGCCAT
CTTCTCCGGTAATCTGAGCCTACTTTCTAATATGTTAGATGTCTGGGTATATAT
GCGCTTGGGGTCGGTCCAGGACATTTCTTCTATGCGAAATTCATCGAAAGA
TGTCGATTTGTGGTCAAACAGAAAGGATATAATGCCCTTCCTATAAGTGCGT
ATTTCCCCTGGCTGGAACGCCTTAAGACGAAGGGACATTGTGATCCCTTGA
GGAGGACGATGATATCGTTTCCCCGGAATCCCTTAGGTGCTATGGAAATACT
TGGCGATGCACACCCCTATGGTATGGTTTCCTCGGATAGGCTCCGGAGGCA
GGATCACCCGGTGCGAAGGTCATGTCTGATCTTTTCGAGCATCCATCCTGA
AGCAGGGTCATAAGCCGTAGGCCAGCTTTTGTCGCGGTCACACCAGCAAC
TTTCCCTTCATATAGGGGCAGGTTCTCGTCTTTGAACACTGCATTAAATGTC
GATTTATATTCTCGGCACACGCCTTGAAAGAGTGATGTGTCGTTGAACTTCT
ACCACCGGAATCTTTCCTTGAACCCAGACTCGAAACTCGGAGATATGAAAG
GTTTCCAAGCTGTAGCTTTATCTACTTTCTCATAGAGTGTAGCTAGGACGAA
TACCTTCGTAGATAGGACTCCATCGGCGGCAGGGTGTTGTTCAATTGGCTTC
CACGGTACACCAATCCTCTTAACATCGATAAATCCGTACTTCCATGAGTTTG
TCTTACTCACAACACGATGTTGCGCCAGTTTGGGGTCACAGGTCCTCCAAT
CAGGCGTCCACGAGGGGATATTGGACGAAACCGTATGAGATGCTGCAAAG
TCAAGAATATCCAATGATCCTGTTCTGGCGATTTCATACTTGACAATCTTGAT
ATACCTCGGATACAGAACGGCTGTAGTCAACTTTCAATGTAGGTTGACTTAT
ACTTATCCCCGTCTGCGATATAGGAACTGTCGTGAAGCCGAGTATCCGTTAA

ACCAGATCACGAGGGTCAGTCACCCCAAAGGACGAAGAAAATATCAGTGC
GTCTAGAAGTGAGACGGGTCCATTCACTAAAGCCTGGTCACTTTTCACCTC
GCATTCTGCGATCTTCTGATAGTCTTCGTGAACTAACGTCATGATCGCATTG
AAGGGAGAGCTTCAGGTAGACGGCATCAGTAAAATCTACAATGGAAGATTC
AGGCCTCGCGTCTGCGTGCTGTCTAATTGACGCAATGAAATCCCTAATGCG
TTCATAGTCTTTCCACGATATCTGGAGTCCGCTAAACTGCATTACCATATCTC
TAGCAGCGAAAGCCTCCTGTCTCACCCAGGTTCGCTTAAATAAGGGAGGCT
GGCACGCTTTTATAAGGAATTGATACAATTTCTTAAACCGTGCAAGGCAGTC
CTCGCTATGTTTTTGACTGCTAGAAATATTTGCGAGGACCGTAGACAGGAC
GGTGCTGTTCTCTGCCGACCATCCGGAATTCCACGCAATACTCTCCGGGCC
TTTTTGTAGATCGAGAACATCCGCCCGACCTGCACTGACTTCTCCTTGGTAT
CAAACTGGTTGATACAGAGCGCGTCGACCCACAAATAGCGCTTAGAGTCA
GTCAAACGTAGGTGACGCAGGGCCGAGTCGAGGTTCTCAGTAATGAAGAA
CTCAATGCCGTTATTTGAACGGGAAGCGAGAAGACTGGCGCACCCCACGT
GTACGAAAGTGCTTCGTATGGAATCTCTTGGGGCCGTCTTCCTCGACTACG
CCTAGACCCTCGTAGGCGATGACATCTGCCACTAAAAGGGTGCAAATGACC
GGCTTATCGAATGAGTTGCGGGATCGATACGTAGAATCCGAGTTTGCCAAG
GCTTGATACTATGGTATAGCTTTGCAGCAGCAGCCTCCCGAATCTTCTCATC
GACCTGAAGGTGGGCTTCCCTGAGTATTGGATAACGCGTGCTCTTGCTCGT
TGGGCAGCGAAAGGCTCCGAACCCAACAAGACTCAGAACATCCTTTCACT
TTGAGTGCTTGTTCCCAGGCCGCCAATTGCTTGGCTTGCTCATTGAAAATAT
ATTGGCTTTGAATAGGTACGTGATTATCTAGAGTTCAACAAAAGAATGTGA
AGAGACTAGCAATCAAGAGAGAGCGGGGCCCCGCTGCATCGGAACTGAAT
TGGGTCTGCTGGTAGATACGGCCCCCAAGCCTAGCAACGGCAGCAACGGT
CACTAGTGAGGTCATCGGCTTACTTGAGATCTATGTATAGAACATCCCAGCT
ATCCATATGTATTTTGATGTATCTAGCAGGTCAGAGCTCTGTCAATTACTTAA
TTCCTACAATATGTCCTCTGTGGCACAGGCTGGTGTGGCTCCCAAAGCAAA
ATGTCCTCTAACCTTGCTCCGTACGTAGCATTGGACATCTTGAACTCAATGG
AGTTTGCTGGCTTGTCGCAAGGTTTCCCACGTCGCGTACGCGGAAGCATTC
AAACTCACTCGTCCGGATTGATACTTCCACTTGAATACATCGTCTAGCCCGC
AAACCGCGTAGTACACAGCTACCGCATCCCAAACATAGTGCTTCTGGCCAT
ACCCGATGTTGGCACGCAGGGCATAGCCGACAGGGTTCTTCGTCACGTCTA
ACTCAGTAGTGATACGGGTACCGATGACGGTGTTCTCGCCGACATCATCCG
ATGCGAAAGTCAGCTTTCCGGGCCACCAATTAGTACGGCTTCGGCAAACGT
TGCGTTATGATAATCGGTGTTGTATGAGTACCCCTCGTCGTTAGCTTGCACA
ACGAGCTCGCGAACTTTGGAAGCGAGCAACTCCGCGCCTGTGTACGGAGA
TATCTGATCTGGCCCGGAGTTCATGAGGTGATACATGTTGTCAAAGAATCCG
ATTACGGCGATAGTGATGGAATTGTCAGCCGATGTTGACAAGAGATATCGGT

ATGACGATACTGAAACACCGAAGTTGAACGTCAGATTAGATTCTCGGCCAA
CAAACAAGTTTTATTCACAAGAAGTCCAACATACCCGGGTCCGGAGTCTTA
CTCCCGTCTCGGATATCTTCGGTGAAATTGTAAGTCAACCCGGTGAGGTATG
TCTCCGTATTCAGGGAATTCTGGCCATTGTGTCTGTGATGTATGAGCATTCTA
GATAATTTGGTCACAGGTTCAATTATCATTGTCGCATAGAACATACTAGGTTA
TCAACGGGCTTCTGGACTGCCGTTGGCACATCTTCACACATTTAGCTCATGC
TGGAACATTACGACTGAGAATCAACGTACCTGGATGCCCAAAGTAGGTATT
TATAGCATCAAATCCCGGACAAGCGTAACGACTATTGATACCTTCACAATAC
TATTAGTGATAATGTATGTAGTGGTACGATTGCTGGATTCATATACTTGACAT
GACGCCGATTAACTCTACTTCACCCCAGTTTTCCAAGATATTCGCCAATCCG
ATTGCTAGTGGGTCATCGTCGAAATTCAACATATCTGTGTCGATAATGAGTC
GTTTCTTTCCAGCCAGTGCTCCCGAGAGCGCGGCCAGCCCTGCTAGCAATG
CGAGAGCATCATGTGAAAAAATTTTCAGTCGAAAAACCGAAATTCTGTAAA
CTTGGGATGAGAGAAAATAGCGGTCTGTCCACACAGTGCTTCGACAAGTG
ATTCAGAATGCGAATTTTCGCACAAAAAAGAGCGAGTCTAACAGCTAGCG
GCGAGATCGCATAGATCGAGGACGCCTAGCAAGGACTTATCAATTCTTGCT
GGGGTGAGATATTTGCAAGATTTATACCGGTGTGATCGTGGACAAGTGGGG
GCCGACCGACCACCCCCATTCTCGACGGCCCTTACTGATAGGAAACCAAAT
CATATCCCTCTTTGGAAAGCAAAAATTATCTACCGATTTAATTATGCAATATT
TAGCCCAAATTACGGTGCATATTTTAAATACATTATTGTATAGGCAGTGTACA
GTGTAAGGTTCTAATTCTTGTGCTAGTTTTATCGAAAGCACCAAGTATACGT
ACGTAGTATATAGCGACTCTTCATATATACGCTTGCTACGTTTGAATACCTAG
TCCGAGTAATCTGATCGCTCTTCCTCTATATGTATTTGGTTTCTAAGGTTTCC
TGTATAGACCCCGATATGCCCAAGTAAACCATAATAGTAGGGTCTAGGTGAC
ATCGTCGTAATATGACCGTCAGATAAAGCGGACGCTGAAGTTGTTGCAAAT
ATCACAGTGGAGCAAGTTCAATACATGTATCTGTCAGAGCGAAAGGATAAC
AACTTACTAACTGGGGCAAGGCAAAAATAAGAGAATAAGAAAAGTTTCTA
ATAGTTGACCACACGTTTTGATTGCTACCTAAATACCCGGATATGCTAATAGT
AATTGCCGACATGGATAAGATAAAGTAGAGTAACTAGCCGCTGAAGACTAG
AAAAGAAAATATTTCTATAAACTGCACAAAATGGTTTACTGTAAACTGGAAT
GTTTATAAACGACGCTATATGTCTACTACAGTGCTACGGGAAACTGGGTAGG
TAGTGAAAAGGGGTGATTGCTTATCTAATACTTGTGTAACTCGACGTTAGGA
TGGAAGGCGTGTCAACAACAGTACTCCTTTTGGGAAGAGAACTGTATAAA
ACTTCTGAAAATTTTCAAGCTTAGGCCTCTTAAATTCTCTTTTGTATTCATAA
TCCTACAGTACAGTGCGAATTCTTAGTCCGTAGAATGCGTAGGCGCGATAC
ACACTGGCTAGTCTACGGTGTCTCTACCTGTAGAATACTTAATGCTCTAATAT
TTATATCTAGAACACTTTCCTTTTAAAACTGTTTCTTCATCTCATGTCATATAA
CATAAGGAGCCAACAGATCATCAAGAAATATTCATGAGCCACCATCATACTT

CTCATCTTACTACTCTGGTTAACCGATATCCTTTACTTACCCAACAAGATATA
TCTCAGCCCAATCATTTTCCATCTTCTCCGAAAGCATTATCATGGCAGACAC
TTCCGCTAGTGCGTACCTTTCGTATAAGAACGACACTATGGTATTTCTCTCG
TGGCTCGGCAATCGTCTGAGAGATATGGCTGGAAGTCGCGTCAAGCACGA
AAAGAAGCTGTACCCACAGTTCAGATAACAGTCAAGTCATCCGGGAGGCT
CAAAGGAAAGGAAAGAAAGAAAGTTAAGGATGCAGCAGAATTGGAGGCC
AAGAGATTATTAGAAAAGGCCCGAAAGGCTGCTTCCAAGAAAATACTCTCA
GTGGCCGAGGTGGCCCAACAGATTGAGCTTGTCGATCGAGCCATCACAGA
AGAAAAAGAGGAACAAATGCCCTTCAACGTATGGAAAGCTCTCCAGCGTT
CCATAAAAGCTAGAGAGCGATTTCTCGGTGGTATAAAGCCTCCAATCCTGT
CCTAAAGACAAGCTGGAATCGCACGATTACTTCATTGATGCATTGAAGAAG
GCGACGTGCCTAGCCCGCGGAGAATATGATAAGAACCGTCTAAAGAAACC
CTTCTCTAACCAGACAAATCGAAACAATACATGACAAATTTGTGAGTTATAC
CACCGAGATTGAAACTGTTCAAAACTAACTTAATATAGAAATGTATTTGAGT
ATCTCGAAGTGGACGATCTATCTGGACTAGATGAACCCAATATTCCCGGTAA
CGAAGGCGATCAGACAAATATTTCCGGAAGATAAGAACTGTCAAGTTTGAT
ATTGAACCGATGGTTGCCGAAATGGGATTCGCCTTTTTCGCCGTCTTTGAA
GATATTCGCCGTGTCCGGGCAGAGACCCAGCGCGTGTTCCGCAGACTCATG
GCTGATGAGATTTGTCTTCTAAATGCAACAGTCTCTATTGGTGTGGCATTTG
ATCTCATCCGAAAAGCCGAGGACGAAGCCATGAGAGTAGCTGATATGGTCA
CCTCTGTCCATTCCGAGTCTGAGAAACTATTTACAGAATTTGGTGCTTACAG
CGCCATGTCCCAAATGTTCATGGCCAATAGAGAAACCGGAAAGGAAGATG
GTGTAACGATCGTTTAGTATCTTCGGACGAATTCGACGAATTTATTCTTTATC
CGCTTGGACTCACGTTAATGGACATTCGTCTGCGCCCGCCAAACCTTAGAG
CATTCCAATTCGGTAGATTACCCTTTTGCGACTCTTGCAAAGCACTACGGAG
ATTCTTTAGAGGAGCTCAATAAGCCCCGAGTCCAGAAAGGTTTAAAGGAA
GATCAGATTCTCAAACATTTTTATTATGAGATGAAAATCATAGAAGATATTGG
GAAGGGTCGTGTTAATTTCTGTACGCCTACTTACAAGACGAAAGATTATTCG
GACAACGATATAGACAAATTCATCCTGCCTTTTTACGACCTTTTCCCCAATG
CCCTTCGTCCGATATGGCAGCAGAAGAAATCAGCTTCGAATCCGCCTTCGC
TGCTAGGGTGATGCTTGATATGTGCGAGATCTGTGGTACTTCGCTACACAAC
TCATTATTACAACCGAAGGAGGTCGCTATCTACGCTAGCAGGTACGGTGTTG
GGGGAGCGAAAGCGAAACTCGAGATCCCTTGCGAAGACAAAGACCTCTC
AGAAAAGCTGTTTCAGATTCAGGTGGAGAACAAGTGTTGAGTTGAGTGAC
AAGCCATTTTGGGTAATCACAAGATCGTGTATCACGAAACGTTGGAAGTCA
GAACGGCAAGAAAGGGTAAAAAAAGAGAAAGACGTCAT

>Mosaic_sequence_75

TGAATTCCAGCCCAAAACAGAGCTCGCGTTCTCTAACTTAACACGATTTGA
AGGGTTGTCAGGCGTTGCTATTCAATTGACAGCTTTCAAATGCGGAGGATA
TGCTGTGAGCATCAAGCTCACACACTGCCTTTCCGATGCTTTGTGTCTCATA
CAATTCGCTCACGTGTGGGCGGCGCATTCACGATCCCTTTTCGAGGACGCC
CCGAATGGCGTCCAGGTCGATGGCGAAGAGTGTGCAGCTCCATTGTTTGAC
CCTCGGCAGCTAGACCAGTATGCCAGCTTGACAGCTAGCCCAGATTCTGAC
AAGATTGAGAAAGCGAGAGCCCTACCCAGGCATCGATACGATTGGTGGGC
CAAAGATGCGCCTGGCTATCCTTCCTGGGCCGAACCATCAACAAACGGCAC
AAAACCATCAGCGGAAGAGCTGGCTCATATTGAACTAAGTCCTTCCACTTT
TCCTCCGTGGCCGACATGGGATATGGCAGCTCCGGTAGAACATGTTCAAAT
CCGATTCAAGGCCGAAGCTGTCGCGAAAATGAAAAGATCCGCCGAATCTA
GCCTTCCGGAGTCTCTGAAGGGTCAGCGGGTCTCCCGTCTCGATTCTGCAT
TGGCACACATATGGATCCTCATCAACCGCGCTCGTCGGCACGAGAACACGC
AAGAGGATGTGTACATGACATTACTCTCGGCGTCCGTGGCCGCGTCGATCC
GCCACTACCAGATACCTTTGCAGGCTCTCCGATCCTATTAGGCTACATCTCC
AAACCCGGCTCAGAAGCAACTACTACGACTATCGGTGCAGTAGCCGGTTCA
ATACGACAGATGATGTCTCGATTTACGCCAGATGCCGTATCTGCGTATATGC
ACGACGTAGCATACGAAATCAGCCCCCAAAGGTTATGGCAGGCTTTTCTCG
GCTCTAGACATACCCTTGTCACATCTTGGGTGCGCGCTCGTGCTTACGAGAT
GGACTTTAGCGGTACGCGAGACTTAGCTCGGTATGTTCAAGGAGTTATGCC
TCGAATGGATGGATTGGTCCAAGTTATGGATATTGCGGACACCGGGGATTTT
GACATCAGTGTATGCCTGGAAAAGGAGACGATGCAGCGATTTCTTCAGGAC
CCTATGTTGAGTGTCTACGGATTATGAGACAAGTAGGTACCTAAACGAATTG
CTTACCCGACTAAGGCGCAGCTCAGTAGGCGTCGGGGCGGGCGTTTTTGGT
ATGTCCAATAGCGAGCGGGTGTGTTCGCGCTACTTAACGTCGCTCGCACCG
TACTGAAGTAGGTAGATAGGCCAATAAAATTGAATATACCTTTCATCTTCGC
GATGAGCTGGAATGTAATTTCTGAGGTAAGCCAATAAAATTACATGGAACTT
ATGCCTTGGCAATTACCGTCGAATCTTTGCACTGTTAAGATGCCCTGTTAAA
AGTATCATGAGCCGTAATTTTTGAGGCATTGCCAAAGTCACTGTAAGCCTAA
GCCATGCGAGTGACGTCTTGAAGCAGTACTGCCTCCAAAATTACGGCTTAG
AGATTCGTACACAAGCGTGGCTGAGTTTCGAACACAAACTTGATAATTGCG
AAGGCATAGATTTCTTTTATTCTCATTGGGCGGCGTAGAATTCATGAATTTCA
ATACCTCGTAGCCAAGATGTTCTCACCGAAATTCCGTTGTCTGGCTGTCAGC
ACCTAGACCGGTATAGGCTAGGCTGCTCCCTACCATGCTCGCTTACAGGCA
GGGGGTAATCCGTGAGCTTCAGGGAACCTTAGGACCTAGAAGTCACCAATC
TTTATCATGTTCAAAGTCCCCTTACAGCGCTAGATGCGAGTTCATCGTCAAT
AGCACCTGAATCGCTAACATTGCCATTCCCTTCGCGCATTTAAGGCAGACTT
CGTGGGCCTCGTCCGTGCTAGCTGCCGCAGCGAATCGAGGGTTATGGATTG

CGATGCCTGGAGCCCGTTGGGACCCCAAATGCGCCGTGGAAACTGGGCAC
GATTTGAGAAACGTTACCCATATCAGTGGACGCCGTGTAAGGCTTTTCTCTC
CATAACATTATTTTTCTCCAATGGCCGACATCTCTTCCACATATGCGCTACA
GAGAGACTTGTTCGAGATCAAAATTTGCGTATGTCGGTGCGCTATTGGTGTT
AGATTAATGTATTTGCGCATTAGTTCAGGACTTACGTGATGTAGTTCAACTC
GCATCCTGTAGCCGTCGCAGCGGCCCCTATAGTTGCCTTCACGCGGGAAAG
CAATTTGTCTGCATCGGCGATATTGGGACTACGTACGTTCCAGTTCATGCGA
GTGTAGTCTGGAATGACGTTTGGTACTGTACCTCCGACTTCGATGACGGCG
TGGATTCGTTCATCCGGTTTGATTTGCTGTCGGAGTAGTCCGATATTACTGTA
TGCCGCGACAGCAGCGTCTAGAGCATTAACGCCATTCCAGGGCTCGTTAGC
GGCGTGGGCAGTCTTGCCACGAAACTCGGTTTGGAATTTGTGACTTGCGAT
AATTTCATCGCCGCCAAACCAGACCACTCGTTAGAGTCCTTAGAGAGCTGG
TCTTTTGGCATAGGATGAGCCACTGCAATAATGGTCAGCAATTGCTCTTTGA
AATAGGGGCCTTAGAAAGTTCGGCATGCCCCGATGGATGTCTTTTCATGAA
GTGAGACGTACTAATAGCAGCCGAAACACCGTTGAATGCTCCAGCATCAAT
CAACTTCGCCTTACCACCCCCACCCTCCTCAGCCGGAGTCCCGAGTAAGCG
GAGTCGCCCGGGACGACCGCTATGTTTCAAAGCCGCCGCGGCTCCGAGAA
AAGCGGCGATAGATGAGGTCGCAATTAGATTATGACCGCATGAATGTCCAAT
CTGTGGTAAGGCATCATACTCAGCACAGATGACTACGAGACGTCCTCCAGA
GCCGATTTCGGCCTCAAAGCTTGTCTCCAGTCCATAGGTGCTGCGCTTCAC
GCTGAAGCCTTGGTCTTCGAGAAACTTCGTGATGGTAGCGTGTGCGAACAC
TTCTTCATAGGCGGTCTCTGGATGTTCGTGTAGTGCTTGGTTGATGCCTGTC
CTCAGATCTCGATTGGCGCCATCAATCGTTGAATTGACAATTGATCGCAAGC
CTTCTATATCAACAGCCCCCATGTTCTCTTTTCTCTGCCAAAGCCTTCACTTA
GTGTATTGCAATATAGGTAATCTGAATATCGTCATTAACTTGCCAATTGAACT
TCGTATTTGTAGATTATTCGAGACGGCTTTGGTCCGACGCTCAACGCTAAAC
TGATAGCATGAAACCGGTAGCATGACAGCCCTAGATCTTGCAGTGGATAGT
GCGGGGTATTATCCACCCCATGCTACACGATCGAGTATAAATCATCGCCATC
GCCACAGTCGCGATCGTACATGGGGTAGGTTTTGGGAAAGATTGATGGTTT
CACAAGAATATAGGGGCAATAGAGAGAGCGTTATAGCTGGTTCCATGAAGG
TTTTCGAATATATACAAATATAGATCCCTTCAACCGATTATATACAGTCAAAT
CTTCCTATAGTATTTTTATGCTTCCTTATGTCCATTCAAATTTTGAATATCCCG
AGTCTTTATCCTATCGTTTTTAACATACACCTGGTCCTGCCTTTAGAGCATCT
GCGACACCTTGAGCGACGACGTCGTGGATAGGGTAAGTCGGATGCAAGTT
GTTGAGCCAGAAGTATTCGTTTACGGGAACTCCGCAGCTCGCAGTAAGGGT
ATCTTCGTTCGGCGTGCCACTGCGATTATTAGTACTCTATCTAGATTTTCATA
TCATCATACTCACTTTTGATAAGCAGTGCAGTAGCTAGTCGTGTTCTTAAGA
CCAGAAGTCTGCTCATACGTGCTTGGCTTGTCCAAAACTTCCCCGAAAACA

TTGTTCGAGTCGTAGACCCAGACATTGACCTCAGCCGTGTGGTTCTTCTTG
AGCGCGGCCGCCCGTGTCCGATACCCTCTTATTCCAATCCGCGATAGCGGCT
TTCTCCAAGTCTACTGAAGCCTTCCCTTGGCCCACAGTTAAGGGACTTCGA
TCTACTGGGGGAACGTTAATGAATACGAAATTCCTCCCGCCACTGCTGTAC
ATCTTATTAACCAATTCGGAGTAAAGGTCAAATATTTTGGTGTATAGCGGGC
CTGAAGAATCTGGCCCGGCTCCGTATGAGTTGCCAACATCATTGATACCGAT
CCAAATGGCGAAGACGAGTCTTCCCCGGTCCATTTTGGAGCTGATGGAGCT
TTAGGCGAGTCGCCGGTATAACCAGGAACGAATTCGTCCTCTACTTGTTGC
TTGACAGAGAGAACAGTGGGTTGGTATGGCTTAACAAGATCAGAATCGAC
GGTAGCACCTCCGTAAGCGAAGTTGTAAGTCTGTAAATGGACGCATTGTGC
TTGACCGTCAGGAAATCGACCCAATTGGGGCCATTTGATGATGTATATCCGG
GGTATGCTGGATTTCCAAGTGGCTTGGCCTCTGAAGGCGCTGCCTGTGTGT
AATTGAAGCCTATGATAAACTGTAAGTTGATTTGTCTCGATTTTAGTGATGT
CTCATTCGTGTCAAAGTACCTGTCGCCGTATAGCTGTCTCCACTAGGCATAA
GTTAGTACATGCTGTGCGTAAATTTCTTAGTGGGACTAACAAGATAAAGGC
GTGCTTAATATCCTTCCACGCTGTCCATTGAGCCGTCGCTGCGCATGACCCT
TTTGGCGCCGAAGGTAGCGGACAGGCTCCAACCACGGCGCATGTCAATGC
TACTGCGGTCAAGTAAGTCATCATCGCTCAGACGCTATTTTGAAACTGCGG
AACTGCTGCGGAACAATTTCAATTGATTTTTCGGGTATCTCAACTCAAAGA
GTCCGGACTTCGACTTCGCACCGGTGTATTGGTGATAAACTTGGAACAGGC
CTTACCGAGACCAAAACAAAGCGATACGGAGCCCAGGAAAGCAATGTAAA
GCCTCGAGCTGGTATTTATCATGAAATCGGGGTTTAAATTCATGGGGGCCGT
AATATATATCGGCGACATTGACTGCTTCTTTGTGGTAGCTGAATACGGTGGC
TTTTCACAGCCGGCATGCAACCCTCCATTGGGGTGGAGCAGTTTTTTTGCA
GCTCATCTCAGCATGGCGTGGTTCAGATTGGGAAGCGAGGATTGGCTAATA
GACGAGTTGTGCGCTTTTTCACATCCTTACAACTCAATATCGTAGGGCCAGC
TTAATGCTTCCAAAATAAGACTTCACAATGTTGGAGAGGACGGCCATCGAG
CCCCGTACTTCTCCATCCAATGGTCTTATTCTAATGCACACTCCGCGTTCTAG
CCCAATTGCAGGCGAATTGTCCCTCATTCGAAGTCGCCCATTAACTCTAGGC
GGAGGCAGGAGTTCCGAACTAGAATGGGATGGGTACTCAGGAGGTACTCT
AAGGCGCACACATTGGAGTTGCGCTGCTACGCCGGCGGCAATCTTATCCCA
GCTTCACGTGTTAGAAGCTGGCATTTGCCTAGAGTAAGAAAGTAAGTATTG
TCTCGGGCGATTTTTCCGTGTAAAAATAGAAATTTACATTGGCTTATTTAGCT
GCCTAGAGTACGCGATATGTCAATTATACGACAGGATTCAATAGCGGAATAC
ACGAGCCACGCTCAGCCGTATGCACGTAATCAACAACACTACTAGGTATGG
AGATCACCATCCTCGATAGCCTTTGACGTCATATATATCTGACAGCTGTGTAA
CAGGTAGCGTGGTTCCACCTGAATTGAGTACGGGTAGGTCGAATAGGAATA
TACCTCGTATATTCCAACCTTTTCGGACTTCGCTAGTTCTGTCAAGGAGAAG

GAATTGTAAATAATGGTTTGTTTTTGAATGCAACTCATCGATTCACCTTTCTG
TTTCACCCCCAAATCGATCTGTTTAGATAAGTCAGTCTAGGAGATGACATTC
GAATACAAGGTTGTTAGATTTACCGGTTGTCATTTGGGTCGTTGAAGAAGG
GGTTGGCGATAGAGCTCATCAGTCAACTGAGAGATCCTATCATCGGTGCTT
GATAGGACCACGGATGTGAATCTATCCAGCATGAATTTCACCAGAGGTGAA
ATGCTAGTTCCATCTCCCAAATCTCTAAAGCCGTTGAAAAACTCGATTCGAC
ACCGGAACTCCTCGAATATGTAGCGATTATGGCAAATCACATTCACACACTC
TACCAGCTTTGCCCAATTTTTCCATACAGATCCCCATCGGCGTTTCACTATG
AACTCGGCAGTACAACGAATAAGCCTGAGGGGCGGTGAGAATGGTTTTCT
GTTATGGAGGACCCTGTAGGCCATGTACCGTTGCCATTGTCGATAGGTTTTT
AGCTTGTCTCGCCGGATTGCAGCCCTAGTGTCCCTGTTTTCAAGGAGGCGT
TTCTCGAAGATTATCCGTTCAGCGACAGCATTCCACTGTCTGTTGACAGAC
CGAAGTCGAGCTGCCCATTTGTAATCTTCATCCCCTATTACGTTAAGTAGGA
TCTTGTTGAGAATCTCGTATGGGAGATGCATTTCAAGTTCCTATATACTCTTA
TAATATCAGCATTGCACGATGATATTTGCTATAGTATCAATAATATGAACCTG
CTTACTGATAGGTAGGCAAATGCCTACGGAAAGCTGATCCAAGACACGATA
GCGATCAAGTCTGGGGCATGAGGTAAGTAGTCAACGTAGTGATTCAAGATC
TTCCTATGTGTCTTATCTCAACCTAGGTAGGTACCTACCTACCTAAACAATCT
AGCGGTAGGTAATTTTTGAACGTCGAAACAATTGAACATCAGCCATTGTCG
ATATGTAGTGCCTAGGCAATATGATGCCATATTCTTATTGGTATCTGACGCCT
GGTAGGGCTTATATGAGTATGCTATGACACGCATATTAATCAGCCTTTAGTTG
AAAACTTTATGAGAGCTCTGAGTTAATTGTAAGTGGTTGTGTGGTGAGTTG
AAAGTATGGCATCTCAAGAGACCTAAACACGTTATTTTGAGGTCCTACATTT
AACAAACTTGAAGCATCACAACTCAACTAAGAAATGACAGCATCCAAACT
AGGTAACCACGACAAAGCGAATGTGTAATAGAACACGTGCGAGTACTAATA
GCTTAGCTGGAGACTGTGCCATTAATAATTCGAATCAAGGGAGACTGGGCA
TACTAAGGGGATTTGGTTGAGCCTACAACAAAGTAAAATATTTCAAGTAAC
CTTAGCCTTGCGTCTAGGGAGCAACTAATCTGACAGAACTTCGAAGAACCA
AACTCCACGCGAATATTTACTTGTGCTTGCCTAGTTAAATTGACCGATAGCT
GAAATTCTATTCAGTTTTGACAGCCACGTGAATATTCCGAAGTAGTCAACGC
CATGGAGAGTAATAAACCTACGCTTCATTTTATGCAGGTCAGTGCAATCTTT
TAGGCTACTGTGTGTACCTTAGGATATCGAATGCCATAATAAAAACTAACCT
CGATAATAGAACTCGGGTTCTCAGCAGAGTCTATGGTTGCTCGAGGAAATT
AGGTATAGAATACGACCTAGTGCTACATGAGCGCCCAGGCGGACGAGCCCC
AGCGGCCCTCAAAGACACGCACCCTCTCGGAAAGGCGCCACAGTTAGTGA
CGGCCTCAGGTCGCGTCATTGCGGAACGGAGCGCGATCGCATATTATCTGA
TCGAGACTTATGATAGTGATGGCCAGTTCAAAGTCCCTGCGGCGGGTACTC
CCGGTTGGGATAGTGTCAACAATGACAAGATCCGTGAAGAGCAACTTTTCT

CAATAAGTCAAACAGCACTAAATCAATTGTTCGCGCTCAAGACGACATTTA
GCGCATTCGCATATGCGACTCCGTTTTTCATCCGGCCTTTTGTCTTAGGCCT
CAAACTGACACTGGAAAAAGCGTTTCTTGATGCGGAAATCGACAATGTGTT
AAAATTCCTAGATGGTGAGCTCGAAGGTCGGGAATATATCAACGGCACGGC
GCGACCGACTCGGCTTGATTTCATTCTACAATGGTACGTGGATTTCGGCGGT
TCAAAGCAGCGACCTCGACCTAGACAATATCCGAGAGTGAAGGGATGGTAT
GACAGGTGTATGTCGCGCGACGCTTGGAAGAGGGCAATGGAGAAGGGAA
ACGGTTACGACCTGGCTTTTTGGAAGAAATGATGGCGAAAGGACGAAGTT
GAAGAGTTGGAGTGCTGTGGTCGAGGAAGTTTAGTGGCCAAGACACTAAA
ACTGGAGGTGATCGGGACTGGGGCTACCTAGCTATCAGCGTGTGGATTAGA
GTTGCCATCCAGTTGAATTCCCTACAATCAGCGCTCTTAGTGCGCCAATAGC
ACTCATTTAATCTCTCCGATCCCGAGTGTTGAGGTATCTTCTTCAGGACAAT
ATCAAGTTACACACATGAAATACCTGGAGATCGGAAAAGAGGTGAAGTAG
CAGTATTAACCGAATTATTGGCTTAGATCAAACATTGAAGTCCTGACCGACA
TCACAATGGAGGGATAAGATCAGATGCTTCAGCGGATTTCAATGTCTTCATA
GCCACAACGCCGAGACTACCTCAATCTACCTGTAAAAGTGCCTACGGCGCC
TTTTGATTACAGATAAACGTAGCTCATGACGTATCCGTACATGAACATTAAA
ATTTATAGTCGCATACGTGGTAGGATCCGTCATTCGATTGATCTCCTTTTTTC
TCCCAAAAAGATTGTTAAATTGTACTACACACCCAGTCTATTCCTATGATGA
AAACCTGTTCCGCTCTTGGTACACTTATCTCCGCTAATTCATTCCGCGTGGT
GATTTGGAATACCTGCGTAGTTTGCTAGGAGGTACCTAATGTAAATAGCATT
GATAGAGAATGTAGGGGGTTTAGTGCTTTATATCGCTATAATTATGCCAAGCC
ATAGCATATACATACAGCATATACCTTCCTAGACCGTACGCTAGCAAGTTGC
AACGTTTAACTCCTAGTAAACGCCGAAGTCGAGTATGCAGTCTAGGATGCT
ATCTATGGATATATGTACTATGTAGGTAGGGTTGTATTATAATAGTTTTATATTT
GGAGTGGTGGACAATTGTAAAATAGATCTTGCAAACGGAGCACCGCCCGTA
GCCAATATGAGAGGTGCCAATTTTCCGGGAGACACGAGAAACACGAGAGG
TTTTCTCATACTGACGTCAACCTAGCGAGAAACGGAAGACACTGTGGTTTG
CTAGAGCCAAAACCTGATCTCATAAGCGATCAATCACTATCCCAACGCGGG
GGAGTCCCGTGCGTACGGTCCCCTATGTGTTCCTTAACAGATAGACCCAAA
TATCCAGTACCACGGTACGAACGTCTCTAACCGAGACAGATCCTGCTCAGG
GGAATTCTGGTCATCCACGAGCAAGTGAAAGAACTTGTAGCAATGATCAAC
TGTCCCTCCATAAAGCAACGAATAGCAATGGCCGGTATGTCCTCCGACGTA
ACCCTTAACGCGTTAGACTGCTTAATAATAGCTGCTACTCTATGTCAGTTGG
CCCCTATGGATTTACAGCAAAGAGTGTCGTCATACTATATTTGTAGTTCGAC
GACCAATTTTATCCCACGTAGATAGGGGCTACCTACATAGCGTGCGACAATG
AATTACCGTGTTCGTAAAACCTCCATGCGAATCAACTTGTCCATCTAGATTT
ATCCATGTGTATGTGTAAATTCCATGCTTGCCACTCCGCTCGTCGCTGATTG

CCATTCGGGTACTTAAAAAGGGGGGCATAAGCGTAGAAATATTTCATAGAA
ACGCGTCCAAACCAACTCCCCAGCAGGATTTCTGAGACTCAAATTGCGAC
CAATTCGTATTATTCAGCATAGTTTCTTACACTCATCAATCCACGATGCAAGC
TCAAGGTTCACAAGATGTCACTGGCACCAACTACGTGGTAAGCCCAGTGG
AATTGGAGAAGCCAAATGTCCAAGGCATCCCAGAGATACCAGACATGCAG
GCACCTGCGCCTTATCAACTTCTCACACCAGCTAGCATAGTACGTGAACTAT
TCCCACGAATGATTTTAAGGACGACCTTACTAACACATTAAGGCCGAGGAG
CTACTCAACGACTTCAATGAATCTGACTTCTCTGACATGAGTAAGCCCTATG
GATTCCCCCCTCCCCGTTTTCCGCCATCTCTTACTAAGTAGAGTTGAGACTC
AGAGCTTAGAGAGGGGTGGTATTTACTAAATACAACCTCAAAAAAATGCCC
CGGATAAATTCAGTCTGTTGACTATTGCAGCCTACAATTTGCAAGGGCAGA
ATGGTCAGAGTTCACTAGATCCCATCCCCCTTGACAACACCATCACCTCCTC
CACCCCCGCCGCCATCCCCAGCACTTCCCAACCCCAATCCCAATCCAAGTG
CAAGAAAGAGAGGAACCGAAAAGCGGCCCACAAATGTCGACAGAAATCT
AAGGCGAACATCGAGCAACTCAAGAAGAAAGAGCAGGAGCTATCACAGA
GGAATCAAGAGCTGAAAGACACTGCCTATGGCCTTCGGAATGAAGTCCTGT
GCCTCAAGAACGAGGTTCTAATGCATGGTGCTACCTGCGATAATGGACCGA
TACAAAGCTATCTCTCCAGAGTTGTCAACGGCCTAACCAACAGGGAGCCTA
CCTGATTATAATCTCCTTATGCTGTTTCGATCGTCCTACTTATAGCAGGGGCT
GTCTTCCTTTTGTGGAACCTCAGCGAGATTTCCTTGAGCGTCGCGCAAGAC
ATGCCAGGTCCTTAAGATTGTCACAGTTTTATGTTTGGGACTTTTGATTATTT
TCGCGAAATTGGAATTGGGAGAGGAGGAACGCTTCATAACCCATATCAAAA
TTCAGTCTTATTGTCACCCATCTTCAGATCACCCTTGATTAGGTATGCATCTA
CCCCAACCTCCCATACCTACCAAGGTACGTTACCTCAAGCTAGTCGGCAATT
TGAGACTGATTTGCTAGAGACGCCAACACAATATCCTTCACCAGCTCGCAA
TTCAGTACTGCGACCCCTCTATTCACAGCTGGAATGGACTTCCAGGCATATA
GTTGCTTGGTTGTAATATGGTTATCGCCAGCAGAATTGTTACAGTAAACCCC
TATCTTTGCATGCCTGGACTTCTTTCAGGTCGGGAGCAGCTGCGAGGTTATG
CTCTCTAGGAAGGTTCAAGACGTAGATGGATAGACTGCCGGTTCCGGTCCT
AGCAGTACTAGCACCGCTGCCGCCACCATTGTCACCATCGCTACTAGGTATG
CCGGCAAGACCCTAGCCTCCGTGATTCTATCTTGAAGCATCAGTATTGTTGC
TGTTGACAGGTGCTACGAGTTTATACCACCTCTTGGGGGTTTAACGTACGA
CATTTTGAACATGAATTCTAGGGGGAGAAGAATATAATGTGGATACTTACGA
AGTGTAGGAATACTGTCCGGAAGACAAGAGGTTGCCACAAAGGTCGATAA
TATTGCAATTGCAAGACATGAATAATAGGATATTGTTAATGTAGCTACGGCGT
TGAGCTACGTAGAGGTACCTAGGTACATAAGAAATAACAGAAGTAGCCATG
GACTTCAACTCGTCACCGACCCTCTTAACCACAGTGCCAACAGTCAATAAG
TCCGTCAGGCAACAGTAAAACAAAGGTTAAATAGCAAAGTATTGCACCAG

ACTTCCATCGGCGGATCCTAACTGCTTGACCGTACCATTCGGATGGTCAAGT
GGTGTGTGTTTCCACAGACACCTTCTCGTTCCTGTTTGACAACTGCTCTGA
AACGGGTGGTTTAGGGTTCGAGTCCCAAAGAGTGCATGCGCATTCGCACC
ATGGGTCTTTGAAAAAGGGTTGGCAAAGAAATACCCAATAGCGGGGCGCA
GTAGCTAGGAGCCGATCAACAGATCCTAGGCTGGCCTTTTAAAAGTTGTTG
ATTCAACTTTTTGTTGTTTGCCATTTCTTTTGAATCGTGATACCCATCGATGA
TGGAAGCATTCGCCATACAACAAACACATACTAATTATTCTCAATTGACGAA
ACTTGACGAGATTCAACTCGGAGAATTCTATTCGGCTGATTGCACTTCCAA
CCGCAATTTCGCGTCTCCTTTATTGTCTCCGAATATCTTCTACATCTCGTCGA
TGGATTTGAATTCCCCCCACTTTATTATATATATCCTCGCTTGACTTATCTAAA
CCAAGAACGCAGCAATTTGTAGATGCGAGTGAATATCTTCCGCGGACAGTC
GTTCGCAGATTCAAGCCCGATGATACAGGTGTCCTTCGATATGACTATCAGC
ACTTGTCACTGCTAGAAGTTCGCCTCTACCAATATAAGATGTCGTTCATACC
TAGATAATCATTTACACGTAATGTAGACACCCCATGCTAACATGCGCCCTTTA
TAGGACTATGAACTTCGCAAAACAAGCCGCACTGGTAACAAAAGCCTGCC
GTCGATTATTTCAATCAAACAGTTCAGCGTCAGGGTTCAATTGATCCATTCG
CCGCCTCTGGTCCTCGTCCCAGCTCCTCTCCAGCTGCAGCAACTCCATCCT
ACATTGCTCCTTATCGAGGTGATCTGGCCACCCATGACGAAGATACGTATTA
TAAACGTCCCCAGCATGTTTTGCTGAATCGTCATCTGGAAGATGACATGGCT
CTAACAGGTCTTGCTGCAGAAGTCTGTTTTCCTCAGTTTTTGATCGGGCGC
GGCTATAAAATCTACCCGTTTTGCGGCTGGCCAATTGGGTTTGGAAGTACC
GTCCAAGCCAATTTCTCATACTTCCGTCTCCAGGTGTCCAGCAATTCCGCAG
CAGGTGTTGTGCGATGGGCCTTCCATTTCTCTGATTCGGGAAGAGCTTCGT
ATTCCTCGTGAGGAAGTACGATGTTAGTTCCAACGTGGGTATACTCAGTTAT
GGTGCCCTCGTTAGTATCAAGAATCAACGCTGTTCCTTCTCGATCGAGACTT
CGTGTCAGATAGACGCAATGGGCTGGAAGGGGTATATCTCTTCTGCGATATT
ATAGTTTTCAGGGTCGCAGTAACAGATCGGAATTGTCTCCCAGTGAATCAG
AAGTCGTTTGTACGGATCCTCGATGTGGAGGTATGGGAGGTGACGAAGTAA
GTCGAGGGACAGCTTCGTTCTTCCCTTCGATACTAATGGTATCCCAACCGTT
CGGAGGTGGTAGAATGAGCACCTCATCGTCCATATACGGGTGCCGTATGAC
TTCTTCATATAATCTCAACACGGCGCTGACGATGCCATCACGGGATACGTCA
TAATCAAGGTTAGACTGGTGAAATAGCTGTGGTCTTGGCTCAGGGGTTGCC
GAGCTTGGGTAATGGCGGATGGCGGGGAGCCCTGGTCCGCCCCACCCCCC
ACTCCTCCCCAAGATCGAATATCCGTTTACGTGATCGCGACCTCGACAGTG
GATTCCTTGATGAGATCAACCTCGGATTAAGGTAGTACCGAGAAGGTGGGT
AGCGTTTCTGACATATCTCAGGAGTGTTATAGGTGGATGTTATTAAGCTAAG
ACCAAGCACCCAAGACCAAGCCAATAGAAACACTATCCGTATCCACCATCT
TTGAAAGCGCACTTTGAAGTTGCATCACTTACACAACAACCGGCCAGCATA

CGATGATATATGATATATACCTAGGTGTGGTATACCATGGCTAGACTTTGCGA
TCATGTCCCCCAATGACAGAGCATTCTGGACTCTTATCATCCTCGAATCAGC
CTTCAAATCTTGCCCATTTTACCCCACTTCATTTCTTAGGTAGGTATATCGCC
AGGTTCGCTGAATGGTAGACGGTGATCTATCGGTTTTGGAGACAGCGAATG
AAATATAGGGATATTTACGTACCACATCACTCACACCTATTGACTCGATAAC
AACTCACCTGGTCTAGTGTACCACATCCGACACACACAGTGCTACTCTTCA
CTAATCAGAATCCGGTTTATTGTGGGTTTCGGTGGGTTATTAAGCAGGGTGG
TATGTTTTCGGACTGGTCTATACCAGGTAGGTAGATCTAGGAGATTGGCGAG
ATGGAAGGATATAAATACAGGCAATATCGCGGAATTGCGGTCAATACATAGT
CTATGTGCTACCAACACCACTGCGCGAGCATACAGCCTCATGTCCAACCAG
ACGGATCACTTGGGCGAGAGAAACCAAATCTGACCCCTTTAATTCCAACAC
ATGGTGTAGCTATCTCTTCACCATGGATTACGCGGAATTGTTAACGATTCGC
CACTTCATCGCAGCCCTGTCTGCTTCTGCGACAATATGGGTTGTATATAAAC
TGCTTGAGATGGTGTACAACCTATCCCCGCTTCATCCCCTGAGCCACATACC
GGGGCCCTGGCTCGCGAGAGGGACATATCTGCCAGAATTCTACTACGACTT
GATCAAGTATGGCAAATATACAAAACAGATCCAGAAAATGCATGAGAAATA
TGGTAATTCCCTGTGTGAAGACCAATGTATTATATGTACTCCGCTAACATTTG
GTACTAGGACCTATCATTCGCATCAGTCCCGATGAGGTACATTGTAGTGACA
TAAACTTCGTCGACGAGATATATGCCGTTGGCAATCGTAAGCGGGATAAGC
CACTGCACCTGATCAGCGGCTCAGCGCACACTGAATCCGGCTTTGGCACTA
TAGACCACAACTTGCACAAGCTACGTCGAGGACCCGTAGCCAAGTTCTTCG
CTCGTGGGATGATCTCGCGGTTGGAGGGTGACATCCACAACTTGACCCAGG
CTCTTTGCGACAAGTTGTTACGCTGTGGCGATAGTTCTATCGATGTTGTCGT
TGGCATACAGCTGTTTTTCTTCAGATGTTATCTCATCTTACTGTTTTGGCGAG
AGCTTCGGCTTTCTAGACCAAGAAGGATGGTACCCTAACTTTCACGTTCCC
GAGATGGCCATACTCAAGCCTACATTCGCCCTTCGCTTTTTCCCTATTCTGA
GGCATCTATTCAATCTTGGTGATTAGTTAGTTACCCCCTTGCTCACAAATTG
ACTCCCAGCTAGATTTTCTAACAGGGAAAAAAGCTTAATAGACTATCTTCCT
GCTGACTTTGCGCTACTCATACGAACTCTGAGAGTCGAGATGCCCAAAAAG
GTCAAGGCCATGGAAGCTGACCTTAGCCAAGGAATAACAACAGAGAAGCC
CACCATATTTAGCCACCTACTGCAATTGTCACCTGAGCAAAGGCAGCCACT
GAATTTCGGCGACGAGGCAGCGGGTCTTCTCGGTGCAGGAACAGTAACTG
CGAGCTGGGCTCTTTCCATCATCACGTTCCACTTGCTCACGAAGCCCGGAA
TGCTGGCAAAATTGACGAAGGAACTAGAAGAAGCTATTGATGATCCTCGAC
ATCTACCAGCCTGGAACATCCTGGAGACTCTACCTTACCTTTACGGGGTTGT
TCAAGAAGGATTGCGTCTCTCCTATGGTAACTCGATTGTTTTCCTATGTTTCC
CTGAACAATGAGCCAGGGTTGCTGATACAGTGTACAATAGGTGTTTCCGCC
CGTTCACCGCGAGTTGCAACTGAGGAAAACTTGGTATACCGCGGTGAATG

GAACAAGAAGCCGGTGGAGTACGTCATCCCCAAAGGCTACGCTATCGGGA
TGTCGTGTGTTATATCCCACCATAACGAAGACATATTCCCCGACTCTCACGC
CTTTATTCCAGAACGTTGGGCGGACGTCGAAAATAGAAAGAATCTCGACCG
TGGCATGTTCGCATTCTCGAAAGGCGGCAGGTCTTGCCTAGGAATGAAGTA
AGCTGCTATCTCTTTATCCGTACCCTACGTGACATAGCTAACGGAACATCCA
AAGCCTCGCATTATGCGAAATGTATGTATCTCTTGCGGCGCTTACACTACGC
GTTTTCCCACAAGCGCAACTCTTTGAGACAACAGAGGAGGATATAATCTAC
GACCATGATATGTTTATTCCATGTCCCAAGGCTGGAAGTAAAGGGGTTCGC
ATTGCTATATCAGCCAAGTAAATGATCTTATTGACCCGGCTTCCATTTGTACT
ATCCTATGTATGGCTTACCTGGAACGGAGTACATGAGGTAAAAGGATGTGG
AACAACCGCGTGGATCGACCAAAAAGCAACCGTACAACAAAGGGGGCAA
AAATAATGTGAACATCTTTTGATAATTGTGTGAGGTCTTTATTAATATTGATA
CGCTCCATAATGCCGCTTCGCGAATTTTGCCATGAAATCTGGCACTTTTCAT
TGATCTTGAAGTATGGAATAAGCTCCCGAGATCTTGATACCTAAACACTAAA
CATCCAAGCAATAAACACCTACGAAGTACTCACCTAGTACCTACCTACATCT
CATCACACGAGAAATACCTAAAACGTAGCACCCTGCTGCTCCTCATCAAAC
TCAAACCAATCATCCACCTCCTCGCGGTAGTTAACGCTCATAAAGAAGAAC
GAGCCTTTCTTGATAGGGCCATATCAAGACAGCTATCGGTCTTCAGGGTAGC
ATATCGGGTCTCTGCCTGACAAACCTCACACATCGTGCCTGAGGAGGAGGG
GCTTTAATGATGAACTCACCTGGAGACCATTTCGAGATGTTAGTGCCGAAA
TATGTGTCTAACTCTCCCAGACTAGGTCTTCTGCGACAGGCCTCTTCGTCGA
GTTGCCGCGCCGCCTCTCTCTGCGCCTCGCGCACAGCTGCCTTCTCGCCCG
CGACTCCTCTGTTATAGCGCGTCATTATTGATAGGTAGATGAGTTCTCGCTG
ACTAGCCTGGATAGATAAGCAGGTAATTTTCAAGTCACCGTGGTTCTCTTTT
GACGTTGGGTATCATTGAAATTCCTCCACTCAATGGAAGTTGTCTTCGTCTA
TTTGGTACGACCGATGGAGGCTTTTTCAGGTAATCGGAAATAATGAAGAAA
TACTACATTTAAATACGTTTCATGCACCTTAACATTCAACAAACAGCATTGT
GTGAGTTGAAAGAACATCAATCTATTGTCCCTCAACGCTAGTATGGTTATAA
AGGCTCTTGTTCCGGATTTTTCATTGAAGGGCTAAACTCTTCTCAGGTGCCT
GTCCAGGTATGTACTAGATCGGCATGAAAAGGGGAACATGGGAAAAGGGA
ACATGGGAAAAGGAAATATGTTTGAAGATTTTCAATCAGTATGAACAGCAA
CAGGCACGTCGATAGCAGACACTTTGATCACATAATCTTAAGACTTGATCAT
GGTTAGAAGCTATTTTCATATTCGTATATATGATCGATAGATATGGAATAAGAT
AAAGGAATCGTATAAGGTATATGTGATGAAGTAGATTCATTAGATCATCATG
ACGTTTCGCCTCAGGAAATCGATATCCCCAAGGCGAGAGTTGGACCACAAC
CAAAATAATAGATCAAGATCAATGATGAATAAGATCACCAAAGCCAATAAC
CCAGTCTCCAAATAGAGGACTACCCTTTAGCCAAATTGTGCGTAATGAACT
CCCCTTGACCTCCGCCAACACCAAAAGTTCGCCAGAAAGATTGTGCAGAA

CGCCGCGCACCAATCTATAACATGGACTCGTGAATACCTTGTTTTAGTATAA
TCGTAATATGGGCAATTCAATAAATCATTTCACATCGTCACTGGTTCCTAAC
ATCGTCGCTATCGGTAGCCCGGTGAACAGGGCGAGGCAATTAGGTGAGGGT
TGCTAGCATAGCCCTCACGAGACCAGGTGGGGGTCCTGTCAGAAATTGATT
GTTAGCCGAGGCTCCTATCCAGAAAATACTGTGGTTCAAGGCGAGGAAGC
GCACCTTCACCTAGCGATACTTAGGCACGCTGATGGACATCCTACCTTTCTT
GTCCTTCTCGATGCGCACCCCGCGCCTTGGCTCCTCAATCCTCTCTTCCTGC
GGAAGCTGGTCAGCGAATCTGACTCCCGGCATCTTGCGGTACATGCGCACT
GCTGGAGTCACCGGTCAGCAGGAATAACTGCGTCCGAGGCGACTACGCCC
CGAATGCCACAGCGGGCGAAAGTGAGAGTCGCACTCGATCAGCAACACAT
ACCACAAAGTCGCAGGTCACGCCAACTTGTTCTCTATGGTCCTCGTCATTT
GTGAGGCACCTAGATGGACCGCGCCCGCTCCACAAATCAGCAACACTGCG
CCAGTAGAGCGCTGCCGAGACGCTGCCGACTCGGGCGGGCATGCGCGTCG
GGCGGTGACCTGATAAGCAGATAAACGCGAGGTGACACGACGACCGAAAA
TGCCGACGACTCGCGAGTATATAGAGCCCGCATGTTGGCCTCCATCATCTTC
ACGACTGCTACTCTCTACTCTCCCCGTTACTCTTGCTCCCGGGGCAGTCTCC
AGGTCAAGCAACCCTCGATACACTGCAGCCCGCCGTTCTATCTGGACATCG
CCTCGTCATGAAAGCCTTTCTCCTCCTTGCTCTCCTCGCTGGCATCGTCGCC
TCTCCCGGCCCCGCCATATACTCCAAGTGAGCTCGAGCTTCGTCTCTTGGTT
GTCTCCTATCCGCATGGTCACGATACTGAATCTCAGGCGTGACCTATTTTGC
GATCCCGAAGACCCCTCTCCCTGCCCGGGGATTGCGAATGCACCGTGTTGC
CACTCCTCGGTGTCGGAGTTTGCTTTGAACCTGGACCGGGACCTCCTGGAC
TGCATTCTGGAACCGCCGTGCGTCACTCTTGCCTCCAACAGCGTAGAGAAG
GTACTTATGACCAGTCCCACCGCAAGTGCCAGTACCACCAGTACCGCGCTC
GGCGTTGGCGCGACCGCTTCGGTCTCGGTGGCTTGAGCCCCATTGCTTTGC
TCCTGCTTTTGTAGCGTCTGCCATTTCTTGATAGTCGTCCGGTTTGACCCGT
CTATAGTCCTCAAGAAGTTTCATATGCCTCGACATGCATGTGCATCACCTCC
CCGCCCTCCTTGACTTCGTGAACAAGGCAAATACACTCGCTATTGCGATAC
ACTAGTGGGCCAGGCCTACATTGAGGTGTAGACGTTCCACGCTCAAGTAGG
TTTCACCTGATGTGCCGAATGAAGGCCAGCCATGCAGAATCCCTCGTATACT
GCCGTGCCGCGGCCTTGCGGAGTGAAGAGGCCCTGAACGTGGACCTCAGT
CCCGCAATGGGTACAGGTGTCTCCCATCTTTCCGGTGAGGTCGTAGCCACC
TGTGGGATGAGGTGACGACCGACCACTTCCGGTACCATGAGGGGAGCATG
TCCTCTGTGTGCGAAACGCACAAGTTGTCGTACTGCTGCTTGACCCTTCCC
CATGCCAATCAATCCACGCACCGCTTTCGCCCATGCGAGCCGATGTGCGGA
AGGGGCCGATCTATGTTTGCCTTGCCCCACGTTGGTCACCAGACCACTCCG
CCTGCTGCCGAGGCCAAGGCCTTCGTCTGTGTGGCCGAGACCCCGAACGT
GTGCTCATTCAGTCCACCTACCCAGCAGGCTCCACCTACCAGCAGGCATGG

TAAGATGTCAGATATCCTCCGCAATGCGCACCGCTCCGCTCACACTCCTCGT
TTTCGCAGCCGGGTCAGGCCTTGTTGCGCCGGGGTCCACTATCATGTGAGT
TGGTCAGAATCCCGGACTTGAGACGCCTGACCAGTGCAGCATTCGCAATAA
TGACTGCAACCCTTTCCAGGATACCGACACATGTGGCCCAGGCTGCAACTG
TGTACTGGTCCCACTGGGCGTTTCAAATGTGTATGGGGTGTGCGTGACGCC
CGAGAACAACCCGTGCGGTTCCAGCGCGACGTGAGTGCTCCACCTCTCCA
TGACCGCGCATGCCCCTGACACCACGGCCAGCCTGAATACGACAGAGGCA
TTCCTCACGACGTGTATGTGGAGATTCGGGGTTGATGGGTCTAGCGTGACA
AGCTAAGGGACGTCATTCAGCCATTCATTGAAATAGATGCGTGCAGTCTACT
TTGCCTCATGAGGTGAAGCACATCTCGTTCCCGTACTCTCAGGAGCCTTTG
GTTTCAATAATGTCCCCTTCAGATCCTTCTCGCCGGAGTTGATCTGACTGCT
GAGACCGATTCGACCTGTTCCGTGCAAGGTCATGCCCTCTGACCCATTGAT
CCGGTTCGCATTGCCAATATATGTACTGTGAGATTCCCAATTGCTCGGGCGC
GATGTTGCCTCGCCGCGGACGAGGAGGCGATCGGTTCGCGATCGCTGCGCT
GGGCGATAGGGCATCGAAAGGAGTGTCCGCCTTTTATCGACTCAGCTTGTG
GTAAAATCAGGACGGCTCATCCATCAGCTTGCGTACAGTAGTACGGGCGAG
GGGCGGCTCCTTCATTAGCGTTGCACACTGCCCCTCACAGGAGCCCCGTCT
GAGCACGAACTGTATTTCACATCGTCAGCCGGGCTTCGGCCATTTCAATGG
TTGGAAGAATTCGTACTATGAATGTAGTCTCGTACGTTGCAGAGCCAATGAT
CAAGCTATTTCCTCTCAGAGGATCGCCACCGGGCGACGCCCAGCGCTGAGC
GCGCTACGTCCTATCTTGGACAAGAAGTTATTCACTGGCGGAGTGACTTCC
ATTCACTGCATCTCCCGGTCCGGGGGCCGGCGGAAGCCTGCAGTCAGTAGC
TATGTATTTGAAGGCTCTTGTCCGATGTCAGATCCCATCCCCATCCCCATAGC
CCCATTTAGTCTCTCACAGCTCTTGGACCTCCGCCTTCGCGTCACTTCTGAC
TCCATCATGAAGGTCCTCGCCGTACTCGCACTCTTGGCTGCCCTCGCCTCG
GCGACCCCATCGCCGCACGTCAAGTGAGTGCCGCGTGCCGCGTCTGCTTCT
CATCTTCGTGGCTCATGACGCAGACGCGTGTCCGTGTCGACTCTTATCGGC
AAAATACCCTGCTTGACCGATGCAAACTGCTCTGGCGGGTGCAAGTGTCTC
AAGTTCACGACCTACGATGAGTTCACTCAAAAGATTGGGATCTGCTCGGAC
CCCAAGGGTAACAACCCGGCGTGCACCTCTTGCTTGGCCTCTGCCTCGTGA
GTCCTGACGCGATGCTAGTCACCGACTCGGAGTGCTGACCCAACAGGACA
CGCGACCTGTTCGAAGGGGAGGCGGAGGCGTATGATCTGAAGGCTAGGGG
CCAGTCCGATGCGGCCGACTGGTGAGACTTGGACAGCAACCCGTTCAGAT
CCGCCTCTTCCGAGTTTCCGTTTGCTAGTGCTCTCTCTCAGCTCGTAAAGCT
CAAACTGTGCGTAGTCCTCGCGGATCTGATTTAGGGGGGTCTATCACCAGG
CATGAATGTTCATCATCTCCCCGCACTCGCACCTAGTTGCATGACTTGCTCA
GCATCAGTTGGCGTCCTCGATCACCATCACTGTCTGTCTCTTGGCCAGACC
GTGGATCAGCGATACTGGTCTCGAGACTCGTGTACCTTCTGCAAACCAGAT

CTCCCGCACACACCGTGACTATCACGGACAGGTGTCACGCACATATGGTCA
CTCTGCGAGGACACTCCGATTGCAGTGTCGGCGAGATGCCTCTGCGATGTT
CAGCGGCCGAAATCTGCCTCGGTACCGAGGAGAACATGATCTGGGGAAGC
GTCGCAACATGCACTCACAAACACGCTCGCCCGCGGCGGTGCAGGTCCCA
TCAGGCGGCCCATGTGACTCCATGTCGGGGACAAGCGAGGCTTGACGAGG
TGGTTCTTTCACAGCTGGAAATAGAAGAGGCAGGATCCTGCATATGGAGTA
GAAACGGTCACACAAGCCCTTCCTTCTTCTCCCTTGCTTCTCTCTTCTACTT
CTAGGTCTGCAATCCGCGACGTGCAACGGTAAAGCCCAGGCAAGATGAGG
TGCGCCACCTTTCTGATCCTGCTCGCCATGACGGTTTGGGCGATGCCCCAA
GGTTGAAGTGAGTGAGTTCTCATTGACCTCGTTTTGTATCACAGCCCATGG
AGCTCAATGTCAGACTCGATCAAAGAACTGAGCTCCCTATCCTTACTCGCG
GAGACACATGCAACGACGAGCCGTGTCCAGCCAGCTGCGAATGCAAGCTC
CTGGGAAGTAATCTTGGGGGAGCCCAAGTACAGATGCGAATGCCCCGACA
AGCACAATGTAGGTCTGACCCATCCGTCGCCACGAATCACGCTTAAGGAGC
AGCGCGACCAGATTGAGAGCAGCTAAGATGGCTTGAGGGGAGGACCGGAC
CTTCCAAGGGACTGGAGCCCAACCGTCAGGCGGGAGTCATGAAGTGTACT
GCTTCTCGGCTTGGGTATAGCAGTCTTCGTCAAGGGGCATGACATGTATTAC
GAGTCGAACCACCAAACTGCTCGTCGTTGCGGCTCAACAGCTCTCCTCTG
ACGAGTTTGGGTGACTACCTGCTCTGCCTGAGTCACCGATCGCTGGACGCT
TTCGGAAAGAGAGCTGCGAGCCATGAAATTGGCTTCTTGTTTCGAGAAATT
GCCTTTACTGGCTGCTGTGTGCTTCGAGAACCTCGTCAAAGCTCTGGAGCA
ACACCCAATACAGCTTCGGGGCACTATAATTGTCGTTGTTCACAGTCTGAG
AAACGTCGCCCGAGCCCATTCATCTTCGCGCGACGACAAGGCCTATTGCGC
GTGCGCACAGAGAGAGAGCAATCCCTAAAACAGGGAGCTGAAGTCAAAG
TCGAAAGACTGCTCGCCGATGGAGGACTGCAAGAACTCCGGAAACGCAA
GGTGCGTAAAAGTGTCCCCAAGGTCTCCCCATCCCGCCGTGTTGTCCAAAC
TGGCCACTTCAGGCATCTCTGCTTCTTGGCATGTCGAGCGGCAGCTTTGGA
CCGATCCAGCATCTGCCTCAAGACCCTGGCACAAAACTTTCCAGGAACGTC
TTCAAGAGCTTGATGCTCTCCTCTAAGATTGGCTCGACGTCTACTAGGCGAT
ACTCCAACGGTAACAGGTGCGTGATCTTGATAAGGAAGCTGGCGCAGTAG
GCAAAGGAGACGTTTGTGACTTCGGACACATAAGCGATGATCGGCGTTTGA
AAGTGTGTGATCGCGGTCGTGAGCTGGTTCTTGGCTGCATGCACTGCAAGC
ATTAGCGCGTCGTGGTGTGAAGCATGATGATCCCATTCACCAGCCTTGATCA
GGCCGTCTTCCTTCCAGGAGTCACATCAGCAGGCGATTGGATGTTTTCCAG
ATCGGATAGCAGATCTGCAGCTTCCCAAGCCAATAGTGCGTAGTGATGCTG
GCCGTCCCGCTGAAAACACCATCTTCCTCGGACAAGCTGTCTGGTACTGTT
CAACACGACCACTCCCGGCGCACTCCCTCTTGATGACACTCACCTGCCCAT
AATTGCCATTTGTGATGCCAGCGATCCAGAAACGTTTCATACAGTTCTAACT

GCCTTGTCGTGGCGAATGCATCACTTGAGAGACTGGTCTCCCGCCTTTGCA
TTTCCGATTGCATTCGTGCTAGACAGCCATTCAGCCACCAGTCGCTAATTTG
GTCGGGGGATCTCGCTTACAGACAGCGAGGCACAGCTCTAGTTGTGCCACT
ATACGGTAATCTTGGAAGCGGGGTGATGGGATGGCGAGCAGTCGTTGCGCG
TCTATCGTCATCTCGTCATCGTGTATCAATCGAGGTCTCGCGGACGTTATCG
AGTATCTAGGGAGCTTCAATGTCCAGCGTCTAACATGCGTTCGCATTGAGG
GTGACGATGACTTACGCCACGTCGCAGAAGACGATCTGGGCCCATAACCTG
AAGTTGTCGATCCTCATTTTGTCGTATCCAAGCGCAGTCAGATCATCTCCAC
TTCGATGGAGATTGAGCTCATAAGCCATGGCAACGAGGTGACCCGGCGGTC
TGACGGGTTGCAGCCATGGGTGACAAGCATGCACCCCTTAATGTCATCCAT
CGTCGGCAGAGATCCTCCCAGCGTTTTCCGGACATTCTCGACGGCGAGACT
GGCGGCAAATCTGTATGTCGCTGCTAATTCGGCCGCCACACGAGACCCAAC
GGCAATGATTGCCCAGTAGAGGGTCTCGGAACTGGACCTGATGGCTTCCAT
GTCTGTTTCTGCCAATCAAAGTCGATGTTGACGCTGCGACTGCACCGCGCC
CGGAATCTGCAGAGAGATCAGCAAAAGTCTTGATAACCTTCTGGGTTGGAA
AGGAAATTTGCGTGGAGAAGAAGATGGCGTACATATCGAAAGCCGTTTTTT
GCAGATCCTGCGAGGGCTCGCCGCTGTCCCCGCTGCCCATCTGCCATTCGT
TTCCTCTTCCTCGAGTGCGGGGACAGGGCCGATGGCCGAGATAATGACAGA
TTGGTGCGGATGAAATCGGGTGAAGCGGGATGGTCAAGATTCAACGTCTCT
TCGCCCACTGCAATCTCCTCGTCCAAGTCGTCTGCACTCTCCGATGCGGAC
GTGGCAGCTTCATGCTGGACAGGTAGCGTTGACGGATCTGGCGATTGTCCG
ATCAGAGCATCATAGAGATCCCGGTTGTCGTCATCCGTTGCACGTGTCGTTG
CCGCCAGACTGGATCGTGACTCCAGAGCGAACAATCGTTGTTCGAGGTTCT
GGACTTGCTTTTGCAGTGTGTTGATCTGTGTCTCGTGGTCACGCAATCGAC
TGTGACTCTCAGCACACTTGCACCTCCCTCACGTCATTGTTGATACACTCAC
GGGACTGGGACGGGGGCAACAGGGGCTTCAGGTGTCCGACATTCATGCCT
ATCATCACACGATCAGTGTCGCGTCCTCTTGGGAAGTCCGGTCTTTCAGAG
CTTTATGCTGAGGTACGTGCGTCGACTTACTGCGTTTCCTCGCATCTCTGGC
AAGGATGAGCCCGACCTGTGCTTCCCGGCCGACACTTTGTCTTGAGTCTAA
GACACCTCAAGCAGGCGCGGCCACTCAGAGCTGTGCTCCCGGAAGCGGCA
AGGACTGAGATACCTGACTGGGAGTTGGTATGCGGTAGGCTCATGAGAATA
CTGTCTATCTGGCTTGACAGGATAATGACTGAGCTATGGGACATTGTTGATC
ACCCGTATATCTGTTACGTAAGTGCTCCGTGCAGCAACGGGCAGTGGCGGG
GAAGCCACAATGTTTTCTTGCGCAGGAGCACCTTTTTTGTGAGAATTAATC
ACCGGGATTGTCGAGGACAAAGAGGCGGCCACATCCACGTCGGGGAGCTA
GCTTTGCTCCAAGAACACGCCCGGCCGAGACGTTGGAAATCACTCCATCTC
TCATCAAATTCTGCCAAATCCCGAAAACAATGGTGATCCATCTTGAGCCTTA
TGGGAGGCGGTGGATTCATCGCGCTTGGGCCTTGAAAGCAAGTGCATATGC

GCGCTGCATACACCAGGCCTGAAGAATTTGGTCCACAAATAGCAGCGGATA
ACGGAGCCTGACAGTGATACACGACCGACAGCCACTCTCTGACAGTTGCT
AATTGCTATCGGGCAGAACGGTCGCTATCGCCTGTCTCACCACATCACCAA
GAAAGACAGCTCTTCCTCCGTCACGGTGTAAGGCGGACTCAACAACACAT
GATCACCCAAAATCCCATCCGCCTGAGCAGTCAACCTCGCTGCGGGTATGC
AATTGGCTTGACGACCTACCGCTCCTTTCATTCCGGGATAGATGACCGCCCC
CAGTCCGGATGCTCTCCTCCGCTAAAAGGTCGGCCAGAGGGAAATCAGCG
GCAAACGGCTCTTTGGTCTCCTTGCTCTTGACAAATTCAATACCCCAGAAA
AGACCCATGCCCCTGTGAAACCATGAGCAAGCCGAGAGGACCCAATGACA
AATCGGACGCACCGCACGTCTCCCACATTGGGAAGTGGCAGGATCCGCTC
CACCAGCAGCTTCTCGAGTGCCTTGCCGCTTTCGTGGCAGCGATCTATCCT
ATTCCCTGTCAGTCCAAGCTGCCTCCCTTGCACGTCCGGATAGACTGACAG
ACTGTGCTGATTGATAAACTTGTAACACTCCAGCGCCGCTCGGCATCCCATC
GCACTGGACTGGAAGGTGTAGCCGTTCATGAACGCTCCGCTCCCTTTCCTG
AATCCGTCTACCACCTTTTCGCTAATCAGAACGGCCGACAAGGGCGAGTAA
CCGCTGTTAAGACCTTTGCCGAGGACTTGAACATCCGGTCGACAGTCCTCC
CATTGCCAGGCGTGAAGTTTGCCGGTACGCCCAAACCCACACATGATCTTT
CAGACATTCAGCTCCGGTGTCCTCTTGATCACGACGAACACAGAACACAC
CTCGTCGGTCACGAAGAGAGCCCCGTGTTTGTGACAAACTTCTCGCATTGC
CTGCATATATCCTGGAACAAAGGGCACGCATCCAGTGGCAGCGCCAACAAC
TGTTCACATCAGCTCTGTCCGTACAGAAACACGACGTCGACTGACCCGGCT
CCATGAAAAATGCTGCAACGGTATCCGGACCAAGCTCAAGGAACTTGCTGT
CCAGCTCGTTTGCCAGTCTCCCCACATACTGATCCATTGACTCGCCCGGCTT
GGCGTGTCTGTAGTAGTAACATGGAGATACCTCGTGGAAAACCGGAGCGA
AGAGGGGCAAGAACAGTGCCCGTCGCCGGGTATGTCGACCTAGCTCCAAA
GCTACGTCTGTCAGTCTTTCCCTTCGGCGAAGACTCGAAACAACTTGCCTC
CAAGACTCGTTCCGTGATACGACTTTTGCCTTGCGATGAAGTTGACTCGCT
GAGGCTCGCCCTTCTCCACATGGTACTGCCTGCACAGCTACACAATCAGCC
GACATTCGGCCAAGAAGGGGAAGGGCAAGCCTCACCTTGATCGCCGACTC
AATCGACTCGCTGCCGCCGTTGAGGAACAACGCTTTCGACATCCCCGACCC
TTCGCACAACATGTCTGCAAGCTCTTCGGCAGGCTATGGAGGCCAGCTTCA
GCATCATTTCGGCTGACGGCAAAGTCTGCGACTAACCTGATGGGCAAATGT
AGCGGAATTAAGATAAGGAAGTGGGCGACTTGCTGCGATATGCCTTCTACG
ATCTGCGGAACACAGTGCCCAACACTGACCACGGCTGCTCCTCCCACAGC
CGTCCAAGATCTTCCGGCCGGTGTCCTGCTCATACAGGTAGTTGCCTTGGG
CTCTTGCAATAAAGATCGGCTCATGCGAAAGCTTTCGGTGGAACACCTTCG
AATCTGCCATGGTCTCTCCGCTCACCATCTTGCCGTTGATCTGCCCAGCTTC
AGTCTCGACGCTTGTGTGGGAAGCCGGAATGGGGATCGACATCTTTGGGCT

GACCAGTATGGAGGCGGTCTGGAGCTGTCCATCACCTTTTGTAAATCCACA
GGCGCATCATTAGCACAATCCGGCATTGCCTGGCCCCGTCGGTGTGATACC
GGCGCCGTCTTCGCCGACGGCACGTTCCTGCTCCGAGCGCCGTAGGGTTCC
GGTCGGACATGCGCGGTCCCGTTTCTCAAAGCTCGGCACCTCCCTTGGCCG
ATGCCGTTGTTGGCGCAACTGAGCGACAGGCGGTGCTACAAAGACACGTC
CAGCTATGGTAGGCGGTACTCAATTTGCCGGCCAGATCTGTGGAAAAATGA
CAAATCCAGTCAACGATTTGGGCGGCTACGAGAAAACCACTTGGAAGATT
GAAGGATTGGGCGAGATCATAGGATGGAACAACCCGGATAGAACAATCCA
GTCAGTCGTCTCTCATCTCGCTCGACTTGCCATCGAACGATAACACCAGGT
TCTTCGGCGTCCCGTATGCCGATCTGGAAGCCCGATTTCGTCACTCCACGCT
CAAGACCACATGGCGCGCGAATGCTTGCGACTGCACCAAACTTCGAGGCT
ATTGTCCTCAGCCCACACCGGGCATTCTACCCGTTCTTGATTGCCGATCGCC
CCAACGCCGGCGAGATGTATATTGACGAATGGGACAGCCTGAACATGCAGA
TTACCTTGCCCCCAGGCACGACCCCCGCGGCCAAGCTGCCTGTGTTAGTGT
ATATTCATGGCGGCGCGTTCATCTTCGGCACCGGCAACTACAGCATACTCGA
CGGCAAGGCCTTGGCCAAACTGGTAAATCAACCATCATAGAGGGGCCCTC
GAGCTGAGCAGTCCAGTCGGCGGCACTCGAGATGCCCACCATCATCATTTC
GATCAACTACAGAACCGGCTTCTTCGGCGTCGGTTGCGATTCGAGCCACGC
TATCATCTCTGGCTGACAAGATGCAGTTCCTGTGCTCTGACGACATTGCGGC
GGACAACCTCGCCTGTGGCTACAATCCCGGCAATTACGCTCTGAATGACCA
AAAGGAATGCCCTCTTGTGGATCCACAACGTGCGTGTCACTTGACGCGGC
GCGAATCTGATACTGATCCTCCTGAAGTATATCGGCAGCCATGGTGGGGACC
CCTCAAGAGTGTTGGTCGCCGGCCAGTCCGCAGGCGCGGGTAGGTTTTTC
GTGAGCTTTACAGTCGTGCCGCTTACTCTCCCAGCTTCGATGGACGTCCATC
TTCGCGCACCGCACAACAATCTCTTCTCGTCTGTCGTCCTGCTTTCAGGTAC
TGCACCAATCTGCGGTATGTATACGCCCAAGGAGTACGAAGTATGTACTACA
AGCTCCTCCGCGTACTTGGGATCTCTCAAAACCTATCCCCAGAAAAACGGC
TCAGTCAGCTACGTGCTGCGCCCACCAGGCACTCAGCAGAGCCGCATATAT
CGTCTTTCAAGGTCTAGACGGACCGCAATTCGGTATCGGCACCGACGTTTC
TGTGTTTGGCCCAAGATTTTGAGCTGCCCAGACCGTCCCAGTATCCCGGCA
GTAAGCTGCCTTTCAACGGCAAGCTGATAATGGGGGACTGCAAGCGTATGT
TTGTCTTCTTCCACTTCCTAGCGCCGACCCTGATGGTCTAGACGAAGGAATT
ATCTACTCCCAGGCATTTGAGCATCACACGGGAGCTACAGCTATCGAGACG
ATCAGCAAGTATCTCAAAGAACCGTTCTTATCTGAGTACCTTGCGCACTACG
GCATCAACGACAAGCAGGACGAGAGGAAGGTCATGCATGCATTGAAGCTC
TGCTCTCCGACAGCATGTTCATTGCTCCGCCGTACTTCATCGCCAAGGCCA
ACCCCCAACAGTGCTACGCCTACCACTTCAATCAAGTTTCCCGGTTTGACA
ATGCCTGGGGCGGGAAGGCACACCATTCTCTCGACTACATGTGTGCGTTCT

CAAACCGTTCTGCTCCCAGAGCACGCCATGACTGGACCTTGCAGTCACTTT
TGGCAACCTCGCCGAGCGGCTCACAGAGCCGGAGAACGCACTGGCACGC
CAGATGGGCAGCGACTTCATTTCGTTCGCCAACGGAGTCGACCCATATCCG
CCCGCCGGGGAGGCGCTTCGGGTTTACGGGCCTAACGAGGTTTGCGAGAC
CTCTCGGGTCAGCCCGAGGGACACCGAATGGTTCGACAAGATGGCGCCTC
ACATGGACGAGCTCATGAAGATGGCTGCAGACTTCAATGTCCGGCAGCCC
GAGCTGCTGAGCGAGGAGTTTGGTCCGGGGACAAAGAACGAGCGGGAAG
TGCCTCAACTGAAGTTTGAGCCAGAGTCAATCGCATAAGTAAGACATAGAG
CTTCGGTCTGTCATCCGTCGTGGCTGATGGATGCATCAATTGCAGCCATCTC
GGTCTGAGATGCTGCCTTCGCAAAGCTCCTTTGTGGATTTGGTGCGGGTCT
AGACTGGTGAACATCTCTGCGAGCGCTTTCACCTGACTGTACAGAGGTAAG
CCAGAGAATAGCACGAAGAGCGTCATTCTCGGCGGACCATGGGCGTCCTT
GACGAGCGTGCTAACGAGGTGGACGGGGCAGACTTGCATCACTCAGAGCG
AGGCCACACTCATTGTCTGTCCGCCTCCATCACTTGACATACGAGTCTAAA
CCGCTATCGTCAAGCCAGCATGGACGTCCAGCGCAATATTAGAGCGAGTAC
CAAATCATTCATGAAGCATCTAATAAGAGAAAATCTATGTATCCAATTTGAA
TCTGGTCAACGTGTTCTAAGACCGTCCGCCCTTCAACGATTGATGTCTCCG
CCCCCGCCTCGTTCGCAGTCCCAGACTGGCTTCCTCATCCCGCGGTAGAGC
TAGCCCCATCGGTCAGTACATATAACGGAACAGTGGTGACTTGTCCCCCATC
TCCTCCAGCTCTTCGGAGGAGAGTGGAGTCCACCATCGTCCTTTTCCTCTT
ATTGTCCCTGACACAGATGTACCAGTTCAGCAAGGCAGCCAAAATCGCGA
GAACAGCCGCCGCCTGTTCGAGGTCAGGTATGCCCGACAATCCCATACACC
ATGGTCACTCACGATGGAGATGCTATACCCGGTCTTGTAGGCGGGGGCTTC
TCTTCCCAGATAGATGTTGGTGCCGACTGCCGCAGAGATGTTCCGATACTG
AGCGCCAAAGCAGTCGAGATAGCCCTCTTGCCTCGGCCTGCCGCATTATTC
GCTGAGAACGAGTCAGTCCAACTTTGATGAGAGACTACCAATGAGGGCAT
ACTCACCTTGCCATGCGAGCGAGGCTGGGTAGCCCGCATAAAATCCAGCCG
GGATGAAAAACAGGAACCCGTATCTGGTCGCAGCCATACGGTCCTTCGGCA
GGGCGAACTCGGCAATCAAGCCAATCGTCCCAGAGTAAAAGGAATGATGA
CCGCAGGCGCACGTCTCCCGACCTTGTCCGAGATGAAGGCCGTGGTGATG
GTGACCACCGCCCCGACTGCGTAGAGCGGAACAGTCATAAGTTGAGCGAT
CTCCGCCGTGTAGCCGAGCTGAACGAGGACCGTTGGAAGCGAGTACGAGA
AACTTATAGTTAGCGCCTGGTAACGGACAGGCGGGAGGAGGCGCACCCAT
AGATGGGCACATTGGCGCCGACGCCGAGGATCCCACATAACCAAATTTGA
TATCCTTGACAGCGTCCAAGAAGTCCTTCTTGCGAAAAGGCGTGTG

>Mosaic_sequence_76

ACAAAGTACTGAGTGGGTCAGTGAGGGCGCACAAGCGCAAACATTGACGA

AGCTTTCGACTGTTATCGCTGACAAGTGGGCGGGAATTCTGAACGACGGCC
GTGGTCAATCTTCAGCTGAGGCCTGTGTGCGAAAAGCAACACTGGGTGAC
AACAAGCGAAGCCGGAAACAAGTACCAAAATTGACTCAGTCAGAGGTGAT
ACAGTCCCTCTTGCAGACTCTGTCTGATGCCATCAAACAACGTGTGTCCAA
CAAAAGGCTGGCTAGGGACTTCCGAGTTGGGTACTATCCCAAGCAGACTTT
TGATAGCCTAGCATCCAGCCGCTCAAATCTTGAGTGTGCCTATACCGATCCC
CTCTTCCAAGGAGTCGGAGAGAAGCCTATCTAGTCGAAACGTATAGTCATG
GCACTTGTACATGACCTCTGCCGTTCTCCAGACTCCATGTTTATCTACTTGT
AGATGAATCAATATGTATATCTCATTTGACAATTTAAAGCTCAGAGCCAAGC
TGGCGGGCCCGCATGATTCTAAGCAAGACGTGGCGTCAGGAAGATCCAGG
ACGCAAATTTCGCAAGGAATGCATTTTGGCGATCCTGGCAGCAACCCCACA
AAATGGCGCAAGCATCCCCTGACTATCTTGCCCGCAGCAAACCGACACGGC
CATTGGCTCAACGCCAACGCCATCACCTGAAACCACTCAGGTTGTTGGCAC
GGAAACAGGTCATCTCGCGCATGCGGAATCTTGCACAGCTTTGATAATGCG
TAAAAGCGAGTCGAGAGGGACATGAGGTCCTGACACCTAAGCATCCGCTC
TCCGGACAGGCCAGGCTGGGAACTTTAGGAGACCGCACGGATCTCACGTG
AACCTCTCAATCTGCACCTCGCCTTGACACCCCATCTCCCCCAAGCACCGC
CTCGGTACACAGCACTGAATGGAGATCACCCCTCAGACATCACCTTGGGCA
GGCGATGAAAGCAGCATACACCCTTCATGGGCCATGACGACTCGCAAGTGT
AGCGCATGACTGAATATGGGCTCCACGTCACAGGCTTGTCGCCCGCACTCA
GCTGTCCGATAACAGTGCTTCTGCATCATGCGTCCAGGATGCGACAAGTAC
AGTACGTCTTGTTAGTAGCCATATGCAGCCCGAAGAAAGGGACCCGCAGTG
CCAAAAGCTGGGCGCCCGTTTCCTTTCAACTCGTTGCCGTGGCGGAGAGA
ATGACGCAAAGGAGTCTCGCCCCTTACTGGACCCGCTTCGAGCGGACCTC
ACTGAAGCCACTTCGAGCGGATGGCTGGCCAAATCGACAAAATCAGCAGA
GCTGTTCACCTGCAATCTTCGCTAAGCATGGCGCCCACCGAGGCGAATTCG
CGTTTTTACGCCAAGGGCAAAGGCCTCTGGGACAAGTACCAAGATATATCC
GACACGGACGACAAGGCTGCTTTTCTCGATCGGCTAATAGAAGATGATCGG
CTCGCCATGACCTATGCCGGCGGGTTCTTCGGACGAAACAAGCTTGTCGAT
TTCGAGGACAATGGAGACGAGGACAAAGTGCGCTTTTGGTTGGCGGAGGG
GATAAGACGTGGACGGGGGCATACCCGATCGACCATCGCGCAGTGGAAGG
AGACAGGCGAGCTGGATACATCGCAGGATAGCGAGACTGAGTCTCGAGAG
AGAAGGTGGTATACTTCTTGGCTTGTCCTCGGCGGGTCGGACATCAGTCGG
AGGAGTAGTCTCCCCTCGATAATCACTGCAAGGTTGAGTGGCACAGAGAA
CGTGAGAGACGTAGGATCATTTGGGTATTCATGATGCGGTATGTAGAACGTA
TAATACCTGGCCTCCGCCACAGGCCAGCGCTTACTCAGATCCTCAAGAGGT
AAATATGAGATTGATAGGGCTCAGCGGGCACTTATGGCTCGCGTAGAAGGC
CCGAGCCCGAGGCGATACACCATATTTTCACATCGTTCGCTGCCAACGATTC

CTCAAACTTGGGATAGCTTGAGCAGATCTTCATTGCCAAAGAAGCGCGCAA
CATTTGAGACGGAGCTTCAATCCTCATGGCAGGTCTGTCGCAAGCGAGCTT
GAGAAGCCATTTGCAAACGCTTGAGAGTGTGTGGAGCGCGCATAAAGGTC
TGCATGTCGCGAGGCAAAGCACTCCTTTCACTCCACTCTGTTCGAATGATG
AGTCTGAGAGCACATATAGAGTCTTCGGCCACCCTGGTCTTTGACTTTGCC
ACTTCATCAGCGACATCCGGCAACATCTTTTCGTTCTCCCAATGACAATCCT
TTTGAATACCCCTGTAGAGCCCAAAGACATGGGTAATCCATCTTCATTGGAT
GCAAGAAAACGACTTGTGGCTGCGCATATATCCAATTTGGAATATGGACCG
ATCACCTATGGTCCATCAATCATCGCTCTCGGTACAGAGGCAAAGCTTGAC
GAGATGACAAATGTGACGCATGTGTACGCCAAGGGCTCCCTCGGCGTGCAT
CAAGACTACGACAAAGAGCGTCGCATCCCATGGGAGTCCCAGAAGGGCAG
CTGCATCCGGGAGGCTTGGGAAGCGTACAACAGTCAAGTTAGCAGATTTGT
AAACGTAGTGCAAGAGGCAATCAACAGAACTCACAACACCCCAGATTACA
AAGAGCAATTTGACGATAATCTCATGTCGGTGTTGCGCATTGAGTTTGACA
GGTATGTCAAGAGTAGCAAGACGTACCGTGAGCTATTCCAAGGGGTCGATC
CTTCCAGCCTCTACGGAATTGCTGTGTTTCCCTCCGACAGCCCCGTCTTTGG
TCCCATTGACAACACGGGACCGGCCCGTTGTCTGGATTACAGCGCCTATGA
GAACCATCTAAAGCCTGAGAACACTCTAAGAACACAGGAGTTAAGACAGA
CAGATTGGTCCGAAGCTCTGCAAGGCCTGAAAGATCGAAGCCATACCTTGC
GTGAAATTGCCAGTGATAAGGTTACTCTTGGACCAAAGACTGCATCAGGGT
ATCCCACCTTTCTTGCTGTGACCTTGACAGTGAGAACGGACGCTGTCACCC
AAGATGTGATGGTACGACTCGAGCACTGTATAGGTCAAAGTGCGGAGTTTG
GGGATGAAGAAGGCGGGCAAGCACCACCAGTGACCCGGCTCTCAACCCTT
GTCACGGAGCAGTGGAGGACAATGGTGTGCAAAGGCGGCGGTAGATCTCT
GGCTGAGGCCTGTGTGCGAAAGGCGATACTGGATGAACGACTGGACAGGA
GTCAAACGCCCAAATCGACTCAATCAGAGGTGATTCAGTCCCTCTTGTCCA
TTCTCTCAAACGACGTCAGAAAATACTTGTCCAAACACGCTACAATGGTCG
CGAGAGACTTTCGAGTTGTGTACTATCCCAAGGAGACGTTCGATAGCCTGG
TATCCAGCCGCTTGAACCTTGAGTGTGCATATACCGATCCCCTCTTCCAAGG
GATTGGAGAGAAGCGGCCTAGCTAGTCGAAACGTATGGTTATGGCTCTTGT
ACATGACCTCTGCCACTCTCCAGATTCCATATTTGTCTAGAGGAATCAATAT
GTACTGTATATCTTGACTGACAATTTAAACCTCAGAGCCGAGCAGGCGGGC
CAGCAAACCCTCGAGCAAGACGTGGTGTCAGGAAGACCCAGATCGCAGAT
TTTGAAAATGATGCATTCTGGCGATACTGCGTAAAAGCGAGGAGGGCTCGA
GTCACGAGTGACATGGGGTCTGGCATCTAAGCATCCGCTCTCGGGAACGCC
TCTCACCGTAAGAAATCCATCTTGAATTCCAAGAGATAGGGCTGACGCGGA
ACCGCCAACTGACAAGGACCATGTCGAACGCCCGGCCATCAGCTCAAGCA
GACCGCACGGACCTCACGTGAACCTCACAATGTGGACCTCGCCTTGACCAT

CCATCTCCGCAAGCACCGCCTCGGTACACAGCACTGAATAGAGATCACCTT
TCAGACATCACCGGGCTCGGCGATGAAAGCAGCATTCACCCTTCAAGGGC
CATGACGACTCGCAAGTGTAGCGCATGACATGAACATGGGGTCCAGGTCAC
AGGCTTGTCGCCCGCACTCAGCTGTCCGACAACAGTGCTTCTGGATCGTGC
GTCCTGGCTGCAAACAGTACAATCAGCGCTTCTACGCGAAGGGCAAGGGC
CTCTGGGACGAGTACCAAGACATATCCGACACGGACGACAAGGCCGCTTT
CCTCGATCGGCTGATAGAAGATGATCGGCTCGCCATGACCTATGCCGGCAG
TTTCTTCGGAAGGAACGAGCTCGTCGATCTGGAGGACGATGGAGACGTTG
ACGAAGTGCGCTTTCGGTTGGCGGAGGGGATAAGGCGTGGACAGAATCAT
GCCCGATCGACCATCGCGCAGTGGAAGGAGACAGGCGAGCTGGATACGGC
ACAGGATAGCGAGACGGAGTCTCGGGAAAGAAGGTGGCATACTTCCTGGC
TTGTGCTGGGCGGGTCGGACATCAATCGGAGAAGTAGTCTCCCTTCGATAC
TCACTGCGAGGTCGAGTGGCACAAAGGCTTCATCGGCGAGTAGCACAAAC
ACAAGCAACACGATAAGCAGCAACACAACGGCCTCACCTTCACGGCCCAT
AACACCTCGCACACTTCAGACACATGATTCGCCAACTCTCGAGCAGGAAA
CGCCCTCCGTCCGACCGAAATGGACGAGCGATTCGAGATCGCCCTCACTGG
ATCCCGATCGAGCATCGGTGTCCTCAGCTTCGCAACCTCAGTCAACAATGA
CACGCCAAAGTACAGATACCGGGGTCGAGCCGGACTGTCACGACGATCGA
TGTGAAGAAGCCTCCGTCACTTCGTAGCCTGGCAAGGTGGACTTGTTGTGC
GGCTTGACCGTGGGCCAGTCCTGGGTATTGGTCGTCGGGTTAATGCTTCCA
TAATGCTATGTGCTCTTGTGTATGCGTGGAAATGATGCAGATTGCCAAGACA
ACCGGTTAGGTCTTTCATGCTTTGGAGAAAAAGATGAGCGGGATAAGCAG
GACCGCGCCGAGTGCACAGCACCCCTTACAGTCAGCAGAGCAGAGGTGAC
ATGTGATGTCAAACTCACTCCGGCTACGAGGAAGAGCAGTTCGGGCTTGCC
CGCTTCCACCAGAATGGCGTACAGACTACCCAGTAGGAAAGGCGAGAGTA
GGCGGGCCGCATTCCACACCATATCGAGGGCTGCAAGTGCGCTGCCTGTAC
AATCAGCGGGGTGATCGTGCTTTTCTGAGGACGTACTAGCTCGACTCGCCG
GAACTAGGCTGGTGAAGACGGACTTGTATGTCGGGTTGTCTCCAGATCCGA
GAGCCAGTAGTGCGTAGCCTAGAGGCCTGTTAGAGTGTGGTCATTCTAGCG
GGCCCGATACTCACTGACCAGAGCCTGCTTGTAGCTCTTGGACAAGGAGA
CCAGTATAAGGCCAACGGCGTCGAAGCACACGGATACGAAGAGCAGGATG
ACCTGGAGCATGGGCTTTAGAGCGCTTGGAGCTATGTAGACTCACGTCAAA
GTGATTGGCCTCTTCTGGCGTGTTGCCCACACTGTCCATCCTCGACAAGAG
AGGCTTGGTCTCGCCACGCTTCTTAGAATTATACCAGTGGTATGCCCGTCGA
CCTGAGCGCTGCAAGAGGGGAAAGACGAGGACGATATAGACGAACTTCGA
GAACGTGAGGAAGGCCAGAACCCAAGCATTCCCTTCTGGCTGAAAGTCGA
ACTTGTCGCTCAGGAAGAGGAGGGAGGCCGTAGCGATGAAGATGGTCTGG
ACGTCAGGCCTATCCCCGACCTTGGCGAGGACGTACACCGAATGTGGTCGA

GAGAAGACTCAGTGTCAAGACGCTCAAACTCCACACGACCTTTCCTGTGT
CTTCGTCGCGCCGTGGAAGCAAGAGGCCCAGCGGCTTTATGGGCTCGACA
GCGATCTCGATGACGTCTTCCACCACGCCGACGATCCCTTCGTTCTCCTCTC
CGTCCTGGCTGCCCTCCTCACTCTCCTCTTCCTCCCCTCGAGTTTTCGAGTC
TCTTTTAAGCGTCTCGGGCATGAATACGGCCGAAAAGATCGAGGCGAGAG
CTGCGAACCCAGGGCGATGAAGTAGGGGAAGAGGACGATGGGGATGTATC
GCGAGGACAGAGCCGAGATGACTTGGGGTCAGCAGGCGACTCAGGAAGT
GACGGGACAGACGGAATGCGAAACCTGGTCCAAGCAGACTGAGAGCCTC
TAGACGGCTATAGAACCGGGTTCTGATGCTCAGCTCAAGCGTCGATTGATG
GTCCCGACACTTACTTCTGCTGCTCGTTGGCTACATCTGCCACGACCAGGG
TGGGAATGAAGACACATTTCGTCGATGCGGCAACAAAGATTCCTGTAGCAG
TCAAGAAGACCCAATCAACCGTCTATGAGTCAGCAACCTGATCTCGGATCA
ATCCACATGCGACCGACAGGGTTGCGAATGATATCTGCGGTCAGTGACGGA
GTGGAGAATGAGCAGCGACTCACATGCCAACATCATCGATGCCGTTGAGAC
CATAGCCAAGAGCGGCAGAGTGAGCATGACTGGCCTTCTGCCATACTTGTC
TGCCAGTGTCTGCACTGCCGACGAGGAGAGGAATGCTTCGAGTTAGCGCA
GTGCCTCCTACTCGAGTGTACCAACATGTTACGCCATCGAGCGTGGCGAGA
CCGGTCATGATGCTGGCCGTGAGGGCGCCGACATCGGGATTGCGGCACAC
ATCATCCCCGGCCTGGGACCCGGGATATCGTACGCGACAGGAGACCACACG
CTGCTCGTCAATGCTGTTCTGAACGACTGAAGCGATACGGACGGCGATGTA
GAGCGCGGTAGCGGCTGTGACGCTGGGCCCGATAGTGCATAAGAATGCATA
AGGGAGCAGTCTCCTGACAGCTCGAGTAGCCTTCGCTGAGGCTTTGCTAG
GAGACCGGGATGGCATGTGGTCGAGGAGGCGAGAAGAGATGAGATGAAG
ACGGAGCGCGAAACGAAATCTCACTGCGAGCGATCACTACTCGAGTACGT
CATTCATTATGCCTGGTACGAGTGACCCAGTACATAAGTTACTCGTACTCGC
TATAACATGATTATGTTTGAATGCTATGCTCATGCAAACACGAGAGGTGAAT
GCAGCTCCCCATGCGTGGTTTTCCCGACATTGTCATACTCTGGTGGGTGGA
CCGGCCGGACGGTGAACGGAGGGCCGCGGTACTAGAGAAGTACACAGTAC
TGTTGTAGCTAGTCTCGGTCAGGAAGGCAAGAACTGCGATTGTTGGGTCAG
ACTCAATTGAGCGACTGTTCAGGGGAGGAAAGAACGCTTTCATTGCTCAA
GGTGTCCGAAGTCGTCCTCAAGAACTCATTCAGTCACGGTCTCGGTCGCCG
CGGTCGGGATCTTGCCTCTTGCCTGGATCTCAACCTGGGCTTTCATCCCGTC
ACCAAATCCAGCAAACCTTCATTCGTGTGCCGTGTTCTGAACGTTACGAGT
AGAAAAGAGCGTCATCCTGGATGTACGCCTACCGATGACGGGCTTGCTGAC
CTCTGGACGTGCTAATCAACCGGCTGGGCTGGCCAAGACGGAGCGTCGAT
GATGTCAGCACGGGTGGGGGAACATGCTGATGCAATCCGGAAACGCTCATT
CGTCGTGATTCAAGTCATGAGTTGTATCGGTCCATGACCAGGAGGATCGGG
CATCTTGTCCAATCGCGCCGCGCGACTTCTCCGGCCAGTCTGTCAATACCG

CCCTCATCACCTCCTAGAGCCGCCCACGCGACCCCCGCCTTGCCTGGATAT
CATCGCTCCCGTCCAACGTATCATGTCGGGCACGCACACTTCAGGCGCGAC
CAAGCCCGGTAAGCACGCTCCACGGCTGTGTGATACAAGCGGGCTAACCA
GGCAGTGTGGCTTGAAAGAGACGACCTGCTGACCTCTGATGCCGGACCGA
CGTACCCGTCATTGAAGCAGGACGCGTCGACCGACGTGCTGGTCGTCGGT
GGCGGGATGGCCGGGCTCCACATCGCCTACGAGTTGCTGAAGAGCGGGAA
GAAGGTGGTGCTCATCGAGGATGGGAGTGAGTCTTCACTGTACCTGCAGCC
CCCGAGAAGTTTGCTCAGAGGGATAGAGATCGGATCGGGCGAGACAGGTC
GCACGACGGGCCACCTGTCGGTGGACAACGAGTACAACGACTTTCTGGCA
AGTTGTCCTTTCACACGCAGATGGTGAGCGACTAGGCGCTGAGTGTGCAG
AGGGTACATGGGTCGCAACAGACGGCACACATCGCCGCGGCACAGCAGGC
CGCGATCGAGCGCATCGCGGGCATTGTACATGAGCATGGGATTCAGTGCGA
CTTTGCGCGCGTGCCGGGCTACATGTTCCAGGGGCTTCCTCCGGGCGACGA
GAAGTACGAGCTCGACAGGCTGCGGCAGGTGTACGACGCCGCCAAGGGTT
CCCAGGCGCTCGATGTCCGCGTCGTGGAGGACGCCGGGATTTCTGGGTGG
AAGAGCGGTCACGCGATACGGTTCGGGAACCAGGCGACGTTCCATCCGAC
GCAGTACGTGCGCGGGCTCGCGCGCGTGGTCGCTGAGGAGCTGGGCGGCA
AGGGGTACGAGCAGACGCGTTACATGAGCCACGAGAAGACGGATGGAGG
CGTCAAGGCCACGCTCGCCGATGGGAAGACGGTCAACGCAGGGGCGCTG
GTGATGGCGACAAACGTCCCGCTCCAGAAGGTGAGCGGTATTACTGCATTT
TGGTAGTTTAGCCTGGGCTGAAGCTGACGGGCAGCTGATCATGATCGACCG
CCTGGAGGGTTTCAGGACATATGCCATCGCGCTCAAGATCGCCACGTCAGA
CGTGTCGACCACGGACGACGAGGCGATGTGGTGGGATATGGCCGACCCGT
ACCACTATGCGCGGGTAACGCCTCACGAGGAACAAGGGCACAGCCTGCTG
GTTGTCGGTGGTGAGGACGAAAAGGTAGGCCAGCACGACGATTATGAGGA
GCGGTACAAGCGGCTCGAAGACTGGACGCGGGAGCGTTGGGCCAAGGCC
GGCAAGGTCGAGTATCGATGGAGCGGACAGGTGCTGGACTCGCAGGACGG
GTCAGTGACGCTCATTGATGCGAGCGGGCGCGAGGGGAGCTGAAGGCCAG
TATCGCGTACATCGGCAAGAACCCCGGCGAGACAAACGTCTACGTACACA
GTAAGTGGACATGACCAGAATATGGCGGTGCCAGAGGTCGACGAGCTGAC
GTGCGCGCAGCGGGGGACAACGGCGACGGCCTCACGTACGCCGCTATCGG
CGGGATGGTCCTGGTCGACATGATCAACGGCCGTGAGAATCCGTGGGCTCA
CACGTTCTCGCCTGCGCGACAGCACAGTACGGGACACTTGCGGCAGCTGG
TCCAGTCGGTGCCGAGTATGATCAAGGAGAACCTGTCTGATCAGGTGTACT
ACACCAAGTGGCTCACGACGTGCACGCGGAGCATCGGGGACATTGAAGAC
CTCGTCCCGGGCGACGGGGCCGTCGTGAGGGATGGCCTCTCGCCCGTGGC
CGTGTACAGGGACGACACGGGCCAAGTGCACAAGATGACAGCGGTGTGCC
CGTGGGTGTTTCACTTCCGCGCTTGCGGGTCATGGCTGATTCACAGGCATAT

GAAGGGGATAGTCGGGTGGAATGGGAGCGAAAAGACGTTTGACTGCCCCA
TCCATGCGTCGAGGTTCACCTGCACGGGTCAGGTTGTCAATGGACCGGCCA
AGGCGCCGCTGGCGAGCATGTGAGTGGATCGGATGTGTTGGTACTTGGTGA
ATGTTGTATGGTATGCAGTCTTCTCACAGCTCGTGTCGCTTTTGCTGTGGCT
TTGCTTGGTGATCGGGAGGGCATGTTCTTGATGAGGCGAGCTGAGAAAGTC
GGAATAGAGCACGAAATCTCACAGTGACCATCAATACGTCATCGCAGGGAC
TGAGCGAAACACCAAAATGTTTGTCGCGTCCCCCTTCCATCTCCAGGGCTG
CGAGGCTGTCAGAGAGTTGACACGGCCTTCTGACAACATAATCAGAAAGA
GGCTAATGGACTCGTTGACGACATTGGAAGCATGAATGCCACAAGACAACT
GATGAGGCGGGCGCAAACGGACCACTACATGGACATGGTATGAAATGCTAA
GTGCTGCAGTTGGAACAAGCACCTACTTCCCAATGCTGCAACAACAGTCA
AGTCACAGGGTACGGTTTGACAGTAATAACAAGTCCAAATGCCCACAGGAT
GTGACATTGCACTTTGCCGCAACTTGCTCTCGCTTGTCAAGTTCACCTACCC
TCTTGCCAAGTTCAGCGAGTCCCTTGCCCCATCCCTCACTCGACCCGCTCA
CATCTTTCACCTCCTCAAGCTCAGCACATCCTCTGCCAAGCTCCTTGCCCAT
CTTCCCCAGTCCTACCAGGCTCAACGAGCCCCTCGCTGATTGTCTGGTGCA
CATGCTTGTCAGGCCCAGGACGGAGAAGGTGGACGGTTGTGACACCATGA
TTGTACATACTACAAGATGCTAATAGCAATCTCCGCCAATGGTATGCACCTC
ATCTTTTGCTAATGACACACCTCTGCCTCAGCTGGCTGACTCAACAACTGC
TCCGTAGCGCAAGAAAGATTCATCGGCAAAGACATATTTAAATCCTCAAA
GGCAAATTGGGGTCAATAGGGGAACGGGGAAAGTAAAACCCCTATGCTTG
GTCTGGAGGTTCTCGAGTTCAGATATTGATCTCTTAGTGGTAGTCTTCATAG
GTTTCCATTTGACTTTCCCTTTTGCTTTTTGGCATAGCTTCACTCATCCCCCT
TTGGCTGAACTCTACACTGAGATTCTTGTCTACTACACACCAGGCAGACAC
CCAGTGAGCCTTTGCCGTCTATGTTGTCTCTGCCAGCTGAACCTGCCAAAG
ACAAGTGCCAAAAACATGGTGCCAATTCCTCAGAGTGTAAATCTATGACAT
ACCCTTTGGTAAAAACAGAAAGAAATTGCATACCCTCTCATGACCATTCCC
AAAGCCTGTTTTGGCAGCGTAACCCCAGCAGCTAGCTCCCTTGAGCCAAG
AGCTCAGTGAACAAAAAAACCAAATTCAAGTTCCTTTGGGTCTGTTTGGG
CAGATATAAAAGCAGTATTGGACTGCAGAAGCTGCAGTCTTACATTGCTTCT
TCACTCTTGTGAGCAGGTAGAGATACACTTGTTAGCAGAAAGCATGACAGA
TGGGACTTGGTGCCAACCAACAACCTCCCCTACACTAGGGTCAGGAAGAA
ACCAAGACCAGTCAACCACAATCATAAATACCGTTGAGGAGTTGGCAGAC
AAAATCACTTTGTCTGCTTTCAAATGCCATAGCGTGGCCAGAGATGCCAGT
ACTTATTCAGAGCTTCCTGAAAAGGTAAGACAGATTATGAAAGAGGGCCTA
TCTAGTATATGCTTCCCACCTGCTACAGGTCATGTTGTCTGTGCCACAACAT
CCGTTGAGGAAACATGCCCTCCAACATACACTTTTGAATGGCAGGGAGTGG
CAATTGATGACACCTTCATAGTGACTACAATCCCTTTTCAAGAGGCAATAAT

GACGATGATTGCAATGAAGTGTCCAATATTGCAATTAAGAAGATTCATACCT
TGGCCAGCAGGGACTCATATAAAATCTCAGCTTGCAATGAGGTAGGTGACA
AGCTTTTGGATGTAGTCTACGAGGAACCTCCTAAGGAGGGATCTTGGATGG
TAGAAGGGTCTATCCAAGGCTGCCTTGAGTCAGACCTGTATTCACCGCCCC
CTAAGCTGCCCAGCCTTGAGTATGCCCTTGCTGCAAGCTATATATTTGGATC
GCATTGTCTAGCAAACACTGGCAAATGGTCTTGCACTTTTCTTCGCGCAGG
CCTCAACAGCAATCAAGAAGCCTATGTGAACTATTCTCATGGATGCTTCAC
AGTTGCAGCAAAGAAGTCCTGCAAGTACCTTTGCACACCTCTTGACTCATC
CGATGGTGCTGGTCAAGCATAAGACAAGCATTGCCACAATCAGCTCAAAGT
TGTGGACCAGCTTGTTTTTGACCTCACCCTACAATCCGGCCATATAAGTGAT
TTGCTGTACAGTTTGGACCCAATGTTTGACCATGAATTTGCTGCCTAGAATG
GTGTCATGACTGATACAAGCCAAGCACTTACAAGGATGTACAACCTAGCCT
TGCTAGCAGTGGTGTCATGACGGGGTCGTGACACCTGGCCAATTCTCAGCC
TATCTAGCTGGGATTCGAGCATAGATTGCTATGAGTTGGATCATTGTACCCTT
TTGGTTCAATGGTTAGCAGCCAGCTGTGCAATCCGGCGTGACAGATTAGAA
CCAACTGTGTGACTGTTGATGGAAAGAAAGGATGAGATTCATTGCTGGAG
AAAAGAGAAATAAGGAAGAAAGTACAGGGAATATATCTTTTTCACGCATCC
ATGTTTTTCCTGTTCATCCAACTGTTTTCCATATCAGACCAAACTTACATCGG
GATCCTCATATTGCAATCACTTCTTACAGTAACAGTTGGAGCTTAATCTCATT
GATAGCAAGGAAGTGATAACATCAGTCGATGTCTGACATCGTAGGTCGAAG
AAGTGATGTTGTCGAGTTGATAACCCAAAGTAATAGGTTGGGCTGGGATCT
GATCATAGTCATGACATCATGCAGCTGTTCTGCCTGAGGTGCAGTCATGACA
CCTCACCCCCAGGGCTCCCTGGTTGTCAAAAGTGATCCTCAGGACCAAGTT
GTGAAATCTGAGACCCTGCAGACTGCACCTTGGTTCCATATGATCCTGTAC
AACAAATGTGCATGTTTCCAAGGGCAAACCAGACAGAGTGTACCTTGATG
GTGCCTGCAAAAGGATTCTCTGCACTTTGAACATGTTCCACTGATGATACA
GGTGGATGTCATGACAAGCCAAGCCAGCAACCAGCCAAAACGGCCTGTGC
GCTCCGTGCGCTCCATGTGCGCTCTTGTGCGCTCCAAGTGCGGTCCTGTGC
GCCCCATGTGCGGTCATGTGCGGTCCGCTGTGCGGTCCTGTGCGCTCCTCT
GTGCGCTCCGGTGCGCTCCCATCTGTAACTTGGATTTCTGGTCGATCGACA
AGATCGACAAATATTCTCTGGCCAACCGACACAATCGACAATGGGTCGACA
TGATCGACAAAGTGTCCCCACTCAATCGACAGAGTCGACAGAGCGTTCCC
ACTCAACCGACAGGGTCGACAATGTACCACTTGGCAGTCGACATGATCGAC
AACTCTTCCTAGACCTGTCATTCAGGTCAACCACTTAGGTTTGATAGCGCAC
GCTTCACTTATTCTAGTTTCTCGTGTTCTCGTATTCTCGCGTTCTCGTGTTCC
CGTGATCTAGGTACATCAGAGTTAGGCTTCAAGCCATCTCAAATATACAAAA
GTACTCAGATATACGAAGGTACCAAACCAAGTATATACGGGATACACGCTCT
TGTACCTCCACTTGCTTGATAAGCGATACTCTTTATTTAGTCCCTCCTGTAGT

CATGTGAAGGGGATATAAACCCAGAGGTTTTGCCTCTGACCCCCTTCTTCTC
TAGCGATGGATCTCTACTGTACTTTCTTCTACAGTTAAGTCACACAGTTGGT
TTCGCCCAACACTCTCGTCCTAAGGCAGACTTCTAGGCTTTGCGAGCCAGA
GACTAAGCCCCCGGGCAACCTCGTTGTACCTTCGCGTACATTCTCGCCACA
CCGCTCTTCAGGTGTGGAACCCCCCCGTGACACCGTGACAGTGGAAGTCC
AGGCTGTTCAGTATATCTGCTGTCAAAGAGTAATGAAAACTTTTGCACAGC
TTGAATCAAATCCACACAGACTGATGTTGCTGTTTCCACCACTTCTTGTTGG
TAAGAGGCTTTGACACCAGATCTGACATCTGCGAAGACTGCCTGTAGTGAG
GGCATCAACCTTTCAAATTCTTGGGTGGCTGACCATATTTGCCCATTGCTCC
AGCACAGTGCCTGACCAACCGCATCACACATCTTTGTTGGGTCAAGCAAGT
CACCCAAGGGTGTTTGGCTGAGCCACGGATGATGACATAGTTGCTTTTGGC
ACTCAGGCTTGCCCTAGAAGAACGCTTGCCTTGCTTCCTCAGTTGTGTTCT
TGATATTTGATGCGGCAAAGTCAGAGTCAAAGGCACTTTGAAACACCATAA
GGTCATTATTGGAAAGGATGGGCCTCAAAGGATTATCTGGATTGGAGTCGT
ATGTGGCCGAATAGCATGAATATGCGTTTTGCGCTAAACTCCACTTTGAATA
CACATTGATTTCCCATTGATCCCCATTGGGGCCAGTGTAGGACTTACACTTC
ACCCTAGAGACCGGGGGAACTCCCACCTCATAAGTGACATTGCAAGTGGA
AATGTCTGGATCAGAACCGGCAGTGGGACTCATTGATTTCACACACAAAGC
TGCTGCTTGTGTGACAATTCCCCATTCCAAGGTTCTCGTTTCCGTGTAGACA
GTATCTAGTGCATTCCACAATGAAGGGATCAGCTCTTGTACTTCTTCTATCA
GTTCCAACTGGTCACGTCTTTGAGACTCGCATATTCTCACAATACCTGCAGT
GAGCTGATTTGGGTCAAAAGTTTCAATATGGCAGGGGGTCCCATGCAAGGG
AGCTGAGGCGCTGGAGGATCTTCTGTCATGCTGCTTGTGATGTTGAATGTAT
GTGAGTGTTGAGAAGAGTTGCCACTTGGATCATGAATATATATGCTCACAGA
ATGGCAATGCCCAGGCCAAGCCATGATCAAAAGGAGGTGTGTGAATTTGC
AAACACGAGTTGCCATTCTGAGCTTGCAAAATGGTGATATGTAAACAAGCC
AGATTGGCAGTTGCGCTTGCAGAGCAGTTGAGCATTTGACGTAAATTGTAT
TGCTCTTCTTGCATTGTAAATTTTGGTCGTCATGCATTGCATTATCGAATCGT
ATCATTTAAGTAGACCATCATTGTACTTCTACAGCATTCTCTAAATTTGTCCC
ATGGATCACCATTGACAATCTCACCATCTCCATAGGTATGCAGCGTGGCTGG
TTTGCACTCAAACTTGTATGCTCAACTACTTGTACCTTGACAAGCTGATTCT
GACCTTTTCCCTTATTCCTGCATCTGATATCATACAAGAGGGCCTCTACCGAT
GGCAGAGTGCAGCATTGCGCCAAAGAGGCAAGACTGGCCCATAGATGCTG
TCAACAAGTGCAAACGCCCATGGCATGTGATATTACCCTCCTTCAGCAGATT
GGCAAAACACCATTTGGAAAAGACACTCATTTTCCACATCACACAGGCATC
TCCTTTGATGTTCCTCACCCGATCAATATATAGAGGACCCAAGCGGGGCAG
ATGTGTCCCTCTCCTGTGCCCAAGAATCCCTGCTGCCATCCAAGGCTTTCA
GCTGCTTTTACCTGCTGAACAGCGCATGAGCAATTGATACTGTTTCTGCTTA

CTTCAGCCCCAGCCTGCCATTCCTGAGTGCAACACCTTGGGATCCTACCAC
TACTGCATTCTGGCTGTCCCTGGTCACCATTCCTTAACACCATCAAGTTGGA
TTAAAAGAGGTGCAATACTAGAAGTGTCAGAGTGCTGTACTCAATGGAAG
ACATAAGTCACTTGATAGCACTAAAACAAAGCTTCAGCTGGTGCACTGAGT
TGATGGAGGTCGAAATTGCTACCAAGAACCTCAAAGAGCTGTACAGAGAG
CATACACGCACATTCTTGAAGAGTGTCACTGATGAGACCACTGAACAAGA
GATACAAGAGACCATGAGGTGCCAAGACCTATCCATTCTTTTAGATGAAGT
ATCAAATGATGCTCATTCATTGAGCGTGCAGTTCCTTTTCTCAGGATGGGGC
CCAACACTCCCGTGCCATTCACAGCAAGCTCAAATTGCACAGGATACTCTT
GGAGAGATTGGCCCTATTGAGCAGATACATAGCGCTTTCAAGCCGGCCTGG
AACAGTTTCCAAAGCAGTCAAGCAAAACTAGATGAAGAGATTGGAGATAT
GAAAAAGAAAAAGCCCATACAGCCAAGTGGGATATAATGGCAGACGAGTG
GTTGTAAAGGTTCAGGTCAGCTGTGTAAGGAAACAGCTTACCTTCTTCTTA
ACATATAATAAAGCCAGCCTCTGTCTGTAAGACTGATAGCTGTGTTGGGTTC
CTTCGCGATAGTCAATGTCACACAGAGCAACACTAGTAAGTAAGTCAGTTA
GGAAAAGTAGAGGGCAGAGCCAAGACTCTGAGAGAGAGAGGAGTAGAGA
GGAGTGGGGAGTCGGGAGTGAGAGGAATAGAAGAAGAGGAGGTGATGAT
GGGTTCTCTCTCTCAGGCCTGGTTTATATACATGAAAGTCAGGCTGAGGAAT
CTATGGAGTACGTACGGTGTACGTACGATATCGTGGCGCGGCTGAGAAGTA
CGGAAGGAGAATCTACTACAAGAGGAAACTATTCTACGGGAGACACGGGG
AGGAAACGGGCGTGGCTTAAGGGTCTGCTACGGATTGTCCAGACATCAGC
CGTATTAGTGTGAGTGGAGCACGATTGTCGAATGGTGCAGGATCAGGCATG
AGAGCTGAGGATCCTAGGTCGGATGATGTGTCGGGTAAGGTCGTATTGTAT
CGTTGGTGGGTCCGAGGTGAGCCAGGCTGTGATTTGGGCATCAGGCCTGA
GGATGGTGGATCGGATACGGCGTCGGGTGTAGTCGTGGTTATGTCGGTTGA
TGTCGGATTGAATGTCGGCTGAGGGGCAAAGGCAGAGGCCGTAGGGCTTT
CTCCTAGTCATGTAGGGGGCAGGCAGAGCGTCAGAGGGGCGGTCCACTAC
AGTGGTGGGCCACATATATTGACACGAAAGCAAAATTGGTCCTGGCCACAA
GTCACACTCATGGATCTGACAGTGCTAGCCAAGTTGCATGAATGGCTTGAT
GGCTGGGTGCAGCAAGGCAAGTTACCCCCATTGAGTGCTGCGGTGTAGCA
GCCACTGGCAGGACAAAAGGAGATGGAAGCGAGGAGGTCCAGCTGTGCA
GTGCCGCTGGCCGCTGACGCGTGTCTCGACGTGACTACCTCGTGCAAGGC
AGGCTTGCATGCGGAGGCGGTCGCCTGTGCATAGGTATCTCATGGATTTTAT
GCATTTGCTCCATGCTTACCGACATCACTTGGTCTTCGTCGGACTGTTGGCG
GAGAGCGTTACATCGAGAGCGATGTGCGCTCCCTCGCCCGCAAACCCGAC
AAAGACCTGATACAGCGTCTCCAACACCTCGCCGGCGTCCCCTCTGAGCTT
CGTGCAAAAGTTCTTGGTACGCTCTAGCGTGCCCGAGTCTGCGATGAAGTC
TATGGCGGCGTAGATGTCGGACATGTAGCCGTGTGTCTCGCCCAACGGATA

GTATGCGAACTGAGCAGCGACCAACTGCCCCGTCGTGGTCGCTCTCACGA
CCGCACGCTTTGCCCTCGCTATCCGCTCGTCCCGAGTCTCGTCCTCTGCCTC
TGCCAACCTCGCCTCGCCAAGCTCCTCTGCCTGTCGCTCGCAGAACGCCAC
GTCCTCGCCTTGACCACCCGGACATCCACGCGAGATCAACGCGTGTAGACA
TACATGGTCCCCGCTCTTCGATGCGGCCACAAATAGGTCGCGTATAGCCGG
GAAGAGAACGCCCGGCGGGCCTATGAGCAGTGTGGAGATGTCGTCCGTGT
CGATCTTGAGGTGTGGGCGGTACGGATTGAGCGCGCTGAGCGAGGAGAGG
ATGAGCGGGACGAAGTCCGAGGTCATGGGGTACAGCGAGATCTGGGCGCC
CGAATACATCTCGGCTGTGGCTTGCTTAGGCTGGTCTGAGCTGCTCAAGCG
AGGTATGATGGGTCGGAGGTGCGCGATGTTCTGTCAGAGAGCATTTGCGAG
TAAAGATAGGAAGACGAGTTGCCGAAGAGCTATCAGTATTGGATGCGGCTG
GCGGGGCGGCAGTTGATGCGGTAGAAATGTCGATATTGGCACCGCCATGCA
TCAAACTTAGGACTAAACCTTCAAGACGGTCAATACGATTCTGCATATCATC
AGGAGTCGAAGAAGCTCCAGCACTACTTTGGTTTTTCCTCCGGCTTGTTGG
AGTAGCGTAAACGCATGACTGTGTGTCACCTCGTTTTACGCAATTCTCGCA
GCTCGGAGTACCCCTATTACATTTCAACCTGTTGAGCAGGTCAGCGACGCC
GTACTATACAGTGAACTGCAACGCGTTGGATTAGGGAATTGAAATGGCAAT
TTCAAGGGCCGGCTTTACTCACTTTCTTGTACGGCACGGATAGCAGCTGAG
CGGCACCCTGTTTCGGCGACGAACCACCCTGAACTGCTCCTCGGGCGAAT
GGCCCGAGGATGATGTCGTAGCAGACGGAGGTGTGGGGGTCATTTTCGAA
ACGATTTAAACCAAATAGGTAAGTAAGAAATGTCTTTTTCAGGGTGCTCGC
TCTTTTGTCAAAGCTGTATGCCGGACTTAGGAAGTTTGAATGTTATAAGGGG
CCAGCCTCTTTTTCAATCCACGTCAGCCACTTCCTAGAATAGTGTTCTAGGA
CTGTATGGATCGATAGAGACCGGAAACCAATGAGTAAGCGTCGATGGCAAC
TTGAAAGGCAGTAGGCTCCTGTCAAGGATTTGTATATTTCGGAGATAGAGA
GGCTCTTGCAGCAAGTTCAGGGGTGTGGCGACCACAGTGTTACCAAGGGC
GATGCAATACCCGCTCAACCGCTAGCCGCGATAGCCAATGAGACCCAATAG
CCGTAGAAAGAGGCCCAACAAGCAAGACGATCTCGACTGAGCGATCTCGC
GATCGTATACACCTTAGCCGAGGATAAGCTAAGGCGACCTCGCTGCTCGAA
GCGGAAACTCGTAGAGAATCTGACGTAGTTTGATATCCCGATCCAATACGTA
TACGTAACCGAATAGATGGTTGTATAGGTATCCAGGGTATTGGTGCGCAACG
TCCGGATCGGTCAAGTGAATCGGTTGGGTGGATGTAGCTATAAAGGACCTT
AACCCTCCCTGGTATACTCGAAAGTGGGCTGGGATCTGATCGCAATCTACC
GACTAGAATCGAAAGACTAAGATTATGGTCGCCTAGTTCCTCCCGTAAAGC
AAAACGCGGGTTCTCTGGGGGAGAGTGGCTATAGCGCACAGGCGACTTCG
AGAGGGGTTGTTGGTACGGACGACGATACTCTATTTTTTGGTAAGCAAATG
CTTTACTACTTGTAATTACTACTTCGGCGATACAAAAGAGATCCGGGTTCTC
CTACAAGTCTATAGAGTGTCGTCTAGCGCAGTTATGGAGGTGGTTACATTTG

GTACTCCAATGGTAGAATTGAGTGTAAGACAATATAGGCCTTGTTTGTAACG
GTTGGTAATAATATACATAAAGTGGCGAATACGTTCACCTTGACTGGTTAAG
GTAATAAAGAAATTGAAACTAGTCCTATCCTCTAGGAGAGTGTCGAAGAGG
TAGAATGGGGTGAAGGGTGGAAGTGGAAGGGGAAGAGAAGACACCGTTT
CGAAGGTGTTTAGGTAAGATGAAAGTGGTGAGGATTGCGACGGCAGCGTC
TTGGAGGGCAAAAGATTCTAAGGTTGACCGCAGATAGTGGGCAAGGTGGG
AACCTCAGTGGAGGCATTCAGTGGAGGCACCGGAAAGAATGGCGTTAAAC
TGAAAAGAGATAGTGGGGGAGACCTTGGCAGACCTTGGGAAACTTCCGGG
CGTCTTAGTCCGGCTTAGTTCTCTCAGCTTCTCAGCTAACTGCTAGAAAATT
AAAACCTCTCCTGATCTTTTTTCCTCTCATTCCATACTGGACGACCAACCAA
TTGTAACATCTGATAGAGTGTATAATAGTTATTTCCATGGTATTACCAATTGAT
GCACAGGAATTTGACCCTATTCTTCCGATAAGCATGTTTCTAATACGGATAA
ATAATTCGACGAGAGCCAATCTGCCTTTGTCTACAAAGTACATAAAGATAAT
ATATTATATTCTGTGAAGAGTCAAAAACCGAGAATACTAGCATTTCTACGCA
TAAAGTTGCTTCATGCAAGGCTACCAACCAGGGAAATTGGTATGTTCTCCAT
ATGACTCATAGATGCTGACATGCCTCTTACCCAAATAGGATGTGGGATAACG
TCGTAAGCGATACGTAGGTGTGCTGCCTATTCTCTGTTATGAAGATCTAATAT
TTATGAAAAAATATTCACAGTTGGCGGGTTTTAGCCACTATCTCCGCATTCA
AATATACGACTGTTTTCTTAACACCTAAAAGTTAAAATTTACTTTTGCTTTTT
CTTGGCTTGATGTCTCACGGAAGAGAAACCCTTGGCATCTCGAGTATCACA
GCATTGAAGCTCACAAAGTACTACATGTGAATATATGCTATATACCACATATG
GTTAGTAAGTAGACTCGATTTAGCCAACAGGGGACAAAGAATATGGTTCAA
ATTCTATCTGGAAATCGCGGTGACGTAAATTATACCATGACCATCATTTCAA
GGCACGCTAATTGATCTAGCTTAAGCTTAAGCTTAACTTAATGACTGCCAGC
CTTACCTGCCACATGAAAATTCACCGACCAAAGTTTTCCAGGTTGTTACTA
ATCTCACCTCAAAGTATCATGATGACTCCCACTAGCTCTCATCAGCCCAATA
TTCTGCATACTATCACATTGAACGATGTCTTCAGATAATTTTACACAGCCCG
CTTTGTTGCGCAAGAAAGTAGCAACATACGAAGGTATCCATATCCTCATAGA
TCTCTTTACAGTTGCTTGCTAGCTATTCTCGCTAGCTATTTGCCAGGTAAGC
AAGGGGTTACACGGTCCGTCTTCCGAAGCGGATCGCACCGAACCTTAGAC
AGAGCACGGAAGGCAACATTGGAAACGGAGGGGCATCAACGGAGAAACG
GTAGCCGAAACCTAACCTCAGGTTAACCCTTCCACAACGTCCTGTTCTTAC
TAAAGCCCATGATCGGTGCCCCGAACAAACGGTGCATTTCGGCGATTAGCG
CGCCGGACCGATGCATTTACCGCGGCAGGAACGAGATGAGTAACGGCTAG
ATACAGCTAAGATACAGTAGTCGCGCTTGCATGAACTGGGTTTCTGGGATC
GACTGCGACCCAATCAGATTGTCACGAGGGTTACACTAGATTCCGAATTTG
GTTAATGGTCAATTTTGAGGCTTAGGTACCAAAGTATCTAAAGATAGTAATC
GCGCTTGAGGTCGTATTTTCTATACTCTTCATCATCTCAACACCATCAGCAAT

ATACCAAGTATGAGATGAGAACTCGTTTTTTAAGTTGGAGAAACGTCTTA
GCGCTCGTATGAATCATCTTGTGACTGTTCTCTAGATGCTTGGATCAATTTTC
CACCTGTCGTCCTACTCCTCCTAGTCCCCTTTAGTCAGCTCAGTTACAAATT
GCCAAGACGCGATGCCTCAATGAAACTCCGGATACTCCTGATGCTGCTAGG
AGACAACA

>Mosaic_sequence_77

TCGGAGATGTGACCCCTGCTCCCGCCTCAGCGGATGGGTCCGACAGCCAC
CACTCGAGCAACTCCAGCTCCTCGGTCCGGCATCAAGTCGAAAAGTATATC
GAAGCTCACAACTCTCACTACGACCAAGAGAGCATTCAGTGGATGAGCTC
TCCCCAAACCTACAGACGGCGCTCAAGACCGATTTCACCTGGGTATCAAGA
GCTGCTTCAACGCGCCCTTTGATGAACACAGCCCGGTCCGCTATGAACCCC
CGTCACCGACCCACGAATATGTCAAAGAGTTCTTTGACGAAGAGGGTGTTG
CATCCGTACTGCAGGCCGCCATGGATCCTCAGGTTCGCGCCGCTCCTCTCTT
CAAAAGGTACTTAGAGGAGAGGTCAATCCAGATGCAGTGGTTGGACGACG
GGCGGGGTGGACGCTTCTTTCAGGTGCACAAGTCGAGAGAGCCCACAGTT
CGAACGCATGAGGAGAGTGACAGGGGGTGTGATCACACATATGACGAGGA
TGAGCCTTGTGTCACATCGTCGGACGATGGACAGACTCAACCTGACTGGG
CTTCGGAGTGGTATCAATAGGCGGGGAAATGCCCATGTGAATATCTCAACA
CTGCGCCTACGTTCGAGACTCCCAAACGTAGCATGCTCTAGTCCAACCGCT
CCATGAGATTCACGGCCTAGTGATTGCGTTTCAAGGCTTACGAGCCCCCTA
CTGCTGTCGATGGTCTGTACTGTAACGCCGAAAGCAGCCGTTGAGGTGTGG
AGTGCTTCATCTGTCCCCTTGGCACTGTCAGGGGCCTACCGCTAGTATCGCC
GAGGCTTCGTCGATTCCCTCACAGAATCGACTGGTCCCAAGGAAAGGGGC
GTTGCCTGTCCTTGCCTACTCGCTCAGGGCTAACGACTTCTGATGCGGACT
TATCTCTTGGGCACCCCTTCACCCATCGCTCCGTTTTGAAGTACAGCTTCAT
CACAGGGGGACGGCCTATTATTTTCGGTATTCCGGCAGGACCACTGCATAC
GAACCGGAGCCCAATTCCGGTGCGGCAAAAGACTCAGGGCTTTGGGTCAC
CAGAGGTGTTTCAGGCTCATAAGCCAGAGGTGATTCAGGCTCATAAGTATA
AAGATCCCCTCTGTCAACGGAGGCAGCCGCGAAAAGTTTTCGAAGGCAAG
ATGCGACGAGCTGTTTGCAAGTCTATTTCCTTTGTTCATCTGCACTTGCCTT
GTCCCCGTAGCCTCAGCCCTCCCCCTAGTTCCTTCGATGGGCTCAGAGCGG
GTATAAGTGCGTGCAAACGCACCCTGTCGGGGACCACACCAGTTTGCACTC
TTCCAACTAACACTGCGGCCTCACAGTCGCTGCGCACCCCATGTCTCAGTC
AGATACAACGCGCGACGCTGGACACACCGCACTACCAGAAGATGAGCGAT
TCTATGACGATCTACTGTCGCCGACGTGGGCAACGGCTTATGACACCGATG
GGAAGCAGATGAAGATGACACTGCACGACGACCAGACTAGACTGGATTTC
TTGTACTTCGATGTCGACTTTGCGACCTGGTCCAGGGTAGCTATCACCCTTG

CTGACCAGTCTGGCTCCGCCACTCAAGGGTTCGAGTCATTCGTGAGCCTCT
TCATGTTCACAATGAAAGAATCAGACTGGACCTTGACCCGCCTGAACGCA
AACGAGGTTGGATCTCTGCAGGTCGCCAATGACGAGGAGAAAATGATGCT
CGAACACGACGTTCGCAGGCTCACAACAATGTCGAAGAGGGGACTGACAT
CGATCTGGAGGCATTTTGTCACTGGAAGAATTCGCCTTATACACTTGGACG
ACGACGGGAAGAACACCACAGTAATAGAACATGGTCGTATGTCCTGTGATG
AGCCCCTGTCTTGGCTTACCTGTACCAGCAAACAAGGTGGATGGGGAGGC
TGGTGCAACAATCATAGGGTCAGGGCATATGCCAGGTGGGGCGATATTTATC
AACGAGGTCTGCACAGTGGTTTGATTACACCCGCTTTCAGTGAGTCATCGT
CTGCAGGCACATAGGCAATCACTGCATGGATCTTATCCTGGGCGATTTGCTC
GAGGATTCGTGATGCATTTGTGGGCAGCATGAACTTGCCGCGCGAGCAGA
AAGAATACTATCATAAAGCCCATACAATTCCTTGAATTCGACGTCTGGGGTA
ATCACACATGTCACAGCAAAGGTAATGGACGCTGGCATCTTCGACCCAAAA
CAGCGAGAAGCTTCAGCATGTCCAGATTCACGATAGCACATGCTGACAGAC
CGGACTCGACGCAGGGCGTCTTACTCACAAGTGCTGGGTCACCTGGTGTC
GATTCCGTGTGCCAGATCCCCTTGGTCCGAATCCTCTCGTGAAGGCGCGCA
TCAGTGTTCACATGCGACTTGTGTCACCCTCGGCCTGATCCGCAGAAGGAT
GTTTGTGAGATCAGGCCCCGTGGTCCGGCTGGCGGCTCAAATGAATGAGTG
AGCCTTCAGACGGCCTTCATGGTGTATTTTGACGTCCTACAAGGGATATGAA
GAACCAAAAGATCAAGAGACGCGCATACCGAGGGAGAAGGCGAAAGAGG
TGAGGGAAACGATCCCCCCCGGGGGGGGGGGGGGGGGGGGGCTGCGAGC
GCACGGACCGCCACATTGCCAATTCAAGGTCCTACAAGTATGGAGCGTCGT
GTACGTGTACGCTCCTGCTACGTCCCATGTGAATCTTCCCTGCAACTAGTCC
TCATTCTTCGTGGAGAAGAAATTTCCATCCGGGAGTGCTCTGCCCAGTCGC
CCTTCTCAGCGTACCACTAGCAAGAAGGTGCACGGATAGACACTGTGCGGT
CGACCACATGATCAATGACGTTGACTCCGCATGAATGGCTCTTACGTCTGCC
CAAGCTGGGCTGATGGATTCCGGGAATCAGATTGCTGGTCGTTCTCGATCA
TTTCTACCGTTATAAGAGATGCATGGACCAGCTGTACACTATTGTAGACGGC
GTCAGTGGAATCATGGATCGTGGAAACACCTTGCCCAGTTCACTGGCTGCC
CTTGCCAAGTTCAGATACCTCCTCCCCAAAGAGTTCATGAGAAAGTTGACC
GAGCTCACGATTCTTCTTCGCGATATCATCCGCCTGGTGCACGCCAACGGT
GAAGCAGAACGTGCTCAAGGCCGTGAGGAACCACGGGTCAAGCACTAAAT
CGGAGCCGCGCAGTCTATTGGCTTCGTGCTTCTCAATCCGTCCTTCGTGCTC
TTCTTGAACAGCCCGGGCCGATAAATACCTTCCCGCAGCCACATACTTGCT
GATGCTACTCGGGCGAATACCTGATTCTCCCCCATGGATAAGAAGCTCTTTT
GTATCAGCACCGCAACGACTGCACCAATAGGTCTTACCGCTCAGCGTGTCC
ACTACTGCCGTACATACTGCGAAGTAGGCCTCATGTGCCGCCAGGTGGTTC
CCATGCTCCGAAGATTAGAGGTCAGTGGGTCTACGACGCCCAAGATTCTCA

GATCTGAACCAGTCGTTCGTGCGTTGCAGCTTTCGAAACGTCAATTCCTGT
CGGTAGCGCAGCGAAAAGGGCGCGATGCACGCAAGAACGTCAGTTGACCC
TCCTCAAACATCCTCGACACCTGTTCGCTTTCCGTGCGGGCATGTAACGCT
GTGCGTGCGCCATCACATCAGATCGTCTTTTCCAAAGTGGATTAGAGTCTC
ATTCGTGGTCTCAGCAAGGGAAGACGTGGACCGTTCATCATCTTTCGTCAG
CTGCGGGCCCAGGCAATGACTGTTGGCGTTGGCAAGATTATCGTCTGGAAT
CCGCACGGAGGCTAGCGAGGTGATTTCGCCCTGTGCTGTGCGCGCGAAAT
GTCTAGGGGATATTTCTTACGCAACAGACAAGGGCTTCGCCTTCGCATTTCT
GACCCACTCTGACCTTTCTCCCCGACGAATGCCTTCCACTGAGAAACGAAG
TATAGCTCCTTGAAACAATGAGCGCCCGAAAGGATCAGCATCGGGCCCTTC
TGGATGCTGGGAAGGCCTCATAGGCCGAGCACAAAGGTTACTCGACTAGA
GTCTATCTGAATAAGCCGTTTCGCTTCTTAGCTTGAGCGCGCTTCAGGCCGA
GTGCCGCTCGACTGAAGGGCTGAGCTCCTATATCTGCATAGGACAAAGGGC
CGCGAAGCACATTGCTTTGTCGGCTGTGGGCTTATTTGTAGCTCTTAAGACT
GTCTTGCCACTCAGGGCCTTACGCATTCAAGCGCATACCAGACTCCCGATC
CATATCCAGAACCTTCCCAGGCATCTGTTCAAGCATGTCCGTGAGAGACCC
CCCAAATTGTTCTCGATCAGGAGCGTTCGACGTCAACGGTCGGTCGATCGA
TTTGACCGTTCATGATGACAACACAGGGTGGGATTTGATGGGACAGCCCGT
AACGAGCGCGTACTGGAACAGAATGACTCTCATCTCGGATGCAACAAGGG
CACAGGAGCACCCCGGCTGGAAAAGCTTTCACAAGCTCAAAGACTTCACA
GAAACAAATGAAGAGTGGGAGACAATCAAGAAGACGCCTGAGAACATCG
GGGAAGGCGCGCTGAGGCCTCCCACTTCTTTGGGCGAACTAGACTTCATAA
ACAAGACCCTCACTGATGCCAAAGCCCTTGAATCAACTTGGGTTTTCCGCA
ATCTGACAACCGACCGTTTGCGCATTATCCATCTCGGCGAGAAGCCCTTTGT
AGTTGAGAGTGGCAAGCTGTGGGAAACTAACTATCACACGAACTGGCTTT
GCGCTCACAAGGATCAGGGCGGCTGGGGTGGTTGGACTAGGGATCATCGG
CTTCAAGTGGACTACACGCTCGAGCCCGATGGTGAGGTCCTAAGCACTCCT
CTTGTCTGGGTTCAGATACGAGACAACCTGTTTGCATCTCGTCAGCGATCG
GACTGATCTTGCAAGACGGCCGACACCATTCAACATATTTTCTCGGCAGCC
AAGCTTCCATAACTTCTATGCACTAGAATGTTGATACATATTATCCGGACGGT
TCTAGTGCGACCTGAGAGAACTCGTCGATGCCACTGGCATGGATGTGACTA
CGAGCTCCCATCACCGCCAATTCATATTCGTATCAATGTTGGCGACGATTGC
ATACACTGCGGGACGTGGTCTCGAAAGGATTTTTGAACAGATTCATCTTGC
TGGGGGCGGCTACATAAGATTTATTCTGCCGCCCTCTATCCGTGTAAGAAAG
GGAATGGCGCGCATGACGGAAGGCTCGGCGACGGGAGACAGGGCAACGA
CCCCCGCCTCGCAAGCGGAGGTCCGGTTCCTGTTGTATCGCTCAGGCGTCT
GCTATTTTACGCGCGCTGTGCACTTCTTCTTATCAAGTGGACGAGGTGTGAC
CAACCCTTAACGTTCCTTACAAACCTCTTGAGCCGCGCCTGACAGTGGTGA

ACATGGTATAGAATACCAGAAATGTCATGAGCATAATATAGCCCGTACCCAG
GTCGAGAGTGGACTGGCCCTCCTCGCAGACTTGCACGGAAAGTGTACTGG
TACAACTCCTAGGAGAGGGCACACGAGTCAACGCCGTTGACAAAACAATG
ACACGCCACTCCAGCATCACTCAGTGGCAGACCCAATCATGATGGTGTCGC
CATTCAAAGTGCAACCAGAATTCAGCCCCAGGGTAGCGGACAGCTCGAGA
GAAGTCGCGTATCTGGAGCCGTCGGTAAGGCAGGACTTGGATATCGACTTG
CCAAACCGTGACATGATATGATCAGCGAGATTGAGGCACTTTGCCGCCAAC
AAAAGCTCCAAGCTTTGGAGCTTGTCGACGTCTCTCAAAGTCTCTGGGAG
GATCTGCGTGATGGGAGCACCCATATCCAGCTCGAGACTAGCTGCAGCGCG
TCCGAAAACAACGCTGGCCTCCGCATAATTGCGTACCAGGGTATATTCGCG
ATCAGCCGTGCTTATGGCGCGGAGTCGCTCAGCTGAATGTCAGCATCGAGT
CCAAGCTGATGTGAGTGATCTGGGCCAATCACGCAAGCGGAGGAAAAGAA
AGAAGTTACGAACGGGTGACATCCTTCAAAGCCTCGTCGACCTGTCTCATC
CATTCATCGACTTCATCAGTCGCGTCTAAGGTCACCCGTTGACCCGTGACCT
TCTCGTGGAGGGCTGCGCGATCGTGGTCCTCTTGGCGCCTCTTCAGTCCGA
CTGATCTCATCTCTGAACCCATTCAGCTCCTCAATCGTATCAAGGTGTCGTC
TCACCCATCAGTGTGTCCCAGACGATAGCGCCCGAATCTGGGTAGATCTCC
TTGGTCAAAGTAGGAGGACCGTCGCACGCTCGCTGACGAAGACGCCGGAC
GGCGCGACTAGCGACAACGGGCCAGTTGGGCTTTGTCTTGGAATCCTCCA
GCAGCTTCTCAATCCTCTTGAGCTCCTCGTGTCTCTTCTCACTGACGGATTG
GTCTGTCTCTGACATCAAGAATAGCAGCTTGTTCAACCTCCTCGAAGTAAG
GAAAGAGTGGGTATGAGTGCCTCAATGGCCACTCGTCTGTCCGGCAGGGA
GTCACATATATACTGTACCACAGCACACGACTGTGCACACCATGGTCTCGG
CCTCTGCTGAGAATACGTCACGGTCTAGACCTTGCGGGCATCGGCGACATT
GGGTAGCATTGCATCGTCCTGCATCATCAGTCCCCCTCAGCTCGCTCACGAT
CTGAGCGGGACGTATGGGTCTTCTTGCCTGTACAGTCTTCTTGGTCGTCGTT
GCAGACTCCTGCGGATGACGCGTGATCCGGATCATGGTCGTCCAAGAGTGG
CGGAGCAGACTCATGTCCGACCAGCGAATGGATGCGTCATCGACAGAACG
AAATGATGCGTGTTACTCTCGTGGCTGATGCTTTTGAGACCCAAGGAGCCC
GTAAGAGTTTTGCCAGGCTTGACCGAGGCTTCTCCAAGTATTTGTACCCCC
CCGCTCTTCACGGAGGCACTAAGCGAGCTCGATGACGAGCGCAGTCGGAA
GAGCGGGGTCGAGTTCTGGCGGAGAAGTCATGGAGGCGACATTTAGAAAG
GGCTGTCTCGGCGCAACGGTGATGATGACGATTTAACAACCATACAAAAGT
CACAAGAGAGACGCAACTCCAGTCTGCACTTCGTCACTTAGATTCACCCTT
CCCTTATACATACATACAAAATGCTCATGCCCGGCCTCATTCCATCGTTGTGC
TCTGATCAAATTCAGCTTCACAGCCGACTTGACTGACTATTGCTATTTTGAC
TCTACAACCCAAGCGTCCTCATGTCCCTACCCGTCCCCTCCAATGCTAATCC
TACCGAACGTGACGGCTACAAGTCCGTAACCATTGCCGAGACAGGACATC

AGGCGCTCGTGCAAATCAAGGATGAGGAGACCAGTATCGATCTTTTGGACG
CGAGATTCCAAATTGCGGAATGGAACGAGATTACGATCATCGCCAAAACGG
CAGAGTCACACGTCGCTCAACAGTGCATCCGACGCCTGGCAGCTTACACC
GGCAGGGATTCCGATTGGTCGTATCATGAGCCCGATAGCGTCAAGCGTAGA
CGCTCGCAGGGCGCAGACCCTTCGAAACGGTCCGCGACAGAACAGTCTGA
GAAGCCGACATATCAATACTGGATGCTGTCGGGGGCCAAGAGAACGCGGA
AGTTCTACCTGAGCCGCGACGAGGCCAAGCTGGTCGAATCTGGCAAGTCG
AGCATAGCTGCGCTGAGCTGGCTGCGCAAGACACCCGAGGACGGTGGCTG
GCAAGGGTGGTCTTTCGAACACGAGATTGAGGCAAGGATGCGGGAGGGA
AAGACTTGCAGGACGGTCAGCTTGAAGCCGAAGCTGCGGTTCCTTACGGA
CAACTCTCCGCCCGCATGATCATTCGCACCTCTTTCCTAGCCCATACTTCGT
CGTTGGGGTTCCGAAGGTACAGTACTGAGGTAAATGTGCCTTCCTGTATAC
ACAGAGCATCTGTGACCCCTCTTTATTCGACTTGCTTCGCCAGTCATCCGGA
TTTCAATTTCCGTTGTTCTGATCTGTGCCTTTTTCCTGTCTATATGCATTCATT
TGCCCATCTTTTGCACGGACGAAGCATTGCAAGGTAGCATTTATAAAGTAG
GATCTACATCAGCTACAGATGACTCGCTGTCGTCGTCCTCAATCCATTGGCT
CCTTCTGATTTTTGAGTCTCGACCAGACTCAGAGGTTGGTCCGTGCGCCAT
GACTGTGTCCACCGGCCTGATCATAGGCTAGAATCAGGAGGGAGAGATGC
GCATGAGGATCGATCCACATCGCCAGAACCACGCGTGTATGTGTGTAGCGC
ATCGAGGCGGCCGAGCGTCCCCTTCTTCCAGGGTGTCAAACGTGGCGCAC
TCGCGTGAAGGGATATTCCCCCATCTCCGCCGCAGAAGGTTGTCCGGGCGT
TGCTGACTGCAGCAGAGATTTGGGGAGTATGGGGGCGAATCTTGTGAGCA
CAGATCAAGTCTCAGCGCCGTGCTGCCGTGCGCATGGAAAGCGGTGCGCG
CACTGCCAAGCGAGACATTCAGATATACATACGTATGTGATTCACAGAGAG
TGGAAAGAAGTGGTGAACCGAGCGCCGCCCGCCTCTTACCTACTGGGACA
GGACGGACAATAGAAAGATCTGCATAGGTTCGACAAGCAGCCGGTCATGC
CTGTCGGGATCGCGGAGTGAGATAATCCCGGATTTGATCGGAGAGCTATTC
GCGAAAACGCGACGTGGCCGACCGACGTCGGTCATCGAGAGGCTCATGTC
CTTCATTCTGGCTCTGTCCGCCTTGATTTGTAGGGAATCCATCATCAGACTC
GAACATGGTAGTGCTACCATCTTCTGCCAGTGTCAAGCTCCCAGTGCTCTG
GGTGCGTGCCAAGCTTTCCAGGAAACGTCCCGCTTTCGCCTTGAGGAGTAT
ATCGTCGGCAAACCACACGCTCCACATGTGGAAACAGGTGCTCAGGTCGT
GCAAAATCCCGGGGTTTCGCAAGGGGTCTTGGACGGATCCTGCCCCAGATT
CTACGTCTGCGGCGTCTGCCAGCTTCTGAAAGTCGCGGGCGGCCGGTGTAT
ACCTCACGAGCTCGAACGGATTCTCGGGGAAGCGGTGAGGATCTCCCAGA
ACGTCTACGAATCAGTCCTGTAAAATACCACCAACATTCGTCTGAGGAGGG
TCTGAGAGCGGTGTGGATGTTTTCCGAAAGAAATGGAACGGGACAGAGAA
CCTACCAGATATCGCCCGTCCCATGCGCCCAAATTTGGCCCCCCCCTGACCC

TCGGACACACTGCTCGCGACCCCACCAGTGTTCTGAGCCTGGGTGGCGTC
CTCCGATCGCGATGTCCGCAAGCTTGCGATGAACCGCATGTACTCGATATTC
CCTTGAAATTGGAACGGCGCCTGGATGGTCTCAAGCAGCTCCTTGGTGATG
CCATGCTCTAGAGCAAACATCTCTGGCGTTTGACTTCGATAAGTGGCGAGT
TCGTCCGCGGTGTACTCCCTCAGACCGTCTTCGGCGAGGATCCTGCACTCC
TCGTGGCGGGCTCGCCACCATCCGGCGCACTGTCGCCCTCAGAATGGCTTC
TGCGTCAGTCCTTGTCAACCGATCCCCGAAGTCCGCCATGTTGGGTGTGAC
TGGGTGCTAGGGCCACTTGAGGCGAGCGGGCAGCAGACGAAACGATAGA
ATCACACCAACAAGGCTTGAGCTCTACTGTGCTGACCAAAGTCGAGCAAA
GGAGGATGGGGCGGCGAGGACAATGGATACTCGAGTGAGACTGCCGTGTT
CCAGAGCTACCTTTGCCGCACTAGCGACGTTTCCCGTCGCCCCAAACCCCC
TTGCCTGTCTTTCATGATAAGAGTAGCGCTCCATTCATTTCCCGAGGCGTGG
AAGGCAGGCGAGCATACGACGTACGGGAAGGCGAGGTTGCGTATACACGG
GTCCGTGCGCCCTGTCAACCCTCGAATGTGTTTTGTGGACGGGCTGAATGC
GGGATTCCACTGGTTTATCTCGTTGATGTATGCCCATGCAAATATCTACATGT
GTGGCCCATAATGCAATGCAAAATTGACTCAACCGACAACACGGCCTCATG
TCACCGTCAAGTTGAGCTTGATGCCCATGCAAGCCATCTCGGCACACAACT
GCTCGTCAGCTCGACTCTCGGACGACCTCTCGCATATCAGGGATCGGATAA
GAACTCACATAACGGAACACATCTGTCTCGTCAGCTTCGATCTACGAATTA
AGCGGGAGAGAAGCTCACAAGGAACAGCGACAACGTCCATCAAGCCCTT
CTTCGCGCCTTGGCCCTTGACGACCATCCCCACTCTATCTCGCTTCTCGTCT
TTGTGCTCGCTCTTAACCAGCGCGGCGTCCGGTCCGGGATCGCATATCCTG
CAATACTCTTTCTGGCCTTCTCCATTGATTTCCTCGAACCCGAGCGTGATGA
GACTTCCACATGTCCGGGCATACCCAGGCTGTCGAGTAGTCGGAGCAATTC
ATCAGTCGGTCTTGCAGCAAGAACGAGGTCCCATGGGCGATAAGGGCATCT
CGCTCCATCTCTCCAAAACGGATACCACCTGCGCGCTTCCGGCCCTGTTCC
ATAAGCACTGGTCTGTCAAACTCTTGCCTAGGAATGACTCACCTTCACAGG
CTGTCTTGTCAAGGGGTCGACAGGCCCCGTCGTCCGCACTTGGAATTTGTC
GTTGACCATGTGTCGCAATCGCTGGTAGTACACGACACCAATATAAATGTCG
GCATGGAACTCCTCTCCGGTGATACCCGAGTACATCGGCTCGTTGCCGTGG
TAGTTGTATCCGGCAGCTTTGAGCTGTTCGCCAAAGTAGTCGACCGGTCGA
TCGAAATCGCTGAATCGGAAGGGCGTCGCATCCTGCGCCAAGCCGTGCAA
GGCCCCGGCCTTACCAGCCAGACTCTCGACGAACATGCCGATGGTCATACG
CGACGGGAAAGCATGGGGGTTGATGATGACATCCGGCTGCATACCGCTCTC
GGTGAACGGCATATCGATCGCCGGGTACTTCTGCGAGCACACACCCTTTTG
CCCGTGTCGCGAGGAGAACTTGTCGCCAATGACGGGTGCACGGGGGATAC
GAAGCTTGATGTGGACTTTTTGGAGCTCGCTATCCCCCGCATCGCTGCCGA
GGAGACGGACCTCGTCGATATATGCCACTTCGTCGCCTTTGTACTTGTGAAC

GCGGGTCTTGCCTGTGACATCGTCGATATATGCACAGAGCGGGTCGCCCGA
CTTGACCTGGACGCCGAGCTTGGGGAGACCATCGTCAGAGATGTAGTCGT
GGTAATTGCTATTTGGCTTGACGTCCCTCCCAAGACCGAAGTGGAGGGTAG
GCTTGGACCGCTTGGGTGCACCGGGAGTGGAGGCGAGGTCGAAAATATCA
GACTTGTAAATTGTGCCGTATCCGAAGCCCCGCTCGTGCGCCGACTTGTTC
AAGATCATAGCGTCCTCCATGTCGTATCCGGTGTAGGAAATGACGGCGACG
ACGGCATTTGTACCATTGGGGAAGTGGTCGAAGCCATAGTGATTGTGCAGA
TTGGGCCGGACAACGGGGGTTTGTCCAGACTGCAACCTGTACATCTTGTTG
TCTGTCCGTTTCGAGAGGGCGGTAGAGGGTGTTCCCATGGACTGCTTGCCC
ATCTGGCACTGGTACATATTTCTTGGGGACTGGTTGAAATCGCTGAAAGGC
GTGAGATTGGCGATGACGGACAAGATGGACGTCGGGTCCAGCTCGACATG
CGTCGAGACGCCCTTCTCGATCTCTGCAGGAGTGCAGGCGATGTCCATGTA
GACCTGCTCGAAGGAACCGACATGGTCGAGTTTGCCATTCTCGAGGTACGT
CACGGGCCGCATCATGCGGGAGCGTGAGGTGAAGAGGTATAGACCGGGGT
ACTGACCACCCTTCGAGACGGGGACGTAACCGACCTCCATGTCGAGCGGG
ACACGGGAGTCGCCTTCTGTCTTGAGACGACGCAGTAGTTGGGCAAGCTG
TTTAGACTTGGAGGGCGACGCCCAACCAATGACTTTGCCGTCGAGCTGGA
CTACGACCATATTCCTGCCGTCGATGGTCGACGCAAAGACCTGAGTCATCC
CATGGGCGGTGAGGAGAGCTGGAATGTGTGATGCGTCGAGCTGAGAGGTG
ATGAGTTTGCAAGTGTGCGAAAGATGGTTGAGCAGACCACACGGTGAGCC
GTCTGGGGTGTGCACGGGACACAAGAAACCCCATGATTCCGGGAGAAGCT
TTCGGACAGTGGTCGTCTTGAGCTCGGCGAAGAAGGCGCCTCGGTGGATG
CAGCGGAAATGGGAGAGGTAGCGATAAAAGTTGAGCTTTTCAGCGACGAT
GGTGTACCCGGATGTCTGTTGCAGGTCGAGACCGGTGGGAGAGATGAGGT
TGCCGGTAGCGAGGAAATAAGCGAGCTTCTGGCCGATATCCCAGTTGGTCT
TGGACAAGACCGAGGTGAAGTAGCGGGCTACGATGTCAGCTTTAGCAGGC
TAGTCATGTATCTAACTCGCGATTTGAAAACTCCCTCGCCTTGCCTTCCCGC
AAGTCCTTCTGGAACTGCGCCTTGACAGCCAGGAGGCAATCGTCAAATTTC
TCTTTGATGATCATGCCGTAGAGGAATCCCGGCAACAGGACCTCGTGATGC
TGAGGCGAATCGGGGTTGTCTGCACCGGTAGTGCCTGACACCTGGGAGTAT
AGTTTCCGAAGCATAAATCTGAAGTCAGTGCTGCTCTTTGTACATCCAAGC
GATAGCAGCAGACTCACATCAACATGCGGAATTTGTCACGGGGCTGGGGC
AGATGCACAAGGACCACCTTATTGAGCAAAAAGCTGCCGACTTGGGATATT
GCTCCAGTCCTCCGGGCACCCGAGCACGACTCTGAACTTGTCGCCAAGGT
AGTCCAAACTTTGCGTCCCCGTATGCAATCCCCAAGCCTTCTGCCCACGCA
GCAGTAGCTCCACACGATCCGTCAAGAAGGTATTGTCGTATTCGCCCTGAA
TGAGACCCTCAAAGACCTCCTTGTCCGAAGCATTGACCAGCGCCTTGAGA
ATGAGCATGAGCGGGATCATGTATTCCACTTTGCGCCACGCAAAGCGAAGC

GTCGCCCGCCGTTCGAGAGGTAGTGGATGGTGTTAGTGCATGCCGACTGGT
CCGGTCGGACACAGCGTATTTGGCATCCATAAGGGGTGTAGGATACGCCGC
GCTTGGCGAACGAGGGGCGGTATAGGTTGAGTGGGTGATGCCGACGGGGA
ACGACGAGATATCGGATGAGCTTCTCATTACCGTTGACGACGAAGTAGCCG
CCGAACGAGGATGATTCCTCGCCGCGCTTGATAAGCTCGGACGAGGGAAG
GTCGCGGAGGTTGCACCGTGCCGACTGTCATAAGCGTCGTCGCCTTTGGCA
AGCTGAATGGGCTCACTCTGACCATGACAGGAAGAAGACCGCAATCTTTC
ACTTCCTCATGCTCAATGCTCTGGCCATCGTCGCCAGTGTACGTCCAGCGA
ATGGTCGCTGTGAGTCTCGAGCGGTAGGTAGTGAGTCGCTCGCGGGCCTAG
ACGTGAGCTCCGGGCAACCATTACTGCCAAGGAAGCTCACCTCGGAGGGG
AAGATGCGTCGTTCTGCCGCAAGCTTGTCTTTCTCCGGGACCATCGGTCGT
CCAATGGCGACTTTGTCTATCCGGTCTAGTATATCAGTAACAAAAACAACAT
ACGGAGAAAAGTAGAGTCGCTGAGCAGGAGGAACCTTGGGCAAGCGGGA
CCAGGCTCACAGCTGATCTTGTTTCCCCAGGGCATGGCCTCAGACGCCTTT
CCATCAAAAACGACTTTACTCCCTACATCCTCCACGCCCAGCGCCAACAGC
CCTTTGTTGCCCTGATCATCCTCCAGCAGAGCATTGAACGACTCGATGTGC
GGCTTGACAAGCTCGTCGAGGGCGGGAATGTCGGATTGCGCGGTTGGGGG
ATGTCGGAACTGTCTCTCGCGGGTGAGGGTGCGGAAGGTGGTGTGGTCTT
TCGAGGACGAGGGTCCCGGGGCGAGAGGATCGGAGAGGGACATGGTGCT
CTGACCAGCTCGACAGCTACTGAATGCTCGCTGCCTGCTGCTGCTATGCGT
ACTCCTGAGTAGAAATGTATACGACAGAGAGACAGAGACGAAGGGTTGAA
GAGGAGCAACTTTTTGCTTCCGTCCCTGCTGGTCGACCTCCACTTTTTCAC
CTCGACCCTCTCTACATGGGAGTACAAGCCCATGGGCCATGCAACATGCAG
TCTACTCTACCCAAACAGCACCTACACCCCGGCTCCCAGCTACCACGCGAT
CTGCACGCCCCACTTTCTCAGCTGCTCGCACGCCGGACTCTTCTCCCGCCG
TATATCTCGGATCTTATCATGATCCGCCCCAAACCCAAAGAAACCCGCCTCG
TTGGCTATACGTTGCTGCTGACGATGACTGAACATCTCGCACTGCCAGTCA
GCCCGTAGTACCACCGACCCACGTAAAGACTTACCTCGTTGCGCAACGTGT
TGACGTAAACATTCTCCACGCCCCGCTCAAACGACCGTACTTTGCTGCTGT
ACATCGCGGCGTCCTTGCTCTCCCGCCTCGACTCTGGCACAGACTGCCATA
TCCTGCTTCCCTCCCACTCGTCTTTGTTGACATAATAGTCGACATTCCAATTC
CACGTGGTGCGGTGTTGGTCGTACTGCGTGCTCGGTGTAAATGCGTAGGTC
GGATCGGGGTGGACGCTCGAAAAGATGGACGGGAGGACGGAGACAAGAC
CAAAGAGGAAGAGGATGAGCAGCGGGAGGAGGGCAACAAATGGCGAAG
ACGGTTCGGCGTTCTGGGCTTGTTGGCCAGGTCGACGAGTCTGGAACGTC
CGGAAACCGCCCGGTCCACCGAACGTAAAGACATTCGCTTGACCACCGAA
CGGGCCGCCGCCCAACCCCCCTCCGCCGAAGAACATGTTGAACAGGTCTT
CCGGGTTGATCTCGCCCTGAAAGCCACCGCCACCTTGGGGGAAGTGCATG

CCGCCTCCACGGAACATGTTGGCCGCTGCTGCAGAGGCACCTCCGCCGCC
GCCTCGCTGTGTCGGGTCGGCGGTGGGATTGCTGTCGAATACTTGCCGCAG
GTTTGTGTCCGACAAGACCTGGAATGCTTTGGAGACCACTATCCGTAAGCT
GTCACTCCAAGTAGACAATGTTGGGCGTACTCTTGAACGCCTCATCTGCGC
CTGGTGCACCACTGCTTGTGAGCTGCAGCCTGGTGGGACGTTTCGTAGCTC
ACTTCTTGTCCGGATGCAATGCCAGCGCCAGCTTCTTGTACGCCTTCTTGAC
ATCATTCTCCGTGCAGGTTTCTCCACTTCTCATCAGCACACCACTCCGCCAA
GCCAAGAACGGACACTCACACGAGAGGATCTCATAGTACTGGTGGTGCTT
GCACATCTTGACCCGCTTGACCACCTCCATCTGCTTGGCCGTATACTCCCTC
TTCTCCTTCGGTTTCTCCCCAGCCCCCGCTCTACTCGGCCCACTTGCACCTG
AGGCCTCATGGCCCGGACGCCGATGCACACCGGCCGCACTCGCATGTTCCT
CCACCCCACTCGCCCTGGCCGTCCCGTTCGGCGTAGCCGAGCCATTCGGCG
TGGGGGTCGAAGGACCCGCGGACGACTCGGACTCGGTCGAGGTGGATGCG
GAGATGATAATTTCTCGCTGGATGATGGTGAGCAGCGCCTCGCCCTCTGGA
GTCGAGTAGAGCGATACGCTCTTTTTGGCGAATTTCAGCGCTGATGGGAGG
TTGGAGGAGGAGCGGTGTCGCTGGGCTATGGACAGGCATCGGATCGCCTCT
TCGCGGTTGACCTCCATTCTGAGTCGAGCGCGGCTTGTCCGCCGGGAGTCG
AGCGCGCCGAGGAGTGCTTTAGATGGTGTTTGTGAGCGTTGAGGGGGGAC
AGATAGTCGCTGAGAGAGGCCACGTATGGGCAAGAGGACCGTCACATGAC
CAGCAACGGAAGCAATTCTAGCAAGGGAGCGGTTACGTAACAAGCACTTC
TCTCACCATCTTGCCACCGATGCTACTCTGCAACCTGCTCTTCAATACTCTT
TGCCTATATAGCGCAACGCCATGCCATCCCTCTCAGCCATCGAGCCAGCGAT
GGACGCGCCGCAGACCGTCTTCCTCCGACTCCCTCACCCTATCGGCTCGCA
TCAACTCCGGGCGTCACCCTCCACTCAACTGATCGACCTCCTCCCTCCTCG
GCTCAAAGGCCAGAATGTCTATATCCGATCGAGCACATCCATCCTTGACCCT
TCCTCATCCCTGTCCAGCCTCCCCAACGATCCCTCTCACCCTGTATTTTTGG
AAGTATGTCCCCGTCTGCTCGGTGGGAAAGGAGGCTTCGGCACCCAGCTC
CGAGCGGCAGGAGGCAGAATGGCCAGTGGAAAAGACACGAACAAAGACG
CATGCCGCGACCTCTCCGGGCGGCGAATAGCGACGATCAAAGAGGCCCAA
CGTACCGCCGAGCTGCTCGAGTCCGCTCCTGCTATCAAGGCCCAAGCGGCC
GCCGCCGAGCGCGCCAAGCTCGAAGCGCTCGAGAGGAAGCTCGGGATCTC
CCCCGCCGGGAACGGGGGCGAGTCGAGTCGCTCCGTCGCGCCGGGCGCTG
AGGAGGGAGAGAAGAAGCGCGTGGCGGATGTGGACCTGGAAGAGCTGGC
GCGGAAGAAGCACAAGTTCGAAGACAACGAGTTTCTCGAGCAGAGCCGG
GAGATCAACGAGAGCGTCAGGTCGGCGGTCAGTAAGGGTGAGTCTTTGGT
GCTTAAGCAGTGAGGTAGAGGGGTAAGGCTGACGAGCAGCCATGTTGCTG
AAGAAGAAGAAAAAGGCCAAGGTCGGCTCGGAGATGCAGAAGGACAAGG
ATAAAGTGGCCATGCCCCCGCCGGCGATGAAGGCCGCGGCGGCCTAGCTT

GACGACTAGCAGTGTGAACAGCGTTGGGGGTGTTGTACTAGAGATGCTTTG
AGCCATGTACAGTTGGCCTACTGCTTATTGAATTGCATGTCCTGGTCGGCGG
CGTTCCGTGCTGCGCCGGCGTAGAAGAACAGGCACGGCGAAGTGATGAGA
TGAAGAGATGTTTACCCCGGTGCTCAATGGGAAACCTTGCCTCTGCTCTTC
CCAATTATCGACACATCCCATCCCTCGTCCTCATGCCAAAGCATCACAAACT
CCTCGGTTCCACCCCGCCCAGGCAGTCGCGTACACACCCCATGCCCATGCC
CACCCGACAGGCTGCGCCGAGAGGACGAAACAGTCCAGGCCAAACATGT
CGTCGATCAGCATGACATGTCCCACCGGACAGACCGCCAACGATGCTCGTC
GGACCGGTCTTCGGAGCTTGGGCCAGCGTACGCCGTGTTTCTGGTCTGACC
ACTGACAGCATGTCGTACGGCGGCGGTGCATGGGATATGTATGGTCGGCGG
CGCCGAACGAACCTTCTGACGGGTCGGGCGTGTTTGGGTGATTGTTGAGC
ACTTACGAACTGACTCGGAGGCGTGGCATATGATAGGCAGTACACCGCGTT
GTTGTCTCTCCCCATAACAACGTATAGAGCAGCTGGAGCACTCGACACTGA
GAACGCAACGCTCGACACTCGACACTAGTGTTCTGCTGGACGCCTTCGCCT
ACACCTGGACACCGACATCGCACCCTCGCTGACGCCCGCCCACTCGCCAC
GATGCACCTCCCACCTCTCCTCCTCTGCCTACTAGCAGCGCACGCCCTCGC
GCGCAAAGCCGCCCCGCCCACTCGAGAGATCCCCACGCTGCAATACCTCA
AACCCAAGTCGAACCGCTCGCGACTGATCGACCAGCGCGCCGCCTTCTAC
GGCGAAGGGGACCGGCTCAACCCCGGCGCGGCGCGGCGCAGCACCGCAG
GCGCCGGGTCGGCCGCCCGTCGTGGGCGGAACAGCCCCAAGCAGACCATC
GAAGAAGTCAGCTTCCCCCTCGCCGACGTGGACGTGGGCACCGAAGACCC
TCTCCCCGAAATCGGCGTTGCGCACGGGCACGAGGTGGACCCCGACGCGC
TCGAGGCGCTCGAAGTGGCCGATGCGCGGCGGGAAGCAGAGGAGGACGC
CATGCTCATGGCGCACCGCGAGGACGAGGCGGCGCGCGAGGTCCGAGCGG
TGGAAGAAGCAGAGAGGGAGGAACGCGCGCTGCGAAAGCGCGAAAAGG
AGCACGAGCGCGCGCTGATGGACCGCGAGGGGCCGCTGGGGCTCGCGGA
GGAGATGGCCTCGTACGCGCGGGCGAAGAGGGGCGTCAGGCAGCGGCGG
AGAAAGCAGGGCAAGGGCAAGGCCAGGACGGGCAAAGGCGGCGCCGTC
ACCGCCGGGGACAGAGCCAAGGCGAAGGCCGAGGCGATGGCCAAGGGGA
TACATTTGAAGATGTAGCAGTGAGAAGGACAAAACTAACTTCGCTGGCCA
GGCCTTTACCGGAATTCACATTGGAATGATAATTGCAACGACATGGATCTTA
TTACTTAATGCTGTCGTGGGATACCAAGTTCTCGATGATGGAACGCCTCTTT
CGATAGGGCTAATGCTCATCTCCGGCGCCATCTTATTCATCGGAACTGGTTA
CATCACGCTTGATACGGGGTATCACTGGACAAACGAGTTTGTTCCGGATCC
TACCGACCACTATCGAAATATCGCTCTATACGTTCTGTACCAGCTCGCACCG
CTCGTCTTCCTCGTAGCTTTCTACGTTTTAGAATCTGTGCTCGTGCTCCGAA
TCCTTGGCGAAGTTCGACCGATGATCTACCTCACAGGTGCTGCTCTTCTCTT
TGCACTAGGGCAGATATTCACCTACGTCATTAGCCCTTACATATGCAACGGA

ACCGCTGGCAAGATCGACGGTGCCTTGTTCGAGACGCTCTTCACTCTGCTT
GCAGTGGTGATGATCTGGGTATTCTGGTCGAGTATCACAGAAGATGACTGG
CCGATTGCGAGCCAGCCCGCGGGATACCCTTAAATCTCGTCCTTCATTTTGA
GTACGGAGTCGGCGTTCAGGTTTACCAATATACCCGTTCATGTTCGTTTCTT
TTCATTCCTTGGATGCGTCCTATAAATCCACCGTCTCGAGGGAAACGGGGAT
AAAAATACATCGAAGATTTAATCCACGTAGAAAAAAAAATAGTTACCGTTG
GGAAGGGGTAAGCGTCGAAGGAGCACGATGGCATGCGCTACACGATGCGA
CGTTAGTACGCTTGGGGAGGGGATGAGTTATATTCATATCCAATACATGTGG
TGGCAGATGTGCCCTTCGCTGGTTAGCGGAGAATTTGGAGTGTGGTTGGTG
GTTGGTGGTTGGTGGTTGATAGTATACCCAGGAGATGGTGATATGCGGCTAA
TGTATATGATGTCGTATGATGATAGTTGAATAGTCTCGAACCAGACGAGTCG
AGTCAGTAATGTGTTTGTTCGAATTCCAACGCGTTGAAATGATTGCATTATG
AGCTTGCATGTGAACTTGAGCATCGCCTAGTTTGATATTCTCATGTATGTGA
CATATGATCGGGAGTGTACCTAGGTACCTTATCCGTAAAAGTATTATCTATCG
GATCTCCCATTGGCCAAAGCTTATGATGCTAAATACAAGGCTGCGCAATTGC
GGCAGTCGCATTCAACAAAGACAAGCTACTAGTAGCCTCGCCTTATAATAA
AATCTTAATCATTACACATTCCTCACTCATTGAGATACCTCCTTTCGGTCGCG
ATCTTTTGGGCGTTTCTAGGTAGCCTACTGGGGTTATGCTGCCCTTTGACTT
GCCGGTTAACAATGTCTTGAGGTGTTGTAAAAAAAAATCGGTCGCGCGTCA
AGTCCTAGCTACAAGCTAAGATTTCTTCTCTTCTTTGCCCTTTCTTTATTCTC
TTCCCCTCTATACATAATACCCGCATTGGCGACCCCGCAACGATAGGAAGGA
CTTTTTACTAAGTCAAAAGGCTGACAATTGTAAAAACCTTTGATATTGGACC
TCGACGAAGGTATATCCCGACCGCGACTCAACCATGGCCGACTCCGACGGC
GAATACCACGATGGTAACGAGAGCGACGACTTCGCGGACCACCAGGTGAC
CTCCGGCTCTAAGCATGGTACTCGATCGCGCTCCCAACCTGATAAGAACAA
GAAGGGGAACCGAAAGGGCGCTAAGGCCGCCTGGGAAGACATTCAACGT
TCATGGGACACAGTTGTCGAAGATGCAGATGGCACGCTTACTGGAGTCATC
GAGGGCCGGGGCGAAGCAGAGAAGAGGGCGAGGCTGCTTAGAGACACCA
CACCCCTTCAGCGCGGTATCATACGGCACTTGGTTCTGGTCATAGATATGTC
GTTTCGCCATGTTGGAAAAGGATATGCTGCCTAGCCGATACGAATTGACGAT
AGCGTACTCCTCACAATTCGTTCGAGAATACTTCGAGCAGAACCCGATCTC
GCAGCTTGCCGTCATAGGAATGCGGGACGGTGTCGCGGTCAGGATCAGTG
ATATGAGTGGCAATCCGGCGGATCATCTTGAGAAGCTTCAAGGCTGGATCA
AGCAGGATCCTCAGGGAAACCCTAGTTTACAAAATGCGCTGGAGATGTGTC
CGAGGTTCTCTATAGTATGTTTTTTTACTAATTTAATAAGAACGGAGGAACT
TGGATTTGAATATGGGCATAAGCATGGACAAAGACGAATTTAGTAGCTAAC
ACCCGTCCAACAGTCACACCCCCTCCCACGGCACGCGCGAAGTCGTAATC
GTATACGGCGCCCTAATGTCCAGCGACCCCGGCGACATTCACGAGACGATC

GCCAGCCTAGTCCGCGACCGGATCCGCGTCTCAATCGTCGGCCTCGCAGCC
CAAGTCGCCATTTGCGACGAACTATGCTCCAAGACCAACGGAGGCGACAA
CTCCAACTACTCGATCGCCCTCCATGAAGACCACTTCCGCAGCTTACTCCTC
GAAGTCACAACTCCCCCGTAACCCGCACCGCGGAACAGAGCAATGCAAGC
CTCCTCATGATGGGCTTTCCCTCGCGTACGCTTGCTGCTGGAGGCGGCATAC
ACTACTGCGCGTGCCATAACCGGCCCACAAAAGAAGGATACAAGTGCACG
CGCTGCGGCAGCAGGGTCTGCCGCCTCCCCGCCGAGTGTCCCGGCTGCGG
CCTCACCCTCATTCTCTCCACGCATCTCGCGCGCTCGTACCATCACCTGTTC
CCCCTCAAAAACTGGGTCGAGGTGCCGTGGGCAGAAGCGAAGAGAAGCA
AGGCATGCTACTCCTGCCTAACGCCGTTCCCGGAGGCCCCGAGGCCGGACT
CGGGTAAGGGAAAGACGCCGGATAAGGGGAAAGGGCGGAGCAACAGCAA
CGCCGTGGCTGGGGGCGGTGGCGGTGGCGTGGTCATACCGACGCCTCTGA
AAGGACTTAGCGAGTCCGGACGGTACGCATGCGAGGTGTGCCGGAATCAC
TTCTGCATCGATTGCGATTTGTTCGCTCATGAGGTGTTACACAACTGCCCTG
GATGCCAGAGTGATACGAGAGGCTTGGGCGGGGGTAAGCAGAATGGGAAA
AGCAATGGCAATGGCAACGGGACGAGTAACGGGAACGCGAATGGGGCTAT
GGAAATAGACTCCTGAGTTTGGATTGAGGCAGGTGGTCGTTTTTGAGAACG
TACTCTAGGCATAACAGTCACGTCACTTTTACATATCACATTACAGCATGGA
CAACAAAAAGGGAGAGGCGGCGTTGGCGGTATTTCACATACAGGATAAGG
CAATTGACTATGACGCGTCCTCAGTTGAGGTACGGAGTTCTTTTTTTCTCTT
AACTTTTCTTCGCATAGAAATTTTGGAAGGGAGGGTGTTCCTTTTTTCTCTA
CAAGCATAGGTATACACGATAAAACGATGTAGGCATCAAGACAATGATGTAT
AGAGAAAGGCATTCAAATGAATGACAGAAAACTATCTAGCTAGGTGCCTGC
TCTACTGTTATGTGAGTAACAGATGGTTGCTTCTAATAGCATATGCTTCAAA
CTATGATATGATGATGTCCGCACTCAGGACTATATGATGGGTTTACCAAGGC
CCTCCAGCATGCTTCCGTGCAATGCAATAGGACCGGAACCCGCCGACGCTA
TGTCGTGATCTCGTTAAGATAGATATTATACATCTTCTAATTTCTCCGACTTG
CCCCCTTCTTTCCTTAGGGGCAAGTCACATCCAAATTTCATCTCCCATTCAC
AAGTTTCCTCTCTCAGCTTGCTCGCGCTCGATAGCCTCGAACAAGCTCTTG
AAGTTCCCAGCGCCGAAGCCGTCAAAATCGTGGCGCTGGATGATCTCGATA
AACACCGTTGGTCGATCCATCAACGGCTTAGTGAACAGCTGAAGCAGGTA
CCCGCCTTCATCGTAATCAATCAATATATTCAACCTTTGCAAAGTCTCAAAG
CTCTCCTCGAGCTCCCAGTTCCTCTTCTCGGTCTTCAGGCGCTCCTTCATAG
TCTGGTAGTACGTCTCCGGTACATTAATAAACTCGACGCCTCTGGCGCGCAT
TCCGGAGACGGTGGCGATGATGTCGTGGGTTAGAAGCGCGACATGCTGGA
CGCCGGCGCCGGAGTTGAAGATGACGAATTCTTCGATTTGGGACTTCTTCT
TGCCGAGAGCGGGCTCGTTGATCGGCATCTTAACTACGCCGTTTTCGCTGC
TCATGACAATGGAGGATAGGGCGCTGAACTCGGTGCAGATCTGGCTGTCGT

CGACGGACCAGAAGCGGTGGAAGCCAAGGCACCGCTCGTAGAACTCGCA
CGCGGATACCATTTCGTTCCAGGACTGGTTGCCGACGCAGTGGTCGATTCG
CGCTAGGTCGATTGGCGTAGGCTCACGTTCGAGGGCGCGGGGGACCGCGC
TACGAAGCCTGGGAGGAAGGGGCCCTTATAATCGCGCCGCGAGATTAGGGT
GTGCGTTGTATCGCCATATGTGCGAATCACAGCTGTCATCACCTCGCCGTGT
AGCTTATCCCTCGTTATCCTAGGCGCTTGGACGGCATCAGCACCTTGCTCTA
CAGCCTTCTCGTACACGCCTTCTACGCTGTCGACCTCGAAGGCTACGTCCT
TGACCGCATCGCCGTGCCTCTCGAGATGATCATGCATCTCTTTGAGTAATTT
GCGCTCATCCGCCGTTATGGGCTCGTCGGCGGGGAGGTGTGCGAGCGATCG
GATCGGCGAGGTAAAGACGAAGCGGATAGCGCCGTTTGCTACGACATAGG
ATGTGAAATACCGGCTACCCGTCTCGAGGCCGCGGTACGCGACCGTTGTGA
AGCCGAAAAGCGTGTTGTAGTATGAGGCGGCTTGCTTGGCGTTACCTACCC
ACCAGGTAACGTGGTCATAGCCCTGAAACTCGGGCACGCCGGCCTGGACG
GCGTAGGAGTCTGATGATGTATGAGGGGGTGGAAGGTAGCATCTCTTGGGG
GAGACTCTGAGATCGCTGAGGGAGCCATGGCTGTTTGTGGTATGTCGGGTT
AGTTACTCAACGATAGATATCTTGATAACCTGATATCACCGAGACCGAGGGT
AGGTAGATGAGATTAGACGGAATAGGTAGGGAAATAGAGACTAGCTAAGTG
CTGAGATACTTGATGTTTAAGTGATTCTATGATATAAGACTTATATAGATGAA
CTTAAACTAAGGAGATATAAGCACGAAGATATAGACAGTTAGCCTACCTTTA
GACGTCCCTTACCTACATTATACTTACGCCTACCTCTAACTTTATGGCGGGTA
ACTATGGAAAGGTCGGGGCCGGATTTGATTCCGCGTTCTTGGATGGCTTGG
ACGGCTTTCTTAAAGCCCACAGTCATACGTAAGATCTCCAAAAGTTAGGTC
CCGGTAATAACTTCCTATGCGTGGGACTGAGATTACCGAACTCTGATTTGCA
ATTGGCAACATTGAACCAAGGAAGCAGGTGGCAGGTGGCAGGTGGCAGG
GTGTCTTGACCGCGAGAAAAAGGGTGATGGAGTATGGAGCCTCGATAGTG
ACCAAATGGGTCTCGAGCGGCTGCGGAAAACAAAGACGTCAGATGGCTTG
CCGAAGCTAGCCGTACCCGAAATCCACGCTTCCGCCCTGCAGCTATCCGAC
GCCACCCACTCAGAGCCAAGGAATATCCAATAAGGGCAAATGGCTTTACGC
GGTTAGAGGGGACGAAAGCTTGTTTTCCTCAGGTTATTCCCTTATCGTTGGC
CATCACTGACATATCACTGACCTTAGGTAGCTGCAGGTGAACCATTCAAAC
TTGTCGGCATTGGAAATAGGACCCGAGGTGGAGAAGAGGCCGTTTTCTTTA
GTGGCATTTGCATGGCAAATATAACCAGTAGTTGACCATCAGTCTGTCGCTG
TGAAGCTTAAATGTCAGCCCTTATTCATGTGCATCTCTGCTGTGTAATTCTTT
TACCTCTAGTCTACTTATCTAGCTCGTCTCACCAAGATTGCCGATATCGTTGC
TTGTCTCATCCTACCTCGCTACCGCTTGTCGTCCTCAAGATTCCGAAAAGTT
CCAGCAGGCCATCTACATCGACGAATTAAACTACTCTTCATAGATTGTAGAC
ATGGCGGCGGCAAGCTCTCTCAGTCGGCGCGACTTTAACGTGCTCGAGAA
AATCAAAGACCCAGAATCAAATCCATTGACGGCTGTTATCGTAGATACGGC

ATTACCTAAAGATCCGAACATAACGGACACATCCGTATATGACCGTGTCTCT
GGGAAAGAACGAGAGATTGTGTTAACTATGCAACAACTGGAGATGCAACT
AGCCGGCCTGAAACCTGCGACGACGACAGAGCCGATAGAAGAATATCGCC
AGTGCGTATCGAGATTAGGCGAAACTCATATCGGAGTACCCCGATTATGCTA
GCGCTCGCAACAATAGAGCACAAGCCTTTAGAAGGCTGTATGGAGATACAA
TGCTCTTGACTGGCGTCCATGACTCAAACCGGCTACTGAGGGATGTTGACG
ATTCCGAAAAATCGCAAGCGGCAGTATCGGCCCTCTCAGATCTTGACAAAG
CTATCGCATTACTGACACCGAGGAGTCTCTTTTCTTCGATTTCGCCCCAAGC
TGGAAAGACCTTATCCCTAGCGCACACCCAACGAGCAGCGATATATCATATG
ACTGCAAAGTCCTTCCAAGCTAGCAATGTGGCTGGTGTTTCTGAAAGGAA
GGAATCCACATGGACAAAGATTGAATTCGAAGAGGCAGCCTCGAGAGACT
TTGCTCTGGGAGGGAGATACGGTAATGAGATCGCAAAGGGGTTGGCTGTG
AGTACTAACCCAACCGCCAAGCTGTGCGGCCAGATGGTTAGGGAAGCTATG
AAGAAAGAATATGGCCCGGCGTATGCGGAATAATGGCTCGTCTTTAGAATG
TTACTATTACTCATTGCGGAGAACTTCAAATATAAAGACCGCTACGTTGTCT
AGCATTGTCTCTAGTATCGTCCATTACCCCTCGGCTTATGGAACTCGGTAGA
ATATCATTATGTTGACTTGAAGAGATTGCCTAGTCGTCTAGAGAAAAGACCA
TATTAGACAATTTCAAACAGTGCTGTGGATAGTTCGTAGTATATACCGTTTTC
CAGCTTCGAAGATCTGCTGCGTTGTTTTACCTTTGTCTTTCCCTTCGCGGTT
GATTCTCGTAGCAGCGGCTCCGAGCCCGATGAGCACTGCGAAGATTGGGTC
CGCTACACGGGAGATGATATATGTCGGAGGAGCCATTGTCATTCGATCTAGG
TTCTGTTGTTCGCAGCGCGTCTTTGGGTCCTATTCTTTCAATATGTAGAATAC
ATAGACGAAGGTAGATGTTCTGTGTGTTGTAGAAAGTTAATATTGGTCCTTG
GAAGTATCGTTCCAGGTTGTTACCCGCACTTGATGTTCTAGCAGTTCTAGCA
TTATTCCGCAACTGCCCCACCGTTAATTGTACAACGCATTGACCACCTTCAA
TAATGATAGAATGCGACCCCACTAGCTAAATTTTGTCCAATCAAAAATAAAA
CCCTTAGGCTCGCTTAATTTTCTCAGGCACAACTTGGATTTTCTTAGCCATC
GCGAACTTGCGAACTTACCTCAATTCTGAACTATAATCTTCAAACGACCAAT
TACGCCAACCCACTTCCCAACTTCCCCGACTTACTTCGCCCTTTCGACAAA
CACGACAGCGAAGAAATCATCATGAAGTTCCTTAAGGTCGGCCGAGTTGCC
ATCATCACCCGCGGCAGATATGCCGGTAAGAAGGTACGTTGTCGAGAAGGG
AAAAAAATCGATGATTGAGAAAATACGCCGACATGTGTGATGGATTGGAGA
TTGAATATTCGATCACAACACTACATACACTATCTACACCTTTCGATATGTCC
GATTTCGAGTCCCTTTGAAAACAAATTTCTACATTATCCTACAGTCCTTGCG
AAATTGAAATCTACACCTAGAACAATCATTTTGATATAGATATGGAACAAGG
AACGAATCTTAACTAATCTCCCTCTTCCACAGGTCGTCATCATCCAGCCCGT
CGACAGCGGCAACAAGGCACACCCCTACGGCCACGCTCTAGTCGCCGGTA
TCGAGCGCTACCCCTCCAAGATCACCCGACGCATGTCCAAGACGCGCCAA

GAGAAGCGCTCCAAGATCAAGCCTTTCATCAAGGCCATCAACTACAACCAC
TTGATGCCCACACGATACACTCTCGAGCTTGAGGGTCTTAAGGGTGCTATTT
CTGGCGAGACCTTCAAGGAGGTCAGCCAACGGGAGGATGCTAAGAAGAA
CGTCAAGAAGGTCTTGGAGGAGCGCTACACAAGTGGAAAGAACCGATGGT
TCTTCACCCCTCTGAGTATGTTTCCCTTTCTTTTTGTTACCATATGTATGGTCT
CTACTACGAAAGCACGTGCTAATGATGTCCTAACAGAATTCTAATCCTCTCG
TCTGCATACTTCTGTCGTTCCGGTTCTAGGGGTTCGGTCTGGGATGTAAATC
GCATCATCAATGGCATTAGGAGCCTCAAGGGTCTTGCTCTGAATTCAGAATC
ATGAAAATGAAACACACACTTGACGAATGAACTCACATGCTTCGGGTATAT
ACATGTGACTTGCGTGCTAGTCTCCTGGATTCTCTCAATTACCTACAACTCG
CCTCTTGATTAGGTGCCTGGGACATGGACTGGAAGTCTACTTGACAATATG
ATTAACGCCAGAACTTAGAGTGTGGCATATTCGGCTTGGGGAAGAACAAGA
ACTGGAATCTGGAAATAAGACGGAGGGAAACTATCCATGGATGCCTTCAAC
AAGCCCAAGTCTTCCAGCGTCTACCCCTTGTAACCTTAGGCCTGTAGATATT
TGAACTATACGGAGAATAGATCAAAACCATTCCCTCTATAACATCAAGTGGT
TACCAATTCGTATGTGATAATGTCACCTCAGATATTCAGAACCTATAATGAAC
CTGTGTCGTAGCCATGCGTTCCCGAAGTAACCTATTGATACGCCGAATTCTT
AACCTTTCGGACATGTAAGTAGCGATGATCAGTTCCTGGACCGTCACTTGT
ATTTTGTCACTTTCTGGATACAAGTCTGACCGAGGGGACGGCTCCGAACCA
TCAACGATTGGAAAGAAATATACCAGATCTACTCTCGACATCCCACGAAAT
GCTATCTCATCCGATCTGCTTCGGGGATATACGGCCGATCAACTCATGTTCC
AGGCAATTCTGCTACTACTCCGTCACGAAGGATCATATTACCCTGACGACCC
AGAATTGAATCCTATAGGCTAAATGAACATCTTCCCCGACCTAGAACTTCAC
ATATTTAGTATTTCCACCATTCAAGCAGGCCACTCAATCACACAATGGACTA
AGAGTGCACAGATCGATAATAGACAAGCAAATGTTTAAACTTTAAGTCTCA
TTTTCATTCAGGCCCGGTTTCCCTTGTGACCTTTCTAAGCTACATGAATTAG
CCCTATCGTGATACGAGCAGATGCAACCCGGCCCTGTGCAATTGACGCCTT
GTCTGCTCTCTATAGCCGTACAAAAGTAAAAAAAGAACCATGTGGCTACCT
ATCTCATTCAGTGCCCACGGCCACAAAGAAAAATGTCTCTCCAATTACTAC
CACCCCCGGCGAAGATGAATTGGGAAATGTGCAAGTATGTACTCGAAAATT
CAGGGAGAACGCATACTAGGTCATCACGTAGCGTTCACCCGTCATCATGCT
TAGCTCTATAAAGCTTTAGTTTAACCCCCGTCCACCCAGAATATTACAAATG
TGTACGGTGAAAGGGAGGTCTAGTCTTGTCACATATTTGCTTAAGAGGAGA
CGCCGAATTTGCCAGCCGCGCTGGAGAGGTATTGCGACGGGTCGTAGTCG
GGGGCGTATGTAGGGGCCAAGCCACCGAAGAGGATGAAGACGAATAGTAC
GACTGCGGCAACGACGATGATCTTGCGAAGAGCAGGACGAGCGGCACCG
AACTCAGCCCACTGTCGGTTAGCCTCACCGGCGTAACCCTTGAGAGCTTCG
AATCCAAGCTTGTAGTAGACCATACCGCCAAGAGCAATGGTGTAACCAAAG

GCCTGGAGACCCGAAACGGGAGTGCCCCAGATAAGCATGGAGGCAACGAC
CAACATGACATCCTTGAGGACACCGCAGAGGGTCATGATGACAGCAGAAG
TCTTGCCAATCTGTATAATTATTAGCAGCTGAAATGTATATGCAGCGCGTGG
ATTAACAACTTACCAAGAAGACGACGGACACGTTCAACAGGAAGGCGCAA
AGACCGTTCAGGAAAAAGGTGAAGAGACCAACGTGGTACACTTCAGCCAT
GGACACCTTAGGAACTTCCCAGGCAAGCGCAACAACACCGTTCATGGCAG
CGCAGATAGGGGCAAAGTAGTACAGGGACACGAGGGGGTCCATCTTGTAC
TCGGCAGAGCTGAGGAGAGCTTGGACCAAGTTGAGACGGATAGCCTCGAA
CATAAGCCCACCTAGCTGGTAGAGGAAACCAATCCAGACGAACTTGATTTC
ACCGAAAGAGGCAATCATGACACCAACGACGATGGCAGACACGTTCGCAA
GCTGCTTCAAGTTGGGGGGGGCAAGACCCATAGCCCAGCTAGAAAGGAGG
ACAGCAACGGGGGTAGTAGCCTATAACATTCAACGTCAGCACCGTGTTACT
TCCAAATTGCGACCGTGATGGATGTTCGTACCTTGATCATCTGGATGAAAGC
AACACTGAGGTAGAGGTAGGTCAAGTTACCGCAGATCAAGCTCAAGCTGA
AGAAGACACCAATAGGGACGATAGCACGGAGGTAGACGCGACCGGTCATC
TTGACAGACTTGCGACCGTCAAGGAGGGTAGTGTAGCGGGCCATCAATTGT
GTCGCAACGGTTGCGAAGGACAGATGGTAGGTGGTGAGGATAACAGGGTA
ACCTGGGACATCGTTAGATCGTGCCCACGGTCGCAACTTCGACTTCAATAA
TTTAAAGGGGCTTCCTTACGGAACTCGAGAGTGTCTAGAATCCATTTGTTG
AAGAGAATGACAGATGACGACAAACTGATCCATACTCTGTAACGATTCGGT
GTCAGCTAGCTATCCTTCCGTGTCTCGACAGTGATCGTGGTTTTAGGATTCG
ACGTACGCAACGTAGAGAGCCGGGTGAATGGCCGGCTTAGGAGGTTGGGG
CTTGTCAGCCTCAGGGTTCGTAGTGGGCAGAATAGGTCCCTTGGGCTCAGG
TCGAGGGACCTCGCCAGATGTACGACTCTTTTCGTCCGCCATTGTTGACTT
GATTAATCTTGGGTTAAAAACGTGAATGTAATCGCGAGGTGCGATATAATTG
TAAAGGGGGTGGTAGTGGTAGTGAAAGTGAATGAATTGAAGATGACACGA
TAGAATGAGTGACTAGGCCAAGCCTGCTGAGACCAATGAAAGGTTTAGGT
AATTTTTGGGGGTGGCGTTTACGATTCAAGAATTCACAATGCGAGATGAAC
TGCGAATATGAACTCTTGTTCTCTCTTCCGTGAAGCTTCTAAAAAAGAGGC
TGTTCGGTATGTGCGGAGGAGAGAAAGGTCGTCGACTCGTGGAGGGACAG
AAGTTGACGAGAAGAAAAAAGGACAAAAAGAGAAGGGTGAGAAAGAGA
TGTACGGCTAATAAATTTTCAAATTGGAAAGGTACCATGAAAGGTACCGAG
TCGCCTTCGCGTTACGACAAGGCACACCTAGCAATTTTGGGATACAGGGTA
TACAAGGGGTGCAGGGGGGTTTAACGGGGGGAGATACGCAGACGCAGTGT
ACATACATACAAAGAGATAGCGGAGGTAGTGGCATGGGCAGGTATAGGTAC
AGGGAGTTGAGCTTATCCCGCACAATTTTAGCCCCCAAAACCCGCCCCAAT
GAAATCAATACTTTCAGACACGGTCGCATCAATTATCAGTGCGTGCCGGTG
TACTGTAATCACTGTAATCAGTCGAACTTGCTGAATTCTACAAACAATTACT

GCAAGCACAGCCCGCATAGTTCCCAGGGCTGGCTATCAGTCACCCTTAAAG
TGCTTTGAATTTTTAACAGCGCTGGGCAAGACCCCACAATGTAAACCGTCC
TGACCATTGTACACCCAGATTAATGTACCGGTACATAATCGCGTCCCATTTG
ATCTTGCAAAAACCCCCACTACAATAACCCACTAGCTCATTCCCTGGAATTC
CTGCGCTCAGGGGAAAGGGGGCAGCAATTGCGTAGTGATACTCTAATAGGA
ATAGCATGCGATTGGTAAAATAGCTAAAACCTGAAATCTAGCGGGAATCTA
GTAATGACTAGCCCTGGCGGTAGCAAGTAACACAAGCATAACACAGGCAAT
AGCCACTGATGTCGTTTTAAGAGGCTCTCATTTCATCACGGGCGCTCAGCAT
TCATAGCAAGTACCCACTGAATATCCCTTTTTCAGGTCCTCTAACAAGTCCC
TTCCCTTCCATACGGCCAGCCACAGGATACCAAGGCTTGGATCGTCAGCAC
GTTTTCTTCTCTCTCATCCAATCCAACCAATCCAATTTTAGATCTGTTACCGG
CACTGCTTTCTACTGTGTATTAGCATCTGTTGATGTGCGGTTTTATTTTAAAG
AGTTTGTTTCATCATAACACTGAACACTTAAAAGGGGCTCTTTCAGCCCTCT
TCGCCCTTCCACAATCTTGGTCCTTGGTCCCTGGTAGAAGAACGTGAGAAG
ACGCGGGAAGACGTGGAAGACTACGCATACCAGCAGCTTGGGCCGAATAA
CAAATAGCATTCCTCTTCCCAATATCGAAATTCGTAAGTCAACTCTATCTGTC
TAACATGAAGGCATGCAAGATTCGGTTTAGGCTTTCTACATGCTTCAAATTC
CTGTTTTGTAATTGGCCACCCGCCTAAGACTCGTATACATACGTATACGCAC
CAGGCACACCTACGTCGCACAACACAAGAAAAACATCGACGAGGACAGAT
GCGGGATGTTTGCCGTTCACTAAAACCACAATAACCGCAATGTCAGGATGT
TTCATCTCCTGCCCGTCATATTTAGAAGCCGCTTGAATCTAAGACTAAGTAA
AGCCGAGAGATTTGAAGCCAGGGCTTATTGGGCTAATCGACGATTATCGAT
GATTATGATTGTGCTCCGTGCCATTATATCTCCTCTGTGGCGTGGACGATGG
AGAAAAAAAAGTACATATCCTATGTAAAAAAGAAAAACCTCCGAGTTCCA
ACATTTCATCTCGGATGGGACTAGCCTTGACTAGGCTATTAAAAGAAACCAT
CGTGAACTTGAGGCTCCGCGGGGTGGTAAGTGGCTAGTCTTCCCGGATACC
CCCCTTACCTGTTGAAGGTTTGCTCGTATGAAACGTCGTCATCATGTGGTTT
CTCAGCTAAAATACGAGGTGCAAAAGCAGGATATATACTTCGGAGCCACGT
ATCACGTCCTTTTCACCACTTTAGCAACTTTTTACAGCTTGCGGTTCGCCAT
TGTCATTCACTCTTCGAGTTCCTTGGAAACACACTGCTCGAAGGTTTCTTC
ATTGCCGGTTACATCTGTTATGTACTCGGGTTACATGAATCGTTGGTAACCT
GTTGTTTGTAATATCTTCCTATCCATTACTTAGCCCATTACTTAGCTGAGGCA
GCATCCTACGTCCCAAGCGAATGGTTGCTCATCTTGCAGATGGGTAGATAA
ACCTACGGCGCGGCATATTCACCCCGCTAGTTGACAGCTTTCACTCAACCC
AAATAGGGCTTCTATGGCGCGTCAGCTGCATGTCAGCTAACCGCGAACGCT
GATTAGGGTCCGTGTCATACGTTAGTAGCCGAAGGTTGGTAGCCACGTGCG
GTAAGAGGCTTGTCTGACCTAGGCCGACCAGACGGCTCGTGTCGTTTATAT
GCAGTTATCAATAGTGCATAGGAATGGCAAGCCATTTTTACAATAGTAGGTG

AAGTGTTCAACTCTTACGGCAGGATCATGCAAGATGATGAAATGCTTCCAA
GAGGATTGTCTTCGGATCGTCATAATGTGCTTAGTTAGATTAGCAAAAATAT
GTATAATACTTTTATTATACCTATTTCTGATTTGATTTTGCGATTTTTAGCAGT
TGATTTCAATGATCGTCTTCTAGTTATCGTTTTGGCTGACTCTCGTCTCATTG
AATGCACTACGAAAATGGTTAACCCCTATTATGGCGTAATACCTATGCCCAC
TTCATTTTGATGACAAGTATTATAGTAGCCACCCAATTAGTGCTAAGCCATC
GGCAAGATGATAAGATCTATGTATCTATGCAAGAAATCATTTCTCGCTTTGTA
CCTACGTTGTATCCAATTCTCTCTTTTTTCTTGGCTCATGTACCGATTCGGAC
TTTCTACAAATACAGGAACCGGCCGAGTGTCAGCCTTTTCACTTTAGGTAC
GTATACGACAGATATGATAAATAGGTAGGCTCGCTCAAGAGGGAACTATTCT
CAACTTAGCCTCGACGGAACTGATCAACTCGGCATTTCCACTTGGGGGATT
TGGTTTTGAGCCGCCCGGTGGTCCCGTGGGCCTAAACATCAACTTGAGACG
GCGGCTTTGGCGACTCTGCAAATCTTCACTGTCGATCTCGCGGAGCTAACC
GGTCCTGAGGCTCCAACTAGAAGTCGAGTTCGAGAAGGGATAGAGTATTAC
CGACCCAAACTCGGTAGTATTGAGAGCGGAAAACAAGATCGTCGGAGGTG
GATGTACAAGTACATAACTACACATGTGGGTAGAAATAGCCATCGACCTGG
TCGCGGACGGGAAACGACCTTATCGAAATTAAACGATCTTCGAGACCCAAT
TTGATTGACGAATGCAACACATTAATCGCGAGTGATAAAGTGGGAGTAGCA
TAGTAGCACGCCTACTATGTAAAAACTAAGGTAAGAGAAGTTCCCTACTCC
GATATTAGTTATTTTAATTGGTAGGTAGATTCAGCACTACTATGCTACTATGTA
CACTACGTAGTACACTCGAACTACACCAAGTTGAGGTGCCGGGATTCTAGA
GCTGGCTAGCATTTGGTAGATAATACTCTCATCAGCGTTCCCACAACTTGAC
TCGCGCACCCGTTGCCACACTCTTTCGACAAAATCGCCGTCACACAGTTTT
AGTATAACACATATTAACACATAACATATAAAAATATTGCAAAAGAAGGGAT
GCGTTTAAGGGGGAGCATTGAAACGAACAGACTCGGCGAAGCTTACGAGT
AAAATCCTTGAATCATATGAATAATAGTCGTCTCGCCTTACGTACATACTGCC
TTAGTCAAATGCTACTAAGGCTCGGCACCTCAGAAGAGACGGCTTGTCTAC
AACATAATGTGTGTAATGCCTCTTGCGTAGGCAAAGACAGTATACGCATACC
CACCTGGGCAGTCAGCCAATATGTCGTGCTCGATATCTAAGATAGCTTGAGG
ACGACGTTGTGCAAGGGACGGGTATGAGCTTCATCTTAATTATGAAATTGC
GAGCAGGGGGGGTTTCCTGGGTGGTGCTGGTGGCCGCCAGTTGGTGGTAA
ATCAACTCTCTTTATGCTCTTAATTGCGCTTGCGCCAATTGCAATGGCACAC
CGAATTTGCTCCTATCAGAAGTTGACCACCTTTCACATGCGGAACGTCTAA
CGTCTGTCTAATGTCGGTTATGCATTTTACGCTGTTCTACGCACAGTATGTCG
ATGAAATAAACCTAGTAATACCCAGTAGAAGGGAGTGATCGTCATAATCGTC
CATATCTCACCTATTAGGGAAGTTAGAGGAGAAGTCTTCCTAAGTCAACCC
AACTTTACCCCTGACTTGATGGTGTTCAGTGGCTTTCTAAAGGAGGGGTGG
GAAACTAGCGGTAACTAAGCGGTGTTTACTATTCGTGCTACTTGGTCGAATC

ATGACGACGAGGCTACTTTGCTGCTTTCAGGGACATGCCCAGGAGCATGCC
TCTAAAGACTAACATAAAGTTGTTAACTTTGGTCCCTGCAGCCGCCTCGAA
TCCGTTTTTTAATAGCGAGGGGAAAAAAGGTAGATTGAGTAGATTGATCAA
ACTGATCTAGAATAACAGCCAACCTAATCTTTACTTCAAATCCTAATTTAGA
AACAGAGCATCGTTTGACTAAAAAGTCTCGATTGTTCGAACATCTTAATTGT
CATGTTTCATGCATATCTCTCTCGGGACATAAGACATGGAATACGCTGGAAT
TCATATGATTTGTTGGTTCGATGTGCTTCAAGTTTTGGGACGAACTTTCTCA
TTCGAGGCTCCAGATTCTCCCTCAATCAATACCTCTCTAGCTTACCTACCTTA
CTTGGGTAGTATGGAGGTATGGGGAGGTACTGTAGGTAACTGTGGTGATAAT
AATAATAAACAATCAAATATTACCTTGGAACAGCACTACTACTAGGTAAGCT
GGTAGAATATAACAAAGGATGCTGCAGTCAAGTCAAGACTGTCAAGTTGA
GGACTTTTCATATGCAGGTATAATGTAGCGTCTTGACCGTATCTGGTATAAGA
CCCCCTCTAATTGGACGGATGAAGATTGGAAATGTTGCCAAGTTGCATGAC
GGAGAGACAGGGGTGGCGGATATGTGGGTCGACGCACACACACTACCTGG
CCACTACATGGCCACTACTATGCGGTACAGCATGGTCTACACGGCCTACCA
GGAGTCTACGCAGGTTTTAGCATGGTAGCTCCTAGTACTCCTGGTACATACC
TAGTACACTCCCTAGCTTGATACCCCCAGCTAACACAATTCCAGATGTCCAC
AATTTTTTTAGTCTAGTACCCCTCCAAAATCTCCAAAATAACCACCTGGGCA
CTCTGCAGTGGGCAAAATATTATACATAGAAAAACACCACGCTTCTCTCCTG
ACCACCGGTCTCTTTTTCTCGAGACTTTTCGTCTGCCACACGAACTTCCTTC
GCGTAAACCATAGACATAGACAGACTTTATCCTTCAGCCTTCGTTGACTTGG
ATAGTCTATCCATTCATTTTCGTCTTTCAGACAACCTTTTGCCTATAGAGCTT
CACTTGACCCGGCCACACTTCCCCGCCTTGTGTTGAAACAACTAAGACAA
GATGTGTGCAGACATGTAAGTTTATTTGATCCTTTGAACATCGGATCACATAT
TTCCATGTCAAGCTAGCAAGTACACCTTCATAAAAGTTCAATTGACTAATAT
ATCCACTTACAGCAACGTCGACGTCCTCGTTATCGGTGCTGGCCCTACCGG
TTTGGGTGCTGCGAAGCGCCTCCACCAAGTTGTATGCCACACCGCCATTCA
CTCACTAGATTATCGTGGGCTAACATTATGGTGTAGAATGGACCTTCGTGGT
TAATCATTGACTCCAACGAGAAGGCTGGTGGTCTTGCCTCCACTGACGTCA
CTCCTGAGGGCTTTGTAAGTCTATATTCACCTCAAGGTCGTTTCCGCCCAGC
AATAAGCTAACCGTCGACTCTGCTTAACAGCTCTACGATGTCGGTGGTCAC
GTCATCTTCTCCCACTATAAGTACTTCGACGACTGTATCGACGAGGCTCTCC
CTAAGGAAGATGACTGGTACACTCACCAGCGTATCTCCTACGTCCGCTACA
AGGGCCTCTGGGTCCCTTACCCCTTCCAGAACAACATCTCTATGCTGCCCA
AGGAGGACCAGGTTGAGGCCCTTGATGGACTAATTGATGCTTCCTTGGAAG
TTCGTGTTGCGAACACTAAGCCCAAGGACTTCGATGAGTGGATCTTGCGTA
TGACTGGTAAGGGTATTGCCGACATGTTCATGCGACCGTACAACTACAAGG
TCTGGGCTGTTCCTACCAGCAAGGTAAGTCGTAAAATATTCCCAAAATACC

GAGGAAGAAGTTAGCTAATTTGCGGTCTCTAGATGCAATGCCAATGGCTCG
GTGAGCGTGTGGCTGCCCCTGACGTCAAGAATGTTGTGAAGAACGTCGTT
CTAAACAAGGTTGCTGGTAATTGGGGCCCTAACGCCACTTTCCGCTTCCCT
GCCCGTGACGGTACCGGTGGTATCTGGATTGCCGTTGCCGACACCCTCCCA
AAGAGAAGAAGCGCTTCGGCAAGAAGGGTGAGGTTACCAAGGTCGATGC
CAACAAGAAGGTCGTCACTCTCCAGGACGGCACCACAATTGGCTACGACA
AGCTCATCTCCACCATGAACGTTGACCAATTGGTCGAGTCCATGGGCAACC
AGGAGCTAGTCACCCTTAGCAAGGGTCTCTTCTACTCTTCTACTCACGTCAT
CGGTGTTGGTCTACGTGGCGCACGACCCGAGCGTATCGGTGATAAGTGCTG
GGTAAGTTATCAAAGAATTGAGTCGCATTGAACTGTCGCTAATTATCCTTAG
CTTTACTTCCCCGAAGACAACTGCCCCGTTCTACCGTGCAACCATCTTCTCT
AACTACTCCCCCTACAACCAGCCCCAGGCTGATGTTAAACTCCCGACGATT
CAGATCGCCGATGGTAGCAAGCCTGCCAGCTCAGAGCCCAAGGAGGGCCC
TTACTGGTCTATCATGTTGGAGGTTTCTCAATCTTCCATGAAGCCGGTTGAC
GAGGCCAACCTCCTGAAGGACTGCATTCAGGGCTTAGTCAACACCGACAT
GATCAAGTCTACTGACGAGATTGTCTCCACTTACCACCGCAAGTTCGACCA
CGGTTACCCCACACCTACTCTAGAGCGTGAAGGTGTTCTCAAGCAACTTCT
TCCCAAGCTCCAGGACCTCGACATCTACTCTCGTGGCCGTTTCGGCAGCTG
GAGATACGAGGTTGGTAACCAGGACCACTCGTTTATGTTGGGTGTTGAGGC
CGCCGACCACATCGTCAACGGTGCCGTCGAACTTACGCTTAACTACCCCGA
CTTCGTCAACACCCGCCAGAACACCGAGCGTCGTCTCGACCAAGGCCCGT
TGGCGGCTAAGAAGAGCATTCCTTCGCACCCCAAATAAATGCCGGTCTCAA
TGGCAATTGGCCCTTGAATGGCGCATAGGCTCGCTTAATCTTGCGTAGCCTG
GAGGCCCCTATGAAATAGTAGGGAGCTTTTAAGAGTTATGTTGGGCATATGG
CAGTTCACGTTTGATGAGTGCGACGGTAATGGAATGTTAGGATGATTCTAAT
GCGGTCGTGTGATGATATGGCATTATAAACCCATGTTTCTCTGCGTTAGAGA
GCCTGATGAATAGAAAGAAAGAAATAGAATTATGAACCCATTGCTTTTTTG
ATTGTACAAATAGTGAGTGTGCATAATGTACGGTGATGTAACTCGGTTCACT
CGACCAATGAGTTTGGGCGAGGTTGAAAATCCACCTATCTCTAAGTGAAGA
GTCGCATGAAATATTGATAGCCTTGCAGCTGTTAAAATCTTACATGCCTCCG
GCTGCTACATAGTTTACGTAGGTGGCTCATAGAGATCTCTTGGTTCTCGATT
GCAAATGACAAATCTGTTCATGTAGGCAAGTAGGCACTGTACCTGATAAAA
CATTTGCCCTTAATACGACTTGGCGGCATATACTTAGTGGTATCGCTGACGTA
TCTGGCGTCCTGTAATACCATTTTGTGTGCAGTTGAAAAAATGTTGTCAAA
AGAAGAGTCGTGGGATTGCTTTTTAGTAAGTGTCTAGATAAACTAATTATGC
CACTTGTATCGACT

>Mosaic_sequence_78

AAGTCTTGATCGGCCGACAGGATGTCGTCGAAAGTGAGGGCAAGGCGTTC
AACCGGGGTGGGGCTTGGCAGGCGTACCGGAAACAGATCGAGCGTCGTTG
TGGACCCTTGGCAGCTTCTGGCCAGGAACAGCCAGGTCGCCCAGTGATGC
CATCATGACGGGCATGTGGAGCCTCTTTGGCACTGCCAGCGGGCGAGCCG
CGGACAGCTCGCCCAAGAGCCGAGTCAGATGACCCGCTTCAATCCAGTGG
CCGAACGTGAGTGCGTCCAGAGGTCCGCCTAGCGGCCCCACCCCTGCGAG
CCAAGCGGCGAGGAGGATATGTGCCGGAACCTCTTCGTCGATATACAGCTG
CACAACTTCAGCCACATCCCATGTGTCCAGCAGGGCGTCCCTCAGATCCAA
CGCGCCACGGTCGACTTCCATGGCTTCCATCTCGCTCGACTCTGAAGATGA
ATCCGGTATCGGGCTGTCCTTGTGTTTCAAGAACAGCACAGGTCTGTGGTA
CAGGACAACTTGGGGAGCTGGTCTTGCCACTCCACGGACGGCATCGCCAG
AAGTGCGAGCAGGGCGCGAGATCACGCGCAGGCTGTCTCCAGGCCAATCG
CGCGCCATCCTCATGTAGAGCAGCGCACGCAGATTGGGCAGATGCTCCACT
GCGTTGCGCACCTCGAACCCGGCCCACCTGCCGCGGCCCTGCGCGATCAG
CCGAGGTATCGTAGATAGTGCCTGGACGCAGTCTGACTTACCGAGTCTCTG
CTCAAGCGCACGGTCTGGCTGAGGTTATTCCAGCTGACCTTGGCGTACAGT
CTACGGACCGCGCAGTCGAAGATATCCCGCGAGACTCGCATCGCGCGGAC
GAGGTCCTGTCGATCCAGGTACCCCACGATCCTGCCGCATATCGGCACGAC
TGCGAACGCTCGCTCCGCCGCAGTCTGCGGCAGATCTGCCGCCCATGTCGT
CATGCTGCGCGAGAGAGATGCGGTCGCAATCAGACGCAGTGACTGATTTG
TGCAACGAGCACCTCAAGTCAGCCAACAAGGCTTGCGCTGGGCCCTCTTT
TCAGCTCGCGCATGCCCGATACTCTAGCGCAAAGGAAGTCTGATTGGTGGC
GTCCCGTTTCATCTCACCGCATTGACCGCGGCGATGCCATCGCAGCTGTCTC
GCGCCAAGAGTCCCACTGACTGGCAGGTGAGCCAGGTTAAAGACGAAGCC
CTAGGCTCCGCACAGGGCCGAGAGGCGTCTCATCGCTGAAATGGGGTTAA
ACCGGCCTGACGCGTGCGTCCGGGGAATTGCTCTGCAGCAAGAATAACAG
GGAGCACCGCTTTGCGCCAAGGTTTGACTCTTGGCATCGATCCTGCGGCGA
GTGCTCTCGACGTTACGCAAGGCAGGGGTGAAGAGTGCCAGGGCTGGGCT
GCTGGGATTTATGCTTGTTGCACGTCTCCTGGATATCGTCCCGGCGGGAAGC
GGACCACTTGCTTCAGGAGACGGCGATGGATTCGAGATATTTCAGACTGCT
GGCGCCCAGGTAGTAGTATTGGCAGTGTCCAGCCCGATCAGAAGCGCCGAT
GCAAGTGCAAAGGATGCTTGCATCGCCGACTGACCGGACGCGACCCACCT
CTCCGTGTTGCACACACGAGCCAGGCAACCTCGCGCTGGGCGACGACGTT
TGCCCATCCTCTCCATCGAGCTACAGTCGGTCGCTTGAGGGAGCACGATGC
GCTTCAGTCCGCTATCGCGAGCAGCTTCACTCGCACAGGGGTACGGGCAC
AGCCTGGCGCGCAGAGCGCTCGCACGACCGCGATTGACGCTGCTTGTGGT
CCTTCCCATCCTCGTCCTGTGGGCATGGTACGGGCCTCTACCGCCTGCCCA
GGGGCCCGTGGGGGCGTTGGTACGGAACTACGTCGTGGGCATGGTGCCAC

CGCTCGCAAACGTCCTCCCGGCAGCCTGGGACCCCGTGCAGAACGAGCCT
CGGTTCATCCCCGAGATCTTCGACGCGCCTTGGATGGGCACATGGACGCTG
CCCGAGCTCTCGGCGCCGGTGCGCAACGCCTCGTGGCCGCACCACACGTA
TCCTATCACCTGTCGCACTTTGTCTCGCCCAGCGCGTACATCTCGCCCAGCA
TCATCAAGCTGCACGTCTTTTCCAAGGCGTGCAAGCCGGACGAGGGTGCA
GGTACAAAGAGGCAGCTCATCAGGGTGCTGCACCCTGCGCGCAATGTCCC
CCCTGAATTGCGGCACCTCATCGAGCTCAAGTTCATCGTTGGACATGCGAG
ACGGCCAGATGGGACGCCCGACGAGGAGGTTCAGCGCTCTCTCGATGCGG
AGCAGGCAGAGTATGGCGACCTGGTCCGTCTCAATCTCAAGAACGGAGAG
AACCTCAGAGAAGGCAAGATCCTCGATTGGGTCCACGCTGTGGGGAGCGG
TCGTGATGGGGGCGGCCCTCCTGGTGGCTGTTCAAGGTGGACGACGATGT
GAGTGATCGCGAGGCGGAGAGTCAACTCGGCTGACGGAACAGACCGTCAT
GCGCCTCCCAAACTTCCTCGACTCGCTTCTCACCTTCAATGCTCATGACCC
GTATTACCTGGGCACATCGCTCAACCGATGGCCATCGTATCATGCACACTTT
ACAGGTATGCTTACGGGCTTCAGCTGGGGCGTGGTGAGCAGTCCCAACAC
GAGCATACGTACATTGCTGATTGCGCTGTAGGTCAAGACGATGGCGCACGG
TATAGGTCGCATGACGCGCGAAGAGATCGAGAAGTCGTGGGACGACGACG
TCATGACTGGCGAGATCATGGTGGGTAGTTGCGATCCGGTCAACTCCCTGT
AGAGACGCTGACGAAACGTAACAGTTCAACCTCCCTTCCGGGCCAAATTG
TCGTCGGTCATACCATCCATTCGACGTCACGCTGCCCAAGTACTGCAACCC
ACACCAGCCCCCTCGATGGGGCTACCAATCCGTGGCCCCCACCTTTAGACC
TGCGATCCGGCTTGCCGTCGACACGATTATCTCCGCCATATGGGTGACTCGG
AGGTGTGGTTCATCAAGGGTGATATCGCGCGGCATACCTGGTGGTACAAGA
CCCTTGAAGGGTACGAGCAGGAATGGCGCAGACATGTCGAGGGCAGGATG
TGGGTGCCACCAAAAGCGTTGCAGTGTACGAGGAGGTCCTGCAGCGAGAG
CACGAGCATGGTGATCCTCTGGAGGACCAGACATAGTCAACCACAGGTGAT
GGGTTGGCGATATTGCCGTCACGTCCGTGAGGTCTGTCTCGCTCCATGCATT
AGGGTGTATAATGCCGATCTAGGCGAACGTGATCTGCGCTGCGGAACGCAG
ATTCGGCGGCAAGCCGTTCCACCACTCGCCAACATCCATCTCGTTCTTCTTG
GATTTCTTGCCGCTTCCTCGAACCTTGAGGATCTCGAGCCAAGTTTGGTCT
CCACTTTCGTCTTTGGCGCACGCAACAAGCAAGCGTCGATGCTTGCCTTGC
TTGTGGGGGATGGCCGTCCCAGGTGCCAAGTCGGAGTCGGGACCAGGCGT
ACTGAGCTCGCAAAGGTCCCAGGAGCTGCAGATCGGAATCCAAGGCTGGC
ACACTAGTCCACAGTGGCACCTGACCATCAACGTTGGGAGCATGTCGACGT
GATGCGACTTGCCTGGTGCGCGATAGCTCTATTCAGACGTGTGATTTGCTTG
GCGTTCTGATCAGGCCACCGTATCCTTGCGGTCTCGTGTGAGACCTTTGGG
GCTCGGGTTGCTCGAGCAGCATCTTGCCGCTGGGCTGTTACCTTGAAGGTC
ATCAGAAAGTTACCAAAGAGATGTAAGTGGCGCTTACAGTCCCATCTCGCA

TATCTCTAAGGACCTGAACAAGTAAGTCCCCACCGATGCTCTCCAGCTCGC
CCAAAAGATGATGGTAGTCTTTATCCCCACTCTCTCAGACATCAGTCCAAA
GCCTTTACACGCGCACAGAGACTGACGACGCTCCCGAGTGCACCTAGGAT
ATCACCCCCGTCTATCCCCTTGCCTTTCTCCACGAGCCGCTGCACGGATACA
CCAGTTTCCACGTCCCCATTTGCGACGGTCCATTGAACAGGAGCAGCGCCG
CGGTACTGCGGCAGCAGTGATGGATGGACGTTGAGGCGCTGATCCGGAGG
GAACAGCTCAAGGAAATCGTTCGGGATGATGTGGCCAAAGACGCTGTCAG
GAGCAGATTGTCGGCTCGCAGAGCATTGAAGGGAGCGTTCGGCTGGACGA
TGAGGACTCGAAGCGGCTCACAGGATGCTTATACGCACAGTGAAGCCCTT
GAGTCCTTCCTGGGGAACTTCGGTTGTTGGGATATCATGCTGCTCAGCGAA
CAGGCTCAGTTCGGCTGCTGGTCAGCCATATTCTTGTGTACGAGGGTGCAG
AGCTAACGACGGTACCGCTCCTTGCCCTTACGTCCGACATTACGCTTCCGG
GGGACAAGCACCTGCACCGAGCTCCATACATCTGCGGAATGAGCATGACTG
TGGGACTCGCTCTGCCTGTCATCGTGCTCACCCTTTGCTTTGAGGACGGCC
GAGAGAGACGCGACGGAGAAACTGTCGAACCGCAAAACAGGATATCAAAT
GGCCCCTTGCCGCTGCTCGTGCTGAGCGAAGCGAACGACCTTGACAAAGA
GGTGCCGTGCTTCCATGAGTTGCTCCTCTGCAGCACTTGCAGAAGCATCGA
AGCCCTCCACGCTCAAGAGTGAGAGATGTGAATGCATGATACCGAAGTGA
GGTCTCTCGGACGGTTCCGCCGAAGCCGCCTCGGTTTTAACGTTGCCGCCG
AAGTTGCCGTCAGCGGTCCACGAGCAACACCCATCCATCTCACAATCACAT
CGCCACCATCATCGTCCCATCGACGTCCATCGATATACGATCCCAAGAACCA
GACCTCGAGCTACACTCCCCTCACTCACCACCGCAAAGGAGCCGGACACC
TGCTGGCCGACCGTCTGGACGGGTAGTCGCCTGCCTCGAAGTTGACTTTGT
GTGTTTGGGAAAACAGACTGCGCACGCCCCGTTGCCCGTTGCAGCTCGGA
GCGTCCAGACGTCTTATCTCTTCCATCATCATGATGGTTTCCTCTAGCCGAG
CGTGACTTGCTTCTCGTCTCGTCAGCATCAGAAGCCCGTCGTAGCTCTCGG
CTAGCCCTATAACCCCTATAAGGTCGTCCGACCCGTCCGACCAAGTCCACTA
ACAAGGCGACATGCCGCCGACCCTCCGCTCCCTCCTCATCATCCCAGGGAT
ATGTACGACCATCCTCACGTCGCTACTGGCCCATGCGTACATACCGGCCGTC
GCTGTCAACGACACTACAGGCTTGAACCTGACCGACTCGTCCACCATTAGC
ATATCGTGGCAAGATCGGCAGGCGTGTTCTCGGGCCCTGTGTGAGTCTATA
ACGCCCGCTTGTTCCAGGAATTCTTCGCAGTCGGGTCGCTGCTCTTTCAAT
GAGGTCGCTGACCCTATAGGTCCTACCAGCTTCGGGCAGATGTCGCAACGG
GAGGCACGACAGCAGGAGCGTTGGTTCATTTCACCGAGTCGGACATGGGA
CAGAATCTCACAACGTCGACGCCATGGATAGCATACATCAGCTGTGATGCC
AACGAGACGAATGCTAGTATGGAGTGGGGTACGTCCTGGCAATAAGGGTAC
AGCGCGGACTGGCTGATGATCACAGACATCTCACCCTTGCTCGAGATCGAG
GCGCAGTATCAGCACTGTTATACTCGGCCACGTCCCAAGCCTGTCTTCTCA

ACATCGAGTACATTACCGATTTCGAGAAGCCCCTGGATATCTTTGCGACCAA
GAACATCTCACCGCAACTCTGCTCGATTCGCAGTTCCAGCATACCAATGCG
ACCTTCCTCAACTACAACGGCACTCTGCTCAACGACTCGGGCACAATTGTG
AACAACTCGCTGGCGGGAAACCCTCCGTCCAGCAAGACCTTCCTGATCGG
CACCCTTGTTGCGCGTGACACCTCTGGACAAGCCACAATGACTTCGATTCC
AAATGCGGCGACCAGCCACTGCATCATCGAAGAGCGACAAGAACACCTCA
CCCAGCCATGATTGTCCTATATGTGATCACCGGCTGTATCGTCACCATGTTCC
TCACCATGCTCGTCATGGGCGCTCGTCGCGCACGCAGACATCCAGAAATGT
ACGGGCGCAGGGAAGGCGACGACTCACAAGGGGCGCAGACCACGGCGGG
GAGGGATCGCCCAGGCCATCTTGGACACATTCCCTGTCATCAAATTCAACC
GCAGGTCCAGAGGCCCGGCAGATGAGTCAAGCCAAAGCGGTTCCCTCCGA
AAACGATCTCAACCGTGGCTATGATCTCAGTGTCATACCGAGTCTGCGGGA
CCCGAGGATTCGGCAATCTGGTGATATGGAAAGAGGTCGTTCGAGTGCTAC
AACCGAGACCGGTTCCTTCCATTCCGCCAGATCAGCCGTCGGGTTAGATGA
CGATGATGGATACGGGAAGCCTGTGGCAGGCTCGTCAGCCTCTCATTCGCT
TTTGGGCGATGCGCTCAGATCCGCTGGCCCACCCGTTGACGACGATATTGG
CCAAGACCAATGCCCTATTTGCCTGATCGATTTTGAAGAGGGCGACGATTT
GCGAGTGCTACCATGCGAGAGGAGCACGTGTACCATCAAACTTGTATAGAC
CCTTGGTAAGTCAAGTGGGGTCGACGCATACCTGATACAGACTAATGTGCA
GGCTGCTGAACGTATCATCAAGTTGCCCTCTCTGCCGAAAAGGCATGTTCA
GTACTTCTCGTTGCTTGATGAATGCTGACGATGAAGATTTCAATGCCCAGCT
GCTCCGCCCATCAATCCCGTTCCACGGCCATTGTCAGGCCCAGGTGGTCCA
GGAAGTGCCTCTCCGACTCACGTGCCGCCAACCCAAGGTGGCTTTGCCAA
ATATCTGCACTTGATGCGGCGACAAGGAGGGGAACGTAATCGTGACCGTCG
GTCCGCGCCGGCGCGCCGACACCCGCTCGAGCGCCATGAGTGCTGGCGCG
GAGTCTTCTTTTGGCCTGGTCGGGCCCGGGCGAAGTCGGGAGGCTGACCA
GACCGGTCCGGGCGGTTATTGATTCAAGGCAGTCCTTGGTGCGTCTTGCCC
ATAGATGGTGGCGGTCATTGACTTGCTGTGGCTTCCCGTTCGTACACTGGA
CCACGTATGTATCGCTAGCACCCGTCGTCGTGGGGCTTGGCCTCTCGTCTTG
TGATATCGGTCCTTTATGGCGGGTCGCCGATTGCGTGCGCATCCTTTGACAT
CCTTTAAATCAAGGTCCTTTGCTCCGGGCAAGATGGCGATTCGCCATACTG
ACATCGATCCCGGGATTGCCGCGCAGCCCTTGAGAGGCCCCCAAGGACGG
CTTACTGGTAGACAGTCTCACAAAGCCGGGGTCGTCTCGGGAAATGGGGC
TTTCATGGCGCATTGCTGCCTTTGTGGGCGTAGTAGCACATGGAACAAAAC
AGTGAATGCGCGGCACCTCGGCCCATCCTGCGAGCTATGCTTCTTCGCGCC
CTCGGTGGCTTCCTGTCCTTCACCGACCCATGTCAGATCCTGTCCAAGTGT
CCTCGTATTCGTCTCTCCGGTTTCTCTCTTCGCCTCTTTGAGACCGGGTTTC
TCCTCCCCGACTTTGTCACCTGCGCTCACCAGCTTGCCTGAGCTGGCGGCG

ACCCCCGATGACGTCCCCATCGACCAACAGCTTCTCCCTCCTAGTGTTCAG
TGACCACTGGCACGGCGCAGACGCGGCAAGCTGACGCTTCAGCGTCATCT
CGGGCTGGATGTTTGTCATGTGGCATACGTGGCACTACGACCACTTTCCGG
TGAGTGTCGCGGACGGGCAGGGGAACGTGGAGGCTCACGCTCATGTCAGA
TGCCTCATTCCCACTCGGCAGAACTGGTCACGGCCATCATGTGCCATGTCC
TGATCCTGTCCATCGCCTTTTCTTCGTGGGCATCGGGATACAGGTCCACATC
TCGTACAAGGAGGTCAGTCGGATGTCCCGGCCGGCGGCGTTTCTGATCCGA
GCCGCAACGTGCCCTCAGCTCACCTGATCAGTTTTATATAGAGCTGCTCGGC
ACAGTTCAGCCGGCGCCGTGGCAAGTCTGGTCCGAGCACTATCAGCAGCT
GTGGCGGCGCTCCATCTACGTCTTCTGCGCCGGATGGGTCTGCCTTCAGGC
CGTACACCTGGAAGAGGTGGTCGTGTCGCACGCACGGTCGGACGTACACA
AGCTTACGGCTGCGGCAGGTCCTGTACTGTGTCGTACCTTATCACAAGCATT
CGGTCGCCTGGAGGGCCCAACCGCACATGGCTTGGTTCGCACTACTTCAAG
GTCTTCGTCGGGAGCGTCATCACCGGCTGCGCGCTGCTCTTTGCCGCTCTG
CAGATCGAGACGACCAATCTGGACATGATGCGCGCGTACCTCTTCTGCGTC
GGCGGGGTTATGAGCTGCTGGTGGGCGATCCGTCATGTTCAGGGAGTGTCC
GTCTGAATCAGTGTAGCTTGGCCATTGGCTCGGTCGTGCTTTGCGTTGTCTT
CCCGTCTTTCATCCGCTCTGTCAAGGCGATGGGGGCGACATTCGAGGTGCT
CGAGCGCCTGTCGTTTTTCAAGGAGCTCAACGAGATACGGACCGTCTTCCG
CCTGATCTACGCTCTCGCATTTGTGAGTCCCGCGCGACACAGACGGCATCA
GCTGATGCACGCAGACCATCCTGGCCGCCGACGGTCTGAGCAAGACTCAG
CGCATCAACAGGTCTTCGTTCGGTGCAGACTTTTGCTATTGCCTTGGCGAG
CTGGCCTTGTTCATCTCGACATGCCTGAGCGTTATCGTAAGCTCTGCCAGGC
GACGACGAGCCAGCAGGTCATTGCTGAGGTACAGATTCTTCTGCCGCGCA
ATATGGACGCCGAGTCGCAGCCGTCCAAGGTCGGTGGGCTGGCGGAAGCA
GGCGCAGGAAGCCAAGAAGGCAACTTTCACCCGATGGTGCCGTACAAACG
GCCACTCCCATCTCCCCACTCGAGCAAAGGCAACGCGCACCCGTCGACTC
CGCACTTCATGGAGCTCGGCGAGCGACTCAATATTGAGCGCGAGACGATGG
CAGCGGGACTACTGGCGCCCGTCATAGAAGAGCACGAGATGTCGCCCACG
TCGCCGAGCACGCGGTGGCGGCAGCAAGACGACGAGGCGCCGTTTGCGG
ATGTGATCGACAGGAGCAGGCGCGCGCGCATGAGCGAGTGACCAATCTGC
CGAGGGCCATACAGAAGTTCAAGTCCCGATTGATGTTGGCATGCCGCAGAT
CCAGGGCCCGCCGCAGGTGCTGGTGACGACCAGTCATCAGGTGGTCAGGG
ACGATGAATATGACCATAAATGAGAGGGGCGAGCGGAGGCCACATGGTCA
ATCCTCGCGAGTTGGGTCAGCCATCACGGACATTGGAATCTACTGTACATGT
CTTGTCAGACCAGATGAATCCAGCCTGTTGTCCGCATACGTCCCCTCGCCG
AATGAGCGCTGTCATGCCTAAGCCTAGACCTGGCAGTAGCGATTCGAAAGT
CCGTATCGCTCATGAACCCCTCTCCTTCTTGACGGCCACCTCTGCGACTGCT

ATCCCGCTCATGAACGCTTGAAGTACTGGCTGGTGACTGCTTGGCCCGCAA
TGATATTGTAGATGGTTTCGCCTGCCGAAAAGTAGCCATCCGCAAGGGACT
TGATCGACGAGTCGGGAATGAGGTCGGTCGAGGGGCTACATGTCAGCGTG
TGTGGCCATAATATGGAGTGTCTGGCCGGAGCTGGCCCGGAGGACAAGAG
ATTCGTGCGGTCGCACGACTCACGCAAAGCCCGTGATTTCTTGACACCCGC
CAGAATGGCCGACACTGCCAACGCGTCAAAGGTGTAGTGAACGAGTCTGC
CGAGAACCATGATGACGAGGAGACCAAGTTGGCTGGAGTCGTGTGTTGGG
CACCGCTCTGATGCAGCAGGTACTCGCAGATGAGACGCGCTCGAGGAGAT
GGATCAGACGCAAAGACGAGTGGGAAGGTGATGCGTCGGCCCGGCTGGTC
GAGCAGCACGCGGGAGAGCAATGGCCACTCAGGCACCGACAACACCGAC
AGGTCCGATAAGATGACCAAATGGCTCATTTTCGAAGCATCTTCGTCTCATC
TCGCTCGTTGCACGACATTCGAGGCCGCCATGAGCGTGCTTGTGGGCCCCT
CGAGGGCTCACATGCTATCCAGCAGGATAGGTATTCGACAAAGCTGCCGGG
TCGTCGACTACTGCATGGTGAGCCCTCCTGATCCCCGCGGCTGACGCGACT
GACTTGTATAGGTCCCGATCTGAGGAAGGCGCATGTACAGAAATCGCTAGC
ATTCGCATTCGACATCGTGCGTCTGGTCACAGGGTAGTAGATGCGGCTTCG
CTGATGCCGAGGACGGCGTGCTCAAGCAGGGCGAGCATGTCTTGCCGCAG
GCACAACGAGTATTGAAGCTCTTGTCGGGACAAGACGGCCGATTGTCAAG
GTTGGTGTAATTTCATCATCCACAACATAACCACGCCTGACAGCTAGACCTG
TACCGTTCCTGCTCATCACGAATGGAGGCGGTATGACCGAGGCGGACCGCT
GTCGTGCCTTGTCCCAAGACTTGGGTGTCGAAGTATGAGACCCCTGCCCTT
TACAATCACCGAGCGACGTGCTGAGGGCAGATCGGCGCCGACCAGCTCGT
ACAATCCCACACGCCCCTCAGAGACTATGTAGGGGGACTACGCCGACAAG
CCGGTCCTGGTCGTAGGCGGCGACGGGGATAGCGCGCCGCATCGCGGA
ATCGTTAGCCGTTTTCATACGCCCACCTGCTATGAACTTATGGACAGGTATG
GACTGCGCCGCGCGTACCTGCTACAGGACATCGTGGCTTGGCGTCCAAGTG
TATGGGACCGATACACGCTCTCAGAAGCCGAGCGGGCGTTCGCAAAGGCC
AGTCTCGCTTCATTCATATCGCAGCTGCGCTCATTTGGGTGTACGCTGACAG
ACAGGCTGATGTGGACTTCAGTACCACACCGCTGCACGCCATCTTCGTCAT
CCATGACAAGGGACTCGACTGGGGTCTCGCCACGCAAATCATCACCGAGC
TCCTGGCATCCGACGGCGGACGTATGGGGACAAGACGCCCACCGCCCATTT
GGCCACGAAGACGGCAAGGAAGTCCCGCTAGTGATGACCAACCCCGACCT
TGTATGGGGCACGTGAGCAATGCGCGTCCCATGTGCCCCATGTTGCTGATG
CGGAAGCGACTATCCCCTGCCGCGATACGGCATGGGTGCGTTCAAAGAAG
CTCTCGGGGCAATTTACAAGATGAGCAGTCTCGCGATATCGAGGGGCGATG
GTTGGGCTGATTGTAGACTGTGACCGGTCTCGAGCTACCTGTTACACAGTA
CGGCAAGCCTCATACTGTCACGTATGCATTTGCGGAGAAGATGCTCCGGCA
CCATCTCGGCAAGATGGGCCGGGATTCGGAGGGCGAGTTGAATGTGTACAT

GGTCGGAGGTGCGTGCTTGCCTTTGACCACGGAGGTGTCTGGTTGACAGT
CAGACAATCCAGCGTCTGATATCGCAGGCGCCAATGGCCACGGGTGGAAAT
CGCTCCTGGTCCGAACCGGCGTTTATAAAGACGAGGACGGGCCACCAGCA
CATAAGCCGACCAGGATCGTCGACGATGTCGAGCAGGGAGTTTTGTGGGCT
TTAGCACAAGAGAGTGAAGCCTAGCTGCCAGCTCCGTTTCGTCTTTTGATC
ATAGACATCCCTAGATCTTCAGGTTGTGCACACATGCGGGGCAATCTGGCC
CAAAAGTGCAACATAGGCCATGCATGTCATGTCGATGTCGACCGGCGCCGG
TTCCCTTCCTCACATATGCAGCTTGAATAGCCCTTCGCCAATGTATCCGGCC
GTAAGGCTCCTGCCGCCAAAGAAACGCCCATTAAGACCCTTGACAGCTCG
TTCTGCTGCACCGGTGCCCTCAAACTCCATGTAGACATCACCCTATTGTCAG
ATTACCCCTGTGATGGACAAAGATGCACTCACAGCAGACATCTTGTCCACC
TTGATCCGCTTGACACGTCCGTACTTGCTCTCGACCTCGCCTTTGACGTCCT
CAGCGAGATCCAGATCCCAGTTACGCTCCGTCTCCCTGAACTGTCAGTGCC
ATACACAGCACCCTGTGGACAGAAAGCGCACTGCTCCGGGTTGAACATGT
TGTTGACGAGAACGAATTGAGTCGGCGCGGTGGACGATGTCGTCGGACTG
CAGTTAACACACCGCTTGTCTTCTTGAATGCGATACTCACCCAGACGAACG
AGGCGCCGGACTGGCCGAACCTGCTCCGTTGCCCTGGTCTGTGCGGGCCA
ATTTCTGCATGAGGGACATGCGGGTGGAGTGGTCCATTCTCATTCCTTGATT
GCGGTTTTCGCGCGAGTCACGACCGGCTTCTTCAAGCGTTTCACCGATAGT
CGCAGCTCCTGAATGGTCTGCACCGAGACTGCAAGATCAGCTCCACACCTC
GTCAAGCCAGGAAGGAGGGATAAACTGACTTGGCTTCCCGCCAGATCAAA
ACTGTTCATGGCATCCAAAGCAGTCTGTGCGGCGGCCAGGTCTTTGAATTG
GACGTAGGCAGTGCCTTTGCTTGATCCCGACTGCCAGTGAATACAGAATCA
ACTTGATTCGCGACACTTACAAAGTCCGCGGTGCAGATCCACAAAGGCGAT
CTCTCCGAAGGGATCGAAGACCTGTTTAAGATCGTCCGCGGTCAGGCTGAA
AGCCAAGTTGGAGATGTAAAGCCTGAAGTACGGTACGGACGCATCGCGATT
GGCGTGCACATCGACTCCTGGTGGTATGTACAGGTCCTGAGACAGGGCTGG
GAATCGCGTTTGATATCGGATTTCTGGACGAGCCTTTGATGCAGACAGCTTT
GCGATCGTAGAAGCAAGGTCCATGCCCTCCCGGTTGCGCTCGGATTCGTCA
AAACGACTTGTATCGGCATACCCATGACCAGGGTGCCGGAGAGCTACGCGT
CAGCAAGGACAACGTCTCCTAACAAGACGTACAGCTAGAGCCCGATTGAC
TAGTTCGACGGCGTCCAGTTCGACGTAACCAATACTACGGTCTTTCAGCAA
AGTGTGGTGTTCCGGATGATTGACTCACCCCTTGGATCGGCCGGACCCTCG
GTCTGTAACGACTCGGGCATCTCGCACGGCACCTCTGCCAGCATGTCCTCG
AAAAACATGCCAAGATCCTGAGAAGTCATGCGGCTCGAAATTTGGCTGATG
AAGACGGATCGAGATTCAGAGTCCACCTCGTCAAGCAGCTCGGTCACGGG
ATCCTTCTCTTTAGGCGGCGGAGGCGGAGGTGTACGCCGGCCGCGCGGGC
TGTTCATCTCATACGAGGTGAAGGACGTCGACGACGGGGCGATACGTCTCT

GCTGATTTCCCGGACACGCCTTTCGCGAGCTCGCTCCCGGTCCATCTGCAT
GTCAACACACGTGGACCTGAGAACAATGGGGATGAGCACCTGCTCAAAGC
GCTCATCTCGTCTTCTAGCAGCCTCCCGACGCTCCGCAGCCATCTCCCGATC
GCGCCTCTCTCGCTCAGCTTCTTTCTCTTCGCGAGACATTTCGTGCCGAGGT
GCTACATCTCTCGAACGGTGTGATCGGTGCGACTCGCGGGAGCGATCCCTC
TCACGGTGATCCCGGGAAGGACGCTCGTCAACGCCGTGACTATCGCCATTC
CTCACTCTGCGATCGTCCCGTCCCCGATCGTCATGCTCGCGGTCCCTATGCC
TGTCCCGGTCACCTCGCTCGGACCTGGAGTGACCATCGCCATTGGCATCGC
GGTCTCGCTCCTTCTCCTTTTCTCTCTCTTCGCGGCGTCGACGATGCCTTTC
GGCACGCTCTTCCTCAGTCTCCTCTTCTCGCTGACGCCGCCGACGCTCGTG
CCTCTCGCGTCGCTCCTCTTCAGTCTCATCGGGACGTCTCTGCTCACGCTCC
TCGCGGTGTCGGCGATGTCTTCGCTCGCGCTCGTCCTCGTCCCCTCTGTCG
CGGTCGTCACGCTCTCGTTCTCGGCGTCGATGAGACCTGTCACGGTCATCT
TCGTCGTCCTGACGCCTCTCACGTTTCCTGCGCCGTCAGCTTTGAACTTCG
CATCTGCCGGCCGATAGGTCGGCGATCTCACTGCGACAGCGATCATCAAGT
AGTGGGCGGATGCCTCTTGATCAGCGCGGGCAGGCGACCCCTTGTGACAC
CCAGTGTGATGGAAAGGTGTAAGAGCTGGAAGGAGAAAAAGGACTCACG
AGGTGAGCTCCGGGGTACGGTCTCTATCCCCATGCAGCGCGTCGAGCGGTG
GCGTGTCGGACATCGTTGGACTGCTCGGAAAAGCAAGACCTACCCGTTGC
CTGCTCCACTTGATGACCTGCCCCCAGCTGAGCAGATGGGGGAAAACGAT
GGAATGAGTGAGCACTGGGTGTCTTTCCTTTCTCGTGACTAGATGGCGCGC
TCTAGCGAATTCGCCTTGGGAGAAAATCACGGTAATCCTCGGACAGAATAT
TGGATCTGGCCCTCCACTCGAGAGGTCCATTTTGTGTTGCTTCACGACAAC
CTCTCGCATCAGTGTCCAGAAAAGAGCACAGCCGCAAGACGCTCGAGGGC
TGGACCTGCAACATGACCCATCCGCACCCTCTCGCCACGTTGGCGCAGGTG
ACGTCGACGCCATCGAGCCAAGACGGGCTCCCGGCGAAGTCGAAACCGAC
CTCCGAGTTGCTGGGTGCATGTTGATACAGGAAGCCGGTGTCATGCTGAAC
CTGAATGTCCGAATTTCCGCAAGGTAGACCAATTGGCTGACCAGCAGACCC
AAAGCACGATGGCGACCGCCGAAGTTCTCTTTGCACCGCTTCTACTATGTC
AGCTCGATGCTCTCTTTTGGCGTCACAGATATCTCGGTTGCGGCATTGTACC
TCTCAACCAAGCTCAACGAGACACCTTGTCGCTTGAGGGATATCATCAACA
CGTATATCTTTTTGCTTGCTCGCATAAGACACCTTCTGGCCCTGCCGTCGGA
GCAAGTTTTAGCCGGCCCTTCGCCTGCATCAAGGGCACTTGATACTGGAAG
CACGGGCAGCGGGATCGCGAAAGGAAAAGAGAAGGCGGAGCAAGACTCA
TGGAAAGGGTTCATGTTTGAGCCTCCTGGATTCCATGACGAAATCTTCTGG
GATTGTAAGCTAGATTGCATGTGCAGCAAATGATTCAGCTGAGCAACACAG
GGAAGGACGTGATCGTCAGCTCGGAAATGCAGATCCTCAAACGCCTGGGC
TTCAACATGCAGGTATATCAACAAGATCGTGTCACGGTTGCTCATAGCTGAC

ATTGAGGTCGACTTGCCGTATTCACATGTCATCAACTACCTCAAAATCCTTG
ATCTCGTGTTCGAGCCAGATGTCGCTCAGACCTGCTGGTCCATCCTCAATG
ACGCGTACGTGATCGCTGTACATTCGTCAGTAGGATGTGAGGAGCTGATATT
GCAGCCTTCTCACGCCTATATACGTATTCCACCCACCCCACGTCCTTGCGTG
TGCGTCCTATATTGCTCTGCACGCGCATGCTGCGCATACCGTTACCGACAAC
TGGTGGATCCTCTTCGACGCGACATTTGAAGACATGTGGTCTTGCAGCGGT
CACGTCACATCGCTCTGGTCTGTATGGGGTATGGCGCCTAGGCCAGACCTTT
TGCCAAACCAGAATGAGGATGCCGGAATCGAAAGACAGAGAGAGAATCGC
TGGCGACGTGCTTGGATCCTGTGCGACTCGCGGAAAGCTGTGCGGAGATG
GGTCGAGGAGAATCGTGAGCTCGGGCAAAAGCCCATGGCGGAATCGAGAT
CGTGACGGGTACGCCTGGGACGGGATTGTCGCGGTAAGATACTCTGCCACT
AATTGCTGTTGTATGCAAAGTGCCACATTAGCTCCGCCGGGTCTAATCTCCG
GGCCCGCGTTGTTATTTCCGCTTCCTTGGGACCCCTTCCGTTCTTAATGGGA
TTGCTACCGGTGGCTTAATCGACCCCTGTTTCCCCTCTTCAGAGTTGTCGTC
TATTCCACTCTTCTTCCTAATCCCATTTCAACCCATTCCACCTTGTCTTCCAT
CCGTTCAATAGGTGAGTGGAGGTGGTCACCCTGCCATCCCTGCCTCTTTCA
ACCATCTCACTCGCATCGCCACTCCCAACCCCAAGACTCGCGCCCGTCATC
TTCGCCGCATCGCTCCCCATGCTCCCCAGCTCCCCCCAACAGAGGAATGCT
GCCTGTTCACTGTCCGACTTTTCTCGACTGCTCTGGATCCCCCCTTGATCAT
GTTACTGATTCCTGCAGCCTCGATTCAGCGTCATGTCTGGTCCGTCATGGGA
GGAGATGGAGGCCAAGCGCCAAGAGGTGAGCCATCGGCTCCAAAGCCCAT
CCCGCTTCTGTCCCATCATCGTCATCACGCATCTGAACGCCTTTCTCCAACT
CAGAACTGACTCTCCCAGATGATCGCCTCATTCCGTGATCTCGCGGCCGTC
ATGACCCGCTGCGTCTCCATCATCGAAGACTACGCACGCCTGTCACCCAGC
ACTCTCGGTACGACCGCTACGAGCTATCCCCTCATACCCAACTTGTCCGCGT
CCGAACTCACTGCGCTCGCGCAGGCCATGCCGTCCTCGCTGGCCGAGGCG
CCGTTGGCACTCAACGGCCTTTCTGACACCAAGAAGGAGCGCAAGAAGCG
CGAGAAGAAACCGAAAGACCCCAACGCGCCCAAACGCCCTCCAAGCGCG
TATATTCTGTTCCAGAACGAAGTCCGAGATGCTTTGCGCTCGAGCCAGCCG
CAGTTGACCTACCGCGAGGTTCTGGATCACATTGCAGAGCGTTGGAAGCAC
CTGACCGACGCACAGAAGAAGGTAGGTCCGCCCACTTGTGGACGTCTGCA
AGCGGCAACAGCTTGGCTGATTTGCCAGGTCTACCAGGACAAGTACAATG
CCGCAATCGAGGTTTACAGGCTGGAGGAGCAAAAGTACAACCATCCTGGC
GGTGCTGTACCTGTGTGTGCCGATTGGAGACGGTACTAGAAAACTCGCTAA
ATATCAGTCATTCGGTGTGACCGTCCCTGTCGCCGCCGCTGCTCCCCTCGAG
TCAGACGAAGAGAGCTCGGACGACTCGGATGACTCCGATGATTCGGAACC
CGTGAGTGCCCTCTAGACATGAATGCGAACCGGATGCTGATCGCCAGACTC
CGCCCGCCAAAACCAAGGTAGCTAAGCCAGTCCCTGCTGCCCCGACCGCC

GCGGCTATAGCGACCCCTGCCCCGGCGTCCAGCAAAAAGGAAAAGAAGCG
AAAGAGCAAAGAGGAGGAGGGCGCCGTCGCAACGATGCTCGACCCGACT
TCAGACAAGAAGGTAAGTTCTATGTCACCTCAATGCTTGATTCTGACATGC
AGAAGAAGAAGAAGGCCAAGGAGTAATGGTCGTGGCTCACATAGATGTCT
TATATTTTGTGTTCCGGATGCCTCATGCGTATACCACTACCCCTATCCCCGCC
AGTTGCGGCCTGATTCCATACGTATATCCCGCTGCCAGAATCTGTTTATGCA
CTCTTGTATTGGACTGGAGCAGTGCAGTGACGCCGAAACCGTAATGCCTCG
AATTTCCTAATGAGGCAAAGGGTTGAAAGGGCTTGGCTAATGTCGCACCGC
CTTTTGATCCGGCGCGGCGGCGGCGGCGAGAGAAACCAGACGCGAGCCAC
TGACACCGTCCACTATGATAGCATCGCATCTTTCCCTCCCTCTTTGTAACAC
ATGATCACTGGCTGGACAACTTTCTCGCTATCTGCCAACGTTGCTCCGACTC
TTCCGTCCTCCTTACCATAGTAAATCTGCGACTGGGAGTCGAGCGACAAAG
GACGACCTCTGAACCGACTTGACGACCGACCGACCATTCCGACCTCTGTTG
TTGGACCGAGCTCGGGGCGAAAACGGCGCTGGTCGCCTTCACGAAGCTGA
GGAGAGGAAGTGCAATCTGGTGAAAGCGGAGGAGACTGAGGAGCATCAC
TCGTCATGGCTGTCCGATATCGCTCGGCCACGCCGGGCACCCTTCTATCGCT
TGCAGCTGCCATCATCCTCGCTCTCGTCTCGTTCAACACGCCTCTATTGAAG
AGCCAGTACTTTCTCTCCGCGACTTATGACTCGTCGAGCATCTCTGGCACCC
TTAAGCTGGGGACACTGGGATACTGCCTGGACAGCAATTGCACTGGCCCG
ACGGTGGGGTACGAGTTTGGTGAGCCGCCCTCTTGTCCCTCACGTCTGCTC
CTCGACCGCATCGGTGTGGGCTGATGCACAGACCCAATACTGTATTCGGCG
TCTCCGTCTTCAGCATACCCAAGACAGTCACGAAGTACCTCACTTACACGC
TCATCCTGCACATTATCGCACTGGCGTTTGCAGTCTTCTCGACGGTCTTTGG
ACTCCTATCGCACATCTCATCCCTCTCCGTCCTGTGCTTCCCGACGTGTTTC
GCATCGCTTGGGTCATCCGTGTCGCTTCTCGCGCTCATCTTCGACCTCGTCA
TTTCTACATCGCCAAGGCGCGCATAGACGATGTGGACGGCGCTTCCGCCTC
CATCGGCATCTCCGTGTGGCTCACACTTGCCGCCTGGCTCTTGTGTGGCTTT
GCCGGTTGCGCGTACGGTATAGGGCGGTGCTGTATTGGACGCAGGAACACG
CGCGAGAGCGGCGACCCGAAGAATGCATACCAGCCCGCGTCTTCGCAGGG
GACCGGACGACCTTCGCCTGCAGGCGATCCGCGACGAGCAGCTCAGGAAG
AAAGAGCAGGGGCCTCCCTAGCTTCCAAGAGCTTGAGCGCACGCCTCTTA
CGGCCAAAGACGACGAGGAGAACAAATATCTGTACGACGACACTCAGAAT
ATGCCCGGCGGTCTGAGGCGAGATGGGAGGATGAGCGCTGGTGGGTCGCC
CGGTACTTCGGAATTCCAGATTCTGGGCGTAAGACGGGTGGTTGGCAAGG
GTGATGGGAGGCCAAGGACCAATCACGTCCTCCTCTCGACTCGATACGGTA
TGTAGTACTGTATGAGAACACAGTGCTATACCCACCTCGAAGGATCCACAG
CCTTGGTTACTTGGCTGTTTGGGTCACACTCTAGAACAAGGAGGGCGCGG
GAGATCGTCCGGGTTGGTTAAATTGACCGAAGTTCAAGTCGAACCCGAAAT

CGAAAATCGAAATCGAGGACGAAGGTAGACAGTTATCTGTCTGCTGTGATC
TCATTTTGGTTTGAGGTTGATAAGTTCTAGACCTACGTATGACGGCAATGTC
TTGAATAGGTACATATCTTGAGCCTTGAGCGAAAGACCCCCTAAAAAGGAT
GTAGGGGGGTAACGCATTTACTGCATTTACTGGATCACTAGAGGATTACTAG
GGGTTTATGCAGCCTATGCAGCTCAGCCATAGCCTAGCATTTTGTGTTGCGT
GGGTCGTGGGTCTTAAAACGTGTAGGTCTTCGTGGCTCTGGCTTGTGATAG
GTAACGATAAACAAAACGGAGCTGACCAGCGACAGGTTACGTAGTGTTTC
GTGGTGTTTCTTTGGTCAGCTCCGGTCCCTCCAAAGTCCCCTCGACGAGAA
TGGCAGCCACTTACATCTAGCCAGCTGGAACGCATCTCCGTACAATTGTCTT
GGTGTTACCTTATACAGTATGTACTAACAGCTAACTTGGACTGACCGTCAAA
AAGTTTCGACTATCTTAGACAATATTGATATCCACATTCTTGGAAAGGGTAG
AGGATAATCGACTTAATGTCGTAAGACAGACGGCATAATATGTGGGCTTAAA
AGACTCAGCCTATGGTGGCTTAAGTTTACGCGGCTACCGCTATATTCGGACG
ATACGACGCCAAATACATATATCTTATGCAACCCGTAAGAGGCAGTCAGAA
GGCAGCACTACGTACGAAATTTAGACAGGTATACTCATCCCCTTGCATTGGG
TACCGAGATGATTATGACCTGGCGGATTCGATCGTGGACCTTGCATTACGGC
CGAAGACAATGCATTATGAATGAGATCGTGGAAGATTGTTGTACTTTACATG
TGAACGGACAGTCTACAAACTCTAGGCCTTGAGCCATTCAGCCAAAGTGG
CGGGCTATTGCTGATGCTGACGGCATCTCCGCCAGGTATTAAATCAATAATA
TTGAGAAAACGTGAGCTTCATTTAAGCAATAATTTATTCTGGGAACCCGTG
GCACGTGGGAGTCAGGTTAACATTAAGACAATTATTCCGAGGTTGCGATGA
TCGACGATCATCTGATTTGATAACAAATAAACATCTTGCAATTCTTGAAACA
ACCTCGAAGACATTCCATCACCGATGATGTAGATATATAAATATGGCAATGTC
TATTGTCTCGATCGATCGCTCAAGACCACTCTCACCAACCTTCGCATCAAGT
TCATGTCTTCAAGATTGCATCAATAACAAGGTAATTGGCTAGTCGGAATGCT
TCATTGAATAACTAATTACCAGACCCTTGTTTCAGTATAACAACCCTCGATG
CTGAATCTTGATACTCAGCTGTCCGTCATACCGACCAGGGGCGACTGGGTA
GGTATAAATCCATGTCTAGTCAACATGGAAAGTAGTTCTCGGCGAAAACCA
TGCTCGTGAGCGTCGGCCGAGACCTCATTATTCAATGCATATCCAATAGAAG
CCAATTTTCCGGCCATCCCGCAAAAAGTGGACGATGCCATCTCTTTAATAGA
AACTCTCCACCATGGTATCCTTAAGTTTACCGCCAATATTGCCTGGTGCCGC
ACCCTCGCCTAGTCGGAAGACGCTCTCGATCACGCTGAGTTCTGTCGCCGT
AGTGTCCAATCCTGTGACTGCCAGCCAGTCGTTGACTACCATGCCGGCTCC
AACTACATTGCTTCCCCGGTTTTACACTTCCCGCACTAGAGGTACTTGGAGC
AAGCTGCTGAGCTCGTCTTGGTCCTGGATGCTGGTCTTGGGATGTACCAGA
CCGCCTTGGTTGCTAAGGCTCATGTAACTGCCCACCAACACGTTGTCTGCA
ACTGTCTGTCGGAAAACCTCGACACCTAAAACATCTGCGATAATCTCCTCT
GTCTCGCGCTCCAGGTCGGGATGAACTAGAGCTATGTGGTCATTCGATACG

ATGACATTGCCTAGGGCAGACAGACGTTCTTCTATTCGTTGGATCTTTGACC
TCATCTGGTAAAGAATTTCGCAGATGTTGAAGCTCTTGATCGGTTGTGGTTG
TAGGGACGAGAAGGCCTTTGCGGTTTCTAAAAACAAGGAGAACTGATCAG
ACCTAATACCAGCATATAGAAGCTACCGATGCTCAGAGGTTTCTTACCCTGC
CGTTAATCGACCGATGATGCGCGTGCCCGCAATTGTAGTTCGGCAAATAGG
AATGACATCTTGCAATTCAGATTCGAAGACACTGTAAAAGTTTTCGCTTGC
GCCGACGGCAGTAAGGGCATAGGAATTGGTGAGTGTCGCGAAGACGCCGA
CCCTATAAGATTAAAATCGCATCGTCAGTCTCAATCTCCTATGCCTCAGACC
TCGCATCTACCACACAAATTTGTTTCTTTCGCGGGGTCGTGGGTCGTGAGC
TAAATGGCGGGGCTTACTCGTTGGAGTTTTCGAATTGTGCGCGCACAGACA
TGGCTATCGTTGTGGTATTACCCGTCACAGTTGATGTTAGAATTTTGACGAA
AAGGAATTCGATCTCTGGGAGAATTGAGATTAAGTAAACAGCCCAGATGCT
AGTAGTTGTTTATAATAAAAGAATTAAAAGTTGATGCTCGTTGCTGATGGGG
ATTATACTCTGTAAGGCAGCGGATAATGCAGGCTATTAATCTAAAATGTCCA
AGTGGGGCTGGTCAAGTACAATCTTCTTATCCGGTACTCAGCCAAGCCAGC
CCACTGGCCCTCCAGGGCTATCCTCCGGCAGTGACTCAACTATAAACTCTG
CTTCGCCACAGGCTATGACTCTACACAAGGGTAACTTCGATGTACCCAGTA
CCTCCCATAATTATCATTCGGTCCCAGATGTCGTTGCCATTATGATTCATTTC
AATCATCGTGGCTTCTGCTAACTAAATCTGCCGTCAGCTAACACCCTAACCC
CTTTTCGTATGCTGTTTGTTAGCTTCGACTTGACTCGACACTAGAGCGAATA
AGAACTTGAAAGAGCTCTATGTTGATTACCTTCAGCTTCAAAGAGCTGCTT
TCATACTGAAGCATGTATCTACATATACTTGAGTTATAGGTACCTTATGAATA
GGATTGTCTTGCTACTAGGTACCTAGTTAACATAAATATATTTAAACAAAGA
GCAATATCACCGCTTCTCTTCCATGTTGGTGCCAGGAAGATCTTTTCATCGC
TTATAGATATGTTCTTGAATTTATCAGGTCGTAAACCCTTTGGGAATACCACC
GCCACTACTCCTAACCCCATTATCGTAATTCTAAAGATGGGAAATACCTTGG
TCCGGCAAACCCTTTAGCAATAACCGCCTGTATGTCAATGTTTGCGCCCGG
GTCGCGATGTGGGCTCCACATCTTGAGTAATATGAACCGTCCAGATACCGG
AGGATCGAATTTTATAGTGCACTTGCTCTTGTTCCGTTCTATGAAGAATCGT
GCGTGAGGAACCATGAGCTCTCCGCCAACCGCTCGAACTGCCTCCTGGGT
AGCTACCTGTGCTGCCTCCACCGCTTCGGCTAATGCTGTGTTCGATCGATTG
CGACGTCGCCGAATTCTGTGGTACGTCTCTCTGAATGAATAGCCATTGAGG
TCGCCCTCATCCTCACTAGTCTCACTACTTTCAAATGGTAAAGAGCCGATCC
TATTTGGGGCTCGCCAATCGCCCCTGGGAATTGGGTCCTCCCCTTCGTCATC
GCTGCATTCCGTCGTCACCCTAAATGGTGGTGGCACATTAAATTCAGAGGG
TATCTGGGCAACTCGAACATTATTGTCTTCATCTTCCATCCATATGTTGTATA
GACGTCTCGCCCTGATCTGCGCATGTGTCATAGTGCTTCCGTCTTCGTTATG
TCTTATGGATATAATAGGTGTCAGGGGTTGGGACTCGCGCTCCGTACGCCTG

GGTGAATTTCTAGAGGTCGAATACTGGATTTGGTATTGGGCAGTACGTGTAA
GGAGATGATCAGATGTCATCGAAACAAAGACCATGCCTTCTTGTACACTATA
AATTTTGTCAACAAGAGAAATGTTTTAGGGAAAGGATATACTGACGGTGAG
GTGAAATTGGACCTGGGCGCCTTGATAACCAGTTCCTTCAGCGTGAAGACG
GTGGCGCCCTGGTGCCTTAGGATAATGTTACACCTTGGTCCTTCGGTGCAAT
AGACAGTTTGGTCATTTTTAAGAATATTTTCTGCAGCATACCTCTGGCCGTC
GTCTGAGTAGATGCCGCCGTCGCAGCTTACCAATTCCATAGTCAGCTGACC
TGGTTCCACCTGACCATATTTGCCATAGCGGAAGCCTTGGAAGCCGCTAGC
TAAACGGTCTGAATCTAATTTGCGCCTCTTAGCCCTTCGATTATCTTCCGTCG
GATCTTGTGGAGGTGGAGGCGGCGTTGGTGAAGAGAAAGCAAGTTCCGAA
TATGTATGGCTAGATGAATTGGTGAATTCTAGAAGTGCTTCTAGTTGTGAGT
TGGTCTCTCGTTCATCGGATATTTGTGTATCAGGAATTTGTGTATCGGGTAGT
TGGGTGTCGGGTATTTGAAATGAGGCAGACCGCCGGCTACCGCGCGAGGA
CCGTTCAAAAGGTTGTGGTTCGAGGCGCTCCCAAAACTCTCCAGCTTCTTC
GGCGGCTTCGTTCCTATTTTCATGCGCACCACGGAGTCTAGCCGAGTATCGA
TCTGAGCGCGTTCGCGCGTGTCGCCGCCCGGGTACACCGCCAGACACAGG
CATAGCTACGGACCGTCGGGTGCTAGTTAAGGAGCGTAAAGGTGGTAGGTA
CGGTGCTGGGGCTGAAGGAGGTAGATTGTACAGAGGCGATGCGTCTGGGT
TTTGTGATCCTCTTGAAATTCTTCGCTGCGCTTCCGAAATAGGCTCCAATGG
CATACTGTTCCGCAAGGAAGACCGCTGGCTTAAACGACCGAGGTGATTCCG
GGCAATGTCCGGTCGCGTATTGCTGTTAACGGGCCGAGGTAAGGATAATAC
CTCTCTATAGCCCGATATAACTCTCCGGAAGGACTGATGTCGGAGGGTGAA
GCATCACCATCGAGTATGGATCGTACAATTTCGTTGGCAGTGTGATAAGGTC
CTGGACTCGAAGGGGGCTGTCCGGAAGAATCGCCCGACTATCGGATGATGT
TATAATTAGTCCATATGACGATCAAAAGTCAAGCGAGAACATAGAAGAAAT
ATCTAAACTGATTGTTTGATGGTAATGTTTAAAAAGAGCGTGCTAAGGGCG
GAATATGGAAATATGTTATGGGCTATGTTGGTGGGCTCAGCTGACGCTCACC
ATTAGAGACAAAGGACCCAAGCGTGTGCGTCGAAACGATAGGCGGCACGT
TAGTGCCAGATGGCAAACAGGTATAAAATCGGACTAGAAATGGAGAGATAG
GGCTGTAAGGATAAAGTATGTATTCGAAAAACTCGGATGGCGGTGATATTG
GTAGTATGGTAGTATGGTAAGATGATCAAGATAATAGTTGATGGGTTGACGG
TTGATTGAATTGTTAGGTGGAGGAAGATCAATCAATTATGGTTGGAGGTTG
AGTAGCAAGAGTGGTTGAATGGGAAATCCTATGGGAATATTAGGAAGGATA
ATGGATGGGAATGGAAATGTAGCGCGGGATGGTGTGAAGGAAGTTCGAAG
CGGGTAGGTCAGTTCTTTAAGTTTAGGCGGGGTCAAAGGTTACAAGTTAAG
CACAGTCTAAAAAGCTAACGGGGAAATGATGAGATATACAAGGCTTATGCT
AGAAAACCACGTGTAAATTCATGTAAATTCATGTACCTCTTTAACAGGCATT
ACCCGCCCACATGCCCACACCGGGGGCACTTGCAGCCCACGCAAACCTTC

CTCTAGAGACACAGCGTACCGCAGTACGTACGATACCTGGTAACTAGTTAC
GCGGTGGCTTACCTCTTTCGTATCTTCAGCTGTACCTATTCAGGTTCCGTCG
CAACCTGTTGTGTAGCCTCTCAATCTCCTTCAACCAATGACACCAATCAGG
ATTTCGATAAGAGGATCGGGTTTTCCGGACTTGTGTGCTACCGAGTGCATTC
CCTTACCTTTTAAGGGGTGGTCAGCCATCAAAGCGATTGCCTTAAGGCTTCT
GCAGGTGTACTAGGGATATCTTGGTATCTTGGTATGTCGAAATGTTGGAAGT
GACTGGTAAAGTTTCAATGTTAGACGGTATTGCCTAATTGAGTTTTACTTGG
GGTGTGTAATTGGTGCAACTGCCAGGTGCTAATTATACTATGCATATAATAGA
TGGAAATGACGACCCGTCAGCCTCGGTCCCACAAATAACGCAAGATTATTC
TATTATCAGCATGAAAACATCTATTTAACGGACTTTAATGCACGTTACAGCTT
TAGTGGTCCTTTAGTGGATCCCGAGCGTTACCTAAAAGAGAGACGAGGCTG
GCTCATCGCATCGCATCTGCATCTGCATCTGTACCAAGTCACATTCATTCCAT
CTACATCTACAAACAAATGAAAAAGGTACATCATCAAGCGACGTGGGCGA
GGGCGGTGCAGTAATCTTATCAGATCCATTGGCCATCTTTATGTTTCTTTCTT
AATACCATATTGATTTTAGAGAGAGAAGGAAAAGAAAGGAAACAAAAATC
AATTTCGTCTCTCACAGATTCACAAATACCACGGATGCCATAAATACCAGGC
AACAGCGGCGGCAACCTCGACTTTTTCTCTCATCGTACATATCCGCCTCGCC
TCATCTTTCCACATCTCACTTGTGTTTCTCATCCCAACCGCCAGGATAAACG
CATTGATAACACTTCATCAAATTCGTCTCAAACAAGATTAGGTGAACTTTTG
CTAGCACACTCATTTATTCTTCTTGTTCTTCATACCGACTTCTACGTTTTGAT
CTCTTCACTACTGCAAGATTCACGATCTGTCGGAAATGGAGACTCAAGACC
AGCAAGCCGCTGTCTCGAATCAGCAACAGCAGCAAACCAATCAAGGCGGA
CCACAAACTTCTGAGACGACTCAGCCAGTTCAAGACGGCAATATTACCGG
ACATGAGGCACAATCATTACCAACCCACGGAATATCCCTACCTGGACATTCC
ATTCCAAACAACGGCCTCCCAATCTTTCCCCATCCCGATAATACATTCGCCA
CTCTTCTACCACAGCCAGTCTTCGGCAATGGAGCGTTTGCTAACCCCGCCG
TCATAATACCTCCAAACCAGCAAGCGCTGGGGTTTAACAACTTAAGTATCG
GTCAGCCATCCAACGGAAATAGCTTAAGCGCAGGTAAAACCATCCCATGTC
CGACACAGTTACTTATAAGACTTTTATTAATGTGAACATTAGATGACATTGCT
CTCTATGACCGTCAGCTACGGCTGTGGGGCATGGAAGCCCAACAAAAGATT
CAGAGCGCCAACATCGTTATCATAACTATGAAGGCACTGGCGAATGAAATC
GCCAAGAATCTTGTCCTAGCAGGTATCGGTTCTCTAACCGTCGTCGACGATC
AAATCGTCACTGAAGCTGATCTCGGGGCCCAATTTTTCCTCACGGAAGAGA
ATATCGGCCAAAGCCGTGCCGAAGCGGCTGTTGGTCGAATTCAAAAGCTG
AATCCTCGAGTTAAGGTTATCGCTGATTCTGGAAGCATCATGACCAAAGGT
GCATCATTCTTCGGTGCCTTCGATATCGTCATCGCAACAGACCTCAGCCCCA
CGCTCCTTGCTTTCATCAACACCGCGACTCGTCTTCACAATCGACAATTCTA
TGCAGCCGGAACCTATGGGTTGTACGGCTACATATTCTGCGACCTGATCGA

ACACGATTATGTCGTACAGCGTGACAAGTCTAACGTACCGACGAATATCGG
TCCAGAAACACAGGACTCGTAGTATTGTTAAGGTGGAAAGCCAGAAGGAA
GGCGGCAAAGTTATCGAGAAGGTCCAGAAACGTGAGCTGTACTCCACTTG
GGATCTCGCCTCCGAGACTAGTCTCCTACCCCATGATTATACCATAAGCAAG
CGTCGCCTGAAGAACGTCACGCCGGCACTGTCTTGCCTCCGCGCATTGTGG
GCTTTCCAGCAGGCCCATGAAGACCGTTTTCCCGAAAACAAGCAAGACCT
GGAAACGTTCACGAGATCAGCTACCCACAATCACACGCTATTATCCCTTCCC
GTGGAGACACTCAGGTCCGAATTTCTGCGCAGCTTCCTCCAGAATATTGGA
AGCGAAATTGCGCCTGTGACGGCAATTCTCGGAGGTCAGCTCGCCAGGAC
GTCATCAACGTGCGCGGTCAACGCCAGCAACCATTCAGAATATGGTCGTCT
TCGACGGAGACAAGATGGAAGCCGAGATGTACCCGCTTCATCCCGAAGGC
ATCCTCGGCAGAGCTCAGCTGGAGCTAGCTACCCCTATGATACCGCTACCTC
CGGTCGATCCTTCTCAGATGATTCCCATGGACCAGTCGGGCATGATGATGG
GCACACCCGGGATGCAATAGTATGCGTAATTACCGGACGGTTTGGTTTGTG
CGTGGTCGGACATCGGGTTCGGGGAGATACATCGACACCGATACCGATATC
GATATCTGGGGCGTTTATTTGCGGATTATGCGTCTGTATGAGCGGTTAGGCAT
AGCAGGTGGCGACGATTTGAATTTGGCGGTCGAGCAGAGTCACTTTGATTT
TCTCATCTCCCCTAACTACGTTTTTTATTAGCTGGGCGGATATCACTAAAGAC
GAATGCCGTTAAGTTTTGAAGTTTCAAAGTTCCATCCCCATCAATGGTAACG
AAAAGGTGCTGTTGGGTAGAAGAAAAATACCCTCGCCCGCGGTTGAGTGT
GCGTTTTTTTTACATTGCTTTTTCCACTTTCTATTTCTCTTAATAACAAGGAA
GTCGGGGCTTCGCGAATAGCTGGCTCTCGGCTCGGTTCTGCACGAAAACAT
GAAAAATGAAATGAAATGAAACGAATAGGGAAGAAGACTACATAGCAACA
AAAATTAGAGGGTGGCGAAGAGATGAGATGAAAAAGAAAAGAAAAGATG
GCGGCGTGAATGTGTATAATATTGACCTGGTGATTGGTACTGCTCATAGATG
ATTCATGAAGCTGCTGAGGTGGTATTTACGGAATCGATCGCATTGCATTTAC
TTTTATGATTTGAGTTCTGTGTTTGCGATATATTAGTTGTGAGCTTGATACGT
CTGAAACTTTCTGTGAGATACAGCAGTATGGTGTGCGGACAGGTCTCAGGA
TCAAGATATGTCACTTTGTGATATGTGATGTGAGACAAGAAATGCTCATTCT
ACGACCCACGTACAATCCATGGCGGCTGTGCCCCATTGCTGTCGAACTGAT
GATGAAGGGCAGTTTCCATACAGGTTTTTGTATGTTGACCTTGAGGCATTTC
CTCCAACTAATAATGACGCACGGTGTGCAATGACGGTGGCTGGGAAGAGG
GCCTACATCTCTGGAGAAAACATGACTCTACATCGCGTCGACTAGGTAACG
GGTAGCCTAGGTAATAAAAGTAATAAAATCATACAAAGCATTCACAATCACT
AATAGAGCAGATCATCAAGTCAAGTCAAAGTGTACAATCAGTGTCGTTGAA
TTATGTGCAGAAGCGCCGATATATCTACAGCGCACCACCCGCCCTTCGAAG
CGGCTCACACCTCATCCGACCCCTTCATGCAATGCCATTTCGTCCCCTTCCT
ATGCGTCTCCATCCACCTCTCCCCTCGGAACTAGTTCCTTCGACAGTCGCGC

ATACGAGACCGTCCTATGCAAACAAGCAAACAAATCTTAACGGGAAAAGC
CCGTCCGTCAGAACCATCCCGCTACCCAAGCCGCGCCGGGCCCGCCATGAT
AAAAAAACACAAGACGATGCGAATTATAGACGAAAGAGACAAAAGTCCTT
CCCGAACTCCGTTGTCCCATGCAATTTTTTTTCGAGGGCGCCTTTGGAAAT
TATGCCTATTCGGACGCCTCTCTCCCTCTCCGATGCTTCCACGATGCTATGCT
GTGCGCCAAGAATACAATAGTACAATGGGGTATAACGACCGTTAATAGCC
GTAATCATAATCGTAGTTCGTGATTTAATAGGTATAATATGGTAGTTAAACGC
GGAGTGCGAGTGCGGAGTGCGAGAATACGTGATAAAGGTTATGAGTTATTT
GGGTATCGTTGTCAAAAAATCGTATGCAAGTCCTCTTCCTCTCTATGTAATC
GTCCACTTTATTAACGCGGGTTCCTTATGTCGTATGTAATAGGTGCGTGTATA
CAATTAATTAATCGTTTGACCCGTTTAATCGTTCAATTGCGGGTTTATTCGTG
TACTATGTACTAATCGCCTCAGTTTCTGTCTGAGGCATTCCACTTGCCAATAT
ATGGCGATATCGTGCTGTGATGGCAAACGTCAATACGACAGCCTTTTAATAG
GAGGCGTCTTAGGGGCCTCTTTAGGGACCTACTTGGGAGAATCCTTCTCCT
CGGAATCCTCCTTTCTGGCGCCTTTGTTGCGCCCCTTGGTCGCGACGCTCTT
CATCTGCTGCCAGCCCCTAATGTTGATGGAGCCGGCGCGTGGAGGGGGCTC
GTTTGCGCGGTAATCCTTGATGGCCTCTTTATGGTCGTAGTGGGCGAGTCTT
AGCTCGGTCCCACCGTTAGTTTGCCCACGGGCGGTTGTTAGACGCCGGGGC
AGCCGCGATTCTGCGGAACTGTCTACCAGCTCCGTTGCCATTCGATGACGC
CGCCGTTCGCAGTCGCGTCCCATGAGCGGGAGGAGAAGGAGCTAACATGG
GCGGGCCAGGTGGTGATACGGAAGGTGCGGGAGGCTCCATTTGCGGCATC
GGGGCTAGCTGCGCCTGCCCATGCATAGGTGACATACTGGGATGATAGGTA
GGAGCATGGTGGTTAGGCATCATGCTCATGGGTTGGCTTTGAATAGGCATTG
GCATCATAGGCGGATCGTGATAAGAGTGTGGCCTGATGATAAGCTCGAGTC
GGGGCTGGAGCATAGTAGGTCCAGAGTTAGTGTGAACCAACCCATGAGGA
GATACGAACTCCTGTGGTTGGTGAAGAGGAGGTGGTAGAGGTATGGAGTT
GCGGAGATAAGTGGGATTTTCCTAAGCACTCGGATCTGTTAGGTCTGAGCG
GTGGGAGACCGGGGCTATCCCTGATTCCGTTAGTGAACGGGTTTCTGTGTG
GTGGTGCCGGTGGTGCCTGTGGTGCCTGTGGTGCGACGTCTGTCGGATGG
GGTGGCGCTGGCGCGATAGGCATGTATGTGGAGGTAGGATTCAAGGCAGTC
GCAAGAAAGTTGTTGATGACGGTAATTGCTGACTATGTCCCGCATAGCCGC
GCGGTTCCATCTCGGTTCGATCGACTCTCGATTGACTGCCCTTAGAACGAC
GTTCATTTCCAGATCGAGAGACGTCTTCAGCTGCACTTGCGAGAACATTTA
AGGGGCTGGTAGAAGCCTCCGAGTACTGCTCTGATCGAGCAGGAGCCGGC
TGTGTGCCGGTAAAGACGTCCCGAACCGCGTCATAAGGGCGAGAATGTCG
TGGGCTAGGCGGATCGGGCGGTGCGGCTGGCTGTTCACCAGCCGCTTCAG
CGGCAGCTAACAGAATATTCACACATTCTCTAAAGAGATTGTCCGAGACAT
GCTGGGGCTGATCCGAGTTCTCCAACGGAACTCCGTCTTCATCAGGAACGG

CCAGTCTCTCGCGAGCTTGGGTCTCTCGTTCGCGCATCTCGGAAGTAACAA
TGTCTTCTTGGTCGATGTCTTCGATCTTGCAAAAGCTACCTAGCAGCGATGT
CATGCGCGATTAGTGCCGTCTTCCTGTAACATGCGATCCATCTTCACAGCTT
TCACTGATCGGGATGTATGTTCAGTAGTATTCTGATCGGGAACATGAACAAT
AGGTCGTGTCCCATCGACGAGCTTGTTAGGTGGTTCTGGCTCGTTGACAGA
TCCAGCTAGGAAGAATCCATACTTGGGATGCAGTCTATGCTTCCTTACCAAC
TCAGGATCTAGGGTTTCATCCTTGAAATCGCGGACATCGTAAGACCTAGCA
GTATGGTCCATGAACCAGGCATTAGAATTGGGAGTGTTGTAAAATTTGGCC
GCGGTCTTGTTGCGTAACCTTTTTAACAAAGTCGTTGGTGGAATCACGAAA
ACCCAATCTCGTCATCATCCCATTGAAAGTATGGAGGAACGACGAATCGAT
TATGCGCGTGTTTACCCGTCTCTTGACTTTTCTCACGCCAAACTCATCGGGT
TCACCAGTGCCGGAGTGGAAGGGCGGTATGTCGGTACTATCGACCGGCGC
ACCTCAAACTCCTCGTTCGCCGAAGGTGTTGGTGCGTTTGACTCAGAGAC
GCCGTCGAGTTGCTGCTCTGAAGCAAGCAGACCGCCAGTGTGTCGAGGTC
TCTTGGGCTCAGACTCCTCTTCAGGCAGACCATCCGCCTTGCGCTTGGCGG
CAAGCCGCTCCTGGCGAGCGCTCTCGCGGGCATGTGCCTGGTCGAGAATT
GATTCTTCCTGGCAGCAGGATACATAGGGTATGTGGTCAACATAAGTGTAGG
TGAGGTCAGGTGTCTGAAGAGATTGTTAGTCATGTTGGCCGCGAAATATCG
AAAGGTGAGAAGTGTGTTAGGTAGATAACTCACAGTGAGCGGGCATCCTTC
GTTGCGAAGCTCCTCCAATATGCTAGTGCGGTTAAGGGCACCGTCCATAAA
TTCCTCGGTGTGGTAATTATAATGAGTCCTATGAAGAACGTTAGTAAATGAT
GAAATCATGAGTTGGCGTGAGATGAAGATGAAGTTTGGATATCAAAAAGGA
TAGAACATACGGTGAAGCATTTGTTAATGCGATCGGTCTCTAGCTCGTACTC
GCGTCCGGCGATGCCGTTTCTAATATCGAACTCCCGTTCAATTGATTTGACA
TTGTTGTCGTACAATTGTTTCAACTCGGCTTGAAGTATCTCATATTCTTCGAC
CTCCTTGTGAACATCTTCCTTGGTGGCTAACTCCTTGAGAGAGTGGTCTGC
GAGTGCCTCGAGACCTGGCTTGACGGCATTGGCCACTTGGGAGTAAGCTCT
AGGAGCTCCTTCTGGCGCTCATGGGCAGCTTGGGTAACGGCGTCAGGGTAT
GTCTTTTTGTGACCACGACCTTTACCTCGAGTAGGCTTCTTAGCAGTAGTTT
TTGGACCAGCTTGCTTGGTCACACCTTTCGGCTTCTTAGCGGGGGGTCGGC
CTTTGCCTTTGACTTTCCCTTTGCCTTTGTAGCAGGGCTTGCCGCGGGGGAT
TGGGTAGAACTACCCATCTTGGCGACGGGTTTGATGAATGTGATTATGAGG
AGTGATGATATGCGTGATATGAGGAATGTAAAATAAGTAGAGGGTATCGGAT
ATGTAGAATAGTTTTATGAAGAGTGCGTGGGAAAATATGTAGGAAGACCAG
CGTTGAGTGAAATTCTTAGGTAGCGAATTACACAAAAAACAAGAACGCGG
TAGTCTTGGCTACGGATAGAACTAAGAAGTAATTATAGCGGCAGTAGTAAAT
ATCCTACCGTGTGCTATGCGAGAAGATGATGTAGAAAAATAGAATCATGGA
AAAAAGGTGGACTTGGAAATGTAAGTGTGTGCATCCGGGTGTCCAGTAAG

TGTAGCTTGTGGGTGGAAAAGTGAAGGATGGCAGGTTTTTGGTAATGTTGT
AAGGTGTAAGGTAAAAAAAAGTCAAGGTCAGAAAAGGTTGGAAGGAGAC
GTTGACGTTGACGAGTAACGAATAACAAAAACAATTAACCAACAGGCGGG
TGGCTGCTGAAGGAAGAGAAGAATTAAGGTTTAGATGATTAAGACTGAGG
CTCAACGAGATATCATATCCTAGAGCGCACTCGTACCGGGTACTGTGTACTG
TGTGTTTTTTTTCCAAGGTGAAGTCGTCGTGGTATCGTGGTATCGTAATAGG
GAGAAATTTAGAGGCTCACACGCTTCAGCGCTGGTAATCCGCTGGTAATCC
ACCAAAGGCCACTGCCAGCATCAGATTTTCTCGGCCAGCACAATTAAAACA
CCCTGGACTTCCCGATACAGTCAAGCACCATGCGCTGTCTAGTGGTACTTA
GACTTAGTATTACTTTTTTATCCTTTTTGTGCCTTCTCATTGGTTCGGACAAA
CACTAGTCCGTTTCCGTTTCCGGTTCAGTCGGGGGTCCACCTGGTACAAAA
CCAACAACACGTACCAAAACTTTAGGTACTTTAGTGACTTTAGCGACTATG
CCTAATAAAGGAGATTCATCTGCGACTTATTTGTATTTCAAGTTGTTGATATA
GATTCTATCTGCGTGTAGTATATAGGATCATAGGCAATACAAATGTGGTGAC
GTAGAACTATGAATTTACTTATATAGAATACGTGCCTATACGTCTAGGTACAC
ATTTAATCGATGATCGATGTTGCCATTCGAGATATAGTACCTGTCAGCGTATC
TAAGTACCTACCTACATATATAAGATCTGTCGGTGCACGTATGTATGAATGTA
TGTACATTGTACCTCAACTCAGACAACTCAGGTGCCACGGTTATATGTACGT
AACCAACGTGTTTGTATCTCTCACTTACATGATGGGGTTCCCTTACGCCACT
CAGCCCCTTGCGCTTGGAGATCTCTTCATCATTAGCCAACGGTATATTAACA
AATACATATACGTGCGTAAATCGCGGTACGTGGTGCCTACTATTTGACCATTC
GCTGAAGCGGCGGGTCACGAGCGGTATCTTGGAGCACCCGGCATCTCGAA
TTGCCAAGTACCTACATATGTCGGTACTCGTACAAGATGCTGTGCGATCACC
GTCTACTACCGGACAGTTTGATATTGTACCTAGGCGTTCATTTGTACATAGA
AGATTAGCCCTAGGTGCGCCATCCCGCCTCAAGGTAAATTTGAACGCCTCG
ACCGAATCCTGTTATTCCGCATCAACTAAACCCGATCGGACGGAAGGCAAC
TTTCTTACGGCTCATACCTGCCTTCATAATACTAATATTGTATAAGAAAAAAG
TTTGTCCACTGTGTACTATATACACTGACATTTCCGCTATCTAGGTGTATTTA
CCTATGTACCTAGGTATGCAATTCGCGATTGGCAATTATTCTGACCGTTTATT
ATGTGTATAGCTAGTTGCCTAGGCAAGAGCAGGAATTTCCAGCCTTACCACT
TACGTACTACGTAAGGCACACGATAGCTTCGAATATCATTCGTCATACTTGG
TAGACCGCTTGCTTACATGTGGGCTGCAAACATGACCTGCAGCAGGTACCG
ATGGAAATATCCGCAAACTCCGTAGACGTGACAAGTGTCTACCATGCTACT
GTGGAAGCGGTATTGTCGATTAAGTATAAAAAGGGCACCATCTGCAACGGA
ATTATCGCAACAAATTATAAGATCGATCATCTCTAACATTTTACACATATATAT
AAAGTACCAACGTCATCCCTATCCCGAACAGCTTCGGTTGTGGTTCAGCGC
ATGTCTCATGTGCCTCATACATATGCTGCGTACCTTAGTTAGAGTTAGGTACC
GTACAGTAATCCAATGATGTACCGTGCTTCCTAAAAGGGGAACTCCATGTAC

ATATTATGATGTACCTCATGTACTTAGTAGCACTTAGTCGCACCTTCCTCTGA
TTGGTCCAAAATACCCCAATCCATCTGAGGACCCCGGAATTTCTAAAATAA
GACTAGCCTCGCAACTTACCCTCGTCTCGTCCCGCAGAAGGCTGTAGCCGC
AGCGAGTGGGATTGTCCCGCGTCTACGGAGCAATCGCGAGCCACTAATTTT
TTTTTTTAATCTTTTTCCCCTTCCCTATCGGCGGGAGCCACAGCTCCAGGCG
CGACCTCACATCATACATCACCTCACCTCTCACCCAAACTTATTTTTCTTTCC
CCGCGCACCTTCCACCGTAACCCGTAATCCGAATTTTCTTCCACATCACGAG
AGCGGTCCCACCAAGATTACCCACAACTAAACTCCGGGAGAGAAAAAGAG
AGGGGTTTCGGTACCCTGCATTACATAACGAAACTACTGTACTGTAGCCCA
GCCGCTTTCGCATCCCCCGCTCTCTTACGCGAAAGCCCGTCTTCGGCCGCG
GCTGCATATGCGACATGACGCGCCGCCCGTAAGCAGCGCAACGGGAAGTA
AGCAGTGAATATGGGGAAACGGCGGAAGGTCGCGCACGGCGCCGACCAC
GCGAAGCCGAAACAAAAACCTGGCGGCGGCAGCCAGAAGCCGCACGCGC
AGGGCAAAGCGACACAAGGCCATCATCAACAACAAAACCAATCGCCGAA
GTCCCACAAGCAACAGCAGAAGAAGAGTAACGCGCCGCAACAACACGAC
GAACCCACGATCCCCTTCTCCCCCGACCACAAGATCCTGCTGATCGGCGAC
GGCGACCTAAGCTTCGCCGCATCCCTCGTCGCGCACCACTACTGCGGCTCT
GTCACCGCCACAGTCCTCGAGAAGAGTCCCGAGGAATTAGCGGAAAAGTA
CCCCCTTGCACCCGATCACATCGCCAAGATCGAAGCCGAGGACGGGTGCC
AGTCCTCTACAACGTCGACGCGACCAAGATGTCGCCCTTCATACGACGCAA
AGGCAAGGACCGGGAAGGCGTCATGGATCGCATCATCTTCAACTTCCCACA
CGTCGGCGGAAAGTCCACGGATGTGGAATAGGCAGGTGCGGTATAACCAG
GAGTTGCTAGTCGAGTTCTTTAAGCGCGCGGCGCCGTCGCTGGCGCCGGG
GGGAAAGATCGTGGTTACGCTGTTTGAGGGAGAGCCGTATACCCTGTGGAA
TATAAGGGACCTGGCGAGACATAGTGGGCTGCAGGTCGAGCGCAGCTTCC
GGTTCCAGGCCGCCGCATATCCGGGATACCACCACGCCCGCACGTTGGGCG
TTGTGAAGAGTAAGAGTGGTGAGGGTGGGTGGTGGGTGGAAGGGTGAGG
ATCGCCCAGCCAGGAGTTATGTCTTTGTGGCAAAGGGTGATGAAGTGAGGC
CGGTGGCGCAAAATGGGAACGGGAAT

>Mosaic_sequence_79

GTAGCTTGAGCCTTGTGACGCCTCCTCGTGCCTACGTCACTATGCTTTAATT
AGGACAATGGGCAGCGACTCTTACGATACGTTATTTTATATGTAAAATCTCA
CATAATAAGAAGTATTCTTCTTAATAAATACACTAAATCTCCAAACCAGTAA
CGATATTCGAAATTTAATATATCGGTTAACCTTCGGAGATGTAGCAGTCACTA
CCCATTTTCTTAATTAAAACGCAGTGCCGTACGCACGAGGAGACGCCACAC
ACCAGTGCTAGTACAGTGGAAAGAGAGAATATGCTGCTAATATATGTGTCTC
GGCAGCGGCCCAGCCTCATTACTGACGGCAGAGCCAGATAACCTATATATAT

CTCACCTTTTCTCTCAAGGTTTGGCAAGTTCATCAAATGTTTTCCAACCAAG
TTTCGGGACTATTTCGTCAAAAAAAATTCTGAGAACTGCCCACCCCTCTTA
ACAAAACTTTCGAACAGCGATGTCACTTGTAGATCTAAGTAATGTCTCTCCT
GACCAGGAAAACAACTTCTCAAAGAGCCAGCCTTGGAAGCACCACCTAAC
ATCACACCCAACTTCCAAAACCCGGTTAATAGTAATGCCGAGTCACGTGGA
TTTCTTATGTTCTGCATTATTTTCACCTCATTTCTTGCCTTAGTTCGTTATTAT
GGCACTTGGACGAGTTCAAGGAGGTTCTTCCTAAGTGATGGTATTAAATGA
CACTAATTCCATTCCTTTTCTTTTCGCTGTGAACTTACGCTAATGTTGTGCTG
GTGAAGTGTTATTTACCATTGCACTAGTAAGTACTATTTTGTCCCCTGCTTTG
GCAGCGGTCTCACAACATTCATTGAAACAGGGTGCATATATGGTATATATGA
GCACGGCACTCGAATCTATCCATCGAGTAGGCTTTTTGATGCATCAATGGGA
TATCACCCTCGAACAGATGATATATATGAATTCTGTGAGTCAAGCCTCTCCC
CATAATTGGTACTCTACGCTAAACATCCAAAAAGACCTTCATTATCAGTCGG
ATATCATTCGCTCTGGCATTCCTGTTCACAAAGTTGGCTATCCTGCTCGAGT
GGCTTCGTATTTTCAACCCAACCAACCATCGAAATTACTTTTTTTGGGTAAC
TTGGGGCATGATAGGCGCAAATGTGACTTATTGTTCTTTGGCAACATTTCTT
CTAGCTTTTGCCTGCAGACCATTCAAAAAACATTACGATCCATTTGTGGATG
GTAGCTGTTTCGATAGCCGCCCACTCGCTGTAGTATCGGCAGTCCTGGATGT
GGTTCTAGGCATTGGGATCTTGGTTCTGCCTCAACAGATCATCTGGAAATTG
CAAATGTCACCGAAGAGAAAGTTTGGCATTGGTATTGTATTCGCGTTTGGT
CTCTTGTAAGTCTTCAGCGAGACTCCCCCATCCGAGCTGCTGGCTGAACCA
TCGAGTATCTAGCACTATCCTGTGTGCTATTTATCGAGTCCCTGCCACGATAA
AATGGATCAAATCAGCCGATACTCTGTATGAGTTTTCTCTCTACAGCCTCGG
GAATTCGGCGGAGATGACGTGCGGGTTTGTGGGTCTTTTGTATACCTTCAAT
TCCTAAAGCGTTCATAGATCTGGATTGTCTCAATTCTTCACAACTTTCAAGT
TATACTTCCCATTTGGAAAAGGGAATTGTTGGAGAGAATTCACCATCAGAC
CAAAATGATTACCATATGCTACGTGGGATCTAGTGTCGTTGCAGACTATTGG
AGGGCTCATCAAAATACAAGTTACGCCAACTCACAAAGCCGAAACACTATT
TAGATAATTCCATCTTACGTACGACGCAATTCACCGCGGAATCTACAGAGGC
CCTTAACAAGGAGTCGACACAGGAGCAATTTAGGGCCCAGCACCCATGGG
TTGGCAATCCATAACTATGGCGAAATGAATTTGAGGCTGTATTCGACCCAAT
TACAGATAGCGCACTTAGGCTGAATTAGGTGGATTCATAGCTGTAATACAGG
GAAGCAGAGCTAAAAGCGACAGAGGACGTTTCCACTGTATTATAGTGGGTA
AGAGGTAAGCCGGCTGTCTATTAGGGTGGGTCTTCTTAGCTTTGTATCACTT
GATAGTTTTGTAGTCTCAAATCTAATAGTGTAGTGCTTAGTGGATGAGCTGC
TATAATTTAATGGATAGTTGCTATTGCTCAGGCAATTCATCCACTCAAAAATC
TCGTATAGTGCTCTTTGCCTCGGTTTCTAGGGGAACAATTGTCTATTAGCTA
ACGTCCGGACCGTCGAATGATCCTATGCTGCCAGCCATCGGGAAGCTTATA

GTCTTCTGGAACGTGTTCGACGAGTTTCGCAAAACCAGTACCTAGGTAGTA
ATGTACTGAGCTGCACATGGCATGATGGGTCTAGACGAAAATAACCATCAG
TAGTTGCAAAGACTAGAAACCATCTGCCAAAGGATGGCCTCTTTTTGTACG
TTTATTACTCGTTTTTACTCATCAAAAAAAAAAAAAACATCTCAGCTACTGTC
TCTCACGAAAACATTCACATTACGCTAGATACGAAACAGATTTAGAAAAGA
TGTCTGCGCAAAAGTCCGGTTTGAGGTAATGCCATGAGGCCTAGATCTAAT
AGTGATCTAGTCGCTATATTTAGATAGTCGCTACAATTTAGGAGGTAAGACG
CTATAGCTTAAGCATATATGATGAGTTAATGCAAAGTGGCTGGAGGATGCAT
AAAATTCATTATAGTCCGTGCCATGCTGCACTGTGGCTGGGATGCCCTCATC
GAGTTTAGGCCAAATTTGAGGTTACACCCGAGGTGACCCTATTTTTCTACA
AATAGAAGACACCGACATATTTAGAAAAGGTCGAGCCCTTCCATCAGACTC
ACAATTATGACAAATCATATTCTCGGACACTGCTTTTATATATCACTCAGCAC
TCACAAAAATTGGTGAGGCATGAAATGCGCTTCACTATTGGGTCGTGTTAC
TTTTCTTCGAGGTAGACGATCATATGATCCTCTCCTATCACGGCCAAGCCAC
AGCGCATCGTGATTCAGATCCTGGCAAAAAATGAAAACGAGGAATACGAA
GATAATCCAGGTAAAGACTAGGTGCATAGATACTATGAGATGCCATGTGAAA
GGCGCTCTAATAAGTACTATAAGCTCACGCATTGTCATATGAATACGATTCTC
GCAAGACTTATAGAAAGTTTACCTGATAATTTTTGACAACCCAGAATTCCTC
TGTTAATTCGAAAGACTGCAGTAAGTCCGTCATAGACAACTAACCTCTTTCT
ATTCTAACTGAAATATGAGGATAGCCTTAATTTGCCTTGTATTCACCTTCCTA
CAGTCCTACCTAGATAAACTTGCTTTTATCTTTTCCTGCAGCCATGCCTTCTA
ATGGAAACAATGGACGGAAAACGAGTACCAATTTACAGGGAGGTAGCTATA
TTCCGAAAACCAGCTCAGCGAGCCCGCCAGAAGAAGTATTTCCAGGAAAG
CTTCTTCCAAGAGTTATCTGTCGTTCCTGACAGTCCGCATATAGCGCCGACC
AACGTTCAATTATCGCCACAAAATGGAGAACCTATGGTGTCCACCATCATG
ACCCCTGTCTTCCTGACGCACATACTCCGTGCAGTCCTAACCGATATCCTAC
CTCCATCATAAATATCTGGTAGAGAAGCACCTACGGTATCGAATCGAATCTT
AACTTCGGTAGGGATCCTAGCAATCAAAGACAATATACCGAGTTTTGCACT
CTAACAGCGTATATACAAATATAGTTGTCATTGCGGGAGGCATATCCACACT
TACTTCTTAGGCGGCAATTTTTCGAGAACTACCTCATCCCCCGTGATCTCAA
AATCCCACACCGCACGATCATCAACATCTAGAACCAATGTGTACTTCCCCG
GCCATAACGTCAAGGCACCTTCCCGCGTCACTACGCGAAATCGCACCTAGC
TTGACATCCAGAGTCGCTGTCCCTGTCTCGCCTGGTTTGATGTCGTGGATCC
GATCAAACTCCACTAAGCTCTTGATCGGATACGGCTCGGGTCCATATTCTCC
TTTTAAAAACGCCAGCACAACATAATCAGACGTCGTATTCCCCCGTGTTTGC
CACAGACACAGGGATCGAAGTAAATGGAGCCAGATCAGGATAGTTGCCTG
AGGCGACTGGTCCAGTGTCCGTGAATGAGACGTTTGATGTGAGGTCTGCG
GTTGAGAAGGTAGTAGGAAGGTCTCCAGGCAACGAGACGTTGAACTTCGT

GTAGTGTAATCCGTGGCCGAAGGGAAATACGGGTTCTTTGGTGTACCATTTA
TAAGTTCGACTGTGAGCATTGTTAGTCAATTTCATCGCACGCATAACATGAT
GAAGTCAGGAATGTGGAATTGGCTTACCCAGGGTTTCCGGTACCGGATTGT
AAGTTCATATCCGTCATCGGGACCTGGTTGACGTAGTCGCCCGGGTACTGT
GTCACGGGCAACCTACCAGCAGGCGCCATTTCACCAGTTAGAATGCTCACC
ACTGCCGCTCCTCCATCTTGTCCCGGATAGCCAGCCCATAATAATGAGTCGA
CGCCTGCCTCGGCGACAATTTCCGAGCTATCAATCATAGTACCCATCTGCAC
CACGACGAAAGGTTTTGTGCCTTTCCCAGCCGCTATCTGCTTGATCAGGGG
TATCTGTTGCCCCGGCCAAGTGATCTGCTCACGGTCCCTGTCCTCCGCTTCG
ATGCTTGTATCAATACCCCCGACAAATACCACCGCATCTGCCGCCTTGGCTG
CGTTTACGGCAGCAGTGAATCCCGAGTTCGACGTTCCGGTAATGGCACATC
CCTGCACATTAGTTACATCGTAGCCTGCCGTCTTGAACGCCGAGACTACTGT
CGAGATACTCTGCGCAATACCGTAGTAATTCCCCTGCATCTGCGTTGTGGCG
GTACCCAGAGGTCCGATAACAGCAATTGTCTGAATTGACTCAGCGAGAGGC
AGGGCGCTACCGTTATTCTTCAAGAGAACGAGGCCCTCCTGCGCAGCGCG
AAGCGCTAGGATTTTGGCTTCCGGCTTGGCGACCGTATCCCAACCTAATTG
GCGATATGGCTGTTCCGCTGGGGGGTCAAACCAGCCCAGTCGTACCATCGG
AAGCGTACCGTCGGATGAGCGAACGGTCTAACACCGTGTCGTTGAACATCT
TCGAATTATACGCCGAGGGAAGATTTTGAGGCCAGAATTGACCGCAGTCAA
GGTCTGTTCCAGCATTTAAACTTGCCGCCGCGGCCCACGCAGCTGAGGAGC
CGTAACGGTGGTCCGTCCAGACGTTCTGAAGCGCGTCGCAATCGGCCGTG
ACCCACCGGTCTTCACTAGTAAATCCATACAACCCGCGCAGCACATCTTCA
AGTAACCAAGGATCAGCGCACGTCGGCACGCCGTTGACGGCGTTATAACTA
CACATTGCGGACTGCGCATTAGCGTCGCGAATACACGCACGGAACGGCTG
GAGATAGTAATCCTGCAGATCCTGGCTACTGATCTTAGCGTCAAACGCGTAT
CTCCGATTCCCCTTCCAATTCTCCATATCGTAACCCGCGTAATGCTTGCACGT
CGCAGCGAGCTTATAGTACGGGTCCGCGCTTAGCCCGCCCTGGAGCCCCGG
GATTAGTTGCATCACGTACTGGCTCAGGTGGTACGGATCCTCCGACGGTAC
TTCCTGGCCCCGGCCCCACCGTGGTCCCGGAAGGGGTTTATGTTCGGCGTC
CAGAAATTTAGACCGCATCTCCCCGCATTGCTGAAGGCGCGCGCTTCGGTG
CTGGTGGTTTCCGCCACGGAGCGGATTAGCGGCATGTCGAAAGCGGCGCC
CATGGTAATCGGCTGTGGGAAGGATGTCGCGTAGCTGAAGTTACCCGAGTT
TGCAAATTGGACTCCGCGGGAGCGGGCCACCCCATGAGAGCTGAGGAATA
ATTGTCAACTTAAGCACCCTATTTTGTCTCCACTTTTTATAAATAGGGGAGG
GGATCAAATTGCCTACCTTCGTTCCACCATTCGTAAGATGGAACTCCTAAAC
GCGAGACACCCGGCGAGTTATCCCAGGAATTTGATATTTTCTCGCTGCTGCT
GAATTGTGCTACTAACTGGATAATAGAATTAGACAAAGTGATCGAGGCATGT
TAGGTGATAGTGAAGATCTTACCGCTGCTGCCCTAGACCACGGATCAAGTG

TCGTGTTACATACTTGATTAGAGGCAAGCTATTTCATCGTAAGCTTATAGCAT
ATATGACGAAGGGTGATGTGATCCTTCCGGTACTCACAGGTCCCGATTGGC
AATTCGGAAAATCCCTAGCGATTGCCCCTTTTGCAGCTATGAGTACGATCAC
GCACAAAGCAGCCCCGTACGCCATTATCCTAGGTTGTAATTTTGAATTACAA
GTTCCAAGCCAGTTCAGATAGAAGTAGGGGATTCTTCGATCTTTATAGATAC
CTACCCATGTGGAATCTAGAATAGTTCGGCTAAGATGGAGAATGTCTCGAG
TGCCCTAAATGCTACATTAATACACAGATAATATGAGTAAAGCGGGGAGATA
TTATTTATAGGATTAAGTATAATGGGGCAGAAGCTATCCGACCATTCTTGAG
ACTAAATTATTCAGGCTAATCAGTCCATAACACGACCAGGATGTATAAATAC
GGTTGATCACGTGGATATATTTACAGCACAATACCTGTACCCCTTTCCATTAC
CGATCCACGCTGACATGTATACCTAGGTACCTATGCACCTATCCACTGAGAA
TTAATCAGGACGCCATCCCACGCCTTCAGTGCTACTCTTCAATAAGGTGGA
GAGACCAGGAAAAACGAGGGCGAGATAAATTCATATCAAGCTAAATAAAG
TGTAGAATATTAGTCTTAAAATCAGTAAACTTGTAACAAGAAGAAAAACAA
TATTTATGCGACAAAACCTACACTAAATATCAGTATATAAAAGTCAGTAAA
CAAGTCAGTGGTAATGTCTAGTACTTACCAAAGCACCTCACTATCCTCCATT
TTTATATCATAGCTCTTACGAACATTTTAGAACTCCATGCCCGACCCGGTCTA
TTGAATTTGTTCACCTCCAACGAGCAAAAGCCCCAATCCAGCCCCGGATTC
CGAATAAAGCGCTAAGCGCAACCTGGGAACGTTCTTCAGGTATTCTTTCAG
TCATAATTGCAATGGCCTCCTTCAGTTCTCAACGTCGTTGCCAAGATAATTG
ACGGCAACTGCTGAGTTGCTCCTGTTGTTACCAGGTAACTGGTAAGTTCCC
CTATGCAAAGCGATTACTACTCCGAAGTAATCAACGACAGGCCCACCAGAG
GCGCCTATGATAAATTATATTAGTTAGTACTAGATAGAAACAAGTTGGGAGA
GATATATCTCATTAAGCACCTTCATATGTATCTCCATTGTGGTCAAGTTCGTC
GTAAGGGTTAGGGATCACTCAACCGTGGCACAGCTATAAGAGAGATAAGG
AAGCCAGTTGTTATTCCGGTCCCTAGGCATATCGCTAGAGAATCCATAGACG
TGGGCATTGGTAGTGGCAAGAGCAACTGGTAACTCGGTATAGCGCATAGGC
CTGATCCCACTTTTTGAATCGAGTTGAAACATGGAGAATAGCGAAGTCATT
CTTAGTGTCCAGTGCATTGGCCCATCCGAAAGTAATAGCACCGGAATCCAC
ATGGTAAACATCTGAACCAGCGCGATTATCTTGATAAAGTTTGATAGACACT
GGCAAGACCGAGCCTGCGATCCCAGATTATATGTCCAACAGTCATGAAATA
CTCGTCATCAATGGCCACTCCTGAGCCTTTGGTAGCCCATGATCGAGAAGA
ATAGTCAATGAACGTTGCAACAACTCTCAATATCCCTAGACGACGTAAGTA
ACGCTCAGATACGTTAGGTAAGGATTATATACCGCTATGAGACCCGTCATTT
TGAACATGGGCAACGGGAATGACCTCCCTAGGGTGTATGCCGAGCACAGC
CTCTTCAGATTCTGGCGAAAGCGGTAGCCTGAACACAACTGCTCCAGGTGC
TCGTCCATTGAACCATGGATGGTGTGGTAGGTCGACCCTGTTCTGGAAGAT
AGTATTTCCATCCCAGATTTGATGAGGGCCGAATGACTCAGATCTTCTGAAT

ACGTTCTCATCAAGGTCCGTATTGGTAGGAACAGGTCTTAAAAGGGACCTA
AAGAAATTCGCAACGAACCTCATTATAGTTTGCAATGTCTAAGGGCAGATC
TTTTAACTGGATGGGATGGCCTAGTATCATACTTGAAAACATCCGGGCGTCG
GGGCTCCTTAAATAGCATTGTGGGGGAAGTAATAAGAATAGTTATACTTTTC
ATCATAATAGGAGCGCTCTTGCAACCTACCTACCTATTAAAATGATAACCTTT
GTATAGGGTGATGATCTTTGCCTTTGCCAGTGCTGTGAGTACCACGATGTTT
ACTGCATCCAATGGGGCAGTAAAACTGCAAAGAATCTGGAACCTAGGAAC
AGCTAATTAATATCCAATCGCTTTTTTTCACAATCTATCGAGAGTCCCGAAA
CTAACCTCTCGCGGAGGCACCGAAGATGCAACTCTAATGGCCGCTCAATTG
AAGAGTCATAATCGTTTTGTTCCTATCGATGATTCGATATTTCATCTATCTACA
TAAGCTACCTACCTACCTCATGAGACAGGGGGGCTTTCGTTGTGTTACTCTA
GAATTGCACTTTGCCTTCATCAAGTGGTCACGCATGATGTACCAGAGCTGA
GCCACGGGTACAATGTCCTGAGAACGCATTTAGGGATCGTTAAGCCTATTTC
TCGAATAGGTAATTGGCCCTGATTGAACTTATCCGCTCGAATGAAGACAGC
CATTGATGATCCGGACGCCAACAGACCTGCACGCACACCGCATTTCTTCGG
CGCCGATGAGTGTGGAAAGAGCGCGCCGTGCGTCGCGTTTCTGCGTTGAA
AGAGCGCGACAGGCATCGTGTCTATCCTTTCCCTGCAGCATCGGATAAGTG
ATGTCCCCGCGCAGTATCGTAACACGAAATGGCGGAGTAAAAGTACCTCT
AGTGCCATACAAACAGAGGTGGTAGTGGATGTACTCGAGGCTCAGCCTGC
ATGATGTTGTAGCATGTAGTCATTGGTCAAAACTTCTATAATCCGGTTCAATA
GAAAATGATAGATCATTTGTTGCAAGTGTCATACGACGAGGGGTGCATCAG
GTTTTATACTCATCGATCAAGATTAGTTCATTGGCTAAGCTCGCAAACGTGG
CACTGAGACCACCTCAGATTTCATCCAATATACGCCTTTGAACGCGTGAGC
GCGTTAACAAATGATGCCACGCGGCTATAAGTTCACACCTATAGTGTGACGT
TAGGCACGGCTCTGATATATGAGCGTTGGCTTTGGAGTGGAAGATTTCTGG
GCCTCCCTTGGTGAACGGTGTAGATATAAAATTATGTCGTAGACCTCTATAAT
TAATAGCATTTCTCCCACTGAATTACATAATAATGCGTGGGCTTACATAATTC
GTACAAAATTGTTGCCGGTATCCCTGATGAGCTGAAACTGTGAATCGGTGG
GTTCATGTTCATGCACTATGGGACGTTTTCACGACCGGTATTGGCCGCATTA
TTGTCTGAGCCAGAAGAATTGTCCACTAAAGTATCGCAGTCTGTTGGCATA
GATAAATGATGCTGTACAGCTAAAGCAAATACAAAACCGGTGGTGATTCCT
AGAGTTGTGGCACACGTACGTACTAGGTAGTTACGTAAGTCTATCTTATGTA
ATATCCATGTAATATAGTGATCAATTGTTACTTAGAATTAATATCTGGAGTAA
GCTTTACTCGGTAACGCAGTTGTGTGGTACATACTTCTATGAGAAGAAGTTT
ATAATAATATCTACCTACCTAGGTAGATACTTAGCCTCCATGTAGAAATTTTA
CCACATGTTTGTTGTCAAATGAGTAGGTGGCTCTTGCCACTTTGACAGTTTG
CTGTGTAAGTTGCTACTGCAGGTACAAACGGACAGCAACCGATCGATGAG
GTCACCATTTGGTAGCCAAGTAACTCAAGATCCAAACCATCATAGATCTAA

AATAATCCAATCAATCAACCCATTCTCTAAAACATCTAACGTTTCTCAGTTA
GTCTTGAGACCCTGGAATCTAGGAGAGGAAAGAAATAAAACTCCCAAATC
GTCTTCCTGTCGACAGGTGAGCATTCTAATAACTCTCGCAAACAGTTCATG
AGCTCAGAACGTAAAACCATGTCTCGGTCCATACTCACCAAAGCTTTCAGG
CTACCAAACGTCAATCCAATCATTTCGCCCTACCGACCGGCCATCCAGAAA
CTCTCCATCATGGGTGACCGAACCGTTTCCTCGAAAGTGATCTCCGAGATT
ACCGCCAAAGAGAAAGAGCTCACGAACGAGAGCCGACCTGTGGCTAGTG
GCCCCACGGCTCAAGCTCAGAAGCATGCCAATGAGCCCATTACAGGAAAA
GTCATCTCCGACATCACCCAGGGCGAGAAGCACGTCACTAGTCGTGATGTG
CCCGTCCACGGTGGTCCAGCAGCCCTTGCTCAGAGTATCGCAGCATCCGTA
AGAGATATCAATTCCTCCTCGCCATCTCTTAATAAAGCATTTAGCTTATTTTT
ATTTTAGAAAAACAACCCCGTATCATCCTCACACACGGGACGCTTAGACGC
GGACACTATCTCGGCGATTTCATCCGCGGAGGCGGCAATCACGGGTAAATC
CGAGCCCGTCAAGGGCGGCCCCACAGCACAGGCTCAAAGCCACGCCGGC
GAGCCCATTAACTCTCAGACCCTGCACGACATCACCGAGGGTGAGAAAGT
GGTCACCGGCGGCGCCCGCGTCAAGGGAGGGCCCACTTCGACAGCTCAGA
GTGAATTAAGCAAGAGTCAGGCTCAGAACAAATCCTAAACGCACGACGGT
ACTTCGATGATGGGCTATGACGCATGCATATCAATCTATCTGTGATTCGGAGT
CAATCGGAAGGATGAGATGACGACATTTACGATAGAGGAATAAAGGATTAG
ACGGTAGTTGAGGTGGGCTGTGCATCTAATATGGAGTAGGTGAGCACGGCT
CATGATGGAAGGTCAAAAAGCCTATTTTATCCTCATGTATGATTAGACTTTT
GAGACTCTCTTTAAAGTGAAACATACCATGAAATAGATATCCACTCACTGGC
TTCGAATAATTCCACTCGTGCAATGTGATCCAGGCAGATGCTTCCCTGAATG
AATTGACGATTGATCTATGAGGGGATGAGTCAGCATAAAATAAGCGTGAGA
TTTCAACTTGCAACTCATCGCTCTCATATTTTCTTGGGAAATTTCATGAATTT
GAATTTCAATTGAATGGTTTTATGGTACAAGGCTCTGGATATCTCATAATATA
CGCCTTATCAAATCCTACCGTCAAACCTCGCCCCCCACGATGAACTCAAAA
CGCCTAAGAAGCAGTATCAAAGTTAATCAGCACAGCGCTAGAATATATGTC
GGGATGCCCCATCGTGTCATTAGTAGTAGTTGCGTTTTTCAAATAGCAAAAT
CCAGAGTTGACTTTATGCAACGTCACACCCATGCAGTAGCCGCCACCGACG
CCTGTGTTATTCGCTAGATTGGCGGAGTACGCTTCGTTATACTATAAGGACTT
CTGTTAGTTATTTGTTTACTCATTACCACCATCTTTAGAATCGAGGTTCTAAG
ATAAGGGCATTATGAGAGAAAACTTACCGCAGCGCAAGCCATAATGCAATT
CTCTAGCGTTGGCATGTAGATCTTTAATATATCATGTGTCTGAGTTGTGGCAG
ATGAGGTGTAGCTGGTGTAGCATTGTTGCTTGAATTGCTGGCCCTCCTTTCC
GGGCTCCAGAGGGATAGATGTGCCGTCCACGGCGTAAGGTGTGTATGTTTG
GTTATTAATCAACGGGCACCCGCTATCGGAAGGTAAGGGACCCGTCGAAGT
GACAGTTGTCGTAGCTGTAGGTGACGTTGATGTAGGTGTTGAGCTACTATCT

GATGATGATGTTCCATTTGTTGCCGTATCGCTGGTTAATTATTTAGTAAATTG
AAAGACCCAATTAAAATGCTATGAGCATCATAGATTGAGACAAACCTCGAA
GCATTGTCGGCGAGCTTCTGTTGTTGCTGTGCCTTCAAACCACCCCCAATT
CCGCCGCCCAGGGCCGCTGCGAGAATAATAAATACGATCACGACCAGCGTC
CAAAATATCGGAACGGTGAGTCCCAGCACCCGTCGCTTCTCTTCCTTCCCC
TTATGACTTTCTATATCCGAAGGCGGCGATGGAGGTAATGGTGGTAATGGCA
GTGCCGTCGCTGCCGTTGCTGCCGATCTAGGCGCATCACTCCACCCTCTCG
AACTCGGAGACCACTTCTCGCTCTCTACATCCGGGGCATCCCCAATCACAT
GGTTCGGGGCGCGCGCAGGCAACGGGTACACCGGCGTTGCGTGAGGGAC
GACCTCGAGGCCATCGTGAAATCCAGCCGGGTACTTCGGCGCAACGTTCTC
GCTGTAGTTCACGCGATACCGGAAACTCGAATCGTCTTCCATCGATGCTCTC
AGCTAGGAGGACTCGTCCTAGTCATAGGTATTATGATTCTACGTAGTTATGTT
GCATACGCACGTCGTCTCGTCCATGATCCCACGTGGGAATGTGTAGAATGG
AGACCAGAAAGCAAAATCTAGAGCTGGGTCCATTGGGGAGGGGAGAGCA
ATGCATATCATATATGGGGAGAGGACATCCCACAGGCGCATCTCCCCCATTT
CCACTTTCCTCCTCCAGCCCCCCTTGCGGACGCCACTCATCTCGTTATGTTT
CTGATAGGTGACACTACGACTCCTAATCGAAACGGAATTCCGAATCTGGCC
AAGGACGGTGACATGGTTCGTGTGCTAAAATAACTCCTTGGTCGAAAACA
GCTGCCAGTACGACTGTGAAGTCGAAGCCGACGTCTGCAGGGGAAATGCC
TGAGTGCGGCACAGCTCAAGCTTAAAGCTCGCCGAATCTATCGAATCCCCT
ATGTAACGGCATCAGCAAGGCACATCCATGTAACGGCGAGAACGTGGCTAT
CGATCGGGCTGGCTGAACGGACAGACAGGTTGCACTCTAGCGTGGTCAGG
GCAAGGCGGAGCTGGTGTCTCAAGAGAAAATCTTGGACCGTCGAAAGATC
TCCGAAGATCTAACCCGATTCGCAAGGAACGGCTCCGCTTCTTCTGCTAAG
TATTGCATGCAGCTATTCGTCGATGTAACCACGTGCAGGATTGGCGAACTAA
ACGAACCAATGGCGGGGCAGAAACACATCACGAATCCTATGAAATGAATGT
CTCTCTTTGGGGGATTCGATTCCCTCCTTCAAACCATTTCAAGATTGGCTAT
TTGGACAGGCACCGAGAATTCTGAAACCTGTACATCACGTATGTCCGTAGC
TACATCGGACGATAAGCACACTAATCCAACCTGTGCGACGTATTCAGCAGG
CAAATAATGTCGCATTGACTATTCCCCAAATAACTCTTAGTGAGACGTCGAC
GCTCGATAGAGGCAAGTAGAAAATGATTAAAGAAGGATCGTACGTCCCTCC
AGTTGCTTCTAGATTACCTCGCCTATTTGCTTTTGCTTCACAACCTTGGAATT
GGGAAAGCTGAAGTTGAAGGAATCGTCTAACAATTGTTGCTAGGTAGAAG
AGGGGCTAGCTATCGATACTATCGTGAATTTCGCCAACCTTGGTGGCGTTCA
GCACGGACCATGATGCCTCGGTATAGCTTCGAGCTTTAAATAGTGGGACGG
TAAATTCCGATTGAAAGATCAGAAAACTCAAATAACTTAAAAATCACTCTG
GTTAAGAGTCTCGGGATCCATCAGATGAAATTCCTGTCTACACTTGCTGGTC
ATCGAACCAACACTATTACTGAGACCATCGTCAACAACGCCGGCATTCAAC

TTCCCTCATTGATTCCTAATCGATCCCGGACGCCCACGAGTTTCAAGGTCCA
CTGAATTCAACCAGCATGCCTGTACCACCATGAGCTTGATCTCAAGTCACC
TACCTAATATAGCACTTACGTTACTCTTTCCTCAGTCTCGCCACATGGGGGT
TCGCCGCTAAGAATCCTTCGCGATCATAGCGCATCACAAAGGTCAATACATA
TCGCCAGAGCACTAACGACTAAACAGGAAGGATTCGAAGAGCAATGCCAA
TGCCTCTTTTCACTGAGCCATGTCGGTTTTAAGTTTAGCATACCTGTCTGCT
AGTTATTAGTCCCTCTAGTGGCGCCTTTCAAAACGCGCCGCCGTCGTCTATG
TTAGCCGCGGGAGATGCTAGCTCGAAAGCGGCATAATTGCGCGAGCTTATT
CTTCTCATACGGCGGATGCCGTGCAGATCATCGGTATTGACAGCATGTTGTC
ATTCTTATCAAAGAGAAATTCTCCTAACTATTGATATGCTATAGTGGTAGTAT
CAAAGGTCCGGAAAGGCAATTGGCCAATTTCCCGTAGTACGATCTATTCAA
GTTCCTGCATACAGAGCACATTATCCCACTTGTATAGGGTGGGATAGACCCG
CCTTAGCGCCGGTTCAACCGAGGATGTTCATCCAGGTTGGCGTGACAAACG
AAACAGAGAAATTATATAACACGTCGGATTTGGAATCGGCGCCCAAAGACA
AGGCATATGCGGAGACCAAGCTGTTTCAAGACAATAGCGATTCCCTTCGGC
ATGAATTCCACACTCTCCCCAGCTGGCATCTTCATGAACACCTGGCGGACA
ATACGCTTGGTGATAGAATCCACATTGCGTACATTTGCCCAGGTCCCTGTTC
TGCTAAGCGCGTCGAACAAGACCAGGACCTTATCCTCAGACCCCTGTTTCC
GCGTATTGAGTATCCTGATCTTCATGTGCTCCAGCTTCTTCTGCAAGAGCCA
GAGACAGTCTTTGGGCTACATATTCGAGAATATTAGCTCAGTGAAGCGCCC
CCGCAGACCTTCGTTCGTGACCATCAGCCGACGCGTCCATTTCCCCTTTATA
GCCGGCGAGCATGATGACGAGTTTGTGTGCGAACCGCTCATTCGCCATGCA
GTGAACAGCTTCCTATACGAACTAATTAATAAATACCCCGGTCAAGTCCGTC
CGCGCCATGCGATAGGCCTCGTCCACGAATGGAACTTTGCCTTGTGCTTGC
TTGAGCAGTTTCTACGTCTCGGGGCCGGTTTGACCCATACCGGTCCCGACG
AGGTCTATCACCGAGCAATCCATGACTTCGGTAGTCGAGAAGAGTCCCATG
CTATAGTAGATCTTGCCGAAAGCCCGGGCGGTGGCGGCCTTGCATCAACTT
CCTGAAGCACTGGAGGAGAGCGTGGATGCCGCCGGTCGTGCTCCGAGAGT
CCAGACCTTCACTTGCACGCTCAATTTCTTTCCTGAACTTTGCGACCGTGA
GAGCTTCCTTTGAAATTATGGCCGGTACCTTCCACGTGATACCGTCCGCGGC
GAGCCTTCAATCCCCCTTTTCCCAGTGTCTCCAGGTGTAGGAGAATGTACT
GTACCTAGGTTCATTCTTGTCGTCGTATATGTTGCTATCCTGCCTTTCATGTG
AGCATACTGTCACAAACCATACAAAATAAGCAACCATCCTATAGGACCCAC
TCTAACATCAGACCAATCACGGCTAAAAACCGGACCTTAGGATCTGTCTCA
TCTGTATGATCTGCTCACGCTATGCTACCAGATCAACCAGAACACCCAGATC
CTAAGGATATGTCGCCAGATCAACCAGATCATGACAACCTCAACCAAGAAA
GTAGGAGGGAAGGATATCTATATAATCATAACTGGCTCTCATGTATCTAGTCA
TTCATTTAGCTCTTTAGCTATCAATCTAAGTCTATTACTTCAACCGCATCGGA

CTAATAGGGAGAGGACTCCAAGCCTCTCTTACGAACAGACTTAACTTTGAT
CTGATCTCTACGAGTACTGCTAACAAACAAGGAAACCCACGAAGCACCCC
GTATACCTGAGTACCTAGGTATAGGTTCGTAACACATACCTTAAGAACGTAC
AACACTCAACGAGGCCATTGTTTGTTATCAAGCTTCGATAGAACAAGACCG
TACCCGTCCTCGGAAGGATTATCCTGCAAACGATTGAAGGCGCCTGACCAG
TCTAGTTGATTCTAGGCCATATATGTGATCAGCAAACGAAATATGTAGAAAC
TGAACGCAGGGGCGGTAAAGTATGTTGACAATGATAGAGCCACGCGGGAA
TCCAGTTAGCCTTTTATAGACATGTTATAGGGTTGGGCATAGTGTGTAATCCA
TACATGGCTGCGAGGCACCAAGGACGCATTGTCACGGACTAACATATAGAG
ACCTCTCAGAGCAAGGCATAATTGCACGTCATTACACATGCTGAGTGATGA
CCATACATCAGCTGAAAAGTAACATATAAGCAGATTCAGGCCTATCAAGTCC
ACTAATACAATGGCAGAGAATCGAGCCTGCTTTTGCAGACAATCAATTTCC
ATTTTCTTTCGTCAATCTCCAATCAAAATGGAAACATCAGACCAGATGACTC
GCTATATAACTTATACGAACTTCTTCAACCTCCGAAATTTTTCTTGCCGTGGC
GTGCGCAGCAGGCAGGCTGATACGTACACTCTTCACCACCAATGTAGAAGC
ACTGTCAATCCAAAGCCCGACACCCAAACCCCGAAGAATATGCAAAGATG
AGGGTCATGCGCGCGAACATCCATTGAGGCACTCTCTCATTTCTATATTCCT
TAGTTCTCCAAACTTACCATAGCTTTGCTGGGTATACCTTCAAAATCACCAA
GCATGTCGTCGCCTCCTTCAGCCGTATACCTAATCTAGCACTTAAGAAAGTA
AGGTTTACGGAACTCCCAAAAAAGAATTCCTATTCATCATGCAGGTCCCAA
ATGATTATAACCTTACCGAAGACCAGGTAAAGCATCAATATCAATATCATCA
ATTACCCTCCAAAACGTCCTTTCGCATCATGGAACTACTGCCTGGCGAGAA
GAGCGCACCAATCTCATATCGTCTTCAAATTGCTGACTGGTCAAGATCTACC
CGGTTCGAGTCCGTGTCGTATGCCTGGGGTGATGTCAATCGCAAGATCATAT
CCACATGCGACGGTCTTAATCTAATGATCACAAACAACTTGAGAGACTGTC
TTGTTGCTATGCGATACACAGACCAATCTAGATTCTTATGGGTGGATGCAAT
CTGTATAGATCAAACCAATACAGAAGAACGAGGATACCAAGTGAGCCACAT
GGGCGATTTCTACCGAAGAGCCACCAGAGTTGTTGTTTGGCTTGGTAGGGA
TGAAAATGACCAGGTTCAACGCTCCATTGCTGCTATAAAGGAAATCGTGGC
GCAATGTCTTGGGCAAGAGGAATCAGTTTTAAGGTCTAGGGATGAACTATG
GCGCCTTATCCCGGAACACTTGCCGGAAGGACTCCAACATTACGATGAGGA
CTCGTTGAAAGCTCTGTCTTGGTTCTTTTCACGTTCATGGTTTAGCCGCCTC
TGGGTTATTCAAGAGGTAATCTCCAATCCAAATGTGGAAGTGCTTTGTGGA
GGCAGTCGTATCAGCTGGGATGTGGTAGCATTAGCAGCGACTTATATCTGGC
GTCACCGTCACACGGAAGTCTATAGACGCTTGGTCCTGGATCATTCACTTAC
ATTTAATATCTATTATATGCGGCGTCGCTTTTGGCACCGACATGTCAGCTTGC
CGTCATTACTTAATTGGGGCCGATTCTTCAACGCCACAGAATCCTTAGACAG
AGTGTATGGCTTGATGGCATTGCCTTCTTTCGCTAACTCGGATCTCTGCTGG

ATAGCTGATTATACGAAGTCGAAACTAGATCTATACCAAGACGTCGCGTACA
AGTGTATTTCGAAATTGGGAGCCTCCGAATCCTTTGCTATAGCCAACACCAT
GGAGACGACAAGACCTTCCCATCTTGGGTTCCGCAATGGGACCGTAAAGC
ACGCTATCAAGCAATAGATGAGTCACTCACCAAGGCCCAGTGGAAGAGCT
CAGGAAACGTAAAGCTATCAGCAGATATTGACATGAAGTATGGGGTATTAA
AACTAACAGGCATTATTCTAGACGCCATCACGTCTTTTTACCCACTCTCCGG
CAACCTGGACATAGAAAATGGACAGGTTTCACAAGACAATCTTACCTTTGA
CTTCTGGGAAAGACAGCAGCAGCAACCATCTAAATATTTTACAGGAGAATC
GCAACTGGAAGCGTTCTCAGTAGTGATGACTGCTGGTTTAAACATTAACCG
CCGCAAGGCATCAGAAACTCCTAAAGAGTTTTACAACCACTTCAATGCCTA
CCTAAGTCACCTTCTACAAGTTAGAGGGCGGGTTCAGGGCAAGGGCGAGC
CGTTCGAAAGGATCAAGGAGCAAGGGGAAAGTGATGCATACCAAGGTCTG
GCTCAATGTAAATGTCGCAACAGATCCTTTTTCTTTACGCAGACGGGATATA
TGGGATTAGGCCCTGATGTCCAGACCGGAGATCTCGTCTGTATCTTTTTTGG
CGCTGAAGTTCCATTCGTACTCCGCCCAAAAAGTGCCTGCTACGAGTTCAT
AGGGGATGCCTATGTGCATGGCGTAATGGAAGGGGAAGCAGTGGGAATGC
AAAGGCCTGGGATACTAAAGAAAACGACATTTGAAATACATTGATTATAAC
TTACCAACTTGCGACAACAATATGTTCTAGGTAGGTGTATTGTTCTATAGAA
CTACTTCTTCTAAGACCAACATCTCTCATCAAACTTGAGGCATACTCTCTAA
AACGACATTCTGTGTCAAGAATATCAAATACTAAGAAAGGTACCTATTTAGG
TAATTCAGCTAGAAATGATCTATCGAAGCTTTTACGCACTTGGAACATCATA
ACATTCATACGAGATAAAATTATGCATCTGCTAAGAAGGTACATCCAACTTA
GCACCGATTCATATGAGAGTATTTACCCAGATTGGCGTCATAAAACGAAACC
GATAAATCATAAATGTAGGTAGTGCAGTAGACTGACGCCTCAGGCAGAAGA
TCGCCGCCGATGCCAACCAGCAGCTTCAGGTCGCCCTCGCTCCTGCCAAA
GACCTCTCGCTCTCGTTGTCCACACATTTGGACAAGTACTCCAAAGCGCTT
GTCAAGTACGGCAACCAGGGGCAGCAGCGGGTGGACACAGTTGTTCGGGC
GATCCGCAATGTCAGTCCCACCCTCAACCTACATCCGACGTGTTCTGATTAC
AGGCCCTGTCGACGCCCGAGCACATCATCGTCGCTGCCATCTCCCTCGAGC
TGGTCGCGCTCTTCTTCCACGTCGTGCAGTTCTACGACCACACCATCTGGTT
CCCCCCCGCCGCCCGGATCCAAGGGTACCATCAGCTCGCTGTTGCACTCGG
CATTCTCCTGGGCGCCTCAGATCCAGCTCAACGTCCGATGGGCAGACGTGC
ATTCGTTCCGCACCTCGGGCGAGATCTACAACGCCGTCGTATGGTGGTGGG
GTCGCCTCGTCTACTCCCATGTACACTCAGCTGACGGCTAGGTTCTTCTCGA
CCGTGCTCCCGCCTCTGGCAGCATCGACCGTCGTCTCATTCATCCCGCAAA
AAGGAGCGCCGCGTACTCACGCCACGCGCCACGCGACCGCTCACCCCGCC
ACACCCACCGTGGACCCCCTGGCATTCTCCATCTTCCGTCTCGCGCTGCTC
GTCTTCCCCCTCACCACCGCCGCCCCGAGCGCGATGGTCGATGCTCTCGAG

ATGAGCGGTAACCTCCAGGGTCGCGCACTCGGTGCTGCTCTCCTGGCGGGC
CTCATGCTCGCCGATCGACTCGGCTCCCTCTGAGCGAGGCGTAAAACCGTG
CATGGCGGGGAGATGCGTATGCGGCAAGAGAGGACGGGCGCGCGGATGGA
CATTGGGAAGGCGGGAGCTGGGCGGGATGTGTGTGCTCTTCAAAGGGGAC
TATGCCTATGTTTATGTCGAGGTGGGCGGAGGTGCCGAGTGCCCGAGGGAG
TGTACGAGAGGAGATCTGGGACTGAGATATCTATATACCTGTGACCTGTGCT
GTTTACCCTGCTTTGTGAGATGTTCGTATCGATACCCCATAGCCTGGTAGTC
CTGCGTGTAAATAATGCTACCTGCGCTGCAGTACACCACCGTCCAAACAGG
AGATCCAGGCTAACAACGACGAGACATGCCCGGCCAAGCGATGCATGGAC
ATGCAAATACAAACATAGCTACGTGAGTCATGACAGATCGTATCACCCGAG
GTAAGAGATGCCCGCTCACGAGATCACGCCCGAGCCGCGATTGGACTGCC
CGTATCCTTGCCCCGCTCAAGAATCTCATAGAGGGCCGTGGCGGCCTGCGC
AACGTCCAAACTGGGGCTGGAGCGCAAGTCTGCGGTGCGCGATTTGAGGT
GGTACGGCTGAAGAACTTCGAGGATCGCCTGCCGAGGTTTGTGCGTCGAG
GGCAGCTCGAGCAGATGGCGGTAGGCACGGAGGGCGGGGACTTTGGGCG
GGTCCGTCGGTGCGTTCTGCCCGTGTCAGTTGCAGCGCAAGGGAAAATGG
GCGAAACATGGTGGAGACGACTGACGAGGGCTTCTGACAGAGCCTCGAGC
AGGCCGACCCGCGCGACGATGGCGGACCGCATGCGGTCCCCACCCAGGGC
GAGGTTCGACACGACGTACAGCGCCGGCACCTTGACGTCCCATCCTGCCTC
CCTGTCTTTCTCCCCGCGGCCCTTGCTGGACGTGGACAGAGACAGGAGATT
CGAGCGGGGTCGTACGGCCTCCTCGACGCTGTCCAGAAATGGCGTCTCGC
CCAGTCCGTCAACGACGCGCGCAATCTCGGCAGATGTGCCGTCTGCCAAA
AGATTCTGAGTGATCTCCATCGCCTGTACTCGCAGAGAGGGTGGCGTGGTC
GCTGCAAGCCAGATCTTGCAGTGCGGGAAAGGTGAGACAATGCGTGATCT
GCACTTTCAGAGGGTCGAGAGCATGGAATGACAGGTTCTTGAGCGCCCAC
AAGGCGTTGAGGGCGAGGGGCTCGTGAGGCGAGTGAGTCAAGAGGGCAA
GGAGGGGAAGGCCATTCTCCTTGAGCAAGACCTGGAGGTCAGAACACCGT
TTATACGTTGAAGGCTGACATACCGCCTTGAGGGGTGAAAAATCGGCAATG
AGGTTGCACAGCGTGGCCGTCGCAGCCACCTCGACCGTCCATGCTCGTTCG
CCGACAGTGCCCGCGCCAACGCCGGTCCTTCCAGCCGCCTTCGCTTCCACC
TCCCTCCGCCTGTGAGAGTCGACCTCACGTTTAAGGCACTCGAGAATCTCA
GCACCTACGCCGCTGTCTACGAGTGACGTCCGCAGTATAGCCACTGTTCGG
CTGAGTGCTCGGGCTAGCTGACAGGCGGCCGCCCGTACACCGTACGAGCT
GTGCGATAGGGCCCTCTTGAGCACGGGAAGGACGGGCGGTTCGGCCTCAG
ATATAGTGGAGCGAGTGGGCTCGTGCTGCATTGCACATGCCGCCAGTGCCA
GCAGAATGGCTTCGAGCGAGCGAGACGAAAAGTCGGGGCTGACATCACTG
GATCCAGGCGCGGGTTGCGGTATGGCGTGTAGCAGATCGATCAGTTGTGAC
GGAACCCCCCGGTCATGCGCAACCTTTTGAAGGTGGGCGTCATCGGAGAC

AAGTGAGGCTGGAGATGAGACACTTCAGGTGAAGGATAACCGATCGTACC
TAGGACGAAGCATAGCTTGACATGTTCTTCGGTACCTTCCGTGCGCAACAA
TGTCGATACGTCATCTAGTAGTGACCGATTGTGCACGGAGATCCGAATCATT
TCCGTCGGTGTAGCACCTTTTGCGGCTTTCAGGATGTTCACGCAGCTAGCG
TCTCAGCAATAGCAAGAAAGCAGATTGGGCAACAGACCACCCGGCGGCAG
CTATCCGCACTCCTGTTGGTCCGCCCTTTATGAGCTCTCCCAAGACTGAGA
GCACTTCGTCCTTTGCCTCCTTACCGCGCAGAGATGGATGTATGAGCTGGTC
CGCTTCATCCTCTTCGTCGGCGCTGGAGCCAGTGACCAGCCTCGAACCAGG
ACTGCCAGTTCAGTCTCTCCGTTGATCAACGACGCTAACAGATCCAGGGCC
GCGTCGAGCAATTTGGGGTTTGGTCGGGGCCCGCCGCCAGGTATCTGTCTC
GTTGGTGAAGCGTACGATACCGTCAGAAGCAGATGCTCGAGAAGATAGGG
CGGTGAGGTGGAAGCGCTCGAGGAGTGTGGTCGCCATTCAGAAAGAGCGC
GGCGATGTGCAGGTAGGATCGTAAGACGGGCATACAGTTGATACAGCGGCA
AAAGAGTCTGCGTCTCGGGATGTAATTCCATCAAGGATAGGAGTGTGTCGA
GATTGTCCTCCTTGGCGTCATAAGCACAAAACAAGGCTGACTTCTTCGCCT
TACCTCAAAGACCATATTCAACGAAGTTGAGGCCTGGACCTCCCAATACTG
GCCACGGCCAGCGACTGCCTTGCCTTTGCCATGATATGGGCCATGTGCTCC
GAGAGACCGTGCGTCCTCGATACCGGTGGAAACAACCCTCTTCTCCGCCCC
GACTCCCCACATATGGCCCCATAGCAGATCAGCCGTTGAAATGAGAAGGTT
ACGCAGCGCGCGCAGGAGAGGCGCAAGCAGGCGCGACACTTGCCCCGGA
GGAAAAGTCGGCGAGGTGATCGCTTTGATTGCCTGTAAGAGGCTTTGAGG
CGTCTGGGCGACAAGCAGGGGTCGCAATGTCAAAGATCCCACTGCTCCCT
CAGTTCACTCTTCCAGGGCAAGGACCCAGCTCACCATTGCTCAACGCCCCT
ACAATTACCCCGGCCTCGCTCACAATCTCGAGCGTGTCCTTCGTTTCATGAT
AGCCAAAAGAGCTCGGGATGCGTAAGAGTCCCAACAGGCTACACGTCAGG
CCCATCTCGGACACCACGACCGTTCCTCACAAGTCCAACAGCTGCTCATCC
TCGGCCACCTCCACCTTCTTCCAAGTGTTCCCAATGACCGCGTTTTGAGAT
CTTTGAGCACGCGAAGTAGCTCGAGCGTGCTTGAAGCCGCCTCAAGCTCA
CGCCACTCCTGTTGTCGTGGTCATGGTGTTCTGGTTTGGGTGAAGGACATG
CTTCACACTGCAAATGAAGAGGCAAAAGGGCGGAAAGAGCCCGGCTTCC
AAAGATGACGGCGGCGAACGATCACGTGACGACCGGCAACCTTTTTCTG
GAAACCAATGATCTCGCTCTTCTGCACTCTTTACTTGGTCGCCAGCAAGAC
GGCGATATCACCAAAGACTGCGCGGCAGGACGGAGCGGACAGGAACTTTG
GACACGTGATAGTAGCCCCCTTGCCTGGCTCACGTACACAGGCCCGGACGG
CTCAGGTCGTCTGACCTCCCGCCGTCGATCTGGCGATGTTGAGGCTCGTCG
CCTCCCGATCTCGCTCCGCCGCCTGTCGAGTATGTCAGGCAACAGCAGTCT
CGTCAGGGACGAGTGAAGCGCTCATACGTGTCCCCTTCGGCTTCCCGGTCA
GCTCCGCAGCCGCTGCTCAGCTCCGAGAACCTGAACATGATGAGCTCCCAT

CATTCCATTACCAAAACCCGACTTTGGAAGCCTCGTCGTCCACCTCTACTC
AACATGTCGCCTCGCGCCATCACCCTCTTCAGATGCCTCGGCCTCTTCCTGG
TGACATCCCCGCTCCGAGTGATTCGCCTCATCATGTTCTCTACCCGTCTACC
GGCGTAATAGACGCCATATCCATGATCTCCATCTGCCTCCGAAGGCCAGAAC
TCATCCCACGGGCGTATCAGATCTTCAAGCAGGTGCTGGAAGATTCTCGTA
CGGGCGTACGAAGAATTCCCGAGGCGGAGCTGTGGTCTCGGGTTGTGGAA
GGAGTTGCATCTTTGGGTAAAGTTGGCGGCAACAACTGGAAAATTTGGAG
GAAACGAGCAGAGACTGTGGTTGGACAATGGGAGTCGGCACACGGACGC
AAGGGAGAACCTGCCGGCCTAGAGCAAGGCGGAGAGAAGGTTTATCAGG
GGTGGTTTGCCGGTCTGATCCAGTCAGTGACAGGTGGACGCAGTTCGGCC
AGCAACTGATGTGCAGATCTCAATCCCCCTTGACGCCAATGATCCCTTATCT
GGCGCACCCGGATCTTCCCCTATCCTACCTACTTTCAGGACTACGACCATCG
GACCTCCCTGTCGCTTTTGACGCCCTCCTTGAGTTATCCCAGAGGGAGAAT
CTGCCCCAATTGGGAGAAATCGCACGCCAGCTGCAGGATTCAGAGAAGGA
GAGGCGAGAAACGCAAGCGCGTGAGATGGTAGAGGAAGTTCATCCTGTGC
TTGAGGTCAGTTTCCCATGATCCCGGGATGAAGACTAACCTGCTAGTCCGC
CGGGGAAGGCGCTCAAGCGAAAGCCACCGAATGCGACAGAGAGCACCGC
GCCCGACCAAATACGCTTCGCCGTGCAGAACCTGAGAAAGGCTCTCTCGT
CGATATCCTCCGCCAATGTCCCCATCAATCGCCAGCACAGCTTGGAACTAG
CATCACTCGAAGCAGCACGAGCAGAGATCGCTCATTCGGCGAAACTCATG
GAGGAAAACCGACTGACTTCGGTGATAGAATCACAGGTCCTTCAGAAGGA
GCAATTGCAGAGCTGGATGCACTCGTGGCTAGGGGCTCTAAGCACTCACTT
GCGAGAGGATATCGAGTCGATGCGAGAACGGCAGTCATCGGTTCAGGACT
CGGTGTTCGATCGAGGTCTACCTACGCATACCAACACCAACGGCATGGCCG
AGTACAACCTACTATTGTACCTCACCCTACTTCCGCCAGAGAAACTCGCTTT
GATCACTATTCTCGAGGTCATGCGCATGAGTGGGTCTGGTGGGATCGCGGA
CGGCATGAAGGCAACGCGAGGGATGTTGGCGGTTGCCAAAGGTGTGGAAA
GAGAGTATCGGGCAGAGACGATCCGATCAGTTGCCGGCATCGACTCGCCAT
TGTGGCTACGCACGATCGATCCTCAAACTCAGAAGCCCAGTCCCCTGTTGA
TTCAGCAAGTCTGGGAACAGGTAGGGAAGATTGGAGAAGGTGACAACAA
GCGCGCACGGAATCAGCGCATTGGCAGTCACGATGCTGCACTGGAGGAAA
GCTTGAAAGCAGTGTGGACTCCGCCCTGGTCGGAACTTGTGCATGTCGCC
GTCGGCGGTTACCTCCTGAAGGCCCTCATTTCCGCTGCCAAAGTCACACGA
TCTGGTATAGACCCAGATACCGGAGCGAAGACATACGAAGAGCAGTTGGC
GTTCTCTCACGAACTCGACTACGTCCATGGCAAACAACTTGGATTGGTCCG
TCTCAACCCGGTGGTGGCCGCACGACTGGCTCGAGACAGCACAAGAATCG
CTATCCATCCGAAGCATCTCCCCATGCTTGTGCCCCCCAATCCTTGGAAGGG
ACCGGAAGATGGTGGTACTTGCTTCATCGGGTACCCATCATGCGTTTCAAA

AAATCCGCCGAGCACATGCTGTATCTGGACGAGGCCGCCAAACAAGGACA
TTTGGAACCCGTCTATCATGGCCTCGATGTGCTTTCGGGAACCCCCTGGACT
ATCAATCGCAAAGTCTTCGACATCGTCTTGTCCGCATGGAATTCTGAAGAA
GCAATCGCCGACATTCCTGCATCTCGAGAAAAGTCTAACTACGACATTCCT
ATCCGACCGCAGAAAGCCGACGAGGATCCTGCTGCTCGGGCGTCATATCTT
CAGAGGGTCAAAGCCGTTCTCGCCCCAGCAGCGGAAGGATCACGCTGAAC
GATGCAAGTTCAACTACAACATCGAGATTGCTCGTTCCTACTTGCACGATAC
CTTCTATCTGCCACACAACATGGATTTCCGAGGGCGAGCGTATCCGATACCA
CCCCATCTGAGCCCTGTCGGCGATGACTTGTGTCGTGGTCTTCTCCAATTTG
GCGTGAAGCGACCTCTCGGCAAAACCGGAATGAGATGGCTCCAGATTCATT
TGGCAAATGTGTATGGGTACGACAAGGCGTCCTTTGAGGAACGAGCAAGG
TTTGCCCTGGACCATCAAGACGACATTTTCGATTCTGCCGACAATCCGCTC
AATGGGCAGCGATGGTGGTTGAAGGCAGAAGATCCATGGCAATGTCTTGC
GACTTGTTTTGATCTCGCCGCCGCTCTTCGCTCGCCTGACTCGGAGGCTTAC
GAGTCCTCTCTACCTATCCACCAGGACGGCACCTGCAACGGGATGCAGCAT
TACGCAGCCCTCGGCGGAGATGTCAAGGGAGCCAGAGCGGTCAACCTGGA
AAATGGAGACCGTCCGGCCGATATCTATACGCGAGTCGCCGATATTGTGAA
CAAGGTCATCGAAGAAGATCGCGCTGCTGGTCTTCCTGTTGCTCAGCTTTT
CAAAGGACCGGTCGGTCGCAAGGTCGTCAAGCAGACGGTCATGACCACGG
TCTACGGCGTGACTTTTATCGGTGCTCGAGCACAAATCGCCAAACAATTGA
CAGCCCGAGGTGATGTGGCCGCTGAAGACCTGTTCTCGGTGTCTGCCTACT
TGGCGAGAACGGTGCGCGATCGCCTCCCATTGTGCACAAAAGTGGGCTTAT
TTTCAGGTACTCAAGTCGATTGGCGACCTCTTCGCGGGCGCGAAAGGCATC
ATGGAGTGGCTTACGACATCGGCTAGACTCATCGCTCGATCTATACCGCCTG
AGCGGGTGCATGACGCGACGACTCCTGTGCAGCGCAAAGACGCCTCTGGC
GTTATACAAACCACCTCCCGTCTCAATAAGGAGTTGATGTCCTCTGTCATTT
GGACGACCCCGCTCGGTCTTCCCGTGGTCCAGCCTTACCGAAAACTCGCCA
AGAAACAAGTCATGACCGCCCTACAGAGCGTATATATCACCGACCCCAACG
CTCCTGCCGAAGTCTCTCCGCAGAAACAGGCCTCGGCTTTCCCTCCAAACT
TCATCCACTCGCTTGACGCCACACACATGCTGCTCACCGCCTACAAGTGCG
CTCAGAGCAACATCACCTTCGCCTCGGTGCACGACTCGTACTGGACCCACG
CATCGAGTGTCGAGTCCATGTCCGACCTCATCCGCGACCAATTTGTGCACC
TGCATTCGCAGGATCTCATCGGCGGTCTGCGCGACGAGTTCCTCGACCGTT
ATGGCGATCACATGATCCCCGTTCAGAGCTACCAGAACATCTTCAAGGCTT
CCGAGATCAAGAAGAGCAGATTGGTTTCTGGGGCCGAGATCGACGAAGTG
ATGCAGGACATCGGGTCGACTTCAGCCGAAAGTATCGCGGACGGGTTGGA
GGACGAGCTCTCAGGGGAACCCGAAGAAGATGAGCCTGGGACACGTCGG
ATGTCGAGACTTTCGGTGGATCTTCCGATCATCCTGAGATCATGAAGGTCA

ACAAACAGAAGTTTGTCAGATTTAGGGACGTCCTGCCGCGCACCCCGGAG
AGGGGTATGTTCGAAGTTGACAGGGTGAGGGAGTCGGCATATTTCTTCTCA
TGATTCTTGGGTCCCTGCCATCTCAATGTTGTATGCTTGTAAACACTTTAGC
ATTGCCACCAGACAGCATCATCACATCCATCACAGCTCAACTTGCATGCATC
GTCAGAGGGCCGTCAGCATATGCCAAGAAGGCGAGTTCGCCACCCTGACC
TGTCACGTGATGACAGTCTAAGCCGTCCACAAAAAGTTGATGAACCGACC
AGTGTTAGCACTCTCTCATCTCAATCTCCCATTTACCGCTGTGGACAGCAGA
ATAAGCCGACCAGAACTTGCGGTCTGGCGATCACGAGCGACTTCAGCTTGC
TGGATCAAAAATGGCACCCGCCGAGCATCACCACCGGGCGACGCTCAAGC
AGGTATGTCCCCCTTCCAAGAGAACCGAGCGCCAGGATGCTCATGGGTAGT
CCAACAAGCCTTACAAATCCAAACACTCCTCGAAGGGCTCTCTTAAAGCG
GCTGCCAAAGGAAAGACCGATGCACCGGTCAAGTCGGCCTCGAAACCCTC
TCAGCTCGGCGGGCAGACCAAGCTCGCCCGCCTGAATGCCCAAGCGCAGA
AACGCGCGCTAAAGGCTAAATCGGTGAACGAGGACGTCAAGTTCTTCTCG
ACCTCTTCATCCGGCTCGGGAGCACCTCGAATCGTCTCCGTCGTCCCCCTG
CTCCCATCTCCCTCTGTATCACCTGCGCGATGGCTGCGAGACCTTTTGCCAA
GCTTGGGGTTGTCCGAGGGGAAAGTCGAAGAGACTCTCGACCACTTGCGC
TCCGCCGGGGACGTCGGGACGTACCTTCTGCGAGCGCCGCGATTCAAGAC
AACCCTTCAGATCAATCTTCTCCCTGCAATCGACGTGTATGCAAGCTTGGAC
GCCGCCCTCGTCAGCGATTATGTCGTTTCCTGCTGAGCTCGAAGGACGAGG
TGCAGCTGGACGGTGAGGGGGTTCTGCGGTGTCTCCAAGGACAGGCGGGA
GGCGTGGAGGTGGTGTCTTGTGTACAGGTACAGCTCCATTACATTGTGCTG
CGGCTCAGCTGACGACAGGCGCCGGAAGATGATCCCATACAACCTACCAC
GAAAAACCTCATCCACAAATCCCTACTGTCCTTCACGCGCTACTTCTTTCCT
GCAGTGGAGAAGATTCACTCGGCAGACACGGGACCTGAGTCTGCTCTGCT
CGCTCGGGCCTTGTGCGAAGCGGCACCTAGAACCACTCGCTCTGAAGGCG
ACCGCGCATACATCGTCGCGGAAGGCAGCAACGCGGTGCGCTTCACGCCT
TCGGTGTCAGACGACCTGGGACGCTTGGAGGTGACTGGGACAATTCGAGG
TGGACGCGTGTCAGCGGATCGGCTCGTACACATTCCCGGTCGTGGAGACTT
CCAAGTGGAGTCGGTCAGTGCCTCGCAGGAAAGCGTGAGCTATGTCGCTG
ATGGGAGCCAGATCTGCGCCGCTGGACCTTCGAGTCTTACTCTCGACACAA
AGCCACATCAGCGTGCGGCTATGATGGCCGTCGAAGAATCAGAAGCC

>Mosaic_sequence_80

CAGGCGGCCCTAACCTCAACCTCCGTGGCGTCGAGCGGCGAGTCAAGCTC
GGCCTTCGAAGCAGAGCCCGGCCAGTGGCCCGACCATCCGCCTGTCAGTT
CTCAATCAACGATCCCCTCACCAATTTTTACTGACCCTCAGACGAACACGC
TTCTTGTTCCGCCTCCTAGTGTGACCCGCGCAGCGCCCTTCCCATTCATGTC

CTTTGTGATGAGACTGGTGGTCCAGAACCCTCGATCGACACGCCGTACTTG
CCGCCGGCATCCCCTTCGCCAGACGAGTCTGAAGACCGGTTGTGCCTCGG
AGCATATATCGCGAGTGGGCGCATGTGGGACGTGTGGTCCGGCATCCTGTAT
TTCTCCAACTCGGATGATGGTAGATCACGCCCATCACCCTTGCCCGTCGTCA
TCAAGATTGCCTCTGCCGACCCGGCCTATGAGCGCCCCTGCGGCATCTCTC
CCCCGGACCGATTGGGAGGAAATCGACCGCAATTCCTGGTCAGAGGCCGA
GCTCTATCGTACCTGCCTGGCGCCATTGCAGGGAGCGGTCGTACCGCGCTG
GCTGGGTCTGTATGACGGCCAGGGCGTATTGCGTAGCTTGTGCGTCAGTAG
GGAAACCGGCAGTCAACTTCCGGCCTGGCAGGTGCGCTGTGGATTTCGCG
TCATGGTCATGGAGAATGTTGGGGCGGAGATGAACATCCAGGAGATAAGG
GAGTAGGTATTGCTCGTTCAGCGTCCCGCTCTTTCGATTCTCGCTCGCTCGC
CCGCCCCGCCACTGTTCGCTGACCCCAGCCATCGACCCCAAATTCTGTCCG
CATACCAAGCCCTTCATGCTCGAGGGGTCATTCACGGAGACGTCGCCATCC
GGCACATCCGCCTGTGCGGGGACCCCGAACGCGATCGCAACCAAGGTCGG
GTCGTGTTGTTGGATTTTGGGGAGGCGAGTCGGGCGAGCGAAGGGGACGG
ACACGATGCCATAGCGGAGGAAGATGACAAAGTACAAGATTTGCTCGTCTT
GGTCGGGAGGGACAAATGAGCGGGCCGAGTAGCCCCTGTTCAATGTATTGT
TTGCCCCGCTTCGTGTATTCCCGCGTCGTCACTGCCCGATGATATCGCCAGC
TCCCCGTTCCCATCCTCTCCTCGACAGTAGTCGCCTCGACCGTCCGCCCGA
CAATTGGTTCTATTGAGTCGGCCGAGAGCTGTCATGGGACGTGGCGGCCAA
AGTGAATGGTCCACTGGTGACTTCTCAATGAACGATGGCTGCTCACCCAAA
CGCTTCCTCTTCGCGTCTCCTGGAACGGTCAGCTCGTGAAGGGGACTGAA
GGGTGGTGTGGTAGGGGTCGATGGTGCTTTTCTCGTGCTTCCGTAGCAAGT
CGATCTAATGGCTGGCTAGTACCTCCATCTTCCTTTCGCCCACATTGTTGTC
GAAGCTGTGAGACGGTGTTACAGCAAAGATAAACATCATTTACAGACAGA
CTGCTGCTCCCATTGAGCGAGACAAGCTCGACACCTTACAGTAAGGTACAG
TCTCAAGCTGTTTGCCTCGCTGTTTGTTGCGTCCTGGCATCATTCGATGATT
GCGACGAGTGCGATGACTAGTGGGTGTCATTTTTGACGTCCTTTCAGGCAC
CAGTTGTTTGTCGTATATTTTCTTTCCCAAAGCGGTAATTCCTGATAGATGTA
TCTCTATCAACTATCAGCGGTGGATGCTCGATGATAAGTAGTGGGTGTCATT
AGTGGGTGTCATTTTTGTTTTCCTTATAGTTTTGGCTGCGAATCTGTAAATGA
CACCCCCGAGGCAGTTTTGGGGATAAAGCACGTGTAAACCACGTGGACCA
CATGGAGAGACAGTCGCGTCGTGTTTGTTGAACGTCTACATGTCAACACCT
CATCTTGGTTGCCTTATTGCTCGACACAGAAGCACTTCCAAGCCCTCTCCC
GCCGTCAGAATGCCCAAGACCAGTCAAAGATTGGTATTATTGCGAAAGATT
GACGCCCGCATACTAGAACTTGAGCCAATTCGCTTCATTCCTTCAGCCCGTC
GACATCTGGCCTATTTGGTGTCATATCGGCACAAGCTGCTCAAAACCCGGT
ACCTTGGCCGGTTTCAATACCTCCCAGTGACTGCTGTTCACCATCCCAACTC

GGCACCTGTGATGTACATGGAAATGAGGGCTGTGGATCACTGTCACGCCTT
CCGAATGACGCCCAGCGAACTCGATCGTCTAGTCAACATGTTTGGTGATCA
TGAGGTCTTTATGTCAACCGGCAGGCATCCTCAAGCCCCACCAAGGGTCCA
GCTGGGACTCCTGGTCCACAGGCTAGCCCATGGACACTGTACGGCTACAAT
TGCACGCAATTTTGGGCTCTCTCGTGCGTCCAGAAACTCTCTGCGGTCTGC
CACTGTGATTATGTTGCTGACGCAGTCAGGTGGCTCTGTGCACCAATTTAC
GGTTCGGGCGTCCATCGCGATTGTCTCTAGACTCAACAGCATCATAGCCTG
GCCTGATCGCGACCGCCGAGAGGTCATAAAGCAGAGGATTCTCGACGATTA
CGGGATACCTCATTGCCTAGGCTACCTCGACGGGGTGAACATCAATCTCGA
CCGTGCTCCGTCGCAGGGCGCAGCCTCTGGGAGCTATCACAGCCGCAAGG
AGCGCTATGGGTTCAATCTACTGGCAGTCTGCGATCACCAAAAGCGCTTTA
CCTTCATCCATTGGGGCTTCTCGGCCAGATCGTCCGACATGCGACTGCAGG
CCGCATGCGGCCTCGAGAACAAACCCGAGCAGTTCTTCTCTCCCGCAGAG
ATTGTCCTTGCCGACCAACGGAAATAGGTGGTACGCTCACATGAGCTTGAC
TCATCCTCGAGTAGCCTCGTCGAGGCACACACATCCTCGACGAGGTTGTGC
CTCGACGAGGTTCAACCTCGACGAGGCAGAGAAGCCGCCACCAACGATAA
CTCCGCATATAACGCCGACACCCTCGACGACCTTGTAGAATGCCGCCGCCT
GTTGCCCTTCTCATCACTCATAGCAAGTGCTGAGCGGGCAACAGGTGTCTG
GAATCCCATTGCCTCAGAGCTAAGATTCCGTCGGTCCCAAATATAGGTGGG
ATGACTCTCGCATGCATCCCTGACTTCACGTAAAACGGTCCAAGGGGGACC
CTTGGAGCTCATATGGCCCAAGGGGGGGACCCTTGGAATTTGCTGGTTGTG
AGTGAAATTATTTTTGTGATGTGAAGGGTGCTACTATACTCTATTTGACGAG
CTGATTCTGAATCTCTAACCCATTCCCAGATCAGAGCGAAACTGGCGGAGT
TGATCCCCCCGAAAGTCCCGTACCTCTGCCCAAGGGGGGACCCTTGTAGCT
TTTTCCAGCGCTGGATAAATTTATTCTGCAAGTCTAATGGTCGGTACTATACA
CTGTTCAACGAGCTGATTAAGAAACTCCAACCCATTCCCAGATCAGAGCGA
AACTGGCGGAGTTAATCCCCCCAAAAGTCCCGTATGTCTGCCCAAGGGGG
GACCCTTGTAGCTTTTTCCAGCGCTTACTATATAGCAAAAATAGCAGGGGGG
TGCTATGTATCCCGCGGTATTGCCCATTAGCACGTGGGGTCAGCGCGGTTAC
TCAGCCTATCCGCGAGTTGTGTTGTGGTATTGGACAATTTTGGGGGGTGGG
AGGTCTCTCCTCACCCGCAAACGGGGCGCGGTAATGCACAATTTCGGCGG
GAAAGACCTCTCTCATCCCAAAAACGGGGCGCGTTAATGGACGATTTTGGT
GAGTGAGGCCTCTCCCACCCCGAACACAGGGCGGAACGCGGTTTCGCAGG
AACCGTCAGGAACGAGACGGTCCTTTATCGACTTAGTCGTTGCAGAGACC
GGTTGGTCAGTCGGGCGGGACATCCCCGTGGGTGACGTCGTGGGGAAGCA
TAGCTTCGCTATTATGGACGATTGTGGTATGGTAATGGACAGTTTTTGGCGG
GGGGAGGCCTCTCCTCACCCCCAAACGGGGCGCGTTAACGGACAATTTGG
GTGGGAGAGGCCTCTCCCATCCCGAACACGGGGCGGAACACGGTTTCGCA

GGAACCGTCAGGAACAGGAGGGTCCTTTATCGACTTTGTCGTTGCGGAGA
CTGTTCGGTCAGTCCGGTCAGACGTCCCCGCGGGCCACGGACAGTCTTTG
GCGGTGGGAGGTCTCTCCTCACACGTATACGGGGCGCAATAATGGACCATT
TCGGCTGGAGAGACCGCTCTCATCCCGAACGCGGGGCGCGTTAATGGACG
ATTTGGGTGGGAGAGGCCTCTCCCATCCCGAACACGGGGCGGAACGCTGT
TTTGCAGGAACCGTCAGGAACAGGAGGGTCCTTTATCAACTTTGTTGTCGC
AGATACTCGTCGGTCAGTCGGGCACAATGACCCCGCATGCGATGTCATGGG
CGCATCATCGCGTCGCACAAGTGCAGATCGTGATACGGTCTGGGATGATTG
TTGGGAGTAGGTGGTCTCTCCTTACCCGTTCACGGGCGCGGTAACAAACAA
ACTCCGGTCAGCGAGGCCTCTCCCGAGCTGTGGTCGACACGCTACTCCGTA
CCACGGAGACCCTCAGGTATGAGACGGTCCTTGAATGACTTCGAGGTCACT
CAACCTCGTCGGCCAGTTGACCGCGGTGTCTTGGACGAGCCCTGCTTCCCA
CCGGAATCAATCAACTCTCGGTTATCGCTTGACGTGAAGCGGCTCAAGAAT
ACGATGCATACCAACCAGGCGAGCGACAGGTACACGAGGCCCGATTTAAA
GAACACTCTCTGCGGATGACCGTTTCTTCCTCGCTTCCCGCACAGGCCAA
CTTAGCATCGGACCGCATGTCCGAATCCATTGTCCCTCGAGTCTCCATGAGT
TGAGACGCTCGGCTTCGATTTTGATTGCCGGTCATATGGCAGTGCGGTGGTT
CTTGCTGCATATCAGCAGGGTATATAGCATCGGAAGTAGCTTCACGCGCCTA
CATCCACGGGCACTGAATCGACGAGTCTTGATACCAGGGAAAGCCGAACG
GACGGCCTTCTCAAGAGTACCATATCAAGCCCATCGTCGCGGGATTGTTTC
GGTTGAGAGCATGTACGAGTGGTACAGAGCTATCCATGAGAAAGACTCAG
CACGGACCATCTACTCACCCAAGCCGCACGCGCCTTATCCTGCTAATTTGTG
GTCAGGATTGAGTTGACCACCTCCGCTGGAGCCGTGGGTTCTTTGCGAACA
ATTGCTTGTCGCTCAGCCGCGGTGCCATGCAGTTTGACCGATCCCGAGGCG
AGCATGTCAAACAAAGCGTACTTTTTAAGGCTCATGGTAGAGCTGCGTCGC
GCACGGGACAGCATTGGCCGTGGGGCGGGGCTGGCCTGAAGACGAGATG
AAGTGGAGGCAATGGAAGAGATGCGGCGGTGGTGCAGGGACATGCGGTTG
AAGCCGCCAGTAAGCGCCTGCGAGGTGGACCACGGGGTCGGCTGGGTACT
GTACGAAGTTGAGGCCGATACCAGATCGGCGAGGTCTTCGTCTTCGGATCT
GAGACTTCAGCTTCGAGTCAAGGCAGCGAGAAGAGAAAGCTGACTGGAG
GGACACGAAGACGGATCGCCGACGCAGGTAGGGCATGGCCCTACTGGCGC
TGCGTGTCGTGTTTATGGCGCGGCCCAGACCTTGCGTGTGGACCTCGAGCT
GCTGCGTGACATGACGGAAAGGCATGGTGTTCTCGCCAAAATGCTGGTAGC
TTGGCTGGCCGACTGCGAGGGCGGCGGTGGCGGAAGGATCATACCGTTGG
ACGTGTAAGTGGGCATGGCGTTGAAGGTAGATGTGCGGAGATGGTACTTCG
TATCAAATTCTTGGAGAGAGAAAGTGGACGAGGTTCGCGAGTGGGAGCGA
GATTGTCACGATACCTCTCTGTCTCTGTCCAACGTCTCTGTCCAACGGCCCG
TCACTGGGTGACCGAGACCCTTCGACTTTGGCGAGGCTGGGCGAGGCTGG

GGGAGAAGGGGCAAGGGCTAGGGCTTGGAGGACTCGTAGAGTCTCAACG
AGTCTCGACGAGGATAGCCGAGGTTGGCTAGGGCAAGGCGAGGCAAGAC
AAGGCGAACCGACAAGTGCTAGGCCTTAGCAGCTGTCGGCGGGACGGAGC
GAGAGAGAGTAAGTGAGGGAGTAGTCCCATGCAACTCAATTGCGAGAGTA
CTGGAGTTCTACGAGGACTTCTGCAAGTCTTAAGTATCCTACAGCCCGTCT
CATTGTCTTTGTGACTGTCCTGCGAGCTCGGCGAGCCCTTGGCTCATCCAC
GTCGAGCCCATTGCAACTGTCTCGTTTCCCTTGCCGAGTTCGCCGCATCCG
GCAAACTCGCTGAGTAAGCATCAAGGAGCCCTATTGTTATTTGGCGGCGGG
CATGGGTGGCGTGGCAAGTTTTGGAGAGCTCCTTGCTGTCGACGAGAGCT
GCGGTGACGGCGGGACTTGGCTGTGCGAGAGATGTATCCTTTTGTCCGACG
ATCCGACGAATGCGGTAATTTTGTGTGGGTCGGCCTCGACGAGTTGCGATG
AGCTTCGGCGAGCGCCGACAAGTGCTTGACTCACGTTCCTTTGGCTCGGTC
TTGCTCGGTCTTGGTGCTGCTTTGTGTCATTCGTGACAGAGATGCGGGCTG
GCTGAGAGGGAGAGTGAACCGCTCAGGAAGATAGGATTGGACAAGCAATC
CAAACGAGGCGGGGCCAGTAGCCGAGAAGGAGGAGAGAATATTGAGGGA
GGACGTCGATGAAGCGTGCGACAGCATGAACGGGTGAACATGGGCATTGG
AGTCAAAGACGCCCGTCTGGAAGGCGGGCTGCAGGTGCGCATTGTCCAAG
GATCCTAAGAGTCAGATTGCCGACAGCCTGAGGAAGGAAATGCTCACCCG
TCCCATTGTATGGAACGCTTCAACGTCCGGGGGAGAGGAAGACCAGAGCC
CAGACGAGTTGTTGTTGGTCAATGAGGAGGCCCACAACGCAGCGAGGTCG
TGCTCGGGGGAAGACCACCATCGATTGCAGGCGGGTGCTTGCCAAGAGGG
CTCGTGCAAGACGTCGAGGTGGGGTCGACGAACGAGAAGCCCAGTTGGTC
AAATGAGTCAGCCATCACGGTGACAGGCTACAGGTGACGAGGCTGCAAAG
GCGACTAGATGGAGGGAAATGGGTGAGGGTCTCCCGGGGGTTAGGGGGTT
ACAGGCAGGTCCCGGGGGGGGGGGGGGACTTTCTTGCGCATCAAGATTT
TCCGATTTTTTCTTTTGGATTCAAAAAGTTTGACAAAAAAGCTACTCGTAA
ATGTAATGATCTTCCCAATCGGGTAAAAACTGACGGATTTACGGGCATCCAA
AGTTGGCTGGGTATTTGACTTTGATTGGCTGTGACGCCGCCAATAATTGGA
ATATTGAGTTGATTGTCAAAGAGAGTAGCCTAATATGGTCCCCAATATATGT
GGGATGACTTAGGGGACCCCTAAGCTTTTGTGATTGGTGAAAACTGTGATT
TCGCTTGATCCTTCGAGGTAGGGTTGCCAATGTTTGAATGCTTTGACAATTG
CGAGCATTTCTTTGTCGTATATTTTGTAGTTGAGTTCGGCTGGTTGGAGTTT
CCTGGAGTAGAAGGCGATCGGATTAAGGTTGTTGGAACTGTTGGCAGCTG
GGTAGGATAGGACACCCGCTATGGCATAGTCGGAGGCGTCGGTTTCAAGAA
TGAGAGATTTGCCGGGCTCAAAGTGTTGTAGGATTGGTGCCGAAGTGAAG
TCGGACTTGAGTGATTCAAAGGCTTCGGTTTCCTTCTCTCCCCATTCAAACT
TCACTTCCTTTTGGAGGAGTCGGGTGAGCGGAGCGGTGACCTTTGAATATG
ACTTGATGAATCGCCGGTAGAAATTGGCGAAGCCGAGGAAAACTTGAATAT

CGTGGACCGAAGTTGGTTTTGGCCAAGAAGTGACTGCTTCAACTTTGCTTG
GGTCCATCCTGATTCCCTTGTCCGAGACAAGATAACCAAGGAAGTCCACTT
CATGTCGGAAGAAGGTGCACTTCTCTGCCTTTGCCCAGAGTTGATGTTTTC
GGAGGCGATCCAGGACTTGTTGGACGTGTCGAACATGATCTTCGCGGGTTT
TGCTGAAGATCAAGATGTCGTCCAGATAGACAATGACAAAGATGTCGAGG
AAGTCGCGAAGAGTGTCGTTGATGAGATTTTGGAATGATGACGGGGCATTT
GTAAGGCCGAAGGGCATGACCAAGTATTCGAAGAGGCCGTAGCGTGTGCG
GAAGGCGGTTTTCCACTCATCGCCAGGTTTGATCCGGATGAGGTTGTAGGC
GGCTCGAAGATCGATTTTGGTGAACCAGGATGCGCCAGAGAGGCGGTCGA
GTGACTCTTGAATGAGCGGGAGTGGGTATCGGTTCTTTCGAGTGATCGAGT
TGAGTGAGCGGTAGTCGACACAGAGTCGGAGGCTGCCGTCGGATTTCTTG
ACAAGAGGATTGGAGAGCCAGCTGGGCTGGTGCTGGGTCGGATAAATCCT
TTCTTGAGGTTCTCCTTGAGGTATTCGTCCAGAGCTCAGAGCTCAACTGCA
GACAAGGAGTAGATCGGTCCAAATTTTGGTTGGGCGTCCCCTTCCAAAGGG
ATTGCGTGATCGTATGGTCCATGGTCTGGGAGCTTGTTGGAGTTGGATTTGT
TGAAGACGTCGAAGTAGAAGTGAAACTCGGTTGGGATTTGGGTTCGGAGT
GAAGCAAGGTCGGGAATGGTATTGTCGTCATCGTCGCCTGTCAATGGAGTT
GGATCGATGAGACCTGAGACTTGATTGGCGGATGTAATGCCAAAGATGCTA
GCGGTTTTGATCGCTTGGCGGAAGGCTTTCCCGCTGATTAGGGATACCTGA
ATCGGTCGACTAGATGATTGATCGTAGCGCCAAATGAATGGAAGAGGCGGA
CTTGAAGCGGAGCTGCTTGGCAAGTTGCGGACTGGATTTGTCAACATGTCG
AAGATGTCGAAGATGTATTTTGCGTACCCAAAGTGTCAATACTTGGCGGGT
TGCGGACTCGGAGAGGCGATGTTGCCTGTGACTGGAATGGCGTATCTGCAG
CTTTGCTGGACAGATTACCGGAACTAGGACTGGGATCTCGACCCTCAGGCT
TGCCTTGCCTGATGCACTGTGCCAGACAGTACTGGGAGGAGAAGACGATG
GTCTGGCGGGACCCATCAACGACTGGATTGTGCTGGGATAGCCATGGAAGG
CCAAGGATGACTGGATAATTGCCAACTGAAGTGATGTCGAAGGAAATCACT
TCGGAGTGATCGAGAATGGCGAGTCGGACTGGAGTTGTGTGATGGGTGAT
GGCGCCAGATTTGATCGGTCGACCATCGATAACTTGGATTGGAACAGGTTG
TTCCTTTTGGACTTGTTGGATGTTTGTGGTGGAGAGGAACGACTTGTTGAT
GACGCTGCCGGTAGCTCCAGAGTCGATCATAGCGTAGGTGTCGAAGGAGG
TGTTGTTGAGCGAGAGTGTGATCGGAACACACAGATGCGGGATAGAGTGG
TTGGAGGTAGTGGACAGAAGCGTTGTAGCCGATATTTGATGACCAGATTGG
ATTGAACCGGAGGTATGGTGCCCAAGTGTGAGCCCCAGAGGTGGGGGCAC
ATCTAGGCCTGGGGCTTGGTCTTTTTTGTCGGTTCGGAGGTGAATCGTGCG
CTGATCACATTGACTTGAGTGGATCGATTGTTGCTTCGATTTGGCCGGATTG
GACATTGGCCTGCGCTGTGACCAGGTTTGCCACAGTAGAGGCAAAGATTGT
TCTTGAAATCGATGTTCCTTTTCCTGAGGAGTGAGGGGCGTGAATCGTGGT

CAAACAGCATCGATTTCCATTGGAGTTGGAGTGAAGGTCGATGTGTTTTGG
GTCGAAGATATCGGAGCGCGAGTGAAGTTGTTGGTCCGTGGCATTGAGTTG
ATGTTTGTGAGGGTTAGGGGTTAGGAACCCCCTAACCCCTAACCCTTCTGT
CCATAGGACTTCAGCATAAGGGTGGGGCATCTGACAGCTGTCAGACAGTGC
ACAAACACTGACACAGGGATGGGACTGGACCCATATGATGACACCCATTTG
TCATCTGCAGGCTGCACAGTCCTCCCACTTCTTCAGCTCTCTGTAGAACCC
ATCCTTGTAGACAGGCTCCACCCAGAGCTGATGATTGCACTCTGCCAAGAG
TAACCAATCACTAAAGTGATGACCCCTGTCACCCTCCTTGGAGGGCTTGAC
TTTGGGTGTGTGCGATGACAGTCTGACAGTAGCCAGGGGAGTGTTGAACT
CTCTGCCATAATGGTGGGGGGACATGCTGACTTCCCACTTGTCCACATCCA
GGGAGGAGCTGCTTACAGTGGTCAGGACCAGGGGGAGCTGATCAGCAGTC
AGCAGGGACAAGGGCCAGCTGTGGGACCAGAAGGGAGGTGGCCAGTGAG
CCTGCTGACCATCCTGCATAAGTCAGCATAAGCATAAGTTGACATGCTGAC
ATGCTGACGTTTGGCTGGGCAGGTGGAGGTGCAGGAGTCTGAGATTGCTG
ACGGATGACATCTTGCTGACCATAATGCTGAAGTCAGCAGTCAGCATTGCT
GACAGGGATGTATACTCACTGAGTGAGCACTCCTGCTGACCATTCTAGGTG
ATGAAAGGGCCACTGTAGGCACTAGTGACACAGAGGGTGACAGGGCTTGA
AACCTCTTGGATCTCCTTCCAGTCTGCCTGTCAGCAACTCTGTCAGCATCAT
CAGTGCACTCACAGCCCTGGGAGTGTCTGAGTCATCTGACTCTCCTTCAGA
TTGCGCATAGACATCCTGACTAGTCTCACTGCCAGACTCAGACTGTCATCA
GCCCCACATCAGACCTGTTTGACTCACTGTGACTGGAGAAGGAGATCTCTG
GACTGTCAAGATGGGTGACACAGATGGTTCCAGGCCTTGAAGTCTCTTGG
AGAGTCTGACTCTAGGCTGGATGTCAGGACTGTCAGCCTGAGTGTGCTCAC
CTGAGGTGTAAGCCAGAGTGGTGAGAGAGAGGGGGAGTGGCCCCTCAGC
CTCCTTGACTGTCTGAGTGGAGCCATGATGAGAGTGATGAGGCAATGTCAA
CAGGCTTGACAGCCCTGACCTGTCCCTATGGTCAGAGGTCAGCAGACTCA
GCCAATGTAAGCAGTCAGCAGTCAGCTGGAGCAAGCAGCAGAGTAAGGAT
GAGCAGGAGCGTAAGCAGAAGCATCAGTCATCAGGGTAGGCAGGAGGTTC
AGCACCAGCATCTGTCAGCGTCAGCAATACGTCAGCAGAGAGTCATCATTT
GGTCATCCATCTGTCATCAATCTGTCATGAAGCGGTCAGCAATCTGTCAGCA
ATCTGTCAGCAATCCGTCAGCAAAGCCAGCTTTTTTTCTCTAAGTTGCTTA
TTGCGCACCAGCTTTTCAAAAATGCGCACCTTGAGAATGATGACCTCAAAG
GCCACTTTTTGACCACTCTTTCCCAATTATCTCATGAACCGTTGCAGCTACA
GCTCCACAAAAAATTCCTTATGATCAGGAGACTAGCAGCTACATAATGGTAC
CACTCACACACCTGGGGGAGCAGCGGATCAAAACCCCCCACTCCCCAGAC
CCCCCTATAACCCCCTAACCCCCTAACCCCCAAGGCTTGAGGGCCGTCCGG
CGGGTGGGGGCGGACCTCGGGATCTCCACCTCCGGGACGGCCCTCGGCCT
GGGACGGTTCGGGGCGGACACGGAGGCCCGTACGGGAGGCCGTGCCGGA

CCGTCCGACTCGGAGTCTGACCCGGATTCGGACTCGGAGCCGGATCCGTCT
TCAGGCTGGCTCTCAGGGACCAAGGTCTGCGCTGGGAGGCATTGCCAT
GGGCTGCGCGGCCAACGGGTACACACCGGCCGGCGACCCGGGGTTCATGT
CCATGATGGCCCGCTCGAGCAGCCTGGAGGTCTCGGGGCTGAAGACGTCC
GGGTACGGGCCGACGGGGGCGGGTGTACCCATCTGAAAGGGGGGCTGGG
GCTGGAACACGTCGGGCGGAAGGACAGGGACGGCGTCCCATTGCTGTCGT
TGGTCTTCGTTGACAAACCCGGGCAGCTCGGCCGGAGCAATGGTCTGCAG
CAGCTCAAGCTGCGCTGGGGTCCACACTGGCAGCTGCGGGGGGACATACG
GCAGCTGCGGTGCCATGTATCTGGAGTGTGAGCCTGAGACATCTCGCACGG
GGCCAACTCGCGGATTGAACTGCAGGGGAGTGTGTACGTCGGGTGCAGCT
GCACCCAGAGCGGGGCCCGGGGCGAACGGCTGCCAAGAGGGCGTTGGCG
CAGGCTCTTCTTGAGGAGCAGAGGGGCCTGGAGTGGCTGCTTGCGGGGTT
GCCCCAGGGCTGAAGCTGCAGTAGTCTGGGAAAAAAGAGGACATGGTGG
GTGGGCTCTATGTGTGCTGTTGGTGAGGTCTATGTGAATGTTGGTGAGGTCT
ATGTGAATGTTGGTGTTGGTGGCAAGGGGGAGCAGGGGCCCAGGGTGATG
ACTTATATAGACAGGCCGTGCGTTGTTTCGTCATTTCGTCTTGCGTCGTTTC
GTCACTGCGTCATCACGTCATCACGTCGTTTCGTCGTTGCCAGCGTCATCTG
ACTGCTTCCGGCGTCCTGCGTCATCACGTCGTGCGTCATCACGTCGTGCGT
CATCACATCGTGCGTCATCGCGTCGTTTCGTCATCACGTCGTTTCGTCATCA
CGTCGTTTCGTCATTGCAGCTTGATCTGGCTGCTGCCGGCGTCTTCCGTCAT
CAAGTCAGAGCTTCAAGGGAGCGACGTAGGCGGAGCAGGCACAGCCAGG
GGACGGAAGCGGCCACAGGTACGGCCGGCGAGGCCCCGGTGCGCCCCCA
AGTACGCCCCTCGGCCCCGGGTGCAGAGCACAGCCAGAGGACCCCCCATA
TCTGGGTCGAGGTCTGCCCCCCAGTTCAGAGGCCTCGACCCCCGGTCAAA
CATTTGGGCCACCCCCAGGTAAAATGACGAAACCCCGGGTCGAATTTTGGG
TACCCCCCAGTAAAATGACGAAACCCCCGGTCGGAACATGTACCCGGCCCC
CCGGTCCAGGATTTCATACTGTTAGGGCAACCTGCACCAAGGATGTCCCCC
AGTGAGGGACAACCTGCACCAAAGATGTCCCCCAGTGGGGGACAGCTGG
GGGACAGCTGGGGGACAACCTCCACCAAAAGTGTCCCCCAGTGAGGGAC
ACATGGGGGACAGCCTTGGGGGACAGGTCTGACCATCCCATGTGACAGTG
GGGGACATCTTGTCCACCATGGGGGACAGCCTGGGGGACAGCCTTGACTC
AAATGCCTTGAAAATCTCTAAGTTGAATGCTGTGACTTAGACATTTTCAGA
GGCCATGAAATCCCCCCAGCCTCCGACCATTTTGACCATAGCTCAAGAACT
ATGCATCCCCCAGCCCAGGGGAGGGCAGGGGAATGATCAGCTCGGTCATAT
CTATCGGTAGGTACCTTCAGATTTGCTGGGGGTGGCCCGTAGCAAAAACCC
CAGGTTGGGAAACCCCATATAACCCCCTAACCCCCTAACCCCCGTACACTT
ACGTACTGGGCCCCCCGAGTGAGGATTTCATACTGTTAGAAATCATGAGGG
CATTTTTCCAGACCCAGATTGTCTGTTGGCAAGCCCGCACAGCGTTCATTC

ACAAGCAAGAGCAGAGGGAACAAGAAATTGTGTGCGGTGGTTCATGAGG
GCCACTTGGAGCATTTTTCCGGTCCCAGACTGTCTGTTGGCAACCCGCACG
GCTGTTCATTCACAAGCAAGAGCAGATGGAGAAGCGACGGAATCTTAGCT
CTGAGGCAATGGGATTCCGGTCACCTGTTGCCCGCTCAGCACTTGCTATGA
GTGATGAGAAGGGCAACAGGCGGCGGCATTCTACAAGGTTGTCGAGGGTG
TCGGCGTTATATGCGGAGTTATCGTTGGTGGCTGCTTCCCTGCCTCGTCGAG
GTTGAACCTTGTTGAGGCACAACCTCGTCGAGGATGTGTGTGCCTCGACG
AGGCTACTCGAGGATGAGTCAAGCTCATGTGAGCGTACCACCTATTTCCGT
TGAGAAGAACGGTTCATCACGGTTGTTTGGAGCATTTTTGCGGTCCCAGAT
TGTGTGTTGGCAAGCCCGCACAGCCGTTCATTCACAAGCAAGAGCAGAGG
GAAGAAGAAATTGTGTGCGGTGGTTCAATAGGGCCATTCGGAGCATTTTTC
CGGTCCTAGACTGTATGTTGGCAATTCCCACGGCTGTTGATTCACAAGCAA
GAGCAGAGGGAGGAGAATGGTTCATCAAGGTCGCTCGGGGCATTTTTCCA
GACCCAGATTGTCTGTTGGCAAGCCCGCACGGCCGTTCACTCACAAGCAA
GAGCAGATGGAGAAGACGATTCATCACGGTCGTTTGGAGGATTTTGCGGTC
CCAGATTGTCTGTTCCCAAGCCCGCACAACCGTTCATTCACAAGCAAGAGC
AGAGGGAACGAGAGATCGTGTGCGGTGGTTCACTAGGGCCTTTCGGTGCA
TTCTCTTGCTGCCAGATGGTCTGTCAGAGAGCCCGTACAGTTTTCCAATGA
CAAGCAAGAGCAGCAGTAGATGTGCTCCCCGTTCATCAGCCTCAAATGGG
GAGGTAGTCCGACAGTGTCTGGTCTTGCGATAGGTGAAGAGAACAACGAG
ACTGTGAACCGAGACTGTGGACCGAGTCTTTGGCTATAGGTTGCCCGCTCA
ACAGGTGGCAACATGCTGTTGCCATCGGGCCGATGGGGAGCAGGGGGGCA
GGGGGGGGCGGGGGGGGGGGGGGGCGCGGATGTAAAATGGTGGTATGA
GCAGGGTTTGACTTGAGTGTGCGATTTGCTGGAGTTGATGAGAACTCACCG
AGGCAGCATGTTAAGCTCCAGACTTTTCAAGACAACTCGTCAGAGGTCTCA
CTGCCGCACGGTACTCACTCTGGGCCTGAAAGCACCCACGAGACAGCATT
TGGGCCACTACAGTCCAGATGGTGGTCTACCATGACTGCGTGGCAGAATCT
TGCTTAGTCGATCAGTTAGGTGCTATCACCGTGGCAGCCGTAGGAAGCAGC
CAAGTGCACATAGTATGCAGTACCTACGAGACGGTATTTGACAATTGCTGAT
GGCAAAATGCATGACGTCCGCTGCGGCGCAACTTGATGCTCAACAAACAG
CTGCCACCAATGGTCCTAGGACTGTCCGTGGTCCACATGGTCCCGTTCATTT
GACTGTACCATCTAGCTGATAGGTATGCCCGCCGAGCTGAAGCTCAGGGGG
TCCGGGGACGTCGAGGGCAGACGAAGCAATGAGAAGGGCTGCTTTGCCCG
GCCGCCAATTTGGTCTTGGTCATTTGTCTCGGGCGACATGAGCGGGTGGAT
TGCAGAGGTTGGGGATATGCCGGGCGTCGGTGTCGATATGGAGTCTGACGT
CGAGGAGATGCCGGTAAAGTCGGAAGGGGCCGAGGACGAGGAAGAAGAG
GAGGCAGGAGAGGAGGAGGCAGGAGAGGTGGGAACCGGTGCCGCTGCAC
GAGATCTATCAGGCGAGCCCGCGCTGCCCCAAATGTCATGCAAAGACGCCT

GGCCATCGACCACCCGGTCCATGTTGGCCGTGCTGATCAACAAGACGGCTA
CAGGAGGGGCATCGGGGCCGGTAAAAAAACTTCGACACCGAGTCCACGCC
AAGGGGTCGCCGGCCGGTGTTGATCCGGGTGATGACGATTCCTGGCTGGG
GAAGGACGCCGATGATGGGGAAACTGGGATTGGTCGCAGAAAATTGGATG
CGTCTTCATTGATTCATCAGCACAAGCCATCCTCTTTGATCGTCTCACGAAC
CTTTGCCAAGACGCGGAGGGATGAACCCAGAGCACGTTTCGCCAAGGGGG
GCGTACGTCTCGTCCTGAAGAGGGGTATCTGCGCCTGTAGCGCCTGGATGT
CGGATGTGACTGCCTTGGGTACCGGCTGCGCCGTAATCAGCTTGAGCGTGG
CTTTCCCAGCCCTGGCCACTTCCTTGAGCACCTTCTTGTCCCCCGCGACCG
CCGCCACTCGGATGAGGACCTCCTCCCCCTCCAGGTGGACCACCGCCTCCC
CTCGCCTTGACACGCGACTTGGCATTCTCATACGACTTCTTATATTCGTCCCT
GTGCCAAGCCATGGCAGCAATGATGCGATCCGTTGATTCCAATGACAGCAA
GGAGGGTGCGGGCTGGGATTGAGACTCGGATTGAGCACCAGAGGCGTCCC
CGAGCTCGGCGAGCATATCCTTGTATGCCCTTCGGATCGCATCCGTCTGCAA
CGCGATCATCCTGTGGAACCCCAGCTCGCTCCCTATGCTCGTTCCGAAGCG
TTCGGTCGACCAGAACGCGTCGAGTGCGTCGAGCTCTCTGCACCTTAAGG
GGACCGCTATTTGATCATCGTCGAGATTATGCTCCACATAGAGCCGGCTCCG
GTCGGGATCCAGGGACAGGCGTACCAACCGATCATTGACAATGGTGGCGA
CGACGTGCAAGCGGCTGTTGCTTGGGCTGAGGCTGATCATCCACAGGTACC
AGACGGTCTGCCAGAAGCCTTCTTTCCGCTTGAAGATATGGAGCTGCTCGA
TGTTGGACATATGCTCGCGCGCCGCATATGACGCAAGTTTGAACTCGAAGA
ACAGGTATATGAGTGTCGCGCCGTCGTTTTGGCGATGCATGACCAGGGCGG
TATCGGGCGTTGTGGGACCGAATTTCTACTTTGGTTCGAGGGGCCAGACCT
GCTTTGAGGGTTGCTCGCGCTTGGCGGTCGCGGATGTGATACGGCGTCGGA
GCCCGAGTCGGAAGGGACGTGAGCGACGCTCAGATGAGTGTCGGAGGAG
CGAGAACGAGGCGTGAGCGTCGGCCGTCGAATTGAGTCGAGGCTTGGAAC
GTTGTTGGCCGGAGTAGCAGCCAGGATGAGGGGCGAGGGCTGACGGGTAG
AGGACGAGCCACGCGCAAGATCCGTGGACGTGGGGGCTGAGATTGGCGG
GGGCGTGGCAGATCTCGCCAAGGCGATGCGTGCGCTGCGACGAACGGACT
GGTCGGACGGTCCAGAGGGGTCTGCTGCGATAGATACGACCTGAGGGATG
AGCTCTAGAAAACCAGCCGAGACGGTAGCATGTGAGGGGATGGCAGGGAC
GAAGGCGCTCACAGGCATCGCAGACACTCGTACACTAGCGGCTCGCGCCG
TGCGGTCCTCTTGCATTTGTTCGAATACCTCGTGGCCGTTATGCATGCCGGC
CAGTTCAAGGTTCAGCAAGTTGTCCTGTATCCCCGATTGTGGGCCCAAGTC
GACGACAGCAGTAGCCGCAACTGTACTGTCCACTTGACTTCCCGGCTGAA
GCTCAGCCTGGACGCTGGCCATCTCCTGCTCGTACAGGTCCTCCAGCTCGC
CCGGAACATCGAGGGCTTGGGCGAGCTCAATGGCCGCTTGACGCCTTTGT
GGATGGATGGCGAACAGCGTGGCCAAGGCGGTGCGGTAGGTGGAGGCGGT

GATGATGGTGGAGGTGTCTGTATGATCAGCGATCGATAGATGGGTACATTTC
GTGCTCACAACTCGAAGACGTTCCGCTCTGGTTGCCGCGTGACAAGGCCA
GTTGTCTCCTCACCAGCGACAATTGCGCCTTCCAGGCCTGGTCCAACCTCC
TGACCTCCTTGTCGGCGTTGTGAATGCGTCTGAGCGCGACGACGCTCTCGT
GGCTGGTCCACCAGGTTGAAGCCTCTTGCAGCTCAGCCATGAGGTTTAGCT
CGTCCAGGAGCTCGGGCAGGACGGCGAGTCGAGTGACGACCGCAGAGGT
CGGCAAAGCTATTCTTTCGACGAGACGGCTGGATCTGACGAATCGCATTAC
CTGCGGATCGAGCGGGAGGTCTGGGTCCAATACATCGGCGAGGATGGTCC
ATGGGGTGATGAACATGGCGAGGGCTTGGTGGATGAGTCGTTGCAGGTCTT
TCTCATGCTTGACAGCGCCGAGGGGGAGAGGAAGAGTCGCTGAGCGCTGC
TTCAGAAGGAAACGAGGCGGATGTGGTCGCCACTTGACGGATGATATCCCG
AAGGGCCGCCTGGACGCGAGGTGTGAATGACTGGATGGTGGATCGGACAA
CGCATACGATCTGATCACCACCAGCACCAGCACCACCATCACCATCACCTG
TCACGTAGAAGGCGGACATTCGCACTTGGGTAGTCGTGATGGACACGAGTA
GCTGACGGCGGGTATCGGAAGGGCTCGAGTGACGGCGAGAAAGGCGAAG
AGAAATAGCCCACAGCCAACGGAAACTGCAGAGTGACGATGAAGACTTGA
TAAGGGTACCCGCACGGCGTGATCGGTCGTGGCATTTTGATCAGTGATCTT
CATCTTCTATTGTTCACTTGCCTTTCCGTCCATTGCATTCATATCACATATTCT
CGCATCTACAAGCATGACACAGGCTTCTCTTCTTGCCGCTCTTGAGACCTG
CCATGCTCATCAGGGTCCTGCTCGTCCCAATTTCTCTGTGATTGCCAAGCAG
CACAGTGTTGACTGCAACACCCTACAACGCCAATATGAGAAGGCACTCCA
GCCCGATCCTCACTCCAATGACCATCGGGCCCTGCTCACACAAGAGCAAG
AGGGTGTCTTACTTGATCACCTGAACGACTTGGAAAGCGACACCTATTCAT
CACCCCAATCATACTTCGCAACATGGTCTACGAGATGATTGGCCATCGACCC
TGGGAGAGTTGGGCTCGACAATTTGTCAAGAAGAACGAGGCGCAAGTAAA
GTGCATGGTGGTGGGAGGAATGGAAGCAAGCCGACACTCTGCAGAGTATA
GGCCTGTGTTTGAGGCCTATTTTGACCTGGTGAGGGGCTTGGTCCTTTGTC
ACACCGTGTTTAGAAAGCTGATGGAAGCTCAGCTTGAAGAAAAGTGTCGA
GATCTCAACCTTGGACCCGAGTCAATCTACAATATGGACGAGAAGGGCTTC
ATGATGGGCAGGACAAACAAGCAGCGGAGGATTGTGCCCATTGCTCAGGC
CAATGAGCCAGGCAGAACGCAGAGCAGGATGGATGGAAGCCGAGAATGG
GTCAGCCTGATTGCCTGCATTTGTGCTAATGGGACTGCCCTCACCCCTTCCG
TGATCTTCTCTGGCAAACATGACAAGCCTTGCAGGATGGATGATCAAGAAT
ATCAACATGACAAGAAACATCAAAGCCCCATCAAACACGTAAATGAAGCTT
TCATGAGAAATAACATATCCTCCCTCTCCTTGTAAGTATTCGATCGCGCGAA
CGACAGCTCGCAGAATTAAAAGTATGGCTACGAGGCCGATACTATAGAAAT
GGAACTGCCACCTGATACTGGGATCTTGGAGGAGGACTATAGTTAAGTCTT
GCTTGATACGTTTATAAAGGAAGCCAACCTGCAACTCGAAAAAAGAGAGC

GCGACAAACTGGGTTATGATACCGGAAATGAGTATGATTTTGGAAGTTTCG
ATTGCTGAGGGGTCACCGGATGCTGGGAGTATGGAACCGATGAGCTGCGTA
AAGATACAGCCAAAGTCTATCAGGACATAAAGCCTAGTCATCCAGCGTAAG
CTAAGTATTGAATGATGCCTGGCGTCTAACACGGTCGCGATTCGACCCAGA
CTCATATATACCGATGCTGCGAGGAATGGAGCGCCTGCGAGGATAAGAAGA
TTTTGTAAGATGAACCATTTTACAACTGTAGGCTCATCGTGGGCCATGGCGC
GAGCATAATAACCGAATAGTTCCGCTAGGTAGGCAAAGTTAATAGCTAGATT
CAAATCATTACCTAGGGAGGTACCTGGCAGAGGCAACGGAGTAGAAACTA
ACCGATTCCACCTAGCAAGAAGGGAATGAAGAACCATGTTCTTAGCCAAA
AGAAATATATCAAATGACCAAGAGTCGCAAGCGCAAATAATGCCATGAACG
CGAAGCCGGCGGTCTTACTCGGTCGATATTGATAGAAAACGATCTGCGTGC
CATTTACAACAGGACCGAAAGGGCCGTCACCTGTATTGTAAAGAGTCGAGT
TCAGGTCCATTGTCCGTCTAATCCCAACGATGGCGTAGTTTGAACTTCGCG
AGGCAAAGTCGATCAAGGTTCGAGAAAAGAAATGAGAGTACGGAGGTAG
AGAGGAGTAACGAAAACGATAGAAAAAAACAAAGATAGAAGTGACAGAA
GATTCATTATGAATATTTCTGAATAAGAAGCCATTTTCATTAGTATTATAATAT
AGGGAAATATCGTACTGGTATAATGGCGATTTTTCGTATAAACTCAAACCAA
ATCGTATACGACGATACGAAAATTGATAAGGTCGCCCTGACGGTGTGTGGT
GTTTCAACTTGCCTACCTACTTTTAATAACGGGTCAGCATAAACTTCCATAA
GTAAGCATCAGGACATGACTTATAATGAAAGTAACTACCAACATTGAAAAT
CATATATCTTAGCGAATAATTAAAGTAAAACATATATCTATTGATGTATGGTCT
CTTAAAAATAATAGTCATGAATATTTTATCCACGTTTTTCAAATGGTACTTATT
TTAGCGCTTTGTCAATTACTTGTTTAGGTTGGTACTGCCCCCGTAAAGTCAA
GCAAAACGTGGTTGCTAGGTATTAGATACCTACCTTAACCCTACACTGCCCA
CTAAGTAAGTACCTACCTACACAACGACGTAATGACAAACGTAAAATAATA
ATCTTTATTCCCAACACATTCATTGTGGTGTAATATATGGCTGACTTACATATT
ATGACCAATCTATTTTCAACCTACCTAGGTATTCAGTAAATAGCTGATCGAC
CATACCTTAAAATCATTCTTTTTGTCAAGCCTTCTACGAAGATTCCCATGAG
CTAGGATCGACCCGTAGAGGAGTTTCCTAGCAGCATCCCTCACTGCTCAGG
TAGTTCTTAGATAGACAAAGAGATCCTTCCGTATATAGTAGGTCTCCTGATA
CGAGGTAGCTTAGAGCTCCTCGTGTAACAATAAAGTCTTTTCCATGCTTCTT
GGTTTACATCGTGACAAAACTACAGGATAGAGGCACCAATAGAAAAGCTTA
CTTAAGTACTTAAGTATAGGTATATTTGAATTTGATCAGGCTATTACCTACTT
GTTAATTAGGTAGTATATTATCATTTAGTTGTAGATGATTGCTATTGAAAAGA
AAGAGAAGTATTTTACTTTTAATGTAAATATTTACTGACATATCACCTTTGAC
TTTTATATCAACGATCAACGGTGCATGTTATATATGGATAATGACCTTATACTA
ATCAAAATGACTAAGTGTATTCTTAAGAGCTATTATACTACCTCGCTGAAGT
CTTACGTACCCCCATTAACTCGACTTAATCTGCCAACTAATATTTTACACTGG

CAGTACTGTTTGGAATTAGAAATGATTTGCGCCCGAAACCGCTACATCCATT
GCCAAGGCTGCTAACGGCTGGCGTCGAAATAGCTGAGGTCATAGCATGGGT
TATATCATCACGTAAAGTGGGTTTCTCATGCTGTAAATTTTGATTTTAAATCT
ACTCCGCCCAATTGCAAACAAATCGCTGTTCTCAGAGGTTTTGTAACGTCT
ACTCAGGTTTTGACACAGAAACTTTAATGCGGTTTTTTATAAGCGCTGGAG
GTAAACATGTTAGGTACAAAATAGCACTGTTTTGAAATCTCTCAAATGATTC
GGTTATGAAGTTTTCATCTTTCGCCAATGTTGTGGATTTAAGATAGAGCTAA
TTAGATGTCATAGTATTAACCTAGAAAAAGGGGTAGTTACGCGCGTCTAAA
AACCCCACATTGCGTGTTCGAACCCACACGCCTATTTTGACGATCGACGTG
CAATGAGTCGTGCTTCGATCCTGTCACTGTAACCCATACAGCTCATCCTTTC
GGTACGGCGAATCTGATTCGAGTGGCAAGAACTAGCTTACGCAACTTTCAC
CAGGTTCTAACCCCTGTCGATAGAACAAGAGGCTTGTTTTTCGGAATGGCT
TAGATATTGGGAAATTATCATTGGCGGGTGCCAAGCGGTAGTTGTTTTGAAC
ATGACGGCCCCCCTAGCTTGGGTACTAATTCTGGGGTTGCGGCATCACGAA
TACCGGGCCTATCACGGCCCTGTTCCCCTTTGAGCAAAGTGCAGCCCTGTC
AGAAGCTGCAGTTTACATATATTTGATCGAATTTTTGTGAGCCTTGCCCTTG
TGCCCAAGTGAGTGTCCAGTAATATTAGACCTATTTGCACACGTAACCACCT
GCCGCCAAGAGCTCACGATAATGACCCTGGACCCACAGACTCTTGAGAAT
GGCCAAAAAAACGAGCACTGCCGATAGTGATGATGATTCGTAACACAGATA
TAGAGATCAGGAATTGGAGTATAATAAGGCGGCGATGGTATCGAAAATTCTT
CCATTCCGTCTGAGTAGGTACCAACGCCGATAGCTTTCACTCTTTCATACTC
AGAACTATCTACCTACCTACATACCTAGGTTCCGCTTCTCTCGGTTCCAGCA
TCATTCATTTTCCCCATTCCAAGCCGACTATACAGAATGAGGTTTAACAGTT
ATGTCGTGCCTGGCTTAGCCATTCTATCAGGTCCCGTCCTGGCGCATCCGGG
CCACGATATCTCTCATGAGATCTTGGAGCGGCGCGAATTCCTCAACTCTATC
AAGCGTACTAACCTCGATCACTGCGCCCCGAGCTAAGGGCGCGCGGTGTC
GAGAAACGTAACGTTGAGCGCCGAGCTGCTTTACTTGAGAAAGCGCGAGC
GAAGAGTGAGTACCTTTCTATCGGAAGCCTATTAATATTCCATCGATGAAAA
TGTTCTAAACGTATAATGAACAGGAGGCCTTAATAAGAAGCGCGATCTCGA
CACTGTCTTAGCTACAAGCCACAATGATACAGGCTTGGGATATACCCCAAA
CACTGATGCTACCACCCTATTTGCTGGCTATAATGCCTGCCTTCTGACCCCC
GAGGTCACTCAGGGCCCCTACTGTAAGGCACCCTGCTTGCGATGATGGGTG
ACGCGACTAACAGCGATATCAAGACGTCGGCGGCGAGTATGTTCGAGAGA
ATCTTGTCGAAGAACAGCCTGGCGTTGACACGATTCTTGATTATCAAGTAAT
CGATGTCAATACTTGCGAGCCCGTCAACGATGTCTACGTCGAGATGTGGCA
TTGCAACGCTACCGGTGTATATGGAGGTATCGTGGCGAGCGGTAATGGTGA
CTCCTCTGATGAGACCAACATTGACAATACTTGGCTTCGAGGCATCCAGAA
GACCGACTCGGACGGCGTAGCACAATTCGAATCGATCTTTCCTGGCCATTA

CACAGGTCGTGCTACTCACATTCATATCATGATCCATCAGAATGCTATCCTCT
TCGCTAATTCGACCCTCGGCAACAACATCAGCGCCAGCCATGTCGGGCAAG
CCTTTTTTGACCAGGATTTAATCAGCTCTGTCGAGCTTACCGAGCCCTACAA
TACCAATACACAGGAACTCACTGCGAACGCTGATGACAGTATCCTATCTGA
AGAGGCCGCCACAGACGGTGTTGACCCCTTCTTTGAGTATACTCTCTTAGG
CAATGAGATAACCGACGGACTCTTTGCCTGGATAGCTTTCGGTATTAACTCT
ACCCAGACTCAATCTATAACTCCCGCAGCATTCTATTACAAGGAAGGAGGT
GTCGCCAACCCGAACTCAGGCGCCGGTGGTCCCGGTGGCGCCCCGCCCAA
CGGGACTGCGTCACCTTCGGTTTCCGCTTCCGCGTCTGCCGCCGCATGAGG
GAAGCGCCACTAGTATAGAAGATCCTACATAGGCACAGCATAGCATGAGCG
AAATGAGCATCGAGTCTTCTTCGATTTGGGAATATTGCAGTAAAGTCATATT
CTAAGACAAAACACCTGCTTCAACTCATTGTGCCGTGAATTAATCTTTAAGT
ACCTACCTAACTATTGTCATCACCTGCTATTTATCAAACTTTTTGGGTATGTG
CGTGCCTTATGTCAAAGAGTTTAGGGCAGAAGCGAGTATCTAGAATAAATT
ACTGTATATTGAACTGCCACTTCATTAGAATGTATTTTAGATCATTGACTTTA
CTCCGTGTGGGTTGATAATATCTATTAATATGCTAGGTTCACGACAGAAAGG
GAACCAAGGAGTCGATGTCTTGTGTAACATACCAATATTGTAGGTACCTACC
TAGTAGGTACCTTTACAAAGCACAGTTTTATTTTGCCCATCAAACCTGAGAT
CATAAGGAGCCGGAGGGTGGGGCTTACTAGGCTCGCCGGTCGAGACGAAA
CGCATAATTGATCAATGATATCTGGACTTGCCAATGCTGCACGATGTCTTGC
ATTGCAGTCGGGTAATATCTTGATTCAGCAGACATCCAACATTAATGCTGTG
GGACCAAGCAATTCCATACCAGGGATAAGATAACTAGTAGTAGAAGAAACG
AAATAGGTAATTCCCTGTAAGTTTTGTCAACTAGTATCTTACAGTTTCTACCA
CGGTATCACCTCAGTTGTGATACCCCCTCATCTACTCTCTAGGTACCTAGGT
ATGTATAAGCCAGTGCTAGTGTTCATGTAACAACAAGACCAGAAAAACAGG
CCTCCAACCAAGTATTAATACTTCGATTTAATAAGTTTGCTATTCTACTATTC
CCTGACCGGTCACGAAAGCTATCGGTATTTGGTTGTACCTAGGTACTAGGTA
GAGAGACCTGTTCCGTTAACGATGCTCTATACGAAGTACAAGCGTAGAATC
CACTCTGCTTGAGGACCCACATATTTTCTACCTTGAACAACTGAGGCTTGTC
AACCTGAATCTGTGCAAATTGCCAACAGCAACCTGATTTGGGCTTGGCCCG
CGAAAAACTCACATTGTAGATCATTATCAAGGAGGCAGTAAGCTTCTTCAC
GTCAAATATACCAATGTGTCTACCAAGGCGGACTCGCCTCCTAGCTCCAAA
CTAAGGGCTACTTAGCCTCCGAAACGCAAACGTATAATATGGATCTTGACCA
TGGCTAAGATTTCTGGTATATTATAGAGGTCAGAAGGTAGATGTACTTATCGT
GATCATCGACTGTT

>Mosaic_sequence_81

GGGCTCCTTATCCCCCTCTACCAGCCTCACGCCCCCGCGAAAATATCTTCGG

ATACCACGACTTCTGCCCCGCCTGCTCCTGCTTCTCATTCCCCTCCGCACGG
CTACAGCGCGTTGATTCCTCGCCGAAGAAGGGATCGGAGTAGGTTGCCGG
GGAACCGGCCGCGGTGGCGCGATACTCGGCCAGAACGTCGTTGTTGAACG
CGCGAGCGCGGAGGAGACCAGGGGTGAGGAACTGGCGTCGGCGCGGTGG
CGGAGAGAGGCTTGTCGTTTGCCTTCTTCCACGCCCTTGCGGAATGCGGCT
TCGATGTTTAGTTGGCATTGCTCGGGGTGGCCTGGGTTTGGGATGGCGGTG
GTGGGCAGGTGTAGGGACGACCAGCCTAGACCTTGTAGATCGGTTTGCGTG
TCCGCGTGGCTTGGGTGGAGGTTTTTGGGTCTTGGGTCTGTGGGGGACTGG
GAGTGAGGTGGTTGAGATGTGGTGGTCGTTTAGGGACATTTGGTGCTGGAA
GCGTGTGGGGAGGTCGGCGTCGCTGCGGTCTCGCACGGATAGCCGTCGGT
TGAAGTCCCTGGATAGCTGTTCTACGGTGGGCGCGTGGCTTTCTCTCCGCT
GATCTGTGTTGCTAACATGCGATCTAGCTCTTTCATGCTGACTTCGGGCTGA
TGGTGGTCGCGCTGGTAGTGATTGGGAAGTGCCGTTGCGGTCTACGGACCC
GACCACTGCGCTAGTCGAGCTCGTCGAGGTTCGTCTAGAGTATTGACTAGT
CCCCGAAACGCTCTGGCTGGTTGGTAGCTTCTCTTCTTTGGGAAGATGAAG
GGCCTTGGCTTGATCTCTGGCGGTGGGTGGGTTCGCGTCTTCCGTCTATTTC
CATATCATCGTCCGTCATCGAGGAATTCTATTCGAACCGAGTATCGTTTGCG
ACGCTTAGGCTCATCAACGGTTGACCTGGAGGAGTGGGAGCCTGATCCGT
GCGATGATGATGATGCCATTTCTTCCTATCCTTATGAATTCATCCGGTCAATA
AGGACATCGGCCGACTCTTGCAGGTACTTAACCTCGTCAAACCTCATGTAA
ACCTCAAAGGTGGGCTTGGGAGCGGTCGCCTATTTGAAGTCTACATAAATG
GCACATTGTATAGGTAGGCACCTACTTATACGGTTAATCCACAGATCTCTCA
AAGAAGGCCTTATTCTAGGTAAGGATCCATCTACATCCGTCTACTTTGATCT
ATGCGTTATTGAGAACTAGCCTACTAGACATGTTTGAGATCTGGTTTACACC
TACCTATTTATGCTGAGACATCACGTAGATACGTCTTCAACTCTTTGTACGTT
CATAAAATGCACTAACCTTCTTAGGAATATGGCGATTCTAACTAGAACCAGC
GAGACTATCTCGACCCAAAATAACTCCATATACCTACCTACTAGGTACGTAT
CTAGATACATACCTCCTAGGTGGGTAAGCTTCTTCGTGAGTGAAGAGGGAA
GCCTGACTTATATATTCCTACCTGTAGGTAGGTACCTAAGTAGGCACCGACA
CAATATTGTCTTATGCCTTCTGCTTTACCTGACAGCAGTCCCTTAACCGGTT
GTATTAACAATCAGTTCTCATATACAACCAGTCCTCATTGGTACATCCCAGA
GGTCAAGGAAGTTTCTAATTCATAGCCTGATCATGGCGGAGGGCAAGTATC
AGCGAAGTACAGCGAAGTAAATGCACGTCAAATCCTACCTTAAGTTAGTAC
TAAGTATCTTTTTCTCGATTAAAACGTCTTATTCGCTGTAAGCAATGCTGACT
ACTTGCATGCAAGAGTCATTTTGATATGTCGTATAGAAAGAGGGGCTCAAC
CGCATCATAGCACCATTCTGGGCTTGATCTACGGGCTGTGTTGATTTGCCGT
TCAACAGCATTATATAACGGTGCATGGGACATGAAGGCCTGATAAAATTGTC
GTCCTATACGCCACTATCGCGTACACGTGTGTGCAGATTCTATAGACTAGGC

AGCACTTCTTCTAGACGTAAAAGACCTCATTATAATCATCATCATTTCACCAT
TCCATTTCTTTTGGCTTCGGGATGCCCTTCTTTCATACCTCGCATCATCTTCC
ATTTCAATTCAACCCGATTCCTAATAATAAGAAATGACAGAAAAAACCGAG
TTCGCAAGGCTCTGGAGCGATGCTGTCACCGAATACTTCAACAGGATGGGC
AGGACGTTCGATACAGGGAAGCTGACGTCACGAGTAACTAATTCCGCCGA
CGAAGTCGAGATGTTGATTGAAGACCACAAATCCAATTTCGCGCAATTTCG
AGCTACACATGGGAAGCTTATCAAGGTATTCTATTCTGTAACGAAGCACCTG
CAAAATCTCAGCAAGATTGCCCAGGCGGGTATTGGCCTGACGCCATTCGCA
CCAGCGTCCATTATCATTGAAGCCGCGTGTTTCCTCATAGATGCTAGCTCAG
CCGTATCGGAGACATACGACTCTCTTGAAATTCTATTCCAAAAGATCCAAGA
CATTATTGATCGGTTGGAGGAGTATCTAAAAGGGACGATCGACGACAAATT
TCGCAAGGTAATAGTGAATCTTCTTTGCTCACTTCTCGAAGTTTTCGGCGAA
GCCGAGCAAGCTGTCAAACGTGGCAGAGGAAAAGAGATGATGAGACGCG
TGGCAGGCAAAGAAAACAAAGTTCAAAGCGCGTTGGACCGCCTTGACCG
AATGTTGCAAACTGAATTGGGTCTTGTTGCCGCGAAGACGTACGCTACAGC
AAGAATGATTGATGAAAAGATGGATAATACCAGCAACCAAAGTCTTCTCGA
TGAAGCCCTTTCCGCCAATATCTCGTACGATATGAACCGGTTATATACTGAG
ATTGAGGAAACTAGACTTCCACAGTCCGGTCAGTGGCTTCTCAATCAGCAG
CGTTTTCGCCGATGGGTCGAGATGAAGTTTCCTGTTCTGTGGATATTGGGTA
AACCTGGAACGGGCAAGACGTACTTGGCATCACGAATCATTACGTTTTTAA
GAGAGTTGCAACCGTGTCATGGTTATGTCGGCTATTTTTACATCAGAGAGA
GCATGCAGACTCAGTATACGCCTAGCATCATTCTAACGAGCATTGCCTGTCA
GATTACGAAACAATCTGATGCATATAGGAAGAAAGCTATTTCCCTGTGCAA
AGACGGTAGTGGCCTACTAAGTCCACGCCTGATATGGGAATCTTTATTTGTT
GAATACTTCAGTAGAGACAATTCCCGTTCCATCTTCATTATTATCGATGGAGT
AGATGAACTTCTACCTATACACCAAAACGCGCTTCTCGTAATGGCCAAGCA
GCTTTCCGACTTACGGGTCAGTCAAAGTACCCTTTAATACAAATATCTGTTG
CTTGGACGTCCTGATCTCGAGTTTGGCCTATCAAATGTTTGGGCTCGGTCAA
AAAAACGGCCGAAAGTCATACAGGTCCAATCATCCAAGTCGCGAGATGATC
TTGAGAAGTTTGTTCGTACAGGAGTTGATGGTATAAGTCTTCTACAAAAGA
TGAAAATGAGCCAGAAGAAATCTATCAGGCGCCATGCCAAGGGTCTCAGG
AGCGAAGTCATATCGACACTCTGCCAGAATGCCGACGGAATGTTCATGTTA
GCTAAACTTATGCTTGCTGAGATCAAGGATATGAATAAGCCAGAATTGATAA
AGCGGACACTCAACAATTGTCCGAGGGTTTGGATGATATGTTTAAACGCGT
TATCGAAAAGTTAGCGGTCATTGGGGGTTTCGATAAACATGAACCTGAACG
AGATCATTATGTGGGTTGCTTGCGCTAAACGCGACCTGTTGCTTGGCGAAA
TTGATCTAGTTATCAAGCTGAGAGATCTTCACAGGGTGGTCTTTATACTCTA
GAAGATGAGCTTAGAACTAGATTTGGGTCTTTCTTCACAATAGTGGCGTCG

ATTCCAATGCTGTCAATGAAGACGAGGACGAATCAGTAGTAGGCGAAAAC
CCTACAGACGTATTTCCTGAAACCGCAGAAAGTTATTGGTAACGTGGATCA
AGAAGTCGAAGACGACGAGATTGAAAACCAAGATACAGATACCAGCAACT
GGACTGAGACTGACAGCGAGGAAGGCGATGGCGATGATGGCGACAGTGA
AGAAGATATCCCATCAAATTTCTCAATCGCAAACTGTCAAGTTTAGCCACG
CAAGCGTTGCCAGTACTTTCGTACACAGGGTTTTCACCAAAGGTATAGGCA
TAGACCTTAACGTGGCACGAGCCCATGTTCTACAGACTTGCCTTTTATTCCT
CACTGACCACATTCCCAAGAAAAATGGAGAGGAATGGTCTCCCCCTGACC
TACTTGGATATTCAGCAGATCATCTTTTAGATCACCTCGTTGAAGTTGACATT
GAAGCTCTCCGAAACTCACAACCTAAGAAATTCAGTAATATATCTGATGAA
GTATTCTTCCTTTTTAGGAATTCAGACGCCCTCGGGCGCTGGTACTCGAAGC
TTTCAGACAAGCAAAAACTCATATATCTGTTACGAACCTATACCTAGGTACT
TAGGTATACGGGTTGATTCGTGGGTTTCCTTGTTTGTTAACAGTACTCGTAG
AGATCAGATCAAAAGTTAGTCTGTTCGTAAGAGAGGCTTGGAGTCCTCTCT
CTATTGTTCGATGCGGTTGAAGTAATAGACTTAGATTGATAGCTGGTGAGCT
AAATGAATGACTAGATACATGAGAGACGTTTGTAGTTATATAAGGATGCCTT
CCCTCCTCCTTGTTACTGAAGCCATCGTGATGTGTTGTGATGTGGTCCTTCC
TTAACACATCGAGATGCGTTTGATGTGTTTTGATGTGTTTAGATCACAACAC
ATCGTTCCGAGGATTAACCGTGATTGGTTAAAGTAGGTATCCGCTGCCTGGA
TCTTAAAGTCGGTTGTATGTTAGTTGGTTTGTGACATTATACCCCTCCTTCTA
GGATCTTTGTCCCAAAGATAAAGAGGTAAGCGTTCTTCTTTTTCCTTCCTCC
TTCTTTTTGTGTCGTCCTTGCTGACTTATTGAATATGTCGGGAAGGGTTTCG
AAAGTCGAGTATTCGTCCTAATCAGCGACAAGAGCGTCATACTCGTGCTGA
TTACATTGCGGCCCACGGTTTCCCTGTTTTCCTCTGTTCGAATTGTAACCAT
CTTGGCGAAGGCGACTGCCTCATTGCTGTTGGCTACGAGTGTTGTAATCATT
GTACCCGTTGGAATCGAAAACATTGCGACGTTTTGGAGATGAATGACCTTG
ACCGTGTTTCTCGAGCTCGCACTCGTCTTGAGCAGGAGCGCAAGGATGCA
GAGGATGAGTTGGTCCGGTTACAACAAGAATACCAGTCTAAGATATCCGAA
AAAATTGGTAAATTGCAACGGCTTCGTCGCCAAGAGGACTTGCTGAAGAA
GCGTGGTTTGGATTTGTTGAGACTTGGCGATGAACAGGATGTTGAGGAAGA
AAGAGTTCGTTTGGAGGCGCAGGCTTCTGTAGAGCGTGAGCAAGCTCTCC
TCAATTCTGGGGTTTCTTCCTCGGCCTTCCCCTCTTTCTCGGGATAAATTTTG
ATTTGGGTCCGTTGTCGCCGTCGACTGCTGCTGCTCTGGGTGTGGTCGATC
AGGGTTCTGGCGATGAAAATCGTCAAGCATCCACTTCACATTCTGGAGGTG
CTTAATGGGTTCCCAGGTATTTTCTGCGTCCTTGTATCCTTGCCACTTGATGT
AATATTTTATCTGACCATCCTGAATAGCTGAGTCCATGATTTCTCGACTTCGT
ATTCGTCAGGGTCTTCTTCGAGTTCTAGATTCTTGTCAAGTTCGGGCGTTTC
TGGTGCGGGTTCTAACATGGAAATATGAAAAACATTTGTTCGTAATTGCATG

GATTTGGGTAGTGACAGTCGATAGTTGGTTTTGGATAAAACTTCTTTGATTT
TAAATGCTCCAAGCTTTTTGTATTCCAATTTGTCGCTTGGCCTTTTGGTTTTG
ATTTGCGATTGTTTTCAGAGTTGAGTGCTGTTTATCGCGTAGTAGGTAGACC
TTGTCTCCTACTTTGAGGGTTGGTCCTCAATCCTTTTCTTATTGTAGTAATGT
GCCATTCTGTTTTGTAGGAATTGAAGTTCTTGCTGCAACCGTTGTTGTGTTG
CTTTAATTTTGGTTGCCATCATTTCCGCTCTAGGTACTGAGCTTGTGGCTTCT
GGTGCTTCTGTGTCGGATATTTCTTGCCTTGTTGTTACTGGCTTCTGACCGT
AGACTGCTTCGTGTGGTGTCATCTTCATTGTTTCATTTACTGAGGTGTTGTAT
GCGTATTGTGCTAGTGGTAGCAGTTCTACCCAGTTGTCTTGTTGGAAATTGA
CATATCCCCTCAGATATTGCTCCAGTGTTTGGTTTAGACGTTCTGTCTGTCCA
TCGGTTTGTGGATGTGCTGTAGTGGAGAGTTTCTGCTTCGTTCCTAATCTTT
TCATTAAGGCTGTCCAAAACTTTGAGGTAAAGAGCTTGTCTCTGTCTGAGA
TGATCTGCTCTGGTAGCCCGTGTCTGCTAAATACTTCGCGTATGAAAGCGAA
TGCTAGCTGTTCTGAAGTAGCGGTTGATAAGAAAGGTATGAAGTAGGCATA
TTTGGTAAGTTTGTCCACTATTACCCATATGTTGTCGTATTTAGCTTCAGTGG
TTGGGTCAGTCGACACTGGTAATGCCATGATAAAATCCCAAGCTATGGTACT
CCATGCTTGATCCGGGATTTGGATTGCTTGAAGAAGCCCATATGGCTTATGT
CTGGCTCCTTTAGTTTTGTTGCAGATGTCGCAACTCTTGACTACCTTTTTGA
CTACTGTTTTGAGTCCGGGGAAATCGTACTTTTCCTGGATTCTTTTGGTAGT
TTTGAATATTCCTTGGTGTCCTGCTAGAGGGTGAGAATGAATTTCTTGAACG
AATTCTTCCTGAATGCTCGGGGGAACGTAGATTTTTCCTTTATAAGATACCG
TGTCTGACTTATCGAAGTTTTCGACAGGTAAGGAGTCTCGTATGGCGTAAG
CTTCATTTTGTATTCGTTGTTCCATTCTTCTTCTGGCTTGGCGAAGAAATTGC
GGATAGTACAAGTGCTCCATCTGGTGTTTCCTTGAAGACTGCTTTTTCTTCT
CTGACTTCCTTAGTACTGAGACCTTCTGGTCTGCTTAGGATGTCTGCTTGTA
TGTTTTCTTTTTCCTTTGATGTGGTAGATTTTGAAATTGAAATGTGTGAGGT
CGTCTACCCATCTGGATTGTCGGTTGTTGAGGACCTTAGTTGACATTAGTGT
TCTTAGGTTGAGGTGGTCAGAGAATATGGTGATGGGTTCCGTAGTTCCGGA
AACCCATGGTCTCCATTCCATCAATGCTTCATGGATTGCCATTAACTCTTGGT
CATAGGTCTGATACCTAGTTTCGGGTCCTTTAAATGTCTTTGAGAAGTATGC
AACTGGATGCCACTTCCCATCTTCTTCTAGTTGTTCTAGAGTGGCTCCCATA
GCACCGTTGGATGCGTCCGTATGCATCCTCTTCTTCTTGTCGGGATGCGGTA
ATCTCATGACTGGGTCACTGGTGACGATTTCCTTGATCTTTTGAAATGCTTC
TTCTGCTTCTGGTGTCCACTTGAAATTTTTAGGTTTGGTAGGTTTTCTTATGG
TCGCCATAATAGGTTCCATTATTTTTCCGAAGTCTTTGACTAAGGCTCGGCT
GTAGTTGACAAAACATGGAATGATCGTACTCCGGTTATGGTTTCAGGGGTTT
TCCAATCTCGTACTGCGGCGATTTTTGAGTCCTGTATCTTGATATGTCCTGGT
GTGATAGTATAGCCCAGGTAGTCAAGTTTCTGGACGAAGAAGTCGCTCTTT

TCTGGGTCGACAAGGAGTTTGGCTTTTGCTAAGGTTTTAATACTGCCTTGA
CATCCCTTTCGTGTTGTTTTCTGGTTTTCGAGTAGATCAAGATATCGTCCAG
ATACACAAACACGCTTTTTCCGAGGAGTCTGCGTAGAACAGTTTGTATCATT
CGCTGAAACGTAGCTGGTGCGTTGGTTAAGCCGAACGGCATCACCAGGCA
CTCGAAATGTCCGTGCTCTGTTCTAATTGCAGTCTTCCATTCGTCTCCTGCT
GCTATGCGTATGAGGTTGAATGCTGCTTTCAGATCTATCGAGGTAAACCATT
CGCTGTCTCCAATTTGTTCCTTGAGACTTTCAATGTGTGGTAGTGGAAATCC
GTCTTTGAGTGTGATGTCGTTCAACTGTCGATAATCATACACATGTCGGTAG
ACTGGTTTTCCTTCTTTGTCCAGTCTGGGTGTCCGTCTTTGTTCTTTTTGGG
AACTAGGAATACTGGGTATCCTACTGGTGATTCTGATTTTCGTATGTATCCTT
TTGCTTCTTCGTTTGCAAGGATAACTCGTAGTGCTTCTGATTCGTGCTTGTT
GAGTCGATAGACTTGGTTAAGCGTAGGAAATTTTCCTTCCTGTAATTGAATC
CGGTGATCCCAATGACTATGGTCAGGGATTCCTGATTCCGTGTCTGGTGTTT
CTTGAAATAATTTAGGAAATAACTTAAGGAATCGTGTTGGTACTTTTGAGAG
GCGTTTTTCCTTCTCGGGTCTAATCATTAGACAAAGAGGTACCGTTTCTTCG
TCTAGGCATAGCATATTTAATTGCTATTTTGATCCGTATGAGTCGCTGTCTGG
GCGATGCTGAGGGACTGGATCTTTCTTTTCTGGCTCATCCCAGGACAGCTG
ATTGGTACTCCAATCTATCTTGGGGTTGCTAGCCTTGAGCCACGCTCTACCC
AAAACCATATCGTAGTTTGTGTCGACAAGGTCGAAAGAGACTTTTTCCGTG
TGTCCATGGATCAGAACAGAGAGTTCTGCAGTTTCTCTTCTGATCTTTCCTC
CATCGTATTGGATAGGTGTTCCGTCGATATTGTTGACTTGGTAGGGCGTCTT
TTTGTCAATCCATGGTATATCATGTTTGTTGACAAGTCGTGGGTGAATGACA
TTGACTGCGCATCCTGAATCTACTAGGGCGTGGAATTGGTGTCCGTTCCAA
ACGAGCTTGGTGAATAGTTGATGTTTGCTGTTTGAGGTCGCCGAGCATAAT
GGTCTGGCTTCTTCTTCCGATTCAGTTGGTGCCGGTTGGCACAATTTGTCAA
GTATTCTTTTCATTATTTCGGACATATCCCGAACTGCTTGTCTGGTTGTTTTC
GAGGTCCTTTCTTTGGTTTGTTGCTTCTATCTGTTGTTGGGTGAGAGCAACG
AGCATTGTTTTCTGTGATGCCATCATATCTCTTATGGTGGCGTTTTGAGTTTC
CCAAGCGTTGACTAAGTGTTGGTTCGAGCGAGCAAGTTTTGTTATCTGCTT
TAACATTATCAGGTTGTCATTAGTGAGCCTTTGGTTGGTGACCTCCTGGTAT
TCGTTGAATTTGGCTTGGACACTGTGATGAAATGCGTGGAGCCAGTCCAGG
ATGTTTGTCGAATTTCTTGTTCTCCTATTAGCCACGGGATCATGTAGTAGAG
CCTTGTGTATTGTCGTCTATGTTTTTGTCGTGCCACTTAAGATCCCGGTTCCA
GGCACCTGGTGGGTAGTTTCCAGTGCTGCTCCAGGTGTGTTGAAAAGATGG
GCGACAAAGCTTGGTCGAAACCTGAGTTTTCGAAGTAAGTTCACGGCTTG
TTTTAATGTCTTTCGGTGAACCATCTTGTCCCATCGTTCCCTGCTAGTAAGC
GCGCTTCTCCATCTGGGGAAATCGCATTCCTCTAGTTCGGTTCTTTGGAGTA
GCCATCCACCCAGGGAATGTCCTTGGTTTCTGGTAGCTGTTGAATTGCTTCC

CTTATGTTTGACGCGAAGAAAGAGCCGGAATGATCAGTACATGATCCTTCT
TCCGTGTAGGTGAATTGGAATGCCCAAAGGTCTTGGGATAGGGTAATCTGG
TGACCTGTGTCGTGGGACTGAAGCAATTTCTGCTCCAACACTCATGTGAGT
GTCCACTGAGTCTGCCACAGATTCCGGCATAGATTCGGCTAACTGATTCTGC
GTCTGCTTCCGATTCGGATGAATCATTGATTGACGTCTGGGGGAAGATGTTG
AATTCCGCGAGGGTCTGCGTTTGTGCTTCATTGTATGTGATGTTTTCCGCAG
ATGGTTGTTCGCTATCCGATTCCTCTCGTCCTTCAATGATTGGAGATATATAT
GCTGCTCCTAAGGTGAGTGCTCCCAATGTTTCTGAGGTCGCGGCGGCGGGG
TCGTCCTTCCGGAACTCTGGGTTGCTTTTTAGGGGATTTGCACTCGTTTTTT
ATAGTGTCCCTTCTTCTGGCAGTTATAGCATGTGATGTGACCTTTGTCCTTCC
TCCTTTTCTCAGGGGTGGCCGCGTTAAGTTCCTTGACATCCTCTTTCTGAAT
TATGTTGTGTCCTTTACGAGAGTTCTGACTTTTGGTTCCTTTGACTTGCGGT
CCTCTAGGTATCGACCGTAGGATCGTCGGTCCATTTGAATGGCGTAATTGAT
GTAGTCATTGATGCCGTAGCTATTGCGGTCAATCCTGGAGAGTTCATCCTTG
ACCCATTTTGCACATCCTCTATAGAATAATTCGAACCTTGAGGCTTCTGGGA
TCTGTGCTTCTGCTGCGAGTCGTTTGAACTCGACGGCATAGTCCGAGGTTC
TCTTGCTTTGTTTGAGGGTGTACAGTTGTCTTTCTGCTTCTTCCTTCTTTAGG
GGGTTCCCAAACATGTCTCTCACTTTGGCGATGAAGGCTTCGTAGTCCTCG
AAGGTTTCTCGGGTTTCCTGCTGAAGGGTATCTTTTCCCTTGTACGCATTAA
CCACAAAATCTTGGAGGAGTGGCTGGAACCAGGTCATTGCCTTTCGGTGA
GGAACTGAGATGCTGCCACAATTCTCTGCGTATCGTTGGTGATACTTTGACT
ATGGAACATTATGTAGACCGCCAGCTGGGTAACGAATGCATCAAGGTCTTC
GGTCTTTTCTCCGCTGTAAGGCCTAGGATTGACTGGTTTGTACGCCTTGATT
GGTGGGAGTCCTATGTGTGTTATTCCTTCTGGAGGTGGCAACGATACATGTC
TCTCGGGTCCTTTTCCCTTTGGGTCATCATCGGGGTCATTGTTGTCATCGTC
ATCCGGGTTCCCTGGAGGTCCAGAGGTGGATCCCGTAGGTCCCACTGGGGT
GGTGGTTGAAGGCCGTTCGGTAGTCGGAACATGAATTGTGTCTCGTGCCGG
CGCTCCTTGCGCGGAACCGGGTGCTGCGTGTGAACGGGTCTCGACTGGGG
CCATGGTGTCAAGAAATCTTAGTACTTTCTCAATAGGGGGAAGTTCGTTAAT
TCCTAGAAGTTCGGGCGTAGGAGATCGTGTCTCTTCGCGATTCTTTTTATAA
AGAGAATAATCACTCTGACTTTCGTCGAAAATCAGTTCGTCCCAGGTCCAG
TGGTAGTATCTATAAAACGGTAATTCTTCCAAAATAGAGGATAAGAGATCTT
CAGATTGGCTGCTCATGCCATAAGCTTGATCCGATCCGTAGGGGTACTGATC
AAGGAGCTATCAAATGTTACGAACCTATACCTAGGTACTTAGGTATACGGGT
TGATTCGTGGGTTTCTTGTTTGTTAACAGTACTCGTAGAGATCAGATCAAAA
GTTAGTCTGTTCGTAAGAGAGGCTTGGAGTCCTCTCTCTATTAGTTCGATGC
GGTTGAAGTAATAGACTTAGATTGATAGCTGGTGAGCTAAATGAATGACTA
GATACATGAGAGACGTTTGTAGTTATATAAGGAATGCCTTCCCTCCTCCTTG

TTACTGAAGCCATCGTGATGTGTTGTGATGTGGTCCTTCCTTAACACATCGA
GATGCGTTTGATGTGTTTTGATGTGTTTAGATCACAACACATCGTTCCGAGG
ATTAACCGTGATTGGTTAAAGTAGGTATCCGCTGCCTGGATCTTAAAGTCGG
TTGTATGTTAGTTGGTTTGTGACAATATCAGCTCTTCGGTAGGACAGATATAT
GTTCTCGCATTCAAGAGTGGATACCCGGACATACGGCTGCTTCCAGGTCTG
AGGCTCGATGGCTGAAGCAGGCGAAGTCTTCGCCACAGGCTTTTGCTCAG
GCCATTGCGCAATTTATTGCTAAATTGTGGCTTGGGTATAATCGCATGGATG
GATTTATACCAATCGTTTTTTTAGATGGATATATATCTATGGTAAGATTCCTAA
AATCGAAGTTATTCATCCTATTTCCTGTCAATAGCTTAACACGTCTAATTGTC
AAAGCTAAGAAGTAATATATTCTAGAATGACCAGTCTCGGCCTCTATGGGTA
TCAGGTCTCAAGCGGCCCTTGAAACAAATAGTACAGAAAATGTCTCCAATA
CATATTCGTCGGATAGCTTCATATGGTAATTTGAAAAGAACCATCCTTTGGC
ATCTCTATCTCGGTACCACATTGATGCATGTTGCGACTCCCCAGCACATTATT
GCAGCTATACGAGAGTTTAGGCGAGCCAAGCGTAAGCATGGATTAATCTGG
ATACTCCATTTTGAAGAAGCTGTTGGACTGTATTGTCTCCAGCTGTATCGAG
AATCGATTGAGGCAGCTGCGCACGCTTTGGAACAAGAAGAATTTCAAGAT
GAGGAATCGAAGTGTGCTCTTCTGGGCTTGTCCGACTTTCAAACCTGGAAT
TAGGAGACTTTGAGTCTGCTATAAAGGCAGCCGAGGAAGTATGCAGAATTG
CTCCACGGTCGCCGCTTTCCGTGCTCAAAATGATCGGCACATACTATACGAC
CGGCCGCTTTTTGGAAAGTGCCCGATACATCCAGTCTATAATGAACCACGA
GGGACTTCTTGTTGACGCCATAAATGCGAATCCTCTGAGCAGCCAATTGAT
ATCCACGACTTGTATGGAATCGGGGCAGTTAGATCTTGCGCAGGATATATTT
ACTAGGGCTGCTACGGAAGCCCGAAGCGTCTGGAGACACCGACAGAGCCG
TATCGGCAAAGATGGCACTCTCCAACTTGTATCTTCGGTATTACGATGCTGA
CAAAGCGATAGAGTCTTGGGAGAGCCTTGCAATAGACCCTTTAAGCACCAA
CGAAGGTGTACAAGTGGCATGCCTACTAGCTTCCTTGTACTATACGAAAGC
TATTGATAGGAATGAGGATAATGAATATTGGACTGCTAAATTACAAACGATG
AACGGGAGGTTGAATAGCAGGTTAATGGGAAACGAAACGCACGACCAGAC
AAGGTACGATTTGTTGGGCGCGCTGCGTTGCTAGGTCGGTGGTATATGGAA
CACGGCGACATGCAAAAGGCACGCTTAGTGATCCAACCTCTAATACAGATA
GGCGTAGATGAACTAACCGATAGAGACGATGCCAATGACATGGTCGCATAT
TCAACCCTGGGTAGGGGACTAATTAGCTTAGGAGATCGCCAAAATGCAGAG
ATCGCTTGGGCCTTCGGATATCCGCTCCAAAAGTCGAATGAACTTCAGGAA
ATAGAAATGCGCGAAGCTAGTAGGAACTCCCCGATTGCAGAAGACCAAGG
AGAATCCACTAGACACGGTAATCGAACGACCAAAACAGTCAAAACTTCGC
TTGAAAATGCAGAAAAAAATCTTTTCTACTTCGATGGAGAATGCGACGGTT
TATGTAGCCGGAGAACAGTGCATTTCAGATCGTTCAGTATATGTGAGATATG
CGTCGATTTAGGATTTTGCGATGGGTGCCTTCAAAAGCTTAGAGACGGCAA

ATTCCCTTACCGAATTTGCGACGCAAAACATCCCTTTGTCGAGGTTTACCCA
CCAAAGGGATTGGTCACCAAGGTAGATGGGGAGTTTAAGGTTCAGAGCGT
AGAGGGCGGTATAATTGGCGTTGATGAGTGGCTTAGCACTATAAGTCGGCG
GTGGCTAACTGGGTAGGCGGGCATTTTAAGGGGGGCGATAACACTGCCACT
ATATAGCCCTGGAGCCCCGCTCTTCTTTGCAACCAACTATCGAGCCCTACTC
ACCAAGTGAAGATGGTATTTCCCTCAGATTCACCTTGAAAGAATATGATATT
TTCATAAAGGTACTTGAAAAGTCTCTGATTCTTTTGAAATCTTCATGTTTTAC
CTGGGGAGTCAGGCTTCCCAAGTATATTATGCCAAGACTGGTTGATCCGTAC
GATCGCGTAGTGCATACTAATATGGAGTATTCAAGCCGCTGCTATTGGTTCTA
TACTAGGTGCATATATACGTGGCTGATTTCTAACGGATATTAGTTGCTCACTA
GATCATGCTATGATTGAAATGGGGCTCATCTGTCAACCTTAAAGGGCATTCT
CGACGTCTACAATCGCTAAGGCACAAGGAAGGCATTTTCTCTGCGTAGAAT
GGTCGCTCTTATTAAAAGGCTATCCTGATAAATCTTTCTCAGATGGAACCTT
CATCCATGGCCATCAGTAAAATCAAGTCAACGATGCTTAAAAAATGCGAAG
GTTGCTCCGACGTGGATATGATTATGTGGAAGAACGGTATGAGAAAGATGA
ATCTCCTTCATATTTCGAGTTTGTAAAGTTAACAGTATCATAGGTGATAAGAT
GGGTCAAAATAAGAAAGGTTAAATTGAACGATTTTAATGGTTTCTAATGCA
GAAAATGCCTTGTGGCCATTTGATCTTGAAAGCAAAGTTGTTATTATGTAGG
TTCGCGAATCAATTTATTTAAGAGCTTTATTCTAATTTTACCTATCAGAGTAG
CCAAAGCTATGAGATTCGCTACTGTGACGTCTGCCGAAACATCCTTACCGA
AAGACTCCGCATGCCTCGATTCTGGTGGTTAGATACGTACAAGAGAATGAA
CGGGTATTTTGGATCTGAATCCACGATAAACTCAGAGGGGTCTATCGAAAG
CTGCTGTGAGTGTCGTTGATACTTGAAAAACGTAGTTTGCTGAATTTGAATA
AAGCAACGTGGTCTCGTGCTCTTGTCAAGCACTTCGATACATTACCAGCCG
TGGACGCTGAACCAGATATAAAATATTGCTGGGCTCAAATTCAACTTATTCA
CACGATGGATCATGCCTGACAAGATGGTAGTCCTCGCTTTCGATCCAGTGC
CGATTATTAAGGAATGGTTATGCTTACCATTTATGGAACAATTGAACGACGA
AGAGCTTCAGGACCGTATTGGCCCTATACACACATCCTTGAGAAGCTTGTG
TCTCTCCATGGAACGTCCGTTTGGAGTGTCCGAAACTTAATAAGAAACACA
GAACTCTCCCCATCGAGAGATCACAGAAATAAGAAGGATAAACCAAAACA
TAATTATACTCGTCTGCATGACATTTCTCGGCATGCTATTCATGTTTTGGAAA
CTTTGGATCTATCCGTTAAGCTGTCAGAGAGTATCTTCCGGGAGCACAAAA
CAATTAGCGTCTTCAAACTCTAGTGTTGATCTAGGATTTGTCATTGGAAGGT
CTCGGAGTTCTAACAAGCGTATTATAATACGCGTTCCCGACTCGCCTTCTTC
AATAATGCACTCTTCAGTTTACGGTGCCGTTCTTTCTCCAACCACCAACGAT
TAAATAATGAGATACAACTTAGATTCCACACGGACTCTCAATTCGATTCCCA
GACCTCCATCGAAATCAACCATTCGACAAAATCTGATAGCGCGTCCATGAG
ATGGATTGCTATCGTGAGTCTCGTGTTTCTTCCAGCAAACATGGTATCATCG

ATCTTTAGCACAACATTTTTCAATGGCTCTGAAGCATTGTCAGTTTCCAACG
ACTTTTGGATTTATTGGGTTGTGCTCGTTGTAGTCACAATAGTAACTATGCTC
TTCTGGTTTTATTTACCCCGTCTTTCTCCTGAAGCGAATACGAATCACAATG
ACTCGGCGGGGGAGGAGGCAAAGAGATCGCTTCAAGAACATATGCGCAGC
TTTAATCAAGAGCATACTGGCCATGGATTTTTCCTTCACGGAAATAGTGTAT
GAGATGTAGAGAAACTACAAATGATACTTAAAAAGGGTTTGCCCCTGAAAC
GACTTGATCAAGTAAGCAACAATCGAGCTAGGTGGTGGATTATGTAATCAA
GACCTAGTTTGAATTACTTGGCCGCTGATATAATACTTTACATTCCGGATACA
TAAGAGTCCACAGGTTTATAAGTAAGTCACAGTTATAAATCTATGCTGATTT
AGAACTTAAATTTGCGAGCTCCCATCCTCCTCCCCACAACTAAGTACCCTTA
AGGAGGCAAGTAAGTCGATGGGAGGCGTCTACTATCTATTGCTTGTTTTGC
GCAAACCTCTGCCATTAATACACAGAGAAGCACAGAATGATATGGCTGTAA
AAATAGTAGATGCGGCAAAAGAGTATTTCAGCCCATCATAATATACCATTCG
TGCTTTGAGTTGAATATCAGGGGGAAGTTGTCGTAATGACTCGGCCGAATG
CCTAAGATCATCGATAATCTATTGAATTCACAATTAGCATTCACATGAGTAAC
TATACACATAGATTTACTTATTAACAGGGCCATACTCTCCATTTATCTGGTATG
TCACTTAAGGCGGTCGGAAGTTGAAAACTGAGGGTGGTCTGTACGATGGC
GGAACTAATTGATACTCCCCACACGGTTCCTAGAGACCTAATGAGGTAAAC
TGTAGACGCCGATACTGCGTGGTCTGTGCAATGTTAAAGACTGTTAGCGTT
AAGAAGGATTCTTGGGAAACCCACCTGCATGCTCGAAAGTGGCTAAGGAG
GTGAATAGGGATACCAGGGTATATGATCCCCTGTCCCAAGTTGGCAGGGAA
TATATAAACAAAATACTTCCAGTTAGCATCTACGAAATCTAGCGAGGTCACC
AAACCGTTTCCAATCATCATCAAGATGGCCCCAGCTCGAACGAGAGTAACA
AGCTTTCCCCAGCGTGACATAACGATACCAGCGATAAGACCACCGATAGGG
GTTGCTAATGATGGAATAGCTAGTCTAGCTCCCGCAGTCGTTGCTGTGTCAA
GTAAAACGATTTGGAAAAACAGGGGTAGGAGGAAAAGATACTACAGGACG
AAGATTAGTTCGATTTCATTTGAGGTAGGAGTCTTCAATACAAACCGCGTAA
GCAGAGATACCCGCGCAAACATTTGCCAATTGTGTAAAAAGGGGTAAACG
CCCCTTTAGTAGACGTAAAGGAATGATAGGAATTGCTTTGGTCCTAGGCCTC
AACCCAAATGAAAAGCCCAAATAAAGTGAGACTTCCAATCAGGGATATGAT
TACCCAAGGGCTGCTCCAAGCAATTCGTTTCCGCCTAGACTCAACCAACTA
ACTGTGTAGAAATTGCTGTGACGAGAATAAAGGATCCTGAGAAGTCGACTA
TTCGCCAAGTCTCCTTGAAACTTGAGCCTCGACTGAAATCTGGGAACTGAT
TTTTTATGACAATATACCCAAATAAAAGTGCTATGGCTGATATTGGTACTTGC
TATAAGATGTTAACCAGGTAAGTTTGCAACGGTACGAAAGGTGATAGGATAT
GCTGACTAGTAGAAAACACCAACGCCAACCGATAGTGTCAGCTATTGAGCC
ACCAAATGAAGCACCACATATTGCACCGAACCCAAAGATGCCGTTTTGCAT
CGCTTGGTACATTCCACGCCGCCTGAATGGAATCATATCCGAGTTGACGATA

GTAGCTAAGATCTAATCAGCAGATACGACACACAGTAATCTAGGTGACCTG
CCTACCCATCGTCATAAGTCCTCCACCGCCAAAGCCGGCCAAAGCTCTCAT
ACAGTTGAGAAATATTATATCGTTAGCGATACCACATCCAAGACATCCTATG
GCAAACGTAATAGTAGAGATGAAGAAGCAGATGCGACGTCCGAAAATATCT
GATACACGTCCGTATAGGGGCTGGAAAGGCCGTGCTTGTGATGAGATATGA
AGTTGTGAGCCAAGACGCAGTGTTGGTCGCATCAAATTCAGAGCTGATCAC
TGCGTATGTAGAAGCGGTAATAGTCCCGTCAAACCCGTAAAGAAAGACATT
GGACATTATCGCTAGCTCAATCCAAGCAAAGTGACGAGGAGCGACCCAGC
CATCCTCTTTTGGTGGGTTCACGCCTATTAGAGGTTGACATTCCCCAGATTC
TATATCGTGGGAATGTTCCCCCAGCACCTGACTGTTTCCTTCGCTGCTATCC
GTCTCTGGGTTGCCCTGGGCTGGAACCAGAGCACTTGGATCTGTATAAGAT
TCATTAGAAGCGGTCATCTTGATAATGTTGACTTGGGCAACTTTGGGTAGTG
TCAAATATTCAAATTTTAGATAGCAGGTAGGCTCAGTGAAAGAAGTGGTGA
CAGAATGTCGGGGCGATACGAAAATTTGTAGCGTTTACCAAACTCGGCGAT
ATACAGTGCAGTACATGTAACTCTTTTATGCAGTACACATACTCAAGAATTG
TAGCGGCAATGAGGAACTCTACCTAGATTGTTGTCAAAGTGACATAAGAAC
AAGGGCAATCCCCGAACTCGGCCTCCTTTTCTAGGAATCGACCTATAATGC
GGAGCCACGGTATCCTTGCATGTCTACTACTACCTAGTAGTTAATGGTATGT
AGGCTAAAATGATGCAATAGTGGATTCTTGAGTTTCTATTCGACTTGACGCG
ATGCAAATTCGTCGTATCTTGGGCCGATATAGCTGTAGCGCATGGAGGTTGC
AGAGACTGGTACTAATCTAGATGCCGGTTGGTCAGTTTCTCTGGCTCATGCC
CGAAATCGTGATTTGGATGGATTGTTAGATGATCGTAGCGACCGATGTGATG
GTATAGGGTACCCAAGACTTGGTGTGGTATATATAGATAGGCTCAAACATCG
CTTCATTCCATGTACCCCGACCCCTATCTTTATCCACTTCAGTTTGGTCAATA
TCGAGCCAACGTCAACTTCATCAATAAACAATTGGAAGGGCTATCTAGGGT
TCTTGTATTCAACCAAGGTTAATGTACCCTCCTTTAACATTGAATGATGGAG
TCGACTTCTAACGCAGCCAGTATCACCCCTTTCCTCCAACCGCCATGAAATC
CTCCTTGTTATTAGGGGTGGCCTTGGGTCTACCACGCCCAATCGTAGCAGAT
TGGCAATTCAAATCCCGCTCAGACTTGGCACCACCAAAATTAAATATCACTA
TCCCAGCGACCGAGGATGTCGAAAAGGGGTACCTGTTTGTTGCTCCTTTGC
AGGCTTCCCTGACACTCCTACGGAGCAACACGGCCCTAGGCAGGCTGGTC
CATATATCTTCCGCGATAATGGAGACCTCGTCTGGTCTGGTTACGGTATCTAT
AGCATTTGGTCAACCAACTTCCAAGCAGGCCGCTGGCGGGGCAAAGACAT
CCTTTTTCAGCTTTGAAGGCGACCACAATCCAGGATATGGTCATGGACATG
GTCATATCACCATAATGGACAATCATTATGAGACGACTCGAGAACTTAGAGC
GGGAAACCATAAGCTTGTAGATAAGCATGAGTTCCACATCATCAACGAGGA
AACTGGTCTTATCCAGATTTACCAGCCAGTCGCTCGTGACCTTACGGCGTG
GGCGGGAGTGCTGAGCAACAGTGGATAGTAAATTCTATCTTCCAAGGTAAC

GCGTCTTCAAATAAATGCCCACTTGCCAAGGGATTTACTAATCAAGGCTTA
AGAACTCGATATTGCGACTGGCAAGTTGCTGTTTGAGTGGGCAAGTCTGGA
TCATGTTTCACCTGACGGTATGACCTCCCGAAGCTCAGCCCGATCCTGCCA
AGGCTGCGGAGCTACTCAAGTCTCTGCCCGATATCAGTAAAGGGCTGCTCC
AGCGGGTCAATCGGATAGTTGCTGCGGAAGAGAACGAAAAGCGATGAGGA
CGAACGCGGGTGGAAGTGCGGTATCTGCCTGGAGGGCATGCAGGAAGGCG
ACGACGACAACATTGGTATCAAGGGGCTTCCGTGCAATCACCTTTTCCACG
CGAAGTGCATCGAGCCGTGGTTCGCCACAAAACATACTTGTCCGACTTGTC
GCCTCGATCTTGACCCGCTCAAGACTCTGACTGGAAGCGATGAATCCAGAC
GGAACCCTCTTCGTCCGAGTCAAACGGGAGCCGACCGTCAGGACAACAGT
CGTCCGTACGACAGACCTGCGAGTGCGGCCCGCCCAACGGCGCCCGATCT
GGATGCTTCCTTCACCGTGATCTGGCGGATGGTTACCCCACAGACACAGTC
AAACATTGGCGCGGTTGAGGCAACGCCTGTGGCGCCTCAGACAGCATCTG
CAGAGCAAGGGAACCTCAACGCCAATACCCTCTTCATCGCCTTTTCCTCAG
CCGACACCGACTTCCACTCCTCCATCTCTCCCGCGACCCATGTTTGGATCGT
TCATGCACACCTCACCGATTCCCATGGAGAGAAGCATCAGCGCTCCTCCCC
CACCTTTGGTTTCTGACTCCCCATCTCCCGGCCCACTCGACCCTGCTCGGC
CGGTTCCAGAGCGTCGCCAACACATAACTGTCATCAGGCAAGGCGGTCCA
GCGCAAGTCATGCATGACGGCACGCAGTTGCATCCCTCCGCCAACCCTCTA
CATCACCACCACCACATTGTGACGCATCATCACCACCATCATCACCATCACC
ATCATGCAGTTCAGCAGCCATCTCGCACGCCAACAGCCACCCAACCTCCCC
CACCGACCCTTCCGGCCGAGCAGCCAACGGTACACTTCTTCTTCAACCACG
GTCCGGGCTCGAACACCCCGCAAGTGCATCCCGACCCGGCCACAGAGCCA
AAGAAGTCTAACTTTGTACCGCAAACGCTTGAAAGCTGGGCCGAGGTTCG
AGAGAAAGCGCTTGGGTGGAGATGCGATGCTCCCGAATGTGGTCTGGCGC
CTTCCGACGATTGGCACGAGGACGCCGACATGACGGCATCAGACGGGAAC
AAGCATAACAACGAGATGTTGTCCATCTACGCGCCCTCGCAGCCGGCACAT
CATGAGGCTACCACCGGAAAGGAATTCATGCGTCAAGCCTGTCCACATCGC
TGGCATCGCTCTTGTTTGGAGACGCTCGAGCGCGTGTCAGGACGCACGGA
CCTTGCAGGGGATGAGCAGGGTCGACTCGCCGTCCGCTGTGCTTTGTGTAG
GAAAGACGGATGGGTCATGGCAGCGCCACCAACCTCCGCTTAGGACACTA
TTACCATGTAGCCCGTATTCATCATTTCTGTAGCCACCCATACGGTACCTTGC
ATTTGCCGATACCCGGAATACTTATGAATCTGCATTTGCATCATCTGCCAGA
AAAGAGCACTCAGCGTCGTATGTAATGTTCAAGCAGGATGTGAAGTTCCTC
GTAGACGCACTTGCAGTCGAAGCCCGTAATCTCGCCGATCAGAGGCGAGAT
CGGATGGAGGTTGGCCGGAAGCAGCGCTCGACCGTCGTATGCTACTCGAC
ATGGATGCAGATGGTGTATTTGGGCGCAGCTCCGGCGGCTGCCAACGCGGG
GGTCGGAGCACAATGCGAAACCCACCTGCAGTACTCGCTGGGCGTCCGTG

AGGTGGGCTCGGCTCTTGACTTGGGTATCGGGGTGACAGGGGCTGCCGGT
ACTGTAAGAGGATTGGTCTGCAGATGGCGAGTAGTCCACGTCACAGTAATA
CCAGTCGTCAGAGCCTGACCACCGCCAAGATATGGCCTTCTTGTTTCAATA
CGATGACGAAGGGGGTCCTCGTCTGTCAAGTAGTCTGTGCTGACAGTGCTG
TCGCTGTAGTCGCTGAAGATCTCACCCAAACTCGGGCTACAATACGGGCAT
GGACCGACCACGAGCAGATCGTCAGGCTGATCCGCCTCGTCTTCTTCGTAG
TCGAGACGCTGTACGGGCCAATGGAGGGCCGTGGCCCAGGGGACGCCGGA
TTCATCCTGGAGCGCCTGGATGCGGTCGTACAGCTCTCTCGATTCCGGCGT
CCACGCCTCTGCGTTCTCGCCGGTCGGAACGTCAACACTGCTTGGTAGAGG
CTCGAGCCGCTGTGTGAGAGAACGGAGATCAACGACGATCTCTTCGAAGG
CATGCCAGTCGTGTTCGTGAGATGGATCGGGCGCCGCGGGCTGGGGGTTCC
TCAAAGACCTGAGAGGGCGCTACGCTGCCGACGACAGTCTCGTCTTCGCT
ACCCCAATGCAGGCTGGACGGCATTGCTCACTTTGAAGGCTGACGCTTAAC
GGCGGGCACTTGAGATGCAGACAATTTATAGGGGTTGAGCCGTCTCTTTTG
GAAGGCTACCCTAAAACCCTTTGTGAGTTCTATGTGTTGGTTTGGCGAGGA
TCGCGAGCCGCAGGGAGTGTGCAAATCGTGTTCCCTCCCTTGTTCTATATAT
CGATTTGGTGCTAAGACCTGCATAAATGATTTCCAACTATACTTTTGAAGTAT
CCCCCATTGTTTTTCGCCCGCAGTTGGAAGACACTGTTGGTAGAGGGGAGA
AGATCCCATCCCGCCCACGGAGACACCGATAGGGCACCCTTGGAAGATCTG
GCGTATTTTTATGCGCAAAGTACGAGCTATCGCCGCACGTTGGGGATGTGTA
AGCGACCTCGACTCTCGTTCACATGCTGTATACGTATGTTCAGCGGTACAGA
CAAATGCATGATGGTAGATTGCCTAGATGAACACAGTGGTGAGCAGCATCA
GCGGTATGAGTGGTAAGAGAGCTGAGAGCCGGATGCAGCCGAGCTATCGG
GATACTCATACGCTGCGCCTGGAAATTGCCCTCCAGCGTAGGGCACGGTTC
TCACCGAGGGCCTGTTTGGGTCAAAACGCTGACGAAGCCTTGTCTTGGCTT
CTTCGTCTTCTTCGTATTGGTCTTCAGCGATCGAAGCAGGCGGGAGTGCTC
GACCTAGGACGATGGCGACTGCCGGGACTGTGCGCGCTGGGCGGAGCAGC
TGTGTAGTCTGTAGACCGAACACGCGATACCGACCCCCCTGACGGATGTCT
GTGACGATGCCTGCTCGTGGGTGACGTTGGATCAACGTCTCTGCTTCCCGC
TGTGCGCTGCTCGAGTGCTGCGTAGCTATCGCCTCCCGCGGGGAGAGGAG
GTCTGTGCACAGACCGATGCCGCGTGGGAGACTGTCTGGGAGTACTGGTT
GCAGCTGAAGACTTCTCTCGAGGTGTGCGAGCCTTCGCGCCGACTTGGTC
GGCGACTTGGATTCCGTATCGGCGAGGGAGCAGTTAAGTCTCTGTGGAGA
GAGTAGGCACTTTGATGGGCGAAGCGAGCCTGCCCCTGCCCTTTTCAGTAG
TAGAATGGCCGGCAGTCTTCTGACTCCGCGATCGACCTCGCGTGTGGTAGG
CGGCTTCGGATCCTCGGGATCGGTGTCGAAGTCGATGGGGTAGGACTTACC
GTCATCGGACGAGAGTCATTGTCGATACGCGGCTCTCGCTTGCATACAATG
CGTTTGGACCCACTTGATTGGCTGTCCACGGCCCATGCGATCTGGTACTTCT

TGAAGGGACTCAGTGACAGTACGATCAGCTAACATGTCGCTAACGTCATAT
GTCGTTCCACGACTTGCGTAAGCTGCGCGGAGAAGCAGACCTAGAAATTC
CAGCTCATCGGACTGTGGTTTCCATATGCCCCATTCTCCTGGTTCTGTTATAG
TGTGCTGGGCCTTAAGCGCAGAGTATCTCATCTGCATTCCGTTTATCCCGTC
GAGGACTATACCGTTCGGGAGTTCGATCTTGGATCGAGCCAGAGCTTCTCC
ATCCAACTTGTCGTCAGCATCAGGAAGATTTCCTGGTAGCGTGAACTGAGA
CCTTCGTTCGGATCGTCGATCCTTATGGTGTTCTTTGACCATGACAGCACAA
TGCTGATGAGTTGAGCGCTGTCTGCAAGAGGTGCTGCCGCCCTTCTTCTTG
TACTGTTGTGCCCAAAGGTGTGCGACACGCGCAGCTCCGAGCTTTTACAGA
TATGTCTTGGCGGTTCAGTGCACCTCCATTGCCCACGGTGTGAGTGTCAAG
GGAACATACCTTACACCTGAAGCAGAGAAGGCGTCAGTGATATCACATCGG
CAGTCATGTCTTGCGCGAAGCAAGATATGAAATTGGCTCAAACCTGTACCG
AGTGGGACAGGAAAGGAGCGCCTCCTGAATGTATCAGTGTTCGAATAACC
AAAGGATAAAGAAAGGATTCCCGTTGCGCTTGAGGATGATCACTCGATGCC
AAAGTCAGTAGCCTTCCCATCGCCGATAAACCGAGCGCTTGACGAGGAGG
CTCGGTCCGACATCGGGCGGCATGCGGATGGTCTGCGAGGGGTGTCCCGA
AGGCGCGTTTGACGCACAGGGGTCCACTCGCCACGAGCAGGAAGCACTGT
CGACAGCTAAAAGGGTTTGTTCGACACTTGTGATGCGCGACTTAGAAGATC
GACCATTCCACTCAGGTGCAGTACGCTACTCGTAGCGTCACCGTTCGGGTC
CACCTCCACCTCAGATAACCAACAAAGCAACCTACTCGTAATCTCTTCGTC
GACTTACTCTCCTCCCTCACAACACGACAAGGATTGCAGCTACGATGCGGT
CATTCCTGGTCGTGTCCACACTCGCAGTGGCGCTCTCGCGTGCATTGCAGC
TTCCTCACATTCCCTCTGCCCAAGACGCTATCAACACTGCCGAGGCCCTTCT
GAACGCCCACTCGTGGCCAGTGTCGGATACCGTCCTCAGCCCTGCGTCCGA
CTTCACGCTCGCCAGTATCCCAGGCGACGGCCATGTGACCATTACGAGCGC
CCACTATCCCGTAGGTTCTCCTGATCCTGCTGTCACTTTGGTCCCTGCTAAC
CAAAGTAGCAACATCGGATCAGGATCAAGTCAACCGAGGGGTGGTGTGAC
CCGACTGTGCGGTCATACTCTGGCTATCTCGATGTCGGTCATGGCAAGGAC
CTATTCTTCTACTTCTTCGAGAGCAGAGATAAGCCCTCCGAGGATCCCGTGG
TCATGTGGATCAACGGTGGACCGGGATGCTCCTCCTCACTTGGCTTGTTTAT
GGAGTTGGGTAGGTTATAAGCCTACCCTTGCGAACCGTTACTGACGCTTAG
GTCCTGCTCGGTGAAGGACGACCCCAAGTCAGCCAATGACACCAAGTTTA
ATCCTCACGCCTGGAACTCGAATGTGAGCATGAGCTTTAGCCCGTTCCACA
ATCTGACGATAGGCAAACATCTTCTTTGTGGACGAGCCGATCAACGTGGGC
TTCAGCAAAGCGCAGCATGGTCAGATAGTCTCGACGACGGAACAGGCAGC
TACAGATATCCAGGCTTTTGTCGCAATGGTTTGTATATATATACCTTATTATTC
CTTGGCTAACATTCAGTTCTTCGATGCCTTCAAGGAGTTCGAGGGAGGGAG
TTTCACATGCCGGGGAAAGCTATGGCGTCAGGGTCAACCTCTATCTGTGTG

TCTCAAGTGGCTGACGATGACAGGGCCGATATCTGCCTGTCTTCGCCTCGG
CCGTCGTAGATGGCAACAAGGCTCTGAAAGCCAGCGGCCGAACGCCGATC
AACCTGAAAAGTGTCATGATCGGCAATGGCGCCACTGATCAAGTTGGTCTG
ATGTGCTGTCCCGTGATTATGACTGAAGCCGGCAGAGACTCTGATCGAGTC
ATACTATCCCTACGTAAGCACCCGTCCCCTATAGCTCTAGTAGTTGATCCTCA
CACTCCAGACTTGCACCATTCAAGGGGGTCTCAACGAAACCGTCCAAACG
ATTGGGGCTTGCGCCGAGATGGCACTCGCCGTGCCCAAGTGTCACAAGTTC
TTCCGAGCCGGCTGTGTCGACACAAATGACCAGACGACATGCGACATGGC
GGTGGCATACTGCGAGCAGGTGCTTGGAGACTCATTCCTATCGGCTGGTGT
CAATCCGTGAGCACACAAAACCGTGGGAATCGCCGTACTAAGTTCAGATAT
GATGTGAGCAAGCCTTGTACACTTCAGGAACTGGCAGACTCTCTCTGCTAT
CCTGAGACGTAAGGGCCCAACATCGAGTGCTCACGAAGCACACTGACTGG
ATAGAGACAAGAAATCAAGACGTACCTCGATCTGCCTGATGTCCGAGGAGT
TCTTGGCGTGAACGACAAGGCACCCTGGGCGGCCTGCGACAACGGCGTTG
GAGTGGCTTTCGCGATGACCCAGGATTCGTATCACGGGCAGACGATCTATT
ACGTTCGAGGACTGCTGGAACGCGGCATCCGAGTTCTCAACTACGCCGGG
TAAGCGCAGCTGGCCATACATCGTGGTCACGAGCATGGTCTGACGGCAGCA
CTTTCGACTTCATCTGCAATCACATCGGGAACGAAGTGTGGACGCATCGCT
TAGAGTGGACTGGTCAAGCGGGTTTCGTCGAAGCAAAATGGCAAGATTGG
GAGGTCGATGGCAAAGTTGCCGGATCTTACAAGACTTTCGGCAAACTATCG
GTGGGTTTGAAGACTCATGTCGCCGGAAGAACGCGTTTCCTGACTGCTAGC
TGCTCAAGATTCTCTACGCTGGCCACATGGTGCCGTTTGACAAGCCAAAGG
AGGCACTGCAGATGCTGAACAGCTGGATCAAGGCAGGTCTCTAAGGCTTG
TGCATACACATAGCCCATGCACTTCATGTCAGAAGAGTGCTTCGTCTCACTG
ATCCTACACCTGTAGCACTGATAGAGAATATGACCTTCCGACCTTATGGGCG
ACATGTTTCATGCGAAAGCGGTCTTGTTTGCCCCTTGCAACCCGAAGACGT
TGCACTTTGAGTGTCCGTCGACAACAAAGCCAGCATCAAAAAGACTTCAT
CGCCGTTCGTACATCTCCCCGCAAGCCTGGAGTCGATCTTCTCCTTCCTGAC
GGACCTCCTGGCCAGACCAAGCATCACACCGTCAACCCGAAAATGTCAGA
TCCATCGGGCATGATAGAACAGTCAACGGTGGAAGAGAGGATAGATGAAA
ATATCAAGGGAGAGCCTGCTCTGGGGATGGCCTGGGCCTGCGACATGCACC
CAGATTTTCCTGTGACGTTGTTCGCAACAGAGAGATTCAGATCCCTCAATA
GGAACAGGAAGGAACGCCGAGCAGAGTGGATTCCATGGACTTCACTTATAT
GTGAGGTCAACTTTTATGCCCGGAAGGCTGCGGGCGATGGTCAGGTAGTTA
TGAACCTCGCAGTCACCGCGAGGACAAAACATCAGAACACCAACACACCT
TGGCCAGCATGTTTGACTGCCCTGACATTACAGTCCTAACGTGGGTTCTTG
AGGCCGTCCGGCCACTCAGCTGATGTGTCAGTGCAGACAGGTATTTCGCCG
TGAGTGTCACCGTGCCGGGCCATCTGGAACCTGCAAATGAGCAGATCGCG

AGGTGGTCAACATTGAATGAATGCTATGTACAAGATGTGCTTCATCCTGTTC
ATTTGGACCTCTGCCTTTTCAACAAAGCCACGCCGGAACCAAGGATGACTT
TGCCTCCTGCGGAAGCGGGGTCCAGCACTGCGCCTCAGTATAAGCAACTG
GATCAGGATGACTGGGCCTTGATGTACCTGCTGAGTCGTCGACTGCTGGGC
GTCAAAGACAAGTACGTTTCGCTTACGACACTTCGATGCTGTCTCTGCAAC
AAGCGCAGTATATACTCTCTACGACGGCGCCACAACCTCCTGTGTCTCCAG
GTCCACTAGCCCCCACAGGCATGAAGGTCACGCCTTTTGAGCTTCTTCCAG
GGGAGCAGGGTGCATCGAACCAGATCGATCGAGGCTTCCACTCCCGCGGA
CCAGGGACAGTGACTAATCTCACCAATGATCTGACGCAAGGCACATGCATG
GACCCCAATACGCGGGAAGGGGGCCAAGCCGCTCAGGCGTCAGCCATCGA
GACAGACTTCACGGAGCCGAGTGAAGCATCCACGAGTAGCCACCAGCAGG
CTTCTGGAGAAGGAGAAGGATCACGCACGCATCGCCGGCAACCTCCTACC
GGTCGGTGCTGGTTCTGGAAAGGCAAGGGAAACCGCCAAGTATCCGTCAG
CAATCTCGCATCGACCCTCAGGACTCACGGGGTGCCACGCAGTCCACACA
CATCATTTGTAGTACCAGTTTATGACTTAGGTCTCCACAGCATTACATGACA
ACCCCTTTTGCCCTAAGGAGCCTCTGGACAGAGTCGCTGTGCTACGTATGA
CAGAGCCTGGTTGCGTATGCAGAGTCTCGCGGTGCTGCCACTCAATCCTTT
AAGGATAGCGTGAGACACGCAGCATGATCCTCCTCTGAGTGCACTCCCTTG
AAATTCTTGCTCAGAAAGGGTCTCAGAGCTTTTGCAGGAAGATACTTAATG
TGCGCCTTGCTGCGGGCCTCTCTCTGTTTCCCCTATCAAAGACCGCATCCTG
AGCTTATCTCCCTGCAGTGTACGCGAAGGGGTTCACTACTCTCAATGTCAC
ACGCAACGACCTCGACGGGGGATGAACGCTGGGAGATATCTCAGTCCCCC
TCCACGATACGCCTGGGGCGCCAATGGACTTGCCCAAGTCATGGTATTACG
CGGGCGGCAATCAGCATACCGCGCACAGTGAAGCTAAACGGTCTCTCCTAC
TCCTTTCCCTTGGCAAGAGCAGTGCACACTATCCGCTTTGCGTGCCCTCAA
CTCGGCTGCCAATGGGAGGGCAGGCAAACTTACAACCCATTTTTCGAGAG
GCGCGATGGGCTAACCAGCAAGAGGGTCGCGGACTTCCTTTTCGGACCAA
GCTGTGAGCCACTAGAGGAATGGGAAGAGAAAGCGGGGAGAGACAAAGG
GTATCGTTTCTTCCCCGGCTCGGTATTCAGACGCGCGACCGAGGAGCCTTT
GCAGTATATATTAAGCGTTTTGGCTCACCCTCAGTCGGAGCAGATTGATGAG
TGGCTTGGCCACTATCCGATAGATGCTCAGATGACCGCTGACGCTGGTCAG
ACCACCTTGGGCTCTGTCGGACAGCAGATACTCTCAGTGAGGGCGAGACT
AGAGGCTATATATCGAATTGGTTCTCACGTGTTACTATCCGCCACTATGACTG
GCCCAGTGGAACTCTATGATGTCTGCGGAGCAAGCTGCGTTTGCAGGAGA
GGAGGACGAGCCAGAGGCACATAGCGGAAGGCACGGGAGGAGATGTTCC
ATGGACAGCGCAAGCTCAGGGAGTGGTGTTTCTCACGAGCTCGGCACCCC
CAGAACGCCTGCCGCACGATATGGAGAGCTACCGTCAAGCAATGTATCGCA
AATCCGCGATGGGCCTAGAGGTTATGACGATGGAGCAGCTCTCCGGGAGCC

TCAATGAGAACTCAGATCCGTACAAGCAGGTGTTCTTCCATGACAACGCCG
GCAATTCTGGTGATGAGGTTGTGATGGTGGACGGAGTACTTGAAGGTCCGG
AAGAGAGGGAACGCCGTGCGCAAACCCGAGATCTGAGCAGTAGAATTCGC
CAAGGAACTTACCCGCTACAACTCACGGGCGATTCTCCCTCCGTCAGCCCA
ATTTTGGCTTTCTGGCAACTGGGTACAGTCATAGACCCCCAAGCCCCTTCT
GGTGCTGGGGTTGACATCACTGCCGGTTCAGCTCAAGATCAGCCGGCTACT
GAGTGGCAGAAACGGCGAAAGAGAGACTGGCTCAAGGAGAGATATGACA
AGGCCCGCTCCCATTGTTCCCCTAACTGAACTCTTAACTTGTTTCAGTGACT
TGATAATCTAGCTGTGTATGCATTTCCGATCAGTCACAATTTTCGCCACATTC
GCCAAGTGCCACATTGATTCCTGCGCGTCGACGACATCGCTGTCTTTTTTG
CCCAAGCGAGTTGAGTTGATATAATCGCAGTGCGTCCGGTAGCATCGGAAC
AGGAGCTCAAAGACGCGCTCTTTAATCTACATCTTGACATCTAGTACACGA
GGACACACCAAGGGAGACCGGCAAAGATGGGCGCAGACGAGATTCACAG
CTTGTACGACACGGTCCTCATTCTCGACTTTGGGTCGCAGGTAGGCGTCTT
GGCGTCAAGTTCGAAAGAGTCAAAGCTCACAAGATAGTACTCGCATCTGAT
CACACGAAGATGTCGAGAGCTGAACGTGAGGACGCACCTCTGGACTTGCG
AGACACTCCTCTGACCCTCAGGTCTACTGCGAGATGCTGCCGTGCACCCAG
AAGATGGCAGACCTCAAGTGGAAGCCCAAAGGTTGGTCGGCAGCAACGG
CTTGCCTGTCCGGGAGCTGACCTCCAGCTATCATTCTCTCCGGCTCCCGTAC
TCGGTTTACGCTGCCGATGCTCCTCACGTCGATCCAGCTGTCTTCGAGGCG
GGCGTCCCGGTCATGGGTATCTGCTATGGTCTCAGGAGATCGCCCGGACTC
ATGGTGGCAACGTCGAGGCTCACACGCACCGCGAGTATGGCTATGCCAAG
ATCAAGGTGGAGAAGACGGGACACGAGCATGCGGACAAGCTATTCGATGG
CATCGAGATGGAAGAGACGGCGTCTGCAGGCATGTTTCCACCGGTATCCCC
GAGTCCGCACAGCTCATCCCGGTTAGGTCTGGATGTCGCACCGGTGACCAG
CTCACCTCCCTCCCTCCCCACTTCATCACCATCGCCTCGACACCCACATCCC
CTTACACCGCAATCGCCCACGAGTCCAAGAGATCTACGGCGTCCAGTTCCA
CCCGGAAGTATCACACAGCCCGAAAGGCAAGGAGGTCATTGCTGGCTTCA
TCAAGAATGTGTGCGGTATCAAAGGCGGCTGGAGCATGGTGAGACCTCGAT
CCCACTCAATGCCCGTATGTGCTCACATTAGGACTCGTTCATCCCCAAAGA
GATTGCTCGTATCAGGGAGATCTGCGGCGAGAAGGGTCAGGTCATTGGCGC
AGTCAGCGGGGGCGTTGATTCGACCGTCGCCGCCAAGCTCATGCACGAGG
CAATCGGAGACCGGTGCGCTCCTCACTTGCTCCTCCCGGCATCCCGCTGAT
CTGCCAGATTTCACGCCATCATGGTGGACAACGGTGTCCTACGGCAAGACG
AGGGCTTGAAGGTGCACAAATGCTTACGGAGGATCTCGGCGTCAACCTTAC
CGTCGTGGACGCATCCGAGCTGTTCCTGTCGCGATTGAAGGGTGTGGAGG
ACCCGAGAGGAAGCGAAAGATCATCGGCAACACATTCATTGAGGTTTTCG
AGGCGGAAGCGCTCAAGTTGGAAGCGGCTGCGGAGAAGGAGGTCGAGGA

GAGGGCGGCGAGGCCAAGGCAAGATTGAGTGGCTGCTGCAAGGAACCCT
CTACCCGGATGTCATCGAGTCGATCAGCTTCAAGGGACCGAGTGCGACTAT
CAAGACGCATCACAACGTTGGCGGGCTGCTTGAGGACATGAAGCTCAAGC
TCATCGAGCCGCTCCGGGAGTTGTTCAAAGGTGAGAATCTCTCCACGTCTC
ATCACGCCAGCTTATATCCAGATGAGGTCAGGCACTGGGTCGCCTTCTTGG
CATTGCACCTCATCTCGTGGGACGTCACCCTTTCCCCGGTCCTGGTCTGGC
CATCCGCATTCTTGGCGAGGTTACTCGAGAGCAGGTCAAAATCCTGCAACA
TGCCGATGACATTTACATCGAGGAGGTTCGCGCAGCAGGCCTGTACGACCA
AATCTCTCAGGCATTCGTCGCCCTTCTTCCCGTCAAAGCAGTCGGTGTCGC
GGGAGATGCGAGGACATACGAGCAGGTGGTGGCATTGCGGGCAGTGTCGA
CCGAGGACTTCATGACTGCGGACTGGTTCGTGTTCCGCCGCAGGTATTGAA
GCGGATCTCGTCGCGGATAACGAACGAGGTGAGCTGCATCGACCTGACTTG
AAGATTCAGCTGACAGGGTAGGTCAAGGGTGTAAACCGGGTCGTGTACGA
CATTACGTCGAAGCCTCCTGGAACGTGAGTTTTTGTCGTCGTATCTGAATTT
GTGGCTGACGAGAAGTGTGGAGTGGCTATAGTGTGTGTTCGGGCATAGATG
CATCTAGATCTGTTTGTATGACGAACCGTGCCTCAGCGCCTCACACACTAAC
CCAGGATGCCCACGCATGGAGCCTCTCTCCCCTTCCACCTCACTCATATACG
CAAGATGCTTCGAGCTTCAGGAGATACTGGTCCGGGACGCGATCGTAGTCT
GGTCAGTCTCAAAGGGCAAGACAAGCAATGGAGCATCAGCGCACACATCT
TGTCGGTGCACAGGTACGGATCTCGTCAGCCACACTGGCAGAGTCGACTA
ACGGTCCAGCGCAGTCTTCAAGGATACATTCGAGCTGGAACACGGGGCAG
TGACGGACGGCGTGGAGGGGCTTGTCATCGAAACAGGGGCGAGCGACGA
GGATCTGCATCACCTCATCGACATGCTGCTCGGGGGGAACAATGGCGCAAC
CTCGATGCGAAGGCGTACTTCTCTCTCATCGAGCTGTGCGACAAATGGGGG
TTCGAGGCGCTAGTCGAGCGCGTTGTCCTGCGGCTCTTCCCACACGCCGCT
TTCGATCCGTGGAGCACTTTCTGCATCGCTGCAAAGTACGACGTCGCGCAG
CTCGCCAAGGCGGCGATCGCTGAATTCGGTCGCAATCCCAAGACGAACAG
GCTGAACCTGAGACGTTCCCGACGGCCATGGGTCGTGAAGTGCCGACCAA
TACCTCCTGGCGTTGGTACGGGCGATGGCGCGTAAAAATGCCTGTGCAGCT
TGACCGTGCGGGGCTGGATTGGGACTGTTCGAATGGAATGGAGAGCGGCT
GGGAGAGGGTCTCGTACGTGTATAACGTTGAGGAAGACGCGAAGTAGACC
CCTCGTCTTCGAGTCTTGCATAATATCGATACATTTTGAACTTGAGCTACATA
CACCAGATCACTGTCAGATTCCGTTTATCGACCATCACGTCTCCCGCCCGGT
AAAGCTCGAGATGTCCTGGCGAGGCTGCCCCCTTTGAGGGGCATAGCATGC
GCCAATCTCAGATACGCCTCGAACGACGTCGACCCATCTTGTCCTCCCTCA
GTTCCTGGGGATAGTCCGCCCGAGCTTGTACGCTGCAGCCTGCCCGTCAGC
CAAGCACGCGTGGAGGAGAGTTCAGCAAGGACTGACCATGCCGCTGATGA
TGGCCACCTCGTGCGCATTGACCATCGTCCACGCCGCGGCGTAGGTGGTAT

GGGGCGACTTGTTGATGTACCGCATGAACGGCACGACGAACGCATAGTGG
GTCCACGAGTGGCATAGCTGGTGCCACCAGTCCTCGCGAATAACCTTGTCC
TTGTTGATTTCATCTTTAGTCCAGAGTGTTTGTGCTCCTTGTTCAGGAAGAT
GGTCTGGAAGACGTGGTCTTCGAGCGGCCGGTTGTTGTCGAGCTGGAACT
GGAAGTTGTTGCAGTCGAAGCACATTTCCTACATGTCAGCTGCGATCCGCT
TATAAAACAGCTCGACGGACCAGCTTGCGGGGGTCTTTCTCCTCGGCAATG
AGGTACATCCTGCTTCTCAGCGAAGGTATGGCTGGTGGGGAAGAGAGACG
CACGGCTTGAATTCGCCCTGGCCCTTCTTGATCCGGCTCGACTCGTCTCGC
CCGCCGAGCGAATGCACTGCCTGGGTGTAGTCAAAGTCGACAGTGTAGTG
CTTGCGAATAAGTCGCGTCCTTCCCGTCAGCCCAACCTTGTATGAAATACCA
ATACGCACATTGTGCGTGACGGTGATGTCGTCTGACCACTTGGTGTTGCCC
AGCACCCATTTCTCCAGTCTTCCCGCCTGCGGCCCCAAGACCCGCTTCGCA
GTATCCGCCAGCACACAGAGCACAATCTCGTCAAACTCCTCGGCGACAGG
GGGAATATCCCTGTCTGCATCATTGGGATTATGCAGATCTTCCTGCTGCCGC
CTAGGGCGGAGGAGGACCTTGACAGTCGGCTCGCGCGACTGGACACTCAA
GAGCTCGGTGTTCAGGCGCACGGACACGCGCCGATCTTCGAGCTGCCGCT
GCCACTTGGTGTAGAACGCGCTCGTCTCGGGGAACACGACCATCGGCGGG
AGGTTTGACGACAGCGATTTCTGGTCGATCGGGTACCACATGCCGTACGTT
GGAGATGTGTATAGGCGCTCGAGCATGATAGTGGGCAGGTTGGGCGTGGCG
TTGCCCGTCCCGAGGAAAAGAGCAAGCCTGCACTCAGCAATGCACAAGGA
ATTTGAGAAGGACGGGAAGCTCACGATGGGTAGATCATGTAGTTGATAAAG
TCTTCGCTGAGGCCCCATAGCTTGAGCGAGATCTTGATTGGTATCACCGCCC
AGAGCATCTCTGTACGTCGGAACCACTTGACGGCCGTTTCGAATCTAGCGA
TATCGCGGGCGTGGCTAGCGACCAGGTTGGTGGGGAAGACGTTGGTCCAG
AACTGCAATCAGCGGAGCACTTAGAGCCATCTATTGAGGATGGATACCTTG
TCGCCCTTGCCAAATGACACCATCAAGTCCACTCTGCCCGTCAGCGCCTCC
TCGTGAGACATCTCCGAGGCCGCTCACGGCTCGGCTGCATAGCCCAACTTG
CGGAAGTGATGAAATGTATGCCTGCTCATCAGCCCCGACTGATCATGACTA
CCATACTCACGCATAGATGTTGCTGCCACCCTGTACACCCTGATTCATCCAC
GGCGCGCCAGCCTTGCTCCCCGGCCCCGACGCGGGAAGGGGATCGAGAAC
GCCTGTCCGCCGCAGTACGCCTGGGCATCTGATATCAGCCCACAGCCTGCT
GCACTCTGATGTTGGCAGCGTACCGATGAGCGTCACCTCGAACCGGTCCGG
GTGTTGCGAGAGCGAGTCGGCACATGCCATGCCCGCCGCCCCTGCACCGA
CAACCAACACTCTCTTTCGCTTGCCTTGTGATGCGGACGACATTTTGCTCTG
ATCAGTTCTCAGTTGACGGGTGGGCATATACTCGATGCATCTGCCATGTATAT
GGATGGACGCCGAGACGCGAGAGAGAGAGAGAGATGATATCATCGTGCCA
TGTCTCAATTGTGCTGCCCGATCGGCGATGCGATGCGATGGGACCGCGACG
GGAGAGATGTCGACAAGATGTCAGCAGCGATGGAGACATCGGGGACCCGC

GATCGTCAGTTTGACGCGTCACGTGATGGAGAAGCAAGGCAAACAAGCGC
TCTTGAGCAGCTATGAGGATGGTGTTGCTTCCAGCAGGCACTCCCTAGGAG
AGAGTTACATGCAGCATCATGTCGTGAATCTTAGCTTCTCGACTGCACGAG
CGATCATCGCGTGACTTGGGCGGCCCCGTTTACGCTAGCGAGAGATTTTGC
TAAGACGTTCCGTGTTGTTTCATCCTTATCACTCCGTCATGCTTCACCCGCG
TGACGATCTCGTTAGCATAGCCGCGGTGCGAGTGCTTTGACGGTCCTGAAG
AGGGCGAGCACAACCTGAAACGGGAGCTGGCGAATACGGTGTCACCGTCC
ATTTGCTGCCATTTGCCGGAGCGGCCATGAACTGAGGCGGTGTCCGTTCTT
CATCTGCCATTTCACTCTCTGCAGCTTGAAGCATACTTCTCCATTCGACCAC
CGCCCAATGCGAAAACAAGATGTTTGCTTCCAGCTGTGACCGAGATGTCCG
TTTCCGATCCGCCGCCGCCTCCGCCGCCCCTTGTCCTATCGGACGGGCATCG
TTCCTACGTTCCTACTTCAGGAAGACCCAGGGGGAGAGTGACCGCCCACT
GCGGATCAAGCGAGCGGGGTCCACCAGGGTCCTCGGCGATGCAAACAGCG
GCGACCACTCACGAGGGCTGACATGATTCCGGCAGCGCTAACGAGAGGCA
AAGGGAGAGGGGCAGAGCGGCCGTACGGCGTCGGATGTGGTGGGAGATG
GATAAGCTTCTAGGCTTGGGCTGGCGGCCTGGGGCATCGGATCGGACAGAC
AGGACAGACCAACAACTTCTTGCTTGCACTGCACTGCTTGGATCAAACATT
CGTCTCCTGCTTCTTCCACTACATTATAGTCTCTCCCCTGCCCCCTCTCCCCC
TCTCGCCTCAACACTCTCTGTCGTCGACGTTAAACGGACAACGCTCGTCGC
CATGAGCTCCTCGCGCAGACTTGCGATCGCCTCGCTCGGCCTGGGCATGGG
TGCCCTCGCCCAATACACCGCAACGTACAGCCCGACAGACATGCCGACGA
GACGCAACAGGGGCAGTACGGGACCAACGACTGCGGCACCACCTCGAGC
CAGTCCAGCAACTGCCAGAACGCATTCATCAACTCTGTGGACGACTTTTGT
CTCTGGGGGCCACCGGCCTCGACATCGCAGTACGGCCCGCCGCTGATCGG
GAACGTCGAGGAGATCGTCGTGAGCTACTGCCTCAGGACGGGTACGGGAC
TCGTATCATACCCTCCGGCACGATCCAGGGGGCGCACTTCGTCAAGGTCAT
GAGCGACGAGGTGTCGTATGTCCAAGTGACGGGCTATGGCGATGTGAGTCC
ATTGCGAATTGCAAGAGGAGGGAGGGACTGATGTGTAGATGACACAGATG
GGATTGGTCGCGGGCGACGAGGGCGGCGAGCTGGATGTAAGTGATCACTG
TCGTGAGAAGTAAAGGCAGATACTGATCGACAGCCTCACTGTGTGTATCAG
TCCTTTGGCTCGGCAGCACCGAGCGGAGAGTGTGGCGCGCAGACAAGGA
GCTGACGGACAGCGTGGACGGGGCTCGGCAATCCGCAAGGCGGAGTAGTA
TTCACCAACGCCTTCACTGGCGCATGGGAGCAGACACACGAGTGGACGTC
CTTCATGTCCGCCAAC

>Mosaic_sequence_82

GACATGGTTGAATGGCGATAGTTTGCAGGCTATAGTTGCTGGAAGGTAAAT
TTACAAGACTCAACTTTCTATTGGGCATACATTCTAATAATTGTCATTTTCCC

AGTGCGCCGATAGCCAACACACTTCTTGGGCTTTTAACTGAGCTTGGCAGA
GCTCCTGTACACTCTCGAAAAATTTACGAGGAGCTCGAAAACATCAATAAT
GTCTCTGATGTAAAAGAACTAGCCTACTGCCTCACCTAAATGCATGCATCAC
GGAAACCTTGCGCATATATCCCGCACTTGTTACGGGCGGCCAACGGAAGTC
TGGAAAGAACAGCGTCACGATCGCGGGCACCGTGATTCCTCCATACACGA
CGATCGTATCGCCTCAGTGGGTAATTGCAAGAAGTATGTGCTGTCTTTATTT
GATCATGCCTAGCTGTCTACCCATTCTCCTATGTATTTATTGAATAAGGCTAA
CCAATTGAGAGAGATATAGGAGAAGACTGTTTCGTTCGGGCCGAAGAGTTC
ATTCCAGAACGCTGGACGACAACACCGGAACTCGTATTAAACCGTGCAGC
CACAGCACCATTCGGTACAGGCAAATACAGCTGTCTAGGGCGACATTTGGC
CCTCGACACCATACGACTCACGGTTGCCCGTCTTGTTCAGAAGTACACATT
CGAATTCGCTCCCGGTGAAGATTGGTAGTGGCATGACCGAAGGGCTGCGTG
ACCACTTCATCACGAACCCAGGCGGTTTGAATTTACAATTTGAGCTGCGTA
AACTGGGAAAAGACGAACGTCCATGTATAGGAGGACTAAAGTAGTCTCGA
CCTATTGATAGGTACTGTAGGTATAGATTTGATAACTAGGATTAATAACCTAG
CAGGCAATTCTCTCTTGCTTATCAGACATCGAAGCAATCCCTACAGGAATG
AACCACCTGTTAAGCCTTCTATGAAGAACCCGTAATAGCTGCATGGCTGGG
GTCGGCCCATAGAGGAGTTCCTTGGCAGAGCATCTCCGAGAGTTTAGATAG
ATAAAGAGGTATTTCCATATATATCGTGTTTCCCGGTGCGAGCTAGCTTAGA
GCTCCTCGTGCAACAATAGAGTGTTTTCCTTGCTTCTAGGTTCGCATCGTGA
TACAACCTAAAAATTATCTTGTAGTTCTCAATTGTACCTAGTCCTGAATGTTG
AATCTTACTCATTCATTCTTGACTAGGGTCCTCACAGTGTAGCCTGCCCCGA
CAGGTTGGATGGATGCGTGAAATGGCAATCAATTGTATTAAGCATTATGAGC
ATCTGTGGCATTTCATGGGCTTGTCCGGTAGTAGACTCAACCCTAACCCGCA
TTGCCTACCTAGGTAGGTACTCAAAAGCATAGGACGTCCAATGGGACGTTG
TTACCTTCATTCATATCACCTCTTATGTTCGGATGATCTCATCCTTTCATGCAT
ATAATTTAAGACTCTCTACTCGATTCATAAGGAATCTGCAATAAGAGGATGT
TGGTGCATCATATGGAGAAAAGTAAACAACACCTTAGTGGCGCAGCCGCTT
TATACTAACCACTGGTCACCTAAGGTGCCAACTGGTAACCTGAGGTCGTAA
CGAAGGTAACCTTGGATGTAATGGAACGCATTCATAAACCAACGTTATCGC
ACTGACCGACTATGTACACGCCTCATTATTCTAAACCGTAAAAATACCTACC
TAAGTAGGTATCTACTTTTAATACATACAACTTGACTAAGAAGAGTTAAGTA
AGGCGTAACACTCACAGAAATGTTACTTTAGAAGACCCGAAAATAGAGGT
GAGTGATTGAGAAGCTCCACATACACAATGAGAGCTTTAGAAATATTCTTG
TCCCGCTTTGAGAACCGACTCAGAGCGTGGGGATCCCCTACCCGGATAACA
CCCAATTGTTTCCAACGATGTCTATAAGGGGTAATAGATAGAAACAGCTATG
CCATAAACACCAAGTTTGCCACGTCCAGCGATCCTCCTTTACGCCTCGCCTT
ACAAGCACCCTCCATATAACCAGGCCGCTGAGTGCTGTCATCGGCAAGCAA

ACCCAGAGCGGCGAACCCCAGAACCAGATGCATATGCAATACCAGAAGCA
CACGAAGAAGGCAATGGACCAACGCGAGAAACGCTCTCCAAGAAACAGG
GCAATGGTCCTACGACCTCGAAGTCGATCCCCCTCTTGGTCCTTCAAATCTT
GTATTTGCATCGTTGTTAAGACCACCCCACTTATGATCGTAATCCATATGCTA
CCTTTGGTGTTGACGTGGGCCTGGCTGCCGATGGCAATACGGAGCGAACCG
AGATTAAACACGCCGTAGCCGACAGCTATAAGCGTTTCCCGGAGAAATATC
TCGTCTCCGCCACCTAGCTCGTTGTACATCCAGATGCCCGTCGCGATGCCTA
ATCCATATTCCCACAGCCCGAGGAGGCGGTGGAAGAATAGGCTGATTGGGA
TAACGACAAGCATAGCACGCCGCGTTTGATCCGGTGTAATTCTGGAGCTTG
GAATAGGCCTCCATGGTTTGTTGGTAGCATCCTCAATAACCGACATTGCAGA
CCGCTGGTTTGCCATGTCGAAGATCAAAAGGTTGCACCAATTGAACGCTAG
AATTAATGGTAGGCGTAATGCGATGTCATGAAAGGACGAAGGAGGAACTCC
TTCATCAGAGGTAGCGAAGGCCATCGATAGTGCTCAAAGAACACCGATGGC
TGTGTTAGGCAGGGCAAAGGTAGCAAAATCGCTCTCAGTGAATCCCAGAG
GACCAATATCAGACTACGAAGCCCCATAGTCCCTTGTCGGGAGGCTTGAAC
GCGTCGTAGGTGAGGCATTTTGAGTAGTCCTCATGTTAGGTAATGAAAATC
ATATAATATAAGGTACTAGGTAGGTAACGATGATAGTATGTTTGATGGTTCCG
AATTTAGCCCTGGAGTGTATAGCGTAGAGCGAGCCTTGAACGTTGTTTTTAT
AACGAAATAATATCCAGTCTTGTTTTAACTTAACCAGCCAGCTAATCATGAC
TCGAAAGCTAATAACAAGCTATTATTCATTTCTAGATATAGACCCCTCAATGC
CCTTTACATTGACATCATATGAGGGAGGTACCTATTAGGTACCTCGTACCTA
GTTATCAGCGTTAATCTCCATCTGCAAAGGTTGGTTGGATACAATTTTGGCT
TCACTAATCATTGCATGTTTCGAAAACTGGACATACAAAGCTAACAATCAA
ATGCATTACTACCTTATGTAGGCCGACATGACCACCAACAATCGCGTTTGAG
GGCTCCAGAGGTTTGTTACGACGACAACCCCAGTGGAAGGTACATAGCTA
ATTATGTAGTGCACGAGACAGAATCCTAACCTGTCGTATACCCAACCTTTGC
GCCCTCATCTTGCAGGTGCTTTAGCTAACGTAGTGTGAACTTAATCAAGGA
GAAGGCTCGGAATGTGACCCTAAATATTGGGCGGATTTTGGCTGGCGAATA
TCCTAAGTGTTAACAGAATAATCAATTGCTCTGGTGCTTCTCGATATCTGTC
GCACGCTTTCCCGTTTCTCGAAAAGCGAGGGAATAGGTATGCTTTACCTCG
TACGAAGGTCAACTTTCGGTTTTTCATTTCAATAAAGATGGCAAATTTCAGC
TCTAGTGGCCTCACACTTTTCATTATTTTCAATTATCTTCACCATCCTGTGCG
GTATCTTCGTGCTACTACGCTTTCTTTCCGCCAAAATATCGAAGCGCTCATTA
TATCCGGACGATGGCTTCATTCTCTTTGCATACGTAAATACTACTGTAGAAG
ACGATTTGACTTTGCAGTAGAAATCCTGGGAGCGCTTGAAATATGGCTAAC
CCGCCTTTTGAGCAGATCAACATGGTGGCCTGGCGGGTGTGGGTATCTGGG
CGAGCGTCAACGGCATCGGAAAGTCTGTCGCTGAGTTGACGCAGGAGGAA
ATTGCCGTGAATGCAAAGGTGCGACAATAACATCGAGAGCCCTAAGATCGC

CCTATTTACTTCAGAACCGATAAAACTGACCTAGCCGAATTAAACAGCTGAT
AATGGCATCGAATGTGTGCTGGCTTCTAGGAAGTGTCTTCGTTAAGCTATCC
ATCCTTTGGCTTTATCATAGACTCTTCGCTACCAAGGAGTTTCGACGGTGGT
CGTGGCCCATGTTGGCACTCGTAAATTGCTACGGCATCGCCTTTCTTGTCGT
CTTCCTTACAAATTGCCACCCCGGATCAACTGTGGAATCCACGACCTAATG
GACATTGTAGAGACATGCAGTATAGCGATTATGCGACTGTCAGCATCAACCT
ATTCCTAGATTTAGCTATCTTGATCCTACCTATGCCAACACTTTGGAGCCTCC
AGCTTCCAGTCAGGAAGAAGGTTATCGTGACTGTAATGTTCAGCTTTGGAT
TCGCGTGAGTATTTACAATTCATGGTATCGGTCTTTACCCCATCCCACATAGT
CGATATTTGGACATTTTATATTAACTTGTATGTACATATATAGGACTATTGTTG
TGATGTTGTACAGGATCATTCTGACTATAAAATTCCGAGGTGATCTTGACTT
CACGAAACACTTGGCTCTTATTGGTCTGGTGAGCGGCTTTGAAGTCTGGTT
TGGTATCATCATTGCCTTGTCTGCCAACATTGGCGCCGCTAGTGAGACTGAA
TATGGGCAAGTTCAGAACTGGCTCCTCTGGCTCCCGACCCTCTGGCTACAG
CGCAACTACAATACCACTGAAAATATTCGGCACCAAAAATCGCACACCGGG
AAGATACGACCAGATTAGTAGTAATAGCCAGGCACGACTCAATAACAACTA
TGAAGGCGCGCAGCAGTTAGACACGGAGCCGGGCGTCACTACGGAGGTCA
CCTATGACCCACGGAATCTGCTAGGGTATCGCCTGACGGACCCTAACAAGA
TATATGTGAGAAGCGAAATAGAGTCCAGGGTCTGATATCGAGGAACTCTTG
ATAATATGGGTCTGTTTGTGTACCTACTTTTCAGTATTATGACTGCTTGAAAT
GAGTGTATTACTTACGTACCTACCCGAATAGTTAATACCTTGTTATATGGATA
GGTAGATCAATAGTGGATCAGCAGTGGATGATTTGCCCCCGCAGATGCGCG
CTACGACTCACAGGGCTTTGAGCGCTGTAATTAATGTAAGCTACAAGGAAT
GCCTCTTGTGCGAGCACTGGAGGTTTAAGCTAGGTACCTAATCAAATGGAT
AGTGATAAAGATCGTAAAGGAACGTAAACAGGTACAATAGTTACTGGTGCC
GTCTACAATCACTCTCGACGGCACCAGTATAGAGGTTTGCAATTGAAAAGC
TACCTAGATAGGTGCCTAGTAGCTAGCACCCGTGCCCAGGTGGACATAACA
CGATTTTCTTATAGGGAAGTATGCGCTATAATAGTTACCTAATTGGTCGGCGC
ATCAGTTGGTGGAAACCCCTTGTCTGCAACCATCTTTTCTTCATGTCCACCC
CCACCCACTGATATCAGAACAGGGTAGATGTGGTTACTTCTGTTGGTTACCG
GAGTCATTCCCAATTTCGGATGGTATGTTAATAATACCCGGAAGAGTGTTTG
TAAATTCTTAATCTAAGCTGACTACTAATATCCCTTTCTATATTCGTATAGCTA
CGCCTGTCATTATGTAGGTCTAGGGAGCTCTATGTATTAGTTAACGAGCAAG
GCGCTTCAAAGTATTTTACCTACCAATAGATCAACCAACATGGTTATTCACG
TCAGATTCAGATTAGCAGTCCGTCCCATAAATTGATAACCGGCTGTAATAGA
ATTTATCTTGTTATACAGTTGTACAACCATACAGCACCCAGCCCATCATGGC
CATTGCCCTATTTCCTCTCATTGCCGCTACTCTTAGCATAGCACTTAATATGA
TACGGCAGCCAGCAGTACTCTTCCCGAATCGGTACGTTATTCGGGTGAAAC

GAGCAATTCGAGTGACTGCCGATGCTTCCCTGGCGATGCGTGTTGGCCGGG
AAGCACCGAATGGGCAGCCTTTAACCAGACCGTAGGCGGCCGACTTATAGC
GACCGTGCCCATAGCCTCCGTCTGCCACTACGATAATCTCACAGCGTATGAC
CCTGTTGCCTGCGCCGACCTTCAGGCTGTCTGGAACTACCCACAGACACAC
TACGTGACGTCTTCCTCTCCCATGGCACCTTTTTTTGCTAATGCAAGCTGTG
ACCCCTTCACAACTCCTGCCGATCAATGCGTGGTCGGAGCCTATGTTCAATA
CGCCGTCAAAGCGAGGTGTATCGAAGATTACAAGGCGACTATAGCCTTTGC
GTCAAAGAATAATATCAGATTAGTTATCCGGAATACTGGTCACGACTACTTC
GGCAAGTCGACGGGGCCGGAGGGTTGGCACTGTGGACGCATCATCTCAAG
GATATCGAGTTTATCGCGAAGTACATGTCGCCATCATACGTCGGCAGAGCGT
TCAAGTTCGGCGCGGGTGTTCAAATATTTGAAGCCTACAGGGCTGCTAATG
CTGCCGGAGTGACGGTTGCCGGCGGAGGTTGTGACACGGTCGGCCTTGTG
GGTGGGTTCACTCAGGGCGGCGGACACGGCCCCTTGAACTCCCGAGTAGG
CCTCGGAGCCGACCAAGTACTCGAGTGGGAGGTTATTACCGCTGCTGGAG
AGCACTTAATTGCAACCCCTACAGTAAATGACGACTTATATTGGGCATTGTC
TGGCGGCGGTGGCGGTACCTATGCGGCCGTTCTGTCTGTGACATTAAGGGC
TCACGAGGACTTGAAAGTTTCAGGTGCCAATCTCAACTTTACCAACATAGA
AGGGTTCGACGACGTATTCTATAGTGTCGTAAAGACGTTTCTCATGTCCCTT
CCAGCGCTAGCTGACGCAGGAAGTTGGAGCAGTTGGCAACTTGCCCAAGA
CTTTTTCATATTACAGCCTCTTTTCGCTCCCGGCTTAGATAAGGAATCCCTTC
AGCATCTACTTAATCCCACTCTTGCCTCTCTGAACGAGAGTGGCATTCCATA
CGGTAAGGTTTTAGTCACTGGCGGGTCAAATCAACATACGCACGTGCTGAC
ATTAGATCAAGAGGCTAATGAGTAGAAATAGACTATCAGATCTACGAGTTTG
ATACATTTTACGGTGGTTTTAAAACACCTTGTTCCCTTTAATAACATCAGTG
GGTATAACTTTTGGAGGTCGATTAATTCCTCGTTCGCTAGTCGAGACTAGTA
GCTCGGCCACTCGACTGGTGGATGTGCTGAAATTCATCGCCGACAACGGTG
GCGGCATTGGTGGAATCATTTCAACGTCTCAAACTTTCCCACTGGGGGAAT
ACAGAACTCTATCAATCCAATCTGGCGAACATCCATCTTCAACGCTGTATTA
GGCTTGTAAGCTGTTGCCGCCTACCTATTATTTTACTTTTTATATTCCTTTGAA
CCCCTTATTCTATATATGCATCGTCTAACAACTGAGAACAATAGGCCGTATAA
TTACGAGAACTTTACAGCCAACTTTGATATACAACAAAAGATGACCGATATG
CTGATGCCAGCGTTAGAGGCTGTAACACCAGGTGGCGGTTCGTATCTCAAC
GAGGCTGATTTTAGACAGCAGGACTGGCAGACAGCATTCTATGGAGCGAAT
TATGAGGCTTTACAAATCATAAAAAGAGAAGTACGATCCTTACGATCTGTTT
TACGCACTTACGGCTGTGGGAGTGATAAATGGGTGATTCGACCAGATGGGC
GTCTCTGCCGAAGCCATAATACTAGGAGGTAGATACCCCTAGACTCGTCAAT
GAAATGATTTCATCTGGGACCAATTCAGTGCAAATATCTAGCGACCAAGGC
AACTTTCTCCATCCCGAGCATATCATTCCATGAGCGAAAGTTGCAAGTTGCA

TATTAACATAGGTAGGTACGTAGGTAGGTACGTAGGTAGGTACCTACCTAGT
ATGTAGGTAGATTGACCTATTTCTTGCCAAAGAAGCTTCTCTGTGCTTGGAA
CACAACCTGCGGGGACATTCCGACAATACTTTCGTAGATCTTGGAATCAGT
TGCTCCCTCTAGGCTGAATATCAATACGTGGCTATCGTTGTCTATACCAAGTT
TTTCTCTGAGAGGCTCGTCGACTCTAGTCTTGAGCAAGAGACCATGTTTGC
AGCTGACGATTCACCACAGACAACTGGAATATCGCCTTGGTAACCTGATGC
AAGCGCCCTCATTCCTTCAACAGCGACGGAGTCGGGCACAGAGACAAAGT
CGCTGGCGAGCCAAGACAGAATTTTCCACGCTCCTGGAGAGGGCATACGG
CAAGCTAGGCCAGCCATGAACGTGTTGAGGGTTCCCGCCGACGGTGTTGG
CTCACCTTTTGAGTGCTTTGAAAAAGGCAATCAGCTTCCTTAGGCTCTACC
ACGATGAAGCGAGGGAGTTCGCAAGTTCTATGAAGCTCGCGGAGACGATG
GGTGATTCCAAGGAAGACTGCCGCTGCGATACTTCCCACTCCGCCGCACAC
CAAGACATGTGTAATGTCCGTTATTCGTGAGACCATGTTCACAGCTTCGTCA
ACAACAACCATATAAGCATTCATGACGTTTTGGGGGTGCCATGGTCAAAAA
TCGTCCCAAGACGTGCTGCTAACAAAATACCATCCATTCATACGCGCATCCT
CCTTAGCTCGCTCCACTGATAATTCGTACTCTCCATCAACACGAATGACGGT
GGCACCCAAGTCTTTCATCGCGTCTGCGCGACCTTCACTGACATGTTCGTG
AATGTAATCAACGCACCGACAACCAAATATCTTGGCGCCATATGCAAGGCC
TCTGCCCTGGTTGCCGTCAGTAGCAACACATACAGTAACGCGCTCTGTAAT
TTTGCTGTACTGTCCACTGCGCAGCTGAGTCTCGACGTCGGTCTCACGCCG
GTTTTGGTAAATACTTCATCAGCCAGGATCTTGTAAACAGCAAAGGGAGCG
CCCAATGCTTTGAAGGACCCCAGACTACTGCCGAATCGTTGAGATCATCTT
TGACAAGTAAACTCACAATCCCAAGTTCCTTGGCTGAATTCGCTAACGAAT
GCAGAGGCTGAGGCTTGTATTCGGGCCAAGCCTTAATTTCTTCACAGCATG
GTCCCAACTTGAGAGGTTACGAACACATGCTGCAGCGTTTGACTCGGCGTT
TTGCTGAGGCGCCGCCTGTTGGAAACGTGGGAGATGCTTTCCATTCCGAAT
ATATTGGCCAATATATCAATCGAAGGGATTATCGTTGGGTAAATTTACTACGA
TATCAACGATTTCTGTTGCCATTTTATAGTATACAAGGTCATCCATAGATGAG
ATACCGGCTCACTTACACAACGGCCACACCGGCGGCAATAGTTGATGCTCT
ACAACTGGCTTAGCGTTCGGCGCGCAAACTAGCGGCCGAATAGTGGTCCA
CCTAACCTTAGATCGCTAAAGAGAGAATAGCAGGAGAGTTCGGCGTGCTAA
CGATTCCGCCGATCAATTTACCATTAGCTCGTCTCTCGCTCTCTACCGCTTAA
TTAAAGGATACTAAAGGTCCCAATTGATTCAATGTTTTGTATGTAGAAAAAT
CTTATCCACTAAAGTAGGTAATGACTGACGTTTTGGAAGGTCCCAGTTTTCA
TCGTCGTCATTTTCTTCTTCTCACTAAATGGTACTGAACGCTCAGTGGGAAC
TCGGAACAATTCACCCACTACGCCTGCTGGAATGCTGGAACTGCCCCGCCC
CCGCACTGCCCTGCCCCTTTTACCACTGAACATTTCCCTCAAGGTCCTCTGA
TTGAGACTATTCTCAACTATTTCCTGAACGATGCCATCAGATGTAGATGAGA

TAACCTCACTTTCCGCTCTATCCAAACCCAAGATCGTCGCGTAAGAGTCTC
ATATTCGAATTGAGCAAACTGAAATGACTAACCGTATGACGCACGTTCAAG
TTGCCGGAAATGCCATGCGAAGAAAGTAAAATGTTTAGGAGGTCGACCATG
TCAGTCCTGTGTGCAATTAAAATGCGAATCCGAATGCGTATATCCCAGAAGA
GATCGACAGATCAAGATTAGCCAAAGGTAATTAGATGATTTTCCTATTTGAT
TTAACCAACTTCTAGCTAAAAGCTGCCAATTCTATCAGCTACATAGAACGGA
TTTTGAAGGAAAACCAATCCTTGAAAGCCCGCCTCGAGCAAGCCGGGCAA
TCTCGGACTACGTTTGAATCAGACCCCCAGCGCTCTACAGAACATATAGAA
CACGGCAGTTCAGCGGAAGAACCAAGTAAAAACCCCTTACTCGATGAAAG
ACCGTGGTTTCTTCCCCAATCACCTCATCTAAGATTCCGATATTTATTGGTGA
ATGGCCGACCCGGCATTTGCGACTCGGTTCCGTCAAATCATCTCGAAGGAC
CCGTTGAATCACATTCCGAGAACCAACTATCCTAATAGCGATCAGATAATGG
TCCTTATGGACTCTGGCCTACCGAAACTCAGCCACACGCATGCTCAATTTCT
CCTACGAACAGCATTGTCCTACCTCGAAGGACACTATCACATCGTGAGAAA
AGTGCTGTGTCGGTGCTACTTGATAGATATATGCATGAACCTGCTCTACTTG
ATTTACTTTCACGATGCAAGATCCATGCTTTATTCGCCCTTGGGGAGCTTTTT
CAAGTCGTTATGGTTCATCGACGAAAGAGGCTCCTGGATTATCTTTCTTTAG
CTACGCGACCAAGGCCTATGGTTTGCTCTACGAAAGACCAAGTATCGATTG
TATCGAAGTCTGTTTATTACTAGTATGTTTCAAAAATATGAACCCGAAGTGT
GTCTTTCTAGAATATAGTATGCCTAGCTTACAATTTGCCAGTCTCTATATTCG
CTATCAATTAATAGACGTCACTCAGCCTATTTCTTAGCAAGCTCGGCCGTTC
GCCATTGTCTTGTTTCGGGTTTACATTTCAACCTCTCAGAATCCCAATTTAC
CGACTCGGGAACTCGTGAACATCTCAGCCGTCTATGGTGGAGTTCGTATCT
ACTCGACCGCATGAACGCGATGATTAGCAGCCAAGTCCCTTCAATTTCCGA
CGATGAAACATTTGCGGACCTTCCGTCGAATGCAAGGGTCTCGCCATTCGA
TCGTGAAGACTTTTCGAATACAGATTATTTAGCTTCGAAAGTATCACTTGCC
AGATTAATATCCAAAGTAGCCAGACGTATTTATAGTCGCAATGCACATAACG
AACCATTCTTACGCCGGGTCCAAAGTTCGTTGAAAGAACTCGAACGATGG
AATCGCGACCTCCCAGAACCTCTCCAAGTTCGGTCGCCAAAAGCACTCCC
CAAAACCCGAGGTGCTCAACCTAAACCTCTTCTTTAATCAGGTCGTTACTG
ATTCCAGCGAAAGACCACTGCTACCTCCTAGTCTTTAATACTGGCTACCCGA
CCAATTCTTCTCCACGTACTTCGCCTCCAAAAGAAGGCAGCTCAAGAAGGC
TTCCGAGAAAATCATTTTCCTTCTGATAATACTAAAGCTCTCGCAGAAGTAT
GCATCCAATGTGCCCGCCACTCATATACCATAGTTACAGACTCCTGGATCGC
GGGCACATTCTATACATACGATTACTTCAATACCCAGTACCTGTTTTCCGCG
ACAACTATTCTTGCGATAGCCAGTCTCATCAATAATCTGACCGCGCAAAAG
GATCGAGAAATGTTCGAACTCGCAGGCCAACTCATGCGCAAACTTCGAGA
CGCCGGGAGTTATCCCGCTACCGAATTCTGCCGACACTTAGATGGTATCGA

GGCCGATATTCAGAACCTCGAACAAGTCGACCAAACATCGCAAGTCGCGA
ATAACGAGCAGCAAGTCGAAAATCAACCAGAAGTGCCGTATTTCACGCCC
AGCGGTATGATATTCACGGATCCCTCGATATGCTCATTCTTATCCCAAAATGA
GTTTAGTCCAGAACAGCCGGGCGACTTTCTTGATAATCTGACGTTGGAAGG
AATATACTGGCCAACTCCAGACGCTGCAGTCTAATGATTACTTGGTATCTAA
ATCCCAATACTGTGCTGGGCAATTCCCGGATTAAATTGTTAGGTATCCACAA
TCGAATTGCTCATGACTGGCCATATTCCGTCAGTATCCGTGGCGTATCGCAT
GAAGGGCGAGTATATTACAACCCACCTCCTCATACATTAAGGATTTTATCAG
TTTCGAGATACTTCTTACGGCTGATTGTCCCGAATATAAATCGAGCATCACA
TTTTAACTTCCACGCTATAAAGCGTCGTTGCGAATAAGTGTTAGGTCTTCGG
CTTAGTGTTGTAGTCGCCGGCGGCGTAGCAGAAAATTCGCCGAGCGCAAC
AATACAATAACAGCCCCCCACTACTAGTTTTGACGAACGATCTTACAACGC
CGATGAAAAGAAAAGATTGGAAGTACATTAATCTGTATAAATATGTGGAATG
GAAGTTTGAAACTGAGTAAGAGACCGAACCGGTCGAAGAATAGTATTAAA
TACAGCCTTTAATAATCTCCATCCCCATCACACAGTGGGTCTAATCACGAGA
TTTTAAGCACCGGCGACCACGGCTTTCCCGAACAGCTTTTCACGCTTCATA
AGCAGTGCAGCGACCAACATGACACCTCCAGCAACAGGCACCAACACAA
ATGTCATCTGCATTGCTCTGGTGATGGCCTCAATGGCTTTACTCCGAAGTTC
GCCGTCTAATTCTTGGAATAGAGTGCTTTGCGCGCCGCTACGGCACCCGAA
ATATCTTGGTCTGAAAACCCATGACCAGCTAAAGTTGCCGACAAGTTTCTA
GTGGCAGTAGATTGATATATCTGCCCAGCAACTGCAAGTGAGATTACCTGTC
CACCAATCTGGGCCACACTCTGCAAGCTGATCCCGGCCCCTGCGTCTTCCG
GCGTCAAGGTCAGCGTAGAGACCGTAAACGAGGTAACCATAGATAGCCCG
CTTCCAACCGCGATAATTACAGTCAGGCCGTAGATAGTTCCCGTCGTCGAG
TTGGGATCGAGAAATACCACTAGTGGTCCACCTCCAGCTATCAAGAAAACA
CTAGCTATCAGGTATAGAGCCATGTATACCTTAATGAGGTGGAGAAAGGACC
CAGAAACGAGGTTTACCGTGACGGCAACAACAACGCAGGGAGTAGTCGA
ACGGCTGCCATGAGTGCCTGATCGTTGTTGACAAATTGAAAGTATAACGGG
ATATAATATACGACGACGAAAAGTGTTGTAGTGCCAGCTGCCGTTACTATAT
ATAGGAGCACTTGAGTACGATTGCGTAGCAGATGTCCTGGGAATAGGCGCT
GATCTATAGTAGTGAAGAGAGAAAAGTATTGTTGCAGAACATAGATGGCTA
AGACCACGCCGAATACAACAAAGGCGGCGATGGTGCGCCCATCGTTCCAT
GGCCACTGACCACCCGCCATTGTAAATGCCAAGAGAAAGGTAACCCAAAC
GCCGGCACCCAGGATAAAGCCGACGAAGTCAATGGTCGCAAGACGGCCTC
TGATACTAACACCCCGTATCGGATGGATGGACGGAAGATAGAAGAGAAGA
GGTGGAGCCGAAATTGCGCCAATGACCAAATTAATGTAGAACGCCCAACG
CCAAGTTGCGGCAGACACAGCAAAAGCACCGCCGATAACAGGTCCTAAGA
CAGCACCGATGCCCCAAAACACGCCAACAAGAGCCATATAGAAGCCGCGT

TCCTTCCTCAGCCGTCATTGCTGTGAAATAGTGGAGTGATCCGAGGTATATG
CCGCTTCCCCAGCACCAGCGATCACGCGGCCGACAATCAAGGCATTCATGC
TCGGCGCAGCACCACAAATTGCCGACCCAACCTCAAAGAGGACTGTTGTC
GTGATGAAGTTCCACTTCATATTGAAGGTTCCATAAAGCCGTCCGAGCAAG
AGTATGACGCAGACCGATCCTAAAGGAAAGCCGGCTCCAACCCAGGCGAG
CTGCTCAACGTGGCCCAGATCTTGGACCACGGGGCCTTGGACGTCCGCTG
CAATCGTCGTGTCAAGACCATAGAGTGTGCAAGTTATGTAGAGTGAGACGC
AGATCATGAACCAGCGAATTCCTCGAATAGTCCTACTCTTGTCCTCTTGCTC
TGAATTGTTCGATGAGCTGTCTTGTAGCTGTTGTCTGGCCGTCTCGTCCTTC
TCAACATCTCGAGTATGGTCGCCTGTGTCGTTTTGGTTATCGCTGGCGGATG
ATCGAGATCAGGTCGATTCGATGACATTCTATCTACCATTGATGGTATTATGT
CAGTCGGTCTTGAAAGGGTAAATCTAGGGTTGAAATAATGAGTGTGAAGCC
TTCTGTTTATTTATCAAATTTAGCAGTTTAACTATCCTCGACAACGTGGATGC
CACGAGGGATACTAGCGATGAACGTCGACAGATAAACAAATCTAGCTGAAT
GAAGTGGAAAAAACTAATTCTTTTGGTGTATTGAATAATAAAAGACTTCAA
GCTACATCTTATTTATATGACTATAGGTTTTGGAAGGCACTTGGGTTCGTAAT
AGATACCTACGAGTTAGGATCGACAATCCGGTTAGGTATTTTCCAGAACCAT
TTGTAATACAATTAGGTACTCGTACATCTTGAGATTATTAAGTATATAAGATAT
GGAGAGTTATGTATTTATTTATTTACTCAGGTACGTTCTTCTTGTTGCTTCCA
CGGAAGCTTTGAGTGTCATCCTACCTCTGTCACATCGTTCGGGACTTTGATA
GGGCTTCAGCGCTTTTCCTTAGCGCTGCATTTGCGGAGGTTAGTGGAGCTT
CCGCCCCACTTAGTCCCTACATCGTCTATTTGTCACCGGAGCCTCCGCGGAA
GCAATCCAATTATTTTAACCTCCGTGTCGTCACTCTCTAACTTTTCTAATATC
CTCCTCTCCATAGTAGCTTTCATATGCTCTACATTACTTGAATACTATTACCTA
CTAAATAAGTATCATATCATTGCAACATTCCGTACCTCCGTGTTTCCAACCGA
GAGATTTTGCACAATCCATTTTAGCCAGGGTGTCCTTTATCATCTCGTTAGA
GATGGAACAGCAACGTAAGGCATGTGACCTCTGCTATCGCAAGAAGATCA
AATGTGACGGGCAATCGCCCCGCTGCTCTACTTGTATAGTGTACAATACCGA
CTGCACTTTTCAGGCAGCTAGTCGAAAGACACTGTCACGGAAGCAGGCTG
CCGCCCAGCGGCATCTAAAGGAGGATGCATTACAATCCCGAGTTAAGACCC
TCGAAGATCAACTTACTTCAGTCCTACAGAAAGTCGAAAATCTAGAAAGAC
TCTCGGACTCTAGACAAGATGTTGCAAGCCCGATGCCGCCAACTATTCGTG
AGGTTGCAATAGACTAGCAATCAGGCCTCCCTAGCACTCCATCTAGAGCTA
CCTCCATATCAAGATATCATTCCAATAATCGAGTATTACATTGCCAATTTCAA
TTCAGTTTTTCCTCTATTTCATCCAGAGACGCTATTACAAGACGTGAGGAAC
TGGTATCGGGATTCGCACACAAGAGATCCAGTTCTATGGGCCATGATCAATG
TTGTTCTTGCCCTTGCTCTTCGTACAAGCCGCCCAGGCGAAATAGCCCCGA
TTGGAAGCACCGAGATCTATCTCAACAACGCCCAATCAGTTTTAACACAAG

CTATCATGTGCGAGACGACTTTGATGAATATTCAGATTCTTCTTGGAATCGC
CATTCTATTTTGGACGGCAGATGATTTAAGACCAGCCTTGGTTCTTATCGCA
ACAGCTATGCGTCTATCCCATCAGCTCGGATTCCACAGCAGAAAATGTTCC
GAACACTTGAGTCAGGCCGCCGCCTTGCAACACAACCGGGTATTTTGGATA
GCATATATCCTTGACAGGGACATCAGCTTGTATTGTAAACTGGCCCCGATCC
AGCTCGACAGCGAAATTGAATTGGATTTGCCTCCGCTTGATGCTGAAGATG
ACCACACTGGCTTCGTCCTTCGCACCTGACGGACGCGCTAAACTAAACTTC
TTCCGTGCCCGTGTGGAGCTAGCAATGATTCAAGGGAAAGTCTACGAATGT
GCTTATTCAGCCTCAGCGCAGAAGTTCACTTCAGAGGAGAGATCTCGTAAA
GCGAGACAAATCTTCGGTATGCTTGACGAGTGGACTTTGAAGATACCGCCT
ATTTTCAGTGCGTCGACCCTAAGACAGGCAAGTATGCCTGCATTATCAAGAT
ATTTCTGTATTCTTTACTCTACTAGCTTATCATGCCGAACTTTGATCAGCTAT
GCTAGCGCCTTGGATTCATTCCACTACTCTAGATGGATGCGAAATGTTCAGG
ATTATGGTAGAAAGGTGGTTGAGGGACAAGGGGCGTCAGAGATACCTATAC
CCCAAAAGTGGCAGGAACTTGTTAGTGAATGTCGCGAATATATGAGGCTAT
ACGCAACAGTCGTTCCGAGCGATGATTTCTTCGTTAAGTCCGTAATTCTCTG
AACATCCCACAGTTTATCCTTCAGTTCTCTAATAGAAATTACAGAATGACAT
TATGCGGACACAATTCAAGCTTGATAACTTTGACAGCTAACCGAATATTTGA
AGGACATCATGGAATCATTGCGTTGGATATGGATCTGATGAGAGCTGGTATG
CTGTACTTGGAGGATATGGTAAAGCAGACAGATCGCAAGATGCTTCAAAAC
ACATGGGATTCTATCATGCGATTACGTTCCTATGCCGACCTTATAGCTCACAG
TTCTCACCCACCAACGTGGCCCAACACCAATCGACTAGATGGAGATACACA
GTCCGAATGTTCTGGAGTTTCTAGAGTGCCACCACACTGTTGATGCGAACT
GGACTCCGTCCGACACAAGTACTTGGGAAATCGATCAAGTTTAACATGTAT
CAATGATACAATTACCTTCGCGATTCACTAAACGATAATACATCAAGACGAC
TTTTCTACTAGCGACTAAAAAGAGAAATGATATATATTTTCCAACTCATGTTT
GCTTTCTTTCAGTTCAGGAATGAGAGTCGCTCAGTGTTACGGAACGGCATG
GCAGCTCCACTAGGCGAAGAGGCCCTGCAACTGTGAAGTTACAGCGCAGT
AGGCCACTGTAGGCGGGCTAATTTGTAGCGAGTGTTAACCACTCCACACTA
AAAAACGTTTCATGCCCCGCGATTACCCAATAAATCACCCAATTCGACCGC
GATGACTCAATCTAGTCAATTAGTGATCATACATAGAACCAGTGTTTAGTTT
AGGATAACACATACCTGTTAGGGACGATTCGGAATAGATTGCTATTAAACAT
CAGATATTTTAAAGTGGTTTACGCTGTCTCTACAAAGAGCTTTCATGGGCTC
CACCCCCGCTCTACGTGACGATCATGGCGGGGTAATCGCCATATTAGAAAG
AGAACCTATGTTAAGATCGATCTGAAAGAGGAATACAAGAACTGCCATTAG
AATACTGGGGGTGTCGTACCACAAATTCAATTGCGTCCTTTCCCGTGTACTT
CATGTTTTCCCTAGGTACCTAGATAATGTTTAGGATTTAGGATTTCGCTAGCA
TAAGAGCTTGGTTGATATCTTTGATTGCCTTGTCGGCTAGGAGGCCGCTAAC

GCTAATCTTGACTTCGAAGTTCTTCAATTTCCCTGAGAGCATCCTTTCCTCT
TATGGGACTTATGTCAAGGAGCAGAAGCAGAACTACACTGCCTTAGAGCCT
CAGACTTCTGCTTGCGTATCTCGGTATATATCGTTTACTAATCAGCTACGAGA
AGCCGCTGTTACACAAAATTTCTTATGGGTCTACGCACCGGCCTCGATCAA
CCAATCAGCGTTTTCATATCCACCCAATTAGGCAATAGAATATCCAAAGACA
ACCTTAAAAGACGATGATTGGTTAGAATAGACTTTACGTACCTACTTAGGCA
GGTATTACTTGCTTAATAGCGTCTAAAGCGCTAATATATCACTTAGTCTAGTA
CCTAGGCGCCTACAGAGGTACCTACAGGGCTATCACATAAACAACTTATTTG
GTAAACAAATAAACTCCTTTAATTAAACAATACAACAAATATTATCTAGAAA
CAAGTATTTTGTAAAATAATTGCCTTGATCTAGTTCAGTTGGTCCAGTTTCA
ATTCAGAATGTCTTCATGGAAACGACGACTACTTAGTAGACTTTTACCAGG
ACGCTTTTTAGGGCATCGAGGACGACTGGCAATTCTGACTGACCGCCTCAA
ACCTTCCTCGTGGTTCGAGAAAGCGATGCGGTGCTTTTCTTTATTAGCCACC
GCCAGTTCACGGTACTTGGCTCGAATCTCTTGTGATTCATCTGCCAACTCAA
ACCGATGACTTTGGTCGCGTACTGGGTCGTGGGTAGCCTGGTCGTATTTTCA
ATGAAGCCTAGTCGAATCAGCTCATCGGTCAAAGCCTTGTATGCTTTCACAT
AAAGGTTAAATGCGTTCAGTGGGGCCTTCACTGTGCCATCCTTGTGAACGT
CTTCCCTCAGCCGCTGCTTATTTCCACGATTCACATATGTAGATATATCTAGG
ATAAGCATATGTGGAATATGCTTTGATAGTAAAGTAAGAGATCGGTGAGATT
CTTAGATGTACTTACAGCCGTTGGGGTTTCGGGCTCGCCAGTTACACTCTCG
GAGGATACGAAAATTACGCTCTCAGCTGAAGGACTAGCCACAGTCTCCTCC
TGTTGATCAACTACCATCTCGGGTTGGCTGTTTACCTGAGGCAGATATGGCA
TGTCGCCATAAGATGCATTATACGCGTCAGGCGAGAAATGAGGTACATCGG
CGTATATAGAGAATTGTTCATTGAGAAGTCTTGATCATCATGTGATGGAGAA
TAGATATTCGTAGACTGGTCAATAGCAGGAGGCATACCCATACGGTAAGAGT
TTAGGTCAGCTACCATCCCCGCTTGATCAAAAAATTGCGGTTCATAGTTAGG
AACCATTTCTGGAAGCATCGCGTTAAATCCTTGAGATGGGTCAACATAGGA
TGGTTCTGGAAACTGGAAGCTATTCTCGCCAAATTCCTGGAACGGTGTAGG
AGCGAAGTTTTGAGCAGCTTGCATGTCCATATAGTTTCAGGGAACATTGAC
ATATCAGGAGACATATCGTAAAGCCTTGCCGGATAGTCTCCGGACTCAACTT
GATGCATCCACTGAGGCTTTTTTGGAATCCATATTTGCCATGTTAACGCTT
GTCGAATGGATGTTACCAAAAGAAATATCATTACTGGGAACGTCGCTTCTAT
GGTTATTATGTACTTTTTCAAATTCTCGAGGATCCAAGAACAATACCCTTGT
TCTTTTGTAATTTTGTTGCTATTTATTTATTCTTTGTCCCTAATTGTTGAATATC
CGGGAGGAATAATACATTGATGGTATATAACAAAGTAGGCATTTTATAAATAT
ATGGTAACAAAAGAGCTGAAATTGGTTCTTCGGTGGTCACTCGCAGATTTC
TGGATTTAAACTACCTACTTACTTTTAATAGAAAAACATCTAGTTCTTTCACC
ATACATAAGAACATATCATAAGCCCAGAATACCTATGGGGTCTCTAGGACGT

TACCAAACATGTCTTCGGCCCTTCGGGACCCAGGTTAAATGGGCCAGAATT
AGAATAAGAAATGAATTAATGCTACCCAATTAAGTTGTGATCTGATATTGCA
ACAGCCTTCCGATATTATAACTAGACGCTGTGTTTCTACTAGGTACTCTATCA
ATATCTAAGACTGTACCTGATAAATGGAGTTGACATCCGAGAAAATTGCAAT
ATGCGCTATAATAGACATTTATGAATCTCTGCACCCAGTTCGTGAACTTAGAT
TCTTTCTTACAATTACAATATCATATCCACCCTTAATTTCTTTTCTAACACACT
TCCATTTTAGTATGTTAATATATTATTTCTTGTTCTACGGTTTTCAAGAAATCA
TATAGATCGCTACTTACCATCACGGGATTGTAACACCTCTCGTGCCTCTGGT
ACCGCACTTTAATTAAGAAAACGAGTAGTCAATACTACGTCTCCGAGGGTT
AATCGGTATACCGGATTTTATTATAATCTTCATCATTTTAAACACCATAAATAC
TCACTAATTTGGCTTGTTTCTCGCACAGATATCCCTTATTATATAAGATGTCTA
ATGCGAAACGGGATGTTGTCGCAATAACTACCCGTTTCCATTGGTCCTTTGG
GTCCGTGCGCGCTCTGCGACTTACCTCTGTTTTGCTCCATGAACCTCTTAAC
GGCCAAGTTCTCCTTCCCGTAGTCTGCCCGCACCCTGCTCGGAAGCCCCAT
ACGCTCCCACAGCGTCTCTGAAGCAGTCAAACACCGTCTCGGCCCGGTTAT
TGGTGCTTGCTTTGAGAAAGGCGATTTTTCGGGACTTGCCGTCTATACCGC
CGTGTATGACGATTTTGACGTCTATGAGCTTGTGATGCCCATCAATATGCCA
CAGGCTGTTGGCGAAGGGTACCTCATACACCCTGCGAACACAGGTGACCG
CAAAACGTCGTCGGGCATCGTCGGTGTCGAGATCTCGGATGGCCTTTCGGA
CCGCCTGCCTAGGAACTTTGAGGTTTTTCCTCTTGAAAGCGCCCAGCACAT
AGCGGTACCCTTCCATGCCTCCACCCTCCTTACGACACCGTTGTACTTCCTG
CGTGTCAGGTACCGAGACGTGCAGGAACTTTTCGCGGCTAAAAGACAGAG
AGACGAACCGCCAGCACCTCTTCGAGCGACAGTCGGTCCCTATCGCTCCGC
CGCAGCCCGTGCTTCTTCTTCCACCTCTGCAGGGTGCTCTCGGACACCTGT
AGGTGCTTCATGAGCTTCTTATCGCTCATACCAAGCCTGTGTAGATCTCGAA
GCTTTGTCTCTGGACATTCAAGTCGCCACCGGCCGAGATCGCCCCTTCTCC
CAGGCTCTGCCCGTTTGCGAGGGAGAGACAGGGCCCATGAGGGTCTCCGG
GGTCCTCTGTGTTGCCTTGAGCATGTCCTGAACGTGTGTGAGGTGATATGG
AGGTGCGCAAAGACCTGAGCGGGGATGGAGAGTTCGAGGCCTCGAAGCT
CGATGATAGTCTGGGTCACAGAGGCGACGAGGAGCGGCAGGGCCGTTTGG
ATCCGATGGGAGGGGTCCGTGGCCACCCAGGCTAGAGCTTGGGACGATATA
GAGGCAACATTACGCCTCATATTGACCAGATCGTCATCGGAAAGACCTTGA
ACATTGGGCTGGAAGTCATTTACGACGTTCGCGTAGATCGTCAATGAACGT
ACCATGGTTTGAGGCTTCCGTGGGGTCTATGCATGAATGGCGCGTCTCAGT
ACGTGTAATCGATGGCGCTGAGTCGATTGGTCGTGGTTTGAGAGCTCTATCT
TCAAAACTGAAAGAGATACATGGATATGTAGAAAAGTCCTGTCGCGAAGGT
TGTAAATGAGAGCTGAAATTCATGCTGACAGTTCCGGCTCGTCATAAACCG
GTCGTCATAACCGTCGCCGACGACCATCCAGAATCCTTGTCAGACTTTCTTT

TGTCTCCTCATTCACAGCGTCACAGCGTCCCTGACTTCCTCTCTCGAGATG
GAGGTGGAGATGGCGGAGGGAAGTAGTGGCGGTCCGACAGGTCAAAGTAT
CGAGAAGGCCGCCGATGAGTTGGGCGAGATCCTAGCTCACCTCGAGAGCA
CCCTCAAAGTGTGCCGTATCTTCGACGACAAGTCCGCCTTCTGCAGCTCCT
TGGTCTGGCCGCCGAGGCGCGAGATGCGATACTCAAACTCTCCTCGCTGGT
AATGCTGGACGAGGGTGATGCCAAGCTCAAGCCCTATGCTCTGCGCTCATG
AACGCAGATACATGGCTCACTTTCTTCGACATCCTGCTTGAAGCGGCGCCT
AGGCCGTTGACGCTGGATTCCTTCGTTGATATTCTCGAGAGATTCGATCAAG
CCGAGCGCGAACTACCTCTGTGAGCGACGGCAAGCCCAGCCCGCGAGTCT
TCAGGCTGACTGCAGCCACGCCCATCCTTTTGCGACATCTCGAATTCATCTT
ACTGTGCGCACACTCTGGCTCCCCAGGTGTGGTGACGACCGAGGGCACAG
ACTCCCTGGACATAGACAGCGAGATCGCTGAGTTTCTCACGCTCGAGACCG
TAAGATCAATGGCGAAAGCAATCGTGTTTCAACGAGCGCACGGCCTGCTCG
GAGACAGTCATCTTCTCTGGCAGCCTTGGATAGATTGGGAGATGGCTCAAT
TGGATGCCGCCAGTGACAAGTGATCGAATCACCCTGTTAGTTCATGAAGCT
GATGAGAGGTAGGCGCGAGCAGGTGGGAGCAGATTCACGCTCTTTACCTG
GAGAGGCTGAGGATTCCACACTCCGGTCGGTTCACACCATCACCTTTGCAG
TACATGCTGAGATCTAGCCATTGAAACGACCCTGGGCGCATATTCGACTTTC
TGCTCTGAACACGCACCCAATTCCTATGAGCGCCATATGGTCGAGGCCACG
GCCGCCTCTCAAAGTGCCAAGTTCAAGTACAATCAGGAGAAGCGATATGG
GAAAACGAGGGAAGATCTCGAGCAGGAGCTAGTGAACATTGTCCTGTTTG
GGTTATGGGACGCGACTGAACAGCAGGCCTATGCTGGTGACAGCAGTGTG
CAACTTCAGATATTTGCGACTTACATCGACTGGGAAACAGATCAGCGAGCC
CGTAATCCGAAACATCCTGGCAAATCTCCCGAGACCGATCAAGCACTAACT
CGAGCCATTTTCGAAAGAACGGTCGCCCTCTATTCCCGGATCGTCGCTCCG
CTGGTGGAGTCATTGTCCTCCTTGGAACTGGTGATACCCAAGCAGACGAGC
AGGAAGGGGAAGGACAAGGCGCAGGATCAAAAAGCCGCAGAAGCCCTGT
CACGACAATCTCTTCATGCTTATAAGCAAGCCGAAGCTGTGGCATGGGAGA
AATATGCTTCATGGGTGGCAAGTGGCTTTCTTCGTTTCAGAGCGAGTCTGA
GGGCCAGGAAGAGACCGAGTCAAGAGAAACGGCACTACTCGTCAGGAGA
CGGGCTGTACGCGCATGTCCGCAAATCGGCTCGGCCTGGTCAGGATTGCTG
ACGTCTATGTGAGCCGAGACGATAAACCTGGCTGACGACAGGAGCAGGAA
AGTGGTGACCTTGAGGAAAGCACCTCGGTCTTCGAGCAAGCCCTGTCCTT
TGGGCTGCTTGACGCAGCCGAGGCCCCAACCACGGACATTGTAGATCTTTT
CCTCGCCCGAGCCGCCTCAGAAGTCCGCACTGGGGAGGATGTGGACGGGG
AGACTCATCCGGTACTCTTGACGATAAGTCGGGCTTTCCAGGTGATCGAAG
CCGGTGCGTATTGATGCTCTATGAGCTTGATGCTATTGCTCATTTTCTAGTCA
ACCGGGCTGGTGATCCTTCCCTGCGATTGGAAAAATTCTTGCTGGACTGGG

TAAATGCTCAAAATCAATCCAGGCTACGAGCTGACAACAAGGCTGAAACT
CGGGCGCCTGACTTCTTGGCGCAGGCGATCGCACTCATCGACAAGCCGAA
CAAAAGTCGGCAGGCCTCATATATCATGGTCACTATGCGGACCGGCGTAGA
AACGTCAGTTTGGCTCCACTGCGGGTGTCAGTCTCAGAATCGCTGAATTCT
CAGGCGCGGTGGGAACGTAGAGCAATCCCGCAAGGTGTTTCTTGCCGCAA
TTCAGCGTTCGGACCTTGATTGGCCCGAAGCCGTCTACGAGGCTTTCCTGC
AGTTTGAAAGTGTTCATGGAGACCTGGACAGCCTAAGTCCAAGCTCAAAG
CTTGATTCGCAAGGAACAGGAGAAGGTTACAAAGCGGCGGGAAAAGGCG
GCACAAAACAGGCCGCACAATATCAAATCGAAACCGCTATGCCTGGGTCTG
AGGTCGTTGCCGCAGTAGCTGTCGCGAACGGTGGTCTTGACGAGGCACCG
GCAGCTGATCCTCCACCTCAAGATGAGGATAGTCCATTTGAAACGGTAGGT
GTCACAAACAGTTAGTCATGACTACTTACCTCTTTAGCGATCGCGAGCACA
CTACCGTGCTAGTCACTGGACTCAAACATGGAATTGAGTCGTCTCAGCTGG
ACTCGTTCTTCGCCGAGGTGGGTCGAGTCCGACTTGTATGCGGTCGCTAAT
CCTGAAGAATGGGCGGGTCCGCGAAATCACAATTCTCGCAGACGTGGCGG
AGCAATCAGACGCGGCTTTGATCGAGTTCCGGAGCACAGATTCCATCCCAG
GTGCTCTGGGTAGAGACCGGAAGAAATTGGGTGGTTCTGAAATACACGTTT
CAATGCTGTGGCGTTCCACACTTTTCGTGACCAACTTCCCTCGAGATATGG
ACGATGCCGCGCTACGCAAACTTCTCAGTGACGTGAGTTGCGGACACGCC
CAGAATAGCGAACTGATCACAGTACGGAAACGTGCTCCAGACTCGTTGGC
CCAGCCGCAAATACGCCGACAACAGAAGGTTCTGCTATGTCACGATGGACT
CGCCAGCTTCTGCTGAGAACGCGTTGCAACTGCATGGACAGCCGACTAAG
GAAGGATTCGCCCTGAGTGTCCTTATTAGCGATCCCTCCAAGAAGGTCAAA
CGCACCGATGCTTCGAACACAACTGTGTTCGTCAACGGCTTGACTGCCAA
GACGACTGAGACGGACGTTCAGACCTATTTAGCCAGGTCAGCGCGCGGCT
CACGTATGAATCGCCGAGCTGACTGCCAGCGTGGGCAGGTCAAGGCTGTC
AAACTGGGATGGAATGCAGAGAAGCAGATTTGCAAAGGCTTTGCTTTTGTC
GAGATGGCGACCGAGGTGAGCGCTTGTTCCGATCTGGTTCAAATTGCTCAC
ATCAGGCGGACGCCAAGGAAGCATTGAATTTACATGGGTTCCATGTCAATG
GCAGATACCTGAAGGTCGAGATGAGCGATCCCAACATCGCGAACAAGAAG
CGAGGACCAAATCCGTGGAATATGTCCGACCTTGATTATATGCTGCAGCTCA
CCCTCTTAGCGGACCAAGGAGGCGATGCTGCTGTGCAGGCCGCCGAGAAG
CGCGCTCGGACGGTACGGTTGCACAACCTACCCGAAGACACGCAAGAGGG
CTTGCTGCAACAAGCCTTGGAGAAGCTGGTTCCTGTGAGGCGACTGGAGC
TGTTTGCCTCCAGTCATGAAGCAATCGCCGAGCTCGAAAGCCAAGCAGTC
AGTCCCCTCTTGATCGGGACTGGCCCGAATTGACCATGCAGGATGCCGGCA
GATTGCTTTTGAGATCCGAGCCATTCTTGTTCAATGGCGTTCCAGTTGAGAT
TCGCGAGCAACAGCGTCGACCACCCGCGCCCGTCCCGAAGCGCGAAGAC

GGCCGCAGTGCTACAACGAATGCAGCTTCCTCTTCTGCGTCTGCAGCTGCG
GGACCGAGTTCATTATCATTTGCACCGCGCGCCGCGCGTCGAGGCGGACGA
GGCATCGGCAGAGGTCGACAAGCGCCAACCTCGGACCGAGCGCTACCCAA
TTTCGTGCCGGCGTCTGCTAATGGCCAAGGTGGTCAGGATGCGTTCCGAGC
GCTGGTGGCTGCGAAGAATAAGCAGCGCGAGGAGAATCTGGTGAACAAGC
AGAGCTCAGCGGCGGATGCTCCATCTGGAACGGCCAGCAAGCGTCCCGCC
GAGGAAGGAGAGGTCGAGATGGGGGCGAAGCGGGTCAAAAAAGATGAAC
AGCAAGCAGAATGAGGAAGTGCGATACATACTGCACATGTCACATCAGCCA
TGCACATCTTTCATGCCTTCCATTTTGGTCGAGTACCTGTTGTCGATCAGAT
CCGAAGCCATTGTTGTTCGTCATGTTCGTCTAGTTGACTATGGCAGTCCATT
AGGTGCATCCACCTTCACAGAGATCTGCAATGTCGGGAGCTGCAGGATGTA
TCATACATTGCAAGCGTCGGATTTGTCAATACCGCCGCCGAGGGTATCAAAT
GCTATGCAAGGTATCTCGACACGCTCGCCCTCTCCTCAGCCGCGCCCAAAC
AGTCTCCTCCACCATCTCTTGAACCTGAACCAGCCCCCGCCTTCCAGCCAG
GATCTCGTTTCTCCAGCCCGACCCACATCCTGCCTGTCCCACTTCTCACCAC
CCCTTCTTCAGCCTCCATCTCAGTTTGGGGCACCTTTGCCACTTCCTCGCCA
TCCAAGACGACAGACTCCCAATTTCCAGCAGTGACGTTCCCGCTCTCGAG
GTCTTTGATGCCTCTGCCCGGGCGGACAAACGGAAGCGTACATCTTTGGGT
TGATCTCTTCGAGGACGAGTGACAGACAGAGGGAAGAGCCGCATCAACGA
CTCGGCTTCCTCGTCGGTGATGTGTGCGGGGAAGACCCATAGACGATATCG
TGGACGATTCAACTCGGTCTCTTCAGCGGCGATATCGTCTTCGGTCGGGTC
GTCCTCCTCGTCATCTTCGGCGATGGCGTTTTCCGGTGCAGGAGCAGGTGT
CCGGCCGAAAGCCAGGCCGGGATCGCTGTCTTCACTCATCGTCCGGCGGC
CCAAAAGCACGACTCGGTCATCTGAGATGTTCGACCCGCCTCTGGGCCTGT
GCTCCAGCTGAGCGCTCCTCTGGGCAGCGGCTTCGAGAACGCGAGCAACA
CTGTCAGAAGAGTCGACGTGTTCTGGCAAAAGGGCGAATTAACATTGCTAC
TAGGCTCAGGGGGATGCAGAGGCGTTGGAATGTCAGAGGGGCTCTGCAAG
GCTTGCAAAGGGTTCGGAATGGATGTCGCGGAGTATCCAGCACCGAGGGT
ACCGGAAACCTGGCGCCGCGAAGGGGGAGGTGGAGGTGCAGGACCTCTG
CCGGATGTGGATCGTTTCTGTGGGGAGCCAGTTCGAGGGTCGATGAAGGG
ATCCGAGGTTGCTCGCGAGCGAAGGTAAGGGTCGTTGCGTGCTGGCAGGG
GAGGTGGCCCTCCGTTGAGGAGGCTGATGTATCGATTCCGGAGCCAATATC
TCCTCGGCTTGGGGCTGAGAAGCGAGATGTCCGGTCAGCCCACGAGAGAA
TGAACCTGATCCTTGCGTCCGTTCTTGCGATCCGAGAGAATGCTGGGAACG
TCCCATTCGCGCGTGCGAAAAGGACCGAGGGATCGTTCGGATGCGTCGCA
GTTTGGGCGGAGTGGTCAGAGGCTGATCGACGAAGCCCAACACTCCCTTG
AGCATGATGACTGCTTCATGCATGAACTCCGCTCCCCTGCTTGTCAGACGAT
GTGGCAGAAAGACTTTGATATCAACGTACCCCGACTCTAGAAAGACGGCGT

CGTCGAGCTCCCCACACCACCCTGCGAGACCCTGCGCCTCCTCTGCTGTCG
TCTCCTCTGCTCTGGGCGCGCAGACACGACCAAAGACCCCGAAAGAATGG
TTCGACACCGGGATTGTTGTTGCCCCAAATGGCGACGAAGCGCGAGAGCT
TGTCACGTCTCTCTAGTCTTGCCTGCTTGCTGTTGAGTTGCGAACGTGACA
GAGGCGTAAGCGAAGCAGAGACTACCGTGGCCGAAATCAGCGCGTGAAA
CGAGGAGATTCGCAGTCGTAAGTGGTGTAGATGTGCGAGGGACCGCATAG
ACGCCTGAGGCGATGTAGATGGCTCGATGTGCCGGTGGAAGAGAGGCCAG
CGCGCTCGAGATGTACGAGGCGGACTCTTCAGGTGGGCTAGGGGAGAAGT
AAAAGCGGCGGCAGTGATGCGCAAAGTTTTGCAGGTCCTGAGAAGCGCGT
CAGCGAGGCTCTACCGAGGCCAGGTCAAGACAGGACATACGTGAGGATTT
GGACTGATAACACCCTCTGTCCTTGCTCCGTGAACATTGCCCTCGCGTCCG
CTGTAAACAGAGGTCCCTCCTGCCGTCCGAGGGACACCGCCCGGCGGCGG
AGTTGCACGACCGCGAGCCGCCTGCGTCCACGCCGGGCCGGCCAAAGCAT
GATCTGAGCCTCGTCGACCCACATTGGGCCTGACGACAGTGTCCTCGGAGC
CGCGTCGCGTCCATCCAGGTCGGTTCTGCACGAGGTGAGTCGAGGGAGTC
GAAGGCGGCGATCTAACGACAGGGGAGGTAGTGCCGGGCCGATTGGCCAT
ACCCATGGCGGCCAGTCCGCCGGGTCGAGGACGCGCACTGATGGGCTGAG
GCTGGAGAGTGGTCGTGCCCAGTGATCCTGGTATATCGTACAGACGGGGAA
GACGGGGATCAGAGCCTGTCACTGCCGGTGCCGGGCTCTGCTCTGTCGAG
CGACGTGGGGCGTGAGATGACCGCTGTACGCGGTATCTATGCGCGGGGGCA
CTCGGGCGGGACGCATGGTGTCAGAGTGACGGGCAGGGGTGGAGGGAGG
GTAGTTGAAGGTCGATGGCAAGGTCGAGGTCGAGGTGGCCGCATCAGCGT
GAAAGTGCGGTTCGGGCATCAAAGATCAAAGGAGCGAGAGCGCGTGATGG
ACTCGTTGAACCGATGCCATTACGTCTAGTCAGCCGGCCGCCGAGAGGAG
GGAGAGAAAGGAGTGTCGTGTGAGCCACGATGCTGCGGCTGCGGCCGCTG
TTGCTGCTGCCTGTGGAGAGTGTAGATGTCGAATGTCGAATGATGAAGGTT
GAATCAACGCCGAAACACGAAACGCGATGACTTTGACACGCGACGAGTTT
CATCTTACTCCCTTCCTCATCTACATGTACAACTCAAGTCGACGACAGGCCT
AGATGATGCATCCGGGTTACATTTATTCAACACGCTGAGCTAGCAGAGATAT
GTGCATACTTGCTGGATACTGGTAGATGAAGCGGCTTAGCGGCGGTCCCTG
GCGCGTCAGAATGGACTGATCCCACCCAATCACACAAGTTCACCTGTTCAG
CCAGACGTAACCGCCGTTGAGGTAGGTTGCTGCAGCGTCAACGGGTCTTTT
CCAAGTACCCGACCTCCCTGGACTCACCATAGCCCAGCCAGGCGCAGTACG
GGGCGAGGAACCATGTGGTGCTGAACGCCGTGTTCAAGTTGTGCATCTTG
ACCTGCAGAAGACTTCAGCCGATGGTAACAAAGACGCGAGTCGACTCACG
GTCATGGTGCCCACAAGACCCGTCAGAGCAAGAATGTTGGCAAATGCCAG
CTCCTTCTGCTTGGCTGGAGAGTCAGCCGATACTCTACTTAGAGGAGAGAG
CCCTGGTGACTCACCGCCGAAGAAAAGCGGGGTCCAGATCATGTTCACCG

CCAGTTGGGCATAGTACAGCTGGAGAGCCTTGTCCGCCTCGGCCCTGCAGC
GACGCTTCAGTTCCTCACACTGTGCTAGAGGCGCAGCTCACGTGCCAGCC
GGGGTGACGGCGCTGTCAAATGCCTGAACGGTCATGTGCGAAGCCCTGCG
CGCTGTCAACCGCGCATGTCACGGGTTGAAGCAGTTGGTACTCACCAACCC
ATGAGTCCGTACAAGACTGTCCAAACGGGGCCGAACACCTCGCGAGGGGG
ATTGCCGGGAGGCGGAGACATGGTCTGCAACTGGTTAGTCGTCGAGGGCG
AGAGAAGGAAGGAGGACTGACGGTGAACCAGTTGCTCCTGGAGTCTGAC
CAGTGATAATCCCACTGATCGCACCTAGGCCGATTGGCAGCCCGACTGCGC
GTGAGCTTGGGTTGCTGTTAGCGTCGAGGGCGCAAGGAAGATTTACCCGC
GAGGACGGGGTTGCGAGCCACATCAAAGAGGACTTCGGGCAGGGGAGAC
ATGATGTGTGAGTGAGTCTTGGGCACGAAACTGTTGGCAGTAGATAGGGTG
TTTGGTGAAGAGGGGCTGGACCGGGTAATGACATGCATTCATATATATAGGG
GGGAGAGAGGGAGAAGGAAGGGAGACAGCAATGGTTCCGGGTGGCAAAC
ACACTCTCGGCCCGGTTCATGCGTTGGCGGCGATCACCGCCTCTCTATCAC
CGGCCAAATGCTGAGGTGGAGGAAAGACGGCACTCGAGGTCAGCTTTCGT
AGAGATATATATACAACCTCGAGGGTACTGGCGACGGATCCTTCCTGAAGA
CCGAAGCGACAGCCGAATAGAGCCTCGCAATCTCCCAGGCAGCGCTCTGG
TCGACCAAGGTGACTGCCCGTCCAGCCTGGCATCCCCAACATGAGCTCGCC
ACTCGCCCATCCGTCCGTGTCCCTTGGCCGACGCCCCTTGTTCGGAGGAGC
CATCTCGATCGAGCTGCCCGAGGACTACCTGGACGCGAGGTACGTCCCCTC
ACACCAGAGCCTGGCCTCGCTGAGAGACAGCGACCTGCGCCAGATACCCG
ACAACCAAGAGGTCTTCCTGTCCAGCGGGTCCGAGACGAGCCTCGTGGTG
GAAGTGCTCGGCATGGTCGAGGAGGGGCTCGCCTCGACCGACCTATGGGA
GGCGGTCAAGTCAGTCATATCACGCAGAGCTTGCGAACCTCGGGCTGATGG
TGTTCTGCAGGTTTCACTTTGGGTCCATTGCACACGACAACGCCTCGCTCT
CGCACGACATCCTCTCCCCACCCCCTTCGTCCCCTCTGCCCTCTCAGCCCC
GATCGGCCATCCCGACCGACCCCTCCAACGCCACTCCACAGCCTGTCATAC
TCACCGGCCAACAAAAGATACACAAGTTCTCGCACGACCCCACGGGTGCC
CCGAGGCCCGGCCACGAGAGCGACGAGCCGGACACGGTGTGGATTGGCG
TGGCACTGTGGCGCTGCTGGGTCGAGCAGGCTCGCCCGACAGAGGATGTG
CCCGTGGACGATACGCATATGCACGGAGGAGGAGCGGGTCAAGGTGCAGG
TGTGCGCAAGAAAAAGGCAGACGTGGTGTGCAGCGTCAATGTCAACCTGT
CCGCCAAGGACGGTACGGGTCAGAGCGAGAGGGAACAGGTAGAGGCGTG
GTGGTTGCGGTCCGTGGGCAGTCTGCGGATCGCCGACTGGTCCCTCTTTGG
CGACGTCGAGTGAGGTGCCGCGACAACCACAGCACAAAGAAGTAGAACA
GCAGATCTTGTACACAACCCATTGTAACACAAAATCAGTCGAAGGCTCGAC
TGCGTGCGCCTGTCTGTACACATGCCCGCCGAGGGCGTGCGGTATGCGGTA
TGCGATCCATCATACACAGCACCGTGGTGCCCTATGCGCTCGTCAAGACTG

AAGACGGAAGACCCCAGCACCCCCTCATGCCTCGATGGCGGCCACCTTCTT
CCCCTTGCGCGAGCTCGTCACGTCCAGGTCCTTTGCACGGGGGACCTCGG
CAGACGGGGTGGGACAGGAAGATCTCGATGTGGCACGGGTGGCCCTGGTA
CGGGTTGCTACGAGTCAGCTTCCTCTTCCTGCGCCGCACATCAGGCGGGCG
ACGCACATTCGGCCGTGGGCGCGGAAAGTCCTGCGGCGGGTTTTAGGGGC
CTGCTGCACGACGATGTTTTTGATGATGAGGTCCTCGACCGCGAGGTCCTT
GGCATCGGCGTTGGACTCGGCGTTCTTCAGGAGACGGAGGATGAACGCGG
ACCGACTTGACGGGCCATCGACCTGTCCGTCAGCCTCTGCCTCCCCACACC
CCATCCATAACCCGACCCACCCTTGGTCGTCTTGAACTGCTTGGCCTGGCC
GGTACGTCGATGGCGCCTGCGAACCGACGGAAGGGATGACCTGCTTGTGC
TCCTGCACGTCGCCGAGGTAAGTGTAGGCCTTCTTCAAGTTCATGCCTGAC
CCGTCAGCCCTCTCCCCTATGTACATGTCTCCAATCCGACCCACCAGAGATG
GCAGCGGCGACCTCGCGCATGTTCTTGAAGTGCGTCCGCACATACTCGCCC
CGGGCCTTGGCGACTGCTTGTCAGTGTCCGTTTCCCCCATCGCATCCTCCTC
ATATCCCCCCGAAAACCTCCATCTTGCTCCGCCAAGTCTCCCGCAGACATCC
TCCAGTATCCTTCCCTCACACAGCGACGAAACACCACGCACACTTTTCCGG
GTTGGCCGAAGCGATGTGAGCCGACGAGTAGCGGACCATGTCGAACGGAG
AGTCGAGAGAGTGGATGAAAGAGAGAGAAGGAACAGCAATACCTCGAGA
CGCAGAAAAGCCAGCCAGCAACCAACGAATTCAGGGTCCACAACCTCTGA
CCGCTCAGCCAGCGGAGTTTCGCGGGAATTTCTCGTCAGTCACCTTGTAAA
TGCACCTGATTGAGTGTGGCGTAATTTGCTTCCGCTGTCCCGCTTTGCTAAA
CTACTCTGACACGTAGATTCGTACAACGCTGTCCTACATGACTGACTACCCA
TTCACGCCGCTGCAGCCTGCCGTGCCCCACTCTCTTCTCCCGCCTCTGCATC
CGCCGCATCAACCACGTCATCCTCCTGTTTGCTCGATGCAGTCTTCTCGATA
CTGGCCGATGCGAGGCTGACCCGTGGTGAATGGCGCAGATCAGCAGGGTT
CGTCTTGCGCAGTGGCCGAGCCAGACGTCTTCTGGCCACCATGACCGGCA
CAGAGGATTTTTGCACGAGATAGTGCGAGGTGGAACCGAGCAAGATGCTG
GTCATCAGTTTGAGCCGCTCATCGCTTCACACACTCTCACCCCTTGATCTCG
CCCAAGCCGCGAGAACCGACATAGCCATGGTCGGCTCGAGAAAGTCGATC
ATGTCCAACAACATGTGTCTCGCGTTCTTGGCATGCAGATACTGACATGTCG
CCGTGATCTGGAGCCGTGTGCGCTGTAGCAGACTGGTAACTTGCCGGACCA
GCTGCTGCACGCCGCCCTGACGTTCTCGTTGATTTCGCAATTTCTGATTCCT
ATCAGAGTTGGACCATTGCTTCGGATCGACTATACATCAGTTCGGGCCTACA
CATACTTAGAATCCCACGTACCCTTGCTCTCGTCCTCTTTGACCGAGATGAC
AAAGAGCTCGTCACCGTCCCGTGCGACAGTACCGATAGCCCACTCCAAAG
CATACCTGCTCTCGTCGCTCAAATCGCTCAGAACGACATACCGCCTGAGAC
GCCTTCCGCTCTTCTCCAGAGCATCGTCCGGGTCGCCCTGGGTGATGGTGA
TCGTACAGCGGTCCCGTGCAAAGGTCGGGATGGTTGTGGTCATCTCGAATC

CAGTCTTGAGCGACTTTGAGCGTTTCGGCGCATGAAGCGAGCTGGCGAAA
GCGCTTGGGGGAGGCTCGATGACATTCGGCCCTTCCCCCAGAACGTCTTGT
TCAATCTCCTCGCCATACGCATCGAGGCGGTCATCGCCGGCAGTCCCATCA
GGAGCTTTACCGCCGACCACCGCTTGCATCGCCCTCGTAGAAGAAGGCGTT
GGCCTCGGTATTCTTCTCGGTCTCTTCGTCAAAGATGATCGGCTCGTCTTCT
GCAACCCGACGTCGTCCATATCTTCCACCTCGCTATCGTCGCTCTCTGCGTC
GGTCGTGGCATCAGCGTCCAGACCAGGTTCGGCATCGCTGACGCCGTCCTC
GTACGCCGAGGCTTGAGGTCGGAATTTGCGCACGGCGCCCACTGATTCAGT
ATCACTTTCGGCATCGCGGTCTTTGCGCAGAGGTCCGACCGTGGGACTAGC
CGCTCTGGCCCTGGCCTTATCGCGAGATCGGCGTGCGCGGAAGAACGGGC
TAGGTGCTCGAGGCTGCCTGCCACGATCGTCGCGGTCGGACGCAGGAGCA
GGGGTCGAGCCGCTGACGCTGCCAAGTGGACGGGAAGGAAGGAGGAACG
AGGCCTTGGACACTGCGGACGCCGAAGCAGAGGCAGGCGGGGGTGAGGG
GACCGAGTCGAGCGTCCGCGGAGGTGTGATCGGACGCGGGGACGAGGTG
CCGTAGCCAGACGTCTTGAGGCCGATGGCGGCAAGAGCGGCACCTGTGCT
GACTCGCTTTGAGTCGGACGACTTGACGTCGCCGGGCTTGCGGCCCTTCAT
AGCGCCCAGTATGAGGTTGGAGGCCTGTCCGTCCAAGTCGAGCGTGGGGA
TAGGGTCGGAAGGCCGGCGAGAGGTGGTAGGGCGGGGAGTGAGGGTGGG
TGTGGAAGATGGGGAATCCTGTGTCAGCGCTACTGAAGCTTCGCTCGGCGA
CCTCTCTCCCGCCGATCTGGGGCCCGCACCAGCAGGCCCATAACCGTCATC
TGAAGCGGACCTGCCCTTGTCTTTGCTATCCCTGCGGCCCATGTCGAGGGA
CGTGCGGGTCTTGGACACAAAGGCCCTCAACGAGCTGCGACGCGGCTTCT
TGCCCTGCCCGCTCCCCGCTGCCGTGGGATCTTTGGACGTGCTCGGGGAGA
GTGCAGCAGGGGTGGGGGCAAGGTGGTGGGGAGCGACGCGGCCGCCGCG
GTGGACGTGAGGGGAGAAGGGGGTTCCGGGACCTGGCCCCAAGGCGCCG
AGACGACCTGGACACCCTCGGGCACAGGCCGGAGCGGGTTGAGGGGGGA
AGACACGCGTCTGAGAGATCCAGTGCCAGAGCCGGCGGTATCGCTTACCT
GCACGTGTCAGCGGGTGTCACTCCTACAGCTCAAGCAGGGGGGAGGGGAC
TCGCCTCATCTTTGCCCGCCGCACCGGAGAAGACTTTGAGTCGGGACACG
CGGTCGGGCAGGGAGATGGGCAGGGAGAAGGAGCGCGAGAGCGGGGCCA
TCGTGTCGATGTCGAGCGAAGCGAGGGCGAGGGCGAGCGGGCGAGGGCG
AGCGGGCGAGCGTGTACGAGTGCGATTGCGTGTGGCGTGTAGCGAGTGGT
TTGGTTTGTGTGTCGTATGGGATGGCGAGCGTCGGGAGACGGATGCCAAGA
GCCAAGAGATGCGCGAGAGACAAAGGGTTGTAGCGTGTGGGTGGGTGGC
GGTGTGGTGAGCAGAGAGGAAAGGATGGCGTCAGCCCTTGTATACTTGCG
CTGGACGAACGTGGTGCGAGAGTGAGTGAGAGAGAGAGTTGGTGACCTC
TGATGGGAGAGGAACGAGGGGTCAAGGGGAAGACACGAGATCATCACCG
CCATCACCGCCATGACCACCACCACCACCCCCGTCCGGCCATCGGACGCTA

CGGGTGACGCTCGGACACCACACCTCATGCACCATTCATTGTTGTAACTCA
CCATAATCAGCTGCTTGTTGGACATTGTTATCTGTTCTCTACCACATCAGCTC
CTCTCCGTCGGGCCGGAAGACAACGATAAGGACTCACCGCCAAGGCGTAG
GCTGCCGAGCGTTCGGATATGCCCAGATCCTTCAGCTGCGCTATGAGCTCT
GGCCTCGGTGTCGAAGTCATCTCTCCCTCCTCCAAGTCAGCCAAGCAAAGC
AGTGAACAGCGAGCAAATGTATGTACAAGCTGAGCTGCCAAGCTCCATCTC
GGGTACTGACGGCGGCGACGGCGAGAACGGGAATGACGTTTACCCTGAAT
CACCACGACTGAACCACGAAGAGTACTCGTAAATGCAGACATGCATGCGT
GTATAGTGCAACAACGACAGTGACCGCGCGGCAAGAGAAAGCAGGCAGC
TCCGATGGGGTGCACACCTCTATGCAGGGTACTCTAAGACCACTAGGAAGG
AGAAAAGAAACAACAACACTGCGTGTATGGGTGTTGCGTGCGCAGAGACG
GACGGACGGATGGACAGTTGAAATGCTCGGAATGACTCGACTTGATGCGTT
GCGTGTATATGTGCTTGATGGACAGAGACCGTGTGTTTGGATGATTCAATGA
TTGATTTATAGTAAGATATCGATGCTGATTTGTGTTGCTTGTTGTTGGTTGAT
CGTTCAGACCGCGGCGTAGCGGTGGACGTTCGAGTAGAGTTGGCGCGGTG
GCGAGGTCGCGGCGGACGAGAATGGCTGGCGGCCGCTGGATGAGGCGTCC
ACCTTCTCCCATTCGGCAATGAGCTGAGCTCCCACATCTGTCACAGTCAGC
TTCTATGTTTGGCACTGGAAGCATCCGAGTCGACTCACTTGGCTCGACCTC
GGTGCCATCTCGGCACACCTTGACCTCACGATCTTGGTTCCATGGGTTACG
AAGATGCCTCGTGCGATTGAAAGGCAGAGGGGTGGCCTTGATCCACTCGAT
CTTGAAAGGCTTGCCCCACCCTTCCGGCGAAAACTCATCTTCCGGTCGATC
AGGATGAGGGATCGGTCCGTGAGGCTGGGGCAGGCTCAGATCTCGCTGCG
CTTTCTCCGTTTGGCGTCAAGATGGTATCCTTTCGCAGAACGCCGTCATCGC
CTCTGATCTGCGGCTTGTGAGAACCACCGAGAGCCTCCTGCCTTCCAGCTT
CGTCGGATCCTCCGCTGATGGGTACAGGCGGGAAGTACGGATTCGGCAAG
GCTTTCGGGTCGAGGGTCTGCGCTCGGGCACCTCCAGCTCCTCCGCCTCCT
GCTGCAGGAGCAGACAGGTTCGATCGCTCGTCAGTGGCCCGCTTGTCGTAC
CGCGTTGGATCTGTGTTGCGCTTGGCAACTTCGGCTTGGTACTTGACCTCCT
CGCCGGGCGAGAACTCTCCAGGAGACGAAGAGGCTATTCGCTGCTGTGAA
GGGGAAAGGAAGTACGCGGGGCGTGCAGGCGGATCGGATCCAGCTCTCGC
TCGGTCGTCTTCGTCTTCTTCTTTGATGCGAGCAGGCTGCGGCTGTGACTT
GGTCTTGAGACTCGGGCCCGAGCCGCCAGACAGAGAGCCTGAATTGGAGG
ATCCTGACAATTTCGACTGCAATGACACTGAGCGAGAGGCCGCGTGCTGG
GCCGCCTTCTCCTTGTCGATTGGCTCGATCATCCTGGCATAGCCAAAGAACT
CGCCAGAGCGATTGGCGCCAAAAATGAGTATGACCTCTTGCGAATTGCGGA
ATGCCTGGTCCAGAATCCCCTCATTATGATACTGTGTTTTCCACGTACCCTTG
CGATAGCTCTCCTCCAATTCAACGGTCGTGAGGGATTTGAGAACGAAGTAG
CGCTTGGGGAAGTGCTTGGCGAGAAAGCTTGAATTGGTGCTGGCGAATGA

AGGCGAGGACTTGTGTTTCGTGAGAGCAGCCTGGGCATCGTTCGCTTGGA
CAATCGAATCCCGTCGTCGCCCTTCACCTTCAGGCGGGTGCTCAAAGATAG
CAGGCGACGGAGGCTCCATCGAAGAGGCGCTCTGAGGGGCAGAGATGAG
TGTTTCTGAGGCCTTCTGCACTTCGATCGGCTGGGGAAGCGGCACGTTGGT
GGGGGTTCCTTCGGCCTTCTGCTTCTCCCTCTTGCTCCTTGATCCACGTGCG
GTGCATGCCAGTGCCTCTTTGAGCGCCCACTCCAGCTCGAAGATCATCGTC
CTTTCTGCGTATGCGACAGACCATTCTCGGGCAGTAAGGGTCAAAGGGGCG
CAAGGAGCGGTTGTTGAAGAAAGCGACGGCACGTTCAAGGTCCAATTCCG
ACGAAAGATTCACAAAGGCGCACGACGAGCGAGAAATAAGGAAGATCGA
GGACGGACCGCGCCATGGCTCGGCATCTTCAGGCTCGTCAAGTCGAGTCG
GCACAGTCGAATTGAAGTATCGCCATAGCTCTTCATGAGAGGTATTGTTGGG
TCTAAGGCACGTCAGTGAGGCTATCATTATGACACAATGGGTTGACTCACA
CGTTGCAACCCACATGACCCAGTCACTCCTCCTCGCCGGTGGTTGGGGGTG
ATACGGTCTCCTCTCCTTTTCCTTCTCCGGGACAGCCCCTCCCTGATCTGCT
CCTGGTTGATCGCCTGCGAGAAAGAGGGCCGCGACGGCCCGCTCCATGAC
ATTCTGTTCGGCTGCTTGTTGCCTCTAGTGGGGCCGTTCCATGAGCCTCGCT
GCTGCTGTTGCTGCCATGCACCAGGCGCAGAGCATTTCCGGACCAATCTGA
AGGACCGGCTCTGGGCGGTCCATCCATCGTCATCCCGCCGGCTCCTGGATA
GGGCGACGGGTAGAACGCATATTGGTTGTGCCCAGCTGGAGTGTTCCAGG
CGGAAGGTGGTGCACTCCCAAACATCATCCCTCTGCCGGCGCCATATTCGT
CCATGAACGAGCCATGCCGCGAGTGGTTGGCGCCCACATCTCCCACCGGA
CCGTAGGGGATCTGCCCGGGCCCTAGGATCGGACTCATCGGCGGACTGCTC
CAGCTCGGAGCGTAGGTCGCTTGCGTCTGAGCCGTGTTGGGACGGGAGTG
TGGGTGATAGTTTGGTAATTGGTGTGGGTACGGGGGCGATGGATTGAGCGG
TGAGGTCTGGGACGATCGACTGGCCTGGGGATGCATTGAACCGTACTGCGA
TTGATCGGGATGTACGGCCTCCGGAAGGTATTCGTGCGAATACTGAGGCCC
CTGCGGATGCTGCTGCTGGTAAGGAGAGTTGAATCCAGATGCCGAGGAAG
TGACGAAAGAATGACCGAATTGCGGATGGGGAGGGGCATAGGGCGAGTAG
TCCTGCTGAGGGGTGTAGGGAGAAAAGTCGCCTTGTGGACCATGGGATAG
GTACGGCTGCTGCTGCTGCTGATGTTGCTGCTGGTGCAGTTGTCCCGGCGG
CTGCGGGTAGCGCTGAGGGGGCAAATGTCCATCGGGGGGCCCGTACGAGT
ACCGCGGGTTCACGCTCATGCCTCTGAACTGGGCCTCGAGGTGATAGTCGT
CCGCACCTTCCCCCTCATCCGGTACGCCATCGGGAAGCTGCCGCTCAGGCA
TAGTGGGCGATGCTGACGCTGCGTGCGCGTCAGCAAGGGAGTTATAGATGG
AGCTAGTCAGATGAAATTCACCCTGCTTCGGCTCCTTCTGCGGGTGAGGGT
GGTGAGATCGATGATCGGACGCGGCGGCAGCGGTTCCACCCAAATTTCCAT
CCGAAGCCGATCGGATGACCATGTCACTGTGGCCGTGGGCGTGAGCGTGT
GTAGGGTCCGCGAGGCGAGCTTCGTTTCCGCCCAACGGGCTCTCCGCCTG

ACCTCGGGGGAGGGCCTCGGTGGTGTGTGCCGTGTGCGCCGAGCTGGTCG
AGGACTGCGTGTGGGGCGAGGGGCGATACGTGTGGGCAGCCGAAGAGAG
CGAAAAAGAATGGTGAGGGGTCTGGGGAGGGACGAGTGGGGGTGAGAGT
GGTGGGAGGGGGGATGAGGATAAGAGTGAGAGTGGGCCGGTGGACGAGG
TTGAGCTCGGTCGGTCATGCTGATGTTGCCTCGTTGGGCCAGCTGGCTCGG
GCGACCATGAGAGGGCGGCGGGGCGCGGTCCGGTACGGCGGCGAGGGGC
GAGGGAGGGAGGAAGAAGGGGAGAGAGGGGGGAGACGACGAGGTGAGT
GACTGCTGGATTGTCGGAAAGGGAGGGGAGAAGGATTCAATTTCGCGGGA
CGGGCGTCATGCACGGATGTCGGGGAGTCGCTACCTCTGCGATGATGGCGG
CGAGGGGAGCGGGTCTTGTGCTTGTACGCCTCGTCGTCCTCGCCGCCGTTA
CCGCTGCCGCCGTTGACGAAAGAGACAAGGGGAGAGAGAGGGAGAGAAA
TTACGAGAACGTAGGGAACTACGGCAAAGGGTTCAAAGACCAAGGGACTC
TATCATCCCGGTCGGAGTCCCTCCATGGCAGGCGGTCTAGCAAGACCCAGA
CAGCTTCTAGAGCCCCAGAACATGCATATGTCGGGTCAAAGATGTCAGCCC
AAGGCCTCGCCCCGGTCCACGCTCGAGCCACGCATCAAACAAGTCGAACA
TGATCAGATGTGATCTGATCTACACTGCCCCTTCCCCTTCCTTCCCCCTCGT
CTACAGTACCTCCTTTCATAGTCTCTCGCCTCTCTCCTTCTGGCTTCTGGACT
CACTCGCCTCTTCTCCGTGATTCCAGCAGATCTACTCTAGTCTTGGTCCCTC
GTTTCATATCCCCGCGTAGCTCAGCCACCAAGCTAAGGAGATATAAGAGAC
CACCTGCACCCTGTGCCTCGGAGGTGCCTCTGTACAATGCCCTTTCCTAAA
GACTCTTGCTCTCCGAGGCTCCGGACTTCAGGCTGTTGACGGATGCATCAC
TATCGAAGCTGAAAGACACATGTGATTGGTGGGATGTGAGACACTGTGTCT
GTCAGACGAATCGTGGTCTCGCTGCAGCATCAACGGTCTCCTGTCCAACTG
ATCAGGATGCTCGGCATGGTCAGGTCAACGACTGAGCCAACACATTACCGA
AGCGTGAACCATGGGGCAAGCTGGGCCGTGCGCAGAACCATACACAACCA
TGCAGACATGCGATCGGACAAGTACAAAACAAGCAACAACAAACACAAG
ACACGCAACAAGTACGAGACGCGCAACGACAAGTACAGGCAACGGTAAC
ACAGACAGAAGACCACAAAGACACTGCGTCAGGTTGGCCGAACCCGCCG
CCTGCGTTGAAGGTACGAGGGTGTCCAGTCGGGCTTGACGTATCTCGCATC
AAAGGCGATATGGTCGAACGGGCGGCTTTTGCGGGACAAACGTGAGGGGC
TGTCGAGATCGAGAGCGCCGTGAGGGTTGAGCAGGATACTGGTTTTGCGC
GGCGGAGTCAGGATACTGTCGACGAGCGTTGATGCGCTGTCGAAGATGTC
GAGCGATGAGCGTGAACGGGGACAAGGGGTCTCAAGGACGAGGGTGGAC
GAGGACGAGCTTGGCGTCGAGGGCGGCGAGTCGACGAGGGTCGCAGGCG
CAACGCATGCGTCCTCATTGTCCAGCATGGGCTCGTCGGTCGAGAACAGCC
GAGTGGTACAGTCCAAGTTGACGATGGCAACGGTAGCTGGCCGAGAGAGG
CGCTTCCACTTGAATGGTGGGGGGACGAATGGCGTGTGGGACAGGTCGCG
CAGGCCGGGAGTATTGAGCTCGGCGAGCGTCTTGACGATGAAAGGGTCCT

GGTGCTCAGCTCACATATACTTACCTGTCAACATATCTCACCGTGTTGTAAG
TATGACGAGACCGCGCTTCATTCGCCATCCAAGCCGTCCACCAGGCCTTGC
GCGTACACTCGGGACACTGGTACTCCCCGTCAAAGTGCAAATATTCATGGC
GCGCATCTTCCATGGCCCGACAGAGACGCACGCCTTCAAGGTATAGCCACG
CGTTGACCTCGTCGGAAAACCCATACTGATCGTCGTCTGAAGCGGGATCGA
GGTTGATCACCTCGGCATGCCGAATGACGCGGTCGAGCTGGCTTTTGAGGA
GAGACATGCGACGGCGGTAGAGGATGTACTGGCACAGCATGGCGAGGAGC
GGTAGGACGATGTGCGTGTGGATGAAGCGGATGCCGTGGAGGATGTGGGT
GATGTGTTGCAGGATTTGCGGGGAAGAGGGGCAGTCGTGTCCAGCACGAC
GAGGAGGCGACATGACGCGCGTCGTTTCTGGTTTCTGCGATGAGGTCAGA
GCAGGAATCTGTCTAGGAGCAACGAGGAGCGATAGGGTGTTCGGCGGGTG
GAAGAAGTAGGCTAATGAGGAGTGGATGTACGTCAAGCACAACTGAGCAC
ATACTCGGCAAGATGGACAGGCAGGCAATGGCATCGACGCGTCCGATCACT
GCACGCCGCGCGACTAGGCCGGGCCCACCCATCACAGTCTCAACTCTGCC
ATGCATGCTGAACCAGCCGCTCACCGCTGACAAGGAACGAGGAATGGGAC
CAAAAAAAAGACGGGAAGACGATGACAAGAGGAATGTAAAGGTGACAAC
GCGCTAAGATTGACGCCGTCGATGATCGACGCCAAGGGCCCAGTCTTCCCA
TCGCCATCTCCTCGCTATCTCTTCCCGCTGCGCCGCTCTTCGCCTCACTTGC
GCTCCCGCTGACCGCTTCTTGCCCCTTTCTCGCAGCTGTTCATGG

>Mosaic_sequence_83

TTGTACTTTCCCTTGAATTCCTTCTGGAAGTGGTTGACTAAGGCTAGGTTGA
AGTCTCGGCCACCGAAGTGACGGTCATATGCCGTCGACTTGACACTGAGCT
CGCCCTTCTTAAACTCGACGATCGAGCAGGAATAGTCGGCGTGGCCGATAT
CGACAAAGGCAACTCGACGAGGTTTCTCTTCGGCCGAAGAAGATCTAGCT
TAGTGATACCGTAGCCCAAAGCCGCGGCAGTAGTGTCGTTCATAACACGGA
GAAGCTTTAGACCGGCGATCTCGGCCGCATCTAGAAGACTTCGTCGTTGAA
CATCAGTAAACCATGCCGGCACGCTGATGACGAGATCCGACACAGGCAAC
TTCAGCTCATTCTGTGTTGTTTGTTTGATCTTGCTCAGGAACATGGCCGTCA
GTTGAGTGGCCGAGAACTTCTCCTTCTTGCCTAGGTACGATACTTCGGCAC
CAACTTGGCCGCCAACATCAACGAGCGCGGCCGAGACATATTGCTGCTCGA
TCTGAATGTCGGGGTCGTTGATTGATCGGCCACCCAAGCGCTTTAAGAGCT
GACAGTGTTCTTGAGGTTAGAGATCTCCTGCGTCTTTGCTGGTTCGCCGAG
GTATCGGGACTTAGGTCCGAATCCAACTAAGGAACTGAAGGTTCAAAATGT
CAGCAAGTGGTATACGAAGCAAAGGATCAAGGGTTTCAACTTACGGGGTA
GCTCGATTCGAGACTTCATTTGTGATCTATGCACCTTGTTAGCGTTTCCATCT
TCGACCTCATATGGACTCCACATGCAGATATATAGACGAAGAACATAGGGTA
TTACATACAACATCAACACCTCGATTTCTGGCGACTGCAATAACAGTGTTCA

GGGTTCCGAAATCGACACCTATACTCATATGCGGTTAGTAAAGAGCTATTCG
CCGTAAAAAAGAGGCAGGCCTGTGCGGGCTGGGAGGACATACCAACTACA
CTCATTTTGGTGGGATTTGTGGAAGAAGCACGATAAACAAATAAAAGTTGA
TATTTGATTGCGGGGATACCAGGAAACGTCGCAAGCCGTTACCCTGTTGCC
AGATTAGAATTGCGAGAAAAATTCGTATCTGTCGAAAAAGTAAAATTTAAT
GTAAAACACGGCCTCAGTTTGGCAGGGACGAAATCCGTTGATATTTCGTTG
GAATGTCGTGTTTGCGGCGAAGAGAAAGGGTGAAAAAAAAAGATTCTTAA
GTAAAAAAGCTGCCCGAAAATGCGAAGTGGGAGATCGCGAAGGACTTTCA
ACCTTAGCGTTTTCACTATAATCAGAGAACCCCTCACTTATTACACGCCCGG
CGTGTGATTCAGCCTTCCCGAAAGTTCTGGACATTTCGACATTCGTCCATGC
CTAGGTGGGTACTTACCACACTTACTTACCACCTGTAAGGCCTTTAAGGTTC
CAAGGTCCAATGCTCTTGACCTTTTTCAGTTGTACTACGCCACGCAAAGCC
TCATTTGGCTGAGACCTAATAGATTAGGAGGTACATAAATGTGTATTATGCA
GTGACATCTTTCCAGTTATGTAATATGATTCGACTATCCTTCCTATACAACTC
ATATGTGATCATGCATAAATATTTCCAGGTTTTTCGAGTCCAATCTATCACGT
AAAATTGGTGGCTGCCAATGCCAAACAAGAAAAAAGGAAATCCAAAAAG
GTACATCGAAAATGACCCAATTCAGCAAGCCCAATTTTATATCTACCCTCGT
CATGCTATCTATCTACCCCGCCAAATTGCTAGGTACCTACCCACCAAAATCC
CCCCACCCACGACTAATGCCAACCTTGCCAACCAACGATAGTGGGGTCTTT
CTCATGAATATGGGATTTGACCGCCTTAAATCTTGCCGCCTTGCCGCCCCGC
GACATAAAAATTTACATATGATGGTGTTCTATACCATACCAAGGATACGTGG
ACAAAGGTCGTGACACTTTTTATAACAGAAATACCGGATATGGACGATCATT
ACGGATGACGAAGACTGGGCAGAAGGCTAGGTCATTAGTAACTAGGTATGC
ACAAGGGTATTTGTAGTTTGTACCTTTTACTTTGAGTATCATCCCAACTATGG
ACTCTTGCATAGAATGTTTACTTTCTATATCTCATGTCTAGTAAGGGCAATAT
GTTACTCATCATATATGACGGTGAACACAGCGCATATATCATGGATATCTCAT
CATATGGTGACTAACAATATAGAGAGCCACGGTTTATTCATATCTAAGAGAA
ACAATGCAATTCTATGATAACCAATCCATCCGACAGCGACATATTTCAAGCT
CTGCTTGTTGTCTATCATTCAAGACGCTCACGCTCCTTCATCTAGTCGACAA
CAGGCTTATTATCCGCCCATGACGGCTCAATACTTCGACAAACGCTTTCATA
GTCTCGGAACCTCCGCCCATCTAAAGCCAAGTTAGGTGTCGTCCAGAACAT
AGCCCAGTTCATATCCTTGAACTTTAGTGACCCTCTCGAGCGTTCGAAATAC
CACTTGTTGACGGGGTTGTCCTTCCTGCTTCTCCAGCAAACCCTTCTGGTA
AGCAACCACGAACCATGGCGTTAAATACGATATCAGCCACCCCTCCAGCGC
CTTGGCTTTTGCGCAAGACCGCAAACTTGTCCAAGTATGGTACGAGACGGC
TCGGATCATCTGCTTCTGATTTATCAAGGCCAGGAGGCTTCTCCCATGTCAG
TAGTGCGCCTCCTTCATACTCTCCTGCGATGATGACACCGGCCAGGTTCCCC
TGTATCCTCTTTAGATAATGGTCCACATCAAGCTTCCTACCGAAAGAATCCT

CTATCAAGTTTACGAGTCTAGGAAGATCAATACACGGGTCGGTGAGATTTA
ATCCCCTCTCCCCGAATTTGGGTGGTTGCCATGGAGTAACTCGAGGGTCGG
GGTATACAGTCAAAGGCATCCCACGCTTAGCCAGTGTAGTTAGTGAGCCTA
TTTGGGCCATACCGGTCGACGATGTGACGGGTCTGAATCGCTCAGAGGGTA
ATGACGAAGACTGGACGGGCTTGTCGGTGAGAAGACTGTGTATCAACGGG
TTCTGGCCACGTCTTGTGCGACAAGGGCCCAATCCACACTATTGTCTTGTA
GGCCACGTTCATTTGCCACCTCTTGTGGGGTGTTATGACTGCGGAAGAAGT
TGGTGGCAGTAGAGATAATACGTTCTTCGTTAATTCGGCATTTTCCAAATGC
TGTAAATTCTCCGCGCTTGGTATTGATGTCAAATCATTTCGGACCTCCGGCA
GCTCTTGTTCTAGGTTTAGAAATAAATGCCTCCCGGTAGCTCGATTTTTCGC
AGGAATTCCTCCTAAGGGATCTAGGACAATGAGTCGATATACCTCGGCTAAC
CTTGACATTTGTATTGATTTTGAATCCTCAACTGGTTGGCCTAGAAACTGAA
GGCCGGACAGCTGCCTTGTAAGGGCCAGTATAGCATCATTGACGTTCACTG
GTTTAACTGTGCAAGCGTCGGAAGTGTACCCAAATGATGATATAATAGGGA
CTACCTCCTCCTGCAATGCAGCCATAAGATCGTTTGGATTGCCGACGAATAG
TCCACGGGAGGTGAATGGGGAGATTCTCCTTCAGTTGGAGTGTCAATGGTA
ATCGGTGAGTCGATGACTCGTGCGCCAGGAGCATCGTATTTGTCTATTGCCA
TAGCAATGCGAATGGCCTGTATGGAGGCCATTTGTCGCTGAGCAGCCTCAT
TTTGCTCGGAAGTACCGCAGTCAACCACAACGATACTAATCAATCCAAGCT
TTCGCAATTGGCCTAGAGTTTTCCCCACGCCATTGAGAGTGGCATCGTTGA
CTTCCTGGGGAGCACGAAATTTAACCAGAGCAACGTACAACGGCAGTTGA
ATAGCAACATCAGTTTCGAGTTTAGGTGCCTGGGTGGTGTCCTTTTTTACGA
CGGCAGCATCGACAATGACGGCCTCGTCGGTAATATTTTTATTACTCGCTGG
GCGAGTCAGTGTTGCACGCTCCTTGAAACCTGTCGCCCCTTTCTTCTCCAA
CAGCGGGACAAATTTGTTCATATAAGCCTTAGCATCTCGTTTAGTGACCGAA
GTCTCCAGAACAGAGAGGAAGAAATCCTGACAAAAGGTCAGGACAATTTA
ATGCAACGAGGAGGGGAGTAGTGAATACTCTGTCAAGACTTTTCTTGTGTT
TTTGAGATTTGGAGATAGAGGTATACGAAGAAGTAAATGTTACCGAGTACC
AACCGCTATTTGCGGTGCTCCGCGCGGACCAGAATGACAATGAATTTGAGC
CATTGATCCTGCTTAGATCTTTGGTAGACGGCGTCTATCGTATATGGGTTAGC
TAATATTACTCTTAATCTTTATTGAGAATTTCGACGTGTTTGGGGCTGAGTAA
GATATTGCCATCCGCTCGTTCGTAAAATTCGAATATAGGGTCGGGGCAGAGC
GATTTCTAGAGTACTCACGTTCAAGTTTGAAGCTGCCCTCTTCCAGGCTGT
GAGTTGCATTGTCAAAGCGCGCGAAGCATTTTGGAGATGAGGAAGTTCAA
CAAGCGATGCACACTACCACCGCATGTTAAAAAAAAGCAGATACGGACTA
GAGTCAGCTAAATGAAAAGTGAATATAAAAATGGAATTTGAAATCGGTGGG
AATGGGATTTCAAGGTTGGACCTCAATATATCCAAATTCCAGTGAGTCAAAT
ACTAGATAATTAGTGGATCAATCTGATAACAATCGATCCATTTATTCTTGGGA

CCTTAGCCACGCACTAACAAAGCCGCCACGGGAAGGATAGACCGCTATCG
GCAGACCCGATCACCCCGTACACCTGCCCTATGTGCCCGCATCTTCAGGGT
AGTCTAGACCCCACGAAGGCCGTGTGACATCAGAAAATGCGAAGACTTTT
CTCCCAGGTGGAAGCGACGTCCGCCGGGTTTCCTTTCTCGTCTTATTGTTGT
CGCATCGATCTTTCTCTCGTCGAACGGAAGGCGTCAATCGGCAATTCAAAC
TGTATCATCTTTACGCAACCAACCAGATGATAGGGATTGTTTGGTTTAGTTT
GGTTCTTGTTACAGTTTTCAATTGAACATACATTTGTGCTACATACGAGTCT
CCGATTCATGGTCGGATTTGCTTCCGACTTATCCACTTCTAATACTTCTCATC
AATTGCGTAAAGGGAGGATTCGTATACATCGAAATGCTAACACACCCGTAA
GCATAAGTTCTCCTAGATCTTCTACTCATTTGACCGAGGAAGAAGTCCGCC
GCCTTCCCAATCCGGAAGCGCTTTCTATCATCGCAGCTCTCGGACGAACGC
CAACGCGTCTCTACGGAGCAGCCAATGCATATAAGTTAAGTTAAATATGTTT
AGCTTCTTTCAACAACTAGGCAATTACGATATCGATATTCATAGATGGTTATT
TTCACATGTGCATAGCTCTCCCCAGTAACCTAAAAAGCTTGAATGACATATG
AAAACGACATGAACCGGGTCTTAATAGAGACCAGCTTTTCCAAGAACAGC
AATCTCTCCGAAGGAGATATGGGTTATTATCGTGACTCCGGATTATTATCATT
AGTCCTATTTCAACTACATAATACCTCACACATAGCGCTGAGGCAATTTCAC
TCGAAAAATTAATGAGTAATAACAACTCACTAAGTACATAAATGGAATATGG
TAGCTTTAATCATTGCTTCAATTACTCGTGGTTGAGACATTGACACTGAATT
TTCTACGGAGTCAAAAGAGCAAAATTAAAAATGACATCAATAATAGCCCTG
CTTTGCAGCTAACTGGAACCTATACGTTCTACTACGACTATGAGACAAATAC
GTTTTGAACATCATATAGTAGGAGAGGATCGTGAACTTGTCATTTCACCAAC
CAATAGGATCTGGCACATCTCGCCCGTAAGGAATATAGTGTAGATTATAGAT
ATAGATAATTAAAACTTCTGACTGAACATCAGTTATGACAACATCAAAGTGA
TAATCCACCAGCTAGTCGTGCGAGTCTACAGTGTCAAGAAGATGTTTTCGA
GTTTGGTAGGTGCCGACAAATGGTGGGGTGCACAGTGGCATTTTTGATAAG
TAGGCGCATTGGTACAAGGAAGAAAACAATTGTATGAATTGAAAATATCGT
TGATACCAAGTTATCTAATTCACGAACTTAAGAAAAAATTAAATCAACGGTC
TAAATCCGTCACCAGACATACACATCTTGTCTTTCTGCGGCTGTTTGGCTTA
TCAACATCAACGCTAACAAGGTCTGCGAACCTCCCGCGAAACTCACGCTT
GCTTCACTTTTTATGTTGACAGTGCTTCGACACTTGCGACATCTCATTCTGT
TTTCGCCTCTATATCACATTGGCTTTGAATTCGACTTCGATGAGGTTCAGCG
CAACCACTGTCGCGGCGCTTGCTGCTAGCCTTTTAGTTTCGCTCACCTCAA
GCATACATATCCTACCAAGTCCCGATGGCACCTTATTGCTTCAAAACAGCTA
TGACTACATCGTCGTGGGTGCGGGAATAGGTGGTCTAGTCGTTGCGAATCG
CCTGTCGGAAGATCCCTTGGGTATGTTACACCCACCAGAGCTACGATGTTTT
CAGTACTGACATGCGTCAATAGTATCAGTGTTGGTGATTGAGGCCGGAGAC
TTGTAAGTACTGTCGCAATCACTCGTGGGGACGGGCGCGCCAGTCTGATAA

CACATCCCCCCCAAGCCTTTATCATCACTAACAGCGCGTTACACTTAGGGAT
GATCGCGCTGAGGATGTCTCGATTCCGGGAAATATTGGGCTTGAAGATCCA
CGCCGATATAAGGTTAAGCTGGATCTAGAGCCTCAGGAGTTTCTCGACGGT
AAGCCCCGAGTCATCAGTCAGGGTAAAGTCGTCGGAGGAAGTACTATCGT
GAACGGTCTTGTCTGGACGAGAGGATCGGAAGCCAACTTCGACGCTTGGG
AGCGCTTGGGGAATCCAAATTGGGGGTGGGCCGATATGCTGCCTTACTTCA
AAAAGTGAGTCCCTCGGTAGAACTTGCTATCCATTCCGTGTCATAATGTTGG
TGATCTAATGAATAAAATGTATAGTCCGAGAAGTACACTACACCGTCTAATA
AGGAGTCGACGCGTAATTTCCATACGTCTATCCACCCAGTTATCGAAAAAC
ATGGCGACGAAGGGCTGGTGGAGGTCGGGTATCCAAACTACTATTATAGCC
AGTCTCGTATGTCATCTCTATTCGTCGTTTGTTGAGGATCGTACTAATCGGTC
CCAAATGAAGACAACTTCCTTGATGGCGTCCAAGAACTCGCGATACCTATC
AACAAAGACCCAAACAGTGGCAATGCCACAGGGGCTAGTATCATTGCTAG
CAGTATGACGGCAGACAACCAGTCTCGTTCCGATGCGCGTATCGCATATCTA
GATCCTGTATTGGACCGCCCGAATTTACACCTCGTTACTGGCTATACCGTAA
CTCGCGTCCTTCATGATAGCGGCGGGCGACTTTTTTAATTCGACCTATAAC
CCTGCAGGCGACGGGCTGGGCATAATTGGTGTTGAGGTATGTTTTGGTACT
CCTAGAATAAACATTGATGTATGTGGTTGCTAATCATACATATTTCTTGTAGT
TTGCGGCTAATTCGACTGCTAATAAGACTAGTATCAAGTGCGAAAAGGAGG
TAATTCTTGCCGCTGGCGCGATGTTCTCTCCTGTTTTGCTGCAGATCTCTGG
AATCGGACCAGCTAAAGTCCTGGGAAATCTGGGAATAGACGTCGCCGTGA
CCTTCCAGGGGTAGGTAGCAATCTCCAGGACCATCCTACACTAGAGCCCGT
TTATAATTGTGAGTATCAACAGTCCCCGAGTGCCTGATAATAACTAACTAGG
TAGACACTTCACCGAATGTATTTTCTGCATGGGATATTGTAGGGAGGACTCG
CGATGCTGTTCGGGAAGAATATCTCACCAATCGTACAGGTCCGTCGTGCTT
CTAGTAATATTCAACTTCCAAGTACTTACAGGTTATAGGGCCCTGGACAGCG
CCGATGGTCGACGTCGTCGCATTGCCTGCTCTAAGTTGGGTAATAGATGAC
ATCCAAGAGTGGCTGGATGAGACGATGAACACTACTGACAACTTACCATCT
TCGTATGACCATACTCTCCACACAGGATACCTCGCGCAGCGAGATGAGATG
ATATCCTTACTTGCACGATTTGACACGCCCGCGTATGAGATTATGTCGACAT
CTTGGGGTCAGCTCGCGGTAAGTGCCATGCAGGTATTCTCCGAGGTACGGT
CCAGGCGAAGTCCTCATGCATTTTTGAGAGCCAGCCTATCATCGACCCGCG
ATTTTGCTCTCATCCTGTCGACTGCAAACTGCTAGTTCTCGGACTTCAGCTC
AACAACCGCCTGATTCAGGACATCGTCAATGGCAGCGCTGATGCCAGTGCC
GCCACCCGGCTTTAGTATCGTTGACGCCAGAAATCAGACGGCACTGGATGA
GGCAATGAGGCCTTTGATCACCTCTGGATACCACCCAAGTGGGACAAACAT
CTATGTTACCACTCGAATTGGGTGGAGTGGTAGACCCATCGCTCAAGGTAC
ACGGCACGAGGAACTTAAGAGTCGTGGATGCAGGGGTCATTCCATTGCTGC

CCTCAGCACATATACAGGCCATATTATACGCCGTTGCTGAAAAGGTACGTCT
TTAAATCGAGGCTAGGCCGTATGTCAATGTAAAGGAAATCAGGAATACTGA
TCATGAAGGAAAACAGGCCACGGATATCATTAAGGCTCAAAACTCGAGAG
TTCTCCCCGGCGTGAACCCGTTGTCTGGGGTTTCGATGGCTCATGAATGAA
GCCTCTTAAGAGTCTTGTCTAACTTAATTTTCTCCTCTAACAAAGCTGGATC
GTTATCCTTGGAATAACAGGCTGAATTGACCGAAATAAGGGTGATGCCGTA
CGCCGCATTCAGATACTCGGTATCGGATGCCATCAGGTGGTAACTTCTAAGA
ACAATCAATGCGGCAAAGAAACTATTCTTTAGAATAGAGCATGAACTGGAT
TGGCGGGAATTATTCTGCCAGTGTATGCAGTCATGGTCTTTACAGGTGTTAA
TAGTAATGAAGGACTGAGCATGAGATTGAAATTAGTAAAAAGTAGAGATTT
GCTTCGTACATGTACTGTGGAAAAATAATCACCCCGAAGTACAAAATTGGA
AGTCTCCATACTTCGTAAGATGAGTCAGCAAGTGTGGCTAGTGACATTCGG
ACTACCTACCCTGCTACCTAGGCAAGCATAAAAAGTAATGCTTGGATTCATA
AAAGATCTAAGCCGTATTCGTAAAGTGTGCCTAAATTTTGAGAACTCCTTCA
TATTTGCTGACCATCACTTGATTCGATAGTTTCAATTGACCAAGCCAGGTTT
CAGTTACACCTTACCTTAAAGAGAATGGCGCACGGCAAAAATACGAATGCG
TCGGCTCCACAGCATCCGAAATCGCAGGACGCCATTACCCCCTGTCCCCCA
TCAATGAGATTTCTTGCCATTGCATAAAAGAATGCATTTCCCATCTTCAATAT
CGATATTGAACTTGATCGATCCCCGGGCCCTTCGAAGCTATCTCGACTTTTG
GGAAGACTACGATGACTCAGGGAAAAAAAAAAGAAGGGCTACTCGAAAA
GGCTGCACATCGCACGATTGAGTTTTCTTCCCTGCCGAATTTGATGTTACTA
TCATGAGCGTTGCTTTATGCGATGCCGTCGAGAAGATCACCAAGGACTTCC
CTTCAGGATGATGGATTCAGCCGTATAACATTCCGTCTGGCTACTCCATATAT
CACCCTGATACTATGGATAGGTTCACGGTTGTCTGATATTTATAGCTAGAGAC
CAGTTTTCGAACAGTTCCAAGTCAACATGTTTGCGTGTTTCTGCAACCATC
CGAGCGTGCGTAAGCAAATGTGTCGGATCGACCACTATAAACGTACGTAGT
AGTTACATCACTGCTTAGCTCTCTTCCTCGCTACTTTAATGCACAGACGCAC
AAGCATCAGGGATTTACTATCCTGGTCTAGTCTTTCCTTGCGTTCGGAATCG
CGAAGTCGCACCAAACTGTAGCTAGCAGTATGATTCAACGGCAACTGGCGT
CTAGGTTACATTCAGATGTGATACGGCACGGCTACTATGTAGCTAATACGCG
CGACTGATGTCGGCTCTCGTTTCCTTCTCCGATCATGGATCAAATATATAGAT
TTATTTATATATATGGAGAATCTAGGCTTTTGCGTTTAAGAGGTCTGAGAACG
TCGAAGGCAACGCTGACTCCGATCTTAATATTGGTTAGACACGCACAGTTG
CGGAAGTGTTCTCGTCACAACTCTGAGTTTTGCCTAGTTCATAGCCGAGGA
ATAATGGCGACTCGCAAAGCTACGGTGACCTAACGGGCTGAAGCTCATTGG
CGCAACTCCAAGGTCCTCCCCTACTTTGAAAGGATTCATGATTGGGCGCGT
GAGATCAGACTGCAAGCATAGCCACGATGTCATGCAGTTTTGGATCGGGAT
GCGACCAACGAAGGCTACCAAGACCTTGTCACCTAAGCATCGGATCTTGGT

CAAAACGTTGAAGCACCTCTGCCGTATGTAGACGGATGGAATAATAGCAGA
TAGAGAGAGGCGGTCAAAAAGCAAATTCCAAGAGACTCATGTGCTTTCTG
CGACGAAGCGAGGCTCAGTCATCTTATCCGCGAATCATAAAGTTTTCAATAT
CAATCACACGATCATCTTGCAGGAAGAATGCCGCAAGTCCGGGACAAACA
CAGCCAAACGGAATATCGAATCCTTTTAGGTTTGTATGTCAGGGTCCCTCCA
GCAAACTACTGGGTACCCAATTGTGCGGACGTGGGAAGATAATCCGGAATC
TTTCCCCCGCTTTCATAGCAAGACCTACTAAGTATCAACTGTTAATTACCTAT
GTAAAGCTTCAGTTCAACCTGCTCAGCGAGTTTCATATTAGAAGCCTAGATT
TCTGAGGTCGTATTTTATTTTTGCGATAGTTCGCCCCTAATCACTTAGCGACA
ACAACTACAACAACCTGATAATATACCATTATTAAAAAGGTATAGATCATTAC
CTATAAGTCGGAGAATTTTCATCACTAAAGGTTTAGTAGTACCCAGTCGAAC
TCCACTGTGATAGTGCGTATGCATTGTACATACATCCATGGAGCAACTGCAT
ATTAGTACGTTATGTACAAGGAAATATGGGATTATGTTTACCGTATTTCATTT
ACTACAGCTAAATAAAAACCGTTTCTTACCATGCTGCTTGATTCTACCGTCA
CAGGGAGGTCCTAGTGACTGTCCCCCGCTCTTCAGACAATTCAGGTTTTCA
AAACCACGAGCTGACCAGTGAGGTGGGATTCCCAGGACCACTGCCACACA
CTGTGTCAAGGATAACTTCGATGCTGACAGGCAGACAGAACAAAGCTACC
TCCGCTGATTCTTCCGTCATAACATAAATTCTTTCCCCTCATATTACAAGCTT
GTACGGAATACCTACCTGCGAAGACCATTATCATCCACAGCTATGTGTCTCT
GTGCCATTTCTACCCATCGTCCACTAAAGACCTAGCAGATTGGGTTTTTCTC
TCTTGATCTTCTCGTCACCACAATACGTTCATATCTGTTTCTCAATAGAGATG
GCCTTCGACTTTTGGAATACACCTATTAGTACCCTTTTCAATAATGATCTCGA
GGGGACCAGAACCAGGAGATCACCTTTTTAATATACCCCTTATGAAATTACT
CCTCAACCTCACATTCCGACTCGGTCACCTCGCTACGAAAAGTGACAGCCC
TTCCTATGACGCTAAAATATTTCTCAGCCGTGGTGATCGAAACCGGAATCGA
ATTCTAGAGTGCTTGACACAAAAAGTGGTTGCAGGAAGTACGAAAGTATA
AGTGAACAATCTTATTGGTCGAATTTTATGCGCGGCAGTCTATCCTGTTTCT
GGCTTAGCCCTTGTCTCGAGCATTGCCCAATGCGCAAGAAGGCTGCAATGC
ATTCCAATGTGAGTGGCTTCCTTCTTTCTCCTCAGTGACACTTGCTCCTCCT
GTTCCCCTCGCGCGTACTTTCCCATTTTCCCCTCGTCCTTTCAACTCTTCATG
CTTTTTAATAGCCTAGTGCAATGTCGTCTAGGTCGTCTAGTCATCCTAAAAC
TCCCCTTGCTCTTATCGTTATCTTTTATTATTTTTTGCTCTTGGTATGTCTTCG
TCTACTAATGAAAAGTGTACAACAGGCAGAACATGTTGGTTGTGTCTCGAC
TTTCTTTCATTTCCCTTCCGCTTCCGCTCCCCTCTCCTTTTCCTATTTTGAGT
CTATACCCACTTTTAATTGGTCGCAAGTGCTGCATCTTTCATCAGCGTCATG
GAAAGCCCAGGAGAGTTTTTAAAGACGGGTCCTTTTTTTTGGTGGTTCCCA
GCACCAAATCTTTCAATGTGGCCCCGCATGGCCGGAACAGAACGCACCAG
CCAGCTTCGAGAACAGCAGCAGCCACACATGAGCGCAATGGGGCACTGCT

GCTGCACCACAGCAATCTTTTTTCTTATTCTCTTGCCCCCTTGTACCGGTAC
ATGTACATTAGTATGTACATTATATATCTTACCTCATGTACATGTGTACATCAA
ATAACATCAAATACGTGCTAGTCTTCATAGGCACTATTTTCCCGCTTTTGCTC
CCATCGCTCCTACTACTTCCCACTACGCACCACCCCAAGAGCCACGCCAAT
TGGTGCTGATGCACGCACCACTACTCAAAATTGTCAGTGGGAAGCTGCAA
GTGCCCTGCAACGCTCGCGCTCCCACTCCGCTCTTGCAGGGCCAGCCCAG
CAACCCGCACCTAGCCCAGCACCCAGTACCCAGCACCCAGCACCTAGCAG
GTAAGGAAAGAGTGAGGGACTTGGACTGGGACTGACTAGGACTGGATTTG
CCTAGGTGGAAGGTAGAAGGTACCTAGGGTAGGTAATCTCTTTGGCGCGCA
TTCTCTCTTTCCCACCCGCCAACTCTTTTGGCTTTGGTCTACCCGGGGCCTT
CTAAGTTCTAACTCTCTCCCTTCTCATACTCCATTACCCATTACCCATTACTC
CATACTCCTCAATTCTCTCTCTCCCCCATCCTCACCCTCATTTTTTCCCGCGA
CCAGTTCAACACGAATTACTTTCTCCTTGAGTCCCAAGCGTCGAATCTCTC
GAGATACTCTTAAACTTAACTTAATTCCGAACTCGCCATTCACGCCCGATCC
GCTCTTCTGCCATTTTTCTACGAGGAGCATTCTTCCCTAAAACAAGGAAGA
AGGAAAAAACCGAAAAAGCTCGAAATTGTCATTTCCCGTCGCCACGCCCT
TTTCGCGAACACGCCTTCAACTCTTCATTTCGCTATTCTACTCAACACACAC
CATCGTGACGGAATCTTTTCTTAGTATTTGTGATCAGACGATTATATACTTTC
GTCTGCCCTACAAAAAGCACCGATTTTCCTTTTGTCCACCGTGCTGTCCTTT
GTGTGCTCATCCCATGCCTGTATTTCGAGTCCGGACGTGTCAAACGTGCTG
AACAACAACAACAATTATTCCTCGACGCGCCGCGCTTTTGATTTGCTATCTC
CTCGACAACCACGAATGATATTCCTACGAGACGATCTCTTCAAATTCAAATA
CTTTGGAACCGGTCTCAGTCACGTCGAACCCGGATTCGTCGTCATGCCATC
CGGGCTTCTACGATAAACCGCCATCTCCTTTCACATACCTCACCGACAATCA
CCTCGTGAGGTGCATGTTAGATTATGACTGACAGGCTCTCATCCTTGCCGAC
GCAATCAGTGACTCCGAGTCAGACGGCCGTCCTCAGTCCCAAATCCACAC
CTGCCTGGCCTCATCATGTCTCACACAAGGTCCAGGCGCCGATCGAGGAAG
GAACCCTATACGATCAAGTGTATTTGTGACTTCGCTGACGATGACGGGAAC
ACTATTTATTGCGAGACGTGCGATACCTGGCAGCATATCGAATGTTACTACC
CCCATACAATCGACGAGGCCCTACGAGAAGATTTCGCTCACTCCTGCAATG
ATTGCAAACCTCGACCACTCGATCGCCAAGGTGCCCTTGACCGACAGCGAT
TACGAAGGAACAAACCCATACGTCGGAGGCTCCGAGAGGAAAGTCAAAC
GGCCTCCGACTAAGAATACAAAAAAGAAGACCAAACCAACAGACTTAATA
CCGAATGGGCACACTACTTCTGATTCGAACCCTAAACACTCCCATGACCAC
ACCTCCACTCACCAACACAAAAAAACTAAGAGCACCCACCGGCCGAGCCA
ATCCATCTCCTCGCAAAACCCGAAACGTAGCCCTTCCTATGGCCAAAAGGC
TGGTAATCCCCACGGTCACCCACCCAGCCCTGCTACAACACCCCCGGATGT
TCCACACGAGTTCGAGATGCAATACTCGAATGCCTTCCTAAACCTTTACAA

AGACGATCGTGACATAGCCATTGTCCAAAACAAATTCTTTCGCCAGCCTTT
CTATTTCAAACGTTCTGTCGTTGTGGTTGCGAGACCACGACAGATTACGAG
CCGAAGCAGGATGCGCTTTTGACGATGTTTTCCAATCTTTGCCTGCTAACAT
TGATTCCATCAAGGCCTCCCCTCGAGTGGAGTCGAAGAAAGTCGCTGTTGG
ACCAGATGCTTTCGTGCAATCACAAAATCTAACAACTTCCGAAATAATCGA
AAAGGATGTGCCCTTAATAGAATTAAATGGCCAGATTGGGATACAAAAAAG
ACTACTGCGAAGATCCTGAGAATCGTTGGCAGGAGTTAAGCTCCCCGCTCC
CATTTGTATTTTTTCCATCCGATGCTCCCGATTTACATCGATACCAGAAAGGA
AGGCTCCCTCGCTCGCTATGTACGACGAAGTTGCAAACCGAATGCGGTATT
AGATACTTATTTGTCCGATAGCTCTGAGTACCATTTTTGGCTTGTAAGCGAC
CGGCGTATACTACCGTCAGAACAGATTACTATTCCGTGGGAATTTAACCTCC
AGAAAGACGTGGCACGAAGATGGGAACAGCTGCTTGGCCTCGGTGACGA
CGAAGCTAGCACCCAACCAGCTTACAACGAAGAAGAGACAGATACATACC
AAAACATCTCATCTTGGGTCCATGCCATACTCACCGAATATGGTGGGTGTGC
ATGTGATTTGGCAGAGGGCTGCGCGTTCACTCGATTCCATCGAAATTACATA
TTGAAACCTCAGCAGCAGGCTAAGGGATCGAAAGGTGCCAAGAAGAAGC
CTCGAAAGACCAAGACGAATGCTCTATCACCGACTAGCACTGGCCACGCC
ACAAATAGCCGTGCCCCAAGTGAAGGCCACGGCGATGATGGAGCGGACAA
CGACTCGGGCTCCTCTCGCAGTAAACCACCCAGCCGAGATATGACTCCTGC
GCCACGGCAAGGATCTTTTGATCAATTAGGTGTATTAACAGAACTCACTGA
CAGAGACAAGCGGAAGGTTCAAATGGTCGAAGATACATTCCGCGAATGGA
GCAGCAACAGCCCCCGCGGAAGAAGAAGCGAGTTTCGGATGGTACTACTA
ATTCCACTTCAACTACAAAGTCGAAACCGAGAGGTTCGTCTATTCATTCGA
CCGGTGCCCCTAATGGCGCACCTGAGCGTCGCTACGTCGATGCTGGTACCT
CTCGAAGTATGTCGGGCTCTCCGTCAAGTGCTATTTCGCCAAATACAATCAG
CCATTATAAATTTGATCAGCGGAACGGTTCAACGCAAGATCCATCACGGCG
CTCTTCGCAAGGACCACGTCCAGAATACCGTGACGTATCTGTCCAGACAGA
TCCGGTTGATGGGGAATGGTTTAGCAGCCCCAGGCAGGCTTCGAAACCAA
AGCGACGAGTAGTCTCCCTTTCGAAGCGATTGCTAGAATCGCGGCATCGTT
TACGAGCGGATGAGGATCGTAGAAGAGCGACCCAAACTGCCTCACCTACAT
CAGTAGTGTCTGCTCTAGTGAAGATGGACTCGAATCCCCCACTACCGAACA
TAGCCCTGGACCAGCAGTCCCAGAAGCCCCAGAAATCTTGAAGCAAGATA
ACCCAACACCAACACCGACACCAGCAGACGACTCGACTACCGACACGCCC
ATGACTGATGCCCCATCTATATCTCCTATAGATACTAAACCTGCTCTGGTCAC
CGTTCCGACAAGCGTACCGACCAAAAATAAATCGCCAGAATTACGTGTTCA
AATGCCTCCTGTTCCTGCTTTCAATAGCTCCGCGTCACCGAGTGTTACTACT
CCAATGTCCGCAAACGGAACTCTAACACAGTCGCCCTTCTCTACTAGGCAA
TATGCCCAGCCCCTTTGCTCAGATATCAGTAAACGGTGTCGCCGTTCAGCCC

AGCCCCGTCAAGAAGAAGCTGAGCTTGAGCGATTACACGAAAAGCAGGAT
GAATAAAGCAGCTGCAGCGAAACCTGTAGGGCAATCGCTAAAGCCTCCAG
CTACAGTTATGGAAGAGGCCAAGTCACCTACGTCTACGGATACGGTGATGC
TCGAGCCTCCAGCTACCGAGAAGAGTGTCGACTAAGCCGCTAATACGAGT
GTTGTATCGAAGACATTCGTGGTATATAGAGTGGGATACCCGATGCATGGCT
TTGTTATCACCGTCGCTATTGCTCTATGAAGTTTTGCCAATCCGTTGTCGGG
TATATCTTGATCTCACCATGATCAAGAATGGTCATAAATGTCATTGGTGCCTG
GAGGTGGATGAGCATGTCGAATGGGCTGCATTATTCTCTGCATTAGGCGTTA
GGGAGGGTATTATCAAATGGGGTTACCGGGTGGTTCGTGACGGGGAATTGT
CATGAGGTTTTTCGCAAAAGATATCGGTTTATTTCGCAATCGACAGCTTCA
TATCGAATCGAACTTGTTCACGTGGTCGGATGTGAGGACTCTTATGTGGTTT
GGCTTTGCTTCACTCTGGCTGGATTGGATGTTGATGACTATATCATCATTCCG
ATCAGCAAGAGATTTACTACTCGATGGGTAACGCGGCTCTCGAAGGACCAG
ACGTTCGTAAAGGCCGAAGGGGAAAACGGAGTCCAAAAGGTTGTAGAGAT
TCGTATATCGTATATGTGTACTACTACTAGAAGCCGAAATGCTTTGGTCGCG
GGGTGTAGGGGCTGGGGAAAGGTGACGAACGCCTACAACTGCATGACTAG
ATGAGGGCCGCAGTCCGGATTCTGCGGGGATCCCGTTAAGGGGATAATCAA
TACCAACCAAGCAAGATTTGTTAAAGACTACGCCGCACCTCCGTGCTTCCA
TTGGTAGAGTGATGTGAACCCTTATGTTCCTAACTTGTGCTGCTAATGTTTT
GAAGTCAGCTCTAGGGCTGGTCTACTAATGGGTGAATATGAAAGCGTGTAT
GTCTGAGTCAAGCAATGGTGGGTTGTGATGATAGTCGTACGACCCAGTTTG
ACTGTTTACTAATGAGTCCGTTGTATGTGCGTGAGGATACTTAAATTAATAA
GAGGTGAGAAAGGCGGTGACAATATCTTTTATAAAATGGTGTTCAAAGTGT
GGATGCCGTCAAGGTGTGGCTGGACCCTGGGGGTTGGATTTGTGTGTAGTA
AAATACCTACCACGGCGCTTGCATCGATCTCTATCCACATATGCCGAGGACT
AACCGTAACCTCTTGGTTAACCAAGTGATAATATTGTAGCTGCACATTTGTG
GGCTCGTCTACACAAGCAAGACATTTCGTGCGTATTGTAGCGTTAATAGCAC
AAGTGATATAGTAAATCAAGAAAGTTAGTCTCACGACCATCGCCTGACGGC
TAGTTTGTAAATTTTGTACATACTATATCAAGCACAATTGAGCATGGGCTACG
TATCAGATCCGAAAGATCAAGGCTTGACCTATCATATAATATCATCCAATCAA
ATCAGACTACGCGCAATCTCCTACGCACACTTACCTTTGTACACAGGAGGA
TCCGAACTCCGAGTCTTGAGGCTTAGACTAAATCTCGATTGGAACGAGGAA
TCGAGACTTGTTTCTATTATTGGTGGATGACGATCCTAGGTCCTGGGGCGTA
AAATGTGAGAAGTCACTTTTGGGATAGGTAATTGTTTTCTTTCGTAACACAG
CGACCTGGTTTTGTCCAGGCCTCGCATTTCATTGCGAAATATTTAATTTATGT
ACACGAGATAGCGATGGGCGTCATATGCCAGCTACCTTTCCGTAATCTTCAT
TTGTACAGCAACATTGGGGTATCTCCGAAAAATCCATCCCAGTCTAGCTCTC
GCGGCTAGACTGAGCCTTGTAATATTGAGGTTGTCCTCTCTGAAGATGAGG

AGTGCTAGTCGGTGGCGCACGAAGAGACCGAAGAGGCCTAGGACTGCTTG
CATGGCCGTGATCTCCATGGCGAGTCTGTCTTCGCGCATCGGTCCTCGCCA
GACTAGCCAGAGGTTCAGGATTGTAAAGGTTGCTCGTTTCTGCGATTCGGC
CTTCCTTATCCTCCGTAAGACGTAGTAGCTTGAACTGCGCCAGTAATCGCAA
GACCTTCTCGATAATAAGGCGGGGTTGCTTATCCTCCTCCCACTTCGTCACG
CTGGGCTCGAGCAATCCGGTTCGCGCGTTGAAGTGCGGCAGGCGGTGTCG
TGGGCACGGGATCAGCCCGAACAGCTGAGGCGTGCTGTAGACGAAGTTGA
AGATCTGAGGTACCAGGAGTAGGATCAGGGTCTTCGAGAAATGGCCTAGG
ATACTAACCACCACGAAAACCATGCCGGCGAAGTAACAATAGGTATCGCCG
CAGAAGACGCGTGCAGGATACCAATTGTGTATAAGTAGCGCGAAGCTGAC
GCCGAGGAAGGGCAGGAGGAAGTAGAGCGAGAACAGATGTGAGTCCATC
GCCGGTGGGGGTACGGCGTCAGTAGGTATAGGGCATCGTTGAAGGCGAGC
AGGAGGGCGATCACGATGCTCTGGCTAACTTCGATCCCATTGATTCCAGCT
AGAATGTTAATGCTGTTGGGACTGAAAATAGAGAACGCCGACATGTAGCCA
TAGTAGAGGACACCTAGGTCAAATAATTCGCGGCCTACGTAGGGTTGTAGG
AAGGTCGGGAGGACGATCGATGTAACGCCGAAGTCGATGAAGTAGATGAC
GAGGAGGGGGATGGAGGCAAAGGCTGGGATGAAGAACTTGTGTCGCCAG
CGAATATCGAACAGGTCGTCGCCAATGCCGAGTAGGGTGATCGATTGGAGG
AGATGATCGCAGAGAGGAAAGACGCGAGCTGTAATTGGTCAGTTTGACGT
CTTACAAGCTAATTGGTTGTAGCGGAGTAGCGAAAGCTATCTTACCTTGTTA
TGAGGGAACCTGTGAAGCAGTCGTCCCTGCTGAACATACTCGAGTTCTTCA
CGACATCACGGTTTCCACCACCACTAGTAGCGACCACAATATCCTTGAAGA
ACGCAAACGGGAGGAATACGATGAGGGTTAGGATGAAGACCACGGCGCAA
ATGGCTCCCATACATTCCGGAATGTAGTGGCTATTGTTCACCTTTGAAAGAT
CGCGACCCTTCAATCCGGCCTTGATGAATGTAGGACCAAGCCATCGTATCAT
AGCATAAGTACTGGCGAATGCCAGACCGCTAAGGGCTGAGACTGCGATGA
GTGGTTCACCCTCGCCATCGAAGGTTCGGACGAGGACGGCAGCGCAGGCT
ATGGTGAGGCAGGAGAGGTTTAAGGTCTCGGTGTTAGAGAGATGGGTAGA
TTGCGCGGTGGTCGGCATCTTTGCGTGTGAAAGGCGTAGGCGTCCAATGCC
TTAAGGCATCCTTCGTGTTTTTTTGTCAGGCGAAGGAATCGGCAGAAAAAC
CGTTGTCCGCCGATATTTCATCGCATTGGAATGATTGGAATGGATACCCCGG
GTGAAATGTGCGTCGTGGCCTTAAGTGGTGGGTGTCGTGGATGTTGACGAA
TAAGGCTGAGTGATAAGATAAACGCGACGTTTATTTCAGGGGACGGAAACG
AGACCACGGAAAAGTTGAAGCCACATTGGCGGATAGGTACAAAGTACATC
ACTGTACTTGGCAGCTTCGTAATGGGGACAGAATCAGAAGCTACGAACCTT
GAAGTGTAAGGGTCGTCGCAAAATTCCATAGCCAATCACATCCGATCACAT
GCTTCTAAGACAGCCTAAGAAAGCTTCATTTATGGAACTTAGCGTCACTGC
TTAGGCACCCTTGCCCCCTAACGAATTACGTACAGCGGTCGCCACAGGTCT

CATGTCTAAGCCCTTCAAAGGCTTGCGGGGAGGTGACCTCGTGAACGAGG
TGACGATCGATGTAAAGAAGCGTGTCGCCCTCGCCCGAACGGACCACATG
GTCGTCAAAGACCTTGTCGTAGAGGGTGCGGGGAGCTGAAGTTGGGGCGG
GCATGACGGGGCGAGTGATGGTTTTCAAAAAGCAGTCGAGTGAATGATGT
AGACACGAGATATTCCTGCTCGCTGGAGCGAGATATATGTGAAAGAAGGGG
TAAGAAAGAGATGTACACGACTCGACGTTATGATGTAGAGAAAGTGGACC
AACTCTTGCGTTTTCGGGCGAATCTGGTTGGTCAGTAGGCTCATCGGACAT
GGCCATGCCGGCCGACCTCGATCCTTCGCAAATTTCGGCCGACGAAGAGCT
AGGCGCCCGTGGTCAGTTACTATTAAGACTGTTGTGCGCCTAATCTGTTGTT
GGGCTGTCTTAATCCCAGCCCGTTCAAGAGACGTTGCATCTGGCTATACACT
CTGTATTCCAAGCCCGGCCTGTAGCTGCCATGCTGCCATACATCACATGCCT
CATGCATCACTGAGCCTGGCAGTCGGTGATGCGGTCCTTTGGGTCGTCTTC
GATCAATGCGGAGCCCCACGGCGACCACGAGTCGATTGGTGCCTGTTGCA
GACTGTCACCTGACAATTTTTGCGAGCGATGAGATGAACGAGGCAGGTGG
CGTCGTCCAATCAACAGGCAAGACCGGAAACGGCCCAATCACCCACTCTG
GCCCAATCTCAGCCCCTCCACCAACCATGTTTTCCTACCTGCGGAGCCCATT
TGGGTCACCGAAATACCAAAGATGCATCGCTCGTACAATTTTAACAACGAC
TCTTGTGTGAACAGCGCGACCAACTTGACAAACGCTCGAAACCCAAAGCA
GAGTGACGAAAGATAGCGTCCCGCGGCGCCTTATCGCCCCCTTCCGACGCC
TTATCTGGGGTGACCTCTCGAGGCTGCGTGGGTCAAAGATAAGGTATTTGC
GGGGGAGAGAGAAAATAGACAGGCTACCCCGAGGAGAGGCCGAGGAGGA
TGGTCAACGTGACGTATGGCGTCCTGTACACCAACAGCGATGGGACCATCA
CACTTGACCCCCTGGGCGCGGACATTGTCAGCACTATCAATGGGACGGCGA
CTTGTTTGTGTCTCCCGTCAGAGGGCTGGGACGAGCTCATGTAGCAGCTTT
TGGCAGCGGAAGACGTCGCATGGGTGTTGACATGCGGCGCCTTGATAATTT
TCATGGTGAGTCGAGGTCATCATATGCGGGAGCTTGTGATGACAGTAGCTT
CCCGGTCTGGGCTACCTCTACTCTGGACTGGTACGGCGTAAAAACGCCCCT
CTCTCTCCTATTCCTATCCATAGTCTCGCTCGCCATCGTGTCTTTCCAATGGT
GGTTCATCGGCTACTCATTGGTTTTCTCAGAGACGGGCGGCTCATTCCTGG
GCGATGGCAGGTCAGTCCATTTCTGTCCAAACTAGTGTATGCTGACAAGCG
AGAAATGTGTTCCTCCGAAACGTCCTGGACACACCGAAAGCGGAGAGCAA
TGACCGCCTTCCCGAGATCGTCTTTGCCATGTACCAACTCTGCTTCGCGGC
ACTGGTACCCGCCATCCTGCTCGGTGCCGCCGCAGAACGGAGTCGCGTGTT
TCCAGCCATGATCTTCATCTTCTGCTGGACGACCTTGGTCTACGACCCATTA
GCACACTGGATCTGGAGCGTGCACGGGTGGGCAGCAAAGTGGGGCGTTCT
CGAGTGAGTGGTGCCAGACTTCAGATGGCGAGCCGTGCTGACTCACAGCT
ATGCGGGCGGGGTGCCCATCGAGATCTGCTCCGGTACCGCTGGTCTCGCCT
ACTCGTACTTCATCGGCAAGCGCCGAGGATATGGCACGGAACAGCTCGTAT

TCAAGCCGTCCAACGTGTCGCAAGTTGTGCTGGGCACCATGTTCCTGTGGG
TCGGCTGGCTGGGATTCAACGGCGGGTCGTGCTTTGCCGCCTCGCTCAAGG
CGGCCTTTGCCGTCTTCAACACCAACCTCGCCGGCTCGACGGGCGGTATCG
TATGGCTCTTTATGGATTACCGTCTGGAGCGGAAGTGGTCCGTCGTGGGATT
CTGCACAGGTGCCATCGCGGGTCTCGTGGCAATCACGCCCGCGGCGGGCT
TTGTCGGTACACCTGTGAGTACTCCCCGCCCACAAATATATATGAAAGCTGA
CCACAGGCCGCTGCGCTGATCGGTGTTGTCTCTGCTGCCGTGTCCAACTTG
TCAACCCGCGTCAAAACACTATGCGAGTGGACGACCCGTGAGTTCACGCA
ACAGCACATGGCCGAAGCGTGGCTAAGGGCAGAATGGATATCTTCGCCGTG
CACGCCCTAGCTGGCGTCGTGGGGCGTGATCATGACTGGCCTGTTCGCGCA
GTCATCCGTCGCTGCAAACGACGGCTACCTGGACATACCCGGCGGGTGGCT
CGATCGACACTGGGTGCAGCTGGGGAAGCAAGTCGCCTGGGCCGCGGTGG
GGATCGCATGGACTTCGTCACCACATACGCGCTCATGTTCGCCATCAACCTC
ATCCCCGCTGCCGGTTCCGCTCGACCGAAGAGGCCGAGATCGTCGGCATG
GACGTGAGCCCTCATCGGCTCCGAGCTGAATTGATCGCTGACATGAGCAGG
AGGTCGAGCTGGGCGAGTACGTCGCCGACTACGCCTTCATCGAGCGAGAC
CTCGAGGGCAACTACTCGCCGCCCTCGTCCGACGGTCCCGGCGCGCGCGG
CAAGTCCCTCATGGTGCCCATGCTGGACGAGCCGACCAAAGGCTTGCACA
GCGCGTCCGACATGTCCACCGGCACCGGCATCGACAGCGTCCAGACGGCC
ACGCAGACGATCATCACGCCGCGCGACGCGTCGCGCAGCCGGTCGCGGAG
CCGCGCGCGCACAAAGGGCATGCCCGAGGTGACCGAGGAGGTGGAGCTG
AGCGATATTGCGCGCATGCGCAGTCGGCGCCTGAGCGAGATCATGATGGAG
GAAGCGGGCCTCGGCGGTCCTGGCGGGCGGGGGAGGACCATGACGATGA
AGAGGGAGCATCAGGATTGAGCAGCATCAATATCGGCATCATGGCATCGCA
TCGTTCGTATCTCAGCACGTCCGTGGCATTGGCAGCACATGTAATGCATCAC
ACAGCATACGCATATCAAAGCACGCTCCTCCCGCGGCACGCGTCCCAGACC
GGCATTGACCGCGTAGTTGCAAAGTCAAGTCCGCGCGATTGGAGGTAATCG
ATGACAGCCACGCTTGAGGTGAGGTTGTTTAACCATATCCGCTCTGGACGA
GCGACCGACTTTCAAGCATGTATGCTAGGTGCTTTTGGCTACATGTCAGCTG
CCTAAATGAGTCGCAGACAGCTTGCTCACAAGAGAGGGAAGATGAGTCCG
TAGTCCACAGACAGGTTGACAAACTGCTCAACGGCATGTGAGGGAGTCTG
AGTGTACCCTGCAAAGAGTGCAGGACTGGGACGTCAGCTTGTACTTACACT
GCCCGCAGCTACTCACTTGGCCAGCAAGCCCCTTGCCGCCATCACGCCTTG
GACCTCCCGTCTCTTCTCTCATCCTTCTTGCGTCGTCCAACGTCCACAGATC
TCCGTTCGCGACAGTGGGCACCTCTCCTCTTACACATTCGACAGCAAACTT
GAGCGAGGGTAGATTGACAGGTTCGGTCGAAGCCTGATGTCGGGTCCGAC
CGTGTATAGTGATATGCGACACGTTGGTCTGCAAAGCCGTGCGTATGAGTT
GGTCGGTCAATCTGCGAGTCAGCATCGCCTGTCAAGCTGCTTGTCTCGGCT

CACTTGAGATCTGGGTCGACCCGTATCTTGATGCTGACCGGGAACTCGTCT
CCCAGCCGAGCCTTGACCGCCCGCACCATGTCTCTCACCAGATCGGGCTTG
CGCAGCAGCGCGCAGCCGATACCCTCATTGTACGCCCACTTCTGGGGACAG
CCGCAGTTGATGTCGATCCCATCGACATACGGGCGGATGAGCTCGGCAGCA
TCTGCGAGACACTCCCCATTCGGCGACGCGAACTGCGCAATGAGCTTGCC
GCGGACAAGCTGAGACTGCTTGGTACGCGGCTCGGAAGTCGGTGGCAGTC
TTCGTCTTGCGCTCCTGGAGCTCTCGCCGTTCTTCTGCAGCTCAGTCCCCGT
CCTCGGGATGCGGCTGGAAGGTGGGTGTCGAGCCGGCGTGCCGGGGCTGC
ATCCAGAACACCCCGCGCTCCGTTGCCGAGGTGCTGAAGTCGCTCATGCGC
GCTGTTTGGGATCGCGAGAACTCCTTGTGCGAGCATCATGGGCGTGTGCGT
GACGTGGGTCTCATATAAGCTGACGAGGTGCCGAAAGGGCAGCTTGGAGC
ATCGCACGAGCGGCGCCAGGACGTTGACGGTCTCGTAGCTGTTGAGCAGG
TCGGCCGTCGAGATTGTGAGCGGCTCGGGAAGGTCGTCGAAGGCGGACAG
GTCGACATCGTGCTCAAAGTCCTCGTAGGCCATGTGTGGGGCAGGATATCT
GAAGGTGAAGTGTAGATGTTACTGCGAAAAAAGTCTGGCAGGTGGACGAC
TCAAGTGGAGGCTGATCTTCACGAGATCGCATCTTGGCTGCACCATATGAC
TGGAACGGAACTCGTAGAGATCCCAGAGCACGGTGCTAGCGCACTCGTGG
ACATGAGAGCAGTGTATGCGATACGTGACCCAATGATGATGCAGAATGGCT
AGAATACAAATGACGCCCTCTTCTCTCTTCCTCCCTCCAACGAATTGTCGGT
CGAACCGCCTGTCATCGCCTCACTTCTTTGCTGCTTGATCAGACTTATATCA
CAAGTGATGGGACCCGACACCTACTCAAGAAGAGCTTTCGCCTCGTTGAC
CTTTCCTGCTAGGTATGGTGCACCTCCCCTATCAGGATGGTTGGCGAGCATC
AGTCTCCGGTGCGCATCCTTCAGGCGATTCGATGTCAACGGATCCCTGCAG
AGTCAGCATTGCTGCGCGCTCCGCTCTCGGCAAAAGCACGAGACCCTCAA
CTGCTCCAGCTCTGCATCCTGCAGGGGGAGAATGGAGAGAACATCGAGGG
TATCCTGCGACATCTAGCCCAAGAGACTCACTTCAGCCCGAGAATCTGCAT
CGCCTCCACTCGATCCATCTTGCCCTTGAAGCCACCTCGTAGGAACTTGTCT
GCCGGTCCTCGTGCCCGGAAGAGCTGCACGACCACACGTCCTGCCAATCC
GGCGCCCAGGAGACCAAGACCGAGGGTGAGGGCCGAGGCCATCGTGAAG
CGTCTTTCGGGATTCGTCTCAGACAATCTGGTGTGGCTTCGACTATCCGCTG
CTCGGCACGTTTGGAGGGCGCTGGCACTGCTGATGTAGATTGTGGGGAATG
CAAACTGTGTATTGCGAGCGCTGTAGACGAAGCCGAACCGACTTTTGCCGC
CGGACCATCAGCTCAAAGGGATCCTCCAAAAAATGCCACATTCGGCATTAC
ACCCCTTCATCAAATTCTCCGCAGCCCAAGAAACTTGCGAGGGTTCCCCAT
CTCCAACCTCGAATTCCCAGCTGCTTGCTCTCTTATCCGTCAAAGTCAGACC
ACACGAACCCGACACGATGGCCCCCACGAAGAAGAAGACAAGCAAGACC
TCCGAGAACATCAACTCGCGATTGCAGTTGGTTGTGAAGAGCGGAAAGTA
CTCTCTCGGGTACAAGCAGGCCTTGAAGCAGCTCCGTGTTGGCCGGTGTG

AGTGCTCCTCTCCTTGGGACCGAGGAGGCGCTTGGGAGATGGATCGAGGA
GCACGACAGCGTAGCTCTGGATGTCAAAGACTGCTTGGAAGCGAAGAAGA
GGAAGTTGATGGGGGTGAAGAAGAGGATTGGCGGAATGGTGTCCAACGAA
TGCTGACCCTGCAGCCAAGCTCATCATCATCTCCCGCAACTGCCCCCCTCT
CCGCAAATCCGAGCTCGAGTACTACGCCATGCTGTGAGTGACGACTGTTAC
GCCCTCGATGGAGTGTCGAGAAGGAGAGTGAATCGGAATCTGACTTGAAG
TTCGAAGACCAACGTCCACCACTACGACGGCACAAACGTCGACCTCGGCA
CCGCCGCCGGCAAGCTTTTCCGTGCGGGCATCATGTCCATCCAGGACGCTG
GTGACTCGGACTTGGTGAGTGCCGCAACGACACTCATGCGCAAGTGCTGA
TATGCGTAGCTCCAGGTTGAGGCTTAGGGCGATAGCATCATGCATTGTATTA
TACGGCACTATGATGATTGGCTGGACGGGGCCAGGCATATGCGCATGATCTC
TACGGGACACTGATTGTCGAGCGCCGTGTCGAAAAATGTTGGATGTAAGGA
CTTGGAGGTGGAAGATCGAGCGTGTCTACAAGGAGGCCACGCCGTGCTGC
TCGGGGTCAGCCGATGTGTCTCGGCAGGAAGGGGTTGCGCACCACATGCC
TGTTTGAAGAGGTCGAGCGTGCGGGGATTGCGGACAGTCGCTGCTATCAA
GGCGAAGGTCTGGTGTGCCAAGAGGACCGCGATGGTCTCGACAAGCAATG
GCACGATGTCGGGTCGTATATCTAATCGTGCCAGCTCTGGCAGCGACTTTCC
ATGTCCAGCTCACCACATCGCAGCAACGATGACGCGGGGTTTTAAGATGAG
GTCTGACCATACCGCCGGTCGGTCAGAGGGGGACAAAGAAGCATCAGACC
AGTCGAGTGCTCTGGACTCCAGGTGGATGTTTGAGCTCATCTACCGGTCAG
ACCCAAAATGTCATCTACGAGAGACGCACTGAGCGCTACGTTATGTCGTAG
CCGCGCGAGTGGTGCTGCGCGCGTCTCCCCCTCTTCTGCCTCGAGGTCGGT
GGCCAAGACTGACCAGCCCAGTTGGGCCAGTAGGATCGAAAGGAATCCGG
CGCCGGCGCCCAGTTCGACCAAACCCGTTGTGTCGTGAGCCGGCAAGAGT
GCGGGTTGTTGCAGTACATGATGTGCGAGGTGCAGTCTAGCTATCAGCACT
GTGCTCACAGTCCTGTGGTCAGACTGACGCAGCTGTCCATGTGCGCAGGC
CCGTTGTTCCTGCTTGTATGACTGCCTGGTCCTCTAGCAATGAGACGTGGG
ATTTCTTTTGCCTGTCTCCGGATTGACACGCCCAGATCGGCGCATGGTAGAT
AAAACGTCCCGGTTCGAAGTGCCGCTGGATGTCAGCTCTTCCTTCTTGGGC
TGCTCATGGGCCACTTACGGATCGTCGCCCCCGTCACTGGGCAACGTCATG
AGCTCCGCCACCGCCCCGTAGATTCTTTCGTCCACCTCGATCTCATCCCTTC
CGCCGACTGCGCATTGCACTCGCACACGCCGCGCTCCAGCTGACCCACGA
GATACCGCCAAAAAGACTTGCGATACCCCGCACACGGCGCGGCGTACCGG
TCTTCCTCCAACAGACGGTCGACAAGGTATCGCTGCGACTCGGGCTGCGCC
AGTGCCGCACTACTGGGCAGACGGAGCAGGTACGGCGGCACGAGGGCAA
AGTACTGCGCACGAAGGCGTTCGAGCTCGGGCCCGGTGGCTGGGTCCGTC
ATGTCATTTTGACTGCTCTATATCCTCGGTCATGAACTGAGAGAATTTTGCG
GAATACGAGACACTAGAGCGGAGCGAGGGTGGGGTCAAAGTGGGGGAAC

GCCGGCGAGGCCGTTTGGCGTGGCCGGGTCAAGCTAAGCCCAGGCGGATC
TGCTGCATGTTGATAGATGCATGGAAGCAGCGACGATATCTCGCCATGCTCT
CCGCTCGTGTCATGTCGAAACGCGACGACGAGACGAGGGAGGTGTAGGGC
CATCCTTTTGCGGCCGAATTGGGAATACTTTGCTATCCGAGGCCTTTTGGCG
GAGTGCGCTCCATCTGCTCCGTTGTTCCTGTCCTGCACTTTCGCGGACAGC
CTTGGCGACCCCAACCCATTCATATTTTGACTTCGGGACAGCTTCAGACGC
GACTCTATATATACTCGTACGGCCAGACCCACGACAGCATTCCAACTTCTTA
TTCTCACTCTCTCACACAGTACCGTTCAGTCCAGTACCCTCAGACTTTTACT
TCAATAGCTCGTCTCACCATGTCTGCGTGAGTCTACCATATCTGCCCTGTTC
GAAAAGCCCACTGACTTCCCAGCCCAGCCCCAGCTTCCGGCTCTTGCTGC
GACAACAAGCCCGCTCAAGCCGTTCACGACCAAACATGCGGGTACGTACT
GCACACCGCTCAATCCTCTTTCCCCAGGTCCAACCCCAGCGACCGCCCAGC
CGCCGGCACAGGCCACACATCTTCTGCGCACTCGGCACCGCATCCCTTCGC
CTCGCTAACGCTCAGATGCACCAGCACCGGCAACTGCACGTGCGAGCCAG
GCAAGTGCGCATGCAGCAACTGCCCCGCGGACAGCGAGAAGAAGGTCGG
GACAGAGGCCTGCGCGTGCGGCGAGAGCTGCTCGTGTCCCCCGGGAGAGT
GTACCTGCGCGGGCTGCCCCAACTCCAAGGCGAAGAAAGAGGGCGAGGG
CGATGCCAAGTGAGTCGGCGCGACTTGCACGCGCTGTGTTGTGGTGACCG
ATAGATGCTGTGAGGGAGGATGCACCTGCGCACCGGGCGAGTGCACTTGC
GCCAACTGCCCGGGTGCCAAGGCAGCGAGCTCGTGCACGTAAGTCCCAAG
GAACGAAAGAATTTCAAGTGCTGAAGTGCAGATGCGGCTCGAGCTGCTCT
TGTGCCCCTGGCGAGTGCTCATGCGCCGGCTGCCCGAAGAACACCAACAA
GGCCTCGTCCTGCGGCTGCTCGGCCAGCAAGGCTTGAGCCCCTAGCCACAT
CTGTCTGTACATTTGCCACCCAGTCCAGTCCAGTCTAGTTCTGTAGTCAGTC
GCGTTTGCCATCAATGGGTATCTGGACTTGGCGCATGTCTTGCCAGCAGCAT
AGCACCTATGCATATTGCTCGCTCATCGACTCTGGATCGTGAAAGGGAAAC
AGGAAGCAGCTCAGCCGGGCCTCTTGATCTCCATGCTCGCCCCATCTGCAT
GTGCAGCGGGCGAGTCGGGCGCTGTCCTTGCGGAGTGCGCTTATCCTCGA
GGACGGTCATCATCGCTGGAACACCATCCGCAGCGCAAGCAAGTCAACCC
CATGTTCTCCACCATAGCCACGGTCCTACGACCTCCTCGAGTCTTCGTCAGT
ATTAGAAAAGTCTGACCATTGGTCTCAGGATCACCGGTCTAGTCATACTATT
GAGTGATGCATGAGAAGGGGTGTTGATACAGCCTAAATGATCTGACTGTCA
GCTGGTCGCCTGTATGCCGAGCTGACACCAACCCGTCTGAAGAGAAACTG
CCCGAATCCATCATTCTCTCATGCTAGCGGTGTAATGAGCCTGGAAGTCAGC
TGTGAAACGGCGAAACAGCAGAAGAGGCCGACTTGATGAGCGATGGCTG
AGGTGACGAGTTGCGGTATCCGACCCAGATGATCATCGGGATCCAGCCCCA
CTGCACAATCGTCTGCGGATCAGTTACCCATCCTTGACACCGTCCTGATCCT
CCATGATAGCCCGACATCTCCTGAGTGTCTGCAGCAATTGACACTTCTCGA

GGCCCATCCGACACTCTCCAGATGATCTCGTTTGATGGGACGTCGAAACCA
GGAAGCGGGTGACGGTGCTCGGCCGGAGACTCACCTTCCCCACGCCGAGC
GCGGCGTTGAACCGGTCTTTTGTCTCGTCTGAGAAGGCCATGTTGTTCGCA
GTAGAGAGGGTTTGCAGACTGACATTTCGATCTTCGTCGCCGTTCAACTTC
TCTGAGCCGCTATCACCTGACGTGTAATTGTGTGTCAGTGTTCGGCTGGCC
GGGCGGATCTTGCCACTGATAACCGGATTGCAGTCTCTCTAAGAGGAATGC
GCCAAGCGCGCTTATACCCCTTTGTGCTCTCCTCGTGTTTCCTCTGCTCACC
GGCTACGTATCGCATCTACATACCTGCCATGCTCGCATGAGAACCACTTCGG
ATCGTTAACCGCGGCTAATCGAAGGTGGTATCGTCGACAAGGACAAAGAC
AAGAACTCGCAATCCGACATTTGATCTGCTGATCGACCCTTTACGTGCTTTC
TCTTGGCTGACACTCACGTCCGCAAGACCACCCTCATCACCCACCATCCTT
CTCTGATGTAGCTACACTTCTCTCATATCCAGCACCCAGCAGCAACATGACA
CTTCTTCGCACCGCGCATTTGCCACTCGGCGCGCGCGTCCGTCTCAGCCTC
GTCCAGAGGCGATTCCTAACCTTGGGCATACGTCGAGAAGATCCGAGCAG
GGTATGGGAACGACGCGCTCCGCTCTCGCCACCGGCAATATCCTCTCTCCT
CTCTTCTGGGGTGCCGGACCTCAATGTGGAGGTGGAGAGCTGTCATCGAC
GATGCTTCCCGGACGCCCACTACGCTCAGGCAGGCGCCAAGATCGTGCCC
GAGCTCTCTGGCGCTGTGGACGTGGTCCTCGGTATAAAGGAGCCAAGACT
GGAGACGTGCAGAGGCTGAGCGAGAGGAGTCGGAAGTGGATGATGTTCTC
GCATACCCACAAGGGCCAGGTGAGCTAATGTCCAACGTGTACGACAAGCT
GACAGGCAAGGAGTACAACATGGCCTTGCTTGGCGCGCTTGTCAATGGCG
GGCATACGGTCATCGATCACGAGCTCTTGACACATGAGCATGGGAAGAGAG
TCGCAGCATTCGGCTGGTATGCGGGAGGTCAGCAGTTGATAAGTCTGTTTT
GGCAACAGCTGAAGATACAGCTGCTGGCGCAGGAGAGGCGTTATGCCTTA
CTGCACTCGCCTTACTCAAAAGAGGCGTCGCCACGCCTCTGCTGGCAAGTT
TCCACGGGGACGTGTTTGGGATAATGACTCACAGAACAGCACCTGCCGCG
CCCATACACGTTCCGCTCGCTCGAGAAGTTCAAGAAATCTCTGCGGAAGCT
CAGCAAACGTCTCGCTGAAACTCACGGGGACTGGGGCAAAGACGGACCTA
TTGTCATCGGTCTGACGGGCTCGGGAAACGTCGGACAAGGCCAAGGCTAT
GTTGGAAGCCATGAACGTCGAGTGGGTGTCACATGATCAGTTGCCACAGG
TGTGTAAGAAGGGTGAGCCGCACATTGAAACCCCACATGGACCAGCTGAT
GTCGCAGGCGAGGCGAAAGTTTACGCATGTGCCGTTCCTCCATCGGCTTAT
CTCGAGCGAACGGACTGTCGGCCATACGACCGGTCCGACTACTTCAGTAAC
CCTGCAAGCTACCGATCGGTCTTTGCCACCAAGGTGGGCGTTTATCCTTCTC
CAATGGACGTACCTTAATGACAGATTGCCCCGTACTTGACCACGCTCATCA
ACGGTGCTGGATGGAGTTCCAGCTTTCCTCGTCTCATGTCCAATCAAGATAT
GGCTGCTCTCCTCGAACGCTCTCAATCTCAGCAGAAGCTAGCAGTAGTCCA
AGACGTCACGTGTGACATGAATGTGAGTCAGAATCTTAGACCGTAGGCCTT

GCTGACATCGCAGGGGGGCCTGGAGTTCGTGACGAAACACACGACTATTG
ACCAGCCGTATTTCACTGGTCCTGGCGGTATCCTCATCAGCACGATTGACAT
ACTGCCCACTGAGCTCCGTGAGTGACAATGAACGTCGATAAGAGCTCATCG
CAGCGATCGACGCATCTGAGCACTTCTCCGAACTGCTGGTGCCCTATGTCC
GCCGAAACCTTGAGCAGCTGTGTGCCCCCACGAGCAGAGCGGACAAGGC
GAACGATATCGACAAAACGCTCGAGCGAGCTACAATACTCGCAACGGCGA
GCTACAGGATCGACATCGCTGGTTGCTGCCGCGGATAGCTGCGACTCCTCC
ACCGCGAGCCGTGCCATCTTCGACTGGGTCGCACGCCGAGTCCACTGACT
CCACCACACCCTCTTCCTCAACATCCAATCTCGCTTCTTCGTATGGGGTTAA
ACCAAAGAAGAAGGTTTTGCTGCTGGGGAGCGGTCTCGTCGCCGGTCCTG
CAGTCGATGTTTTTGTGCAGCGTGGCGACGTGGAGCTCGCCATTGGCAAGT
CACTGTCTGTTCATGAGTCCCCGGCTGACCGTACAGCTAGCAACAACATTG
GGGAGGCACGAGCCCTTGCTGCGTCCCGTCAGGGCGTGACTGCTGTCGCG
CTAGACGTGGCGGACCATCAAGCAGTCCGCGAAGCTGTCTCTGCCGCTGAT
GTTGTGGTCAGGTCAGTATCCTGAAGGCATTATGGCATGGGCTGACACGCA
CAGTCTTCTGCCCGCCCCAATGCACCCGCCGTAGCGAAACTTTGCATCGAA
TATGAGAAACATCTTGTGACAGCCTCATATGTTTCGCCGGAGATGAAGGAG
CTGCACGAACGGTAAGCTCTCGTCTTCCGCCGCTTTGAAAGCTGAGTCAAT
AGGGCTGCTGCAAGAGACGTGTTGCTGCTCAATGAGTGCGGACTTGATCCT
GGCATTGACTCGATGTGCGTACTGTCAAATCATCATTGTTTGGTGCTGACGC
GTAAGGGCGGCTATGCGAATCCTGGATCAAGCGAAGCGACAAGGTCTCAG
ACCCACGTCATTCGTATCCTGGTGTGGGGGCCTGCCTTCGCCGGAGTGCGC
AAATGCAAGTCTCCCTTAGAGAAAACCGTCAGCTCTGACGGATAGGTGCC
ACTAGGTTACAAGTTCTCATGGTCGCCCAAAGGCGTCTTGACAGCTGCTCT
TAACCCGGCGACATACAAATTGAACAACCGTGTCCACTCCATACCACCGCG
TCGGGCTCTTACGGCACCCACATACCGACCTGAATCATCTATGGCGGGGCC
TCAATCTCGAAGGTCTTCCCAACAGAGACTCCGCTCCCTTACTCCTGAGAA
GTATGGCATGGGATCGGTAGAAGGCATGCGCGACGTATTCAGAGGCACTCT
GCGATACTCCCGGCTTTACCAAAATCATGGACGGCTTTGAGCGGATGGGTC
TGCTTGGCACAGATGCGCTGGGGACAAGGCCAGCATCGTGGAGAGAGTTC
CTGAGCATGTGCGTGGAGGGACAGCGATCTCGAGTGGATCAGCTGGAGAA
TAAAGTGATGCGGCCTGAAGACTTGCAGGCGGCCAAGGAGGCGTATGCTT
GGTGAGTCGAGGTGTTGTCTCAGGGGAAGTATTCTCGCTAACGTCAGGCTT
ACTGAAGAAGGGACAGGAAGAACTCCAGATCCCCGGCGGTGCTCAGACG
CCCATCGATATCCTCGCTTACCTGATGTCTCGCCGACTCGCATACGCGCCCC
ATGAGCGAGACATGGTCCTGCTTCACCACTCGTTCGGCTTCACCACCCCCG
GACAGGATCCAGATGGGCCAGAGAGGAAGGTGACGGCGTCTCTCGTGCAC
TATGGGGATGACAAGGCCTCCGCCATGTCAGTGACGGTCGGCAAGACCCTC

GCGTTCGCCGCTGGTCGGGTGTTGGACGGGGAAGTCAAGGAGAGGGGCG
TGACGGGTCCGTATGCGCGCGAGGTCTGGGAGGGGACGTTGGAGAGGCTG
GAAGAGGCGGGTGTGCGGGTGGAGAGCGGTGGGCGTAGGTACAGGAGGC
GACTTTGCATACAGCACGATACCACATAGCACTTGACCATGTTGCATTGAAG
GTGGACTCGCCGGTGACCGGATTTACCGGGTGAAGAGACTCCGAGGCGAC
GAACAATAAAAGGTTGGACCGACAGGTCTGTGCGTGATGCATCTCATCGCT
CGCCATGTAGCGTAGCAGCAGCGGCGCAAGGGGTCAAGAAGAGACAGGG
GCAAGATGTCGCAGGTATATCAGTACGACCCCGGCGGTCCTCTCGAAGAGG
TTCACCATCCTCTGTCCGAGACGAACGGTACCGATATCGACATAGAGATGG
CACCGAGTCCGGCCTCGAGCATTGGGGACGAAGGCATCAAGTATATCTGCC
TCGATACGAATATCCTCGTGCACCACCTGCCCCTCGTCGCGCAGCTGCATTC
GCTCTTCAGCAGCGGCGGCGAGAGCAGCAGCGGCAGCACTGAAGCTGCG
CGGGGGTATGGAGGGTGGTGTCTCCTCATCCCGACCGTGGTGATCAACGGT
CAGTCGCCCTCACTGTAGAGGGCAGAGATGCGGGCTGACCCCACCCAGAA
CTCGACAAGCTGTCTTCGTCCTCTGCGCCGGCACTGCAGGGCCACTTTGAC
GCTCCGCTCACGATTGGAGACCTGACGCAGGCCGCCACGCAATGGCTAGG
CCGAGTCGGCCCCTCTGCCTCACCACCTGCGCCGTTCGAGTCTCGCTCTGA
TGGCGCTCGCCCTGCCCACGCCCGCCTCCGCCTCCAGGCATGGGACGAGC
GCCTGCACCCCCCGTCCACTCTGACCAAAGCCGACGACCGCGTGCTCGAC
TGCTGCCTCTTCTACCGGCAACACCGCGCCGCCCGCGTGGTGCTCTGGACG
CAAGACAAGAACCTCGCCATACTCGCCACTGCCAATCGCGTCGAGGTGCT
GAGAGTGCCCCACCTCTCCCTGGCAGGCGTGGCCATGGCCCTAAAGCTCG
ACTTGCCGGCCAGATTGCCTCTCGACAACGGCCATACGAGCGGGCACATGG
CGCATGGCATGGACCTCGACCACGACCTCCACGCCGACGACCCGGCCAAC
GACCACCTGCGTGCCCTCCTCCCTGACAATACTATCTTCGCCGCCCCCACG
CCCGACCTGCCTTCTCGCATTGCGCCGCATGCGCGTTCTCGCTCGCATTCGC
ACAAACCGACACTGCCATCCACACCACCCTCCGCCCGCCAGATTCCCCTCG
ATATCCTGCGCCTACTCGACGCCATCTTCTTCCCGCTCGCATCGTACCTCGC
CCGCCTCTCCGATCGCCCACCCCCACTGCCCCCATCACACGACGACAGGCA
GCTCGCTCCCCTCGACCCCGCAGAGACGACCACACGCCCACAACCACGGC
TACTGCCGCGCTGCTCCAGCACGCGTACGACTCGTTCGACCACCTCGACGC
GACCCGCTTCCCGCCCCATCGTGCCCGGCCACACCACGATGACCCCGGACC
GAGTGGCCCTCTTGCGCGCGATGGCGTGTCTCCGAACGCTCTCCTTGTTCC
TCGTGCCGAGAGACGCCCGGCGGCCTCGGACGGGAGAAGTGGTAGAGGC
TTTACAGACCCTGGTTGAGCAGCTGGCCTTCTTGCACGTACCCATAGACGC
ACCCGCTGTAGCACGACTTGTAGACGGTGTCCAGCATTTATCATAAACCAG
CAGTACGAGTATCACATACCCCGCATCGCGCCATACCCTGCCCACTGTCAA
ACACACAGATATGCATACAGGTCCGCCGAGGCGTAGCGTTTAAAATGTCAT

GAGCCCCGTCCCGGGTGCGGTTTTTGTTTTCCCGACTCCCTCCCTCCGTAC
ACCTCATCGTCCAACACACCACTTACACCTTGTCGCGCAGAGAAGGGGTG
GAGCTGCCATCAGCACAATCTTACGACATGAAGCTTTCCAAAACTCACAAT
CTGCTCGTGCAGCGCCTTGCCGGCGGGCACCATGGGGTACACGTGCTCGCT
GCTGACCAGGCACTCCATGACGATCGGGCGCGTGTTGTCGTACTCGAGGA
ACTCCTTCATCATGGCGGGCAGGTCCTCAAGGCTGCCGCAGCGCATGGCCT
TGGCGCCCATGGCCTCGGACAGCTTGACAAAGTCCGGGTTGTGCATCTTGG
TGTGCGAGTAGCGGTTCTCGTAGAACAGGTCCTGCCATTGCTCCACCATGC
CCTGGAACTCATTGTTGAACAGCAGCACTTTGACGCCGATGTTGTACTGAT
GCGCGGTGGCGAGCTCCATGGCGGTCATGGAGAACGAGGCGTCGCCGTCG
ACGTCGACGACGATCTTCTCGGGAGCAGCGACCTTGGCGCCGATGGCGGA
TGGAAGACCAAAGCCCATAGTCTGGCAGTCAGCTAAGTTCCAGGATTACCT
AGGTGACTCACGCCAAGACCGCCGGACGAAACCCATGACCGCGGCTCAGT
CCATCGGTAGTATTGGCACGCCCACATCTGGTGCTGGCCGACACCGGTCGA
AACGACAAACTTGCCCTTGCCCATCTCCTCGGCCTGCTTTGTTCAACTCCTT
CATAACCTCTTGCGGCTTGAGCTTCTGGCCAGGCTGGCTGGGGGTGTACGA
GAACGGCAGCTTCTCCTTGTTGGCGACACAGCGGGCGATCCACTCTTTCCG
GTCGTTCTGCTTGATCTGCGGGACGAGCTCGGCGAGAGAGGCAACGACAT
CGCCGAGGATCGGAAGCTGGGCCTCGACAATCTTGTTGATGTTTTTAGGTT
GGATCTCAAAGTGGATGATACCGCCAGTGCCCTGGAGAGCCGCCTGTCTTG
CGCCGGGAGCAAAGGTGTCGACCTTGCCAGTGACACGGTCATCGAATCGG
GCACCGAGCGCAATCAGAACGTCGGCCTCCTGCATGGCGATGTTGGCATAG
GCAGCGCCGTGCATCCCGAGCATGTGGAGCGACTTTTCATCTCGCTCGTCG
AAAGCACCGAGACCTTGCAGGGTGGTGGTGACGGGGATACCGGCCTTGTC
GGACAGCTCCTTGAGGAGCTTAGGACCGTCAGGAGACGACAAAACCACCG
TTGCCGGCGTAGATGACGGGTCGTTTTTGCCCCGTTGATCATCTTGGCGGC
GGCCGAGATGAGCGATTGCTCTCCGGGTTGAGGTCGAGCCGGAATGCTGG
CCGGGTTGAGGGGGGACGAGGGTAGATAGGGTGCCGATCCTGGCTGAGTG
GATTTTGTGGGGATAGGGGATCTAAGGATGGCGGCAGTCACGTCTTTCGGC
AGGTCAACGAGGACGGGGCCGGGTCGGCCGGTGGTGGCAATCTTGAAAG
CCTCGTTGATTCTTCTCGGCAGCTCGGCGACGTCCTTGACGACAACATTCC
ACTTTGTGCAGCTCCGCGAGATACCGACGACATCGGCCTCCTGGAACGCGT
CGCTACCGATCAGGTTGGTGGCGACCTGGCCACAGAAAACGACCATCGGG
ACACCGTCCGACAGGGCGTCCTGCATGGGTGTAATGACATTGGTTGCTCCG
GGGCCAGATGTCACGAGGACGACACCGGGTTTGCCGGACACACGGGCATA
ACCCTCGGCCATGTGACCGGCACCCTGCTCGTGTCTGGGGAGGACAAACT
CAAAATGGGGCGAGTTGTAGATGGCGTCAAAGACGGGGAGGATAGCACCA
CCGGGGTAGCCAAAGACCTGAGAGACGCCCTGTCGGAGCATCATCTCGTG

GAAGATCTGACCGCCGGAGAGACCGATGAAGCTGGAGATCAGTTTGATGT
CAAGGCTGCCGAGAAGGGCGACTCACGAGTAGTCGAGTCCATCGCGGATT
TTCGAGTCCTCGGGGTTCTCGAATGCAGGTGTCTGAGTGGGAAACTGGGC
AGGCGACGCCTGGAAGTGTTGTGCCGGCTCGGGTCTTGTGGCCTGTGCGA
CGGCTGCTCCACTGGAAGCGCTCGACTGGATTCTCCTAACGTGCTCCTGGA
CGCGAGCCTTGCCTTCGAGCGAGATGCCGCGCTCTCTGGTTATGTCGGCTG
CCCTGGCGTTGGCCGTGGTCGATCTGAGCCTGGCCTGGGCTGCGATGGACC
GTGCCGAGCCGATATTTCTGAGGATGCGAGTATTTCTTGACAGCATGTGGGT
GATGGGATGGTGATGAGGGGGAGCGATGGATACTCGAGTACAGGCTAAGTA
GTTGATCTATCCAGGACTTTTCGACTCAAAAGGCATGTCGGAACCTCTCCT
CCGATCCCTCCACTGGGATGCCCGAGCCAGGCAAGATTCGCCACTTGCACA
GAAGGAACGGTGTTTCCGGGAATATGAGAACTGCCTGATCGGGTAAGAGG
GTTGTGATGGCCAAGAAGTGACGTGTTAATTTCCCTCTTACCCGATCGGGA
CAATGAAGCTGTGGCCCCCCAGTTCGCCTCTGTTCTATCCTTTGCCCATAAC
GCGATTGCGCACCTCCCCGCCTATTCCTCACTGTCTCCTCCGTGCCAACCTC
AGGAAGCGAGCGACCCAGACCTTTTTTCCTCCACCCGGTCCAACCCGGT
CAAACATCATCTGGTAGACAAGCCGAAGGCCGAGCAAGGGAGAGCAGAG
CGCAGAGGAAAGGAAAAAGAAAAGAAGACGCGCATCACCTGAAACCCTC
GAGGTCCCCAGACTCCCCAATCGCGGTCGCCGTCTACTGTACCAGGCAGTC
ACGAGTTATCAGCCGCGCAGCCGGGACACGTTAACTCCGATCCTCTCCCCT
ACGCCGCGCCGTCACTCTCACTCTCTAGCAGACGACGTGGGTCCAGGCCC
ACTATACGGACTGGGCAGTGATCGACGTGGCTTCCCCGGTCGACCCCTTAT
CACGAGTCTATCAGCATATCGAGCGAGGGACGCGTCTCATTCCACCGCCCA
GATACCAGCTACTCCCATCTCACGTCACGTCGGCCATTCGTCAGGTGGAGG
TGGTCCACTCGTATGTCCCCCTCTCTGTCCCCTTTGTCCCCCGGCTGACCCG
ACCTAAGATGGTCTAAGAGGCCATGTCCACTTCTGTGAGCGGTGCGAACGG
AGTAACCCAACCTACCCCTGCCCCGTCCCTCCCTCAGCACCCTTCACCTTC
GCCCTCCATCCCTGCCACTCAGCTCCCTACACTTGCTCCGTCAAATGGGAC
AGACTCGCCTGGCCCCTCGGCAAACCCTCGTCCGCCAGCGACCCCTCATCA
GCTCAGCGTCAACGGCAATGGCTCGTCTCATCCCTCGGGACCCATGGACGC
TCAGGCGATGCACACCCGACTCGTCCTCGCTCAGCAGGCAATGCAGGCCA
ACATGCGACAGCAGCAGCCGCAAACCAATGATGAACGACCGAGCTCTTCA
GGCGGACCGACAGAGAGGAGATCATCGGGCTCGGGAACCGACTGGACGG
CAATGGTCAACCAGCTGATTGGCGAAGTGCCGGGCGCCACACCAGAGGCT
ATCATGAAGCAGGTCCGTCTTTTGCTCTGTCTGGCCTCTGCCTATCTGATCA
AACAGTTGCAGGCGTTGCAGAACTCAAAGGCACGTCTCGCTGCGGCAGGC
AACACTCCGATCCCGAGCATTCCTCCTCTTCCCACTCCTACAGCTGCCTCGA
CTTCTCTGCCCCTGGAGCAGCAGAACGCGACCGCCGGGCCTTCACGCTCA

CCGTCTGCCCAACCTCCAGCCTCGCTCCCTGAAGGAGACGATGCAGGAGC
TGCCTCGACGTCGAATGGCGCTCCGCCCACAGCGACGCCCGCTGCTCCCTC
TCCGGTGCCCAACGATGCCCAGCCAGTGCTCGTCGCCCAGCAGGAAAAGG
CGCGCCAACCTCCGCCAGCTCGACCTCCACCTTCTGCTCCTGCGAATTCCT
CAGCCTCTCAACCACGCCCACCACCAGCTCCAGGTCAAGCAGGTCCGGGC
AGACCTTCGGGCCGTATGTCGGTTGCCGATCAACGCAAGCAGTTCTTGCAT
ACTCTGGTCGGGATGCATAAGCAGAATAACACCGCACCGCCCCGAGCTATT
TTCAATGGCGAGCGCGATGGCAGTTTCAAGATCGGCGACGTCTGGCTCGAT
GCGATGGAGCTCTTCATGGCCGTTTTGAAGACTGGCGGTGTCTCCAAGGTC
GCATCGCTTCCTCCAGATAGCCCATTTTGGACAACATTCCTGACAGCGAAA
ACATCCCGAATCCCTTGGCAGCACCGTTACCTCTCCCTCGAGCTGCCAATAT
GGATCCGAATGCACCCCGCATATGACCAATAACCCCATCACTTATCTTCTCG
GTGCCTATGCAGCCTGGCTCCAGCCATTCGAGGAGCACATGCAGAGGCAA
CGTACAGCTGTCATGCAGCGTCAAGCTGCAGCTGGTGGTGCACGGCCCCCT
ACTGCCAGTGCGAACGGAGCGCCACAAGCTTCGACACCGGTCTCCGCGCC
CTCACCCGCCCTACCGATGACGCCTGCATCAGCGCAAGCACCTACTCCTGG
TCCTCTGTCAGCCCCAACGCCGTCAGCCATGAATCATGTATCGCCCGCACC
ACTGGCAGGTTCACCGGCTCCGATGAACGTTGATATCAATCAAGCACCGAG
GCCGAAAAGTGCTTCGAAGCCGATTCCTTCACCCATCAAAGTCGAGACGA
ACGGAGTCTCCCCAGCGCCTCATTCGGCGACCTCAGCGAGCGGCAAAAAG
CGCAAGCGCGACAAGTCTGGTGTCAAACCGAGCGATGCCTCTGCACCGCC
GACGCCGCTGGTCTTACCTATCCCGACCCTTCAGCACCTCCTCCCCGGCCT
CCAACGCCCAAGCGAGCTCGCTATAAGGTAGAATACCGCCCTTTCCATTTC
CCGCAACCGGCCCTTGGTGGTTGGGACGAAAGAGCCGTAGCATCGACCTT
TCCGAAACACAACCTACATCAACCGCCAAAATCGATACATGAACTCGGCAT
AGTCGACCTAGAGGCCGTGCTCATGGGTTTGCGATCTCGCTTGCCACAGCA
ACTTGGCTACGCGTTGACTGTATTATCCATGCTGTCCATGCCGCTACACGAG
GAGGGCCTGTCTGGATTGCCTCTGGTGCATATTCGCGATATCCTCGTGGAGG
TCCTCGAGTTGTTGCAAGAGGCTGCTTTCGGAGAT

>Mosaic_sequence_84

GACGTACCTTCAGAGAATGACGTTTCTCAGGTTCCAAACCAACGTCTCAGC
TCAATTCGAATATTCCGAATATTTATTATCAAATTTCTCGCGCGACTATAGGC
CAAAAGCTAAAGACGTAAGATATTCGAGATAATCGACGTTGACAGGTGCAA
ATCCGGTTGCAATGACGCGTGCGCGAAAAGATCAGATGAAAGCTAAAGCG
CTGTCAAGATTTTGGCAGACAGTTGACAGGTCGGTCTAATGTTGAGATTGG
AGTCAAATGAATTGTAGGGACGGGTACGGGACGGTACGAGGTAGCTTAAC
GAGATACAGTGCGCGGTCGAAGATAAAATGTTGCTGATATGGCTGATTTGA

ACTGTGCAACGCGAAAAAAAAAAAAAGGGAACAACGGAGCTAAACAATCC
CAAGGTCCAAGGTTGTCACACGAAAGATACGCGACTTAGGCGACCGAAAA
AAGATTTAGTACGGTTTAAGCTTCAACAAAAAAAGGTACCAAGGTAACCTG
TGCCGTGGCTATCGTATCACAGATAGCTTCAATTAGCTTTGGCTATAGTGAAT
CAATAGCTGTAGCTTGGTAAAAATTCACGTAGGAAGACTTTCGAATTGAAT
TGAATCGGACTGGATCGGCGCATCGACAGAAGACGAGTTGAGATGGATTTT
TCGGATTGTCGCGATTGTTGTTTTGTTGAAAACGTCAAGATTACCTAGGTAC
TGGCAGAAACAAAGGTACTTGAGGTACGTAAGTATTAACAATTAGATAAAA
CAAAGTTCAAGTTCCTTCGGGAAAGAAAAAGGTAGAAACCGGGTTTATCG
CGATTAATCCAGTAATTGTCTTGAATTTTGGGTTTGATATTTTTTTTCTTAGG
AAAAAGGAGGAAATAAAAAAAAGACGTATAGACGAAATTTGGTGAGTGAA
CGGTGGGTTAAGTTAGCGAGTGTGAATTGTCCGAATAAGTGACCAAACGA
GTGTACTGGTTAATAAACGCAGGTATTTTGCATGGTAACTATTAACAAAATA
ATAGTGTACCCAGGTATGCGATCGACCGGCTGCCTCGTATTGAAACGACGC
GCCTTGTCCTGCTTAAGAAGAAACAATGAACCCGACGGGAATCTGGGAAT
CTGAGATCCTGAGAATTTGGAGATGGGAAATTCGTCAGATAACAAAATCTG
CCGAATTCCTGAATTGGCTGGAATTGTCTCACAAATAATAGGAACGGGATC
CAGAGATCACACGTGAAAGAGGGTTACAGAAGAAACGAGAGGGAGAGGA
TATGGATAAGGATAGGGATAGGGATAAAGGGTAAAAGGGTTAAAGGGTTAA
AGGGTAAGGGTAGAGGACGGGAAAGGTGTTGACAATGTCGAAGACAGAG
GATGGTAAATCTGTATTAAACAATTGCAACACCGCGCATGCTAGCATGGAG
GTTTGCGGTGACGAAGTGTGGATTCAGGACTGAGCGGACTGAGCTACGTC
AAGGATCTAACGTGGTGTATTTCTTGGGTTCGTCCCAGGTCTGACTGACCTT
CCTAGCGACTACTTGCTCGATGTACAGGCTAGGTTTACGGGCAGGTGAGGG
TTCGTGTAATATCCCGAAAACAGTGTGCAAAGCATAACTTAATGTATTGACA
CGTGCTTACCCGCAACGTTTTACCTGAGATTGGACAGTGCGACGGACACAT
ATAATCACTATATACAGACCAGGTAAGAGGAATCGACAAGTCAATAATACCT
GAATATTATTGACCCTGATTCACCTTAGACATATAACCTACCCACTCACTATT
TAAAGAAATAAGACAATTATGTACACACTTTTACCTCCCTTCTTTCTTTAGA
GGCTTAAGGTTCTATTTTTTGCAGCTTCAAGATTGTTATCGATTTGCAAAAG
CAAGCAAGTGATACGATGAAGCCACGATGAGGCCACGATGAAGCTTGACA
CAAAAACGGGCATTTGTTTACCTCACTAGCAAGAGCGGTAAACCATTGGAC
CACATCAATTGCACATCAATGTATCGATGTTACCTAAGTACGCGCTGTTACG
CATGAACCCAATTTTAGGAGCTGAGGGGTTCCCTGTTTCCCTGTTGATTTAA
ACTCCCGCTAGGCGAGACAGGCGAGATAACAACAAGGCTGAGAATTGTGA
GAATTGAAAGAGCAATAACTGGGTATGCCACACGTCGCACTGTATTCATCC
CGAAGCTAGTACGGATAAGGTCTAGAATATGCAAGAATGCCAACCAATAAA
CTCCCGTTCAGCATTCCGACGCTCCAAATCTGTACAACCCTTAGTAATATAT

CACTTATGCAATATAATTTCTAGAACATTTCCATTACAAAGATTTCGAGATTC
TAATCTCCATGACGCAGATTTGTTTCGTTCATATTATTTACCCTTGACTTAAC
AAAGAACTGTGAACCCGGCAGCATCCCGGGAAGGAATTAATCAAACATAA
TTCCCGTCCTCACATAATGGCCGACGAGAACGCTTCCAACAGCCCAGCAAC
TACCGACACTCCAATTGCTCCGGTCGCCATTTTCAAGAAACGAGGTGCGAA
AGGGAAGGCAAATATTAGAAAACGGCCAGCGACGCCACCACCAGCCAACA
GCGACAGCGACGATGACTACACGTCATCCGAAGATGAAACAGGTCAACGA
GTGAAAAGGCGCAAGAAGAATACGGCAGTCGTAACAGCTTCATCCAAAAA
CAACACAACGCACACCGACAAAAACCACGGCGCCACCATCTTCGAAGCTA
ATCGCAGCGTGCCCATCACAAGCTCAAACGATGCCACGAAACAGACGAAC
TGGTTCGATGAAGATGCGAACCTTCTAGGCAAAACACGACCTCTCCCCAAG
CCCAAAGATGCGAGCGATACTCCATCGCAGCCGGACGGGACCTACAAAGG
TCTAGCCAACCAGACCAAGTTCATACAAAGGAATCCGGACGCGCCCAATC
GGACAGTCGGCCCCATAAAAGCAGCGACCAACATCCGAACCATCACAGTC
ACCGACTTCGCGCCCGACGTGTGCAAGGACTACAAGCAAACCGGCTTCTG
CGGTTTCGGCGACAACTGCAAGTTCCTGCACGCTCGCGAGGACTATAAGC
AAGGCTGGCAACTAGACAAGGAGTGGGAGAATGTGACGAAAGGAAAGAA
GAACATAGGAGGGAAGGTCGTCGCAGGTCGTGGTGCCAATGCCGATGAGG
ATGACTTCGACGCGGCTGAGGAAGCTCTGCTGGAGAAGATACCATTCGCGT
GTATCATATGCAAGGGCCCGTACAAGGAACCTATCGTGACGAGATGTCAAC
ACTACTTTTGCCTACCATGCGCGTTGCAGAGGTATAGGAAAGATCCGAGTT
GCGCCAACTGCGGCGCGGGGACGGGAGGAGTGTTCAATACGGCCAAGAC
GTTGAGCAAGTTGTTGGAGAAGAAAAGAGATCGCCAAGCGAAGAAGCGG
CAGAAGGCTATTGAAGCAGGAGAGGAAGTGTCTGAGGAAGACGAACCCG
AACAATGAGGCGTGGAGATTGATGACCAGCCAGGATGATACCAAATATGAT
ATGAACTTTTCAACGTTAGGACACGCTAGTGATGTCAAGACTATAATTCTAA
GTCTCAAAGATTCGACTATTCTAGCTTCCGCGCCGTTGGGTCACTACACTTA
TGCTCAAGTACGGCCTCGGCTGCTCGAACTAGCTAGTAAGAATATAGTCCA
ACAGGTGGCATCGCTAGACATAGATATGCAAACTAATAGTAATCACTAGAAG
CCCGAGCATATCGCCAGAGGAACTGATCTGCCGTAGGCTGGAACATGTTCA
TTTAAGTGAAACTTACAATACACATCTTCACACGTGGAATTTAGAAATCTAT
TCCCATCCCTTACCTCGGGTATCTCAATCATGTCCTGCCTATCTACTATAACT
AATAGTATAGGGAATTAGAATTGATCGGACATCTAATTCCTAACACTCGCTA
GTTACAGAAAATATCCACAGACAGTGTATTATATGTTGCTCGCTTCGGTGCC
TTCATAACTCGCGGGAAGCGTCCGTCTAGATAATAACTATCTCAGTGTCACC
GCTGCGTAAGACAACAGCAACTTACCCATACTTGGTAAGTAGGTACATGAT
AAATTGAGTAACTTCGTATCATATAGAATAGAATCTTCGCACTTAAAAGAAA
CGAGCACATCTGCGAGGGAATTCCGATAAGAACAATGTCATATTCTTATATC

TTATACATGCAAAGCTCATCGACCCATACACCACCTACTTCCATGGATAAAA
TTGCACATAAAGCACATGGGTAAATAATATTCTGAAAGAAAGTCCGGCACT
TCATCCTGCGGCGTTCTTGAAAGCCTGGCACTGCCACCGGATTCCACGCCA
TGCATGCATGTCAGATCCATCTATGCATGTAGCCATGACTGCATGAGACCCA
CAATCTTCGTTTGATCCAATAGTAAGAGTTTGCAGAGAAGACTCTAAACTTT
TCGAAGACAAAATTCCTGGCGTATTCGAAGAAAATAGAGGAATAAAACGAT
GGTGTTTTTGTAAGTTGAGGACTCGAAGAGCCACTTCTAAAATTTAGACTT
ATAGGCACCCCTCAGAGTGCGTATAGCACCTTCCTCAGTAAGACTACATCTT
TCGAAATCTAGATATTGCATTGTATACAGTATTTTGTACCGTTACCGAGGTAT
AATTCGTACTACATACTGTATTTTACAGGGGGCTTAGCGCTCAATAGCCCTG
TACGAAGGCCCTATAAAAAAAGCCCTCCCTAAAGTTACAGTGCATACACGC
CCTATAAGCCCTTATACGTAACAACCTTACACACCTATTTCAACGCTTTATTA
TCAAGCCATCCACCGTCGCAATACACTTAATTTAACACCCTGGATATTCACC
AAACCCCCTGTCATGCGTGCTAGGTCACGTGGCGGTTGGGTCATATTCCCG
TTCCCATTATTTTAAACTACTAGGTACTAACCAATCATCTCGCCAAACTGCTC
AAGTTTCTGCTGATACTCGTCATACTCGTCATCTGCGTTCACGAATCCTCCT
TTTGCAAGTTGAATCTTGCGGTGCTGTCTTGACTGTACGACCTAGGGCGAT
ATTTTCATTAGTGTAAATTCTACCATTTCTCCCGTACTAAATGGCTTACTTCT
TCAATTGATCCATGCATCACGTAACGGATAACAGTGACTCTACTAGTTTGGT
CAATGCGAAGAAATCTTCCAACAGCCTGATTCTCTGCTGCCGGGTTCCACT
GCGGTTCAAGAATGTGGATTCTGTTGGCAATTGTAAGGCCATTCAACCTAC
CCGAGATTTCAAGTCAGTTACGTGCAGTTAGTAATACGTGTGCTATCAAGTT
CTTACCCAGTAGCGCCTGTGGAGAATGTCAAGAGCAAAACTCGTGAAGCA
TTTCGAGATTGAAATTCATTCAGAACAGCGTGCCGCTTGCCCGATGCAATG
CTGCCATCAATGCGTTGGTGCTTGATGCCTTTCTCATCCAAGGCACTTGAAA
CGATATCTAGAGTTTTCTTCCAGAACGAAAATATCACGCTGTTACCAGTTAG
TCAACCAGAAAAATACCTACCTAGGTAAGTCTCCAGGGTTCACAACTCACC
ATTTATCTTGGAGATAATGCATTTCAACATGTCGTACAAGGGTTTCGATTTTC
GAGGGGTATTGTCTTTTTTCAGAACCTGAAAATGCTGCGTTATCGTTACCTA
GCCCTTCAATAACATGTTGGCTTTTGCAAAGGGGACATATTTCTGAGCCAG
ACGCTTGATGCTCTATTCGATATTGTGTCGTGCACTCACGACAGACAAGTCG
ACGACATCGGGTCAGATATCCTTCTTGGGAGTCCACTTGTCTACTAGCTGAC
AAGATATCGCAAGAACAGTATGAACATATCGCCTCATCGCTTTGTTGCAACA
TGCTGAGGATCTCGTCAGACTGCGAATCGGAACCAAGCACGCTATTAGTTG
GACGATCCTTCTAGCCCGTTGTTGCAAAATATACGCAATCGAAGTAGAGCTT
CCATTACCCTGTTGTGATTGAGTCTGTTTGCAGTCCCCTTTGTGGCAAAAGC
CATTGCCCTTTTGAAGGATAGCTCTAGCATTTCGTATTGTTGCCTCTCATGTA
GTGTAAAGCTTATTCGGCAATCTTCGTTCATAAATCCAGGGTTTAGAAGGAC

AGACTTATTCCTTCGCAGACATATAGAGCCTAGGAGTAATCGAAGGTTGTCG
AACTGTTCCTTTGCTGAACTTTTATATAGCCCCATTCTAGATATGTGCTTTCG
GAAGGTTGCGCTATTATCCATAATAGGAAATTTTTAGAAATCTAACTAACGA
ACCGAGGTCATCTAAAGAGTTTTGAATTGGTGTCCCCGTTATGCACCATCTA
ATCTTAGAAGAAATACTATTGACACAGTGGAATTGCTTGCTAGAGGAATTCC
TAACTGTGTGGGCTTTTGAGGGTTAGCAGTATTTCTATTCCTAATAAATGGC
AATCATACCTTCGTCTAAAACCACCCTATACCATTCTATATCATAAAGAGGGT
TAGGGCGCCTTCGAAATTCTGCCATCACAGTGGCGTATTTTGTGAGAATGAT
GTCGTAGGCTGAGAGAGTTGCATCATATTCCCTTCTTTCTGACCCATGGTAT
ACGATGGAGCGTATAGCGCCAGGGTATATGTGTCTATGATAGGAGTAATTAG
TTTATATAAATGAAGTTTGAGCGGCCGCCCACTTTGATATTTCTTGTCTCCAC
ATATTCAAAAGTACTGAAACACCTTTTGTTAATGGAATGGCTTCAGATATAC
GGGGATATTGATACACTTGCACTCAGACGGCACAATAACTATGGTTGACTTT
GCGAATCTTTCCGCGTTCTTCAGTGAGAAGAGAGCCGCGGAATCCATAGAG
TTGATGATGGAAGATAGAACTGTCAAGGTCTTTCCAAGCCCCATATCATCGG
CAAGAATCCCACCTATGCAATCCGCAGCTATTCGACTCTTTGCGCCTGTTAT
GACATGCTGATAGCTATAGAAGATAGTTAAATTTAAACGCCACCATTGGAAA
TGTTTCACGTACTATACTTCTCCCTGATCGGACGCATTGGATTGCCATAGGC
TATGGTTAGGGTGAATCTCGTGGCTTTCTCTCCGAGCAATGAAGTCAACAC
CGGTTCGCTGGTGACTTAAATAGGCTGGTTAGCAATAGGTATCGATGATCTT
ATAAAGTATGTACCTCAATAATTCAGTGACTATTCTTTTATCGATTTCGCGTT
GGGAAAGATAGTCATGGGTTGGAAGATCACTGAAAAGGAATCGATGTCCA
CTGGCATGGCGGCATCATTTGCCTGAGCTACCACCTCTCCGTCCTCAAATGT
CTGCTTATCTTTCACGTCAAAACTAGAAACCCCATCGCTGCCAGACAAAAT
TAACCCTTGTGCGTGGAATGATTGGGGGTTACAATATTGGCACTGAAAAGT
CCCCATGTACGGATCCTGAAGATAAAGGTCCGAAACGCAAAGTTTCTGCGC
AACATTCTCAGAATGACGTCTTGCACCACAAAGCAGAAGACTGATTTTCAA
GTGCACTGGCATGTTTTTGGATTTATTCTTGACAAGGGAGTGAACCTTCTCA
TCCCATTCTTTGGACTCTATCCATGGTTGATAGCTAATACCTTCGAGGTCATC
CAAACTAGAAATTGCCCGAAATGTATGGTTGCTGAGAACTCCGACGGGTTC
CTCGTTTGTAGTTTGGAGAAATCCAACGTCTTGCTGCATATTTAGAGCCATG
CGGATTAAGGACTGGCCGTCAGGAAGCACTACAGGGAACAAACTCCGGTC
CTGGGTTTCTGGGCGCCACGTTAATTGGGTAGTAATCCCATGAAGCTTGGTT
ATTAAATTCTTAGCAGGCATTTCAATTCATATATAATGGTGGGTGGTTACCAT
GCCAAAACATATTTCTTCCTCTTGTGGGGAGCGAAGACCTTCTTTCTGCAC
CTCGTGAGGAATCTCACGTTGGGCATCCATTGGAGAAGGAAGAAGTAGTT
GTGATACTATCATTGATGATGAGGTAACGCTTTGGGGGGCATGGAGCCCTTC
TGATGGCATCGTCAGCTTTAGCCGCTTCTCACATCGACTGTTGTTGTGTGTC

GCATGATGCTTTCTCCAAACGGACTCGGGCTTCATGACTCATGAAAATATGC
AATAGCACCTTGGGAATAGGATATCTTTCGAAGATTCTATCCCTTGAGGCTG
CCTACCTAGCCAGATGTATGGAAACTAATTAATTTTAACGGCAATTTATTTGT
GTAAATTACTTAAGTGCCTACCTTAGAACATTCTTAGCCTTGATACTGCCTCC
TGCAGGCTCCGGTGGCGGACCAGCTGATGCGATCAGATTTCTAATCAGAGG
GCCTAGAAGGCTGACTGATAGCAAGTACTAGCACTGCCACCGCCACTCATC
TTGTACCTAGGTAGGACTCTAAGCGAAAGCTAGGATTCTATTCAACCAACA
ATTTCCAGTATAATAACGGCTATGACTACGCAAGTAGTGTCAGGTATCGTGG
AGGAGTTTGAACAGGCTGTGAAAGACTTCAAAGCGAATGCAGCTCTAACA
CAGAAGGAACTACAAGATTTCCAATTTACAAAGCTTGACGATGTTAGGAGG
GAAATGTTGAAGGTTCAGAGCCAGCAAGCTCAAGCAAAACAACTGAGGTA
TATGAAGAGGCTCGAGCCATTTATTGAGGCCATGCTCGAGTACGGAAAAGT
AATCGAGGTCTTTGTGAATGTATCGGAAGTTCCGGCCTTCGTCTGGGTGAG
TGTTCACATATCTCGGTACTAACCTATTGTCAGAAAAGGGTAGGTGTTGACG
TTCTTTTAGGGGCCGATGAAATATGTCTTACTGGTGAGCTCTATAAACTTGC
GATATCAGATCCTCTTGATTTAAGATTCTCACTCTTGCTAGACCGCTTCCTCT
CTTTCGAAGGCTTTCGGTTTACTCCTAGATACATATGAGTATATTGGAAAGC
AAATACCATTGCTCTCATCTTATCAATCTCTATTTTCTGAGAATATTCATATGA
GATCTTTACTAGCTATGATATTCAGGGATATTCTAGAGTTTCATAAGGACGCA
CTGAGGGTTTTTCAGACAACGATGTTCGTCTTACTCTCGCTCTCAGTGCGTA
CATGGTACCTAATTAGTGCCTATTTGGCTGACATGAAAATGAACACCTAGTG
TGGAAACAAATGTTTGATGCAACATGGAAAGGTTTCGCATCTAAGATTGAC
AACCTGAAAGAATCTATGAACTGTCATCGCCGATTGATCGAGAACCGGGCC
ACATTAGTGGAATTTGAAGAGGTCCAGAATCTTCGACGAATGGCAGAAAC
AAATTTGCGCGATTCGTATAAAGCGCAGCTTAGTCGCCAAAGAGAGGCTGT
CCTAAGATGGCTATCCCCTGGGCCTCTTGAATGTATTCACGAACGCCACGTT
GTGACGAGATCCAGCAATCCAGCTGCTGGAAGATGGCTCATCGGTAATGAA
AACTTCCAAAAGTGGTTTGATCCATTGTGCTGTTCAACCCCTCTTGTATGGC
TGAACGGTAAGCCGGGCGCAGGTAGGCAAGGAATGACCCCATCAGTTTTT
GGTATAACTACTAACTACCATAAGGTAAATCCGTTCTCGCGTCAGTGATAAT
CGACGAAACTAGAACGATTCAAGACACTTCGACAGCATTTTTTATTGTGC
TGAGAATGACCCACTTAGAAATAGCTTTATCTCTATCGCTCGAGGGATGTTA
TCCCAGCTTTTAATCCAGGACCAAGATCTCGTTCTGTTCTTCCACGATAAAA
TGTCGACAGTGAGCGGGGATGTTATATTATCTACTATAACGCTAGCGGAAGA
ATTATTGGAAGTTGCTTTGAAGAGACGAAAGACATACGTTATTCTAGACGG
TATTGACGAATGTGCCAGAAACGAGCGAAAGAAAATCTGCACTTGGTTTCG
CAACGTGGTAGATTCACTCTCCCGTGCCAATATGGATGAAATTCGCTGTCTC
TTCATCAGTCAAGACGATGGGATTGCCCGCAAAGATTTGTCGATGCTCTCG

TCTATTAAAATTACTACTCAAGATAATAAATGCGACATCGAAACTTTTGCAA
ACGCGTGGAAAATCCGAATCGAGGAGAAATTCGGTGTCTTCACCAAGAAA
GAACTCGATATACCTCTGGTGGTCACGGCAAAATCGCAAGGTCAGTCACAA
AAATGCTCACATTCAGGTAGAAGCTAATAACTTCACCAGGCATGTTTATTTT
CGCGAAGTGCGTTCTGGAAGAGCTCTACCTACAACGTTTAAGATATTCACT
CTTGGCAGAATGGAAAGCGGATAATTTCCCTAACGAACTTGATGAAGTGTA
TGTCACCTAATAGATACAGTATGGTCGAAAAAGTAATATACTCACCAAGTGC
ACATAGATACCGTCGTATTATCCGCCGTGTCTTAGATAGTGGTCTGCACTCG
CAGCATGAAATATCTAAGAGGCTGCTGGCGTGGATATCGAGTTCAAAACGG
CCATTAAAATGGTATGAAATACAAGCTGTGGTATCAATTGATCTGAACGAAC
CAAGTATAAACAATGAGAGTCGACGCTTGGCTCGCAACTCGAAAGATATCT
GTTTATCTCTGGTGGAAATACACTCCGACCAGACAATAGAGTTGGTTCATCT
AACCCTTAAGGAGTGAGCGATAGTCTAAAGATACTTCAAACACAAAAACT
GATTTGATATGCTAGGTTTCTTGTCCGAGAGAAGATAGTAAACTTAGCCGAG
GTTGACCGAGAACTCTGCTCTCTAAGTATGGCCTATCTATGCTTCCCTAGTAT
AGATTCGGAACTCGAATCCCCCATTGTCAATGATTACCTCCTCACAGGAGC
ATATTCCTTTTATGAGTATGCTGTGGCATGCTGGGCGCCACATTTACAGTCAT
GGCTTCCTCAGGCAACACCAGAGGAAGTCCAAGATTGGAGCGAAAAATTT
GAGGTCTTCCTAGACGCACATTATTCAGAACCGGGACTTCGTCAACTAGTG
TCGAAGTCTGTTGGAAATAAGCTCAAGATATTGGAGTCTCTAGATACCTATG
ATGACCTTGTCCAGTGCATTGTATGGATGCAGAAATTGCTCATGGCTGACAA
GGTAGAGGAATCTAATGATATGATGGATTTTCCGAGGATCACGACGATGATA
AGGTCGGCATCAGAAAATATATCACTATCCAGCCTTTCGACAGAGACCAGC
GAGAAATTGAAACGTTATTACGGGGTAGGATGGTTCAAATGTCCCCGAATT
TACTGCCAACATTTTTATAGCGGATTCCGAAAGCCCGAAGATAGAGATGCA
CACGATAATCGACATGATAGACCATTCATGTGCACAATCAGCAGTTGTCCTA
CTGCAACATTGGTTGCGTATCTAAGCCTGACCTGGACAAGCACATGTTAAA
TTCTCATGGCATATGCGACGATACCGAGCAGTTCCAATCCTACCCGATCGAC
ACGATGACGTGCGAAAAGGAGACGGTGAATTCCAATGCCCATCCTGTCCTA
GGTGTTTCCAATACGCATATCTCTTCAGACGCCACGTCAAGTCTCACCTACG
TGATCGTCGTTTCAAGTGCACTGTGTGCGGTAAAGACTTTTTTCGGGATTAT
GATAGGAAGAGACACGAGAACCTACACGGAGAAAAGAAATTTATATGCCA
GGGAAACAGAGTCAAATGGGGATGTGGTAAGCGCTTTGCAAGGGAGGATG
CCCTTAGGGAACATCTCAAGACAAAGTTAGGTTCCTCTTGCCTGAAGGCAT
TAGCCCAAGAATATCCCGGAAGCCATCAAAACGATTTGGTTGACCTGGCAC
TTCGGGGAATAGAATGAATGTGATCATGCCTATGGGCACACCCGGGTACTT
GGGATAGATGCGTGGAGAGCATGTAGGGATATGTTAAGAGGGCCGTTATTC
TCAAACTGCAATGAAATGGAATCTCGTGCAATTATTTCCCACCCTGCAGGTA

GGCAGCTCGCAATCGTGGTCAGTAGGTAGCTGTAAAGTAACTTCGATATTTC
AATAAACTGATCCTATCTCATATTACTTCACTTCATAAGCCAGCAAATAAAAT
AAACATCTTCCACCGGGGTATGTAAGTAAAAGTACCTAACAGCGTAGAACA
GAAGAAACAACAAAAGCGTAGATGAAGTATACCAAGGTGTATCCGCTCCG
CCCCGCCAGACATAAATAAAGATTGAAAATCTCCAAAAACCGACGAGGAG
GAAAGATGTAAACCGATTAGATCTCAATCCTTATATCTTCAATCCAGATTACC
GCCTGCGTGAAATATGCTAAATGTGCTATCCCCCTTTTCTCCGTCGCTTAGC
TTCGTCTGCTTCATACCTATCCATGTCCTTCAATACGTCAAAAATATCCTCCA
GATATCCTTCCAATATCCTTGAAATGCGATAATTAGGCACCTAGTAGTAGGTA
TACGTCGGCATATTTAATCCCAATCCCCAAACTCGCCCGCCCCGTAAGGATT
CCACTCCTGTCCGGGATACGACGTAGCGCTCATAACAGGGATACCATCATC
GTCGTCGTTGACAGCAGCGAGTTCGACGGGTTGCTCGACCAGGATCTTCTC
TTCAGAGTCCATGTTGCTCGAGACCTGCTTCTCCGAAAACGACGAGTCGTC
CGAGCCCCTCTGGCTCCCCGCGGCATCGACGCCCAGTCCGACACCGAGCC
CGACTCCGGCTCCGGCGCCAATCCCGGCGGCAGTTGCGAGACTCGCGTTC
GACGCGACCCCGTTTATCGTAGAGCGACTGCGTTCGGAACCCGCGTACTTA
ATATTCTCATCCTGGCCTCCTCCCCCTCCAACCTCCATCCCCCCACCAATCT
GCTGTCCCTGTCCCTGCCCCCCGTACATCCCCATCGCACTCCCCATCCTCGG
CGTCGCATCATACAAATCCTCATCCGGCACGCCTCCCCTCGCCTCATTCGCC
CTCTCCACGTTAACACTCATCACGTTTAGACTCGTGCTCGAGACCTGGTGG
AAGGGCGCGCGCGGGGGCCCGGGAGGCTCCGGACTCACGCTTGCGGCGC
GCGCGGCGCTCATGTCTGGGCTGAGCGAGGCTTGGCTGCGCAGGGGCTCC
GTCGCGCTCATTTTGCTTACGAGGCGTGTGGCTGGCGTGTCTGGGTCCAGC
GGGGTGCTTACTTGGCCGTGTTGACTTATGCTTAGGCTCGGCGAACCCTGC
TGGTGTTGATGTTGTTGCATGGGTGCACTTGTAGGCGAGAAAGCGCGTTGA
GCATTCGCGGGGCCGCTTTGCGGCGTTCCCGGTTGCAGGATAGAGGGGTG
AGGGGGGCTGTTGCTCATAGCCGTGACGATGTGCTGGTGGTGGTGCTGCTG
CGCGGGACGCTGGATGTCGTCGTTGTCGCTGGGCGGCGAGACGTTCGGGT
CCGAGGGCTTCGATTGCAGGATCCCGCCACCGCCGGCGGCTATGAGAGCA
GCGCTGCGGGCGCTGTCTGGTGCGCTGCCCCAGTTCGAGCTGCTCGGCGT
CTGCGTCTGTACTTCTGGTCTTGGCGGTTGTTGGGGTCCGTTTTGTGGCTGA
GCCTGCTGGCGTAGGAATTGGGGTGGTACAGGAGGCGGCGCTTGACGGTC
GTATCTGTACTCGTCTTGTGACGGTCGGGGCGGTGGCGTCGGCGCCATATC
CAGGAATGTGCCTTGGTCGGATTGCGTCGGCGTACTCGACTCCGAACCATC
GTTCCTCTTAATAGACGGTGGCTGACCGGGCTGTAATTGCCCATTGTTCCAC
TGTTGCTGCTGTTGTGCCCCCTGCGGTCCAAAAGACTGGAATTGTTGATGC
GACGCGGGAGGCGGACTGGGCCTTCCGACGTTATACGGCGAGGGTGCTAT
CGTACCTGCATTTCCGTATCCGTGAACAGCCTCGTAGCCACGCGGAATAGG

CACTTGATCGTACTGCGGCTCTAAGTTCTGTGCTGATCTTCGTCGCTGCTGG
ACTACTTGCTGCGGTTTCAGATTTCCTGCCTGAGGCTCCTGTCCCCGACCTG
CCTGTCCCTGAGGTGGCGGTGCACCTCGCCCAGCTCCTGACATCATCGGCG
GATTCATCGGTCCGTACTCCGGCGCTCCACGACCCTGGCCAGGTTGCATCG
TGCCCTGCGGCGGTAACTTGCTCATCGGGCCTGCCGGTGATGGCCCGCTAA
TCTGCGGCCTCGTCATGATTGTTTGTGGTTGCTGTTGCTGCTGCTGCTGAGT
CATCGGTTGCCCCGATTGCTGCGGCGACCTCATCGACGGCGGAGTCGAATG
TCCAGGCGGCGGCTTCTGATGCTGCGCCTTCTCCGGCCTCGGCGAGCCGCC
CGACTCCGACCCAGTCTGTTTGCTGACCCTCTTGAACGCGCCGAGGAATTT
TTCCTTAGCGCTCTTCTCCTTCGTCTCTTTCGGCGGCTGCGTCGGGATGTTC
TTATCCCTGGGATCGGTGCCGAAGAACTTATTTATGGTCTTCTGCTGGTCCG
GCGGCAGCGCCGGGGTCCCGACGAAGCGCGGCCCGTTGGGGTGGTGGTG
GTGGAGCGGTTGGCCTGGACGTGGCTGGTTAGCGAAGGAATAGTTGGGGC
CCCAGCCGGGAGGGATCTGGCCCGGGTTGTGCTGGGGGCTAAATCCGGAC
GGAGAAGAAATGTAGCCCATCGGCCCCTGAACCGGCTGTGACATGCCGCG
TGGTCGCTGGGGGCCATCGTTCGGGTGGCCCTGGAACTGGTCCGCAAGGG
TGACGTCGGCCCCTGCGGGGAGATTGGGGTGTTGCCGCCCGCGATAGGCT
GGAAGTTCACCTGGCCGGGCTGAGATGTGATAAGGGGACGTCCCGGGGAT
GGAAGAGACGTCTGCCGCACGTGACCTAACTTACCAAGAGTAGGGCTTTG
CTGACCAGATGGCAGTTGACTAGACGGTACCTCCTCATCCTCGACACTGTC
AGGCTGAGATGGGTCGTGAGATTTCGCGGGCTTTTTGGTTTGGGGTGAGGC
AGTAGGCGAAGCTCCGTCTTGCCGCGAGAACCCGGAGCCGACAGGCTTTC
GGTTAGGAGTGATTCGGGTTGCGATTTGCGATCCACCAACGTCTGGGGAAG
ACGGCCGTGAGCGGTGGGGCGAATCTCTTGAAAGTGCGCTAGGACCCGAG
AAAACGTCATCTCCAGGTTGTTTGCCTATATTTCTATTCTCCGCATCTTGCGC
AGTAGCTGGTCGAATGTTGACTTGTGGTGGTCCTTGCTGAAACCCGAATTG
CGATCGTTGGCTAGGCGATATTGGTGTTCCGACTGCCTGGTTTTGGGAAGA
CACCGTTATTTCCGAAGGTCTCCGCATCATCACTTTTTGCGCGGTCCCAAGA
AATTGAGGCGATAAAGGCGACGTCATAGTCGGCTGAGCAGGTCCCGCGGG
TTGTCGTCCCTGTTGTCCTGGTATACCTTCCTGGACAGGAGGTTGTTGACCG
GCAAATATCGGATGCGGACCAAACTGCTGCCGGGAGGTGGCATCTGACCC
GGCCTAAACTGACTTGGTTGCATGACAAATTGCTGCGGCGGTCGTTGAGGC
TGCGGTGGTGCTTGTTGATTCTGCTGCTTCGACTCAGGAGGTTTGGATCCTT
GCTTATTGAAAAGTCCACCTAAAAATCCACCCATAGGTTTCTTCTCACGTTC
CTCCTCGACATTTTGGAGCTGCTGAGTTTCCGGAGGTCGCATTCCGCCGGT
ACTGCTTCGCACTATCCCCAGTGGCGGGCCTCCTAACGGACTTGGCTGCAT
CTGTCCTGTCTGTGTTCGCACCGGACCTTGCATTGAAGGTCTCGAAGGATA
CGCTGTGCTTGATTTAGCGGGTTGATCATCCTTCGCTCCCTCTTTCGCATTCC

CAACTCCCTTAATCATCCCCGTGAGCTCAGATAGCCGCTTCTTCATCGACAC
ATGCTCTGAGGTGTCATCGTGGTCAAACGAAGAACCAAGGTTGGACTTCT
GCTTCGGCTGGAATTTGTTATGGTTAGATGCCCCACCGAAGAACGAGCTCT
TTTCCTTCCCAGATGCACTTTGCTCGGACCCTTGATCTCCTGTGTTGCGACG
ATTAGTTAGACTAAAGAGTCCCGAACCTCGTCTGCCATCTTTAGGCCTTTCC
TCGCTAGCAGTACTTGAGACCGATACCTCATCAACATGAACTAATTGAAAAT
ACATTAGATACGTGGTCGGCCTCGAATCAGGTCAACTTACCGTCCATCTCCT
TACTAGGTCCGGCATCATGCGACCCCCTTCCAGCTTGGTTGGCTGCGTTATT
GGGTCCGCCCCGGAAAAGAGGATGGCCGGCTTGGTGTGGAGGTCCAGCTT
GACCTTGGTCATGGCCATGAGGAACAAAAATGTTATGCTTACGAAATCGCT
CCGCTGAAACCGGAGGTATACTATTGCTATTATTTCTCGGACGCGGTCCCGA
CGTACCTTGCCCCCTCCCTGACGGCAACGGATCCTCAGTTTCCTTGTCGTAC
GCTGCATAATAGCTAGGTTGTTGCTGCGATGGGCTATTTGCGCCCGGGCGG
GGTTTGTGCTGAATTAAAGGTTCTGAGAGATGAGACTCTTCTAGCTTCCATT
GACTGTTGGGAGGAAATTTCTGTATTGGGTTGCCGACCATTTGTGGGTTAA
AGGGTTGGCTGGGGTTTTGCATAGGCGGTGCGCCTGGTTGGCCTTGGAATT
GACCTTGAAATTGGTGATGCGGGCCAGCTGGTCCTCCACGCCCCATGAACA
TTTGCTGCGGGAATCCATTTGGCTGAGGTGAGCCTCTACCAACTGCATATCC
GTTGGGATTTTGAGGCTGACCATTTACATGATTAGGATGCTGCTGAGGCGCT
[truncated: 209,587 more chars]
